# Identification of Combinatorial Patterns of Post-Translational Modifications on Individual Histones in the Mouse Brain

**DOI:** 10.1371/journal.pone.0036980

**Published:** 2012-05-31

**Authors:** Ry Y. Tweedie-Cullen, Andrea M. Brunner, Jonas Grossmann, Safa Mohanna, David Sichau, Paolo Nanni, Christian Panse, Isabelle M. Mansuy

**Affiliations:** 1 Medical Faculty, Brain Research Institute, University of Zürich and Department of Biology, ETH Zürich, Zürich, Switzerland; 2 Functional Genomics Centre Zürich, University of Zürich/ETH Zürich, Zürich, Switzerland; Ludwig-Maximilians-Universität München, Germany

## Abstract

Post-translational modifications (PTMs) of proteins are biochemical processes required for cellular functions and signalling that occur in every sub-cellular compartment. Multiple protein PTMs exist, and are established by specific enzymes that can act in basal conditions and upon cellular activity. In the nucleus, histone proteins are subjected to numerous PTMs that together form a histone code that contributes to regulate transcriptional activity and gene expression. Despite their importance however, histone PTMs have remained poorly characterised in most tissues, in particular the brain where they are thought to be required for complex functions such as learning and memory formation. Here, we report the comprehensive identification of histone PTMs, of their combinatorial patterns, and of the rules that govern these patterns in the adult mouse brain. Based on liquid chromatography, electron transfer, and collision-induced dissociation mass spectrometry, we generated a dataset containing a total of 10,646 peptides from H1, H2A, H2B, H3, H4, and variants in the adult brain. 1475 of these peptides carried one or more PTMs, including 141 unique sites and a total of 58 novel sites not described before. We observed that these PTMs are not only classical modifications such as serine/threonine (Ser/Thr) phosphorylation, lysine (Lys) acetylation, and Lys/arginine (Arg) methylation, but also include several atypical modifications such as Ser/Thr acetylation, and Lys butyrylation, crotonylation, and propionylation. Using synthetic peptides, we validated the presence of these atypical novel PTMs in the mouse brain. The application of data-mining algorithms further revealed that histone PTMs occur in specific combinations with different ratios. Overall, the present data newly identify a specific histone code in the mouse brain and reveal its level of complexity, suggesting its potential relevance for higher-order brain functions.

## Introduction

The acquisition and storage of information in memory require specific long-lasting changes in gene expression. These changes have been proposed to depend upon chromatin remodelling, and on site-specific and dynamic post-translational modifications (PTMs) of histone proteins in brain cells. In the chromatin, histones and DNA are tightly associated and form nucleosomes. Each nucleosome contains a histone octamer composed of two heterodimers of the core histones H2A and H2B, and a tetramer of the core histones H3 and H4, around which 146 base pairs of DNA are wrapped [1]. Nucleosomes are separated from each other by a short stretch of internucleosomal DNA bound to the linker histone H1. All core histones, their variants and linker H1 are known to be subjected to PTMs [2], which are covalent modifications that can occur on selective amino acids and that can be induced and erased by complexes of chromatin-modifying enzymes. Whilst over 200 histone PTMs have been identified in the brain [3], only few have been well characterised and shown to be linked to specific brain functions [4]. The role/s of most, however, remain unknown.

Some of the best characterised PTMs on histones have been studied in the brain in relation to memory formation and include Lys acetylation, Ser/Thr phosphorylation, and Lys/Arg methylation. In particular, the level of phosphorylation on H3S10, acetylation on H3K9, H3K14, H4K5, H4K8 and methylation on residues including H3K4 and H3K9, have all been shown to be correlated with some forms of memory [5]–[7]. These PTMs are established by an ensemble of enzymes comprising histone acetyltransferases (HATs), protein kinases and histone methyltransferases (HMTs), and are erased by histone deacetylases (HDACs), protein phosphatases and histone demethylases (HDMs) [4], [8]. Further to being induced directly by specific enzymes, histone PTMs are also subjected to multiple *cis* and *trans* regulatory cross-talk. This results in the establishment of specific combinations of PTMs thought to form a gene-specific ‘histone code’ that determines the level of transcriptional activity [1], [9]. The impact of histone PTMs on gene activity is, in part, mediated by specific reader and effector proteins that can bind in the presence (or absence) of specific PTMs. For instance, the HDM JMJD2A associates with chromatin only when H4K20 is methylated, but not when neighbouring sites are phosphorylated or acetylated [10]. Selective interactions between neighbouring histones are also regulated by PTMs. Thus, residues 16–20 of H4 can interact with two acidic patches on the adjacent C-terminus of H2A, but this interaction is prevented by H4K16 acetylation by KAT8, leading to an increase in the local accessibility of the DNA to the transcriptional machinery [4], [11].

Determining the ensemble of histone PTMs and identifying their different combinations and cross-talk are essential steps for the understanding of gene regulation. This is particularly relevant to the brain, because many brain functions are regulated by gene expression. Histone PTMs contribute to this dynamic regulation of gene expression, as they can alter the accessibility of DNA to the transcriptional machinery by opening or closing the chromatin [12]. Over the past decade, great progress has been made in the identification and mapping of histone PTMs, and in the characterisation of the enzymes that catalyse them [13]. Mass spectrometry (MS) has been particularly instrumental [14], [15], and led to the detection of many PTMs on individual histones, and to the generation of comprehensive maps of histone PTMs in several species [2], [16], [17]. However, a drawback of conventional ‘bottom-up’ MS methods is that proteins are typically digested into short peptides prior to MS. This generates complex biological samples that contain a mixture of short peptides coming from independent copies of the same proteins. This means that PTMs occurring simultaneously on a given histone cannot be determined because the peptides generated from this histone cannot be identified [18]. Recently, however, this limitation has been circumvented by new MS techniques, specifically electron transfer dissociation (ETD) and electron capture dissociation [19], [20]. These techniques have allowed the analysis of long peptides (>20 aa), and have led to the analyses of PTMs co-occurring on individual histones [21]–[23]. However despite these techniques, little progress has been made in the identification of PTMs *in vivo*. Most studies to date have been carried out in cultured cells. They have therefore been limited by *in vitro* conditions that often do not fully reflect the *in vivo* situation, particularly in relation to the adult brain, where most neurons are postmitotic.

Here, we report on a novel approach that captures the status of histone PTMs and their combinatorial patterns directly in the adult brain in mice. This approach is based on ETD and collision induced dissociation (CID) high mass accuracy MS/MS using an Orbitrap XL-ETD. It allowed us to detect and identify multiple PTMs on individual histones in the adult mouse brain, and determine their combinations and association rules. Furthermore, this approach newly revealed the presence of atypical PTMs such as Ser and Thr acetylation, and Lys propionylation, butyrylation and crotonylation on specific histones, and of several novel motifs flanking phosphorylation, methylation and acetylation sites. The ensemble of these data provides important new insight into the histone code in the adult brain that may be relevant for complex brain functions.

## Materials and Methods

### C57BL/6 mice

Brain tissue was isolated from adult C57BL/6 mice, from a wide range of age (3–24 months) to optimise PTM coverage. Mice were maintained in standard conditions under a reversed light cycle (dark phase, 7:00 to 19:00). All experiments were carried out in accordance with the guidelines and regulations of the Cantonal Veterinary Office, Zürich.

### Isolation of nuclei and histones from the mouse brain

Histones were isolated from the adult mouse brain as previously described [2]. Briefly, nuclei were obtained by homogenising brain tissue in lysis buffer (Sigma Nuclei Pure isolation kit) containing 1× protease cocktail inhibitor and phosphatase inhibitor cocktail I and II (Sigma). Homogenates were mixed with 2 volumes of 1.8 M sucrose and layered on top a 1.8 M sucrose gradient. Nuclei were pelleted by centrifugation at 30,000 g for 45 min, and then snap frozen at −80°C until analysed. Isolated nuclei were resuspended in 0.2 M H_2_SO_4_ and incubated for >2 h at 4°C with end-over-end rotation, prior to centrifugation at 16,000 g for 10 min to remove nuclear debris. The supernatant was transferred to a new tube and histones were precipitated by the drop-wise addition of 1/3 volume trichloroacetic acid (TCA) followed by a 30-min incubation on ice. After centrifugation at 16,000 g for 10 min, the pellet containing histones was washed twice with ice-cold acetone and centrifuged a second time. The pellet was then dissolved in H_2_O, sonicated for 10 min, spun at 16,000 g for 10 min and the supernatant loaded on an Agilent C8 column attached to an Agilent HP1100 binary HPLC system. Histone variants were separated and eluted with the following gradient: 0–5 min, 0% solvent B; 5–15 min, 0–35% Buffer B; 15–25 min, 35% Buffer B, 25–75 min, 35–65% Buffer B. Buffer A was 5% acetonitrile (ACN) in 0.1% TFA and Buffer B was 90% ACN in 0.1% TFA.

### In-solution digestion of histone proteins

For digestion, RP-HPLC fractions of histones were collected, lyophilised and dissolved in the appropriate buffer; they were not alkylated with iodoacetamide prior to digestion. Glu-C digestion was carried out in 25 mM ammonium carbonate, pH 7.8, for 18 h with Glu-C (Roche, Switzerland) at 24°C (1∶20 enzyme∶substrate). CNBr digestion was carried out in 0.1 M HCl for 18 h with CNBr (100× molar excess of CNBr, Sigma) at 24°C. Semi-tryptic digestion was carried out in 50 mM ammonium bicarbonate, pH 8.0. The samples were heated at 60°C for 15 min prior to digestion with trypsin (Promega, USA) at 37°C (1∶200 enzyme∶substrate) for 2 h. AspN and chymotrypsin digestion was carried out in 100 mM Tris-HCl, 10 mM CaCl_2_, pH 7.8, for 25 h with AspN (Roche, Switzerland) or chymotrypsin (Roche, Switzerland) at 25°C (1∶200 enzyme:substrate). Digestions were stopped by lowering the pH<3 by the addition of TFA to a final concentration of 0.1%.

### SCX fractionation of peptides (prior to ETD/CID-MS/MS analysis)

ACN was added to peptide digests to a final concentration of 25% and loaded onto a 4.6×200 mm polySULFOETHYL aspartamide A column (PolyLC, USA) on an Agilent HP1100 binary HPLC system. Peptides were eluted with an increasing KCl gradient (0 to 105 mM over 30 min and 105 mM to 350 mM over the following 20 min) in 10 mM KH_2_PO_4_, 25% ACN, pH 3 [24]. Fractions were then lyophilised to remove ACN, desalted with Sep-Pak reversed-phase cartridges (Waters, UK) and lyophilised dry prior to analysis by ETD/CID-MS/MS.

### Phosphopeptide enrichment

Peptides from semi-tryptic digests were enriched for phosphopeptides with immobilised metal affinity chromatography (IMAC) or titanium dioxide (TiO_2_) as previously described [2], [24].

### Synthetic peptides

Synthetic peptides were ordered from 21^st^ Century Biochemicals, Boston, MA, USA. Lyophilised peptides were re-suspended in 50% ACN, 0.1% FA and analysed by direct infusion.

### MS analysis

Samples were analysed on a calibrated hybrid LTQ-Orbitrap XL-ETD mass spectrometer (Thermo Scientific). Peptides were resuspended in 3% ACN and 0.2% formic acid and loaded on a 10 cm fused silica column packed with 3 µm 200 Å pore size C18 resin. Peptides were eluted via an ACN gradient of 5–30% ACN over 35 min and 30–80% ACN over the subsequent 13 min in a buffer containing 0.2% formic acid at flow rate of 200 nl/min. One scan cycle comprised of a full scan MS survey spectrum, followed by up to 6 sequential CID and ETD MS/MS scans on the 3 most intense signals above a threshold of 500. Full-scan MS spectra (300–2000 m/z) were acquired in the FT-Orbitrap at a resolution of 60 000 at 400 m/z, while CID and ETD MS/MS spectra were recorded in the linear ion trap. CID was performed with a target value of 1e4 in the linear trap, collision energy at 35 V, Q value at 0.25 and activation time at 30 ms. AGC target values were 5e5 for full FTMS scans and 1e4 for ion trap MSn scans. The ETD anion target value was set at 1e6 and activation time at 100 ms. Supplementary activation was employed to enhance the fragmentation efficiency for 2+ precursors and charge state dependent ETD time enabled. The ETD reaction time was 120 ms and isolation width was 2 m/z. For all experiments, dynamic exclusion was used with 1 repeat count, 30 s repeat duration, and 10 s exclusion duration. Samples were acquired using internal lock mass calibration set on m/z 429.0887 and 445.1200. The synthetic peptides were analysed by direct infusion on an LTQ-Orbitrap XL-ETD and a LTQ-Orbitrap Velos-ETD. For every peptide, fragmentation was performed by CID and ETD, and spectra acquired in both the linear ion trap and the FT-Orbitrap. Twenty scans were collected for every acquisition. For CID/ETD fragmentation and for the acquisition of ion trap MS/MS spectra the same parameters used for LC-MS analysis were applied. For FT MS/MS spectra an AGC target value of 5E5 and an injection time of 200 ms were set.

### Database analysis and identification of modified residues

MS and MS/MS data were processed into the Mascot generic format (mgfs) files and searched using Mascot version 2.2. The monoisotopic masses of 2+ or more charged peptides were searched with a peptide tolerance of 8 ppm and a MS/MS tolerance of 0.6 Da for fragment ions using a mouse protein database from the European Bioinformatics Institute (EBI, 48,564 sequences) concatenated to the same database in reverse to allow calculation of the false discovery rate. PTMs searched included phosphorylation (STY, variable), acetylation (N-term protein, S, T, Y and K, variable), mono-, di- and tri-methylation (R and K, variable) and GG and LRGG tags characteristic of ubiquitination (K, variable, semi-tryptic search). Only peptides with a maximum of 2 (3 for semi-tryptic digests) missed cleavage sites were allowed in database searches. Positive identification of phosphorylated, acetylated, methylated or ubiquitinated peptides was performed using a variety of strict criteria including manual inspection of spectra. Only peptides with the Mascot parameters bold-red, rank 1, and a peptide expect score of less than 0.05 were considered. All single hit peptides containing a PTM only observed once were discarded. Spectra from all peptides containing PTMs were manually validated for site placement and ambiguous sites indicated by parentheses. The confirmation of modification sites was primarily based on the presence of site-specific singly or doubly charged b/y and c/z type fragment ions (b/c and y/z ions generated by fragmentation between two potentially modified residues). Relative intensity of essential diagnostic fragment ions was checked in MS/MS spectra. Rules for increased or decreased peptide fragmentation probability were taken into account (enhanced fragmentation on the N-terminal side of proline and the C-terminal side of aspartic acid; reduced fragmentation on the C-terminal side of proline). All identified peptides with PTMs and their respective spectra are listed in Table S1, Table S2 and Figure S5.

### Bioinformatics

Motif-X [25] was used to extract PTM motifs and align peptides. WebLogo [26] was used to generate frequency plots of amino acids flanking a given PTM. Predmod was used to predict sites of histone acetylation [27]. A script written in Matlab was used to align peptides. This allowed the generation of tables with PTM counts for each modified residue on each histone, and of figures showing PTM patterns. For computational pattern analysis, data was pre-processed, and analysed using the Apriori algorithm and plotted as heat charts using in-house written software. Specifically, the list of all histone peptides with PTMs was parsed to extract the minimal consensus length for each peptide. In addition, all peptides shorter than 21 amino acids were disregarded for the analyses of long-range combinatorial PTM patterns. Peptides were then converted to a text-numeric format with PTMs indicated by specific letters, and amino acids by a combination of the number of the residue in the protein sequence and its corresponding standard amino acid abbreviation. In the next step, only residues with unambiguous PTMs were extracted, and those without precise site assignment were ignored and the respective residues treated as unmodified. The resulting data was stored in a matrix where each row corresponded to an identified peptide and each column to a residue (with or without PTMs). The matrix was then hashed to integrate values and fed into the Apriori algorithm [28]. The Apriori algorithm produced output containing ‘frequent itemsets’, which in our case were frequent combinations of modified residues. For these itemsets, the support was calculated by dividing the number of peptides containing a certain combination of modified and unmodified residues through the total number of peptides. Itemsets were classified as frequent if their minimal support was greater than 20%. Frequent itsemsets were written to an output file and then used to generate association rules. Association rules reflect the probability that the occurrence of an itemset X, i.e. a certain combination of modified and unmodified residues, in a transaction, i.e. a given histone peptide, leads to the occurrence of itemset Y∶X → Y. This probability is called confidence, and is calculated as follows: conf(X → Y) = {supp(X) U supp(Y)}/{sup(X)}. The itemset is split into two sub itemsets X and Y, the support of the itemset X and (X U Y) is then looked up. The confidence of a rule is then calculated by dividing the support of (X U Y) by the support of X. Software written in C++ was used to re-apply the Apriori implementation by Ferenc Bodon to our needs [29]. All source code of the software is available at http://histone-modifications.origo.ethz.ch. To visualise patterns of PTMs, the generated association rules were plotted using heatmaps and line diagrams.

## Results and Discussion

### An ETD workflow to determine combinatorial patterns of PTMs on histones

**Figure 1:**
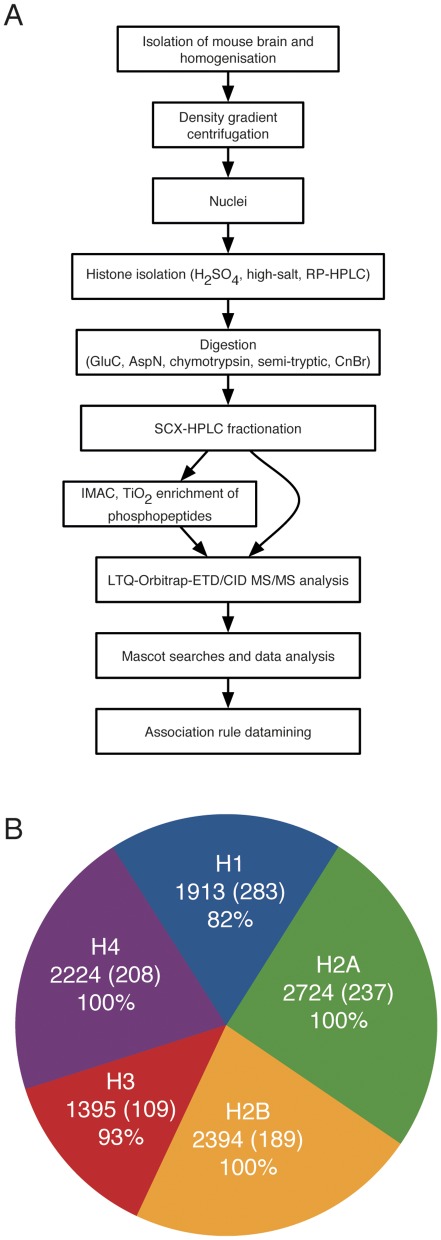
Proportion of detected histone peptides in the adult mouse brain. **A**) Workflow for the isolation and analysis of long histone peptides from the mouse brain. **B**) Number of peptides identified for each histone subtype, in brackets the number of unique peptides identified and typical sequence coverage observed.

Using a ‘bottom-up’ approach, we recently generated a map of PTMs that exist on histone proteins in the adult mouse brain [2]. To expand this map and examine the combinatorial patterns of PTMs on individual histones, we developed a proteomic approach that exploits liquid chromatography and CID/ETD-MS. A workflow was established consisting of the isolation of histone subtypes by RP-HPLC, followed by their digestion into large peptide fragments and the separation of the peptides by on-line RP-HPLC directly into a quadrupole ion trap MS (Orbitrap-XL) with ETD and CID fragmentation (Figure 1A). To obtain long histone peptides needed to identify PTMs occurring simultaneously on individual histones, a multi-enzyme strategy based on GluC, AspN and chymotrypsin was used. These enzymes cut, respectively, C-terminally of Asp and Glu, N-terminally of Asp, and C-terminally of Tyr, Phe and Trp. They generated peptides of an average length of 25 aa for GluC, 27 aa for AspN, and 26 aa for chymotrypsin, which greatly facilitated the analysis of combinatorial patterns of PTMs. Such analysis is otherwise not possible to the same degree with small peptides generated by classical enzymes such as trypsin or by cyanogen bromide (CNBr). PTMs on these peptides were analysed using ETD, a method of peptide fragmentation based on the transfer of electrons to positively charged peptides, providing better backbone fragmentation and thereby allowing the analysis of longer peptides than classical CID MS (typically 14 aa versus 26 for ETD). ETD was used on a hybrid LTQ-Orbitrap-XL machine, which records the mass of each eluting peptide with high accuracy, and thus makes the assignment of overall PTM states possible. Peptide fragmentation via ETD/CID also allowed us to determine the exact residues with precision, and the high accuracy data meant that the FDR rate at the peptide level was less than 2%. Furthermore, only PTMs observed on at least two independent instances were kept for analysis. A total of 160 sites could not be localised to a specific residue and are indicated by parentheses in Table S1.

A total of 10,646 non-unique peptides (1020 unique) derived from all histone subtypes were identified. Of these peptides, a comparable number were derived from H1, H2B and H4 (1913–2394), while there was ∼25% more peptides (2724) derived from H2A and ∼25% less (1395) from H3. These different proportions of peptides likely reflect the workflow we utilised and differences in the amenability of the derived peptides to MS/MS analysis rather than the different size of the histones, since most histones are approximately 120 aa long (except H1 subtypes which are about 210 aa). Of the detected peptides, 1475 contained between one and seven PTMs, which represented a total of 141 unique sites distributed across the different isoforms as follow: 27 on H1, 28 on H2A, 25 on H2B, 30 on H3, and 31 on H4 respectively. While 83 of these sites had previously been reported [2], 58 were novel. Modification site occupancy was observed to vary greatly, with some residues being modified by a certain PTM on all peptides, whilst others were modified on less than 1% of observed peptides (see Table 1). This is consistent with the varying role these PTMs play and the idea that some histone PTMs in the brain are activity-dependent [30].

**Table 1:** Summary of PTM abundance.

A) H1 canonical																					
Residue	-	S1	T3	S4	K16	S/T17	K21	K25	S28	K33	T34	S35	K45	S50	K51	K96	K109	S112	K167	S171	S186
**N-term Acetylation**	58%																				
**Phosphorylation**		22%	12%	27%		24%			7%		5%	6%								100%	100%
**Acetylation**					1%							<1%	1%	<1%	<1%			2%	3%		
**Monomethylation**							2%	1%							1%						
**Dimethylation**					2%					1%						1%	2%				
**Crotonylation**															1%						

B) H2A canonical																					
Residue	-	S1	R3	K5	K9	R71	K74	T79	K95	K99	K119	K125	K127	K129							
**N-term Acetylation**	87%																				
**Phosphorylation**		3%																			
**Acetylation**				18%	4%		1%	100%	<1%		<1%		3%	11%							
**Monomethylation**						2%			<1%	1%	<1%										
**Dimethylation**					2%				1%	16%											
**Trimethylation**			12%																		
**Butyrylation**									<1%												
**Crotonylation**									<1%			1%									

C) H2B canonical																					
Residue	K5	S6	T7	K11	K12	K15	K16	T19	K20	K34	K57	S75	K108	S114	S114	K116	K120	K125			
**Phosphorylation**		5%	2%									4%		2%							
**Acetylation**	1%			1%	27%	28%	16%	5%	22%		1%		1%		<1%	<1%	2%	1%			
**Monomethylation**									1%	1%											
**Trimethylation**					2%																
**Propionylation**																					
**Crotonylation**													1%								

D) H3 canonical																					
Residue	K9	S10	K14	K18	K23	K27	S31	K36	K79	T80	R83	T107	K115	K122	R134						
**Phosphorylation**		10%					11%					13%									
**Acetylation**	28%		81%	9%	43%	11%				1%			13%	4%							
**Monomethylation**	22%							11%	21%		5%				1%						
**Dimethylation**	39%					74%		74%	12%		2%										
**Trimethylation**			10%			11%		5%													
**Butyrylation**													31%								
**Crotonylation**														3%							

D) H4																					
Residue	-	S1	R3	K5	K8	K12	K16	R17	R19	K20	R23	S47	K77	K79	K91						
**N-term Acetylation**	93%																				
**Phosphorylation**		2%										5%									
**Acetylation**				13%	22%	27%	46%			4%			2%	2%	1%						
**Monomethylation**			8%				1%	1%	3%	12%	3%										
**Dimethylation**			2%					1%	5%	46%	4%										
**Trimethylation**			3%	1%			3%		1%	9%	1%										
**Propionylation**							1%														
**Crotonylation**															1%						

The site, type and level of modification site occupancy of each PTM is indicated for each histone. For each PTM, the level of modification site occupancy is calculated by dividing the number of instances that a PTM was detected by the number of instances that a given amino acid was observed, providing an estimation of the relative abundance of each PTM. Sites of phosphorylation, which were detected only in IMAC/TiO_2_ enriched fractions, are not listed. Residues are numbered starting with the first residue after the cleaved methionine. Canonical H1 (**1A**), H2A (**1B**), H2B (**1C**), **H3** (**1D**), and H4 (**1E**) histones are shown which represent sequences common across all subtypes.

### General patterns of PTMs

**Figure 2:**
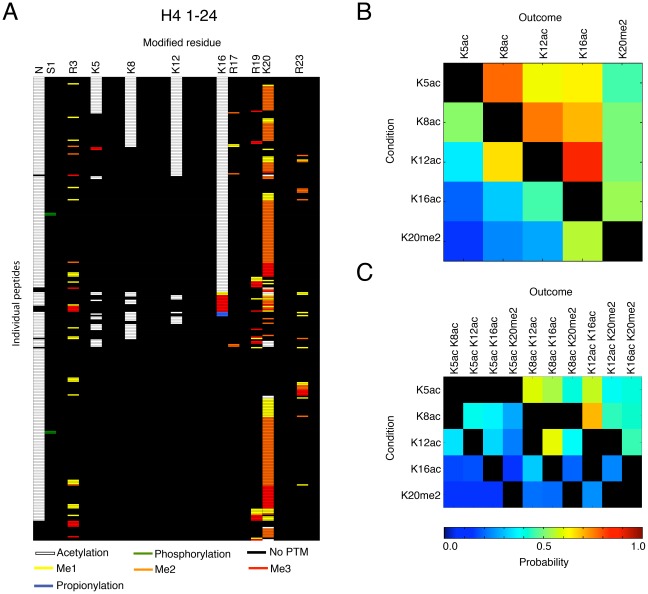
Multiple PTMs occur in distinct combinations on H4. **A**) Table depicting each of the 304 combinatorial codes identified on H4_1–24_ by ETD-MS. The number of each residue carrying a PTM is indicated at the top and each line represents an individual peptide. Probability of co-occurrence of (**B**) individual PTMs and (**C**) individual PTMs with groups of PTMs, on H4_1–23_ determined by an association rule data-mining algorithm. The condition (left rows) is when a specific PTM is observed on H4, and the outcome (top columns) is the probability (indicated by a heat plot) that a second or several PTM(s) are observed at the same time on the same histone molecule.

**Figure 3:**
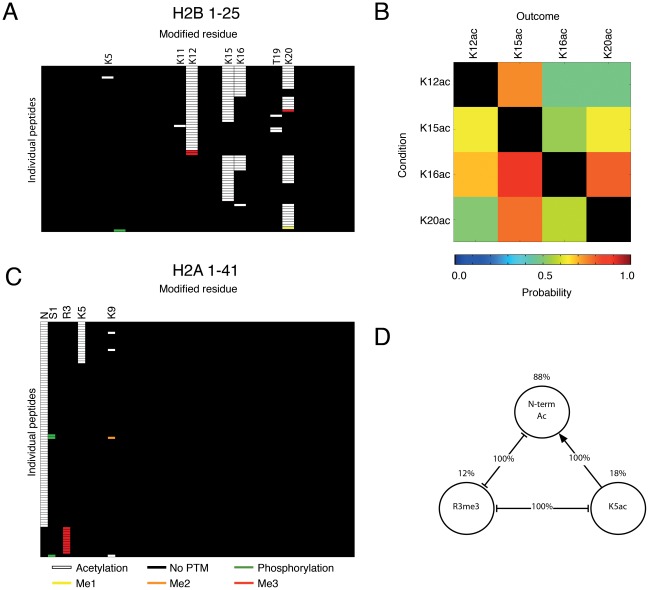
Combinatorial patterns on H2A and H2B. Summary of N-terminal H2B and H2A combinatorial codes identified using ETD-MS. Each line represents an individual H2B_1–25_ (**A**) or H2A_1–41_ (**C**) peptide. Probability of co-occurrence of individual PTMs on H2B_1–25_ determined by an association rule data-mining algorithm (**B**). The condition (left rows) is when a specific PTM is observed on H2A/H2B, and the outcome (top columns) is the probability (indicated by a heat plot) that a second or several PTM(s) are observed at the same time on the same histone molecule. Diagram depicting the relationship between three PTMs on H2A_1–41_ (**D**). N-term Ac, K5ac and R3Me3 were either mutually exclusive or always seen in combination. The frequency of each PTM is indicated by the % above the circle, connections indicate the derived rule and its % occurrence. For instance, when K5ac was present, N-term ac was also observed in 100% of cases (connected by arrow), but R3me3 was never observed. When R3me3 was observed, N-term acetylation or K5ac was never observed (100% of cases), suggesting mutual exclusion of N-term acetylation and R3me3, potentially due to steric hindrance or conformational changes induced by each PTM.

We next determined the general pattern of histone PTMs. We chose to examine H4, H2A and H2B because these core histones are known to be implicated in memory processes and can yield almost the entire N-terminal tails with our methodology. The ensemble of site occupancy (defined by the residues carrying a PTM) of N-terminal tails corresponding to the first 24, 25 and 41 residues of H4, H2A and H2B (H4_1–24_, H2A_1–25_ and H2B_1–41_ respectively) was determined (Figure 2 and 3). For each of these tails, site occupancy showed a high degree of specificity. Thus, acetylated amino acids on H4_1–24_, H2A_1–25_ and H2B_1–41_ were not randomly distributed, but were localised in an ordered manner (see white bars indicating acetylation on Figure 2A and 3A, C). For instance, H4_1–24_ peptides carrying an acetylated K16 were found to be systematically acetylated on K12, K8 and K5, but peptides without K16 were rarely acetylated on other lysines (see below section on H4).

### Histone H4 combinatorial patterns

**Figure 4:**
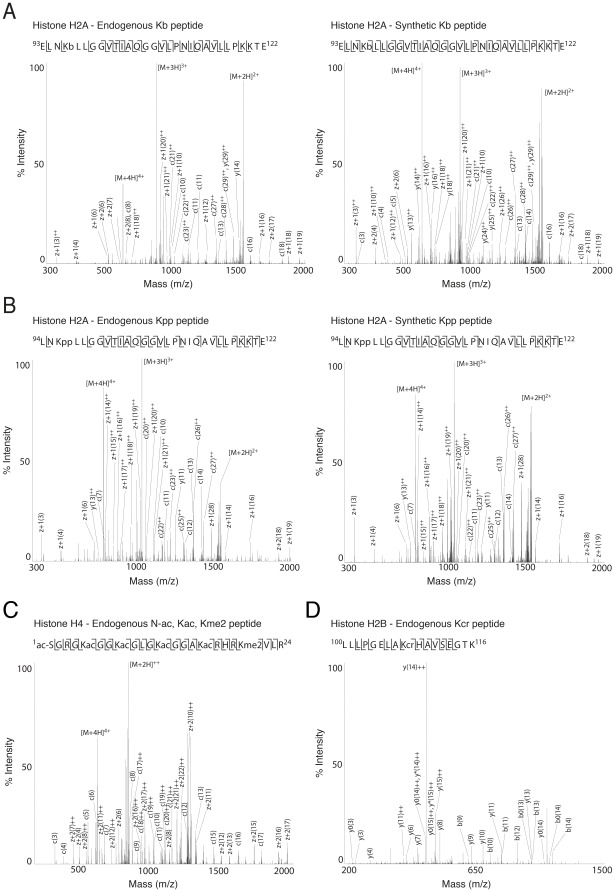
A,B) Mass spectra of identified endogenous peptides with lysine propionylation and butyrylation and their synthetic counterparts. Major peaks are labelled in the mass spectra and the fragment ions indicated in the peptide sequence using standard Mascot nomenclature [56]. **A**) A novel site of lysine butyrylation on residue K95 of H2A. **B**) A novel site of lysine propionylation on residue K95 of H2A. **C**) Highly modified peptides that were detected using ETD-MS/MS included the N-terminal peptide from H4 ac-SGRGKacGGKacGLGKacGGAKacRHRKme2VLR, which contains 5 sites of acetylation and 1 site of dimethylation **E**) A novel site of lysine crotonylation on residue K108 of H2B.

Histone H4 is the most amenable to detailed MS analysis because it has only one variant, and is therefore easier to study and analyse. The analyses of the combinatorial patterns of PTMs on H4 showed that 97% of all PTMs are in the first 20aa N-terminal fragment (Table 1A), consistent with previous observations [1], [2]. To look at this region more closely, we next analysed H4 peptides obtained from digestion with GluC, a protease that can preserve almost the entire H4 tail (see Figure 4C). A total of 304 distinct H4_1–24_ peptides could be identified. On these peptides, 10 novel PTMs could be detected in addition to all other already known PTMs. Furthermore, while every lysine and arginine in this region was found to be modified at least once, a subset of these residues was more frequently modified, consistent with a previous report [23]. The most frequent PTM was lysine acetylation, which occurred most commonly on K16 (46%), K12 (27%), K8 (22%) and K5 (13%). Acetylation was present within the tail, and also at its N-terminus. While only 7% of modified H4 tails were not acetylated at the N-terminus, lysines within the tail were acetylated even in the absence of N-terminus acetylation. Interestingly, methylation of these residues was also observed but in only 1-4% of the detected peptides. In contrast, K20 was the most highly methylated residue (67% methylated), followed by R3 (13%) and R19 (9%). K20 was only acetylated in 4% of the cases (Table 1 and Figure 2A). Figure 2A shows the combination of PTMs on H4 in a map depicting each detected peptide and its modifications.

To derive rules for the combinatorial pattern of PTMs on H4_1–24_ peptides, we next applied frequent itemset mining using the Apriori algorithm [31] and association rule mining (Figure 2B). The goal was to determine whether existing rules [1] could be confirmed and/or whether new rules could be extracted. Upon first observation, an ordered and hierarchical profile of acetylation could be identified on residues K5, K8, K12 and K16 (Figure 2A), and was confirmed by association rules extracted from the analyses. For instance, the presence of K5ac (left column Figure 2B) was highly predictive of K8ac, K12ac, K16ac or K20me2 (top row Figure 2B). Further, the analysis of the probability of certain PTMs to predict groups of other PTMs showed that K8ac (left column, Figure 2C) is more likely to exist when K12 and K16 are both acetylated (top row Figure 2C). This rule corroborates a zip model of acetylation suggested for H4 in earlier MS and Edman sequencing analyses [32], [33]. In this initial model, H4 acetylation was proposed to occur from C- to N-terminus, while de-acetylation would occur in the opposite direction (from N- to C-terminus). Our data extend this model by revealing new combinations, and also by showing that the combinatorial patterns of PTMs on H4 are more flexible than initially proposed. For instance, they revealed that acetylation of K5, K8 and K12 can occur in the absence of other PTMs, albeit at low level. They also demonstrated that these residues can be acetylated in the absence of K20me2, and that K20me1/2/3 can occur without any neighbouring acetylation. This therefore suggests that K20 methylation does not interfere with the enzymes that regulate other PTMs such as HATs or HDACs in agreement with other studies of the H4 interactor Suv4-20 [34]. Finally, our analyses also showed that acetylation of H4 is influenced by serine phosphorylation. Thus, peptides with phosphorylated S1 do not carry any acetylation on neighbouring K5, K8 or K12 residues, but only at more distal residues such as K16, which is acetylated in 50% of H4pS1 peptides. These results corroborate previous findings in yeast suggesting that S1 phosphorylation antagonises overall H4 acetylation [35].

### Histone H2A and H2B combinatorial patterns

We next assessed whether similar patterns of PTMs also exist on other core histones, and examined in particular H2 subtypes. Using the same approach, we looked at the first 41 residues on the N-terminus of H2A (H2A_1–41_), and the first 25 residues of H2B (H2B_1–25_), which are known to carry a large proportion of PTMs [2]. A total of 104 peptides carrying PTMs were detected for H2A, and 77 peptides for H2B. In addition, 207 modified peptides corresponding to the C-terminus proximal end of histone H2A (H2A_93–123_) were identified. In 86% of the cases in which a modified H2A_93–123_ peptide was observed, K99 was dimethylated. However, no other residue was modified at a frequency high enough for pattern analysis (Figure S4). In contrast to H4, only four residues were found highly modified on H2B_1–25_ peptides, specifically K15 (62%), K20 (52%), K12 (49%) and K16 (31%), which were acetylated. Acetylation and phosphorylation were also observed on other residues, however only at low frequency (Figure 3A and Table 1). Moreover unlike H4, H2B had a lower level of specificity of site occupancy reflected by less ordered positioning of acetyl groups. Nevertheless, it appears that one of the central residues K12, K15 and K20 needs to be modified first, for flanking sites at residue K5, K11, K23 or K24 to be modified as well, consistent with previous observations [33]. Multiple associations between the acetylated residues could also be observed (Figure 3A/B). For instance, the presence of K16ac was highly predictive of K15ac and K20ac on the same peptide, and of K12ac as well, but to a lower degree. The presence of acetylated K12, K15 or K20 however did not necessarily imply the presence of K16ac, suggesting a unidirectional interaction.

When compared to H2B and H4, H2A was modified mainly within the first 10 N-terminal residues (Table 1). N-terminal acetylation was particularly abundant and occurred on 88% of all modified H2A_1–41_ peptides, followed by K5 acetylation (18%) and R3 trimethylation (12%). The main associations that could be extracted on H2A were between N-terminal acetylation, K5ac and R3me3. K5ac was only detected on N-terminally acetylated peptides. In contrast, acetylation at either or both of these sites and R3me3 were mutually exclusive (See Figure 3CD). Interestingly, no PTM was observed on any residue between R17 and R71 (Table 1 and Figure 3C), suggesting that this part of the protein may be inaccessible to modifying enzymes potentially due to conformational hindrance.

### Analysis of acetylation, methylation and phosphorylation motifs

We next analysed the sequences surrounding the identified acetylation, methylation and phosphorylation sites, and examined over-represented motifs using Motif-X [25]. Because of the high proportion of lysine and arginine in histones, we used a histone-specific reference database for normalisation. Three major motifs for lysine acetylation sites could be identified and included kAXXK, GkXXXK (the GK motif, where X is any residue), and kXXS (Table 2 and Figure S2). In addition to these motifs, the amino acids surrounding acetylation sites were found to be preferentially glycine, lysine and alanine, as well as serine and threonine, two residues that can be phosphorylated (Figure S1), in line with what has been previously suggested [36], [37]. Subsequently, we compared the identified acetylation sites with those predicted by the recently released prediction tool PredMod [27]. No significant match between the experimentally identified and the predicted acetylation sites could be seen (Figure S3), suggesting that the current tools are limited in their predictive capacity and thus, that other tools may be needed. This could be due to the fact that acetylation depends on the secondary structure of proteins and not solely on primary sequence, as is the case, for instance, for ubiquitination [38], and/or that the motifs governing a given PTM need to be better understood to implement more realistic models [39].

**Table 2:** Summary of overrepresented motifs at PTM sites.

PTM	Motif	Motif in literature	Binding motif in literature
pS	.....sP....	ERK1, ERK2 Kinase substrate motif	WW domain binding motif
pS	.E…s.....	Unknown	Unknown
pT	...E.t.....	Unknown	Unknown
Kac	.....kA..K.	Enrichment for small residues [1], k...K, K...k [2]	In yeast, Gcn5 binds to the H3 peptide K-S-T-G-G-K-A-P-R-K-Q [3]
Kac	.....k..S..	Enrichment for phosphorylatable residues [1]	H3K14 acetylation by Gcn5 is increased when H3S10 is phosphorylated [4]
Kac	....Gk...K.	Gk [5], [6], Enrichment for small residues [1], k...K, K...k [2]	In yeast, Gcn5 binds to the H3 peptide K-S-T-G-G-K-A-P-R-K-Q [3]
Kme	.....k...R.	RG-rich motif [7], [8]	Unknown
Kme	.....kG....	RG-rich motif [7], [8]	Unknown
Kme	.....k..S..	Serine at +2 position [8]	Unknown
Kme	...G.k.....	RG-rich motif [7], [8]	Unknown

A total of ten motifs were detected, flanking either acetylation, methylation or phosphorylation sites. For each motif, bibliographic references for the same or similar motifs are listed, as well as whether it is known to function as a binding motif. Unknown motifs were novel at the time of writing. Many of the identified motifs are novel and distinct from human motifs in the human protein reference database (HPRD). In support of our dataset many of the sites also matched known motifs for the enzymes that catalyse these PTMs, and/or known binding motifs that require modified residues.

In addition to acetylation, lysine methylation was also found to be enriched in specific motifs, in particular in KmeXXR, KmeG, KmeXXXS and GXXKme (Figure S2). These motifs are comparable to those described in previous large-scale methylation studies, which revealed a preference for methylation on arginine in glycine/arginine-rich (GR-rich) protein domains [40], [41]. This apparent overlap of lysine and arginine methylation motifs is intriguing since these PTMs are induced by distinct histone methyltransferases belonging to different families of enzymes, the PRMT1 family, and the SET-DOMAIN-containing protein family and the non-SET-domain proteins DOT1/DOT1L, respectively [42], [43]. In addition to the three GR-rich sequence motifs above, serine upstream of acetylation (+3 position) was also found to be enriched. Such enrichment may be comparable to the over-representation of serine in the +2 position previously reported which is proposed to enable hydrogen bonding between substrate and enzyme during methylation [41]. Thus taken together, these results suggest that both the +2 and +3 positions may be favourable for the action of methyltransferases, and promote histone methylation. Finally, in addition to acetylation and methylation motifs, 3 phosphorylation motifs were also detected, two of which are novel (EXXXpS, EXpT) (Table 2 and Figure S2).

### Novel PTMs on brain histones: Acetylation of serine/threonine, and propionylation, butyrylation and crotonylation of lysine residues

**Figure 5:**
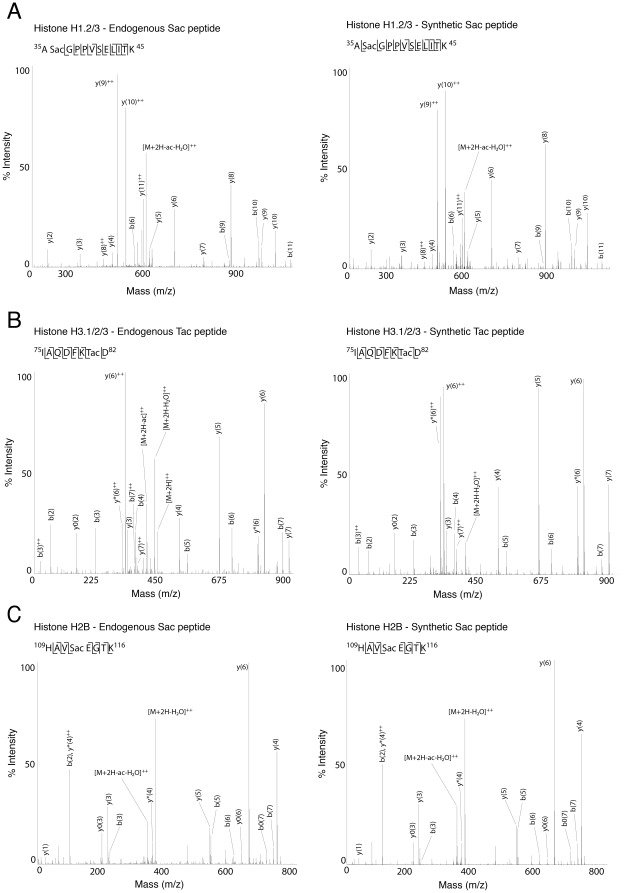
Mass spectra of identified endogenous peptides with serine and threonine acetylation and their synthetic counterparts. Major peaks are labelled in the mass spectra and the fragment ions indicated in the peptide sequence using standard Mascot nomenclature [56]. **A**) A novel site of serine acetylation on residue S35 of H1. **B**) A novel site of threonine acetylation on residue T80 of H3. The reporter ion characteristic of the loss of an acetyl group (−80 Da) from the parent ion is indicated. **C**) A novel site of serine acetylation on S112 histone H1.

**Figure 6:**
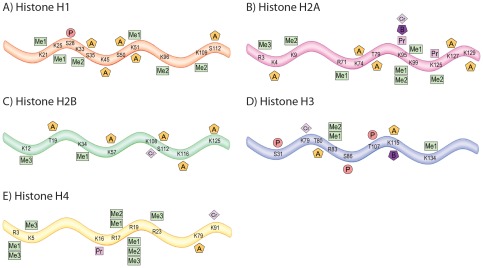
All novel histone PTMs. Summary of all novel PTMs identified on H1 (**A**), H2A (**B**), H2B (**C**), H3 (**D**) **and H4** (**E**). Sites of PTMs are indicated by A for acetylation, B for butyrylation, Cr for crotonylation, Me1, Me2 and Me3 for mono-, di- and trimethylation, P for phosphorylation and Pr for propionylation. Residues are numbered starting with the first residue after the cleaved methionine. Canonical H1, H2A, H2B and H3 histones are shown which represent sequences common across all subtypes.

In addition to already known PTMs, our analyses revealed the existence of other less common PTMs on histones. One of these novel PTMs is Ser/Thr acetylation, a modification that can prevent the phosphorylation of these residues and thus interfere with kinase/phosphatase pathways [44]. A total of 3 serines were found to be acetylated on H1 isoforms (S35/S36 on H1.2/H1.3, S50/51 on H1.4/H1.2/H1.3, S112/113 on H1.4/H1.5/1.3), and 1 on H2B (S112). Furthermore, 4 sites of threonine acetylation were also detected (T79 on H2A, T19 and S112 on H2B, and T80 on H3) (Figure 5, Figure 6, and Table S1). Overall, this data suggests that Ser/Thr acetylation is a fairly common PTM on histone proteins and potentially plays key functions in chromatin remodelling. Because it competes phosphorylation at Ser/Thr, it is likely to have a significant impact on brain functions given the critical role of phosphorylation throughout the cell in complex brain processes [45]. On histones the phosphorylation of H3S10 is a key phosphosite associated with learning and memory. Although not seen here, it will be particularly interesting as to whether this residue is a target of acetylation [6], [46].

Our data further revealed the existence of three atypical PTMs, propionylation, butyrylation and crotonylation of lysine residues on histones in the mouse brain. Originally discovered in human HeLa cells [47], [48], some of these PTMs have subsequently been reported on histones in a range of cell lines [49], but have not yet been described *in vivo*. Our data revealed that H4 (K16) and H2A (K95 (see Figure 4B) and K125 in the isoform H2AV) are propionylated in the mouse brain (Table 1 and Table S1). Our analyses also showed sites of butyrylation on H2A (K95 (see Figure 4A)), and potentially on all H3 isoforms (K115). Crotonylation was also seen on H1 (K51), H2A (K95 and K125), H2B (K108, see Figure 4D), H3 (K122) and H4 (K91). H2AK125cr was previously shown in a human cell line. Our data demonstrates that along with several others, it also occurs in the mouse brain. Very little is known about these atypical PTMs, aside from a potential role for crotonylation in modulating X-linked gene expression in germ cells [48].

To further validate the presence of these novel PTMs, we synthesised peptides corresponding to S35ac on H1 (^35^ASacGPPVSELITK^45^), T80ac on H3 (^75^IAQDKTacD^82^), S112ac on H2B (^109^HAVSacEGTK^116^), K96b and K96pp on H2A (^93^ELNKbLLGGVTIAQGGVLPNIQAVLLPKKTE^122^ and ^94^LNKppLLGGVTIAQGGVLPNIQAVLLPKKTE^122^). Peptides were analysed by CID or ETD and their MS/MS spectra compared with those of the endogenous peptides (see Figure 5ABC and Figure 4AB). In each case, the spectrum of the synthetic peptide was highly similar to those from the endogenous peptide, strengthening the evidence that these novel PTM sites on histones are valid.

### Analysing memory-specific codes

The idea that epigenetic mechanisms play a role in higher-order brain functions such as memory formation was first proposed in 1984 by Francis Crick [50]. Since then, numerous studies have linked specific histone marks to different forms of learning in several species [51], [52]. These learning-induced codes are reminiscent of developmental processes such as the differentiation of stem cells, in that they are associated with the ability of cells to respond and adapt to their environment while at the same time, keeping a cellular memory of their previous activity [53]. In the brain, histone PTMs have been proposed to mark specific genes required for learning and memory formation. They may be selectively induced upon neuronal activation, and when needed, maintained over different phases of memory processing or reinstated to control gene expression. Furthermore, it is possible that different ‘codes’ are in use at different stages of development. The present approach provides a unique means to identify such memory-specific histone codes in different brain areas, and developmental stages, and determine the genes that they control. It may help better delineate the mechanisms underlying memory processes, and possibly bring novel evidence in favour of a potential involvement of epigenetic marks as memory tags. One potential means to achieve this goal would be the purification of synaptic plasticity genes [54] and their associated histones, before and after learning, followed by the determination of the combinatorial pattern/s of PTMs on the tails using the workflow described here. Such an *in vivo* approach would be highly valuable to connect the wealth of known histone PTMs to functional memory states. However, distinguishing biologically distinct states of chromatin from the data may be difficult due to the high complexity of PTMs. Thus while some PTMs may be associated with a gene activating state when combined with a certain set of modifications, they may be repressive when combined with another set [55]. To clarify such complexity, several recent studies have begun to use large-scale methods based on histone PTM-specific antibodies and chromatin immunoprecipitation sequencing (ChiP-Seq). They have allowed the identification of dozens of marks across the genome, and computationally found recurring combinations that could be turned into particular chromatin states. Similarly, the strategy here was to identify histones PTMs occurring in basal conditions in the mouse brain, and group these PTMs into common core codes. However, a major advantage of our proteomic approach over ChiP-Seq is that it allows the sampling of all histone PTMs in an unbiased fashion, and is not limited to only one PTM since it does not rely on any antibody. It therefore holds great promises for a thorough and complete delineation of the epigenetic underpinning of memory processes, and also of other functions of the nervous system.

### Conclusions

The present study reports the identification and analyses of histone PTMs in the adult mouse brain, and describes new rules for the combination of these PTMs on individual histones. Using MS, we detected 10,646 peptides from histones H1, H2A, H2B, H3, and H4, and identified 58 novel PTMs on these histones. While the majority of these PTMs were known modifications such as Ser/Thr phosphorylation, Lys acetylation or Lys/Arg methylation, several novel or atypical modifications were identified, specifically Ser/Thr acetylation, and Lys propionylation, butyrylation and crotonylation. Several of these novel sites of atypical PTMs were validated using synthetic peptides. The use of bioinformatic tools and algorithms further allowed us to determine the combinations of PTMs, and revealed novel combinatorial rules and possible cross-talk between PTMs on H2A, H2B and H4. The detection of these sets of known and novel PTMs and their combination was made possible by using GluC to digest histones into long peptides, and thereby maintain adjacent PTMs that co-exist on the same peptide. The identification of the PTMs on all resulting peptides relied on the use of ETD, an adapted mode of fragmentation for MS that is appropriate for analysing large peptides. Overall, the data provide important new insight into the nature of PTMs that constitute the histone code in the adult brain, and the rules that govern the various combinations of these PTMs. They represent an important step forward in the understanding of the epigenetic marking of brain cells.

## Supporting Information

Figure S1 **Frequency plot of the amino acids surrounding lysines (A) or arginines (B) across all histones in the mouse.** Frequency plot of the amino acids surrounding sites of modification detected in this study; acetylation of lysine (C), methylation of lysine (D) and arginine (E), and phosphorylation of Ser/Thr/Tyr (F). The frequency of amino acids surrounding the modified residue was different for each type of modification, and different to the typical amino acid frequency seen surrounding lysines and arginines across all histones. These different frequencies likely reflect the different enzymes that act on the sites.
(PDF)

Figure S2 **Using the web-based tool Motif-X we analysed the motifs surrounding modification sites on histones, revealing over-represented acetylation, methylation and phosphorylation motifs.** For the motif extraction, pS, pT or pY-centered 11 amino acid sequences were used. To minimise extraction of randomly occurring patterns, we utilised the Motif-X algorithm, which takes the background dataset into account for pattern extraction. Because the amino acid composition in histones is different to the average across all proteins, we used a reference database only containing histones.
(PDF)

Figure S3 **Comparison of predicted and experimentally observed acetylation sites.** Putative lysine acetylation sites (1st column) were predicted by the acetylation prediction tool PredMod to be acetylated (ac) or not (unmodified) (2nd column). The predicted results were compared to what we observed in our CID/ETD MS experiments (3rd column). The overlap of predicted and experimentally verified sites was calculated in percentage.
(PDF)

Figure S4 **207 peptides corresponding to the C-terminal proximal end of Histone H2A (H2A_93–121_) were identified via ETD MS.** In most cases only K99 was dimethylated, preventing the analysis of combinatorial patterns.
(PDF)

Figure S5 **MS/MS spectra and ion tables of all modified peptides, i.e. those with one or more PTMs, obtained from histone digests across all experiments.** The type of modification is indicated after the residue modified e.g. Sphospho for a phosphorylated serine. N-terminal acetylation is indicated by square brackets e.g. [Acetyl]. Ambiguous residues are indicated by parentheses e.g. (acetyl).
(PDF)

Table S1 A full list of all modified peptides derived from histones that were found in all experiments. In the peptide sequence the site/s of N-terminal acetylation are designated by “ac-”, phosphorylation by “p” before the modified residue, acetylation by “ac”, , mono-/di-/tri-methylation by “me1, me2 or me3” respectively, propionylation by pp, and butyrylation by b after the modified residue. If the site of modification is ambiguous and cannot be assigned to a single residue, the modification is enclosed in parenthesis e.g. (p)S. Numbering of residues for the sites of modifications is as listed in Uniprot where the cleaved Met is residue 1. Modifications in manuscript text follow the usual published standard of not counting the cleaved Met residue.
(PDF)

Table S2 A full list of all unmodified peptides derived from histones that were found in all experiments.
(PDF)
