# Supplementary material for: Identification of Combinatorial Patterns of Post-Translational Modifications on Individual Histones in the Mouse Brain
Source: PLoS One. 2012 May 31;7(5):e36980. doi: 10.1371/journal.pone.0036980 (PMC3365036; doi:10.1371/journal.pone.0036980)
Supplement: Figure S1 — Frequency plot of the amino acids surrounding lysines (A) or arginines (B) across all histones in the mouse. Frequency plot of the amino acids surrounding sites of modification detected in this study; acetylation of lysine (C), methylation of lysine (D) and arginine (E), and phosphorylation of Ser/Thr/Tyr (F). The frequency of amino acids surrounding the modified residue was different for each type of modification, and different to the typical amino acid frequency seen surrounding lysines and arginines across all histones. These different frequencies likely reflect the different enzymes that act on the sites. (PDF) [file pone.0036980.s001.pdf]

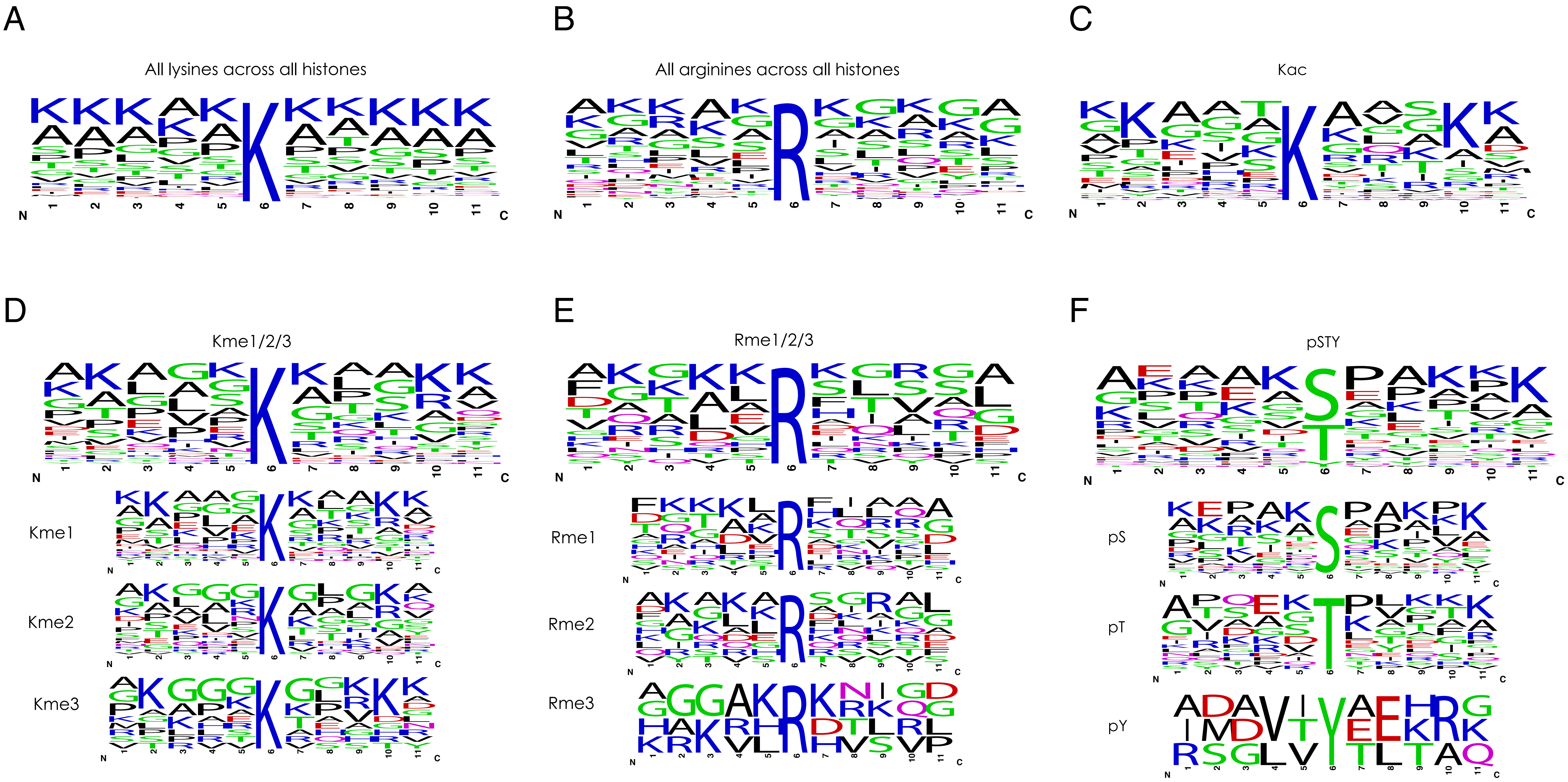
A
All lysines across all histones
B
All arginines across all histones
C
Kac
D
Kme1/2/3
Kme1
Kme2
Kme3
E
Rme1/2/3
Rme1
Rme2
Rme3
F
pSTY
pS
pT
pY

Figure S1
