# Supplementary material for: Identification of Combinatorial Patterns of Post-Translational Modifications on Individual Histones in the Mouse Brain
Source: PLoS One. 2012 May 31;7(5):e36980. doi: 10.1371/journal.pone.0036980 (PMC3365036; doi:10.1371/journal.pone.0036980)
Supplement: Figure S2 — Using the web-based tool Motif-X we analysed the motifs surrounding modification sites on histones, revealing over-represented acetylation, methylation and phosphorylation motifs. For the motif extraction, pS, pT or pY-centered 11 amino acid sequences were used. To minimise extraction of randomly occurring patterns, we utilised the Motif-X algorithm, which takes the background dataset into account for pattern extraction. Because the amino acid composition in histones is different to the average across all proteins, we used a reference database only containing histones. (PDF) [file pone.0036980.s002.pdf]

pS motifs

#	Motif	Motif Score	Foreground Matches	Foreground Size	Background Matches	Background Size	Fold Increase
1	.....sP....	3.42	7	27	18	344	4.95
3	.E...s.....	2.4	5	20	18	326	4.53

#1

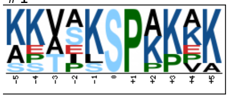

#2

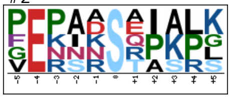

pT motifs

#	Motif	Motif Score	Foreground Matches	Foreground Size	Background Matches	Background Size	Fold Increase
1	...E.t.....	2.9	5	18	11	230	5.81

#1

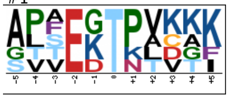

Kme1 motifs

#	Motif	Motif Score	Foreground Matches	Foreground Size	Background Matches	Background Size	Fold Increase
1	....Gk.....	1.63	5	29	40	694	2.99

#1

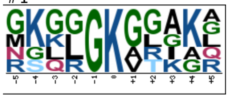

Kme2 motifs

#	Motif	Motif Score	Foreground Matches	Foreground Size	Background Matches	Background Size	Fold Increase
1	.....kG....	3.28	7	26	40	694	4.67
2	.....k...R.	2.68	5	19	33	654	5.22

#1

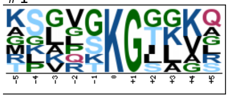

#2

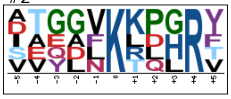

Kme motifs

#	Motif	Motif Score	Foreground Matches	Foreground Size	Background Matches	Background Size	Fold Increase
1	.....k...R.	2.34	7	43	34	694	3.32
2	.....kG....	2.34	7	36	39	660	3.29
3	.....k..S..	1.71	6	29	47	621	2.73
4	...G.k.....	1.72	5	23	40	574	3.12

#1

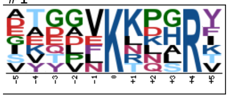

#2

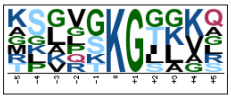

#3

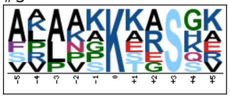

#4

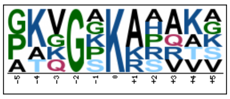

Kac motifs

#	Motif	Motif Score	Foreground Matches	Foreground Size	Background Matches	Background Size	Fold Increase
1	.....kA..K.	5.06	11	52	41	694	3.58
2	.....k..S..	1.94	8	41	50	653	2.55
3	....Gk...K.	4.24	5	33	9	603	10.15

#1

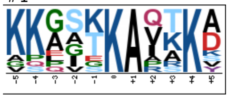

#2

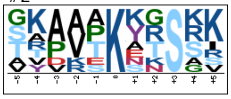

#3

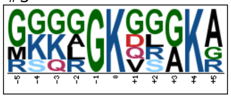

Figure S2
