# Supplementary material for: Identification of Combinatorial Patterns of Post-Translational Modifications on Individual Histones in the Mouse Brain
Source: PLoS One. 2012 May 31;7(5):e36980. doi: 10.1371/journal.pone.0036980 (PMC3365036; doi:10.1371/journal.pone.0036980)
Supplement: Figure S3 — Comparison of predicted and experimentally observed acetylation sites. Putative lysine acetylation sites (1st column) were predicted by the acetylation prediction tool PredMod to be acetylated (ac) or not (unmodified) (2nd column). The predicted results were compared to what we observed in our CID/ETD MS experiments (3rd column). The overlap of predicted and experimentally verified sites was calculated in percentage. (PDF) [file pone.0036980.s003.pdf]

H2A

Residue	Predicted by Predmod	Observed in our CID/ETD MS experiments
K5	ac	ac
K9	ac	ac
K13	ac	unmodified
K15	ac	unmodified
K36	unmodified	unmodified
K74	unmodified	ac
K75	unmodified	unmodified
K95	unmodified	ac
K118	unmodified	unmodified
K119	unmodified	ac
K125	ac	unmodified
K127	ac	ac
K129	ac	ac

Overlap between predicted and detected: 46%

H3

Residue	Predicted by Predmod	Observed in our CID/ETD MS experiments
K4	ac	unmodified
K9	ac	ac
K14	ac	ac
K18	ac	ac
K23	ac	ac
K27	ac	ac
K36	ac	unmodified
K37	ac	unmodified
K56	ac	unmodified
K64	unmodified	unmodified
K79	unmodified	unmodified
K115	unmodified	ac
K122	unmodified	ac

Overlap between predicted and detected: 54%

H2B

Residue	Predicted	Observed
K5	ac	ac
K11	ac	ac
K12	ac	ac
K15	unmodified	ac
K16	ac	ac
K20	ac	ac
K21	ac	unmodified
K23	ac	unmodified
K24	ac	unmodified
K27	unmodified	unmodified
K28	unmodified	unmodified
K30	unmodified	unmodified
K34	unmodified	unmodified
K43	unmodified	unmodified
K46	unmodified	unmodified
K57	unmodified	ac
K85	unmodified	unmodified
K108	unmodified	ac
K116	unmodified	ac
K120	unmodified	ac
K125	unmodified	ac

Overlap between predicted and detected: 57%

H4

Residue	Predicted	Observed
K5	ac	ac
K8	ac	ac
K12	ac	ac
K16	ac	ac
K20	unmodified	ac
K31	ac	unmodified
K44	unmodified	unmodified
K59	unmodified	unmodified
K77	unmodified	ac
K79	unmodified	ac
K91	unmodified	ac

Overlap between predicted and detected: 55%

Figure S3
