# Supplementary material for: Identification of Combinatorial Patterns of Post-Translational Modifications on Individual Histones in the Mouse Brain
Source: PLoS One. 2012 May 31;7(5):e36980. doi: 10.1371/journal.pone.0036980 (PMC3365036; doi:10.1371/journal.pone.0036980)
Supplement: Figure S4 — 207 peptides corresponding to the C-terminal proximal end of Histone H2A (H2A93–121) were identified via ETD MS. In most cases only K99 was dimethylated, preventing the analysis of combinatorial patterns. (PDF) [file pone.0036980.s004.pdf]

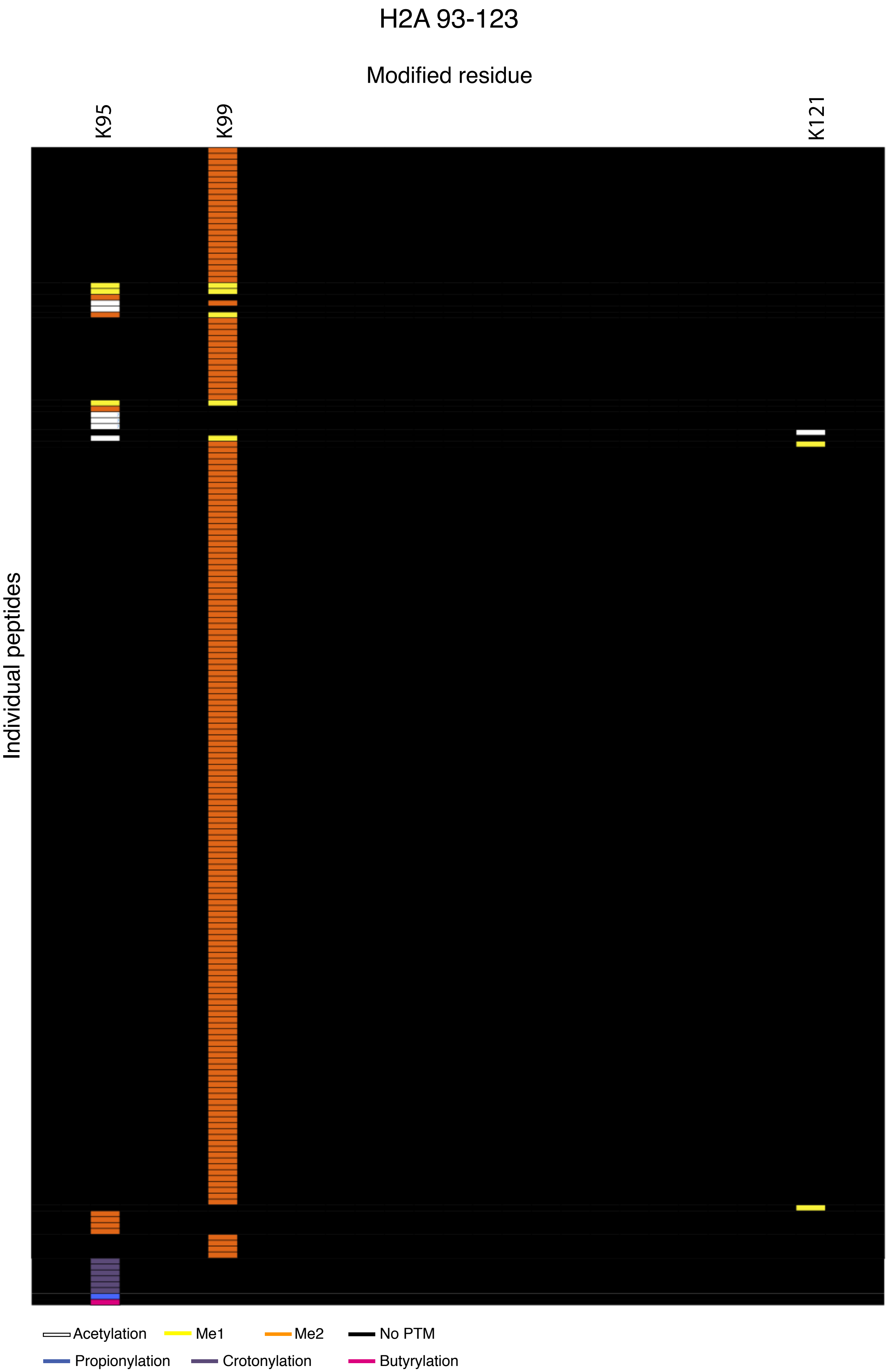
H2A 93-123
Modified residue
K95
K99
K121
Individual peptides
Acetylation
Me1
Me2
No PTM
Propionylation
Crotonylation
Butyrylation

Figure S4
