# Supplementary material for: Identification of Combinatorial Patterns of Post-Translational Modifications on Individual Histones in the Mouse Brain
Source: PLoS One. 2012 May 31;7(5):e36980. doi: 10.1371/journal.pone.0036980 (PMC3365036; doi:10.1371/journal.pone.0036980)
Supplement: Table S1 — A full list of all modified peptides derived from histones that were found in all experiments. In the peptide sequence the site/s of N-terminal acetylation are designated by “ac-”, phosphorylation by “p” before the modified residue, acetylation by “ac”, , mono-/di-/tri-methylation by “me1, me2 or me3” respectively, propionylation by pp, and butyrylation by b after the modified residue. If the site of modification is ambiguous and cannot be assigned to a single residue, the modification is enclosed in parenthesis e.g. (p)S. Numbering of residues for the sites of modifications is as listed in Uniprot where the cleaved Met is residue 1. Modifications in manuscript text follow the usual published standard of not counting the cleaved Met residue. (PDF) [file pone.0036980.s006.pdf]

uniprot accession/s for all possible isoforms	histone type	pep exp mz	pep exp mr	pep exp z	pep delta	pep length	pep miss	pep expect	peptide and site of PTM	enzyme
H10_MOUSE	Histone H1	646.3185	2581.2450	4	0.0091	22	3	1.20E-04	KSTDHPKYpSDMIVAAIQAEKNR	Semi-tryptic
H10_MOUSE	Histone H1	614.2946	2453.1493	4	0.0083	21	2	4.20E-06	STDHPKYpSDMIVAAIQAEKNR	Semi-tryptic
H10_MOUSE	Histone H1	737.8638	1473.7130	2	-0.0003	14	0	6.00E-03	ac-MTENSTSAPAAKPK	Semi-tryptic
H10_MOUSE	Histone H1	500.5992	1498.7758	3	0.0019	14	1	1.60E-04	ac-TENSTSAPAAKPKR	Semi-tryptic
H10_MOUSE	Histone H1	500.5992	1498.7758	3	0.0019	14	1	1.30E-04	ac-TENSTSAPAAKPKR	Semi-tryptic
H10_MOUSE	Histone H1	790.3808	1578.7470	2	0.0068	14	1	3.30E-04	ac-TENpSTSAPAAKPKR	Semi-tryptic
H10_MOUSE	Histone H1	527.2556	1578.7451	3	0.0048	14	1	4.70E-03	ac-TENpSTSAPAAKPKR	Semi-tryptic
H10_MOUSE	Histone H1	513.2496	1536.7269	3	-0.0027	14	1	4.10E-02	TENpSTSAPAAKPKR	Semi-tryptic
H10_MOUSE	Histone H1	672.3439	1342.6733	2	0.0005	13	0	5.60E-07	ac-TENSTSAPAAKPK	Semi-tryptic
H10_MOUSE	Histone H1	672.3439	1342.6732	2	0.0004	13	0	3.60E-04	ac-TENSTSAPAAKPK	Semi-tryptic
H10_MOUSE	Histone H1	672.3448	1342.6750	2	0.0022	13	0	5.20E-04	ac-TENSTSAPAAKPK	Semi-tryptic
H10_MOUSE	Histone H1	672.3441	1342.6736	2	0.0008	13	0	3.90E-03	ac-TENSTSAPAAKPK	Semi-tryptic
H10_MOUSE	Histone H1	672.3439	1342.6733	2	0.0005	13	0	5.60E-07	ac-TENSTSAPAAKPK	Semi-tryptic
H10_MOUSE	Histone H1	672.3439	1342.6732	2	0.0004	13	0	3.20E-04	ac-TENSTSAPAAKPK	Semi-tryptic
H10_MOUSE	Histone H1	672.3448	1342.6750	2	0.0022	13	0	5.30E-04	ac-TENSTSAPAAKPK	Semi-tryptic
H10_MOUSE	Histone H1	672.3441	1342.6736	2	0.0008	13	0	3.70E-03	ac-TENSTSAPAAKPK	Semi-tryptic
H10_MOUSE	Histone H1	712.3237	1422.6329	2	-0.0062	13	0	1.00E-02	ac-TENpSTSAPAAKPK	Semi-tryptic
H10_MOUSE	Histone H1	691.3224	1380.6303	2	0.0017	13	0	5.90E-07	TENpSTSAPAAKPK	Semi-tryptic
H10_MOUSE	Histone H1	691.3225	1380.6304	2	0.0018	13	0	2.80E-06	TENpSTSAPAAKPK	Semi-tryptic
H10_MOUSE	Histone H1	691.3199	1380.6253	2	-0.0033	13	0	7.90E-06	TENpSTSAPAAKPK	Semi-tryptic
H10_MOUSE	Histone H1	461.2169	1380.6290	3	0.0004	13	0	2.00E-02	TENpSTSAPAAKPK	Semi-tryptic
H10_MOUSE	Histone H1	712.3274	1422.6402	2	0.0011	13	0	7.20E-05	ac-TENpSTSAPAAKPK	Semi-tryptic
H10_MOUSE	Histone H1	672.3437	1342.6729	2	0.0001	13	0	1.90E-05	ac-TENSTSAPAAKPK	Semi-tryptic
H10_MOUSE	Histone H1	672.3445	1342.6744	2	0.0015	13	0	2.10E-04	ac-TENSTSAPAAKPK	Semi-tryptic
H10_MOUSE	Histone H1	672.3450	1342.6754	2	0.0026	13	0	2.40E-04	ac-TENSTSAPAAKPK	Semi-tryptic
H10_MOUSE	Histone H1	672.3440	1342.6734	2	0.0006	12	0	7.20E-03	ac-TENSTSAPAAKPK	Semi-tryptic
H10_MOUSE	Histone H1	672.3450	1342.6754	2	0.0026	12	0	1.80E-05	ac-TENSTSAPAAKPK	Semi-tryptic
H11_MOUSE	Histone H1	877.4964	4382.4458	5	0.0173	44	0	6.30E-04	ac-SETAPVAQAASTATEKPAAAKKTKKPAKAAAPRKKPAGPSVSEL	Chymotrypsin
H11_MOUSE	Histone H1	877.4961	4382.4440	5	0.0155	44	0	8.90E-04	ac-SETAPVAQAASTATEKPAAAKKTKKPAKAAAPRKKPAGPSVSEL	Chymotrypsin
H11_MOUSE	Histone H1	572.7687	2287.0455	4	-0.0042	22	1	1.40E-06	pSEpTAPVAQAASTATEKPAAAKK	Semi-tryptic
H11_MOUSE	Histone H1	1125.5553	2249.0960	2	0.0021	22	1	3.70E-03	ac-pSETAPVAQAASTATEKPAAAKK	Semi-tryptic
H11_MOUSE	Histone H1	720.6589	2158.9549	3	0.0001	21	0	3.60E-04	pSEpTAPVAQAASTATEKPAAAK	Semi-tryptic
H11_MOUSE	Histone H1	1021.5252	2041.0358	2	0.0031	21	0	1.10E-08	ac-SETAPVAQAASTATEKPAAAK	Semi-tryptic
H11_MOUSE	Histone H1	1061.5075	2121.0004	2	0.0014	21	0	7.00E-05	ac-pSETAPVAQAASTATEKPAAAK	Semi-tryptic
H11_MOUSE	Histone H1	1061.5085	2121.0024	2	0.0034	21	0	9.80E-03	ac-pSETAPVAQAASTATEKPAAAK	Semi-tryptic
H12_MOUSE	Histone H1	823.6756	4113.3417	5	0.0243	42	0	2.40E-02	ac-SEAAPAAPAAAPPAEKAPAKKKAAKKPAGVRRKASGPPVSEL	Chymotrypsin
H12_MOUSE	Histone H1	823.6732	4113.3294	5	0.0121	42	0	4.00E-02	ac-SEAAPAAPAAAPPAEKAPAKKKAAKKPAGVRRKASGPPVSEL	Chymotrypsin
H12_MOUSE	Histone H1	801.0591	4000.2591	5	0.0258	41	2	7.70E-03	ac-SEAAPAAPAAAPPAEKAPAKKKAAKKPAGVRRKASGPPVSE	GluC
H12_MOUSE	Histone H1	801.0552	4000.2397	5	0.0064	41	2	9.00E-03	ac-SEAAPAAPAAAPPAEKAPAKKKAAKKPAGVRRKASGPPVSE	GluC
H12_MOUSE	Histone H1	667.7169	4000.2577	6	0.0245	41	2	2.00E-02	ac-SEAAPAAPAAAPPAEKAPAKKKAAKKPAGVRRKASGPPVSE	GluC
H12_MOUSE	Histone H1	662.6945	1985.0618	3	0.0037	21	2	7.30E-05	ac-SEAAPAAPAAAPPAEKAPAKK	Semi-tryptic
H12_MOUSE	Histone H1	662.6938	1985.0595	3	0.0014	21	2	8.30E-03	ac-SEAAPAAPAAAPPAEKAPAKK	Semi-tryptic
H12_MOUSE	Histone H1	662.6939	1985.0599	3	0.0018	21	2	3.60E-02	ac-SEAAPAAPAAAPPAEKAPAKK	Semi-tryptic
H12_MOUSE	Histone H1	662.6945	1985.0618	3	0.0037	21	2	4.40E-05	ac-SEAAPAAPAAAPPAEKAPAKK	Semi-tryptic
H12_MOUSE	Histone H1	662.6938	1985.0595	3	0.0014	21	2	4.90E-03	ac-SEAAPAAPAAAPPAEKAPAKK	Semi-tryptic
H12_MOUSE	Histone H1	662.6939	1985.0599	3	0.0018	21	2	1.70E-02	ac-SEAAPAAPAAAPPAEKAPAKK	Semi-tryptic
H12_MOUSE	Histone H1	689.3510	2065.0312	3	0.0067	21	2	3.60E-02	ac-pSEAAPAAPAAAPPAEKAPAKK	Semi-tryptic
H12_MOUSE	Histone H1	689.3493	2065.0262	3	0.0017	21	2	3.30E-03	ac-pSEAAPAAPAAAPPAEKAPAKK	Semi-tryptic
H12_MOUSE	Histone H1	929.4914	1856.9682	2	0.0051	20	1	1.70E-03	ac-SEAAPAAPAAAPPAEKAPAK	Semi-tryptic
H12_MOUSE	Histone H1	929.4914	1856.9682	2	0.0051	20	1	1.20E-03	ac-SEAAPAAPAAAPPAEKAPAK	Semi-tryptic
H12_MOUSE	Histone H1	929.4905	1856.9664	2	0.0032	20	1	3.60E-04	ac-SEAAPAAPAAAPPAEKAPAK	Semi-tryptic
H12_MOUSE	Histone H1	969.4734	1936.9322	2	0.0027	20	1	3.10E-03	ac-pSEAAPAAPAAAPPAEKAPAK	Semi-tryptic
H12_MOUSE	Histone H1	745.8784	1489.7423	2	0.0011	16	0	4.00E-04	ac-SEAAPAAPAAAPPAEK	Semi-tryptic
H12_MOUSE, H13_MOUSE	Histone H1	555.5527	2218.1819	4	0.0097	21	3	4.40E-08	KApSGPPVSELITKAVAASKER	Semi-tryptic
H12_MOUSE, H13_MOUSE	Histone H1	521.6149	1561.8230	3	0.0001	14	2	2.50E-05	RKApSGPPVSELITK	Semi-tryptic
H12_MOUSE, H13_MOUSE	Histone H1	781.9201	1561.8257	2	0.0028	14	2	2.10E-03	RKApSGPPVSELITK	Semi-tryptic
H12_MOUSE, H13_MOUSE	Histone H1	521.6151	1561.8234	3	0.0005	14	2	6.10E-07	RKApSGPPVSELITK	Semi-tryptic
H12_MOUSE, H13_MOUSE	Histone H1	521.6159	1561.8260	3	0.0031	14	2	8.80E-07	RKApSGPPVSELITK	Semi-tryptic
H12_MOUSE, H13_MOUSE	Histone H1	521.6152	1561.8239	3	0.0010	14	2	1.00E-05	RKApSGPPVSELITK	Semi-tryptic
H12_MOUSE, H13_MOUSE	Histone H1	781.9197	1561.8248	2	0.0020	14	2	3.50E-04	RKApSGPPVSELITK	Semi-tryptic
H12_MOUSE, H13_MOUSE	Histone H1	499.6313	1495.8722	3	0.0000	14	2	8.60E-03	RK(me1)ASGPPVSELITK	Semi-tryptic
H12_MOUSE, H13_MOUSE	Histone H1	684.8907	1367.7669	2	0.0009	13	1	2.10E-07	KASGPPVSacELITK	Semi-tryptic
H12_MOUSE, H13_MOUSE	Histone H1	703.8689	1405.7233	2	0.0015	13	1	1.30E-07	KApSGPPVSELITK	Semi-tryptic
H12_MOUSE, H13_MOUSE	Histone H1	469.5813	1405.7222	3	0.0004	13	1	7.60E-05	KApSGPPVSELITK	Semi-tryptic
H12_MOUSE, H13_MOUSE	Histone H1	469.5825	1405.7256	3	0.0038	13	1	1.20E-03	KApSGPPVSELITK	Semi-tryptic
H12_MOUSE, H13_MOUSE	Histone H1	469.5807	1405.7202	3	-0.0016	13	1	8.20E-03	KApSGPPVSELITK	Semi-tryptic
H12_MOUSE, H13_MOUSE	Histone H1	703.8688	1405.7231	2	0.0013	13	1	4.80E-07	KApSGPPVSELITK	Semi-tryptic
H12_MOUSE, H13_MOUSE	Histone H1	703.8690	1405.7235	2	0.0017	13	1	1.50E-04	KApSGPPVSELITK	Semi-tryptic
H12_MOUSE, H13_MOUSE	Histone H1	703.8686	1405.7226	2	0.0008	13	1	3.40E-03	KApSGPPVSELITK	Semi-tryptic
H12_MOUSE, H13_MOUSE	Histone H1	620.8436	1239.6726	2	0.0015	12	0	1.30E-06	ASacGPPVSELITK	Semi-tryptic
H12_MOUSE, H13_MOUSE, H14_MOUSE	Histone H1	687.8430	1373.6715	2	0.0011	13	2	6.80E-03	GTGApSGSFKLNKK	Semi-tryptic
H12_MOUSE, H13_MOUSE, H14_MOUSE	Histone H1	458.8974	1373.6703	3	-0.0001	13	2	6.50E-09	GTGASGpSFKLNKK	Semi-tryptic
H12_MOUSE, H13_MOUSE, H14_MOUSE	Histone H1	687.8443	1373.6741	2	0.0037	13	2	2.50E-05	GTGASGpSFKLNKK	Semi-tryptic

uniprot accession/s for all possible isoforms	histone type	pep exp mz	pep exp mr	pep exp z	pep delta	pep length	pep miss	pep expect	peptide and site of PTM	enzyme
H12_MOUSE, H13_MOUSE, H14_MOUSE	Histone H1	687.8428	1373.6711	2	0.0007	13	2	2.70E-03	GTGApSGSFKLNKK	Semi-tryptic
H12_MOUSE, H13_MOUSE, H14_MOUSE	Histone H1	623.7952	1245.5758	2	0.0004	12	1	2.90E-03	GTGASGpSFKLNK	Semi-tryptic
H12_MOUSE, H13_MOUSE, H14_MOUSE	Histone H1	382.2352	1143.6838	3	-0.0025	11	0	1.70E-04	LITKAVAASKme1E	GluC
H12_MOUSE, H13_MOUSE, H14_MOUSE	Histone H1	586.8490	1171.6835	2	0.0023	11	0	6.80E-04	LITKAVAASKacE	GluC
H12_MOUSE, H13_MOUSE, H14_MOUSE	Histone H1	586.8474	1171.6803	2	-0.0009	11	0	1.70E-03	LITKAVAASKacE	GluC
H12_MOUSE, H13_MOUSE, H14_MOUSE	Histone H1	586.8492	1171.6838	2	0.0026	11	0	4.60E-07	LITKacAVAASKE	GluC
H12_MOUSE, H13_MOUSE, H14_MOUSE	Histone H1	586.8492	1171.6838	2	0.0026	11	0	1.00E-02	LITKacAVAASKE	GluC
H12_MOUSE, H13_MOUSE, H14_MOUSE	Histone H1	382.2372	1143.6898	3	0.0035	11	0	5.00E-05	LITKAVAASKme1E	GluC
H12_MOUSE, H13_MOUSE, H14_MOUSE	Histone H1	572.8518	1143.6891	2	0.0028	11	0	2.70E-04	LITKAVAASKme1E	GluC
H12_MOUSE, H13_MOUSE, H14_MOUSE	Histone H1	586.8516	1171.6886	2	0.0074	11	0	4.20E-03	LITKAVAASKacE	GluC
H12_MOUSE, H13_MOUSE, H14_MOUSE	Histone H1	586.8512	1171.6878	2	0.0066	11	0	6.40E-03	LITKacAVAASKE	GluC
H12_MOUSE, H13_MOUSE, H14_MOUSE	Histone H1	391.5702	1171.6889	3	0.0077	11	0	3.20E-05	LITKAVAASacKE	GluC
H12_MOUSE, H13_MOUSE, H14_MOUSE	Histone H1	391.5676	1171.6809	3	-0.0003	11	0	1.60E-04	LITKAVAASacKE	GluC
H12_MOUSE, H13_MOUSE, H14_MOUSE	Histone H1	599.8567	1197.6988	2	0.0020	10	0	1.10E-05	LITKAVAASKcrE	GluC
H12_MOUSE, H13_MOUSE, H14_MOUSE	Histone H1	599.8567	1197.6988	2	0.0020	10	0	4.20E-04	LITKAVAASKcrE	GluC
H12_MOUSE, H13_MOUSE, H14_MOUSE	Histone H1	607.8538	1213.6930	2	0.0012	10	0	1.40E-02	LITKAVAASKcrE	GluC
H12_MOUSE, H13_MOUSE, H14_MOUSE, H15_MOUSE, H1T_MOUSE	Histone H1	446.1840	890.3535	2	0.0000	9	0	3.50E-02	GTGASG(p)SFK	Semi-tryptic
H12_MOUSE, H15_MOUSE	Histone H1	346.2021	1035.5844	3	0.0002	9	3	3.00E-02	VAKpSPKKAK	Semi-tryptic
H12_MOUSE, H15_MOUSE	Histone H1	483.2805	964.5464	2	-0.0006	8	3	4.90E-03	KVAKpSPKK	Semi-tryptic
H13_MOUSE	Histone H1	713.0643	4272.3422	6	0.0064	43	0	6.50E-04	(p)SETAPAAPAAPAPVEKTPVKKKAKKTGAAAGKRKASGPPVSEL	Chymotrypsin
H13_MOUSE	Histone H1	706.7410	4234.4023	6	0.0222	43	0	4.20E-03	ac-SETAPAAPAAPAPVEKTPVKKKAKKTGAAAGKRKASGPPVSEL	Chymotrypsin
H13_MOUSE	Histone H1	706.7404	4234.3987	6	0.0186	43	0	5.80E-03	ac-SETAPAAPAAPAPVEKTPVKKKAKKTGAAAGKRKASGPPVSEL	Chymotrypsin
H13_MOUSE	Histone H1	1059.6084	4234.4045	4	0.0244	43	0	3.50E-02	ac-SETAPAAPAAPAPVEKTPVKKKAKKTGAAAGKRKASGPPVSEL	Chymotrypsin
H13_MOUSE	Histone H1	847.8879	4234.4033	5	0.0233	43	0	4.00E-02	ac-SETAPAAPAAPAPVEKTPVKKKAKKTGAAAGKRKASGPPVSEL	Chymotrypsin
H13_MOUSE	Histone H1	706.7397	4234.3947	6	0.0147	43	0	5.90E-05	ac-SETAPAAPAAPAPVEKTPVKKKAKKTGAAAGKRKASGPPVSEL	Chymotrypsin
H13_MOUSE	Histone H1	847.8857	4234.3920	5	0.0119	43	0	1.40E-03	ac-SETAPAAPAAPAPVEKTPVKKKAKKTGAAAGKRKASGPPVSEL	Chymotrypsin
H13_MOUSE	Histone H1	847.8862	4234.3945	5	0.0145	43	0	2.80E-02	ac-SETAPAAPAAPAPVEKTPVKKKAKKTGAAAGKRKASGPPVSEL	Chymotrypsin
H13_MOUSE	Histone H1	847.8845	4234.3863	5	0.0063	43	0	1.90E-04	ac-SETAPAAPAAPAPVEKTPVKKKAKKTGAAAGKRKASGPPVSEL	Chymotrypsin
H13_MOUSE	Histone H1	720.0656	4314.3496	6	0.0033	43	0	8.90E-03	ac-(p)SETAPAAPAAPAPVEKTPVKKKAKKTGAAAGKRKASGPPVSEL	Chymotrypsin
H13_MOUSE	Histone H1	825.2714	4121.3206	5	0.0246	42	2	3.70E-04	ac-SETAPAAPAAPAPVEKTPVKKKAKKTGAAAGKRKASGPPVSE	GluC
H13_MOUSE	Histone H1	825.2666	4121.2967	5	0.0007	42	2	5.60E-06	ac-SETAPAAPAAPAPVEKTPVKKKAKKTGAAAGKRKASGPPVSE	GluC
H13_MOUSE	Histone H1	841.2658	4201.2924	5	0.0301	42	2	7.20E-05	ac-SETAPAAPAAPAPVEKpTPVKKKAKKTGAAAGKRKASGPPVSE	GluC
H13_MOUSE	Histone H1	832.8582	4159.2545	5	0.0028	42	2	1.50E-04	(p)SETAPAAPAAPAPVEKTPVKKKAKKTGAAAGKRKASGPPVSE	GluC
H13_MOUSE	Histone H1	694.2172	4159.2598	6	0.0080	42	2	1.60E-04	SEpTAPAAPAAPAPVEKTPVKKKAKKTGAAAGKRKASGPPVSE	GluC
H13_MOUSE	Histone H1	825.2667	4121.2972	5	0.0012	42	2	3.30E-04	ac-SETAPAAPAAPAPVEKTPVKKKAKKTGAAAGKRKASGPPVSE	GluC
H13_MOUSE	Histone H1	825.2664	4121.2955	5	-0.0005	42	2	7.90E-04	ac-SETAPAAPAAPAPVEKTPVKKKAKKTGAAAGKRKASGPPVSE	GluC
H13_MOUSE	Histone H1	825.2669	4121.2982	5	0.0022	42	2	1.00E-03	ac-SETAPAAPAAPAPVEKTPVKKKAKKTGAAAGKRKASGPPVSE	GluC
H13_MOUSE	Histone H1	825.2672	4121.2995	5	0.0035	42	2	1.50E-03	ac-SETAPAAPAAPAPVEKTPVKKKAKKTGAAAGKRKASGPPVSE	GluC
H13_MOUSE	Histone H1	1031.3364	4121.3165	4	0.0205	42	2	2.20E-03	ac-SETAPAAPAAPAPVEKTPVKKKAKKTGAAAGKRKASGPPVSE	GluC
H13_MOUSE	Histone H1	825.2687	4121.3072	5	0.0112	42	2	5.30E-03	ac-SETAPAAPAAPAPVEKTPVKKKAKKTGAAAGKRKASGPPVSE	GluC
H13_MOUSE	Histone H1	687.8894	4121.2926	6	-0.0034	42	2	6.50E-03	ac-SETAPAAPAAPAPVEKTPVKKKAKKTGAAAGKRKASGPPVSE	GluC
H13_MOUSE	Histone H1	687.8900	4121.2965	6	0.0005	42	2	3.30E-02	ac-SETAPAAPAAPAPVEKTPVKKKAKKTGAAAGKRKASGPPVSE	GluC
H13_MOUSE	Histone H1	1051.3306	4201.2933	4	0.0310	42	2	4.90E-02	ac-SETAPAAPAAPAPVEKpTPVKKKAKKTGAAAGKRKASGPPVSE	GluC
H13_MOUSE	Histone H1	825.2681	4121.3040	5	0.0080	42	2	6.10E-04	ac-SETAPAAPAAPAPVEKTPVKKKAKKTGAAAGKRKASGPPVSE	GluC
H13_MOUSE	Histone H1	825.2680	4121.3038	5	0.0078	42	2	3.30E-03	ac-SETAPAAPAAPAPVEKTPVKKKAKKTGAAAGKRKASGPPVSE	GluC
H13_MOUSE	Histone H1	740.6947	2219.0624	3	-0.0015	21	2	4.80E-02	pSEpTAPAAPAAPAPVEKTPVKK	Semi-tryptic
H13_MOUSE	Histone H1	1008.5412	2015.0678	2	0.0104	20	1	2.30E-07	ac-SETAPAAPAAPAPVEKTPVK	Semi-tryptic
H13_MOUSE	Histone H1	1046.4889	2090.9632	2	-0.0058	20	1	3.20E-04	pSEpTAPAAPAAPAPVEKTPVK	Semi-tryptic
H13_MOUSE	Histone H1	503.7570	2010.9989	4	-0.0037	20	1	1.20E-02	SE(p)TAPAAPAAPAPVEKTPVK	Semi-tryptic
H13_MOUSE	Histone H1	1006.5107	2011.0068	2	0.0042	20	1	1.50E-03	pSETAPAAPAAPAPVEKTPVK	Semi-tryptic
H13_MOUSE	Histone H1	1046.4883	2090.9620	2	-0.0070	20	1	2.20E-03	pSEpTAPAAPAAPAPVEKTPVK	Semi-tryptic
H13_MOUSE	Histone H1	793.8779	1585.7413	2	0.0024	16	0	6.50E-05	pSETAPAAPAAPAPVEK	Semi-tryptic
H13_MOUSE	Histone H1	556.2394	1665.6963	3	-0.0089	16	0	6.20E-05	pSEpTAPAAPAAPAPVEK	Semi-tryptic
H13_MOUSE	Histone H1	556.2395	1665.6967	3	-0.0085	16	0	1.10E-04	pSEpTAPAAPAAPAPVEK	Semi-tryptic
H13_MOUSE	Histone H1	529.5834	1585.7284	3	-0.0105	16	0	4.30E-03	SE(p)TAPAAPAAPAPVEK	Semi-tryptic
H13_MOUSE	Histone H1	556.2397	1665.6972	3	-0.0080	16	0	6.20E-03	pSEpTAPAAPAAPAPVEK	Semi-tryptic
H13_MOUSE	Histone H1	529.5851	1585.7333	3	-0.0055	16	0	4.10E-02	SE(p)TAPAAPAAPAPVEK	Semi-tryptic
H13_MOUSE	Histone H1	793.8782	1585.7418	2	0.0030	16	0	9.30E-05	SE(p)TAPAAPAAPAPVEK	Semi-tryptic
H13_MOUSE	Histone H1	774.9004	1547.7863	2	0.0032	16	0	1.50E-04	ac-SETAPAAPAAPAPVEK	Semi-tryptic
H13_MOUSE, H14_MOUSE	Histone H1	477.7902	953.5658	2	0.0000	10	1	4.80E-02	KPAAAAGAKacK	Semi-tryptic
H13_MOUSE, H14_MOUSE	Histone H1	477.7902	953.5658	2	0.0000	10	1	2.40E-02	KPAAAAGAKacK	Semi-tryptic
H13_MOUSE, H14_MOUSE, H15_MOUSE	Histone H1	713.0837	4272.4584	6	0.0303	41	0	5.60E-10	KNNSRIKLGLKSLVSKGTLVQTKGTGASGSFKLNKKme2AASacGE	GluC
H13_MOUSE, H14_MOUSE, H15_MOUSE	Histone H1	611.3586	4272.4592	7	0.0311	41	0	4.70E-04	KNNSRIKLGLKSLVSKGTLVQTKGTGASGSFKLNKKme2AASacGE	GluC
H13_MOUSE, H14_MOUSE, H15_MOUSE	Histone H1	1069.1230	4272.4629	4	-0.0016	41	0	1.10E-02	KNNSRIKLGLKSLVSKGTLVQTKGTGASGSFKLNK(me2)K(me3)AASGE	GluC
H13_MOUSE, H14_MOUSE, H15_MOUSE	Histone H1	847.0888	4230.4079	5	-0.0097	41	0	7.40E-06	KNNSRIKLGLKSLVSKGTLVQTKme2GTGASGSFKLNKKAASGE	GluC
H13_MOUSE, H14_MOUSE, H15_MOUSE	Histone H1	706.0747	4230.4046	6	-0.0129	41	0	1.80E-05	KNNSRIKLGLKSLVSKGTLVQTKme2GTGASGSFKLNKKAASGE	GluC
H13_MOUSE, H14_MOUSE, H15_MOUSE	Histone H1	855.4996	4272.4617	5	0.0336	41	0	5.60E-06	KNNSRIKLGLKSLVSKGTLVQTKGTGASGSFKLNK(me2)K(me3)AASGE	GluC
H14_MOUSE	Histone H1	849.6684	4243.3056	5	0.0215	42	0	8.50E-04	ac-SETAPAAPAAPAPAEKpTPVKKKARKAAGGAKRKTSGPPVSEL	Chymotrypsin
H14_MOUSE	Histone H1	833.6751	4163.3389	5	0.0211	42	0	2.70E-03	ac-SETAPAAPAAPAPAEKTPVKKKARKAAGGAKRKTSGPPVSEL	Chymotrypsin
H14_MOUSE	Histone H1	841.2632	4201.2796	5	0.0061	42	0	4.90E-03	(p)SETAPAAPAAPAPAEKTPVKKKARKAAGGAKRKTSGPPVSEL	Chymotrypsin
H14_MOUSE	Histone H1	833.6723	4163.3251	5	0.0073	42	0	3.70E-03	ac-SETAPAAPAAPAPAEKTPVKKKARKAAGGAKRKTSGPPVSEL	Chymotrypsin
H14_MOUSE	Histone H1	811.0591	4050.2589	5	0.0252	41	2	2.50E-06	ac-SETAPAAPAAPAPAEKTPVKKKARKAAGGAKRKTSGPPVSE	GluC
H14_MOUSE	Histone H1	816.6567	4078.2473	5	-0.0177	41	2	2.30E-07	ac-SETAPAAPAAPAPAEKTPVKKKARKAAGGAKRKme2TSGPPVSE	GluC

uniprot accession/s for all possible isoforms	histone type	pep exp mz	pep exp mr	pep exp z	pep delta	pep length	pep miss	pep expect	peptide and site of PTM	enzyme
H14_MOUSE	Histone H1	811.0543	4050.2350	5	0.0013	41	2	3.50E-05	ac-SETAPAAPAAPAPAEKTPVKKKARKAAGGAKRKTSGPPVSE	GluC
H14_MOUSE	Histone H1	811.0558	4050.2426	5	0.0089	41	2	5.00E-05	ac-SETAPAAPAAPAPAEKTPVKKKARKAAGGAKRKTSGPPVSE	GluC
H14_MOUSE	Histone H1	811.0543	4050.2350	5	0.0013	41	2	1.10E-04	ac-SETAPAAPAAPAPAEKTPVKKKARKAAGGAKRKTSGPPVSE	GluC
H14_MOUSE	Histone H1	811.0554	4050.2408	5	0.0071	41	2	1.30E-03	ac-SETAPAAPAAPAPAEKTPVKKKARKAAGGAKRKTSGPPVSE	GluC
H14_MOUSE	Histone H1	811.0543	4050.2350	5	0.0013	41	2	4.30E-03	ac-SETAPAAPAAPAPAEKTPVKKKARKAAGGAKRKTSGPPVSE	GluC
H14_MOUSE	Histone H1	811.0547	4050.2373	5	0.0036	41	2	4.70E-03	ac-SETAPAAPAAPAPAEKTPVKKKARKAAGGAKRKTSGPPVSE	GluC
H14_MOUSE	Histone H1	811.0579	4050.2532	5	0.0195	41	2	9.20E-03	ac-SETAPAAPAAPAPAEKTPVKKKARKAAGGAKRKTSGPPVSE	GluC
H14_MOUSE	Histone H1	827.0518	4130.2224	5	0.0224	41	2	1.30E-02	ac-(p)SETAPAAPAAPAPAEKTPVKKKARKAAGGAKRKTSGPPVSE	GluC
H14_MOUSE	Histone H1	811.0569	4050.2481	5	0.0144	41	2	1.40E-02	ac-SETAPAAPAAPAPAEKTPVKKKARKAAGGAKRKTSGPPVSE	GluC
H14_MOUSE	Histone H1	952.5512	3806.1759	4	0.0117	39	1	8.10E-03	TAPAAPAAPAPAEKTPVKKKARK(me1)AAGGAKRKTSGPPVSE	GluC
H14_MOUSE	Histone H1	765.0399	3820.1633	5	-0.0166	39	1	1.60E-04	TAPAAPAAPAPAEKTPVKKKARKAAGGAKRKme2TSGPPVSE	GluC
H14_MOUSE	Histone H1	673.6581	2690.6033	4	0.0002	26	0	5.50E-04	KTPVKKKARKme1AAGGAKRKTSGPPVSE	GluC
H14_MOUSE	Histone H1	673.6586	2690.6054	4	0.0023	26	0	7.80E-04	KTPVKKKARKme1AAGGAKRKTSGPPVSE	GluC
H14_MOUSE	Histone H1	747.3948	2239.1625	3	0.0012	22	3	8.40E-03	SETAPAAPAAPAPAEKpTPVKKK	Semi-tryptic
H14_MOUSE	Histone H1	560.7977	2239.1616	4	0.0003	22	3	1.00E-02	SETAPAAPAAPAPAEKpTPVKKK	Semi-tryptic
H14_MOUSE	Histone H1	551.3084	2201.2047	4	-0.0009	22	3	1.00E-02	ac-SETAPAAPAAPAPAEKTPVKKK	Semi-tryptic
H14_MOUSE	Histone H1	571.3020	2281.1789	4	0.0071	22	3	2.40E-04	ac-SETAPAAPAAPAPAEKpTPVKKK	Semi-tryptic
H14_MOUSE	Histone H1	551.3084	2201.2047	4	-0.0009	22	3	8.00E-04	ac-SETAPAAPAAPAPAEKTPVKKK	Semi-tryptic
H14_MOUSE	Histone H1	580.7888	2319.1263	4	-0.0014	22	3	6.50E-05	(p)SETAPAAPAAPAPAEKpTPVKKK	Semi-tryptic
H14_MOUSE	Histone H1	747.3951	2239.1636	3	0.0023	22	3	2.40E-03	pSETAPAAPAAPAPAEKTPVKKK	Semi-tryptic
H14_MOUSE	Histone H1	560.7980	2239.1628	4	0.0015	22	3	3.40E-03	SETAPAAPAAPAPAEKpTPVKKK	Semi-tryptic
H14_MOUSE	Histone H1	704.6965	2111.0678	3	0.0015	21	2	1.30E-03	pSETAPAAPAAPAPAEKTPVKK	Semi-tryptic
H14_MOUSE	Histone H1	692.0435	2073.1086	3	-0.0020	21	2	2.30E-02	ac-SETAPAAPAAPAPAEKTPVKK	Semi-tryptic
H14_MOUSE	Histone H1	692.0466	2073.1181	3	0.0076	21	2	3.70E-02	ac-SETAPAAPAAPAPAEKTPVKK	Semi-tryptic
H14_MOUSE	Histone H1	692.0435	2073.1086	3	-0.0020	21	2	1.40E-03	ac-SETAPAAPAAPAPAEKTPVKK	Semi-tryptic
H14_MOUSE	Histone H1	718.7015	2153.0826	3	0.0057	21	2	1.80E-03	ac-SETAPAAPAAPAPAEKpTPVKK	Semi-tryptic
H14_MOUSE	Histone H1	718.7001	2153.0784	3	0.0016	21	2	5.20E-03	ac-SETAPAAPAAPAPAEKpTPVKK	Semi-tryptic
H14_MOUSE	Histone H1	692.0466	2073.1181	3	0.0076	21	2	6.20E-03	ac-SETAPAAPAAPAPAEKTPVKK	Semi-tryptic
H14_MOUSE	Histone H1	548.7634	2191.0244	4	-0.0082	21	2	8.90E-06	SEpTAPAAPAAPAPAEKpTPVKK	Semi-tryptic
H14_MOUSE	Histone H1	548.7639	2191.0267	4	-0.0060	21	2	7.40E-04	SEpTAPAAPAAPAPAEKpTPVKK	Semi-tryptic
H14_MOUSE	Histone H1	548.7630	2191.0229	4	-0.0097	21	2	2.80E-03	pSEpTAPAAPAAPAPAEKTPVKK	Semi-tryptic
H14_MOUSE	Histone H1	548.7635	2191.0249	4	-0.0078	21	2	4.00E-03	(p)SETAPAAPAAPAPAEKpTPVKK	Semi-tryptic
H14_MOUSE	Histone H1	704.6967	2111.0682	3	0.0019	21	2	3.00E-03	pSETAPAAPAAPAPAEKTPVKK	Semi-tryptic
H14_MOUSE	Histone H1	1077.5471	2153.0796	2	0.0028	21	2	4.30E-05	ac-pSETAPAAPAAPAPAEKTPVKK	Semi-tryptic
H14_MOUSE	Histone H1	992.4951	1982.9757	2	0.0044	20	1	1.40E-04	pSETAPAAPAAPAPAEKTPVK	Semi-tryptic
H14_MOUSE	Histone H1	1032.4718	2062.9290	2	-0.0086	20	1	2.00E-02	pSEpTAPAAPAAPAPAEKTPVK	Semi-tryptic
H14_MOUSE	Histone H1	676.0018	2024.9835	3	0.0015	20	1	3.10E-03	ac-SETAPAAPAAPAPAEKpTPVK	Semi-tryptic
H14_MOUSE	Histone H1	516.7399	2062.9307	4	-0.0070	20	1	2.60E-03	pSEpTAPAAPAAPAPAEKTPVK	Semi-tryptic
H14_MOUSE	Histone H1	661.9997	1982.9772	3	0.0058	20	1	4.70E-03	pSETAPAAPAAPAPAEKTPVK	Semi-tryptic
H14_MOUSE	Histone H1	688.6528	2062.9365	3	-0.0012	20	1	3.70E-02	pSEpTAPAAPAAPAPAEKTPVK	Semi-tryptic
H14_MOUSE	Histone H1	992.4945	1982.9744	2	0.0030	20	1	4.80E-05	pSETAPAAPAAPAPAEKTPVK	Semi-tryptic
H14_MOUSE	Histone H1	661.9984	1982.9734	3	0.0020	20	1	1.40E-02	pSETAPAAPAAPAPAEKTPVK	Semi-tryptic
H14_MOUSE	Histone H1	973.5164	1945.0182	2	0.0026	20	1	1.50E-07	ac-SETAPAAPAAPAPAEKTPVK	Semi-tryptic
H14_MOUSE	Histone H1	973.5169	1945.0193	2	0.0037	20	1	6.40E-07	ac-SETAPAAPAAPAPAEKTPVK	Semi-tryptic
H14_MOUSE	Histone H1	973.5152	1945.0159	2	0.0003	20	1	3.00E-06	ac-SETAPAAPAAPAPAEKTPVK	Semi-tryptic
H14_MOUSE	Histone H1	1013.5000	2024.9854	2	0.0035	20	1	4.80E-05	ac-SETAPAAPAAPAPAEKpTPVK	Semi-tryptic
H14_MOUSE	Histone H1	973.5163	1945.0181	2	0.0025	20	1	6.00E-05	ac-SETAPAAPAAPAPAEKTPVK	Semi-tryptic
H14_MOUSE	Histone H1	1013.4993	2024.9840	2	0.0021	20	1	2.60E-04	ac-SETAPAAPAAPAPAEKpTPVK	Semi-tryptic
H14_MOUSE	Histone H1	1013.4997	2024.9848	2	0.0029	20	1	3.40E-04	ac-pSETAPAAPAAPAPAEKTPVK	Semi-tryptic
H14_MOUSE	Histone H1	692.0440	2073.1103	3	-0.0003	20	2	1.20E-02	ac-SETAPAAPAAPAPAEKTPVKK	Semi-tryptic
H14_MOUSE	Histone H1	779.8627	1557.7108	2	0.0033	16	0	6.70E-05	pSETAPAAPAAPAPAEK	Semi-tryptic
H14_MOUSE	Histone H1	819.8401	1637.6656	2	-0.0082	16	0	2.80E-02	pSEpTAPAAPAAPAPAEK	Semi-tryptic
H14_MOUSE	Histone H1	819.8406	1637.6667	2	-0.0072	16	0	4.10E-02	pSEpTAPAAPAAPAPAEK	Semi-tryptic
H14_MOUSE	Histone H1	819.8409	1637.6673	2	-0.0065	16	0	4.30E-02	pSEpTAPAAPAAPAPAEK	Semi-tryptic
H14_MOUSE	Histone H1	774.9021	1547.7897	2	0.0066	16	0	8.30E-03	ac-SETAPAAPAAPAPAEKme2	Semi-tryptic
H14_MOUSE	Histone H1	774.9021	1547.7897	2	0.0066	16	0	6.50E-03	ac-SETAPAAPAAPAPAEKme2	Semi-tryptic
H14_MOUSE	Histone H1	520.2398	1557.6977	3	-0.0099	16	0	6.40E-03	SE(p)TAPAAPAAPAPAEK	Semi-tryptic
H14_MOUSE	Histone H1	520.2403	1557.6990	3	-0.0086	16	0	1.40E-02	SEAAPAAPAAAPPAEKacAPAKKKAAKKPAGVRRKASGPPVSELITKAVAASKER	Semi-tryptic
H14_MOUSE	Histone H1	520.2414	1557.7025	3	-0.0051	16	0	1.80E-02	SEAAPAAPAAAPPAEKacAPAKKKAAKKPAGVRRKASGPPVSELITKAVAASKER	Semi-tryptic
H14_MOUSE	Histone H1	546.8955	1637.6648	3	-0.0091	16	0	2.60E-02	pSEpTAPAAPAAPAPAEK	Semi-tryptic
H14_MOUSE	Histone H1	779.8624	1557.7102	2	0.0027	16	0	5.00E-03	pSETAPAAPAAPAPAEK	Semi-tryptic
H14_MOUSE	Histone H1	819.8406	1637.6667	2	-0.0071	16	0	1.10E-02	pSEpTAPAAPAAPAPAEK	Semi-tryptic
H14_MOUSE	Histone H1	774.9002	1547.7858	2	0.0027	16	0	2.40E-03	ac-SETAPAAPAAPAPAEKme2	Semi-tryptic
H14_MOUSE	Histone H1	814.8841	1627.7536	2	0.0042	16	0	7.00E-03	ac-SE(p)TAPAAPAAPAPAEKme2	Semi-tryptic
H14_MOUSE	Histone H1	814.8833	1627.7520	2	0.0026	16	0	7.20E-03	ac-SE(p)TAPAAPAAPAPAEKme2	Semi-tryptic
H14_MOUSE	Histone H1	814.8833	1627.7520	2	0.0026	16	0	2.10E-02	ac-SE(p)TAPAAPAAPAPAEKme2	Semi-tryptic
H14_MOUSE	Histone H1	800.8681	1599.7217	2	0.0036	16	0	1.60E-03	ac-(p)SETAPAAPAAPAPAEK	Semi-tryptic
H14_MOUSE	Histone H1	800.8686	1599.7226	2	0.0045	16	0	3.70E-03	ac-(p)SETAPAAPAAPAPAEK	Semi-tryptic
H14_MOUSE	Histone H1	800.8677	1599.7209	2	0.0028	16	0	3.70E-03	ac-(p)SETAPAAPAAPAPAEK	Semi-tryptic
H14_MOUSE	Histone H1	800.8681	1599.7216	2	0.0035	16	0	6.80E-03	ac-(p)SETAPAAPAAPAPAEK	Semi-tryptic
H14_MOUSE	Histone H1	760.8843	1519.7540	2	0.0023	16	0	1.20E-03	ac-SETAPAAPAAPAPAEK	Semi-tryptic
H14_MOUSE	Histone H1	796.9251	1591.8357	2	0.0022	14	2	1.60E-03	RKpTSGPPVSELITK	Semi-tryptic
H14_MOUSE	Histone H1	531.6184	1591.8334	3	0.0000	14	2	1.90E-03	RKpTSGPPVSELITK	Semi-tryptic

uniprot accession/s for all possible isoforms	histone type	pep exp mz	pep exp mr	pep exp z	pep delta	pep length	pep miss	pep expect	peptide and site of PTM	enzyme
H14_MOUSE	Histone H1	531.6195	1591.8366	3	0.0032	14	2	2.00E-05	RKpTSGPPVSELITK	Semi-tryptic
H14_MOUSE	Histone H1	531.6180	1591.8322	3	-0.0013	14	2	8.70E-04	RKpTSGPPVSELITK	Semi-tryptic
H14_MOUSE	Histone H1	796.9254	1591.8363	2	0.0029	14	2	5.20E-05	RKpTSGPPVSELITK	Semi-tryptic
H14_MOUSE	Histone H1	531.6184	1591.8333	3	-0.0002	14	2	2.70E-04	RKpTSGPPVSELITK	Semi-tryptic
H14_MOUSE	Histone H1	718.8746	1435.7347	2	0.0023	13	1	3.60E-06	KTpSGPPVSELITK	Semi-tryptic
H14_MOUSE	Histone H1	479.5846	1435.7319	3	-0.0004	13	1	7.60E-04	KTpSGPPVSELITK	Semi-tryptic
H14_MOUSE	Histone H1	479.5852	1435.7339	3	0.0015	13	1	1.30E-05	KTpSGPPVSELITK	Semi-tryptic
H14_MOUSE	Histone H1	718.8742	1435.7339	2	0.0015	13	1	1.20E-06	KTpSGPPVSELITK	Semi-tryptic
H14_MOUSE	Histone H1	479.5847	1435.7322	3	-0.0001	13	1	1.50E-02	KTpSGPPVSELITK	Semi-tryptic
H14_MOUSE	Histone H1	553.3101	1104.6055	2	0.0000	10	3	2.00E-04	KAPKpSPAKAK	Semi-tryptic
H14_MOUSE	Histone H1	553.3104	1104.6063	2	0.0007	10	3	3.10E-04	KAPKpSPAKAK	Semi-tryptic
H14_MOUSE	Histone H1	553.3101	1104.6056	2	0.0000	10	3	3.60E-04	AKKAPKpSPAK	Semi-tryptic
H14_MOUSE	Histone H1	553.3103	1104.6060	2	0.0005	10	3	9.20E-04	AKKAPKpSPAK	Semi-tryptic
H14_MOUSE	Histone H1	369.2092	1104.6058	3	0.0002	10	3	2.10E-03	KAPKpSPAKAK	Semi-tryptic
H14_MOUSE	Histone H1	369.2094	1104.6064	3	0.0008	10	3	2.50E-02	KAPKpSPAKAK	Semi-tryptic
H14_MOUSE	Histone H1	553.3101	1104.6057	2	0.0001	10	3	2.90E-02	KAPKpSPAKAK	Semi-tryptic
H14_MOUSE	Histone H1	369.2093	1104.6062	3	0.0006	10	3	3.60E-02	KAPKpSPAKAK	Semi-tryptic
H14_MOUSE	Histone H1	369.2093	1104.6060	3	0.0005	10	3	4.80E-03	KAPKpSPAKAK	Semi-tryptic
H14_MOUSE	Histone H1	553.3098	1104.6051	2	-0.0005	10	3	3.10E-02	KAPKpSPAKAK	Semi-tryptic
H14_MOUSE	Histone H1	553.3100	1104.6055	2	-0.0001	10	3	2.70E-04	KAPKpSPAKAK	Semi-tryptic
H14_MOUSE	Histone H1	553.3100	1104.6055	2	-0.0001	10	3	3.10E-02	KAPKpSPAKAK	Semi-tryptic
H14_MOUSE	Histone H1	489.2626	976.5106	2	-0.0001	9	2	2.60E-02	APKpSPAKAK	Semi-tryptic
H14_MOUSE	Histone H1	453.7427	905.4708	2	-0.0028	8	2	6.50E-04	KAPKpSPAK	Semi-tryptic
H14_MOUSE	Histone H1	453.7439	905.4733	2	-0.0002	8	2	1.40E-03	KAPKpSPAK	Semi-tryptic
H14_MOUSE	Histone H1	453.7437	905.4728	2	-0.0007	8	2	3.50E-02	KAPKpSPAK	Semi-tryptic
H14_MOUSE	Histone H1	453.7441	905.4737	2	0.0002	8	2	2.10E-04	KAPKpSPAK	Semi-tryptic
H15_MOUSE	Histone H1	865.2730	4321.3286	5	0.0240	42	0	1.50E-06	ac-SETAPAETAAPAPVEKpSPAKKKTTKKAGAAKRKATGPPVSEL	Chymotrypsin
H15_MOUSE	Histone H1	849.2788	4241.3576	5	0.0194	42	0	5.50E-05	ac-SETAPAETAAPAPVEKSPAKKKTTKKAGAAKRKATGPPVSEL	Chymotrypsin
H15_MOUSE	Histone H1	849.2765	4241.3461	5	0.0078	42	0	4.60E-07	ac-SETAPAETAAPAPVEKSPAKKKTTKKAGAAKRKATGPPVSEL	Chymotrypsin
H15_MOUSE	Histone H1	865.2705	4321.3160	5	0.0114	42	0	7.80E-07	ac-SETAPAETAAPAPVEKpSPAKKKTTKKAGAAKRKATGPPVSEL	Chymotrypsin
H15_MOUSE	Histone H1	1081.3369	4321.3185	4	0.0139	42	0	1.10E-02	ac-SETAPAETAAPAPVEKpSPAKKKTTKKAGAAKRKATGPPVSEL	Chymotrypsin
H15_MOUSE	Histone H1	707.8989	4241.3499	6	0.0116	42	0	4.50E-02	ac-SETAPAETAAPAPVEKSPAKKKTTKKAGAAKRKATGPPVSEL	Chymotrypsin
H15_MOUSE	Histone H1	777.8486	3884.2066	5	0.0220	39	2	1.90E-06	TAPAETAAPAPVEKSPAKKme1KTTKKAGAAKRKATGPPVSE	GluC
H15_MOUSE	Histone H1	697.1947	3480.9373	5	0.0192	34	1	3.70E-05	TAAPAPVEKpSPAKKKTTKKAGAAKRKATGPPVSE	GluC
H15_MOUSE	Histone H1	581.1631	3480.9347	6	0.0166	34	1	1.70E-03	TAAPAPVEKpSPAKKKTTKKAGAAKRKATGPPVSE	GluC
H15_MOUSE	Histone H1	871.2424	3480.9406	4	0.0225	34	1	1.90E-03	TAAPAPVEKpSPAKKKTTKKAGAAKRKATGPPVSE	GluC
H15_MOUSE	Histone H1	854.7553	3414.9920	4	0.0246	34	1	2.10E-03	TAAPAPVEKSPAKKme1KTTKKAGAAKRKATGPPVSE	GluC
H15_MOUSE	Histone H1	683.9996	3414.9617	5	-0.0057	34	1	1.70E-02	TAAPAPVEKSPAKKme1KTTKKAGAAKRKATGPPVSE	GluC
H15_MOUSE	Histone H1	763.3897	2287.1472	3	0.0012	22	3	1.70E-04	SETAPAETAAPAPVEKpSPAKKK	Semi-tryptic
H15_MOUSE	Histone H1	583.2975	2329.1609	4	0.0043	22	3	5.80E-05	ac-SETAPAETAAPAPVEKpSPAKKK	Semi-tryptic
H15_MOUSE	Histone H1	572.7944	2287.1485	4	0.0024	22	3	2.50E-06	SETAPAETAAPAPVEKpSPAKKK	Semi-tryptic
H15_MOUSE	Histone H1	592.7836	2367.1054	4	-0.0070	22	3	2.20E-03	SE(p)TAPAETAAPAPVEKpSPAKKK	Semi-tryptic
H15_MOUSE	Histone H1	790.0414	2367.1023	3	-0.0100	22	3	1.60E-02	SE(p)TAPAETAAPAPVEKpSPAKKK	Semi-tryptic
H15_MOUSE	Histone H1	612.7760	2447.0751	4	-0.0036	22	3	3.20E-02	pSEpTAPAETAAPAPVEKpSPAKKK	Semi-tryptic
H15_MOUSE	Histone H1	763.3894	2287.1463	3	0.0003	22	3	4.40E-04	SETAPAETAAPAPVEKpSPAKKK	Semi-tryptic
H15_MOUSE	Histone H1	782.0635	2343.1687	3	-0.0036	22	3	3.40E-04	ac-SETAPAETAAPAPVEKpSPAKK(me1)K	Semi-tryptic
H15_MOUSE	Histone H1	777.3936	2329.1589	3	0.0023	22	3	8.70E-03	ac-SETAPAETAAPAPVEKpSPAKKK	Semi-tryptic
H15_MOUSE	Histone H1	786.7362	2357.1867	3	-0.0011	22	3	1.20E-02	ac-SETAPAETAAPAPVEKpSPAKK(me2)K	Semi-tryptic
H15_MOUSE	Histone H1	720.6915	2159.0526	3	0.0016	21	2	2.30E-03	SETAPAETAAPAPVEKpSPAKK	Semi-tryptic
H15_MOUSE	Histone H1	560.7592	2239.0079	4	-0.0095	21	2	2.00E-02	pSETAPAETAAPAPVEKpSPAKK	Semi-tryptic
H15_MOUSE	Histone H1	708.0410	2121.1011	3	0.0058	21	2	4.30E-03	ac-SETAPAETAAPAPVEKSPAKK	Semi-tryptic
H15_MOUSE	Histone H1	708.0410	2121.1011	3	0.0058	21	2	1.50E-03	ac-SETAPAETAAPAPVEKSPAKK	Semi-tryptic
H15_MOUSE	Histone H1	734.6952	2201.0637	3	0.0021	21	2	2.50E-02	ac-SETAPAETAAPAPVEKpSPAKK	Semi-tryptic
H15_MOUSE	Histone H1	560.7610	2239.0148	4	-0.0026	21	2	4.70E-04	SE(p)TAPAETAAPAPVEKpSPAKK	Semi-tryptic
H15_MOUSE	Histone H1	720.6928	2159.0565	3	0.0054	21	2	6.20E-03	SETAPAETAAPAPVEKpSPAKK	Semi-tryptic
H15_MOUSE	Histone H1	720.6918	2159.0536	3	0.0025	21	2	1.10E-05	SETAPAETAAPAPVEKpSPAKK	Semi-tryptic
H15_MOUSE	Histone H1	708.0397	2121.0972	3	0.0019	21	2	5.20E-05	ac-SETAPAETAAPAPVEKSPAKK	Semi-tryptic
H15_MOUSE	Histone H1	734.6960	2201.0661	3	0.0044	21	2	4.90E-02	ac-SETAPAETAAPAPVEKpSPAKK	Semi-tryptic
H15_MOUSE	Histone H1	1016.4870	2030.9594	2	0.0033	20	1	3.80E-05	SETAPAETAAPAPVEKpSPAK	Semi-tryptic
H15_MOUSE	Histone H1	1056.4647	2110.9148	2	-0.0076	20	1	1.40E-02	pSEpTAPAETAAPAPVEKSPAK	Semi-tryptic
H15_MOUSE	Histone H1	677.9939	2030.9600	3	0.0039	20	1	1.50E-02	SETAPAETAAPAPVEKpSPAK	Semi-tryptic
H15_MOUSE	Histone H1	997.5084	1993.0022	2	0.0018	20	1	2.60E-03	ac-SETAPAETAAPAPVEKSPAK	Semi-tryptic
H15_MOUSE	Histone H1	997.5111	1993.0076	2	0.0073	20	1	7.00E-03	ac-SETAPAETAAPAPVEKSPAK	Semi-tryptic
H15_MOUSE	Histone H1	997.5084	1993.0022	2	0.0018	20	1	2.40E-03	ac-SETAPAETAAPAPVEKSPAK	Semi-tryptic
H15_MOUSE	Histone H1	691.9991	2072.9755	3	0.0088	20	1	2.00E-04	ac-SETAPAETAAPAPVEKpSPAK	Semi-tryptic
H15_MOUSE	Histone H1	691.9966	2072.9679	3	0.0012	20	1	3.90E-03	ac-SETAPAETAAPAPVEKpSPAK	Semi-tryptic
H15_MOUSE	Histone H1	997.5111	1993.0076	2	0.0073	20	1	6.30E-03	ac-SETAPAETAAPAPVEKSPAK	Semi-tryptic
H15_MOUSE	Histone H1	677.9946	2030.9621	3	0.0060	20	1	5.60E-06	SETAPAETAAPAPVEKpSPAK	Semi-tryptic
H15_MOUSE	Histone H1	528.7364	2110.9165	4	-0.0059	20	1	2.40E-05	pSEpTAPAETAAPAPVEKSPAK	Semi-tryptic
H15_MOUSE	Histone H1	704.6462	2110.9169	3	-0.0056	20	1	6.70E-04	SE(p)TAPAETAAPAPVEKpSPAK	Semi-tryptic
H15_MOUSE	Histone H1	731.3032	2190.8878	3	-0.0009	20	1	7.30E-03	pSEpTAPAETAAPAPVEKpSPAK	Semi-tryptic
H15_MOUSE	Histone H1	704.6482	2110.9228	3	0.0003	20	1	9.60E-03	SE(p)TAPAETAAPAPVEK	Semi-tryptic
H15_MOUSE	Histone H1	704.6461	2110.9165	3	-0.0060	20	1	1.70E-02	SE(p)TAPAETAAPAPVEK	Semi-tryptic

uniprot accession/s for all possible isoforms	histone type	pep exp mz	pep exp mr	pep exp z	pep delta	pep length	pep miss	pep expect	peptide and site of PTM	enzyme
H15_MOUSE	Histone H1	704.6455	2110.9148	3	-0.0077	20	1	1.90E-02	SE(p)TAPAETAAPAPVEKpSPAKK	Semi-tryptic
H15_MOUSE	Histone H1	1056.4638	2110.9130	2	-0.0094	20	1	2.80E-02	pSEpTAPAETAAPAPVEKSPAK	Semi-tryptic
H15_MOUSE	Histone H1	997.5084	1993.0023	2	0.0020	20	1	1.60E-05	ac-SETAPAETAAPAPVEKSPAK	Semi-tryptic
H15_MOUSE	Histone H1	1037.4920	2072.9694	2	0.0028	20	1	1.90E-05	ac-SETAPAETAAPAPVEKpSPAK	Semi-tryptic
H15_MOUSE	Histone H1	1037.4926	2072.9706	2	0.0040	20	1	3.10E-05	ac-SETAPAETAAPAPVEKpSPAK	Semi-tryptic
H15_MOUSE	Histone H1	691.9981	2072.9725	3	0.0058	20	1	1.70E-04	ac-SETAPAETAAPAPVEKpSPAK	Semi-tryptic
H15_MOUSE	Histone H1	1077.4749	2152.9352	2	0.0022	20	1	1.80E-04	ac-pSETAPAETAAPAPVEKpSPAK	Semi-tryptic
H15_MOUSE	Histone H1	1037.4917	2072.9688	2	0.0022	20	1	8.20E-04	ac-SETAPAETAAPAPVEKpSPAK	Semi-tryptic
H15_MOUSE	Histone H1	1037.4922	2072.9698	2	0.0032	20	1	6.20E-03	ac-SETAPAETAAPAPVEKpSPAK	Semi-tryptic
H15_MOUSE	Histone H1	824.8773	1647.7400	2	0.0008	16	0	3.00E-06	(p)SETAPAETAAPAPVEK	Semi-tryptic
H15_MOUSE	Histone H1	550.2521	1647.7344	3	-0.0048	16	0	3.10E-03	SE(p)TAPAETAAPAPVEK	Semi-tryptic
H15_MOUSE	Histone H1	576.9059	1727.6958	3	-0.0097	16	0	1.30E-02	pSEpTAPAETAAPAPVEK	Semi-tryptic
H15_MOUSE	Histone H1	576.9070	1727.6990	3	-0.0066	16	0	2.20E-02	pSEpTAPAETAAPAPVEK	Semi-tryptic
H15_MOUSE	Histone H1	845.8831	1689.7517	2	0.0019	16	0	1.70E-04	ac-SEpTAPAETAAPAPVEK	Semi-tryptic
H15_MOUSE	Histone H1	845.8841	1689.7536	2	0.0038	16	0	6.80E-04	ac-pSETAPAETAAPAPVEK	Semi-tryptic
H15_MOUSE	Histone H1	845.8831	1689.7516	2	0.0018	16	0	1.10E-03	ac-pSETAPAETAAPAPVEK	Semi-tryptic
H15_MOUSE	Histone H1	845.8833	1689.7521	2	0.0023	16	0	3.30E-02	ac-pSETAPAETAAPAPVEK	Semi-tryptic
H15_MOUSE	Histone H1	805.9014	1609.7882	2	0.0047	16	0	2.20E-02	ac-SETAPAETAAPAPVEK	Semi-tryptic
H2A1_MOUSE, H2A2A_MOUSE, H2A3_MOUSE	Histone H2A	467.7652	933.5159	2	0.0015	8	0	3.30E-03	SHHKAKacGK	GluC
H2A1_MOUSE, H2A2A_MOUSE, H2A3_MOUSE	Histone H2A	312.1789	933.5150	3	0.0005	8	0	3.80E-03	SHHKAKGKac	GluC
H2A1_MOUSE, H2A2A_MOUSE, H2A3_MOUSE	Histone H2A	467.7649	933.5152	2	0.0007	8	0	4.40E-03	SHHKAKacGK	GluC
H2A1_MOUSE, H2A2A_MOUSE, H2A3_MOUSE	Histone H2A	312.1796	933.5168	3	0.0024	8	0	8.30E-03	SHHKAKacGK	GluC
H2A1_MOUSE, H2A2A_MOUSE, H2A3_MOUSE	Histone H2A	467.7636	933.5125	2	-0.0019	8	0	1.60E-02	SHHKAKGKac	GluC
H2A1_MOUSE, H2A2A_MOUSE, H2A3_MOUSE	Histone H2A	312.1789	933.5150	3	0.0005	8	0	8.60E-04	SHHKAKGKac	GluC
H2A1_MOUSE, H2A2A_MOUSE, H2A3_MOUSE	Histone H2A	467.7649	933.5153	2	0.0009	8	0	3.90E-02	SHHKAKGKac	GluC
H2A1_MOUSE, H2A2A_MOUSE, H2A3_MOUSE	Histone H2A	312.1790	933.5151	3	0.0007	8	0	2.50E-03	SHHKAKacGK	GluC
H2A1_MOUSE, H2A2A_MOUSE, H2A3_MOUSE	Histone H2A	467.7642	933.5139	2	-0.0005	8	0	6.40E-03	SHHKAKGKac	GluC
H2A1_MOUSE, H2A2A_MOUSE, H2A3_MOUSE	Histone H2A	467.7643	933.5141	2	-0.0003	8	0	1.90E-02	SHHKAKGKac	GluC
H2A1_MOUSE, H2A2A_MOUSE, H2A3_MOUSE	Histone H2A	312.1789	933.5148	3	0.0003	8	0	2.00E-02	SHHKAKGKac	GluC
H2A1_MOUSE, H2A2A_MOUSE, H2A3_MOUSE	Histone H2A	467.7638	933.5131	2	-0.0013	8	0	3.60E-02	SHHKAKGKac	GluC
H2A1_MOUSE, H2A2A_MOUSE, H2A3_MOUSE	Histone H2A	312.1789	933.5149	3	0.0005	8	0	3.40E-03	SHHKAKGKac	GluC
H2A1_MOUSE, H2A2A_MOUSE, H2A3_MOUSE	Histone H2A	312.1790	933.5152	3	0.0008	8	0	4.40E-03	SHHKAKacGK	GluC
H2A1_MOUSE, H2A2A_MOUSE, H2A3_MOUSE	Histone H2A	467.7647	933.5149	2	0.0005	8	0	4.80E-03	SHHKAKGKac	GluC
H2A1_MOUSE, H2A2A_MOUSE, H2A3_MOUSE	Histone H2A	312.1793	933.5160	3	0.0016	8	0	1.10E-02	SHHKAKGKac	GluC
H2A1_MOUSE, H2A2A_MOUSE, H2A3_MOUSE	Histone H2A	312.1796	933.5169	3	0.0024	8	0	4.40E-02	SHHKAKGKac	GluC
H2A1_MOUSE, H2A2A_MOUSE, H2A3_MOUSE	Histone H2A	320.8512	959.5316	3	0.0015	7	0	2.90E-02	SHHKcrAKGK	GluC
H2A1_MOUSE, H2A2A_MOUSE, H2A3_MOUSE	Histone H2A	320.8513	959.5321	3	0.0020	7	0	1.40E-03	SHHKcrAKGK	GluC
H2A1F_MOUSE	Histone H2A	320.8516	959.5329	3	0.0028	8	0	3.20E-02	SHHKPKGKac	GluC
H2A1F_MOUSE, H2A1H_MOUSE, H2A1_MOUSE, H2A1K_MOUSE	Histone H2A	752.0961	4506.5327	6	0.0194	41	0	1.00E-03	ac-SGRGKQGGKARAKAKTRSSRAGLQFPVGRVHRLLRKGNYSE	GluC
H2A1F_MOUSE, H2A1H_MOUSE, H2A1_MOUSE, H2A1K_MOUSE	Histone H2A	759.0995	4548.5533	6	0.0295	41	0	1.70E-04	ac-SGRGKacQGGKARAKAKTRSSRAGLQFPVGRVHRLLRKGNYSE	GluC
H2A1F_MOUSE, H2A1H_MOUSE, H2A1_MOUSE, H2A1K_MOUSE	Histone H2A	752.0977	4506.5424	6	0.0291	41	0	2.00E-04	ac-SGRGKQGGKARAKAKTRSSRAGLQFPVGRVHRLLRKGNYSE	GluC
H2A1F_MOUSE, H2A1H_MOUSE, H2A1_MOUSE, H2A1K_MOUSE	Histone H2A	650.7997	4548.5470	7	0.0232	41	0	7.40E-03	ac-SGRGKacQGGKARAKAKTRSSRAGLQFPVGRVHRLLRKGNYSE	GluC
H2A1F_MOUSE, H2A1H_MOUSE, H2A1_MOUSE, H2A1K_MOUSE	Histone H2A	644.7991	4506.5430	7	0.0298	41	0	4.20E-02	ac-SGRGKQGGKARAKAKTRSSRAGLQFPVGRVHRLLRKGNYSE	GluC
H2A1F_MOUSE, H2A1H_MOUSE, H2A1_MOUSE, H2A1K_MOUSE	Histone H2A	902.3154	4506.5405	5	-0.0092	41	0	9.00E-09	SGRme3GKQGGKARAKAKTRSSRAGLQFPVGRVHRLLRKGNYSE	GluC
H2A1F_MOUSE, H2A1H_MOUSE, H2A1_MOUSE, H2A1K_MOUSE	Histone H2A	644.7987	4506.5398	7	-0.0098	41	0	5.00E-03	SGRme3GKQGGKARAKAKTRSSRAGLQFPVGRVHRLLRKGNYSE	GluC
H2A1F_MOUSE, H2A1H_MOUSE, H2A1_MOUSE, H2A1K_MOUSE	Histone H2A	644.7976	4506.5321	7	-0.0175	41	0	1.00E-02	SGRme3GKQGGKARAKAKTRSSRAGLQFPVGRVHRLLRKGNYSE	GluC
H2A1F_MOUSE, H2A1H_MOUSE, H2A1_MOUSE, H2A1K_MOUSE	Histone H2A	752.0959	4506.5316	6	-0.0180	41	0	8.20E-03	SGRme3GKQGGKARAKAKTRSSRAGLQFPVGRVHRLLRKGNYSE	GluC
H2A1F_MOUSE, H2A1H_MOUSE, H2A1_MOUSE, H2A1K_MOUSE	Histone H2A	644.7976	4506.5325	7	-0.0172	41	0	1.10E-02	SGRme3GKQGGKARAKAKTRSSRAGLQFPVGRVHRLLRKGNYSE	GluC
H2A1F_MOUSE, H2A1H_MOUSE, H2A1_MOUSE, H2A1K_MOUSE	Histone H2A	902.3111	4506.5194	5	0.0061	41	0	9.30E-06	ac-SGRGKQGGKARAKAKTRSSRAGLQFPVGRVHRLLRKGNYSE	GluC
H2A1F_MOUSE, H2A1H_MOUSE, H2A1_MOUSE, H2A1K_MOUSE	Histone H2A	902.3107	4506.5170	5	0.0038	41	0	1.40E-05	ac-SGRGKQGGKARAKAKTRSSRAGLQFPVGRVHRLLRKGNYSE	GluC
H2A1F_MOUSE, H2A1H_MOUSE, H2A1_MOUSE, H2A1K_MOUSE	Histone H2A	910.7135	4548.5309	5	0.0071	41	0	4.70E-04	ac-SGRGKacQGGKARAKAKTRSSRAGLQFPVGRVHRLLRKGNYSE	GluC
H2A1F_MOUSE, H2A1H_MOUSE, H2A1_MOUSE, H2A1K_MOUSE	Histone H2A	902.3103	4506.5151	5	0.0018	41	0	6.10E-04	ac-SGRGKQGGKARAKAKTRSSRAGLQFPVGRVHRLLRKGNYSE	GluC
H2A1F_MOUSE, H2A1H_MOUSE, H2A1_MOUSE, H2A1K_MOUSE	Histone H2A	902.3120	4506.5239	5	0.0106	41	0	9.40E-04	ac-SGRGKQGGKARAKAKTRSSRAGLQFPVGRVHRLLRKGNYSE	GluC
H2A1F_MOUSE, H2A1H_MOUSE, H2A1_MOUSE, H2A1K_MOUSE	Histone H2A	1127.6374	4506.5205	4	0.0072	41	0	2.40E-03	ac-SGRGKQGGKARAKAKTRSSRAGLQFPVGRVHRLLRKGNYSE	GluC
H2A1F_MOUSE, H2A1H_MOUSE, H2A1_MOUSE, H2A1K_MOUSE	Histone H2A	644.7953	4506.5162	7	0.0029	41	0	3.00E-03	ac-SGRGKQGGKARAKAKTRSSRAGLQFPVGRVHRLLRKGNYSE	GluC
H2A1F_MOUSE, H2A1H_MOUSE, H2A1_MOUSE, H2A1K_MOUSE	Histone H2A	902.3101	4506.5143	5	0.0010	41	0	3.50E-03	ac-SGRGKQGGKARAKAKTRSSRAGLQFPVGRVHRLLRKGNYSE	GluC
H2A1F_MOUSE, H2A1H_MOUSE, H2A1_MOUSE, H2A1K_MOUSE	Histone H2A	752.0932	4506.5154	6	0.0021	41	0	5.00E-03	ac-SGRGKQGGKARAKAKTRSSRAGLQFPVGRVHRLLRKGNYSE	GluC
H2A1F_MOUSE, H2A1H_MOUSE, H2A1_MOUSE, H2A1K_MOUSE	Histone H2A	752.0933	4506.5164	6	0.0031	41	0	5.10E-03	ac-SGRGKQGGKARAKAKTRSSRAGLQFPVGRVHRLLRKGNYSE	GluC
H2A1F_MOUSE, H2A1H_MOUSE, H2A1_MOUSE, H2A1K_MOUSE	Histone H2A	752.0934	4506.5166	6	0.0033	41	0	5.40E-03	ac-SGRGKQGGKARAKAKTRSSRAGLQFPVGRVHRLLRKGNYSE	GluC
H2A1F_MOUSE, H2A1H_MOUSE, H2A1_MOUSE, H2A1K_MOUSE	Histone H2A	644.7931	4506.5011	7	-0.0121	41	0	6.50E-03	ac-SGRGKQGGKARAKAKTRSSRAGLQFPVGRVHRLLRKGNYSE	GluC
H2A1F_MOUSE, H2A1H_MOUSE, H2A1_MOUSE, H2A1K_MOUSE	Histone H2A	752.0934	4506.5169	6	0.0036	41	0	7.90E-03	ac-SGRGKQGGKARAKAKTRSSRAGLQFPVGRVHRLLRKGNYSE	GluC
H2A1F_MOUSE, H2A1H_MOUSE, H2A1_MOUSE, H2A1K_MOUSE	Histone H2A	902.3110	4506.5187	5	0.0055	41	0	8.40E-03	ac-SGRGKQGGKARAKAKTRSSRAGLQFPVGRVHRLLRKGNYSE	GluC
H2A1F_MOUSE, H2A1H_MOUSE, H2A1_MOUSE, H2A1K_MOUSE	Histone H2A	752.0925	4506.5115	6	-0.0017	41	0	1.20E-02	ac-SGRGKQGGKARAKAKTRSSRAGLQFPVGRVHRLLRKGNYSE	GluC
H2A1F_MOUSE, H2A1H_MOUSE, H2A1_MOUSE, H2A1K_MOUSE	Histone H2A	644.7954	4506.5167	7	0.0034	41	0	1.30E-02	ac-SGRGKQGGKARAKAKTRSSRAGLQFPVGRVHRLLRKGNYSE	GluC
H2A1F_MOUSE, H2A1H_MOUSE, H2A1_MOUSE, H2A1K_MOUSE	Histone H2A	752.0939	4506.5195	6	0.0062	41	0	1.30E-02	ac-SGRGKQGGKARAKAKTRSSRAGLQFPVGRVHRLLRKGNYSE	GluC
H2A1F_MOUSE, H2A1H_MOUSE, H2A1_MOUSE, H2A1K_MOUSE	Histone H2A	759.0947	4548.5247	6	0.0008	41	0	2.70E-02	ac-SGRGKacQGGKARAKAKTRSSRAGLQFPVGRVHRLLRKGNYSE	GluC
H2A1F_MOUSE, H2A1H_MOUSE, H2A1_MOUSE, H2A1K_MOUSE	Histone H2A	752.0938	4506.5192	6	0.0059	41	0	3.00E-02	ac-SGRGKQGGKARAKAKTRSSRAGLQFPVGRVHRLLRKGNYSE	GluC
H2A1F_MOUSE, H2A1H_MOUSE, H2A1_MOUSE, H2A1K_MOUSE	Histone H2A	644.7952	4506.5152	7	0.0019	41	0	3.20E-02	ac-SGRGKQGGKARAKAKTRSSRAGLQFPVGRVHRLLRKGNYSE	GluC
H2A1F_MOUSE, H2A1H_MOUSE, H2A1_MOUSE, H2A1K_MOUSE	Histone H2A	752.0936	4506.5180	6	0.0047	41	0	4.10E-02	ac-SGRGKQGGKARAKAKTRSSRAGLQFPVGRVHRLLRKGNYSE	GluC
H2A1F_MOUSE, H2A1H_MOUSE, H2A1_MOUSE, H2A1K_MOUSE	Histone H2A	752.0953	4506.5281	6	0.0148	41	0	2.30E-04	ac-SGRGKQGGKARAKAKTRSSRAGLQFPVGRVHRLLRKGNYSE	GluC
H2A1F_MOUSE, H2A1H_MOUSE, H2A1_MOUSE, H2A1K_MOUSE	Histone H2A	752.0957	4506.5305	6	0.0173	41	0	2.60E-03	ac-SGRGKQGGKARAKAKTRSSRAGLQFPVGRVHRLLRKGNYSE	GluC
H2A1F_MOUSE, H2A1H_MOUSE, H2A1_MOUSE, H2A1K_MOUSE	Histone H2A	752.0959	4506.5319	6	0.0187	41	0	3.20E-03	ac-SGRGKQGGKARAKAKTRSSRAGLQFPVGRVHRLLRKGNYSE	GluC
H2A1F_MOUSE, H2A1H_MOUSE, H2A1_MOUSE, H2A1K_MOUSE	Histone H2A	644.7967	4506.5262	7	0.0130	41	0	5.50E-03	ac-SGRGKQGGKARAKAKTRSSRAGLQFPVGRVHRLLRKGNYSE	GluC

uniprot accession/s for all possible isoforms	histone type	pep exp mz	pep exp mr	pep exp z	pep delta	pep length	pep miss	pep expect	peptide and site of PTM	enzyme
H2A1F_MOUSE, H2A1H_MOUSE, H2A1_MOUSE, H2A1K_MOUSE	Histone H2A	752.0958	4506.5311	6	0.0179	41	0	4.90E-02	ac-SGRGKQGGKARAKAKTRSSRAGLQFPVGRVHRLLRKGNYSE	GluC
H2A1F_MOUSE, H2A1H_MOUSE, H2A1_MOUSE, H2A1K_MOUSE	Histone H2A	752.0938	4506.5191	6	0.0059	41	0	1.40E-02	ac-SGRGKQGGKARAKAKTRSSRAGLQFPVGRVHRLLRKGNYSE	GluC
H2A1F_MOUSE, H2A1H_MOUSE, H2A1_MOUSE, H2A1K_MOUSE	Histone H2A	752.0934	4506.5165	6	0.0032	41	0	1.90E-02	ac-SGRGKQGGKARAKAKTRSSRAGLQFPVGRVHRLLRKGNYSE	GluC
H2A1F_MOUSE, H2A1H_MOUSE, H2A1_MOUSE, H2A2A_MOUSE, H2A2B_MOUSE, H2A3_MOUSE, H2A2C_MOUSE, H2AJ_MOUSE, H2AX_MOUSE, H2A1K_MOUSE, H2AY_MOUSE, H2AW_MOUSE	Histone H2A	401.2144	800.4143	2	0.0002	8	0	5.90E-04	LAGNAARme1D	GluC
H2A1F_MOUSE, H2A1H_MOUSE, H2A1_MOUSE, H2A2A_MOUSE, H2A2B_MOUSE, H2A3_MOUSE, H2A2C_MOUSE, H2AJ_MOUSE, H2AX_MOUSE, H2A1K_MOUSE, H2AY_MOUSE, H2AW_MOUSE	Histone H2A	401.2144	800.4143	2	0.0002	8	0	4.40E-02	LAGNAARme1D	GluC
H2A1F_MOUSE, H2A1H_MOUSE, H2A1_MOUSE, H2A2A_MOUSE, H2A2B_MOUSE, H2A3_MOUSE, H2A2C_MOUSE, H2AJ_MOUSE, H2AX_MOUSE, H2A1K_MOUSE, H2AY_MOUSE, H2AW_MOUSE	Histone H2A	401.2149	800.4153	2	0.0013	8	0	1.40E-03	LAGNAARme1D	GluC
H2A1F_MOUSE, H2A1H_MOUSE, H2A1_MOUSE, H2A2A_MOUSE, H2A3_MOUSE, H2A2C_MOUSE, H2AJ_MOUSE, H2AX_MOUSE, H2A1K_MOUSE	Histone H2A	557.8344	2227.3084	4	0.0059	18	0	2.70E-05	DNKacKTRIIPRHLQLAIRN	AspN
H2A1F_MOUSE, H2A1H_MOUSE, H2A1_MOUSE, H2A2A_MOUSE, H2A3_MOUSE, H2A2C_MOUSE, H2AJ_MOUSE, H2AX_MOUSE, H2A1K_MOUSE	Histone H2A	743.4436	2227.3091	3	0.0066	18	0	2.20E-03	DNKacKTRIIPRHLQLAIRN	AspN
H2A1F_MOUSE, H2A1H_MOUSE, H2A1_MOUSE, H2A3_MOUSE, H2AJ_MOUSE, H2A1K_MOUSE	Histone H2A	778.7251	3110.8714	4	-0.0193	29	0	4.30E-05	LNKme2LLGRVTIAQGGVLPNIQAVLLPKKTE	GluC
H2A1F_MOUSE, H2A1H_MOUSE, H2A1_MOUSE, H2A3_MOUSE, H2AJ_MOUSE, H2A1K_MOUSE	Histone H2A	782.2260	3124.8751	4	0.0051	29	0	9.10E-04	LNKLLGRVTIAQGGVLPNIQAVLLPKKacTE	GluC
H2A1F_MOUSE, H2A1H_MOUSE, H2A1_MOUSE, H2A3_MOUSE, H2AJ_MOUSE, H2A1K_MOUSE	Histone H2A	782.2260	3124.8751	4	0.0051	29	0	1.20E-02	LNKLLGRVTIAQGGVLPNIQAVLLPKKacTE	GluC
H2A1K_MOUSE	Histone H2A	316.8504	947.5295	3	-0.0006	8	0	4.70E-03	THHKAKacGK	GluC
H2A2A_MOUSE	Histone H2A	866.9051	4329.4893	5	-0.0006	40	0	8.80E-09	DEELNKLLGKme2VTIAQGGVLPNIQAVLLPKKTESHHKAKGK	AspN
H2A2A_MOUSE	Histone H2A	1083.3818	4329.4981	4	0.0081	40	0	3.00E-06	DEELNKLLGKme2VTIAQGGVLPNIQAVLLPKKTESHHKAKGK	AspN
H2A2A_MOUSE	Histone H2A	722.5890	4329.4904	6	0.0004	40	0	1.80E-03	DEELNKLLGKme2VTIAQGGVLPNIQAVLLPKKTESHHKAKGK	AspN
H2A2A_MOUSE	Histone H2A	1083.3800	4329.4909	4	0.0009	40	0	1.90E-03	DEELNKLLGKme2VTIAQGGVLPNIQAVLLPKKTESHHKAKGK	AspN
H2A2A_MOUSE	Histone H2A	875.3083	4371.5053	5	0.0048	40	0	4.90E-03	DEELNKLLGKme2VTIAQGGVLPNIQAVLLPKKTESHHKAKGKac	AspN
H2A2A_MOUSE	Histone H2A	866.9045	4329.4860	5	-0.0039	40	0	2.70E-08	DEELNKLLGKme2VTIAQGGVLPNIQAVLLPKKTESHHKAKGK	AspN
H2A2A_MOUSE	Histone H2A	1083.3799	4329.4905	4	0.0005	40	0	7.30E-06	DEELNKLLGKme2VTIAQGGVLPNIQAVLLPKKTESHHKAKGK	AspN
H2A2A_MOUSE	Histone H2A	869.7057	4343.4923	5	0.0230	40	0	5.20E-05	DEELNKLLGKVTIAQGGVLPNIQAVLLPKKTESHHKAKGK(ac)	AspN
H2A2A_MOUSE	Histone H2A	619.5050	4329.4844	7	-0.0055	40	0	6.10E-03	DEELNKLLGKme2VTIAQGGVLPNIQAVLLPKKTESHHKAKGK	AspN
H2A2A_MOUSE	Histone H2A	619.5062	4329.4927	7	0.0027	40	0	5.00E-04	DEELNKme1LLGKme1VTIAQGGVLPNIQAVLLPKKTESHHKAKGK	AspN
H2A2A_MOUSE	Histone H2A	722.5886	4329.4878	6	-0.0022	40	0	9.10E-04	DEELNKme1LLGKme1VTIAQGGVLPNIQAVLLPKKTESHHKAKGK	AspN
H2A2A_MOUSE	Histone H2A	869.7024	4343.4758	5	0.0065	40	0	1.50E-06	DEELNKLLGKVTIAQGGVLPNIQAVLLPKKTESHHKAKGK(ac)	AspN
H2A2A_MOUSE	Histone H2A	866.9002	4329.4648	5	-0.0251	40	0	3.00E-06	DEELNKLLGKme2VTIAQGGVLPNIQAVLLPKKTESHHKAKGK	AspN
H2A2A_MOUSE	Histone H2A	869.7032	4343.4797	5	0.0105	40	0	1.70E-05	DEELNKLLGKVTIAQGGVLPNIQAVLLPKKTESHHKAKGK(ac)	AspN
H2A2A_MOUSE	Histone H2A	869.7028	4343.4778	5	0.0085	40	0	2.70E-05	DEELNKLLGKVTIAQGGVLPNIQAVLLPKKTESHHKAKGK(ac)	AspN
H2A2A_MOUSE	Histone H2A	866.9005	4329.4661	5	-0.0239	40	0	2.90E-05	DEELNKLLGKme2VTIAQGGVLPNIQAVLLPKKTESHHKAKGK	AspN
H2A2A_MOUSE	Histone H2A	724.9205	4343.4793	6	0.0101	40	0	6.00E-05	DEELNKLLGKVTIAQGGVLPNIQAVLLPKKTESHHKAKGK(ac)	AspN
H2A2A_MOUSE	Histone H2A	866.9016	4329.4718	5	-0.0182	40	0	9.20E-05	DEELNKLLGKme2VTIAQGGVLPNIQAVLLPKKTESHHKAKGK	AspN
H2A2A_MOUSE	Histone H2A	1093.8777	4371.4817	4	-0.0188	40	0	1.70E-04	DEELNKLLGKme2VTIAQGGVLPNIQAVLLPKKTESHHKAKGKac	AspN
H2A2A_MOUSE	Histone H2A	869.7028	4343.4778	5	0.0085	40	0	2.40E-04	DEELNKLLGKVTIAQGGVLPNIQAVLLPKKTESHHKAKGK(ac)	AspN
H2A2A_MOUSE	Histone H2A	866.9010	4329.4687	5	-0.0212	40	0	4.70E-04	DEELNKLLGKme2VTIAQGGVLPNIQAVLLPKKTESHHKAKGK	AspN
H2A2A_MOUSE	Histone H2A	1086.8766	4343.4773	4	0.0081	40	0	5.80E-04	DEELNKLLGKVTIAQGGVLPNIQAVLLPKKTESHHKAKGK(ac)	AspN
H2A2A_MOUSE	Histone H2A	1083.3755	4329.4729	4	-0.0171	40	0	7.90E-04	DEELNKLLGKme2VTIAQGGVLPNIQAVLLPKKTESHHKAKGK	AspN
H2A2A_MOUSE	Histone H2A	866.9009	4329.4680	5	-0.0220	40	0	1.50E-03	DEELNKLLGKme2VTIAQGGVLPNIQAVLLPKKTESHHKAKGK	AspN
H2A2A_MOUSE	Histone H2A	1444.1645	4329.4717	3	-0.0183	40	0	1.90E-03	DEELNKLLGKme2VTIAQGGVLPNIQAVLLPKKTESHHKAKGK	AspN
H2A2A_MOUSE	Histone H2A	866.9002	4329.4648	5	-0.0251	40	0	3.40E-03	DEELNKLLGKme2VTIAQGGVLPNIQAVLLPKKTESHHKAKGK	AspN
H2A2A_MOUSE	Histone H2A	869.7024	4343.4758	5	0.0065	40	0	6.60E-03	DEELNKLLGKVTIAQGGVLPNIQAVLLPKKTESHHKAKGK(ac)	AspN
H2A2A_MOUSE	Histone H2A	875.3028	4371.4776	5	-0.0229	40	0	8.60E-03	DEELNKLLGKme2VTIAQGGVLPNIQAVLLPKKTESHHKAKGKac	AspN
H2A2A_MOUSE	Histone H2A	1083.3753	4329.4721	4	-0.0179	40	0	1.10E-02	DEELNKLLGKme2VTIAQGGVLPNIQAVLLPKKTESHHKAKGK	AspN
H2A2A_MOUSE	Histone H2A	724.9206	4343.4800	6	0.0108	40	0	1.10E-02	DEELNKLLGKVTIAQGGVLPNIQAVLLPKKTESHHKAKGK(ac)	AspN
H2A2A_MOUSE	Histone H2A	875.3048	4371.4877	5	-0.0128	40	0	1.50E-02	DEELNKLLGKme2VTIAQGGVLPNIQAVLLPKKTESHHKAKGKac	AspN
H2A2A_MOUSE	Histone H2A	1083.3747	4329.4697	4	-0.0203	40	0	2.20E-02	DEELNKLLGKme2VTIAQGGVLPNIQAVLLPKKTESHHKAKGK	AspN
H2A2A_MOUSE	Histone H2A	722.5853	4329.4684	6	-0.0216	40	0	3.10E-02	DEELNKLLGKme2VTIAQGGVLPNIQAVLLPKKTESHHKAKGK	AspN
H2A2A_MOUSE	Histone H2A	866.9014	4329.4708	5	-0.0192	40	0	4.40E-02	DEELNKLLGKme2VTIAQGGVLPNIQAVLLPKKTESHHKAKGK	AspN
H2A2A_MOUSE	Histone H2A	619.5025	4329.4666	7	-0.0233	40	0	4.90E-02	DEELNKme2LLGKVTIAQGGVLPNIQAVLLPKKTESHHKAKGK	AspN
H2A2A_MOUSE	Histone H2A	792.2820	3956.3734	5	-0.0045	37	1	1.30E-08	LNKLLGKme2VTIAQGGVLPNIQAVLLPKKTESHHKAKGK	GluC
H2A2A_MOUSE	Histone H2A	660.4025	3956.3714	6	-0.0064	37	1	1.10E-04	LNKLLGKme2VTIAQGGVLPNIQAVLLPKKTESHHKAKGK	GluC
H2A2A_MOUSE, H2A2B_MOUSE, H2A2C_MOUSE	Histone H2A	655.9390	4584.5223	7	0.0220	41	0	3.30E-04	ac-pSGRGKQGGKme2ARAKAKSRSSRAGLQFPVGRVHRLLRKGNYAE	GluC
H2A2A_MOUSE, H2A2B_MOUSE, H2A2C_MOUSE	Histone H2A	912.3056	4556.4918	5	0.0228	41	0	5.10E-04	pSGRGKQGGKacARAKAKSRSSRAGLQFPVGRVHRLLRKGNYAE	GluC
H2A2A_MOUSE, H2A2B_MOUSE, H2A2C_MOUSE	Histone H2A	758.7686	4546.5678	6	0.0232	41	0	5.40E-04	ac-SGRGKacQGGKme2ARAKAKSRSSRAGLQFPVGRVHRLLRKGNYAE	GluC
H2A2A_MOUSE, H2A2B_MOUSE, H2A2C_MOUSE	Histone H2A	760.4239	4556.4998	6	0.0308	41	0	2.30E-03	ac-pSGRGKQGGKARAKAKSRSSRAGLQFPVGRVHRLLRKGNYAE	GluC
H2A2A_MOUSE, H2A2B_MOUSE, H2A2C_MOUSE	Histone H2A	747.0957	4476.5307	6	-0.0084	41	0	3.50E-03	SGRme3GKQGGKARAKAKSRSSRAGLQFPVGRVHRLLRKGNYAE	GluC
H2A2A_MOUSE, H2A2B_MOUSE, H2A2C_MOUSE	Histone H2A	747.0950	4476.5264	6	-0.0127	41	0	8.60E-05	SGRme3GKQGGKARAKAKSRSSRAGLQFPVGRVHRLLRKGNYAE	GluC
H2A2A_MOUSE, H2A2B_MOUSE, H2A2C_MOUSE	Histone H2A	747.0955	4476.5293	6	-0.0097	41	0	3.30E-03	SGRme3GKQGGKARAKAKSRSSRAGLQFPVGRVHRLLRKGNYAE	GluC
H2A2A_MOUSE, H2A2B_MOUSE, H2A2C_MOUSE	Histone H2A	896.3076	4476.5015	5	-0.0012	41	0	2.50E-05	ac-SGRGKQGGKARAKAKSRSSRAGLQFPVGRVHRLLRKGNYAE	GluC
H2A2A_MOUSE, H2A2B_MOUSE, H2A2C_MOUSE	Histone H2A	913.1134	4560.5309	5	0.0071	41	0	2.60E-05	ac-SGRGKacQGGKacARAKAKSRSSRAGLQFPVGRVHRLLRKGNYAE	GluC
H2A2A_MOUSE, H2A2B_MOUSE, H2A2C_MOUSE	Histone H2A	896.3097	4476.5120	5	0.0093	41	0	2.70E-05	ac-SGRGKQGGKARAKAKSRSSRAGLQFPVGRVHRLLRKGNYAE	GluC
H2A2A_MOUSE, H2A2B_MOUSE, H2A2C_MOUSE	Histone H2A	896.3088	4476.5077	5	0.0050	41	0	8.70E-05	ac-SGRGKQGGKARAKAKSRSSRAGLQFPVGRVHRLLRKGNYAE	GluC
H2A2A_MOUSE, H2A2B_MOUSE, H2A2C_MOUSE	Histone H2A	896.3093	4476.5099	5	0.0072	41	0	1.10E-04	ac-SGRGKQGGKARAKAKSRSSRAGLQFPVGRVHRLLRKGNYAE	GluC
H2A2A_MOUSE, H2A2B_MOUSE, H2A2C_MOUSE	Histone H2A	896.3097	4476.5121	5	0.0094	41	0	1.50E-04	ac-SGRGKQGGKARAKAKSRSSRAGLQFPVGRVHRLLRKGNYAE	GluC

uniprot accession/s for all possible isoforms	histone type	pep exp mz	pep exp mr	pep exp z	pep delta	pep length	pep miss	pep expect	peptide and site of PTM	enzyme
H2A2A_MOUSE, H2A2B_MOUSE, H2A2C_MOUSE	Histone H2A	896.3087	4476.5071	5	0.0044	41	0	5.40E-04	ac-SGRGKQGGKARAKAKSRSSRAGLQFPVGRVHRLLRKGNYAE	GluC
H2A2A_MOUSE, H2A2B_MOUSE, H2A2C_MOUSE	Histone H2A	747.0919	4476.5079	6	0.0052	41	0	6.00E-04	ac-SGRGKQGGKARAKAKSRSSRAGLQFPVGRVHRLLRKGNYAE	GluC
H2A2A_MOUSE, H2A2B_MOUSE, H2A2C_MOUSE	Histone H2A	896.3088	4476.5076	5	0.0049	41	0	6.50E-04	ac-SGRGKQGGKARAKAKSRSSRAGLQFPVGRVHRLLRKGNYAE	GluC
H2A2A_MOUSE, H2A2B_MOUSE, H2A2C_MOUSE	Histone H2A	904.7103	4518.5153	5	0.0020	41	0	1.80E-03	ac-SGRGKacQGGKARAKAKSRSSRAGLQFPVGRVHRLLRKGNYAE	GluC
H2A2A_MOUSE, H2A2B_MOUSE, H2A2C_MOUSE	Histone H2A	747.0930	4476.5146	6	0.0119	41	0	2.20E-03	ac-SGRGKQGGKARAKAKSRSSRAGLQFPVGRVHRLLRKGNYAE	GluC
H2A2A_MOUSE, H2A2B_MOUSE, H2A2C_MOUSE	Histone H2A	747.0925	4476.5116	6	0.0089	41	0	5.70E-03	ac-SGRGKQGGKARAKAKSRSSRAGLQFPVGRVHRLLRKGNYAE	GluC
H2A2A_MOUSE, H2A2B_MOUSE, H2A2C_MOUSE	Histone H2A	747.0944	4476.5227	6	0.0200	41	0	7.50E-03	ac-SGRGKQGGKARAKAKSRSSRAGLQFPVGRVHRLLRKGNYAE	GluC
H2A2A_MOUSE, H2A2B_MOUSE, H2A2C_MOUSE	Histone H2A	747.0917	4476.5067	6	0.0040	41	0	1.60E-02	ac-SGRGKQGGKARAKAKSRSSRAGLQFPVGRVHRLLRKGNYAE	GluC
H2A2A_MOUSE, H2A2B_MOUSE, H2A2C_MOUSE	Histone H2A	765.7674	4588.5608	6	0.0057	41	0	2.10E-02	ac-SGRGKacQGGKacARAKAKSRSSRAGLQFPVGRVHRLLRKGNYAE	GluC
H2A2A_MOUSE, H2A2B_MOUSE, H2A2C_MOUSE	Histone H2A	747.0910	4476.5025	6	-0.0002	41	0	2.50E-02	ac-SGRGKQGGKARAKAKSRSSRAGLQFPVGRVHRLLRKGNYAE	GluC
H2A2A_MOUSE, H2A2B_MOUSE, H2A2C_MOUSE	Histone H2A	747.0900	4476.4963	6	-0.0063	41	0	2.70E-02	ac-SGRGKQGGKARAKAKSRSSRAGLQFPVGRVHRLLRKGNYAE	GluC
H2A2A_MOUSE, H2A2B_MOUSE, H2A2C_MOUSE	Histone H2A	747.0913	4476.5043	6	0.0016	41	0	3.60E-02	ac-SGRGKQGGKARAKAKSRSSRAGLQFPVGRVHRLLRKGNYAE	GluC
H2A2A_MOUSE, H2A2B_MOUSE, H2A2C_MOUSE	Histone H2A	896.3127	4476.5271	5	0.0244	41	0	1.30E-06	ac-SGRGKQGGKARAKAKSRSSRAGLQFPVGRVHRLLRKGNYAE	GluC
H2A2A_MOUSE, H2A2B_MOUSE, H2A2C_MOUSE	Histone H2A	904.7152	4518.5397	5	0.0265	41	0	1.90E-06	ac-SGRGKacQGGKARAKAKSRSSRAGLQFPVGRVHRLLRKGNYAE	GluC
H2A2A_MOUSE, H2A2B_MOUSE, H2A2C_MOUSE	Histone H2A	896.3132	4476.5294	5	0.0267	41	0	3.30E-04	ac-SGRGKQGGKARAKAKSRSSRAGLQFPVGRVHRLLRKGNYAE	GluC
H2A2A_MOUSE, H2A2B_MOUSE, H2A2C_MOUSE	Histone H2A	904.7146	4518.5367	5	0.0234	41	0	8.70E-04	ac-SGRGKacQGGKARAKAKSRSSRAGLQFPVGRVHRLLRKGNYAE	GluC
H2A2A_MOUSE, H2A2B_MOUSE, H2A2C_MOUSE	Histone H2A	747.0940	4476.5203	6	0.0176	41	0	2.90E-03	ac-SGRGKQGGKARAKAKSRSSRAGLQFPVGRVHRLLRKGNYAE	GluC
H2A2A_MOUSE, H2A2B_MOUSE, H2A2C_MOUSE	Histone H2A	747.0934	4476.5165	6	0.0138	41	0	1.20E-02	ac-SGRGKQGGKARAKAKSRSSRAGLQFPVGRVHRLLRKGNYAE	GluC
H2A2A_MOUSE, H2A2B_MOUSE, H2A2C_MOUSE	Histone H2A	646.5089	4518.5113	7	-0.0019	41	0	3.40E-02	ac-SGRGKacQGGKARAKAKSRSSRAGLQFPVGRVHRLLRKGNYAE	GluC
H2A2A_MOUSE, H2A2B_MOUSE, H2A2C_MOUSE	Histone H2A	747.0912	4476.5038	6	0.0012	41	0	4.50E-02	ac-SGRGKQGGKARAKAKSRSSRAGLQFPVGRVHRLLRKGNYAE	GluC
H2A2A_MOUSE, H2A2C_MOUSE	Histone H2A	839.7486	3354.9655	4	0.0165	31	2	2.40E-05	EELNKacLLGKVTIAQGGVLPNIQAVLLPKKTE	GluC
H2A2A_MOUSE, H2A2C_MOUSE	Histone H2A	839.7486	3354.9655	4	-0.0199	31	2	1.20E-04	EELNKme2LLGKme1VTIAQGGVLPNIQAVLLPKKTE	GluC
H2A2A_MOUSE, H2A2C_MOUSE	Histone H2A	677.6006	3382.9666	5	-0.0137	31	2	4.60E-02	EELNKacLLGKme2VTIAQGGVLPNIQAVLLPKKTE	GluC
H2A2A_MOUSE, H2A2C_MOUSE	Histone H2A	803.9870	3211.9189	4	-0.0082	30	1	2.10E-09	ELNKLLGKme2VTIAQGGVLPNIQAVLLPKKTE	GluC
H2A2A_MOUSE, H2A2C_MOUSE	Histone H2A	643.3913	3211.9200	5	-0.0072	30	1	2.20E-05	ELNKLLGKme2VTIAQGGVLPNIQAVLLPKKTE	GluC
H2A2A_MOUSE, H2A2C_MOUSE	Histone H2A	1071.6479	3211.9219	3	-0.0053	30	1	2.00E-02	ELNKLLGKme2VTIAQGGVLPNIQAVLLPKKTE	GluC
H2A2A_MOUSE, H2A2C_MOUSE	Histone H2A	803.9887	3211.9255	4	-0.0016	30	1	8.10E-06	ELNKLLGKme2VTIAQGGVLPNIQAVLLPKKTE	GluC
H2A2A_MOUSE, H2A2C_MOUSE	Histone H2A	803.9880	3211.9230	4	-0.0041	30	1	1.10E-12	ELNKLLGKme2VTIAQGGVLPNIQAVLLPKKTE	GluC
H2A2A_MOUSE, H2A2C_MOUSE	Histone H2A	1071.6472	3211.9198	3	-0.0074	30	1	1.50E-02	ELNKLLGKme2VTIAQGGVLPNIQAVLLPKKTE	GluC
H2A2A_MOUSE, H2A2C_MOUSE	Histone H2A	803.9839	3211.9065	4	-0.0206	30	1	4.20E-02	ELNKLLGKme2VTIAQGGVLPNIQAVLLPKKTE	GluC
H2A2A_MOUSE, H2A2C_MOUSE	Histone H2A	803.9867	3211.9176	4	-0.0095	30	1	2.00E-10	ELNKLLGKme2VTIAQGGVLPNIQAVLLPKKTE	GluC
H2A2A_MOUSE, H2A2C_MOUSE	Histone H2A	803.9849	3211.9104	4	-0.0167	30	1	2.70E-03	ELNKme1LLGKme1VTIAQGGVLPNIQAVLLPKKTE	GluC
H2A2A_MOUSE, H2A2C_MOUSE	Histone H2A	803.9851	3211.9113	4	-0.0158	30	1	1.10E-08	ELNKLLGKme2VTIAQGGVLPNIQAVLLPKKTE	GluC
H2A2A_MOUSE, H2A2C_MOUSE	Histone H2A	803.9872	3211.9197	4	-0.0074	30	1	1.70E-05	ELNKLLGKme2VTIAQGGVLPNIQAVLLPKKTE	GluC
H2A2A_MOUSE, H2A2C_MOUSE	Histone H2A	1071.6453	3211.9141	3	-0.0131	30	1	3.50E-04	ELNKLLGKme2VTIAQGGVLPNIQAVLLPKKTE	GluC
H2A2A_MOUSE, H2A2C_MOUSE	Histone H2A	803.9872	3211.9197	4	-0.0074	30	1	2.00E-02	ELNKme2LLGKVTIAQGGVLPNIQAVLLPKKTE	GluC
H2A2A_MOUSE, H2A2C_MOUSE	Histone H2A	1071.6453	3211.9141	3	-0.0131	30	1	2.10E-02	ELNKLLGKme2VTIAQGGVLPNIQAVLLPKKTE	GluC
H2A2A_MOUSE, H2A2C_MOUSE	Histone H2A	803.9847	3211.9096	4	-0.0175	30	1	1.50E-07	ELNKLLGKme2VTIAQGGVLPNIQAVLLPKKTE	GluC
H2A2A_MOUSE, H2A2C_MOUSE	Histone H2A	803.9847	3211.9096	4	-0.0175	30	1	3.10E-03	ELNKLLGKme2VTIAQGGVLPNIQAVLLPKKTE	GluC
H2A2A_MOUSE, H2A2C_MOUSE	Histone H2A	775.2316	3096.8972	4	-0.0030	29	0	7.60E-03	LNKLLGKme2VTIAQGGVLPNIQAVLLPKKme1TE	GluC
H2A2A_MOUSE, H2A2C_MOUSE	Histone H2A	771.7268	3082.8782	4	-0.0064	29	0	1.60E-10	LNKLLGKme2VTIAQGGVLPNIQAVLLPKKTE	GluC
H2A2A_MOUSE, H2A2C_MOUSE	Histone H2A	617.5833	3082.8801	5	-0.0044	29	0	5.50E-10	LNKLLGKme2VTIAQGGVLPNIQAVLLPKKTE	GluC
H2A2A_MOUSE, H2A2C_MOUSE	Histone H2A	771.7261	3082.8755	4	-0.0091	29	0	3.00E-08	LNKLLGKme2VTIAQGGVLPNIQAVLLPKKTE	GluC
H2A2A_MOUSE, H2A2C_MOUSE	Histone H2A	771.7249	3082.8704	4	-0.0141	29	0	1.20E-07	LNKLLGKme2VTIAQGGVLPNIQAVLLPKKTE	GluC
H2A2A_MOUSE, H2A2C_MOUSE	Histone H2A	771.7250	3082.8710	4	-0.0135	29	0	9.80E-06	LNKLLGKme2VTIAQGGVLPNIQAVLLPKKTE	GluC
H2A2A_MOUSE, H2A2C_MOUSE	Histone H2A	771.7278	3082.8823	4	-0.0022	29	0	2.70E-03	LNKLLGKme2VTIAQGGVLPNIQAVLLPKKTE	GluC
H2A2A_MOUSE, H2A2C_MOUSE	Histone H2A	1028.6336	3082.8790	3	-0.0056	29	0	7.70E-03	LNKLLGKme2VTIAQGGVLPNIQAVLLPKKTE	GluC
H2A2A_MOUSE, H2A2C_MOUSE	Histone H2A	1028.6328	3082.8766	3	-0.0080	29	0	3.70E-02	LNKLLGKme2VTIAQGGVLPNIQAVLLPKKTE	GluC
H2A2A_MOUSE, H2A2C_MOUSE	Histone H2A	771.7244	3082.8685	4	-0.0160	29	0	2.10E-07	LNKLLGKme2VTIAQGGVLPNIQAVLLPKKTE	GluC
H2A2A_MOUSE, H2A2C_MOUSE	Histone H2A	771.7246	3082.8694	4	-0.0151	29	0	1.70E-02	LNKme2LLGKVTIAQGGVLPNIQAVLLPKKTE	GluC
H2A2A_MOUSE, H2A2C_MOUSE	Histone H2A	617.5821	3082.8743	5	-0.0103	29	0	5.80E-13	LNKLLGKme2VTIAQGGVLPNIQAVLLPKKTE	GluC
H2A2A_MOUSE, H2A2C_MOUSE	Histone H2A	617.5827	3082.8773	5	-0.0072	29	0	3.40E-12	LNKLLGKme2VTIAQGGVLPNIQAVLLPKKTE	GluC
H2A2A_MOUSE, H2A2C_MOUSE	Histone H2A	771.7262	3082.8755	4	-0.0090	29	0	2.70E-10	LNKLLGKme2VTIAQGGVLPNIQAVLLPKKTE	GluC
H2A2A_MOUSE, H2A2C_MOUSE	Histone H2A	771.7265	3082.8769	4	-0.0077	29	0	3.40E-10	LNKLLGKme2VTIAQGGVLPNIQAVLLPKKTE	GluC
H2A2A_MOUSE, H2A2C_MOUSE	Histone H2A	771.7255	3082.8729	4	-0.0116	29	0	1.20E-09	LNKLLGKme2VTIAQGGVLPNIQAVLLPKKTE	GluC
H2A2A_MOUSE, H2A2C_MOUSE	Histone H2A	771.7257	3082.8736	4	-0.0110	29	0	1.20E-09	LNKLLGKme2VTIAQGGVLPNIQAVLLPKKTE	GluC
H2A2A_MOUSE, H2A2C_MOUSE	Histone H2A	617.5819	3082.8729	5	-0.0116	29	0	1.30E-09	LNKLLGKme2VTIAQGGVLPNIQAVLLPKKTE	GluC
H2A2A_MOUSE, H2A2C_MOUSE	Histone H2A	617.5821	3082.8741	5	-0.0105	29	0	3.70E-09	LNKLLGKme2VTIAQGGVLPNIQAVLLPKKTE	GluC
H2A2A_MOUSE, H2A2C_MOUSE	Histone H2A	617.5827	3082.8771	5	-0.0074	29	0	7.70E-09	LNKLLGKme2VTIAQGGVLPNIQAVLLPKKTE	GluC
H2A2A_MOUSE, H2A2C_MOUSE	Histone H2A	771.7229	3082.8626	4	-0.0219	29	0	1.10E-07	LNKLLGKme2VTIAQGGVLPNIQAVLLPKKTE	GluC
H2A2A_MOUSE, H2A2C_MOUSE	Histone H2A	771.7249	3082.8705	4	-0.0140	29	0	3.60E-07	LNKLLGKme2VTIAQGGVLPNIQAVLLPKKTE	GluC
H2A2A_MOUSE, H2A2C_MOUSE	Histone H2A	771.7265	3082.8769	4	-0.0077	29	0	3.90E-07	LNKLLGKme2VTIAQGGVLPNIQAVLLPKKTE	GluC
H2A2A_MOUSE, H2A2C_MOUSE	Histone H2A	1028.6319	3082.8739	3	-0.0107	29	0	9.40E-04	LNKLLGKme2VTIAQGGVLPNIQAVLLPKKTE	GluC
H2A2A_MOUSE, H2A2C_MOUSE	Histone H2A	1028.6333	3082.8781	3	-0.0065	29	0	2.60E-03	LNKLLGKme2VTIAQGGVLPNIQAVLLPKKTE	GluC
H2A2A_MOUSE, H2A2C_MOUSE	Histone H2A	771.7262	3082.8758	4	-0.0087	29	0	1.40E-02	LNKLLGKme2VTIAQGGVLPNIQAVLLPKKTE	GluC
H2A2A_MOUSE, H2A2C_MOUSE	Histone H2A	1028.6326	3082.8760	3	-0.0086	29	0	3.30E-02	LNKLLGKme2VTIAQGGVLPNIQAVLLPKKTE	GluC
H2A2A_MOUSE, H2A2C_MOUSE	Histone H2A	771.7247	3082.8695	4	-0.0150	29	0	3.40E-08	LNKLLGKme2VTIAQGGVLPNIQAVLLPKKTE	GluC
H2A2A_MOUSE, H2A2C_MOUSE	Histone H2A	771.7245	3082.8687	4	-0.0158	29	0	4.40E-03	LNKLLGKme2VTIAQGGVLPNIQAVLLPKKTE	GluC
H2A2A_MOUSE, H2A2C_MOUSE	Histone H2A	1028.6326	3082.8760	3	-0.0086	29	0	6.00E-03	LNKLLGKme2VTIAQGGVLPNIQAVLLPKKTE	GluC
H2A2A_MOUSE, H2A2C_MOUSE	Histone H2A	617.5832	3082.8795	5	-0.0051	29	0	3.90E-12	LNKLLGKme2VTIAQGGVLPNIQAVLLPKKTE	GluC
H2A2A_MOUSE, H2A2C_MOUSE	Histone H2A	771.7271	3082.8791	4	-0.0054	29	0	5.10E-10	LNKLLGKme2VTIAQGGVLPNIQAVLLPKKTE	GluC
H2A2A_MOUSE, H2A2C_MOUSE	Histone H2A	771.7277	3082.8815	4	-0.0030	29	0	4.00E-09	LNKLLGKme2VTIAQGGVLPNIQAVLLPKKTE	GluC
H2A2A_MOUSE, H2A2C_MOUSE	Histone H2A	771.7260	3082.8747	4	-0.0098	29	0	7.90E-09	LNKLLGKme2VTIAQGGVLPNIQAVLLPKKTE	GluC

uniprot accession/s for all possible isoforms	histone type	pep exp mz	pep exp mr	pep exp z	pep delta	pep length	pep miss	pep expect	peptide and site of PTM	enzyme
H2A2A_MOUSE, H2A2C_MOUSE	Histone H2A	771.7265	3082.8771	4	-0.0074	29	0	9.00E-09	LNKLLGKme2VTIAQGGVLPNIQAVLLPKKTE	GluC
H2A2A_MOUSE, H2A2C_MOUSE	Histone H2A	771.7263	3082.8760	4	-0.0085	29	0	5.40E-08	LNKLLGKme2VTIAQGGVLPNIQAVLLPKKTE	GluC
H2A2A_MOUSE, H2A2C_MOUSE	Histone H2A	1028.6348	3082.8826	3	-0.0020	29	0	5.40E-05	LNKLLGKme2VTIAQGGVLPNIQAVLLPKKTE	GluC
H2A2A_MOUSE, H2A2C_MOUSE	Histone H2A	771.7249	3082.8706	4	-0.0140	29	0	3.60E-04	LNKLLGKme2VTIAQGGVLPNIQAVLLPKKTE	GluC
H2A2A_MOUSE, H2A2C_MOUSE	Histone H2A	617.5835	3082.8813	5	-0.0032	29	0	1.10E-03	LNKLLGKme2VTIAQGGVLPNIQAVLLPKKTE	GluC
H2A2A_MOUSE, H2A2C_MOUSE	Histone H2A	1028.6314	3082.8724	3	-0.0122	29	0	2.50E-03	LNKLLGKme2VTIAQGGVLPNIQAVLLPKKTE	GluC
H2A2A_MOUSE, H2A2C_MOUSE	Histone H2A	771.7273	3082.8802	4	-0.0043	29	0	1.00E-02	LNKLLGKme2VTIAQGGVLPNIQAVLLPKKTE	GluC
H2A2A_MOUSE, H2A2C_MOUSE	Histone H2A	1028.6346	3082.8820	3	-0.0026	29	0	1.10E-02	LNKLLGKme2VTIAQGGVLPNIQAVLLPKKTE	GluC
H2A2A_MOUSE, H2A2C_MOUSE	Histone H2A	1028.6334	3082.8784	3	-0.0062	29	0	1.80E-02	LNKLLGKme2VTIAQGGVLPNIQAVLLPKKTE	GluC
H2A2A_MOUSE, H2A2C_MOUSE	Histone H2A	771.7284	3082.8845	4	-0.0001	29	0	3.50E-02	LNKme2LLGKVTIAQGGVLPNIQAVLLPKKTE	GluC
H2A2A_MOUSE, H2A2C_MOUSE	Histone H2A	1028.6342	3082.8808	3	-0.0038	29	0	3.70E-02	LNKLLGKme2VTIAQGGVLPNIQAVLLPKKTE	GluC
H2A2A_MOUSE, H2A2C_MOUSE	Histone H2A	771.7262	3082.8757	4	-0.0088	29	0	5.50E-10	LNKLLGKme2VTIAQGGVLPNIQAVLLPKKTE	GluC
H2A2A_MOUSE, H2A2C_MOUSE	Histone H2A	771.7245	3082.8687	4	-0.0158	29	0	1.10E-11	LNKLLGKme2VTIAQGGVLPNIQAVLLPKKTE	GluC
H2A2A_MOUSE, H2A2C_MOUSE	Histone H2A	771.7228	3082.8619	4	-0.0226	29	0	6.20E-07	LNKLLGKme2VTIAQGGVLPNIQAVLLPKKTE	GluC
H2A2A_MOUSE, H2A2C_MOUSE	Histone H2A	771.7239	3082.8667	4	-0.0179	29	0	1.40E-05	LNKLLGKme2VTIAQGGVLPNIQAVLLPKKTE	GluC
H2A2A_MOUSE, H2A2C_MOUSE	Histone H2A	1028.6306	3082.8700	3	-0.0146	29	0	1.50E-02	LNKLLGKme2VTIAQGGVLPNIQAVLLPKKTE	GluC
H2A2A_MOUSE, H2A2C_MOUSE	Histone H2A	771.7288	3082.8860	4	0.0015	29	0	8.70E-08	LNKLLGKme2VTIAQGGVLPNIQAVLLPKKTE	GluC
H2A2A_MOUSE, H2A2C_MOUSE	Histone H2A	617.5843	3082.8850	5	0.0005	29	0	1.40E-06	LNKLLGKme2VTIAQGGVLPNIQAVLLPKKTE	GluC
H2A2A_MOUSE, H2A2C_MOUSE	Histone H2A	1028.6342	3082.8808	3	-0.0038	29	0	1.20E-02	LNKLLGKme2VTIAQGGVLPNIQAVLLPKKTE	GluC
H2A2A_MOUSE, H2A2C_MOUSE	Histone H2A	771.7242	3082.8677	4	-0.0168	29	0	4.70E-08	LNKLLGKme2VTIAQGGVLPNIQAVLLPKKTE	GluC
H2A2A_MOUSE, H2A2C_MOUSE	Histone H2A	1028.6328	3082.8766	3	-0.0080	29	0	4.20E-02	LNKLLGKme2VTIAQGGVLPNIQAVLLPKKTE	GluC
H2A2A_MOUSE, H2A2C_MOUSE	Histone H2A	1028.6359	3082.8859	3	0.0013	29	0	2.00E-02	LNKLLGKme2VTIAQGGVLPNIQAVLLPKKTE	GluC
H2A2A_MOUSE, H2A2C_MOUSE	Histone H2A	617.5828	3082.8777	5	-0.0068	29	0	9.10E-11	LNKLLGKme2VTIAQGGVLPNIQAVLLPKKTE	GluC
H2A2A_MOUSE, H2A2C_MOUSE	Histone H2A	771.7242	3082.8675	4	-0.0170	29	0	5.60E-10	LNKLLGKme2VTIAQGGVLPNIQAVLLPKKTE	GluC
H2A2A_MOUSE, H2A2C_MOUSE	Histone H2A	771.7272	3082.8796	4	-0.0050	29	0	3.00E-08	LNKLLGKme2VTIAQGGVLPNIQAVLLPKKTE	GluC
H2A2A_MOUSE, H2A2C_MOUSE	Histone H2A	1028.6283	3082.8631	3	-0.0215	29	0	1.90E-02	LNKLLGKme2VTIAQGGVLPNIQAVLLPKKTE	GluC
H2A2A_MOUSE, H2A2C_MOUSE	Histone H2A	1028.6280	3082.8622	3	-0.0224	29	0	3.90E-02	LNKLLGKme2VTIAQGGVLPNIQAVLLPKKTE	GluC
H2A2A_MOUSE, H2A2C_MOUSE	Histone H2A	768.2297	3068.8897	4	0.0208	29	0	7.10E-03	LNKLLGKVTIAQGGVLPNIQAVLLPKKme1TE	GluC
H2A2A_MOUSE, H2A2C_MOUSE	Histone H2A	771.7236	3082.8653	4	-0.0192	29	0	1.80E-09	LNKLLGKme2VTIAQGGVLPNIQAVLLPKKTE	GluC
H2A2A_MOUSE, H2A2C_MOUSE	Histone H2A	771.7258	3082.8742	4	-0.0104	29	0	2.10E-09	LNKLLGKme2VTIAQGGVLPNIQAVLLPKKTE	GluC
H2A2A_MOUSE, H2A2C_MOUSE	Histone H2A	1028.6332	3082.8778	3	-0.0068	29	0	1.60E-02	LNKLLGKme2VTIAQGGVLPNIQAVLLPKKTE	GluC
H2A2A_MOUSE, H2A2C_MOUSE	Histone H2A	778.7250	3110.8710	4	-0.0084	29	0	1.70E-03	LNKacLLGKme1VTIAQGGVLPNIQAVLLPKKTE	GluC
H2A2A_MOUSE, H2A2C_MOUSE	Histone H2A	771.7239	3082.8665	4	-0.0180	29	0	1.00E-06	LNKLLGKme2VTIAQGGVLPNIQAVLLPKKTE	GluC
H2A2A_MOUSE, H2A2C_MOUSE	Histone H2A	771.7239	3082.8665	4	-0.0180	29	0	1.40E-05	LNKLLGKme2VTIAQGGVLPNIQAVLLPKKTE	GluC
H2A2A_MOUSE, H2A2C_MOUSE	Histone H2A	1542.4403	3082.8660	2	-0.0185	29	0	1.20E-08	LNKLLGKme2VTIAQGGVLPNIQAVLLPKKTE	GluC
H2A2A_MOUSE, H2A2C_MOUSE	Histone H2A	1028.6302	3082.8688	3	-0.0158	29	0	9.70E-08	LNKLLGKme2VTIAQGGVLPNIQAVLLPKKTE	GluC
H2A2A_MOUSE, H2A2C_MOUSE	Histone H2A	1028.6295	3082.8667	3	-0.0179	29	0	1.10E-07	LNKLLGKme2VTIAQGGVLPNIQAVLLPKKTE	GluC
H2A2A_MOUSE, H2A2C_MOUSE	Histone H2A	617.5808	3082.8678	5	-0.0167	29	0	1.00E-06	LNKLLGKme2VTIAQGGVLPNIQAVLLPKKTE	GluC
H2A2A_MOUSE, H2A2C_MOUSE	Histone H2A	617.5806	3082.8665	5	-0.0181	29	0	1.60E-06	LNKLLGKme2VTIAQGGVLPNIQAVLLPKKTE	GluC
H2A2A_MOUSE, H2A2C_MOUSE	Histone H2A	1542.4403	3082.8660	2	-0.0185	29	0	1.90E-06	LNKLLGKme2VTIAQGGVLPNIQAVLLPKKTE	GluC
H2A2A_MOUSE, H2A2C_MOUSE	Histone H2A	1542.4421	3082.8696	2	-0.0149	29	0	2.80E-06	LNKLLGKme2VTIAQGGVLPNIQAVLLPKKTE	GluC
H2A2A_MOUSE, H2A2C_MOUSE	Histone H2A	617.5805	3082.8662	5	-0.0184	29	0	1.10E-04	LNKLLGKme2VTIAQGGVLPNIQAVLLPKKTE	GluC
H2A2A_MOUSE, H2A2C_MOUSE	Histone H2A	617.5806	3082.8665	5	-0.0181	29	0	1.20E-03	LNKLLGKme2VTIAQGGVLPNIQAVLLPKKTE	GluC
H2A2A_MOUSE, H2A2C_MOUSE	Histone H2A	617.5808	3082.8678	5	-0.0167	29	0	2.10E-03	LNKLLGKme2VTIAQGGVLPNIQAVLLPKKTE	GluC
H2A2A_MOUSE, H2A2C_MOUSE	Histone H2A	1028.6302	3082.8688	3	-0.0158	29	0	3.40E-03	LNKLLGKme2VTIAQGGVLPNIQAVLLPKKTE	GluC
H2A2A_MOUSE, H2A2C_MOUSE	Histone H2A	771.7241	3082.8672	4	-0.0174	29	0	7.50E-03	LNKLLGKme2VTIAQGGVLPNIQAVLLPKKTE	GluC
H2A2A_MOUSE, H2A2C_MOUSE	Histone H2A	771.7244	3082.8684	4	-0.0161	29	0	1.10E-02	LNKLLGKme2VTIAQGGVLPNIQAVLLPKKTE	GluC
H2A2A_MOUSE, H2A2C_MOUSE	Histone H2A	1028.6295	3082.8667	3	-0.0179	29	0	1.20E-02	LNKLLGKme2VTIAQGGVLPNIQAVLLPKKTE	GluC
H2A2A_MOUSE, H2A2C_MOUSE	Histone H2A	771.7241	3082.8672	4	-0.0174	29	0	1.30E-02	LNKLLGKme2VTIAQGGVLPNIQAVLLPKKTE	GluC
H2A2A_MOUSE, H2A2C_MOUSE	Histone H2A	617.5805	3082.8662	5	-0.0184	29	0	3.00E-02	LNKLLGKme2VTIAQGGVLPNIQAVLLPKKTE	GluC
H2A2A_MOUSE, H2A2C_MOUSE	Histone H2A	771.7241	3082.8672	4	-0.0173	29	0	4.50E-02	LNKLLGKme2VTIAQGGVLPNIQAVLLPKKTE	GluC
H2A2A_MOUSE, H2A2C_MOUSE	Histone H2A	617.5820	3082.8736	5	-0.0109	29	0	3.60E-09	LNKLLGKme2VTIAQGGVLPNIQAVLLPKKTE	GluC
H2A2A_MOUSE, H2A2C_MOUSE	Histone H2A	771.7261	3082.8755	4	-0.0090	29	0	1.30E-08	LNKLLGKme2VTIAQGGVLPNIQAVLLPKKTE	GluC
H2A2A_MOUSE, H2A2C_MOUSE	Histone H2A	771.7243	3082.8680	4	-0.0166	29	0	1.30E-08	LNKLLGKme2VTIAQGGVLPNIQAVLLPKKTE	GluC
H2A2A_MOUSE, H2A2C_MOUSE	Histone H2A	617.5818	3082.8725	5	-0.0121	29	0	1.50E-08	LNKLLGKme2VTIAQGGVLPNIQAVLLPKKTE	GluC
H2A2A_MOUSE, H2A2C_MOUSE	Histone H2A	771.7258	3082.8739	4	-0.0106	29	0	1.60E-08	LNKLLGKme2VTIAQGGVLPNIQAVLLPKKTE	GluC
H2A2A_MOUSE, H2A2C_MOUSE	Histone H2A	771.7246	3082.8694	4	-0.0152	29	0	4.10E-08	LNKLLGKme2VTIAQGGVLPNIQAVLLPKKTE	GluC
H2A2A_MOUSE, H2A2C_MOUSE	Histone H2A	617.5820	3082.8738	5	-0.0108	29	0	4.40E-08	LNKLLGKme2VTIAQGGVLPNIQAVLLPKKTE	GluC
H2A2A_MOUSE, H2A2C_MOUSE	Histone H2A	771.7242	3082.8675	4	-0.0170	29	0	4.60E-08	LNKLLGKme2VTIAQGGVLPNIQAVLLPKKTE	GluC
H2A2A_MOUSE, H2A2C_MOUSE	Histone H2A	771.7254	3082.8724	4	-0.0122	29	0	7.40E-08	LNKLLGKme2VTIAQGGVLPNIQAVLLPKKTE	GluC
H2A2A_MOUSE, H2A2C_MOUSE	Histone H2A	771.7255	3082.8729	4	-0.0117	29	0	8.40E-08	LNKLLGKme2VTIAQGGVLPNIQAVLLPKKTE	GluC
H2A2A_MOUSE, H2A2C_MOUSE	Histone H2A	771.7249	3082.8703	4	-0.0142	29	0	8.60E-08	LNKLLGKme2VTIAQGGVLPNIQAVLLPKKTE	GluC
H2A2A_MOUSE, H2A2C_MOUSE	Histone H2A	771.7238	3082.8661	4	-0.0184	29	0	1.20E-07	LNKLLGKme2VTIAQGGVLPNIQAVLLPKKTE	GluC
H2A2A_MOUSE, H2A2C_MOUSE	Histone H2A	1028.6299	3082.8679	3	-0.0167	29	0	1.50E-07	LNKLLGKme2VTIAQGGVLPNIQAVLLPKKTE	GluC
H2A2A_MOUSE, H2A2C_MOUSE	Histone H2A	771.7247	3082.8697	4	-0.0149	29	0	1.80E-07	LNKLLGKme2VTIAQGGVLPNIQAVLLPKKTE	GluC
H2A2A_MOUSE, H2A2C_MOUSE	Histone H2A	1028.6314	3082.8724	3	-0.0122	29	0	2.10E-07	LNKme2LLGKVTIAQGGVLPNIQAVLLPKKTE	GluC
H2A2A_MOUSE, H2A2C_MOUSE	Histone H2A	1028.6302	3082.8688	3	-0.0158	29	0	2.30E-07	LNKLLGKme2VTIAQGGVLPNIQAVLLPKKTE	GluC
H2A2A_MOUSE, H2A2C_MOUSE	Histone H2A	771.7252	3082.8715	4	-0.0130	29	0	4.60E-07	LNKLLGKme2VTIAQGGVLPNIQAVLLPKKTE	GluC
H2A2A_MOUSE, H2A2C_MOUSE	Histone H2A	1028.6315	3082.8727	3	-0.0119	29	0	1.40E-06	LNKLLGKme2VTIAQGGVLPNIQAVLLPKKTE	GluC
H2A2A_MOUSE, H2A2C_MOUSE	Histone H2A	617.5806	3082.8668	5	-0.0178	29	0	1.50E-06	LNKLLGKme2VTIAQGGVLPNIQAVLLPKKTE	GluC
H2A2A_MOUSE, H2A2C_MOUSE	Histone H2A	771.7254	3082.8725	4	-0.0121	29	0	1.90E-06	LNKLLGKme2VTIAQGGVLPNIQAVLLPKKTE	GluC
H2A2A_MOUSE, H2A2C_MOUSE	Histone H2A	617.5818	3082.8724	5	-0.0122	29	0	2.00E-06	LNKLLGKme2VTIAQGGVLPNIQAVLLPKKTE	GluC
H2A2A_MOUSE, H2A2C_MOUSE	Histone H2A	617.5816	3082.8715	5	-0.0131	29	0	2.60E-06	LNKLLGKme2VTIAQGGVLPNIQAVLLPKKTE	GluC

uniprot accession/s for all possible isoforms	histone type	pep exp mz	pep exp mr	pep exp z	pep delta	pep length	pep miss	pep expect	peptide and site of PTM	enzyme
H2A2A_MOUSE, H2A2C_MOUSE	Histone H2A	1542.4444	3082.8742	2	-0.0103	29	0	4.50E-06	LNKme2LLGKVTIAQGGVLPNIQAVLLPKKTE	GluC
H2A2A_MOUSE, H2A2C_MOUSE	Histone H2A	771.7255	3082.8729	4	-0.0117	29	0	4.60E-06	LNKLLGKme2VTIAQGGVLPNIQAVLLPKKTE	GluC
H2A2A_MOUSE, H2A2C_MOUSE	Histone H2A	1542.4417	3082.8688	2	-0.0157	29	0	5.80E-06	LNKLLGKme2VTIAQGGVLPNIQAVLLPKKTE	GluC
H2A2A_MOUSE, H2A2C_MOUSE	Histone H2A	1542.4416	3082.8686	2	-0.0159	29	0	8.40E-06	LNKLLGKme2VTIAQGGVLPNIQAVLLPKKTE	GluC
H2A2A_MOUSE, H2A2C_MOUSE	Histone H2A	771.7243	3082.8680	4	-0.0166	29	0	1.00E-05	LNKLLGKme2VTIAQGGVLPNIQAVLLPKKTE	GluC
H2A2A_MOUSE, H2A2C_MOUSE	Histone H2A	1028.6308	3082.8706	3	-0.0140	29	0	1.70E-05	LNKLLGKme2VTIAQGGVLPNIQAVLLPKKTE	GluC
H2A2A_MOUSE, H2A2C_MOUSE	Histone H2A	771.7254	3082.8725	4	-0.0121	29	0	1.90E-05	LNKLLGKme2VTIAQGGVLPNIQAVLLPKKTE	GluC
H2A2A_MOUSE, H2A2C_MOUSE	Histone H2A	771.7252	3082.8715	4	-0.0130	29	0	2.30E-05	LNKLLGKme2VTIAQGGVLPNIQAVLLPKKTE	GluC
H2A2A_MOUSE, H2A2C_MOUSE	Histone H2A	771.7246	3082.8694	4	-0.0152	29	0	4.30E-05	LNKLLGKme2VTIAQGGVLPNIQAVLLPKKTE	GluC
H2A2A_MOUSE, H2A2C_MOUSE	Histone H2A	771.7255	3082.8728	4	-0.0117	29	0	7.30E-05	LNKLLGKme2VTIAQGGVLPNIQAVLLPKKTE	GluC
H2A2A_MOUSE, H2A2C_MOUSE	Histone H2A	617.5812	3082.8697	5	-0.0148	29	0	1.20E-04	LNKLLGKme2VTIAQGGVLPNIQAVLLPKKTE	GluC
H2A2A_MOUSE, H2A2C_MOUSE	Histone H2A	771.7242	3082.8675	4	-0.0170	29	0	1.20E-04	LNKLLGKme2VTIAQGGVLPNIQAVLLPKKTE	GluC
H2A2A_MOUSE, H2A2C_MOUSE	Histone H2A	617.5808	3082.8675	5	-0.0170	29	0	2.10E-04	LNKLLGKme2VTIAQGGVLPNIQAVLLPKKTE	GluC
H2A2A_MOUSE, H2A2C_MOUSE	Histone H2A	771.7258	3082.8739	4	-0.0106	29	0	2.10E-04	LNKLLGKme2VTIAQGGVLPNIQAVLLPKKTE	GluC
H2A2A_MOUSE, H2A2C_MOUSE	Histone H2A	771.7238	3082.8661	4	-0.0184	29	0	4.60E-04	LNKLLGKme2VTIAQGGVLPNIQAVLLPKKTE	GluC
H2A2A_MOUSE, H2A2C_MOUSE	Histone H2A	617.5812	3082.8695	5	-0.0150	29	0	5.10E-04	LNKLLGKme2VTIAQGGVLPNIQAVLLPKKTE	GluC
H2A2A_MOUSE, H2A2C_MOUSE	Histone H2A	617.5815	3082.8712	5	-0.0134	29	0	5.40E-04	LNKLLGKme2VTIAQGGVLPNIQAVLLPKKTE	GluC
H2A2A_MOUSE, H2A2C_MOUSE	Histone H2A	771.7262	3082.8757	4	-0.0089	29	0	1.40E-03	LNKLLGKme2VTIAQGGVLPNIQAVLLPKKTE	GluC
H2A2A_MOUSE, H2A2C_MOUSE	Histone H2A	617.5805	3082.8663	5	-0.0183	29	0	2.10E-03	LNKLLGKme2VTIAQGGVLPNIQAVLLPKKTE	GluC
H2A2A_MOUSE, H2A2C_MOUSE	Histone H2A	771.7258	3082.8743	4	-0.0103	29	0	2.10E-03	LNKLLGKme2VTIAQGGVLPNIQAVLLPKKTE	GluC
H2A2A_MOUSE, H2A2C_MOUSE	Histone H2A	617.5805	3082.8663	5	-0.0183	29	0	2.40E-03	LNKLLGKme2VTIAQGGVLPNIQAVLLPKKTE	GluC
H2A2A_MOUSE, H2A2C_MOUSE	Histone H2A	617.5808	3082.8674	5	-0.0171	29	0	3.60E-03	LNKLLGKme2VTIAQGGVLPNIQAVLLPKKTE	GluC
H2A2A_MOUSE, H2A2C_MOUSE	Histone H2A	1028.6312	3082.8718	3	-0.0128	29	0	7.70E-03	LNKLLGKme2VTIAQGGVLPNIQAVLLPKKTE	GluC
H2A2A_MOUSE, H2A2C_MOUSE	Histone H2A	1028.6304	3082.8694	3	-0.0152	29	0	8.10E-03	LNKLLGKme2VTIAQGGVLPNIQAVLLPKKTE	GluC
H2A2A_MOUSE, H2A2C_MOUSE	Histone H2A	617.5815	3082.8712	5	-0.0134	29	0	9.40E-03	LNKLLGKme2VTIAQGGVLPNIQAVLLPKKTE	GluC
H2A2A_MOUSE, H2A2C_MOUSE	Histone H2A	617.5808	3082.8675	5	-0.0170	29	0	2.20E-02	LNKLLGKme2VTIAQGGVLPNIQAVLLPKKTE	GluC
H2A2A_MOUSE, H2A2C_MOUSE	Histone H2A	617.5806	3082.8668	5	-0.0178	29	0	3.30E-02	LNKLLGKme2VTIAQGGVLPNIQAVLLPKKTE	GluC
H2A2A_MOUSE, H2A2C_MOUSE	Histone H2A	1028.6295	3082.8667	3	-0.0179	29	0	3.70E-02	LNKLLGKme2VTIAQGGVLPNIQAVLLPKKTE	GluC
H2A2A_MOUSE, H2A2C_MOUSE	Histone H2A	1028.6299	3082.8679	3	-0.0167	29	0	4.40E-02	LNKLLGKme2VTIAQGGVLPNIQAVLLPKKTE	GluC
H2A2A_MOUSE, H2A2C_MOUSE	Histone H2A	1028.6294	3082.8664	3	-0.0182	29	0	5.80E-06	LNKLLGKme2VTIAQGGVLPNIQAVLLPKKTE	GluC
H2A2A_MOUSE, H2A2C_MOUSE	Histone H2A	1028.6294	3082.8664	3	-0.0182	29	0	2.10E-02	LNKLLGKme2VTIAQGGVLPNIQAVLLPKKTE	GluC
H2A2A_MOUSE, H2A2C_MOUSE	Histone H2A	778.7239	3110.8666	4	-0.0128	29	0	9.40E-06	LNKacLLGKme1VTIAQGGVLPNIQAVLLPKKTE	GluC
H2A2A_MOUSE, H2A2C_MOUSE	Histone H2A	781.7283	3122.8843	4	0.0048	28	0	1.20E-04	LNKcrLLGKVTIAQGGVLPNIQAVLLPKKTE	GluC
H2A2A_MOUSE, H2A2C_MOUSE	Histone H2A	781.7283	3122.8843	4	0.0048	28	0	7.00E-04	LNKcrLLGKVTIAQGGVLPNIQAVLLPKKTE	GluC
H2A2A_MOUSE, H2A2C_MOUSE	Histone H2A	788.7304	3150.8924	4	0.0068	28	0	3.50E-04	LNKcrLLGKVTIAQGGVLPNIQAVLLPKKTE	GluC
H2A2A_MOUSE, H2A2C_MOUSE	Histone H2A	788.7304	3150.8924	4	0.0068	28	0	3.80E-04	LNKcrLLGKVTIAQGGVLPNIQAVLLPKKTE	GluC
H2A2A_MOUSE, H2A2C_MOUSE	Histone H2A	788.7306	3150.8935	4	0.0079	28	0	4.80E-02	LNKcrLLGKVTIAQGGVLPNIQAVLLPKKTE	GluC
H2A2A_MOUSE, H2A2C_MOUSE	Histone H2A	788.7306	3150.8935	4	0.0079	28	0	7.20E-05	LNKcrLLGKVTIAQGGVLPNIQAVLLPKKTE	GluC
H2A2A_MOUSE, H2A2C_MOUSE	Histone H2A	598.0282	1791.0627	3	0.0009	18	0	1.40E-07	GKme1VTIAQGGVLPNIQAVL	Chymotrypsin
H2A2A_MOUSE, H2A2C_MOUSE	Histone H2A	896.5389	1791.0633	2	0.0015	18	0	2.90E-04	GKme1VTIAQGGVLPNIQAVL	Chymotrypsin
H2A2B_MOUSE	Histone H2A	796.7270	3182.8789	4	0.0147	30	1	1.60E-03	ELNKbLLGGVTIAQGGVLPNIQAVLLPKKTE	GluC
H2A2B_MOUSE	Histone H2A	796.7261	3182.8754	4	0.0112	30	1	2.00E-03	ELNKbLLGGVTIAQGGVLPNIQAVLLPKKTE	GluC
H2A2B_MOUSE	Histone H2A	796.7261	3182.8754	4	0.0112	30	1	2.60E-03	ELNKbLLGGVTIAQGGVLPNIQAVLLPKKTE	GluC
H2A2B_MOUSE	Histone H2A	760.9600	3039.8110	4	0.0050	29	0	3.20E-03	LNKppLLGGVTIAQGGVLPNIQAVLLPKKTE	GluC
H2A2C_MOUSE	Histone H2A	414.2403	826.4661	2	0.0001	7	0	6.50E-03	SHKAKacSK	GluC
H2A3_MOUSE	Histone H2A	749.7607	4492.5204	6	0.0228	41	0	1.90E-02	ac-SGRGKQGGKARAKAKSRSSRAGLQFPVGRVHRLLRKGNYSE	GluC
H2A3_MOUSE	Histone H2A	756.7630	4534.5345	6	0.0264	41	0	2.00E-04	ac-SGRGKacQGGKARAKAKSRSSRAGLQFPVGRVHRLLRKGNYSE	GluC
H2A3_MOUSE	Histone H2A	749.7617	4492.5265	6	-0.0075	41	0	2.00E-03	SGRme3GKQGGKARAKAKSRSSRAGLQFPVGRVHRLLRKGNYSE	GluC
H2A3_MOUSE	Histone H2A	899.5058	4492.4926	5	-0.0050	41	0	3.10E-04	ac-SGRGKQGGKARAKAKSRSSRAGLQFPVGRVHRLLRKGNYSE	GluC
H2A3_MOUSE	Histone H2A	907.9111	4534.5191	5	0.0110	41	0	1.30E-03	ac-SGRGKacQGGKARAKAKSRSSRAGLQFPVGRVHRLLRKGNYSE	GluC
H2A3_MOUSE	Histone H2A	749.7576	4492.5018	6	0.0042	41	0	2.80E-03	ac-SGRGKQGGKARAKAKSRSSRAGLQFPVGRVHRLLRKGNYSE	GluC
H2A3_MOUSE	Histone H2A	916.3121	4576.5240	5	0.0052	41	0	5.60E-03	ac-SGRGKacQGGKacARAKAKSRSSRAGLQFPVGRVHRLLRKGNYSE	GluC
H2A3_MOUSE	Histone H2A	899.5069	4492.4984	5	0.0008	41	0	1.20E-02	ac-SGRGKQGGKARAKAKSRSSRAGLQFPVGRVHRLLRKGNYSE	GluC
H2A3_MOUSE	Histone H2A	749.7574	4492.5010	6	0.0034	41	0	3.00E-02	ac-SGRGKQGGKARAKAKSRSSRAGLQFPVGRVHRLLRKGNYSE	GluC
H2A3_MOUSE	Histone H2A	756.7602	4534.5178	6	0.0096	41	0	3.90E-02	ac-SGRGKacQGGKARAKAKSRSSRAGLQFPVGRVHRLLRKGNYSE	GluC
H2A3_MOUSE	Histone H2A	899.5108	4492.5176	5	0.0200	41	0	1.20E-05	ac-SGRGKQGGKARAKAKSRSSRAGLQFPVGRVHRLLRKGNYSE	GluC
H2A3_MOUSE	Histone H2A	749.7607	4492.5204	6	0.0228	41	0	1.90E-05	ac-SGRGKQGGKARAKAKSRSSRAGLQFPVGRVHRLLRKGNYSE	GluC
H2A3_MOUSE	Histone H2A	899.5110	4492.5188	5	0.0212	41	0	4.20E-05	ac-SGRGKQGGKARAKAKSRSSRAGLQFPVGRVHRLLRKGNYSE	GluC
H2A3_MOUSE	Histone H2A	749.7600	4492.5166	6	0.0190	41	0	3.40E-02	ac-SGRGKQGGKARAKAKSRSSRAGLQFPVGRVHRLLRKGNYSE	GluC
H2A3_MOUSE	Histone H2A	899.5065	4492.4962	5	-0.0014	41	0	2.80E-03	ac-SGRGKQGGKARAKAKSRSSRAGLQFPVGRVHRLLRKGNYSE	GluC
H2AJ_MOUSE	Histone H2A	857.7048	4283.4875	5	0.0295	39	0	1.50E-08	DEELNKLLGRVTIAQGGVLPNIQAVLLPKKTESQKVKSKac	AspN
H2AJ_MOUSE	Histone H2A	423.7558	845.4970	2	0.0000	7	0	5.50E-04	SQKVKSKac	GluC
H2AJ_MOUSE	Histone H2A	423.7552	845.4959	2	-0.0011	7	0	2.50E-03	SQKVKSKac	GluC
H2AJ_MOUSE	Histone H2A	423.7562	845.4978	2	0.0007	7	0	5.70E-04	SQKVKSKac	GluC
H2AJ_MOUSE	Histone H2A	423.7562	845.4978	2	0.0007	7	0	1.60E-02	SQKVKSKac	GluC
H2AV_MOUSE	Histone H2A	662.3997	3306.9623	5	0.0120	32	1	3.80E-07	LDSLIKATIAGGGVIPHIHKSLIGKKGQQKme2TA	GluC
H2AV_MOUSE	Histone H2A	1103.3290	3306.9652	3	0.0148	32	1	5.60E-05	LDSLIKATIAGGGVIPHIHKSLIGKKGQQKme2TA	GluC
H2AV_MOUSE	Histone H2A	827.7469	3306.9586	4	0.0082	32	1	1.70E-02	LDSLIKATIAGGGVIPHIHKSLIGKKGQQKme2TA	GluC
H2AV_MOUSE	Histone H2A	827.7457	3306.9537	4	0.0033	32	1	7.00E-05	LDSLIKATIAGGGVIPHIHKSLIGKKGQQKme2TA	GluC
H2AV_MOUSE	Histone H2A	1065.6349	3193.8829	3	0.0166	31	0	4.80E-02	DSLIKATIAGGGVIPHIHKSLIGKKGQQKme2TA	AspN
H2AV_MOUSE	Histone H2A	1065.6323	3193.8751	3	0.0088	31	0	5.90E-05	DSLIKATIAGGGVIPHIHKSLIGKKGQQKme2TA	AspN
H2AV_MOUSE	Histone H2A	639.7815	3193.8710	5	0.0047	31	0	3.00E-03	DSLIKATIAGGGVIPHIHKSLIGKKGQQKme2TA	AspN
H2AV_MOUSE	Histone H2A	622.3746	3106.8365	5	0.0023	30	0	3.50E-09	SLIKATIAGGGVIPHIHKSLIGKKGQQKppTA	GluC

uniprot accession/s for all possible isoforms	histone type	pep exp mz	pep exp mr	pep exp z	pep delta	pep length	pep miss	pep expect	peptide and site of PTM	enzyme
H2AV_MOUSE	Histone H2A	777.7155	3106.8329	4	-0.0014	30	0	2.50E-08	SLIKATIAGGGVIPHIHKSLIGKKGQQKppTA	GluC
H2AV_MOUSE	Histone H2A	770.7177	3078.8417	4	0.0023	30	0	5.90E-06	SLIKATIAGGGVIPHIHKSLIGKKGQQKme2TA	GluC
H2AV_MOUSE	Histone H2A	799.4742	3193.8679	4	0.0016	30	0	1.40E-02	DSLIKATIAGGGVIPHIHKSLIG(Kme2)KGQQKTA	AspN
H2AV_MOUSE, H2AZ_MOUSE	Histone H2A	650.8471	1299.6797	2	0.0015	13	3	1.30E-03	AGGKacAGKacDSGKacAK	Semi-tryptic
H2AV_MOUSE, H2AZ_MOUSE	Histone H2A	650.8471	1299.6797	2	0.0015	13	3	1.00E-03	AGGKacAGKacDSGKacAK	Semi-tryptic
H2AV_MOUSE, H2AZ_MOUSE	Histone H2A	373.1957	744.3768	2	0.0002	8	0	9.30E-03	AGGKAGKacD	GluC
H2AX_MOUSE	Histone H2A	746.4291	4472.5308	6	-0.0134	41	0	1.60E-04	SGRme3GKTGGKARAKAKSRSSRAGLQFPVGRVHRLLRKGHYAE	GluC
H2AX_MOUSE	Histone H2A	895.5163	4472.5453	5	0.0011	41	0	1.50E-04	SGRme3GKTGGKARAKAKSRSSRAGLQFPVGRVHRLLRKGHYAE	GluC
H2AX_MOUSE	Histone H2A	895.5099	4472.5133	5	0.0055	41	0	5.10E-05	ac-SGRGKTGGKARAKAKSRSSRAGLQFPVGRVHRLLRKGHYAE	GluC
H2AX_MOUSE	Histone H2A	895.5133	4472.5302	5	0.0224	41	0	6.10E-04	ac-SGRGKTGGKARAKAKSRSSRAGLQFPVGRVHRLLRKGHYAE	GluC
H2AX_MOUSE	Histone H2A	753.4288	4514.5294	6	0.0110	41	0	1.90E-03	ac-SGRGKacTGGKARAKAKSRSSRAGLQFPVGRVHRLLRKGHYAE	GluC
H2AX_MOUSE	Histone H2A	746.4286	4472.5277	6	0.0199	41	0	3.90E-03	ac-SGRGKTGGKARAKAKSRSSRAGLQFPVGRVHRLLRKGHYAE	GluC
H2AX_MOUSE	Histone H2A	746.4292	4472.5317	6	0.0239	41	0	8.60E-03	ac-SGRGKTGGKARAKAKSRSSRAGLQFPVGRVHRLLRKGHYAE	GluC
H2AX_MOUSE	Histone H2A	753.4291	4514.5311	6	0.0127	41	0	1.90E-02	ac-SGRGKacTGGKARAKAKSRSSRAGLQFPVGRVHRLLRKGHYAE	GluC
H2AX_MOUSE	Histone H2A	746.4259	4472.5120	6	0.0043	41	0	2.60E-02	ac-SGRGKTGGKARAKAKSRSSRAGLQFPVGRVHRLLRKGHYAE	GluC
H2AX_MOUSE	Histone H2A	807.8806	1613.7466	2	0.0016	15	2	4.80E-06	APAVGKKApSQASQEY	Semi-tryptic
H2AX_MOUSE	Histone H2A	546.2237	1090.4328	2	-0.0003	9	1	4.40E-05	KApSQASQEY	Semi-tryptic
H2AX_MOUSE	Histone H2A	546.2245	1090.4345	2	0.0013	9	1	1.10E-02	KASQApSQEY	Semi-tryptic
H2AX_MOUSE	Histone H2A	546.2240	1090.4334	2	0.0002	9	1	9.50E-03	KApSQASQEY	Semi-tryptic
H2AX_MOUSE	Histone H2A	546.2241	1090.4337	2	0.0005	9	1	3.40E-04	KASQApSQEY	Semi-tryptic
H2AX_MOUSE	Histone H2A	463.1984	924.3823	2	-0.0001	8	0	2.00E-04	ASacQASQEY	Semi-tryptic
H2B1A_MOUSE, H2B1B_MOUSE, H2B1C_MOUSE, H2B1F_MOUSE, H2B1H_MOUSE, H2B1K_MOUSE, H2B1M_MOUSE, H2B1P_MOUSE, H2B2B_MOUSE	Histone H2B	514.5941	1540.7605	3	-0.0005	13	1	4.80E-04	STITS(p)REIQTAVR	Semi-tryptic
H2B1A_MOUSE, H2B1B_MOUSE, H2B1C_MOUSE, H2B1F_MOUSE, H2B1H_MOUSE, H2B1K_MOUSE, H2B1M_MOUSE, H2B1P_MOUSE, H2B2B_MOUSE	Histone H2B	771.3883	1540.7620	2	0.0010	13	1	5.20E-03	STIT(p)SREIQTAVR	Semi-tryptic
H2B1A_MOUSE, H2B1B_MOUSE, H2B1C_MOUSE, H2B1F_MOUSE, H2B1H_MOUSE, H2B1K_MOUSE, H2B1M_MOUSE, H2B1P_MOUSE, H2B2B_MOUSE	Histone H2B	514.5940	1540.7601	3	-0.0010	13	1	6.10E-06	STI(p)TSREIQTAVR	Semi-tryptic
H2B1A_MOUSE, H2B1B_MOUSE, H2B1C_MOUSE, H2B1F_MOUSE, H2B1H_MOUSE, H2B1K_MOUSE, H2B1M_MOUSE, H2B1P_MOUSE, H2B2B_MOUSE	Histone H2B	514.5942	1540.7609	3	-0.0002	13	1	6.40E-04	STIT(p)SREIQTAVR	Semi-tryptic
H2B1A_MOUSE, H2B1B_MOUSE, H2B1C_MOUSE, H2B1F_MOUSE, H2B1H_MOUSE, H2B1K_MOUSE, H2B1M_MOUSE, H2B1P_MOUSE, H2B2B_MOUSE	Histone H2B	771.3883	1540.7620	2	0.0010	13	1	9.90E-04	STIT(p)SREIQTAVR	Semi-tryptic
H2B1A_MOUSE, H2B1B_MOUSE, H2B1C_MOUSE, H2B1F_MOUSE, H2B1H_MOUSE, H2B1K_MOUSE, H2B1M_MOUSE, H2B1P_MOUSE, H2B2B_MOUSE, H2B2E_MOUSE, H2B3A_MOUSE, H2B3B_MOUSE	Histone H2B	690.6838	2069.0296	3	0.0129	17	0	8.30E-06	ASRLAHYNKRSTI(p)TSRE	GluC
H2B1A_MOUSE, H2B1B_MOUSE, H2B1C_MOUSE, H2B1F_MOUSE, H2B1H_MOUSE, H2B1K_MOUSE, H2B1M_MOUSE, H2B1P_MOUSE, H2B2B_MOUSE, H2B2E_MOUSE, H2B3A_MOUSE, H2B3B_MOUSE	Histone H2B	518.2640	2069.0270	4	0.0103	17	0	8.60E-04	ASRLAHYNKRSTITpSRE	GluC
H2B1A_MOUSE, H2B1B_MOUSE, H2B1C_MOUSE, H2B1F_MOUSE, H2B1H_MOUSE, H2B1K_MOUSE, H2B1M_MOUSE, H2B1P_MOUSE, H2B2B_MOUSE, H2B2E_MOUSE, H2B3A_MOUSE, H2B3B_MOUSE	Histone H2B	916.0208	1830.0270	2	0.0020	16	1	3.80E-05	LLLPGELAKcrHAVSEGTK	Semi-tryptic
H2B1A_MOUSE, H2B1B_MOUSE, H2B1C_MOUSE, H2B1F_MOUSE, H2B1H_MOUSE, H2B1K_MOUSE, H2B1M_MOUSE, H2B1P_MOUSE, H2B2B_MOUSE, H2B2E_MOUSE, H2B3A_MOUSE, H2B3B_MOUSE	Histone H2B	916.0218	1830.0291	2	0.0040	16	1	2.00E-05	LLLPGELAKcrHAVSEGTK	Semi-tryptic
H2B1A_MOUSE, H2B1B_MOUSE, H2B1C_MOUSE, H2B1F_MOUSE, H2B1H_MOUSE, H2B1K_MOUSE, H2B1M_MOUSE, H2B1P_MOUSE, H2B2B_MOUSE, H2B2E_MOUSE, H2B3A_MOUSE, H2B3B_MOUSE	Histone H2B	436.5500	1306.6281	3	-0.0002	12	1	4.90E-03	HAVSEGpTKAVTK	Semi-tryptic
H2B1A_MOUSE, H2B1B_MOUSE, H2B1C_MOUSE, H2B1F_MOUSE, H2B1H_MOUSE, H2B1K_MOUSE, H2B1M_MOUSE, H2B1P_MOUSE, H2B2B_MOUSE, H2B2E_MOUSE, H2B3A_MOUSE, H2B3B_MOUSE	Histone H2B	416.8591	1247.5556	3	0.0008	11	0	2.30E-05	QVHPDTGISpSK	Semi-tryptic
H2B1A_MOUSE, H2B1B_MOUSE, H2B1C_MOUSE, H2B1F_MOUSE, H2B1H_MOUSE, H2B1K_MOUSE, H2B1M_MOUSE, H2B1P_MOUSE, H2B2B_MOUSE, H2B2E_MOUSE, H2B3A_MOUSE, H2B3B_MOUSE	Histone H2B	624.7849	1247.5552	2	0.0005	11	0	3.80E-02	QVHPDTGISpSK	Semi-tryptic
H2B1A_MOUSE, H2B1B_MOUSE, H2B1C_MOUSE, H2B1F_MOUSE, H2B1H_MOUSE, H2B1K_MOUSE, H2B1M_MOUSE, H2B1P_MOUSE, H2B2B_MOUSE, H2B2E_MOUSE, H2B3A_MOUSE, H2B3B_MOUSE	Histone H2B	435.7193	869.4241	2	-0.0002	8	0	2.00E-03	HAVSacEGTK	Semi-tryptic
H2B1A_MOUSE, H2B1B_MOUSE, H2B1C_MOUSE, H2B1F_MOUSE, H2B1H_MOUSE, H2B1K_MOUSE, H2B1M_MOUSE, H2B1P_MOUSE, H2B2B_MOUSE, H2B2E_MOUSE, H2B3A_MOUSE, H2B3B_MOUSE	Histone H2B	454.6974	907.3802	2	0.0001	8	0	1.20E-03	HAVpSEGTK	Semi-tryptic
H2B1A_MOUSE, H2B1B_MOUSE, H2B1C_MOUSE, H2B1F_MOUSE, H2B1H_MOUSE, H2B1K_MOUSE, H2B1M_MOUSE, H2B1P_MOUSE, H2B2B_MOUSE, H2B2E_MOUSE, H2B3A_MOUSE, H2B3B_MOUSE	Histone H2B	454.6971	907.3797	2	-0.0003	8	0	1.10E-02	HAVpSEGTK	Semi-tryptic
H2B1A_MOUSE, H2B1B_MOUSE, H2B1C_MOUSE, H2B1F_MOUSE, H2B1H_MOUSE, H2B1K_MOUSE, H2B1M_MOUSE, H2B1P_MOUSE, H2B2B_MOUSE, H2B2E_MOUSE, H2B3A_MOUSE, H2B3B_MOUSE	Histone H2B	454.6973	907.3800	2	0.0000	8	0	2.00E-02	HAVpSEGTK	Semi-tryptic
H2B1A_MOUSE, H2B1B_MOUSE, H2B1C_MOUSE, H2B1F_MOUSE, H2B1H_MOUSE, H2B1K_MOUSE, H2B1M_MOUSE, H2B1P_MOUSE, H2B2B_MOUSE, H2B2E_MOUSE, H2B3A_MOUSE, H2B3B_MOUSE	Histone H2B	448.7455	895.4765	2	0.0002	8	0	2.20E-02	LAKacHAVSE	GluC
H2B1A_MOUSE, H2B1B_MOUSE, H2B1C_MOUSE, H2B1F_MOUSE, H2B1H_MOUSE, H2B1K_MOUSE, H2B1M_MOUSE, H2B1P_MOUSE, H2B2B_MOUSE, H2B2E_MOUSE, H2B3A_MOUSE, H2B3B_MOUSE	Histone H2B	454.6974	907.3802	2	0.0002	8	0	6.70E-03	HAVpSEGTK	Semi-tryptic

uniprot accession/s for all possible isoforms	histone type	pep exp mz	pep exp mr	pep exp z	pep delta	pep length	pep miss	pep expect	peptide and site of PTM	enzyme
H2B1A_MOUSE, H2B1B_MOUSE, H2B1C_MOUSE, H2B1F_MOUSE, H2B1H_MOUSE, H2B1K_MOUSE, H2B1M_MOUSE, H2B1P_MOUSE, H2B2B_MOUSE, H2B2E_MOUSE, H2B3A_MOUSE, H2B3B_MOUSE	Histone H2B	454.6974	907.3803	2	0.0002	8	0	1.60E-02	HAVpSEGTK	Semi-tryptic
H2B1A_MOUSE, H2B1B_MOUSE, H2B1C_MOUSE, H2B1F_MOUSE, H2B1H_MOUSE, H2B1K_MOUSE, H2B1M_MOUSE, H2B1P_MOUSE, H2B2B_MOUSE, H2B2E_MOUSE, H2B3A_MOUSE, H2B3B_MOUSE	Histone H2B	448.7452	895.4759	2	-0.0004	8	0	8.50E-03	LAKacHAVSE	GluC
H2B1A_MOUSE, H2B1B_MOUSE, H2B1C_MOUSE, H2B1F_MOUSE, H2B1H_MOUSE, H2B1K_MOUSE, H2B1M_MOUSE, H2B1P_MOUSE, H2B2B_MOUSE, H2B2E_MOUSE, H2B3A_MOUSE, H2B3B_MOUSE	Histone H2B	448.7452	895.4759	2	-0.0004	8	0	3.80E-02	LAKacHAVSE	GluC
H2B1A_MOUSE, H2B1B_MOUSE, H2B1C_MOUSE, H2B1F_MOUSE, H2B1H_MOUSE, H2B1K_MOUSE, H2B1M_MOUSE, H2B1P_MOUSE, H2B2B_MOUSE, H2B2E_MOUSE, H2B3A_MOUSE, H2B3B_MOUSE	Histone H2B	448.7456	895.4767	2	0.0004	8	0	2.10E-03	LAKacHAVSE	GluC
H2B1A_MOUSE, H2B1B_MOUSE, H2B1C_MOUSE, H2B1F_MOUSE, H2B1H_MOUSE, H2B1K_MOUSE, H2B1M_MOUSE, H2B1P_MOUSE, H2B2B_MOUSE, H2B2E_MOUSE, H2B3A_MOUSE, H2B3B_MOUSE	Histone H2B	448.7456	895.4767	2	0.0004	8	0	2.30E-02	LAKacHAVSE	GluC
H2B1A_MOUSE, H2B1B_MOUSE, H2B1C_MOUSE, H2B1F_MOUSE, H2B1H_MOUSE, H2B1K_MOUSE, H2B1M_MOUSE, H2B1P_MOUSE, H2B2B_MOUSE, H2B2E_MOUSE, H2B3A_MOUSE, H2B3B_MOUSE	Histone H2B	454.6974	907.3803	2	0.0002	8	0	3.00E-04	HAVpSEGTK	Semi-tryptic
H2B1A_MOUSE, H2B1B_MOUSE, H2B1C_MOUSE, H2B1F_MOUSE, H2B1H_MOUSE, H2B1K_MOUSE, H2B1M_MOUSE, H2B1P_MOUSE, H2B2B_MOUSE, H2B2E_MOUSE, H2B3A_MOUSE, H2B3B_MOUSE	Histone H2B	454.6972	907.3799	2	-0.0001	8	0	6.00E-04	HAVpSEGTK	Semi-tryptic
H2B1A_MOUSE, H2B1B_MOUSE, H2B1C_MOUSE, H2B1F_MOUSE, H2B1H_MOUSE, H2B1K_MOUSE, H2B1M_MOUSE, H2B1P_MOUSE, H2B2B_MOUSE, H2B2E_MOUSE, H2B3A_MOUSE, H2B3B_MOUSE	Histone H2B	461.7535	921.4925	2	0.0005	7	0	1.10E-04	LAKcrHAVSE	GluC
H2B1A_MOUSE, H2B1B_MOUSE, H2B1C_MOUSE, H2B1F_MOUSE, H2B1H_MOUSE, H2B1K_MOUSE, H2B1M_MOUSE, H2B1P_MOUSE, H2B2B_MOUSE, H2B2E_MOUSE, H2B3A_MOUSE, H2B3B_MOUSE	Histone H2B	461.7534	921.4922	2	0.0003	7	0	3.60E-02	LAKcrHAVSE	GluC
H2B1A_MOUSE, H2B1B_MOUSE, H2B1C_MOUSE, H2B1F_MOUSE, H2B1H_MOUSE, H2B1K_MOUSE, H2B1M_MOUSE, H2B1P_MOUSE, H2B2B_MOUSE, H2B2E_MOUSE, H2B3A_MOUSE, H2B3B_MOUSE	Histone H2B	461.7534	921.4922	2	0.0003	7	0	6.20E-05	LAKcrHAVSE	GluC
H2B1A_MOUSE, H2B1B_MOUSE, H2B1C_MOUSE, H2B1F_MOUSE, H2B1H_MOUSE, H2B1M_MOUSE, H2B1P_MOUSE, H2B2B_MOUSE, H2B3A_MOUSE, H2B3B_MOUSE	Histone H2B	625.3069	1872.8988	3	0.0005	17	2	3.40E-02	HAVSEGpTKAVTKYTSSK	Semi-tryptic
H2B1A_MOUSE, H2B1B_MOUSE, H2B1C_MOUSE, H2B1F_MOUSE, H2B1H_MOUSE, H2B1M_MOUSE, H2B1P_MOUSE, H2B2B_MOUSE, H2B3A_MOUSE, H2B3B_MOUSE	Histone H2B	656.8595	1311.7045	2	0.0011	12	0	3.10E-02	GTKacAVTKYTSSK	GluC
H2B1A_MOUSE, H2B1B_MOUSE, H2B1C_MOUSE, H2B1F_MOUSE, H2B1H_MOUSE, H2B1M_MOUSE, H2B1P_MOUSE, H2B2B_MOUSE, H2B3A_MOUSE, H2B3B_MOUSE	Histone H2B	656.8599	1311.7053	2	0.0019	12	0	3.30E-06	GTKAVTKacYTSSK	GluC
H2B1A_MOUSE, H2B1B_MOUSE, H2B1C_MOUSE, H2B1F_MOUSE, H2B1H_MOUSE, H2B1M_MOUSE, H2B1P_MOUSE, H2B2B_MOUSE, H2B3A_MOUSE, H2B3B_MOUSE	Histone H2B	438.2417	1311.7033	3	-0.0001	12	0	1.90E-05	GTKacAVTKYTSSK	GluC
H2B1A_MOUSE, H2B1B_MOUSE, H2B1C_MOUSE, H2B1F_MOUSE, H2B1H_MOUSE, H2B1M_MOUSE, H2B1P_MOUSE, H2B2B_MOUSE, H2B3A_MOUSE, H2B3B_MOUSE	Histone H2B	656.8594	1311.7042	2	0.0008	12	0	3.90E-04	GTKAVTKacYTSSK	GluC
H2B1A_MOUSE, H2B1B_MOUSE, H2B1C_MOUSE, H2B1F_MOUSE, H2B1H_MOUSE, H2B1M_MOUSE, H2B1P_MOUSE, H2B2B_MOUSE, H2B3A_MOUSE, H2B3B_MOUSE	Histone H2B	438.2416	1311.7029	3	-0.0006	12	0	1.50E-04	GTKAVTKacYTSSK	GluC
H2B1A_MOUSE, H2B1B_MOUSE, H2B1C_MOUSE, H2B1F_MOUSE, H2B1H_MOUSE, H2B1M_MOUSE, H2B1P_MOUSE, H2B2B_MOUSE, H2B3A_MOUSE, H2B3B_MOUSE	Histone H2B	438.2409	1311.7009	3	-0.0025	12	0	1.90E-02	GTKAVTKacYTSSK	GluC
H2B1A_MOUSE, H2B1B_MOUSE, H2B1C_MOUSE, H2B1F_MOUSE, H2B1H_MOUSE, H2B1M_MOUSE, H2B1P_MOUSE, H2B2B_MOUSE, H2B3A_MOUSE, H2B3B_MOUSE	Histone H2B	656.8600	1311.7055	2	0.0021	12	0	9.80E-08	GTKAVTKYTSSKac	GluC
H2B1A_MOUSE, H2B1B_MOUSE, H2B1C_MOUSE, H2B1F_MOUSE, H2B1H_MOUSE, H2B1M_MOUSE, H2B1P_MOUSE, H2B2B_MOUSE, H2B3A_MOUSE, H2B3B_MOUSE	Histone H2B	656.8599	1311.7053	2	0.0019	12	0	4.10E-06	GTKAVTKYTSSKac	GluC
H2B1A_MOUSE, H2B1B_MOUSE, H2B1C_MOUSE, H2B1F_MOUSE, H2B1H_MOUSE, H2B1M_MOUSE, H2B1P_MOUSE, H2B2B_MOUSE, H2B3A_MOUSE, H2B3B_MOUSE	Histone H2B	656.8595	1311.7045	2	0.0011	12	0	6.70E-06	GTKAVTKYTSSKac	GluC
H2B1A_MOUSE, H2B1B_MOUSE, H2B1C_MOUSE, H2B1F_MOUSE, H2B1H_MOUSE, H2B1M_MOUSE, H2B1P_MOUSE, H2B2B_MOUSE, H2B3A_MOUSE, H2B3B_MOUSE	Histone H2B	656.8598	1311.7050	2	0.0016	12	0	1.70E-05	GTKAVTKYTSSKac	GluC
H2B1A_MOUSE, H2B1B_MOUSE, H2B1C_MOUSE, H2B1F_MOUSE, H2B1H_MOUSE, H2B1M_MOUSE, H2B1P_MOUSE, H2B2B_MOUSE, H2B3A_MOUSE, H2B3B_MOUSE	Histone H2B	656.8593	1311.7040	2	0.0006	12	0	3.20E-05	GTKAVTKacYTSSK	GluC
H2B1A_MOUSE, H2B1B_MOUSE, H2B1C_MOUSE, H2B1F_MOUSE, H2B1H_MOUSE, H2B1M_MOUSE, H2B1P_MOUSE, H2B2B_MOUSE, H2B3A_MOUSE, H2B3B_MOUSE	Histone H2B	438.2412	1311.7018	3	-0.0016	12	0	9.20E-05	GTKAVTKacYTSSK	GluC
H2B1A_MOUSE, H2B1B_MOUSE, H2B1C_MOUSE, H2B1F_MOUSE, H2B1H_MOUSE, H2B1M_MOUSE, H2B1P_MOUSE, H2B2B_MOUSE, H2B3A_MOUSE, H2B3B_MOUSE	Histone H2B	438.2408	1311.7006	3	-0.0028	12	0	3.90E-04	GTKAVTKYTSSKac	GluC
H2B1A_MOUSE, H2B1B_MOUSE, H2B1C_MOUSE, H2B1F_MOUSE, H2B1H_MOUSE, H2B1M_MOUSE, H2B1P_MOUSE, H2B2B_MOUSE, H2B3A_MOUSE, H2B3B_MOUSE	Histone H2B	656.8599	1311.7053	2	0.0019	12	0	3.00E-03	GTKAVTKYTSSKac	GluC
H2B1A_MOUSE, H2B1B_MOUSE, H2B1C_MOUSE, H2B1F_MOUSE, H2B1H_MOUSE, H2B1M_MOUSE, H2B1P_MOUSE, H2B2B_MOUSE, H2B3A_MOUSE, H2B3B_MOUSE	Histone H2B	656.8600	1311.7055	2	0.0021	12	0	3.60E-03	GTKAVTKYTSSKac	GluC
H2B1A_MOUSE, H2B1B_MOUSE, H2B1C_MOUSE, H2B1F_MOUSE, H2B1H_MOUSE, H2B1M_MOUSE, H2B1P_MOUSE, H2B2B_MOUSE, H2B3A_MOUSE, H2B3B_MOUSE	Histone H2B	656.8593	1311.7040	2	0.0006	12	0	4.20E-03	GTKAVTKacYTSSK	GluC
H2B1A_MOUSE, H2B1B_MOUSE, H2B1C_MOUSE, H2B1F_MOUSE, H2B1H_MOUSE, H2B1M_MOUSE, H2B1P_MOUSE, H2B2B_MOUSE, H2B3A_MOUSE, H2B3B_MOUSE	Histone H2B	438.2423	1311.7052	3	0.0017	12	0	1.00E-02	GTKAVTKYTSSKac	GluC
H2B1A_MOUSE, H2B1B_MOUSE, H2B1C_MOUSE, H2B1F_MOUSE, H2B1H_MOUSE, H2B1M_MOUSE, H2B1P_MOUSE, H2B2B_MOUSE, H2B3A_MOUSE, H2B3B_MOUSE	Histone H2B	656.8595	1311.7045	2	0.0011	12	0	1.60E-02	GTKAVTKacYTSSK	GluC
H2B1A_MOUSE, H2B1B_MOUSE, H2B1C_MOUSE, H2B1F_MOUSE, H2B1H_MOUSE, H2B1M_MOUSE, H2B1P_MOUSE, H2B2B_MOUSE, H2B3A_MOUSE, H2B3B_MOUSE	Histone H2B	656.8596	1311.7047	2	0.0013	12	0	6.00E-05	GTKAVTKacYTSSK	GluC
H2B1A_MOUSE, H2B1B_MOUSE, H2B1C_MOUSE, H2B1F_MOUSE, H2B1H_MOUSE, H2B1M_MOUSE, H2B1P_MOUSE, H2B2B_MOUSE, H2B3A_MOUSE, H2B3B_MOUSE	Histone H2B	328.9334	1311.7044	4	0.0010	12	0	3.10E-04	GTKAVTKYTSSKac	GluC

uniprot accession/s for all possible isoforms	histone type	pep exp mz	pep exp mr	pep exp z	pep delta	pep length	pep miss	pep expect	peptide and site of PTM	enzyme
H2B1A_MOUSE, H2B1B_MOUSE, H2B1F_MOUSE, H2B1K_MOUSE, H2B1P_MOUSE, H2B3A_MOUSE, H2B3B_MOUSE	Histone H2B	407.1782	812.3418	2	-0.0011	7	0	1.50E-03	IApSEASR	Semi-tryptic
H2B1A_MOUSE, H2B1B_MOUSE, H2B1F_MOUSE, H2B1K_MOUSE, H2B1P_MOUSE, H2B3A_MOUSE, H2B3B_MOUSE	Histone H2B	407.1778	812.3409	2	-0.0020	7	0	2.60E-02	IApSEASR	Semi-tryptic
H2B1A_MOUSE, H2B1B_MOUSE, H2B1F_MOUSE, H2B1K_MOUSE, H2B1P_MOUSE, H2B3A_MOUSE, H2B3B_MOUSE	Histone H2B	407.1791	812.3436	2	0.0007	7	0	5.30E-04	IApSEASR	Semi-tryptic
H2B1A_MOUSE, H2B1B_MOUSE, H2B1F_MOUSE, H2B1K_MOUSE, H2B1P_MOUSE, H2B3A_MOUSE, H2B3B_MOUSE	Histone H2B	407.1789	812.3433	2	0.0004	7	0	3.50E-02	IApSEASR	Semi-tryptic
H2B1A_MOUSE, H2B1B_MOUSE, H2B1F_MOUSE, H2B1K_MOUSE, H2B1P_MOUSE, H2B3A_MOUSE, H2B3B_MOUSE	Histone H2B	407.1789	812.3433	2	0.0004	7	0	4.50E-05	IApSEASR	Semi-tryptic
H2B1A_MOUSE, H2B1B_MOUSE, H2B1F_MOUSE, H2B1K_MOUSE, H2B1P_MOUSE, H2B3A_MOUSE, H2B3B_MOUSE	Histone H2B	407.1790	812.3435	2	0.0006	7	0	9.90E-04	IApSEASR	Semi-tryptic
H2B1B_MOUSE	Histone H2B	558.3379	3901.3143	7	-0.0033	35	1	2.20E-03	PEPSKSAPAPKKGSKKAISKme1AQKKDGKKRKRSRKE	GluC
H2B1B_MOUSE	Histone H2B	683.8889	2731.5266	4	0.0034	25	0	2.80E-05	PEPSKSAPAPKKacGSKacKacAISKAQKKD	GluC
H2B1B_MOUSE	Histone H2B	694.3908	2773.5339	4	0.0002	25	0	9.10E-05	PEPSKSAPAPKKacGSKacKacAISKacAQKKD	GluC
H2B1B_MOUSE	Histone H2B	555.7139	2773.5332	5	-0.0005	25	0	1.30E-03	PEPSKSAPAPKKacGSKacKacAISKacAQKKD	GluC
H2B1B_MOUSE, H2B1C_MOUSE, H2B1F_MOUSE, H2B1H_MOUSE, H2B1K_MOUSE, H2B1M_MOUSE, H2B1P_MOUSE, H2B2B_MOUSE	Histone H2B	530.2515	1587.7328	3	-0.0006	12	2	4.30E-05	SRKEpSYSVYVYK	Semi-tryptic
H2B1B_MOUSE, H2B1C_MOUSE, H2B1F_MOUSE, H2B1H_MOUSE, H2B1K_MOUSE, H2B1M_MOUSE, H2B1P_MOUSE, H2B2B_MOUSE	Histone H2B	673.3083	1344.6020	2	0.0018	10	1	1.30E-03	KEpSYSVYVYK	Semi-tryptic
H2B1B_MOUSE, H2B1C_MOUSE, H2B1F_MOUSE, H2B1H_MOUSE, H2B1K_MOUSE, H2B1M_MOUSE, H2B1P_MOUSE, H2B2B_MOUSE	Histone H2B	673.3081	1344.6016	2	0.0014	10	1	1.60E-05	KEpSYSVYVYK	Semi-tryptic
H2B1B_MOUSE, H2B1C_MOUSE, H2B1F_MOUSE, H2B1H_MOUSE, H2B1K_MOUSE, H2B1M_MOUSE, H2B1P_MOUSE, H2B2B_MOUSE, H2B2E_MOUSE	Histone H2B	429.6089	1285.8050	3	-0.0004	10	0	1.70E-03	GKKRKRSRKme1E	GluC
H2B1B_MOUSE, H2B1C_MOUSE, H2B1F_MOUSE, H2B1H_MOUSE, H2B1K_MOUSE, H2B1M_MOUSE, H2B1P_MOUSE, H2B2B_MOUSE, H2B2E_MOUSE	Histone H2B	322.4585	1285.8049	4	-0.0005	10	0	1.00E-02	GKKRKRSRKme1E	GluC
H2B1B_MOUSE, H2B1C_MOUSE, H2B1F_MOUSE, H2B1H_MOUSE, H2B1K_MOUSE, H2B1M_MOUSE, H2B1P_MOUSE, H2B2B_MOUSE, H2B2E_MOUSE, H2B3A_MOUSE, H2B3B_MOUSE	Histone H2B	915.4246	1828.8346	2	0.0011	17	0	1.90E-03	TGISSKacAMGIMNSFVND	GluC
H2B1B_MOUSE, H2B1C_MOUSE, H2B1F_MOUSE, H2B1H_MOUSE, H2B1K_MOUSE, H2B1M_MOUSE, H2B1P_MOUSE, H2B2B_MOUSE, H2B2E_MOUSE, H2B3A_MOUSE, H2B3B_MOUSE	Histone H2B	923.4210	1844.8274	2	-0.0010	17	0	2.40E-02	TGISSKacAMGIMNSFVND	GluC
H2B1C_MOUSE, H2B1F_MOUSE, H2B1H_MOUSE, H2B1K_MOUSE	Histone H2B	586.7893	1171.5640	2	0.0002	11	1	2.80E-03	PEPAKpSAPAPK	Semi-tryptic
H2B1C_MOUSE, H2B1F_MOUSE, H2B1H_MOUSE, H2B1K_MOUSE	Histone H2B	586.7894	1171.5643	2	0.0006	11	1	5.00E-03	PEPAKpSAPAPK	Semi-tryptic
H2B1C_MOUSE, H2B1F_MOUSE, H2B1H_MOUSE, H2B1K_MOUSE	Histone H2B	586.7895	1171.5644	2	0.0006	11	1	2.20E-04	PEPAKpSAPAPK	Semi-tryptic
H2B1C_MOUSE, H2B1F_MOUSE, H2B1K_MOUSE	Histone H2B	778.0678	3885.3026	5	-0.0202	35	1	2.80E-06	PEPAKSAPAPKKme3GSKKAVTKAQKKDGKKRKRSRKE	GluC
H2B1C_MOUSE, H2B1F_MOUSE, H2B1K_MOUSE	Histone H2B	556.0501	3885.2996	7	-0.0231	35	1	5.10E-05	PEPAKSAPAPKKme3GSKKAVTKAQKKDGKKRKRSRKE	GluC
H2B1C_MOUSE, H2B1F_MOUSE, H2B1K_MOUSE	Histone H2B	552.0514	3857.3089	7	0.0174	35	1	2.60E-03	PEPAKSAPAPKKGSKKAVTKme1AQKKDGKKRKRSRKE	GluC
H2B1C_MOUSE, H2B1F_MOUSE, H2B1K_MOUSE	Histone H2B	778.0635	3885.2809	5	-0.0054	35	1	3.90E-05	PEPAKSAPAPKKGSKacKAVTKAQKKDGKKRKRSRKE	GluC
H2B1C_MOUSE, H2B1F_MOUSE, H2B1K_MOUSE	Histone H2B	794.8675	3969.3011	5	-0.0063	35	1	5.10E-05	PEPAKSAPAPKKGSK(ac)KacAVTKacAQKKDGKKRKRSRKE	GluC
H2B1C_MOUSE, H2B1F_MOUSE, H2B1K_MOUSE	Histone H2B	648.5548	3885.2853	6	-0.0011	35	1	3.60E-04	PEPAKSAPAPKKGSKacKAVTKAQKKDGKKRKRSRKE	GluC
H2B1C_MOUSE, H2B1F_MOUSE, H2B1K_MOUSE	Histone H2B	658.8860	2631.5148	4	0.0077	25	0	5.60E-07	PEPAKSAPAPKKacGSKKAVTKAQKKD	GluC
H2B1C_MOUSE, H2B1F_MOUSE, H2B1K_MOUSE	Histone H2B	878.1814	2631.5224	3	0.0153	25	0	1.90E-03	PEPAKSAPAPKKacGSKKAVTKAQKKD	GluC
H2B1C_MOUSE, H2B1F_MOUSE, H2B1K_MOUSE	Histone H2B	892.1835	2673.5287	3	0.0110	25	0	2.00E-03	PEPAKSAPAPKKacGSKacKAVTKAQKKD	GluC
H2B1C_MOUSE, H2B1F_MOUSE, H2B1K_MOUSE	Histone H2B	669.3868	2673.5179	4	0.0003	25	0	3.70E-05	PEPAKSAPAPKKacGSKacKAVTKAQKKD	GluC
H2B1C_MOUSE, H2B1F_MOUSE, H2B1K_MOUSE	Histone H2B	679.8890	2715.5271	4	-0.0011	25	0	5.50E-04	PEPAKSAPAPKKGSKacKacAVTKacAQKKD	GluC
H2B1C_MOUSE, H2B1F_MOUSE, H2B1K_MOUSE	Histone H2B	658.8830	2631.5029	4	-0.0042	25	0	2.30E-03	PEPAKSAPAPKKGSKacKAVTKAQKKD	GluC
H2B1C_MOUSE, H2B1F_MOUSE, H2B1K_MOUSE	Histone H2B	544.1128	2715.5277	5	-0.0006	25	0	1.20E-02	PEPAKSAPAPKKacGSKacKacAVTKAQKKD	GluC
H2B1C_MOUSE, H2B1F_MOUSE, H2B1K_MOUSE	Histone H2B	658.8807	2631.4936	4	-0.0135	25	0	2.30E-05	PEPAKSAPAPKKacGSKKAVTKAQKKD	GluC
H2B1C_MOUSE, H2B1F_MOUSE, H2B1K_MOUSE	Histone H2B	544.1129	2715.5280	5	-0.0002	25	0	7.40E-04	PEPAKSAPAPKKacGSKacKAVTKacAQKKD	GluC
H2B1C_MOUSE, H2B1F_MOUSE, H2B1K_MOUSE	Histone H2B	934.1920	2799.5542	3	0.0049	25	0	5.70E-04	PEPAKSAPAPKKacGSKacKacAVTKacAQKKD	GluC
H2B1C_MOUSE, H2B1F_MOUSE, H2B1K_MOUSE	Histone H2B	679.8897	2715.5297	4	0.0015	25	0	1.50E-02	PEPAKSAPAPKKGSKacKacAVTKacAQKKD	GluC
H2B1C_MOUSE, H2B1F_MOUSE, H2B1K_MOUSE	Histone H2B	906.1847	2715.5323	3	0.0040	25	0	4.90E-02	PEPAKSAPAPKKGSKacKacAVTKacAQKKD	GluC
H2B1C_MOUSE, H2B1F_MOUSE, H2B1K_MOUSE	Histone H2B	878.1746	2631.5021	3	-0.0050	25	0	1.20E-03	PEPAKSAPAPKKacGSKKAVTKAQKKD	GluC
H2B1C_MOUSE, H2B1F_MOUSE, H2B1K_MOUSE	Histone H2B	690.3912	2757.5359	4	-0.0029	25	0	1.20E-07	PEPAKSAPAPKKacGSKacKacAVTKacAQKKD	GluC
H2B1C_MOUSE, H2B1F_MOUSE, H2B1K_MOUSE	Histone H2B	527.3086	2631.5065	5	-0.0006	25	0	8.50E-07	PEPAKSAPAPKKacGSKKAVTKAQKKD	GluC
H2B1C_MOUSE, H2B1F_MOUSE, H2B1K_MOUSE	Histone H2B	690.3924	2757.5405	4	0.0017	25	0	9.00E-03	PEPAKSAPAPKKacGSKacKacAVTKacAQKKD	GluC
H2B1C_MOUSE, H2B1F_MOUSE, H2B1K_MOUSE	Histone H2B	658.8854	2631.5123	4	0.0052	25	0	2.40E-02	PEPAKSAPAPKKGSKKAVTKacAQKKD	GluC
H2B1C_MOUSE, H2B1F_MOUSE, H2B1K_MOUSE	Histone H2B	690.3957	2757.5536	4	0.0148	25	0	2.70E-02	PEPAKSAPAPKKacGSKacKacAVTKacAQKKD	GluC
H2B1C_MOUSE, H2B1F_MOUSE, H2B1K_MOUSE	Histone H2B	867.8470	2600.5191	3	0.0179	24	0	2.30E-02	PEPAKSAPAPKKacGSKacKAVTKacAQKK	AspN
H2B1C_MOUSE, H2B1F_MOUSE, H2B1K_MOUSE	Histone H2B	504.3064	2516.4955	5	0.0154	24	0	6.40E-05	PEPAKSAPAPKKacGSKKAVTKAQKK	AspN
H2B1C_MOUSE, H2B1F_MOUSE, H2B1K_MOUSE	Histone H2B	852.4833	2554.4282	3	-0.0077	24	0	1.50E-02	PEPAKpSAPAPKKGSKKAVTKAQKK	AspN
H2B1C_MOUSE, H2B1F_MOUSE, H2B1K_MOUSE	Histone H2B	434.4238	2600.4993	6	-0.0020	24	0	4.10E-06	PEPAKSAPAPKKacGSKacKAVTKacAQKK	AspN
H2B1C_MOUSE, H2B1F_MOUSE, H2B1K_MOUSE	Histone H2B	672.1387	2684.5259	4	0.0035	24	0	1.70E-04	PEPAKacSAPAPKKacGSKacKacAVTKacAQKK	AspN
H2B1C_MOUSE, H2B1F_MOUSE, H2B1K_MOUSE	Histone H2B	651.1321	2600.4991	4	-0.0021	24	0	5.10E-04	PEPAKSAPAPKKacGSKacKAVTKacAQKK	AspN
H2B1C_MOUSE, H2B1F_MOUSE, H2B1K_MOUSE	Histone H2B	630.1276	2516.4813	4	0.0012	24	0	1.60E-06	PEPAKSAPAPKKGSKacKAVTKAQKK	AspN
H2B1C_MOUSE, H2B1F_MOUSE, H2B1K_MOUSE	Histone H2B	521.1074	2600.5008	5	-0.0005	24	0	1.10E-03	PEPAKSAPAPKKacGSKacKacAVTKAQKK	AspN
H2B1C_MOUSE, H2B1F_MOUSE, H2B1K_MOUSE	Histone H2B	853.8395	2558.4966	3	0.0059	24	0	3.00E-03	PEPAKSAPAPKacKacGSKKAVTKAQKK	AspN
H2B1C_MOUSE, H2B1F_MOUSE, H2B1K_MOUSE	Histone H2B	640.6306	2558.4933	4	0.0026	24	0	8.00E-07	PEPAKSAPAPKKacGSKKAVTacKAQKK	AspN
H2B1C_MOUSE, H2B1F_MOUSE, H2B1K_MOUSE	Histone H2B	651.1313	2600.4962	4	-0.0051	24	0	5.10E-06	PEPAKSAPAPKKacGSKacKAVTacKAQKK	AspN
H2B1C_MOUSE, H2B1F_MOUSE, H2B1K_MOUSE	Histone H2B	640.6309	2558.4944	4	0.0037	24	0	2.30E-05	PEPAKSAPAPKKacGSKKAVTacKAQKK	AspN
H2B1C_MOUSE, H2B1F_MOUSE, H2B1K_MOUSE	Histone H2B	672.8911	2687.5353	4	0.0020	24	0	5.60E-03	PDPAKSAPAPKKacGSKKAVTacKVQKKD	GluC
H2B1C_MOUSE, H2B1F_MOUSE, H2B1K_MOUSE	Histone H2B	544.3147	2716.5371	5	1.0088	24	0	6.80E-05	PEPAKSAPAPKKacG(Sac)KKAVTacKAQKKD	GluC

uniprot accession/s for all possible isoforms	histone type	pep exp mz	pep exp mr	pep exp z	pep delta	pep length	pep miss	pep expect	peptide and site of PTM	enzyme
H2B1C_MOUSE, H2B1F_MOUSE, H2B1K_MOUSE	Histone H2B	544.3147	2716.5371	5	1.0088	24	0	6.80E-05	PEPAKSAPAPKKacG(Sac)KKAVTacKAQKKD	GluC
H2B1C_MOUSE, H2B1F_MOUSE, H2B1K_MOUSE	Histone H2B	448.9294	2687.5329	6	-0.0005	24	0	1.10E-02	PDPAKSAPAPKKacGSKKAV(Tac)KVQKKD	GluC
H2B1C_MOUSE, H2B1F_MOUSE, H2B1K_MOUSE	Histone H2B	658.8810	2631.4948	4	-0.0123	24	0	4.80E-02	PEPAKSAPAPKKGSKKAVTacKAQKKD	GluC
H2B1C_MOUSE, H2B1F_MOUSE, H2B1K_MOUSE	Histone H2B	651.1327	2600.5018	4	0.0005	23	0	4.10E-05	PEPAKSAPAPKKacGSKacKAVTacKAQKK	AspN
H2B1C_MOUSE, H2B1F_MOUSE, H2B1K_MOUSE	Histone H2B	651.1327	2600.5018	4	0.0005	23	0	3.30E-05	PEPAKSAPAPKKacGSKacKAVTacKAQKK	AspN
H2B1C_MOUSE, H2B1F_MOUSE, H2B1K_MOUSE, H2B1M_MOUSE, H2B1P_MOUSE, H2B2B_MOUSE, H2B2E_MOUSE, H2B3B_MOUSE	Histone H2B	536.8216	1071.6287	2	0.0000	9	3	4.00E-02	KacGSKacKacAVTK	Semi-tryptic
H2B1C_MOUSE, H2B1F_MOUSE, H2B1K_MOUSE, H2B1M_MOUSE, H2B1P_MOUSE, H2B2B_MOUSE, H2B2E_MOUSE, H2B3B_MOUSE	Histone H2B	536.8215	1071.6285	2	-0.0003	9	3	8.00E-03	KacGSKacKacAVTK	Semi-tryptic
H2B1C_MOUSE, H2B1F_MOUSE, H2B1K_MOUSE, H2B1M_MOUSE, H2B1P_MOUSE, H2B2E_MOUSE, H2B3B_MOUSE	Histone H2B	458.2852	914.5559	2	0.0010	8	2	2.80E-02	KAVTKacAQK	Semi-tryptic
H2B1K_MOUSE, H2B2E_MOUSE	Histone H2B	544.3013	2173.1763	4	0.0021	20	1	4.40E-02	LAKHAVSEGTKacAVTKacYTSAK	GluC
H2B1K_MOUSE, H2B2E_MOUSE	Histone H2B	669.8667	1337.7188	2	-0.0002	12	0	2.90E-02	GTKAVTKacYTS(ac)AK	GluC
H2B1K_MOUSE, H2B2E_MOUSE	Histone H2B	669.8675	1337.7204	2	0.0013	12	0	3.10E-02	GTKAVTKacYTS(ac)AK	GluC
H2B1K_MOUSE, H2B2E_MOUSE	Histone H2B	432.9103	1295.7091	3	0.0006	12	0	6.50E-05	GTKAVTKacYTSAK	GluC
H2B2B_MOUSE	Histone H2B	789.2700	3941.3136	5	0.0010	35	1	4.00E-03	PDPAKSAPAPKKGSKKacAVTKacVQKKDGKKRKRSRKE	GluC
H2B2B_MOUSE	Histone H2B	797.6711	3983.3191	5	-0.0040	35	1	9.70E-04	PDPAKSAPAPKK(ac)GSK(ac)KAVTKacVQKKDGKKRKRSRKE	GluC
H2B2B_MOUSE	Histone H2B	650.8908	3899.3013	6	-0.0007	35	1	3.70E-03	PDPAKSAPAPKKGSKacKAVTKVQKKDGKKRKRSRKE	GluC
H2B2B_MOUSE	Histone H2B	558.0498	3899.2979	7	-0.0041	35	1	7.30E-03	PDPAKSAPAPKKacGSKKAVTKVQKKDGKKRKRSRKE	GluC
H2B2B_MOUSE	Histone H2B	650.8916	3899.3061	6	0.0041	35	1	7.70E-03	PDPAKSAPAPKKGSKacKAVTKVQKKDGKKRKRSRKE	GluC
H2B2B_MOUSE	Histone H2B	780.8672	3899.2996	5	-0.0024	35	1	1.10E-02	PDPAKSAPAPKKGSKacKAVTKVQKKDGKKRKRSRKE	GluC
H2B2B_MOUSE	Histone H2B	657.8913	3941.3042	6	-0.0084	35	1	2.10E-02	PDPAKSAPAPKKacGSKacKAVTKVQKKDGKKRKRSRKE	GluC
H2B2B_MOUSE	Histone H2B	558.0517	3899.3108	7	0.0088	35	1	7.70E-03	PDPAKSAPAPKKGSKKAVTKacVQKKDGKKRKRSRKE	GluC
H2B2B_MOUSE	Histone H2B	672.8939	2687.5465	4	0.0131	25	0	7.10E-08	PDPAKSAPAPKKGSKacKAVTKacVQKKD	GluC
H2B2B_MOUSE	Histone H2B	672.8915	2687.5369	4	0.0035	25	0	1.70E-06	PDPAKSAPAPKKGSKacKAVTKacVQKKD	GluC
H2B2B_MOUSE	Histone H2B	448.9300	2687.5366	6	0.0033	25	0	1.80E-05	PDPAKSAPAPKKGSKacKAVTKacVQKKD	GluC
H2B2B_MOUSE	Histone H2B	882.8522	2645.5348	3	0.0121	25	0	4.90E-02	PDPAKSAPAPKKGSKKAVTKacVQKKD	GluC
H2B2B_MOUSE	Histone H2B	546.9158	2729.5425	5	-0.0014	25	0	3.00E-04	PDPAKSAPAPKKacGSKacKAVTKacVQKKD	GluC
H2B2B_MOUSE	Histone H2B	662.3888	2645.5259	4	0.0032	25	0	1.50E-04	PDPAKSAPAPKKGSKKAVTKacVQKKD	GluC
H2B2B_MOUSE	Histone H2B	441.9275	2645.5215	6	-0.0013	25	0	4.20E-02	PDPAKSAPAPKKGSKKAVTKacVQKKD	GluC
H2B2B_MOUSE	Histone H2B	693.8967	2771.5578	4	0.0034	25	0	7.60E-06	PDPAKSAPAPKKacGSKacKacAVTKacVQKKD	GluC
H2B2B_MOUSE	Histone H2B	693.8970	2771.5590	4	0.0046	25	0	1.20E-03	PDPAKSAPAPKKacGSKacKacAVTKacVQKKD	GluC
H2B2B_MOUSE	Histone H2B	704.3986	2813.5654	4	0.0004	25	0	9.90E-03	PDPAKacSAPAPKKacGSKacKacAVTKacVQKKD	GluC
H2B2B_MOUSE	Histone H2B	683.3947	2729.5497	4	0.0058	25	0	1.30E-02	PDPAKSAPAPKKGSKacKacAVTKacVQKKD	GluC
H2B2B_MOUSE	Histone H2B	555.3184	2771.5556	5	0.0012	25	0	2.10E-02	PDPAKSAPAPKKacGSKacKacAVTKacVQKKD	GluC
H2B2B_MOUSE	Histone H2B	882.8450	2645.5131	3	-0.0096	25	0	2.30E-02	PDPAKSAPAPKKGSKKAVTKacVQKKD	GluC
H2B2B_MOUSE	Histone H2B	683.3939	2729.5465	4	0.0026	25	0	2.70E-04	PDPAKSAPAPKKacGSKacKacAVTKVQKKD	GluC
H2B2B_MOUSE	Histone H2B	683.3954	2729.5526	4	0.0087	25	0	2.20E-03	PDPAKSAPAPKKGSKacKacAVTKacVQKKD	GluC
H2B2B_MOUSE	Histone H2B	546.9178	2729.5525	5	0.0086	25	0	3.10E-02	PDPAKSAPAPKKGSKacKacAVTKacVQKKD	GluC
H2B2B_MOUSE	Histone H2B	672.8911	2687.5353	4	0.0020	25	0	2.80E-02	PDPAKSAPAPKKGSKacKAVTKacVQKKD	GluC
H2B2B_MOUSE	Histone H2B	672.8911	2687.5352	4	0.0019	25	0	6.60E-03	PDPAKSAPAPKKGSKacKAVTKacVQKKD	GluC
H2B2B_MOUSE	Histone H2B	579.7814	1157.5483	2	0.0001	11	1	1.50E-02	PDPAKpSAPAPK	Semi-tryptic
H2B2B_MOUSE	Histone H2B	472.3004	942.5863	2	0.0001	8	2	1.60E-03	KAVTKacVQK	Semi-tryptic
H2B3A_MOUSE	Histone H2B	658.2242	3943.3013	6	-0.0018	35	1	6.30E-06	PEPSRSTPAPKKacGSKKAITKAQKKDGKKRKRGRKE	GluC
H2B3A_MOUSE	Histone H2B	570.1201	2845.5641	5	-0.0020	25	0	3.30E-05	PEPSRSTPAPKKacGSKacKacAITKacAQKKD	GluC
H2B3A_MOUSE	Histone H2B	701.8958	2803.5539	4	-0.0016	25	0	1.00E-04	PEPSRSTPAPKKacGSKacKacAITKAQKKD	GluC
H2B3A_MOUSE	Histone H2B	712.3986	2845.5655	4	-0.0006	25	0	1.90E-04	PEPSRSTPAPKKacGSKacKacAITKacAQKKD	GluC
H2B3A_MOUSE	Histone H2B	683.6425	2730.5409	4	0.0018	24	0	1.70E-06	PEPSRSTPAPKKacGSKacKacAITKacAQKK	AspN
H2B3A_MOUSE	Histone H2B	530.3113	2646.5201	5	0.0021	24	0	1.40E-03	PEPSRSTPAPKKacGSKacKAITKAQKK	AspN
H2B3A_MOUSE	Histone H2B	683.6425	2730.5409	4	0.0018	24	0	2.70E-02	PEPSRSTPAPKKacGSKacKacAITKacAQKK	AspN
H2B3A_MOUSE	Histone H2B	577.6203	1729.8392	3	-0.0008	15	3	1.10E-02	PEPSRSpTPAPKacKacGSK	Semi-tryptic
H2B3A_MOUSE	Histone H2B	623.7961	1245.5776	2	0.0021	11	1	3.90E-03	PEPSRpSTPAPK	Semi-tryptic
H2B3A_MOUSE	Histone H2B	623.7950	1245.5754	2	0.0000	11	1	1.70E-02	PEPSRSpTPAPK	Semi-tryptic
H2B3A_MOUSE	Histone H2B	623.7947	1245.5749	2	-0.0006	11	1	2.60E-02	PEPSRSpTPAPK	Semi-tryptic
H2B3A_MOUSE	Histone H2B	623.7955	1245.5764	2	0.0009	11	1	3.20E-02	PEPSRpSTPAPK	Semi-tryptic
H2B3A_MOUSE	Histone H2B	416.2001	1245.5784	3	0.0029	11	1	2.70E-05	PEPSRSpTPAPK	Semi-tryptic
H2B3A_MOUSE	Histone H2B	416.1994	1245.5764	3	0.0009	11	1	5.10E-04	PEPSRSpTPAPK	Semi-tryptic
H2B3A_MOUSE	Histone H2B	623.7952	1245.5758	2	0.0004	11	1	9.40E-03	PEPSRpSTPAPK	Semi-tryptic
H2B3A_MOUSE	Histone H2B	623.7952	1245.5758	2	0.0003	11	1	1.90E-02	PEPSRpSTPAPK	Semi-tryptic
H2B3A_MOUSE	Histone H2B	623.7958	1245.5770	2	0.0016	11	1	2.10E-02	PEPSRpSTPAPK	Semi-tryptic
H2B3A_MOUSE	Histone H2B	623.7956	1245.5766	2	0.0012	11	1	4.50E-02	PEPSRpSTPAPK	Semi-tryptic
H2B3A_MOUSE	Histone H2B	623.7954	1245.5763	2	0.0009	11	1	4.70E-02	PEPSRSpTPAPK	Semi-tryptic
H2B3A_MOUSE, H2B3B_MOUSE	Histone H2B	764.3796	1526.7446	2	-0.0007	13	1	4.90E-02	STITpSREVQTAVR	Semi-tryptic
H2B3B_MOUSE	Histone H2B	527.7128	2633.5275	5	0.0047	25	0	1.60E-02	PDPSKSAPAPKKme3GSKKAVTKAQKKD	GluC
H31_MOUSE, H32_MOUSE	Histone H3	810.0782	2427.2127	3	0.0023	21	2	4.10E-03	IAQDFKTDLRme1FQSSAVMALQE	GluC
H31_MOUSE, H32_MOUSE	Histone H3	804.7439	2411.2098	3	-0.0057	21	2	3.00E-05	IAQDFKTDLRme1FQSSAVMALQE	GluC
H31_MOUSE, H32_MOUSE	Histone H3	804.7452	2411.2137	3	-0.0018	21	2	1.10E-04	IAQDFKTDLRme1FQSSAVMALQE	GluC
H31_MOUSE, H32_MOUSE	Histone H3	804.7441	2411.2104	3	-0.0051	21	2	1.40E-04	IAQDFKTDLRme1FQSSAVMALQE	GluC
H31_MOUSE, H32_MOUSE	Histone H3	804.7415	2411.2028	3	-0.0127	21	2	4.50E-04	IAQDFKTDLRme1FQSSAVMALQE	GluC
H31_MOUSE, H32_MOUSE	Histone H3	804.7468	2411.2185	3	0.0030	21	2	7.50E-04	IAQDFKTDLRme1FQSSAVMALQE	GluC
H31_MOUSE, H32_MOUSE	Histone H3	804.7453	2411.2141	3	-0.0014	21	2	1.60E-03	IAQDFKTDLRme1FQSSAVMALQE	GluC
H31_MOUSE, H32_MOUSE	Histone H3	804.7453	2411.2142	3	-0.0013	21	2	7.30E-03	IAQDFKTDLRme1FQSSAVMALQE	GluC
H31_MOUSE, H32_MOUSE	Histone H3	804.7442	2411.2107	3	-0.0048	21	2	1.30E-02	IAQDFKme1TDLRFQSSAVMALQE	GluC

uniprot accession/s for all possible isoforms	histone type	pep exp mz	pep exp mr	pep exp z	pep delta	pep length	pep miss	pep expect	peptide and site of PTM	enzyme
H31_MOUSE, H32_MOUSE	Histone H3	804.7442	2411.2107	3	-0.0048	21	2	1.70E-02	IAQDFKTDLRme1FQSSAVMALQE	GluC
H31_MOUSE, H32_MOUSE	Histone H3	804.7453	2411.2142	3	-0.0013	21	2	1.80E-02	IAQDFKme1TDLRFQSSAVMALQE	GluC
H31_MOUSE, H32_MOUSE	Histone H3	373.2296	1488.8894	4	0.0005	14	2	8.60E-07	Kme2SAPATGGVKme2KPHR	Semi-tryptic
H31_MOUSE, H32_MOUSE	Histone H3	373.2300	1488.8908	4	0.0019	14	2	9.80E-03	Kme2SAPATGGVKme2KPHR	Semi-tryptic
H31_MOUSE, H32_MOUSE	Histone H3	369.7258	1474.8742	4	0.0010	14	2	5.80E-09	Kme2SAPATGGVKme1KPHR	Semi-tryptic
H31_MOUSE, H32_MOUSE	Histone H3	373.2296	1488.8894	4	0.0005	14	2	4.10E-07	Kme2SAPATGGVKme2KPHR	Semi-tryptic
H31_MOUSE, H32_MOUSE	Histone H3	373.2300	1488.8908	4	0.0019	14	2	3.70E-03	Kme2SAPATGGVKme2KPHR	Semi-tryptic
H31_MOUSE, H32_MOUSE	Histone H3	492.6321	1474.8745	3	0.0013	14	2	4.40E-08	Kme2SAPATGGVKme1KPHR	Semi-tryptic
H31_MOUSE, H32_MOUSE	Histone H3	497.3036	1488.8889	3	0.0001	14	2	3.10E-02	Kme2SAPATGGVKme2KPHR	Semi-tryptic
H31_MOUSE, H32_MOUSE	Histone H3	472.2824	942.5502	2	0.0004	10	1	1.60E-03	Kme2SAPATGGVK	Semi-tryptic
H31_MOUSE, H32_MOUSE	Histone H3	479.2900	956.5655	2	0.0000	9	1	8.90E-03	Kme3SAPATGGVK	Semi-tryptic
H31_MOUSE, H32_MOUSE, H33_MOUSE	Histone H3	660.5505	3297.7162	5	-0.0153	28	2	4.80E-03	DpTNLCAIHAKRVTIMPKDIQLARRIRGE	GluC
H31_MOUSE, H32_MOUSE, H33_MOUSE	Histone H3	651.6895	1952.0467	3	0.0131	17	0	3.60E-02	DTNLCAIHAKacRVTIMPK	AspN
H31_MOUSE, H32_MOUSE, H33_MOUSE	Histone H3	496.0262	1980.0756	4	0.0108	17	0	1.70E-04	DTNLCAIHAKbRVTIMPK	AspN
H31_MOUSE, H32_MOUSE, H33_MOUSE	Histone H3	661.0328	1980.0766	3	0.0118	17	0	2.00E-02	DTNLCAIHAKbRVTIMPK	AspN
H31_MOUSE, H32_MOUSE, H33_MOUSE	Histone H3	664.3336	1989.9791	3	-0.0102	17	0	4.10E-04	DpTNLCAIHAKRVTIMPK	AspN
H31_MOUSE, H32_MOUSE, H33_MOUSE	Histone H3	496.0241	1980.0673	4	0.0024	17	0	2.20E-03	DTNLCAIHAKbRVTIMPK	AspN
H31_MOUSE, H32_MOUSE, H33_MOUSE	Histone H3	661.0292	1980.0657	3	0.0009	17	0	3.10E-03	DTNLCAIHAKbRVTIMPK	AspN
H31_MOUSE, H32_MOUSE, H33_MOUSE	Histone H3	651.6855	1952.0347	3	0.0011	17	0	4.80E-02	DTNLCAIHAKacRVTIMPK	AspN
H31_MOUSE, H32_MOUSE, H33_MOUSE	Histone H3	496.2743	1981.0680	4	1.0031	16	0	5.00E-02	DTNLCAIHAKbRVTIMPK	AspN
H31_MOUSE, H32_MOUSE, H33_MOUSE	Histone H3	598.3583	1792.0531	3	-0.0012	15	0	3.80E-05	PKDIQLARRIRGERme1A	CnBr
H31_MOUSE, H32_MOUSE, H33_MOUSE	Histone H3	449.0208	1792.0543	4	-0.0001	15	0	4.00E-05	PKDIQLARRIRGERme1A	CnBr
H31_MOUSE, H32_MOUSE, H33_MOUSE	Histone H3	607.6898	1820.0475	3	-0.0017	15	0	1.90E-06	PKacDIQLARRIRGERA	CnBr
H31_MOUSE, H32_MOUSE, H33_MOUSE	Histone H3	456.0193	1820.0483	4	-0.0010	15	0	1.40E-05	PKacDIQLARRIRGERA	CnBr
H31_MOUSE, H32_MOUSE, H33_MOUSE	Histone H3	607.6903	1820.0491	3	-0.0001	15	0	1.90E-05	PKacDIQLARRIRGERA	CnBr
H31_MOUSE, H32_MOUSE, H33_MOUSE	Histone H3	456.0190	1820.0467	4	-0.0025	15	0	5.50E-05	PKacDIQLARRIRGERA	CnBr
H31_MOUSE, H32_MOUSE, H33_MOUSE	Histone H3	365.0166	1820.0466	5	-0.0027	15	0	3.40E-04	PKacDIQLARRIRGERA	CnBr
H31_MOUSE, H32_MOUSE, H33_MOUSE	Histone H3	607.6908	1820.0505	3	0.0013	15	0	1.70E-03	PKacDIQLARRIRGERA	CnBr
H31_MOUSE, H32_MOUSE, H33_MOUSE	Histone H3	365.0167	1820.0472	5	-0.0020	15	0	3.80E-03	PKacDIQLARRIRGERA	CnBr
H31_MOUSE, H32_MOUSE, H33_MOUSE	Histone H3	607.6904	1820.0493	3	0.0000	15	0	6.00E-03	PKacDIQLARRIRGERA	CnBr
H31_MOUSE, H32_MOUSE, H33_MOUSE	Histone H3	433.7481	1730.9635	4	-0.0044	14	2	6.10E-05	LVREIAQDFKme2TDLR	Semi-tryptic
H31_MOUSE, H32_MOUSE, H33_MOUSE	Histone H3	577.9947	1730.9624	3	-0.0055	14	2	8.50E-04	LVREIAQDFKme2TDLR	Semi-tryptic
H31_MOUSE, H32_MOUSE, H33_MOUSE	Histone H3	433.7481	1730.9635	4	-0.0044	14	2	1.20E-06	LVREIAQDFKme2TDLR	Semi-tryptic
H31_MOUSE, H32_MOUSE, H33_MOUSE	Histone H3	577.9947	1730.9624	3	-0.0055	14	2	2.50E-04	LVREIAQDFKme2TDLR	Semi-tryptic
H31_MOUSE, H32_MOUSE, H33_MOUSE	Histone H3	573.3234	1716.9484	3	-0.0039	14	2	6.00E-06	LVREIAQDFKme1TDLR	Semi-tryptic
H31_MOUSE, H32_MOUSE, H33_MOUSE	Histone H3	606.6366	1816.8880	3	0.0007	14	2	2.30E-06	VREIAQDFKpTDLRF	Chymotrypsin
H31_MOUSE, H32_MOUSE, H33_MOUSE	Histone H3	462.5223	1846.0601	4	-0.0048	14	0	1.10E-04	PKcrDIQLARRIRGERA	CNBr
H31_MOUSE, H32_MOUSE, H33_MOUSE	Histone H3	462.5225	1846.0607	4	-0.0042	14	0	7.60E-06	PKcrDIQLARRIRGERA	CNBr
H31_MOUSE, H32_MOUSE, H33_MOUSE	Histone H3	462.5230	1846.0629	4	-0.0020	14	0	1.00E-04	PKcrDIQLARRIRGERA	CNBr
H31_MOUSE, H32_MOUSE, H33_MOUSE	Histone H3	370.2201	1846.0641	5	-0.0008	14	0	1.40E-02	PKcrDIQLARRIRGERA	CNBr
H31_MOUSE, H32_MOUSE, H33_MOUSE	Histone H3	462.5234	1846.0645	4	-0.0004	14	0	5.70E-06	PKcrDIQLARRIRGERA	CNBr
H31_MOUSE, H32_MOUSE, H33_MOUSE	Histone H3	616.3624	1846.0653	3	0.0004	14	0	9.70E-06	PKcrDIQLARRIRGERA	CNBr
H31_MOUSE, H32_MOUSE, H33_MOUSE	Histone H3	483.5966	1447.7681	3	0.0010	12	1	6.10E-03	VREIAQDFKme1TDL	Chymotrypsin
H31_MOUSE, H32_MOUSE, H33_MOUSE	Histone H3	483.5966	1447.7681	3	0.0010	12	1	2.90E-02	VREIAQDFKme1TDL	Chymotrypsin
H31_MOUSE, H32_MOUSE, H33_MOUSE	Histone H3	708.3320	1414.6493	2	0.0000	11	1	9.40E-04	EIAQDFKpTDLR	Semi-tryptic
H31_MOUSE, H32_MOUSE, H33_MOUSE	Histone H3	708.3313	1414.6481	2	-0.0012	11	1	4.40E-02	EIAQDFKpTDLR	Semi-tryptic
H31_MOUSE, H32_MOUSE, H33_MOUSE	Histone H3	455.2438	1362.7095	3	-0.0048	11	1	2.40E-04	EIAQDFKme2TDLR	Semi-tryptic
H31_MOUSE, H32_MOUSE, H33_MOUSE	Histone H3	455.2452	1362.7139	3	-0.0004	11	1	2.30E-03	EIAQDFKme2TDLR	Semi-tryptic
H31_MOUSE, H32_MOUSE, H33_MOUSE	Histone H3	455.2438	1362.7095	3	-0.0048	11	1	1.20E-04	EIAQDFKme2TDLR	Semi-tryptic
H31_MOUSE, H32_MOUSE, H33_MOUSE	Histone H3	455.2452	1362.7139	3	-0.0004	11	1	1.50E-03	EIAQDFKme2TDLR	Semi-tryptic
H31_MOUSE, H32_MOUSE, H33_MOUSE	Histone H3	450.5734	1348.6984	3	-0.0003	11	1	1.30E-04	EIAQDFKme1TDLR	Semi-tryptic
H31_MOUSE, H32_MOUSE, H33_MOUSE	Histone H3	450.5750	1348.7031	3	0.0044	11	1	7.30E-03	EIAQDFKme1TDLR	Semi-tryptic
H31_MOUSE, H32_MOUSE, H33_MOUSE	Histone H3	675.3564	1348.6983	2	-0.0004	11	1	1.50E-02	EIAQDFKTDLR(me1)	Semi-tryptic
H31_MOUSE, H32_MOUSE, H33_MOUSE	Histone H3	450.5734	1348.6983	3	-0.0004	11	1	2.00E-02	EIAQDFKme1TDLR	Semi-tryptic
H31_MOUSE, H32_MOUSE, H33_MOUSE	Histone H3	472.5582	1414.6529	3	0.0036	11	1	4.30E-06	EIAQDFKpTDLR	Semi-tryptic
H31_MOUSE, H32_MOUSE, H33_MOUSE	Histone H3	496.2639	1485.7698	3	-0.0006	11	2	1.60E-04	RpYQKSTELLIR	Semi-tryptic
H31_MOUSE, H32_MOUSE, H33_MOUSE	Histone H3	708.3326	1414.6506	2	0.0013	11	1	8.40E-03	EIAQDFKpTDLR	Semi-tryptic
H31_MOUSE, H32_MOUSE, H33_MOUSE	Histone H3	708.3320	1414.6495	2	0.0001	11	1	1.40E-02	EIAQDFKpTDLR	Semi-tryptic
H31_MOUSE, H32_MOUSE, H33_MOUSE	Histone H3	665.8432	1329.6719	2	0.0026	10	1	7.30E-05	YQKSpTELLIR	Semi-tryptic
H31_MOUSE, H32_MOUSE, H33_MOUSE	Histone H3	665.8424	1329.6702	2	0.0008	10	1	3.40E-04	pYQKSTELLIR	Semi-tryptic
H31_MOUSE, H32_MOUSE, H33_MOUSE	Histone H3	444.2300	1329.6681	3	-0.0013	10	1	8.40E-04	YQKSpTELLIR	Semi-tryptic
H31_MOUSE, H32_MOUSE, H33_MOUSE	Histone H3	324.5260	970.5562	3	0.0003	9	2	1.40E-03	Kme2STGGKacAPR	Semi-tryptic
H31_MOUSE, H32_MOUSE, H33_MOUSE	Histone H3	343.5457	1027.6152	3	0.0014	9	2	5.70E-03	KQLATKacAAR	Semi-tryptic
H31_MOUSE, H32_MOUSE, H33_MOUSE	Histone H3	343.5449	1027.6129	3	-0.0009	9	2	2.10E-02	KQLATKacAAR	Semi-tryptic
H31_MOUSE, H32_MOUSE, H33_MOUSE	Histone H3	493.2745	984.5344	2	-0.0008	9	2	4.10E-02	KacSTGGKacAPR	Semi-tryptic
H31_MOUSE, H32_MOUSE, H33_MOUSE	Histone H3	479.2774	956.5403	2	0.0000	9	2	1.40E-03	Kme1STGGKacAPR	Semi-tryptic
H31_MOUSE, H32_MOUSE, H33_MOUSE	Histone H3	329.1981	984.5725	3	0.0009	9	2	3.50E-03	KacSTGGKme3APR	Semi-tryptic
H31_MOUSE, H32_MOUSE, H33_MOUSE	Histone H3	319.8547	956.5422	3	0.0019	9	2	4.00E-02	Kme1STGGKacAPR	Semi-tryptic
H31_MOUSE, H32_MOUSE, H33_MOUSE	Histone H3	324.5260	970.5562	3	0.0003	9	2	9.40E-04	Kme2STGGKacAPR	Semi-tryptic
H31_MOUSE, H32_MOUSE, H33_MOUSE	Histone H3	343.5457	1027.6152	3	0.0014	9	2	3.80E-03	KQLATKacAAR	Semi-tryptic
H31_MOUSE, H32_MOUSE, H33_MOUSE	Histone H3	343.5449	1027.6129	3	-0.0009	9	2	6.20E-03	KQLATKacAAR	Semi-tryptic
H31_MOUSE, H32_MOUSE, H33_MOUSE	Histone H3	526.2686	1050.5227	2	0.0004	9	2	1.90E-02	Kme2pSTGGKacAPR	Semi-tryptic
H31_MOUSE, H32_MOUSE, H33_MOUSE	Histone H3	493.2745	984.5344	2	-0.0008	9	2	3.60E-02	KacSTGGKacAPR	Semi-tryptic

uniprot accession/s for all possible isoforms	histone type	pep exp mz	pep exp mr	pep exp z	pep delta	pep length	pep miss	pep expect	peptide and site of PTM	enzyme
H31_MOUSE, H32_MOUSE, H33_MOUSE	Histone H3	512.2521	1022.4897	2	-0.0013	9	2	3.60E-02	KpSTGGKacAPR	Semi-tryptic
H31_MOUSE, H32_MOUSE, H33_MOUSE	Histone H3	486.2851	970.5557	2	-0.0002	9	2	4.30E-02	Kme2STGGKacAPR	Semi-tryptic
H31_MOUSE, H32_MOUSE, H33_MOUSE	Histone H3	535.8194	1069.6243	2	-0.0001	9	2	6.90E-06	KacQLATKacAAR	Semi-tryptic
H31_MOUSE, H32_MOUSE, H33_MOUSE	Histone H3	514.8142	1027.6137	2	0.0000	9	2	1.30E-04	KQLATKacAAR	Semi-tryptic
H31_MOUSE, H32_MOUSE, H33_MOUSE	Histone H3	493.2748	984.5350	2	-0.0002	9	2	2.10E-04	KacSTGGKacAPR	Semi-tryptic
H31_MOUSE, H32_MOUSE, H33_MOUSE	Histone H3	514.8138	1027.6131	2	-0.0007	9	2	4.00E-04	KQLATKacAAR	Semi-tryptic
H31_MOUSE, H32_MOUSE, H33_MOUSE	Histone H3	486.2851	970.5556	2	-0.0003	9	2	4.50E-04	Kme2STGGKacAPR	Semi-tryptic
H31_MOUSE, H32_MOUSE, H33_MOUSE	Histone H3	486.2852	970.5558	2	-0.0001	9	2	1.10E-03	Kme2STGGKacAPR	Semi-tryptic
H31_MOUSE, H32_MOUSE, H33_MOUSE	Histone H3	479.2781	956.5417	2	0.0014	9	2	1.50E-03	Kme1STGGKacAPR	Semi-tryptic
H31_MOUSE, H32_MOUSE, H33_MOUSE	Histone H3	472.2697	942.5248	2	0.0001	9	2	2.40E-03	KSTGGKacAPR	Semi-tryptic
H31_MOUSE, H32_MOUSE, H33_MOUSE	Histone H3	493.2927	984.5709	2	-0.0007	9	2	3.10E-03	KacSTGGKme3APR	Semi-tryptic
H31_MOUSE, H32_MOUSE, H33_MOUSE	Histone H3	479.2781	956.5417	2	0.0014	9	2	2.50E-02	Kme1STGGKacAPR	Semi-tryptic
H31_MOUSE, H32_MOUSE, H33_MOUSE	Histone H3	324.5263	970.5572	3	0.0012	9	2	2.90E-02	Kme2STGGKacAPR	Semi-tryptic
H31_MOUSE, H32_MOUSE, H33_MOUSE	Histone H3	420.8748	1259.6025	3	0.0002	9	0	2.90E-03	IRRpYQKSTE	GluC
H31_MOUSE, H32_MOUSE, H33_MOUSE	Histone H3	630.8085	1259.6024	2	0.0001	9	0	1.20E-02	IRRpYQKSTE	GluC
H31_MOUSE, H32_MOUSE, H33_MOUSE	Histone H3	535.8197	1069.6248	2	0.0005	9	2	1.00E-03	KacQLATKacAAR	Semi-tryptic
H31_MOUSE, H32_MOUSE, H33_MOUSE	Histone H3	343.5453	1027.6141	3	0.0003	9	2	1.80E-03	KQLATKacAAR	Semi-tryptic
H31_MOUSE, H32_MOUSE, H33_MOUSE	Histone H3	343.5453	1027.6141	3	0.0003	9	2	2.10E-02	KQLATKacAAR	Semi-tryptic
H31_MOUSE, H32_MOUSE, H33_MOUSE	Histone H3	483.2518	964.4891	2	0.0026	8	1	2.00E-03	IAQDFKme2TD	GluC
H31_MOUSE, H32_MOUSE, H33_MOUSE	Histone H3	483.2502	964.4859	2	-0.0007	8	1	4.50E-04	IAQDFKme2TD	GluC
H31_MOUSE, H32_MOUSE, H33_MOUSE	Histone H3	483.2504	964.4863	2	-0.0003	8	1	1.00E-02	IAQDFKme2TD	GluC
H31_MOUSE, H32_MOUSE, H33_MOUSE	Histone H3	476.2447	950.4748	2	0.0039	8	1	4.30E-02	IAQDFKme1TD	GluC
H31_MOUSE, H32_MOUSE, H33_MOUSE	Histone H3	476.2420	950.4695	2	-0.0014	8	1	4.40E-04	IAQDFKme1TD	GluC
H31_MOUSE, H32_MOUSE, H33_MOUSE	Histone H3	476.2428	950.4710	2	0.0001	8	1	2.50E-03	IAQDFKme1TD	GluC
H31_MOUSE, H32_MOUSE, H33_MOUSE	Histone H3	476.2443	950.4741	2	0.0032	8	1	2.10E-03	IAQDFKme1TD	GluC
H31_MOUSE, H32_MOUSE, H33_MOUSE	Histone H3	476.2420	950.4694	2	-0.0015	8	1	1.70E-02	IAQDFKme1TD	GluC
H31_MOUSE, H32_MOUSE, H33_MOUSE	Histone H3	476.2423	950.4700	2	-0.0009	8	1	3.80E-02	IAQDFKme1TD	GluC
H31_MOUSE, H32_MOUSE, H33_MOUSE	Histone H3	476.2427	950.4708	2	-0.0001	8	1	4.90E-02	IAQDFKme1TD	GluC
H31_MOUSE, H32_MOUSE, H33_MOUSE	Histone H3	476.2415	950.4685	2	-0.0024	8	1	2.00E-04	IAQDFKme1TD	GluC
H31_MOUSE, H32_MOUSE, H33_MOUSE	Histone H3	476.2425	950.4704	2	-0.0005	8	1	3.40E-04	IAQDFKme1TD	GluC
H31_MOUSE, H32_MOUSE, H33_MOUSE	Histone H3	476.2415	950.4685	2	-0.0024	8	1	7.90E-04	IAQDFKme1TD	GluC
H31_MOUSE, H32_MOUSE, H33_MOUSE	Histone H3	476.2424	950.4702	2	-0.0007	8	1	1.60E-03	IAQDFKme1TD	GluC
H31_MOUSE, H32_MOUSE, H33_MOUSE	Histone H3	476.2427	950.4709	2	0.0000	8	1	1.10E-03	IAQDFKme1TD	GluC
H31_MOUSE, H32_MOUSE, H33_MOUSE	Histone H3	476.2426	950.4707	2	-0.0002	8	1	4.20E-03	IAQDFKme1TD	GluC
H31_MOUSE, H32_MOUSE, H33_MOUSE	Histone H3	476.2427	950.4709	2	0.0000	8	1	1.40E-02	IAQDFKme1TD	GluC
H31_MOUSE, H32_MOUSE, H33_MOUSE	Histone H3	476.2427	950.4709	2	0.0000	8	1	4.50E-02	IAQDFKme1TD	GluC
H31_MOUSE, H32_MOUSE, H33_MOUSE	Histone H3	476.2420	950.4695	2	-0.0014	8	1	1.40E-04	IAQDFKme1TD	GluC
H31_MOUSE, H32_MOUSE, H33_MOUSE	Histone H3	476.2426	950.4707	2	-0.0002	8	1	7.00E-05	IAQDFKme1TD	GluC
H31_MOUSE, H32_MOUSE, H33_MOUSE	Histone H3	476.2427	950.4708	2	-0.0001	8	1	2.00E-02	IAQDFKme1TD	GluC
H31_MOUSE, H32_MOUSE, H33_MOUSE	Histone H3	483.2501	964.4857	2	-0.0008	8	1	3.50E-03	IAQDFKme2TD	GluC
H31_MOUSE, H32_MOUSE, H33_MOUSE	Histone H3	483.2492	964.4839	2	-0.0026	8	1	9.40E-05	IAQDFKme2TD	GluC
H31_MOUSE, H32_MOUSE, H33_MOUSE	Histone H3	483.2496	964.4847	2	-0.0018	8	1	4.70E-03	IAQDFKme2TD	GluC
H31_MOUSE, H32_MOUSE, H33_MOUSE	Histone H3	483.2492	964.4839	2	-0.0026	8	1	2.00E-02	IAQDFKme2TD	GluC
H31_MOUSE, H32_MOUSE, H33_MOUSE	Histone H3	476.2432	950.4717	2	0.0008	8	1	2.70E-05	IAQDFKme1TD	GluC
H31_MOUSE, H32_MOUSE, H33_MOUSE	Histone H3	476.2421	950.4697	2	-0.0012	8	1	3.10E-05	IAQDFKme1TD	GluC
H31_MOUSE, H32_MOUSE, H33_MOUSE	Histone H3	483.2498	964.4851	2	-0.0014	8	1	1.80E-04	IAQDFKme2TD	GluC
H31_MOUSE, H32_MOUSE, H33_MOUSE	Histone H3	483.2507	964.4868	2	0.0002	8	1	2.50E-04	IAQDFKme2TD	GluC
H31_MOUSE, H32_MOUSE, H33_MOUSE	Histone H3	483.2504	964.4863	2	-0.0003	8	1	6.30E-04	IAQDFKme2TD	GluC
H31_MOUSE, H32_MOUSE, H33_MOUSE	Histone H3	476.2430	950.4715	2	0.0006	8	1	7.30E-04	IAQDFKme1TD	GluC
H31_MOUSE, H32_MOUSE, H33_MOUSE	Histone H3	476.2421	950.4697	2	-0.0012	8	1	3.60E-03	IAQDFKme1TD	GluC
H31_MOUSE, H32_MOUSE, H33_MOUSE	Histone H3	483.2498	964.4851	2	-0.0014	8	1	4.20E-03	IAQDFKme2TD	GluC
H31_MOUSE, H32_MOUSE, H33_MOUSE	Histone H3	476.2432	950.4717	2	0.0008	8	1	6.20E-03	IAQDFKme1TD	GluC
H31_MOUSE, H32_MOUSE, H33_MOUSE	Histone H3	476.2427	950.4708	2	-0.0001	8	1	6.80E-03	IAQDFKme1TD	GluC
H31_MOUSE, H32_MOUSE, H33_MOUSE	Histone H3	483.2507	964.4868	2	0.0002	8	1	8.00E-03	IAQDFKme2TD	GluC
H31_MOUSE, H32_MOUSE, H33_MOUSE	Histone H3	476.2428	950.4709	2	0.0000	8	1	1.10E-02	IAQDFKme1TD	GluC
H31_MOUSE, H32_MOUSE, H33_MOUSE	Histone H3	476.2416	950.4687	2	-0.0022	8	1	4.30E-02	IAQDFKme1TD	GluC
H31_MOUSE, H32_MOUSE, H33_MOUSE	Histone H3	483.2516	964.4887	2	0.0021	8	1	3.30E-04	IAQDFKme2TD	GluC
H31_MOUSE, H32_MOUSE, H33_MOUSE	Histone H3	476.2439	950.4732	2	0.0023	8	1	3.40E-04	IAQDFKme1TD	GluC
H31_MOUSE, H32_MOUSE, H33_MOUSE	Histone H3	483.2516	964.4887	2	0.0021	8	1	6.10E-04	IAQDFKme2TD	GluC
H31_MOUSE, H32_MOUSE, H33_MOUSE	Histone H3	476.2439	950.4732	2	0.0023	8	1	6.70E-03	IAQDFKme1TD	GluC
H31_MOUSE, H32_MOUSE, H33_MOUSE	Histone H3	408.2225	814.4305	2	0.0008	8	1	5.70E-03	STGGKacAPR	Semi-tryptic
H31_MOUSE, H32_MOUSE, H33_MOUSE	Histone H3	476.2425	950.4704	2	-0.0005	8	1	2.20E-05	IAQDFKme1TD	GluC
H31_MOUSE, H32_MOUSE, H33_MOUSE	Histone H3	483.2506	964.4867	2	0.0001	8	1	4.20E-05	IAQDFKme2TD	GluC
H31_MOUSE, H32_MOUSE, H33_MOUSE	Histone H3	483.2507	964.4867	2	0.0002	8	1	5.10E-05	IAQDFKme2TD	GluC
H31_MOUSE, H32_MOUSE, H33_MOUSE	Histone H3	476.2439	950.4733	2	0.0024	8	1	6.40E-05	IAQDFKme1TD	GluC
H31_MOUSE, H32_MOUSE, H33_MOUSE	Histone H3	483.2512	964.4879	2	0.0013	8	1	7.60E-05	IAQDFKme2TD	GluC
H31_MOUSE, H32_MOUSE, H33_MOUSE	Histone H3	483.2496	964.4847	2	-0.0018	8	1	8.30E-05	IAQDFKme2TD	GluC
H31_MOUSE, H32_MOUSE, H33_MOUSE	Histone H3	483.2495	964.4844	2	-0.0022	8	1	9.80E-05	IAQDFKme2TD	GluC
H31_MOUSE, H32_MOUSE, H33_MOUSE	Histone H3	476.2438	950.4730	2	0.0021	8	1	1.40E-04	IAQDFKme1TD	GluC
H31_MOUSE, H32_MOUSE, H33_MOUSE	Histone H3	476.2426	950.4706	2	-0.0003	8	1	1.70E-04	IAQDFKme1TD	GluC
H31_MOUSE, H32_MOUSE, H33_MOUSE	Histone H3	476.2426	950.4707	2	-0.0002	8	1	2.50E-04	IAQDFKme1TD	GluC
H31_MOUSE, H32_MOUSE, H33_MOUSE	Histone H3	476.2427	950.4708	2	-0.0001	8	1	6.50E-04	IAQDFKme1TD	GluC
H31_MOUSE, H32_MOUSE, H33_MOUSE	Histone H3	476.2428	950.4711	2	0.0002	8	1	6.70E-04	IAQDFKme1TD	GluC

uniprot accession/s for all possible isoforms	histone type	pep exp mz	pep exp mr	pep exp z	pep delta	pep length	pep miss	pep expect	peptide and site of PTM	enzyme
H31_MOUSE, H32_MOUSE, H33_MOUSE	Histone H3	476.2425	950.4705	2	-0.0004	8	1	8.70E-04	IAQDFKme1TD	GluC
H31_MOUSE, H32_MOUSE, H33_MOUSE	Histone H3	476.2425	950.4704	2	-0.0005	8	1	1.00E-03	IAQDFKme1TD	GluC
H31_MOUSE, H32_MOUSE, H33_MOUSE	Histone H3	476.2426	950.4707	2	-0.0002	8	1	2.30E-03	IAQDFKme1TD	GluC
H31_MOUSE, H32_MOUSE, H33_MOUSE	Histone H3	476.2433	950.4721	2	0.0012	8	1	2.40E-03	IAQDFKme1TD	GluC
H31_MOUSE, H32_MOUSE, H33_MOUSE	Histone H3	483.2506	964.4867	2	0.0001	8	1	2.60E-03	IAQDFKme2TD	GluC
H31_MOUSE, H32_MOUSE, H33_MOUSE	Histone H3	483.2508	964.4870	2	0.0004	8	1	3.00E-03	IAQDFKme2TD	GluC
H31_MOUSE, H32_MOUSE, H33_MOUSE	Histone H3	483.2496	964.4847	2	-0.0018	8	1	4.50E-03	IAQDFKme2TD	GluC
H31_MOUSE, H32_MOUSE, H33_MOUSE	Histone H3	476.2426	950.4706	2	-0.0003	8	1	5.00E-03	IAQDFKme1TD	GluC
H31_MOUSE, H32_MOUSE, H33_MOUSE	Histone H3	483.2514	964.4881	2	0.0016	8	1	5.90E-03	IAQDFKme2TD	GluC
H31_MOUSE, H32_MOUSE, H33_MOUSE	Histone H3	476.2427	950.4707	2	-0.0002	8	1	6.00E-03	IAQDFKme1TD	GluC
H31_MOUSE, H32_MOUSE, H33_MOUSE	Histone H3	476.2430	950.4714	2	0.0005	8	1	6.50E-03	IAQDFKme1TD	GluC
H31_MOUSE, H32_MOUSE, H33_MOUSE	Histone H3	476.2426	950.4707	2	-0.0002	8	1	1.10E-02	IAQDFKme1TD	GluC
H31_MOUSE, H32_MOUSE, H33_MOUSE	Histone H3	483.2512	964.4879	2	0.0013	8	1	1.20E-02	IAQDFKme2TD	GluC
H31_MOUSE, H32_MOUSE, H33_MOUSE	Histone H3	476.2427	950.4708	2	-0.0001	8	1	1.30E-02	IAQDFKme1TD	GluC
H31_MOUSE, H32_MOUSE, H33_MOUSE	Histone H3	476.2425	950.4704	2	-0.0005	8	1	1.40E-02	IAQDFKme1TD	GluC
H31_MOUSE, H32_MOUSE, H33_MOUSE	Histone H3	476.2436	950.4726	2	0.0017	8	1	1.50E-02	IAQDFKme1TD	GluC
H31_MOUSE, H32_MOUSE, H33_MOUSE	Histone H3	483.2506	964.4867	2	0.0001	8	1	1.60E-02	IAQDFKme2TD	GluC
H31_MOUSE, H32_MOUSE, H33_MOUSE	Histone H3	476.2436	950.4727	2	0.0018	8	1	1.80E-02	IAQDFKme1TD	GluC
H31_MOUSE, H32_MOUSE, H33_MOUSE	Histone H3	483.2515	964.4885	2	0.0019	8	1	2.20E-02	IAQDFKme2TD	GluC
H31_MOUSE, H32_MOUSE, H33_MOUSE	Histone H3	476.2437	950.4728	2	0.0019	8	1	2.60E-02	IAQDFKme1TD	GluC
H31_MOUSE, H32_MOUSE, H33_MOUSE	Histone H3	476.2431	950.4717	2	0.0008	8	1	4.50E-02	IAQDFKme1TD	GluC
H31_MOUSE, H32_MOUSE, H33_MOUSE	Histone H3	476.2426	950.4707	2	-0.0002	8	1	4.90E-02	IAQDFKme1TD	GluC
H31_MOUSE, H32_MOUSE, H33_MOUSE	Histone H3	483.2505	964.4865	2	0.0000	8	1	9.10E-04	IAQDFKme2TD	GluC
H31_MOUSE, H32_MOUSE, H33_MOUSE	Histone H3	483.2507	964.4868	2	0.0002	8	1	1.50E-03	IAQDFKme2TD	GluC
H31_MOUSE, H32_MOUSE, H33_MOUSE	Histone H3	483.2507	964.4868	2	0.0002	8	1	1.50E-03	IAQDFKme2TD	GluC
H31_MOUSE, H32_MOUSE, H33_MOUSE	Histone H3	483.2505	964.4864	2	-0.0001	8	1	1.00E-02	IAQDFKme2TD	GluC
H31_MOUSE, H32_MOUSE, H33_MOUSE	Histone H3	483.2505	964.4864	2	-0.0001	8	1	1.30E-02	IAQDFKme2TD	GluC
H31_MOUSE, H32_MOUSE, H33_MOUSE	Histone H3	476.2431	950.4717	2	0.0008	8	1	8.50E-04	IAQDFKme1TD	GluC
H31_MOUSE, H32_MOUSE, H33_MOUSE	Histone H3	476.2431	950.4717	2	0.0008	8	1	1.10E-03	IAQDFKme1TD	GluC
H31_MOUSE, H32_MOUSE, H33_MOUSE	Histone H3	476.2427	950.4709	2	0.0000	8	1	3.90E-03	IAQDFKme1TD	GluC
H31_MOUSE, H32_MOUSE, H33_MOUSE	Histone H3	476.2428	950.4711	2	0.0002	8	1	5.20E-03	IAQDFKme1TD	GluC
H31_MOUSE, H32_MOUSE, H33_MOUSE	Histone H3	476.2427	950.4709	2	0.0000	8	1	4.20E-03	IAQDFKme1TD	GluC
H31_MOUSE, H32_MOUSE, H33_MOUSE	Histone H3	476.2435	950.4725	2	0.0016	8	1	1.40E-04	IAQDFKme1TD	GluC
H31_MOUSE, H32_MOUSE, H33_MOUSE	Histone H3	483.2519	964.4893	2	0.0028	8	1	1.60E-03	IAQDFKme2TD	GluC
H31_MOUSE, H32_MOUSE, H33_MOUSE	Histone H3	476.2418	950.4691	2	-0.0018	8	1	2.00E-04	IAQDFKme1TD	GluC
H31_MOUSE, H32_MOUSE, H33_MOUSE	Histone H3	490.2405	978.4664	2	0.0006	8	1	3.20E-03	IAQDFKTacD	GluC
H31_MOUSE, H32_MOUSE, H33_MOUSE	Histone H3	483.2510	964.4875	2	0.0009	8	1	4.50E-02	IAQDFKme2TD	GluC
H31_MOUSE, H32_MOUSE, H33_MOUSE	Histone H3	456.2345	910.4544	2	0.0019	7	0	2.00E-02	S(p)TELLIR	Semi-tryptic
H31_MOUSE, H32_MOUSE, H33_MOUSE	Histone H3	456.2331	910.4516	2	-0.0009	7	0	1.90E-03	S(p)TELLIR	Semi-tryptic
H31_MOUSE, H32_MOUSE, H33_MOUSE	Histone H3	404.2394	806.4643	2	-0.0007	6	1	3.00E-02	Kme2TDLRF	Chymotrypsin
H31_MOUSE, H32_MOUSE, H33_MOUSE	Histone H3	397.2324	792.4503	2	0.0010	6	1	4.00E-02	Kme1TDLRF	Chymotrypsin
H33_MOUSE	Histone H3	779.4160	2335.2262	3	0.0090	21	2	3.30E-04	IAQDFKme1TDLRFQSAAIGALQE	GluC
H33_MOUSE	Histone H3	779.4135	2335.2187	3	0.0015	21	2	4.30E-03	IAQDFKme1TDLRFQSAAIGALQE	GluC
H33_MOUSE	Histone H3	779.4138	2335.2196	3	0.0024	21	2	1.30E-03	IAQDFKme1TDLRFQSAAIGALQE	GluC
H33_MOUSE	Histone H3	779.4143	2335.2211	3	0.0039	21	2	4.60E-03	IAQDFKme1TDLRFQSAAIGALQE	GluC
H33_MOUSE	Histone H3	784.0849	2349.2328	3	-0.0001	21	2	1.20E-03	IAQDFKme2TDLRFQSAAIGALQE	GluC
H33_MOUSE	Histone H3	784.0859	2349.2357	3	0.0029	21	2	3.00E-04	IAQDFKme2TDLRFQSAAIGALQE	GluC
H33_MOUSE	Histone H3	784.0855	2349.2346	3	0.0018	21	2	8.00E-03	IAQDFKme2TDLRFQSAAIGALQE	GluC
H33_MOUSE	Histone H3	779.4141	2335.2204	3	0.0032	21	2	9.60E-03	IAQDFKme1TDLRFQSAAIGALQE	GluC
H33_MOUSE	Histone H3	779.4141	2335.2206	3	0.0034	21	2	4.40E-02	IAQDFKme1TDLRFQSAAIGALQE	GluC
H33_MOUSE	Histone H3	779.4138	2335.2197	3	0.0025	21	2	2.60E-03	IAQDFKme1TDLRFQSAAIGALQE	GluC
H33_MOUSE	Histone H3	779.4138	2335.2197	3	0.0025	21	2	4.30E-03	IAQDFKme1TDLRFQSAAIGALQE	GluC
H33_MOUSE	Histone H3	779.4146	2335.2219	3	0.0047	21	2	5.90E-03	IAQDFKme1TDLRFQSAAIGALQE	GluC
H33_MOUSE	Histone H3	779.4145	2335.2217	3	0.0045	21	2	6.80E-03	IAQDFKme1TDLRFQSAAIGALQE	GluC
H33_MOUSE	Histone H3	784.0858	2349.2356	3	0.0027	21	2	4.90E-04	IAQDFKTDLRme2FQSAAIGALQE	GluC
H33_MOUSE	Histone H3	784.0858	2349.2356	3	0.0027	21	2	1.60E-03	IAQDFKme2TDLRFQSAAIGALQE	GluC
H33_MOUSE	Histone H3	779.4156	2335.2248	3	0.0076	21	2	1.90E-05	IAQDFKTDLRme1FQSAAIGALQE	GluC
H33_MOUSE	Histone H3	784.0856	2349.2349	3	0.0021	21	2	2.30E-05	IAQDFKTDLRme2FQSAAIGALQE	GluC
H33_MOUSE	Histone H3	784.0865	2349.2377	3	0.0049	21	2	3.90E-05	IAQDFKTDLRme2FQSAAIGALQE	GluC
H33_MOUSE	Histone H3	784.0861	2349.2366	3	0.0038	21	2	4.10E-05	IAQDFKTDLRme2FQSAAIGALQE	GluC
H33_MOUSE	Histone H3	784.0855	2349.2346	3	0.0018	21	2	4.20E-05	IAQDFKTDLRme2FQSAAIGALQE	GluC
H33_MOUSE	Histone H3	784.0863	2349.2370	3	0.0041	21	2	4.20E-05	IAQDFKTDLRme2FQSAAIGALQE	GluC
H33_MOUSE	Histone H3	784.0862	2349.2367	3	0.0039	21	2	4.60E-05	IAQDFKme1TDLRme1FQSAAIGALQE	GluC
H33_MOUSE	Histone H3	779.4146	2335.2220	3	0.0048	21	2	9.50E-05	IAQDFKTDLRme1FQSAAIGALQE	GluC
H33_MOUSE	Histone H3	779.4138	2335.2195	3	0.0023	21	2	1.10E-04	IAQDFKTDLRme1FQSAAIGALQE	GluC
H33_MOUSE	Histone H3	779.4142	2335.2208	3	0.0036	21	2	1.60E-04	IAQDFKTDLRme1FQSAAIGALQE	GluC
H33_MOUSE	Histone H3	784.0858	2349.2355	3	0.0026	21	2	1.90E-04	IAQDFKTDLRme2FQSAAIGALQE	GluC
H33_MOUSE	Histone H3	779.4132	2335.2177	3	0.0005	21	2	2.30E-04	IAQDFKTDLRme1FQSAAIGALQE	GluC
H33_MOUSE	Histone H3	779.4148	2335.2225	3	0.0053	21	2	3.30E-04	IAQDFKTDLRme1FQSAAIGALQE	GluC
H33_MOUSE	Histone H3	784.0853	2349.2340	3	0.0011	21	2	3.60E-04	IAQDFKme2TDLRFQSAAIGALQE	GluC
H33_MOUSE	Histone H3	779.4138	2335.2195	3	0.0023	21	2	4.00E-04	IAQDFKTDLRme1FQSAAIGALQE	GluC
H33_MOUSE	Histone H3	779.4138	2335.2196	3	0.0024	21	2	4.50E-04	IAQDFKme1TDLRFQSAAIGALQE	GluC
H33_MOUSE	Histone H3	1168.6184	2335.2222	2	0.0051	21	2	5.40E-04	IAQDFKme1TDLRFQSAAIGALQE	GluC

uniprot accession/s for all possible isoforms	histone type	pep exp mz	pep exp mr	pep exp z	pep delta	pep length	pep miss	pep expect	peptide and site of PTM	enzyme
H33_MOUSE	Histone H3	779.4130	2335.2173	3	0.0001	21	2	8.50E-04	IAQDFKTDLRme1FQSAAIGALQE	GluC
H33_MOUSE	Histone H3	779.4143	2335.2211	3	0.0039	21	2	1.20E-03	IAQDFKme1TDLRFQSAAIGALQE	GluC
H33_MOUSE	Histone H3	779.4149	2335.2229	3	0.0057	21	2	1.30E-03	IAQDFKTDLRme1FQSAAIGALQE	GluC
H33_MOUSE	Histone H3	784.0858	2349.2356	3	0.0028	21	2	1.80E-03	IAQDFKTDLRme2FQSAAIGALQE	GluC
H33_MOUSE	Histone H3	784.0856	2349.2351	3	0.0023	21	2	2.10E-03	IAQDFKme1TDLRme1FQSAAIGALQE	GluC
H33_MOUSE	Histone H3	779.4146	2335.2221	3	0.0049	21	2	2.30E-03	IAQDFKme1TDLRFQSAAIGALQE	GluC
H33_MOUSE	Histone H3	784.0856	2349.2349	3	0.0021	21	2	2.50E-03	IAQDFKme2TDLRFQSAAIGALQE	GluC
H33_MOUSE	Histone H3	779.4130	2335.2173	3	0.0001	21	2	3.00E-03	IAQDFKme1TDLRFQSAAIGALQE	GluC
H33_MOUSE	Histone H3	779.4144	2335.2214	3	0.0042	21	2	4.30E-03	IAQDFKTDLRme1FQSAAIGALQE	GluC
H33_MOUSE	Histone H3	779.4150	2335.2233	3	0.0061	21	2	4.90E-03	IAQDFKTDLRme1FQSAAIGALQE	GluC
H33_MOUSE	Histone H3	784.0855	2349.2346	3	0.0018	21	2	5.30E-03	IAQDFKme2TDLRFQSAAIGALQE	GluC
H33_MOUSE	Histone H3	784.0858	2349.2355	3	0.0026	21	2	7.00E-03	IAQDFKme2TDLRFQSAAIGALQE	GluC
H33_MOUSE	Histone H3	784.0853	2349.2340	3	0.0011	21	2	1.30E-02	IAQDFKTDLRme2FQSAAIGALQE	GluC
H33_MOUSE	Histone H3	779.4144	2335.2214	3	0.0042	21	2	1.50E-02	IAQDFKme1TDLRFQSAAIGALQE	GluC
H33_MOUSE	Histone H3	784.0863	2349.2370	3	0.0041	21	2	1.60E-02	IAQDFKme2TDLRFQSAAIGALQE	GluC
H33_MOUSE	Histone H3	779.4144	2335.2215	3	0.0043	21	2	2.50E-02	IAQDFKme1TDLRFQSAAIGALQE	GluC
H33_MOUSE	Histone H3	779.4146	2335.2219	3	0.0047	21	2	2.50E-02	IAQDFKme1TDLRFQSAAIGALQE	GluC
H33_MOUSE	Histone H3	779.4144	2335.2215	3	0.0043	21	2	4.70E-02	IAQDFKme1TDLRFQSAAIGALQE	GluC
H33_MOUSE	Histone H3	784.0861	2349.2366	3	0.0038	21	2	4.80E-02	IAQDFKme2TDLRFQSAAIGALQE	GluC
H33_MOUSE	Histone H3	784.0873	2349.2401	3	0.0073	21	2	4.80E-02	IAQDFKTDLRme2FQSAAIGALQE	GluC
H33_MOUSE	Histone H3	801.3976	2401.1711	3	0.0032	21	2	6.30E-04	IAQDFKTDLRFQpSAAIGALQE	GluC
H33_MOUSE	Histone H3	801.3976	2401.1711	3	0.0032	21	2	1.00E-02	IAQDFKTDLRFQpSAAIGALQE	GluC
H33_MOUSE	Histone H3	637.0129	1908.0169	3	0.0064	17	1	7.70E-04	FKme1TDLRFQSAAIGALQE	GluC
H33_MOUSE	Histone H3	377.2281	1504.8834	4	-0.0004	14	2	1.40E-05	Kme2SAPSTGGVKme2KPHR	Semi-tryptic
H33_MOUSE	Histone H3	380.7230	1518.8629	4	-0.0002	14	2	4.70E-04	KacSAPSTGGVKme2KPHR	Semi-tryptic
H33_MOUSE	Histone H3	380.7302	1518.8916	4	-0.0079	14	2	2.90E-07	Kme3SAPSTGGVKme2KPHR	Semi-tryptic
H33_MOUSE	Histone H3	377.2281	1504.8834	4	-0.0004	14	2	5.20E-06	Kme2SAPSTGGVKme2KPHR	Semi-tryptic
H33_MOUSE	Histone H3	380.7230	1518.8629	4	-0.0002	14	2	1.90E-04	KacSAPSTGGVKme2KPHR	Semi-tryptic
H33_MOUSE	Histone H3	529.2905	1584.8498	3	-0.0003	14	2	1.70E-02	Kme2SAPpSTGGVKme2KPHR	Semi-tryptic
H33_MOUSE	Histone H3	397.2198	1584.8502	4	0.0001	14	2	2.40E-02	Kme2SAPpSTGGVKme2KPHR	Semi-tryptic
H33_MOUSE	Histone H3	502.6357	1504.8853	3	0.0015	14	2	2.00E-04	Kme2SAPSTGGVKme2KPHR	Semi-tryptic
H33_MOUSE	Histone H3	377.2283	1504.8839	4	0.0002	14	2	7.50E-04	Kme2SAPSTGGVKme2KPHR	Semi-tryptic
H33_MOUSE	Histone H3	742.3724	1482.7303	2	0.0071	13	0	9.50E-03	LRFQpSAAIGALQE	GluC
H4_MOUSE	Histone H4	658.1382	2628.5238	4	0.0078	24	0	8.60E-08	ac-SGRGKacGGKGLGKGGAKacRHRKme2VLRD	GluC
H4_MOUSE	Histone H4	651.1405	2600.5331	4	0.0119	24	0	1.70E-11	SGRme3GKGGKGLGKGGAKacRHRKacVLRD	GluC
H4_MOUSE	Histone H4	640.6389	2558.5263	4	0.0158	24	0	1.90E-07	ac-SGRGKGGKGLGKGGAKRHRKme3VLRD	GluC
H4_MOUSE	Histone H4	532.3119	2656.5229	5	0.0120	24	0	5.00E-07	ac-SGRGKGGKacGLGKacGGAKacRHRKme1VLRD	GluC
H4_MOUSE	Histone H4	633.6306	2530.4935	4	0.0142	24	0	1.20E-10	ac-SGRGKGGKGLGKGGAKRHRKme1VLRD	GluC
H4_MOUSE	Histone H4	507.1058	2530.4927	5	0.0135	24	0	8.20E-10	ac-SGRGKGGKGLGKGGAKRHRKme1VLRD	GluC
H4_MOUSE	Histone H4	515.5067	2572.4973	5	0.0075	24	0	4.90E-09	ac-SGRGKGGKGLGKGGAKacRHRKme1VLRD	GluC
H4_MOUSE	Histone H4	512.7105	2558.5160	5	0.0054	24	0	1.10E-08	ac-SGRme1GKGGKGLGKGGAKRHRme2KVLRD	GluC
H4_MOUSE	Histone H4	640.6378	2558.5220	4	0.0115	24	0	1.90E-08	ac-SGRme1GKGGKGLGKGGAKRHRKme2VLRD	GluC
H4_MOUSE	Histone H4	654.6441	2614.5472	4	0.0104	24	0	5.80E-08	ac-SGRme1GKGGKGLGKGGAKacRHRKme3VLRD	GluC
H4_MOUSE	Histone H4	867.8525	2600.5357	3	0.0146	24	0	1.30E-03	ac-SGRme1GKGGKGLGKGGAKacRHRK(me2)VLRD	GluC
H4_MOUSE	Histone H4	512.7095	2558.5111	5	0.0005	24	0	1.90E-05	ac-SGRGKGGKGLGKGGAKRHRKme3VLRD	GluC
H4_MOUSE	Histone H4	647.6365	2586.5168	4	0.0113	24	0	4.40E-14	ac-SGRGKGGKGLGKGGAKacRHRKme2VLRD	GluC
H4_MOUSE	Histone H4	668.6418	2670.5379	4	0.0113	24	0	4.50E-11	ac-SGRGKGGKacGLGKacGGAKacRHRKme2VLRD	GluC
H4_MOUSE	Histone H4	679.1448	2712.5501	4	0.0130	24	0	1.20E-08	ac-SGRGKacGGKacGLGKacGGAKacRHRme2KVLRD	GluC
H4_MOUSE	Histone H4	654.6441	2614.5472	4	0.0104	24	0	1.90E-08	ac-SGRme2GKGGKGLGKGGAKacRHRKme2VLRD	GluC
H4_MOUSE	Histone H4	534.3034	2666.4808	5	0.0090	24	0	7.40E-06	ac-pSGRGKGGKGLGKGGAKacRHRKme2VLRD	GluC
H4_MOUSE	Histone H4	849.1767	2544.5084	3	0.0135	24	0	1.40E-05	ac-SGRGKGGKGLGKGGAKRHRKme2VLRD	GluC
H4_MOUSE	Histone H4	863.1797	2586.5173	3	0.0119	24	0	1.70E-05	ac-SGRGKGGKGLGKGGAKRHR(me2)K(ac)VLRD	GluC
H4_MOUSE	Histone H4	668.6417	2670.5375	4	0.0109	24	0	4.90E-04	ac-SGRGKacGGKacGLGKacGGAKRHRKme2VLRD	GluC
H4_MOUSE	Histone H4	647.6368	2586.5181	4	0.0127	24	0	2.10E-10	ac-SGRGKGGKGLGKGGAKacRHRKme2VLRD	GluC
H4_MOUSE	Histone H4	637.1346	2544.5093	4	0.0144	24	0	2.10E-09	ac-SGRGKGGKGLGKGGAKRHRKme2VLRD	GluC
H4_MOUSE	Histone H4	668.6420	2670.5390	4	0.0124	24	0	8.20E-09	ac-SGRGKGGKacGLGKacGGAKacRHRKme2VLRD	GluC
H4_MOUSE	Histone H4	535.1151	2670.5393	5	0.0127	24	0	5.80E-08	ac-SGRGKGGKacGLGKacGGAKacRHRKme2VLRD	GluC
H4_MOUSE	Histone H4	658.1393	2628.5279	4	0.0119	24	0	1.60E-06	ac-SGRGKGGKGLGKacGGAKacRHRKme2VLRD	GluC
H4_MOUSE	Histone H4	667.6284	2666.4844	4	0.0126	24	0	3.00E-06	ac-pSGRGKGGKGLGKGGAKacRHRKme2VLRD	GluC
H4_MOUSE	Histone H4	877.1829	2628.5269	3	0.0109	24	0	1.00E-05	ac-SGRGKGGKGLGKacGGAKacRHRKme2VLRD	GluC
H4_MOUSE	Histone H4	512.7061	2558.4940	5	0.0199	24	0	4.40E-05	ac-SGRGKGGKGLGKGGAKRHRKacVLRD	GluC
H4_MOUSE	Histone H4	863.1800	2586.5181	3	0.0126	24	0	5.30E-05	ac-SGRGKGGKGLGKGGAKacRHRKme2VLRD	GluC
H4_MOUSE	Histone H4	849.1784	2544.5133	3	0.0184	24	0	8.80E-05	ac-SGRGKGGKGLGKGGAKRHRKme2VLRD	GluC
H4_MOUSE	Histone H4	637.1333	2544.5041	4	0.0092	24	0	6.40E-11	ac-SGRGKGGKGLGKGGAKRHRKme2VLRD	GluC
H4_MOUSE	Histone H4	509.9071	2544.4990	5	0.0041	24	0	6.50E-11	ac-SGRGKGGKGLGKGGAKRHRKme2VLRD	GluC
H4_MOUSE	Histone H4	637.1332	2544.5036	4	0.0087	24	0	6.40E-13	ac-SGRGKGGKGLGKGGAKRHRKme2VLRD	GluC
H4_MOUSE	Histone H4	640.6361	2558.5154	4	0.0048	24	0	3.00E-10	ac-SGRGKGGKGLGKGGAKRHRKme3VLRD	GluC
H4_MOUSE	Histone H4	651.1368	2600.5182	4	-0.0029	24	0	1.20E-09	ac-SGRGKGGKGLGKGGAKacRHRKme3VLRD	GluC
H4_MOUSE	Histone H4	521.1114	2600.5207	5	-0.0004	24	0	7.00E-09	ac-SGRGKGGKGLGKGGAKacRHRme3KVLRD	GluC
H4_MOUSE	Histone H4	507.1053	2530.4902	5	0.0110	24	0	9.20E-08	ac-SGRGKGGKGLGKGGAKRHRKme1VLRD	GluC
H4_MOUSE	Histone H4	654.6325	2614.5011	4	0.0007	24	0	2.70E-06	ac-SGRGKGGKGLGKacGGAKacRHRKme1VLRD	GluC
H4_MOUSE	Histone H4	661.6402	2642.5317	4	0.0001	24	0	1.20E-04	ac-SGRGKGGKGLGKacGGAKacRme2HRK(me1)VLRD	GluC
H4_MOUSE	Histone H4	637.1306	2544.4932	4	-0.0017	24	0	1.90E-11	ac-SGRGKGGKGLGKGGAKRHRKme2VLRD	GluC

uniprot accession/s for all possible isoforms	histone type	pep exp mz	pep exp mr	pep exp z	pep delta	pep length	pep miss	pep expect	peptide and site of PTM	enzyme
H4_MOUSE	Histone H4	647.6336	2586.5055	4	0.0000	24	0	2.60E-11	ac-SGRGKGGKGLGKGGAKacRHRKme2VLRD	GluC
H4_MOUSE	Histone H4	647.6330	2586.5030	4	-0.0025	24	0	2.00E-10	ac-SGRGKGGKGLGKGGAKacRHRKme2VLRD	GluC
H4_MOUSE	Histone H4	658.1368	2628.5183	4	0.0022	24	0	1.10E-08	ac-SGRGKGGKGLGKacGGAKacRHRKme2VLRD	GluC
H4_MOUSE	Histone H4	518.3110	2586.5187	5	0.0133	24	0	1.80E-08	ac-SGRGKGGKGLGKGGAKacRHRKme2VLRD	GluC
H4_MOUSE	Histone H4	679.1415	2712.5368	4	-0.0003	24	0	4.50E-08	ac-SGRGKacGGKacGLGKacGGAKacRHRKme2VLRD	GluC
H4_MOUSE	Histone H4	657.1226	2624.4612	4	0.0000	24	0	1.50E-07	ac-pSGRGKGGKGLGKGGAKRHRKme2VLRD	GluC
H4_MOUSE	Histone H4	668.6393	2670.5281	4	0.0016	24	0	3.50E-07	ac-SGRGKacGGKacGLGKGGAKRHR(me2)K(ac)VLRD	GluC
H4_MOUSE	Histone H4	658.1360	2628.5148	4	-0.0012	24	0	1.70E-06	ac-SGRGKacGGKGLGKGGAKRHRme2KacVLRD	GluC
H4_MOUSE	Histone H4	526.7102	2628.5148	5	-0.0012	24	0	7.20E-06	ac-SGRGKGGKGLGKacGGAKacRHRKme2VLRD	GluC
H4_MOUSE	Histone H4	637.1304	2544.4927	4	-0.0022	24	0	1.10E-05	SGRGKGGKGLGKGGAKacRHRKme2VLRD	GluC
H4_MOUSE	Histone H4	543.5143	2712.5352	5	-0.0019	24	0	5.10E-05	ac-SGRGKacGGKacGLGKacGGAKacRHRKme2VLRD	GluC
H4_MOUSE	Histone H4	863.1774	2586.5103	3	0.0049	24	0	7.30E-05	ac-SGRGKGGKGLGKGGAKacRHRKme2VLRD	GluC
H4_MOUSE	Histone H4	849.1717	2544.4934	3	-0.0015	24	0	1.50E-03	ac-SGRGKGGKGLGKGGAKRHRKme2VLRD	GluC
H4_MOUSE	Histone H4	644.1380	2572.5229	4	-0.0033	24	0	2.60E-11	ac-SGRme1GKGGKGLGKGGAKRHRKme3VLRD	GluC
H4_MOUSE	Histone H4	647.6484	2586.5643	4	-0.0140	24	0	7.00E-10	SGRme3GKGGKGLGKGGAKme3RHRKme2VLRD	GluC
H4_MOUSE	Histone H4	654.6429	2614.5425	4	0.0057	24	0	1.10E-09	ac-SGRme1GKGGKGLGKGGAKacRHRKme3VLRD	GluC
H4_MOUSE	Histone H4	640.6374	2558.5207	4	0.0101	24	0	3.90E-09	ac-SGRGKGGKGLGKGGAKRHRKme3VLRD	GluC
H4_MOUSE	Histone H4	651.1376	2600.5212	4	0.0001	24	0	7.00E-09	ac-SGRGKGGKGLGKGGAKacRHRKme3VLRD	GluC
H4_MOUSE	Histone H4	640.6344	2558.5083	4	-0.0022	24	0	6.10E-08	ac-SGRGKGGKGLGKGGAKRHRKme3VLRD	GluC
H4_MOUSE	Histone H4	515.5170	2572.5487	5	-0.0139	24	0	2.50E-07	SGRme3GKGGKGLGKGGAKme3RHRKme1VLRD	GluC
H4_MOUSE	Histone H4	651.1528	2600.5823	4	-0.0116	24	0	5.40E-07	SGRme3GKGGKGLGKGGAKme3RHRKme3VLRD	GluC
H4_MOUSE	Histone H4	523.9221	2614.5741	5	0.0009	24	0	6.90E-07	ac-SGRme1GKGGKGLGKGGAKme3RHRKme3VLRD	GluC
H4_MOUSE	Histone H4	521.1234	2600.5808	5	-0.0131	24	0	4.10E-05	SGRme3GKGGKGLGKGGAKme3RHRme2Kme1VLRD	GluC
H4_MOUSE	Histone H4	640.6355	2558.5127	4	0.0022	24	0	1.10E-03	ac-SGRGKGGKGLGKGGAKRHRKme3VLRD	GluC
H4_MOUSE	Histone H4	863.1967	2586.5682	3	-0.0101	24	0	1.10E-02	SGRme3GKGGKGLGKGGAKR(me3)HRK(me2)VLRD	GluC
H4_MOUSE	Histone H4	651.1383	2600.5243	4	0.0031	24	0	1.50E-02	ac-SGRGKGGKGLGKGGAKacRHRKme3VLRD	GluC
H4_MOUSE	Histone H4	512.7114	2558.5207	5	0.0102	24	0	4.90E-10	ac-SGRGKGGKGLGKGGAKRHRKme3VLRD	GluC
H4_MOUSE	Histone H4	644.1405	2572.5331	4	0.0068	24	0	3.30E-09	ac-SGRme1GKGGKGLGKGGAKRHRKme3VLRD	GluC
H4_MOUSE	Histone H4	640.6366	2558.5171	4	0.0065	24	0	1.50E-08	ac-SGRGKGGKGLGKGGAKRHRKme3VLRD	GluC
H4_MOUSE	Histone H4	640.6369	2558.5186	4	0.0080	24	0	4.10E-07	ac-SGRGKGGKGLGKGGAKRHRKme3VLRD	GluC
H4_MOUSE	Histone H4	651.1382	2600.5239	4	0.0027	24	0	7.40E-09	ac-SGRGKGGKGLGKGGAKacRHRKme3VLRD	GluC
H4_MOUSE	Histone H4	512.7093	2558.5101	5	-0.0004	24	0	1.10E-07	ac-SGRGKGGKGLGKGGAKRHRKme3VLRD	GluC
H4_MOUSE	Histone H4	366.5066	2558.4953	7	-0.0152	24	0	9.60E-04	ac-SGRGKGGKGLGKGGAKRHRKVLRme3D	GluC
H4_MOUSE	Histone H4	529.5119	2642.5234	5	-0.0083	24	0	1.10E-03	SGRme3GKGGKGLGKacGGAKacRHRKacVLRD	GluC
H4_MOUSE	Histone H4	518.3167	2586.5472	5	0.0054	24	0	3.70E-08	ac-SGRme2GKGGKGLGKGGAKRHRKme2VLRme1D	GluC
H4_MOUSE	Histone H4	644.1301	2572.4915	4	0.0017	24	0	1.30E-11	ac-SGRGKGGKGLGKGGAKacRHRKme1VLRD	GluC
H4_MOUSE	Histone H4	633.6266	2530.4773	4	-0.0020	24	0	7.80E-10	ac-SGRGKGGKGLGKGGAKRHRKme1VLRD	GluC
H4_MOUSE	Histone H4	507.1030	2530.4787	5	-0.0006	24	0	1.30E-08	ac-SGRGKGGKGLGKGGAKRHRKme1VLRD	GluC
H4_MOUSE	Histone H4	512.7087	2558.5071	5	-0.0035	24	0	1.30E-07	ac-SGRme1GKGGKGLGKGGAKRHRKme2VLRD	GluC
H4_MOUSE	Histone H4	507.1035	2530.4811	5	0.0018	24	0	2.20E-07	ac-SGRGKGGKGLGKGGAKRHRKme1VLRD	GluC
H4_MOUSE	Histone H4	507.1041	2530.4842	5	0.0049	24	0	2.30E-07	ac-SGRGKGGKGLGKGGAKRHR(me1)KVLRD	GluC
H4_MOUSE	Histone H4	521.1110	2600.5187	5	-0.0024	24	0	1.20E-05	ac-SGRGKGGKGLGKGGAKacRHRK(me1)VLRme2D	GluC
H4_MOUSE	Histone H4	515.5057	2572.4921	5	0.0022	24	0	1.20E-05	ac-SGRGKGGKGLGKGGAKacRHRme1KVLRD	GluC
H4_MOUSE	Histone H4	512.7086	2558.5067	5	-0.0038	24	0	1.40E-05	ac-SGRGKGGKGLGKGGAKRHRme2Kme1VLRD	GluC
H4_MOUSE	Histone H4	872.5101	2614.5085	3	0.0081	24	0	4.70E-05	ac-SGRGKGGKGLGKacGGAKacRHRK(me1)VLRD	GluC
H4_MOUSE	Histone H4	853.8440	2558.5103	3	-0.0003	24	0	8.30E-05	ac-SGRGKGGKGLGKGGAKRHRK(me2)VLR(me1)D	GluC
H4_MOUSE	Histone H4	858.5038	2572.4896	3	-0.0002	24	0	1.60E-04	ac-SGRGKGGKGLGKGGAKacRHRK(me1)VLRD	GluC
H4_MOUSE	Histone H4	844.5009	2530.4808	3	0.0015	24	0	2.70E-04	ac-SGRGKGGKGLGKGGAKRHRK(me1)VLRD	GluC
H4_MOUSE	Histone H4	881.8534	2642.5384	3	0.0067	24	0	4.20E-04	ac-SGRGKGGKGLGKacGGAKacRHRKme1VLRme2D	GluC
H4_MOUSE	Histone H4	633.6294	2530.4886	4	0.0093	24	0	4.60E-04	ac-SGRGKGGKGLGKGGAKRHRKme1VLRD	GluC
H4_MOUSE	Histone H4	867.8491	2600.5255	3	0.0044	24	0	8.50E-04	ac-SGRGKGGKGLGKGGAKacRHRK(me2)VLR(me1)D	GluC
H4_MOUSE	Histone H4	867.8624	2600.5654	3	0.0079	24	0	1.20E-03	ac-SGRme1GKGGKGLGKGGAK(me2)R(me1)HRK(me2)VLRD	GluC
H4_MOUSE	Histone H4	633.6276	2530.4811	4	0.0019	24	0	2.40E-02	ac-SGRGKGGKGLGKGGAKRHRK(me1)VLRD	GluC
H4_MOUSE	Histone H4	515.5128	2572.5278	5	0.0016	24	0	4.60E-02	SGRme1GKGGKacGLGKGGAKRHRme1KVLRme2D	GluC
H4_MOUSE	Histone H4	633.6279	2530.4825	4	0.0033	24	0	5.30E-10	ac-SGRGKGGKGLGKGGAKRHRKme1VLRD	GluC
H4_MOUSE	Histone H4	532.3100	2656.5138	5	0.0029	24	0	3.40E-09	ac-SGRGKGGKacGLGKacGGAKacRHRKme1VLRD	GluC
H4_MOUSE	Histone H4	644.1306	2572.4933	4	0.0034	24	0	1.20E-08	ac-SGRGKGGKGLGKGGAKacRHRKme1VLRD	GluC
H4_MOUSE	Histone H4	658.1357	2628.5136	4	-0.0024	24	0	1.90E-08	ac-SGRGKacGGKGLGKGGAKacRHRKme2VLRD	GluC
H4_MOUSE	Histone H4	633.6280	2530.4827	4	0.0035	24	0	2.90E-08	ac-SGRGKGGKGLGKGGAKRHRK(me1)VLRD	GluC
H4_MOUSE	Histone H4	640.6357	2558.5135	4	0.0030	24	0	1.10E-07	ac-SGRGKGGKGLGKGGAKRHRme1Kme2VLRD	GluC
H4_MOUSE	Histone H4	654.6325	2614.5010	4	0.0006	24	0	1.30E-06	ac-SGRGKGGKGLGKacGGAKacRHRKme1VLRD	GluC
H4_MOUSE	Histone H4	675.6389	2698.5266	4	0.0051	24	0	1.70E-06	ac-SGRGKacGGKacGLGKacGGAKacRHRKme1VLRD	GluC
H4_MOUSE	Histone H4	867.8479	2600.5219	3	0.0008	24	0	1.90E-05	ac-SGRme1GKGGKGLGKGGAKacRHRme2KVLRD	GluC
H4_MOUSE	Histone H4	853.8463	2558.5169	3	0.0064	24	0	3.90E-04	ac-SGRGKGGKGLGKGGAKRHR(me2)K(me1)VLRD	GluC
H4_MOUSE	Histone H4	512.7100	2558.5138	5	0.0032	24	0	1.60E-03	ac-SGRGKGGKGLGKGGAKRHRme2Kme1VLRD	GluC
H4_MOUSE	Histone H4	507.1018	2530.4726	5	-0.0067	24	0	3.60E-03	ac-SGRGKGGKGLGKGGAKRHRKme1VLRD	GluC
H4_MOUSE	Histone H4	647.6341	2586.5074	4	0.0019	24	0	1.20E-11	ac-SGRGKGGKGLGKGGAKacRHRKme2VLRD	GluC
H4_MOUSE	Histone H4	637.1312	2544.4956	4	0.0007	24	0	1.80E-11	ac-SGRGKGGKGLGKGGAKRHRKme2VLRD	GluC
H4_MOUSE	Histone H4	637.1342	2544.5079	4	0.0130	24	0	2.50E-11	ac-SGRGKGGKGLGKGGAKRHRKme2VLRD	GluC
H4_MOUSE	Histone H4	647.6316	2586.4975	4	-0.0080	24	0	8.70E-11	ac-SGRGKGGKGLGKGGAKacRHRKme2VLRD	GluC
H4_MOUSE	Histone H4	637.1315	2544.4967	4	0.0018	24	0	2.90E-10	ac-SGRGKGGKGLGKGGAKRHRKme2VLRD	GluC
H4_MOUSE	Histone H4	626.6284	2502.4846	4	0.0002	24	0	9.40E-10	SGRGKGGKGLGKGGAKRHRKme2VLRD	GluC
H4_MOUSE	Histone H4	518.3081	2586.5041	5	-0.0014	24	0	1.50E-08	ac-SGRGKGGKGLGKGGAKacRHRKme2VLRD	GluC

uniprot accession/s for all possible isoforms	histone type	pep exp mz	pep exp mr	pep exp z	pep delta	pep length	pep miss	pep expect	peptide and site of PTM	enzyme
H4_MOUSE	Histone H4	509.9069	2544.4983	5	0.0034	24	0	1.70E-08	ac-SGRGKGGKGLGKGGAKRHRKme2VLRD	GluC
H4_MOUSE	Histone H4	518.3086	2586.5068	5	0.0013	24	0	1.90E-08	ac-SGRGKGGKGLGKGGAKacRHRKme2VLRD	GluC
H4_MOUSE	Histone H4	509.9058	2544.4927	5	-0.0022	24	0	2.70E-08	ac-SGRGKGGKGLGKGGAKRHRKme2VLRD	GluC
H4_MOUSE	Histone H4	543.5148	2712.5374	5	0.0003	24	0	6.70E-08	ac-SGRGKacGGKacGLGKacGGAKacRHRKme2VLRD	GluC
H4_MOUSE	Histone H4	647.6344	2586.5083	4	0.0028	24	0	8.70E-08	ac-SGRGKGGKGLGKGGAKacRHRKme2VLRD	GluC
H4_MOUSE	Histone H4	637.1312	2544.4959	4	0.0010	24	0	1.10E-07	ac-SGRGKGGKGLGKGGAKRHRKme2VLRD	GluC
H4_MOUSE	Histone H4	518.3083	2586.5052	5	-0.0002	24	0	2.50E-07	ac-SGRGKGGKGLGKGGAKacRHRKme2VLRD	GluC
H4_MOUSE	Histone H4	679.1404	2712.5325	4	-0.0046	24	0	3.00E-07	ac-SGRGKacGGKacGLGKacGGAKacRHRKme2VLRD	GluC
H4_MOUSE	Histone H4	679.1421	2712.5394	4	0.0023	24	0	1.80E-06	ac-SGRGKacGGKacGLGKacGGAKacRHRKme2VLRD	GluC
H4_MOUSE	Histone H4	509.9062	2544.4944	5	-0.0005	24	0	1.80E-06	ac-SGRGKGGKGLGKGGAKRHR(me2)KVLRD	GluC
H4_MOUSE	Histone H4	647.6339	2586.5064	4	0.0010	24	0	1.00E-05	ac-SGRGKGGKGLGKGGAKacRHRKme2VLRD	GluC
H4_MOUSE	Histone H4	647.6346	2586.5093	4	0.0039	24	0	3.50E-05	ac-SGRGKGGKGLGKGGAKacRHRKme2VLRD	GluC
H4_MOUSE	Histone H4	518.3081	2586.5043	5	-0.0011	24	0	8.80E-05	ac-SGRGKGGKacGLGKGGAKRHRKme2VLRD	GluC
H4_MOUSE	Histone H4	849.1726	2544.4958	3	0.0009	24	0	2.00E-04	ac-SGRGKGGKGLGKGGAKRHRKme2VLRD	GluC
H4_MOUSE	Histone H4	863.1765	2586.5076	3	0.0021	24	0	2.40E-04	ac-SGRGKGGKGLGKGGAKacRHRKme2VLRD	GluC
H4_MOUSE	Histone H4	364.5067	2544.4959	7	0.0010	24	0	1.10E-02	ac-SGRGKGGKGLGKGGAKRHRKme2VLRD	GluC
H4_MOUSE	Histone H4	637.1332	2544.5037	4	0.0088	24	0	4.00E-12	ac-SGRGKGGKGLGKGGAKRHRKme2VLRD	GluC
H4_MOUSE	Histone H4	637.1330	2544.5028	4	0.0079	24	0	1.30E-11	ac-SGRGKGGKGLGKGGAKRHRKme2VLRD	GluC
H4_MOUSE	Histone H4	637.1281	2544.4833	4	-0.0116	24	0	7.50E-11	ac-SGRGKGGKGLGKGGAKRHRKme2VLRD	GluC
H4_MOUSE	Histone H4	647.6363	2586.5161	4	0.0106	24	0	1.80E-10	ac-SGRGKGGKGLGKGGAKacRHRKme2VLRD	GluC
H4_MOUSE	Histone H4	668.6403	2670.5320	4	0.0054	24	0	2.10E-09	ac-SGRGKGGKacGLGKacGGAKacRHRKme2VLRD	GluC
H4_MOUSE	Histone H4	679.1436	2712.5455	4	0.0083	24	0	6.20E-09	ac-SGRGKacGGKacGLGKacGGAKacRHRKme2VLRD	GluC
H4_MOUSE	Histone H4	679.1429	2712.5424	4	0.0053	24	0	8.20E-09	ac-SGRGKacGGKacGLGKacGGAKacRHRKme2VLRD	GluC
H4_MOUSE	Histone H4	668.6419	2670.5385	4	0.0120	24	0	2.00E-08	ac-SGRGKGGKacGLGKacGGAKacRHRKme2VLRD	GluC
H4_MOUSE	Histone H4	679.1438	2712.5462	4	0.0090	24	0	4.80E-08	ac-SGRGKacGGKacGLGKacGGAKacRHRKme2VLRD	GluC
H4_MOUSE	Histone H4	543.5158	2712.5425	5	0.0054	24	0	1.80E-07	ac-SGRGKacGGKacGLGKacGGAKacRHRKme2VLRD	GluC
H4_MOUSE	Histone H4	518.3101	2586.5139	5	0.0084	24	0	2.20E-06	ac-SGRGKGGKGLGKGGAKacRHRKme2VLRD	GluC
H4_MOUSE	Histone H4	849.1757	2544.5052	3	0.0103	24	0	8.30E-06	ac-SGRGKGGKGLGKGGAKRHRKme2VLRD	GluC
H4_MOUSE	Histone H4	849.1749	2544.5028	3	0.0079	24	0	2.00E-05	ac-SGRGKGGKGLGKGGAKRHRKme2VLRD	GluC
H4_MOUSE	Histone H4	535.1143	2670.5354	5	0.0088	24	0	4.10E-05	ac-SGRGKGGKacGLGKacGGAKacRHRme2KVLRD	GluC
H4_MOUSE	Histone H4	526.7107	2628.5170	5	0.0010	24	0	4.90E-04	ac-SGRGKacGGKGLGKGGAKRme2HRKacVLRD	GluC
H4_MOUSE	Histone H4	535.1146	2670.5365	5	0.0099	24	0	7.20E-04	ac-SGRGKGGKacGLGKacGGAKacRHRme2KVLRD	GluC
H4_MOUSE	Histone H4	658.1384	2628.5247	4	0.0086	24	0	1.20E-03	ac-SGRGKacGGKGLGKGGAKRHRme2KacVLRD	GluC
H4_MOUSE	Histone H4	647.6340	2586.5067	4	0.0012	24	0	3.70E-12	ac-SGRGKGGKGLGKGGAKacRHRKme2VLRD	GluC
H4_MOUSE	Histone H4	647.6342	2586.5077	4	0.0022	24	0	5.60E-12	ac-SGRGKGGKGLGKGGAKacRHRKme2VLRD	GluC
H4_MOUSE	Histone H4	637.1319	2544.4985	4	0.0036	24	0	9.70E-12	ac-SGRGKGGKGLGKGGAKRHRKme2VLRD	GluC
H4_MOUSE	Histone H4	637.1318	2544.4980	4	0.0031	24	0	5.30E-11	ac-SGRGKGGKGLGKGGAKRHRKme2VLRD	GluC
H4_MOUSE	Histone H4	637.1314	2544.4965	4	0.0016	24	0	1.30E-09	ac-SGRGKGGKGLGKGGAKRHRKme2VLRD	GluC
H4_MOUSE	Histone H4	647.6338	2586.5062	4	0.0007	24	0	1.40E-09	ac-SGRGKGGKGLGKGGAKacRHRKme2VLRD	GluC
H4_MOUSE	Histone H4	637.1318	2544.4980	4	0.0031	24	0	5.60E-09	ac-SGRGKGGKGLGKGGAKRHRKme2VLRD	GluC
H4_MOUSE	Histone H4	668.6393	2670.5281	4	0.0015	24	0	1.20E-08	ac-SGRGKGGKacGLGKacGGAKacRHRKme2VLRD	GluC
H4_MOUSE	Histone H4	626.6293	2502.4881	4	0.0037	24	0	3.50E-08	SGRGKGGKGLGKGGAKRHRme2KVLRD	GluC
H4_MOUSE	Histone H4	501.5035	2502.4813	5	-0.0031	24	0	1.00E-07	SGRGKGGKGLGKGGAKRHRKme2VLRD	GluC
H4_MOUSE	Histone H4	509.9060	2544.4939	5	-0.0010	24	0	1.70E-07	ac-SGRGKGGKGLGKGGAKRHRKme2VLRD	GluC
H4_MOUSE	Histone H4	626.6291	2502.4871	4	0.0028	24	0	1.00E-06	SGRGKGGKGLGKGGAKRHRKme2VLRD	GluC
H4_MOUSE	Histone H4	658.1372	2628.5197	4	0.0037	24	0	1.10E-06	ac-SGRGKGGKGLGKacGGAKacRHRKme2VLRD	GluC
H4_MOUSE	Histone H4	647.6339	2586.5065	4	0.0010	24	0	1.80E-06	ac-SGRGKGGKGLGKGGAKacRHRKme2VLRD	GluC
H4_MOUSE	Histone H4	432.0913	2586.5040	6	-0.0014	24	0	3.60E-06	ac-SGRGKGGKGLGKGGAKacRHRKme2VLRD	GluC
H4_MOUSE	Histone H4	509.9058	2544.4924	5	-0.0025	24	0	4.30E-06	ac-SGRGKGGKGLGKGGAKRHR(me2)KVLRD	GluC
H4_MOUSE	Histone H4	849.1734	2544.4984	3	0.0035	24	0	6.40E-06	ac-SGRGKGGKGLGKGGAKRHRKme2VLRD	GluC
H4_MOUSE	Histone H4	863.1767	2586.5083	3	0.0029	24	0	1.20E-05	ac-SGRGKGGKGLGKGGAKacRHRKme2VLRD	GluC
H4_MOUSE	Histone H4	518.3088	2586.5077	5	0.0022	24	0	1.40E-05	ac-SGRGKGGKGLGKGGAKacRHRKme2VLRD	GluC
H4_MOUSE	Histone H4	526.7109	2628.5181	5	0.0021	24	0	2.10E-05	ac-SGRGKGGKGLGKacGGAKacRHRKme2VLRD	GluC
H4_MOUSE	Histone H4	849.1732	2544.4977	3	0.0028	24	0	2.20E-04	ac-SGRGKGGKGLGKGGAKRHRKme2VLRD	GluC
H4_MOUSE	Histone H4	358.5054	2502.4866	7	0.0022	24	0	5.50E-04	SGRGKGGKGLGKGGAKRHRKme2VLRD	GluC
H4_MOUSE	Histone H4	432.0915	2586.5054	6	-0.0001	24	0	5.60E-04	ac-SGRGKGGKGLGKGGAKacRHRKVLRme2D	GluC
H4_MOUSE	Histone H4	905.1889	2712.5450	3	0.0079	24	0	7.80E-04	ac-SGRGKacGGKacGLGKacGGAKacRHRKme2VLRD	GluC
H4_MOUSE	Histone H4	535.1138	2670.5326	5	0.0060	24	0	8.20E-04	ac-SGRGKGGKacGLGKacGGAKacRHRKme2VLRD	GluC
H4_MOUSE	Histone H4	509.9062	2544.4947	5	-0.0002	24	0	2.10E-03	ac-SGRGKGGKGLGKGGAKRHRKme2VLRD	GluC
H4_MOUSE	Histone H4	370.5076	2586.5025	7	-0.0029	24	0	2.50E-03	ac-SGRGKGGKGLGKGGAKacRHRKme2VLRD	GluC
H4_MOUSE	Histone H4	439.0924	2628.5104	6	-0.0056	24	0	3.20E-03	ac-SGRGKGGKacGLGKGGAKRHRK(pp)VLRD	GluC
H4_MOUSE	Histone H4	509.9064	2544.4956	5	0.0007	24	0	2.50E-02	ac-SGRGKGGKGLGKGGAKRHR(me2)KVLRD	GluC
H4_MOUSE	Histone H4	501.5037	2502.4820	5	-0.0024	24	0	3.70E-02	SGRGKGGKGLGKGGAKRHRKme2VLRD	GluC
H4_MOUSE	Histone H4	637.1375	2544.5210	4	-0.0103	24	0	8.00E-07	SGRme3GKGGKGLGKGGAKRHRKme2VLRD	GluC
H4_MOUSE	Histone H4	647.6380	2586.5229	4	0.0174	24	0	1.00E-07	ac-SGRGKGGKGLGKacGGAKRHRKme2VLRD	GluC
H4_MOUSE	Histone H4	658.1381	2628.5233	4	0.0073	24	0	8.10E-07	ac-SGRGKacGGKGLGKGGAK(me1)R(me1)HRK(ac)VLRD	GluC
H4_MOUSE	Histone H4	518.3098	2586.5129	5	0.0074	24	0	2.80E-06	ac-SGRGKGGKGLGKGGAKacRHRKme2VLRD	GluC
H4_MOUSE	Histone H4	532.3100	2656.5134	5	0.0025	24	0	5.40E-06	ac-SGRGKGGKacGLGKacGGAKacRHRKme1VLRD	GluC
H4_MOUSE	Histone H4	637.1336	2544.5055	4	0.0106	24	0	6.90E-06	ac-SGRGKGGKGLGKGGAKRHRme1Kme1VLRD	GluC
H4_MOUSE	Histone H4	647.6369	2586.5185	4	0.0130	24	0	7.40E-06	ac-SGRGKGGKGLGKGGAKacRHRKme2VLRD	GluC
H4_MOUSE	Histone H4	637.1339	2544.5064	4	0.0115	24	0	1.00E-05	ac-SGRGKGGKGLGKGGAK(me1)RHRKVLR(me1)D	GluC
H4_MOUSE	Histone H4	654.6444	2614.5486	4	0.0118	24	0	1.80E-05	ac-SGRme1GKGGKGLGKGGAKRHRK(ac)VLR(me3)D	GluC
H4_MOUSE	Histone H4	640.6369	2558.5185	4	0.0079	24	0	1.00E-04	ac-SGRGKGGKGLGKGGAKRHRKVLRme3D	GluC

uniprot accession/s for all possible isoforms	histone type	pep exp mz	pep exp mr	pep exp z	pep delta	pep length	pep miss	pep expect	peptide and site of PTM	enzyme
H4_MOUSE	Histone H4	521.1144	2600.5354	5	0.0143	24	0	1.10E-04	ac-SGRGKGGKGLGKGGAKacRHRme2Kme1VLRD	GluC
H4_MOUSE	Histone H4	654.6348	2614.5102	4	0.0098	24	0	1.60E-04	ac-SGRGKGGKGLGKacGGAKacRHRKVLR(me1)D	GluC
H4_MOUSE	Histone H4	665.1459	2656.5547	4	0.0073	24	0	1.80E-04	ac-SGRme2GKGGKGLGKacGGAKacRHRKVLR(me2)D	GluC
H4_MOUSE	Histone H4	640.6398	2558.5302	4	-0.0167	24	0	1.80E-04	SGRme3GKGGKGLGKGGAKRHRK(me1)VLR(me2)D	GluC
H4_MOUSE	Histone H4	665.1365	2656.5167	4	0.0058	24	0	1.90E-04	ac-SGRGKGGKacGLGKacGGAKacRme1HRKVLRD	GluC
H4_MOUSE	Histone H4	644.1332	2572.5039	4	0.0140	24	0	3.50E-04	ac-SGRGKGGKGLGKGGAKRHRKacVLRme1D	GluC
H4_MOUSE	Histone H4	668.6415	2670.5370	4	0.0104	24	0	8.70E-04	ac-SGRGKGGKacGLGKacGGAKacRHRKVLR(me2)D	GluC
H4_MOUSE	Histone H4	658.1401	2628.5315	4	0.0154	24	0	1.70E-03	ac-SGRGKGGKGLGKacGGAKacRHRKVLRme2D	GluC
H4_MOUSE	Histone H4	668.6401	2670.5312	4	0.0046	24	0	1.80E-03	ac-SGRGKacGGKacGLGKGGAKme1RHRKacVLRme1D	GluC
H4_MOUSE	Histone H4	535.1128	2670.5277	5	0.0011	24	0	1.90E-03	ac-SGRGKGGKacGLGKacGGAKacRHRKme2VLRD	GluC
H4_MOUSE	Histone H4	543.5166	2712.5467	5	0.0095	24	0	2.10E-02	ac-SGRGKacGGKacGLGKacGGAKacRHRKme2VLRD	GluC
H4_MOUSE	Histone H4	668.6403	2670.5322	4	0.0056	24	0	2.80E-02	ac-SGRGKGGKacGLGKacGGAKacRHR(me1)K(me1)VLRD	GluC
H4_MOUSE	Histone H4	529.5136	2642.5316	5	-0.0001	24	0	4.70E-02	ac-SGRGKGGKGLGKacGGAKacRHRKme3VLRD	GluC
H4_MOUSE	Histone H4	509.9078	2544.5027	5	0.0078	24	0	7.00E-09	ac-SGRGKGGKGLGKGGAKRHR(me2)KVLRD	GluC
H4_MOUSE	Histone H4	644.1328	2572.5023	4	0.0125	24	0	9.30E-09	ac-SGRGKGGKGLGKGGAKacRHRme1KVLRD	GluC
H4_MOUSE	Histone H4	633.6300	2530.4911	4	0.0118	24	0	3.20E-08	ac-SGRGKGGKGLGKGGAKRHRKVLR(me1)D	GluC
H4_MOUSE	Histone H4	633.6312	2530.4955	4	0.0162	24	0	3.70E-08	ac-SGRGKGGKGLGKGGAKRHRKVLR(me1)D	GluC
H4_MOUSE	Histone H4	633.6293	2530.4882	4	0.0089	24	0	1.80E-07	SGRGKGGKGLGKGGAKRHR(me1)KacVLRD	GluC
H4_MOUSE	Histone H4	637.1324	2544.5005	4	0.0056	24	0	2.30E-07	ac-SGRGKGGKGLGKGGAK(me1)RHRKVLR(me1)D	GluC
H4_MOUSE	Histone H4	637.1335	2544.5047	4	0.0098	24	0	4.20E-07	ac-SGRGKGGKGLGKGGAKRHRKme2VLRD	GluC
H4_MOUSE	Histone H4	637.1328	2544.5019	4	0.0070	24	0	5.80E-07	ac-SGRGKGGKGLGKGGAKRHRKVLRme2D	GluC
H4_MOUSE	Histone H4	637.1327	2544.5015	4	0.0066	24	0	1.20E-06	ac-SGRGKGGKGLGKGGAKRHRKVLRme2D	GluC
H4_MOUSE	Histone H4	647.6346	2586.5095	4	0.0040	24	0	1.80E-06	ac-SGRGKGGKGLGKGGAKacRHRKme2VLRD	GluC
H4_MOUSE	Histone H4	644.1409	2572.5347	4	0.0085	24	0	3.90E-06	ac-SGRme1GKGGKGLGKGGAKRHRKVLRme3D	GluC
H4_MOUSE	Histone H4	658.1391	2628.5273	4	0.0113	24	0	1.00E-05	ac-SGRGKacGGKGLGKGGAKRme2HRKacVLRD	GluC
H4_MOUSE	Histone H4	521.1124	2600.5259	5	0.0048	24	0	1.00E-05	ac-SGRGKGGKGLGKGGAKacRHRme3KVLRD	GluC
H4_MOUSE	Histone H4	679.1452	2712.5517	4	0.0146	24	0	2.80E-05	ac-SGRGKacGGKacGLGKacGGAKacRHRKVLR(me2)D	GluC
H4_MOUSE	Histone H4	647.6367	2586.5175	4	0.0120	24	0	5.30E-05	ac-SGRGKGGKGLGKGGAKRHRK(ac)VLRme2D	GluC
H4_MOUSE	Histone H4	651.1399	2600.5305	4	0.0094	24	0	6.80E-05	ac-SGRme1GKGGKGLGKGGAKR(me2)HRK(ac)VLRD	GluC
H4_MOUSE	Histone H4	658.1391	2628.5272	4	0.0111	24	0	7.60E-05	ac-SGRGKGGKGLGKacGGAKacRHRKme1VLRme1D	GluC
H4_MOUSE	Histone H4	675.6500	2698.5711	4	0.0132	24	0	1.20E-04	ac-SGRme2GKGGKacGLGKacGGAKacRme1HR(me1)KVLRD	GluC
H4_MOUSE	Histone H4	672.1447	2684.5496	4	0.0074	24	0	2.10E-04	ac-SGRme1GKGGKacGLGKacGGAKacRHR(me1)KVLR(me1)D	GluC
H4_MOUSE	Histone H4	675.6386	2698.5254	4	0.0039	24	0	2.20E-04	ac-SGRGKacGGKacGLGKacGGAKacRHRKVLR(me1)D	GluC
H4_MOUSE	Histone H4	512.7105	2558.5162	5	0.0056	24	0	3.00E-04	ac-SGRGKGGKGLGKGGAKRHRK(me2)VLR(me1)D	GluC
H4_MOUSE	Histone H4	518.3101	2586.5139	5	0.0085	24	0	3.60E-04	ac-SGRGKGGKGLGKGGAKacRHRKme2VLRD	GluC
H4_MOUSE	Histone H4	640.6354	2558.5127	4	0.0021	24	0	5.00E-04	ac-SGRGKGGKGLGKGGAKR(me1)HR(me1)KVLR(me1)D	GluC
H4_MOUSE	Histone H4	675.6412	2698.5356	4	0.0141	24	0	6.30E-04	ac-SGRGKacGGKacGLGKacGGAKacRHRKVLR(me1)D	GluC
H4_MOUSE	Histone H4	521.1114	2600.5205	5	-0.0006	24	0	9.60E-04	ac-SGRGKGGKGLGKGGAKacRHRK(me1)VLRme2D	GluC
H4_MOUSE	Histone H4	537.9169	2684.5480	5	0.0058	24	0	1.30E-03	ac-SGRme1GKGGKacGLGKacGGAKacRHRKme2VLRD	GluC
H4_MOUSE	Histone H4	682.6480	2726.5628	4	0.0100	24	0	4.20E-03	ac-SGRGKacGGKacGLGKacGGAKacRme2HRKVLR(me1)D	GluC
H4_MOUSE	Histone H4	679.1418	2712.5379	4	0.0008	24	0	6.80E-03	ac-SGRGKacGGKacGLGKacGGAKacRHRKVLR(me2)D	GluC
H4_MOUSE	Histone H4	668.6390	2670.5269	4	0.0003	24	0	6.80E-03	ac-SGRGKacGGKacGLGKGGAKR(me1)HR(me1)K(ac)VLRD	GluC
H4_MOUSE	Histone H4	668.6409	2670.5346	4	0.0080	24	0	7.90E-03	ac-SGRGKacGGKacGLGKGGAKme1RHRKacVLRme1D	GluC
H4_MOUSE	Histone H4	651.1409	2600.5343	4	0.0132	24	0	1.10E-02	ac-SGRme1GKGGKacGLGKGGAKRHRme2KVLRD	GluC
H4_MOUSE	Histone H4	507.1050	2530.4886	5	0.0093	24	0	1.30E-02	ac-SGRGKGGKGLGKGGAKRHR(me1)KVLRD	GluC
H4_MOUSE	Histone H4	647.6373	2586.5202	4	0.0147	24	0	1.90E-02	ac-SGRGKGGKGLGKacGGAKRHRKVLR(me2)D	GluC
H4_MOUSE	Histone H4	518.3101	2586.5143	5	0.0088	24	0	1.90E-02	ac-SGRGKGGKGLGKGGAKacRHRme1Kme1VLRD	GluC
H4_MOUSE	Histone H4	535.1124	2670.5255	5	-0.0011	24	0	2.70E-02	ac-SGRGKGGKacGLGKacGGAKacRHRKme2VLRD	GluC
H4_MOUSE	Histone H4	668.6409	2670.5345	4	0.0079	24	0	2.80E-02	ac-SGRGKGGKacGLGKacGGAK(ac)RHR(me1)KVLR(me1)D	GluC
H4_MOUSE	Histone H4	672.1449	2684.5507	4	0.0084	24	0	3.10E-02	ac-SGRme1GKacGGKacGLGKGGAKR(me2)HRK(ac)VLRD	GluC
H4_MOUSE	Histone H4	665.1378	2656.5223	4	0.0114	24	0	4.00E-02	ac-SGRGKacGGKacGLGKacGGAKR(me1)HRKVLRD	GluC
H4_MOUSE	Histone H4	637.1362	2544.5157	4	-0.0156	24	0	1.30E-05	SGRme3GKGGKGLGKGGAKRHR(me1)K(me1)VLRD	GluC
H4_MOUSE	Histone H4	526.7153	2628.5399	5	-0.0125	24	0	1.70E-05	ac-SGRGKGGKGLGKacGGAKme3RHRme1Kme1VLRD	GluC
H4_MOUSE	Histone H4	509.9103	2544.5149	5	-0.0164	24	0	8.10E-04	SGRme3GKGGKGLGKGGAKRHR(me1)K(me1)VLRD	GluC
H4_MOUSE	Histone H4	425.0897	2544.4945	6	-0.0004	24	0	2.60E-02	ac-SGRGKGGKGLGKGGAKRHRme1Kme1VLRD	GluC
H4_MOUSE	Histone H4	512.7100	2558.5135	5	0.0030	24	0	7.40E-07	SGRGKGGKGLGKGGAKacRHRKme3VLRD	GluC
H4_MOUSE	Histone H4	637.1333	2544.5041	4	0.0092	24	0	2.70E-06	ac-SGRGKGGKGLGKGGAKRHR(me1)KVLRme1D	GluC
H4_MOUSE	Histone H4	651.1403	2600.5319	4	0.0108	24	0	1.10E-05	ac-SGRGKGGKGLGKGGAK(ac)R(me2)HRKVLR(me1)D	GluC
H4_MOUSE	Histone H4	640.6369	2558.5186	4	0.0080	24	0	3.80E-05	SGRGKGGKGLGKGGAKRHRme3KacVLRD	GluC
H4_MOUSE	Histone H4	640.6376	2558.5212	4	0.0107	24	0	2.80E-04	ac-SGRGKGGKGLGKGGAKRHRKVLRme3D	GluC
H4_MOUSE	Histone H4	525.9012	2624.4697	5	0.0084	24	0	3.70E-02	ac-pSGRGKGGKGLGKGGAKRHRKme2VLRD	GluC
H4_MOUSE	Histone H4	654.6336	2614.5053	4	0.0049	24	0	1.50E-06	ac-SGRGKGGKacGLGKGGAKRHRK(pp)VLRD	GluC
H4_MOUSE	Histone H4	668.6399	2670.5305	4	0.0039	24	0	1.10E-05	ac-SGRGKacGGKacGLGKGGAKppRHRK(me1)VLRD	GluC
H4_MOUSE	Histone H4	858.5055	2572.4946	3	0.0048	24	0	4.70E-05	ac-SGRGKGGKGLGKGGAKacRHRK(me1)VLRD	GluC
H4_MOUSE	Histone H4	537.9159	2684.5429	5	0.0007	24	0	5.40E-05	ac-SGRGKacGGKacGLGKGGAKme1Rme2HRKacVLRD	GluC
H4_MOUSE	Histone H4	877.1790	2628.5152	3	-0.0008	24	0	7.00E-05	ac-SGRGKGGKacGLGKGGAK(pp)RHRK(me1)VLRD	GluC
H4_MOUSE	Histone H4	608.3786	2429.4851	4	0.0171	23	0	1.90E-14	ac-SGRGKGGKGLGKGGAKRHRKme2VLR	AspN
H4_MOUSE	Histone H4	629.3854	2513.5127	4	-0.0128	23	0	2.10E-12	ac-SGRGKGGKacGLGKGGAKme3RHRKme2VLR	AspN
H4_MOUSE	Histone H4	615.3864	2457.5166	4	0.0173	23	0	1.50E-11	ac-SGRme1GKGGKGLGKGGAKRHRKme3VLR	AspN
H4_MOUSE	Histone H4	611.8817	2443.4976	4	0.0140	23	0	1.70E-10	ac-SGRGKGGKGLGKGGAKRHRKme3VLR	AspN
H4_MOUSE	Histone H4	636.3830	2541.5027	4	-0.0176	23	0	1.60E-08	ac-SGRGKme3GGKGLGKacGGAKacRHRKme1VLR	AspN
H4_MOUSE	Histone H4	503.7094	2513.5108	5	-0.0147	23	0	1.80E-08	ac-SGRGKGGKGLGKacGGAKme3RHRKme2VLR	AspN
H4_MOUSE	Histone H4	500.9115	2499.5214	5	0.0115	23	0	5.20E-08	ac-SGRme1GKGGKGLGKGGAKacRHRKme3VLR	AspN

uniprot accession/s for all possible isoforms	histone type	pep exp mz	pep exp mr	pep exp z	pep delta	pep length	pep miss	pep expect	peptide and site of PTM	enzyme
H4_MOUSE	Histone H4	838.8439	2513.5098	3	-0.0157	23	0	1.50E-05	ac-SGRGKGGKacGLGKGGAKme3RHRKme2VLR	AspN
H4_MOUSE	Histone H4	417.5947	2499.5246	6	0.0148	23	0	1.90E-05	ac-SGRme1GKGGKGLGKGGAKacRHRKme1VLRme2	AspN
H4_MOUSE	Histone H4	650.3892	2597.5278	4	0.0176	23	0	2.30E-12	ac-SGRGKacGGKacGLGKacGGAKacRHRKme2VLR	AspN
H4_MOUSE	Histone H4	639.8869	2555.5184	4	0.0187	23	0	2.00E-09	ac-SGRGKGGKacGLGKacGGAKacRHRKme2VLR	AspN
H4_MOUSE	Histone H4	618.8811	2471.4954	4	0.0169	23	0	3.40E-09	ac-SGRGKGGKGLGKGGAKacRHRKme2VLR	AspN
H4_MOUSE	Histone H4	824.8393	2471.4961	3	0.0176	23	0	1.20E-06	ac-SGRGKGGKGLGKGGAKacRHRKme2VLR	AspN
H4_MOUSE	Histone H4	866.8509	2597.5308	3	0.0206	23	0	1.70E-04	ac-SGRGKacGGKacGLGKacGGAKacRHRKme2VLR	AspN
H4_MOUSE	Histone H4	639.8881	2555.5233	4	-0.0128	23	0	6.40E-11	ac-SGRGKacGGKacGLGKGGAKme3RHRKme2VLR	AspN
H4_MOUSE	Histone H4	622.3802	2485.4917	4	-0.0025	23	0	1.50E-08	ac-SGRGKGGKGLGKGGAKacRHRKme3VLR	AspN
H4_MOUSE	Histone H4	611.8786	2443.4855	4	0.0019	23	0	2.60E-08	ac-SGRGKGGKGLGKGGAKRHRKme3VLR	AspN
H4_MOUSE	Histone H4	848.1764	2541.5074	3	-0.0130	23	0	2.40E-04	ac-SGRGKGGKacGLGKacGGAKme3RHRKme1VLR	AspN
H4_MOUSE	Histone H4	852.8485	2555.5237	3	-0.0123	23	0	1.00E-03	ac-SGRGKme3GGKGLGKacGGAKacRHRKVLR(me2)	AspN
H4_MOUSE	Histone H4	408.2567	2443.4963	6	0.0127	23	0	8.40E-09	ac-SGRGKGGKGLGKGGAKRHRKme1VLRme2	AspN
H4_MOUSE	Histone H4	484.0989	2415.4580	5	0.0057	23	0	1.70E-08	ac-SGRGKGGKGLGKGGAKRHRKme1VLR	AspN
H4_MOUSE	Histone H4	622.3811	2485.4951	4	0.0010	23	0	1.90E-08	ac-SGRGKGGKGLGKGGAKacRHR(me2)K(me1)VLR	AspN
H4_MOUSE	Histone H4	815.5024	2443.4854	3	0.0018	23	0	3.00E-06	ac-SGRme1GKGGKGLGKGGAKRHRKme2VLR	AspN
H4_MOUSE	Histone H4	492.5006	2457.4665	5	0.0036	23	0	4.30E-06	ac-SGRGKGGKGLGKGGAKacRHRKme1VLR	AspN
H4_MOUSE	Histone H4	806.1594	2415.4563	3	0.0040	23	0	4.90E-05	ac-SGRGKGGKGLGKGGAKRHRKVLRme1	AspN
H4_MOUSE	Histone H4	484.0976	2415.4515	5	-0.0008	23	0	1.50E-04	ac-SGRGKGGKGLGKGGAKRHRKme1VLR	AspN
H4_MOUSE	Histone H4	653.8895	2611.5289	4	0.0031	23	0	9.50E-04	ac-SGRme1GKacGGKacGLGKacGGAKacRHRK(me2)VLR	AspN
H4_MOUSE	Histone H4	405.9181	2429.4651	6	-0.0028	23	0	2.10E-02	ac-SGRGKGGKGLGKGGAKRHRme1Kme1VLR	AspN
H4_MOUSE	Histone H4	829.5061	2485.4964	3	0.0022	23	0	4.90E-02	ac-SGRGKGGKGLGKGGAKacRHRK(me2)VLR(me1)	AspN
H4_MOUSE	Histone H4	608.3744	2429.4683	4	0.0003	23	0	1.80E-12	ac-SGRGKGGKGLGKGGAKRHRKme2VLR	AspN
H4_MOUSE	Histone H4	608.3742	2429.4676	4	-0.0003	23	0	8.30E-12	ac-SGRGKGGKGLGKGGAKRHRKme2VLR	AspN
H4_MOUSE	Histone H4	618.8771	2471.4792	4	0.0007	23	0	3.80E-11	ac-SGRGKGGKGLGKGGAKacRHRKme2VLR	AspN
H4_MOUSE	Histone H4	650.3855	2597.5128	4	0.0026	23	0	2.30E-10	ac-SGRGKacGGKacGLGKacGGAKacRHRKme2VLR	AspN
H4_MOUSE	Histone H4	486.9011	2429.4691	5	0.0012	23	0	6.10E-09	ac-SGRGKGGKGLGKGGAKRHRKme2VLR	AspN
H4_MOUSE	Histone H4	608.3768	2429.4780	4	0.0100	23	0	1.60E-08	ac-SGRGKGGKGLGKGGAKRHRKme2VLR	AspN
H4_MOUSE	Histone H4	810.8305	2429.4698	3	0.0018	23	0	3.50E-08	ac-SGRGKGGKGLGKGGAKRHRKme2VLR	AspN
H4_MOUSE	Histone H4	512.1056	2555.4919	5	-0.0078	23	0	3.70E-08	ac-SGRGKGGKacGLGKacGGAKacRHRKme2VLR	AspN
H4_MOUSE	Histone H4	495.3029	2471.4783	5	-0.0002	23	0	6.80E-08	ac-SGRGKGGKGLGKGGAKacRHRKme2VLR	AspN
H4_MOUSE	Histone H4	618.8774	2471.4805	4	0.0020	23	0	6.80E-07	ac-SGRGKGGKGLGKGGAKacRHRKme2VLR	AspN
H4_MOUSE	Histone H4	495.3025	2471.4762	5	-0.0023	23	0	8.70E-07	ac-SGRGKGGKGLGKGGAKacRHRKme2VLR	AspN
H4_MOUSE	Histone H4	486.9011	2429.4689	5	0.0010	23	0	2.40E-05	ac-SGRGKGGKGLGKGGAKRHRKme2VLR	AspN
H4_MOUSE	Histone H4	520.5091	2597.5089	5	-0.0013	23	0	4.00E-05	ac-SGRGKacGGKacGLGKacGGAKacRHRKme2VLR	AspN
H4_MOUSE	Histone H4	824.8343	2471.4810	3	0.0024	23	0	1.20E-04	ac-SGRGKGGKGLGKGGAKacRHRKme2VLR	AspN
H4_MOUSE	Histone H4	866.8457	2597.5153	3	0.0051	23	0	1.90E-04	ac-SGRGKacGGKacGLGKacGGAKacRHRKme2VLR	AspN
H4_MOUSE	Histone H4	1215.7427	2429.4708	2	0.0029	23	0	7.20E-04	ac-SGRGKGGKGLGKGGAKRHRKme2VLR	AspN
H4_MOUSE	Histone H4	412.9208	2471.4813	6	0.0028	23	0	4.30E-03	ac-SGRGKGGKGLGKGGAKacRHRKme2VLR	AspN
H4_MOUSE	Histone H4	415.2558	2485.4910	6	-0.0032	23	0	3.40E-03	ac-SGRGKGGKGLGKGGAKacRHRKme3VLR	AspN
H4_MOUSE	Histone H4	439.2603	2629.5183	6	1.0023	23	0	1.30E-03	ac-SGRGKGGKacGLGKGGAKppRHRKVLRme1D	GluC
H4_MOUSE	Histone H4	382.2374	2287.3810	6	-0.0015	22	1	2.50E-02	ac-SGRGKGGKGLGKGGAKRHRme2Kme1VL	Chymotrypsin
H4_MOUSE	Histone H4	569.3489	2273.3663	4	-0.0005	22	1	1.20E-11	ac-SGRGKGGKGLGKGGAKRHRKme2VL	Chymotrypsin
H4_MOUSE	Histone H4	350.2194	2444.4848	7	1.0012	22	0	3.80E-03	SGRGKppGGKGLGKGGAKRHRme1KVLRme1	AspN
H4_MOUSE	Histone H4	694.0182	2079.0329	3	0.0026	18	0	8.30E-05	DVVpYALKRQGRTLYGFGG	AspN
H4_MOUSE	Histone H4	694.0182	2079.0329	3	0.0026	18	0	6.10E-03	DVVpYALKRQGRTLYGFGG	AspN
H4_MOUSE	Histone H4	694.0172	2079.0299	3	-0.0004	18	0	2.00E-03	DVVpYALKRQGRTLYGFGG	CnBr
H4_MOUSE	Histone H4	681.3673	2041.0800	3	0.0055	18	0	1.50E-04	DVVYALKacRQGRTLYGFGG	AspN
H4_MOUSE	Histone H4	681.3660	2041.0763	3	0.0018	18	0	2.40E-02	DVVYALKacRQGRTLYGFGG	AspN
H4_MOUSE	Histone H4	1021.5469	2041.0792	2	0.0047	18	0	4.70E-02	DVVYALKacRQGRTLYGFGG	AspN
H4_MOUSE	Histone H4	681.3646	2041.0721	3	-0.0024	18	0	9.10E-05	DVVYALKacRQGRTLYGFGG	CnBr
H4_MOUSE	Histone H4	655.3389	1962.9948	3	0.0115	17	0	1.30E-04	DAVTYTEHAKRKacTVTAM	AspN
H4_MOUSE	Histone H4	655.3393	1962.9960	3	0.0127	17	0	7.60E-03	DAVTYTEHAKacRKTVTAM	AspN
H4_MOUSE	Histone H4	660.6678	1978.9815	3	0.0032	17	0	2.10E-05	DAVTYTEHAKRKacTVTAM	AspN
H4_MOUSE	Histone H4	990.4981	1978.9815	2	0.0033	17	0	1.70E-03	DAVTYTEHAKacRKTVTAM	AspN
H4_MOUSE	Histone H4	990.4981	1978.9815	2	0.0033	17	0	1.80E-03	DAVTYTEHAKacRKTVTAM	AspN
H4_MOUSE	Histone H4	990.4979	1978.9812	2	0.0030	17	0	4.20E-02	DAVTYTEHAKRKacTVTAM	AspN
H4_MOUSE	Histone H4	655.3367	1962.9884	3	0.0051	17	0	8.30E-06	DAVTYTEHAKacRKTVTAM	AspN
H4_MOUSE	Histone H4	982.5007	1962.9868	2	0.0035	17	0	9.20E-05	DAVTYTEHAKacRKTVTAM	AspN
H4_MOUSE	Histone H4	655.3357	1962.9852	3	0.0019	17	0	4.70E-04	DAVTYTEHAKRKacTVTAM	AspN
H4_MOUSE	Histone H4	982.5007	1962.9868	2	0.0036	17	0	6.80E-03	DAVTYTEHAKRKacTVTAM	AspN
H4_MOUSE	Histone H4	502.3150	2005.2308	4	0.0036	17	3	9.90E-03	Kme2VLRDNIQGITKPAIRR	Semi-tryptic
H4_MOUSE	Histone H4	690.0377	2067.0913	3	0.0011	17	0	1.90E-03	DVVYALKcrRQGRTLYGFGG	CNBr
H4_MOUSE	Histone H4	1034.5533	2067.0920	2	0.0019	17	0	2.60E-02	DVVYALKcrRQGRTLYGFGG	CNBr
H4_MOUSE	Histone H4	605.3240	1812.9503	3	0.0004	15	2	1.20E-03	RIpSGLIYEETRGVLK	Semi-tryptic
H4_MOUSE	Histone H4	605.3231	1812.9474	3	-0.0024	15	2	7.80E-08	RIpSGLIYEETRGVLK	Semi-tryptic
H4_MOUSE	Histone H4	605.3246	1812.9520	3	0.0022	15	2	3.50E-03	RIpSGLIYEETRGVLK	Semi-tryptic
H4_MOUSE	Histone H4	459.6251	1375.8535	3	0.0011	12	0	1.80E-02	GKGGAKacRHRKme2VL	Chymotrypsin
H4_MOUSE	Histone H4	606.3467	1210.6789	2	0.0007	12	3	5.90E-06	GGKacGLGKacGGAKacR	Semi-tryptic
H4_MOUSE	Histone H4	606.3459	1210.6773	2	-0.0009	12	3	1.50E-04	GGKacGLGKacGGAKacR	Semi-tryptic
H4_MOUSE	Histone H4	606.3467	1210.6789	2	0.0007	12	3	4.60E-06	GGKacGLGKacGGAKacR	Semi-tryptic
H4_MOUSE	Histone H4	606.3459	1210.6773	2	-0.0009	12	3	5.40E-05	GGKacGLGKacGGAKacR	Semi-tryptic
H4_MOUSE	Histone H4	606.3468	1210.6790	2	0.0008	12	3	2.30E-04	GGKacGLGKacGGAKacR	Semi-tryptic

uniprot accession/s for all possible isoforms	histone type	pep exp mz	pep exp mr	pep exp z	pep delta	pep length	pep miss	pep expect	peptide and site of PTM	enzyme
H4_MOUSE	Histone H4	708.8460	1415.6775	2	-0.0034	11	1	7.40E-07	RIpSGLIYEETR	Semi-tryptic
H4_MOUSE	Histone H4	708.8470	1415.6794	2	-0.0016	11	1	1.40E-06	RIpSGLIYEETR	Semi-tryptic
H4_MOUSE	Histone H4	472.9015	1415.6825	3	0.0016	11	1	7.40E-06	RIpSGLIYEETR	Semi-tryptic
H4_MOUSE	Histone H4	708.8490	1415.6834	2	0.0024	11	1	3.90E-02	RIpSGLIYEETR	Semi-tryptic
H4_MOUSE	Histone H4	708.8472	1415.6798	2	-0.0012	11	1	2.80E-07	RIpSGLIYEETR	Semi-tryptic
H4_MOUSE	Histone H4	708.8488	1415.6831	2	0.0021	11	1	2.60E-03	RIpSGLIYEETR	Semi-tryptic
H4_MOUSE	Histone H4	320.1858	957.5355	3	-0.0001	10	0	4.00E-02	ac-SGRGKGGKGL	Chymotrypsin
H4_MOUSE	Histone H4	500.7803	999.5461	2	-0.0001	10	0	1.60E-03	ac-SGRGKacGGKGL	Chymotrypsin
H4_MOUSE	Histone H4	507.7874	1013.5603	2	-0.0014	10	0	3.70E-03	ac-SGRme1GKacGGKGL	Chymotrypsin
H4_MOUSE	Histone H4	324.8582	971.5529	3	0.0017	10	0	5.70E-03	ac-SGRme1GKGGKGL	Chymotrypsin
H4_MOUSE	Histone H4	507.7876	1013.5606	2	-0.0012	10	0	6.80E-04	ac-SGRme1GKGGKacGL	Chymotrypsin
H4_MOUSE	Histone H4	479.7753	957.5360	2	0.0004	10	0	1.20E-03	ac-SGRGKGGKGL	Chymotrypsin
H4_MOUSE	Histone H4	320.1868	957.5385	3	0.0029	10	0	1.50E-03	ac-SGRGKGGKGL	Chymotrypsin
H4_MOUSE	Histone H4	479.7751	957.5355	2	0.0000	10	0	3.50E-03	ac-SGRGKGGKGL	Chymotrypsin
H4_MOUSE	Histone H4	479.7748	957.5351	2	-0.0005	10	0	2.80E-02	ac-SGRGKGGKGL	Chymotrypsin
H4_MOUSE	Histone H4	479.7751	957.5356	2	0.0000	10	0	3.60E-02	ac-SGRGKGGKGL	Chymotrypsin
H4_MOUSE	Histone H4	320.1866	957.5379	3	0.0024	10	0	8.00E-05	ac-SGRGKGGKGL	Chymotrypsin
H4_MOUSE	Histone H4	500.7809	999.5473	2	0.0012	10	0	3.70E-03	ac-SGRGKGGKacGL	Chymotrypsin
H4_MOUSE	Histone H4	479.7761	957.5377	2	0.0021	10	0	7.60E-03	ac-SGRGKGGKGL	Chymotrypsin
H4_MOUSE	Histone H4	320.1866	957.5380	3	0.0024	10	0	5.20E-05	ac-SGRGKGGKGL	Chymotrypsin
H4_MOUSE	Histone H4	500.7811	999.5476	2	0.0014	10	0	2.10E-04	ac-SGRGKacGGKGL	Chymotrypsin
H4_MOUSE	Histone H4	514.7953	1027.5761	2	-0.0014	10	0	6.40E-03	ac-SGRme2GKGGKacGL	Chymotrypsin
H4_MOUSE	Histone H4	479.7752	957.5358	2	0.0002	10	0	3.40E-02	ac-SGRGKGGKGL	Chymotrypsin
H4_MOUSE	Histone H4	320.1850	957.5331	3	-0.0024	10	0	6.20E-04	ac-SGRGKGGKGL	Chymotrypsin
H4_MOUSE	Histone H4	479.7751	957.5356	2	0.0000	10	0	9.30E-04	ac-SGRGKGGKGL	Chymotrypsin
H4_MOUSE	Histone H4	479.7753	957.5360	2	0.0004	10	0	5.90E-03	ac-SGRGKGGKGL	Chymotrypsin
H4_MOUSE	Histone H4	320.1863	957.5371	3	0.0015	10	0	2.90E-02	ac-SGRGKGGKGL	Chymotrypsin
H4_MOUSE	Histone H4	630.7977	1259.5809	2	0.0010	10	0	2.70E-03	IpSGLIYEETR	Semi-tryptic
H4_MOUSE	Histone H4	320.1860	957.5362	3	0.0007	10	0	7.10E-04	ac-SGRGKGGKGL	Chymotrypsin
H4_MOUSE	Histone H4	519.7588	1037.5031	2	0.0012	10	0	2.40E-02	ac-pSGRGKGGKGL	Chymotrypsin
H4_MOUSE	Histone H4	464.2722	926.5299	2	0.0001	9	2	2.80E-03	GLGKacGGAKacR	Semi-tryptic
H4_MOUSE	Histone H4	464.2722	926.5298	2	0.0000	9	2	4.80E-03	GLGKacGGAKacR	Semi-tryptic
H4_MOUSE	Histone H4	464.2721	926.5296	2	-0.0001	9	2	9.20E-03	GLGKacGGAKacR	Semi-tryptic
H4_MOUSE	Histone H4	464.2722	926.5299	2	0.0001	9	2	2.20E-03	GLGKacGGAKacR	Semi-tryptic
H4_MOUSE	Histone H4	464.2722	926.5298	2	0.0000	9	2	3.80E-03	GLGKacGGAKacR	Semi-tryptic
H4_MOUSE	Histone H4	464.2721	926.5296	2	-0.0001	9	2	1.50E-02	GLGKacGGAKacR	Semi-tryptic
H4_MOUSE	Histone H4	464.2720	926.5295	2	-0.0002	9	2	6.10E-05	GLGKacGGAKacR	Semi-tryptic
H4_MOUSE	Histone H4	464.2723	926.5300	2	0.0002	9	2	1.30E-04	GLGKacGGAKacR	Semi-tryptic
H4_MOUSE	Histone H4	365.2167	728.4189	2	0.0008	8	1	3.30E-02	GLGKacGGAK	Semi-tryptic
H4_MOUSE	Histone H4	365.2167	728.4188	2	0.0008	8	1	4.60E-02	GLGKacGGAK	Semi-tryptic
H4_MOUSE	Histone H4	365.2168	728.4191	2	0.0010	8	1	1.30E-02	GLGKacGGAK	Semi-tryptic
Q80ZM5_MOUSE	Histone H1	668.5381	3337.6540	5	0.0151	33	2	3.20E-05	GGAERRGASAApSSPAPKARTAAADRTPARPQPE	GluC
Q80ZM5_MOUSE	Histone H1	756.8906	3023.5335	4	0.0173	29	1	1.80E-04	RRGASAASpSPAPKARTAAADRTPARPQPE	GluC
Q8CGP4_MOUSE	Histone H2A	554.8240	2215.2669	4	0.0008	18	0	2.50E-02	DNKKTRITacPRHLQLAIRN	AspN
Q8CGP4_MOUSE	Histone H2A	554.8248	2215.2699	4	0.0038	18	0	3.10E-02	DNKKTRITacPRHLQLAIRN	AspN
