# Supplementary material for: Identification of Combinatorial Patterns of Post-Translational Modifications on Individual Histones in the Mouse Brain
Source: PLoS One. 2012 May 31;7(5):e36980. doi: 10.1371/journal.pone.0036980 (PMC3365036; doi:10.1371/journal.pone.0036980)
Supplement: Table S2 — A full list of all unmodified peptides derived from histones that were found in all experiments. (PDF) [file pone.0036980.s007.pdf]

uniprot accession/s for all possible isoforms	histone type	peptide sequence	spectral count	pep expect	pep exp mz	pep exp mr	pep exp z	pep delta	pep length	pep miss	enzyme
H10_MOUSE	Histone H1	KSTDHPKYSDMIVAAIQAEKNR	4	3.40E-06	626.326	2501.2751	4	0.0054	22	3	Semi-tryptic
H10_MOUSE	Histone H1	KSTDHPKYSDMIVAAIQAEKNR	4	6.10E-08	626.326	2501.2751	4	0.0054	22	3	Semi-tryptic
H10_MOUSE	Histone H1	KSTDHPKYSDMIVAAIQAEKNR	4	4.90E-10	834.7637	2501.2693	3	-0.0003	22	3	Semi-tryptic
H10_MOUSE	Histone H1	KSTDHPKYSDMIVAAIQAEKNR	4	3.00E-05	626.324	2501.2671	4	-0.0026	22	3	Semi-tryptic
H10_MOUSE	Histone H1	STDHPKYSDMIVAAIQAEKNR	6	7.40E-05	594.3025	2373.1811	4	0.0064	21	2	Semi-tryptic
H10_MOUSE	Histone H1	STDHPKYSDMIVAAIQAEKNR	6	3.80E-06	594.3025	2373.1811	4	0.0064	21	2	Semi-tryptic
H10_MOUSE	Histone H1	STDHPKYSDMIVAAIQAEKNR	6	9.30E-05	594.3013	2373.1762	4	0.0015	21	2	Semi-tryptic
H10_MOUSE	Histone H1	STDHPKYSDMIVAAIQAEKNR	6	2.40E-06	594.3013	2373.1762	4	0.0015	21	2	Semi-tryptic
H10_MOUSE	Histone H1	STDHPKYSDMIVAAIQAEKNR	6	3.90E-07	792.0656	2373.1751	3	0.0004	21	2	Semi-tryptic
H10_MOUSE	Histone H1	STDHPKYSDMIVAAIQAEKNR	6	1.40E-07	594.3004	2373.1725	4	-0.0022	21	2	Semi-tryptic
H10_MOUSE	Histone H1	KSTDHPKYSDMIVAAIQAEK	4	1.90E-07	744.7189	2231.1348	3	0.0092	20	2	Semi-tryptic
H10_MOUSE	Histone H1	KSTDHPKYSDMIVAAIQAEK	4	4.50E-09	744.7189	2231.1348	3	0.0092	20	2	Semi-tryptic
H10_MOUSE	Histone H1	KSTDHPKYSDMIVAAIQAEK	4	1.60E-09	744.7157	2231.1252	3	-0.0003	20	2	Semi-tryptic
H10_MOUSE	Histone H1	KSTDHPKYSDMIVAAIQAEK	4	1.70E-03	558.788	2231.1230	4	-0.0026	20	2	Semi-tryptic
H10_MOUSE	Histone H1	STDHPKYSDMIVAAIQAEK	4	1.80E-03	702.0195	2103.0366	3	0.0060	19	1	Semi-tryptic
H10_MOUSE	Histone H1	STDHPKYSDMIVAAIQAEK	4	7.10E-05	702.0195	2103.0366	3	0.0060	19	1	Semi-tryptic
H10_MOUSE	Histone H1	STDHPKYSDMIVAAIQAEK	4	7.80E-05	526.7659	2103.0343	4	0.0037	19	1	Semi-tryptic
H10_MOUSE	Histone H1	STDHPKYSDMIVAAIQAEK	4	2.10E-10	702.018	2103.0321	3	0.0014	19	1	Semi-tryptic
H10_MOUSE	Histone H1	YSDMIVAAIQAEKNR	5	3.70E-03	570.296	1707.8661	3	0.0048	15	1	Semi-tryptic
H10_MOUSE	Histone H1	YSDMIVAAIQAEKNR	5	2.40E-04	570.296	1707.8661	3	0.0048	15	1	Semi-tryptic
H10_MOUSE	Histone H1	YSDMIVAAIQAEKNR	5	4.10E-03	570.2957	1707.8652	3	0.0038	15	1	Semi-tryptic
H10_MOUSE	Histone H1	YSDMIVAAIQAEKNR	5	6.40E-07	854.938	1707.8615	2	0.0001	15	1	Semi-tryptic
H10_MOUSE	Histone H1	YSDMIVAAIQAEKNR	5	2.40E-05	570.2941	1707.8604	3	-0.0009	15	1	Semi-tryptic
H10_MOUSE	Histone H1	TENSTSAPAAKPKR	2	3.70E-05	486.595	1456.7632	3	-0.0001	14	1	Semi-tryptic
H10_MOUSE	Histone H1	TENSTSAPAAKPKR	2	3.30E-06	486.595	1456.7632	3	-0.0001	14	1	Semi-tryptic
H10_MOUSE	Histone H1	VGENADSQIKLSIK	3	2.00E-05	501.2809	1500.8209	3	0.0062	14	1	Semi-tryptic
H10_MOUSE	Histone H1	VGENADSQIKLSIK	3	1.30E-06	501.2809	1500.8209	3	0.0062	14	1	Semi-tryptic
H10_MOUSE	Histone H1	VGENADSQIKLSIK	3	6.70E-06	501.2796	1500.8170	3	0.0023	14	1	Semi-tryptic
H10_MOUSE	Histone H1	SHYKVGENADSQIK	11	3.90E-06	525.9312	1574.7717	3	0.0029	14	1	Semi-tryptic
H10_MOUSE	Histone H1	SHYKVGENADSQIK	11	1.50E-07	525.9312	1574.7717	3	0.0029	14	1	Semi-tryptic
H10_MOUSE	Histone H1	SHYKVGENADSQIK	11	7.90E-04	525.931	1574.7713	3	0.0024	14	1	Semi-tryptic
H10_MOUSE	Histone H1	SHYKVGENADSQIK	11	1.90E-06	525.9304	1574.7695	3	0.0006	14	1	Semi-tryptic
H10_MOUSE	Histone H1	SHYKVGENADSQIK	11	2.00E-07	525.9304	1574.7695	3	0.0006	14	1	Semi-tryptic
H10_MOUSE	Histone H1	SHYKVGENADSQIK	11	2.70E-09	788.3919	1574.7692	2	0.0003	14	1	Semi-tryptic
H10_MOUSE	Histone H1	SHYKVGENADSQIK	11	5.70E-04	525.9302	1574.7689	3	0.0000	14	1	Semi-tryptic
H10_MOUSE	Histone H1	SHYKVGENADSQIK	11	8.90E-04	525.9301	1574.7684	3	-0.0005	14	1	Semi-tryptic
H10_MOUSE	Histone H1	SHYKVGENADSQIK	11	2.80E-06	525.9301	1574.7683	3	-0.0005	14	1	Semi-tryptic
H10_MOUSE	Histone H1	SHYKVGENADSQIK	11	3.00E-08	525.9301	1574.7683	3	-0.0005	14	1	Semi-tryptic
H10_MOUSE	Histone H1	SHYKVGENADSQIK	11	8.70E-04	525.9299	1574.7680	3	-0.0008	14	1	Semi-tryptic
H10_MOUSE	Histone H1	YSDMIVAAIQAEK	4	1.90E-03	719.8694	1437.7241	2	0.0068	13	0	Semi-tryptic
H10_MOUSE	Histone H1	YSDMIVAAIQAEK	4	1.10E-03	719.8692	1437.7238	2	0.0065	13	0	Semi-tryptic
H10_MOUSE	Histone H1	YSDMIVAAIQAEK	4	6.10E-08	719.869	1437.7235	2	0.0062	13	0	Semi-tryptic
H10_MOUSE	Histone H1	YSDMIVAAIQAEK	4	1.10E-07	719.8669	1437.7192	2	0.0018	13	0	Semi-tryptic
H10_MOUSE	Histone H1	TENSTSAPAAKPK	6	7.80E-04	651.3392	1300.6638	2	0.0015	13	0	Semi-tryptic
H10_MOUSE	Histone H1	TENSTSAPAAKPK	6	7.20E-05	651.3392	1300.6638	2	0.0015	13	0	Semi-tryptic
H10_MOUSE	Histone H1	TENSTSAPAAKPK	6	4.70E-06	651.339	1300.6634	2	0.0011	13	0	Semi-tryptic
H10_MOUSE	Histone H1	TENSTSAPAAKPK	6	2.10E-06	651.3387	1300.6628	2	0.0005	13	0	Semi-tryptic
H10_MOUSE	Histone H1	TENSTSAPAAKPK	6	3.60E-03	434.5614	1300.6623	3	0.0000	13	0	Semi-tryptic
H10_MOUSE	Histone H1	TENSTSAPAAKPK	6	6.60E-03	434.5612	1300.6619	3	-0.0004	13	0	Semi-tryptic
H10_MOUSE	Histone H1	KAASKAPSKKPK	4	1.40E-04	414.2631	1239.7674	3	0.0011	12	3	Semi-tryptic
H10_MOUSE	Histone H1	KAASKAPSKKPK	4	4.50E-04	620.8907	1239.7669	2	0.0006	12	3	Semi-tryptic
H10_MOUSE	Histone H1	KAASKAPSKKPK	4	4.60E-05	414.2626	1239.7659	3	-0.0004	12	3	Semi-tryptic
H10_MOUSE	Histone H1	RLVTTGVLKQTK	5	3.60E-07	448.6177	1342.8313	3	0.0016	12	2	Semi-tryptic
H10_MOUSE	Histone H1	RLVTTGVLKQTK	5	8.50E-09	448.6177	1342.8313	3	0.0016	12	2	Semi-tryptic
H10_MOUSE	Histone H1	RLVTTGVLKQTK	5	3.60E-05	448.6175	1342.8308	3	0.0012	12	2	Semi-tryptic
H10_MOUSE	Histone H1	RLVTTGVLKQTK	5	6.70E-07	448.617	1342.8292	3	-0.0005	12	2	Semi-tryptic
H10_MOUSE	Histone H1	RLVTTGVLKQTK	5	2.20E-08	448.617	1342.8292	3	-0.0005	12	2	Semi-tryptic
H10_MOUSE	Histone H1	KAASKAPSKKPK	4	7.90E-03	620.8882	1239.7618	2	-0.0045	12	3	Semi-tryptic
H10_MOUSE	Histone H1	LVTTGVLKQTK	11	8.00E-05	594.3729	1186.7313	2	0.0028	11	1	Semi-tryptic
H10_MOUSE	Histone H1	LVTTGVLKQTK	11	5.20E-06	594.3729	1186.7313	2	0.0028	11	1	Semi-tryptic
H10_MOUSE	Histone H1	LVTTGVLKQTK	11	5.30E-07	396.5842	1186.7307	3	0.0022	11	1	Semi-tryptic
H10_MOUSE	Histone H1	LVTTGVLKQTK	11	8.90E-08	396.5842	1186.7307	3	0.0022	11	1	Semi-tryptic
H10_MOUSE	Histone H1	LVTTGVLKQTK	11	1.00E-05	396.5839	1186.7298	3	0.0013	11	1	Semi-tryptic
H10_MOUSE	Histone H1	LVTTGVLKQTK	11	1.70E-07	396.5839	1186.7298	3	0.0013	11	1	Semi-tryptic
H10_MOUSE	Histone H1	LVTTGVLKQTK	11	9.70E-08	396.5838	1186.7297	3	0.0011	11	1	Semi-tryptic
H10_MOUSE	Histone H1	LVTTGVLKQTK	11	1.60E-04	594.3717	1186.7288	2	0.0003	11	1	Semi-tryptic
H10_MOUSE	Histone H1	LVTTGVLKQTK	11	9.70E-06	594.3717	1186.7288	2	0.0003	11	1	Semi-tryptic
H10_MOUSE	Histone H1	LVTTGVLKQTK	11	1.10E-05	396.5834	1186.7285	3	-0.0001	11	1	Semi-tryptic
H10_MOUSE	Histone H1	LVTTGVLKQTK	11	2.00E-06	396.5834	1186.7285	3	-0.0001	11	1	Semi-tryptic
H10_MOUSE	Histone H1	VKPVKASKPK	4	5.70E-03	361.2425	1080.7058	3	0.0039	10	1	Semi-tryptic
H10_MOUSE	Histone H1	VKPVKASKPK	4	2.10E-06	361.2421	1080.7045	3	0.0026	10	1	Semi-tryptic
H10_MOUSE	Histone H1	VKPVKASKPK	4	1.80E-04	541.359	1080.7035	2	0.0016	10	1	Semi-tryptic

H10_MOUSE	Histone H1	VKPVKASKPK	4	3.30E-03	361.2416	1080.7029	3	0.0010	10	1	Semi-tryptic
H10_MOUSE	Histone H1	VGENADSQIK	13	1.60E-03	530.7682	1059.5218	2	0.0022	10	0	Semi-tryptic
H10_MOUSE	Histone H1	VGENADSQIK	13	2.50E-06	530.7682	1059.5218	2	0.0022	10	0	Semi-tryptic
H10_MOUSE	Histone H1	VGENADSQIK	13	7.00E-05	530.7674	1059.5203	2	0.0006	10	0	Semi-tryptic
H10_MOUSE	Histone H1	VGENADSQIK	13	8.80E-03	530.7673	1059.5201	2	0.0005	10	0	Semi-tryptic
H10_MOUSE	Histone H1	VGENADSQIK	13	2.80E-05	530.7673	1059.5201	2	0.0005	10	0	Semi-tryptic
H10_MOUSE	Histone H1	VGENADSQIK	13	2.20E-03	530.7672	1059.5199	2	0.0003	10	0	Semi-tryptic
H10_MOUSE	Histone H1	VGENADSQIK	13	2.00E-06	530.7672	1059.5199	2	0.0002	10	0	Semi-tryptic
H10_MOUSE	Histone H1	VGENADSQIK	13	1.90E-03	530.7671	1059.5197	2	0.0001	10	0	Semi-tryptic
H10_MOUSE	Histone H1	VGENADSQIK	13	3.10E-04	530.7671	1059.5197	2	0.0001	10	0	Semi-tryptic
H10_MOUSE	Histone H1	VGENADSQIK	13	3.40E-03	530.7671	1059.5196	2	-0.0001	10	0	Semi-tryptic
H10_MOUSE	Histone H1	VGENADSQIK	13	3.60E-05	530.7671	1059.5196	2	-0.0001	10	0	Semi-tryptic
H10_MOUSE	Histone H1	VGENADSQIK	13	8.10E-03	530.7664	1059.5183	2	-0.0014	10	0	Semi-tryptic
H10_MOUSE	Histone H1	VGENADSQIK	13	8.10E-04	530.7664	1059.5183	2	-0.0014	10	0	Semi-tryptic
H10_MOUSE	Histone H1	RLVTTGVLK	5	5.10E-06	493.8221	985.6296	2	0.0012	9	1	Semi-tryptic
H10_MOUSE	Histone H1	RLVTTGVLK	5	2.10E-04	493.822	985.6293	2	0.0009	9	1	Semi-tryptic
H10_MOUSE	Histone H1	RLVTTGVLK	5	8.10E-03	493.8214	985.6282	2	-0.0002	9	1	Semi-tryptic
H10_MOUSE	Histone H1	RLVTTGVLK	5	3.80E-04	493.8214	985.6282	2	-0.0002	9	1	Semi-tryptic
H10_MOUSE	Histone H1	RLVTTGVLK	5	2.10E-05	493.8212	985.6279	2	-0.0005	9	1	Semi-tryptic
H10_MOUSE	Histone H1	LAKGDEPKR	11	1.40E-04	507.2914	1012.5683	2	0.0018	9	2	Semi-tryptic
H10_MOUSE	Histone H1	LAKGDEPKR	11	7.90E-03	338.5299	1012.5679	3	0.0014	9	2	Semi-tryptic
H10_MOUSE	Histone H1	LAKGDEPKR	11	1.90E-05	338.5299	1012.5677	3	0.0012	9	2	Semi-tryptic
H10_MOUSE	Histone H1	LAKGDEPKR	11	1.20E-03	338.5297	1012.5673	3	0.0008	9	2	Semi-tryptic
H10_MOUSE	Histone H1	LAKGDEPKR	11	9.40E-03	507.2909	1012.5672	2	0.0007	9	2	Semi-tryptic
H10_MOUSE	Histone H1	LAKGDEPKR	11	2.60E-03	338.5297	1012.5672	3	0.0007	9	2	Semi-tryptic
H10_MOUSE	Histone H1	LAKGDEPKR	11	2.60E-04	338.5297	1012.5672	3	0.0007	9	2	Semi-tryptic
H10_MOUSE	Histone H1	LAKGDEPKR	11	6.40E-04	338.5296	1012.5669	3	0.0004	9	2	Semi-tryptic
H10_MOUSE	Histone H1	LAKGDEPKR	11	1.00E-02	338.5295	1012.5667	3	0.0002	9	2	Semi-tryptic
H10_MOUSE	Histone H1	LAKGDEPKR	11	8.80E-03	338.5295	1012.5667	3	0.0002	9	2	Semi-tryptic
H10_MOUSE	Histone H1	LAKGDEPKR	11	6.10E-05	507.2904	1012.5663	2	-0.0002	9	2	Semi-tryptic
H10_MOUSE	Histone H1	GVGASGSFR	22	5.10E-03	419.2158	836.4170	2	0.0030	9	0	Semi-tryptic
H10_MOUSE	Histone H1	GVGASGSFR	22	3.20E-03	419.2158	836.4170	2	0.0030	9	0	Semi-tryptic
H10_MOUSE	Histone H1	GVGASGSFR	22	3.90E-04	419.2154	836.4162	2	0.0021	9	0	Semi-tryptic
H10_MOUSE	Histone H1	GVGASGSFR	22	2.30E-05	419.2153	836.4161	2	0.0020	9	0	Semi-tryptic
H10_MOUSE	Histone H1	GVGASGSFR	22	4.40E-06	419.2153	836.4161	2	0.0020	9	0	Semi-tryptic
H10_MOUSE	Histone H1	GVGASGSFR	22	6.10E-04	419.2151	836.4156	2	0.0015	9	0	Semi-tryptic
H10_MOUSE	Histone H1	GVGASGSFR	22	4.60E-03	419.215	836.4155	2	0.0014	9	0	Semi-tryptic
H10_MOUSE	Histone H1	GVGASGSFR	22	5.10E-03	419.2149	836.4153	2	0.0012	9	0	Semi-tryptic
H10_MOUSE	Histone H1	GVGASGSFR	22	5.20E-05	419.2149	836.4153	2	0.0012	9	0	Semi-tryptic
H10_MOUSE	Histone H1	GVGASGSFR	22	3.40E-04	419.2148	836.4151	2	0.0010	9	0	Semi-tryptic
H10_MOUSE	Histone H1	GVGASGSFR	22	2.10E-04	419.2148	836.4151	2	0.0010	9	0	Semi-tryptic
H10_MOUSE	Histone H1	GVGASGSFR	22	2.20E-04	419.2146	836.4147	2	0.0006	9	0	Semi-tryptic
H10_MOUSE	Histone H1	GVGASGSFR	22	4.90E-05	419.2146	836.4147	2	0.0006	9	0	Semi-tryptic
H10_MOUSE	Histone H1	GVGASGSFR	22	2.20E-05	419.2145	836.4145	2	0.0004	9	0	Semi-tryptic
H10_MOUSE	Histone H1	GVGASGSFR	22	5.10E-06	419.2145	836.4145	2	0.0004	9	0	Semi-tryptic
H10_MOUSE	Histone H1	GVGASGSFR	22	3.10E-04	419.2144	836.4143	2	0.0002	9	0	Semi-tryptic
H10_MOUSE	Histone H1	GVGASGSFR	22	3.40E-05	419.2144	836.4143	2	0.0002	9	0	Semi-tryptic
H10_MOUSE	Histone H1	GVGASGSFR	22	7.70E-06	419.2144	836.4143	2	0.0002	9	0	Semi-tryptic
H10_MOUSE	Histone H1	GVGASGSFR	22	2.70E-03	419.2143	836.4141	2	0.0000	9	0	Semi-tryptic
H10_MOUSE	Histone H1	GVGASGSFR	22	6.30E-04	419.2143	836.4140	2	-0.0001	9	0	Semi-tryptic
H10_MOUSE	Histone H1	GVGASGSFR	22	7.50E-03	419.214	836.4134	2	-0.0007	9	0	Semi-tryptic
H10_MOUSE	Histone H1	GVGASGSFR	22	1.20E-04	419.214	836.4134	2	-0.0007	9	0	Semi-tryptic
H10_MOUSE	Histone H1	LVTTGVLK	2	1.00E-03	415.7724	829.5302	2	0.0029	8	0	Semi-tryptic
H10_MOUSE	Histone H1	LVTTGVLK	2	2.40E-03	415.772	829.5295	2	0.0022	8	0	Semi-tryptic
H10_MOUSE	Histone H1	LAKGDEPK	2	4.70E-03	429.2399	856.4653	2	-0.0001	8	1	Semi-tryptic
H10_MOUSE	Histone H1	LAKGDEPK	2	9.50E-03	429.2398	856.4650	2	-0.0004	8	1	Semi-tryptic
H10_MOUSE	Histone H1	RSVAFKK	2	7.20E-03	418.2618	834.5091	2	0.0016	7	2	Semi-tryptic
H10_MOUSE	Histone H1	RSVAFKK	2	1.20E-03	418.2618	834.5091	2	0.0016	7	2	Semi-tryptic
H11_MOUSE	Histone H1	KNNSRIKLGLKSLVNKGTLVQTKGTGAAGSFKLNKKAE	10	1.40E-07	800.6733	3998.3299	5	0.0183	38	0	GluC
H11_MOUSE	Histone H1	KNNSRIKLGLKSLVNKGTLVQTKGTGAAGSFKLNKKAE	10	1.10E-05	800.6722	3998.3248	5	0.0132	38	0	GluC
H11_MOUSE	Histone H1	KNNSRIKLGLKSLVNKGTLVQTKGTGAAGSFKLNKKAE	10	2.70E-04	667.3947	3998.3246	6	0.0130	38	0	GluC
H11_MOUSE	Histone H1	KNNSRIKLGLKSLVNKGTLVQTKGTGAAGSFKLNKKAE	10	2.10E-08	800.6717	3998.3223	5	0.0107	38	0	GluC
H11_MOUSE	Histone H1	KNNSRIKLGLKSLVNKGTLVQTKGTGAAGSFKLNKKAE	10	9.40E-07	572.1961	3998.3218	7	0.0102	38	0	GluC
H11_MOUSE	Histone H1	KNNSRIKLGLKSLVNKGTLVQTKGTGAAGSFKLNKKAE	10	2.80E-05	800.67	3998.3137	5	0.0021	38	0	GluC
H11_MOUSE	Histone H1	KNNSRIKLGLKSLVNKGTLVQTKGTGAAGSFKLNKKAE	10	1.10E-08	667.3926	3998.3119	6	0.0003	38	0	GluC
H11_MOUSE	Histone H1	KNNSRIKLGLKSLVNKGTLVQTKGTGAAGSFKLNKKAE	10	1.20E-03	800.6694	3998.3108	5	-0.0008	38	0	GluC
H11_MOUSE	Histone H1	KNNSRIKLGLKSLVNKGTLVQTKGTGAAGSFKLNKKAE	10	1.20E-10	800.6694	3998.3108	5	-0.0008	38	0	GluC
H11_MOUSE	Histone H1	KNNSRIKLGLKSLVNKGTLVQTKGTGAAGSFKLNKKAE	10	4.50E-10	800.6692	3998.3095	5	-0.0021	38	0	GluC
H11_MOUSE	Histone H1	SGVSLAALKKSLAAAGYDVEKNNSR	2	1.30E-08	850.4636	2548.3689	3	0.0080	25	3	Semi-tryptic
H11_MOUSE	Histone H1	SGVSLAALKKSLAAAGYDVEKNNSR	2	1.10E-05	638.0985	2548.3648	4	0.0039	25	3	Semi-tryptic
H11_MOUSE	Histone H1	KKPAGPSVSELIVQAVSSSKER	4	1.20E-03	766.4351	2296.2833	3	0.0083	22	2	Semi-tryptic
H11_MOUSE	Histone H1	KKPAGPSVSELIVQAVSSSKER	4	4.00E-05	766.4351	2296.2833	3	0.0083	22	2	Semi-tryptic
H11_MOUSE	Histone H1	KKPAGPSVSELIVQAVSSSKER	4	4.10E-07	575.0776	2296.2813	4	0.0063	22	2	Semi-tryptic
H11_MOUSE	Histone H1	KKPAGPSVSELIVQAVSSSKER	4	3.00E-09	575.0776	2296.2813	4	0.0063	22	2	Semi-tryptic

H11_MOUSE	Histone H1	RSGVSLAALKKSLAAAGYDVE	21	7.20E-10	702.7287	2105.1644	3	0.0164	21	1	GluC
H11_MOUSE	Histone H1	RSGVSLAALKKSLAAAGYDVE	21	2.70E-09	702.7278	2105.1614	3	0.0134	21	1	GluC
H11_MOUSE	Histone H1	RSGVSLAALKKSLAAAGYDVE	21	1.30E-07	702.7272	2105.1598	3	0.0118	21	1	GluC
H11_MOUSE	Histone H1	RSGVSLAALKKSLAAAGYDVE	21	5.70E-03	702.727	2105.1592	3	0.0112	21	1	GluC
H11_MOUSE	Histone H1	RSGVSLAALKKSLAAAGYDVE	21	6.30E-06	702.7269	2105.1587	3	0.0107	21	1	GluC
H11_MOUSE	Histone H1	RSGVSLAALKKSLAAAGYDVE	21	1.30E-08	702.7267	2105.1582	3	0.0102	21	1	GluC
H11_MOUSE	Histone H1	RSGVSLAALKKSLAAAGYDVE	21	8.90E-04	702.7242	2105.1508	3	0.0028	21	1	GluC
H11_MOUSE	Histone H1	RSGVSLAALKKSLAAAGYDVE	21	4.50E-05	702.7241	2105.1505	3	0.0024	21	1	GluC
H11_MOUSE	Histone H1	RSGVSLAALKKSLAAAGYDVE	21	3.40E-05	702.7241	2105.1505	3	0.0024	21	1	GluC
H11_MOUSE	Histone H1	RSGVSLAALKKSLAAAGYDVE	21	1.00E-10	1053.5825	2105.1504	2	0.0024	21	1	GluC
H11_MOUSE	Histone H1	RSGVSLAALKKSLAAAGYDVE	21	3.30E-05	702.724	2105.1502	3	0.0022	21	1	GluC
H11_MOUSE	Histone H1	RSGVSLAALKKSLAAAGYDVE	21	1.20E-05	702.724	2105.1502	3	0.0022	21	1	GluC
H11_MOUSE	Histone H1	RSGVSLAALKKSLAAAGYDVE	21	8.60E-03	527.2947	2105.1498	4	0.0018	21	1	GluC
H11_MOUSE	Histone H1	RSGVSLAALKKSLAAAGYDVE	21	1.70E-05	702.7239	2105.1498	3	0.0018	21	1	GluC
H11_MOUSE	Histone H1	RSGVSLAALKKSLAAAGYDVE	21	8.50E-09	1053.5822	2105.1498	2	0.0018	21	1	GluC
H11_MOUSE	Histone H1	RSGVSLAALKKSLAAAGYDVE	21	1.30E-03	702.7237	2105.1491	3	0.0011	21	1	GluC
H11_MOUSE	Histone H1	RSGVSLAALKKSLAAAGYDVE	21	1.80E-04	702.7237	2105.1491	3	0.0011	21	1	GluC
H11_MOUSE	Histone H1	RSGVSLAALKKSLAAAGYDVE	21	2.10E-08	527.2945	2105.1491	4	0.0010	21	1	GluC
H11_MOUSE	Histone H1	RSGVSLAALKKSLAAAGYDVE	21	6.10E-06	527.2942	2105.1478	4	-0.0002	21	1	GluC
H11_MOUSE	Histone H1	RSGVSLAALKKSLAAAGYDVE	21	1.90E-05	702.7229	2105.1470	3	-0.0010	21	1	GluC
H11_MOUSE	Histone H1	RSGVSLAALKKSLAAAGYDVE	21	6.30E-07	702.7229	2105.1470	3	-0.0010	21	1	GluC
H11_MOUSE	Histone H1	KKPAGPSVSELIVQAVSSSK	11	5.20E-08	671.3831	2011.1274	3	-0.0040	20	1	Semi-tryptic
H11_MOUSE	Histone H1	KKPAGPSVSELIVQAVSSSK	11	1.20E-04	671.3864	2011.1374	3	0.0061	20	1	Semi-tryptic
H11_MOUSE	Histone H1	KKPAGPSVSELIVQAVSSSK	11	3.30E-06	671.3864	2011.1374	3	0.0061	20	1	Semi-tryptic
H11_MOUSE	Histone H1	KKPAGPSVSELIVQAVSSSK	11	5.50E-08	671.3852	2011.1337	3	0.0024	20	1	Semi-tryptic
H11_MOUSE	Histone H1	KKPAGPSVSELIVQAVSSSK	11	1.30E-09	671.3852	2011.1338	3	0.0024	20	1	Semi-tryptic
H11_MOUSE	Histone H1	KKPAGPSVSELIVQAVSSSK	11	3.50E-10	671.3852	2011.1337	3	0.0023	20	1	Semi-tryptic
H11_MOUSE	Histone H1	KKPAGPSVSELIVQAVSSSK	11	5.70E-09	671.3848	2011.1324	3	0.0011	20	1	Semi-tryptic
H11_MOUSE	Histone H1	KKPAGPSVSELIVQAVSSSK	11	7.70E-03	503.7902	2011.1317	4	0.0003	20	1	Semi-tryptic
H11_MOUSE	Histone H1	KKPAGPSVSELIVQAVSSSK	11	1.30E-03	503.7901	2011.1315	4	0.0002	20	1	Semi-tryptic
H11_MOUSE	Histone H1	KKPAGPSVSELIVQAVSSSK	11	4.00E-10	671.3844	2011.1313	3	-0.0001	20	1	Semi-tryptic
H11_MOUSE	Histone H1	KKPAGPSVSELIVQAVSSSK	11	1.90E-03	503.7899	2011.1306	4	-0.0007	20	1	Semi-tryptic
H11_MOUSE	Histone H1	RSGVSLAALKKSLAAAGYD	2	4.80E-06	626.6868	1877.0386	3	0.0016	19	0	GluC
H11_MOUSE	Histone H1	RSGVSLAALKKSLAAAGYD	2	2.30E-07	626.6868	1877.0386	3	0.0016	19	0	GluC
H11_MOUSE	Histone H1	KSLAAAGYDVEKNNSR	3	4.90E-10	574.9645	1721.8716	3	0.0019	16	2	Semi-tryptic
H11_MOUSE	Histone H1	KSLAAAGYDVEKNNSR	3	3.00E-11	574.9645	1721.8716	3	0.0019	16	2	Semi-tryptic
H11_MOUSE	Histone H1	KSLAAAGYDVEKNNSR	3	1.50E-05	574.9645	1721.8710	3	0.0014	16	2	Semi-tryptic
H11_MOUSE	Histone H1	IVQAVSSSKERSGVSL	4	8.30E-05	823.9612	1645.9079	2	0.0080	16	0	Chymotrypsin
H11_MOUSE	Histone H1	IVQAVSSSKERSGVSL	4	4.80E-05	549.643	1645.9070	3	0.0071	16	0	Chymotrypsin
H11_MOUSE	Histone H1	IVQAVSSSKERSGVSL	4	3.70E-03	823.9607	1645.9069	2	0.0070	16	0	Chymotrypsin
H11_MOUSE	Histone H1	IVQAVSSSKERSGVSL	4	3.00E-06	549.6417	1645.9033	3	0.0034	16	0	Chymotrypsin
H11_MOUSE	Histone H1	SLAAAGYDVEKNNSR	12	5.30E-03	532.2647	1593.7724	3	-0.0023	15	1	Semi-tryptic
H11_MOUSE	Histone H1	SLAAAGYDVEKNNSR	12	7.40E-04	797.8975	1593.7804	2	0.0057	15	1	Semi-tryptic
H11_MOUSE	Histone H1	SLAAAGYDVEKNNSR	12	5.10E-05	797.8975	1593.7804	2	0.0057	15	1	Semi-tryptic
H11_MOUSE	Histone H1	SLAAAGYDVEKNNSR	12	3.20E-05	532.267	1593.7792	3	0.0045	15	1	Semi-tryptic
H11_MOUSE	Histone H1	SLAAAGYDVEKNNSR	12	2.30E-06	532.267	1593.7792	3	0.0045	15	1	Semi-tryptic
H11_MOUSE	Histone H1	SLAAAGYDVEKNNSR	12	1.20E-10	797.8957	1593.7768	2	0.0022	15	1	Semi-tryptic
H11_MOUSE	Histone H1	SLAAAGYDVEKNNSR	12	5.00E-08	797.8957	1593.7767	2	0.0021	15	1	Semi-tryptic
H11_MOUSE	Histone H1	SLAAAGYDVEKNNSR	12	1.00E-03	797.8956	1593.7766	2	0.0019	15	1	Semi-tryptic
H11_MOUSE	Histone H1	SLAAAGYDVEKNNSR	12	2.60E-03	532.2654	1593.7745	3	-0.0002	15	1	Semi-tryptic
H11_MOUSE	Histone H1	SLAAAGYDVEKNNSR	12	1.80E-11	797.8942	1593.7739	2	-0.0008	15	1	Semi-tryptic
H11_MOUSE	Histone H1	SLAAAGYDVEKNNSR	12	2.80E-05	532.2652	1593.7737	3	-0.0009	15	1	Semi-tryptic
H11_MOUSE	Histone H1	SLAAAGYDVEKNNSR	12	1.20E-06	532.2652	1593.7737	3	-0.0009	15	1	Semi-tryptic
H11_MOUSE	Histone H1	AALKKSLAAAGY	2	6.80E-06	388.5658	1162.6755	3	0.0045	12	2	Chymotrypsin
H11_MOUSE	Histone H1	VQTKGTGAAGSF	2	8.70E-04	562.2927	1122.5709	2	0.0039	12	0	Chymotrypsin
H11_MOUSE	Histone H1	AALKKSLAAAGY	2	1.60E-05	582.3437	1162.6728	2	0.0018	12	2	Chymotrypsin
H11_MOUSE	Histone H1	VQTKGTGAAGSF	2	5.10E-03	562.2914	1122.5682	2	0.0013	12	0	Chymotrypsin
H11_MOUSE	Histone H1	SLVNKGTLVQTK	6	2.80E-03	644.3868	1286.7591	2	0.0033	12	1	Semi-tryptic
H11_MOUSE	Histone H1	SLVNKGTLVQTK	6	2.10E-05	429.9269	1286.7588	3	0.0030	12	1	Semi-tryptic
H11_MOUSE	Histone H1	SLVNKGTLVQTK	6	8.90E-07	429.9269	1286.7588	3	0.0030	12	1	Semi-tryptic
H11_MOUSE	Histone H1	SLVNKGTLVQTK	6	5.90E-09	644.3857	1286.7568	2	0.0010	12	1	Semi-tryptic
H11_MOUSE	Histone H1	SLVNKGTLVQTK	6	1.20E-08	644.3856	1286.7566	2	0.0008	12	1	Semi-tryptic
H11_MOUSE	Histone H1	SLVNKGTLVQTK	6	3.60E-08	644.3848	1286.7551	2	-0.0007	12	1	Semi-tryptic
H11_MOUSE	Histone H1	GLKSLVNKGTL	2	3.10E-06	565.3536	1128.6926	2	0.0060	11	2	Chymotrypsin
H11_MOUSE	Histone H1	GLKSLVNKGTL	2	1.10E-03	565.3525	1128.6905	2	0.0038	11	2	Chymotrypsin
H11_MOUSE	Histone H1	LIVQAVSSSKE	25	9.80E-07	580.8285	1159.6425	2	-0.0023	11	0	GluC
H11_MOUSE	Histone H1	LIVQAVSSSKE	25	1.80E-03	580.8281	1159.6416	2	-0.0032	11	0	GluC
H11_MOUSE	Histone H1	LIVQAVSSSKE	25	1.90E-06	580.8281	1159.6416	2	-0.0032	11	0	GluC
H11_MOUSE	Histone H1	LIVQAVSSSKE	25	2.60E-05	580.8324	1159.6503	2	0.0055	11	0	GluC
H11_MOUSE	Histone H1	LIVQAVSSSKE	25	1.30E-03	580.832	1159.6495	2	0.0046	11	0	GluC
H11_MOUSE	Histone H1	LIVQAVSSSKE	25	4.50E-07	580.8318	1159.6491	2	0.0043	11	0	GluC
H11_MOUSE	Histone H1	LIVQAVSSSKE	25	1.00E-03	387.557	1159.6491	3	0.0042	11	0	GluC
H11_MOUSE	Histone H1	LIVQAVSSSKE	25	1.00E-02	580.8312	1159.6478	2	0.0030	11	0	GluC
H11_MOUSE	Histone H1	LIVQAVSSSKE	25	4.40E-08	580.8312	1159.6478	2	0.0030	11	0	GluC

H11_MOUSE	Histone H1	LIVQAVSSSKE	25	4.10E-06	580.8306	1159.6467	2	0.0019	11	0	GluC
H11_MOUSE	Histone H1	LIVQAVSSSKE	25	1.30E-06	580.8305	1159.6464	2	0.0016	11	0	GluC
H11_MOUSE	Histone H1	LIVQAVSSSKE	25	3.10E-07	580.8305	1159.6464	2	0.0015	11	0	GluC
H11_MOUSE	Histone H1	LIVQAVSSSKE	25	1.30E-05	580.8303	1159.6460	2	0.0012	11	0	GluC
H11_MOUSE	Histone H1	LIVQAVSSSKE	25	1.10E-03	580.8303	1159.6460	2	0.0011	11	0	GluC
H11_MOUSE	Histone H1	LIVQAVSSSKE	25	1.40E-04	580.8302	1159.6458	2	0.0010	11	0	GluC
H11_MOUSE	Histone H1	LIVQAVSSSKE	25	2.60E-05	580.8301	1159.6457	2	0.0009	11	0	GluC
H11_MOUSE	Histone H1	LIVQAVSSSKE	25	3.30E-04	580.8301	1159.6456	2	0.0008	11	0	GluC
H11_MOUSE	Histone H1	LIVQAVSSSKE	25	1.60E-07	580.8301	1159.6457	2	0.0008	11	0	GluC
H11_MOUSE	Histone H1	LIVQAVSSSKE	25	6.10E-06	580.8301	1159.6456	2	0.0007	11	0	GluC
H11_MOUSE	Histone H1	LIVQAVSSSKE	25	2.00E-04	580.83	1159.6455	2	0.0006	11	0	GluC
H11_MOUSE	Histone H1	LIVQAVSSSKE	25	1.50E-07	580.83	1159.6454	2	0.0005	11	0	GluC
H11_MOUSE	Histone H1	LIVQAVSSSKE	25	9.80E-03	580.8298	1159.6451	2	0.0003	11	0	GluC
H11_MOUSE	Histone H1	LIVQAVSSSKE	25	3.10E-03	580.8296	1159.6446	2	-0.0003	11	0	GluC
H11_MOUSE	Histone H1	LIVQAVSSSKE	25	6.30E-06	580.8294	1159.6443	2	-0.0005	11	0	GluC
H11_MOUSE	Histone H1	LIVQAVSSSKE	25	8.60E-06	580.8293	1159.6440	2	-0.0008	11	0	GluC
H11_MOUSE	Histone H1	KKSLAAAGY	2	1.30E-03	454.7656	907.5167	2	0.0040	9	1	Chymotrypsin
H11_MOUSE	Histone H1	KSLVNKGTL	2	2.80E-04	480.2995	958.5844	2	0.0033	9	1	Chymotrypsin
H11_MOUSE	Histone H1	KPKKPAVSK	2	6.60E-04	491.8254	981.6363	2	0.0028	9	1	Semi-tryptic
H11_MOUSE	Histone H1	KKSLAAAGY	2	2.30E-04	454.7647	907.5148	2	0.0021	9	1	Chymotrypsin
H11_MOUSE	Histone H1	KPKKPAVSK	2	6.40E-03	328.2188	981.6346	3	0.0011	9	1	Semi-tryptic
H11_MOUSE	Histone H1	KSLVNKGTL	2	7.10E-04	480.2982	958.5818	2	0.0007	9	1	Chymotrypsin
H11_MOUSE	Histone H1	AALKKSL	2	1.30E-03	365.7472	729.4799	2	0.0050	7	1	Chymotrypsin
H11_MOUSE	Histone H1	AALKKSL	2	1.70E-03	365.7456	729.4766	2	0.0017	7	1	Chymotrypsin
H11_MOUSE, H12_MOUSE, H13_MOUSE, H14_MOUSE	Histone H1	ERSGVSLAALKK	9	3.00E-07	420.2555	1257.7447	3	0.0043	12	2	Semi-tryptic
H11_MOUSE, H12_MOUSE, H13_MOUSE, H14_MOUSE	Histone H1	ERSGVSLAALKK	9	6.20E-08	420.2555	1257.7447	3	0.0043	12	2	Semi-tryptic
H11_MOUSE, H12_MOUSE, H13_MOUSE, H14_MOUSE	Histone H1	ERSGVSLAALKK	9	1.30E-05	420.2544	1257.7414	3	0.0010	12	2	Semi-tryptic
H11_MOUSE, H12_MOUSE, H13_MOUSE, H14_MOUSE	Histone H1	ERSGVSLAALKK	9	5.60E-07	420.2544	1257.7414	3	0.0010	12	2	Semi-tryptic
H11_MOUSE, H12_MOUSE, H13_MOUSE, H14_MOUSE	Histone H1	ERSGVSLAALKK	9	1.40E-05	420.2542	1257.7407	3	0.0003	12	2	Semi-tryptic
H11_MOUSE, H12_MOUSE, H13_MOUSE, H14_MOUSE	Histone H1	ERSGVSLAALKK	9	2.50E-03	629.8776	1257.7406	2	0.0002	12	2	Semi-tryptic
H11_MOUSE, H12_MOUSE, H13_MOUSE, H14_MOUSE	Histone H1	ERSGVSLAALKK	9	1.40E-05	420.254	1257.7403	3	-0.0002	12	2	Semi-tryptic
H11_MOUSE, H12_MOUSE, H13_MOUSE, H14_MOUSE	Histone H1	ERSGVSLAALKK	9	5.10E-05	420.2539	1257.7400	3	-0.0004	12	2	Semi-tryptic
H11_MOUSE, H12_MOUSE, H13_MOUSE, H14_MOUSE	Histone H1	ERSGVSLAALKK	9	6.90E-03	420.2539	1257.7398	3	-0.0006	12	2	Semi-tryptic
H11_MOUSE, H12_MOUSE, H13_MOUSE, H14_MOUSE	Histone H1	ERSGVSLAALK	3	1.30E-03	565.8321	1129.6497	2	0.0042	11	1	Semi-tryptic
H11_MOUSE, H12_MOUSE, H13_MOUSE, H14_MOUSE	Histone H1	ERSGVSLAALK	3	6.30E-06	565.83	1129.6455	2	0.0000	11	1	Semi-tryptic
H11_MOUSE, H12_MOUSE, H13_MOUSE, H14_MOUSE	Histone H1	ERSGVSLAALK	3	3.80E-04	565.8297	1129.6449	2	-0.0006	11	1	Semi-tryptic
H11_MOUSE, H12_MOUSE, H13_MOUSE, H14_MOUSE	Histone H1	SGVSLAALKK	20	2.40E-06	487.3043	972.5940	2	-0.0028	10	1	Semi-tryptic
H11_MOUSE, H12_MOUSE, H13_MOUSE, H14_MOUSE	Histone H1	SGVSLAALKK	20	5.80E-03	325.2066	972.5981	3	0.0013	10	1	Semi-tryptic
H11_MOUSE, H12_MOUSE, H13_MOUSE, H14_MOUSE	Histone H1	SGVSLAALKK	20	4.80E-06	325.2065	972.5978	3	0.0011	10	1	Semi-tryptic
H11_MOUSE, H12_MOUSE, H13_MOUSE, H14_MOUSE	Histone H1	SGVSLAALKK	20	3.30E-07	325.2065	972.5978	3	0.0011	10	1	Semi-tryptic
H11_MOUSE, H12_MOUSE, H13_MOUSE, H14_MOUSE	Histone H1	SGVSLAALKK	20	1.30E-03	325.2065	972.5976	3	0.0009	10	1	Semi-tryptic
H11_MOUSE, H12_MOUSE, H13_MOUSE, H14_MOUSE	Histone H1	SGVSLAALKK	20	9.00E-05	325.2065	972.5976	3	0.0009	10	1	Semi-tryptic
H11_MOUSE, H12_MOUSE, H13_MOUSE, H14_MOUSE	Histone H1	SGVSLAALKK	20	4.00E-06	487.306	972.5974	2	0.0007	10	1	Semi-tryptic
H11_MOUSE, H12_MOUSE, H13_MOUSE, H14_MOUSE	Histone H1	SGVSLAALKK	20	8.20E-04	487.3058	972.5970	2	0.0003	10	1	Semi-tryptic
H11_MOUSE, H12_MOUSE, H13_MOUSE, H14_MOUSE	Histone H1	SGVSLAALKK	20	1.50E-05	487.3058	972.5970	2	0.0003	10	1	Semi-tryptic
H11_MOUSE, H12_MOUSE, H13_MOUSE, H14_MOUSE	Histone H1	SGVSLAALKK	20	1.90E-04	487.3057	972.5968	2	0.0001	10	1	Semi-tryptic
H11_MOUSE, H12_MOUSE, H13_MOUSE, H14_MOUSE	Histone H1	SGVSLAALKK	20	1.30E-05	487.3057	972.5968	2	0.0001	10	1	Semi-tryptic
H11_MOUSE, H12_MOUSE, H13_MOUSE, H14_MOUSE	Histone H1	SGVSLAALKK	20	1.10E-06	487.3057	972.5968	2	0.0001	10	1	Semi-tryptic
H11_MOUSE, H12_MOUSE, H13_MOUSE, H14_MOUSE	Histone H1	SGVSLAALKK	20	7.80E-07	487.3056	972.5967	2	0.0000	10	1	Semi-tryptic
H11_MOUSE, H12_MOUSE, H13_MOUSE, H14_MOUSE	Histone H1	SGVSLAALKK	20	1.10E-06	487.3056	972.5966	2	-0.0001	10	1	Semi-tryptic
H11_MOUSE, H12_MOUSE, H13_MOUSE, H14_MOUSE	Histone H1	SGVSLAALKK	20	3.40E-03	487.3055	972.5965	2	-0.0002	10	1	Semi-tryptic
H11_MOUSE, H12_MOUSE, H13_MOUSE, H14_MOUSE	Histone H1	SGVSLAALKK	20	4.80E-04	487.3055	972.5965	2	-0.0002	10	1	Semi-tryptic
H11_MOUSE, H12_MOUSE, H13_MOUSE, H14_MOUSE	Histone H1	SGVSLAALKK	20	2.30E-04	487.3055	972.5965	2	-0.0002	10	1	Semi-tryptic
H11_MOUSE, H12_MOUSE, H13_MOUSE, H14_MOUSE	Histone H1	SGVSLAALKK	20	1.40E-06	487.3054	972.5963	2	-0.0005	10	1	Semi-tryptic
H11_MOUSE, H12_MOUSE, H13_MOUSE, H14_MOUSE	Histone H1	SGVSLAALKK	20	2.50E-07	487.3054	972.5962	2	-0.0005	10	1	Semi-tryptic
H11_MOUSE, H12_MOUSE, H13_MOUSE, H14_MOUSE	Histone H1	SGVSLAALKK	20	9.80E-07	487.3051	972.5956	2	-0.0012	10	1	Semi-tryptic
H11_MOUSE, H12_MOUSE, H13_MOUSE, H14_MOUSE	Histone H1	SGVSLAALK	12	1.10E-03	423.2595	844.5045	2	0.0027	9	0	Semi-tryptic
H11_MOUSE, H12_MOUSE, H13_MOUSE, H14_MOUSE	Histone H1	SGVSLAALK	12	1.20E-03	423.2589	844.5032	2	0.0014	9	0	Semi-tryptic
H11_MOUSE, H12_MOUSE, H13_MOUSE, H14_MOUSE	Histone H1	SGVSLAALK	12	1.70E-04	423.2589	844.5032	2	0.0014	9	0	Semi-tryptic
H11_MOUSE, H12_MOUSE, H13_MOUSE, H14_MOUSE	Histone H1	SGVSLAALK	12	7.80E-03	423.2585	844.5025	2	0.0007	9	0	Semi-tryptic
H11_MOUSE, H12_MOUSE, H13_MOUSE, H14_MOUSE	Histone H1	SGVSLAALK	12	7.10E-07	423.2584	844.5022	2	0.0004	9	0	Semi-tryptic
H11_MOUSE, H12_MOUSE, H13_MOUSE, H14_MOUSE	Histone H1	SGVSLAALK	12	1.20E-03	423.2583	844.5020	2	0.0002	9	0	Semi-tryptic
H11_MOUSE, H12_MOUSE, H13_MOUSE, H14_MOUSE	Histone H1	SGVSLAALK	12	4.30E-08	423.2583	844.5020	2	0.0002	9	0	Semi-tryptic
H11_MOUSE, H12_MOUSE, H13_MOUSE, H14_MOUSE	Histone H1	SGVSLAALK	12	4.60E-08	423.2582	844.5019	2	0.0001	9	0	Semi-tryptic
H11_MOUSE, H12_MOUSE, H13_MOUSE, H14_MOUSE	Histone H1	SGVSLAALK	12	5.20E-07	423.258	844.5014	2	-0.0004	9	0	Semi-tryptic
H11_MOUSE, H12_MOUSE, H13_MOUSE, H14_MOUSE	Histone H1	SGVSLAALK	12	2.30E-07	423.2579	844.5013	2	-0.0005	9	0	Semi-tryptic
H11_MOUSE, H12_MOUSE, H13_MOUSE, H14_MOUSE	Histone H1	SGVSLAALK	12	4.30E-03	423.2576	844.5007	2	-0.0011	9	0	Semi-tryptic
H11_MOUSE, H12_MOUSE, H13_MOUSE, H14_MOUSE	Histone H1	SGVSLAALK	12	7.20E-05	423.2573	844.5001	2	-0.0017	9	0	Semi-tryptic
H11_MOUSE, H12_MOUSE, H13_MOUSE, H14_MOUSE, H15_MOUSE	Histone H1	DVEKNNSRIKLGL	3	1.50E-04	743.4265	1484.8384	2	0.0074	13	1	Chymotrypsin
H11_MOUSE, H12_MOUSE, H13_MOUSE, H14_MOUSE, H15_MOUSE	Histone H1	DVEKNNSRIKLGL	3	7.80E-05	495.9517	1484.8333	3	0.0022	13	1	Chymotrypsin
H11_MOUSE, H12_MOUSE, H13_MOUSE, H14_MOUSE, H15_MOUSE	Histone H1	DVEKNNSRIKLGL	3	3.70E-05	495.9507	1484.8304	3	-0.0007	13	1	Chymotrypsin
H11_MOUSE, H13_MOUSE, H14_MOUSE, H15_MOUSE	Histone H1	GTLVQTK	10	5.10E-03	373.7255	745.4364	2	0.0030	7	0	Semi-tryptic
H11_MOUSE, H13_MOUSE, H14_MOUSE, H15_MOUSE	Histone H1	GTLVQTK	10	8.20E-03	373.7254	745.4363	2	0.0029	7	0	Semi-tryptic
H11_MOUSE, H13_MOUSE, H14_MOUSE, H15_MOUSE	Histone H1	GTLVQTK	10	4.10E-03	373.7252	745.4358	2	0.0024	7	0	Semi-tryptic

H11_MOUSE, H13_MOUSE, H14_MOUSE, H15_MOUSE	Histone H1	GTLVQTK	10	6.80E-03	373.7251	745.4357	2	0.0023	7	0	Semi-tryptic
H11_MOUSE, H13_MOUSE, H14_MOUSE, H15_MOUSE	Histone H1	GTLVQTK	10	4.90E-03	373.7251	745.4356	2	0.0022	7	0	Semi-tryptic
H11_MOUSE, H13_MOUSE, H14_MOUSE, H15_MOUSE	Histone H1	GTLVQTK	10	6.60E-03	373.7247	745.4349	2	0.0015	7	0	Semi-tryptic
H11_MOUSE, H13_MOUSE, H14_MOUSE, H15_MOUSE	Histone H1	GTLVQTK	10	5.50E-03	373.7247	745.4348	2	0.0014	7	0	Semi-tryptic
H11_MOUSE, H13_MOUSE, H14_MOUSE, H15_MOUSE	Histone H1	GTLVQTK	10	5.70E-03	373.7241	745.4337	2	0.0003	7	0	Semi-tryptic
H11_MOUSE, H13_MOUSE, H14_MOUSE, H15_MOUSE	Histone H1	GTLVQTK	10	6.50E-03	373.724	745.4335	2	0.0001	7	0	Semi-tryptic
H11_MOUSE, H13_MOUSE, H14_MOUSE, H15_MOUSE	Histone H1	GTLVQTK	10	5.10E-03	373.724	745.4334	2	0.0000	7	0	Semi-tryptic
H12_MOUSE	Histone H1	KNNSRIKLGLKSLVSKGILVQTKGTGASGSFKLNKKAASGE	19	1.30E-05	603.0671	4214.4191	7	-0.0035	41	0	GluC
H12_MOUSE	Histone H1	KNNSRIKLGLKSLVSKGILVQTKGTGASGSFKLNKKAASGE	19	6.80E-06	703.4102	4214.4177	6	-0.0049	41	0	GluC
H12_MOUSE	Histone H1	KNNSRIKLGLKSLVSKGILVQTKGTGASGSFKLNKKAASGE	19	3.50E-05	703.4102	4214.4174	6	-0.0052	41	0	GluC
H12_MOUSE	Histone H1	KNNSRIKLGLKSLVSKGILVQTKGTGASGSFKLNKKAASGE	19	1.60E-03	703.4144	4214.4424	6	0.0198	41	0	GluC
H12_MOUSE	Histone H1	KNNSRIKLGLKSLVSKGILVQTKGTGASGSFKLNKKAASGE	19	1.60E-06	843.8955	4214.4413	5	0.0187	41	0	GluC
H12_MOUSE	Histone H1	KNNSRIKLGLKSLVSKGILVQTKGTGASGSFKLNKKAASGE	19	5.10E-08	603.0702	4214.4406	7	0.0180	41	0	GluC
H12_MOUSE	Histone H1	KNNSRIKLGLKSLVSKGILVQTKGTGASGSFKLNKKAASGE	19	8.30E-08	703.414	4214.4401	6	0.0175	41	0	GluC
H12_MOUSE	Histone H1	KNNSRIKLGLKSLVSKGILVQTKGTGASGSFKLNKKAASGE	19	3.50E-07	603.0701	4214.4397	7	0.0171	41	0	GluC
H12_MOUSE	Histone H1	KNNSRIKLGLKSLVSKGILVQTKGTGASGSFKLNKKAASGE	19	4.00E-04	1054.6159	4214.4345	4	0.0119	41	0	GluC
H12_MOUSE	Histone H1	KNNSRIKLGLKSLVSKGILVQTKGTGASGSFKLNKKAASGE	19	9.50E-07	843.8939	4214.4330	5	0.0104	41	0	GluC
H12_MOUSE	Histone H1	KNNSRIKLGLKSLVSKGILVQTKGTGASGSFKLNKKAASGE	19	2.30E-04	603.0688	4214.4309	7	0.0083	41	0	GluC
H12_MOUSE	Histone H1	KNNSRIKLGLKSLVSKGILVQTKGTGASGSFKLNKKAASGE	19	1.40E-03	843.8925	4214.4260	5	0.0034	41	0	GluC
H12_MOUSE	Histone H1	KNNSRIKLGLKSLVSKGILVQTKGTGASGSFKLNKKAASGE	19	9.50E-04	843.8925	4214.4260	5	0.0034	41	0	GluC
H12_MOUSE	Histone H1	KNNSRIKLGLKSLVSKGILVQTKGTGASGSFKLNKKAASGE	19	5.40E-03	703.4115	4214.4255	6	0.0029	41	0	GluC
H12_MOUSE	Histone H1	KNNSRIKLGLKSLVSKGILVQTKGTGASGSFKLNKKAASGE	19	9.10E-04	843.8919	4214.4233	5	0.0007	41	0	GluC
H12_MOUSE	Histone H1	KNNSRIKLGLKSLVSKGILVQTKGTGASGSFKLNKKAASGE	19	2.10E-04	843.8919	4214.4233	5	0.0007	41	0	GluC
H12_MOUSE	Histone H1	KNNSRIKLGLKSLVSKGILVQTKGTGASGSFKLNKKAASGE	19	3.10E-03	843.8916	4214.4218	5	-0.0008	41	0	GluC
H12_MOUSE	Histone H1	KNNSRIKLGLKSLVSKGILVQTKGTGASGSFKLNKKAASGE	19	3.60E-04	843.8916	4214.4218	5	-0.0008	41	0	GluC
H12_MOUSE	Histone H1	KNNSRIKLGLKSLVSKGILVQTKGTGASGSFKLNKKAASGE	19	7.90E-04	703.4108	4214.4211	6	-0.0015	41	0	GluC
H12_MOUSE	Histone H1	KAPAKKKAAKKPAGVRRKASGPPVSE	11	1.70E-03	886.5445	2656.6118	3	0.0142	26	0	GluC
H12_MOUSE	Histone H1	KAPAKKKAAKKPAGVRRKASGPPVSE	11	2.50E-06	665.1599	2656.6103	4	0.0127	26	0	GluC
H12_MOUSE	Histone H1	KAPAKKKAAKKPAGVRRKASGPPVSE	11	7.10E-05	886.5426	2656.6060	3	0.0084	26	0	GluC
H12_MOUSE	Histone H1	KAPAKKKAAKKPAGVRRKASGPPVSE	11	1.40E-06	532.3284	2656.6057	5	0.0081	26	0	GluC
H12_MOUSE	Histone H1	KAPAKKKAAKKPAGVRRKASGPPVSE	11	1.30E-03	532.3284	2656.6056	5	0.0080	26	0	GluC
H12_MOUSE	Histone H1	KAPAKKKAAKKPAGVRRKASGPPVSE	11	3.40E-06	665.1582	2656.6037	4	0.0061	26	0	GluC
H12_MOUSE	Histone H1	KAPAKKKAAKKPAGVRRKASGPPVSE	11	3.60E-08	665.1578	2656.6021	4	0.0045	26	0	GluC
H12_MOUSE	Histone H1	KAPAKKKAAKKPAGVRRKASGPPVSE	11	6.70E-07	532.3274	2656.6008	5	0.0032	26	0	GluC
H12_MOUSE	Histone H1	KAPAKKKAAKKPAGVRRKASGPPVSE	11	6.70E-03	532.3273	2656.6002	5	0.0026	26	0	GluC
H12_MOUSE	Histone H1	KAPAKKKAAKKPAGVRRKASGPPVSE	11	2.10E-05	665.1572	2656.5997	4	0.0021	26	0	GluC
H12_MOUSE	Histone H1	KAPAKKKAAKKPAGVRRKASGPPVSE	11	6.50E-05	665.157	2656.5990	4	0.0014	26	0	GluC
H12_MOUSE	Histone H1	KSLVSKGILVQTKGTGASGSF	2	4.90E-13	689.0639	2064.1698	3	0.0118	21	2	Chymotrypsin
H12_MOUSE	Histone H1	KSLVSKGILVQTKGTGASGSF	2	2.90E-08	689.0614	2064.1623	3	0.0044	21	2	Chymotrypsin
H12_MOUSE	Histone H1	VSKGILVQTKGTGASGSF	2	1.30E-07	579.6588	1735.9547	3	0.0078	18	1	Chymotrypsin
H12_MOUSE	Histone H1	VSKGILVQTKGTGASGSF	2	7.40E-09	579.6576	1735.9509	3	0.0041	18	1	Chymotrypsin
H12_MOUSE	Histone H1	IKLGLKSLVSKGILVQTK	2	2.10E-04	642.4224	1924.2453	3	0.0005	18	3	Semi-tryptic
H12_MOUSE	Histone H1	IKLGLKSLVSKGILVQTK	2	4.80E-08	642.4223	1924.2450	3	0.0002	18	3	Semi-tryptic
H12_MOUSE	Histone H1	KAKKPAAAAVTKK	6	3.30E-09	437.9547	1310.8421	3	0.0024	13	3	Semi-tryptic
H12_MOUSE	Histone H1	KAKKPAAAAVTKK	6	8.10E-08	437.9545	1310.8418	3	0.0020	13	3	Semi-tryptic
H12_MOUSE	Histone H1	KAKKPAAAAVTKK	6	9.70E-07	437.954	1310.8401	3	0.0004	13	3	Semi-tryptic
H12_MOUSE	Histone H1	KAKKPAAAAVTKK	6	8.00E-03	437.9539	1310.8399	3	0.0002	13	3	Semi-tryptic
H12_MOUSE	Histone H1	KAKKPAAAAVTKK	6	3.90E-04	437.9539	1310.8399	3	0.0002	13	3	Semi-tryptic
H12_MOUSE	Histone H1	KAKKPAAAAVTKK	6	7.00E-06	437.9539	1310.8400	3	0.0002	13	3	Semi-tryptic
H12_MOUSE	Histone H1	AKKPAAAAVTKK	3	1.10E-05	395.257	1182.7491	3	0.0043	12	2	Semi-tryptic
H12_MOUSE	Histone H1	AKKPAAAAVTKK	3	1.80E-06	592.3814	1182.7482	2	0.0035	12	2	Semi-tryptic
H12_MOUSE	Histone H1	AKKPAAAAVTKK	3	9.40E-07	395.2564	1182.7473	3	0.0025	12	2	Semi-tryptic
H12_MOUSE	Histone H1	SLVSKGILVQTK	3	8.50E-04	424.9348	1271.7825	3	0.0012	12	1	Semi-tryptic
H12_MOUSE	Histone H1	SLVSKGILVQTK	3	2.80E-08	636.8985	1271.7823	2	0.0011	12	1	Semi-tryptic
H12_MOUSE	Histone H1	SLVSKGILVQTK	3	4.00E-08	636.8982	1271.7819	2	0.0006	12	1	Semi-tryptic
H12_MOUSE	Histone H1	KAASGEAKPQAK	8	4.70E-03	395.8922	1184.6548	3	0.0035	12	1	Semi-tryptic
H12_MOUSE	Histone H1	KAASGEAKPQAK	8	5.40E-05	593.3336	1184.6526	2	0.0013	12	1	Semi-tryptic
H12_MOUSE	Histone H1	KAASGEAKPQAK	8	2.10E-03	395.8912	1184.6518	3	0.0005	12	1	Semi-tryptic
H12_MOUSE	Histone H1	KAASGEAKPQAK	8	2.60E-07	593.3331	1184.6516	2	0.0004	12	1	Semi-tryptic
H12_MOUSE	Histone H1	KAASGEAKPQAK	8	1.90E-06	395.8911	1184.6515	3	0.0002	12	1	Semi-tryptic
H12_MOUSE	Histone H1	KAASGEAKPQAK	8	1.10E-03	395.8911	1184.6514	3	0.0001	12	1	Semi-tryptic
H12_MOUSE	Histone H1	KAASGEAKPQAK	8	9.10E-06	593.3329	1184.6513	2	0.0000	12	1	Semi-tryptic
H12_MOUSE	Histone H1	KAASGEAKPQAK	8	4.90E-03	395.891	1184.6512	3	-0.0001	12	1	Semi-tryptic
H12_MOUSE	Histone H1	AKKPAAAAVTK	2	1.20E-04	528.3336	1054.6527	2	0.0029	11	1	Semi-tryptic
H12_MOUSE	Histone H1	AKKPAAAAVTK	2	5.70E-04	528.3335	1054.6525	2	0.0026	11	1	Semi-tryptic
H12_MOUSE	Histone H1	GLKSLVSKGIL	7	6.30E-03	557.8663	1113.7180	2	0.0059	11	2	Chymotrypsin
H12_MOUSE	Histone H1	GLKSLVSKGIL	7	7.00E-07	372.246	1113.7162	3	0.0040	11	2	Chymotrypsin
H12_MOUSE	Histone H1	GLKSLVSKGIL	7	3.50E-05	557.8651	1113.7157	2	0.0036	11	2	Chymotrypsin
H12_MOUSE	Histone H1	GLKSLVSKGIL	7	8.20E-03	372.2451	1113.7135	3	0.0014	11	2	Chymotrypsin
H12_MOUSE	Histone H1	GLKSLVSKGIL	7	3.70E-04	372.2451	1113.7135	3	0.0014	11	2	Chymotrypsin
H12_MOUSE	Histone H1	GLKSLVSKGIL	7	4.90E-05	557.8634	1113.7122	2	0.0001	11	2	Chymotrypsin
H12_MOUSE	Histone H1	GLKSLVSKGIL	7	1.20E-05	557.8634	1113.7122	2	0.0001	11	2	Chymotrypsin
H12_MOUSE	Histone H1	AASGEAKPQAK	8	5.10E-05	529.2861	1056.5576	2	0.0013	11	0	Semi-tryptic
H12_MOUSE	Histone H1	AASGEAKPQAK	8	6.10E-05	529.2857	1056.5569	2	0.0006	11	0	Semi-tryptic

H12_MOUSE	Histone H1	AASGEAKPQAK	8	2.70E-03	529.2856	1056.5566	2	0.0003	11	0	Semi-tryptic
H12_MOUSE	Histone H1	AASGEAKPQAK	8	2.30E-04	529.2855	1056.5564	2	0.0001	11	0	Semi-tryptic
H12_MOUSE	Histone H1	AASGEAKPQAK	8	8.30E-03	529.2853	1056.5561	2	-0.0002	11	0	Semi-tryptic
H12_MOUSE	Histone H1	AASGEAKPQAK	8	9.90E-04	529.2853	1056.5560	2	-0.0003	11	0	Semi-tryptic
H12_MOUSE	Histone H1	AASGEAKPQAK	8	9.40E-05	529.2853	1056.5560	2	-0.0003	11	0	Semi-tryptic
H12_MOUSE	Histone H1	AASGEAKPQAK	8	8.30E-05	529.2847	1056.5548	2	-0.0015	11	0	Semi-tryptic
H12_MOUSE	Histone H1	AAKKPAGVRR	5	8.20E-03	351.8945	1052.6616	3	0.0050	10	2	Semi-tryptic
H12_MOUSE	Histone H1	AAKKPAGVRR	5	2.90E-05	351.8941	1052.6604	3	0.0037	10	2	Semi-tryptic
H12_MOUSE	Histone H1	AAKKPAGVRR	5	3.60E-03	527.3374	1052.6603	2	0.0036	10	2	Semi-tryptic
H12_MOUSE	Histone H1	AAKKPAGVRR	5	6.90E-03	351.8939	1052.6600	3	0.0033	10	2	Semi-tryptic
H12_MOUSE	Histone H1	AAKKPAGVRR	5	2.30E-04	351.8934	1052.6585	3	0.0018	10	2	Semi-tryptic
H12_MOUSE	Histone H1	KPAAAAVTKK	12	8.50E-04	328.8775	983.6108	3	-0.0019	10	1	Semi-tryptic
H12_MOUSE	Histone H1	KPAAAAVTKK	12	3.30E-06	492.8154	983.6163	2	0.0036	10	1	Semi-tryptic
H12_MOUSE	Histone H1	KPAAAAVTKK	12	7.50E-05	492.8152	983.6159	2	0.0032	10	1	Semi-tryptic
H12_MOUSE	Histone H1	KPAAAAVTKK	12	1.10E-06	492.8152	983.6158	2	0.0030	10	1	Semi-tryptic
H12_MOUSE	Histone H1	KPAAAAVTKK	12	4.80E-07	492.8151	983.6157	2	0.0030	10	1	Semi-tryptic
H12_MOUSE	Histone H1	KPAAAAVTKK	12	3.30E-03	328.8791	983.6156	3	0.0028	10	1	Semi-tryptic
H12_MOUSE	Histone H1	KPAAAAVTKK	12	1.70E-05	328.8791	983.6154	3	0.0026	10	1	Semi-tryptic
H12_MOUSE	Histone H1	KPAAAAVTKK	12	6.00E-03	492.8148	983.6149	2	0.0022	10	1	Semi-tryptic
H12_MOUSE	Histone H1	KPAAAAVTKK	12	6.80E-04	492.8142	983.6138	2	0.0010	10	1	Semi-tryptic
H12_MOUSE	Histone H1	KPAAAAVTKK	12	8.50E-03	328.8785	983.6137	3	0.0009	10	1	Semi-tryptic
H12_MOUSE	Histone H1	KPAAAAVTKK	12	2.70E-04	492.8136	983.6127	2	0.0000	10	1	Semi-tryptic
H12_MOUSE	Histone H1	KPAAAAVTKK	12	2.70E-06	492.8136	983.6127	2	0.0000	10	1	Semi-tryptic
H12_MOUSE	Histone H1	KSLVSKGIL	2	1.70E-04	472.8123	943.6101	2	0.0035	9	1	Chymotrypsin
H12_MOUSE	Histone H1	KSLVSKGIL	2	9.30E-03	472.8115	943.6085	2	0.0019	9	1	Chymotrypsin
H12_MOUSE	Histone H1	KPAAAAVTK	6	3.40E-04	428.7677	855.5208	2	0.0030	9	0	Semi-tryptic
H12_MOUSE	Histone H1	KPAAAAVTK	6	2.70E-04	428.7667	855.5188	2	0.0011	9	0	Semi-tryptic
H12_MOUSE	Histone H1	KPAAAAVTK	6	1.80E-03	428.7665	855.5185	2	0.0007	9	0	Semi-tryptic
H12_MOUSE	Histone H1	KPAAAAVTK	6	1.40E-04	428.7665	855.5185	2	0.0007	9	0	Semi-tryptic
H12_MOUSE	Histone H1	KPAAAAVTK	6	3.50E-04	428.7664	855.5182	2	0.0005	9	0	Semi-tryptic
H12_MOUSE	Histone H1	KPAAAAVTK	6	2.10E-04	428.7661	855.5176	2	-0.0002	9	0	Semi-tryptic
H12_MOUSE	Histone H1	GILVQTK	3	5.50E-03	379.7426	757.4706	2	0.0008	7	0	Semi-tryptic
H12_MOUSE	Histone H1	GILVQTK	3	6.70E-03	379.7423	757.4701	2	0.0003	7	0	Semi-tryptic
H12_MOUSE	Histone H1	GILVQTK	3	5.20E-03	379.742	757.4695	2	-0.0003	7	0	Semi-tryptic
H12_MOUSE, H13_MOUSE	Histone H1	KASGPPVSELITKAVAASKER	4	4.00E-09	713.7438	2138.2095	3	0.0036	21	3	Semi-tryptic
H12_MOUSE, H13_MOUSE	Histone H1	KASGPPVSELITKAVAASKER	4	3.60E-09	535.5589	2138.2065	4	0.0006	21	3	Semi-tryptic
H12_MOUSE, H13_MOUSE	Histone H1	KASGPPVSELITKAVAASKER	4	3.60E-06	535.5589	2138.2064	4	0.0005	21	3	Semi-tryptic
H12_MOUSE, H13_MOUSE	Histone H1	KASGPPVSELITKAVAASKER	4	8.70E-09	535.5587	2138.2058	4	-0.0001	21	3	Semi-tryptic
H12_MOUSE, H13_MOUSE	Histone H1	KASGPPVSELITKAVAASK	2	2.10E-09	618.6965	1853.0676	3	0.0054	19	2	Semi-tryptic
H12_MOUSE, H13_MOUSE	Histone H1	KASGPPVSELITKAVAASK	2	1.40E-03	618.6947	1853.0623	3	0.0001	19	2	Semi-tryptic
H12_MOUSE, H13_MOUSE	Histone H1	ASGPPVSELITKAVAASK	2	5.20E-09	863.4918	1724.9690	2	0.0018	18	1	Semi-tryptic
H12_MOUSE, H13_MOUSE	Histone H1	ASGPPVSELITKAVAASK	2	1.80E-03	575.9964	1724.9675	3	0.0003	18	1	Semi-tryptic
H12_MOUSE, H13_MOUSE	Histone H1	RKASGPPVSELITK	10	1.20E-05	494.9623	1481.8652	3	0.0086	14	2	Semi-tryptic
H12_MOUSE, H13_MOUSE	Histone H1	RKASGPPVSELITK	10	4.20E-04	494.9602	1481.8587	3	0.0021	14	2	Semi-tryptic
H12_MOUSE, H13_MOUSE	Histone H1	RKASGPPVSELITK	10	1.90E-05	494.9602	1481.8587	3	0.0021	14	2	Semi-tryptic
H12_MOUSE, H13_MOUSE	Histone H1	RKASGPPVSELITK	10	4.90E-08	741.9363	1481.8581	2	0.0016	14	2	Semi-tryptic
H12_MOUSE, H13_MOUSE	Histone H1	RKASGPPVSELITK	10	2.20E-05	494.9597	1481.8573	3	0.0008	14	2	Semi-tryptic
H12_MOUSE, H13_MOUSE	Histone H1	RKASGPPVSELITK	10	6.30E-06	494.9595	1481.8566	3	0.0001	14	2	Semi-tryptic
H12_MOUSE, H13_MOUSE	Histone H1	RKASGPPVSELITK	10	1.20E-06	494.9593	1481.8560	3	-0.0006	14	2	Semi-tryptic
H12_MOUSE, H13_MOUSE	Histone H1	RKASGPPVSELITK	10	2.90E-03	494.9584	1481.8534	3	-0.0032	14	2	Semi-tryptic
H12_MOUSE, H13_MOUSE	Histone H1	RKASGPPVSELITK	10	1.00E-04	494.9584	1481.8534	3	-0.0032	14	2	Semi-tryptic
H12_MOUSE, H13_MOUSE	Histone H1	RKASGPPVSELITK	10	3.10E-05	494.9577	1481.8512	3	-0.0053	14	2	Semi-tryptic
H12_MOUSE, H13_MOUSE	Histone H1	KASGPPVSELITK	29	4.10E-03	442.9245	1325.7516	3	-0.0039	13	1	Semi-tryptic
H12_MOUSE, H13_MOUSE	Histone H1	KASGPPVSELITK	29	5.50E-05	442.9245	1325.7516	3	-0.0039	13	1	Semi-tryptic
H12_MOUSE, H13_MOUSE	Histone H1	KASGPPVSELITK	29	3.10E-04	663.8814	1325.7482	2	-0.0072	13	1	Semi-tryptic
H12_MOUSE, H13_MOUSE	Histone H1	KASGPPVSELITK	29	2.20E-05	442.9233	1325.7481	3	-0.0073	13	1	Semi-tryptic
H12_MOUSE, H13_MOUSE	Histone H1	KASGPPVSELITK	29	1.90E-06	663.8883	1325.7621	2	0.0067	13	1	Semi-tryptic
H12_MOUSE, H13_MOUSE	Histone H1	KASGPPVSELITK	29	1.60E-05	442.9274	1325.7603	3	0.0048	13	1	Semi-tryptic
H12_MOUSE, H13_MOUSE	Histone H1	KASGPPVSELITK	29	2.00E-06	442.9274	1325.7603	3	0.0048	13	1	Semi-tryptic
H12_MOUSE, H13_MOUSE	Histone H1	KASGPPVSELITK	29	7.00E-06	442.9272	1325.7597	3	0.0042	13	1	Semi-tryptic
H12_MOUSE, H13_MOUSE	Histone H1	KASGPPVSELITK	29	3.50E-06	442.9267	1325.7584	3	0.0029	13	1	Semi-tryptic
H12_MOUSE, H13_MOUSE	Histone H1	KASGPPVSELITK	29	2.50E-06	442.9266	1325.7581	3	0.0026	13	1	Semi-tryptic
H12_MOUSE, H13_MOUSE	Histone H1	KASGPPVSELITK	29	3.10E-07	663.886	1325.7575	2	0.0021	13	1	Semi-tryptic
H12_MOUSE, H13_MOUSE	Histone H1	KASGPPVSELITK	29	4.70E-06	442.9264	1325.7575	3	0.0020	13	1	Semi-tryptic
H12_MOUSE, H13_MOUSE	Histone H1	KASGPPVSELITK	29	1.70E-04	442.9264	1325.7573	3	0.0018	13	1	Semi-tryptic
H12_MOUSE, H13_MOUSE	Histone H1	KASGPPVSELITK	29	1.60E-06	663.8858	1325.7571	2	0.0017	13	1	Semi-tryptic
H12_MOUSE, H13_MOUSE	Histone H1	KASGPPVSELITK	29	7.60E-09	663.8857	1325.7568	2	0.0014	13	1	Semi-tryptic
H12_MOUSE, H13_MOUSE	Histone H1	KASGPPVSELITK	29	7.80E-09	663.8856	1325.7566	2	0.0012	13	1	Semi-tryptic
H12_MOUSE, H13_MOUSE	Histone H1	KASGPPVSELITK	29	1.10E-04	663.8856	1325.7566	2	0.0011	13	1	Semi-tryptic
H12_MOUSE, H13_MOUSE	Histone H1	KASGPPVSELITK	29	6.50E-03	663.8855	1325.7565	2	0.0010	13	1	Semi-tryptic
H12_MOUSE, H13_MOUSE	Histone H1	KASGPPVSELITK	29	1.20E-08	663.8854	1325.7562	2	0.0008	13	1	Semi-tryptic
H12_MOUSE, H13_MOUSE	Histone H1	KASGPPVSELITK	29	1.70E-06	442.926	1325.7561	3	0.0007	13	1	Semi-tryptic
H12_MOUSE, H13_MOUSE	Histone H1	KASGPPVSELITK	29	2.70E-11	663.8853	1325.7560	2	0.0005	13	1	Semi-tryptic
H12_MOUSE, H13_MOUSE	Histone H1	KASGPPVSELITK	29	4.90E-08	663.8852	1325.7558	2	0.0003	13	1	Semi-tryptic

H12_MOUSE, H13_MOUSE	Histone H1	KASGPPVSELITK	29	6.50E-06	442.9258	1325.7557	3	0.0002	13	1	Semi-tryptic
H12_MOUSE, H13_MOUSE	Histone H1	KASGPPVSELITK	29	1.00E-09	663.885	1325.7554	2	0.0000	13	1	Semi-tryptic
H12_MOUSE, H13_MOUSE	Histone H1	KASGPPVSELITK	29	1.80E-05	442.9257	1325.7553	3	-0.0001	13	1	Semi-tryptic
H12_MOUSE, H13_MOUSE	Histone H1	KASGPPVSELITK	29	1.20E-09	663.8849	1325.7553	2	-0.0001	13	1	Semi-tryptic
H12_MOUSE, H13_MOUSE	Histone H1	KASGPPVSELITK	29	3.30E-05	442.9257	1325.7552	3	-0.0002	13	1	Semi-tryptic
H12_MOUSE, H13_MOUSE	Histone H1	KASGPPVSELITK	29	7.80E-05	663.8847	1325.7549	2	-0.0006	13	1	Semi-tryptic
H12_MOUSE, H13_MOUSE	Histone H1	KASGPPVSELITK	29	3.50E-03	442.9251	1325.7535	3	-0.0019	13	1	Semi-tryptic
H12_MOUSE, H13_MOUSE	Histone H1	ASGPPVSELITK	13	4.50E-07	599.8392	1197.6638	2	0.0034	12	0	Semi-tryptic
H12_MOUSE, H13_MOUSE	Histone H1	ASGPPVSELITK	13	2.30E-07	599.8389	1197.6632	2	0.0027	12	0	Semi-tryptic
H12_MOUSE, H13_MOUSE	Histone H1	ASGPPVSELITK	13	3.00E-03	599.8383	1197.6621	2	0.0016	12	0	Semi-tryptic
H12_MOUSE, H13_MOUSE	Histone H1	ASGPPVSELITK	13	7.40E-07	599.838	1197.6614	2	0.0010	12	0	Semi-tryptic
H12_MOUSE, H13_MOUSE	Histone H1	ASGPPVSELITK	13	4.20E-04	599.8378	1197.6611	2	0.0006	12	0	Semi-tryptic
H12_MOUSE, H13_MOUSE	Histone H1	ASGPPVSELITK	13	2.90E-05	599.8378	1197.6611	2	0.0006	12	0	Semi-tryptic
H12_MOUSE, H13_MOUSE	Histone H1	ASGPPVSELITK	13	5.70E-05	599.8378	1197.6610	2	0.0005	12	0	Semi-tryptic
H12_MOUSE, H13_MOUSE	Histone H1	ASGPPVSELITK	13	4.70E-07	599.8377	1197.6608	2	0.0003	12	0	Semi-tryptic
H12_MOUSE, H13_MOUSE	Histone H1	ASGPPVSELITK	13	5.00E-07	599.8376	1197.6606	2	0.0001	12	0	Semi-tryptic
H12_MOUSE, H13_MOUSE	Histone H1	ASGPPVSELITK	13	1.60E-06	599.8374	1197.6602	2	-0.0002	12	0	Semi-tryptic
H12_MOUSE, H13_MOUSE	Histone H1	ASGPPVSELITK	13	2.20E-07	599.8374	1197.6602	2	-0.0003	12	0	Semi-tryptic
H12_MOUSE, H13_MOUSE	Histone H1	ASGPPVSELITK	13	3.90E-04	599.837	1197.6595	2	-0.0009	12	0	Semi-tryptic
H12_MOUSE, H13_MOUSE	Histone H1	ASGPPVSELITK	13	2.80E-06	599.837	1197.6594	2	-0.0011	12	0	Semi-tryptic
H12_MOUSE, H13_MOUSE	Histone H1	KATGAATPK	10	2.90E-03	422.7489	843.4833	2	0.0019	9	1	Semi-tryptic
H12_MOUSE, H13_MOUSE	Histone H1	KATGAATPK	10	9.80E-03	422.7489	843.4832	2	0.0018	9	1	Semi-tryptic
H12_MOUSE, H13_MOUSE	Histone H1	KATGAATPK	10	2.00E-03	422.7489	843.4831	2	0.0017	9	1	Semi-tryptic
H12_MOUSE, H13_MOUSE	Histone H1	KATGAATPK	10	2.90E-03	422.7488	843.4830	2	0.0016	9	1	Semi-tryptic
H12_MOUSE, H13_MOUSE	Histone H1	KATGAATPK	10	1.60E-04	422.7482	843.4819	2	0.0005	9	1	Semi-tryptic
H12_MOUSE, H13_MOUSE	Histone H1	KATGAATPK	10	4.80E-03	422.7482	843.4818	2	0.0004	9	1	Semi-tryptic
H12_MOUSE, H13_MOUSE	Histone H1	KATGAATPK	10	1.20E-03	422.7482	843.4818	2	0.0004	9	1	Semi-tryptic
H12_MOUSE, H13_MOUSE	Histone H1	KATGAATPK	10	1.70E-03	422.7481	843.4817	2	0.0003	9	1	Semi-tryptic
H12_MOUSE, H13_MOUSE	Histone H1	KATGAATPK	10	5.10E-05	422.7482	843.4817	2	0.0003	9	1	Semi-tryptic
H12_MOUSE, H13_MOUSE	Histone H1	KATGAATPK	10	1.80E-03	422.747	843.4795	2	-0.0019	9	1	Semi-tryptic
H12_MOUSE, H13_MOUSE, H14_MOUSE	Histone H1	LITKAVAASKERSGVSLAALKKALAAAGYDVE	7	2.30E-09	801.2155	3200.8329	4	0.0197	32	2	GluC
H12_MOUSE, H13_MOUSE, H14_MOUSE	Histone H1	LITKAVAASKERSGVSLAALKKALAAAGYDVE	7	2.50E-04	1067.951	3200.8312	3	0.0180	32	2	GluC
H12_MOUSE, H13_MOUSE, H14_MOUSE	Histone H1	LITKAVAASKERSGVSLAALKKALAAAGYDVE	7	6.40E-09	801.2147	3200.8299	4	0.0167	32	2	GluC
H12_MOUSE, H13_MOUSE, H14_MOUSE	Histone H1	LITKAVAASKERSGVSLAALKKALAAAGYDVE	7	2.00E-13	801.2117	3200.8176	4	0.0044	32	2	GluC
H12_MOUSE, H13_MOUSE, H14_MOUSE	Histone H1	LITKAVAASKERSGVSLAALKKALAAAGYDVE	7	1.90E-10	801.2114	3200.8166	4	0.0034	32	2	GluC
H12_MOUSE, H13_MOUSE, H14_MOUSE	Histone H1	LITKAVAASKERSGVSLAALKKALAAAGYDVE	7	6.00E-05	641.1702	3200.8145	5	0.0013	32	2	GluC
H12_MOUSE, H13_MOUSE, H14_MOUSE	Histone H1	LITKAVAASKERSGVSLAALKKALAAAGYDVE	7	3.20E-07	801.2085	3200.8048	4	-0.0084	32	2	GluC
H12_MOUSE, H13_MOUSE, H14_MOUSE	Histone H1	LITKAVAASKERSGVSLAALKKALAAAGYD	2	7.50E-10	744.1877	2972.7216	4	0.0194	30	1	GluC
H12_MOUSE, H13_MOUSE, H14_MOUSE	Histone H1	LITKAVAASKERSGVSLAALKKALAAAGYD	2	9.60E-07	595.5513	2972.7201	5	0.0179	30	1	GluC
H12_MOUSE, H13_MOUSE, H14_MOUSE	Histone H1	SGVSLAALKKALAAAGYDVEKNNSR	9	6.70E-04	634.1005	2532.3730	4	0.0071	25	3	Semi-tryptic
H12_MOUSE, H13_MOUSE, H14_MOUSE	Histone H1	SGVSLAALKKALAAAGYDVEKNNSR	9	1.10E-07	634.1003	2532.3721	4	0.0061	25	3	Semi-tryptic
H12_MOUSE, H13_MOUSE, H14_MOUSE	Histone H1	SGVSLAALKKALAAAGYDVEKNNSR	9	2.50E-11	634.1003	2532.3721	4	0.0061	25	3	Semi-tryptic
H12_MOUSE, H13_MOUSE, H14_MOUSE	Histone H1	SGVSLAALKKALAAAGYDVEKNNSR	9	5.90E-10	845.1308	2532.3706	3	0.0047	25	3	Semi-tryptic
H12_MOUSE, H13_MOUSE, H14_MOUSE	Histone H1	SGVSLAALKKALAAAGYDVEKNNSR	9	9.60E-10	845.1304	2532.3692	3	0.0033	25	3	Semi-tryptic
H12_MOUSE, H13_MOUSE, H14_MOUSE	Histone H1	SGVSLAALKKALAAAGYDVEKNNSR	9	7.00E-10	845.1303	2532.3691	3	0.0031	25	3	Semi-tryptic
H12_MOUSE, H13_MOUSE, H14_MOUSE	Histone H1	SGVSLAALKKALAAAGYDVEKNNSR	9	2.40E-04	634.099	2532.3669	4	0.0009	25	3	Semi-tryptic
H12_MOUSE, H13_MOUSE, H14_MOUSE	Histone H1	SGVSLAALKKALAAAGYDVEKNNSR	9	6.50E-03	634.0987	2532.3658	4	-0.0002	25	3	Semi-tryptic
H12_MOUSE, H13_MOUSE, H14_MOUSE	Histone H1	SGVSLAALKKALAAAGYDVEKNNSR	9	1.80E-04	634.0986	2532.3651	4	-0.0008	25	3	Semi-tryptic
H12_MOUSE, H13_MOUSE, H14_MOUSE	Histone H1	SGVSLAALKKALAAAGYDVEK	2	2.40E-06	688.057	2061.1492	3	0.0022	21	2	Semi-tryptic
H12_MOUSE, H13_MOUSE, H14_MOUSE	Histone H1	SGVSLAALKKALAAAGYDVEK	2	3.70E-03	688.0564	2061.1474	3	0.0005	21	2	Semi-tryptic
H12_MOUSE, H13_MOUSE, H14_MOUSE	Histone H1	RSGVSLAALKKALAAAGYDVE	105	2.80E-03	697.3869	2089.1389	3	-0.0142	21	1	GluC
H12_MOUSE, H13_MOUSE, H14_MOUSE	Histone H1	RSGVSLAALKKALAAAGYDVE	105	6.00E-10	697.3969	2089.1689	3	0.0158	21	1	GluC
H12_MOUSE, H13_MOUSE, H14_MOUSE	Histone H1	RSGVSLAALKKALAAAGYDVE	105	3.80E-03	697.3962	2089.1669	3	0.0138	21	1	GluC
H12_MOUSE, H13_MOUSE, H14_MOUSE	Histone H1	RSGVSLAALKKALAAAGYDVE	105	1.80E-03	523.299	2089.1668	4	0.0137	21	1	GluC
H12_MOUSE, H13_MOUSE, H14_MOUSE	Histone H1	RSGVSLAALKKALAAAGYDVE	105	5.80E-08	697.3962	2089.1667	3	0.0136	21	1	GluC
H12_MOUSE, H13_MOUSE, H14_MOUSE	Histone H1	RSGVSLAALKKALAAAGYDVE	105	1.50E-08	697.3962	2089.1667	3	0.0136	21	1	GluC
H12_MOUSE, H13_MOUSE, H14_MOUSE	Histone H1	RSGVSLAALKKALAAAGYDVE	105	1.40E-08	697.3958	2089.1656	3	0.0125	21	1	GluC
H12_MOUSE, H13_MOUSE, H14_MOUSE	Histone H1	RSGVSLAALKKALAAAGYDVE	105	7.60E-03	697.3958	2089.1655	3	0.0124	21	1	GluC
H12_MOUSE, H13_MOUSE, H14_MOUSE	Histone H1	RSGVSLAALKKALAAAGYDVE	105	9.40E-08	697.3957	2089.1652	3	0.0121	21	1	GluC
H12_MOUSE, H13_MOUSE, H14_MOUSE	Histone H1	RSGVSLAALKKALAAAGYDVE	105	2.40E-08	697.3957	2089.1651	3	0.0120	21	1	GluC
H12_MOUSE, H13_MOUSE, H14_MOUSE	Histone H1	RSGVSLAALKKALAAAGYDVE	105	1.10E-08	697.3955	2089.1646	3	0.0114	21	1	GluC
H12_MOUSE, H13_MOUSE, H14_MOUSE	Histone H1	RSGVSLAALKKALAAAGYDVE	105	1.10E-09	697.3954	2089.1644	3	0.0113	21	1	GluC
H12_MOUSE, H13_MOUSE, H14_MOUSE	Histone H1	RSGVSLAALKKALAAAGYDVE	105	1.50E-07	697.3954	2089.1642	3	0.0111	21	1	GluC
H12_MOUSE, H13_MOUSE, H14_MOUSE	Histone H1	RSGVSLAALKKALAAAGYDVE	105	1.10E-09	523.2983	2089.1641	4	0.0110	21	1	GluC
H12_MOUSE, H13_MOUSE, H14_MOUSE	Histone H1	RSGVSLAALKKALAAAGYDVE	105	1.90E-10	523.2983	2089.1640	4	0.0109	21	1	GluC
H12_MOUSE, H13_MOUSE, H14_MOUSE	Histone H1	RSGVSLAALKKALAAAGYDVE	105	1.50E-08	697.3953	2089.1639	3	0.0108	21	1	GluC
H12_MOUSE, H13_MOUSE, H14_MOUSE	Histone H1	RSGVSLAALKKALAAAGYDVE	105	4.00E-11	523.2982	2089.1636	4	0.0105	21	1	GluC
H12_MOUSE, H13_MOUSE, H14_MOUSE	Histone H1	RSGVSLAALKKALAAAGYDVE	105	1.20E-08	697.3951	2089.1635	3	0.0104	21	1	GluC
H12_MOUSE, H13_MOUSE, H14_MOUSE	Histone H1	RSGVSLAALKKALAAAGYDVE	105	9.20E-09	697.3951	2089.1634	3	0.0103	21	1	GluC
H12_MOUSE, H13_MOUSE, H14_MOUSE	Histone H1	RSGVSLAALKKALAAAGYDVE	105	4.50E-11	523.298	2089.1631	4	0.0100	21	1	GluC
H12_MOUSE, H13_MOUSE, H14_MOUSE	Histone H1	RSGVSLAALKKALAAAGYDVE	105	3.80E-08	697.3949	2089.1630	3	0.0099	21	1	GluC
H12_MOUSE, H13_MOUSE, H14_MOUSE	Histone H1	RSGVSLAALKKALAAAGYDVE	105	3.30E-12	523.2979	2089.1625	4	0.0093	21	1	GluC
H12_MOUSE, H13_MOUSE, H14_MOUSE	Histone H1	RSGVSLAALKKALAAAGYDVE	105	4.20E-03	697.3946	2089.1619	3	0.0088	21	1	GluC
H12_MOUSE, H13_MOUSE, H14_MOUSE	Histone H1	RSGVSLAALKKALAAAGYDVE	105	2.90E-11	523.2976	2089.1614	4	0.0083	21	1	GluC

H12_MOUSE, H13_MOUSE, H14_MOUSE	Histone H1	RSGVSLAALKKALAAAGYDVE	105	3.10E-08	523.2976	2089.1612	4	0.0081	21	1	GluC
H12_MOUSE, H13_MOUSE, H14_MOUSE	Histone H1	RSGVSLAALKKALAAAGYDVE	105	5.00E-03	697.3939	2089.1600	3	0.0069	21	1	GluC
H12_MOUSE, H13_MOUSE, H14_MOUSE	Histone H1	RSGVSLAALKKALAAAGYDVE	105	1.00E-03	697.3939	2089.1597	3	0.0066	21	1	GluC
H12_MOUSE, H13_MOUSE, H14_MOUSE	Histone H1	RSGVSLAALKKALAAAGYDVE	105	1.10E-04	697.3938	2089.1597	3	0.0066	21	1	GluC
H12_MOUSE, H13_MOUSE, H14_MOUSE	Histone H1	RSGVSLAALKKALAAAGYDVE	105	8.00E-05	697.3938	2089.1595	3	0.0064	21	1	GluC
H12_MOUSE, H13_MOUSE, H14_MOUSE	Histone H1	RSGVSLAALKKALAAAGYDVE	105	1.60E-09	1045.5869	2089.1592	2	0.0061	21	1	GluC
H12_MOUSE, H13_MOUSE, H14_MOUSE	Histone H1	RSGVSLAALKKALAAAGYDVE	105	3.50E-03	697.3936	2089.1591	3	0.0060	21	1	GluC
H12_MOUSE, H13_MOUSE, H14_MOUSE	Histone H1	RSGVSLAALKKALAAAGYDVE	105	1.50E-04	697.3936	2089.1590	3	0.0059	21	1	GluC
H12_MOUSE, H13_MOUSE, H14_MOUSE	Histone H1	RSGVSLAALKKALAAAGYDVE	105	8.70E-06	697.3936	2089.1590	3	0.0059	21	1	GluC
H12_MOUSE, H13_MOUSE, H14_MOUSE	Histone H1	RSGVSLAALKKALAAAGYDVE	105	1.70E-03	697.3934	2089.1583	3	0.0051	21	1	GluC
H12_MOUSE, H13_MOUSE, H14_MOUSE	Histone H1	RSGVSLAALKKALAAAGYDVE	105	1.50E-05	697.3934	2089.1583	3	0.0051	21	1	GluC
H12_MOUSE, H13_MOUSE, H14_MOUSE	Histone H1	RSGVSLAALKKALAAAGYDVE	105	5.40E-07	697.3932	2089.1579	3	0.0048	21	1	GluC
H12_MOUSE, H13_MOUSE, H14_MOUSE	Histone H1	RSGVSLAALKKALAAAGYDVE	105	5.00E-08	697.3932	2089.1579	3	0.0048	21	1	GluC
H12_MOUSE, H13_MOUSE, H14_MOUSE	Histone H1	RSGVSLAALKKALAAAGYDVE	105	7.10E-07	697.3931	2089.1576	3	0.0045	21	1	GluC
H12_MOUSE, H13_MOUSE, H14_MOUSE	Histone H1	RSGVSLAALKKALAAAGYDVE	105	1.00E-07	697.3931	2089.1576	3	0.0045	21	1	GluC
H12_MOUSE, H13_MOUSE, H14_MOUSE	Histone H1	RSGVSLAALKKALAAAGYDVE	105	4.10E-10	1045.5858	2089.1570	2	0.0039	21	1	GluC
H12_MOUSE, H13_MOUSE, H14_MOUSE	Histone H1	RSGVSLAALKKALAAAGYDVE	105	1.50E-05	697.3929	2089.1568	3	0.0037	21	1	GluC
H12_MOUSE, H13_MOUSE, H14_MOUSE	Histone H1	RSGVSLAALKKALAAAGYDVE	105	8.60E-08	697.3929	2089.1568	3	0.0037	21	1	GluC
H12_MOUSE, H13_MOUSE, H14_MOUSE	Histone H1	RSGVSLAALKKALAAAGYDVE	105	3.80E-09	1045.5856	2089.1566	2	0.0035	21	1	GluC
H12_MOUSE, H13_MOUSE, H14_MOUSE	Histone H1	RSGVSLAALKKALAAAGYDVE	105	3.30E-03	697.3927	2089.1564	3	0.0033	21	1	GluC
H12_MOUSE, H13_MOUSE, H14_MOUSE	Histone H1	RSGVSLAALKKALAAAGYDVE	105	3.10E-07	697.3928	2089.1565	3	0.0033	21	1	GluC
H12_MOUSE, H13_MOUSE, H14_MOUSE	Histone H1	RSGVSLAALKKALAAAGYDVE	105	4.10E-08	697.3928	2089.1565	3	0.0033	21	1	GluC
H12_MOUSE, H13_MOUSE, H14_MOUSE	Histone H1	RSGVSLAALKKALAAAGYDVE	105	6.00E-10	1045.5855	2089.1564	2	0.0033	21	1	GluC
H12_MOUSE, H13_MOUSE, H14_MOUSE	Histone H1	RSGVSLAALKKALAAAGYDVE	105	4.50E-04	697.3927	2089.1562	3	0.0031	21	1	GluC
H12_MOUSE, H13_MOUSE, H14_MOUSE	Histone H1	RSGVSLAALKKALAAAGYDVE	105	5.70E-05	697.3927	2089.1561	3	0.0030	21	1	GluC
H12_MOUSE, H13_MOUSE, H14_MOUSE	Histone H1	RSGVSLAALKKALAAAGYDVE	105	3.80E-05	697.3927	2089.1561	3	0.0030	21	1	GluC
H12_MOUSE, H13_MOUSE, H14_MOUSE	Histone H1	RSGVSLAALKKALAAAGYDVE	105	4.80E-03	697.3926	2089.1560	3	0.0029	21	1	GluC
H12_MOUSE, H13_MOUSE, H14_MOUSE	Histone H1	RSGVSLAALKKALAAAGYDVE	105	2.70E-07	697.3926	2089.1560	3	0.0029	21	1	GluC
H12_MOUSE, H13_MOUSE, H14_MOUSE	Histone H1	RSGVSLAALKKALAAAGYDVE	105	1.50E-07	697.3926	2089.1560	3	0.0029	21	1	GluC
H12_MOUSE, H13_MOUSE, H14_MOUSE	Histone H1	RSGVSLAALKKALAAAGYDVE	105	7.30E-08	697.3926	2089.1560	3	0.0029	21	1	GluC
H12_MOUSE, H13_MOUSE, H14_MOUSE	Histone H1	RSGVSLAALKKALAAAGYDVE	105	5.20E-08	697.3926	2089.1560	3	0.0029	21	1	GluC
H12_MOUSE, H13_MOUSE, H14_MOUSE	Histone H1	RSGVSLAALKKALAAAGYDVE	105	3.20E-08	697.3926	2089.1560	3	0.0029	21	1	GluC
H12_MOUSE, H13_MOUSE, H14_MOUSE	Histone H1	RSGVSLAALKKALAAAGYDVE	105	1.80E-09	697.3926	2089.1560	3	0.0029	21	1	GluC
H12_MOUSE, H13_MOUSE, H14_MOUSE	Histone H1	RSGVSLAALKKALAAAGYDVE	105	1.60E-05	697.3926	2089.1558	3	0.0027	21	1	GluC
H12_MOUSE, H13_MOUSE, H14_MOUSE	Histone H1	RSGVSLAALKKALAAAGYDVE	105	1.00E-05	697.3926	2089.1558	3	0.0027	21	1	GluC
H12_MOUSE, H13_MOUSE, H14_MOUSE	Histone H1	RSGVSLAALKKALAAAGYDVE	105	3.40E-03	697.3924	2089.1554	3	0.0023	21	1	GluC
H12_MOUSE, H13_MOUSE, H14_MOUSE	Histone H1	RSGVSLAALKKALAAAGYDVE	105	8.30E-04	697.3924	2089.1554	3	0.0023	21	1	GluC
H12_MOUSE, H13_MOUSE, H14_MOUSE	Histone H1	RSGVSLAALKKALAAAGYDVE	105	3.60E-12	523.2961	2089.1551	4	0.0020	21	1	GluC
H12_MOUSE, H13_MOUSE, H14_MOUSE	Histone H1	RSGVSLAALKKALAAAGYDVE	105	6.60E-06	697.3923	2089.1550	3	0.0019	21	1	GluC
H12_MOUSE, H13_MOUSE, H14_MOUSE	Histone H1	RSGVSLAALKKALAAAGYDVE	105	4.70E-06	697.3922	2089.1547	3	0.0016	21	1	GluC
H12_MOUSE, H13_MOUSE, H14_MOUSE	Histone H1	RSGVSLAALKKALAAAGYDVE	105	1.20E-06	697.3922	2089.1547	3	0.0016	21	1	GluC
H12_MOUSE, H13_MOUSE, H14_MOUSE	Histone H1	RSGVSLAALKKALAAAGYDVE	105	9.40E-07	697.3922	2089.1547	3	0.0016	21	1	GluC
H12_MOUSE, H13_MOUSE, H14_MOUSE	Histone H1	RSGVSLAALKKALAAAGYDVE	105	8.30E-09	697.3922	2089.1547	3	0.0015	21	1	GluC
H12_MOUSE, H13_MOUSE, H14_MOUSE	Histone H1	RSGVSLAALKKALAAAGYDVE	105	2.70E-03	697.3921	2089.1545	3	0.0014	21	1	GluC
H12_MOUSE, H13_MOUSE, H14_MOUSE	Histone H1	RSGVSLAALKKALAAAGYDVE	105	6.50E-07	697.3921	2089.1544	3	0.0013	21	1	GluC
H12_MOUSE, H13_MOUSE, H14_MOUSE	Histone H1	RSGVSLAALKKALAAAGYDVE	105	5.10E-03	697.3921	2089.1544	3	0.0012	21	1	GluC
H12_MOUSE, H13_MOUSE, H14_MOUSE	Histone H1	RSGVSLAALKKALAAAGYDVE	105	1.40E-05	697.3921	2089.1544	3	0.0012	21	1	GluC
H12_MOUSE, H13_MOUSE, H14_MOUSE	Histone H1	RSGVSLAALKKALAAAGYDVE	105	1.10E-05	697.392	2089.1543	3	0.0012	21	1	GluC
H12_MOUSE, H13_MOUSE, H14_MOUSE	Histone H1	RSGVSLAALKKALAAAGYDVE	105	3.80E-06	697.392	2089.1543	3	0.0012	21	1	GluC
H12_MOUSE, H13_MOUSE, H14_MOUSE	Histone H1	RSGVSLAALKKALAAAGYDVE	105	2.70E-03	697.392	2089.1541	3	0.0010	21	1	GluC
H12_MOUSE, H13_MOUSE, H14_MOUSE	Histone H1	RSGVSLAALKKALAAAGYDVE	105	1.40E-03	697.392	2089.1541	3	0.0010	21	1	GluC
H12_MOUSE, H13_MOUSE, H14_MOUSE	Histone H1	RSGVSLAALKKALAAAGYDVE	105	2.20E-05	697.392	2089.1541	3	0.0010	21	1	GluC
H12_MOUSE, H13_MOUSE, H14_MOUSE	Histone H1	RSGVSLAALKKALAAAGYDVE	105	1.30E-06	697.3919	2089.1540	3	0.0009	21	1	GluC
H12_MOUSE, H13_MOUSE, H14_MOUSE	Histone H1	RSGVSLAALKKALAAAGYDVE	105	3.00E-07	697.3919	2089.1540	3	0.0009	21	1	GluC
H12_MOUSE, H13_MOUSE, H14_MOUSE	Histone H1	RSGVSLAALKKALAAAGYDVE	105	1.10E-04	697.3919	2089.1539	3	0.0008	21	1	GluC
H12_MOUSE, H13_MOUSE, H14_MOUSE	Histone H1	RSGVSLAALKKALAAAGYDVE	105	7.40E-08	697.3919	2089.1539	3	0.0008	21	1	GluC
H12_MOUSE, H13_MOUSE, H14_MOUSE	Histone H1	RSGVSLAALKKALAAAGYDVE	105	1.80E-05	697.3918	2089.1537	3	0.0006	21	1	GluC
H12_MOUSE, H13_MOUSE, H14_MOUSE	Histone H1	RSGVSLAALKKALAAAGYDVE	105	5.70E-07	697.3918	2089.1537	3	0.0006	21	1	GluC
H12_MOUSE, H13_MOUSE, H14_MOUSE	Histone H1	RSGVSLAALKKALAAAGYDVE	105	5.60E-07	697.3918	2089.1537	3	0.0006	21	1	GluC
H12_MOUSE, H13_MOUSE, H14_MOUSE	Histone H1	RSGVSLAALKKALAAAGYDVE	105	1.10E-07	697.3918	2089.1537	3	0.0006	21	1	GluC
H12_MOUSE, H13_MOUSE, H14_MOUSE	Histone H1	RSGVSLAALKKALAAAGYDVE	105	9.60E-08	697.3918	2089.1537	3	0.0006	21	1	GluC
H12_MOUSE, H13_MOUSE, H14_MOUSE	Histone H1	RSGVSLAALKKALAAAGYDVE	105	6.90E-08	697.3918	2089.1537	3	0.0006	21	1	GluC
H12_MOUSE, H13_MOUSE, H14_MOUSE	Histone H1	RSGVSLAALKKALAAAGYDVE	105	6.30E-04	697.3918	2089.1536	3	0.0005	21	1	GluC
H12_MOUSE, H13_MOUSE, H14_MOUSE	Histone H1	RSGVSLAALKKALAAAGYDVE	105	1.10E-03	697.3918	2089.1535	3	0.0004	21	1	GluC
H12_MOUSE, H13_MOUSE, H14_MOUSE	Histone H1	RSGVSLAALKKALAAAGYDVE	105	1.10E-04	523.2957	2089.1535	4	0.0004	21	1	GluC
H12_MOUSE, H13_MOUSE, H14_MOUSE	Histone H1	RSGVSLAALKKALAAAGYDVE	105	2.40E-06	697.3918	2089.1535	3	0.0004	21	1	GluC
H12_MOUSE, H13_MOUSE, H14_MOUSE	Histone H1	RSGVSLAALKKALAAAGYDVE	105	5.00E-08	697.3918	2089.1535	3	0.0004	21	1	GluC
H12_MOUSE, H13_MOUSE, H14_MOUSE	Histone H1	RSGVSLAALKKALAAAGYDVE	105	2.30E-05	697.3917	2089.1534	3	0.0003	21	1	GluC
H12_MOUSE, H13_MOUSE, H14_MOUSE	Histone H1	RSGVSLAALKKALAAAGYDVE	105	4.30E-07	697.3918	2089.1535	3	0.0003	21	1	GluC
H12_MOUSE, H13_MOUSE, H14_MOUSE	Histone H1	RSGVSLAALKKALAAAGYDVE	105	1.00E-02	523.2956	2089.1533	4	0.0001	21	1	GluC
H12_MOUSE, H13_MOUSE, H14_MOUSE	Histone H1	RSGVSLAALKKALAAAGYDVE	105	7.80E-04	523.2956	2089.1532	4	0.0001	21	1	GluC
H12_MOUSE, H13_MOUSE, H14_MOUSE	Histone H1	RSGVSLAALKKALAAAGYDVE	105	6.10E-05	697.3916	2089.1530	3	-0.0001	21	1	GluC
H12_MOUSE, H13_MOUSE, H14_MOUSE	Histone H1	RSGVSLAALKKALAAAGYDVE	105	4.00E-05	523.2953	2089.1522	4	-0.0009	21	1	GluC
H12_MOUSE, H13_MOUSE, H14_MOUSE	Histone H1	RSGVSLAALKKALAAAGYDVE	105	2.00E-10	523.2953	2089.1522	4	-0.0009	21	1	GluC

H12_MOUSE, H13_MOUSE, H14_MOUSE	Histone H1	RSGVSLAALKKALAAAGYDVE	105	6.40E-03	697.3913	2089.1521	3	-0.0010	21	1	GluC
H12_MOUSE, H13_MOUSE, H14_MOUSE	Histone H1	RSGVSLAALKKALAAAGYDVE	105	1.50E-03	697.3913	2089.1521	3	-0.0010	21	1	GluC
H12_MOUSE, H13_MOUSE, H14_MOUSE	Histone H1	RSGVSLAALKKALAAAGYDVE	105	1.60E-04	697.3913	2089.1521	3	-0.0010	21	1	GluC
H12_MOUSE, H13_MOUSE, H14_MOUSE	Histone H1	RSGVSLAALKKALAAAGYDVE	105	8.50E-05	697.3913	2089.1521	3	-0.0010	21	1	GluC
H12_MOUSE, H13_MOUSE, H14_MOUSE	Histone H1	RSGVSLAALKKALAAAGYDVE	105	4.70E-07	697.3913	2089.1519	3	-0.0012	21	1	GluC
H12_MOUSE, H13_MOUSE, H14_MOUSE	Histone H1	RSGVSLAALKKALAAAGYDVE	105	3.70E-07	523.2952	2089.1518	4	-0.0013	21	1	GluC
H12_MOUSE, H13_MOUSE, H14_MOUSE	Histone H1	RSGVSLAALKKALAAAGYDVE	105	4.10E-04	697.3911	2089.1514	3	-0.0017	21	1	GluC
H12_MOUSE, H13_MOUSE, H14_MOUSE	Histone H1	RSGVSLAALKKALAAAGYD	14	4.10E-11	621.3592	1861.0559	3	0.0138	19	0	GluC
H12_MOUSE, H13_MOUSE, H14_MOUSE	Histone H1	RSGVSLAALKKALAAAGYD	14	1.10E-07	621.3592	1861.0558	3	0.0137	19	0	GluC
H12_MOUSE, H13_MOUSE, H14_MOUSE	Histone H1	RSGVSLAALKKALAAAGYD	14	2.30E-14	621.3591	1861.0554	3	0.0133	19	0	GluC
H12_MOUSE, H13_MOUSE, H14_MOUSE	Histone H1	RSGVSLAALKKALAAAGYD	14	5.90E-04	621.359	1861.0552	3	0.0131	19	0	GluC
H12_MOUSE, H13_MOUSE, H14_MOUSE	Histone H1	RSGVSLAALKKALAAAGYD	14	5.70E-06	621.3583	1861.0530	3	0.0109	19	0	GluC
H12_MOUSE, H13_MOUSE, H14_MOUSE	Histone H1	RSGVSLAALKKALAAAGYD	14	9.60E-03	466.2698	1861.0501	4	0.0080	19	0	GluC
H12_MOUSE, H13_MOUSE, H14_MOUSE	Histone H1	RSGVSLAALKKALAAAGYD	14	1.90E-07	931.53	1861.0454	2	0.0033	19	0	GluC
H12_MOUSE, H13_MOUSE, H14_MOUSE	Histone H1	RSGVSLAALKKALAAAGYD	14	3.00E-05	621.3557	1861.0453	3	0.0032	19	0	GluC
H12_MOUSE, H13_MOUSE, H14_MOUSE	Histone H1	RSGVSLAALKKALAAAGYD	14	1.90E-05	621.3557	1861.0453	3	0.0032	19	0	GluC
H12_MOUSE, H13_MOUSE, H14_MOUSE	Histone H1	RSGVSLAALKKALAAAGYD	14	2.00E-05	621.3553	1861.0442	3	0.0021	19	0	GluC
H12_MOUSE, H13_MOUSE, H14_MOUSE	Histone H1	RSGVSLAALKKALAAAGYD	14	8.50E-09	621.3553	1861.0442	3	0.0021	19	0	GluC
H12_MOUSE, H13_MOUSE, H14_MOUSE	Histone H1	RSGVSLAALKKALAAAGYD	14	3.20E-03	621.3549	1861.0428	3	0.0007	19	0	GluC
H12_MOUSE, H13_MOUSE, H14_MOUSE	Histone H1	RSGVSLAALKKALAAAGYD	14	2.30E-06	621.3549	1861.0428	3	0.0007	19	0	GluC
H12_MOUSE, H13_MOUSE, H14_MOUSE	Histone H1	RSGVSLAALKKALAAAGYD	14	8.20E-08	621.3544	1861.0413	3	-0.0008	19	0	GluC
H12_MOUSE, H13_MOUSE, H14_MOUSE	Histone H1	AVAASKERSGVSLAALKK	3	6.70E-04	596.0232	1785.0477	3	0.0006	18	3	Semi-tryptic
H12_MOUSE, H13_MOUSE, H14_MOUSE	Histone H1	AVAASKERSGVSLAALKK	3	5.70E-04	596.023	1785.0470	3	-0.0002	18	3	Semi-tryptic
H12_MOUSE, H13_MOUSE, H14_MOUSE	Histone H1	AVAASKERSGVSLAALKK	3	9.20E-03	447.2689	1785.0464	4	-0.0008	18	3	Semi-tryptic
H12_MOUSE, H13_MOUSE, H14_MOUSE	Histone H1	ITKAVAASKERSGVSL	48	9.30E-06	404.9882	1615.9236	4	-0.0021	16	0	Chymotrypsin
H12_MOUSE, H13_MOUSE, H14_MOUSE	Histone H1	ITKAVAASKERSGVSL	48	3.30E-07	404.9918	1615.9382	4	0.0125	16	0	Chymotrypsin
H12_MOUSE, H13_MOUSE, H14_MOUSE	Histone H1	ITKAVAASKERSGVSL	48	4.70E-04	539.6525	1615.9357	3	0.0100	16	0	Chymotrypsin
H12_MOUSE, H13_MOUSE, H14_MOUSE	Histone H1	ITKAVAASKERSGVSL	48	4.20E-08	404.9912	1615.9357	4	0.0100	16	0	Chymotrypsin
H12_MOUSE, H13_MOUSE, H14_MOUSE	Histone H1	ITKAVAASKERSGVSL	48	6.00E-05	539.6524	1615.9353	3	0.0096	16	0	Chymotrypsin
H12_MOUSE, H13_MOUSE, H14_MOUSE	Histone H1	ITKAVAASKERSGVSL	48	1.10E-04	404.991	1615.9351	4	0.0094	16	0	Chymotrypsin
H12_MOUSE, H13_MOUSE, H14_MOUSE	Histone H1	ITKAVAASKERSGVSL	48	6.80E-03	808.9748	1615.9350	2	0.0093	16	0	Chymotrypsin
H12_MOUSE, H13_MOUSE, H14_MOUSE	Histone H1	ITKAVAASKERSGVSL	48	2.20E-04	808.9746	1615.9347	2	0.0090	16	0	Chymotrypsin
H12_MOUSE, H13_MOUSE, H14_MOUSE	Histone H1	ITKAVAASKERSGVSL	48	3.10E-04	808.9745	1615.9345	2	0.0088	16	0	Chymotrypsin
H12_MOUSE, H13_MOUSE, H14_MOUSE	Histone H1	ITKAVAASKERSGVSL	48	1.30E-06	539.6521	1615.9345	3	0.0088	16	0	Chymotrypsin
H12_MOUSE, H13_MOUSE, H14_MOUSE	Histone H1	ITKAVAASKERSGVSL	48	1.30E-07	404.9908	1615.9340	4	0.0083	16	0	Chymotrypsin
H12_MOUSE, H13_MOUSE, H14_MOUSE	Histone H1	ITKAVAASKERSGVSL	48	5.10E-09	404.9908	1615.9340	4	0.0083	16	0	Chymotrypsin
H12_MOUSE, H13_MOUSE, H14_MOUSE	Histone H1	ITKAVAASKERSGVSL	48	4.70E-06	404.9907	1615.9337	4	0.0080	16	0	Chymotrypsin
H12_MOUSE, H13_MOUSE, H14_MOUSE	Histone H1	ITKAVAASKERSGVSL	48	1.90E-09	404.9907	1615.9336	4	0.0079	16	0	Chymotrypsin
H12_MOUSE, H13_MOUSE, H14_MOUSE	Histone H1	ITKAVAASKERSGVSL	48	3.70E-04	404.9905	1615.9331	4	0.0074	16	0	Chymotrypsin
H12_MOUSE, H13_MOUSE, H14_MOUSE	Histone H1	ITKAVAASKERSGVSL	48	2.80E-03	404.9905	1615.9329	4	0.0072	16	0	Chymotrypsin
H12_MOUSE, H13_MOUSE, H14_MOUSE	Histone H1	ITKAVAASKERSGVSL	48	7.40E-03	539.6515	1615.9327	3	0.0070	16	0	Chymotrypsin
H12_MOUSE, H13_MOUSE, H14_MOUSE	Histone H1	ITKAVAASKERSGVSL	48	8.00E-04	539.6515	1615.9326	3	0.0069	16	0	Chymotrypsin
H12_MOUSE, H13_MOUSE, H14_MOUSE	Histone H1	ITKAVAASKERSGVSL	48	4.50E-08	404.9903	1615.9323	4	0.0066	16	0	Chymotrypsin
H12_MOUSE, H13_MOUSE, H14_MOUSE	Histone H1	ITKAVAASKERSGVSL	48	6.20E-06	404.9902	1615.9317	4	0.0060	16	0	Chymotrypsin
H12_MOUSE, H13_MOUSE, H14_MOUSE	Histone H1	ITKAVAASKERSGVSL	48	5.80E-11	539.6512	1615.9317	3	0.0060	16	0	Chymotrypsin
H12_MOUSE, H13_MOUSE, H14_MOUSE	Histone H1	ITKAVAASKERSGVSL	48	2.00E-03	539.6511	1615.9315	3	0.0058	16	0	Chymotrypsin
H12_MOUSE, H13_MOUSE, H14_MOUSE	Histone H1	ITKAVAASKERSGVSL	48	2.00E-04	404.9901	1615.9313	4	0.0056	16	0	Chymotrypsin
H12_MOUSE, H13_MOUSE, H14_MOUSE	Histone H1	ITKAVAASKERSGVSL	48	1.20E-07	539.651	1615.9312	3	0.0055	16	0	Chymotrypsin
H12_MOUSE, H13_MOUSE, H14_MOUSE	Histone H1	ITKAVAASKERSGVSL	48	6.60E-05	808.9728	1615.9311	2	0.0054	16	0	Chymotrypsin
H12_MOUSE, H13_MOUSE, H14_MOUSE	Histone H1	ITKAVAASKERSGVSL	48	2.70E-10	539.6509	1615.9310	3	0.0053	16	0	Chymotrypsin
H12_MOUSE, H13_MOUSE, H14_MOUSE	Histone H1	ITKAVAASKERSGVSL	48	1.90E-03	539.6509	1615.9309	3	0.0052	16	0	Chymotrypsin
H12_MOUSE, H13_MOUSE, H14_MOUSE	Histone H1	ITKAVAASKERSGVSL	48	1.40E-05	539.6509	1615.9308	3	0.0052	16	0	Chymotrypsin
H12_MOUSE, H13_MOUSE, H14_MOUSE	Histone H1	ITKAVAASKERSGVSL	48	1.50E-04	404.9899	1615.9306	4	0.0049	16	0	Chymotrypsin
H12_MOUSE, H13_MOUSE, H14_MOUSE	Histone H1	ITKAVAASKERSGVSL	48	1.00E-10	539.6508	1615.9306	3	0.0049	16	0	Chymotrypsin
H12_MOUSE, H13_MOUSE, H14_MOUSE	Histone H1	ITKAVAASKERSGVSL	48	5.20E-05	808.972	1615.9294	2	0.0038	16	0	Chymotrypsin
H12_MOUSE, H13_MOUSE, H14_MOUSE	Histone H1	ITKAVAASKERSGVSL	48	9.80E-08	539.6504	1615.9295	3	0.0038	16	0	Chymotrypsin
H12_MOUSE, H13_MOUSE, H14_MOUSE	Histone H1	ITKAVAASKERSGVSL	48	9.50E-03	539.6504	1615.9293	3	0.0037	16	0	Chymotrypsin
H12_MOUSE, H13_MOUSE, H14_MOUSE	Histone H1	ITKAVAASKERSGVSL	48	5.20E-03	539.6504	1615.9294	3	0.0037	16	0	Chymotrypsin
H12_MOUSE, H13_MOUSE, H14_MOUSE	Histone H1	ITKAVAASKERSGVSL	48	5.60E-04	808.9713	1615.9280	2	0.0023	16	0	Chymotrypsin
H12_MOUSE, H13_MOUSE, H14_MOUSE	Histone H1	ITKAVAASKERSGVSL	48	9.90E-07	808.9713	1615.9280	2	0.0023	16	0	Chymotrypsin
H12_MOUSE, H13_MOUSE, H14_MOUSE	Histone H1	ITKAVAASKERSGVSL	48	7.20E-07	404.9893	1615.9280	4	0.0023	16	0	Chymotrypsin
H12_MOUSE, H13_MOUSE, H14_MOUSE	Histone H1	ITKAVAASKERSGVSL	48	1.40E-06	404.989	1615.9270	4	0.0013	16	0	Chymotrypsin
H12_MOUSE, H13_MOUSE, H14_MOUSE	Histone H1	ITKAVAASKERSGVSL	48	6.30E-06	539.6494	1615.9265	3	0.0008	16	0	Chymotrypsin
H12_MOUSE, H13_MOUSE, H14_MOUSE	Histone H1	ITKAVAASKERSGVSL	48	4.00E-10	539.6494	1615.9265	3	0.0008	16	0	Chymotrypsin
H12_MOUSE, H13_MOUSE, H14_MOUSE	Histone H1	ITKAVAASKERSGVSL	48	1.40E-07	539.6494	1615.9263	3	0.0006	16	0	Chymotrypsin
H12_MOUSE, H13_MOUSE, H14_MOUSE	Histone H1	ITKAVAASKERSGVSL	48	4.80E-03	539.6493	1615.9260	3	0.0003	16	0	Chymotrypsin
H12_MOUSE, H13_MOUSE, H14_MOUSE	Histone H1	ITKAVAASKERSGVSL	48	1.10E-04	539.6491	1615.9256	3	-0.0001	16	0	Chymotrypsin
H12_MOUSE, H13_MOUSE, H14_MOUSE	Histone H1	ITKAVAASKERSGVSL	48	6.20E-08	539.6491	1615.9256	3	-0.0001	16	0	Chymotrypsin
H12_MOUSE, H13_MOUSE, H14_MOUSE	Histone H1	ITKAVAASKERSGVSL	48	6.40E-10	539.6491	1615.9256	3	-0.0001	16	0	Chymotrypsin
H12_MOUSE, H13_MOUSE, H14_MOUSE	Histone H1	ITKAVAASKERSGVSL	48	2.30E-08	404.9887	1615.9255	4	-0.0002	16	0	Chymotrypsin
H12_MOUSE, H13_MOUSE, H14_MOUSE	Histone H1	ITKAVAASKERSGVSL	48	1.30E-08	539.649	1615.9252	3	-0.0005	16	0	Chymotrypsin
H12_MOUSE, H13_MOUSE, H14_MOUSE	Histone H1	ITKAVAASKERSGVSL	48	3.20E-04	404.9886	1615.9251	4	-0.0006	16	0	Chymotrypsin
H12_MOUSE, H13_MOUSE, H14_MOUSE	Histone H1	AKKPAGAAKKPK	4	4.90E-03	597.8881	1193.7616	2	0.0008	12	2	Semi-tryptic
H12_MOUSE, H13_MOUSE, H14_MOUSE	Histone H1	AKKPAGAAKKPK	4	6.40E-03	398.9275	1193.7607	3	0.0000	12	2	Semi-tryptic

H12_MOUSE, H13_MOUSE, H14_MOUSE	Histone H1	AKKPAGAAKKPK	4	9.10E-06	398.9274	1193.7604	3	-0.0004	12	2	Semi-tryptic
H12_MOUSE, H13_MOUSE, H14_MOUSE	Histone H1	AKKPAGAAKKPK	4	1.30E-05	398.9262	1193.7566	3	-0.0042	12	2	Semi-tryptic
H12_MOUSE, H13_MOUSE, H14_MOUSE	Histone H1	LITKAVAASKE	215	1.70E-05	565.8417	1129.6688	2	-0.0019	11	0	GluC
H12_MOUSE, H13_MOUSE, H14_MOUSE	Histone H1	LITKAVAASKE	215	3.00E-03	377.5635	1129.6687	3	-0.0020	11	0	GluC
H12_MOUSE, H13_MOUSE, H14_MOUSE	Histone H1	LITKAVAASKE	215	9.60E-05	377.5635	1129.6687	3	-0.0020	11	0	GluC
H12_MOUSE, H13_MOUSE, H14_MOUSE	Histone H1	LITKAVAASKE	215	1.30E-05	565.8415	1129.6685	2	-0.0022	11	0	GluC
H12_MOUSE, H13_MOUSE, H14_MOUSE	Histone H1	LITKAVAASKE	215	8.90E-06	565.8415	1129.6685	2	-0.0022	11	0	GluC
H12_MOUSE, H13_MOUSE, H14_MOUSE	Histone H1	LITKAVAASKE	215	1.80E-03	377.5634	1129.6683	3	-0.0024	11	0	GluC
H12_MOUSE, H13_MOUSE, H14_MOUSE	Histone H1	LITKAVAASKE	215	2.20E-06	377.5634	1129.6683	3	-0.0024	11	0	GluC
H12_MOUSE, H13_MOUSE, H14_MOUSE	Histone H1	LITKAVAASKE	215	8.10E-09	377.567	1129.6792	3	0.0085	11	0	GluC
H12_MOUSE, H13_MOUSE, H14_MOUSE	Histone H1	LITKAVAASKE	215	9.70E-06	565.8465	1129.6785	2	0.0079	11	0	GluC
H12_MOUSE, H13_MOUSE, H14_MOUSE	Histone H1	LITKAVAASKE	215	9.80E-04	565.8463	1129.6780	2	0.0074	11	0	GluC
H12_MOUSE, H13_MOUSE, H14_MOUSE	Histone H1	LITKAVAASKE	215	1.10E-07	377.5666	1129.6779	3	0.0073	11	0	GluC
H12_MOUSE, H13_MOUSE, H14_MOUSE	Histone H1	LITKAVAASKE	215	6.90E-04	565.8462	1129.6778	2	0.0072	11	0	GluC
H12_MOUSE, H13_MOUSE, H14_MOUSE	Histone H1	LITKAVAASKE	215	9.10E-08	377.5665	1129.6776	3	0.0069	11	0	GluC
H12_MOUSE, H13_MOUSE, H14_MOUSE	Histone H1	LITKAVAASKE	215	6.40E-06	377.5664	1129.6774	3	0.0068	11	0	GluC
H12_MOUSE, H13_MOUSE, H14_MOUSE	Histone H1	LITKAVAASKE	215	4.50E-03	565.846	1129.6774	2	0.0067	11	0	GluC
H12_MOUSE, H13_MOUSE, H14_MOUSE	Histone H1	LITKAVAASKE	215	7.50E-09	377.5662	1129.6769	3	0.0062	11	0	GluC
H12_MOUSE, H13_MOUSE, H14_MOUSE	Histone H1	LITKAVAASKE	215	2.00E-03	565.8456	1129.6765	2	0.0059	11	0	GluC
H12_MOUSE, H13_MOUSE, H14_MOUSE	Histone H1	LITKAVAASKE	215	3.60E-03	377.5661	1129.6765	3	0.0058	11	0	GluC
H12_MOUSE, H13_MOUSE, H14_MOUSE	Histone H1	LITKAVAASKE	215	1.70E-08	377.566	1129.6763	3	0.0057	11	0	GluC
H12_MOUSE, H13_MOUSE, H14_MOUSE	Histone H1	LITKAVAASKE	215	2.50E-08	377.5658	1129.6756	3	0.0050	11	0	GluC
H12_MOUSE, H13_MOUSE, H14_MOUSE	Histone H1	LITKAVAASKE	215	2.10E-04	377.5657	1129.6752	3	0.0045	11	0	GluC
H12_MOUSE, H13_MOUSE, H14_MOUSE	Histone H1	LITKAVAASKE	215	1.70E-03	565.8448	1129.6750	2	0.0043	11	0	GluC
H12_MOUSE, H13_MOUSE, H14_MOUSE	Histone H1	LITKAVAASKE	215	3.50E-04	565.8447	1129.6748	2	0.0041	11	0	GluC
H12_MOUSE, H13_MOUSE, H14_MOUSE	Histone H1	LITKAVAASKE	215	5.50E-08	377.5654	1129.6745	3	0.0039	11	0	GluC
H12_MOUSE, H13_MOUSE, H14_MOUSE	Histone H1	LITKAVAASKE	215	1.40E-03	565.8445	1129.6744	2	0.0037	11	0	GluC
H12_MOUSE, H13_MOUSE, H14_MOUSE	Histone H1	LITKAVAASKE	215	3.30E-03	565.8444	1129.6742	2	0.0036	11	0	GluC
H12_MOUSE, H13_MOUSE, H14_MOUSE	Histone H1	LITKAVAASKE	215	4.70E-08	377.5653	1129.6741	3	0.0035	11	0	GluC
H12_MOUSE, H13_MOUSE, H14_MOUSE	Histone H1	LITKAVAASKE	215	4.40E-04	565.8443	1129.6740	2	0.0034	11	0	GluC
H12_MOUSE, H13_MOUSE, H14_MOUSE	Histone H1	LITKAVAASKE	215	8.00E-03	565.8442	1129.6737	2	0.0031	11	0	GluC
H12_MOUSE, H13_MOUSE, H14_MOUSE	Histone H1	LITKAVAASKE	215	2.00E-04	565.8442	1129.6738	2	0.0031	11	0	GluC
H12_MOUSE, H13_MOUSE, H14_MOUSE	Histone H1	LITKAVAASKE	215	8.50E-06	565.8442	1129.6738	2	0.0031	11	0	GluC
H12_MOUSE, H13_MOUSE, H14_MOUSE	Histone H1	LITKAVAASKE	215	8.40E-04	565.8441	1129.6737	2	0.0030	11	0	GluC
H12_MOUSE, H13_MOUSE, H14_MOUSE	Histone H1	LITKAVAASKE	215	2.60E-04	377.5651	1129.6736	3	0.0029	11	0	GluC
H12_MOUSE, H13_MOUSE, H14_MOUSE	Histone H1	LITKAVAASKE	215	1.50E-08	377.5651	1129.6735	3	0.0029	11	0	GluC
H12_MOUSE, H13_MOUSE, H14_MOUSE	Histone H1	LITKAVAASKE	215	1.90E-08	377.5651	1129.6734	3	0.0028	11	0	GluC
H12_MOUSE, H13_MOUSE, H14_MOUSE	Histone H1	LITKAVAASKE	215	6.20E-03	377.565	1129.6733	3	0.0026	11	0	GluC
H12_MOUSE, H13_MOUSE, H14_MOUSE	Histone H1	LITKAVAASKE	215	5.30E-05	377.565	1129.6731	3	0.0024	11	0	GluC
H12_MOUSE, H13_MOUSE, H14_MOUSE	Histone H1	LITKAVAASKE	215	7.10E-06	565.8438	1129.6730	2	0.0024	11	0	GluC
H12_MOUSE, H13_MOUSE, H14_MOUSE	Histone H1	LITKAVAASKE	215	1.80E-07	565.8438	1129.6730	2	0.0024	11	0	GluC
H12_MOUSE, H13_MOUSE, H14_MOUSE	Histone H1	LITKAVAASKE	215	2.10E-05	377.5649	1129.6730	3	0.0023	11	0	GluC
H12_MOUSE, H13_MOUSE, H14_MOUSE	Histone H1	LITKAVAASKE	215	2.10E-03	565.8436	1129.6727	2	0.0021	11	0	GluC
H12_MOUSE, H13_MOUSE, H14_MOUSE	Histone H1	LITKAVAASKE	215	2.10E-04	377.5648	1129.6727	3	0.0021	11	0	GluC
H12_MOUSE, H13_MOUSE, H14_MOUSE	Histone H1	LITKAVAASKE	215	1.50E-05	377.5648	1129.6727	3	0.0021	11	0	GluC
H12_MOUSE, H13_MOUSE, H14_MOUSE	Histone H1	LITKAVAASKE	215	6.10E-08	377.5648	1129.6727	3	0.0021	11	0	GluC
H12_MOUSE, H13_MOUSE, H14_MOUSE	Histone H1	LITKAVAASKE	215	3.80E-08	377.5648	1129.6727	3	0.0021	11	0	GluC
H12_MOUSE, H13_MOUSE, H14_MOUSE	Histone H1	LITKAVAASKE	215	3.90E-04	377.5648	1129.6726	3	0.0019	11	0	GluC
H12_MOUSE, H13_MOUSE, H14_MOUSE	Histone H1	LITKAVAASKE	215	4.00E-03	377.5648	1129.6724	3	0.0018	11	0	GluC
H12_MOUSE, H13_MOUSE, H14_MOUSE	Histone H1	LITKAVAASKE	215	1.90E-03	565.8435	1129.6725	2	0.0018	11	0	GluC
H12_MOUSE, H13_MOUSE, H14_MOUSE	Histone H1	LITKAVAASKE	215	2.50E-05	565.8435	1129.6724	2	0.0018	11	0	GluC
H12_MOUSE, H13_MOUSE, H14_MOUSE	Histone H1	LITKAVAASKE	215	3.00E-03	565.8434	1129.6723	2	0.0017	11	0	GluC
H12_MOUSE, H13_MOUSE, H14_MOUSE	Histone H1	LITKAVAASKE	215	2.70E-05	377.5647	1129.6724	3	0.0017	11	0	GluC
H12_MOUSE, H13_MOUSE, H14_MOUSE	Histone H1	LITKAVAASKE	215	5.90E-06	565.8434	1129.6723	2	0.0017	11	0	GluC
H12_MOUSE, H13_MOUSE, H14_MOUSE	Histone H1	LITKAVAASKE	215	2.30E-09	377.5647	1129.6724	3	0.0017	11	0	GluC
H12_MOUSE, H13_MOUSE, H14_MOUSE	Histone H1	LITKAVAASKE	215	8.20E-03	377.5647	1129.6722	3	0.0016	11	0	GluC
H12_MOUSE, H13_MOUSE, H14_MOUSE	Histone H1	LITKAVAASKE	215	1.80E-03	565.8434	1129.6722	2	0.0016	11	0	GluC
H12_MOUSE, H13_MOUSE, H14_MOUSE	Histone H1	LITKAVAASKE	215	1.80E-05	565.8434	1129.6723	2	0.0016	11	0	GluC
H12_MOUSE, H13_MOUSE, H14_MOUSE	Histone H1	LITKAVAASKE	215	1.30E-07	565.8434	1129.6723	2	0.0016	11	0	GluC
H12_MOUSE, H13_MOUSE, H14_MOUSE	Histone H1	LITKAVAASKE	215	4.90E-08	377.5647	1129.6722	3	0.0016	11	0	GluC
H12_MOUSE, H13_MOUSE, H14_MOUSE	Histone H1	LITKAVAASKE	215	1.00E-04	565.8433	1129.6721	2	0.0015	11	0	GluC
H12_MOUSE, H13_MOUSE, H14_MOUSE	Histone H1	LITKAVAASKE	215	4.80E-07	377.5647	1129.6722	3	0.0015	11	0	GluC
H12_MOUSE, H13_MOUSE, H14_MOUSE	Histone H1	LITKAVAASKE	215	7.40E-03	377.5646	1129.6720	3	0.0014	11	0	GluC
H12_MOUSE, H13_MOUSE, H14_MOUSE	Histone H1	LITKAVAASKE	215	4.20E-04	377.5646	1129.6721	3	0.0014	11	0	GluC
H12_MOUSE, H13_MOUSE, H14_MOUSE	Histone H1	LITKAVAASKE	215	3.50E-05	377.5646	1129.6721	3	0.0014	11	0	GluC
H12_MOUSE, H13_MOUSE, H14_MOUSE	Histone H1	LITKAVAASKE	215	1.40E-05	565.8433	1129.6721	2	0.0014	11	0	GluC
H12_MOUSE, H13_MOUSE, H14_MOUSE	Histone H1	LITKAVAASKE	215	1.30E-05	565.8433	1129.6720	2	0.0014	11	0	GluC
H12_MOUSE, H13_MOUSE, H14_MOUSE	Histone H1	LITKAVAASKE	215	1.80E-03	565.8432	1129.6719	2	0.0013	11	0	GluC
H12_MOUSE, H13_MOUSE, H14_MOUSE	Histone H1	LITKAVAASKE	215	1.80E-05	565.8433	1129.6720	2	0.0013	11	0	GluC
H12_MOUSE, H13_MOUSE, H14_MOUSE	Histone H1	LITKAVAASKE	215	4.50E-07	377.5646	1129.6719	3	0.0013	11	0	GluC
H12_MOUSE, H13_MOUSE, H14_MOUSE	Histone H1	LITKAVAASKE	215	2.00E-07	377.5646	1129.6720	3	0.0013	11	0	GluC
H12_MOUSE, H13_MOUSE, H14_MOUSE	Histone H1	LITKAVAASKE	215	1.40E-03	565.8432	1129.6718	2	0.0012	11	0	GluC
H12_MOUSE, H13_MOUSE, H14_MOUSE	Histone H1	LITKAVAASKE	215	2.90E-05	565.8432	1129.6718	2	0.0012	11	0	GluC
H12_MOUSE, H13_MOUSE, H14_MOUSE	Histone H1	LITKAVAASKE	215	3.50E-07	565.8432	1129.6718	2	0.0012	11	0	GluC

H12_MOUSE, H13_MOUSE, H14_MOUSE	Histone H1	LITKAVAASKE	215	8.10E-03	565.8432	1129.6718	2	0.0011	11	0	GluC
H12_MOUSE, H13_MOUSE, H14_MOUSE	Histone H1	LITKAVAASKE	215	6.70E-03	565.8431	1129.6717	2	0.0011	11	0	GluC
H12_MOUSE, H13_MOUSE, H14_MOUSE	Histone H1	LITKAVAASKE	215	1.00E-04	565.8431	1129.6717	2	0.0011	11	0	GluC
H12_MOUSE, H13_MOUSE, H14_MOUSE	Histone H1	LITKAVAASKE	215	4.00E-05	565.8431	1129.6717	2	0.0011	11	0	GluC
H12_MOUSE, H13_MOUSE, H14_MOUSE	Histone H1	LITKAVAASKE	215	8.00E-06	565.8432	1129.6718	2	0.0011	11	0	GluC
H12_MOUSE, H13_MOUSE, H14_MOUSE	Histone H1	LITKAVAASKE	215	3.20E-06	377.5645	1129.6716	3	0.0010	11	0	GluC
H12_MOUSE, H13_MOUSE, H14_MOUSE	Histone H1	LITKAVAASKE	215	1.10E-07	377.5645	1129.6716	3	0.0010	11	0	GluC
H12_MOUSE, H13_MOUSE, H14_MOUSE	Histone H1	LITKAVAASKE	215	7.50E-03	377.5644	1129.6715	3	0.0009	11	0	GluC
H12_MOUSE, H13_MOUSE, H14_MOUSE	Histone H1	LITKAVAASKE	215	1.60E-03	565.8431	1129.6716	2	0.0009	11	0	GluC
H12_MOUSE, H13_MOUSE, H14_MOUSE	Histone H1	LITKAVAASKE	215	3.90E-04	565.843	1129.6715	2	0.0009	11	0	GluC
H12_MOUSE, H13_MOUSE, H14_MOUSE	Histone H1	LITKAVAASKE	215	2.20E-05	377.5645	1129.6715	3	0.0009	11	0	GluC
H12_MOUSE, H13_MOUSE, H14_MOUSE	Histone H1	LITKAVAASKE	215	4.60E-07	377.5644	1129.6715	3	0.0009	11	0	GluC
H12_MOUSE, H13_MOUSE, H14_MOUSE	Histone H1	LITKAVAASKE	215	2.00E-07	565.843	1129.6715	2	0.0009	11	0	GluC
H12_MOUSE, H13_MOUSE, H14_MOUSE	Histone H1	LITKAVAASKE	215	1.00E-02	565.843	1129.6714	2	0.0008	11	0	GluC
H12_MOUSE, H13_MOUSE, H14_MOUSE	Histone H1	LITKAVAASKE	215	1.00E-02	565.843	1129.6714	2	0.0008	11	0	GluC
H12_MOUSE, H13_MOUSE, H14_MOUSE	Histone H1	LITKAVAASKE	215	8.70E-03	565.843	1129.6714	2	0.0008	11	0	GluC
H12_MOUSE, H13_MOUSE, H14_MOUSE	Histone H1	LITKAVAASKE	215	3.60E-03	377.5644	1129.6715	3	0.0008	11	0	GluC
H12_MOUSE, H13_MOUSE, H14_MOUSE	Histone H1	LITKAVAASKE	215	2.60E-04	565.843	1129.6714	2	0.0008	11	0	GluC
H12_MOUSE, H13_MOUSE, H14_MOUSE	Histone H1	LITKAVAASKE	215	5.40E-05	377.5644	1129.6715	3	0.0008	11	0	GluC
H12_MOUSE, H13_MOUSE, H14_MOUSE	Histone H1	LITKAVAASKE	215	8.30E-06	377.5644	1129.6715	3	0.0008	11	0	GluC
H12_MOUSE, H13_MOUSE, H14_MOUSE	Histone H1	LITKAVAASKE	215	4.20E-06	565.843	1129.6714	2	0.0008	11	0	GluC
H12_MOUSE, H13_MOUSE, H14_MOUSE	Histone H1	LITKAVAASKE	215	4.00E-06	565.843	1129.6714	2	0.0008	11	0	GluC
H12_MOUSE, H13_MOUSE, H14_MOUSE	Histone H1	LITKAVAASKE	215	7.90E-07	377.5644	1129.6715	3	0.0008	11	0	GluC
H12_MOUSE, H13_MOUSE, H14_MOUSE	Histone H1	LITKAVAASKE	215	1.20E-08	377.5644	1129.6715	3	0.0008	11	0	GluC
H12_MOUSE, H13_MOUSE, H14_MOUSE	Histone H1	LITKAVAASKE	215	9.40E-03	565.8429	1129.6713	2	0.0007	11	0	GluC
H12_MOUSE, H13_MOUSE, H14_MOUSE	Histone H1	LITKAVAASKE	215	9.20E-03	565.8429	1129.6713	2	0.0007	11	0	GluC
H12_MOUSE, H13_MOUSE, H14_MOUSE	Histone H1	LITKAVAASKE	215	8.50E-03	565.843	1129.6714	2	0.0007	11	0	GluC
H12_MOUSE, H13_MOUSE, H14_MOUSE	Histone H1	LITKAVAASKE	215	5.60E-03	377.5644	1129.6714	3	0.0007	11	0	GluC
H12_MOUSE, H13_MOUSE, H14_MOUSE	Histone H1	LITKAVAASKE	215	1.90E-03	565.8429	1129.6713	2	0.0007	11	0	GluC
H12_MOUSE, H13_MOUSE, H14_MOUSE	Histone H1	LITKAVAASKE	215	9.30E-04	377.5644	1129.6714	3	0.0007	11	0	GluC
H12_MOUSE, H13_MOUSE, H14_MOUSE	Histone H1	LITKAVAASKE	215	4.20E-06	565.8429	1129.6713	2	0.0007	11	0	GluC
H12_MOUSE, H13_MOUSE, H14_MOUSE	Histone H1	LITKAVAASKE	215	5.30E-07	565.8429	1129.6713	2	0.0007	11	0	GluC
H12_MOUSE, H13_MOUSE, H14_MOUSE	Histone H1	LITKAVAASKE	215	3.40E-08	377.5644	1129.6714	3	0.0007	11	0	GluC
H12_MOUSE, H13_MOUSE, H14_MOUSE	Histone H1	LITKAVAASKE	215	2.20E-03	565.8429	1129.6712	2	0.0006	11	0	GluC
H12_MOUSE, H13_MOUSE, H14_MOUSE	Histone H1	LITKAVAASKE	215	1.70E-04	377.5643	1129.6712	3	0.0006	11	0	GluC
H12_MOUSE, H13_MOUSE, H14_MOUSE	Histone H1	LITKAVAASKE	215	1.50E-04	565.8429	1129.6712	2	0.0006	11	0	GluC
H12_MOUSE, H13_MOUSE, H14_MOUSE	Histone H1	LITKAVAASKE	215	8.30E-05	377.5643	1129.6712	3	0.0006	11	0	GluC
H12_MOUSE, H13_MOUSE, H14_MOUSE	Histone H1	LITKAVAASKE	215	5.10E-05	565.8429	1129.6713	2	0.0006	11	0	GluC
H12_MOUSE, H13_MOUSE, H14_MOUSE	Histone H1	LITKAVAASKE	215	2.20E-05	565.8429	1129.6713	2	0.0006	11	0	GluC
H12_MOUSE, H13_MOUSE, H14_MOUSE	Histone H1	LITKAVAASKE	215	3.40E-09	377.5643	1129.6712	3	0.0006	11	0	GluC
H12_MOUSE, H13_MOUSE, H14_MOUSE	Histone H1	LITKAVAASKE	215	3.90E-03	565.8428	1129.6711	2	0.0005	11	0	GluC
H12_MOUSE, H13_MOUSE, H14_MOUSE	Histone H1	LITKAVAASKE	215	2.70E-03	377.5643	1129.6711	3	0.0005	11	0	GluC
H12_MOUSE, H13_MOUSE, H14_MOUSE	Histone H1	LITKAVAASKE	215	6.20E-04	377.5643	1129.6711	3	0.0005	11	0	GluC
H12_MOUSE, H13_MOUSE, H14_MOUSE	Histone H1	LITKAVAASKE	215	9.10E-05	565.8429	1129.6712	2	0.0005	11	0	GluC
H12_MOUSE, H13_MOUSE, H14_MOUSE	Histone H1	LITKAVAASKE	215	3.40E-05	377.5643	1129.6712	3	0.0005	11	0	GluC
H12_MOUSE, H13_MOUSE, H14_MOUSE	Histone H1	LITKAVAASKE	215	5.80E-06	565.8428	1129.6711	2	0.0005	11	0	GluC
H12_MOUSE, H13_MOUSE, H14_MOUSE	Histone H1	LITKAVAASKE	215	6.10E-03	377.5643	1129.6711	3	0.0004	11	0	GluC
H12_MOUSE, H13_MOUSE, H14_MOUSE	Histone H1	LITKAVAASKE	215	1.00E-03	565.8428	1129.6710	2	0.0004	11	0	GluC
H12_MOUSE, H13_MOUSE, H14_MOUSE	Histone H1	LITKAVAASKE	215	1.10E-04	565.8428	1129.6710	2	0.0004	11	0	GluC
H12_MOUSE, H13_MOUSE, H14_MOUSE	Histone H1	LITKAVAASKE	215	2.10E-05	377.5643	1129.6710	3	0.0004	11	0	GluC
H12_MOUSE, H13_MOUSE, H14_MOUSE	Histone H1	LITKAVAASKE	215	1.10E-05	377.5643	1129.6710	3	0.0004	11	0	GluC
H12_MOUSE, H13_MOUSE, H14_MOUSE	Histone H1	LITKAVAASKE	215	6.60E-07	377.5643	1129.6711	3	0.0004	11	0	GluC
H12_MOUSE, H13_MOUSE, H14_MOUSE	Histone H1	LITKAVAASKE	215	1.90E-07	377.5643	1129.6711	3	0.0004	11	0	GluC
H12_MOUSE, H13_MOUSE, H14_MOUSE	Histone H1	LITKAVAASKE	215	1.80E-08	377.5643	1129.6710	3	0.0004	11	0	GluC
H12_MOUSE, H13_MOUSE, H14_MOUSE	Histone H1	LITKAVAASKE	215	6.50E-03	565.8427	1129.6709	2	0.0003	11	0	GluC
H12_MOUSE, H13_MOUSE, H14_MOUSE	Histone H1	LITKAVAASKE	215	4.10E-03	377.5642	1129.6709	3	0.0003	11	0	GluC
H12_MOUSE, H13_MOUSE, H14_MOUSE	Histone H1	LITKAVAASKE	215	4.00E-03	377.5643	1129.6710	3	0.0003	11	0	GluC
H12_MOUSE, H13_MOUSE, H14_MOUSE	Histone H1	LITKAVAASKE	215	3.90E-03	377.5643	1129.6710	3	0.0003	11	0	GluC
H12_MOUSE, H13_MOUSE, H14_MOUSE	Histone H1	LITKAVAASKE	215	1.20E-03	377.5643	1129.6710	3	0.0003	11	0	GluC
H12_MOUSE, H13_MOUSE, H14_MOUSE	Histone H1	LITKAVAASKE	215	1.20E-03	377.5643	1129.6709	3	0.0003	11	0	GluC
H12_MOUSE, H13_MOUSE, H14_MOUSE	Histone H1	LITKAVAASKE	215	8.10E-04	565.8428	1129.6709	2	0.0003	11	0	GluC
H12_MOUSE, H13_MOUSE, H14_MOUSE	Histone H1	LITKAVAASKE	215	3.90E-04	377.5643	1129.6709	3	0.0003	11	0	GluC
H12_MOUSE, H13_MOUSE, H14_MOUSE	Histone H1	LITKAVAASKE	215	3.50E-04	565.8428	1129.6710	2	0.0003	11	0	GluC
H12_MOUSE, H13_MOUSE, H14_MOUSE	Histone H1	LITKAVAASKE	215	1.60E-04	377.5642	1129.6709	3	0.0003	11	0	GluC
H12_MOUSE, H13_MOUSE, H14_MOUSE	Histone H1	LITKAVAASKE	215	1.30E-04	565.8428	1129.6709	2	0.0003	11	0	GluC
H12_MOUSE, H13_MOUSE, H14_MOUSE	Histone H1	LITKAVAASKE	215	1.20E-04	377.5643	1129.6709	3	0.0003	11	0	GluC
H12_MOUSE, H13_MOUSE, H14_MOUSE	Histone H1	LITKAVAASKE	215	8.50E-06	377.5643	1129.6710	3	0.0003	11	0	GluC
H12_MOUSE, H13_MOUSE, H14_MOUSE	Histone H1	LITKAVAASKE	215	8.00E-06	377.5643	1129.6709	3	0.0003	11	0	GluC
H12_MOUSE, H13_MOUSE, H14_MOUSE	Histone H1	LITKAVAASKE	215	3.70E-06	565.8427	1129.6709	2	0.0003	11	0	GluC
H12_MOUSE, H13_MOUSE, H14_MOUSE	Histone H1	LITKAVAASKE	215	3.50E-07	377.5643	1129.6710	3	0.0003	11	0	GluC
H12_MOUSE, H13_MOUSE, H14_MOUSE	Histone H1	LITKAVAASKE	215	3.30E-07	565.8427	1129.6709	2	0.0003	11	0	GluC
H12_MOUSE, H13_MOUSE, H14_MOUSE	Histone H1	LITKAVAASKE	215	8.90E-03	377.5642	1129.6708	3	0.0002	11	0	GluC
H12_MOUSE, H13_MOUSE, H14_MOUSE	Histone H1	LITKAVAASKE	215	8.50E-03	565.8427	1129.6709	2	0.0002	11	0	GluC
H12_MOUSE, H13_MOUSE, H14_MOUSE	Histone H1	LITKAVAASKE	215	3.70E-04	565.8427	1129.6709	2	0.0002	11	0	GluC

H12_MOUSE, H13_MOUSE, H14_MOUSE	Histone H1	LITKAVAASKE	215	1.30E-04	377.5642	1129.6708	3	0.0002	11	0	GluC
H12_MOUSE, H13_MOUSE, H14_MOUSE	Histone H1	LITKAVAASKE	215	9.80E-05	377.5642	1129.6708	3	0.0002	11	0	GluC
H12_MOUSE, H13_MOUSE, H14_MOUSE	Histone H1	LITKAVAASKE	215	4.40E-06	565.8427	1129.6708	2	0.0002	11	0	GluC
H12_MOUSE, H13_MOUSE, H14_MOUSE	Histone H1	LITKAVAASKE	215	5.90E-07	377.5642	1129.6708	3	0.0002	11	0	GluC
H12_MOUSE, H13_MOUSE, H14_MOUSE	Histone H1	LITKAVAASKE	215	2.70E-07	377.5642	1129.6708	3	0.0002	11	0	GluC
H12_MOUSE, H13_MOUSE, H14_MOUSE	Histone H1	LITKAVAASKE	215	2.10E-07	377.5642	1129.6709	3	0.0002	11	0	GluC
H12_MOUSE, H13_MOUSE, H14_MOUSE	Histone H1	LITKAVAASKE	215	3.70E-08	377.5642	1129.6709	3	0.0002	11	0	GluC
H12_MOUSE, H13_MOUSE, H14_MOUSE	Histone H1	LITKAVAASKE	215	7.00E-03	377.5642	1129.6707	3	0.0001	11	0	GluC
H12_MOUSE, H13_MOUSE, H14_MOUSE	Histone H1	LITKAVAASKE	215	1.50E-03	377.5642	1129.6707	3	0.0001	11	0	GluC
H12_MOUSE, H13_MOUSE, H14_MOUSE	Histone H1	LITKAVAASKE	215	4.10E-08	377.5642	1129.6707	3	0.0001	11	0	GluC
H12_MOUSE, H13_MOUSE, H14_MOUSE	Histone H1	LITKAVAASKE	215	3.20E-03	565.8426	1129.6706	2	0.0000	11	0	GluC
H12_MOUSE, H13_MOUSE, H14_MOUSE	Histone H1	LITKAVAASKE	215	3.00E-03	565.8426	1129.6706	2	0.0000	11	0	GluC
H12_MOUSE, H13_MOUSE, H14_MOUSE	Histone H1	LITKAVAASKE	215	1.80E-03	377.5642	1129.6707	3	0.0000	11	0	GluC
H12_MOUSE, H13_MOUSE, H14_MOUSE	Histone H1	LITKAVAASKE	215	9.20E-04	565.8426	1129.6706	2	0.0000	11	0	GluC
H12_MOUSE, H13_MOUSE, H14_MOUSE	Histone H1	LITKAVAASKE	215	8.20E-04	565.8426	1129.6706	2	0.0000	11	0	GluC
H12_MOUSE, H13_MOUSE, H14_MOUSE	Histone H1	LITKAVAASKE	215	2.30E-04	377.5642	1129.6707	3	0.0000	11	0	GluC
H12_MOUSE, H13_MOUSE, H14_MOUSE	Histone H1	LITKAVAASKE	215	1.70E-04	565.8426	1129.6707	2	0.0000	11	0	GluC
H12_MOUSE, H13_MOUSE, H14_MOUSE	Histone H1	LITKAVAASKE	215	1.00E-04	377.5641	1129.6706	3	0.0000	11	0	GluC
H12_MOUSE, H13_MOUSE, H14_MOUSE	Histone H1	LITKAVAASKE	215	1.50E-06	377.5641	1129.6706	3	0.0000	11	0	GluC
H12_MOUSE, H13_MOUSE, H14_MOUSE	Histone H1	LITKAVAASKE	215	5.90E-07	565.8426	1129.6706	2	0.0000	11	0	GluC
H12_MOUSE, H13_MOUSE, H14_MOUSE	Histone H1	LITKAVAASKE	215	1.10E-08	377.5641	1129.6706	3	0.0000	11	0	GluC
H12_MOUSE, H13_MOUSE, H14_MOUSE	Histone H1	LITKAVAASKE	215	2.20E-03	377.5641	1129.6706	3	-0.0001	11	0	GluC
H12_MOUSE, H13_MOUSE, H14_MOUSE	Histone H1	LITKAVAASKE	215	1.50E-04	377.5641	1129.6706	3	-0.0001	11	0	GluC
H12_MOUSE, H13_MOUSE, H14_MOUSE	Histone H1	LITKAVAASKE	215	5.40E-06	377.5641	1129.6706	3	-0.0001	11	0	GluC
H12_MOUSE, H13_MOUSE, H14_MOUSE	Histone H1	LITKAVAASKE	215	1.20E-06	377.5641	1129.6705	3	-0.0001	11	0	GluC
H12_MOUSE, H13_MOUSE, H14_MOUSE	Histone H1	LITKAVAASKE	215	1.20E-06	377.5641	1129.6705	3	-0.0001	11	0	GluC
H12_MOUSE, H13_MOUSE, H14_MOUSE	Histone H1	LITKAVAASKE	215	4.40E-07	377.5641	1129.6705	3	-0.0001	11	0	GluC
H12_MOUSE, H13_MOUSE, H14_MOUSE	Histone H1	LITKAVAASKE	215	3.40E-07	565.8426	1129.6706	2	-0.0001	11	0	GluC
H12_MOUSE, H13_MOUSE, H14_MOUSE	Histone H1	LITKAVAASKE	215	2.70E-04	377.5641	1129.6704	3	-0.0002	11	0	GluC
H12_MOUSE, H13_MOUSE, H14_MOUSE	Histone H1	LITKAVAASKE	215	8.30E-05	565.8425	1129.6705	2	-0.0002	11	0	GluC
H12_MOUSE, H13_MOUSE, H14_MOUSE	Histone H1	LITKAVAASKE	215	5.80E-05	377.5641	1129.6704	3	-0.0002	11	0	GluC
H12_MOUSE, H13_MOUSE, H14_MOUSE	Histone H1	LITKAVAASKE	215	1.30E-07	377.5641	1129.6705	3	-0.0002	11	0	GluC
H12_MOUSE, H13_MOUSE, H14_MOUSE	Histone H1	LITKAVAASKE	215	3.20E-09	377.5641	1129.6705	3	-0.0002	11	0	GluC
H12_MOUSE, H13_MOUSE, H14_MOUSE	Histone H1	LITKAVAASKE	215	3.80E-03	565.8424	1129.6703	2	-0.0003	11	0	GluC
H12_MOUSE, H13_MOUSE, H14_MOUSE	Histone H1	LITKAVAASKE	215	3.40E-05	377.5641	1129.6704	3	-0.0003	11	0	GluC
H12_MOUSE, H13_MOUSE, H14_MOUSE	Histone H1	LITKAVAASKE	215	1.10E-05	377.5641	1129.6704	3	-0.0003	11	0	GluC
H12_MOUSE, H13_MOUSE, H14_MOUSE	Histone H1	LITKAVAASKE	215	4.30E-06	377.5641	1129.6703	3	-0.0003	11	0	GluC
H12_MOUSE, H13_MOUSE, H14_MOUSE	Histone H1	LITKAVAASKE	215	1.40E-06	565.8424	1129.6703	2	-0.0003	11	0	GluC
H12_MOUSE, H13_MOUSE, H14_MOUSE	Histone H1	LITKAVAASKE	215	2.00E-04	565.8424	1129.6702	2	-0.0004	11	0	GluC
H12_MOUSE, H13_MOUSE, H14_MOUSE	Histone H1	LITKAVAASKE	215	6.80E-05	377.564	1129.6702	3	-0.0004	11	0	GluC
H12_MOUSE, H13_MOUSE, H14_MOUSE	Histone H1	LITKAVAASKE	215	1.80E-05	565.8424	1129.6702	2	-0.0004	11	0	GluC
H12_MOUSE, H13_MOUSE, H14_MOUSE	Histone H1	LITKAVAASKE	215	1.40E-05	377.564	1129.6703	3	-0.0004	11	0	GluC
H12_MOUSE, H13_MOUSE, H14_MOUSE	Histone H1	LITKAVAASKE	215	9.50E-07	377.564	1129.6702	3	-0.0004	11	0	GluC
H12_MOUSE, H13_MOUSE, H14_MOUSE	Histone H1	LITKAVAASKE	215	4.70E-07	377.564	1129.6703	3	-0.0004	11	0	GluC
H12_MOUSE, H13_MOUSE, H14_MOUSE	Histone H1	LITKAVAASKE	215	2.30E-07	377.564	1129.6703	3	-0.0004	11	0	GluC
H12_MOUSE, H13_MOUSE, H14_MOUSE	Histone H1	LITKAVAASKE	215	7.30E-03	565.8424	1129.6702	2	-0.0005	11	0	GluC
H12_MOUSE, H13_MOUSE, H14_MOUSE	Histone H1	LITKAVAASKE	215	4.20E-05	377.564	1129.6701	3	-0.0005	11	0	GluC
H12_MOUSE, H13_MOUSE, H14_MOUSE	Histone H1	LITKAVAASKE	215	2.30E-05	377.564	1129.6701	3	-0.0005	11	0	GluC
H12_MOUSE, H13_MOUSE, H14_MOUSE	Histone H1	LITKAVAASKE	215	3.40E-07	377.564	1129.6702	3	-0.0005	11	0	GluC
H12_MOUSE, H13_MOUSE, H14_MOUSE	Histone H1	LITKAVAASKE	215	3.90E-03	565.8423	1129.6700	2	-0.0006	11	0	GluC
H12_MOUSE, H13_MOUSE, H14_MOUSE	Histone H1	LITKAVAASKE	215	1.10E-03	377.5639	1129.6700	3	-0.0006	11	0	GluC
H12_MOUSE, H13_MOUSE, H14_MOUSE	Histone H1	LITKAVAASKE	215	3.10E-05	565.8423	1129.6700	2	-0.0006	11	0	GluC
H12_MOUSE, H13_MOUSE, H14_MOUSE	Histone H1	LITKAVAASKE	215	2.00E-05	565.8423	1129.6701	2	-0.0006	11	0	GluC
H12_MOUSE, H13_MOUSE, H14_MOUSE	Histone H1	LITKAVAASKE	215	1.40E-05	565.8423	1129.6701	2	-0.0006	11	0	GluC
H12_MOUSE, H13_MOUSE, H14_MOUSE	Histone H1	LITKAVAASKE	215	4.60E-03	377.5639	1129.6700	3	-0.0007	11	0	GluC
H12_MOUSE, H13_MOUSE, H14_MOUSE	Histone H1	LITKAVAASKE	215	9.10E-06	377.5639	1129.6699	3	-0.0007	11	0	GluC
H12_MOUSE, H13_MOUSE, H14_MOUSE	Histone H1	LITKAVAASKE	215	1.30E-03	565.8422	1129.6699	2	-0.0008	11	0	GluC
H12_MOUSE, H13_MOUSE, H14_MOUSE	Histone H1	LITKAVAASKE	215	4.50E-04	377.5639	1129.6698	3	-0.0008	11	0	GluC
H12_MOUSE, H13_MOUSE, H14_MOUSE	Histone H1	LITKAVAASKE	215	5.40E-03	377.5638	1129.6697	3	-0.0009	11	0	GluC
H12_MOUSE, H13_MOUSE, H14_MOUSE	Histone H1	LITKAVAASKE	215	7.20E-04	565.8421	1129.6697	2	-0.0009	11	0	GluC
H12_MOUSE, H13_MOUSE, H14_MOUSE	Histone H1	LITKAVAASKE	215	5.30E-05	377.5639	1129.6697	3	-0.0009	11	0	GluC
H12_MOUSE, H13_MOUSE, H14_MOUSE	Histone H1	LITKAVAASKE	215	1.60E-04	565.8421	1129.6696	2	-0.0010	11	0	GluC
H12_MOUSE, H13_MOUSE, H14_MOUSE	Histone H1	LITKAVAASKE	215	5.60E-05	565.8421	1129.6696	2	-0.0010	11	0	GluC
H12_MOUSE, H13_MOUSE, H14_MOUSE	Histone H1	LITKAVAASKE	215	2.30E-08	377.5638	1129.6695	3	-0.0012	11	0	GluC
H12_MOUSE, H13_MOUSE, H14_MOUSE	Histone H1	LITKAVAASKE	215	1.40E-05	377.5637	1129.6693	3	-0.0014	11	0	GluC
H12_MOUSE, H13_MOUSE, H14_MOUSE	Histone H1	LITKAVAASKE	215	3.50E-03	565.8418	1129.6691	2	-0.0015	11	0	GluC
H12_MOUSE, H13_MOUSE, H14_MOUSE	Histone H1	LITKAVAASKE	215	8.00E-06	377.5637	1129.6692	3	-0.0015	11	0	GluC
H12_MOUSE, H13_MOUSE, H14_MOUSE	Histone H1	LITKAVAASKE	215	1.50E-06	565.8418	1129.6691	2	-0.0015	11	0	GluC
H12_MOUSE, H13_MOUSE, H14_MOUSE	Histone H1	LITKAVAASKE	215	2.50E-05	565.8417	1129.6688	2	-0.0018	11	0	GluC
H12_MOUSE, H13_MOUSE, H14_MOUSE	Histone H1	KPAGAAKKPK	2	1.40E-03	332.5511	994.6315	3	0.0028	10	1	Semi-tryptic
H12_MOUSE, H13_MOUSE, H14_MOUSE	Histone H1	KPAGAAKKPK	2	1.10E-03	498.3219	994.6293	2	0.0006	10	1	Semi-tryptic
H12_MOUSE, H13_MOUSE, H14_MOUSE	Histone H1	AKKPAGAAK	10	6.80E-04	421.2684	840.5222	2	0.0041	9	1	Semi-tryptic
H12_MOUSE, H13_MOUSE, H14_MOUSE	Histone H1	AKKPAGAAK	10	6.60E-04	421.2682	840.5218	2	0.0037	9	1	Semi-tryptic
H12_MOUSE, H13_MOUSE, H14_MOUSE	Histone H1	AKKPAGAAK	10	6.70E-04	421.268	840.5214	2	0.0033	9	1	Semi-tryptic

H12_MOUSE, H13_MOUSE, H14_MOUSE	Histone H1	AKKPAGAAK	10	3.50E-04	421.2679	840.5212	2	0.0032	9	1	Semi-tryptic
H12_MOUSE, H13_MOUSE, H14_MOUSE	Histone H1	AKKPAGAAK	10	1.50E-03	421.2671	840.5197	2	0.0016	9	1	Semi-tryptic
H12_MOUSE, H13_MOUSE, H14_MOUSE	Histone H1	AKKPAGAAK	10	1.30E-03	421.2669	840.5192	2	0.0011	9	1	Semi-tryptic
H12_MOUSE, H13_MOUSE, H14_MOUSE	Histone H1	AKKPAGAAK	10	7.40E-05	421.2668	840.5190	2	0.0010	9	1	Semi-tryptic
H12_MOUSE, H13_MOUSE, H14_MOUSE	Histone H1	AKKPAGAAK	10	7.40E-05	421.2668	840.5191	2	0.0010	9	1	Semi-tryptic
H12_MOUSE, H13_MOUSE, H14_MOUSE	Histone H1	AKKPAGAAK	10	7.10E-03	421.2665	840.5185	2	0.0004	9	1	Semi-tryptic
H12_MOUSE, H13_MOUSE, H14_MOUSE	Histone H1	AKKPAGAAK	10	4.30E-04	421.2663	840.5179	2	-0.0001	9	1	Semi-tryptic
H12_MOUSE, H13_MOUSE, H14_MOUSE	Histone H1	AVAASKER	12	1.90E-04	416.2382	830.4618	2	0.0008	8	1	Semi-tryptic
H12_MOUSE, H13_MOUSE, H14_MOUSE	Histone H1	AVAASKER	12	1.30E-04	416.238	830.4614	2	0.0005	8	1	Semi-tryptic
H12_MOUSE, H13_MOUSE, H14_MOUSE	Histone H1	AVAASKER	12	9.90E-04	416.238	830.4614	2	0.0004	8	1	Semi-tryptic
H12_MOUSE, H13_MOUSE, H14_MOUSE	Histone H1	AVAASKER	12	1.30E-04	416.238	830.4614	2	0.0004	8	1	Semi-tryptic
H12_MOUSE, H13_MOUSE, H14_MOUSE	Histone H1	AVAASKER	12	3.00E-03	416.2379	830.4613	2	0.0003	8	1	Semi-tryptic
H12_MOUSE, H13_MOUSE, H14_MOUSE	Histone H1	AVAASKER	12	5.90E-03	416.2379	830.4612	2	0.0002	8	1	Semi-tryptic
H12_MOUSE, H13_MOUSE, H14_MOUSE	Histone H1	AVAASKER	12	1.70E-06	416.2378	830.4611	2	0.0001	8	1	Semi-tryptic
H12_MOUSE, H13_MOUSE, H14_MOUSE	Histone H1	AVAASKER	12	1.10E-03	416.2378	830.4610	2	0.0000	8	1	Semi-tryptic
H12_MOUSE, H13_MOUSE, H14_MOUSE	Histone H1	AVAASKER	12	1.00E-02	416.2377	830.4609	2	-0.0001	8	1	Semi-tryptic
H12_MOUSE, H13_MOUSE, H14_MOUSE	Histone H1	AVAASKER	12	3.00E-04	416.2377	830.4609	2	-0.0001	8	1	Semi-tryptic
H12_MOUSE, H13_MOUSE, H14_MOUSE	Histone H1	AVAASKER	12	1.30E-05	416.2377	830.4608	2	-0.0001	8	1	Semi-tryptic
H12_MOUSE, H13_MOUSE, H14_MOUSE	Histone H1	AVAASKER	12	4.30E-05	416.2377	830.4608	2	-0.0002	8	1	Semi-tryptic
H12_MOUSE, H13_MOUSE, H14_MOUSE, H15_MOUSE	Histone H1	GTGASGSFKLNKK	14	6.70E-05	647.8604	1293.7063	2	0.0022	13	2	Semi-tryptic
H12_MOUSE, H13_MOUSE, H14_MOUSE, H15_MOUSE	Histone H1	GTGASGSFKLNKK	14	5.40E-06	647.8604	1293.7063	2	0.0022	13	2	Semi-tryptic
H12_MOUSE, H13_MOUSE, H14_MOUSE, H15_MOUSE	Histone H1	GTGASGSFKLNKK	14	5.10E-04	647.8603	1293.7060	2	0.0019	13	2	Semi-tryptic
H12_MOUSE, H13_MOUSE, H14_MOUSE, H15_MOUSE	Histone H1	GTGASGSFKLNKK	14	6.30E-06	647.8601	1293.7056	2	0.0015	13	2	Semi-tryptic
H12_MOUSE, H13_MOUSE, H14_MOUSE, H15_MOUSE	Histone H1	GTGASGSFKLNKK	14	4.60E-08	432.2425	1293.7056	3	0.0015	13	2	Semi-tryptic
H12_MOUSE, H13_MOUSE, H14_MOUSE, H15_MOUSE	Histone H1	GTGASGSFKLNKK	14	3.70E-09	432.2425	1293.7056	3	0.0015	13	2	Semi-tryptic
H12_MOUSE, H13_MOUSE, H14_MOUSE, H15_MOUSE	Histone H1	GTGASGSFKLNKK	14	6.50E-05	647.86	1293.7054	2	0.0013	13	2	Semi-tryptic
H12_MOUSE, H13_MOUSE, H14_MOUSE, H15_MOUSE	Histone H1	GTGASGSFKLNKK	14	1.70E-06	432.2421	1293.7046	3	0.0005	13	2	Semi-tryptic
H12_MOUSE, H13_MOUSE, H14_MOUSE, H15_MOUSE	Histone H1	GTGASGSFKLNKK	14	1.40E-07	432.2421	1293.7046	3	0.0005	13	2	Semi-tryptic
H12_MOUSE, H13_MOUSE, H14_MOUSE, H15_MOUSE	Histone H1	GTGASGSFKLNKK	14	1.10E-03	432.2421	1293.7044	3	0.0004	13	2	Semi-tryptic
H12_MOUSE, H13_MOUSE, H14_MOUSE, H15_MOUSE	Histone H1	GTGASGSFKLNKK	14	8.80E-06	432.2421	1293.7044	3	0.0004	13	2	Semi-tryptic
H12_MOUSE, H13_MOUSE, H14_MOUSE, H15_MOUSE	Histone H1	GTGASGSFKLNKK	14	4.80E-03	647.8591	1293.7037	2	-0.0003	13	2	Semi-tryptic
H12_MOUSE, H13_MOUSE, H14_MOUSE, H15_MOUSE	Histone H1	GTGASGSFKLNKK	14	1.40E-04	647.8591	1293.7037	2	-0.0003	13	2	Semi-tryptic
H12_MOUSE, H13_MOUSE, H14_MOUSE, H15_MOUSE	Histone H1	GTGASGSFKLNKK	14	3.20E-06	647.859	1293.7035	2	-0.0006	13	2	Semi-tryptic
H12_MOUSE, H13_MOUSE, H14_MOUSE, H15_MOUSE	Histone H1	GTGASGSFKLNK	18	6.40E-06	583.8104	1165.6062	2	-0.0029	12	1	Semi-tryptic
H12_MOUSE, H13_MOUSE, H14_MOUSE, H15_MOUSE	Histone H1	GTGASGSFKLNK	18	6.30E-07	583.8104	1165.6062	2	-0.0029	12	1	Semi-tryptic
H12_MOUSE, H13_MOUSE, H14_MOUSE, H15_MOUSE	Histone H1	GTGASGSFKLNK	18	2.00E-06	583.8102	1165.6058	2	-0.0033	12	1	Semi-tryptic
H12_MOUSE, H13_MOUSE, H14_MOUSE, H15_MOUSE	Histone H1	GTGASGSFKLNK	18	6.10E-07	389.5443	1165.6110	3	0.0019	12	1	Semi-tryptic
H12_MOUSE, H13_MOUSE, H14_MOUSE, H15_MOUSE	Histone H1	GTGASGSFKLNK	18	1.60E-07	389.5443	1165.6111	3	0.0019	12	1	Semi-tryptic
H12_MOUSE, H13_MOUSE, H14_MOUSE, H15_MOUSE	Histone H1	GTGASGSFKLNK	18	9.80E-08	389.5443	1165.6110	3	0.0019	12	1	Semi-tryptic
H12_MOUSE, H13_MOUSE, H14_MOUSE, H15_MOUSE	Histone H1	GTGASGSFKLNK	18	2.80E-08	583.8128	1165.6111	2	0.0019	12	1	Semi-tryptic
H12_MOUSE, H13_MOUSE, H14_MOUSE, H15_MOUSE	Histone H1	GTGASGSFKLNK	18	1.90E-08	389.5443	1165.6111	3	0.0019	12	1	Semi-tryptic
H12_MOUSE, H13_MOUSE, H14_MOUSE, H15_MOUSE	Histone H1	GTGASGSFKLNK	18	4.90E-05	583.8121	1165.6096	2	0.0005	12	1	Semi-tryptic
H12_MOUSE, H13_MOUSE, H14_MOUSE, H15_MOUSE	Histone H1	GTGASGSFKLNK	18	8.50E-07	583.812	1165.6094	2	0.0003	12	1	Semi-tryptic
H12_MOUSE, H13_MOUSE, H14_MOUSE, H15_MOUSE	Histone H1	GTGASGSFKLNK	18	3.70E-05	583.8119	1165.6093	2	0.0001	12	1	Semi-tryptic
H12_MOUSE, H13_MOUSE, H14_MOUSE, H15_MOUSE	Histone H1	GTGASGSFKLNK	18	8.70E-07	583.8119	1165.6093	2	0.0001	12	1	Semi-tryptic
H12_MOUSE, H13_MOUSE, H14_MOUSE, H15_MOUSE	Histone H1	GTGASGSFKLNK	18	2.60E-03	389.5436	1165.6089	3	-0.0003	12	1	Semi-tryptic
H12_MOUSE, H13_MOUSE, H14_MOUSE, H15_MOUSE	Histone H1	GTGASGSFKLNK	18	2.00E-04	583.8116	1165.6087	2	-0.0004	12	1	Semi-tryptic
H12_MOUSE, H13_MOUSE, H14_MOUSE, H15_MOUSE	Histone H1	GTGASGSFKLNK	18	4.30E-08	583.8116	1165.6087	2	-0.0004	12	1	Semi-tryptic
H12_MOUSE, H13_MOUSE, H14_MOUSE, H15_MOUSE	Histone H1	GTGASGSFKLNK	18	4.70E-05	583.8116	1165.6086	2	-0.0006	12	1	Semi-tryptic
H12_MOUSE, H13_MOUSE, H14_MOUSE, H15_MOUSE	Histone H1	GTGASGSFKLNK	18	4.90E-06	583.8116	1165.6086	2	-0.0006	12	1	Semi-tryptic
H12_MOUSE, H13_MOUSE, H14_MOUSE, H15_MOUSE	Histone H1	GTGASGSFKLNK	18	3.30E-06	583.8113	1165.6080	2	-0.0012	12	1	Semi-tryptic
H12_MOUSE, H13_MOUSE, H14_MOUSE, H15_MOUSE	Histone H1	IKLGLKSLVSK	7	9.90E-04	593.4021	1184.7896	2	0.0040	11	2	Semi-tryptic
H12_MOUSE, H13_MOUSE, H14_MOUSE, H15_MOUSE	Histone H1	IKLGLKSLVSK	7	5.40E-07	593.4005	1184.7864	2	0.0008	11	2	Semi-tryptic
H12_MOUSE, H13_MOUSE, H14_MOUSE, H15_MOUSE	Histone H1	IKLGLKSLVSK	7	9.00E-04	395.936	1184.7863	3	0.0007	11	2	Semi-tryptic
H12_MOUSE, H13_MOUSE, H14_MOUSE, H15_MOUSE	Histone H1	IKLGLKSLVSK	7	3.20E-03	395.9359	1184.7860	3	0.0003	11	2	Semi-tryptic
H12_MOUSE, H13_MOUSE, H14_MOUSE, H15_MOUSE	Histone H1	IKLGLKSLVSK	7	3.50E-06	593.4001	1184.7856	2	0.0000	11	2	Semi-tryptic
H12_MOUSE, H13_MOUSE, H14_MOUSE, H15_MOUSE	Histone H1	IKLGLKSLVSK	7	7.50E-05	395.9358	1184.7855	3	-0.0001	11	2	Semi-tryptic
H12_MOUSE, H13_MOUSE, H14_MOUSE, H15_MOUSE	Histone H1	IKLGLKSLVSK	7	3.80E-07	593.3995	1184.7844	2	-0.0012	11	2	Semi-tryptic
H12_MOUSE, H13_MOUSE, H14_MOUSE, H15_MOUSE	Histone H1	LGLKSLVSK	2	7.70E-06	472.8121	943.6096	2	0.0030	9	1	Semi-tryptic
H12_MOUSE, H13_MOUSE, H14_MOUSE, H15_MOUSE	Histone H1	LGLKSLVSK	2	1.20E-07	472.8104	943.6063	2	-0.0003	9	1	Semi-tryptic
H13_MOUSE	Histone H1	TAPAAPAAPAPVEKTPVKKKAKKTGAAAGKRKASGPPVSE	6	5.10E-05	966.8149	3863.2305	4	0.0197	40	1	GluC
H13_MOUSE	Histone H1	TAPAAPAAPAPVEKTPVKKKAKKTGAAAGKRKASGPPVSE	6	6.90E-03	773.6533	3863.2303	5	0.0195	40	1	GluC
H13_MOUSE	Histone H1	TAPAAPAAPAPVEKTPVKKKAKKTGAAAGKRKASGPPVSE	6	3.90E-04	773.6532	3863.2299	5	0.0191	40	1	GluC
H13_MOUSE	Histone H1	TAPAAPAAPAPVEKTPVKKKAKKTGAAAGKRKASGPPVSE	6	2.30E-03	552.897	3863.2281	7	0.0173	40	1	GluC
H13_MOUSE	Histone H1	TAPAAPAAPAPVEKTPVKKKAKKTGAAAGKRKASGPPVSE	6	4.70E-04	644.8785	3863.2276	6	0.0168	40	1	GluC
H13_MOUSE	Histone H1	TAPAAPAAPAPVEKTPVKKKAKKTGAAAGKRKASGPPVSE	6	7.50E-03	644.8779	3863.2236	6	0.0127	40	1	GluC
H13_MOUSE	Histone H1	KTPVKKKAKKTGAAAGKRKASGPPVSE	20	5.60E-03	680.9113	2719.6163	4	-0.0021	27	0	GluC
H13_MOUSE	Histone H1	KTPVKKKAKKTGAAAGKRKASGPPVSE	20	6.00E-04	680.9112	2719.6156	4	-0.0028	27	0	GluC
H13_MOUSE	Histone H1	KTPVKKKAKKTGAAAGKRKASGPPVSE	20	1.70E-05	907.552	2719.6342	3	0.0158	27	0	GluC
H13_MOUSE	Histone H1	KTPVKKKAKKTGAAAGKRKASGPPVSE	20	2.80E-08	680.9149	2719.6305	4	0.0121	27	0	GluC
H13_MOUSE	Histone H1	KTPVKKKAKKTGAAAGKRKASGPPVSE	20	1.30E-08	544.9333	2719.6299	5	0.0115	27	0	GluC
H13_MOUSE	Histone H1	KTPVKKKAKKTGAAAGKRKASGPPVSE	20	4.30E-04	680.9147	2719.6298	4	0.0114	27	0	GluC
H13_MOUSE	Histone H1	KTPVKKKAKKTGAAAGKRKASGPPVSE	20	1.80E-03	680.9147	2719.6297	4	0.0113	27	0	GluC
H13_MOUSE	Histone H1	KTPVKKKAKKTGAAAGKRKASGPPVSE	20	9.10E-08	680.9143	2719.6281	4	0.0096	27	0	GluC

H13_MOUSE	Histone H1	KTPVKKKAKKTGAAAGKRKASGPPVSE	20	1.00E-03	680.9129	2719.6226	4	0.0042	27	0	GluC
H13_MOUSE	Histone H1	KTPVKKKAKKTGAAAGKRKASGPPVSE	20	5.00E-03	544.9311	2719.6192	5	0.0008	27	0	GluC
H13_MOUSE	Histone H1	KTPVKKKAKKTGAAAGKRKASGPPVSE	20	5.70E-04	544.9311	2719.6192	5	0.0008	27	0	GluC
H13_MOUSE	Histone H1	KTPVKKKAKKTGAAAGKRKASGPPVSE	20	2.10E-03	544.931	2719.6186	5	0.0002	27	0	GluC
H13_MOUSE	Histone H1	KTPVKKKAKKTGAAAGKRKASGPPVSE	20	1.20E-04	544.9309	2719.6182	5	-0.0002	27	0	GluC
H13_MOUSE	Histone H1	KTPVKKKAKKTGAAAGKRKASGPPVSE	20	1.00E-05	544.9309	2719.6181	5	-0.0003	27	0	GluC
H13_MOUSE	Histone H1	KTPVKKKAKKTGAAAGKRKASGPPVSE	20	1.60E-06	544.9309	2719.6181	5	-0.0003	27	0	GluC
H13_MOUSE	Histone H1	KTPVKKKAKKTGAAAGKRKASGPPVSE	20	1.60E-03	544.9309	2719.6180	5	-0.0004	27	0	GluC
H13_MOUSE	Histone H1	KTPVKKKAKKTGAAAGKRKASGPPVSE	20	9.40E-03	544.9308	2719.6178	5	-0.0006	27	0	GluC
H13_MOUSE	Histone H1	KTPVKKKAKKTGAAAGKRKASGPPVSE	20	5.70E-04	544.9308	2719.6178	5	-0.0006	27	0	GluC
H13_MOUSE	Histone H1	KTPVKKKAKKTGAAAGKRKASGPPVSE	20	1.60E-05	544.9307	2719.6170	5	-0.0014	27	0	GluC
H13_MOUSE	Histone H1	KTPVKKKAKKTGAAAGKRKASGPPVSE	20	2.80E-07	544.9307	2719.6170	5	-0.0014	27	0	GluC
H13_MOUSE	Histone H1	SETAPAAPAAPAPVEKTPVK	2	7.70E-05	644.688	1931.0423	3	0.0059	20	1	Semi-tryptic
H13_MOUSE	Histone H1	SETAPAAPAAPAPVEKTPVK	2	2.40E-08	966.5266	1931.0387	2	0.0023	20	1	Semi-tryptic
H13_MOUSE	Histone H1	SETAPAAPAAPAPVEK	3	3.00E-06	753.897	1505.7795	2	0.0069	16	0	Semi-tryptic
H13_MOUSE	Histone H1	SETAPAAPAAPAPVEK	3	1.20E-06	753.8947	1505.7749	2	0.0024	16	0	Semi-tryptic
H13_MOUSE	Histone H1	SETAPAAPAAPAPVEK	3	1.50E-03	753.8947	1505.7748	2	0.0023	16	0	Semi-tryptic
H13_MOUSE	Histone H1	TGAAAGKR	4	3.90E-05	366.2121	730.4096	2	0.0010	8	1	Semi-tryptic
H13_MOUSE	Histone H1	TGAAAGKR	4	6.00E-06	366.212	730.4095	2	0.0009	8	1	Semi-tryptic
H13_MOUSE	Histone H1	TGAAAGKR	4	4.80E-03	366.2118	730.4091	2	0.0005	8	1	Semi-tryptic
H13_MOUSE	Histone H1	TGAAAGKR	4	1.80E-06	366.2111	730.4077	2	-0.0008	8	1	Semi-tryptic
H13_MOUSE, H14_MOUSE	Histone H1	KAKKPAAAAGAK	6	6.10E-06	556.3537	1110.6928	2	0.0055	12	2	Semi-tryptic
H13_MOUSE, H14_MOUSE	Histone H1	KAKKPAAAAGAK	6	6.10E-04	371.2375	1110.6908	3	0.0036	12	2	Semi-tryptic
H13_MOUSE, H14_MOUSE	Histone H1	KAKKPAAAAGAK	6	1.10E-07	556.3527	1110.6908	2	0.0035	12	2	Semi-tryptic
H13_MOUSE, H14_MOUSE	Histone H1	KAKKPAAAAGAK	6	5.00E-05	371.2364	1110.6875	3	0.0003	12	2	Semi-tryptic
H13_MOUSE, H14_MOUSE	Histone H1	KAKKPAAAAGAK	6	5.30E-07	556.3508	1110.6870	2	-0.0003	12	2	Semi-tryptic
H13_MOUSE, H14_MOUSE	Histone H1	KAKKPAAAAGAK	6	2.00E-04	556.3505	1110.6865	2	-0.0007	12	2	Semi-tryptic
H13_MOUSE, H14_MOUSE	Histone H1	AKKPAAAAGAK	19	1.40E-03	328.5371	982.5894	3	-0.0029	11	1	Semi-tryptic
H13_MOUSE, H14_MOUSE	Histone H1	AKKPAAAAGAK	19	7.30E-04	492.3054	982.5962	2	0.0039	11	1	Semi-tryptic
H13_MOUSE, H14_MOUSE	Histone H1	AKKPAAAAGAK	19	5.50E-05	328.5389	982.5950	3	0.0027	11	1	Semi-tryptic
H13_MOUSE, H14_MOUSE	Histone H1	AKKPAAAAGAK	19	2.90E-06	328.5389	982.5950	3	0.0027	11	1	Semi-tryptic
H13_MOUSE, H14_MOUSE	Histone H1	AKKPAAAAGAK	19	1.20E-04	328.5388	982.5945	3	0.0022	11	1	Semi-tryptic
H13_MOUSE, H14_MOUSE	Histone H1	AKKPAAAAGAK	19	1.10E-05	328.5386	982.5940	3	0.0017	11	1	Semi-tryptic
H13_MOUSE, H14_MOUSE	Histone H1	AKKPAAAAGAK	19	4.60E-07	328.5386	982.5939	3	0.0016	11	1	Semi-tryptic
H13_MOUSE, H14_MOUSE	Histone H1	AKKPAAAAGAK	19	1.10E-04	328.5385	982.5938	3	0.0014	11	1	Semi-tryptic
H13_MOUSE, H14_MOUSE	Histone H1	AKKPAAAAGAK	19	6.80E-06	328.5385	982.5937	3	0.0014	11	1	Semi-tryptic
H13_MOUSE, H14_MOUSE	Histone H1	AKKPAAAAGAK	19	7.10E-07	328.5385	982.5937	3	0.0014	11	1	Semi-tryptic
H13_MOUSE, H14_MOUSE	Histone H1	AKKPAAAAGAK	19	1.00E-06	328.5384	982.5933	3	0.0010	11	1	Semi-tryptic
H13_MOUSE, H14_MOUSE	Histone H1	AKKPAAAAGAK	19	5.50E-04	492.3035	982.5925	2	0.0002	11	1	Semi-tryptic
H13_MOUSE, H14_MOUSE	Histone H1	AKKPAAAAGAK	19	8.10E-04	492.3034	982.5922	2	-0.0001	11	1	Semi-tryptic
H13_MOUSE, H14_MOUSE	Histone H1	AKKPAAAAGAK	19	1.90E-04	492.3034	982.5921	2	-0.0002	11	1	Semi-tryptic
H13_MOUSE, H14_MOUSE	Histone H1	AKKPAAAAGAK	19	1.30E-03	492.3033	982.5920	2	-0.0003	11	1	Semi-tryptic
H13_MOUSE, H14_MOUSE	Histone H1	AKKPAAAAGAK	19	1.00E-06	328.5378	982.5916	3	-0.0007	11	1	Semi-tryptic
H13_MOUSE, H14_MOUSE	Histone H1	AKKPAAAAGAK	19	4.40E-03	492.3028	982.5911	2	-0.0012	11	1	Semi-tryptic
H13_MOUSE, H14_MOUSE	Histone H1	AKKPAAAAGAK	19	1.20E-06	328.5376	982.5908	3	-0.0015	11	1	Semi-tryptic
H13_MOUSE, H14_MOUSE	Histone H1	AKKPAAAAGAK	19	1.70E-04	492.3026	982.5906	2	-0.0017	11	1	Semi-tryptic
H13_MOUSE, H14_MOUSE	Histone H1	KPAAAAGAK	11	1.40E-03	392.7391	783.4636	2	0.0034	9	0	Semi-tryptic
H13_MOUSE, H14_MOUSE	Histone H1	KPAAAAGAK	11	8.30E-03	392.7391	783.4636	2	0.0033	9	0	Semi-tryptic
H13_MOUSE, H14_MOUSE	Histone H1	KPAAAAGAK	11	1.10E-03	392.7389	783.4632	2	0.0030	9	0	Semi-tryptic
H13_MOUSE, H14_MOUSE	Histone H1	KPAAAAGAK	11	5.90E-03	392.7387	783.4629	2	0.0026	9	0	Semi-tryptic
H13_MOUSE, H14_MOUSE	Histone H1	KPAAAAGAK	11	3.40E-03	392.7381	783.4617	2	0.0015	9	0	Semi-tryptic
H13_MOUSE, H14_MOUSE	Histone H1	KPAAAAGAK	11	1.30E-03	392.7378	783.4611	2	0.0009	9	0	Semi-tryptic
H13_MOUSE, H14_MOUSE	Histone H1	KPAAAAGAK	11	6.70E-03	392.7377	783.4608	2	0.0006	9	0	Semi-tryptic
H13_MOUSE, H14_MOUSE	Histone H1	KPAAAAGAK	11	4.30E-04	392.7377	783.4608	2	0.0005	9	0	Semi-tryptic
H13_MOUSE, H14_MOUSE	Histone H1	KPAAAAGAK	11	7.80E-03	392.7375	783.4604	2	0.0002	9	0	Semi-tryptic
H13_MOUSE, H14_MOUSE	Histone H1	KPAAAAGAK	11	7.00E-03	392.7375	783.4604	2	0.0002	9	0	Semi-tryptic
H13_MOUSE, H14_MOUSE	Histone H1	KPAAAAGAK	11	6.40E-03	392.7374	783.4603	2	0.0000	9	0	Semi-tryptic
H13_MOUSE, H14_MOUSE, H15_MOUSE	Histone H1	KNNSRIKLGLKSLVSKGTLVQTKGTGASGSFKLNKKAASGE	49	2.50E-04	701.4046	4202.3840	6	-0.0022	41	0	GluC
H13_MOUSE, H14_MOUSE, H15_MOUSE	Histone H1	KNNSRIKLGLKSLVSKGTLVQTKGTGASGSFKLNKKAASGE	49	5.70E-04	701.4045	4202.3832	6	-0.0030	41	0	GluC
H13_MOUSE, H14_MOUSE, H15_MOUSE	Histone H1	KNNSRIKLGLKSLVSKGTLVQTKGTGASGSFKLNKKAASGE	49	8.80E-04	701.4041	4202.3808	6	-0.0055	41	0	GluC
H13_MOUSE, H14_MOUSE, H15_MOUSE	Histone H1	KNNSRIKLGLKSLVSKGTLVQTKGTGASGSFKLNKKAASGE	49	4.60E-05	601.3473	4202.3800	7	-0.0063	41	0	GluC
H13_MOUSE, H14_MOUSE, H15_MOUSE	Histone H1	KNNSRIKLGLKSLVSKGTLVQTKGTGASGSFKLNKKAASGE	49	9.30E-05	841.4884	4202.4055	5	0.0192	41	0	GluC
H13_MOUSE, H14_MOUSE, H15_MOUSE	Histone H1	KNNSRIKLGLKSLVSKGTLVQTKGTGASGSFKLNKKAASGE	49	2.70E-09	601.3509	4202.4054	7	0.0192	41	0	GluC
H13_MOUSE, H14_MOUSE, H15_MOUSE	Histone H1	KNNSRIKLGLKSLVSKGTLVQTKGTGASGSFKLNKKAASGE	49	5.90E-06	701.4081	4202.4049	6	0.0187	41	0	GluC
H13_MOUSE, H14_MOUSE, H15_MOUSE	Histone H1	KNNSRIKLGLKSLVSKGTLVQTKGTGASGSFKLNKKAASGE	49	7.40E-05	601.3508	4202.4047	7	0.0184	41	0	GluC
H13_MOUSE, H14_MOUSE, H15_MOUSE	Histone H1	KNNSRIKLGLKSLVSKGTLVQTKGTGASGSFKLNKKAASGE	49	1.60E-04	1051.6084	4202.4045	4	0.0183	41	0	GluC
H13_MOUSE, H14_MOUSE, H15_MOUSE	Histone H1	KNNSRIKLGLKSLVSKGTLVQTKGTGASGSFKLNKKAASGE	49	9.00E-03	841.4874	4202.4006	5	0.0143	41	0	GluC
H13_MOUSE, H14_MOUSE, H15_MOUSE	Histone H1	KNNSRIKLGLKSLVSKGTLVQTKGTGASGSFKLNKKAASGE	49	8.40E-03	701.4072	4202.3997	6	0.0134	41	0	GluC
H13_MOUSE, H14_MOUSE, H15_MOUSE	Histone H1	KNNSRIKLGLKSLVSKGTLVQTKGTGASGSFKLNKKAASGE	49	2.80E-05	701.4066	4202.3961	6	0.0099	41	0	GluC
H13_MOUSE, H14_MOUSE, H15_MOUSE	Histone H1	KNNSRIKLGLKSLVSKGTLVQTKGTGASGSFKLNKKAASGE	49	2.50E-03	601.3495	4202.3957	7	0.0095	41	0	GluC
H13_MOUSE, H14_MOUSE, H15_MOUSE	Histone H1	KNNSRIKLGLKSLVSKGTLVQTKGTGASGSFKLNKKAASGE	49	6.30E-05	841.4864	4202.3957	5	0.0095	41	0	GluC
H13_MOUSE, H14_MOUSE, H15_MOUSE	Histone H1	KNNSRIKLGLKSLVSKGTLVQTKGTGASGSFKLNKKAASGE	49	1.00E-03	701.4065	4202.3955	6	0.0093	41	0	GluC
H13_MOUSE, H14_MOUSE, H15_MOUSE	Histone H1	KNNSRIKLGLKSLVSKGTLVQTKGTGASGSFKLNKKAASGE	49	2.80E-04	701.4065	4202.3952	6	0.0090	41	0	GluC
H13_MOUSE, H14_MOUSE, H15_MOUSE	Histone H1	KNNSRIKLGLKSLVSKGTLVQTKGTGASGSFKLNKKAASGE	49	8.40E-06	701.406	4202.3923	6	0.0061	41	0	GluC

H13_MOUSE, H14_MOUSE, H15_MOUSE	Histone H1	KNNSRIKLGLKSLVSKGTLVQTKGTGASGSFKLNKKAASGE	49	7.10E-04	841.4856	4202.3917	5	0.0055	41	0	GluC
H13_MOUSE, H14_MOUSE, H15_MOUSE	Histone H1	KNNSRIKLGLKSLVSKGTLVQTKGTGASGSFKLNKKAASGE	49	1.60E-05	601.3489	4202.3912	7	0.0050	41	0	GluC
H13_MOUSE, H14_MOUSE, H15_MOUSE	Histone H1	KNNSRIKLGLKSLVSKGTLVQTKGTGASGSFKLNKKAASGE	49	4.40E-03	841.4854	4202.3907	5	0.0045	41	0	GluC
H13_MOUSE, H14_MOUSE, H15_MOUSE	Histone H1	KNNSRIKLGLKSLVSKGTLVQTKGTGASGSFKLNKKAASGE	49	2.20E-08	601.3488	4202.3907	7	0.0044	41	0	GluC
H13_MOUSE, H14_MOUSE, H15_MOUSE	Histone H1	KNNSRIKLGLKSLVSKGTLVQTKGTGASGSFKLNKKAASGE	49	5.50E-04	1051.6048	4202.3901	4	0.0039	41	0	GluC
H13_MOUSE, H14_MOUSE, H15_MOUSE	Histone H1	KNNSRIKLGLKSLVSKGTLVQTKGTGASGSFKLNKKAASGE	49	1.80E-04	841.4853	4202.3899	5	0.0037	41	0	GluC
H13_MOUSE, H14_MOUSE, H15_MOUSE	Histone H1	KNNSRIKLGLKSLVSKGTLVQTKGTGASGSFKLNKKAASGE	49	8.10E-03	841.4852	4202.3899	5	0.0036	41	0	GluC
H13_MOUSE, H14_MOUSE, H15_MOUSE	Histone H1	KNNSRIKLGLKSLVSKGTLVQTKGTGASGSFKLNKKAASGE	49	7.40E-06	841.4852	4202.3897	5	0.0034	41	0	GluC
H13_MOUSE, H14_MOUSE, H15_MOUSE	Histone H1	KNNSRIKLGLKSLVSKGTLVQTKGTGASGSFKLNKKAASGE	49	2.40E-04	701.4055	4202.3893	6	0.0031	41	0	GluC
H13_MOUSE, H14_MOUSE, H15_MOUSE	Histone H1	KNNSRIKLGLKSLVSKGTLVQTKGTGASGSFKLNKKAASGE	49	5.10E-03	701.4055	4202.3892	6	0.0030	41	0	GluC
H13_MOUSE, H14_MOUSE, H15_MOUSE	Histone H1	KNNSRIKLGLKSLVSKGTLVQTKGTGASGSFKLNKKAASGE	49	9.10E-03	701.4054	4202.3890	6	0.0028	41	0	GluC
H13_MOUSE, H14_MOUSE, H15_MOUSE	Histone H1	KNNSRIKLGLKSLVSKGTLVQTKGTGASGSFKLNKKAASGE	49	8.50E-05	701.4054	4202.3890	6	0.0028	41	0	GluC
H13_MOUSE, H14_MOUSE, H15_MOUSE	Histone H1	KNNSRIKLGLKSLVSKGTLVQTKGTGASGSFKLNKKAASGE	49	8.70E-04	701.4054	4202.3889	6	0.0026	41	0	GluC
H13_MOUSE, H14_MOUSE, H15_MOUSE	Histone H1	KNNSRIKLGLKSLVSKGTLVQTKGTGASGSFKLNKKAASGE	49	1.40E-03	601.3485	4202.3885	7	0.0023	41	0	GluC
H13_MOUSE, H14_MOUSE, H15_MOUSE	Histone H1	KNNSRIKLGLKSLVSKGTLVQTKGTGASGSFKLNKKAASGE	49	5.50E-04	701.4053	4202.3884	6	0.0022	41	0	GluC
H13_MOUSE, H14_MOUSE, H15_MOUSE	Histone H1	KNNSRIKLGLKSLVSKGTLVQTKGTGASGSFKLNKKAASGE	49	2.00E-03	701.4053	4202.3880	6	0.0017	41	0	GluC
H13_MOUSE, H14_MOUSE, H15_MOUSE	Histone H1	KNNSRIKLGLKSLVSKGTLVQTKGTGASGSFKLNKKAASGE	49	6.30E-04	841.4849	4202.3879	5	0.0017	41	0	GluC
H13_MOUSE, H14_MOUSE, H15_MOUSE	Histone H1	KNNSRIKLGLKSLVSKGTLVQTKGTGASGSFKLNKKAASGE	49	3.90E-04	1051.6042	4202.3877	4	0.0015	41	0	GluC
H13_MOUSE, H14_MOUSE, H15_MOUSE	Histone H1	KNNSRIKLGLKSLVSKGTLVQTKGTGASGSFKLNKKAASGE	49	5.30E-06	1051.6042	4202.3877	4	0.0015	41	0	GluC
H13_MOUSE, H14_MOUSE, H15_MOUSE	Histone H1	KNNSRIKLGLKSLVSKGTLVQTKGTGASGSFKLNKKAASGE	49	6.60E-04	601.3484	4202.3876	7	0.0014	41	0	GluC
H13_MOUSE, H14_MOUSE, H15_MOUSE	Histone H1	KNNSRIKLGLKSLVSKGTLVQTKGTGASGSFKLNKKAASGE	49	1.30E-03	701.4052	4202.3875	6	0.0013	41	0	GluC
H13_MOUSE, H14_MOUSE, H15_MOUSE	Histone H1	KNNSRIKLGLKSLVSKGTLVQTKGTGASGSFKLNKKAASGE	49	5.10E-04	841.4848	4202.3875	5	0.0012	41	0	GluC
H13_MOUSE, H14_MOUSE, H15_MOUSE	Histone H1	KNNSRIKLGLKSLVSKGTLVQTKGTGASGSFKLNKKAASGE	49	5.80E-03	841.4847	4202.3870	5	0.0008	41	0	GluC
H13_MOUSE, H14_MOUSE, H15_MOUSE	Histone H1	KNNSRIKLGLKSLVSKGTLVQTKGTGASGSFKLNKKAASGE	49	5.70E-04	841.4847	4202.3870	5	0.0008	41	0	GluC
H13_MOUSE, H14_MOUSE, H15_MOUSE	Histone H1	KNNSRIKLGLKSLVSKGTLVQTKGTGASGSFKLNKKAASGE	49	6.00E-04	701.4051	4202.3869	6	0.0007	41	0	GluC
H13_MOUSE, H14_MOUSE, H15_MOUSE	Histone H1	KNNSRIKLGLKSLVSKGTLVQTKGTGASGSFKLNKKAASGE	49	7.10E-05	701.4049	4202.3856	6	-0.0006	41	0	GluC
H13_MOUSE, H14_MOUSE, H15_MOUSE	Histone H1	KNNSRIKLGLKSLVSKGTLVQTKGTGASGSFKLNKKAASGE	49	3.30E-04	701.4049	4202.3854	6	-0.0008	41	0	GluC
H13_MOUSE, H14_MOUSE, H15_MOUSE	Histone H1	KNNSRIKLGLKSLVSKGTLVQTKGTGASGSFKLNKKAASGE	49	2.50E-05	601.348	4202.3854	7	-0.0008	41	0	GluC
H13_MOUSE, H14_MOUSE, H15_MOUSE	Histone H1	KNNSRIKLGLKSLVSKGTLVQTKGTGASGSFKLNKKAASGE	49	7.70E-04	841.4843	4202.3852	5	-0.0011	41	0	GluC
H13_MOUSE, H14_MOUSE, H15_MOUSE	Histone H1	KNNSRIKLGLKSLVSKGTLVQTKGTGASGSFKLNKKAASGE	49	6.80E-05	601.348	4202.3850	7	-0.0012	41	0	GluC
H13_MOUSE, H14_MOUSE, H15_MOUSE	Histone H1	KNNSRIKLGLKSLVSKGTLVQTKGTGASGSFKLNKKAASGE	49	4.30E-05	1051.6035	4202.3849	4	-0.0013	41	0	GluC
H13_MOUSE, H14_MOUSE, H15_MOUSE	Histone H1	KNNSRIKLGLKSLVSKGTLVQTKGTGASGSFKLNKKAASGE	49	1.00E-02	701.4047	4202.3847	6	-0.0015	41	0	GluC
H13_MOUSE, H14_MOUSE, H15_MOUSE	Histone H1	KSLVSKGTLVQTKGTGASGSF	3	3.40E-11	685.0507	2052.1304	3	0.0089	21	2	Chymotrypsin
H13_MOUSE, H14_MOUSE, H15_MOUSE	Histone H1	KSLVSKGTLVQTKGTGASGSF	3	2.10E-11	514.0397	2052.1297	4	0.0082	21	2	Chymotrypsin
H13_MOUSE, H14_MOUSE, H15_MOUSE	Histone H1	KSLVSKGTLVQTKGTGASGSF	3	1.60E-09	685.0494	2052.1263	3	0.0048	21	2	Chymotrypsin
H13_MOUSE, H14_MOUSE, H15_MOUSE	Histone H1	VSKGTLVQTKGTGASGSF	2	4.80E-03	862.9665	1723.9185	2	0.0080	18	1	Chymotrypsin
H13_MOUSE, H14_MOUSE, H15_MOUSE	Histone H1	VSKGTLVQTKGTGASGSF	2	7.30E-08	575.6462	1723.9167	3	0.0062	18	1	Chymotrypsin
H13_MOUSE, H14_MOUSE, H15_MOUSE	Histone H1	IKLGLKSLVSKGTLVQTK	8	1.30E-06	638.4106	1912.2100	3	0.0015	18	3	Semi-tryptic
H13_MOUSE, H14_MOUSE, H15_MOUSE	Histone H1	IKLGLKSLVSKGTLVQTK	8	9.80E-03	638.4105	1912.2097	3	0.0013	18	3	Semi-tryptic
H13_MOUSE, H14_MOUSE, H15_MOUSE	Histone H1	IKLGLKSLVSKGTLVQTK	8	2.40E-08	638.4105	1912.2097	3	0.0012	18	3	Semi-tryptic
H13_MOUSE, H14_MOUSE, H15_MOUSE	Histone H1	IKLGLKSLVSKGTLVQTK	8	7.80E-08	638.4104	1912.2095	3	0.0010	18	3	Semi-tryptic
H13_MOUSE, H14_MOUSE, H15_MOUSE	Histone H1	IKLGLKSLVSKGTLVQTK	8	4.00E-03	638.4102	1912.2089	3	0.0004	18	3	Semi-tryptic
H13_MOUSE, H14_MOUSE, H15_MOUSE	Histone H1	IKLGLKSLVSKGTLVQTK	8	1.50E-05	638.4102	1912.2086	3	0.0002	18	3	Semi-tryptic
H13_MOUSE, H14_MOUSE, H15_MOUSE	Histone H1	IKLGLKSLVSKGTLVQTK	8	6.10E-06	638.4101	1912.2085	3	0.0000	18	3	Semi-tryptic
H13_MOUSE, H14_MOUSE, H15_MOUSE	Histone H1	IKLGLKSLVSKGTLVQTK	8	2.60E-03	479.0591	1912.2072	4	-0.0013	18	3	Semi-tryptic
H13_MOUSE, H14_MOUSE, H15_MOUSE	Histone H1	LGLKSLVSKGTLVQTK	7	1.90E-09	836.523	1671.0314	2	0.0019	16	2	Semi-tryptic
H13_MOUSE, H14_MOUSE, H15_MOUSE	Histone H1	LGLKSLVSKGTLVQTK	7	7.40E-08	558.0174	1671.0305	3	0.0010	16	2	Semi-tryptic
H13_MOUSE, H14_MOUSE, H15_MOUSE	Histone H1	LGLKSLVSKGTLVQTK	7	2.10E-04	418.7647	1671.0299	4	0.0004	16	2	Semi-tryptic
H13_MOUSE, H14_MOUSE, H15_MOUSE	Histone H1	LGLKSLVSKGTLVQTK	7	1.50E-06	558.0171	1671.0294	3	-0.0001	16	2	Semi-tryptic
H13_MOUSE, H14_MOUSE, H15_MOUSE	Histone H1	LGLKSLVSKGTLVQTK	7	1.10E-03	418.7645	1671.0287	4	-0.0007	16	2	Semi-tryptic
H13_MOUSE, H14_MOUSE, H15_MOUSE	Histone H1	LGLKSLVSKGTLVQTK	7	6.90E-06	558.0168	1671.0285	3	-0.0009	16	2	Semi-tryptic
H13_MOUSE, H14_MOUSE, H15_MOUSE	Histone H1	LGLKSLVSKGTLVQTK	7	4.20E-07	558.0162	1671.0267	3	-0.0028	16	2	Semi-tryptic
H13_MOUSE, H14_MOUSE, H15_MOUSE	Histone H1	SLVSKGTLVQTK	20	3.50E-06	630.8809	1259.7472	2	0.0023	12	1	Semi-tryptic
H13_MOUSE, H14_MOUSE, H15_MOUSE	Histone H1	SLVSKGTLVQTK	20	2.90E-08	420.9228	1259.7466	3	0.0017	12	1	Semi-tryptic
H13_MOUSE, H14_MOUSE, H15_MOUSE	Histone H1	SLVSKGTLVQTK	20	2.80E-09	420.9228	1259.7466	3	0.0017	12	1	Semi-tryptic
H13_MOUSE, H14_MOUSE, H15_MOUSE	Histone H1	SLVSKGTLVQTK	20	3.10E-07	630.8805	1259.7465	2	0.0016	12	1	Semi-tryptic
H13_MOUSE, H14_MOUSE, H15_MOUSE	Histone H1	SLVSKGTLVQTK	20	4.80E-03	630.8804	1259.7463	2	0.0014	12	1	Semi-tryptic
H13_MOUSE, H14_MOUSE, H15_MOUSE	Histone H1	SLVSKGTLVQTK	20	1.60E-04	630.8804	1259.7463	2	0.0014	12	1	Semi-tryptic
H13_MOUSE, H14_MOUSE, H15_MOUSE	Histone H1	SLVSKGTLVQTK	20	2.50E-07	630.8804	1259.7462	2	0.0013	12	1	Semi-tryptic
H13_MOUSE, H14_MOUSE, H15_MOUSE	Histone H1	SLVSKGTLVQTK	20	3.20E-03	630.8803	1259.7460	2	0.0011	12	1	Semi-tryptic
H13_MOUSE, H14_MOUSE, H15_MOUSE	Histone H1	SLVSKGTLVQTK	20	9.20E-09	630.8802	1259.7458	2	0.0009	12	1	Semi-tryptic
H13_MOUSE, H14_MOUSE, H15_MOUSE	Histone H1	SLVSKGTLVQTK	20	1.10E-05	630.8801	1259.7457	2	0.0008	12	1	Semi-tryptic
H13_MOUSE, H14_MOUSE, H15_MOUSE	Histone H1	SLVSKGTLVQTK	20	2.80E-05	420.9224	1259.7455	3	0.0006	12	1	Semi-tryptic
H13_MOUSE, H14_MOUSE, H15_MOUSE	Histone H1	SLVSKGTLVQTK	20	1.10E-06	420.9224	1259.7455	3	0.0006	12	1	Semi-tryptic
H13_MOUSE, H14_MOUSE, H15_MOUSE	Histone H1	SLVSKGTLVQTK	20	7.50E-03	420.9224	1259.7454	3	0.0005	12	1	Semi-tryptic
H13_MOUSE, H14_MOUSE, H15_MOUSE	Histone H1	SLVSKGTLVQTK	20	3.90E-08	630.88	1259.7454	2	0.0005	12	1	Semi-tryptic
H13_MOUSE, H14_MOUSE, H15_MOUSE	Histone H1	SLVSKGTLVQTK	20	1.60E-09	630.8799	1259.7453	2	0.0004	12	1	Semi-tryptic
H13_MOUSE, H14_MOUSE, H15_MOUSE	Histone H1	SLVSKGTLVQTK	20	1.50E-03	420.9223	1259.7452	3	0.0003	12	1	Semi-tryptic
H13_MOUSE, H14_MOUSE, H15_MOUSE	Histone H1	SLVSKGTLVQTK	20	3.30E-07	630.8798	1259.7450	2	0.0001	12	1	Semi-tryptic
H13_MOUSE, H14_MOUSE, H15_MOUSE	Histone H1	SLVSKGTLVQTK	20	1.20E-06	630.8797	1259.7449	2	0.0000	12	1	Semi-tryptic
H13_MOUSE, H14_MOUSE, H15_MOUSE	Histone H1	SLVSKGTLVQTK	20	1.60E-09	630.8795	1259.7445	2	-0.0004	12	1	Semi-tryptic
H13_MOUSE, H14_MOUSE, H15_MOUSE	Histone H1	SLVSKGTLVQTK	20	2.90E-08	630.8791	1259.7436	2	-0.0013	12	1	Semi-tryptic
H13_MOUSE, H14_MOUSE, H15_MOUSE	Histone H1	GLKSLVSKGTL	5	1.50E-04	368.2334	1101.6784	3	0.0026	11	2	Chymotrypsin
H13_MOUSE, H14_MOUSE, H15_MOUSE	Histone H1	GLKSLVSKGTL	5	1.40E-04	551.8454	1101.6762	2	0.0004	11	2	Chymotrypsin

H13_MOUSE, H14_MOUSE, H15_MOUSE	Histone H1	GLKSLVSKGTL	5	1.50E-06	551.8454	1101.6762	2	0.0004	11	2	Chymotrypsin
H13_MOUSE, H14_MOUSE, H15_MOUSE	Histone H1	GLKSLVSKGTL	5	3.40E-03	368.2325	1101.6757	3	0.0000	11	2	Chymotrypsin
H13_MOUSE, H14_MOUSE, H15_MOUSE	Histone H1	GLKSLVSKGTL	5	5.40E-04	368.2325	1101.6757	3	0.0000	11	2	Chymotrypsin
H13_MOUSE, H14_MOUSE, H15_MOUSE	Histone H1	KAASGEAKPK	22	3.30E-03	493.7871	985.5596	2	0.0040	10	1	Semi-tryptic
H13_MOUSE, H14_MOUSE, H15_MOUSE	Histone H1	KAASGEAKPK	22	7.00E-03	329.5268	985.5587	3	0.0031	10	1	Semi-tryptic
H13_MOUSE, H14_MOUSE, H15_MOUSE	Histone H1	KAASGEAKPK	22	5.50E-04	493.7863	985.5581	2	0.0025	10	1	Semi-tryptic
H13_MOUSE, H14_MOUSE, H15_MOUSE	Histone H1	KAASGEAKPK	22	8.20E-04	329.5264	985.5573	3	0.0017	10	1	Semi-tryptic
H13_MOUSE, H14_MOUSE, H15_MOUSE	Histone H1	KAASGEAKPK	22	3.20E-05	329.5263	985.5571	3	0.0015	10	1	Semi-tryptic
H13_MOUSE, H14_MOUSE, H15_MOUSE	Histone H1	KAASGEAKPK	22	3.00E-03	493.7855	985.5563	2	0.0007	10	1	Semi-tryptic
H13_MOUSE, H14_MOUSE, H15_MOUSE	Histone H1	KAASGEAKPK	22	9.70E-04	493.7854	985.5563	2	0.0007	10	1	Semi-tryptic
H13_MOUSE, H14_MOUSE, H15_MOUSE	Histone H1	KAASGEAKPK	22	2.60E-03	329.526	985.5562	3	0.0006	10	1	Semi-tryptic
H13_MOUSE, H14_MOUSE, H15_MOUSE	Histone H1	KAASGEAKPK	22	1.70E-04	329.526	985.5561	3	0.0004	10	1	Semi-tryptic
H13_MOUSE, H14_MOUSE, H15_MOUSE	Histone H1	KAASGEAKPK	22	7.40E-03	493.7853	985.5559	2	0.0003	10	1	Semi-tryptic
H13_MOUSE, H14_MOUSE, H15_MOUSE	Histone H1	KAASGEAKPK	22	6.90E-04	493.7852	985.5558	2	0.0002	10	1	Semi-tryptic
H13_MOUSE, H14_MOUSE, H15_MOUSE	Histone H1	KAASGEAKPK	22	2.50E-03	493.7851	985.5557	2	0.0001	10	1	Semi-tryptic
H13_MOUSE, H14_MOUSE, H15_MOUSE	Histone H1	KAASGEAKPK	22	4.60E-04	493.7851	985.5556	2	0.0000	10	1	Semi-tryptic
H13_MOUSE, H14_MOUSE, H15_MOUSE	Histone H1	KAASGEAKPK	22	9.50E-05	493.7851	985.5556	2	0.0000	10	1	Semi-tryptic
H13_MOUSE, H14_MOUSE, H15_MOUSE	Histone H1	KAASGEAKPK	22	9.00E-08	493.785	985.5555	2	-0.0001	10	1	Semi-tryptic
H13_MOUSE, H14_MOUSE, H15_MOUSE	Histone H1	KAASGEAKPK	22	6.40E-04	493.785	985.5554	2	-0.0002	10	1	Semi-tryptic
H13_MOUSE, H14_MOUSE, H15_MOUSE	Histone H1	KAASGEAKPK	22	7.60E-06	493.785	985.5554	2	-0.0002	10	1	Semi-tryptic
H13_MOUSE, H14_MOUSE, H15_MOUSE	Histone H1	KAASGEAKPK	22	2.10E-04	493.7849	985.5553	2	-0.0003	10	1	Semi-tryptic
H13_MOUSE, H14_MOUSE, H15_MOUSE	Histone H1	KAASGEAKPK	22	1.70E-04	493.7849	985.5552	2	-0.0004	10	1	Semi-tryptic
H13_MOUSE, H14_MOUSE, H15_MOUSE	Histone H1	KAASGEAKPK	22	1.50E-06	493.7846	985.5547	2	-0.0009	10	1	Semi-tryptic
H13_MOUSE, H14_MOUSE, H15_MOUSE	Histone H1	KAASGEAKPK	22	8.70E-04	493.7845	985.5545	2	-0.0011	10	1	Semi-tryptic
H13_MOUSE, H14_MOUSE, H15_MOUSE	Histone H1	KAASGEAKPK	22	1.30E-06	493.7845	985.5545	2	-0.0011	10	1	Semi-tryptic
H13_MOUSE, H14_MOUSE, H15_MOUSE	Histone H1	KSLVSKGTL	2	1.50E-03	466.7939	931.5732	2	0.0030	9	1	Chymotrypsin
H13_MOUSE, H14_MOUSE, H15_MOUSE	Histone H1	KSLVSKGTL	2	1.20E-03	466.7931	931.5716	2	0.0014	9	1	Chymotrypsin
H13_MOUSE, H14_MOUSE, H15_MOUSE	Histone H1	AASGEAKPK	5	2.00E-04	429.7386	857.4626	2	0.0019	9	0	Semi-tryptic
H13_MOUSE, H14_MOUSE, H15_MOUSE	Histone H1	AASGEAKPK	5	1.30E-04	429.7377	857.4609	2	0.0003	9	0	Semi-tryptic
H13_MOUSE, H14_MOUSE, H15_MOUSE	Histone H1	AASGEAKPK	5	5.20E-05	429.7376	857.4606	2	0.0000	9	0	Semi-tryptic
H13_MOUSE, H14_MOUSE, H15_MOUSE	Histone H1	AASGEAKPK	5	3.10E-05	429.7375	857.4605	2	-0.0002	9	0	Semi-tryptic
H13_MOUSE, H14_MOUSE, H15_MOUSE	Histone H1	AASGEAKPK	5	7.60E-04	429.7373	857.4601	2	-0.0005	9	0	Semi-tryptic
H14_MOUSE	Histone H1	TAPAAPAAPAPAEKTPVKKKARKAAGGAKRKTSGPPVSE	4	8.30E-05	949.0494	3792.1685	4	0.0199	39	1	GluC
H14_MOUSE	Histone H1	TAPAAPAAPAPAEKTPVKKKARKAAGGAKRKTSGPPVSE	4	7.10E-03	633.0353	3792.1683	6	0.0198	39	1	GluC
H14_MOUSE	Histone H1	TAPAAPAAPAPAEKTPVKKKARKAAGGAKRKTSGPPVSE	4	9.30E-03	949.0485	3792.1647	4	0.0162	39	1	GluC
H14_MOUSE	Histone H1	TAPAAPAAPAPAEKTPVKKKARKAAGGAKRKTSGPPVSE	4	8.80E-03	759.4374	3792.1507	5	0.0021	39	1	GluC
H14_MOUSE	Histone H1	KTPVKKKARKAAGGAKRKTSGPPVSE	23	1.40E-03	536.3244	2676.5855	5	-0.0020	26	0	GluC
H14_MOUSE	Histone H1	KTPVKKKARKAAGGAKRKTSGPPVSE	23	5.50E-04	536.3244	2676.5855	5	-0.0020	26	0	GluC
H14_MOUSE	Histone H1	KTPVKKKARKAAGGAKRKTSGPPVSE	23	3.70E-07	670.1528	2676.5822	4	-0.0053	26	0	GluC
H14_MOUSE	Histone H1	KTPVKKKARKAAGGAKRKTSGPPVSE	23	2.60E-08	670.1586	2676.6053	4	0.0178	26	0	GluC
H14_MOUSE	Histone H1	KTPVKKKARKAAGGAKRKTSGPPVSE	23	7.00E-08	670.1561	2676.5951	4	0.0077	26	0	GluC
H14_MOUSE	Histone H1	KTPVKKKARKAAGGAKRKTSGPPVSE	23	2.70E-03	893.2056	2676.5950	3	0.0076	26	0	GluC
H14_MOUSE	Histone H1	KTPVKKKARKAAGGAKRKTSGPPVSE	23	5.60E-04	536.3262	2676.5948	5	0.0073	26	0	GluC
H14_MOUSE	Histone H1	KTPVKKKARKAAGGAKRKTSGPPVSE	23	1.10E-03	670.1557	2676.5938	4	0.0063	26	0	GluC
H14_MOUSE	Histone H1	KTPVKKKARKAAGGAKRKTSGPPVSE	23	7.00E-05	670.155	2676.5910	4	0.0036	26	0	GluC
H14_MOUSE	Histone H1	KTPVKKKARKAAGGAKRKTSGPPVSE	23	5.50E-05	670.1549	2676.5907	4	0.0032	26	0	GluC
H14_MOUSE	Histone H1	KTPVKKKARKAAGGAKRKTSGPPVSE	23	1.90E-03	670.1549	2676.5905	4	0.0030	26	0	GluC
H14_MOUSE	Histone H1	KTPVKKKARKAAGGAKRKTSGPPVSE	23	1.90E-03	670.1546	2676.5893	4	0.0019	26	0	GluC
H14_MOUSE	Histone H1	KTPVKKKARKAAGGAKRKTSGPPVSE	23	2.40E-03	536.3251	2676.5889	5	0.0014	26	0	GluC
H14_MOUSE	Histone H1	KTPVKKKARKAAGGAKRKTSGPPVSE	23	5.20E-03	670.1544	2676.5887	4	0.0012	26	0	GluC
H14_MOUSE	Histone H1	KTPVKKKARKAAGGAKRKTSGPPVSE	23	7.00E-03	670.1544	2676.5884	4	0.0010	26	0	GluC
H14_MOUSE	Histone H1	KTPVKKKARKAAGGAKRKTSGPPVSE	23	1.70E-03	670.1543	2676.5881	4	0.0006	26	0	GluC
H14_MOUSE	Histone H1	KTPVKKKARKAAGGAKRKTSGPPVSE	23	3.00E-03	536.3248	2676.5879	5	0.0004	26	0	GluC
H14_MOUSE	Histone H1	KTPVKKKARKAAGGAKRKTSGPPVSE	23	1.20E-03	536.3248	2676.5879	5	0.0004	26	0	GluC
H14_MOUSE	Histone H1	KTPVKKKARKAAGGAKRKTSGPPVSE	23	9.90E-06	670.1542	2676.5878	4	0.0004	26	0	GluC
H14_MOUSE	Histone H1	KTPVKKKARKAAGGAKRKTSGPPVSE	23	8.30E-04	670.1542	2676.5877	4	0.0002	26	0	GluC
H14_MOUSE	Histone H1	KTPVKKKARKAAGGAKRKTSGPPVSE	23	3.50E-05	536.3247	2676.5874	5	-0.0001	26	0	GluC
H14_MOUSE	Histone H1	KTPVKKKARKAAGGAKRKTSGPPVSE	23	5.00E-04	536.3247	2676.5870	5	-0.0005	26	0	GluC
H14_MOUSE	Histone H1	KTPVKKKARKAAGGAKRKTSGPPVSE	23	9.00E-03	536.3244	2676.5857	5	-0.0018	26	0	GluC
H14_MOUSE	Histone H1	SETAPAAPAAPAPAEKTPVKKK	2	1.00E-04	720.7408	2159.2006	3	0.0056	22	3	Semi-tryptic
H14_MOUSE	Histone H1	SETAPAAPAAPAPAEKTPVKKK	2	9.30E-04	720.7395	2159.1966	3	0.0017	22	3	Semi-tryptic
H14_MOUSE	Histone H1	SETAPAAPAAPAPAEKTPVKK	2	1.00E-02	678.0417	2031.1032	3	0.0032	21	2	Semi-tryptic
H14_MOUSE	Histone H1	SETAPAAPAAPAPAEKTPVKK	2	5.10E-03	678.0402	2031.0987	3	-0.0013	21	2	Semi-tryptic
H14_MOUSE	Histone H1	SETAPAAPAAPAPAEKTPVK	4	3.50E-03	635.3438	1903.0097	3	0.0047	20	1	Semi-tryptic
H14_MOUSE	Histone H1	SETAPAAPAAPAPAEKTPVK	4	2.10E-04	635.3438	1903.0097	3	0.0047	20	1	Semi-tryptic
H14_MOUSE	Histone H1	SETAPAAPAAPAPAEKTPVK	4	7.30E-08	952.51	1903.0055	2	0.0005	20	1	Semi-tryptic
H14_MOUSE	Histone H1	SETAPAAPAAPAPAEKTPVK	4	8.20E-03	635.3422	1903.0047	3	-0.0004	20	1	Semi-tryptic
H14_MOUSE	Histone H1	SETAPAAPAAPAPAEK	3	1.10E-04	739.8809	1477.7473	2	0.0061	16	0	Semi-tryptic
H14_MOUSE	Histone H1	SETAPAAPAAPAPAEK	3	2.20E-04	739.878	1477.7415	2	0.0003	16	0	Semi-tryptic
H14_MOUSE	Histone H1	SETAPAAPAAPAPAEK	3	4.90E-05	739.8779	1477.7412	2	0.0000	16	0	Semi-tryptic
H14_MOUSE	Histone H1	RKTSGPPVSELITK	21	2.00E-04	504.9622	1511.8649	3	-0.0022	14	2	Semi-tryptic
H14_MOUSE	Histone H1	RKTSGPPVSELITK	21	1.20E-04	504.9617	1511.8632	3	-0.0040	14	2	Semi-tryptic
H14_MOUSE	Histone H1	RKTSGPPVSELITK	21	1.40E-05	504.9616	1511.8631	3	-0.0040	14	2	Semi-tryptic
H14_MOUSE	Histone H1	RKTSGPPVSELITK	21	5.50E-03	504.9651	1511.8733	3	0.0062	14	2	Semi-tryptic

H14_MOUSE	Histone H1	RKTSGPPVSELITK	21	3.10E-05	504.9645	1511.8718	3	0.0047	14	2	Semi-tryptic
H14_MOUSE	Histone H1	RKTSGPPVSELITK	21	5.60E-06	504.9645	1511.8716	3	0.0044	14	2	Semi-tryptic
H14_MOUSE	Histone H1	RKTSGPPVSELITK	21	4.20E-04	504.9643	1511.8710	3	0.0039	14	2	Semi-tryptic
H14_MOUSE	Histone H1	RKTSGPPVSELITK	21	1.40E-04	504.9643	1511.8710	3	0.0039	14	2	Semi-tryptic
H14_MOUSE	Histone H1	RKTSGPPVSELITK	21	1.90E-04	756.9415	1511.8684	2	0.0013	14	2	Semi-tryptic
H14_MOUSE	Histone H1	RKTSGPPVSELITK	21	4.80E-05	504.9633	1511.8681	3	0.0009	14	2	Semi-tryptic
H14_MOUSE	Histone H1	RKTSGPPVSELITK	21	3.20E-03	504.9632	1511.8677	3	0.0006	14	2	Semi-tryptic
H14_MOUSE	Histone H1	RKTSGPPVSELITK	21	4.80E-06	504.9632	1511.8677	3	0.0006	14	2	Semi-tryptic
H14_MOUSE	Histone H1	RKTSGPPVSELITK	21	2.70E-03	504.9631	1511.8675	3	0.0003	14	2	Semi-tryptic
H14_MOUSE	Histone H1	RKTSGPPVSELITK	21	1.40E-07	756.941	1511.8674	2	0.0003	14	2	Semi-tryptic
H14_MOUSE	Histone H1	RKTSGPPVSELITK	21	2.70E-05	504.9629	1511.8670	3	-0.0002	14	2	Semi-tryptic
H14_MOUSE	Histone H1	RKTSGPPVSELITK	21	2.50E-05	504.9629	1511.8668	3	-0.0003	14	2	Semi-tryptic
H14_MOUSE	Histone H1	RKTSGPPVSELITK	21	1.40E-06	756.9407	1511.8668	2	-0.0003	14	2	Semi-tryptic
H14_MOUSE	Histone H1	RKTSGPPVSELITK	21	1.60E-04	504.9627	1511.8662	3	-0.0009	14	2	Semi-tryptic
H14_MOUSE	Histone H1	RKTSGPPVSELITK	21	1.00E-04	504.9626	1511.8661	3	-0.0010	14	2	Semi-tryptic
H14_MOUSE	Histone H1	RKTSGPPVSELITK	21	2.00E-05	504.9626	1511.8661	3	-0.0010	14	2	Semi-tryptic
H14_MOUSE	Histone H1	RKTSGPPVSELITK	21	2.90E-04	504.9626	1511.8659	3	-0.0012	14	2	Semi-tryptic
H14_MOUSE	Histone H1	TAPAAPAAPAPAE	2	7.30E-03	567.7934	1133.5722	2	0.0006	13	0	GluC
H14_MOUSE	Histone H1	TAPAAPAAPAPAE	2	7.00E-03	567.7933	1133.5720	2	0.0004	13	0	GluC
H14_MOUSE	Histone H1	KTSGPPVSELITK	22	7.80E-05	678.889	1355.7634	2	-0.0026	13	1	Semi-tryptic
H14_MOUSE	Histone H1	KTSGPPVSELITK	22	8.20E-07	452.9279	1355.7620	3	-0.0041	13	1	Semi-tryptic
H14_MOUSE	Histone H1	KTSGPPVSELITK	22	9.50E-09	678.8933	1355.7720	2	0.0060	13	1	Semi-tryptic
H14_MOUSE	Histone H1	KTSGPPVSELITK	22	3.30E-07	678.8918	1355.7691	2	0.0031	13	1	Semi-tryptic
H14_MOUSE	Histone H1	KTSGPPVSELITK	22	1.70E-06	452.93	1355.7683	3	0.0023	13	1	Semi-tryptic
H14_MOUSE	Histone H1	KTSGPPVSELITK	22	4.80E-04	452.93	1355.7682	3	0.0022	13	1	Semi-tryptic
H14_MOUSE	Histone H1	KTSGPPVSELITK	22	3.10E-05	452.93	1355.7682	3	0.0022	13	1	Semi-tryptic
H14_MOUSE	Histone H1	KTSGPPVSELITK	22	8.20E-07	452.93	1355.7680	3	0.0020	13	1	Semi-tryptic
H14_MOUSE	Histone H1	KTSGPPVSELITK	22	2.10E-06	678.8912	1355.7679	2	0.0019	13	1	Semi-tryptic
H14_MOUSE	Histone H1	KTSGPPVSELITK	22	3.30E-08	678.8912	1355.7679	2	0.0019	13	1	Semi-tryptic
H14_MOUSE	Histone H1	KTSGPPVSELITK	22	4.50E-09	678.8912	1355.7679	2	0.0019	13	1	Semi-tryptic
H14_MOUSE	Histone H1	KTSGPPVSELITK	22	6.00E-09	678.891	1355.7674	2	0.0014	13	1	Semi-tryptic
H14_MOUSE	Histone H1	KTSGPPVSELITK	22	9.30E-03	452.9297	1355.7673	3	0.0013	13	1	Semi-tryptic
H14_MOUSE	Histone H1	KTSGPPVSELITK	22	1.30E-03	452.9297	1355.7673	3	0.0013	13	1	Semi-tryptic
H14_MOUSE	Histone H1	KTSGPPVSELITK	22	9.90E-09	678.891	1355.7674	2	0.0013	13	1	Semi-tryptic
H14_MOUSE	Histone H1	KTSGPPVSELITK	22	3.40E-07	678.8907	1355.7669	2	0.0009	13	1	Semi-tryptic
H14_MOUSE	Histone H1	KTSGPPVSELITK	22	2.20E-04	452.9295	1355.7668	3	0.0007	13	1	Semi-tryptic
H14_MOUSE	Histone H1	KTSGPPVSELITK	22	1.00E-02	452.9291	1355.7656	3	-0.0005	13	1	Semi-tryptic
H14_MOUSE	Histone H1	KTSGPPVSELITK	22	8.70E-10	678.8901	1355.7656	2	-0.0005	13	1	Semi-tryptic
H14_MOUSE	Histone H1	KTSGPPVSELITK	22	7.40E-07	452.9291	1355.7655	3	-0.0006	13	1	Semi-tryptic
H14_MOUSE	Histone H1	KTSGPPVSELITK	22	8.40E-07	452.9289	1355.7648	3	-0.0012	13	1	Semi-tryptic
H14_MOUSE	Histone H1	KTSGPPVSELITK	22	4.70E-07	452.9287	1355.7644	3	-0.0016	13	1	Semi-tryptic
H14_MOUSE	Histone H1	TSGPPVSELITK	20	8.00E-08	614.8416	1227.6687	2	-0.0024	12	0	Semi-tryptic
H14_MOUSE	Histone H1	TSGPPVSELITK	20	2.20E-08	614.8415	1227.6683	2	-0.0027	12	0	Semi-tryptic
H14_MOUSE	Histone H1	TSGPPVSELITK	20	3.90E-08	614.844	1227.6735	2	0.0024	12	0	Semi-tryptic
H14_MOUSE	Histone H1	TSGPPVSELITK	20	1.50E-07	614.8435	1227.6724	2	0.0014	12	0	Semi-tryptic
H14_MOUSE	Histone H1	TSGPPVSELITK	20	2.80E-03	614.8434	1227.6722	2	0.0011	12	0	Semi-tryptic
H14_MOUSE	Histone H1	TSGPPVSELITK	20	7.70E-08	614.8433	1227.6720	2	0.0010	12	0	Semi-tryptic
H14_MOUSE	Histone H1	TSGPPVSELITK	20	8.40E-05	614.8432	1227.6717	2	0.0007	12	0	Semi-tryptic
H14_MOUSE	Histone H1	TSGPPVSELITK	20	7.90E-08	614.8432	1227.6718	2	0.0007	12	0	Semi-tryptic
H14_MOUSE	Histone H1	TSGPPVSELITK	20	8.90E-04	614.8431	1227.6717	2	0.0006	12	0	Semi-tryptic
H14_MOUSE	Histone H1	TSGPPVSELITK	20	2.00E-04	614.8431	1227.6716	2	0.0006	12	0	Semi-tryptic
H14_MOUSE	Histone H1	TSGPPVSELITK	20	2.00E-08	614.8431	1227.6717	2	0.0006	12	0	Semi-tryptic
H14_MOUSE	Histone H1	TSGPPVSELITK	20	8.30E-08	614.843	1227.6714	2	0.0003	12	0	Semi-tryptic
H14_MOUSE	Histone H1	TSGPPVSELITK	20	1.00E-05	614.8427	1227.6707	2	-0.0003	12	0	Semi-tryptic
H14_MOUSE	Histone H1	TSGPPVSELITK	20	6.90E-07	614.8425	1227.6705	2	-0.0005	12	0	Semi-tryptic
H14_MOUSE	Histone H1	TSGPPVSELITK	20	7.90E-08	614.8426	1227.6706	2	-0.0005	12	0	Semi-tryptic
H14_MOUSE	Histone H1	TSGPPVSELITK	20	1.10E-03	614.8425	1227.6705	2	-0.0006	12	0	Semi-tryptic
H14_MOUSE	Histone H1	TSGPPVSELITK	20	7.90E-09	614.8424	1227.6703	2	-0.0008	12	0	Semi-tryptic
H14_MOUSE	Histone H1	TSGPPVSELITK	20	2.90E-04	614.8423	1227.6701	2	-0.0010	12	0	Semi-tryptic
H14_MOUSE	Histone H1	TSGPPVSELITK	20	8.30E-08	614.8422	1227.6698	2	-0.0012	12	0	Semi-tryptic
H14_MOUSE	Histone H1	TSGPPVSELITK	20	1.10E-03	614.8421	1227.6697	2	-0.0013	12	0	Semi-tryptic
H14_MOUSE	Histone H1	KAAGTATAKK	7	6.70E-05	316.1944	945.5615	3	0.0008	10	2	Semi-tryptic
H14_MOUSE	Histone H1	KAAGTATAKK	7	1.40E-03	473.788	945.5614	2	0.0007	10	2	Semi-tryptic
H14_MOUSE	Histone H1	KAAGTATAKK	7	1.20E-05	473.7877	945.5608	2	0.0001	10	2	Semi-tryptic
H14_MOUSE	Histone H1	KAAGTATAKK	7	1.20E-04	473.7876	945.5606	2	-0.0001	10	2	Semi-tryptic
H14_MOUSE	Histone H1	KAAGTATAKK	7	3.40E-03	473.7875	945.5605	2	-0.0002	10	2	Semi-tryptic
H14_MOUSE	Histone H1	KAAGTATAKK	7	3.20E-03	473.7876	945.5605	2	-0.0002	10	2	Semi-tryptic
H14_MOUSE	Histone H1	KAAGTATAKK	7	2.00E-03	473.7875	945.5604	2	-0.0003	10	2	Semi-tryptic
H14_MOUSE	Histone H1	KAAGTATAK	13	8.50E-03	409.7416	817.4686	2	0.0029	9	1	Semi-tryptic
H14_MOUSE	Histone H1	KAAGTATAK	13	1.30E-03	409.7409	817.4673	2	0.0016	9	1	Semi-tryptic
H14_MOUSE	Histone H1	KAAGTATAK	13	6.30E-03	409.7407	817.4669	2	0.0012	9	1	Semi-tryptic
H14_MOUSE	Histone H1	KAAGTATAK	13	2.30E-03	409.7407	817.4669	2	0.0012	9	1	Semi-tryptic
H14_MOUSE	Histone H1	KAAGTATAK	13	1.30E-03	409.7407	817.4669	2	0.0012	9	1	Semi-tryptic
H14_MOUSE	Histone H1	KAAGTATAK	13	3.50E-04	409.7407	817.4669	2	0.0012	9	1	Semi-tryptic

H14_MOUSE	Histone H1	KAAGTATAK	13	9.70E-03	409.7407	817.4667	2	0.0010	9	1	Semi-tryptic
H14_MOUSE	Histone H1	KAAGTATAK	13	5.40E-03	409.7405	817.4665	2	0.0008	9	1	Semi-tryptic
H14_MOUSE	Histone H1	KAAGTATAK	13	7.90E-04	409.7404	817.4663	2	0.0005	9	1	Semi-tryptic
H14_MOUSE	Histone H1	KAAGTATAK	13	3.50E-03	409.7403	817.4661	2	0.0004	9	1	Semi-tryptic
H14_MOUSE	Histone H1	KAAGTATAK	13	3.00E-03	409.7403	817.4661	2	0.0004	9	1	Semi-tryptic
H14_MOUSE	Histone H1	KAAGTATAK	13	4.20E-03	409.7403	817.4660	2	0.0003	9	1	Semi-tryptic
H14_MOUSE	Histone H1	KAAGTATAK	13	6.90E-04	409.7402	817.4658	2	0.0000	9	1	Semi-tryptic
H15_MOUSE	Histone H1	ITKAVSASKERGGVSLPALKKALAAGGY	3	9.00E-06	915.2008	2742.5805	3	0.0049	28	2	Chymotrypsin
H15_MOUSE	Histone H1	ITKAVSASKERGGVSLPALKKALAAGGY	3	3.30E-09	686.6507	2742.5737	4	-0.0019	28	2	Chymotrypsin
H15_MOUSE	Histone H1	ITKAVSASKERGGVSLPALKKALAAGGY	3	3.60E-03	549.5197	2742.5622	5	-0.0134	28	2	Chymotrypsin
H15_MOUSE	Histone H1	KSPAKKKTTKKAGAAKRKATGPPVSE	11	5.80E-03	533.9216	2664.5717	5	-0.0045	26	0	GluC
H15_MOUSE	Histone H1	KSPAKKKTTKKAGAAKRKATGPPVSE	11	1.60E-05	889.2059	2664.5960	3	0.0198	26	0	GluC
H15_MOUSE	Histone H1	KSPAKKKTTKKAGAAKRKATGPPVSE	11	6.50E-03	889.2033	2664.5880	3	0.0118	26	0	GluC
H15_MOUSE	Histone H1	KSPAKKKTTKKAGAAKRKATGPPVSE	11	3.50E-03	889.2033	2664.5880	3	0.0118	26	0	GluC
H15_MOUSE	Histone H1	KSPAKKKTTKKAGAAKRKATGPPVSE	11	1.20E-04	533.9248	2664.5874	5	0.0112	26	0	GluC
H15_MOUSE	Histone H1	KSPAKKKTTKKAGAAKRKATGPPVSE	11	2.10E-06	667.1535	2664.5847	4	0.0085	26	0	GluC
H15_MOUSE	Histone H1	KSPAKKKTTKKAGAAKRKATGPPVSE	11	1.30E-04	667.1527	2664.5817	4	0.0055	26	0	GluC
H15_MOUSE	Histone H1	KSPAKKKTTKKAGAAKRKATGPPVSE	11	1.30E-05	667.1525	2664.5807	4	0.0045	26	0	GluC
H15_MOUSE	Histone H1	KSPAKKKTTKKAGAAKRKATGPPVSE	11	1.90E-03	667.152	2664.5790	4	0.0028	26	0	GluC
H15_MOUSE	Histone H1	KSPAKKKTTKKAGAAKRKATGPPVSE	11	4.10E-06	667.1511	2664.5752	4	-0.0010	26	0	GluC
H15_MOUSE	Histone H1	KSPAKKKTTKKAGAAKRKATGPPVSE	11	8.90E-08	533.9222	2664.5745	5	-0.0017	26	0	GluC
H15_MOUSE	Histone H1	ITKAVSASKERGGVSLPALKKAL	5	5.30E-04	775.4765	2323.4078	3	0.0128	23	1	Chymotrypsin
H15_MOUSE	Histone H1	ITKAVSASKERGGVSLPALKKAL	5	7.40E-03	581.8585	2323.4050	4	0.0100	23	1	Chymotrypsin
H15_MOUSE	Histone H1	ITKAVSASKERGGVSLPALKKAL	5	1.10E-08	775.4751	2323.4034	3	0.0083	23	1	Chymotrypsin
H15_MOUSE	Histone H1	ITKAVSASKERGGVSLPALKKAL	5	1.80E-09	581.8578	2323.4019	4	0.0069	23	1	Chymotrypsin
H15_MOUSE	Histone H1	ITKAVSASKERGGVSLPALKKAL	5	7.90E-06	465.6873	2323.4003	5	0.0052	23	1	Chymotrypsin
H15_MOUSE	Histone H1	RGGVSLPALKKALAAGGYDVE	25	6.80E-03	691.3934	2071.1585	3	0.0159	21	1	GluC
H15_MOUSE	Histone H1	RGGVSLPALKKALAAGGYDVE	25	1.60E-06	691.3922	2071.1547	3	0.0122	21	1	GluC
H15_MOUSE	Histone H1	RGGVSLPALKKALAAGGYDVE	25	3.80E-04	691.3904	2071.1493	3	0.0067	21	1	GluC
H15_MOUSE	Histone H1	RGGVSLPALKKALAAGGYDVE	25	2.20E-04	691.3901	2071.1485	3	0.0060	21	1	GluC
H15_MOUSE	Histone H1	RGGVSLPALKKALAAGGYDVE	25	7.30E-06	691.3901	2071.1485	3	0.0060	21	1	GluC
H15_MOUSE	Histone H1	RGGVSLPALKKALAAGGYDVE	25	2.00E-04	691.3898	2071.1475	3	0.0049	21	1	GluC
H15_MOUSE	Histone H1	RGGVSLPALKKALAAGGYDVE	25	1.80E-03	691.3897	2071.1472	3	0.0047	21	1	GluC
H15_MOUSE	Histone H1	RGGVSLPALKKALAAGGYDVE	25	3.10E-03	691.3892	2071.1459	3	0.0033	21	1	GluC
H15_MOUSE	Histone H1	RGGVSLPALKKALAAGGYDVE	25	1.40E-06	691.3892	2071.1459	3	0.0033	21	1	GluC
H15_MOUSE	Histone H1	RGGVSLPALKKALAAGGYDVE	25	2.10E-09	1036.5802	2071.1458	2	0.0033	21	1	GluC
H15_MOUSE	Histone H1	RGGVSLPALKKALAAGGYDVE	25	4.50E-04	691.3889	2071.1449	3	0.0023	21	1	GluC
H15_MOUSE	Histone H1	RGGVSLPALKKALAAGGYDVE	25	1.10E-03	691.3889	2071.1448	3	0.0022	21	1	GluC
H15_MOUSE	Histone H1	RGGVSLPALKKALAAGGYDVE	25	4.30E-05	691.3889	2071.1447	3	0.0022	21	1	GluC
H15_MOUSE	Histone H1	RGGVSLPALKKALAAGGYDVE	25	4.10E-04	691.3887	2071.1443	3	0.0018	21	1	GluC
H15_MOUSE	Histone H1	RGGVSLPALKKALAAGGYDVE	25	8.60E-05	691.3887	2071.1444	3	0.0018	21	1	GluC
H15_MOUSE	Histone H1	RGGVSLPALKKALAAGGYDVE	25	1.80E-08	691.3887	2071.1443	3	0.0018	21	1	GluC
H15_MOUSE	Histone H1	RGGVSLPALKKALAAGGYDVE	25	1.30E-04	691.3886	2071.1440	3	0.0014	21	1	GluC
H15_MOUSE	Histone H1	RGGVSLPALKKALAAGGYDVE	25	6.70E-07	691.3886	2071.1440	3	0.0014	21	1	GluC
H15_MOUSE	Histone H1	RGGVSLPALKKALAAGGYDVE	25	4.70E-03	691.3884	2071.1435	3	0.0009	21	1	GluC
H15_MOUSE	Histone H1	RGGVSLPALKKALAAGGYDVE	25	9.00E-06	691.3883	2071.1432	3	0.0006	21	1	GluC
H15_MOUSE	Histone H1	RGGVSLPALKKALAAGGYDVE	25	1.60E-07	691.3883	2071.1432	3	0.0006	21	1	GluC
H15_MOUSE	Histone H1	RGGVSLPALKKALAAGGYDVE	25	8.70E-04	691.3883	2071.1431	3	0.0005	21	1	GluC
H15_MOUSE	Histone H1	RGGVSLPALKKALAAGGYDVE	25	9.80E-10	1036.5785	2071.1424	2	-0.0001	21	1	GluC
H15_MOUSE	Histone H1	RGGVSLPALKKALAAGGYDVE	25	7.70E-05	691.388	2071.1423	3	-0.0003	21	1	GluC
H15_MOUSE	Histone H1	RGGVSLPALKKALAAGGYDVE	25	3.20E-06	691.388	2071.1423	3	-0.0003	21	1	GluC
H15_MOUSE	Histone H1	ITKAVSASKERGGVSLPAL	2	2.20E-05	628.7052	1883.0937	3	0.0097	19	0	Chymotrypsin
H15_MOUSE	Histone H1	ITKAVSASKERGGVSLPAL	2	1.10E-07	628.7032	1883.0877	3	0.0037	19	0	Chymotrypsin
H15_MOUSE	Histone H1	RGGVSLPALKKALAAGGYD	3	9.50E-09	615.3541	1843.0405	3	0.0089	19	0	GluC
H15_MOUSE	Histone H1	RGGVSLPALKKALAAGGYD	3	7.60E-03	615.3512	1843.0318	3	0.0002	19	0	GluC
H15_MOUSE	Histone H1	RGGVSLPALKKALAAGGYD	3	5.10E-03	615.3512	1843.0318	3	0.0002	19	0	GluC
H15_MOUSE	Histone H1	AAGGYDVEKNNSRIKL	4	1.60E-06	578.9791	1733.9154	3	0.0094	16	1	Chymotrypsin
H15_MOUSE	Histone H1	AAGGYDVEKNNSRIKL	4	8.20E-07	867.9642	1733.9138	2	0.0078	16	1	Chymotrypsin
H15_MOUSE	Histone H1	AAGGYDVEKNNSRIKL	4	8.20E-05	434.4849	1733.9104	4	0.0044	16	1	Chymotrypsin
H15_MOUSE	Histone H1	AAGGYDVEKNNSRIKL	4	1.80E-05	867.962	1733.9095	2	0.0035	16	1	Chymotrypsin
H15_MOUSE	Histone H1	KALAAGGYDVEKNNSR	14	1.90E-07	564.9595	1691.8566	3	-0.0024	16	2	Semi-tryptic
H15_MOUSE	Histone H1	KALAAGGYDVEKNNSR	14	2.90E-04	846.9388	1691.8631	2	0.0040	16	2	Semi-tryptic
H15_MOUSE	Histone H1	KALAAGGYDVEKNNSR	14	1.70E-05	846.9388	1691.8631	2	0.0040	16	2	Semi-tryptic
H15_MOUSE	Histone H1	KALAAGGYDVEKNNSR	14	2.30E-10	564.9612	1691.8617	3	0.0027	16	2	Semi-tryptic
H15_MOUSE	Histone H1	KALAAGGYDVEKNNSR	14	8.50E-12	564.9612	1691.8617	3	0.0027	16	2	Semi-tryptic
H15_MOUSE	Histone H1	KALAAGGYDVEKNNSR	14	1.30E-07	564.961	1691.8610	3	0.0020	16	2	Semi-tryptic
H15_MOUSE	Histone H1	KALAAGGYDVEKNNSR	14	1.60E-08	846.9378	1691.8611	2	0.0020	16	2	Semi-tryptic
H15_MOUSE	Histone H1	KALAAGGYDVEKNNSR	14	1.30E-03	564.9609	1691.8609	3	0.0018	16	2	Semi-tryptic
H15_MOUSE	Histone H1	KALAAGGYDVEKNNSR	14	7.90E-10	846.9375	1691.8604	2	0.0013	16	2	Semi-tryptic
H15_MOUSE	Histone H1	KALAAGGYDVEKNNSR	14	8.60E-08	564.9605	1691.8597	3	0.0006	16	2	Semi-tryptic
H15_MOUSE	Histone H1	KALAAGGYDVEKNNSR	14	8.10E-08	564.9605	1691.8596	3	0.0006	16	2	Semi-tryptic
H15_MOUSE	Histone H1	KALAAGGYDVEKNNSR	14	3.80E-10	564.9605	1691.8596	3	0.0006	16	2	Semi-tryptic
H15_MOUSE	Histone H1	KALAAGGYDVEKNNSR	14	3.20E-06	564.9605	1691.8595	3	0.0005	16	2	Semi-tryptic
H15_MOUSE	Histone H1	KALAAGGYDVEKNNSR	14	9.60E-06	564.9603	1691.8590	3	0.0000	16	2	Semi-tryptic

H15_MOUSE	Histone H1	ALAAGGYDVEKNNSR	16	5.10E-05	522.2605	1563.7596	3	-0.0045	15	1	Semi-tryptic
H15_MOUSE	Histone H1	ALAAGGYDVEKNNSR	16	2.50E-07	522.2605	1563.7596	3	-0.0045	15	1	Semi-tryptic
H15_MOUSE	Histone H1	ALAAGGYDVEKNNSR	16	1.80E-12	782.8919	1563.7693	2	0.0052	15	1	Semi-tryptic
H15_MOUSE	Histone H1	ALAAGGYDVEKNNSR	16	9.00E-03	782.8914	1563.7683	2	0.0042	15	1	Semi-tryptic
H15_MOUSE	Histone H1	ALAAGGYDVEKNNSR	16	5.40E-04	782.8914	1563.7683	2	0.0042	15	1	Semi-tryptic
H15_MOUSE	Histone H1	ALAAGGYDVEKNNSR	16	8.90E-03	522.2631	1563.7676	3	0.0035	15	1	Semi-tryptic
H15_MOUSE	Histone H1	ALAAGGYDVEKNNSR	16	2.00E-12	782.8911	1563.7676	2	0.0035	15	1	Semi-tryptic
H15_MOUSE	Histone H1	ALAAGGYDVEKNNSR	16	4.20E-07	522.2628	1563.7665	3	0.0024	15	1	Semi-tryptic
H15_MOUSE	Histone H1	ALAAGGYDVEKNNSR	16	2.80E-08	522.2628	1563.7665	3	0.0024	15	1	Semi-tryptic
H15_MOUSE	Histone H1	ALAAGGYDVEKNNSR	16	7.80E-04	782.8905	1563.7664	2	0.0023	15	1	Semi-tryptic
H15_MOUSE	Histone H1	ALAAGGYDVEKNNSR	16	1.80E-06	522.262	1563.7643	3	0.0002	15	1	Semi-tryptic
H15_MOUSE	Histone H1	ALAAGGYDVEKNNSR	16	1.10E-07	522.262	1563.7643	3	0.0002	15	1	Semi-tryptic
H15_MOUSE	Histone H1	ALAAGGYDVEKNNSR	16	1.80E-09	782.8895	1563.7643	2	0.0002	15	1	Semi-tryptic
H15_MOUSE	Histone H1	ALAAGGYDVEKNNSR	16	5.80E-07	522.2619	1563.7639	3	-0.0002	15	1	Semi-tryptic
H15_MOUSE	Histone H1	ALAAGGYDVEKNNSR	16	6.20E-10	782.8892	1563.7638	2	-0.0003	15	1	Semi-tryptic
H15_MOUSE	Histone H1	ALAAGGYDVEKNNSR	16	6.90E-04	782.8886	1563.7627	2	-0.0014	15	1	Semi-tryptic
H15_MOUSE	Histone H1	RKATGPPVSELITK	9	8.60E-07	748.9469	1495.8792	2	0.0070	14	2	Semi-tryptic
H15_MOUSE	Histone H1	RKATGPPVSELITK	9	5.90E-04	499.6326	1495.8760	3	0.0038	14	2	Semi-tryptic
H15_MOUSE	Histone H1	RKATGPPVSELITK	9	1.00E-04	499.6321	1495.8746	3	0.0024	14	2	Semi-tryptic
H15_MOUSE	Histone H1	RKATGPPVSELITK	9	9.90E-06	499.6321	1495.8746	3	0.0024	14	2	Semi-tryptic
H15_MOUSE	Histone H1	RKATGPPVSELITK	9	9.80E-05	499.6315	1495.8726	3	0.0004	14	2	Semi-tryptic
H15_MOUSE	Histone H1	RKATGPPVSELITK	9	2.50E-04	499.6315	1495.8726	3	0.0003	14	2	Semi-tryptic
H15_MOUSE	Histone H1	RKATGPPVSELITK	9	1.20E-07	748.943	1495.8715	2	-0.0007	14	2	Semi-tryptic
H15_MOUSE	Histone H1	RKATGPPVSELITK	9	2.70E-04	499.6307	1495.8704	3	-0.0018	14	2	Semi-tryptic
H15_MOUSE	Histone H1	RKATGPPVSELITK	9	9.80E-05	499.6303	1495.8690	3	-0.0032	14	2	Semi-tryptic
H15_MOUSE	Histone H1	AKKPAGATPKKPK	3	5.60E-06	441.2831	1320.8276	3	0.0035	13	2	Semi-tryptic
H15_MOUSE	Histone H1	AKKPAGATPKKPK	3	9.20E-03	441.2831	1320.8274	3	0.0033	13	2	Semi-tryptic
H15_MOUSE	Histone H1	AKKPAGATPKKPK	3	1.50E-05	331.2139	1320.8264	4	0.0023	13	2	Semi-tryptic
H15_MOUSE	Histone H1	KATGPPVSELITK	14	1.50E-06	447.5996	1339.7771	3	0.0060	13	1	Semi-tryptic
H15_MOUSE	Histone H1	KATGPPVSELITK	14	4.60E-08	670.8953	1339.7761	2	0.0050	13	1	Semi-tryptic
H15_MOUSE	Histone H1	KATGPPVSELITK	14	5.30E-04	447.5991	1339.7754	3	0.0043	13	1	Semi-tryptic
H15_MOUSE	Histone H1	KATGPPVSELITK	14	9.80E-10	670.8939	1339.7733	2	0.0022	13	1	Semi-tryptic
H15_MOUSE	Histone H1	KATGPPVSELITK	14	1.40E-04	447.598	1339.7722	3	0.0011	13	1	Semi-tryptic
H15_MOUSE	Histone H1	KATGPPVSELITK	14	7.60E-05	447.598	1339.7722	3	0.0011	13	1	Semi-tryptic
H15_MOUSE	Histone H1	KATGPPVSELITK	14	8.50E-10	670.8933	1339.7721	2	0.0010	13	1	Semi-tryptic
H15_MOUSE	Histone H1	KATGPPVSELITK	14	4.50E-05	447.598	1339.7721	3	0.0009	13	1	Semi-tryptic
H15_MOUSE	Histone H1	KATGPPVSELITK	14	2.90E-08	670.8933	1339.7720	2	0.0009	13	1	Semi-tryptic
H15_MOUSE	Histone H1	KATGPPVSELITK	14	6.70E-09	670.8932	1339.7719	2	0.0008	13	1	Semi-tryptic
H15_MOUSE	Histone H1	KATGPPVSELITK	14	3.20E-04	447.5978	1339.7714	3	0.0003	13	1	Semi-tryptic
H15_MOUSE	Histone H1	KATGPPVSELITK	14	4.30E-06	447.5977	1339.7713	3	0.0002	13	1	Semi-tryptic
H15_MOUSE	Histone H1	KATGPPVSELITK	14	2.90E-06	447.5976	1339.7711	3	0.0000	13	1	Semi-tryptic
H15_MOUSE	Histone H1	KATGPPVSELITK	14	4.40E-06	447.5975	1339.7708	3	-0.0003	13	1	Semi-tryptic
H15_MOUSE	Histone H1	KALAAGGYDVEK	2	1.80E-09	611.3294	1220.6442	2	0.0041	12	1	Semi-tryptic
H15_MOUSE	Histone H1	KALAAGGYDVEK	2	6.70E-04	407.8879	1220.6420	3	0.0019	12	1	Semi-tryptic
H15_MOUSE	Histone H1	KAKKPAAAGVKK	3	5.10E-05	399.5999	1195.7779	3	0.0015	12	3	Semi-tryptic
H15_MOUSE	Histone H1	KAKKPAAAGVKK	3	4.80E-04	399.5994	1195.7765	3	0.0001	12	3	Semi-tryptic
H15_MOUSE	Histone H1	KAKKPAAAGVKK	3	8.20E-05	399.5994	1195.7764	3	-0.0001	12	3	Semi-tryptic
H15_MOUSE	Histone H1	ATGPPVSELITK	5	5.90E-05	606.8487	1211.6828	2	0.0066	12	0	Semi-tryptic
H15_MOUSE	Histone H1	ATGPPVSELITK	5	1.40E-06	606.8469	1211.6791	2	0.0030	12	0	Semi-tryptic
H15_MOUSE	Histone H1	ERGGVSLPALKK	5	1.00E-03	418.9232	1253.7478	3	0.0023	12	2	Semi-tryptic
H15_MOUSE	Histone H1	ERGGVSLPALKK	5	3.80E-05	418.9232	1253.7478	3	0.0023	12	2	Semi-tryptic
H15_MOUSE	Histone H1	ATGPPVSELITK	5	3.90E-03	606.8463	1211.6780	2	0.0019	12	0	Semi-tryptic
H15_MOUSE	Histone H1	ERGGVSLPALKK	5	1.00E-02	418.9227	1253.7464	3	0.0008	12	2	Semi-tryptic
H15_MOUSE	Histone H1	ERGGVSLPALKK	5	7.60E-05	418.9227	1253.7462	3	0.0007	12	2	Semi-tryptic
H15_MOUSE	Histone H1	ATGPPVSELITK	5	3.70E-07	606.8456	1211.6767	2	0.0006	12	0	Semi-tryptic
H15_MOUSE	Histone H1	ERGGVSLPALKK	5	9.10E-04	418.9224	1253.7455	3	-0.0001	12	2	Semi-tryptic
H15_MOUSE	Histone H1	ATGPPVSELITK	5	8.40E-07	606.8448	1211.6750	2	-0.0011	12	0	Semi-tryptic
H15_MOUSE	Histone H1	ERGGVSLPALK	3	4.10E-04	563.8347	1125.6549	2	0.0043	11	1	Semi-tryptic
H15_MOUSE	Histone H1	ERGGVSLPALK	3	9.80E-06	376.2242	1125.6509	3	0.0003	11	1	Semi-tryptic
H15_MOUSE	Histone H1	ERGGVSLPALK	3	5.50E-03	563.8326	1125.6506	2	0.0000	11	1	Semi-tryptic
H15_MOUSE	Histone H1	ALAAGGYDVEK	6	1.60E-03	547.2806	1092.5467	2	0.0016	11	0	Semi-tryptic
H15_MOUSE	Histone H1	ALAAGGYDVEK	6	1.30E-08	547.2803	1092.5459	2	0.0008	11	0	Semi-tryptic
H15_MOUSE	Histone H1	ALAAGGYDVEK	6	2.90E-03	547.2802	1092.5458	2	0.0007	11	0	Semi-tryptic
H15_MOUSE	Histone H1	ALAAGGYDVEK	6	4.40E-06	547.2802	1092.5458	2	0.0007	11	0	Semi-tryptic
H15_MOUSE	Histone H1	ALAAGGYDVEK	6	3.90E-06	547.2798	1092.5451	2	-0.0001	11	0	Semi-tryptic
H15_MOUSE	Histone H1	ALAAGGYDVEK	6	3.10E-07	547.2796	1092.5447	2	-0.0004	11	0	Semi-tryptic
H15_MOUSE	Histone H1	LITKAVSASKE	55	5.70E-05	573.839	1145.6635	2	-0.0021	11	0	GluC
H15_MOUSE	Histone H1	LITKAVSASKE	55	9.90E-06	382.8944	1145.6614	3	-0.0042	11	0	GluC
H15_MOUSE	Histone H1	LITKAVSASKE	55	1.70E-03	573.8433	1145.6721	2	0.0066	11	0	GluC
H15_MOUSE	Histone H1	LITKAVSASKE	55	2.50E-08	382.8977	1145.6712	3	0.0056	11	0	GluC
H15_MOUSE	Histone H1	LITKAVSASKE	55	3.70E-03	573.8425	1145.6705	2	0.0049	11	0	GluC
H15_MOUSE	Histone H1	LITKAVSASKE	55	9.90E-09	382.8973	1145.6700	3	0.0044	11	0	GluC
H15_MOUSE	Histone H1	LITKAVSASKE	55	5.30E-06	382.8972	1145.6699	3	0.0043	11	0	GluC
H15_MOUSE	Histone H1	LITKAVSASKE	55	2.50E-03	573.8418	1145.6691	2	0.0036	11	0	GluC

H15_MOUSE	Histone H1	LITKAVSASKE	55	1.80E-07	382.8969	1145.6689	3	0.0033	11	0	GluC
H15_MOUSE	Histone H1	LITKAVSASKE	55	1.70E-07	382.8967	1145.6682	3	0.0027	11	0	GluC
H15_MOUSE	Histone H1	LITKAVSASKE	55	4.40E-03	382.8967	1145.6681	3	0.0026	11	0	GluC
H15_MOUSE	Histone H1	LITKAVSASKE	55	7.20E-06	382.8967	1145.6682	3	0.0026	11	0	GluC
H15_MOUSE	Histone H1	LITKAVSASKE	55	6.50E-06	573.8411	1145.6677	2	0.0022	11	0	GluC
H15_MOUSE	Histone H1	LITKAVSASKE	55	7.90E-05	382.8965	1145.6677	3	0.0021	11	0	GluC
H15_MOUSE	Histone H1	LITKAVSASKE	55	2.20E-06	382.8965	1145.6676	3	0.0021	11	0	GluC
H15_MOUSE	Histone H1	LITKAVSASKE	55	2.80E-04	382.8964	1145.6674	3	0.0019	11	0	GluC
H15_MOUSE	Histone H1	LITKAVSASKE	55	5.60E-06	382.8964	1145.6674	3	0.0019	11	0	GluC
H15_MOUSE	Histone H1	LITKAVSASKE	55	1.80E-03	382.8963	1145.6671	3	0.0015	11	0	GluC
H15_MOUSE	Histone H1	LITKAVSASKE	55	3.80E-04	382.8963	1145.6671	3	0.0015	11	0	GluC
H15_MOUSE	Histone H1	LITKAVSASKE	55	4.50E-03	382.8962	1145.6667	3	0.0012	11	0	GluC
H15_MOUSE	Histone H1	LITKAVSASKE	55	1.20E-07	382.8962	1145.6667	3	0.0012	11	0	GluC
H15_MOUSE	Histone H1	LITKAVSASKE	55	2.90E-08	382.8962	1145.6667	3	0.0012	11	0	GluC
H15_MOUSE	Histone H1	LITKAVSASKE	55	8.80E-06	573.8406	1145.6666	2	0.0011	11	0	GluC
H15_MOUSE	Histone H1	LITKAVSASKE	55	9.70E-05	382.8961	1145.6665	3	0.0010	11	0	GluC
H15_MOUSE	Histone H1	LITKAVSASKE	55	5.40E-06	382.8961	1145.6666	3	0.0010	11	0	GluC
H15_MOUSE	Histone H1	LITKAVSASKE	55	6.80E-08	382.8961	1145.6665	3	0.0010	11	0	GluC
H15_MOUSE	Histone H1	LITKAVSASKE	55	1.10E-03	573.8404	1145.6662	2	0.0006	11	0	GluC
H15_MOUSE	Histone H1	LITKAVSASKE	55	3.10E-05	382.896	1145.6662	3	0.0006	11	0	GluC
H15_MOUSE	Histone H1	LITKAVSASKE	55	1.90E-06	382.896	1145.6662	3	0.0006	11	0	GluC
H15_MOUSE	Histone H1	LITKAVSASKE	55	2.30E-07	573.8404	1145.6662	2	0.0006	11	0	GluC
H15_MOUSE	Histone H1	LITKAVSASKE	55	1.30E-08	382.896	1145.6662	3	0.0006	11	0	GluC
H15_MOUSE	Histone H1	LITKAVSASKE	55	1.00E-02	382.896	1145.6661	3	0.0005	11	0	GluC
H15_MOUSE	Histone H1	LITKAVSASKE	55	4.20E-03	573.8403	1145.6660	2	0.0005	11	0	GluC
H15_MOUSE	Histone H1	LITKAVSASKE	55	1.80E-06	573.8403	1145.6660	2	0.0005	11	0	GluC
H15_MOUSE	Histone H1	LITKAVSASKE	55	1.50E-07	382.8959	1145.6660	3	0.0004	11	0	GluC
H15_MOUSE	Histone H1	LITKAVSASKE	55	5.20E-03	382.8959	1145.6659	3	0.0003	11	0	GluC
H15_MOUSE	Histone H1	LITKAVSASKE	55	4.30E-07	382.8959	1145.6659	3	0.0003	11	0	GluC
H15_MOUSE	Histone H1	LITKAVSASKE	55	8.60E-03	573.8401	1145.6657	2	0.0002	11	0	GluC
H15_MOUSE	Histone H1	LITKAVSASKE	55	5.60E-03	573.8402	1145.6658	2	0.0002	11	0	GluC
H15_MOUSE	Histone H1	LITKAVSASKE	55	2.80E-03	573.8401	1145.6657	2	0.0002	11	0	GluC
H15_MOUSE	Histone H1	LITKAVSASKE	55	9.00E-04	573.8401	1145.6657	2	0.0002	11	0	GluC
H15_MOUSE	Histone H1	LITKAVSASKE	55	1.10E-06	573.8401	1145.6657	2	0.0002	11	0	GluC
H15_MOUSE	Histone H1	LITKAVSASKE	55	7.90E-07	382.8959	1145.6658	3	0.0002	11	0	GluC
H15_MOUSE	Histone H1	LITKAVSASKE	55	3.50E-07	382.8959	1145.6658	3	0.0002	11	0	GluC
H15_MOUSE	Histone H1	LITKAVSASKE	55	1.90E-03	382.8958	1145.6655	3	-0.0001	11	0	GluC
H15_MOUSE	Histone H1	LITKAVSASKE	55	8.20E-04	382.8958	1145.6655	3	-0.0001	11	0	GluC
H15_MOUSE	Histone H1	LITKAVSASKE	55	1.50E-05	382.8958	1145.6655	3	-0.0001	11	0	GluC
H15_MOUSE	Histone H1	LITKAVSASKE	55	1.20E-06	573.84	1145.6655	2	-0.0001	11	0	GluC
H15_MOUSE	Histone H1	LITKAVSASKE	55	6.70E-03	573.8399	1145.6653	2	-0.0002	11	0	GluC
H15_MOUSE	Histone H1	LITKAVSASKE	55	1.00E-03	573.84	1145.6654	2	-0.0002	11	0	GluC
H15_MOUSE	Histone H1	LITKAVSASKE	55	2.80E-05	573.84	1145.6654	2	-0.0002	11	0	GluC
H15_MOUSE	Histone H1	LITKAVSASKE	55	9.40E-06	573.8399	1145.6653	2	-0.0002	11	0	GluC
H15_MOUSE	Histone H1	LITKAVSASKE	55	1.20E-03	382.8957	1145.6652	3	-0.0004	11	0	GluC
H15_MOUSE	Histone H1	LITKAVSASKE	55	7.20E-03	382.8952	1145.6637	3	-0.0018	11	0	GluC
H15_MOUSE	Histone H1	LITKAVSASKE	55	2.00E-07	382.8952	1145.6637	3	-0.0018	11	0	GluC
H15_MOUSE	Histone H1	AKKPAGATPK	2	4.30E-03	484.8003	967.5861	2	0.0047	10	1	Semi-tryptic
H15_MOUSE	Histone H1	AKKPAGATPK	2	2.90E-04	323.535	967.5831	3	0.0017	10	1	Semi-tryptic
H15_MOUSE	Histone H1	AKKPAAAGVK	3	2.10E-04	314.2035	939.5887	3	0.0022	10	1	Semi-tryptic
H15_MOUSE	Histone H1	AKKPAAAGVK	3	5.60E-03	314.2032	939.5877	3	0.0011	10	1	Semi-tryptic
H15_MOUSE	Histone H1	AKKPAAAGVK	3	1.10E-03	314.2032	939.5877	3	0.0011	10	1	Semi-tryptic
H15_MOUSE	Histone H1	GGVSLPALKK	8	8.50E-04	323.8756	968.6050	3	0.0032	10	1	Semi-tryptic
H15_MOUSE	Histone H1	GGVSLPALKK	8	3.00E-04	485.3095	968.6044	2	0.0026	10	1	Semi-tryptic
H15_MOUSE	Histone H1	GGVSLPALKK	8	2.20E-03	485.309	968.6034	2	0.0015	10	1	Semi-tryptic
H15_MOUSE	Histone H1	GGVSLPALKK	8	1.40E-03	485.3082	968.6019	2	0.0001	10	1	Semi-tryptic
H15_MOUSE	Histone H1	GGVSLPALKK	8	7.20E-03	323.8746	968.6019	3	0.0000	10	1	Semi-tryptic
H15_MOUSE	Histone H1	GGVSLPALKK	8	2.70E-04	485.3081	968.6017	2	-0.0002	10	1	Semi-tryptic
H15_MOUSE	Histone H1	GGVSLPALKK	8	7.40E-04	323.8745	968.6015	3	-0.0003	10	1	Semi-tryptic
H15_MOUSE	Histone H1	GGVSLPALKK	8	2.20E-04	485.3076	968.6007	2	-0.0011	10	1	Semi-tryptic
H15_MOUSE	Histone H1	GGVSLPALK	5	5.50E-03	421.262	840.5095	2	0.0026	9	0	Semi-tryptic
H15_MOUSE	Histone H1	GGVSLPALK	5	2.70E-05	421.261	840.5074	2	0.0005	9	0	Semi-tryptic
H15_MOUSE	Histone H1	GGVSLPALK	5	3.00E-03	421.2607	840.5069	2	0.0000	9	0	Semi-tryptic
H15_MOUSE	Histone H1	GGVSLPALK	5	2.50E-03	421.2607	840.5069	2	0.0000	9	0	Semi-tryptic
H15_MOUSE	Histone H1	GGVSLPALK	5	1.30E-03	421.2601	840.5057	2	-0.0012	9	0	Semi-tryptic
H15_MOUSE	Histone H1	AVSASKER	2	9.60E-05	424.2351	846.4557	2	-0.0002	8	1	Semi-tryptic
H15_MOUSE	Histone H1	AVSASKER	2	2.50E-05	424.2346	846.4547	2	-0.0012	8	1	Semi-tryptic
H1T_MOUSE, H11_MOUSE, H12_MOUSE, H13_MOUSE, H14_MOUSE	Histone H1	AAAGYDVEKNNSRIKL	3	2.60E-07	583.6502	1747.9286	3	0.0070	16	1	Chymotrypsin
H1T_MOUSE, H11_MOUSE, H12_MOUSE, H13_MOUSE, H14_MOUSE	Histone H1	AAAGYDVEKNNSRIKL	3	3.10E-03	874.9714	1747.9282	2	0.0066	16	1	Chymotrypsin
H1T_MOUSE, H11_MOUSE, H12_MOUSE, H13_MOUSE, H14_MOUSE	Histone H1	AAAGYDVEKNNSRIKL	3	5.10E-06	583.6489	1747.9249	3	0.0033	16	1	Chymotrypsin
H1T_MOUSE, H11_MOUSE, H12_MOUSE, H13_MOUSE, H14_MOUSE, H15_MOUSE	Histone H1	DVEKNNSRIKL	10	4.30E-04	658.3729	1314.7313	2	0.0058	11	0	Chymotrypsin
H1T_MOUSE, H11_MOUSE, H12_MOUSE, H13_MOUSE, H14_MOUSE, H15_MOUSE	Histone H1	DVEKNNSRIKL	10	4.80E-05	439.2508	1314.7305	3	0.0050	11	0	Chymotrypsin

H1T_MOUSE, H11_MOUSE, H12_MOUSE, H13_MOUSE, H14_MOUSE, H15_MOUSE	Histone H1	DVEKNNSRIKL	10	2.50E-04	658.3718	1314.7290	2	0.0034	11	0	Chymotrypsin
H1T_MOUSE, H11_MOUSE, H12_MOUSE, H13_MOUSE, H14_MOUSE, H15_MOUSE	Histone H1	DVEKNNSRIKL	10	1.50E-03	439.2501	1314.7286	3	0.0031	11	0	Chymotrypsin
H1T_MOUSE, H11_MOUSE, H12_MOUSE, H13_MOUSE, H14_MOUSE, H15_MOUSE	Histone H1	DVEKNNSRIKL	10	6.40E-04	658.3715	1314.7285	2	0.0030	11	0	Chymotrypsin
H1T_MOUSE, H11_MOUSE, H12_MOUSE, H13_MOUSE, H14_MOUSE, H15_MOUSE	Histone H1	DVEKNNSRIKL	10	2.00E-03	658.3711	1314.7276	2	0.0021	11	0	Chymotrypsin
H1T_MOUSE, H11_MOUSE, H12_MOUSE, H13_MOUSE, H14_MOUSE, H15_MOUSE	Histone H1	DVEKNNSRIKL	10	1.10E-03	658.3711	1314.7276	2	0.0021	11	0	Chymotrypsin
H1T_MOUSE, H11_MOUSE, H12_MOUSE, H13_MOUSE, H14_MOUSE, H15_MOUSE	Histone H1	DVEKNNSRIKL	10	2.00E-05	439.2493	1314.7260	3	0.0005	11	0	Chymotrypsin
H1T_MOUSE, H11_MOUSE, H12_MOUSE, H13_MOUSE, H14_MOUSE, H15_MOUSE	Histone H1	DVEKNNSRIKL	10	3.20E-03	439.249	1314.7251	3	-0.0004	11	0	Chymotrypsin
H1T_MOUSE, H11_MOUSE, H12_MOUSE, H13_MOUSE, H14_MOUSE, H15_MOUSE	Histone H1	DVEKNNSRIKL	10	2.00E-04	439.2488	1314.7247	3	-0.0008	11	0	Chymotrypsin
H1T_MOUSE, H12_MOUSE, H13_MOUSE, H14_MOUSE	Histone H1	KALAAAGYDVEKNNSR	24	4.60E-09	569.6337	1705.8793	3	0.0046	16	2	Semi-tryptic
H1T_MOUSE, H12_MOUSE, H13_MOUSE, H14_MOUSE	Histone H1	KALAAAGYDVEKNNSR	24	1.60E-03	853.9466	1705.8786	2	0.0039	16	2	Semi-tryptic
H1T_MOUSE, H12_MOUSE, H13_MOUSE, H14_MOUSE	Histone H1	KALAAAGYDVEKNNSR	24	7.40E-05	853.9466	1705.8786	2	0.0039	16	2	Semi-tryptic
H1T_MOUSE, H12_MOUSE, H13_MOUSE, H14_MOUSE	Histone H1	KALAAAGYDVEKNNSR	24	6.00E-03	853.9461	1705.8775	2	0.0029	16	2	Semi-tryptic
H1T_MOUSE, H12_MOUSE, H13_MOUSE, H14_MOUSE	Histone H1	KALAAAGYDVEKNNSR	24	2.80E-04	853.9461	1705.8775	2	0.0029	16	2	Semi-tryptic
H1T_MOUSE, H12_MOUSE, H13_MOUSE, H14_MOUSE	Histone H1	KALAAAGYDVEKNNSR	24	2.50E-09	569.6329	1705.8770	3	0.0023	16	2	Semi-tryptic
H1T_MOUSE, H12_MOUSE, H13_MOUSE, H14_MOUSE	Histone H1	KALAAAGYDVEKNNSR	24	1.20E-10	569.6329	1705.8770	3	0.0023	16	2	Semi-tryptic
H1T_MOUSE, H12_MOUSE, H13_MOUSE, H14_MOUSE	Histone H1	KALAAAGYDVEKNNSR	24	6.60E-08	569.6328	1705.8766	3	0.0019	16	2	Semi-tryptic
H1T_MOUSE, H12_MOUSE, H13_MOUSE, H14_MOUSE	Histone H1	KALAAAGYDVEKNNSR	24	8.10E-10	853.9455	1705.8765	2	0.0018	16	2	Semi-tryptic
H1T_MOUSE, H12_MOUSE, H13_MOUSE, H14_MOUSE	Histone H1	KALAAAGYDVEKNNSR	24	3.30E-09	427.4764	1705.8764	4	0.0017	16	2	Semi-tryptic
H1T_MOUSE, H12_MOUSE, H13_MOUSE, H14_MOUSE	Histone H1	KALAAAGYDVEKNNSR	24	1.60E-10	427.4764	1705.8764	4	0.0017	16	2	Semi-tryptic
H1T_MOUSE, H12_MOUSE, H13_MOUSE, H14_MOUSE	Histone H1	KALAAAGYDVEKNNSR	24	4.60E-08	569.6327	1705.8762	3	0.0015	16	2	Semi-tryptic
H1T_MOUSE, H12_MOUSE, H13_MOUSE, H14_MOUSE	Histone H1	KALAAAGYDVEKNNSR	24	9.50E-08	569.6327	1705.8761	3	0.0014	16	2	Semi-tryptic
H1T_MOUSE, H12_MOUSE, H13_MOUSE, H14_MOUSE	Histone H1	KALAAAGYDVEKNNSR	24	6.00E-08	569.6325	1705.8757	3	0.0010	16	2	Semi-tryptic
H1T_MOUSE, H12_MOUSE, H13_MOUSE, H14_MOUSE	Histone H1	KALAAAGYDVEKNNSR	24	6.90E-07	569.6324	1705.8755	3	0.0008	16	2	Semi-tryptic
H1T_MOUSE, H12_MOUSE, H13_MOUSE, H14_MOUSE	Histone H1	KALAAAGYDVEKNNSR	24	1.10E-09	853.9449	1705.8753	2	0.0007	16	2	Semi-tryptic
H1T_MOUSE, H12_MOUSE, H13_MOUSE, H14_MOUSE	Histone H1	KALAAAGYDVEKNNSR	24	1.10E-11	853.9449	1705.8752	2	0.0005	16	2	Semi-tryptic
H1T_MOUSE, H12_MOUSE, H13_MOUSE, H14_MOUSE	Histone H1	KALAAAGYDVEKNNSR	24	4.50E-09	569.6323	1705.8751	3	0.0004	16	2	Semi-tryptic
H1T_MOUSE, H12_MOUSE, H13_MOUSE, H14_MOUSE	Histone H1	KALAAAGYDVEKNNSR	24	7.30E-08	569.6323	1705.8750	3	0.0003	16	2	Semi-tryptic
H1T_MOUSE, H12_MOUSE, H13_MOUSE, H14_MOUSE	Histone H1	KALAAAGYDVEKNNSR	24	1.20E-09	569.6323	1705.8750	3	0.0003	16	2	Semi-tryptic
H1T_MOUSE, H12_MOUSE, H13_MOUSE, H14_MOUSE	Histone H1	KALAAAGYDVEKNNSR	24	8.30E-09	569.6322	1705.8749	3	0.0002	16	2	Semi-tryptic
H1T_MOUSE, H12_MOUSE, H13_MOUSE, H14_MOUSE	Histone H1	KALAAAGYDVEKNNSR	24	8.90E-09	569.6322	1705.8748	3	0.0001	16	2	Semi-tryptic
H1T_MOUSE, H12_MOUSE, H13_MOUSE, H14_MOUSE	Histone H1	KALAAAGYDVEKNNSR	24	1.60E-07	569.6319	1705.8740	3	-0.0007	16	2	Semi-tryptic
H1T_MOUSE, H12_MOUSE, H13_MOUSE, H14_MOUSE	Histone H1	KALAAAGYDVEKNNSR	24	2.30E-08	569.6318	1705.8735	3	-0.0012	16	2	Semi-tryptic
H1T_MOUSE, H12_MOUSE, H13_MOUSE, H14_MOUSE	Histone H1	ALAAAGYDVEKNNSR	29	1.70E-06	789.8943	1577.7739	2	-0.0058	15	1	Semi-tryptic
H1T_MOUSE, H12_MOUSE, H13_MOUSE, H14_MOUSE	Histone H1	ALAAAGYDVEKNNSR	29	8.10E-10	789.8989	1577.7833	2	0.0036	15	1	Semi-tryptic
H1T_MOUSE, H12_MOUSE, H13_MOUSE, H14_MOUSE	Histone H1	ALAAAGYDVEKNNSR	29	1.10E-05	526.9347	1577.7824	3	0.0027	15	1	Semi-tryptic
H1T_MOUSE, H12_MOUSE, H13_MOUSE, H14_MOUSE	Histone H1	ALAAAGYDVEKNNSR	29	4.60E-07	526.9347	1577.7824	3	0.0027	15	1	Semi-tryptic
H1T_MOUSE, H12_MOUSE, H13_MOUSE, H14_MOUSE	Histone H1	ALAAAGYDVEKNNSR	29	4.40E-09	789.8983	1577.7821	2	0.0024	15	1	Semi-tryptic
H1T_MOUSE, H12_MOUSE, H13_MOUSE, H14_MOUSE	Histone H1	ALAAAGYDVEKNNSR	29	5.30E-11	789.8983	1577.7821	2	0.0023	15	1	Semi-tryptic
H1T_MOUSE, H12_MOUSE, H13_MOUSE, H14_MOUSE	Histone H1	ALAAAGYDVEKNNSR	29	1.50E-03	789.8982	1577.7819	2	0.0021	15	1	Semi-tryptic
H1T_MOUSE, H12_MOUSE, H13_MOUSE, H14_MOUSE	Histone H1	ALAAAGYDVEKNNSR	29	1.20E-04	789.8982	1577.7819	2	0.0021	15	1	Semi-tryptic
H1T_MOUSE, H12_MOUSE, H13_MOUSE, H14_MOUSE	Histone H1	ALAAAGYDVEKNNSR	29	7.80E-05	526.9344	1577.7815	3	0.0018	15	1	Semi-tryptic
H1T_MOUSE, H12_MOUSE, H13_MOUSE, H14_MOUSE	Histone H1	ALAAAGYDVEKNNSR	29	1.10E-06	526.9344	1577.7815	3	0.0018	15	1	Semi-tryptic
H1T_MOUSE, H12_MOUSE, H13_MOUSE, H14_MOUSE	Histone H1	ALAAAGYDVEKNNSR	29	1.80E-10	789.898	1577.7814	2	0.0017	15	1	Semi-tryptic
H1T_MOUSE, H12_MOUSE, H13_MOUSE, H14_MOUSE	Histone H1	ALAAAGYDVEKNNSR	29	1.50E-04	789.8979	1577.7812	2	0.0014	15	1	Semi-tryptic
H1T_MOUSE, H12_MOUSE, H13_MOUSE, H14_MOUSE	Histone H1	ALAAAGYDVEKNNSR	29	1.20E-05	789.8979	1577.7812	2	0.0014	15	1	Semi-tryptic
H1T_MOUSE, H12_MOUSE, H13_MOUSE, H14_MOUSE	Histone H1	ALAAAGYDVEKNNSR	29	1.80E-09	789.8978	1577.7810	2	0.0013	15	1	Semi-tryptic
H1T_MOUSE, H12_MOUSE, H13_MOUSE, H14_MOUSE	Histone H1	ALAAAGYDVEKNNSR	29	1.90E-09	789.8977	1577.7808	2	0.0011	15	1	Semi-tryptic
H1T_MOUSE, H12_MOUSE, H13_MOUSE, H14_MOUSE	Histone H1	ALAAAGYDVEKNNSR	29	2.10E-10	789.8977	1577.7808	2	0.0011	15	1	Semi-tryptic
H1T_MOUSE, H12_MOUSE, H13_MOUSE, H14_MOUSE	Histone H1	ALAAAGYDVEKNNSR	29	6.20E-04	789.8976	1577.7807	2	0.0009	15	1	Semi-tryptic
H1T_MOUSE, H12_MOUSE, H13_MOUSE, H14_MOUSE	Histone H1	ALAAAGYDVEKNNSR	29	4.70E-05	789.8976	1577.7807	2	0.0009	15	1	Semi-tryptic
H1T_MOUSE, H12_MOUSE, H13_MOUSE, H14_MOUSE	Histone H1	ALAAAGYDVEKNNSR	29	2.20E-10	789.8976	1577.7806	2	0.0009	15	1	Semi-tryptic
H1T_MOUSE, H12_MOUSE, H13_MOUSE, H14_MOUSE	Histone H1	ALAAAGYDVEKNNSR	29	8.80E-10	789.8975	1577.7804	2	0.0007	15	1	Semi-tryptic
H1T_MOUSE, H12_MOUSE, H13_MOUSE, H14_MOUSE	Histone H1	ALAAAGYDVEKNNSR	29	1.90E-10	789.897	1577.7795	2	-0.0002	15	1	Semi-tryptic
H1T_MOUSE, H12_MOUSE, H13_MOUSE, H14_MOUSE	Histone H1	ALAAAGYDVEKNNSR	29	3.80E-10	789.897	1577.7795	2	-0.0003	15	1	Semi-tryptic
H1T_MOUSE, H12_MOUSE, H13_MOUSE, H14_MOUSE	Histone H1	ALAAAGYDVEKNNSR	29	8.90E-06	526.9337	1577.7793	3	-0.0004	15	1	Semi-tryptic
H1T_MOUSE, H12_MOUSE, H13_MOUSE, H14_MOUSE	Histone H1	ALAAAGYDVEKNNSR	29	2.20E-08	526.9337	1577.7793	3	-0.0004	15	1	Semi-tryptic
H1T_MOUSE, H12_MOUSE, H13_MOUSE, H14_MOUSE	Histone H1	ALAAAGYDVEKNNSR	29	1.90E-09	789.8969	1577.7793	2	-0.0004	15	1	Semi-tryptic
H1T_MOUSE, H12_MOUSE, H13_MOUSE, H14_MOUSE	Histone H1	ALAAAGYDVEKNNSR	29	1.70E-03	526.9337	1577.7792	3	-0.0005	15	1	Semi-tryptic
H1T_MOUSE, H12_MOUSE, H13_MOUSE, H14_MOUSE	Histone H1	ALAAAGYDVEKNNSR	29	8.80E-10	789.8969	1577.7793	2	-0.0005	15	1	Semi-tryptic
H1T_MOUSE, H12_MOUSE, H13_MOUSE, H14_MOUSE	Histone H1	ALAAAGYDVEKNNSR	29	7.00E-06	526.9333	1577.7781	3	-0.0016	15	1	Semi-tryptic
H1T_MOUSE, H12_MOUSE, H13_MOUSE, H14_MOUSE	Histone H1	ALAAAGYDVEKNNSR	29	1.80E-07	526.9333	1577.7781	3	-0.0016	15	1	Semi-tryptic
H1T_MOUSE, H12_MOUSE, H13_MOUSE, H14_MOUSE	Histone H1	AALKKALAAAGY	3	1.00E-07	383.2343	1146.6811	3	0.0050	12	2	Chymotrypsin
H1T_MOUSE, H12_MOUSE, H13_MOUSE, H14_MOUSE	Histone H1	AALKKALAAAGY	3	7.70E-05	574.3475	1146.6805	2	0.0045	12	2	Chymotrypsin
H1T_MOUSE, H12_MOUSE, H13_MOUSE, H14_MOUSE	Histone H1	AALKKALAAAGY	3	4.90E-05	574.347	1146.6795	2	0.0034	12	2	Chymotrypsin
H1T_MOUSE, H12_MOUSE, H13_MOUSE, H14_MOUSE	Histone H1	KALAAAGYDVEK	9	8.50E-10	618.338	1234.6615	2	0.0058	12	1	Semi-tryptic
H1T_MOUSE, H12_MOUSE, H13_MOUSE, H14_MOUSE	Histone H1	KALAAAGYDVEK	9	9.70E-07	412.56	1234.6583	3	0.0026	12	1	Semi-tryptic

H1T_MOUSE, H12_MOUSE, H13_MOUSE, H14_MOUSE	Histone H1	KALAAAGYDVEK	9	8.20E-08	412.56	1234.6583	3	0.0026	12	1	Semi-tryptic
H1T_MOUSE, H12_MOUSE, H13_MOUSE, H14_MOUSE	Histone H1	KALAAAGYDVEK	9	1.20E-08	618.336	1234.6574	2	0.0017	12	1	Semi-tryptic
H1T_MOUSE, H12_MOUSE, H13_MOUSE, H14_MOUSE	Histone H1	KALAAAGYDVEK	9	3.50E-03	618.3358	1234.6570	2	0.0013	12	1	Semi-tryptic
H1T_MOUSE, H12_MOUSE, H13_MOUSE, H14_MOUSE	Histone H1	KALAAAGYDVEK	9	1.20E-07	618.3356	1234.6567	2	0.0010	12	1	Semi-tryptic
H1T_MOUSE, H12_MOUSE, H13_MOUSE, H14_MOUSE	Histone H1	KALAAAGYDVEK	9	8.30E-07	618.3354	1234.6563	2	0.0006	12	1	Semi-tryptic
H1T_MOUSE, H12_MOUSE, H13_MOUSE, H14_MOUSE	Histone H1	KALAAAGYDVEK	9	2.20E-06	412.5592	1234.6559	3	0.0001	12	1	Semi-tryptic
H1T_MOUSE, H12_MOUSE, H13_MOUSE, H14_MOUSE	Histone H1	KALAAAGYDVEK	9	3.10E-07	412.5592	1234.6559	3	0.0001	12	1	Semi-tryptic
H1T_MOUSE, H12_MOUSE, H13_MOUSE, H14_MOUSE	Histone H1	ALAAAGYDVEK	18	3.30E-07	554.2897	1106.5649	2	0.0041	11	0	Semi-tryptic
H1T_MOUSE, H12_MOUSE, H13_MOUSE, H14_MOUSE	Histone H1	ALAAAGYDVEK	18	1.60E-06	554.2895	1106.5645	2	0.0038	11	0	Semi-tryptic
H1T_MOUSE, H12_MOUSE, H13_MOUSE, H14_MOUSE	Histone H1	ALAAAGYDVEK	18	5.80E-08	554.2895	1106.5644	2	0.0037	11	0	Semi-tryptic
H1T_MOUSE, H12_MOUSE, H13_MOUSE, H14_MOUSE	Histone H1	ALAAAGYDVEK	18	4.90E-03	554.2894	1106.5641	2	0.0034	11	0	Semi-tryptic
H1T_MOUSE, H12_MOUSE, H13_MOUSE, H14_MOUSE	Histone H1	ALAAAGYDVEK	18	4.60E-04	554.2894	1106.5641	2	0.0034	11	0	Semi-tryptic
H1T_MOUSE, H12_MOUSE, H13_MOUSE, H14_MOUSE	Histone H1	ALAAAGYDVEK	18	3.00E-07	554.2888	1106.5630	2	0.0023	11	0	Semi-tryptic
H1T_MOUSE, H12_MOUSE, H13_MOUSE, H14_MOUSE	Histone H1	ALAAAGYDVEK	18	1.60E-07	554.2882	1106.5618	2	0.0010	11	0	Semi-tryptic
H1T_MOUSE, H12_MOUSE, H13_MOUSE, H14_MOUSE	Histone H1	ALAAAGYDVEK	18	7.10E-03	554.288	1106.5615	2	0.0008	11	0	Semi-tryptic
H1T_MOUSE, H12_MOUSE, H13_MOUSE, H14_MOUSE	Histone H1	ALAAAGYDVEK	18	7.90E-08	554.288	1106.5615	2	0.0007	11	0	Semi-tryptic
H1T_MOUSE, H12_MOUSE, H13_MOUSE, H14_MOUSE	Histone H1	ALAAAGYDVEK	18	5.30E-06	554.288	1106.5613	2	0.0006	11	0	Semi-tryptic
H1T_MOUSE, H12_MOUSE, H13_MOUSE, H14_MOUSE	Histone H1	ALAAAGYDVEK	18	2.60E-08	554.2879	1106.5613	2	0.0005	11	0	Semi-tryptic
H1T_MOUSE, H12_MOUSE, H13_MOUSE, H14_MOUSE	Histone H1	ALAAAGYDVEK	18	2.50E-08	554.2878	1106.5611	2	0.0004	11	0	Semi-tryptic
H1T_MOUSE, H12_MOUSE, H13_MOUSE, H14_MOUSE	Histone H1	ALAAAGYDVEK	18	1.10E-05	554.2877	1106.5609	2	0.0001	11	0	Semi-tryptic
H1T_MOUSE, H12_MOUSE, H13_MOUSE, H14_MOUSE	Histone H1	ALAAAGYDVEK	18	1.40E-06	554.2877	1106.5608	2	0.0000	11	0	Semi-tryptic
H1T_MOUSE, H12_MOUSE, H13_MOUSE, H14_MOUSE	Histone H1	ALAAAGYDVEK	18	5.70E-07	554.2877	1106.5608	2	0.0000	11	0	Semi-tryptic
H1T_MOUSE, H12_MOUSE, H13_MOUSE, H14_MOUSE	Histone H1	ALAAAGYDVEK	18	3.50E-08	554.2876	1106.5607	2	0.0000	11	0	Semi-tryptic
H1T_MOUSE, H12_MOUSE, H13_MOUSE, H14_MOUSE	Histone H1	ALAAAGYDVEK	18	1.20E-08	554.2875	1106.5604	2	-0.0003	11	0	Semi-tryptic
H1T_MOUSE, H12_MOUSE, H13_MOUSE, H14_MOUSE	Histone H1	ALAAAGYDVEK	18	9.50E-07	554.2875	1106.5604	2	-0.0004	11	0	Semi-tryptic
H1T_MOUSE, H12_MOUSE, H13_MOUSE, H14_MOUSE	Histone H1	KKALAAAGY	2	1.00E-03	446.7678	891.5210	2	0.0032	9	1	Chymotrypsin
H1T_MOUSE, H12_MOUSE, H13_MOUSE, H14_MOUSE	Histone H1	KKALAAAGY	2	3.20E-04	446.7673	891.5201	2	0.0024	9	1	Chymotrypsin
H1T_MOUSE, H12_MOUSE, H13_MOUSE, H14_MOUSE	Histone H1	AALKKAL	2	7.00E-04	357.7488	713.4831	2	0.0032	7	1	Chymotrypsin
H1T_MOUSE, H12_MOUSE, H13_MOUSE, H14_MOUSE	Histone H1	AALKKAL	2	1.30E-03	357.7478	713.4810	2	0.0011	7	1	Chymotrypsin
H1T_MOUSE, H12_MOUSE, H13_MOUSE, H14_MOUSE, H15_MOUSE	Histone H1	VQTKGTGASGSF	10	3.30E-03	570.2902	1138.5658	2	0.0040	12	0	Chymotrypsin
H1T_MOUSE, H12_MOUSE, H13_MOUSE, H14_MOUSE, H15_MOUSE	Histone H1	VQTKGTGASGSF	10	5.30E-08	570.2892	1138.5638	2	0.0019	12	0	Chymotrypsin
H1T_MOUSE, H12_MOUSE, H13_MOUSE, H14_MOUSE, H15_MOUSE	Histone H1	VQTKGTGASGSF	10	2.10E-04	570.2888	1138.5631	2	0.0012	12	0	Chymotrypsin
H1T_MOUSE, H12_MOUSE, H13_MOUSE, H14_MOUSE, H15_MOUSE	Histone H1	VQTKGTGASGSF	10	3.60E-03	570.2888	1138.5630	2	0.0011	12	0	Chymotrypsin
H1T_MOUSE, H12_MOUSE, H13_MOUSE, H14_MOUSE, H15_MOUSE	Histone H1	VQTKGTGASGSF	10	1.80E-03	570.2887	1138.5629	2	0.0010	12	0	Chymotrypsin
H1T_MOUSE, H12_MOUSE, H13_MOUSE, H14_MOUSE, H15_MOUSE	Histone H1	VQTKGTGASGSF	10	1.30E-03	570.2886	1138.5626	2	0.0007	12	0	Chymotrypsin
H1T_MOUSE, H12_MOUSE, H13_MOUSE, H14_MOUSE, H15_MOUSE	Histone H1	VQTKGTGASGSF	10	1.10E-03	570.2886	1138.5626	2	0.0007	12	0	Chymotrypsin
H1T_MOUSE, H12_MOUSE, H13_MOUSE, H14_MOUSE, H15_MOUSE	Histone H1	VQTKGTGASGSF	10	5.40E-03	570.2884	1138.5622	2	0.0003	12	0	Chymotrypsin
H1T_MOUSE, H12_MOUSE, H13_MOUSE, H14_MOUSE, H15_MOUSE	Histone H1	VQTKGTGASGSF	10	1.80E-03	570.2882	1138.5619	2	0.0000	12	0	Chymotrypsin
H1T_MOUSE, H12_MOUSE, H13_MOUSE, H14_MOUSE, H15_MOUSE	Histone H1	VQTKGTGASGSF	10	3.50E-07	570.2882	1138.5619	2	0.0000	12	0	Chymotrypsin
H1T_MOUSE, H12_MOUSE, H13_MOUSE, H14_MOUSE, H15_MOUSE	Histone H1	GTGASGSFK	12	9.00E-05	406.2029	810.3913	2	0.0041	9	0	Semi-tryptic
H1T_MOUSE, H12_MOUSE, H13_MOUSE, H14_MOUSE, H15_MOUSE	Histone H1	GTGASGSFK	12	9.20E-05	406.2025	810.3904	2	0.0032	9	0	Semi-tryptic
H1T_MOUSE, H12_MOUSE, H13_MOUSE, H14_MOUSE, H15_MOUSE	Histone H1	GTGASGSFK	12	5.10E-04	406.2017	810.3889	2	0.0017	9	0	Semi-tryptic
H1T_MOUSE, H12_MOUSE, H13_MOUSE, H14_MOUSE, H15_MOUSE	Histone H1	GTGASGSFK	12	7.40E-05	406.2017	810.3889	2	0.0017	9	0	Semi-tryptic
H1T_MOUSE, H12_MOUSE, H13_MOUSE, H14_MOUSE, H15_MOUSE	Histone H1	GTGASGSFK	12	9.50E-03	406.2017	810.3888	2	0.0016	9	0	Semi-tryptic
H1T_MOUSE, H12_MOUSE, H13_MOUSE, H14_MOUSE, H15_MOUSE	Histone H1	GTGASGSFK	12	6.20E-03	406.2011	810.3876	2	0.0005	9	0	Semi-tryptic
H1T_MOUSE, H12_MOUSE, H13_MOUSE, H14_MOUSE, H15_MOUSE	Histone H1	GTGASGSFK	12	1.20E-03	406.2011	810.3876	2	0.0005	9	0	Semi-tryptic
H1T_MOUSE, H12_MOUSE, H13_MOUSE, H14_MOUSE, H15_MOUSE	Histone H1	GTGASGSFK	12	2.90E-03	406.2011	810.3876	2	0.0004	9	0	Semi-tryptic
H1T_MOUSE, H12_MOUSE, H13_MOUSE, H14_MOUSE, H15_MOUSE	Histone H1	GTGASGSFK	12	2.40E-03	406.2009	810.3873	2	0.0002	9	0	Semi-tryptic
H1T_MOUSE, H12_MOUSE, H13_MOUSE, H14_MOUSE, H15_MOUSE	Histone H1	GTGASGSFK	12	3.30E-03	406.2007	810.3868	2	-0.0003	9	0	Semi-tryptic
H1T_MOUSE, H12_MOUSE, H13_MOUSE, H14_MOUSE, H15_MOUSE	Histone H1	GTGASGSFK	12	6.40E-04	406.2007	810.3868	2	-0.0003	9	0	Semi-tryptic
H1T_MOUSE, H12_MOUSE, H13_MOUSE, H14_MOUSE, H15_MOUSE	Histone H1	GTGASGSFK	12	6.90E-04	406.2004	810.3863	2	-0.0009	9	0	Semi-tryptic
Q80ZM5_MOUSE	Histone H1	RGGSSLARIYAE	4	8.90E-03	427.2304	1278.6694	3	0.0014	12	0	GluC
Q80ZM5_MOUSE	Histone H1	RGGSSLARIYAE	4	4.60E-03	427.23	1278.6681	3	0.0001	12	0	GluC
Q80ZM5_MOUSE	Histone H1	RGGSSLARIYAE	4	4.80E-04	427.2294	1278.6663	3	-0.0017	12	0	GluC
Q80ZM5_MOUSE	Histone H1	RGGSSLARIYAE	4	2.10E-03	640.3395	1278.6645	2	-0.0035	12	0	GluC
Q80ZM5_MOUSE	Histone H1	SQLVVETIRKL	4	1.90E-04	643.3981	1284.7816	2	0.0051	11	1	Chymotrypsin
Q80ZM5_MOUSE	Histone H1	SQLVVETIRKL	4	3.40E-04	429.2676	1284.7809	3	0.0044	11	1	Chymotrypsin
Q80ZM5_MOUSE	Histone H1	SQLVVETIRKL	4	2.10E-03	643.3953	1284.7760	2	-0.0005	11	1	Chymotrypsin
Q80ZM5_MOUSE	Histone H1	SQLVVETIRKL	4	4.10E-03	429.2658	1284.7755	3	-0.0010	11	1	Chymotrypsin
H2A1_MOUSE, H2A2A_MOUSE, H2A3_MOUSE	Histone H2A	LPKKTESHHKAKGK	18	2.70E-06	397.9866	1587.9175	4	-0.0034	14	0	GluC
H2A1_MOUSE, H2A2A_MOUSE, H2A3_MOUSE	Histone H2A	LPKKTESHHKAKGK	18	1.40E-05	530.313	1587.9172	3	-0.0037	14	0	GluC
H2A1_MOUSE, H2A2A_MOUSE, H2A3_MOUSE	Histone H2A	LPKKTESHHKAKGK	18	1.10E-06	397.9866	1587.9172	4	-0.0037	14	0	GluC
H2A1_MOUSE, H2A2A_MOUSE, H2A3_MOUSE	Histone H2A	LPKKTESHHKAKGK	18	5.70E-08	397.986	1587.9150	4	-0.0059	14	0	GluC
H2A1_MOUSE, H2A2A_MOUSE, H2A3_MOUSE	Histone H2A	LPKKTESHHKAKGK	18	1.60E-06	397.9896	1587.9293	4	0.0085	14	0	GluC
H2A1_MOUSE, H2A2A_MOUSE, H2A3_MOUSE	Histone H2A	LPKKTESHHKAKGK	18	1.10E-03	318.5922	1587.9248	5	0.0039	14	0	GluC
H2A1_MOUSE, H2A2A_MOUSE, H2A3_MOUSE	Histone H2A	LPKKTESHHKAKGK	18	1.90E-07	397.988	1587.9229	4	0.0020	14	0	GluC
H2A1_MOUSE, H2A2A_MOUSE, H2A3_MOUSE	Histone H2A	LPKKTESHHKAKGK	18	4.40E-10	397.9878	1587.9221	4	0.0013	14	0	Chymotrypsin
H2A1_MOUSE, H2A2A_MOUSE, H2A3_MOUSE	Histone H2A	LPKKTESHHKAKGK	18	3.90E-03	794.9683	1587.9220	2	0.0012	14	0	Chymotrypsin
H2A1_MOUSE, H2A2A_MOUSE, H2A3_MOUSE	Histone H2A	LPKKTESHHKAKGK	18	8.30E-04	318.5916	1587.9218	5	0.0010	14	0	GluC
H2A1_MOUSE, H2A2A_MOUSE, H2A3_MOUSE	Histone H2A	LPKKTESHHKAKGK	18	1.80E-03	530.3144	1587.9214	3	0.0005	14	0	Chymotrypsin
H2A1_MOUSE, H2A2A_MOUSE, H2A3_MOUSE	Histone H2A	LPKKTESHHKAKGK	18	1.10E-06	530.3144	1587.9214	3	0.0005	14	0	Chymotrypsin
H2A1_MOUSE, H2A2A_MOUSE, H2A3_MOUSE	Histone H2A	LPKKTESHHKAKGK	18	4.60E-07	397.9876	1587.9214	4	0.0005	14	0	Chymotrypsin
H2A1_MOUSE, H2A2A_MOUSE, H2A3_MOUSE	Histone H2A	LPKKTESHHKAKGK	18	1.00E-08	397.9875	1587.9210	4	0.0001	14	0	Chymotrypsin
H2A1_MOUSE, H2A2A_MOUSE, H2A3_MOUSE	Histone H2A	LPKKTESHHKAKGK	18	2.30E-07	530.3142	1587.9207	3	-0.0002	14	0	GluC

H2A1_MOUSE, H2A2A_MOUSE, H2A3_MOUSE	Histone H2A	LPKKTESHHKAKGK	18	1.00E-08	530.314	1587.9201	3	-0.0008	14	0	GluC
H2A1_MOUSE, H2A2A_MOUSE, H2A3_MOUSE	Histone H2A	LPKKTESHHKAKGK	18	3.50E-08	530.3139	1587.9199	3	-0.0009	14	0	Chymotrypsin
H2A1_MOUSE, H2A2A_MOUSE, H2A3_MOUSE	Histone H2A	LPKKTESHHKAKGK	18	2.30E-07	530.3137	1587.9193	3	-0.0016	14	0	GluC
H2A1_MOUSE, H2A2A_MOUSE, H2A3_MOUSE	Histone H2A	SHHKAKGK	16	7.20E-03	446.7596	891.5046	2	0.0008	8	0	GluC
H2A1_MOUSE, H2A2A_MOUSE, H2A3_MOUSE	Histone H2A	SHHKAKGK	16	3.50E-03	446.7596	891.5046	2	0.0007	8	0	GluC
H2A1_MOUSE, H2A2A_MOUSE, H2A3_MOUSE	Histone H2A	SHHKAKGK	16	6.60E-03	446.7595	891.5044	2	0.0006	8	0	GluC
H2A1_MOUSE, H2A2A_MOUSE, H2A3_MOUSE	Histone H2A	SHHKAKGK	16	1.00E-02	446.7594	891.5043	2	0.0004	8	0	GluC
H2A1_MOUSE, H2A2A_MOUSE, H2A3_MOUSE	Histone H2A	SHHKAKGK	16	6.70E-03	446.7594	891.5043	2	0.0004	8	0	GluC
H2A1_MOUSE, H2A2A_MOUSE, H2A3_MOUSE	Histone H2A	SHHKAKGK	16	2.60E-03	446.7593	891.5041	2	0.0002	8	0	GluC
H2A1_MOUSE, H2A2A_MOUSE, H2A3_MOUSE	Histone H2A	SHHKAKGK	16	8.70E-03	446.7592	891.5039	2	0.0000	8	0	GluC
H2A1_MOUSE, H2A2A_MOUSE, H2A3_MOUSE	Histone H2A	SHHKAKGK	16	7.50E-03	446.7592	891.5039	2	0.0000	8	0	GluC
H2A1_MOUSE, H2A2A_MOUSE, H2A3_MOUSE	Histone H2A	SHHKAKGK	16	2.10E-03	446.7592	891.5039	2	0.0000	8	0	GluC
H2A1_MOUSE, H2A2A_MOUSE, H2A3_MOUSE	Histone H2A	SHHKAKGK	16	5.60E-03	446.7592	891.5038	2	-0.0001	8	0	GluC
H2A1_MOUSE, H2A2A_MOUSE, H2A3_MOUSE	Histone H2A	SHHKAKGK	16	3.40E-03	446.7591	891.5036	2	-0.0003	8	0	GluC
H2A1_MOUSE, H2A2A_MOUSE, H2A3_MOUSE	Histone H2A	SHHKAKGK	16	3.00E-03	446.7591	891.5035	2	-0.0003	8	0	GluC
H2A1_MOUSE, H2A2A_MOUSE, H2A3_MOUSE	Histone H2A	SHHKAKGK	16	9.10E-03	446.759	891.5034	2	-0.0004	8	0	GluC
H2A1_MOUSE, H2A2A_MOUSE, H2A3_MOUSE	Histone H2A	SHHKAKGK	16	7.80E-03	446.759	891.5035	2	-0.0004	8	0	GluC
H2A1_MOUSE, H2A2A_MOUSE, H2A3_MOUSE	Histone H2A	SHHKAKGK	16	5.10E-03	446.759	891.5035	2	-0.0004	8	0	GluC
H2A1_MOUSE, H2A2A_MOUSE, H2A3_MOUSE	Histone H2A	SHHKAKGK	16	3.40E-03	446.759	891.5034	2	-0.0005	8	0	GluC
H2A1_MOUSE, H2A3_MOUSE	Histone H2A	DEELNKLLGRVTIAQGGVLPNIQAVLLPKKTESHHKAKGK	10	1.10E-04	1083.3753	4329.4721	4	0.0073	40	0	AspN
H2A1_MOUSE, H2A3_MOUSE	Histone H2A	DEELNKLLGRVTIAQGGVLPNIQAVLLPKKTESHHKAKGK	10	3.90E-03	866.9016	4329.4718	5	0.0070	40	0	AspN
H2A1_MOUSE, H2A3_MOUSE	Histone H2A	DEELNKLLGRVTIAQGGVLPNIQAVLLPKKTESHHKAKGK	10	7.80E-03	866.9014	4329.4708	5	0.0060	40	0	AspN
H2A1_MOUSE, H2A3_MOUSE	Histone H2A	DEELNKLLGRVTIAQGGVLPNIQAVLLPKKTESHHKAKGK	10	1.70E-04	1444.1639	4329.4699	3	0.0051	40	0	AspN
H2A1_MOUSE, H2A3_MOUSE	Histone H2A	DEELNKLLGRVTIAQGGVLPNIQAVLLPKKTESHHKAKGK	10	6.10E-03	1083.374	4329.4669	4	0.0021	40	0	AspN
H2A1_MOUSE, H2A3_MOUSE	Histone H2A	DEELNKLLGRVTIAQGGVLPNIQAVLLPKKTESHHKAKGK	10	2.80E-03	1083.374	4329.4669	4	0.0021	40	0	AspN
H2A1_MOUSE, H2A3_MOUSE	Histone H2A	DEELNKLLGRVTIAQGGVLPNIQAVLLPKKTESHHKAKGK	10	9.10E-03	722.5844	4329.4627	6	-0.0021	40	0	AspN
H2A1_MOUSE, H2A3_MOUSE	Histone H2A	DEELNKLLGRVTIAQGGVLPNIQAVLLPKKTESHHKAKGK	10	1.10E-04	866.8997	4329.4623	5	-0.0025	40	0	AspN
H2A1_MOUSE, H2A3_MOUSE	Histone H2A	DEELNKLLGRVTIAQGGVLPNIQAVLLPKKTESHHKAKGK	10	5.90E-07	866.8997	4329.4623	5	-0.0025	40	0	AspN
H2A1_MOUSE, H2A3_MOUSE	Histone H2A	DEELNKLLGRVTIAQGGVLPNIQAVLLPKKTESHHKAKGK	10	4.20E-07	866.8997	4329.4621	5	-0.0027	40	0	AspN
H2A1F_MOUSE	Histone H2A	LPKKTESHHKPKGK	4	5.80E-04	404.4923	1613.9399	4	0.0034	14	0	Chymotrypsin
H2A1F_MOUSE	Histone H2A	LPKKTESHHKPKGK	4	2.90E-06	404.492	1613.9388	4	0.0023	14	0	GluC
H2A1F_MOUSE	Histone H2A	LPKKTESHHKPKGK	4	7.90E-04	323.7948	1613.9376	5	0.0010	14	0	GluC
H2A1F_MOUSE	Histone H2A	LPKKTESHHKPKGK	4	1.30E-06	538.9863	1613.9370	3	0.0004	14	0	GluC
H2A1F_MOUSE	Histone H2A	SHHKPKGK	3	9.60E-04	306.8476	917.5211	3	0.0016	8	0	GluC
H2A1F_MOUSE	Histone H2A	SHHKPKGK	3	4.50E-03	306.8472	917.5197	3	0.0001	8	0	GluC
H2A1F_MOUSE	Histone H2A	SHHKPKGK	3	1.30E-03	306.847	917.5191	3	-0.0004	8	0	GluC
H2A1F_MOUSE, H2A1H_MOUSE, H2A1_MOUSE, H2A1K_MOUSE	Histone H2A	TRSSRAGLQFPVGR	2	2.40E-03	511.286	1530.8361	3	-0.0018	14	2	Semi-tryptic
H2A1F_MOUSE, H2A1H_MOUSE, H2A1_MOUSE, H2A1K_MOUSE	Histone H2A	TRSSRAGLQFPVGR	2	4.50E-03	511.2855	1530.8346	3	-0.0034	14	2	Semi-tryptic
H2A1F_MOUSE, H2A1H_MOUSE, H2A1_MOUSE, H2A1K_MOUSE, H2A2A_MOUSE, H2A2B_MOUSE, H2A2C_MOUSE, H2A3_MOUSE, H2AJ_MOUSE, H2AV_MOUSE, H2AX_MOUSE, H2AZ_MOUSE	Histone H2A	AGLQFPVGR	26	6.50E-04	472.7682	943.5218	2	-0.0022	9	0	Semi-tryptic
H2A1F_MOUSE, H2A1H_MOUSE, H2A1_MOUSE, H2A1K_MOUSE, H2A2A_MOUSE, H2A2B_MOUSE, H2A2C_MOUSE, H2A3_MOUSE, H2AJ_MOUSE, H2AV_MOUSE, H2AX_MOUSE, H2AZ_MOUSE	Histone H2A	AGLQFPVGR	26	6.20E-04	472.7679	943.5213	2	-0.0027	9	0	Semi-tryptic
H2A1F_MOUSE, H2A1H_MOUSE, H2A1_MOUSE, H2A1K_MOUSE, H2A2A_MOUSE, H2A2B_MOUSE, H2A2C_MOUSE, H2A3_MOUSE, H2AJ_MOUSE, H2AV_MOUSE, H2AX_MOUSE, H2AZ_MOUSE	Histone H2A	AGLQFPVGR	26	3.30E-03	472.7677	943.5209	2	-0.0030	9	0	Semi-tryptic
H2A1F_MOUSE, H2A1H_MOUSE, H2A1_MOUSE, H2A1K_MOUSE, H2A2A_MOUSE, H2A2B_MOUSE, H2A2C_MOUSE, H2A3_MOUSE, H2AJ_MOUSE, H2AV_MOUSE, H2AX_MOUSE, H2AZ_MOUSE	Histone H2A	AGLQFPVGR	26	5.20E-04	472.7677	943.5209	2	-0.0030	9	0	Semi-tryptic
H2A1F_MOUSE, H2A1H_MOUSE, H2A1_MOUSE, H2A1K_MOUSE, H2A2A_MOUSE, H2A2B_MOUSE, H2A2C_MOUSE, H2A3_MOUSE, H2AJ_MOUSE, H2AV_MOUSE, H2AX_MOUSE, H2AZ_MOUSE	Histone H2A	AGLQFPVGR	26	6.30E-04	472.7676	943.5206	2	-0.0034	9	0	Semi-tryptic
H2A1F_MOUSE, H2A1H_MOUSE, H2A1_MOUSE, H2A1K_MOUSE, H2A2A_MOUSE, H2A2B_MOUSE, H2A2C_MOUSE, H2A3_MOUSE, H2AJ_MOUSE, H2AV_MOUSE, H2AX_MOUSE, H2AZ_MOUSE	Histone H2A	AGLQFPVGR	26	5.10E-04	472.7711	943.5277	2	0.0037	9	0	Semi-tryptic
H2A1F_MOUSE, H2A1H_MOUSE, H2A1_MOUSE, H2A1K_MOUSE, H2A2A_MOUSE, H2A2B_MOUSE, H2A2C_MOUSE, H2A3_MOUSE, H2AJ_MOUSE, H2AV_MOUSE, H2AX_MOUSE, H2AZ_MOUSE	Histone H2A	AGLQFPVGR	26	5.20E-03	472.7697	943.5248	2	0.0009	9	0	Semi-tryptic
H2A1F_MOUSE, H2A1H_MOUSE, H2A1_MOUSE, H2A1K_MOUSE, H2A2A_MOUSE, H2A2B_MOUSE, H2A2C_MOUSE, H2A3_MOUSE, H2AJ_MOUSE, H2AV_MOUSE, H2AX_MOUSE, H2AZ_MOUSE	Histone H2A	AGLQFPVGR	26	4.80E-03	472.7696	943.5247	2	0.0008	9	0	Semi-tryptic
H2A1F_MOUSE, H2A1H_MOUSE, H2A1_MOUSE, H2A1K_MOUSE, H2A2A_MOUSE, H2A2B_MOUSE, H2A2C_MOUSE, H2A3_MOUSE, H2AJ_MOUSE, H2AV_MOUSE, H2AX_MOUSE, H2AZ_MOUSE	Histone H2A	AGLQFPVGR	26	6.40E-04	472.7696	943.5246	2	0.0007	9	0	Semi-tryptic
H2A1F_MOUSE, H2A1H_MOUSE, H2A1_MOUSE, H2A1K_MOUSE, H2A2A_MOUSE, H2A2B_MOUSE, H2A2C_MOUSE, H2A3_MOUSE, H2AJ_MOUSE, H2AV_MOUSE, H2AX_MOUSE, H2AZ_MOUSE	Histone H2A	AGLQFPVGR	26	6.30E-04	472.7693	943.5241	2	0.0002	9	0	Semi-tryptic
H2A1F_MOUSE, H2A1H_MOUSE, H2A1_MOUSE, H2A1K_MOUSE, H2A2A_MOUSE, H2A2B_MOUSE, H2A2C_MOUSE, H2A3_MOUSE, H2AJ_MOUSE, H2AV_MOUSE, H2AX_MOUSE, H2AZ_MOUSE	Histone H2A	AGLQFPVGR	26	5.10E-04	472.7693	943.5241	2	0.0001	9	0	Semi-tryptic
H2A1F_MOUSE, H2A1H_MOUSE, H2A1_MOUSE, H2A1K_MOUSE, H2A2A_MOUSE, H2A2B_MOUSE, H2A2C_MOUSE, H2A3_MOUSE, H2AJ_MOUSE, H2AV_MOUSE, H2AX_MOUSE, H2AZ_MOUSE	Histone H2A	AGLQFPVGR	26	3.10E-04	472.7693	943.5241	2	0.0001	9	0	Semi-tryptic

H2A1F_MOUSE, H2A1H_MOUSE, H2A1_MOUSE, H2A1K_MOUSE, H2A2A_MOUSE, H2A2B_MOUSE, H2A2C_MOUSE, H2A3_MOUSE, H2AJ_MOUSE, H2AV_MOUSE, H2AX_MOUSE, H2AZ_MOUSE	Histone H2A	AGLQFPVGR	26	6.90E-03	472.7693	943.5240	2	0.0000	9	0	Semi-tryptic
H2A1F_MOUSE, H2A1H_MOUSE, H2A1_MOUSE, H2A1K_MOUSE, H2A2A_MOUSE, H2A2B_MOUSE, H2A2C_MOUSE, H2A3_MOUSE, H2AJ_MOUSE, H2AV_MOUSE, H2AX_MOUSE, H2AZ_MOUSE	Histone H2A	AGLQFPVGR	26	5.00E-04	472.7693	943.5240	2	0.0000	9	0	Semi-tryptic
H2A1F_MOUSE, H2A1H_MOUSE, H2A1_MOUSE, H2A1K_MOUSE, H2A2A_MOUSE, H2A2B_MOUSE, H2A2C_MOUSE, H2A3_MOUSE, H2AJ_MOUSE, H2AV_MOUSE, H2AX_MOUSE, H2AZ_MOUSE	Histone H2A	AGLQFPVGR	26	5.30E-03	472.7692	943.5238	2	-0.0001	9	0	Semi-tryptic
H2A1F_MOUSE, H2A1H_MOUSE, H2A1_MOUSE, H2A1K_MOUSE, H2A2A_MOUSE, H2A2B_MOUSE, H2A2C_MOUSE, H2A3_MOUSE, H2AJ_MOUSE, H2AV_MOUSE, H2AX_MOUSE, H2AZ_MOUSE	Histone H2A	AGLQFPVGR	26	2.80E-03	472.7692	943.5238	2	-0.0001	9	0	Semi-tryptic
H2A1F_MOUSE, H2A1H_MOUSE, H2A1_MOUSE, H2A1K_MOUSE, H2A2A_MOUSE, H2A2B_MOUSE, H2A2C_MOUSE, H2A3_MOUSE, H2AJ_MOUSE, H2AV_MOUSE, H2AX_MOUSE, H2AZ_MOUSE	Histone H2A	AGLQFPVGR	26	6.30E-04	472.7692	943.5238	2	-0.0001	9	0	Semi-tryptic
H2A1F_MOUSE, H2A1H_MOUSE, H2A1_MOUSE, H2A1K_MOUSE, H2A2A_MOUSE, H2A2B_MOUSE, H2A2C_MOUSE, H2A3_MOUSE, H2AJ_MOUSE, H2AV_MOUSE, H2AX_MOUSE, H2AZ_MOUSE	Histone H2A	AGLQFPVGR	26	3.10E-04	472.7692	943.5238	2	-0.0001	9	0	Semi-tryptic
H2A1F_MOUSE, H2A1H_MOUSE, H2A1_MOUSE, H2A1K_MOUSE, H2A2A_MOUSE, H2A2B_MOUSE, H2A2C_MOUSE, H2A3_MOUSE, H2AJ_MOUSE, H2AV_MOUSE, H2AX_MOUSE, H2AZ_MOUSE	Histone H2A	AGLQFPVGR	26	5.10E-04	472.7691	943.5237	2	-0.0003	9	0	Semi-tryptic
H2A1F_MOUSE, H2A1H_MOUSE, H2A1_MOUSE, H2A1K_MOUSE, H2A2A_MOUSE, H2A2B_MOUSE, H2A2C_MOUSE, H2A3_MOUSE, H2AJ_MOUSE, H2AV_MOUSE, H2AX_MOUSE, H2AZ_MOUSE	Histone H2A	AGLQFPVGR	26	6.80E-04	472.769	943.5235	2	-0.0004	9	0	Semi-tryptic
H2A1F_MOUSE, H2A1H_MOUSE, H2A1_MOUSE, H2A1K_MOUSE, H2A2A_MOUSE, H2A2B_MOUSE, H2A2C_MOUSE, H2A3_MOUSE, H2AJ_MOUSE, H2AV_MOUSE, H2AX_MOUSE, H2AZ_MOUSE	Histone H2A	AGLQFPVGR	26	1.80E-03	472.769	943.5235	2	-0.0005	9	0	Semi-tryptic
H2A1F_MOUSE, H2A1H_MOUSE, H2A1_MOUSE, H2A1K_MOUSE, H2A2A_MOUSE, H2A2B_MOUSE, H2A2C_MOUSE, H2A3_MOUSE, H2AJ_MOUSE, H2AV_MOUSE, H2AX_MOUSE, H2AZ_MOUSE	Histone H2A	AGLQFPVGR	26	6.60E-04	472.769	943.5234	2	-0.0006	9	0	Semi-tryptic
H2A1F_MOUSE, H2A1H_MOUSE, H2A1_MOUSE, H2A1K_MOUSE, H2A2A_MOUSE, H2A2B_MOUSE, H2A2C_MOUSE, H2A3_MOUSE, H2AJ_MOUSE, H2AV_MOUSE, H2AX_MOUSE, H2AZ_MOUSE	Histone H2A	AGLQFPVGR	26	6.50E-04	472.7686	943.5225	2	-0.0014	9	0	Semi-tryptic
H2A1F_MOUSE, H2A1H_MOUSE, H2A1_MOUSE, H2A1K_MOUSE, H2A2A_MOUSE, H2A2B_MOUSE, H2A2C_MOUSE, H2A3_MOUSE, H2AJ_MOUSE, H2AV_MOUSE, H2AX_MOUSE, H2AZ_MOUSE	Histone H2A	AGLQFPVGR	26	7.30E-04	472.7685	943.5225	2	-0.0015	9	0	Semi-tryptic
H2A1F_MOUSE, H2A1H_MOUSE, H2A1_MOUSE, H2A1K_MOUSE, H2A2A_MOUSE, H2A2B_MOUSE, H2A2C_MOUSE, H2A3_MOUSE, H2AJ_MOUSE, H2AV_MOUSE, H2AX_MOUSE, H2AZ_MOUSE	Histone H2A	AGLQFPVGR	26	6.50E-04	472.7684	943.5223	2	-0.0016	9	0	Semi-tryptic
H2A1F_MOUSE, H2A1H_MOUSE, H2A1_MOUSE, H2A1K_MOUSE, H2A2A_MOUSE, H2A2B_MOUSE, H2A2C_MOUSE, H2A3_MOUSE, H2AJ_MOUSE, H2AV_MOUSE, H2AX_MOUSE, H2AZ_MOUSE	Histone H2A	AGLQFPVGR	26	2.00E-04	472.7683	943.5221	2	-0.0019	9	0	Semi-tryptic
H2A1F_MOUSE, H2A1H_MOUSE, H2A1_MOUSE, H2A1K_MOUSE, H2A2A_MOUSE, H2A2B_MOUSE, H2A2C_MOUSE, H2A3_MOUSE, H2AJ_MOUSE, H2AX_MOUSE	Histone H2A	VTIAQGGVLPNIQAVLLPKK	6	1.50E-11	1030.1378	2058.2610	2	0.0046	20	1	Semi-tryptic
H2A1F_MOUSE, H2A1H_MOUSE, H2A1_MOUSE, H2A1K_MOUSE, H2A2A_MOUSE, H2A2B_MOUSE, H2A2C_MOUSE, H2A3_MOUSE, H2AJ_MOUSE, H2AX_MOUSE	Histone H2A	VTIAQGGVLPNIQAVLLPKK	6	7.90E-07	687.0939	2058.2599	3	0.0034	20	1	Semi-tryptic
H2A1F_MOUSE, H2A1H_MOUSE, H2A1_MOUSE, H2A1K_MOUSE, H2A2A_MOUSE, H2A2B_MOUSE, H2A2C_MOUSE, H2A3_MOUSE, H2AJ_MOUSE, H2AX_MOUSE	Histone H2A	VTIAQGGVLPNIQAVLLPKK	6	7.50E-08	687.0936	2058.2590	3	0.0025	20	1	Semi-tryptic
H2A1F_MOUSE, H2A1H_MOUSE, H2A1_MOUSE, H2A1K_MOUSE, H2A2A_MOUSE, H2A2B_MOUSE, H2A2C_MOUSE, H2A3_MOUSE, H2AJ_MOUSE, H2AX_MOUSE	Histone H2A	VTIAQGGVLPNIQAVLLPKK	6	1.10E-05	687.0935	2058.2586	3	0.0021	20	1	Semi-tryptic
H2A1F_MOUSE, H2A1H_MOUSE, H2A1_MOUSE, H2A1K_MOUSE, H2A2A_MOUSE, H2A2B_MOUSE, H2A2C_MOUSE, H2A3_MOUSE, H2AJ_MOUSE, H2AX_MOUSE	Histone H2A	VTIAQGGVLPNIQAVLLPKK	6	1.30E-06	687.0929	2058.2568	3	0.0003	20	1	Semi-tryptic
H2A1F_MOUSE, H2A1H_MOUSE, H2A1_MOUSE, H2A1K_MOUSE, H2A2A_MOUSE, H2A2B_MOUSE, H2A2C_MOUSE, H2A3_MOUSE, H2AJ_MOUSE, H2AX_MOUSE	Histone H2A	VTIAQGGVLPNIQAVLLPKK	6	2.20E-07	687.0928	2058.2565	3	0.0000	20	1	Semi-tryptic
H2A1F_MOUSE, H2A1H_MOUSE, H2A1_MOUSE, H2A1K_MOUSE, H2A2A_MOUSE, H2A2B_MOUSE, H2A2C_MOUSE, H2A3_MOUSE, H2AJ_MOUSE, H2AX_MOUSE	Histone H2A	VTIAQGGVLPNIQAVLLPK	50	8.20E-09	966.0867	1930.1589	2	-0.0026	19	0	Semi-tryptic
H2A1F_MOUSE, H2A1H_MOUSE, H2A1_MOUSE, H2A1K_MOUSE, H2A2A_MOUSE, H2A2B_MOUSE, H2A2C_MOUSE, H2A3_MOUSE, H2AJ_MOUSE, H2AX_MOUSE	Histone H2A	VTIAQGGVLPNIQAVLLPK	50	6.50E-04	966.0929	1930.1712	2	0.0097	19	0	Semi-tryptic
H2A1F_MOUSE, H2A1H_MOUSE, H2A1_MOUSE, H2A1K_MOUSE, H2A2A_MOUSE, H2A2B_MOUSE, H2A2C_MOUSE, H2A3_MOUSE, H2AJ_MOUSE, H2AX_MOUSE	Histone H2A	VTIAQGGVLPNIQAVLLPK	50	8.90E-05	966.0929	1930.1712	2	0.0097	19	0	Semi-tryptic
H2A1F_MOUSE, H2A1H_MOUSE, H2A1_MOUSE, H2A1K_MOUSE, H2A2A_MOUSE, H2A2B_MOUSE, H2A2C_MOUSE, H2A3_MOUSE, H2AJ_MOUSE, H2AX_MOUSE	Histone H2A	VTIAQGGVLPNIQAVLLPK	50	2.20E-05	644.3974	1930.1703	3	0.0087	19	0	Semi-tryptic

H2A1F_MOUSE, H2A1H_MOUSE, H2A1_MOUSE, H2A1K_MOUSE, H2A2A_MOUSE, H2A2B_MOUSE, H2A2C_MOUSE, H2A3_MOUSE, H2AJ_MOUSE, H2AX_MOUSE	Histone H2A	VTIAQGGVLPNIQAVLLPK	50	8.80E-07	644.3974	1930.1703	3	0.0087	19	0	Semi-tryptic
H2A1F_MOUSE, H2A1H_MOUSE, H2A1_MOUSE, H2A1K_MOUSE, H2A2A_MOUSE, H2A2B_MOUSE, H2A2C_MOUSE, H2A3_MOUSE, H2AJ_MOUSE, H2AX_MOUSE	Histone H2A	VTIAQGGVLPNIQAVLLPK	50	1.50E-07	644.3963	1930.1670	3	0.0054	19	0	Semi-tryptic
H2A1F_MOUSE, H2A1H_MOUSE, H2A1_MOUSE, H2A1K_MOUSE, H2A2A_MOUSE, H2A2B_MOUSE, H2A2C_MOUSE, H2A3_MOUSE, H2AJ_MOUSE, H2AX_MOUSE	Histone H2A	VTIAQGGVLPNIQAVLLPK	50	4.70E-09	644.3963	1930.1670	3	0.0054	19	0	Semi-tryptic
H2A1F_MOUSE, H2A1H_MOUSE, H2A1_MOUSE, H2A1K_MOUSE, H2A2A_MOUSE, H2A2B_MOUSE, H2A2C_MOUSE, H2A3_MOUSE, H2AJ_MOUSE, H2AX_MOUSE	Histone H2A	VTIAQGGVLPNIQAVLLPK	50	3.60E-03	966.0904	1930.1663	2	0.0048	19	0	Semi-tryptic
H2A1F_MOUSE, H2A1H_MOUSE, H2A1_MOUSE, H2A1K_MOUSE, H2A2A_MOUSE, H2A2B_MOUSE, H2A2C_MOUSE, H2A3_MOUSE, H2AJ_MOUSE, H2AX_MOUSE	Histone H2A	VTIAQGGVLPNIQAVLLPK	50	4.90E-05	966.0904	1930.1663	2	0.0048	19	0	Semi-tryptic
H2A1F_MOUSE, H2A1H_MOUSE, H2A1_MOUSE, H2A1K_MOUSE, H2A2A_MOUSE, H2A2B_MOUSE, H2A2C_MOUSE, H2A3_MOUSE, H2AJ_MOUSE, H2AX_MOUSE	Histone H2A	VTIAQGGVLPNIQAVLLPK	50	1.40E-03	966.0901	1930.1656	2	0.0040	19	0	Semi-tryptic
H2A1F_MOUSE, H2A1H_MOUSE, H2A1_MOUSE, H2A1K_MOUSE, H2A2A_MOUSE, H2A2B_MOUSE, H2A2C_MOUSE, H2A3_MOUSE, H2AJ_MOUSE, H2AX_MOUSE	Histone H2A	VTIAQGGVLPNIQAVLLPK	50	1.60E-03	966.0896	1930.1647	2	0.0032	19	0	Semi-tryptic
H2A1F_MOUSE, H2A1H_MOUSE, H2A1_MOUSE, H2A1K_MOUSE, H2A2A_MOUSE, H2A2B_MOUSE, H2A2C_MOUSE, H2A3_MOUSE, H2AJ_MOUSE, H2AX_MOUSE	Histone H2A	VTIAQGGVLPNIQAVLLPK	50	6.50E-07	966.0896	1930.1646	2	0.0031	19	0	Semi-tryptic
H2A1F_MOUSE, H2A1H_MOUSE, H2A1_MOUSE, H2A1K_MOUSE, H2A2A_MOUSE, H2A2B_MOUSE, H2A2C_MOUSE, H2A3_MOUSE, H2AJ_MOUSE, H2AX_MOUSE	Histone H2A	VTIAQGGVLPNIQAVLLPK	50	2.60E-09	966.0895	1930.1645	2	0.0030	19	0	Semi-tryptic
H2A1F_MOUSE, H2A1H_MOUSE, H2A1_MOUSE, H2A1K_MOUSE, H2A2A_MOUSE, H2A2B_MOUSE, H2A2C_MOUSE, H2A3_MOUSE, H2AJ_MOUSE, H2AX_MOUSE	Histone H2A	VTIAQGGVLPNIQAVLLPK	50	2.40E-03	644.3954	1930.1645	3	0.0029	19	0	Semi-tryptic
H2A1F_MOUSE, H2A1H_MOUSE, H2A1_MOUSE, H2A1K_MOUSE, H2A2A_MOUSE, H2A2B_MOUSE, H2A2C_MOUSE, H2A3_MOUSE, H2AJ_MOUSE, H2AX_MOUSE	Histone H2A	VTIAQGGVLPNIQAVLLPK	50	1.00E-08	966.0895	1930.1644	2	0.0029	19	0	Semi-tryptic
H2A1F_MOUSE, H2A1H_MOUSE, H2A1_MOUSE, H2A1K_MOUSE, H2A2A_MOUSE, H2A2B_MOUSE, H2A2C_MOUSE, H2A3_MOUSE, H2AJ_MOUSE, H2AX_MOUSE	Histone H2A	VTIAQGGVLPNIQAVLLPK	50	2.00E-08	966.0894	1930.1643	2	0.0027	19	0	Semi-tryptic
H2A1F_MOUSE, H2A1H_MOUSE, H2A1_MOUSE, H2A1K_MOUSE, H2A2A_MOUSE, H2A2B_MOUSE, H2A2C_MOUSE, H2A3_MOUSE, H2AJ_MOUSE, H2AX_MOUSE	Histone H2A	VTIAQGGVLPNIQAVLLPK	50	1.80E-08	966.0893	1930.1640	2	0.0025	19	0	Semi-tryptic
H2A1F_MOUSE, H2A1H_MOUSE, H2A1_MOUSE, H2A1K_MOUSE, H2A2A_MOUSE, H2A2B_MOUSE, H2A2C_MOUSE, H2A3_MOUSE, H2AJ_MOUSE, H2AX_MOUSE	Histone H2A	VTIAQGGVLPNIQAVLLPK	50	8.40E-07	644.3952	1930.1639	3	0.0024	19	0	Semi-tryptic
H2A1F_MOUSE, H2A1H_MOUSE, H2A1_MOUSE, H2A1K_MOUSE, H2A2A_MOUSE, H2A2B_MOUSE, H2A2C_MOUSE, H2A3_MOUSE, H2AJ_MOUSE, H2AX_MOUSE	Histone H2A	VTIAQGGVLPNIQAVLLPK	50	1.90E-07	966.0892	1930.1639	2	0.0024	19	0	Semi-tryptic
H2A1F_MOUSE, H2A1H_MOUSE, H2A1_MOUSE, H2A1K_MOUSE, H2A2A_MOUSE, H2A2B_MOUSE, H2A2C_MOUSE, H2A3_MOUSE, H2AJ_MOUSE, H2AX_MOUSE	Histone H2A	VTIAQGGVLPNIQAVLLPK	50	9.70E-09	966.0892	1930.1638	2	0.0023	19	0	Semi-tryptic
H2A1F_MOUSE, H2A1H_MOUSE, H2A1_MOUSE, H2A1K_MOUSE, H2A2A_MOUSE, H2A2B_MOUSE, H2A2C_MOUSE, H2A3_MOUSE, H2AJ_MOUSE, H2AX_MOUSE	Histone H2A	VTIAQGGVLPNIQAVLLPK	50	6.10E-09	966.0892	1930.1638	2	0.0023	19	0	Semi-tryptic
H2A1F_MOUSE, H2A1H_MOUSE, H2A1_MOUSE, H2A1K_MOUSE, H2A2A_MOUSE, H2A2B_MOUSE, H2A2C_MOUSE, H2A3_MOUSE, H2AJ_MOUSE, H2AX_MOUSE	Histone H2A	VTIAQGGVLPNIQAVLLPK	50	5.70E-09	966.0892	1930.1638	2	0.0023	19	0	Semi-tryptic
H2A1F_MOUSE, H2A1H_MOUSE, H2A1_MOUSE, H2A1K_MOUSE, H2A2A_MOUSE, H2A2B_MOUSE, H2A2C_MOUSE, H2A3_MOUSE, H2AJ_MOUSE, H2AX_MOUSE	Histone H2A	VTIAQGGVLPNIQAVLLPK	50	9.20E-07	644.3952	1930.1638	3	0.0022	19	0	Semi-tryptic
H2A1F_MOUSE, H2A1H_MOUSE, H2A1_MOUSE, H2A1K_MOUSE, H2A2A_MOUSE, H2A2B_MOUSE, H2A2C_MOUSE, H2A3_MOUSE, H2AJ_MOUSE, H2AX_MOUSE	Histone H2A	VTIAQGGVLPNIQAVLLPK	50	7.70E-03	966.0891	1930.1637	2	0.0021	19	0	Semi-tryptic
H2A1F_MOUSE, H2A1H_MOUSE, H2A1_MOUSE, H2A1K_MOUSE, H2A2A_MOUSE, H2A2B_MOUSE, H2A2C_MOUSE, H2A3_MOUSE, H2AJ_MOUSE, H2AX_MOUSE	Histone H2A	VTIAQGGVLPNIQAVLLPK	50	1.50E-06	644.3951	1930.1635	3	0.0020	19	0	Semi-tryptic
H2A1F_MOUSE, H2A1H_MOUSE, H2A1_MOUSE, H2A1K_MOUSE, H2A2A_MOUSE, H2A2B_MOUSE, H2A2C_MOUSE, H2A3_MOUSE, H2AJ_MOUSE, H2AX_MOUSE	Histone H2A	VTIAQGGVLPNIQAVLLPK	50	4.30E-07	644.3951	1930.1636	3	0.0020	19	0	Semi-tryptic
H2A1F_MOUSE, H2A1H_MOUSE, H2A1_MOUSE, H2A1K_MOUSE, H2A2A_MOUSE, H2A2B_MOUSE, H2A2C_MOUSE, H2A3_MOUSE, H2AJ_MOUSE, H2AX_MOUSE	Histone H2A	VTIAQGGVLPNIQAVLLPK	50	2.10E-08	644.3951	1930.1636	3	0.0020	19	0	Semi-tryptic
H2A1F_MOUSE, H2A1H_MOUSE, H2A1_MOUSE, H2A1K_MOUSE, H2A2A_MOUSE, H2A2B_MOUSE, H2A2C_MOUSE, H2A3_MOUSE, H2AJ_MOUSE, H2AX_MOUSE	Histone H2A	VTIAQGGVLPNIQAVLLPK	50	1.30E-09	966.089	1930.1635	2	0.0020	19	0	Semi-tryptic

H2A1F_MOUSE, H2A1H_MOUSE, H2A1_MOUSE, H2A1K_MOUSE, H2A2A_MOUSE, H2A2B_MOUSE, H2A2C_MOUSE, H2A3_MOUSE, H2AJ_MOUSE, H2AX_MOUSE	Histone H2A	VTIAQGGVLPNIQAVLLPK	50	2.40E-03	644.395	1930.1633	3	0.0018	19	0	Semi-tryptic
H2A1F_MOUSE, H2A1H_MOUSE, H2A1_MOUSE, H2A1K_MOUSE, H2A2A_MOUSE, H2A2B_MOUSE, H2A2C_MOUSE, H2A3_MOUSE, H2AJ_MOUSE, H2AX_MOUSE	Histone H2A	VTIAQGGVLPNIQAVLLPK	50	4.90E-07	644.395	1930.1632	3	0.0017	19	0	Semi-tryptic
H2A1F_MOUSE, H2A1H_MOUSE, H2A1_MOUSE, H2A1K_MOUSE, H2A2A_MOUSE, H2A2B_MOUSE, H2A2C_MOUSE, H2A3_MOUSE, H2AJ_MOUSE, H2AX_MOUSE	Histone H2A	VTIAQGGVLPNIQAVLLPK	50	1.50E-07	644.395	1930.1632	3	0.0017	19	0	Semi-tryptic
H2A1F_MOUSE, H2A1H_MOUSE, H2A1_MOUSE, H2A1K_MOUSE, H2A2A_MOUSE, H2A2B_MOUSE, H2A2C_MOUSE, H2A3_MOUSE, H2AJ_MOUSE, H2AX_MOUSE	Histone H2A	VTIAQGGVLPNIQAVLLPK	50	4.40E-08	644.395	1930.1633	3	0.0017	19	0	Semi-tryptic
H2A1F_MOUSE, H2A1H_MOUSE, H2A1_MOUSE, H2A1K_MOUSE, H2A2A_MOUSE, H2A2B_MOUSE, H2A2C_MOUSE, H2A3_MOUSE, H2AJ_MOUSE, H2AX_MOUSE	Histone H2A	VTIAQGGVLPNIQAVLLPK	50	3.40E-08	966.0889	1930.1632	2	0.0017	19	0	Semi-tryptic
H2A1F_MOUSE, H2A1H_MOUSE, H2A1_MOUSE, H2A1K_MOUSE, H2A2A_MOUSE, H2A2B_MOUSE, H2A2C_MOUSE, H2A3_MOUSE, H2AJ_MOUSE, H2AX_MOUSE	Histone H2A	VTIAQGGVLPNIQAVLLPK	50	1.70E-06	644.3949	1930.1630	3	0.0015	19	0	Semi-tryptic
H2A1F_MOUSE, H2A1H_MOUSE, H2A1_MOUSE, H2A1K_MOUSE, H2A2A_MOUSE, H2A2B_MOUSE, H2A2C_MOUSE, H2A3_MOUSE, H2AJ_MOUSE, H2AX_MOUSE	Histone H2A	VTIAQGGVLPNIQAVLLPK	50	1.90E-06	966.0887	1930.1629	2	0.0013	19	0	Semi-tryptic
H2A1F_MOUSE, H2A1H_MOUSE, H2A1_MOUSE, H2A1K_MOUSE, H2A2A_MOUSE, H2A2B_MOUSE, H2A2C_MOUSE, H2A3_MOUSE, H2AJ_MOUSE, H2AX_MOUSE	Histone H2A	VTIAQGGVLPNIQAVLLPK	50	1.70E-07	644.3949	1930.1628	3	0.0013	19	0	Semi-tryptic
H2A1F_MOUSE, H2A1H_MOUSE, H2A1_MOUSE, H2A1K_MOUSE, H2A2A_MOUSE, H2A2B_MOUSE, H2A2C_MOUSE, H2A3_MOUSE, H2AJ_MOUSE, H2AX_MOUSE	Histone H2A	VTIAQGGVLPNIQAVLLPK	50	4.60E-06	966.0886	1930.1627	2	0.0012	19	0	Semi-tryptic
H2A1F_MOUSE, H2A1H_MOUSE, H2A1_MOUSE, H2A1K_MOUSE, H2A2A_MOUSE, H2A2B_MOUSE, H2A2C_MOUSE, H2A3_MOUSE, H2AJ_MOUSE, H2AX_MOUSE	Histone H2A	VTIAQGGVLPNIQAVLLPK	50	5.60E-04	966.0885	1930.1625	2	0.0010	19	0	Semi-tryptic
H2A1F_MOUSE, H2A1H_MOUSE, H2A1_MOUSE, H2A1K_MOUSE, H2A2A_MOUSE, H2A2B_MOUSE, H2A2C_MOUSE, H2A3_MOUSE, H2AJ_MOUSE, H2AX_MOUSE	Histone H2A	VTIAQGGVLPNIQAVLLPK	50	1.70E-08	966.0885	1930.1624	2	0.0009	19	0	Semi-tryptic
H2A1F_MOUSE, H2A1H_MOUSE, H2A1_MOUSE, H2A1K_MOUSE, H2A2A_MOUSE, H2A2B_MOUSE, H2A2C_MOUSE, H2A3_MOUSE, H2AJ_MOUSE, H2AX_MOUSE	Histone H2A	VTIAQGGVLPNIQAVLLPK	50	9.10E-04	644.3947	1930.1623	3	0.0008	19	0	Semi-tryptic
H2A1F_MOUSE, H2A1H_MOUSE, H2A1_MOUSE, H2A1K_MOUSE, H2A2A_MOUSE, H2A2B_MOUSE, H2A2C_MOUSE, H2A3_MOUSE, H2AJ_MOUSE, H2AX_MOUSE	Histone H2A	VTIAQGGVLPNIQAVLLPK	50	2.40E-05	644.3947	1930.1623	3	0.0008	19	0	Semi-tryptic
H2A1F_MOUSE, H2A1H_MOUSE, H2A1_MOUSE, H2A1K_MOUSE, H2A2A_MOUSE, H2A2B_MOUSE, H2A2C_MOUSE, H2A3_MOUSE, H2AJ_MOUSE, H2AX_MOUSE	Histone H2A	VTIAQGGVLPNIQAVLLPK	50	9.00E-09	966.0884	1930.1623	2	0.0008	19	0	Semi-tryptic
H2A1F_MOUSE, H2A1H_MOUSE, H2A1_MOUSE, H2A1K_MOUSE, H2A2A_MOUSE, H2A2B_MOUSE, H2A2C_MOUSE, H2A3_MOUSE, H2AJ_MOUSE, H2AX_MOUSE	Histone H2A	VTIAQGGVLPNIQAVLLPK	50	1.50E-08	966.0884	1930.1623	2	0.0007	19	0	Semi-tryptic
H2A1F_MOUSE, H2A1H_MOUSE, H2A1_MOUSE, H2A1K_MOUSE, H2A2A_MOUSE, H2A2B_MOUSE, H2A2C_MOUSE, H2A3_MOUSE, H2AJ_MOUSE, H2AX_MOUSE	Histone H2A	VTIAQGGVLPNIQAVLLPK	50	2.80E-07	644.3946	1930.1619	3	0.0004	19	0	Semi-tryptic
H2A1F_MOUSE, H2A1H_MOUSE, H2A1_MOUSE, H2A1K_MOUSE, H2A2A_MOUSE, H2A2B_MOUSE, H2A2C_MOUSE, H2A3_MOUSE, H2AJ_MOUSE, H2AX_MOUSE	Histone H2A	VTIAQGGVLPNIQAVLLPK	50	4.40E-03	644.3945	1930.1617	3	0.0002	19	0	Semi-tryptic
H2A1F_MOUSE, H2A1H_MOUSE, H2A1_MOUSE, H2A1K_MOUSE, H2A2A_MOUSE, H2A2B_MOUSE, H2A2C_MOUSE, H2A3_MOUSE, H2AJ_MOUSE, H2AX_MOUSE	Histone H2A	VTIAQGGVLPNIQAVLLPK	50	1.90E-09	644.3945	1930.1618	3	0.0002	19	0	Semi-tryptic
H2A1F_MOUSE, H2A1H_MOUSE, H2A1_MOUSE, H2A1K_MOUSE, H2A2A_MOUSE, H2A2B_MOUSE, H2A2C_MOUSE, H2A3_MOUSE, H2AJ_MOUSE, H2AX_MOUSE	Histone H2A	VTIAQGGVLPNIQAVLLPK	50	3.30E-08	966.088	1930.1615	2	-0.0001	19	0	Semi-tryptic
H2A1F_MOUSE, H2A1H_MOUSE, H2A1_MOUSE, H2A1K_MOUSE, H2A2A_MOUSE, H2A2B_MOUSE, H2A2C_MOUSE, H2A3_MOUSE, H2AJ_MOUSE, H2AX_MOUSE	Histone H2A	VTIAQGGVLPNIQAVLLPK	50	1.40E-07	966.0879	1930.1612	2	-0.0003	19	0	Semi-tryptic
H2A1F_MOUSE, H2A1H_MOUSE, H2A1_MOUSE, H2A1K_MOUSE, H2A2A_MOUSE, H2A2B_MOUSE, H2A2C_MOUSE, H2A3_MOUSE, H2AJ_MOUSE, H2AX_MOUSE	Histone H2A	VTIAQGGVLPNIQAVLLPK	50	4.30E-08	644.3942	1930.1608	3	-0.0007	19	0	Semi-tryptic
H2A1F_MOUSE, H2A1H_MOUSE, H2A1_MOUSE, H2A1K_MOUSE, H2A2A_MOUSE, H2A2B_MOUSE, H2A2C_MOUSE, H2A3_MOUSE, H2AJ_MOUSE, H2AX_MOUSE	Histone H2A	VTIAQGGVLPNIQAVLLPK	50	9.20E-09	644.3942	1930.1609	3	-0.0007	19	0	Semi-tryptic
H2A1F_MOUSE, H2A1H_MOUSE, H2A1_MOUSE, H2A1K_MOUSE, H2A2A_MOUSE, H2A2B_MOUSE, H2A2C_MOUSE, H2A3_MOUSE, H2AJ_MOUSE, H2AX_MOUSE	Histone H2A	AGLQFPVGRVHRLLR	2	1.60E-03	573.6814	1718.0225	3	0.0009	15	2	Semi-tryptic
H2A1F_MOUSE, H2A1H_MOUSE, H2A1_MOUSE, H2A1K_MOUSE, H2A2A_MOUSE, H2A2B_MOUSE, H2A2C_MOUSE, H2A3_MOUSE, H2AJ_MOUSE, H2AX_MOUSE	Histone H2A	AGLQFPVGRVHRLLR	2	2.00E-03	573.6812	1718.0219	3	0.0002	15	2	Semi-tryptic

H2A1F_MOUSE, H2A1H_MOUSE, H2A1_MOUSE, H2A1K_MOUSE, H2A2A_MOUSE, H2A2B_MOUSE, H2A2C_MOUSE, H2A3_MOUSE, H2AJ_MOUSE, H2AX_MOUSE	Histone H2A	SSRAGLQFPVGRVHR	4	1.20E-03	556.3139	1665.9198	3	0.0022	15	2	Semi-tryptic
H2A1F_MOUSE, H2A1H_MOUSE, H2A1_MOUSE, H2A1K_MOUSE, H2A2A_MOUSE, H2A2B_MOUSE, H2A2C_MOUSE, H2A3_MOUSE, H2AJ_MOUSE, H2AX_MOUSE	Histone H2A	SSRAGLQFPVGRVHR	4	2.70E-03	556.3131	1665.9174	3	-0.0001	15	2	Semi-tryptic
H2A1F_MOUSE, H2A1H_MOUSE, H2A1_MOUSE, H2A1K_MOUSE, H2A2A_MOUSE, H2A2B_MOUSE, H2A2C_MOUSE, H2A3_MOUSE, H2AJ_MOUSE, H2AX_MOUSE	Histone H2A	SSRAGLQFPVGRVHR	4	3.10E-04	556.3128	1665.9164	3	-0.0011	15	2	Semi-tryptic
H2A1F_MOUSE, H2A1H_MOUSE, H2A1_MOUSE, H2A1K_MOUSE, H2A2A_MOUSE, H2A2B_MOUSE, H2A2C_MOUSE, H2A3_MOUSE, H2AJ_MOUSE, H2AX_MOUSE	Histone H2A	SSRAGLQFPVGRVHR	4	8.20E-04	556.3127	1665.9162	3	-0.0013	15	2	Semi-tryptic
H2A1F_MOUSE, H2A1H_MOUSE, H2A1_MOUSE, H2A1K_MOUSE, H2A2A_MOUSE, H2A2B_MOUSE, H2A2C_MOUSE, H2A3_MOUSE, H2AJ_MOUSE, H2AX_MOUSE	Histone H2A	AGLQFPVGRVHR	9	3.90E-03	334.9457	1335.7537	4	0.0013	12	1	Semi-tryptic
H2A1F_MOUSE, H2A1H_MOUSE, H2A1_MOUSE, H2A1K_MOUSE, H2A2A_MOUSE, H2A2B_MOUSE, H2A2C_MOUSE, H2A3_MOUSE, H2AJ_MOUSE, H2AX_MOUSE	Histone H2A	AGLQFPVGRVHR	9	6.80E-03	334.9454	1335.7527	4	0.0003	12	1	Semi-tryptic
H2A1F_MOUSE, H2A1H_MOUSE, H2A1_MOUSE, H2A1K_MOUSE, H2A2A_MOUSE, H2A2B_MOUSE, H2A2C_MOUSE, H2A3_MOUSE, H2AJ_MOUSE, H2AX_MOUSE	Histone H2A	AGLQFPVGRVHR	9	2.30E-03	334.9454	1335.7527	4	0.0003	12	1	Semi-tryptic
H2A1F_MOUSE, H2A1H_MOUSE, H2A1_MOUSE, H2A1K_MOUSE, H2A2A_MOUSE, H2A2B_MOUSE, H2A2C_MOUSE, H2A3_MOUSE, H2AJ_MOUSE, H2AX_MOUSE	Histone H2A	AGLQFPVGRVHR	9	7.40E-03	668.8836	1335.7526	2	0.0002	12	1	Semi-tryptic
H2A1F_MOUSE, H2A1H_MOUSE, H2A1_MOUSE, H2A1K_MOUSE, H2A2A_MOUSE, H2A2B_MOUSE, H2A2C_MOUSE, H2A3_MOUSE, H2AJ_MOUSE, H2AX_MOUSE	Histone H2A	AGLQFPVGRVHR	9	2.60E-03	446.2579	1335.7518	3	-0.0006	12	1	Semi-tryptic
H2A1F_MOUSE, H2A1H_MOUSE, H2A1_MOUSE, H2A1K_MOUSE, H2A2A_MOUSE, H2A2B_MOUSE, H2A2C_MOUSE, H2A3_MOUSE, H2AJ_MOUSE, H2AX_MOUSE	Histone H2A	AGLQFPVGRVHR	9	4.20E-03	334.9451	1335.7513	4	-0.0011	12	1	Semi-tryptic
H2A1F_MOUSE, H2A1H_MOUSE, H2A1_MOUSE, H2A1K_MOUSE, H2A2A_MOUSE, H2A2B_MOUSE, H2A2C_MOUSE, H2A3_MOUSE, H2AJ_MOUSE, H2AX_MOUSE	Histone H2A	AGLQFPVGRVHR	9	9.00E-03	446.2577	1335.7512	3	-0.0012	12	1	Semi-tryptic
H2A1F_MOUSE, H2A1H_MOUSE, H2A1_MOUSE, H2A1K_MOUSE, H2A2A_MOUSE, H2A2B_MOUSE, H2A2C_MOUSE, H2A3_MOUSE, H2AJ_MOUSE, H2AX_MOUSE	Histone H2A	AGLQFPVGRVHR	9	1.90E-03	446.2577	1335.7511	3	-0.0013	12	1	Semi-tryptic
H2A1F_MOUSE, H2A1H_MOUSE, H2A1_MOUSE, H2A1K_MOUSE, H2A2A_MOUSE, H2A2B_MOUSE, H2A2C_MOUSE, H2A3_MOUSE, H2AJ_MOUSE, H2AX_MOUSE	Histone H2A	AGLQFPVGRVHR	9	5.90E-03	446.2576	1335.7510	3	-0.0014	12	1	Semi-tryptic
H2A1F_MOUSE, H2A1H_MOUSE, H2A1_MOUSE, H2A1K_MOUSE, H2A2A_MOUSE, H2A2B_MOUSE, H2A2C_MOUSE, H2A3_MOUSE, H2AJ_MOUSE, H2AX_MOUSE	Histone H2A	SSRAGLQFPVGR	12	4.80E-06	637.8526	1273.6907	2	0.0016	12	1	Semi-tryptic
H2A1F_MOUSE, H2A1H_MOUSE, H2A1_MOUSE, H2A1K_MOUSE, H2A2A_MOUSE, H2A2B_MOUSE, H2A2C_MOUSE, H2A3_MOUSE, H2AJ_MOUSE, H2AX_MOUSE	Histone H2A	SSRAGLQFPVGR	12	6.20E-04	425.5705	1273.6897	3	0.0006	12	1	Semi-tryptic
H2A1F_MOUSE, H2A1H_MOUSE, H2A1_MOUSE, H2A1K_MOUSE, H2A2A_MOUSE, H2A2B_MOUSE, H2A2C_MOUSE, H2A3_MOUSE, H2AJ_MOUSE, H2AX_MOUSE	Histone H2A	SSRAGLQFPVGR	12	9.60E-03	425.5705	1273.6896	3	0.0005	12	1	Semi-tryptic
H2A1F_MOUSE, H2A1H_MOUSE, H2A1_MOUSE, H2A1K_MOUSE, H2A2A_MOUSE, H2A2B_MOUSE, H2A2C_MOUSE, H2A3_MOUSE, H2AJ_MOUSE, H2AX_MOUSE	Histone H2A	SSRAGLQFPVGR	12	1.00E-03	425.5704	1273.6895	3	0.0004	12	1	Semi-tryptic
H2A1F_MOUSE, H2A1H_MOUSE, H2A1_MOUSE, H2A1K_MOUSE, H2A2A_MOUSE, H2A2B_MOUSE, H2A2C_MOUSE, H2A3_MOUSE, H2AJ_MOUSE, H2AX_MOUSE	Histone H2A	SSRAGLQFPVGR	12	2.00E-05	637.852	1273.6895	2	0.0004	12	1	Semi-tryptic
H2A1F_MOUSE, H2A1H_MOUSE, H2A1_MOUSE, H2A1K_MOUSE, H2A2A_MOUSE, H2A2B_MOUSE, H2A2C_MOUSE, H2A3_MOUSE, H2AJ_MOUSE, H2AX_MOUSE	Histone H2A	SSRAGLQFPVGR	12	2.10E-06	637.8518	1273.6891	2	0.0000	12	1	Semi-tryptic
H2A1F_MOUSE, H2A1H_MOUSE, H2A1_MOUSE, H2A1K_MOUSE, H2A2A_MOUSE, H2A2B_MOUSE, H2A2C_MOUSE, H2A3_MOUSE, H2AJ_MOUSE, H2AX_MOUSE	Histone H2A	SSRAGLQFPVGR	12	2.20E-03	425.5702	1273.6889	3	-0.0002	12	1	Semi-tryptic
H2A1F_MOUSE, H2A1H_MOUSE, H2A1_MOUSE, H2A1K_MOUSE, H2A2A_MOUSE, H2A2B_MOUSE, H2A2C_MOUSE, H2A3_MOUSE, H2AJ_MOUSE, H2AX_MOUSE	Histone H2A	SSRAGLQFPVGR	12	3.20E-06	637.8517	1273.6889	2	-0.0002	12	1	Semi-tryptic
H2A1F_MOUSE, H2A1H_MOUSE, H2A1_MOUSE, H2A1K_MOUSE, H2A2A_MOUSE, H2A2B_MOUSE, H2A2C_MOUSE, H2A3_MOUSE, H2AJ_MOUSE, H2AX_MOUSE	Histone H2A	SSRAGLQFPVGR	12	1.90E-05	637.8515	1273.6885	2	-0.0006	12	1	Semi-tryptic
H2A1F_MOUSE, H2A1H_MOUSE, H2A1_MOUSE, H2A1K_MOUSE, H2A2A_MOUSE, H2A2B_MOUSE, H2A2C_MOUSE, H2A3_MOUSE, H2AJ_MOUSE, H2AX_MOUSE	Histone H2A	SSRAGLQFPVGR	12	2.00E-03	425.5701	1273.6884	3	-0.0007	12	1	Semi-tryptic
H2A1F_MOUSE, H2A1H_MOUSE, H2A1_MOUSE, H2A1K_MOUSE, H2A2A_MOUSE, H2A2B_MOUSE, H2A2C_MOUSE, H2A3_MOUSE, H2AJ_MOUSE, H2AX_MOUSE	Histone H2A	SSRAGLQFPVGR	12	6.30E-06	637.8514	1273.6883	2	-0.0008	12	1	Semi-tryptic

H2A1F_MOUSE, H2A1H_MOUSE, H2A1_MOUSE, H2A1K_MOUSE, H2A2A_MOUSE, H2A2B_MOUSE, H2A2C_MOUSE, H2A3_MOUSE, H2AJ_MOUSE, H2AX_MOUSE	Histone H2A	SSRAGLQFPVGR	12	3.50E-03	425.5699	1273.6879	3	-0.0012	12	1	Semi-tryptic
H2A1F_MOUSE, H2A1H_MOUSE, H2A1_MOUSE, H2A1K_MOUSE, H2A2A_MOUSE, H2A2B_MOUSE, H2A2C_MOUSE, H2A3_MOUSE, H2AJ_MOUSE, H2AX_MOUSE	Histone H2A	NDEELNK	32	6.50E-03	431.2	860.3855	2	-0.0021	7	0	Semi-tryptic
H2A1F_MOUSE, H2A1H_MOUSE, H2A1_MOUSE, H2A1K_MOUSE, H2A2A_MOUSE, H2A2B_MOUSE, H2A2C_MOUSE, H2A3_MOUSE, H2AJ_MOUSE, H2AX_MOUSE	Histone H2A	NDEELNK	32	2.70E-03	431.2024	860.3903	2	0.0027	7	0	Semi-tryptic
H2A1F_MOUSE, H2A1H_MOUSE, H2A1_MOUSE, H2A1K_MOUSE, H2A2A_MOUSE, H2A2B_MOUSE, H2A2C_MOUSE, H2A3_MOUSE, H2AJ_MOUSE, H2AX_MOUSE	Histone H2A	NDEELNK	32	5.50E-03	431.202	860.3895	2	0.0020	7	0	Semi-tryptic
H2A1F_MOUSE, H2A1H_MOUSE, H2A1_MOUSE, H2A1K_MOUSE, H2A2A_MOUSE, H2A2B_MOUSE, H2A2C_MOUSE, H2A3_MOUSE, H2AJ_MOUSE, H2AX_MOUSE	Histone H2A	NDEELNK	32	5.90E-04	431.202	860.3895	2	0.0020	7	0	Semi-tryptic
H2A1F_MOUSE, H2A1H_MOUSE, H2A1_MOUSE, H2A1K_MOUSE, H2A2A_MOUSE, H2A2B_MOUSE, H2A2C_MOUSE, H2A3_MOUSE, H2AJ_MOUSE, H2AX_MOUSE	Histone H2A	NDEELNK	32	2.30E-04	431.202	860.3894	2	0.0019	7	0	Semi-tryptic
H2A1F_MOUSE, H2A1H_MOUSE, H2A1_MOUSE, H2A1K_MOUSE, H2A2A_MOUSE, H2A2B_MOUSE, H2A2C_MOUSE, H2A3_MOUSE, H2AJ_MOUSE, H2AX_MOUSE	Histone H2A	NDEELNK	32	2.30E-04	431.202	860.3895	2	0.0019	7	0	Semi-tryptic
H2A1F_MOUSE, H2A1H_MOUSE, H2A1_MOUSE, H2A1K_MOUSE, H2A2A_MOUSE, H2A2B_MOUSE, H2A2C_MOUSE, H2A3_MOUSE, H2AJ_MOUSE, H2AX_MOUSE	Histone H2A	NDEELNK	32	1.20E-03	431.202	860.3893	2	0.0018	7	0	Semi-tryptic
H2A1F_MOUSE, H2A1H_MOUSE, H2A1_MOUSE, H2A1K_MOUSE, H2A2A_MOUSE, H2A2B_MOUSE, H2A2C_MOUSE, H2A3_MOUSE, H2AJ_MOUSE, H2AX_MOUSE	Histone H2A	NDEELNK	32	4.20E-04	431.202	860.3894	2	0.0018	7	0	Semi-tryptic
H2A1F_MOUSE, H2A1H_MOUSE, H2A1_MOUSE, H2A1K_MOUSE, H2A2A_MOUSE, H2A2B_MOUSE, H2A2C_MOUSE, H2A3_MOUSE, H2AJ_MOUSE, H2AX_MOUSE	Histone H2A	NDEELNK	32	2.10E-04	431.2019	860.3893	2	0.0018	7	0	Semi-tryptic
H2A1F_MOUSE, H2A1H_MOUSE, H2A1_MOUSE, H2A1K_MOUSE, H2A2A_MOUSE, H2A2B_MOUSE, H2A2C_MOUSE, H2A3_MOUSE, H2AJ_MOUSE, H2AX_MOUSE	Histone H2A	NDEELNK	32	2.70E-04	431.2019	860.3892	2	0.0017	7	0	Semi-tryptic
H2A1F_MOUSE, H2A1H_MOUSE, H2A1_MOUSE, H2A1K_MOUSE, H2A2A_MOUSE, H2A2B_MOUSE, H2A2C_MOUSE, H2A3_MOUSE, H2AJ_MOUSE, H2AX_MOUSE	Histone H2A	NDEELNK	32	1.80E-04	431.2019	860.3893	2	0.0017	7	0	Semi-tryptic
H2A1F_MOUSE, H2A1H_MOUSE, H2A1_MOUSE, H2A1K_MOUSE, H2A2A_MOUSE, H2A2B_MOUSE, H2A2C_MOUSE, H2A3_MOUSE, H2AJ_MOUSE, H2AX_MOUSE	Histone H2A	NDEELNK	32	3.60E-04	431.2017	860.3888	2	0.0013	7	0	Semi-tryptic
H2A1F_MOUSE, H2A1H_MOUSE, H2A1_MOUSE, H2A1K_MOUSE, H2A2A_MOUSE, H2A2B_MOUSE, H2A2C_MOUSE, H2A3_MOUSE, H2AJ_MOUSE, H2AX_MOUSE	Histone H2A	NDEELNK	32	5.80E-04	431.2016	860.3886	2	0.0010	7	0	Semi-tryptic
H2A1F_MOUSE, H2A1H_MOUSE, H2A1_MOUSE, H2A1K_MOUSE, H2A2A_MOUSE, H2A2B_MOUSE, H2A2C_MOUSE, H2A3_MOUSE, H2AJ_MOUSE, H2AX_MOUSE	Histone H2A	NDEELNK	32	6.80E-04	431.2014	860.3883	2	0.0008	7	0	Semi-tryptic
H2A1F_MOUSE, H2A1H_MOUSE, H2A1_MOUSE, H2A1K_MOUSE, H2A2A_MOUSE, H2A2B_MOUSE, H2A2C_MOUSE, H2A3_MOUSE, H2AJ_MOUSE, H2AX_MOUSE	Histone H2A	NDEELNK	32	7.80E-04	431.2013	860.3881	2	0.0006	7	0	Semi-tryptic
H2A1F_MOUSE, H2A1H_MOUSE, H2A1_MOUSE, H2A1K_MOUSE, H2A2A_MOUSE, H2A2B_MOUSE, H2A2C_MOUSE, H2A3_MOUSE, H2AJ_MOUSE, H2AX_MOUSE	Histone H2A	NDEELNK	32	2.90E-04	431.2013	860.3881	2	0.0006	7	0	Semi-tryptic
H2A1F_MOUSE, H2A1H_MOUSE, H2A1_MOUSE, H2A1K_MOUSE, H2A2A_MOUSE, H2A2B_MOUSE, H2A2C_MOUSE, H2A3_MOUSE, H2AJ_MOUSE, H2AX_MOUSE	Histone H2A	NDEELNK	32	1.50E-03	431.2013	860.3880	2	0.0005	7	0	Semi-tryptic
H2A1F_MOUSE, H2A1H_MOUSE, H2A1_MOUSE, H2A1K_MOUSE, H2A2A_MOUSE, H2A2B_MOUSE, H2A2C_MOUSE, H2A3_MOUSE, H2AJ_MOUSE, H2AX_MOUSE	Histone H2A	NDEELNK	32	1.90E-03	431.2012	860.3879	2	0.0004	7	0	Semi-tryptic
H2A1F_MOUSE, H2A1H_MOUSE, H2A1_MOUSE, H2A1K_MOUSE, H2A2A_MOUSE, H2A2B_MOUSE, H2A2C_MOUSE, H2A3_MOUSE, H2AJ_MOUSE, H2AX_MOUSE	Histone H2A	NDEELNK	32	4.30E-04	431.2013	860.3880	2	0.0004	7	0	Semi-tryptic
H2A1F_MOUSE, H2A1H_MOUSE, H2A1_MOUSE, H2A1K_MOUSE, H2A2A_MOUSE, H2A2B_MOUSE, H2A2C_MOUSE, H2A3_MOUSE, H2AJ_MOUSE, H2AX_MOUSE	Histone H2A	NDEELNK	32	5.30E-03	431.2012	860.3878	2	0.0003	7	0	Semi-tryptic
H2A1F_MOUSE, H2A1H_MOUSE, H2A1_MOUSE, H2A1K_MOUSE, H2A2A_MOUSE, H2A2B_MOUSE, H2A2C_MOUSE, H2A3_MOUSE, H2AJ_MOUSE, H2AX_MOUSE	Histone H2A	NDEELNK	32	3.00E-04	431.2012	860.3878	2	0.0003	7	0	Semi-tryptic
H2A1F_MOUSE, H2A1H_MOUSE, H2A1_MOUSE, H2A1K_MOUSE, H2A2A_MOUSE, H2A2B_MOUSE, H2A2C_MOUSE, H2A3_MOUSE, H2AJ_MOUSE, H2AX_MOUSE	Histone H2A	NDEELNK	32	1.30E-03	431.2011	860.3877	2	0.0002	7	0	Semi-tryptic
H2A1F_MOUSE, H2A1H_MOUSE, H2A1_MOUSE, H2A1K_MOUSE, H2A2A_MOUSE, H2A2B_MOUSE, H2A2C_MOUSE, H2A3_MOUSE, H2AJ_MOUSE, H2AX_MOUSE	Histone H2A	NDEELNK	32	7.70E-04	431.2011	860.3877	2	0.0001	7	0	Semi-tryptic

H2A1F_MOUSE, H2A1H_MOUSE, H2A1_MOUSE, H2A1K_MOUSE, H2A2A_MOUSE, H2A2B_MOUSE, H2A2C_MOUSE, H2A3_MOUSE, H2AJ_MOUSE, H2AX_MOUSE	Histone H2A	NDEELNK	32	1.80E-04	431.2011	860.3876	2	0.0001	7	0	Semi-tryptic
H2A1F_MOUSE, H2A1H_MOUSE, H2A1_MOUSE, H2A1K_MOUSE, H2A2A_MOUSE, H2A2B_MOUSE, H2A2C_MOUSE, H2A3_MOUSE, H2AJ_MOUSE, H2AX_MOUSE	Histone H2A	NDEELNK	32	2.60E-04	431.2011	860.3876	2	0.0000	7	0	Semi-tryptic
H2A1F_MOUSE, H2A1H_MOUSE, H2A1_MOUSE, H2A1K_MOUSE, H2A2A_MOUSE, H2A2B_MOUSE, H2A2C_MOUSE, H2A3_MOUSE, H2AJ_MOUSE, H2AX_MOUSE	Histone H2A	NDEELNK	32	7.40E-03	431.201	860.3875	2	-0.0001	7	0	Semi-tryptic
H2A1F_MOUSE, H2A1H_MOUSE, H2A1_MOUSE, H2A1K_MOUSE, H2A2A_MOUSE, H2A2B_MOUSE, H2A2C_MOUSE, H2A3_MOUSE, H2AJ_MOUSE, H2AX_MOUSE	Histone H2A	NDEELNK	32	6.30E-03	431.201	860.3874	2	-0.0001	7	0	Semi-tryptic
H2A1F_MOUSE, H2A1H_MOUSE, H2A1_MOUSE, H2A1K_MOUSE, H2A2A_MOUSE, H2A2B_MOUSE, H2A2C_MOUSE, H2A3_MOUSE, H2AJ_MOUSE, H2AX_MOUSE	Histone H2A	NDEELNK	32	7.20E-04	431.2009	860.3873	2	-0.0003	7	0	Semi-tryptic
H2A1F_MOUSE, H2A1H_MOUSE, H2A1_MOUSE, H2A1K_MOUSE, H2A2A_MOUSE, H2A2B_MOUSE, H2A2C_MOUSE, H2A3_MOUSE, H2AJ_MOUSE, H2AX_MOUSE	Histone H2A	NDEELNK	32	1.00E-02	431.2008	860.3870	2	-0.0005	7	0	Semi-tryptic
H2A1F_MOUSE, H2A1H_MOUSE, H2A1_MOUSE, H2A1K_MOUSE, H2A2A_MOUSE, H2A2B_MOUSE, H2A2C_MOUSE, H2A3_MOUSE, H2AJ_MOUSE, H2AX_MOUSE	Histone H2A	NDEELNK	32	4.90E-04	431.2008	860.3871	2	-0.0005	7	0	Semi-tryptic
H2A1F_MOUSE, H2A1H_MOUSE, H2A1_MOUSE, H2A1K_MOUSE, H2A2A_MOUSE, H2A2B_MOUSE, H2A2C_MOUSE, H2A3_MOUSE, H2AJ_MOUSE, H2AX_MOUSE	Histone H2A	NDEELNK	32	1.00E-03	431.2007	860.3868	2	-0.0007	7	0	Semi-tryptic
H2A1F_MOUSE, H2A1H_MOUSE, H2A1_MOUSE, H2A1K_MOUSE, H2A2A_MOUSE, H2A2B_MOUSE, H2A2C_MOUSE, H2A3_MOUSE, H2AJ_MOUSE, H2AX_MOUSE	Histone H2A	NDEELNK	32	1.70E-03	431.2006	860.3867	2	-0.0008	7	0	Semi-tryptic
H2A1F_MOUSE, H2A1H_MOUSE, H2A1_MOUSE, H2A1K_MOUSE, H2A2A_MOUSE, H2A2B_MOUSE, H2A2C_MOUSE, H2A3_MOUSE, H2AJ_MOUSE, H2AX_MOUSE, H2AY_MOUSE, H2AW_MOUSE, Q8CA90_MOUSE	Histone H2A	ILELAGNAARD	3	5.70E-03	571.812	1141.6094	2	0.0003	11	1	GluC
H2A1F_MOUSE, H2A1H_MOUSE, H2A1_MOUSE, H2A1K_MOUSE, H2A2A_MOUSE, H2A2B_MOUSE, H2A2C_MOUSE, H2A3_MOUSE, H2AJ_MOUSE, H2AX_MOUSE, H2AY_MOUSE, H2AW_MOUSE, Q8CA90_MOUSE	Histone H2A	ILELAGNAARD	3	4.80E-06	571.812	1141.6094	2	0.0003	11	1	GluC
H2A1F_MOUSE, H2A1H_MOUSE, H2A1_MOUSE, H2A1K_MOUSE, H2A2A_MOUSE, H2A2B_MOUSE, H2A2C_MOUSE, H2A3_MOUSE, H2AJ_MOUSE, H2AX_MOUSE, H2AY_MOUSE, H2AW_MOUSE, Q8CA90_MOUSE	Histone H2A	ILELAGNAARD	3	3.60E-04	571.8116	1141.6087	2	-0.0004	11	1	GluC
H2A1F_MOUSE, H2A1H_MOUSE, H2A1_MOUSE, H2A1K_MOUSE, H2A2A_MOUSE, H2A2B_MOUSE, H2A2C_MOUSE, H2A3_MOUSE, H2AJ_MOUSE, H2AX_MOUSE, H2AY_MOUSE, H2AW_MOUSE, Q8CA90_MOUSE	Histone H2A	LAGNAARD	150	5.20E-05	394.2082	786.4018	2	0.0035	8	0	GluC
H2A1F_MOUSE, H2A1H_MOUSE, H2A1_MOUSE, H2A1K_MOUSE, H2A2A_MOUSE, H2A2B_MOUSE, H2A2C_MOUSE, H2A3_MOUSE, H2AJ_MOUSE, H2AX_MOUSE, H2AY_MOUSE, H2AW_MOUSE, Q8CA90_MOUSE	Histone H2A	LAGNAARD	150	8.10E-04	394.2082	786.4018	2	0.0034	8	0	GluC
H2A1F_MOUSE, H2A1H_MOUSE, H2A1_MOUSE, H2A1K_MOUSE, H2A2A_MOUSE, H2A2B_MOUSE, H2A2C_MOUSE, H2A3_MOUSE, H2AJ_MOUSE, H2AX_MOUSE, H2AY_MOUSE, H2AW_MOUSE, Q8CA90_MOUSE	Histone H2A	LAGNAARD	150	1.10E-05	394.2082	786.4018	2	0.0034	8	0	GluC
H2A1F_MOUSE, H2A1H_MOUSE, H2A1_MOUSE, H2A1K_MOUSE, H2A2A_MOUSE, H2A2B_MOUSE, H2A2C_MOUSE, H2A3_MOUSE, H2AJ_MOUSE, H2AX_MOUSE, H2AY_MOUSE, H2AW_MOUSE, Q8CA90_MOUSE	Histone H2A	LAGNAARD	150	9.10E-04	394.208	786.4014	2	0.0031	8	0	GluC
H2A1F_MOUSE, H2A1H_MOUSE, H2A1_MOUSE, H2A1K_MOUSE, H2A2A_MOUSE, H2A2B_MOUSE, H2A2C_MOUSE, H2A3_MOUSE, H2AJ_MOUSE, H2AX_MOUSE, H2AY_MOUSE, H2AW_MOUSE, Q8CA90_MOUSE	Histone H2A	LAGNAARD	150	7.60E-03	394.2077	786.4008	2	0.0025	8	0	GluC
H2A1F_MOUSE, H2A1H_MOUSE, H2A1_MOUSE, H2A1K_MOUSE, H2A2A_MOUSE, H2A2B_MOUSE, H2A2C_MOUSE, H2A3_MOUSE, H2AJ_MOUSE, H2AX_MOUSE, H2AY_MOUSE, H2AW_MOUSE, Q8CA90_MOUSE	Histone H2A	LAGNAARD	150	4.30E-04	394.2075	786.4004	2	0.0021	8	0	GluC
H2A1F_MOUSE, H2A1H_MOUSE, H2A1_MOUSE, H2A1K_MOUSE, H2A2A_MOUSE, H2A2B_MOUSE, H2A2C_MOUSE, H2A3_MOUSE, H2AJ_MOUSE, H2AX_MOUSE, H2AY_MOUSE, H2AW_MOUSE, Q8CA90_MOUSE	Histone H2A	LAGNAARD	150	2.50E-04	394.2075	786.4004	2	0.0020	8	0	GluC
H2A1F_MOUSE, H2A1H_MOUSE, H2A1_MOUSE, H2A1K_MOUSE, H2A2A_MOUSE, H2A2B_MOUSE, H2A2C_MOUSE, H2A3_MOUSE, H2AJ_MOUSE, H2AX_MOUSE, H2AY_MOUSE, H2AW_MOUSE, Q8CA90_MOUSE	Histone H2A	LAGNAARD	150	3.30E-03	394.2074	786.4002	2	0.0018	8	0	GluC

H2A1F_MOUSE, H2A1H_MOUSE, H2A1_MOUSE, H2A1K_MOUSE, H2A2A_MOUSE, H2A2B_MOUSE, H2A2C_MOUSE, H2A3_MOUSE, H2AJ_MOUSE, H2AX_MOUSE, H2AY_MOUSE, H2AW_MOUSE, Q8CA90_MOUSE	Histone H2A	LAGNAARD	150	1.50E-04	394.2073	786.4001	2	0.0017	8	0	GluC
H2A1F_MOUSE, H2A1H_MOUSE, H2A1_MOUSE, H2A1K_MOUSE, H2A2A_MOUSE, H2A2B_MOUSE, H2A2C_MOUSE, H2A3_MOUSE, H2AJ_MOUSE, H2AX_MOUSE, H2AY_MOUSE, H2AW_MOUSE, Q8CA90_MOUSE	Histone H2A	LAGNAARD	150	1.30E-04	394.2073	786.4000	2	0.0017	8	0	GluC
H2A1F_MOUSE, H2A1H_MOUSE, H2A1_MOUSE, H2A1K_MOUSE, H2A2A_MOUSE, H2A2B_MOUSE, H2A2C_MOUSE, H2A3_MOUSE, H2AJ_MOUSE, H2AX_MOUSE, H2AY_MOUSE, H2AW_MOUSE, Q8CA90_MOUSE	Histone H2A	LAGNAARD	150	5.20E-05	394.2073	786.4000	2	0.0017	8	0	GluC
H2A1F_MOUSE, H2A1H_MOUSE, H2A1_MOUSE, H2A1K_MOUSE, H2A2A_MOUSE, H2A2B_MOUSE, H2A2C_MOUSE, H2A3_MOUSE, H2AJ_MOUSE, H2AX_MOUSE, H2AY_MOUSE, H2AW_MOUSE, Q8CA90_MOUSE	Histone H2A	LAGNAARD	150	7.30E-03	394.2072	786.3999	2	0.0016	8	0	GluC
H2A1F_MOUSE, H2A1H_MOUSE, H2A1_MOUSE, H2A1K_MOUSE, H2A2A_MOUSE, H2A2B_MOUSE, H2A2C_MOUSE, H2A3_MOUSE, H2AJ_MOUSE, H2AX_MOUSE, H2AY_MOUSE, H2AW_MOUSE, Q8CA90_MOUSE	Histone H2A	LAGNAARD	150	2.40E-03	394.2072	786.3999	2	0.0016	8	0	GluC
H2A1F_MOUSE, H2A1H_MOUSE, H2A1_MOUSE, H2A1K_MOUSE, H2A2A_MOUSE, H2A2B_MOUSE, H2A2C_MOUSE, H2A3_MOUSE, H2AJ_MOUSE, H2AX_MOUSE, H2AY_MOUSE, H2AW_MOUSE, Q8CA90_MOUSE	Histone H2A	LAGNAARD	150	1.60E-03	394.2072	786.3999	2	0.0016	8	0	GluC
H2A1F_MOUSE, H2A1H_MOUSE, H2A1_MOUSE, H2A1K_MOUSE, H2A2A_MOUSE, H2A2B_MOUSE, H2A2C_MOUSE, H2A3_MOUSE, H2AJ_MOUSE, H2AX_MOUSE, H2AY_MOUSE, H2AW_MOUSE, Q8CA90_MOUSE	Histone H2A	LAGNAARD	150	9.90E-04	394.2073	786.4000	2	0.0016	8	0	GluC
H2A1F_MOUSE, H2A1H_MOUSE, H2A1_MOUSE, H2A1K_MOUSE, H2A2A_MOUSE, H2A2B_MOUSE, H2A2C_MOUSE, H2A3_MOUSE, H2AJ_MOUSE, H2AX_MOUSE, H2AY_MOUSE, H2AW_MOUSE, Q8CA90_MOUSE	Histone H2A	LAGNAARD	150	6.50E-04	394.2072	786.3999	2	0.0016	8	0	GluC
H2A1F_MOUSE, H2A1H_MOUSE, H2A1_MOUSE, H2A1K_MOUSE, H2A2A_MOUSE, H2A2B_MOUSE, H2A2C_MOUSE, H2A3_MOUSE, H2AJ_MOUSE, H2AX_MOUSE, H2AY_MOUSE, H2AW_MOUSE, Q8CA90_MOUSE	Histone H2A	LAGNAARD	150	2.80E-05	394.2073	786.4000	2	0.0016	8	0	GluC
H2A1F_MOUSE, H2A1H_MOUSE, H2A1_MOUSE, H2A1K_MOUSE, H2A2A_MOUSE, H2A2B_MOUSE, H2A2C_MOUSE, H2A3_MOUSE, H2AJ_MOUSE, H2AX_MOUSE, H2AY_MOUSE, H2AW_MOUSE, Q8CA90_MOUSE	Histone H2A	LAGNAARD	150	2.20E-03	394.2072	786.3999	2	0.0015	8	0	GluC
H2A1F_MOUSE, H2A1H_MOUSE, H2A1_MOUSE, H2A1K_MOUSE, H2A2A_MOUSE, H2A2B_MOUSE, H2A2C_MOUSE, H2A3_MOUSE, H2AJ_MOUSE, H2AX_MOUSE, H2AY_MOUSE, H2AW_MOUSE, Q8CA90_MOUSE	Histone H2A	LAGNAARD	150	1.50E-05	394.2072	786.3999	2	0.0015	8	0	GluC
H2A1F_MOUSE, H2A1H_MOUSE, H2A1_MOUSE, H2A1K_MOUSE, H2A2A_MOUSE, H2A2B_MOUSE, H2A2C_MOUSE, H2A3_MOUSE, H2AJ_MOUSE, H2AX_MOUSE, H2AY_MOUSE, H2AW_MOUSE, Q8CA90_MOUSE	Histone H2A	LAGNAARD	150	2.10E-03	394.2072	786.3998	2	0.0014	8	0	GluC
H2A1F_MOUSE, H2A1H_MOUSE, H2A1_MOUSE, H2A1K_MOUSE, H2A2A_MOUSE, H2A2B_MOUSE, H2A2C_MOUSE, H2A3_MOUSE, H2AJ_MOUSE, H2AX_MOUSE, H2AY_MOUSE, H2AW_MOUSE, Q8CA90_MOUSE	Histone H2A	LAGNAARD	150	1.40E-03	394.2072	786.3998	2	0.0014	8	0	GluC
H2A1F_MOUSE, H2A1H_MOUSE, H2A1_MOUSE, H2A1K_MOUSE, H2A2A_MOUSE, H2A2B_MOUSE, H2A2C_MOUSE, H2A3_MOUSE, H2AJ_MOUSE, H2AX_MOUSE, H2AY_MOUSE, H2AW_MOUSE, Q8CA90_MOUSE	Histone H2A	LAGNAARD	150	5.20E-03	394.2071	786.3997	2	0.0013	8	0	GluC
H2A1F_MOUSE, H2A1H_MOUSE, H2A1_MOUSE, H2A1K_MOUSE, H2A2A_MOUSE, H2A2B_MOUSE, H2A2C_MOUSE, H2A3_MOUSE, H2AJ_MOUSE, H2AX_MOUSE, H2AY_MOUSE, H2AW_MOUSE, Q8CA90_MOUSE	Histone H2A	LAGNAARD	150	1.60E-03	394.2071	786.3996	2	0.0013	8	0	GluC
H2A1F_MOUSE, H2A1H_MOUSE, H2A1_MOUSE, H2A1K_MOUSE, H2A2A_MOUSE, H2A2B_MOUSE, H2A2C_MOUSE, H2A3_MOUSE, H2AJ_MOUSE, H2AX_MOUSE, H2AY_MOUSE, H2AW_MOUSE, Q8CA90_MOUSE	Histone H2A	LAGNAARD	150	2.20E-04	394.2071	786.3997	2	0.0013	8	0	GluC
H2A1F_MOUSE, H2A1H_MOUSE, H2A1_MOUSE, H2A1K_MOUSE, H2A2A_MOUSE, H2A2B_MOUSE, H2A2C_MOUSE, H2A3_MOUSE, H2AJ_MOUSE, H2AX_MOUSE, H2AY_MOUSE, H2AW_MOUSE, Q8CA90_MOUSE	Histone H2A	LAGNAARD	150	3.70E-05	394.2071	786.3997	2	0.0013	8	0	GluC
H2A1F_MOUSE, H2A1H_MOUSE, H2A1_MOUSE, H2A1K_MOUSE, H2A2A_MOUSE, H2A2B_MOUSE, H2A2C_MOUSE, H2A3_MOUSE, H2AJ_MOUSE, H2AX_MOUSE, H2AY_MOUSE, H2AW_MOUSE, Q8CA90_MOUSE	Histone H2A	LAGNAARD	150	3.80E-07	394.2071	786.3996	2	0.0013	8	0	GluC

H2A1F_MOUSE, H2A1H_MOUSE, H2A1_MOUSE, H2A1K_MOUSE, H2A2A_MOUSE, H2A2B_MOUSE, H2A2C_MOUSE, H2A3_MOUSE, H2AJ_MOUSE, H2AX_MOUSE, H2AY_MOUSE, H2AW_MOUSE, Q8CA90_MOUSE	Histone H2A	LAGNAARD	150	7.20E-03	394.207	786.3995	2	0.0012	8	0	GluC
H2A1F_MOUSE, H2A1H_MOUSE, H2A1_MOUSE, H2A1K_MOUSE, H2A2A_MOUSE, H2A2B_MOUSE, H2A2C_MOUSE, H2A3_MOUSE, H2AJ_MOUSE, H2AX_MOUSE, H2AY_MOUSE, H2AW_MOUSE, Q8CA90_MOUSE	Histone H2A	LAGNAARD	150	3.00E-03	394.2071	786.3996	2	0.0012	8	0	GluC
H2A1F_MOUSE, H2A1H_MOUSE, H2A1_MOUSE, H2A1K_MOUSE, H2A2A_MOUSE, H2A2B_MOUSE, H2A2C_MOUSE, H2A3_MOUSE, H2AJ_MOUSE, H2AX_MOUSE, H2AY_MOUSE, H2AW_MOUSE, Q8CA90_MOUSE	Histone H2A	LAGNAARD	150	3.40E-03	394.207	786.3994	2	0.0011	8	0	GluC
H2A1F_MOUSE, H2A1H_MOUSE, H2A1_MOUSE, H2A1K_MOUSE, H2A2A_MOUSE, H2A2B_MOUSE, H2A2C_MOUSE, H2A3_MOUSE, H2AJ_MOUSE, H2AX_MOUSE, H2AY_MOUSE, H2AW_MOUSE, Q8CA90_MOUSE	Histone H2A	LAGNAARD	150	2.20E-03	394.207	786.3995	2	0.0011	8	0	GluC
H2A1F_MOUSE, H2A1H_MOUSE, H2A1_MOUSE, H2A1K_MOUSE, H2A2A_MOUSE, H2A2B_MOUSE, H2A2C_MOUSE, H2A3_MOUSE, H2AJ_MOUSE, H2AX_MOUSE, H2AY_MOUSE, H2AW_MOUSE, Q8CA90_MOUSE	Histone H2A	LAGNAARD	150	1.40E-05	394.207	786.3995	2	0.0011	8	0	GluC
H2A1F_MOUSE, H2A1H_MOUSE, H2A1_MOUSE, H2A1K_MOUSE, H2A2A_MOUSE, H2A2B_MOUSE, H2A2C_MOUSE, H2A3_MOUSE, H2AJ_MOUSE, H2AX_MOUSE, H2AY_MOUSE, H2AW_MOUSE, Q8CA90_MOUSE	Histone H2A	LAGNAARD	150	2.70E-03	394.207	786.3993	2	0.0010	8	0	GluC
H2A1F_MOUSE, H2A1H_MOUSE, H2A1_MOUSE, H2A1K_MOUSE, H2A2A_MOUSE, H2A2B_MOUSE, H2A2C_MOUSE, H2A3_MOUSE, H2AJ_MOUSE, H2AX_MOUSE, H2AY_MOUSE, H2AW_MOUSE, Q8CA90_MOUSE	Histone H2A	LAGNAARD	150	2.80E-03	394.2069	786.3993	2	0.0009	8	0	GluC
H2A1F_MOUSE, H2A1H_MOUSE, H2A1_MOUSE, H2A1K_MOUSE, H2A2A_MOUSE, H2A2B_MOUSE, H2A2C_MOUSE, H2A3_MOUSE, H2AJ_MOUSE, H2AX_MOUSE, H2AY_MOUSE, H2AW_MOUSE, Q8CA90_MOUSE	Histone H2A	LAGNAARD	150	1.30E-04	394.2069	786.3993	2	0.0009	8	0	GluC
H2A1F_MOUSE, H2A1H_MOUSE, H2A1_MOUSE, H2A1K_MOUSE, H2A2A_MOUSE, H2A2B_MOUSE, H2A2C_MOUSE, H2A3_MOUSE, H2AJ_MOUSE, H2AX_MOUSE, H2AY_MOUSE, H2AW_MOUSE, Q8CA90_MOUSE	Histone H2A	LAGNAARD	150	2.20E-03	394.2069	786.3992	2	0.0008	8	0	GluC
H2A1F_MOUSE, H2A1H_MOUSE, H2A1_MOUSE, H2A1K_MOUSE, H2A2A_MOUSE, H2A2B_MOUSE, H2A2C_MOUSE, H2A3_MOUSE, H2AJ_MOUSE, H2AX_MOUSE, H2AY_MOUSE, H2AW_MOUSE, Q8CA90_MOUSE	Histone H2A	LAGNAARD	150	6.20E-04	394.2069	786.3992	2	0.0008	8	0	GluC
H2A1F_MOUSE, H2A1H_MOUSE, H2A1_MOUSE, H2A1K_MOUSE, H2A2A_MOUSE, H2A2B_MOUSE, H2A2C_MOUSE, H2A3_MOUSE, H2AJ_MOUSE, H2AX_MOUSE, H2AY_MOUSE, H2AW_MOUSE, Q8CA90_MOUSE	Histone H2A	LAGNAARD	150	5.10E-05	394.2069	786.3992	2	0.0008	8	0	GluC
H2A1F_MOUSE, H2A1H_MOUSE, H2A1_MOUSE, H2A1K_MOUSE, H2A2A_MOUSE, H2A2B_MOUSE, H2A2C_MOUSE, H2A3_MOUSE, H2AJ_MOUSE, H2AX_MOUSE, H2AY_MOUSE, H2AW_MOUSE, Q8CA90_MOUSE	Histone H2A	LAGNAARD	150	4.70E-05	394.2068	786.3991	2	0.0007	8	0	GluC
H2A1F_MOUSE, H2A1H_MOUSE, H2A1_MOUSE, H2A1K_MOUSE, H2A2A_MOUSE, H2A2B_MOUSE, H2A2C_MOUSE, H2A3_MOUSE, H2AJ_MOUSE, H2AX_MOUSE, H2AY_MOUSE, H2AW_MOUSE, Q8CA90_MOUSE	Histone H2A	LAGNAARD	150	9.20E-03	394.2067	786.3989	2	0.0005	8	0	GluC
H2A1F_MOUSE, H2A1H_MOUSE, H2A1_MOUSE, H2A1K_MOUSE, H2A2A_MOUSE, H2A2B_MOUSE, H2A2C_MOUSE, H2A3_MOUSE, H2AJ_MOUSE, H2AX_MOUSE, H2AY_MOUSE, H2AW_MOUSE, Q8CA90_MOUSE	Histone H2A	LAGNAARD	150	1.10E-03	394.2067	786.3989	2	0.0005	8	0	GluC
H2A1F_MOUSE, H2A1H_MOUSE, H2A1_MOUSE, H2A1K_MOUSE, H2A2A_MOUSE, H2A2B_MOUSE, H2A2C_MOUSE, H2A3_MOUSE, H2AJ_MOUSE, H2AX_MOUSE, H2AY_MOUSE, H2AW_MOUSE, Q8CA90_MOUSE	Histone H2A	LAGNAARD	150	2.60E-04	394.2067	786.3989	2	0.0005	8	0	GluC
H2A1F_MOUSE, H2A1H_MOUSE, H2A1_MOUSE, H2A1K_MOUSE, H2A2A_MOUSE, H2A2B_MOUSE, H2A2C_MOUSE, H2A3_MOUSE, H2AJ_MOUSE, H2AX_MOUSE, H2AY_MOUSE, H2AW_MOUSE, Q8CA90_MOUSE	Histone H2A	LAGNAARD	150	1.40E-04	394.2067	786.3989	2	0.0005	8	0	GluC
H2A1F_MOUSE, H2A1H_MOUSE, H2A1_MOUSE, H2A1K_MOUSE, H2A2A_MOUSE, H2A2B_MOUSE, H2A2C_MOUSE, H2A3_MOUSE, H2AJ_MOUSE, H2AX_MOUSE, H2AY_MOUSE, H2AW_MOUSE, Q8CA90_MOUSE	Histone H2A	LAGNAARD	150	1.10E-04	394.2067	786.3989	2	0.0005	8	0	GluC
H2A1F_MOUSE, H2A1H_MOUSE, H2A1_MOUSE, H2A1K_MOUSE, H2A2A_MOUSE, H2A2B_MOUSE, H2A2C_MOUSE, H2A3_MOUSE, H2AJ_MOUSE, H2AX_MOUSE, H2AY_MOUSE, H2AW_MOUSE, Q8CA90_MOUSE	Histone H2A	LAGNAARD	150	7.50E-05	394.2067	786.3989	2	0.0005	8	0	GluC

H2A1F_MOUSE, H2A1H_MOUSE, H2A1_MOUSE, H2A1K_MOUSE, H2A2A_MOUSE, H2A2B_MOUSE, H2A2C_MOUSE, H2A3_MOUSE, H2AJ_MOUSE, H2AX_MOUSE, H2AY_MOUSE, H2AW_MOUSE, Q8CA90_MOUSE	Histone H2A	LAGNAARD	150	2.20E-05	394.2067	786.3989	2	0.0005	8	0	GluC
H2A1F_MOUSE, H2A1H_MOUSE, H2A1_MOUSE, H2A1K_MOUSE, H2A2A_MOUSE, H2A2B_MOUSE, H2A2C_MOUSE, H2A3_MOUSE, H2AJ_MOUSE, H2AX_MOUSE, H2AY_MOUSE, H2AW_MOUSE, Q8CA90_MOUSE	Histone H2A	LAGNAARD	150	9.60E-03	394.2067	786.3988	2	0.0004	8	0	GluC
H2A1F_MOUSE, H2A1H_MOUSE, H2A1_MOUSE, H2A1K_MOUSE, H2A2A_MOUSE, H2A2B_MOUSE, H2A2C_MOUSE, H2A3_MOUSE, H2AJ_MOUSE, H2AX_MOUSE, H2AY_MOUSE, H2AW_MOUSE, Q8CA90_MOUSE	Histone H2A	LAGNAARD	150	6.20E-03	394.2067	786.3988	2	0.0004	8	0	GluC
H2A1F_MOUSE, H2A1H_MOUSE, H2A1_MOUSE, H2A1K_MOUSE, H2A2A_MOUSE, H2A2B_MOUSE, H2A2C_MOUSE, H2A3_MOUSE, H2AJ_MOUSE, H2AX_MOUSE, H2AY_MOUSE, H2AW_MOUSE, Q8CA90_MOUSE	Histone H2A	LAGNAARD	150	4.30E-03	394.2066	786.3987	2	0.0003	8	0	GluC
H2A1F_MOUSE, H2A1H_MOUSE, H2A1_MOUSE, H2A1K_MOUSE, H2A2A_MOUSE, H2A2B_MOUSE, H2A2C_MOUSE, H2A3_MOUSE, H2AJ_MOUSE, H2AX_MOUSE, H2AY_MOUSE, H2AW_MOUSE, Q8CA90_MOUSE	Histone H2A	LAGNAARD	150	4.10E-03	394.2066	786.3987	2	0.0003	8	0	GluC
H2A1F_MOUSE, H2A1H_MOUSE, H2A1_MOUSE, H2A1K_MOUSE, H2A2A_MOUSE, H2A2B_MOUSE, H2A2C_MOUSE, H2A3_MOUSE, H2AJ_MOUSE, H2AX_MOUSE, H2AY_MOUSE, H2AW_MOUSE, Q8CA90_MOUSE	Histone H2A	LAGNAARD	150	1.90E-03	394.2066	786.3987	2	0.0003	8	0	GluC
H2A1F_MOUSE, H2A1H_MOUSE, H2A1_MOUSE, H2A1K_MOUSE, H2A2A_MOUSE, H2A2B_MOUSE, H2A2C_MOUSE, H2A3_MOUSE, H2AJ_MOUSE, H2AX_MOUSE, H2AY_MOUSE, H2AW_MOUSE, Q8CA90_MOUSE	Histone H2A	LAGNAARD	150	8.70E-04	394.2066	786.3987	2	0.0003	8	0	GluC
H2A1F_MOUSE, H2A1H_MOUSE, H2A1_MOUSE, H2A1K_MOUSE, H2A2A_MOUSE, H2A2B_MOUSE, H2A2C_MOUSE, H2A3_MOUSE, H2AJ_MOUSE, H2AX_MOUSE, H2AY_MOUSE, H2AW_MOUSE, Q8CA90_MOUSE	Histone H2A	LAGNAARD	150	1.30E-04	394.2066	786.3987	2	0.0003	8	0	GluC
H2A1F_MOUSE, H2A1H_MOUSE, H2A1_MOUSE, H2A1K_MOUSE, H2A2A_MOUSE, H2A2B_MOUSE, H2A2C_MOUSE, H2A3_MOUSE, H2AJ_MOUSE, H2AX_MOUSE, H2AY_MOUSE, H2AW_MOUSE, Q8CA90_MOUSE	Histone H2A	LAGNAARD	150	1.00E-04	394.2066	786.3987	2	0.0003	8	0	GluC
H2A1F_MOUSE, H2A1H_MOUSE, H2A1_MOUSE, H2A1K_MOUSE, H2A2A_MOUSE, H2A2B_MOUSE, H2A2C_MOUSE, H2A3_MOUSE, H2AJ_MOUSE, H2AX_MOUSE, H2AY_MOUSE, H2AW_MOUSE, Q8CA90_MOUSE	Histone H2A	LAGNAARD	150	5.90E-05	394.2066	786.3987	2	0.0003	8	0	GluC
H2A1F_MOUSE, H2A1H_MOUSE, H2A1_MOUSE, H2A1K_MOUSE, H2A2A_MOUSE, H2A2B_MOUSE, H2A2C_MOUSE, H2A3_MOUSE, H2AJ_MOUSE, H2AX_MOUSE, H2AY_MOUSE, H2AW_MOUSE, Q8CA90_MOUSE	Histone H2A	LAGNAARD	150	2.60E-05	394.2066	786.3987	2	0.0003	8	0	GluC
H2A1F_MOUSE, H2A1H_MOUSE, H2A1_MOUSE, H2A1K_MOUSE, H2A2A_MOUSE, H2A2B_MOUSE, H2A2C_MOUSE, H2A3_MOUSE, H2AJ_MOUSE, H2AX_MOUSE, H2AY_MOUSE, H2AW_MOUSE, Q8CA90_MOUSE	Histone H2A	LAGNAARD	150	5.50E-03	394.2065	786.3985	2	0.0002	8	0	GluC
H2A1F_MOUSE, H2A1H_MOUSE, H2A1_MOUSE, H2A1K_MOUSE, H2A2A_MOUSE, H2A2B_MOUSE, H2A2C_MOUSE, H2A3_MOUSE, H2AJ_MOUSE, H2AX_MOUSE, H2AY_MOUSE, H2AW_MOUSE, Q8CA90_MOUSE	Histone H2A	LAGNAARD	150	4.60E-03	394.2066	786.3986	2	0.0002	8	0	GluC
H2A1F_MOUSE, H2A1H_MOUSE, H2A1_MOUSE, H2A1K_MOUSE, H2A2A_MOUSE, H2A2B_MOUSE, H2A2C_MOUSE, H2A3_MOUSE, H2AJ_MOUSE, H2AX_MOUSE, H2AY_MOUSE, H2AW_MOUSE, Q8CA90_MOUSE	Histone H2A	LAGNAARD	150	4.50E-03	394.2066	786.3986	2	0.0002	8	0	GluC
H2A1F_MOUSE, H2A1H_MOUSE, H2A1_MOUSE, H2A1K_MOUSE, H2A2A_MOUSE, H2A2B_MOUSE, H2A2C_MOUSE, H2A3_MOUSE, H2AJ_MOUSE, H2AX_MOUSE, H2AY_MOUSE, H2AW_MOUSE, Q8CA90_MOUSE	Histone H2A	LAGNAARD	150	2.00E-03	394.2065	786.3985	2	0.0002	8	0	GluC
H2A1F_MOUSE, H2A1H_MOUSE, H2A1_MOUSE, H2A1K_MOUSE, H2A2A_MOUSE, H2A2B_MOUSE, H2A2C_MOUSE, H2A3_MOUSE, H2AJ_MOUSE, H2AX_MOUSE, H2AY_MOUSE, H2AW_MOUSE, Q8CA90_MOUSE	Histone H2A	LAGNAARD	150	8.60E-04	394.2066	786.3986	2	0.0002	8	0	GluC
H2A1F_MOUSE, H2A1H_MOUSE, H2A1_MOUSE, H2A1K_MOUSE, H2A2A_MOUSE, H2A2B_MOUSE, H2A2C_MOUSE, H2A3_MOUSE, H2AJ_MOUSE, H2AX_MOUSE, H2AY_MOUSE, H2AW_MOUSE, Q8CA90_MOUSE	Histone H2A	LAGNAARD	150	8.00E-04	394.2066	786.3986	2	0.0002	8	0	GluC
H2A1F_MOUSE, H2A1H_MOUSE, H2A1_MOUSE, H2A1K_MOUSE, H2A2A_MOUSE, H2A2B_MOUSE, H2A2C_MOUSE, H2A3_MOUSE, H2AJ_MOUSE, H2AX_MOUSE, H2AY_MOUSE, H2AW_MOUSE, Q8CA90_MOUSE	Histone H2A	LAGNAARD	150	4.90E-04	394.2066	786.3986	2	0.0002	8	0	GluC

H2A1F_MOUSE, H2A1H_MOUSE, H2A1_MOUSE, H2A1K_MOUSE, H2A2A_MOUSE, H2A2B_MOUSE, H2A2C_MOUSE, H2A3_MOUSE, H2AJ_MOUSE, H2AX_MOUSE, H2AY_MOUSE, H2AW_MOUSE, Q8CA90_MOUSE	Histone H2A	LAGNAARD	150	3.70E-04	394.2066	786.3986	2	0.0002	8	0	GluC
H2A1F_MOUSE, H2A1H_MOUSE, H2A1_MOUSE, H2A1K_MOUSE, H2A2A_MOUSE, H2A2B_MOUSE, H2A2C_MOUSE, H2A3_MOUSE, H2AJ_MOUSE, H2AX_MOUSE, H2AY_MOUSE, H2AW_MOUSE, Q8CA90_MOUSE	Histone H2A	LAGNAARD	150	2.60E-04	394.2066	786.3986	2	0.0002	8	0	GluC
H2A1F_MOUSE, H2A1H_MOUSE, H2A1_MOUSE, H2A1K_MOUSE, H2A2A_MOUSE, H2A2B_MOUSE, H2A2C_MOUSE, H2A3_MOUSE, H2AJ_MOUSE, H2AX_MOUSE, H2AY_MOUSE, H2AW_MOUSE, Q8CA90_MOUSE	Histone H2A	LAGNAARD	150	1.70E-04	394.2066	786.3986	2	0.0002	8	0	GluC
H2A1F_MOUSE, H2A1H_MOUSE, H2A1_MOUSE, H2A1K_MOUSE, H2A2A_MOUSE, H2A2B_MOUSE, H2A2C_MOUSE, H2A3_MOUSE, H2AJ_MOUSE, H2AX_MOUSE, H2AY_MOUSE, H2AW_MOUSE, Q8CA90_MOUSE	Histone H2A	LAGNAARD	150	1.50E-04	394.2065	786.3985	2	0.0002	8	0	GluC
H2A1F_MOUSE, H2A1H_MOUSE, H2A1_MOUSE, H2A1K_MOUSE, H2A2A_MOUSE, H2A2B_MOUSE, H2A2C_MOUSE, H2A3_MOUSE, H2AJ_MOUSE, H2AX_MOUSE, H2AY_MOUSE, H2AW_MOUSE, Q8CA90_MOUSE	Histone H2A	LAGNAARD	150	6.80E-05	394.2065	786.3985	2	0.0002	8	0	GluC
H2A1F_MOUSE, H2A1H_MOUSE, H2A1_MOUSE, H2A1K_MOUSE, H2A2A_MOUSE, H2A2B_MOUSE, H2A2C_MOUSE, H2A3_MOUSE, H2AJ_MOUSE, H2AX_MOUSE, H2AY_MOUSE, H2AW_MOUSE, Q8CA90_MOUSE	Histone H2A	LAGNAARD	150	6.20E-05	394.2065	786.3985	2	0.0002	8	0	GluC
H2A1F_MOUSE, H2A1H_MOUSE, H2A1_MOUSE, H2A1K_MOUSE, H2A2A_MOUSE, H2A2B_MOUSE, H2A2C_MOUSE, H2A3_MOUSE, H2AJ_MOUSE, H2AX_MOUSE, H2AY_MOUSE, H2AW_MOUSE, Q8CA90_MOUSE	Histone H2A	LAGNAARD	150	4.50E-05	394.2066	786.3986	2	0.0002	8	0	GluC
H2A1F_MOUSE, H2A1H_MOUSE, H2A1_MOUSE, H2A1K_MOUSE, H2A2A_MOUSE, H2A2B_MOUSE, H2A2C_MOUSE, H2A3_MOUSE, H2AJ_MOUSE, H2AX_MOUSE, H2AY_MOUSE, H2AW_MOUSE, Q8CA90_MOUSE	Histone H2A	LAGNAARD	150	3.80E-05	394.2066	786.3986	2	0.0002	8	0	GluC
H2A1F_MOUSE, H2A1H_MOUSE, H2A1_MOUSE, H2A1K_MOUSE, H2A2A_MOUSE, H2A2B_MOUSE, H2A2C_MOUSE, H2A3_MOUSE, H2AJ_MOUSE, H2AX_MOUSE, H2AY_MOUSE, H2AW_MOUSE, Q8CA90_MOUSE	Histone H2A	LAGNAARD	150	3.60E-05	394.2066	786.3986	2	0.0002	8	0	GluC
H2A1F_MOUSE, H2A1H_MOUSE, H2A1_MOUSE, H2A1K_MOUSE, H2A2A_MOUSE, H2A2B_MOUSE, H2A2C_MOUSE, H2A3_MOUSE, H2AJ_MOUSE, H2AX_MOUSE, H2AY_MOUSE, H2AW_MOUSE, Q8CA90_MOUSE	Histone H2A	LAGNAARD	150	4.20E-03	394.2065	786.3984	2	0.0001	8	0	GluC
H2A1F_MOUSE, H2A1H_MOUSE, H2A1_MOUSE, H2A1K_MOUSE, H2A2A_MOUSE, H2A2B_MOUSE, H2A2C_MOUSE, H2A3_MOUSE, H2AJ_MOUSE, H2AX_MOUSE, H2AY_MOUSE, H2AW_MOUSE, Q8CA90_MOUSE	Histone H2A	LAGNAARD	150	2.70E-03	394.2065	786.3984	2	0.0001	8	0	GluC
H2A1F_MOUSE, H2A1H_MOUSE, H2A1_MOUSE, H2A1K_MOUSE, H2A2A_MOUSE, H2A2B_MOUSE, H2A2C_MOUSE, H2A3_MOUSE, H2AJ_MOUSE, H2AX_MOUSE, H2AY_MOUSE, H2AW_MOUSE, Q8CA90_MOUSE	Histone H2A	LAGNAARD	150	2.20E-03	394.2065	786.3985	2	0.0001	8	0	GluC
H2A1F_MOUSE, H2A1H_MOUSE, H2A1_MOUSE, H2A1K_MOUSE, H2A2A_MOUSE, H2A2B_MOUSE, H2A2C_MOUSE, H2A3_MOUSE, H2AJ_MOUSE, H2AX_MOUSE, H2AY_MOUSE, H2AW_MOUSE, Q8CA90_MOUSE	Histone H2A	LAGNAARD	150	2.20E-03	394.2065	786.3985	2	0.0001	8	0	GluC
H2A1F_MOUSE, H2A1H_MOUSE, H2A1_MOUSE, H2A1K_MOUSE, H2A2A_MOUSE, H2A2B_MOUSE, H2A2C_MOUSE, H2A3_MOUSE, H2AJ_MOUSE, H2AX_MOUSE, H2AY_MOUSE, H2AW_MOUSE, Q8CA90_MOUSE	Histone H2A	LAGNAARD	150	2.20E-03	394.2065	786.3985	2	0.0001	8	0	GluC
H2A1F_MOUSE, H2A1H_MOUSE, H2A1_MOUSE, H2A1K_MOUSE, H2A2A_MOUSE, H2A2B_MOUSE, H2A2C_MOUSE, H2A3_MOUSE, H2AJ_MOUSE, H2AX_MOUSE, H2AY_MOUSE, H2AW_MOUSE, Q8CA90_MOUSE	Histone H2A	LAGNAARD	150	9.10E-04	394.2065	786.3985	2	0.0001	8	0	GluC
H2A1F_MOUSE, H2A1H_MOUSE, H2A1_MOUSE, H2A1K_MOUSE, H2A2A_MOUSE, H2A2B_MOUSE, H2A2C_MOUSE, H2A3_MOUSE, H2AJ_MOUSE, H2AX_MOUSE, H2AY_MOUSE, H2AW_MOUSE, Q8CA90_MOUSE	Histone H2A	LAGNAARD	150	4.50E-04	394.2065	786.3984	2	0.0001	8	0	GluC
H2A1F_MOUSE, H2A1H_MOUSE, H2A1_MOUSE, H2A1K_MOUSE, H2A2A_MOUSE, H2A2B_MOUSE, H2A2C_MOUSE, H2A3_MOUSE, H2AJ_MOUSE, H2AX_MOUSE, H2AY_MOUSE, H2AW_MOUSE, Q8CA90_MOUSE	Histone H2A	LAGNAARD	150	1.20E-04	394.2065	786.3985	2	0.0001	8	0	GluC
H2A1F_MOUSE, H2A1H_MOUSE, H2A1_MOUSE, H2A1K_MOUSE, H2A2A_MOUSE, H2A2B_MOUSE, H2A2C_MOUSE, H2A3_MOUSE, H2AJ_MOUSE, H2AX_MOUSE, H2AY_MOUSE, H2AW_MOUSE, Q8CA90_MOUSE	Histone H2A	LAGNAARD	150	4.90E-05	394.2065	786.3985	2	0.0001	8	0	GluC

H2A1F_MOUSE, H2A1H_MOUSE, H2A1_MOUSE, H2A1K_MOUSE, H2A2A_MOUSE, H2A2B_MOUSE, H2A2C_MOUSE, H2A3_MOUSE, H2AJ_MOUSE, H2AX_MOUSE, H2AY_MOUSE, H2AW_MOUSE, Q8CA90_MOUSE	Histone H2A	LAGNAARD	150	4.70E-05	394.2065	786.3985	2	0.0001	8	0	GluC
H2A1F_MOUSE, H2A1H_MOUSE, H2A1_MOUSE, H2A1K_MOUSE, H2A2A_MOUSE, H2A2B_MOUSE, H2A2C_MOUSE, H2A3_MOUSE, H2AJ_MOUSE, H2AX_MOUSE, H2AY_MOUSE, H2AW_MOUSE, Q8CA90_MOUSE	Histone H2A	LAGNAARD	150	3.40E-05	394.2065	786.3984	2	0.0001	8	0	GluC
H2A1F_MOUSE, H2A1H_MOUSE, H2A1_MOUSE, H2A1K_MOUSE, H2A2A_MOUSE, H2A2B_MOUSE, H2A2C_MOUSE, H2A3_MOUSE, H2AJ_MOUSE, H2AX_MOUSE, H2AY_MOUSE, H2AW_MOUSE, Q8CA90_MOUSE	Histone H2A	LAGNAARD	150	3.10E-05	394.2065	786.3984	2	0.0001	8	0	GluC
H2A1F_MOUSE, H2A1H_MOUSE, H2A1_MOUSE, H2A1K_MOUSE, H2A2A_MOUSE, H2A2B_MOUSE, H2A2C_MOUSE, H2A3_MOUSE, H2AJ_MOUSE, H2AX_MOUSE, H2AY_MOUSE, H2AW_MOUSE, Q8CA90_MOUSE	Histone H2A	LAGNAARD	150	4.40E-03	394.2065	786.3983	2	0.0000	8	0	GluC
H2A1F_MOUSE, H2A1H_MOUSE, H2A1_MOUSE, H2A1K_MOUSE, H2A2A_MOUSE, H2A2B_MOUSE, H2A2C_MOUSE, H2A3_MOUSE, H2AJ_MOUSE, H2AX_MOUSE, H2AY_MOUSE, H2AW_MOUSE, Q8CA90_MOUSE	Histone H2A	LAGNAARD	150	3.90E-03	394.2065	786.3984	2	0.0000	8	0	GluC
H2A1F_MOUSE, H2A1H_MOUSE, H2A1_MOUSE, H2A1K_MOUSE, H2A2A_MOUSE, H2A2B_MOUSE, H2A2C_MOUSE, H2A3_MOUSE, H2AJ_MOUSE, H2AX_MOUSE, H2AY_MOUSE, H2AW_MOUSE, Q8CA90_MOUSE	Histone H2A	LAGNAARD	150	2.40E-03	394.2065	786.3984	2	0.0000	8	0	GluC
H2A1F_MOUSE, H2A1H_MOUSE, H2A1_MOUSE, H2A1K_MOUSE, H2A2A_MOUSE, H2A2B_MOUSE, H2A2C_MOUSE, H2A3_MOUSE, H2AJ_MOUSE, H2AX_MOUSE, H2AY_MOUSE, H2AW_MOUSE, Q8CA90_MOUSE	Histone H2A	LAGNAARD	150	1.90E-03	394.2065	786.3984	2	0.0000	8	0	GluC
H2A1F_MOUSE, H2A1H_MOUSE, H2A1_MOUSE, H2A1K_MOUSE, H2A2A_MOUSE, H2A2B_MOUSE, H2A2C_MOUSE, H2A3_MOUSE, H2AJ_MOUSE, H2AX_MOUSE, H2AY_MOUSE, H2AW_MOUSE, Q8CA90_MOUSE	Histone H2A	LAGNAARD	150	1.20E-03	394.2065	786.3983	2	0.0000	8	0	GluC
H2A1F_MOUSE, H2A1H_MOUSE, H2A1_MOUSE, H2A1K_MOUSE, H2A2A_MOUSE, H2A2B_MOUSE, H2A2C_MOUSE, H2A3_MOUSE, H2AJ_MOUSE, H2AX_MOUSE, H2AY_MOUSE, H2AW_MOUSE, Q8CA90_MOUSE	Histone H2A	LAGNAARD	150	3.00E-04	394.2065	786.3983	2	0.0000	8	0	GluC
H2A1F_MOUSE, H2A1H_MOUSE, H2A1_MOUSE, H2A1K_MOUSE, H2A2A_MOUSE, H2A2B_MOUSE, H2A2C_MOUSE, H2A3_MOUSE, H2AJ_MOUSE, H2AX_MOUSE, H2AY_MOUSE, H2AW_MOUSE, Q8CA90_MOUSE	Histone H2A	LAGNAARD	150	1.40E-04	394.2065	786.3984	2	0.0000	8	0	GluC
H2A1F_MOUSE, H2A1H_MOUSE, H2A1_MOUSE, H2A1K_MOUSE, H2A2A_MOUSE, H2A2B_MOUSE, H2A2C_MOUSE, H2A3_MOUSE, H2AJ_MOUSE, H2AX_MOUSE, H2AY_MOUSE, H2AW_MOUSE, Q8CA90_MOUSE	Histone H2A	LAGNAARD	150	9.80E-05	394.2065	786.3984	2	0.0000	8	0	GluC
H2A1F_MOUSE, H2A1H_MOUSE, H2A1_MOUSE, H2A1K_MOUSE, H2A2A_MOUSE, H2A2B_MOUSE, H2A2C_MOUSE, H2A3_MOUSE, H2AJ_MOUSE, H2AX_MOUSE, H2AY_MOUSE, H2AW_MOUSE, Q8CA90_MOUSE	Histone H2A	LAGNAARD	150	9.50E-05	394.2065	786.3984	2	0.0000	8	0	GluC
H2A1F_MOUSE, H2A1H_MOUSE, H2A1_MOUSE, H2A1K_MOUSE, H2A2A_MOUSE, H2A2B_MOUSE, H2A2C_MOUSE, H2A3_MOUSE, H2AJ_MOUSE, H2AX_MOUSE, H2AY_MOUSE, H2AW_MOUSE, Q8CA90_MOUSE	Histone H2A	LAGNAARD	150	6.20E-05	394.2065	786.3984	2	0.0000	8	0	GluC
H2A1F_MOUSE, H2A1H_MOUSE, H2A1_MOUSE, H2A1K_MOUSE, H2A2A_MOUSE, H2A2B_MOUSE, H2A2C_MOUSE, H2A3_MOUSE, H2AJ_MOUSE, H2AX_MOUSE, H2AY_MOUSE, H2AW_MOUSE, Q8CA90_MOUSE	Histone H2A	LAGNAARD	150	4.10E-03	394.2064	786.3983	2	-0.0001	8	0	GluC
H2A1F_MOUSE, H2A1H_MOUSE, H2A1_MOUSE, H2A1K_MOUSE, H2A2A_MOUSE, H2A2B_MOUSE, H2A2C_MOUSE, H2A3_MOUSE, H2AJ_MOUSE, H2AX_MOUSE, H2AY_MOUSE, H2AW_MOUSE, Q8CA90_MOUSE	Histone H2A	LAGNAARD	150	3.80E-03	394.2064	786.3983	2	-0.0001	8	0	GluC
H2A1F_MOUSE, H2A1H_MOUSE, H2A1_MOUSE, H2A1K_MOUSE, H2A2A_MOUSE, H2A2B_MOUSE, H2A2C_MOUSE, H2A3_MOUSE, H2AJ_MOUSE, H2AX_MOUSE, H2AY_MOUSE, H2AW_MOUSE, Q8CA90_MOUSE	Histone H2A	LAGNAARD	150	3.60E-03	394.2064	786.3983	2	-0.0001	8	0	GluC
H2A1F_MOUSE, H2A1H_MOUSE, H2A1_MOUSE, H2A1K_MOUSE, H2A2A_MOUSE, H2A2B_MOUSE, H2A2C_MOUSE, H2A3_MOUSE, H2AJ_MOUSE, H2AX_MOUSE, H2AY_MOUSE, H2AW_MOUSE, Q8CA90_MOUSE	Histone H2A	LAGNAARD	150	3.60E-03	394.2064	786.3983	2	-0.0001	8	0	GluC
H2A1F_MOUSE, H2A1H_MOUSE, H2A1_MOUSE, H2A1K_MOUSE, H2A2A_MOUSE, H2A2B_MOUSE, H2A2C_MOUSE, H2A3_MOUSE, H2AJ_MOUSE, H2AX_MOUSE, H2AY_MOUSE, H2AW_MOUSE, Q8CA90_MOUSE	Histone H2A	LAGNAARD	150	2.20E-03	394.2064	786.3983	2	-0.0001	8	0	GluC

H2A1F_MOUSE, H2A1H_MOUSE, H2A1_MOUSE, H2A1K_MOUSE, H2A2A_MOUSE, H2A2B_MOUSE, H2A2C_MOUSE, H2A3_MOUSE, H2AJ_MOUSE, H2AX_MOUSE, H2AY_MOUSE, H2AW_MOUSE, Q8CA90_MOUSE	Histone H2A	LAGNAARD	150	2.20E-03	394.2064	786.3983	2	-0.0001	8	0	GluC
H2A1F_MOUSE, H2A1H_MOUSE, H2A1_MOUSE, H2A1K_MOUSE, H2A2A_MOUSE, H2A2B_MOUSE, H2A2C_MOUSE, H2A3_MOUSE, H2AJ_MOUSE, H2AX_MOUSE, H2AY_MOUSE, H2AW_MOUSE, Q8CA90_MOUSE	Histone H2A	LAGNAARD	150	1.80E-03	394.2064	786.3983	2	-0.0001	8	0	GluC
H2A1F_MOUSE, H2A1H_MOUSE, H2A1_MOUSE, H2A1K_MOUSE, H2A2A_MOUSE, H2A2B_MOUSE, H2A2C_MOUSE, H2A3_MOUSE, H2AJ_MOUSE, H2AX_MOUSE, H2AY_MOUSE, H2AW_MOUSE, Q8CA90_MOUSE	Histone H2A	LAGNAARD	150	8.80E-04	394.2064	786.3983	2	-0.0001	8	0	GluC
H2A1F_MOUSE, H2A1H_MOUSE, H2A1_MOUSE, H2A1K_MOUSE, H2A2A_MOUSE, H2A2B_MOUSE, H2A2C_MOUSE, H2A3_MOUSE, H2AJ_MOUSE, H2AX_MOUSE, H2AY_MOUSE, H2AW_MOUSE, Q8CA90_MOUSE	Histone H2A	LAGNAARD	150	6.20E-04	394.2064	786.3983	2	-0.0001	8	0	GluC
H2A1F_MOUSE, H2A1H_MOUSE, H2A1_MOUSE, H2A1K_MOUSE, H2A2A_MOUSE, H2A2B_MOUSE, H2A2C_MOUSE, H2A3_MOUSE, H2AJ_MOUSE, H2AX_MOUSE, H2AY_MOUSE, H2AW_MOUSE, Q8CA90_MOUSE	Histone H2A	LAGNAARD	150	5.10E-04	394.2064	786.3983	2	-0.0001	8	0	GluC
H2A1F_MOUSE, H2A1H_MOUSE, H2A1_MOUSE, H2A1K_MOUSE, H2A2A_MOUSE, H2A2B_MOUSE, H2A2C_MOUSE, H2A3_MOUSE, H2AJ_MOUSE, H2AX_MOUSE, H2AY_MOUSE, H2AW_MOUSE, Q8CA90_MOUSE	Histone H2A	LAGNAARD	150	5.00E-04	394.2064	786.3982	2	-0.0001	8	0	GluC
H2A1F_MOUSE, H2A1H_MOUSE, H2A1_MOUSE, H2A1K_MOUSE, H2A2A_MOUSE, H2A2B_MOUSE, H2A2C_MOUSE, H2A3_MOUSE, H2AJ_MOUSE, H2AX_MOUSE, H2AY_MOUSE, H2AW_MOUSE, Q8CA90_MOUSE	Histone H2A	LAGNAARD	150	2.10E-04	394.2064	786.3983	2	-0.0001	8	0	GluC
H2A1F_MOUSE, H2A1H_MOUSE, H2A1_MOUSE, H2A1K_MOUSE, H2A2A_MOUSE, H2A2B_MOUSE, H2A2C_MOUSE, H2A3_MOUSE, H2AJ_MOUSE, H2AX_MOUSE, H2AY_MOUSE, H2AW_MOUSE, Q8CA90_MOUSE	Histone H2A	LAGNAARD	150	8.70E-05	394.2064	786.3983	2	-0.0001	8	0	GluC
H2A1F_MOUSE, H2A1H_MOUSE, H2A1_MOUSE, H2A1K_MOUSE, H2A2A_MOUSE, H2A2B_MOUSE, H2A2C_MOUSE, H2A3_MOUSE, H2AJ_MOUSE, H2AX_MOUSE, H2AY_MOUSE, H2AW_MOUSE, Q8CA90_MOUSE	Histone H2A	LAGNAARD	150	8.50E-05	394.2064	786.3983	2	-0.0001	8	0	GluC
H2A1F_MOUSE, H2A1H_MOUSE, H2A1_MOUSE, H2A1K_MOUSE, H2A2A_MOUSE, H2A2B_MOUSE, H2A2C_MOUSE, H2A3_MOUSE, H2AJ_MOUSE, H2AX_MOUSE, H2AY_MOUSE, H2AW_MOUSE, Q8CA90_MOUSE	Histone H2A	LAGNAARD	150	2.70E-05	394.2064	786.3983	2	-0.0001	8	0	GluC
H2A1F_MOUSE, H2A1H_MOUSE, H2A1_MOUSE, H2A1K_MOUSE, H2A2A_MOUSE, H2A2B_MOUSE, H2A2C_MOUSE, H2A3_MOUSE, H2AJ_MOUSE, H2AX_MOUSE, H2AY_MOUSE, H2AW_MOUSE, Q8CA90_MOUSE	Histone H2A	LAGNAARD	150	2.60E-05	394.2064	786.3983	2	-0.0001	8	0	GluC
H2A1F_MOUSE, H2A1H_MOUSE, H2A1_MOUSE, H2A1K_MOUSE, H2A2A_MOUSE, H2A2B_MOUSE, H2A2C_MOUSE, H2A3_MOUSE, H2AJ_MOUSE, H2AX_MOUSE, H2AY_MOUSE, H2AW_MOUSE, Q8CA90_MOUSE	Histone H2A	LAGNAARD	150	8.20E-06	394.2064	786.3983	2	-0.0001	8	0	GluC
H2A1F_MOUSE, H2A1H_MOUSE, H2A1_MOUSE, H2A1K_MOUSE, H2A2A_MOUSE, H2A2B_MOUSE, H2A2C_MOUSE, H2A3_MOUSE, H2AJ_MOUSE, H2AX_MOUSE, H2AY_MOUSE, H2AW_MOUSE, Q8CA90_MOUSE	Histone H2A	LAGNAARD	150	3.50E-03	394.2064	786.3982	2	-0.0002	8	0	GluC
H2A1F_MOUSE, H2A1H_MOUSE, H2A1_MOUSE, H2A1K_MOUSE, H2A2A_MOUSE, H2A2B_MOUSE, H2A2C_MOUSE, H2A3_MOUSE, H2AJ_MOUSE, H2AX_MOUSE, H2AY_MOUSE, H2AW_MOUSE, Q8CA90_MOUSE	Histone H2A	LAGNAARD	150	2.40E-03	394.2064	786.3982	2	-0.0002	8	0	GluC
H2A1F_MOUSE, H2A1H_MOUSE, H2A1_MOUSE, H2A1K_MOUSE, H2A2A_MOUSE, H2A2B_MOUSE, H2A2C_MOUSE, H2A3_MOUSE, H2AJ_MOUSE, H2AX_MOUSE, H2AY_MOUSE, H2AW_MOUSE, Q8CA90_MOUSE	Histone H2A	LAGNAARD	150	7.80E-04	394.2064	786.3982	2	-0.0002	8	0	GluC
H2A1F_MOUSE, H2A1H_MOUSE, H2A1_MOUSE, H2A1K_MOUSE, H2A2A_MOUSE, H2A2B_MOUSE, H2A2C_MOUSE, H2A3_MOUSE, H2AJ_MOUSE, H2AX_MOUSE, H2AY_MOUSE, H2AW_MOUSE, Q8CA90_MOUSE	Histone H2A	LAGNAARD	150	3.80E-04	394.2064	786.3982	2	-0.0002	8	0	GluC
H2A1F_MOUSE, H2A1H_MOUSE, H2A1_MOUSE, H2A1K_MOUSE, H2A2A_MOUSE, H2A2B_MOUSE, H2A2C_MOUSE, H2A3_MOUSE, H2AJ_MOUSE, H2AX_MOUSE, H2AY_MOUSE, H2AW_MOUSE, Q8CA90_MOUSE	Histone H2A	LAGNAARD	150	3.60E-04	394.2064	786.3982	2	-0.0002	8	0	GluC
H2A1F_MOUSE, H2A1H_MOUSE, H2A1_MOUSE, H2A1K_MOUSE, H2A2A_MOUSE, H2A2B_MOUSE, H2A2C_MOUSE, H2A3_MOUSE, H2AJ_MOUSE, H2AX_MOUSE, H2AY_MOUSE, H2AW_MOUSE, Q8CA90_MOUSE	Histone H2A	LAGNAARD	150	2.20E-04	394.2064	786.3982	2	-0.0002	8	0	GluC

H2A1F_MOUSE, H2A1H_MOUSE, H2A1_MOUSE, H2A1K_MOUSE, H2A2A_MOUSE, H2A2B_MOUSE, H2A2C_MOUSE, H2A3_MOUSE, H2AJ_MOUSE, H2AX_MOUSE, H2AY_MOUSE, H2AW_MOUSE, Q8CA90_MOUSE	Histone H2A	LAGNAARD	150	1.40E-04	394.2064	786.3982	2	-0.0002	8	0	GluC
H2A1F_MOUSE, H2A1H_MOUSE, H2A1_MOUSE, H2A1K_MOUSE, H2A2A_MOUSE, H2A2B_MOUSE, H2A2C_MOUSE, H2A3_MOUSE, H2AJ_MOUSE, H2AX_MOUSE, H2AY_MOUSE, H2AW_MOUSE, Q8CA90_MOUSE	Histone H2A	LAGNAARD	150	1.10E-04	394.2064	786.3982	2	-0.0002	8	0	GluC
H2A1F_MOUSE, H2A1H_MOUSE, H2A1_MOUSE, H2A1K_MOUSE, H2A2A_MOUSE, H2A2B_MOUSE, H2A2C_MOUSE, H2A3_MOUSE, H2AJ_MOUSE, H2AX_MOUSE, H2AY_MOUSE, H2AW_MOUSE, Q8CA90_MOUSE	Histone H2A	LAGNAARD	150	5.00E-05	394.2064	786.3982	2	-0.0002	8	0	GluC
H2A1F_MOUSE, H2A1H_MOUSE, H2A1_MOUSE, H2A1K_MOUSE, H2A2A_MOUSE, H2A2B_MOUSE, H2A2C_MOUSE, H2A3_MOUSE, H2AJ_MOUSE, H2AX_MOUSE, H2AY_MOUSE, H2AW_MOUSE, Q8CA90_MOUSE	Histone H2A	LAGNAARD	150	3.00E-05	394.2064	786.3982	2	-0.0002	8	0	GluC
H2A1F_MOUSE, H2A1H_MOUSE, H2A1_MOUSE, H2A1K_MOUSE, H2A2A_MOUSE, H2A2B_MOUSE, H2A2C_MOUSE, H2A3_MOUSE, H2AJ_MOUSE, H2AX_MOUSE, H2AY_MOUSE, H2AW_MOUSE, Q8CA90_MOUSE	Histone H2A	LAGNAARD	150	2.60E-05	394.2064	786.3982	2	-0.0002	8	0	GluC
H2A1F_MOUSE, H2A1H_MOUSE, H2A1_MOUSE, H2A1K_MOUSE, H2A2A_MOUSE, H2A2B_MOUSE, H2A2C_MOUSE, H2A3_MOUSE, H2AJ_MOUSE, H2AX_MOUSE, H2AY_MOUSE, H2AW_MOUSE, Q8CA90_MOUSE	Histone H2A	LAGNAARD	150	6.10E-03	394.2063	786.3981	2	-0.0003	8	0	GluC
H2A1F_MOUSE, H2A1H_MOUSE, H2A1_MOUSE, H2A1K_MOUSE, H2A2A_MOUSE, H2A2B_MOUSE, H2A2C_MOUSE, H2A3_MOUSE, H2AJ_MOUSE, H2AX_MOUSE, H2AY_MOUSE, H2AW_MOUSE, Q8CA90_MOUSE	Histone H2A	LAGNAARD	150	2.60E-03	394.2063	786.3981	2	-0.0003	8	0	GluC
H2A1F_MOUSE, H2A1H_MOUSE, H2A1_MOUSE, H2A1K_MOUSE, H2A2A_MOUSE, H2A2B_MOUSE, H2A2C_MOUSE, H2A3_MOUSE, H2AJ_MOUSE, H2AX_MOUSE, H2AY_MOUSE, H2AW_MOUSE, Q8CA90_MOUSE	Histone H2A	LAGNAARD	150	1.10E-04	394.2063	786.3981	2	-0.0003	8	0	GluC
H2A1F_MOUSE, H2A1H_MOUSE, H2A1_MOUSE, H2A1K_MOUSE, H2A2A_MOUSE, H2A2B_MOUSE, H2A2C_MOUSE, H2A3_MOUSE, H2AJ_MOUSE, H2AX_MOUSE, H2AY_MOUSE, H2AW_MOUSE, Q8CA90_MOUSE	Histone H2A	LAGNAARD	150	9.10E-05	394.2063	786.3981	2	-0.0003	8	0	GluC
H2A1F_MOUSE, H2A1H_MOUSE, H2A1_MOUSE, H2A1K_MOUSE, H2A2A_MOUSE, H2A2B_MOUSE, H2A2C_MOUSE, H2A3_MOUSE, H2AJ_MOUSE, H2AX_MOUSE, H2AY_MOUSE, H2AW_MOUSE, Q8CA90_MOUSE	Histone H2A	LAGNAARD	150	6.80E-05	394.2063	786.3981	2	-0.0003	8	0	GluC
H2A1F_MOUSE, H2A1H_MOUSE, H2A1_MOUSE, H2A1K_MOUSE, H2A2A_MOUSE, H2A2B_MOUSE, H2A2C_MOUSE, H2A3_MOUSE, H2AJ_MOUSE, H2AX_MOUSE, H2AY_MOUSE, H2AW_MOUSE, Q8CA90_MOUSE	Histone H2A	LAGNAARD	150	3.60E-05	394.2063	786.3981	2	-0.0003	8	0	GluC
H2A1F_MOUSE, H2A1H_MOUSE, H2A1_MOUSE, H2A1K_MOUSE, H2A2A_MOUSE, H2A2B_MOUSE, H2A2C_MOUSE, H2A3_MOUSE, H2AJ_MOUSE, H2AX_MOUSE, H2AY_MOUSE, H2AW_MOUSE, Q8CA90_MOUSE	Histone H2A	LAGNAARD	150	1.00E-02	394.2063	786.3980	2	-0.0004	8	0	GluC
H2A1F_MOUSE, H2A1H_MOUSE, H2A1_MOUSE, H2A1K_MOUSE, H2A2A_MOUSE, H2A2B_MOUSE, H2A2C_MOUSE, H2A3_MOUSE, H2AJ_MOUSE, H2AX_MOUSE, H2AY_MOUSE, H2AW_MOUSE, Q8CA90_MOUSE	Histone H2A	LAGNAARD	150	8.60E-03	394.2063	786.3980	2	-0.0004	8	0	GluC
H2A1F_MOUSE, H2A1H_MOUSE, H2A1_MOUSE, H2A1K_MOUSE, H2A2A_MOUSE, H2A2B_MOUSE, H2A2C_MOUSE, H2A3_MOUSE, H2AJ_MOUSE, H2AX_MOUSE, H2AY_MOUSE, H2AW_MOUSE, Q8CA90_MOUSE	Histone H2A	LAGNAARD	150	1.60E-04	394.2063	786.3979	2	-0.0004	8	0	GluC
H2A1F_MOUSE, H2A1H_MOUSE, H2A1_MOUSE, H2A1K_MOUSE, H2A2A_MOUSE, H2A2B_MOUSE, H2A2C_MOUSE, H2A3_MOUSE, H2AJ_MOUSE, H2AX_MOUSE, H2AY_MOUSE, H2AW_MOUSE, Q8CA90_MOUSE	Histone H2A	LAGNAARD	150	9.80E-03	394.2062	786.3978	2	-0.0005	8	0	GluC
H2A1F_MOUSE, H2A1H_MOUSE, H2A1_MOUSE, H2A1K_MOUSE, H2A2A_MOUSE, H2A2B_MOUSE, H2A2C_MOUSE, H2A3_MOUSE, H2AJ_MOUSE, H2AX_MOUSE, H2AY_MOUSE, H2AW_MOUSE, Q8CA90_MOUSE	Histone H2A	LAGNAARD	150	6.60E-03	394.2062	786.3979	2	-0.0005	8	0	GluC
H2A1F_MOUSE, H2A1H_MOUSE, H2A1_MOUSE, H2A1K_MOUSE, H2A2A_MOUSE, H2A2B_MOUSE, H2A2C_MOUSE, H2A3_MOUSE, H2AJ_MOUSE, H2AX_MOUSE, H2AY_MOUSE, H2AW_MOUSE, Q8CA90_MOUSE	Histone H2A	LAGNAARD	150	4.70E-03	394.2062	786.3978	2	-0.0005	8	0	GluC
H2A1F_MOUSE, H2A1H_MOUSE, H2A1_MOUSE, H2A1K_MOUSE, H2A2A_MOUSE, H2A2B_MOUSE, H2A2C_MOUSE, H2A3_MOUSE, H2AJ_MOUSE, H2AX_MOUSE, H2AY_MOUSE, H2AW_MOUSE, Q8CA90_MOUSE	Histone H2A	LAGNAARD	150	3.90E-04	394.2062	786.3979	2	-0.0005	8	0	GluC

H2A1F_MOUSE, H2A1H_MOUSE, H2A1_MOUSE, H2A1K_MOUSE, H2A2A_MOUSE, H2A2B_MOUSE, H2A2C_MOUSE, H2A3_MOUSE, H2AJ_MOUSE, H2AX_MOUSE, H2AY_MOUSE, H2AW_MOUSE, Q8CA90_MOUSE	Histone H2A	LAGNAARD	150	3.10E-04	394.2062	786.3979	2	-0.0005	8	0	GluC
H2A1F_MOUSE, H2A1H_MOUSE, H2A1_MOUSE, H2A1K_MOUSE, H2A2A_MOUSE, H2A2B_MOUSE, H2A2C_MOUSE, H2A3_MOUSE, H2AJ_MOUSE, H2AX_MOUSE, H2AY_MOUSE, H2AW_MOUSE, Q8CA90_MOUSE	Histone H2A	LAGNAARD	150	7.20E-05	394.2062	786.3979	2	-0.0005	8	0	GluC
H2A1F_MOUSE, H2A1H_MOUSE, H2A1_MOUSE, H2A1K_MOUSE, H2A2A_MOUSE, H2A2B_MOUSE, H2A2C_MOUSE, H2A3_MOUSE, H2AJ_MOUSE, H2AX_MOUSE, H2AY_MOUSE, H2AW_MOUSE, Q8CA90_MOUSE	Histone H2A	LAGNAARD	150	2.60E-03	394.2062	786.3978	2	-0.0006	8	0	GluC
H2A1F_MOUSE, H2A1H_MOUSE, H2A1_MOUSE, H2A1K_MOUSE, H2A2A_MOUSE, H2A2B_MOUSE, H2A2C_MOUSE, H2A3_MOUSE, H2AJ_MOUSE, H2AX_MOUSE, H2AY_MOUSE, H2AW_MOUSE, Q8CA90_MOUSE	Histone H2A	LAGNAARD	150	7.20E-04	394.2062	786.3978	2	-0.0006	8	0	GluC
H2A1F_MOUSE, H2A1H_MOUSE, H2A1_MOUSE, H2A1K_MOUSE, H2A2A_MOUSE, H2A2B_MOUSE, H2A2C_MOUSE, H2A3_MOUSE, H2AJ_MOUSE, H2AX_MOUSE, H2AY_MOUSE, H2AW_MOUSE, Q8CA90_MOUSE	Histone H2A	LAGNAARD	150	2.20E-03	394.2061	786.3975	2	-0.0008	8	0	GluC
H2A1F_MOUSE, H2A1H_MOUSE, H2A1_MOUSE, H2A1K_MOUSE, H2A2A_MOUSE, H2A2B_MOUSE, H2A2C_MOUSE, H2A3_MOUSE, H2AJ_MOUSE, H2AX_MOUSE, H2AY_MOUSE, H2AW_MOUSE, Q8CA90_MOUSE	Histone H2A	LAGNAARD	150	3.50E-04	394.206	786.3975	2	-0.0009	8	0	GluC
H2A1F_MOUSE, H2A1H_MOUSE, H2A1_MOUSE, H2A1K_MOUSE, H2A2A_MOUSE, H2A2B_MOUSE, H2A2C_MOUSE, H2A3_MOUSE, H2AJ_MOUSE, H2AX_MOUSE, H2AY_MOUSE, H2AW_MOUSE, Q8CA90_MOUSE	Histone H2A	LAGNAARD	150	2.90E-04	394.206	786.3975	2	-0.0009	8	0	GluC
H2A1F_MOUSE, H2A1H_MOUSE, H2A1_MOUSE, H2A1K_MOUSE, H2A2A_MOUSE, H2A2B_MOUSE, H2A2C_MOUSE, H2A3_MOUSE, H2AJ_MOUSE, H2AX_MOUSE, H2AY_MOUSE, H2AW_MOUSE, Q8CA90_MOUSE	Histone H2A	LAGNAARD	150	1.70E-03	394.2059	786.3973	2	-0.0010	8	0	GluC
H2A1F_MOUSE, H2A1H_MOUSE, H2A1_MOUSE, H2A1K_MOUSE, H2A2A_MOUSE, H2A2B_MOUSE, H2A2C_MOUSE, H2A3_MOUSE, H2AJ_MOUSE, H2AX_MOUSE, H2AY_MOUSE, H2AW_MOUSE, Q8CA90_MOUSE	Histone H2A	LAGNAARD	150	3.90E-03	394.2059	786.3972	2	-0.0011	8	0	GluC
H2A1F_MOUSE, H2A1H_MOUSE, H2A1_MOUSE, H2A1K_MOUSE, H2A2A_MOUSE, H2A2B_MOUSE, H2A2C_MOUSE, H2A3_MOUSE, H2AJ_MOUSE, H2AX_MOUSE, H2AY_MOUSE, H2AW_MOUSE, Q8CA90_MOUSE	Histone H2A	LAGNAARD	150	6.20E-03	394.2058	786.3971	2	-0.0013	8	0	GluC
H2A1F_MOUSE, H2A1H_MOUSE, H2A1_MOUSE, H2A1K_MOUSE, H2A2A_MOUSE, H2A2B_MOUSE, H2A2C_MOUSE, H2A3_MOUSE, H2AJ_MOUSE, H2AX_MOUSE, H2AY_MOUSE, H2AW_MOUSE, Q8CA90_MOUSE	Histone H2A	LAGNAARD	150	5.50E-05	394.2058	786.3971	2	-0.0013	8	0	GluC
H2A1F_MOUSE, H2A1H_MOUSE, H2A1_MOUSE, H2A1K_MOUSE, H2A2A_MOUSE, H2A2B_MOUSE, H2A2C_MOUSE, H2A3_MOUSE, H2AJ_MOUSE, H2AX_MOUSE, H2AY_MOUSE, H2AW_MOUSE, Q8CA90_MOUSE	Histone H2A	LAGNAARD	150	2.10E-03	394.2057	786.3969	2	-0.0015	8	0	GluC
H2A1F_MOUSE, H2A1H_MOUSE, H2A1_MOUSE, H2A1K_MOUSE, H2A2A_MOUSE, H2A2B_MOUSE, H2A2C_MOUSE, H2A3_MOUSE, H2AJ_MOUSE, H2AX_MOUSE, H2AY_MOUSE, H2AW_MOUSE, Q8CA90_MOUSE	Histone H2A	LAGNAARD	150	2.50E-05	394.2057	786.3969	2	-0.0015	8	0	GluC
H2A1F_MOUSE, H2A1H_MOUSE, H2A1_MOUSE, H2A1K_MOUSE, H2A2A_MOUSE, H2A2B_MOUSE, H2A2C_MOUSE, H2A3_MOUSE, H2AJ_MOUSE, H2AX_MOUSE, H2AY_MOUSE, H2AW_MOUSE, Q8CA90_MOUSE	Histone H2A	LAGNAARD	150	1.20E-04	394.2056	786.3967	2	-0.0017	8	0	GluC
H2A1F_MOUSE, H2A1H_MOUSE, H2A1_MOUSE, H2A1K_MOUSE, H2A2A_MOUSE, H2A2B_MOUSE, H2A2C_MOUSE, H2A3_MOUSE, H2AJ_MOUSE, H2AX_MOUSE, H2AY_MOUSE, H2AW_MOUSE, Q8CA90_MOUSE	Histone H2A	LAGNAARD	150	4.80E-05	394.2056	786.3967	2	-0.0017	8	0	GluC
H2A1F_MOUSE, H2A1H_MOUSE, H2A1_MOUSE, H2A1K_MOUSE, H2A2A_MOUSE, H2A2B_MOUSE, H2A2C_MOUSE, H2A3_MOUSE, H2AJ_MOUSE, H2AX_MOUSE, H2AY_MOUSE, H2AW_MOUSE, Q8CA90_MOUSE	Histone H2A	LAGNAARD	150	1.60E-04	394.2056	786.3965	2	-0.0018	8	0	GluC
H2A1F_MOUSE, H2A1H_MOUSE, H2A1_MOUSE, H2A1K_MOUSE, H2A2A_MOUSE, H2A2C_MOUSE, H2A3_MOUSE, H2AJ_MOUSE, H2AV_MOUSE, H2AZ_MOUSE	Histone H2A	HLQLAIR	23	8.00E-05	425.7687	849.5229	2	0.0045	7	0	Semi-tryptic
H2A1F_MOUSE, H2A1H_MOUSE, H2A1_MOUSE, H2A1K_MOUSE, H2A2A_MOUSE, H2A2C_MOUSE, H2A3_MOUSE, H2AJ_MOUSE, H2AV_MOUSE, H2AZ_MOUSE	Histone H2A	HLQLAIR	23	6.30E-05	425.7684	849.5223	2	0.0039	7	0	Semi-tryptic
H2A1F_MOUSE, H2A1H_MOUSE, H2A1_MOUSE, H2A1K_MOUSE, H2A2A_MOUSE, H2A2C_MOUSE, H2A3_MOUSE, H2AJ_MOUSE, H2AV_MOUSE, H2AZ_MOUSE	Histone H2A	HLQLAIR	23	2.60E-03	425.7683	849.5220	2	0.0035	7	0	Semi-tryptic

H2A1F_MOUSE, H2A1H_MOUSE, H2A1_MOUSE, H2A1K_MOUSE, H2A2A_MOUSE, H2A2C_MOUSE, H2A3_MOUSE, H2AJ_MOUSE, H2AV_MOUSE, H2AZ_MOUSE	Histone H2A	HLQLAIR	23	3.00E-03	425.7679	849.5212	2	0.0028	7	0	Semi-tryptic
H2A1F_MOUSE, H2A1H_MOUSE, H2A1_MOUSE, H2A1K_MOUSE, H2A2A_MOUSE, H2A2C_MOUSE, H2A3_MOUSE, H2AJ_MOUSE, H2AV_MOUSE, H2AZ_MOUSE	Histone H2A	HLQLAIR	23	3.20E-05	425.7676	849.5206	2	0.0021	7	0	Semi-tryptic
H2A1F_MOUSE, H2A1H_MOUSE, H2A1_MOUSE, H2A1K_MOUSE, H2A2A_MOUSE, H2A2C_MOUSE, H2A3_MOUSE, H2AJ_MOUSE, H2AV_MOUSE, H2AZ_MOUSE	Histone H2A	HLQLAIR	23	2.40E-05	425.7668	849.5191	2	0.0007	7	0	Semi-tryptic
H2A1F_MOUSE, H2A1H_MOUSE, H2A1_MOUSE, H2A1K_MOUSE, H2A2A_MOUSE, H2A2C_MOUSE, H2A3_MOUSE, H2AJ_MOUSE, H2AV_MOUSE, H2AZ_MOUSE	Histone H2A	HLQLAIR	23	1.70E-05	425.7668	849.5190	2	0.0006	7	0	Semi-tryptic
H2A1F_MOUSE, H2A1H_MOUSE, H2A1_MOUSE, H2A1K_MOUSE, H2A2A_MOUSE, H2A2C_MOUSE, H2A3_MOUSE, H2AJ_MOUSE, H2AV_MOUSE, H2AZ_MOUSE	Histone H2A	HLQLAIR	23	2.90E-03	425.7668	849.5190	2	0.0005	7	0	Semi-tryptic
H2A1F_MOUSE, H2A1H_MOUSE, H2A1_MOUSE, H2A1K_MOUSE, H2A2A_MOUSE, H2A2C_MOUSE, H2A3_MOUSE, H2AJ_MOUSE, H2AV_MOUSE, H2AZ_MOUSE	Histone H2A	HLQLAIR	23	8.70E-05	425.7668	849.5190	2	0.0005	7	0	Semi-tryptic
H2A1F_MOUSE, H2A1H_MOUSE, H2A1_MOUSE, H2A1K_MOUSE, H2A2A_MOUSE, H2A2C_MOUSE, H2A3_MOUSE, H2AJ_MOUSE, H2AV_MOUSE, H2AZ_MOUSE	Histone H2A	HLQLAIR	23	9.40E-04	425.7667	849.5188	2	0.0004	7	0	Semi-tryptic
H2A1F_MOUSE, H2A1H_MOUSE, H2A1_MOUSE, H2A1K_MOUSE, H2A2A_MOUSE, H2A2C_MOUSE, H2A3_MOUSE, H2AJ_MOUSE, H2AV_MOUSE, H2AZ_MOUSE	Histone H2A	HLQLAIR	23	6.40E-05	425.7666	849.5187	2	0.0003	7	0	Semi-tryptic
H2A1F_MOUSE, H2A1H_MOUSE, H2A1_MOUSE, H2A1K_MOUSE, H2A2A_MOUSE, H2A2C_MOUSE, H2A3_MOUSE, H2AJ_MOUSE, H2AV_MOUSE, H2AZ_MOUSE	Histone H2A	HLQLAIR	23	5.90E-05	425.7667	849.5188	2	0.0003	7	0	Semi-tryptic
H2A1F_MOUSE, H2A1H_MOUSE, H2A1_MOUSE, H2A1K_MOUSE, H2A2A_MOUSE, H2A2C_MOUSE, H2A3_MOUSE, H2AJ_MOUSE, H2AV_MOUSE, H2AZ_MOUSE	Histone H2A	HLQLAIR	23	3.80E-05	425.7666	849.5186	2	0.0002	7	0	Semi-tryptic
H2A1F_MOUSE, H2A1H_MOUSE, H2A1_MOUSE, H2A1K_MOUSE, H2A2A_MOUSE, H2A2C_MOUSE, H2A3_MOUSE, H2AJ_MOUSE, H2AV_MOUSE, H2AZ_MOUSE	Histone H2A	HLQLAIR	23	8.70E-05	425.7665	849.5184	2	0.0000	7	0	Semi-tryptic
H2A1F_MOUSE, H2A1H_MOUSE, H2A1_MOUSE, H2A1K_MOUSE, H2A2A_MOUSE, H2A2C_MOUSE, H2A3_MOUSE, H2AJ_MOUSE, H2AV_MOUSE, H2AZ_MOUSE	Histone H2A	HLQLAIR	23	6.20E-05	425.7665	849.5185	2	0.0000	7	0	Semi-tryptic
H2A1F_MOUSE, H2A1H_MOUSE, H2A1_MOUSE, H2A1K_MOUSE, H2A2A_MOUSE, H2A2C_MOUSE, H2A3_MOUSE, H2AJ_MOUSE, H2AV_MOUSE, H2AZ_MOUSE	Histone H2A	HLQLAIR	23	1.20E-04	425.7665	849.5184	2	-0.0001	7	0	Semi-tryptic
H2A1F_MOUSE, H2A1H_MOUSE, H2A1_MOUSE, H2A1K_MOUSE, H2A2A_MOUSE, H2A2C_MOUSE, H2A3_MOUSE, H2AJ_MOUSE, H2AV_MOUSE, H2AZ_MOUSE	Histone H2A	HLQLAIR	23	6.60E-03	425.7664	849.5183	2	-0.0002	7	0	Semi-tryptic
H2A1F_MOUSE, H2A1H_MOUSE, H2A1_MOUSE, H2A1K_MOUSE, H2A2A_MOUSE, H2A2C_MOUSE, H2A3_MOUSE, H2AJ_MOUSE, H2AV_MOUSE, H2AZ_MOUSE	Histone H2A	HLQLAIR	23	5.70E-05	425.7664	849.5183	2	-0.0002	7	0	Semi-tryptic
H2A1F_MOUSE, H2A1H_MOUSE, H2A1_MOUSE, H2A1K_MOUSE, H2A2A_MOUSE, H2A2C_MOUSE, H2A3_MOUSE, H2AJ_MOUSE, H2AV_MOUSE, H2AZ_MOUSE	Histone H2A	HLQLAIR	23	1.50E-05	425.7662	849.5178	2	-0.0006	7	0	Semi-tryptic
H2A1F_MOUSE, H2A1H_MOUSE, H2A1_MOUSE, H2A1K_MOUSE, H2A2A_MOUSE, H2A2C_MOUSE, H2A3_MOUSE, H2AJ_MOUSE, H2AV_MOUSE, H2AZ_MOUSE	Histone H2A	HLQLAIR	23	2.10E-05	425.7662	849.5178	2	-0.0007	7	0	Semi-tryptic
H2A1F_MOUSE, H2A1H_MOUSE, H2A1_MOUSE, H2A1K_MOUSE, H2A2A_MOUSE, H2A2C_MOUSE, H2A3_MOUSE, H2AJ_MOUSE, H2AV_MOUSE, H2AZ_MOUSE	Histone H2A	HLQLAIR	23	3.40E-05	425.766	849.5175	2	-0.0010	7	0	Semi-tryptic
H2A1F_MOUSE, H2A1H_MOUSE, H2A1_MOUSE, H2A1K_MOUSE, H2A2A_MOUSE, H2A2C_MOUSE, H2A3_MOUSE, H2AJ_MOUSE, H2AV_MOUSE, H2AZ_MOUSE	Histone H2A	HLQLAIR	23	4.10E-03	425.7659	849.5172	2	-0.0012	7	0	Semi-tryptic
H2A1F_MOUSE, H2A1H_MOUSE, H2A1_MOUSE, H2A1K_MOUSE, H2A2A_MOUSE, H2A2C_MOUSE, H2A3_MOUSE, H2AJ_MOUSE, H2AV_MOUSE, H2AZ_MOUSE	Histone H2A	HLQLAIR	23	1.50E-04	425.7658	849.5171	2	-0.0014	7	0	Semi-tryptic
H2A1F_MOUSE, H2A1H_MOUSE, H2A1_MOUSE, H2A1K_MOUSE, H2A2A_MOUSE, H2A2C_MOUSE, H2A3_MOUSE, H2AJ_MOUSE, H2AX_MOUSE	Histone H2A	LAGNAARDNKKTRIIPRHLQLAIRNDE	7	1.80E-05	771.6924	3082.7403	4	0.0179	27	2	GluC
H2A1F_MOUSE, H2A1H_MOUSE, H2A1_MOUSE, H2A1K_MOUSE, H2A2A_MOUSE, H2A2C_MOUSE, H2A3_MOUSE, H2AJ_MOUSE, H2AX_MOUSE	Histone H2A	LAGNAARDNKKTRIIPRHLQLAIRNDE	7	3.40E-05	617.5537	3082.7323	5	0.0100	27	2	GluC
H2A1F_MOUSE, H2A1H_MOUSE, H2A1_MOUSE, H2A1K_MOUSE, H2A2A_MOUSE, H2A2C_MOUSE, H2A3_MOUSE, H2AJ_MOUSE, H2AX_MOUSE	Histone H2A	LAGNAARDNKKTRIIPRHLQLAIRNDE	7	8.30E-03	617.5529	3082.7283	5	0.0060	27	2	GluC
H2A1F_MOUSE, H2A1H_MOUSE, H2A1_MOUSE, H2A1K_MOUSE, H2A2A_MOUSE, H2A2C_MOUSE, H2A3_MOUSE, H2AJ_MOUSE, H2AX_MOUSE	Histone H2A	LAGNAARDNKKTRIIPRHLQLAIRNDE	7	2.20E-05	771.6885	3082.7247	4	0.0024	27	2	GluC

H2A1F_MOUSE, H2A1H_MOUSE, H2A1_MOUSE, H2A1K_MOUSE, H2A2A_MOUSE, H2A2C_MOUSE, H2A3_MOUSE, H2AJ_MOUSE, H2AX_MOUSE	Histone H2A	LAGNAARDNKKTRIIPRHLQLAIRNDE	7	7.60E-07	771.6871	3082.7193	4	-0.0030	27	2	GluC
H2A1F_MOUSE, H2A1H_MOUSE, H2A1_MOUSE, H2A1K_MOUSE, H2A2A_MOUSE, H2A2C_MOUSE, H2A3_MOUSE, H2AJ_MOUSE, H2AX_MOUSE	Histone H2A	LAGNAARDNKKTRIIPRHLQLAIRNDE	7	1.90E-04	617.551	3082.7185	5	-0.0038	27	2	GluC
H2A1F_MOUSE, H2A1H_MOUSE, H2A1_MOUSE, H2A1K_MOUSE, H2A2A_MOUSE, H2A2C_MOUSE, H2A3_MOUSE, H2AJ_MOUSE, H2AX_MOUSE	Histone H2A	LAGNAARDNKKTRIIPRHLQLAIRNDE	7	1.70E-08	771.6859	3082.7146	4	-0.0077	27	2	GluC
H2A1F_MOUSE, H2A1H_MOUSE, H2A1_MOUSE, H2A1K_MOUSE, H2A2A_MOUSE, H2A2C_MOUSE, H2A3_MOUSE, H2AJ_MOUSE, H2AX_MOUSE	Histone H2A	NKKTRIIPRHLQLAIRNDEE	58	1.50E-03	611.8509	2443.3745	4	-0.0027	20	2	GluC
H2A1F_MOUSE, H2A1H_MOUSE, H2A1_MOUSE, H2A1K_MOUSE, H2A2A_MOUSE, H2A2C_MOUSE, H2A3_MOUSE, H2AJ_MOUSE, H2AX_MOUSE	Histone H2A	NKKTRIIPRHLQLAIRNDEE	58	5.20E-03	611.8509	2443.3743	4	-0.0028	20	2	GluC
H2A1F_MOUSE, H2A1H_MOUSE, H2A1_MOUSE, H2A1K_MOUSE, H2A2A_MOUSE, H2A2C_MOUSE, H2A3_MOUSE, H2AJ_MOUSE, H2AX_MOUSE	Histone H2A	NKKTRIIPRHLQLAIRNDEE	58	6.30E-04	611.8508	2443.3742	4	-0.0029	20	2	GluC
H2A1F_MOUSE, H2A1H_MOUSE, H2A1_MOUSE, H2A1K_MOUSE, H2A2A_MOUSE, H2A2C_MOUSE, H2A3_MOUSE, H2AJ_MOUSE, H2AX_MOUSE	Histone H2A	NKKTRIIPRHLQLAIRNDEE	58	3.30E-03	815.4653	2443.3740	3	-0.0032	20	2	GluC
H2A1F_MOUSE, H2A1H_MOUSE, H2A1_MOUSE, H2A1K_MOUSE, H2A2A_MOUSE, H2A2C_MOUSE, H2A3_MOUSE, H2AJ_MOUSE, H2AX_MOUSE	Histone H2A	NKKTRIIPRHLQLAIRNDEE	58	7.30E-04	815.4653	2443.3740	3	-0.0032	20	2	GluC
H2A1F_MOUSE, H2A1H_MOUSE, H2A1_MOUSE, H2A1K_MOUSE, H2A2A_MOUSE, H2A2C_MOUSE, H2A3_MOUSE, H2AJ_MOUSE, H2AX_MOUSE	Histone H2A	NKKTRIIPRHLQLAIRNDEE	58	3.00E-06	611.8507	2443.3735	4	-0.0036	20	2	GluC
H2A1F_MOUSE, H2A1H_MOUSE, H2A1_MOUSE, H2A1K_MOUSE, H2A2A_MOUSE, H2A2C_MOUSE, H2A3_MOUSE, H2AJ_MOUSE, H2AX_MOUSE	Histone H2A	NKKTRIIPRHLQLAIRNDEE	58	1.00E-04	611.8502	2443.3717	4	-0.0054	20	2	GluC
H2A1F_MOUSE, H2A1H_MOUSE, H2A1_MOUSE, H2A1K_MOUSE, H2A2A_MOUSE, H2A2C_MOUSE, H2A3_MOUSE, H2AJ_MOUSE, H2AX_MOUSE	Histone H2A	NKKTRIIPRHLQLAIRNDEE	58	5.30E-04	611.8502	2443.3716	4	-0.0055	20	2	GluC
H2A1F_MOUSE, H2A1H_MOUSE, H2A1_MOUSE, H2A1K_MOUSE, H2A2A_MOUSE, H2A2C_MOUSE, H2A3_MOUSE, H2AJ_MOUSE, H2AX_MOUSE	Histone H2A	NKKTRIIPRHLQLAIRNDEE	58	2.70E-04	611.8487	2443.3657	4	-0.0114	20	2	GluC
H2A1F_MOUSE, H2A1H_MOUSE, H2A1_MOUSE, H2A1K_MOUSE, H2A2A_MOUSE, H2A2C_MOUSE, H2A3_MOUSE, H2AJ_MOUSE, H2AX_MOUSE	Histone H2A	NKKTRIIPRHLQLAIRNDEE	58	4.00E-04	611.8562	2443.3959	4	0.0187	20	2	GluC
H2A1F_MOUSE, H2A1H_MOUSE, H2A1_MOUSE, H2A1K_MOUSE, H2A2A_MOUSE, H2A2C_MOUSE, H2A3_MOUSE, H2AJ_MOUSE, H2AX_MOUSE	Histone H2A	NKKTRIIPRHLQLAIRNDEE	58	2.70E-04	815.4718	2443.3935	3	0.0164	20	2	GluC
H2A1F_MOUSE, H2A1H_MOUSE, H2A1_MOUSE, H2A1K_MOUSE, H2A2A_MOUSE, H2A2C_MOUSE, H2A3_MOUSE, H2AJ_MOUSE, H2AX_MOUSE	Histone H2A	NKKTRIIPRHLQLAIRNDEE	58	1.40E-03	815.4718	2443.3935	3	0.0163	20	2	GluC
H2A1F_MOUSE, H2A1H_MOUSE, H2A1_MOUSE, H2A1K_MOUSE, H2A2A_MOUSE, H2A2C_MOUSE, H2A3_MOUSE, H2AJ_MOUSE, H2AX_MOUSE	Histone H2A	NKKTRIIPRHLQLAIRNDEE	58	1.10E-04	815.4713	2443.3922	3	0.0151	20	2	GluC
H2A1F_MOUSE, H2A1H_MOUSE, H2A1_MOUSE, H2A1K_MOUSE, H2A2A_MOUSE, H2A2C_MOUSE, H2A3_MOUSE, H2AJ_MOUSE, H2AX_MOUSE	Histone H2A	NKKTRIIPRHLQLAIRNDEE	58	9.40E-04	815.4713	2443.3920	3	0.0149	20	2	GluC
H2A1F_MOUSE, H2A1H_MOUSE, H2A1_MOUSE, H2A1K_MOUSE, H2A2A_MOUSE, H2A2C_MOUSE, H2A3_MOUSE, H2AJ_MOUSE, H2AX_MOUSE	Histone H2A	NKKTRIIPRHLQLAIRNDEE	58	3.30E-09	611.8553	2443.3919	4	0.0148	20	2	GluC
H2A1F_MOUSE, H2A1H_MOUSE, H2A1_MOUSE, H2A1K_MOUSE, H2A2A_MOUSE, H2A2C_MOUSE, H2A3_MOUSE, H2AJ_MOUSE, H2AX_MOUSE	Histone H2A	NKKTRIIPRHLQLAIRNDEE	58	1.20E-03	815.4712	2443.3917	3	0.0146	20	2	GluC
H2A1F_MOUSE, H2A1H_MOUSE, H2A1_MOUSE, H2A1K_MOUSE, H2A2A_MOUSE, H2A2C_MOUSE, H2A3_MOUSE, H2AJ_MOUSE, H2AX_MOUSE	Histone H2A	NKKTRIIPRHLQLAIRNDEE	58	7.90E-07	611.8551	2443.3914	4	0.0143	20	2	GluC
H2A1F_MOUSE, H2A1H_MOUSE, H2A1_MOUSE, H2A1K_MOUSE, H2A2A_MOUSE, H2A2C_MOUSE, H2A3_MOUSE, H2AJ_MOUSE, H2AX_MOUSE	Histone H2A	NKKTRIIPRHLQLAIRNDEE	58	3.20E-06	611.8551	2443.3913	4	0.0142	20	2	GluC
H2A1F_MOUSE, H2A1H_MOUSE, H2A1_MOUSE, H2A1K_MOUSE, H2A2A_MOUSE, H2A2C_MOUSE, H2A3_MOUSE, H2AJ_MOUSE, H2AX_MOUSE	Histone H2A	NKKTRIIPRHLQLAIRNDEE	58	5.40E-07	611.8548	2443.3899	4	0.0128	20	2	GluC
H2A1F_MOUSE, H2A1H_MOUSE, H2A1_MOUSE, H2A1K_MOUSE, H2A2A_MOUSE, H2A2C_MOUSE, H2A3_MOUSE, H2AJ_MOUSE, H2AX_MOUSE	Histone H2A	NKKTRIIPRHLQLAIRNDEE	58	1.50E-04	815.4705	2443.3898	3	0.0127	20	2	GluC
H2A1F_MOUSE, H2A1H_MOUSE, H2A1_MOUSE, H2A1K_MOUSE, H2A2A_MOUSE, H2A2C_MOUSE, H2A3_MOUSE, H2AJ_MOUSE, H2AX_MOUSE	Histone H2A	NKKTRIIPRHLQLAIRNDEE	58	5.40E-04	815.4703	2443.3892	3	0.0121	20	2	GluC

H2A1F_MOUSE, H2A1H_MOUSE, H2A1_MOUSE, H2A1K_MOUSE, H2A2A_MOUSE, H2A2C_MOUSE, H2A3_MOUSE, H2AJ_MOUSE, H2AX_MOUSE	Histone H2A	NKKTRIIPRHLQLAIRNDEE	58	3.10E-04	815.47	2443.3882	3	0.0111	20	2	GluC
H2A1F_MOUSE, H2A1H_MOUSE, H2A1_MOUSE, H2A1K_MOUSE, H2A2A_MOUSE, H2A2C_MOUSE, H2A3_MOUSE, H2AJ_MOUSE, H2AX_MOUSE	Histone H2A	NKKTRIIPRHLQLAIRNDEE	58	9.40E-09	611.8542	2443.3877	4	0.0106	20	2	GluC
H2A1F_MOUSE, H2A1H_MOUSE, H2A1_MOUSE, H2A1K_MOUSE, H2A2A_MOUSE, H2A2C_MOUSE, H2A3_MOUSE, H2AJ_MOUSE, H2AX_MOUSE	Histone H2A	NKKTRIIPRHLQLAIRNDEE	58	7.60E-06	611.8541	2443.3872	4	0.0101	20	2	GluC
H2A1F_MOUSE, H2A1H_MOUSE, H2A1_MOUSE, H2A1K_MOUSE, H2A2A_MOUSE, H2A2C_MOUSE, H2A3_MOUSE, H2AJ_MOUSE, H2AX_MOUSE	Histone H2A	NKKTRIIPRHLQLAIRNDEE	58	1.30E-03	815.4693	2443.3861	3	0.0090	20	2	GluC
H2A1F_MOUSE, H2A1H_MOUSE, H2A1_MOUSE, H2A1K_MOUSE, H2A2A_MOUSE, H2A2C_MOUSE, H2A3_MOUSE, H2AJ_MOUSE, H2AX_MOUSE	Histone H2A	NKKTRIIPRHLQLAIRNDEE	58	1.00E-02	815.4685	2443.3837	3	0.0066	20	2	GluC
H2A1F_MOUSE, H2A1H_MOUSE, H2A1_MOUSE, H2A1K_MOUSE, H2A2A_MOUSE, H2A2C_MOUSE, H2A3_MOUSE, H2AJ_MOUSE, H2AX_MOUSE	Histone H2A	NKKTRIIPRHLQLAIRNDEE	58	1.50E-04	611.8532	2443.3835	4	0.0064	20	2	GluC
H2A1F_MOUSE, H2A1H_MOUSE, H2A1_MOUSE, H2A1K_MOUSE, H2A2A_MOUSE, H2A2C_MOUSE, H2A3_MOUSE, H2AJ_MOUSE, H2AX_MOUSE	Histone H2A	NKKTRIIPRHLQLAIRNDEE	58	2.00E-04	611.8531	2443.3832	4	0.0061	20	2	GluC
H2A1F_MOUSE, H2A1H_MOUSE, H2A1_MOUSE, H2A1K_MOUSE, H2A2A_MOUSE, H2A2C_MOUSE, H2A3_MOUSE, H2AJ_MOUSE, H2AX_MOUSE	Histone H2A	NKKTRIIPRHLQLAIRNDEE	58	2.10E-04	611.8531	2443.3831	4	0.0060	20	2	GluC
H2A1F_MOUSE, H2A1H_MOUSE, H2A1_MOUSE, H2A1K_MOUSE, H2A2A_MOUSE, H2A2C_MOUSE, H2A3_MOUSE, H2AJ_MOUSE, H2AX_MOUSE	Histone H2A	NKKTRIIPRHLQLAIRNDEE	58	1.40E-04	611.853	2443.3830	4	0.0059	20	2	GluC
H2A1F_MOUSE, H2A1H_MOUSE, H2A1_MOUSE, H2A1K_MOUSE, H2A2A_MOUSE, H2A2C_MOUSE, H2A3_MOUSE, H2AJ_MOUSE, H2AX_MOUSE	Histone H2A	NKKTRIIPRHLQLAIRNDEE	58	7.50E-03	815.468	2443.3822	3	0.0051	20	2	GluC
H2A1F_MOUSE, H2A1H_MOUSE, H2A1_MOUSE, H2A1K_MOUSE, H2A2A_MOUSE, H2A2C_MOUSE, H2A3_MOUSE, H2AJ_MOUSE, H2AX_MOUSE	Histone H2A	NKKTRIIPRHLQLAIRNDEE	58	1.40E-04	611.8527	2443.3815	4	0.0044	20	2	GluC
H2A1F_MOUSE, H2A1H_MOUSE, H2A1_MOUSE, H2A1K_MOUSE, H2A2A_MOUSE, H2A2C_MOUSE, H2A3_MOUSE, H2AJ_MOUSE, H2AX_MOUSE	Histone H2A	NKKTRIIPRHLQLAIRNDEE	58	8.20E-05	611.8526	2443.3814	4	0.0043	20	2	GluC
H2A1F_MOUSE, H2A1H_MOUSE, H2A1_MOUSE, H2A1K_MOUSE, H2A2A_MOUSE, H2A2C_MOUSE, H2A3_MOUSE, H2AJ_MOUSE, H2AX_MOUSE	Histone H2A	NKKTRIIPRHLQLAIRNDEE	58	1.00E-02	611.8525	2443.3811	4	0.0040	20	2	GluC
H2A1F_MOUSE, H2A1H_MOUSE, H2A1_MOUSE, H2A1K_MOUSE, H2A2A_MOUSE, H2A2C_MOUSE, H2A3_MOUSE, H2AJ_MOUSE, H2AX_MOUSE	Histone H2A	NKKTRIIPRHLQLAIRNDEE	58	1.50E-04	611.8524	2443.3807	4	0.0036	20	2	GluC
H2A1F_MOUSE, H2A1H_MOUSE, H2A1_MOUSE, H2A1K_MOUSE, H2A2A_MOUSE, H2A2C_MOUSE, H2A3_MOUSE, H2AJ_MOUSE, H2AX_MOUSE	Histone H2A	NKKTRIIPRHLQLAIRNDEE	58	2.40E-04	611.8524	2443.3806	4	0.0035	20	2	GluC
H2A1F_MOUSE, H2A1H_MOUSE, H2A1_MOUSE, H2A1K_MOUSE, H2A2A_MOUSE, H2A2C_MOUSE, H2A3_MOUSE, H2AJ_MOUSE, H2AX_MOUSE	Histone H2A	NKKTRIIPRHLQLAIRNDEE	58	2.00E-03	611.8522	2443.3796	4	0.0025	20	2	GluC
H2A1F_MOUSE, H2A1H_MOUSE, H2A1_MOUSE, H2A1K_MOUSE, H2A2A_MOUSE, H2A2C_MOUSE, H2A3_MOUSE, H2AJ_MOUSE, H2AX_MOUSE	Histone H2A	NKKTRIIPRHLQLAIRNDEE	58	6.70E-03	611.8521	2443.3793	4	0.0022	20	2	GluC
H2A1F_MOUSE, H2A1H_MOUSE, H2A1_MOUSE, H2A1K_MOUSE, H2A2A_MOUSE, H2A2C_MOUSE, H2A3_MOUSE, H2AJ_MOUSE, H2AX_MOUSE	Histone H2A	NKKTRIIPRHLQLAIRNDEE	58	2.00E-04	611.8521	2443.3793	4	0.0022	20	2	GluC
H2A1F_MOUSE, H2A1H_MOUSE, H2A1_MOUSE, H2A1K_MOUSE, H2A2A_MOUSE, H2A2C_MOUSE, H2A3_MOUSE, H2AJ_MOUSE, H2AX_MOUSE	Histone H2A	NKKTRIIPRHLQLAIRNDEE	58	1.40E-03	611.852	2443.3791	4	0.0020	20	2	GluC
H2A1F_MOUSE, H2A1H_MOUSE, H2A1_MOUSE, H2A1K_MOUSE, H2A2A_MOUSE, H2A2C_MOUSE, H2A3_MOUSE, H2AJ_MOUSE, H2AX_MOUSE	Histone H2A	NKKTRIIPRHLQLAIRNDEE	58	2.00E-03	611.8519	2443.3787	4	0.0016	20	2	GluC
H2A1F_MOUSE, H2A1H_MOUSE, H2A1_MOUSE, H2A1K_MOUSE, H2A2A_MOUSE, H2A2C_MOUSE, H2A3_MOUSE, H2AJ_MOUSE, H2AX_MOUSE	Histone H2A	NKKTRIIPRHLQLAIRNDEE	58	6.90E-03	611.8519	2443.3786	4	0.0015	20	2	GluC
H2A1F_MOUSE, H2A1H_MOUSE, H2A1_MOUSE, H2A1K_MOUSE, H2A2A_MOUSE, H2A2C_MOUSE, H2A3_MOUSE, H2AJ_MOUSE, H2AX_MOUSE	Histone H2A	NKKTRIIPRHLQLAIRNDEE	58	2.10E-04	611.8519	2443.3783	4	0.0012	20	2	GluC
H2A1F_MOUSE, H2A1H_MOUSE, H2A1_MOUSE, H2A1K_MOUSE, H2A2A_MOUSE, H2A2C_MOUSE, H2A3_MOUSE, H2AJ_MOUSE, H2AX_MOUSE	Histone H2A	NKKTRIIPRHLQLAIRNDEE	58	3.30E-03	611.8518	2443.3780	4	0.0009	20	2	GluC
H2A1F_MOUSE, H2A1H_MOUSE, H2A1_MOUSE, H2A1K_MOUSE, H2A2A_MOUSE, H2A2C_MOUSE, H2A3_MOUSE, H2AJ_MOUSE, H2AX_MOUSE	Histone H2A	NKKTRIIPRHLQLAIRNDEE	58	5.10E-04	611.8518	2443.3780	4	0.0009	20	2	GluC

H2A1F_MOUSE, H2A1H_MOUSE, H2A1_MOUSE, H2A1K_MOUSE, H2A2A_MOUSE, H2A2C_MOUSE, H2A3_MOUSE, H2AJ_MOUSE, H2AX_MOUSE	Histone H2A	NKKTRIIPRHLQLAIRNDEE	58	3.20E-07	611.8518	2443.3781	4	0.0009	20	2	GluC
H2A1F_MOUSE, H2A1H_MOUSE, H2A1_MOUSE, H2A1K_MOUSE, H2A2A_MOUSE, H2A2C_MOUSE, H2A3_MOUSE, H2AJ_MOUSE, H2AX_MOUSE	Histone H2A	NKKTRIIPRHLQLAIRNDEE	58	1.00E-03	611.8518	2443.3779	4	0.0008	20	2	GluC
H2A1F_MOUSE, H2A1H_MOUSE, H2A1_MOUSE, H2A1K_MOUSE, H2A2A_MOUSE, H2A2C_MOUSE, H2A3_MOUSE, H2AJ_MOUSE, H2AX_MOUSE	Histone H2A	NKKTRIIPRHLQLAIRNDEE	58	2.60E-03	611.8517	2443.3777	4	0.0006	20	2	GluC
H2A1F_MOUSE, H2A1H_MOUSE, H2A1_MOUSE, H2A1K_MOUSE, H2A2A_MOUSE, H2A2C_MOUSE, H2A3_MOUSE, H2AJ_MOUSE, H2AX_MOUSE	Histone H2A	NKKTRIIPRHLQLAIRNDEE	58	1.90E-03	611.8517	2443.3777	4	0.0006	20	2	GluC
H2A1F_MOUSE, H2A1H_MOUSE, H2A1_MOUSE, H2A1K_MOUSE, H2A2A_MOUSE, H2A2C_MOUSE, H2A3_MOUSE, H2AJ_MOUSE, H2AX_MOUSE	Histone H2A	NKKTRIIPRHLQLAIRNDEE	58	3.60E-04	611.8517	2443.3777	4	0.0006	20	2	GluC
H2A1F_MOUSE, H2A1H_MOUSE, H2A1_MOUSE, H2A1K_MOUSE, H2A2A_MOUSE, H2A2C_MOUSE, H2A3_MOUSE, H2AJ_MOUSE, H2AX_MOUSE	Histone H2A	NKKTRIIPRHLQLAIRNDEE	58	1.30E-04	611.8517	2443.3777	4	0.0006	20	2	GluC
H2A1F_MOUSE, H2A1H_MOUSE, H2A1_MOUSE, H2A1K_MOUSE, H2A2A_MOUSE, H2A2C_MOUSE, H2A3_MOUSE, H2AJ_MOUSE, H2AX_MOUSE	Histone H2A	NKKTRIIPRHLQLAIRNDEE	58	3.60E-03	611.8516	2443.3774	4	0.0003	20	2	GluC
H2A1F_MOUSE, H2A1H_MOUSE, H2A1_MOUSE, H2A1K_MOUSE, H2A2A_MOUSE, H2A2C_MOUSE, H2A3_MOUSE, H2AJ_MOUSE, H2AX_MOUSE	Histone H2A	NKKTRIIPRHLQLAIRNDEE	58	3.20E-05	611.8516	2443.3772	4	0.0001	20	2	GluC
H2A1F_MOUSE, H2A1H_MOUSE, H2A1_MOUSE, H2A1K_MOUSE, H2A2A_MOUSE, H2A2C_MOUSE, H2A3_MOUSE, H2AJ_MOUSE, H2AX_MOUSE	Histone H2A	NKKTRIIPRHLQLAIRNDEE	58	7.60E-05	611.8516	2443.3771	4	0.0000	20	2	GluC
H2A1F_MOUSE, H2A1H_MOUSE, H2A1_MOUSE, H2A1K_MOUSE, H2A2A_MOUSE, H2A2C_MOUSE, H2A3_MOUSE, H2AJ_MOUSE, H2AX_MOUSE	Histone H2A	NKKTRIIPRHLQLAIRNDEE	58	1.30E-04	611.8515	2443.3769	4	-0.0002	20	2	GluC
H2A1F_MOUSE, H2A1H_MOUSE, H2A1_MOUSE, H2A1K_MOUSE, H2A2A_MOUSE, H2A2C_MOUSE, H2A3_MOUSE, H2AJ_MOUSE, H2AX_MOUSE	Histone H2A	NKKTRIIPRHLQLAIRNDEE	58	1.50E-04	489.6825	2443.3763	5	-0.0008	20	2	GluC
H2A1F_MOUSE, H2A1H_MOUSE, H2A1_MOUSE, H2A1K_MOUSE, H2A2A_MOUSE, H2A2C_MOUSE, H2A3_MOUSE, H2AJ_MOUSE, H2AX_MOUSE	Histone H2A	NKKTRIIPRHLQLAIRNDEE	58	6.10E-04	611.8513	2443.3759	4	-0.0012	20	2	GluC
H2A1F_MOUSE, H2A1H_MOUSE, H2A1_MOUSE, H2A1K_MOUSE, H2A2A_MOUSE, H2A2C_MOUSE, H2A3_MOUSE, H2AJ_MOUSE, H2AX_MOUSE	Histone H2A	NKKTRIIPRHLQLAIRNDEE	58	8.90E-06	611.8512	2443.3759	4	-0.0013	20	2	GluC
H2A1F_MOUSE, H2A1H_MOUSE, H2A1_MOUSE, H2A1K_MOUSE, H2A2A_MOUSE, H2A2C_MOUSE, H2A3_MOUSE, H2AJ_MOUSE, H2AX_MOUSE	Histone H2A	NKKTRIIPRHLQLAIRNDE	14	3.60E-05	579.5902	2314.3319	4	-0.0026	19	1	GluC
H2A1F_MOUSE, H2A1H_MOUSE, H2A1_MOUSE, H2A1K_MOUSE, H2A2A_MOUSE, H2A2C_MOUSE, H2A3_MOUSE, H2AJ_MOUSE, H2AX_MOUSE	Histone H2A	NKKTRIIPRHLQLAIRNDE	14	2.50E-03	772.4511	2314.3316	3	-0.0029	19	1	GluC
H2A1F_MOUSE, H2A1H_MOUSE, H2A1_MOUSE, H2A1K_MOUSE, H2A2A_MOUSE, H2A2C_MOUSE, H2A3_MOUSE, H2AJ_MOUSE, H2AX_MOUSE	Histone H2A	NKKTRIIPRHLQLAIRNDE	14	2.20E-06	579.5896	2314.3294	4	-0.0051	19	1	GluC
H2A1F_MOUSE, H2A1H_MOUSE, H2A1_MOUSE, H2A1K_MOUSE, H2A2A_MOUSE, H2A2C_MOUSE, H2A3_MOUSE, H2AJ_MOUSE, H2AX_MOUSE	Histone H2A	NKKTRIIPRHLQLAIRNDE	14	7.00E-03	463.873	2314.3285	5	-0.0060	19	1	GluC
H2A1F_MOUSE, H2A1H_MOUSE, H2A1_MOUSE, H2A1K_MOUSE, H2A2A_MOUSE, H2A2C_MOUSE, H2A3_MOUSE, H2AJ_MOUSE, H2AX_MOUSE	Histone H2A	NKKTRIIPRHLQLAIRNDE	14	2.60E-08	579.5948	2314.3501	4	0.0156	19	1	GluC
H2A1F_MOUSE, H2A1H_MOUSE, H2A1_MOUSE, H2A1K_MOUSE, H2A2A_MOUSE, H2A2C_MOUSE, H2A3_MOUSE, H2AJ_MOUSE, H2AX_MOUSE	Histone H2A	NKKTRIIPRHLQLAIRNDE	14	8.70E-04	772.4568	2314.3486	3	0.0141	19	1	GluC
H2A1F_MOUSE, H2A1H_MOUSE, H2A1_MOUSE, H2A1K_MOUSE, H2A2A_MOUSE, H2A2C_MOUSE, H2A3_MOUSE, H2AJ_MOUSE, H2AX_MOUSE	Histone H2A	NKKTRIIPRHLQLAIRNDE	14	1.00E-03	463.8768	2314.3474	5	0.0129	19	1	GluC
H2A1F_MOUSE, H2A1H_MOUSE, H2A1_MOUSE, H2A1K_MOUSE, H2A2A_MOUSE, H2A2C_MOUSE, H2A3_MOUSE, H2AJ_MOUSE, H2AX_MOUSE	Histone H2A	NKKTRIIPRHLQLAIRNDE	14	3.40E-05	772.4556	2314.3451	3	0.0106	19	1	GluC
H2A1F_MOUSE, H2A1H_MOUSE, H2A1_MOUSE, H2A1K_MOUSE, H2A2A_MOUSE, H2A2C_MOUSE, H2A3_MOUSE, H2AJ_MOUSE, H2AX_MOUSE	Histone H2A	NKKTRIIPRHLQLAIRNDE	14	4.70E-07	579.5934	2314.3447	4	0.0102	19	1	GluC
H2A1F_MOUSE, H2A1H_MOUSE, H2A1_MOUSE, H2A1K_MOUSE, H2A2A_MOUSE, H2A2C_MOUSE, H2A3_MOUSE, H2AJ_MOUSE, H2AX_MOUSE	Histone H2A	NKKTRIIPRHLQLAIRNDE	14	3.40E-07	579.5926	2314.3411	4	0.0066	19	1	GluC
H2A1F_MOUSE, H2A1H_MOUSE, H2A1_MOUSE, H2A1K_MOUSE, H2A2A_MOUSE, H2A2C_MOUSE, H2A3_MOUSE, H2AJ_MOUSE, H2AX_MOUSE	Histone H2A	NKKTRIIPRHLQLAIRNDE	14	1.00E-02	772.4542	2314.3407	3	0.0062	19	1	GluC

H2A1F_MOUSE, H2A1H_MOUSE, H2A1_MOUSE, H2A1K_MOUSE, H2A2A_MOUSE, H2A2C_MOUSE, H2A3_MOUSE, H2AJ_MOUSE, H2AX_MOUSE	Histone H2A	NKKTRIIPRHLQLAIRNDE	14	4.20E-06	772.4542	2314.3407	3	0.0062	19	1	GluC
H2A1F_MOUSE, H2A1H_MOUSE, H2A1_MOUSE, H2A1K_MOUSE, H2A2A_MOUSE, H2A2C_MOUSE, H2A3_MOUSE, H2AJ_MOUSE, H2AX_MOUSE	Histone H2A	NKKTRIIPRHLQLAIRNDE	14	4.00E-04	579.5912	2314.3356	4	0.0011	19	1	GluC
H2A1F_MOUSE, H2A1H_MOUSE, H2A1_MOUSE, H2A1K_MOUSE, H2A2A_MOUSE, H2A2C_MOUSE, H2A3_MOUSE, H2AJ_MOUSE, H2AX_MOUSE	Histone H2A	NKKTRIIPRHLQLAIRNDE	14	8.40E-04	579.5909	2314.3343	4	-0.0002	19	1	GluC
H2A1F_MOUSE, H2A1H_MOUSE, H2A1_MOUSE, H2A1K_MOUSE, H2A2A_MOUSE, H2A2C_MOUSE, H2A3_MOUSE, H2AJ_MOUSE, H2AX_MOUSE	Histone H2A	NKKTRIIPRHLQLAIRND	5	1.90E-04	729.4429	2185.3068	3	0.0149	18	0	GluC
H2A1F_MOUSE, H2A1H_MOUSE, H2A1_MOUSE, H2A1K_MOUSE, H2A2A_MOUSE, H2A2C_MOUSE, H2A3_MOUSE, H2AJ_MOUSE, H2AX_MOUSE	Histone H2A	NKKTRIIPRHLQLAIRND	5	2.60E-03	729.4384	2185.2935	3	0.0016	18	0	GluC
H2A1F_MOUSE, H2A1H_MOUSE, H2A1_MOUSE, H2A1K_MOUSE, H2A2A_MOUSE, H2A2C_MOUSE, H2A3_MOUSE, H2AJ_MOUSE, H2AX_MOUSE	Histone H2A	NKKTRIIPRHLQLAIRND	5	6.50E-03	547.3306	2185.2933	4	0.0013	18	0	GluC
H2A1F_MOUSE, H2A1H_MOUSE, H2A1_MOUSE, H2A1K_MOUSE, H2A2A_MOUSE, H2A2C_MOUSE, H2A3_MOUSE, H2AJ_MOUSE, H2AX_MOUSE	Histone H2A	NKKTRIIPRHLQLAIRND	5	2.00E-05	547.3301	2185.2914	4	-0.0006	18	0	GluC
H2A1F_MOUSE, H2A1H_MOUSE, H2A1_MOUSE, H2A1K_MOUSE, H2A2A_MOUSE, H2A2C_MOUSE, H2A3_MOUSE, H2AJ_MOUSE, H2AX_MOUSE	Histone H2A	DNKKTRIIPRHLQLAIRN	23	6.00E-05	729.4371	2185.2894	3	-0.0025	18	0	AspN
H2A1F_MOUSE, H2A1H_MOUSE, H2A1_MOUSE, H2A1K_MOUSE, H2A2A_MOUSE, H2A2C_MOUSE, H2A3_MOUSE, H2AJ_MOUSE, H2AX_MOUSE	Histone H2A	DNKKTRIIPRHLQLAIRN	23	3.30E-06	547.3296	2185.2895	4	-0.0025	18	0	AspN
H2A1F_MOUSE, H2A1H_MOUSE, H2A1_MOUSE, H2A1K_MOUSE, H2A2A_MOUSE, H2A2C_MOUSE, H2A3_MOUSE, H2AJ_MOUSE, H2AX_MOUSE	Histone H2A	DNKKTRIIPRHLQLAIRN	23	6.30E-06	547.3296	2185.2893	4	-0.0026	18	0	AspN
H2A1F_MOUSE, H2A1H_MOUSE, H2A1_MOUSE, H2A1K_MOUSE, H2A2A_MOUSE, H2A2C_MOUSE, H2A3_MOUSE, H2AJ_MOUSE, H2AX_MOUSE	Histone H2A	NKKTRIIPRHLQLAIRND	5	3.80E-05	729.4369	2185.2888	3	-0.0031	18	0	GluC
H2A1F_MOUSE, H2A1H_MOUSE, H2A1_MOUSE, H2A1K_MOUSE, H2A2A_MOUSE, H2A2C_MOUSE, H2A3_MOUSE, H2AJ_MOUSE, H2AX_MOUSE	Histone H2A	DNKKTRIIPRHLQLAIRN	23	3.40E-06	547.3293	2185.2880	4	-0.0039	18	0	AspN
H2A1F_MOUSE, H2A1H_MOUSE, H2A1_MOUSE, H2A1K_MOUSE, H2A2A_MOUSE, H2A2C_MOUSE, H2A3_MOUSE, H2AJ_MOUSE, H2AX_MOUSE	Histone H2A	DNKKTRIIPRHLQLAIRN	23	6.20E-06	547.3292	2185.2877	4	-0.0042	18	0	AspN
H2A1F_MOUSE, H2A1H_MOUSE, H2A1_MOUSE, H2A1K_MOUSE, H2A2A_MOUSE, H2A2C_MOUSE, H2A3_MOUSE, H2AJ_MOUSE, H2AX_MOUSE	Histone H2A	DNKKTRIIPRHLQLAIRN	23	7.90E-04	729.4427	2185.3064	3	0.0144	18	0	AspN
H2A1F_MOUSE, H2A1H_MOUSE, H2A1_MOUSE, H2A1K_MOUSE, H2A2A_MOUSE, H2A2C_MOUSE, H2A3_MOUSE, H2AJ_MOUSE, H2AX_MOUSE	Histone H2A	DNKKTRIIPRHLQLAIRN	23	2.00E-05	729.4427	2185.3063	3	0.0144	18	0	AspN
H2A1F_MOUSE, H2A1H_MOUSE, H2A1_MOUSE, H2A1K_MOUSE, H2A2A_MOUSE, H2A2C_MOUSE, H2A3_MOUSE, H2AJ_MOUSE, H2AX_MOUSE	Histone H2A	DNKKTRIIPRHLQLAIRN	23	2.10E-05	729.4426	2185.3061	3	0.0142	18	0	AspN
H2A1F_MOUSE, H2A1H_MOUSE, H2A1_MOUSE, H2A1K_MOUSE, H2A2A_MOUSE, H2A2C_MOUSE, H2A3_MOUSE, H2AJ_MOUSE, H2AX_MOUSE	Histone H2A	DNKKTRIIPRHLQLAIRN	23	6.30E-05	729.4419	2185.3038	3	0.0119	18	0	AspN
H2A1F_MOUSE, H2A1H_MOUSE, H2A1_MOUSE, H2A1K_MOUSE, H2A2A_MOUSE, H2A2C_MOUSE, H2A3_MOUSE, H2AJ_MOUSE, H2AX_MOUSE	Histone H2A	DNKKTRIIPRHLQLAIRN	23	1.70E-06	547.3332	2185.3037	4	0.0117	18	0	AspN
H2A1F_MOUSE, H2A1H_MOUSE, H2A1_MOUSE, H2A1K_MOUSE, H2A2A_MOUSE, H2A2C_MOUSE, H2A3_MOUSE, H2AJ_MOUSE, H2AX_MOUSE	Histone H2A	DNKKTRIIPRHLQLAIRN	23	9.60E-08	547.333	2185.3029	4	0.0110	18	0	AspN
H2A1F_MOUSE, H2A1H_MOUSE, H2A1_MOUSE, H2A1K_MOUSE, H2A2A_MOUSE, H2A2C_MOUSE, H2A3_MOUSE, H2AJ_MOUSE, H2AX_MOUSE	Histone H2A	DNKKTRIIPRHLQLAIRN	23	2.20E-04	547.3328	2185.3023	4	0.0103	18	0	AspN
H2A1F_MOUSE, H2A1H_MOUSE, H2A1_MOUSE, H2A1K_MOUSE, H2A2A_MOUSE, H2A2C_MOUSE, H2A3_MOUSE, H2AJ_MOUSE, H2AX_MOUSE	Histone H2A	DNKKTRIIPRHLQLAIRN	23	6.70E-04	729.4396	2185.2969	3	0.0050	18	0	AspN
H2A1F_MOUSE, H2A1H_MOUSE, H2A1_MOUSE, H2A1K_MOUSE, H2A2A_MOUSE, H2A2C_MOUSE, H2A3_MOUSE, H2AJ_MOUSE, H2AX_MOUSE	Histone H2A	DNKKTRIIPRHLQLAIRN	23	1.10E-05	729.4395	2185.2967	3	0.0048	18	0	AspN
H2A1F_MOUSE, H2A1H_MOUSE, H2A1_MOUSE, H2A1K_MOUSE, H2A2A_MOUSE, H2A2C_MOUSE, H2A3_MOUSE, H2AJ_MOUSE, H2AX_MOUSE	Histone H2A	DNKKTRIIPRHLQLAIRN	23	6.20E-03	547.331	2185.2950	4	0.0031	18	0	AspN
H2A1F_MOUSE, H2A1H_MOUSE, H2A1_MOUSE, H2A1K_MOUSE, H2A2A_MOUSE, H2A2C_MOUSE, H2A3_MOUSE, H2AJ_MOUSE, H2AX_MOUSE	Histone H2A	DNKKTRIIPRHLQLAIRN	23	1.50E-04	729.4387	2185.2944	3	0.0024	18	0	AspN

H2A1F_MOUSE, H2A1H_MOUSE, H2A1_MOUSE, H2A1K_MOUSE, H2A2A_MOUSE, H2A2C_MOUSE, H2A3_MOUSE, H2AJ_MOUSE, H2AX_MOUSE	Histone H2A	DNKKTRIIPRHLQLAIRN	23	6.70E-06	547.3307	2185.2935	4	0.0016	18	0	AspN
H2A1F_MOUSE, H2A1H_MOUSE, H2A1_MOUSE, H2A1K_MOUSE, H2A2A_MOUSE, H2A2C_MOUSE, H2A3_MOUSE, H2AJ_MOUSE, H2AX_MOUSE	Histone H2A	DNKKTRIIPRHLQLAIRN	23	1.40E-04	547.3306	2185.2935	4	0.0015	18	0	AspN
H2A1F_MOUSE, H2A1H_MOUSE, H2A1_MOUSE, H2A1K_MOUSE, H2A2A_MOUSE, H2A2C_MOUSE, H2A3_MOUSE, H2AJ_MOUSE, H2AX_MOUSE	Histone H2A	DNKKTRIIPRHLQLAIRN	23	3.60E-06	547.3306	2185.2933	4	0.0013	18	0	AspN
H2A1F_MOUSE, H2A1H_MOUSE, H2A1_MOUSE, H2A1K_MOUSE, H2A2A_MOUSE, H2A2C_MOUSE, H2A3_MOUSE, H2AJ_MOUSE, H2AX_MOUSE	Histone H2A	DNKKTRIIPRHLQLAIRN	23	1.50E-05	729.4381	2185.2926	3	0.0007	18	0	AspN
H2A1F_MOUSE, H2A1H_MOUSE, H2A1_MOUSE, H2A1K_MOUSE, H2A2A_MOUSE, H2A2C_MOUSE, H2A3_MOUSE, H2AJ_MOUSE, H2AX_MOUSE	Histone H2A	DNKKTRIIPRHLQLAIRN	23	3.50E-06	547.3304	2185.2926	4	0.0007	18	0	AspN
H2A1F_MOUSE, H2A1H_MOUSE, H2A1_MOUSE, H2A1K_MOUSE, H2A2A_MOUSE, H2A2C_MOUSE, H2A3_MOUSE, H2AJ_MOUSE, H2AX_MOUSE	Histone H2A	DNKKTRIIPRHLQLAIRN	23	7.90E-05	729.4378	2185.2916	3	-0.0003	18	0	AspN
H2A1F_MOUSE, H2A1H_MOUSE, H2A1_MOUSE, H2A1K_MOUSE, H2A2A_MOUSE, H2A2C_MOUSE, H2A3_MOUSE, H2AJ_MOUSE, H2AX_MOUSE	Histone H2A	DNKKTRIIPRHLQLAIRN	23	1.80E-05	729.4376	2185.2910	3	-0.0010	18	0	AspN
H2A1F_MOUSE, H2A1H_MOUSE, H2A1_MOUSE, H2A1K_MOUSE, H2A2A_MOUSE, H2A2C_MOUSE, H2A3_MOUSE, H2AJ_MOUSE, H2AX_MOUSE	Histone H2A	HLQLAIRNDEELNK	13	9.80E-10	846.9565	1691.8984	2	0.0029	14	1	Semi-tryptic
H2A1F_MOUSE, H2A1H_MOUSE, H2A1_MOUSE, H2A1K_MOUSE, H2A2A_MOUSE, H2A2C_MOUSE, H2A3_MOUSE, H2AJ_MOUSE, H2AX_MOUSE	Histone H2A	HLQLAIRNDEELNK	13	7.70E-04	564.9733	1691.8982	3	0.0028	14	1	Semi-tryptic
H2A1F_MOUSE, H2A1H_MOUSE, H2A1_MOUSE, H2A1K_MOUSE, H2A2A_MOUSE, H2A2C_MOUSE, H2A3_MOUSE, H2AJ_MOUSE, H2AX_MOUSE	Histone H2A	HLQLAIRNDEELNK	13	2.30E-06	564.9733	1691.8982	3	0.0028	14	1	Semi-tryptic
H2A1F_MOUSE, H2A1H_MOUSE, H2A1_MOUSE, H2A1K_MOUSE, H2A2A_MOUSE, H2A2C_MOUSE, H2A3_MOUSE, H2AJ_MOUSE, H2AX_MOUSE	Histone H2A	HLQLAIRNDEELNK	13	4.60E-09	846.9562	1691.8979	2	0.0025	14	1	Semi-tryptic
H2A1F_MOUSE, H2A1H_MOUSE, H2A1_MOUSE, H2A1K_MOUSE, H2A2A_MOUSE, H2A2C_MOUSE, H2A3_MOUSE, H2AJ_MOUSE, H2AX_MOUSE	Histone H2A	HLQLAIRNDEELNK	13	5.30E-03	564.973	1691.8971	3	0.0017	14	1	Semi-tryptic
H2A1F_MOUSE, H2A1H_MOUSE, H2A1_MOUSE, H2A1K_MOUSE, H2A2A_MOUSE, H2A2C_MOUSE, H2A3_MOUSE, H2AJ_MOUSE, H2AX_MOUSE	Histone H2A	HLQLAIRNDEELNK	13	3.20E-03	564.9729	1691.8970	3	0.0015	14	1	Semi-tryptic
H2A1F_MOUSE, H2A1H_MOUSE, H2A1_MOUSE, H2A1K_MOUSE, H2A2A_MOUSE, H2A2C_MOUSE, H2A3_MOUSE, H2AJ_MOUSE, H2AX_MOUSE	Histone H2A	HLQLAIRNDEELNK	13	1.80E-09	846.9556	1691.8966	2	0.0012	14	1	Semi-tryptic
H2A1F_MOUSE, H2A1H_MOUSE, H2A1_MOUSE, H2A1K_MOUSE, H2A2A_MOUSE, H2A2C_MOUSE, H2A3_MOUSE, H2AJ_MOUSE, H2AX_MOUSE	Histone H2A	HLQLAIRNDEELNK	13	7.10E-04	564.9727	1691.8964	3	0.0009	14	1	Semi-tryptic
H2A1F_MOUSE, H2A1H_MOUSE, H2A1_MOUSE, H2A1K_MOUSE, H2A2A_MOUSE, H2A2C_MOUSE, H2A3_MOUSE, H2AJ_MOUSE, H2AX_MOUSE	Histone H2A	HLQLAIRNDEELNK	13	6.20E-03	564.9726	1691.8961	3	0.0006	14	1	Semi-tryptic
H2A1F_MOUSE, H2A1H_MOUSE, H2A1_MOUSE, H2A1K_MOUSE, H2A2A_MOUSE, H2A2C_MOUSE, H2A3_MOUSE, H2AJ_MOUSE, H2AX_MOUSE	Histone H2A	HLQLAIRNDEELNK	13	3.50E-03	564.9726	1691.8959	3	0.0004	14	1	Semi-tryptic
H2A1F_MOUSE, H2A1H_MOUSE, H2A1_MOUSE, H2A1K_MOUSE, H2A2A_MOUSE, H2A2C_MOUSE, H2A3_MOUSE, H2AJ_MOUSE, H2AX_MOUSE	Histone H2A	HLQLAIRNDEELNK	13	7.10E-04	564.9723	1691.8950	3	-0.0005	14	1	Semi-tryptic
H2A1F_MOUSE, H2A1H_MOUSE, H2A1_MOUSE, H2A1K_MOUSE, H2A2A_MOUSE, H2A2C_MOUSE, H2A3_MOUSE, H2AJ_MOUSE, H2AX_MOUSE	Histone H2A	HLQLAIRNDEELNK	13	6.10E-04	564.9721	1691.8944	3	-0.0010	14	1	Semi-tryptic
H2A1F_MOUSE, H2A1H_MOUSE, H2A1_MOUSE, H2A1K_MOUSE, H2A2A_MOUSE, H2A2C_MOUSE, H2A3_MOUSE, H2AJ_MOUSE, H2AX_MOUSE	Histone H2A	HLQLAIRNDEELNK	13	5.70E-06	564.9721	1691.8944	3	-0.0010	14	1	Semi-tryptic
H2A1F_MOUSE, H2A1H_MOUSE, H2A1_MOUSE, H2A1K_MOUSE, H2A2A_MOUSE, H2A2C_MOUSE, H2A3_MOUSE, H2AJ_MOUSE, H2AX_MOUSE	Histone H2A	QLAIRNDEELNKL	6	4.20E-06	519.287	1554.8391	3	0.0026	13	2	Chymotrypsin
H2A1F_MOUSE, H2A1H_MOUSE, H2A1_MOUSE, H2A1K_MOUSE, H2A2A_MOUSE, H2A2C_MOUSE, H2A3_MOUSE, H2AJ_MOUSE, H2AX_MOUSE	Histone H2A	QLAIRNDEELNKL	6	3.90E-03	519.2867	1554.8384	3	0.0019	13	2	Chymotrypsin
H2A1F_MOUSE, H2A1H_MOUSE, H2A1_MOUSE, H2A1K_MOUSE, H2A2A_MOUSE, H2A2C_MOUSE, H2A3_MOUSE, H2AJ_MOUSE, H2AX_MOUSE	Histone H2A	QLAIRNDEELNKL	6	4.30E-03	519.2866	1554.8381	3	0.0015	13	2	Chymotrypsin
H2A1F_MOUSE, H2A1H_MOUSE, H2A1_MOUSE, H2A1K_MOUSE, H2A2A_MOUSE, H2A2C_MOUSE, H2A3_MOUSE, H2AJ_MOUSE, H2AX_MOUSE	Histone H2A	QLAIRNDEELNKL	6	6.10E-06	519.2864	1554.8373	3	0.0008	13	2	Chymotrypsin

H2A1F_MOUSE, H2A1H_MOUSE, H2A1_MOUSE, H2A1K_MOUSE, H2A2A_MOUSE, H2A2C_MOUSE, H2A3_MOUSE, H2AJ_MOUSE, H2AX_MOUSE	Histone H2A	QLAIRNDEELNKL	6	1.40E-03	778.4256	1554.8366	2	0.0001	13	2	Chymotrypsin
H2A1F_MOUSE, H2A1H_MOUSE, H2A1_MOUSE, H2A1K_MOUSE, H2A2A_MOUSE, H2A2C_MOUSE, H2A3_MOUSE, H2AJ_MOUSE, H2AX_MOUSE	Histone H2A	QLAIRNDEELNKL	6	5.90E-07	519.2858	1554.8355	3	-0.0010	13	2	Chymotrypsin
H2A1F_MOUSE, H2A1H_MOUSE, H2A1_MOUSE, H2A1K_MOUSE, H2A2A_MOUSE, H2A2C_MOUSE, H2A3_MOUSE, H2AJ_MOUSE, H2AX_MOUSE	Histone H2A	AIRNDEELNKLL	13	8.70E-04	476.5988	1426.7746	3	-0.0034	12	2	Chymotrypsin
H2A1F_MOUSE, H2A1H_MOUSE, H2A1_MOUSE, H2A1K_MOUSE, H2A2A_MOUSE, H2A2C_MOUSE, H2A3_MOUSE, H2AJ_MOUSE, H2AX_MOUSE	Histone H2A	AIRNDEELNKLL	13	5.70E-04	476.5988	1426.7746	3	-0.0034	12	2	Chymotrypsin
H2A1F_MOUSE, H2A1H_MOUSE, H2A1_MOUSE, H2A1K_MOUSE, H2A2A_MOUSE, H2A2C_MOUSE, H2A3_MOUSE, H2AJ_MOUSE, H2AX_MOUSE	Histone H2A	AIRNDEELNKLL	13	5.00E-04	476.6017	1426.7832	3	0.0053	12	2	Chymotrypsin
H2A1F_MOUSE, H2A1H_MOUSE, H2A1_MOUSE, H2A1K_MOUSE, H2A2A_MOUSE, H2A2C_MOUSE, H2A3_MOUSE, H2AJ_MOUSE, H2AX_MOUSE	Histone H2A	AIRNDEELNKLL	13	4.80E-04	714.3986	1426.7827	2	0.0047	12	2	Chymotrypsin
H2A1F_MOUSE, H2A1H_MOUSE, H2A1_MOUSE, H2A1K_MOUSE, H2A2A_MOUSE, H2A2C_MOUSE, H2A3_MOUSE, H2AJ_MOUSE, H2AX_MOUSE	Histone H2A	AIRNDEELNKLL	13	1.00E-06	476.6006	1426.7800	3	0.0020	12	2	Chymotrypsin
H2A1F_MOUSE, H2A1H_MOUSE, H2A1_MOUSE, H2A1K_MOUSE, H2A2A_MOUSE, H2A2C_MOUSE, H2A3_MOUSE, H2AJ_MOUSE, H2AX_MOUSE	Histone H2A	AIRNDEELNKLL	13	7.60E-03	714.3964	1426.7782	2	0.0003	12	2	Chymotrypsin
H2A1F_MOUSE, H2A1H_MOUSE, H2A1_MOUSE, H2A1K_MOUSE, H2A2A_MOUSE, H2A2C_MOUSE, H2A3_MOUSE, H2AJ_MOUSE, H2AX_MOUSE	Histone H2A	AIRNDEELNKLL	13	4.10E-03	714.3964	1426.7783	2	0.0003	12	2	Chymotrypsin
H2A1F_MOUSE, H2A1H_MOUSE, H2A1_MOUSE, H2A1K_MOUSE, H2A2A_MOUSE, H2A2C_MOUSE, H2A3_MOUSE, H2AJ_MOUSE, H2AX_MOUSE	Histone H2A	AIRNDEELNKLL	13	3.50E-04	714.3964	1426.7782	2	0.0003	12	2	Chymotrypsin
H2A1F_MOUSE, H2A1H_MOUSE, H2A1_MOUSE, H2A1K_MOUSE, H2A2A_MOUSE, H2A2C_MOUSE, H2A3_MOUSE, H2AJ_MOUSE, H2AX_MOUSE	Histone H2A	AIRNDEELNKLL	13	2.90E-06	476.6	1426.7782	3	0.0002	12	2	Chymotrypsin
H2A1F_MOUSE, H2A1H_MOUSE, H2A1_MOUSE, H2A1K_MOUSE, H2A2A_MOUSE, H2A2C_MOUSE, H2A3_MOUSE, H2AJ_MOUSE, H2AX_MOUSE	Histone H2A	AIRNDEELNKLL	13	1.10E-03	476.5999	1426.7780	3	0.0001	12	2	Chymotrypsin
H2A1F_MOUSE, H2A1H_MOUSE, H2A1_MOUSE, H2A1K_MOUSE, H2A2A_MOUSE, H2A2C_MOUSE, H2A3_MOUSE, H2AJ_MOUSE, H2AX_MOUSE	Histone H2A	AIRNDEELNKLL	13	6.20E-05	714.3963	1426.7780	2	0.0001	12	2	Chymotrypsin
H2A1F_MOUSE, H2A1H_MOUSE, H2A1_MOUSE, H2A1K_MOUSE, H2A2A_MOUSE, H2A2C_MOUSE, H2A3_MOUSE, H2AJ_MOUSE, H2AX_MOUSE	Histone H2A	AIRNDEELNKLL	13	3.70E-03	476.5998	1426.7775	3	-0.0004	12	2	Chymotrypsin
H2A1F_MOUSE, H2A1H_MOUSE, H2A1_MOUSE, H2A1K_MOUSE, H2A2A_MOUSE, H2A2C_MOUSE, H2A3_MOUSE, H2AJ_MOUSE, H2AX_MOUSE	Histone H2A	AIRNDEELNKLL	13	4.90E-06	476.5998	1426.7775	3	-0.0005	12	2	Chymotrypsin
H2A1F_MOUSE, H2A1H_MOUSE, H2A1_MOUSE, H2A1K_MOUSE, H2A2A_MOUSE, H2A2C_MOUSE, H2A3_MOUSE, H2AJ_MOUSE, H2AX_MOUSE	Histone H2A	AIRNDEELNKL	15	1.00E-03	438.9068	1313.6985	3	0.0047	11	1	Chymotrypsin
H2A1F_MOUSE, H2A1H_MOUSE, H2A1_MOUSE, H2A1K_MOUSE, H2A2A_MOUSE, H2A2C_MOUSE, H2A3_MOUSE, H2AJ_MOUSE, H2AX_MOUSE	Histone H2A	AIRNDEELNKL	15	8.80E-03	657.8555	1313.6963	2	0.0025	11	1	Chymotrypsin
H2A1F_MOUSE, H2A1H_MOUSE, H2A1_MOUSE, H2A1K_MOUSE, H2A2A_MOUSE, H2A2C_MOUSE, H2A3_MOUSE, H2AJ_MOUSE, H2AX_MOUSE	Histone H2A	AIRNDEELNKL	15	6.70E-06	438.9058	1313.6957	3	0.0018	11	1	Chymotrypsin
H2A1F_MOUSE, H2A1H_MOUSE, H2A1_MOUSE, H2A1K_MOUSE, H2A2A_MOUSE, H2A2C_MOUSE, H2A3_MOUSE, H2AJ_MOUSE, H2AX_MOUSE	Histone H2A	AIRNDEELNKL	15	5.00E-04	438.9058	1313.6955	3	0.0017	11	1	Chymotrypsin
H2A1F_MOUSE, H2A1H_MOUSE, H2A1_MOUSE, H2A1K_MOUSE, H2A2A_MOUSE, H2A2C_MOUSE, H2A3_MOUSE, H2AJ_MOUSE, H2AX_MOUSE	Histone H2A	AIRNDEELNKL	15	3.70E-04	657.855	1313.6955	2	0.0016	11	1	Chymotrypsin
H2A1F_MOUSE, H2A1H_MOUSE, H2A1_MOUSE, H2A1K_MOUSE, H2A2A_MOUSE, H2A2C_MOUSE, H2A3_MOUSE, H2AJ_MOUSE, H2AX_MOUSE	Histone H2A	AIRNDEELNKL	15	2.90E-03	438.9057	1313.6954	3	0.0015	11	1	Chymotrypsin
H2A1F_MOUSE, H2A1H_MOUSE, H2A1_MOUSE, H2A1K_MOUSE, H2A2A_MOUSE, H2A2C_MOUSE, H2A3_MOUSE, H2AJ_MOUSE, H2AX_MOUSE	Histone H2A	AIRNDEELNKL	15	6.70E-05	438.9057	1313.6954	3	0.0015	11	1	Chymotrypsin
H2A1F_MOUSE, H2A1H_MOUSE, H2A1_MOUSE, H2A1K_MOUSE, H2A2A_MOUSE, H2A2C_MOUSE, H2A3_MOUSE, H2AJ_MOUSE, H2AX_MOUSE	Histone H2A	AIRNDEELNKL	15	1.10E-03	657.8549	1313.6952	2	0.0014	11	1	GluC
H2A1F_MOUSE, H2A1H_MOUSE, H2A1_MOUSE, H2A1K_MOUSE, H2A2A_MOUSE, H2A2C_MOUSE, H2A3_MOUSE, H2AJ_MOUSE, H2AX_MOUSE	Histone H2A	AIRNDEELNKL	15	1.40E-04	657.8547	1313.6948	2	0.0009	11	1	Chymotrypsin

H2A1F_MOUSE, H2A1H_MOUSE, H2A1_MOUSE, H2A1K_MOUSE, H2A2A_MOUSE, H2A2C_MOUSE, H2A3_MOUSE, H2AJ_MOUSE, H2AX_MOUSE	Histone H2A	AIRNDEELNKL	15	6.00E-06	438.9055	1313.6946	3	0.0007	11	1	Chymotrypsin
H2A1F_MOUSE, H2A1H_MOUSE, H2A1_MOUSE, H2A1K_MOUSE, H2A2A_MOUSE, H2A2C_MOUSE, H2A3_MOUSE, H2AJ_MOUSE, H2AX_MOUSE	Histone H2A	AIRNDEELNKL	15	7.30E-03	438.9053	1313.6942	3	0.0003	11	1	Chymotrypsin
H2A1F_MOUSE, H2A1H_MOUSE, H2A1_MOUSE, H2A1K_MOUSE, H2A2A_MOUSE, H2A2C_MOUSE, H2A3_MOUSE, H2AJ_MOUSE, H2AX_MOUSE	Histone H2A	AIRNDEELNKL	15	6.10E-03	438.9053	1313.6942	3	0.0003	11	1	GluC
H2A1F_MOUSE, H2A1H_MOUSE, H2A1_MOUSE, H2A1K_MOUSE, H2A2A_MOUSE, H2A2C_MOUSE, H2A3_MOUSE, H2AJ_MOUSE, H2AX_MOUSE	Histone H2A	AIRNDEELNKL	15	2.00E-04	438.9053	1313.6941	3	0.0003	11	1	Chymotrypsin
H2A1F_MOUSE, H2A1H_MOUSE, H2A1_MOUSE, H2A1K_MOUSE, H2A2A_MOUSE, H2A2C_MOUSE, H2A3_MOUSE, H2AJ_MOUSE, H2AX_MOUSE	Histone H2A	AIRNDEELNKL	15	1.80E-04	438.9053	1313.6941	3	0.0003	11	1	Chymotrypsin
H2A1F_MOUSE, H2A1H_MOUSE, H2A1_MOUSE, H2A1K_MOUSE, H2A2A_MOUSE, H2A2C_MOUSE, H2A3_MOUSE, H2AJ_MOUSE, H2AX_MOUSE	Histone H2A	AIRNDEELNKL	15	1.80E-04	438.905	1313.6933	3	-0.0006	11	1	Chymotrypsin
H2A1F_MOUSE, H2A1H_MOUSE, H2A1_MOUSE, H2A1K_MOUSE, H2A3_MOUSE	Histone H2A	SERVGAGAPVYL	5	9.30E-05	609.8286	1217.6427	2	0.0023	12	1	Chymotrypsin
H2A1F_MOUSE, H2A1H_MOUSE, H2A1_MOUSE, H2A1K_MOUSE, H2A3_MOUSE	Histone H2A	SERVGAGAPVYL	5	6.40E-05	609.828	1217.6414	2	0.0010	12	1	Chymotrypsin
H2A1F_MOUSE, H2A1H_MOUSE, H2A1_MOUSE, H2A1K_MOUSE, H2A3_MOUSE	Histone H2A	SERVGAGAPVYL	5	4.60E-05	609.828	1217.6414	2	0.0010	12	1	Chymotrypsin
H2A1F_MOUSE, H2A1H_MOUSE, H2A1_MOUSE, H2A1K_MOUSE, H2A3_MOUSE	Histone H2A	SERVGAGAPVYL	5	7.80E-03	609.8269	1217.6392	2	-0.0012	12	1	Chymotrypsin
H2A1F_MOUSE, H2A1H_MOUSE, H2A1_MOUSE, H2A1K_MOUSE, H2A3_MOUSE	Histone H2A	SERVGAGAPVYL	5	1.40E-03	609.8269	1217.6392	2	-0.0012	12	1	Chymotrypsin
H2A1F_MOUSE, H2A1H_MOUSE, H2A1_MOUSE, H2A1K_MOUSE, H2A3_MOUSE	Histone H2A	SERVGAGAPVY	3	4.50E-06	553.2859	1104.5572	2	0.0008	11	0	Chymotrypsin
H2A1F_MOUSE, H2A1H_MOUSE, H2A1_MOUSE, H2A1K_MOUSE, H2A3_MOUSE	Histone H2A	SERVGAGAPVY	3	1.00E-05	553.2856	1104.5566	2	0.0003	11	0	Chymotrypsin
H2A1F_MOUSE, H2A1H_MOUSE, H2A1_MOUSE, H2A1K_MOUSE, H2A3_MOUSE	Histone H2A	SERVGAGAPVY	3	1.50E-04	553.284	1104.5535	2	-0.0028	11	0	Chymotrypsin
H2A1F_MOUSE, H2A1H_MOUSE, H2A1_MOUSE, H2A1K_MOUSE, H2A3_MOUSE	Histone H2A	LLRKGNYSER	8	4.80E-04	618.3472	1234.6799	2	0.0017	10	2	Semi-tryptic
H2A1F_MOUSE, H2A1H_MOUSE, H2A1_MOUSE, H2A1K_MOUSE, H2A3_MOUSE	Histone H2A	LLRKGNYSER	8	2.10E-03	412.567	1234.6793	3	0.0011	10	2	Semi-tryptic
H2A1F_MOUSE, H2A1H_MOUSE, H2A1_MOUSE, H2A1K_MOUSE, H2A3_MOUSE	Histone H2A	LLRKGNYSER	8	3.10E-03	412.5669	1234.6788	3	0.0007	10	2	Semi-tryptic
H2A1F_MOUSE, H2A1H_MOUSE, H2A1_MOUSE, H2A1K_MOUSE, H2A3_MOUSE	Histone H2A	LLRKGNYSER	8	3.10E-03	412.5668	1234.6787	3	0.0005	10	2	Semi-tryptic
H2A1F_MOUSE, H2A1H_MOUSE, H2A1_MOUSE, H2A1K_MOUSE, H2A3_MOUSE	Histone H2A	LLRKGNYSER	8	2.00E-03	412.5668	1234.6787	3	0.0005	10	2	Semi-tryptic
H2A1F_MOUSE, H2A1H_MOUSE, H2A1_MOUSE, H2A1K_MOUSE, H2A3_MOUSE	Histone H2A	LLRKGNYSER	8	1.10E-04	618.3465	1234.6785	2	0.0004	10	2	Semi-tryptic
H2A1F_MOUSE, H2A1H_MOUSE, H2A1_MOUSE, H2A1K_MOUSE, H2A3_MOUSE	Histone H2A	LLRKGNYSER	8	8.00E-04	412.5668	1234.6785	3	0.0003	10	2	Semi-tryptic
H2A1F_MOUSE, H2A1H_MOUSE, H2A1_MOUSE, H2A1K_MOUSE, H2A3_MOUSE	Histone H2A	LLRKGNYSER	8	2.10E-03	412.5662	1234.6767	3	-0.0014	10	2	Semi-tryptic
H2A1F_MOUSE, H2A1H_MOUSE, H2A1_MOUSE, H2A1K_MOUSE, H2A3_MOUSE	Histone H2A	GNYSER	2	2.20E-03	363.1647	724.3148	2	0.0008	6	0	Semi-tryptic
H2A1F_MOUSE, H2A1H_MOUSE, H2A1_MOUSE, H2A1K_MOUSE, H2A3_MOUSE	Histone H2A	GNYSER	2	3.90E-04	363.1641	724.3136	2	-0.0004	6	0	Semi-tryptic
H2A1F_MOUSE, H2A1H_MOUSE, H2A1_MOUSE, H2A1K_MOUSE, H2A3_MOUSE, H2AJ_MOUSE	Histone H2A	HLQLAIRNDEELNKLLGRVTIAQGGVLPNIQAVLLPK	2	1.90E-05	1011.8439	4043.3465	4	0.0094	37	3	Semi-tryptic
H2A1F_MOUSE, H2A1H_MOUSE, H2A1_MOUSE, H2A1K_MOUSE, H2A3_MOUSE, H2AJ_MOUSE	Histone H2A	HLQLAIRNDEELNKLLGRVTIAQGGVLPNIQAVLLPK	2	4.60E-03	1011.8416	4043.3373	4	0.0002	37	3	Semi-tryptic
H2A1F_MOUSE, H2A1H_MOUSE, H2A1_MOUSE, H2A1K_MOUSE, H2A3_MOUSE, H2AJ_MOUSE	Histone H2A	ELNKLLGRVTIAQGGVLPNIQAVLLPKKTE	6	1.30E-05	643.3886	3211.9066	5	0.0046	30	1	GluC
H2A1F_MOUSE, H2A1H_MOUSE, H2A1_MOUSE, H2A1K_MOUSE, H2A3_MOUSE, H2AJ_MOUSE	Histone H2A	ELNKLLGRVTIAQGGVLPNIQAVLLPKKTE	6	2.60E-08	803.9839	3211.9064	4	0.0044	30	1	GluC
H2A1F_MOUSE, H2A1H_MOUSE, H2A1_MOUSE, H2A1K_MOUSE, H2A3_MOUSE, H2AJ_MOUSE	Histone H2A	ELNKLLGRVTIAQGGVLPNIQAVLLPKKTE	6	3.20E-06	1071.6423	3211.9051	3	0.0031	30	1	GluC
H2A1F_MOUSE, H2A1H_MOUSE, H2A1_MOUSE, H2A1K_MOUSE, H2A3_MOUSE, H2AJ_MOUSE	Histone H2A	ELNKLLGRVTIAQGGVLPNIQAVLLPKKTE	6	5.60E-03	803.9831	3211.9034	4	0.0014	30	1	GluC
H2A1F_MOUSE, H2A1H_MOUSE, H2A1_MOUSE, H2A1K_MOUSE, H2A3_MOUSE, H2AJ_MOUSE	Histone H2A	ELNKLLGRVTIAQGGVLPNIQAVLLPKKTE	6	1.10E-08	803.9831	3211.9034	4	0.0014	30	1	GluC
H2A1F_MOUSE, H2A1H_MOUSE, H2A1_MOUSE, H2A1K_MOUSE, H2A3_MOUSE, H2AJ_MOUSE	Histone H2A	ELNKLLGRVTIAQGGVLPNIQAVLLPKKTE	6	4.80E-03	643.3877	3211.9021	5	0.0001	30	1	GluC
H2A1F_MOUSE, H2A1H_MOUSE, H2A1_MOUSE, H2A1K_MOUSE, H2A3_MOUSE, H2AJ_MOUSE	Histone H2A	LNKLLGRVTIAQGGVLPNIQAVLLPKKTE	301	1.10E-06	771.7216	3082.8573	4	-0.0021	29	0	GluC
H2A1F_MOUSE, H2A1H_MOUSE, H2A1_MOUSE, H2A1K_MOUSE, H2A3_MOUSE, H2AJ_MOUSE	Histone H2A	LNKLLGRVTIAQGGVLPNIQAVLLPKKTE	301	5.20E-03	617.5787	3082.8572	5	-0.0022	29	0	GluC

H2A1F_MOUSE, H2A1H_MOUSE, H2A1_MOUSE, H2A1K_MOUSE, H2A3_MOUSE, H2AJ_MOUSE	Histone H2A	LNKLLGRVTIAQGGVLPNIQAVLLPKKTE	301	1.40E-03	617.5787	3082.8572	5	-0.0022	29	0	GluC
H2A1F_MOUSE, H2A1H_MOUSE, H2A1_MOUSE, H2A1K_MOUSE, H2A3_MOUSE, H2AJ_MOUSE	Histone H2A	LNKLLGRVTIAQGGVLPNIQAVLLPKKTE	301	7.70E-06	771.7215	3082.8570	4	-0.0024	29	0	GluC
H2A1F_MOUSE, H2A1H_MOUSE, H2A1_MOUSE, H2A1K_MOUSE, H2A3_MOUSE, H2AJ_MOUSE	Histone H2A	LNKLLGRVTIAQGGVLPNIQAVLLPKKTE	301	2.10E-04	771.7215	3082.8567	4	-0.0027	29	0	GluC
H2A1F_MOUSE, H2A1H_MOUSE, H2A1_MOUSE, H2A1K_MOUSE, H2A3_MOUSE, H2AJ_MOUSE	Histone H2A	LNKLLGRVTIAQGGVLPNIQAVLLPKKTE	301	1.30E-04	771.7214	3082.8565	4	-0.0029	29	0	GluC
H2A1F_MOUSE, H2A1H_MOUSE, H2A1_MOUSE, H2A1K_MOUSE, H2A3_MOUSE, H2AJ_MOUSE	Histone H2A	LNKLLGRVTIAQGGVLPNIQAVLLPKKTE	301	7.70E-05	771.7214	3082.8565	4	-0.0029	29	0	GluC
H2A1F_MOUSE, H2A1H_MOUSE, H2A1_MOUSE, H2A1K_MOUSE, H2A3_MOUSE, H2AJ_MOUSE	Histone H2A	LNKLLGRVTIAQGGVLPNIQAVLLPKKTE	301	1.80E-04	771.7212	3082.8557	4	-0.0037	29	0	GluC
H2A1F_MOUSE, H2A1H_MOUSE, H2A1_MOUSE, H2A1K_MOUSE, H2A3_MOUSE, H2AJ_MOUSE	Histone H2A	LNKLLGRVTIAQGGVLPNIQAVLLPKKTE	301	8.20E-03	771.7211	3082.8555	4	-0.0039	29	0	GluC
H2A1F_MOUSE, H2A1H_MOUSE, H2A1_MOUSE, H2A1K_MOUSE, H2A3_MOUSE, H2AJ_MOUSE	Histone H2A	LNKLLGRVTIAQGGVLPNIQAVLLPKKTE	301	1.50E-09	771.7211	3082.8555	4	-0.0039	29	0	GluC
H2A1F_MOUSE, H2A1H_MOUSE, H2A1_MOUSE, H2A1K_MOUSE, H2A3_MOUSE, H2AJ_MOUSE	Histone H2A	LNKLLGRVTIAQGGVLPNIQAVLLPKKTE	301	1.30E-08	1028.6257	3082.8553	3	-0.0041	29	0	GluC
H2A1F_MOUSE, H2A1H_MOUSE, H2A1_MOUSE, H2A1K_MOUSE, H2A3_MOUSE, H2AJ_MOUSE	Histone H2A	LNKLLGRVTIAQGGVLPNIQAVLLPKKTE	301	1.80E-05	771.721	3082.8548	4	-0.0046	29	0	GluC
H2A1F_MOUSE, H2A1H_MOUSE, H2A1_MOUSE, H2A1K_MOUSE, H2A3_MOUSE, H2AJ_MOUSE	Histone H2A	LNKLLGRVTIAQGGVLPNIQAVLLPKKTE	301	7.20E-08	771.721	3082.8548	4	-0.0046	29	0	GluC
H2A1F_MOUSE, H2A1H_MOUSE, H2A1_MOUSE, H2A1K_MOUSE, H2A3_MOUSE, H2AJ_MOUSE	Histone H2A	LNKLLGRVTIAQGGVLPNIQAVLLPKKTE	301	2.90E-04	771.721	3082.8547	4	-0.0047	29	0	GluC
H2A1F_MOUSE, H2A1H_MOUSE, H2A1_MOUSE, H2A1K_MOUSE, H2A3_MOUSE, H2AJ_MOUSE	Histone H2A	LNKLLGRVTIAQGGVLPNIQAVLLPKKTE	301	2.10E-05	771.721	3082.8547	4	-0.0047	29	0	GluC
H2A1F_MOUSE, H2A1H_MOUSE, H2A1_MOUSE, H2A1K_MOUSE, H2A3_MOUSE, H2AJ_MOUSE	Histone H2A	LNKLLGRVTIAQGGVLPNIQAVLLPKKTE	301	2.80E-04	771.7209	3082.8545	4	-0.0049	29	0	GluC
H2A1F_MOUSE, H2A1H_MOUSE, H2A1_MOUSE, H2A1K_MOUSE, H2A3_MOUSE, H2AJ_MOUSE	Histone H2A	LNKLLGRVTIAQGGVLPNIQAVLLPKKTE	301	7.70E-05	771.7209	3082.8544	4	-0.0050	29	0	GluC
H2A1F_MOUSE, H2A1H_MOUSE, H2A1_MOUSE, H2A1K_MOUSE, H2A3_MOUSE, H2AJ_MOUSE	Histone H2A	LNKLLGRVTIAQGGVLPNIQAVLLPKKTE	301	5.30E-05	617.5782	3082.8544	5	-0.0050	29	0	GluC
H2A1F_MOUSE, H2A1H_MOUSE, H2A1_MOUSE, H2A1K_MOUSE, H2A3_MOUSE, H2AJ_MOUSE	Histone H2A	LNKLLGRVTIAQGGVLPNIQAVLLPKKTE	301	5.70E-11	771.7209	3082.8544	4	-0.0050	29	0	GluC
H2A1F_MOUSE, H2A1H_MOUSE, H2A1_MOUSE, H2A1K_MOUSE, H2A3_MOUSE, H2AJ_MOUSE	Histone H2A	LNKLLGRVTIAQGGVLPNIQAVLLPKKTE	301	3.40E-06	1028.6253	3082.8541	3	-0.0053	29	0	GluC
H2A1F_MOUSE, H2A1H_MOUSE, H2A1_MOUSE, H2A1K_MOUSE, H2A3_MOUSE, H2AJ_MOUSE	Histone H2A	LNKLLGRVTIAQGGVLPNIQAVLLPKKTE	301	3.10E-05	771.7205	3082.8529	4	-0.0065	29	0	GluC
H2A1F_MOUSE, H2A1H_MOUSE, H2A1_MOUSE, H2A1K_MOUSE, H2A3_MOUSE, H2AJ_MOUSE	Histone H2A	LNKLLGRVTIAQGGVLPNIQAVLLPKKTE	301	4.10E-05	617.5778	3082.8527	5	-0.0067	29	0	GluC
H2A1F_MOUSE, H2A1H_MOUSE, H2A1_MOUSE, H2A1K_MOUSE, H2A3_MOUSE, H2AJ_MOUSE	Histone H2A	LNKLLGRVTIAQGGVLPNIQAVLLPKKTE	301	1.40E-04	617.5776	3082.8519	5	-0.0075	29	0	GluC
H2A1F_MOUSE, H2A1H_MOUSE, H2A1_MOUSE, H2A1K_MOUSE, H2A3_MOUSE, H2AJ_MOUSE	Histone H2A	LNKLLGRVTIAQGGVLPNIQAVLLPKKTE	301	7.50E-12	617.5776	3082.8519	5	-0.0075	29	0	GluC
H2A1F_MOUSE, H2A1H_MOUSE, H2A1_MOUSE, H2A1K_MOUSE, H2A3_MOUSE, H2AJ_MOUSE	Histone H2A	LNKLLGRVTIAQGGVLPNIQAVLLPKKTE	301	6.30E-03	771.7201	3082.8515	4	-0.0079	29	0	GluC
H2A1F_MOUSE, H2A1H_MOUSE, H2A1_MOUSE, H2A1K_MOUSE, H2A3_MOUSE, H2AJ_MOUSE	Histone H2A	LNKLLGRVTIAQGGVLPNIQAVLLPKKTE	301	3.10E-03	771.7197	3082.8495	4	-0.0099	29	0	GluC
H2A1F_MOUSE, H2A1H_MOUSE, H2A1_MOUSE, H2A1K_MOUSE, H2A3_MOUSE, H2AJ_MOUSE	Histone H2A	LNKLLGRVTIAQGGVLPNIQAVLLPKKTE	301	1.40E-04	771.7197	3082.8495	4	-0.0099	29	0	GluC
H2A1F_MOUSE, H2A1H_MOUSE, H2A1_MOUSE, H2A1K_MOUSE, H2A3_MOUSE, H2AJ_MOUSE	Histone H2A	LNKLLGRVTIAQGGVLPNIQAVLLPKKTE	301	5.10E-04	771.7196	3082.8491	4	-0.0103	29	0	GluC
H2A1F_MOUSE, H2A1H_MOUSE, H2A1_MOUSE, H2A1K_MOUSE, H2A3_MOUSE, H2AJ_MOUSE	Histone H2A	LNKLLGRVTIAQGGVLPNIQAVLLPKKTE	301	1.10E-06	771.7196	3082.8491	4	-0.0103	29	0	GluC
H2A1F_MOUSE, H2A1H_MOUSE, H2A1_MOUSE, H2A1K_MOUSE, H2A3_MOUSE, H2AJ_MOUSE	Histone H2A	LNKLLGRVTIAQGGVLPNIQAVLLPKKTE	301	9.80E-06	771.719	3082.8469	4	-0.0125	29	0	GluC
H2A1F_MOUSE, H2A1H_MOUSE, H2A1_MOUSE, H2A1K_MOUSE, H2A3_MOUSE, H2AJ_MOUSE	Histone H2A	LNKLLGRVTIAQGGVLPNIQAVLLPKKTE	301	1.80E-04	1028.6309	3082.8709	3	0.0115	29	0	GluC
H2A1F_MOUSE, H2A1H_MOUSE, H2A1_MOUSE, H2A1K_MOUSE, H2A3_MOUSE, H2AJ_MOUSE	Histone H2A	LNKLLGRVTIAQGGVLPNIQAVLLPKKTE	301	8.00E-05	771.7247	3082.8696	4	0.0102	29	0	GluC
H2A1F_MOUSE, H2A1H_MOUSE, H2A1_MOUSE, H2A1K_MOUSE, H2A3_MOUSE, H2AJ_MOUSE	Histone H2A	LNKLLGRVTIAQGGVLPNIQAVLLPKKTE	301	1.30E-04	1028.6304	3082.8694	3	0.0100	29	0	GluC
H2A1F_MOUSE, H2A1H_MOUSE, H2A1_MOUSE, H2A1K_MOUSE, H2A3_MOUSE, H2AJ_MOUSE	Histone H2A	LNKLLGRVTIAQGGVLPNIQAVLLPKKTE	301	2.90E-05	1028.6304	3082.8694	3	0.0100	29	0	GluC
H2A1F_MOUSE, H2A1H_MOUSE, H2A1_MOUSE, H2A1K_MOUSE, H2A3_MOUSE, H2AJ_MOUSE	Histone H2A	LNKLLGRVTIAQGGVLPNIQAVLLPKKTE	301	4.40E-04	771.7242	3082.8677	4	0.0083	29	0	GluC
H2A1F_MOUSE, H2A1H_MOUSE, H2A1_MOUSE, H2A1K_MOUSE, H2A3_MOUSE, H2AJ_MOUSE	Histone H2A	LNKLLGRVTIAQGGVLPNIQAVLLPKKTE	301	1.00E-04	617.5808	3082.8675	5	0.0081	29	0	GluC
H2A1F_MOUSE, H2A1H_MOUSE, H2A1_MOUSE, H2A1K_MOUSE, H2A3_MOUSE, H2AJ_MOUSE	Histone H2A	LNKLLGRVTIAQGGVLPNIQAVLLPKKTE	301	2.60E-05	771.7242	3082.8675	4	0.0081	29	0	GluC
H2A1F_MOUSE, H2A1H_MOUSE, H2A1_MOUSE, H2A1K_MOUSE, H2A3_MOUSE, H2AJ_MOUSE	Histone H2A	LNKLLGRVTIAQGGVLPNIQAVLLPKKTE	301	1.60E-06	771.7242	3082.8675	4	0.0081	29	0	GluC
H2A1F_MOUSE, H2A1H_MOUSE, H2A1_MOUSE, H2A1K_MOUSE, H2A3_MOUSE, H2AJ_MOUSE	Histone H2A	LNKLLGRVTIAQGGVLPNIQAVLLPKKTE	301	3.40E-06	1028.6296	3082.8670	3	0.0076	29	0	GluC

H2A1F_MOUSE, H2A1H_MOUSE, H2A1_MOUSE, H2A1K_MOUSE, H2A3_MOUSE, H2AJ_MOUSE	Histone H2A	LNKLLGRVTIAQGGVLPNIQAVLLPKKTE	301	8.10E-07	617.5806	3082.8664	5	0.0070	29	0	GluC
H2A1F_MOUSE, H2A1H_MOUSE, H2A1_MOUSE, H2A1K_MOUSE, H2A3_MOUSE, H2AJ_MOUSE	Histone H2A	LNKLLGRVTIAQGGVLPNIQAVLLPKKTE	301	1.80E-03	771.7238	3082.8662	4	0.0068	29	0	GluC
H2A1F_MOUSE, H2A1H_MOUSE, H2A1_MOUSE, H2A1K_MOUSE, H2A3_MOUSE, H2AJ_MOUSE	Histone H2A	LNKLLGRVTIAQGGVLPNIQAVLLPKKTE	301	1.00E-04	1028.6293	3082.8661	3	0.0067	29	0	GluC
H2A1F_MOUSE, H2A1H_MOUSE, H2A1_MOUSE, H2A1K_MOUSE, H2A3_MOUSE, H2AJ_MOUSE	Histone H2A	LNKLLGRVTIAQGGVLPNIQAVLLPKKTE	301	1.70E-06	617.5805	3082.8661	5	0.0067	29	0	GluC
H2A1F_MOUSE, H2A1H_MOUSE, H2A1_MOUSE, H2A1K_MOUSE, H2A3_MOUSE, H2AJ_MOUSE	Histone H2A	LNKLLGRVTIAQGGVLPNIQAVLLPKKTE	301	5.90E-03	771.7238	3082.8660	4	0.0066	29	0	GluC
H2A1F_MOUSE, H2A1H_MOUSE, H2A1_MOUSE, H2A1K_MOUSE, H2A3_MOUSE, H2AJ_MOUSE	Histone H2A	LNKLLGRVTIAQGGVLPNIQAVLLPKKTE	301	9.40E-05	771.7238	3082.8660	4	0.0066	29	0	GluC
H2A1F_MOUSE, H2A1H_MOUSE, H2A1_MOUSE, H2A1K_MOUSE, H2A3_MOUSE, H2AJ_MOUSE	Histone H2A	LNKLLGRVTIAQGGVLPNIQAVLLPKKTE	301	4.20E-05	771.7238	3082.8660	4	0.0066	29	0	GluC
H2A1F_MOUSE, H2A1H_MOUSE, H2A1_MOUSE, H2A1K_MOUSE, H2A3_MOUSE, H2AJ_MOUSE	Histone H2A	LNKLLGRVTIAQGGVLPNIQAVLLPKKTE	301	1.00E-02	771.7238	3082.8659	4	0.0065	29	0	GluC
H2A1F_MOUSE, H2A1H_MOUSE, H2A1_MOUSE, H2A1K_MOUSE, H2A3_MOUSE, H2AJ_MOUSE	Histone H2A	LNKLLGRVTIAQGGVLPNIQAVLLPKKTE	301	1.70E-05	1542.4402	3082.8658	2	0.0065	29	0	GluC
H2A1F_MOUSE, H2A1H_MOUSE, H2A1_MOUSE, H2A1K_MOUSE, H2A3_MOUSE, H2AJ_MOUSE	Histone H2A	LNKLLGRVTIAQGGVLPNIQAVLLPKKTE	301	5.60E-08	771.7238	3082.8659	4	0.0065	29	0	GluC
H2A1F_MOUSE, H2A1H_MOUSE, H2A1_MOUSE, H2A1K_MOUSE, H2A3_MOUSE, H2AJ_MOUSE	Histone H2A	LNKLLGRVTIAQGGVLPNIQAVLLPKKTE	301	1.30E-03	771.7237	3082.8658	4	0.0064	29	0	GluC
H2A1F_MOUSE, H2A1H_MOUSE, H2A1_MOUSE, H2A1K_MOUSE, H2A3_MOUSE, H2AJ_MOUSE	Histone H2A	LNKLLGRVTIAQGGVLPNIQAVLLPKKTE	301	3.50E-04	1028.6292	3082.8658	3	0.0064	29	0	GluC
H2A1F_MOUSE, H2A1H_MOUSE, H2A1_MOUSE, H2A1K_MOUSE, H2A3_MOUSE, H2AJ_MOUSE	Histone H2A	LNKLLGRVTIAQGGVLPNIQAVLLPKKTE	301	3.20E-04	1028.6292	3082.8658	3	0.0064	29	0	GluC
H2A1F_MOUSE, H2A1H_MOUSE, H2A1_MOUSE, H2A1K_MOUSE, H2A3_MOUSE, H2AJ_MOUSE	Histone H2A	LNKLLGRVTIAQGGVLPNIQAVLLPKKTE	301	4.20E-07	1028.6292	3082.8658	3	0.0064	29	0	GluC
H2A1F_MOUSE, H2A1H_MOUSE, H2A1_MOUSE, H2A1K_MOUSE, H2A3_MOUSE, H2AJ_MOUSE	Histone H2A	LNKLLGRVTIAQGGVLPNIQAVLLPKKTE	301	1.70E-07	771.7237	3082.8658	4	0.0064	29	0	GluC
H2A1F_MOUSE, H2A1H_MOUSE, H2A1_MOUSE, H2A1K_MOUSE, H2A3_MOUSE, H2AJ_MOUSE	Histone H2A	LNKLLGRVTIAQGGVLPNIQAVLLPKKTE	301	7.80E-03	771.7237	3082.8657	4	0.0063	29	0	GluC
H2A1F_MOUSE, H2A1H_MOUSE, H2A1_MOUSE, H2A1K_MOUSE, H2A3_MOUSE, H2AJ_MOUSE	Histone H2A	LNKLLGRVTIAQGGVLPNIQAVLLPKKTE	301	3.20E-03	771.7237	3082.8656	4	0.0062	29	0	GluC
H2A1F_MOUSE, H2A1H_MOUSE, H2A1_MOUSE, H2A1K_MOUSE, H2A3_MOUSE, H2AJ_MOUSE	Histone H2A	LNKLLGRVTIAQGGVLPNIQAVLLPKKTE	301	8.40E-06	771.7237	3082.8656	4	0.0062	29	0	GluC
H2A1F_MOUSE, H2A1H_MOUSE, H2A1_MOUSE, H2A1K_MOUSE, H2A3_MOUSE, H2AJ_MOUSE	Histone H2A	LNKLLGRVTIAQGGVLPNIQAVLLPKKTE	301	9.20E-03	1028.6291	3082.8655	3	0.0061	29	0	GluC
H2A1F_MOUSE, H2A1H_MOUSE, H2A1_MOUSE, H2A1K_MOUSE, H2A3_MOUSE, H2AJ_MOUSE	Histone H2A	LNKLLGRVTIAQGGVLPNIQAVLLPKKTE	301	9.70E-04	1028.6291	3082.8655	3	0.0061	29	0	GluC
H2A1F_MOUSE, H2A1H_MOUSE, H2A1_MOUSE, H2A1K_MOUSE, H2A3_MOUSE, H2AJ_MOUSE	Histone H2A	LNKLLGRVTIAQGGVLPNIQAVLLPKKTE	301	1.90E-04	1028.6291	3082.8655	3	0.0061	29	0	GluC
H2A1F_MOUSE, H2A1H_MOUSE, H2A1_MOUSE, H2A1K_MOUSE, H2A3_MOUSE, H2AJ_MOUSE	Histone H2A	LNKLLGRVTIAQGGVLPNIQAVLLPKKTE	301	7.40E-04	771.7236	3082.8653	4	0.0059	29	0	GluC
H2A1F_MOUSE, H2A1H_MOUSE, H2A1_MOUSE, H2A1K_MOUSE, H2A3_MOUSE, H2AJ_MOUSE	Histone H2A	LNKLLGRVTIAQGGVLPNIQAVLLPKKTE	301	5.00E-05	771.7236	3082.8653	4	0.0059	29	0	GluC
H2A1F_MOUSE, H2A1H_MOUSE, H2A1_MOUSE, H2A1K_MOUSE, H2A3_MOUSE, H2AJ_MOUSE	Histone H2A	LNKLLGRVTIAQGGVLPNIQAVLLPKKTE	301	4.60E-03	771.7236	3082.8652	4	0.0058	29	0	GluC
H2A1F_MOUSE, H2A1H_MOUSE, H2A1_MOUSE, H2A1K_MOUSE, H2A3_MOUSE, H2AJ_MOUSE	Histone H2A	LNKLLGRVTIAQGGVLPNIQAVLLPKKTE	301	9.00E-04	771.7236	3082.8652	4	0.0058	29	0	GluC
H2A1F_MOUSE, H2A1H_MOUSE, H2A1_MOUSE, H2A1K_MOUSE, H2A3_MOUSE, H2AJ_MOUSE	Histone H2A	LNKLLGRVTIAQGGVLPNIQAVLLPKKTE	301	1.60E-05	771.7236	3082.8652	4	0.0058	29	0	GluC
H2A1F_MOUSE, H2A1H_MOUSE, H2A1_MOUSE, H2A1K_MOUSE, H2A3_MOUSE, H2AJ_MOUSE	Histone H2A	LNKLLGRVTIAQGGVLPNIQAVLLPKKTE	301	9.70E-06	1028.629	3082.8652	3	0.0058	29	0	GluC
H2A1F_MOUSE, H2A1H_MOUSE, H2A1_MOUSE, H2A1K_MOUSE, H2A3_MOUSE, H2AJ_MOUSE	Histone H2A	LNKLLGRVTIAQGGVLPNIQAVLLPKKTE	301	6.60E-07	771.7236	3082.8652	4	0.0058	29	0	GluC
H2A1F_MOUSE, H2A1H_MOUSE, H2A1_MOUSE, H2A1K_MOUSE, H2A3_MOUSE, H2AJ_MOUSE	Histone H2A	LNKLLGRVTIAQGGVLPNIQAVLLPKKTE	301	5.70E-04	771.7236	3082.8651	4	0.0057	29	0	GluC
H2A1F_MOUSE, H2A1H_MOUSE, H2A1_MOUSE, H2A1K_MOUSE, H2A3_MOUSE, H2AJ_MOUSE	Histone H2A	LNKLLGRVTIAQGGVLPNIQAVLLPKKTE	301	2.50E-04	617.5803	3082.8651	5	0.0057	29	0	GluC
H2A1F_MOUSE, H2A1H_MOUSE, H2A1_MOUSE, H2A1K_MOUSE, H2A3_MOUSE, H2AJ_MOUSE	Histone H2A	LNKLLGRVTIAQGGVLPNIQAVLLPKKTE	301	2.10E-04	771.7236	3082.8651	4	0.0057	29	0	GluC
H2A1F_MOUSE, H2A1H_MOUSE, H2A1_MOUSE, H2A1K_MOUSE, H2A3_MOUSE, H2AJ_MOUSE	Histone H2A	LNKLLGRVTIAQGGVLPNIQAVLLPKKTE	301	4.70E-05	771.7236	3082.8651	4	0.0057	29	0	GluC
H2A1F_MOUSE, H2A1H_MOUSE, H2A1_MOUSE, H2A1K_MOUSE, H2A3_MOUSE, H2AJ_MOUSE	Histone H2A	LNKLLGRVTIAQGGVLPNIQAVLLPKKTE	301	6.30E-06	617.5803	3082.8651	5	0.0057	29	0	GluC
H2A1F_MOUSE, H2A1H_MOUSE, H2A1_MOUSE, H2A1K_MOUSE, H2A3_MOUSE, H2AJ_MOUSE	Histone H2A	LNKLLGRVTIAQGGVLPNIQAVLLPKKTE	301	5.50E-06	771.7236	3082.8651	4	0.0057	29	0	GluC
H2A1F_MOUSE, H2A1H_MOUSE, H2A1_MOUSE, H2A1K_MOUSE, H2A3_MOUSE, H2AJ_MOUSE	Histone H2A	LNKLLGRVTIAQGGVLPNIQAVLLPKKTE	301	7.30E-04	771.7235	3082.8648	4	0.0054	29	0	GluC
H2A1F_MOUSE, H2A1H_MOUSE, H2A1_MOUSE, H2A1K_MOUSE, H2A3_MOUSE, H2AJ_MOUSE	Histone H2A	LNKLLGRVTIAQGGVLPNIQAVLLPKKTE	301	4.50E-04	771.7235	3082.8648	4	0.0054	29	0	GluC
H2A1F_MOUSE, H2A1H_MOUSE, H2A1_MOUSE, H2A1K_MOUSE, H2A3_MOUSE, H2AJ_MOUSE	Histone H2A	LNKLLGRVTIAQGGVLPNIQAVLLPKKTE	301	4.10E-08	771.7235	3082.8648	4	0.0054	29	0	GluC

H2A1F_MOUSE, H2A1H_MOUSE, H2A1_MOUSE, H2A1K_MOUSE, H2A3_MOUSE, H2AJ_MOUSE	Histone H2A	LNKLLGRVTIAQGGVLPNIQAVLLPKKTE	301	3.30E-03	771.7234	3082.8647	4	0.0053	29	0	GluC
H2A1F_MOUSE, H2A1H_MOUSE, H2A1_MOUSE, H2A1K_MOUSE, H2A3_MOUSE, H2AJ_MOUSE	Histone H2A	LNKLLGRVTIAQGGVLPNIQAVLLPKKTE	301	3.10E-03	617.5802	3082.8647	5	0.0053	29	0	GluC
H2A1F_MOUSE, H2A1H_MOUSE, H2A1_MOUSE, H2A1K_MOUSE, H2A3_MOUSE, H2AJ_MOUSE	Histone H2A	LNKLLGRVTIAQGGVLPNIQAVLLPKKTE	301	3.90E-04	771.7234	3082.8647	4	0.0053	29	0	GluC
H2A1F_MOUSE, H2A1H_MOUSE, H2A1_MOUSE, H2A1K_MOUSE, H2A3_MOUSE, H2AJ_MOUSE	Histone H2A	LNKLLGRVTIAQGGVLPNIQAVLLPKKTE	301	1.20E-04	771.7234	3082.8647	4	0.0053	29	0	GluC
H2A1F_MOUSE, H2A1H_MOUSE, H2A1_MOUSE, H2A1K_MOUSE, H2A3_MOUSE, H2AJ_MOUSE	Histone H2A	LNKLLGRVTIAQGGVLPNIQAVLLPKKTE	301	6.60E-04	1028.6288	3082.8646	3	0.0052	29	0	GluC
H2A1F_MOUSE, H2A1H_MOUSE, H2A1_MOUSE, H2A1K_MOUSE, H2A3_MOUSE, H2AJ_MOUSE	Histone H2A	LNKLLGRVTIAQGGVLPNIQAVLLPKKTE	301	4.40E-05	617.5802	3082.8646	5	0.0052	29	0	GluC
H2A1F_MOUSE, H2A1H_MOUSE, H2A1_MOUSE, H2A1K_MOUSE, H2A3_MOUSE, H2AJ_MOUSE	Histone H2A	LNKLLGRVTIAQGGVLPNIQAVLLPKKTE	301	2.60E-05	1028.6288	3082.8646	3	0.0052	29	0	GluC
H2A1F_MOUSE, H2A1H_MOUSE, H2A1_MOUSE, H2A1K_MOUSE, H2A3_MOUSE, H2AJ_MOUSE	Histone H2A	LNKLLGRVTIAQGGVLPNIQAVLLPKKTE	301	1.60E-05	1028.6288	3082.8646	3	0.0052	29	0	GluC
H2A1F_MOUSE, H2A1H_MOUSE, H2A1_MOUSE, H2A1K_MOUSE, H2A3_MOUSE, H2AJ_MOUSE	Histone H2A	LNKLLGRVTIAQGGVLPNIQAVLLPKKTE	301	4.70E-06	1028.6288	3082.8646	3	0.0052	29	0	GluC
H2A1F_MOUSE, H2A1H_MOUSE, H2A1_MOUSE, H2A1K_MOUSE, H2A3_MOUSE, H2AJ_MOUSE	Histone H2A	LNKLLGRVTIAQGGVLPNIQAVLLPKKTE	301	8.40E-03	771.7234	3082.8645	4	0.0051	29	0	GluC
H2A1F_MOUSE, H2A1H_MOUSE, H2A1_MOUSE, H2A1K_MOUSE, H2A3_MOUSE, H2AJ_MOUSE	Histone H2A	LNKLLGRVTIAQGGVLPNIQAVLLPKKTE	301	5.30E-03	617.5802	3082.8645	5	0.0051	29	0	GluC
H2A1F_MOUSE, H2A1H_MOUSE, H2A1_MOUSE, H2A1K_MOUSE, H2A3_MOUSE, H2AJ_MOUSE	Histone H2A	LNKLLGRVTIAQGGVLPNIQAVLLPKKTE	301	3.90E-04	771.7234	3082.8645	4	0.0051	29	0	GluC
H2A1F_MOUSE, H2A1H_MOUSE, H2A1_MOUSE, H2A1K_MOUSE, H2A3_MOUSE, H2AJ_MOUSE	Histone H2A	LNKLLGRVTIAQGGVLPNIQAVLLPKKTE	301	9.20E-06	771.7234	3082.8645	4	0.0051	29	0	GluC
H2A1F_MOUSE, H2A1H_MOUSE, H2A1_MOUSE, H2A1K_MOUSE, H2A3_MOUSE, H2AJ_MOUSE	Histone H2A	LNKLLGRVTIAQGGVLPNIQAVLLPKKTE	301	3.00E-06	617.5802	3082.8645	5	0.0051	29	0	GluC
H2A1F_MOUSE, H2A1H_MOUSE, H2A1_MOUSE, H2A1K_MOUSE, H2A3_MOUSE, H2AJ_MOUSE	Histone H2A	LNKLLGRVTIAQGGVLPNIQAVLLPKKTE	301	1.10E-07	617.5802	3082.8645	5	0.0051	29	0	GluC
H2A1F_MOUSE, H2A1H_MOUSE, H2A1_MOUSE, H2A1K_MOUSE, H2A3_MOUSE, H2AJ_MOUSE	Histone H2A	LNKLLGRVTIAQGGVLPNIQAVLLPKKTE	301	9.00E-03	771.7233	3082.8643	4	0.0049	29	0	GluC
H2A1F_MOUSE, H2A1H_MOUSE, H2A1_MOUSE, H2A1K_MOUSE, H2A3_MOUSE, H2AJ_MOUSE	Histone H2A	LNKLLGRVTIAQGGVLPNIQAVLLPKKTE	301	3.70E-03	771.7233	3082.8643	4	0.0049	29	0	GluC
H2A1F_MOUSE, H2A1H_MOUSE, H2A1_MOUSE, H2A1K_MOUSE, H2A3_MOUSE, H2AJ_MOUSE	Histone H2A	LNKLLGRVTIAQGGVLPNIQAVLLPKKTE	301	1.60E-03	771.7233	3082.8643	4	0.0049	29	0	GluC
H2A1F_MOUSE, H2A1H_MOUSE, H2A1_MOUSE, H2A1K_MOUSE, H2A3_MOUSE, H2AJ_MOUSE	Histone H2A	LNKLLGRVTIAQGGVLPNIQAVLLPKKTE	301	1.30E-04	771.7233	3082.8643	4	0.0049	29	0	GluC
H2A1F_MOUSE, H2A1H_MOUSE, H2A1_MOUSE, H2A1K_MOUSE, H2A3_MOUSE, H2AJ_MOUSE	Histone H2A	LNKLLGRVTIAQGGVLPNIQAVLLPKKTE	301	2.70E-05	771.7234	3082.8643	4	0.0049	29	0	GluC
H2A1F_MOUSE, H2A1H_MOUSE, H2A1_MOUSE, H2A1K_MOUSE, H2A3_MOUSE, H2AJ_MOUSE	Histone H2A	LNKLLGRVTIAQGGVLPNIQAVLLPKKTE	301	1.10E-05	1028.6287	3082.8643	3	0.0049	29	0	GluC
H2A1F_MOUSE, H2A1H_MOUSE, H2A1_MOUSE, H2A1K_MOUSE, H2A3_MOUSE, H2AJ_MOUSE	Histone H2A	LNKLLGRVTIAQGGVLPNIQAVLLPKKTE	301	6.10E-07	771.7234	3082.8643	4	0.0049	29	0	GluC
H2A1F_MOUSE, H2A1H_MOUSE, H2A1_MOUSE, H2A1K_MOUSE, H2A3_MOUSE, H2AJ_MOUSE	Histone H2A	LNKLLGRVTIAQGGVLPNIQAVLLPKKTE	301	2.90E-07	771.7234	3082.8643	4	0.0049	29	0	GluC
H2A1F_MOUSE, H2A1H_MOUSE, H2A1_MOUSE, H2A1K_MOUSE, H2A3_MOUSE, H2AJ_MOUSE	Histone H2A	LNKLLGRVTIAQGGVLPNIQAVLLPKKTE	301	2.40E-07	771.7234	3082.8643	4	0.0049	29	0	GluC
H2A1F_MOUSE, H2A1H_MOUSE, H2A1_MOUSE, H2A1K_MOUSE, H2A3_MOUSE, H2AJ_MOUSE	Histone H2A	LNKLLGRVTIAQGGVLPNIQAVLLPKKTE	301	8.30E-05	1028.6286	3082.8640	3	0.0046	29	0	GluC
H2A1F_MOUSE, H2A1H_MOUSE, H2A1_MOUSE, H2A1K_MOUSE, H2A3_MOUSE, H2AJ_MOUSE	Histone H2A	LNKLLGRVTIAQGGVLPNIQAVLLPKKTE	301	9.70E-06	1028.6286	3082.8640	3	0.0046	29	0	GluC
H2A1F_MOUSE, H2A1H_MOUSE, H2A1_MOUSE, H2A1K_MOUSE, H2A3_MOUSE, H2AJ_MOUSE	Histone H2A	LNKLLGRVTIAQGGVLPNIQAVLLPKKTE	301	4.70E-07	1028.6286	3082.8640	3	0.0046	29	0	GluC
H2A1F_MOUSE, H2A1H_MOUSE, H2A1_MOUSE, H2A1K_MOUSE, H2A3_MOUSE, H2AJ_MOUSE	Histone H2A	LNKLLGRVTIAQGGVLPNIQAVLLPKKTE	301	6.30E-04	1542.4392	3082.8638	2	0.0045	29	0	GluC
H2A1F_MOUSE, H2A1H_MOUSE, H2A1_MOUSE, H2A1K_MOUSE, H2A3_MOUSE, H2AJ_MOUSE	Histone H2A	LNKLLGRVTIAQGGVLPNIQAVLLPKKTE	301	2.00E-04	771.7233	3082.8639	4	0.0045	29	0	GluC
H2A1F_MOUSE, H2A1H_MOUSE, H2A1_MOUSE, H2A1K_MOUSE, H2A3_MOUSE, H2AJ_MOUSE	Histone H2A	LNKLLGRVTIAQGGVLPNIQAVLLPKKTE	301	3.20E-06	771.7233	3082.8639	4	0.0045	29	0	GluC
H2A1F_MOUSE, H2A1H_MOUSE, H2A1_MOUSE, H2A1K_MOUSE, H2A3_MOUSE, H2AJ_MOUSE	Histone H2A	LNKLLGRVTIAQGGVLPNIQAVLLPKKTE	301	2.00E-06	771.7233	3082.8639	4	0.0045	29	0	GluC
H2A1F_MOUSE, H2A1H_MOUSE, H2A1_MOUSE, H2A1K_MOUSE, H2A3_MOUSE, H2AJ_MOUSE	Histone H2A	LNKLLGRVTIAQGGVLPNIQAVLLPKKTE	301	2.20E-07	771.7233	3082.8639	4	0.0045	29	0	GluC
H2A1F_MOUSE, H2A1H_MOUSE, H2A1_MOUSE, H2A1K_MOUSE, H2A3_MOUSE, H2AJ_MOUSE	Histone H2A	LNKLLGRVTIAQGGVLPNIQAVLLPKKTE	301	2.60E-03	771.7232	3082.8638	4	0.0044	29	0	GluC
H2A1F_MOUSE, H2A1H_MOUSE, H2A1_MOUSE, H2A1K_MOUSE, H2A3_MOUSE, H2AJ_MOUSE	Histone H2A	LNKLLGRVTIAQGGVLPNIQAVLLPKKTE	301	5.40E-04	771.7232	3082.8638	4	0.0044	29	0	GluC
H2A1F_MOUSE, H2A1H_MOUSE, H2A1_MOUSE, H2A1K_MOUSE, H2A3_MOUSE, H2AJ_MOUSE	Histone H2A	LNKLLGRVTIAQGGVLPNIQAVLLPKKTE	301	2.50E-07	771.7232	3082.8638	4	0.0044	29	0	GluC
H2A1F_MOUSE, H2A1H_MOUSE, H2A1_MOUSE, H2A1K_MOUSE, H2A3_MOUSE, H2AJ_MOUSE	Histone H2A	LNKLLGRVTIAQGGVLPNIQAVLLPKKTE	301	5.00E-08	771.7232	3082.8638	4	0.0044	29	0	GluC
H2A1F_MOUSE, H2A1H_MOUSE, H2A1_MOUSE, H2A1K_MOUSE, H2A3_MOUSE, H2AJ_MOUSE	Histone H2A	LNKLLGRVTIAQGGVLPNIQAVLLPKKTE	301	6.40E-04	1028.6285	3082.8637	3	0.0043	29	0	GluC

H2A1F_MOUSE, H2A1H_MOUSE, H2A1_MOUSE, H2A1K_MOUSE, H2A3_MOUSE, H2AJ_MOUSE	Histone H2A	LNKLLGRVTIAQGGVLPNIQAVLLPKKTE	301	3.60E-04	1028.6285	3082.8637	3	0.0043	29	0	GluC
H2A1F_MOUSE, H2A1H_MOUSE, H2A1_MOUSE, H2A1K_MOUSE, H2A3_MOUSE, H2AJ_MOUSE	Histone H2A	LNKLLGRVTIAQGGVLPNIQAVLLPKKTE	301	2.40E-04	1028.6285	3082.8637	3	0.0043	29	0	GluC
H2A1F_MOUSE, H2A1H_MOUSE, H2A1_MOUSE, H2A1K_MOUSE, H2A3_MOUSE, H2AJ_MOUSE	Histone H2A	LNKLLGRVTIAQGGVLPNIQAVLLPKKTE	301	2.20E-04	1028.6285	3082.8637	3	0.0043	29	0	GluC
H2A1F_MOUSE, H2A1H_MOUSE, H2A1_MOUSE, H2A1K_MOUSE, H2A3_MOUSE, H2AJ_MOUSE	Histone H2A	LNKLLGRVTIAQGGVLPNIQAVLLPKKTE	301	5.40E-05	771.7232	3082.8637	4	0.0043	29	0	GluC
H2A1F_MOUSE, H2A1H_MOUSE, H2A1_MOUSE, H2A1K_MOUSE, H2A3_MOUSE, H2AJ_MOUSE	Histone H2A	LNKLLGRVTIAQGGVLPNIQAVLLPKKTE	301	1.60E-05	1028.6285	3082.8637	3	0.0043	29	0	GluC
H2A1F_MOUSE, H2A1H_MOUSE, H2A1_MOUSE, H2A1K_MOUSE, H2A3_MOUSE, H2AJ_MOUSE	Histone H2A	LNKLLGRVTIAQGGVLPNIQAVLLPKKTE	301	1.50E-07	771.7232	3082.8637	4	0.0043	29	0	GluC
H2A1F_MOUSE, H2A1H_MOUSE, H2A1_MOUSE, H2A1K_MOUSE, H2A3_MOUSE, H2AJ_MOUSE	Histone H2A	LNKLLGRVTIAQGGVLPNIQAVLLPKKTE	301	7.20E-04	771.7232	3082.8635	4	0.0041	29	0	GluC
H2A1F_MOUSE, H2A1H_MOUSE, H2A1_MOUSE, H2A1K_MOUSE, H2A3_MOUSE, H2AJ_MOUSE	Histone H2A	LNKLLGRVTIAQGGVLPNIQAVLLPKKTE	301	5.30E-04	771.7232	3082.8635	4	0.0041	29	0	GluC
H2A1F_MOUSE, H2A1H_MOUSE, H2A1_MOUSE, H2A1K_MOUSE, H2A3_MOUSE, H2AJ_MOUSE	Histone H2A	LNKLLGRVTIAQGGVLPNIQAVLLPKKTE	301	2.30E-04	771.7232	3082.8635	4	0.0041	29	0	GluC
H2A1F_MOUSE, H2A1H_MOUSE, H2A1_MOUSE, H2A1K_MOUSE, H2A3_MOUSE, H2AJ_MOUSE	Histone H2A	LNKLLGRVTIAQGGVLPNIQAVLLPKKTE	301	1.70E-04	771.7232	3082.8635	4	0.0041	29	0	GluC
H2A1F_MOUSE, H2A1H_MOUSE, H2A1_MOUSE, H2A1K_MOUSE, H2A3_MOUSE, H2AJ_MOUSE	Histone H2A	LNKLLGRVTIAQGGVLPNIQAVLLPKKTE	301	4.10E-05	771.7232	3082.8635	4	0.0041	29	0	GluC
H2A1F_MOUSE, H2A1H_MOUSE, H2A1_MOUSE, H2A1K_MOUSE, H2A3_MOUSE, H2AJ_MOUSE	Histone H2A	LNKLLGRVTIAQGGVLPNIQAVLLPKKTE	301	2.90E-05	771.7232	3082.8635	4	0.0041	29	0	GluC
H2A1F_MOUSE, H2A1H_MOUSE, H2A1_MOUSE, H2A1K_MOUSE, H2A3_MOUSE, H2AJ_MOUSE	Histone H2A	LNKLLGRVTIAQGGVLPNIQAVLLPKKTE	301	6.90E-06	771.7231	3082.8635	4	0.0041	29	0	GluC
H2A1F_MOUSE, H2A1H_MOUSE, H2A1_MOUSE, H2A1K_MOUSE, H2A3_MOUSE, H2AJ_MOUSE	Histone H2A	LNKLLGRVTIAQGGVLPNIQAVLLPKKTE	301	1.70E-03	771.7231	3082.8634	4	0.0040	29	0	GluC
H2A1F_MOUSE, H2A1H_MOUSE, H2A1_MOUSE, H2A1K_MOUSE, H2A3_MOUSE, H2AJ_MOUSE	Histone H2A	LNKLLGRVTIAQGGVLPNIQAVLLPKKTE	301	2.90E-04	1028.6284	3082.8634	3	0.0040	29	0	GluC
H2A1F_MOUSE, H2A1H_MOUSE, H2A1_MOUSE, H2A1K_MOUSE, H2A3_MOUSE, H2AJ_MOUSE	Histone H2A	LNKLLGRVTIAQGGVLPNIQAVLLPKKTE	301	2.90E-04	1028.6284	3082.8634	3	0.0040	29	0	GluC
H2A1F_MOUSE, H2A1H_MOUSE, H2A1_MOUSE, H2A1K_MOUSE, H2A3_MOUSE, H2AJ_MOUSE	Histone H2A	LNKLLGRVTIAQGGVLPNIQAVLLPKKTE	301	1.70E-04	771.7231	3082.8634	4	0.0040	29	0	GluC
H2A1F_MOUSE, H2A1H_MOUSE, H2A1_MOUSE, H2A1K_MOUSE, H2A3_MOUSE, H2AJ_MOUSE	Histone H2A	LNKLLGRVTIAQGGVLPNIQAVLLPKKTE	301	1.20E-04	771.7231	3082.8634	4	0.0040	29	0	GluC
H2A1F_MOUSE, H2A1H_MOUSE, H2A1_MOUSE, H2A1K_MOUSE, H2A3_MOUSE, H2AJ_MOUSE	Histone H2A	LNKLLGRVTIAQGGVLPNIQAVLLPKKTE	301	1.10E-04	1028.6284	3082.8634	3	0.0040	29	0	GluC
H2A1F_MOUSE, H2A1H_MOUSE, H2A1_MOUSE, H2A1K_MOUSE, H2A3_MOUSE, H2AJ_MOUSE	Histone H2A	LNKLLGRVTIAQGGVLPNIQAVLLPKKTE	301	1.00E-07	771.7231	3082.8634	4	0.0040	29	0	GluC
H2A1F_MOUSE, H2A1H_MOUSE, H2A1_MOUSE, H2A1K_MOUSE, H2A3_MOUSE, H2AJ_MOUSE	Histone H2A	LNKLLGRVTIAQGGVLPNIQAVLLPKKTE	301	1.30E-05	771.7231	3082.8633	4	0.0039	29	0	GluC
H2A1F_MOUSE, H2A1H_MOUSE, H2A1_MOUSE, H2A1K_MOUSE, H2A3_MOUSE, H2AJ_MOUSE	Histone H2A	LNKLLGRVTIAQGGVLPNIQAVLLPKKTE	301	5.20E-07	771.7231	3082.8633	4	0.0039	29	0	GluC
H2A1F_MOUSE, H2A1H_MOUSE, H2A1_MOUSE, H2A1K_MOUSE, H2A3_MOUSE, H2AJ_MOUSE	Histone H2A	LNKLLGRVTIAQGGVLPNIQAVLLPKKTE	301	1.00E-02	771.7231	3082.8632	4	0.0038	29	0	GluC
H2A1F_MOUSE, H2A1H_MOUSE, H2A1_MOUSE, H2A1K_MOUSE, H2A3_MOUSE, H2AJ_MOUSE	Histone H2A	LNKLLGRVTIAQGGVLPNIQAVLLPKKTE	301	2.00E-06	771.7231	3082.8632	4	0.0038	29	0	GluC
H2A1F_MOUSE, H2A1H_MOUSE, H2A1_MOUSE, H2A1K_MOUSE, H2A3_MOUSE, H2AJ_MOUSE	Histone H2A	LNKLLGRVTIAQGGVLPNIQAVLLPKKTE	301	9.40E-08	771.7231	3082.8632	4	0.0038	29	0	GluC
H2A1F_MOUSE, H2A1H_MOUSE, H2A1_MOUSE, H2A1K_MOUSE, H2A3_MOUSE, H2AJ_MOUSE	Histone H2A	LNKLLGRVTIAQGGVLPNIQAVLLPKKTE	301	3.20E-08	771.7231	3082.8632	4	0.0038	29	0	GluC
H2A1F_MOUSE, H2A1H_MOUSE, H2A1_MOUSE, H2A1K_MOUSE, H2A3_MOUSE, H2AJ_MOUSE	Histone H2A	LNKLLGRVTIAQGGVLPNIQAVLLPKKTE	301	2.90E-09	771.7231	3082.8632	4	0.0038	29	0	GluC
H2A1F_MOUSE, H2A1H_MOUSE, H2A1_MOUSE, H2A1K_MOUSE, H2A3_MOUSE, H2AJ_MOUSE	Histone H2A	LNKLLGRVTIAQGGVLPNIQAVLLPKKTE	301	9.40E-03	771.7231	3082.8631	4	0.0037	29	0	GluC
H2A1F_MOUSE, H2A1H_MOUSE, H2A1_MOUSE, H2A1K_MOUSE, H2A3_MOUSE, H2AJ_MOUSE	Histone H2A	LNKLLGRVTIAQGGVLPNIQAVLLPKKTE	301	6.80E-03	514.8178	3082.8631	6	0.0037	29	0	GluC
H2A1F_MOUSE, H2A1H_MOUSE, H2A1_MOUSE, H2A1K_MOUSE, H2A3_MOUSE, H2AJ_MOUSE	Histone H2A	LNKLLGRVTIAQGGVLPNIQAVLLPKKTE	301	3.00E-03	1028.6283	3082.8631	3	0.0037	29	0	GluC
H2A1F_MOUSE, H2A1H_MOUSE, H2A1_MOUSE, H2A1K_MOUSE, H2A3_MOUSE, H2AJ_MOUSE	Histone H2A	LNKLLGRVTIAQGGVLPNIQAVLLPKKTE	301	8.80E-04	1028.6283	3082.8631	3	0.0037	29	0	GluC
H2A1F_MOUSE, H2A1H_MOUSE, H2A1_MOUSE, H2A1K_MOUSE, H2A3_MOUSE, H2AJ_MOUSE	Histone H2A	LNKLLGRVTIAQGGVLPNIQAVLLPKKTE	301	9.90E-05	771.7231	3082.8631	4	0.0037	29	0	GluC
H2A1F_MOUSE, H2A1H_MOUSE, H2A1_MOUSE, H2A1K_MOUSE, H2A3_MOUSE, H2AJ_MOUSE	Histone H2A	LNKLLGRVTIAQGGVLPNIQAVLLPKKTE	301	4.20E-06	1028.6283	3082.8631	3	0.0037	29	0	GluC
H2A1F_MOUSE, H2A1H_MOUSE, H2A1_MOUSE, H2A1K_MOUSE, H2A3_MOUSE, H2AJ_MOUSE	Histone H2A	LNKLLGRVTIAQGGVLPNIQAVLLPKKTE	301	4.00E-07	1028.6283	3082.8631	3	0.0037	29	0	GluC
H2A1F_MOUSE, H2A1H_MOUSE, H2A1_MOUSE, H2A1K_MOUSE, H2A3_MOUSE, H2AJ_MOUSE	Histone H2A	LNKLLGRVTIAQGGVLPNIQAVLLPKKTE	301	4.70E-08	617.5799	3082.8630	5	0.0036	29	0	GluC
H2A1F_MOUSE, H2A1H_MOUSE, H2A1_MOUSE, H2A1K_MOUSE, H2A3_MOUSE, H2AJ_MOUSE	Histone H2A	LNKLLGRVTIAQGGVLPNIQAVLLPKKTE	301	5.40E-03	771.723	3082.8629	4	0.0035	29	0	GluC
H2A1F_MOUSE, H2A1H_MOUSE, H2A1_MOUSE, H2A1K_MOUSE, H2A3_MOUSE, H2AJ_MOUSE	Histone H2A	LNKLLGRVTIAQGGVLPNIQAVLLPKKTE	301	6.90E-04	771.723	3082.8629	4	0.0035	29	0	GluC

H2A1F_MOUSE, H2A1H_MOUSE, H2A1_MOUSE, H2A1K_MOUSE, H2A3_MOUSE, H2AJ_MOUSE	Histone H2A	LNKLLGRVTIAQGGVLPNIQAVLLPKKTE	301	2.20E-04	771.723	3082.8629	4	0.0035	29	0	GluC
H2A1F_MOUSE, H2A1H_MOUSE, H2A1_MOUSE, H2A1K_MOUSE, H2A3_MOUSE, H2AJ_MOUSE	Histone H2A	LNKLLGRVTIAQGGVLPNIQAVLLPKKTE	301	3.40E-05	771.723	3082.8629	4	0.0035	29	0	GluC
H2A1F_MOUSE, H2A1H_MOUSE, H2A1_MOUSE, H2A1K_MOUSE, H2A3_MOUSE, H2AJ_MOUSE	Histone H2A	LNKLLGRVTIAQGGVLPNIQAVLLPKKTE	301	1.80E-06	771.723	3082.8629	4	0.0035	29	0	GluC
H2A1F_MOUSE, H2A1H_MOUSE, H2A1_MOUSE, H2A1K_MOUSE, H2A3_MOUSE, H2AJ_MOUSE	Histone H2A	LNKLLGRVTIAQGGVLPNIQAVLLPKKTE	301	3.70E-07	771.723	3082.8629	4	0.0035	29	0	GluC
H2A1F_MOUSE, H2A1H_MOUSE, H2A1_MOUSE, H2A1K_MOUSE, H2A3_MOUSE, H2AJ_MOUSE	Histone H2A	LNKLLGRVTIAQGGVLPNIQAVLLPKKTE	301	2.10E-06	617.5798	3082.8628	5	0.0034	29	0	GluC
H2A1F_MOUSE, H2A1H_MOUSE, H2A1_MOUSE, H2A1K_MOUSE, H2A3_MOUSE, H2AJ_MOUSE	Histone H2A	LNKLLGRVTIAQGGVLPNIQAVLLPKKTE	301	1.50E-06	771.723	3082.8628	4	0.0034	29	0	GluC
H2A1F_MOUSE, H2A1H_MOUSE, H2A1_MOUSE, H2A1K_MOUSE, H2A3_MOUSE, H2AJ_MOUSE	Histone H2A	LNKLLGRVTIAQGGVLPNIQAVLLPKKTE	301	3.20E-07	1028.6282	3082.8628	3	0.0034	29	0	GluC
H2A1F_MOUSE, H2A1H_MOUSE, H2A1_MOUSE, H2A1K_MOUSE, H2A3_MOUSE, H2AJ_MOUSE	Histone H2A	LNKLLGRVTIAQGGVLPNIQAVLLPKKTE	301	2.40E-08	771.723	3082.8628	4	0.0034	29	0	GluC
H2A1F_MOUSE, H2A1H_MOUSE, H2A1_MOUSE, H2A1K_MOUSE, H2A3_MOUSE, H2AJ_MOUSE	Histone H2A	LNKLLGRVTIAQGGVLPNIQAVLLPKKTE	301	4.30E-03	771.7229	3082.8627	4	0.0033	29	0	GluC
H2A1F_MOUSE, H2A1H_MOUSE, H2A1_MOUSE, H2A1K_MOUSE, H2A3_MOUSE, H2AJ_MOUSE	Histone H2A	LNKLLGRVTIAQGGVLPNIQAVLLPKKTE	301	1.20E-03	771.7229	3082.8627	4	0.0033	29	0	GluC
H2A1F_MOUSE, H2A1H_MOUSE, H2A1_MOUSE, H2A1K_MOUSE, H2A3_MOUSE, H2AJ_MOUSE	Histone H2A	LNKLLGRVTIAQGGVLPNIQAVLLPKKTE	301	2.50E-04	771.7229	3082.8627	4	0.0033	29	0	GluC
H2A1F_MOUSE, H2A1H_MOUSE, H2A1_MOUSE, H2A1K_MOUSE, H2A3_MOUSE, H2AJ_MOUSE	Histone H2A	LNKLLGRVTIAQGGVLPNIQAVLLPKKTE	301	1.10E-04	771.7229	3082.8627	4	0.0033	29	0	GluC
H2A1F_MOUSE, H2A1H_MOUSE, H2A1_MOUSE, H2A1K_MOUSE, H2A3_MOUSE, H2AJ_MOUSE	Histone H2A	LNKLLGRVTIAQGGVLPNIQAVLLPKKTE	301	7.20E-05	771.7229	3082.8627	4	0.0033	29	0	GluC
H2A1F_MOUSE, H2A1H_MOUSE, H2A1_MOUSE, H2A1K_MOUSE, H2A3_MOUSE, H2AJ_MOUSE	Histone H2A	LNKLLGRVTIAQGGVLPNIQAVLLPKKTE	301	7.10E-06	771.7229	3082.8627	4	0.0033	29	0	GluC
H2A1F_MOUSE, H2A1H_MOUSE, H2A1_MOUSE, H2A1K_MOUSE, H2A3_MOUSE, H2AJ_MOUSE	Histone H2A	LNKLLGRVTIAQGGVLPNIQAVLLPKKTE	301	1.50E-06	771.7229	3082.8627	4	0.0033	29	0	GluC
H2A1F_MOUSE, H2A1H_MOUSE, H2A1_MOUSE, H2A1K_MOUSE, H2A3_MOUSE, H2AJ_MOUSE	Histone H2A	LNKLLGRVTIAQGGVLPNIQAVLLPKKTE	301	7.10E-07	771.7229	3082.8627	4	0.0033	29	0	GluC
H2A1F_MOUSE, H2A1H_MOUSE, H2A1_MOUSE, H2A1K_MOUSE, H2A3_MOUSE, H2AJ_MOUSE	Histone H2A	LNKLLGRVTIAQGGVLPNIQAVLLPKKTE	301	1.80E-07	617.5798	3082.8626	5	0.0032	29	0	GluC
H2A1F_MOUSE, H2A1H_MOUSE, H2A1_MOUSE, H2A1K_MOUSE, H2A3_MOUSE, H2AJ_MOUSE	Histone H2A	LNKLLGRVTIAQGGVLPNIQAVLLPKKTE	301	7.40E-04	1028.6281	3082.8625	3	0.0031	29	0	GluC
H2A1F_MOUSE, H2A1H_MOUSE, H2A1_MOUSE, H2A1K_MOUSE, H2A3_MOUSE, H2AJ_MOUSE	Histone H2A	LNKLLGRVTIAQGGVLPNIQAVLLPKKTE	301	7.20E-04	1028.6281	3082.8625	3	0.0031	29	0	GluC
H2A1F_MOUSE, H2A1H_MOUSE, H2A1_MOUSE, H2A1K_MOUSE, H2A3_MOUSE, H2AJ_MOUSE	Histone H2A	LNKLLGRVTIAQGGVLPNIQAVLLPKKTE	301	5.80E-04	1028.6281	3082.8625	3	0.0031	29	0	GluC
H2A1F_MOUSE, H2A1H_MOUSE, H2A1_MOUSE, H2A1K_MOUSE, H2A3_MOUSE, H2AJ_MOUSE	Histone H2A	LNKLLGRVTIAQGGVLPNIQAVLLPKKTE	301	3.10E-04	771.7229	3082.8625	4	0.0031	29	0	GluC
H2A1F_MOUSE, H2A1H_MOUSE, H2A1_MOUSE, H2A1K_MOUSE, H2A3_MOUSE, H2AJ_MOUSE	Histone H2A	LNKLLGRVTIAQGGVLPNIQAVLLPKKTE	301	2.60E-04	771.7229	3082.8625	4	0.0031	29	0	GluC
H2A1F_MOUSE, H2A1H_MOUSE, H2A1_MOUSE, H2A1K_MOUSE, H2A3_MOUSE, H2AJ_MOUSE	Histone H2A	LNKLLGRVTIAQGGVLPNIQAVLLPKKTE	301	5.30E-05	1028.6281	3082.8625	3	0.0031	29	0	GluC
H2A1F_MOUSE, H2A1H_MOUSE, H2A1_MOUSE, H2A1K_MOUSE, H2A3_MOUSE, H2AJ_MOUSE	Histone H2A	LNKLLGRVTIAQGGVLPNIQAVLLPKKTE	301	5.80E-06	771.7229	3082.8625	4	0.0031	29	0	GluC
H2A1F_MOUSE, H2A1H_MOUSE, H2A1_MOUSE, H2A1K_MOUSE, H2A3_MOUSE, H2AJ_MOUSE	Histone H2A	LNKLLGRVTIAQGGVLPNIQAVLLPKKTE	301	2.30E-06	771.7229	3082.8625	4	0.0031	29	0	GluC
H2A1F_MOUSE, H2A1H_MOUSE, H2A1_MOUSE, H2A1K_MOUSE, H2A3_MOUSE, H2AJ_MOUSE	Histone H2A	LNKLLGRVTIAQGGVLPNIQAVLLPKKTE	301	3.90E-09	617.5798	3082.8625	5	0.0031	29	0	GluC
H2A1F_MOUSE, H2A1H_MOUSE, H2A1_MOUSE, H2A1K_MOUSE, H2A3_MOUSE, H2AJ_MOUSE	Histone H2A	LNKLLGRVTIAQGGVLPNIQAVLLPKKTE	301	7.40E-03	617.5797	3082.8622	5	0.0028	29	0	GluC
H2A1F_MOUSE, H2A1H_MOUSE, H2A1_MOUSE, H2A1K_MOUSE, H2A3_MOUSE, H2AJ_MOUSE	Histone H2A	LNKLLGRVTIAQGGVLPNIQAVLLPKKTE	301	8.70E-05	617.5797	3082.8622	5	0.0028	29	0	GluC
H2A1F_MOUSE, H2A1H_MOUSE, H2A1_MOUSE, H2A1K_MOUSE, H2A3_MOUSE, H2AJ_MOUSE	Histone H2A	LNKLLGRVTIAQGGVLPNIQAVLLPKKTE	301	4.00E-06	771.7228	3082.8622	4	0.0028	29	0	GluC
H2A1F_MOUSE, H2A1H_MOUSE, H2A1_MOUSE, H2A1K_MOUSE, H2A3_MOUSE, H2AJ_MOUSE	Histone H2A	LNKLLGRVTIAQGGVLPNIQAVLLPKKTE	301	5.10E-06	771.7228	3082.8621	4	0.0027	29	0	GluC
H2A1F_MOUSE, H2A1H_MOUSE, H2A1_MOUSE, H2A1K_MOUSE, H2A3_MOUSE, H2AJ_MOUSE	Histone H2A	LNKLLGRVTIAQGGVLPNIQAVLLPKKTE	301	2.30E-06	771.7228	3082.8621	4	0.0027	29	0	GluC
H2A1F_MOUSE, H2A1H_MOUSE, H2A1_MOUSE, H2A1K_MOUSE, H2A3_MOUSE, H2AJ_MOUSE	Histone H2A	LNKLLGRVTIAQGGVLPNIQAVLLPKKTE	301	3.60E-03	771.7228	3082.8620	4	0.0026	29	0	GluC
H2A1F_MOUSE, H2A1H_MOUSE, H2A1_MOUSE, H2A1K_MOUSE, H2A3_MOUSE, H2AJ_MOUSE	Histone H2A	LNKLLGRVTIAQGGVLPNIQAVLLPKKTE	301	1.60E-04	771.7228	3082.8620	4	0.0026	29	0	GluC
H2A1F_MOUSE, H2A1H_MOUSE, H2A1_MOUSE, H2A1K_MOUSE, H2A3_MOUSE, H2AJ_MOUSE	Histone H2A	LNKLLGRVTIAQGGVLPNIQAVLLPKKTE	301	9.60E-07	771.7228	3082.8620	4	0.0026	29	0	GluC
H2A1F_MOUSE, H2A1H_MOUSE, H2A1_MOUSE, H2A1K_MOUSE, H2A3_MOUSE, H2AJ_MOUSE	Histone H2A	LNKLLGRVTIAQGGVLPNIQAVLLPKKTE	301	4.40E-03	771.7227	3082.8619	4	0.0025	29	0	GluC
H2A1F_MOUSE, H2A1H_MOUSE, H2A1_MOUSE, H2A1K_MOUSE, H2A3_MOUSE, H2AJ_MOUSE	Histone H2A	LNKLLGRVTIAQGGVLPNIQAVLLPKKTE	301	5.80E-06	771.7227	3082.8619	4	0.0025	29	0	GluC
H2A1F_MOUSE, H2A1H_MOUSE, H2A1_MOUSE, H2A1K_MOUSE, H2A3_MOUSE, H2AJ_MOUSE	Histone H2A	LNKLLGRVTIAQGGVLPNIQAVLLPKKTE	301	3.10E-09	771.7227	3082.8619	4	0.0025	29	0	GluC

H2A1F_MOUSE, H2A1H_MOUSE, H2A1_MOUSE, H2A1K_MOUSE, H2A3_MOUSE, H2AJ_MOUSE	Histone H2A	LNKLLGRVTIAQGGVLPNIQAVLLPKKTE	301	1.50E-05	771.7227	3082.8617	4	0.0023	29	0	GluC
H2A1F_MOUSE, H2A1H_MOUSE, H2A1_MOUSE, H2A1K_MOUSE, H2A3_MOUSE, H2AJ_MOUSE	Histone H2A	LNKLLGRVTIAQGGVLPNIQAVLLPKKTE	301	2.70E-06	617.5796	3082.8617	5	0.0023	29	0	GluC
H2A1F_MOUSE, H2A1H_MOUSE, H2A1_MOUSE, H2A1K_MOUSE, H2A3_MOUSE, H2AJ_MOUSE	Histone H2A	LNKLLGRVTIAQGGVLPNIQAVLLPKKTE	301	6.40E-03	1028.6278	3082.8616	3	0.0022	29	0	GluC
H2A1F_MOUSE, H2A1H_MOUSE, H2A1_MOUSE, H2A1K_MOUSE, H2A3_MOUSE, H2AJ_MOUSE	Histone H2A	LNKLLGRVTIAQGGVLPNIQAVLLPKKTE	301	4.80E-03	771.7227	3082.8616	4	0.0022	29	0	GluC
H2A1F_MOUSE, H2A1H_MOUSE, H2A1_MOUSE, H2A1K_MOUSE, H2A3_MOUSE, H2AJ_MOUSE	Histone H2A	LNKLLGRVTIAQGGVLPNIQAVLLPKKTE	301	2.90E-03	1028.6278	3082.8616	3	0.0022	29	0	GluC
H2A1F_MOUSE, H2A1H_MOUSE, H2A1_MOUSE, H2A1K_MOUSE, H2A3_MOUSE, H2AJ_MOUSE	Histone H2A	LNKLLGRVTIAQGGVLPNIQAVLLPKKTE	301	6.50E-07	771.7227	3082.8616	4	0.0022	29	0	GluC
H2A1F_MOUSE, H2A1H_MOUSE, H2A1_MOUSE, H2A1K_MOUSE, H2A3_MOUSE, H2AJ_MOUSE	Histone H2A	LNKLLGRVTIAQGGVLPNIQAVLLPKKTE	301	3.70E-07	1028.6278	3082.8616	3	0.0022	29	0	GluC
H2A1F_MOUSE, H2A1H_MOUSE, H2A1_MOUSE, H2A1K_MOUSE, H2A3_MOUSE, H2AJ_MOUSE	Histone H2A	LNKLLGRVTIAQGGVLPNIQAVLLPKKTE	301	8.40E-10	617.5796	3082.8616	5	0.0022	29	0	GluC
H2A1F_MOUSE, H2A1H_MOUSE, H2A1_MOUSE, H2A1K_MOUSE, H2A3_MOUSE, H2AJ_MOUSE	Histone H2A	LNKLLGRVTIAQGGVLPNIQAVLLPKKTE	301	6.20E-04	771.7227	3082.8615	4	0.0021	29	0	GluC
H2A1F_MOUSE, H2A1H_MOUSE, H2A1_MOUSE, H2A1K_MOUSE, H2A3_MOUSE, H2AJ_MOUSE	Histone H2A	LNKLLGRVTIAQGGVLPNIQAVLLPKKTE	301	6.70E-03	617.5796	3082.8614	5	0.0020	29	0	GluC
H2A1F_MOUSE, H2A1H_MOUSE, H2A1_MOUSE, H2A1K_MOUSE, H2A3_MOUSE, H2AJ_MOUSE	Histone H2A	LNKLLGRVTIAQGGVLPNIQAVLLPKKTE	301	1.40E-06	617.5796	3082.8614	5	0.0020	29	0	GluC
H2A1F_MOUSE, H2A1H_MOUSE, H2A1_MOUSE, H2A1K_MOUSE, H2A3_MOUSE, H2AJ_MOUSE	Histone H2A	LNKLLGRVTIAQGGVLPNIQAVLLPKKTE	301	2.60E-07	617.5796	3082.8614	5	0.0020	29	0	GluC
H2A1F_MOUSE, H2A1H_MOUSE, H2A1_MOUSE, H2A1K_MOUSE, H2A3_MOUSE, H2AJ_MOUSE	Histone H2A	LNKLLGRVTIAQGGVLPNIQAVLLPKKTE	301	5.70E-05	771.7226	3082.8613	4	0.0019	29	0	GluC
H2A1F_MOUSE, H2A1H_MOUSE, H2A1_MOUSE, H2A1K_MOUSE, H2A3_MOUSE, H2AJ_MOUSE	Histone H2A	LNKLLGRVTIAQGGVLPNIQAVLLPKKTE	301	2.50E-05	1028.6277	3082.8613	3	0.0019	29	0	GluC
H2A1F_MOUSE, H2A1H_MOUSE, H2A1_MOUSE, H2A1K_MOUSE, H2A3_MOUSE, H2AJ_MOUSE	Histone H2A	LNKLLGRVTIAQGGVLPNIQAVLLPKKTE	301	1.20E-05	771.7226	3082.8613	4	0.0019	29	0	GluC
H2A1F_MOUSE, H2A1H_MOUSE, H2A1_MOUSE, H2A1K_MOUSE, H2A3_MOUSE, H2AJ_MOUSE	Histone H2A	LNKLLGRVTIAQGGVLPNIQAVLLPKKTE	301	6.30E-06	771.7226	3082.8613	4	0.0019	29	0	GluC
H2A1F_MOUSE, H2A1H_MOUSE, H2A1_MOUSE, H2A1K_MOUSE, H2A3_MOUSE, H2AJ_MOUSE	Histone H2A	LNKLLGRVTIAQGGVLPNIQAVLLPKKTE	301	1.10E-06	1028.6277	3082.8613	3	0.0019	29	0	GluC
H2A1F_MOUSE, H2A1H_MOUSE, H2A1_MOUSE, H2A1K_MOUSE, H2A3_MOUSE, H2AJ_MOUSE	Histone H2A	LNKLLGRVTIAQGGVLPNIQAVLLPKKTE	301	2.90E-07	771.7226	3082.8613	4	0.0019	29	0	GluC
H2A1F_MOUSE, H2A1H_MOUSE, H2A1_MOUSE, H2A1K_MOUSE, H2A3_MOUSE, H2AJ_MOUSE	Histone H2A	LNKLLGRVTIAQGGVLPNIQAVLLPKKTE	301	1.30E-07	771.7226	3082.8613	4	0.0019	29	0	GluC
H2A1F_MOUSE, H2A1H_MOUSE, H2A1_MOUSE, H2A1K_MOUSE, H2A3_MOUSE, H2AJ_MOUSE	Histone H2A	LNKLLGRVTIAQGGVLPNIQAVLLPKKTE	301	8.90E-10	771.7226	3082.8613	4	0.0019	29	0	GluC
H2A1F_MOUSE, H2A1H_MOUSE, H2A1_MOUSE, H2A1K_MOUSE, H2A3_MOUSE, H2AJ_MOUSE	Histone H2A	LNKLLGRVTIAQGGVLPNIQAVLLPKKTE	301	3.10E-05	771.7226	3082.8612	4	0.0018	29	0	GluC
H2A1F_MOUSE, H2A1H_MOUSE, H2A1_MOUSE, H2A1K_MOUSE, H2A3_MOUSE, H2AJ_MOUSE	Histone H2A	LNKLLGRVTIAQGGVLPNIQAVLLPKKTE	301	1.10E-05	771.7226	3082.8612	4	0.0018	29	0	GluC
H2A1F_MOUSE, H2A1H_MOUSE, H2A1_MOUSE, H2A1K_MOUSE, H2A3_MOUSE, H2AJ_MOUSE	Histone H2A	LNKLLGRVTIAQGGVLPNIQAVLLPKKTE	301	2.10E-03	771.7225	3082.8611	4	0.0017	29	0	GluC
H2A1F_MOUSE, H2A1H_MOUSE, H2A1_MOUSE, H2A1K_MOUSE, H2A3_MOUSE, H2AJ_MOUSE	Histone H2A	LNKLLGRVTIAQGGVLPNIQAVLLPKKTE	301	3.10E-04	771.7225	3082.8611	4	0.0017	29	0	GluC
H2A1F_MOUSE, H2A1H_MOUSE, H2A1_MOUSE, H2A1K_MOUSE, H2A3_MOUSE, H2AJ_MOUSE	Histone H2A	LNKLLGRVTIAQGGVLPNIQAVLLPKKTE	301	4.30E-05	771.7225	3082.8611	4	0.0017	29	0	GluC
H2A1F_MOUSE, H2A1H_MOUSE, H2A1_MOUSE, H2A1K_MOUSE, H2A3_MOUSE, H2AJ_MOUSE	Histone H2A	LNKLLGRVTIAQGGVLPNIQAVLLPKKTE	301	5.60E-08	771.7225	3082.8611	4	0.0017	29	0	GluC
H2A1F_MOUSE, H2A1H_MOUSE, H2A1_MOUSE, H2A1K_MOUSE, H2A3_MOUSE, H2AJ_MOUSE	Histone H2A	LNKLLGRVTIAQGGVLPNIQAVLLPKKTE	301	5.70E-03	617.5795	3082.8610	5	0.0016	29	0	GluC
H2A1F_MOUSE, H2A1H_MOUSE, H2A1_MOUSE, H2A1K_MOUSE, H2A3_MOUSE, H2AJ_MOUSE	Histone H2A	LNKLLGRVTIAQGGVLPNIQAVLLPKKTE	301	3.70E-03	617.5795	3082.8610	5	0.0016	29	0	GluC
H2A1F_MOUSE, H2A1H_MOUSE, H2A1_MOUSE, H2A1K_MOUSE, H2A3_MOUSE, H2AJ_MOUSE	Histone H2A	LNKLLGRVTIAQGGVLPNIQAVLLPKKTE	301	1.10E-05	771.7225	3082.8610	4	0.0016	29	0	GluC
H2A1F_MOUSE, H2A1H_MOUSE, H2A1_MOUSE, H2A1K_MOUSE, H2A3_MOUSE, H2AJ_MOUSE	Histone H2A	LNKLLGRVTIAQGGVLPNIQAVLLPKKTE	301	9.80E-08	771.7225	3082.8610	4	0.0016	29	0	GluC
H2A1F_MOUSE, H2A1H_MOUSE, H2A1_MOUSE, H2A1K_MOUSE, H2A3_MOUSE, H2AJ_MOUSE	Histone H2A	LNKLLGRVTIAQGGVLPNIQAVLLPKKTE	301	2.30E-11	617.5795	3082.8610	5	0.0016	29	0	GluC
H2A1F_MOUSE, H2A1H_MOUSE, H2A1_MOUSE, H2A1K_MOUSE, H2A3_MOUSE, H2AJ_MOUSE	Histone H2A	LNKLLGRVTIAQGGVLPNIQAVLLPKKTE	301	2.90E-03	771.7225	3082.8609	4	0.0015	29	0	GluC
H2A1F_MOUSE, H2A1H_MOUSE, H2A1_MOUSE, H2A1K_MOUSE, H2A3_MOUSE, H2AJ_MOUSE	Histone H2A	LNKLLGRVTIAQGGVLPNIQAVLLPKKTE	301	1.80E-03	771.7225	3082.8609	4	0.0015	29	0	GluC
H2A1F_MOUSE, H2A1H_MOUSE, H2A1_MOUSE, H2A1K_MOUSE, H2A3_MOUSE, H2AJ_MOUSE	Histone H2A	LNKLLGRVTIAQGGVLPNIQAVLLPKKTE	301	1.00E-04	771.7225	3082.8609	4	0.0015	29	0	GluC
H2A1F_MOUSE, H2A1H_MOUSE, H2A1_MOUSE, H2A1K_MOUSE, H2A3_MOUSE, H2AJ_MOUSE	Histone H2A	LNKLLGRVTIAQGGVLPNIQAVLLPKKTE	301	2.60E-05	771.7225	3082.8609	4	0.0015	29	0	GluC
H2A1F_MOUSE, H2A1H_MOUSE, H2A1_MOUSE, H2A1K_MOUSE, H2A3_MOUSE, H2AJ_MOUSE	Histone H2A	LNKLLGRVTIAQGGVLPNIQAVLLPKKTE	301	7.40E-06	771.7225	3082.8609	4	0.0015	29	0	GluC
H2A1F_MOUSE, H2A1H_MOUSE, H2A1_MOUSE, H2A1K_MOUSE, H2A3_MOUSE, H2AJ_MOUSE	Histone H2A	LNKLLGRVTIAQGGVLPNIQAVLLPKKTE	301	5.30E-06	771.7225	3082.8609	4	0.0015	29	0	GluC

H2A1F_MOUSE, H2A1H_MOUSE, H2A1_MOUSE, H2A1K_MOUSE, H2A3_MOUSE, H2AJ_MOUSE	Histone H2A	LNKLLGRVTIAQGGVLPNIQAVLLPKKTE	301	5.00E-06	771.7225	3082.8609	4	0.0015	29	0	GluC
H2A1F_MOUSE, H2A1H_MOUSE, H2A1_MOUSE, H2A1K_MOUSE, H2A3_MOUSE, H2AJ_MOUSE	Histone H2A	LNKLLGRVTIAQGGVLPNIQAVLLPKKTE	301	6.40E-07	617.5794	3082.8609	5	0.0015	29	0	GluC
H2A1F_MOUSE, H2A1H_MOUSE, H2A1_MOUSE, H2A1K_MOUSE, H2A3_MOUSE, H2AJ_MOUSE	Histone H2A	LNKLLGRVTIAQGGVLPNIQAVLLPKKTE	301	3.20E-07	617.5795	3082.8609	5	0.0015	29	0	GluC
H2A1F_MOUSE, H2A1H_MOUSE, H2A1_MOUSE, H2A1K_MOUSE, H2A3_MOUSE, H2AJ_MOUSE	Histone H2A	LNKLLGRVTIAQGGVLPNIQAVLLPKKTE	301	2.40E-08	771.7225	3082.8609	4	0.0015	29	0	GluC
H2A1F_MOUSE, H2A1H_MOUSE, H2A1_MOUSE, H2A1K_MOUSE, H2A3_MOUSE, H2AJ_MOUSE	Histone H2A	LNKLLGRVTIAQGGVLPNIQAVLLPKKTE	301	1.70E-04	771.7225	3082.8608	4	0.0014	29	0	GluC
H2A1F_MOUSE, H2A1H_MOUSE, H2A1_MOUSE, H2A1K_MOUSE, H2A3_MOUSE, H2AJ_MOUSE	Histone H2A	LNKLLGRVTIAQGGVLPNIQAVLLPKKTE	301	1.50E-05	771.7225	3082.8608	4	0.0014	29	0	GluC
H2A1F_MOUSE, H2A1H_MOUSE, H2A1_MOUSE, H2A1K_MOUSE, H2A3_MOUSE, H2AJ_MOUSE	Histone H2A	LNKLLGRVTIAQGGVLPNIQAVLLPKKTE	301	2.40E-03	771.7224	3082.8607	4	0.0013	29	0	GluC
H2A1F_MOUSE, H2A1H_MOUSE, H2A1_MOUSE, H2A1K_MOUSE, H2A3_MOUSE, H2AJ_MOUSE	Histone H2A	LNKLLGRVTIAQGGVLPNIQAVLLPKKTE	301	1.30E-03	771.7225	3082.8607	4	0.0013	29	0	GluC
H2A1F_MOUSE, H2A1H_MOUSE, H2A1_MOUSE, H2A1K_MOUSE, H2A3_MOUSE, H2AJ_MOUSE	Histone H2A	LNKLLGRVTIAQGGVLPNIQAVLLPKKTE	301	5.20E-04	771.7224	3082.8607	4	0.0013	29	0	GluC
H2A1F_MOUSE, H2A1H_MOUSE, H2A1_MOUSE, H2A1K_MOUSE, H2A3_MOUSE, H2AJ_MOUSE	Histone H2A	LNKLLGRVTIAQGGVLPNIQAVLLPKKTE	301	2.70E-04	771.7225	3082.8607	4	0.0013	29	0	GluC
H2A1F_MOUSE, H2A1H_MOUSE, H2A1_MOUSE, H2A1K_MOUSE, H2A3_MOUSE, H2AJ_MOUSE	Histone H2A	LNKLLGRVTIAQGGVLPNIQAVLLPKKTE	301	1.50E-05	771.7225	3082.8607	4	0.0013	29	0	GluC
H2A1F_MOUSE, H2A1H_MOUSE, H2A1_MOUSE, H2A1K_MOUSE, H2A3_MOUSE, H2AJ_MOUSE	Histone H2A	LNKLLGRVTIAQGGVLPNIQAVLLPKKTE	301	9.50E-07	771.7224	3082.8607	4	0.0013	29	0	GluC
H2A1F_MOUSE, H2A1H_MOUSE, H2A1_MOUSE, H2A1K_MOUSE, H2A3_MOUSE, H2AJ_MOUSE	Histone H2A	LNKLLGRVTIAQGGVLPNIQAVLLPKKTE	301	1.60E-07	1028.6275	3082.8607	3	0.0013	29	0	GluC
H2A1F_MOUSE, H2A1H_MOUSE, H2A1_MOUSE, H2A1K_MOUSE, H2A3_MOUSE, H2AJ_MOUSE	Histone H2A	LNKLLGRVTIAQGGVLPNIQAVLLPKKTE	301	2.80E-08	771.7224	3082.8607	4	0.0013	29	0	GluC
H2A1F_MOUSE, H2A1H_MOUSE, H2A1_MOUSE, H2A1K_MOUSE, H2A3_MOUSE, H2AJ_MOUSE	Histone H2A	LNKLLGRVTIAQGGVLPNIQAVLLPKKTE	301	8.60E-03	617.5794	3082.8606	5	0.0012	29	0	GluC
H2A1F_MOUSE, H2A1H_MOUSE, H2A1_MOUSE, H2A1K_MOUSE, H2A3_MOUSE, H2AJ_MOUSE	Histone H2A	LNKLLGRVTIAQGGVLPNIQAVLLPKKTE	301	3.50E-03	514.8174	3082.8606	6	0.0012	29	0	GluC
H2A1F_MOUSE, H2A1H_MOUSE, H2A1_MOUSE, H2A1K_MOUSE, H2A3_MOUSE, H2AJ_MOUSE	Histone H2A	LNKLLGRVTIAQGGVLPNIQAVLLPKKTE	301	1.90E-04	771.7224	3082.8606	4	0.0012	29	0	GluC
H2A1F_MOUSE, H2A1H_MOUSE, H2A1_MOUSE, H2A1K_MOUSE, H2A3_MOUSE, H2AJ_MOUSE	Histone H2A	LNKLLGRVTIAQGGVLPNIQAVLLPKKTE	301	5.10E-06	617.5794	3082.8606	5	0.0012	29	0	GluC
H2A1F_MOUSE, H2A1H_MOUSE, H2A1_MOUSE, H2A1K_MOUSE, H2A3_MOUSE, H2AJ_MOUSE	Histone H2A	LNKLLGRVTIAQGGVLPNIQAVLLPKKTE	301	2.00E-07	771.7224	3082.8606	4	0.0012	29	0	GluC
H2A1F_MOUSE, H2A1H_MOUSE, H2A1_MOUSE, H2A1K_MOUSE, H2A3_MOUSE, H2AJ_MOUSE	Histone H2A	LNKLLGRVTIAQGGVLPNIQAVLLPKKTE	301	1.30E-07	771.7224	3082.8606	4	0.0012	29	0	GluC
H2A1F_MOUSE, H2A1H_MOUSE, H2A1_MOUSE, H2A1K_MOUSE, H2A3_MOUSE, H2AJ_MOUSE	Histone H2A	LNKLLGRVTIAQGGVLPNIQAVLLPKKTE	301	1.50E-08	771.7224	3082.8606	4	0.0012	29	0	GluC
H2A1F_MOUSE, H2A1H_MOUSE, H2A1_MOUSE, H2A1K_MOUSE, H2A3_MOUSE, H2AJ_MOUSE	Histone H2A	LNKLLGRVTIAQGGVLPNIQAVLLPKKTE	301	5.50E-05	1028.6274	3082.8604	3	0.0010	29	0	GluC
H2A1F_MOUSE, H2A1H_MOUSE, H2A1_MOUSE, H2A1K_MOUSE, H2A3_MOUSE, H2AJ_MOUSE	Histone H2A	LNKLLGRVTIAQGGVLPNIQAVLLPKKTE	301	5.40E-07	1028.6274	3082.8604	3	0.0010	29	0	GluC
H2A1F_MOUSE, H2A1H_MOUSE, H2A1_MOUSE, H2A1K_MOUSE, H2A3_MOUSE, H2AJ_MOUSE	Histone H2A	LNKLLGRVTIAQGGVLPNIQAVLLPKKTE	301	2.20E-08	617.5793	3082.8604	5	0.0010	29	0	GluC
H2A1F_MOUSE, H2A1H_MOUSE, H2A1_MOUSE, H2A1K_MOUSE, H2A3_MOUSE, H2AJ_MOUSE	Histone H2A	LNKLLGRVTIAQGGVLPNIQAVLLPKKTE	301	1.50E-03	771.7223	3082.8603	4	0.0009	29	0	GluC
H2A1F_MOUSE, H2A1H_MOUSE, H2A1_MOUSE, H2A1K_MOUSE, H2A3_MOUSE, H2AJ_MOUSE	Histone H2A	LNKLLGRVTIAQGGVLPNIQAVLLPKKTE	301	1.50E-04	617.5793	3082.8603	5	0.0009	29	0	GluC
H2A1F_MOUSE, H2A1H_MOUSE, H2A1_MOUSE, H2A1K_MOUSE, H2A3_MOUSE, H2AJ_MOUSE	Histone H2A	LNKLLGRVTIAQGGVLPNIQAVLLPKKTE	301	2.60E-05	771.7223	3082.8603	4	0.0009	29	0	GluC
H2A1F_MOUSE, H2A1H_MOUSE, H2A1_MOUSE, H2A1K_MOUSE, H2A3_MOUSE, H2AJ_MOUSE	Histone H2A	LNKLLGRVTIAQGGVLPNIQAVLLPKKTE	301	6.30E-06	771.7223	3082.8603	4	0.0009	29	0	GluC
H2A1F_MOUSE, H2A1H_MOUSE, H2A1_MOUSE, H2A1K_MOUSE, H2A3_MOUSE, H2AJ_MOUSE	Histone H2A	LNKLLGRVTIAQGGVLPNIQAVLLPKKTE	301	2.20E-07	771.7223	3082.8603	4	0.0009	29	0	GluC
H2A1F_MOUSE, H2A1H_MOUSE, H2A1_MOUSE, H2A1K_MOUSE, H2A3_MOUSE, H2AJ_MOUSE	Histone H2A	LNKLLGRVTIAQGGVLPNIQAVLLPKKTE	301	3.10E-04	617.5793	3082.8602	5	0.0008	29	0	GluC
H2A1F_MOUSE, H2A1H_MOUSE, H2A1_MOUSE, H2A1K_MOUSE, H2A3_MOUSE, H2AJ_MOUSE	Histone H2A	LNKLLGRVTIAQGGVLPNIQAVLLPKKTE	301	2.20E-05	617.5793	3082.8602	5	0.0008	29	0	GluC
H2A1F_MOUSE, H2A1H_MOUSE, H2A1_MOUSE, H2A1K_MOUSE, H2A3_MOUSE, H2AJ_MOUSE	Histone H2A	LNKLLGRVTIAQGGVLPNIQAVLLPKKTE	301	1.10E-03	771.7223	3082.8601	4	0.0007	29	0	GluC
H2A1F_MOUSE, H2A1H_MOUSE, H2A1_MOUSE, H2A1K_MOUSE, H2A3_MOUSE, H2AJ_MOUSE	Histone H2A	LNKLLGRVTIAQGGVLPNIQAVLLPKKTE	301	6.10E-04	771.7223	3082.8601	4	0.0007	29	0	GluC
H2A1F_MOUSE, H2A1H_MOUSE, H2A1_MOUSE, H2A1K_MOUSE, H2A3_MOUSE, H2AJ_MOUSE	Histone H2A	LNKLLGRVTIAQGGVLPNIQAVLLPKKTE	301	4.30E-04	771.7223	3082.8601	4	0.0007	29	0	GluC
H2A1F_MOUSE, H2A1H_MOUSE, H2A1_MOUSE, H2A1K_MOUSE, H2A3_MOUSE, H2AJ_MOUSE	Histone H2A	LNKLLGRVTIAQGGVLPNIQAVLLPKKTE	301	8.10E-05	771.7223	3082.8601	4	0.0007	29	0	GluC
H2A1F_MOUSE, H2A1H_MOUSE, H2A1_MOUSE, H2A1K_MOUSE, H2A3_MOUSE, H2AJ_MOUSE	Histone H2A	LNKLLGRVTIAQGGVLPNIQAVLLPKKTE	301	4.50E-06	771.7223	3082.8601	4	0.0007	29	0	GluC
H2A1F_MOUSE, H2A1H_MOUSE, H2A1_MOUSE, H2A1K_MOUSE, H2A3_MOUSE, H2AJ_MOUSE	Histone H2A	LNKLLGRVTIAQGGVLPNIQAVLLPKKTE	301	5.60E-07	771.7223	3082.8601	4	0.0007	29	0	GluC

H2A1F_MOUSE, H2A1H_MOUSE, H2A1_MOUSE, H2A1K_MOUSE, H2A3_MOUSE, H2AJ_MOUSE	Histone H2A	LNKLLGRVTIAQGGVLPNIQAVLLPKKTE	301	3.90E-04	771.7223	3082.8600	4	0.0006	29	0	GluC
H2A1F_MOUSE, H2A1H_MOUSE, H2A1_MOUSE, H2A1K_MOUSE, H2A3_MOUSE, H2AJ_MOUSE	Histone H2A	LNKLLGRVTIAQGGVLPNIQAVLLPKKTE	301	1.10E-04	771.7223	3082.8600	4	0.0006	29	0	GluC
H2A1F_MOUSE, H2A1H_MOUSE, H2A1_MOUSE, H2A1K_MOUSE, H2A3_MOUSE, H2AJ_MOUSE	Histone H2A	LNKLLGRVTIAQGGVLPNIQAVLLPKKTE	301	9.80E-04	771.7223	3082.8599	4	0.0005	29	0	GluC
H2A1F_MOUSE, H2A1H_MOUSE, H2A1_MOUSE, H2A1K_MOUSE, H2A3_MOUSE, H2AJ_MOUSE	Histone H2A	LNKLLGRVTIAQGGVLPNIQAVLLPKKTE	301	2.00E-06	771.7223	3082.8599	4	0.0005	29	0	GluC
H2A1F_MOUSE, H2A1H_MOUSE, H2A1_MOUSE, H2A1K_MOUSE, H2A3_MOUSE, H2AJ_MOUSE	Histone H2A	LNKLLGRVTIAQGGVLPNIQAVLLPKKTE	301	2.00E-06	617.5792	3082.8598	5	0.0004	29	0	GluC
H2A1F_MOUSE, H2A1H_MOUSE, H2A1_MOUSE, H2A1K_MOUSE, H2A3_MOUSE, H2AJ_MOUSE	Histone H2A	LNKLLGRVTIAQGGVLPNIQAVLLPKKTE	301	1.00E-08	617.5792	3082.8598	5	0.0004	29	0	GluC
H2A1F_MOUSE, H2A1H_MOUSE, H2A1_MOUSE, H2A1K_MOUSE, H2A3_MOUSE, H2AJ_MOUSE	Histone H2A	LNKLLGRVTIAQGGVLPNIQAVLLPKKTE	301	5.80E-04	771.7222	3082.8597	4	0.0003	29	0	GluC
H2A1F_MOUSE, H2A1H_MOUSE, H2A1_MOUSE, H2A1K_MOUSE, H2A3_MOUSE, H2AJ_MOUSE	Histone H2A	LNKLLGRVTIAQGGVLPNIQAVLLPKKTE	301	6.50E-04	771.7222	3082.8596	4	0.0002	29	0	GluC
H2A1F_MOUSE, H2A1H_MOUSE, H2A1_MOUSE, H2A1K_MOUSE, H2A3_MOUSE, H2AJ_MOUSE	Histone H2A	LNKLLGRVTIAQGGVLPNIQAVLLPKKTE	301	7.90E-05	617.5792	3082.8596	5	0.0002	29	0	GluC
H2A1F_MOUSE, H2A1H_MOUSE, H2A1_MOUSE, H2A1K_MOUSE, H2A3_MOUSE, H2AJ_MOUSE	Histone H2A	LNKLLGRVTIAQGGVLPNIQAVLLPKKTE	301	3.20E-06	771.7222	3082.8596	4	0.0002	29	0	GluC
H2A1F_MOUSE, H2A1H_MOUSE, H2A1_MOUSE, H2A1K_MOUSE, H2A3_MOUSE, H2AJ_MOUSE	Histone H2A	LNKLLGRVTIAQGGVLPNIQAVLLPKKTE	301	1.10E-03	771.7222	3082.8595	4	0.0001	29	0	GluC
H2A1F_MOUSE, H2A1H_MOUSE, H2A1_MOUSE, H2A1K_MOUSE, H2A3_MOUSE, H2AJ_MOUSE	Histone H2A	LNKLLGRVTIAQGGVLPNIQAVLLPKKTE	301	3.00E-04	771.7222	3082.8595	4	0.0001	29	0	GluC
H2A1F_MOUSE, H2A1H_MOUSE, H2A1_MOUSE, H2A1K_MOUSE, H2A3_MOUSE, H2AJ_MOUSE	Histone H2A	LNKLLGRVTIAQGGVLPNIQAVLLPKKTE	301	7.00E-07	771.7222	3082.8595	4	0.0001	29	0	GluC
H2A1F_MOUSE, H2A1H_MOUSE, H2A1_MOUSE, H2A1K_MOUSE, H2A3_MOUSE, H2AJ_MOUSE	Histone H2A	LNKLLGRVTIAQGGVLPNIQAVLLPKKTE	301	5.50E-07	771.7222	3082.8595	4	0.0001	29	0	GluC
H2A1F_MOUSE, H2A1H_MOUSE, H2A1_MOUSE, H2A1K_MOUSE, H2A3_MOUSE, H2AJ_MOUSE	Histone H2A	LNKLLGRVTIAQGGVLPNIQAVLLPKKTE	301	3.90E-04	771.7221	3082.8594	4	0.0000	29	0	GluC
H2A1F_MOUSE, H2A1H_MOUSE, H2A1_MOUSE, H2A1K_MOUSE, H2A3_MOUSE, H2AJ_MOUSE	Histone H2A	LNKLLGRVTIAQGGVLPNIQAVLLPKKTE	301	8.10E-03	514.8172	3082.8593	6	-0.0001	29	0	GluC
H2A1F_MOUSE, H2A1H_MOUSE, H2A1_MOUSE, H2A1K_MOUSE, H2A3_MOUSE, H2AJ_MOUSE	Histone H2A	LNKLLGRVTIAQGGVLPNIQAVLLPKKTE	301	3.40E-06	617.5791	3082.8593	5	-0.0001	29	0	GluC
H2A1F_MOUSE, H2A1H_MOUSE, H2A1_MOUSE, H2A1K_MOUSE, H2A3_MOUSE, H2AJ_MOUSE	Histone H2A	LNKLLGRVTIAQGGVLPNIQAVLLPKKTE	301	4.40E-08	617.5791	3082.8593	5	-0.0001	29	0	GluC
H2A1F_MOUSE, H2A1H_MOUSE, H2A1_MOUSE, H2A1K_MOUSE, H2A3_MOUSE, H2AJ_MOUSE	Histone H2A	LNKLLGRVTIAQGGVLPNIQAVLLPKKTE	301	3.10E-03	771.7221	3082.8592	4	-0.0002	29	0	GluC
H2A1F_MOUSE, H2A1H_MOUSE, H2A1_MOUSE, H2A1K_MOUSE, H2A3_MOUSE, H2AJ_MOUSE	Histone H2A	LNKLLGRVTIAQGGVLPNIQAVLLPKKTE	301	9.00E-05	771.7221	3082.8592	4	-0.0002	29	0	GluC
H2A1F_MOUSE, H2A1H_MOUSE, H2A1_MOUSE, H2A1K_MOUSE, H2A3_MOUSE, H2AJ_MOUSE	Histone H2A	LNKLLGRVTIAQGGVLPNIQAVLLPKKTE	301	3.90E-06	771.7221	3082.8592	4	-0.0002	29	0	GluC
H2A1F_MOUSE, H2A1H_MOUSE, H2A1_MOUSE, H2A1K_MOUSE, H2A3_MOUSE, H2AJ_MOUSE	Histone H2A	LNKLLGRVTIAQGGVLPNIQAVLLPKKTE	301	1.80E-07	617.5791	3082.8592	5	-0.0002	29	0	GluC
H2A1F_MOUSE, H2A1H_MOUSE, H2A1_MOUSE, H2A1K_MOUSE, H2A3_MOUSE, H2AJ_MOUSE	Histone H2A	LNKLLGRVTIAQGGVLPNIQAVLLPKKTE	301	1.00E-03	771.7221	3082.8591	4	-0.0003	29	0	GluC
H2A1F_MOUSE, H2A1H_MOUSE, H2A1_MOUSE, H2A1K_MOUSE, H2A3_MOUSE, H2AJ_MOUSE	Histone H2A	LNKLLGRVTIAQGGVLPNIQAVLLPKKTE	301	1.90E-06	771.7221	3082.8591	4	-0.0003	29	0	GluC
H2A1F_MOUSE, H2A1H_MOUSE, H2A1_MOUSE, H2A1K_MOUSE, H2A3_MOUSE, H2AJ_MOUSE	Histone H2A	LNKLLGRVTIAQGGVLPNIQAVLLPKKTE	301	1.50E-04	771.722	3082.8590	4	-0.0004	29	0	GluC
H2A1F_MOUSE, H2A1H_MOUSE, H2A1_MOUSE, H2A1K_MOUSE, H2A3_MOUSE, H2AJ_MOUSE	Histone H2A	LNKLLGRVTIAQGGVLPNIQAVLLPKKTE	301	7.90E-07	771.722	3082.8590	4	-0.0004	29	0	GluC
H2A1F_MOUSE, H2A1H_MOUSE, H2A1_MOUSE, H2A1K_MOUSE, H2A3_MOUSE, H2AJ_MOUSE	Histone H2A	LNKLLGRVTIAQGGVLPNIQAVLLPKKTE	301	7.80E-05	1542.4367	3082.8588	2	-0.0005	29	0	GluC
H2A1F_MOUSE, H2A1H_MOUSE, H2A1_MOUSE, H2A1K_MOUSE, H2A3_MOUSE, H2AJ_MOUSE	Histone H2A	LNKLLGRVTIAQGGVLPNIQAVLLPKKTE	301	6.60E-06	1028.6269	3082.8589	3	-0.0005	29	0	GluC
H2A1F_MOUSE, H2A1H_MOUSE, H2A1_MOUSE, H2A1K_MOUSE, H2A3_MOUSE, H2AJ_MOUSE	Histone H2A	LNKLLGRVTIAQGGVLPNIQAVLLPKKTE	301	4.60E-03	771.722	3082.8588	4	-0.0006	29	0	GluC
H2A1F_MOUSE, H2A1H_MOUSE, H2A1_MOUSE, H2A1K_MOUSE, H2A3_MOUSE, H2AJ_MOUSE	Histone H2A	LNKLLGRVTIAQGGVLPNIQAVLLPKKTE	301	2.30E-04	771.722	3082.8588	4	-0.0006	29	0	GluC
H2A1F_MOUSE, H2A1H_MOUSE, H2A1_MOUSE, H2A1K_MOUSE, H2A3_MOUSE, H2AJ_MOUSE	Histone H2A	LNKLLGRVTIAQGGVLPNIQAVLLPKKTE	301	6.90E-08	771.7219	3082.8587	4	-0.0007	29	0	GluC
H2A1F_MOUSE, H2A1H_MOUSE, H2A1_MOUSE, H2A1K_MOUSE, H2A3_MOUSE, H2AJ_MOUSE	Histone H2A	LNKLLGRVTIAQGGVLPNIQAVLLPKKTE	301	9.60E-07	617.5789	3082.8584	5	-0.0010	29	0	GluC
H2A1F_MOUSE, H2A1H_MOUSE, H2A1_MOUSE, H2A1K_MOUSE, H2A3_MOUSE, H2AJ_MOUSE	Histone H2A	LNKLLGRVTIAQGGVLPNIQAVLLPKKTE	301	2.30E-09	617.5789	3082.8583	5	-0.0011	29	0	GluC
H2A1F_MOUSE, H2A1H_MOUSE, H2A1_MOUSE, H2A1K_MOUSE, H2A3_MOUSE, H2AJ_MOUSE	Histone H2A	LNKLLGRVTIAQGGVLPNIQAVLLPKKTE	301	1.30E-05	771.7218	3082.8581	4	-0.0013	29	0	GluC
H2A1F_MOUSE, H2A1H_MOUSE, H2A1_MOUSE, H2A1K_MOUSE, H2A3_MOUSE, H2AJ_MOUSE	Histone H2A	LNKLLGRVTIAQGGVLPNIQAVLLPKKTE	301	1.10E-06	771.7218	3082.8581	4	-0.0013	29	0	GluC
H2A1F_MOUSE, H2A1H_MOUSE, H2A1_MOUSE, H2A1K_MOUSE, H2A3_MOUSE, H2AJ_MOUSE	Histone H2A	LNKLLGRVTIAQGGVLPNIQAVLLPKKTE	301	4.30E-03	1028.6266	3082.8580	3	-0.0014	29	0	GluC
H2A1F_MOUSE, H2A1H_MOUSE, H2A1_MOUSE, H2A1K_MOUSE, H2A3_MOUSE, H2AJ_MOUSE	Histone H2A	LNKLLGRVTIAQGGVLPNIQAVLLPKKTE	301	3.80E-05	1028.6266	3082.8580	3	-0.0014	29	0	GluC

H2A1F_MOUSE, H2A1H_MOUSE, H2A1_MOUSE, H2A1K_MOUSE, H2A3_MOUSE, H2AJ_MOUSE	Histone H2A	LNKLLGRVTIAQGGVLPNIQAVLLPKKTE	301	7.00E-04	771.7217	3082.8578	4	-0.0016	29	0	GluC
H2A1F_MOUSE, H2A1H_MOUSE, H2A1_MOUSE, H2A1K_MOUSE, H2A3_MOUSE, H2AJ_MOUSE	Histone H2A	LNKLLGRVTIAQGGVLPNIQAVLLPKKTE	301	3.20E-04	1028.6265	3082.8577	3	-0.0017	29	0	GluC
H2A1F_MOUSE, H2A1H_MOUSE, H2A1_MOUSE, H2A1K_MOUSE, H2A3_MOUSE, H2AJ_MOUSE	Histone H2A	LNKLLGRVTIAQGGVLPNIQAVLLPKKTE	301	1.20E-04	771.7217	3082.8577	4	-0.0017	29	0	GluC
H2A1F_MOUSE, H2A1H_MOUSE, H2A1_MOUSE, H2A1K_MOUSE, H2A3_MOUSE, H2AJ_MOUSE	Histone H2A	IIPRHLQLAIRNDEELNKLLGR	3	5.30E-04	653.6344	2610.5084	4	0.0003	22	3	Semi-tryptic
H2A1F_MOUSE, H2A1H_MOUSE, H2A1_MOUSE, H2A1K_MOUSE, H2A3_MOUSE, H2AJ_MOUSE	Histone H2A	IIPRHLQLAIRNDEELNKLLGR	3	3.40E-06	871.1763	2610.5072	3	-0.0009	22	3	Semi-tryptic
H2A1F_MOUSE, H2A1H_MOUSE, H2A1_MOUSE, H2A1K_MOUSE, H2A3_MOUSE, H2AJ_MOUSE	Histone H2A	IIPRHLQLAIRNDEELNKLLGR	3	5.00E-04	653.6337	2610.5056	4	-0.0025	22	3	Semi-tryptic
H2A1F_MOUSE, H2A1H_MOUSE, H2A1_MOUSE, H2A1K_MOUSE, H2A3_MOUSE, H2AJ_MOUSE	Histone H2A	LGRVTIAQGGVLPNIQAVL	6	2.40E-03	960.0767	1918.1389	2	0.0025	19	1	Chymotrypsin
H2A1F_MOUSE, H2A1H_MOUSE, H2A1_MOUSE, H2A1K_MOUSE, H2A3_MOUSE, H2AJ_MOUSE	Histone H2A	LGRVTIAQGGVLPNIQAVL	6	1.00E-04	960.0767	1918.1389	2	0.0025	19	1	Chymotrypsin
H2A1F_MOUSE, H2A1H_MOUSE, H2A1_MOUSE, H2A1K_MOUSE, H2A3_MOUSE, H2AJ_MOUSE	Histone H2A	LGRVTIAQGGVLPNIQAVL	6	4.70E-03	640.3866	1918.1381	3	0.0017	19	1	Chymotrypsin
H2A1F_MOUSE, H2A1H_MOUSE, H2A1_MOUSE, H2A1K_MOUSE, H2A3_MOUSE, H2AJ_MOUSE	Histone H2A	LGRVTIAQGGVLPNIQAVL	6	8.80E-03	960.0761	1918.1377	2	0.0014	19	1	Chymotrypsin
H2A1F_MOUSE, H2A1H_MOUSE, H2A1_MOUSE, H2A1K_MOUSE, H2A3_MOUSE, H2AJ_MOUSE	Histone H2A	LGRVTIAQGGVLPNIQAVL	6	1.40E-05	960.075	1918.1354	2	-0.0010	19	1	Chymotrypsin
H2A1F_MOUSE, H2A1H_MOUSE, H2A1_MOUSE, H2A1K_MOUSE, H2A3_MOUSE, H2AJ_MOUSE	Histone H2A	LGRVTIAQGGVLPNIQAVL	6	7.50E-04	960.0709	1918.1273	2	-0.0091	19	1	Chymotrypsin
H2A1F_MOUSE, H2A1H_MOUSE, H2A1_MOUSE, H2A1K_MOUSE, H2A3_MOUSE, H2AJ_MOUSE	Histone H2A	GRVTIAQGGVLPNIQAVL	8	4.30E-03	903.5364	1805.0583	2	0.0060	18	0	Chymotrypsin
H2A1F_MOUSE, H2A1H_MOUSE, H2A1_MOUSE, H2A1K_MOUSE, H2A3_MOUSE, H2AJ_MOUSE	Histone H2A	GRVTIAQGGVLPNIQAVL	8	1.20E-04	903.5352	1805.0558	2	0.0035	18	0	Chymotrypsin
H2A1F_MOUSE, H2A1H_MOUSE, H2A1_MOUSE, H2A1K_MOUSE, H2A3_MOUSE, H2AJ_MOUSE	Histone H2A	GRVTIAQGGVLPNIQAVL	8	1.80E-09	602.6925	1805.0556	3	0.0032	18	0	Chymotrypsin
H2A1F_MOUSE, H2A1H_MOUSE, H2A1_MOUSE, H2A1K_MOUSE, H2A3_MOUSE, H2AJ_MOUSE	Histone H2A	GRVTIAQGGVLPNIQAVL	8	2.90E-03	903.5344	1805.0541	2	0.0018	18	0	Chymotrypsin
H2A1F_MOUSE, H2A1H_MOUSE, H2A1_MOUSE, H2A1K_MOUSE, H2A3_MOUSE, H2AJ_MOUSE	Histone H2A	GRVTIAQGGVLPNIQAVL	8	7.90E-05	903.5344	1805.0541	2	0.0018	18	0	Chymotrypsin
H2A1F_MOUSE, H2A1H_MOUSE, H2A1_MOUSE, H2A1K_MOUSE, H2A3_MOUSE, H2AJ_MOUSE	Histone H2A	GRVTIAQGGVLPNIQAVL	8	7.60E-04	602.6918	1805.0535	3	0.0012	18	0	Chymotrypsin
H2A1F_MOUSE, H2A1H_MOUSE, H2A1_MOUSE, H2A1K_MOUSE, H2A3_MOUSE, H2AJ_MOUSE	Histone H2A	GRVTIAQGGVLPNIQAVL	8	1.10E-04	903.5338	1805.0531	2	0.0008	18	0	Chymotrypsin
H2A1F_MOUSE, H2A1H_MOUSE, H2A1_MOUSE, H2A1K_MOUSE, H2A3_MOUSE, H2AJ_MOUSE	Histone H2A	GRVTIAQGGVLPNIQAVL	8	7.00E-04	903.5331	1805.0517	2	-0.0006	18	0	Chymotrypsin
H2A1F_MOUSE, H2A1H_MOUSE, H2A1_MOUSE, H2A1K_MOUSE, H2A3_MOUSE, H2AJ_MOUSE	Histone H2A	HLQLAIRNDEELNKLLGR	17	4.90E-08	1066.5993	2131.1840	2	-0.0021	18	2	Semi-tryptic
H2A1F_MOUSE, H2A1H_MOUSE, H2A1_MOUSE, H2A1K_MOUSE, H2A3_MOUSE, H2AJ_MOUSE	Histone H2A	HLQLAIRNDEELNKLLGR	17	4.70E-03	533.803	2131.1829	4	-0.0032	18	2	Semi-tryptic
H2A1F_MOUSE, H2A1H_MOUSE, H2A1_MOUSE, H2A1K_MOUSE, H2A3_MOUSE, H2AJ_MOUSE	Histone H2A	HLQLAIRNDEELNKLLGR	17	3.00E-09	711.4014	2131.1823	3	-0.0038	18	2	Semi-tryptic
H2A1F_MOUSE, H2A1H_MOUSE, H2A1_MOUSE, H2A1K_MOUSE, H2A3_MOUSE, H2AJ_MOUSE	Histone H2A	HLQLAIRNDEELNKLLGR	17	1.70E-03	533.8028	2131.1820	4	-0.0041	18	2	Semi-tryptic
H2A1F_MOUSE, H2A1H_MOUSE, H2A1_MOUSE, H2A1K_MOUSE, H2A3_MOUSE, H2AJ_MOUSE	Histone H2A	HLQLAIRNDEELNKLLGR	17	1.20E-06	711.4013	2131.1820	3	-0.0041	18	2	Semi-tryptic
H2A1F_MOUSE, H2A1H_MOUSE, H2A1_MOUSE, H2A1K_MOUSE, H2A3_MOUSE, H2AJ_MOUSE	Histone H2A	HLQLAIRNDEELNKLLGR	17	8.30E-04	533.8027	2131.1816	4	-0.0045	18	2	Semi-tryptic
H2A1F_MOUSE, H2A1H_MOUSE, H2A1_MOUSE, H2A1K_MOUSE, H2A3_MOUSE, H2AJ_MOUSE	Histone H2A	HLQLAIRNDEELNKLLGR	17	1.80E-07	711.4034	2131.1883	3	0.0022	18	2	Semi-tryptic
H2A1F_MOUSE, H2A1H_MOUSE, H2A1_MOUSE, H2A1K_MOUSE, H2A3_MOUSE, H2AJ_MOUSE	Histone H2A	HLQLAIRNDEELNKLLGR	17	1.10E-05	711.403	2131.1872	3	0.0011	18	2	Semi-tryptic
H2A1F_MOUSE, H2A1H_MOUSE, H2A1_MOUSE, H2A1K_MOUSE, H2A3_MOUSE, H2AJ_MOUSE	Histone H2A	HLQLAIRNDEELNKLLGR	17	7.00E-07	711.403	2131.1872	3	0.0011	18	2	Semi-tryptic
H2A1F_MOUSE, H2A1H_MOUSE, H2A1_MOUSE, H2A1K_MOUSE, H2A3_MOUSE, H2AJ_MOUSE	Histone H2A	HLQLAIRNDEELNKLLGR	17	2.00E-06	711.403	2131.1871	3	0.0010	18	2	Semi-tryptic
H2A1F_MOUSE, H2A1H_MOUSE, H2A1_MOUSE, H2A1K_MOUSE, H2A3_MOUSE, H2AJ_MOUSE	Histone H2A	HLQLAIRNDEELNKLLGR	17	1.90E-03	533.804	2131.1870	4	0.0009	18	2	Semi-tryptic
H2A1F_MOUSE, H2A1H_MOUSE, H2A1_MOUSE, H2A1K_MOUSE, H2A3_MOUSE, H2AJ_MOUSE	Histone H2A	HLQLAIRNDEELNKLLGR	17	2.30E-07	711.403	2131.1870	3	0.0009	18	2	Semi-tryptic
H2A1F_MOUSE, H2A1H_MOUSE, H2A1_MOUSE, H2A1K_MOUSE, H2A3_MOUSE, H2AJ_MOUSE	Histone H2A	HLQLAIRNDEELNKLLGR	17	4.90E-03	533.804	2131.1868	4	0.0007	18	2	Semi-tryptic
H2A1F_MOUSE, H2A1H_MOUSE, H2A1_MOUSE, H2A1K_MOUSE, H2A3_MOUSE, H2AJ_MOUSE	Histone H2A	HLQLAIRNDEELNKLLGR	17	7.60E-04	533.8039	2131.1865	4	0.0004	18	2	Semi-tryptic
H2A1F_MOUSE, H2A1H_MOUSE, H2A1_MOUSE, H2A1K_MOUSE, H2A3_MOUSE, H2AJ_MOUSE	Histone H2A	HLQLAIRNDEELNKLLGR	17	5.90E-03	533.8039	2131.1865	4	0.0003	18	2	Semi-tryptic
H2A1F_MOUSE, H2A1H_MOUSE, H2A1_MOUSE, H2A1K_MOUSE, H2A3_MOUSE, H2AJ_MOUSE	Histone H2A	HLQLAIRNDEELNKLLGR	17	4.90E-07	711.4028	2131.1865	3	0.0003	18	2	Semi-tryptic
H2A1F_MOUSE, H2A1H_MOUSE, H2A1_MOUSE, H2A1K_MOUSE, H2A3_MOUSE, H2AJ_MOUSE	Histone H2A	HLQLAIRNDEELNKLLGR	17	1.90E-07	711.4027	2131.1864	3	0.0003	18	2	Semi-tryptic

H2A1F_MOUSE, H2A1H_MOUSE, H2A1_MOUSE, H2A1K_MOUSE, H2A3_MOUSE, H2AJ_MOUSE	Histone H2A	NDEELNKLLGR	26	1.20E-07	650.8454	1299.6763	2	-0.0019	11	1	Semi-tryptic
H2A1F_MOUSE, H2A1H_MOUSE, H2A1_MOUSE, H2A1K_MOUSE, H2A3_MOUSE, H2AJ_MOUSE	Histone H2A	NDEELNKLLGR	26	2.30E-03	434.2326	1299.6761	3	-0.0021	11	1	Semi-tryptic
H2A1F_MOUSE, H2A1H_MOUSE, H2A1_MOUSE, H2A1K_MOUSE, H2A3_MOUSE, H2AJ_MOUSE	Histone H2A	NDEELNKLLGR	26	2.00E-06	650.8453	1299.6761	2	-0.0021	11	1	Semi-tryptic
H2A1F_MOUSE, H2A1H_MOUSE, H2A1_MOUSE, H2A1K_MOUSE, H2A3_MOUSE, H2AJ_MOUSE	Histone H2A	NDEELNKLLGR	26	7.70E-07	650.8449	1299.6752	2	-0.0030	11	1	Semi-tryptic
H2A1F_MOUSE, H2A1H_MOUSE, H2A1_MOUSE, H2A1K_MOUSE, H2A3_MOUSE, H2AJ_MOUSE	Histone H2A	NDEELNKLLGR	26	4.50E-06	650.8448	1299.6750	2	-0.0032	11	1	Semi-tryptic
H2A1F_MOUSE, H2A1H_MOUSE, H2A1_MOUSE, H2A1K_MOUSE, H2A3_MOUSE, H2AJ_MOUSE	Histone H2A	NDEELNKLLGR	26	7.40E-06	650.8497	1299.6849	2	0.0067	11	1	Semi-tryptic
H2A1F_MOUSE, H2A1H_MOUSE, H2A1_MOUSE, H2A1K_MOUSE, H2A3_MOUSE, H2AJ_MOUSE	Histone H2A	NDEELNKLLGR	26	1.60E-03	650.8486	1299.6826	2	0.0044	11	1	Semi-tryptic
H2A1F_MOUSE, H2A1H_MOUSE, H2A1_MOUSE, H2A1K_MOUSE, H2A3_MOUSE, H2AJ_MOUSE	Histone H2A	NDEELNKLLGR	26	9.80E-04	650.8472	1299.6798	2	0.0015	11	1	Semi-tryptic
H2A1F_MOUSE, H2A1H_MOUSE, H2A1_MOUSE, H2A1K_MOUSE, H2A3_MOUSE, H2AJ_MOUSE	Histone H2A	NDEELNKLLGR	26	4.20E-05	434.2338	1299.6795	3	0.0013	11	1	Semi-tryptic
H2A1F_MOUSE, H2A1H_MOUSE, H2A1_MOUSE, H2A1K_MOUSE, H2A3_MOUSE, H2AJ_MOUSE	Histone H2A	NDEELNKLLGR	26	5.50E-06	434.2338	1299.6795	3	0.0013	11	1	Semi-tryptic
H2A1F_MOUSE, H2A1H_MOUSE, H2A1_MOUSE, H2A1K_MOUSE, H2A3_MOUSE, H2AJ_MOUSE	Histone H2A	NDEELNKLLGR	26	4.30E-07	650.847	1299.6794	2	0.0012	11	1	Semi-tryptic
H2A1F_MOUSE, H2A1H_MOUSE, H2A1_MOUSE, H2A1K_MOUSE, H2A3_MOUSE, H2AJ_MOUSE	Histone H2A	NDEELNKLLGR	26	3.50E-05	650.847	1299.6794	2	0.0011	11	1	Semi-tryptic
H2A1F_MOUSE, H2A1H_MOUSE, H2A1_MOUSE, H2A1K_MOUSE, H2A3_MOUSE, H2AJ_MOUSE	Histone H2A	NDEELNKLLGR	26	1.30E-05	650.8469	1299.6792	2	0.0010	11	1	Semi-tryptic
H2A1F_MOUSE, H2A1H_MOUSE, H2A1_MOUSE, H2A1K_MOUSE, H2A3_MOUSE, H2AJ_MOUSE	Histone H2A	NDEELNKLLGR	26	6.80E-06	650.8466	1299.6787	2	0.0005	11	1	Semi-tryptic
H2A1F_MOUSE, H2A1H_MOUSE, H2A1_MOUSE, H2A1K_MOUSE, H2A3_MOUSE, H2AJ_MOUSE	Histone H2A	NDEELNKLLGR	26	8.70E-08	650.8465	1299.6784	2	0.0001	11	1	Semi-tryptic
H2A1F_MOUSE, H2A1H_MOUSE, H2A1_MOUSE, H2A1K_MOUSE, H2A3_MOUSE, H2AJ_MOUSE	Histone H2A	NDEELNKLLGR	26	4.60E-04	434.2334	1299.6783	3	0.0000	11	1	Semi-tryptic
H2A1F_MOUSE, H2A1H_MOUSE, H2A1_MOUSE, H2A1K_MOUSE, H2A3_MOUSE, H2AJ_MOUSE	Histone H2A	NDEELNKLLGR	26	8.20E-08	650.8464	1299.6782	2	-0.0001	11	1	Semi-tryptic
H2A1F_MOUSE, H2A1H_MOUSE, H2A1_MOUSE, H2A1K_MOUSE, H2A3_MOUSE, H2AJ_MOUSE	Histone H2A	NDEELNKLLGR	26	8.50E-04	434.2333	1299.6781	3	-0.0002	11	1	Semi-tryptic
H2A1F_MOUSE, H2A1H_MOUSE, H2A1_MOUSE, H2A1K_MOUSE, H2A3_MOUSE, H2AJ_MOUSE	Histone H2A	NDEELNKLLGR	26	6.70E-05	434.2331	1299.6776	3	-0.0006	11	1	Semi-tryptic
H2A1F_MOUSE, H2A1H_MOUSE, H2A1_MOUSE, H2A1K_MOUSE, H2A3_MOUSE, H2AJ_MOUSE	Histone H2A	NDEELNKLLGR	26	1.80E-06	650.8459	1299.6773	2	-0.0009	11	1	Semi-tryptic
H2A1F_MOUSE, H2A1H_MOUSE, H2A1_MOUSE, H2A1K_MOUSE, H2A3_MOUSE, H2AJ_MOUSE	Histone H2A	NDEELNKLLGR	26	7.10E-05	434.233	1299.6772	3	-0.0011	11	1	Semi-tryptic
H2A1F_MOUSE, H2A1H_MOUSE, H2A1_MOUSE, H2A1K_MOUSE, H2A3_MOUSE, H2AJ_MOUSE	Histone H2A	NDEELNKLLGR	26	1.30E-05	434.233	1299.6772	3	-0.0011	11	1	Semi-tryptic
H2A1F_MOUSE, H2A1H_MOUSE, H2A1_MOUSE, H2A1K_MOUSE, H2A3_MOUSE, H2AJ_MOUSE	Histone H2A	NDEELNKLLGR	26	6.00E-07	650.8458	1299.6770	2	-0.0012	11	1	Semi-tryptic
H2A1F_MOUSE, H2A1H_MOUSE, H2A1_MOUSE, H2A1K_MOUSE, H2A3_MOUSE, H2AJ_MOUSE	Histone H2A	NDEELNKLLGR	26	1.10E-03	650.8456	1299.6767	2	-0.0015	11	1	Semi-tryptic
H2A1F_MOUSE, H2A1H_MOUSE, H2A1_MOUSE, H2A1K_MOUSE, H2A3_MOUSE, H2AJ_MOUSE	Histone H2A	NDEELNKLLGR	26	5.90E-07	650.8457	1299.6768	2	-0.0015	11	1	Semi-tryptic
H2A1F_MOUSE, H2A1H_MOUSE, H2A1_MOUSE, H2A1K_MOUSE, H2A3_MOUSE, H2AJ_MOUSE	Histone H2A	NDEELNKLLGR	26	3.50E-05	434.2328	1299.6765	3	-0.0017	11	1	Semi-tryptic
H2A1F_MOUSE, H2A1H_MOUSE, H2A1_MOUSE, H2A1K_MOUSE, H2A3_MOUSE, H2AJ_MOUSE, H2AX_MOUSE	Histone H2A	VGAGAPVYLAAVLEYLTAEILELAGNAARDNKK	2	3.20E-06	850.9686	3399.8452	4	0.0051	33	2	Semi-tryptic
H2A1F_MOUSE, H2A1H_MOUSE, H2A1_MOUSE, H2A1K_MOUSE, H2A3_MOUSE, H2AJ_MOUSE, H2AX_MOUSE	Histone H2A	VGAGAPVYLAAVLEYLTAEILELAGNAARDNKK	2	7.90E-06	850.9679	3399.8426	4	0.0024	33	2	Semi-tryptic
H2A1F_MOUSE, H2A1H_MOUSE, H2A1_MOUSE, H2A1K_MOUSE, H2A3_MOUSE, H2AJ_MOUSE, H2AX_MOUSE	Histone H2A	VGAGAPVYLAAVLEYLTAEILELAGNAAR	8	6.60E-09	972.5355	2914.5846	3	0.0043	29	0	Semi-tryptic
H2A1F_MOUSE, H2A1H_MOUSE, H2A1_MOUSE, H2A1K_MOUSE, H2A3_MOUSE, H2AJ_MOUSE, H2AX_MOUSE	Histone H2A	VGAGAPVYLAAVLEYLTAEILELAGNAAR	8	4.80E-04	729.6529	2914.5826	4	0.0023	29	0	Semi-tryptic
H2A1F_MOUSE, H2A1H_MOUSE, H2A1_MOUSE, H2A1K_MOUSE, H2A3_MOUSE, H2AJ_MOUSE, H2AX_MOUSE	Histone H2A	VGAGAPVYLAAVLEYLTAEILELAGNAAR	8	5.70E-11	729.6529	2914.5824	4	0.0021	29	0	Semi-tryptic
H2A1F_MOUSE, H2A1H_MOUSE, H2A1_MOUSE, H2A1K_MOUSE, H2A3_MOUSE, H2AJ_MOUSE, H2AX_MOUSE	Histone H2A	VGAGAPVYLAAVLEYLTAEILELAGNAAR	8	1.40E-09	972.5347	2914.5822	3	0.0019	29	0	Semi-tryptic
H2A1F_MOUSE, H2A1H_MOUSE, H2A1_MOUSE, H2A1K_MOUSE, H2A3_MOUSE, H2AJ_MOUSE, H2AX_MOUSE	Histone H2A	VGAGAPVYLAAVLEYLTAEILELAGNAAR	8	2.80E-12	1458.2983	2914.5820	2	0.0017	29	0	Semi-tryptic
H2A1F_MOUSE, H2A1H_MOUSE, H2A1_MOUSE, H2A1K_MOUSE, H2A3_MOUSE, H2AJ_MOUSE, H2AX_MOUSE	Histone H2A	VGAGAPVYLAAVLEYLTAEILELAGNAAR	8	6.30E-03	972.5345	2914.5817	3	0.0013	29	0	Semi-tryptic
H2A1F_MOUSE, H2A1H_MOUSE, H2A1_MOUSE, H2A1K_MOUSE, H2A3_MOUSE, H2AJ_MOUSE, H2AX_MOUSE	Histone H2A	VGAGAPVYLAAVLEYLTAEILELAGNAAR	8	3.60E-06	729.652	2914.5788	4	-0.0015	29	0	Semi-tryptic
H2A1F_MOUSE, H2A1H_MOUSE, H2A1_MOUSE, H2A1K_MOUSE, H2A3_MOUSE, H2AJ_MOUSE, H2AX_MOUSE	Histone H2A	VGAGAPVYLAAVLEYLTAEILELAGNAAR	8	2.50E-09	729.652	2914.5788	4	-0.0015	29	0	Semi-tryptic
H2A1F_MOUSE, H2A1H_MOUSE, H2A1_MOUSE, H2A1K_MOUSE, H2A3_MOUSE, H2AJ_MOUSE, H2AX_MOUSE	Histone H2A	RVGAGAPVYLAAVLE	111	6.10E-04	495.9515	1484.8328	3	-0.0023	15	0	GluC

H2A1F_MOUSE, H2A1H_MOUSE, H2A1_MOUSE, H2A1K_MOUSE, H2A3_MOUSE, H2AJ_MOUSE, H2AX_MOUSE	Histone H2A	RVGAGAPVYLAAVLE	111	6.00E-03	743.4307	1484.8468	2	0.0118	15	0	GluC
H2A1F_MOUSE, H2A1H_MOUSE, H2A1_MOUSE, H2A1K_MOUSE, H2A3_MOUSE, H2AJ_MOUSE, H2AX_MOUSE	Histone H2A	RVGAGAPVYLAAVLE	111	6.10E-03	743.4301	1484.8457	2	0.0106	15	0	GluC
H2A1F_MOUSE, H2A1H_MOUSE, H2A1_MOUSE, H2A1K_MOUSE, H2A3_MOUSE, H2AJ_MOUSE, H2AX_MOUSE	Histone H2A	RVGAGAPVYLAAVLE	111	4.90E-04	743.4301	1484.8455	2	0.0104	15	0	GluC
H2A1F_MOUSE, H2A1H_MOUSE, H2A1_MOUSE, H2A1K_MOUSE, H2A3_MOUSE, H2AJ_MOUSE, H2AX_MOUSE	Histone H2A	RVGAGAPVYLAAVLE	111	9.70E-03	743.4299	1484.8453	2	0.0103	15	0	GluC
H2A1F_MOUSE, H2A1H_MOUSE, H2A1_MOUSE, H2A1K_MOUSE, H2A3_MOUSE, H2AJ_MOUSE, H2AX_MOUSE	Histone H2A	RVGAGAPVYLAAVLE	111	2.70E-03	743.4295	1484.8444	2	0.0094	15	0	GluC
H2A1F_MOUSE, H2A1H_MOUSE, H2A1_MOUSE, H2A1K_MOUSE, H2A3_MOUSE, H2AJ_MOUSE, H2AX_MOUSE	Histone H2A	RVGAGAPVYLAAVLE	111	9.30E-03	743.4291	1484.8436	2	0.0085	15	0	GluC
H2A1F_MOUSE, H2A1H_MOUSE, H2A1_MOUSE, H2A1K_MOUSE, H2A3_MOUSE, H2AJ_MOUSE, H2AX_MOUSE	Histone H2A	RVGAGAPVYLAAVLE	111	9.60E-03	743.4288	1484.8431	2	0.0080	15	0	GluC
H2A1F_MOUSE, H2A1H_MOUSE, H2A1_MOUSE, H2A1K_MOUSE, H2A3_MOUSE, H2AJ_MOUSE, H2AX_MOUSE	Histone H2A	RVGAGAPVYLAAVLE	111	1.50E-03	743.4287	1484.8428	2	0.0077	15	0	GluC
H2A1F_MOUSE, H2A1H_MOUSE, H2A1_MOUSE, H2A1K_MOUSE, H2A3_MOUSE, H2AJ_MOUSE, H2AX_MOUSE	Histone H2A	RVGAGAPVYLAAVLE	111	5.30E-03	743.4285	1484.8424	2	0.0073	15	0	GluC
H2A1F_MOUSE, H2A1H_MOUSE, H2A1_MOUSE, H2A1K_MOUSE, H2A3_MOUSE, H2AJ_MOUSE, H2AX_MOUSE	Histone H2A	RVGAGAPVYLAAVLE	111	5.10E-04	743.4275	1484.8404	2	0.0053	15	0	GluC
H2A1F_MOUSE, H2A1H_MOUSE, H2A1_MOUSE, H2A1K_MOUSE, H2A3_MOUSE, H2AJ_MOUSE, H2AX_MOUSE	Histone H2A	RVGAGAPVYLAAVLE	111	6.40E-04	743.4273	1484.8400	2	0.0050	15	0	GluC
H2A1F_MOUSE, H2A1H_MOUSE, H2A1_MOUSE, H2A1K_MOUSE, H2A3_MOUSE, H2AJ_MOUSE, H2AX_MOUSE	Histone H2A	RVGAGAPVYLAAVLE	111	5.60E-04	743.4273	1484.8401	2	0.0050	15	0	GluC
H2A1F_MOUSE, H2A1H_MOUSE, H2A1_MOUSE, H2A1K_MOUSE, H2A3_MOUSE, H2AJ_MOUSE, H2AX_MOUSE	Histone H2A	RVGAGAPVYLAAVLE	111	4.40E-03	743.4272	1484.8399	2	0.0048	15	0	GluC
H2A1F_MOUSE, H2A1H_MOUSE, H2A1_MOUSE, H2A1K_MOUSE, H2A3_MOUSE, H2AJ_MOUSE, H2AX_MOUSE	Histone H2A	RVGAGAPVYLAAVLE	111	3.30E-03	743.4272	1484.8399	2	0.0048	15	0	GluC
H2A1F_MOUSE, H2A1H_MOUSE, H2A1_MOUSE, H2A1K_MOUSE, H2A3_MOUSE, H2AJ_MOUSE, H2AX_MOUSE	Histone H2A	RVGAGAPVYLAAVLE	111	4.00E-03	743.4268	1484.8391	2	0.0040	15	0	GluC
H2A1F_MOUSE, H2A1H_MOUSE, H2A1_MOUSE, H2A1K_MOUSE, H2A3_MOUSE, H2AJ_MOUSE, H2AX_MOUSE	Histone H2A	RVGAGAPVYLAAVLE	111	5.30E-05	743.4268	1484.8391	2	0.0040	15	0	GluC
H2A1F_MOUSE, H2A1H_MOUSE, H2A1_MOUSE, H2A1K_MOUSE, H2A3_MOUSE, H2AJ_MOUSE, H2AX_MOUSE	Histone H2A	RVGAGAPVYLAAVLE	111	7.60E-03	743.4266	1484.8387	2	0.0037	15	0	GluC
H2A1F_MOUSE, H2A1H_MOUSE, H2A1_MOUSE, H2A1K_MOUSE, H2A3_MOUSE, H2AJ_MOUSE, H2AX_MOUSE	Histone H2A	RVGAGAPVYLAAVLE	111	4.40E-03	743.4267	1484.8388	2	0.0037	15	0	GluC
H2A1F_MOUSE, H2A1H_MOUSE, H2A1_MOUSE, H2A1K_MOUSE, H2A3_MOUSE, H2AJ_MOUSE, H2AX_MOUSE	Histone H2A	RVGAGAPVYLAAVLE	111	6.50E-04	743.4266	1484.8387	2	0.0037	15	0	GluC
H2A1F_MOUSE, H2A1H_MOUSE, H2A1_MOUSE, H2A1K_MOUSE, H2A3_MOUSE, H2AJ_MOUSE, H2AX_MOUSE	Histone H2A	RVGAGAPVYLAAVLE	111	5.40E-05	743.4266	1484.8387	2	0.0037	15	0	GluC
H2A1F_MOUSE, H2A1H_MOUSE, H2A1_MOUSE, H2A1K_MOUSE, H2A3_MOUSE, H2AJ_MOUSE, H2AX_MOUSE	Histone H2A	RVGAGAPVYLAAVLE	111	9.90E-03	743.4266	1484.8387	2	0.0036	15	0	GluC
H2A1F_MOUSE, H2A1H_MOUSE, H2A1_MOUSE, H2A1K_MOUSE, H2A3_MOUSE, H2AJ_MOUSE, H2AX_MOUSE	Histone H2A	RVGAGAPVYLAAVLE	111	6.10E-03	743.4266	1484.8387	2	0.0036	15	0	GluC
H2A1F_MOUSE, H2A1H_MOUSE, H2A1_MOUSE, H2A1K_MOUSE, H2A3_MOUSE, H2AJ_MOUSE, H2AX_MOUSE	Histone H2A	RVGAGAPVYLAAVLE	111	1.40E-04	743.4266	1484.8387	2	0.0036	15	0	GluC
H2A1F_MOUSE, H2A1H_MOUSE, H2A1_MOUSE, H2A1K_MOUSE, H2A3_MOUSE, H2AJ_MOUSE, H2AX_MOUSE	Histone H2A	RVGAGAPVYLAAVLE	111	8.60E-04	743.4266	1484.8386	2	0.0035	15	0	GluC
H2A1F_MOUSE, H2A1H_MOUSE, H2A1_MOUSE, H2A1K_MOUSE, H2A3_MOUSE, H2AJ_MOUSE, H2AX_MOUSE	Histone H2A	RVGAGAPVYLAAVLE	111	2.00E-04	743.4266	1484.8386	2	0.0035	15	0	GluC
H2A1F_MOUSE, H2A1H_MOUSE, H2A1_MOUSE, H2A1K_MOUSE, H2A3_MOUSE, H2AJ_MOUSE, H2AX_MOUSE	Histone H2A	RVGAGAPVYLAAVLE	111	1.50E-03	743.4265	1484.8384	2	0.0033	15	0	GluC
H2A1F_MOUSE, H2A1H_MOUSE, H2A1_MOUSE, H2A1K_MOUSE, H2A3_MOUSE, H2AJ_MOUSE, H2AX_MOUSE	Histone H2A	RVGAGAPVYLAAVLE	111	1.40E-03	743.4265	1484.8384	2	0.0033	15	0	GluC
H2A1F_MOUSE, H2A1H_MOUSE, H2A1_MOUSE, H2A1K_MOUSE, H2A3_MOUSE, H2AJ_MOUSE, H2AX_MOUSE	Histone H2A	RVGAGAPVYLAAVLE	111	9.30E-03	743.4265	1484.8383	2	0.0032	15	0	GluC
H2A1F_MOUSE, H2A1H_MOUSE, H2A1_MOUSE, H2A1K_MOUSE, H2A3_MOUSE, H2AJ_MOUSE, H2AX_MOUSE	Histone H2A	RVGAGAPVYLAAVLE	111	2.80E-03	743.4264	1484.8383	2	0.0032	15	0	GluC
H2A1F_MOUSE, H2A1H_MOUSE, H2A1_MOUSE, H2A1K_MOUSE, H2A3_MOUSE, H2AJ_MOUSE, H2AX_MOUSE	Histone H2A	RVGAGAPVYLAAVLE	111	8.00E-04	743.4264	1484.8383	2	0.0032	15	0	GluC
H2A1F_MOUSE, H2A1H_MOUSE, H2A1_MOUSE, H2A1K_MOUSE, H2A3_MOUSE, H2AJ_MOUSE, H2AX_MOUSE	Histone H2A	RVGAGAPVYLAAVLE	111	2.50E-04	743.4265	1484.8383	2	0.0032	15	0	GluC
H2A1F_MOUSE, H2A1H_MOUSE, H2A1_MOUSE, H2A1K_MOUSE, H2A3_MOUSE, H2AJ_MOUSE, H2AX_MOUSE	Histone H2A	RVGAGAPVYLAAVLE	111	8.50E-04	743.4263	1484.8381	2	0.0030	15	0	GluC
H2A1F_MOUSE, H2A1H_MOUSE, H2A1_MOUSE, H2A1K_MOUSE, H2A3_MOUSE, H2AJ_MOUSE, H2AX_MOUSE	Histone H2A	RVGAGAPVYLAAVLE	111	4.60E-04	743.4263	1484.8381	2	0.0030	15	0	GluC
H2A1F_MOUSE, H2A1H_MOUSE, H2A1_MOUSE, H2A1K_MOUSE, H2A3_MOUSE, H2AJ_MOUSE, H2AX_MOUSE	Histone H2A	RVGAGAPVYLAAVLE	111	2.00E-03	743.4263	1484.8380	2	0.0029	15	0	GluC
H2A1F_MOUSE, H2A1H_MOUSE, H2A1_MOUSE, H2A1K_MOUSE, H2A3_MOUSE, H2AJ_MOUSE, H2AX_MOUSE	Histone H2A	RVGAGAPVYLAAVLE	111	3.40E-05	743.4263	1484.8380	2	0.0029	15	0	GluC
H2A1F_MOUSE, H2A1H_MOUSE, H2A1_MOUSE, H2A1K_MOUSE, H2A3_MOUSE, H2AJ_MOUSE, H2AX_MOUSE	Histone H2A	RVGAGAPVYLAAVLE	111	5.10E-03	743.4262	1484.8378	2	0.0027	15	0	GluC
H2A1F_MOUSE, H2A1H_MOUSE, H2A1_MOUSE, H2A1K_MOUSE, H2A3_MOUSE, H2AJ_MOUSE, H2AX_MOUSE	Histone H2A	RVGAGAPVYLAAVLE	111	1.40E-03	743.4262	1484.8378	2	0.0027	15	0	GluC

H2A1F_MOUSE, H2A1H_MOUSE, H2A1_MOUSE, H2A1K_MOUSE, H2A3_MOUSE, H2AJ_MOUSE, H2AX_MOUSE	Histone H2A	RVGAGAPVYLAAVLE	111	7.80E-04	743.4262	1484.8377	2	0.0027	15	0	GluC
H2A1F_MOUSE, H2A1H_MOUSE, H2A1_MOUSE, H2A1K_MOUSE, H2A3_MOUSE, H2AJ_MOUSE, H2AX_MOUSE	Histone H2A	RVGAGAPVYLAAVLE	111	6.40E-03	743.4261	1484.8376	2	0.0025	15	0	GluC
H2A1F_MOUSE, H2A1H_MOUSE, H2A1_MOUSE, H2A1K_MOUSE, H2A3_MOUSE, H2AJ_MOUSE, H2AX_MOUSE	Histone H2A	RVGAGAPVYLAAVLE	111	5.70E-03	743.4261	1484.8376	2	0.0025	15	0	GluC
H2A1F_MOUSE, H2A1H_MOUSE, H2A1_MOUSE, H2A1K_MOUSE, H2A3_MOUSE, H2AJ_MOUSE, H2AX_MOUSE	Histone H2A	RVGAGAPVYLAAVLE	111	1.00E-02	743.426	1484.8375	2	0.0024	15	0	GluC
H2A1F_MOUSE, H2A1H_MOUSE, H2A1_MOUSE, H2A1K_MOUSE, H2A3_MOUSE, H2AJ_MOUSE, H2AX_MOUSE	Histone H2A	RVGAGAPVYLAAVLE	111	4.20E-03	743.426	1484.8375	2	0.0024	15	0	GluC
H2A1F_MOUSE, H2A1H_MOUSE, H2A1_MOUSE, H2A1K_MOUSE, H2A3_MOUSE, H2AJ_MOUSE, H2AX_MOUSE	Histone H2A	RVGAGAPVYLAAVLE	111	4.10E-03	743.426	1484.8375	2	0.0024	15	0	GluC
H2A1F_MOUSE, H2A1H_MOUSE, H2A1_MOUSE, H2A1K_MOUSE, H2A3_MOUSE, H2AJ_MOUSE, H2AX_MOUSE	Histone H2A	RVGAGAPVYLAAVLE	111	8.80E-04	743.426	1484.8375	2	0.0024	15	0	GluC
H2A1F_MOUSE, H2A1H_MOUSE, H2A1_MOUSE, H2A1K_MOUSE, H2A3_MOUSE, H2AJ_MOUSE, H2AX_MOUSE	Histone H2A	RVGAGAPVYLAAVLE	111	1.00E-02	743.426	1484.8374	2	0.0023	15	0	GluC
H2A1F_MOUSE, H2A1H_MOUSE, H2A1_MOUSE, H2A1K_MOUSE, H2A3_MOUSE, H2AJ_MOUSE, H2AX_MOUSE	Histone H2A	RVGAGAPVYLAAVLE	111	8.50E-03	743.426	1484.8374	2	0.0023	15	0	GluC
H2A1F_MOUSE, H2A1H_MOUSE, H2A1_MOUSE, H2A1K_MOUSE, H2A3_MOUSE, H2AJ_MOUSE, H2AX_MOUSE	Histone H2A	RVGAGAPVYLAAVLE	111	1.20E-03	743.426	1484.8374	2	0.0023	15	0	GluC
H2A1F_MOUSE, H2A1H_MOUSE, H2A1_MOUSE, H2A1K_MOUSE, H2A3_MOUSE, H2AJ_MOUSE, H2AX_MOUSE	Histone H2A	RVGAGAPVYLAAVLE	111	5.80E-05	495.9531	1484.8374	3	0.0023	15	0	GluC
H2A1F_MOUSE, H2A1H_MOUSE, H2A1_MOUSE, H2A1K_MOUSE, H2A3_MOUSE, H2AJ_MOUSE, H2AX_MOUSE	Histone H2A	RVGAGAPVYLAAVLE	111	4.10E-05	743.426	1484.8374	2	0.0023	15	0	GluC
H2A1F_MOUSE, H2A1H_MOUSE, H2A1_MOUSE, H2A1K_MOUSE, H2A3_MOUSE, H2AJ_MOUSE, H2AX_MOUSE	Histone H2A	RVGAGAPVYLAAVLE	111	8.40E-03	743.4259	1484.8373	2	0.0022	15	0	GluC
H2A1F_MOUSE, H2A1H_MOUSE, H2A1_MOUSE, H2A1K_MOUSE, H2A3_MOUSE, H2AJ_MOUSE, H2AX_MOUSE	Histone H2A	RVGAGAPVYLAAVLE	111	6.70E-03	743.4259	1484.8372	2	0.0022	15	0	GluC
H2A1F_MOUSE, H2A1H_MOUSE, H2A1_MOUSE, H2A1K_MOUSE, H2A3_MOUSE, H2AJ_MOUSE, H2AX_MOUSE	Histone H2A	RVGAGAPVYLAAVLE	111	4.80E-03	743.4259	1484.8373	2	0.0022	15	0	GluC
H2A1F_MOUSE, H2A1H_MOUSE, H2A1_MOUSE, H2A1K_MOUSE, H2A3_MOUSE, H2AJ_MOUSE, H2AX_MOUSE	Histone H2A	RVGAGAPVYLAAVLE	111	2.50E-03	743.4259	1484.8372	2	0.0022	15	0	GluC
H2A1F_MOUSE, H2A1H_MOUSE, H2A1_MOUSE, H2A1K_MOUSE, H2A3_MOUSE, H2AJ_MOUSE, H2AX_MOUSE	Histone H2A	RVGAGAPVYLAAVLE	111	2.40E-03	743.4259	1484.8372	2	0.0022	15	0	GluC
H2A1F_MOUSE, H2A1H_MOUSE, H2A1_MOUSE, H2A1K_MOUSE, H2A3_MOUSE, H2AJ_MOUSE, H2AX_MOUSE	Histone H2A	RVGAGAPVYLAAVLE	111	9.00E-04	743.4259	1484.8373	2	0.0022	15	0	GluC
H2A1F_MOUSE, H2A1H_MOUSE, H2A1_MOUSE, H2A1K_MOUSE, H2A3_MOUSE, H2AJ_MOUSE, H2AX_MOUSE	Histone H2A	RVGAGAPVYLAAVLE	111	8.30E-04	743.4259	1484.8372	2	0.0022	15	0	GluC
H2A1F_MOUSE, H2A1H_MOUSE, H2A1_MOUSE, H2A1K_MOUSE, H2A3_MOUSE, H2AJ_MOUSE, H2AX_MOUSE	Histone H2A	RVGAGAPVYLAAVLE	111	5.10E-03	743.4259	1484.8372	2	0.0021	15	0	GluC
H2A1F_MOUSE, H2A1H_MOUSE, H2A1_MOUSE, H2A1K_MOUSE, H2A3_MOUSE, H2AJ_MOUSE, H2AX_MOUSE	Histone H2A	RVGAGAPVYLAAVLE	111	2.50E-03	743.4259	1484.8372	2	0.0021	15	0	GluC
H2A1F_MOUSE, H2A1H_MOUSE, H2A1_MOUSE, H2A1K_MOUSE, H2A3_MOUSE, H2AJ_MOUSE, H2AX_MOUSE	Histone H2A	RVGAGAPVYLAAVLE	111	1.40E-03	743.4259	1484.8372	2	0.0021	15	0	GluC
H2A1F_MOUSE, H2A1H_MOUSE, H2A1_MOUSE, H2A1K_MOUSE, H2A3_MOUSE, H2AJ_MOUSE, H2AX_MOUSE	Histone H2A	RVGAGAPVYLAAVLE	111	1.20E-03	743.4259	1484.8372	2	0.0021	15	0	GluC
H2A1F_MOUSE, H2A1H_MOUSE, H2A1_MOUSE, H2A1K_MOUSE, H2A3_MOUSE, H2AJ_MOUSE, H2AX_MOUSE	Histone H2A	RVGAGAPVYLAAVLE	111	8.00E-03	743.4258	1484.8370	2	0.0019	15	0	GluC
H2A1F_MOUSE, H2A1H_MOUSE, H2A1_MOUSE, H2A1K_MOUSE, H2A3_MOUSE, H2AJ_MOUSE, H2AX_MOUSE	Histone H2A	RVGAGAPVYLAAVLE	111	6.40E-03	743.4258	1484.8370	2	0.0019	15	0	GluC
H2A1F_MOUSE, H2A1H_MOUSE, H2A1_MOUSE, H2A1K_MOUSE, H2A3_MOUSE, H2AJ_MOUSE, H2AX_MOUSE	Histone H2A	RVGAGAPVYLAAVLE	111	4.50E-03	743.4258	1484.8370	2	0.0019	15	0	GluC
H2A1F_MOUSE, H2A1H_MOUSE, H2A1_MOUSE, H2A1K_MOUSE, H2A3_MOUSE, H2AJ_MOUSE, H2AX_MOUSE	Histone H2A	RVGAGAPVYLAAVLE	111	3.00E-03	743.4258	1484.8370	2	0.0019	15	0	GluC
H2A1F_MOUSE, H2A1H_MOUSE, H2A1_MOUSE, H2A1K_MOUSE, H2A3_MOUSE, H2AJ_MOUSE, H2AX_MOUSE	Histone H2A	RVGAGAPVYLAAVLE	111	2.10E-03	743.4258	1484.8370	2	0.0019	15	0	GluC
H2A1F_MOUSE, H2A1H_MOUSE, H2A1_MOUSE, H2A1K_MOUSE, H2A3_MOUSE, H2AJ_MOUSE, H2AX_MOUSE	Histone H2A	RVGAGAPVYLAAVLE	111	9.10E-04	743.4258	1484.8370	2	0.0019	15	0	GluC
H2A1F_MOUSE, H2A1H_MOUSE, H2A1_MOUSE, H2A1K_MOUSE, H2A3_MOUSE, H2AJ_MOUSE, H2AX_MOUSE	Histone H2A	RVGAGAPVYLAAVLE	111	7.70E-04	743.4257	1484.8369	2	0.0019	15	0	GluC
H2A1F_MOUSE, H2A1H_MOUSE, H2A1_MOUSE, H2A1K_MOUSE, H2A3_MOUSE, H2AJ_MOUSE, H2AX_MOUSE	Histone H2A	RVGAGAPVYLAAVLE	111	3.60E-04	743.4258	1484.8370	2	0.0019	15	0	GluC
H2A1F_MOUSE, H2A1H_MOUSE, H2A1_MOUSE, H2A1K_MOUSE, H2A3_MOUSE, H2AJ_MOUSE, H2AX_MOUSE	Histone H2A	RVGAGAPVYLAAVLE	111	8.70E-03	743.4257	1484.8369	2	0.0018	15	0	GluC
H2A1F_MOUSE, H2A1H_MOUSE, H2A1_MOUSE, H2A1K_MOUSE, H2A3_MOUSE, H2AJ_MOUSE, H2AX_MOUSE	Histone H2A	RVGAGAPVYLAAVLE	111	3.10E-03	743.4257	1484.8368	2	0.0018	15	0	GluC
H2A1F_MOUSE, H2A1H_MOUSE, H2A1_MOUSE, H2A1K_MOUSE, H2A3_MOUSE, H2AJ_MOUSE, H2AX_MOUSE	Histone H2A	RVGAGAPVYLAAVLE	111	7.00E-04	743.4257	1484.8369	2	0.0018	15	0	GluC
H2A1F_MOUSE, H2A1H_MOUSE, H2A1_MOUSE, H2A1K_MOUSE, H2A3_MOUSE, H2AJ_MOUSE, H2AX_MOUSE	Histone H2A	RVGAGAPVYLAAVLE	111	3.50E-05	743.4257	1484.8369	2	0.0018	15	0	GluC
H2A1F_MOUSE, H2A1H_MOUSE, H2A1_MOUSE, H2A1K_MOUSE, H2A3_MOUSE, H2AJ_MOUSE, H2AX_MOUSE	Histone H2A	RVGAGAPVYLAAVLE	111	9.80E-03	743.4257	1484.8368	2	0.0017	15	0	GluC
H2A1F_MOUSE, H2A1H_MOUSE, H2A1_MOUSE, H2A1K_MOUSE, H2A3_MOUSE, H2AJ_MOUSE, H2AX_MOUSE	Histone H2A	RVGAGAPVYLAAVLE	111	9.30E-03	743.4257	1484.8368	2	0.0017	15	0	GluC

H2A1F_MOUSE, H2A1H_MOUSE, H2A1_MOUSE, H2A1K_MOUSE, H2A3_MOUSE, H2AJ_MOUSE, H2AX_MOUSE	Histone H2A	RVGAGAPVYLAAVLE	111	5.10E-04	743.4257	1484.8368	2	0.0017	15	0	GluC
H2A1F_MOUSE, H2A1H_MOUSE, H2A1_MOUSE, H2A1K_MOUSE, H2A3_MOUSE, H2AJ_MOUSE, H2AX_MOUSE	Histone H2A	RVGAGAPVYLAAVLE	111	7.50E-03	743.4256	1484.8367	2	0.0016	15	0	GluC
H2A1F_MOUSE, H2A1H_MOUSE, H2A1_MOUSE, H2A1K_MOUSE, H2A3_MOUSE, H2AJ_MOUSE, H2AX_MOUSE	Histone H2A	RVGAGAPVYLAAVLE	111	7.30E-03	743.4256	1484.8367	2	0.0016	15	0	GluC
H2A1F_MOUSE, H2A1H_MOUSE, H2A1_MOUSE, H2A1K_MOUSE, H2A3_MOUSE, H2AJ_MOUSE, H2AX_MOUSE	Histone H2A	RVGAGAPVYLAAVLE	111	2.20E-03	743.4257	1484.8367	2	0.0016	15	0	GluC
H2A1F_MOUSE, H2A1H_MOUSE, H2A1_MOUSE, H2A1K_MOUSE, H2A3_MOUSE, H2AJ_MOUSE, H2AX_MOUSE	Histone H2A	RVGAGAPVYLAAVLE	111	2.00E-03	743.4257	1484.8367	2	0.0016	15	0	GluC
H2A1F_MOUSE, H2A1H_MOUSE, H2A1_MOUSE, H2A1K_MOUSE, H2A3_MOUSE, H2AJ_MOUSE, H2AX_MOUSE	Histone H2A	RVGAGAPVYLAAVLE	111	7.10E-03	743.4256	1484.8366	2	0.0015	15	0	GluC
H2A1F_MOUSE, H2A1H_MOUSE, H2A1_MOUSE, H2A1K_MOUSE, H2A3_MOUSE, H2AJ_MOUSE, H2AX_MOUSE	Histone H2A	RVGAGAPVYLAAVLE	111	8.50E-04	743.4256	1484.8366	2	0.0015	15	0	GluC
H2A1F_MOUSE, H2A1H_MOUSE, H2A1_MOUSE, H2A1K_MOUSE, H2A3_MOUSE, H2AJ_MOUSE, H2AX_MOUSE	Histone H2A	RVGAGAPVYLAAVLE	111	5.00E-03	743.4256	1484.8365	2	0.0014	15	0	GluC
H2A1F_MOUSE, H2A1H_MOUSE, H2A1_MOUSE, H2A1K_MOUSE, H2A3_MOUSE, H2AJ_MOUSE, H2AX_MOUSE	Histone H2A	RVGAGAPVYLAAVLE	111	4.70E-03	743.4256	1484.8365	2	0.0014	15	0	GluC
H2A1F_MOUSE, H2A1H_MOUSE, H2A1_MOUSE, H2A1K_MOUSE, H2A3_MOUSE, H2AJ_MOUSE, H2AX_MOUSE	Histone H2A	RVGAGAPVYLAAVLE	111	4.10E-03	743.4256	1484.8365	2	0.0014	15	0	GluC
H2A1F_MOUSE, H2A1H_MOUSE, H2A1_MOUSE, H2A1K_MOUSE, H2A3_MOUSE, H2AJ_MOUSE, H2AX_MOUSE	Histone H2A	RVGAGAPVYLAAVLE	111	1.90E-03	743.4255	1484.8365	2	0.0014	15	0	GluC
H2A1F_MOUSE, H2A1H_MOUSE, H2A1_MOUSE, H2A1K_MOUSE, H2A3_MOUSE, H2AJ_MOUSE, H2AX_MOUSE	Histone H2A	RVGAGAPVYLAAVLE	111	1.00E-05	743.4255	1484.8365	2	0.0014	15	0	GluC
H2A1F_MOUSE, H2A1H_MOUSE, H2A1_MOUSE, H2A1K_MOUSE, H2A3_MOUSE, H2AJ_MOUSE, H2AX_MOUSE	Histone H2A	RVGAGAPVYLAAVLE	111	6.70E-03	743.4255	1484.8364	2	0.0013	15	0	GluC
H2A1F_MOUSE, H2A1H_MOUSE, H2A1_MOUSE, H2A1K_MOUSE, H2A3_MOUSE, H2AJ_MOUSE, H2AX_MOUSE	Histone H2A	RVGAGAPVYLAAVLE	111	4.90E-03	743.4255	1484.8364	2	0.0013	15	0	GluC
H2A1F_MOUSE, H2A1H_MOUSE, H2A1_MOUSE, H2A1K_MOUSE, H2A3_MOUSE, H2AJ_MOUSE, H2AX_MOUSE	Histone H2A	RVGAGAPVYLAAVLE	111	3.00E-03	743.4255	1484.8364	2	0.0013	15	0	GluC
H2A1F_MOUSE, H2A1H_MOUSE, H2A1_MOUSE, H2A1K_MOUSE, H2A3_MOUSE, H2AJ_MOUSE, H2AX_MOUSE	Histone H2A	RVGAGAPVYLAAVLE	111	1.90E-03	743.4255	1484.8364	2	0.0013	15	0	GluC
H2A1F_MOUSE, H2A1H_MOUSE, H2A1_MOUSE, H2A1K_MOUSE, H2A3_MOUSE, H2AJ_MOUSE, H2AX_MOUSE	Histone H2A	RVGAGAPVYLAAVLE	111	4.90E-03	743.4254	1484.8362	2	0.0011	15	0	GluC
H2A1F_MOUSE, H2A1H_MOUSE, H2A1_MOUSE, H2A1K_MOUSE, H2A3_MOUSE, H2AJ_MOUSE, H2AX_MOUSE	Histone H2A	RVGAGAPVYLAAVLE	111	1.90E-04	743.4254	1484.8362	2	0.0011	15	0	GluC
H2A1F_MOUSE, H2A1H_MOUSE, H2A1_MOUSE, H2A1K_MOUSE, H2A3_MOUSE, H2AJ_MOUSE, H2AX_MOUSE	Histone H2A	RVGAGAPVYLAAVLE	111	2.40E-03	743.4253	1484.8361	2	0.0010	15	0	GluC
H2A1F_MOUSE, H2A1H_MOUSE, H2A1_MOUSE, H2A1K_MOUSE, H2A3_MOUSE, H2AJ_MOUSE, H2AX_MOUSE	Histone H2A	RVGAGAPVYLAAVLE	111	5.30E-04	743.4253	1484.8361	2	0.0010	15	0	GluC
H2A1F_MOUSE, H2A1H_MOUSE, H2A1_MOUSE, H2A1K_MOUSE, H2A3_MOUSE, H2AJ_MOUSE, H2AX_MOUSE	Histone H2A	RVGAGAPVYLAAVLE	111	9.90E-05	743.4253	1484.8361	2	0.0010	15	0	GluC
H2A1F_MOUSE, H2A1H_MOUSE, H2A1_MOUSE, H2A1K_MOUSE, H2A3_MOUSE, H2AJ_MOUSE, H2AX_MOUSE	Histone H2A	RVGAGAPVYLAAVLE	111	9.20E-05	495.9527	1484.8361	3	0.0010	15	0	GluC
H2A1F_MOUSE, H2A1H_MOUSE, H2A1_MOUSE, H2A1K_MOUSE, H2A3_MOUSE, H2AJ_MOUSE, H2AX_MOUSE	Histone H2A	RVGAGAPVYLAAVLE	111	6.20E-05	743.4253	1484.8361	2	0.0010	15	0	GluC
H2A1F_MOUSE, H2A1H_MOUSE, H2A1_MOUSE, H2A1K_MOUSE, H2A3_MOUSE, H2AJ_MOUSE, H2AX_MOUSE	Histone H2A	RVGAGAPVYLAAVLE	111	6.00E-05	495.9526	1484.8361	3	0.0010	15	0	GluC
H2A1F_MOUSE, H2A1H_MOUSE, H2A1_MOUSE, H2A1K_MOUSE, H2A3_MOUSE, H2AJ_MOUSE, H2AX_MOUSE	Histone H2A	RVGAGAPVYLAAVLE	111	4.40E-03	743.4253	1484.8360	2	0.0009	15	0	GluC
H2A1F_MOUSE, H2A1H_MOUSE, H2A1_MOUSE, H2A1K_MOUSE, H2A3_MOUSE, H2AJ_MOUSE, H2AX_MOUSE	Histone H2A	RVGAGAPVYLAAVLE	111	7.30E-04	743.4253	1484.8360	2	0.0009	15	0	GluC
H2A1F_MOUSE, H2A1H_MOUSE, H2A1_MOUSE, H2A1K_MOUSE, H2A3_MOUSE, H2AJ_MOUSE, H2AX_MOUSE	Histone H2A	RVGAGAPVYLAAVLE	111	4.50E-04	495.9526	1484.8360	3	0.0009	15	0	GluC
H2A1F_MOUSE, H2A1H_MOUSE, H2A1_MOUSE, H2A1K_MOUSE, H2A3_MOUSE, H2AJ_MOUSE, H2AX_MOUSE	Histone H2A	RVGAGAPVYLAAVLE	111	1.70E-03	743.4252	1484.8359	2	0.0008	15	0	GluC
H2A1F_MOUSE, H2A1H_MOUSE, H2A1_MOUSE, H2A1K_MOUSE, H2A3_MOUSE, H2AJ_MOUSE, H2AX_MOUSE	Histone H2A	RVGAGAPVYLAAVLE	111	1.90E-04	743.4252	1484.8358	2	0.0007	15	0	GluC
H2A1F_MOUSE, H2A1H_MOUSE, H2A1_MOUSE, H2A1K_MOUSE, H2A3_MOUSE, H2AJ_MOUSE, H2AX_MOUSE	Histone H2A	RVGAGAPVYLAAVLE	111	3.10E-03	743.4251	1484.8357	2	0.0006	15	0	GluC
H2A1F_MOUSE, H2A1H_MOUSE, H2A1_MOUSE, H2A1K_MOUSE, H2A3_MOUSE, H2AJ_MOUSE, H2AX_MOUSE	Histone H2A	RVGAGAPVYLAAVLE	111	1.90E-05	743.4251	1484.8356	2	0.0005	15	0	GluC
H2A1F_MOUSE, H2A1H_MOUSE, H2A1_MOUSE, H2A1K_MOUSE, H2A3_MOUSE, H2AJ_MOUSE, H2AX_MOUSE	Histone H2A	RVGAGAPVYLAAVLE	111	6.90E-04	743.425	1484.8355	2	0.0004	15	0	GluC
H2A1F_MOUSE, H2A1H_MOUSE, H2A1_MOUSE, H2A1K_MOUSE, H2A3_MOUSE, H2AJ_MOUSE, H2AX_MOUSE	Histone H2A	RVGAGAPVYLAAVLE	111	6.10E-04	743.425	1484.8355	2	0.0004	15	0	GluC
H2A1F_MOUSE, H2A1H_MOUSE, H2A1_MOUSE, H2A1K_MOUSE, H2A3_MOUSE, H2AJ_MOUSE, H2AX_MOUSE	Histone H2A	RVGAGAPVYLAAVLE	111	2.30E-03	743.425	1484.8354	2	0.0003	15	0	GluC
H2A1F_MOUSE, H2A1H_MOUSE, H2A1_MOUSE, H2A1K_MOUSE, H2A3_MOUSE, H2AJ_MOUSE, H2AX_MOUSE	Histone H2A	RVGAGAPVYLAAVLE	111	3.60E-03	743.4249	1484.8352	2	0.0001	15	0	GluC
H2A1F_MOUSE, H2A1H_MOUSE, H2A1_MOUSE, H2A1K_MOUSE, H2A3_MOUSE, H2AJ_MOUSE, H2AX_MOUSE	Histone H2A	RVGAGAPVYLAAVLE	111	5.60E-04	495.9518	1484.8337	3	-0.0014	15	0	GluC
H2A1F_MOUSE, H2A1H_MOUSE, H2A1_MOUSE, H2A2A_MOUSE, H2A3_MOUSE	Histone H2A	KTESHHK	2	1.50E-03	433.7278	865.4411	2	0.0005	7	1	Semi-tryptic

H2A1F_MOUSE, H2A1H_MOUSE, H2A1_MOUSE, H2A2A_MOUSE, H2A3_MOUSE	Histone H2A	KTESHHK	2	8.80E-04	433.7277	865.4408	2	0.0002	7	1	Semi-tryptic
H2A1H_MOUSE, H2A1_MOUSE, H2A2A_MOUSE, H2A3_MOUSE	Histone H2A	LPKKTESHHKAK	20	4.90E-06	468.6079	1402.8019	3	-0.0025	12	0	GluC
H2A1H_MOUSE, H2A1_MOUSE, H2A2A_MOUSE, H2A3_MOUSE	Histone H2A	LPKKTESHHKAK	20	1.30E-05	351.7071	1402.7995	4	-0.0050	12	0	GluC
H2A1H_MOUSE, H2A1_MOUSE, H2A2A_MOUSE, H2A3_MOUSE	Histone H2A	LPKKTESHHKAK	20	1.20E-07	351.7099	1402.8107	4	0.0063	12	0	Chymotrypsin
H2A1H_MOUSE, H2A1_MOUSE, H2A2A_MOUSE, H2A3_MOUSE	Histone H2A	LPKKTESHHKAK	20	4.40E-05	702.4115	1402.8084	2	0.0040	12	0	Chymotrypsin
H2A1H_MOUSE, H2A1_MOUSE, H2A2A_MOUSE, H2A3_MOUSE	Histone H2A	LPKKTESHHKAK	20	9.10E-03	702.4107	1402.8068	2	0.0024	12	0	Chymotrypsin
H2A1H_MOUSE, H2A1_MOUSE, H2A2A_MOUSE, H2A3_MOUSE	Histone H2A	LPKKTESHHKAK	20	1.80E-04	702.4107	1402.8068	2	0.0024	12	0	Chymotrypsin
H2A1H_MOUSE, H2A1_MOUSE, H2A2A_MOUSE, H2A3_MOUSE	Histone H2A	LPKKTESHHKAK	20	1.30E-06	468.6093	1402.8062	3	0.0017	12	0	Chymotrypsin
H2A1H_MOUSE, H2A1_MOUSE, H2A2A_MOUSE, H2A3_MOUSE	Histone H2A	LPKKTESHHKAK	20	2.30E-03	702.4099	1402.8051	2	0.0007	12	0	GluC
H2A1H_MOUSE, H2A1_MOUSE, H2A2A_MOUSE, H2A3_MOUSE	Histone H2A	LPKKTESHHKAK	20	4.40E-04	702.4099	1402.8051	2	0.0007	12	0	GluC
H2A1H_MOUSE, H2A1_MOUSE, H2A2A_MOUSE, H2A3_MOUSE	Histone H2A	LPKKTESHHKAK	20	3.70E-05	351.7085	1402.8048	4	0.0004	12	0	Chymotrypsin
H2A1H_MOUSE, H2A1_MOUSE, H2A2A_MOUSE, H2A3_MOUSE	Histone H2A	LPKKTESHHKAK	20	2.60E-06	468.6089	1402.8048	3	0.0003	12	0	Chymotrypsin
H2A1H_MOUSE, H2A1_MOUSE, H2A2A_MOUSE, H2A3_MOUSE	Histone H2A	LPKKTESHHKAK	20	4.30E-05	468.6087	1402.8044	3	0.0000	12	0	Chymotrypsin
H2A1H_MOUSE, H2A1_MOUSE, H2A2A_MOUSE, H2A3_MOUSE	Histone H2A	LPKKTESHHKAK	20	1.90E-06	468.6087	1402.8042	3	-0.0002	12	0	Chymotrypsin
H2A1H_MOUSE, H2A1_MOUSE, H2A2A_MOUSE, H2A3_MOUSE	Histone H2A	LPKKTESHHKAK	20	1.90E-06	351.7083	1402.8042	4	-0.0002	12	0	GluC
H2A1H_MOUSE, H2A1_MOUSE, H2A2A_MOUSE, H2A3_MOUSE	Histone H2A	LPKKTESHHKAK	20	9.90E-07	468.6086	1402.8041	3	-0.0004	12	0	GluC
H2A1H_MOUSE, H2A1_MOUSE, H2A2A_MOUSE, H2A3_MOUSE	Histone H2A	LPKKTESHHKAK	20	3.80E-06	351.7083	1402.8039	4	-0.0005	12	0	Chymotrypsin
H2A1H_MOUSE, H2A1_MOUSE, H2A2A_MOUSE, H2A3_MOUSE	Histone H2A	LPKKTESHHKAK	20	8.40E-07	351.7082	1402.8037	4	-0.0008	12	0	GluC
H2A1H_MOUSE, H2A1_MOUSE, H2A2A_MOUSE, H2A3_MOUSE	Histone H2A	LPKKTESHHKAK	20	2.80E-08	351.7081	1402.8035	4	-0.0009	12	0	GluC
H2A1H_MOUSE, H2A1_MOUSE, H2A2A_MOUSE, H2A3_MOUSE	Histone H2A	LPKKTESHHKAK	20	4.00E-05	468.6084	1402.8035	3	-0.0010	12	0	GluC
H2A1H_MOUSE, H2A1_MOUSE, H2A2A_MOUSE, H2A3_MOUSE	Histone H2A	LPKKTESHHKAK	20	7.20E-06	468.6084	1402.8033	3	-0.0012	12	0	GluC
H2A1H_MOUSE, H2A1_MOUSE, H2A3_MOUSE, H2A3_MOUSE	Histone H2A	DEELNKLLGRVTIAQGGVLPNIQAVLLPKKTESHHKAK	2	7.90E-03	829.8782	4144.3545	5	0.0061	38	0	AspN
H2A1H_MOUSE, H2A1_MOUSE, H2A3_MOUSE, H2A3_MOUSE	Histone H2A	DEELNKLLGRVTIAQGGVLPNIQAVLLPKKTESHHKAK	2	8.70E-07	829.8782	4144.3545	5	0.0061	38	0	AspN
H2A1K_MOUSE	Histone H2A	THHKAKGK	9	7.00E-06	302.8474	905.5203	3	0.0008	8	0	GluC
H2A1K_MOUSE	Histone H2A	THHKAKGK	9	1.00E-02	453.7671	905.5197	2	0.0002	8	0	GluC
H2A1K_MOUSE	Histone H2A	THHKAKGK	9	2.10E-03	453.767	905.5194	2	-0.0001	8	0	GluC
H2A1K_MOUSE	Histone H2A	THHKAKGK	9	8.10E-06	302.847	905.5193	3	-0.0003	8	0	GluC
H2A1K_MOUSE	Histone H2A	THHKAKGK	9	1.00E-04	302.8469	905.5188	3	-0.0007	8	0	GluC
H2A1K_MOUSE	Histone H2A	THHKAKGK	9	1.70E-05	302.8469	905.5188	3	-0.0007	8	0	GluC
H2A1K_MOUSE	Histone H2A	THHKAKGK	9	2.40E-04	302.8468	905.5185	3	-0.0011	8	0	GluC
H2A1K_MOUSE	Histone H2A	THHKAKGK	9	1.40E-04	302.8467	905.5182	3	-0.0013	8	0	GluC
H2A1K_MOUSE	Histone H2A	THHKAKGK	9	2.10E-03	453.7654	905.5161	2	-0.0034	8	0	GluC
H2A2A_MOUSE	Histone H2A	DEELNKLLGKVTIAQGGVLPNIQAVLLPKKTESHHKAKGK	28	2.60E-04	861.3017	4301.4721	5	0.0135	40	0	AspN
H2A2A_MOUSE	Histone H2A	DEELNKLLGKVTIAQGGVLPNIQAVLLPKKTESHHKAKGK	28	5.70E-03	615.5027	4301.4682	7	0.0095	40	0	AspN
H2A2A_MOUSE	Histone H2A	DEELNKLLGKVTIAQGGVLPNIQAVLLPKKTESHHKAKGK	28	2.60E-03	615.5027	4301.4682	7	0.0095	40	0	AspN
H2A2A_MOUSE	Histone H2A	DEELNKLLGKVTIAQGGVLPNIQAVLLPKKTESHHKAKGK	28	1.10E-05	861.3008	4301.4676	5	0.0089	40	0	AspN
H2A2A_MOUSE	Histone H2A	DEELNKLLGKVTIAQGGVLPNIQAVLLPKKTESHHKAKGK	28	1.60E-07	861.3008	4301.4676	5	0.0089	40	0	AspN
H2A2A_MOUSE	Histone H2A	DEELNKLLGKVTIAQGGVLPNIQAVLLPKKTESHHKAKGK	28	4.10E-03	717.9184	4301.4664	6	0.0078	40	0	AspN
H2A2A_MOUSE	Histone H2A	DEELNKLLGKVTIAQGGVLPNIQAVLLPKKTESHHKAKGK	28	2.80E-03	615.5024	4301.4662	7	0.0076	40	0	AspN
H2A2A_MOUSE	Histone H2A	DEELNKLLGKVTIAQGGVLPNIQAVLLPKKTESHHKAKGK	28	1.90E-03	1434.8292	4301.4658	3	0.0071	40	0	AspN
H2A2A_MOUSE	Histone H2A	DEELNKLLGKVTIAQGGVLPNIQAVLLPKKTESHHKAKGK	28	5.50E-04	615.5023	4301.4651	7	0.0064	40	0	AspN
H2A2A_MOUSE	Histone H2A	DEELNKLLGKVTIAQGGVLPNIQAVLLPKKTESHHKAKGK	28	1.60E-03	1076.3734	4301.4645	4	0.0058	40	0	AspN
H2A2A_MOUSE	Histone H2A	DEELNKLLGKVTIAQGGVLPNIQAVLLPKKTESHHKAKGK	28	6.60E-05	1076.3734	4301.4645	4	0.0058	40	0	AspN
H2A2A_MOUSE	Histone H2A	DEELNKLLGKVTIAQGGVLPNIQAVLLPKKTESHHKAKGK	28	5.20E-03	861.3001	4301.4640	5	0.0054	40	0	AspN
H2A2A_MOUSE	Histone H2A	DEELNKLLGKVTIAQGGVLPNIQAVLLPKKTESHHKAKGK	28	5.90E-08	861.3001	4301.4640	5	0.0054	40	0	AspN
H2A2A_MOUSE	Histone H2A	DEELNKLLGKVTIAQGGVLPNIQAVLLPKKTESHHKAKGK	28	3.60E-03	861.3	4301.4637	5	0.0051	40	0	AspN
H2A2A_MOUSE	Histone H2A	DEELNKLLGKVTIAQGGVLPNIQAVLLPKKTESHHKAKGK	28	1.70E-05	861.3	4301.4637	5	0.0051	40	0	AspN
H2A2A_MOUSE	Histone H2A	DEELNKLLGKVTIAQGGVLPNIQAVLLPKKTESHHKAKGK	28	6.70E-04	861.3	4301.4636	5	0.0049	40	0	AspN
H2A2A_MOUSE	Histone H2A	DEELNKLLGKVTIAQGGVLPNIQAVLLPKKTESHHKAKGK	28	7.10E-05	861.3	4301.4636	5	0.0049	40	0	AspN
H2A2A_MOUSE	Histone H2A	DEELNKLLGKVTIAQGGVLPNIQAVLLPKKTESHHKAKGK	28	1.80E-03	717.9178	4301.4631	6	0.0044	40	0	AspN
H2A2A_MOUSE	Histone H2A	DEELNKLLGKVTIAQGGVLPNIQAVLLPKKTESHHKAKGK	28	2.30E-04	1434.8283	4301.4631	3	0.0044	40	0	AspN
H2A2A_MOUSE	Histone H2A	DEELNKLLGKVTIAQGGVLPNIQAVLLPKKTESHHKAKGK	28	6.70E-03	1076.3729	4301.4625	4	0.0038	40	0	AspN
H2A2A_MOUSE	Histone H2A	DEELNKLLGKVTIAQGGVLPNIQAVLLPKKTESHHKAKGK	28	3.60E-03	717.9177	4301.4625	6	0.0038	40	0	AspN
H2A2A_MOUSE	Histone H2A	DEELNKLLGKVTIAQGGVLPNIQAVLLPKKTESHHKAKGK	28	7.70E-04	1076.3729	4301.4625	4	0.0038	40	0	AspN
H2A2A_MOUSE	Histone H2A	DEELNKLLGKVTIAQGGVLPNIQAVLLPKKTESHHKAKGK	28	6.70E-06	861.2995	4301.4609	5	0.0023	40	0	AspN
H2A2A_MOUSE	Histone H2A	DEELNKLLGKVTIAQGGVLPNIQAVLLPKKTESHHKAKGK	28	1.40E-08	861.2995	4301.4609	5	0.0023	40	0	AspN
H2A2A_MOUSE	Histone H2A	DEELNKLLGKVTIAQGGVLPNIQAVLLPKKTESHHKAKGK	28	4.40E-03	615.5017	4301.4608	7	0.0022	40	0	AspN
H2A2A_MOUSE	Histone H2A	DEELNKLLGKVTIAQGGVLPNIQAVLLPKKTESHHKAKGK	28	4.80E-04	615.5017	4301.4608	7	0.0022	40	0	AspN
H2A2A_MOUSE	Histone H2A	DEELNKLLGKVTIAQGGVLPNIQAVLLPKKTESHHKAKGK	28	1.60E-03	1076.3724	4301.4605	4	0.0018	40	0	AspN
H2A2A_MOUSE	Histone H2A	DEELNKLLGKVTIAQGGVLPNIQAVLLPKKTESHHKAKGK	28	2.10E-04	1076.3724	4301.4605	4	0.0018	40	0	AspN
H2A2A_MOUSE	Histone H2A	LNKLLGKVTIAQGGVLPNIQAVLLPKKTESHHKAKGK	2	1.10E-06	655.7341	3928.3609	6	0.0144	37	1	GluC
H2A2A_MOUSE	Histone H2A	LNKLLGKVTIAQGGVLPNIQAVLLPKKTESHHKAKGK	2	6.50E-03	786.6758	3928.3424	5	-0.0041	37	1	GluC
H2A2A_MOUSE, H2A2B_MOUSE, H2A2C_MOUSE	Histone H2A	VGAGAPVYMAAVLEYLTAEILELAGNAAR	2	4.40E-07	978.5207	2932.5402	3	0.0034	29	0	Semi-tryptic
H2A2A_MOUSE, H2A2B_MOUSE, H2A2C_MOUSE	Histone H2A	VGAGAPVYMAAVLEYLTAEILELAGNAAR	2	2.10E-04	734.1418	2932.5380	4	0.0012	29	0	Semi-tryptic
H2A2A_MOUSE, H2A2B_MOUSE, H2A2C_MOUSE	Histone H2A	RVGAGAPVYMAAVLE	89	7.80E-04	752.402	1502.7894	2	-0.0021	15	0	GluC
H2A2A_MOUSE, H2A2B_MOUSE, H2A2C_MOUSE	Histone H2A	RVGAGAPVYMAAVLE	89	3.10E-03	752.402	1502.7894	2	-0.0022	15	0	GluC
H2A2A_MOUSE, H2A2B_MOUSE, H2A2C_MOUSE	Histone H2A	RVGAGAPVYMAAVLE	89	5.10E-04	501.937	1502.7892	3	-0.0023	15	0	GluC
H2A2A_MOUSE, H2A2B_MOUSE, H2A2C_MOUSE	Histone H2A	RVGAGAPVYMAAVLE	89	2.00E-03	752.4017	1502.7888	2	-0.0028	15	0	GluC
H2A2A_MOUSE, H2A2B_MOUSE, H2A2C_MOUSE	Histone H2A	RVGAGAPVYMAAVLE	89	1.30E-03	752.4016	1502.7886	2	-0.0029	15	0	GluC
H2A2A_MOUSE, H2A2B_MOUSE, H2A2C_MOUSE	Histone H2A	RVGAGAPVYMAAVLE	89	3.20E-03	752.4085	1502.8023	2	0.0108	15	0	GluC
H2A2A_MOUSE, H2A2B_MOUSE, H2A2C_MOUSE	Histone H2A	RVGAGAPVYMAAVLE	89	8.80E-04	752.4079	1502.8013	2	0.0098	15	0	GluC
H2A2A_MOUSE, H2A2B_MOUSE, H2A2C_MOUSE	Histone H2A	RVGAGAPVYMAAVLE	89	2.00E-03	752.4077	1502.8008	2	0.0092	15	0	GluC
H2A2A_MOUSE, H2A2B_MOUSE, H2A2C_MOUSE	Histone H2A	RVGAGAPVYMAAVLE	89	9.40E-03	752.407	1502.7994	2	0.0078	15	0	GluC

H2A2A_MOUSE, H2A2B_MOUSE, H2A2C_MOUSE	Histone H2A	RVGAGAPVYMAAVLE	89	1.60E-03	752.4066	1502.7986	2	0.0071	15	0	GluC
H2A2A_MOUSE, H2A2B_MOUSE, H2A2C_MOUSE	Histone H2A	RVGAGAPVYMAAVLE	89	5.90E-03	752.4054	1502.7963	2	0.0048	15	0	GluC
H2A2A_MOUSE, H2A2B_MOUSE, H2A2C_MOUSE	Histone H2A	RVGAGAPVYMAAVLE	89	7.80E-04	752.4053	1502.7961	2	0.0046	15	0	GluC
H2A2A_MOUSE, H2A2B_MOUSE, H2A2C_MOUSE	Histone H2A	RVGAGAPVYMAAVLE	89	1.20E-03	752.405	1502.7954	2	0.0038	15	0	GluC
H2A2A_MOUSE, H2A2B_MOUSE, H2A2C_MOUSE	Histone H2A	RVGAGAPVYMAAVLE	89	9.10E-03	752.4049	1502.7953	2	0.0037	15	0	GluC
H2A2A_MOUSE, H2A2B_MOUSE, H2A2C_MOUSE	Histone H2A	RVGAGAPVYMAAVLE	89	3.80E-03	752.4047	1502.7949	2	0.0034	15	0	GluC
H2A2A_MOUSE, H2A2B_MOUSE, H2A2C_MOUSE	Histone H2A	RVGAGAPVYMAAVLE	89	4.30E-04	752.4047	1502.7949	2	0.0034	15	0	GluC
H2A2A_MOUSE, H2A2B_MOUSE, H2A2C_MOUSE	Histone H2A	RVGAGAPVYMAAVLE	89	6.80E-05	752.4047	1502.7949	2	0.0034	15	0	GluC
H2A2A_MOUSE, H2A2B_MOUSE, H2A2C_MOUSE	Histone H2A	RVGAGAPVYMAAVLE	89	4.70E-04	752.4047	1502.7948	2	0.0033	15	0	GluC
H2A2A_MOUSE, H2A2B_MOUSE, H2A2C_MOUSE	Histone H2A	RVGAGAPVYMAAVLE	89	1.20E-03	752.4047	1502.7949	2	0.0033	15	0	GluC
H2A2A_MOUSE, H2A2B_MOUSE, H2A2C_MOUSE	Histone H2A	RVGAGAPVYMAAVLE	89	7.30E-04	752.4046	1502.7947	2	0.0032	15	0	GluC
H2A2A_MOUSE, H2A2B_MOUSE, H2A2C_MOUSE	Histone H2A	RVGAGAPVYMAAVLE	89	7.30E-03	752.4046	1502.7946	2	0.0031	15	0	GluC
H2A2A_MOUSE, H2A2B_MOUSE, H2A2C_MOUSE	Histone H2A	RVGAGAPVYMAAVLE	89	5.30E-03	752.4046	1502.7946	2	0.0031	15	0	GluC
H2A2A_MOUSE, H2A2B_MOUSE, H2A2C_MOUSE	Histone H2A	RVGAGAPVYMAAVLE	89	1.80E-03	752.4046	1502.7946	2	0.0031	15	0	GluC
H2A2A_MOUSE, H2A2B_MOUSE, H2A2C_MOUSE	Histone H2A	RVGAGAPVYMAAVLE	89	1.50E-03	752.4045	1502.7945	2	0.0029	15	0	GluC
H2A2A_MOUSE, H2A2B_MOUSE, H2A2C_MOUSE	Histone H2A	RVGAGAPVYMAAVLE	89	1.10E-05	752.4045	1502.7944	2	0.0029	15	0	GluC
H2A2A_MOUSE, H2A2B_MOUSE, H2A2C_MOUSE	Histone H2A	RVGAGAPVYMAAVLE	89	7.30E-03	752.4044	1502.7943	2	0.0027	15	0	GluC
H2A2A_MOUSE, H2A2B_MOUSE, H2A2C_MOUSE	Histone H2A	RVGAGAPVYMAAVLE	89	1.30E-03	752.4044	1502.7942	2	0.0027	15	0	GluC
H2A2A_MOUSE, H2A2B_MOUSE, H2A2C_MOUSE	Histone H2A	RVGAGAPVYMAAVLE	89	8.10E-04	752.4044	1502.7942	2	0.0027	15	0	GluC
H2A2A_MOUSE, H2A2B_MOUSE, H2A2C_MOUSE	Histone H2A	RVGAGAPVYMAAVLE	89	4.90E-04	752.4044	1502.7942	2	0.0027	15	0	GluC
H2A2A_MOUSE, H2A2B_MOUSE, H2A2C_MOUSE	Histone H2A	RVGAGAPVYMAAVLE	89	3.50E-03	752.4044	1502.7942	2	0.0026	15	0	GluC
H2A2A_MOUSE, H2A2B_MOUSE, H2A2C_MOUSE	Histone H2A	RVGAGAPVYMAAVLE	89	3.20E-03	752.4043	1502.7941	2	0.0026	15	0	GluC
H2A2A_MOUSE, H2A2B_MOUSE, H2A2C_MOUSE	Histone H2A	RVGAGAPVYMAAVLE	89	3.10E-05	752.4044	1502.7942	2	0.0026	15	0	GluC
H2A2A_MOUSE, H2A2B_MOUSE, H2A2C_MOUSE	Histone H2A	RVGAGAPVYMAAVLE	89	6.00E-04	752.4043	1502.7940	2	0.0025	15	0	GluC
H2A2A_MOUSE, H2A2B_MOUSE, H2A2C_MOUSE	Histone H2A	RVGAGAPVYMAAVLE	89	2.80E-03	752.4042	1502.7939	2	0.0024	15	0	GluC
H2A2A_MOUSE, H2A2B_MOUSE, H2A2C_MOUSE	Histone H2A	RVGAGAPVYMAAVLE	89	7.20E-04	752.4042	1502.7939	2	0.0024	15	0	GluC
H2A2A_MOUSE, H2A2B_MOUSE, H2A2C_MOUSE	Histone H2A	RVGAGAPVYMAAVLE	89	7.20E-04	752.4042	1502.7938	2	0.0023	15	0	GluC
H2A2A_MOUSE, H2A2B_MOUSE, H2A2C_MOUSE	Histone H2A	RVGAGAPVYMAAVLE	89	3.40E-03	752.4041	1502.7937	2	0.0022	15	0	GluC
H2A2A_MOUSE, H2A2B_MOUSE, H2A2C_MOUSE	Histone H2A	RVGAGAPVYMAAVLE	89	2.40E-03	752.4042	1502.7938	2	0.0022	15	0	GluC
H2A2A_MOUSE, H2A2B_MOUSE, H2A2C_MOUSE	Histone H2A	RVGAGAPVYMAAVLE	89	1.30E-03	752.4041	1502.7937	2	0.0022	15	0	GluC
H2A2A_MOUSE, H2A2B_MOUSE, H2A2C_MOUSE	Histone H2A	RVGAGAPVYMAAVLE	89	2.10E-04	752.4041	1502.7937	2	0.0022	15	0	GluC
H2A2A_MOUSE, H2A2B_MOUSE, H2A2C_MOUSE	Histone H2A	RVGAGAPVYMAAVLE	89	6.10E-05	752.4042	1502.7938	2	0.0022	15	0	GluC
H2A2A_MOUSE, H2A2B_MOUSE, H2A2C_MOUSE	Histone H2A	RVGAGAPVYMAAVLE	89	8.70E-03	752.4041	1502.7936	2	0.0021	15	0	GluC
H2A2A_MOUSE, H2A2B_MOUSE, H2A2C_MOUSE	Histone H2A	RVGAGAPVYMAAVLE	89	7.90E-03	752.4041	1502.7936	2	0.0021	15	0	GluC
H2A2A_MOUSE, H2A2B_MOUSE, H2A2C_MOUSE	Histone H2A	RVGAGAPVYMAAVLE	89	7.90E-04	752.4041	1502.7936	2	0.0021	15	0	GluC
H2A2A_MOUSE, H2A2B_MOUSE, H2A2C_MOUSE	Histone H2A	RVGAGAPVYMAAVLE	89	6.80E-03	752.404	1502.7935	2	0.0020	15	0	GluC
H2A2A_MOUSE, H2A2B_MOUSE, H2A2C_MOUSE	Histone H2A	RVGAGAPVYMAAVLE	89	4.20E-03	752.4041	1502.7936	2	0.0020	15	0	GluC
H2A2A_MOUSE, H2A2B_MOUSE, H2A2C_MOUSE	Histone H2A	RVGAGAPVYMAAVLE	89	3.30E-04	752.4041	1502.7936	2	0.0020	15	0	GluC
H2A2A_MOUSE, H2A2B_MOUSE, H2A2C_MOUSE	Histone H2A	RVGAGAPVYMAAVLE	89	8.40E-03	752.404	1502.7934	2	0.0019	15	0	GluC
H2A2A_MOUSE, H2A2B_MOUSE, H2A2C_MOUSE	Histone H2A	RVGAGAPVYMAAVLE	89	6.90E-03	752.404	1502.7934	2	0.0019	15	0	GluC
H2A2A_MOUSE, H2A2B_MOUSE, H2A2C_MOUSE	Histone H2A	RVGAGAPVYMAAVLE	89	1.30E-03	752.404	1502.7934	2	0.0019	15	0	GluC
H2A2A_MOUSE, H2A2B_MOUSE, H2A2C_MOUSE	Histone H2A	RVGAGAPVYMAAVLE	89	4.60E-04	752.404	1502.7934	2	0.0019	15	0	GluC
H2A2A_MOUSE, H2A2B_MOUSE, H2A2C_MOUSE	Histone H2A	RVGAGAPVYMAAVLE	89	8.20E-03	752.4039	1502.7933	2	0.0018	15	0	GluC
H2A2A_MOUSE, H2A2B_MOUSE, H2A2C_MOUSE	Histone H2A	RVGAGAPVYMAAVLE	89	1.00E-03	752.404	1502.7934	2	0.0018	15	0	GluC
H2A2A_MOUSE, H2A2B_MOUSE, H2A2C_MOUSE	Histone H2A	RVGAGAPVYMAAVLE	89	7.40E-04	752.404	1502.7934	2	0.0018	15	0	GluC
H2A2A_MOUSE, H2A2B_MOUSE, H2A2C_MOUSE	Histone H2A	RVGAGAPVYMAAVLE	89	8.40E-03	752.4039	1502.7932	2	0.0017	15	0	GluC
H2A2A_MOUSE, H2A2B_MOUSE, H2A2C_MOUSE	Histone H2A	RVGAGAPVYMAAVLE	89	2.70E-03	752.4039	1502.7932	2	0.0017	15	0	GluC
H2A2A_MOUSE, H2A2B_MOUSE, H2A2C_MOUSE	Histone H2A	RVGAGAPVYMAAVLE	89	3.80E-03	752.4038	1502.7931	2	0.0016	15	0	GluC
H2A2A_MOUSE, H2A2B_MOUSE, H2A2C_MOUSE	Histone H2A	RVGAGAPVYMAAVLE	89	2.40E-03	752.4038	1502.7931	2	0.0016	15	0	GluC
H2A2A_MOUSE, H2A2B_MOUSE, H2A2C_MOUSE	Histone H2A	RVGAGAPVYMAAVLE	89	2.30E-03	752.4039	1502.7931	2	0.0016	15	0	GluC
H2A2A_MOUSE, H2A2B_MOUSE, H2A2C_MOUSE	Histone H2A	RVGAGAPVYMAAVLE	89	1.50E-03	752.4038	1502.7931	2	0.0016	15	0	GluC
H2A2A_MOUSE, H2A2B_MOUSE, H2A2C_MOUSE	Histone H2A	RVGAGAPVYMAAVLE	89	7.00E-04	752.4038	1502.7931	2	0.0016	15	0	GluC
H2A2A_MOUSE, H2A2B_MOUSE, H2A2C_MOUSE	Histone H2A	RVGAGAPVYMAAVLE	89	3.80E-03	752.4037	1502.7929	2	0.0014	15	0	GluC
H2A2A_MOUSE, H2A2B_MOUSE, H2A2C_MOUSE	Histone H2A	RVGAGAPVYMAAVLE	89	3.70E-03	752.4037	1502.7929	2	0.0014	15	0	GluC
H2A2A_MOUSE, H2A2B_MOUSE, H2A2C_MOUSE	Histone H2A	RVGAGAPVYMAAVLE	89	8.00E-04	752.4037	1502.7929	2	0.0014	15	0	GluC
H2A2A_MOUSE, H2A2B_MOUSE, H2A2C_MOUSE	Histone H2A	RVGAGAPVYMAAVLE	89	7.60E-04	752.4038	1502.7930	2	0.0014	15	0	GluC
H2A2A_MOUSE, H2A2B_MOUSE, H2A2C_MOUSE	Histone H2A	RVGAGAPVYMAAVLE	89	1.40E-06	752.4037	1502.7929	2	0.0014	15	0	GluC
H2A2A_MOUSE, H2A2B_MOUSE, H2A2C_MOUSE	Histone H2A	RVGAGAPVYMAAVLE	89	2.30E-04	752.4037	1502.7928	2	0.0013	15	0	GluC
H2A2A_MOUSE, H2A2B_MOUSE, H2A2C_MOUSE	Histone H2A	RVGAGAPVYMAAVLE	89	1.00E-02	752.4036	1502.7927	2	0.0012	15	0	GluC
H2A2A_MOUSE, H2A2B_MOUSE, H2A2C_MOUSE	Histone H2A	RVGAGAPVYMAAVLE	89	1.30E-03	752.4036	1502.7927	2	0.0012	15	0	GluC
H2A2A_MOUSE, H2A2B_MOUSE, H2A2C_MOUSE	Histone H2A	RVGAGAPVYMAAVLE	89	8.70E-03	752.4036	1502.7926	2	0.0011	15	0	GluC
H2A2A_MOUSE, H2A2B_MOUSE, H2A2C_MOUSE	Histone H2A	RVGAGAPVYMAAVLE	89	4.40E-04	752.4036	1502.7927	2	0.0011	15	0	GluC
H2A2A_MOUSE, H2A2B_MOUSE, H2A2C_MOUSE	Histone H2A	RVGAGAPVYMAAVLE	89	1.50E-04	752.4036	1502.7926	2	0.0011	15	0	GluC
H2A2A_MOUSE, H2A2B_MOUSE, H2A2C_MOUSE	Histone H2A	RVGAGAPVYMAAVLE	89	2.60E-04	752.4035	1502.7925	2	0.0010	15	0	GluC
H2A2A_MOUSE, H2A2B_MOUSE, H2A2C_MOUSE	Histone H2A	RVGAGAPVYMAAVLE	89	7.00E-04	752.4035	1502.7925	2	0.0009	15	0	GluC
H2A2A_MOUSE, H2A2B_MOUSE, H2A2C_MOUSE	Histone H2A	RVGAGAPVYMAAVLE	89	2.60E-03	752.4034	1502.7923	2	0.0007	15	0	GluC
H2A2A_MOUSE, H2A2B_MOUSE, H2A2C_MOUSE	Histone H2A	RVGAGAPVYMAAVLE	89	2.70E-04	752.4034	1502.7922	2	0.0007	15	0	GluC
H2A2A_MOUSE, H2A2B_MOUSE, H2A2C_MOUSE	Histone H2A	RVGAGAPVYMAAVLE	89	4.90E-04	752.4034	1502.7922	2	0.0006	15	0	GluC
H2A2A_MOUSE, H2A2B_MOUSE, H2A2C_MOUSE	Histone H2A	RVGAGAPVYMAAVLE	89	4.30E-03	752.4032	1502.7919	2	0.0004	15	0	GluC
H2A2A_MOUSE, H2A2B_MOUSE, H2A2C_MOUSE	Histone H2A	RVGAGAPVYMAAVLE	89	6.80E-04	752.4032	1502.7918	2	0.0003	15	0	GluC
H2A2A_MOUSE, H2A2B_MOUSE, H2A2C_MOUSE	Histone H2A	RVGAGAPVYMAAVLE	89	2.10E-03	752.4031	1502.7917	2	0.0002	15	0	GluC
H2A2A_MOUSE, H2A2B_MOUSE, H2A2C_MOUSE	Histone H2A	RVGAGAPVYMAAVLE	89	2.60E-03	752.403	1502.7915	2	0.0000	15	0	GluC
H2A2A_MOUSE, H2A2B_MOUSE, H2A2C_MOUSE	Histone H2A	RVGAGAPVYMAAVLE	89	1.10E-03	501.9378	1502.7915	3	0.0000	15	0	GluC
H2A2A_MOUSE, H2A2B_MOUSE, H2A2C_MOUSE	Histone H2A	RVGAGAPVYMAAVLE	89	9.40E-03	752.4029	1502.7913	2	-0.0002	15	0	GluC

H2A2A_MOUSE, H2A2B_MOUSE, H2A2C_MOUSE	Histone H2A	RVGAGAPVYMAAVLE	89	4.80E-03	752.4029	1502.7912	2	-0.0003	15	0	GluC
H2A2A_MOUSE, H2A2B_MOUSE, H2A2C_MOUSE	Histone H2A	RVGAGAPVYMAAVLE	89	1.70E-03	752.4028	1502.7910	2	-0.0005	15	0	GluC
H2A2A_MOUSE, H2A2B_MOUSE, H2A2C_MOUSE	Histone H2A	RVGAGAPVYMAAVLE	89	7.10E-04	752.4025	1502.7903	2	-0.0012	15	0	GluC
H2A2A_MOUSE, H2A2B_MOUSE, H2A2C_MOUSE	Histone H2A	RVGAGAPVYMAAVLE	89	3.10E-03	752.4024	1502.7902	2	-0.0013	15	0	GluC
H2A2A_MOUSE, H2A2B_MOUSE, H2A2C_MOUSE	Histone H2A	RVGAGAPVYMAAVLE	89	9.40E-03	501.9373	1502.7900	3	-0.0015	15	0	GluC
H2A2A_MOUSE, H2A2B_MOUSE, H2A2C_MOUSE	Histone H2A	RVGAGAPVYMAAVLE	89	4.40E-04	752.4021	1502.7897	2	-0.0019	15	0	GluC
H2A2A_MOUSE, H2A2B_MOUSE, H2A2C_MOUSE, H2A3_MOUSE, H2AJ_MOUSE, H2AX_MOUSE	Histone H2A	SRSSRAGLQFPVGRVHR	3	9.70E-03	478.2705	1909.0528	4	0.0021	17	3	Semi-tryptic
H2A2A_MOUSE, H2A2B_MOUSE, H2A2C_MOUSE, H2A3_MOUSE, H2AJ_MOUSE, H2AX_MOUSE	Histone H2A	SRSSRAGLQFPVGRVHR	3	3.50E-03	478.2699	1909.0506	4	-0.0001	17	3	Semi-tryptic
H2A2A_MOUSE, H2A2B_MOUSE, H2A2C_MOUSE, H2A3_MOUSE, H2AJ_MOUSE, H2AX_MOUSE	Histone H2A	SRSSRAGLQFPVGRVHR	3	4.00E-03	478.2698	1909.0499	4	-0.0008	17	3	Semi-tryptic
H2A2A_MOUSE, H2A2B_MOUSE, H2A2C_MOUSE, H2A3_MOUSE, H2AJ_MOUSE, H2AX_MOUSE	Histone H2A	SRSSRAGLQFPVGR	3	3.60E-05	506.6148	1516.8226	3	0.0004	14	2	Semi-tryptic
H2A2A_MOUSE, H2A2B_MOUSE, H2A2C_MOUSE, H2A3_MOUSE, H2AJ_MOUSE, H2AX_MOUSE	Histone H2A	SRSSRAGLQFPVGR	3	1.80E-06	506.6144	1516.8215	3	-0.0007	14	2	Semi-tryptic
H2A2A_MOUSE, H2A2B_MOUSE, H2A2C_MOUSE, H2A3_MOUSE, H2AJ_MOUSE, H2AX_MOUSE	Histone H2A	SRSSRAGLQFPVGR	3	2.50E-05	506.6141	1516.8204	3	-0.0019	14	2	Semi-tryptic
H2A2A_MOUSE, H2A2B_MOUSE, H2A2C_MOUSE, H2AJ_MOUSE	Histone H2A	LLRKGNYAER	9	9.80E-03	407.2361	1218.6864	3	0.0032	10	2	Semi-tryptic
H2A2A_MOUSE, H2A2B_MOUSE, H2A2C_MOUSE, H2AJ_MOUSE	Histone H2A	LLRKGNYAER	9	7.90E-03	407.2357	1218.6853	3	0.0020	10	2	Semi-tryptic
H2A2A_MOUSE, H2A2B_MOUSE, H2A2C_MOUSE, H2AJ_MOUSE	Histone H2A	LLRKGNYAER	9	3.90E-04	610.3497	1218.6848	2	0.0015	10	2	Semi-tryptic
H2A2A_MOUSE, H2A2B_MOUSE, H2A2C_MOUSE, H2AJ_MOUSE	Histone H2A	LLRKGNYAER	9	2.80E-05	610.3496	1218.6846	2	0.0014	10	2	Semi-tryptic
H2A2A_MOUSE, H2A2B_MOUSE, H2A2C_MOUSE, H2AJ_MOUSE	Histone H2A	LLRKGNYAER	9	2.30E-04	610.3494	1218.6843	2	0.0010	10	2	Semi-tryptic
H2A2A_MOUSE, H2A2B_MOUSE, H2A2C_MOUSE, H2AJ_MOUSE	Histone H2A	LLRKGNYAER	9	3.00E-05	610.3494	1218.6842	2	0.0009	10	2	Semi-tryptic
H2A2A_MOUSE, H2A2B_MOUSE, H2A2C_MOUSE, H2AJ_MOUSE	Histone H2A	LLRKGNYAER	9	4.20E-03	407.2353	1218.6839	3	0.0007	10	2	Semi-tryptic
H2A2A_MOUSE, H2A2B_MOUSE, H2A2C_MOUSE, H2AJ_MOUSE	Histone H2A	LLRKGNYAER	9	7.40E-04	407.2351	1218.6836	3	0.0003	10	2	Semi-tryptic
H2A2A_MOUSE, H2A2B_MOUSE, H2A2C_MOUSE, H2AJ_MOUSE	Histone H2A	LLRKGNYAER	9	4.70E-04	407.2348	1218.6825	3	-0.0008	10	2	Semi-tryptic
H2A2A_MOUSE, H2A2B_MOUSE, H2A2C_MOUSE, H2AJ_MOUSE	Histone H2A	KGNYAER	21	1.90E-04	419.2154	836.4162	2	0.0022	7	1	Semi-tryptic
H2A2A_MOUSE, H2A2B_MOUSE, H2A2C_MOUSE, H2AJ_MOUSE	Histone H2A	KGNYAER	21	8.40E-05	419.2152	836.4158	2	0.0018	7	1	Semi-tryptic
H2A2A_MOUSE, H2A2B_MOUSE, H2A2C_MOUSE, H2AJ_MOUSE	Histone H2A	KGNYAER	21	6.70E-05	419.2149	836.4152	2	0.0012	7	1	Semi-tryptic
H2A2A_MOUSE, H2A2B_MOUSE, H2A2C_MOUSE, H2AJ_MOUSE	Histone H2A	KGNYAER	21	2.00E-05	419.2148	836.4150	2	0.0010	7	1	Semi-tryptic
H2A2A_MOUSE, H2A2B_MOUSE, H2A2C_MOUSE, H2AJ_MOUSE	Histone H2A	KGNYAER	21	2.10E-04	419.2147	836.4148	2	0.0008	7	1	Semi-tryptic
H2A2A_MOUSE, H2A2B_MOUSE, H2A2C_MOUSE, H2AJ_MOUSE	Histone H2A	KGNYAER	21	1.90E-04	419.2146	836.4147	2	0.0007	7	1	Semi-tryptic
H2A2A_MOUSE, H2A2B_MOUSE, H2A2C_MOUSE, H2AJ_MOUSE	Histone H2A	KGNYAER	21	5.20E-04	419.2146	836.4147	2	0.0006	7	1	Semi-tryptic
H2A2A_MOUSE, H2A2B_MOUSE, H2A2C_MOUSE, H2AJ_MOUSE	Histone H2A	KGNYAER	21	2.10E-04	419.2146	836.4146	2	0.0006	7	1	Semi-tryptic
H2A2A_MOUSE, H2A2B_MOUSE, H2A2C_MOUSE, H2AJ_MOUSE	Histone H2A	KGNYAER	21	7.90E-06	419.2146	836.4147	2	0.0006	7	1	Semi-tryptic
H2A2A_MOUSE, H2A2B_MOUSE, H2A2C_MOUSE, H2AJ_MOUSE	Histone H2A	KGNYAER	21	1.90E-04	419.2145	836.4145	2	0.0004	7	1	Semi-tryptic
H2A2A_MOUSE, H2A2B_MOUSE, H2A2C_MOUSE, H2AJ_MOUSE	Histone H2A	KGNYAER	21	2.90E-04	419.2144	836.4143	2	0.0003	7	1	Semi-tryptic
H2A2A_MOUSE, H2A2B_MOUSE, H2A2C_MOUSE, H2AJ_MOUSE	Histone H2A	KGNYAER	21	6.40E-05	419.2144	836.4143	2	0.0002	7	1	Semi-tryptic
H2A2A_MOUSE, H2A2B_MOUSE, H2A2C_MOUSE, H2AJ_MOUSE	Histone H2A	KGNYAER	21	1.90E-05	419.2144	836.4142	2	0.0002	7	1	Semi-tryptic
H2A2A_MOUSE, H2A2B_MOUSE, H2A2C_MOUSE, H2AJ_MOUSE	Histone H2A	KGNYAER	21	7.60E-06	419.2143	836.4141	2	0.0000	7	1	Semi-tryptic
H2A2A_MOUSE, H2A2B_MOUSE, H2A2C_MOUSE, H2AJ_MOUSE	Histone H2A	KGNYAER	21	3.70E-06	419.2143	836.4140	2	0.0000	7	1	Semi-tryptic
H2A2A_MOUSE, H2A2B_MOUSE, H2A2C_MOUSE, H2AJ_MOUSE	Histone H2A	KGNYAER	21	3.80E-05	419.2142	836.4139	2	-0.0001	7	1	Semi-tryptic
H2A2A_MOUSE, H2A2B_MOUSE, H2A2C_MOUSE, H2AJ_MOUSE	Histone H2A	KGNYAER	21	8.10E-06	419.2142	836.4137	2	-0.0003	7	1	Semi-tryptic
H2A2A_MOUSE, H2A2B_MOUSE, H2A2C_MOUSE, H2AJ_MOUSE	Histone H2A	KGNYAER	21	2.50E-06	419.214	836.4135	2	-0.0005	7	1	Semi-tryptic
H2A2A_MOUSE, H2A2B_MOUSE, H2A2C_MOUSE, H2AJ_MOUSE	Histone H2A	KGNYAER	21	2.20E-04	419.214	836.4134	2	-0.0006	7	1	Semi-tryptic
H2A2A_MOUSE, H2A2B_MOUSE, H2A2C_MOUSE, H2AJ_MOUSE	Histone H2A	KGNYAER	21	2.00E-04	419.2139	836.4132	2	-0.0008	7	1	Semi-tryptic
H2A2A_MOUSE, H2A2B_MOUSE, H2A2C_MOUSE, H2AJ_MOUSE	Histone H2A	KGNYAER	21	6.30E-05	419.2137	836.4128	2	-0.0012	7	1	Semi-tryptic
H2A2A_MOUSE, H2A2B_MOUSE, H2A2C_MOUSE, H2AJ_MOUSE, H2AX_MOUSE	Histone H2A	AERVGAGAPVY	2	5.10E-04	545.2883	1088.5620	2	0.0005	11	0	Chymotrypsin
H2A2A_MOUSE, H2A2B_MOUSE, H2A2C_MOUSE, H2AJ_MOUSE, H2AX_MOUSE	Histone H2A	AERVGAGAPVY	2	1.80E-05	545.2878	1088.5610	2	-0.0005	11	0	Chymotrypsin
H2A2A_MOUSE, H2A2C_MOUSE	Histone H2A	HLQLAIRNDEELNKLLGKVTIAQGGVLPNIQAVLLPK	2	4.90E-03	1004.8414	4015.3365	4	0.0055	37	3	Semi-tryptic
H2A2A_MOUSE, H2A2C_MOUSE	Histone H2A	HLQLAIRNDEELNKLLGKVTIAQGGVLPNIQAVLLPK	2	1.80E-04	1004.8411	4015.3353	4	0.0043	37	3	Semi-tryptic
H2A2A_MOUSE, H2A2C_MOUSE	Histone H2A	NDEELNKLLGKVTIAQGGVLPNIQAVLLPK	2	2.30E-08	1062.2838	3183.8296	3	0.0065	30	2	Semi-tryptic
H2A2A_MOUSE, H2A2C_MOUSE	Histone H2A	NDEELNKLLGKVTIAQGGVLPNIQAVLLPK	2	1.50E-05	796.9645	3183.8288	4	0.0057	30	2	Semi-tryptic
H2A2A_MOUSE, H2A2C_MOUSE	Histone H2A	ELNKLLGKVTIAQGGVLPNIQAVLLPKKTE	20	3.10E-05	637.7904	3183.9156	5	0.0197	30	1	GluC
H2A2A_MOUSE, H2A2C_MOUSE	Histone H2A	ELNKLLGKVTIAQGGVLPNIQAVLLPKKTE	20	1.50E-08	796.9855	3183.9129	4	0.0170	30	1	GluC
H2A2A_MOUSE, H2A2C_MOUSE	Histone H2A	ELNKLLGKVTIAQGGVLPNIQAVLLPKKTE	20	3.50E-05	1062.3108	3183.9106	3	0.0147	30	1	GluC
H2A2A_MOUSE, H2A2C_MOUSE	Histone H2A	ELNKLLGKVTIAQGGVLPNIQAVLLPKKTE	20	2.20E-06	796.9849	3183.9103	4	0.0145	30	1	GluC
H2A2A_MOUSE, H2A2C_MOUSE	Histone H2A	ELNKLLGKVTIAQGGVLPNIQAVLLPKKTE	20	1.40E-07	637.7892	3183.9095	5	0.0136	30	1	GluC
H2A2A_MOUSE, H2A2C_MOUSE	Histone H2A	ELNKLLGKVTIAQGGVLPNIQAVLLPKKTE	20	4.10E-04	796.9843	3183.9083	4	0.0125	30	1	GluC
H2A2A_MOUSE, H2A2C_MOUSE	Histone H2A	ELNKLLGKVTIAQGGVLPNIQAVLLPKKTE	20	1.60E-07	796.9843	3183.9083	4	0.0125	30	1	GluC
H2A2A_MOUSE, H2A2C_MOUSE	Histone H2A	ELNKLLGKVTIAQGGVLPNIQAVLLPKKTE	20	7.20E-03	637.7881	3183.9042	5	0.0083	30	1	GluC
H2A2A_MOUSE, H2A2C_MOUSE	Histone H2A	ELNKLLGKVTIAQGGVLPNIQAVLLPKKTE	20	5.40E-04	796.9831	3183.9033	4	0.0075	30	1	GluC
H2A2A_MOUSE, H2A2C_MOUSE	Histone H2A	ELNKLLGKVTIAQGGVLPNIQAVLLPKKTE	20	2.70E-11	796.9831	3183.9033	4	0.0075	30	1	GluC
H2A2A_MOUSE, H2A2C_MOUSE	Histone H2A	ELNKLLGKVTIAQGGVLPNIQAVLLPKKTE	20	2.70E-05	796.9828	3183.9020	4	0.0061	30	1	GluC
H2A2A_MOUSE, H2A2C_MOUSE	Histone H2A	ELNKLLGKVTIAQGGVLPNIQAVLLPKKTE	20	2.60E-11	796.9828	3183.9020	4	0.0061	30	1	GluC
H2A2A_MOUSE, H2A2C_MOUSE	Histone H2A	ELNKLLGKVTIAQGGVLPNIQAVLLPKKTE	20	9.00E-07	1062.3072	3183.8998	3	0.0039	30	1	GluC
H2A2A_MOUSE, H2A2C_MOUSE	Histone H2A	ELNKLLGKVTIAQGGVLPNIQAVLLPKKTE	20	5.50E-04	796.9821	3183.8992	4	0.0033	30	1	GluC
H2A2A_MOUSE, H2A2C_MOUSE	Histone H2A	ELNKLLGKVTIAQGGVLPNIQAVLLPKKTE	20	7.60E-06	796.982	3183.8991	4	0.0033	30	1	GluC
H2A2A_MOUSE, H2A2C_MOUSE	Histone H2A	ELNKLLGKVTIAQGGVLPNIQAVLLPKKTE	20	2.90E-10	796.982	3183.8991	4	0.0033	30	1	GluC
H2A2A_MOUSE, H2A2C_MOUSE	Histone H2A	ELNKLLGKVTIAQGGVLPNIQAVLLPKKTE	20	8.70E-04	796.9816	3183.8974	4	0.0015	30	1	GluC
H2A2A_MOUSE, H2A2C_MOUSE	Histone H2A	ELNKLLGKVTIAQGGVLPNIQAVLLPKKTE	20	1.30E-05	796.9815	3183.8971	4	0.0012	30	1	GluC

H2A2A_MOUSE, H2A2C_MOUSE	Histone H2A	ELNKLLGKVTIAQGGVLPNIQAVLLPKKTE	20	3.20E-10	796.9815	3183.8971	4	0.0012	30	1	GluC
H2A2A_MOUSE, H2A2C_MOUSE	Histone H2A	ELNKLLGKVTIAQGGVLPNIQAVLLPKKTE	20	5.50E-04	796.9814	3183.8966	4	0.0007	30	1	GluC
H2A2A_MOUSE, H2A2C_MOUSE	Histone H2A	LNKLLGKVTIAQGGVLPNIQAVLLPKKTE	372	6.30E-04	611.9773	3054.8501	5	-0.0031	29	0	GluC
H2A2A_MOUSE, H2A2C_MOUSE	Histone H2A	LNKLLGKVTIAQGGVLPNIQAVLLPKKTE	372	7.60E-11	611.9773	3054.8501	5	-0.0031	29	0	GluC
H2A2A_MOUSE, H2A2C_MOUSE	Histone H2A	LNKLLGKVTIAQGGVLPNIQAVLLPKKTE	372	3.60E-07	764.7188	3054.8463	4	-0.0070	29	0	GluC
H2A2A_MOUSE, H2A2C_MOUSE	Histone H2A	LNKLLGKVTIAQGGVLPNIQAVLLPKKTE	372	8.20E-03	764.7255	3054.8731	4	0.0198	29	0	GluC
H2A2A_MOUSE, H2A2C_MOUSE	Histone H2A	LNKLLGKVTIAQGGVLPNIQAVLLPKKTE	372	5.00E-13	611.9819	3054.8729	5	0.0196	29	0	GluC
H2A2A_MOUSE, H2A2C_MOUSE	Histone H2A	LNKLLGKVTIAQGGVLPNIQAVLLPKKTE	372	2.40E-10	764.7254	3054.8727	4	0.0194	29	0	GluC
H2A2A_MOUSE, H2A2C_MOUSE	Histone H2A	LNKLLGKVTIAQGGVLPNIQAVLLPKKTE	372	7.30E-11	764.7254	3054.8724	4	0.0191	29	0	GluC
H2A2A_MOUSE, H2A2C_MOUSE	Histone H2A	LNKLLGKVTIAQGGVLPNIQAVLLPKKTE	372	1.60E-05	1019.2978	3054.8716	3	0.0183	29	0	GluC
H2A2A_MOUSE, H2A2C_MOUSE	Histone H2A	LNKLLGKVTIAQGGVLPNIQAVLLPKKTE	372	5.80E-12	764.7252	3054.8715	4	0.0183	29	0	GluC
H2A2A_MOUSE, H2A2C_MOUSE	Histone H2A	LNKLLGKVTIAQGGVLPNIQAVLLPKKTE	372	7.70E-11	764.7251	3054.8712	4	0.0180	29	0	GluC
H2A2A_MOUSE, H2A2C_MOUSE	Histone H2A	LNKLLGKVTIAQGGVLPNIQAVLLPKKTE	372	3.30E-04	764.7251	3054.8711	4	0.0179	29	0	GluC
H2A2A_MOUSE, H2A2C_MOUSE	Histone H2A	LNKLLGKVTIAQGGVLPNIQAVLLPKKTE	372	6.00E-05	764.725	3054.8707	4	0.0175	29	0	GluC
H2A2A_MOUSE, H2A2C_MOUSE	Histone H2A	LNKLLGKVTIAQGGVLPNIQAVLLPKKTE	372	1.10E-10	764.7249	3054.8706	4	0.0174	29	0	GluC
H2A2A_MOUSE, H2A2C_MOUSE	Histone H2A	LNKLLGKVTIAQGGVLPNIQAVLLPKKTE	372	6.00E-03	611.9814	3054.8704	5	0.0172	29	0	GluC
H2A2A_MOUSE, H2A2C_MOUSE	Histone H2A	LNKLLGKVTIAQGGVLPNIQAVLLPKKTE	372	3.00E-07	764.7248	3054.8701	4	0.0169	29	0	GluC
H2A2A_MOUSE, H2A2C_MOUSE	Histone H2A	LNKLLGKVTIAQGGVLPNIQAVLLPKKTE	372	3.40E-08	764.7248	3054.8702	4	0.0169	29	0	GluC
H2A2A_MOUSE, H2A2C_MOUSE	Histone H2A	LNKLLGKVTIAQGGVLPNIQAVLLPKKTE	372	9.70E-11	764.7248	3054.8702	4	0.0169	29	0	GluC
H2A2A_MOUSE, H2A2C_MOUSE	Histone H2A	LNKLLGKVTIAQGGVLPNIQAVLLPKKTE	372	2.10E-11	764.7248	3054.8702	4	0.0169	29	0	GluC
H2A2A_MOUSE, H2A2C_MOUSE	Histone H2A	LNKLLGKVTIAQGGVLPNIQAVLLPKKTE	372	6.40E-03	1019.2973	3054.8701	3	0.0168	29	0	GluC
H2A2A_MOUSE, H2A2C_MOUSE	Histone H2A	LNKLLGKVTIAQGGVLPNIQAVLLPKKTE	372	5.00E-10	764.7248	3054.8700	4	0.0168	29	0	GluC
H2A2A_MOUSE, H2A2C_MOUSE	Histone H2A	LNKLLGKVTIAQGGVLPNIQAVLLPKKTE	372	2.10E-10	764.7248	3054.8700	4	0.0168	29	0	GluC
H2A2A_MOUSE, H2A2C_MOUSE	Histone H2A	LNKLLGKVTIAQGGVLPNIQAVLLPKKTE	372	1.00E-06	764.7247	3054.8699	4	0.0167	29	0	GluC
H2A2A_MOUSE, H2A2C_MOUSE	Histone H2A	LNKLLGKVTIAQGGVLPNIQAVLLPKKTE	372	1.00E-09	764.7247	3054.8698	4	0.0165	29	0	GluC
H2A2A_MOUSE, H2A2C_MOUSE	Histone H2A	LNKLLGKVTIAQGGVLPNIQAVLLPKKTE	372	4.60E-10	764.7247	3054.8697	4	0.0165	29	0	GluC
H2A2A_MOUSE, H2A2C_MOUSE	Histone H2A	LNKLLGKVTIAQGGVLPNIQAVLLPKKTE	372	9.80E-06	611.9812	3054.8696	5	0.0164	29	0	GluC
H2A2A_MOUSE, H2A2C_MOUSE	Histone H2A	LNKLLGKVTIAQGGVLPNIQAVLLPKKTE	372	7.40E-06	611.9812	3054.8696	5	0.0164	29	0	GluC
H2A2A_MOUSE, H2A2C_MOUSE	Histone H2A	LNKLLGKVTIAQGGVLPNIQAVLLPKKTE	372	4.40E-06	764.7247	3054.8696	4	0.0164	29	0	GluC
H2A2A_MOUSE, H2A2C_MOUSE	Histone H2A	LNKLLGKVTIAQGGVLPNIQAVLLPKKTE	372	1.50E-04	764.7246	3054.8695	4	0.0163	29	0	GluC
H2A2A_MOUSE, H2A2C_MOUSE	Histone H2A	LNKLLGKVTIAQGGVLPNIQAVLLPKKTE	372	9.60E-12	764.7246	3054.8692	4	0.0159	29	0	GluC
H2A2A_MOUSE, H2A2C_MOUSE	Histone H2A	LNKLLGKVTIAQGGVLPNIQAVLLPKKTE	372	2.30E-06	611.9811	3054.8691	5	0.0158	29	0	GluC
H2A2A_MOUSE, H2A2C_MOUSE	Histone H2A	LNKLLGKVTIAQGGVLPNIQAVLLPKKTE	372	8.50E-04	764.7245	3054.8690	4	0.0157	29	0	GluC
H2A2A_MOUSE, H2A2C_MOUSE	Histone H2A	LNKLLGKVTIAQGGVLPNIQAVLLPKKTE	372	2.20E-05	764.7245	3054.8689	4	0.0157	29	0	GluC
H2A2A_MOUSE, H2A2C_MOUSE	Histone H2A	LNKLLGKVTIAQGGVLPNIQAVLLPKKTE	372	3.70E-06	764.7245	3054.8689	4	0.0157	29	0	GluC
H2A2A_MOUSE, H2A2C_MOUSE	Histone H2A	LNKLLGKVTIAQGGVLPNIQAVLLPKKTE	372	5.80E-08	764.7245	3054.8690	4	0.0157	29	0	GluC
H2A2A_MOUSE, H2A2C_MOUSE	Histone H2A	LNKLLGKVTIAQGGVLPNIQAVLLPKKTE	372	1.20E-08	764.7245	3054.8689	4	0.0157	29	0	GluC
H2A2A_MOUSE, H2A2C_MOUSE	Histone H2A	LNKLLGKVTIAQGGVLPNIQAVLLPKKTE	372	4.10E-09	611.9811	3054.8689	5	0.0157	29	0	GluC
H2A2A_MOUSE, H2A2C_MOUSE	Histone H2A	LNKLLGKVTIAQGGVLPNIQAVLLPKKTE	372	1.50E-03	764.7244	3054.8685	4	0.0152	29	0	GluC
H2A2A_MOUSE, H2A2C_MOUSE	Histone H2A	LNKLLGKVTIAQGGVLPNIQAVLLPKKTE	372	3.60E-04	764.7244	3054.8683	4	0.0151	29	0	GluC
H2A2A_MOUSE, H2A2C_MOUSE	Histone H2A	LNKLLGKVTIAQGGVLPNIQAVLLPKKTE	372	6.80E-08	764.7244	3054.8683	4	0.0151	29	0	GluC
H2A2A_MOUSE, H2A2C_MOUSE	Histone H2A	LNKLLGKVTIAQGGVLPNIQAVLLPKKTE	372	4.70E-07	611.9809	3054.8682	5	0.0150	29	0	GluC
H2A2A_MOUSE, H2A2C_MOUSE	Histone H2A	LNKLLGKVTIAQGGVLPNIQAVLLPKKTE	372	8.90E-11	611.9809	3054.8681	5	0.0148	29	0	GluC
H2A2A_MOUSE, H2A2C_MOUSE	Histone H2A	LNKLLGKVTIAQGGVLPNIQAVLLPKKTE	372	8.20E-11	611.9809	3054.8679	5	0.0146	29	0	GluC
H2A2A_MOUSE, H2A2C_MOUSE	Histone H2A	LNKLLGKVTIAQGGVLPNIQAVLLPKKTE	372	2.10E-08	764.7242	3054.8677	4	0.0145	29	0	GluC
H2A2A_MOUSE, H2A2C_MOUSE	Histone H2A	LNKLLGKVTIAQGGVLPNIQAVLLPKKTE	372	3.40E-03	764.7242	3054.8677	4	0.0144	29	0	GluC
H2A2A_MOUSE, H2A2C_MOUSE	Histone H2A	LNKLLGKVTIAQGGVLPNIQAVLLPKKTE	372	1.80E-08	1019.2965	3054.8677	3	0.0144	29	0	GluC
H2A2A_MOUSE, H2A2C_MOUSE	Histone H2A	LNKLLGKVTIAQGGVLPNIQAVLLPKKTE	372	2.40E-09	764.7242	3054.8676	4	0.0144	29	0	GluC
H2A2A_MOUSE, H2A2C_MOUSE	Histone H2A	LNKLLGKVTIAQGGVLPNIQAVLLPKKTE	372	3.60E-08	611.9808	3054.8675	5	0.0142	29	0	GluC
H2A2A_MOUSE, H2A2C_MOUSE	Histone H2A	LNKLLGKVTIAQGGVLPNIQAVLLPKKTE	372	6.70E-04	764.7241	3054.8673	4	0.0141	29	0	GluC
H2A2A_MOUSE, H2A2C_MOUSE	Histone H2A	LNKLLGKVTIAQGGVLPNIQAVLLPKKTE	372	1.40E-04	1019.2964	3054.8674	3	0.0141	29	0	GluC
H2A2A_MOUSE, H2A2C_MOUSE	Histone H2A	LNKLLGKVTIAQGGVLPNIQAVLLPKKTE	372	5.40E-04	611.9807	3054.8672	5	0.0140	29	0	GluC
H2A2A_MOUSE, H2A2C_MOUSE	Histone H2A	LNKLLGKVTIAQGGVLPNIQAVLLPKKTE	372	5.60E-04	764.724	3054.8671	4	0.0138	29	0	GluC
H2A2A_MOUSE, H2A2C_MOUSE	Histone H2A	LNKLLGKVTIAQGGVLPNIQAVLLPKKTE	372	3.80E-08	764.724	3054.8670	4	0.0138	29	0	GluC
H2A2A_MOUSE, H2A2C_MOUSE	Histone H2A	LNKLLGKVTIAQGGVLPNIQAVLLPKKTE	372	2.10E-08	764.724	3054.8671	4	0.0138	29	0	GluC
H2A2A_MOUSE, H2A2C_MOUSE	Histone H2A	LNKLLGKVTIAQGGVLPNIQAVLLPKKTE	372	8.40E-08	611.9807	3054.8670	5	0.0137	29	0	GluC
H2A2A_MOUSE, H2A2C_MOUSE	Histone H2A	LNKLLGKVTIAQGGVLPNIQAVLLPKKTE	372	1.00E-04	764.724	3054.8668	4	0.0136	29	0	GluC
H2A2A_MOUSE, H2A2C_MOUSE	Histone H2A	LNKLLGKVTIAQGGVLPNIQAVLLPKKTE	372	2.40E-07	764.724	3054.8669	4	0.0136	29	0	GluC
H2A2A_MOUSE, H2A2C_MOUSE	Histone H2A	LNKLLGKVTIAQGGVLPNIQAVLLPKKTE	372	2.60E-08	764.724	3054.8668	4	0.0136	29	0	GluC
H2A2A_MOUSE, H2A2C_MOUSE	Histone H2A	LNKLLGKVTIAQGGVLPNIQAVLLPKKTE	372	1.60E-03	764.724	3054.8667	4	0.0135	29	0	GluC
H2A2A_MOUSE, H2A2C_MOUSE	Histone H2A	LNKLLGKVTIAQGGVLPNIQAVLLPKKTE	372	2.00E-03	764.7239	3054.8665	4	0.0133	29	0	GluC
H2A2A_MOUSE, H2A2C_MOUSE	Histone H2A	LNKLLGKVTIAQGGVLPNIQAVLLPKKTE	372	2.30E-07	764.7239	3054.8665	4	0.0133	29	0	GluC
H2A2A_MOUSE, H2A2C_MOUSE	Histone H2A	LNKLLGKVTIAQGGVLPNIQAVLLPKKTE	372	3.80E-07	1019.2961	3054.8665	3	0.0132	29	0	GluC
H2A2A_MOUSE, H2A2C_MOUSE	Histone H2A	LNKLLGKVTIAQGGVLPNIQAVLLPKKTE	372	6.90E-06	1019.296	3054.8662	3	0.0129	29	0	GluC
H2A2A_MOUSE, H2A2C_MOUSE	Histone H2A	LNKLLGKVTIAQGGVLPNIQAVLLPKKTE	372	4.00E-07	611.9804	3054.8656	5	0.0123	29	0	GluC
H2A2A_MOUSE, H2A2C_MOUSE	Histone H2A	LNKLLGKVTIAQGGVLPNIQAVLLPKKTE	372	1.00E-08	1528.44	3054.8654	2	0.0122	29	0	GluC
H2A2A_MOUSE, H2A2C_MOUSE	Histone H2A	LNKLLGKVTIAQGGVLPNIQAVLLPKKTE	372	2.20E-07	764.7236	3054.8653	4	0.0121	29	0	GluC
H2A2A_MOUSE, H2A2C_MOUSE	Histone H2A	LNKLLGKVTIAQGGVLPNIQAVLLPKKTE	372	7.60E-07	1019.2957	3054.8653	3	0.0120	29	0	GluC
H2A2A_MOUSE, H2A2C_MOUSE	Histone H2A	LNKLLGKVTIAQGGVLPNIQAVLLPKKTE	372	3.80E-09	1528.4399	3054.8652	2	0.0120	29	0	GluC
H2A2A_MOUSE, H2A2C_MOUSE	Histone H2A	LNKLLGKVTIAQGGVLPNIQAVLLPKKTE	372	6.20E-03	764.7236	3054.8651	4	0.0119	29	0	GluC
H2A2A_MOUSE, H2A2C_MOUSE	Histone H2A	LNKLLGKVTIAQGGVLPNIQAVLLPKKTE	372	6.50E-07	764.7236	3054.8651	4	0.0119	29	0	GluC
H2A2A_MOUSE, H2A2C_MOUSE	Histone H2A	LNKLLGKVTIAQGGVLPNIQAVLLPKKTE	372	6.90E-05	764.7235	3054.8651	4	0.0118	29	0	GluC
H2A2A_MOUSE, H2A2C_MOUSE	Histone H2A	LNKLLGKVTIAQGGVLPNIQAVLLPKKTE	372	4.20E-08	764.7235	3054.8651	4	0.0118	29	0	GluC
H2A2A_MOUSE, H2A2C_MOUSE	Histone H2A	LNKLLGKVTIAQGGVLPNIQAVLLPKKTE	372	7.10E-03	764.7235	3054.8649	4	0.0116	29	0	GluC

H2A2A_MOUSE, H2A2C_MOUSE	Histone H2A	LNKLLGKVTIAQGGVLPNIQAVLLPKKTE	372	4.00E-08	764.7235	3054.8649	4	0.0116	29	0	GluC
H2A2A_MOUSE, H2A2C_MOUSE	Histone H2A	LNKLLGKVTIAQGGVLPNIQAVLLPKKTE	372	6.70E-09	1528.4397	3054.8648	2	0.0116	29	0	GluC
H2A2A_MOUSE, H2A2C_MOUSE	Histone H2A	LNKLLGKVTIAQGGVLPNIQAVLLPKKTE	372	9.80E-06	611.9801	3054.8643	5	0.0111	29	0	GluC
H2A2A_MOUSE, H2A2C_MOUSE	Histone H2A	LNKLLGKVTIAQGGVLPNIQAVLLPKKTE	372	2.40E-04	764.7232	3054.8637	4	0.0104	29	0	GluC
H2A2A_MOUSE, H2A2C_MOUSE	Histone H2A	LNKLLGKVTIAQGGVLPNIQAVLLPKKTE	372	1.90E-06	764.7232	3054.8637	4	0.0104	29	0	GluC
H2A2A_MOUSE, H2A2C_MOUSE	Histone H2A	LNKLLGKVTIAQGGVLPNIQAVLLPKKTE	372	4.20E-04	611.98	3054.8635	5	0.0103	29	0	GluC
H2A2A_MOUSE, H2A2C_MOUSE	Histone H2A	LNKLLGKVTIAQGGVLPNIQAVLLPKKTE	372	9.70E-07	764.7232	3054.8635	4	0.0103	29	0	GluC
H2A2A_MOUSE, H2A2C_MOUSE	Histone H2A	LNKLLGKVTIAQGGVLPNIQAVLLPKKTE	372	4.80E-05	764.7231	3054.8633	4	0.0100	29	0	GluC
H2A2A_MOUSE, H2A2C_MOUSE	Histone H2A	LNKLLGKVTIAQGGVLPNIQAVLLPKKTE	372	5.40E-07	764.7231	3054.8633	4	0.0100	29	0	GluC
H2A2A_MOUSE, H2A2C_MOUSE	Histone H2A	LNKLLGKVTIAQGGVLPNIQAVLLPKKTE	372	4.20E-03	764.723	3054.8630	4	0.0098	29	0	GluC
H2A2A_MOUSE, H2A2C_MOUSE	Histone H2A	LNKLLGKVTIAQGGVLPNIQAVLLPKKTE	372	1.70E-08	764.723	3054.8630	4	0.0098	29	0	GluC
H2A2A_MOUSE, H2A2C_MOUSE	Histone H2A	LNKLLGKVTIAQGGVLPNIQAVLLPKKTE	372	8.80E-06	1019.2949	3054.8629	3	0.0096	29	0	GluC
H2A2A_MOUSE, H2A2C_MOUSE	Histone H2A	LNKLLGKVTIAQGGVLPNIQAVLLPKKTE	372	3.80E-04	764.723	3054.8627	4	0.0095	29	0	GluC
H2A2A_MOUSE, H2A2C_MOUSE	Histone H2A	LNKLLGKVTIAQGGVLPNIQAVLLPKKTE	372	5.30E-09	764.723	3054.8627	4	0.0095	29	0	GluC
H2A2A_MOUSE, H2A2C_MOUSE	Histone H2A	LNKLLGKVTIAQGGVLPNIQAVLLPKKTE	372	6.40E-03	611.9798	3054.8625	5	0.0093	29	0	GluC
H2A2A_MOUSE, H2A2C_MOUSE	Histone H2A	LNKLLGKVTIAQGGVLPNIQAVLLPKKTE	372	5.70E-03	611.9798	3054.8625	5	0.0093	29	0	GluC
H2A2A_MOUSE, H2A2C_MOUSE	Histone H2A	LNKLLGKVTIAQGGVLPNIQAVLLPKKTE	372	5.70E-05	764.7229	3054.8626	4	0.0093	29	0	GluC
H2A2A_MOUSE, H2A2C_MOUSE	Histone H2A	LNKLLGKVTIAQGGVLPNIQAVLLPKKTE	372	5.10E-06	764.7229	3054.8626	4	0.0093	29	0	GluC
H2A2A_MOUSE, H2A2C_MOUSE	Histone H2A	LNKLLGKVTIAQGGVLPNIQAVLLPKKTE	372	1.70E-06	611.9797	3054.8622	5	0.0089	29	0	GluC
H2A2A_MOUSE, H2A2C_MOUSE	Histone H2A	LNKLLGKVTIAQGGVLPNIQAVLLPKKTE	372	8.20E-05	764.7228	3054.8621	4	0.0088	29	0	GluC
H2A2A_MOUSE, H2A2C_MOUSE	Histone H2A	LNKLLGKVTIAQGGVLPNIQAVLLPKKTE	372	7.90E-06	764.7228	3054.8621	4	0.0088	29	0	GluC
H2A2A_MOUSE, H2A2C_MOUSE	Histone H2A	LNKLLGKVTIAQGGVLPNIQAVLLPKKTE	372	3.20E-10	764.7228	3054.8620	4	0.0088	29	0	GluC
H2A2A_MOUSE, H2A2C_MOUSE	Histone H2A	LNKLLGKVTIAQGGVLPNIQAVLLPKKTE	372	2.60E-10	1528.4383	3054.8620	2	0.0088	29	0	GluC
H2A2A_MOUSE, H2A2C_MOUSE	Histone H2A	LNKLLGKVTIAQGGVLPNIQAVLLPKKTE	372	2.90E-04	764.7228	3054.8619	4	0.0087	29	0	GluC
H2A2A_MOUSE, H2A2C_MOUSE	Histone H2A	LNKLLGKVTIAQGGVLPNIQAVLLPKKTE	372	8.10E-06	764.7228	3054.8619	4	0.0087	29	0	GluC
H2A2A_MOUSE, H2A2C_MOUSE	Histone H2A	LNKLLGKVTIAQGGVLPNIQAVLLPKKTE	372	3.50E-06	764.7227	3054.8618	4	0.0086	29	0	GluC
H2A2A_MOUSE, H2A2C_MOUSE	Histone H2A	LNKLLGKVTIAQGGVLPNIQAVLLPKKTE	372	1.20E-05	764.7227	3054.8617	4	0.0085	29	0	GluC
H2A2A_MOUSE, H2A2C_MOUSE	Histone H2A	LNKLLGKVTIAQGGVLPNIQAVLLPKKTE	372	2.50E-07	764.7227	3054.8617	4	0.0085	29	0	GluC
H2A2A_MOUSE, H2A2C_MOUSE	Histone H2A	LNKLLGKVTIAQGGVLPNIQAVLLPKKTE	372	1.00E-05	1019.2945	3054.8617	3	0.0084	29	0	GluC
H2A2A_MOUSE, H2A2C_MOUSE	Histone H2A	LNKLLGKVTIAQGGVLPNIQAVLLPKKTE	372	3.20E-03	764.7226	3054.8614	4	0.0081	29	0	GluC
H2A2A_MOUSE, H2A2C_MOUSE	Histone H2A	LNKLLGKVTIAQGGVLPNIQAVLLPKKTE	372	3.50E-05	1019.2944	3054.8614	3	0.0081	29	0	GluC
H2A2A_MOUSE, H2A2C_MOUSE	Histone H2A	LNKLLGKVTIAQGGVLPNIQAVLLPKKTE	372	2.50E-06	1019.2944	3054.8614	3	0.0081	29	0	GluC
H2A2A_MOUSE, H2A2C_MOUSE	Histone H2A	LNKLLGKVTIAQGGVLPNIQAVLLPKKTE	372	8.80E-07	1019.2943	3054.8611	3	0.0078	29	0	GluC
H2A2A_MOUSE, H2A2C_MOUSE	Histone H2A	LNKLLGKVTIAQGGVLPNIQAVLLPKKTE	372	9.90E-09	1528.4378	3054.8610	2	0.0078	29	0	GluC
H2A2A_MOUSE, H2A2C_MOUSE	Histone H2A	LNKLLGKVTIAQGGVLPNIQAVLLPKKTE	372	1.10E-06	764.7225	3054.8608	4	0.0076	29	0	GluC
H2A2A_MOUSE, H2A2C_MOUSE	Histone H2A	LNKLLGKVTIAQGGVLPNIQAVLLPKKTE	372	2.20E-03	764.7224	3054.8607	4	0.0075	29	0	GluC
H2A2A_MOUSE, H2A2C_MOUSE	Histone H2A	LNKLLGKVTIAQGGVLPNIQAVLLPKKTE	372	7.10E-04	764.7225	3054.8607	4	0.0075	29	0	GluC
H2A2A_MOUSE, H2A2C_MOUSE	Histone H2A	LNKLLGKVTIAQGGVLPNIQAVLLPKKTE	372	1.50E-04	1019.2942	3054.8608	3	0.0075	29	0	GluC
H2A2A_MOUSE, H2A2C_MOUSE	Histone H2A	LNKLLGKVTIAQGGVLPNIQAVLLPKKTE	372	2.00E-05	764.7224	3054.8607	4	0.0075	29	0	GluC
H2A2A_MOUSE, H2A2C_MOUSE	Histone H2A	LNKLLGKVTIAQGGVLPNIQAVLLPKKTE	372	1.20E-11	764.7225	3054.8607	4	0.0075	29	0	GluC
H2A2A_MOUSE, H2A2C_MOUSE	Histone H2A	LNKLLGKVTIAQGGVLPNIQAVLLPKKTE	372	8.50E-03	611.9794	3054.8605	5	0.0073	29	0	GluC
H2A2A_MOUSE, H2A2C_MOUSE	Histone H2A	LNKLLGKVTIAQGGVLPNIQAVLLPKKTE	372	3.60E-03	611.9794	3054.8605	5	0.0073	29	0	GluC
H2A2A_MOUSE, H2A2C_MOUSE	Histone H2A	LNKLLGKVTIAQGGVLPNIQAVLLPKKTE	372	8.60E-04	764.7224	3054.8605	4	0.0073	29	0	GluC
H2A2A_MOUSE, H2A2C_MOUSE	Histone H2A	LNKLLGKVTIAQGGVLPNIQAVLLPKKTE	372	2.80E-05	764.7224	3054.8605	4	0.0073	29	0	GluC
H2A2A_MOUSE, H2A2C_MOUSE	Histone H2A	LNKLLGKVTIAQGGVLPNIQAVLLPKKTE	372	1.30E-05	611.9794	3054.8605	5	0.0073	29	0	GluC
H2A2A_MOUSE, H2A2C_MOUSE	Histone H2A	LNKLLGKVTIAQGGVLPNIQAVLLPKKTE	372	2.00E-07	764.7224	3054.8605	4	0.0073	29	0	GluC
H2A2A_MOUSE, H2A2C_MOUSE	Histone H2A	LNKLLGKVTIAQGGVLPNIQAVLLPKKTE	372	5.70E-09	764.7224	3054.8605	4	0.0073	29	0	GluC
H2A2A_MOUSE, H2A2C_MOUSE	Histone H2A	LNKLLGKVTIAQGGVLPNIQAVLLPKKTE	372	8.40E-06	1019.2941	3054.8605	3	0.0072	29	0	GluC
H2A2A_MOUSE, H2A2C_MOUSE	Histone H2A	LNKLLGKVTIAQGGVLPNIQAVLLPKKTE	372	1.50E-06	1019.2941	3054.8605	3	0.0072	29	0	GluC
H2A2A_MOUSE, H2A2C_MOUSE	Histone H2A	LNKLLGKVTIAQGGVLPNIQAVLLPKKTE	372	2.30E-04	764.7223	3054.8603	4	0.0070	29	0	GluC
H2A2A_MOUSE, H2A2C_MOUSE	Histone H2A	LNKLLGKVTIAQGGVLPNIQAVLLPKKTE	372	1.20E-04	764.7223	3054.8603	4	0.0070	29	0	GluC
H2A2A_MOUSE, H2A2C_MOUSE	Histone H2A	LNKLLGKVTIAQGGVLPNIQAVLLPKKTE	372	1.10E-04	764.7223	3054.8603	4	0.0070	29	0	GluC
H2A2A_MOUSE, H2A2C_MOUSE	Histone H2A	LNKLLGKVTIAQGGVLPNIQAVLLPKKTE	372	4.00E-06	764.7223	3054.8603	4	0.0070	29	0	GluC
H2A2A_MOUSE, H2A2C_MOUSE	Histone H2A	LNKLLGKVTIAQGGVLPNIQAVLLPKKTE	372	4.20E-08	764.7223	3054.8603	4	0.0070	29	0	GluC
H2A2A_MOUSE, H2A2C_MOUSE	Histone H2A	LNKLLGKVTIAQGGVLPNIQAVLLPKKTE	372	5.80E-09	764.7223	3054.8603	4	0.0070	29	0	GluC
H2A2A_MOUSE, H2A2C_MOUSE	Histone H2A	LNKLLGKVTIAQGGVLPNIQAVLLPKKTE	372	1.70E-06	1019.294	3054.8602	3	0.0069	29	0	GluC
H2A2A_MOUSE, H2A2C_MOUSE	Histone H2A	LNKLLGKVTIAQGGVLPNIQAVLLPKKTE	372	6.50E-04	764.7223	3054.8599	4	0.0067	29	0	GluC
H2A2A_MOUSE, H2A2C_MOUSE	Histone H2A	LNKLLGKVTIAQGGVLPNIQAVLLPKKTE	372	4.70E-07	1019.2939	3054.8599	3	0.0066	29	0	GluC
H2A2A_MOUSE, H2A2C_MOUSE	Histone H2A	LNKLLGKVTIAQGGVLPNIQAVLLPKKTE	372	4.70E-03	764.7222	3054.8597	4	0.0065	29	0	GluC
H2A2A_MOUSE, H2A2C_MOUSE	Histone H2A	LNKLLGKVTIAQGGVLPNIQAVLLPKKTE	372	1.60E-05	764.7222	3054.8597	4	0.0065	29	0	GluC
H2A2A_MOUSE, H2A2C_MOUSE	Histone H2A	LNKLLGKVTIAQGGVLPNIQAVLLPKKTE	372	2.50E-03	764.7222	3054.8597	4	0.0064	29	0	GluC
H2A2A_MOUSE, H2A2C_MOUSE	Histone H2A	LNKLLGKVTIAQGGVLPNIQAVLLPKKTE	372	9.60E-04	764.7222	3054.8597	4	0.0064	29	0	GluC
H2A2A_MOUSE, H2A2C_MOUSE	Histone H2A	LNKLLGKVTIAQGGVLPNIQAVLLPKKTE	372	5.80E-04	764.7222	3054.8596	4	0.0064	29	0	GluC
H2A2A_MOUSE, H2A2C_MOUSE	Histone H2A	LNKLLGKVTIAQGGVLPNIQAVLLPKKTE	372	2.10E-05	611.9792	3054.8597	5	0.0064	29	0	GluC
H2A2A_MOUSE, H2A2C_MOUSE	Histone H2A	LNKLLGKVTIAQGGVLPNIQAVLLPKKTE	372	1.80E-07	764.7222	3054.8597	4	0.0064	29	0	GluC
H2A2A_MOUSE, H2A2C_MOUSE	Histone H2A	LNKLLGKVTIAQGGVLPNIQAVLLPKKTE	372	1.20E-05	1019.2938	3054.8596	3	0.0063	29	0	GluC
H2A2A_MOUSE, H2A2C_MOUSE	Histone H2A	LNKLLGKVTIAQGGVLPNIQAVLLPKKTE	372	3.90E-06	1019.2938	3054.8596	3	0.0063	29	0	GluC
H2A2A_MOUSE, H2A2C_MOUSE	Histone H2A	LNKLLGKVTIAQGGVLPNIQAVLLPKKTE	372	2.80E-07	1019.2938	3054.8596	3	0.0063	29	0	GluC
H2A2A_MOUSE, H2A2C_MOUSE	Histone H2A	LNKLLGKVTIAQGGVLPNIQAVLLPKKTE	372	2.10E-04	764.7221	3054.8595	4	0.0062	29	0	GluC
H2A2A_MOUSE, H2A2C_MOUSE	Histone H2A	LNKLLGKVTIAQGGVLPNIQAVLLPKKTE	372	1.70E-07	764.7221	3054.8595	4	0.0062	29	0	GluC
H2A2A_MOUSE, H2A2C_MOUSE	Histone H2A	LNKLLGKVTIAQGGVLPNIQAVLLPKKTE	372	9.80E-03	764.7221	3054.8593	4	0.0061	29	0	GluC
H2A2A_MOUSE, H2A2C_MOUSE	Histone H2A	LNKLLGKVTIAQGGVLPNIQAVLLPKKTE	372	1.50E-05	764.7221	3054.8593	4	0.0061	29	0	GluC
H2A2A_MOUSE, H2A2C_MOUSE	Histone H2A	LNKLLGKVTIAQGGVLPNIQAVLLPKKTE	372	3.10E-10	764.7221	3054.8593	4	0.0061	29	0	GluC
H2A2A_MOUSE, H2A2C_MOUSE	Histone H2A	LNKLLGKVTIAQGGVLPNIQAVLLPKKTE	372	3.00E-03	611.9791	3054.8592	5	0.0060	29	0	GluC

H2A2A_MOUSE, H2A2C_MOUSE	Histone H2A	LNKLLGKVTIAQGGVLPNIQAVLLPKKTE	372	7.00E-06	611.9791	3054.8593	5	0.0060	29	0	GluC
H2A2A_MOUSE, H2A2C_MOUSE	Histone H2A	LNKLLGKVTIAQGGVLPNIQAVLLPKKTE	372	2.30E-07	1019.2937	3054.8593	3	0.0060	29	0	GluC
H2A2A_MOUSE, H2A2C_MOUSE	Histone H2A	LNKLLGKVTIAQGGVLPNIQAVLLPKKTE	372	7.70E-05	764.722	3054.8591	4	0.0059	29	0	GluC
H2A2A_MOUSE, H2A2C_MOUSE	Histone H2A	LNKLLGKVTIAQGGVLPNIQAVLLPKKTE	372	2.80E-05	764.722	3054.8591	4	0.0059	29	0	GluC
H2A2A_MOUSE, H2A2C_MOUSE	Histone H2A	LNKLLGKVTIAQGGVLPNIQAVLLPKKTE	372	2.20E-03	764.722	3054.8591	4	0.0058	29	0	GluC
H2A2A_MOUSE, H2A2C_MOUSE	Histone H2A	LNKLLGKVTIAQGGVLPNIQAVLLPKKTE	372	1.90E-03	764.722	3054.8591	4	0.0058	29	0	GluC
H2A2A_MOUSE, H2A2C_MOUSE	Histone H2A	LNKLLGKVTIAQGGVLPNIQAVLLPKKTE	372	4.30E-05	764.722	3054.8590	4	0.0058	29	0	GluC
H2A2A_MOUSE, H2A2C_MOUSE	Histone H2A	LNKLLGKVTIAQGGVLPNIQAVLLPKKTE	372	1.70E-07	764.722	3054.8591	4	0.0058	29	0	GluC
H2A2A_MOUSE, H2A2C_MOUSE	Histone H2A	LNKLLGKVTIAQGGVLPNIQAVLLPKKTE	372	7.30E-04	764.722	3054.8589	4	0.0057	29	0	GluC
H2A2A_MOUSE, H2A2C_MOUSE	Histone H2A	LNKLLGKVTIAQGGVLPNIQAVLLPKKTE	372	3.50E-04	764.722	3054.8589	4	0.0057	29	0	GluC
H2A2A_MOUSE, H2A2C_MOUSE	Histone H2A	LNKLLGKVTIAQGGVLPNIQAVLLPKKTE	372	5.40E-07	1019.2936	3054.8590	3	0.0057	29	0	GluC
H2A2A_MOUSE, H2A2C_MOUSE	Histone H2A	LNKLLGKVTIAQGGVLPNIQAVLLPKKTE	372	3.50E-09	1019.2936	3054.8590	3	0.0057	29	0	GluC
H2A2A_MOUSE, H2A2C_MOUSE	Histone H2A	LNKLLGKVTIAQGGVLPNIQAVLLPKKTE	372	2.30E-10	764.722	3054.8589	4	0.0057	29	0	GluC
H2A2A_MOUSE, H2A2C_MOUSE	Histone H2A	LNKLLGKVTIAQGGVLPNIQAVLLPKKTE	372	1.30E-10	764.722	3054.8589	4	0.0057	29	0	GluC
H2A2A_MOUSE, H2A2C_MOUSE	Histone H2A	LNKLLGKVTIAQGGVLPNIQAVLLPKKTE	372	6.30E-07	611.9791	3054.8589	5	0.0056	29	0	GluC
H2A2A_MOUSE, H2A2C_MOUSE	Histone H2A	LNKLLGKVTIAQGGVLPNIQAVLLPKKTE	372	1.80E-08	1528.4367	3054.8588	2	0.0056	29	0	GluC
H2A2A_MOUSE, H2A2C_MOUSE	Histone H2A	LNKLLGKVTIAQGGVLPNIQAVLLPKKTE	372	8.60E-04	764.7219	3054.8587	4	0.0055	29	0	GluC
H2A2A_MOUSE, H2A2C_MOUSE	Histone H2A	LNKLLGKVTIAQGGVLPNIQAVLLPKKTE	372	3.30E-04	764.722	3054.8587	4	0.0055	29	0	GluC
H2A2A_MOUSE, H2A2C_MOUSE	Histone H2A	LNKLLGKVTIAQGGVLPNIQAVLLPKKTE	372	2.90E-04	764.7219	3054.8587	4	0.0055	29	0	GluC
H2A2A_MOUSE, H2A2C_MOUSE	Histone H2A	LNKLLGKVTIAQGGVLPNIQAVLLPKKTE	372	3.00E-03	764.7219	3054.8586	4	0.0054	29	0	GluC
H2A2A_MOUSE, H2A2C_MOUSE	Histone H2A	LNKLLGKVTIAQGGVLPNIQAVLLPKKTE	372	7.00E-04	764.7219	3054.8587	4	0.0054	29	0	GluC
H2A2A_MOUSE, H2A2C_MOUSE	Histone H2A	LNKLLGKVTIAQGGVLPNIQAVLLPKKTE	372	5.30E-07	764.7219	3054.8586	4	0.0054	29	0	GluC
H2A2A_MOUSE, H2A2C_MOUSE	Histone H2A	LNKLLGKVTIAQGGVLPNIQAVLLPKKTE	372	1.90E-07	764.7219	3054.8587	4	0.0054	29	0	GluC
H2A2A_MOUSE, H2A2C_MOUSE	Histone H2A	LNKLLGKVTIAQGGVLPNIQAVLLPKKTE	372	1.10E-07	1019.2935	3054.8587	3	0.0054	29	0	GluC
H2A2A_MOUSE, H2A2C_MOUSE	Histone H2A	LNKLLGKVTIAQGGVLPNIQAVLLPKKTE	372	4.80E-08	1019.2935	3054.8587	3	0.0054	29	0	GluC
H2A2A_MOUSE, H2A2C_MOUSE	Histone H2A	LNKLLGKVTIAQGGVLPNIQAVLLPKKTE	372	4.60E-08	1019.2935	3054.8587	3	0.0054	29	0	GluC
H2A2A_MOUSE, H2A2C_MOUSE	Histone H2A	LNKLLGKVTIAQGGVLPNIQAVLLPKKTE	372	2.90E-08	1019.2935	3054.8587	3	0.0054	29	0	GluC
H2A2A_MOUSE, H2A2C_MOUSE	Histone H2A	LNKLLGKVTIAQGGVLPNIQAVLLPKKTE	372	9.40E-10	1019.2935	3054.8587	3	0.0054	29	0	GluC
H2A2A_MOUSE, H2A2C_MOUSE	Histone H2A	LNKLLGKVTIAQGGVLPNIQAVLLPKKTE	372	3.10E-11	1528.4366	3054.8586	2	0.0054	29	0	GluC
H2A2A_MOUSE, H2A2C_MOUSE	Histone H2A	LNKLLGKVTIAQGGVLPNIQAVLLPKKTE	372	6.90E-03	764.7219	3054.8585	4	0.0053	29	0	GluC
H2A2A_MOUSE, H2A2C_MOUSE	Histone H2A	LNKLLGKVTIAQGGVLPNIQAVLLPKKTE	372	2.90E-04	764.7219	3054.8585	4	0.0053	29	0	GluC
H2A2A_MOUSE, H2A2C_MOUSE	Histone H2A	LNKLLGKVTIAQGGVLPNIQAVLLPKKTE	372	5.10E-05	764.7219	3054.8586	4	0.0053	29	0	GluC
H2A2A_MOUSE, H2A2C_MOUSE	Histone H2A	LNKLLGKVTIAQGGVLPNIQAVLLPKKTE	372	5.60E-07	764.7219	3054.8585	4	0.0053	29	0	GluC
H2A2A_MOUSE, H2A2C_MOUSE	Histone H2A	LNKLLGKVTIAQGGVLPNIQAVLLPKKTE	372	4.10E-07	764.7219	3054.8585	4	0.0053	29	0	GluC
H2A2A_MOUSE, H2A2C_MOUSE	Histone H2A	LNKLLGKVTIAQGGVLPNIQAVLLPKKTE	372	5.50E-08	764.7219	3054.8586	4	0.0053	29	0	GluC
H2A2A_MOUSE, H2A2C_MOUSE	Histone H2A	LNKLLGKVTIAQGGVLPNIQAVLLPKKTE	372	1.80E-03	764.7219	3054.8584	4	0.0052	29	0	GluC
H2A2A_MOUSE, H2A2C_MOUSE	Histone H2A	LNKLLGKVTIAQGGVLPNIQAVLLPKKTE	372	3.00E-09	1528.4365	3054.8584	2	0.0052	29	0	GluC
H2A2A_MOUSE, H2A2C_MOUSE	Histone H2A	LNKLLGKVTIAQGGVLPNIQAVLLPKKTE	372	8.70E-03	764.7219	3054.8583	4	0.0051	29	0	GluC
H2A2A_MOUSE, H2A2C_MOUSE	Histone H2A	LNKLLGKVTIAQGGVLPNIQAVLLPKKTE	372	5.40E-03	764.7219	3054.8583	4	0.0051	29	0	GluC
H2A2A_MOUSE, H2A2C_MOUSE	Histone H2A	LNKLLGKVTIAQGGVLPNIQAVLLPKKTE	372	3.70E-03	764.7219	3054.8583	4	0.0051	29	0	GluC
H2A2A_MOUSE, H2A2C_MOUSE	Histone H2A	LNKLLGKVTIAQGGVLPNIQAVLLPKKTE	372	1.20E-03	764.7219	3054.8583	4	0.0051	29	0	GluC
H2A2A_MOUSE, H2A2C_MOUSE	Histone H2A	LNKLLGKVTIAQGGVLPNIQAVLLPKKTE	372	1.10E-04	1019.2934	3054.8584	3	0.0051	29	0	GluC
H2A2A_MOUSE, H2A2C_MOUSE	Histone H2A	LNKLLGKVTIAQGGVLPNIQAVLLPKKTE	372	3.60E-06	1019.2934	3054.8584	3	0.0051	29	0	GluC
H2A2A_MOUSE, H2A2C_MOUSE	Histone H2A	LNKLLGKVTIAQGGVLPNIQAVLLPKKTE	372	4.30E-07	1019.2934	3054.8584	3	0.0051	29	0	GluC
H2A2A_MOUSE, H2A2C_MOUSE	Histone H2A	LNKLLGKVTIAQGGVLPNIQAVLLPKKTE	372	2.20E-09	1019.2934	3054.8584	3	0.0051	29	0	GluC
H2A2A_MOUSE, H2A2C_MOUSE	Histone H2A	LNKLLGKVTIAQGGVLPNIQAVLLPKKTE	372	1.30E-04	764.7218	3054.8582	4	0.0050	29	0	GluC
H2A2A_MOUSE, H2A2C_MOUSE	Histone H2A	LNKLLGKVTIAQGGVLPNIQAVLLPKKTE	372	2.70E-07	764.7218	3054.8582	4	0.0050	29	0	GluC
H2A2A_MOUSE, H2A2C_MOUSE	Histone H2A	LNKLLGKVTIAQGGVLPNIQAVLLPKKTE	372	7.10E-04	764.7218	3054.8581	4	0.0049	29	0	GluC
H2A2A_MOUSE, H2A2C_MOUSE	Histone H2A	LNKLLGKVTIAQGGVLPNIQAVLLPKKTE	372	1.60E-04	764.7218	3054.8581	4	0.0049	29	0	GluC
H2A2A_MOUSE, H2A2C_MOUSE	Histone H2A	LNKLLGKVTIAQGGVLPNIQAVLLPKKTE	372	8.70E-08	764.7218	3054.8581	4	0.0049	29	0	GluC
H2A2A_MOUSE, H2A2C_MOUSE	Histone H2A	LNKLLGKVTIAQGGVLPNIQAVLLPKKTE	372	1.30E-09	764.7218	3054.8581	4	0.0049	29	0	GluC
H2A2A_MOUSE, H2A2C_MOUSE	Histone H2A	LNKLLGKVTIAQGGVLPNIQAVLLPKKTE	372	3.80E-03	1019.2933	3054.8581	3	0.0048	29	0	GluC
H2A2A_MOUSE, H2A2C_MOUSE	Histone H2A	LNKLLGKVTIAQGGVLPNIQAVLLPKKTE	372	4.80E-04	764.7218	3054.8581	4	0.0048	29	0	GluC
H2A2A_MOUSE, H2A2C_MOUSE	Histone H2A	LNKLLGKVTIAQGGVLPNIQAVLLPKKTE	372	2.20E-04	764.7218	3054.8581	4	0.0048	29	0	GluC
H2A2A_MOUSE, H2A2C_MOUSE	Histone H2A	LNKLLGKVTIAQGGVLPNIQAVLLPKKTE	372	6.70E-05	611.9789	3054.8581	5	0.0048	29	0	GluC
H2A2A_MOUSE, H2A2C_MOUSE	Histone H2A	LNKLLGKVTIAQGGVLPNIQAVLLPKKTE	372	3.00E-05	764.7218	3054.8581	4	0.0048	29	0	GluC
H2A2A_MOUSE, H2A2C_MOUSE	Histone H2A	LNKLLGKVTIAQGGVLPNIQAVLLPKKTE	372	9.90E-06	764.7218	3054.8581	4	0.0048	29	0	GluC
H2A2A_MOUSE, H2A2C_MOUSE	Histone H2A	LNKLLGKVTIAQGGVLPNIQAVLLPKKTE	372	7.30E-07	1019.2933	3054.8581	3	0.0048	29	0	GluC
H2A2A_MOUSE, H2A2C_MOUSE	Histone H2A	LNKLLGKVTIAQGGVLPNIQAVLLPKKTE	372	1.20E-07	611.9789	3054.8581	5	0.0048	29	0	GluC
H2A2A_MOUSE, H2A2C_MOUSE	Histone H2A	LNKLLGKVTIAQGGVLPNIQAVLLPKKTE	372	1.10E-09	764.7218	3054.8581	4	0.0048	29	0	GluC
H2A2A_MOUSE, H2A2C_MOUSE	Histone H2A	LNKLLGKVTIAQGGVLPNIQAVLLPKKTE	372	9.50E-05	764.7218	3054.8580	4	0.0047	29	0	GluC
H2A2A_MOUSE, H2A2C_MOUSE	Histone H2A	LNKLLGKVTIAQGGVLPNIQAVLLPKKTE	372	6.30E-04	764.7217	3054.8578	4	0.0046	29	0	GluC
H2A2A_MOUSE, H2A2C_MOUSE	Histone H2A	LNKLLGKVTIAQGGVLPNIQAVLLPKKTE	372	3.60E-04	764.7217	3054.8578	4	0.0046	29	0	GluC
H2A2A_MOUSE, H2A2C_MOUSE	Histone H2A	LNKLLGKVTIAQGGVLPNIQAVLLPKKTE	372	1.40E-04	764.7217	3054.8579	4	0.0046	29	0	GluC
H2A2A_MOUSE, H2A2C_MOUSE	Histone H2A	LNKLLGKVTIAQGGVLPNIQAVLLPKKTE	372	1.60E-05	764.7217	3054.8578	4	0.0046	29	0	GluC
H2A2A_MOUSE, H2A2C_MOUSE	Histone H2A	LNKLLGKVTIAQGGVLPNIQAVLLPKKTE	372	3.30E-07	764.7217	3054.8578	4	0.0046	29	0	GluC
H2A2A_MOUSE, H2A2C_MOUSE	Histone H2A	LNKLLGKVTIAQGGVLPNIQAVLLPKKTE	372	2.00E-09	764.7217	3054.8579	4	0.0046	29	0	GluC
H2A2A_MOUSE, H2A2C_MOUSE	Histone H2A	LNKLLGKVTIAQGGVLPNIQAVLLPKKTE	372	6.60E-05	1019.2932	3054.8578	3	0.0045	29	0	GluC
H2A2A_MOUSE, H2A2C_MOUSE	Histone H2A	LNKLLGKVTIAQGGVLPNIQAVLLPKKTE	372	6.20E-05	764.7217	3054.8577	4	0.0045	29	0	GluC
H2A2A_MOUSE, H2A2C_MOUSE	Histone H2A	LNKLLGKVTIAQGGVLPNIQAVLLPKKTE	372	7.20E-06	1019.2932	3054.8578	3	0.0045	29	0	GluC
H2A2A_MOUSE, H2A2C_MOUSE	Histone H2A	LNKLLGKVTIAQGGVLPNIQAVLLPKKTE	372	5.90E-07	1019.2932	3054.8578	3	0.0045	29	0	GluC
H2A2A_MOUSE, H2A2C_MOUSE	Histone H2A	LNKLLGKVTIAQGGVLPNIQAVLLPKKTE	372	5.00E-07	611.9788	3054.8578	5	0.0045	29	0	GluC
H2A2A_MOUSE, H2A2C_MOUSE	Histone H2A	LNKLLGKVTIAQGGVLPNIQAVLLPKKTE	372	2.40E-07	1019.2932	3054.8578	3	0.0045	29	0	GluC
H2A2A_MOUSE, H2A2C_MOUSE	Histone H2A	LNKLLGKVTIAQGGVLPNIQAVLLPKKTE	372	4.30E-08	764.7217	3054.8577	4	0.0045	29	0	GluC

H2A2A_MOUSE, H2A2C_MOUSE	Histone H2A	LNKLLGKVTIAQGGVLPNIQAVLLPKKTE	372	2.00E-08	1019.2932	3054.8578	3	0.0045	29	0	GluC
H2A2A_MOUSE, H2A2C_MOUSE	Histone H2A	LNKLLGKVTIAQGGVLPNIQAVLLPKKTE	372	5.50E-03	764.7216	3054.8575	4	0.0043	29	0	GluC
H2A2A_MOUSE, H2A2C_MOUSE	Histone H2A	LNKLLGKVTIAQGGVLPNIQAVLLPKKTE	372	5.40E-03	764.7216	3054.8575	4	0.0043	29	0	GluC
H2A2A_MOUSE, H2A2C_MOUSE	Histone H2A	LNKLLGKVTIAQGGVLPNIQAVLLPKKTE	372	1.70E-03	764.7217	3054.8576	4	0.0043	29	0	GluC
H2A2A_MOUSE, H2A2C_MOUSE	Histone H2A	LNKLLGKVTIAQGGVLPNIQAVLLPKKTE	372	2.20E-04	764.7216	3054.8575	4	0.0043	29	0	GluC
H2A2A_MOUSE, H2A2C_MOUSE	Histone H2A	LNKLLGKVTIAQGGVLPNIQAVLLPKKTE	372	6.20E-05	764.7216	3054.8575	4	0.0043	29	0	GluC
H2A2A_MOUSE, H2A2C_MOUSE	Histone H2A	LNKLLGKVTIAQGGVLPNIQAVLLPKKTE	372	3.50E-06	764.7216	3054.8575	4	0.0043	29	0	GluC
H2A2A_MOUSE, H2A2C_MOUSE	Histone H2A	LNKLLGKVTIAQGGVLPNIQAVLLPKKTE	372	3.00E-04	764.7216	3054.8575	4	0.0042	29	0	GluC
H2A2A_MOUSE, H2A2C_MOUSE	Histone H2A	LNKLLGKVTIAQGGVLPNIQAVLLPKKTE	372	6.40E-05	1019.2931	3054.8575	3	0.0042	29	0	GluC
H2A2A_MOUSE, H2A2C_MOUSE	Histone H2A	LNKLLGKVTIAQGGVLPNIQAVLLPKKTE	372	6.00E-05	1019.2931	3054.8575	3	0.0042	29	0	GluC
H2A2A_MOUSE, H2A2C_MOUSE	Histone H2A	LNKLLGKVTIAQGGVLPNIQAVLLPKKTE	372	2.10E-05	764.7216	3054.8575	4	0.0042	29	0	GluC
H2A2A_MOUSE, H2A2C_MOUSE	Histone H2A	LNKLLGKVTIAQGGVLPNIQAVLLPKKTE	372	2.00E-05	764.7216	3054.8574	4	0.0042	29	0	GluC
H2A2A_MOUSE, H2A2C_MOUSE	Histone H2A	LNKLLGKVTIAQGGVLPNIQAVLLPKKTE	372	1.40E-05	1019.2931	3054.8575	3	0.0042	29	0	GluC
H2A2A_MOUSE, H2A2C_MOUSE	Histone H2A	LNKLLGKVTIAQGGVLPNIQAVLLPKKTE	372	1.60E-06	1019.2931	3054.8575	3	0.0042	29	0	GluC
H2A2A_MOUSE, H2A2C_MOUSE	Histone H2A	LNKLLGKVTIAQGGVLPNIQAVLLPKKTE	372	1.60E-07	764.7216	3054.8575	4	0.0042	29	0	GluC
H2A2A_MOUSE, H2A2C_MOUSE	Histone H2A	LNKLLGKVTIAQGGVLPNIQAVLLPKKTE	372	1.50E-07	764.7216	3054.8575	4	0.0042	29	0	GluC
H2A2A_MOUSE, H2A2C_MOUSE	Histone H2A	LNKLLGKVTIAQGGVLPNIQAVLLPKKTE	372	8.80E-08	764.7216	3054.8574	4	0.0042	29	0	GluC
H2A2A_MOUSE, H2A2C_MOUSE	Histone H2A	LNKLLGKVTIAQGGVLPNIQAVLLPKKTE	372	3.80E-04	764.7216	3054.8573	4	0.0041	29	0	GluC
H2A2A_MOUSE, H2A2C_MOUSE	Histone H2A	LNKLLGKVTIAQGGVLPNIQAVLLPKKTE	372	1.20E-04	764.7216	3054.8573	4	0.0041	29	0	GluC
H2A2A_MOUSE, H2A2C_MOUSE	Histone H2A	LNKLLGKVTIAQGGVLPNIQAVLLPKKTE	372	1.10E-04	764.7216	3054.8573	4	0.0041	29	0	GluC
H2A2A_MOUSE, H2A2C_MOUSE	Histone H2A	LNKLLGKVTIAQGGVLPNIQAVLLPKKTE	372	3.30E-05	764.7216	3054.8573	4	0.0041	29	0	GluC
H2A2A_MOUSE, H2A2C_MOUSE	Histone H2A	LNKLLGKVTIAQGGVLPNIQAVLLPKKTE	372	3.60E-03	764.7216	3054.8573	4	0.0040	29	0	GluC
H2A2A_MOUSE, H2A2C_MOUSE	Histone H2A	LNKLLGKVTIAQGGVLPNIQAVLLPKKTE	372	2.80E-04	764.7216	3054.8573	4	0.0040	29	0	GluC
H2A2A_MOUSE, H2A2C_MOUSE	Histone H2A	LNKLLGKVTIAQGGVLPNIQAVLLPKKTE	372	3.60E-06	764.7216	3054.8572	4	0.0040	29	0	GluC
H2A2A_MOUSE, H2A2C_MOUSE	Histone H2A	LNKLLGKVTIAQGGVLPNIQAVLLPKKTE	372	5.90E-08	764.7216	3054.8572	4	0.0040	29	0	GluC
H2A2A_MOUSE, H2A2C_MOUSE	Histone H2A	LNKLLGKVTIAQGGVLPNIQAVLLPKKTE	372	3.00E-03	764.7215	3054.8571	4	0.0039	29	0	GluC
H2A2A_MOUSE, H2A2C_MOUSE	Histone H2A	LNKLLGKVTIAQGGVLPNIQAVLLPKKTE	372	6.40E-05	1019.293	3054.8572	3	0.0039	29	0	GluC
H2A2A_MOUSE, H2A2C_MOUSE	Histone H2A	LNKLLGKVTIAQGGVLPNIQAVLLPKKTE	372	5.10E-05	764.7216	3054.8572	4	0.0039	29	0	GluC
H2A2A_MOUSE, H2A2C_MOUSE	Histone H2A	LNKLLGKVTIAQGGVLPNIQAVLLPKKTE	372	1.30E-06	1019.293	3054.8572	3	0.0039	29	0	GluC
H2A2A_MOUSE, H2A2C_MOUSE	Histone H2A	LNKLLGKVTIAQGGVLPNIQAVLLPKKTE	372	6.20E-07	1019.293	3054.8572	3	0.0039	29	0	GluC
H2A2A_MOUSE, H2A2C_MOUSE	Histone H2A	LNKLLGKVTIAQGGVLPNIQAVLLPKKTE	372	4.00E-07	1019.293	3054.8572	3	0.0039	29	0	GluC
H2A2A_MOUSE, H2A2C_MOUSE	Histone H2A	LNKLLGKVTIAQGGVLPNIQAVLLPKKTE	372	5.80E-08	1019.293	3054.8572	3	0.0039	29	0	GluC
H2A2A_MOUSE, H2A2C_MOUSE	Histone H2A	LNKLLGKVTIAQGGVLPNIQAVLLPKKTE	372	6.70E-09	764.7216	3054.8572	4	0.0039	29	0	GluC
H2A2A_MOUSE, H2A2C_MOUSE	Histone H2A	LNKLLGKVTIAQGGVLPNIQAVLLPKKTE	372	1.40E-04	764.7215	3054.8571	4	0.0038	29	0	GluC
H2A2A_MOUSE, H2A2C_MOUSE	Histone H2A	LNKLLGKVTIAQGGVLPNIQAVLLPKKTE	372	1.90E-08	764.7215	3054.8571	4	0.0038	29	0	GluC
H2A2A_MOUSE, H2A2C_MOUSE	Histone H2A	LNKLLGKVTIAQGGVLPNIQAVLLPKKTE	372	1.10E-09	611.9787	3054.8571	5	0.0038	29	0	GluC
H2A2A_MOUSE, H2A2C_MOUSE	Histone H2A	LNKLLGKVTIAQGGVLPNIQAVLLPKKTE	372	4.20E-03	764.7215	3054.8570	4	0.0037	29	0	GluC
H2A2A_MOUSE, H2A2C_MOUSE	Histone H2A	LNKLLGKVTIAQGGVLPNIQAVLLPKKTE	372	1.90E-03	764.7215	3054.8569	4	0.0037	29	0	GluC
H2A2A_MOUSE, H2A2C_MOUSE	Histone H2A	LNKLLGKVTIAQGGVLPNIQAVLLPKKTE	372	7.30E-05	764.7215	3054.8569	4	0.0037	29	0	GluC
H2A2A_MOUSE, H2A2C_MOUSE	Histone H2A	LNKLLGKVTIAQGGVLPNIQAVLLPKKTE	372	1.30E-05	764.7215	3054.8569	4	0.0037	29	0	GluC
H2A2A_MOUSE, H2A2C_MOUSE	Histone H2A	LNKLLGKVTIAQGGVLPNIQAVLLPKKTE	372	1.10E-05	764.7215	3054.8569	4	0.0037	29	0	GluC
H2A2A_MOUSE, H2A2C_MOUSE	Histone H2A	LNKLLGKVTIAQGGVLPNIQAVLLPKKTE	372	6.60E-06	611.9787	3054.8569	5	0.0037	29	0	GluC
H2A2A_MOUSE, H2A2C_MOUSE	Histone H2A	LNKLLGKVTIAQGGVLPNIQAVLLPKKTE	372	4.40E-06	764.7215	3054.8569	4	0.0037	29	0	GluC
H2A2A_MOUSE, H2A2C_MOUSE	Histone H2A	LNKLLGKVTIAQGGVLPNIQAVLLPKKTE	372	1.10E-06	764.7215	3054.8569	4	0.0037	29	0	GluC
H2A2A_MOUSE, H2A2C_MOUSE	Histone H2A	LNKLLGKVTIAQGGVLPNIQAVLLPKKTE	372	5.90E-07	764.7215	3054.8570	4	0.0037	29	0	GluC
H2A2A_MOUSE, H2A2C_MOUSE	Histone H2A	LNKLLGKVTIAQGGVLPNIQAVLLPKKTE	372	4.60E-07	611.9787	3054.8570	5	0.0037	29	0	GluC
H2A2A_MOUSE, H2A2C_MOUSE	Histone H2A	LNKLLGKVTIAQGGVLPNIQAVLLPKKTE	372	6.70E-03	1019.2929	3054.8569	3	0.0036	29	0	GluC
H2A2A_MOUSE, H2A2C_MOUSE	Histone H2A	LNKLLGKVTIAQGGVLPNIQAVLLPKKTE	372	5.10E-04	1019.2929	3054.8569	3	0.0036	29	0	GluC
H2A2A_MOUSE, H2A2C_MOUSE	Histone H2A	LNKLLGKVTIAQGGVLPNIQAVLLPKKTE	372	6.00E-03	764.7215	3054.8567	4	0.0035	29	0	GluC
H2A2A_MOUSE, H2A2C_MOUSE	Histone H2A	LNKLLGKVTIAQGGVLPNIQAVLLPKKTE	372	1.80E-03	764.7215	3054.8568	4	0.0035	29	0	GluC
H2A2A_MOUSE, H2A2C_MOUSE	Histone H2A	LNKLLGKVTIAQGGVLPNIQAVLLPKKTE	372	1.50E-03	764.7215	3054.8568	4	0.0035	29	0	GluC
H2A2A_MOUSE, H2A2C_MOUSE	Histone H2A	LNKLLGKVTIAQGGVLPNIQAVLLPKKTE	372	1.60E-04	764.7215	3054.8568	4	0.0035	29	0	GluC
H2A2A_MOUSE, H2A2C_MOUSE	Histone H2A	LNKLLGKVTIAQGGVLPNIQAVLLPKKTE	372	3.20E-05	764.7215	3054.8568	4	0.0035	29	0	GluC
H2A2A_MOUSE, H2A2C_MOUSE	Histone H2A	LNKLLGKVTIAQGGVLPNIQAVLLPKKTE	372	2.40E-07	764.7215	3054.8568	4	0.0035	29	0	GluC
H2A2A_MOUSE, H2A2C_MOUSE	Histone H2A	LNKLLGKVTIAQGGVLPNIQAVLLPKKTE	372	4.70E-09	764.7215	3054.8568	4	0.0035	29	0	GluC
H2A2A_MOUSE, H2A2C_MOUSE	Histone H2A	LNKLLGKVTIAQGGVLPNIQAVLLPKKTE	372	5.70E-03	764.7214	3054.8567	4	0.0034	29	0	GluC
H2A2A_MOUSE, H2A2C_MOUSE	Histone H2A	LNKLLGKVTIAQGGVLPNIQAVLLPKKTE	372	5.60E-06	764.7214	3054.8567	4	0.0034	29	0	GluC
H2A2A_MOUSE, H2A2C_MOUSE	Histone H2A	LNKLLGKVTIAQGGVLPNIQAVLLPKKTE	372	2.40E-08	764.7214	3054.8567	4	0.0034	29	0	GluC
H2A2A_MOUSE, H2A2C_MOUSE	Histone H2A	LNKLLGKVTIAQGGVLPNIQAVLLPKKTE	372	9.70E-04	764.7214	3054.8565	4	0.0033	29	0	GluC
H2A2A_MOUSE, H2A2C_MOUSE	Histone H2A	LNKLLGKVTIAQGGVLPNIQAVLLPKKTE	372	6.10E-04	1019.2928	3054.8566	3	0.0033	29	0	GluC
H2A2A_MOUSE, H2A2C_MOUSE	Histone H2A	LNKLLGKVTIAQGGVLPNIQAVLLPKKTE	372	2.70E-05	1019.2928	3054.8566	3	0.0033	29	0	GluC
H2A2A_MOUSE, H2A2C_MOUSE	Histone H2A	LNKLLGKVTIAQGGVLPNIQAVLLPKKTE	372	1.20E-05	611.9786	3054.8565	5	0.0033	29	0	GluC
H2A2A_MOUSE, H2A2C_MOUSE	Histone H2A	LNKLLGKVTIAQGGVLPNIQAVLLPKKTE	372	2.00E-06	611.9786	3054.8565	5	0.0033	29	0	GluC
H2A2A_MOUSE, H2A2C_MOUSE	Histone H2A	LNKLLGKVTIAQGGVLPNIQAVLLPKKTE	372	1.50E-06	1019.2928	3054.8566	3	0.0033	29	0	GluC
H2A2A_MOUSE, H2A2C_MOUSE	Histone H2A	LNKLLGKVTIAQGGVLPNIQAVLLPKKTE	372	3.80E-07	764.7214	3054.8565	4	0.0033	29	0	GluC
H2A2A_MOUSE, H2A2C_MOUSE	Histone H2A	LNKLLGKVTIAQGGVLPNIQAVLLPKKTE	372	2.70E-08	1019.2928	3054.8566	3	0.0033	29	0	GluC
H2A2A_MOUSE, H2A2C_MOUSE	Histone H2A	LNKLLGKVTIAQGGVLPNIQAVLLPKKTE	372	9.90E-03	611.9786	3054.8564	5	0.0032	29	0	GluC
H2A2A_MOUSE, H2A2C_MOUSE	Histone H2A	LNKLLGKVTIAQGGVLPNIQAVLLPKKTE	372	5.60E-04	764.7214	3054.8564	4	0.0032	29	0	GluC
H2A2A_MOUSE, H2A2C_MOUSE	Histone H2A	LNKLLGKVTIAQGGVLPNIQAVLLPKKTE	372	1.00E-05	764.7214	3054.8565	4	0.0032	29	0	GluC
H2A2A_MOUSE, H2A2C_MOUSE	Histone H2A	LNKLLGKVTIAQGGVLPNIQAVLLPKKTE	372	1.30E-06	764.7214	3054.8564	4	0.0032	29	0	GluC
H2A2A_MOUSE, H2A2C_MOUSE	Histone H2A	LNKLLGKVTIAQGGVLPNIQAVLLPKKTE	372	1.70E-07	764.7214	3054.8564	4	0.0032	29	0	GluC
H2A2A_MOUSE, H2A2C_MOUSE	Histone H2A	LNKLLGKVTIAQGGVLPNIQAVLLPKKTE	372	7.70E-08	611.9786	3054.8564	5	0.0032	29	0	GluC
H2A2A_MOUSE, H2A2C_MOUSE	Histone H2A	LNKLLGKVTIAQGGVLPNIQAVLLPKKTE	372	1.60E-08	764.7214	3054.8564	4	0.0032	29	0	GluC
H2A2A_MOUSE, H2A2C_MOUSE	Histone H2A	LNKLLGKVTIAQGGVLPNIQAVLLPKKTE	372	6.50E-03	764.7214	3054.8563	4	0.0031	29	0	GluC

H2A2A_MOUSE, H2A2C_MOUSE	Histone H2A	LNKLLGKVTIAQGGVLPNIQAVLLPKKTE	372	3.50E-03	764.7214	3054.8563	4	0.0031	29	0	GluC
H2A2A_MOUSE, H2A2C_MOUSE	Histone H2A	LNKLLGKVTIAQGGVLPNIQAVLLPKKTE	372	7.40E-04	764.7214	3054.8563	4	0.0031	29	0	GluC
H2A2A_MOUSE, H2A2C_MOUSE	Histone H2A	LNKLLGKVTIAQGGVLPNIQAVLLPKKTE	372	7.80E-05	764.7214	3054.8563	4	0.0031	29	0	GluC
H2A2A_MOUSE, H2A2C_MOUSE	Histone H2A	LNKLLGKVTIAQGGVLPNIQAVLLPKKTE	372	3.70E-05	764.7214	3054.8563	4	0.0031	29	0	GluC
H2A2A_MOUSE, H2A2C_MOUSE	Histone H2A	LNKLLGKVTIAQGGVLPNIQAVLLPKKTE	372	2.10E-03	764.7213	3054.8563	4	0.0030	29	0	GluC
H2A2A_MOUSE, H2A2C_MOUSE	Histone H2A	LNKLLGKVTIAQGGVLPNIQAVLLPKKTE	372	3.80E-07	764.7213	3054.8563	4	0.0030	29	0	GluC
H2A2A_MOUSE, H2A2C_MOUSE	Histone H2A	LNKLLGKVTIAQGGVLPNIQAVLLPKKTE	372	5.70E-03	764.7213	3054.8561	4	0.0029	29	0	GluC
H2A2A_MOUSE, H2A2C_MOUSE	Histone H2A	LNKLLGKVTIAQGGVLPNIQAVLLPKKTE	372	1.80E-03	764.7213	3054.8561	4	0.0029	29	0	GluC
H2A2A_MOUSE, H2A2C_MOUSE	Histone H2A	LNKLLGKVTIAQGGVLPNIQAVLLPKKTE	372	1.50E-05	764.7213	3054.8561	4	0.0029	29	0	GluC
H2A2A_MOUSE, H2A2C_MOUSE	Histone H2A	LNKLLGKVTIAQGGVLPNIQAVLLPKKTE	372	8.10E-06	764.7213	3054.8561	4	0.0029	29	0	GluC
H2A2A_MOUSE, H2A2C_MOUSE	Histone H2A	LNKLLGKVTIAQGGVLPNIQAVLLPKKTE	372	1.50E-06	764.7213	3054.8562	4	0.0029	29	0	GluC
H2A2A_MOUSE, H2A2C_MOUSE	Histone H2A	LNKLLGKVTIAQGGVLPNIQAVLLPKKTE	372	3.80E-04	764.7213	3054.8561	4	0.0028	29	0	GluC
H2A2A_MOUSE, H2A2C_MOUSE	Histone H2A	LNKLLGKVTIAQGGVLPNIQAVLLPKKTE	372	3.50E-04	764.7213	3054.8561	4	0.0028	29	0	GluC
H2A2A_MOUSE, H2A2C_MOUSE	Histone H2A	LNKLLGKVTIAQGGVLPNIQAVLLPKKTE	372	3.70E-06	764.7213	3054.8561	4	0.0028	29	0	GluC
H2A2A_MOUSE, H2A2C_MOUSE	Histone H2A	LNKLLGKVTIAQGGVLPNIQAVLLPKKTE	372	1.50E-08	764.7213	3054.8561	4	0.0028	29	0	GluC
H2A2A_MOUSE, H2A2C_MOUSE	Histone H2A	LNKLLGKVTIAQGGVLPNIQAVLLPKKTE	372	2.30E-12	611.9785	3054.8560	5	0.0028	29	0	GluC
H2A2A_MOUSE, H2A2C_MOUSE	Histone H2A	LNKLLGKVTIAQGGVLPNIQAVLLPKKTE	372	2.90E-03	611.9785	3054.8559	5	0.0027	29	0	GluC
H2A2A_MOUSE, H2A2C_MOUSE	Histone H2A	LNKLLGKVTIAQGGVLPNIQAVLLPKKTE	372	2.50E-06	611.9785	3054.8559	5	0.0027	29	0	GluC
H2A2A_MOUSE, H2A2C_MOUSE	Histone H2A	LNKLLGKVTIAQGGVLPNIQAVLLPKKTE	372	6.60E-07	611.9785	3054.8559	5	0.0027	29	0	GluC
H2A2A_MOUSE, H2A2C_MOUSE	Histone H2A	LNKLLGKVTIAQGGVLPNIQAVLLPKKTE	372	2.50E-03	764.7212	3054.8558	4	0.0026	29	0	GluC
H2A2A_MOUSE, H2A2C_MOUSE	Histone H2A	LNKLLGKVTIAQGGVLPNIQAVLLPKKTE	372	5.50E-06	764.7212	3054.8558	4	0.0026	29	0	GluC
H2A2A_MOUSE, H2A2C_MOUSE	Histone H2A	LNKLLGKVTIAQGGVLPNIQAVLLPKKTE	372	1.30E-07	764.7212	3054.8558	4	0.0026	29	0	GluC
H2A2A_MOUSE, H2A2C_MOUSE	Histone H2A	LNKLLGKVTIAQGGVLPNIQAVLLPKKTE	372	1.00E-03	764.7212	3054.8557	4	0.0025	29	0	GluC
H2A2A_MOUSE, H2A2C_MOUSE	Histone H2A	LNKLLGKVTIAQGGVLPNIQAVLLPKKTE	372	1.10E-08	764.7212	3054.8557	4	0.0025	29	0	GluC
H2A2A_MOUSE, H2A2C_MOUSE	Histone H2A	LNKLLGKVTIAQGGVLPNIQAVLLPKKTE	372	2.50E-06	611.9784	3054.8556	5	0.0024	29	0	GluC
H2A2A_MOUSE, H2A2C_MOUSE	Histone H2A	LNKLLGKVTIAQGGVLPNIQAVLLPKKTE	372	7.80E-07	764.7212	3054.8557	4	0.0024	29	0	GluC
H2A2A_MOUSE, H2A2C_MOUSE	Histone H2A	LNKLLGKVTIAQGGVLPNIQAVLLPKKTE	372	2.30E-09	1528.4351	3054.8556	2	0.0024	29	0	GluC
H2A2A_MOUSE, H2A2C_MOUSE	Histone H2A	LNKLLGKVTIAQGGVLPNIQAVLLPKKTE	372	4.70E-03	764.7211	3054.8555	4	0.0023	29	0	GluC
H2A2A_MOUSE, H2A2C_MOUSE	Histone H2A	LNKLLGKVTIAQGGVLPNIQAVLLPKKTE	372	4.10E-03	764.7211	3054.8555	4	0.0023	29	0	GluC
H2A2A_MOUSE, H2A2C_MOUSE	Histone H2A	LNKLLGKVTIAQGGVLPNIQAVLLPKKTE	372	5.00E-07	764.7211	3054.8555	4	0.0023	29	0	GluC
H2A2A_MOUSE, H2A2C_MOUSE	Histone H2A	LNKLLGKVTIAQGGVLPNIQAVLLPKKTE	372	4.90E-05	611.9784	3054.8555	5	0.0022	29	0	GluC
H2A2A_MOUSE, H2A2C_MOUSE	Histone H2A	LNKLLGKVTIAQGGVLPNIQAVLLPKKTE	372	3.50E-04	764.7211	3054.8553	4	0.0020	29	0	GluC
H2A2A_MOUSE, H2A2C_MOUSE	Histone H2A	LNKLLGKVTIAQGGVLPNIQAVLLPKKTE	372	2.80E-04	764.7211	3054.8553	4	0.0020	29	0	GluC
H2A2A_MOUSE, H2A2C_MOUSE	Histone H2A	LNKLLGKVTIAQGGVLPNIQAVLLPKKTE	372	1.70E-04	611.9783	3054.8552	5	0.0020	29	0	GluC
H2A2A_MOUSE, H2A2C_MOUSE	Histone H2A	LNKLLGKVTIAQGGVLPNIQAVLLPKKTE	372	3.90E-05	611.9783	3054.8553	5	0.0020	29	0	GluC
H2A2A_MOUSE, H2A2C_MOUSE	Histone H2A	LNKLLGKVTIAQGGVLPNIQAVLLPKKTE	372	1.00E-08	764.7211	3054.8553	4	0.0020	29	0	GluC
H2A2A_MOUSE, H2A2C_MOUSE	Histone H2A	LNKLLGKVTIAQGGVLPNIQAVLLPKKTE	372	2.80E-09	764.7211	3054.8553	4	0.0020	29	0	GluC
H2A2A_MOUSE, H2A2C_MOUSE	Histone H2A	LNKLLGKVTIAQGGVLPNIQAVLLPKKTE	372	6.40E-04	764.7211	3054.8552	4	0.0019	29	0	GluC
H2A2A_MOUSE, H2A2C_MOUSE	Histone H2A	LNKLLGKVTIAQGGVLPNIQAVLLPKKTE	372	5.10E-06	764.7211	3054.8551	4	0.0019	29	0	GluC
H2A2A_MOUSE, H2A2C_MOUSE	Histone H2A	LNKLLGKVTIAQGGVLPNIQAVLLPKKTE	372	6.70E-10	764.7211	3054.8551	4	0.0019	29	0	GluC
H2A2A_MOUSE, H2A2C_MOUSE	Histone H2A	LNKLLGKVTIAQGGVLPNIQAVLLPKKTE	372	5.10E-08	764.721	3054.8550	4	0.0017	29	0	GluC
H2A2A_MOUSE, H2A2C_MOUSE	Histone H2A	LNKLLGKVTIAQGGVLPNIQAVLLPKKTE	372	2.80E-03	764.721	3054.8548	4	0.0015	29	0	GluC
H2A2A_MOUSE, H2A2C_MOUSE	Histone H2A	LNKLLGKVTIAQGGVLPNIQAVLLPKKTE	372	4.00E-04	764.721	3054.8547	4	0.0015	29	0	GluC
H2A2A_MOUSE, H2A2C_MOUSE	Histone H2A	LNKLLGKVTIAQGGVLPNIQAVLLPKKTE	372	4.00E-06	611.9782	3054.8547	5	0.0015	29	0	GluC
H2A2A_MOUSE, H2A2C_MOUSE	Histone H2A	LNKLLGKVTIAQGGVLPNIQAVLLPKKTE	372	2.00E-06	764.721	3054.8548	4	0.0015	29	0	GluC
H2A2A_MOUSE, H2A2C_MOUSE	Histone H2A	LNKLLGKVTIAQGGVLPNIQAVLLPKKTE	372	1.40E-06	764.721	3054.8547	4	0.0015	29	0	GluC
H2A2A_MOUSE, H2A2C_MOUSE	Histone H2A	LNKLLGKVTIAQGGVLPNIQAVLLPKKTE	372	2.20E-07	764.721	3054.8548	4	0.0015	29	0	GluC
H2A2A_MOUSE, H2A2C_MOUSE	Histone H2A	LNKLLGKVTIAQGGVLPNIQAVLLPKKTE	372	9.80E-05	611.9782	3054.8546	5	0.0014	29	0	GluC
H2A2A_MOUSE, H2A2C_MOUSE	Histone H2A	LNKLLGKVTIAQGGVLPNIQAVLLPKKTE	372	1.20E-05	764.7209	3054.8547	4	0.0014	29	0	GluC
H2A2A_MOUSE, H2A2C_MOUSE	Histone H2A	LNKLLGKVTIAQGGVLPNIQAVLLPKKTE	372	7.20E-06	764.7209	3054.8547	4	0.0014	29	0	GluC
H2A2A_MOUSE, H2A2C_MOUSE	Histone H2A	LNKLLGKVTIAQGGVLPNIQAVLLPKKTE	372	7.80E-11	1528.4346	3054.8546	2	0.0014	29	0	GluC
H2A2A_MOUSE, H2A2C_MOUSE	Histone H2A	LNKLLGKVTIAQGGVLPNIQAVLLPKKTE	372	8.80E-03	764.7209	3054.8546	4	0.0013	29	0	GluC
H2A2A_MOUSE, H2A2C_MOUSE	Histone H2A	LNKLLGKVTIAQGGVLPNIQAVLLPKKTE	372	3.30E-03	611.9782	3054.8545	5	0.0013	29	0	GluC
H2A2A_MOUSE, H2A2C_MOUSE	Histone H2A	LNKLLGKVTIAQGGVLPNIQAVLLPKKTE	372	1.60E-03	764.7209	3054.8546	4	0.0013	29	0	GluC
H2A2A_MOUSE, H2A2C_MOUSE	Histone H2A	LNKLLGKVTIAQGGVLPNIQAVLLPKKTE	372	2.60E-04	764.7209	3054.8546	4	0.0013	29	0	GluC
H2A2A_MOUSE, H2A2C_MOUSE	Histone H2A	LNKLLGKVTIAQGGVLPNIQAVLLPKKTE	372	1.40E-04	764.7209	3054.8546	4	0.0013	29	0	GluC
H2A2A_MOUSE, H2A2C_MOUSE	Histone H2A	LNKLLGKVTIAQGGVLPNIQAVLLPKKTE	372	8.20E-10	764.7209	3054.8546	4	0.0013	29	0	GluC
H2A2A_MOUSE, H2A2C_MOUSE	Histone H2A	LNKLLGKVTIAQGGVLPNIQAVLLPKKTE	372	2.40E-11	611.9782	3054.8545	5	0.0013	29	0	GluC
H2A2A_MOUSE, H2A2C_MOUSE	Histone H2A	LNKLLGKVTIAQGGVLPNIQAVLLPKKTE	372	1.70E-07	1019.2921	3054.8545	3	0.0012	29	0	GluC
H2A2A_MOUSE, H2A2C_MOUSE	Histone H2A	LNKLLGKVTIAQGGVLPNIQAVLLPKKTE	372	5.40E-08	1019.2921	3054.8545	3	0.0012	29	0	GluC
H2A2A_MOUSE, H2A2C_MOUSE	Histone H2A	LNKLLGKVTIAQGGVLPNIQAVLLPKKTE	372	9.70E-06	611.9781	3054.8543	5	0.0010	29	0	GluC
H2A2A_MOUSE, H2A2C_MOUSE	Histone H2A	LNKLLGKVTIAQGGVLPNIQAVLLPKKTE	372	9.30E-07	764.7208	3054.8543	4	0.0010	29	0	GluC
H2A2A_MOUSE, H2A2C_MOUSE	Histone H2A	LNKLLGKVTIAQGGVLPNIQAVLLPKKTE	372	1.00E-08	764.7208	3054.8543	4	0.0010	29	0	GluC
H2A2A_MOUSE, H2A2C_MOUSE	Histone H2A	LNKLLGKVTIAQGGVLPNIQAVLLPKKTE	372	2.30E-03	764.7208	3054.8541	4	0.0009	29	0	GluC
H2A2A_MOUSE, H2A2C_MOUSE	Histone H2A	LNKLLGKVTIAQGGVLPNIQAVLLPKKTE	372	1.80E-03	1019.292	3054.8542	3	0.0009	29	0	GluC
H2A2A_MOUSE, H2A2C_MOUSE	Histone H2A	LNKLLGKVTIAQGGVLPNIQAVLLPKKTE	372	9.00E-05	764.7208	3054.8541	4	0.0009	29	0	GluC
H2A2A_MOUSE, H2A2C_MOUSE	Histone H2A	LNKLLGKVTIAQGGVLPNIQAVLLPKKTE	372	4.70E-07	764.7208	3054.8541	4	0.0009	29	0	GluC
H2A2A_MOUSE, H2A2C_MOUSE	Histone H2A	LNKLLGKVTIAQGGVLPNIQAVLLPKKTE	372	3.00E-07	611.9781	3054.8542	5	0.0009	29	0	GluC
H2A2A_MOUSE, H2A2C_MOUSE	Histone H2A	LNKLLGKVTIAQGGVLPNIQAVLLPKKTE	372	1.30E-08	764.7208	3054.8541	4	0.0009	29	0	GluC
H2A2A_MOUSE, H2A2C_MOUSE	Histone H2A	LNKLLGKVTIAQGGVLPNIQAVLLPKKTE	372	3.20E-04	1019.2919	3054.8539	3	0.0006	29	0	GluC
H2A2A_MOUSE, H2A2C_MOUSE	Histone H2A	LNKLLGKVTIAQGGVLPNIQAVLLPKKTE	372	2.60E-04	1019.2919	3054.8539	3	0.0006	29	0	GluC
H2A2A_MOUSE, H2A2C_MOUSE	Histone H2A	LNKLLGKVTIAQGGVLPNIQAVLLPKKTE	372	1.40E-08	1019.2919	3054.8539	3	0.0006	29	0	GluC
H2A2A_MOUSE, H2A2C_MOUSE	Histone H2A	LNKLLGKVTIAQGGVLPNIQAVLLPKKTE	372	3.00E-05	1019.2918	3054.8536	3	0.0003	29	0	GluC
H2A2A_MOUSE, H2A2C_MOUSE	Histone H2A	LNKLLGKVTIAQGGVLPNIQAVLLPKKTE	372	9.10E-05	611.9779	3054.8530	5	-0.0002	29	0	GluC

H2A2A_MOUSE, H2A2C_MOUSE	Histone H2A	LNKLLGKVTIAQGGVLPNIQAVLLPKKTE	372	3.10E-06	611.9779	3054.8530	5	-0.0002	29	0	GluC
H2A2A_MOUSE, H2A2C_MOUSE	Histone H2A	LNKLLGKVTIAQGGVLPNIQAVLLPKKTE	372	7.30E-03	611.9778	3054.8526	5	-0.0006	29	0	GluC
H2A2A_MOUSE, H2A2C_MOUSE	Histone H2A	LNKLLGKVTIAQGGVLPNIQAVLLPKKTE	372	4.60E-08	611.9778	3054.8526	5	-0.0006	29	0	GluC
H2A2A_MOUSE, H2A2C_MOUSE	Histone H2A	LNKLLGKVTIAQGGVLPNIQAVLLPKKTE	372	1.00E-03	611.9777	3054.8522	5	-0.0011	29	0	GluC
H2A2A_MOUSE, H2A2C_MOUSE	Histone H2A	IIPRHLQLAIRNDEELNKLLGK	2	3.60E-05	646.6333	2582.5042	4	0.0022	22	3	Semi-tryptic
H2A2A_MOUSE, H2A2C_MOUSE	Histone H2A	IIPRHLQLAIRNDEELNKLLGK	2	6.10E-03	646.6315	2582.4968	4	-0.0052	22	3	Semi-tryptic
H2A2A_MOUSE, H2A2C_MOUSE	Histone H2A	LGKVTIAQGGVLPNIQAVL	9	2.70E-03	946.0786	1890.1426	2	0.0124	19	1	Chymotrypsin
H2A2A_MOUSE, H2A2C_MOUSE	Histone H2A	LGKVTIAQGGVLPNIQAVL	9	7.70E-03	946.0738	1890.1330	2	0.0028	19	1	Chymotrypsin
H2A2A_MOUSE, H2A2C_MOUSE	Histone H2A	LGKVTIAQGGVLPNIQAVL	9	1.60E-08	946.0734	1890.1321	2	0.0019	19	1	Chymotrypsin
H2A2A_MOUSE, H2A2C_MOUSE	Histone H2A	LGKVTIAQGGVLPNIQAVL	9	3.00E-04	946.0733	1890.1320	2	0.0018	19	1	Chymotrypsin
H2A2A_MOUSE, H2A2C_MOUSE	Histone H2A	LGKVTIAQGGVLPNIQAVL	9	3.50E-07	946.0732	1890.1318	2	0.0016	19	1	Chymotrypsin
H2A2A_MOUSE, H2A2C_MOUSE	Histone H2A	LGKVTIAQGGVLPNIQAVL	9	2.30E-06	946.0731	1890.1316	2	0.0014	19	1	Chymotrypsin
H2A2A_MOUSE, H2A2C_MOUSE	Histone H2A	LGKVTIAQGGVLPNIQAVL	9	1.10E-03	631.0509	1890.1310	3	0.0007	19	1	Chymotrypsin
H2A2A_MOUSE, H2A2C_MOUSE	Histone H2A	LGKVTIAQGGVLPNIQAVL	9	1.00E-07	631.0509	1890.1310	3	0.0007	19	1	Chymotrypsin
H2A2A_MOUSE, H2A2C_MOUSE	Histone H2A	LGKVTIAQGGVLPNIQAVL	9	2.60E-03	631.0507	1890.1304	3	0.0002	19	1	Chymotrypsin
H2A2A_MOUSE, H2A2C_MOUSE	Histone H2A	GKVTIAQGGVLPNIQAVL	6	2.00E-03	889.5354	1777.0563	2	0.0101	18	0	Chymotrypsin
H2A2A_MOUSE, H2A2C_MOUSE	Histone H2A	GKVTIAQGGVLPNIQAVL	6	2.50E-08	593.3585	1777.0536	3	0.0075	18	0	Chymotrypsin
H2A2A_MOUSE, H2A2C_MOUSE	Histone H2A	GKVTIAQGGVLPNIQAVL	6	9.70E-03	889.5317	1777.0489	2	0.0027	18	0	Chymotrypsin
H2A2A_MOUSE, H2A2C_MOUSE	Histone H2A	GKVTIAQGGVLPNIQAVL	6	1.80E-03	889.5317	1777.0489	2	0.0027	18	0	Chymotrypsin
H2A2A_MOUSE, H2A2C_MOUSE	Histone H2A	GKVTIAQGGVLPNIQAVL	6	3.40E-05	889.5317	1777.0488	2	0.0027	18	0	Chymotrypsin
H2A2A_MOUSE, H2A2C_MOUSE	Histone H2A	GKVTIAQGGVLPNIQAVL	6	1.10E-05	889.5317	1777.0489	2	0.0027	18	0	Chymotrypsin
H2A2A_MOUSE, H2A2C_MOUSE	Histone H2A	HLQLAIRNDEELNKLLGK	17	6.10E-05	702.0666	2103.1778	3	-0.0022	18	2	Semi-tryptic
H2A2A_MOUSE, H2A2C_MOUSE	Histone H2A	HLQLAIRNDEELNKLLGK	17	3.20E-03	526.8014	2103.1763	4	-0.0036	18	2	Semi-tryptic
H2A2A_MOUSE, H2A2C_MOUSE	Histone H2A	HLQLAIRNDEELNKLLGK	17	2.50E-03	526.8013	2103.1760	4	-0.0040	18	2	Semi-tryptic
H2A2A_MOUSE, H2A2C_MOUSE	Histone H2A	HLQLAIRNDEELNKLLGK	17	2.90E-05	526.8037	2103.1857	4	0.0057	18	2	Semi-tryptic
H2A2A_MOUSE, H2A2C_MOUSE	Histone H2A	HLQLAIRNDEELNKLLGK	17	2.90E-06	702.0682	2103.1827	3	0.0027	18	2	Semi-tryptic
H2A2A_MOUSE, H2A2C_MOUSE	Histone H2A	HLQLAIRNDEELNKLLGK	17	1.60E-05	702.0681	2103.1824	3	0.0024	18	2	Semi-tryptic
H2A2A_MOUSE, H2A2C_MOUSE	Histone H2A	HLQLAIRNDEELNKLLGK	17	1.30E-05	702.0681	2103.1824	3	0.0024	18	2	Semi-tryptic
H2A2A_MOUSE, H2A2C_MOUSE	Histone H2A	HLQLAIRNDEELNKLLGK	17	2.70E-05	702.068	2103.1821	3	0.0021	18	2	Semi-tryptic
H2A2A_MOUSE, H2A2C_MOUSE	Histone H2A	HLQLAIRNDEELNKLLGK	17	7.60E-03	526.8026	2103.1813	4	0.0013	18	2	Semi-tryptic
H2A2A_MOUSE, H2A2C_MOUSE	Histone H2A	HLQLAIRNDEELNKLLGK	17	1.50E-04	526.8026	2103.1813	4	0.0013	18	2	Semi-tryptic
H2A2A_MOUSE, H2A2C_MOUSE	Histone H2A	HLQLAIRNDEELNKLLGK	17	3.80E-07	526.8026	2103.1813	4	0.0013	18	2	Semi-tryptic
H2A2A_MOUSE, H2A2C_MOUSE	Histone H2A	HLQLAIRNDEELNKLLGK	17	4.80E-05	702.0676	2103.1810	3	0.0010	18	2	Semi-tryptic
H2A2A_MOUSE, H2A2C_MOUSE	Histone H2A	HLQLAIRNDEELNKLLGK	17	7.10E-03	526.8024	2103.1806	4	0.0006	18	2	Semi-tryptic
H2A2A_MOUSE, H2A2C_MOUSE	Histone H2A	HLQLAIRNDEELNKLLGK	17	6.20E-03	702.0669	2103.1789	3	-0.0011	18	2	Semi-tryptic
H2A2A_MOUSE, H2A2C_MOUSE	Histone H2A	HLQLAIRNDEELNKLLGK	17	6.50E-03	526.802	2103.1787	4	-0.0013	18	2	Semi-tryptic
H2A2A_MOUSE, H2A2C_MOUSE	Histone H2A	HLQLAIRNDEELNKLLGK	17	7.90E-07	702.0667	2103.1783	3	-0.0016	18	2	Semi-tryptic
H2A2A_MOUSE, H2A2C_MOUSE	Histone H2A	HLQLAIRNDEELNKLLGK	17	5.90E-05	702.0667	2103.1782	3	-0.0018	18	2	Semi-tryptic
H2A2A_MOUSE, H2A2C_MOUSE	Histone H2A	NDEELNKLLGK	21	4.80E-05	636.8423	1271.6701	2	-0.0020	11	1	Semi-tryptic
H2A2A_MOUSE, H2A2C_MOUSE	Histone H2A	NDEELNKLLGK	21	2.00E-06	636.8423	1271.6701	2	-0.0020	11	1	Semi-tryptic
H2A2A_MOUSE, H2A2C_MOUSE	Histone H2A	NDEELNKLLGK	21	1.30E-03	424.8972	1271.6698	3	-0.0023	11	1	Semi-tryptic
H2A2A_MOUSE, H2A2C_MOUSE	Histone H2A	NDEELNKLLGK	21	1.30E-05	424.8972	1271.6698	3	-0.0023	11	1	Semi-tryptic
H2A2A_MOUSE, H2A2C_MOUSE	Histone H2A	NDEELNKLLGK	21	4.30E-06	636.842	1271.6695	2	-0.0026	11	1	Semi-tryptic
H2A2A_MOUSE, H2A2C_MOUSE	Histone H2A	NDEELNKLLGK	21	1.80E-07	636.8442	1271.6738	2	0.0017	11	1	Semi-tryptic
H2A2A_MOUSE, H2A2C_MOUSE	Histone H2A	NDEELNKLLGK	21	6.70E-04	636.8441	1271.6736	2	0.0015	11	1	Semi-tryptic
H2A2A_MOUSE, H2A2C_MOUSE	Histone H2A	NDEELNKLLGK	21	4.80E-05	424.8985	1271.6736	3	0.0015	11	1	Semi-tryptic
H2A2A_MOUSE, H2A2C_MOUSE	Histone H2A	NDEELNKLLGK	21	2.90E-06	424.8985	1271.6736	3	0.0015	11	1	Semi-tryptic
H2A2A_MOUSE, H2A2C_MOUSE	Histone H2A	NDEELNKLLGK	21	4.50E-05	636.8438	1271.6731	2	0.0010	11	1	Semi-tryptic
H2A2A_MOUSE, H2A2C_MOUSE	Histone H2A	NDEELNKLLGK	21	6.70E-05	636.8438	1271.6730	2	0.0009	11	1	Semi-tryptic
H2A2A_MOUSE, H2A2C_MOUSE	Histone H2A	NDEELNKLLGK	21	1.60E-05	636.8437	1271.6729	2	0.0008	11	1	Semi-tryptic
H2A2A_MOUSE, H2A2C_MOUSE	Histone H2A	NDEELNKLLGK	21	1.90E-06	636.8437	1271.6728	2	0.0008	11	1	Semi-tryptic
H2A2A_MOUSE, H2A2C_MOUSE	Histone H2A	NDEELNKLLGK	21	2.10E-06	636.8437	1271.6728	2	0.0007	11	1	Semi-tryptic
H2A2A_MOUSE, H2A2C_MOUSE	Histone H2A	NDEELNKLLGK	21	7.80E-08	636.8437	1271.6728	2	0.0007	11	1	Semi-tryptic
H2A2A_MOUSE, H2A2C_MOUSE	Histone H2A	NDEELNKLLGK	21	7.70E-07	636.8436	1271.6727	2	0.0006	11	1	Semi-tryptic
H2A2A_MOUSE, H2A2C_MOUSE	Histone H2A	NDEELNKLLGK	21	1.10E-06	636.8436	1271.6725	2	0.0005	11	1	Semi-tryptic
H2A2A_MOUSE, H2A2C_MOUSE	Histone H2A	NDEELNKLLGK	21	1.20E-03	636.8434	1271.6722	2	0.0001	11	1	Semi-tryptic
H2A2A_MOUSE, H2A2C_MOUSE	Histone H2A	NDEELNKLLGK	21	3.10E-07	636.8429	1271.6712	2	-0.0009	11	1	Semi-tryptic
H2A2A_MOUSE, H2A2C_MOUSE	Histone H2A	NDEELNKLLGK	21	7.80E-07	636.8426	1271.6707	2	-0.0014	11	1	Semi-tryptic
H2A2A_MOUSE, H2A2C_MOUSE	Histone H2A	NDEELNKLLGK	21	3.10E-07	636.8426	1271.6707	2	-0.0014	11	1	Semi-tryptic
H2A2B_MOUSE	Histone H2A	DEELNKLLGGVTIAQGGVLPNIQAVLLPKKTESHKPGKNK	8	3.80E-04	1059.3574	4233.4005	4	0.0157	40	0	AspN
H2A2B_MOUSE	Histone H2A	DEELNKLLGGVTIAQGGVLPNIQAVLLPKKTESHKPGKNK	8	3.50E-06	706.5738	4233.3992	6	0.0144	40	0	AspN
H2A2B_MOUSE	Histone H2A	DEELNKLLGGVTIAQGGVLPNIQAVLLPKKTESHKPGKNK	8	6.00E-04	605.7785	4233.3986	7	0.0137	40	0	AspN
H2A2B_MOUSE	Histone H2A	DEELNKLLGGVTIAQGGVLPNIQAVLLPKKTESHKPGKNK	8	1.00E-04	847.687	4233.3985	5	0.0136	40	0	AspN
H2A2B_MOUSE	Histone H2A	DEELNKLLGGVTIAQGGVLPNIQAVLLPKKTESHKPGKNK	8	2.20E-03	706.5736	4233.3982	6	0.0133	40	0	AspN
H2A2B_MOUSE	Histone H2A	DEELNKLLGGVTIAQGGVLPNIQAVLLPKKTESHKPGKNK	8	1.20E-03	706.5736	4233.3982	6	0.0133	40	0	AspN
H2A2B_MOUSE	Histone H2A	DEELNKLLGGVTIAQGGVLPNIQAVLLPKKTESHKPGKNK	8	6.00E-04	605.7783	4233.3973	7	0.0125	40	0	AspN
H2A2B_MOUSE	Histone H2A	DEELNKLLGGVTIAQGGVLPNIQAVLLPKKTESHKPGKNK	8	2.20E-04	847.6867	4233.3972	5	0.0124	40	0	AspN
H2A2B_MOUSE	Histone H2A	ELNKLLGGVTIAQGGVLPNIQAVLLPKKTE	2	2.40E-03	779.2133	3112.8243	4	0.0020	30	1	GluC
H2A2B_MOUSE	Histone H2A	ELNKLLGGVTIAQGGVLPNIQAVLLPKKTE	2	4.60E-04	779.2133	3112.8243	4	0.0020	30	1	GluC
H2A2B_MOUSE	Histone H2A	LNKLLGGVTIAQGGVLPNIQAVLLPKKTE	42	2.00E-03	995.607	2983.7993	3	0.0195	29	0	GluC
H2A2B_MOUSE	Histone H2A	LNKLLGGVTIAQGGVLPNIQAVLLPKKTE	42	3.10E-03	995.6069	2983.7990	3	0.0192	29	0	GluC
H2A2B_MOUSE	Histone H2A	LNKLLGGVTIAQGGVLPNIQAVLLPKKTE	42	8.80E-08	746.957	2983.7988	4	0.0190	29	0	GluC
H2A2B_MOUSE	Histone H2A	LNKLLGGVTIAQGGVLPNIQAVLLPKKTE	42	2.50E-13	746.9567	2983.7977	4	0.0179	29	0	GluC
H2A2B_MOUSE	Histone H2A	LNKLLGGVTIAQGGVLPNIQAVLLPKKTE	42	5.00E-08	746.9555	2983.7929	4	0.0132	29	0	GluC

H2A2B_MOUSE	Histone H2A	LNKLLGGVTIAQGGVLPNIQAVLLPKKTE	42	1.50E-03	746.9552	2983.7917	4	0.0120	29	0	GluC
H2A2B_MOUSE	Histone H2A	LNKLLGGVTIAQGGVLPNIQAVLLPKKTE	42	1.20E-03	995.6027	2983.7861	3	0.0064	29	0	GluC
H2A2B_MOUSE	Histone H2A	LNKLLGGVTIAQGGVLPNIQAVLLPKKTE	42	6.50E-05	746.9538	2983.7861	4	0.0064	29	0	GluC
H2A2B_MOUSE	Histone H2A	LNKLLGGVTIAQGGVLPNIQAVLLPKKTE	42	2.80E-06	995.6027	2983.7861	3	0.0064	29	0	GluC
H2A2B_MOUSE	Histone H2A	LNKLLGGVTIAQGGVLPNIQAVLLPKKTE	42	1.70E-11	746.9538	2983.7861	4	0.0064	29	0	GluC
H2A2B_MOUSE	Histone H2A	LNKLLGGVTIAQGGVLPNIQAVLLPKKTE	42	3.50E-08	1492.9001	2983.7856	2	0.0059	29	0	GluC
H2A2B_MOUSE	Histone H2A	LNKLLGGVTIAQGGVLPNIQAVLLPKKTE	42	9.80E-06	995.6019	2983.7838	3	0.0041	29	0	GluC
H2A2B_MOUSE	Histone H2A	LNKLLGGVTIAQGGVLPNIQAVLLPKKTE	42	5.20E-04	995.6019	2983.7837	3	0.0040	29	0	GluC
H2A2B_MOUSE	Histone H2A	LNKLLGGVTIAQGGVLPNIQAVLLPKKTE	42	1.20E-04	746.9532	2983.7837	4	0.0039	29	0	GluC
H2A2B_MOUSE	Histone H2A	LNKLLGGVTIAQGGVLPNIQAVLLPKKTE	42	1.70E-09	746.9531	2983.7835	4	0.0037	29	0	GluC
H2A2B_MOUSE	Histone H2A	LNKLLGGVTIAQGGVLPNIQAVLLPKKTE	42	1.60E-06	995.6017	2983.7833	3	0.0036	29	0	GluC
H2A2B_MOUSE	Histone H2A	LNKLLGGVTIAQGGVLPNIQAVLLPKKTE	42	2.10E-03	746.9531	2983.7831	4	0.0034	29	0	GluC
H2A2B_MOUSE	Histone H2A	LNKLLGGVTIAQGGVLPNIQAVLLPKKTE	42	3.40E-10	746.9531	2983.7831	4	0.0034	29	0	GluC
H2A2B_MOUSE	Histone H2A	LNKLLGGVTIAQGGVLPNIQAVLLPKKTE	42	4.30E-12	746.953	2983.7830	4	0.0033	29	0	GluC
H2A2B_MOUSE	Histone H2A	LNKLLGGVTIAQGGVLPNIQAVLLPKKTE	42	6.50E-03	746.953	2983.7829	4	0.0032	29	0	GluC
H2A2B_MOUSE	Histone H2A	LNKLLGGVTIAQGGVLPNIQAVLLPKKTE	42	3.20E-05	995.6016	2983.7830	3	0.0032	29	0	GluC
H2A2B_MOUSE	Histone H2A	LNKLLGGVTIAQGGVLPNIQAVLLPKKTE	42	3.00E-09	746.953	2983.7829	4	0.0032	29	0	GluC
H2A2B_MOUSE	Histone H2A	LNKLLGGVTIAQGGVLPNIQAVLLPKKTE	42	3.10E-10	995.6016	2983.7830	3	0.0032	29	0	GluC
H2A2B_MOUSE	Histone H2A	LNKLLGGVTIAQGGVLPNIQAVLLPKKTE	42	8.80E-10	746.953	2983.7829	4	0.0031	29	0	GluC
H2A2B_MOUSE	Histone H2A	LNKLLGGVTIAQGGVLPNIQAVLLPKKTE	42	5.30E-03	746.953	2983.7828	4	0.0030	29	0	GluC
H2A2B_MOUSE	Histone H2A	LNKLLGGVTIAQGGVLPNIQAVLLPKKTE	42	9.60E-06	746.953	2983.7828	4	0.0030	29	0	GluC
H2A2B_MOUSE	Histone H2A	LNKLLGGVTIAQGGVLPNIQAVLLPKKTE	42	3.50E-06	746.953	2983.7827	4	0.0030	29	0	GluC
H2A2B_MOUSE	Histone H2A	LNKLLGGVTIAQGGVLPNIQAVLLPKKTE	42	1.50E-03	746.9527	2983.7817	4	0.0020	29	0	GluC
H2A2B_MOUSE	Histone H2A	LNKLLGGVTIAQGGVLPNIQAVLLPKKTE	42	4.10E-06	746.9527	2983.7817	4	0.0020	29	0	GluC
H2A2B_MOUSE	Histone H2A	LNKLLGGVTIAQGGVLPNIQAVLLPKKTE	42	7.70E-04	746.9527	2983.7817	4	0.0019	29	0	GluC
H2A2B_MOUSE	Histone H2A	LNKLLGGVTIAQGGVLPNIQAVLLPKKTE	42	1.30E-08	995.601	2983.7811	3	0.0014	29	0	GluC
H2A2B_MOUSE	Histone H2A	LNKLLGGVTIAQGGVLPNIQAVLLPKKTE	42	5.60E-03	746.9525	2983.7810	4	0.0013	29	0	GluC
H2A2B_MOUSE	Histone H2A	LNKLLGGVTIAQGGVLPNIQAVLLPKKTE	42	3.70E-05	597.7634	2983.7806	5	0.0008	29	0	GluC
H2A2B_MOUSE	Histone H2A	LNKLLGGVTIAQGGVLPNIQAVLLPKKTE	42	6.90E-07	995.6008	2983.7805	3	0.0008	29	0	GluC
H2A2B_MOUSE	Histone H2A	LNKLLGGVTIAQGGVLPNIQAVLLPKKTE	42	1.50E-03	746.9523	2983.7801	4	0.0004	29	0	GluC
H2A2B_MOUSE	Histone H2A	LNKLLGGVTIAQGGVLPNIQAVLLPKKTE	42	5.70E-06	746.9523	2983.7801	4	0.0004	29	0	GluC
H2A2B_MOUSE	Histone H2A	LNKLLGGVTIAQGGVLPNIQAVLLPKKTE	42	2.60E-09	1492.8973	2983.7800	2	0.0003	29	0	GluC
H2A2B_MOUSE	Histone H2A	LNKLLGGVTIAQGGVLPNIQAVLLPKKTE	42	2.30E-10	746.9522	2983.7797	4	-0.0001	29	0	GluC
H2A2B_MOUSE	Histone H2A	LNKLLGGVTIAQGGVLPNIQAVLLPKKTE	42	4.90E-03	597.7631	2983.7793	5	-0.0005	29	0	GluC
H2A2B_MOUSE	Histone H2A	LNKLLGGVTIAQGGVLPNIQAVLLPKKTE	42	4.20E-14	597.7631	2983.7793	5	-0.0005	29	0	GluC
H2A2B_MOUSE	Histone H2A	LNKLLGGVTIAQGGVLPNIQAVLLPKKTE	42	2.90E-14	597.7631	2983.7793	5	-0.0005	29	0	GluC
H2A2B_MOUSE	Histone H2A	LNKLLGGVTIAQGGVLPNIQAVLLPKKTE	42	1.40E-10	746.952	2983.7789	4	-0.0009	29	0	GluC
H2A2B_MOUSE	Histone H2A	NKKTRIIPRHLQLAVRNDEE	12	1.50E-04	810.7936	2429.3589	3	-0.0026	20	2	GluC
H2A2B_MOUSE	Histone H2A	NKKTRIIPRHLQLAVRNDEE	12	2.10E-03	810.7986	2429.3740	3	0.0125	20	2	GluC
H2A2B_MOUSE	Histone H2A	NKKTRIIPRHLQLAVRNDEE	12	3.30E-06	608.3506	2429.3732	4	0.0117	20	2	GluC
H2A2B_MOUSE	Histone H2A	NKKTRIIPRHLQLAVRNDEE	12	4.30E-03	810.7983	2429.3730	3	0.0115	20	2	GluC
H2A2B_MOUSE	Histone H2A	NKKTRIIPRHLQLAVRNDEE	12	3.30E-06	608.3498	2429.3700	4	0.0086	20	2	GluC
H2A2B_MOUSE	Histone H2A	NKKTRIIPRHLQLAVRNDEE	12	4.30E-05	608.3489	2429.3664	4	0.0049	20	2	GluC
H2A2B_MOUSE	Histone H2A	NKKTRIIPRHLQLAVRNDEE	12	5.90E-04	608.3482	2429.3637	4	0.0022	20	2	GluC
H2A2B_MOUSE	Histone H2A	NKKTRIIPRHLQLAVRNDEE	12	1.30E-03	608.348	2429.3631	4	0.0016	20	2	GluC
H2A2B_MOUSE	Histone H2A	NKKTRIIPRHLQLAVRNDEE	12	9.00E-03	608.3477	2429.3617	4	0.0002	20	2	GluC
H2A2B_MOUSE	Histone H2A	NKKTRIIPRHLQLAVRNDEE	12	3.40E-04	608.3474	2429.3607	4	-0.0008	20	2	GluC
H2A2B_MOUSE	Histone H2A	NKKTRIIPRHLQLAVRNDEE	12	3.80E-03	608.3474	2429.3604	4	-0.0010	20	2	GluC
H2A2B_MOUSE	Histone H2A	NKKTRIIPRHLQLAVRNDEE	12	6.30E-03	608.3472	2429.3596	4	-0.0019	20	2	GluC
H2A2B_MOUSE	Histone H2A	DNKKTRIIPRHLQLAVRN	4	6.90E-04	724.7715	2171.2927	3	0.0164	18	0	AspN
H2A2B_MOUSE	Histone H2A	DNKKTRIIPRHLQLAVRN	4	2.70E-05	724.7662	2171.2767	3	0.0004	18	0	AspN
H2A2B_MOUSE	Histone H2A	DNKKTRIIPRHLQLAVRN	4	8.20E-03	543.8257	2171.2739	4	-0.0024	18	0	AspN
H2A2B_MOUSE	Histone H2A	DNKKTRIIPRHLQLAVRN	4	3.30E-07	543.8254	2171.2725	4	-0.0038	18	0	AspN
H2A2B_MOUSE	Histone H2A	LPKKTESHKPGKNK	3	1.50E-03	796.4684	1590.9223	2	0.0018	14	0	Chymotrypsin
H2A2B_MOUSE	Histone H2A	LPKKTESHKPGKNK	3	9.80E-04	531.314	1590.9201	3	-0.0004	14	0	Chymotrypsin
H2A2B_MOUSE	Histone H2A	LPKKTESHKPGKNK	3	2.70E-05	398.7373	1590.9201	4	-0.0005	14	0	Chymotrypsin
H2A2B_MOUSE	Histone H2A	HLQLAVR	9	2.30E-05	418.7593	835.5040	2	0.0012	7	0	Semi-tryptic
H2A2B_MOUSE	Histone H2A	HLQLAVR	9	1.10E-03	418.7591	835.5036	2	0.0008	7	0	Semi-tryptic
H2A2B_MOUSE	Histone H2A	HLQLAVR	9	1.10E-04	418.7588	835.5031	2	0.0003	7	0	Semi-tryptic
H2A2B_MOUSE	Histone H2A	HLQLAVR	9	1.90E-04	418.7587	835.5029	2	0.0001	7	0	Semi-tryptic
H2A2B_MOUSE	Histone H2A	HLQLAVR	9	6.30E-05	418.7587	835.5029	2	0.0001	7	0	Semi-tryptic
H2A2B_MOUSE	Histone H2A	HLQLAVR	9	5.50E-05	418.7586	835.5027	2	-0.0001	7	0	Semi-tryptic
H2A2B_MOUSE	Histone H2A	HLQLAVR	9	4.00E-05	418.7586	835.5026	2	-0.0002	7	0	Semi-tryptic
H2A2B_MOUSE	Histone H2A	HLQLAVR	9	3.80E-03	418.7586	835.5025	2	-0.0003	7	0	Semi-tryptic
H2A2B_MOUSE	Histone H2A	HLQLAVR	9	5.20E-03	418.7583	835.5020	2	-0.0008	7	0	Semi-tryptic
H2A2B_MOUSE, H2AX_MOUSE	Histone H2A	NDEELNKLLGGVTIAQGGVLPNIQAVLLPK	6	9.20E-05	1038.5933	3112.7581	3	0.0085	30	1	Semi-tryptic
H2A2B_MOUSE, H2AX_MOUSE	Histone H2A	NDEELNKLLGGVTIAQGGVLPNIQAVLLPK	6	1.80E-04	1038.5928	3112.7566	3	0.0070	30	1	Semi-tryptic
H2A2B_MOUSE, H2AX_MOUSE	Histone H2A	NDEELNKLLGGVTIAQGGVLPNIQAVLLPK	6	2.90E-07	1038.5924	3112.7554	3	0.0058	30	1	Semi-tryptic
H2A2B_MOUSE, H2AX_MOUSE	Histone H2A	NDEELNKLLGGVTIAQGGVLPNIQAVLLPK	6	2.60E-04	1038.5923	3112.7551	3	0.0055	30	1	Semi-tryptic
H2A2B_MOUSE, H2AX_MOUSE	Histone H2A	NDEELNKLLGGVTIAQGGVLPNIQAVLLPK	6	1.30E-05	1038.5922	3112.7548	3	0.0052	30	1	Semi-tryptic
H2A2B_MOUSE, H2AX_MOUSE	Histone H2A	NDEELNKLLGGVTIAQGGVLPNIQAVLLPK	6	3.70E-03	1038.5917	3112.7533	3	0.0037	30	1	Semi-tryptic
H2A2B_MOUSE, H2AX_MOUSE	Histone H2A	LLGGVTIAQGGVLPNIQAVLLPK	22	1.60E-03	757.8022	2270.3847	3	0.0121	23	0	Semi-tryptic
H2A2B_MOUSE, H2AX_MOUSE	Histone H2A	LLGGVTIAQGGVLPNIQAVLLPK	22	2.30E-05	757.8022	2270.3847	3	0.0121	23	0	Semi-tryptic
H2A2B_MOUSE, H2AX_MOUSE	Histone H2A	LLGGVTIAQGGVLPNIQAVLLPK	22	4.80E-07	757.7997	2270.3773	3	0.0047	23	0	Semi-tryptic

H2A2B_MOUSE, H2AX_MOUSE	Histone H2A	LLGGVTIAQGGVLPNIQAVLLPK	22	5.50E-09	1136.1959	2270.3772	2	0.0047	23	0	Semi-tryptic
H2A2B_MOUSE, H2AX_MOUSE	Histone H2A	LLGGVTIAQGGVLPNIQAVLLPK	22	8.80E-09	1136.1957	2270.3768	2	0.0043	23	0	Semi-tryptic
H2A2B_MOUSE, H2AX_MOUSE	Histone H2A	LLGGVTIAQGGVLPNIQAVLLPK	22	5.10E-10	1136.1957	2270.3768	2	0.0043	23	0	Semi-tryptic
H2A2B_MOUSE, H2AX_MOUSE	Histone H2A	LLGGVTIAQGGVLPNIQAVLLPK	22	8.70E-12	1136.1955	2270.3764	2	0.0039	23	0	Semi-tryptic
H2A2B_MOUSE, H2AX_MOUSE	Histone H2A	LLGGVTIAQGGVLPNIQAVLLPK	22	1.10E-09	1136.1954	2270.3762	2	0.0037	23	0	Semi-tryptic
H2A2B_MOUSE, H2AX_MOUSE	Histone H2A	LLGGVTIAQGGVLPNIQAVLLPK	22	4.40E-11	1136.1954	2270.3762	2	0.0037	23	0	Semi-tryptic
H2A2B_MOUSE, H2AX_MOUSE	Histone H2A	LLGGVTIAQGGVLPNIQAVLLPK	22	2.10E-10	1136.1953	2270.3760	2	0.0035	23	0	Semi-tryptic
H2A2B_MOUSE, H2AX_MOUSE	Histone H2A	LLGGVTIAQGGVLPNIQAVLLPK	22	5.60E-05	757.7992	2270.3759	3	0.0033	23	0	Semi-tryptic
H2A2B_MOUSE, H2AX_MOUSE	Histone H2A	LLGGVTIAQGGVLPNIQAVLLPK	22	1.00E-10	1136.1952	2270.3758	2	0.0033	23	0	Semi-tryptic
H2A2B_MOUSE, H2AX_MOUSE	Histone H2A	LLGGVTIAQGGVLPNIQAVLLPK	22	3.80E-11	1136.1948	2270.3750	2	0.0025	23	0	Semi-tryptic
H2A2B_MOUSE, H2AX_MOUSE	Histone H2A	LLGGVTIAQGGVLPNIQAVLLPK	22	2.80E-11	1136.1948	2270.3750	2	0.0025	23	0	Semi-tryptic
H2A2B_MOUSE, H2AX_MOUSE	Histone H2A	LLGGVTIAQGGVLPNIQAVLLPK	22	2.70E-07	757.7989	2270.3750	3	0.0024	23	0	Semi-tryptic
H2A2B_MOUSE, H2AX_MOUSE	Histone H2A	LLGGVTIAQGGVLPNIQAVLLPK	22	1.40E-10	1136.1947	2270.3748	2	0.0023	23	0	Semi-tryptic
H2A2B_MOUSE, H2AX_MOUSE	Histone H2A	LLGGVTIAQGGVLPNIQAVLLPK	22	5.30E-11	1136.1946	2270.3746	2	0.0021	23	0	Semi-tryptic
H2A2B_MOUSE, H2AX_MOUSE	Histone H2A	LLGGVTIAQGGVLPNIQAVLLPK	22	2.20E-08	757.7988	2270.3745	3	0.0019	23	0	Semi-tryptic
H2A2B_MOUSE, H2AX_MOUSE	Histone H2A	LLGGVTIAQGGVLPNIQAVLLPK	22	7.70E-05	757.7986	2270.3741	3	0.0015	23	0	Semi-tryptic
H2A2B_MOUSE, H2AX_MOUSE	Histone H2A	LLGGVTIAQGGVLPNIQAVLLPK	22	1.90E-06	757.7986	2270.3740	3	0.0014	23	0	Semi-tryptic
H2A2B_MOUSE, H2AX_MOUSE	Histone H2A	LLGGVTIAQGGVLPNIQAVLLPK	22	1.20E-07	757.7986	2270.3740	3	0.0014	23	0	Semi-tryptic
H2A2B_MOUSE, H2AX_MOUSE	Histone H2A	LLGGVTIAQGGVLPNIQAVLLPK	22	3.90E-11	1136.1941	2270.3736	2	0.0011	23	0	Semi-tryptic
H2A2C_MOUSE	Histone H2A	DEELNKLLGKVTIAQGGVLPNIQAVLLPKKTESHKAKSK	13	8.90E-04	1399.1501	4194.4285	3	0.0182	39	0	AspN
H2A2C_MOUSE	Histone H2A	DEELNKLLGKVTIAQGGVLPNIQAVLLPKKTESHKAKSK	13	7.00E-03	600.2107	4194.4238	7	0.0134	39	0	AspN
H2A2C_MOUSE	Histone H2A	DEELNKLLGKVTIAQGGVLPNIQAVLLPKKTESHKAKSK	13	2.40E-04	600.2107	4194.4238	7	0.0134	39	0	AspN
H2A2C_MOUSE	Histone H2A	DEELNKLLGKVTIAQGGVLPNIQAVLLPKKTESHKAKSK	13	1.50E-03	839.8919	4194.4232	5	0.0129	39	0	AspN
H2A2C_MOUSE	Histone H2A	DEELNKLLGKVTIAQGGVLPNIQAVLLPKKTESHKAKSK	13	3.20E-05	839.8919	4194.4232	5	0.0129	39	0	AspN
H2A2C_MOUSE	Histone H2A	DEELNKLLGKVTIAQGGVLPNIQAVLLPKKTESHKAKSK	13	3.30E-06	839.8916	4194.4215	5	0.0112	39	0	AspN
H2A2C_MOUSE	Histone H2A	DEELNKLLGKVTIAQGGVLPNIQAVLLPKKTESHKAKSK	13	7.30E-03	700.0775	4194.4214	6	0.0111	39	0	AspN
H2A2C_MOUSE	Histone H2A	DEELNKLLGKVTIAQGGVLPNIQAVLLPKKTESHKAKSK	13	1.50E-05	700.0775	4194.4214	6	0.0111	39	0	AspN
H2A2C_MOUSE	Histone H2A	DEELNKLLGKVTIAQGGVLPNIQAVLLPKKTESHKAKSK	13	1.80E-04	1049.6126	4194.4213	4	0.0110	39	0	AspN
H2A2C_MOUSE	Histone H2A	DEELNKLLGKVTIAQGGVLPNIQAVLLPKKTESHKAKSK	13	5.10E-07	600.2102	4194.4207	7	0.0104	39	0	AspN
H2A2C_MOUSE	Histone H2A	DEELNKLLGKVTIAQGGVLPNIQAVLLPKKTESHKAKSK	13	7.10E-04	839.8905	4194.4160	5	0.0057	39	0	AspN
H2A2C_MOUSE	Histone H2A	DEELNKLLGKVTIAQGGVLPNIQAVLLPKKTESHKAKSK	13	5.50E-05	839.8904	4194.4157	5	0.0054	39	0	AspN
H2A2C_MOUSE	Histone H2A	DEELNKLLGKVTIAQGGVLPNIQAVLLPKKTESHKAKSK	13	4.40E-05	700.0761	4194.4132	6	0.0029	39	0	AspN
H2A2C_MOUSE	Histone H2A	SHKAKSK	2	1.00E-03	393.2351	784.4557	2	0.0002	7	0	GluC
H2A2C_MOUSE	Histone H2A	SHKAKSK	2	1.40E-03	393.2342	784.4539	2	-0.0016	7	0	GluC
H2AJ_MOUSE	Histone H2A	DEELNKLLGRVTIAQGGVLPNIQAVLLPKKTESQKVKSK	8	5.00E-06	707.9174	4241.4607	6	0.0133	39	0	AspN
H2AJ_MOUSE	Histone H2A	DEELNKLLGRVTIAQGGVLPNIQAVLLPKKTESQKVKSK	8	6.80E-06	849.2992	4241.4598	5	0.0124	39	0	AspN
H2AJ_MOUSE	Histone H2A	DEELNKLLGRVTIAQGGVLPNIQAVLLPKKTESQKVKSK	8	6.80E-06	849.2991	4241.4593	5	0.0118	39	0	AspN
H2AJ_MOUSE	Histone H2A	DEELNKLLGRVTIAQGGVLPNIQAVLLPKKTESQKVKSK	8	1.90E-07	606.9299	4241.4581	7	0.0107	39	0	AspN
H2AJ_MOUSE	Histone H2A	DEELNKLLGRVTIAQGGVLPNIQAVLLPKKTESQKVKSK	8	2.10E-05	606.9297	4241.4570	7	0.0096	39	0	AspN
H2AJ_MOUSE	Histone H2A	DEELNKLLGRVTIAQGGVLPNIQAVLLPKKTESQKVKSK	8	6.20E-03	1414.8258	4241.4556	3	0.0081	39	0	AspN
H2AJ_MOUSE	Histone H2A	DEELNKLLGRVTIAQGGVLPNIQAVLLPKKTESQKVKSK	8	9.30E-04	1061.3705	4241.4529	4	0.0055	39	0	AspN
H2AJ_MOUSE	Histone H2A	DEELNKLLGRVTIAQGGVLPNIQAVLLPKKTESQKVKSK	8	1.40E-05	707.9159	4241.4518	6	0.0044	39	0	AspN
H2AJ_MOUSE	Histone H2A	KTESQKVKSK	7	1.00E-02	388.2314	1161.6725	3	0.0008	10	3	Semi-tryptic
H2AJ_MOUSE	Histone H2A	KTESQKVKSK	7	3.90E-03	581.8435	1161.6724	2	0.0007	10	3	Semi-tryptic
H2AJ_MOUSE	Histone H2A	KTESQKVKSK	7	3.00E-03	581.8434	1161.6722	2	0.0005	10	3	Semi-tryptic
H2AJ_MOUSE	Histone H2A	KTESQKVKSK	7	4.20E-03	581.8432	1161.6719	2	0.0002	10	3	Semi-tryptic
H2AJ_MOUSE	Histone H2A	KTESQKVKSK	7	2.40E-03	581.8433	1161.6719	2	0.0002	10	3	Semi-tryptic
H2AJ_MOUSE	Histone H2A	KTESQKVKSK	7	3.60E-03	388.2311	1161.6715	3	-0.0002	10	3	Semi-tryptic
H2AJ_MOUSE	Histone H2A	KTESQKVKSK	7	6.00E-04	581.8429	1161.6713	2	-0.0004	10	3	Semi-tryptic
H2AJ_MOUSE	Histone H2A	SQKVKSK	6	8.50E-03	402.7511	803.4876	2	0.0011	7	0	GluC
H2AJ_MOUSE	Histone H2A	SQKVKSK	6	4.40E-03	402.7506	803.4866	2	0.0001	7	0	GluC
H2AJ_MOUSE	Histone H2A	SQKVKSK	6	1.40E-03	402.7505	803.4865	2	0.0000	7	0	GluC
H2AJ_MOUSE	Histone H2A	SQKVKSK	6	7.80E-04	402.7505	803.4864	2	0.0000	7	0	GluC
H2AJ_MOUSE	Histone H2A	SQKVKSK	6	7.70E-03	402.7503	803.4861	2	-0.0004	7	0	GluC
H2AJ_MOUSE	Histone H2A	SQKVKSK	6	1.30E-03	402.7503	803.4860	2	-0.0005	7	0	GluC
H2AJ_MOUSE, H2AX_MOUSE	Histone H2A	AERVGAGAPVYL	2	3.50E-03	601.8305	1201.6464	2	0.0009	12	1	Chymotrypsin
H2AJ_MOUSE, H2AX_MOUSE	Histone H2A	AERVGAGAPVYL	2	7.10E-04	601.83	1201.6455	2	0.0000	12	1	Chymotrypsin
H2AV_MOUSE	Histone H2A	LDSLIKATIAGGGVIPHIHKSLIGKKGQQKTA	19	2.90E-04	1093.9866	3278.9380	3	0.0189	32	1	GluC
H2AV_MOUSE	Histone H2A	LDSLIKATIAGGGVIPHIHKSLIGKKGQQKTA	19	1.30E-09	820.7416	3278.9375	4	0.0184	32	1	GluC
H2AV_MOUSE	Histone H2A	LDSLIKATIAGGGVIPHIHKSLIGKKGQQKTA	19	1.30E-04	656.7943	3278.9350	5	0.0160	32	1	GluC
H2AV_MOUSE	Histone H2A	LDSLIKATIAGGGVIPHIHKSLIGKKGQQKTA	19	4.80E-04	1093.9844	3278.9314	3	0.0123	32	1	GluC
H2AV_MOUSE	Histone H2A	LDSLIKATIAGGGVIPHIHKSLIGKKGQQKTA	19	1.20E-08	820.74	3278.9309	4	0.0119	32	1	GluC
H2AV_MOUSE	Histone H2A	LDSLIKATIAGGGVIPHIHKSLIGKKGQQKTA	19	2.70E-05	1093.9842	3278.9308	3	0.0117	32	1	GluC
H2AV_MOUSE	Histone H2A	LDSLIKATIAGGGVIPHIHKSLIGKKGQQKTA	19	4.70E-03	656.793	3278.9287	5	0.0096	32	1	GluC
H2AV_MOUSE	Histone H2A	LDSLIKATIAGGGVIPHIHKSLIGKKGQQKTA	19	2.60E-03	820.7388	3278.9263	4	0.0072	32	1	GluC
H2AV_MOUSE	Histone H2A	LDSLIKATIAGGGVIPHIHKSLIGKKGQQKTA	19	1.10E-07	820.7387	3278.9258	4	0.0068	32	1	GluC
H2AV_MOUSE	Histone H2A	LDSLIKATIAGGGVIPHIHKSLIGKKGQQKTA	19	2.80E-09	820.7384	3278.9247	4	0.0056	32	1	GluC
H2AV_MOUSE	Histone H2A	LDSLIKATIAGGGVIPHIHKSLIGKKGQQKTA	19	7.50E-07	656.7919	3278.9230	5	0.0039	32	1	GluC
H2AV_MOUSE	Histone H2A	LDSLIKATIAGGGVIPHIHKSLIGKKGQQKTA	19	2.10E-04	469.4246	3278.9215	7	0.0024	32	1	GluC
H2AV_MOUSE	Histone H2A	LDSLIKATIAGGGVIPHIHKSLIGKKGQQKTA	19	6.00E-03	820.7375	3278.9210	4	0.0019	32	1	GluC
H2AV_MOUSE	Histone H2A	LDSLIKATIAGGGVIPHIHKSLIGKKGQQKTA	19	1.30E-08	820.7375	3278.9210	4	0.0019	32	1	GluC
H2AV_MOUSE	Histone H2A	LDSLIKATIAGGGVIPHIHKSLIGKKGQQKTA	19	4.20E-05	656.7911	3278.9192	5	0.0001	32	1	GluC
H2AV_MOUSE	Histone H2A	LDSLIKATIAGGGVIPHIHKSLIGKKGQQKTA	19	1.20E-05	1093.9802	3278.9188	3	-0.0003	32	1	GluC
H2AV_MOUSE	Histone H2A	LDSLIKATIAGGGVIPHIHKSLIGKKGQQKTA	19	4.80E-09	1093.9802	3278.9188	3	-0.0003	32	1	GluC

H2AV_MOUSE	Histone H2A	LDSLIKATIAGGGVIPHIHKSLIGKKGQQKTA	19	5.20E-05	656.7909	3278.9180	5	-0.0010	32	1	GluC
H2AV_MOUSE	Histone H2A	LDSLIKATIAGGGVIPHIHKSLIGKKGQQKTA	19	2.30E-09	547.4936	3278.9180	6	-0.0010	32	1	GluC
H2AV_MOUSE	Histone H2A	DSLIKATIAGGGVIPHIHKSLIGKKGQQKTA	10	8.00E-08	792.4704	3165.8525	4	0.0175	31	0	AspN
H2AV_MOUSE	Histone H2A	DSLIKATIAGGGVIPHIHKSLIGKKGQQKTA	10	3.00E-05	1056.2884	3165.8434	3	0.0084	31	0	AspN
H2AV_MOUSE	Histone H2A	DSLIKATIAGGGVIPHIHKSLIGKKGQQKTA	10	7.70E-08	1056.2884	3165.8434	3	0.0084	31	0	AspN
H2AV_MOUSE	Histone H2A	DSLIKATIAGGGVIPHIHKSLIGKKGQQKTA	10	1.50E-03	1056.2883	3165.8431	3	0.0081	31	0	AspN
H2AV_MOUSE	Histone H2A	DSLIKATIAGGGVIPHIHKSLIGKKGQQKTA	10	4.20E-08	634.1756	3165.8416	5	0.0066	31	0	AspN
H2AV_MOUSE	Histone H2A	DSLIKATIAGGGVIPHIHKSLIGKKGQQKTA	10	1.70E-04	792.4677	3165.8415	4	0.0065	31	0	AspN
H2AV_MOUSE	Histone H2A	DSLIKATIAGGGVIPHIHKSLIGKKGQQKTA	10	1.50E-08	792.4676	3165.8415	4	0.0065	31	0	AspN
H2AV_MOUSE	Histone H2A	DSLIKATIAGGGVIPHIHKSLIGKKGQQKTA	10	1.40E-08	792.4677	3165.8415	4	0.0065	31	0	AspN
H2AV_MOUSE	Histone H2A	DSLIKATIAGGGVIPHIHKSLIGKKGQQKTA	10	6.00E-07	634.1756	3165.8414	5	0.0064	31	0	AspN
H2AV_MOUSE	Histone H2A	DSLIKATIAGGGVIPHIHKSLIGKKGQQKTA	10	4.90E-03	792.4669	3165.8383	4	0.0034	31	0	AspN
H2AV_MOUSE	Histone H2A	SLIKATIAGGGVIPHIHKSLIGKKGQQKTA	6	6.20E-06	763.712	3050.8190	4	0.0109	30	0	GluC
H2AV_MOUSE	Histone H2A	SLIKATIAGGGVIPHIHKSLIGKKGQQKTA	6	1.00E-06	763.7115	3050.8167	4	0.0087	30	0	GluC
H2AV_MOUSE	Histone H2A	SLIKATIAGGGVIPHIHKSLIGKKGQQKTA	6	1.10E-04	763.7098	3050.8103	4	0.0023	30	0	GluC
H2AV_MOUSE	Histone H2A	SLIKATIAGGGVIPHIHKSLIGKKGQQKTA	6	1.50E-06	763.7098	3050.8103	4	0.0023	30	0	GluC
H2AV_MOUSE	Histone H2A	SLIKATIAGGGVIPHIHKSLIGKKGQQKTA	6	4.50E-08	763.7095	3050.8088	4	0.0007	30	0	GluC
H2AV_MOUSE	Histone H2A	SLIKATIAGGGVIPHIHKSLIGKKGQQKTA	6	4.00E-08	611.1688	3050.8077	5	-0.0004	30	0	GluC
H2AV_MOUSE	Histone H2A	IGKKGQQKTA	2	2.20E-04	353.5488	1057.6247	3	0.0003	10	0	Chymotrypsin
H2AV_MOUSE	Histone H2A	IGKKGQQKTA	2	3.40E-03	529.8195	1057.6245	2	0.0001	10	0	Chymotrypsin
H2AV_MOUSE, H2AZ_MOUSE	Histone H2A	TTSHGRVGATAAVYSAAILEYLTAEVLELAGNASK	4	2.50E-04	884.468	3533.8429	4	0.0063	35	1	Semi-tryptic
H2AV_MOUSE, H2AZ_MOUSE	Histone H2A	TTSHGRVGATAAVYSAAILEYLTAEVLELAGNASK	4	2.90E-10	884.4677	3533.8415	4	0.0050	35	1	Semi-tryptic
H2AV_MOUSE, H2AZ_MOUSE	Histone H2A	TTSHGRVGATAAVYSAAILEYLTAEVLELAGNASK	4	9.00E-08	884.4676	3533.8414	4	0.0049	35	1	Semi-tryptic
H2AV_MOUSE, H2AZ_MOUSE	Histone H2A	TTSHGRVGATAAVYSAAILEYLTAEVLELAGNASK	4	5.90E-15	1178.9544	3533.8414	3	0.0048	35	1	Semi-tryptic
H2AV_MOUSE, H2AZ_MOUSE	Histone H2A	VGATAAVYSAAILEYLTAEVLELAGNASKDLK	2	1.90E-06	1084.5858	3250.7356	3	0.0020	32	1	Semi-tryptic
H2AV_MOUSE, H2AZ_MOUSE	Histone H2A	VGATAAVYSAAILEYLTAEVLELAGNASKDLK	2	9.10E-07	1084.5858	3250.7356	3	0.0020	32	1	Semi-tryptic
H2AV_MOUSE, H2AZ_MOUSE	Histone H2A	HLQLAIRGDEELDSLIKATIAGGGVIPHIHK	4	1.50E-05	661.1744	3300.8355	5	0.0049	31	2	Semi-tryptic
H2AV_MOUSE, H2AZ_MOUSE	Histone H2A	HLQLAIRGDEELDSLIKATIAGGGVIPHIHK	4	4.10E-07	826.2155	3300.8328	4	0.0022	31	2	Semi-tryptic
H2AV_MOUSE, H2AZ_MOUSE	Histone H2A	HLQLAIRGDEELDSLIKATIAGGGVIPHIHK	4	5.60E-05	661.1738	3300.8327	5	0.0021	31	2	Semi-tryptic
H2AV_MOUSE, H2AZ_MOUSE	Histone H2A	HLQLAIRGDEELDSLIKATIAGGGVIPHIHK	4	1.90E-05	661.1736	3300.8315	5	0.0009	31	2	Semi-tryptic
H2AV_MOUSE, H2AZ_MOUSE	Histone H2A	VGATAAVYSAAILEYLTAEVLELAGNASK	18	3.00E-03	965.8537	2894.5394	3	0.0117	29	0	Semi-tryptic
H2AV_MOUSE, H2AZ_MOUSE	Histone H2A	VGATAAVYSAAILEYLTAEVLELAGNASK	18	3.50E-09	965.853	2894.5371	3	0.0094	29	0	Semi-tryptic
H2AV_MOUSE, H2AZ_MOUSE	Histone H2A	VGATAAVYSAAILEYLTAEVLELAGNASK	18	8.40E-13	1448.2748	2894.5350	2	0.0074	29	0	Semi-tryptic
H2AV_MOUSE, H2AZ_MOUSE	Histone H2A	VGATAAVYSAAILEYLTAEVLELAGNASK	18	3.20E-10	965.8522	2894.5347	3	0.0070	29	0	Semi-tryptic
H2AV_MOUSE, H2AZ_MOUSE	Histone H2A	VGATAAVYSAAILEYLTAEVLELAGNASK	18	1.10E-08	965.8518	2894.5336	3	0.0059	29	0	Semi-tryptic
H2AV_MOUSE, H2AZ_MOUSE	Histone H2A	VGATAAVYSAAILEYLTAEVLELAGNASK	18	1.00E-05	965.8516	2894.5331	3	0.0054	29	0	Semi-tryptic
H2AV_MOUSE, H2AZ_MOUSE	Histone H2A	VGATAAVYSAAILEYLTAEVLELAGNASK	18	2.70E-09	965.8515	2894.5327	3	0.0051	29	0	Semi-tryptic
H2AV_MOUSE, H2AZ_MOUSE	Histone H2A	VGATAAVYSAAILEYLTAEVLELAGNASK	18	1.70E-09	965.8514	2894.5325	3	0.0048	29	0	Semi-tryptic
H2AV_MOUSE, H2AZ_MOUSE	Histone H2A	VGATAAVYSAAILEYLTAEVLELAGNASK	18	1.70E-13	1448.2735	2894.5324	2	0.0048	29	0	Semi-tryptic
H2AV_MOUSE, H2AZ_MOUSE	Histone H2A	VGATAAVYSAAILEYLTAEVLELAGNASK	18	1.90E-10	1448.2734	2894.5322	2	0.0046	29	0	Semi-tryptic
H2AV_MOUSE, H2AZ_MOUSE	Histone H2A	VGATAAVYSAAILEYLTAEVLELAGNASK	18	1.30E-10	965.8513	2894.5322	3	0.0045	29	0	Semi-tryptic
H2AV_MOUSE, H2AZ_MOUSE	Histone H2A	VGATAAVYSAAILEYLTAEVLELAGNASK	18	1.50E-06	965.8513	2894.5320	3	0.0043	29	0	Semi-tryptic
H2AV_MOUSE, H2AZ_MOUSE	Histone H2A	VGATAAVYSAAILEYLTAEVLELAGNASK	18	4.90E-12	965.8512	2894.5317	3	0.0041	29	0	Semi-tryptic
H2AV_MOUSE, H2AZ_MOUSE	Histone H2A	VGATAAVYSAAILEYLTAEVLELAGNASK	18	3.00E-12	1448.2731	2894.5316	2	0.0040	29	0	Semi-tryptic
H2AV_MOUSE, H2AZ_MOUSE	Histone H2A	VGATAAVYSAAILEYLTAEVLELAGNASK	18	3.30E-10	965.8511	2894.5316	3	0.0039	29	0	Semi-tryptic
H2AV_MOUSE, H2AZ_MOUSE	Histone H2A	VGATAAVYSAAILEYLTAEVLELAGNASK	18	2.30E-05	965.851	2894.5312	3	0.0035	29	0	Semi-tryptic
H2AV_MOUSE, H2AZ_MOUSE	Histone H2A	VGATAAVYSAAILEYLTAEVLELAGNASK	18	1.90E-12	965.8508	2894.5305	3	0.0028	29	0	Semi-tryptic
H2AV_MOUSE, H2AZ_MOUSE	Histone H2A	VGATAAVYSAAILEYLTAEVLELAGNASK	18	2.20E-09	965.8503	2894.5291	3	0.0014	29	0	Semi-tryptic
H2AV_MOUSE, H2AZ_MOUSE	Histone H2A	LKVKRITPRHLQLAIRGDEE	16	1.50E-03	791.4662	2371.3767	3	-0.0045	20	2	GluC
H2AV_MOUSE, H2AZ_MOUSE	Histone H2A	LKVKRITPRHLQLAIRGDEE	16	1.00E-02	475.2824	2371.3757	5	-0.0054	20	2	GluC
H2AV_MOUSE, H2AZ_MOUSE	Histone H2A	LKVKRITPRHLQLAIRGDEE	16	5.40E-04	791.4727	2371.3964	3	0.0152	20	2	GluC
H2AV_MOUSE, H2AZ_MOUSE	Histone H2A	LKVKRITPRHLQLAIRGDEE	16	3.90E-04	791.472	2371.3941	3	0.0130	20	2	GluC
H2AV_MOUSE, H2AZ_MOUSE	Histone H2A	LKVKRITPRHLQLAIRGDEE	16	3.90E-07	593.8558	2371.3941	4	0.0129	20	2	GluC
H2AV_MOUSE, H2AZ_MOUSE	Histone H2A	LKVKRITPRHLQLAIRGDEE	16	6.90E-03	791.4716	2371.3930	3	0.0119	20	2	GluC
H2AV_MOUSE, H2AZ_MOUSE	Histone H2A	LKVKRITPRHLQLAIRGDEE	16	7.80E-09	593.8552	2371.3916	4	0.0104	20	2	GluC
H2AV_MOUSE, H2AZ_MOUSE	Histone H2A	LKVKRITPRHLQLAIRGDEE	16	2.90E-08	593.855	2371.3909	4	0.0098	20	2	GluC
H2AV_MOUSE, H2AZ_MOUSE	Histone H2A	LKVKRITPRHLQLAIRGDEE	16	6.20E-04	593.855	2371.3908	4	0.0096	20	2	GluC
H2AV_MOUSE, H2AZ_MOUSE	Histone H2A	LKVKRITPRHLQLAIRGDEE	16	5.80E-03	593.8532	2371.3836	4	0.0025	20	2	GluC
H2AV_MOUSE, H2AZ_MOUSE	Histone H2A	LKVKRITPRHLQLAIRGDEE	16	1.20E-03	791.4684	2371.3834	3	0.0023	20	2	GluC
H2AV_MOUSE, H2AZ_MOUSE	Histone H2A	LKVKRITPRHLQLAIRGDEE	16	1.50E-06	593.8529	2371.3827	4	0.0015	20	2	GluC
H2AV_MOUSE, H2AZ_MOUSE	Histone H2A	LKVKRITPRHLQLAIRGDEE	16	3.80E-07	593.8529	2371.3825	4	0.0014	20	2	GluC
H2AV_MOUSE, H2AZ_MOUSE	Histone H2A	LKVKRITPRHLQLAIRGDEE	16	1.70E-03	791.468	2371.3821	3	0.0010	20	2	GluC
H2AV_MOUSE, H2AZ_MOUSE	Histone H2A	LKVKRITPRHLQLAIRGDEE	16	2.80E-04	593.8527	2371.3818	4	0.0006	20	2	GluC
H2AV_MOUSE, H2AZ_MOUSE	Histone H2A	LKVKRITPRHLQLAIRGDEE	16	3.80E-07	593.8525	2371.3809	4	-0.0003	20	2	GluC
H2AV_MOUSE, H2AZ_MOUSE	Histone H2A	LKVKRITPRHLQLAIRGDE	2	1.50E-03	748.4541	2242.3404	3	0.0018	19	1	GluC
H2AV_MOUSE, H2AZ_MOUSE	Histone H2A	LKVKRITPRHLQLAIRGDE	2	1.10E-05	561.5918	2242.3382	4	-0.0003	19	1	GluC
H2AV_MOUSE, H2AZ_MOUSE	Histone H2A	DLKVKRITPRHLQLAIRG	8	2.40E-05	705.4429	2113.3068	3	0.0108	18	0	AspN
H2AV_MOUSE, H2AZ_MOUSE	Histone H2A	DLKVKRITPRHLQLAIRG	8	9.50E-04	529.3321	2113.2995	4	0.0035	18	0	AspN
H2AV_MOUSE, H2AZ_MOUSE	Histone H2A	DLKVKRITPRHLQLAIRG	8	2.40E-06	529.332	2113.2991	4	0.0031	18	0	AspN
H2AV_MOUSE, H2AZ_MOUSE	Histone H2A	DLKVKRITPRHLQLAIRG	8	2.10E-03	423.6664	2113.2957	5	-0.0003	18	0	AspN
H2AV_MOUSE, H2AZ_MOUSE	Histone H2A	DLKVKRITPRHLQLAIRG	8	7.00E-03	705.439	2113.2952	3	-0.0007	18	0	AspN
H2AV_MOUSE, H2AZ_MOUSE	Histone H2A	DLKVKRITPRHLQLAIRG	8	9.50E-05	705.439	2113.2952	3	-0.0007	18	0	AspN
H2AV_MOUSE, H2AZ_MOUSE	Histone H2A	DLKVKRITPRHLQLAIRG	8	3.10E-08	529.331	2113.2951	4	-0.0009	18	0	AspN
H2AV_MOUSE, H2AZ_MOUSE	Histone H2A	DLKVKRITPRHLQLAIRG	8	1.50E-03	353.2227	2113.2927	6	-0.0033	18	0	AspN

H2AV_MOUSE, H2AZ_MOUSE	Histone H2A	HLQLAIRGDEELDSLIK	5	7.60E-03	650.694	1949.0601	3	0.0020	17	1	Semi-tryptic
H2AV_MOUSE, H2AZ_MOUSE	Histone H2A	HLQLAIRGDEELDSLIK	5	4.30E-06	650.6936	1949.0590	3	0.0008	17	1	Semi-tryptic
H2AV_MOUSE, H2AZ_MOUSE	Histone H2A	HLQLAIRGDEELDSLIK	5	2.80E-06	650.6933	1949.0581	3	0.0000	17	1	Semi-tryptic
H2AV_MOUSE, H2AZ_MOUSE	Histone H2A	HLQLAIRGDEELDSLIK	5	1.50E-06	650.6931	1949.0574	3	-0.0007	17	1	Semi-tryptic
H2AV_MOUSE, H2AZ_MOUSE	Histone H2A	HLQLAIRGDEELDSLIK	5	2.60E-05	650.693	1949.0571	3	-0.0010	17	1	Semi-tryptic
H2AV_MOUSE, H2AZ_MOUSE	Histone H2A	ATIAGGGVIPHIHK	21	1.10E-07	685.9019	1369.7893	2	0.0063	14	0	Semi-tryptic
H2AV_MOUSE, H2AZ_MOUSE	Histone H2A	ATIAGGGVIPHIHK	21	9.30E-09	685.902	1369.7893	2	0.0063	14	0	Semi-tryptic
H2AV_MOUSE, H2AZ_MOUSE	Histone H2A	ATIAGGGVIPHIHK	21	1.70E-08	685.9013	1369.7880	2	0.0050	14	0	Semi-tryptic
H2AV_MOUSE, H2AZ_MOUSE	Histone H2A	ATIAGGGVIPHIHK	21	3.80E-03	457.603	1369.7873	3	0.0043	14	0	Semi-tryptic
H2AV_MOUSE, H2AZ_MOUSE	Histone H2A	ATIAGGGVIPHIHK	21	1.20E-05	685.9006	1369.7866	2	0.0035	14	0	Semi-tryptic
H2AV_MOUSE, H2AZ_MOUSE	Histone H2A	ATIAGGGVIPHIHK	21	5.00E-05	457.6026	1369.7859	3	0.0029	14	0	Semi-tryptic
H2AV_MOUSE, H2AZ_MOUSE	Histone H2A	ATIAGGGVIPHIHK	21	2.90E-05	457.6024	1369.7854	3	0.0024	14	0	Semi-tryptic
H2AV_MOUSE, H2AZ_MOUSE	Histone H2A	ATIAGGGVIPHIHK	21	5.70E-06	457.6024	1369.7853	3	0.0023	14	0	Semi-tryptic
H2AV_MOUSE, H2AZ_MOUSE	Histone H2A	ATIAGGGVIPHIHK	21	7.30E-07	685.8994	1369.7842	2	0.0012	14	0	Semi-tryptic
H2AV_MOUSE, H2AZ_MOUSE	Histone H2A	ATIAGGGVIPHIHK	21	2.70E-05	685.8993	1369.7840	2	0.0010	14	0	Semi-tryptic
H2AV_MOUSE, H2AZ_MOUSE	Histone H2A	ATIAGGGVIPHIHK	21	1.30E-04	343.4532	1369.7837	4	0.0006	14	0	Semi-tryptic
H2AV_MOUSE, H2AZ_MOUSE	Histone H2A	ATIAGGGVIPHIHK	21	4.60E-06	343.4532	1369.7837	4	0.0006	14	0	Semi-tryptic
H2AV_MOUSE, H2AZ_MOUSE	Histone H2A	ATIAGGGVIPHIHK	21	3.80E-08	685.899	1369.7834	2	0.0004	14	0	Semi-tryptic
H2AV_MOUSE, H2AZ_MOUSE	Histone H2A	ATIAGGGVIPHIHK	21	1.40E-08	685.8988	1369.7830	2	0.0000	14	0	Semi-tryptic
H2AV_MOUSE, H2AZ_MOUSE	Histone H2A	ATIAGGGVIPHIHK	21	9.50E-05	457.6015	1369.7828	3	-0.0002	14	0	Semi-tryptic
H2AV_MOUSE, H2AZ_MOUSE	Histone H2A	ATIAGGGVIPHIHK	21	4.20E-08	685.8986	1369.7827	2	-0.0003	14	0	Semi-tryptic
H2AV_MOUSE, H2AZ_MOUSE	Histone H2A	ATIAGGGVIPHIHK	21	1.80E-05	457.6015	1369.7826	3	-0.0004	14	0	Semi-tryptic
H2AV_MOUSE, H2AZ_MOUSE	Histone H2A	ATIAGGGVIPHIHK	21	3.20E-07	457.6015	1369.7826	3	-0.0004	14	0	Semi-tryptic
H2AV_MOUSE, H2AZ_MOUSE	Histone H2A	ATIAGGGVIPHIHK	21	3.70E-07	685.8985	1369.7824	2	-0.0006	14	0	Semi-tryptic
H2AV_MOUSE, H2AZ_MOUSE	Histone H2A	ATIAGGGVIPHIHK	21	2.00E-03	457.6013	1369.7822	3	-0.0009	14	0	Semi-tryptic
H2AV_MOUSE, H2AZ_MOUSE	Histone H2A	ATIAGGGVIPHIHK	21	1.40E-04	685.8983	1369.7821	2	-0.0009	14	0	Semi-tryptic
H2AV_MOUSE, H2AZ_MOUSE	Histone H2A	AGLQFPVGRIHR	2	6.00E-03	450.9298	1349.7676	3	-0.0005	12	1	Semi-tryptic
H2AV_MOUSE, H2AZ_MOUSE	Histone H2A	AGLQFPVGRIHR	2	6.90E-04	450.9298	1349.7675	3	-0.0006	12	1	Semi-tryptic
H2AV_MOUSE, H2AZ_MOUSE	Histone H2A	SQRAGLQFPVGR	4	1.00E-02	439.2472	1314.7197	3	0.0040	12	1	Semi-tryptic
H2AV_MOUSE, H2AZ_MOUSE	Histone H2A	SQRAGLQFPVGR	4	6.80E-05	658.3652	1314.7159	2	0.0002	12	1	Semi-tryptic
H2AV_MOUSE, H2AZ_MOUSE	Histone H2A	SQRAGLQFPVGR	4	2.70E-06	658.3644	1314.7142	2	-0.0014	12	1	Semi-tryptic
H2AV_MOUSE, H2AZ_MOUSE	Histone H2A	SQRAGLQFPVGR	4	1.30E-06	658.3641	1314.7136	2	-0.0021	12	1	Semi-tryptic
H2AV_MOUSE, H2AZ_MOUSE	Histone H2A	ELAGNASKDL	5	1.80E-03	509.2653	1016.5160	2	0.0022	10	1	Chymotrypsin
H2AV_MOUSE, H2AZ_MOUSE	Histone H2A	ELAGNASKDL	5	6.40E-03	509.2643	1016.5141	2	0.0003	10	1	Chymotrypsin
H2AV_MOUSE, H2AZ_MOUSE	Histone H2A	ELAGNASKDL	5	1.20E-06	509.2643	1016.5141	2	0.0003	10	1	Chymotrypsin
H2AV_MOUSE, H2AZ_MOUSE	Histone H2A	ELAGNASKDL	5	4.90E-06	509.2641	1016.5137	2	-0.0001	10	1	Chymotrypsin
H2AV_MOUSE, H2AZ_MOUSE	Histone H2A	ELAGNASKDL	5	3.20E-05	509.264	1016.5135	2	-0.0003	10	1	Chymotrypsin
H2AV_MOUSE, H2AZ_MOUSE	Histone H2A	GDEELDSLIK	11	1.00E-07	559.7812	1117.5478	2	-0.0024	10	0	Semi-tryptic
H2AV_MOUSE, H2AZ_MOUSE	Histone H2A	GDEELDSLIK	11	4.40E-07	559.784	1117.5533	2	0.0031	10	0	GluC
H2AV_MOUSE, H2AZ_MOUSE	Histone H2A	GDEELDSLIK	11	1.20E-07	559.7829	1117.5512	2	0.0010	10	0	Semi-tryptic
H2AV_MOUSE, H2AZ_MOUSE	Histone H2A	GDEELDSLIK	11	4.10E-03	559.7828	1117.5510	2	0.0008	10	0	Semi-tryptic
H2AV_MOUSE, H2AZ_MOUSE	Histone H2A	GDEELDSLIK	11	1.40E-06	559.7826	1117.5506	2	0.0003	10	0	Semi-tryptic
H2AV_MOUSE, H2AZ_MOUSE	Histone H2A	GDEELDSLIK	11	1.10E-05	559.7825	1117.5504	2	0.0001	10	0	Semi-tryptic
H2AV_MOUSE, H2AZ_MOUSE	Histone H2A	GDEELDSLIK	11	5.20E-06	559.7824	1117.5503	2	0.0000	10	0	Semi-tryptic
H2AV_MOUSE, H2AZ_MOUSE	Histone H2A	GDEELDSLIK	11	8.80E-03	559.7824	1117.5502	2	-0.0001	10	0	Semi-tryptic
H2AV_MOUSE, H2AZ_MOUSE	Histone H2A	GDEELDSLIK	11	9.30E-03	559.7823	1117.5500	2	-0.0002	10	0	Semi-tryptic
H2AV_MOUSE, H2AZ_MOUSE	Histone H2A	GDEELDSLIK	11	5.50E-06	559.7823	1117.5501	2	-0.0002	10	0	Semi-tryptic
H2AV_MOUSE, H2AZ_MOUSE	Histone H2A	GDEELDSLIK	11	2.90E-05	559.7819	1117.5492	2	-0.0011	10	0	Semi-tryptic
H2AV_MOUSE, H2AZ_MOUSE	Histone H2A	LAIRGDEE	6	1.60E-03	451.7329	901.4512	2	0.0008	8	2	GluC
H2AV_MOUSE, H2AZ_MOUSE	Histone H2A	LAIRGDEE	6	3.90E-03	451.7326	901.4506	2	0.0001	8	2	GluC
H2AV_MOUSE, H2AZ_MOUSE	Histone H2A	LAIRGDEE	6	3.90E-03	451.7325	901.4505	2	0.0000	8	2	GluC
H2AV_MOUSE, H2AZ_MOUSE	Histone H2A	LAIRGDEE	6	3.80E-03	451.7325	901.4505	2	0.0000	8	2	GluC
H2AV_MOUSE, H2AZ_MOUSE	Histone H2A	LAIRGDEE	6	5.40E-04	451.7324	901.4503	2	-0.0002	8	2	GluC
H2AV_MOUSE, H2AZ_MOUSE	Histone H2A	LAIRGDEE	6	1.80E-04	451.7324	901.4503	2	-0.0002	8	2	GluC
H2AV_MOUSE, H2AZ_MOUSE	Histone H2A	LAGNASKD	9	3.40E-03	388.2011	774.3877	2	0.0006	8	0	GluC
H2AV_MOUSE, H2AZ_MOUSE	Histone H2A	LAGNASKD	9	2.00E-05	388.201	774.3874	2	0.0002	8	0	GluC
H2AV_MOUSE, H2AZ_MOUSE	Histone H2A	LAGNASKD	9	1.30E-06	388.201	774.3874	2	0.0002	8	0	GluC
H2AV_MOUSE, H2AZ_MOUSE	Histone H2A	LAGNASKD	9	2.20E-03	388.2009	774.3873	2	0.0001	8	0	GluC
H2AV_MOUSE, H2AZ_MOUSE	Histone H2A	LAGNASKD	9	2.10E-05	388.2008	774.3871	2	0.0000	8	0	GluC
H2AV_MOUSE, H2AZ_MOUSE	Histone H2A	LAGNASKD	9	2.10E-05	388.2008	774.3870	2	-0.0002	8	0	GluC
H2AV_MOUSE, H2AZ_MOUSE	Histone H2A	LAGNASKD	9	9.90E-03	388.2004	774.3862	2	-0.0009	8	0	GluC
H2AV_MOUSE, H2AZ_MOUSE	Histone H2A	LAGNASKD	9	2.60E-06	388.2004	774.3862	2	-0.0009	8	0	GluC
H2AV_MOUSE, H2AZ_MOUSE	Histone H2A	LAGNASKD	9	5.20E-03	388.1993	774.3839	2	-0.0032	8	0	GluC
H2AW_MOUSE	Histone H2A	AKDSDKEGTSNSTSEDGPGDGFTILSSK	4	4.40E-08	944.1006	2829.2799	3	0.0011	28	2	Semi-tryptic
H2AW_MOUSE	Histone H2A	AKDSDKEGTSNSTSEDGPGDGFTILSSK	4	4.30E-08	944.1004	2829.2794	3	0.0005	28	2	Semi-tryptic
H2AW_MOUSE	Histone H2A	AKDSDKEGTSNSTSEDGPGDGFTILSSK	4	2.80E-07	944.1001	2829.2786	3	-0.0002	28	2	Semi-tryptic
H2AW_MOUSE	Histone H2A	AKDSDKEGTSNSTSEDGPGDGFTILSSK	4	5.40E-03	944.0993	2829.2760	3	-0.0028	28	2	Semi-tryptic
H2AW_MOUSE	Histone H2A	DSDKEGTSNSTSEDGPGDGFTILSSK	7	4.60E-08	877.7249	2630.1529	3	0.0061	26	1	Semi-tryptic
H2AW_MOUSE	Histone H2A	DSDKEGTSNSTSEDGPGDGFTILSSK	7	1.90E-03	877.7244	2630.1514	3	0.0047	26	1	Semi-tryptic
H2AW_MOUSE	Histone H2A	DSDKEGTSNSTSEDGPGDGFTILSSK	7	8.80E-10	877.724	2630.1501	3	0.0033	26	1	Semi-tryptic
H2AW_MOUSE	Histone H2A	DSDKEGTSNSTSEDGPGDGFTILSSK	7	2.20E-11	1316.0822	2630.1498	2	0.0031	26	1	Semi-tryptic
H2AW_MOUSE	Histone H2A	DSDKEGTSNSTSEDGPGDGFTILSSK	7	7.60E-10	877.7223	2630.1450	3	-0.0018	26	1	Semi-tryptic
H2AW_MOUSE	Histone H2A	DSDKEGTSNSTSEDGPGDGFTILSSK	7	8.60E-05	1316.0797	2630.1448	2	-0.0019	26	1	Semi-tryptic
H2AW_MOUSE	Histone H2A	DSDKEGTSNSTSEDGPGDGFTILSSK	7	3.60E-08	877.7211	2630.1416	3	-0.0051	26	1	Semi-tryptic

H2AW_MOUSE	Histone H2A	KSQGPLEVAEAAVSQSSGLAAK	3	3.00E-03	710.0474	2127.1203	3	0.0031	22	1	Semi-tryptic
H2AW_MOUSE	Histone H2A	KSQGPLEVAEAAVSQSSGLAAK	3	7.80E-04	710.0469	2127.1190	3	0.0019	22	1	Semi-tryptic
H2AW_MOUSE	Histone H2A	KSQGPLEVAEAAVSQSSGLAAK	3	3.50E-03	710.0465	2127.1178	3	0.0007	22	1	Semi-tryptic
H2AW_MOUSE	Histone H2A	EGTSNSTSEDGPGDGFTILSSK	2	1.90E-08	1093.4877	2184.9608	2	-0.0050	22	0	Semi-tryptic
H2AW_MOUSE	Histone H2A	EGTSNSTSEDGPGDGFTILSSK	2	3.60E-10	1093.4869	2184.9592	2	-0.0066	22	0	Semi-tryptic
H2AW_MOUSE	Histone H2A	LNQLLKGVTIASGGVLPRIHPE	16	8.50E-04	771.4588	2311.3545	3	0.0169	22	0	GluC
H2AW_MOUSE	Histone H2A	LNQLLKGVTIASGGVLPRIHPE	16	8.60E-03	771.4579	2311.3520	3	0.0144	22	0	GluC
H2AW_MOUSE	Histone H2A	LNQLLKGVTIASGGVLPRIHPE	16	3.30E-03	771.4579	2311.3517	3	0.0141	22	0	GluC
H2AW_MOUSE	Histone H2A	LNQLLKGVTIASGGVLPRIHPE	16	5.30E-11	578.845	2311.3510	4	0.0134	22	0	GluC
H2AW_MOUSE	Histone H2A	LNQLLKGVTIASGGVLPRIHPE	16	1.00E-03	771.4574	2311.3505	3	0.0129	22	0	GluC
H2AW_MOUSE	Histone H2A	LNQLLKGVTIASGGVLPRIHPE	16	1.60E-12	578.8445	2311.3490	4	0.0114	22	0	GluC
H2AW_MOUSE	Histone H2A	LNQLLKGVTIASGGVLPRIHPE	16	3.60E-03	771.4566	2311.3481	3	0.0105	22	0	GluC
H2AW_MOUSE	Histone H2A	LNQLLKGVTIASGGVLPRIHPE	16	1.70E-05	771.4547	2311.3421	3	0.0045	22	0	GluC
H2AW_MOUSE	Histone H2A	LNQLLKGVTIASGGVLPRIHPE	16	8.50E-07	771.4547	2311.3421	3	0.0045	22	0	GluC
H2AW_MOUSE	Histone H2A	LNQLLKGVTIASGGVLPRIHPE	16	3.50E-05	578.8425	2311.3409	4	0.0033	22	0	GluC
H2AW_MOUSE	Histone H2A	LNQLLKGVTIASGGVLPRIHPE	16	3.00E-08	578.8425	2311.3409	4	0.0033	22	0	GluC
H2AW_MOUSE	Histone H2A	LNQLLKGVTIASGGVLPRIHPE	16	2.50E-04	771.4541	2311.3406	3	0.0030	22	0	GluC
H2AW_MOUSE	Histone H2A	LNQLLKGVTIASGGVLPRIHPE	16	1.50E-04	771.4541	2311.3406	3	0.0030	22	0	GluC
H2AW_MOUSE	Histone H2A	LNQLLKGVTIASGGVLPRIHPE	16	8.10E-12	578.8424	2311.3405	4	0.0029	22	0	GluC
H2AW_MOUSE	Histone H2A	LNQLLKGVTIASGGVLPRIHPE	16	1.60E-11	578.8424	2311.3404	4	0.0028	22	0	GluC
H2AW_MOUSE	Histone H2A	LNQLLKGVTIASGGVLPRIHPE	16	1.50E-04	771.454	2311.3402	3	0.0026	22	0	GluC
H2AW_MOUSE	Histone H2A	DISHIGSMRVEGIVHPTTAEI	2	2.10E-03	754.7231	2261.1476	3	0.0001	21	0	AspN
H2AW_MOUSE	Histone H2A	DISHIGSMRVEGIVHPTTAEI	2	7.80E-04	754.7231	2261.1476	3	0.0001	21	0	AspN
H2AW_MOUSE	Histone H2A	VEGIVHPTTAEIDLKEEIGK	2	5.50E-05	726.7273	2177.1600	3	0.0020	20	1	Semi-tryptic
H2AW_MOUSE	Histone H2A	VEGIVHPTTAEIDLKEEIGK	2	1.80E-06	726.7264	2177.1573	3	-0.0006	20	1	Semi-tryptic
H2AW_MOUSE	Histone H2A	NKKARIAPRHILLAVANDEE	8	3.70E-05	753.4335	2257.2786	3	0.0132	20	2	GluC
H2AW_MOUSE	Histone H2A	NKKARIAPRHILLAVANDEE	8	7.90E-08	565.3269	2257.2785	4	0.0131	20	2	GluC
H2AW_MOUSE	Histone H2A	NKKARIAPRHILLAVANDEE	8	5.90E-05	753.4332	2257.2779	3	0.0125	20	2	GluC
H2AW_MOUSE	Histone H2A	NKKARIAPRHILLAVANDEE	8	5.80E-03	753.4321	2257.2744	3	0.0090	20	2	GluC
H2AW_MOUSE	Histone H2A	NKKARIAPRHILLAVANDEE	8	3.50E-03	753.4321	2257.2744	3	0.0090	20	2	GluC
H2AW_MOUSE	Histone H2A	NKKARIAPRHILLAVANDEE	8	2.80E-04	753.4293	2257.2662	3	0.0008	20	2	GluC
H2AW_MOUSE	Histone H2A	NKKARIAPRHILLAVANDEE	8	1.00E-04	753.4293	2257.2662	3	0.0008	20	2	GluC
H2AW_MOUSE	Histone H2A	NKKARIAPRHILLAVANDEE	8	1.40E-06	565.3229	2257.2626	4	-0.0028	20	2	GluC
H2AW_MOUSE	Histone H2A	KGVTIASGGVLPRIHPELL	3	1.80E-11	653.0615	1956.1625	3	0.0105	19	1	Chymotrypsin
H2AW_MOUSE	Histone H2A	KGVTIASGGVLPRIHPELL	3	1.10E-10	653.0601	1956.1584	3	0.0064	19	1	Chymotrypsin
H2AW_MOUSE	Histone H2A	KGVTIASGGVLPRIHPELL	3	1.50E-05	653.0581	1956.1526	3	0.0006	19	1	Chymotrypsin
H2AW_MOUSE	Histone H2A	DNKKARIAPRHILLAVAN	6	1.30E-03	667.4044	1999.1913	3	0.0110	18	0	AspN
H2AW_MOUSE	Histone H2A	DNKKARIAPRHILLAVAN	6	1.40E-03	1000.6	1999.1854	2	0.0052	18	0	AspN
H2AW_MOUSE	Histone H2A	DNKKARIAPRHILLAVAN	6	3.40E-03	400.8442	1999.1848	5	0.0046	18	0	AspN
H2AW_MOUSE	Histone H2A	DNKKARIAPRHILLAVAN	6	1.90E-06	667.4018	1999.1837	3	0.0034	18	0	AspN
H2AW_MOUSE	Histone H2A	DNKKARIAPRHILLAVAN	6	1.30E-03	500.8028	1999.1823	4	0.0020	18	0	AspN
H2AW_MOUSE	Histone H2A	DNKKARIAPRHILLAVAN	6	1.20E-04	667.4011	1999.1814	3	0.0012	18	0	AspN
H2AW_MOUSE	Histone H2A	VEGIVHPTTAEIDLK	2	1.00E-07	811.4441	1620.8736	2	0.0013	15	0	Semi-tryptic
H2AW_MOUSE	Histone H2A	EKAGGKEFLETVKEL	2	3.40E-03	559.9737	1676.8993	3	0.0008	15	2	Chymotrypsin
H2AW_MOUSE	Histone H2A	EKAGGKEFLETVKEL	2	3.80E-04	559.9737	1676.8993	3	0.0008	15	2	Chymotrypsin
H2AW_MOUSE	Histone H2A	VEGIVHPTTAEIDLK	2	2.30E-08	811.4429	1620.8713	2	-0.0010	15	0	Semi-tryptic
H2AW_MOUSE	Histone H2A	AISAHFDDSSSSLK	3	2.60E-08	776.3713	1550.7281	2	0.0069	15	0	Semi-tryptic
H2AW_MOUSE	Histone H2A	AISAHFDDSSSSLK	3	2.70E-07	776.37	1550.7254	2	0.0042	15	0	Semi-tryptic
H2AW_MOUSE	Histone H2A	LSLTQSDISHIGSMR	3	3.20E-10	822.9235	1643.8325	2	0.0024	15	0	Semi-tryptic
H2AW_MOUSE	Histone H2A	AISAHFDDSSSSLK	3	3.40E-04	776.3689	1550.7232	2	0.0019	15	0	Semi-tryptic
H2AW_MOUSE	Histone H2A	LSLTQSDISHIGSMR	3	6.40E-04	548.9506	1643.8299	3	-0.0002	15	0	Semi-tryptic
H2AW_MOUSE	Histone H2A	LSLTQSDISHIGSMR	3	1.40E-04	548.9489	1643.8249	3	-0.0052	15	0	Semi-tryptic
H2AW_MOUSE	Histone H2A	GKSETILSPPPEKR	5	1.60E-05	769.9329	1537.8512	2	0.0048	14	2	Semi-tryptic
H2AW_MOUSE	Histone H2A	GKSETILSPPPEKR	5	1.70E-03	513.6237	1537.8492	3	0.0028	14	2	Semi-tryptic
H2AW_MOUSE	Histone H2A	GKSETILSPPPEKR	5	1.90E-03	513.6233	1537.8480	3	0.0016	14	2	Semi-tryptic
H2AW_MOUSE	Histone H2A	GKSETILSPPPEKR	5	1.50E-06	769.9311	1537.8477	2	0.0013	14	2	Semi-tryptic
H2AW_MOUSE	Histone H2A	GKSETILSPPPEKR	5	8.20E-08	769.9305	1537.8465	2	0.0001	14	2	Semi-tryptic
H2AW_MOUSE	Histone H2A	LLAKKRGTKGKSE	7	2.30E-05	708.4402	1414.8659	2	0.0040	13	0	GluC
H2AW_MOUSE	Histone H2A	LLAKKRGTKGKSE	7	6.00E-03	708.4392	1414.8638	2	0.0019	13	0	GluC
H2AW_MOUSE	Histone H2A	LLAKKRGTKGKSE	7	2.00E-08	472.6286	1414.8639	3	0.0019	13	0	GluC
H2AW_MOUSE	Histone H2A	LLAKKRGTKGKSE	7	6.90E-03	472.6276	1414.8609	3	-0.0010	13	0	GluC
H2AW_MOUSE	Histone H2A	LLAKKRGTKGKSE	7	5.00E-04	472.6276	1414.8609	3	-0.0010	13	0	GluC
H2AW_MOUSE	Histone H2A	LLAKKRGTKGKSE	7	1.40E-06	472.6275	1414.8605	3	-0.0014	13	0	GluC
H2AW_MOUSE	Histone H2A	LLAKKRGTKGKSE	7	2.10E-06	354.7223	1414.8601	4	-0.0019	13	0	GluC
H2AW_MOUSE	Histone H2A	LRKSQGPLEVAE	2	8.40E-06	663.8738	1325.7331	2	0.0028	12	1	GluC
H2AW_MOUSE	Histone H2A	LRKSQGPLEVAE	2	9.50E-03	663.8727	1325.7308	2	0.0005	12	1	GluC
H2AW_MOUSE	Histone H2A	SETILSPPPEKR	3	1.20E-03	677.3755	1352.7365	2	0.0065	12	1	Semi-tryptic
H2AW_MOUSE	Histone H2A	SETILSPPPEKR	3	2.20E-03	677.3745	1352.7345	2	0.0045	12	1	Semi-tryptic
H2AW_MOUSE	Histone H2A	SETILSPPPEKR	3	1.00E-02	677.3728	1352.7311	2	0.0012	12	1	Semi-tryptic
H2AW_MOUSE	Histone H2A	GVTIASGGVLPR	6	6.60E-08	563.833	1125.6514	2	0.0007	12	0	Semi-tryptic
H2AW_MOUSE	Histone H2A	GVTIASGGVLPR	6	1.10E-07	563.8325	1125.6505	2	-0.0002	12	0	Semi-tryptic
H2AW_MOUSE	Histone H2A	GVTIASGGVLPR	6	3.90E-06	563.8324	1125.6503	2	-0.0003	12	0	Semi-tryptic
H2AW_MOUSE	Histone H2A	GVTIASGGVLPR	6	4.60E-08	563.8325	1125.6504	2	-0.0003	12	0	Semi-tryptic
H2AW_MOUSE	Histone H2A	GVTIASGGVLPR	6	4.10E-03	563.8317	1125.6488	2	-0.0018	12	0	Semi-tryptic

H2AW_MOUSE	Histone H2A	GVTIASGGVLPR	6	4.10E-05	563.8317	1125.6488	2	-0.0018	12	0	Semi-tryptic
H2AW_MOUSE	Histone H2A	AGGKEFLETVK	3	4.70E-06	589.8274	1177.6402	2	0.0059	11	1	Semi-tryptic
H2AW_MOUSE	Histone H2A	AGGKEFLETVK	3	5.20E-03	393.5533	1177.6380	3	0.0038	11	1	Semi-tryptic
H2AW_MOUSE	Histone H2A	AGGKEFLETVK	3	1.20E-06	589.8258	1177.6370	2	0.0027	11	1	Semi-tryptic
H2AW_MOUSE	Histone H2A	SVAFPPFPSGR	3	3.60E-03	581.3065	1160.5985	2	0.0006	11	0	Semi-tryptic
H2AW_MOUSE	Histone H2A	SVAFPPFPSGR	3	4.80E-03	581.3063	1160.5980	2	0.0002	11	0	Semi-tryptic
H2AW_MOUSE	Histone H2A	SVAFPPFPSGR	3	3.30E-04	581.3057	1160.5968	2	-0.0011	11	0	Semi-tryptic
H2AW_MOUSE	Histone H2A	ISHIGSMRVE	6	1.10E-03	376.8672	1127.5797	3	0.0040	10	0	GluC
H2AW_MOUSE	Histone H2A	ISHIGSMRVE	6	2.30E-03	564.797	1127.5795	2	0.0038	10	0	GluC
H2AW_MOUSE	Histone H2A	ISHIGSMRVE	6	2.80E-05	376.8671	1127.5794	3	0.0036	10	0	GluC
H2AW_MOUSE	Histone H2A	ISHIGSMRVE	6	2.60E-07	376.8671	1127.5794	3	0.0036	10	0	GluC
H2AW_MOUSE	Histone H2A	ISHIGSMRVE	6	3.20E-03	376.8661	1127.5766	3	0.0008	10	0	GluC
H2AW_MOUSE	Histone H2A	ISHIGSMRVE	6	4.60E-04	564.7956	1127.5765	2	0.0008	10	0	GluC
H2AW_MOUSE	Histone H2A	QTAAQVTLK	2	9.90E-03	480.2813	958.5481	2	0.0034	9	0	Semi-tryptic
H2AW_MOUSE	Histone H2A	QTAAQVTLK	2	1.80E-04	480.2798	958.5450	2	0.0002	9	0	Semi-tryptic
H2AW_MOUSE	Histone H2A	LRKSQGPLE	3	8.00E-04	514.2996	1026.5847	2	0.0025	9	0	GluC
H2AW_MOUSE	Histone H2A	LRKSQGPLE	3	9.70E-03	343.2012	1026.5819	3	-0.0003	9	0	GluC
H2AW_MOUSE	Histone H2A	LRKSQGPLE	3	6.30E-03	514.2979	1026.5813	2	-0.0009	9	0	GluC
H2AW_MOUSE	Histone H2A	KAGGKEFLE	8	5.20E-03	489.7679	977.5212	2	0.0030	9	1	GluC
H2AW_MOUSE	Histone H2A	GIVHPTTAE	8	4.40E-03	462.7443	923.4741	2	0.0028	9	0	GluC
H2AW_MOUSE	Histone H2A	GIVHPTTAE	8	8.50E-06	462.7443	923.4741	2	0.0028	9	0	GluC
H2AW_MOUSE	Histone H2A	GIVHPTTAE	8	8.10E-03	462.7438	923.4731	2	0.0019	9	0	GluC
H2AW_MOUSE	Histone H2A	KAGGKEFLE	8	5.20E-03	489.7673	977.5201	2	0.0019	9	1	GluC
H2AW_MOUSE	Histone H2A	KAGGKEFLE	8	2.80E-03	489.7673	977.5201	2	0.0019	9	1	GluC
H2AW_MOUSE	Histone H2A	KAGGKEFLE	8	1.70E-03	489.7669	977.5192	2	0.0011	9	1	GluC
H2AW_MOUSE	Histone H2A	KAGGKEFLE	8	2.50E-03	489.7669	977.5192	2	0.0010	9	1	GluC
H2AW_MOUSE	Histone H2A	GIVHPTTAE	8	1.50E-05	462.7432	923.4719	2	0.0006	9	0	GluC
H2AW_MOUSE	Histone H2A	GIVHPTTAE	8	3.30E-03	462.743	923.4714	2	0.0002	9	0	GluC
H2AW_MOUSE	Histone H2A	GIVHPTTAE	8	1.30E-03	462.743	923.4715	2	0.0002	9	0	GluC
H2AW_MOUSE	Histone H2A	GIVHPTTAE	8	6.50E-05	462.743	923.4714	2	0.0002	9	0	GluC
H2AW_MOUSE	Histone H2A	KAGGKEFLE	8	2.20E-03	489.7661	977.5176	2	-0.0006	9	1	GluC
H2AW_MOUSE	Histone H2A	KAGGKEFLE	8	1.50E-03	489.7659	977.5172	2	-0.0010	9	1	GluC
H2AW_MOUSE	Histone H2A	KAGGKEFLE	8	1.30E-03	489.7659	977.5172	2	-0.0010	9	1	GluC
H2AW_MOUSE	Histone H2A	GIVHPTTAE	8	5.00E-04	462.7424	923.4702	2	-0.0011	9	0	GluC
H2AW_MOUSE	Histone H2A	SLVLGQK	2	8.80E-03	372.7342	743.4538	2	-0.0003	7	0	Semi-tryptic
H2AW_MOUSE	Histone H2A	SLVLGQK	2	3.70E-03	372.734	743.4535	2	-0.0006	7	0	Semi-tryptic
H2AX_MOUSE	Histone H2A	LNKLLGGVTIAQGGVLPNIQAVLLPKKSSATVGPKAPAVGKKASQASQEY	7	4.80E-03	1250.2222	4996.8597	4	0.0156	50	1	GluC
H2AX_MOUSE	Histone H2A	LNKLLGGVTIAQGGVLPNIQAVLLPKKSSATVGPKAPAVGKKASQASQEY	7	1.30E-03	1000.3781	4996.8541	5	0.0100	50	1	GluC
H2AX_MOUSE	Histone H2A	LNKLLGGVTIAQGGVLPNIQAVLLPKKSSATVGPKAPAVGKKASQASQEY	7	3.30E-04	833.8162	4996.8536	6	0.0095	50	1	GluC
H2AX_MOUSE	Histone H2A	LNKLLGGVTIAQGGVLPNIQAVLLPKKSSATVGPKAPAVGKKASQASQEY	7	7.30E-04	1000.3769	4996.8481	5	0.0040	50	1	GluC
H2AX_MOUSE	Histone H2A	LNKLLGGVTIAQGGVLPNIQAVLLPKKSSATVGPKAPAVGKKASQASQEY	7	5.80E-03	1250.2191	4996.8473	4	0.0032	50	1	GluC
H2AX_MOUSE	Histone H2A	LNKLLGGVTIAQGGVLPNIQAVLLPKKSSATVGPKAPAVGKKASQASQEY	7	6.20E-05	833.8152	4996.8472	6	0.0032	50	1	GluC
H2AX_MOUSE	Histone H2A	LNKLLGGVTIAQGGVLPNIQAVLLPKKSSATVGPKAPAVGKKASQASQEY	7	3.10E-04	833.8136	4996.8382	6	-0.0059	50	1	GluC
H2AX_MOUSE	Histone H2A	LNKLLGGVTIAQGGVLPNIQAVLLPKKSSATVGPKAPAVGKKASQASQE	2	3.40E-04	806.6379	4833.7837	6	0.0030	49	0	GluC
H2AX_MOUSE	Histone H2A	LNKLLGGVTIAQGGVLPNIQAVLLPKKSSATVGPKAPAVGKKASQASQE	2	3.80E-03	967.7639	4833.7830	5	0.0023	49	0	GluC
H2AX_MOUSE	Histone H2A	HLQLAIRNDEELNKLLGGVTIAQGGVLPNIQAVLLPKK	3	6.20E-10	1019.097	4072.3589	4	0.0065	38	3	Semi-tryptic
H2AX_MOUSE	Histone H2A	HLQLAIRNDEELNKLLGGVTIAQGGVLPNIQAVLLPKK	3	2.10E-03	815.4787	4072.3573	5	0.0049	38	3	Semi-tryptic
H2AX_MOUSE	Histone H2A	HLQLAIRNDEELNKLLGGVTIAQGGVLPNIQAVLLPKK	3	2.60E-04	815.4782	4072.3548	5	0.0024	38	3	Semi-tryptic
H2AX_MOUSE	Histone H2A	HLQLAIRNDEELNKLLGGVTIAQGGVLPNIQAVLLPK	2	6.90E-11	1315.7621	3944.2645	3	0.0070	37	2	Semi-tryptic
H2AX_MOUSE	Histone H2A	HLQLAIRNDEELNKLLGGVTIAQGGVLPNIQAVLLPK	2	8.70E-03	789.86	3944.2635	5	0.0060	37	2	Semi-tryptic
H2AX_MOUSE	Histone H2A	LPKKSSATVGPKAPAVGKKASQASQEY	8	1.30E-06	910.177	2727.5092	3	0.0174	27	0	Chymotrypsin
H2AX_MOUSE	Histone H2A	LPKKSSATVGPKAPAVGKKASQASQEY	8	1.90E-09	682.8824	2727.5006	4	0.0087	27	0	Chymotrypsin
H2AX_MOUSE	Histone H2A	LPKKSSATVGPKAPAVGKKASQASQEY	8	2.10E-07	910.1736	2727.4989	3	0.0070	27	0	Chymotrypsin
H2AX_MOUSE	Histone H2A	LPKKSSATVGPKAPAVGKKASQASQEY	8	3.40E-09	682.8805	2727.4931	4	0.0012	27	0	GluC
H2AX_MOUSE	Histone H2A	LPKKSSATVGPKAPAVGKKASQASQEY	8	2.00E-07	682.8802	2727.4915	4	-0.0003	27	0	Chymotrypsin
H2AX_MOUSE	Histone H2A	LPKKSSATVGPKAPAVGKKASQASQEY	8	9.40E-03	910.1711	2727.4914	3	-0.0005	27	0	Chymotrypsin
H2AX_MOUSE	Histone H2A	LPKKSSATVGPKAPAVGKKASQASQEY	8	1.20E-05	910.1711	2727.4914	3	-0.0005	27	0	Chymotrypsin
H2AX_MOUSE	Histone H2A	LPKKSSATVGPKAPAVGKKASQASQEY	8	2.20E-07	682.8798	2727.4900	4	-0.0019	27	0	Chymotrypsin
H2AX_MOUSE	Histone H2A	SSATVGPKAPAVGKKASQASQEY	3	2.80E-03	754.7323	2261.1751	3	0.0100	23	3	Semi-tryptic
H2AX_MOUSE	Histone H2A	SSATVGPKAPAVGKKASQASQEY	3	4.20E-05	754.7299	2261.1678	3	0.0027	23	3	Semi-tryptic
H2AX_MOUSE	Histone H2A	SSATVGPKAPAVGKKASQASQEY	3	2.70E-04	754.7295	2261.1668	3	0.0016	23	3	Semi-tryptic
H2AX_MOUSE	Histone H2A	KSSATVGPKAPAVGKK	16	8.70E-03	382.233	1524.9029	4	0.0041	16	3	Semi-tryptic
H2AX_MOUSE	Histone H2A	KSSATVGPKAPAVGKK	16	4.60E-05	382.233	1524.9028	4	0.0040	16	3	Semi-tryptic
H2AX_MOUSE	Histone H2A	KSSATVGPKAPAVGKK	16	1.40E-03	382.2328	1524.9021	4	0.0034	16	3	Semi-tryptic
H2AX_MOUSE	Histone H2A	KSSATVGPKAPAVGKK	16	7.20E-05	763.4582	1524.9019	2	0.0031	16	3	Semi-tryptic
H2AX_MOUSE	Histone H2A	KSSATVGPKAPAVGKK	16	3.00E-04	382.2326	1524.9013	4	0.0025	16	3	Semi-tryptic
H2AX_MOUSE	Histone H2A	KSSATVGPKAPAVGKK	16	1.30E-03	382.2325	1524.9010	4	0.0022	16	3	Semi-tryptic
H2AX_MOUSE	Histone H2A	KSSATVGPKAPAVGKK	16	1.00E-03	382.2324	1524.9006	4	0.0019	16	3	Semi-tryptic
H2AX_MOUSE	Histone H2A	KSSATVGPKAPAVGKK	16	8.10E-06	763.4574	1524.9003	2	0.0016	16	3	Semi-tryptic
H2AX_MOUSE	Histone H2A	KSSATVGPKAPAVGKK	16	4.20E-04	382.2323	1524.9000	4	0.0013	16	3	Semi-tryptic
H2AX_MOUSE	Histone H2A	KSSATVGPKAPAVGKK	16	8.80E-03	382.2322	1524.8999	4	0.0011	16	3	Semi-tryptic
H2AX_MOUSE	Histone H2A	KSSATVGPKAPAVGKK	16	3.10E-03	509.3071	1524.8996	3	0.0009	16	3	Semi-tryptic
H2AX_MOUSE	Histone H2A	KSSATVGPKAPAVGKK	16	3.20E-03	509.3071	1524.8996	3	0.0008	16	3	Semi-tryptic
H2AX_MOUSE	Histone H2A	KSSATVGPKAPAVGKK	16	4.20E-04	382.2321	1524.8995	4	0.0007	16	3	Semi-tryptic

H2AX_MOUSE	Histone H2A	KSSATVGPKAPAVGKK	16	2.20E-03	509.3069	1524.8990	3	0.0002	16	3	Semi-tryptic
H2AX_MOUSE	Histone H2A	KSSATVGPKAPAVGKK	16	4.20E-05	382.232	1524.8989	4	0.0002	16	3	Semi-tryptic
H2AX_MOUSE	Histone H2A	KSSATVGPKAPAVGKK	16	9.60E-03	382.2319	1524.8985	4	-0.0002	16	3	Semi-tryptic
H2AX_MOUSE	Histone H2A	APAVGKKASQASQEY	5	6.20E-08	767.8978	1533.7810	2	0.0024	15	2	Semi-tryptic
H2AX_MOUSE	Histone H2A	APAVGKKASQASQEY	5	5.50E-07	767.8976	1533.7807	2	0.0020	15	2	Semi-tryptic
H2AX_MOUSE	Histone H2A	APAVGKKASQASQEY	5	1.00E-05	767.8975	1533.7804	2	0.0017	15	2	Semi-tryptic
H2AX_MOUSE	Histone H2A	APAVGKKASQASQEY	5	4.50E-07	767.8972	1533.7799	2	0.0013	15	2	Semi-tryptic
H2AX_MOUSE	Histone H2A	APAVGKKASQASQEY	5	1.90E-07	767.8973	1533.7800	2	0.0013	15	2	Semi-tryptic
H2AX_MOUSE	Histone H2A	SSATVGPKAPAVGKK	6	1.10E-04	466.6096	1396.8071	3	0.0033	15	2	Semi-tryptic
H2AX_MOUSE	Histone H2A	SSATVGPKAPAVGKK	6	3.90E-05	699.4095	1396.8044	2	0.0006	15	2	Semi-tryptic
H2AX_MOUSE	Histone H2A	SSATVGPKAPAVGKK	6	1.30E-04	466.6085	1396.8037	3	-0.0001	15	2	Semi-tryptic
H2AX_MOUSE	Histone H2A	SSATVGPKAPAVGKK	6	1.80E-04	466.6084	1396.8034	3	-0.0004	15	2	Semi-tryptic
H2AX_MOUSE	Histone H2A	SSATVGPKAPAVGKK	6	2.50E-05	466.6082	1396.8028	3	-0.0010	15	2	Semi-tryptic
H2AX_MOUSE	Histone H2A	SSATVGPKAPAVGKK	6	2.50E-04	466.6081	1396.8026	3	-0.0012	15	2	Semi-tryptic
H2AX_MOUSE	Histone H2A	SSATVGPKAPAVGK	3	1.70E-04	635.3622	1268.7099	2	0.0011	14	1	Semi-tryptic
H2AX_MOUSE	Histone H2A	SSATVGPKAPAVGK	3	1.30E-06	635.3619	1268.7092	2	0.0004	14	1	Semi-tryptic
H2AX_MOUSE	Histone H2A	SSATVGPKAPAVGK	3	9.80E-06	635.3616	1268.7086	2	-0.0002	14	1	Semi-tryptic
H2AX_MOUSE	Histone H2A	KASQASQEY	8	9.00E-06	506.2413	1010.4681	2	0.0012	9	1	Semi-tryptic
H2AX_MOUSE	Histone H2A	KASQASQEY	8	4.70E-05	506.241	1010.4674	2	0.0006	9	1	Semi-tryptic
H2AX_MOUSE	Histone H2A	KASQASQEY	8	5.00E-06	506.2409	1010.4673	2	0.0005	9	1	Semi-tryptic
H2AX_MOUSE	Histone H2A	KASQASQEY	8	1.10E-04	506.2409	1010.4673	2	0.0004	9	1	Semi-tryptic
H2AX_MOUSE	Histone H2A	KASQASQEY	8	5.10E-06	506.2409	1010.4673	2	0.0004	9	1	Semi-tryptic
H2AX_MOUSE	Histone H2A	KASQASQEY	8	3.40E-06	506.2408	1010.4670	2	0.0001	9	1	Semi-tryptic
H2AX_MOUSE	Histone H2A	KASQASQEY	8	9.90E-06	506.2407	1010.4668	2	0.0000	9	1	Semi-tryptic
H2AX_MOUSE	Histone H2A	KASQASQEY	8	5.60E-06	506.2404	1010.4662	2	-0.0007	9	1	Semi-tryptic
H2AX_MOUSE	Histone H2A	KSSATVGPK	9	8.60E-03	437.7543	873.4941	2	0.0021	9	1	Semi-tryptic
H2AX_MOUSE	Histone H2A	KSSATVGPK	9	7.50E-04	437.7541	873.4936	2	0.0016	9	1	Semi-tryptic
H2AX_MOUSE	Histone H2A	KSSATVGPK	9	8.50E-03	437.7536	873.4926	2	0.0006	9	1	Semi-tryptic
H2AX_MOUSE	Histone H2A	KSSATVGPK	9	9.00E-04	437.7534	873.4923	2	0.0004	9	1	Semi-tryptic
H2AX_MOUSE	Histone H2A	KSSATVGPK	9	6.40E-04	437.7533	873.4920	2	0.0001	9	1	Semi-tryptic
H2AX_MOUSE	Histone H2A	KSSATVGPK	9	5.20E-04	437.7532	873.4919	2	0.0000	9	1	Semi-tryptic
H2AX_MOUSE	Histone H2A	KSSATVGPK	9	8.70E-03	437.7532	873.4917	2	-0.0002	9	1	Semi-tryptic
H2AX_MOUSE	Histone H2A	KSSATVGPK	9	7.30E-03	437.7531	873.4916	2	-0.0004	9	1	Semi-tryptic
H2AX_MOUSE	Histone H2A	KSSATVGPK	9	4.00E-03	437.7528	873.4910	2	-0.0010	9	1	Semi-tryptic
H2AX_MOUSE	Histone H2A	SSATVGPK	2	6.10E-06	373.706	745.3974	2	0.0004	8	0	Semi-tryptic
H2AX_MOUSE	Histone H2A	SSATVGPK	2	2.20E-03	373.7059	745.3972	2	0.0002	8	0	Semi-tryptic
H2AX_MOUSE	Histone H2A	ASQASQEY	8	3.50E-03	442.1937	882.3728	2	0.0009	8	0	Semi-tryptic
H2AX_MOUSE	Histone H2A	ASQASQEY	8	8.80E-03	442.1935	882.3725	2	0.0006	8	0	Semi-tryptic
H2AX_MOUSE	Histone H2A	ASQASQEY	8	1.10E-03	442.1934	882.3723	2	0.0004	8	0	Semi-tryptic
H2AX_MOUSE	Histone H2A	ASQASQEY	8	3.20E-03	442.1934	882.3722	2	0.0003	8	0	Semi-tryptic
H2AX_MOUSE	Histone H2A	ASQASQEY	8	4.10E-03	442.1933	882.3721	2	0.0002	8	0	Semi-tryptic
H2AX_MOUSE	Histone H2A	ASQASQEY	8	4.00E-03	442.1933	882.3721	2	0.0002	8	0	Semi-tryptic
H2AX_MOUSE	Histone H2A	ASQASQEY	8	9.50E-03	442.1932	882.3719	2	0.0000	8	0	Semi-tryptic
H2AX_MOUSE	Histone H2A	ASQASQEY	8	4.40E-03	442.1932	882.3718	2	-0.0001	8	0	Semi-tryptic
H2AX_MOUSE	Histone H2A	KGHYAER	2	3.50E-03	430.7223	859.4300	2	0.0000	7	1	Semi-tryptic
H2AX_MOUSE	Histone H2A	KGHYAER	2	9.30E-03	430.7219	859.4292	2	-0.0008	7	1	Semi-tryptic
H2AY_MOUSE	Histone H2A	GTPTDGFTVLSTKSLFLGQKLNLIHSE	4	1.60E-04	726.6475	2902.5607	4	0.0167	27	1	GluC
H2AY_MOUSE	Histone H2A	GTPTDGFTVLSTKSLFLGQKLNLIHSE	4	9.50E-03	968.5241	2902.5506	3	0.0065	27	1	GluC
H2AY_MOUSE	Histone H2A	GTPTDGFTVLSTKSLFLGQKLNLIHSE	4	9.10E-03	968.5241	2902.5506	3	0.0065	27	1	GluC
H2AY_MOUSE	Histone H2A	GTPTDGFTVLSTKSLFLGQKLNLIHSE	4	1.60E-03	968.5233	2902.5481	3	0.0041	27	1	GluC
H2AY_MOUSE	Histone H2A	NGPLEVAGAAISAGHGLPAK	4	1.70E-14	915.4979	1828.9812	2	0.0017	20	0	Semi-tryptic
H2AY_MOUSE	Histone H2A	NGPLEVAGAAISAGHGLPAK	4	1.30E-12	915.4977	1828.9809	2	0.0014	20	0	Semi-tryptic
H2AY_MOUSE	Histone H2A	NGPLEVAGAAISAGHGLPAK	4	1.50E-05	610.6675	1828.9806	3	0.0011	20	0	Semi-tryptic
H2AY_MOUSE	Histone H2A	NGPLEVAGAAISAGHGLPAK	4	8.10E-06	610.6667	1828.9781	3	-0.0014	20	0	Semi-tryptic
H2AY_MOUSE	Histone H2A	AVVHPTNTDFYTGGE	3	2.70E-04	804.3725	1606.7304	2	0.0040	15	1	GluC
H2AY_MOUSE	Histone H2A	AVVHPTNTDFYTGGE	3	6.60E-05	804.3719	1606.7292	2	0.0028	15	1	GluC
H2AY_MOUSE	Histone H2A	AVVHPTNTDFYTGGE	3	4.30E-06	804.3714	1606.7283	2	0.0019	15	1	GluC
H2AY_MOUSE	Histone H2A	EKKGGKEFVEAVL	3	1.30E-07	478.6051	1432.7935	3	0.0009	13	1	Chymotrypsin
H2AY_MOUSE	Histone H2A	EKKGGKEFVEAVL	3	6.20E-04	359.2055	1432.7927	4	0.0002	13	1	Chymotrypsin
H2AY_MOUSE	Histone H2A	EKKGGKEFVEAVL	3	2.00E-04	478.6042	1432.7908	3	-0.0018	13	1	Chymotrypsin
H2AY_MOUSE	Histone H2A	LKDDLGNTLE	3	8.40E-03	559.2915	1116.5683	2	0.0021	10	2	GluC
H2AY_MOUSE	Histone H2A	LKDDLGNTLE	3	1.10E-06	559.2908	1116.5671	2	0.0009	10	2	GluC
H2AY_MOUSE	Histone H2A	LKDDLGNTLE	3	5.20E-03	559.2907	1116.5669	2	0.0007	10	2	GluC
H2AY_MOUSE	Histone H2A	KKGGKEFVE	2	8.10E-03	511.2878	1020.5609	2	0.0006	9	1	GluC
H2AY_MOUSE	Histone H2A	KKGGKEFVE	2	1.30E-03	341.1942	1020.5608	3	0.0004	9	1	GluC
H2AY_MOUSE	Histone H2A	LRKKNGPLE	7	4.30E-05	352.2189	1053.6349	3	0.0055	9	0	GluC
H2AY_MOUSE	Histone H2A	LRKKNGPLE	7	8.00E-03	527.8239	1053.6333	2	0.0039	9	0	GluC
H2AY_MOUSE	Histone H2A	LRKKNGPLE	7	5.50E-04	352.2175	1053.6307	3	0.0012	9	0	GluC
H2AY_MOUSE	Histone H2A	LRKKNGPLE	7	5.20E-03	352.2174	1053.6303	3	0.0009	9	0	GluC
H2AY_MOUSE	Histone H2A	LRKKNGPLE	7	2.00E-03	352.2172	1053.6297	3	0.0002	9	0	GluC
H2AY_MOUSE	Histone H2A	LRKKNGPLE	7	8.80E-03	527.8219	1053.6291	2	-0.0003	9	0	GluC
H2AY_MOUSE	Histone H2A	LRKKNGPLE	7	7.40E-04	352.2167	1053.6283	3	-0.0011	9	0	GluC
H2AY_MOUSE	Histone H2A	MAKLDAN	3	4.90E-04	381.695	761.3754	2	0.0012	7	1	GluC
H2AY_MOUSE	Histone H2A	MAKLDAN	3	4.20E-04	381.695	761.3754	2	0.0012	7	1	GluC

H2AY_MOUSE	Histone H2A	MAKLDAN	3	6.50E-03	381.6941	761.3737	2	-0.0005	7	1	GluC
H2AY_MOUSE, H2AW_MOUSE	Histone H2A	HILLAVANDEELNQLLK	7	3.80E-10	967.0465	1932.0784	2	0.0104	17	0	Semi-tryptic
H2AY_MOUSE, H2AW_MOUSE	Histone H2A	HILLAVANDEELNQLLK	7	4.60E-05	967.0459	1932.0773	2	0.0093	17	0	Semi-tryptic
H2AY_MOUSE, H2AW_MOUSE	Histone H2A	HILLAVANDEELNQLLK	7	4.00E-03	645.0315	1932.0727	3	0.0047	17	0	Semi-tryptic
H2AY_MOUSE, H2AW_MOUSE	Histone H2A	HILLAVANDEELNQLLK	7	1.50E-04	645.0315	1932.0727	3	0.0047	17	0	Semi-tryptic
H2AY_MOUSE, H2AW_MOUSE	Histone H2A	HILLAVANDEELNQLLK	7	1.10E-13	967.0424	1932.0702	2	0.0022	17	0	Semi-tryptic
H2AY_MOUSE, H2AW_MOUSE	Histone H2A	HILLAVANDEELNQLLK	7	3.80E-05	645.0304	1932.0693	3	0.0013	17	0	Semi-tryptic
H2AY_MOUSE, H2AW_MOUSE	Histone H2A	HILLAVANDEELNQLLK	7	1.00E-10	967.0415	1932.0685	2	0.0005	17	0	Semi-tryptic
H2AY_MOUSE, H2AW_MOUSE	Histone H2A	AGVIFPVGR	2	5.80E-03	458.2761	914.5376	2	0.0038	9	0	Semi-tryptic
H2AY_MOUSE, H2AW_MOUSE	Histone H2A	AGVIFPVGR	2	1.80E-03	458.2741	914.5336	2	-0.0002	9	0	Semi-tryptic
H2AY_MOUSE, Q8CA90_MOUSE	Histone H2A	HILLAVANDEELNQLLKGVTIASGGVLPNIHPELLAK	2	4.70E-04	975.8061	3899.1954	4	0.0070	37	1	Semi-tryptic
H2AY_MOUSE, Q8CA90_MOUSE	Histone H2A	HILLAVANDEELNQLLKGVTIASGGVLPNIHPELLAK	2	1.10E-03	975.8058	3899.1941	4	0.0058	37	1	Semi-tryptic
H2AY_MOUSE, Q8CA90_MOUSE	Histone H2A	AASADSTTEGTPTDGFTVLSTK	3	1.70E-12	1079.0158	2156.0170	2	0.0049	22	0	Semi-tryptic
H2AY_MOUSE, Q8CA90_MOUSE	Histone H2A	AASADSTTEGTPTDGFTVLSTK	3	1.20E-10	1079.0151	2156.0156	2	0.0035	22	0	Semi-tryptic
H2AY_MOUSE, Q8CA90_MOUSE	Histone H2A	AASADSTTEGTPTDGFTVLSTK	3	2.90E-08	1079.015	2156.0154	2	0.0033	22	0	Semi-tryptic
H2AY_MOUSE, Q8CA90_MOUSE	Histone H2A	LNQLLKGVTIASGGVLPNIHPE	29	3.40E-04	757.433	2269.2771	3	-0.0023	22	0	GluC
H2AY_MOUSE, Q8CA90_MOUSE	Histone H2A	LNQLLKGVTIASGGVLPNIHPE	29	7.20E-06	757.4324	2269.2755	3	-0.0039	22	0	GluC
H2AY_MOUSE, Q8CA90_MOUSE	Histone H2A	LNQLLKGVTIASGGVLPNIHPE	29	2.20E-04	757.4322	2269.2748	3	-0.0046	22	0	GluC
H2AY_MOUSE, Q8CA90_MOUSE	Histone H2A	LNQLLKGVTIASGGVLPNIHPE	29	1.60E-08	757.4389	2269.2948	3	0.0153	22	0	GluC
H2AY_MOUSE, Q8CA90_MOUSE	Histone H2A	LNQLLKGVTIASGGVLPNIHPE	29	7.60E-11	757.4387	2269.2943	3	0.0149	22	0	GluC
H2AY_MOUSE, Q8CA90_MOUSE	Histone H2A	LNQLLKGVTIASGGVLPNIHPE	29	5.10E-08	757.4383	2269.2930	3	0.0136	22	0	GluC
H2AY_MOUSE, Q8CA90_MOUSE	Histone H2A	LNQLLKGVTIASGGVLPNIHPE	29	5.90E-07	757.4383	2269.2930	3	0.0135	22	0	GluC
H2AY_MOUSE, Q8CA90_MOUSE	Histone H2A	LNQLLKGVTIASGGVLPNIHPE	29	3.30E-07	757.4378	2269.2915	3	0.0121	22	0	GluC
H2AY_MOUSE, Q8CA90_MOUSE	Histone H2A	LNQLLKGVTIASGGVLPNIHPE	29	7.70E-09	757.437	2269.2892	3	0.0098	22	0	GluC
H2AY_MOUSE, Q8CA90_MOUSE	Histone H2A	LNQLLKGVTIASGGVLPNIHPE	29	2.50E-11	1135.6506	2269.2866	2	0.0072	22	0	GluC
H2AY_MOUSE, Q8CA90_MOUSE	Histone H2A	LNQLLKGVTIASGGVLPNIHPE	29	2.80E-08	1135.6502	2269.2858	2	0.0064	22	0	GluC
H2AY_MOUSE, Q8CA90_MOUSE	Histone H2A	LNQLLKGVTIASGGVLPNIHPE	29	8.50E-06	757.4355	2269.2846	3	0.0052	22	0	GluC
H2AY_MOUSE, Q8CA90_MOUSE	Histone H2A	LNQLLKGVTIASGGVLPNIHPE	29	6.70E-07	757.4355	2269.2846	3	0.0052	22	0	GluC
H2AY_MOUSE, Q8CA90_MOUSE	Histone H2A	LNQLLKGVTIASGGVLPNIHPE	29	1.50E-08	1135.6496	2269.2846	2	0.0052	22	0	GluC
H2AY_MOUSE, Q8CA90_MOUSE	Histone H2A	LNQLLKGVTIASGGVLPNIHPE	29	3.80E-09	1135.6495	2269.2844	2	0.0050	22	0	GluC
H2AY_MOUSE, Q8CA90_MOUSE	Histone H2A	LNQLLKGVTIASGGVLPNIHPE	29	2.10E-11	1135.6494	2269.2842	2	0.0048	22	0	GluC
H2AY_MOUSE, Q8CA90_MOUSE	Histone H2A	LNQLLKGVTIASGGVLPNIHPE	29	4.00E-06	757.4351	2269.2836	3	0.0042	22	0	GluC
H2AY_MOUSE, Q8CA90_MOUSE	Histone H2A	LNQLLKGVTIASGGVLPNIHPE	29	2.60E-03	568.328	2269.2829	4	0.0035	22	0	GluC
H2AY_MOUSE, Q8CA90_MOUSE	Histone H2A	LNQLLKGVTIASGGVLPNIHPE	29	4.00E-03	757.4349	2269.2828	3	0.0033	22	0	GluC
H2AY_MOUSE, Q8CA90_MOUSE	Histone H2A	LNQLLKGVTIASGGVLPNIHPE	29	4.90E-08	1135.6484	2269.2822	2	0.0028	22	0	GluC
H2AY_MOUSE, Q8CA90_MOUSE	Histone H2A	LNQLLKGVTIASGGVLPNIHPE	29	9.70E-08	757.4347	2269.2822	3	0.0027	22	0	GluC
H2AY_MOUSE, Q8CA90_MOUSE	Histone H2A	LNQLLKGVTIASGGVLPNIHPE	29	1.00E-05	757.4345	2269.2817	3	0.0023	22	0	GluC
H2AY_MOUSE, Q8CA90_MOUSE	Histone H2A	LNQLLKGVTIASGGVLPNIHPE	29	1.40E-03	757.4344	2269.2815	3	0.0021	22	0	GluC
H2AY_MOUSE, Q8CA90_MOUSE	Histone H2A	LNQLLKGVTIASGGVLPNIHPE	29	1.40E-07	757.4344	2269.2815	3	0.0021	22	0	GluC
H2AY_MOUSE, Q8CA90_MOUSE	Histone H2A	LNQLLKGVTIASGGVLPNIHPE	29	6.80E-05	757.4343	2269.2812	3	0.0018	22	0	GluC
H2AY_MOUSE, Q8CA90_MOUSE	Histone H2A	LNQLLKGVTIASGGVLPNIHPE	29	7.20E-09	1135.6479	2269.2812	2	0.0018	22	0	GluC
H2AY_MOUSE, Q8CA90_MOUSE	Histone H2A	LNQLLKGVTIASGGVLPNIHPE	29	4.40E-03	757.4341	2269.2806	3	0.0012	22	0	GluC
H2AY_MOUSE, Q8CA90_MOUSE	Histone H2A	LNQLLKGVTIASGGVLPNIHPE	29	1.10E-04	757.4337	2269.2793	3	-0.0001	22	0	GluC
H2AY_MOUSE, Q8CA90_MOUSE	Histone H2A	LNQLLKGVTIASGGVLPNIHPE	29	1.10E-07	757.4337	2269.2793	3	-0.0001	22	0	GluC
H2AY_MOUSE, Q8CA90_MOUSE	Histone H2A	NKKGRVTPRHILLAVANDEE	3	5.40E-04	754.0933	2259.2580	3	0.0133	20	2	GluC
H2AY_MOUSE, Q8CA90_MOUSE	Histone H2A	NKKGRVTPRHILLAVANDEE	3	1.40E-04	754.0931	2259.2575	3	0.0128	20	2	GluC
H2AY_MOUSE, Q8CA90_MOUSE	Histone H2A	NKKGRVTPRHILLAVANDEE	3	5.50E-04	754.093	2259.2571	3	0.0124	20	2	GluC
H2AY_MOUSE, Q8CA90_MOUSE	Histone H2A	GVTIASGGVLPNIHPELLAK	10	2.40E-08	993.5744	1985.1342	2	0.0033	20	0	Semi-tryptic
H2AY_MOUSE, Q8CA90_MOUSE	Histone H2A	GVTIASGGVLPNIHPELLAK	10	2.60E-03	662.7185	1985.1337	3	0.0028	20	0	Semi-tryptic
H2AY_MOUSE, Q8CA90_MOUSE	Histone H2A	GVTIASGGVLPNIHPELLAK	10	3.70E-05	662.7182	1985.1328	3	0.0018	20	0	Semi-tryptic
H2AY_MOUSE, Q8CA90_MOUSE	Histone H2A	GVTIASGGVLPNIHPELLAK	10	2.40E-04	662.7181	1985.1324	3	0.0015	20	0	Semi-tryptic
H2AY_MOUSE, Q8CA90_MOUSE	Histone H2A	GVTIASGGVLPNIHPELLAK	10	1.70E-10	993.5734	1985.1323	2	0.0014	20	0	Semi-tryptic
H2AY_MOUSE, Q8CA90_MOUSE	Histone H2A	GVTIASGGVLPNIHPELLAK	10	1.10E-11	993.5734	1985.1323	2	0.0014	20	0	Semi-tryptic
H2AY_MOUSE, Q8CA90_MOUSE	Histone H2A	GVTIASGGVLPNIHPELLAK	10	1.00E-06	662.7176	1985.1311	3	0.0001	20	0	Semi-tryptic
H2AY_MOUSE, Q8CA90_MOUSE	Histone H2A	GVTIASGGVLPNIHPELLAK	10	2.60E-06	662.7176	1985.1309	3	-0.0001	20	0	Semi-tryptic
H2AY_MOUSE, Q8CA90_MOUSE	Histone H2A	GVTIASGGVLPNIHPELLAK	10	4.10E-03	662.7169	1985.1289	3	-0.0020	20	0	Semi-tryptic
H2AY_MOUSE, Q8CA90_MOUSE	Histone H2A	GVTIASGGVLPNIHPELLAK	10	1.40E-04	662.7169	1985.1289	3	-0.0020	20	0	Semi-tryptic
H2AY_MOUSE, Q8CA90_MOUSE	Histone H2A	KGVTIASGGVLPNIHPELL	10	1.00E-02	958.0599	1914.1053	2	0.0114	19	1	Chymotrypsin
H2AY_MOUSE, Q8CA90_MOUSE	Histone H2A	KGVTIASGGVLPNIHPELL	10	3.10E-06	639.0406	1914.0999	3	0.0060	19	1	Chymotrypsin
H2AY_MOUSE, Q8CA90_MOUSE	Histone H2A	KGVTIASGGVLPNIHPELL	10	1.30E-05	958.0568	1914.0991	2	0.0052	19	1	Chymotrypsin
H2AY_MOUSE, Q8CA90_MOUSE	Histone H2A	KGVTIASGGVLPNIHPELL	10	1.00E-02	958.0554	1914.0963	2	0.0024	19	1	Chymotrypsin
H2AY_MOUSE, Q8CA90_MOUSE	Histone H2A	KGVTIASGGVLPNIHPELL	10	3.30E-03	639.0394	1914.0963	3	0.0024	19	1	Chymotrypsin
H2AY_MOUSE, Q8CA90_MOUSE	Histone H2A	KGVTIASGGVLPNIHPELL	10	1.80E-03	639.0391	1914.0956	3	0.0018	19	1	Chymotrypsin
H2AY_MOUSE, Q8CA90_MOUSE	Histone H2A	KGVTIASGGVLPNIHPELL	10	2.90E-03	958.0551	1914.0956	2	0.0017	19	1	Chymotrypsin
H2AY_MOUSE, Q8CA90_MOUSE	Histone H2A	KGVTIASGGVLPNIHPELL	10	1.10E-03	639.0389	1914.0949	3	0.0011	19	1	Chymotrypsin
H2AY_MOUSE, Q8CA90_MOUSE	Histone H2A	KGVTIASGGVLPNIHPELL	10	1.60E-04	958.0547	1914.0949	2	0.0010	19	1	Chymotrypsin
H2AY_MOUSE, Q8CA90_MOUSE	Histone H2A	KGVTIASGGVLPNIHPELL	10	1.70E-04	639.0378	1914.0916	3	-0.0023	19	1	Chymotrypsin
H2AY_MOUSE, Q8CA90_MOUSE	Histone H2A	DNKKGRVTPRHILLAVAN	7	4.90E-04	1001.5896	2001.1646	2	0.0051	18	0	AspN
H2AY_MOUSE, Q8CA90_MOUSE	Histone H2A	DNKKGRVTPRHILLAVAN	7	1.20E-04	668.0617	2001.1632	3	0.0036	18	0	AspN
H2AY_MOUSE, Q8CA90_MOUSE	Histone H2A	DNKKGRVTPRHILLAVAN	7	3.70E-03	668.0615	2001.1625	3	0.0030	18	0	AspN
H2AY_MOUSE, Q8CA90_MOUSE	Histone H2A	DNKKGRVTPRHILLAVAN	7	1.30E-06	668.0615	2001.1625	3	0.0030	18	0	AspN
H2AY_MOUSE, Q8CA90_MOUSE	Histone H2A	DNKKGRVTPRHILLAVAN	7	1.20E-06	501.2973	2001.1603	4	0.0007	18	0	AspN
H2AY_MOUSE, Q8CA90_MOUSE	Histone H2A	DNKKGRVTPRHILLAVAN	7	1.30E-06	501.2973	2001.1599	4	0.0004	18	0	AspN
H2AY_MOUSE, Q8CA90_MOUSE	Histone H2A	DNKKGRVTPRHILLAVAN	7	2.50E-05	501.2965	2001.1568	4	-0.0028	18	0	AspN

H2AY_MOUSE, Q8CA90_MOUSE	Histone H2A	GKLEAIITPPPAKK	3	8.30E-06	731.9551	1461.8955	2	0.0037	14	2	Semi-tryptic
H2AY_MOUSE, Q8CA90_MOUSE	Histone H2A	GKLEAIITPPPAKK	3	2.80E-07	731.9542	1461.8938	2	0.0020	14	2	Semi-tryptic
H2AY_MOUSE, Q8CA90_MOUSE	Histone H2A	GKLEAIITPPPAKK	3	2.10E-03	488.3041	1461.8905	3	-0.0014	14	2	Semi-tryptic
H2AY_MOUSE, Q8CA90_MOUSE	Histone H2A	GKLEAIITPPPAK	2	2.00E-06	667.9063	1333.7981	2	0.0012	13	1	Semi-tryptic
H2AY_MOUSE, Q8CA90_MOUSE	Histone H2A	GKLEAIITPPPAK	2	1.40E-06	667.9059	1333.7972	2	0.0003	13	1	Semi-tryptic
H2AY_MOUSE, Q8CA90_MOUSE	Histone H2A	LLAKKRGSKGKLE	27	1.50E-05	476.6392	1426.8958	3	-0.0025	13	0	GluC
H2AY_MOUSE, Q8CA90_MOUSE	Histone H2A	LLAKKRGSKGKLE	27	2.00E-04	476.6428	1426.9065	3	0.0081	13	0	GluC
H2AY_MOUSE, Q8CA90_MOUSE	Histone H2A	LLAKKRGSKGKLE	27	3.70E-08	476.6427	1426.9062	3	0.0079	13	0	GluC
H2AY_MOUSE, Q8CA90_MOUSE	Histone H2A	LLAKKRGSKGKLE	27	1.60E-05	714.4597	1426.9049	2	0.0066	13	0	GluC
H2AY_MOUSE, Q8CA90_MOUSE	Histone H2A	LLAKKRGSKGKLE	27	1.70E-05	714.4593	1426.9040	2	0.0056	13	0	GluC
H2AY_MOUSE, Q8CA90_MOUSE	Histone H2A	LLAKKRGSKGKLE	27	1.00E-08	357.7331	1426.9033	4	0.0050	13	0	GluC
H2AY_MOUSE, Q8CA90_MOUSE	Histone H2A	LLAKKRGSKGKLE	27	1.10E-05	357.733	1426.9031	4	0.0048	13	0	GluC
H2AY_MOUSE, Q8CA90_MOUSE	Histone H2A	LLAKKRGSKGKLE	27	1.40E-08	476.6416	1426.9031	3	0.0048	13	0	GluC
H2AY_MOUSE, Q8CA90_MOUSE	Histone H2A	LLAKKRGSKGKLE	27	3.50E-03	357.7329	1426.9025	4	0.0042	13	0	GluC
H2AY_MOUSE, Q8CA90_MOUSE	Histone H2A	LLAKKRGSKGKLE	27	7.90E-04	357.7328	1426.9019	4	0.0036	13	0	GluC
H2AY_MOUSE, Q8CA90_MOUSE	Histone H2A	LLAKKRGSKGKLE	27	1.50E-08	476.6411	1426.9015	3	0.0032	13	0	GluC
H2AY_MOUSE, Q8CA90_MOUSE	Histone H2A	LLAKKRGSKGKLE	27	2.90E-03	357.7326	1426.9011	4	0.0028	13	0	GluC
H2AY_MOUSE, Q8CA90_MOUSE	Histone H2A	LLAKKRGSKGKLE	27	2.20E-03	714.4577	1426.9008	2	0.0025	13	0	GluC
H2AY_MOUSE, Q8CA90_MOUSE	Histone H2A	LLAKKRGSKGKLE	27	2.50E-04	476.6408	1426.9006	3	0.0023	13	0	GluC
H2AY_MOUSE, Q8CA90_MOUSE	Histone H2A	LLAKKRGSKGKLE	27	3.30E-03	714.4575	1426.9004	2	0.0020	13	0	GluC
H2AY_MOUSE, Q8CA90_MOUSE	Histone H2A	LLAKKRGSKGKLE	27	3.50E-04	357.7322	1426.8998	4	0.0014	13	0	GluC
H2AY_MOUSE, Q8CA90_MOUSE	Histone H2A	LLAKKRGSKGKLE	27	5.80E-07	357.7322	1426.8996	4	0.0012	13	0	GluC
H2AY_MOUSE, Q8CA90_MOUSE	Histone H2A	LLAKKRGSKGKLE	27	4.30E-04	476.6404	1426.8994	3	0.0011	13	0	GluC
H2AY_MOUSE, Q8CA90_MOUSE	Histone H2A	LLAKKRGSKGKLE	27	4.70E-09	476.6404	1426.8994	3	0.0011	13	0	GluC
H2AY_MOUSE, Q8CA90_MOUSE	Histone H2A	LLAKKRGSKGKLE	27	4.60E-05	357.7321	1426.8992	4	0.0009	13	0	GluC
H2AY_MOUSE, Q8CA90_MOUSE	Histone H2A	LLAKKRGSKGKLE	27	1.00E-03	476.6403	1426.8991	3	0.0008	13	0	GluC
H2AY_MOUSE, Q8CA90_MOUSE	Histone H2A	LLAKKRGSKGKLE	27	3.10E-03	476.6401	1426.8984	3	0.0001	13	0	GluC
H2AY_MOUSE, Q8CA90_MOUSE	Histone H2A	LLAKKRGSKGKLE	27	3.50E-04	357.7319	1426.8983	4	0.0000	13	0	GluC
H2AY_MOUSE, Q8CA90_MOUSE	Histone H2A	LLAKKRGSKGKLE	27	9.90E-05	357.7319	1426.8983	4	0.0000	13	0	GluC
H2AY_MOUSE, Q8CA90_MOUSE	Histone H2A	LLAKKRGSKGKLE	27	7.90E-03	357.7318	1426.8981	4	-0.0002	13	0	GluC
H2AY_MOUSE, Q8CA90_MOUSE	Histone H2A	LLAKKRGSKGKLE	27	8.30E-07	357.7317	1426.8977	4	-0.0007	13	0	GluC
H2AY_MOUSE, Q8CA90_MOUSE	Histone H2A	LLAKKRGSKGKLE	27	2.50E-04	357.7315	1426.8969	4	-0.0014	13	0	GluC
H2AY_MOUSE, Q8CA90_MOUSE	Histone H2A	SAKAGVIFPVGR	2	1.80E-05	601.3586	1200.7026	2	0.0047	12	1	Semi-tryptic
H2AY_MOUSE, Q8CA90_MOUSE	Histone H2A	SAKAGVIFPVGR	2	1.20E-03	601.3573	1200.7001	2	0.0023	12	1	Semi-tryptic
H2AY_MOUSE, Q8CA90_MOUSE	Histone H2A	VSKAASADSTTE	8	4.60E-09	583.7825	1165.5505	2	0.0043	12	1	GluC
H2AY_MOUSE, Q8CA90_MOUSE	Histone H2A	VSKAASADSTTE	8	1.00E-08	583.7817	1165.5488	2	0.0025	12	1	GluC
H2AY_MOUSE, Q8CA90_MOUSE	Histone H2A	VSKAASADSTTE	8	2.90E-07	583.7812	1165.5479	2	0.0017	12	1	GluC
H2AY_MOUSE, Q8CA90_MOUSE	Histone H2A	VSKAASADSTTE	8	4.20E-04	583.7811	1165.5477	2	0.0014	12	1	GluC
H2AY_MOUSE, Q8CA90_MOUSE	Histone H2A	VSKAASADSTTE	8	1.20E-06	583.781	1165.5474	2	0.0011	12	1	GluC
H2AY_MOUSE, Q8CA90_MOUSE	Histone H2A	VSKAASADSTTE	8	3.10E-09	583.7809	1165.5472	2	0.0010	12	1	GluC
H2AY_MOUSE, Q8CA90_MOUSE	Histone H2A	VSKAASADSTTE	8	2.10E-09	583.7807	1165.5469	2	0.0007	12	1	GluC
H2AY_MOUSE, Q8CA90_MOUSE	Histone H2A	VSKAASADSTTE	8	4.70E-05	583.7805	1165.5465	2	0.0002	12	1	GluC
H2AZ_MOUSE	Histone H2A	LDSLIKATIAGGGVIPHIHKSLIGKKGQQKTV	7	1.20E-05	662.4009	3306.9681	5	0.0178	32	1	GluC
H2AZ_MOUSE	Histone H2A	LDSLIKATIAGGGVIPHIHKSLIGKKGQQKTV	7	4.80E-04	827.7488	3306.9661	4	0.0157	32	1	GluC
H2AZ_MOUSE	Histone H2A	LDSLIKATIAGGGVIPHIHKSLIGKKGQQKTV	7	1.60E-04	827.7483	3306.9643	4	0.0139	32	1	GluC
H2AZ_MOUSE	Histone H2A	LDSLIKATIAGGGVIPHIHKSLIGKKGQQKTV	7	2.20E-04	662.3978	3306.9524	5	0.0021	32	1	GluC
H2AZ_MOUSE	Histone H2A	LDSLIKATIAGGGVIPHIHKSLIGKKGQQKTV	7	1.90E-03	662.3976	3306.9515	5	0.0012	32	1	GluC
H2AZ_MOUSE	Histone H2A	LDSLIKATIAGGGVIPHIHKSLIGKKGQQKTV	7	5.10E-03	827.7449	3306.9504	4	0.0000	32	1	GluC
H2AZ_MOUSE	Histone H2A	LDSLIKATIAGGGVIPHIHKSLIGKKGQQKTV	7	1.10E-05	827.7449	3306.9504	4	0.0000	32	1	GluC
H2AZ_MOUSE	Histone H2A	DSLIKATIAGGGVIPHIHKSLIGKKGQQKTV	11	1.80E-07	799.473	3193.8631	4	-0.0032	31	0	AspN
H2AZ_MOUSE	Histone H2A	DSLIKATIAGGGVIPHIHKSLIGKKGQQKTV	11	3.10E-03	799.4782	3193.8838	4	0.0175	31	0	AspN
H2AZ_MOUSE	Histone H2A	DSLIKATIAGGGVIPHIHKSLIGKKGQQKTV	11	4.40E-03	1065.6333	3193.8781	3	0.0118	31	0	AspN
H2AZ_MOUSE	Histone H2A	DSLIKATIAGGGVIPHIHKSLIGKKGQQKTV	11	2.90E-07	799.4762	3193.8759	4	0.0096	31	0	AspN
H2AZ_MOUSE	Histone H2A	DSLIKATIAGGGVIPHIHKSLIGKKGQQKTV	11	7.30E-04	639.7823	3193.8751	5	0.0088	31	0	AspN
H2AZ_MOUSE	Histone H2A	DSLIKATIAGGGVIPHIHKSLIGKKGQQKTV	11	2.20E-04	1065.6323	3193.8751	3	0.0088	31	0	AspN
H2AZ_MOUSE	Histone H2A	DSLIKATIAGGGVIPHIHKSLIGKKGQQKTV	11	6.10E-03	639.7823	3193.8750	5	0.0087	31	0	AspN
H2AZ_MOUSE	Histone H2A	DSLIKATIAGGGVIPHIHKSLIGKKGQQKTV	11	3.50E-05	639.7823	3193.8750	5	0.0087	31	0	AspN
H2AZ_MOUSE	Histone H2A	DSLIKATIAGGGVIPHIHKSLIGKKGQQKTV	11	2.90E-06	799.4755	3193.8730	4	0.0067	31	0	AspN
H2AZ_MOUSE	Histone H2A	DSLIKATIAGGGVIPHIHKSLIGKKGQQKTV	11	7.60E-03	799.4745	3193.8691	4	0.0028	31	0	AspN
H2AZ_MOUSE	Histone H2A	DSLIKATIAGGGVIPHIHKSLIGKKGQQKTV	11	3.80E-07	799.4745	3193.8691	4	0.0028	31	0	AspN
H2AZ_MOUSE	Histone H2A	SLIKATIAGGGVIPHIHKSLIGKKGQQKTV	7	9.40E-07	616.7766	3078.8467	5	0.0073	30	0	GluC
H2AZ_MOUSE	Histone H2A	SLIKATIAGGGVIPHIHKSLIGKKGQQKTV	7	1.70E-03	1027.2885	3078.8437	3	0.0043	30	0	GluC
H2AZ_MOUSE	Histone H2A	SLIKATIAGGGVIPHIHKSLIGKKGQQKTV	7	1.10E-08	770.7177	3078.8418	4	0.0025	30	0	GluC
H2AZ_MOUSE	Histone H2A	SLIKATIAGGGVIPHIHKSLIGKKGQQKTV	7	1.10E-03	770.7177	3078.8417	4	0.0023	30	0	GluC
H2AZ_MOUSE	Histone H2A	SLIKATIAGGGVIPHIHKSLIGKKGQQKTV	7	6.90E-10	616.775	3078.8386	5	-0.0008	30	0	GluC
H2AZ_MOUSE	Histone H2A	SLIKATIAGGGVIPHIHKSLIGKKGQQKTV	7	5.60E-03	770.7166	3078.8373	4	-0.0020	30	0	GluC
H2AZ_MOUSE	Histone H2A	SLIKATIAGGGVIPHIHKSLIGKKGQQKTV	7	2.40E-06	770.7166	3078.8373	4	-0.0020	30	0	GluC
Q8CA90_MOUSE	Histone H2A	AIITPPPAKKAKSPSQKKPVAKKTGGKKGARKSKKQGE	2	1.60E-03	791.6785	3953.3563	5	-0.0178	38	0	GluC
Q8CA90_MOUSE	Histone H2A	AIITPPPAKKAKSPSQKKPVAKKTGGKKGARKSKKQGE	2	5.30E-04	659.8994	3953.3527	6	-0.0214	38	0	GluC
Q8CA90_MOUSE	Histone H2A	DGFTVLSTKSLFLGQKPPQ	2	6.00E-06	688.3787	2062.1143	3	0.0044	19	0	AspN
Q8CA90_MOUSE	Histone H2A	DGFTVLSTKSLFLGQKPPQ	2	4.30E-07	1032.0638	2062.1130	2	0.0031	19	0	AspN
Q8CA90_MOUSE	Histone H2A	SLFLGQKPPQ	4	9.60E-03	557.8182	1113.6219	2	0.0036	10	0	Semi-tryptic
Q8CA90_MOUSE	Histone H2A	SLFLGQKPPQ	4	2.40E-05	557.8168	1113.6190	2	0.0008	10	0	Semi-tryptic
Q8CA90_MOUSE	Histone H2A	SLFLGQKPPQ	4	1.40E-04	557.8166	1113.6186	2	0.0004	10	0	Semi-tryptic

Q8CA90_MOUSE	Histone H2A	SLFLGQKPPQ	4	3.70E-06	557.816	1113.6173	2	-0.0009	10	0	Semi-tryptic
Q8CA90_MOUSE	Histone H2A	LGQKPPQ	2	2.00E-03	384.2244	766.4341	2	0.0004	7	1	Chymotrypsin
Q8CA90_MOUSE	Histone H2A	LGQKPPQ	2	6.70E-04	384.2243	766.4341	2	0.0004	7	1	Chymotrypsin
H2B1A_MOUSE, H2B1B_MOUSE, H2B1C_MOUSE, H2B1F_MOUSE, H2B1H_MOUSE, H2B1K_MOUSE, H2B1M_MOUSE, H2B1P_MOUSE, H2B2B_MOUSE	Histone H2B	ASRLAHYNKRSTITSREIQTAVRLLLPGELAKHAVSE	10	1.30E-08	824.0651	4115.2891	5	0.0176	37	2	GluC
H2B1A_MOUSE, H2B1B_MOUSE, H2B1C_MOUSE, H2B1F_MOUSE, H2B1H_MOUSE, H2B1K_MOUSE, H2B1M_MOUSE, H2B1P_MOUSE, H2B2B_MOUSE	Histone H2B	ASRLAHYNKRSTITSREIQTAVRLLLPGELAKHAVSE	10	1.40E-05	686.8888	4115.2889	6	0.0174	37	2	GluC
H2B1A_MOUSE, H2B1B_MOUSE, H2B1C_MOUSE, H2B1F_MOUSE, H2B1H_MOUSE, H2B1K_MOUSE, H2B1M_MOUSE, H2B1P_MOUSE, H2B2B_MOUSE	Histone H2B	ASRLAHYNKRSTITSREIQTAVRLLLPGELAKHAVSE	10	4.70E-04	824.0649	4115.2881	5	0.0166	37	2	GluC
H2B1A_MOUSE, H2B1B_MOUSE, H2B1C_MOUSE, H2B1F_MOUSE, H2B1H_MOUSE, H2B1K_MOUSE, H2B1M_MOUSE, H2B1P_MOUSE, H2B2B_MOUSE	Histone H2B	ASRLAHYNKRSTITSREIQTAVRLLLPGELAKHAVSE	10	3.80E-03	588.9055	4115.2876	7	0.0161	37	2	GluC
H2B1A_MOUSE, H2B1B_MOUSE, H2B1C_MOUSE, H2B1F_MOUSE, H2B1H_MOUSE, H2B1K_MOUSE, H2B1M_MOUSE, H2B1P_MOUSE, H2B2B_MOUSE	Histone H2B	ASRLAHYNKRSTITSREIQTAVRLLLPGELAKHAVSE	10	1.80E-03	686.8882	4115.2856	6	0.0141	37	2	GluC
H2B1A_MOUSE, H2B1B_MOUSE, H2B1C_MOUSE, H2B1F_MOUSE, H2B1H_MOUSE, H2B1K_MOUSE, H2B1M_MOUSE, H2B1P_MOUSE, H2B2B_MOUSE	Histone H2B	ASRLAHYNKRSTITSREIQTAVRLLLPGELAKHAVSE	10	5.70E-06	824.0643	4115.2849	5	0.0134	37	2	GluC
H2B1A_MOUSE, H2B1B_MOUSE, H2B1C_MOUSE, H2B1F_MOUSE, H2B1H_MOUSE, H2B1K_MOUSE, H2B1M_MOUSE, H2B1P_MOUSE, H2B2B_MOUSE	Histone H2B	ASRLAHYNKRSTITSREIQTAVRLLLPGELAKHAVSE	10	1.40E-05	824.0625	4115.2762	5	0.0047	37	2	GluC
H2B1A_MOUSE, H2B1B_MOUSE, H2B1C_MOUSE, H2B1F_MOUSE, H2B1H_MOUSE, H2B1K_MOUSE, H2B1M_MOUSE, H2B1P_MOUSE, H2B2B_MOUSE	Histone H2B	ASRLAHYNKRSTITSREIQTAVRLLLPGELAKHAVSE	10	5.10E-03	824.062	4115.2736	5	0.0021	37	2	GluC
H2B1A_MOUSE, H2B1B_MOUSE, H2B1C_MOUSE, H2B1F_MOUSE, H2B1H_MOUSE, H2B1K_MOUSE, H2B1M_MOUSE, H2B1P_MOUSE, H2B2B_MOUSE	Histone H2B	ASRLAHYNKRSTITSREIQTAVRLLLPGELAKHAVSE	10	2.50E-03	686.8858	4115.2713	6	-0.0002	37	2	GluC
H2B1A_MOUSE, H2B1B_MOUSE, H2B1C_MOUSE, H2B1F_MOUSE, H2B1H_MOUSE, H2B1K_MOUSE, H2B1M_MOUSE, H2B1P_MOUSE, H2B2B_MOUSE	Histone H2B	ASRLAHYNKRSTITSREIQTAVRLLLPGELAKHAVSE	10	1.40E-06	824.0615	4115.2711	5	-0.0004	37	2	GluC
H2B1A_MOUSE, H2B1B_MOUSE, H2B1C_MOUSE, H2B1F_MOUSE, H2B1H_MOUSE, H2B1K_MOUSE, H2B1M_MOUSE, H2B1P_MOUSE, H2B2B_MOUSE	Histone H2B	ASRLAHYNKRSTITSREIQTAVRLLLPGE	14	8.10E-03	820.9662	3279.8358	4	0.0195	29	1	GluC
H2B1A_MOUSE, H2B1B_MOUSE, H2B1C_MOUSE, H2B1F_MOUSE, H2B1H_MOUSE, H2B1K_MOUSE, H2B1M_MOUSE, H2B1P_MOUSE, H2B2B_MOUSE	Histone H2B	ASRLAHYNKRSTITSREIQTAVRLLLPGE	14	2.90E-04	656.9744	3279.8355	5	0.0191	29	1	GluC
H2B1A_MOUSE, H2B1B_MOUSE, H2B1C_MOUSE, H2B1F_MOUSE, H2B1H_MOUSE, H2B1K_MOUSE, H2B1M_MOUSE, H2B1P_MOUSE, H2B2B_MOUSE	Histone H2B	ASRLAHYNKRSTITSREIQTAVRLLLPGE	14	5.20E-06	820.9657	3279.8337	4	0.0174	29	1	GluC
H2B1A_MOUSE, H2B1B_MOUSE, H2B1C_MOUSE, H2B1F_MOUSE, H2B1H_MOUSE, H2B1K_MOUSE, H2B1M_MOUSE, H2B1P_MOUSE, H2B2B_MOUSE	Histone H2B	ASRLAHYNKRSTITSREIQTAVRLLLPGE	14	1.60E-06	820.9657	3279.8335	4	0.0172	29	1	GluC
H2B1A_MOUSE, H2B1B_MOUSE, H2B1C_MOUSE, H2B1F_MOUSE, H2B1H_MOUSE, H2B1K_MOUSE, H2B1M_MOUSE, H2B1P_MOUSE, H2B2B_MOUSE	Histone H2B	ASRLAHYNKRSTITSREIQTAVRLLLPGE	14	1.70E-07	820.9652	3279.8319	4	0.0156	29	1	GluC
H2B1A_MOUSE, H2B1B_MOUSE, H2B1C_MOUSE, H2B1F_MOUSE, H2B1H_MOUSE, H2B1K_MOUSE, H2B1M_MOUSE, H2B1P_MOUSE, H2B2B_MOUSE	Histone H2B	ASRLAHYNKRSTITSREIQTAVRLLLPGE	14	8.20E-04	820.9651	3279.8315	4	0.0152	29	1	GluC
H2B1A_MOUSE, H2B1B_MOUSE, H2B1C_MOUSE, H2B1F_MOUSE, H2B1H_MOUSE, H2B1K_MOUSE, H2B1M_MOUSE, H2B1P_MOUSE, H2B2B_MOUSE	Histone H2B	ASRLAHYNKRSTITSREIQTAVRLLLPGE	14	3.90E-05	656.9736	3279.8315	5	0.0151	29	1	GluC
H2B1A_MOUSE, H2B1B_MOUSE, H2B1C_MOUSE, H2B1F_MOUSE, H2B1H_MOUSE, H2B1K_MOUSE, H2B1M_MOUSE, H2B1P_MOUSE, H2B2B_MOUSE	Histone H2B	ASRLAHYNKRSTITSREIQTAVRLLLPGE	14	5.50E-07	820.9647	3279.8297	4	0.0134	29	1	GluC
H2B1A_MOUSE, H2B1B_MOUSE, H2B1C_MOUSE, H2B1F_MOUSE, H2B1H_MOUSE, H2B1K_MOUSE, H2B1M_MOUSE, H2B1P_MOUSE, H2B2B_MOUSE	Histone H2B	ASRLAHYNKRSTITSREIQTAVRLLLPGE	14	1.50E-06	656.9731	3279.8290	5	0.0127	29	1	GluC
H2B1A_MOUSE, H2B1B_MOUSE, H2B1C_MOUSE, H2B1F_MOUSE, H2B1H_MOUSE, H2B1K_MOUSE, H2B1M_MOUSE, H2B1P_MOUSE, H2B2B_MOUSE	Histone H2B	ASRLAHYNKRSTITSREIQTAVRLLLPGE	14	4.40E-06	820.9643	3279.8280	4	0.0117	29	1	GluC
H2B1A_MOUSE, H2B1B_MOUSE, H2B1C_MOUSE, H2B1F_MOUSE, H2B1H_MOUSE, H2B1K_MOUSE, H2B1M_MOUSE, H2B1P_MOUSE, H2B2B_MOUSE	Histone H2B	ASRLAHYNKRSTITSREIQTAVRLLLPGE	14	8.90E-06	820.9642	3279.8276	4	0.0112	29	1	GluC
H2B1A_MOUSE, H2B1B_MOUSE, H2B1C_MOUSE, H2B1F_MOUSE, H2B1H_MOUSE, H2B1K_MOUSE, H2B1M_MOUSE, H2B1P_MOUSE, H2B2B_MOUSE	Histone H2B	ASRLAHYNKRSTITSREIQTAVRLLLPGE	14	1.90E-03	820.9638	3279.8261	4	0.0098	29	1	GluC
H2B1A_MOUSE, H2B1B_MOUSE, H2B1C_MOUSE, H2B1F_MOUSE, H2B1H_MOUSE, H2B1K_MOUSE, H2B1M_MOUSE, H2B1P_MOUSE, H2B2B_MOUSE	Histone H2B	ASRLAHYNKRSTITSREIQTAVRLLLPGE	14	8.80E-04	820.9634	3279.8245	4	0.0082	29	1	GluC

H2B1A_MOUSE, H2B1B_MOUSE, H2B1C_MOUSE, H2B1F_MOUSE, H2B1H_MOUSE, H2B1K_MOUSE, H2B1M_MOUSE, H2B1P_MOUSE, H2B2B_MOUSE	Histone H2B	ASRLAHYNKRSTITSREIQTAVRLLLPGE	14	4.80E-06	820.9624	3279.8205	4	0.0042	29	1	GluC
H2B1A_MOUSE, H2B1B_MOUSE, H2B1C_MOUSE, H2B1F_MOUSE, H2B1H_MOUSE, H2B1K_MOUSE, H2B1M_MOUSE, H2B1P_MOUSE, H2B2B_MOUSE	Histone H2B	EIQTAVRLLLPGELAKHAVSEGTKAVTK	2	7.90E-03	740.6799	2958.6903	4	0.0038	28	3	Semi-tryptic
H2B1A_MOUSE, H2B1B_MOUSE, H2B1C_MOUSE, H2B1F_MOUSE, H2B1H_MOUSE, H2B1K_MOUSE, H2B1M_MOUSE, H2B1P_MOUSE, H2B2B_MOUSE	Histone H2B	EIQTAVRLLLPGELAKHAVSEGTKAVTK	2	4.90E-04	740.6794	2958.6885	4	0.0020	28	3	Semi-tryptic
H2B1A_MOUSE, H2B1B_MOUSE, H2B1C_MOUSE, H2B1F_MOUSE, H2B1H_MOUSE, H2B1K_MOUSE, H2B1M_MOUSE, H2B1P_MOUSE, H2B2B_MOUSE	Histone H2B	EIQTAVRLLLPGELAKHAVSEGTK	2	1.80E-05	854.1551	2559.4435	3	0.0051	24	2	Semi-tryptic
H2B1A_MOUSE, H2B1B_MOUSE, H2B1C_MOUSE, H2B1F_MOUSE, H2B1H_MOUSE, H2B1K_MOUSE, H2B1M_MOUSE, H2B1P_MOUSE, H2B2B_MOUSE	Histone H2B	EIQTAVRLLLPGELAKHAVSEGTK	2	7.80E-07	854.1546	2559.4419	3	0.0035	24	2	Semi-tryptic
H2B1A_MOUSE, H2B1B_MOUSE, H2B1C_MOUSE, H2B1F_MOUSE, H2B1H_MOUSE, H2B1K_MOUSE, H2B1M_MOUSE, H2B1P_MOUSE, H2B2B_MOUSE	Histone H2B	STITSREIQTAVRLLLPGELAK	12	3.90E-04	799.4693	2395.3862	3	0.0064	22	2	Semi-tryptic
H2B1A_MOUSE, H2B1B_MOUSE, H2B1C_MOUSE, H2B1F_MOUSE, H2B1H_MOUSE, H2B1K_MOUSE, H2B1M_MOUSE, H2B1P_MOUSE, H2B2B_MOUSE	Histone H2B	STITSREIQTAVRLLLPGELAK	12	5.80E-06	799.4693	2395.3862	3	0.0064	22	2	Semi-tryptic
H2B1A_MOUSE, H2B1B_MOUSE, H2B1C_MOUSE, H2B1F_MOUSE, H2B1H_MOUSE, H2B1K_MOUSE, H2B1M_MOUSE, H2B1P_MOUSE, H2B2B_MOUSE	Histone H2B	STITSREIQTAVRLLLPGELAK	12	6.50E-06	799.469	2395.3852	3	0.0054	22	2	Semi-tryptic
H2B1A_MOUSE, H2B1B_MOUSE, H2B1C_MOUSE, H2B1F_MOUSE, H2B1H_MOUSE, H2B1K_MOUSE, H2B1M_MOUSE, H2B1P_MOUSE, H2B2B_MOUSE	Histone H2B	STITSREIQTAVRLLLPGELAK	12	4.80E-03	799.4687	2395.3842	3	0.0044	22	2	Semi-tryptic
H2B1A_MOUSE, H2B1B_MOUSE, H2B1C_MOUSE, H2B1F_MOUSE, H2B1H_MOUSE, H2B1K_MOUSE, H2B1M_MOUSE, H2B1P_MOUSE, H2B2B_MOUSE	Histone H2B	STITSREIQTAVRLLLPGELAK	12	2.40E-06	599.8532	2395.3835	4	0.0037	22	2	Semi-tryptic
H2B1A_MOUSE, H2B1B_MOUSE, H2B1C_MOUSE, H2B1F_MOUSE, H2B1H_MOUSE, H2B1K_MOUSE, H2B1M_MOUSE, H2B1P_MOUSE, H2B2B_MOUSE	Histone H2B	STITSREIQTAVRLLLPGELAK	12	3.90E-08	599.8532	2395.3835	4	0.0037	22	2	Semi-tryptic
H2B1A_MOUSE, H2B1B_MOUSE, H2B1C_MOUSE, H2B1F_MOUSE, H2B1H_MOUSE, H2B1K_MOUSE, H2B1M_MOUSE, H2B1P_MOUSE, H2B2B_MOUSE	Histone H2B	STITSREIQTAVRLLLPGELAK	12	9.90E-05	799.4683	2395.3832	3	0.0034	22	2	Semi-tryptic
H2B1A_MOUSE, H2B1B_MOUSE, H2B1C_MOUSE, H2B1F_MOUSE, H2B1H_MOUSE, H2B1K_MOUSE, H2B1M_MOUSE, H2B1P_MOUSE, H2B2B_MOUSE	Histone H2B	STITSREIQTAVRLLLPGELAK	12	3.60E-05	799.4681	2395.3824	3	0.0026	22	2	Semi-tryptic
H2B1A_MOUSE, H2B1B_MOUSE, H2B1C_MOUSE, H2B1F_MOUSE, H2B1H_MOUSE, H2B1K_MOUSE, H2B1M_MOUSE, H2B1P_MOUSE, H2B2B_MOUSE	Histone H2B	STITSREIQTAVRLLLPGELAK	12	1.20E-05	799.4681	2395.3824	3	0.0025	22	2	Semi-tryptic
H2B1A_MOUSE, H2B1B_MOUSE, H2B1C_MOUSE, H2B1F_MOUSE, H2B1H_MOUSE, H2B1K_MOUSE, H2B1M_MOUSE, H2B1P_MOUSE, H2B2B_MOUSE	Histone H2B	STITSREIQTAVRLLLPGELAK	12	2.10E-04	799.4678	2395.3815	3	0.0017	22	2	Semi-tryptic
H2B1A_MOUSE, H2B1B_MOUSE, H2B1C_MOUSE, H2B1F_MOUSE, H2B1H_MOUSE, H2B1K_MOUSE, H2B1M_MOUSE, H2B1P_MOUSE, H2B2B_MOUSE	Histone H2B	STITSREIQTAVRLLLPGELAK	12	5.80E-05	799.4676	2395.3810	3	0.0012	22	2	Semi-tryptic
H2B1A_MOUSE, H2B1B_MOUSE, H2B1C_MOUSE, H2B1F_MOUSE, H2B1H_MOUSE, H2B1K_MOUSE, H2B1M_MOUSE, H2B1P_MOUSE, H2B2B_MOUSE	Histone H2B	STITSREIQTAVRLLLPGELAK	12	7.70E-03	799.4675	2395.3807	3	0.0009	22	2	Semi-tryptic
H2B1A_MOUSE, H2B1B_MOUSE, H2B1C_MOUSE, H2B1F_MOUSE, H2B1H_MOUSE, H2B1K_MOUSE, H2B1M_MOUSE, H2B1P_MOUSE, H2B2B_MOUSE	Histone H2B	LAHYNKRSTITSREIQTAVR	2	2.60E-07	586.8267	2343.2776	4	0.0005	20	3	Semi-tryptic
H2B1A_MOUSE, H2B1B_MOUSE, H2B1C_MOUSE, H2B1F_MOUSE, H2B1H_MOUSE, H2B1K_MOUSE, H2B1M_MOUSE, H2B1P_MOUSE, H2B2B_MOUSE	Histone H2B	LAHYNKRSTITSREIQTAVR	2	1.50E-08	586.8267	2343.2776	4	0.0005	20	3	Semi-tryptic
H2B1A_MOUSE, H2B1B_MOUSE, H2B1C_MOUSE, H2B1F_MOUSE, H2B1H_MOUSE, H2B1K_MOUSE, H2B1M_MOUSE, H2B1P_MOUSE, H2B2B_MOUSE	Histone H2B	IQTAVRLLLPGELAKHAVSE	35	3.90E-07	537.0637	2144.2258	4	-0.0059	20	1	GluC
H2B1A_MOUSE, H2B1B_MOUSE, H2B1C_MOUSE, H2B1F_MOUSE, H2B1H_MOUSE, H2B1K_MOUSE, H2B1M_MOUSE, H2B1P_MOUSE, H2B2B_MOUSE	Histone H2B	IQTAVRLLLPGELAKHAVSE	35	5.60E-05	715.7559	2144.2458	3	0.0141	20	1	GluC
H2B1A_MOUSE, H2B1B_MOUSE, H2B1C_MOUSE, H2B1F_MOUSE, H2B1H_MOUSE, H2B1K_MOUSE, H2B1M_MOUSE, H2B1P_MOUSE, H2B2B_MOUSE	Histone H2B	IQTAVRLLLPGELAKHAVSE	35	4.40E-10	537.0685	2144.2448	4	0.0131	20	1	GluC
H2B1A_MOUSE, H2B1B_MOUSE, H2B1C_MOUSE, H2B1F_MOUSE, H2B1H_MOUSE, H2B1K_MOUSE, H2B1M_MOUSE, H2B1P_MOUSE, H2B2B_MOUSE	Histone H2B	IQTAVRLLLPGELAKHAVSE	35	6.60E-05	715.7553	2144.2442	3	0.0125	20	1	GluC
H2B1A_MOUSE, H2B1B_MOUSE, H2B1C_MOUSE, H2B1F_MOUSE, H2B1H_MOUSE, H2B1K_MOUSE, H2B1M_MOUSE, H2B1P_MOUSE, H2B2B_MOUSE	Histone H2B	IQTAVRLLLPGELAKHAVSE	35	1.10E-05	715.7548	2144.2426	3	0.0109	20	1	GluC

H2B1A_MOUSE, H2B1B_MOUSE, H2B1C_MOUSE, H2B1F_MOUSE, H2B1H_MOUSE, H2B1K_MOUSE, H2B1M_MOUSE, H2B1P_MOUSE, H2B2B_MOUSE	Histone H2B	IQTAVRLLLPGELAKHAVSE	35	4.00E-06	715.7545	2144.2417	3	0.0100	20	1	GluC
H2B1A_MOUSE, H2B1B_MOUSE, H2B1C_MOUSE, H2B1F_MOUSE, H2B1H_MOUSE, H2B1K_MOUSE, H2B1M_MOUSE, H2B1P_MOUSE, H2B2B_MOUSE	Histone H2B	IQTAVRLLLPGELAKHAVSE	35	4.40E-04	537.0675	2144.2409	4	0.0092	20	1	GluC
H2B1A_MOUSE, H2B1B_MOUSE, H2B1C_MOUSE, H2B1F_MOUSE, H2B1H_MOUSE, H2B1K_MOUSE, H2B1M_MOUSE, H2B1P_MOUSE, H2B2B_MOUSE	Histone H2B	IQTAVRLLLPGELAKHAVSE	35	2.30E-08	715.754	2144.2402	3	0.0085	20	1	GluC
H2B1A_MOUSE, H2B1B_MOUSE, H2B1C_MOUSE, H2B1F_MOUSE, H2B1H_MOUSE, H2B1K_MOUSE, H2B1M_MOUSE, H2B1P_MOUSE, H2B2B_MOUSE	Histone H2B	IQTAVRLLLPGELAKHAVSE	35	1.10E-03	715.7539	2144.2400	3	0.0083	20	1	GluC
H2B1A_MOUSE, H2B1B_MOUSE, H2B1C_MOUSE, H2B1F_MOUSE, H2B1H_MOUSE, H2B1K_MOUSE, H2B1M_MOUSE, H2B1P_MOUSE, H2B2B_MOUSE	Histone H2B	IQTAVRLLLPGELAKHAVSE	35	3.30E-09	537.0672	2144.2399	4	0.0082	20	1	GluC
H2B1A_MOUSE, H2B1B_MOUSE, H2B1C_MOUSE, H2B1F_MOUSE, H2B1H_MOUSE, H2B1K_MOUSE, H2B1M_MOUSE, H2B1P_MOUSE, H2B2B_MOUSE	Histone H2B	IQTAVRLLLPGELAKHAVSE	35	3.80E-11	537.0672	2144.2399	4	0.0082	20	1	GluC
H2B1A_MOUSE, H2B1B_MOUSE, H2B1C_MOUSE, H2B1F_MOUSE, H2B1H_MOUSE, H2B1K_MOUSE, H2B1M_MOUSE, H2B1P_MOUSE, H2B2B_MOUSE	Histone H2B	IQTAVRLLLPGELAKHAVSE	35	9.80E-08	715.7539	2144.2398	3	0.0081	20	1	GluC
H2B1A_MOUSE, H2B1B_MOUSE, H2B1C_MOUSE, H2B1F_MOUSE, H2B1H_MOUSE, H2B1K_MOUSE, H2B1M_MOUSE, H2B1P_MOUSE, H2B2B_MOUSE	Histone H2B	IQTAVRLLLPGELAKHAVSE	35	2.10E-04	1073.1271	2144.2396	2	0.0079	20	1	GluC
H2B1A_MOUSE, H2B1B_MOUSE, H2B1C_MOUSE, H2B1F_MOUSE, H2B1H_MOUSE, H2B1K_MOUSE, H2B1M_MOUSE, H2B1P_MOUSE, H2B2B_MOUSE	Histone H2B	IQTAVRLLLPGELAKHAVSE	35	3.20E-06	537.0671	2144.2395	4	0.0078	20	1	GluC
H2B1A_MOUSE, H2B1B_MOUSE, H2B1C_MOUSE, H2B1F_MOUSE, H2B1H_MOUSE, H2B1K_MOUSE, H2B1M_MOUSE, H2B1P_MOUSE, H2B2B_MOUSE	Histone H2B	IQTAVRLLLPGELAKHAVSE	35	3.00E-05	715.7537	2144.2392	3	0.0074	20	1	GluC
H2B1A_MOUSE, H2B1B_MOUSE, H2B1C_MOUSE, H2B1F_MOUSE, H2B1H_MOUSE, H2B1K_MOUSE, H2B1M_MOUSE, H2B1P_MOUSE, H2B2B_MOUSE	Histone H2B	IQTAVRLLLPGELAKHAVSE	35	9.70E-10	537.0666	2144.2375	4	0.0058	20	1	GluC
H2B1A_MOUSE, H2B1B_MOUSE, H2B1C_MOUSE, H2B1F_MOUSE, H2B1H_MOUSE, H2B1K_MOUSE, H2B1M_MOUSE, H2B1P_MOUSE, H2B2B_MOUSE	Histone H2B	IQTAVRLLLPGELAKHAVSE	35	8.30E-05	1073.1253	2144.2360	2	0.0043	20	1	GluC
H2B1A_MOUSE, H2B1B_MOUSE, H2B1C_MOUSE, H2B1F_MOUSE, H2B1H_MOUSE, H2B1K_MOUSE, H2B1M_MOUSE, H2B1P_MOUSE, H2B2B_MOUSE	Histone H2B	IQTAVRLLLPGELAKHAVSE	35	4.00E-03	1073.1249	2144.2352	2	0.0035	20	1	GluC
H2B1A_MOUSE, H2B1B_MOUSE, H2B1C_MOUSE, H2B1F_MOUSE, H2B1H_MOUSE, H2B1K_MOUSE, H2B1M_MOUSE, H2B1P_MOUSE, H2B2B_MOUSE	Histone H2B	IQTAVRLLLPGELAKHAVSE	35	3.50E-03	715.7522	2144.2347	3	0.0029	20	1	GluC
H2B1A_MOUSE, H2B1B_MOUSE, H2B1C_MOUSE, H2B1F_MOUSE, H2B1H_MOUSE, H2B1K_MOUSE, H2B1M_MOUSE, H2B1P_MOUSE, H2B2B_MOUSE	Histone H2B	IQTAVRLLLPGELAKHAVSE	35	5.10E-04	715.7522	2144.2347	3	0.0029	20	1	GluC
H2B1A_MOUSE, H2B1B_MOUSE, H2B1C_MOUSE, H2B1F_MOUSE, H2B1H_MOUSE, H2B1K_MOUSE, H2B1M_MOUSE, H2B1P_MOUSE, H2B2B_MOUSE	Histone H2B	IQTAVRLLLPGELAKHAVSE	35	2.80E-04	715.7521	2144.2344	3	0.0027	20	1	GluC
H2B1A_MOUSE, H2B1B_MOUSE, H2B1C_MOUSE, H2B1F_MOUSE, H2B1H_MOUSE, H2B1K_MOUSE, H2B1M_MOUSE, H2B1P_MOUSE, H2B2B_MOUSE	Histone H2B	IQTAVRLLLPGELAKHAVSE	35	1.10E-04	1073.1245	2144.2344	2	0.0027	20	1	GluC
H2B1A_MOUSE, H2B1B_MOUSE, H2B1C_MOUSE, H2B1F_MOUSE, H2B1H_MOUSE, H2B1K_MOUSE, H2B1M_MOUSE, H2B1P_MOUSE, H2B2B_MOUSE	Histone H2B	IQTAVRLLLPGELAKHAVSE	35	1.20E-05	715.7521	2144.2344	3	0.0027	20	1	GluC
H2B1A_MOUSE, H2B1B_MOUSE, H2B1C_MOUSE, H2B1F_MOUSE, H2B1H_MOUSE, H2B1K_MOUSE, H2B1M_MOUSE, H2B1P_MOUSE, H2B2B_MOUSE	Histone H2B	IQTAVRLLLPGELAKHAVSE	35	1.20E-07	715.7521	2144.2344	3	0.0027	20	1	GluC
H2B1A_MOUSE, H2B1B_MOUSE, H2B1C_MOUSE, H2B1F_MOUSE, H2B1H_MOUSE, H2B1K_MOUSE, H2B1M_MOUSE, H2B1P_MOUSE, H2B2B_MOUSE	Histone H2B	IQTAVRLLLPGELAKHAVSE	35	4.00E-04	715.7518	2144.2335	3	0.0018	20	1	GluC
H2B1A_MOUSE, H2B1B_MOUSE, H2B1C_MOUSE, H2B1F_MOUSE, H2B1H_MOUSE, H2B1K_MOUSE, H2B1M_MOUSE, H2B1P_MOUSE, H2B2B_MOUSE	Histone H2B	IQTAVRLLLPGELAKHAVSE	35	9.70E-05	715.7518	2144.2335	3	0.0018	20	1	GluC
H2B1A_MOUSE, H2B1B_MOUSE, H2B1C_MOUSE, H2B1F_MOUSE, H2B1H_MOUSE, H2B1K_MOUSE, H2B1M_MOUSE, H2B1P_MOUSE, H2B2B_MOUSE	Histone H2B	IQTAVRLLLPGELAKHAVSE	35	2.10E-04	715.7517	2144.2333	3	0.0016	20	1	GluC
H2B1A_MOUSE, H2B1B_MOUSE, H2B1C_MOUSE, H2B1F_MOUSE, H2B1H_MOUSE, H2B1K_MOUSE, H2B1M_MOUSE, H2B1P_MOUSE, H2B2B_MOUSE	Histone H2B	IQTAVRLLLPGELAKHAVSE	35	2.70E-03	715.7516	2144.2331	3	0.0014	20	1	GluC
H2B1A_MOUSE, H2B1B_MOUSE, H2B1C_MOUSE, H2B1F_MOUSE, H2B1H_MOUSE, H2B1K_MOUSE, H2B1M_MOUSE, H2B1P_MOUSE, H2B2B_MOUSE	Histone H2B	IQTAVRLLLPGELAKHAVSE	35	1.80E-05	715.7516	2144.2331	3	0.0014	20	1	GluC

H2B1A_MOUSE, H2B1B_MOUSE, H2B1C_MOUSE, H2B1F_MOUSE, H2B1H_MOUSE, H2B1K_MOUSE, H2B1M_MOUSE, H2B1P_MOUSE, H2B2B_MOUSE	Histone H2B	IQTAVRLLLPGELAKHAVSE	35	6.10E-06	715.7516	2144.2331	3	0.0014	20	1	GluC
H2B1A_MOUSE, H2B1B_MOUSE, H2B1C_MOUSE, H2B1F_MOUSE, H2B1H_MOUSE, H2B1K_MOUSE, H2B1M_MOUSE, H2B1P_MOUSE, H2B2B_MOUSE	Histone H2B	IQTAVRLLLPGELAKHAVSE	35	5.80E-06	715.7516	2144.2331	3	0.0014	20	1	GluC
H2B1A_MOUSE, H2B1B_MOUSE, H2B1C_MOUSE, H2B1F_MOUSE, H2B1H_MOUSE, H2B1K_MOUSE, H2B1M_MOUSE, H2B1P_MOUSE, H2B2B_MOUSE	Histone H2B	IQTAVRLLLPGELAKHAVSE	35	1.50E-07	537.0653	2144.2321	4	0.0004	20	1	GluC
H2B1A_MOUSE, H2B1B_MOUSE, H2B1C_MOUSE, H2B1F_MOUSE, H2B1H_MOUSE, H2B1K_MOUSE, H2B1M_MOUSE, H2B1P_MOUSE, H2B2B_MOUSE	Histone H2B	IQTAVRLLLPGELAKHAVSE	35	2.10E-04	537.065	2144.2310	4	-0.0007	20	1	GluC
H2B1A_MOUSE, H2B1B_MOUSE, H2B1C_MOUSE, H2B1F_MOUSE, H2B1H_MOUSE, H2B1K_MOUSE, H2B1M_MOUSE, H2B1P_MOUSE, H2B2B_MOUSE	Histone H2B	IQTAVRLLLPGELAKHAVSE	35	6.40E-06	537.065	2144.2310	4	-0.0007	20	1	GluC
H2B1A_MOUSE, H2B1B_MOUSE, H2B1C_MOUSE, H2B1F_MOUSE, H2B1H_MOUSE, H2B1K_MOUSE, H2B1M_MOUSE, H2B1P_MOUSE, H2B2B_MOUSE	Histone H2B	IQTAVRLLLPGELAKHAVSE	35	3.30E-07	537.065	2144.2307	4	-0.0010	20	1	GluC
H2B1A_MOUSE, H2B1B_MOUSE, H2B1C_MOUSE, H2B1F_MOUSE, H2B1H_MOUSE, H2B1K_MOUSE, H2B1M_MOUSE, H2B1P_MOUSE, H2B2B_MOUSE	Histone H2B	NKRSTITSREIQTAVRL	7	3.90E-06	658.3831	1972.1273	3	0.0096	17	0	Chymotrypsin
H2B1A_MOUSE, H2B1B_MOUSE, H2B1C_MOUSE, H2B1F_MOUSE, H2B1H_MOUSE, H2B1K_MOUSE, H2B1M_MOUSE, H2B1P_MOUSE, H2B2B_MOUSE	Histone H2B	NKRSTITSREIQTAVRL	7	3.70E-10	658.383	1972.1271	3	0.0094	17	0	Chymotrypsin
H2B1A_MOUSE, H2B1B_MOUSE, H2B1C_MOUSE, H2B1F_MOUSE, H2B1H_MOUSE, H2B1K_MOUSE, H2B1M_MOUSE, H2B1P_MOUSE, H2B2B_MOUSE	Histone H2B	NKRSTITSREIQTAVRL	7	6.40E-06	658.3829	1972.1270	3	0.0093	17	0	Chymotrypsin
H2B1A_MOUSE, H2B1B_MOUSE, H2B1C_MOUSE, H2B1F_MOUSE, H2B1H_MOUSE, H2B1K_MOUSE, H2B1M_MOUSE, H2B1P_MOUSE, H2B2B_MOUSE	Histone H2B	NKRSTITSREIQTAVRL	7	3.90E-07	658.3821	1972.1245	3	0.0067	17	0	Chymotrypsin
H2B1A_MOUSE, H2B1B_MOUSE, H2B1C_MOUSE, H2B1F_MOUSE, H2B1H_MOUSE, H2B1K_MOUSE, H2B1M_MOUSE, H2B1P_MOUSE, H2B2B_MOUSE	Histone H2B	NKRSTITSREIQTAVRL	7	2.30E-07	494.0376	1972.1215	4	0.0038	17	0	Chymotrypsin
H2B1A_MOUSE, H2B1B_MOUSE, H2B1C_MOUSE, H2B1F_MOUSE, H2B1H_MOUSE, H2B1K_MOUSE, H2B1M_MOUSE, H2B1P_MOUSE, H2B2B_MOUSE	Histone H2B	NKRSTITSREIQTAVRL	7	2.20E-04	494.0376	1972.1213	4	0.0035	17	0	Chymotrypsin
H2B1A_MOUSE, H2B1B_MOUSE, H2B1C_MOUSE, H2B1F_MOUSE, H2B1H_MOUSE, H2B1K_MOUSE, H2B1M_MOUSE, H2B1P_MOUSE, H2B2B_MOUSE	Histone H2B	NKRSTITSREIQTAVRL	7	6.60E-04	658.3798	1972.1176	3	-0.0001	17	0	Chymotrypsin
H2B1A_MOUSE, H2B1B_MOUSE, H2B1C_MOUSE, H2B1F_MOUSE, H2B1H_MOUSE, H2B1K_MOUSE, H2B1M_MOUSE, H2B1P_MOUSE, H2B2B_MOUSE	Histone H2B	EIQTAVRLLLPGELAK	28	5.60E-03	876.0283	1750.0420	2	0.0067	16	1	Semi-tryptic
H2B1A_MOUSE, H2B1B_MOUSE, H2B1C_MOUSE, H2B1F_MOUSE, H2B1H_MOUSE, H2B1K_MOUSE, H2B1M_MOUSE, H2B1P_MOUSE, H2B2B_MOUSE	Histone H2B	EIQTAVRLLLPGELAK	28	3.00E-03	876.0277	1750.0408	2	0.0056	16	1	Semi-tryptic
H2B1A_MOUSE, H2B1B_MOUSE, H2B1C_MOUSE, H2B1F_MOUSE, H2B1H_MOUSE, H2B1K_MOUSE, H2B1M_MOUSE, H2B1P_MOUSE, H2B2B_MOUSE	Histone H2B	EIQTAVRLLLPGELAK	28	4.20E-03	584.3538	1750.0397	3	0.0045	16	1	Semi-tryptic
H2B1A_MOUSE, H2B1B_MOUSE, H2B1C_MOUSE, H2B1F_MOUSE, H2B1H_MOUSE, H2B1K_MOUSE, H2B1M_MOUSE, H2B1P_MOUSE, H2B2B_MOUSE	Histone H2B	EIQTAVRLLLPGELAK	28	1.30E-07	876.0265	1750.0384	2	0.0032	16	1	Semi-tryptic
H2B1A_MOUSE, H2B1B_MOUSE, H2B1C_MOUSE, H2B1F_MOUSE, H2B1H_MOUSE, H2B1K_MOUSE, H2B1M_MOUSE, H2B1P_MOUSE, H2B2B_MOUSE	Histone H2B	EIQTAVRLLLPGELAK	28	1.60E-07	876.0263	1750.0381	2	0.0029	16	1	Semi-tryptic
H2B1A_MOUSE, H2B1B_MOUSE, H2B1C_MOUSE, H2B1F_MOUSE, H2B1H_MOUSE, H2B1K_MOUSE, H2B1M_MOUSE, H2B1P_MOUSE, H2B2B_MOUSE	Histone H2B	EIQTAVRLLLPGELAK	28	5.10E-03	876.0261	1750.0377	2	0.0025	16	1	Semi-tryptic
H2B1A_MOUSE, H2B1B_MOUSE, H2B1C_MOUSE, H2B1F_MOUSE, H2B1H_MOUSE, H2B1K_MOUSE, H2B1M_MOUSE, H2B1P_MOUSE, H2B2B_MOUSE	Histone H2B	EIQTAVRLLLPGELAK	28	1.40E-04	584.3532	1750.0377	3	0.0025	16	1	Semi-tryptic
H2B1A_MOUSE, H2B1B_MOUSE, H2B1C_MOUSE, H2B1F_MOUSE, H2B1H_MOUSE, H2B1K_MOUSE, H2B1M_MOUSE, H2B1P_MOUSE, H2B2B_MOUSE	Histone H2B	EIQTAVRLLLPGELAK	28	8.10E-04	584.3531	1750.0373	3	0.0021	16	1	Semi-tryptic
H2B1A_MOUSE, H2B1B_MOUSE, H2B1C_MOUSE, H2B1F_MOUSE, H2B1H_MOUSE, H2B1K_MOUSE, H2B1M_MOUSE, H2B1P_MOUSE, H2B2B_MOUSE	Histone H2B	EIQTAVRLLLPGELAK	28	8.10E-07	584.3531	1750.0373	3	0.0021	16	1	Semi-tryptic
H2B1A_MOUSE, H2B1B_MOUSE, H2B1C_MOUSE, H2B1F_MOUSE, H2B1H_MOUSE, H2B1K_MOUSE, H2B1M_MOUSE, H2B1P_MOUSE, H2B2B_MOUSE	Histone H2B	EIQTAVRLLLPGELAK	28	1.70E-07	876.0258	1750.0371	2	0.0019	16	1	Semi-tryptic
H2B1A_MOUSE, H2B1B_MOUSE, H2B1C_MOUSE, H2B1F_MOUSE, H2B1H_MOUSE, H2B1K_MOUSE, H2B1M_MOUSE, H2B1P_MOUSE, H2B2B_MOUSE	Histone H2B	EIQTAVRLLLPGELAK	28	1.30E-07	876.0258	1750.0371	2	0.0019	16	1	Semi-tryptic

H2B1A_MOUSE, H2B1B_MOUSE, H2B1C_MOUSE, H2B1F_MOUSE, H2B1H_MOUSE, H2B1K_MOUSE, H2B1M_MOUSE, H2B1P_MOUSE, H2B2B_MOUSE	Histone H2B	EIQTAVRLLLPGELAK	28	6.40E-05	584.3529	1750.0370	3	0.0017	16	1	Semi-tryptic
H2B1A_MOUSE, H2B1B_MOUSE, H2B1C_MOUSE, H2B1F_MOUSE, H2B1H_MOUSE, H2B1K_MOUSE, H2B1M_MOUSE, H2B1P_MOUSE, H2B2B_MOUSE	Histone H2B	EIQTAVRLLLPGELAK	28	5.10E-07	876.0257	1750.0369	2	0.0017	16	1	Semi-tryptic
H2B1A_MOUSE, H2B1B_MOUSE, H2B1C_MOUSE, H2B1F_MOUSE, H2B1H_MOUSE, H2B1K_MOUSE, H2B1M_MOUSE, H2B1P_MOUSE, H2B2B_MOUSE	Histone H2B	EIQTAVRLLLPGELAK	28	1.40E-03	584.3529	1750.0368	3	0.0016	16	1	Semi-tryptic
H2B1A_MOUSE, H2B1B_MOUSE, H2B1C_MOUSE, H2B1F_MOUSE, H2B1H_MOUSE, H2B1K_MOUSE, H2B1M_MOUSE, H2B1P_MOUSE, H2B2B_MOUSE	Histone H2B	EIQTAVRLLLPGELAK	28	8.30E-08	876.0257	1750.0368	2	0.0016	16	1	Semi-tryptic
H2B1A_MOUSE, H2B1B_MOUSE, H2B1C_MOUSE, H2B1F_MOUSE, H2B1H_MOUSE, H2B1K_MOUSE, H2B1M_MOUSE, H2B1P_MOUSE, H2B2B_MOUSE	Histone H2B	EIQTAVRLLLPGELAK	28	5.10E-04	584.3529	1750.0368	3	0.0015	16	1	Semi-tryptic
H2B1A_MOUSE, H2B1B_MOUSE, H2B1C_MOUSE, H2B1F_MOUSE, H2B1H_MOUSE, H2B1K_MOUSE, H2B1M_MOUSE, H2B1P_MOUSE, H2B2B_MOUSE	Histone H2B	EIQTAVRLLLPGELAK	28	7.20E-04	584.3527	1750.0364	3	0.0011	16	1	Semi-tryptic
H2B1A_MOUSE, H2B1B_MOUSE, H2B1C_MOUSE, H2B1F_MOUSE, H2B1H_MOUSE, H2B1K_MOUSE, H2B1M_MOUSE, H2B1P_MOUSE, H2B2B_MOUSE	Histone H2B	EIQTAVRLLLPGELAK	28	7.70E-06	584.3527	1750.0362	3	0.0010	16	1	Semi-tryptic
H2B1A_MOUSE, H2B1B_MOUSE, H2B1C_MOUSE, H2B1F_MOUSE, H2B1H_MOUSE, H2B1K_MOUSE, H2B1M_MOUSE, H2B1P_MOUSE, H2B2B_MOUSE	Histone H2B	EIQTAVRLLLPGELAK	28	1.00E-08	876.0254	1750.0362	2	0.0010	16	1	Semi-tryptic
H2B1A_MOUSE, H2B1B_MOUSE, H2B1C_MOUSE, H2B1F_MOUSE, H2B1H_MOUSE, H2B1K_MOUSE, H2B1M_MOUSE, H2B1P_MOUSE, H2B2B_MOUSE	Histone H2B	EIQTAVRLLLPGELAK	28	9.00E-04	584.3526	1750.0360	3	0.0007	16	1	Semi-tryptic
H2B1A_MOUSE, H2B1B_MOUSE, H2B1C_MOUSE, H2B1F_MOUSE, H2B1H_MOUSE, H2B1K_MOUSE, H2B1M_MOUSE, H2B1P_MOUSE, H2B2B_MOUSE	Histone H2B	EIQTAVRLLLPGELAK	28	1.70E-04	584.3526	1750.0359	3	0.0006	16	1	Semi-tryptic
H2B1A_MOUSE, H2B1B_MOUSE, H2B1C_MOUSE, H2B1F_MOUSE, H2B1H_MOUSE, H2B1K_MOUSE, H2B1M_MOUSE, H2B1P_MOUSE, H2B2B_MOUSE	Histone H2B	EIQTAVRLLLPGELAK	28	6.30E-05	584.3526	1750.0359	3	0.0006	16	1	Semi-tryptic
H2B1A_MOUSE, H2B1B_MOUSE, H2B1C_MOUSE, H2B1F_MOUSE, H2B1H_MOUSE, H2B1K_MOUSE, H2B1M_MOUSE, H2B1P_MOUSE, H2B2B_MOUSE	Histone H2B	EIQTAVRLLLPGELAK	28	7.40E-06	584.3525	1750.0356	3	0.0004	16	1	Semi-tryptic
H2B1A_MOUSE, H2B1B_MOUSE, H2B1C_MOUSE, H2B1F_MOUSE, H2B1H_MOUSE, H2B1K_MOUSE, H2B1M_MOUSE, H2B1P_MOUSE, H2B2B_MOUSE	Histone H2B	EIQTAVRLLLPGELAK	28	1.70E-06	584.3525	1750.0356	3	0.0003	16	1	Semi-tryptic
H2B1A_MOUSE, H2B1B_MOUSE, H2B1C_MOUSE, H2B1F_MOUSE, H2B1H_MOUSE, H2B1K_MOUSE, H2B1M_MOUSE, H2B1P_MOUSE, H2B2B_MOUSE	Histone H2B	EIQTAVRLLLPGELAK	28	1.20E-05	584.3523	1750.0350	3	-0.0002	16	1	Semi-tryptic
H2B1A_MOUSE, H2B1B_MOUSE, H2B1C_MOUSE, H2B1F_MOUSE, H2B1H_MOUSE, H2B1K_MOUSE, H2B1M_MOUSE, H2B1P_MOUSE, H2B2B_MOUSE	Histone H2B	EIQTAVRLLLPGELAK	28	7.60E-06	584.3523	1750.0351	3	-0.0002	16	1	Semi-tryptic
H2B1A_MOUSE, H2B1B_MOUSE, H2B1C_MOUSE, H2B1F_MOUSE, H2B1H_MOUSE, H2B1K_MOUSE, H2B1M_MOUSE, H2B1P_MOUSE, H2B2B_MOUSE	Histone H2B	EIQTAVRLLLPGELAK	28	3.80E-04	584.3522	1750.0349	3	-0.0003	16	1	Semi-tryptic
H2B1A_MOUSE, H2B1B_MOUSE, H2B1C_MOUSE, H2B1F_MOUSE, H2B1H_MOUSE, H2B1K_MOUSE, H2B1M_MOUSE, H2B1P_MOUSE, H2B2B_MOUSE	Histone H2B	EIQTAVRLLLPGELAK	28	9.30E-05	584.3519	1750.0338	3	-0.0014	16	1	Semi-tryptic
H2B1A_MOUSE, H2B1B_MOUSE, H2B1C_MOUSE, H2B1F_MOUSE, H2B1H_MOUSE, H2B1K_MOUSE, H2B1M_MOUSE, H2B1P_MOUSE, H2B2B_MOUSE	Histone H2B	RSTITSREIQTAVR	8	6.90E-04	539.9726	1616.8961	3	0.0003	14	2	Semi-tryptic
H2B1A_MOUSE, H2B1B_MOUSE, H2B1C_MOUSE, H2B1F_MOUSE, H2B1H_MOUSE, H2B1K_MOUSE, H2B1M_MOUSE, H2B1P_MOUSE, H2B2B_MOUSE	Histone H2B	RSTITSREIQTAVR	8	3.10E-05	539.9726	1616.8961	3	0.0003	14	2	Semi-tryptic
H2B1A_MOUSE, H2B1B_MOUSE, H2B1C_MOUSE, H2B1F_MOUSE, H2B1H_MOUSE, H2B1K_MOUSE, H2B1M_MOUSE, H2B1P_MOUSE, H2B2B_MOUSE	Histone H2B	RSTITSREIQTAVR	8	8.60E-07	539.9726	1616.8961	3	0.0003	14	2	Semi-tryptic
H2B1A_MOUSE, H2B1B_MOUSE, H2B1C_MOUSE, H2B1F_MOUSE, H2B1H_MOUSE, H2B1K_MOUSE, H2B1M_MOUSE, H2B1P_MOUSE, H2B2B_MOUSE	Histone H2B	RSTITSREIQTAVR	8	5.60E-04	539.9726	1616.8959	3	0.0001	14	2	Semi-tryptic
H2B1A_MOUSE, H2B1B_MOUSE, H2B1C_MOUSE, H2B1F_MOUSE, H2B1H_MOUSE, H2B1K_MOUSE, H2B1M_MOUSE, H2B1P_MOUSE, H2B2B_MOUSE	Histone H2B	RSTITSREIQTAVR	8	1.10E-03	539.9724	1616.8955	3	-0.0003	14	2	Semi-tryptic
H2B1A_MOUSE, H2B1B_MOUSE, H2B1C_MOUSE, H2B1F_MOUSE, H2B1H_MOUSE, H2B1K_MOUSE, H2B1M_MOUSE, H2B1P_MOUSE, H2B2B_MOUSE	Histone H2B	RSTITSREIQTAVR	8	7.40E-04	539.9723	1616.8952	3	-0.0006	14	2	Semi-tryptic
H2B1A_MOUSE, H2B1B_MOUSE, H2B1C_MOUSE, H2B1F_MOUSE, H2B1H_MOUSE, H2B1K_MOUSE, H2B1M_MOUSE, H2B1P_MOUSE, H2B2B_MOUSE	Histone H2B	RSTITSREIQTAVR	8	3.60E-03	539.9722	1616.8948	3	-0.0010	14	2	Semi-tryptic

H2B1A_MOUSE, H2B1B_MOUSE, H2B1C_MOUSE, H2B1F_MOUSE, H2B1H_MOUSE, H2B1K_MOUSE, H2B1M_MOUSE, H2B1P_MOUSE, H2B2B_MOUSE	Histone H2B	RSTITSREIQTAVR	8	5.30E-03	539.9721	1616.8946	3	-0.0012	14	2	Semi-tryptic
H2B1A_MOUSE, H2B1B_MOUSE, H2B1C_MOUSE, H2B1F_MOUSE, H2B1H_MOUSE, H2B1K_MOUSE, H2B1M_MOUSE, H2B1P_MOUSE, H2B2B_MOUSE	Histone H2B	STITSREIQTAVR	29	2.30E-06	731.4073	1460.8001	2	0.0054	13	1	Semi-tryptic
H2B1A_MOUSE, H2B1B_MOUSE, H2B1C_MOUSE, H2B1F_MOUSE, H2B1H_MOUSE, H2B1K_MOUSE, H2B1M_MOUSE, H2B1P_MOUSE, H2B2B_MOUSE	Histone H2B	STITSREIQTAVR	29	1.70E-03	487.9403	1460.7992	3	0.0045	13	1	Semi-tryptic
H2B1A_MOUSE, H2B1B_MOUSE, H2B1C_MOUSE, H2B1F_MOUSE, H2B1H_MOUSE, H2B1K_MOUSE, H2B1M_MOUSE, H2B1P_MOUSE, H2B2B_MOUSE	Histone H2B	STITSREIQTAVR	29	1.10E-03	487.9402	1460.7988	3	0.0041	13	1	Semi-tryptic
H2B1A_MOUSE, H2B1B_MOUSE, H2B1C_MOUSE, H2B1F_MOUSE, H2B1H_MOUSE, H2B1K_MOUSE, H2B1M_MOUSE, H2B1P_MOUSE, H2B2B_MOUSE	Histone H2B	STITSREIQTAVR	29	7.10E-05	731.4058	1460.7971	2	0.0025	13	1	Semi-tryptic
H2B1A_MOUSE, H2B1B_MOUSE, H2B1C_MOUSE, H2B1F_MOUSE, H2B1H_MOUSE, H2B1K_MOUSE, H2B1M_MOUSE, H2B1P_MOUSE, H2B2B_MOUSE	Histone H2B	STITSREIQTAVR	29	5.30E-03	487.9396	1460.7969	3	0.0022	13	1	Semi-tryptic
H2B1A_MOUSE, H2B1B_MOUSE, H2B1C_MOUSE, H2B1F_MOUSE, H2B1H_MOUSE, H2B1K_MOUSE, H2B1M_MOUSE, H2B1P_MOUSE, H2B2B_MOUSE	Histone H2B	STITSREIQTAVR	29	5.70E-05	731.4056	1460.7966	2	0.0020	13	1	Semi-tryptic
H2B1A_MOUSE, H2B1B_MOUSE, H2B1C_MOUSE, H2B1F_MOUSE, H2B1H_MOUSE, H2B1K_MOUSE, H2B1M_MOUSE, H2B1P_MOUSE, H2B2B_MOUSE	Histone H2B	STITSREIQTAVR	29	1.50E-03	731.4055	1460.7965	2	0.0018	13	1	Semi-tryptic
H2B1A_MOUSE, H2B1B_MOUSE, H2B1C_MOUSE, H2B1F_MOUSE, H2B1H_MOUSE, H2B1K_MOUSE, H2B1M_MOUSE, H2B1P_MOUSE, H2B2B_MOUSE	Histone H2B	STITSREIQTAVR	29	4.30E-05	731.4052	1460.7958	2	0.0011	13	1	Semi-tryptic
H2B1A_MOUSE, H2B1B_MOUSE, H2B1C_MOUSE, H2B1F_MOUSE, H2B1H_MOUSE, H2B1K_MOUSE, H2B1M_MOUSE, H2B1P_MOUSE, H2B2B_MOUSE	Histone H2B	STITSREIQTAVR	29	4.20E-06	731.4052	1460.7957	2	0.0011	13	1	Semi-tryptic
H2B1A_MOUSE, H2B1B_MOUSE, H2B1C_MOUSE, H2B1F_MOUSE, H2B1H_MOUSE, H2B1K_MOUSE, H2B1M_MOUSE, H2B1P_MOUSE, H2B2B_MOUSE	Histone H2B	STITSREIQTAVR	29	3.60E-03	731.4051	1460.7956	2	0.0010	13	1	Semi-tryptic
H2B1A_MOUSE, H2B1B_MOUSE, H2B1C_MOUSE, H2B1F_MOUSE, H2B1H_MOUSE, H2B1K_MOUSE, H2B1M_MOUSE, H2B1P_MOUSE, H2B2B_MOUSE	Histone H2B	STITSREIQTAVR	29	9.50E-06	731.4051	1460.7957	2	0.0010	13	1	Semi-tryptic
H2B1A_MOUSE, H2B1B_MOUSE, H2B1C_MOUSE, H2B1F_MOUSE, H2B1H_MOUSE, H2B1K_MOUSE, H2B1M_MOUSE, H2B1P_MOUSE, H2B2B_MOUSE	Histone H2B	STITSREIQTAVR	29	1.40E-05	731.405	1460.7955	2	0.0009	13	1	Semi-tryptic
H2B1A_MOUSE, H2B1B_MOUSE, H2B1C_MOUSE, H2B1F_MOUSE, H2B1H_MOUSE, H2B1K_MOUSE, H2B1M_MOUSE, H2B1P_MOUSE, H2B2B_MOUSE	Histone H2B	STITSREIQTAVR	29	4.00E-04	731.4048	1460.7950	2	0.0004	13	1	Semi-tryptic
H2B1A_MOUSE, H2B1B_MOUSE, H2B1C_MOUSE, H2B1F_MOUSE, H2B1H_MOUSE, H2B1K_MOUSE, H2B1M_MOUSE, H2B1P_MOUSE, H2B2B_MOUSE	Histone H2B	STITSREIQTAVR	29	1.80E-05	731.4048	1460.7951	2	0.0004	13	1	Semi-tryptic
H2B1A_MOUSE, H2B1B_MOUSE, H2B1C_MOUSE, H2B1F_MOUSE, H2B1H_MOUSE, H2B1K_MOUSE, H2B1M_MOUSE, H2B1P_MOUSE, H2B2B_MOUSE	Histone H2B	STITSREIQTAVR	29	4.30E-06	731.4048	1460.7950	2	0.0003	13	1	Semi-tryptic
H2B1A_MOUSE, H2B1B_MOUSE, H2B1C_MOUSE, H2B1F_MOUSE, H2B1H_MOUSE, H2B1K_MOUSE, H2B1M_MOUSE, H2B1P_MOUSE, H2B2B_MOUSE	Histone H2B	STITSREIQTAVR	29	2.90E-03	487.9389	1460.7949	3	0.0002	13	1	Semi-tryptic
H2B1A_MOUSE, H2B1B_MOUSE, H2B1C_MOUSE, H2B1F_MOUSE, H2B1H_MOUSE, H2B1K_MOUSE, H2B1M_MOUSE, H2B1P_MOUSE, H2B2B_MOUSE	Histone H2B	STITSREIQTAVR	29	1.00E-04	731.4047	1460.7949	2	0.0002	13	1	Semi-tryptic
H2B1A_MOUSE, H2B1B_MOUSE, H2B1C_MOUSE, H2B1F_MOUSE, H2B1H_MOUSE, H2B1K_MOUSE, H2B1M_MOUSE, H2B1P_MOUSE, H2B2B_MOUSE	Histone H2B	STITSREIQTAVR	29	3.00E-03	487.9389	1460.7948	3	0.0001	13	1	Semi-tryptic
H2B1A_MOUSE, H2B1B_MOUSE, H2B1C_MOUSE, H2B1F_MOUSE, H2B1H_MOUSE, H2B1K_MOUSE, H2B1M_MOUSE, H2B1P_MOUSE, H2B2B_MOUSE	Histone H2B	STITSREIQTAVR	29	4.00E-03	487.9388	1460.7945	3	-0.0002	13	1	Semi-tryptic
H2B1A_MOUSE, H2B1B_MOUSE, H2B1C_MOUSE, H2B1F_MOUSE, H2B1H_MOUSE, H2B1K_MOUSE, H2B1M_MOUSE, H2B1P_MOUSE, H2B2B_MOUSE	Histone H2B	STITSREIQTAVR	29	1.50E-03	487.9388	1460.7945	3	-0.0002	13	1	Semi-tryptic
H2B1A_MOUSE, H2B1B_MOUSE, H2B1C_MOUSE, H2B1F_MOUSE, H2B1H_MOUSE, H2B1K_MOUSE, H2B1M_MOUSE, H2B1P_MOUSE, H2B2B_MOUSE	Histone H2B	STITSREIQTAVR	29	9.30E-04	487.9387	1460.7944	3	-0.0003	13	1	Semi-tryptic
H2B1A_MOUSE, H2B1B_MOUSE, H2B1C_MOUSE, H2B1F_MOUSE, H2B1H_MOUSE, H2B1K_MOUSE, H2B1M_MOUSE, H2B1P_MOUSE, H2B2B_MOUSE	Histone H2B	STITSREIQTAVR	29	1.40E-04	487.9387	1460.7942	3	-0.0005	13	1	Semi-tryptic
H2B1A_MOUSE, H2B1B_MOUSE, H2B1C_MOUSE, H2B1F_MOUSE, H2B1H_MOUSE, H2B1K_MOUSE, H2B1M_MOUSE, H2B1P_MOUSE, H2B2B_MOUSE	Histone H2B	STITSREIQTAVR	29	2.50E-05	487.9387	1460.7942	3	-0.0005	13	1	Semi-tryptic

H2B1A_MOUSE, H2B1B_MOUSE, H2B1C_MOUSE, H2B1F_MOUSE, H2B1H_MOUSE, H2B1K_MOUSE, H2B1M_MOUSE, H2B1P_MOUSE, H2B2B_MOUSE	Histone H2B	STITSREIQTAVR	29	7.20E-03	487.9386	1460.7941	3	-0.0006	13	1	Semi-tryptic
H2B1A_MOUSE, H2B1B_MOUSE, H2B1C_MOUSE, H2B1F_MOUSE, H2B1H_MOUSE, H2B1K_MOUSE, H2B1M_MOUSE, H2B1P_MOUSE, H2B2B_MOUSE	Histone H2B	STITSREIQTAVR	29	4.00E-03	487.9386	1460.7939	3	-0.0008	13	1	Semi-tryptic
H2B1A_MOUSE, H2B1B_MOUSE, H2B1C_MOUSE, H2B1F_MOUSE, H2B1H_MOUSE, H2B1K_MOUSE, H2B1M_MOUSE, H2B1P_MOUSE, H2B2B_MOUSE	Histone H2B	STITSREIQTAVR	29	9.10E-03	487.9386	1460.7938	3	-0.0009	13	1	Semi-tryptic
H2B1A_MOUSE, H2B1B_MOUSE, H2B1C_MOUSE, H2B1F_MOUSE, H2B1H_MOUSE, H2B1K_MOUSE, H2B1M_MOUSE, H2B1P_MOUSE, H2B2B_MOUSE	Histone H2B	STITSREIQTAVR	29	5.30E-04	487.9385	1460.7938	3	-0.0009	13	1	Semi-tryptic
H2B1A_MOUSE, H2B1B_MOUSE, H2B1C_MOUSE, H2B1F_MOUSE, H2B1H_MOUSE, H2B1K_MOUSE, H2B1M_MOUSE, H2B1P_MOUSE, H2B2B_MOUSE	Histone H2B	STITSREIQTAVR	29	4.00E-05	487.9385	1460.7936	3	-0.0011	13	1	Semi-tryptic
H2B1A_MOUSE, H2B1B_MOUSE, H2B1C_MOUSE, H2B1F_MOUSE, H2B1H_MOUSE, H2B1K_MOUSE, H2B1M_MOUSE, H2B1P_MOUSE, H2B2B_MOUSE	Histone H2B	STITSREIQTAVR	29	2.20E-06	487.9385	1460.7936	3	-0.0011	13	1	Semi-tryptic
H2B1A_MOUSE, H2B1B_MOUSE, H2B1C_MOUSE, H2B1F_MOUSE, H2B1H_MOUSE, H2B1K_MOUSE, H2B1M_MOUSE, H2B1P_MOUSE, H2B2B_MOUSE	Histone H2B	IQTAVRLLLPGE	96	2.20E-03	655.3945	1308.7745	2	-0.0020	12	0	GluC
H2B1A_MOUSE, H2B1B_MOUSE, H2B1C_MOUSE, H2B1F_MOUSE, H2B1H_MOUSE, H2B1K_MOUSE, H2B1M_MOUSE, H2B1P_MOUSE, H2B2B_MOUSE	Histone H2B	IQTAVRLLLPGE	96	3.80E-04	655.3945	1308.7743	2	-0.0022	12	0	GluC
H2B1A_MOUSE, H2B1B_MOUSE, H2B1C_MOUSE, H2B1F_MOUSE, H2B1H_MOUSE, H2B1K_MOUSE, H2B1M_MOUSE, H2B1P_MOUSE, H2B2B_MOUSE	Histone H2B	IQTAVRLLLPGE	96	2.50E-03	655.3944	1308.7742	2	-0.0024	12	0	GluC
H2B1A_MOUSE, H2B1B_MOUSE, H2B1C_MOUSE, H2B1F_MOUSE, H2B1H_MOUSE, H2B1K_MOUSE, H2B1M_MOUSE, H2B1P_MOUSE, H2B2B_MOUSE	Histone H2B	IQTAVRLLLPGE	96	7.30E-04	655.394	1308.7735	2	-0.0031	12	0	GluC
H2B1A_MOUSE, H2B1B_MOUSE, H2B1C_MOUSE, H2B1F_MOUSE, H2B1H_MOUSE, H2B1K_MOUSE, H2B1M_MOUSE, H2B1P_MOUSE, H2B2B_MOUSE	Histone H2B	IQTAVRLLLPGE	96	6.20E-03	437.265	1308.7732	3	-0.0033	12	0	GluC
H2B1A_MOUSE, H2B1B_MOUSE, H2B1C_MOUSE, H2B1F_MOUSE, H2B1H_MOUSE, H2B1K_MOUSE, H2B1M_MOUSE, H2B1P_MOUSE, H2B2B_MOUSE	Histone H2B	IQTAVRLLLPGE	96	3.00E-05	437.265	1308.7732	3	-0.0033	12	0	GluC
H2B1A_MOUSE, H2B1B_MOUSE, H2B1C_MOUSE, H2B1F_MOUSE, H2B1H_MOUSE, H2B1K_MOUSE, H2B1M_MOUSE, H2B1P_MOUSE, H2B2B_MOUSE	Histone H2B	IQTAVRLLLPGE	96	9.60E-03	437.2648	1308.7725	3	-0.0040	12	0	GluC
H2B1A_MOUSE, H2B1B_MOUSE, H2B1C_MOUSE, H2B1F_MOUSE, H2B1H_MOUSE, H2B1K_MOUSE, H2B1M_MOUSE, H2B1P_MOUSE, H2B2B_MOUSE	Histone H2B	IQTAVRLLLPGE	96	4.30E-05	437.2648	1308.7725	3	-0.0040	12	0	GluC
H2B1A_MOUSE, H2B1B_MOUSE, H2B1C_MOUSE, H2B1F_MOUSE, H2B1H_MOUSE, H2B1K_MOUSE, H2B1M_MOUSE, H2B1P_MOUSE, H2B2B_MOUSE	Histone H2B	IQTAVRLLLPGE	96	6.60E-03	437.2644	1308.7714	3	-0.0051	12	0	GluC
H2B1A_MOUSE, H2B1B_MOUSE, H2B1C_MOUSE, H2B1F_MOUSE, H2B1H_MOUSE, H2B1K_MOUSE, H2B1M_MOUSE, H2B1P_MOUSE, H2B2B_MOUSE	Histone H2B	IQTAVRLLLPGE	96	1.80E-03	655.4004	1308.7863	2	0.0098	12	0	GluC
H2B1A_MOUSE, H2B1B_MOUSE, H2B1C_MOUSE, H2B1F_MOUSE, H2B1H_MOUSE, H2B1K_MOUSE, H2B1M_MOUSE, H2B1P_MOUSE, H2B2B_MOUSE	Histone H2B	IQTAVRLLLPGE	96	2.00E-03	655.4003	1308.7860	2	0.0095	12	0	GluC
H2B1A_MOUSE, H2B1B_MOUSE, H2B1C_MOUSE, H2B1F_MOUSE, H2B1H_MOUSE, H2B1K_MOUSE, H2B1M_MOUSE, H2B1P_MOUSE, H2B2B_MOUSE	Histone H2B	IQTAVRLLLPGE	96	5.10E-03	655.3998	1308.7850	2	0.0084	12	0	GluC
H2B1A_MOUSE, H2B1B_MOUSE, H2B1C_MOUSE, H2B1F_MOUSE, H2B1H_MOUSE, H2B1K_MOUSE, H2B1M_MOUSE, H2B1P_MOUSE, H2B2B_MOUSE	Histone H2B	IQTAVRLLLPGE	96	7.70E-03	655.3994	1308.7842	2	0.0076	12	0	GluC
H2B1A_MOUSE, H2B1B_MOUSE, H2B1C_MOUSE, H2B1F_MOUSE, H2B1H_MOUSE, H2B1K_MOUSE, H2B1M_MOUSE, H2B1P_MOUSE, H2B2B_MOUSE	Histone H2B	IQTAVRLLLPGE	96	5.00E-03	655.3993	1308.7840	2	0.0075	12	0	GluC
H2B1A_MOUSE, H2B1B_MOUSE, H2B1C_MOUSE, H2B1F_MOUSE, H2B1H_MOUSE, H2B1K_MOUSE, H2B1M_MOUSE, H2B1P_MOUSE, H2B2B_MOUSE	Histone H2B	IQTAVRLLLPGE	96	8.70E-03	655.3993	1308.7839	2	0.0074	12	0	GluC
H2B1A_MOUSE, H2B1B_MOUSE, H2B1C_MOUSE, H2B1F_MOUSE, H2B1H_MOUSE, H2B1K_MOUSE, H2B1M_MOUSE, H2B1P_MOUSE, H2B2B_MOUSE	Histone H2B	IQTAVRLLLPGE	96	3.40E-04	655.3988	1308.7831	2	0.0065	12	0	GluC
H2B1A_MOUSE, H2B1B_MOUSE, H2B1C_MOUSE, H2B1F_MOUSE, H2B1H_MOUSE, H2B1K_MOUSE, H2B1M_MOUSE, H2B1P_MOUSE, H2B2B_MOUSE	Histone H2B	IQTAVRLLLPGE	96	7.70E-03	655.3986	1308.7826	2	0.0060	12	0	GluC
H2B1A_MOUSE, H2B1B_MOUSE, H2B1C_MOUSE, H2B1F_MOUSE, H2B1H_MOUSE, H2B1K_MOUSE, H2B1M_MOUSE, H2B1P_MOUSE, H2B2B_MOUSE	Histone H2B	IQTAVRLLLPGE	96	2.00E-03	655.3985	1308.7823	2	0.0058	12	0	GluC

H2B1A_MOUSE, H2B1B_MOUSE, H2B1C_MOUSE, H2B1F_MOUSE, H2B1H_MOUSE, H2B1K_MOUSE, H2B1M_MOUSE, H2B1P_MOUSE, H2B2B_MOUSE	Histone H2B	IQTAVRLLLPGE	96	6.10E-05	437.2677	1308.7811	3	0.0046	12	0	GluC
H2B1A_MOUSE, H2B1B_MOUSE, H2B1C_MOUSE, H2B1F_MOUSE, H2B1H_MOUSE, H2B1K_MOUSE, H2B1M_MOUSE, H2B1P_MOUSE, H2B2B_MOUSE	Histone H2B	IQTAVRLLLPGE	96	4.70E-03	655.3976	1308.7807	2	0.0042	12	0	GluC
H2B1A_MOUSE, H2B1B_MOUSE, H2B1C_MOUSE, H2B1F_MOUSE, H2B1H_MOUSE, H2B1K_MOUSE, H2B1M_MOUSE, H2B1P_MOUSE, H2B2B_MOUSE	Histone H2B	IQTAVRLLLPGE	96	1.30E-03	655.3974	1308.7802	2	0.0037	12	0	GluC
H2B1A_MOUSE, H2B1B_MOUSE, H2B1C_MOUSE, H2B1F_MOUSE, H2B1H_MOUSE, H2B1K_MOUSE, H2B1M_MOUSE, H2B1P_MOUSE, H2B2B_MOUSE	Histone H2B	IQTAVRLLLPGE	96	2.50E-05	655.3972	1308.7799	2	0.0034	12	0	GluC
H2B1A_MOUSE, H2B1B_MOUSE, H2B1C_MOUSE, H2B1F_MOUSE, H2B1H_MOUSE, H2B1K_MOUSE, H2B1M_MOUSE, H2B1P_MOUSE, H2B2B_MOUSE	Histone H2B	IQTAVRLLLPGE	96	1.20E-04	655.397	1308.7794	2	0.0029	12	0	GluC
H2B1A_MOUSE, H2B1B_MOUSE, H2B1C_MOUSE, H2B1F_MOUSE, H2B1H_MOUSE, H2B1K_MOUSE, H2B1M_MOUSE, H2B1P_MOUSE, H2B2B_MOUSE	Histone H2B	IQTAVRLLLPGE	96	2.50E-08	655.397	1308.7794	2	0.0029	12	0	GluC
H2B1A_MOUSE, H2B1B_MOUSE, H2B1C_MOUSE, H2B1F_MOUSE, H2B1H_MOUSE, H2B1K_MOUSE, H2B1M_MOUSE, H2B1P_MOUSE, H2B2B_MOUSE	Histone H2B	IQTAVRLLLPGE	96	9.60E-03	655.3968	1308.7791	2	0.0026	12	0	GluC
H2B1A_MOUSE, H2B1B_MOUSE, H2B1C_MOUSE, H2B1F_MOUSE, H2B1H_MOUSE, H2B1K_MOUSE, H2B1M_MOUSE, H2B1P_MOUSE, H2B2B_MOUSE	Histone H2B	IQTAVRLLLPGE	96	9.40E-03	655.3968	1308.7791	2	0.0026	12	0	GluC
H2B1A_MOUSE, H2B1B_MOUSE, H2B1C_MOUSE, H2B1F_MOUSE, H2B1H_MOUSE, H2B1K_MOUSE, H2B1M_MOUSE, H2B1P_MOUSE, H2B2B_MOUSE	Histone H2B	IQTAVRLLLPGE	96	2.20E-03	655.3968	1308.7791	2	0.0026	12	0	GluC
H2B1A_MOUSE, H2B1B_MOUSE, H2B1C_MOUSE, H2B1F_MOUSE, H2B1H_MOUSE, H2B1K_MOUSE, H2B1M_MOUSE, H2B1P_MOUSE, H2B2B_MOUSE	Histone H2B	IQTAVRLLLPGE	96	4.20E-04	437.267	1308.7790	3	0.0025	12	0	GluC
H2B1A_MOUSE, H2B1B_MOUSE, H2B1C_MOUSE, H2B1F_MOUSE, H2B1H_MOUSE, H2B1K_MOUSE, H2B1M_MOUSE, H2B1P_MOUSE, H2B2B_MOUSE	Histone H2B	IQTAVRLLLPGE	96	1.40E-04	437.267	1308.7790	3	0.0025	12	0	GluC
H2B1A_MOUSE, H2B1B_MOUSE, H2B1C_MOUSE, H2B1F_MOUSE, H2B1H_MOUSE, H2B1K_MOUSE, H2B1M_MOUSE, H2B1P_MOUSE, H2B2B_MOUSE	Histone H2B	IQTAVRLLLPGE	96	7.20E-03	437.2669	1308.7789	3	0.0024	12	0	GluC
H2B1A_MOUSE, H2B1B_MOUSE, H2B1C_MOUSE, H2B1F_MOUSE, H2B1H_MOUSE, H2B1K_MOUSE, H2B1M_MOUSE, H2B1P_MOUSE, H2B2B_MOUSE	Histone H2B	IQTAVRLLLPGE	96	1.00E-03	437.2669	1308.7789	3	0.0024	12	0	GluC
H2B1A_MOUSE, H2B1B_MOUSE, H2B1C_MOUSE, H2B1F_MOUSE, H2B1H_MOUSE, H2B1K_MOUSE, H2B1M_MOUSE, H2B1P_MOUSE, H2B2B_MOUSE	Histone H2B	IQTAVRLLLPGE	96	1.50E-04	437.2669	1308.7789	3	0.0024	12	0	GluC
H2B1A_MOUSE, H2B1B_MOUSE, H2B1C_MOUSE, H2B1F_MOUSE, H2B1H_MOUSE, H2B1K_MOUSE, H2B1M_MOUSE, H2B1P_MOUSE, H2B2B_MOUSE	Histone H2B	IQTAVRLLLPGE	96	1.00E-05	437.2669	1308.7789	3	0.0024	12	0	GluC
H2B1A_MOUSE, H2B1B_MOUSE, H2B1C_MOUSE, H2B1F_MOUSE, H2B1H_MOUSE, H2B1K_MOUSE, H2B1M_MOUSE, H2B1P_MOUSE, H2B2B_MOUSE	Histone H2B	IQTAVRLLLPGE	96	2.30E-03	655.3967	1308.7788	2	0.0023	12	0	GluC
H2B1A_MOUSE, H2B1B_MOUSE, H2B1C_MOUSE, H2B1F_MOUSE, H2B1H_MOUSE, H2B1K_MOUSE, H2B1M_MOUSE, H2B1P_MOUSE, H2B2B_MOUSE	Histone H2B	IQTAVRLLLPGE	96	8.90E-04	655.3967	1308.7788	2	0.0023	12	0	GluC
H2B1A_MOUSE, H2B1B_MOUSE, H2B1C_MOUSE, H2B1F_MOUSE, H2B1H_MOUSE, H2B1K_MOUSE, H2B1M_MOUSE, H2B1P_MOUSE, H2B2B_MOUSE	Histone H2B	IQTAVRLLLPGE	96	2.70E-04	437.2668	1308.7787	3	0.0022	12	0	GluC
H2B1A_MOUSE, H2B1B_MOUSE, H2B1C_MOUSE, H2B1F_MOUSE, H2B1H_MOUSE, H2B1K_MOUSE, H2B1M_MOUSE, H2B1P_MOUSE, H2B2B_MOUSE	Histone H2B	IQTAVRLLLPGE	96	3.40E-05	437.2668	1308.7787	3	0.0022	12	0	GluC
H2B1A_MOUSE, H2B1B_MOUSE, H2B1C_MOUSE, H2B1F_MOUSE, H2B1H_MOUSE, H2B1K_MOUSE, H2B1M_MOUSE, H2B1P_MOUSE, H2B2B_MOUSE	Histone H2B	IQTAVRLLLPGE	96	4.00E-03	655.3964	1308.7783	2	0.0018	12	0	GluC
H2B1A_MOUSE, H2B1B_MOUSE, H2B1C_MOUSE, H2B1F_MOUSE, H2B1H_MOUSE, H2B1K_MOUSE, H2B1M_MOUSE, H2B1P_MOUSE, H2B2B_MOUSE	Histone H2B	IQTAVRLLLPGE	96	9.30E-03	655.3964	1308.7782	2	0.0017	12	0	GluC
H2B1A_MOUSE, H2B1B_MOUSE, H2B1C_MOUSE, H2B1F_MOUSE, H2B1H_MOUSE, H2B1K_MOUSE, H2B1M_MOUSE, H2B1P_MOUSE, H2B2B_MOUSE	Histone H2B	IQTAVRLLLPGE	96	3.40E-04	655.3964	1308.7782	2	0.0017	12	0	GluC
H2B1A_MOUSE, H2B1B_MOUSE, H2B1C_MOUSE, H2B1F_MOUSE, H2B1H_MOUSE, H2B1K_MOUSE, H2B1M_MOUSE, H2B1P_MOUSE, H2B2B_MOUSE	Histone H2B	IQTAVRLLLPGE	96	9.20E-03	655.3963	1308.7781	2	0.0016	12	0	GluC
H2B1A_MOUSE, H2B1B_MOUSE, H2B1C_MOUSE, H2B1F_MOUSE, H2B1H_MOUSE, H2B1K_MOUSE, H2B1M_MOUSE, H2B1P_MOUSE, H2B2B_MOUSE	Histone H2B	IQTAVRLLLPGE	96	1.50E-03	655.3964	1308.7782	2	0.0016	12	0	GluC

H2B1A_MOUSE, H2B1B_MOUSE, H2B1C_MOUSE, H2B1F_MOUSE, H2B1H_MOUSE, H2B1K_MOUSE, H2B1M_MOUSE, H2B1P_MOUSE, H2B2B_MOUSE	Histone H2B	IQTAVRLLLPGE	96	6.20E-03	655.3963	1308.7780	2	0.0015	12	0	GluC
H2B1A_MOUSE, H2B1B_MOUSE, H2B1C_MOUSE, H2B1F_MOUSE, H2B1H_MOUSE, H2B1K_MOUSE, H2B1M_MOUSE, H2B1P_MOUSE, H2B2B_MOUSE	Histone H2B	IQTAVRLLLPGE	96	3.20E-04	655.3963	1308.7780	2	0.0015	12	0	GluC
H2B1A_MOUSE, H2B1B_MOUSE, H2B1C_MOUSE, H2B1F_MOUSE, H2B1H_MOUSE, H2B1K_MOUSE, H2B1M_MOUSE, H2B1P_MOUSE, H2B2B_MOUSE	Histone H2B	IQTAVRLLLPGE	96	1.40E-04	655.3963	1308.7780	2	0.0015	12	0	GluC
H2B1A_MOUSE, H2B1B_MOUSE, H2B1C_MOUSE, H2B1F_MOUSE, H2B1H_MOUSE, H2B1K_MOUSE, H2B1M_MOUSE, H2B1P_MOUSE, H2B2B_MOUSE	Histone H2B	IQTAVRLLLPGE	96	4.60E-04	655.3962	1308.7779	2	0.0014	12	0	GluC
H2B1A_MOUSE, H2B1B_MOUSE, H2B1C_MOUSE, H2B1F_MOUSE, H2B1H_MOUSE, H2B1K_MOUSE, H2B1M_MOUSE, H2B1P_MOUSE, H2B2B_MOUSE	Histone H2B	IQTAVRLLLPGE	96	1.90E-04	655.3962	1308.7779	2	0.0014	12	0	GluC
H2B1A_MOUSE, H2B1B_MOUSE, H2B1C_MOUSE, H2B1F_MOUSE, H2B1H_MOUSE, H2B1K_MOUSE, H2B1M_MOUSE, H2B1P_MOUSE, H2B2B_MOUSE	Histone H2B	IQTAVRLLLPGE	96	5.70E-03	655.3962	1308.7778	2	0.0013	12	0	GluC
H2B1A_MOUSE, H2B1B_MOUSE, H2B1C_MOUSE, H2B1F_MOUSE, H2B1H_MOUSE, H2B1K_MOUSE, H2B1M_MOUSE, H2B1P_MOUSE, H2B2B_MOUSE	Histone H2B	IQTAVRLLLPGE	96	2.40E-03	655.3962	1308.7778	2	0.0013	12	0	GluC
H2B1A_MOUSE, H2B1B_MOUSE, H2B1C_MOUSE, H2B1F_MOUSE, H2B1H_MOUSE, H2B1K_MOUSE, H2B1M_MOUSE, H2B1P_MOUSE, H2B2B_MOUSE	Histone H2B	IQTAVRLLLPGE	96	2.00E-03	655.3962	1308.7778	2	0.0013	12	0	GluC
H2B1A_MOUSE, H2B1B_MOUSE, H2B1C_MOUSE, H2B1F_MOUSE, H2B1H_MOUSE, H2B1K_MOUSE, H2B1M_MOUSE, H2B1P_MOUSE, H2B2B_MOUSE	Histone H2B	IQTAVRLLLPGE	96	4.40E-04	437.2665	1308.7778	3	0.0013	12	0	GluC
H2B1A_MOUSE, H2B1B_MOUSE, H2B1C_MOUSE, H2B1F_MOUSE, H2B1H_MOUSE, H2B1K_MOUSE, H2B1M_MOUSE, H2B1P_MOUSE, H2B2B_MOUSE	Histone H2B	IQTAVRLLLPGE	96	3.50E-04	437.2665	1308.7778	3	0.0013	12	0	GluC
H2B1A_MOUSE, H2B1B_MOUSE, H2B1C_MOUSE, H2B1F_MOUSE, H2B1H_MOUSE, H2B1K_MOUSE, H2B1M_MOUSE, H2B1P_MOUSE, H2B2B_MOUSE	Histone H2B	IQTAVRLLLPGE	96	6.70E-05	655.3962	1308.7778	2	0.0013	12	0	GluC
H2B1A_MOUSE, H2B1B_MOUSE, H2B1C_MOUSE, H2B1F_MOUSE, H2B1H_MOUSE, H2B1K_MOUSE, H2B1M_MOUSE, H2B1P_MOUSE, H2B2B_MOUSE	Histone H2B	IQTAVRLLLPGE	96	6.60E-05	655.3962	1308.7778	2	0.0013	12	0	GluC
H2B1A_MOUSE, H2B1B_MOUSE, H2B1C_MOUSE, H2B1F_MOUSE, H2B1H_MOUSE, H2B1K_MOUSE, H2B1M_MOUSE, H2B1P_MOUSE, H2B2B_MOUSE	Histone H2B	IQTAVRLLLPGE	96	5.90E-03	655.3961	1308.7777	2	0.0012	12	0	GluC
H2B1A_MOUSE, H2B1B_MOUSE, H2B1C_MOUSE, H2B1F_MOUSE, H2B1H_MOUSE, H2B1K_MOUSE, H2B1M_MOUSE, H2B1P_MOUSE, H2B2B_MOUSE	Histone H2B	IQTAVRLLLPGE	96	3.90E-03	655.3962	1308.7777	2	0.0012	12	0	GluC
H2B1A_MOUSE, H2B1B_MOUSE, H2B1C_MOUSE, H2B1F_MOUSE, H2B1H_MOUSE, H2B1K_MOUSE, H2B1M_MOUSE, H2B1P_MOUSE, H2B2B_MOUSE	Histone H2B	IQTAVRLLLPGE	96	3.70E-03	655.3961	1308.7777	2	0.0012	12	0	GluC
H2B1A_MOUSE, H2B1B_MOUSE, H2B1C_MOUSE, H2B1F_MOUSE, H2B1H_MOUSE, H2B1K_MOUSE, H2B1M_MOUSE, H2B1P_MOUSE, H2B2B_MOUSE	Histone H2B	IQTAVRLLLPGE	96	4.80E-04	655.3962	1308.7777	2	0.0012	12	0	GluC
H2B1A_MOUSE, H2B1B_MOUSE, H2B1C_MOUSE, H2B1F_MOUSE, H2B1H_MOUSE, H2B1K_MOUSE, H2B1M_MOUSE, H2B1P_MOUSE, H2B2B_MOUSE	Histone H2B	IQTAVRLLLPGE	96	6.80E-03	437.2665	1308.7776	3	0.0011	12	0	GluC
H2B1A_MOUSE, H2B1B_MOUSE, H2B1C_MOUSE, H2B1F_MOUSE, H2B1H_MOUSE, H2B1K_MOUSE, H2B1M_MOUSE, H2B1P_MOUSE, H2B2B_MOUSE	Histone H2B	IQTAVRLLLPGE	96	4.00E-03	655.3961	1308.7776	2	0.0011	12	0	GluC
H2B1A_MOUSE, H2B1B_MOUSE, H2B1C_MOUSE, H2B1F_MOUSE, H2B1H_MOUSE, H2B1K_MOUSE, H2B1M_MOUSE, H2B1P_MOUSE, H2B2B_MOUSE	Histone H2B	IQTAVRLLLPGE	96	3.50E-03	655.3961	1308.7776	2	0.0011	12	0	GluC
H2B1A_MOUSE, H2B1B_MOUSE, H2B1C_MOUSE, H2B1F_MOUSE, H2B1H_MOUSE, H2B1K_MOUSE, H2B1M_MOUSE, H2B1P_MOUSE, H2B2B_MOUSE	Histone H2B	IQTAVRLLLPGE	96	7.10E-04	655.3961	1308.7776	2	0.0011	12	0	GluC
H2B1A_MOUSE, H2B1B_MOUSE, H2B1C_MOUSE, H2B1F_MOUSE, H2B1H_MOUSE, H2B1K_MOUSE, H2B1M_MOUSE, H2B1P_MOUSE, H2B2B_MOUSE	Histone H2B	IQTAVRLLLPGE	96	3.80E-04	655.3961	1308.7776	2	0.0011	12	0	GluC
H2B1A_MOUSE, H2B1B_MOUSE, H2B1C_MOUSE, H2B1F_MOUSE, H2B1H_MOUSE, H2B1K_MOUSE, H2B1M_MOUSE, H2B1P_MOUSE, H2B2B_MOUSE	Histone H2B	IQTAVRLLLPGE	96	3.70E-04	655.3961	1308.7776	2	0.0011	12	0	GluC
H2B1A_MOUSE, H2B1B_MOUSE, H2B1C_MOUSE, H2B1F_MOUSE, H2B1H_MOUSE, H2B1K_MOUSE, H2B1M_MOUSE, H2B1P_MOUSE, H2B2B_MOUSE	Histone H2B	IQTAVRLLLPGE	96	1.30E-04	655.3961	1308.7777	2	0.0011	12	0	GluC
H2B1A_MOUSE, H2B1B_MOUSE, H2B1C_MOUSE, H2B1F_MOUSE, H2B1H_MOUSE, H2B1K_MOUSE, H2B1M_MOUSE, H2B1P_MOUSE, H2B2B_MOUSE	Histone H2B	IQTAVRLLLPGE	96	4.50E-03	655.396	1308.7775	2	0.0010	12	0	GluC

H2B1A_MOUSE, H2B1B_MOUSE, H2B1C_MOUSE, H2B1F_MOUSE, H2B1H_MOUSE, H2B1K_MOUSE, H2B1M_MOUSE, H2B1P_MOUSE, H2B2B_MOUSE	Histone H2B	IQTAVRLLLPGE	96	7.60E-04	655.396	1308.7774	2	0.0009	12	0	GluC
H2B1A_MOUSE, H2B1B_MOUSE, H2B1C_MOUSE, H2B1F_MOUSE, H2B1H_MOUSE, H2B1K_MOUSE, H2B1M_MOUSE, H2B1P_MOUSE, H2B2B_MOUSE	Histone H2B	IQTAVRLLLPGE	96	6.90E-05	655.396	1308.7774	2	0.0009	12	0	GluC
H2B1A_MOUSE, H2B1B_MOUSE, H2B1C_MOUSE, H2B1F_MOUSE, H2B1H_MOUSE, H2B1K_MOUSE, H2B1M_MOUSE, H2B1P_MOUSE, H2B2B_MOUSE	Histone H2B	IQTAVRLLLPGE	96	6.90E-03	655.396	1308.7774	2	0.0008	12	0	GluC
H2B1A_MOUSE, H2B1B_MOUSE, H2B1C_MOUSE, H2B1F_MOUSE, H2B1H_MOUSE, H2B1K_MOUSE, H2B1M_MOUSE, H2B1P_MOUSE, H2B2B_MOUSE	Histone H2B	IQTAVRLLLPGE	96	4.80E-03	655.396	1308.7774	2	0.0008	12	0	GluC
H2B1A_MOUSE, H2B1B_MOUSE, H2B1C_MOUSE, H2B1F_MOUSE, H2B1H_MOUSE, H2B1K_MOUSE, H2B1M_MOUSE, H2B1P_MOUSE, H2B2B_MOUSE	Histone H2B	IQTAVRLLLPGE	96	2.30E-03	655.3959	1308.7773	2	0.0008	12	0	GluC
H2B1A_MOUSE, H2B1B_MOUSE, H2B1C_MOUSE, H2B1F_MOUSE, H2B1H_MOUSE, H2B1K_MOUSE, H2B1M_MOUSE, H2B1P_MOUSE, H2B2B_MOUSE	Histone H2B	IQTAVRLLLPGE	96	1.30E-03	655.3959	1308.7773	2	0.0008	12	0	GluC
H2B1A_MOUSE, H2B1B_MOUSE, H2B1C_MOUSE, H2B1F_MOUSE, H2B1H_MOUSE, H2B1K_MOUSE, H2B1M_MOUSE, H2B1P_MOUSE, H2B2B_MOUSE	Histone H2B	IQTAVRLLLPGE	96	6.90E-04	655.396	1308.7774	2	0.0008	12	0	GluC
H2B1A_MOUSE, H2B1B_MOUSE, H2B1C_MOUSE, H2B1F_MOUSE, H2B1H_MOUSE, H2B1K_MOUSE, H2B1M_MOUSE, H2B1P_MOUSE, H2B2B_MOUSE	Histone H2B	IQTAVRLLLPGE	96	1.10E-04	437.2664	1308.7773	3	0.0007	12	0	GluC
H2B1A_MOUSE, H2B1B_MOUSE, H2B1C_MOUSE, H2B1F_MOUSE, H2B1H_MOUSE, H2B1K_MOUSE, H2B1M_MOUSE, H2B1P_MOUSE, H2B2B_MOUSE	Histone H2B	IQTAVRLLLPGE	96	2.70E-05	655.3958	1308.7771	2	0.0006	12	0	GluC
H2B1A_MOUSE, H2B1B_MOUSE, H2B1C_MOUSE, H2B1F_MOUSE, H2B1H_MOUSE, H2B1K_MOUSE, H2B1M_MOUSE, H2B1P_MOUSE, H2B2B_MOUSE	Histone H2B	IQTAVRLLLPGE	96	3.70E-03	437.2663	1308.7770	3	0.0005	12	0	GluC
H2B1A_MOUSE, H2B1B_MOUSE, H2B1C_MOUSE, H2B1F_MOUSE, H2B1H_MOUSE, H2B1K_MOUSE, H2B1M_MOUSE, H2B1P_MOUSE, H2B2B_MOUSE	Histone H2B	IQTAVRLLLPGE	96	2.90E-03	437.2663	1308.7770	3	0.0005	12	0	GluC
H2B1A_MOUSE, H2B1B_MOUSE, H2B1C_MOUSE, H2B1F_MOUSE, H2B1H_MOUSE, H2B1K_MOUSE, H2B1M_MOUSE, H2B1P_MOUSE, H2B2B_MOUSE	Histone H2B	IQTAVRLLLPGE	96	5.60E-04	655.3958	1308.7771	2	0.0005	12	0	GluC
H2B1A_MOUSE, H2B1B_MOUSE, H2B1C_MOUSE, H2B1F_MOUSE, H2B1H_MOUSE, H2B1K_MOUSE, H2B1M_MOUSE, H2B1P_MOUSE, H2B2B_MOUSE	Histone H2B	IQTAVRLLLPGE	96	2.70E-04	437.2663	1308.7770	3	0.0005	12	0	GluC
H2B1A_MOUSE, H2B1B_MOUSE, H2B1C_MOUSE, H2B1F_MOUSE, H2B1H_MOUSE, H2B1K_MOUSE, H2B1M_MOUSE, H2B1P_MOUSE, H2B2B_MOUSE	Histone H2B	IQTAVRLLLPGE	96	2.80E-03	655.3958	1308.7769	2	0.0004	12	0	GluC
H2B1A_MOUSE, H2B1B_MOUSE, H2B1C_MOUSE, H2B1F_MOUSE, H2B1H_MOUSE, H2B1K_MOUSE, H2B1M_MOUSE, H2B1P_MOUSE, H2B2B_MOUSE	Histone H2B	IQTAVRLLLPGE	96	2.30E-04	655.3957	1308.7769	2	0.0003	12	0	GluC
H2B1A_MOUSE, H2B1B_MOUSE, H2B1C_MOUSE, H2B1F_MOUSE, H2B1H_MOUSE, H2B1K_MOUSE, H2B1M_MOUSE, H2B1P_MOUSE, H2B2B_MOUSE	Histone H2B	IQTAVRLLLPGE	96	7.40E-03	437.2662	1308.7767	3	0.0002	12	0	GluC
H2B1A_MOUSE, H2B1B_MOUSE, H2B1C_MOUSE, H2B1F_MOUSE, H2B1H_MOUSE, H2B1K_MOUSE, H2B1M_MOUSE, H2B1P_MOUSE, H2B2B_MOUSE	Histone H2B	IQTAVRLLLPGE	96	2.40E-03	655.3956	1308.7767	2	0.0002	12	0	GluC
H2B1A_MOUSE, H2B1B_MOUSE, H2B1C_MOUSE, H2B1F_MOUSE, H2B1H_MOUSE, H2B1K_MOUSE, H2B1M_MOUSE, H2B1P_MOUSE, H2B2B_MOUSE	Histone H2B	IQTAVRLLLPGE	96	1.20E-03	655.3956	1308.7767	2	0.0002	12	0	GluC
H2B1A_MOUSE, H2B1B_MOUSE, H2B1C_MOUSE, H2B1F_MOUSE, H2B1H_MOUSE, H2B1K_MOUSE, H2B1M_MOUSE, H2B1P_MOUSE, H2B2B_MOUSE	Histone H2B	IQTAVRLLLPGE	96	3.60E-05	437.2662	1308.7767	3	0.0002	12	0	GluC
H2B1A_MOUSE, H2B1B_MOUSE, H2B1C_MOUSE, H2B1F_MOUSE, H2B1H_MOUSE, H2B1K_MOUSE, H2B1M_MOUSE, H2B1P_MOUSE, H2B2B_MOUSE	Histone H2B	IQTAVRLLLPGE	96	1.40E-03	437.2661	1308.7766	3	0.0001	12	0	GluC
H2B1A_MOUSE, H2B1B_MOUSE, H2B1C_MOUSE, H2B1F_MOUSE, H2B1H_MOUSE, H2B1K_MOUSE, H2B1M_MOUSE, H2B1P_MOUSE, H2B2B_MOUSE	Histone H2B	IQTAVRLLLPGE	96	2.00E-06	437.2661	1308.7766	3	0.0001	12	0	GluC
H2B1A_MOUSE, H2B1B_MOUSE, H2B1C_MOUSE, H2B1F_MOUSE, H2B1H_MOUSE, H2B1K_MOUSE, H2B1M_MOUSE, H2B1P_MOUSE, H2B2B_MOUSE	Histone H2B	IQTAVRLLLPGE	96	1.60E-03	437.2661	1308.7764	3	-0.0001	12	0	GluC
H2B1A_MOUSE, H2B1B_MOUSE, H2B1C_MOUSE, H2B1F_MOUSE, H2B1H_MOUSE, H2B1K_MOUSE, H2B1M_MOUSE, H2B1P_MOUSE, H2B2B_MOUSE	Histone H2B	IQTAVRLLLPGE	96	1.10E-04	437.2661	1308.7764	3	-0.0001	12	0	GluC
H2B1A_MOUSE, H2B1B_MOUSE, H2B1C_MOUSE, H2B1F_MOUSE, H2B1H_MOUSE, H2B1K_MOUSE, H2B1M_MOUSE, H2B1P_MOUSE, H2B2B_MOUSE	Histone H2B	IQTAVRLLLPGE	96	4.70E-03	655.3954	1308.7763	2	-0.0002	12	0	GluC

H2B1A_MOUSE, H2B1B_MOUSE, H2B1C_MOUSE, H2B1F_MOUSE, H2B1H_MOUSE, H2B1K_MOUSE, H2B1M_MOUSE, H2B1P_MOUSE, H2B2B_MOUSE	Histone H2B	IQTAVRLLLPGE	96	1.60E-04	437.2661	1308.7763	3	-0.0002	12	0	GluC
H2B1A_MOUSE, H2B1B_MOUSE, H2B1C_MOUSE, H2B1F_MOUSE, H2B1H_MOUSE, H2B1K_MOUSE, H2B1M_MOUSE, H2B1P_MOUSE, H2B2B_MOUSE	Histone H2B	IQTAVRLLLPGE	96	7.60E-04	655.3953	1308.7760	2	-0.0006	12	0	GluC
H2B1A_MOUSE, H2B1B_MOUSE, H2B1C_MOUSE, H2B1F_MOUSE, H2B1H_MOUSE, H2B1K_MOUSE, H2B1M_MOUSE, H2B1P_MOUSE, H2B2B_MOUSE	Histone H2B	IQTAVRLLLPGE	96	2.30E-04	655.3953	1308.7760	2	-0.0006	12	0	GluC
H2B1A_MOUSE, H2B1B_MOUSE, H2B1C_MOUSE, H2B1F_MOUSE, H2B1H_MOUSE, H2B1K_MOUSE, H2B1M_MOUSE, H2B1P_MOUSE, H2B2B_MOUSE	Histone H2B	IQTAVRLLLPGE	96	5.60E-03	655.3951	1308.7756	2	-0.0009	12	0	GluC
H2B1A_MOUSE, H2B1B_MOUSE, H2B1C_MOUSE, H2B1F_MOUSE, H2B1H_MOUSE, H2B1K_MOUSE, H2B1M_MOUSE, H2B1P_MOUSE, H2B2B_MOUSE	Histone H2B	IQTAVRLLLPGE	96	2.40E-04	437.2658	1308.7755	3	-0.0010	12	0	GluC
H2B1A_MOUSE, H2B1B_MOUSE, H2B1C_MOUSE, H2B1F_MOUSE, H2B1H_MOUSE, H2B1K_MOUSE, H2B1M_MOUSE, H2B1P_MOUSE, H2B2B_MOUSE	Histone H2B	IQTAVRLLLPGE	96	2.20E-06	437.2658	1308.7755	3	-0.0010	12	0	GluC
H2B1A_MOUSE, H2B1B_MOUSE, H2B1C_MOUSE, H2B1F_MOUSE, H2B1H_MOUSE, H2B1K_MOUSE, H2B1M_MOUSE, H2B1P_MOUSE, H2B2B_MOUSE	Histone H2B	EIQTAVR	7	3.10E-04	408.7338	815.4530	2	0.0029	7	0	Semi-tryptic
H2B1A_MOUSE, H2B1B_MOUSE, H2B1C_MOUSE, H2B1F_MOUSE, H2B1H_MOUSE, H2B1K_MOUSE, H2B1M_MOUSE, H2B1P_MOUSE, H2B2B_MOUSE	Histone H2B	EIQTAVR	7	6.60E-03	408.733	815.4515	2	0.0014	7	0	Semi-tryptic
H2B1A_MOUSE, H2B1B_MOUSE, H2B1C_MOUSE, H2B1F_MOUSE, H2B1H_MOUSE, H2B1K_MOUSE, H2B1M_MOUSE, H2B1P_MOUSE, H2B2B_MOUSE	Histone H2B	EIQTAVR	7	1.20E-03	408.733	815.4514	2	0.0013	7	0	Semi-tryptic
H2B1A_MOUSE, H2B1B_MOUSE, H2B1C_MOUSE, H2B1F_MOUSE, H2B1H_MOUSE, H2B1K_MOUSE, H2B1M_MOUSE, H2B1P_MOUSE, H2B2B_MOUSE	Histone H2B	EIQTAVR	7	9.20E-03	408.7326	815.4507	2	0.0006	7	0	Semi-tryptic
H2B1A_MOUSE, H2B1B_MOUSE, H2B1C_MOUSE, H2B1F_MOUSE, H2B1H_MOUSE, H2B1K_MOUSE, H2B1M_MOUSE, H2B1P_MOUSE, H2B2B_MOUSE	Histone H2B	EIQTAVR	7	1.00E-03	408.7326	815.4507	2	0.0006	7	0	Semi-tryptic
H2B1A_MOUSE, H2B1B_MOUSE, H2B1C_MOUSE, H2B1F_MOUSE, H2B1H_MOUSE, H2B1K_MOUSE, H2B1M_MOUSE, H2B1P_MOUSE, H2B2B_MOUSE	Histone H2B	EIQTAVR	7	3.80E-03	408.7324	815.4503	2	0.0002	7	0	Semi-tryptic
H2B1A_MOUSE, H2B1B_MOUSE, H2B1C_MOUSE, H2B1F_MOUSE, H2B1H_MOUSE, H2B1K_MOUSE, H2B1M_MOUSE, H2B1P_MOUSE, H2B2B_MOUSE	Histone H2B	EIQTAVR	7	6.00E-04	408.7324	815.4502	2	0.0001	7	0	Semi-tryptic
H2B1A_MOUSE, H2B1B_MOUSE, H2B1C_MOUSE, H2B1F_MOUSE, H2B1H_MOUSE, H2B1K_MOUSE, H2B1M_MOUSE, H2B1P_MOUSE, H2B2B_MOUSE, H2B2E_MOUSE, H2B3A_MOUSE, H2B3B_MOUSE	Histone H2B	LLLPGELAKHAVSEGTKAVTK	4	5.70E-03	721.4237	2161.2492	3	0.0022	21	2	Semi-tryptic
H2B1A_MOUSE, H2B1B_MOUSE, H2B1C_MOUSE, H2B1F_MOUSE, H2B1H_MOUSE, H2B1K_MOUSE, H2B1M_MOUSE, H2B1P_MOUSE, H2B2B_MOUSE, H2B2E_MOUSE, H2B3A_MOUSE, H2B3B_MOUSE	Histone H2B	LLLPGELAKHAVSEGTKAVTK	4	9.80E-03	721.4236	2161.2489	3	0.0019	21	2	Semi-tryptic
H2B1A_MOUSE, H2B1B_MOUSE, H2B1C_MOUSE, H2B1F_MOUSE, H2B1H_MOUSE, H2B1K_MOUSE, H2B1M_MOUSE, H2B1P_MOUSE, H2B2B_MOUSE, H2B2E_MOUSE, H2B3A_MOUSE, H2B3B_MOUSE	Histone H2B	LLLPGELAKHAVSEGTKAVTK	4	3.40E-03	721.4228	2161.2465	3	-0.0005	21	2	Semi-tryptic
H2B1A_MOUSE, H2B1B_MOUSE, H2B1C_MOUSE, H2B1F_MOUSE, H2B1H_MOUSE, H2B1K_MOUSE, H2B1M_MOUSE, H2B1P_MOUSE, H2B2B_MOUSE, H2B2E_MOUSE, H2B3A_MOUSE, H2B3B_MOUSE	Histone H2B	LLPGELAKHAVSEGTKAVTKY	10	2.20E-03	738.0877	2211.2411	3	0.0148	21	2	Chymotrypsin
H2B1A_MOUSE, H2B1B_MOUSE, H2B1C_MOUSE, H2B1F_MOUSE, H2B1H_MOUSE, H2B1K_MOUSE, H2B1M_MOUSE, H2B1P_MOUSE, H2B2B_MOUSE, H2B2E_MOUSE, H2B3A_MOUSE, H2B3B_MOUSE	Histone H2B	LLPGELAKHAVSEGTKAVTKY	10	9.50E-03	738.0859	2211.2358	3	0.0095	21	2	Chymotrypsin
H2B1A_MOUSE, H2B1B_MOUSE, H2B1C_MOUSE, H2B1F_MOUSE, H2B1H_MOUSE, H2B1K_MOUSE, H2B1M_MOUSE, H2B1P_MOUSE, H2B2B_MOUSE, H2B2E_MOUSE, H2B3A_MOUSE, H2B3B_MOUSE	Histone H2B	LLPGELAKHAVSEGTKAVTKY	10	2.80E-03	738.0838	2211.2297	3	0.0034	21	2	Chymotrypsin
H2B1A_MOUSE, H2B1B_MOUSE, H2B1C_MOUSE, H2B1F_MOUSE, H2B1H_MOUSE, H2B1K_MOUSE, H2B1M_MOUSE, H2B1P_MOUSE, H2B2B_MOUSE, H2B2E_MOUSE, H2B3A_MOUSE, H2B3B_MOUSE	Histone H2B	LLPGELAKHAVSEGTKAVTKY	10	7.90E-03	443.253	2211.2288	5	0.0025	21	2	Chymotrypsin
H2B1A_MOUSE, H2B1B_MOUSE, H2B1C_MOUSE, H2B1F_MOUSE, H2B1H_MOUSE, H2B1K_MOUSE, H2B1M_MOUSE, H2B1P_MOUSE, H2B2B_MOUSE, H2B2E_MOUSE, H2B3A_MOUSE, H2B3B_MOUSE	Histone H2B	LLPGELAKHAVSEGTKAVTKY	10	2.00E-03	738.0835	2211.2286	3	0.0023	21	2	Chymotrypsin
H2B1A_MOUSE, H2B1B_MOUSE, H2B1C_MOUSE, H2B1F_MOUSE, H2B1H_MOUSE, H2B1K_MOUSE, H2B1M_MOUSE, H2B1P_MOUSE, H2B2B_MOUSE, H2B2E_MOUSE, H2B3A_MOUSE, H2B3B_MOUSE	Histone H2B	LLPGELAKHAVSEGTKAVTKY	10	5.40E-03	738.0834	2211.2283	3	0.0020	21	2	Chymotrypsin
H2B1A_MOUSE, H2B1B_MOUSE, H2B1C_MOUSE, H2B1F_MOUSE, H2B1H_MOUSE, H2B1K_MOUSE, H2B1M_MOUSE, H2B1P_MOUSE, H2B2B_MOUSE, H2B2E_MOUSE, H2B3A_MOUSE, H2B3B_MOUSE	Histone H2B	LLPGELAKHAVSEGTKAVTKY	10	3.30E-05	738.0834	2211.2283	3	0.0020	21	2	Chymotrypsin
H2B1A_MOUSE, H2B1B_MOUSE, H2B1C_MOUSE, H2B1F_MOUSE, H2B1H_MOUSE, H2B1K_MOUSE, H2B1M_MOUSE, H2B1P_MOUSE, H2B2B_MOUSE, H2B2E_MOUSE, H2B3A_MOUSE, H2B3B_MOUSE	Histone H2B	LLPGELAKHAVSEGTKAVTKY	10	6.60E-07	553.8142	2211.2275	4	0.0012	21	2	Chymotrypsin

H2B1A_MOUSE, H2B1B_MOUSE, H2B1C_MOUSE, H2B1F_MOUSE, H2B1H_MOUSE, H2B1K_MOUSE, H2B1M_MOUSE, H2B1P_MOUSE, H2B2B_MOUSE, H2B2E_MOUSE, H2B3A_MOUSE, H2B3B_MOUSE	Histone H2B	LLPGELAKHAVSEGTKAVTKY	10	1.00E-03	443.2525	2211.2262	5	-0.0001	21	2	Chymotrypsin
H2B1A_MOUSE, H2B1B_MOUSE, H2B1C_MOUSE, H2B1F_MOUSE, H2B1H_MOUSE, H2B1K_MOUSE, H2B1M_MOUSE, H2B1P_MOUSE, H2B2B_MOUSE, H2B2E_MOUSE, H2B3A_MOUSE, H2B3B_MOUSE	Histone H2B	LLPGELAKHAVSEGTKAVTKY	10	2.40E-07	553.8135	2211.2248	4	-0.0015	21	2	Chymotrypsin
H2B1A_MOUSE, H2B1B_MOUSE, H2B1C_MOUSE, H2B1F_MOUSE, H2B1H_MOUSE, H2B1K_MOUSE, H2B1M_MOUSE, H2B1P_MOUSE, H2B2B_MOUSE, H2B2E_MOUSE, H2B3A_MOUSE, H2B3B_MOUSE	Histone H2B	LLLPGELAKHAVSEGTKAVTK	4	4.90E-03	721.4214	2161.2425	3	-0.0045	21	2	Semi-tryptic
H2B1A_MOUSE, H2B1B_MOUSE, H2B1C_MOUSE, H2B1F_MOUSE, H2B1H_MOUSE, H2B1K_MOUSE, H2B1M_MOUSE, H2B1P_MOUSE, H2B2B_MOUSE, H2B2E_MOUSE, H2B3A_MOUSE, H2B3B_MOUSE	Histone H2B	ASRLAHYNKRSTITSRE	140	8.40E-09	664.0234	1989.0484	3	-0.0019	17	0	GluC
H2B1A_MOUSE, H2B1B_MOUSE, H2B1C_MOUSE, H2B1F_MOUSE, H2B1H_MOUSE, H2B1K_MOUSE, H2B1M_MOUSE, H2B1P_MOUSE, H2B2B_MOUSE, H2B2E_MOUSE, H2B3A_MOUSE, H2B3B_MOUSE	Histone H2B	ASRLAHYNKRSTITSRE	140	8.50E-03	498.2694	1989.0484	4	-0.0020	17	0	GluC
H2B1A_MOUSE, H2B1B_MOUSE, H2B1C_MOUSE, H2B1F_MOUSE, H2B1H_MOUSE, H2B1K_MOUSE, H2B1M_MOUSE, H2B1P_MOUSE, H2B2B_MOUSE, H2B2E_MOUSE, H2B3A_MOUSE, H2B3B_MOUSE	Histone H2B	ASRLAHYNKRSTITSRE	140	1.70E-04	398.8169	1989.0483	5	-0.0020	17	0	GluC
H2B1A_MOUSE, H2B1B_MOUSE, H2B1C_MOUSE, H2B1F_MOUSE, H2B1H_MOUSE, H2B1K_MOUSE, H2B1M_MOUSE, H2B1P_MOUSE, H2B2B_MOUSE, H2B2E_MOUSE, H2B3A_MOUSE, H2B3B_MOUSE	Histone H2B	ASRLAHYNKRSTITSRE	140	3.80E-04	398.8169	1989.0482	5	-0.0022	17	0	GluC
H2B1A_MOUSE, H2B1B_MOUSE, H2B1C_MOUSE, H2B1F_MOUSE, H2B1H_MOUSE, H2B1K_MOUSE, H2B1M_MOUSE, H2B1P_MOUSE, H2B2B_MOUSE, H2B2E_MOUSE, H2B3A_MOUSE, H2B3B_MOUSE	Histone H2B	ASRLAHYNKRSTITSRE	140	1.70E-04	498.2693	1989.0482	4	-0.0022	17	0	GluC
H2B1A_MOUSE, H2B1B_MOUSE, H2B1C_MOUSE, H2B1F_MOUSE, H2B1H_MOUSE, H2B1K_MOUSE, H2B1M_MOUSE, H2B1P_MOUSE, H2B2B_MOUSE, H2B2E_MOUSE, H2B3A_MOUSE, H2B3B_MOUSE	Histone H2B	ASRLAHYNKRSTITSRE	140	1.80E-05	498.2693	1989.0480	4	-0.0024	17	0	GluC
H2B1A_MOUSE, H2B1B_MOUSE, H2B1C_MOUSE, H2B1F_MOUSE, H2B1H_MOUSE, H2B1K_MOUSE, H2B1M_MOUSE, H2B1P_MOUSE, H2B2B_MOUSE, H2B2E_MOUSE, H2B3A_MOUSE, H2B3B_MOUSE	Histone H2B	LLLPGELAKHAVSEGTK	19	6.60E-03	588.3393	1761.9962	3	-0.0027	17	1	Semi-tryptic
H2B1A_MOUSE, H2B1B_MOUSE, H2B1C_MOUSE, H2B1F_MOUSE, H2B1H_MOUSE, H2B1K_MOUSE, H2B1M_MOUSE, H2B1P_MOUSE, H2B2B_MOUSE, H2B2E_MOUSE, H2B3A_MOUSE, H2B3B_MOUSE	Histone H2B	ASRLAHYNKRSTITSRE	140	1.80E-05	498.2692	1989.0477	4	-0.0027	17	0	GluC
H2B1A_MOUSE, H2B1B_MOUSE, H2B1C_MOUSE, H2B1F_MOUSE, H2B1H_MOUSE, H2B1K_MOUSE, H2B1M_MOUSE, H2B1P_MOUSE, H2B2B_MOUSE, H2B2E_MOUSE, H2B3A_MOUSE, H2B3B_MOUSE	Histone H2B	ASRLAHYNKRSTITSRE	140	2.30E-08	498.2692	1989.0477	4	-0.0027	17	0	GluC
H2B1A_MOUSE, H2B1B_MOUSE, H2B1C_MOUSE, H2B1F_MOUSE, H2B1H_MOUSE, H2B1K_MOUSE, H2B1M_MOUSE, H2B1P_MOUSE, H2B2B_MOUSE, H2B2E_MOUSE, H2B3A_MOUSE, H2B3B_MOUSE	Histone H2B	ASRLAHYNKRSTITSRE	140	3.10E-03	498.2691	1989.0475	4	-0.0029	17	0	GluC
H2B1A_MOUSE, H2B1B_MOUSE, H2B1C_MOUSE, H2B1F_MOUSE, H2B1H_MOUSE, H2B1K_MOUSE, H2B1M_MOUSE, H2B1P_MOUSE, H2B2B_MOUSE, H2B2E_MOUSE, H2B3A_MOUSE, H2B3B_MOUSE	Histone H2B	ASRLAHYNKRSTITSRE	140	3.00E-04	498.2692	1989.0475	4	-0.0029	17	0	GluC
H2B1A_MOUSE, H2B1B_MOUSE, H2B1C_MOUSE, H2B1F_MOUSE, H2B1H_MOUSE, H2B1K_MOUSE, H2B1M_MOUSE, H2B1P_MOUSE, H2B2B_MOUSE, H2B2E_MOUSE, H2B3A_MOUSE, H2B3B_MOUSE	Histone H2B	LLLPGELAKHAVSEGTK	19	1.90E-04	588.3393	1761.9960	3	-0.0029	17	1	Semi-tryptic
H2B1A_MOUSE, H2B1B_MOUSE, H2B1C_MOUSE, H2B1F_MOUSE, H2B1H_MOUSE, H2B1K_MOUSE, H2B1M_MOUSE, H2B1P_MOUSE, H2B2B_MOUSE, H2B2E_MOUSE, H2B3A_MOUSE, H2B3B_MOUSE	Histone H2B	ASRLAHYNKRSTITSRE	140	9.20E-06	498.2691	1989.0475	4	-0.0029	17	0	GluC
H2B1A_MOUSE, H2B1B_MOUSE, H2B1C_MOUSE, H2B1F_MOUSE, H2B1H_MOUSE, H2B1K_MOUSE, H2B1M_MOUSE, H2B1P_MOUSE, H2B2B_MOUSE, H2B2E_MOUSE, H2B3A_MOUSE, H2B3B_MOUSE	Histone H2B	LLLPGELAKHAVSEGTK	19	1.80E-03	588.3392	1761.9958	3	-0.0030	17	1	Semi-tryptic
H2B1A_MOUSE, H2B1B_MOUSE, H2B1C_MOUSE, H2B1F_MOUSE, H2B1H_MOUSE, H2B1K_MOUSE, H2B1M_MOUSE, H2B1P_MOUSE, H2B2B_MOUSE, H2B2E_MOUSE, H2B3A_MOUSE, H2B3B_MOUSE	Histone H2B	ASRLAHYNKRSTITSRE	140	3.80E-05	498.2691	1989.0473	4	-0.0030	17	0	GluC
H2B1A_MOUSE, H2B1B_MOUSE, H2B1C_MOUSE, H2B1F_MOUSE, H2B1H_MOUSE, H2B1K_MOUSE, H2B1M_MOUSE, H2B1P_MOUSE, H2B2B_MOUSE, H2B2E_MOUSE, H2B3A_MOUSE, H2B3B_MOUSE	Histone H2B	ASRLAHYNKRSTITSRE	140	9.40E-07	498.269	1989.0471	4	-0.0033	17	0	GluC
H2B1A_MOUSE, H2B1B_MOUSE, H2B1C_MOUSE, H2B1F_MOUSE, H2B1H_MOUSE, H2B1K_MOUSE, H2B1M_MOUSE, H2B1P_MOUSE, H2B2B_MOUSE, H2B2E_MOUSE, H2B3A_MOUSE, H2B3B_MOUSE	Histone H2B	ASRLAHYNKRSTITSRE	140	1.50E-09	664.0227	1989.0463	3	-0.0040	17	0	GluC
H2B1A_MOUSE, H2B1B_MOUSE, H2B1C_MOUSE, H2B1F_MOUSE, H2B1H_MOUSE, H2B1K_MOUSE, H2B1M_MOUSE, H2B1P_MOUSE, H2B2B_MOUSE, H2B2E_MOUSE, H2B3A_MOUSE, H2B3B_MOUSE	Histone H2B	LLLPGELAKHAVSEGTK	19	7.70E-03	588.3389	1761.9948	3	-0.0041	17	1	Semi-tryptic
H2B1A_MOUSE, H2B1B_MOUSE, H2B1C_MOUSE, H2B1F_MOUSE, H2B1H_MOUSE, H2B1K_MOUSE, H2B1M_MOUSE, H2B1P_MOUSE, H2B2B_MOUSE, H2B2E_MOUSE, H2B3A_MOUSE, H2B3B_MOUSE	Histone H2B	LLLPGELAKHAVSEGTK	19	3.10E-03	588.3389	1761.9948	3	-0.0041	17	1	Semi-tryptic
H2B1A_MOUSE, H2B1B_MOUSE, H2B1C_MOUSE, H2B1F_MOUSE, H2B1H_MOUSE, H2B1K_MOUSE, H2B1M_MOUSE, H2B1P_MOUSE, H2B2B_MOUSE, H2B2E_MOUSE, H2B3A_MOUSE, H2B3B_MOUSE	Histone H2B	ASRLAHYNKRSTITSRE	140	1.60E-10	664.0226	1989.0460	3	-0.0044	17	0	GluC
H2B1A_MOUSE, H2B1B_MOUSE, H2B1C_MOUSE, H2B1F_MOUSE, H2B1H_MOUSE, H2B1K_MOUSE, H2B1M_MOUSE, H2B1P_MOUSE, H2B2B_MOUSE, H2B2E_MOUSE, H2B3A_MOUSE, H2B3B_MOUSE	Histone H2B	LLLPGELAKHAVSEGTK	19	1.40E-03	588.3386	1761.9938	3	-0.0050	17	1	Semi-tryptic

H2B1A_MOUSE, H2B1B_MOUSE, H2B1C_MOUSE, H2B1F_MOUSE, H2B1H_MOUSE, H2B1K_MOUSE, H2B1M_MOUSE, H2B1P_MOUSE, H2B2B_MOUSE, H2B2E_MOUSE, H2B3A_MOUSE, H2B3B_MOUSE	Histone H2B	ASRLAHYNKRSTITSRE	140	7.60E-07	498.2686	1989.0453	4	-0.0050	17	0	GluC
H2B1A_MOUSE, H2B1B_MOUSE, H2B1C_MOUSE, H2B1F_MOUSE, H2B1H_MOUSE, H2B1K_MOUSE, H2B1M_MOUSE, H2B1P_MOUSE, H2B2B_MOUSE, H2B2E_MOUSE, H2B3A_MOUSE, H2B3B_MOUSE	Histone H2B	LLLPGELAKHAVSEGTK	19	5.90E-06	588.3386	1761.9938	3	-0.0050	17	1	Semi-tryptic
H2B1A_MOUSE, H2B1B_MOUSE, H2B1C_MOUSE, H2B1F_MOUSE, H2B1H_MOUSE, H2B1K_MOUSE, H2B1M_MOUSE, H2B1P_MOUSE, H2B2B_MOUSE, H2B2E_MOUSE, H2B3A_MOUSE, H2B3B_MOUSE	Histone H2B	LLLPGELAKHAVSEGTK	19	9.00E-08	882.0041	1761.9937	2	-0.0051	17	1	Semi-tryptic
H2B1A_MOUSE, H2B1B_MOUSE, H2B1C_MOUSE, H2B1F_MOUSE, H2B1H_MOUSE, H2B1K_MOUSE, H2B1M_MOUSE, H2B1P_MOUSE, H2B2B_MOUSE, H2B2E_MOUSE, H2B3A_MOUSE, H2B3B_MOUSE	Histone H2B	ASRLAHYNKRSTITSRE	140	1.00E-02	498.2685	1989.0450	4	-0.0054	17	0	GluC
H2B1A_MOUSE, H2B1B_MOUSE, H2B1C_MOUSE, H2B1F_MOUSE, H2B1H_MOUSE, H2B1K_MOUSE, H2B1M_MOUSE, H2B1P_MOUSE, H2B2B_MOUSE, H2B2E_MOUSE, H2B3A_MOUSE, H2B3B_MOUSE	Histone H2B	LLLPGELAKHAVSEGTK	19	1.20E-04	441.5056	1761.9933	4	-0.0055	17	1	Semi-tryptic
H2B1A_MOUSE, H2B1B_MOUSE, H2B1C_MOUSE, H2B1F_MOUSE, H2B1H_MOUSE, H2B1K_MOUSE, H2B1M_MOUSE, H2B1P_MOUSE, H2B2B_MOUSE, H2B2E_MOUSE, H2B3A_MOUSE, H2B3B_MOUSE	Histone H2B	LLLPGELAKHAVSEGTK	19	9.10E-08	441.5056	1761.9933	4	-0.0055	17	1	Semi-tryptic
H2B1A_MOUSE, H2B1B_MOUSE, H2B1C_MOUSE, H2B1F_MOUSE, H2B1H_MOUSE, H2B1K_MOUSE, H2B1M_MOUSE, H2B1P_MOUSE, H2B2B_MOUSE, H2B2E_MOUSE, H2B3A_MOUSE, H2B3B_MOUSE	Histone H2B	ASRLAHYNKRSTITSRE	140	3.20E-04	498.2684	1989.0443	4	-0.0060	17	0	GluC
H2B1A_MOUSE, H2B1B_MOUSE, H2B1C_MOUSE, H2B1F_MOUSE, H2B1H_MOUSE, H2B1K_MOUSE, H2B1M_MOUSE, H2B1P_MOUSE, H2B2B_MOUSE, H2B2E_MOUSE, H2B3A_MOUSE, H2B3B_MOUSE	Histone H2B	ASRLAHYNKRSTITSRE	140	1.80E-10	664.0215	1989.0427	3	-0.0076	17	0	GluC
H2B1A_MOUSE, H2B1B_MOUSE, H2B1C_MOUSE, H2B1F_MOUSE, H2B1H_MOUSE, H2B1K_MOUSE, H2B1M_MOUSE, H2B1P_MOUSE, H2B2B_MOUSE, H2B2E_MOUSE, H2B3A_MOUSE, H2B3B_MOUSE	Histone H2B	ASRLAHYNKRSTITSRE	140	8.90E-09	498.2676	1989.0413	4	-0.0091	17	0	GluC
H2B1A_MOUSE, H2B1B_MOUSE, H2B1C_MOUSE, H2B1F_MOUSE, H2B1H_MOUSE, H2B1K_MOUSE, H2B1M_MOUSE, H2B1P_MOUSE, H2B2B_MOUSE, H2B2E_MOUSE, H2B3A_MOUSE, H2B3B_MOUSE	Histone H2B	ASRLAHYNKRSTITSRE	140	1.50E-10	664.0203	1989.0391	3	-0.0113	17	0	GluC
H2B1A_MOUSE, H2B1B_MOUSE, H2B1C_MOUSE, H2B1F_MOUSE, H2B1H_MOUSE, H2B1K_MOUSE, H2B1M_MOUSE, H2B1P_MOUSE, H2B2B_MOUSE, H2B2E_MOUSE, H2B3A_MOUSE, H2B3B_MOUSE	Histone H2B	ASRLAHYNKRSTITSRE	140	1.30E-04	398.815	1989.0384	5	-0.0119	17	0	GluC
H2B1A_MOUSE, H2B1B_MOUSE, H2B1C_MOUSE, H2B1F_MOUSE, H2B1H_MOUSE, H2B1K_MOUSE, H2B1M_MOUSE, H2B1P_MOUSE, H2B2B_MOUSE, H2B2E_MOUSE, H2B3A_MOUSE, H2B3B_MOUSE	Histone H2B	ASRLAHYNKRSTITSRE	140	7.10E-08	498.2668	1989.0380	4	-0.0124	17	0	GluC
H2B1A_MOUSE, H2B1B_MOUSE, H2B1C_MOUSE, H2B1F_MOUSE, H2B1H_MOUSE, H2B1K_MOUSE, H2B1M_MOUSE, H2B1P_MOUSE, H2B2B_MOUSE, H2B2E_MOUSE, H2B3A_MOUSE, H2B3B_MOUSE	Histone H2B	ASRLAHYNKRSTITSRE	140	2.20E-03	498.2667	1989.0376	4	-0.0128	17	0	GluC
H2B1A_MOUSE, H2B1B_MOUSE, H2B1C_MOUSE, H2B1F_MOUSE, H2B1H_MOUSE, H2B1K_MOUSE, H2B1M_MOUSE, H2B1P_MOUSE, H2B2B_MOUSE, H2B2E_MOUSE, H2B3A_MOUSE, H2B3B_MOUSE	Histone H2B	LLLPGELAKHAVSEGTK	19	5.10E-03	588.3436	1762.0090	3	0.0101	17	1	Semi-tryptic
H2B1A_MOUSE, H2B1B_MOUSE, H2B1C_MOUSE, H2B1F_MOUSE, H2B1H_MOUSE, H2B1K_MOUSE, H2B1M_MOUSE, H2B1P_MOUSE, H2B2B_MOUSE, H2B2E_MOUSE, H2B3A_MOUSE, H2B3B_MOUSE	Histone H2B	LLLPGELAKHAVSEGTK	19	8.50E-08	882.009	1762.0034	2	0.0046	17	1	Semi-tryptic
H2B1A_MOUSE, H2B1B_MOUSE, H2B1C_MOUSE, H2B1F_MOUSE, H2B1H_MOUSE, H2B1K_MOUSE, H2B1M_MOUSE, H2B1P_MOUSE, H2B2B_MOUSE, H2B2E_MOUSE, H2B3A_MOUSE, H2B3B_MOUSE	Histone H2B	LLLPGELAKHAVSEGTK	19	7.90E-05	588.3412	1762.0017	3	0.0029	17	1	Semi-tryptic
H2B1A_MOUSE, H2B1B_MOUSE, H2B1C_MOUSE, H2B1F_MOUSE, H2B1H_MOUSE, H2B1K_MOUSE, H2B1M_MOUSE, H2B1P_MOUSE, H2B2B_MOUSE, H2B2E_MOUSE, H2B3A_MOUSE, H2B3B_MOUSE	Histone H2B	LLLPGELAKHAVSEGTK	19	2.40E-06	588.3412	1762.0017	3	0.0029	17	1	Semi-tryptic
H2B1A_MOUSE, H2B1B_MOUSE, H2B1C_MOUSE, H2B1F_MOUSE, H2B1H_MOUSE, H2B1K_MOUSE, H2B1M_MOUSE, H2B1P_MOUSE, H2B2B_MOUSE, H2B2E_MOUSE, H2B3A_MOUSE, H2B3B_MOUSE	Histone H2B	LLLPGELAKHAVSEGTK	19	1.30E-07	882.0078	1762.0010	2	0.0021	17	1	Semi-tryptic
H2B1A_MOUSE, H2B1B_MOUSE, H2B1C_MOUSE, H2B1F_MOUSE, H2B1H_MOUSE, H2B1K_MOUSE, H2B1M_MOUSE, H2B1P_MOUSE, H2B2B_MOUSE, H2B2E_MOUSE, H2B3A_MOUSE, H2B3B_MOUSE	Histone H2B	LLLPGELAKHAVSEGTK	19	4.80E-08	882.007	1761.9995	2	0.0007	17	1	Semi-tryptic
H2B1A_MOUSE, H2B1B_MOUSE, H2B1C_MOUSE, H2B1F_MOUSE, H2B1H_MOUSE, H2B1K_MOUSE, H2B1M_MOUSE, H2B1P_MOUSE, H2B2B_MOUSE, H2B2E_MOUSE, H2B3A_MOUSE, H2B3B_MOUSE	Histone H2B	LLLPGELAKHAVSEGTK	19	4.80E-07	441.5071	1761.9992	4	0.0003	17	1	Semi-tryptic
H2B1A_MOUSE, H2B1B_MOUSE, H2B1C_MOUSE, H2B1F_MOUSE, H2B1H_MOUSE, H2B1K_MOUSE, H2B1M_MOUSE, H2B1P_MOUSE, H2B2B_MOUSE, H2B2E_MOUSE, H2B3A_MOUSE, H2B3B_MOUSE	Histone H2B	LLLPGELAKHAVSEGTK	19	1.40E-08	441.5071	1761.9992	4	0.0003	17	1	Semi-tryptic
H2B1A_MOUSE, H2B1B_MOUSE, H2B1C_MOUSE, H2B1F_MOUSE, H2B1H_MOUSE, H2B1K_MOUSE, H2B1M_MOUSE, H2B1P_MOUSE, H2B2B_MOUSE, H2B2E_MOUSE, H2B3A_MOUSE, H2B3B_MOUSE	Histone H2B	LLLPGELAKHAVSEGTK	19	1.00E-02	588.3401	1761.9985	3	-0.0004	17	1	Semi-tryptic
H2B1A_MOUSE, H2B1B_MOUSE, H2B1C_MOUSE, H2B1F_MOUSE, H2B1H_MOUSE, H2B1K_MOUSE, H2B1M_MOUSE, H2B1P_MOUSE, H2B2B_MOUSE, H2B2E_MOUSE, H2B3A_MOUSE, H2B3B_MOUSE	Histone H2B	ASRLAHYNKRSTITSRE	140	4.20E-07	498.2732	1989.0638	4	0.0134	17	0	GluC
H2B1A_MOUSE, H2B1B_MOUSE, H2B1C_MOUSE, H2B1F_MOUSE, H2B1H_MOUSE, H2B1K_MOUSE, H2B1M_MOUSE, H2B1P_MOUSE, H2B2B_MOUSE, H2B2E_MOUSE, H2B3A_MOUSE, H2B3B_MOUSE	Histone H2B	ASRLAHYNKRSTITSRE	140	4.10E-07	498.2729	1989.0623	4	0.0119	17	0	GluC

H2B1A_MOUSE, H2B1B_MOUSE, H2B1C_MOUSE, H2B1F_MOUSE, H2B1H_MOUSE, H2B1K_MOUSE, H2B1M_MOUSE, H2B1P_MOUSE, H2B2B_MOUSE, H2B2E_MOUSE, H2B3A_MOUSE, H2B3B_MOUSE	Histone H2B	ASRLAHYNKRSTITSRE	140	1.20E-07	498.2727	1989.0618	4	0.0114	17	0	GluC
H2B1A_MOUSE, H2B1B_MOUSE, H2B1C_MOUSE, H2B1F_MOUSE, H2B1H_MOUSE, H2B1K_MOUSE, H2B1M_MOUSE, H2B1P_MOUSE, H2B2B_MOUSE, H2B2E_MOUSE, H2B3A_MOUSE, H2B3B_MOUSE	Histone H2B	ASRLAHYNKRSTITSRE	140	1.90E-04	498.2727	1989.0617	4	0.0113	17	0	GluC
H2B1A_MOUSE, H2B1B_MOUSE, H2B1C_MOUSE, H2B1F_MOUSE, H2B1H_MOUSE, H2B1K_MOUSE, H2B1M_MOUSE, H2B1P_MOUSE, H2B2B_MOUSE, H2B2E_MOUSE, H2B3A_MOUSE, H2B3B_MOUSE	Histone H2B	ASRLAHYNKRSTITSRE	140	4.00E-04	398.8194	1989.0607	5	0.0103	17	0	GluC
H2B1A_MOUSE, H2B1B_MOUSE, H2B1C_MOUSE, H2B1F_MOUSE, H2B1H_MOUSE, H2B1K_MOUSE, H2B1M_MOUSE, H2B1P_MOUSE, H2B2B_MOUSE, H2B2E_MOUSE, H2B3A_MOUSE, H2B3B_MOUSE	Histone H2B	ASRLAHYNKRSTITSRE	140	3.20E-10	664.0274	1989.0604	3	0.0100	17	0	GluC
H2B1A_MOUSE, H2B1B_MOUSE, H2B1C_MOUSE, H2B1F_MOUSE, H2B1H_MOUSE, H2B1K_MOUSE, H2B1M_MOUSE, H2B1P_MOUSE, H2B2B_MOUSE, H2B2E_MOUSE, H2B3A_MOUSE, H2B3B_MOUSE	Histone H2B	ASRLAHYNKRSTITSRE	140	4.80E-05	498.2723	1989.0601	4	0.0098	17	0	GluC
H2B1A_MOUSE, H2B1B_MOUSE, H2B1C_MOUSE, H2B1F_MOUSE, H2B1H_MOUSE, H2B1K_MOUSE, H2B1M_MOUSE, H2B1P_MOUSE, H2B2B_MOUSE, H2B2E_MOUSE, H2B3A_MOUSE, H2B3B_MOUSE	Histone H2B	ASRLAHYNKRSTITSRE	140	2.20E-04	498.2723	1989.0601	4	0.0097	17	0	GluC
H2B1A_MOUSE, H2B1B_MOUSE, H2B1C_MOUSE, H2B1F_MOUSE, H2B1H_MOUSE, H2B1K_MOUSE, H2B1M_MOUSE, H2B1P_MOUSE, H2B2B_MOUSE, H2B2E_MOUSE, H2B3A_MOUSE, H2B3B_MOUSE	Histone H2B	ASRLAHYNKRSTITSRE	140	6.30E-06	498.2722	1989.0597	4	0.0094	17	0	GluC
H2B1A_MOUSE, H2B1B_MOUSE, H2B1C_MOUSE, H2B1F_MOUSE, H2B1H_MOUSE, H2B1K_MOUSE, H2B1M_MOUSE, H2B1P_MOUSE, H2B2B_MOUSE, H2B2E_MOUSE, H2B3A_MOUSE, H2B3B_MOUSE	Histone H2B	ASRLAHYNKRSTITSRE	140	5.10E-06	498.2721	1989.0593	4	0.0089	17	0	GluC
H2B1A_MOUSE, H2B1B_MOUSE, H2B1C_MOUSE, H2B1F_MOUSE, H2B1H_MOUSE, H2B1K_MOUSE, H2B1M_MOUSE, H2B1P_MOUSE, H2B2B_MOUSE, H2B2E_MOUSE, H2B3A_MOUSE, H2B3B_MOUSE	Histone H2B	ASRLAHYNKRSTITSRE	140	4.10E-08	498.2721	1989.0593	4	0.0089	17	0	GluC
H2B1A_MOUSE, H2B1B_MOUSE, H2B1C_MOUSE, H2B1F_MOUSE, H2B1H_MOUSE, H2B1K_MOUSE, H2B1M_MOUSE, H2B1P_MOUSE, H2B2B_MOUSE, H2B2E_MOUSE, H2B3A_MOUSE, H2B3B_MOUSE	Histone H2B	ASRLAHYNKRSTITSRE	140	4.60E-03	498.2721	1989.0591	4	0.0088	17	0	GluC
H2B1A_MOUSE, H2B1B_MOUSE, H2B1C_MOUSE, H2B1F_MOUSE, H2B1H_MOUSE, H2B1K_MOUSE, H2B1M_MOUSE, H2B1P_MOUSE, H2B2B_MOUSE, H2B2E_MOUSE, H2B3A_MOUSE, H2B3B_MOUSE	Histone H2B	ASRLAHYNKRSTITSRE	140	4.00E-05	498.272	1989.0591	4	0.0087	17	0	GluC
H2B1A_MOUSE, H2B1B_MOUSE, H2B1C_MOUSE, H2B1F_MOUSE, H2B1H_MOUSE, H2B1K_MOUSE, H2B1M_MOUSE, H2B1P_MOUSE, H2B2B_MOUSE, H2B2E_MOUSE, H2B3A_MOUSE, H2B3B_MOUSE	Histone H2B	ASRLAHYNKRSTITSRE	140	7.40E-08	498.272	1989.0588	4	0.0084	17	0	GluC
H2B1A_MOUSE, H2B1B_MOUSE, H2B1C_MOUSE, H2B1F_MOUSE, H2B1H_MOUSE, H2B1K_MOUSE, H2B1M_MOUSE, H2B1P_MOUSE, H2B2B_MOUSE, H2B2E_MOUSE, H2B3A_MOUSE, H2B3B_MOUSE	Histone H2B	ASRLAHYNKRSTITSRE	140	1.20E-04	398.819	1989.0586	5	0.0083	17	0	GluC
H2B1A_MOUSE, H2B1B_MOUSE, H2B1C_MOUSE, H2B1F_MOUSE, H2B1H_MOUSE, H2B1K_MOUSE, H2B1M_MOUSE, H2B1P_MOUSE, H2B2B_MOUSE, H2B2E_MOUSE, H2B3A_MOUSE, H2B3B_MOUSE	Histone H2B	ASRLAHYNKRSTITSRE	140	1.50E-05	498.2719	1989.0587	4	0.0083	17	0	GluC
H2B1A_MOUSE, H2B1B_MOUSE, H2B1C_MOUSE, H2B1F_MOUSE, H2B1H_MOUSE, H2B1K_MOUSE, H2B1M_MOUSE, H2B1P_MOUSE, H2B2B_MOUSE, H2B2E_MOUSE, H2B3A_MOUSE, H2B3B_MOUSE	Histone H2B	ASRLAHYNKRSTITSRE	140	3.40E-05	498.2719	1989.0585	4	0.0082	17	0	GluC
H2B1A_MOUSE, H2B1B_MOUSE, H2B1C_MOUSE, H2B1F_MOUSE, H2B1H_MOUSE, H2B1K_MOUSE, H2B1M_MOUSE, H2B1P_MOUSE, H2B2B_MOUSE, H2B2E_MOUSE, H2B3A_MOUSE, H2B3B_MOUSE	Histone H2B	ASRLAHYNKRSTITSRE	140	4.40E-06	498.2719	1989.0583	4	0.0080	17	0	GluC
H2B1A_MOUSE, H2B1B_MOUSE, H2B1C_MOUSE, H2B1F_MOUSE, H2B1H_MOUSE, H2B1K_MOUSE, H2B1M_MOUSE, H2B1P_MOUSE, H2B2B_MOUSE, H2B2E_MOUSE, H2B3A_MOUSE, H2B3B_MOUSE	Histone H2B	ASRLAHYNKRSTITSRE	140	4.50E-04	498.2718	1989.0582	4	0.0078	17	0	GluC
H2B1A_MOUSE, H2B1B_MOUSE, H2B1C_MOUSE, H2B1F_MOUSE, H2B1H_MOUSE, H2B1K_MOUSE, H2B1M_MOUSE, H2B1P_MOUSE, H2B2B_MOUSE, H2B2E_MOUSE, H2B3A_MOUSE, H2B3B_MOUSE	Histone H2B	ASRLAHYNKRSTITSRE	140	1.30E-06	498.2718	1989.0581	4	0.0077	17	0	GluC
H2B1A_MOUSE, H2B1B_MOUSE, H2B1C_MOUSE, H2B1F_MOUSE, H2B1H_MOUSE, H2B1K_MOUSE, H2B1M_MOUSE, H2B1P_MOUSE, H2B2B_MOUSE, H2B2E_MOUSE, H2B3A_MOUSE, H2B3B_MOUSE	Histone H2B	ASRLAHYNKRSTITSRE	140	2.10E-03	498.2717	1989.0579	4	0.0075	17	0	GluC
H2B1A_MOUSE, H2B1B_MOUSE, H2B1C_MOUSE, H2B1F_MOUSE, H2B1H_MOUSE, H2B1K_MOUSE, H2B1M_MOUSE, H2B1P_MOUSE, H2B2B_MOUSE, H2B2E_MOUSE, H2B3A_MOUSE, H2B3B_MOUSE	Histone H2B	ASRLAHYNKRSTITSRE	140	8.80E-05	498.2717	1989.0579	4	0.0075	17	0	GluC
H2B1A_MOUSE, H2B1B_MOUSE, H2B1C_MOUSE, H2B1F_MOUSE, H2B1H_MOUSE, H2B1K_MOUSE, H2B1M_MOUSE, H2B1P_MOUSE, H2B2B_MOUSE, H2B2E_MOUSE, H2B3A_MOUSE, H2B3B_MOUSE	Histone H2B	ASRLAHYNKRSTITSRE	140	3.10E-11	664.0266	1989.0578	3	0.0075	17	0	GluC
H2B1A_MOUSE, H2B1B_MOUSE, H2B1C_MOUSE, H2B1F_MOUSE, H2B1H_MOUSE, H2B1K_MOUSE, H2B1M_MOUSE, H2B1P_MOUSE, H2B2B_MOUSE, H2B2E_MOUSE, H2B3A_MOUSE, H2B3B_MOUSE	Histone H2B	ASRLAHYNKRSTITSRE	140	5.80E-06	498.2717	1989.0578	4	0.0074	17	0	GluC
H2B1A_MOUSE, H2B1B_MOUSE, H2B1C_MOUSE, H2B1F_MOUSE, H2B1H_MOUSE, H2B1K_MOUSE, H2B1M_MOUSE, H2B1P_MOUSE, H2B2B_MOUSE, H2B2E_MOUSE, H2B3A_MOUSE, H2B3B_MOUSE	Histone H2B	ASRLAHYNKRSTITSRE	140	1.70E-06	498.2717	1989.0577	4	0.0074	17	0	GluC
H2B1A_MOUSE, H2B1B_MOUSE, H2B1C_MOUSE, H2B1F_MOUSE, H2B1H_MOUSE, H2B1K_MOUSE, H2B1M_MOUSE, H2B1P_MOUSE, H2B2B_MOUSE, H2B2E_MOUSE, H2B3A_MOUSE, H2B3B_MOUSE	Histone H2B	ASRLAHYNKRSTITSRE	140	1.30E-06	498.2717	1989.0577	4	0.0074	17	0	GluC

H2B1A_MOUSE, H2B1B_MOUSE, H2B1C_MOUSE, H2B1F_MOUSE, H2B1H_MOUSE, H2B1K_MOUSE, H2B1M_MOUSE, H2B1P_MOUSE, H2B2B_MOUSE, H2B2E_MOUSE, H2B3A_MOUSE, H2B3B_MOUSE	Histone H2B	ASRLAHYNKRSTITSRE	140	3.70E-06	498.2717	1989.0575	4	0.0072	17	0	GluC
H2B1A_MOUSE, H2B1B_MOUSE, H2B1C_MOUSE, H2B1F_MOUSE, H2B1H_MOUSE, H2B1K_MOUSE, H2B1M_MOUSE, H2B1P_MOUSE, H2B2B_MOUSE, H2B2E_MOUSE, H2B3A_MOUSE, H2B3B_MOUSE	Histone H2B	ASRLAHYNKRSTITSRE	140	8.20E-06	498.2717	1989.0575	4	0.0071	17	0	GluC
H2B1A_MOUSE, H2B1B_MOUSE, H2B1C_MOUSE, H2B1F_MOUSE, H2B1H_MOUSE, H2B1K_MOUSE, H2B1M_MOUSE, H2B1P_MOUSE, H2B2B_MOUSE, H2B2E_MOUSE, H2B3A_MOUSE, H2B3B_MOUSE	Histone H2B	ASRLAHYNKRSTITSRE	140	1.80E-04	498.2716	1989.0573	4	0.0070	17	0	GluC
H2B1A_MOUSE, H2B1B_MOUSE, H2B1C_MOUSE, H2B1F_MOUSE, H2B1H_MOUSE, H2B1K_MOUSE, H2B1M_MOUSE, H2B1P_MOUSE, H2B2B_MOUSE, H2B2E_MOUSE, H2B3A_MOUSE, H2B3B_MOUSE	Histone H2B	ASRLAHYNKRSTITSRE	140	8.10E-04	498.2716	1989.0573	4	0.0069	17	0	GluC
H2B1A_MOUSE, H2B1B_MOUSE, H2B1C_MOUSE, H2B1F_MOUSE, H2B1H_MOUSE, H2B1K_MOUSE, H2B1M_MOUSE, H2B1P_MOUSE, H2B2B_MOUSE, H2B2E_MOUSE, H2B3A_MOUSE, H2B3B_MOUSE	Histone H2B	ASRLAHYNKRSTITSRE	140	8.60E-03	498.2716	1989.0571	4	0.0068	17	0	GluC
H2B1A_MOUSE, H2B1B_MOUSE, H2B1C_MOUSE, H2B1F_MOUSE, H2B1H_MOUSE, H2B1K_MOUSE, H2B1M_MOUSE, H2B1P_MOUSE, H2B2B_MOUSE, H2B2E_MOUSE, H2B3A_MOUSE, H2B3B_MOUSE	Histone H2B	ASRLAHYNKRSTITSRE	140	5.40E-04	498.2716	1989.0572	4	0.0068	17	0	GluC
H2B1A_MOUSE, H2B1B_MOUSE, H2B1C_MOUSE, H2B1F_MOUSE, H2B1H_MOUSE, H2B1K_MOUSE, H2B1M_MOUSE, H2B1P_MOUSE, H2B2B_MOUSE, H2B2E_MOUSE, H2B3A_MOUSE, H2B3B_MOUSE	Histone H2B	ASRLAHYNKRSTITSRE	140	1.80E-05	498.2716	1989.0572	4	0.0068	17	0	GluC
H2B1A_MOUSE, H2B1B_MOUSE, H2B1C_MOUSE, H2B1F_MOUSE, H2B1H_MOUSE, H2B1K_MOUSE, H2B1M_MOUSE, H2B1P_MOUSE, H2B2B_MOUSE, H2B2E_MOUSE, H2B3A_MOUSE, H2B3B_MOUSE	Histone H2B	ASRLAHYNKRSTITSRE	140	5.10E-11	664.0263	1989.0572	3	0.0068	17	0	GluC
H2B1A_MOUSE, H2B1B_MOUSE, H2B1C_MOUSE, H2B1F_MOUSE, H2B1H_MOUSE, H2B1K_MOUSE, H2B1M_MOUSE, H2B1P_MOUSE, H2B2B_MOUSE, H2B2E_MOUSE, H2B3A_MOUSE, H2B3B_MOUSE	Histone H2B	ASRLAHYNKRSTITSRE	140	4.30E-06	498.2715	1989.0571	4	0.0067	17	0	GluC
H2B1A_MOUSE, H2B1B_MOUSE, H2B1C_MOUSE, H2B1F_MOUSE, H2B1H_MOUSE, H2B1K_MOUSE, H2B1M_MOUSE, H2B1P_MOUSE, H2B2B_MOUSE, H2B2E_MOUSE, H2B3A_MOUSE, H2B3B_MOUSE	Histone H2B	ASRLAHYNKRSTITSRE	140	6.00E-05	498.2715	1989.0570	4	0.0066	17	0	GluC
H2B1A_MOUSE, H2B1B_MOUSE, H2B1C_MOUSE, H2B1F_MOUSE, H2B1H_MOUSE, H2B1K_MOUSE, H2B1M_MOUSE, H2B1P_MOUSE, H2B2B_MOUSE, H2B2E_MOUSE, H2B3A_MOUSE, H2B3B_MOUSE	Histone H2B	ASRLAHYNKRSTITSRE	140	1.80E-04	498.2715	1989.0569	4	0.0065	17	0	GluC
H2B1A_MOUSE, H2B1B_MOUSE, H2B1C_MOUSE, H2B1F_MOUSE, H2B1H_MOUSE, H2B1K_MOUSE, H2B1M_MOUSE, H2B1P_MOUSE, H2B2B_MOUSE, H2B2E_MOUSE, H2B3A_MOUSE, H2B3B_MOUSE	Histone H2B	ASRLAHYNKRSTITSRE	140	1.00E-05	498.2715	1989.0569	4	0.0065	17	0	GluC
H2B1A_MOUSE, H2B1B_MOUSE, H2B1C_MOUSE, H2B1F_MOUSE, H2B1H_MOUSE, H2B1K_MOUSE, H2B1M_MOUSE, H2B1P_MOUSE, H2B2B_MOUSE, H2B2E_MOUSE, H2B3A_MOUSE, H2B3B_MOUSE	Histone H2B	ASRLAHYNKRSTITSRE	140	4.50E-07	498.2715	1989.0569	4	0.0065	17	0	GluC
H2B1A_MOUSE, H2B1B_MOUSE, H2B1C_MOUSE, H2B1F_MOUSE, H2B1H_MOUSE, H2B1K_MOUSE, H2B1M_MOUSE, H2B1P_MOUSE, H2B2B_MOUSE, H2B2E_MOUSE, H2B3A_MOUSE, H2B3B_MOUSE	Histone H2B	ASRLAHYNKRSTITSRE	140	2.00E-05	498.2714	1989.0566	4	0.0062	17	0	GluC
H2B1A_MOUSE, H2B1B_MOUSE, H2B1C_MOUSE, H2B1F_MOUSE, H2B1H_MOUSE, H2B1K_MOUSE, H2B1M_MOUSE, H2B1P_MOUSE, H2B2B_MOUSE, H2B2E_MOUSE, H2B3A_MOUSE, H2B3B_MOUSE	Histone H2B	ASRLAHYNKRSTITSRE	140	2.40E-10	664.0261	1989.0563	3	0.0060	17	0	GluC
H2B1A_MOUSE, H2B1B_MOUSE, H2B1C_MOUSE, H2B1F_MOUSE, H2B1H_MOUSE, H2B1K_MOUSE, H2B1M_MOUSE, H2B1P_MOUSE, H2B2B_MOUSE, H2B2E_MOUSE, H2B3A_MOUSE, H2B3B_MOUSE	Histone H2B	ASRLAHYNKRSTITSRE	140	6.80E-03	498.2713	1989.0561	4	0.0057	17	0	GluC
H2B1A_MOUSE, H2B1B_MOUSE, H2B1C_MOUSE, H2B1F_MOUSE, H2B1H_MOUSE, H2B1K_MOUSE, H2B1M_MOUSE, H2B1P_MOUSE, H2B2B_MOUSE, H2B2E_MOUSE, H2B3A_MOUSE, H2B3B_MOUSE	Histone H2B	ASRLAHYNKRSTITSRE	140	9.70E-05	498.2713	1989.0561	4	0.0057	17	0	GluC
H2B1A_MOUSE, H2B1B_MOUSE, H2B1C_MOUSE, H2B1F_MOUSE, H2B1H_MOUSE, H2B1K_MOUSE, H2B1M_MOUSE, H2B1P_MOUSE, H2B2B_MOUSE, H2B2E_MOUSE, H2B3A_MOUSE, H2B3B_MOUSE	Histone H2B	ASRLAHYNKRSTITSRE	140	2.10E-05	498.2713	1989.0559	4	0.0056	17	0	GluC
H2B1A_MOUSE, H2B1B_MOUSE, H2B1C_MOUSE, H2B1F_MOUSE, H2B1H_MOUSE, H2B1K_MOUSE, H2B1M_MOUSE, H2B1P_MOUSE, H2B2B_MOUSE, H2B2E_MOUSE, H2B3A_MOUSE, H2B3B_MOUSE	Histone H2B	ASRLAHYNKRSTITSRE	140	5.30E-08	498.2713	1989.0559	4	0.0056	17	0	GluC
H2B1A_MOUSE, H2B1B_MOUSE, H2B1C_MOUSE, H2B1F_MOUSE, H2B1H_MOUSE, H2B1K_MOUSE, H2B1M_MOUSE, H2B1P_MOUSE, H2B2B_MOUSE, H2B2E_MOUSE, H2B3A_MOUSE, H2B3B_MOUSE	Histone H2B	ASRLAHYNKRSTITSRE	140	3.20E-06	498.2712	1989.0559	4	0.0055	17	0	GluC
H2B1A_MOUSE, H2B1B_MOUSE, H2B1C_MOUSE, H2B1F_MOUSE, H2B1H_MOUSE, H2B1K_MOUSE, H2B1M_MOUSE, H2B1P_MOUSE, H2B2B_MOUSE, H2B2E_MOUSE, H2B3A_MOUSE, H2B3B_MOUSE	Histone H2B	ASRLAHYNKRSTITSRE	140	6.90E-06	498.2712	1989.0558	4	0.0054	17	0	GluC
H2B1A_MOUSE, H2B1B_MOUSE, H2B1C_MOUSE, H2B1F_MOUSE, H2B1H_MOUSE, H2B1K_MOUSE, H2B1M_MOUSE, H2B1P_MOUSE, H2B2B_MOUSE, H2B2E_MOUSE, H2B3A_MOUSE, H2B3B_MOUSE	Histone H2B	ASRLAHYNKRSTITSRE	140	5.10E-07	664.0259	1989.0558	3	0.0054	17	0	GluC
H2B1A_MOUSE, H2B1B_MOUSE, H2B1C_MOUSE, H2B1F_MOUSE, H2B1H_MOUSE, H2B1K_MOUSE, H2B1M_MOUSE, H2B1P_MOUSE, H2B2B_MOUSE, H2B2E_MOUSE, H2B3A_MOUSE, H2B3B_MOUSE	Histone H2B	ASRLAHYNKRSTITSRE	140	7.80E-04	664.0258	1989.0557	3	0.0053	17	0	GluC
H2B1A_MOUSE, H2B1B_MOUSE, H2B1C_MOUSE, H2B1F_MOUSE, H2B1H_MOUSE, H2B1K_MOUSE, H2B1M_MOUSE, H2B1P_MOUSE, H2B2B_MOUSE, H2B2E_MOUSE, H2B3A_MOUSE, H2B3B_MOUSE	Histone H2B	ASRLAHYNKRSTITSRE	140	4.60E-06	498.2712	1989.0557	4	0.0053	17	0	GluC

H2B1A_MOUSE, H2B1B_MOUSE, H2B1C_MOUSE, H2B1F_MOUSE, H2B1H_MOUSE, H2B1K_MOUSE, H2B1M_MOUSE, H2B1P_MOUSE, H2B2B_MOUSE, H2B2E_MOUSE, H2B3A_MOUSE, H2B3B_MOUSE	Histone H2B	ASRLAHYNKRSTITSRE	140	1.20E-03	498.2711	1989.0553	4	0.0049	17	0	GluC
H2B1A_MOUSE, H2B1B_MOUSE, H2B1C_MOUSE, H2B1F_MOUSE, H2B1H_MOUSE, H2B1K_MOUSE, H2B1M_MOUSE, H2B1P_MOUSE, H2B2B_MOUSE, H2B2E_MOUSE, H2B3A_MOUSE, H2B3B_MOUSE	Histone H2B	ASRLAHYNKRSTITSRE	140	2.40E-05	498.2711	1989.0553	4	0.0049	17	0	GluC
H2B1A_MOUSE, H2B1B_MOUSE, H2B1C_MOUSE, H2B1F_MOUSE, H2B1H_MOUSE, H2B1K_MOUSE, H2B1M_MOUSE, H2B1P_MOUSE, H2B2B_MOUSE, H2B2E_MOUSE, H2B3A_MOUSE, H2B3B_MOUSE	Histone H2B	ASRLAHYNKRSTITSRE	140	1.40E-09	498.2711	1989.0553	4	0.0049	17	0	GluC
H2B1A_MOUSE, H2B1B_MOUSE, H2B1C_MOUSE, H2B1F_MOUSE, H2B1H_MOUSE, H2B1K_MOUSE, H2B1M_MOUSE, H2B1P_MOUSE, H2B2B_MOUSE, H2B2E_MOUSE, H2B3A_MOUSE, H2B3B_MOUSE	Histone H2B	ASRLAHYNKRSTITSRE	140	1.60E-03	664.0256	1989.0550	3	0.0047	17	0	GluC
H2B1A_MOUSE, H2B1B_MOUSE, H2B1C_MOUSE, H2B1F_MOUSE, H2B1H_MOUSE, H2B1K_MOUSE, H2B1M_MOUSE, H2B1P_MOUSE, H2B2B_MOUSE, H2B2E_MOUSE, H2B3A_MOUSE, H2B3B_MOUSE	Histone H2B	ASRLAHYNKRSTITSRE	140	4.40E-05	498.271	1989.0549	4	0.0045	17	0	GluC
H2B1A_MOUSE, H2B1B_MOUSE, H2B1C_MOUSE, H2B1F_MOUSE, H2B1H_MOUSE, H2B1K_MOUSE, H2B1M_MOUSE, H2B1P_MOUSE, H2B2B_MOUSE, H2B2E_MOUSE, H2B3A_MOUSE, H2B3B_MOUSE	Histone H2B	ASRLAHYNKRSTITSRE	140	4.10E-06	664.0256	1989.0549	3	0.0045	17	0	GluC
H2B1A_MOUSE, H2B1B_MOUSE, H2B1C_MOUSE, H2B1F_MOUSE, H2B1H_MOUSE, H2B1K_MOUSE, H2B1M_MOUSE, H2B1P_MOUSE, H2B2B_MOUSE, H2B2E_MOUSE, H2B3A_MOUSE, H2B3B_MOUSE	Histone H2B	ASRLAHYNKRSTITSRE	140	6.10E-05	498.271	1989.0548	4	0.0044	17	0	GluC
H2B1A_MOUSE, H2B1B_MOUSE, H2B1C_MOUSE, H2B1F_MOUSE, H2B1H_MOUSE, H2B1K_MOUSE, H2B1M_MOUSE, H2B1P_MOUSE, H2B2B_MOUSE, H2B2E_MOUSE, H2B3A_MOUSE, H2B3B_MOUSE	Histone H2B	ASRLAHYNKRSTITSRE	140	1.40E-06	498.271	1989.0548	4	0.0044	17	0	GluC
H2B1A_MOUSE, H2B1B_MOUSE, H2B1C_MOUSE, H2B1F_MOUSE, H2B1H_MOUSE, H2B1K_MOUSE, H2B1M_MOUSE, H2B1P_MOUSE, H2B2B_MOUSE, H2B2E_MOUSE, H2B3A_MOUSE, H2B3B_MOUSE	Histone H2B	ASRLAHYNKRSTITSRE	140	8.50E-03	498.2708	1989.0543	4	0.0039	17	0	GluC
H2B1A_MOUSE, H2B1B_MOUSE, H2B1C_MOUSE, H2B1F_MOUSE, H2B1H_MOUSE, H2B1K_MOUSE, H2B1M_MOUSE, H2B1P_MOUSE, H2B2B_MOUSE, H2B2E_MOUSE, H2B3A_MOUSE, H2B3B_MOUSE	Histone H2B	ASRLAHYNKRSTITSRE	140	3.90E-09	664.0253	1989.0542	3	0.0038	17	0	GluC
H2B1A_MOUSE, H2B1B_MOUSE, H2B1C_MOUSE, H2B1F_MOUSE, H2B1H_MOUSE, H2B1K_MOUSE, H2B1M_MOUSE, H2B1P_MOUSE, H2B2B_MOUSE, H2B2E_MOUSE, H2B3A_MOUSE, H2B3B_MOUSE	Histone H2B	ASRLAHYNKRSTITSRE	140	2.50E-05	498.2708	1989.0541	4	0.0037	17	0	GluC
H2B1A_MOUSE, H2B1B_MOUSE, H2B1C_MOUSE, H2B1F_MOUSE, H2B1H_MOUSE, H2B1K_MOUSE, H2B1M_MOUSE, H2B1P_MOUSE, H2B2B_MOUSE, H2B2E_MOUSE, H2B3A_MOUSE, H2B3B_MOUSE	Histone H2B	ASRLAHYNKRSTITSRE	140	3.50E-07	498.2707	1989.0538	4	0.0034	17	0	GluC
H2B1A_MOUSE, H2B1B_MOUSE, H2B1C_MOUSE, H2B1F_MOUSE, H2B1H_MOUSE, H2B1K_MOUSE, H2B1M_MOUSE, H2B1P_MOUSE, H2B2B_MOUSE, H2B2E_MOUSE, H2B3A_MOUSE, H2B3B_MOUSE	Histone H2B	ASRLAHYNKRSTITSRE	140	3.10E-03	498.2707	1989.0536	4	0.0032	17	0	GluC
H2B1A_MOUSE, H2B1B_MOUSE, H2B1C_MOUSE, H2B1F_MOUSE, H2B1H_MOUSE, H2B1K_MOUSE, H2B1M_MOUSE, H2B1P_MOUSE, H2B2B_MOUSE, H2B2E_MOUSE, H2B3A_MOUSE, H2B3B_MOUSE	Histone H2B	ASRLAHYNKRSTITSRE	140	5.00E-05	664.0251	1989.0534	3	0.0030	17	0	GluC
H2B1A_MOUSE, H2B1B_MOUSE, H2B1C_MOUSE, H2B1F_MOUSE, H2B1H_MOUSE, H2B1K_MOUSE, H2B1M_MOUSE, H2B1P_MOUSE, H2B2B_MOUSE, H2B2E_MOUSE, H2B3A_MOUSE, H2B3B_MOUSE	Histone H2B	ASRLAHYNKRSTITSRE	140	1.70E-04	498.2706	1989.0531	4	0.0027	17	0	GluC
H2B1A_MOUSE, H2B1B_MOUSE, H2B1C_MOUSE, H2B1F_MOUSE, H2B1H_MOUSE, H2B1K_MOUSE, H2B1M_MOUSE, H2B1P_MOUSE, H2B2B_MOUSE, H2B2E_MOUSE, H2B3A_MOUSE, H2B3B_MOUSE	Histone H2B	ASRLAHYNKRSTITSRE	140	1.70E-06	498.2705	1989.0529	4	0.0025	17	0	GluC
H2B1A_MOUSE, H2B1B_MOUSE, H2B1C_MOUSE, H2B1F_MOUSE, H2B1H_MOUSE, H2B1K_MOUSE, H2B1M_MOUSE, H2B1P_MOUSE, H2B2B_MOUSE, H2B2E_MOUSE, H2B3A_MOUSE, H2B3B_MOUSE	Histone H2B	ASRLAHYNKRSTITSRE	140	6.00E-07	498.2704	1989.0525	4	0.0021	17	0	GluC
H2B1A_MOUSE, H2B1B_MOUSE, H2B1C_MOUSE, H2B1F_MOUSE, H2B1H_MOUSE, H2B1K_MOUSE, H2B1M_MOUSE, H2B1P_MOUSE, H2B2B_MOUSE, H2B2E_MOUSE, H2B3A_MOUSE, H2B3B_MOUSE	Histone H2B	ASRLAHYNKRSTITSRE	140	2.10E-06	664.0247	1989.0522	3	0.0018	17	0	GluC
H2B1A_MOUSE, H2B1B_MOUSE, H2B1C_MOUSE, H2B1F_MOUSE, H2B1H_MOUSE, H2B1K_MOUSE, H2B1M_MOUSE, H2B1P_MOUSE, H2B2B_MOUSE, H2B2E_MOUSE, H2B3A_MOUSE, H2B3B_MOUSE	Histone H2B	ASRLAHYNKRSTITSRE	140	1.30E-03	498.2703	1989.0521	4	0.0017	17	0	GluC
H2B1A_MOUSE, H2B1B_MOUSE, H2B1C_MOUSE, H2B1F_MOUSE, H2B1H_MOUSE, H2B1K_MOUSE, H2B1M_MOUSE, H2B1P_MOUSE, H2B2B_MOUSE, H2B2E_MOUSE, H2B3A_MOUSE, H2B3B_MOUSE	Histone H2B	ASRLAHYNKRSTITSRE	140	4.60E-04	498.2703	1989.0521	4	0.0017	17	0	GluC
H2B1A_MOUSE, H2B1B_MOUSE, H2B1C_MOUSE, H2B1F_MOUSE, H2B1H_MOUSE, H2B1K_MOUSE, H2B1M_MOUSE, H2B1P_MOUSE, H2B2B_MOUSE, H2B2E_MOUSE, H2B3A_MOUSE, H2B3B_MOUSE	Histone H2B	ASRLAHYNKRSTITSRE	140	9.70E-08	664.0246	1989.0520	3	0.0017	17	0	GluC
H2B1A_MOUSE, H2B1B_MOUSE, H2B1C_MOUSE, H2B1F_MOUSE, H2B1H_MOUSE, H2B1K_MOUSE, H2B1M_MOUSE, H2B1P_MOUSE, H2B2B_MOUSE, H2B2E_MOUSE, H2B3A_MOUSE, H2B3B_MOUSE	Histone H2B	ASRLAHYNKRSTITSRE	140	9.00E-09	664.0246	1989.0520	3	0.0017	17	0	GluC
H2B1A_MOUSE, H2B1B_MOUSE, H2B1C_MOUSE, H2B1F_MOUSE, H2B1H_MOUSE, H2B1K_MOUSE, H2B1M_MOUSE, H2B1P_MOUSE, H2B2B_MOUSE, H2B2E_MOUSE, H2B3A_MOUSE, H2B3B_MOUSE	Histone H2B	ASRLAHYNKRSTITSRE	140	5.70E-09	498.2702	1989.0519	4	0.0015	17	0	GluC
H2B1A_MOUSE, H2B1B_MOUSE, H2B1C_MOUSE, H2B1F_MOUSE, H2B1H_MOUSE, H2B1K_MOUSE, H2B1M_MOUSE, H2B1P_MOUSE, H2B2B_MOUSE, H2B2E_MOUSE, H2B3A_MOUSE, H2B3B_MOUSE	Histone H2B	ASRLAHYNKRSTITSRE	140	6.20E-09	664.0245	1989.0517	3	0.0013	17	0	GluC

H2B1A_MOUSE, H2B1B_MOUSE, H2B1C_MOUSE, H2B1F_MOUSE, H2B1H_MOUSE, H2B1K_MOUSE, H2B1M_MOUSE, H2B1P_MOUSE, H2B2B_MOUSE, H2B2E_MOUSE, H2B3A_MOUSE, H2B3B_MOUSE	Histone H2B	ASRLAHYNKRSTITSRE	140	1.10E-03	498.2702	1989.0516	4	0.0012	17	0	GluC
H2B1A_MOUSE, H2B1B_MOUSE, H2B1C_MOUSE, H2B1F_MOUSE, H2B1H_MOUSE, H2B1K_MOUSE, H2B1M_MOUSE, H2B1P_MOUSE, H2B2B_MOUSE, H2B2E_MOUSE, H2B3A_MOUSE, H2B3B_MOUSE	Histone H2B	ASRLAHYNKRSTITSRE	140	7.20E-05	498.2701	1989.0515	4	0.0011	17	0	GluC
H2B1A_MOUSE, H2B1B_MOUSE, H2B1C_MOUSE, H2B1F_MOUSE, H2B1H_MOUSE, H2B1K_MOUSE, H2B1M_MOUSE, H2B1P_MOUSE, H2B2B_MOUSE, H2B2E_MOUSE, H2B3A_MOUSE, H2B3B_MOUSE	Histone H2B	ASRLAHYNKRSTITSRE	140	9.20E-07	498.2701	1989.0514	4	0.0010	17	0	GluC
H2B1A_MOUSE, H2B1B_MOUSE, H2B1C_MOUSE, H2B1F_MOUSE, H2B1H_MOUSE, H2B1K_MOUSE, H2B1M_MOUSE, H2B1P_MOUSE, H2B2B_MOUSE, H2B2E_MOUSE, H2B3A_MOUSE, H2B3B_MOUSE	Histone H2B	ASRLAHYNKRSTITSRE	140	7.20E-04	498.2701	1989.0513	4	0.0009	17	0	GluC
H2B1A_MOUSE, H2B1B_MOUSE, H2B1C_MOUSE, H2B1F_MOUSE, H2B1H_MOUSE, H2B1K_MOUSE, H2B1M_MOUSE, H2B1P_MOUSE, H2B2B_MOUSE, H2B2E_MOUSE, H2B3A_MOUSE, H2B3B_MOUSE	Histone H2B	ASRLAHYNKRSTITSRE	140	9.30E-04	498.2701	1989.0511	4	0.0008	17	0	GluC
H2B1A_MOUSE, H2B1B_MOUSE, H2B1C_MOUSE, H2B1F_MOUSE, H2B1H_MOUSE, H2B1K_MOUSE, H2B1M_MOUSE, H2B1P_MOUSE, H2B2B_MOUSE, H2B2E_MOUSE, H2B3A_MOUSE, H2B3B_MOUSE	Histone H2B	ASRLAHYNKRSTITSRE	140	7.40E-05	498.2701	1989.0511	4	0.0007	17	0	GluC
H2B1A_MOUSE, H2B1B_MOUSE, H2B1C_MOUSE, H2B1F_MOUSE, H2B1H_MOUSE, H2B1K_MOUSE, H2B1M_MOUSE, H2B1P_MOUSE, H2B2B_MOUSE, H2B2E_MOUSE, H2B3A_MOUSE, H2B3B_MOUSE	Histone H2B	ASRLAHYNKRSTITSRE	140	3.30E-03	498.27	1989.0510	4	0.0006	17	0	GluC
H2B1A_MOUSE, H2B1B_MOUSE, H2B1C_MOUSE, H2B1F_MOUSE, H2B1H_MOUSE, H2B1K_MOUSE, H2B1M_MOUSE, H2B1P_MOUSE, H2B2B_MOUSE, H2B2E_MOUSE, H2B3A_MOUSE, H2B3B_MOUSE	Histone H2B	ASRLAHYNKRSTITSRE	140	8.50E-05	498.27	1989.0510	4	0.0006	17	0	GluC
H2B1A_MOUSE, H2B1B_MOUSE, H2B1C_MOUSE, H2B1F_MOUSE, H2B1H_MOUSE, H2B1K_MOUSE, H2B1M_MOUSE, H2B1P_MOUSE, H2B2B_MOUSE, H2B2E_MOUSE, H2B3A_MOUSE, H2B3B_MOUSE	Histone H2B	ASRLAHYNKRSTITSRE	140	5.30E-05	498.27	1989.0509	4	0.0005	17	0	GluC
H2B1A_MOUSE, H2B1B_MOUSE, H2B1C_MOUSE, H2B1F_MOUSE, H2B1H_MOUSE, H2B1K_MOUSE, H2B1M_MOUSE, H2B1P_MOUSE, H2B2B_MOUSE, H2B2E_MOUSE, H2B3A_MOUSE, H2B3B_MOUSE	Histone H2B	ASRLAHYNKRSTITSRE	140	2.00E-05	498.27	1989.0509	4	0.0005	17	0	GluC
H2B1A_MOUSE, H2B1B_MOUSE, H2B1C_MOUSE, H2B1F_MOUSE, H2B1H_MOUSE, H2B1K_MOUSE, H2B1M_MOUSE, H2B1P_MOUSE, H2B2B_MOUSE, H2B2E_MOUSE, H2B3A_MOUSE, H2B3B_MOUSE	Histone H2B	ASRLAHYNKRSTITSRE	140	3.60E-05	498.27	1989.0508	4	0.0004	17	0	GluC
H2B1A_MOUSE, H2B1B_MOUSE, H2B1C_MOUSE, H2B1F_MOUSE, H2B1H_MOUSE, H2B1K_MOUSE, H2B1M_MOUSE, H2B1P_MOUSE, H2B2B_MOUSE, H2B2E_MOUSE, H2B3A_MOUSE, H2B3B_MOUSE	Histone H2B	ASRLAHYNKRSTITSRE	140	7.60E-05	498.2699	1989.0506	4	0.0002	17	0	GluC
H2B1A_MOUSE, H2B1B_MOUSE, H2B1C_MOUSE, H2B1F_MOUSE, H2B1H_MOUSE, H2B1K_MOUSE, H2B1M_MOUSE, H2B1P_MOUSE, H2B2B_MOUSE, H2B2E_MOUSE, H2B3A_MOUSE, H2B3B_MOUSE	Histone H2B	ASRLAHYNKRSTITSRE	140	1.20E-06	664.0241	1989.0505	3	0.0002	17	0	GluC
H2B1A_MOUSE, H2B1B_MOUSE, H2B1C_MOUSE, H2B1F_MOUSE, H2B1H_MOUSE, H2B1K_MOUSE, H2B1M_MOUSE, H2B1P_MOUSE, H2B2B_MOUSE, H2B2E_MOUSE, H2B3A_MOUSE, H2B3B_MOUSE	Histone H2B	ASRLAHYNKRSTITSRE	140	2.10E-08	498.2699	1989.0505	4	0.0002	17	0	GluC
H2B1A_MOUSE, H2B1B_MOUSE, H2B1C_MOUSE, H2B1F_MOUSE, H2B1H_MOUSE, H2B1K_MOUSE, H2B1M_MOUSE, H2B1P_MOUSE, H2B2B_MOUSE, H2B2E_MOUSE, H2B3A_MOUSE, H2B3B_MOUSE	Histone H2B	ASRLAHYNKRSTITSRE	140	7.10E-08	498.2699	1989.0505	4	0.0001	17	0	GluC
H2B1A_MOUSE, H2B1B_MOUSE, H2B1C_MOUSE, H2B1F_MOUSE, H2B1H_MOUSE, H2B1K_MOUSE, H2B1M_MOUSE, H2B1P_MOUSE, H2B2B_MOUSE, H2B2E_MOUSE, H2B3A_MOUSE, H2B3B_MOUSE	Histone H2B	ASRLAHYNKRSTITSRE	140	5.60E-10	664.0241	1989.0505	3	0.0001	17	0	GluC
H2B1A_MOUSE, H2B1B_MOUSE, H2B1C_MOUSE, H2B1F_MOUSE, H2B1H_MOUSE, H2B1K_MOUSE, H2B1M_MOUSE, H2B1P_MOUSE, H2B2B_MOUSE, H2B2E_MOUSE, H2B3A_MOUSE, H2B3B_MOUSE	Histone H2B	ASRLAHYNKRSTITSRE	140	1.30E-06	498.2699	1989.0503	4	-0.0001	17	0	GluC
H2B1A_MOUSE, H2B1B_MOUSE, H2B1C_MOUSE, H2B1F_MOUSE, H2B1H_MOUSE, H2B1K_MOUSE, H2B1M_MOUSE, H2B1P_MOUSE, H2B2B_MOUSE, H2B2E_MOUSE, H2B3A_MOUSE, H2B3B_MOUSE	Histone H2B	ASRLAHYNKRSTITSRE	140	3.80E-10	664.024	1989.0503	3	-0.0001	17	0	GluC
H2B1A_MOUSE, H2B1B_MOUSE, H2B1C_MOUSE, H2B1F_MOUSE, H2B1H_MOUSE, H2B1K_MOUSE, H2B1M_MOUSE, H2B1P_MOUSE, H2B2B_MOUSE, H2B2E_MOUSE, H2B3A_MOUSE, H2B3B_MOUSE	Histone H2B	ASRLAHYNKRSTITSRE	140	2.50E-04	498.2698	1989.0501	4	-0.0002	17	0	GluC
H2B1A_MOUSE, H2B1B_MOUSE, H2B1C_MOUSE, H2B1F_MOUSE, H2B1H_MOUSE, H2B1K_MOUSE, H2B1M_MOUSE, H2B1P_MOUSE, H2B2B_MOUSE, H2B2E_MOUSE, H2B3A_MOUSE, H2B3B_MOUSE	Histone H2B	ASRLAHYNKRSTITSRE	140	3.20E-05	498.2698	1989.0501	4	-0.0002	17	0	GluC
H2B1A_MOUSE, H2B1B_MOUSE, H2B1C_MOUSE, H2B1F_MOUSE, H2B1H_MOUSE, H2B1K_MOUSE, H2B1M_MOUSE, H2B1P_MOUSE, H2B2B_MOUSE, H2B2E_MOUSE, H2B3A_MOUSE, H2B3B_MOUSE	Histone H2B	ASRLAHYNKRSTITSRE	140	1.20E-05	498.2698	1989.0502	4	-0.0002	17	0	GluC
H2B1A_MOUSE, H2B1B_MOUSE, H2B1C_MOUSE, H2B1F_MOUSE, H2B1H_MOUSE, H2B1K_MOUSE, H2B1M_MOUSE, H2B1P_MOUSE, H2B2B_MOUSE, H2B2E_MOUSE, H2B3A_MOUSE, H2B3B_MOUSE	Histone H2B	ASRLAHYNKRSTITSRE	140	3.50E-09	498.2698	1989.0502	4	-0.0002	17	0	GluC
H2B1A_MOUSE, H2B1B_MOUSE, H2B1C_MOUSE, H2B1F_MOUSE, H2B1H_MOUSE, H2B1K_MOUSE, H2B1M_MOUSE, H2B1P_MOUSE, H2B2B_MOUSE, H2B2E_MOUSE, H2B3A_MOUSE, H2B3B_MOUSE	Histone H2B	ASRLAHYNKRSTITSRE	140	4.30E-03	498.2698	1989.0501	4	-0.0003	17	0	GluC
H2B1A_MOUSE, H2B1B_MOUSE, H2B1C_MOUSE, H2B1F_MOUSE, H2B1H_MOUSE, H2B1K_MOUSE, H2B1M_MOUSE, H2B1P_MOUSE, H2B2B_MOUSE, H2B2E_MOUSE, H2B3A_MOUSE, H2B3B_MOUSE	Histone H2B	ASRLAHYNKRSTITSRE	140	3.80E-04	498.2698	1989.0499	4	-0.0004	17	0	GluC

H2B1A_MOUSE, H2B1B_MOUSE, H2B1C_MOUSE, H2B1F_MOUSE, H2B1H_MOUSE, H2B1K_MOUSE, H2B1M_MOUSE, H2B1P_MOUSE, H2B2B_MOUSE, H2B2E_MOUSE, H2B3A_MOUSE, H2B3B_MOUSE	Histone H2B	ASRLAHYNKRSTITSRE	140	8.40E-03	498.2697	1989.0499	4	-0.0005	17	0	GluC
H2B1A_MOUSE, H2B1B_MOUSE, H2B1C_MOUSE, H2B1F_MOUSE, H2B1H_MOUSE, H2B1K_MOUSE, H2B1M_MOUSE, H2B1P_MOUSE, H2B2B_MOUSE, H2B2E_MOUSE, H2B3A_MOUSE, H2B3B_MOUSE	Histone H2B	ASRLAHYNKRSTITSRE	140	4.90E-03	398.8173	1989.0499	5	-0.0005	17	0	GluC
H2B1A_MOUSE, H2B1B_MOUSE, H2B1C_MOUSE, H2B1F_MOUSE, H2B1H_MOUSE, H2B1K_MOUSE, H2B1M_MOUSE, H2B1P_MOUSE, H2B2B_MOUSE, H2B2E_MOUSE, H2B3A_MOUSE, H2B3B_MOUSE	Histone H2B	ASRLAHYNKRSTITSRE	140	8.80E-06	498.2697	1989.0499	4	-0.0005	17	0	GluC
H2B1A_MOUSE, H2B1B_MOUSE, H2B1C_MOUSE, H2B1F_MOUSE, H2B1H_MOUSE, H2B1K_MOUSE, H2B1M_MOUSE, H2B1P_MOUSE, H2B2B_MOUSE, H2B2E_MOUSE, H2B3A_MOUSE, H2B3B_MOUSE	Histone H2B	ASRLAHYNKRSTITSRE	140	6.70E-04	498.2697	1989.0498	4	-0.0006	17	0	GluC
H2B1A_MOUSE, H2B1B_MOUSE, H2B1C_MOUSE, H2B1F_MOUSE, H2B1H_MOUSE, H2B1K_MOUSE, H2B1M_MOUSE, H2B1P_MOUSE, H2B2B_MOUSE, H2B2E_MOUSE, H2B3A_MOUSE, H2B3B_MOUSE	Histone H2B	ASRLAHYNKRSTITSRE	140	2.00E-06	498.2697	1989.0498	4	-0.0006	17	0	GluC
H2B1A_MOUSE, H2B1B_MOUSE, H2B1C_MOUSE, H2B1F_MOUSE, H2B1H_MOUSE, H2B1K_MOUSE, H2B1M_MOUSE, H2B1P_MOUSE, H2B2B_MOUSE, H2B2E_MOUSE, H2B3A_MOUSE, H2B3B_MOUSE	Histone H2B	ASRLAHYNKRSTITSRE	140	1.50E-08	498.2697	1989.0497	4	-0.0006	17	0	GluC
H2B1A_MOUSE, H2B1B_MOUSE, H2B1C_MOUSE, H2B1F_MOUSE, H2B1H_MOUSE, H2B1K_MOUSE, H2B1M_MOUSE, H2B1P_MOUSE, H2B2B_MOUSE, H2B2E_MOUSE, H2B3A_MOUSE, H2B3B_MOUSE	Histone H2B	ASRLAHYNKRSTITSRE	140	1.50E-06	498.2697	1989.0497	4	-0.0007	17	0	GluC
H2B1A_MOUSE, H2B1B_MOUSE, H2B1C_MOUSE, H2B1F_MOUSE, H2B1H_MOUSE, H2B1K_MOUSE, H2B1M_MOUSE, H2B1P_MOUSE, H2B2B_MOUSE, H2B2E_MOUSE, H2B3A_MOUSE, H2B3B_MOUSE	Histone H2B	ASRLAHYNKRSTITSRE	140	2.20E-05	498.2697	1989.0496	4	-0.0008	17	0	GluC
H2B1A_MOUSE, H2B1B_MOUSE, H2B1C_MOUSE, H2B1F_MOUSE, H2B1H_MOUSE, H2B1K_MOUSE, H2B1M_MOUSE, H2B1P_MOUSE, H2B2B_MOUSE, H2B2E_MOUSE, H2B3A_MOUSE, H2B3B_MOUSE	Histone H2B	ASRLAHYNKRSTITSRE	140	3.70E-06	498.2697	1989.0495	4	-0.0009	17	0	GluC
H2B1A_MOUSE, H2B1B_MOUSE, H2B1C_MOUSE, H2B1F_MOUSE, H2B1H_MOUSE, H2B1K_MOUSE, H2B1M_MOUSE, H2B1P_MOUSE, H2B2B_MOUSE, H2B2E_MOUSE, H2B3A_MOUSE, H2B3B_MOUSE	Histone H2B	ASRLAHYNKRSTITSRE	140	1.60E-05	498.2696	1989.0494	4	-0.0010	17	0	GluC
H2B1A_MOUSE, H2B1B_MOUSE, H2B1C_MOUSE, H2B1F_MOUSE, H2B1H_MOUSE, H2B1K_MOUSE, H2B1M_MOUSE, H2B1P_MOUSE, H2B2B_MOUSE, H2B2E_MOUSE, H2B3A_MOUSE, H2B3B_MOUSE	Histone H2B	ASRLAHYNKRSTITSRE	140	8.60E-04	398.8171	1989.0493	5	-0.0011	17	0	GluC
H2B1A_MOUSE, H2B1B_MOUSE, H2B1C_MOUSE, H2B1F_MOUSE, H2B1H_MOUSE, H2B1K_MOUSE, H2B1M_MOUSE, H2B1P_MOUSE, H2B2B_MOUSE, H2B2E_MOUSE, H2B3A_MOUSE, H2B3B_MOUSE	Histone H2B	ASRLAHYNKRSTITSRE	140	1.60E-06	498.2696	1989.0493	4	-0.0011	17	0	GluC
H2B1A_MOUSE, H2B1B_MOUSE, H2B1C_MOUSE, H2B1F_MOUSE, H2B1H_MOUSE, H2B1K_MOUSE, H2B1M_MOUSE, H2B1P_MOUSE, H2B2B_MOUSE, H2B2E_MOUSE, H2B3A_MOUSE, H2B3B_MOUSE	Histone H2B	ASRLAHYNKRSTITSRE	140	3.90E-04	498.2696	1989.0492	4	-0.0012	17	0	GluC
H2B1A_MOUSE, H2B1B_MOUSE, H2B1C_MOUSE, H2B1F_MOUSE, H2B1H_MOUSE, H2B1K_MOUSE, H2B1M_MOUSE, H2B1P_MOUSE, H2B2B_MOUSE, H2B2E_MOUSE, H2B3A_MOUSE, H2B3B_MOUSE	Histone H2B	ASRLAHYNKRSTITSRE	140	3.50E-05	498.2695	1989.0491	4	-0.0013	17	0	GluC
H2B1A_MOUSE, H2B1B_MOUSE, H2B1C_MOUSE, H2B1F_MOUSE, H2B1H_MOUSE, H2B1K_MOUSE, H2B1M_MOUSE, H2B1P_MOUSE, H2B2B_MOUSE, H2B2E_MOUSE, H2B3A_MOUSE, H2B3B_MOUSE	Histone H2B	ASRLAHYNKRSTITSRE	140	1.20E-03	498.2695	1989.0489	4	-0.0014	17	0	GluC
H2B1A_MOUSE, H2B1B_MOUSE, H2B1C_MOUSE, H2B1F_MOUSE, H2B1H_MOUSE, H2B1K_MOUSE, H2B1M_MOUSE, H2B1P_MOUSE, H2B2B_MOUSE, H2B2E_MOUSE, H2B3A_MOUSE, H2B3B_MOUSE	Histone H2B	ASRLAHYNKRSTITSRE	140	1.90E-06	498.2695	1989.0490	4	-0.0014	17	0	GluC
H2B1A_MOUSE, H2B1B_MOUSE, H2B1C_MOUSE, H2B1F_MOUSE, H2B1H_MOUSE, H2B1K_MOUSE, H2B1M_MOUSE, H2B1P_MOUSE, H2B2B_MOUSE, H2B2E_MOUSE, H2B3A_MOUSE, H2B3B_MOUSE	Histone H2B	ASRLAHYNKRSTITSRE	140	6.90E-03	498.2695	1989.0488	4	-0.0016	17	0	GluC
H2B1A_MOUSE, H2B1B_MOUSE, H2B1C_MOUSE, H2B1F_MOUSE, H2B1H_MOUSE, H2B1K_MOUSE, H2B1M_MOUSE, H2B1P_MOUSE, H2B2B_MOUSE, H2B2E_MOUSE, H2B3A_MOUSE, H2B3B_MOUSE	Histone H2B	ASRLAHYNKRSTITSRE	140	4.60E-10	664.0235	1989.0487	3	-0.0017	17	0	GluC
H2B1A_MOUSE, H2B1B_MOUSE, H2B1C_MOUSE, H2B1F_MOUSE, H2B1H_MOUSE, H2B1K_MOUSE, H2B1M_MOUSE, H2B1P_MOUSE, H2B2B_MOUSE, H2B2E_MOUSE, H2B3A_MOUSE, H2B3B_MOUSE	Histone H2B	AKHAVSEGTKAVTKY	22	1.00E-02	795.4407	1588.8669	2	0.0096	15	0	Chymotrypsin
H2B1A_MOUSE, H2B1B_MOUSE, H2B1C_MOUSE, H2B1F_MOUSE, H2B1H_MOUSE, H2B1K_MOUSE, H2B1M_MOUSE, H2B1P_MOUSE, H2B2B_MOUSE, H2B2E_MOUSE, H2B3A_MOUSE, H2B3B_MOUSE	Histone H2B	AKHAVSEGTKAVTKY	22	1.90E-10	530.629	1588.8650	3	0.0077	15	0	Chymotrypsin
H2B1A_MOUSE, H2B1B_MOUSE, H2B1C_MOUSE, H2B1F_MOUSE, H2B1H_MOUSE, H2B1K_MOUSE, H2B1M_MOUSE, H2B1P_MOUSE, H2B2B_MOUSE, H2B2E_MOUSE, H2B3A_MOUSE, H2B3B_MOUSE	Histone H2B	AKHAVSEGTKAVTKY	22	3.30E-08	398.2225	1588.8608	4	0.0035	15	0	Chymotrypsin
H2B1A_MOUSE, H2B1B_MOUSE, H2B1C_MOUSE, H2B1F_MOUSE, H2B1H_MOUSE, H2B1K_MOUSE, H2B1M_MOUSE, H2B1P_MOUSE, H2B2B_MOUSE, H2B2E_MOUSE, H2B3A_MOUSE, H2B3B_MOUSE	Histone H2B	AKHAVSEGTKAVTKY	22	1.70E-04	795.4372	1588.8599	2	0.0026	15	0	Chymotrypsin
H2B1A_MOUSE, H2B1B_MOUSE, H2B1C_MOUSE, H2B1F_MOUSE, H2B1H_MOUSE, H2B1K_MOUSE, H2B1M_MOUSE, H2B1P_MOUSE, H2B2B_MOUSE, H2B2E_MOUSE, H2B3A_MOUSE, H2B3B_MOUSE	Histone H2B	AKHAVSEGTKAVTKY	22	3.70E-07	398.2221	1588.8591	4	0.0018	15	0	Chymotrypsin
H2B1A_MOUSE, H2B1B_MOUSE, H2B1C_MOUSE, H2B1F_MOUSE, H2B1H_MOUSE, H2B1K_MOUSE, H2B1M_MOUSE, H2B1P_MOUSE, H2B2B_MOUSE, H2B2E_MOUSE, H2B3A_MOUSE, H2B3B_MOUSE	Histone H2B	AKHAVSEGTKAVTKY	22	1.30E-05	795.4366	1588.8587	2	0.0014	15	0	Chymotrypsin

H2B1A_MOUSE, H2B1B_MOUSE, H2B1C_MOUSE, H2B1F_MOUSE, H2B1H_MOUSE, H2B1K_MOUSE, H2B1M_MOUSE, H2B1P_MOUSE, H2B2B_MOUSE, H2B2E_MOUSE, H2B3A_MOUSE, H2B3B_MOUSE	Histone H2B	AKHAVSEGTKAVTKY	22	1.90E-07	530.6268	1588.8587	3	0.0014	15	0	Chymotrypsin
H2B1A_MOUSE, H2B1B_MOUSE, H2B1C_MOUSE, H2B1F_MOUSE, H2B1H_MOUSE, H2B1K_MOUSE, H2B1M_MOUSE, H2B1P_MOUSE, H2B2B_MOUSE, H2B2E_MOUSE, H2B3A_MOUSE, H2B3B_MOUSE	Histone H2B	AKHAVSEGTKAVTKY	22	9.40E-08	530.6268	1588.8585	3	0.0012	15	0	Chymotrypsin
H2B1A_MOUSE, H2B1B_MOUSE, H2B1C_MOUSE, H2B1F_MOUSE, H2B1H_MOUSE, H2B1K_MOUSE, H2B1M_MOUSE, H2B1P_MOUSE, H2B2B_MOUSE, H2B2E_MOUSE, H2B3A_MOUSE, H2B3B_MOUSE	Histone H2B	AKHAVSEGTKAVTKY	22	1.50E-08	530.6267	1588.8583	3	0.0010	15	0	Chymotrypsin
H2B1A_MOUSE, H2B1B_MOUSE, H2B1C_MOUSE, H2B1F_MOUSE, H2B1H_MOUSE, H2B1K_MOUSE, H2B1M_MOUSE, H2B1P_MOUSE, H2B2B_MOUSE, H2B2E_MOUSE, H2B3A_MOUSE, H2B3B_MOUSE	Histone H2B	AKHAVSEGTKAVTKY	22	1.00E-02	398.2217	1588.8577	4	0.0004	15	0	Chymotrypsin
H2B1A_MOUSE, H2B1B_MOUSE, H2B1C_MOUSE, H2B1F_MOUSE, H2B1H_MOUSE, H2B1K_MOUSE, H2B1M_MOUSE, H2B1P_MOUSE, H2B2B_MOUSE, H2B2E_MOUSE, H2B3A_MOUSE, H2B3B_MOUSE	Histone H2B	AKHAVSEGTKAVTKY	22	1.70E-05	398.2217	1588.8577	4	0.0004	15	0	Chymotrypsin
H2B1A_MOUSE, H2B1B_MOUSE, H2B1C_MOUSE, H2B1F_MOUSE, H2B1H_MOUSE, H2B1K_MOUSE, H2B1M_MOUSE, H2B1P_MOUSE, H2B2B_MOUSE, H2B2E_MOUSE, H2B3A_MOUSE, H2B3B_MOUSE	Histone H2B	AKHAVSEGTKAVTKY	22	6.10E-05	398.2216	1588.8574	4	0.0001	15	0	GluC
H2B1A_MOUSE, H2B1B_MOUSE, H2B1C_MOUSE, H2B1F_MOUSE, H2B1H_MOUSE, H2B1K_MOUSE, H2B1M_MOUSE, H2B1P_MOUSE, H2B2B_MOUSE, H2B2E_MOUSE, H2B3A_MOUSE, H2B3B_MOUSE	Histone H2B	AKHAVSEGTKAVTKY	22	9.70E-04	530.6263	1588.8570	3	-0.0003	15	0	Chymotrypsin
H2B1A_MOUSE, H2B1B_MOUSE, H2B1C_MOUSE, H2B1F_MOUSE, H2B1H_MOUSE, H2B1K_MOUSE, H2B1M_MOUSE, H2B1P_MOUSE, H2B2B_MOUSE, H2B2E_MOUSE, H2B3A_MOUSE, H2B3B_MOUSE	Histone H2B	AKHAVSEGTKAVTKY	22	2.80E-03	530.6262	1588.8569	3	-0.0004	15	0	Chymotrypsin
H2B1A_MOUSE, H2B1B_MOUSE, H2B1C_MOUSE, H2B1F_MOUSE, H2B1H_MOUSE, H2B1K_MOUSE, H2B1M_MOUSE, H2B1P_MOUSE, H2B2B_MOUSE, H2B2E_MOUSE, H2B3A_MOUSE, H2B3B_MOUSE	Histone H2B	AKHAVSEGTKAVTKY	22	9.20E-07	530.6263	1588.8569	3	-0.0004	15	0	GluC
H2B1A_MOUSE, H2B1B_MOUSE, H2B1C_MOUSE, H2B1F_MOUSE, H2B1H_MOUSE, H2B1K_MOUSE, H2B1M_MOUSE, H2B1P_MOUSE, H2B2B_MOUSE, H2B2E_MOUSE, H2B3A_MOUSE, H2B3B_MOUSE	Histone H2B	AKHAVSEGTKAVTKY	22	1.70E-07	398.2215	1588.8568	4	-0.0005	15	0	Chymotrypsin
H2B1A_MOUSE, H2B1B_MOUSE, H2B1C_MOUSE, H2B1F_MOUSE, H2B1H_MOUSE, H2B1K_MOUSE, H2B1M_MOUSE, H2B1P_MOUSE, H2B2B_MOUSE, H2B2E_MOUSE, H2B3A_MOUSE, H2B3B_MOUSE	Histone H2B	AKHAVSEGTKAVTKY	22	3.10E-03	530.6261	1588.8566	3	-0.0007	15	0	Chymotrypsin
H2B1A_MOUSE, H2B1B_MOUSE, H2B1C_MOUSE, H2B1F_MOUSE, H2B1H_MOUSE, H2B1K_MOUSE, H2B1M_MOUSE, H2B1P_MOUSE, H2B2B_MOUSE, H2B2E_MOUSE, H2B3A_MOUSE, H2B3B_MOUSE	Histone H2B	AKHAVSEGTKAVTKY	22	2.00E-09	530.6261	1588.8566	3	-0.0007	15	0	Chymotrypsin
H2B1A_MOUSE, H2B1B_MOUSE, H2B1C_MOUSE, H2B1F_MOUSE, H2B1H_MOUSE, H2B1K_MOUSE, H2B1M_MOUSE, H2B1P_MOUSE, H2B2B_MOUSE, H2B2E_MOUSE, H2B3A_MOUSE, H2B3B_MOUSE	Histone H2B	AKHAVSEGTKAVTKY	22	1.00E-02	530.6261	1588.8564	3	-0.0008	15	0	Chymotrypsin
H2B1A_MOUSE, H2B1B_MOUSE, H2B1C_MOUSE, H2B1F_MOUSE, H2B1H_MOUSE, H2B1K_MOUSE, H2B1M_MOUSE, H2B1P_MOUSE, H2B2B_MOUSE, H2B2E_MOUSE, H2B3A_MOUSE, H2B3B_MOUSE	Histone H2B	AKHAVSEGTKAVTKY	22	2.40E-09	530.6261	1588.8564	3	-0.0008	15	0	Chymotrypsin
H2B1A_MOUSE, H2B1B_MOUSE, H2B1C_MOUSE, H2B1F_MOUSE, H2B1H_MOUSE, H2B1K_MOUSE, H2B1M_MOUSE, H2B1P_MOUSE, H2B2B_MOUSE, H2B2E_MOUSE, H2B3A_MOUSE, H2B3B_MOUSE	Histone H2B	AKHAVSEGTKAVTKY	22	5.70E-03	530.6261	1588.8564	3	-0.0009	15	0	Chymotrypsin
H2B1A_MOUSE, H2B1B_MOUSE, H2B1C_MOUSE, H2B1F_MOUSE, H2B1H_MOUSE, H2B1K_MOUSE, H2B1M_MOUSE, H2B1P_MOUSE, H2B2B_MOUSE, H2B2E_MOUSE, H2B3A_MOUSE, H2B3B_MOUSE	Histone H2B	AKHAVSEGTKAVTKY	22	1.70E-08	398.2213	1588.8563	4	-0.0010	15	0	Chymotrypsin
H2B1A_MOUSE, H2B1B_MOUSE, H2B1C_MOUSE, H2B1F_MOUSE, H2B1H_MOUSE, H2B1K_MOUSE, H2B1M_MOUSE, H2B1P_MOUSE, H2B2B_MOUSE, H2B2E_MOUSE, H2B3A_MOUSE, H2B3B_MOUSE	Histone H2B	VLKQVHPDTGISSK	28	5.80E-03	503.6185	1507.8336	3	-0.0023	14	1	Semi-tryptic
H2B1A_MOUSE, H2B1B_MOUSE, H2B1C_MOUSE, H2B1F_MOUSE, H2B1H_MOUSE, H2B1K_MOUSE, H2B1M_MOUSE, H2B1P_MOUSE, H2B2B_MOUSE, H2B2E_MOUSE, H2B3A_MOUSE, H2B3B_MOUSE	Histone H2B	VLKQVHPDTGISSK	28	2.60E-06	754.929	1507.8434	2	0.0076	14	1	Semi-tryptic
H2B1A_MOUSE, H2B1B_MOUSE, H2B1C_MOUSE, H2B1F_MOUSE, H2B1H_MOUSE, H2B1K_MOUSE, H2B1M_MOUSE, H2B1P_MOUSE, H2B2B_MOUSE, H2B2E_MOUSE, H2B3A_MOUSE, H2B3B_MOUSE	Histone H2B	VLKQVHPDTGISSK	28	2.10E-05	754.9288	1507.8430	2	0.0072	14	1	Semi-tryptic
H2B1A_MOUSE, H2B1B_MOUSE, H2B1C_MOUSE, H2B1F_MOUSE, H2B1H_MOUSE, H2B1K_MOUSE, H2B1M_MOUSE, H2B1P_MOUSE, H2B2B_MOUSE, H2B2E_MOUSE, H2B3A_MOUSE, H2B3B_MOUSE	Histone H2B	VLKQVHPDTGISSK	28	6.90E-06	754.9274	1507.8403	2	0.0044	14	1	Semi-tryptic
H2B1A_MOUSE, H2B1B_MOUSE, H2B1C_MOUSE, H2B1F_MOUSE, H2B1H_MOUSE, H2B1K_MOUSE, H2B1M_MOUSE, H2B1P_MOUSE, H2B2B_MOUSE, H2B2E_MOUSE, H2B3A_MOUSE, H2B3B_MOUSE	Histone H2B	VLKQVHPDTGISSK	28	2.50E-05	503.6199	1507.8380	3	0.0021	14	1	Semi-tryptic
H2B1A_MOUSE, H2B1B_MOUSE, H2B1C_MOUSE, H2B1F_MOUSE, H2B1H_MOUSE, H2B1K_MOUSE, H2B1M_MOUSE, H2B1P_MOUSE, H2B2B_MOUSE, H2B2E_MOUSE, H2B3A_MOUSE, H2B3B_MOUSE	Histone H2B	VLKQVHPDTGISSK	28	3.90E-04	754.9262	1507.8378	2	0.0020	14	1	Semi-tryptic
H2B1A_MOUSE, H2B1B_MOUSE, H2B1C_MOUSE, H2B1F_MOUSE, H2B1H_MOUSE, H2B1K_MOUSE, H2B1M_MOUSE, H2B1P_MOUSE, H2B2B_MOUSE, H2B2E_MOUSE, H2B3A_MOUSE, H2B3B_MOUSE	Histone H2B	VLKQVHPDTGISSK	28	4.30E-06	754.926	1507.8375	2	0.0017	14	1	Semi-tryptic
H2B1A_MOUSE, H2B1B_MOUSE, H2B1C_MOUSE, H2B1F_MOUSE, H2B1H_MOUSE, H2B1K_MOUSE, H2B1M_MOUSE, H2B1P_MOUSE, H2B2B_MOUSE, H2B2E_MOUSE, H2B3A_MOUSE, H2B3B_MOUSE	Histone H2B	VLKQVHPDTGISSK	28	8.90E-04	503.6198	1507.8374	3	0.0016	14	1	Semi-tryptic

H2B1A_MOUSE, H2B1B_MOUSE, H2B1C_MOUSE, H2B1F_MOUSE, H2B1H_MOUSE, H2B1K_MOUSE, H2B1M_MOUSE, H2B1P_MOUSE, H2B2B_MOUSE, H2B2E_MOUSE, H2B3A_MOUSE, H2B3B_MOUSE	Histone H2B	VLKQVHPDTGISSK	28	1.80E-05	754.926	1507.8374	2	0.0016	14	1	Semi-tryptic
H2B1A_MOUSE, H2B1B_MOUSE, H2B1C_MOUSE, H2B1F_MOUSE, H2B1H_MOUSE, H2B1K_MOUSE, H2B1M_MOUSE, H2B1P_MOUSE, H2B2B_MOUSE, H2B2E_MOUSE, H2B3A_MOUSE, H2B3B_MOUSE	Histone H2B	VLKQVHPDTGISSK	28	1.30E-07	754.9258	1507.8370	2	0.0012	14	1	Semi-tryptic
H2B1A_MOUSE, H2B1B_MOUSE, H2B1C_MOUSE, H2B1F_MOUSE, H2B1H_MOUSE, H2B1K_MOUSE, H2B1M_MOUSE, H2B1P_MOUSE, H2B2B_MOUSE, H2B2E_MOUSE, H2B3A_MOUSE, H2B3B_MOUSE	Histone H2B	VLKQVHPDTGISSK	28	3.50E-06	754.9257	1507.8369	2	0.0011	14	1	Semi-tryptic
H2B1A_MOUSE, H2B1B_MOUSE, H2B1C_MOUSE, H2B1F_MOUSE, H2B1H_MOUSE, H2B1K_MOUSE, H2B1M_MOUSE, H2B1P_MOUSE, H2B2B_MOUSE, H2B2E_MOUSE, H2B3A_MOUSE, H2B3B_MOUSE	Histone H2B	VLKQVHPDTGISSK	28	5.90E-04	503.6196	1507.8369	3	0.0010	14	1	Semi-tryptic
H2B1A_MOUSE, H2B1B_MOUSE, H2B1C_MOUSE, H2B1F_MOUSE, H2B1H_MOUSE, H2B1K_MOUSE, H2B1M_MOUSE, H2B1P_MOUSE, H2B2B_MOUSE, H2B2E_MOUSE, H2B3A_MOUSE, H2B3B_MOUSE	Histone H2B	VLKQVHPDTGISSK	28	3.80E-05	754.9257	1507.8368	2	0.0010	14	1	Semi-tryptic
H2B1A_MOUSE, H2B1B_MOUSE, H2B1C_MOUSE, H2B1F_MOUSE, H2B1H_MOUSE, H2B1K_MOUSE, H2B1M_MOUSE, H2B1P_MOUSE, H2B2B_MOUSE, H2B2E_MOUSE, H2B3A_MOUSE, H2B3B_MOUSE	Histone H2B	VLKQVHPDTGISSK	28	4.10E-06	754.9256	1507.8366	2	0.0007	14	1	Semi-tryptic
H2B1A_MOUSE, H2B1B_MOUSE, H2B1C_MOUSE, H2B1F_MOUSE, H2B1H_MOUSE, H2B1K_MOUSE, H2B1M_MOUSE, H2B1P_MOUSE, H2B2B_MOUSE, H2B2E_MOUSE, H2B3A_MOUSE, H2B3B_MOUSE	Histone H2B	VLKQVHPDTGISSK	28	8.80E-03	503.6194	1507.8365	3	0.0006	14	1	Semi-tryptic
H2B1A_MOUSE, H2B1B_MOUSE, H2B1C_MOUSE, H2B1F_MOUSE, H2B1H_MOUSE, H2B1K_MOUSE, H2B1M_MOUSE, H2B1P_MOUSE, H2B2B_MOUSE, H2B2E_MOUSE, H2B3A_MOUSE, H2B3B_MOUSE	Histone H2B	VLKQVHPDTGISSK	28	2.20E-05	503.6194	1507.8365	3	0.0006	14	1	Semi-tryptic
H2B1A_MOUSE, H2B1B_MOUSE, H2B1C_MOUSE, H2B1F_MOUSE, H2B1H_MOUSE, H2B1K_MOUSE, H2B1M_MOUSE, H2B1P_MOUSE, H2B2B_MOUSE, H2B2E_MOUSE, H2B3A_MOUSE, H2B3B_MOUSE	Histone H2B	VLKQVHPDTGISSK	28	1.70E-04	377.9663	1507.8362	4	0.0004	14	1	Semi-tryptic
H2B1A_MOUSE, H2B1B_MOUSE, H2B1C_MOUSE, H2B1F_MOUSE, H2B1H_MOUSE, H2B1K_MOUSE, H2B1M_MOUSE, H2B1P_MOUSE, H2B2B_MOUSE, H2B2E_MOUSE, H2B3A_MOUSE, H2B3B_MOUSE	Histone H2B	VLKQVHPDTGISSK	28	6.60E-06	377.9663	1507.8362	4	0.0004	14	1	Semi-tryptic
H2B1A_MOUSE, H2B1B_MOUSE, H2B1C_MOUSE, H2B1F_MOUSE, H2B1H_MOUSE, H2B1K_MOUSE, H2B1M_MOUSE, H2B1P_MOUSE, H2B2B_MOUSE, H2B2E_MOUSE, H2B3A_MOUSE, H2B3B_MOUSE	Histone H2B	VLKQVHPDTGISSK	28	1.90E-03	503.6193	1507.8361	3	0.0003	14	1	Semi-tryptic
H2B1A_MOUSE, H2B1B_MOUSE, H2B1C_MOUSE, H2B1F_MOUSE, H2B1H_MOUSE, H2B1K_MOUSE, H2B1M_MOUSE, H2B1P_MOUSE, H2B2B_MOUSE, H2B2E_MOUSE, H2B3A_MOUSE, H2B3B_MOUSE	Histone H2B	VLKQVHPDTGISSK	28	6.00E-04	503.6193	1507.8360	3	0.0001	14	1	Semi-tryptic
H2B1A_MOUSE, H2B1B_MOUSE, H2B1C_MOUSE, H2B1F_MOUSE, H2B1H_MOUSE, H2B1K_MOUSE, H2B1M_MOUSE, H2B1P_MOUSE, H2B2B_MOUSE, H2B2E_MOUSE, H2B3A_MOUSE, H2B3B_MOUSE	Histone H2B	VLKQVHPDTGISSK	28	2.40E-05	503.6193	1507.8360	3	0.0001	14	1	Semi-tryptic
H2B1A_MOUSE, H2B1B_MOUSE, H2B1C_MOUSE, H2B1F_MOUSE, H2B1H_MOUSE, H2B1K_MOUSE, H2B1M_MOUSE, H2B1P_MOUSE, H2B2B_MOUSE, H2B2E_MOUSE, H2B3A_MOUSE, H2B3B_MOUSE	Histone H2B	VLKQVHPDTGISSK	28	4.80E-04	503.6192	1507.8358	3	-0.0001	14	1	Semi-tryptic
H2B1A_MOUSE, H2B1B_MOUSE, H2B1C_MOUSE, H2B1F_MOUSE, H2B1H_MOUSE, H2B1K_MOUSE, H2B1M_MOUSE, H2B1P_MOUSE, H2B2B_MOUSE, H2B2E_MOUSE, H2B3A_MOUSE, H2B3B_MOUSE	Histone H2B	VLKQVHPDTGISSK	28	4.00E-04	503.6191	1507.8353	3	-0.0005	14	1	Semi-tryptic
H2B1A_MOUSE, H2B1B_MOUSE, H2B1C_MOUSE, H2B1F_MOUSE, H2B1H_MOUSE, H2B1K_MOUSE, H2B1M_MOUSE, H2B1P_MOUSE, H2B2B_MOUSE, H2B2E_MOUSE, H2B3A_MOUSE, H2B3B_MOUSE	Histone H2B	VLKQVHPDTGISSK	28	1.50E-03	503.619	1507.8352	3	-0.0006	14	1	Semi-tryptic
H2B1A_MOUSE, H2B1B_MOUSE, H2B1C_MOUSE, H2B1F_MOUSE, H2B1H_MOUSE, H2B1K_MOUSE, H2B1M_MOUSE, H2B1P_MOUSE, H2B2B_MOUSE, H2B2E_MOUSE, H2B3A_MOUSE, H2B3B_MOUSE	Histone H2B	VLKQVHPDTGISSK	28	7.70E-04	503.619	1507.8352	3	-0.0006	14	1	Semi-tryptic
H2B1A_MOUSE, H2B1B_MOUSE, H2B1C_MOUSE, H2B1F_MOUSE, H2B1H_MOUSE, H2B1K_MOUSE, H2B1M_MOUSE, H2B1P_MOUSE, H2B2B_MOUSE, H2B2E_MOUSE, H2B3A_MOUSE, H2B3B_MOUSE	Histone H2B	VLKQVHPDTGISSK	28	1.40E-05	503.6189	1507.8349	3	-0.0010	14	1	Semi-tryptic
H2B1A_MOUSE, H2B1B_MOUSE, H2B1C_MOUSE, H2B1F_MOUSE, H2B1H_MOUSE, H2B1K_MOUSE, H2B1M_MOUSE, H2B1P_MOUSE, H2B2B_MOUSE, H2B2E_MOUSE, H2B3A_MOUSE, H2B3B_MOUSE	Histone H2B	VLKQVHPDTGISSK	28	8.20E-07	503.6189	1507.8349	3	-0.0010	14	1	Semi-tryptic
H2B1A_MOUSE, H2B1B_MOUSE, H2B1C_MOUSE, H2B1F_MOUSE, H2B1H_MOUSE, H2B1K_MOUSE, H2B1M_MOUSE, H2B1P_MOUSE, H2B2B_MOUSE, H2B2E_MOUSE, H2B3A_MOUSE, H2B3B_MOUSE	Histone H2B	VLKQVHPDTGISSK	28	1.50E-03	503.6189	1507.8347	3	-0.0011	14	1	Semi-tryptic
H2B1A_MOUSE, H2B1B_MOUSE, H2B1C_MOUSE, H2B1F_MOUSE, H2B1H_MOUSE, H2B1K_MOUSE, H2B1M_MOUSE, H2B1P_MOUSE, H2B2B_MOUSE, H2B2E_MOUSE, H2B3A_MOUSE, H2B3B_MOUSE	Histone H2B	LAHYNKRSTITSR	5	2.70E-03	387.4671	1545.8393	4	0.0018	13	2	Semi-tryptic
H2B1A_MOUSE, H2B1B_MOUSE, H2B1C_MOUSE, H2B1F_MOUSE, H2B1H_MOUSE, H2B1K_MOUSE, H2B1M_MOUSE, H2B1P_MOUSE, H2B2B_MOUSE, H2B2E_MOUSE, H2B3A_MOUSE, H2B3B_MOUSE	Histone H2B	LAHYNKRSTITSR	5	9.50E-04	387.4668	1545.8381	4	0.0006	13	2	Semi-tryptic
H2B1A_MOUSE, H2B1B_MOUSE, H2B1C_MOUSE, H2B1F_MOUSE, H2B1H_MOUSE, H2B1K_MOUSE, H2B1M_MOUSE, H2B1P_MOUSE, H2B2B_MOUSE, H2B2E_MOUSE, H2B3A_MOUSE, H2B3B_MOUSE	Histone H2B	LAHYNKRSTITSR	5	6.50E-06	387.4668	1545.8381	4	0.0006	13	2	Semi-tryptic
H2B1A_MOUSE, H2B1B_MOUSE, H2B1C_MOUSE, H2B1F_MOUSE, H2B1H_MOUSE, H2B1K_MOUSE, H2B1M_MOUSE, H2B1P_MOUSE, H2B2B_MOUSE, H2B2E_MOUSE, H2B3A_MOUSE, H2B3B_MOUSE	Histone H2B	LAHYNKRSTITSR	5	2.20E-06	516.2867	1545.8382	3	0.0006	13	2	Semi-tryptic

H2B1A_MOUSE, H2B1B_MOUSE, H2B1C_MOUSE, H2B1F_MOUSE, H2B1H_MOUSE, H2B1K_MOUSE, H2B1M_MOUSE, H2B1P_MOUSE, H2B2B_MOUSE, H2B2E_MOUSE, H2B3A_MOUSE, H2B3B_MOUSE	Histone H2B	LAHYNKRSTITSR	5	2.60E-07	516.2867	1545.8382	3	0.0006	13	2	Semi-tryptic
H2B1A_MOUSE, H2B1B_MOUSE, H2B1C_MOUSE, H2B1F_MOUSE, H2B1H_MOUSE, H2B1K_MOUSE, H2B1M_MOUSE, H2B1P_MOUSE, H2B2B_MOUSE, H2B2E_MOUSE, H2B3A_MOUSE, H2B3B_MOUSE	Histone H2B	HAVSEGTKAVTK	16	8.70E-04	409.8938	1226.6594	3	-0.0025	12	1	Semi-tryptic
H2B1A_MOUSE, H2B1B_MOUSE, H2B1C_MOUSE, H2B1F_MOUSE, H2B1H_MOUSE, H2B1K_MOUSE, H2B1M_MOUSE, H2B1P_MOUSE, H2B2B_MOUSE, H2B2E_MOUSE, H2B3A_MOUSE, H2B3B_MOUSE	Histone H2B	HAVSEGTKAVTK	16	1.40E-08	614.3403	1226.6661	2	0.0043	12	1	Semi-tryptic
H2B1A_MOUSE, H2B1B_MOUSE, H2B1C_MOUSE, H2B1F_MOUSE, H2B1H_MOUSE, H2B1K_MOUSE, H2B1M_MOUSE, H2B1P_MOUSE, H2B2B_MOUSE, H2B2E_MOUSE, H2B3A_MOUSE, H2B3B_MOUSE	Histone H2B	HAVSEGTKAVTK	16	5.20E-07	409.896	1226.6661	3	0.0042	12	1	Semi-tryptic
H2B1A_MOUSE, H2B1B_MOUSE, H2B1C_MOUSE, H2B1F_MOUSE, H2B1H_MOUSE, H2B1K_MOUSE, H2B1M_MOUSE, H2B1P_MOUSE, H2B2B_MOUSE, H2B2E_MOUSE, H2B3A_MOUSE, H2B3B_MOUSE	Histone H2B	HAVSEGTKAVTK	16	4.90E-09	409.896	1226.6661	3	0.0042	12	1	Semi-tryptic
H2B1A_MOUSE, H2B1B_MOUSE, H2B1C_MOUSE, H2B1F_MOUSE, H2B1H_MOUSE, H2B1K_MOUSE, H2B1M_MOUSE, H2B1P_MOUSE, H2B2B_MOUSE, H2B2E_MOUSE, H2B3A_MOUSE, H2B3B_MOUSE	Histone H2B	HAVSEGTKAVTK	16	3.90E-05	409.8959	1226.6659	3	0.0040	12	1	Semi-tryptic
H2B1A_MOUSE, H2B1B_MOUSE, H2B1C_MOUSE, H2B1F_MOUSE, H2B1H_MOUSE, H2B1K_MOUSE, H2B1M_MOUSE, H2B1P_MOUSE, H2B2B_MOUSE, H2B2E_MOUSE, H2B3A_MOUSE, H2B3B_MOUSE	Histone H2B	HAVSEGTKAVTK	16	6.10E-06	409.8957	1226.6653	3	0.0034	12	1	Semi-tryptic
H2B1A_MOUSE, H2B1B_MOUSE, H2B1C_MOUSE, H2B1F_MOUSE, H2B1H_MOUSE, H2B1K_MOUSE, H2B1M_MOUSE, H2B1P_MOUSE, H2B2B_MOUSE, H2B2E_MOUSE, H2B3A_MOUSE, H2B3B_MOUSE	Histone H2B	HAVSEGTKAVTK	16	2.70E-05	409.8955	1226.6646	3	0.0027	12	1	Semi-tryptic
H2B1A_MOUSE, H2B1B_MOUSE, H2B1C_MOUSE, H2B1F_MOUSE, H2B1H_MOUSE, H2B1K_MOUSE, H2B1M_MOUSE, H2B1P_MOUSE, H2B2B_MOUSE, H2B2E_MOUSE, H2B3A_MOUSE, H2B3B_MOUSE	Histone H2B	HAVSEGTKAVTK	16	9.40E-09	614.3396	1226.6646	2	0.0027	12	1	Semi-tryptic
H2B1A_MOUSE, H2B1B_MOUSE, H2B1C_MOUSE, H2B1F_MOUSE, H2B1H_MOUSE, H2B1K_MOUSE, H2B1M_MOUSE, H2B1P_MOUSE, H2B2B_MOUSE, H2B2E_MOUSE, H2B3A_MOUSE, H2B3B_MOUSE	Histone H2B	HAVSEGTKAVTK	16	1.50E-04	409.8949	1226.6629	3	0.0010	12	1	Semi-tryptic
H2B1A_MOUSE, H2B1B_MOUSE, H2B1C_MOUSE, H2B1F_MOUSE, H2B1H_MOUSE, H2B1K_MOUSE, H2B1M_MOUSE, H2B1P_MOUSE, H2B2B_MOUSE, H2B2E_MOUSE, H2B3A_MOUSE, H2B3B_MOUSE	Histone H2B	HAVSEGTKAVTK	16	4.00E-08	614.3386	1226.6625	2	0.0007	12	1	Semi-tryptic
H2B1A_MOUSE, H2B1B_MOUSE, H2B1C_MOUSE, H2B1F_MOUSE, H2B1H_MOUSE, H2B1K_MOUSE, H2B1M_MOUSE, H2B1P_MOUSE, H2B2B_MOUSE, H2B2E_MOUSE, H2B3A_MOUSE, H2B3B_MOUSE	Histone H2B	HAVSEGTKAVTK	16	6.70E-09	614.3386	1226.6626	2	0.0007	12	1	Semi-tryptic
H2B1A_MOUSE, H2B1B_MOUSE, H2B1C_MOUSE, H2B1F_MOUSE, H2B1H_MOUSE, H2B1K_MOUSE, H2B1M_MOUSE, H2B1P_MOUSE, H2B2B_MOUSE, H2B2E_MOUSE, H2B3A_MOUSE, H2B3B_MOUSE	Histone H2B	HAVSEGTKAVTK	16	5.30E-04	409.8948	1226.6625	3	0.0006	12	1	Semi-tryptic
H2B1A_MOUSE, H2B1B_MOUSE, H2B1C_MOUSE, H2B1F_MOUSE, H2B1H_MOUSE, H2B1K_MOUSE, H2B1M_MOUSE, H2B1P_MOUSE, H2B2B_MOUSE, H2B2E_MOUSE, H2B3A_MOUSE, H2B3B_MOUSE	Histone H2B	HAVSEGTKAVTK	16	1.20E-08	614.3385	1226.6625	2	0.0006	12	1	Semi-tryptic
H2B1A_MOUSE, H2B1B_MOUSE, H2B1C_MOUSE, H2B1F_MOUSE, H2B1H_MOUSE, H2B1K_MOUSE, H2B1M_MOUSE, H2B1P_MOUSE, H2B2B_MOUSE, H2B2E_MOUSE, H2B3A_MOUSE, H2B3B_MOUSE	Histone H2B	HAVSEGTKAVTK	16	5.30E-08	614.3381	1226.6617	2	-0.0002	12	1	Semi-tryptic
H2B1A_MOUSE, H2B1B_MOUSE, H2B1C_MOUSE, H2B1F_MOUSE, H2B1H_MOUSE, H2B1K_MOUSE, H2B1M_MOUSE, H2B1P_MOUSE, H2B2B_MOUSE, H2B2E_MOUSE, H2B3A_MOUSE, H2B3B_MOUSE	Histone H2B	HAVSEGTKAVTK	16	3.20E-05	409.8945	1226.6616	3	-0.0003	12	1	Semi-tryptic
H2B1A_MOUSE, H2B1B_MOUSE, H2B1C_MOUSE, H2B1F_MOUSE, H2B1H_MOUSE, H2B1K_MOUSE, H2B1M_MOUSE, H2B1P_MOUSE, H2B2B_MOUSE, H2B2E_MOUSE, H2B3A_MOUSE, H2B3B_MOUSE	Histone H2B	HAVSEGTKAVTK	16	7.40E-06	409.8943	1226.6611	3	-0.0008	12	1	Semi-tryptic
H2B1A_MOUSE, H2B1B_MOUSE, H2B1C_MOUSE, H2B1F_MOUSE, H2B1H_MOUSE, H2B1K_MOUSE, H2B1M_MOUSE, H2B1P_MOUSE, H2B2B_MOUSE, H2B2E_MOUSE, H2B3A_MOUSE, H2B3B_MOUSE	Histone H2B	QVHPDTGISSK	23	3.80E-03	584.798	1167.5815	2	-0.0069	11	0	Semi-tryptic
H2B1A_MOUSE, H2B1B_MOUSE, H2B1C_MOUSE, H2B1F_MOUSE, H2B1H_MOUSE, H2B1K_MOUSE, H2B1M_MOUSE, H2B1P_MOUSE, H2B2B_MOUSE, H2B2E_MOUSE, H2B3A_MOUSE, H2B3B_MOUSE	Histone H2B	QVHPDTGISSK	23	1.20E-03	584.8037	1167.5928	2	0.0044	11	0	Semi-tryptic
H2B1A_MOUSE, H2B1B_MOUSE, H2B1C_MOUSE, H2B1F_MOUSE, H2B1H_MOUSE, H2B1K_MOUSE, H2B1M_MOUSE, H2B1P_MOUSE, H2B2B_MOUSE, H2B2E_MOUSE, H2B3A_MOUSE, H2B3B_MOUSE	Histone H2B	QVHPDTGISSK	23	2.10E-03	584.8036	1167.5926	2	0.0042	11	0	Semi-tryptic
H2B1A_MOUSE, H2B1B_MOUSE, H2B1C_MOUSE, H2B1F_MOUSE, H2B1H_MOUSE, H2B1K_MOUSE, H2B1M_MOUSE, H2B1P_MOUSE, H2B2B_MOUSE, H2B2E_MOUSE, H2B3A_MOUSE, H2B3B_MOUSE	Histone H2B	QVHPDTGISSK	23	3.00E-04	584.8022	1167.5898	2	0.0014	11	0	Semi-tryptic
H2B1A_MOUSE, H2B1B_MOUSE, H2B1C_MOUSE, H2B1F_MOUSE, H2B1H_MOUSE, H2B1K_MOUSE, H2B1M_MOUSE, H2B1P_MOUSE, H2B2B_MOUSE, H2B2E_MOUSE, H2B3A_MOUSE, H2B3B_MOUSE	Histone H2B	QVHPDTGISSK	23	1.00E-03	584.8021	1167.5897	2	0.0013	11	0	Semi-tryptic
H2B1A_MOUSE, H2B1B_MOUSE, H2B1C_MOUSE, H2B1F_MOUSE, H2B1H_MOUSE, H2B1K_MOUSE, H2B1M_MOUSE, H2B1P_MOUSE, H2B2B_MOUSE, H2B2E_MOUSE, H2B3A_MOUSE, H2B3B_MOUSE	Histone H2B	QVHPDTGISSK	23	5.70E-04	584.8021	1167.5897	2	0.0013	11	0	Semi-tryptic
H2B1A_MOUSE, H2B1B_MOUSE, H2B1C_MOUSE, H2B1F_MOUSE, H2B1H_MOUSE, H2B1K_MOUSE, H2B1M_MOUSE, H2B1P_MOUSE, H2B2B_MOUSE, H2B2E_MOUSE, H2B3A_MOUSE, H2B3B_MOUSE	Histone H2B	QVHPDTGISSK	23	4.10E-03	584.802	1167.5894	2	0.0010	11	0	Semi-tryptic

H2B1A_MOUSE, H2B1B_MOUSE, H2B1C_MOUSE, H2B1F_MOUSE, H2B1H_MOUSE, H2B1K_MOUSE, H2B1M_MOUSE, H2B1P_MOUSE, H2B2B_MOUSE, H2B2E_MOUSE, H2B3A_MOUSE, H2B3B_MOUSE	Histone H2B	QVHPDTGISSK	23	8.10E-03	390.2037	1167.5893	3	0.0009	11	0	Semi-tryptic
H2B1A_MOUSE, H2B1B_MOUSE, H2B1C_MOUSE, H2B1F_MOUSE, H2B1H_MOUSE, H2B1K_MOUSE, H2B1M_MOUSE, H2B1P_MOUSE, H2B2B_MOUSE, H2B2E_MOUSE, H2B3A_MOUSE, H2B3B_MOUSE	Histone H2B	QVHPDTGISSK	23	7.90E-03	584.8019	1167.5892	2	0.0008	11	0	Semi-tryptic
H2B1A_MOUSE, H2B1B_MOUSE, H2B1C_MOUSE, H2B1F_MOUSE, H2B1H_MOUSE, H2B1K_MOUSE, H2B1M_MOUSE, H2B1P_MOUSE, H2B2B_MOUSE, H2B2E_MOUSE, H2B3A_MOUSE, H2B3B_MOUSE	Histone H2B	QVHPDTGISSK	23	9.80E-03	584.8018	1167.5891	2	0.0007	11	0	Semi-tryptic
H2B1A_MOUSE, H2B1B_MOUSE, H2B1C_MOUSE, H2B1F_MOUSE, H2B1H_MOUSE, H2B1K_MOUSE, H2B1M_MOUSE, H2B1P_MOUSE, H2B2B_MOUSE, H2B2E_MOUSE, H2B3A_MOUSE, H2B3B_MOUSE	Histone H2B	QVHPDTGISSK	23	9.50E-03	584.8018	1167.5890	2	0.0006	11	0	Semi-tryptic
H2B1A_MOUSE, H2B1B_MOUSE, H2B1C_MOUSE, H2B1F_MOUSE, H2B1H_MOUSE, H2B1K_MOUSE, H2B1M_MOUSE, H2B1P_MOUSE, H2B2B_MOUSE, H2B2E_MOUSE, H2B3A_MOUSE, H2B3B_MOUSE	Histone H2B	QVHPDTGISSK	23	9.50E-04	390.2036	1167.5890	3	0.0006	11	0	Semi-tryptic
H2B1A_MOUSE, H2B1B_MOUSE, H2B1C_MOUSE, H2B1F_MOUSE, H2B1H_MOUSE, H2B1K_MOUSE, H2B1M_MOUSE, H2B1P_MOUSE, H2B2B_MOUSE, H2B2E_MOUSE, H2B3A_MOUSE, H2B3B_MOUSE	Histone H2B	QVHPDTGISSK	23	6.00E-05	390.2036	1167.5890	3	0.0006	11	0	Semi-tryptic
H2B1A_MOUSE, H2B1B_MOUSE, H2B1C_MOUSE, H2B1F_MOUSE, H2B1H_MOUSE, H2B1K_MOUSE, H2B1M_MOUSE, H2B1P_MOUSE, H2B2B_MOUSE, H2B2E_MOUSE, H2B3A_MOUSE, H2B3B_MOUSE	Histone H2B	QVHPDTGISSK	23	6.20E-03	584.8017	1167.5889	2	0.0005	11	0	Semi-tryptic
H2B1A_MOUSE, H2B1B_MOUSE, H2B1C_MOUSE, H2B1F_MOUSE, H2B1H_MOUSE, H2B1K_MOUSE, H2B1M_MOUSE, H2B1P_MOUSE, H2B2B_MOUSE, H2B2E_MOUSE, H2B3A_MOUSE, H2B3B_MOUSE	Histone H2B	QVHPDTGISSK	23	2.30E-03	584.8016	1167.5887	2	0.0003	11	0	Semi-tryptic
H2B1A_MOUSE, H2B1B_MOUSE, H2B1C_MOUSE, H2B1F_MOUSE, H2B1H_MOUSE, H2B1K_MOUSE, H2B1M_MOUSE, H2B1P_MOUSE, H2B2B_MOUSE, H2B2E_MOUSE, H2B3A_MOUSE, H2B3B_MOUSE	Histone H2B	QVHPDTGISSK	23	1.70E-03	390.2035	1167.5887	3	0.0003	11	0	Semi-tryptic
H2B1A_MOUSE, H2B1B_MOUSE, H2B1C_MOUSE, H2B1F_MOUSE, H2B1H_MOUSE, H2B1K_MOUSE, H2B1M_MOUSE, H2B1P_MOUSE, H2B2B_MOUSE, H2B2E_MOUSE, H2B3A_MOUSE, H2B3B_MOUSE	Histone H2B	QVHPDTGISSK	23	1.50E-04	390.2035	1167.5887	3	0.0003	11	0	Semi-tryptic
H2B1A_MOUSE, H2B1B_MOUSE, H2B1C_MOUSE, H2B1F_MOUSE, H2B1H_MOUSE, H2B1K_MOUSE, H2B1M_MOUSE, H2B1P_MOUSE, H2B2B_MOUSE, H2B2E_MOUSE, H2B3A_MOUSE, H2B3B_MOUSE	Histone H2B	QVHPDTGISSK	23	5.50E-03	390.2034	1167.5884	3	0.0000	11	0	Semi-tryptic
H2B1A_MOUSE, H2B1B_MOUSE, H2B1C_MOUSE, H2B1F_MOUSE, H2B1H_MOUSE, H2B1K_MOUSE, H2B1M_MOUSE, H2B1P_MOUSE, H2B2B_MOUSE, H2B2E_MOUSE, H2B3A_MOUSE, H2B3B_MOUSE	Histone H2B	QVHPDTGISSK	23	5.20E-03	390.2034	1167.5884	3	0.0000	11	0	Semi-tryptic
H2B1A_MOUSE, H2B1B_MOUSE, H2B1C_MOUSE, H2B1F_MOUSE, H2B1H_MOUSE, H2B1K_MOUSE, H2B1M_MOUSE, H2B1P_MOUSE, H2B2B_MOUSE, H2B2E_MOUSE, H2B3A_MOUSE, H2B3B_MOUSE	Histone H2B	QVHPDTGISSK	23	3.30E-04	390.2034	1167.5884	3	0.0000	11	0	Semi-tryptic
H2B1A_MOUSE, H2B1B_MOUSE, H2B1C_MOUSE, H2B1F_MOUSE, H2B1H_MOUSE, H2B1K_MOUSE, H2B1M_MOUSE, H2B1P_MOUSE, H2B2B_MOUSE, H2B2E_MOUSE, H2B3A_MOUSE, H2B3B_MOUSE	Histone H2B	QVHPDTGISSK	23	5.80E-03	584.801	1167.5874	2	-0.0010	11	0	Semi-tryptic
H2B1A_MOUSE, H2B1B_MOUSE, H2B1C_MOUSE, H2B1F_MOUSE, H2B1H_MOUSE, H2B1K_MOUSE, H2B1M_MOUSE, H2B1P_MOUSE, H2B2B_MOUSE, H2B2E_MOUSE, H2B3A_MOUSE, H2B3B_MOUSE	Histone H2B	QVHPDTGISSK	23	4.10E-04	390.2029	1167.5868	3	-0.0016	11	0	Semi-tryptic
H2B1A_MOUSE, H2B1B_MOUSE, H2B1C_MOUSE, H2B1F_MOUSE, H2B1H_MOUSE, H2B1K_MOUSE, H2B1M_MOUSE, H2B1P_MOUSE, H2B2B_MOUSE, H2B2E_MOUSE, H2B3A_MOUSE, H2B3B_MOUSE	Histone H2B	QVHPDTGISSK	23	4.00E-05	390.2029	1167.5868	3	-0.0016	11	0	Semi-tryptic
H2B1A_MOUSE, H2B1B_MOUSE, H2B1C_MOUSE, H2B1F_MOUSE, H2B1H_MOUSE, H2B1K_MOUSE, H2B1M_MOUSE, H2B1P_MOUSE, H2B2B_MOUSE, H2B2E_MOUSE, H2B3A_MOUSE, H2B3B_MOUSE	Histone H2B	LLLPGELAK	3	4.90E-03	477.307	952.5994	2	0.0037	9	0	Semi-tryptic
H2B1A_MOUSE, H2B1B_MOUSE, H2B1C_MOUSE, H2B1F_MOUSE, H2B1H_MOUSE, H2B1K_MOUSE, H2B1M_MOUSE, H2B1P_MOUSE, H2B2B_MOUSE, H2B2E_MOUSE, H2B3A_MOUSE, H2B3B_MOUSE	Histone H2B	LLLPGELAK	3	2.10E-03	477.3054	952.5962	2	0.0005	9	0	Semi-tryptic
H2B1A_MOUSE, H2B1B_MOUSE, H2B1C_MOUSE, H2B1F_MOUSE, H2B1H_MOUSE, H2B1K_MOUSE, H2B1M_MOUSE, H2B1P_MOUSE, H2B2B_MOUSE, H2B2E_MOUSE, H2B3A_MOUSE, H2B3B_MOUSE	Histone H2B	LLLPGELAK	3	9.00E-03	477.305	952.5953	2	-0.0003	9	0	Semi-tryptic
H2B1A_MOUSE, H2B1B_MOUSE, H2B1C_MOUSE, H2B1F_MOUSE, H2B1H_MOUSE, H2B1K_MOUSE, H2B1M_MOUSE, H2B1P_MOUSE, H2B2B_MOUSE, H2B2E_MOUSE, H2B3A_MOUSE, H2B3B_MOUSE	Histone H2B	HAVSEGTK	90	4.10E-04	414.7128	827.4110	2	-0.0027	8	0	Semi-tryptic
H2B1A_MOUSE, H2B1B_MOUSE, H2B1C_MOUSE, H2B1F_MOUSE, H2B1H_MOUSE, H2B1K_MOUSE, H2B1M_MOUSE, H2B1P_MOUSE, H2B2B_MOUSE, H2B2E_MOUSE, H2B3A_MOUSE, H2B3B_MOUSE	Histone H2B	HAVSEGTK	90	2.40E-04	414.7165	827.4184	2	0.0047	8	0	Semi-tryptic
H2B1A_MOUSE, H2B1B_MOUSE, H2B1C_MOUSE, H2B1F_MOUSE, H2B1H_MOUSE, H2B1K_MOUSE, H2B1M_MOUSE, H2B1P_MOUSE, H2B2B_MOUSE, H2B2E_MOUSE, H2B3A_MOUSE, H2B3B_MOUSE	Histone H2B	HAVSEGTK	90	8.40E-05	414.7158	827.4170	2	0.0032	8	0	Semi-tryptic
H2B1A_MOUSE, H2B1B_MOUSE, H2B1C_MOUSE, H2B1F_MOUSE, H2B1H_MOUSE, H2B1K_MOUSE, H2B1M_MOUSE, H2B1P_MOUSE, H2B2B_MOUSE, H2B2E_MOUSE, H2B3A_MOUSE, H2B3B_MOUSE	Histone H2B	HAVSEGTK	90	8.50E-05	414.7154	827.4163	2	0.0026	8	0	Semi-tryptic
H2B1A_MOUSE, H2B1B_MOUSE, H2B1C_MOUSE, H2B1F_MOUSE, H2B1H_MOUSE, H2B1K_MOUSE, H2B1M_MOUSE, H2B1P_MOUSE, H2B2B_MOUSE, H2B2E_MOUSE, H2B3A_MOUSE, H2B3B_MOUSE	Histone H2B	HAVSEGTK	90	6.60E-03	414.715	827.4154	2	0.0017	8	0	Semi-tryptic

H2B1A_MOUSE, H2B1B_MOUSE, H2B1C_MOUSE, H2B1F_MOUSE, H2B1H_MOUSE, H2B1K_MOUSE, H2B1M_MOUSE, H2B1P_MOUSE, H2B2B_MOUSE, H2B2E_MOUSE, H2B3A_MOUSE, H2B3B_MOUSE	Histone H2B	HAVSEGTK	90	1.40E-04	414.715	827.4154	2	0.0017	8	0	Semi-tryptic
H2B1A_MOUSE, H2B1B_MOUSE, H2B1C_MOUSE, H2B1F_MOUSE, H2B1H_MOUSE, H2B1K_MOUSE, H2B1M_MOUSE, H2B1P_MOUSE, H2B2B_MOUSE, H2B2E_MOUSE, H2B3A_MOUSE, H2B3B_MOUSE	Histone H2B	HAVSEGTK	90	2.00E-03	414.7149	827.4153	2	0.0016	8	0	Semi-tryptic
H2B1A_MOUSE, H2B1B_MOUSE, H2B1C_MOUSE, H2B1F_MOUSE, H2B1H_MOUSE, H2B1K_MOUSE, H2B1M_MOUSE, H2B1P_MOUSE, H2B2B_MOUSE, H2B2E_MOUSE, H2B3A_MOUSE, H2B3B_MOUSE	Histone H2B	HAVSEGTK	90	2.40E-04	414.7149	827.4153	2	0.0016	8	0	Semi-tryptic
H2B1A_MOUSE, H2B1B_MOUSE, H2B1C_MOUSE, H2B1F_MOUSE, H2B1H_MOUSE, H2B1K_MOUSE, H2B1M_MOUSE, H2B1P_MOUSE, H2B2B_MOUSE, H2B2E_MOUSE, H2B3A_MOUSE, H2B3B_MOUSE	Histone H2B	HAVSEGTK	90	3.20E-03	414.7149	827.4152	2	0.0015	8	0	Semi-tryptic
H2B1A_MOUSE, H2B1B_MOUSE, H2B1C_MOUSE, H2B1F_MOUSE, H2B1H_MOUSE, H2B1K_MOUSE, H2B1M_MOUSE, H2B1P_MOUSE, H2B2B_MOUSE, H2B2E_MOUSE, H2B3A_MOUSE, H2B3B_MOUSE	Histone H2B	HAVSEGTK	90	3.00E-03	414.7149	827.4153	2	0.0015	8	0	Semi-tryptic
H2B1A_MOUSE, H2B1B_MOUSE, H2B1C_MOUSE, H2B1F_MOUSE, H2B1H_MOUSE, H2B1K_MOUSE, H2B1M_MOUSE, H2B1P_MOUSE, H2B2B_MOUSE, H2B2E_MOUSE, H2B3A_MOUSE, H2B3B_MOUSE	Histone H2B	HAVSEGTK	90	3.90E-04	414.7149	827.4152	2	0.0015	8	0	Semi-tryptic
H2B1A_MOUSE, H2B1B_MOUSE, H2B1C_MOUSE, H2B1F_MOUSE, H2B1H_MOUSE, H2B1K_MOUSE, H2B1M_MOUSE, H2B1P_MOUSE, H2B2B_MOUSE, H2B2E_MOUSE, H2B3A_MOUSE, H2B3B_MOUSE	Histone H2B	HAVSEGTK	90	2.60E-03	414.7148	827.4151	2	0.0014	8	0	Semi-tryptic
H2B1A_MOUSE, H2B1B_MOUSE, H2B1C_MOUSE, H2B1F_MOUSE, H2B1H_MOUSE, H2B1K_MOUSE, H2B1M_MOUSE, H2B1P_MOUSE, H2B2B_MOUSE, H2B2E_MOUSE, H2B3A_MOUSE, H2B3B_MOUSE	Histone H2B	HAVSEGTK	90	3.20E-04	414.7149	827.4152	2	0.0014	8	0	Semi-tryptic
H2B1A_MOUSE, H2B1B_MOUSE, H2B1C_MOUSE, H2B1F_MOUSE, H2B1H_MOUSE, H2B1K_MOUSE, H2B1M_MOUSE, H2B1P_MOUSE, H2B2B_MOUSE, H2B2E_MOUSE, H2B3A_MOUSE, H2B3B_MOUSE	Histone H2B	HAVSEGTK	90	4.60E-05	414.7148	827.4151	2	0.0014	8	0	Semi-tryptic
H2B1A_MOUSE, H2B1B_MOUSE, H2B1C_MOUSE, H2B1F_MOUSE, H2B1H_MOUSE, H2B1K_MOUSE, H2B1M_MOUSE, H2B1P_MOUSE, H2B2B_MOUSE, H2B2E_MOUSE, H2B3A_MOUSE, H2B3B_MOUSE	Histone H2B	HAVSEGTK	90	7.70E-04	414.7148	827.4150	2	0.0013	8	0	Semi-tryptic
H2B1A_MOUSE, H2B1B_MOUSE, H2B1C_MOUSE, H2B1F_MOUSE, H2B1H_MOUSE, H2B1K_MOUSE, H2B1M_MOUSE, H2B1P_MOUSE, H2B2B_MOUSE, H2B2E_MOUSE, H2B3A_MOUSE, H2B3B_MOUSE	Histone H2B	HAVSEGTK	90	6.40E-04	414.7148	827.4150	2	0.0013	8	0	Semi-tryptic
H2B1A_MOUSE, H2B1B_MOUSE, H2B1C_MOUSE, H2B1F_MOUSE, H2B1H_MOUSE, H2B1K_MOUSE, H2B1M_MOUSE, H2B1P_MOUSE, H2B2B_MOUSE, H2B2E_MOUSE, H2B3A_MOUSE, H2B3B_MOUSE	Histone H2B	HAVSEGTK	90	2.40E-04	414.7148	827.4150	2	0.0013	8	0	Semi-tryptic
H2B1A_MOUSE, H2B1B_MOUSE, H2B1C_MOUSE, H2B1F_MOUSE, H2B1H_MOUSE, H2B1K_MOUSE, H2B1M_MOUSE, H2B1P_MOUSE, H2B2B_MOUSE, H2B2E_MOUSE, H2B3A_MOUSE, H2B3B_MOUSE	Histone H2B	HAVSEGTK	90	2.10E-03	414.7148	827.4150	2	0.0012	8	0	Semi-tryptic
H2B1A_MOUSE, H2B1B_MOUSE, H2B1C_MOUSE, H2B1F_MOUSE, H2B1H_MOUSE, H2B1K_MOUSE, H2B1M_MOUSE, H2B1P_MOUSE, H2B2B_MOUSE, H2B2E_MOUSE, H2B3A_MOUSE, H2B3B_MOUSE	Histone H2B	HAVSEGTK	90	1.20E-03	414.7147	827.4149	2	0.0012	8	0	Semi-tryptic
H2B1A_MOUSE, H2B1B_MOUSE, H2B1C_MOUSE, H2B1F_MOUSE, H2B1H_MOUSE, H2B1K_MOUSE, H2B1M_MOUSE, H2B1P_MOUSE, H2B2B_MOUSE, H2B2E_MOUSE, H2B3A_MOUSE, H2B3B_MOUSE	Histone H2B	HAVSEGTK	90	4.00E-04	414.7147	827.4149	2	0.0012	8	0	Semi-tryptic
H2B1A_MOUSE, H2B1B_MOUSE, H2B1C_MOUSE, H2B1F_MOUSE, H2B1H_MOUSE, H2B1K_MOUSE, H2B1M_MOUSE, H2B1P_MOUSE, H2B2B_MOUSE, H2B2E_MOUSE, H2B3A_MOUSE, H2B3B_MOUSE	Histone H2B	HAVSEGTK	90	4.00E-04	414.7147	827.4149	2	0.0012	8	0	Semi-tryptic
H2B1A_MOUSE, H2B1B_MOUSE, H2B1C_MOUSE, H2B1F_MOUSE, H2B1H_MOUSE, H2B1K_MOUSE, H2B1M_MOUSE, H2B1P_MOUSE, H2B2B_MOUSE, H2B2E_MOUSE, H2B3A_MOUSE, H2B3B_MOUSE	Histone H2B	HAVSEGTK	90	7.30E-05	414.7147	827.4149	2	0.0012	8	0	Semi-tryptic
H2B1A_MOUSE, H2B1B_MOUSE, H2B1C_MOUSE, H2B1F_MOUSE, H2B1H_MOUSE, H2B1K_MOUSE, H2B1M_MOUSE, H2B1P_MOUSE, H2B2B_MOUSE, H2B2E_MOUSE, H2B3A_MOUSE, H2B3B_MOUSE	Histone H2B	HAVSEGTK	90	5.40E-03	414.7147	827.4148	2	0.0011	8	0	Semi-tryptic
H2B1A_MOUSE, H2B1B_MOUSE, H2B1C_MOUSE, H2B1F_MOUSE, H2B1H_MOUSE, H2B1K_MOUSE, H2B1M_MOUSE, H2B1P_MOUSE, H2B2B_MOUSE, H2B2E_MOUSE, H2B3A_MOUSE, H2B3B_MOUSE	Histone H2B	HAVSEGTK	90	1.10E-03	414.7147	827.4148	2	0.0011	8	0	Semi-tryptic
H2B1A_MOUSE, H2B1B_MOUSE, H2B1C_MOUSE, H2B1F_MOUSE, H2B1H_MOUSE, H2B1K_MOUSE, H2B1M_MOUSE, H2B1P_MOUSE, H2B2B_MOUSE, H2B2E_MOUSE, H2B3A_MOUSE, H2B3B_MOUSE	Histone H2B	HAVSEGTK	90	3.80E-04	414.7147	827.4148	2	0.0011	8	0	Semi-tryptic
H2B1A_MOUSE, H2B1B_MOUSE, H2B1C_MOUSE, H2B1F_MOUSE, H2B1H_MOUSE, H2B1K_MOUSE, H2B1M_MOUSE, H2B1P_MOUSE, H2B2B_MOUSE, H2B2E_MOUSE, H2B3A_MOUSE, H2B3B_MOUSE	Histone H2B	HAVSEGTK	90	3.50E-04	414.7147	827.4149	2	0.0011	8	0	Semi-tryptic
H2B1A_MOUSE, H2B1B_MOUSE, H2B1C_MOUSE, H2B1F_MOUSE, H2B1H_MOUSE, H2B1K_MOUSE, H2B1M_MOUSE, H2B1P_MOUSE, H2B2B_MOUSE, H2B2E_MOUSE, H2B3A_MOUSE, H2B3B_MOUSE	Histone H2B	HAVSEGTK	90	2.30E-04	414.7147	827.4148	2	0.0011	8	0	Semi-tryptic
H2B1A_MOUSE, H2B1B_MOUSE, H2B1C_MOUSE, H2B1F_MOUSE, H2B1H_MOUSE, H2B1K_MOUSE, H2B1M_MOUSE, H2B1P_MOUSE, H2B2B_MOUSE, H2B2E_MOUSE, H2B3A_MOUSE, H2B3B_MOUSE	Histone H2B	HAVSEGTK	90	1.10E-04	414.7147	827.4148	2	0.0011	8	0	Semi-tryptic
H2B1A_MOUSE, H2B1B_MOUSE, H2B1C_MOUSE, H2B1F_MOUSE, H2B1H_MOUSE, H2B1K_MOUSE, H2B1M_MOUSE, H2B1P_MOUSE, H2B2B_MOUSE, H2B2E_MOUSE, H2B3A_MOUSE, H2B3B_MOUSE	Histone H2B	HAVSEGTK	90	3.20E-03	414.7147	827.4147	2	0.0010	8	0	Semi-tryptic

H2B1A_MOUSE, H2B1B_MOUSE, H2B1C_MOUSE, H2B1F_MOUSE, H2B1H_MOUSE, H2B1K_MOUSE, H2B1M_MOUSE, H2B1P_MOUSE, H2B2B_MOUSE, H2B2E_MOUSE, H2B3A_MOUSE, H2B3B_MOUSE	Histone H2B	HAVSEGTK	90	1.70E-03	414.7146	827.4147	2	0.0010	8	0	Semi-tryptic
H2B1A_MOUSE, H2B1B_MOUSE, H2B1C_MOUSE, H2B1F_MOUSE, H2B1H_MOUSE, H2B1K_MOUSE, H2B1M_MOUSE, H2B1P_MOUSE, H2B2B_MOUSE, H2B2E_MOUSE, H2B3A_MOUSE, H2B3B_MOUSE	Histone H2B	HAVSEGTK	90	1.30E-03	414.7146	827.4147	2	0.0010	8	0	Semi-tryptic
H2B1A_MOUSE, H2B1B_MOUSE, H2B1C_MOUSE, H2B1F_MOUSE, H2B1H_MOUSE, H2B1K_MOUSE, H2B1M_MOUSE, H2B1P_MOUSE, H2B2B_MOUSE, H2B2E_MOUSE, H2B3A_MOUSE, H2B3B_MOUSE	Histone H2B	HAVSEGTK	90	4.90E-05	414.7147	827.4147	2	0.0010	8	0	Semi-tryptic
H2B1A_MOUSE, H2B1B_MOUSE, H2B1C_MOUSE, H2B1F_MOUSE, H2B1H_MOUSE, H2B1K_MOUSE, H2B1M_MOUSE, H2B1P_MOUSE, H2B2B_MOUSE, H2B2E_MOUSE, H2B3A_MOUSE, H2B3B_MOUSE	Histone H2B	HAVSEGTK	90	8.90E-03	414.7146	827.4146	2	0.0009	8	0	Semi-tryptic
H2B1A_MOUSE, H2B1B_MOUSE, H2B1C_MOUSE, H2B1F_MOUSE, H2B1H_MOUSE, H2B1K_MOUSE, H2B1M_MOUSE, H2B1P_MOUSE, H2B2B_MOUSE, H2B2E_MOUSE, H2B3A_MOUSE, H2B3B_MOUSE	Histone H2B	HAVSEGTK	90	1.90E-04	414.7146	827.4147	2	0.0009	8	0	Semi-tryptic
H2B1A_MOUSE, H2B1B_MOUSE, H2B1C_MOUSE, H2B1F_MOUSE, H2B1H_MOUSE, H2B1K_MOUSE, H2B1M_MOUSE, H2B1P_MOUSE, H2B2B_MOUSE, H2B2E_MOUSE, H2B3A_MOUSE, H2B3B_MOUSE	Histone H2B	HAVSEGTK	90	2.40E-03	414.7146	827.4146	2	0.0008	8	0	Semi-tryptic
H2B1A_MOUSE, H2B1B_MOUSE, H2B1C_MOUSE, H2B1F_MOUSE, H2B1H_MOUSE, H2B1K_MOUSE, H2B1M_MOUSE, H2B1P_MOUSE, H2B2B_MOUSE, H2B2E_MOUSE, H2B3A_MOUSE, H2B3B_MOUSE	Histone H2B	HAVSEGTK	90	1.60E-03	414.7145	827.4145	2	0.0008	8	0	Semi-tryptic
H2B1A_MOUSE, H2B1B_MOUSE, H2B1C_MOUSE, H2B1F_MOUSE, H2B1H_MOUSE, H2B1K_MOUSE, H2B1M_MOUSE, H2B1P_MOUSE, H2B2B_MOUSE, H2B2E_MOUSE, H2B3A_MOUSE, H2B3B_MOUSE	Histone H2B	HAVSEGTK	90	1.90E-04	414.7145	827.4145	2	0.0008	8	0	Semi-tryptic
H2B1A_MOUSE, H2B1B_MOUSE, H2B1C_MOUSE, H2B1F_MOUSE, H2B1H_MOUSE, H2B1K_MOUSE, H2B1M_MOUSE, H2B1P_MOUSE, H2B2B_MOUSE, H2B2E_MOUSE, H2B3A_MOUSE, H2B3B_MOUSE	Histone H2B	HAVSEGTK	90	4.80E-03	414.7145	827.4144	2	0.0007	8	0	Semi-tryptic
H2B1A_MOUSE, H2B1B_MOUSE, H2B1C_MOUSE, H2B1F_MOUSE, H2B1H_MOUSE, H2B1K_MOUSE, H2B1M_MOUSE, H2B1P_MOUSE, H2B2B_MOUSE, H2B2E_MOUSE, H2B3A_MOUSE, H2B3B_MOUSE	Histone H2B	HAVSEGTK	90	2.70E-03	414.7145	827.4144	2	0.0007	8	0	Semi-tryptic
H2B1A_MOUSE, H2B1B_MOUSE, H2B1C_MOUSE, H2B1F_MOUSE, H2B1H_MOUSE, H2B1K_MOUSE, H2B1M_MOUSE, H2B1P_MOUSE, H2B2B_MOUSE, H2B2E_MOUSE, H2B3A_MOUSE, H2B3B_MOUSE	Histone H2B	HAVSEGTK	90	1.70E-04	414.7145	827.4144	2	0.0007	8	0	Semi-tryptic
H2B1A_MOUSE, H2B1B_MOUSE, H2B1C_MOUSE, H2B1F_MOUSE, H2B1H_MOUSE, H2B1K_MOUSE, H2B1M_MOUSE, H2B1P_MOUSE, H2B2B_MOUSE, H2B2E_MOUSE, H2B3A_MOUSE, H2B3B_MOUSE	Histone H2B	HAVSEGTK	90	5.00E-05	414.7145	827.4144	2	0.0007	8	0	Semi-tryptic
H2B1A_MOUSE, H2B1B_MOUSE, H2B1C_MOUSE, H2B1F_MOUSE, H2B1H_MOUSE, H2B1K_MOUSE, H2B1M_MOUSE, H2B1P_MOUSE, H2B2B_MOUSE, H2B2E_MOUSE, H2B3A_MOUSE, H2B3B_MOUSE	Histone H2B	HAVSEGTK	90	9.10E-03	414.7144	827.4143	2	0.0006	8	0	Semi-tryptic
H2B1A_MOUSE, H2B1B_MOUSE, H2B1C_MOUSE, H2B1F_MOUSE, H2B1H_MOUSE, H2B1K_MOUSE, H2B1M_MOUSE, H2B1P_MOUSE, H2B2B_MOUSE, H2B2E_MOUSE, H2B3A_MOUSE, H2B3B_MOUSE	Histone H2B	HAVSEGTK	90	1.10E-03	414.7144	827.4143	2	0.0006	8	0	Semi-tryptic
H2B1A_MOUSE, H2B1B_MOUSE, H2B1C_MOUSE, H2B1F_MOUSE, H2B1H_MOUSE, H2B1K_MOUSE, H2B1M_MOUSE, H2B1P_MOUSE, H2B2B_MOUSE, H2B2E_MOUSE, H2B3A_MOUSE, H2B3B_MOUSE	Histone H2B	HAVSEGTK	90	1.00E-03	414.7144	827.4143	2	0.0006	8	0	Semi-tryptic
H2B1A_MOUSE, H2B1B_MOUSE, H2B1C_MOUSE, H2B1F_MOUSE, H2B1H_MOUSE, H2B1K_MOUSE, H2B1M_MOUSE, H2B1P_MOUSE, H2B2B_MOUSE, H2B2E_MOUSE, H2B3A_MOUSE, H2B3B_MOUSE	Histone H2B	HAVSEGTK	90	3.40E-04	414.7144	827.4143	2	0.0006	8	0	Semi-tryptic
H2B1A_MOUSE, H2B1B_MOUSE, H2B1C_MOUSE, H2B1F_MOUSE, H2B1H_MOUSE, H2B1K_MOUSE, H2B1M_MOUSE, H2B1P_MOUSE, H2B2B_MOUSE, H2B2E_MOUSE, H2B3A_MOUSE, H2B3B_MOUSE	Histone H2B	HAVSEGTK	90	2.90E-03	414.7144	827.4142	2	0.0005	8	0	Semi-tryptic
H2B1A_MOUSE, H2B1B_MOUSE, H2B1C_MOUSE, H2B1F_MOUSE, H2B1H_MOUSE, H2B1K_MOUSE, H2B1M_MOUSE, H2B1P_MOUSE, H2B2B_MOUSE, H2B2E_MOUSE, H2B3A_MOUSE, H2B3B_MOUSE	Histone H2B	HAVSEGTK	90	7.40E-04	414.7144	827.4142	2	0.0005	8	0	Semi-tryptic
H2B1A_MOUSE, H2B1B_MOUSE, H2B1C_MOUSE, H2B1F_MOUSE, H2B1H_MOUSE, H2B1K_MOUSE, H2B1M_MOUSE, H2B1P_MOUSE, H2B2B_MOUSE, H2B2E_MOUSE, H2B3A_MOUSE, H2B3B_MOUSE	Histone H2B	HAVSEGTK	90	3.20E-04	414.7144	827.4142	2	0.0005	8	0	Semi-tryptic
H2B1A_MOUSE, H2B1B_MOUSE, H2B1C_MOUSE, H2B1F_MOUSE, H2B1H_MOUSE, H2B1K_MOUSE, H2B1M_MOUSE, H2B1P_MOUSE, H2B2B_MOUSE, H2B2E_MOUSE, H2B3A_MOUSE, H2B3B_MOUSE	Histone H2B	HAVSEGTK	90	7.50E-05	414.7144	827.4143	2	0.0005	8	0	Semi-tryptic
H2B1A_MOUSE, H2B1B_MOUSE, H2B1C_MOUSE, H2B1F_MOUSE, H2B1H_MOUSE, H2B1K_MOUSE, H2B1M_MOUSE, H2B1P_MOUSE, H2B2B_MOUSE, H2B2E_MOUSE, H2B3A_MOUSE, H2B3B_MOUSE	Histone H2B	HAVSEGTK	90	2.80E-03	414.7143	827.4141	2	0.0004	8	0	Semi-tryptic
H2B1A_MOUSE, H2B1B_MOUSE, H2B1C_MOUSE, H2B1F_MOUSE, H2B1H_MOUSE, H2B1K_MOUSE, H2B1M_MOUSE, H2B1P_MOUSE, H2B2B_MOUSE, H2B2E_MOUSE, H2B3A_MOUSE, H2B3B_MOUSE	Histone H2B	HAVSEGTK	90	9.50E-04	414.7143	827.4141	2	0.0004	8	0	Semi-tryptic
H2B1A_MOUSE, H2B1B_MOUSE, H2B1C_MOUSE, H2B1F_MOUSE, H2B1H_MOUSE, H2B1K_MOUSE, H2B1M_MOUSE, H2B1P_MOUSE, H2B2B_MOUSE, H2B2E_MOUSE, H2B3A_MOUSE, H2B3B_MOUSE	Histone H2B	HAVSEGTK	90	3.20E-04	414.7144	827.4141	2	0.0004	8	0	Semi-tryptic
H2B1A_MOUSE, H2B1B_MOUSE, H2B1C_MOUSE, H2B1F_MOUSE, H2B1H_MOUSE, H2B1K_MOUSE, H2B1M_MOUSE, H2B1P_MOUSE, H2B2B_MOUSE, H2B2E_MOUSE, H2B3A_MOUSE, H2B3B_MOUSE	Histone H2B	HAVSEGTK	90	3.10E-04	414.7144	827.4142	2	0.0004	8	0	Semi-tryptic

H2B1A_MOUSE, H2B1B_MOUSE, H2B1C_MOUSE, H2B1F_MOUSE, H2B1H_MOUSE, H2B1K_MOUSE, H2B1M_MOUSE, H2B1P_MOUSE, H2B2B_MOUSE, H2B2E_MOUSE, H2B3A_MOUSE, H2B3B_MOUSE	Histone H2B	HAVSEGTK	90	7.90E-05	414.7143	827.4141	2	0.0004	8	0	Semi-tryptic
H2B1A_MOUSE, H2B1B_MOUSE, H2B1C_MOUSE, H2B1F_MOUSE, H2B1H_MOUSE, H2B1K_MOUSE, H2B1M_MOUSE, H2B1P_MOUSE, H2B2B_MOUSE, H2B2E_MOUSE, H2B3A_MOUSE, H2B3B_MOUSE	Histone H2B	HAVSEGTK	90	2.60E-05	414.7144	827.4142	2	0.0004	8	0	Semi-tryptic
H2B1A_MOUSE, H2B1B_MOUSE, H2B1C_MOUSE, H2B1F_MOUSE, H2B1H_MOUSE, H2B1K_MOUSE, H2B1M_MOUSE, H2B1P_MOUSE, H2B2B_MOUSE, H2B2E_MOUSE, H2B3A_MOUSE, H2B3B_MOUSE	Histone H2B	HAVSEGTK	90	3.30E-03	414.7143	827.4141	2	0.0003	8	0	Semi-tryptic
H2B1A_MOUSE, H2B1B_MOUSE, H2B1C_MOUSE, H2B1F_MOUSE, H2B1H_MOUSE, H2B1K_MOUSE, H2B1M_MOUSE, H2B1P_MOUSE, H2B2B_MOUSE, H2B2E_MOUSE, H2B3A_MOUSE, H2B3B_MOUSE	Histone H2B	HAVSEGTK	90	1.30E-03	414.7143	827.4140	2	0.0003	8	0	Semi-tryptic
H2B1A_MOUSE, H2B1B_MOUSE, H2B1C_MOUSE, H2B1F_MOUSE, H2B1H_MOUSE, H2B1K_MOUSE, H2B1M_MOUSE, H2B1P_MOUSE, H2B2B_MOUSE, H2B2E_MOUSE, H2B3A_MOUSE, H2B3B_MOUSE	Histone H2B	HAVSEGTK	90	9.60E-04	414.7143	827.4140	2	0.0003	8	0	Semi-tryptic
H2B1A_MOUSE, H2B1B_MOUSE, H2B1C_MOUSE, H2B1F_MOUSE, H2B1H_MOUSE, H2B1K_MOUSE, H2B1M_MOUSE, H2B1P_MOUSE, H2B2B_MOUSE, H2B2E_MOUSE, H2B3A_MOUSE, H2B3B_MOUSE	Histone H2B	HAVSEGTK	90	8.10E-04	414.7143	827.4141	2	0.0003	8	0	Semi-tryptic
H2B1A_MOUSE, H2B1B_MOUSE, H2B1C_MOUSE, H2B1F_MOUSE, H2B1H_MOUSE, H2B1K_MOUSE, H2B1M_MOUSE, H2B1P_MOUSE, H2B2B_MOUSE, H2B2E_MOUSE, H2B3A_MOUSE, H2B3B_MOUSE	Histone H2B	HAVSEGTK	90	7.40E-05	414.7143	827.4140	2	0.0003	8	0	Semi-tryptic
H2B1A_MOUSE, H2B1B_MOUSE, H2B1C_MOUSE, H2B1F_MOUSE, H2B1H_MOUSE, H2B1K_MOUSE, H2B1M_MOUSE, H2B1P_MOUSE, H2B2B_MOUSE, H2B2E_MOUSE, H2B3A_MOUSE, H2B3B_MOUSE	Histone H2B	HAVSEGTK	90	2.80E-03	414.7142	827.4139	2	0.0002	8	0	Semi-tryptic
H2B1A_MOUSE, H2B1B_MOUSE, H2B1C_MOUSE, H2B1F_MOUSE, H2B1H_MOUSE, H2B1K_MOUSE, H2B1M_MOUSE, H2B1P_MOUSE, H2B2B_MOUSE, H2B2E_MOUSE, H2B3A_MOUSE, H2B3B_MOUSE	Histone H2B	HAVSEGTK	90	5.40E-04	414.7143	827.4140	2	0.0002	8	0	Semi-tryptic
H2B1A_MOUSE, H2B1B_MOUSE, H2B1C_MOUSE, H2B1F_MOUSE, H2B1H_MOUSE, H2B1K_MOUSE, H2B1M_MOUSE, H2B1P_MOUSE, H2B2B_MOUSE, H2B2E_MOUSE, H2B3A_MOUSE, H2B3B_MOUSE	Histone H2B	HAVSEGTK	90	3.20E-04	414.7142	827.4139	2	0.0002	8	0	Semi-tryptic
H2B1A_MOUSE, H2B1B_MOUSE, H2B1C_MOUSE, H2B1F_MOUSE, H2B1H_MOUSE, H2B1K_MOUSE, H2B1M_MOUSE, H2B1P_MOUSE, H2B2B_MOUSE, H2B2E_MOUSE, H2B3A_MOUSE, H2B3B_MOUSE	Histone H2B	HAVSEGTK	90	2.20E-05	414.7143	827.4140	2	0.0002	8	0	Semi-tryptic
H2B1A_MOUSE, H2B1B_MOUSE, H2B1C_MOUSE, H2B1F_MOUSE, H2B1H_MOUSE, H2B1K_MOUSE, H2B1M_MOUSE, H2B1P_MOUSE, H2B2B_MOUSE, H2B2E_MOUSE, H2B3A_MOUSE, H2B3B_MOUSE	Histone H2B	HAVSEGTK	90	5.10E-03	414.7142	827.4138	2	0.0001	8	0	Semi-tryptic
H2B1A_MOUSE, H2B1B_MOUSE, H2B1C_MOUSE, H2B1F_MOUSE, H2B1H_MOUSE, H2B1K_MOUSE, H2B1M_MOUSE, H2B1P_MOUSE, H2B2B_MOUSE, H2B2E_MOUSE, H2B3A_MOUSE, H2B3B_MOUSE	Histone H2B	HAVSEGTK	90	1.40E-03	414.7142	827.4138	2	0.0001	8	0	Semi-tryptic
H2B1A_MOUSE, H2B1B_MOUSE, H2B1C_MOUSE, H2B1F_MOUSE, H2B1H_MOUSE, H2B1K_MOUSE, H2B1M_MOUSE, H2B1P_MOUSE, H2B2B_MOUSE, H2B2E_MOUSE, H2B3A_MOUSE, H2B3B_MOUSE	Histone H2B	HAVSEGTK	90	1.20E-03	414.7142	827.4138	2	0.0001	8	0	Semi-tryptic
H2B1A_MOUSE, H2B1B_MOUSE, H2B1C_MOUSE, H2B1F_MOUSE, H2B1H_MOUSE, H2B1K_MOUSE, H2B1M_MOUSE, H2B1P_MOUSE, H2B2B_MOUSE, H2B2E_MOUSE, H2B3A_MOUSE, H2B3B_MOUSE	Histone H2B	HAVSEGTK	90	1.10E-03	414.7142	827.4139	2	0.0001	8	0	Semi-tryptic
H2B1A_MOUSE, H2B1B_MOUSE, H2B1C_MOUSE, H2B1F_MOUSE, H2B1H_MOUSE, H2B1K_MOUSE, H2B1M_MOUSE, H2B1P_MOUSE, H2B2B_MOUSE, H2B2E_MOUSE, H2B3A_MOUSE, H2B3B_MOUSE	Histone H2B	HAVSEGTK	90	1.10E-03	414.7142	827.4139	2	0.0001	8	0	Semi-tryptic
H2B1A_MOUSE, H2B1B_MOUSE, H2B1C_MOUSE, H2B1F_MOUSE, H2B1H_MOUSE, H2B1K_MOUSE, H2B1M_MOUSE, H2B1P_MOUSE, H2B2B_MOUSE, H2B2E_MOUSE, H2B3A_MOUSE, H2B3B_MOUSE	Histone H2B	HAVSEGTK	90	1.70E-04	414.7142	827.4139	2	0.0001	8	0	Semi-tryptic
H2B1A_MOUSE, H2B1B_MOUSE, H2B1C_MOUSE, H2B1F_MOUSE, H2B1H_MOUSE, H2B1K_MOUSE, H2B1M_MOUSE, H2B1P_MOUSE, H2B2B_MOUSE, H2B2E_MOUSE, H2B3A_MOUSE, H2B3B_MOUSE	Histone H2B	HAVSEGTK	90	6.30E-05	414.7142	827.4138	2	0.0001	8	0	Semi-tryptic
H2B1A_MOUSE, H2B1B_MOUSE, H2B1C_MOUSE, H2B1F_MOUSE, H2B1H_MOUSE, H2B1K_MOUSE, H2B1M_MOUSE, H2B1P_MOUSE, H2B2B_MOUSE, H2B2E_MOUSE, H2B3A_MOUSE, H2B3B_MOUSE	Histone H2B	HAVSEGTK	90	2.70E-05	414.7142	827.4138	2	0.0001	8	0	Semi-tryptic
H2B1A_MOUSE, H2B1B_MOUSE, H2B1C_MOUSE, H2B1F_MOUSE, H2B1H_MOUSE, H2B1K_MOUSE, H2B1M_MOUSE, H2B1P_MOUSE, H2B2B_MOUSE, H2B2E_MOUSE, H2B3A_MOUSE, H2B3B_MOUSE	Histone H2B	HAVSEGTK	90	1.00E-03	414.7141	827.4137	2	0.0000	8	0	Semi-tryptic
H2B1A_MOUSE, H2B1B_MOUSE, H2B1C_MOUSE, H2B1F_MOUSE, H2B1H_MOUSE, H2B1K_MOUSE, H2B1M_MOUSE, H2B1P_MOUSE, H2B2B_MOUSE, H2B2E_MOUSE, H2B3A_MOUSE, H2B3B_MOUSE	Histone H2B	HAVSEGTK	90	8.00E-04	414.7142	827.4137	2	0.0000	8	0	Semi-tryptic
H2B1A_MOUSE, H2B1B_MOUSE, H2B1C_MOUSE, H2B1F_MOUSE, H2B1H_MOUSE, H2B1K_MOUSE, H2B1M_MOUSE, H2B1P_MOUSE, H2B2B_MOUSE, H2B2E_MOUSE, H2B3A_MOUSE, H2B3B_MOUSE	Histone H2B	HAVSEGTK	90	5.90E-04	414.7141	827.4137	2	0.0000	8	0	Semi-tryptic
H2B1A_MOUSE, H2B1B_MOUSE, H2B1C_MOUSE, H2B1F_MOUSE, H2B1H_MOUSE, H2B1K_MOUSE, H2B1M_MOUSE, H2B1P_MOUSE, H2B2B_MOUSE, H2B2E_MOUSE, H2B3A_MOUSE, H2B3B_MOUSE	Histone H2B	HAVSEGTK	90	4.10E-04	414.7141	827.4137	2	0.0000	8	0	Semi-tryptic
H2B1A_MOUSE, H2B1B_MOUSE, H2B1C_MOUSE, H2B1F_MOUSE, H2B1H_MOUSE, H2B1K_MOUSE, H2B1M_MOUSE, H2B1P_MOUSE, H2B2B_MOUSE, H2B2E_MOUSE, H2B3A_MOUSE, H2B3B_MOUSE	Histone H2B	HAVSEGTK	90	1.10E-04	414.7141	827.4137	2	0.0000	8	0	Semi-tryptic

H2B1A_MOUSE, H2B1B_MOUSE, H2B1C_MOUSE, H2B1F_MOUSE, H2B1H_MOUSE, H2B1K_MOUSE, H2B1M_MOUSE, H2B1P_MOUSE, H2B2B_MOUSE, H2B2E_MOUSE, H2B3A_MOUSE, H2B3B_MOUSE	Histone H2B	HAVSEGTK	90	2.50E-03	414.7141	827.4137	2	-0.0001	8	0	Semi-tryptic
H2B1A_MOUSE, H2B1B_MOUSE, H2B1C_MOUSE, H2B1F_MOUSE, H2B1H_MOUSE, H2B1K_MOUSE, H2B1M_MOUSE, H2B1P_MOUSE, H2B2B_MOUSE, H2B2E_MOUSE, H2B3A_MOUSE, H2B3B_MOUSE	Histone H2B	HAVSEGTK	90	1.60E-03	414.7141	827.4136	2	-0.0001	8	0	Semi-tryptic
H2B1A_MOUSE, H2B1B_MOUSE, H2B1C_MOUSE, H2B1F_MOUSE, H2B1H_MOUSE, H2B1K_MOUSE, H2B1M_MOUSE, H2B1P_MOUSE, H2B2B_MOUSE, H2B2E_MOUSE, H2B3A_MOUSE, H2B3B_MOUSE	Histone H2B	HAVSEGTK	90	1.30E-03	414.7141	827.4137	2	-0.0001	8	0	Semi-tryptic
H2B1A_MOUSE, H2B1B_MOUSE, H2B1C_MOUSE, H2B1F_MOUSE, H2B1H_MOUSE, H2B1K_MOUSE, H2B1M_MOUSE, H2B1P_MOUSE, H2B2B_MOUSE, H2B2E_MOUSE, H2B3A_MOUSE, H2B3B_MOUSE	Histone H2B	HAVSEGTK	90	4.20E-03	414.7141	827.4136	2	-0.0002	8	0	Semi-tryptic
H2B1A_MOUSE, H2B1B_MOUSE, H2B1C_MOUSE, H2B1F_MOUSE, H2B1H_MOUSE, H2B1K_MOUSE, H2B1M_MOUSE, H2B1P_MOUSE, H2B2B_MOUSE, H2B2E_MOUSE, H2B3A_MOUSE, H2B3B_MOUSE	Histone H2B	HAVSEGTK	90	9.30E-05	414.7141	827.4136	2	-0.0002	8	0	Semi-tryptic
H2B1A_MOUSE, H2B1B_MOUSE, H2B1C_MOUSE, H2B1F_MOUSE, H2B1H_MOUSE, H2B1K_MOUSE, H2B1M_MOUSE, H2B1P_MOUSE, H2B2B_MOUSE, H2B2E_MOUSE, H2B3A_MOUSE, H2B3B_MOUSE	Histone H2B	HAVSEGTK	90	4.40E-03	414.714	827.4134	2	-0.0003	8	0	Semi-tryptic
H2B1A_MOUSE, H2B1B_MOUSE, H2B1C_MOUSE, H2B1F_MOUSE, H2B1H_MOUSE, H2B1K_MOUSE, H2B1M_MOUSE, H2B1P_MOUSE, H2B2B_MOUSE, H2B2E_MOUSE, H2B3A_MOUSE, H2B3B_MOUSE	Histone H2B	HAVSEGTK	90	4.40E-04	414.714	827.4134	2	-0.0003	8	0	Semi-tryptic
H2B1A_MOUSE, H2B1B_MOUSE, H2B1C_MOUSE, H2B1F_MOUSE, H2B1H_MOUSE, H2B1K_MOUSE, H2B1M_MOUSE, H2B1P_MOUSE, H2B2B_MOUSE, H2B2E_MOUSE, H2B3A_MOUSE, H2B3B_MOUSE	Histone H2B	HAVSEGTK	90	9.40E-04	414.714	827.4134	2	-0.0004	8	0	Semi-tryptic
H2B1A_MOUSE, H2B1B_MOUSE, H2B1C_MOUSE, H2B1F_MOUSE, H2B1H_MOUSE, H2B1K_MOUSE, H2B1M_MOUSE, H2B1P_MOUSE, H2B2B_MOUSE, H2B2E_MOUSE, H2B3A_MOUSE, H2B3B_MOUSE	Histone H2B	HAVSEGTK	90	4.30E-05	414.714	827.4134	2	-0.0004	8	0	Semi-tryptic
H2B1A_MOUSE, H2B1B_MOUSE, H2B1C_MOUSE, H2B1F_MOUSE, H2B1H_MOUSE, H2B1K_MOUSE, H2B1M_MOUSE, H2B1P_MOUSE, H2B2B_MOUSE, H2B2E_MOUSE, H2B3A_MOUSE, H2B3B_MOUSE	Histone H2B	HAVSEGTK	90	1.20E-03	414.7138	827.4129	2	-0.0008	8	0	Semi-tryptic
H2B1A_MOUSE, H2B1B_MOUSE, H2B1C_MOUSE, H2B1F_MOUSE, H2B1H_MOUSE, H2B1K_MOUSE, H2B1M_MOUSE, H2B1P_MOUSE, H2B2B_MOUSE, H2B2E_MOUSE, H2B3A_MOUSE, H2B3B_MOUSE	Histone H2B	HAVSEGTK	90	3.80E-03	414.7136	827.4126	2	-0.0011	8	0	Semi-tryptic
H2B1A_MOUSE, H2B1B_MOUSE, H2B1C_MOUSE, H2B1F_MOUSE, H2B1H_MOUSE, H2B1K_MOUSE, H2B1M_MOUSE, H2B1P_MOUSE, H2B2B_MOUSE, H2B2E_MOUSE, H2B3A_MOUSE, H2B3B_MOUSE	Histone H2B	HAVSEGTK	90	3.00E-03	414.7135	827.4124	2	-0.0013	8	0	Semi-tryptic
H2B1A_MOUSE, H2B1B_MOUSE, H2B1C_MOUSE, H2B1F_MOUSE, H2B1H_MOUSE, H2B1K_MOUSE, H2B1M_MOUSE, H2B1P_MOUSE, H2B2B_MOUSE, H2B2E_MOUSE, H2B3A_MOUSE, H2B3B_MOUSE	Histone H2B	HAVSEGTK	90	4.80E-04	414.7134	827.4122	2	-0.0016	8	0	Semi-tryptic
H2B1A_MOUSE, H2B1B_MOUSE, H2B1C_MOUSE, H2B1F_MOUSE, H2B1H_MOUSE, H2B1K_MOUSE, H2B1M_MOUSE, H2B1P_MOUSE, H2B2B_MOUSE, H2B2E_MOUSE, H2B3A_MOUSE, H2B3B_MOUSE	Histone H2B	LAKHAVSE	122	3.00E-03	427.7425	853.4705	2	0.0047	8	0	GluC
H2B1A_MOUSE, H2B1B_MOUSE, H2B1C_MOUSE, H2B1F_MOUSE, H2B1H_MOUSE, H2B1K_MOUSE, H2B1M_MOUSE, H2B1P_MOUSE, H2B2B_MOUSE, H2B2E_MOUSE, H2B3A_MOUSE, H2B3B_MOUSE	Histone H2B	LAKHAVSE	122	6.40E-03	427.7424	853.4703	2	0.0046	8	0	GluC
H2B1A_MOUSE, H2B1B_MOUSE, H2B1C_MOUSE, H2B1F_MOUSE, H2B1H_MOUSE, H2B1K_MOUSE, H2B1M_MOUSE, H2B1P_MOUSE, H2B2B_MOUSE, H2B2E_MOUSE, H2B3A_MOUSE, H2B3B_MOUSE	Histone H2B	LAKHAVSE	122	8.50E-04	427.7422	853.4698	2	0.0041	8	0	GluC
H2B1A_MOUSE, H2B1B_MOUSE, H2B1C_MOUSE, H2B1F_MOUSE, H2B1H_MOUSE, H2B1K_MOUSE, H2B1M_MOUSE, H2B1P_MOUSE, H2B2B_MOUSE, H2B2E_MOUSE, H2B3A_MOUSE, H2B3B_MOUSE	Histone H2B	LAKHAVSE	122	3.80E-03	427.7421	853.4696	2	0.0039	8	0	GluC
H2B1A_MOUSE, H2B1B_MOUSE, H2B1C_MOUSE, H2B1F_MOUSE, H2B1H_MOUSE, H2B1K_MOUSE, H2B1M_MOUSE, H2B1P_MOUSE, H2B2B_MOUSE, H2B2E_MOUSE, H2B3A_MOUSE, H2B3B_MOUSE	Histone H2B	LAKHAVSE	122	2.50E-04	427.7421	853.4696	2	0.0039	8	0	GluC
H2B1A_MOUSE, H2B1B_MOUSE, H2B1C_MOUSE, H2B1F_MOUSE, H2B1H_MOUSE, H2B1K_MOUSE, H2B1M_MOUSE, H2B1P_MOUSE, H2B2B_MOUSE, H2B2E_MOUSE, H2B3A_MOUSE, H2B3B_MOUSE	Histone H2B	LAKHAVSE	122	3.00E-03	427.7419	853.4693	2	0.0036	8	0	GluC
H2B1A_MOUSE, H2B1B_MOUSE, H2B1C_MOUSE, H2B1F_MOUSE, H2B1H_MOUSE, H2B1K_MOUSE, H2B1M_MOUSE, H2B1P_MOUSE, H2B2B_MOUSE, H2B2E_MOUSE, H2B3A_MOUSE, H2B3B_MOUSE	Histone H2B	LAKHAVSE	122	1.50E-03	427.7417	853.4689	2	0.0032	8	0	GluC
H2B1A_MOUSE, H2B1B_MOUSE, H2B1C_MOUSE, H2B1F_MOUSE, H2B1H_MOUSE, H2B1K_MOUSE, H2B1M_MOUSE, H2B1P_MOUSE, H2B2B_MOUSE, H2B2E_MOUSE, H2B3A_MOUSE, H2B3B_MOUSE	Histone H2B	LAKHAVSE	122	2.30E-04	427.7417	853.4689	2	0.0031	8	0	GluC
H2B1A_MOUSE, H2B1B_MOUSE, H2B1C_MOUSE, H2B1F_MOUSE, H2B1H_MOUSE, H2B1K_MOUSE, H2B1M_MOUSE, H2B1P_MOUSE, H2B2B_MOUSE, H2B2E_MOUSE, H2B3A_MOUSE, H2B3B_MOUSE	Histone H2B	LAKHAVSE	122	4.50E-03	427.7417	853.4688	2	0.0030	8	0	GluC
H2B1A_MOUSE, H2B1B_MOUSE, H2B1C_MOUSE, H2B1F_MOUSE, H2B1H_MOUSE, H2B1K_MOUSE, H2B1M_MOUSE, H2B1P_MOUSE, H2B2B_MOUSE, H2B2E_MOUSE, H2B3A_MOUSE, H2B3B_MOUSE	Histone H2B	LAKHAVSE	122	3.00E-04	427.7416	853.4687	2	0.0030	8	0	GluC
H2B1A_MOUSE, H2B1B_MOUSE, H2B1C_MOUSE, H2B1F_MOUSE, H2B1H_MOUSE, H2B1K_MOUSE, H2B1M_MOUSE, H2B1P_MOUSE, H2B2B_MOUSE, H2B2E_MOUSE, H2B3A_MOUSE, H2B3B_MOUSE	Histone H2B	LAKHAVSE	122	5.70E-04	427.7416	853.4687	2	0.0029	8	0	GluC

H2B1A_MOUSE, H2B1B_MOUSE, H2B1C_MOUSE, H2B1F_MOUSE, H2B1H_MOUSE, H2B1K_MOUSE, H2B1M_MOUSE, H2B1P_MOUSE, H2B2B_MOUSE, H2B2E_MOUSE, H2B3A_MOUSE, H2B3B_MOUSE	Histone H2B	LAKHAVSE	122	9.30E-04	427.7415	853.4685	2	0.0028	8	0	GluC
H2B1A_MOUSE, H2B1B_MOUSE, H2B1C_MOUSE, H2B1F_MOUSE, H2B1H_MOUSE, H2B1K_MOUSE, H2B1M_MOUSE, H2B1P_MOUSE, H2B2B_MOUSE, H2B2E_MOUSE, H2B3A_MOUSE, H2B3B_MOUSE	Histone H2B	LAKHAVSE	122	2.30E-05	427.7413	853.4681	2	0.0024	8	0	GluC
H2B1A_MOUSE, H2B1B_MOUSE, H2B1C_MOUSE, H2B1F_MOUSE, H2B1H_MOUSE, H2B1K_MOUSE, H2B1M_MOUSE, H2B1P_MOUSE, H2B2B_MOUSE, H2B2E_MOUSE, H2B3A_MOUSE, H2B3B_MOUSE	Histone H2B	LAKHAVSE	122	5.00E-04	427.7413	853.4680	2	0.0023	8	0	GluC
H2B1A_MOUSE, H2B1B_MOUSE, H2B1C_MOUSE, H2B1F_MOUSE, H2B1H_MOUSE, H2B1K_MOUSE, H2B1M_MOUSE, H2B1P_MOUSE, H2B2B_MOUSE, H2B2E_MOUSE, H2B3A_MOUSE, H2B3B_MOUSE	Histone H2B	LAKHAVSE	122	1.10E-04	427.7413	853.4681	2	0.0023	8	0	GluC
H2B1A_MOUSE, H2B1B_MOUSE, H2B1C_MOUSE, H2B1F_MOUSE, H2B1H_MOUSE, H2B1K_MOUSE, H2B1M_MOUSE, H2B1P_MOUSE, H2B2B_MOUSE, H2B2E_MOUSE, H2B3A_MOUSE, H2B3B_MOUSE	Histone H2B	LAKHAVSE	122	5.30E-03	427.7412	853.4679	2	0.0022	8	0	GluC
H2B1A_MOUSE, H2B1B_MOUSE, H2B1C_MOUSE, H2B1F_MOUSE, H2B1H_MOUSE, H2B1K_MOUSE, H2B1M_MOUSE, H2B1P_MOUSE, H2B2B_MOUSE, H2B2E_MOUSE, H2B3A_MOUSE, H2B3B_MOUSE	Histone H2B	LAKHAVSE	122	4.80E-04	427.7412	853.4678	2	0.0021	8	0	GluC
H2B1A_MOUSE, H2B1B_MOUSE, H2B1C_MOUSE, H2B1F_MOUSE, H2B1H_MOUSE, H2B1K_MOUSE, H2B1M_MOUSE, H2B1P_MOUSE, H2B2B_MOUSE, H2B2E_MOUSE, H2B3A_MOUSE, H2B3B_MOUSE	Histone H2B	LAKHAVSE	122	6.70E-03	427.7412	853.4677	2	0.0020	8	0	GluC
H2B1A_MOUSE, H2B1B_MOUSE, H2B1C_MOUSE, H2B1F_MOUSE, H2B1H_MOUSE, H2B1K_MOUSE, H2B1M_MOUSE, H2B1P_MOUSE, H2B2B_MOUSE, H2B2E_MOUSE, H2B3A_MOUSE, H2B3B_MOUSE	Histone H2B	LAKHAVSE	122	5.80E-04	427.7412	853.4678	2	0.0020	8	0	GluC
H2B1A_MOUSE, H2B1B_MOUSE, H2B1C_MOUSE, H2B1F_MOUSE, H2B1H_MOUSE, H2B1K_MOUSE, H2B1M_MOUSE, H2B1P_MOUSE, H2B2B_MOUSE, H2B2E_MOUSE, H2B3A_MOUSE, H2B3B_MOUSE	Histone H2B	LAKHAVSE	122	2.00E-04	427.7411	853.4676	2	0.0019	8	0	GluC
H2B1A_MOUSE, H2B1B_MOUSE, H2B1C_MOUSE, H2B1F_MOUSE, H2B1H_MOUSE, H2B1K_MOUSE, H2B1M_MOUSE, H2B1P_MOUSE, H2B2B_MOUSE, H2B2E_MOUSE, H2B3A_MOUSE, H2B3B_MOUSE	Histone H2B	LAKHAVSE	122	6.10E-04	427.7409	853.4673	2	0.0016	8	0	GluC
H2B1A_MOUSE, H2B1B_MOUSE, H2B1C_MOUSE, H2B1F_MOUSE, H2B1H_MOUSE, H2B1K_MOUSE, H2B1M_MOUSE, H2B1P_MOUSE, H2B2B_MOUSE, H2B2E_MOUSE, H2B3A_MOUSE, H2B3B_MOUSE	Histone H2B	LAKHAVSE	122	4.30E-03	427.7409	853.4673	2	0.0015	8	0	GluC
H2B1A_MOUSE, H2B1B_MOUSE, H2B1C_MOUSE, H2B1F_MOUSE, H2B1H_MOUSE, H2B1K_MOUSE, H2B1M_MOUSE, H2B1P_MOUSE, H2B2B_MOUSE, H2B2E_MOUSE, H2B3A_MOUSE, H2B3B_MOUSE	Histone H2B	LAKHAVSE	122	2.80E-04	427.7409	853.4673	2	0.0015	8	0	GluC
H2B1A_MOUSE, H2B1B_MOUSE, H2B1C_MOUSE, H2B1F_MOUSE, H2B1H_MOUSE, H2B1K_MOUSE, H2B1M_MOUSE, H2B1P_MOUSE, H2B2B_MOUSE, H2B2E_MOUSE, H2B3A_MOUSE, H2B3B_MOUSE	Histone H2B	LAKHAVSE	122	4.90E-03	427.7409	853.4671	2	0.0014	8	0	GluC
H2B1A_MOUSE, H2B1B_MOUSE, H2B1C_MOUSE, H2B1F_MOUSE, H2B1H_MOUSE, H2B1K_MOUSE, H2B1M_MOUSE, H2B1P_MOUSE, H2B2B_MOUSE, H2B2E_MOUSE, H2B3A_MOUSE, H2B3B_MOUSE	Histone H2B	LAKHAVSE	122	4.40E-03	427.7408	853.4671	2	0.0014	8	0	GluC
H2B1A_MOUSE, H2B1B_MOUSE, H2B1C_MOUSE, H2B1F_MOUSE, H2B1H_MOUSE, H2B1K_MOUSE, H2B1M_MOUSE, H2B1P_MOUSE, H2B2B_MOUSE, H2B2E_MOUSE, H2B3A_MOUSE, H2B3B_MOUSE	Histone H2B	LAKHAVSE	122	1.40E-03	427.7408	853.4671	2	0.0014	8	0	GluC
H2B1A_MOUSE, H2B1B_MOUSE, H2B1C_MOUSE, H2B1F_MOUSE, H2B1H_MOUSE, H2B1K_MOUSE, H2B1M_MOUSE, H2B1P_MOUSE, H2B2B_MOUSE, H2B2E_MOUSE, H2B3A_MOUSE, H2B3B_MOUSE	Histone H2B	LAKHAVSE	122	5.10E-06	427.7409	853.4671	2	0.0014	8	0	GluC
H2B1A_MOUSE, H2B1B_MOUSE, H2B1C_MOUSE, H2B1F_MOUSE, H2B1H_MOUSE, H2B1K_MOUSE, H2B1M_MOUSE, H2B1P_MOUSE, H2B2B_MOUSE, H2B2E_MOUSE, H2B3A_MOUSE, H2B3B_MOUSE	Histone H2B	LAKHAVSE	122	6.00E-03	427.7408	853.4671	2	0.0013	8	0	GluC
H2B1A_MOUSE, H2B1B_MOUSE, H2B1C_MOUSE, H2B1F_MOUSE, H2B1H_MOUSE, H2B1K_MOUSE, H2B1M_MOUSE, H2B1P_MOUSE, H2B2B_MOUSE, H2B2E_MOUSE, H2B3A_MOUSE, H2B3B_MOUSE	Histone H2B	LAKHAVSE	122	2.00E-03	427.7408	853.4671	2	0.0013	8	0	GluC
H2B1A_MOUSE, H2B1B_MOUSE, H2B1C_MOUSE, H2B1F_MOUSE, H2B1H_MOUSE, H2B1K_MOUSE, H2B1M_MOUSE, H2B1P_MOUSE, H2B2B_MOUSE, H2B2E_MOUSE, H2B3A_MOUSE, H2B3B_MOUSE	Histone H2B	LAKHAVSE	122	8.50E-04	427.7408	853.4671	2	0.0013	8	0	GluC
H2B1A_MOUSE, H2B1B_MOUSE, H2B1C_MOUSE, H2B1F_MOUSE, H2B1H_MOUSE, H2B1K_MOUSE, H2B1M_MOUSE, H2B1P_MOUSE, H2B2B_MOUSE, H2B2E_MOUSE, H2B3A_MOUSE, H2B3B_MOUSE	Histone H2B	LAKHAVSE	122	6.90E-05	427.7408	853.4671	2	0.0013	8	0	GluC
H2B1A_MOUSE, H2B1B_MOUSE, H2B1C_MOUSE, H2B1F_MOUSE, H2B1H_MOUSE, H2B1K_MOUSE, H2B1M_MOUSE, H2B1P_MOUSE, H2B2B_MOUSE, H2B2E_MOUSE, H2B3A_MOUSE, H2B3B_MOUSE	Histone H2B	LAKHAVSE	122	8.50E-04	427.7408	853.4670	2	0.0012	8	0	GluC
H2B1A_MOUSE, H2B1B_MOUSE, H2B1C_MOUSE, H2B1F_MOUSE, H2B1H_MOUSE, H2B1K_MOUSE, H2B1M_MOUSE, H2B1P_MOUSE, H2B2B_MOUSE, H2B2E_MOUSE, H2B3A_MOUSE, H2B3B_MOUSE	Histone H2B	LAKHAVSE	122	2.70E-06	427.7408	853.4670	2	0.0012	8	0	GluC
H2B1A_MOUSE, H2B1B_MOUSE, H2B1C_MOUSE, H2B1F_MOUSE, H2B1H_MOUSE, H2B1K_MOUSE, H2B1M_MOUSE, H2B1P_MOUSE, H2B2B_MOUSE, H2B2E_MOUSE, H2B3A_MOUSE, H2B3B_MOUSE	Histone H2B	LAKHAVSE	122	9.30E-03	427.7407	853.4669	2	0.0011	8	0	GluC
H2B1A_MOUSE, H2B1B_MOUSE, H2B1C_MOUSE, H2B1F_MOUSE, H2B1H_MOUSE, H2B1K_MOUSE, H2B1M_MOUSE, H2B1P_MOUSE, H2B2B_MOUSE, H2B2E_MOUSE, H2B3A_MOUSE, H2B3B_MOUSE	Histone H2B	LAKHAVSE	122	3.40E-03	427.7406	853.4666	2	0.0009	8	0	GluC

H2B1A_MOUSE, H2B1B_MOUSE, H2B1C_MOUSE, H2B1F_MOUSE, H2B1H_MOUSE, H2B1K_MOUSE, H2B1M_MOUSE, H2B1P_MOUSE, H2B2B_MOUSE, H2B2E_MOUSE, H2B3A_MOUSE, H2B3B_MOUSE	Histone H2B	LAKHAVSE	122	9.50E-03	427.7405	853.4665	2	0.0008	8	0	GluC
H2B1A_MOUSE, H2B1B_MOUSE, H2B1C_MOUSE, H2B1F_MOUSE, H2B1H_MOUSE, H2B1K_MOUSE, H2B1M_MOUSE, H2B1P_MOUSE, H2B2B_MOUSE, H2B2E_MOUSE, H2B3A_MOUSE, H2B3B_MOUSE	Histone H2B	LAKHAVSE	122	8.80E-04	427.7405	853.4665	2	0.0008	8	0	GluC
H2B1A_MOUSE, H2B1B_MOUSE, H2B1C_MOUSE, H2B1F_MOUSE, H2B1H_MOUSE, H2B1K_MOUSE, H2B1M_MOUSE, H2B1P_MOUSE, H2B2B_MOUSE, H2B2E_MOUSE, H2B3A_MOUSE, H2B3B_MOUSE	Histone H2B	LAKHAVSE	122	4.70E-04	427.7405	853.4665	2	0.0008	8	0	GluC
H2B1A_MOUSE, H2B1B_MOUSE, H2B1C_MOUSE, H2B1F_MOUSE, H2B1H_MOUSE, H2B1K_MOUSE, H2B1M_MOUSE, H2B1P_MOUSE, H2B2B_MOUSE, H2B2E_MOUSE, H2B3A_MOUSE, H2B3B_MOUSE	Histone H2B	LAKHAVSE	122	7.40E-03	427.7405	853.4665	2	0.0007	8	0	GluC
H2B1A_MOUSE, H2B1B_MOUSE, H2B1C_MOUSE, H2B1F_MOUSE, H2B1H_MOUSE, H2B1K_MOUSE, H2B1M_MOUSE, H2B1P_MOUSE, H2B2B_MOUSE, H2B2E_MOUSE, H2B3A_MOUSE, H2B3B_MOUSE	Histone H2B	LAKHAVSE	122	7.80E-03	427.7404	853.4663	2	0.0006	8	0	GluC
H2B1A_MOUSE, H2B1B_MOUSE, H2B1C_MOUSE, H2B1F_MOUSE, H2B1H_MOUSE, H2B1K_MOUSE, H2B1M_MOUSE, H2B1P_MOUSE, H2B2B_MOUSE, H2B2E_MOUSE, H2B3A_MOUSE, H2B3B_MOUSE	Histone H2B	LAKHAVSE	122	6.70E-03	427.7405	853.4663	2	0.0006	8	0	GluC
H2B1A_MOUSE, H2B1B_MOUSE, H2B1C_MOUSE, H2B1F_MOUSE, H2B1H_MOUSE, H2B1K_MOUSE, H2B1M_MOUSE, H2B1P_MOUSE, H2B2B_MOUSE, H2B2E_MOUSE, H2B3A_MOUSE, H2B3B_MOUSE	Histone H2B	LAKHAVSE	122	1.50E-03	427.7404	853.4663	2	0.0006	8	0	GluC
H2B1A_MOUSE, H2B1B_MOUSE, H2B1C_MOUSE, H2B1F_MOUSE, H2B1H_MOUSE, H2B1K_MOUSE, H2B1M_MOUSE, H2B1P_MOUSE, H2B2B_MOUSE, H2B2E_MOUSE, H2B3A_MOUSE, H2B3B_MOUSE	Histone H2B	LAKHAVSE	122	5.90E-04	427.7404	853.4662	2	0.0005	8	0	GluC
H2B1A_MOUSE, H2B1B_MOUSE, H2B1C_MOUSE, H2B1F_MOUSE, H2B1H_MOUSE, H2B1K_MOUSE, H2B1M_MOUSE, H2B1P_MOUSE, H2B2B_MOUSE, H2B2E_MOUSE, H2B3A_MOUSE, H2B3B_MOUSE	Histone H2B	LAKHAVSE	122	9.20E-04	427.7404	853.4662	2	0.0004	8	0	GluC
H2B1A_MOUSE, H2B1B_MOUSE, H2B1C_MOUSE, H2B1F_MOUSE, H2B1H_MOUSE, H2B1K_MOUSE, H2B1M_MOUSE, H2B1P_MOUSE, H2B2B_MOUSE, H2B2E_MOUSE, H2B3A_MOUSE, H2B3B_MOUSE	Histone H2B	LAKHAVSE	122	5.10E-03	427.7403	853.4660	2	0.0003	8	0	GluC
H2B1A_MOUSE, H2B1B_MOUSE, H2B1C_MOUSE, H2B1F_MOUSE, H2B1H_MOUSE, H2B1K_MOUSE, H2B1M_MOUSE, H2B1P_MOUSE, H2B2B_MOUSE, H2B2E_MOUSE, H2B3A_MOUSE, H2B3B_MOUSE	Histone H2B	LAKHAVSE	122	1.60E-03	427.7403	853.4660	2	0.0003	8	0	GluC
H2B1A_MOUSE, H2B1B_MOUSE, H2B1C_MOUSE, H2B1F_MOUSE, H2B1H_MOUSE, H2B1K_MOUSE, H2B1M_MOUSE, H2B1P_MOUSE, H2B2B_MOUSE, H2B2E_MOUSE, H2B3A_MOUSE, H2B3B_MOUSE	Histone H2B	LAKHAVSE	122	9.30E-04	427.7403	853.4660	2	0.0003	8	0	GluC
H2B1A_MOUSE, H2B1B_MOUSE, H2B1C_MOUSE, H2B1F_MOUSE, H2B1H_MOUSE, H2B1K_MOUSE, H2B1M_MOUSE, H2B1P_MOUSE, H2B2B_MOUSE, H2B2E_MOUSE, H2B3A_MOUSE, H2B3B_MOUSE	Histone H2B	LAKHAVSE	122	3.40E-04	427.7403	853.4661	2	0.0003	8	0	GluC
H2B1A_MOUSE, H2B1B_MOUSE, H2B1C_MOUSE, H2B1F_MOUSE, H2B1H_MOUSE, H2B1K_MOUSE, H2B1M_MOUSE, H2B1P_MOUSE, H2B2B_MOUSE, H2B2E_MOUSE, H2B3A_MOUSE, H2B3B_MOUSE	Histone H2B	LAKHAVSE	122	9.70E-03	427.7403	853.4659	2	0.0002	8	0	GluC
H2B1A_MOUSE, H2B1B_MOUSE, H2B1C_MOUSE, H2B1F_MOUSE, H2B1H_MOUSE, H2B1K_MOUSE, H2B1M_MOUSE, H2B1P_MOUSE, H2B2B_MOUSE, H2B2E_MOUSE, H2B3A_MOUSE, H2B3B_MOUSE	Histone H2B	LAKHAVSE	122	8.80E-03	427.7402	853.4659	2	0.0002	8	0	GluC
H2B1A_MOUSE, H2B1B_MOUSE, H2B1C_MOUSE, H2B1F_MOUSE, H2B1H_MOUSE, H2B1K_MOUSE, H2B1M_MOUSE, H2B1P_MOUSE, H2B2B_MOUSE, H2B2E_MOUSE, H2B3A_MOUSE, H2B3B_MOUSE	Histone H2B	LAKHAVSE	122	7.10E-03	427.7402	853.4659	2	0.0002	8	0	GluC
H2B1A_MOUSE, H2B1B_MOUSE, H2B1C_MOUSE, H2B1F_MOUSE, H2B1H_MOUSE, H2B1K_MOUSE, H2B1M_MOUSE, H2B1P_MOUSE, H2B2B_MOUSE, H2B2E_MOUSE, H2B3A_MOUSE, H2B3B_MOUSE	Histone H2B	LAKHAVSE	122	1.50E-03	427.7402	853.4659	2	0.0002	8	0	GluC
H2B1A_MOUSE, H2B1B_MOUSE, H2B1C_MOUSE, H2B1F_MOUSE, H2B1H_MOUSE, H2B1K_MOUSE, H2B1M_MOUSE, H2B1P_MOUSE, H2B2B_MOUSE, H2B2E_MOUSE, H2B3A_MOUSE, H2B3B_MOUSE	Histone H2B	LAKHAVSE	122	1.20E-03	427.7402	853.4659	2	0.0002	8	0	GluC
H2B1A_MOUSE, H2B1B_MOUSE, H2B1C_MOUSE, H2B1F_MOUSE, H2B1H_MOUSE, H2B1K_MOUSE, H2B1M_MOUSE, H2B1P_MOUSE, H2B2B_MOUSE, H2B2E_MOUSE, H2B3A_MOUSE, H2B3B_MOUSE	Histone H2B	LAKHAVSE	122	6.30E-04	427.7402	853.4659	2	0.0002	8	0	GluC
H2B1A_MOUSE, H2B1B_MOUSE, H2B1C_MOUSE, H2B1F_MOUSE, H2B1H_MOUSE, H2B1K_MOUSE, H2B1M_MOUSE, H2B1P_MOUSE, H2B2B_MOUSE, H2B2E_MOUSE, H2B3A_MOUSE, H2B3B_MOUSE	Histone H2B	LAKHAVSE	122	3.00E-04	427.7402	853.4659	2	0.0002	8	0	GluC
H2B1A_MOUSE, H2B1B_MOUSE, H2B1C_MOUSE, H2B1F_MOUSE, H2B1H_MOUSE, H2B1K_MOUSE, H2B1M_MOUSE, H2B1P_MOUSE, H2B2B_MOUSE, H2B2E_MOUSE, H2B3A_MOUSE, H2B3B_MOUSE	Histone H2B	LAKHAVSE	122	1.10E-04	427.7402	853.4659	2	0.0002	8	0	GluC
H2B1A_MOUSE, H2B1B_MOUSE, H2B1C_MOUSE, H2B1F_MOUSE, H2B1H_MOUSE, H2B1K_MOUSE, H2B1M_MOUSE, H2B1P_MOUSE, H2B2B_MOUSE, H2B2E_MOUSE, H2B3A_MOUSE, H2B3B_MOUSE	Histone H2B	LAKHAVSE	122	1.10E-04	427.7403	853.4659	2	0.0002	8	0	GluC
H2B1A_MOUSE, H2B1B_MOUSE, H2B1C_MOUSE, H2B1F_MOUSE, H2B1H_MOUSE, H2B1K_MOUSE, H2B1M_MOUSE, H2B1P_MOUSE, H2B2B_MOUSE, H2B2E_MOUSE, H2B3A_MOUSE, H2B3B_MOUSE	Histone H2B	LAKHAVSE	122	6.70E-05	427.7403	853.4659	2	0.0002	8	0	GluC
H2B1A_MOUSE, H2B1B_MOUSE, H2B1C_MOUSE, H2B1F_MOUSE, H2B1H_MOUSE, H2B1K_MOUSE, H2B1M_MOUSE, H2B1P_MOUSE, H2B2B_MOUSE, H2B2E_MOUSE, H2B3A_MOUSE, H2B3B_MOUSE	Histone H2B	LAKHAVSE	122	7.30E-03	427.7402	853.4658	2	0.0001	8	0	GluC

H2B1A_MOUSE, H2B1B_MOUSE, H2B1C_MOUSE, H2B1F_MOUSE, H2B1H_MOUSE, H2B1K_MOUSE, H2B1M_MOUSE, H2B1P_MOUSE, H2B2B_MOUSE, H2B2E_MOUSE, H2B3A_MOUSE, H2B3B_MOUSE	Histone H2B	LAKHAVSE	122	5.00E-03	427.7402	853.4659	2	0.0001	8	0	GluC
H2B1A_MOUSE, H2B1B_MOUSE, H2B1C_MOUSE, H2B1F_MOUSE, H2B1H_MOUSE, H2B1K_MOUSE, H2B1M_MOUSE, H2B1P_MOUSE, H2B2B_MOUSE, H2B2E_MOUSE, H2B3A_MOUSE, H2B3B_MOUSE	Histone H2B	LAKHAVSE	122	1.60E-03	427.7402	853.4658	2	0.0001	8	0	GluC
H2B1A_MOUSE, H2B1B_MOUSE, H2B1C_MOUSE, H2B1F_MOUSE, H2B1H_MOUSE, H2B1K_MOUSE, H2B1M_MOUSE, H2B1P_MOUSE, H2B2B_MOUSE, H2B2E_MOUSE, H2B3A_MOUSE, H2B3B_MOUSE	Histone H2B	LAKHAVSE	122	6.10E-04	427.7402	853.4658	2	0.0001	8	0	GluC
H2B1A_MOUSE, H2B1B_MOUSE, H2B1C_MOUSE, H2B1F_MOUSE, H2B1H_MOUSE, H2B1K_MOUSE, H2B1M_MOUSE, H2B1P_MOUSE, H2B2B_MOUSE, H2B2E_MOUSE, H2B3A_MOUSE, H2B3B_MOUSE	Histone H2B	LAKHAVSE	122	3.10E-04	427.7402	853.4659	2	0.0001	8	0	GluC
H2B1A_MOUSE, H2B1B_MOUSE, H2B1C_MOUSE, H2B1F_MOUSE, H2B1H_MOUSE, H2B1K_MOUSE, H2B1M_MOUSE, H2B1P_MOUSE, H2B2B_MOUSE, H2B2E_MOUSE, H2B3A_MOUSE, H2B3B_MOUSE	Histone H2B	LAKHAVSE	122	2.80E-04	427.7402	853.4658	2	0.0001	8	0	GluC
H2B1A_MOUSE, H2B1B_MOUSE, H2B1C_MOUSE, H2B1F_MOUSE, H2B1H_MOUSE, H2B1K_MOUSE, H2B1M_MOUSE, H2B1P_MOUSE, H2B2B_MOUSE, H2B2E_MOUSE, H2B3A_MOUSE, H2B3B_MOUSE	Histone H2B	LAKHAVSE	122	2.50E-04	427.7402	853.4658	2	0.0001	8	0	GluC
H2B1A_MOUSE, H2B1B_MOUSE, H2B1C_MOUSE, H2B1F_MOUSE, H2B1H_MOUSE, H2B1K_MOUSE, H2B1M_MOUSE, H2B1P_MOUSE, H2B2B_MOUSE, H2B2E_MOUSE, H2B3A_MOUSE, H2B3B_MOUSE	Histone H2B	LAKHAVSE	122	1.30E-04	427.7402	853.4658	2	0.0001	8	0	GluC
H2B1A_MOUSE, H2B1B_MOUSE, H2B1C_MOUSE, H2B1F_MOUSE, H2B1H_MOUSE, H2B1K_MOUSE, H2B1M_MOUSE, H2B1P_MOUSE, H2B2B_MOUSE, H2B2E_MOUSE, H2B3A_MOUSE, H2B3B_MOUSE	Histone H2B	LAKHAVSE	122	1.40E-05	427.7402	853.4658	2	0.0001	8	0	GluC
H2B1A_MOUSE, H2B1B_MOUSE, H2B1C_MOUSE, H2B1F_MOUSE, H2B1H_MOUSE, H2B1K_MOUSE, H2B1M_MOUSE, H2B1P_MOUSE, H2B2B_MOUSE, H2B2E_MOUSE, H2B3A_MOUSE, H2B3B_MOUSE	Histone H2B	LAKHAVSE	122	7.00E-03	427.7401	853.4657	2	0.0000	8	0	GluC
H2B1A_MOUSE, H2B1B_MOUSE, H2B1C_MOUSE, H2B1F_MOUSE, H2B1H_MOUSE, H2B1K_MOUSE, H2B1M_MOUSE, H2B1P_MOUSE, H2B2B_MOUSE, H2B2E_MOUSE, H2B3A_MOUSE, H2B3B_MOUSE	Histone H2B	LAKHAVSE	122	5.50E-03	427.7402	853.4658	2	0.0000	8	0	GluC
H2B1A_MOUSE, H2B1B_MOUSE, H2B1C_MOUSE, H2B1F_MOUSE, H2B1H_MOUSE, H2B1K_MOUSE, H2B1M_MOUSE, H2B1P_MOUSE, H2B2B_MOUSE, H2B2E_MOUSE, H2B3A_MOUSE, H2B3B_MOUSE	Histone H2B	LAKHAVSE	122	4.70E-03	427.7402	853.4658	2	0.0000	8	0	GluC
H2B1A_MOUSE, H2B1B_MOUSE, H2B1C_MOUSE, H2B1F_MOUSE, H2B1H_MOUSE, H2B1K_MOUSE, H2B1M_MOUSE, H2B1P_MOUSE, H2B2B_MOUSE, H2B2E_MOUSE, H2B3A_MOUSE, H2B3B_MOUSE	Histone H2B	LAKHAVSE	122	3.30E-03	427.7402	853.4658	2	0.0000	8	0	GluC
H2B1A_MOUSE, H2B1B_MOUSE, H2B1C_MOUSE, H2B1F_MOUSE, H2B1H_MOUSE, H2B1K_MOUSE, H2B1M_MOUSE, H2B1P_MOUSE, H2B2B_MOUSE, H2B2E_MOUSE, H2B3A_MOUSE, H2B3B_MOUSE	Histone H2B	LAKHAVSE	122	2.20E-03	427.7402	853.4658	2	0.0000	8	0	GluC
H2B1A_MOUSE, H2B1B_MOUSE, H2B1C_MOUSE, H2B1F_MOUSE, H2B1H_MOUSE, H2B1K_MOUSE, H2B1M_MOUSE, H2B1P_MOUSE, H2B2B_MOUSE, H2B2E_MOUSE, H2B3A_MOUSE, H2B3B_MOUSE	Histone H2B	LAKHAVSE	122	1.60E-03	427.7402	853.4658	2	0.0000	8	0	GluC
H2B1A_MOUSE, H2B1B_MOUSE, H2B1C_MOUSE, H2B1F_MOUSE, H2B1H_MOUSE, H2B1K_MOUSE, H2B1M_MOUSE, H2B1P_MOUSE, H2B2B_MOUSE, H2B2E_MOUSE, H2B3A_MOUSE, H2B3B_MOUSE	Histone H2B	LAKHAVSE	122	1.50E-03	427.7401	853.4657	2	0.0000	8	0	GluC
H2B1A_MOUSE, H2B1B_MOUSE, H2B1C_MOUSE, H2B1F_MOUSE, H2B1H_MOUSE, H2B1K_MOUSE, H2B1M_MOUSE, H2B1P_MOUSE, H2B2B_MOUSE, H2B2E_MOUSE, H2B3A_MOUSE, H2B3B_MOUSE	Histone H2B	LAKHAVSE	122	7.30E-04	427.7402	853.4657	2	0.0000	8	0	GluC
H2B1A_MOUSE, H2B1B_MOUSE, H2B1C_MOUSE, H2B1F_MOUSE, H2B1H_MOUSE, H2B1K_MOUSE, H2B1M_MOUSE, H2B1P_MOUSE, H2B2B_MOUSE, H2B2E_MOUSE, H2B3A_MOUSE, H2B3B_MOUSE	Histone H2B	LAKHAVSE	122	3.20E-04	427.7402	853.4658	2	0.0000	8	0	GluC
H2B1A_MOUSE, H2B1B_MOUSE, H2B1C_MOUSE, H2B1F_MOUSE, H2B1H_MOUSE, H2B1K_MOUSE, H2B1M_MOUSE, H2B1P_MOUSE, H2B2B_MOUSE, H2B2E_MOUSE, H2B3A_MOUSE, H2B3B_MOUSE	Histone H2B	LAKHAVSE	122	1.50E-04	427.7402	853.4657	2	0.0000	8	0	GluC
H2B1A_MOUSE, H2B1B_MOUSE, H2B1C_MOUSE, H2B1F_MOUSE, H2B1H_MOUSE, H2B1K_MOUSE, H2B1M_MOUSE, H2B1P_MOUSE, H2B2B_MOUSE, H2B2E_MOUSE, H2B3A_MOUSE, H2B3B_MOUSE	Histone H2B	LAKHAVSE	122	7.80E-05	427.7401	853.4657	2	0.0000	8	0	GluC
H2B1A_MOUSE, H2B1B_MOUSE, H2B1C_MOUSE, H2B1F_MOUSE, H2B1H_MOUSE, H2B1K_MOUSE, H2B1M_MOUSE, H2B1P_MOUSE, H2B2B_MOUSE, H2B2E_MOUSE, H2B3A_MOUSE, H2B3B_MOUSE	Histone H2B	LAKHAVSE	122	8.50E-03	427.7401	853.4656	2	-0.0001	8	0	GluC
H2B1A_MOUSE, H2B1B_MOUSE, H2B1C_MOUSE, H2B1F_MOUSE, H2B1H_MOUSE, H2B1K_MOUSE, H2B1M_MOUSE, H2B1P_MOUSE, H2B2B_MOUSE, H2B2E_MOUSE, H2B3A_MOUSE, H2B3B_MOUSE	Histone H2B	LAKHAVSE	122	7.90E-03	427.7401	853.4657	2	-0.0001	8	0	GluC
H2B1A_MOUSE, H2B1B_MOUSE, H2B1C_MOUSE, H2B1F_MOUSE, H2B1H_MOUSE, H2B1K_MOUSE, H2B1M_MOUSE, H2B1P_MOUSE, H2B2B_MOUSE, H2B2E_MOUSE, H2B3A_MOUSE, H2B3B_MOUSE	Histone H2B	LAKHAVSE	122	7.40E-03	427.7401	853.4657	2	-0.0001	8	0	GluC
H2B1A_MOUSE, H2B1B_MOUSE, H2B1C_MOUSE, H2B1F_MOUSE, H2B1H_MOUSE, H2B1K_MOUSE, H2B1M_MOUSE, H2B1P_MOUSE, H2B2B_MOUSE, H2B2E_MOUSE, H2B3A_MOUSE, H2B3B_MOUSE	Histone H2B	LAKHAVSE	122	4.80E-03	427.7401	853.4656	2	-0.0001	8	0	GluC
H2B1A_MOUSE, H2B1B_MOUSE, H2B1C_MOUSE, H2B1F_MOUSE, H2B1H_MOUSE, H2B1K_MOUSE, H2B1M_MOUSE, H2B1P_MOUSE, H2B2B_MOUSE, H2B2E_MOUSE, H2B3A_MOUSE, H2B3B_MOUSE	Histone H2B	LAKHAVSE	122	4.50E-03	427.7401	853.4656	2	-0.0001	8	0	GluC

H2B1A_MOUSE, H2B1B_MOUSE, H2B1C_MOUSE, H2B1F_MOUSE, H2B1H_MOUSE, H2B1K_MOUSE, H2B1M_MOUSE, H2B1P_MOUSE, H2B2B_MOUSE, H2B2E_MOUSE, H2B3A_MOUSE, H2B3B_MOUSE	Histone H2B	LAKHAVSE	122	3.00E-03	427.7401	853.4656	2	-0.0001	8	0	GluC
H2B1A_MOUSE, H2B1B_MOUSE, H2B1C_MOUSE, H2B1F_MOUSE, H2B1H_MOUSE, H2B1K_MOUSE, H2B1M_MOUSE, H2B1P_MOUSE, H2B2B_MOUSE, H2B2E_MOUSE, H2B3A_MOUSE, H2B3B_MOUSE	Histone H2B	LAKHAVSE	122	2.50E-03	427.7401	853.4657	2	-0.0001	8	0	GluC
H2B1A_MOUSE, H2B1B_MOUSE, H2B1C_MOUSE, H2B1F_MOUSE, H2B1H_MOUSE, H2B1K_MOUSE, H2B1M_MOUSE, H2B1P_MOUSE, H2B2B_MOUSE, H2B2E_MOUSE, H2B3A_MOUSE, H2B3B_MOUSE	Histone H2B	LAKHAVSE	122	2.10E-03	427.7401	853.4657	2	-0.0001	8	0	GluC
H2B1A_MOUSE, H2B1B_MOUSE, H2B1C_MOUSE, H2B1F_MOUSE, H2B1H_MOUSE, H2B1K_MOUSE, H2B1M_MOUSE, H2B1P_MOUSE, H2B2B_MOUSE, H2B2E_MOUSE, H2B3A_MOUSE, H2B3B_MOUSE	Histone H2B	LAKHAVSE	122	3.00E-04	427.7401	853.4657	2	-0.0001	8	0	GluC
H2B1A_MOUSE, H2B1B_MOUSE, H2B1C_MOUSE, H2B1F_MOUSE, H2B1H_MOUSE, H2B1K_MOUSE, H2B1M_MOUSE, H2B1P_MOUSE, H2B2B_MOUSE, H2B2E_MOUSE, H2B3A_MOUSE, H2B3B_MOUSE	Histone H2B	LAKHAVSE	122	7.70E-05	427.7401	853.4657	2	-0.0001	8	0	GluC
H2B1A_MOUSE, H2B1B_MOUSE, H2B1C_MOUSE, H2B1F_MOUSE, H2B1H_MOUSE, H2B1K_MOUSE, H2B1M_MOUSE, H2B1P_MOUSE, H2B2B_MOUSE, H2B2E_MOUSE, H2B3A_MOUSE, H2B3B_MOUSE	Histone H2B	LAKHAVSE	122	5.80E-05	427.7401	853.4657	2	-0.0001	8	0	GluC
H2B1A_MOUSE, H2B1B_MOUSE, H2B1C_MOUSE, H2B1F_MOUSE, H2B1H_MOUSE, H2B1K_MOUSE, H2B1M_MOUSE, H2B1P_MOUSE, H2B2B_MOUSE, H2B2E_MOUSE, H2B3A_MOUSE, H2B3B_MOUSE	Histone H2B	LAKHAVSE	122	2.50E-05	427.7401	853.4657	2	-0.0001	8	0	GluC
H2B1A_MOUSE, H2B1B_MOUSE, H2B1C_MOUSE, H2B1F_MOUSE, H2B1H_MOUSE, H2B1K_MOUSE, H2B1M_MOUSE, H2B1P_MOUSE, H2B2B_MOUSE, H2B2E_MOUSE, H2B3A_MOUSE, H2B3B_MOUSE	Histone H2B	LAKHAVSE	122	8.80E-03	427.74	853.4655	2	-0.0002	8	0	GluC
H2B1A_MOUSE, H2B1B_MOUSE, H2B1C_MOUSE, H2B1F_MOUSE, H2B1H_MOUSE, H2B1K_MOUSE, H2B1M_MOUSE, H2B1P_MOUSE, H2B2B_MOUSE, H2B2E_MOUSE, H2B3A_MOUSE, H2B3B_MOUSE	Histone H2B	LAKHAVSE	122	4.80E-03	427.7401	853.4656	2	-0.0002	8	0	GluC
H2B1A_MOUSE, H2B1B_MOUSE, H2B1C_MOUSE, H2B1F_MOUSE, H2B1H_MOUSE, H2B1K_MOUSE, H2B1M_MOUSE, H2B1P_MOUSE, H2B2B_MOUSE, H2B2E_MOUSE, H2B3A_MOUSE, H2B3B_MOUSE	Histone H2B	LAKHAVSE	122	2.60E-03	427.74	853.4655	2	-0.0002	8	0	GluC
H2B1A_MOUSE, H2B1B_MOUSE, H2B1C_MOUSE, H2B1F_MOUSE, H2B1H_MOUSE, H2B1K_MOUSE, H2B1M_MOUSE, H2B1P_MOUSE, H2B2B_MOUSE, H2B2E_MOUSE, H2B3A_MOUSE, H2B3B_MOUSE	Histone H2B	LAKHAVSE	122	2.60E-03	427.7401	853.4656	2	-0.0002	8	0	GluC
H2B1A_MOUSE, H2B1B_MOUSE, H2B1C_MOUSE, H2B1F_MOUSE, H2B1H_MOUSE, H2B1K_MOUSE, H2B1M_MOUSE, H2B1P_MOUSE, H2B2B_MOUSE, H2B2E_MOUSE, H2B3A_MOUSE, H2B3B_MOUSE	Histone H2B	LAKHAVSE	122	1.00E-03	427.7401	853.4656	2	-0.0002	8	0	GluC
H2B1A_MOUSE, H2B1B_MOUSE, H2B1C_MOUSE, H2B1F_MOUSE, H2B1H_MOUSE, H2B1K_MOUSE, H2B1M_MOUSE, H2B1P_MOUSE, H2B2B_MOUSE, H2B2E_MOUSE, H2B3A_MOUSE, H2B3B_MOUSE	Histone H2B	LAKHAVSE	122	9.70E-03	427.74	853.4655	2	-0.0003	8	0	GluC
H2B1A_MOUSE, H2B1B_MOUSE, H2B1C_MOUSE, H2B1F_MOUSE, H2B1H_MOUSE, H2B1K_MOUSE, H2B1M_MOUSE, H2B1P_MOUSE, H2B2B_MOUSE, H2B2E_MOUSE, H2B3A_MOUSE, H2B3B_MOUSE	Histone H2B	LAKHAVSE	122	6.50E-03	427.74	853.4654	2	-0.0003	8	0	GluC
H2B1A_MOUSE, H2B1B_MOUSE, H2B1C_MOUSE, H2B1F_MOUSE, H2B1H_MOUSE, H2B1K_MOUSE, H2B1M_MOUSE, H2B1P_MOUSE, H2B2B_MOUSE, H2B2E_MOUSE, H2B3A_MOUSE, H2B3B_MOUSE	Histone H2B	LAKHAVSE	122	2.70E-03	427.74	853.4654	2	-0.0003	8	0	GluC
H2B1A_MOUSE, H2B1B_MOUSE, H2B1C_MOUSE, H2B1F_MOUSE, H2B1H_MOUSE, H2B1K_MOUSE, H2B1M_MOUSE, H2B1P_MOUSE, H2B2B_MOUSE, H2B2E_MOUSE, H2B3A_MOUSE, H2B3B_MOUSE	Histone H2B	LAKHAVSE	122	1.40E-03	427.74	853.4655	2	-0.0003	8	0	GluC
H2B1A_MOUSE, H2B1B_MOUSE, H2B1C_MOUSE, H2B1F_MOUSE, H2B1H_MOUSE, H2B1K_MOUSE, H2B1M_MOUSE, H2B1P_MOUSE, H2B2B_MOUSE, H2B2E_MOUSE, H2B3A_MOUSE, H2B3B_MOUSE	Histone H2B	LAKHAVSE	122	1.40E-03	427.74	853.4655	2	-0.0003	8	0	GluC
H2B1A_MOUSE, H2B1B_MOUSE, H2B1C_MOUSE, H2B1F_MOUSE, H2B1H_MOUSE, H2B1K_MOUSE, H2B1M_MOUSE, H2B1P_MOUSE, H2B2B_MOUSE, H2B2E_MOUSE, H2B3A_MOUSE, H2B3B_MOUSE	Histone H2B	LAKHAVSE	122	4.70E-04	427.74	853.4654	2	-0.0003	8	0	GluC
H2B1A_MOUSE, H2B1B_MOUSE, H2B1C_MOUSE, H2B1F_MOUSE, H2B1H_MOUSE, H2B1K_MOUSE, H2B1M_MOUSE, H2B1P_MOUSE, H2B2B_MOUSE, H2B2E_MOUSE, H2B3A_MOUSE, H2B3B_MOUSE	Histone H2B	LAKHAVSE	122	3.10E-04	427.74	853.4654	2	-0.0003	8	0	GluC
H2B1A_MOUSE, H2B1B_MOUSE, H2B1C_MOUSE, H2B1F_MOUSE, H2B1H_MOUSE, H2B1K_MOUSE, H2B1M_MOUSE, H2B1P_MOUSE, H2B2B_MOUSE, H2B2E_MOUSE, H2B3A_MOUSE, H2B3B_MOUSE	Histone H2B	LAKHAVSE	122	7.10E-05	427.74	853.4655	2	-0.0003	8	0	GluC
H2B1A_MOUSE, H2B1B_MOUSE, H2B1C_MOUSE, H2B1F_MOUSE, H2B1H_MOUSE, H2B1K_MOUSE, H2B1M_MOUSE, H2B1P_MOUSE, H2B2B_MOUSE, H2B2E_MOUSE, H2B3A_MOUSE, H2B3B_MOUSE	Histone H2B	LAKHAVSE	122	2.30E-05	427.74	853.4654	2	-0.0003	8	0	GluC
H2B1A_MOUSE, H2B1B_MOUSE, H2B1C_MOUSE, H2B1F_MOUSE, H2B1H_MOUSE, H2B1K_MOUSE, H2B1M_MOUSE, H2B1P_MOUSE, H2B2B_MOUSE, H2B2E_MOUSE, H2B3A_MOUSE, H2B3B_MOUSE	Histone H2B	LAKHAVSE	122	3.40E-03	427.74	853.4654	2	-0.0004	8	0	GluC
H2B1A_MOUSE, H2B1B_MOUSE, H2B1C_MOUSE, H2B1F_MOUSE, H2B1H_MOUSE, H2B1K_MOUSE, H2B1M_MOUSE, H2B1P_MOUSE, H2B2B_MOUSE, H2B2E_MOUSE, H2B3A_MOUSE, H2B3B_MOUSE	Histone H2B	LAKHAVSE	122	2.30E-03	427.74	853.4654	2	-0.0004	8	0	GluC
H2B1A_MOUSE, H2B1B_MOUSE, H2B1C_MOUSE, H2B1F_MOUSE, H2B1H_MOUSE, H2B1K_MOUSE, H2B1M_MOUSE, H2B1P_MOUSE, H2B2B_MOUSE, H2B2E_MOUSE, H2B3A_MOUSE, H2B3B_MOUSE	Histone H2B	LAKHAVSE	122	2.30E-03	427.7399	853.4653	2	-0.0005	8	0	GluC

H2B1A_MOUSE, H2B1B_MOUSE, H2B1C_MOUSE, H2B1F_MOUSE, H2B1H_MOUSE, H2B1K_MOUSE, H2B1M_MOUSE, H2B1P_MOUSE, H2B2B_MOUSE, H2B2E_MOUSE, H2B3A_MOUSE, H2B3B_MOUSE	Histone H2B	LAKHAVSE	122	2.10E-03	427.7399	853.4652	2	-0.0005	8	0	GluC
H2B1A_MOUSE, H2B1B_MOUSE, H2B1C_MOUSE, H2B1F_MOUSE, H2B1H_MOUSE, H2B1K_MOUSE, H2B1M_MOUSE, H2B1P_MOUSE, H2B2B_MOUSE, H2B2E_MOUSE, H2B3A_MOUSE, H2B3B_MOUSE	Histone H2B	LAKHAVSE	122	1.60E-03	427.7399	853.4653	2	-0.0005	8	0	GluC
H2B1A_MOUSE, H2B1B_MOUSE, H2B1C_MOUSE, H2B1F_MOUSE, H2B1H_MOUSE, H2B1K_MOUSE, H2B1M_MOUSE, H2B1P_MOUSE, H2B2B_MOUSE, H2B2E_MOUSE, H2B3A_MOUSE, H2B3B_MOUSE	Histone H2B	LAKHAVSE	122	3.80E-03	427.7398	853.4651	2	-0.0006	8	0	GluC
H2B1A_MOUSE, H2B1B_MOUSE, H2B1C_MOUSE, H2B1F_MOUSE, H2B1H_MOUSE, H2B1K_MOUSE, H2B1M_MOUSE, H2B1P_MOUSE, H2B2B_MOUSE, H2B2E_MOUSE, H2B3A_MOUSE, H2B3B_MOUSE	Histone H2B	LAKHAVSE	122	2.60E-03	427.7398	853.4651	2	-0.0006	8	0	GluC
H2B1A_MOUSE, H2B1B_MOUSE, H2B1C_MOUSE, H2B1F_MOUSE, H2B1H_MOUSE, H2B1K_MOUSE, H2B1M_MOUSE, H2B1P_MOUSE, H2B2B_MOUSE, H2B2E_MOUSE, H2B3A_MOUSE, H2B3B_MOUSE	Histone H2B	LAKHAVSE	122	2.70E-03	427.7398	853.4651	2	-0.0007	8	0	GluC
H2B1A_MOUSE, H2B1B_MOUSE, H2B1C_MOUSE, H2B1F_MOUSE, H2B1H_MOUSE, H2B1K_MOUSE, H2B1M_MOUSE, H2B1P_MOUSE, H2B2B_MOUSE, H2B2E_MOUSE, H2B3A_MOUSE, H2B3B_MOUSE	Histone H2B	LAKHAVSE	122	8.40E-04	427.7398	853.4650	2	-0.0007	8	0	GluC
H2B1A_MOUSE, H2B1B_MOUSE, H2B1C_MOUSE, H2B1F_MOUSE, H2B1H_MOUSE, H2B1K_MOUSE, H2B1M_MOUSE, H2B1P_MOUSE, H2B2B_MOUSE, H2B2E_MOUSE, H2B3A_MOUSE, H2B3B_MOUSE	Histone H2B	LAKHAVSE	122	5.90E-03	427.7398	853.4650	2	-0.0008	8	0	GluC
H2B1A_MOUSE, H2B1B_MOUSE, H2B1C_MOUSE, H2B1F_MOUSE, H2B1H_MOUSE, H2B1K_MOUSE, H2B1M_MOUSE, H2B1P_MOUSE, H2B2B_MOUSE, H2B2E_MOUSE, H2B3A_MOUSE, H2B3B_MOUSE	Histone H2B	LAKHAVSE	122	8.60E-04	427.7398	853.4650	2	-0.0008	8	0	GluC
H2B1A_MOUSE, H2B1B_MOUSE, H2B1C_MOUSE, H2B1F_MOUSE, H2B1H_MOUSE, H2B1K_MOUSE, H2B1M_MOUSE, H2B1P_MOUSE, H2B2B_MOUSE, H2B2E_MOUSE, H2B3A_MOUSE, H2B3B_MOUSE	Histone H2B	LAKHAVSE	122	3.80E-03	427.7396	853.4647	2	-0.0010	8	0	GluC
H2B1A_MOUSE, H2B1B_MOUSE, H2B1C_MOUSE, H2B1F_MOUSE, H2B1H_MOUSE, H2B1K_MOUSE, H2B1M_MOUSE, H2B1P_MOUSE, H2B2B_MOUSE, H2B2E_MOUSE, H2B3A_MOUSE, H2B3B_MOUSE	Histone H2B	LAKHAVSE	122	6.30E-04	427.7396	853.4646	2	-0.0011	8	0	GluC
H2B1A_MOUSE, H2B1B_MOUSE, H2B1C_MOUSE, H2B1F_MOUSE, H2B1H_MOUSE, H2B1K_MOUSE, H2B1M_MOUSE, H2B1P_MOUSE, H2B2B_MOUSE, H2B2E_MOUSE, H2B3A_MOUSE, H2B3B_MOUSE	Histone H2B	LAKHAVSE	122	4.10E-04	427.7396	853.4647	2	-0.0011	8	0	GluC
H2B1A_MOUSE, H2B1B_MOUSE, H2B1C_MOUSE, H2B1F_MOUSE, H2B1H_MOUSE, H2B1K_MOUSE, H2B1M_MOUSE, H2B1P_MOUSE, H2B2B_MOUSE, H2B2E_MOUSE, H2B3A_MOUSE, H2B3B_MOUSE	Histone H2B	LAKHAVSE	122	2.60E-04	427.7395	853.4644	2	-0.0013	8	0	GluC
H2B1A_MOUSE, H2B1B_MOUSE, H2B1C_MOUSE, H2B1F_MOUSE, H2B1H_MOUSE, H2B1K_MOUSE, H2B1M_MOUSE, H2B1P_MOUSE, H2B2B_MOUSE, H2B2E_MOUSE, H2B3A_MOUSE, H2B3B_MOUSE	Histone H2B	LAKHAVSE	122	1.80E-03	427.7394	853.4643	2	-0.0015	8	0	GluC
H2B1A_MOUSE, H2B1B_MOUSE, H2B1C_MOUSE, H2B1F_MOUSE, H2B1H_MOUSE, H2B1K_MOUSE, H2B1M_MOUSE, H2B1P_MOUSE, H2B2B_MOUSE, H2B2E_MOUSE, H2B3A_MOUSE, H2B3B_MOUSE	Histone H2B	LAKHAVSE	122	2.30E-03	427.7393	853.4641	2	-0.0017	8	0	GluC
H2B1A_MOUSE, H2B1B_MOUSE, H2B1C_MOUSE, H2B1F_MOUSE, H2B1H_MOUSE, H2B1K_MOUSE, H2B1M_MOUSE, H2B1P_MOUSE, H2B2B_MOUSE, H2B2E_MOUSE, H2B3A_MOUSE, H2B3B_MOUSE	Histone H2B	LAKHAVSE	122	1.70E-04	427.7393	853.4640	2	-0.0018	8	0	GluC
H2B1A_MOUSE, H2B1B_MOUSE, H2B1C_MOUSE, H2B1F_MOUSE, H2B1H_MOUSE, H2B1K_MOUSE, H2B1M_MOUSE, H2B1P_MOUSE, H2B2B_MOUSE, H2B2E_MOUSE, H2B3A_MOUSE, H2B3B_MOUSE	Histone H2B	LAHYNKR	24	5.30E-04	451.2526	900.4907	2	-0.0023	7	1	Semi-tryptic
H2B1A_MOUSE, H2B1B_MOUSE, H2B1C_MOUSE, H2B1F_MOUSE, H2B1H_MOUSE, H2B1K_MOUSE, H2B1M_MOUSE, H2B1P_MOUSE, H2B2B_MOUSE, H2B2E_MOUSE, H2B3A_MOUSE, H2B3B_MOUSE	Histone H2B	LAHYNKR	24	2.10E-03	451.2525	900.4903	2	-0.0026	7	1	Semi-tryptic
H2B1A_MOUSE, H2B1B_MOUSE, H2B1C_MOUSE, H2B1F_MOUSE, H2B1H_MOUSE, H2B1K_MOUSE, H2B1M_MOUSE, H2B1P_MOUSE, H2B2B_MOUSE, H2B2E_MOUSE, H2B3A_MOUSE, H2B3B_MOUSE	Histone H2B	RSTITSR	11	1.20E-03	410.7365	819.4584	2	0.0022	7	1	Semi-tryptic
H2B1A_MOUSE, H2B1B_MOUSE, H2B1C_MOUSE, H2B1F_MOUSE, H2B1H_MOUSE, H2B1K_MOUSE, H2B1M_MOUSE, H2B1P_MOUSE, H2B2B_MOUSE, H2B2E_MOUSE, H2B3A_MOUSE, H2B3B_MOUSE	Histone H2B	RSTITSR	11	3.20E-03	410.7362	819.4579	2	0.0016	7	1	Semi-tryptic
H2B1A_MOUSE, H2B1B_MOUSE, H2B1C_MOUSE, H2B1F_MOUSE, H2B1H_MOUSE, H2B1K_MOUSE, H2B1M_MOUSE, H2B1P_MOUSE, H2B2B_MOUSE, H2B2E_MOUSE, H2B3A_MOUSE, H2B3B_MOUSE	Histone H2B	RSTITSR	11	6.20E-03	410.736	819.4574	2	0.0011	7	1	Semi-tryptic
H2B1A_MOUSE, H2B1B_MOUSE, H2B1C_MOUSE, H2B1F_MOUSE, H2B1H_MOUSE, H2B1K_MOUSE, H2B1M_MOUSE, H2B1P_MOUSE, H2B2B_MOUSE, H2B2E_MOUSE, H2B3A_MOUSE, H2B3B_MOUSE	Histone H2B	RSTITSR	11	1.50E-03	410.7357	819.4569	2	0.0006	7	1	Semi-tryptic
H2B1A_MOUSE, H2B1B_MOUSE, H2B1C_MOUSE, H2B1F_MOUSE, H2B1H_MOUSE, H2B1K_MOUSE, H2B1M_MOUSE, H2B1P_MOUSE, H2B2B_MOUSE, H2B2E_MOUSE, H2B3A_MOUSE, H2B3B_MOUSE	Histone H2B	RSTITSR	11	5.20E-03	410.7352	819.4559	2	-0.0003	7	1	Semi-tryptic
H2B1A_MOUSE, H2B1B_MOUSE, H2B1C_MOUSE, H2B1F_MOUSE, H2B1H_MOUSE, H2B1K_MOUSE, H2B1M_MOUSE, H2B1P_MOUSE, H2B2B_MOUSE, H2B2E_MOUSE, H2B3A_MOUSE, H2B3B_MOUSE	Histone H2B	RSTITSR	11	1.80E-03	410.7351	819.4557	2	-0.0005	7	1	Semi-tryptic
H2B1A_MOUSE, H2B1B_MOUSE, H2B1C_MOUSE, H2B1F_MOUSE, H2B1H_MOUSE, H2B1K_MOUSE, H2B1M_MOUSE, H2B1P_MOUSE, H2B2B_MOUSE, H2B2E_MOUSE, H2B3A_MOUSE, H2B3B_MOUSE	Histone H2B	RSTITSR	11	9.80E-04	410.735	819.4554	2	-0.0008	7	1	Semi-tryptic

H2B1A_MOUSE, H2B1B_MOUSE, H2B1C_MOUSE, H2B1F_MOUSE, H2B1H_MOUSE, H2B1K_MOUSE, H2B1M_MOUSE, H2B1P_MOUSE, H2B2B_MOUSE, H2B2E_MOUSE, H2B3A_MOUSE, H2B3B_MOUSE	Histone H2B	RSTITSR	11	8.50E-04	410.735	819.4554	2	-0.0008	7	1	Semi-tryptic
H2B1A_MOUSE, H2B1B_MOUSE, H2B1C_MOUSE, H2B1F_MOUSE, H2B1H_MOUSE, H2B1K_MOUSE, H2B1M_MOUSE, H2B1P_MOUSE, H2B2B_MOUSE, H2B2E_MOUSE, H2B3A_MOUSE, H2B3B_MOUSE	Histone H2B	RSTITSR	11	6.10E-04	410.735	819.4554	2	-0.0008	7	1	Semi-tryptic
H2B1A_MOUSE, H2B1B_MOUSE, H2B1C_MOUSE, H2B1F_MOUSE, H2B1H_MOUSE, H2B1K_MOUSE, H2B1M_MOUSE, H2B1P_MOUSE, H2B2B_MOUSE, H2B2E_MOUSE, H2B3A_MOUSE, H2B3B_MOUSE	Histone H2B	RSTITSR	11	6.50E-04	410.7348	819.4550	2	-0.0012	7	1	Semi-tryptic
H2B1A_MOUSE, H2B1B_MOUSE, H2B1C_MOUSE, H2B1F_MOUSE, H2B1H_MOUSE, H2B1K_MOUSE, H2B1M_MOUSE, H2B1P_MOUSE, H2B2B_MOUSE, H2B2E_MOUSE, H2B3A_MOUSE, H2B3B_MOUSE	Histone H2B	RSTITSR	11	6.50E-04	410.7346	819.4547	2	-0.0015	7	1	Semi-tryptic
H2B1A_MOUSE, H2B1B_MOUSE, H2B1C_MOUSE, H2B1F_MOUSE, H2B1H_MOUSE, H2B1K_MOUSE, H2B1M_MOUSE, H2B1P_MOUSE, H2B2B_MOUSE, H2B2E_MOUSE, H2B3A_MOUSE, H2B3B_MOUSE	Histone H2B	LAHYNKR	24	1.10E-03	301.1724	900.4955	3	0.0025	7	1	Semi-tryptic
H2B1A_MOUSE, H2B1B_MOUSE, H2B1C_MOUSE, H2B1F_MOUSE, H2B1H_MOUSE, H2B1K_MOUSE, H2B1M_MOUSE, H2B1P_MOUSE, H2B2B_MOUSE, H2B2E_MOUSE, H2B3A_MOUSE, H2B3B_MOUSE	Histone H2B	LAHYNKR	24	3.30E-03	451.2548	900.4950	2	0.0020	7	1	Semi-tryptic
H2B1A_MOUSE, H2B1B_MOUSE, H2B1C_MOUSE, H2B1F_MOUSE, H2B1H_MOUSE, H2B1K_MOUSE, H2B1M_MOUSE, H2B1P_MOUSE, H2B2B_MOUSE, H2B2E_MOUSE, H2B3A_MOUSE, H2B3B_MOUSE	Histone H2B	LAHYNKR	24	9.30E-03	451.2543	900.4941	2	0.0011	7	1	Semi-tryptic
H2B1A_MOUSE, H2B1B_MOUSE, H2B1C_MOUSE, H2B1F_MOUSE, H2B1H_MOUSE, H2B1K_MOUSE, H2B1M_MOUSE, H2B1P_MOUSE, H2B2B_MOUSE, H2B2E_MOUSE, H2B3A_MOUSE, H2B3B_MOUSE	Histone H2B	LAHYNKR	24	6.90E-04	451.2541	900.4936	2	0.0007	7	1	Semi-tryptic
H2B1A_MOUSE, H2B1B_MOUSE, H2B1C_MOUSE, H2B1F_MOUSE, H2B1H_MOUSE, H2B1K_MOUSE, H2B1M_MOUSE, H2B1P_MOUSE, H2B2B_MOUSE, H2B2E_MOUSE, H2B3A_MOUSE, H2B3B_MOUSE	Histone H2B	LAHYNKR	24	7.00E-03	451.2539	900.4932	2	0.0002	7	1	Semi-tryptic
H2B1A_MOUSE, H2B1B_MOUSE, H2B1C_MOUSE, H2B1F_MOUSE, H2B1H_MOUSE, H2B1K_MOUSE, H2B1M_MOUSE, H2B1P_MOUSE, H2B2B_MOUSE, H2B2E_MOUSE, H2B3A_MOUSE, H2B3B_MOUSE	Histone H2B	LAHYNKR	24	4.00E-03	451.2538	900.4930	2	0.0001	7	1	Semi-tryptic
H2B1A_MOUSE, H2B1B_MOUSE, H2B1C_MOUSE, H2B1F_MOUSE, H2B1H_MOUSE, H2B1K_MOUSE, H2B1M_MOUSE, H2B1P_MOUSE, H2B2B_MOUSE, H2B2E_MOUSE, H2B3A_MOUSE, H2B3B_MOUSE	Histone H2B	LAHYNKR	24	3.70E-03	451.2538	900.4931	2	0.0001	7	1	Semi-tryptic
H2B1A_MOUSE, H2B1B_MOUSE, H2B1C_MOUSE, H2B1F_MOUSE, H2B1H_MOUSE, H2B1K_MOUSE, H2B1M_MOUSE, H2B1P_MOUSE, H2B2B_MOUSE, H2B2E_MOUSE, H2B3A_MOUSE, H2B3B_MOUSE	Histone H2B	LAHYNKR	24	7.10E-03	451.2538	900.4929	2	0.0000	7	1	Semi-tryptic
H2B1A_MOUSE, H2B1B_MOUSE, H2B1C_MOUSE, H2B1F_MOUSE, H2B1H_MOUSE, H2B1K_MOUSE, H2B1M_MOUSE, H2B1P_MOUSE, H2B2B_MOUSE, H2B2E_MOUSE, H2B3A_MOUSE, H2B3B_MOUSE	Histone H2B	LAHYNKR	24	5.90E-03	451.2536	900.4927	2	-0.0003	7	1	Semi-tryptic
H2B1A_MOUSE, H2B1B_MOUSE, H2B1C_MOUSE, H2B1F_MOUSE, H2B1H_MOUSE, H2B1K_MOUSE, H2B1M_MOUSE, H2B1P_MOUSE, H2B2B_MOUSE, H2B2E_MOUSE, H2B3A_MOUSE, H2B3B_MOUSE	Histone H2B	LAHYNKR	24	5.00E-03	451.2536	900.4926	2	-0.0003	7	1	Semi-tryptic
H2B1A_MOUSE, H2B1B_MOUSE, H2B1C_MOUSE, H2B1F_MOUSE, H2B1H_MOUSE, H2B1K_MOUSE, H2B1M_MOUSE, H2B1P_MOUSE, H2B2B_MOUSE, H2B2E_MOUSE, H2B3A_MOUSE, H2B3B_MOUSE	Histone H2B	LAHYNKR	24	3.90E-03	451.2536	900.4926	2	-0.0003	7	1	Semi-tryptic
H2B1A_MOUSE, H2B1B_MOUSE, H2B1C_MOUSE, H2B1F_MOUSE, H2B1H_MOUSE, H2B1K_MOUSE, H2B1M_MOUSE, H2B1P_MOUSE, H2B2B_MOUSE, H2B2E_MOUSE, H2B3A_MOUSE, H2B3B_MOUSE	Histone H2B	LAHYNKR	24	3.60E-03	451.2536	900.4926	2	-0.0003	7	1	Semi-tryptic
H2B1A_MOUSE, H2B1B_MOUSE, H2B1C_MOUSE, H2B1F_MOUSE, H2B1H_MOUSE, H2B1K_MOUSE, H2B1M_MOUSE, H2B1P_MOUSE, H2B2B_MOUSE, H2B2E_MOUSE, H2B3A_MOUSE, H2B3B_MOUSE	Histone H2B	LAHYNKR	24	6.60E-04	451.2536	900.4926	2	-0.0003	7	1	Semi-tryptic
H2B1A_MOUSE, H2B1B_MOUSE, H2B1C_MOUSE, H2B1F_MOUSE, H2B1H_MOUSE, H2B1K_MOUSE, H2B1M_MOUSE, H2B1P_MOUSE, H2B2B_MOUSE, H2B2E_MOUSE, H2B3A_MOUSE, H2B3B_MOUSE	Histone H2B	LAHYNKR	24	3.30E-03	451.2535	900.4924	2	-0.0006	7	1	Semi-tryptic
H2B1A_MOUSE, H2B1B_MOUSE, H2B1C_MOUSE, H2B1F_MOUSE, H2B1H_MOUSE, H2B1K_MOUSE, H2B1M_MOUSE, H2B1P_MOUSE, H2B2B_MOUSE, H2B2E_MOUSE, H2B3A_MOUSE, H2B3B_MOUSE	Histone H2B	LAHYNKR	24	6.40E-04	451.2535	900.4924	2	-0.0006	7	1	Semi-tryptic
H2B1A_MOUSE, H2B1B_MOUSE, H2B1C_MOUSE, H2B1F_MOUSE, H2B1H_MOUSE, H2B1K_MOUSE, H2B1M_MOUSE, H2B1P_MOUSE, H2B2B_MOUSE, H2B2E_MOUSE, H2B3A_MOUSE, H2B3B_MOUSE	Histone H2B	LAHYNKR	24	7.00E-04	451.2534	900.4921	2	-0.0008	7	1	Semi-tryptic
H2B1A_MOUSE, H2B1B_MOUSE, H2B1C_MOUSE, H2B1F_MOUSE, H2B1H_MOUSE, H2B1K_MOUSE, H2B1M_MOUSE, H2B1P_MOUSE, H2B2B_MOUSE, H2B2E_MOUSE, H2B3A_MOUSE, H2B3B_MOUSE	Histone H2B	LAHYNKR	24	2.40E-03	451.2532	900.4919	2	-0.0011	7	1	Semi-tryptic
H2B1A_MOUSE, H2B1B_MOUSE, H2B1C_MOUSE, H2B1F_MOUSE, H2B1H_MOUSE, H2B1K_MOUSE, H2B1M_MOUSE, H2B1P_MOUSE, H2B2B_MOUSE, H2B2E_MOUSE, H2B3A_MOUSE, H2B3B_MOUSE	Histone H2B	LAHYNKR	24	2.00E-03	451.2531	900.4916	2	-0.0014	7	1	Semi-tryptic
H2B1A_MOUSE, H2B1B_MOUSE, H2B1C_MOUSE, H2B1F_MOUSE, H2B1H_MOUSE, H2B1K_MOUSE, H2B1M_MOUSE, H2B1P_MOUSE, H2B2B_MOUSE, H2B2E_MOUSE, H2B3A_MOUSE, H2B3B_MOUSE	Histone H2B	LAHYNKR	24	2.70E-03	451.253	900.4914	2	-0.0015	7	1	Semi-tryptic
H2B1A_MOUSE, H2B1B_MOUSE, H2B1C_MOUSE, H2B1F_MOUSE, H2B1H_MOUSE, H2B1K_MOUSE, H2B1M_MOUSE, H2B1P_MOUSE, H2B2B_MOUSE, H2B2E_MOUSE, H2B3A_MOUSE, H2B3B_MOUSE	Histone H2B	LAHYNKR	24	1.30E-03	451.253	900.4914	2	-0.0015	7	1	Semi-tryptic

H2B1A_MOUSE, H2B1B_MOUSE, H2B1C_MOUSE, H2B1F_MOUSE, H2B1H_MOUSE, H2B1K_MOUSE, H2B1M_MOUSE, H2B1P_MOUSE, H2B2B_MOUSE, H2B2E_MOUSE, H2B3A_MOUSE, H2B3B_MOUSE	Histone H2B	LAHYNKR	24	1.60E-03	451.253	900.4914	2	-0.0016	7	1	Semi-tryptic
H2B1A_MOUSE, H2B1B_MOUSE, H2B1C_MOUSE, H2B1F_MOUSE, H2B1H_MOUSE, H2B1K_MOUSE, H2B1M_MOUSE, H2B1P_MOUSE, H2B2B_MOUSE, H2B2E_MOUSE, H2B3A_MOUSE, H2B3B_MOUSE	Histone H2B	LAHYNKR	24	3.30E-03	451.2528	900.4910	2	-0.0019	7	1	Semi-tryptic
H2B1A_MOUSE, H2B1B_MOUSE, H2B1C_MOUSE, H2B1F_MOUSE, H2B1H_MOUSE, H2B1K_MOUSE, H2B1M_MOUSE, H2B1P_MOUSE, H2B2B_MOUSE, H2B2E_MOUSE, H2B3A_MOUSE, H2B3B_MOUSE	Histone H2B	LAHYNK	6	1.20E-03	373.2042	744.3939	2	0.0021	6	0	Semi-tryptic
H2B1A_MOUSE, H2B1B_MOUSE, H2B1C_MOUSE, H2B1F_MOUSE, H2B1H_MOUSE, H2B1K_MOUSE, H2B1M_MOUSE, H2B1P_MOUSE, H2B2B_MOUSE, H2B2E_MOUSE, H2B3A_MOUSE, H2B3B_MOUSE	Histone H2B	LAHYNK	6	2.90E-03	373.204	744.3935	2	0.0017	6	0	Semi-tryptic
H2B1A_MOUSE, H2B1B_MOUSE, H2B1C_MOUSE, H2B1F_MOUSE, H2B1H_MOUSE, H2B1K_MOUSE, H2B1M_MOUSE, H2B1P_MOUSE, H2B2B_MOUSE, H2B2E_MOUSE, H2B3A_MOUSE, H2B3B_MOUSE	Histone H2B	LAHYNK	6	2.60E-03	373.2038	744.3930	2	0.0012	6	0	Semi-tryptic
H2B1A_MOUSE, H2B1B_MOUSE, H2B1C_MOUSE, H2B1F_MOUSE, H2B1H_MOUSE, H2B1K_MOUSE, H2B1M_MOUSE, H2B1P_MOUSE, H2B2B_MOUSE, H2B2E_MOUSE, H2B3A_MOUSE, H2B3B_MOUSE	Histone H2B	LAHYNK	6	6.30E-03	373.2029	744.3913	2	-0.0006	6	0	Semi-tryptic
H2B1A_MOUSE, H2B1B_MOUSE, H2B1C_MOUSE, H2B1F_MOUSE, H2B1H_MOUSE, H2B1K_MOUSE, H2B1M_MOUSE, H2B1P_MOUSE, H2B2B_MOUSE, H2B2E_MOUSE, H2B3A_MOUSE, H2B3B_MOUSE	Histone H2B	LAHYNK	6	5.30E-03	373.2027	744.3909	2	-0.0010	6	0	Semi-tryptic
H2B1A_MOUSE, H2B1B_MOUSE, H2B1C_MOUSE, H2B1F_MOUSE, H2B1H_MOUSE, H2B1K_MOUSE, H2B1M_MOUSE, H2B1P_MOUSE, H2B2B_MOUSE, H2B2E_MOUSE, H2B3A_MOUSE, H2B3B_MOUSE	Histone H2B	LAHYNK	6	7.30E-03	373.2027	744.3908	2	-0.0011	6	0	Semi-tryptic
H2B1A_MOUSE, H2B1B_MOUSE, H2B1C_MOUSE, H2B1F_MOUSE, H2B1H_MOUSE, H2B1M_MOUSE, H2B1P_MOUSE, H2B2B_MOUSE	Histone H2B	IQTAVRLLLPGELAKHAVSEGTKAVTKYTSSK	14	1.20E-03	849.9902	3395.9317	4	0.0177	32	2	GluC
H2B1A_MOUSE, H2B1B_MOUSE, H2B1C_MOUSE, H2B1F_MOUSE, H2B1H_MOUSE, H2B1M_MOUSE, H2B1P_MOUSE, H2B2B_MOUSE	Histone H2B	IQTAVRLLLPGELAKHAVSEGTKAVTKYTSSK	14	2.20E-03	849.9902	3395.9316	4	0.0176	32	2	GluC
H2B1A_MOUSE, H2B1B_MOUSE, H2B1C_MOUSE, H2B1F_MOUSE, H2B1H_MOUSE, H2B1M_MOUSE, H2B1P_MOUSE, H2B2B_MOUSE	Histone H2B	IQTAVRLLLPGELAKHAVSEGTKAVTKYTSSK	14	8.80E-04	680.1936	3395.9314	5	0.0174	32	2	GluC
H2B1A_MOUSE, H2B1B_MOUSE, H2B1C_MOUSE, H2B1F_MOUSE, H2B1H_MOUSE, H2B1M_MOUSE, H2B1P_MOUSE, H2B2B_MOUSE	Histone H2B	IQTAVRLLLPGELAKHAVSEGTKAVTKYTSSK	14	6.80E-04	849.9897	3395.9297	4	0.0157	32	2	GluC
H2B1A_MOUSE, H2B1B_MOUSE, H2B1C_MOUSE, H2B1F_MOUSE, H2B1H_MOUSE, H2B1M_MOUSE, H2B1P_MOUSE, H2B2B_MOUSE	Histone H2B	IQTAVRLLLPGELAKHAVSEGTKAVTKYTSSK	14	5.40E-10	566.9955	3395.9296	6	0.0156	32	2	GluC
H2B1A_MOUSE, H2B1B_MOUSE, H2B1C_MOUSE, H2B1F_MOUSE, H2B1H_MOUSE, H2B1M_MOUSE, H2B1P_MOUSE, H2B2B_MOUSE	Histone H2B	IQTAVRLLLPGELAKHAVSEGTKAVTKYTSSK	14	8.00E-04	849.9894	3395.9283	4	0.0143	32	2	GluC
H2B1A_MOUSE, H2B1B_MOUSE, H2B1C_MOUSE, H2B1F_MOUSE, H2B1H_MOUSE, H2B1M_MOUSE, H2B1P_MOUSE, H2B2B_MOUSE	Histone H2B	IQTAVRLLLPGELAKHAVSEGTKAVTKYTSSK	14	1.80E-07	566.9953	3395.9281	6	0.0141	32	2	GluC
H2B1A_MOUSE, H2B1B_MOUSE, H2B1C_MOUSE, H2B1F_MOUSE, H2B1H_MOUSE, H2B1M_MOUSE, H2B1P_MOUSE, H2B2B_MOUSE	Histone H2B	IQTAVRLLLPGELAKHAVSEGTKAVTKYTSSK	14	1.30E-03	680.1928	3395.9277	5	0.0137	32	2	GluC
H2B1A_MOUSE, H2B1B_MOUSE, H2B1C_MOUSE, H2B1F_MOUSE, H2B1H_MOUSE, H2B1M_MOUSE, H2B1P_MOUSE, H2B2B_MOUSE	Histone H2B	IQTAVRLLLPGELAKHAVSEGTKAVTKYTSSK	14	1.60E-03	849.9892	3395.9276	4	0.0136	32	2	GluC
H2B1A_MOUSE, H2B1B_MOUSE, H2B1C_MOUSE, H2B1F_MOUSE, H2B1H_MOUSE, H2B1M_MOUSE, H2B1P_MOUSE, H2B2B_MOUSE	Histone H2B	IQTAVRLLLPGELAKHAVSEGTKAVTKYTSSK	14	1.80E-05	566.9941	3395.9208	6	0.0068	32	2	GluC
H2B1A_MOUSE, H2B1B_MOUSE, H2B1C_MOUSE, H2B1F_MOUSE, H2B1H_MOUSE, H2B1M_MOUSE, H2B1P_MOUSE, H2B2B_MOUSE	Histone H2B	IQTAVRLLLPGELAKHAVSEGTKAVTKYTSSK	14	5.90E-06	849.9871	3395.9191	4	0.0051	32	2	GluC
H2B1A_MOUSE, H2B1B_MOUSE, H2B1C_MOUSE, H2B1F_MOUSE, H2B1H_MOUSE, H2B1M_MOUSE, H2B1P_MOUSE, H2B2B_MOUSE	Histone H2B	IQTAVRLLLPGELAKHAVSEGTKAVTKYTSSK	14	3.60E-07	849.9871	3395.9191	4	0.0051	32	2	GluC
H2B1A_MOUSE, H2B1B_MOUSE, H2B1C_MOUSE, H2B1F_MOUSE, H2B1H_MOUSE, H2B1M_MOUSE, H2B1P_MOUSE, H2B2B_MOUSE	Histone H2B	IQTAVRLLLPGELAKHAVSEGTKAVTKYTSSK	14	3.40E-03	680.1908	3395.9176	5	0.0036	32	2	GluC
H2B1A_MOUSE, H2B1B_MOUSE, H2B1C_MOUSE, H2B1F_MOUSE, H2B1H_MOUSE, H2B1M_MOUSE, H2B1P_MOUSE, H2B2B_MOUSE	Histone H2B	IQTAVRLLLPGELAKHAVSEGTKAVTKYTSSK	14	1.30E-04	680.1905	3395.9160	5	0.0020	32	2	GluC
H2B1A_MOUSE, H2B1B_MOUSE, H2B1C_MOUSE, H2B1F_MOUSE, H2B1H_MOUSE, H2B1M_MOUSE, H2B1P_MOUSE, H2B2B_MOUSE, H2B3A_MOUSE, H2B3B_MOUSE	Histone H2B	LLLPGELAKHAVSEGTKAVTKYTSSK	8	1.60E-03	910.1808	2727.5205	3	0.0034	26	3	Semi-tryptic
H2B1A_MOUSE, H2B1B_MOUSE, H2B1C_MOUSE, H2B1F_MOUSE, H2B1H_MOUSE, H2B1M_MOUSE, H2B1P_MOUSE, H2B2B_MOUSE, H2B3A_MOUSE, H2B3B_MOUSE	Histone H2B	LLLPGELAKHAVSEGTKAVTKYTSSK	8	8.90E-05	910.1803	2727.5192	3	0.0022	26	3	Semi-tryptic
H2B1A_MOUSE, H2B1B_MOUSE, H2B1C_MOUSE, H2B1F_MOUSE, H2B1H_MOUSE, H2B1M_MOUSE, H2B1P_MOUSE, H2B2B_MOUSE, H2B3A_MOUSE, H2B3B_MOUSE	Histone H2B	LLLPGELAKHAVSEGTKAVTKYTSSK	8	7.10E-06	910.1804	2727.5193	3	0.0022	26	3	Semi-tryptic
H2B1A_MOUSE, H2B1B_MOUSE, H2B1C_MOUSE, H2B1F_MOUSE, H2B1H_MOUSE, H2B1M_MOUSE, H2B1P_MOUSE, H2B2B_MOUSE, H2B3A_MOUSE, H2B3B_MOUSE	Histone H2B	LLLPGELAKHAVSEGTKAVTKYTSSK	8	9.80E-03	682.8863	2727.5161	4	-0.0009	26	3	Semi-tryptic
H2B1A_MOUSE, H2B1B_MOUSE, H2B1C_MOUSE, H2B1F_MOUSE, H2B1H_MOUSE, H2B1M_MOUSE, H2B1P_MOUSE, H2B2B_MOUSE, H2B3A_MOUSE, H2B3B_MOUSE	Histone H2B	LLLPGELAKHAVSEGTKAVTKYTSSK	8	3.10E-03	682.8863	2727.5161	4	-0.0009	26	3	Semi-tryptic
H2B1A_MOUSE, H2B1B_MOUSE, H2B1C_MOUSE, H2B1F_MOUSE, H2B1H_MOUSE, H2B1M_MOUSE, H2B1P_MOUSE, H2B2B_MOUSE, H2B3A_MOUSE, H2B3B_MOUSE	Histone H2B	LLLPGELAKHAVSEGTKAVTKYTSSK	8	2.70E-07	682.8863	2727.5161	4	-0.0009	26	3	Semi-tryptic
H2B1A_MOUSE, H2B1B_MOUSE, H2B1C_MOUSE, H2B1F_MOUSE, H2B1H_MOUSE, H2B1M_MOUSE, H2B1P_MOUSE, H2B2B_MOUSE, H2B3A_MOUSE, H2B3B_MOUSE	Histone H2B	LLLPGELAKHAVSEGTKAVTKYTSSK	8	3.80E-03	682.8862	2727.5158	4	-0.0012	26	3	Semi-tryptic

H2B1A_MOUSE, H2B1B_MOUSE, H2B1C_MOUSE, H2B1F_MOUSE, H2B1H_MOUSE, H2B1M_MOUSE, H2B1P_MOUSE, H2B2B_MOUSE, H2B3A_MOUSE, H2B3B_MOUSE	Histone H2B	LLLPGELAKHAVSEGTKAVTKYTSSK	8	5.40E-05	682.8862	2727.5158	4	-0.0012	26	3	Semi-tryptic
H2B1A_MOUSE, H2B1B_MOUSE, H2B1C_MOUSE, H2B1F_MOUSE, H2B1H_MOUSE, H2B1M_MOUSE, H2B1P_MOUSE, H2B2B_MOUSE, H2B3A_MOUSE, H2B3B_MOUSE	Histone H2B	LAKHAVSEGTKAVTKYTSSK	26	6.70E-03	527.2937	2105.1457	4	-0.0023	20	1	GluC
H2B1A_MOUSE, H2B1B_MOUSE, H2B1C_MOUSE, H2B1F_MOUSE, H2B1H_MOUSE, H2B1M_MOUSE, H2B1P_MOUSE, H2B2B_MOUSE, H2B3A_MOUSE, H2B3B_MOUSE	Histone H2B	LAKHAVSEGTKAVTKYTSSK	26	1.60E-05	527.2937	2105.1457	4	-0.0023	20	1	GluC
H2B1A_MOUSE, H2B1B_MOUSE, H2B1C_MOUSE, H2B1F_MOUSE, H2B1H_MOUSE, H2B1M_MOUSE, H2B1P_MOUSE, H2B2B_MOUSE, H2B3A_MOUSE, H2B3B_MOUSE	Histone H2B	LAKHAVSEGTKAVTKYTSSK	26	6.00E-08	702.7268	2105.1587	3	0.0106	20	1	GluC
H2B1A_MOUSE, H2B1B_MOUSE, H2B1C_MOUSE, H2B1F_MOUSE, H2B1H_MOUSE, H2B1M_MOUSE, H2B1P_MOUSE, H2B2B_MOUSE, H2B3A_MOUSE, H2B3B_MOUSE	Histone H2B	LAKHAVSEGTKAVTKYTSSK	26	2.60E-09	702.7268	2105.1586	3	0.0106	20	1	GluC
H2B1A_MOUSE, H2B1B_MOUSE, H2B1C_MOUSE, H2B1F_MOUSE, H2B1H_MOUSE, H2B1M_MOUSE, H2B1P_MOUSE, H2B2B_MOUSE, H2B3A_MOUSE, H2B3B_MOUSE	Histone H2B	LAKHAVSEGTKAVTKYTSSK	26	1.20E-06	527.2965	2105.1570	4	0.0090	20	1	GluC
H2B1A_MOUSE, H2B1B_MOUSE, H2B1C_MOUSE, H2B1F_MOUSE, H2B1H_MOUSE, H2B1M_MOUSE, H2B1P_MOUSE, H2B2B_MOUSE, H2B3A_MOUSE, H2B3B_MOUSE	Histone H2B	LAKHAVSEGTKAVTKYTSSK	26	4.30E-04	527.2965	2105.1567	4	0.0087	20	1	GluC
H2B1A_MOUSE, H2B1B_MOUSE, H2B1C_MOUSE, H2B1F_MOUSE, H2B1H_MOUSE, H2B1M_MOUSE, H2B1P_MOUSE, H2B2B_MOUSE, H2B3A_MOUSE, H2B3B_MOUSE	Histone H2B	LAKHAVSEGTKAVTKYTSSK	26	1.10E-05	527.2963	2105.1560	4	0.0080	20	1	GluC
H2B1A_MOUSE, H2B1B_MOUSE, H2B1C_MOUSE, H2B1F_MOUSE, H2B1H_MOUSE, H2B1M_MOUSE, H2B1P_MOUSE, H2B2B_MOUSE, H2B3A_MOUSE, H2B3B_MOUSE	Histone H2B	LAKHAVSEGTKAVTKYTSSK	26	4.00E-07	702.7256	2105.1549	3	0.0068	20	1	GluC
H2B1A_MOUSE, H2B1B_MOUSE, H2B1C_MOUSE, H2B1F_MOUSE, H2B1H_MOUSE, H2B1M_MOUSE, H2B1P_MOUSE, H2B2B_MOUSE, H2B3A_MOUSE, H2B3B_MOUSE	Histone H2B	LAKHAVSEGTKAVTKYTSSK	26	9.00E-10	527.2958	2105.1541	4	0.0061	20	1	GluC
H2B1A_MOUSE, H2B1B_MOUSE, H2B1C_MOUSE, H2B1F_MOUSE, H2B1H_MOUSE, H2B1M_MOUSE, H2B1P_MOUSE, H2B2B_MOUSE, H2B3A_MOUSE, H2B3B_MOUSE	Histone H2B	LAKHAVSEGTKAVTKYTSSK	26	6.00E-10	527.2951	2105.1514	4	0.0034	20	1	GluC
H2B1A_MOUSE, H2B1B_MOUSE, H2B1C_MOUSE, H2B1F_MOUSE, H2B1H_MOUSE, H2B1M_MOUSE, H2B1P_MOUSE, H2B2B_MOUSE, H2B3A_MOUSE, H2B3B_MOUSE	Histone H2B	LAKHAVSEGTKAVTKYTSSK	26	4.30E-10	527.295	2105.1511	4	0.0030	20	1	GluC
H2B1A_MOUSE, H2B1B_MOUSE, H2B1C_MOUSE, H2B1F_MOUSE, H2B1H_MOUSE, H2B1M_MOUSE, H2B1P_MOUSE, H2B2B_MOUSE, H2B3A_MOUSE, H2B3B_MOUSE	Histone H2B	LAKHAVSEGTKAVTKYTSSK	26	1.30E-05	527.2949	2105.1505	4	0.0025	20	1	GluC
H2B1A_MOUSE, H2B1B_MOUSE, H2B1C_MOUSE, H2B1F_MOUSE, H2B1H_MOUSE, H2B1M_MOUSE, H2B1P_MOUSE, H2B2B_MOUSE, H2B3A_MOUSE, H2B3B_MOUSE	Histone H2B	LAKHAVSEGTKAVTKYTSSK	26	3.90E-03	702.724	2105.1502	3	0.0022	20	1	GluC
H2B1A_MOUSE, H2B1B_MOUSE, H2B1C_MOUSE, H2B1F_MOUSE, H2B1H_MOUSE, H2B1M_MOUSE, H2B1P_MOUSE, H2B2B_MOUSE, H2B3A_MOUSE, H2B3B_MOUSE	Histone H2B	LAKHAVSEGTKAVTKYTSSK	26	2.40E-09	702.724	2105.1502	3	0.0022	20	1	GluC
H2B1A_MOUSE, H2B1B_MOUSE, H2B1C_MOUSE, H2B1F_MOUSE, H2B1H_MOUSE, H2B1M_MOUSE, H2B1P_MOUSE, H2B2B_MOUSE, H2B3A_MOUSE, H2B3B_MOUSE	Histone H2B	LAKHAVSEGTKAVTKYTSSK	26	8.30E-04	527.2945	2105.1490	4	0.0010	20	1	GluC
H2B1A_MOUSE, H2B1B_MOUSE, H2B1C_MOUSE, H2B1F_MOUSE, H2B1H_MOUSE, H2B1M_MOUSE, H2B1P_MOUSE, H2B2B_MOUSE, H2B3A_MOUSE, H2B3B_MOUSE	Histone H2B	LAKHAVSEGTKAVTKYTSSK	26	2.70E-13	527.2945	2105.1489	4	0.0009	20	1	GluC
H2B1A_MOUSE, H2B1B_MOUSE, H2B1C_MOUSE, H2B1F_MOUSE, H2B1H_MOUSE, H2B1M_MOUSE, H2B1P_MOUSE, H2B2B_MOUSE, H2B3A_MOUSE, H2B3B_MOUSE	Histone H2B	LAKHAVSEGTKAVTKYTSSK	26	6.40E-06	702.7235	2105.1486	3	0.0005	20	1	GluC
H2B1A_MOUSE, H2B1B_MOUSE, H2B1C_MOUSE, H2B1F_MOUSE, H2B1H_MOUSE, H2B1M_MOUSE, H2B1P_MOUSE, H2B2B_MOUSE, H2B3A_MOUSE, H2B3B_MOUSE	Histone H2B	LAKHAVSEGTKAVTKYTSSK	26	3.70E-07	422.0369	2105.1484	5	0.0003	20	1	GluC
H2B1A_MOUSE, H2B1B_MOUSE, H2B1C_MOUSE, H2B1F_MOUSE, H2B1H_MOUSE, H2B1M_MOUSE, H2B1P_MOUSE, H2B2B_MOUSE, H2B3A_MOUSE, H2B3B_MOUSE	Histone H2B	LAKHAVSEGTKAVTKYTSSK	26	4.80E-07	527.2943	2105.1482	4	0.0001	20	1	GluC
H2B1A_MOUSE, H2B1B_MOUSE, H2B1C_MOUSE, H2B1F_MOUSE, H2B1H_MOUSE, H2B1M_MOUSE, H2B1P_MOUSE, H2B2B_MOUSE, H2B3A_MOUSE, H2B3B_MOUSE	Histone H2B	LAKHAVSEGTKAVTKYTSSK	26	5.60E-07	527.2943	2105.1480	4	-0.0001	20	1	GluC
H2B1A_MOUSE, H2B1B_MOUSE, H2B1C_MOUSE, H2B1F_MOUSE, H2B1H_MOUSE, H2B1M_MOUSE, H2B1P_MOUSE, H2B2B_MOUSE, H2B3A_MOUSE, H2B3B_MOUSE	Histone H2B	LAKHAVSEGTKAVTKYTSSK	26	2.60E-08	702.7232	2105.1477	3	-0.0003	20	1	GluC
H2B1A_MOUSE, H2B1B_MOUSE, H2B1C_MOUSE, H2B1F_MOUSE, H2B1H_MOUSE, H2B1M_MOUSE, H2B1P_MOUSE, H2B2B_MOUSE, H2B3A_MOUSE, H2B3B_MOUSE	Histone H2B	LAKHAVSEGTKAVTKYTSSK	26	7.60E-09	527.2942	2105.1477	4	-0.0003	20	1	GluC
H2B1A_MOUSE, H2B1B_MOUSE, H2B1C_MOUSE, H2B1F_MOUSE, H2B1H_MOUSE, H2B1M_MOUSE, H2B1P_MOUSE, H2B2B_MOUSE, H2B3A_MOUSE, H2B3B_MOUSE	Histone H2B	LAKHAVSEGTKAVTKYTSSK	26	1.70E-03	527.2942	2105.1476	4	-0.0005	20	1	GluC

H2B1A_MOUSE, H2B1B_MOUSE, H2B1C_MOUSE, H2B1F_MOUSE, H2B1H_MOUSE, H2B1M_MOUSE, H2B1P_MOUSE, H2B2B_MOUSE, H2B3A_MOUSE, H2B3B_MOUSE	Histone H2B	LAKHAVSEGTKAVTKYTSSK	26	6.40E-14	527.2941	2105.1473	4	-0.0007	20	1	GluC
H2B1A_MOUSE, H2B1B_MOUSE, H2B1C_MOUSE, H2B1F_MOUSE, H2B1H_MOUSE, H2B1M_MOUSE, H2B1P_MOUSE, H2B2B_MOUSE, H2B3A_MOUSE, H2B3B_MOUSE	Histone H2B	LAKHAVSEGTKAVTKYTSSK	26	1.10E-05	527.2941	2105.1471	4	-0.0009	20	1	GluC
H2B1A_MOUSE, H2B1B_MOUSE, H2B1C_MOUSE, H2B1F_MOUSE, H2B1H_MOUSE, H2B1M_MOUSE, H2B1P_MOUSE, H2B2B_MOUSE, H2B3A_MOUSE, H2B3B_MOUSE	Histone H2B	LAKHAVSEGTKAVTKYTSSK	26	4.70E-06	527.294	2105.1469	4	-0.0011	20	1	GluC
H2B1A_MOUSE, H2B1B_MOUSE, H2B1C_MOUSE, H2B1F_MOUSE, H2B1H_MOUSE, H2B1M_MOUSE, H2B1P_MOUSE, H2B2B_MOUSE, H2B3A_MOUSE, H2B3B_MOUSE	Histone H2B	HAVSEGTKAVTKYTSSK	36	4.20E-03	449.2398	1792.9299	4	-0.0020	17	2	Semi-tryptic
H2B1A_MOUSE, H2B1B_MOUSE, H2B1C_MOUSE, H2B1F_MOUSE, H2B1H_MOUSE, H2B1M_MOUSE, H2B1P_MOUSE, H2B2B_MOUSE, H2B3A_MOUSE, H2B3B_MOUSE	Histone H2B	HAVSEGTKAVTKYTSSK	36	1.30E-05	598.6506	1792.9299	3	-0.0020	17	2	Semi-tryptic
H2B1A_MOUSE, H2B1B_MOUSE, H2B1C_MOUSE, H2B1F_MOUSE, H2B1H_MOUSE, H2B1M_MOUSE, H2B1P_MOUSE, H2B2B_MOUSE, H2B3A_MOUSE, H2B3B_MOUSE	Histone H2B	HAVSEGTKAVTKYTSSK	36	3.50E-03	449.2397	1792.9298	4	-0.0021	17	2	Semi-tryptic
H2B1A_MOUSE, H2B1B_MOUSE, H2B1C_MOUSE, H2B1F_MOUSE, H2B1H_MOUSE, H2B1M_MOUSE, H2B1P_MOUSE, H2B2B_MOUSE, H2B3A_MOUSE, H2B3B_MOUSE	Histone H2B	HAVSEGTKAVTKYTSSK	36	2.40E-06	598.6504	1792.9293	3	-0.0026	17	2	Semi-tryptic
H2B1A_MOUSE, H2B1B_MOUSE, H2B1C_MOUSE, H2B1F_MOUSE, H2B1H_MOUSE, H2B1M_MOUSE, H2B1P_MOUSE, H2B2B_MOUSE, H2B3A_MOUSE, H2B3B_MOUSE	Histone H2B	HAVSEGTKAVTKYTSSK	36	4.10E-10	897.4716	1792.9287	2	-0.0032	17	2	Semi-tryptic
H2B1A_MOUSE, H2B1B_MOUSE, H2B1C_MOUSE, H2B1F_MOUSE, H2B1H_MOUSE, H2B1M_MOUSE, H2B1P_MOUSE, H2B2B_MOUSE, H2B3A_MOUSE, H2B3B_MOUSE	Histone H2B	HAVSEGTKAVTKYTSSK	36	1.60E-06	598.6497	1792.9272	3	-0.0047	17	2	Semi-tryptic
H2B1A_MOUSE, H2B1B_MOUSE, H2B1C_MOUSE, H2B1F_MOUSE, H2B1H_MOUSE, H2B1M_MOUSE, H2B1P_MOUSE, H2B2B_MOUSE, H2B3A_MOUSE, H2B3B_MOUSE	Histone H2B	HAVSEGTKAVTKYTSSK	36	2.10E-03	598.6524	1792.9353	3	0.0034	17	2	Semi-tryptic
H2B1A_MOUSE, H2B1B_MOUSE, H2B1C_MOUSE, H2B1F_MOUSE, H2B1H_MOUSE, H2B1M_MOUSE, H2B1P_MOUSE, H2B2B_MOUSE, H2B3A_MOUSE, H2B3B_MOUSE	Histone H2B	HAVSEGTKAVTKYTSSK	36	1.60E-05	598.6522	1792.9346	3	0.0027	17	2	Semi-tryptic
H2B1A_MOUSE, H2B1B_MOUSE, H2B1C_MOUSE, H2B1F_MOUSE, H2B1H_MOUSE, H2B1M_MOUSE, H2B1P_MOUSE, H2B2B_MOUSE, H2B3A_MOUSE, H2B3B_MOUSE	Histone H2B	HAVSEGTKAVTKYTSSK	36	3.80E-05	598.652	1792.9342	3	0.0023	17	2	Semi-tryptic
H2B1A_MOUSE, H2B1B_MOUSE, H2B1C_MOUSE, H2B1F_MOUSE, H2B1H_MOUSE, H2B1M_MOUSE, H2B1P_MOUSE, H2B2B_MOUSE, H2B3A_MOUSE, H2B3B_MOUSE	Histone H2B	HAVSEGTKAVTKYTSSK	36	2.30E-10	897.4741	1792.9336	2	0.0017	17	2	Semi-tryptic
H2B1A_MOUSE, H2B1B_MOUSE, H2B1C_MOUSE, H2B1F_MOUSE, H2B1H_MOUSE, H2B1M_MOUSE, H2B1P_MOUSE, H2B2B_MOUSE, H2B3A_MOUSE, H2B3B_MOUSE	Histone H2B	HAVSEGTKAVTKYTSSK	36	1.80E-09	897.474	1792.9335	2	0.0016	17	2	Semi-tryptic
H2B1A_MOUSE, H2B1B_MOUSE, H2B1C_MOUSE, H2B1F_MOUSE, H2B1H_MOUSE, H2B1M_MOUSE, H2B1P_MOUSE, H2B2B_MOUSE, H2B3A_MOUSE, H2B3B_MOUSE	Histone H2B	HAVSEGTKAVTKYTSSK	36	1.50E-10	897.4738	1792.9330	2	0.0011	17	2	Semi-tryptic
H2B1A_MOUSE, H2B1B_MOUSE, H2B1C_MOUSE, H2B1F_MOUSE, H2B1H_MOUSE, H2B1M_MOUSE, H2B1P_MOUSE, H2B2B_MOUSE, H2B3A_MOUSE, H2B3B_MOUSE	Histone H2B	HAVSEGTKAVTKYTSSK	36	2.00E-07	598.6516	1792.9329	3	0.0009	17	2	Semi-tryptic
H2B1A_MOUSE, H2B1B_MOUSE, H2B1C_MOUSE, H2B1F_MOUSE, H2B1H_MOUSE, H2B1M_MOUSE, H2B1P_MOUSE, H2B2B_MOUSE, H2B3A_MOUSE, H2B3B_MOUSE	Histone H2B	HAVSEGTKAVTKYTSSK	36	3.30E-03	449.2404	1792.9324	4	0.0005	17	2	Semi-tryptic
H2B1A_MOUSE, H2B1B_MOUSE, H2B1C_MOUSE, H2B1F_MOUSE, H2B1H_MOUSE, H2B1M_MOUSE, H2B1P_MOUSE, H2B2B_MOUSE, H2B3A_MOUSE, H2B3B_MOUSE	Histone H2B	HAVSEGTKAVTKYTSSK	36	2.50E-04	598.6514	1792.9324	3	0.0005	17	2	Semi-tryptic
H2B1A_MOUSE, H2B1B_MOUSE, H2B1C_MOUSE, H2B1F_MOUSE, H2B1H_MOUSE, H2B1M_MOUSE, H2B1P_MOUSE, H2B2B_MOUSE, H2B3A_MOUSE, H2B3B_MOUSE	Histone H2B	HAVSEGTKAVTKYTSSK	36	2.40E-06	598.6514	1792.9324	3	0.0005	17	2	Semi-tryptic
H2B1A_MOUSE, H2B1B_MOUSE, H2B1C_MOUSE, H2B1F_MOUSE, H2B1H_MOUSE, H2B1M_MOUSE, H2B1P_MOUSE, H2B2B_MOUSE, H2B3A_MOUSE, H2B3B_MOUSE	Histone H2B	HAVSEGTKAVTKYTSSK	36	4.80E-09	598.6514	1792.9324	3	0.0005	17	2	Semi-tryptic
H2B1A_MOUSE, H2B1B_MOUSE, H2B1C_MOUSE, H2B1F_MOUSE, H2B1H_MOUSE, H2B1M_MOUSE, H2B1P_MOUSE, H2B2B_MOUSE, H2B3A_MOUSE, H2B3B_MOUSE	Histone H2B	HAVSEGTKAVTKYTSSK	36	2.40E-03	449.2403	1792.9322	4	0.0003	17	2	Semi-tryptic
H2B1A_MOUSE, H2B1B_MOUSE, H2B1C_MOUSE, H2B1F_MOUSE, H2B1H_MOUSE, H2B1M_MOUSE, H2B1P_MOUSE, H2B2B_MOUSE, H2B3A_MOUSE, H2B3B_MOUSE	Histone H2B	HAVSEGTKAVTKYTSSK	36	2.10E-06	598.6513	1792.9321	3	0.0002	17	2	Semi-tryptic
H2B1A_MOUSE, H2B1B_MOUSE, H2B1C_MOUSE, H2B1F_MOUSE, H2B1H_MOUSE, H2B1M_MOUSE, H2B1P_MOUSE, H2B2B_MOUSE, H2B3A_MOUSE, H2B3B_MOUSE	Histone H2B	HAVSEGTKAVTKYTSSK	36	1.00E-06	598.6513	1792.9321	3	0.0002	17	2	Semi-tryptic
H2B1A_MOUSE, H2B1B_MOUSE, H2B1C_MOUSE, H2B1F_MOUSE, H2B1H_MOUSE, H2B1M_MOUSE, H2B1P_MOUSE, H2B2B_MOUSE, H2B3A_MOUSE, H2B3B_MOUSE	Histone H2B	HAVSEGTKAVTKYTSSK	36	6.00E-03	449.2403	1792.9321	4	0.0001	17	2	Semi-tryptic

H2B1A_MOUSE, H2B1B_MOUSE, H2B1C_MOUSE, H2B1F_MOUSE, H2B1H_MOUSE, H2B1M_MOUSE, H2B1P_MOUSE, H2B2B_MOUSE, H2B3A_MOUSE, H2B3B_MOUSE	Histone H2B	HAVSEGTKAVTKYTSSK	36	2.30E-05	598.6512	1792.9319	3	0.0000	17	2	Semi-tryptic
H2B1A_MOUSE, H2B1B_MOUSE, H2B1C_MOUSE, H2B1F_MOUSE, H2B1H_MOUSE, H2B1M_MOUSE, H2B1P_MOUSE, H2B2B_MOUSE, H2B3A_MOUSE, H2B3B_MOUSE	Histone H2B	HAVSEGTKAVTKYTSSK	36	5.20E-04	449.2402	1792.9315	4	-0.0004	17	2	Semi-tryptic
H2B1A_MOUSE, H2B1B_MOUSE, H2B1C_MOUSE, H2B1F_MOUSE, H2B1H_MOUSE, H2B1M_MOUSE, H2B1P_MOUSE, H2B2B_MOUSE, H2B3A_MOUSE, H2B3B_MOUSE	Histone H2B	HAVSEGTKAVTKYTSSK	36	6.00E-06	598.6511	1792.9315	3	-0.0004	17	2	Semi-tryptic
H2B1A_MOUSE, H2B1B_MOUSE, H2B1C_MOUSE, H2B1F_MOUSE, H2B1H_MOUSE, H2B1M_MOUSE, H2B1P_MOUSE, H2B2B_MOUSE, H2B3A_MOUSE, H2B3B_MOUSE	Histone H2B	HAVSEGTKAVTKYTSSK	36	9.50E-14	449.2402	1792.9315	4	-0.0004	17	2	Semi-tryptic
H2B1A_MOUSE, H2B1B_MOUSE, H2B1C_MOUSE, H2B1F_MOUSE, H2B1H_MOUSE, H2B1M_MOUSE, H2B1P_MOUSE, H2B2B_MOUSE, H2B3A_MOUSE, H2B3B_MOUSE	Histone H2B	HAVSEGTKAVTKYTSSK	36	3.30E-15	449.2402	1792.9315	4	-0.0004	17	2	Semi-tryptic
H2B1A_MOUSE, H2B1B_MOUSE, H2B1C_MOUSE, H2B1F_MOUSE, H2B1H_MOUSE, H2B1M_MOUSE, H2B1P_MOUSE, H2B2B_MOUSE, H2B3A_MOUSE, H2B3B_MOUSE	Histone H2B	HAVSEGTKAVTKYTSSK	36	5.70E-03	449.2401	1792.9312	4	-0.0007	17	2	Semi-tryptic
H2B1A_MOUSE, H2B1B_MOUSE, H2B1C_MOUSE, H2B1F_MOUSE, H2B1H_MOUSE, H2B1M_MOUSE, H2B1P_MOUSE, H2B2B_MOUSE, H2B3A_MOUSE, H2B3B_MOUSE	Histone H2B	HAVSEGTKAVTKYTSSK	36	2.50E-03	449.24	1792.9309	4	-0.0010	17	2	Semi-tryptic
H2B1A_MOUSE, H2B1B_MOUSE, H2B1C_MOUSE, H2B1F_MOUSE, H2B1H_MOUSE, H2B1M_MOUSE, H2B1P_MOUSE, H2B2B_MOUSE, H2B3A_MOUSE, H2B3B_MOUSE	Histone H2B	HAVSEGTKAVTKYTSSK	36	1.70E-04	598.6509	1792.9310	3	-0.0010	17	2	Semi-tryptic
H2B1A_MOUSE, H2B1B_MOUSE, H2B1C_MOUSE, H2B1F_MOUSE, H2B1H_MOUSE, H2B1M_MOUSE, H2B1P_MOUSE, H2B2B_MOUSE, H2B3A_MOUSE, H2B3B_MOUSE	Histone H2B	HAVSEGTKAVTKYTSSK	36	6.90E-05	598.6508	1792.9306	3	-0.0013	17	2	Semi-tryptic
H2B1A_MOUSE, H2B1B_MOUSE, H2B1C_MOUSE, H2B1F_MOUSE, H2B1H_MOUSE, H2B1M_MOUSE, H2B1P_MOUSE, H2B2B_MOUSE, H2B3A_MOUSE, H2B3B_MOUSE	Histone H2B	HAVSEGTKAVTKYTSSK	36	2.00E-06	598.6508	1792.9306	3	-0.0013	17	2	Semi-tryptic
H2B1A_MOUSE, H2B1B_MOUSE, H2B1C_MOUSE, H2B1F_MOUSE, H2B1H_MOUSE, H2B1M_MOUSE, H2B1P_MOUSE, H2B2B_MOUSE, H2B3A_MOUSE, H2B3B_MOUSE	Histone H2B	HAVSEGTKAVTKYTSSK	36	1.20E-07	598.6508	1792.9306	3	-0.0013	17	2	Semi-tryptic
H2B1A_MOUSE, H2B1B_MOUSE, H2B1C_MOUSE, H2B1F_MOUSE, H2B1H_MOUSE, H2B1M_MOUSE, H2B1P_MOUSE, H2B2B_MOUSE, H2B3A_MOUSE, H2B3B_MOUSE	Histone H2B	HAVSEGTKAVTKYTSSK	36	3.50E-09	449.2399	1792.9307	4	-0.0013	17	2	Semi-tryptic
H2B1A_MOUSE, H2B1B_MOUSE, H2B1C_MOUSE, H2B1F_MOUSE, H2B1H_MOUSE, H2B1M_MOUSE, H2B1P_MOUSE, H2B2B_MOUSE, H2B3A_MOUSE, H2B3B_MOUSE	Histone H2B	HAVSEGTKAVTKYTSSK	36	6.70E-11	449.2399	1792.9307	4	-0.0013	17	2	Semi-tryptic
H2B1A_MOUSE, H2B1B_MOUSE, H2B1C_MOUSE, H2B1F_MOUSE, H2B1H_MOUSE, H2B1M_MOUSE, H2B1P_MOUSE, H2B2B_MOUSE, H2B3A_MOUSE, H2B3B_MOUSE	Histone H2B	HAVSEGTKAVTKYTSSK	36	6.30E-04	598.6508	1792.9305	3	-0.0015	17	2	Semi-tryptic
H2B1A_MOUSE, H2B1B_MOUSE, H2B1C_MOUSE, H2B1F_MOUSE, H2B1H_MOUSE, H2B1M_MOUSE, H2B1P_MOUSE, H2B2B_MOUSE, H2B3A_MOUSE, H2B3B_MOUSE	Histone H2B	HAVSEGTKAVTKYTSSK	36	2.60E-10	897.4725	1792.9304	2	-0.0015	17	2	Semi-tryptic
H2B1A_MOUSE, H2B1B_MOUSE, H2B1C_MOUSE, H2B1F_MOUSE, H2B1H_MOUSE, H2B1M_MOUSE, H2B1P_MOUSE, H2B2B_MOUSE, H2B3A_MOUSE, H2B3B_MOUSE	Histone H2B	GTKAVTKYTSSK	473	5.70E-06	318.43	1269.6910	4	-0.0019	12	0	GluC
H2B1A_MOUSE, H2B1B_MOUSE, H2B1C_MOUSE, H2B1F_MOUSE, H2B1H_MOUSE, H2B1M_MOUSE, H2B1P_MOUSE, H2B2B_MOUSE, H2B3A_MOUSE, H2B3B_MOUSE	Histone H2B	GTKAVTKYTSSK	473	1.10E-06	424.2376	1269.6910	3	-0.0019	12	0	GluC
H2B1A_MOUSE, H2B1B_MOUSE, H2B1C_MOUSE, H2B1F_MOUSE, H2B1H_MOUSE, H2B1M_MOUSE, H2B1P_MOUSE, H2B2B_MOUSE, H2B3A_MOUSE, H2B3B_MOUSE	Histone H2B	GTKAVTKYTSSK	473	2.70E-08	424.2376	1269.6909	3	-0.0019	12	0	GluC
H2B1A_MOUSE, H2B1B_MOUSE, H2B1C_MOUSE, H2B1F_MOUSE, H2B1H_MOUSE, H2B1M_MOUSE, H2B1P_MOUSE, H2B2B_MOUSE, H2B3A_MOUSE, H2B3B_MOUSE	Histone H2B	GTKAVTKYTSSK	473	1.60E-09	318.43	1269.6909	4	-0.0019	12	0	GluC
H2B1A_MOUSE, H2B1B_MOUSE, H2B1C_MOUSE, H2B1F_MOUSE, H2B1H_MOUSE, H2B1M_MOUSE, H2B1P_MOUSE, H2B2B_MOUSE, H2B3A_MOUSE, H2B3B_MOUSE	Histone H2B	GTKAVTKYTSSK	473	5.70E-05	424.2376	1269.6909	3	-0.0020	12	0	GluC
H2B1A_MOUSE, H2B1B_MOUSE, H2B1C_MOUSE, H2B1F_MOUSE, H2B1H_MOUSE, H2B1M_MOUSE, H2B1P_MOUSE, H2B2B_MOUSE, H2B3A_MOUSE, H2B3B_MOUSE	Histone H2B	GTKAVTKYTSSK	473	1.50E-03	424.2375	1269.6907	3	-0.0021	12	0	GluC
H2B1A_MOUSE, H2B1B_MOUSE, H2B1C_MOUSE, H2B1F_MOUSE, H2B1H_MOUSE, H2B1M_MOUSE, H2B1P_MOUSE, H2B2B_MOUSE, H2B3A_MOUSE, H2B3B_MOUSE	Histone H2B	GTKAVTKYTSSK	473	1.20E-03	424.2375	1269.6908	3	-0.0021	12	0	GluC
H2B1A_MOUSE, H2B1B_MOUSE, H2B1C_MOUSE, H2B1F_MOUSE, H2B1H_MOUSE, H2B1M_MOUSE, H2B1P_MOUSE, H2B2B_MOUSE, H2B3A_MOUSE, H2B3B_MOUSE	Histone H2B	GTKAVTKYTSSK	473	3.90E-08	318.43	1269.6908	4	-0.0021	12	0	GluC
H2B1A_MOUSE, H2B1B_MOUSE, H2B1C_MOUSE, H2B1F_MOUSE, H2B1H_MOUSE, H2B1M_MOUSE, H2B1P_MOUSE, H2B2B_MOUSE, H2B3A_MOUSE, H2B3B_MOUSE	Histone H2B	GTKAVTKYTSSK	473	2.00E-03	318.4299	1269.6906	4	-0.0023	12	0	GluC

H2B1A_MOUSE, H2B1B_MOUSE, H2B1C_MOUSE, H2B1F_MOUSE, H2B1H_MOUSE, H2B1M_MOUSE, H2B1P_MOUSE, H2B2B_MOUSE, H2B3A_MOUSE, H2B3B_MOUSE	Histone H2B	GTKAVTKYTSSK	473	1.00E-04	424.2374	1269.6904	3	-0.0025	12	0	GluC
H2B1A_MOUSE, H2B1B_MOUSE, H2B1C_MOUSE, H2B1F_MOUSE, H2B1H_MOUSE, H2B1M_MOUSE, H2B1P_MOUSE, H2B2B_MOUSE, H2B3A_MOUSE, H2B3B_MOUSE	Histone H2B	GTKAVTKYTSSK	473	1.40E-04	318.4298	1269.6902	4	-0.0027	12	0	GluC
H2B1A_MOUSE, H2B1B_MOUSE, H2B1C_MOUSE, H2B1F_MOUSE, H2B1H_MOUSE, H2B1M_MOUSE, H2B1P_MOUSE, H2B2B_MOUSE, H2B3A_MOUSE, H2B3B_MOUSE	Histone H2B	GTKAVTKYTSSK	473	4.60E-06	318.4298	1269.6899	4	-0.0029	12	0	GluC
H2B1A_MOUSE, H2B1B_MOUSE, H2B1C_MOUSE, H2B1F_MOUSE, H2B1H_MOUSE, H2B1M_MOUSE, H2B1P_MOUSE, H2B2B_MOUSE, H2B3A_MOUSE, H2B3B_MOUSE	Histone H2B	GTKAVTKYTSSK	473	4.80E-05	318.4297	1269.6896	4	-0.0033	12	0	GluC
H2B1A_MOUSE, H2B1B_MOUSE, H2B1C_MOUSE, H2B1F_MOUSE, H2B1H_MOUSE, H2B1M_MOUSE, H2B1P_MOUSE, H2B2B_MOUSE, H2B3A_MOUSE, H2B3B_MOUSE	Histone H2B	GTKAVTKYTSSK	473	1.30E-03	424.2368	1269.6887	3	-0.0042	12	0	GluC
H2B1A_MOUSE, H2B1B_MOUSE, H2B1C_MOUSE, H2B1F_MOUSE, H2B1H_MOUSE, H2B1M_MOUSE, H2B1P_MOUSE, H2B2B_MOUSE, H2B3A_MOUSE, H2B3B_MOUSE	Histone H2B	GTKAVTKYTSSK	473	4.40E-03	635.8516	1269.6886	2	-0.0043	12	0	GluC
H2B1A_MOUSE, H2B1B_MOUSE, H2B1C_MOUSE, H2B1F_MOUSE, H2B1H_MOUSE, H2B1M_MOUSE, H2B1P_MOUSE, H2B2B_MOUSE, H2B3A_MOUSE, H2B3B_MOUSE	Histone H2B	GTKAVTKYTSSK	473	2.70E-05	635.8516	1269.6886	2	-0.0043	12	0	GluC
H2B1A_MOUSE, H2B1B_MOUSE, H2B1C_MOUSE, H2B1F_MOUSE, H2B1H_MOUSE, H2B1M_MOUSE, H2B1P_MOUSE, H2B2B_MOUSE, H2B3A_MOUSE, H2B3B_MOUSE	Histone H2B	GTKAVTKYTSSK	473	5.00E-03	318.4293	1269.6880	4	-0.0049	12	0	GluC
H2B1A_MOUSE, H2B1B_MOUSE, H2B1C_MOUSE, H2B1F_MOUSE, H2B1H_MOUSE, H2B1M_MOUSE, H2B1P_MOUSE, H2B2B_MOUSE, H2B3A_MOUSE, H2B3B_MOUSE	Histone H2B	GTKAVTKYTSSK	473	1.30E-03	318.4291	1269.6875	4	-0.0054	12	0	GluC
H2B1A_MOUSE, H2B1B_MOUSE, H2B1C_MOUSE, H2B1F_MOUSE, H2B1H_MOUSE, H2B1M_MOUSE, H2B1P_MOUSE, H2B2B_MOUSE, H2B3A_MOUSE, H2B3B_MOUSE	Histone H2B	GTKAVTKYTSSK	473	2.10E-04	424.241	1269.7013	3	0.0084	12	0	GluC
H2B1A_MOUSE, H2B1B_MOUSE, H2B1C_MOUSE, H2B1F_MOUSE, H2B1H_MOUSE, H2B1M_MOUSE, H2B1P_MOUSE, H2B2B_MOUSE, H2B3A_MOUSE, H2B3B_MOUSE	Histone H2B	GTKAVTKYTSSK	473	2.30E-06	424.241	1269.7013	3	0.0084	12	0	GluC
H2B1A_MOUSE, H2B1B_MOUSE, H2B1C_MOUSE, H2B1F_MOUSE, H2B1H_MOUSE, H2B1M_MOUSE, H2B1P_MOUSE, H2B2B_MOUSE, H2B3A_MOUSE, H2B3B_MOUSE	Histone H2B	GTKAVTKYTSSK	473	9.90E-05	635.8577	1269.7009	2	0.0081	12	0	GluC
H2B1A_MOUSE, H2B1B_MOUSE, H2B1C_MOUSE, H2B1F_MOUSE, H2B1H_MOUSE, H2B1M_MOUSE, H2B1P_MOUSE, H2B2B_MOUSE, H2B3A_MOUSE, H2B3B_MOUSE	Histone H2B	GTKAVTKYTSSK	473	2.30E-09	424.2409	1269.7008	3	0.0079	12	0	GluC
H2B1A_MOUSE, H2B1B_MOUSE, H2B1C_MOUSE, H2B1F_MOUSE, H2B1H_MOUSE, H2B1M_MOUSE, H2B1P_MOUSE, H2B2B_MOUSE, H2B3A_MOUSE, H2B3B_MOUSE	Histone H2B	GTKAVTKYTSSK	473	9.10E-03	635.8576	1269.7006	2	0.0078	12	0	GluC
H2B1A_MOUSE, H2B1B_MOUSE, H2B1C_MOUSE, H2B1F_MOUSE, H2B1H_MOUSE, H2B1M_MOUSE, H2B1P_MOUSE, H2B2B_MOUSE, H2B3A_MOUSE, H2B3B_MOUSE	Histone H2B	GTKAVTKYTSSK	473	5.70E-08	318.4324	1269.7006	4	0.0078	12	0	GluC
H2B1A_MOUSE, H2B1B_MOUSE, H2B1C_MOUSE, H2B1F_MOUSE, H2B1H_MOUSE, H2B1M_MOUSE, H2B1P_MOUSE, H2B2B_MOUSE, H2B3A_MOUSE, H2B3B_MOUSE	Histone H2B	GTKAVTKYTSSK	473	2.40E-03	635.8575	1269.7005	2	0.0077	12	0	GluC
H2B1A_MOUSE, H2B1B_MOUSE, H2B1C_MOUSE, H2B1F_MOUSE, H2B1H_MOUSE, H2B1M_MOUSE, H2B1P_MOUSE, H2B2B_MOUSE, H2B3A_MOUSE, H2B3B_MOUSE	Histone H2B	GTKAVTKYTSSK	473	1.20E-08	318.4322	1269.6999	4	0.0070	12	0	GluC
H2B1A_MOUSE, H2B1B_MOUSE, H2B1C_MOUSE, H2B1F_MOUSE, H2B1H_MOUSE, H2B1M_MOUSE, H2B1P_MOUSE, H2B2B_MOUSE, H2B3A_MOUSE, H2B3B_MOUSE	Histone H2B	GTKAVTKYTSSK	473	3.50E-04	424.2404	1269.6994	3	0.0066	12	0	GluC
H2B1A_MOUSE, H2B1B_MOUSE, H2B1C_MOUSE, H2B1F_MOUSE, H2B1H_MOUSE, H2B1M_MOUSE, H2B1P_MOUSE, H2B2B_MOUSE, H2B3A_MOUSE, H2B3B_MOUSE	Histone H2B	GTKAVTKYTSSK	473	4.20E-07	424.2404	1269.6994	3	0.0066	12	0	GluC
H2B1A_MOUSE, H2B1B_MOUSE, H2B1C_MOUSE, H2B1F_MOUSE, H2B1H_MOUSE, H2B1M_MOUSE, H2B1P_MOUSE, H2B2B_MOUSE, H2B3A_MOUSE, H2B3B_MOUSE	Histone H2B	GTKAVTKYTSSK	473	9.10E-04	635.8569	1269.6992	2	0.0064	12	0	GluC
H2B1A_MOUSE, H2B1B_MOUSE, H2B1C_MOUSE, H2B1F_MOUSE, H2B1H_MOUSE, H2B1M_MOUSE, H2B1P_MOUSE, H2B2B_MOUSE, H2B3A_MOUSE, H2B3B_MOUSE	Histone H2B	GTKAVTKYTSSK	473	2.00E-03	424.2403	1269.6990	3	0.0061	12	0	GluC
H2B1A_MOUSE, H2B1B_MOUSE, H2B1C_MOUSE, H2B1F_MOUSE, H2B1H_MOUSE, H2B1M_MOUSE, H2B1P_MOUSE, H2B2B_MOUSE, H2B3A_MOUSE, H2B3B_MOUSE	Histone H2B	GTKAVTKYTSSK	473	1.20E-03	424.2402	1269.6987	3	0.0058	12	0	GluC
H2B1A_MOUSE, H2B1B_MOUSE, H2B1C_MOUSE, H2B1F_MOUSE, H2B1H_MOUSE, H2B1M_MOUSE, H2B1P_MOUSE, H2B2B_MOUSE, H2B3A_MOUSE, H2B3B_MOUSE	Histone H2B	GTKAVTKYTSSK	473	3.20E-04	424.2402	1269.6986	3	0.0058	12	0	GluC
H2B1A_MOUSE, H2B1B_MOUSE, H2B1C_MOUSE, H2B1F_MOUSE, H2B1H_MOUSE, H2B1M_MOUSE, H2B1P_MOUSE, H2B2B_MOUSE, H2B3A_MOUSE, H2B3B_MOUSE	Histone H2B	GTKAVTKYTSSK	473	1.50E-06	424.2402	1269.6986	3	0.0058	12	0	GluC

H2B1A_MOUSE, H2B1B_MOUSE, H2B1C_MOUSE, H2B1F_MOUSE, H2B1H_MOUSE, H2B1M_MOUSE, H2B1P_MOUSE, H2B2B_MOUSE, H2B3A_MOUSE, H2B3B_MOUSE	Histone H2B	GTKAVTKYTSSK	473	1.60E-04	424.2401	1269.6986	3	0.0057	12	0	GluC
H2B1A_MOUSE, H2B1B_MOUSE, H2B1C_MOUSE, H2B1F_MOUSE, H2B1H_MOUSE, H2B1M_MOUSE, H2B1P_MOUSE, H2B2B_MOUSE, H2B3A_MOUSE, H2B3B_MOUSE	Histone H2B	GTKAVTKYTSSK	473	3.30E-04	424.2401	1269.6985	3	0.0056	12	0	GluC
H2B1A_MOUSE, H2B1B_MOUSE, H2B1C_MOUSE, H2B1F_MOUSE, H2B1H_MOUSE, H2B1M_MOUSE, H2B1P_MOUSE, H2B2B_MOUSE, H2B3A_MOUSE, H2B3B_MOUSE	Histone H2B	GTKAVTKYTSSK	473	2.70E-04	424.2401	1269.6985	3	0.0056	12	0	GluC
H2B1A_MOUSE, H2B1B_MOUSE, H2B1C_MOUSE, H2B1F_MOUSE, H2B1H_MOUSE, H2B1M_MOUSE, H2B1P_MOUSE, H2B2B_MOUSE, H2B3A_MOUSE, H2B3B_MOUSE	Histone H2B	GTKAVTKYTSSK	473	3.10E-08	424.24	1269.6982	3	0.0054	12	0	GluC
H2B1A_MOUSE, H2B1B_MOUSE, H2B1C_MOUSE, H2B1F_MOUSE, H2B1H_MOUSE, H2B1M_MOUSE, H2B1P_MOUSE, H2B2B_MOUSE, H2B3A_MOUSE, H2B3B_MOUSE	Histone H2B	GTKAVTKYTSSK	473	5.60E-05	424.24	1269.6981	3	0.0053	12	0	GluC
H2B1A_MOUSE, H2B1B_MOUSE, H2B1C_MOUSE, H2B1F_MOUSE, H2B1H_MOUSE, H2B1M_MOUSE, H2B1P_MOUSE, H2B2B_MOUSE, H2B3A_MOUSE, H2B3B_MOUSE	Histone H2B	GTKAVTKYTSSK	473	1.00E-07	424.24	1269.6981	3	0.0053	12	0	GluC
H2B1A_MOUSE, H2B1B_MOUSE, H2B1C_MOUSE, H2B1F_MOUSE, H2B1H_MOUSE, H2B1M_MOUSE, H2B1P_MOUSE, H2B2B_MOUSE, H2B3A_MOUSE, H2B3B_MOUSE	Histone H2B	GTKAVTKYTSSK	473	3.10E-04	424.24	1269.6981	3	0.0052	12	0	GluC
H2B1A_MOUSE, H2B1B_MOUSE, H2B1C_MOUSE, H2B1F_MOUSE, H2B1H_MOUSE, H2B1M_MOUSE, H2B1P_MOUSE, H2B2B_MOUSE, H2B3A_MOUSE, H2B3B_MOUSE	Histone H2B	GTKAVTKYTSSK	473	2.00E-05	424.24	1269.6981	3	0.0052	12	0	GluC
H2B1A_MOUSE, H2B1B_MOUSE, H2B1C_MOUSE, H2B1F_MOUSE, H2B1H_MOUSE, H2B1M_MOUSE, H2B1P_MOUSE, H2B2B_MOUSE, H2B3A_MOUSE, H2B3B_MOUSE	Histone H2B	GTKAVTKYTSSK	473	1.40E-05	635.8562	1269.6979	2	0.0051	12	0	GluC
H2B1A_MOUSE, H2B1B_MOUSE, H2B1C_MOUSE, H2B1F_MOUSE, H2B1H_MOUSE, H2B1M_MOUSE, H2B1P_MOUSE, H2B2B_MOUSE, H2B3A_MOUSE, H2B3B_MOUSE	Histone H2B	GTKAVTKYTSSK	473	4.10E-07	424.2399	1269.6979	3	0.0050	12	0	GluC
H2B1A_MOUSE, H2B1B_MOUSE, H2B1C_MOUSE, H2B1F_MOUSE, H2B1H_MOUSE, H2B1M_MOUSE, H2B1P_MOUSE, H2B2B_MOUSE, H2B3A_MOUSE, H2B3B_MOUSE	Histone H2B	GTKAVTKYTSSK	473	1.20E-03	424.2399	1269.6978	3	0.0049	12	0	GluC
H2B1A_MOUSE, H2B1B_MOUSE, H2B1C_MOUSE, H2B1F_MOUSE, H2B1H_MOUSE, H2B1M_MOUSE, H2B1P_MOUSE, H2B2B_MOUSE, H2B3A_MOUSE, H2B3B_MOUSE	Histone H2B	GTKAVTKYTSSK	473	3.20E-04	635.8561	1269.6977	2	0.0049	12	0	GluC
H2B1A_MOUSE, H2B1B_MOUSE, H2B1C_MOUSE, H2B1F_MOUSE, H2B1H_MOUSE, H2B1M_MOUSE, H2B1P_MOUSE, H2B2B_MOUSE, H2B3A_MOUSE, H2B3B_MOUSE	Histone H2B	GTKAVTKYTSSK	473	4.00E-03	635.8561	1269.6976	2	0.0048	12	0	GluC
H2B1A_MOUSE, H2B1B_MOUSE, H2B1C_MOUSE, H2B1F_MOUSE, H2B1H_MOUSE, H2B1M_MOUSE, H2B1P_MOUSE, H2B2B_MOUSE, H2B3A_MOUSE, H2B3B_MOUSE	Histone H2B	GTKAVTKYTSSK	473	3.70E-03	424.2398	1269.6977	3	0.0048	12	0	GluC
H2B1A_MOUSE, H2B1B_MOUSE, H2B1C_MOUSE, H2B1F_MOUSE, H2B1H_MOUSE, H2B1M_MOUSE, H2B1P_MOUSE, H2B2B_MOUSE, H2B3A_MOUSE, H2B3B_MOUSE	Histone H2B	GTKAVTKYTSSK	473	5.60E-04	635.8561	1269.6977	2	0.0048	12	0	GluC
H2B1A_MOUSE, H2B1B_MOUSE, H2B1C_MOUSE, H2B1F_MOUSE, H2B1H_MOUSE, H2B1M_MOUSE, H2B1P_MOUSE, H2B2B_MOUSE, H2B3A_MOUSE, H2B3B_MOUSE	Histone H2B	GTKAVTKYTSSK	473	3.70E-08	424.2398	1269.6977	3	0.0048	12	0	GluC
H2B1A_MOUSE, H2B1B_MOUSE, H2B1C_MOUSE, H2B1F_MOUSE, H2B1H_MOUSE, H2B1M_MOUSE, H2B1P_MOUSE, H2B2B_MOUSE, H2B3A_MOUSE, H2B3B_MOUSE	Histone H2B	GTKAVTKYTSSK	473	7.70E-07	424.2398	1269.6975	3	0.0047	12	0	GluC
H2B1A_MOUSE, H2B1B_MOUSE, H2B1C_MOUSE, H2B1F_MOUSE, H2B1H_MOUSE, H2B1M_MOUSE, H2B1P_MOUSE, H2B2B_MOUSE, H2B3A_MOUSE, H2B3B_MOUSE	Histone H2B	GTKAVTKYTSSK	473	2.70E-04	424.2398	1269.6974	3	0.0046	12	0	GluC
H2B1A_MOUSE, H2B1B_MOUSE, H2B1C_MOUSE, H2B1F_MOUSE, H2B1H_MOUSE, H2B1M_MOUSE, H2B1P_MOUSE, H2B2B_MOUSE, H2B3A_MOUSE, H2B3B_MOUSE	Histone H2B	GTKAVTKYTSSK	473	1.70E-05	424.2398	1269.6975	3	0.0046	12	0	GluC
H2B1A_MOUSE, H2B1B_MOUSE, H2B1C_MOUSE, H2B1F_MOUSE, H2B1H_MOUSE, H2B1M_MOUSE, H2B1P_MOUSE, H2B2B_MOUSE, H2B3A_MOUSE, H2B3B_MOUSE	Histone H2B	GTKAVTKYTSSK	473	1.90E-06	635.856	1269.6974	2	0.0046	12	0	GluC
H2B1A_MOUSE, H2B1B_MOUSE, H2B1C_MOUSE, H2B1F_MOUSE, H2B1H_MOUSE, H2B1M_MOUSE, H2B1P_MOUSE, H2B2B_MOUSE, H2B3A_MOUSE, H2B3B_MOUSE	Histone H2B	GTKAVTKYTSSK	473	8.40E-04	424.2397	1269.6974	3	0.0045	12	0	GluC
H2B1A_MOUSE, H2B1B_MOUSE, H2B1C_MOUSE, H2B1F_MOUSE, H2B1H_MOUSE, H2B1M_MOUSE, H2B1P_MOUSE, H2B2B_MOUSE, H2B3A_MOUSE, H2B3B_MOUSE	Histone H2B	GTKAVTKYTSSK	473	3.30E-05	424.2397	1269.6974	3	0.0045	12	0	GluC
H2B1A_MOUSE, H2B1B_MOUSE, H2B1C_MOUSE, H2B1F_MOUSE, H2B1H_MOUSE, H2B1M_MOUSE, H2B1P_MOUSE, H2B2B_MOUSE, H2B3A_MOUSE, H2B3B_MOUSE	Histone H2B	GTKAVTKYTSSK	473	5.70E-05	424.2397	1269.6973	3	0.0044	12	0	GluC
H2B1A_MOUSE, H2B1B_MOUSE, H2B1C_MOUSE, H2B1F_MOUSE, H2B1H_MOUSE, H2B1M_MOUSE, H2B1P_MOUSE, H2B2B_MOUSE, H2B3A_MOUSE, H2B3B_MOUSE	Histone H2B	GTKAVTKYTSSK	473	3.80E-05	424.2397	1269.6973	3	0.0044	12	0	GluC

H2B1A_MOUSE, H2B1B_MOUSE, H2B1C_MOUSE, H2B1F_MOUSE, H2B1H_MOUSE, H2B1M_MOUSE, H2B1P_MOUSE, H2B2B_MOUSE, H2B3A_MOUSE, H2B3B_MOUSE	Histone H2B	GTKAVTKYTSSK	473	2.80E-07	424.2397	1269.6973	3	0.0044	12	0	GluC
H2B1A_MOUSE, H2B1B_MOUSE, H2B1C_MOUSE, H2B1F_MOUSE, H2B1H_MOUSE, H2B1M_MOUSE, H2B1P_MOUSE, H2B2B_MOUSE, H2B3A_MOUSE, H2B3B_MOUSE	Histone H2B	GTKAVTKYTSSK	473	3.80E-03	424.2396	1269.6969	3	0.0041	12	0	GluC
H2B1A_MOUSE, H2B1B_MOUSE, H2B1C_MOUSE, H2B1F_MOUSE, H2B1H_MOUSE, H2B1M_MOUSE, H2B1P_MOUSE, H2B2B_MOUSE, H2B3A_MOUSE, H2B3B_MOUSE	Histone H2B	GTKAVTKYTSSK	473	4.50E-06	424.2396	1269.6969	3	0.0041	12	0	GluC
H2B1A_MOUSE, H2B1B_MOUSE, H2B1C_MOUSE, H2B1F_MOUSE, H2B1H_MOUSE, H2B1M_MOUSE, H2B1P_MOUSE, H2B2B_MOUSE, H2B3A_MOUSE, H2B3B_MOUSE	Histone H2B	GTKAVTKYTSSK	473	3.00E-06	424.2396	1269.6969	3	0.0041	12	0	GluC
H2B1A_MOUSE, H2B1B_MOUSE, H2B1C_MOUSE, H2B1F_MOUSE, H2B1H_MOUSE, H2B1M_MOUSE, H2B1P_MOUSE, H2B2B_MOUSE, H2B3A_MOUSE, H2B3B_MOUSE	Histone H2B	GTKAVTKYTSSK	473	4.20E-03	635.8557	1269.6968	2	0.0040	12	0	GluC
H2B1A_MOUSE, H2B1B_MOUSE, H2B1C_MOUSE, H2B1F_MOUSE, H2B1H_MOUSE, H2B1M_MOUSE, H2B1P_MOUSE, H2B2B_MOUSE, H2B3A_MOUSE, H2B3B_MOUSE	Histone H2B	GTKAVTKYTSSK	473	4.50E-04	635.8557	1269.6969	2	0.0040	12	0	GluC
H2B1A_MOUSE, H2B1B_MOUSE, H2B1C_MOUSE, H2B1F_MOUSE, H2B1H_MOUSE, H2B1M_MOUSE, H2B1P_MOUSE, H2B2B_MOUSE, H2B3A_MOUSE, H2B3B_MOUSE	Histone H2B	GTKAVTKYTSSK	473	2.20E-05	635.8557	1269.6968	2	0.0040	12	0	GluC
H2B1A_MOUSE, H2B1B_MOUSE, H2B1C_MOUSE, H2B1F_MOUSE, H2B1H_MOUSE, H2B1M_MOUSE, H2B1P_MOUSE, H2B2B_MOUSE, H2B3A_MOUSE, H2B3B_MOUSE	Histone H2B	GTKAVTKYTSSK	473	3.80E-03	424.2395	1269.6967	3	0.0039	12	0	GluC
H2B1A_MOUSE, H2B1B_MOUSE, H2B1C_MOUSE, H2B1F_MOUSE, H2B1H_MOUSE, H2B1M_MOUSE, H2B1P_MOUSE, H2B2B_MOUSE, H2B3A_MOUSE, H2B3B_MOUSE	Histone H2B	GTKAVTKYTSSK	473	2.20E-05	318.4315	1269.6967	4	0.0039	12	0	GluC
H2B1A_MOUSE, H2B1B_MOUSE, H2B1C_MOUSE, H2B1F_MOUSE, H2B1H_MOUSE, H2B1M_MOUSE, H2B1P_MOUSE, H2B2B_MOUSE, H2B3A_MOUSE, H2B3B_MOUSE	Histone H2B	GTKAVTKYTSSK	473	6.70E-07	635.8556	1269.6967	2	0.0039	12	0	GluC
H2B1A_MOUSE, H2B1B_MOUSE, H2B1C_MOUSE, H2B1F_MOUSE, H2B1H_MOUSE, H2B1M_MOUSE, H2B1P_MOUSE, H2B2B_MOUSE, H2B3A_MOUSE, H2B3B_MOUSE	Histone H2B	GTKAVTKYTSSK	473	9.70E-03	424.2395	1269.6967	3	0.0038	12	0	GluC
H2B1A_MOUSE, H2B1B_MOUSE, H2B1C_MOUSE, H2B1F_MOUSE, H2B1H_MOUSE, H2B1M_MOUSE, H2B1P_MOUSE, H2B2B_MOUSE, H2B3A_MOUSE, H2B3B_MOUSE	Histone H2B	GTKAVTKYTSSK	473	1.00E-03	424.2395	1269.6966	3	0.0038	12	0	GluC
H2B1A_MOUSE, H2B1B_MOUSE, H2B1C_MOUSE, H2B1F_MOUSE, H2B1H_MOUSE, H2B1M_MOUSE, H2B1P_MOUSE, H2B2B_MOUSE, H2B3A_MOUSE, H2B3B_MOUSE	Histone H2B	GTKAVTKYTSSK	473	1.90E-06	424.2395	1269.6967	3	0.0038	12	0	GluC
H2B1A_MOUSE, H2B1B_MOUSE, H2B1C_MOUSE, H2B1F_MOUSE, H2B1H_MOUSE, H2B1M_MOUSE, H2B1P_MOUSE, H2B2B_MOUSE, H2B3A_MOUSE, H2B3B_MOUSE	Histone H2B	GTKAVTKYTSSK	473	1.80E-06	424.2395	1269.6967	3	0.0038	12	0	GluC
H2B1A_MOUSE, H2B1B_MOUSE, H2B1C_MOUSE, H2B1F_MOUSE, H2B1H_MOUSE, H2B1M_MOUSE, H2B1P_MOUSE, H2B2B_MOUSE, H2B3A_MOUSE, H2B3B_MOUSE	Histone H2B	GTKAVTKYTSSK	473	4.60E-07	424.2395	1269.6967	3	0.0038	12	0	GluC
H2B1A_MOUSE, H2B1B_MOUSE, H2B1C_MOUSE, H2B1F_MOUSE, H2B1H_MOUSE, H2B1M_MOUSE, H2B1P_MOUSE, H2B2B_MOUSE, H2B3A_MOUSE, H2B3B_MOUSE	Histone H2B	GTKAVTKYTSSK	473	9.20E-09	318.4314	1269.6966	4	0.0038	12	0	GluC
H2B1A_MOUSE, H2B1B_MOUSE, H2B1C_MOUSE, H2B1F_MOUSE, H2B1H_MOUSE, H2B1M_MOUSE, H2B1P_MOUSE, H2B2B_MOUSE, H2B3A_MOUSE, H2B3B_MOUSE	Histone H2B	GTKAVTKYTSSK	473	2.60E-04	424.2395	1269.6966	3	0.0037	12	0	GluC
H2B1A_MOUSE, H2B1B_MOUSE, H2B1C_MOUSE, H2B1F_MOUSE, H2B1H_MOUSE, H2B1M_MOUSE, H2B1P_MOUSE, H2B2B_MOUSE, H2B3A_MOUSE, H2B3B_MOUSE	Histone H2B	GTKAVTKYTSSK	473	2.50E-03	635.8555	1269.6964	2	0.0036	12	0	GluC
H2B1A_MOUSE, H2B1B_MOUSE, H2B1C_MOUSE, H2B1F_MOUSE, H2B1H_MOUSE, H2B1M_MOUSE, H2B1P_MOUSE, H2B2B_MOUSE, H2B3A_MOUSE, H2B3B_MOUSE	Histone H2B	GTKAVTKYTSSK	473	1.90E-03	635.8555	1269.6964	2	0.0036	12	0	GluC
H2B1A_MOUSE, H2B1B_MOUSE, H2B1C_MOUSE, H2B1F_MOUSE, H2B1H_MOUSE, H2B1M_MOUSE, H2B1P_MOUSE, H2B2B_MOUSE, H2B3A_MOUSE, H2B3B_MOUSE	Histone H2B	GTKAVTKYTSSK	473	2.70E-04	424.2394	1269.6965	3	0.0036	12	0	GluC
H2B1A_MOUSE, H2B1B_MOUSE, H2B1C_MOUSE, H2B1F_MOUSE, H2B1H_MOUSE, H2B1M_MOUSE, H2B1P_MOUSE, H2B2B_MOUSE, H2B3A_MOUSE, H2B3B_MOUSE	Histone H2B	GTKAVTKYTSSK	473	1.80E-04	424.2394	1269.6965	3	0.0036	12	0	GluC
H2B1A_MOUSE, H2B1B_MOUSE, H2B1C_MOUSE, H2B1F_MOUSE, H2B1H_MOUSE, H2B1M_MOUSE, H2B1P_MOUSE, H2B2B_MOUSE, H2B3A_MOUSE, H2B3B_MOUSE	Histone H2B	GTKAVTKYTSSK	473	5.20E-05	635.8555	1269.6964	2	0.0036	12	0	GluC
H2B1A_MOUSE, H2B1B_MOUSE, H2B1C_MOUSE, H2B1F_MOUSE, H2B1H_MOUSE, H2B1M_MOUSE, H2B1P_MOUSE, H2B2B_MOUSE, H2B3A_MOUSE, H2B3B_MOUSE	Histone H2B	GTKAVTKYTSSK	473	3.20E-05	635.8555	1269.6964	2	0.0036	12	0	GluC
H2B1A_MOUSE, H2B1B_MOUSE, H2B1C_MOUSE, H2B1F_MOUSE, H2B1H_MOUSE, H2B1M_MOUSE, H2B1P_MOUSE, H2B2B_MOUSE, H2B3A_MOUSE, H2B3B_MOUSE	Histone H2B	GTKAVTKYTSSK	473	1.50E-06	424.2394	1269.6965	3	0.0036	12	0	GluC

H2B1A_MOUSE, H2B1B_MOUSE, H2B1C_MOUSE, H2B1F_MOUSE, H2B1H_MOUSE, H2B1M_MOUSE, H2B1P_MOUSE, H2B2B_MOUSE, H2B3A_MOUSE, H2B3B_MOUSE	Histone H2B	GTKAVTKYTSSK		473	1.50E-03	424.2394	1269.6964	3	0.0035	12	0	GluC
H2B1A_MOUSE, H2B1B_MOUSE, H2B1C_MOUSE, H2B1F_MOUSE, H2B1H_MOUSE, H2B1M_MOUSE, H2B1P_MOUSE, H2B2B_MOUSE, H2B3A_MOUSE, H2B3B_MOUSE	Histone H2B	GTKAVTKYTSSK		473	5.00E-04	424.2394	1269.6964	3	0.0035	12	0	GluC
H2B1A_MOUSE, H2B1B_MOUSE, H2B1C_MOUSE, H2B1F_MOUSE, H2B1H_MOUSE, H2B1M_MOUSE, H2B1P_MOUSE, H2B2B_MOUSE, H2B3A_MOUSE, H2B3B_MOUSE	Histone H2B	GTKAVTKYTSSK		473	3.90E-04	424.2394	1269.6964	3	0.0035	12	0	GluC
H2B1A_MOUSE, H2B1B_MOUSE, H2B1C_MOUSE, H2B1F_MOUSE, H2B1H_MOUSE, H2B1M_MOUSE, H2B1P_MOUSE, H2B2B_MOUSE, H2B3A_MOUSE, H2B3B_MOUSE	Histone H2B	GTKAVTKYTSSK		473	1.20E-04	318.4314	1269.6964	4	0.0035	12	0	GluC
H2B1A_MOUSE, H2B1B_MOUSE, H2B1C_MOUSE, H2B1F_MOUSE, H2B1H_MOUSE, H2B1M_MOUSE, H2B1P_MOUSE, H2B2B_MOUSE, H2B3A_MOUSE, H2B3B_MOUSE	Histone H2B	GTKAVTKYTSSK		473	3.50E-06	424.2394	1269.6964	3	0.0035	12	0	GluC
H2B1A_MOUSE, H2B1B_MOUSE, H2B1C_MOUSE, H2B1F_MOUSE, H2B1H_MOUSE, H2B1M_MOUSE, H2B1P_MOUSE, H2B2B_MOUSE, H2B3A_MOUSE, H2B3B_MOUSE	Histone H2B	GTKAVTKYTSSK		473	6.20E-07	424.2394	1269.6963	3	0.0035	12	0	GluC
H2B1A_MOUSE, H2B1B_MOUSE, H2B1C_MOUSE, H2B1F_MOUSE, H2B1H_MOUSE, H2B1M_MOUSE, H2B1P_MOUSE, H2B2B_MOUSE, H2B3A_MOUSE, H2B3B_MOUSE	Histone H2B	GTKAVTKYTSSK		473	1.20E-07	424.2394	1269.6963	3	0.0035	12	0	GluC
H2B1A_MOUSE, H2B1B_MOUSE, H2B1C_MOUSE, H2B1F_MOUSE, H2B1H_MOUSE, H2B1M_MOUSE, H2B1P_MOUSE, H2B2B_MOUSE, H2B3A_MOUSE, H2B3B_MOUSE	Histone H2B	GTKAVTKYTSSK		473	6.60E-09	424.2394	1269.6963	3	0.0035	12	0	GluC
H2B1A_MOUSE, H2B1B_MOUSE, H2B1C_MOUSE, H2B1F_MOUSE, H2B1H_MOUSE, H2B1M_MOUSE, H2B1P_MOUSE, H2B2B_MOUSE, H2B3A_MOUSE, H2B3B_MOUSE	Histone H2B	GTKAVTKYTSSK		473	2.20E-04	424.2394	1269.6962	3	0.0034	12	0	GluC
H2B1A_MOUSE, H2B1B_MOUSE, H2B1C_MOUSE, H2B1F_MOUSE, H2B1H_MOUSE, H2B1M_MOUSE, H2B1P_MOUSE, H2B2B_MOUSE, H2B3A_MOUSE, H2B3B_MOUSE	Histone H2B	GTKAVTKYTSSK		473	7.10E-05	318.4314	1269.6963	4	0.0034	12	0	GluC
H2B1A_MOUSE, H2B1B_MOUSE, H2B1C_MOUSE, H2B1F_MOUSE, H2B1H_MOUSE, H2B1M_MOUSE, H2B1P_MOUSE, H2B2B_MOUSE, H2B3A_MOUSE, H2B3B_MOUSE	Histone H2B	GTKAVTKYTSSK		473	5.00E-05	424.2393	1269.6962	3	0.0033	12	0	GluC
H2B1A_MOUSE, H2B1B_MOUSE, H2B1C_MOUSE, H2B1F_MOUSE, H2B1H_MOUSE, H2B1M_MOUSE, H2B1P_MOUSE, H2B2B_MOUSE, H2B3A_MOUSE, H2B3B_MOUSE	Histone H2B	GTKAVTKYTSSK		473	1.20E-04	318.4313	1269.6960	4	0.0032	12	0	GluC
H2B1A_MOUSE, H2B1B_MOUSE, H2B1C_MOUSE, H2B1F_MOUSE, H2B1H_MOUSE, H2B1M_MOUSE, H2B1P_MOUSE, H2B2B_MOUSE, H2B3A_MOUSE, H2B3B_MOUSE	Histone H2B	GTKAVTKYTSSK		473	2.10E-05	424.2393	1269.6960	3	0.0032	12	0	GluC
H2B1A_MOUSE, H2B1B_MOUSE, H2B1C_MOUSE, H2B1F_MOUSE, H2B1H_MOUSE, H2B1M_MOUSE, H2B1P_MOUSE, H2B2B_MOUSE, H2B3A_MOUSE, H2B3B_MOUSE	Histone H2B	GTKAVTKYTSSK		473	1.90E-07	318.4313	1269.6961	4	0.0032	12	0	GluC
H2B1A_MOUSE, H2B1B_MOUSE, H2B1C_MOUSE, H2B1F_MOUSE, H2B1H_MOUSE, H2B1M_MOUSE, H2B1P_MOUSE, H2B2B_MOUSE, H2B3A_MOUSE, H2B3B_MOUSE	Histone H2B	GTKAVTKYTSSK		473	4.60E-03	424.2393	1269.6960	3	0.0031	12	0	GluC
H2B1A_MOUSE, H2B1B_MOUSE, H2B1C_MOUSE, H2B1F_MOUSE, H2B1H_MOUSE, H2B1M_MOUSE, H2B1P_MOUSE, H2B2B_MOUSE, H2B3A_MOUSE, H2B3B_MOUSE	Histone H2B	GTKAVTKYTSSK		473	1.60E-03	424.2393	1269.6959	3	0.0031	12	0	GluC
H2B1A_MOUSE, H2B1B_MOUSE, H2B1C_MOUSE, H2B1F_MOUSE, H2B1H_MOUSE, H2B1M_MOUSE, H2B1P_MOUSE, H2B2B_MOUSE, H2B3A_MOUSE, H2B3B_MOUSE	Histone H2B	GTKAVTKYTSSK		473	1.20E-03	424.2393	1269.6960	3	0.0031	12	0	GluC
H2B1A_MOUSE, H2B1B_MOUSE, H2B1C_MOUSE, H2B1F_MOUSE, H2B1H_MOUSE, H2B1M_MOUSE, H2B1P_MOUSE, H2B2B_MOUSE, H2B3A_MOUSE, H2B3B_MOUSE	Histone H2B	GTKAVTKYTSSK		473	6.20E-04	635.8552	1269.6959	2	0.0031	12	0	GluC
H2B1A_MOUSE, H2B1B_MOUSE, H2B1C_MOUSE, H2B1F_MOUSE, H2B1H_MOUSE, H2B1M_MOUSE, H2B1P_MOUSE, H2B2B_MOUSE, H2B3A_MOUSE, H2B3B_MOUSE	Histone H2B	GTKAVTKYTSSK		473	5.00E-06	424.2393	1269.6960	3	0.0031	12	0	GluC
H2B1A_MOUSE, H2B1B_MOUSE, H2B1C_MOUSE, H2B1F_MOUSE, H2B1H_MOUSE, H2B1M_MOUSE, H2B1P_MOUSE, H2B2B_MOUSE, H2B3A_MOUSE, H2B3B_MOUSE	Histone H2B	GTKAVTKYTSSK		473	5.60E-08	424.2393	1269.6959	3	0.0031	12	0	GluC
H2B1A_MOUSE, H2B1B_MOUSE, H2B1C_MOUSE, H2B1F_MOUSE, H2B1H_MOUSE, H2B1M_MOUSE, H2B1P_MOUSE, H2B2B_MOUSE, H2B3A_MOUSE, H2B3B_MOUSE	Histone H2B	GTKAVTKYTSSK		473	2.70E-03	318.4312	1269.6959	4	0.0030	12	0	GluC
H2B1A_MOUSE, H2B1B_MOUSE, H2B1C_MOUSE, H2B1F_MOUSE, H2B1H_MOUSE, H2B1M_MOUSE, H2B1P_MOUSE, H2B2B_MOUSE, H2B3A_MOUSE, H2B3B_MOUSE	Histone H2B	GTKAVTKYTSSK		473	9.60E-05	424.2392	1269.6959	3	0.0030	12	0	GluC
H2B1A_MOUSE, H2B1B_MOUSE, H2B1C_MOUSE, H2B1F_MOUSE, H2B1H_MOUSE, H2B1M_MOUSE, H2B1P_MOUSE, H2B2B_MOUSE, H2B3A_MOUSE, H2B3B_MOUSE	Histone H2B	GTKAVTKYTSSK		473	3.60E-06	635.8552	1269.6959	2	0.0030	12	0	GluC
H2B1A_MOUSE, H2B1B_MOUSE, H2B1C_MOUSE, H2B1F_MOUSE, H2B1H_MOUSE, H2B1M_MOUSE, H2B1P_MOUSE, H2B2B_MOUSE, H2B3A_MOUSE, H2B3B_MOUSE	Histone H2B	GTKAVTKYTSSK		473	3.90E-03	635.8551	1269.6956	2	0.0028	12	0	GluC

H2B1A_MOUSE, H2B1B_MOUSE, H2B1C_MOUSE, H2B1F_MOUSE, H2B1H_MOUSE, H2B1M_MOUSE, H2B1P_MOUSE, H2B2B_MOUSE, H2B3A_MOUSE, H2B3B_MOUSE	Histone H2B	GTKAVTKYTSSK	473	9.80E-05	424.2392	1269.6957	3	0.0028	12	0	GluC
H2B1A_MOUSE, H2B1B_MOUSE, H2B1C_MOUSE, H2B1F_MOUSE, H2B1H_MOUSE, H2B1M_MOUSE, H2B1P_MOUSE, H2B2B_MOUSE, H2B3A_MOUSE, H2B3B_MOUSE	Histone H2B	GTKAVTKYTSSK	473	8.40E-07	424.2392	1269.6957	3	0.0028	12	0	GluC
H2B1A_MOUSE, H2B1B_MOUSE, H2B1C_MOUSE, H2B1F_MOUSE, H2B1H_MOUSE, H2B1M_MOUSE, H2B1P_MOUSE, H2B2B_MOUSE, H2B3A_MOUSE, H2B3B_MOUSE	Histone H2B	GTKAVTKYTSSK	473	2.80E-04	635.855	1269.6955	2	0.0027	12	0	GluC
H2B1A_MOUSE, H2B1B_MOUSE, H2B1C_MOUSE, H2B1F_MOUSE, H2B1H_MOUSE, H2B1M_MOUSE, H2B1P_MOUSE, H2B2B_MOUSE, H2B3A_MOUSE, H2B3B_MOUSE	Histone H2B	GTKAVTKYTSSK	473	1.50E-04	635.855	1269.6955	2	0.0027	12	0	GluC
H2B1A_MOUSE, H2B1B_MOUSE, H2B1C_MOUSE, H2B1F_MOUSE, H2B1H_MOUSE, H2B1M_MOUSE, H2B1P_MOUSE, H2B2B_MOUSE, H2B3A_MOUSE, H2B3B_MOUSE	Histone H2B	GTKAVTKYTSSK	473	8.00E-06	635.8551	1269.6956	2	0.0027	12	0	GluC
H2B1A_MOUSE, H2B1B_MOUSE, H2B1C_MOUSE, H2B1F_MOUSE, H2B1H_MOUSE, H2B1M_MOUSE, H2B1P_MOUSE, H2B2B_MOUSE, H2B3A_MOUSE, H2B3B_MOUSE	Histone H2B	GTKAVTKYTSSK	473	3.20E-06	424.2391	1269.6955	3	0.0027	12	0	GluC
H2B1A_MOUSE, H2B1B_MOUSE, H2B1C_MOUSE, H2B1F_MOUSE, H2B1H_MOUSE, H2B1M_MOUSE, H2B1P_MOUSE, H2B2B_MOUSE, H2B3A_MOUSE, H2B3B_MOUSE	Histone H2B	GTKAVTKYTSSK	473	8.90E-08	424.2391	1269.6955	3	0.0027	12	0	GluC
H2B1A_MOUSE, H2B1B_MOUSE, H2B1C_MOUSE, H2B1F_MOUSE, H2B1H_MOUSE, H2B1M_MOUSE, H2B1P_MOUSE, H2B2B_MOUSE, H2B3A_MOUSE, H2B3B_MOUSE	Histone H2B	GTKAVTKYTSSK	473	3.30E-03	424.2391	1269.6954	3	0.0026	12	0	GluC
H2B1A_MOUSE, H2B1B_MOUSE, H2B1C_MOUSE, H2B1F_MOUSE, H2B1H_MOUSE, H2B1M_MOUSE, H2B1P_MOUSE, H2B2B_MOUSE, H2B3A_MOUSE, H2B3B_MOUSE	Histone H2B	GTKAVTKYTSSK	473	3.80E-05	318.4312	1269.6955	4	0.0026	12	0	GluC
H2B1A_MOUSE, H2B1B_MOUSE, H2B1C_MOUSE, H2B1F_MOUSE, H2B1H_MOUSE, H2B1M_MOUSE, H2B1P_MOUSE, H2B2B_MOUSE, H2B3A_MOUSE, H2B3B_MOUSE	Histone H2B	GTKAVTKYTSSK	473	4.80E-03	424.2391	1269.6953	3	0.0025	12	0	GluC
H2B1A_MOUSE, H2B1B_MOUSE, H2B1C_MOUSE, H2B1F_MOUSE, H2B1H_MOUSE, H2B1M_MOUSE, H2B1P_MOUSE, H2B2B_MOUSE, H2B3A_MOUSE, H2B3B_MOUSE	Histone H2B	GTKAVTKYTSSK	473	2.40E-03	635.8549	1269.6953	2	0.0025	12	0	GluC
H2B1A_MOUSE, H2B1B_MOUSE, H2B1C_MOUSE, H2B1F_MOUSE, H2B1H_MOUSE, H2B1M_MOUSE, H2B1P_MOUSE, H2B2B_MOUSE, H2B3A_MOUSE, H2B3B_MOUSE	Histone H2B	GTKAVTKYTSSK	473	6.80E-07	424.2391	1269.6953	3	0.0025	12	0	GluC
H2B1A_MOUSE, H2B1B_MOUSE, H2B1C_MOUSE, H2B1F_MOUSE, H2B1H_MOUSE, H2B1M_MOUSE, H2B1P_MOUSE, H2B2B_MOUSE, H2B3A_MOUSE, H2B3B_MOUSE	Histone H2B	GTKAVTKYTSSK	473	1.70E-05	635.8549	1269.6953	2	0.0024	12	0	GluC
H2B1A_MOUSE, H2B1B_MOUSE, H2B1C_MOUSE, H2B1F_MOUSE, H2B1H_MOUSE, H2B1M_MOUSE, H2B1P_MOUSE, H2B2B_MOUSE, H2B3A_MOUSE, H2B3B_MOUSE	Histone H2B	GTKAVTKYTSSK	473	1.70E-04	424.239	1269.6951	3	0.0023	12	0	GluC
H2B1A_MOUSE, H2B1B_MOUSE, H2B1C_MOUSE, H2B1F_MOUSE, H2B1H_MOUSE, H2B1M_MOUSE, H2B1P_MOUSE, H2B2B_MOUSE, H2B3A_MOUSE, H2B3B_MOUSE	Histone H2B	GTKAVTKYTSSK	473	1.60E-05	424.239	1269.6952	3	0.0023	12	0	GluC
H2B1A_MOUSE, H2B1B_MOUSE, H2B1C_MOUSE, H2B1F_MOUSE, H2B1H_MOUSE, H2B1M_MOUSE, H2B1P_MOUSE, H2B2B_MOUSE, H2B3A_MOUSE, H2B3B_MOUSE	Histone H2B	GTKAVTKYTSSK	473	6.10E-06	635.8548	1269.6951	2	0.0023	12	0	GluC
H2B1A_MOUSE, H2B1B_MOUSE, H2B1C_MOUSE, H2B1F_MOUSE, H2B1H_MOUSE, H2B1M_MOUSE, H2B1P_MOUSE, H2B2B_MOUSE, H2B3A_MOUSE, H2B3B_MOUSE	Histone H2B	GTKAVTKYTSSK	473	6.50E-09	424.239	1269.6952	3	0.0023	12	0	GluC
H2B1A_MOUSE, H2B1B_MOUSE, H2B1C_MOUSE, H2B1F_MOUSE, H2B1H_MOUSE, H2B1M_MOUSE, H2B1P_MOUSE, H2B2B_MOUSE, H2B3A_MOUSE, H2B3B_MOUSE	Histone H2B	GTKAVTKYTSSK	473	1.10E-09	424.239	1269.6952	3	0.0023	12	0	GluC
H2B1A_MOUSE, H2B1B_MOUSE, H2B1C_MOUSE, H2B1F_MOUSE, H2B1H_MOUSE, H2B1M_MOUSE, H2B1P_MOUSE, H2B2B_MOUSE, H2B3A_MOUSE, H2B3B_MOUSE	Histone H2B	GTKAVTKYTSSK	473	2.90E-03	318.431	1269.6951	4	0.0022	12	0	GluC
H2B1A_MOUSE, H2B1B_MOUSE, H2B1C_MOUSE, H2B1F_MOUSE, H2B1H_MOUSE, H2B1M_MOUSE, H2B1P_MOUSE, H2B2B_MOUSE, H2B3A_MOUSE, H2B3B_MOUSE	Histone H2B	GTKAVTKYTSSK	473	4.10E-06	635.8548	1269.6950	2	0.0022	12	0	GluC
H2B1A_MOUSE, H2B1B_MOUSE, H2B1C_MOUSE, H2B1F_MOUSE, H2B1H_MOUSE, H2B1M_MOUSE, H2B1P_MOUSE, H2B2B_MOUSE, H2B3A_MOUSE, H2B3B_MOUSE	Histone H2B	GTKAVTKYTSSK	473	3.50E-06	424.239	1269.6951	3	0.0022	12	0	GluC
H2B1A_MOUSE, H2B1B_MOUSE, H2B1C_MOUSE, H2B1F_MOUSE, H2B1H_MOUSE, H2B1M_MOUSE, H2B1P_MOUSE, H2B2B_MOUSE, H2B3A_MOUSE, H2B3B_MOUSE	Histone H2B	GTKAVTKYTSSK	473	2.10E-06	635.8548	1269.6950	2	0.0022	12	0	GluC
H2B1A_MOUSE, H2B1B_MOUSE, H2B1C_MOUSE, H2B1F_MOUSE, H2B1H_MOUSE, H2B1M_MOUSE, H2B1P_MOUSE, H2B2B_MOUSE, H2B3A_MOUSE, H2B3B_MOUSE	Histone H2B	GTKAVTKYTSSK	473	1.30E-06	635.8548	1269.6950	2	0.0022	12	0	GluC
H2B1A_MOUSE, H2B1B_MOUSE, H2B1C_MOUSE, H2B1F_MOUSE, H2B1H_MOUSE, H2B1M_MOUSE, H2B1P_MOUSE, H2B2B_MOUSE, H2B3A_MOUSE, H2B3B_MOUSE	Histone H2B	GTKAVTKYTSSK	473	5.00E-07	424.2389	1269.6950	3	0.0022	12	0	GluC

H2B1A_MOUSE, H2B1B_MOUSE, H2B1C_MOUSE, H2B1F_MOUSE, H2B1H_MOUSE, H2B1M_MOUSE, H2B1P_MOUSE, H2B2B_MOUSE, H2B3A_MOUSE, H2B3B_MOUSE	Histone H2B	GTKAVTKYTSSK	473	8.30E-03	424.2389	1269.6950	3	0.0021	12	0	GluC
H2B1A_MOUSE, H2B1B_MOUSE, H2B1C_MOUSE, H2B1F_MOUSE, H2B1H_MOUSE, H2B1M_MOUSE, H2B1P_MOUSE, H2B2B_MOUSE, H2B3A_MOUSE, H2B3B_MOUSE	Histone H2B	GTKAVTKYTSSK	473	2.90E-04	424.2389	1269.6949	3	0.0021	12	0	GluC
H2B1A_MOUSE, H2B1B_MOUSE, H2B1C_MOUSE, H2B1F_MOUSE, H2B1H_MOUSE, H2B1M_MOUSE, H2B1P_MOUSE, H2B2B_MOUSE, H2B3A_MOUSE, H2B3B_MOUSE	Histone H2B	GTKAVTKYTSSK	473	9.70E-05	318.431	1269.6949	4	0.0021	12	0	GluC
H2B1A_MOUSE, H2B1B_MOUSE, H2B1C_MOUSE, H2B1F_MOUSE, H2B1H_MOUSE, H2B1M_MOUSE, H2B1P_MOUSE, H2B2B_MOUSE, H2B3A_MOUSE, H2B3B_MOUSE	Histone H2B	GTKAVTKYTSSK	473	1.30E-04	318.431	1269.6949	4	0.0020	12	0	GluC
H2B1A_MOUSE, H2B1B_MOUSE, H2B1C_MOUSE, H2B1F_MOUSE, H2B1H_MOUSE, H2B1M_MOUSE, H2B1P_MOUSE, H2B2B_MOUSE, H2B3A_MOUSE, H2B3B_MOUSE	Histone H2B	GTKAVTKYTSSK	473	2.70E-05	424.2389	1269.6948	3	0.0020	12	0	GluC
H2B1A_MOUSE, H2B1B_MOUSE, H2B1C_MOUSE, H2B1F_MOUSE, H2B1H_MOUSE, H2B1M_MOUSE, H2B1P_MOUSE, H2B2B_MOUSE, H2B3A_MOUSE, H2B3B_MOUSE	Histone H2B	GTKAVTKYTSSK	473	1.10E-05	424.2389	1269.6948	3	0.0020	12	0	GluC
H2B1A_MOUSE, H2B1B_MOUSE, H2B1C_MOUSE, H2B1F_MOUSE, H2B1H_MOUSE, H2B1M_MOUSE, H2B1P_MOUSE, H2B2B_MOUSE, H2B3A_MOUSE, H2B3B_MOUSE	Histone H2B	GTKAVTKYTSSK	473	2.20E-03	635.8547	1269.6948	2	0.0019	12	0	GluC
H2B1A_MOUSE, H2B1B_MOUSE, H2B1C_MOUSE, H2B1F_MOUSE, H2B1H_MOUSE, H2B1M_MOUSE, H2B1P_MOUSE, H2B2B_MOUSE, H2B3A_MOUSE, H2B3B_MOUSE	Histone H2B	GTKAVTKYTSSK	473	1.30E-03	635.8546	1269.6947	2	0.0019	12	0	GluC
H2B1A_MOUSE, H2B1B_MOUSE, H2B1C_MOUSE, H2B1F_MOUSE, H2B1H_MOUSE, H2B1M_MOUSE, H2B1P_MOUSE, H2B2B_MOUSE, H2B3A_MOUSE, H2B3B_MOUSE	Histone H2B	GTKAVTKYTSSK	473	3.00E-05	318.431	1269.6947	4	0.0019	12	0	GluC
H2B1A_MOUSE, H2B1B_MOUSE, H2B1C_MOUSE, H2B1F_MOUSE, H2B1H_MOUSE, H2B1M_MOUSE, H2B1P_MOUSE, H2B2B_MOUSE, H2B3A_MOUSE, H2B3B_MOUSE	Histone H2B	GTKAVTKYTSSK	473	1.60E-07	635.8547	1269.6948	2	0.0019	12	0	GluC
H2B1A_MOUSE, H2B1B_MOUSE, H2B1C_MOUSE, H2B1F_MOUSE, H2B1H_MOUSE, H2B1M_MOUSE, H2B1P_MOUSE, H2B2B_MOUSE, H2B3A_MOUSE, H2B3B_MOUSE	Histone H2B	GTKAVTKYTSSK	473	3.30E-03	635.8546	1269.6947	2	0.0018	12	0	GluC
H2B1A_MOUSE, H2B1B_MOUSE, H2B1C_MOUSE, H2B1F_MOUSE, H2B1H_MOUSE, H2B1M_MOUSE, H2B1P_MOUSE, H2B2B_MOUSE, H2B3A_MOUSE, H2B3B_MOUSE	Histone H2B	GTKAVTKYTSSK	473	1.40E-03	424.2388	1269.6947	3	0.0018	12	0	GluC
H2B1A_MOUSE, H2B1B_MOUSE, H2B1C_MOUSE, H2B1F_MOUSE, H2B1H_MOUSE, H2B1M_MOUSE, H2B1P_MOUSE, H2B2B_MOUSE, H2B3A_MOUSE, H2B3B_MOUSE	Histone H2B	GTKAVTKYTSSK	473	4.00E-04	424.2388	1269.6947	3	0.0018	12	0	GluC
H2B1A_MOUSE, H2B1B_MOUSE, H2B1C_MOUSE, H2B1F_MOUSE, H2B1H_MOUSE, H2B1M_MOUSE, H2B1P_MOUSE, H2B2B_MOUSE, H2B3A_MOUSE, H2B3B_MOUSE	Histone H2B	GTKAVTKYTSSK	473	7.50E-05	424.2388	1269.6946	3	0.0018	12	0	GluC
H2B1A_MOUSE, H2B1B_MOUSE, H2B1C_MOUSE, H2B1F_MOUSE, H2B1H_MOUSE, H2B1M_MOUSE, H2B1P_MOUSE, H2B2B_MOUSE, H2B3A_MOUSE, H2B3B_MOUSE	Histone H2B	GTKAVTKYTSSK	473	2.90E-05	318.4309	1269.6946	4	0.0018	12	0	GluC
H2B1A_MOUSE, H2B1B_MOUSE, H2B1C_MOUSE, H2B1F_MOUSE, H2B1H_MOUSE, H2B1M_MOUSE, H2B1P_MOUSE, H2B2B_MOUSE, H2B3A_MOUSE, H2B3B_MOUSE	Histone H2B	GTKAVTKYTSSK	473	1.80E-06	424.2388	1269.6946	3	0.0018	12	0	GluC
H2B1A_MOUSE, H2B1B_MOUSE, H2B1C_MOUSE, H2B1F_MOUSE, H2B1H_MOUSE, H2B1M_MOUSE, H2B1P_MOUSE, H2B2B_MOUSE, H2B3A_MOUSE, H2B3B_MOUSE	Histone H2B	GTKAVTKYTSSK	473	2.70E-07	424.2388	1269.6947	3	0.0018	12	0	GluC
H2B1A_MOUSE, H2B1B_MOUSE, H2B1C_MOUSE, H2B1F_MOUSE, H2B1H_MOUSE, H2B1M_MOUSE, H2B1P_MOUSE, H2B2B_MOUSE, H2B3A_MOUSE, H2B3B_MOUSE	Histone H2B	GTKAVTKYTSSK	473	1.90E-08	424.2388	1269.6947	3	0.0018	12	0	GluC
H2B1A_MOUSE, H2B1B_MOUSE, H2B1C_MOUSE, H2B1F_MOUSE, H2B1H_MOUSE, H2B1M_MOUSE, H2B1P_MOUSE, H2B2B_MOUSE, H2B3A_MOUSE, H2B3B_MOUSE	Histone H2B	GTKAVTKYTSSK	473	1.30E-08	318.4309	1269.6946	4	0.0018	12	0	GluC
H2B1A_MOUSE, H2B1B_MOUSE, H2B1C_MOUSE, H2B1F_MOUSE, H2B1H_MOUSE, H2B1M_MOUSE, H2B1P_MOUSE, H2B2B_MOUSE, H2B3A_MOUSE, H2B3B_MOUSE	Histone H2B	GTKAVTKYTSSK	473	8.40E-03	424.2388	1269.6945	3	0.0017	12	0	GluC
H2B1A_MOUSE, H2B1B_MOUSE, H2B1C_MOUSE, H2B1F_MOUSE, H2B1H_MOUSE, H2B1M_MOUSE, H2B1P_MOUSE, H2B2B_MOUSE, H2B3A_MOUSE, H2B3B_MOUSE	Histone H2B	GTKAVTKYTSSK	473	1.90E-03	424.2388	1269.6945	3	0.0017	12	0	GluC
H2B1A_MOUSE, H2B1B_MOUSE, H2B1C_MOUSE, H2B1F_MOUSE, H2B1H_MOUSE, H2B1M_MOUSE, H2B1P_MOUSE, H2B2B_MOUSE, H2B3A_MOUSE, H2B3B_MOUSE	Histone H2B	GTKAVTKYTSSK	473	1.90E-05	318.4309	1269.6945	4	0.0017	12	0	GluC
H2B1A_MOUSE, H2B1B_MOUSE, H2B1C_MOUSE, H2B1F_MOUSE, H2B1H_MOUSE, H2B1M_MOUSE, H2B1P_MOUSE, H2B2B_MOUSE, H2B3A_MOUSE, H2B3B_MOUSE	Histone H2B	GTKAVTKYTSSK	473	1.20E-07	424.2388	1269.6945	3	0.0017	12	0	GluC
H2B1A_MOUSE, H2B1B_MOUSE, H2B1C_MOUSE, H2B1F_MOUSE, H2B1H_MOUSE, H2B1M_MOUSE, H2B1P_MOUSE, H2B2B_MOUSE, H2B3A_MOUSE, H2B3B_MOUSE	Histone H2B	GTKAVTKYTSSK	473	8.60E-08	424.2388	1269.6946	3	0.0017	12	0	GluC

H2B1A_MOUSE, H2B1B_MOUSE, H2B1C_MOUSE, H2B1F_MOUSE, H2B1H_MOUSE, H2B1M_MOUSE, H2B1P_MOUSE, H2B2B_MOUSE, H2B3A_MOUSE, H2B3B_MOUSE	Histone H2B	GTKAVTKYTSSK	473	1.30E-08	318.4309	1269.6945	4	0.0017	12	0	GluC
H2B1A_MOUSE, H2B1B_MOUSE, H2B1C_MOUSE, H2B1F_MOUSE, H2B1H_MOUSE, H2B1M_MOUSE, H2B1P_MOUSE, H2B2B_MOUSE, H2B3A_MOUSE, H2B3B_MOUSE	Histone H2B	GTKAVTKYTSSK	473	6.30E-03	635.8545	1269.6944	2	0.0016	12	0	GluC
H2B1A_MOUSE, H2B1B_MOUSE, H2B1C_MOUSE, H2B1F_MOUSE, H2B1H_MOUSE, H2B1M_MOUSE, H2B1P_MOUSE, H2B2B_MOUSE, H2B3A_MOUSE, H2B3B_MOUSE	Histone H2B	GTKAVTKYTSSK	473	1.30E-03	424.2387	1269.6944	3	0.0016	12	0	GluC
H2B1A_MOUSE, H2B1B_MOUSE, H2B1C_MOUSE, H2B1F_MOUSE, H2B1H_MOUSE, H2B1M_MOUSE, H2B1P_MOUSE, H2B2B_MOUSE, H2B3A_MOUSE, H2B3B_MOUSE	Histone H2B	GTKAVTKYTSSK	473	3.10E-04	318.4309	1269.6944	4	0.0016	12	0	GluC
H2B1A_MOUSE, H2B1B_MOUSE, H2B1C_MOUSE, H2B1F_MOUSE, H2B1H_MOUSE, H2B1M_MOUSE, H2B1P_MOUSE, H2B2B_MOUSE, H2B3A_MOUSE, H2B3B_MOUSE	Histone H2B	GTKAVTKYTSSK	473	5.40E-05	635.8545	1269.6944	2	0.0016	12	0	GluC
H2B1A_MOUSE, H2B1B_MOUSE, H2B1C_MOUSE, H2B1F_MOUSE, H2B1H_MOUSE, H2B1M_MOUSE, H2B1P_MOUSE, H2B2B_MOUSE, H2B3A_MOUSE, H2B3B_MOUSE	Histone H2B	GTKAVTKYTSSK	473	6.70E-09	318.4309	1269.6944	4	0.0016	12	0	GluC
H2B1A_MOUSE, H2B1B_MOUSE, H2B1C_MOUSE, H2B1F_MOUSE, H2B1H_MOUSE, H2B1M_MOUSE, H2B1P_MOUSE, H2B2B_MOUSE, H2B3A_MOUSE, H2B3B_MOUSE	Histone H2B	GTKAVTKYTSSK	473	5.80E-10	424.2387	1269.6944	3	0.0016	12	0	GluC
H2B1A_MOUSE, H2B1B_MOUSE, H2B1C_MOUSE, H2B1F_MOUSE, H2B1H_MOUSE, H2B1M_MOUSE, H2B1P_MOUSE, H2B2B_MOUSE, H2B3A_MOUSE, H2B3B_MOUSE	Histone H2B	GTKAVTKYTSSK	473	4.10E-03	635.8545	1269.6944	2	0.0015	12	0	GluC
H2B1A_MOUSE, H2B1B_MOUSE, H2B1C_MOUSE, H2B1F_MOUSE, H2B1H_MOUSE, H2B1M_MOUSE, H2B1P_MOUSE, H2B2B_MOUSE, H2B3A_MOUSE, H2B3B_MOUSE	Histone H2B	GTKAVTKYTSSK	473	3.80E-05	424.2387	1269.6944	3	0.0015	12	0	GluC
H2B1A_MOUSE, H2B1B_MOUSE, H2B1C_MOUSE, H2B1F_MOUSE, H2B1H_MOUSE, H2B1M_MOUSE, H2B1P_MOUSE, H2B2B_MOUSE, H2B3A_MOUSE, H2B3B_MOUSE	Histone H2B	GTKAVTKYTSSK	473	1.60E-05	318.4309	1269.6944	4	0.0015	12	0	GluC
H2B1A_MOUSE, H2B1B_MOUSE, H2B1C_MOUSE, H2B1F_MOUSE, H2B1H_MOUSE, H2B1M_MOUSE, H2B1P_MOUSE, H2B2B_MOUSE, H2B3A_MOUSE, H2B3B_MOUSE	Histone H2B	GTKAVTKYTSSK	473	1.20E-05	318.4309	1269.6943	4	0.0015	12	0	GluC
H2B1A_MOUSE, H2B1B_MOUSE, H2B1C_MOUSE, H2B1F_MOUSE, H2B1H_MOUSE, H2B1M_MOUSE, H2B1P_MOUSE, H2B2B_MOUSE, H2B3A_MOUSE, H2B3B_MOUSE	Histone H2B	GTKAVTKYTSSK	473	8.60E-08	424.2387	1269.6944	3	0.0015	12	0	GluC
H2B1A_MOUSE, H2B1B_MOUSE, H2B1C_MOUSE, H2B1F_MOUSE, H2B1H_MOUSE, H2B1M_MOUSE, H2B1P_MOUSE, H2B2B_MOUSE, H2B3A_MOUSE, H2B3B_MOUSE	Histone H2B	GTKAVTKYTSSK	473	2.10E-03	318.4309	1269.6943	4	0.0014	12	0	GluC
H2B1A_MOUSE, H2B1B_MOUSE, H2B1C_MOUSE, H2B1F_MOUSE, H2B1H_MOUSE, H2B1M_MOUSE, H2B1P_MOUSE, H2B2B_MOUSE, H2B3A_MOUSE, H2B3B_MOUSE	Histone H2B	GTKAVTKYTSSK	473	6.80E-05	424.2387	1269.6943	3	0.0014	12	0	GluC
H2B1A_MOUSE, H2B1B_MOUSE, H2B1C_MOUSE, H2B1F_MOUSE, H2B1H_MOUSE, H2B1M_MOUSE, H2B1P_MOUSE, H2B2B_MOUSE, H2B3A_MOUSE, H2B3B_MOUSE	Histone H2B	GTKAVTKYTSSK	473	4.80E-05	635.8544	1269.6942	2	0.0014	12	0	GluC
H2B1A_MOUSE, H2B1B_MOUSE, H2B1C_MOUSE, H2B1F_MOUSE, H2B1H_MOUSE, H2B1M_MOUSE, H2B1P_MOUSE, H2B2B_MOUSE, H2B3A_MOUSE, H2B3B_MOUSE	Histone H2B	GTKAVTKYTSSK	473	2.00E-05	318.4309	1269.6943	4	0.0014	12	0	GluC
H2B1A_MOUSE, H2B1B_MOUSE, H2B1C_MOUSE, H2B1F_MOUSE, H2B1H_MOUSE, H2B1M_MOUSE, H2B1P_MOUSE, H2B2B_MOUSE, H2B3A_MOUSE, H2B3B_MOUSE	Histone H2B	GTKAVTKYTSSK	473	2.50E-06	635.8544	1269.6943	2	0.0014	12	0	GluC
H2B1A_MOUSE, H2B1B_MOUSE, H2B1C_MOUSE, H2B1F_MOUSE, H2B1H_MOUSE, H2B1M_MOUSE, H2B1P_MOUSE, H2B2B_MOUSE, H2B3A_MOUSE, H2B3B_MOUSE	Histone H2B	GTKAVTKYTSSK	473	4.40E-08	318.4308	1269.6943	4	0.0014	12	0	GluC
H2B1A_MOUSE, H2B1B_MOUSE, H2B1C_MOUSE, H2B1F_MOUSE, H2B1H_MOUSE, H2B1M_MOUSE, H2B1P_MOUSE, H2B2B_MOUSE, H2B3A_MOUSE, H2B3B_MOUSE	Histone H2B	GTKAVTKYTSSK	473	9.20E-03	635.8544	1269.6942	2	0.0013	12	0	GluC
H2B1A_MOUSE, H2B1B_MOUSE, H2B1C_MOUSE, H2B1F_MOUSE, H2B1H_MOUSE, H2B1M_MOUSE, H2B1P_MOUSE, H2B2B_MOUSE, H2B3A_MOUSE, H2B3B_MOUSE	Histone H2B	GTKAVTKYTSSK	473	6.30E-04	424.2387	1269.6942	3	0.0013	12	0	GluC
H2B1A_MOUSE, H2B1B_MOUSE, H2B1C_MOUSE, H2B1F_MOUSE, H2B1H_MOUSE, H2B1M_MOUSE, H2B1P_MOUSE, H2B2B_MOUSE, H2B3A_MOUSE, H2B3B_MOUSE	Histone H2B	GTKAVTKYTSSK	473	3.60E-04	424.2387	1269.6942	3	0.0013	12	0	GluC
H2B1A_MOUSE, H2B1B_MOUSE, H2B1C_MOUSE, H2B1F_MOUSE, H2B1H_MOUSE, H2B1M_MOUSE, H2B1P_MOUSE, H2B2B_MOUSE, H2B3A_MOUSE, H2B3B_MOUSE	Histone H2B	GTKAVTKYTSSK	473	2.10E-05	318.4308	1269.6941	4	0.0013	12	0	GluC
H2B1A_MOUSE, H2B1B_MOUSE, H2B1C_MOUSE, H2B1F_MOUSE, H2B1H_MOUSE, H2B1M_MOUSE, H2B1P_MOUSE, H2B2B_MOUSE, H2B3A_MOUSE, H2B3B_MOUSE	Histone H2B	GTKAVTKYTSSK	473	1.30E-05	318.4308	1269.6942	4	0.0013	12	0	GluC
H2B1A_MOUSE, H2B1B_MOUSE, H2B1C_MOUSE, H2B1F_MOUSE, H2B1H_MOUSE, H2B1M_MOUSE, H2B1P_MOUSE, H2B2B_MOUSE, H2B3A_MOUSE, H2B3B_MOUSE	Histone H2B	GTKAVTKYTSSK	473	4.60E-06	424.2387	1269.6942	3	0.0013	12	0	GluC

H2B1A_MOUSE, H2B1B_MOUSE, H2B1C_MOUSE, H2B1F_MOUSE, H2B1H_MOUSE, H2B1M_MOUSE, H2B1P_MOUSE, H2B2B_MOUSE, H2B3A_MOUSE, H2B3B_MOUSE	Histone H2B	GTKAVTKYTSSK	473	4.20E-06	635.8543	1269.6941	2	0.0013	12	0	GluC
H2B1A_MOUSE, H2B1B_MOUSE, H2B1C_MOUSE, H2B1F_MOUSE, H2B1H_MOUSE, H2B1M_MOUSE, H2B1P_MOUSE, H2B2B_MOUSE, H2B3A_MOUSE, H2B3B_MOUSE	Histone H2B	GTKAVTKYTSSK	473	3.30E-06	635.8544	1269.6942	2	0.0013	12	0	GluC
H2B1A_MOUSE, H2B1B_MOUSE, H2B1C_MOUSE, H2B1F_MOUSE, H2B1H_MOUSE, H2B1M_MOUSE, H2B1P_MOUSE, H2B2B_MOUSE, H2B3A_MOUSE, H2B3B_MOUSE	Histone H2B	GTKAVTKYTSSK	473	1.90E-08	424.2387	1269.6942	3	0.0013	12	0	GluC
H2B1A_MOUSE, H2B1B_MOUSE, H2B1C_MOUSE, H2B1F_MOUSE, H2B1H_MOUSE, H2B1M_MOUSE, H2B1P_MOUSE, H2B2B_MOUSE, H2B3A_MOUSE, H2B3B_MOUSE	Histone H2B	GTKAVTKYTSSK	473	2.10E-03	635.8543	1269.6940	2	0.0012	12	0	GluC
H2B1A_MOUSE, H2B1B_MOUSE, H2B1C_MOUSE, H2B1F_MOUSE, H2B1H_MOUSE, H2B1M_MOUSE, H2B1P_MOUSE, H2B2B_MOUSE, H2B3A_MOUSE, H2B3B_MOUSE	Histone H2B	GTKAVTKYTSSK	473	5.30E-04	635.8543	1269.6940	2	0.0012	12	0	GluC
H2B1A_MOUSE, H2B1B_MOUSE, H2B1C_MOUSE, H2B1F_MOUSE, H2B1H_MOUSE, H2B1M_MOUSE, H2B1P_MOUSE, H2B2B_MOUSE, H2B3A_MOUSE, H2B3B_MOUSE	Histone H2B	GTKAVTKYTSSK	473	6.30E-05	424.2386	1269.6940	3	0.0012	12	0	GluC
H2B1A_MOUSE, H2B1B_MOUSE, H2B1C_MOUSE, H2B1F_MOUSE, H2B1H_MOUSE, H2B1M_MOUSE, H2B1P_MOUSE, H2B2B_MOUSE, H2B3A_MOUSE, H2B3B_MOUSE	Histone H2B	GTKAVTKYTSSK	473	5.40E-07	318.4308	1269.6941	4	0.0012	12	0	GluC
H2B1A_MOUSE, H2B1B_MOUSE, H2B1C_MOUSE, H2B1F_MOUSE, H2B1H_MOUSE, H2B1M_MOUSE, H2B1P_MOUSE, H2B2B_MOUSE, H2B3A_MOUSE, H2B3B_MOUSE	Histone H2B	GTKAVTKYTSSK	473	4.80E-08	424.2386	1269.6941	3	0.0012	12	0	GluC
H2B1A_MOUSE, H2B1B_MOUSE, H2B1C_MOUSE, H2B1F_MOUSE, H2B1H_MOUSE, H2B1M_MOUSE, H2B1P_MOUSE, H2B2B_MOUSE, H2B3A_MOUSE, H2B3B_MOUSE	Histone H2B	GTKAVTKYTSSK	473	1.40E-09	318.4308	1269.6941	4	0.0012	12	0	GluC
H2B1A_MOUSE, H2B1B_MOUSE, H2B1C_MOUSE, H2B1F_MOUSE, H2B1H_MOUSE, H2B1M_MOUSE, H2B1P_MOUSE, H2B2B_MOUSE, H2B3A_MOUSE, H2B3B_MOUSE	Histone H2B	GTKAVTKYTSSK	473	5.20E-03	424.2386	1269.6940	3	0.0011	12	0	GluC
H2B1A_MOUSE, H2B1B_MOUSE, H2B1C_MOUSE, H2B1F_MOUSE, H2B1H_MOUSE, H2B1M_MOUSE, H2B1P_MOUSE, H2B2B_MOUSE, H2B3A_MOUSE, H2B3B_MOUSE	Histone H2B	GTKAVTKYTSSK	473	1.10E-03	424.2386	1269.6939	3	0.0011	12	0	GluC
H2B1A_MOUSE, H2B1B_MOUSE, H2B1C_MOUSE, H2B1F_MOUSE, H2B1H_MOUSE, H2B1M_MOUSE, H2B1P_MOUSE, H2B2B_MOUSE, H2B3A_MOUSE, H2B3B_MOUSE	Histone H2B	GTKAVTKYTSSK	473	5.10E-04	424.2386	1269.6939	3	0.0011	12	0	GluC
H2B1A_MOUSE, H2B1B_MOUSE, H2B1C_MOUSE, H2B1F_MOUSE, H2B1H_MOUSE, H2B1M_MOUSE, H2B1P_MOUSE, H2B2B_MOUSE, H2B3A_MOUSE, H2B3B_MOUSE	Histone H2B	GTKAVTKYTSSK	473	1.00E-04	318.4308	1269.6939	4	0.0011	12	0	GluC
H2B1A_MOUSE, H2B1B_MOUSE, H2B1C_MOUSE, H2B1F_MOUSE, H2B1H_MOUSE, H2B1M_MOUSE, H2B1P_MOUSE, H2B2B_MOUSE, H2B3A_MOUSE, H2B3B_MOUSE	Histone H2B	GTKAVTKYTSSK	473	6.00E-05	318.4308	1269.6939	4	0.0011	12	0	GluC
H2B1A_MOUSE, H2B1B_MOUSE, H2B1C_MOUSE, H2B1F_MOUSE, H2B1H_MOUSE, H2B1M_MOUSE, H2B1P_MOUSE, H2B2B_MOUSE, H2B3A_MOUSE, H2B3B_MOUSE	Histone H2B	GTKAVTKYTSSK	473	4.80E-06	635.8543	1269.6940	2	0.0011	12	0	GluC
H2B1A_MOUSE, H2B1B_MOUSE, H2B1C_MOUSE, H2B1F_MOUSE, H2B1H_MOUSE, H2B1M_MOUSE, H2B1P_MOUSE, H2B2B_MOUSE, H2B3A_MOUSE, H2B3B_MOUSE	Histone H2B	GTKAVTKYTSSK	473	2.90E-03	424.2385	1269.6938	3	0.0010	12	0	GluC
H2B1A_MOUSE, H2B1B_MOUSE, H2B1C_MOUSE, H2B1F_MOUSE, H2B1H_MOUSE, H2B1M_MOUSE, H2B1P_MOUSE, H2B2B_MOUSE, H2B3A_MOUSE, H2B3B_MOUSE	Histone H2B	GTKAVTKYTSSK	473	3.30E-04	424.2385	1269.6938	3	0.0010	12	0	GluC
H2B1A_MOUSE, H2B1B_MOUSE, H2B1C_MOUSE, H2B1F_MOUSE, H2B1H_MOUSE, H2B1M_MOUSE, H2B1P_MOUSE, H2B2B_MOUSE, H2B3A_MOUSE, H2B3B_MOUSE	Histone H2B	GTKAVTKYTSSK	473	1.30E-04	424.2386	1269.6939	3	0.0010	12	0	GluC
H2B1A_MOUSE, H2B1B_MOUSE, H2B1C_MOUSE, H2B1F_MOUSE, H2B1H_MOUSE, H2B1M_MOUSE, H2B1P_MOUSE, H2B2B_MOUSE, H2B3A_MOUSE, H2B3B_MOUSE	Histone H2B	GTKAVTKYTSSK	473	7.50E-05	635.8542	1269.6938	2	0.0010	12	0	GluC
H2B1A_MOUSE, H2B1B_MOUSE, H2B1C_MOUSE, H2B1F_MOUSE, H2B1H_MOUSE, H2B1M_MOUSE, H2B1P_MOUSE, H2B2B_MOUSE, H2B3A_MOUSE, H2B3B_MOUSE	Histone H2B	GTKAVTKYTSSK	473	2.70E-05	318.4307	1269.6938	4	0.0010	12	0	GluC
H2B1A_MOUSE, H2B1B_MOUSE, H2B1C_MOUSE, H2B1F_MOUSE, H2B1H_MOUSE, H2B1M_MOUSE, H2B1P_MOUSE, H2B2B_MOUSE, H2B3A_MOUSE, H2B3B_MOUSE	Histone H2B	GTKAVTKYTSSK	473	2.40E-06	424.2386	1269.6939	3	0.0010	12	0	GluC
H2B1A_MOUSE, H2B1B_MOUSE, H2B1C_MOUSE, H2B1F_MOUSE, H2B1H_MOUSE, H2B1M_MOUSE, H2B1P_MOUSE, H2B2B_MOUSE, H2B3A_MOUSE, H2B3B_MOUSE	Histone H2B	GTKAVTKYTSSK	473	6.70E-07	318.4307	1269.6939	4	0.0010	12	0	GluC
H2B1A_MOUSE, H2B1B_MOUSE, H2B1C_MOUSE, H2B1F_MOUSE, H2B1H_MOUSE, H2B1M_MOUSE, H2B1P_MOUSE, H2B2B_MOUSE, H2B3A_MOUSE, H2B3B_MOUSE	Histone H2B	GTKAVTKYTSSK	473	1.90E-07	424.2385	1269.6938	3	0.0010	12	0	GluC
H2B1A_MOUSE, H2B1B_MOUSE, H2B1C_MOUSE, H2B1F_MOUSE, H2B1H_MOUSE, H2B1M_MOUSE, H2B1P_MOUSE, H2B2B_MOUSE, H2B3A_MOUSE, H2B3B_MOUSE	Histone H2B	GTKAVTKYTSSK	473	9.00E-03	424.2385	1269.6937	3	0.0009	12	0	GluC

H2B1A_MOUSE, H2B1B_MOUSE, H2B1C_MOUSE, H2B1F_MOUSE, H2B1H_MOUSE, H2B1M_MOUSE, H2B1P_MOUSE, H2B2B_MOUSE, H2B3A_MOUSE, H2B3B_MOUSE	Histone H2B	GTKAVTKYTSSK	473	7.00E-03	635.8542	1269.6938	2	0.0009	12	0	GluC
H2B1A_MOUSE, H2B1B_MOUSE, H2B1C_MOUSE, H2B1F_MOUSE, H2B1H_MOUSE, H2B1M_MOUSE, H2B1P_MOUSE, H2B2B_MOUSE, H2B3A_MOUSE, H2B3B_MOUSE	Histone H2B	GTKAVTKYTSSK	473	1.30E-03	424.2385	1269.6938	3	0.0009	12	0	GluC
H2B1A_MOUSE, H2B1B_MOUSE, H2B1C_MOUSE, H2B1F_MOUSE, H2B1H_MOUSE, H2B1M_MOUSE, H2B1P_MOUSE, H2B2B_MOUSE, H2B3A_MOUSE, H2B3B_MOUSE	Histone H2B	GTKAVTKYTSSK	473	1.20E-03	424.2385	1269.6938	3	0.0009	12	0	GluC
H2B1A_MOUSE, H2B1B_MOUSE, H2B1C_MOUSE, H2B1F_MOUSE, H2B1H_MOUSE, H2B1M_MOUSE, H2B1P_MOUSE, H2B2B_MOUSE, H2B3A_MOUSE, H2B3B_MOUSE	Histone H2B	GTKAVTKYTSSK	473	5.90E-04	424.2385	1269.6938	3	0.0009	12	0	GluC
H2B1A_MOUSE, H2B1B_MOUSE, H2B1C_MOUSE, H2B1F_MOUSE, H2B1H_MOUSE, H2B1M_MOUSE, H2B1P_MOUSE, H2B2B_MOUSE, H2B3A_MOUSE, H2B3B_MOUSE	Histone H2B	GTKAVTKYTSSK	473	1.20E-05	635.8541	1269.6937	2	0.0009	12	0	GluC
H2B1A_MOUSE, H2B1B_MOUSE, H2B1C_MOUSE, H2B1F_MOUSE, H2B1H_MOUSE, H2B1M_MOUSE, H2B1P_MOUSE, H2B2B_MOUSE, H2B3A_MOUSE, H2B3B_MOUSE	Histone H2B	GTKAVTKYTSSK	473	1.60E-06	424.2385	1269.6938	3	0.0009	12	0	GluC
H2B1A_MOUSE, H2B1B_MOUSE, H2B1C_MOUSE, H2B1F_MOUSE, H2B1H_MOUSE, H2B1M_MOUSE, H2B1P_MOUSE, H2B2B_MOUSE, H2B3A_MOUSE, H2B3B_MOUSE	Histone H2B	GTKAVTKYTSSK	473	5.30E-03	635.8541	1269.6937	2	0.0008	12	0	GluC
H2B1A_MOUSE, H2B1B_MOUSE, H2B1C_MOUSE, H2B1F_MOUSE, H2B1H_MOUSE, H2B1M_MOUSE, H2B1P_MOUSE, H2B2B_MOUSE, H2B3A_MOUSE, H2B3B_MOUSE	Histone H2B	GTKAVTKYTSSK	473	3.00E-03	424.2385	1269.6936	3	0.0008	12	0	GluC
H2B1A_MOUSE, H2B1B_MOUSE, H2B1C_MOUSE, H2B1F_MOUSE, H2B1H_MOUSE, H2B1M_MOUSE, H2B1P_MOUSE, H2B2B_MOUSE, H2B3A_MOUSE, H2B3B_MOUSE	Histone H2B	GTKAVTKYTSSK	473	2.90E-03	635.8541	1269.6936	2	0.0008	12	0	GluC
H2B1A_MOUSE, H2B1B_MOUSE, H2B1C_MOUSE, H2B1F_MOUSE, H2B1H_MOUSE, H2B1M_MOUSE, H2B1P_MOUSE, H2B2B_MOUSE, H2B3A_MOUSE, H2B3B_MOUSE	Histone H2B	GTKAVTKYTSSK	473	1.80E-03	318.4307	1269.6937	4	0.0008	12	0	GluC
H2B1A_MOUSE, H2B1B_MOUSE, H2B1C_MOUSE, H2B1F_MOUSE, H2B1H_MOUSE, H2B1M_MOUSE, H2B1P_MOUSE, H2B2B_MOUSE, H2B3A_MOUSE, H2B3B_MOUSE	Histone H2B	GTKAVTKYTSSK	473	1.40E-03	635.8541	1269.6937	2	0.0008	12	0	GluC
H2B1A_MOUSE, H2B1B_MOUSE, H2B1C_MOUSE, H2B1F_MOUSE, H2B1H_MOUSE, H2B1M_MOUSE, H2B1P_MOUSE, H2B2B_MOUSE, H2B3A_MOUSE, H2B3B_MOUSE	Histone H2B	GTKAVTKYTSSK	473	8.10E-04	424.2385	1269.6936	3	0.0008	12	0	GluC
H2B1A_MOUSE, H2B1B_MOUSE, H2B1C_MOUSE, H2B1F_MOUSE, H2B1H_MOUSE, H2B1M_MOUSE, H2B1P_MOUSE, H2B2B_MOUSE, H2B3A_MOUSE, H2B3B_MOUSE	Histone H2B	GTKAVTKYTSSK	473	3.70E-04	635.8541	1269.6936	2	0.0008	12	0	GluC
H2B1A_MOUSE, H2B1B_MOUSE, H2B1C_MOUSE, H2B1F_MOUSE, H2B1H_MOUSE, H2B1M_MOUSE, H2B1P_MOUSE, H2B2B_MOUSE, H2B3A_MOUSE, H2B3B_MOUSE	Histone H2B	GTKAVTKYTSSK	473	1.40E-05	424.2385	1269.6937	3	0.0008	12	0	GluC
H2B1A_MOUSE, H2B1B_MOUSE, H2B1C_MOUSE, H2B1F_MOUSE, H2B1H_MOUSE, H2B1M_MOUSE, H2B1P_MOUSE, H2B2B_MOUSE, H2B3A_MOUSE, H2B3B_MOUSE	Histone H2B	GTKAVTKYTSSK	473	1.10E-05	424.2385	1269.6937	3	0.0008	12	0	GluC
H2B1A_MOUSE, H2B1B_MOUSE, H2B1C_MOUSE, H2B1F_MOUSE, H2B1H_MOUSE, H2B1M_MOUSE, H2B1P_MOUSE, H2B2B_MOUSE, H2B3A_MOUSE, H2B3B_MOUSE	Histone H2B	GTKAVTKYTSSK	473	2.90E-08	635.8541	1269.6936	2	0.0008	12	0	GluC
H2B1A_MOUSE, H2B1B_MOUSE, H2B1C_MOUSE, H2B1F_MOUSE, H2B1H_MOUSE, H2B1M_MOUSE, H2B1P_MOUSE, H2B2B_MOUSE, H2B3A_MOUSE, H2B3B_MOUSE	Histone H2B	GTKAVTKYTSSK	473	2.30E-09	424.2385	1269.6936	3	0.0008	12	0	GluC
H2B1A_MOUSE, H2B1B_MOUSE, H2B1C_MOUSE, H2B1F_MOUSE, H2B1H_MOUSE, H2B1M_MOUSE, H2B1P_MOUSE, H2B2B_MOUSE, H2B3A_MOUSE, H2B3B_MOUSE	Histone H2B	GTKAVTKYTSSK	473	7.50E-03	424.2385	1269.6936	3	0.0007	12	0	GluC
H2B1A_MOUSE, H2B1B_MOUSE, H2B1C_MOUSE, H2B1F_MOUSE, H2B1H_MOUSE, H2B1M_MOUSE, H2B1P_MOUSE, H2B2B_MOUSE, H2B3A_MOUSE, H2B3B_MOUSE	Histone H2B	GTKAVTKYTSSK	473	4.80E-03	635.8541	1269.6936	2	0.0007	12	0	GluC
H2B1A_MOUSE, H2B1B_MOUSE, H2B1C_MOUSE, H2B1F_MOUSE, H2B1H_MOUSE, H2B1M_MOUSE, H2B1P_MOUSE, H2B2B_MOUSE, H2B3A_MOUSE, H2B3B_MOUSE	Histone H2B	GTKAVTKYTSSK	473	4.80E-04	424.2385	1269.6936	3	0.0007	12	0	GluC
H2B1A_MOUSE, H2B1B_MOUSE, H2B1C_MOUSE, H2B1F_MOUSE, H2B1H_MOUSE, H2B1M_MOUSE, H2B1P_MOUSE, H2B2B_MOUSE, H2B3A_MOUSE, H2B3B_MOUSE	Histone H2B	GTKAVTKYTSSK	473	4.70E-04	424.2385	1269.6936	3	0.0007	12	0	GluC
H2B1A_MOUSE, H2B1B_MOUSE, H2B1C_MOUSE, H2B1F_MOUSE, H2B1H_MOUSE, H2B1M_MOUSE, H2B1P_MOUSE, H2B2B_MOUSE, H2B3A_MOUSE, H2B3B_MOUSE	Histone H2B	GTKAVTKYTSSK	473	4.00E-06	635.8541	1269.6936	2	0.0007	12	0	GluC
H2B1A_MOUSE, H2B1B_MOUSE, H2B1C_MOUSE, H2B1F_MOUSE, H2B1H_MOUSE, H2B1M_MOUSE, H2B1P_MOUSE, H2B2B_MOUSE, H2B3A_MOUSE, H2B3B_MOUSE	Histone H2B	GTKAVTKYTSSK	473	1.20E-06	635.8541	1269.6936	2	0.0007	12	0	GluC
H2B1A_MOUSE, H2B1B_MOUSE, H2B1C_MOUSE, H2B1F_MOUSE, H2B1H_MOUSE, H2B1M_MOUSE, H2B1P_MOUSE, H2B2B_MOUSE, H2B3A_MOUSE, H2B3B_MOUSE	Histone H2B	GTKAVTKYTSSK	473	1.10E-07	424.2385	1269.6936	3	0.0007	12	0	GluC

H2B1A_MOUSE, H2B1B_MOUSE, H2B1C_MOUSE, H2B1F_MOUSE, H2B1H_MOUSE, H2B1M_MOUSE, H2B1P_MOUSE, H2B2B_MOUSE, H2B3A_MOUSE, H2B3B_MOUSE	Histone H2B	GTKAVTKYTSSK	473	9.70E-03	318.4306	1269.6934	4	0.0006	12	0	GluC
H2B1A_MOUSE, H2B1B_MOUSE, H2B1C_MOUSE, H2B1F_MOUSE, H2B1H_MOUSE, H2B1M_MOUSE, H2B1P_MOUSE, H2B2B_MOUSE, H2B3A_MOUSE, H2B3B_MOUSE	Histone H2B	GTKAVTKYTSSK	473	5.60E-03	424.2384	1269.6935	3	0.0006	12	0	GluC
H2B1A_MOUSE, H2B1B_MOUSE, H2B1C_MOUSE, H2B1F_MOUSE, H2B1H_MOUSE, H2B1M_MOUSE, H2B1P_MOUSE, H2B2B_MOUSE, H2B3A_MOUSE, H2B3B_MOUSE	Histone H2B	GTKAVTKYTSSK	473	9.20E-04	635.854	1269.6934	2	0.0006	12	0	GluC
H2B1A_MOUSE, H2B1B_MOUSE, H2B1C_MOUSE, H2B1F_MOUSE, H2B1H_MOUSE, H2B1M_MOUSE, H2B1P_MOUSE, H2B2B_MOUSE, H2B3A_MOUSE, H2B3B_MOUSE	Histone H2B	GTKAVTKYTSSK	473	1.50E-04	424.2384	1269.6935	3	0.0006	12	0	GluC
H2B1A_MOUSE, H2B1B_MOUSE, H2B1C_MOUSE, H2B1F_MOUSE, H2B1H_MOUSE, H2B1M_MOUSE, H2B1P_MOUSE, H2B2B_MOUSE, H2B3A_MOUSE, H2B3B_MOUSE	Histone H2B	GTKAVTKYTSSK	473	1.10E-04	318.4307	1269.6935	4	0.0006	12	0	GluC
H2B1A_MOUSE, H2B1B_MOUSE, H2B1C_MOUSE, H2B1F_MOUSE, H2B1H_MOUSE, H2B1M_MOUSE, H2B1P_MOUSE, H2B2B_MOUSE, H2B3A_MOUSE, H2B3B_MOUSE	Histone H2B	GTKAVTKYTSSK	473	7.50E-05	424.2384	1269.6934	3	0.0006	12	0	GluC
H2B1A_MOUSE, H2B1B_MOUSE, H2B1C_MOUSE, H2B1F_MOUSE, H2B1H_MOUSE, H2B1M_MOUSE, H2B1P_MOUSE, H2B2B_MOUSE, H2B3A_MOUSE, H2B3B_MOUSE	Histone H2B	GTKAVTKYTSSK	473	2.30E-05	424.2384	1269.6935	3	0.0006	12	0	GluC
H2B1A_MOUSE, H2B1B_MOUSE, H2B1C_MOUSE, H2B1F_MOUSE, H2B1H_MOUSE, H2B1M_MOUSE, H2B1P_MOUSE, H2B2B_MOUSE, H2B3A_MOUSE, H2B3B_MOUSE	Histone H2B	GTKAVTKYTSSK	473	2.50E-06	424.2384	1269.6934	3	0.0006	12	0	GluC
H2B1A_MOUSE, H2B1B_MOUSE, H2B1C_MOUSE, H2B1F_MOUSE, H2B1H_MOUSE, H2B1M_MOUSE, H2B1P_MOUSE, H2B2B_MOUSE, H2B3A_MOUSE, H2B3B_MOUSE	Histone H2B	GTKAVTKYTSSK	473	1.80E-06	318.4307	1269.6935	4	0.0006	12	0	GluC
H2B1A_MOUSE, H2B1B_MOUSE, H2B1C_MOUSE, H2B1F_MOUSE, H2B1H_MOUSE, H2B1M_MOUSE, H2B1P_MOUSE, H2B2B_MOUSE, H2B3A_MOUSE, H2B3B_MOUSE	Histone H2B	GTKAVTKYTSSK	473	1.10E-08	424.2384	1269.6935	3	0.0006	12	0	GluC
H2B1A_MOUSE, H2B1B_MOUSE, H2B1C_MOUSE, H2B1F_MOUSE, H2B1H_MOUSE, H2B1M_MOUSE, H2B1P_MOUSE, H2B2B_MOUSE, H2B3A_MOUSE, H2B3B_MOUSE	Histone H2B	GTKAVTKYTSSK	473	2.20E-03	424.2384	1269.6933	3	0.0005	12	0	GluC
H2B1A_MOUSE, H2B1B_MOUSE, H2B1C_MOUSE, H2B1F_MOUSE, H2B1H_MOUSE, H2B1M_MOUSE, H2B1P_MOUSE, H2B2B_MOUSE, H2B3A_MOUSE, H2B3B_MOUSE	Histone H2B	GTKAVTKYTSSK	473	6.10E-04	635.8539	1269.6933	2	0.0005	12	0	GluC
H2B1A_MOUSE, H2B1B_MOUSE, H2B1C_MOUSE, H2B1F_MOUSE, H2B1H_MOUSE, H2B1M_MOUSE, H2B1P_MOUSE, H2B2B_MOUSE, H2B3A_MOUSE, H2B3B_MOUSE	Histone H2B	GTKAVTKYTSSK	473	4.90E-04	424.2384	1269.6933	3	0.0005	12	0	GluC
H2B1A_MOUSE, H2B1B_MOUSE, H2B1C_MOUSE, H2B1F_MOUSE, H2B1H_MOUSE, H2B1M_MOUSE, H2B1P_MOUSE, H2B2B_MOUSE, H2B3A_MOUSE, H2B3B_MOUSE	Histone H2B	GTKAVTKYTSSK	473	4.20E-04	424.2384	1269.6934	3	0.0005	12	0	GluC
H2B1A_MOUSE, H2B1B_MOUSE, H2B1C_MOUSE, H2B1F_MOUSE, H2B1H_MOUSE, H2B1M_MOUSE, H2B1P_MOUSE, H2B2B_MOUSE, H2B3A_MOUSE, H2B3B_MOUSE	Histone H2B	GTKAVTKYTSSK	473	3.70E-05	635.854	1269.6934	2	0.0005	12	0	GluC
H2B1A_MOUSE, H2B1B_MOUSE, H2B1C_MOUSE, H2B1F_MOUSE, H2B1H_MOUSE, H2B1M_MOUSE, H2B1P_MOUSE, H2B2B_MOUSE, H2B3A_MOUSE, H2B3B_MOUSE	Histone H2B	GTKAVTKYTSSK	473	4.50E-07	318.4306	1269.6934	4	0.0005	12	0	GluC
H2B1A_MOUSE, H2B1B_MOUSE, H2B1C_MOUSE, H2B1F_MOUSE, H2B1H_MOUSE, H2B1M_MOUSE, H2B1P_MOUSE, H2B2B_MOUSE, H2B3A_MOUSE, H2B3B_MOUSE	Histone H2B	GTKAVTKYTSSK	473	9.80E-08	424.2384	1269.6934	3	0.0005	12	0	GluC
H2B1A_MOUSE, H2B1B_MOUSE, H2B1C_MOUSE, H2B1F_MOUSE, H2B1H_MOUSE, H2B1M_MOUSE, H2B1P_MOUSE, H2B2B_MOUSE, H2B3A_MOUSE, H2B3B_MOUSE	Histone H2B	GTKAVTKYTSSK	473	9.90E-03	424.2384	1269.6932	3	0.0004	12	0	GluC
H2B1A_MOUSE, H2B1B_MOUSE, H2B1C_MOUSE, H2B1F_MOUSE, H2B1H_MOUSE, H2B1M_MOUSE, H2B1P_MOUSE, H2B2B_MOUSE, H2B3A_MOUSE, H2B3B_MOUSE	Histone H2B	GTKAVTKYTSSK	473	4.80E-03	424.2384	1269.6933	3	0.0004	12	0	GluC
H2B1A_MOUSE, H2B1B_MOUSE, H2B1C_MOUSE, H2B1F_MOUSE, H2B1H_MOUSE, H2B1M_MOUSE, H2B1P_MOUSE, H2B2B_MOUSE, H2B3A_MOUSE, H2B3B_MOUSE	Histone H2B	GTKAVTKYTSSK	473	1.50E-04	424.2384	1269.6933	3	0.0004	12	0	GluC
H2B1A_MOUSE, H2B1B_MOUSE, H2B1C_MOUSE, H2B1F_MOUSE, H2B1H_MOUSE, H2B1M_MOUSE, H2B1P_MOUSE, H2B2B_MOUSE, H2B3A_MOUSE, H2B3B_MOUSE	Histone H2B	GTKAVTKYTSSK	473	3.00E-05	318.4306	1269.6932	4	0.0004	12	0	GluC
H2B1A_MOUSE, H2B1B_MOUSE, H2B1C_MOUSE, H2B1F_MOUSE, H2B1H_MOUSE, H2B1M_MOUSE, H2B1P_MOUSE, H2B2B_MOUSE, H2B3A_MOUSE, H2B3B_MOUSE	Histone H2B	GTKAVTKYTSSK	473	1.90E-05	424.2384	1269.6932	3	0.0004	12	0	GluC
H2B1A_MOUSE, H2B1B_MOUSE, H2B1C_MOUSE, H2B1F_MOUSE, H2B1H_MOUSE, H2B1M_MOUSE, H2B1P_MOUSE, H2B2B_MOUSE, H2B3A_MOUSE, H2B3B_MOUSE	Histone H2B	GTKAVTKYTSSK	473	6.50E-06	424.2384	1269.6933	3	0.0004	12	0	GluC
H2B1A_MOUSE, H2B1B_MOUSE, H2B1C_MOUSE, H2B1F_MOUSE, H2B1H_MOUSE, H2B1M_MOUSE, H2B1P_MOUSE, H2B2B_MOUSE, H2B3A_MOUSE, H2B3B_MOUSE	Histone H2B	GTKAVTKYTSSK	473	1.50E-06	318.4306	1269.6933	4	0.0004	12	0	GluC

H2B1A_MOUSE, H2B1B_MOUSE, H2B1C_MOUSE, H2B1F_MOUSE, H2B1H_MOUSE, H2B1M_MOUSE, H2B1P_MOUSE, H2B2B_MOUSE, H2B3A_MOUSE, H2B3B_MOUSE	Histone H2B	GTKAVTKYTSSK	473	3.50E-07	424.2384	1269.6932	3	0.0004	12	0	GluC
H2B1A_MOUSE, H2B1B_MOUSE, H2B1C_MOUSE, H2B1F_MOUSE, H2B1H_MOUSE, H2B1M_MOUSE, H2B1P_MOUSE, H2B2B_MOUSE, H2B3A_MOUSE, H2B3B_MOUSE	Histone H2B	GTKAVTKYTSSK	473	2.00E-07	318.4306	1269.6933	4	0.0004	12	0	GluC
H2B1A_MOUSE, H2B1B_MOUSE, H2B1C_MOUSE, H2B1F_MOUSE, H2B1H_MOUSE, H2B1M_MOUSE, H2B1P_MOUSE, H2B2B_MOUSE, H2B3A_MOUSE, H2B3B_MOUSE	Histone H2B	GTKAVTKYTSSK	473	9.10E-08	424.2384	1269.6933	3	0.0004	12	0	GluC
H2B1A_MOUSE, H2B1B_MOUSE, H2B1C_MOUSE, H2B1F_MOUSE, H2B1H_MOUSE, H2B1M_MOUSE, H2B1P_MOUSE, H2B2B_MOUSE, H2B3A_MOUSE, H2B3B_MOUSE	Histone H2B	GTKAVTKYTSSK	473	4.90E-08	424.2384	1269.6933	3	0.0004	12	0	GluC
H2B1A_MOUSE, H2B1B_MOUSE, H2B1C_MOUSE, H2B1F_MOUSE, H2B1H_MOUSE, H2B1M_MOUSE, H2B1P_MOUSE, H2B2B_MOUSE, H2B3A_MOUSE, H2B3B_MOUSE	Histone H2B	GTKAVTKYTSSK	473	2.80E-08	318.4306	1269.6932	4	0.0004	12	0	GluC
H2B1A_MOUSE, H2B1B_MOUSE, H2B1C_MOUSE, H2B1F_MOUSE, H2B1H_MOUSE, H2B1M_MOUSE, H2B1P_MOUSE, H2B2B_MOUSE, H2B3A_MOUSE, H2B3B_MOUSE	Histone H2B	GTKAVTKYTSSK	473	8.50E-03	424.2383	1269.6932	3	0.0003	12	0	GluC
H2B1A_MOUSE, H2B1B_MOUSE, H2B1C_MOUSE, H2B1F_MOUSE, H2B1H_MOUSE, H2B1M_MOUSE, H2B1P_MOUSE, H2B2B_MOUSE, H2B3A_MOUSE, H2B3B_MOUSE	Histone H2B	GTKAVTKYTSSK	473	4.20E-03	424.2383	1269.6931	3	0.0003	12	0	GluC
H2B1A_MOUSE, H2B1B_MOUSE, H2B1C_MOUSE, H2B1F_MOUSE, H2B1H_MOUSE, H2B1M_MOUSE, H2B1P_MOUSE, H2B2B_MOUSE, H2B3A_MOUSE, H2B3B_MOUSE	Histone H2B	GTKAVTKYTSSK	473	1.30E-03	424.2383	1269.6931	3	0.0003	12	0	GluC
H2B1A_MOUSE, H2B1B_MOUSE, H2B1C_MOUSE, H2B1F_MOUSE, H2B1H_MOUSE, H2B1M_MOUSE, H2B1P_MOUSE, H2B2B_MOUSE, H2B3A_MOUSE, H2B3B_MOUSE	Histone H2B	GTKAVTKYTSSK	473	9.30E-04	424.2383	1269.6931	3	0.0003	12	0	GluC
H2B1A_MOUSE, H2B1B_MOUSE, H2B1C_MOUSE, H2B1F_MOUSE, H2B1H_MOUSE, H2B1M_MOUSE, H2B1P_MOUSE, H2B2B_MOUSE, H2B3A_MOUSE, H2B3B_MOUSE	Histone H2B	GTKAVTKYTSSK	473	3.70E-04	424.2383	1269.6931	3	0.0003	12	0	GluC
H2B1A_MOUSE, H2B1B_MOUSE, H2B1C_MOUSE, H2B1F_MOUSE, H2B1H_MOUSE, H2B1M_MOUSE, H2B1P_MOUSE, H2B2B_MOUSE, H2B3A_MOUSE, H2B3B_MOUSE	Histone H2B	GTKAVTKYTSSK	473	1.10E-04	424.2383	1269.6932	3	0.0003	12	0	GluC
H2B1A_MOUSE, H2B1B_MOUSE, H2B1C_MOUSE, H2B1F_MOUSE, H2B1H_MOUSE, H2B1M_MOUSE, H2B1P_MOUSE, H2B2B_MOUSE, H2B3A_MOUSE, H2B3B_MOUSE	Histone H2B	GTKAVTKYTSSK	473	9.10E-05	424.2383	1269.6932	3	0.0003	12	0	GluC
H2B1A_MOUSE, H2B1B_MOUSE, H2B1C_MOUSE, H2B1F_MOUSE, H2B1H_MOUSE, H2B1M_MOUSE, H2B1P_MOUSE, H2B2B_MOUSE, H2B3A_MOUSE, H2B3B_MOUSE	Histone H2B	GTKAVTKYTSSK	473	3.60E-06	318.4306	1269.6931	4	0.0003	12	0	GluC
H2B1A_MOUSE, H2B1B_MOUSE, H2B1C_MOUSE, H2B1F_MOUSE, H2B1H_MOUSE, H2B1M_MOUSE, H2B1P_MOUSE, H2B2B_MOUSE, H2B3A_MOUSE, H2B3B_MOUSE	Histone H2B	GTKAVTKYTSSK	473	8.60E-07	424.2383	1269.6931	3	0.0003	12	0	GluC
H2B1A_MOUSE, H2B1B_MOUSE, H2B1C_MOUSE, H2B1F_MOUSE, H2B1H_MOUSE, H2B1M_MOUSE, H2B1P_MOUSE, H2B2B_MOUSE, H2B3A_MOUSE, H2B3B_MOUSE	Histone H2B	GTKAVTKYTSSK	473	5.50E-07	424.2383	1269.6931	3	0.0003	12	0	GluC
H2B1A_MOUSE, H2B1B_MOUSE, H2B1C_MOUSE, H2B1F_MOUSE, H2B1H_MOUSE, H2B1M_MOUSE, H2B1P_MOUSE, H2B2B_MOUSE, H2B3A_MOUSE, H2B3B_MOUSE	Histone H2B	GTKAVTKYTSSK	473	4.30E-07	424.2383	1269.6932	3	0.0003	12	0	GluC
H2B1A_MOUSE, H2B1B_MOUSE, H2B1C_MOUSE, H2B1F_MOUSE, H2B1H_MOUSE, H2B1M_MOUSE, H2B1P_MOUSE, H2B2B_MOUSE, H2B3A_MOUSE, H2B3B_MOUSE	Histone H2B	GTKAVTKYTSSK	473	2.80E-07	424.2383	1269.6932	3	0.0003	12	0	GluC
H2B1A_MOUSE, H2B1B_MOUSE, H2B1C_MOUSE, H2B1F_MOUSE, H2B1H_MOUSE, H2B1M_MOUSE, H2B1P_MOUSE, H2B2B_MOUSE, H2B3A_MOUSE, H2B3B_MOUSE	Histone H2B	GTKAVTKYTSSK	473	1.50E-07	635.8538	1269.6931	2	0.0003	12	0	GluC
H2B1A_MOUSE, H2B1B_MOUSE, H2B1C_MOUSE, H2B1F_MOUSE, H2B1H_MOUSE, H2B1M_MOUSE, H2B1P_MOUSE, H2B2B_MOUSE, H2B3A_MOUSE, H2B3B_MOUSE	Histone H2B	GTKAVTKYTSSK	473	7.00E-03	318.4305	1269.6931	4	0.0002	12	0	GluC
H2B1A_MOUSE, H2B1B_MOUSE, H2B1C_MOUSE, H2B1F_MOUSE, H2B1H_MOUSE, H2B1M_MOUSE, H2B1P_MOUSE, H2B2B_MOUSE, H2B3A_MOUSE, H2B3B_MOUSE	Histone H2B	GTKAVTKYTSSK	473	3.40E-03	424.2383	1269.6930	3	0.0002	12	0	GluC
H2B1A_MOUSE, H2B1B_MOUSE, H2B1C_MOUSE, H2B1F_MOUSE, H2B1H_MOUSE, H2B1M_MOUSE, H2B1P_MOUSE, H2B2B_MOUSE, H2B3A_MOUSE, H2B3B_MOUSE	Histone H2B	GTKAVTKYTSSK	473	1.60E-03	424.2383	1269.6930	3	0.0002	12	0	GluC
H2B1A_MOUSE, H2B1B_MOUSE, H2B1C_MOUSE, H2B1F_MOUSE, H2B1H_MOUSE, H2B1M_MOUSE, H2B1P_MOUSE, H2B2B_MOUSE, H2B3A_MOUSE, H2B3B_MOUSE	Histone H2B	GTKAVTKYTSSK	473	3.30E-04	424.2383	1269.6930	3	0.0002	12	0	GluC
H2B1A_MOUSE, H2B1B_MOUSE, H2B1C_MOUSE, H2B1F_MOUSE, H2B1H_MOUSE, H2B1M_MOUSE, H2B1P_MOUSE, H2B2B_MOUSE, H2B3A_MOUSE, H2B3B_MOUSE	Histone H2B	GTKAVTKYTSSK	473	2.70E-04	424.2383	1269.6931	3	0.0002	12	0	GluC
H2B1A_MOUSE, H2B1B_MOUSE, H2B1C_MOUSE, H2B1F_MOUSE, H2B1H_MOUSE, H2B1M_MOUSE, H2B1P_MOUSE, H2B2B_MOUSE, H2B3A_MOUSE, H2B3B_MOUSE	Histone H2B	GTKAVTKYTSSK	473	1.30E-04	424.2383	1269.6930	3	0.0002	12	0	GluC

H2B1A_MOUSE, H2B1B_MOUSE, H2B1C_MOUSE, H2B1F_MOUSE, H2B1H_MOUSE, H2B1M_MOUSE, H2B1P_MOUSE, H2B2B_MOUSE, H2B3A_MOUSE, H2B3B_MOUSE	Histone H2B	GTKAVTKYTSSK	473	6.50E-05	424.2383	1269.6931	3	0.0002	12	0	GluC
H2B1A_MOUSE, H2B1B_MOUSE, H2B1C_MOUSE, H2B1F_MOUSE, H2B1H_MOUSE, H2B1M_MOUSE, H2B1P_MOUSE, H2B2B_MOUSE, H2B3A_MOUSE, H2B3B_MOUSE	Histone H2B	GTKAVTKYTSSK	473	3.50E-05	424.2383	1269.6930	3	0.0002	12	0	GluC
H2B1A_MOUSE, H2B1B_MOUSE, H2B1C_MOUSE, H2B1F_MOUSE, H2B1H_MOUSE, H2B1M_MOUSE, H2B1P_MOUSE, H2B2B_MOUSE, H2B3A_MOUSE, H2B3B_MOUSE	Histone H2B	GTKAVTKYTSSK	473	3.20E-05	424.2383	1269.6930	3	0.0002	12	0	GluC
H2B1A_MOUSE, H2B1B_MOUSE, H2B1C_MOUSE, H2B1F_MOUSE, H2B1H_MOUSE, H2B1M_MOUSE, H2B1P_MOUSE, H2B2B_MOUSE, H2B3A_MOUSE, H2B3B_MOUSE	Histone H2B	GTKAVTKYTSSK	473	9.00E-06	424.2383	1269.6930	3	0.0002	12	0	GluC
H2B1A_MOUSE, H2B1B_MOUSE, H2B1C_MOUSE, H2B1F_MOUSE, H2B1H_MOUSE, H2B1M_MOUSE, H2B1P_MOUSE, H2B2B_MOUSE, H2B3A_MOUSE, H2B3B_MOUSE	Histone H2B	GTKAVTKYTSSK	473	4.20E-06	635.8538	1269.6930	2	0.0002	12	0	GluC
H2B1A_MOUSE, H2B1B_MOUSE, H2B1C_MOUSE, H2B1F_MOUSE, H2B1H_MOUSE, H2B1M_MOUSE, H2B1P_MOUSE, H2B2B_MOUSE, H2B3A_MOUSE, H2B3B_MOUSE	Histone H2B	GTKAVTKYTSSK	473	2.50E-06	424.2383	1269.6931	3	0.0002	12	0	GluC
H2B1A_MOUSE, H2B1B_MOUSE, H2B1C_MOUSE, H2B1F_MOUSE, H2B1H_MOUSE, H2B1M_MOUSE, H2B1P_MOUSE, H2B2B_MOUSE, H2B3A_MOUSE, H2B3B_MOUSE	Histone H2B	GTKAVTKYTSSK	473	5.10E-07	424.2383	1269.6930	3	0.0002	12	0	GluC
H2B1A_MOUSE, H2B1B_MOUSE, H2B1C_MOUSE, H2B1F_MOUSE, H2B1H_MOUSE, H2B1M_MOUSE, H2B1P_MOUSE, H2B2B_MOUSE, H2B3A_MOUSE, H2B3B_MOUSE	Histone H2B	GTKAVTKYTSSK	473	2.40E-07	424.2383	1269.6930	3	0.0002	12	0	GluC
H2B1A_MOUSE, H2B1B_MOUSE, H2B1C_MOUSE, H2B1F_MOUSE, H2B1H_MOUSE, H2B1M_MOUSE, H2B1P_MOUSE, H2B2B_MOUSE, H2B3A_MOUSE, H2B3B_MOUSE	Histone H2B	GTKAVTKYTSSK	473	8.90E-08	424.2383	1269.6930	3	0.0002	12	0	GluC
H2B1A_MOUSE, H2B1B_MOUSE, H2B1C_MOUSE, H2B1F_MOUSE, H2B1H_MOUSE, H2B1M_MOUSE, H2B1P_MOUSE, H2B2B_MOUSE, H2B3A_MOUSE, H2B3B_MOUSE	Histone H2B	GTKAVTKYTSSK	473	1.00E-08	424.2383	1269.6930	3	0.0002	12	0	GluC
H2B1A_MOUSE, H2B1B_MOUSE, H2B1C_MOUSE, H2B1F_MOUSE, H2B1H_MOUSE, H2B1M_MOUSE, H2B1P_MOUSE, H2B2B_MOUSE, H2B3A_MOUSE, H2B3B_MOUSE	Histone H2B	GTKAVTKYTSSK	473	4.20E-03	424.2383	1269.6930	3	0.0001	12	0	GluC
H2B1A_MOUSE, H2B1B_MOUSE, H2B1C_MOUSE, H2B1F_MOUSE, H2B1H_MOUSE, H2B1M_MOUSE, H2B1P_MOUSE, H2B2B_MOUSE, H2B3A_MOUSE, H2B3B_MOUSE	Histone H2B	GTKAVTKYTSSK	473	8.60E-04	424.2382	1269.6929	3	0.0001	12	0	GluC
H2B1A_MOUSE, H2B1B_MOUSE, H2B1C_MOUSE, H2B1F_MOUSE, H2B1H_MOUSE, H2B1M_MOUSE, H2B1P_MOUSE, H2B2B_MOUSE, H2B3A_MOUSE, H2B3B_MOUSE	Histone H2B	GTKAVTKYTSSK	473	8.00E-04	424.2383	1269.6930	3	0.0001	12	0	GluC
H2B1A_MOUSE, H2B1B_MOUSE, H2B1C_MOUSE, H2B1F_MOUSE, H2B1H_MOUSE, H2B1M_MOUSE, H2B1P_MOUSE, H2B2B_MOUSE, H2B3A_MOUSE, H2B3B_MOUSE	Histone H2B	GTKAVTKYTSSK	473	7.00E-04	424.2382	1269.6929	3	0.0001	12	0	GluC
H2B1A_MOUSE, H2B1B_MOUSE, H2B1C_MOUSE, H2B1F_MOUSE, H2B1H_MOUSE, H2B1M_MOUSE, H2B1P_MOUSE, H2B2B_MOUSE, H2B3A_MOUSE, H2B3B_MOUSE	Histone H2B	GTKAVTKYTSSK	473	4.80E-04	424.2383	1269.6930	3	0.0001	12	0	GluC
H2B1A_MOUSE, H2B1B_MOUSE, H2B1C_MOUSE, H2B1F_MOUSE, H2B1H_MOUSE, H2B1M_MOUSE, H2B1P_MOUSE, H2B2B_MOUSE, H2B3A_MOUSE, H2B3B_MOUSE	Histone H2B	GTKAVTKYTSSK	473	1.70E-04	424.2382	1269.6929	3	0.0001	12	0	GluC
H2B1A_MOUSE, H2B1B_MOUSE, H2B1C_MOUSE, H2B1F_MOUSE, H2B1H_MOUSE, H2B1M_MOUSE, H2B1P_MOUSE, H2B2B_MOUSE, H2B3A_MOUSE, H2B3B_MOUSE	Histone H2B	GTKAVTKYTSSK	473	2.50E-05	424.2383	1269.6930	3	0.0001	12	0	GluC
H2B1A_MOUSE, H2B1B_MOUSE, H2B1C_MOUSE, H2B1F_MOUSE, H2B1H_MOUSE, H2B1M_MOUSE, H2B1P_MOUSE, H2B2B_MOUSE, H2B3A_MOUSE, H2B3B_MOUSE	Histone H2B	GTKAVTKYTSSK	473	9.80E-06	318.4305	1269.6930	4	0.0001	12	0	GluC
H2B1A_MOUSE, H2B1B_MOUSE, H2B1C_MOUSE, H2B1F_MOUSE, H2B1H_MOUSE, H2B1M_MOUSE, H2B1P_MOUSE, H2B2B_MOUSE, H2B3A_MOUSE, H2B3B_MOUSE	Histone H2B	GTKAVTKYTSSK	473	7.80E-06	424.2383	1269.6930	3	0.0001	12	0	GluC
H2B1A_MOUSE, H2B1B_MOUSE, H2B1C_MOUSE, H2B1F_MOUSE, H2B1H_MOUSE, H2B1M_MOUSE, H2B1P_MOUSE, H2B2B_MOUSE, H2B3A_MOUSE, H2B3B_MOUSE	Histone H2B	GTKAVTKYTSSK	473	5.20E-06	424.2383	1269.6930	3	0.0001	12	0	GluC
H2B1A_MOUSE, H2B1B_MOUSE, H2B1C_MOUSE, H2B1F_MOUSE, H2B1H_MOUSE, H2B1M_MOUSE, H2B1P_MOUSE, H2B2B_MOUSE, H2B3A_MOUSE, H2B3B_MOUSE	Histone H2B	GTKAVTKYTSSK	473	3.60E-06	318.4305	1269.6929	4	0.0001	12	0	GluC
H2B1A_MOUSE, H2B1B_MOUSE, H2B1C_MOUSE, H2B1F_MOUSE, H2B1H_MOUSE, H2B1M_MOUSE, H2B1P_MOUSE, H2B2B_MOUSE, H2B3A_MOUSE, H2B3B_MOUSE	Histone H2B	GTKAVTKYTSSK	473	1.90E-06	424.2383	1269.6930	3	0.0001	12	0	GluC
H2B1A_MOUSE, H2B1B_MOUSE, H2B1C_MOUSE, H2B1F_MOUSE, H2B1H_MOUSE, H2B1M_MOUSE, H2B1P_MOUSE, H2B2B_MOUSE, H2B3A_MOUSE, H2B3B_MOUSE	Histone H2B	GTKAVTKYTSSK	473	1.30E-07	424.2382	1269.6929	3	0.0001	12	0	GluC
H2B1A_MOUSE, H2B1B_MOUSE, H2B1C_MOUSE, H2B1F_MOUSE, H2B1H_MOUSE, H2B1M_MOUSE, H2B1P_MOUSE, H2B2B_MOUSE, H2B3A_MOUSE, H2B3B_MOUSE	Histone H2B	GTKAVTKYTSSK	473	1.00E-07	424.2382	1269.6929	3	0.0001	12	0	GluC

H2B1A_MOUSE, H2B1B_MOUSE, H2B1C_MOUSE, H2B1F_MOUSE, H2B1H_MOUSE, H2B1M_MOUSE, H2B1P_MOUSE, H2B2B_MOUSE, H2B3A_MOUSE, H2B3B_MOUSE	Histone H2B	GTKAVTKYTSSK	473	2.20E-08	424.2383	1269.6930	3	0.0001	12	0	GluC
H2B1A_MOUSE, H2B1B_MOUSE, H2B1C_MOUSE, H2B1F_MOUSE, H2B1H_MOUSE, H2B1M_MOUSE, H2B1P_MOUSE, H2B2B_MOUSE, H2B3A_MOUSE, H2B3B_MOUSE	Histone H2B	GTKAVTKYTSSK	473	2.80E-03	424.2382	1269.6929	3	0.0000	12	0	GluC
H2B1A_MOUSE, H2B1B_MOUSE, H2B1C_MOUSE, H2B1F_MOUSE, H2B1H_MOUSE, H2B1M_MOUSE, H2B1P_MOUSE, H2B2B_MOUSE, H2B3A_MOUSE, H2B3B_MOUSE	Histone H2B	GTKAVTKYTSSK	473	2.50E-03	318.4305	1269.6929	4	0.0000	12	0	GluC
H2B1A_MOUSE, H2B1B_MOUSE, H2B1C_MOUSE, H2B1F_MOUSE, H2B1H_MOUSE, H2B1M_MOUSE, H2B1P_MOUSE, H2B2B_MOUSE, H2B3A_MOUSE, H2B3B_MOUSE	Histone H2B	GTKAVTKYTSSK	473	1.50E-03	424.2382	1269.6928	3	0.0000	12	0	GluC
H2B1A_MOUSE, H2B1B_MOUSE, H2B1C_MOUSE, H2B1F_MOUSE, H2B1H_MOUSE, H2B1M_MOUSE, H2B1P_MOUSE, H2B2B_MOUSE, H2B3A_MOUSE, H2B3B_MOUSE	Histone H2B	GTKAVTKYTSSK	473	1.30E-03	318.4305	1269.6929	4	0.0000	12	0	GluC
H2B1A_MOUSE, H2B1B_MOUSE, H2B1C_MOUSE, H2B1F_MOUSE, H2B1H_MOUSE, H2B1M_MOUSE, H2B1P_MOUSE, H2B2B_MOUSE, H2B3A_MOUSE, H2B3B_MOUSE	Histone H2B	GTKAVTKYTSSK	473	1.10E-03	424.2382	1269.6928	3	0.0000	12	0	GluC
H2B1A_MOUSE, H2B1B_MOUSE, H2B1C_MOUSE, H2B1F_MOUSE, H2B1H_MOUSE, H2B1M_MOUSE, H2B1P_MOUSE, H2B2B_MOUSE, H2B3A_MOUSE, H2B3B_MOUSE	Histone H2B	GTKAVTKYTSSK	473	9.00E-04	424.2382	1269.6929	3	0.0000	12	0	GluC
H2B1A_MOUSE, H2B1B_MOUSE, H2B1C_MOUSE, H2B1F_MOUSE, H2B1H_MOUSE, H2B1M_MOUSE, H2B1P_MOUSE, H2B2B_MOUSE, H2B3A_MOUSE, H2B3B_MOUSE	Histone H2B	GTKAVTKYTSSK	473	1.30E-04	318.4305	1269.6929	4	0.0000	12	0	GluC
H2B1A_MOUSE, H2B1B_MOUSE, H2B1C_MOUSE, H2B1F_MOUSE, H2B1H_MOUSE, H2B1M_MOUSE, H2B1P_MOUSE, H2B2B_MOUSE, H2B3A_MOUSE, H2B3B_MOUSE	Histone H2B	GTKAVTKYTSSK	473	1.20E-04	424.2382	1269.6929	3	0.0000	12	0	GluC
H2B1A_MOUSE, H2B1B_MOUSE, H2B1C_MOUSE, H2B1F_MOUSE, H2B1H_MOUSE, H2B1M_MOUSE, H2B1P_MOUSE, H2B2B_MOUSE, H2B3A_MOUSE, H2B3B_MOUSE	Histone H2B	GTKAVTKYTSSK	473	4.70E-06	424.2382	1269.6929	3	0.0000	12	0	GluC
H2B1A_MOUSE, H2B1B_MOUSE, H2B1C_MOUSE, H2B1F_MOUSE, H2B1H_MOUSE, H2B1M_MOUSE, H2B1P_MOUSE, H2B2B_MOUSE, H2B3A_MOUSE, H2B3B_MOUSE	Histone H2B	GTKAVTKYTSSK	473	2.10E-06	424.2382	1269.6929	3	0.0000	12	0	GluC
H2B1A_MOUSE, H2B1B_MOUSE, H2B1C_MOUSE, H2B1F_MOUSE, H2B1H_MOUSE, H2B1M_MOUSE, H2B1P_MOUSE, H2B2B_MOUSE, H2B3A_MOUSE, H2B3B_MOUSE	Histone H2B	GTKAVTKYTSSK	473	1.80E-06	424.2382	1269.6929	3	0.0000	12	0	GluC
H2B1A_MOUSE, H2B1B_MOUSE, H2B1C_MOUSE, H2B1F_MOUSE, H2B1H_MOUSE, H2B1M_MOUSE, H2B1P_MOUSE, H2B2B_MOUSE, H2B3A_MOUSE, H2B3B_MOUSE	Histone H2B	GTKAVTKYTSSK	473	9.70E-07	424.2382	1269.6929	3	0.0000	12	0	GluC
H2B1A_MOUSE, H2B1B_MOUSE, H2B1C_MOUSE, H2B1F_MOUSE, H2B1H_MOUSE, H2B1M_MOUSE, H2B1P_MOUSE, H2B2B_MOUSE, H2B3A_MOUSE, H2B3B_MOUSE	Histone H2B	GTKAVTKYTSSK	473	4.70E-08	424.2382	1269.6929	3	0.0000	12	0	GluC
H2B1A_MOUSE, H2B1B_MOUSE, H2B1C_MOUSE, H2B1F_MOUSE, H2B1H_MOUSE, H2B1M_MOUSE, H2B1P_MOUSE, H2B2B_MOUSE, H2B3A_MOUSE, H2B3B_MOUSE	Histone H2B	GTKAVTKYTSSK	473	8.50E-03	424.2382	1269.6927	3	-0.0001	12	0	GluC
H2B1A_MOUSE, H2B1B_MOUSE, H2B1C_MOUSE, H2B1F_MOUSE, H2B1H_MOUSE, H2B1M_MOUSE, H2B1P_MOUSE, H2B2B_MOUSE, H2B3A_MOUSE, H2B3B_MOUSE	Histone H2B	GTKAVTKYTSSK	473	4.60E-03	424.2382	1269.6927	3	-0.0001	12	0	GluC
H2B1A_MOUSE, H2B1B_MOUSE, H2B1C_MOUSE, H2B1F_MOUSE, H2B1H_MOUSE, H2B1M_MOUSE, H2B1P_MOUSE, H2B2B_MOUSE, H2B3A_MOUSE, H2B3B_MOUSE	Histone H2B	GTKAVTKYTSSK	473	3.10E-03	424.2382	1269.6927	3	-0.0001	12	0	GluC
H2B1A_MOUSE, H2B1B_MOUSE, H2B1C_MOUSE, H2B1F_MOUSE, H2B1H_MOUSE, H2B1M_MOUSE, H2B1P_MOUSE, H2B2B_MOUSE, H2B3A_MOUSE, H2B3B_MOUSE	Histone H2B	GTKAVTKYTSSK	473	2.40E-03	424.2382	1269.6927	3	-0.0001	12	0	GluC
H2B1A_MOUSE, H2B1B_MOUSE, H2B1C_MOUSE, H2B1F_MOUSE, H2B1H_MOUSE, H2B1M_MOUSE, H2B1P_MOUSE, H2B2B_MOUSE, H2B3A_MOUSE, H2B3B_MOUSE	Histone H2B	GTKAVTKYTSSK	473	1.50E-03	424.2382	1269.6927	3	-0.0001	12	0	GluC
H2B1A_MOUSE, H2B1B_MOUSE, H2B1C_MOUSE, H2B1F_MOUSE, H2B1H_MOUSE, H2B1M_MOUSE, H2B1P_MOUSE, H2B2B_MOUSE, H2B3A_MOUSE, H2B3B_MOUSE	Histone H2B	GTKAVTKYTSSK	473	1.30E-03	424.2382	1269.6927	3	-0.0001	12	0	GluC
H2B1A_MOUSE, H2B1B_MOUSE, H2B1C_MOUSE, H2B1F_MOUSE, H2B1H_MOUSE, H2B1M_MOUSE, H2B1P_MOUSE, H2B2B_MOUSE, H2B3A_MOUSE, H2B3B_MOUSE	Histone H2B	GTKAVTKYTSSK	473	1.40E-04	424.2382	1269.6927	3	-0.0001	12	0	GluC
H2B1A_MOUSE, H2B1B_MOUSE, H2B1C_MOUSE, H2B1F_MOUSE, H2B1H_MOUSE, H2B1M_MOUSE, H2B1P_MOUSE, H2B2B_MOUSE, H2B3A_MOUSE, H2B3B_MOUSE	Histone H2B	GTKAVTKYTSSK	473	9.70E-05	424.2382	1269.6927	3	-0.0001	12	0	GluC
H2B1A_MOUSE, H2B1B_MOUSE, H2B1C_MOUSE, H2B1F_MOUSE, H2B1H_MOUSE, H2B1M_MOUSE, H2B1P_MOUSE, H2B2B_MOUSE, H2B3A_MOUSE, H2B3B_MOUSE	Histone H2B	GTKAVTKYTSSK	473	4.30E-05	424.2382	1269.6928	3	-0.0001	12	0	GluC
H2B1A_MOUSE, H2B1B_MOUSE, H2B1C_MOUSE, H2B1F_MOUSE, H2B1H_MOUSE, H2B1M_MOUSE, H2B1P_MOUSE, H2B2B_MOUSE, H2B3A_MOUSE, H2B3B_MOUSE	Histone H2B	GTKAVTKYTSSK	473	4.30E-05	424.2382	1269.6927	3	-0.0001	12	0	GluC

H2B1A_MOUSE, H2B1B_MOUSE, H2B1C_MOUSE, H2B1F_MOUSE, H2B1H_MOUSE, H2B1M_MOUSE, H2B1P_MOUSE, H2B2B_MOUSE, H2B3A_MOUSE, H2B3B_MOUSE	Histone H2B	GTKAVTKYTSSK	473	3.60E-05	424.2382	1269.6927	3	-0.0001	12	0	GluC
H2B1A_MOUSE, H2B1B_MOUSE, H2B1C_MOUSE, H2B1F_MOUSE, H2B1H_MOUSE, H2B1M_MOUSE, H2B1P_MOUSE, H2B2B_MOUSE, H2B3A_MOUSE, H2B3B_MOUSE	Histone H2B	GTKAVTKYTSSK	473	3.20E-05	424.2382	1269.6928	3	-0.0001	12	0	GluC
H2B1A_MOUSE, H2B1B_MOUSE, H2B1C_MOUSE, H2B1F_MOUSE, H2B1H_MOUSE, H2B1M_MOUSE, H2B1P_MOUSE, H2B2B_MOUSE, H2B3A_MOUSE, H2B3B_MOUSE	Histone H2B	GTKAVTKYTSSK	473	2.80E-05	318.4305	1269.6928	4	-0.0001	12	0	GluC
H2B1A_MOUSE, H2B1B_MOUSE, H2B1C_MOUSE, H2B1F_MOUSE, H2B1H_MOUSE, H2B1M_MOUSE, H2B1P_MOUSE, H2B2B_MOUSE, H2B3A_MOUSE, H2B3B_MOUSE	Histone H2B	GTKAVTKYTSSK	473	2.80E-05	318.4305	1269.6928	4	-0.0001	12	0	GluC
H2B1A_MOUSE, H2B1B_MOUSE, H2B1C_MOUSE, H2B1F_MOUSE, H2B1H_MOUSE, H2B1M_MOUSE, H2B1P_MOUSE, H2B2B_MOUSE, H2B3A_MOUSE, H2B3B_MOUSE	Histone H2B	GTKAVTKYTSSK	473	8.00E-06	424.2382	1269.6927	3	-0.0001	12	0	GluC
H2B1A_MOUSE, H2B1B_MOUSE, H2B1C_MOUSE, H2B1F_MOUSE, H2B1H_MOUSE, H2B1M_MOUSE, H2B1P_MOUSE, H2B2B_MOUSE, H2B3A_MOUSE, H2B3B_MOUSE	Histone H2B	GTKAVTKYTSSK	473	1.80E-06	424.2382	1269.6928	3	-0.0001	12	0	GluC
H2B1A_MOUSE, H2B1B_MOUSE, H2B1C_MOUSE, H2B1F_MOUSE, H2B1H_MOUSE, H2B1M_MOUSE, H2B1P_MOUSE, H2B2B_MOUSE, H2B3A_MOUSE, H2B3B_MOUSE	Histone H2B	GTKAVTKYTSSK	473	1.50E-06	424.2382	1269.6927	3	-0.0001	12	0	GluC
H2B1A_MOUSE, H2B1B_MOUSE, H2B1C_MOUSE, H2B1F_MOUSE, H2B1H_MOUSE, H2B1M_MOUSE, H2B1P_MOUSE, H2B2B_MOUSE, H2B3A_MOUSE, H2B3B_MOUSE	Histone H2B	GTKAVTKYTSSK	473	5.80E-07	424.2382	1269.6927	3	-0.0001	12	0	GluC
H2B1A_MOUSE, H2B1B_MOUSE, H2B1C_MOUSE, H2B1F_MOUSE, H2B1H_MOUSE, H2B1M_MOUSE, H2B1P_MOUSE, H2B2B_MOUSE, H2B3A_MOUSE, H2B3B_MOUSE	Histone H2B	GTKAVTKYTSSK	473	6.60E-08	424.2382	1269.6927	3	-0.0001	12	0	GluC
H2B1A_MOUSE, H2B1B_MOUSE, H2B1C_MOUSE, H2B1F_MOUSE, H2B1H_MOUSE, H2B1M_MOUSE, H2B1P_MOUSE, H2B2B_MOUSE, H2B3A_MOUSE, H2B3B_MOUSE	Histone H2B	GTKAVTKYTSSK	473	3.30E-08	424.2382	1269.6927	3	-0.0001	12	0	GluC
H2B1A_MOUSE, H2B1B_MOUSE, H2B1C_MOUSE, H2B1F_MOUSE, H2B1H_MOUSE, H2B1M_MOUSE, H2B1P_MOUSE, H2B2B_MOUSE, H2B3A_MOUSE, H2B3B_MOUSE	Histone H2B	GTKAVTKYTSSK	473	6.20E-03	318.4304	1269.6926	4	-0.0002	12	0	GluC
H2B1A_MOUSE, H2B1B_MOUSE, H2B1C_MOUSE, H2B1F_MOUSE, H2B1H_MOUSE, H2B1M_MOUSE, H2B1P_MOUSE, H2B2B_MOUSE, H2B3A_MOUSE, H2B3B_MOUSE	Histone H2B	GTKAVTKYTSSK	473	4.90E-03	424.2382	1269.6926	3	-0.0002	12	0	GluC
H2B1A_MOUSE, H2B1B_MOUSE, H2B1C_MOUSE, H2B1F_MOUSE, H2B1H_MOUSE, H2B1M_MOUSE, H2B1P_MOUSE, H2B2B_MOUSE, H2B3A_MOUSE, H2B3B_MOUSE	Histone H2B	GTKAVTKYTSSK	473	7.40E-04	424.2382	1269.6927	3	-0.0002	12	0	GluC
H2B1A_MOUSE, H2B1B_MOUSE, H2B1C_MOUSE, H2B1F_MOUSE, H2B1H_MOUSE, H2B1M_MOUSE, H2B1P_MOUSE, H2B2B_MOUSE, H2B3A_MOUSE, H2B3B_MOUSE	Histone H2B	GTKAVTKYTSSK	473	3.30E-04	635.8536	1269.6927	2	-0.0002	12	0	GluC
H2B1A_MOUSE, H2B1B_MOUSE, H2B1C_MOUSE, H2B1F_MOUSE, H2B1H_MOUSE, H2B1M_MOUSE, H2B1P_MOUSE, H2B2B_MOUSE, H2B3A_MOUSE, H2B3B_MOUSE	Histone H2B	GTKAVTKYTSSK	473	5.10E-05	424.2382	1269.6926	3	-0.0002	12	0	GluC
H2B1A_MOUSE, H2B1B_MOUSE, H2B1C_MOUSE, H2B1F_MOUSE, H2B1H_MOUSE, H2B1M_MOUSE, H2B1P_MOUSE, H2B2B_MOUSE, H2B3A_MOUSE, H2B3B_MOUSE	Histone H2B	GTKAVTKYTSSK	473	1.10E-05	424.2382	1269.6926	3	-0.0002	12	0	GluC
H2B1A_MOUSE, H2B1B_MOUSE, H2B1C_MOUSE, H2B1F_MOUSE, H2B1H_MOUSE, H2B1M_MOUSE, H2B1P_MOUSE, H2B2B_MOUSE, H2B3A_MOUSE, H2B3B_MOUSE	Histone H2B	GTKAVTKYTSSK	473	9.10E-06	635.8536	1269.6927	2	-0.0002	12	0	GluC
H2B1A_MOUSE, H2B1B_MOUSE, H2B1C_MOUSE, H2B1F_MOUSE, H2B1H_MOUSE, H2B1M_MOUSE, H2B1P_MOUSE, H2B2B_MOUSE, H2B3A_MOUSE, H2B3B_MOUSE	Histone H2B	GTKAVTKYTSSK	473	1.80E-06	424.2382	1269.6927	3	-0.0002	12	0	GluC
H2B1A_MOUSE, H2B1B_MOUSE, H2B1C_MOUSE, H2B1F_MOUSE, H2B1H_MOUSE, H2B1M_MOUSE, H2B1P_MOUSE, H2B2B_MOUSE, H2B3A_MOUSE, H2B3B_MOUSE	Histone H2B	GTKAVTKYTSSK	473	1.50E-06	318.4304	1269.6926	4	-0.0002	12	0	GluC
H2B1A_MOUSE, H2B1B_MOUSE, H2B1C_MOUSE, H2B1F_MOUSE, H2B1H_MOUSE, H2B1M_MOUSE, H2B1P_MOUSE, H2B2B_MOUSE, H2B3A_MOUSE, H2B3B_MOUSE	Histone H2B	GTKAVTKYTSSK	473	9.20E-07	318.4305	1269.6927	4	-0.0002	12	0	GluC
H2B1A_MOUSE, H2B1B_MOUSE, H2B1C_MOUSE, H2B1F_MOUSE, H2B1H_MOUSE, H2B1M_MOUSE, H2B1P_MOUSE, H2B2B_MOUSE, H2B3A_MOUSE, H2B3B_MOUSE	Histone H2B	GTKAVTKYTSSK	473	7.10E-07	318.4305	1269.6927	4	-0.0002	12	0	GluC
H2B1A_MOUSE, H2B1B_MOUSE, H2B1C_MOUSE, H2B1F_MOUSE, H2B1H_MOUSE, H2B1M_MOUSE, H2B1P_MOUSE, H2B2B_MOUSE, H2B3A_MOUSE, H2B3B_MOUSE	Histone H2B	GTKAVTKYTSSK	473	1.60E-07	424.2382	1269.6926	3	-0.0002	12	0	GluC
H2B1A_MOUSE, H2B1B_MOUSE, H2B1C_MOUSE, H2B1F_MOUSE, H2B1H_MOUSE, H2B1M_MOUSE, H2B1P_MOUSE, H2B2B_MOUSE, H2B3A_MOUSE, H2B3B_MOUSE	Histone H2B	GTKAVTKYTSSK	473	1.40E-07	424.2382	1269.6926	3	-0.0002	12	0	GluC
H2B1A_MOUSE, H2B1B_MOUSE, H2B1C_MOUSE, H2B1F_MOUSE, H2B1H_MOUSE, H2B1M_MOUSE, H2B1P_MOUSE, H2B2B_MOUSE, H2B3A_MOUSE, H2B3B_MOUSE	Histone H2B	GTKAVTKYTSSK	473	1.10E-07	424.2382	1269.6926	3	-0.0002	12	0	GluC

H2B1A_MOUSE, H2B1B_MOUSE, H2B1C_MOUSE, H2B1F_MOUSE, H2B1H_MOUSE, H2B1M_MOUSE, H2B1P_MOUSE, H2B2B_MOUSE, H2B3A_MOUSE, H2B3B_MOUSE	Histone H2B	GTKAVTKYTSSK	473	1.20E-08	424.2382	1269.6926	3	-0.0002	12	0	GluC
H2B1A_MOUSE, H2B1B_MOUSE, H2B1C_MOUSE, H2B1F_MOUSE, H2B1H_MOUSE, H2B1M_MOUSE, H2B1P_MOUSE, H2B2B_MOUSE, H2B3A_MOUSE, H2B3B_MOUSE	Histone H2B	GTKAVTKYTSSK	473	4.40E-04	424.2381	1269.6925	3	-0.0003	12	0	GluC
H2B1A_MOUSE, H2B1B_MOUSE, H2B1C_MOUSE, H2B1F_MOUSE, H2B1H_MOUSE, H2B1M_MOUSE, H2B1P_MOUSE, H2B2B_MOUSE, H2B3A_MOUSE, H2B3B_MOUSE	Histone H2B	GTKAVTKYTSSK	473	2.40E-04	424.2381	1269.6926	3	-0.0003	12	0	GluC
H2B1A_MOUSE, H2B1B_MOUSE, H2B1C_MOUSE, H2B1F_MOUSE, H2B1H_MOUSE, H2B1M_MOUSE, H2B1P_MOUSE, H2B2B_MOUSE, H2B3A_MOUSE, H2B3B_MOUSE	Histone H2B	GTKAVTKYTSSK	473	1.00E-04	424.2381	1269.6926	3	-0.0003	12	0	GluC
H2B1A_MOUSE, H2B1B_MOUSE, H2B1C_MOUSE, H2B1F_MOUSE, H2B1H_MOUSE, H2B1M_MOUSE, H2B1P_MOUSE, H2B2B_MOUSE, H2B3A_MOUSE, H2B3B_MOUSE	Histone H2B	GTKAVTKYTSSK	473	6.90E-05	424.2381	1269.6926	3	-0.0003	12	0	GluC
H2B1A_MOUSE, H2B1B_MOUSE, H2B1C_MOUSE, H2B1F_MOUSE, H2B1H_MOUSE, H2B1M_MOUSE, H2B1P_MOUSE, H2B2B_MOUSE, H2B3A_MOUSE, H2B3B_MOUSE	Histone H2B	GTKAVTKYTSSK	473	1.40E-05	424.2381	1269.6926	3	-0.0003	12	0	GluC
H2B1A_MOUSE, H2B1B_MOUSE, H2B1C_MOUSE, H2B1F_MOUSE, H2B1H_MOUSE, H2B1M_MOUSE, H2B1P_MOUSE, H2B2B_MOUSE, H2B3A_MOUSE, H2B3B_MOUSE	Histone H2B	GTKAVTKYTSSK	473	1.10E-05	424.2381	1269.6926	3	-0.0003	12	0	GluC
H2B1A_MOUSE, H2B1B_MOUSE, H2B1C_MOUSE, H2B1F_MOUSE, H2B1H_MOUSE, H2B1M_MOUSE, H2B1P_MOUSE, H2B2B_MOUSE, H2B3A_MOUSE, H2B3B_MOUSE	Histone H2B	GTKAVTKYTSSK	473	5.80E-03	424.2381	1269.6924	3	-0.0004	12	0	GluC
H2B1A_MOUSE, H2B1B_MOUSE, H2B1C_MOUSE, H2B1F_MOUSE, H2B1H_MOUSE, H2B1M_MOUSE, H2B1P_MOUSE, H2B2B_MOUSE, H2B3A_MOUSE, H2B3B_MOUSE	Histone H2B	GTKAVTKYTSSK	473	3.00E-03	424.2381	1269.6925	3	-0.0004	12	0	GluC
H2B1A_MOUSE, H2B1B_MOUSE, H2B1C_MOUSE, H2B1F_MOUSE, H2B1H_MOUSE, H2B1M_MOUSE, H2B1P_MOUSE, H2B2B_MOUSE, H2B3A_MOUSE, H2B3B_MOUSE	Histone H2B	GTKAVTKYTSSK	473	1.00E-03	424.2381	1269.6924	3	-0.0004	12	0	GluC
H2B1A_MOUSE, H2B1B_MOUSE, H2B1C_MOUSE, H2B1F_MOUSE, H2B1H_MOUSE, H2B1M_MOUSE, H2B1P_MOUSE, H2B2B_MOUSE, H2B3A_MOUSE, H2B3B_MOUSE	Histone H2B	GTKAVTKYTSSK	473	9.20E-04	424.2381	1269.6925	3	-0.0004	12	0	GluC
H2B1A_MOUSE, H2B1B_MOUSE, H2B1C_MOUSE, H2B1F_MOUSE, H2B1H_MOUSE, H2B1M_MOUSE, H2B1P_MOUSE, H2B2B_MOUSE, H2B3A_MOUSE, H2B3B_MOUSE	Histone H2B	GTKAVTKYTSSK	473	8.20E-04	318.4304	1269.6924	4	-0.0004	12	0	GluC
H2B1A_MOUSE, H2B1B_MOUSE, H2B1C_MOUSE, H2B1F_MOUSE, H2B1H_MOUSE, H2B1M_MOUSE, H2B1P_MOUSE, H2B2B_MOUSE, H2B3A_MOUSE, H2B3B_MOUSE	Histone H2B	GTKAVTKYTSSK	473	1.50E-04	424.2381	1269.6925	3	-0.0004	12	0	GluC
H2B1A_MOUSE, H2B1B_MOUSE, H2B1C_MOUSE, H2B1F_MOUSE, H2B1H_MOUSE, H2B1M_MOUSE, H2B1P_MOUSE, H2B2B_MOUSE, H2B3A_MOUSE, H2B3B_MOUSE	Histone H2B	GTKAVTKYTSSK	473	1.30E-04	635.8535	1269.6924	2	-0.0004	12	0	GluC
H2B1A_MOUSE, H2B1B_MOUSE, H2B1C_MOUSE, H2B1F_MOUSE, H2B1H_MOUSE, H2B1M_MOUSE, H2B1P_MOUSE, H2B2B_MOUSE, H2B3A_MOUSE, H2B3B_MOUSE	Histone H2B	GTKAVTKYTSSK	473	9.00E-05	424.2381	1269.6925	3	-0.0004	12	0	GluC
H2B1A_MOUSE, H2B1B_MOUSE, H2B1C_MOUSE, H2B1F_MOUSE, H2B1H_MOUSE, H2B1M_MOUSE, H2B1P_MOUSE, H2B2B_MOUSE, H2B3A_MOUSE, H2B3B_MOUSE	Histone H2B	GTKAVTKYTSSK	473	7.40E-05	424.2381	1269.6924	3	-0.0004	12	0	GluC
H2B1A_MOUSE, H2B1B_MOUSE, H2B1C_MOUSE, H2B1F_MOUSE, H2B1H_MOUSE, H2B1M_MOUSE, H2B1P_MOUSE, H2B2B_MOUSE, H2B3A_MOUSE, H2B3B_MOUSE	Histone H2B	GTKAVTKYTSSK	473	6.60E-05	424.2381	1269.6925	3	-0.0004	12	0	GluC
H2B1A_MOUSE, H2B1B_MOUSE, H2B1C_MOUSE, H2B1F_MOUSE, H2B1H_MOUSE, H2B1M_MOUSE, H2B1P_MOUSE, H2B2B_MOUSE, H2B3A_MOUSE, H2B3B_MOUSE	Histone H2B	GTKAVTKYTSSK	473	5.80E-05	424.2381	1269.6924	3	-0.0004	12	0	GluC
H2B1A_MOUSE, H2B1B_MOUSE, H2B1C_MOUSE, H2B1F_MOUSE, H2B1H_MOUSE, H2B1M_MOUSE, H2B1P_MOUSE, H2B2B_MOUSE, H2B3A_MOUSE, H2B3B_MOUSE	Histone H2B	GTKAVTKYTSSK	473	4.40E-05	424.2381	1269.6924	3	-0.0004	12	0	GluC
H2B1A_MOUSE, H2B1B_MOUSE, H2B1C_MOUSE, H2B1F_MOUSE, H2B1H_MOUSE, H2B1M_MOUSE, H2B1P_MOUSE, H2B2B_MOUSE, H2B3A_MOUSE, H2B3B_MOUSE	Histone H2B	GTKAVTKYTSSK	473	3.40E-05	424.2381	1269.6925	3	-0.0004	12	0	GluC
H2B1A_MOUSE, H2B1B_MOUSE, H2B1C_MOUSE, H2B1F_MOUSE, H2B1H_MOUSE, H2B1M_MOUSE, H2B1P_MOUSE, H2B2B_MOUSE, H2B3A_MOUSE, H2B3B_MOUSE	Histone H2B	GTKAVTKYTSSK	473	1.20E-05	424.2381	1269.6925	3	-0.0004	12	0	GluC
H2B1A_MOUSE, H2B1B_MOUSE, H2B1C_MOUSE, H2B1F_MOUSE, H2B1H_MOUSE, H2B1M_MOUSE, H2B1P_MOUSE, H2B2B_MOUSE, H2B3A_MOUSE, H2B3B_MOUSE	Histone H2B	GTKAVTKYTSSK	473	5.60E-06	318.4304	1269.6924	4	-0.0004	12	0	GluC
H2B1A_MOUSE, H2B1B_MOUSE, H2B1C_MOUSE, H2B1F_MOUSE, H2B1H_MOUSE, H2B1M_MOUSE, H2B1P_MOUSE, H2B2B_MOUSE, H2B3A_MOUSE, H2B3B_MOUSE	Histone H2B	GTKAVTKYTSSK	473	5.30E-06	424.2381	1269.6925	3	-0.0004	12	0	GluC
H2B1A_MOUSE, H2B1B_MOUSE, H2B1C_MOUSE, H2B1F_MOUSE, H2B1H_MOUSE, H2B1M_MOUSE, H2B1P_MOUSE, H2B2B_MOUSE, H2B3A_MOUSE, H2B3B_MOUSE	Histone H2B	GTKAVTKYTSSK	473	3.60E-06	424.2381	1269.6924	3	-0.0004	12	0	GluC

H2B1A_MOUSE, H2B1B_MOUSE, H2B1C_MOUSE, H2B1F_MOUSE, H2B1H_MOUSE, H2B1M_MOUSE, H2B1P_MOUSE, H2B2B_MOUSE, H2B3A_MOUSE, H2B3B_MOUSE	Histone H2B	GTKAVTKYTSSK	473	1.50E-06	424.2381	1269.6924	3	-0.0004	12	0	GluC
H2B1A_MOUSE, H2B1B_MOUSE, H2B1C_MOUSE, H2B1F_MOUSE, H2B1H_MOUSE, H2B1M_MOUSE, H2B1P_MOUSE, H2B2B_MOUSE, H2B3A_MOUSE, H2B3B_MOUSE	Histone H2B	GTKAVTKYTSSK	473	9.00E-07	424.2381	1269.6925	3	-0.0004	12	0	GluC
H2B1A_MOUSE, H2B1B_MOUSE, H2B1C_MOUSE, H2B1F_MOUSE, H2B1H_MOUSE, H2B1M_MOUSE, H2B1P_MOUSE, H2B2B_MOUSE, H2B3A_MOUSE, H2B3B_MOUSE	Histone H2B	GTKAVTKYTSSK	473	6.30E-07	424.2381	1269.6924	3	-0.0004	12	0	GluC
H2B1A_MOUSE, H2B1B_MOUSE, H2B1C_MOUSE, H2B1F_MOUSE, H2B1H_MOUSE, H2B1M_MOUSE, H2B1P_MOUSE, H2B2B_MOUSE, H2B3A_MOUSE, H2B3B_MOUSE	Histone H2B	GTKAVTKYTSSK	473	4.90E-07	424.2381	1269.6924	3	-0.0004	12	0	GluC
H2B1A_MOUSE, H2B1B_MOUSE, H2B1C_MOUSE, H2B1F_MOUSE, H2B1H_MOUSE, H2B1M_MOUSE, H2B1P_MOUSE, H2B2B_MOUSE, H2B3A_MOUSE, H2B3B_MOUSE	Histone H2B	GTKAVTKYTSSK	473	3.30E-07	424.2381	1269.6925	3	-0.0004	12	0	GluC
H2B1A_MOUSE, H2B1B_MOUSE, H2B1C_MOUSE, H2B1F_MOUSE, H2B1H_MOUSE, H2B1M_MOUSE, H2B1P_MOUSE, H2B2B_MOUSE, H2B3A_MOUSE, H2B3B_MOUSE	Histone H2B	GTKAVTKYTSSK	473	1.50E-07	424.2381	1269.6924	3	-0.0004	12	0	GluC
H2B1A_MOUSE, H2B1B_MOUSE, H2B1C_MOUSE, H2B1F_MOUSE, H2B1H_MOUSE, H2B1M_MOUSE, H2B1P_MOUSE, H2B2B_MOUSE, H2B3A_MOUSE, H2B3B_MOUSE	Histone H2B	GTKAVTKYTSSK	473	1.10E-07	424.2381	1269.6925	3	-0.0004	12	0	GluC
H2B1A_MOUSE, H2B1B_MOUSE, H2B1C_MOUSE, H2B1F_MOUSE, H2B1H_MOUSE, H2B1M_MOUSE, H2B1P_MOUSE, H2B2B_MOUSE, H2B3A_MOUSE, H2B3B_MOUSE	Histone H2B	GTKAVTKYTSSK	473	1.10E-07	424.2381	1269.6924	3	-0.0004	12	0	GluC
H2B1A_MOUSE, H2B1B_MOUSE, H2B1C_MOUSE, H2B1F_MOUSE, H2B1H_MOUSE, H2B1M_MOUSE, H2B1P_MOUSE, H2B2B_MOUSE, H2B3A_MOUSE, H2B3B_MOUSE	Histone H2B	GTKAVTKYTSSK	473	8.10E-08	318.4304	1269.6925	4	-0.0004	12	0	GluC
H2B1A_MOUSE, H2B1B_MOUSE, H2B1C_MOUSE, H2B1F_MOUSE, H2B1H_MOUSE, H2B1M_MOUSE, H2B1P_MOUSE, H2B2B_MOUSE, H2B3A_MOUSE, H2B3B_MOUSE	Histone H2B	GTKAVTKYTSSK	473	3.30E-08	318.4304	1269.6925	4	-0.0004	12	0	GluC
H2B1A_MOUSE, H2B1B_MOUSE, H2B1C_MOUSE, H2B1F_MOUSE, H2B1H_MOUSE, H2B1M_MOUSE, H2B1P_MOUSE, H2B2B_MOUSE, H2B3A_MOUSE, H2B3B_MOUSE	Histone H2B	GTKAVTKYTSSK	473	1.40E-08	318.4304	1269.6925	4	-0.0004	12	0	GluC
H2B1A_MOUSE, H2B1B_MOUSE, H2B1C_MOUSE, H2B1F_MOUSE, H2B1H_MOUSE, H2B1M_MOUSE, H2B1P_MOUSE, H2B2B_MOUSE, H2B3A_MOUSE, H2B3B_MOUSE	Histone H2B	GTKAVTKYTSSK	473	3.90E-03	424.2381	1269.6924	3	-0.0005	12	0	GluC
H2B1A_MOUSE, H2B1B_MOUSE, H2B1C_MOUSE, H2B1F_MOUSE, H2B1H_MOUSE, H2B1M_MOUSE, H2B1P_MOUSE, H2B2B_MOUSE, H2B3A_MOUSE, H2B3B_MOUSE	Histone H2B	GTKAVTKYTSSK	473	1.30E-03	424.2381	1269.6924	3	-0.0005	12	0	GluC
H2B1A_MOUSE, H2B1B_MOUSE, H2B1C_MOUSE, H2B1F_MOUSE, H2B1H_MOUSE, H2B1M_MOUSE, H2B1P_MOUSE, H2B2B_MOUSE, H2B3A_MOUSE, H2B3B_MOUSE	Histone H2B	GTKAVTKYTSSK	473	7.50E-04	424.2381	1269.6924	3	-0.0005	12	0	GluC
H2B1A_MOUSE, H2B1B_MOUSE, H2B1C_MOUSE, H2B1F_MOUSE, H2B1H_MOUSE, H2B1M_MOUSE, H2B1P_MOUSE, H2B2B_MOUSE, H2B3A_MOUSE, H2B3B_MOUSE	Histone H2B	GTKAVTKYTSSK	473	6.50E-04	424.238	1269.6923	3	-0.0005	12	0	GluC
H2B1A_MOUSE, H2B1B_MOUSE, H2B1C_MOUSE, H2B1F_MOUSE, H2B1H_MOUSE, H2B1M_MOUSE, H2B1P_MOUSE, H2B2B_MOUSE, H2B3A_MOUSE, H2B3B_MOUSE	Histone H2B	GTKAVTKYTSSK	473	3.40E-04	424.2381	1269.6924	3	-0.0005	12	0	GluC
H2B1A_MOUSE, H2B1B_MOUSE, H2B1C_MOUSE, H2B1F_MOUSE, H2B1H_MOUSE, H2B1M_MOUSE, H2B1P_MOUSE, H2B2B_MOUSE, H2B3A_MOUSE, H2B3B_MOUSE	Histone H2B	GTKAVTKYTSSK	473	1.70E-05	635.8534	1269.6923	2	-0.0005	12	0	GluC
H2B1A_MOUSE, H2B1B_MOUSE, H2B1C_MOUSE, H2B1F_MOUSE, H2B1H_MOUSE, H2B1M_MOUSE, H2B1P_MOUSE, H2B2B_MOUSE, H2B3A_MOUSE, H2B3B_MOUSE	Histone H2B	GTKAVTKYTSSK	473	1.30E-05	424.2381	1269.6924	3	-0.0005	12	0	GluC
H2B1A_MOUSE, H2B1B_MOUSE, H2B1C_MOUSE, H2B1F_MOUSE, H2B1H_MOUSE, H2B1M_MOUSE, H2B1P_MOUSE, H2B2B_MOUSE, H2B3A_MOUSE, H2B3B_MOUSE	Histone H2B	GTKAVTKYTSSK	473	1.10E-05	424.238	1269.6923	3	-0.0005	12	0	GluC
H2B1A_MOUSE, H2B1B_MOUSE, H2B1C_MOUSE, H2B1F_MOUSE, H2B1H_MOUSE, H2B1M_MOUSE, H2B1P_MOUSE, H2B2B_MOUSE, H2B3A_MOUSE, H2B3B_MOUSE	Histone H2B	GTKAVTKYTSSK	473	4.90E-07	424.2381	1269.6924	3	-0.0005	12	0	GluC
H2B1A_MOUSE, H2B1B_MOUSE, H2B1C_MOUSE, H2B1F_MOUSE, H2B1H_MOUSE, H2B1M_MOUSE, H2B1P_MOUSE, H2B2B_MOUSE, H2B3A_MOUSE, H2B3B_MOUSE	Histone H2B	GTKAVTKYTSSK	473	3.40E-07	424.2381	1269.6924	3	-0.0005	12	0	GluC
H2B1A_MOUSE, H2B1B_MOUSE, H2B1C_MOUSE, H2B1F_MOUSE, H2B1H_MOUSE, H2B1M_MOUSE, H2B1P_MOUSE, H2B2B_MOUSE, H2B3A_MOUSE, H2B3B_MOUSE	Histone H2B	GTKAVTKYTSSK	473	2.20E-08	318.4304	1269.6923	4	-0.0005	12	0	GluC
H2B1A_MOUSE, H2B1B_MOUSE, H2B1C_MOUSE, H2B1F_MOUSE, H2B1H_MOUSE, H2B1M_MOUSE, H2B1P_MOUSE, H2B2B_MOUSE, H2B3A_MOUSE, H2B3B_MOUSE	Histone H2B	GTKAVTKYTSSK	473	4.80E-03	424.238	1269.6922	3	-0.0006	12	0	GluC
H2B1A_MOUSE, H2B1B_MOUSE, H2B1C_MOUSE, H2B1F_MOUSE, H2B1H_MOUSE, H2B1M_MOUSE, H2B1P_MOUSE, H2B2B_MOUSE, H2B3A_MOUSE, H2B3B_MOUSE	Histone H2B	GTKAVTKYTSSK	473	1.20E-03	424.238	1269.6922	3	-0.0006	12	0	GluC

H2B1A_MOUSE, H2B1B_MOUSE, H2B1C_MOUSE, H2B1F_MOUSE, H2B1H_MOUSE, H2B1M_MOUSE, H2B1P_MOUSE, H2B2B_MOUSE, H2B3A_MOUSE, H2B3B_MOUSE	Histone H2B	GTKAVTKYTSSK	473	6.70E-04	424.238	1269.6923	3	-0.0006	12	0	GluC
H2B1A_MOUSE, H2B1B_MOUSE, H2B1C_MOUSE, H2B1F_MOUSE, H2B1H_MOUSE, H2B1M_MOUSE, H2B1P_MOUSE, H2B2B_MOUSE, H2B3A_MOUSE, H2B3B_MOUSE	Histone H2B	GTKAVTKYTSSK	473	6.50E-04	635.8534	1269.6922	2	-0.0006	12	0	GluC
H2B1A_MOUSE, H2B1B_MOUSE, H2B1C_MOUSE, H2B1F_MOUSE, H2B1H_MOUSE, H2B1M_MOUSE, H2B1P_MOUSE, H2B2B_MOUSE, H2B3A_MOUSE, H2B3B_MOUSE	Histone H2B	GTKAVTKYTSSK	473	5.80E-04	424.238	1269.6922	3	-0.0006	12	0	GluC
H2B1A_MOUSE, H2B1B_MOUSE, H2B1C_MOUSE, H2B1F_MOUSE, H2B1H_MOUSE, H2B1M_MOUSE, H2B1P_MOUSE, H2B2B_MOUSE, H2B3A_MOUSE, H2B3B_MOUSE	Histone H2B	GTKAVTKYTSSK	473	3.70E-04	424.238	1269.6923	3	-0.0006	12	0	GluC
H2B1A_MOUSE, H2B1B_MOUSE, H2B1C_MOUSE, H2B1F_MOUSE, H2B1H_MOUSE, H2B1M_MOUSE, H2B1P_MOUSE, H2B2B_MOUSE, H2B3A_MOUSE, H2B3B_MOUSE	Histone H2B	GTKAVTKYTSSK	473	2.90E-04	424.238	1269.6923	3	-0.0006	12	0	GluC
H2B1A_MOUSE, H2B1B_MOUSE, H2B1C_MOUSE, H2B1F_MOUSE, H2B1H_MOUSE, H2B1M_MOUSE, H2B1P_MOUSE, H2B2B_MOUSE, H2B3A_MOUSE, H2B3B_MOUSE	Histone H2B	GTKAVTKYTSSK	473	1.60E-04	424.238	1269.6922	3	-0.0006	12	0	GluC
H2B1A_MOUSE, H2B1B_MOUSE, H2B1C_MOUSE, H2B1F_MOUSE, H2B1H_MOUSE, H2B1M_MOUSE, H2B1P_MOUSE, H2B2B_MOUSE, H2B3A_MOUSE, H2B3B_MOUSE	Histone H2B	GTKAVTKYTSSK	473	8.30E-05	318.4303	1269.6923	4	-0.0006	12	0	GluC
H2B1A_MOUSE, H2B1B_MOUSE, H2B1C_MOUSE, H2B1F_MOUSE, H2B1H_MOUSE, H2B1M_MOUSE, H2B1P_MOUSE, H2B2B_MOUSE, H2B3A_MOUSE, H2B3B_MOUSE	Histone H2B	GTKAVTKYTSSK	473	5.10E-06	424.238	1269.6922	3	-0.0006	12	0	GluC
H2B1A_MOUSE, H2B1B_MOUSE, H2B1C_MOUSE, H2B1F_MOUSE, H2B1H_MOUSE, H2B1M_MOUSE, H2B1P_MOUSE, H2B2B_MOUSE, H2B3A_MOUSE, H2B3B_MOUSE	Histone H2B	GTKAVTKYTSSK	473	3.80E-06	424.238	1269.6922	3	-0.0006	12	0	GluC
H2B1A_MOUSE, H2B1B_MOUSE, H2B1C_MOUSE, H2B1F_MOUSE, H2B1H_MOUSE, H2B1M_MOUSE, H2B1P_MOUSE, H2B2B_MOUSE, H2B3A_MOUSE, H2B3B_MOUSE	Histone H2B	GTKAVTKYTSSK	473	9.50E-07	424.238	1269.6923	3	-0.0006	12	0	GluC
H2B1A_MOUSE, H2B1B_MOUSE, H2B1C_MOUSE, H2B1F_MOUSE, H2B1H_MOUSE, H2B1M_MOUSE, H2B1P_MOUSE, H2B2B_MOUSE, H2B3A_MOUSE, H2B3B_MOUSE	Histone H2B	GTKAVTKYTSSK	473	6.90E-03	424.238	1269.6921	3	-0.0007	12	0	GluC
H2B1A_MOUSE, H2B1B_MOUSE, H2B1C_MOUSE, H2B1F_MOUSE, H2B1H_MOUSE, H2B1M_MOUSE, H2B1P_MOUSE, H2B2B_MOUSE, H2B3A_MOUSE, H2B3B_MOUSE	Histone H2B	GTKAVTKYTSSK	473	3.50E-03	424.238	1269.6921	3	-0.0007	12	0	GluC
H2B1A_MOUSE, H2B1B_MOUSE, H2B1C_MOUSE, H2B1F_MOUSE, H2B1H_MOUSE, H2B1M_MOUSE, H2B1P_MOUSE, H2B2B_MOUSE, H2B3A_MOUSE, H2B3B_MOUSE	Histone H2B	GTKAVTKYTSSK	473	2.20E-03	424.238	1269.6921	3	-0.0007	12	0	GluC
H2B1A_MOUSE, H2B1B_MOUSE, H2B1C_MOUSE, H2B1F_MOUSE, H2B1H_MOUSE, H2B1M_MOUSE, H2B1P_MOUSE, H2B2B_MOUSE, H2B3A_MOUSE, H2B3B_MOUSE	Histone H2B	GTKAVTKYTSSK	473	1.80E-03	424.238	1269.6921	3	-0.0007	12	0	GluC
H2B1A_MOUSE, H2B1B_MOUSE, H2B1C_MOUSE, H2B1F_MOUSE, H2B1H_MOUSE, H2B1M_MOUSE, H2B1P_MOUSE, H2B2B_MOUSE, H2B3A_MOUSE, H2B3B_MOUSE	Histone H2B	GTKAVTKYTSSK	473	1.60E-03	424.238	1269.6921	3	-0.0007	12	0	GluC
H2B1A_MOUSE, H2B1B_MOUSE, H2B1C_MOUSE, H2B1F_MOUSE, H2B1H_MOUSE, H2B1M_MOUSE, H2B1P_MOUSE, H2B2B_MOUSE, H2B3A_MOUSE, H2B3B_MOUSE	Histone H2B	GTKAVTKYTSSK	473	9.10E-04	424.238	1269.6922	3	-0.0007	12	0	GluC
H2B1A_MOUSE, H2B1B_MOUSE, H2B1C_MOUSE, H2B1F_MOUSE, H2B1H_MOUSE, H2B1M_MOUSE, H2B1P_MOUSE, H2B2B_MOUSE, H2B3A_MOUSE, H2B3B_MOUSE	Histone H2B	GTKAVTKYTSSK	473	1.40E-04	424.238	1269.6921	3	-0.0007	12	0	GluC
H2B1A_MOUSE, H2B1B_MOUSE, H2B1C_MOUSE, H2B1F_MOUSE, H2B1H_MOUSE, H2B1M_MOUSE, H2B1P_MOUSE, H2B2B_MOUSE, H2B3A_MOUSE, H2B3B_MOUSE	Histone H2B	GTKAVTKYTSSK	473	9.00E-05	424.238	1269.6922	3	-0.0007	12	0	GluC
H2B1A_MOUSE, H2B1B_MOUSE, H2B1C_MOUSE, H2B1F_MOUSE, H2B1H_MOUSE, H2B1M_MOUSE, H2B1P_MOUSE, H2B2B_MOUSE, H2B3A_MOUSE, H2B3B_MOUSE	Histone H2B	GTKAVTKYTSSK	473	5.80E-05	424.238	1269.6921	3	-0.0007	12	0	GluC
H2B1A_MOUSE, H2B1B_MOUSE, H2B1C_MOUSE, H2B1F_MOUSE, H2B1H_MOUSE, H2B1M_MOUSE, H2B1P_MOUSE, H2B2B_MOUSE, H2B3A_MOUSE, H2B3B_MOUSE	Histone H2B	GTKAVTKYTSSK	473	5.70E-05	424.238	1269.6922	3	-0.0007	12	0	GluC
H2B1A_MOUSE, H2B1B_MOUSE, H2B1C_MOUSE, H2B1F_MOUSE, H2B1H_MOUSE, H2B1M_MOUSE, H2B1P_MOUSE, H2B2B_MOUSE, H2B3A_MOUSE, H2B3B_MOUSE	Histone H2B	GTKAVTKYTSSK	473	5.40E-05	424.238	1269.6921	3	-0.0007	12	0	GluC
H2B1A_MOUSE, H2B1B_MOUSE, H2B1C_MOUSE, H2B1F_MOUSE, H2B1H_MOUSE, H2B1M_MOUSE, H2B1P_MOUSE, H2B2B_MOUSE, H2B3A_MOUSE, H2B3B_MOUSE	Histone H2B	GTKAVTKYTSSK	473	2.10E-05	424.238	1269.6922	3	-0.0007	12	0	GluC
H2B1A_MOUSE, H2B1B_MOUSE, H2B1C_MOUSE, H2B1F_MOUSE, H2B1H_MOUSE, H2B1M_MOUSE, H2B1P_MOUSE, H2B2B_MOUSE, H2B3A_MOUSE, H2B3B_MOUSE	Histone H2B	GTKAVTKYTSSK	473	3.30E-06	424.238	1269.6921	3	-0.0007	12	0	GluC
H2B1A_MOUSE, H2B1B_MOUSE, H2B1C_MOUSE, H2B1F_MOUSE, H2B1H_MOUSE, H2B1M_MOUSE, H2B1P_MOUSE, H2B2B_MOUSE, H2B3A_MOUSE, H2B3B_MOUSE	Histone H2B	GTKAVTKYTSSK	473	6.70E-08	424.238	1269.6921	3	-0.0007	12	0	GluC

H2B1A_MOUSE, H2B1B_MOUSE, H2B1C_MOUSE, H2B1F_MOUSE, H2B1H_MOUSE, H2B1M_MOUSE, H2B1P_MOUSE, H2B2B_MOUSE, H2B3A_MOUSE, H2B3B_MOUSE	Histone H2B	GTKAVTKYTSSK	473	1.30E-08	318.4303	1269.6921	4	-0.0007	12	0	GluC
H2B1A_MOUSE, H2B1B_MOUSE, H2B1C_MOUSE, H2B1F_MOUSE, H2B1H_MOUSE, H2B1M_MOUSE, H2B1P_MOUSE, H2B2B_MOUSE, H2B3A_MOUSE, H2B3B_MOUSE	Histone H2B	GTKAVTKYTSSK	473	7.30E-04	424.238	1269.6921	3	-0.0008	12	0	GluC
H2B1A_MOUSE, H2B1B_MOUSE, H2B1C_MOUSE, H2B1F_MOUSE, H2B1H_MOUSE, H2B1M_MOUSE, H2B1P_MOUSE, H2B2B_MOUSE, H2B3A_MOUSE, H2B3B_MOUSE	Histone H2B	GTKAVTKYTSSK	473	8.80E-05	424.238	1269.6920	3	-0.0008	12	0	GluC
H2B1A_MOUSE, H2B1B_MOUSE, H2B1C_MOUSE, H2B1F_MOUSE, H2B1H_MOUSE, H2B1M_MOUSE, H2B1P_MOUSE, H2B2B_MOUSE, H2B3A_MOUSE, H2B3B_MOUSE	Histone H2B	GTKAVTKYTSSK	473	1.50E-05	318.4303	1269.6921	4	-0.0008	12	0	GluC
H2B1A_MOUSE, H2B1B_MOUSE, H2B1C_MOUSE, H2B1F_MOUSE, H2B1H_MOUSE, H2B1M_MOUSE, H2B1P_MOUSE, H2B2B_MOUSE, H2B3A_MOUSE, H2B3B_MOUSE	Histone H2B	GTKAVTKYTSSK	473	1.20E-05	424.238	1269.6921	3	-0.0008	12	0	GluC
H2B1A_MOUSE, H2B1B_MOUSE, H2B1C_MOUSE, H2B1F_MOUSE, H2B1H_MOUSE, H2B1M_MOUSE, H2B1P_MOUSE, H2B2B_MOUSE, H2B3A_MOUSE, H2B3B_MOUSE	Histone H2B	GTKAVTKYTSSK	473	4.70E-06	424.238	1269.6921	3	-0.0008	12	0	GluC
H2B1A_MOUSE, H2B1B_MOUSE, H2B1C_MOUSE, H2B1F_MOUSE, H2B1H_MOUSE, H2B1M_MOUSE, H2B1P_MOUSE, H2B2B_MOUSE, H2B3A_MOUSE, H2B3B_MOUSE	Histone H2B	GTKAVTKYTSSK	473	4.50E-06	424.238	1269.6920	3	-0.0008	12	0	GluC
H2B1A_MOUSE, H2B1B_MOUSE, H2B1C_MOUSE, H2B1F_MOUSE, H2B1H_MOUSE, H2B1M_MOUSE, H2B1P_MOUSE, H2B2B_MOUSE, H2B3A_MOUSE, H2B3B_MOUSE	Histone H2B	GTKAVTKYTSSK	473	6.00E-09	424.238	1269.6921	3	-0.0008	12	0	GluC
H2B1A_MOUSE, H2B1B_MOUSE, H2B1C_MOUSE, H2B1F_MOUSE, H2B1H_MOUSE, H2B1M_MOUSE, H2B1P_MOUSE, H2B2B_MOUSE, H2B3A_MOUSE, H2B3B_MOUSE	Histone H2B	GTKAVTKYTSSK	473	5.70E-03	424.2379	1269.6920	3	-0.0009	12	0	GluC
H2B1A_MOUSE, H2B1B_MOUSE, H2B1C_MOUSE, H2B1F_MOUSE, H2B1H_MOUSE, H2B1M_MOUSE, H2B1P_MOUSE, H2B2B_MOUSE, H2B3A_MOUSE, H2B3B_MOUSE	Histone H2B	GTKAVTKYTSSK	473	3.90E-03	424.2379	1269.6920	3	-0.0009	12	0	GluC
H2B1A_MOUSE, H2B1B_MOUSE, H2B1C_MOUSE, H2B1F_MOUSE, H2B1H_MOUSE, H2B1M_MOUSE, H2B1P_MOUSE, H2B2B_MOUSE, H2B3A_MOUSE, H2B3B_MOUSE	Histone H2B	GTKAVTKYTSSK	473	9.60E-05	318.4303	1269.6920	4	-0.0009	12	0	GluC
H2B1A_MOUSE, H2B1B_MOUSE, H2B1C_MOUSE, H2B1F_MOUSE, H2B1H_MOUSE, H2B1M_MOUSE, H2B1P_MOUSE, H2B2B_MOUSE, H2B3A_MOUSE, H2B3B_MOUSE	Histone H2B	GTKAVTKYTSSK	473	8.60E-05	424.2379	1269.6920	3	-0.0009	12	0	GluC
H2B1A_MOUSE, H2B1B_MOUSE, H2B1C_MOUSE, H2B1F_MOUSE, H2B1H_MOUSE, H2B1M_MOUSE, H2B1P_MOUSE, H2B2B_MOUSE, H2B3A_MOUSE, H2B3B_MOUSE	Histone H2B	GTKAVTKYTSSK	473	3.60E-05	424.2379	1269.6920	3	-0.0009	12	0	GluC
H2B1A_MOUSE, H2B1B_MOUSE, H2B1C_MOUSE, H2B1F_MOUSE, H2B1H_MOUSE, H2B1M_MOUSE, H2B1P_MOUSE, H2B2B_MOUSE, H2B3A_MOUSE, H2B3B_MOUSE	Histone H2B	GTKAVTKYTSSK	473	2.20E-07	424.2379	1269.6919	3	-0.0009	12	0	GluC
H2B1A_MOUSE, H2B1B_MOUSE, H2B1C_MOUSE, H2B1F_MOUSE, H2B1H_MOUSE, H2B1M_MOUSE, H2B1P_MOUSE, H2B2B_MOUSE, H2B3A_MOUSE, H2B3B_MOUSE	Histone H2B	GTKAVTKYTSSK	473	3.40E-03	424.2379	1269.6919	3	-0.0010	12	0	GluC
H2B1A_MOUSE, H2B1B_MOUSE, H2B1C_MOUSE, H2B1F_MOUSE, H2B1H_MOUSE, H2B1M_MOUSE, H2B1P_MOUSE, H2B2B_MOUSE, H2B3A_MOUSE, H2B3B_MOUSE	Histone H2B	GTKAVTKYTSSK	473	2.70E-03	424.2379	1269.6919	3	-0.0010	12	0	GluC
H2B1A_MOUSE, H2B1B_MOUSE, H2B1C_MOUSE, H2B1F_MOUSE, H2B1H_MOUSE, H2B1M_MOUSE, H2B1P_MOUSE, H2B2B_MOUSE, H2B3A_MOUSE, H2B3B_MOUSE	Histone H2B	GTKAVTKYTSSK	473	1.90E-03	635.8532	1269.6919	2	-0.0010	12	0	GluC
H2B1A_MOUSE, H2B1B_MOUSE, H2B1C_MOUSE, H2B1F_MOUSE, H2B1H_MOUSE, H2B1M_MOUSE, H2B1P_MOUSE, H2B2B_MOUSE, H2B3A_MOUSE, H2B3B_MOUSE	Histone H2B	GTKAVTKYTSSK	473	1.00E-03	318.4303	1269.6919	4	-0.0010	12	0	GluC
H2B1A_MOUSE, H2B1B_MOUSE, H2B1C_MOUSE, H2B1F_MOUSE, H2B1H_MOUSE, H2B1M_MOUSE, H2B1P_MOUSE, H2B2B_MOUSE, H2B3A_MOUSE, H2B3B_MOUSE	Histone H2B	GTKAVTKYTSSK	473	6.30E-04	318.4303	1269.6919	4	-0.0010	12	0	GluC
H2B1A_MOUSE, H2B1B_MOUSE, H2B1C_MOUSE, H2B1F_MOUSE, H2B1H_MOUSE, H2B1M_MOUSE, H2B1P_MOUSE, H2B2B_MOUSE, H2B3A_MOUSE, H2B3B_MOUSE	Histone H2B	GTKAVTKYTSSK	473	3.30E-04	424.2379	1269.6918	3	-0.0010	12	0	GluC
H2B1A_MOUSE, H2B1B_MOUSE, H2B1C_MOUSE, H2B1F_MOUSE, H2B1H_MOUSE, H2B1M_MOUSE, H2B1P_MOUSE, H2B2B_MOUSE, H2B3A_MOUSE, H2B3B_MOUSE	Histone H2B	GTKAVTKYTSSK	473	2.10E-04	424.2379	1269.6919	3	-0.0010	12	0	GluC
H2B1A_MOUSE, H2B1B_MOUSE, H2B1C_MOUSE, H2B1F_MOUSE, H2B1H_MOUSE, H2B1M_MOUSE, H2B1P_MOUSE, H2B2B_MOUSE, H2B3A_MOUSE, H2B3B_MOUSE	Histone H2B	GTKAVTKYTSSK	473	1.70E-05	424.2379	1269.6919	3	-0.0010	12	0	GluC
H2B1A_MOUSE, H2B1B_MOUSE, H2B1C_MOUSE, H2B1F_MOUSE, H2B1H_MOUSE, H2B1M_MOUSE, H2B1P_MOUSE, H2B2B_MOUSE, H2B3A_MOUSE, H2B3B_MOUSE	Histone H2B	GTKAVTKYTSSK	473	2.60E-06	424.2379	1269.6919	3	-0.0010	12	0	GluC
H2B1A_MOUSE, H2B1B_MOUSE, H2B1C_MOUSE, H2B1F_MOUSE, H2B1H_MOUSE, H2B1M_MOUSE, H2B1P_MOUSE, H2B2B_MOUSE, H2B3A_MOUSE, H2B3B_MOUSE	Histone H2B	GTKAVTKYTSSK	473	2.90E-08	635.8532	1269.6919	2	-0.0010	12	0	GluC

H2B1A_MOUSE, H2B1B_MOUSE, H2B1C_MOUSE, H2B1F_MOUSE, H2B1H_MOUSE, H2B1M_MOUSE, H2B1P_MOUSE, H2B2B_MOUSE, H2B3A_MOUSE, H2B3B_MOUSE	Histone H2B	GTKAVTKYTSSK	473	1.30E-08	318.4302	1269.6918	4	-0.0010	12	0	GluC
H2B1A_MOUSE, H2B1B_MOUSE, H2B1C_MOUSE, H2B1F_MOUSE, H2B1H_MOUSE, H2B1M_MOUSE, H2B1P_MOUSE, H2B2B_MOUSE, H2B3A_MOUSE, H2B3B_MOUSE	Histone H2B	GTKAVTKYTSSK	473	4.10E-04	424.2379	1269.6918	3	-0.0011	12	0	GluC
H2B1A_MOUSE, H2B1B_MOUSE, H2B1C_MOUSE, H2B1F_MOUSE, H2B1H_MOUSE, H2B1M_MOUSE, H2B1P_MOUSE, H2B2B_MOUSE, H2B3A_MOUSE, H2B3B_MOUSE	Histone H2B	GTKAVTKYTSSK	473	1.70E-04	424.2379	1269.6918	3	-0.0011	12	0	GluC
H2B1A_MOUSE, H2B1B_MOUSE, H2B1C_MOUSE, H2B1F_MOUSE, H2B1H_MOUSE, H2B1M_MOUSE, H2B1P_MOUSE, H2B2B_MOUSE, H2B3A_MOUSE, H2B3B_MOUSE	Histone H2B	GTKAVTKYTSSK	473	1.70E-05	318.4302	1269.6918	4	-0.0011	12	0	GluC
H2B1A_MOUSE, H2B1B_MOUSE, H2B1C_MOUSE, H2B1F_MOUSE, H2B1H_MOUSE, H2B1M_MOUSE, H2B1P_MOUSE, H2B2B_MOUSE, H2B3A_MOUSE, H2B3B_MOUSE	Histone H2B	GTKAVTKYTSSK	473	1.00E-02	424.2378	1269.6917	3	-0.0012	12	0	GluC
H2B1A_MOUSE, H2B1B_MOUSE, H2B1C_MOUSE, H2B1F_MOUSE, H2B1H_MOUSE, H2B1M_MOUSE, H2B1P_MOUSE, H2B2B_MOUSE, H2B3A_MOUSE, H2B3B_MOUSE	Histone H2B	GTKAVTKYTSSK	473	4.00E-06	424.2378	1269.6917	3	-0.0012	12	0	GluC
H2B1A_MOUSE, H2B1B_MOUSE, H2B1C_MOUSE, H2B1F_MOUSE, H2B1H_MOUSE, H2B1M_MOUSE, H2B1P_MOUSE, H2B2B_MOUSE, H2B3A_MOUSE, H2B3B_MOUSE	Histone H2B	GTKAVTKYTSSK	473	7.80E-03	424.2378	1269.6916	3	-0.0013	12	0	GluC
H2B1A_MOUSE, H2B1B_MOUSE, H2B1C_MOUSE, H2B1F_MOUSE, H2B1H_MOUSE, H2B1M_MOUSE, H2B1P_MOUSE, H2B2B_MOUSE, H2B3A_MOUSE, H2B3B_MOUSE	Histone H2B	GTKAVTKYTSSK	473	6.90E-04	424.2378	1269.6916	3	-0.0013	12	0	GluC
H2B1A_MOUSE, H2B1B_MOUSE, H2B1C_MOUSE, H2B1F_MOUSE, H2B1H_MOUSE, H2B1M_MOUSE, H2B1P_MOUSE, H2B2B_MOUSE, H2B3A_MOUSE, H2B3B_MOUSE	Histone H2B	GTKAVTKYTSSK	473	3.00E-06	424.2378	1269.6916	3	-0.0013	12	0	GluC
H2B1A_MOUSE, H2B1B_MOUSE, H2B1C_MOUSE, H2B1F_MOUSE, H2B1H_MOUSE, H2B1M_MOUSE, H2B1P_MOUSE, H2B2B_MOUSE, H2B3A_MOUSE, H2B3B_MOUSE	Histone H2B	GTKAVTKYTSSK	473	3.90E-07	424.2378	1269.6915	3	-0.0013	12	0	GluC
H2B1A_MOUSE, H2B1B_MOUSE, H2B1C_MOUSE, H2B1F_MOUSE, H2B1H_MOUSE, H2B1M_MOUSE, H2B1P_MOUSE, H2B2B_MOUSE, H2B3A_MOUSE, H2B3B_MOUSE	Histone H2B	GTKAVTKYTSSK	473	3.90E-03	424.2378	1269.6914	3	-0.0014	12	0	GluC
H2B1A_MOUSE, H2B1B_MOUSE, H2B1C_MOUSE, H2B1F_MOUSE, H2B1H_MOUSE, H2B1M_MOUSE, H2B1P_MOUSE, H2B2B_MOUSE, H2B3A_MOUSE, H2B3B_MOUSE	Histone H2B	GTKAVTKYTSSK	473	3.90E-03	318.4301	1269.6914	4	-0.0014	12	0	GluC
H2B1A_MOUSE, H2B1B_MOUSE, H2B1C_MOUSE, H2B1F_MOUSE, H2B1H_MOUSE, H2B1M_MOUSE, H2B1P_MOUSE, H2B2B_MOUSE, H2B3A_MOUSE, H2B3B_MOUSE	Histone H2B	GTKAVTKYTSSK	473	2.70E-03	318.4301	1269.6915	4	-0.0014	12	0	GluC
H2B1A_MOUSE, H2B1B_MOUSE, H2B1C_MOUSE, H2B1F_MOUSE, H2B1H_MOUSE, H2B1M_MOUSE, H2B1P_MOUSE, H2B2B_MOUSE, H2B3A_MOUSE, H2B3B_MOUSE	Histone H2B	GTKAVTKYTSSK	473	2.70E-03	424.2378	1269.6914	3	-0.0014	12	0	GluC
H2B1A_MOUSE, H2B1B_MOUSE, H2B1C_MOUSE, H2B1F_MOUSE, H2B1H_MOUSE, H2B1M_MOUSE, H2B1P_MOUSE, H2B2B_MOUSE, H2B3A_MOUSE, H2B3B_MOUSE	Histone H2B	GTKAVTKYTSSK	473	1.70E-03	424.2378	1269.6915	3	-0.0014	12	0	GluC
H2B1A_MOUSE, H2B1B_MOUSE, H2B1C_MOUSE, H2B1F_MOUSE, H2B1H_MOUSE, H2B1M_MOUSE, H2B1P_MOUSE, H2B2B_MOUSE, H2B3A_MOUSE, H2B3B_MOUSE	Histone H2B	GTKAVTKYTSSK	473	8.10E-04	424.2378	1269.6915	3	-0.0014	12	0	GluC
H2B1A_MOUSE, H2B1B_MOUSE, H2B1C_MOUSE, H2B1F_MOUSE, H2B1H_MOUSE, H2B1M_MOUSE, H2B1P_MOUSE, H2B2B_MOUSE, H2B3A_MOUSE, H2B3B_MOUSE	Histone H2B	GTKAVTKYTSSK	473	1.60E-04	318.4301	1269.6914	4	-0.0014	12	0	GluC
H2B1A_MOUSE, H2B1B_MOUSE, H2B1C_MOUSE, H2B1F_MOUSE, H2B1H_MOUSE, H2B1M_MOUSE, H2B1P_MOUSE, H2B2B_MOUSE, H2B3A_MOUSE, H2B3B_MOUSE	Histone H2B	GTKAVTKYTSSK	473	8.30E-06	424.2378	1269.6914	3	-0.0014	12	0	GluC
H2B1A_MOUSE, H2B1B_MOUSE, H2B1C_MOUSE, H2B1F_MOUSE, H2B1H_MOUSE, H2B1M_MOUSE, H2B1P_MOUSE, H2B2B_MOUSE, H2B3A_MOUSE, H2B3B_MOUSE	Histone H2B	GTKAVTKYTSSK	473	6.00E-06	424.2378	1269.6915	3	-0.0014	12	0	GluC
H2B1A_MOUSE, H2B1B_MOUSE, H2B1C_MOUSE, H2B1F_MOUSE, H2B1H_MOUSE, H2B1M_MOUSE, H2B1P_MOUSE, H2B2B_MOUSE, H2B3A_MOUSE, H2B3B_MOUSE	Histone H2B	GTKAVTKYTSSK	473	3.80E-07	424.2378	1269.6914	3	-0.0014	12	0	GluC
H2B1A_MOUSE, H2B1B_MOUSE, H2B1C_MOUSE, H2B1F_MOUSE, H2B1H_MOUSE, H2B1M_MOUSE, H2B1P_MOUSE, H2B2B_MOUSE, H2B3A_MOUSE, H2B3B_MOUSE	Histone H2B	GTKAVTKYTSSK	473	9.40E-08	424.2378	1269.6914	3	-0.0014	12	0	GluC
H2B1A_MOUSE, H2B1B_MOUSE, H2B1C_MOUSE, H2B1F_MOUSE, H2B1H_MOUSE, H2B1M_MOUSE, H2B1P_MOUSE, H2B2B_MOUSE, H2B3A_MOUSE, H2B3B_MOUSE	Histone H2B	GTKAVTKYTSSK	473	1.00E-03	424.2377	1269.6913	3	-0.0015	12	0	GluC
H2B1A_MOUSE, H2B1B_MOUSE, H2B1C_MOUSE, H2B1F_MOUSE, H2B1H_MOUSE, H2B1M_MOUSE, H2B1P_MOUSE, H2B2B_MOUSE, H2B3A_MOUSE, H2B3B_MOUSE	Histone H2B	GTKAVTKYTSSK	473	8.00E-04	424.2377	1269.6914	3	-0.0015	12	0	GluC
H2B1A_MOUSE, H2B1B_MOUSE, H2B1C_MOUSE, H2B1F_MOUSE, H2B1H_MOUSE, H2B1M_MOUSE, H2B1P_MOUSE, H2B2B_MOUSE, H2B3A_MOUSE, H2B3B_MOUSE	Histone H2B	GTKAVTKYTSSK	473	2.30E-04	318.4301	1269.6914	4	-0.0015	12	0	GluC

H2B1A_MOUSE, H2B1B_MOUSE, H2B1C_MOUSE, H2B1F_MOUSE, H2B1H_MOUSE, H2B1M_MOUSE, H2B1P_MOUSE, H2B2B_MOUSE, H2B3A_MOUSE, H2B3B_MOUSE	Histone H2B	GTKAVTKYTSSK	473	2.30E-04	318.4301	1269.6914	4	-0.0015	12	0	GluC
H2B1A_MOUSE, H2B1B_MOUSE, H2B1C_MOUSE, H2B1F_MOUSE, H2B1H_MOUSE, H2B1M_MOUSE, H2B1P_MOUSE, H2B2B_MOUSE, H2B3A_MOUSE, H2B3B_MOUSE	Histone H2B	GTKAVTKYTSSK	473	1.70E-08	424.2377	1269.6914	3	-0.0015	12	0	GluC
H2B1A_MOUSE, H2B1B_MOUSE, H2B1C_MOUSE, H2B1F_MOUSE, H2B1H_MOUSE, H2B1M_MOUSE, H2B1P_MOUSE, H2B2B_MOUSE, H2B3A_MOUSE, H2B3B_MOUSE	Histone H2B	GTKAVTKYTSSK	473	1.20E-03	318.4301	1269.6912	4	-0.0017	12	0	GluC
H2B1A_MOUSE, H2B1B_MOUSE, H2B1C_MOUSE, H2B1F_MOUSE, H2B1H_MOUSE, H2B1M_MOUSE, H2B1P_MOUSE, H2B2B_MOUSE, H2B3A_MOUSE, H2B3B_MOUSE	Histone H2B	GTKAVTKYTSSK	473	4.30E-04	424.2377	1269.6912	3	-0.0017	12	0	GluC
H2B1A_MOUSE, H2B1B_MOUSE, H2B1C_MOUSE, H2B1F_MOUSE, H2B1H_MOUSE, H2B1M_MOUSE, H2B1P_MOUSE, H2B2B_MOUSE, H2B3A_MOUSE, H2B3B_MOUSE	Histone H2B	GTKAVTKYTSSK	473	3.60E-07	424.2377	1269.6912	3	-0.0017	12	0	GluC
H2B1A_MOUSE, H2B1B_MOUSE, H2B1C_MOUSE, H2B1F_MOUSE, H2B1H_MOUSE, H2B1M_MOUSE, H2B1P_MOUSE, H2B2B_MOUSE, H2B3A_MOUSE, H2B3B_MOUSE	Histone H2B	GTKAVTKYTSSK	473	8.20E-09	318.4301	1269.6911	4	-0.0017	12	0	GluC
H2B1A_MOUSE, H2B1B_MOUSE, H2B1C_MOUSE, H2B1F_MOUSE, H2B1H_MOUSE, H2B1M_MOUSE, H2B1P_MOUSE, H2B2B_MOUSE, H2B3A_MOUSE, H2B3B_MOUSE	Histone H2B	GTKAVTKYTSSK	473	1.60E-04	318.43	1269.6911	4	-0.0018	12	0	GluC
H2B1A_MOUSE, H2B1B_MOUSE, H2B1C_MOUSE, H2B1F_MOUSE, H2B1H_MOUSE, H2B1M_MOUSE, H2B1P_MOUSE, H2B2B_MOUSE, H2B3A_MOUSE, H2B3B_MOUSE	Histone H2B	GTKAVTKYTSSK	473	2.10E-03	424.2376	1269.6909	3	-0.0019	12	0	GluC
H2B1A_MOUSE, H2B1B_MOUSE, H2B1C_MOUSE, H2B1F_MOUSE, H2B1H_MOUSE, H2B1M_MOUSE, H2B1P_MOUSE, H2B2B_MOUSE, H2B3A_MOUSE, H2B3B_MOUSE	Histone H2B	AVTKYTSSK	6	1.30E-05	492.7726	983.5305	2	0.0018	9	1	Semi-tryptic
H2B1A_MOUSE, H2B1B_MOUSE, H2B1C_MOUSE, H2B1F_MOUSE, H2B1H_MOUSE, H2B1M_MOUSE, H2B1P_MOUSE, H2B2B_MOUSE, H2B3A_MOUSE, H2B3B_MOUSE	Histone H2B	AVTKYTSSK	6	6.20E-05	492.7722	983.5298	2	0.0011	9	1	Semi-tryptic
H2B1A_MOUSE, H2B1B_MOUSE, H2B1C_MOUSE, H2B1F_MOUSE, H2B1H_MOUSE, H2B1M_MOUSE, H2B1P_MOUSE, H2B2B_MOUSE, H2B3A_MOUSE, H2B3B_MOUSE	Histone H2B	AVTKYTSSK	6	1.80E-04	492.7719	983.5292	2	0.0004	9	1	Semi-tryptic
H2B1A_MOUSE, H2B1B_MOUSE, H2B1C_MOUSE, H2B1F_MOUSE, H2B1H_MOUSE, H2B1M_MOUSE, H2B1P_MOUSE, H2B2B_MOUSE, H2B3A_MOUSE, H2B3B_MOUSE	Histone H2B	AVTKYTSSK	6	2.50E-05	492.7717	983.5288	2	0.0001	9	1	Semi-tryptic
H2B1A_MOUSE, H2B1B_MOUSE, H2B1C_MOUSE, H2B1F_MOUSE, H2B1H_MOUSE, H2B1M_MOUSE, H2B1P_MOUSE, H2B2B_MOUSE, H2B3A_MOUSE, H2B3B_MOUSE	Histone H2B	AVTKYTSSK	6	1.00E-04	492.7716	983.5286	2	-0.0001	9	1	Semi-tryptic
H2B1A_MOUSE, H2B1B_MOUSE, H2B1C_MOUSE, H2B1F_MOUSE, H2B1H_MOUSE, H2B1M_MOUSE, H2B1P_MOUSE, H2B2B_MOUSE, H2B3A_MOUSE, H2B3B_MOUSE	Histone H2B	AVTKYTSSK	6	2.30E-04	492.7715	983.5285	2	-0.0002	9	1	Semi-tryptic
H2B1A_MOUSE, H2B1B_MOUSE, H2B1F_MOUSE, H2B1K_MOUSE, H2B1P_MOUSE, H2B3A_MOUSE, H2B3B_MOUSE	Histone H2B	IASEASRLAHYNKR	13	7.70E-06	404.7228	1614.8619	4	0.0030	14	2	Semi-tryptic
H2B1A_MOUSE, H2B1B_MOUSE, H2B1F_MOUSE, H2B1K_MOUSE, H2B1P_MOUSE, H2B3A_MOUSE, H2B3B_MOUSE	Histone H2B	IASEASRLAHYNKR	13	2.60E-07	404.7228	1614.8619	4	0.0030	14	2	Semi-tryptic
H2B1A_MOUSE, H2B1B_MOUSE, H2B1F_MOUSE, H2B1K_MOUSE, H2B1P_MOUSE, H2B3A_MOUSE, H2B3B_MOUSE	Histone H2B	IASEASRLAHYNKR	13	7.30E-05	539.2946	1614.8619	3	0.0029	14	2	Semi-tryptic
H2B1A_MOUSE, H2B1B_MOUSE, H2B1F_MOUSE, H2B1K_MOUSE, H2B1P_MOUSE, H2B3A_MOUSE, H2B3B_MOUSE	Histone H2B	IASEASRLAHYNKR	13	6.80E-04	539.2941	1614.8604	3	0.0014	14	2	Semi-tryptic
H2B1A_MOUSE, H2B1B_MOUSE, H2B1F_MOUSE, H2B1K_MOUSE, H2B1P_MOUSE, H2B3A_MOUSE, H2B3B_MOUSE	Histone H2B	IASEASRLAHYNKR	13	3.50E-05	539.2941	1614.8604	3	0.0014	14	2	Semi-tryptic
H2B1A_MOUSE, H2B1B_MOUSE, H2B1F_MOUSE, H2B1K_MOUSE, H2B1P_MOUSE, H2B3A_MOUSE, H2B3B_MOUSE	Histone H2B	IASEASRLAHYNKR	13	2.00E-04	539.2938	1614.8597	3	0.0007	14	2	Semi-tryptic
H2B1A_MOUSE, H2B1B_MOUSE, H2B1F_MOUSE, H2B1K_MOUSE, H2B1P_MOUSE, H2B3A_MOUSE, H2B3B_MOUSE	Histone H2B	IASEASRLAHYNKR	13	2.60E-06	404.7222	1614.8597	4	0.0007	14	2	Semi-tryptic
H2B1A_MOUSE, H2B1B_MOUSE, H2B1F_MOUSE, H2B1K_MOUSE, H2B1P_MOUSE, H2B3A_MOUSE, H2B3B_MOUSE	Histone H2B	IASEASRLAHYNKR	13	3.30E-08	404.7222	1614.8597	4	0.0007	14	2	Semi-tryptic
H2B1A_MOUSE, H2B1B_MOUSE, H2B1F_MOUSE, H2B1K_MOUSE, H2B1P_MOUSE, H2B3A_MOUSE, H2B3B_MOUSE	Histone H2B	IASEASRLAHYNKR	13	2.90E-05	539.2938	1614.8596	3	0.0006	14	2	Semi-tryptic
H2B1A_MOUSE, H2B1B_MOUSE, H2B1F_MOUSE, H2B1K_MOUSE, H2B1P_MOUSE, H2B3A_MOUSE, H2B3B_MOUSE	Histone H2B	IASEASRLAHYNKR	13	6.90E-04	539.2935	1614.8586	3	-0.0004	14	2	Semi-tryptic
H2B1A_MOUSE, H2B1B_MOUSE, H2B1F_MOUSE, H2B1K_MOUSE, H2B1P_MOUSE, H2B3A_MOUSE, H2B3B_MOUSE	Histone H2B	IASEASRLAHYNKR	13	9.80E-06	539.2933	1614.8580	3	-0.0010	14	2	Semi-tryptic
H2B1A_MOUSE, H2B1B_MOUSE, H2B1F_MOUSE, H2B1K_MOUSE, H2B1P_MOUSE, H2B3A_MOUSE, H2B3B_MOUSE	Histone H2B	IASEASRLAHYNKR	13	7.00E-05	539.2931	1614.8574	3	-0.0016	14	2	Semi-tryptic
H2B1A_MOUSE, H2B1B_MOUSE, H2B1F_MOUSE, H2B1K_MOUSE, H2B1P_MOUSE, H2B3A_MOUSE, H2B3B_MOUSE	Histone H2B	IASEASRLAHYNKR	13	2.00E-04	539.293	1614.8571	3	-0.0019	14	2	Semi-tryptic
H2B1A_MOUSE, H2B1B_MOUSE, H2B1F_MOUSE, H2B1K_MOUSE, H2B1P_MOUSE, H2B3A_MOUSE, H2B3B_MOUSE	Histone H2B	ERIASEASRL	3	2.20E-04	377.8765	1130.6078	3	0.0034	10	0	Chymotrypsin
H2B1A_MOUSE, H2B1B_MOUSE, H2B1F_MOUSE, H2B1K_MOUSE, H2B1P_MOUSE, H2B3A_MOUSE, H2B3B_MOUSE	Histone H2B	ERIASEASRL	3	1.30E-03	377.8759	1130.6060	3	0.0017	10	0	Chymotrypsin
H2B1A_MOUSE, H2B1B_MOUSE, H2B1F_MOUSE, H2B1K_MOUSE, H2B1P_MOUSE, H2B3A_MOUSE, H2B3B_MOUSE	Histone H2B	ERIASEASRL	3	7.90E-03	566.3098	1130.6050	2	0.0006	10	0	Chymotrypsin

H2B1A_MOUSE, H2B1B_MOUSE, H2B1F_MOUSE, H2B1K_MOUSE, H2B1P_MOUSE, H2B3A_MOUSE, H2B3B_MOUSE	Histone H2B	IFERIASE	9	6.30E-04	482.76	963.5053	2	0.0028	8	1	GluC
H2B1A_MOUSE, H2B1B_MOUSE, H2B1F_MOUSE, H2B1K_MOUSE, H2B1P_MOUSE, H2B3A_MOUSE, H2B3B_MOUSE	Histone H2B	IFERIASE	9	3.50E-03	482.7593	963.5041	2	0.0016	8	1	GluC
H2B1A_MOUSE, H2B1B_MOUSE, H2B1F_MOUSE, H2B1K_MOUSE, H2B1P_MOUSE, H2B3A_MOUSE, H2B3B_MOUSE	Histone H2B	IFERIASE	9	3.20E-05	482.7593	963.5041	2	0.0016	8	1	GluC
H2B1A_MOUSE, H2B1B_MOUSE, H2B1F_MOUSE, H2B1K_MOUSE, H2B1P_MOUSE, H2B3A_MOUSE, H2B3B_MOUSE	Histone H2B	IFERIASE	9	1.40E-05	482.7586	963.5027	2	0.0002	8	1	GluC
H2B1A_MOUSE, H2B1B_MOUSE, H2B1F_MOUSE, H2B1K_MOUSE, H2B1P_MOUSE, H2B3A_MOUSE, H2B3B_MOUSE	Histone H2B	IFERIASE	9	8.20E-04	482.7585	963.5024	2	-0.0001	8	1	GluC
H2B1A_MOUSE, H2B1B_MOUSE, H2B1F_MOUSE, H2B1K_MOUSE, H2B1P_MOUSE, H2B3A_MOUSE, H2B3B_MOUSE	Histone H2B	IFERIASE	9	7.40E-06	482.7584	963.5023	2	-0.0002	8	1	GluC
H2B1A_MOUSE, H2B1B_MOUSE, H2B1F_MOUSE, H2B1K_MOUSE, H2B1P_MOUSE, H2B3A_MOUSE, H2B3B_MOUSE	Histone H2B	IFERIASE	9	8.10E-03	482.7584	963.5022	2	-0.0003	8	1	GluC
H2B1A_MOUSE, H2B1B_MOUSE, H2B1F_MOUSE, H2B1K_MOUSE, H2B1P_MOUSE, H2B3A_MOUSE, H2B3B_MOUSE	Histone H2B	IFERIASE	9	2.10E-05	482.7584	963.5022	2	-0.0003	8	1	GluC
H2B1A_MOUSE, H2B1B_MOUSE, H2B1F_MOUSE, H2B1K_MOUSE, H2B1P_MOUSE, H2B3A_MOUSE, H2B3B_MOUSE	Histone H2B	IFERIASE	9	4.70E-06	482.758	963.5014	2	-0.0011	8	1	GluC
H2B1A_MOUSE, H2B1B_MOUSE, H2B1F_MOUSE, H2B1K_MOUSE, H2B1P_MOUSE, H2B3A_MOUSE, H2B3B_MOUSE	Histone H2B	IASEASR	13	4.90E-03	367.1966	732.3787	2	0.0021	7	0	Semi-tryptic
H2B1A_MOUSE, H2B1B_MOUSE, H2B1F_MOUSE, H2B1K_MOUSE, H2B1P_MOUSE, H2B3A_MOUSE, H2B3B_MOUSE	Histone H2B	IASEASR	13	1.10E-03	367.1964	732.3782	2	0.0016	7	0	Semi-tryptic
H2B1A_MOUSE, H2B1B_MOUSE, H2B1F_MOUSE, H2B1K_MOUSE, H2B1P_MOUSE, H2B3A_MOUSE, H2B3B_MOUSE	Histone H2B	IASEASR	13	5.30E-04	367.1963	732.3781	2	0.0015	7	0	Semi-tryptic
H2B1A_MOUSE, H2B1B_MOUSE, H2B1F_MOUSE, H2B1K_MOUSE, H2B1P_MOUSE, H2B3A_MOUSE, H2B3B_MOUSE	Histone H2B	IASEASR	13	1.80E-03	367.1962	732.3778	2	0.0012	7	0	Semi-tryptic
H2B1A_MOUSE, H2B1B_MOUSE, H2B1F_MOUSE, H2B1K_MOUSE, H2B1P_MOUSE, H2B3A_MOUSE, H2B3B_MOUSE	Histone H2B	IASEASR	13	5.20E-03	367.1961	732.3777	2	0.0011	7	0	Semi-tryptic
H2B1A_MOUSE, H2B1B_MOUSE, H2B1F_MOUSE, H2B1K_MOUSE, H2B1P_MOUSE, H2B3A_MOUSE, H2B3B_MOUSE	Histone H2B	IASEASR	13	4.00E-04	367.1961	732.3777	2	0.0011	7	0	Semi-tryptic
H2B1A_MOUSE, H2B1B_MOUSE, H2B1F_MOUSE, H2B1K_MOUSE, H2B1P_MOUSE, H2B3A_MOUSE, H2B3B_MOUSE	Histone H2B	IASEASR	13	8.80E-03	367.196	732.3774	2	0.0008	7	0	Semi-tryptic
H2B1A_MOUSE, H2B1B_MOUSE, H2B1F_MOUSE, H2B1K_MOUSE, H2B1P_MOUSE, H2B3A_MOUSE, H2B3B_MOUSE	Histone H2B	IASEASR	13	3.50E-03	367.1959	732.3773	2	0.0007	7	0	Semi-tryptic
H2B1A_MOUSE, H2B1B_MOUSE, H2B1F_MOUSE, H2B1K_MOUSE, H2B1P_MOUSE, H2B3A_MOUSE, H2B3B_MOUSE	Histone H2B	IASEASR	13	9.20E-04	367.1959	732.3773	2	0.0007	7	0	Semi-tryptic
H2B1A_MOUSE, H2B1B_MOUSE, H2B1F_MOUSE, H2B1K_MOUSE, H2B1P_MOUSE, H2B3A_MOUSE, H2B3B_MOUSE	Histone H2B	IASEASR	13	9.00E-04	367.1957	732.3768	2	0.0002	7	0	Semi-tryptic
H2B1A_MOUSE, H2B1B_MOUSE, H2B1F_MOUSE, H2B1K_MOUSE, H2B1P_MOUSE, H2B3A_MOUSE, H2B3B_MOUSE	Histone H2B	IASEASR	13	5.00E-03	367.1956	732.3767	2	0.0001	7	0	Semi-tryptic
H2B1A_MOUSE, H2B1B_MOUSE, H2B1F_MOUSE, H2B1K_MOUSE, H2B1P_MOUSE, H2B3A_MOUSE, H2B3B_MOUSE	Histone H2B	IASEASR	13	5.00E-03	367.1955	732.3764	2	-0.0002	7	0	Semi-tryptic
H2B1A_MOUSE, H2B1B_MOUSE, H2B1F_MOUSE, H2B1K_MOUSE, H2B1P_MOUSE, H2B3A_MOUSE, H2B3B_MOUSE	Histone H2B	IASEASR	13	3.90E-03	367.1951	732.3756	2	-0.0010	7	0	Semi-tryptic
H2B1B_MOUSE	Histone H2B	PEPSKSAPAPKKGSKKAISKAQKKDGKKRKRSRKE	2	2.00E-07	772.8617	3859.2719	5	0.0012	35	1	GluC
H2B1B_MOUSE	Histone H2B	PEPSKSAPAPKKGSKKAISKAQKKDGKKRKRSRKE	2	6.80E-04	644.2185	3859.2671	6	-0.0036	35	1	GluC
H2B1B_MOUSE	Histone H2B	PEPSKSAPAPKKGSKKAISKAQKKD	6	1.70E-03	869.509	2605.5052	3	0.0137	25	0	GluC
H2B1B_MOUSE	Histone H2B	PEPSKSAPAPKKGSKKAISKAQKKD	6	2.10E-10	652.3813	2605.4960	4	0.0045	25	0	GluC
H2B1B_MOUSE	Histone H2B	PEPSKSAPAPKKGSKKAISKAQKKD	6	3.10E-10	652.3811	2605.4953	4	0.0038	25	0	GluC
H2B1B_MOUSE	Histone H2B	PEPSKSAPAPKKGSKKAISKAQKKD	6	1.80E-06	652.3808	2605.4940	4	0.0026	25	0	GluC
H2B1B_MOUSE	Histone H2B	PEPSKSAPAPKKGSKKAISKAQKKD	6	1.50E-03	652.3804	2605.4925	4	0.0011	25	0	GluC
H2B1B_MOUSE	Histone H2B	PEPSKSAPAPKKGSKKAISKAQKKD	6	1.10E-04	522.1057	2605.4921	5	0.0007	25	0	GluC
H2B1B_MOUSE	Histone H2B	PEPSKSAPAPK	6	9.00E-04	370.2055	1107.5946	3	0.0022	11	1	Semi-tryptic
H2B1B_MOUSE	Histone H2B	PEPSKSAPAPK	6	8.50E-04	370.2054	1107.5942	3	0.0018	11	1	Semi-tryptic
H2B1B_MOUSE	Histone H2B	PEPSKSAPAPK	6	5.10E-04	370.2051	1107.5936	3	0.0012	11	1	Semi-tryptic
H2B1B_MOUSE	Histone H2B	PEPSKSAPAPK	6	1.10E-05	554.8039	1107.5933	2	0.0009	11	1	Semi-tryptic
H2B1B_MOUSE	Histone H2B	PEPSKSAPAPK	6	1.20E-04	554.8036	1107.5927	2	0.0003	11	1	Semi-tryptic
H2B1B_MOUSE	Histone H2B	PEPSKSAPAPK	6	4.70E-04	554.8034	1107.5923	2	-0.0001	11	1	Semi-tryptic
H2B1B_MOUSE, H2B1C_MOUSE, H2B1F_MOUSE, H2B1H_MOUSE, H2B1K_MOUSE, H2B1M_MOUSE, H2B1P_MOUSE, H2B2B_MOUSE	Histone H2B	SYSVYVYKVLKQVHPDTGISSKAMGIMNSFVND	34	6.00E-04	1226.6225	3676.8457	3	0.0187	33	1	GluC
H2B1B_MOUSE, H2B1C_MOUSE, H2B1F_MOUSE, H2B1H_MOUSE, H2B1K_MOUSE, H2B1M_MOUSE, H2B1P_MOUSE, H2B2B_MOUSE	Histone H2B	SYSVYVYKVLKQVHPDTGISSKAMGIMNSFVND	34	1.20E-04	920.2187	3676.8455	4	0.0186	33	1	GluC
H2B1B_MOUSE, H2B1C_MOUSE, H2B1F_MOUSE, H2B1H_MOUSE, H2B1K_MOUSE, H2B1M_MOUSE, H2B1P_MOUSE, H2B2B_MOUSE	Histone H2B	SYSVYVYKVLKQVHPDTGISSKAMGIMNSFVND	34	1.60E-04	736.3757	3676.8422	5	0.0153	33	1	GluC
H2B1B_MOUSE, H2B1C_MOUSE, H2B1F_MOUSE, H2B1H_MOUSE, H2B1K_MOUSE, H2B1M_MOUSE, H2B1P_MOUSE, H2B2B_MOUSE	Histone H2B	SYSVYVYKVLKQVHPDTGISSKAMGIMNSFVND	34	5.20E-04	920.2178	3676.8419	4	0.0150	33	1	GluC
H2B1B_MOUSE, H2B1C_MOUSE, H2B1F_MOUSE, H2B1H_MOUSE, H2B1K_MOUSE, H2B1M_MOUSE, H2B1P_MOUSE, H2B2B_MOUSE	Histone H2B	SYSVYVYKVLKQVHPDTGISSKAMGIMNSFVND	34	1.70E-03	736.3755	3676.8412	5	0.0143	33	1	GluC
H2B1B_MOUSE, H2B1C_MOUSE, H2B1F_MOUSE, H2B1H_MOUSE, H2B1K_MOUSE, H2B1M_MOUSE, H2B1P_MOUSE, H2B2B_MOUSE	Histone H2B	SYSVYVYKVLKQVHPDTGISSKAMGIMNSFVND	34	3.80E-09	920.2171	3676.8395	4	0.0126	33	1	GluC
H2B1B_MOUSE, H2B1C_MOUSE, H2B1F_MOUSE, H2B1H_MOUSE, H2B1K_MOUSE, H2B1M_MOUSE, H2B1P_MOUSE, H2B2B_MOUSE	Histone H2B	SYSVYVYKVLKQVHPDTGISSKAMGIMNSFVND	34	1.10E-04	1226.62	3676.8382	3	0.0112	33	1	GluC
H2B1B_MOUSE, H2B1C_MOUSE, H2B1F_MOUSE, H2B1H_MOUSE, H2B1K_MOUSE, H2B1M_MOUSE, H2B1P_MOUSE, H2B2B_MOUSE	Histone H2B	SYSVYVYKVLKQVHPDTGISSKAMGIMNSFVND	34	8.90E-04	1226.6199	3676.8379	3	0.0109	33	1	GluC

H2B1B_MOUSE, H2B1C_MOUSE, H2B1F_MOUSE, H2B1H_MOUSE, H2B1K_MOUSE, H2B1M_MOUSE, H2B1P_MOUSE, H2B2B_MOUSE	Histone H2B	SYSVYVYKVLKQVHPDTGISSKAMGIMNSFVND	34	2.60E-06	920.2165	3676.8368	4	0.0098	33	1	GluC
H2B1B_MOUSE, H2B1C_MOUSE, H2B1F_MOUSE, H2B1H_MOUSE, H2B1K_MOUSE, H2B1M_MOUSE, H2B1P_MOUSE, H2B2B_MOUSE	Histone H2B	SYSVYVYKVLKQVHPDTGISSKAMGIMNSFVND	34	2.30E-03	1226.6195	3676.8367	3	0.0097	33	1	GluC
H2B1B_MOUSE, H2B1C_MOUSE, H2B1F_MOUSE, H2B1H_MOUSE, H2B1K_MOUSE, H2B1M_MOUSE, H2B1P_MOUSE, H2B2B_MOUSE	Histone H2B	SYSVYVYKVLKQVHPDTGISSKAMGIMNSFVND	34	2.00E-07	920.2163	3676.8363	4	0.0093	33	1	GluC
H2B1B_MOUSE, H2B1C_MOUSE, H2B1F_MOUSE, H2B1H_MOUSE, H2B1K_MOUSE, H2B1M_MOUSE, H2B1P_MOUSE, H2B2B_MOUSE	Histone H2B	SYSVYVYKVLKQVHPDTGISSKAMGIMNSFVND	34	2.10E-03	1226.6193	3676.8361	3	0.0091	33	1	GluC
H2B1B_MOUSE, H2B1C_MOUSE, H2B1F_MOUSE, H2B1H_MOUSE, H2B1K_MOUSE, H2B1M_MOUSE, H2B1P_MOUSE, H2B2B_MOUSE	Histone H2B	SYSVYVYKVLKQVHPDTGISSKAMGIMNSFVND	34	5.50E-03	1226.6188	3676.8346	3	0.0076	33	1	GluC
H2B1B_MOUSE, H2B1C_MOUSE, H2B1F_MOUSE, H2B1H_MOUSE, H2B1K_MOUSE, H2B1M_MOUSE, H2B1P_MOUSE, H2B2B_MOUSE	Histone H2B	SYSVYVYKVLKQVHPDTGISSKAMGIMNSFVND	34	2.20E-04	1226.6188	3676.8346	3	0.0076	33	1	GluC
H2B1B_MOUSE, H2B1C_MOUSE, H2B1F_MOUSE, H2B1H_MOUSE, H2B1K_MOUSE, H2B1M_MOUSE, H2B1P_MOUSE, H2B2B_MOUSE	Histone H2B	SYSVYVYKVLKQVHPDTGISSKAMGIMNSFVND	34	4.40E-06	920.2159	3676.8345	4	0.0076	33	1	GluC
H2B1B_MOUSE, H2B1C_MOUSE, H2B1F_MOUSE, H2B1H_MOUSE, H2B1K_MOUSE, H2B1M_MOUSE, H2B1P_MOUSE, H2B2B_MOUSE	Histone H2B	SYSVYVYKVLKQVHPDTGISSKAMGIMNSFVND	34	7.00E-04	736.3741	3676.8344	5	0.0074	33	1	GluC
H2B1B_MOUSE, H2B1C_MOUSE, H2B1F_MOUSE, H2B1H_MOUSE, H2B1K_MOUSE, H2B1M_MOUSE, H2B1P_MOUSE, H2B2B_MOUSE	Histone H2B	SYSVYVYKVLKQVHPDTGISSKAMGIMNSFVND	34	1.10E-03	920.2158	3676.8342	4	0.0073	33	1	GluC
H2B1B_MOUSE, H2B1C_MOUSE, H2B1F_MOUSE, H2B1H_MOUSE, H2B1K_MOUSE, H2B1M_MOUSE, H2B1P_MOUSE, H2B2B_MOUSE	Histone H2B	SYSVYVYKVLKQVHPDTGISSKAMGIMNSFVND	34	2.80E-05	1226.6186	3676.8340	3	0.0070	33	1	GluC
H2B1B_MOUSE, H2B1C_MOUSE, H2B1F_MOUSE, H2B1H_MOUSE, H2B1K_MOUSE, H2B1M_MOUSE, H2B1P_MOUSE, H2B2B_MOUSE	Histone H2B	SYSVYVYKVLKQVHPDTGISSKAMGIMNSFVND	34	2.80E-03	920.2157	3676.8338	4	0.0069	33	1	GluC
H2B1B_MOUSE, H2B1C_MOUSE, H2B1F_MOUSE, H2B1H_MOUSE, H2B1K_MOUSE, H2B1M_MOUSE, H2B1P_MOUSE, H2B2B_MOUSE	Histone H2B	SYSVYVYKVLKQVHPDTGISSKAMGIMNSFVND	34	4.60E-03	920.2157	3676.8337	4	0.0068	33	1	GluC
H2B1B_MOUSE, H2B1C_MOUSE, H2B1F_MOUSE, H2B1H_MOUSE, H2B1K_MOUSE, H2B1M_MOUSE, H2B1P_MOUSE, H2B2B_MOUSE	Histone H2B	SYSVYVYKVLKQVHPDTGISSKAMGIMNSFVND	34	7.50E-05	920.2155	3676.8329	4	0.0060	33	1	GluC
H2B1B_MOUSE, H2B1C_MOUSE, H2B1F_MOUSE, H2B1H_MOUSE, H2B1K_MOUSE, H2B1M_MOUSE, H2B1P_MOUSE, H2B2B_MOUSE	Histone H2B	SYSVYVYKVLKQVHPDTGISSKAMGIMNSFVND	34	6.10E-03	920.2154	3676.8326	4	0.0057	33	1	GluC
H2B1B_MOUSE, H2B1C_MOUSE, H2B1F_MOUSE, H2B1H_MOUSE, H2B1K_MOUSE, H2B1M_MOUSE, H2B1P_MOUSE, H2B2B_MOUSE	Histone H2B	SYSVYVYKVLKQVHPDTGISSKAMGIMNSFVND	34	1.60E-04	920.2153	3676.8323	4	0.0053	33	1	GluC
H2B1B_MOUSE, H2B1C_MOUSE, H2B1F_MOUSE, H2B1H_MOUSE, H2B1K_MOUSE, H2B1M_MOUSE, H2B1P_MOUSE, H2B2B_MOUSE	Histone H2B	SYSVYVYKVLKQVHPDTGISSKAMGIMNSFVND	34	4.40E-03	920.2153	3676.8321	4	0.0052	33	1	GluC
H2B1B_MOUSE, H2B1C_MOUSE, H2B1F_MOUSE, H2B1H_MOUSE, H2B1K_MOUSE, H2B1M_MOUSE, H2B1P_MOUSE, H2B2B_MOUSE	Histone H2B	SYSVYVYKVLKQVHPDTGISSKAMGIMNSFVND	34	5.20E-06	1226.618	3676.8322	3	0.0052	33	1	GluC
H2B1B_MOUSE, H2B1C_MOUSE, H2B1F_MOUSE, H2B1H_MOUSE, H2B1K_MOUSE, H2B1M_MOUSE, H2B1P_MOUSE, H2B2B_MOUSE	Histone H2B	SYSVYVYKVLKQVHPDTGISSKAMGIMNSFVND	34	5.50E-03	920.2153	3676.8319	4	0.0050	33	1	GluC
H2B1B_MOUSE, H2B1C_MOUSE, H2B1F_MOUSE, H2B1H_MOUSE, H2B1K_MOUSE, H2B1M_MOUSE, H2B1P_MOUSE, H2B2B_MOUSE	Histone H2B	SYSVYVYKVLKQVHPDTGISSKAMGIMNSFVND	34	8.70E-03	736.3736	3676.8317	5	0.0047	33	1	GluC
H2B1B_MOUSE, H2B1C_MOUSE, H2B1F_MOUSE, H2B1H_MOUSE, H2B1K_MOUSE, H2B1M_MOUSE, H2B1P_MOUSE, H2B2B_MOUSE	Histone H2B	SYSVYVYKVLKQVHPDTGISSKAMGIMNSFVND	34	4.20E-07	920.2152	3676.8315	4	0.0046	33	1	GluC
H2B1B_MOUSE, H2B1C_MOUSE, H2B1F_MOUSE, H2B1H_MOUSE, H2B1K_MOUSE, H2B1M_MOUSE, H2B1P_MOUSE, H2B2B_MOUSE	Histone H2B	SYSVYVYKVLKQVHPDTGISSKAMGIMNSFVND	34	9.00E-04	920.2151	3676.8314	4	0.0045	33	1	GluC
H2B1B_MOUSE, H2B1C_MOUSE, H2B1F_MOUSE, H2B1H_MOUSE, H2B1K_MOUSE, H2B1M_MOUSE, H2B1P_MOUSE, H2B2B_MOUSE	Histone H2B	SYSVYVYKVLKQVHPDTGISSKAMGIMNSFVND	34	3.60E-03	920.2151	3676.8312	4	0.0042	33	1	GluC
H2B1B_MOUSE, H2B1C_MOUSE, H2B1F_MOUSE, H2B1H_MOUSE, H2B1K_MOUSE, H2B1M_MOUSE, H2B1P_MOUSE, H2B2B_MOUSE	Histone H2B	SYSVYVYKVLKQVHPDTGISSKAMGIMNSFVND	34	1.50E-04	920.215	3676.8310	4	0.0040	33	1	GluC
H2B1B_MOUSE, H2B1C_MOUSE, H2B1F_MOUSE, H2B1H_MOUSE, H2B1K_MOUSE, H2B1M_MOUSE, H2B1P_MOUSE, H2B2B_MOUSE	Histone H2B	SYSVYVYKVLKQVHPDTGISSKAMGIMNSFVND	34	1.50E-05	920.215	3676.8309	4	0.0040	33	1	GluC
H2B1B_MOUSE, H2B1C_MOUSE, H2B1F_MOUSE, H2B1H_MOUSE, H2B1K_MOUSE, H2B1M_MOUSE, H2B1P_MOUSE, H2B2B_MOUSE	Histone H2B	SYSVYVYKVLKQVHPDTGISSKAMGIMNSFVND	34	7.50E-04	920.2148	3676.8303	4	0.0033	33	1	GluC
H2B1B_MOUSE, H2B1C_MOUSE, H2B1F_MOUSE, H2B1H_MOUSE, H2B1K_MOUSE, H2B1M_MOUSE, H2B1P_MOUSE, H2B2B_MOUSE	Histone H2B	SYSVYVYKVLKQVHPDTGISSKAMGIMNSFVND	34	1.80E-03	920.2143	3676.8280	4	0.0011	33	1	GluC
H2B1B_MOUSE, H2B1C_MOUSE, H2B1F_MOUSE, H2B1H_MOUSE, H2B1K_MOUSE, H2B1M_MOUSE, H2B1P_MOUSE, H2B2B_MOUSE	Histone H2B	GKKRKRSRKESYSVYVYKVLKQVHPD	2	1.10E-03	795.4581	3177.8035	4	0.0148	26	1	GluC
H2B1B_MOUSE, H2B1C_MOUSE, H2B1F_MOUSE, H2B1H_MOUSE, H2B1K_MOUSE, H2B1M_MOUSE, H2B1P_MOUSE, H2B2B_MOUSE	Histone H2B	GKKRKRSRKESYSVYVYKVLKQVHPD	2	2.00E-07	636.5669	3177.7980	5	0.0093	26	1	GluC
H2B1B_MOUSE, H2B1C_MOUSE, H2B1F_MOUSE, H2B1H_MOUSE, H2B1K_MOUSE, H2B1M_MOUSE, H2B1P_MOUSE, H2B2B_MOUSE	Histone H2B	DGKKRKRSRKESYSVYVYKVLKQVHP	7	8.50E-04	1060.2707	3177.7903	3	0.0016	26	0	AspN
H2B1B_MOUSE, H2B1C_MOUSE, H2B1F_MOUSE, H2B1H_MOUSE, H2B1K_MOUSE, H2B1M_MOUSE, H2B1P_MOUSE, H2B2B_MOUSE	Histone H2B	DGKKRKRSRKESYSVYVYKVLKQVHP	7	1.40E-03	1060.2706	3177.7900	3	0.0013	26	0	AspN
H2B1B_MOUSE, H2B1C_MOUSE, H2B1F_MOUSE, H2B1H_MOUSE, H2B1K_MOUSE, H2B1M_MOUSE, H2B1P_MOUSE, H2B2B_MOUSE	Histone H2B	DGKKRKRSRKESYSVYVYKVLKQVHP	7	1.30E-03	795.4545	3177.7891	4	0.0004	26	0	AspN
H2B1B_MOUSE, H2B1C_MOUSE, H2B1F_MOUSE, H2B1H_MOUSE, H2B1K_MOUSE, H2B1M_MOUSE, H2B1P_MOUSE, H2B2B_MOUSE	Histone H2B	DGKKRKRSRKESYSVYVYKVLKQVHP	7	2.20E-05	795.4546	3177.7891	4	0.0004	26	0	AspN
H2B1B_MOUSE, H2B1C_MOUSE, H2B1F_MOUSE, H2B1H_MOUSE, H2B1K_MOUSE, H2B1M_MOUSE, H2B1P_MOUSE, H2B2B_MOUSE	Histone H2B	DGKKRKRSRKESYSVYVYKVLKQVHP	7	8.30E-09	530.6386	3177.7878	6	-0.0009	26	0	AspN
H2B1B_MOUSE, H2B1C_MOUSE, H2B1F_MOUSE, H2B1H_MOUSE, H2B1K_MOUSE, H2B1M_MOUSE, H2B1P_MOUSE, H2B2B_MOUSE	Histone H2B	DGKKRKRSRKESYSVYVYKVLKQVHP	7	1.40E-10	636.5645	3177.7863	5	-0.0023	26	0	AspN
H2B1B_MOUSE, H2B1C_MOUSE, H2B1F_MOUSE, H2B1H_MOUSE, H2B1K_MOUSE, H2B1M_MOUSE, H2B1P_MOUSE, H2B2B_MOUSE	Histone H2B	DGKKRKRSRKESYSVYVYKVLKQVHP	7	7.60E-07	636.5644	3177.7859	5	-0.0028	26	0	AspN
H2B1B_MOUSE, H2B1C_MOUSE, H2B1F_MOUSE, H2B1H_MOUSE, H2B1K_MOUSE, H2B1M_MOUSE, H2B1P_MOUSE, H2B2B_MOUSE	Histone H2B	KESYSVYVYKVLKQVHPDTGISSK	6	2.70E-08	919.1626	2754.4659	3	0.0067	24	3	Semi-tryptic
H2B1B_MOUSE, H2B1C_MOUSE, H2B1F_MOUSE, H2B1H_MOUSE, H2B1K_MOUSE, H2B1M_MOUSE, H2B1P_MOUSE, H2B2B_MOUSE	Histone H2B	KESYSVYVYKVLKQVHPDTGISSK	6	3.10E-05	551.8998	2754.4624	5	0.0032	24	3	Semi-tryptic

H2B1B_MOUSE, H2B1C_MOUSE, H2B1F_MOUSE, H2B1H_MOUSE, H2B1K_MOUSE, H2B1M_MOUSE, H2B1P_MOUSE, H2B2B_MOUSE	Histone H2B	KESYSVYVYKVLKQVHPDTGISSK	6	2.10E-07	551.8998	2754.4624	5	0.0032	24	3	Semi-tryptic
H2B1B_MOUSE, H2B1C_MOUSE, H2B1F_MOUSE, H2B1H_MOUSE, H2B1K_MOUSE, H2B1M_MOUSE, H2B1P_MOUSE, H2B2B_MOUSE	Histone H2B	KESYSVYVYKVLKQVHPDTGISSK	6	3.70E-04	689.6227	2754.4618	4	0.0026	24	3	Semi-tryptic
H2B1B_MOUSE, H2B1C_MOUSE, H2B1F_MOUSE, H2B1H_MOUSE, H2B1K_MOUSE, H2B1M_MOUSE, H2B1P_MOUSE, H2B2B_MOUSE	Histone H2B	KESYSVYVYKVLKQVHPDTGISSK	6	4.30E-05	689.6227	2754.4617	4	0.0024	24	3	Semi-tryptic
H2B1B_MOUSE, H2B1C_MOUSE, H2B1F_MOUSE, H2B1H_MOUSE, H2B1K_MOUSE, H2B1M_MOUSE, H2B1P_MOUSE, H2B2B_MOUSE	Histone H2B	KESYSVYVYKVLKQVHPDTGISSK	6	6.40E-05	689.6223	2754.4601	4	0.0009	24	3	Semi-tryptic
H2B1B_MOUSE, H2B1C_MOUSE, H2B1F_MOUSE, H2B1H_MOUSE, H2B1K_MOUSE, H2B1M_MOUSE, H2B1P_MOUSE, H2B2B_MOUSE	Histone H2B	ESYSVYVYKVLKQVHPDTGISSK	5	4.50E-07	876.4629	2626.3667	3	0.0025	23	2	Semi-tryptic
H2B1B_MOUSE, H2B1C_MOUSE, H2B1F_MOUSE, H2B1H_MOUSE, H2B1K_MOUSE, H2B1M_MOUSE, H2B1P_MOUSE, H2B2B_MOUSE	Histone H2B	ESYSVYVYKVLKQVHPDTGISSK	5	1.50E-04	876.462	2626.3643	3	0.0001	23	2	Semi-tryptic
H2B1B_MOUSE, H2B1C_MOUSE, H2B1F_MOUSE, H2B1H_MOUSE, H2B1K_MOUSE, H2B1M_MOUSE, H2B1P_MOUSE, H2B2B_MOUSE	Histone H2B	ESYSVYVYKVLKQVHPDTGISSK	5	4.60E-03	876.4616	2626.3630	3	-0.0013	23	2	Semi-tryptic
H2B1B_MOUSE, H2B1C_MOUSE, H2B1F_MOUSE, H2B1H_MOUSE, H2B1K_MOUSE, H2B1M_MOUSE, H2B1P_MOUSE, H2B2B_MOUSE	Histone H2B	ESYSVYVYKVLKQVHPDTGISSK	5	9.00E-08	657.5975	2626.3607	4	-0.0035	23	2	Semi-tryptic
H2B1B_MOUSE, H2B1C_MOUSE, H2B1F_MOUSE, H2B1H_MOUSE, H2B1K_MOUSE, H2B1M_MOUSE, H2B1P_MOUSE, H2B2B_MOUSE	Histone H2B	ESYSVYVYKVLKQVHPDTGISSK	5	1.30E-09	657.5975	2626.3607	4	-0.0035	23	2	Semi-tryptic
H2B1B_MOUSE, H2B1C_MOUSE, H2B1F_MOUSE, H2B1H_MOUSE, H2B1K_MOUSE, H2B1M_MOUSE, H2B1P_MOUSE, H2B2B_MOUSE	Histone H2B	SYSVYVYKVLKQVHPD	80	2.20E-04	963.0194	1924.0243	2	0.0149	16	0	GluC
H2B1B_MOUSE, H2B1C_MOUSE, H2B1F_MOUSE, H2B1H_MOUSE, H2B1K_MOUSE, H2B1M_MOUSE, H2B1P_MOUSE, H2B2B_MOUSE	Histone H2B	SYSVYVYKVLKQVHPD	80	4.10E-03	963.0189	1924.0232	2	0.0138	16	0	GluC
H2B1B_MOUSE, H2B1C_MOUSE, H2B1F_MOUSE, H2B1H_MOUSE, H2B1K_MOUSE, H2B1M_MOUSE, H2B1P_MOUSE, H2B2B_MOUSE	Histone H2B	SYSVYVYKVLKQVHPD	80	3.60E-06	642.3481	1924.0224	3	0.0130	16	0	GluC
H2B1B_MOUSE, H2B1C_MOUSE, H2B1F_MOUSE, H2B1H_MOUSE, H2B1K_MOUSE, H2B1M_MOUSE, H2B1P_MOUSE, H2B2B_MOUSE	Histone H2B	SYSVYVYKVLKQVHPD	80	7.10E-03	963.0173	1924.0201	2	0.0107	16	0	GluC
H2B1B_MOUSE, H2B1C_MOUSE, H2B1F_MOUSE, H2B1H_MOUSE, H2B1K_MOUSE, H2B1M_MOUSE, H2B1P_MOUSE, H2B2B_MOUSE	Histone H2B	SYSVYVYKVLKQVHPD	80	7.00E-05	642.347	1924.0191	3	0.0097	16	0	GluC
H2B1B_MOUSE, H2B1C_MOUSE, H2B1F_MOUSE, H2B1H_MOUSE, H2B1K_MOUSE, H2B1M_MOUSE, H2B1P_MOUSE, H2B2B_MOUSE	Histone H2B	SYSVYVYKVLKQVHPD	80	3.10E-03	963.0167	1924.0189	2	0.0094	16	0	GluC
H2B1B_MOUSE, H2B1C_MOUSE, H2B1F_MOUSE, H2B1H_MOUSE, H2B1K_MOUSE, H2B1M_MOUSE, H2B1P_MOUSE, H2B2B_MOUSE	Histone H2B	SYSVYVYKVLKQVHPD	80	1.00E-08	482.012	1924.0187	4	0.0093	16	0	GluC
H2B1B_MOUSE, H2B1C_MOUSE, H2B1F_MOUSE, H2B1H_MOUSE, H2B1K_MOUSE, H2B1M_MOUSE, H2B1P_MOUSE, H2B2B_MOUSE	Histone H2B	SYSVYVYKVLKQVHPD	80	1.60E-04	642.3468	1924.0184	3	0.0090	16	0	GluC
H2B1B_MOUSE, H2B1C_MOUSE, H2B1F_MOUSE, H2B1H_MOUSE, H2B1K_MOUSE, H2B1M_MOUSE, H2B1P_MOUSE, H2B2B_MOUSE	Histone H2B	SYSVYVYKVLKQVHPD	80	1.40E-05	642.3467	1924.0184	3	0.0089	16	0	GluC
H2B1B_MOUSE, H2B1C_MOUSE, H2B1F_MOUSE, H2B1H_MOUSE, H2B1K_MOUSE, H2B1M_MOUSE, H2B1P_MOUSE, H2B2B_MOUSE	Histone H2B	SYSVYVYKVLKQVHPD	80	1.60E-05	482.0117	1924.0177	4	0.0083	16	0	GluC
H2B1B_MOUSE, H2B1C_MOUSE, H2B1F_MOUSE, H2B1H_MOUSE, H2B1K_MOUSE, H2B1M_MOUSE, H2B1P_MOUSE, H2B2B_MOUSE	Histone H2B	SYSVYVYKVLKQVHPD	80	8.30E-08	482.0117	1924.0177	4	0.0083	16	0	GluC
H2B1B_MOUSE, H2B1C_MOUSE, H2B1F_MOUSE, H2B1H_MOUSE, H2B1K_MOUSE, H2B1M_MOUSE, H2B1P_MOUSE, H2B2B_MOUSE	Histone H2B	SYSVYVYKVLKQVHPD	80	4.90E-05	642.3463	1924.0170	3	0.0075	16	0	GluC
H2B1B_MOUSE, H2B1C_MOUSE, H2B1F_MOUSE, H2B1H_MOUSE, H2B1K_MOUSE, H2B1M_MOUSE, H2B1P_MOUSE, H2B2B_MOUSE	Histone H2B	SYSVYVYKVLKQVHPD	80	2.40E-08	482.0115	1924.0169	4	0.0075	16	0	GluC
H2B1B_MOUSE, H2B1C_MOUSE, H2B1F_MOUSE, H2B1H_MOUSE, H2B1K_MOUSE, H2B1M_MOUSE, H2B1P_MOUSE, H2B2B_MOUSE	Histone H2B	SYSVYVYKVLKQVHPD	80	1.30E-04	642.3458	1924.0156	3	0.0061	16	0	GluC
H2B1B_MOUSE, H2B1C_MOUSE, H2B1F_MOUSE, H2B1H_MOUSE, H2B1K_MOUSE, H2B1M_MOUSE, H2B1P_MOUSE, H2B2B_MOUSE	Histone H2B	SYSVYVYKVLKQVHPD	80	7.50E-06	642.3458	1924.0155	3	0.0061	16	0	GluC
H2B1B_MOUSE, H2B1C_MOUSE, H2B1F_MOUSE, H2B1H_MOUSE, H2B1K_MOUSE, H2B1M_MOUSE, H2B1P_MOUSE, H2B2B_MOUSE	Histone H2B	SYSVYVYKVLKQVHPD	80	1.20E-06	482.0111	1924.0153	4	0.0059	16	0	GluC
H2B1B_MOUSE, H2B1C_MOUSE, H2B1F_MOUSE, H2B1H_MOUSE, H2B1K_MOUSE, H2B1M_MOUSE, H2B1P_MOUSE, H2B2B_MOUSE	Histone H2B	SYSVYVYKVLKQVHPD	80	5.20E-05	642.3457	1924.0152	3	0.0057	16	0	GluC
H2B1B_MOUSE, H2B1C_MOUSE, H2B1F_MOUSE, H2B1H_MOUSE, H2B1K_MOUSE, H2B1M_MOUSE, H2B1P_MOUSE, H2B2B_MOUSE	Histone H2B	SYSVYVYKVLKQVHPD	80	1.20E-03	963.0148	1924.0150	2	0.0056	16	0	GluC
H2B1B_MOUSE, H2B1C_MOUSE, H2B1F_MOUSE, H2B1H_MOUSE, H2B1K_MOUSE, H2B1M_MOUSE, H2B1P_MOUSE, H2B2B_MOUSE	Histone H2B	SYSVYVYKVLKQVHPD	80	4.40E-06	482.011	1924.0151	4	0.0056	16	0	GluC
H2B1B_MOUSE, H2B1C_MOUSE, H2B1F_MOUSE, H2B1H_MOUSE, H2B1K_MOUSE, H2B1M_MOUSE, H2B1P_MOUSE, H2B2B_MOUSE	Histone H2B	SYSVYVYKVLKQVHPD	80	8.20E-03	963.0141	1924.0136	2	0.0041	16	0	GluC
H2B1B_MOUSE, H2B1C_MOUSE, H2B1F_MOUSE, H2B1H_MOUSE, H2B1K_MOUSE, H2B1M_MOUSE, H2B1P_MOUSE, H2B2B_MOUSE	Histone H2B	SYSVYVYKVLKQVHPD	80	5.20E-03	642.3451	1924.0136	3	0.0041	16	0	GluC
H2B1B_MOUSE, H2B1C_MOUSE, H2B1F_MOUSE, H2B1H_MOUSE, H2B1K_MOUSE, H2B1M_MOUSE, H2B1P_MOUSE, H2B2B_MOUSE	Histone H2B	SYSVYVYKVLKQVHPD	80	2.10E-04	963.0141	1924.0136	2	0.0041	16	0	GluC
H2B1B_MOUSE, H2B1C_MOUSE, H2B1F_MOUSE, H2B1H_MOUSE, H2B1K_MOUSE, H2B1M_MOUSE, H2B1P_MOUSE, H2B2B_MOUSE	Histone H2B	SYSVYVYKVLKQVHPD	80	4.80E-05	642.3451	1924.0136	3	0.0041	16	0	GluC
H2B1B_MOUSE, H2B1C_MOUSE, H2B1F_MOUSE, H2B1H_MOUSE, H2B1K_MOUSE, H2B1M_MOUSE, H2B1P_MOUSE, H2B2B_MOUSE	Histone H2B	SYSVYVYKVLKQVHPD	80	3.20E-03	642.3451	1924.0135	3	0.0040	16	0	GluC
H2B1B_MOUSE, H2B1C_MOUSE, H2B1F_MOUSE, H2B1H_MOUSE, H2B1K_MOUSE, H2B1M_MOUSE, H2B1P_MOUSE, H2B2B_MOUSE	Histone H2B	SYSVYVYKVLKQVHPD	80	2.40E-04	963.014	1924.0133	2	0.0039	16	0	GluC
H2B1B_MOUSE, H2B1C_MOUSE, H2B1F_MOUSE, H2B1H_MOUSE, H2B1K_MOUSE, H2B1M_MOUSE, H2B1P_MOUSE, H2B2B_MOUSE	Histone H2B	SYSVYVYKVLKQVHPD	80	5.50E-03	642.345	1924.0131	3	0.0037	16	0	GluC
H2B1B_MOUSE, H2B1C_MOUSE, H2B1F_MOUSE, H2B1H_MOUSE, H2B1K_MOUSE, H2B1M_MOUSE, H2B1P_MOUSE, H2B2B_MOUSE	Histone H2B	SYSVYVYKVLKQVHPD	80	1.30E-05	642.345	1924.0131	3	0.0037	16	0	GluC
H2B1B_MOUSE, H2B1C_MOUSE, H2B1F_MOUSE, H2B1H_MOUSE, H2B1K_MOUSE, H2B1M_MOUSE, H2B1P_MOUSE, H2B2B_MOUSE	Histone H2B	SYSVYVYKVLKQVHPD	80	1.40E-03	642.3449	1924.0129	3	0.0034	16	0	GluC

H2B1B_MOUSE, H2B1C_MOUSE, H2B1F_MOUSE, H2B1H_MOUSE, H2B1K_MOUSE, H2B1M_MOUSE, H2B1P_MOUSE, H2B2B_MOUSE	Histone H2B	SYSVYVYKVLKQVHPD	80	2.00E-04	642.3449	1924.0129	3	0.0034	16	0	GluC
H2B1B_MOUSE, H2B1C_MOUSE, H2B1F_MOUSE, H2B1H_MOUSE, H2B1K_MOUSE, H2B1M_MOUSE, H2B1P_MOUSE, H2B2B_MOUSE	Histone H2B	SYSVYVYKVLKQVHPD	80	1.50E-04	963.0137	1924.0128	2	0.0034	16	0	GluC
H2B1B_MOUSE, H2B1C_MOUSE, H2B1F_MOUSE, H2B1H_MOUSE, H2B1K_MOUSE, H2B1M_MOUSE, H2B1P_MOUSE, H2B2B_MOUSE	Histone H2B	SYSVYVYKVLKQVHPD	80	1.20E-06	482.0105	1924.0129	4	0.0034	16	0	GluC
H2B1B_MOUSE, H2B1C_MOUSE, H2B1F_MOUSE, H2B1H_MOUSE, H2B1K_MOUSE, H2B1M_MOUSE, H2B1P_MOUSE, H2B2B_MOUSE	Histone H2B	SYSVYVYKVLKQVHPD	80	9.30E-06	642.3448	1924.0126	3	0.0031	16	0	GluC
H2B1B_MOUSE, H2B1C_MOUSE, H2B1F_MOUSE, H2B1H_MOUSE, H2B1K_MOUSE, H2B1M_MOUSE, H2B1P_MOUSE, H2B2B_MOUSE	Histone H2B	SYSVYVYKVLKQVHPD	80	1.30E-03	642.3447	1924.0122	3	0.0027	16	0	GluC
H2B1B_MOUSE, H2B1C_MOUSE, H2B1F_MOUSE, H2B1H_MOUSE, H2B1K_MOUSE, H2B1M_MOUSE, H2B1P_MOUSE, H2B2B_MOUSE	Histone H2B	SYSVYVYKVLKQVHPD	80	1.50E-04	963.0132	1924.0119	2	0.0025	16	0	GluC
H2B1B_MOUSE, H2B1C_MOUSE, H2B1F_MOUSE, H2B1H_MOUSE, H2B1K_MOUSE, H2B1M_MOUSE, H2B1P_MOUSE, H2B2B_MOUSE	Histone H2B	SYSVYVYKVLKQVHPD	80	8.70E-03	642.3446	1924.0119	3	0.0024	16	0	GluC
H2B1B_MOUSE, H2B1C_MOUSE, H2B1F_MOUSE, H2B1H_MOUSE, H2B1K_MOUSE, H2B1M_MOUSE, H2B1P_MOUSE, H2B2B_MOUSE	Histone H2B	SYSVYVYKVLKQVHPD	80	6.80E-04	963.0132	1924.0118	2	0.0024	16	0	GluC
H2B1B_MOUSE, H2B1C_MOUSE, H2B1F_MOUSE, H2B1H_MOUSE, H2B1K_MOUSE, H2B1M_MOUSE, H2B1P_MOUSE, H2B2B_MOUSE	Histone H2B	SYSVYVYKVLKQVHPD	80	2.90E-05	642.3446	1924.0119	3	0.0024	16	0	GluC
H2B1B_MOUSE, H2B1C_MOUSE, H2B1F_MOUSE, H2B1H_MOUSE, H2B1K_MOUSE, H2B1M_MOUSE, H2B1P_MOUSE, H2B2B_MOUSE	Histone H2B	SYSVYVYKVLKQVHPD	80	1.90E-07	482.0102	1924.0118	4	0.0024	16	0	GluC
H2B1B_MOUSE, H2B1C_MOUSE, H2B1F_MOUSE, H2B1H_MOUSE, H2B1K_MOUSE, H2B1M_MOUSE, H2B1P_MOUSE, H2B2B_MOUSE	Histone H2B	SYSVYVYKVLKQVHPD	80	1.50E-03	642.3445	1924.0118	3	0.0023	16	0	GluC
H2B1B_MOUSE, H2B1C_MOUSE, H2B1F_MOUSE, H2B1H_MOUSE, H2B1K_MOUSE, H2B1M_MOUSE, H2B1P_MOUSE, H2B2B_MOUSE	Histone H2B	SYSVYVYKVLKQVHPD	80	7.60E-07	482.0102	1924.0117	4	0.0023	16	0	GluC
H2B1B_MOUSE, H2B1C_MOUSE, H2B1F_MOUSE, H2B1H_MOUSE, H2B1K_MOUSE, H2B1M_MOUSE, H2B1P_MOUSE, H2B2B_MOUSE	Histone H2B	SYSVYVYKVLKQVHPD	80	8.70E-08	642.3445	1924.0117	3	0.0023	16	0	GluC
H2B1B_MOUSE, H2B1C_MOUSE, H2B1F_MOUSE, H2B1H_MOUSE, H2B1K_MOUSE, H2B1M_MOUSE, H2B1P_MOUSE, H2B2B_MOUSE	Histone H2B	SYSVYVYKVLKQVHPD	80	7.80E-04	963.0131	1924.0116	2	0.0022	16	0	GluC
H2B1B_MOUSE, H2B1C_MOUSE, H2B1F_MOUSE, H2B1H_MOUSE, H2B1K_MOUSE, H2B1M_MOUSE, H2B1P_MOUSE, H2B2B_MOUSE	Histone H2B	SYSVYVYKVLKQVHPD	80	2.00E-04	963.0131	1924.0116	2	0.0022	16	0	GluC
H2B1B_MOUSE, H2B1C_MOUSE, H2B1F_MOUSE, H2B1H_MOUSE, H2B1K_MOUSE, H2B1M_MOUSE, H2B1P_MOUSE, H2B2B_MOUSE	Histone H2B	SYSVYVYKVLKQVHPD	80	1.80E-04	963.0131	1924.0116	2	0.0022	16	0	GluC
H2B1B_MOUSE, H2B1C_MOUSE, H2B1F_MOUSE, H2B1H_MOUSE, H2B1K_MOUSE, H2B1M_MOUSE, H2B1P_MOUSE, H2B2B_MOUSE	Histone H2B	SYSVYVYKVLKQVHPD	80	6.60E-05	642.3445	1924.0117	3	0.0022	16	0	GluC
H2B1B_MOUSE, H2B1C_MOUSE, H2B1F_MOUSE, H2B1H_MOUSE, H2B1K_MOUSE, H2B1M_MOUSE, H2B1P_MOUSE, H2B2B_MOUSE	Histone H2B	SYSVYVYKVLKQVHPD	80	3.00E-06	482.0101	1924.0115	4	0.0020	16	0	GluC
H2B1B_MOUSE, H2B1C_MOUSE, H2B1F_MOUSE, H2B1H_MOUSE, H2B1K_MOUSE, H2B1M_MOUSE, H2B1P_MOUSE, H2B2B_MOUSE	Histone H2B	SYSVYVYKVLKQVHPD	80	1.20E-03	963.013	1924.0113	2	0.0019	16	0	GluC
H2B1B_MOUSE, H2B1C_MOUSE, H2B1F_MOUSE, H2B1H_MOUSE, H2B1K_MOUSE, H2B1M_MOUSE, H2B1P_MOUSE, H2B2B_MOUSE	Histone H2B	SYSVYVYKVLKQVHPD	80	8.10E-03	642.3444	1924.0113	3	0.0018	16	0	GluC
H2B1B_MOUSE, H2B1C_MOUSE, H2B1F_MOUSE, H2B1H_MOUSE, H2B1K_MOUSE, H2B1M_MOUSE, H2B1P_MOUSE, H2B2B_MOUSE	Histone H2B	SYSVYVYKVLKQVHPD	80	5.10E-05	642.3444	1924.0113	3	0.0018	16	0	GluC
H2B1B_MOUSE, H2B1C_MOUSE, H2B1F_MOUSE, H2B1H_MOUSE, H2B1K_MOUSE, H2B1M_MOUSE, H2B1P_MOUSE, H2B2B_MOUSE	Histone H2B	SYSVYVYKVLKQVHPD	80	2.20E-03	963.0129	1924.0112	2	0.0017	16	0	GluC
H2B1B_MOUSE, H2B1C_MOUSE, H2B1F_MOUSE, H2B1H_MOUSE, H2B1K_MOUSE, H2B1M_MOUSE, H2B1P_MOUSE, H2B2B_MOUSE	Histone H2B	SYSVYVYKVLKQVHPD	80	1.30E-03	642.3443	1924.0112	3	0.0017	16	0	GluC
H2B1B_MOUSE, H2B1C_MOUSE, H2B1F_MOUSE, H2B1H_MOUSE, H2B1K_MOUSE, H2B1M_MOUSE, H2B1P_MOUSE, H2B2B_MOUSE	Histone H2B	SYSVYVYKVLKQVHPD	80	6.70E-04	642.3443	1924.0111	3	0.0016	16	0	GluC
H2B1B_MOUSE, H2B1C_MOUSE, H2B1F_MOUSE, H2B1H_MOUSE, H2B1K_MOUSE, H2B1M_MOUSE, H2B1P_MOUSE, H2B2B_MOUSE	Histone H2B	SYSVYVYKVLKQVHPD	80	3.70E-04	963.0127	1924.0109	2	0.0015	16	0	GluC
H2B1B_MOUSE, H2B1C_MOUSE, H2B1F_MOUSE, H2B1H_MOUSE, H2B1K_MOUSE, H2B1M_MOUSE, H2B1P_MOUSE, H2B2B_MOUSE	Histone H2B	SYSVYVYKVLKQVHPD	80	8.70E-06	482.01	1924.0109	4	0.0015	16	0	GluC
H2B1B_MOUSE, H2B1C_MOUSE, H2B1F_MOUSE, H2B1H_MOUSE, H2B1K_MOUSE, H2B1M_MOUSE, H2B1P_MOUSE, H2B2B_MOUSE	Histone H2B	SYSVYVYKVLKQVHPD	80	8.20E-03	642.3442	1924.0109	3	0.0014	16	0	GluC
H2B1B_MOUSE, H2B1C_MOUSE, H2B1F_MOUSE, H2B1H_MOUSE, H2B1K_MOUSE, H2B1M_MOUSE, H2B1P_MOUSE, H2B2B_MOUSE	Histone H2B	SYSVYVYKVLKQVHPD	80	1.00E-03	642.3442	1924.0108	3	0.0014	16	0	GluC
H2B1B_MOUSE, H2B1C_MOUSE, H2B1F_MOUSE, H2B1H_MOUSE, H2B1K_MOUSE, H2B1M_MOUSE, H2B1P_MOUSE, H2B2B_MOUSE	Histone H2B	SYSVYVYKVLKQVHPD	80	4.80E-04	642.3442	1924.0108	3	0.0014	16	0	GluC
H2B1B_MOUSE, H2B1C_MOUSE, H2B1F_MOUSE, H2B1H_MOUSE, H2B1K_MOUSE, H2B1M_MOUSE, H2B1P_MOUSE, H2B2B_MOUSE	Histone H2B	SYSVYVYKVLKQVHPD	80	3.00E-04	642.3442	1924.0109	3	0.0014	16	0	GluC
H2B1B_MOUSE, H2B1C_MOUSE, H2B1F_MOUSE, H2B1H_MOUSE, H2B1K_MOUSE, H2B1M_MOUSE, H2B1P_MOUSE, H2B2B_MOUSE	Histone H2B	SYSVYVYKVLKQVHPD	80	9.70E-06	482.01	1924.0108	4	0.0014	16	0	GluC
H2B1B_MOUSE, H2B1C_MOUSE, H2B1F_MOUSE, H2B1H_MOUSE, H2B1K_MOUSE, H2B1M_MOUSE, H2B1P_MOUSE, H2B2B_MOUSE	Histone H2B	SYSVYVYKVLKQVHPD	80	2.60E-03	642.3442	1924.0107	3	0.0013	16	0	GluC
H2B1B_MOUSE, H2B1C_MOUSE, H2B1F_MOUSE, H2B1H_MOUSE, H2B1K_MOUSE, H2B1M_MOUSE, H2B1P_MOUSE, H2B2B_MOUSE	Histone H2B	SYSVYVYKVLKQVHPD	80	6.60E-05	642.3442	1924.0107	3	0.0013	16	0	GluC
H2B1B_MOUSE, H2B1C_MOUSE, H2B1F_MOUSE, H2B1H_MOUSE, H2B1K_MOUSE, H2B1M_MOUSE, H2B1P_MOUSE, H2B2B_MOUSE	Histone H2B	SYSVYVYKVLKQVHPD	80	4.50E-04	642.3442	1924.0107	3	0.0012	16	0	GluC
H2B1B_MOUSE, H2B1C_MOUSE, H2B1F_MOUSE, H2B1H_MOUSE, H2B1K_MOUSE, H2B1M_MOUSE, H2B1P_MOUSE, H2B2B_MOUSE	Histone H2B	SYSVYVYKVLKQVHPD	80	3.00E-04	642.3442	1924.0107	3	0.0012	16	0	GluC
H2B1B_MOUSE, H2B1C_MOUSE, H2B1F_MOUSE, H2B1H_MOUSE, H2B1K_MOUSE, H2B1M_MOUSE, H2B1P_MOUSE, H2B2B_MOUSE	Histone H2B	SYSVYVYKVLKQVHPD	80	3.90E-03	642.3441	1924.0105	3	0.0011	16	0	GluC
H2B1B_MOUSE, H2B1C_MOUSE, H2B1F_MOUSE, H2B1H_MOUSE, H2B1K_MOUSE, H2B1M_MOUSE, H2B1P_MOUSE, H2B2B_MOUSE	Histone H2B	SYSVYVYKVLKQVHPD	80	4.70E-05	482.0099	1924.0104	4	0.0009	16	0	GluC

H2B1B_MOUSE, H2B1C_MOUSE, H2B1F_MOUSE, H2B1H_MOUSE, H2B1K_MOUSE, H2B1M_MOUSE, H2B1P_MOUSE, H2B2B_MOUSE	Histone H2B	SYSVYVYKVLKQVHPD	80	2.70E-03	482.0098	1924.0101	4	0.0007	16	0	GluC
H2B1B_MOUSE, H2B1C_MOUSE, H2B1F_MOUSE, H2B1H_MOUSE, H2B1K_MOUSE, H2B1M_MOUSE, H2B1P_MOUSE, H2B2B_MOUSE	Histone H2B	SYSVYVYKVLKQVHPD	80	1.90E-06	482.0098	1924.0101	4	0.0007	16	0	GluC
H2B1B_MOUSE, H2B1C_MOUSE, H2B1F_MOUSE, H2B1H_MOUSE, H2B1K_MOUSE, H2B1M_MOUSE, H2B1P_MOUSE, H2B2B_MOUSE	Histone H2B	SYSVYVYKVLKQVHPD	80	8.80E-03	642.344	1924.0101	3	0.0006	16	0	GluC
H2B1B_MOUSE, H2B1C_MOUSE, H2B1F_MOUSE, H2B1H_MOUSE, H2B1K_MOUSE, H2B1M_MOUSE, H2B1P_MOUSE, H2B2B_MOUSE	Histone H2B	SYSVYVYKVLKQVHPD	80	1.30E-04	642.344	1924.0101	3	0.0006	16	0	GluC
H2B1B_MOUSE, H2B1C_MOUSE, H2B1F_MOUSE, H2B1H_MOUSE, H2B1K_MOUSE, H2B1M_MOUSE, H2B1P_MOUSE, H2B2B_MOUSE	Histone H2B	SYSVYVYKVLKQVHPD	80	6.10E-03	482.0097	1924.0099	4	0.0004	16	0	GluC
H2B1B_MOUSE, H2B1C_MOUSE, H2B1F_MOUSE, H2B1H_MOUSE, H2B1K_MOUSE, H2B1M_MOUSE, H2B1P_MOUSE, H2B2B_MOUSE	Histone H2B	SYSVYVYKVLKQVHPD	80	2.90E-03	642.3439	1924.0098	3	0.0004	16	0	GluC
H2B1B_MOUSE, H2B1C_MOUSE, H2B1F_MOUSE, H2B1H_MOUSE, H2B1K_MOUSE, H2B1M_MOUSE, H2B1P_MOUSE, H2B2B_MOUSE	Histone H2B	SYSVYVYKVLKQVHPD	80	2.80E-03	642.3439	1924.0099	3	0.0004	16	0	GluC
H2B1B_MOUSE, H2B1C_MOUSE, H2B1F_MOUSE, H2B1H_MOUSE, H2B1K_MOUSE, H2B1M_MOUSE, H2B1P_MOUSE, H2B2B_MOUSE	Histone H2B	SYSVYVYKVLKQVHPD	80	2.10E-03	642.3439	1924.0099	3	0.0004	16	0	GluC
H2B1B_MOUSE, H2B1C_MOUSE, H2B1F_MOUSE, H2B1H_MOUSE, H2B1K_MOUSE, H2B1M_MOUSE, H2B1P_MOUSE, H2B2B_MOUSE	Histone H2B	SYSVYVYKVLKQVHPD	80	8.70E-04	482.0097	1924.0095	4	0.0001	16	0	GluC
H2B1B_MOUSE, H2B1C_MOUSE, H2B1F_MOUSE, H2B1H_MOUSE, H2B1K_MOUSE, H2B1M_MOUSE, H2B1P_MOUSE, H2B2B_MOUSE	Histone H2B	SYSVYVYKVLKQVHPD	80	2.10E-05	482.0097	1924.0095	4	0.0001	16	0	GluC
H2B1B_MOUSE, H2B1C_MOUSE, H2B1F_MOUSE, H2B1H_MOUSE, H2B1K_MOUSE, H2B1M_MOUSE, H2B1P_MOUSE, H2B2B_MOUSE	Histone H2B	SYSVYVYKVLKQVHPD	80	7.80E-03	642.3438	1924.0095	3	0.0000	16	0	GluC
H2B1B_MOUSE, H2B1C_MOUSE, H2B1F_MOUSE, H2B1H_MOUSE, H2B1K_MOUSE, H2B1M_MOUSE, H2B1P_MOUSE, H2B2B_MOUSE	Histone H2B	SYSVYVYKVLKQVHPD	80	8.90E-03	642.3436	1924.0091	3	-0.0004	16	0	GluC
H2B1B_MOUSE, H2B1C_MOUSE, H2B1F_MOUSE, H2B1H_MOUSE, H2B1K_MOUSE, H2B1M_MOUSE, H2B1P_MOUSE, H2B2B_MOUSE	Histone H2B	SYSVYVYKVLKQVHPD	80	5.40E-04	482.0095	1924.0089	4	-0.0005	16	0	GluC
H2B1B_MOUSE, H2B1C_MOUSE, H2B1F_MOUSE, H2B1H_MOUSE, H2B1K_MOUSE, H2B1M_MOUSE, H2B1P_MOUSE, H2B2B_MOUSE	Histone H2B	SYSVYVYKVLKQVHPD	80	6.40E-04	482.0093	1924.0082	4	-0.0012	16	0	GluC
H2B1B_MOUSE, H2B1C_MOUSE, H2B1F_MOUSE, H2B1H_MOUSE, H2B1K_MOUSE, H2B1M_MOUSE, H2B1P_MOUSE, H2B2B_MOUSE	Histone H2B	SYSVYVYKVLKQVHPD	80	1.00E-03	642.3433	1924.0080	3	-0.0015	16	0	GluC
H2B1B_MOUSE, H2B1C_MOUSE, H2B1F_MOUSE, H2B1H_MOUSE, H2B1K_MOUSE, H2B1M_MOUSE, H2B1P_MOUSE, H2B2B_MOUSE	Histone H2B	SRKESYSVYVYKVLK	13	7.40E-05	463.0104	1848.0124	4	-0.0021	15	3	Semi-tryptic
H2B1B_MOUSE, H2B1C_MOUSE, H2B1F_MOUSE, H2B1H_MOUSE, H2B1K_MOUSE, H2B1M_MOUSE, H2B1P_MOUSE, H2B2B_MOUSE	Histone H2B	SRKESYSVYVYKVLK	13	2.10E-05	463.0102	1848.0117	4	-0.0028	15	3	Semi-tryptic
H2B1B_MOUSE, H2B1C_MOUSE, H2B1F_MOUSE, H2B1H_MOUSE, H2B1K_MOUSE, H2B1M_MOUSE, H2B1P_MOUSE, H2B2B_MOUSE	Histone H2B	SRKESYSVYVYKVLK	13	2.20E-05	617.0111	1848.0115	3	-0.0030	15	3	Semi-tryptic
H2B1B_MOUSE, H2B1C_MOUSE, H2B1F_MOUSE, H2B1H_MOUSE, H2B1K_MOUSE, H2B1M_MOUSE, H2B1P_MOUSE, H2B2B_MOUSE	Histone H2B	SRKESYSVYVYKVLK	13	6.30E-07	463.0119	1848.0186	4	0.0041	15	3	Semi-tryptic
H2B1B_MOUSE, H2B1C_MOUSE, H2B1F_MOUSE, H2B1H_MOUSE, H2B1K_MOUSE, H2B1M_MOUSE, H2B1P_MOUSE, H2B2B_MOUSE	Histone H2B	SRKESYSVYVYKVLK	13	1.00E-09	463.0119	1848.0186	4	0.0041	15	3	Semi-tryptic
H2B1B_MOUSE, H2B1C_MOUSE, H2B1F_MOUSE, H2B1H_MOUSE, H2B1K_MOUSE, H2B1M_MOUSE, H2B1P_MOUSE, H2B2B_MOUSE	Histone H2B	SRKESYSVYVYKVLK	13	3.80E-06	617.0122	1848.0147	3	0.0002	15	3	Semi-tryptic
H2B1B_MOUSE, H2B1C_MOUSE, H2B1F_MOUSE, H2B1H_MOUSE, H2B1K_MOUSE, H2B1M_MOUSE, H2B1P_MOUSE, H2B2B_MOUSE	Histone H2B	SRKESYSVYVYKVLK	13	1.30E-06	925.0146	1848.0147	2	0.0002	15	3	Semi-tryptic
H2B1B_MOUSE, H2B1C_MOUSE, H2B1F_MOUSE, H2B1H_MOUSE, H2B1K_MOUSE, H2B1M_MOUSE, H2B1P_MOUSE, H2B2B_MOUSE	Histone H2B	SRKESYSVYVYKVLK	13	1.00E-04	463.0109	1848.0144	4	-0.0001	15	3	Semi-tryptic
H2B1B_MOUSE, H2B1C_MOUSE, H2B1F_MOUSE, H2B1H_MOUSE, H2B1K_MOUSE, H2B1M_MOUSE, H2B1P_MOUSE, H2B2B_MOUSE	Histone H2B	SRKESYSVYVYKVLK	13	1.10E-05	463.0109	1848.0144	4	-0.0001	15	3	Semi-tryptic
H2B1B_MOUSE, H2B1C_MOUSE, H2B1F_MOUSE, H2B1H_MOUSE, H2B1K_MOUSE, H2B1M_MOUSE, H2B1P_MOUSE, H2B2B_MOUSE	Histone H2B	SRKESYSVYVYKVLK	13	7.70E-06	617.0121	1848.0144	3	-0.0001	15	3	Semi-tryptic
H2B1B_MOUSE, H2B1C_MOUSE, H2B1F_MOUSE, H2B1H_MOUSE, H2B1K_MOUSE, H2B1M_MOUSE, H2B1P_MOUSE, H2B2B_MOUSE	Histone H2B	SRKESYSVYVYKVLK	13	1.30E-04	463.0107	1848.0138	4	-0.0007	15	3	Semi-tryptic
H2B1B_MOUSE, H2B1C_MOUSE, H2B1F_MOUSE, H2B1H_MOUSE, H2B1K_MOUSE, H2B1M_MOUSE, H2B1P_MOUSE, H2B2B_MOUSE	Histone H2B	SRKESYSVYVYKVLK	13	1.10E-06	617.0116	1848.0130	3	-0.0015	15	3	Semi-tryptic
H2B1B_MOUSE, H2B1C_MOUSE, H2B1F_MOUSE, H2B1H_MOUSE, H2B1K_MOUSE, H2B1M_MOUSE, H2B1P_MOUSE, H2B2B_MOUSE	Histone H2B	SRKESYSVYVYKVLK	13	5.00E-04	463.0104	1848.0127	4	-0.0018	15	3	Semi-tryptic
H2B1B_MOUSE, H2B1C_MOUSE, H2B1F_MOUSE, H2B1H_MOUSE, H2B1K_MOUSE, H2B1M_MOUSE, H2B1P_MOUSE, H2B2B_MOUSE	Histone H2B	KESYSVYVYKVLK	8	4.40E-08	803.4487	1604.8828	2	0.0014	13	2	Semi-tryptic
H2B1B_MOUSE, H2B1C_MOUSE, H2B1F_MOUSE, H2B1H_MOUSE, H2B1K_MOUSE, H2B1M_MOUSE, H2B1P_MOUSE, H2B2B_MOUSE	Histone H2B	KESYSVYVYKVLK	8	5.10E-05	535.9678	1604.8817	3	0.0003	13	2	Semi-tryptic
H2B1B_MOUSE, H2B1C_MOUSE, H2B1F_MOUSE, H2B1H_MOUSE, H2B1K_MOUSE, H2B1M_MOUSE, H2B1P_MOUSE, H2B2B_MOUSE	Histone H2B	KESYSVYVYKVLK	8	4.80E-07	535.9678	1604.8815	3	0.0002	13	2	Semi-tryptic
H2B1B_MOUSE, H2B1C_MOUSE, H2B1F_MOUSE, H2B1H_MOUSE, H2B1K_MOUSE, H2B1M_MOUSE, H2B1P_MOUSE, H2B2B_MOUSE	Histone H2B	KESYSVYVYKVLK	8	7.00E-08	535.9678	1604.8815	3	0.0002	13	2	Semi-tryptic
H2B1B_MOUSE, H2B1C_MOUSE, H2B1F_MOUSE, H2B1H_MOUSE, H2B1K_MOUSE, H2B1M_MOUSE, H2B1P_MOUSE, H2B2B_MOUSE	Histone H2B	KESYSVYVYKVLK	8	3.90E-03	535.9674	1604.8804	3	-0.0009	13	2	Semi-tryptic
H2B1B_MOUSE, H2B1C_MOUSE, H2B1F_MOUSE, H2B1H_MOUSE, H2B1K_MOUSE, H2B1M_MOUSE, H2B1P_MOUSE, H2B2B_MOUSE	Histone H2B	KESYSVYVYKVLK	8	2.70E-04	535.9668	1604.8785	3	-0.0029	13	2	Semi-tryptic
H2B1B_MOUSE, H2B1C_MOUSE, H2B1F_MOUSE, H2B1H_MOUSE, H2B1K_MOUSE, H2B1M_MOUSE, H2B1P_MOUSE, H2B2B_MOUSE	Histone H2B	KESYSVYVYKVLK	8	1.30E-07	803.446	1604.8775	2	-0.0038	13	2	Semi-tryptic
H2B1B_MOUSE, H2B1C_MOUSE, H2B1F_MOUSE, H2B1H_MOUSE, H2B1K_MOUSE, H2B1M_MOUSE, H2B1P_MOUSE, H2B2B_MOUSE	Histone H2B	KESYSVYVYKVLK	8	1.20E-04	535.9661	1604.8766	3	-0.0047	13	2	Semi-tryptic
H2B1B_MOUSE, H2B1C_MOUSE, H2B1F_MOUSE, H2B1H_MOUSE, H2B1K_MOUSE, H2B1M_MOUSE, H2B1P_MOUSE, H2B2B_MOUSE	Histone H2B	ESYSVYVYKVLK	7	1.40E-04	739.4033	1476.7920	2	0.0056	12	1	Semi-tryptic

H2B1B_MOUSE, H2B1C_MOUSE, H2B1F_MOUSE, H2B1H_MOUSE, H2B1K_MOUSE, H2B1M_MOUSE, H2B1P_MOUSE, H2B2B_MOUSE	Histone H2B	ESYSVYVYKVLK	7	9.80E-06	739.4033	1476.7920	2	0.0056	12	1	Semi-tryptic
H2B1B_MOUSE, H2B1C_MOUSE, H2B1F_MOUSE, H2B1H_MOUSE, H2B1K_MOUSE, H2B1M_MOUSE, H2B1P_MOUSE, H2B2B_MOUSE	Histone H2B	ESYSVYVYKVLK	7	1.20E-06	493.2707	1476.7902	3	0.0037	12	1	Semi-tryptic
H2B1B_MOUSE, H2B1C_MOUSE, H2B1F_MOUSE, H2B1H_MOUSE, H2B1K_MOUSE, H2B1M_MOUSE, H2B1P_MOUSE, H2B2B_MOUSE	Histone H2B	ESYSVYVYKVLK	7	7.50E-08	493.2707	1476.7902	3	0.0037	12	1	Semi-tryptic
H2B1B_MOUSE, H2B1C_MOUSE, H2B1F_MOUSE, H2B1H_MOUSE, H2B1K_MOUSE, H2B1M_MOUSE, H2B1P_MOUSE, H2B2B_MOUSE	Histone H2B	ESYSVYVYKVLK	7	1.80E-06	739.4008	1476.7871	2	0.0007	12	1	Semi-tryptic
H2B1B_MOUSE, H2B1C_MOUSE, H2B1F_MOUSE, H2B1H_MOUSE, H2B1K_MOUSE, H2B1M_MOUSE, H2B1P_MOUSE, H2B2B_MOUSE	Histone H2B	ESYSVYVYKVLK	7	2.00E-05	739.4005	1476.7864	2	0.0000	12	1	Semi-tryptic
H2B1B_MOUSE, H2B1C_MOUSE, H2B1F_MOUSE, H2B1H_MOUSE, H2B1K_MOUSE, H2B1M_MOUSE, H2B1P_MOUSE, H2B2B_MOUSE	Histone H2B	SRKESYSVYVYK	9	2.70E-04	754.892	1507.7695	2	0.0024	12	2	Semi-tryptic
H2B1B_MOUSE, H2B1C_MOUSE, H2B1F_MOUSE, H2B1H_MOUSE, H2B1K_MOUSE, H2B1M_MOUSE, H2B1P_MOUSE, H2B2B_MOUSE	Histone H2B	SRKESYSVYVYK	9	2.90E-03	503.5969	1507.7690	3	0.0019	12	2	Semi-tryptic
H2B1B_MOUSE, H2B1C_MOUSE, H2B1F_MOUSE, H2B1H_MOUSE, H2B1K_MOUSE, H2B1M_MOUSE, H2B1P_MOUSE, H2B2B_MOUSE	Histone H2B	SRKESYSVYVYK	9	2.20E-03	754.8917	1507.7689	2	0.0019	12	2	Semi-tryptic
H2B1B_MOUSE, H2B1C_MOUSE, H2B1F_MOUSE, H2B1H_MOUSE, H2B1K_MOUSE, H2B1M_MOUSE, H2B1P_MOUSE, H2B2B_MOUSE	Histone H2B	SRKESYSVYVYK	9	1.10E-03	754.8913	1507.7681	2	0.0010	12	2	Semi-tryptic
H2B1B_MOUSE, H2B1C_MOUSE, H2B1F_MOUSE, H2B1H_MOUSE, H2B1K_MOUSE, H2B1M_MOUSE, H2B1P_MOUSE, H2B2B_MOUSE	Histone H2B	SRKESYSVYVYK	9	9.00E-04	754.8911	1507.7676	2	0.0005	12	2	Semi-tryptic
H2B1B_MOUSE, H2B1C_MOUSE, H2B1F_MOUSE, H2B1H_MOUSE, H2B1K_MOUSE, H2B1M_MOUSE, H2B1P_MOUSE, H2B2B_MOUSE	Histone H2B	SRKESYSVYVYK	9	6.60E-04	503.5963	1507.7670	3	0.0000	12	2	Semi-tryptic
H2B1B_MOUSE, H2B1C_MOUSE, H2B1F_MOUSE, H2B1H_MOUSE, H2B1K_MOUSE, H2B1M_MOUSE, H2B1P_MOUSE, H2B2B_MOUSE	Histone H2B	SRKESYSVYVYK	9	9.00E-04	503.5962	1507.7667	3	-0.0003	12	2	Semi-tryptic
H2B1B_MOUSE, H2B1C_MOUSE, H2B1F_MOUSE, H2B1H_MOUSE, H2B1K_MOUSE, H2B1M_MOUSE, H2B1P_MOUSE, H2B2B_MOUSE	Histone H2B	SRKESYSVYVYK	9	3.70E-03	503.5957	1507.7652	3	-0.0018	12	2	Semi-tryptic
H2B1B_MOUSE, H2B1C_MOUSE, H2B1F_MOUSE, H2B1H_MOUSE, H2B1K_MOUSE, H2B1M_MOUSE, H2B1P_MOUSE, H2B2B_MOUSE	Histone H2B	ESYSVYVYKVLK	7	8.50E-07	739.3992	1476.7838	2	-0.0026	12	1	Semi-tryptic
H2B1B_MOUSE, H2B1C_MOUSE, H2B1F_MOUSE, H2B1H_MOUSE, H2B1K_MOUSE, H2B1M_MOUSE, H2B1P_MOUSE, H2B2B_MOUSE	Histone H2B	SRKESYSVYVYK	9	3.10E-04	503.5945	1507.7618	3	-0.0053	12	2	Semi-tryptic
H2B1B_MOUSE, H2B1C_MOUSE, H2B1F_MOUSE, H2B1H_MOUSE, H2B1K_MOUSE, H2B1M_MOUSE, H2B1P_MOUSE, H2B2B_MOUSE	Histone H2B	KESYSVYVYK	13	3.20E-03	633.3265	1264.6384	2	0.0044	10	1	Semi-tryptic
H2B1B_MOUSE, H2B1C_MOUSE, H2B1F_MOUSE, H2B1H_MOUSE, H2B1K_MOUSE, H2B1M_MOUSE, H2B1P_MOUSE, H2B2B_MOUSE	Histone H2B	KESYSVYVYK	13	2.70E-04	633.3265	1264.6384	2	0.0044	10	1	Semi-tryptic
H2B1B_MOUSE, H2B1C_MOUSE, H2B1F_MOUSE, H2B1H_MOUSE, H2B1K_MOUSE, H2B1M_MOUSE, H2B1P_MOUSE, H2B2B_MOUSE	Histone H2B	KESYSVYVYK	13	1.90E-03	633.3253	1264.6361	2	0.0022	10	1	Semi-tryptic
H2B1B_MOUSE, H2B1C_MOUSE, H2B1F_MOUSE, H2B1H_MOUSE, H2B1K_MOUSE, H2B1M_MOUSE, H2B1P_MOUSE, H2B2B_MOUSE	Histone H2B	KESYSVYVYK	13	3.00E-05	633.3253	1264.6361	2	0.0022	10	1	Semi-tryptic
H2B1B_MOUSE, H2B1C_MOUSE, H2B1F_MOUSE, H2B1H_MOUSE, H2B1K_MOUSE, H2B1M_MOUSE, H2B1P_MOUSE, H2B2B_MOUSE	Histone H2B	KESYSVYVYK	13	3.40E-07	633.3249	1264.6353	2	0.0014	10	1	Semi-tryptic
H2B1B_MOUSE, H2B1C_MOUSE, H2B1F_MOUSE, H2B1H_MOUSE, H2B1K_MOUSE, H2B1M_MOUSE, H2B1P_MOUSE, H2B2B_MOUSE	Histone H2B	KESYSVYVYK	13	3.40E-07	633.3249	1264.6352	2	0.0013	10	1	Semi-tryptic
H2B1B_MOUSE, H2B1C_MOUSE, H2B1F_MOUSE, H2B1H_MOUSE, H2B1K_MOUSE, H2B1M_MOUSE, H2B1P_MOUSE, H2B2B_MOUSE	Histone H2B	KESYSVYVYK	13	5.10E-03	633.3247	1264.6348	2	0.0009	10	1	Semi-tryptic
H2B1B_MOUSE, H2B1C_MOUSE, H2B1F_MOUSE, H2B1H_MOUSE, H2B1K_MOUSE, H2B1M_MOUSE, H2B1P_MOUSE, H2B2B_MOUSE	Histone H2B	KESYSVYVYK	13	6.90E-08	633.3246	1264.6346	2	0.0006	10	1	Semi-tryptic
H2B1B_MOUSE, H2B1C_MOUSE, H2B1F_MOUSE, H2B1H_MOUSE, H2B1K_MOUSE, H2B1M_MOUSE, H2B1P_MOUSE, H2B2B_MOUSE	Histone H2B	KESYSVYVYK	13	6.80E-07	633.3245	1264.6345	2	0.0005	10	1	Semi-tryptic
H2B1B_MOUSE, H2B1C_MOUSE, H2B1F_MOUSE, H2B1H_MOUSE, H2B1K_MOUSE, H2B1M_MOUSE, H2B1P_MOUSE, H2B2B_MOUSE	Histone H2B	KESYSVYVYK	13	6.50E-07	633.3245	1264.6344	2	0.0005	10	1	Semi-tryptic
H2B1B_MOUSE, H2B1C_MOUSE, H2B1F_MOUSE, H2B1H_MOUSE, H2B1K_MOUSE, H2B1M_MOUSE, H2B1P_MOUSE, H2B2B_MOUSE	Histone H2B	KESYSVYVYK	13	4.30E-07	633.3243	1264.6341	2	0.0002	10	1	Semi-tryptic
H2B1B_MOUSE, H2B1C_MOUSE, H2B1F_MOUSE, H2B1H_MOUSE, H2B1K_MOUSE, H2B1M_MOUSE, H2B1P_MOUSE, H2B2B_MOUSE	Histone H2B	KESYSVYVYK	13	2.30E-04	422.5516	1264.6329	3	-0.0010	10	1	Semi-tryptic
H2B1B_MOUSE, H2B1C_MOUSE, H2B1F_MOUSE, H2B1H_MOUSE, H2B1K_MOUSE, H2B1M_MOUSE, H2B1P_MOUSE, H2B2B_MOUSE	Histone H2B	KESYSVYVYK	13	2.20E-05	422.5516	1264.6329	3	-0.0010	10	1	Semi-tryptic
H2B1B_MOUSE, H2B1C_MOUSE, H2B1F_MOUSE, H2B1H_MOUSE, H2B1K_MOUSE, H2B1M_MOUSE, H2B1P_MOUSE, H2B2B_MOUSE	Histone H2B	ESYSVYVYK	6	4.50E-03	569.2791	1136.5437	2	0.0047	9	0	Semi-tryptic
H2B1B_MOUSE, H2B1C_MOUSE, H2B1F_MOUSE, H2B1H_MOUSE, H2B1K_MOUSE, H2B1M_MOUSE, H2B1P_MOUSE, H2B2B_MOUSE	Histone H2B	ESYSVYVYK	6	6.40E-03	569.2772	1136.5398	2	0.0009	9	0	Semi-tryptic
H2B1B_MOUSE, H2B1C_MOUSE, H2B1F_MOUSE, H2B1H_MOUSE, H2B1K_MOUSE, H2B1M_MOUSE, H2B1P_MOUSE, H2B2B_MOUSE	Histone H2B	ESYSVYVYK	6	6.60E-03	569.2771	1136.5396	2	0.0006	9	0	Semi-tryptic
H2B1B_MOUSE, H2B1C_MOUSE, H2B1F_MOUSE, H2B1H_MOUSE, H2B1K_MOUSE, H2B1M_MOUSE, H2B1P_MOUSE, H2B2B_MOUSE	Histone H2B	ESYSVYVYK	6	1.20E-03	569.2768	1136.5391	2	0.0001	9	0	Semi-tryptic
H2B1B_MOUSE, H2B1C_MOUSE, H2B1F_MOUSE, H2B1H_MOUSE, H2B1K_MOUSE, H2B1M_MOUSE, H2B1P_MOUSE, H2B2B_MOUSE	Histone H2B	ESYSVYVYK	6	1.60E-03	569.2766	1136.5387	2	-0.0002	9	0	Semi-tryptic
H2B1B_MOUSE, H2B1C_MOUSE, H2B1F_MOUSE, H2B1H_MOUSE, H2B1K_MOUSE, H2B1M_MOUSE, H2B1P_MOUSE, H2B2B_MOUSE	Histone H2B	ESYSVYVYK	6	5.30E-03	569.2761	1136.5376	2	-0.0013	9	0	Semi-tryptic
H2B1B_MOUSE, H2B1C_MOUSE, H2B1F_MOUSE, H2B1H_MOUSE, H2B1K_MOUSE, H2B1M_MOUSE, H2B1P_MOUSE, H2B2B_MOUSE, H2B2E_MOUSE	Histone H2B	GKKRKRSRKE	44	1.90E-06	424.9359	1271.7859	3	-0.0039	10	0	GluC
H2B1B_MOUSE, H2B1C_MOUSE, H2B1F_MOUSE, H2B1H_MOUSE, H2B1K_MOUSE, H2B1M_MOUSE, H2B1P_MOUSE, H2B2B_MOUSE, H2B2E_MOUSE	Histone H2B	GKKRKRSRKE	44	6.40E-03	424.9385	1271.7937	3	0.0040	10	0	GluC

H2B1B_MOUSE, H2B1C_MOUSE, H2B1F_MOUSE, H2B1H_MOUSE, H2B1K_MOUSE, H2B1M_MOUSE, H2B1P_MOUSE, H2B2B_MOUSE, H2B2E_MOUSE	Histone H2B	GKKRKRSRKE	44	5.00E-04	318.9554	1271.7923	4	0.0026	10	0	GluC
H2B1B_MOUSE, H2B1C_MOUSE, H2B1F_MOUSE, H2B1H_MOUSE, H2B1K_MOUSE, H2B1M_MOUSE, H2B1P_MOUSE, H2B2B_MOUSE, H2B2E_MOUSE	Histone H2B	GKKRKRSRKE	44	3.40E-06	424.938	1271.7923	3	0.0025	10	0	GluC
H2B1B_MOUSE, H2B1C_MOUSE, H2B1F_MOUSE, H2B1H_MOUSE, H2B1K_MOUSE, H2B1M_MOUSE, H2B1P_MOUSE, H2B2B_MOUSE, H2B2E_MOUSE	Histone H2B	GKKRKRSRKE	44	1.30E-03	636.9033	1271.7920	2	0.0023	10	0	GluC
H2B1B_MOUSE, H2B1C_MOUSE, H2B1F_MOUSE, H2B1H_MOUSE, H2B1K_MOUSE, H2B1M_MOUSE, H2B1P_MOUSE, H2B2B_MOUSE, H2B2E_MOUSE	Histone H2B	GKKRKRSRKE	44	1.00E-02	636.903	1271.7915	2	0.0017	10	0	GluC
H2B1B_MOUSE, H2B1C_MOUSE, H2B1F_MOUSE, H2B1H_MOUSE, H2B1K_MOUSE, H2B1M_MOUSE, H2B1P_MOUSE, H2B2B_MOUSE, H2B2E_MOUSE	Histone H2B	GKKRKRSRKE	44	6.90E-03	636.9029	1271.7913	2	0.0015	10	0	GluC
H2B1B_MOUSE, H2B1C_MOUSE, H2B1F_MOUSE, H2B1H_MOUSE, H2B1K_MOUSE, H2B1M_MOUSE, H2B1P_MOUSE, H2B2B_MOUSE, H2B2E_MOUSE	Histone H2B	GKKRKRSRKE	44	3.70E-03	424.9377	1271.7913	3	0.0015	10	0	GluC
H2B1B_MOUSE, H2B1C_MOUSE, H2B1F_MOUSE, H2B1H_MOUSE, H2B1K_MOUSE, H2B1M_MOUSE, H2B1P_MOUSE, H2B2B_MOUSE, H2B2E_MOUSE	Histone H2B	GKKRKRSRKE	44	9.70E-06	424.9377	1271.7913	3	0.0015	10	0	GluC
H2B1B_MOUSE, H2B1C_MOUSE, H2B1F_MOUSE, H2B1H_MOUSE, H2B1K_MOUSE, H2B1M_MOUSE, H2B1P_MOUSE, H2B2B_MOUSE, H2B2E_MOUSE	Histone H2B	GKKRKRSRKE	44	6.40E-06	424.9376	1271.7910	3	0.0012	10	0	GluC
H2B1B_MOUSE, H2B1C_MOUSE, H2B1F_MOUSE, H2B1H_MOUSE, H2B1K_MOUSE, H2B1M_MOUSE, H2B1P_MOUSE, H2B2B_MOUSE, H2B2E_MOUSE	Histone H2B	GKKRKRSRKE	44	6.70E-03	424.9375	1271.7907	3	0.0010	10	0	GluC
H2B1B_MOUSE, H2B1C_MOUSE, H2B1F_MOUSE, H2B1H_MOUSE, H2B1K_MOUSE, H2B1M_MOUSE, H2B1P_MOUSE, H2B2B_MOUSE, H2B2E_MOUSE	Histone H2B	GKKRKRSRKE	44	6.30E-05	424.9375	1271.7908	3	0.0010	10	0	GluC
H2B1B_MOUSE, H2B1C_MOUSE, H2B1F_MOUSE, H2B1H_MOUSE, H2B1K_MOUSE, H2B1M_MOUSE, H2B1P_MOUSE, H2B2B_MOUSE, H2B2E_MOUSE	Histone H2B	GKKRKRSRKE	44	1.30E-03	318.9549	1271.7907	4	0.0009	10	0	GluC
H2B1B_MOUSE, H2B1C_MOUSE, H2B1F_MOUSE, H2B1H_MOUSE, H2B1K_MOUSE, H2B1M_MOUSE, H2B1P_MOUSE, H2B2B_MOUSE, H2B2E_MOUSE	Histone H2B	GKKRKRSRKE	44	7.80E-07	424.9375	1271.7906	3	0.0009	10	0	GluC
H2B1B_MOUSE, H2B1C_MOUSE, H2B1F_MOUSE, H2B1H_MOUSE, H2B1K_MOUSE, H2B1M_MOUSE, H2B1P_MOUSE, H2B2B_MOUSE, H2B2E_MOUSE	Histone H2B	GKKRKRSRKE	44	6.80E-06	424.9375	1271.7906	3	0.0008	10	0	GluC
H2B1B_MOUSE, H2B1C_MOUSE, H2B1F_MOUSE, H2B1H_MOUSE, H2B1K_MOUSE, H2B1M_MOUSE, H2B1P_MOUSE, H2B2B_MOUSE, H2B2E_MOUSE	Histone H2B	GKKRKRSRKE	44	2.50E-03	318.9549	1271.7905	4	0.0007	10	0	GluC
H2B1B_MOUSE, H2B1C_MOUSE, H2B1F_MOUSE, H2B1H_MOUSE, H2B1K_MOUSE, H2B1M_MOUSE, H2B1P_MOUSE, H2B2B_MOUSE, H2B2E_MOUSE	Histone H2B	GKKRKRSRKE	44	4.10E-04	318.9549	1271.7905	4	0.0007	10	0	GluC
H2B1B_MOUSE, H2B1C_MOUSE, H2B1F_MOUSE, H2B1H_MOUSE, H2B1K_MOUSE, H2B1M_MOUSE, H2B1P_MOUSE, H2B2B_MOUSE, H2B2E_MOUSE	Histone H2B	GKKRKRSRKE	44	7.50E-05	424.9374	1271.7904	3	0.0007	10	0	GluC
H2B1B_MOUSE, H2B1C_MOUSE, H2B1F_MOUSE, H2B1H_MOUSE, H2B1K_MOUSE, H2B1M_MOUSE, H2B1P_MOUSE, H2B2B_MOUSE, H2B2E_MOUSE	Histone H2B	GKKRKRSRKE	44	2.70E-05	424.9375	1271.7905	3	0.0007	10	0	GluC
H2B1B_MOUSE, H2B1C_MOUSE, H2B1F_MOUSE, H2B1H_MOUSE, H2B1K_MOUSE, H2B1M_MOUSE, H2B1P_MOUSE, H2B2B_MOUSE, H2B2E_MOUSE	Histone H2B	GKKRKRSRKE	44	4.80E-04	318.9549	1271.7904	4	0.0006	10	0	GluC
H2B1B_MOUSE, H2B1C_MOUSE, H2B1F_MOUSE, H2B1H_MOUSE, H2B1K_MOUSE, H2B1M_MOUSE, H2B1P_MOUSE, H2B2B_MOUSE, H2B2E_MOUSE	Histone H2B	GKKRKRSRKE	44	2.70E-04	318.9549	1271.7904	4	0.0006	10	0	GluC
H2B1B_MOUSE, H2B1C_MOUSE, H2B1F_MOUSE, H2B1H_MOUSE, H2B1K_MOUSE, H2B1M_MOUSE, H2B1P_MOUSE, H2B2B_MOUSE, H2B2E_MOUSE	Histone H2B	GKKRKRSRKE	44	2.30E-03	318.9548	1271.7901	4	0.0003	10	0	GluC
H2B1B_MOUSE, H2B1C_MOUSE, H2B1F_MOUSE, H2B1H_MOUSE, H2B1K_MOUSE, H2B1M_MOUSE, H2B1P_MOUSE, H2B2B_MOUSE, H2B2E_MOUSE	Histone H2B	GKKRKRSRKE	44	8.20E-04	424.9373	1271.7901	3	0.0003	10	0	GluC
H2B1B_MOUSE, H2B1C_MOUSE, H2B1F_MOUSE, H2B1H_MOUSE, H2B1K_MOUSE, H2B1M_MOUSE, H2B1P_MOUSE, H2B2B_MOUSE, H2B2E_MOUSE	Histone H2B	GKKRKRSRKE	44	4.00E-03	318.9547	1271.7899	4	0.0001	10	0	GluC
H2B1B_MOUSE, H2B1C_MOUSE, H2B1F_MOUSE, H2B1H_MOUSE, H2B1K_MOUSE, H2B1M_MOUSE, H2B1P_MOUSE, H2B2B_MOUSE, H2B2E_MOUSE	Histone H2B	GKKRKRSRKE	44	1.50E-03	318.9547	1271.7899	4	0.0001	10	0	GluC
H2B1B_MOUSE, H2B1C_MOUSE, H2B1F_MOUSE, H2B1H_MOUSE, H2B1K_MOUSE, H2B1M_MOUSE, H2B1P_MOUSE, H2B2B_MOUSE, H2B2E_MOUSE	Histone H2B	GKKRKRSRKE	44	6.10E-04	424.9372	1271.7899	3	0.0001	10	0	GluC

H2B1B_MOUSE, H2B1C_MOUSE, H2B1F_MOUSE, H2B1H_MOUSE, H2B1K_MOUSE, H2B1M_MOUSE, H2B1P_MOUSE, H2B2B_MOUSE, H2B2E_MOUSE	Histone H2B	GKKRKRSRKE	44	4.10E-04	424.9372	1271.7898	3	0.0001	10	0	GluC
H2B1B_MOUSE, H2B1C_MOUSE, H2B1F_MOUSE, H2B1H_MOUSE, H2B1K_MOUSE, H2B1M_MOUSE, H2B1P_MOUSE, H2B2B_MOUSE, H2B2E_MOUSE	Histone H2B	GKKRKRSRKE	44	3.80E-04	424.9373	1271.7899	3	0.0001	10	0	GluC
H2B1B_MOUSE, H2B1C_MOUSE, H2B1F_MOUSE, H2B1H_MOUSE, H2B1K_MOUSE, H2B1M_MOUSE, H2B1P_MOUSE, H2B2B_MOUSE, H2B2E_MOUSE	Histone H2B	GKKRKRSRKE	44	8.90E-04	424.9372	1271.7898	3	0.0000	10	0	GluC
H2B1B_MOUSE, H2B1C_MOUSE, H2B1F_MOUSE, H2B1H_MOUSE, H2B1K_MOUSE, H2B1M_MOUSE, H2B1P_MOUSE, H2B2B_MOUSE, H2B2E_MOUSE	Histone H2B	GKKRKRSRKE	44	1.60E-04	424.9372	1271.7897	3	0.0000	10	0	GluC
H2B1B_MOUSE, H2B1C_MOUSE, H2B1F_MOUSE, H2B1H_MOUSE, H2B1K_MOUSE, H2B1M_MOUSE, H2B1P_MOUSE, H2B2B_MOUSE, H2B2E_MOUSE	Histone H2B	GKKRKRSRKE	44	1.00E-04	424.9372	1271.7898	3	0.0000	10	0	GluC
H2B1B_MOUSE, H2B1C_MOUSE, H2B1F_MOUSE, H2B1H_MOUSE, H2B1K_MOUSE, H2B1M_MOUSE, H2B1P_MOUSE, H2B2B_MOUSE, H2B2E_MOUSE	Histone H2B	GKKRKRSRKE	44	9.30E-04	424.9371	1271.7896	3	-0.0002	10	0	GluC
H2B1B_MOUSE, H2B1C_MOUSE, H2B1F_MOUSE, H2B1H_MOUSE, H2B1K_MOUSE, H2B1M_MOUSE, H2B1P_MOUSE, H2B2B_MOUSE, H2B2E_MOUSE	Histone H2B	GKKRKRSRKE	44	8.00E-04	318.9547	1271.7896	4	-0.0002	10	0	GluC
H2B1B_MOUSE, H2B1C_MOUSE, H2B1F_MOUSE, H2B1H_MOUSE, H2B1K_MOUSE, H2B1M_MOUSE, H2B1P_MOUSE, H2B2B_MOUSE, H2B2E_MOUSE	Histone H2B	GKKRKRSRKE	44	2.10E-05	424.9371	1271.7896	3	-0.0002	10	0	GluC
H2B1B_MOUSE, H2B1C_MOUSE, H2B1F_MOUSE, H2B1H_MOUSE, H2B1K_MOUSE, H2B1M_MOUSE, H2B1P_MOUSE, H2B2B_MOUSE, H2B2E_MOUSE	Histone H2B	GKKRKRSRKE	44	1.00E-03	424.9371	1271.7895	3	-0.0003	10	0	GluC
H2B1B_MOUSE, H2B1C_MOUSE, H2B1F_MOUSE, H2B1H_MOUSE, H2B1K_MOUSE, H2B1M_MOUSE, H2B1P_MOUSE, H2B2B_MOUSE, H2B2E_MOUSE	Histone H2B	GKKRKRSRKE	44	4.40E-06	424.9371	1271.7895	3	-0.0003	10	0	GluC
H2B1B_MOUSE, H2B1C_MOUSE, H2B1F_MOUSE, H2B1H_MOUSE, H2B1K_MOUSE, H2B1M_MOUSE, H2B1P_MOUSE, H2B2B_MOUSE, H2B2E_MOUSE	Histone H2B	GKKRKRSRKE	44	6.20E-05	424.9371	1271.7894	3	-0.0004	10	0	GluC
H2B1B_MOUSE, H2B1C_MOUSE, H2B1F_MOUSE, H2B1H_MOUSE, H2B1K_MOUSE, H2B1M_MOUSE, H2B1P_MOUSE, H2B2B_MOUSE, H2B2E_MOUSE	Histone H2B	GKKRKRSRKE	44	7.80E-04	318.9546	1271.7893	4	-0.0005	10	0	GluC
H2B1B_MOUSE, H2B1C_MOUSE, H2B1F_MOUSE, H2B1H_MOUSE, H2B1K_MOUSE, H2B1M_MOUSE, H2B1P_MOUSE, H2B2B_MOUSE, H2B2E_MOUSE	Histone H2B	GKKRKRSRKE	44	3.30E-03	318.9546	1271.7892	4	-0.0006	10	0	GluC
H2B1B_MOUSE, H2B1C_MOUSE, H2B1F_MOUSE, H2B1H_MOUSE, H2B1K_MOUSE, H2B1M_MOUSE, H2B1P_MOUSE, H2B2B_MOUSE, H2B2E_MOUSE	Histone H2B	GKKRKRSRKE	44	3.20E-03	318.9546	1271.7891	4	-0.0006	10	0	GluC
H2B1B_MOUSE, H2B1C_MOUSE, H2B1F_MOUSE, H2B1H_MOUSE, H2B1K_MOUSE, H2B1M_MOUSE, H2B1P_MOUSE, H2B2B_MOUSE, H2B2E_MOUSE	Histone H2B	GKKRKRSRKE	44	2.60E-05	424.937	1271.7891	3	-0.0007	10	0	GluC
H2B1B_MOUSE, H2B1C_MOUSE, H2B1F_MOUSE, H2B1H_MOUSE, H2B1K_MOUSE, H2B1M_MOUSE, H2B1P_MOUSE, H2B2B_MOUSE, H2B2E_MOUSE	Histone H2B	GKKRKRSRKE	44	6.20E-03	318.9545	1271.7888	4	-0.0010	10	0	GluC
H2B1B_MOUSE, H2B1C_MOUSE, H2B1F_MOUSE, H2B1H_MOUSE, H2B1K_MOUSE, H2B1M_MOUSE, H2B1P_MOUSE, H2B2B_MOUSE, H2B2E_MOUSE	Histone H2B	GKKRKRSRKE	44	1.20E-03	424.9368	1271.7886	3	-0.0012	10	0	GluC
H2B1B_MOUSE, H2B1C_MOUSE, H2B1F_MOUSE, H2B1H_MOUSE, H2B1K_MOUSE, H2B1M_MOUSE, H2B1P_MOUSE, H2B2B_MOUSE, H2B2E_MOUSE	Histone H2B	GKKRKRSRKE	44	3.90E-03	318.9544	1271.7884	4	-0.0014	10	0	GluC
H2B1B_MOUSE, H2B1C_MOUSE, H2B1F_MOUSE, H2B1H_MOUSE, H2B1K_MOUSE, H2B1M_MOUSE, H2B1P_MOUSE, H2B2B_MOUSE, H2B2E_MOUSE, H2B3A_MOUSE, H2B3B_MOUSE	Histone H2B	TGISSKAMGIMNSFVNDIFE	14	2.10E-03	721.0207	2160.0402	3	0.0171	20	1	GluC
H2B1B_MOUSE, H2B1C_MOUSE, H2B1F_MOUSE, H2B1H_MOUSE, H2B1K_MOUSE, H2B1M_MOUSE, H2B1P_MOUSE, H2B2B_MOUSE, H2B2E_MOUSE, H2B3A_MOUSE, H2B3B_MOUSE	Histone H2B	TGISSKAMGIMNSFVNDIFE	14	6.30E-08	1081.0249	2160.0352	2	0.0121	20	1	GluC
H2B1B_MOUSE, H2B1C_MOUSE, H2B1F_MOUSE, H2B1H_MOUSE, H2B1K_MOUSE, H2B1M_MOUSE, H2B1P_MOUSE, H2B2B_MOUSE, H2B2E_MOUSE, H2B3A_MOUSE, H2B3B_MOUSE	Histone H2B	TGISSKAMGIMNSFVNDIFE	14	5.70E-08	1081.0222	2160.0298	2	0.0067	20	1	GluC
H2B1B_MOUSE, H2B1C_MOUSE, H2B1F_MOUSE, H2B1H_MOUSE, H2B1K_MOUSE, H2B1M_MOUSE, H2B1P_MOUSE, H2B2B_MOUSE, H2B2E_MOUSE, H2B3A_MOUSE, H2B3B_MOUSE	Histone H2B	TGISSKAMGIMNSFVNDIFE	14	1.60E-09	1081.022	2160.0294	2	0.0063	20	1	GluC
H2B1B_MOUSE, H2B1C_MOUSE, H2B1F_MOUSE, H2B1H_MOUSE, H2B1K_MOUSE, H2B1M_MOUSE, H2B1P_MOUSE, H2B2B_MOUSE, H2B2E_MOUSE, H2B3A_MOUSE, H2B3B_MOUSE	Histone H2B	TGISSKAMGIMNSFVNDIFE	14	1.20E-05	721.0171	2160.0293	3	0.0062	20	1	GluC
H2B1B_MOUSE, H2B1C_MOUSE, H2B1F_MOUSE, H2B1H_MOUSE, H2B1K_MOUSE, H2B1M_MOUSE, H2B1P_MOUSE, H2B2B_MOUSE, H2B2E_MOUSE, H2B3A_MOUSE, H2B3B_MOUSE	Histone H2B	TGISSKAMGIMNSFVNDIFE	14	3.50E-06	721.0171	2160.0293	3	0.0062	20	1	GluC

H2B1B_MOUSE, H2B1C_MOUSE, H2B1F_MOUSE, H2B1H_MOUSE, H2B1K_MOUSE, H2B1M_MOUSE, H2B1P_MOUSE, H2B2B_MOUSE, H2B2E_MOUSE, H2B3A_MOUSE, H2B3B_MOUSE	Histone H2B	TGISSKAMGIMNSFVNDIFE	14	3.20E-07	1081.0213	2160.0280	2	0.0049	20	1	GluC
H2B1B_MOUSE, H2B1C_MOUSE, H2B1F_MOUSE, H2B1H_MOUSE, H2B1K_MOUSE, H2B1M_MOUSE, H2B1P_MOUSE, H2B2B_MOUSE, H2B2E_MOUSE, H2B3A_MOUSE, H2B3B_MOUSE	Histone H2B	TGISSKAMGIMNSFVNDIFE	14	1.10E-07	1081.0211	2160.0276	2	0.0045	20	1	GluC
H2B1B_MOUSE, H2B1C_MOUSE, H2B1F_MOUSE, H2B1H_MOUSE, H2B1K_MOUSE, H2B1M_MOUSE, H2B1P_MOUSE, H2B2B_MOUSE, H2B2E_MOUSE, H2B3A_MOUSE, H2B3B_MOUSE	Histone H2B	TGISSKAMGIMNSFVNDIFE	14	2.20E-06	1081.021	2160.0274	2	0.0043	20	1	GluC
H2B1B_MOUSE, H2B1C_MOUSE, H2B1F_MOUSE, H2B1H_MOUSE, H2B1K_MOUSE, H2B1M_MOUSE, H2B1P_MOUSE, H2B2B_MOUSE, H2B2E_MOUSE, H2B3A_MOUSE, H2B3B_MOUSE	Histone H2B	TGISSKAMGIMNSFVNDIFE	14	1.80E-07	1081.0209	2160.0272	2	0.0041	20	1	GluC
H2B1B_MOUSE, H2B1C_MOUSE, H2B1F_MOUSE, H2B1H_MOUSE, H2B1K_MOUSE, H2B1M_MOUSE, H2B1P_MOUSE, H2B2B_MOUSE, H2B2E_MOUSE, H2B3A_MOUSE, H2B3B_MOUSE	Histone H2B	TGISSKAMGIMNSFVNDIFE	14	8.50E-05	1081.0207	2160.0268	2	0.0037	20	1	GluC
H2B1B_MOUSE, H2B1C_MOUSE, H2B1F_MOUSE, H2B1H_MOUSE, H2B1K_MOUSE, H2B1M_MOUSE, H2B1P_MOUSE, H2B2B_MOUSE, H2B2E_MOUSE, H2B3A_MOUSE, H2B3B_MOUSE	Histone H2B	TGISSKAMGIMNSFVNDIFE	14	1.40E-03	721.0161	2160.0265	3	0.0034	20	1	GluC
H2B1B_MOUSE, H2B1C_MOUSE, H2B1F_MOUSE, H2B1H_MOUSE, H2B1K_MOUSE, H2B1M_MOUSE, H2B1P_MOUSE, H2B2B_MOUSE, H2B2E_MOUSE, H2B3A_MOUSE, H2B3B_MOUSE	Histone H2B	TGISSKAMGIMNSFVNDIFE	14	1.50E-06	1081.0201	2160.0256	2	0.0025	20	1	GluC
H2B1B_MOUSE, H2B1C_MOUSE, H2B1F_MOUSE, H2B1H_MOUSE, H2B1K_MOUSE, H2B1M_MOUSE, H2B1P_MOUSE, H2B2B_MOUSE, H2B2E_MOUSE, H2B3A_MOUSE, H2B3B_MOUSE	Histone H2B	TGISSKAMGIMNSFVNDIFE	14	2.20E-08	1081.0191	2160.0236	2	0.0005	20	1	GluC
H2B1B_MOUSE, H2B1C_MOUSE, H2B1F_MOUSE, H2B1H_MOUSE, H2B1K_MOUSE, H2B1M_MOUSE, H2B1P_MOUSE, H2B2B_MOUSE, H2B2E_MOUSE, H2B3A_MOUSE, H2B3B_MOUSE	Histone H2B	DTGISSKAMGIMNSFVN	7	5.30E-03	886.4278	1770.8411	2	0.0130	17	0	AspN
H2B1B_MOUSE, H2B1C_MOUSE, H2B1F_MOUSE, H2B1H_MOUSE, H2B1K_MOUSE, H2B1M_MOUSE, H2B1P_MOUSE, H2B2B_MOUSE, H2B2E_MOUSE, H2B3A_MOUSE, H2B3B_MOUSE	Histone H2B	DTGISSKAMGIMNSFVN	7	3.10E-08	886.4235	1770.8324	2	0.0044	17	0	AspN
H2B1B_MOUSE, H2B1C_MOUSE, H2B1F_MOUSE, H2B1H_MOUSE, H2B1K_MOUSE, H2B1M_MOUSE, H2B1P_MOUSE, H2B2B_MOUSE, H2B2E_MOUSE, H2B3A_MOUSE, H2B3B_MOUSE	Histone H2B	DTGISSKAMGIMNSFVN	7	2.20E-05	886.4232	1770.8319	2	0.0039	17	0	AspN
H2B1B_MOUSE, H2B1C_MOUSE, H2B1F_MOUSE, H2B1H_MOUSE, H2B1K_MOUSE, H2B1M_MOUSE, H2B1P_MOUSE, H2B2B_MOUSE, H2B2E_MOUSE, H2B3A_MOUSE, H2B3B_MOUSE	Histone H2B	DTGISSKAMGIMNSFVN	7	9.50E-03	886.4229	1770.8313	2	0.0033	17	0	AspN
H2B1B_MOUSE, H2B1C_MOUSE, H2B1F_MOUSE, H2B1H_MOUSE, H2B1K_MOUSE, H2B1M_MOUSE, H2B1P_MOUSE, H2B2B_MOUSE, H2B2E_MOUSE, H2B3A_MOUSE, H2B3B_MOUSE	Histone H2B	DTGISSKAMGIMNSFVN	7	4.10E-03	886.4227	1770.8308	2	0.0028	17	0	AspN
H2B1B_MOUSE, H2B1C_MOUSE, H2B1F_MOUSE, H2B1H_MOUSE, H2B1K_MOUSE, H2B1M_MOUSE, H2B1P_MOUSE, H2B2B_MOUSE, H2B2E_MOUSE, H2B3A_MOUSE, H2B3B_MOUSE	Histone H2B	DTGISSKAMGIMNSFVN	7	2.50E-04	886.4215	1770.8284	2	0.0003	17	0	AspN
H2B1B_MOUSE, H2B1C_MOUSE, H2B1F_MOUSE, H2B1H_MOUSE, H2B1K_MOUSE, H2B1M_MOUSE, H2B1P_MOUSE, H2B2B_MOUSE, H2B2E_MOUSE, H2B3A_MOUSE, H2B3B_MOUSE	Histone H2B	DTGISSKAMGIMNSFVN	7	1.60E-06	886.4205	1770.8265	2	-0.0016	17	0	AspN
H2B1B_MOUSE, H2B1C_MOUSE, H2B1F_MOUSE, H2B1H_MOUSE, H2B1K_MOUSE, H2B1M_MOUSE, H2B1P_MOUSE, H2B2B_MOUSE, H2B2E_MOUSE, H2B3A_MOUSE, H2B3B_MOUSE	Histone H2B	TGISSKAMGIMNSFVND	56	3.70E-07	886.4198	1770.8250	2	-0.0030	17	0	GluC
H2B1B_MOUSE, H2B1C_MOUSE, H2B1F_MOUSE, H2B1H_MOUSE, H2B1K_MOUSE, H2B1M_MOUSE, H2B1P_MOUSE, H2B2B_MOUSE, H2B2E_MOUSE, H2B3A_MOUSE, H2B3B_MOUSE	Histone H2B	TGISSKAMGIMNSFVND	56	2.50E-07	886.418	1770.8213	2	-0.0067	17	0	GluC
H2B1B_MOUSE, H2B1C_MOUSE, H2B1F_MOUSE, H2B1H_MOUSE, H2B1K_MOUSE, H2B1M_MOUSE, H2B1P_MOUSE, H2B2B_MOUSE, H2B2E_MOUSE, H2B3A_MOUSE, H2B3B_MOUSE	Histone H2B	TGISSKAMGIMNSFVND	56	6.00E-03	886.4168	1770.8191	2	-0.0090	17	0	GluC
H2B1B_MOUSE, H2B1C_MOUSE, H2B1F_MOUSE, H2B1H_MOUSE, H2B1K_MOUSE, H2B1M_MOUSE, H2B1P_MOUSE, H2B2B_MOUSE, H2B2E_MOUSE, H2B3A_MOUSE, H2B3B_MOUSE	Histone H2B	TGISSKAMGIMNSFVND	56	2.60E-05	886.4168	1770.8191	2	-0.0090	17	0	GluC
H2B1B_MOUSE, H2B1C_MOUSE, H2B1F_MOUSE, H2B1H_MOUSE, H2B1K_MOUSE, H2B1M_MOUSE, H2B1P_MOUSE, H2B2B_MOUSE, H2B2E_MOUSE, H2B3A_MOUSE, H2B3B_MOUSE	Histone H2B	TGISSKAMGIMNSFVND	56	1.00E-02	886.4278	1770.8411	2	0.0131	17	0	GluC
H2B1B_MOUSE, H2B1C_MOUSE, H2B1F_MOUSE, H2B1H_MOUSE, H2B1K_MOUSE, H2B1M_MOUSE, H2B1P_MOUSE, H2B2B_MOUSE, H2B2E_MOUSE, H2B3A_MOUSE, H2B3B_MOUSE	Histone H2B	TGISSKAMGIMNSFVND	56	1.70E-03	886.4258	1770.8371	2	0.0090	17	0	GluC
H2B1B_MOUSE, H2B1C_MOUSE, H2B1F_MOUSE, H2B1H_MOUSE, H2B1K_MOUSE, H2B1M_MOUSE, H2B1P_MOUSE, H2B2B_MOUSE, H2B2E_MOUSE, H2B3A_MOUSE, H2B3B_MOUSE	Histone H2B	TGISSKAMGIMNSFVND	56	3.40E-06	886.4254	1770.8362	2	0.0081	17	0	GluC
H2B1B_MOUSE, H2B1C_MOUSE, H2B1F_MOUSE, H2B1H_MOUSE, H2B1K_MOUSE, H2B1M_MOUSE, H2B1P_MOUSE, H2B2B_MOUSE, H2B2E_MOUSE, H2B3A_MOUSE, H2B3B_MOUSE	Histone H2B	TGISSKAMGIMNSFVND	56	5.40E-05	886.4244	1770.8343	2	0.0063	17	0	GluC
H2B1B_MOUSE, H2B1C_MOUSE, H2B1F_MOUSE, H2B1H_MOUSE, H2B1K_MOUSE, H2B1M_MOUSE, H2B1P_MOUSE, H2B2B_MOUSE, H2B2E_MOUSE, H2B3A_MOUSE, H2B3B_MOUSE	Histone H2B	TGISSKAMGIMNSFVND	56	4.00E-07	886.4244	1770.8342	2	0.0062	17	0	GluC

H2B1B_MOUSE, H2B1C_MOUSE, H2B1F_MOUSE, H2B1H_MOUSE, H2B1K_MOUSE, H2B1M_MOUSE, H2B1P_MOUSE, H2B2B_MOUSE, H2B2E_MOUSE, H2B3A_MOUSE, H2B3B_MOUSE	Histone H2B	TGISSKAMGIMNSFVND	56	2.50E-07	886.4239	1770.8331	2	0.0051	17	0	GluC
H2B1B_MOUSE, H2B1C_MOUSE, H2B1F_MOUSE, H2B1H_MOUSE, H2B1K_MOUSE, H2B1M_MOUSE, H2B1P_MOUSE, H2B2B_MOUSE, H2B2E_MOUSE, H2B3A_MOUSE, H2B3B_MOUSE	Histone H2B	TGISSKAMGIMNSFVND	56	9.70E-07	886.4238	1770.8330	2	0.0050	17	0	GluC
H2B1B_MOUSE, H2B1C_MOUSE, H2B1F_MOUSE, H2B1H_MOUSE, H2B1K_MOUSE, H2B1M_MOUSE, H2B1P_MOUSE, H2B2B_MOUSE, H2B2E_MOUSE, H2B3A_MOUSE, H2B3B_MOUSE	Histone H2B	TGISSKAMGIMNSFVND	56	6.80E-06	886.4237	1770.8329	2	0.0049	17	0	GluC
H2B1B_MOUSE, H2B1C_MOUSE, H2B1F_MOUSE, H2B1H_MOUSE, H2B1K_MOUSE, H2B1M_MOUSE, H2B1P_MOUSE, H2B2B_MOUSE, H2B2E_MOUSE, H2B3A_MOUSE, H2B3B_MOUSE	Histone H2B	TGISSKAMGIMNSFVND	56	1.10E-06	886.4237	1770.8328	2	0.0048	17	0	GluC
H2B1B_MOUSE, H2B1C_MOUSE, H2B1F_MOUSE, H2B1H_MOUSE, H2B1K_MOUSE, H2B1M_MOUSE, H2B1P_MOUSE, H2B2B_MOUSE, H2B2E_MOUSE, H2B3A_MOUSE, H2B3B_MOUSE	Histone H2B	TGISSKAMGIMNSFVND	56	2.80E-06	886.4237	1770.8328	2	0.0047	17	0	GluC
H2B1B_MOUSE, H2B1C_MOUSE, H2B1F_MOUSE, H2B1H_MOUSE, H2B1K_MOUSE, H2B1M_MOUSE, H2B1P_MOUSE, H2B2B_MOUSE, H2B2E_MOUSE, H2B3A_MOUSE, H2B3B_MOUSE	Histone H2B	TGISSKAMGIMNSFVND	56	5.70E-06	886.4233	1770.8320	2	0.0039	17	0	GluC
H2B1B_MOUSE, H2B1C_MOUSE, H2B1F_MOUSE, H2B1H_MOUSE, H2B1K_MOUSE, H2B1M_MOUSE, H2B1P_MOUSE, H2B2B_MOUSE, H2B2E_MOUSE, H2B3A_MOUSE, H2B3B_MOUSE	Histone H2B	TGISSKAMGIMNSFVND	56	1.60E-06	886.4232	1770.8318	2	0.0038	17	0	GluC
H2B1B_MOUSE, H2B1C_MOUSE, H2B1F_MOUSE, H2B1H_MOUSE, H2B1K_MOUSE, H2B1M_MOUSE, H2B1P_MOUSE, H2B2B_MOUSE, H2B2E_MOUSE, H2B3A_MOUSE, H2B3B_MOUSE	Histone H2B	TGISSKAMGIMNSFVND	56	7.20E-09	886.4232	1770.8318	2	0.0038	17	0	GluC
H2B1B_MOUSE, H2B1C_MOUSE, H2B1F_MOUSE, H2B1H_MOUSE, H2B1K_MOUSE, H2B1M_MOUSE, H2B1P_MOUSE, H2B2B_MOUSE, H2B2E_MOUSE, H2B3A_MOUSE, H2B3B_MOUSE	Histone H2B	TGISSKAMGIMNSFVND	56	3.60E-08	886.4231	1770.8317	2	0.0037	17	0	GluC
H2B1B_MOUSE, H2B1C_MOUSE, H2B1F_MOUSE, H2B1H_MOUSE, H2B1K_MOUSE, H2B1M_MOUSE, H2B1P_MOUSE, H2B2B_MOUSE, H2B2E_MOUSE, H2B3A_MOUSE, H2B3B_MOUSE	Histone H2B	TGISSKAMGIMNSFVND	56	4.10E-09	886.4231	1770.8317	2	0.0037	17	0	GluC
H2B1B_MOUSE, H2B1C_MOUSE, H2B1F_MOUSE, H2B1H_MOUSE, H2B1K_MOUSE, H2B1M_MOUSE, H2B1P_MOUSE, H2B2B_MOUSE, H2B2E_MOUSE, H2B3A_MOUSE, H2B3B_MOUSE	Histone H2B	TGISSKAMGIMNSFVND	56	7.00E-03	886.4231	1770.8316	2	0.0036	17	0	GluC
H2B1B_MOUSE, H2B1C_MOUSE, H2B1F_MOUSE, H2B1H_MOUSE, H2B1K_MOUSE, H2B1M_MOUSE, H2B1P_MOUSE, H2B2B_MOUSE, H2B2E_MOUSE, H2B3A_MOUSE, H2B3B_MOUSE	Histone H2B	TGISSKAMGIMNSFVND	56	2.20E-04	886.4231	1770.8316	2	0.0035	17	0	GluC
H2B1B_MOUSE, H2B1C_MOUSE, H2B1F_MOUSE, H2B1H_MOUSE, H2B1K_MOUSE, H2B1M_MOUSE, H2B1P_MOUSE, H2B2B_MOUSE, H2B2E_MOUSE, H2B3A_MOUSE, H2B3B_MOUSE	Histone H2B	TGISSKAMGIMNSFVND	56	6.40E-06	886.4231	1770.8316	2	0.0035	17	0	GluC
H2B1B_MOUSE, H2B1C_MOUSE, H2B1F_MOUSE, H2B1H_MOUSE, H2B1K_MOUSE, H2B1M_MOUSE, H2B1P_MOUSE, H2B2B_MOUSE, H2B2E_MOUSE, H2B3A_MOUSE, H2B3B_MOUSE	Histone H2B	TGISSKAMGIMNSFVND	56	4.40E-08	886.423	1770.8315	2	0.0035	17	0	GluC
H2B1B_MOUSE, H2B1C_MOUSE, H2B1F_MOUSE, H2B1H_MOUSE, H2B1K_MOUSE, H2B1M_MOUSE, H2B1P_MOUSE, H2B2B_MOUSE, H2B2E_MOUSE, H2B3A_MOUSE, H2B3B_MOUSE	Histone H2B	TGISSKAMGIMNSFVND	56	2.70E-06	886.423	1770.8314	2	0.0034	17	0	GluC
H2B1B_MOUSE, H2B1C_MOUSE, H2B1F_MOUSE, H2B1H_MOUSE, H2B1K_MOUSE, H2B1M_MOUSE, H2B1P_MOUSE, H2B2B_MOUSE, H2B2E_MOUSE, H2B3A_MOUSE, H2B3B_MOUSE	Histone H2B	TGISSKAMGIMNSFVND	56	1.80E-08	886.423	1770.8315	2	0.0034	17	0	GluC
H2B1B_MOUSE, H2B1C_MOUSE, H2B1F_MOUSE, H2B1H_MOUSE, H2B1K_MOUSE, H2B1M_MOUSE, H2B1P_MOUSE, H2B2B_MOUSE, H2B2E_MOUSE, H2B3A_MOUSE, H2B3B_MOUSE	Histone H2B	TGISSKAMGIMNSFVND	56	7.90E-07	886.4229	1770.8313	2	0.0032	17	0	GluC
H2B1B_MOUSE, H2B1C_MOUSE, H2B1F_MOUSE, H2B1H_MOUSE, H2B1K_MOUSE, H2B1M_MOUSE, H2B1P_MOUSE, H2B2B_MOUSE, H2B2E_MOUSE, H2B3A_MOUSE, H2B3B_MOUSE	Histone H2B	TGISSKAMGIMNSFVND	56	7.40E-03	591.2843	1770.8311	3	0.0030	17	0	GluC
H2B1B_MOUSE, H2B1C_MOUSE, H2B1F_MOUSE, H2B1H_MOUSE, H2B1K_MOUSE, H2B1M_MOUSE, H2B1P_MOUSE, H2B2B_MOUSE, H2B2E_MOUSE, H2B3A_MOUSE, H2B3B_MOUSE	Histone H2B	TGISSKAMGIMNSFVND	56	6.30E-07	886.4228	1770.8310	2	0.0030	17	0	GluC
H2B1B_MOUSE, H2B1C_MOUSE, H2B1F_MOUSE, H2B1H_MOUSE, H2B1K_MOUSE, H2B1M_MOUSE, H2B1P_MOUSE, H2B2B_MOUSE, H2B2E_MOUSE, H2B3A_MOUSE, H2B3B_MOUSE	Histone H2B	TGISSKAMGIMNSFVND	56	4.50E-08	886.4228	1770.8310	2	0.0030	17	0	GluC
H2B1B_MOUSE, H2B1C_MOUSE, H2B1F_MOUSE, H2B1H_MOUSE, H2B1K_MOUSE, H2B1M_MOUSE, H2B1P_MOUSE, H2B2B_MOUSE, H2B2E_MOUSE, H2B3A_MOUSE, H2B3B_MOUSE	Histone H2B	TGISSKAMGIMNSFVND	56	2.90E-06	886.4228	1770.8310	2	0.0029	17	0	GluC
H2B1B_MOUSE, H2B1C_MOUSE, H2B1F_MOUSE, H2B1H_MOUSE, H2B1K_MOUSE, H2B1M_MOUSE, H2B1P_MOUSE, H2B2B_MOUSE, H2B2E_MOUSE, H2B3A_MOUSE, H2B3B_MOUSE	Histone H2B	TGISSKAMGIMNSFVND	56	1.20E-07	886.4227	1770.8309	2	0.0029	17	0	GluC
H2B1B_MOUSE, H2B1C_MOUSE, H2B1F_MOUSE, H2B1H_MOUSE, H2B1K_MOUSE, H2B1M_MOUSE, H2B1P_MOUSE, H2B2B_MOUSE, H2B2E_MOUSE, H2B3A_MOUSE, H2B3B_MOUSE	Histone H2B	TGISSKAMGIMNSFVND	56	1.10E-05	886.4227	1770.8309	2	0.0028	17	0	GluC
H2B1B_MOUSE, H2B1C_MOUSE, H2B1F_MOUSE, H2B1H_MOUSE, H2B1K_MOUSE, H2B1M_MOUSE, H2B1P_MOUSE, H2B2B_MOUSE, H2B2E_MOUSE, H2B3A_MOUSE, H2B3B_MOUSE	Histone H2B	TGISSKAMGIMNSFVND	56	7.10E-03	886.4226	1770.8307	2	0.0027	17	0	GluC

H2B1B_MOUSE, H2B1C_MOUSE, H2B1F_MOUSE, H2B1H_MOUSE, H2B1K_MOUSE, H2B1M_MOUSE, H2B1P_MOUSE, H2B2B_MOUSE, H2B2E_MOUSE, H2B3A_MOUSE, H2B3B_MOUSE	Histone H2B	TGISSKAMGIMNSFVND	56	3.90E-06	886.4227	1770.8308	2	0.0027	17	0	GluC
H2B1B_MOUSE, H2B1C_MOUSE, H2B1F_MOUSE, H2B1H_MOUSE, H2B1K_MOUSE, H2B1M_MOUSE, H2B1P_MOUSE, H2B2B_MOUSE, H2B2E_MOUSE, H2B3A_MOUSE, H2B3B_MOUSE	Histone H2B	TGISSKAMGIMNSFVND	56	1.10E-07	886.4227	1770.8308	2	0.0027	17	0	GluC
H2B1B_MOUSE, H2B1C_MOUSE, H2B1F_MOUSE, H2B1H_MOUSE, H2B1K_MOUSE, H2B1M_MOUSE, H2B1P_MOUSE, H2B2B_MOUSE, H2B2E_MOUSE, H2B3A_MOUSE, H2B3B_MOUSE	Histone H2B	TGISSKAMGIMNSFVND	56	1.40E-07	886.4226	1770.8307	2	0.0026	17	0	GluC
H2B1B_MOUSE, H2B1C_MOUSE, H2B1F_MOUSE, H2B1H_MOUSE, H2B1K_MOUSE, H2B1M_MOUSE, H2B1P_MOUSE, H2B2B_MOUSE, H2B2E_MOUSE, H2B3A_MOUSE, H2B3B_MOUSE	Histone H2B	TGISSKAMGIMNSFVND	56	2.20E-05	886.4225	1770.8305	2	0.0025	17	0	GluC
H2B1B_MOUSE, H2B1C_MOUSE, H2B1F_MOUSE, H2B1H_MOUSE, H2B1K_MOUSE, H2B1M_MOUSE, H2B1P_MOUSE, H2B2B_MOUSE, H2B2E_MOUSE, H2B3A_MOUSE, H2B3B_MOUSE	Histone H2B	TGISSKAMGIMNSFVND	56	3.90E-06	886.4226	1770.8305	2	0.0025	17	0	GluC
H2B1B_MOUSE, H2B1C_MOUSE, H2B1F_MOUSE, H2B1H_MOUSE, H2B1K_MOUSE, H2B1M_MOUSE, H2B1P_MOUSE, H2B2B_MOUSE, H2B2E_MOUSE, H2B3A_MOUSE, H2B3B_MOUSE	Histone H2B	TGISSKAMGIMNSFVND	56	1.10E-05	886.4225	1770.8305	2	0.0024	17	0	GluC
H2B1B_MOUSE, H2B1C_MOUSE, H2B1F_MOUSE, H2B1H_MOUSE, H2B1K_MOUSE, H2B1M_MOUSE, H2B1P_MOUSE, H2B2B_MOUSE, H2B2E_MOUSE, H2B3A_MOUSE, H2B3B_MOUSE	Histone H2B	TGISSKAMGIMNSFVND	56	4.10E-08	886.4225	1770.8304	2	0.0024	17	0	GluC
H2B1B_MOUSE, H2B1C_MOUSE, H2B1F_MOUSE, H2B1H_MOUSE, H2B1K_MOUSE, H2B1M_MOUSE, H2B1P_MOUSE, H2B2B_MOUSE, H2B2E_MOUSE, H2B3A_MOUSE, H2B3B_MOUSE	Histone H2B	TGISSKAMGIMNSFVND	56	2.40E-09	886.4225	1770.8304	2	0.0024	17	0	GluC
H2B1B_MOUSE, H2B1C_MOUSE, H2B1F_MOUSE, H2B1H_MOUSE, H2B1K_MOUSE, H2B1M_MOUSE, H2B1P_MOUSE, H2B2B_MOUSE, H2B2E_MOUSE, H2B3A_MOUSE, H2B3B_MOUSE	Histone H2B	TGISSKAMGIMNSFVND	56	6.90E-07	886.4225	1770.8304	2	0.0023	17	0	GluC
H2B1B_MOUSE, H2B1C_MOUSE, H2B1F_MOUSE, H2B1H_MOUSE, H2B1K_MOUSE, H2B1M_MOUSE, H2B1P_MOUSE, H2B2B_MOUSE, H2B2E_MOUSE, H2B3A_MOUSE, H2B3B_MOUSE	Histone H2B	TGISSKAMGIMNSFVND	56	5.40E-08	886.4224	1770.8302	2	0.0022	17	0	GluC
H2B1B_MOUSE, H2B1C_MOUSE, H2B1F_MOUSE, H2B1H_MOUSE, H2B1K_MOUSE, H2B1M_MOUSE, H2B1P_MOUSE, H2B2B_MOUSE, H2B2E_MOUSE, H2B3A_MOUSE, H2B3B_MOUSE	Histone H2B	TGISSKAMGIMNSFVND	56	1.00E-08	886.4224	1770.8302	2	0.0022	17	0	GluC
H2B1B_MOUSE, H2B1C_MOUSE, H2B1F_MOUSE, H2B1H_MOUSE, H2B1K_MOUSE, H2B1M_MOUSE, H2B1P_MOUSE, H2B2B_MOUSE, H2B2E_MOUSE, H2B3A_MOUSE, H2B3B_MOUSE	Histone H2B	TGISSKAMGIMNSFVND	56	1.20E-07	886.4223	1770.8301	2	0.0021	17	0	GluC
H2B1B_MOUSE, H2B1C_MOUSE, H2B1F_MOUSE, H2B1H_MOUSE, H2B1K_MOUSE, H2B1M_MOUSE, H2B1P_MOUSE, H2B2B_MOUSE, H2B2E_MOUSE, H2B3A_MOUSE, H2B3B_MOUSE	Histone H2B	TGISSKAMGIMNSFVND	56	2.10E-05	886.4223	1770.8301	2	0.0020	17	0	GluC
H2B1B_MOUSE, H2B1C_MOUSE, H2B1F_MOUSE, H2B1H_MOUSE, H2B1K_MOUSE, H2B1M_MOUSE, H2B1P_MOUSE, H2B2B_MOUSE, H2B2E_MOUSE, H2B3A_MOUSE, H2B3B_MOUSE	Histone H2B	TGISSKAMGIMNSFVND	56	1.40E-04	886.4222	1770.8299	2	0.0019	17	0	GluC
H2B1B_MOUSE, H2B1C_MOUSE, H2B1F_MOUSE, H2B1H_MOUSE, H2B1K_MOUSE, H2B1M_MOUSE, H2B1P_MOUSE, H2B2B_MOUSE, H2B2E_MOUSE, H2B3A_MOUSE, H2B3B_MOUSE	Histone H2B	TGISSKAMGIMNSFVND	56	5.60E-08	886.4222	1770.8299	2	0.0019	17	0	GluC
H2B1B_MOUSE, H2B1C_MOUSE, H2B1F_MOUSE, H2B1H_MOUSE, H2B1K_MOUSE, H2B1M_MOUSE, H2B1P_MOUSE, H2B2B_MOUSE, H2B2E_MOUSE, H2B3A_MOUSE, H2B3B_MOUSE	Histone H2B	TGISSKAMGIMNSFVND	56	5.00E-04	591.2839	1770.8298	3	0.0018	17	0	GluC
H2B1B_MOUSE, H2B1C_MOUSE, H2B1F_MOUSE, H2B1H_MOUSE, H2B1K_MOUSE, H2B1M_MOUSE, H2B1P_MOUSE, H2B2B_MOUSE, H2B2E_MOUSE, H2B3A_MOUSE, H2B3B_MOUSE	Histone H2B	TGISSKAMGIMNSFVND	56	5.40E-05	591.2839	1770.8298	3	0.0018	17	0	GluC
H2B1B_MOUSE, H2B1C_MOUSE, H2B1F_MOUSE, H2B1H_MOUSE, H2B1K_MOUSE, H2B1M_MOUSE, H2B1P_MOUSE, H2B2B_MOUSE, H2B2E_MOUSE, H2B3A_MOUSE, H2B3B_MOUSE	Histone H2B	TGISSKAMGIMNSFVND	56	4.60E-08	886.4222	1770.8298	2	0.0018	17	0	GluC
H2B1B_MOUSE, H2B1C_MOUSE, H2B1F_MOUSE, H2B1H_MOUSE, H2B1K_MOUSE, H2B1M_MOUSE, H2B1P_MOUSE, H2B2B_MOUSE, H2B2E_MOUSE, H2B3A_MOUSE, H2B3B_MOUSE	Histone H2B	TGISSKAMGIMNSFVND	56	7.60E-07	886.4221	1770.8297	2	0.0016	17	0	GluC
H2B1B_MOUSE, H2B1C_MOUSE, H2B1F_MOUSE, H2B1H_MOUSE, H2B1K_MOUSE, H2B1M_MOUSE, H2B1P_MOUSE, H2B2B_MOUSE, H2B2E_MOUSE, H2B3A_MOUSE, H2B3B_MOUSE	Histone H2B	TGISSKAMGIMNSFVND	56	2.80E-05	886.4219	1770.8293	2	0.0012	17	0	GluC
H2B1B_MOUSE, H2B1C_MOUSE, H2B1F_MOUSE, H2B1H_MOUSE, H2B1K_MOUSE, H2B1M_MOUSE, H2B1P_MOUSE, H2B2B_MOUSE, H2B2E_MOUSE, H2B3A_MOUSE, H2B3B_MOUSE	Histone H2B	TGISSKAMGIMNSFVND	56	2.00E-07	886.4218	1770.8290	2	0.0010	17	0	GluC
H2B1B_MOUSE, H2B1C_MOUSE, H2B1F_MOUSE, H2B1H_MOUSE, H2B1K_MOUSE, H2B1M_MOUSE, H2B1P_MOUSE, H2B2B_MOUSE, H2B2E_MOUSE, H2B3A_MOUSE, H2B3B_MOUSE	Histone H2B	TGISSKAMGIMNSFVND	56	2.70E-04	591.2835	1770.8288	3	0.0007	17	0	GluC
H2B1B_MOUSE, H2B1C_MOUSE, H2B1F_MOUSE, H2B1H_MOUSE, H2B1K_MOUSE, H2B1M_MOUSE, H2B1P_MOUSE, H2B2B_MOUSE, H2B2E_MOUSE, H2B3A_MOUSE, H2B3B_MOUSE	Histone H2B	TGISSKAMGIMNSFVND	56	2.40E-09	886.4216	1770.8286	2	0.0005	17	0	GluC
H2B1B_MOUSE, H2B1C_MOUSE, H2B1F_MOUSE, H2B1H_MOUSE, H2B1K_MOUSE, H2B1M_MOUSE, H2B1P_MOUSE, H2B2B_MOUSE, H2B2E_MOUSE, H2B3A_MOUSE, H2B3B_MOUSE	Histone H2B	AMGIMNSFVNDIFER	20	8.50E-05	872.4183	1742.8221	2	0.0101	15	0	Semi-tryptic

H2B1B_MOUSE, H2B1C_MOUSE, H2B1F_MOUSE, H2B1H_MOUSE, H2B1K_MOUSE, H2B1M_MOUSE, H2B1P_MOUSE, H2B2B_MOUSE, H2B2E_MOUSE, H2B3A_MOUSE, H2B3B_MOUSE	Histone H2B	AMGIMNSFVNDIFER	20	6.80E-06	872.4183	1742.8221	2	0.0101	15	0	Semi-tryptic
H2B1B_MOUSE, H2B1C_MOUSE, H2B1F_MOUSE, H2B1H_MOUSE, H2B1K_MOUSE, H2B1M_MOUSE, H2B1P_MOUSE, H2B2B_MOUSE, H2B2E_MOUSE, H2B3A_MOUSE, H2B3B_MOUSE	Histone H2B	AMGIMNSFVNDIFER	20	9.00E-05	872.4174	1742.8203	2	0.0083	15	0	Semi-tryptic
H2B1B_MOUSE, H2B1C_MOUSE, H2B1F_MOUSE, H2B1H_MOUSE, H2B1K_MOUSE, H2B1M_MOUSE, H2B1P_MOUSE, H2B2B_MOUSE, H2B2E_MOUSE, H2B3A_MOUSE, H2B3B_MOUSE	Histone H2B	AMGIMNSFVNDIFER	20	6.90E-06	872.4174	1742.8203	2	0.0083	15	0	Semi-tryptic
H2B1B_MOUSE, H2B1C_MOUSE, H2B1F_MOUSE, H2B1H_MOUSE, H2B1K_MOUSE, H2B1M_MOUSE, H2B1P_MOUSE, H2B2B_MOUSE, H2B2E_MOUSE, H2B3A_MOUSE, H2B3B_MOUSE	Histone H2B	AMGIMNSFVNDIFER	20	3.20E-05	872.4158	1742.8171	2	0.0052	15	0	Semi-tryptic
H2B1B_MOUSE, H2B1C_MOUSE, H2B1F_MOUSE, H2B1H_MOUSE, H2B1K_MOUSE, H2B1M_MOUSE, H2B1P_MOUSE, H2B2B_MOUSE, H2B2E_MOUSE, H2B3A_MOUSE, H2B3B_MOUSE	Histone H2B	AMGIMNSFVNDIFER	20	4.50E-06	872.4154	1742.8162	2	0.0042	15	0	Semi-tryptic
H2B1B_MOUSE, H2B1C_MOUSE, H2B1F_MOUSE, H2B1H_MOUSE, H2B1K_MOUSE, H2B1M_MOUSE, H2B1P_MOUSE, H2B2B_MOUSE, H2B2E_MOUSE, H2B3A_MOUSE, H2B3B_MOUSE	Histone H2B	AMGIMNSFVNDIFER	20	4.40E-09	872.4151	1742.8156	2	0.0037	15	0	Semi-tryptic
H2B1B_MOUSE, H2B1C_MOUSE, H2B1F_MOUSE, H2B1H_MOUSE, H2B1K_MOUSE, H2B1M_MOUSE, H2B1P_MOUSE, H2B2B_MOUSE, H2B2E_MOUSE, H2B3A_MOUSE, H2B3B_MOUSE	Histone H2B	AMGIMNSFVNDIFER	20	3.60E-09	872.4151	1742.8157	2	0.0037	15	0	Semi-tryptic
H2B1B_MOUSE, H2B1C_MOUSE, H2B1F_MOUSE, H2B1H_MOUSE, H2B1K_MOUSE, H2B1M_MOUSE, H2B1P_MOUSE, H2B2B_MOUSE, H2B2E_MOUSE, H2B3A_MOUSE, H2B3B_MOUSE	Histone H2B	AMGIMNSFVNDIFER	20	9.80E-11	872.4151	1742.8156	2	0.0036	15	0	Semi-tryptic
H2B1B_MOUSE, H2B1C_MOUSE, H2B1F_MOUSE, H2B1H_MOUSE, H2B1K_MOUSE, H2B1M_MOUSE, H2B1P_MOUSE, H2B2B_MOUSE, H2B2E_MOUSE, H2B3A_MOUSE, H2B3B_MOUSE	Histone H2B	AMGIMNSFVNDIFER	20	2.20E-09	872.415	1742.8154	2	0.0035	15	0	Semi-tryptic
H2B1B_MOUSE, H2B1C_MOUSE, H2B1F_MOUSE, H2B1H_MOUSE, H2B1K_MOUSE, H2B1M_MOUSE, H2B1P_MOUSE, H2B2B_MOUSE, H2B2E_MOUSE, H2B3A_MOUSE, H2B3B_MOUSE	Histone H2B	AMGIMNSFVNDIFER	20	1.80E-09	872.4149	1742.8151	2	0.0032	15	0	Semi-tryptic
H2B1B_MOUSE, H2B1C_MOUSE, H2B1F_MOUSE, H2B1H_MOUSE, H2B1K_MOUSE, H2B1M_MOUSE, H2B1P_MOUSE, H2B2B_MOUSE, H2B2E_MOUSE, H2B3A_MOUSE, H2B3B_MOUSE	Histone H2B	AMGIMNSFVNDIFER	20	8.70E-05	872.4147	1742.8149	2	0.0029	15	0	Semi-tryptic
H2B1B_MOUSE, H2B1C_MOUSE, H2B1F_MOUSE, H2B1H_MOUSE, H2B1K_MOUSE, H2B1M_MOUSE, H2B1P_MOUSE, H2B2B_MOUSE, H2B2E_MOUSE, H2B3A_MOUSE, H2B3B_MOUSE	Histone H2B	AMGIMNSFVNDIFER	20	2.30E-09	872.4147	1742.8149	2	0.0029	15	0	Semi-tryptic
H2B1B_MOUSE, H2B1C_MOUSE, H2B1F_MOUSE, H2B1H_MOUSE, H2B1K_MOUSE, H2B1M_MOUSE, H2B1P_MOUSE, H2B2B_MOUSE, H2B2E_MOUSE, H2B3A_MOUSE, H2B3B_MOUSE	Histone H2B	AMGIMNSFVNDIFER	20	1.90E-08	872.4147	1742.8148	2	0.0028	15	0	Semi-tryptic
H2B1B_MOUSE, H2B1C_MOUSE, H2B1F_MOUSE, H2B1H_MOUSE, H2B1K_MOUSE, H2B1M_MOUSE, H2B1P_MOUSE, H2B2B_MOUSE, H2B2E_MOUSE, H2B3A_MOUSE, H2B3B_MOUSE	Histone H2B	AMGIMNSFVNDIFER	20	1.20E-03	872.4145	1742.8145	2	0.0026	15	0	Semi-tryptic
H2B1B_MOUSE, H2B1C_MOUSE, H2B1F_MOUSE, H2B1H_MOUSE, H2B1K_MOUSE, H2B1M_MOUSE, H2B1P_MOUSE, H2B2B_MOUSE, H2B2E_MOUSE, H2B3A_MOUSE, H2B3B_MOUSE	Histone H2B	AMGIMNSFVNDIFER	20	2.30E-08	872.4145	1742.8145	2	0.0025	15	0	Semi-tryptic
H2B1B_MOUSE, H2B1C_MOUSE, H2B1F_MOUSE, H2B1H_MOUSE, H2B1K_MOUSE, H2B1M_MOUSE, H2B1P_MOUSE, H2B2B_MOUSE, H2B2E_MOUSE, H2B3A_MOUSE, H2B3B_MOUSE	Histone H2B	AMGIMNSFVNDIFER	20	1.50E-07	872.4145	1742.8144	2	0.0024	15	0	Semi-tryptic
H2B1B_MOUSE, H2B1C_MOUSE, H2B1F_MOUSE, H2B1H_MOUSE, H2B1K_MOUSE, H2B1M_MOUSE, H2B1P_MOUSE, H2B2B_MOUSE, H2B2E_MOUSE, H2B3A_MOUSE, H2B3B_MOUSE	Histone H2B	AMGIMNSFVNDIFER	20	1.10E-06	872.4144	1742.8143	2	0.0023	15	0	Semi-tryptic
H2B1B_MOUSE, H2B1C_MOUSE, H2B1F_MOUSE, H2B1H_MOUSE, H2B1K_MOUSE, H2B1M_MOUSE, H2B1P_MOUSE, H2B2B_MOUSE, H2B2E_MOUSE, H2B3A_MOUSE, H2B3B_MOUSE	Histone H2B	AMGIMNSFVNDIFER	20	1.30E-03	872.4143	1742.8140	2	0.0020	15	0	Semi-tryptic
H2B1B_MOUSE, H2B1C_MOUSE, H2B1F_MOUSE, H2B1H_MOUSE, H2B1K_MOUSE, H2B1M_MOUSE, H2B1P_MOUSE, H2B2B_MOUSE, H2B2E_MOUSE, H2B3A_MOUSE, H2B3B_MOUSE	Histone H2B	AMGIMNSFVNDIFER	20	1.60E-08	872.4135	1742.8125	2	0.0005	15	0	Semi-tryptic
H2B1B_MOUSE, H2B1F_MOUSE, H2B1K_MOUSE, H2B1P_MOUSE, H2B3B_MOUSE	Histone H2B	AMGIMNSFVNDIFERIASEASR	3	3.30E-06	820.0683	2457.1831	3	0.0051	22	1	Semi-tryptic
H2B1B_MOUSE, H2B1F_MOUSE, H2B1K_MOUSE, H2B1P_MOUSE, H2B3B_MOUSE	Histone H2B	AMGIMNSFVNDIFERIASEASR	3	2.60E-08	820.0679	2457.1819	3	0.0039	22	1	Semi-tryptic
H2B1B_MOUSE, H2B1F_MOUSE, H2B1K_MOUSE, H2B1P_MOUSE, H2B3B_MOUSE	Histone H2B	AMGIMNSFVNDIFERIASEASR	3	4.10E-06	820.0674	2457.1805	3	0.0025	22	1	Semi-tryptic
H2B1B_MOUSE, H2B1F_MOUSE, H2B1K_MOUSE, H2B1P_MOUSE, H2B3B_MOUSE	Histone H2B	VNDIFERIASEASRL	13	1.80E-07	573.974	1718.9002	3	0.0051	15	1	Chymotrypsin
H2B1B_MOUSE, H2B1F_MOUSE, H2B1K_MOUSE, H2B1P_MOUSE, H2B3B_MOUSE	Histone H2B	VNDIFERIASEASRL	13	4.80E-05	860.4565	1718.8984	2	0.0033	15	1	Chymotrypsin
H2B1B_MOUSE, H2B1F_MOUSE, H2B1K_MOUSE, H2B1P_MOUSE, H2B3B_MOUSE	Histone H2B	VNDIFERIASEASRL	13	5.20E-04	860.4563	1718.8981	2	0.0030	15	1	Chymotrypsin
H2B1B_MOUSE, H2B1F_MOUSE, H2B1K_MOUSE, H2B1P_MOUSE, H2B3B_MOUSE	Histone H2B	VNDIFERIASEASRL	13	1.00E-02	860.4562	1718.8978	2	0.0027	15	1	Chymotrypsin
H2B1B_MOUSE, H2B1F_MOUSE, H2B1K_MOUSE, H2B1P_MOUSE, H2B3B_MOUSE	Histone H2B	VNDIFERIASEASRL	13	6.00E-03	573.9731	1718.8975	3	0.0024	15	1	Chymotrypsin

H2B1B_MOUSE, H2B1F_MOUSE, H2B1K_MOUSE, H2B1P_MOUSE, H2B3B_MOUSE	Histone H2B	VNDIFERIASEASRL	13	4.10E-04	573.9729	1718.8968	3	0.0017	15	1	Chymotrypsin
H2B1B_MOUSE, H2B1F_MOUSE, H2B1K_MOUSE, H2B1P_MOUSE, H2B3B_MOUSE	Histone H2B	VNDIFERIASEASRL	13	3.60E-04	573.9729	1718.8968	3	0.0017	15	1	Chymotrypsin
H2B1B_MOUSE, H2B1F_MOUSE, H2B1K_MOUSE, H2B1P_MOUSE, H2B3B_MOUSE	Histone H2B	VNDIFERIASEASRL	13	8.20E-05	573.9726	1718.8960	3	0.0009	15	1	Chymotrypsin
H2B1B_MOUSE, H2B1F_MOUSE, H2B1K_MOUSE, H2B1P_MOUSE, H2B3B_MOUSE	Histone H2B	VNDIFERIASEASRL	13	6.90E-05	573.9726	1718.8960	3	0.0009	15	1	Chymotrypsin
H2B1B_MOUSE, H2B1F_MOUSE, H2B1K_MOUSE, H2B1P_MOUSE, H2B3B_MOUSE	Histone H2B	VNDIFERIASEASRL	13	2.30E-04	573.9725	1718.8957	3	0.0006	15	1	Chymotrypsin
H2B1B_MOUSE, H2B1F_MOUSE, H2B1K_MOUSE, H2B1P_MOUSE, H2B3B_MOUSE	Histone H2B	VNDIFERIASEASRL	13	1.10E-05	573.9725	1718.8957	3	0.0006	15	1	Chymotrypsin
H2B1B_MOUSE, H2B1F_MOUSE, H2B1K_MOUSE, H2B1P_MOUSE, H2B3B_MOUSE	Histone H2B	VNDIFERIASEASRL	13	2.20E-04	573.9722	1718.8948	3	-0.0003	15	1	Chymotrypsin
H2B1B_MOUSE, H2B1F_MOUSE, H2B1K_MOUSE, H2B1P_MOUSE, H2B3B_MOUSE	Histone H2B	VNDIFERIASEASRL	13	2.20E-06	573.9722	1718.8948	3	-0.0003	15	1	Chymotrypsin
H2B1C_MOUSE, H2B1F_MOUSE, H2B1H_MOUSE, H2B1K_MOUSE	Histone H2B	PEPAKSAPAPK	25	5.10E-03	546.8087	1091.6028	2	0.0053	11	1	Semi-tryptic
H2B1C_MOUSE, H2B1F_MOUSE, H2B1H_MOUSE, H2B1K_MOUSE	Histone H2B	PEPAKSAPAPK	25	8.00E-04	364.8744	1091.6014	3	0.0040	11	1	Semi-tryptic
H2B1C_MOUSE, H2B1F_MOUSE, H2B1H_MOUSE, H2B1K_MOUSE	Histone H2B	PEPAKSAPAPK	25	1.60E-05	546.8079	1091.6013	2	0.0038	11	1	Semi-tryptic
H2B1C_MOUSE, H2B1F_MOUSE, H2B1H_MOUSE, H2B1K_MOUSE	Histone H2B	PEPAKSAPAPK	25	9.00E-04	364.8743	1091.6011	3	0.0036	11	1	Semi-tryptic
H2B1C_MOUSE, H2B1F_MOUSE, H2B1H_MOUSE, H2B1K_MOUSE	Histone H2B	PEPAKSAPAPK	25	5.20E-06	546.8074	1091.6003	2	0.0028	11	1	Semi-tryptic
H2B1C_MOUSE, H2B1F_MOUSE, H2B1H_MOUSE, H2B1K_MOUSE	Histone H2B	PEPAKSAPAPK	25	1.90E-04	364.8739	1091.5999	3	0.0024	11	1	Semi-tryptic
H2B1C_MOUSE, H2B1F_MOUSE, H2B1H_MOUSE, H2B1K_MOUSE	Histone H2B	PEPAKSAPAPK	25	2.00E-05	364.8738	1091.5996	3	0.0022	11	1	Semi-tryptic
H2B1C_MOUSE, H2B1F_MOUSE, H2B1H_MOUSE, H2B1K_MOUSE	Histone H2B	PEPAKSAPAPK	25	5.30E-06	364.8738	1091.5996	3	0.0022	11	1	Semi-tryptic
H2B1C_MOUSE, H2B1F_MOUSE, H2B1H_MOUSE, H2B1K_MOUSE	Histone H2B	PEPAKSAPAPK	25	1.90E-04	364.8735	1091.5988	3	0.0013	11	1	Semi-tryptic
H2B1C_MOUSE, H2B1F_MOUSE, H2B1H_MOUSE, H2B1K_MOUSE	Histone H2B	PEPAKSAPAPK	25	1.40E-03	364.8735	1091.5986	3	0.0011	11	1	Semi-tryptic
H2B1C_MOUSE, H2B1F_MOUSE, H2B1H_MOUSE, H2B1K_MOUSE	Histone H2B	PEPAKSAPAPK	25	1.70E-05	546.8066	1091.5986	2	0.0011	11	1	Semi-tryptic
H2B1C_MOUSE, H2B1F_MOUSE, H2B1H_MOUSE, H2B1K_MOUSE	Histone H2B	PEPAKSAPAPK	25	5.40E-03	364.8733	1091.5982	3	0.0007	11	1	Semi-tryptic
H2B1C_MOUSE, H2B1F_MOUSE, H2B1H_MOUSE, H2B1K_MOUSE	Histone H2B	PEPAKSAPAPK	25	3.00E-04	364.8733	1091.5982	3	0.0007	11	1	Semi-tryptic
H2B1C_MOUSE, H2B1F_MOUSE, H2B1H_MOUSE, H2B1K_MOUSE	Histone H2B	PEPAKSAPAPK	25	8.80E-06	546.8064	1091.5982	2	0.0007	11	1	Semi-tryptic
H2B1C_MOUSE, H2B1F_MOUSE, H2B1H_MOUSE, H2B1K_MOUSE	Histone H2B	PEPAKSAPAPK	25	7.40E-04	364.8733	1091.5981	3	0.0006	11	1	Semi-tryptic
H2B1C_MOUSE, H2B1F_MOUSE, H2B1H_MOUSE, H2B1K_MOUSE	Histone H2B	PEPAKSAPAPK	25	2.10E-03	364.8733	1091.5980	3	0.0005	11	1	Semi-tryptic
H2B1C_MOUSE, H2B1F_MOUSE, H2B1H_MOUSE, H2B1K_MOUSE	Histone H2B	PEPAKSAPAPK	25	5.20E-06	546.8062	1091.5979	2	0.0005	11	1	Semi-tryptic
H2B1C_MOUSE, H2B1F_MOUSE, H2B1H_MOUSE, H2B1K_MOUSE	Histone H2B	PEPAKSAPAPK	25	4.60E-06	546.8062	1091.5979	2	0.0005	11	1	Semi-tryptic
H2B1C_MOUSE, H2B1F_MOUSE, H2B1H_MOUSE, H2B1K_MOUSE	Histone H2B	PEPAKSAPAPK	25	1.00E-03	364.8732	1091.5979	3	0.0004	11	1	Semi-tryptic
H2B1C_MOUSE, H2B1F_MOUSE, H2B1H_MOUSE, H2B1K_MOUSE	Histone H2B	PEPAKSAPAPK	25	4.70E-05	546.8062	1091.5978	2	0.0004	11	1	Semi-tryptic
H2B1C_MOUSE, H2B1F_MOUSE, H2B1H_MOUSE, H2B1K_MOUSE	Histone H2B	PEPAKSAPAPK	25	5.20E-06	546.806	1091.5975	2	0.0001	11	1	Semi-tryptic
H2B1C_MOUSE, H2B1F_MOUSE, H2B1H_MOUSE, H2B1K_MOUSE	Histone H2B	PEPAKSAPAPK	25	1.60E-05	546.8057	1091.5969	2	-0.0005	11	1	Semi-tryptic
H2B1C_MOUSE, H2B1F_MOUSE, H2B1H_MOUSE, H2B1K_MOUSE	Histone H2B	PEPAKSAPAPK	25	5.70E-03	364.8729	1091.5968	3	-0.0006	11	1	Semi-tryptic
H2B1C_MOUSE, H2B1F_MOUSE, H2B1H_MOUSE, H2B1K_MOUSE	Histone H2B	PEPAKSAPAPK	25	1.70E-03	546.8057	1091.5968	2	-0.0007	11	1	Semi-tryptic
H2B1C_MOUSE, H2B1F_MOUSE, H2B1H_MOUSE, H2B1K_MOUSE	Histone H2B	PEPAKSAPAPK	25	3.00E-05	546.8054	1091.5962	2	-0.0012	11	1	Semi-tryptic
H2B1C_MOUSE, H2B1F_MOUSE, H2B1K_MOUSE	Histone H2B	PEPAKSAPAPKKGSKKAVTKAQKKDGKKRKRSRKE	14	5.10E-05	769.6659	3843.2934	5	0.0176	35	1	GluC
H2B1C_MOUSE, H2B1F_MOUSE, H2B1K_MOUSE	Histone H2B	PEPAKSAPAPKKGSKKAVTKAQKKDGKKRKRSRKE	14	1.30E-04	641.5558	3843.2912	6	0.0154	35	1	GluC
H2B1C_MOUSE, H2B1F_MOUSE, H2B1K_MOUSE	Histone H2B	PEPAKSAPAPKKGSKKAVTKAQKKDGKKRKRSRKE	14	1.00E-02	550.0483	3843.2870	7	0.0113	35	1	GluC
H2B1C_MOUSE, H2B1F_MOUSE, H2B1K_MOUSE	Histone H2B	PEPAKSAPAPKKGSKKAVTKAQKKDGKKRKRSRKE	14	2.30E-06	769.6635	3843.2812	5	0.0054	35	1	GluC
H2B1C_MOUSE, H2B1F_MOUSE, H2B1K_MOUSE	Histone H2B	PEPAKSAPAPKKGSKKAVTKAQKKDGKKRKRSRKE	14	2.00E-04	641.5538	3843.2793	6	0.0036	35	1	GluC
H2B1C_MOUSE, H2B1F_MOUSE, H2B1K_MOUSE	Histone H2B	PEPAKSAPAPKKGSKKAVTKAQKKDGKKRKRSRKE	14	2.50E-06	769.6631	3843.2790	5	0.0032	35	1	GluC
H2B1C_MOUSE, H2B1F_MOUSE, H2B1K_MOUSE	Histone H2B	PEPAKSAPAPKKGSKKAVTKAQKKDGKKRKRSRKE	14	1.30E-05	769.663	3843.2788	5	0.0030	35	1	GluC
H2B1C_MOUSE, H2B1F_MOUSE, H2B1K_MOUSE	Histone H2B	PEPAKSAPAPKKGSKKAVTKAQKKDGKKRKRSRKE	14	2.20E-03	641.5537	3843.2785	6	0.0028	35	1	GluC
H2B1C_MOUSE, H2B1F_MOUSE, H2B1K_MOUSE	Histone H2B	PEPAKSAPAPKKGSKKAVTKAQKKDGKKRKRSRKE	14	7.30E-04	641.5536	3843.2778	6	0.0021	35	1	GluC
H2B1C_MOUSE, H2B1F_MOUSE, H2B1K_MOUSE	Histone H2B	PEPAKSAPAPKKGSKKAVTKAQKKDGKKRKRSRKE	14	8.40E-03	550.0469	3843.2776	7	0.0019	35	1	GluC
H2B1C_MOUSE, H2B1F_MOUSE, H2B1K_MOUSE	Histone H2B	PEPAKSAPAPKKGSKKAVTKAQKKDGKKRKRSRKE	14	2.10E-04	641.5535	3843.2775	6	0.0017	35	1	GluC
H2B1C_MOUSE, H2B1F_MOUSE, H2B1K_MOUSE	Histone H2B	PEPAKSAPAPKKGSKKAVTKAQKKDGKKRKRSRKE	14	2.40E-05	641.5534	3843.2769	6	0.0012	35	1	GluC
H2B1C_MOUSE, H2B1F_MOUSE, H2B1K_MOUSE	Histone H2B	PEPAKSAPAPKKGSKKAVTKAQKKDGKKRKRSRKE	14	4.60E-03	550.0468	3843.2765	7	0.0007	35	1	GluC
H2B1C_MOUSE, H2B1F_MOUSE, H2B1K_MOUSE	Histone H2B	PEPAKSAPAPKKGSKKAVTKAQKKDGKKRKRSRKE	14	1.30E-04	550.0466	3843.2753	7	-0.0004	35	1	GluC
H2B1C_MOUSE, H2B1F_MOUSE, H2B1K_MOUSE	Histone H2B	PEPAKSAPAPKKGSKKAVTKAQKKD	30	5.40E-07	518.9062	2589.4946	5	-0.0020	25	0	GluC
H2B1C_MOUSE, H2B1F_MOUSE, H2B1K_MOUSE	Histone H2B	PEPAKSAPAPKKGSKKAVTKAQKKD	30	4.40E-03	518.9061	2589.4941	5	-0.0024	25	0	GluC
H2B1C_MOUSE, H2B1F_MOUSE, H2B1K_MOUSE	Histone H2B	PEPAKSAPAPKKGSKKAVTKAQKKD	30	6.20E-06	432.5896	2589.4939	6	-0.0027	25	0	GluC
H2B1C_MOUSE, H2B1F_MOUSE, H2B1K_MOUSE	Histone H2B	PEPAKSAPAPKKGSKKAVTKAQKKD	30	7.60E-04	518.9058	2589.4925	5	-0.0041	25	0	GluC
H2B1C_MOUSE, H2B1F_MOUSE, H2B1K_MOUSE	Histone H2B	PEPAKSAPAPKKGSKKAVTKAQKKD	30	4.90E-06	648.3781	2589.4833	4	-0.0132	25	0	GluC
H2B1C_MOUSE, H2B1F_MOUSE, H2B1K_MOUSE	Histone H2B	PEPAKSAPAPKKGSKKAVTKAQKKD	30	5.00E-06	518.9036	2589.4817	5	-0.0148	25	0	GluC
H2B1C_MOUSE, H2B1F_MOUSE, H2B1K_MOUSE	Histone H2B	PEPAKSAPAPKKGSKKAVTKAQKKD	30	6.10E-03	648.385	2589.5111	4	0.0145	25	0	GluC
H2B1C_MOUSE, H2B1F_MOUSE, H2B1K_MOUSE	Histone H2B	PEPAKSAPAPKKGSKKAVTKAQKKD	30	2.70E-03	648.3848	2589.5100	4	0.0135	25	0	GluC
H2B1C_MOUSE, H2B1F_MOUSE, H2B1K_MOUSE	Histone H2B	PEPAKSAPAPKKGSKKAVTKAQKKD	30	7.70E-05	648.3845	2589.5091	4	0.0125	25	0	GluC
H2B1C_MOUSE, H2B1F_MOUSE, H2B1K_MOUSE	Histone H2B	PEPAKSAPAPKKGSKKAVTKAQKKD	30	1.30E-05	648.3843	2589.5080	4	0.0115	25	0	GluC
H2B1C_MOUSE, H2B1F_MOUSE, H2B1K_MOUSE	Histone H2B	PEPAKSAPAPKKGSKKAVTKAQKKD	30	6.50E-05	864.1761	2589.5066	3	0.0100	25	0	GluC
H2B1C_MOUSE, H2B1F_MOUSE, H2B1K_MOUSE	Histone H2B	PEPAKSAPAPKKGSKKAVTKAQKKD	30	2.70E-03	518.9082	2589.5047	5	0.0081	25	0	GluC
H2B1C_MOUSE, H2B1F_MOUSE, H2B1K_MOUSE	Histone H2B	PEPAKSAPAPKKGSKKAVTKAQKKD	30	2.30E-04	648.383	2589.5029	4	0.0063	25	0	GluC
H2B1C_MOUSE, H2B1F_MOUSE, H2B1K_MOUSE	Histone H2B	PEPAKSAPAPKKGSKKAVTKAQKKD	30	3.30E-05	648.383	2589.5027	4	0.0062	25	0	GluC
H2B1C_MOUSE, H2B1F_MOUSE, H2B1K_MOUSE	Histone H2B	PEPAKSAPAPKKGSKKAVTKAQKKD	30	2.80E-10	648.3828	2589.5021	4	0.0055	25	0	GluC
H2B1C_MOUSE, H2B1F_MOUSE, H2B1K_MOUSE	Histone H2B	PEPAKSAPAPKKGSKKAVTKAQKKD	30	4.10E-04	648.3827	2589.5017	4	0.0051	25	0	GluC
H2B1C_MOUSE, H2B1F_MOUSE, H2B1K_MOUSE	Histone H2B	PEPAKSAPAPKKGSKKAVTKAQKKD	30	8.20E-09	648.3827	2589.5016	4	0.0050	25	0	GluC
H2B1C_MOUSE, H2B1F_MOUSE, H2B1K_MOUSE	Histone H2B	PEPAKSAPAPKKGSKKAVTKAQKKD	30	4.30E-07	518.9075	2589.5012	5	0.0046	25	0	GluC
H2B1C_MOUSE, H2B1F_MOUSE, H2B1K_MOUSE	Histone H2B	PEPAKSAPAPKKGSKKAVTKAQKKD	30	8.90E-07	518.9074	2589.5007	5	0.0042	25	0	GluC

H2B1C_MOUSE, H2B1F_MOUSE, H2B1K_MOUSE	Histone H2B	PEPAKSAPAPKKGSKKAVTKAQKKD	30	8.20E-03	864.174	2589.5003	3	0.0038	25	0	GluC
H2B1C_MOUSE, H2B1F_MOUSE, H2B1K_MOUSE	Histone H2B	PEPAKSAPAPKKGSKKAVTKAQKKD	30	1.40E-04	864.1739	2589.4999	3	0.0033	25	0	GluC
H2B1C_MOUSE, H2B1F_MOUSE, H2B1K_MOUSE	Histone H2B	PEPAKSAPAPKKGSKKAVTKAQKKD	30	2.20E-03	648.382	2589.4988	4	0.0023	25	0	GluC
H2B1C_MOUSE, H2B1F_MOUSE, H2B1K_MOUSE	Histone H2B	PEPAKSAPAPKKGSKKAVTKAQKKD	30	1.90E-04	648.3819	2589.4987	4	0.0021	25	0	GluC
H2B1C_MOUSE, H2B1F_MOUSE, H2B1K_MOUSE	Histone H2B	PEPAKSAPAPKKGSKKAVTKAQKKD	30	5.80E-09	648.3818	2589.4983	4	0.0017	25	0	GluC
H2B1C_MOUSE, H2B1F_MOUSE, H2B1K_MOUSE	Histone H2B	PEPAKSAPAPKKGSKKAVTKAQKKD	30	1.20E-07	518.9069	2589.4982	5	0.0016	25	0	GluC
H2B1C_MOUSE, H2B1F_MOUSE, H2B1K_MOUSE	Histone H2B	PEPAKSAPAPKKGSKKAVTKAQKKD	30	4.30E-10	518.9067	2589.4969	5	0.0003	25	0	GluC
H2B1C_MOUSE, H2B1F_MOUSE, H2B1K_MOUSE	Histone H2B	PEPAKSAPAPKKGSKKAVTKAQKKD	30	2.40E-07	432.5898	2589.4953	6	-0.0012	25	0	GluC
H2B1C_MOUSE, H2B1F_MOUSE, H2B1K_MOUSE	Histone H2B	PEPAKSAPAPKKGSKKAVTKAQKKD	30	4.40E-09	518.9063	2589.4953	5	-0.0013	25	0	GluC
H2B1C_MOUSE, H2B1F_MOUSE, H2B1K_MOUSE	Histone H2B	PEPAKSAPAPKKGSKKAVTKAQKKD	30	7.60E-10	432.5898	2589.4951	6	-0.0014	25	0	GluC
H2B1C_MOUSE, H2B1F_MOUSE, H2B1K_MOUSE	Histone H2B	PEPAKSAPAPKKGSKKAVTKAQKKD	30	2.20E-08	432.5897	2589.4948	6	-0.0017	25	0	GluC
H2B1C_MOUSE, H2B1F_MOUSE, H2B1K_MOUSE	Histone H2B	PEPAKSAPAPKKGSKKAVTKAQKK	9	4.90E-08	495.902	2474.4735	5	0.0039	24	0	AspN
H2B1C_MOUSE, H2B1F_MOUSE, H2B1K_MOUSE	Histone H2B	PEPAKSAPAPKKGSKKAVTKAQKK	9	1.10E-04	495.9017	2474.4719	5	0.0023	24	0	AspN
H2B1C_MOUSE, H2B1F_MOUSE, H2B1K_MOUSE	Histone H2B	PEPAKSAPAPKKGSKKAVTKAQKK	9	4.30E-04	825.8311	2474.4714	3	0.0018	24	0	AspN
H2B1C_MOUSE, H2B1F_MOUSE, H2B1K_MOUSE	Histone H2B	PEPAKSAPAPKKGSKKAVTKAQKK	9	1.50E-04	495.9015	2474.4713	5	0.0017	24	0	AspN
H2B1C_MOUSE, H2B1F_MOUSE, H2B1K_MOUSE	Histone H2B	PEPAKSAPAPKKGSKKAVTKAQKK	9	2.10E-10	619.6249	2474.4706	4	0.0010	24	0	AspN
H2B1C_MOUSE, H2B1F_MOUSE, H2B1K_MOUSE	Histone H2B	PEPAKSAPAPKKGSKKAVTKAQKK	9	3.40E-08	495.9011	2474.4692	5	-0.0004	24	0	AspN
H2B1C_MOUSE, H2B1F_MOUSE, H2B1K_MOUSE	Histone H2B	PEPAKSAPAPKKGSKKAVTKAQKK	9	5.90E-09	619.6245	2474.4688	4	-0.0008	24	0	AspN
H2B1C_MOUSE, H2B1F_MOUSE, H2B1K_MOUSE	Histone H2B	PEPAKSAPAPKKGSKKAVTKAQKK	9	1.00E-09	619.6241	2474.4672	4	-0.0024	24	0	AspN
H2B1C_MOUSE, H2B1F_MOUSE, H2B1K_MOUSE	Histone H2B	PEPAKSAPAPKKGSKKAVTKAQKK	9	5.60E-06	413.4173	2474.4603	6	-0.0093	24	0	AspN
H2B1C_MOUSE, H2B1H_MOUSE, H2B1M_MOUSE, H2B2B_MOUSE	Histone H2B	RIAGEASRLAHYNKRSTITSREIQTAVRLLLPGE	5	5.60E-05	762.2315	3806.1210	5	0.0183	34	2	GluC
H2B1C_MOUSE, H2B1H_MOUSE, H2B1M_MOUSE, H2B2B_MOUSE	Histone H2B	RIAGEASRLAHYNKRSTITSREIQTAVRLLLPGE	5	7.30E-03	762.2313	3806.1202	5	0.0175	34	2	GluC
H2B1C_MOUSE, H2B1H_MOUSE, H2B1M_MOUSE, H2B2B_MOUSE	Histone H2B	RIAGEASRLAHYNKRSTITSREIQTAVRLLLPGE	5	5.70E-03	952.537	3806.1187	4	0.0161	34	2	GluC
H2B1C_MOUSE, H2B1H_MOUSE, H2B1M_MOUSE, H2B2B_MOUSE	Histone H2B	RIAGEASRLAHYNKRSTITSREIQTAVRLLLPGE	5	1.90E-03	762.2309	3806.1180	5	0.0153	34	2	GluC
H2B1C_MOUSE, H2B1H_MOUSE, H2B1M_MOUSE, H2B2B_MOUSE	Histone H2B	RIAGEASRLAHYNKRSTITSREIQTAVRLLLPGE	5	2.10E-04	762.2286	3806.1065	5	0.0038	34	2	GluC
H2B1C_MOUSE, H2B1H_MOUSE, H2B1M_MOUSE, H2B2B_MOUSE	Histone H2B	RIAGEASRLAHYNKRSTITSRE	2	2.30E-10	629.8431	2515.3434	4	0.0067	22	1	GluC
H2B1C_MOUSE, H2B1H_MOUSE, H2B1M_MOUSE, H2B2B_MOUSE	Histone H2B	AMGIMNSFVNDIFERIAGEASR	3	8.90E-09	810.0643	2427.1710	3	0.0036	22	1	Semi-tryptic
H2B1C_MOUSE, H2B1H_MOUSE, H2B1M_MOUSE, H2B2B_MOUSE	Histone H2B	AMGIMNSFVNDIFERIAGEASR	3	1.70E-09	810.064	2427.1701	3	0.0026	22	1	Semi-tryptic
H2B1C_MOUSE, H2B1H_MOUSE, H2B1M_MOUSE, H2B2B_MOUSE	Histone H2B	AMGIMNSFVNDIFERIAGEASR	3	2.30E-09	810.0639	2427.1697	3	0.0023	22	1	Semi-tryptic
H2B1C_MOUSE, H2B1H_MOUSE, H2B1M_MOUSE, H2B2B_MOUSE	Histone H2B	RIAGEASRLAHYNKRSTITSRE	2	1.50E-06	504.072	2515.3234	5	-0.0133	22	1	GluC
H2B1C_MOUSE, H2B1H_MOUSE, H2B1M_MOUSE, H2B2B_MOUSE	Histone H2B	VNDIFERIAGEASRL	16	1.30E-04	563.9715	1688.8926	3	0.0080	15	1	Chymotrypsin
H2B1C_MOUSE, H2B1H_MOUSE, H2B1M_MOUSE, H2B2B_MOUSE	Histone H2B	VNDIFERIAGEASRL	16	3.60E-06	563.971	1688.8913	3	0.0067	15	1	Chymotrypsin
H2B1C_MOUSE, H2B1H_MOUSE, H2B1M_MOUSE, H2B2B_MOUSE	Histone H2B	VNDIFERIAGEASRL	16	3.70E-04	845.4511	1688.8877	2	0.0032	15	1	Chymotrypsin
H2B1C_MOUSE, H2B1H_MOUSE, H2B1M_MOUSE, H2B2B_MOUSE	Histone H2B	VNDIFERIAGEASRL	16	1.90E-05	563.9697	1688.8874	3	0.0029	15	1	Chymotrypsin
H2B1C_MOUSE, H2B1H_MOUSE, H2B1M_MOUSE, H2B2B_MOUSE	Histone H2B	VNDIFERIAGEASRL	16	2.90E-04	845.4505	1688.8864	2	0.0019	15	1	Chymotrypsin
H2B1C_MOUSE, H2B1H_MOUSE, H2B1M_MOUSE, H2B2B_MOUSE	Histone H2B	VNDIFERIAGEASRL	16	1.70E-03	563.9693	1688.8860	3	0.0015	15	1	GluC
H2B1C_MOUSE, H2B1H_MOUSE, H2B1M_MOUSE, H2B2B_MOUSE	Histone H2B	VNDIFERIAGEASRL	16	9.00E-04	563.9693	1688.8860	3	0.0015	15	1	GluC
H2B1C_MOUSE, H2B1H_MOUSE, H2B1M_MOUSE, H2B2B_MOUSE	Histone H2B	VNDIFERIAGEASRL	16	3.20E-05	563.9693	1688.8861	3	0.0015	15	1	Chymotrypsin
H2B1C_MOUSE, H2B1H_MOUSE, H2B1M_MOUSE, H2B2B_MOUSE	Histone H2B	VNDIFERIAGEASRL	16	1.40E-05	563.9693	1688.8861	3	0.0015	15	1	Chymotrypsin
H2B1C_MOUSE, H2B1H_MOUSE, H2B1M_MOUSE, H2B2B_MOUSE	Histone H2B	VNDIFERIAGEASRL	16	9.20E-05	563.9692	1688.8858	3	0.0013	15	1	Chymotrypsin
H2B1C_MOUSE, H2B1H_MOUSE, H2B1M_MOUSE, H2B2B_MOUSE	Histone H2B	VNDIFERIAGEASRL	16	5.60E-05	563.9692	1688.8858	3	0.0013	15	1	Chymotrypsin
H2B1C_MOUSE, H2B1H_MOUSE, H2B1M_MOUSE, H2B2B_MOUSE	Histone H2B	VNDIFERIAGEASRL	16	2.20E-04	845.4502	1688.8858	2	0.0012	15	1	Chymotrypsin
H2B1C_MOUSE, H2B1H_MOUSE, H2B1M_MOUSE, H2B2B_MOUSE	Histone H2B	VNDIFERIAGEASRL	16	6.70E-04	563.9692	1688.8857	3	0.0011	15	1	Chymotrypsin
H2B1C_MOUSE, H2B1H_MOUSE, H2B1M_MOUSE, H2B2B_MOUSE	Histone H2B	VNDIFERIAGEASRL	16	2.50E-04	563.9689	1688.8850	3	0.0005	15	1	Chymotrypsin
H2B1C_MOUSE, H2B1H_MOUSE, H2B1M_MOUSE, H2B2B_MOUSE	Histone H2B	VNDIFERIAGEASRL	16	1.50E-04	563.9689	1688.8850	3	0.0005	15	1	Chymotrypsin
H2B1C_MOUSE, H2B1H_MOUSE, H2B1M_MOUSE, H2B2B_MOUSE	Histone H2B	VNDIFERIAGEASRL	16	4.20E-06	563.9688	1688.8845	3	0.0000	15	1	Chymotrypsin
H2B1C_MOUSE, H2B1H_MOUSE, H2B1M_MOUSE, H2B2B_MOUSE	Histone H2B	IAGEASRLAHYNKR	14	7.60E-08	397.2199	1584.8503	4	0.0019	14	2	Semi-tryptic
H2B1C_MOUSE, H2B1H_MOUSE, H2B1M_MOUSE, H2B2B_MOUSE	Histone H2B	IAGEASRLAHYNKR	14	2.40E-09	397.2199	1584.8503	4	0.0019	14	2	Semi-tryptic
H2B1C_MOUSE, H2B1H_MOUSE, H2B1M_MOUSE, H2B2B_MOUSE	Histone H2B	IAGEASRLAHYNKR	14	2.50E-03	793.4321	1584.8497	2	0.0013	14	2	Semi-tryptic
H2B1C_MOUSE, H2B1H_MOUSE, H2B1M_MOUSE, H2B2B_MOUSE	Histone H2B	IAGEASRLAHYNKR	14	6.70E-05	529.2902	1584.8488	3	0.0004	14	2	Semi-tryptic
H2B1C_MOUSE, H2B1H_MOUSE, H2B1M_MOUSE, H2B2B_MOUSE	Histone H2B	IAGEASRLAHYNKR	14	2.10E-06	529.2902	1584.8488	3	0.0004	14	2	Semi-tryptic
H2B1C_MOUSE, H2B1H_MOUSE, H2B1M_MOUSE, H2B2B_MOUSE	Histone H2B	IAGEASRLAHYNKR	14	1.70E-03	529.29	1584.8482	3	-0.0002	14	2	Semi-tryptic
H2B1C_MOUSE, H2B1H_MOUSE, H2B1M_MOUSE, H2B2B_MOUSE	Histone H2B	IAGEASRLAHYNKR	14	2.40E-05	529.29	1584.8481	3	-0.0003	14	2	Semi-tryptic
H2B1C_MOUSE, H2B1H_MOUSE, H2B1M_MOUSE, H2B2B_MOUSE	Histone H2B	IAGEASRLAHYNKR	14	3.10E-05	529.2899	1584.8478	3	-0.0006	14	2	Semi-tryptic
H2B1C_MOUSE, H2B1H_MOUSE, H2B1M_MOUSE, H2B2B_MOUSE	Histone H2B	IAGEASRLAHYNKR	14	1.30E-05	529.2898	1584.8475	3	-0.0009	14	2	Semi-tryptic
H2B1C_MOUSE, H2B1H_MOUSE, H2B1M_MOUSE, H2B2B_MOUSE	Histone H2B	IAGEASRLAHYNKR	14	4.10E-07	529.2898	1584.8475	3	-0.0009	14	2	Semi-tryptic
H2B1C_MOUSE, H2B1H_MOUSE, H2B1M_MOUSE, H2B2B_MOUSE	Histone H2B	IAGEASRLAHYNKR	14	1.30E-04	529.2898	1584.8475	3	-0.0010	14	2	Semi-tryptic
H2B1C_MOUSE, H2B1H_MOUSE, H2B1M_MOUSE, H2B2B_MOUSE	Histone H2B	IAGEASRLAHYNKR	14	1.50E-04	529.2897	1584.8473	3	-0.0011	14	2	Semi-tryptic
H2B1C_MOUSE, H2B1H_MOUSE, H2B1M_MOUSE, H2B2B_MOUSE	Histone H2B	IAGEASRLAHYNKR	14	1.70E-03	529.2896	1584.8470	3	-0.0014	14	2	Semi-tryptic
H2B1C_MOUSE, H2B1H_MOUSE, H2B1M_MOUSE, H2B2B_MOUSE	Histone H2B	IAGEASRLAHYNKR	14	4.60E-05	529.2895	1584.8467	3	-0.0017	14	2	Semi-tryptic
H2B1C_MOUSE, H2B1H_MOUSE, H2B1M_MOUSE, H2B2B_MOUSE	Histone H2B	IAGEASRLAHYNK	2	9.00E-04	477.2564	1428.7473	3	0.0000	13	1	Semi-tryptic
H2B1C_MOUSE, H2B1H_MOUSE, H2B1M_MOUSE, H2B2B_MOUSE	Histone H2B	IAGEASRLAHYNK	2	8.30E-03	477.2557	1428.7453	3	-0.0020	13	1	Semi-tryptic
H2B1C_MOUSE, H2B1H_MOUSE, H2B1M_MOUSE, H2B2B_MOUSE	Histone H2B	ERIAGEASRL	4	2.40E-04	367.8726	1100.5959	3	0.0021	10	0	Chymotrypsin
H2B1C_MOUSE, H2B1H_MOUSE, H2B1M_MOUSE, H2B2B_MOUSE	Histone H2B	ERIAGEASRL	4	2.30E-04	367.8721	1100.5945	3	0.0007	10	0	Chymotrypsin
H2B1C_MOUSE, H2B1H_MOUSE, H2B1M_MOUSE, H2B2B_MOUSE	Histone H2B	ERIAGEASRL	4	2.10E-03	551.3044	1100.5943	2	0.0005	10	0	Chymotrypsin
H2B1C_MOUSE, H2B1H_MOUSE, H2B1M_MOUSE, H2B2B_MOUSE	Histone H2B	ERIAGEASRL	4	1.90E-03	551.3043	1100.5940	2	0.0002	10	0	Chymotrypsin
H2B1C_MOUSE, H2B1H_MOUSE, H2B1M_MOUSE, H2B2B_MOUSE	Histone H2B	IFERIAGE	11	1.20E-04	467.7543	933.4941	2	0.0021	8	1	GluC
H2B1C_MOUSE, H2B1H_MOUSE, H2B1M_MOUSE, H2B2B_MOUSE	Histone H2B	IFERIAGE	11	8.00E-04	467.7541	933.4936	2	0.0016	8	1	GluC
H2B1C_MOUSE, H2B1H_MOUSE, H2B1M_MOUSE, H2B2B_MOUSE	Histone H2B	IFERIAGE	11	7.90E-04	467.7541	933.4936	2	0.0016	8	1	GluC
H2B1C_MOUSE, H2B1H_MOUSE, H2B1M_MOUSE, H2B2B_MOUSE	Histone H2B	IFERIAGE	11	1.40E-06	467.7541	933.4936	2	0.0016	8	1	GluC
H2B1C_MOUSE, H2B1H_MOUSE, H2B1M_MOUSE, H2B2B_MOUSE	Histone H2B	IFERIAGE	11	1.20E-04	467.7534	933.4922	2	0.0002	8	1	GluC
H2B1C_MOUSE, H2B1H_MOUSE, H2B1M_MOUSE, H2B2B_MOUSE	Histone H2B	IFERIAGE	11	8.40E-03	467.7532	933.4919	2	-0.0001	8	1	GluC
H2B1C_MOUSE, H2B1H_MOUSE, H2B1M_MOUSE, H2B2B_MOUSE	Histone H2B	IFERIAGE	11	5.00E-04	467.7531	933.4917	2	-0.0002	8	1	GluC
H2B1C_MOUSE, H2B1H_MOUSE, H2B1M_MOUSE, H2B2B_MOUSE	Histone H2B	IFERIAGE	11	3.90E-03	467.7528	933.4910	2	-0.0010	8	1	GluC

H2B1C_MOUSE, H2B1H_MOUSE, H2B1M_MOUSE, H2B2B_MOUSE	Histone H2B	IFERIAGE	11	2.40E-05	467.7528	933.4910	2	-0.0010	8	1	GluC
H2B1C_MOUSE, H2B1H_MOUSE, H2B1M_MOUSE, H2B2B_MOUSE	Histone H2B	IFERIAGE	11	1.50E-03	467.7525	933.4904	2	-0.0015	8	1	GluC
H2B1C_MOUSE, H2B1H_MOUSE, H2B1M_MOUSE, H2B2B_MOUSE	Histone H2B	IFERIAGE	11	4.50E-04	467.7525	933.4905	2	-0.0015	8	1	GluC
H2B1C_MOUSE, H2B1H_MOUSE, H2B1M_MOUSE, H2B2B_MOUSE	Histone H2B	IAGEASR	32	1.20E-04	352.1924	702.3702	2	0.0042	7	0	Semi-tryptic
H2B1C_MOUSE, H2B1H_MOUSE, H2B1M_MOUSE, H2B2B_MOUSE	Histone H2B	IAGEASR	32	1.10E-04	352.1923	702.3701	2	0.0041	7	0	Semi-tryptic
H2B1C_MOUSE, H2B1H_MOUSE, H2B1M_MOUSE, H2B2B_MOUSE	Histone H2B	IAGEASR	32	1.10E-04	352.1923	702.3700	2	0.0040	7	0	Semi-tryptic
H2B1C_MOUSE, H2B1H_MOUSE, H2B1M_MOUSE, H2B2B_MOUSE	Histone H2B	IAGEASR	32	5.50E-04	352.1922	702.3698	2	0.0038	7	0	Semi-tryptic
H2B1C_MOUSE, H2B1H_MOUSE, H2B1M_MOUSE, H2B2B_MOUSE	Histone H2B	IAGEASR	32	1.20E-04	352.1922	702.3699	2	0.0038	7	0	Semi-tryptic
H2B1C_MOUSE, H2B1H_MOUSE, H2B1M_MOUSE, H2B2B_MOUSE	Histone H2B	IAGEASR	32	1.20E-04	352.1921	702.3697	2	0.0037	7	0	Semi-tryptic
H2B1C_MOUSE, H2B1H_MOUSE, H2B1M_MOUSE, H2B2B_MOUSE	Histone H2B	IAGEASR	32	3.20E-04	352.192	702.3695	2	0.0035	7	0	Semi-tryptic
H2B1C_MOUSE, H2B1H_MOUSE, H2B1M_MOUSE, H2B2B_MOUSE	Histone H2B	IAGEASR	32	1.10E-04	352.192	702.3694	2	0.0034	7	0	Semi-tryptic
H2B1C_MOUSE, H2B1H_MOUSE, H2B1M_MOUSE, H2B2B_MOUSE	Histone H2B	IAGEASR	32	8.20E-04	352.1918	702.3691	2	0.0031	7	0	Semi-tryptic
H2B1C_MOUSE, H2B1H_MOUSE, H2B1M_MOUSE, H2B2B_MOUSE	Histone H2B	IAGEASR	32	2.90E-04	352.1916	702.3687	2	0.0027	7	0	Semi-tryptic
H2B1C_MOUSE, H2B1H_MOUSE, H2B1M_MOUSE, H2B2B_MOUSE	Histone H2B	IAGEASR	32	4.30E-05	352.1916	702.3687	2	0.0027	7	0	Semi-tryptic
H2B1C_MOUSE, H2B1H_MOUSE, H2B1M_MOUSE, H2B2B_MOUSE	Histone H2B	IAGEASR	32	2.40E-04	352.1914	702.3683	2	0.0023	7	0	Semi-tryptic
H2B1C_MOUSE, H2B1H_MOUSE, H2B1M_MOUSE, H2B2B_MOUSE	Histone H2B	IAGEASR	32	4.50E-03	352.1911	702.3676	2	0.0016	7	0	Semi-tryptic
H2B1C_MOUSE, H2B1H_MOUSE, H2B1M_MOUSE, H2B2B_MOUSE	Histone H2B	IAGEASR	32	1.10E-04	352.191	702.3675	2	0.0015	7	0	Semi-tryptic
H2B1C_MOUSE, H2B1H_MOUSE, H2B1M_MOUSE, H2B2B_MOUSE	Histone H2B	IAGEASR	32	1.10E-04	352.191	702.3674	2	0.0014	7	0	Semi-tryptic
H2B1C_MOUSE, H2B1H_MOUSE, H2B1M_MOUSE, H2B2B_MOUSE	Histone H2B	IAGEASR	32	3.60E-03	352.1909	702.3673	2	0.0013	7	0	Semi-tryptic
H2B1C_MOUSE, H2B1H_MOUSE, H2B1M_MOUSE, H2B2B_MOUSE	Histone H2B	IAGEASR	32	1.90E-03	352.1909	702.3672	2	0.0012	7	0	Semi-tryptic
H2B1C_MOUSE, H2B1H_MOUSE, H2B1M_MOUSE, H2B2B_MOUSE	Histone H2B	IAGEASR	32	1.80E-03	352.1909	702.3673	2	0.0012	7	0	Semi-tryptic
H2B1C_MOUSE, H2B1H_MOUSE, H2B1M_MOUSE, H2B2B_MOUSE	Histone H2B	IAGEASR	32	3.10E-04	352.1909	702.3672	2	0.0012	7	0	Semi-tryptic
H2B1C_MOUSE, H2B1H_MOUSE, H2B1M_MOUSE, H2B2B_MOUSE	Histone H2B	IAGEASR	32	2.20E-05	352.1909	702.3672	2	0.0012	7	0	Semi-tryptic
H2B1C_MOUSE, H2B1H_MOUSE, H2B1M_MOUSE, H2B2B_MOUSE	Histone H2B	IAGEASR	32	7.40E-03	352.1909	702.3671	2	0.0011	7	0	Semi-tryptic
H2B1C_MOUSE, H2B1H_MOUSE, H2B1M_MOUSE, H2B2B_MOUSE	Histone H2B	IAGEASR	32	1.10E-04	352.1908	702.3671	2	0.0011	7	0	Semi-tryptic
H2B1C_MOUSE, H2B1H_MOUSE, H2B1M_MOUSE, H2B2B_MOUSE	Histone H2B	IAGEASR	32	1.00E-04	352.1909	702.3672	2	0.0011	7	0	Semi-tryptic
H2B1C_MOUSE, H2B1H_MOUSE, H2B1M_MOUSE, H2B2B_MOUSE	Histone H2B	IAGEASR	32	8.20E-04	352.1908	702.3670	2	0.0010	7	0	Semi-tryptic
H2B1C_MOUSE, H2B1H_MOUSE, H2B1M_MOUSE, H2B2B_MOUSE	Histone H2B	IAGEASR	32	2.50E-04	352.1907	702.3669	2	0.0009	7	0	Semi-tryptic
H2B1C_MOUSE, H2B1H_MOUSE, H2B1M_MOUSE, H2B2B_MOUSE	Histone H2B	IAGEASR	32	1.00E-04	352.1906	702.3667	2	0.0007	7	0	Semi-tryptic
H2B1C_MOUSE, H2B1H_MOUSE, H2B1M_MOUSE, H2B2B_MOUSE	Histone H2B	IAGEASR	32	1.00E-04	352.1906	702.3667	2	0.0006	7	0	Semi-tryptic
H2B1C_MOUSE, H2B1H_MOUSE, H2B1M_MOUSE, H2B2B_MOUSE	Histone H2B	IAGEASR	32	4.30E-03	352.1905	702.3665	2	0.0005	7	0	Semi-tryptic
H2B1C_MOUSE, H2B1H_MOUSE, H2B1M_MOUSE, H2B2B_MOUSE	Histone H2B	IAGEASR	32	1.20E-03	352.1905	702.3665	2	0.0004	7	0	Semi-tryptic
H2B1C_MOUSE, H2B1H_MOUSE, H2B1M_MOUSE, H2B2B_MOUSE	Histone H2B	IAGEASR	32	5.00E-04	352.1905	702.3664	2	0.0004	7	0	Semi-tryptic
H2B1C_MOUSE, H2B1H_MOUSE, H2B1M_MOUSE, H2B2B_MOUSE	Histone H2B	IAGEASR	32	1.00E-04	352.1904	702.3663	2	0.0003	7	0	Semi-tryptic
H2B1C_MOUSE, H2B1H_MOUSE, H2B1M_MOUSE, H2B2B_MOUSE	Histone H2B	IAGEASR	32	2.20E-04	352.1902	702.3658	2	-0.0002	7	0	Semi-tryptic
H2B1K_MOUSE, H2B2E_MOUSE	Histone H2B	HAVSEGTKAVTKYTSAK	7	1.50E-04	445.2415	1776.9369	4	-0.0001	17	2	Semi-tryptic
H2B1K_MOUSE, H2B2E_MOUSE	Histone H2B	HAVSEGTKAVTKYTSAK	7	7.50E-05	593.3196	1776.9369	3	-0.0001	17	2	Semi-tryptic
H2B1K_MOUSE, H2B2E_MOUSE	Histone H2B	HAVSEGTKAVTKYTSAK	7	3.30E-05	593.3195	1776.9367	3	-0.0003	17	2	Semi-tryptic
H2B1K_MOUSE, H2B2E_MOUSE	Histone H2B	HAVSEGTKAVTKYTSAK	7	2.40E-07	593.3195	1776.9367	3	-0.0003	17	2	Semi-tryptic
H2B1K_MOUSE, H2B2E_MOUSE	Histone H2B	HAVSEGTKAVTKYTSAK	7	4.50E-05	593.3191	1776.9354	3	-0.0016	17	2	Semi-tryptic
H2B1K_MOUSE, H2B2E_MOUSE	Histone H2B	HAVSEGTKAVTKYTSAK	7	1.70E-03	445.241	1776.9348	4	-0.0022	17	2	Semi-tryptic
H2B1K_MOUSE, H2B2E_MOUSE	Histone H2B	HAVSEGTKAVTKYTSAK	7	5.00E-03	445.2408	1776.9343	4	-0.0027	17	2	Semi-tryptic
H2B1K_MOUSE, H2B2E_MOUSE	Histone H2B	GTKAVTKYTSAK	60	8.80E-03	314.4333	1253.7043	4	0.0063	12	0	GluC
H2B1K_MOUSE, H2B2E_MOUSE	Histone H2B	GTKAVTKYTSAK	60	7.90E-03	418.9084	1253.7033	3	0.0054	12	0	GluC
H2B1K_MOUSE, H2B2E_MOUSE	Histone H2B	GTKAVTKYTSAK	60	2.00E-03	627.8588	1253.7030	2	0.0051	12	0	GluC
H2B1K_MOUSE, H2B2E_MOUSE	Histone H2B	GTKAVTKYTSAK	60	7.60E-03	627.8587	1253.7028	2	0.0049	12	0	GluC
H2B1K_MOUSE, H2B2E_MOUSE	Histone H2B	GTKAVTKYTSAK	60	7.50E-04	627.8586	1253.7025	2	0.0046	12	0	GluC
H2B1K_MOUSE, H2B2E_MOUSE	Histone H2B	GTKAVTKYTSAK	60	2.50E-10	418.908	1253.7021	3	0.0042	12	0	GluC
H2B1K_MOUSE, H2B2E_MOUSE	Histone H2B	GTKAVTKYTSAK	60	9.90E-06	418.9079	1253.7019	3	0.0040	12	0	GluC
H2B1K_MOUSE, H2B2E_MOUSE	Histone H2B	GTKAVTKYTSAK	60	9.40E-09	314.4327	1253.7019	4	0.0040	12	0	GluC
H2B1K_MOUSE, H2B2E_MOUSE	Histone H2B	GTKAVTKYTSAK	60	1.70E-05	627.8582	1253.7017	2	0.0038	12	0	GluC
H2B1K_MOUSE, H2B2E_MOUSE	Histone H2B	GTKAVTKYTSAK	60	1.20E-06	418.9078	1253.7015	3	0.0036	12	0	GluC
H2B1K_MOUSE, H2B2E_MOUSE	Histone H2B	GTKAVTKYTSAK	60	7.10E-04	314.4326	1253.7013	4	0.0034	12	0	GluC
H2B1K_MOUSE, H2B2E_MOUSE	Histone H2B	GTKAVTKYTSAK	60	5.10E-08	418.9077	1253.7013	3	0.0034	12	0	GluC
H2B1K_MOUSE, H2B2E_MOUSE	Histone H2B	GTKAVTKYTSAK	60	3.80E-03	418.9076	1253.7010	3	0.0030	12	0	GluC
H2B1K_MOUSE, H2B2E_MOUSE	Histone H2B	GTKAVTKYTSAK	60	1.90E-04	418.9076	1253.7009	3	0.0030	12	0	GluC
H2B1K_MOUSE, H2B2E_MOUSE	Histone H2B	GTKAVTKYTSAK	60	8.50E-05	418.9076	1253.7010	3	0.0030	12	0	GluC
H2B1K_MOUSE, H2B2E_MOUSE	Histone H2B	GTKAVTKYTSAK	60	5.20E-08	418.9076	1253.7009	3	0.0030	12	0	GluC
H2B1K_MOUSE, H2B2E_MOUSE	Histone H2B	GTKAVTKYTSAK	60	6.30E-03	627.8577	1253.7009	2	0.0029	12	0	GluC
H2B1K_MOUSE, H2B2E_MOUSE	Histone H2B	GTKAVTKYTSAK	60	6.20E-04	627.8577	1253.7009	2	0.0029	12	0	GluC
H2B1K_MOUSE, H2B2E_MOUSE	Histone H2B	GTKAVTKYTSAK	60	7.00E-09	418.9075	1253.7008	3	0.0029	12	0	GluC
H2B1K_MOUSE, H2B2E_MOUSE	Histone H2B	GTKAVTKYTSAK	60	8.90E-08	314.4325	1253.7008	4	0.0028	12	0	GluC
H2B1K_MOUSE, H2B2E_MOUSE	Histone H2B	GTKAVTKYTSAK	60	2.00E-03	627.8576	1253.7006	2	0.0027	12	0	GluC
H2B1K_MOUSE, H2B2E_MOUSE	Histone H2B	GTKAVTKYTSAK	60	6.00E-04	418.9074	1253.7003	3	0.0024	12	0	GluC
H2B1K_MOUSE, H2B2E_MOUSE	Histone H2B	GTKAVTKYTSAK	60	6.70E-08	418.9074	1253.7003	3	0.0024	12	0	GluC
H2B1K_MOUSE, H2B2E_MOUSE	Histone H2B	GTKAVTKYTSAK	60	1.50E-03	418.9074	1253.7003	3	0.0023	12	0	GluC
H2B1K_MOUSE, H2B2E_MOUSE	Histone H2B	GTKAVTKYTSAK	60	1.40E-04	627.8573	1253.7000	2	0.0021	12	0	GluC
H2B1K_MOUSE, H2B2E_MOUSE	Histone H2B	GTKAVTKYTSAK	60	4.60E-05	627.8573	1253.7000	2	0.0021	12	0	GluC
H2B1K_MOUSE, H2B2E_MOUSE	Histone H2B	GTKAVTKYTSAK	60	1.40E-06	418.9072	1253.6999	3	0.0019	12	0	GluC
H2B1K_MOUSE, H2B2E_MOUSE	Histone H2B	GTKAVTKYTSAK	60	5.20E-03	418.907	1253.6991	3	0.0012	12	0	GluC
H2B1K_MOUSE, H2B2E_MOUSE	Histone H2B	GTKAVTKYTSAK	60	2.50E-03	418.907	1253.6992	3	0.0012	12	0	GluC
H2B1K_MOUSE, H2B2E_MOUSE	Histone H2B	GTKAVTKYTSAK	60	1.80E-03	627.8569	1253.6991	2	0.0012	12	0	GluC
H2B1K_MOUSE, H2B2E_MOUSE	Histone H2B	GTKAVTKYTSAK	60	6.00E-06	627.8569	1253.6991	2	0.0012	12	0	GluC
H2B1K_MOUSE, H2B2E_MOUSE	Histone H2B	GTKAVTKYTSAK	60	2.50E-03	418.907	1253.6991	3	0.0011	12	0	GluC

H2B1K_MOUSE, H2B2E_MOUSE	Histone H2B	GTKAVTKYTSAK	60	1.30E-03	418.907	1253.6991	3	0.0011	12	0	GluC
H2B1K_MOUSE, H2B2E_MOUSE	Histone H2B	GTKAVTKYTSAK	60	1.80E-04	314.432	1253.6990	4	0.0011	12	0	GluC
H2B1K_MOUSE, H2B2E_MOUSE	Histone H2B	GTKAVTKYTSAK	60	2.70E-08	418.907	1253.6991	3	0.0011	12	0	GluC
H2B1K_MOUSE, H2B2E_MOUSE	Histone H2B	GTKAVTKYTSAK	60	9.10E-06	418.9069	1253.6990	3	0.0010	12	0	GluC
H2B1K_MOUSE, H2B2E_MOUSE	Histone H2B	GTKAVTKYTSAK	60	6.80E-08	418.9069	1253.6990	3	0.0010	12	0	GluC
H2B1K_MOUSE, H2B2E_MOUSE	Histone H2B	GTKAVTKYTSAK	60	4.20E-07	314.432	1253.6988	4	0.0009	12	0	GluC
H2B1K_MOUSE, H2B2E_MOUSE	Histone H2B	GTKAVTKYTSAK	60	8.30E-03	627.8566	1253.6987	2	0.0008	12	0	GluC
H2B1K_MOUSE, H2B2E_MOUSE	Histone H2B	GTKAVTKYTSAK	60	2.40E-05	418.9069	1253.6987	3	0.0008	12	0	GluC
H2B1K_MOUSE, H2B2E_MOUSE	Histone H2B	GTKAVTKYTSAK	60	7.90E-04	418.9068	1253.6987	3	0.0007	12	0	GluC
H2B1K_MOUSE, H2B2E_MOUSE	Histone H2B	GTKAVTKYTSAK	60	1.90E-04	314.4319	1253.6987	4	0.0007	12	0	GluC
H2B1K_MOUSE, H2B2E_MOUSE	Histone H2B	GTKAVTKYTSAK	60	4.20E-05	418.9068	1253.6986	3	0.0007	12	0	GluC
H2B1K_MOUSE, H2B2E_MOUSE	Histone H2B	GTKAVTKYTSAK	60	2.00E-09	418.9068	1253.6987	3	0.0007	12	0	GluC
H2B1K_MOUSE, H2B2E_MOUSE	Histone H2B	GTKAVTKYTSAK	60	2.30E-04	418.9068	1253.6985	3	0.0006	12	0	GluC
H2B1K_MOUSE, H2B2E_MOUSE	Histone H2B	GTKAVTKYTSAK	60	7.70E-04	627.8565	1253.6984	2	0.0005	12	0	GluC
H2B1K_MOUSE, H2B2E_MOUSE	Histone H2B	GTKAVTKYTSAK	60	5.40E-05	627.8565	1253.6984	2	0.0005	12	0	GluC
H2B1K_MOUSE, H2B2E_MOUSE	Histone H2B	GTKAVTKYTSAK	60	3.30E-05	314.4319	1253.6984	4	0.0005	12	0	GluC
H2B1K_MOUSE, H2B2E_MOUSE	Histone H2B	GTKAVTKYTSAK	60	1.80E-05	627.8564	1253.6982	2	0.0003	12	0	GluC
H2B1K_MOUSE, H2B2E_MOUSE	Histone H2B	GTKAVTKYTSAK	60	2.40E-04	418.9066	1253.6979	3	0.0000	12	0	GluC
H2B1K_MOUSE, H2B2E_MOUSE	Histone H2B	GTKAVTKYTSAK	60	3.00E-08	418.9066	1253.6979	3	0.0000	12	0	GluC
H2B1K_MOUSE, H2B2E_MOUSE	Histone H2B	GTKAVTKYTSAK	60	8.50E-09	314.4318	1253.6979	4	0.0000	12	0	GluC
H2B1K_MOUSE, H2B2E_MOUSE	Histone H2B	GTKAVTKYTSAK	60	3.10E-03	418.9065	1253.6978	3	-0.0001	12	0	GluC
H2B1K_MOUSE, H2B2E_MOUSE	Histone H2B	GTKAVTKYTSAK	60	1.80E-06	418.9066	1253.6979	3	-0.0001	12	0	GluC
H2B1K_MOUSE, H2B2E_MOUSE	Histone H2B	GTKAVTKYTSAK	60	9.80E-05	627.8561	1253.6977	2	-0.0002	12	0	GluC
H2B1K_MOUSE, H2B2E_MOUSE	Histone H2B	GTKAVTKYTSAK	60	3.30E-07	418.9065	1253.6977	3	-0.0002	12	0	GluC
H2B1K_MOUSE, H2B2E_MOUSE	Histone H2B	GTKAVTKYTSAK	60	2.30E-04	418.9065	1253.6977	3	-0.0003	12	0	GluC
H2B1K_MOUSE, H2B2E_MOUSE	Histone H2B	GTKAVTKYTSAK	60	5.30E-07	418.9065	1253.6977	3	-0.0003	12	0	GluC
H2B1K_MOUSE, H2B2E_MOUSE	Histone H2B	GTKAVTKYTSAK	60	9.20E-06	418.9063	1253.6970	3	-0.0010	12	0	GluC
H2B1K_MOUSE, H2B2E_MOUSE	Histone H2B	GTKAVTKYTSAK	60	2.30E-05	418.9061	1253.6964	3	-0.0016	12	0	GluC
H2B1K_MOUSE, H2B2E_MOUSE	Histone H2B	AVTKYTSAK	3	2.80E-04	484.7745	967.5345	2	0.0007	9	1	Semi-tryptic
H2B1K_MOUSE, H2B2E_MOUSE	Histone H2B	AVTKYTSAK	3	9.60E-05	484.7744	967.5343	2	0.0005	9	1	Semi-tryptic
H2B1K_MOUSE, H2B2E_MOUSE	Histone H2B	AVTKYTSAK	3	9.30E-03	484.7742	967.5338	2	-0.0001	9	1	Semi-tryptic
H2B1M_MOUSE	Histone H2B	PEPTKSAPAPKKGSKKAVTKAQKKD	1	3.40E-03	524.908	2619.5034	5	-0.0038	25	0	GluC
H2B1M_MOUSE	Histone H2B	PEPTKSAPAPK	2	1.90E-03	374.8769	1121.6088	3	0.0008	11	1	Semi-tryptic
H2B1M_MOUSE	Histone H2B	PEPTKSAPAPK	2	7.60E-06	561.8116	1121.6087	2	0.0006	11	1	Semi-tryptic
H2B2B_MOUSE	Histone H2B	PDPAKSAPAPKKGSKKAVTKVQKKDGKKRKRSRKE	10	4.20E-06	772.4695	3857.3110	5	0.0196	35	1	GluC
H2B2B_MOUSE	Histone H2B	PDPAKSAPAPKKGSKKAVTKVQKKDGKKRKRSRKE	10	5.80E-04	643.8919	3857.3080	6	0.0165	35	1	GluC
H2B2B_MOUSE	Histone H2B	PDPAKSAPAPKKGSKKAVTKVQKKDGKKRKRSRKE	10	3.30E-03	552.0501	3857.2995	7	0.0080	35	1	GluC
H2B2B_MOUSE	Histone H2B	PDPAKSAPAPKKGSKKAVTKVQKKDGKKRKRSRKE	10	5.00E-05	772.4664	3857.2956	5	0.0042	35	1	GluC
H2B2B_MOUSE	Histone H2B	PDPAKSAPAPKKGSKKAVTKVQKKDGKKRKRSRKE	10	7.20E-04	772.4662	3857.2948	5	0.0033	35	1	GluC
H2B2B_MOUSE	Histone H2B	PDPAKSAPAPKKGSKKAVTKVQKKDGKKRKRSRKE	10	7.80E-04	552.0494	3857.2945	7	0.0031	35	1	GluC
H2B2B_MOUSE	Histone H2B	PDPAKSAPAPKKGSKKAVTKVQKKDGKKRKRSRKE	10	2.20E-03	772.4661	3857.2942	5	0.0028	35	1	GluC
H2B2B_MOUSE	Histone H2B	PDPAKSAPAPKKGSKKAVTKVQKKDGKKRKRSRKE	10	1.40E-03	643.8895	3857.2933	6	0.0019	35	1	GluC
H2B2B_MOUSE	Histone H2B	PDPAKSAPAPKKGSKKAVTKVQKKDGKKRKRSRKE	10	6.40E-03	643.8894	3857.2928	6	0.0014	35	1	GluC
H2B2B_MOUSE	Histone H2B	PDPAKSAPAPKKGSKKAVTKVQKKDGKKRKRSRKE	10	2.40E-04	552.0487	3857.2902	7	-0.0013	35	1	GluC
H2B2B_MOUSE	Histone H2B	PDPAKSAPAPKKGSKKAVTKVQKKD	15	3.30E-07	651.8893	2603.5282	4	0.0160	25	0	GluC
H2B2B_MOUSE	Histone H2B	PDPAKSAPAPKKGSKKAVTKVQKKD	15	4.70E-03	868.8492	2603.5258	3	0.0136	25	0	GluC
H2B2B_MOUSE	Histone H2B	PDPAKSAPAPKKGSKKAVTKVQKKD	15	1.40E-06	434.9281	2603.5251	6	0.0129	25	0	GluC
H2B2B_MOUSE	Histone H2B	PDPAKSAPAPKKGSKKAVTKVQKKD	15	4.30E-05	651.8872	2603.5196	4	0.0074	25	0	GluC
H2B2B_MOUSE	Histone H2B	PDPAKSAPAPKKGSKKAVTKVQKKD	15	1.90E-03	521.7109	2603.5181	5	0.0059	25	0	GluC
H2B2B_MOUSE	Histone H2B	PDPAKSAPAPKKGSKKAVTKVQKKD	15	1.20E-04	651.8866	2603.5171	4	0.0049	25	0	GluC
H2B2B_MOUSE	Histone H2B	PDPAKSAPAPKKGSKKAVTKVQKKD	15	3.70E-08	651.8864	2603.5163	4	0.0041	25	0	GluC
H2B2B_MOUSE	Histone H2B	PDPAKSAPAPKKGSKKAVTKVQKKD	15	3.10E-07	521.7104	2603.5158	5	0.0036	25	0	GluC
H2B2B_MOUSE	Histone H2B	PDPAKSAPAPKKGSKKAVTKVQKKD	15	1.20E-08	651.886	2603.5148	4	0.0026	25	0	GluC
H2B2B_MOUSE	Histone H2B	PDPAKSAPAPKKGSKKAVTKVQKKD	15	3.70E-06	651.8859	2603.5144	4	0.0022	25	0	GluC
H2B2B_MOUSE	Histone H2B	PDPAKSAPAPKKGSKKAVTKVQKKD	15	1.10E-03	521.7098	2603.5125	5	0.0002	25	0	GluC
H2B2B_MOUSE	Histone H2B	PDPAKSAPAPKKGSKKAVTKVQKKD	15	6.20E-06	651.8854	2603.5124	4	0.0002	25	0	GluC
H2B2B_MOUSE	Histone H2B	PDPAKSAPAPKKGSKKAVTKVQKKD	15	3.30E-04	521.7097	2603.5120	5	-0.0002	25	0	GluC
H2B2B_MOUSE	Histone H2B	PDPAKSAPAPKKGSKKAVTKVQKKD	15	1.70E-04	434.9258	2603.5111	6	-0.0011	25	0	GluC
H2B2B_MOUSE	Histone H2B	PDPAKSAPAPKKGSKKAVTKVQKKD	15	3.30E-09	651.8849	2603.5104	4	-0.0018	25	0	GluC
H2B2B_MOUSE	Histone H2B	PDPAKSAPAPKKGSKKAVTKVQKK	2	8.10E-03	830.5089	2488.5048	3	0.0196	24	1	AspN
H2B2B_MOUSE	Histone H2B	PDPAKSAPAPKKGSKKAVTKVQKK	2	2.80E-07	623.1319	2488.4985	4	0.0133	24	1	AspN
H2B2B_MOUSE	Histone H2B	PDPAKSAPAPK	30	1.50E-03	360.2027	1077.5862	3	0.0044	11	1	Semi-tryptic
H2B2B_MOUSE	Histone H2B	PDPAKSAPAPK	30	9.20E-06	539.8003	1077.5860	2	0.0042	11	1	Semi-tryptic
H2B2B_MOUSE	Histone H2B	PDPAKSAPAPK	30	2.60E-04	360.2026	1077.5859	3	0.0040	11	1	Semi-tryptic
H2B2B_MOUSE	Histone H2B	PDPAKSAPAPK	30	2.20E-05	539.8	1077.5855	2	0.0036	11	1	Semi-tryptic
H2B2B_MOUSE	Histone H2B	PDPAKSAPAPK	30	4.20E-03	360.202	1077.5842	3	0.0023	11	1	Semi-tryptic
H2B2B_MOUSE	Histone H2B	PDPAKSAPAPK	30	2.00E-03	360.202	1077.5840	3	0.0022	11	1	Semi-tryptic
H2B2B_MOUSE	Histone H2B	PDPAKSAPAPK	30	2.60E-04	360.2018	1077.5837	3	0.0019	11	1	Semi-tryptic
H2B2B_MOUSE	Histone H2B	PDPAKSAPAPK	30	5.00E-03	360.2018	1077.5834	3	0.0016	11	1	Semi-tryptic
H2B2B_MOUSE	Histone H2B	PDPAKSAPAPK	30	2.20E-03	360.2016	1077.5831	3	0.0013	11	1	Semi-tryptic
H2B2B_MOUSE	Histone H2B	PDPAKSAPAPK	30	1.50E-03	539.7986	1077.5827	2	0.0009	11	1	Semi-tryptic
H2B2B_MOUSE	Histone H2B	PDPAKSAPAPK	30	1.40E-03	360.2015	1077.5827	3	0.0009	11	1	Semi-tryptic
H2B2B_MOUSE	Histone H2B	PDPAKSAPAPK	30	1.50E-03	360.2015	1077.5825	3	0.0007	11	1	Semi-tryptic
H2B2B_MOUSE	Histone H2B	PDPAKSAPAPK	30	9.90E-04	360.2015	1077.5826	3	0.0007	11	1	Semi-tryptic

H2B2B_MOUSE	Histone H2B	PDPAKSAPAPK	30	4.70E-04	360.2015	1077.5826	3	0.0007	11	1	Semi-tryptic
H2B2B_MOUSE	Histone H2B	PDPAKSAPAPK	30	2.10E-03	539.7985	1077.5823	2	0.0005	11	1	Semi-tryptic
H2B2B_MOUSE	Histone H2B	PDPAKSAPAPK	30	1.80E-03	360.2014	1077.5824	3	0.0005	11	1	Semi-tryptic
H2B2B_MOUSE	Histone H2B	PDPAKSAPAPK	30	2.40E-04	539.7985	1077.5824	2	0.0005	11	1	Semi-tryptic
H2B2B_MOUSE	Histone H2B	PDPAKSAPAPK	30	9.20E-03	360.2014	1077.5823	3	0.0004	11	1	Semi-tryptic
H2B2B_MOUSE	Histone H2B	PDPAKSAPAPK	30	2.70E-04	539.7983	1077.5821	2	0.0003	11	1	Semi-tryptic
H2B2B_MOUSE	Histone H2B	PDPAKSAPAPK	30	3.40E-03	360.2013	1077.5821	3	0.0002	11	1	Semi-tryptic
H2B2B_MOUSE	Histone H2B	PDPAKSAPAPK	30	9.50E-06	539.7983	1077.5820	2	0.0002	11	1	Semi-tryptic
H2B2B_MOUSE	Histone H2B	PDPAKSAPAPK	30	4.30E-03	360.2012	1077.5819	3	0.0001	11	1	Semi-tryptic
H2B2B_MOUSE	Histone H2B	PDPAKSAPAPK	30	2.30E-03	539.7982	1077.5819	2	0.0001	11	1	Semi-tryptic
H2B2B_MOUSE	Histone H2B	PDPAKSAPAPK	30	1.30E-03	539.7982	1077.5818	2	0.0000	11	1	Semi-tryptic
H2B2B_MOUSE	Histone H2B	PDPAKSAPAPK	30	1.20E-03	360.2012	1077.5818	3	0.0000	11	1	Semi-tryptic
H2B2B_MOUSE	Histone H2B	PDPAKSAPAPK	30	4.90E-03	360.2011	1077.5814	3	-0.0004	11	1	Semi-tryptic
H2B2B_MOUSE	Histone H2B	PDPAKSAPAPK	30	6.80E-03	360.2011	1077.5814	3	-0.0005	11	1	Semi-tryptic
H2B2B_MOUSE	Histone H2B	PDPAKSAPAPK	30	1.20E-03	539.7979	1077.5812	2	-0.0006	11	1	Semi-tryptic
H2B2B_MOUSE	Histone H2B	PDPAKSAPAPK	30	1.40E-04	539.7976	1077.5807	2	-0.0011	11	1	Semi-tryptic
H2B2B_MOUSE	Histone H2B	PDPAKSAPAPK	30	3.20E-05	539.7974	1077.5803	2	-0.0016	11	1	Semi-tryptic
H2B2B_MOUSE	Histone H2B	KAVTKVQKK	5	2.40E-03	343.8977	1028.6713	3	0.0007	9	3	Semi-tryptic
H2B2B_MOUSE	Histone H2B	KAVTKVQKK	5	1.60E-05	515.3427	1028.6708	2	0.0002	9	3	Semi-tryptic
H2B2B_MOUSE	Histone H2B	KAVTKVQKK	5	3.00E-04	515.3426	1028.6706	2	0.0000	9	3	Semi-tryptic
H2B2B_MOUSE	Histone H2B	KAVTKVQKK	5	9.40E-04	515.3424	1028.6702	2	-0.0003	9	3	Semi-tryptic
H2B2B_MOUSE	Histone H2B	KAVTKVQKK	5	4.30E-04	515.3422	1028.6699	2	-0.0007	9	3	Semi-tryptic
H2B2E_MOUSE	Histone H2B	VNDIFERIANEASRL	2	8.10E-03	582.976	1745.9061	3	0.0001	15	1	Chymotrypsin
H2B2E_MOUSE	Histone H2B	VNDIFERIANEASRL	2	5.60E-03	582.976	1745.9061	3	0.0001	15	1	Chymotrypsin
H2B2E_MOUSE	Histone H2B	IANEASR	2	2.00E-04	380.7009	759.3872	2	-0.0003	7	0	Semi-tryptic
H2B2E_MOUSE	Histone H2B	IANEASR	2	4.00E-04	380.7007	759.3869	2	-0.0006	7	0	Semi-tryptic
H2B2E_MOUSE, H2B3A_MOUSE, H2B3B_MOUSE	Histone H2B	SYSIYVYKVLKQVHPDTGISSKAMGIMNSFVND	7	1.20E-05	923.7226	3690.8613	4	0.0187	33	1	GluC
H2B2E_MOUSE, H2B3A_MOUSE, H2B3B_MOUSE	Histone H2B	SYSIYVYKVLKQVHPDTGISSKAMGIMNSFVND	7	5.00E-03	739.1766	3690.8465	5	0.0039	33	1	GluC
H2B2E_MOUSE, H2B3A_MOUSE, H2B3B_MOUSE	Histone H2B	SYSIYVYKVLKQVHPDTGISSKAMGIMNSFVND	7	5.90E-04	1231.2891	3690.8455	3	0.0029	33	1	GluC
H2B2E_MOUSE, H2B3A_MOUSE, H2B3B_MOUSE	Histone H2B	SYSIYVYKVLKQVHPDTGISSKAMGIMNSFVND	7	7.00E-04	923.7183	3690.8441	4	0.0015	33	1	GluC
H2B2E_MOUSE, H2B3A_MOUSE, H2B3B_MOUSE	Histone H2B	SYSIYVYKVLKQVHPDTGISSKAMGIMNSFVND	7	8.90E-04	1231.2886	3690.8440	3	0.0014	33	1	GluC
H2B2E_MOUSE, H2B3A_MOUSE, H2B3B_MOUSE	Histone H2B	SYSIYVYKVLKQVHPDTGISSKAMGIMNSFVND	7	1.60E-04	739.1755	3690.8413	5	-0.0013	33	1	GluC
H2B2E_MOUSE, H2B3A_MOUSE, H2B3B_MOUSE	Histone H2B	SYSIYVYKVLKQVHPDTGISSKAMGIMNSFVND	7	4.80E-04	923.7175	3690.8408	4	-0.0018	33	1	GluC
H2B2E_MOUSE, H2B3A_MOUSE, H2B3B_MOUSE	Histone H2B	KESYSIYVYKVLKQVHPDTGISSK	3	2.90E-06	693.1273	2768.4800	4	0.0052	24	3	Semi-tryptic
H2B2E_MOUSE, H2B3A_MOUSE, H2B3B_MOUSE	Histone H2B	KESYSIYVYKVLKQVHPDTGISSK	3	7.00E-08	693.1273	2768.4800	4	0.0052	24	3	Semi-tryptic
H2B2E_MOUSE, H2B3A_MOUSE, H2B3B_MOUSE	Histone H2B	KESYSIYVYKVLKQVHPDTGISSK	3	4.10E-05	693.1266	2768.4773	4	0.0025	24	3	Semi-tryptic
H2B2E_MOUSE, H2B3A_MOUSE, H2B3B_MOUSE	Histone H2B	ESYSIYVYKVLKQVHPDTGISSK	4	2.50E-05	661.1042	2640.3879	4	0.0080	23	2	Semi-tryptic
H2B2E_MOUSE, H2B3A_MOUSE, H2B3B_MOUSE	Histone H2B	ESYSIYVYKVLKQVHPDTGISSK	4	4.00E-09	661.1042	2640.3879	4	0.0080	23	2	Semi-tryptic
H2B2E_MOUSE, H2B3A_MOUSE, H2B3B_MOUSE	Histone H2B	ESYSIYVYKVLKQVHPDTGISSK	4	2.90E-09	881.1362	2640.3867	3	0.0068	23	2	Semi-tryptic
H2B2E_MOUSE, H2B3A_MOUSE, H2B3B_MOUSE	Histone H2B	ESYSIYVYKVLKQVHPDTGISSK	4	6.80E-07	881.1345	2640.3818	3	0.0019	23	2	Semi-tryptic
H2B2E_MOUSE, H2B3A_MOUSE, H2B3B_MOUSE	Histone H2B	SYSIYVYKVLKQVHPD	23	1.30E-06	647.02	1938.0383	3	0.0132	16	0	GluC
H2B2E_MOUSE, H2B3A_MOUSE, H2B3B_MOUSE	Histone H2B	SYSIYVYKVLKQVHPD	23	5.50E-04	647.0196	1938.0371	3	0.0120	16	0	GluC
H2B2E_MOUSE, H2B3A_MOUSE, H2B3B_MOUSE	Histone H2B	SYSIYVYKVLKQVHPD	23	3.90E-05	485.5164	1938.0365	4	0.0114	16	0	GluC
H2B2E_MOUSE, H2B3A_MOUSE, H2B3B_MOUSE	Histone H2B	SYSIYVYKVLKQVHPD	23	3.00E-04	647.0191	1938.0356	3	0.0105	16	0	GluC
H2B2E_MOUSE, H2B3A_MOUSE, H2B3B_MOUSE	Histone H2B	SYSIYVYKVLKQVHPD	23	8.80E-05	970.0246	1938.0347	2	0.0096	16	0	GluC
H2B2E_MOUSE, H2B3A_MOUSE, H2B3B_MOUSE	Histone H2B	SYSIYVYKVLKQVHPD	23	1.20E-06	647.0186	1938.0339	3	0.0088	16	0	GluC
H2B2E_MOUSE, H2B3A_MOUSE, H2B3B_MOUSE	Histone H2B	SYSIYVYKVLKQVHPD	23	2.20E-08	647.0181	1938.0324	3	0.0073	16	0	GluC
H2B2E_MOUSE, H2B3A_MOUSE, H2B3B_MOUSE	Histone H2B	SYSIYVYKVLKQVHPD	23	4.40E-03	647.018	1938.0323	3	0.0072	16	0	GluC
H2B2E_MOUSE, H2B3A_MOUSE, H2B3B_MOUSE	Histone H2B	SYSIYVYKVLKQVHPD	23	1.30E-07	485.5152	1938.0319	4	0.0068	16	0	GluC
H2B2E_MOUSE, H2B3A_MOUSE, H2B3B_MOUSE	Histone H2B	SYSIYVYKVLKQVHPD	23	1.30E-03	647.0172	1938.0296	3	0.0045	16	0	GluC
H2B2E_MOUSE, H2B3A_MOUSE, H2B3B_MOUSE	Histone H2B	SYSIYVYKVLKQVHPD	23	1.20E-06	647.0172	1938.0296	3	0.0045	16	0	GluC
H2B2E_MOUSE, H2B3A_MOUSE, H2B3B_MOUSE	Histone H2B	SYSIYVYKVLKQVHPD	23	3.30E-04	970.0217	1938.0287	2	0.0037	16	0	GluC
H2B2E_MOUSE, H2B3A_MOUSE, H2B3B_MOUSE	Histone H2B	SYSIYVYKVLKQVHPD	23	9.70E-04	970.0213	1938.0281	2	0.0030	16	0	GluC
H2B2E_MOUSE, H2B3A_MOUSE, H2B3B_MOUSE	Histone H2B	SYSIYVYKVLKQVHPD	23	1.60E-03	647.0163	1938.0270	3	0.0019	16	0	GluC
H2B2E_MOUSE, H2B3A_MOUSE, H2B3B_MOUSE	Histone H2B	SYSIYVYKVLKQVHPD	23	6.60E-03	647.0161	1938.0266	3	0.0015	16	0	GluC
H2B2E_MOUSE, H2B3A_MOUSE, H2B3B_MOUSE	Histone H2B	SYSIYVYKVLKQVHPD	23	7.00E-06	647.0161	1938.0266	3	0.0015	16	0	GluC
H2B2E_MOUSE, H2B3A_MOUSE, H2B3B_MOUSE	Histone H2B	SYSIYVYKVLKQVHPD	23	2.60E-03	485.5138	1938.0259	4	0.0008	16	0	GluC
H2B2E_MOUSE, H2B3A_MOUSE, H2B3B_MOUSE	Histone H2B	SYSIYVYKVLKQVHPD	23	1.40E-03	647.0158	1938.0256	3	0.0005	16	0	GluC
H2B2E_MOUSE, H2B3A_MOUSE, H2B3B_MOUSE	Histone H2B	SYSIYVYKVLKQVHPD	23	4.80E-05	647.0158	1938.0256	3	0.0005	16	0	GluC
H2B2E_MOUSE, H2B3A_MOUSE, H2B3B_MOUSE	Histone H2B	SYSIYVYKVLKQVHPD	23	1.20E-04	647.0157	1938.0253	3	0.0002	16	0	GluC
H2B2E_MOUSE, H2B3A_MOUSE, H2B3B_MOUSE	Histone H2B	SYSIYVYKVLKQVHPD	23	1.00E-02	647.0153	1938.0242	3	-0.0009	16	0	GluC
H2B2E_MOUSE, H2B3A_MOUSE, H2B3B_MOUSE	Histone H2B	SYSIYVYKVLKQVHPD	23	4.40E-05	485.5132	1938.0238	4	-0.0013	16	0	GluC
H2B2E_MOUSE, H2B3A_MOUSE, H2B3B_MOUSE	Histone H2B	SYSIYVYKVLKQVHPD	23	5.40E-07	485.5131	1938.0235	4	-0.0016	16	0	GluC
H2B2E_MOUSE, H2B3A_MOUSE, H2B3B_MOUSE	Histone H2B	KESYSIYVYKVLK	5	3.80E-08	810.457	1618.8995	2	0.0025	13	2	Semi-tryptic
H2B2E_MOUSE, H2B3A_MOUSE, H2B3B_MOUSE	Histone H2B	KESYSIYVYKVLK	5	2.50E-06	540.6396	1618.8971	3	0.0001	13	2	Semi-tryptic
H2B2E_MOUSE, H2B3A_MOUSE, H2B3B_MOUSE	Histone H2B	KESYSIYVYKVLK	5	1.30E-07	540.6396	1618.8971	3	0.0001	13	2	Semi-tryptic
H2B2E_MOUSE, H2B3A_MOUSE, H2B3B_MOUSE	Histone H2B	KESYSIYVYKVLK	5	1.00E-05	540.6388	1618.8946	3	-0.0024	13	2	Semi-tryptic
H2B2E_MOUSE, H2B3A_MOUSE, H2B3B_MOUSE	Histone H2B	KESYSIYVYKVLK	5	1.00E-03	540.6375	1618.8908	3	-0.0062	13	2	Semi-tryptic
H2B2E_MOUSE, H2B3A_MOUSE, H2B3B_MOUSE	Histone H2B	KESYSIYVYK	4	1.80E-05	640.3326	1278.6506	2	0.0010	10	1	Semi-tryptic
H2B2E_MOUSE, H2B3A_MOUSE, H2B3B_MOUSE	Histone H2B	KESYSIYVYK	4	1.50E-06	640.3324	1278.6502	2	0.0007	10	1	Semi-tryptic
H2B2E_MOUSE, H2B3A_MOUSE, H2B3B_MOUSE	Histone H2B	KESYSIYVYK	4	6.60E-06	640.3322	1278.6498	2	0.0003	10	1	Semi-tryptic
H2B2E_MOUSE, H2B3A_MOUSE, H2B3B_MOUSE	Histone H2B	KESYSIYVYK	4	9.30E-04	640.3319	1278.6492	2	-0.0004	10	1	Semi-tryptic
H2B3A_MOUSE	Histone H2B	PEPSRSTPAPKKGSKKAITKAQKKD	5	1.80E-07	670.3911	2677.5352	4	0.0114	25	0	GluC
H2B3A_MOUSE	Histone H2B	PEPSRSTPAPKKGSKKAITKAQKKD	5	4.70E-06	536.513	2677.5286	5	0.0047	25	0	GluC

H2B3A_MOUSE	Histone H2B	PEPSRSTPAPKKGSKKAITKAQKKD	5	4.80E-07	670.3879	2677.5226	4	-0.0013	25	0	GluC
H2B3A_MOUSE	Histone H2B	PEPSRSTPAPKKGSKKAITKAQKKD	5	1.20E-10	670.3878	2677.5221	4	-0.0018	25	0	GluC
H2B3A_MOUSE	Histone H2B	PEPSRSTPAPKKGSKKAITKAQKKD	5	3.10E-06	536.5116	2677.5216	5	-0.0022	25	0	GluC
H2B3A_MOUSE	Histone H2B	PEPSRSTPAPKKGSKKAITKAQKK	5	3.20E-03	855.1789	2562.5150	3	0.0181	24	0	AspN
H2B3A_MOUSE	Histone H2B	PEPSRSTPAPKKGSKKAITKAQKK	5	1.50E-07	428.0921	2562.5091	6	0.0122	24	0	AspN
H2B3A_MOUSE	Histone H2B	PEPSRSTPAPKKGSKKAITKAQKK	5	1.10E-08	513.5089	2562.5079	5	0.0110	24	0	AspN
H2B3A_MOUSE	Histone H2B	PEPSRSTPAPKKGSKKAITKAQKK	5	6.60E-05	513.5065	2562.4959	5	-0.0010	24	0	AspN
H2B3A_MOUSE	Histone H2B	PEPSRSTPAPKKGSKKAITKAQKK	5	3.80E-05	641.6313	2562.4959	4	-0.0010	24	0	AspN
H2B3A_MOUSE, H2B3B_MOUSE	Histone H2B	ASRLAHYNKRSTITSREVQTAVRLLLPGELAKHAVSE	5	7.00E-03	821.2618	4101.2727	5	0.0168	37	2	GluC
H2B3A_MOUSE, H2B3B_MOUSE	Histone H2B	ASRLAHYNKRSTITSREVQTAVRLLLPGELAKHAVSE	5	5.40E-07	821.2614	4101.2708	5	0.0149	37	2	GluC
H2B3A_MOUSE, H2B3B_MOUSE	Histone H2B	ASRLAHYNKRSTITSREVQTAVRLLLPGELAKHAVSE	5	2.60E-06	684.5522	4101.2695	6	0.0136	37	2	GluC
H2B3A_MOUSE, H2B3B_MOUSE	Histone H2B	ASRLAHYNKRSTITSREVQTAVRLLLPGELAKHAVSE	5	2.80E-03	684.5502	4101.2575	6	0.0016	37	2	GluC
H2B3A_MOUSE, H2B3B_MOUSE	Histone H2B	ASRLAHYNKRSTITSREVQTAVRLLLPGELAKHAVSE	5	4.80E-05	821.2582	4101.2546	5	-0.0013	37	2	GluC
H2B3A_MOUSE, H2B3B_MOUSE	Histone H2B	VQTAVRLLLPGELAKHAVSEGTKAVTKYTSSK	5	3.00E-03	677.3906	3381.9165	5	0.0181	32	2	GluC
H2B3A_MOUSE, H2B3B_MOUSE	Histone H2B	VQTAVRLLLPGELAKHAVSEGTKAVTKYTSSK	5	1.80E-03	846.4864	3381.9163	4	0.0180	32	2	GluC
H2B3A_MOUSE, H2B3B_MOUSE	Histone H2B	VQTAVRLLLPGELAKHAVSEGTKAVTKYTSSK	5	4.60E-03	677.3898	3381.9128	5	0.0144	32	2	GluC
H2B3A_MOUSE, H2B3B_MOUSE	Histone H2B	VQTAVRLLLPGELAKHAVSEGTKAVTKYTSSK	5	8.20E-03	846.4846	3381.9092	4	0.0109	32	2	GluC
H2B3A_MOUSE, H2B3B_MOUSE	Histone H2B	VQTAVRLLLPGELAKHAVSEGTKAVTKYTSSK	5	1.50E-03	677.3871	3381.8993	5	0.0010	32	2	GluC
H2B3A_MOUSE, H2B3B_MOUSE	Histone H2B	ASRLAHYNKRSTITSREVQTAVRLLLPGE	4	4.50E-07	817.4621	3265.8192	4	0.0185	29	1	GluC
H2B3A_MOUSE, H2B3B_MOUSE	Histone H2B	ASRLAHYNKRSTITSREVQTAVRLLLPGE	4	3.30E-04	654.1705	3265.8163	5	0.0156	29	1	GluC
H2B3A_MOUSE, H2B3B_MOUSE	Histone H2B	ASRLAHYNKRSTITSREVQTAVRLLLPGE	4	4.00E-07	817.4612	3265.8159	4	0.0152	29	1	GluC
H2B3A_MOUSE, H2B3B_MOUSE	Histone H2B	ASRLAHYNKRSTITSREVQTAVRLLLPGE	4	3.00E-04	817.4596	3265.8094	4	0.0087	29	1	GluC
H2B3A_MOUSE, H2B3B_MOUSE	Histone H2B	GKKRKRGRKESYSIYVYKVLKQVHPD	2	2.30E-07	633.3688	3161.8078	5	0.0141	26	1	GluC
H2B3A_MOUSE, H2B3B_MOUSE	Histone H2B	GKKRKRGRKESYSIYVYKVLKQVHPD	2	2.10E-04	633.3688	3161.8074	5	0.0137	26	1	GluC
H2B3A_MOUSE, H2B3B_MOUSE	Histone H2B	DGKKRKRGRKESYSIYVYKVLKQVHP	5	3.40E-09	633.3667	3161.7972	5	0.0034	26	0	AspN
H2B3A_MOUSE, H2B3B_MOUSE	Histone H2B	DGKKRKRGRKESYSIYVYKVLKQVHP	5	3.00E-08	527.9733	3161.7964	6	0.0026	26	0	AspN
H2B3A_MOUSE, H2B3B_MOUSE	Histone H2B	DGKKRKRGRKESYSIYVYKVLKQVHP	5	1.10E-06	791.456	3161.7950	4	0.0013	26	0	AspN
H2B3A_MOUSE, H2B3B_MOUSE	Histone H2B	DGKKRKRGRKESYSIYVYKVLKQVHP	5	8.80E-03	1054.9388	3161.7946	3	0.0008	26	0	AspN
H2B3A_MOUSE, H2B3B_MOUSE	Histone H2B	DGKKRKRGRKESYSIYVYKVLKQVHP	5	8.70E-04	527.9729	3161.7940	6	0.0002	26	0	AspN
H2B3A_MOUSE, H2B3B_MOUSE	Histone H2B	STITSREVQTAVRLLLPGELAK	8	1.40E-05	596.35	2381.3710	4	0.0068	22	2	Semi-tryptic
H2B3A_MOUSE, H2B3B_MOUSE	Histone H2B	STITSREVQTAVRLLLPGELAK	8	1.40E-07	596.35	2381.3710	4	0.0068	22	2	Semi-tryptic
H2B3A_MOUSE, H2B3B_MOUSE	Histone H2B	STITSREVQTAVRLLLPGELAK	8	3.20E-04	794.7966	2381.3679	3	0.0037	22	2	Semi-tryptic
H2B3A_MOUSE, H2B3B_MOUSE	Histone H2B	STITSREVQTAVRLLLPGELAK	8	7.10E-05	794.7965	2381.3675	3	0.0033	22	2	Semi-tryptic
H2B3A_MOUSE, H2B3B_MOUSE	Histone H2B	STITSREVQTAVRLLLPGELAK	8	3.20E-05	794.7965	2381.3675	3	0.0033	22	2	Semi-tryptic
H2B3A_MOUSE, H2B3B_MOUSE	Histone H2B	STITSREVQTAVRLLLPGELAK	8	1.40E-03	794.7964	2381.3674	3	0.0032	22	2	Semi-tryptic
H2B3A_MOUSE, H2B3B_MOUSE	Histone H2B	STITSREVQTAVRLLLPGELAK	8	9.00E-06	794.7957	2381.3654	3	0.0012	22	2	Semi-tryptic
H2B3A_MOUSE, H2B3B_MOUSE	Histone H2B	STITSREVQTAVRLLLPGELAK	8	5.30E-04	794.7957	2381.3652	3	0.0010	22	2	Semi-tryptic
H2B3A_MOUSE, H2B3B_MOUSE	Histone H2B	VQTAVRLLLPGELAKHAVSE	6	5.20E-05	711.0824	2130.2252	3	0.0092	20	1	GluC
H2B3A_MOUSE, H2B3B_MOUSE	Histone H2B	VQTAVRLLLPGELAKHAVSE	6	7.60E-07	711.0823	2130.2251	3	0.0091	20	1	GluC
H2B3A_MOUSE, H2B3B_MOUSE	Histone H2B	VQTAVRLLLPGELAKHAVSE	6	3.10E-04	711.0821	2130.2245	3	0.0085	20	1	GluC
H2B3A_MOUSE, H2B3B_MOUSE	Histone H2B	VQTAVRLLLPGELAKHAVSE	6	2.40E-04	711.0808	2130.2206	3	0.0046	20	1	GluC
H2B3A_MOUSE, H2B3B_MOUSE	Histone H2B	VQTAVRLLLPGELAKHAVSE	6	4.80E-06	711.0808	2130.2206	3	0.0046	20	1	GluC
H2B3A_MOUSE, H2B3B_MOUSE	Histone H2B	VQTAVRLLLPGELAKHAVSE	6	1.90E-03	711.0779	2130.2117	3	-0.0043	20	1	GluC
H2B3A_MOUSE, H2B3B_MOUSE	Histone H2B	EVQTAVRLLLPGELAK	16	5.40E-03	869.0194	1736.0243	2	0.0047	16	1	Semi-tryptic
H2B3A_MOUSE, H2B3B_MOUSE	Histone H2B	EVQTAVRLLLPGELAK	16	9.80E-04	869.0191	1736.0237	2	0.0041	16	1	Semi-tryptic
H2B3A_MOUSE, H2B3B_MOUSE	Histone H2B	EVQTAVRLLLPGELAK	16	8.70E-07	869.0184	1736.0221	2	0.0026	16	1	Semi-tryptic
H2B3A_MOUSE, H2B3B_MOUSE	Histone H2B	EVQTAVRLLLPGELAK	16	5.20E-04	579.6812	1736.0216	3	0.0020	16	1	Semi-tryptic
H2B3A_MOUSE, H2B3B_MOUSE	Histone H2B	EVQTAVRLLLPGELAK	16	4.40E-03	579.6811	1736.0215	3	0.0019	16	1	Semi-tryptic
H2B3A_MOUSE, H2B3B_MOUSE	Histone H2B	EVQTAVRLLLPGELAK	16	2.30E-07	869.0179	1736.0212	2	0.0017	16	1	Semi-tryptic
H2B3A_MOUSE, H2B3B_MOUSE	Histone H2B	EVQTAVRLLLPGELAK	16	6.00E-04	869.0177	1736.0207	2	0.0012	16	1	Semi-tryptic
H2B3A_MOUSE, H2B3B_MOUSE	Histone H2B	EVQTAVRLLLPGELAK	16	2.40E-06	579.6808	1736.0205	3	0.0009	16	1	Semi-tryptic
H2B3A_MOUSE, H2B3B_MOUSE	Histone H2B	EVQTAVRLLLPGELAK	16	2.10E-06	869.0174	1736.0202	2	0.0006	16	1	Semi-tryptic
H2B3A_MOUSE, H2B3B_MOUSE	Histone H2B	EVQTAVRLLLPGELAK	16	4.20E-07	869.0172	1736.0198	2	0.0003	16	1	Semi-tryptic
H2B3A_MOUSE, H2B3B_MOUSE	Histone H2B	EVQTAVRLLLPGELAK	16	5.80E-06	579.6805	1736.0197	3	0.0001	16	1	Semi-tryptic
H2B3A_MOUSE, H2B3B_MOUSE	Histone H2B	EVQTAVRLLLPGELAK	16	3.20E-06	579.6804	1736.0194	3	-0.0002	16	1	Semi-tryptic
H2B3A_MOUSE, H2B3B_MOUSE	Histone H2B	EVQTAVRLLLPGELAK	16	3.40E-06	579.6803	1736.0192	3	-0.0004	16	1	Semi-tryptic
H2B3A_MOUSE, H2B3B_MOUSE	Histone H2B	EVQTAVRLLLPGELAK	16	2.80E-03	579.6802	1736.0189	3	-0.0007	16	1	Semi-tryptic
H2B3A_MOUSE, H2B3B_MOUSE	Histone H2B	EVQTAVRLLLPGELAK	16	8.20E-05	579.6802	1736.0189	3	-0.0007	16	1	Semi-tryptic
H2B3A_MOUSE, H2B3B_MOUSE	Histone H2B	EVQTAVRLLLPGELAK	16	1.60E-06	579.6802	1736.0189	3	-0.0007	16	1	Semi-tryptic
H2B3A_MOUSE, H2B3B_MOUSE	Histone H2B	GRKESYSIYVYKVLK	4	6.10E-04	459.0124	1832.0205	4	0.0010	15	3	Semi-tryptic
H2B3A_MOUSE, H2B3B_MOUSE	Histone H2B	GRKESYSIYVYKVLK	4	5.00E-06	611.6801	1832.0184	3	-0.0012	15	3	Semi-tryptic
H2B3A_MOUSE, H2B3B_MOUSE	Histone H2B	GRKESYSIYVYKVLK	4	1.20E-04	611.6791	1832.0156	3	-0.0040	15	3	Semi-tryptic
H2B3A_MOUSE, H2B3B_MOUSE	Histone H2B	GRKESYSIYVYKVLK	4	6.60E-04	459.011	1832.0150	4	-0.0046	15	3	Semi-tryptic
H2B3A_MOUSE, H2B3B_MOUSE	Histone H2B	STITSREVQTAVR	18	4.00E-06	724.4011	1446.7875	2	0.0085	13	1	Semi-tryptic
H2B3A_MOUSE, H2B3B_MOUSE	Histone H2B	STITSREVQTAVR	18	2.50E-03	483.269	1446.7851	3	0.0061	13	1	Semi-tryptic
H2B3A_MOUSE, H2B3B_MOUSE	Histone H2B	STITSREVQTAVR	18	1.40E-04	724.3986	1446.7826	2	0.0036	13	1	Semi-tryptic
H2B3A_MOUSE, H2B3B_MOUSE	Histone H2B	STITSREVQTAVR	18	2.00E-06	724.3978	1446.7810	2	0.0020	13	1	Semi-tryptic
H2B3A_MOUSE, H2B3B_MOUSE	Histone H2B	STITSREVQTAVR	18	2.30E-06	724.3977	1446.7808	2	0.0018	13	1	Semi-tryptic
H2B3A_MOUSE, H2B3B_MOUSE	Histone H2B	STITSREVQTAVR	18	7.60E-04	724.3974	1446.7802	2	0.0011	13	1	Semi-tryptic
H2B3A_MOUSE, H2B3B_MOUSE	Histone H2B	STITSREVQTAVR	18	4.90E-03	483.2673	1446.7801	3	0.0010	13	1	Semi-tryptic
H2B3A_MOUSE, H2B3B_MOUSE	Histone H2B	STITSREVQTAVR	18	4.60E-05	724.397	1446.7795	2	0.0004	13	1	Semi-tryptic
H2B3A_MOUSE, H2B3B_MOUSE	Histone H2B	STITSREVQTAVR	18	1.50E-03	483.2671	1446.7794	3	0.0003	13	1	Semi-tryptic
H2B3A_MOUSE, H2B3B_MOUSE	Histone H2B	STITSREVQTAVR	18	2.10E-06	724.3969	1446.7793	2	0.0003	13	1	Semi-tryptic
H2B3A_MOUSE, H2B3B_MOUSE	Histone H2B	STITSREVQTAVR	18	8.10E-04	483.267	1446.7792	3	0.0002	13	1	Semi-tryptic

H2B3A_MOUSE, H2B3B_MOUSE	Histone H2B	STITSREVQTAVR	18	2.10E-03	483.267	1446.7791	3	0.0000	13	1	Semi-tryptic
H2B3A_MOUSE, H2B3B_MOUSE	Histone H2B	STITSREVQTAVR	18	3.20E-05	724.3968	1446.7791	2	0.0000	13	1	Semi-tryptic
H2B3A_MOUSE, H2B3B_MOUSE	Histone H2B	STITSREVQTAVR	18	2.40E-05	483.267	1446.7791	3	0.0000	13	1	Semi-tryptic
H2B3A_MOUSE, H2B3B_MOUSE	Histone H2B	STITSREVQTAVR	18	8.90E-08	483.267	1446.7791	3	0.0000	13	1	Semi-tryptic
H2B3A_MOUSE, H2B3B_MOUSE	Histone H2B	STITSREVQTAVR	18	1.00E-02	724.3967	1446.7788	2	-0.0002	13	1	Semi-tryptic
H2B3A_MOUSE, H2B3B_MOUSE	Histone H2B	STITSREVQTAVR	18	1.70E-03	483.2669	1446.7788	3	-0.0002	13	1	Semi-tryptic
H2B3A_MOUSE, H2B3B_MOUSE	Histone H2B	STITSREVQTAVR	18	7.10E-06	724.3967	1446.7788	2	-0.0002	13	1	Semi-tryptic
H2B3A_MOUSE, H2B3B_MOUSE	Histone H2B	GRKESYSIYVYK	5	1.70E-03	746.8936	1491.7727	2	0.0006	12	2	Semi-tryptic
H2B3A_MOUSE, H2B3B_MOUSE	Histone H2B	GRKESYSIYVYK	5	7.60E-04	498.2644	1491.7713	3	-0.0008	12	2	Semi-tryptic
H2B3A_MOUSE, H2B3B_MOUSE	Histone H2B	GRKESYSIYVYK	5	2.00E-04	498.2642	1491.7709	3	-0.0012	12	2	Semi-tryptic
H2B3A_MOUSE, H2B3B_MOUSE	Histone H2B	GRKESYSIYVYK	5	1.30E-05	498.2642	1491.7709	3	-0.0012	12	2	Semi-tryptic
H2B3A_MOUSE, H2B3B_MOUSE	Histone H2B	VQTAVRLLLPGE	31	2.30E-04	432.593	1294.7573	3	-0.0036	12	0	GluC
H2B3A_MOUSE, H2B3B_MOUSE	Histone H2B	GRKESYSIYVYK	5	5.30E-03	498.263	1491.7673	3	-0.0048	12	2	Semi-tryptic
H2B3A_MOUSE, H2B3B_MOUSE	Histone H2B	VQTAVRLLLPGE	31	8.00E-03	648.3904	1294.7663	2	0.0055	12	0	GluC
H2B3A_MOUSE, H2B3B_MOUSE	Histone H2B	VQTAVRLLLPGE	31	1.30E-03	648.3895	1294.7644	2	0.0035	12	0	GluC
H2B3A_MOUSE, H2B3B_MOUSE	Histone H2B	VQTAVRLLLPGE	31	4.70E-03	648.3892	1294.7639	2	0.0030	12	0	GluC
H2B3A_MOUSE, H2B3B_MOUSE	Histone H2B	VQTAVRLLLPGE	31	6.20E-04	648.3888	1294.7630	2	0.0021	12	0	GluC
H2B3A_MOUSE, H2B3B_MOUSE	Histone H2B	VQTAVRLLLPGE	31	2.00E-04	648.3888	1294.7630	2	0.0021	12	0	GluC
H2B3A_MOUSE, H2B3B_MOUSE	Histone H2B	VQTAVRLLLPGE	31	5.90E-05	648.3885	1294.7624	2	0.0016	12	0	GluC
H2B3A_MOUSE, H2B3B_MOUSE	Histone H2B	VQTAVRLLLPGE	31	1.90E-04	648.3885	1294.7623	2	0.0015	12	0	GluC
H2B3A_MOUSE, H2B3B_MOUSE	Histone H2B	VQTAVRLLLPGE	31	9.60E-03	648.3884	1294.7623	2	0.0014	12	0	GluC
H2B3A_MOUSE, H2B3B_MOUSE	Histone H2B	VQTAVRLLLPGE	31	4.20E-03	648.3884	1294.7623	2	0.0014	12	0	GluC
H2B3A_MOUSE, H2B3B_MOUSE	Histone H2B	VQTAVRLLLPGE	31	1.90E-03	648.3884	1294.7623	2	0.0014	12	0	GluC
H2B3A_MOUSE, H2B3B_MOUSE	Histone H2B	VQTAVRLLLPGE	31	3.50E-04	648.3884	1294.7623	2	0.0014	12	0	GluC
H2B3A_MOUSE, H2B3B_MOUSE	Histone H2B	VQTAVRLLLPGE	31	8.70E-03	648.3883	1294.7621	2	0.0012	12	0	GluC
H2B3A_MOUSE, H2B3B_MOUSE	Histone H2B	VQTAVRLLLPGE	31	2.80E-03	648.3883	1294.7621	2	0.0012	12	0	GluC
H2B3A_MOUSE, H2B3B_MOUSE	Histone H2B	VQTAVRLLLPGE	31	3.30E-05	648.3883	1294.7620	2	0.0011	12	0	GluC
H2B3A_MOUSE, H2B3B_MOUSE	Histone H2B	VQTAVRLLLPGE	31	3.00E-03	648.3882	1294.7619	2	0.0010	12	0	GluC
H2B3A_MOUSE, H2B3B_MOUSE	Histone H2B	VQTAVRLLLPGE	31	7.10E-04	648.3882	1294.7619	2	0.0010	12	0	GluC
H2B3A_MOUSE, H2B3B_MOUSE	Histone H2B	VQTAVRLLLPGE	31	3.50E-04	648.3882	1294.7619	2	0.0010	12	0	GluC
H2B3A_MOUSE, H2B3B_MOUSE	Histone H2B	VQTAVRLLLPGE	31	2.40E-08	648.3882	1294.7619	2	0.0010	12	0	GluC
H2B3A_MOUSE, H2B3B_MOUSE	Histone H2B	VQTAVRLLLPGE	31	9.70E-03	432.5945	1294.7618	3	0.0009	12	0	GluC
H2B3A_MOUSE, H2B3B_MOUSE	Histone H2B	VQTAVRLLLPGE	31	2.00E-07	432.5945	1294.7618	3	0.0009	12	0	GluC
H2B3A_MOUSE, H2B3B_MOUSE	Histone H2B	VQTAVRLLLPGE	31	5.70E-03	648.3879	1294.7613	2	0.0004	12	0	GluC
H2B3A_MOUSE, H2B3B_MOUSE	Histone H2B	VQTAVRLLLPGE	31	1.00E-03	648.3879	1294.7613	2	0.0004	12	0	GluC
H2B3A_MOUSE, H2B3B_MOUSE	Histone H2B	VQTAVRLLLPGE	31	4.00E-03	432.5943	1294.7610	3	0.0001	12	0	GluC
H2B3A_MOUSE, H2B3B_MOUSE	Histone H2B	VQTAVRLLLPGE	31	1.90E-03	432.5941	1294.7604	3	-0.0005	12	0	GluC
H2B3A_MOUSE, H2B3B_MOUSE	Histone H2B	VQTAVRLLLPGE	31	3.20E-04	432.5941	1294.7604	3	-0.0005	12	0	GluC
H2B3A_MOUSE, H2B3B_MOUSE	Histone H2B	VQTAVRLLLPGE	31	3.20E-03	432.594	1294.7602	3	-0.0006	12	0	GluC
H2B3A_MOUSE, H2B3B_MOUSE	Histone H2B	VQTAVRLLLPGE	31	2.90E-04	648.3873	1294.7601	2	-0.0008	12	0	GluC
H2B3A_MOUSE, H2B3B_MOUSE	Histone H2B	VQTAVRLLLPGE	31	4.20E-03	648.3872	1294.7599	2	-0.0009	12	0	GluC
H2B3A_MOUSE, H2B3B_MOUSE	Histone H2B	VQTAVRLLLPGE	31	6.00E-05	432.5938	1294.7597	3	-0.0012	12	0	GluC
H2B3A_MOUSE, H2B3B_MOUSE	Histone H2B	VQTAVRLLLPGE	31	3.00E-04	648.387	1294.7594	2	-0.0015	12	0	GluC
H2B3A_MOUSE, H2B3B_MOUSE	Histone H2B	GKKRKRGRKE	9	1.70E-04	414.9347	1241.7823	3	0.0031	10	0	GluC
H2B3A_MOUSE, H2B3B_MOUSE	Histone H2B	GKKRKRGRKE	9	1.20E-04	414.9347	1241.7822	3	0.0030	10	0	GluC
H2B3A_MOUSE, H2B3B_MOUSE	Histone H2B	GKKRKRGRKE	9	1.30E-03	311.4527	1241.7819	4	0.0026	10	0	GluC
H2B3A_MOUSE, H2B3B_MOUSE	Histone H2B	GKKRKRGRKE	9	6.30E-05	414.9343	1241.7809	3	0.0017	10	0	GluC
H2B3A_MOUSE, H2B3B_MOUSE	Histone H2B	GKKRKRGRKE	9	2.00E-04	311.4523	1241.7803	4	0.0010	10	0	GluC
H2B3A_MOUSE, H2B3B_MOUSE	Histone H2B	GKKRKRGRKE	9	3.90E-04	414.9339	1241.7797	3	0.0005	10	0	GluC
H2B3A_MOUSE, H2B3B_MOUSE	Histone H2B	GKKRKRGRKE	9	1.00E-02	414.9338	1241.7795	3	0.0003	10	0	GluC
H2B3A_MOUSE, H2B3B_MOUSE	Histone H2B	GKKRKRGRKE	9	1.10E-04	414.9336	1241.7789	3	-0.0003	10	0	GluC
H2B3A_MOUSE, H2B3B_MOUSE	Histone H2B	GKKRKRGRKE	9	2.50E-04	414.9334	1241.7785	3	-0.0007	10	0	GluC
H2B3A_MOUSE, H2B3B_MOUSE	Histone H2B	EVQTAVR	2	8.50E-04	401.7242	801.4338	2	-0.0006	7	0	Semi-tryptic
H2B3A_MOUSE, H2B3B_MOUSE	Histone H2B	EVQTAVR	2	7.80E-03	401.7235	801.4324	2	-0.0021	7	0	Semi-tryptic
H2B3B_MOUSE	Histone H2B	PDPSKSAPAPKKGSKKAVTKAQKKD	4	3.10E-05	864.8373	2591.4901	3	0.0143	25	0	GluC
H2B3B_MOUSE	Histone H2B	PDPSKSAPAPKKGSKKAVTKAQKKD	4	1.40E-07	648.8795	2591.4887	4	0.0129	25	0	GluC
H2B3B_MOUSE	Histone H2B	PDPSKSAPAPKKGSKKAVTKAQKKD	4	1.20E-08	519.3043	2591.4854	5	0.0095	25	0	GluC
H2B3B_MOUSE	Histone H2B	PDPSKSAPAPKKGSKKAVTKAQKKD	4	8.90E-03	519.3037	2591.4820	5	0.0062	25	0	GluC
H2B3B_MOUSE	Histone H2B	PDPSKSAPAPK	3	6.20E-03	365.5336	1093.5790	3	0.0022	11	1	Semi-tryptic
H2B3B_MOUSE	Histone H2B	PDPSKSAPAPK	3	3.00E-03	547.7964	1093.5782	2	0.0014	11	1	Semi-tryptic
H2B3B_MOUSE	Histone H2B	PDPSKSAPAPK	3	6.50E-04	547.7959	1093.5772	2	0.0005	11	1	Semi-tryptic
H31_MOUSE, H32_MOUSE	Histone H3	IAQDFKTDLRFQSSAVMALQE	36	9.50E-03	800.08	2397.2181	3	0.0183	21	2	GluC
H31_MOUSE, H32_MOUSE	Histone H3	IAQDFKTDLRFQSSAVMALQE	36	1.10E-03	800.0768	2397.2086	3	0.0088	21	2	GluC
H31_MOUSE, H32_MOUSE	Histone H3	IAQDFKTDLRFQSSAVMALQE	36	6.50E-04	800.0765	2397.2076	3	0.0078	21	2	GluC
H31_MOUSE, H32_MOUSE	Histone H3	IAQDFKTDLRFQSSAVMALQE	36	2.60E-07	1199.6102	2397.2058	2	0.0060	21	2	GluC
H31_MOUSE, H32_MOUSE	Histone H3	IAQDFKTDLRFQSSAVMALQE	36	8.20E-04	800.0757	2397.2054	3	0.0055	21	2	GluC
H31_MOUSE, H32_MOUSE	Histone H3	IAQDFKTDLRFQSSAVMALQE	36	5.50E-04	800.0757	2397.2052	3	0.0053	21	2	GluC
H31_MOUSE, H32_MOUSE	Histone H3	IAQDFKTDLRFQSSAVMALQE	36	3.10E-04	800.0757	2397.2052	3	0.0053	21	2	GluC
H31_MOUSE, H32_MOUSE	Histone H3	IAQDFKTDLRFQSSAVMALQE	36	3.30E-03	800.0752	2397.2037	3	0.0038	21	2	GluC
H31_MOUSE, H32_MOUSE	Histone H3	IAQDFKTDLRFQSSAVMALQE	36	2.70E-04	800.0752	2397.2036	3	0.0038	21	2	GluC
H31_MOUSE, H32_MOUSE	Histone H3	IAQDFKTDLRFQSSAVMALQE	36	1.70E-04	800.0751	2397.2035	3	0.0036	21	2	GluC
H31_MOUSE, H32_MOUSE	Histone H3	IAQDFKTDLRFQSSAVMALQE	36	2.90E-05	1199.609	2397.2034	2	0.0036	21	2	GluC
H31_MOUSE, H32_MOUSE	Histone H3	IAQDFKTDLRFQSSAVMALQE	36	1.30E-05	800.0751	2397.2034	3	0.0036	21	2	GluC
H31_MOUSE, H32_MOUSE	Histone H3	IAQDFKTDLRFQSSAVMALQE	36	1.30E-06	1199.609	2397.2034	2	0.0036	21	2	GluC

H31_MOUSE, H32_MOUSE	Histone H3	IAQDFKTDLRFQSSAVMALQE	36	3.90E-04	800.075	2397.2032	3	0.0034	21	2	GluC
H31_MOUSE, H32_MOUSE	Histone H3	IAQDFKTDLRFQSSAVMALQE	36	4.80E-04	800.075	2397.2032	3	0.0033	21	2	GluC
H31_MOUSE, H32_MOUSE	Histone H3	IAQDFKTDLRFQSSAVMALQE	36	3.00E-04	800.075	2397.2031	3	0.0033	21	2	GluC
H31_MOUSE, H32_MOUSE	Histone H3	IAQDFKTDLRFQSSAVMALQE	36	1.20E-03	800.075	2397.2031	3	0.0032	21	2	GluC
H31_MOUSE, H32_MOUSE	Histone H3	IAQDFKTDLRFQSSAVMALQE	36	1.50E-04	800.075	2397.2031	3	0.0032	21	2	GluC
H31_MOUSE, H32_MOUSE	Histone H3	IAQDFKTDLRFQSSAVMALQE	36	1.50E-03	800.0749	2397.2029	3	0.0031	21	2	GluC
H31_MOUSE, H32_MOUSE	Histone H3	IAQDFKTDLRFQSSAVMALQE	36	2.50E-03	800.0749	2397.2028	3	0.0030	21	2	GluC
H31_MOUSE, H32_MOUSE	Histone H3	IAQDFKTDLRFQSSAVMALQE	36	1.40E-03	800.0748	2397.2027	3	0.0029	21	2	GluC
H31_MOUSE, H32_MOUSE	Histone H3	IAQDFKTDLRFQSSAVMALQE	36	5.60E-05	800.0748	2397.2025	3	0.0027	21	2	GluC
H31_MOUSE, H32_MOUSE	Histone H3	IAQDFKTDLRFQSSAVMALQE	36	1.00E-02	800.0747	2397.2023	3	0.0024	21	2	GluC
H31_MOUSE, H32_MOUSE	Histone H3	IAQDFKTDLRFQSSAVMALQE	36	2.90E-03	800.0747	2397.2022	3	0.0024	21	2	GluC
H31_MOUSE, H32_MOUSE	Histone H3	IAQDFKTDLRFQSSAVMALQE	36	2.60E-04	800.0747	2397.2022	3	0.0024	21	2	GluC
H31_MOUSE, H32_MOUSE	Histone H3	IAQDFKTDLRFQSSAVMALQE	36	1.60E-06	800.0747	2397.2022	3	0.0024	21	2	GluC
H31_MOUSE, H32_MOUSE	Histone H3	IAQDFKTDLRFQSSAVMALQE	36	1.00E-06	800.0747	2397.2021	3	0.0023	21	2	GluC
H31_MOUSE, H32_MOUSE	Histone H3	IAQDFKTDLRFQSSAVMALQE	36	3.60E-03	800.0746	2397.2019	3	0.0020	21	2	GluC
H31_MOUSE, H32_MOUSE	Histone H3	IAQDFKTDLRFQSSAVMALQE	36	9.60E-04	800.0746	2397.2018	3	0.0020	21	2	GluC
H31_MOUSE, H32_MOUSE	Histone H3	IAQDFKTDLRFQSSAVMALQE	36	6.80E-04	800.0746	2397.2018	3	0.0020	21	2	GluC
H31_MOUSE, H32_MOUSE	Histone H3	IAQDFKTDLRFQSSAVMALQE	36	2.10E-04	800.0744	2397.2015	3	0.0017	21	2	GluC
H31_MOUSE, H32_MOUSE	Histone H3	IAQDFKTDLRFQSSAVMALQE	36	2.90E-06	800.0744	2397.2015	3	0.0017	21	2	GluC
H31_MOUSE, H32_MOUSE	Histone H3	IAQDFKTDLRFQSSAVMALQE	36	1.90E-05	800.0744	2397.2015	3	0.0016	21	2	GluC
H31_MOUSE, H32_MOUSE	Histone H3	IAQDFKTDLRFQSSAVMALQE	36	7.20E-05	800.0742	2397.2007	3	0.0009	21	2	GluC
H31_MOUSE, H32_MOUSE	Histone H3	IAQDFKTDLRFQSSAVMALQE	36	7.70E-06	800.0742	2397.2007	3	0.0009	21	2	GluC
H31_MOUSE, H32_MOUSE	Histone H3	IAQDFKTDLRFQSSAVMALQE	36	1.80E-04	800.0739	2397.1999	3	0.0000	21	2	GluC
H31_MOUSE, H32_MOUSE	Histone H3	LRFQSSAVMALQE	33	3.80E-03	740.3817	1478.7487	2	-0.0064	13	0	GluC
H31_MOUSE, H32_MOUSE	Histone H3	LRFQSSAVMALQE	33	3.20E-05	740.3817	1478.7487	2	-0.0064	13	0	GluC
H31_MOUSE, H32_MOUSE	Histone H3	LRFQSSAVMALQE	33	1.70E-05	740.3875	1478.7604	2	0.0052	13	0	GluC
H31_MOUSE, H32_MOUSE	Histone H3	LRFQSSAVMALQE	33	1.00E-07	740.3874	1478.7602	2	0.0050	13	0	GluC
H31_MOUSE, H32_MOUSE	Histone H3	LRFQSSAVMALQE	33	3.60E-05	740.387	1478.7595	2	0.0044	13	0	GluC
H31_MOUSE, H32_MOUSE	Histone H3	LRFQSSAVMALQE	33	2.70E-06	740.3868	1478.7589	2	0.0038	13	0	GluC
H31_MOUSE, H32_MOUSE	Histone H3	LRFQSSAVMALQE	33	6.80E-07	740.3868	1478.7589	2	0.0038	13	0	GluC
H31_MOUSE, H32_MOUSE	Histone H3	LRFQSSAVMALQE	33	2.20E-03	740.3867	1478.7588	2	0.0036	13	0	GluC
H31_MOUSE, H32_MOUSE	Histone H3	LRFQSSAVMALQE	33	3.10E-07	740.3866	1478.7587	2	0.0036	13	0	GluC
H31_MOUSE, H32_MOUSE	Histone H3	LRFQSSAVMALQE	33	6.90E-03	740.3866	1478.7586	2	0.0035	13	0	GluC
H31_MOUSE, H32_MOUSE	Histone H3	LRFQSSAVMALQE	33	3.00E-07	740.3866	1478.7586	2	0.0035	13	0	GluC
H31_MOUSE, H32_MOUSE	Histone H3	LRFQSSAVMALQE	33	2.30E-03	740.3865	1478.7584	2	0.0033	13	0	GluC
H31_MOUSE, H32_MOUSE	Histone H3	LRFQSSAVMALQE	33	1.50E-05	740.3865	1478.7584	2	0.0033	13	0	GluC
H31_MOUSE, H32_MOUSE	Histone H3	LRFQSSAVMALQE	33	6.20E-03	740.3864	1478.7582	2	0.0031	13	0	GluC
H31_MOUSE, H32_MOUSE	Histone H3	LRFQSSAVMALQE	33	5.80E-03	740.3863	1478.7581	2	0.0030	13	0	GluC
H31_MOUSE, H32_MOUSE	Histone H3	LRFQSSAVMALQE	33	1.90E-03	740.3863	1478.7581	2	0.0030	13	0	GluC
H31_MOUSE, H32_MOUSE	Histone H3	LRFQSSAVMALQE	33	5.40E-04	740.3863	1478.7580	2	0.0029	13	0	GluC
H31_MOUSE, H32_MOUSE	Histone H3	LRFQSSAVMALQE	33	2.30E-04	740.3862	1478.7578	2	0.0027	13	0	GluC
H31_MOUSE, H32_MOUSE	Histone H3	LRFQSSAVMALQE	33	8.90E-06	740.3862	1478.7578	2	0.0027	13	0	GluC
H31_MOUSE, H32_MOUSE	Histone H3	LRFQSSAVMALQE	33	3.70E-04	740.3861	1478.7577	2	0.0026	13	0	GluC
H31_MOUSE, H32_MOUSE	Histone H3	LRFQSSAVMALQE	33	1.80E-03	740.3861	1478.7576	2	0.0025	13	0	GluC
H31_MOUSE, H32_MOUSE	Histone H3	LRFQSSAVMALQE	33	5.00E-04	740.386	1478.7574	2	0.0023	13	0	GluC
H31_MOUSE, H32_MOUSE	Histone H3	LRFQSSAVMALQE	33	4.40E-06	740.386	1478.7574	2	0.0023	13	0	GluC
H31_MOUSE, H32_MOUSE	Histone H3	LRFQSSAVMALQE	33	6.20E-07	740.3859	1478.7572	2	0.0021	13	0	GluC
H31_MOUSE, H32_MOUSE	Histone H3	LRFQSSAVMALQE	33	3.00E-06	740.3859	1478.7572	2	0.0020	13	0	GluC
H31_MOUSE, H32_MOUSE	Histone H3	LRFQSSAVMALQE	33	1.20E-07	740.3858	1478.7571	2	0.0020	13	0	GluC
H31_MOUSE, H32_MOUSE	Histone H3	LRFQSSAVMALQE	33	3.60E-06	740.3858	1478.7570	2	0.0019	13	0	GluC
H31_MOUSE, H32_MOUSE	Histone H3	LRFQSSAVMALQE	33	2.10E-05	740.3857	1478.7568	2	0.0017	13	0	GluC
H31_MOUSE, H32_MOUSE	Histone H3	LRFQSSAVMALQE	33	1.30E-03	740.3856	1478.7567	2	0.0016	13	0	GluC
H31_MOUSE, H32_MOUSE	Histone H3	LRFQSSAVMALQE	33	9.00E-06	740.3855	1478.7565	2	0.0014	13	0	GluC
H31_MOUSE, H32_MOUSE	Histone H3	LRFQSSAVMALQE	33	2.40E-04	740.3854	1478.7563	2	0.0012	13	0	GluC
H31_MOUSE, H32_MOUSE	Histone H3	LRFQSSAVMALQE	33	8.70E-07	740.3854	1478.7563	2	0.0012	13	0	GluC
H31_MOUSE, H32_MOUSE	Histone H3	LRFQSSAVMALQE	33	3.00E-04	740.3853	1478.7561	2	0.0010	13	0	GluC
H31_MOUSE, H32_MOUSE, H33_MOUSE	Histone H3	IRRYQKSTELLIRKLPFQRLVRE	2	1.20E-06	589.3594	2941.7606	5	0.0152	23	1	GluC
H31_MOUSE, H32_MOUSE, H33_MOUSE	Histone H3	IRRYQKSTELLIRKLPFQRLVRE	2	3.10E-04	589.3565	2941.7463	5	0.0009	23	1	GluC
H31_MOUSE, H32_MOUSE, H33_MOUSE	Histone H3	DTNLCAIHAKRVTIMPK	2	3.80E-03	956.0253	1910.0360	2	0.0130	17	0	AspN
H31_MOUSE, H32_MOUSE, H33_MOUSE	Histone H3	DTNLCAIHAKRVTIMPK	2	6.90E-06	478.5105	1910.0128	4	-0.0102	17	0	AspN
H31_MOUSE, H32_MOUSE, H33_MOUSE	Histone H3	KPHRYRPGTVALREIR	2	8.00E-04	390.6323	1948.1251	5	0.0020	16	2	Semi-tryptic
H31_MOUSE, H32_MOUSE, H33_MOUSE	Histone H3	YQKSTELLIRKLPFQR	2	6.80E-04	505.7982	2019.1636	4	0.0007	16	3	Semi-tryptic
H31_MOUSE, H32_MOUSE, H33_MOUSE	Histone H3	KPHRYRPGTVALREIR	2	5.40E-04	488.0379	1948.1226	4	-0.0005	16	2	Semi-tryptic
H31_MOUSE, H32_MOUSE, H33_MOUSE	Histone H3	YQKSTELLIRKLPFQR	2	3.00E-05	505.7976	2019.1614	4	-0.0015	16	3	Semi-tryptic
H31_MOUSE, H32_MOUSE, H33_MOUSE	Histone H3	PKDIQLARRIRGERA	116	8.10E-05	356.6146	1778.0368	5	-0.0019	15	0	CnBr
H31_MOUSE, H32_MOUSE, H33_MOUSE	Histone H3	PKDIQLARRIRGERA	116	6.90E-06	445.5165	1778.0368	4	-0.0019	15	0	CnBr
H31_MOUSE, H32_MOUSE, H33_MOUSE	Histone H3	PKDIQLARRIRGERA	116	1.40E-07	593.6862	1778.0368	3	-0.0019	15	0	CnBr
H31_MOUSE, H32_MOUSE, H33_MOUSE	Histone H3	PKDIQLARRIRGERA	116	1.80E-07	593.6862	1778.0367	3	-0.0020	15	0	CnBr
H31_MOUSE, H32_MOUSE, H33_MOUSE	Histone H3	PKDIQLARRIRGERA	116	5.90E-04	356.6146	1778.0366	5	-0.0021	15	0	CnBr
H31_MOUSE, H32_MOUSE, H33_MOUSE	Histone H3	PKDIQLARRIRGERA	116	2.90E-07	593.6861	1778.0366	3	-0.0021	15	0	CnBr
H31_MOUSE, H32_MOUSE, H33_MOUSE	Histone H3	PKDIQLARRIRGERA	116	3.80E-03	356.6145	1778.0363	5	-0.0024	15	0	CnBr
H31_MOUSE, H32_MOUSE, H33_MOUSE	Histone H3	PKDIQLARRIRGERA	116	1.40E-05	445.5163	1778.0363	4	-0.0024	15	0	CnBr
H31_MOUSE, H32_MOUSE, H33_MOUSE	Histone H3	PKDIQLARRIRGERA	116	1.40E-05	593.686	1778.0361	3	-0.0026	15	0	CnBr
H31_MOUSE, H32_MOUSE, H33_MOUSE	Histone H3	PKDIQLARRIRGERA	116	3.80E-06	593.6859	1778.0360	3	-0.0027	15	0	CnBr

H31_MOUSE, H32_MOUSE, H33_MOUSE	Histone H3	PKDIQLARRIRGERA	116	4.30E-04	356.6144	1778.0358	5	-0.0029	15	0	CnBr
H31_MOUSE, H32_MOUSE, H33_MOUSE	Histone H3	PKDIQLARRIRGERA	116	4.30E-07	593.6857	1778.0352	3	-0.0035	15	0	CnBr
H31_MOUSE, H32_MOUSE, H33_MOUSE	Histone H3	PKDIQLARRIRGERA	116	2.50E-08	593.6857	1778.0351	3	-0.0036	15	0	CnBr
H31_MOUSE, H32_MOUSE, H33_MOUSE	Histone H3	PKDIQLARRIRGERA	116	2.40E-03	356.6143	1778.0350	5	-0.0037	15	0	CnBr
H31_MOUSE, H32_MOUSE, H33_MOUSE	Histone H3	PKDIQLARRIRGERA	116	9.50E-03	356.6143	1778.0349	5	-0.0038	15	0	CnBr
H31_MOUSE, H32_MOUSE, H33_MOUSE	Histone H3	PKDIQLARRIRGERA	116	1.20E-04	445.516	1778.0349	4	-0.0038	15	0	CnBr
H31_MOUSE, H32_MOUSE, H33_MOUSE	Histone H3	PKDIQLARRIRGERA	116	5.00E-03	356.6142	1778.0348	5	-0.0039	15	0	CnBr
H31_MOUSE, H32_MOUSE, H33_MOUSE	Histone H3	PKDIQLARRIRGERA	116	5.10E-03	890.0244	1778.0342	2	-0.0045	15	0	CnBr
H31_MOUSE, H32_MOUSE, H33_MOUSE	Histone H3	PKDIQLARRIRGERA	116	3.30E-03	356.6141	1778.0341	5	-0.0046	15	0	CnBr
H31_MOUSE, H32_MOUSE, H33_MOUSE	Histone H3	PKDIQLARRIRGERA	116	3.20E-03	356.614	1778.0335	5	-0.0052	15	0	CnBr
H31_MOUSE, H32_MOUSE, H33_MOUSE	Histone H3	PKDIQLARRIRGERA	116	1.00E-05	445.5156	1778.0334	4	-0.0053	15	0	CnBr
H31_MOUSE, H32_MOUSE, H33_MOUSE	Histone H3	PKDIQLARRIRGERA	116	8.70E-06	593.6849	1778.0329	3	-0.0058	15	0	CnBr
H31_MOUSE, H32_MOUSE, H33_MOUSE	Histone H3	PKDIQLARRIRGERA	116	3.50E-05	445.5154	1778.0323	4	-0.0064	15	0	CnBr
H31_MOUSE, H32_MOUSE, H33_MOUSE	Histone H3	PKDIQLARRIRGERA	116	2.80E-07	593.6846	1778.0320	3	-0.0067	15	0	CnBr
H31_MOUSE, H32_MOUSE, H33_MOUSE	Histone H3	PKDIQLARRIRGERA	116	1.20E-03	356.6136	1778.0314	5	-0.0073	15	0	CnBr
H31_MOUSE, H32_MOUSE, H33_MOUSE	Histone H3	PKDIQLARRIRGERA	116	5.40E-04	356.6135	1778.0314	5	-0.0073	15	0	CnBr
H31_MOUSE, H32_MOUSE, H33_MOUSE	Histone H3	PKDIQLARRIRGERA	116	1.30E-06	593.6841	1778.0306	3	-0.0081	15	0	CnBr
H31_MOUSE, H32_MOUSE, H33_MOUSE	Histone H3	PKDIQLARRIRGERA	116	7.00E-08	593.6839	1778.0299	3	-0.0088	15	0	CnBr
H31_MOUSE, H32_MOUSE, H33_MOUSE	Histone H3	PKDIQLARRIRGERA	116	1.30E-08	593.6885	1778.0436	3	0.0049	15	0	CnBr
H31_MOUSE, H32_MOUSE, H33_MOUSE	Histone H3	PKDIQLARRIRGERA	116	8.90E-08	593.6881	1778.0426	3	0.0039	15	0	CnBr
H31_MOUSE, H32_MOUSE, H33_MOUSE	Histone H3	PKDIQLARRIRGERA	116	1.30E-06	593.6881	1778.0424	3	0.0037	15	0	CnBr
H31_MOUSE, H32_MOUSE, H33_MOUSE	Histone H3	PKDIQLARRIRGERA	116	2.70E-08	593.6876	1778.0411	3	0.0024	15	0	CnBr
H31_MOUSE, H32_MOUSE, H33_MOUSE	Histone H3	PKDIQLARRIRGERA	116	4.10E-03	445.5175	1778.0409	4	0.0022	15	0	CnBr
H31_MOUSE, H32_MOUSE, H33_MOUSE	Histone H3	PKDIQLARRIRGERA	116	8.20E-07	593.6875	1778.0406	3	0.0019	15	0	CnBr
H31_MOUSE, H32_MOUSE, H33_MOUSE	Histone H3	PKDIQLARRIRGERA	116	1.20E-07	593.6874	1778.0405	3	0.0018	15	0	CnBr
H31_MOUSE, H32_MOUSE, H33_MOUSE	Histone H3	PKDIQLARRIRGERA	116	5.30E-07	593.6874	1778.0403	3	0.0017	15	0	CnBr
H31_MOUSE, H32_MOUSE, H33_MOUSE	Histone H3	PKDIQLARRIRGERA	116	3.70E-07	593.6874	1778.0404	3	0.0017	15	0	CnBr
H31_MOUSE, H32_MOUSE, H33_MOUSE	Histone H3	PKDIQLARRIRGERA	116	5.40E-03	890.0274	1778.0403	2	0.0016	15	0	CnBr
H31_MOUSE, H32_MOUSE, H33_MOUSE	Histone H3	PKDIQLARRIRGERA	116	6.40E-04	593.6873	1778.0401	3	0.0015	15	0	CnBr
H31_MOUSE, H32_MOUSE, H33_MOUSE	Histone H3	PKDIQLARRIRGERA	116	1.40E-04	445.5173	1778.0402	4	0.0015	15	0	CnBr
H31_MOUSE, H32_MOUSE, H33_MOUSE	Histone H3	PKDIQLARRIRGERA	116	8.20E-03	593.6873	1778.0400	3	0.0013	15	0	CnBr
H31_MOUSE, H32_MOUSE, H33_MOUSE	Histone H3	PKDIQLARRIRGERA	116	8.00E-03	356.6153	1778.0400	5	0.0013	15	0	CnBr
H31_MOUSE, H32_MOUSE, H33_MOUSE	Histone H3	PKDIQLARRIRGERA	116	2.10E-03	593.6873	1778.0400	3	0.0013	15	0	CnBr
H31_MOUSE, H32_MOUSE, H33_MOUSE	Histone H3	PKDIQLARRIRGERA	116	2.20E-04	593.6872	1778.0398	3	0.0012	15	0	CnBr
H31_MOUSE, H32_MOUSE, H33_MOUSE	Histone H3	PKDIQLARRIRGERA	116	4.80E-05	593.6872	1778.0398	3	0.0011	15	0	CnBr
H31_MOUSE, H32_MOUSE, H33_MOUSE	Histone H3	PKDIQLARRIRGERA	116	2.80E-05	445.5172	1778.0397	4	0.0011	15	0	CnBr
H31_MOUSE, H32_MOUSE, H33_MOUSE	Histone H3	PKDIQLARRIRGERA	116	2.80E-07	593.6872	1778.0398	3	0.0011	15	0	CnBr
H31_MOUSE, H32_MOUSE, H33_MOUSE	Histone H3	PKDIQLARRIRGERA	116	4.30E-03	890.0271	1778.0397	2	0.0010	15	0	CnBr
H31_MOUSE, H32_MOUSE, H33_MOUSE	Histone H3	PKDIQLARRIRGERA	116	4.20E-05	593.6872	1778.0397	3	0.0010	15	0	CnBr
H31_MOUSE, H32_MOUSE, H33_MOUSE	Histone H3	PKDIQLARRIRGERA	116	1.00E-03	593.6871	1778.0395	3	0.0009	15	0	CnBr
H31_MOUSE, H32_MOUSE, H33_MOUSE	Histone H3	PKDIQLARRIRGERA	116	5.00E-07	593.6871	1778.0396	3	0.0009	15	0	CnBr
H31_MOUSE, H32_MOUSE, H33_MOUSE	Histone H3	PKDIQLARRIRGERA	116	8.70E-05	593.6871	1778.0395	3	0.0008	15	0	CnBr
H31_MOUSE, H32_MOUSE, H33_MOUSE	Histone H3	PKDIQLARRIRGERA	116	1.90E-05	593.6871	1778.0395	3	0.0008	15	0	CnBr
H31_MOUSE, H32_MOUSE, H33_MOUSE	Histone H3	PKDIQLARRIRGERA	116	1.50E-05	445.5171	1778.0395	4	0.0008	15	0	CnBr
H31_MOUSE, H32_MOUSE, H33_MOUSE	Histone H3	PKDIQLARRIRGERA	116	1.10E-05	445.5171	1778.0395	4	0.0008	15	0	CnBr
H31_MOUSE, H32_MOUSE, H33_MOUSE	Histone H3	PKDIQLARRIRGERA	116	7.20E-06	593.6871	1778.0395	3	0.0008	15	0	CnBr
H31_MOUSE, H32_MOUSE, H33_MOUSE	Histone H3	PKDIQLARRIRGERA	116	1.20E-06	593.6871	1778.0394	3	0.0008	15	0	CnBr
H31_MOUSE, H32_MOUSE, H33_MOUSE	Histone H3	PKDIQLARRIRGERA	116	1.60E-03	890.027	1778.0394	2	0.0007	15	0	CnBr
H31_MOUSE, H32_MOUSE, H33_MOUSE	Histone H3	PKDIQLARRIRGERA	116	3.40E-06	445.5171	1778.0394	4	0.0007	15	0	CnBr
H31_MOUSE, H32_MOUSE, H33_MOUSE	Histone H3	PKDIQLARRIRGERA	116	8.00E-07	593.6871	1778.0394	3	0.0007	15	0	CnBr
H31_MOUSE, H32_MOUSE, H33_MOUSE	Histone H3	PKDIQLARRIRGERA	116	8.40E-08	593.6871	1778.0394	3	0.0007	15	0	CnBr
H31_MOUSE, H32_MOUSE, H33_MOUSE	Histone H3	PKDIQLARRIRGERA	116	1.50E-08	445.5171	1778.0393	4	0.0007	15	0	CnBr
H31_MOUSE, H32_MOUSE, H33_MOUSE	Histone H3	PKDIQLARRIRGERA	116	4.30E-07	593.687	1778.0393	3	0.0006	15	0	CnBr
H31_MOUSE, H32_MOUSE, H33_MOUSE	Histone H3	PKDIQLARRIRGERA	116	9.10E-03	890.0268	1778.0391	2	0.0005	15	0	CnBr
H31_MOUSE, H32_MOUSE, H33_MOUSE	Histone H3	PKDIQLARRIRGERA	116	8.50E-03	890.0268	1778.0391	2	0.0005	15	0	CnBr
H31_MOUSE, H32_MOUSE, H33_MOUSE	Histone H3	PKDIQLARRIRGERA	116	5.30E-06	445.5171	1778.0392	4	0.0005	15	0	CnBr
H31_MOUSE, H32_MOUSE, H33_MOUSE	Histone H3	PKDIQLARRIRGERA	116	7.70E-04	445.517	1778.0391	4	0.0004	15	0	CnBr
H31_MOUSE, H32_MOUSE, H33_MOUSE	Histone H3	PKDIQLARRIRGERA	116	5.10E-04	593.687	1778.0391	3	0.0004	15	0	CnBr
H31_MOUSE, H32_MOUSE, H33_MOUSE	Histone H3	PKDIQLARRIRGERA	116	2.10E-08	445.517	1778.0391	4	0.0004	15	0	CnBr
H31_MOUSE, H32_MOUSE, H33_MOUSE	Histone H3	PKDIQLARRIRGERA	116	4.50E-05	445.517	1778.0390	4	0.0003	15	0	CnBr
H31_MOUSE, H32_MOUSE, H33_MOUSE	Histone H3	PKDIQLARRIRGERA	116	1.90E-07	593.687	1778.0390	3	0.0003	15	0	CnBr
H31_MOUSE, H32_MOUSE, H33_MOUSE	Histone H3	PKDIQLARRIRGERA	116	4.10E-03	890.0267	1778.0389	2	0.0002	15	0	CnBr
H31_MOUSE, H32_MOUSE, H33_MOUSE	Histone H3	PKDIQLARRIRGERA	116	3.90E-08	593.6869	1778.0389	3	0.0002	15	0	CnBr
H31_MOUSE, H32_MOUSE, H33_MOUSE	Histone H3	PKDIQLARRIRGERA	116	9.70E-03	356.615	1778.0388	5	0.0001	15	0	CnBr
H31_MOUSE, H32_MOUSE, H33_MOUSE	Histone H3	PKDIQLARRIRGERA	116	2.00E-03	445.517	1778.0387	4	0.0001	15	0	CnBr
H31_MOUSE, H32_MOUSE, H33_MOUSE	Histone H3	PKDIQLARRIRGERA	116	1.60E-04	593.6869	1778.0388	3	0.0001	15	0	CnBr
H31_MOUSE, H32_MOUSE, H33_MOUSE	Histone H3	PKDIQLARRIRGERA	116	1.10E-05	593.6869	1778.0388	3	0.0001	15	0	CnBr
H31_MOUSE, H32_MOUSE, H33_MOUSE	Histone H3	PKDIQLARRIRGERA	116	6.50E-07	593.6869	1778.0388	3	0.0001	15	0	CnBr
H31_MOUSE, H32_MOUSE, H33_MOUSE	Histone H3	PKDIQLARRIRGERA	116	7.90E-05	445.5169	1778.0385	4	-0.0001	15	0	CnBr
H31_MOUSE, H32_MOUSE, H33_MOUSE	Histone H3	PKDIQLARRIRGERA	116	1.10E-05	593.6868	1778.0386	3	-0.0001	15	0	CnBr
H31_MOUSE, H32_MOUSE, H33_MOUSE	Histone H3	PKDIQLARRIRGERA	116	6.70E-07	445.5169	1778.0385	4	-0.0001	15	0	CnBr
H31_MOUSE, H32_MOUSE, H33_MOUSE	Histone H3	PKDIQLARRIRGERA	116	6.00E-03	356.615	1778.0384	5	-0.0003	15	0	CnBr
H31_MOUSE, H32_MOUSE, H33_MOUSE	Histone H3	PKDIQLARRIRGERA	116	1.20E-03	356.615	1778.0384	5	-0.0003	15	0	CnBr
H31_MOUSE, H32_MOUSE, H33_MOUSE	Histone H3	PKDIQLARRIRGERA	116	1.10E-06	445.5169	1778.0384	4	-0.0003	15	0	CnBr

H31_MOUSE, H32_MOUSE, H33_MOUSE	Histone H3	PKDIQLARRIRGERA	116	9.10E-08	445.5169	1778.0383	4	-0.0003	15	0	CnBr
H31_MOUSE, H32_MOUSE, H33_MOUSE	Histone H3	PKDIQLARRIRGERA	116	1.30E-08	593.6867	1778.0384	3	-0.0003	15	0	CnBr
H31_MOUSE, H32_MOUSE, H33_MOUSE	Histone H3	PKDIQLARRIRGERA	116	7.10E-06	445.5168	1778.0383	4	-0.0004	15	0	CnBr
H31_MOUSE, H32_MOUSE, H33_MOUSE	Histone H3	PKDIQLARRIRGERA	116	2.70E-08	593.6867	1778.0383	3	-0.0004	15	0	CnBr
H31_MOUSE, H32_MOUSE, H33_MOUSE	Histone H3	PKDIQLARRIRGERA	116	1.60E-04	593.6867	1778.0382	3	-0.0005	15	0	CnBr
H31_MOUSE, H32_MOUSE, H33_MOUSE	Histone H3	PKDIQLARRIRGERA	116	2.00E-04	356.6149	1778.0381	5	-0.0006	15	0	CnBr
H31_MOUSE, H32_MOUSE, H33_MOUSE	Histone H3	PKDIQLARRIRGERA	116	3.30E-05	445.5168	1778.0381	4	-0.0006	15	0	CnBr
H31_MOUSE, H32_MOUSE, H33_MOUSE	Histone H3	PKDIQLARRIRGERA	116	3.60E-06	593.6866	1778.0380	3	-0.0006	15	0	CnBr
H31_MOUSE, H32_MOUSE, H33_MOUSE	Histone H3	PKDIQLARRIRGERA	116	1.50E-03	356.6149	1778.0379	5	-0.0008	15	0	CnBr
H31_MOUSE, H32_MOUSE, H33_MOUSE	Histone H3	PKDIQLARRIRGERA	116	2.90E-05	445.5167	1778.0379	4	-0.0008	15	0	CnBr
H31_MOUSE, H32_MOUSE, H33_MOUSE	Histone H3	PKDIQLARRIRGERA	116	4.00E-04	356.6148	1778.0378	5	-0.0009	15	0	CnBr
H31_MOUSE, H32_MOUSE, H33_MOUSE	Histone H3	PKDIQLARRIRGERA	116	1.80E-05	445.5167	1778.0378	4	-0.0009	15	0	CnBr
H31_MOUSE, H32_MOUSE, H33_MOUSE	Histone H3	PKDIQLARRIRGERA	116	9.20E-07	445.5167	1778.0378	4	-0.0009	15	0	CnBr
H31_MOUSE, H32_MOUSE, H33_MOUSE	Histone H3	PKDIQLARRIRGERA	116	3.40E-07	593.6865	1778.0378	3	-0.0009	15	0	CnBr
H31_MOUSE, H32_MOUSE, H33_MOUSE	Histone H3	PKDIQLARRIRGERA	116	2.50E-05	593.6865	1778.0377	3	-0.0010	15	0	CnBr
H31_MOUSE, H32_MOUSE, H33_MOUSE	Histone H3	PKDIQLARRIRGERA	116	2.20E-05	593.6865	1778.0376	3	-0.0010	15	0	CnBr
H31_MOUSE, H32_MOUSE, H33_MOUSE	Histone H3	PKDIQLARRIRGERA	116	3.80E-06	593.6865	1778.0377	3	-0.0010	15	0	CnBr
H31_MOUSE, H32_MOUSE, H33_MOUSE	Histone H3	PKDIQLARRIRGERA	116	3.70E-06	593.6865	1778.0377	3	-0.0010	15	0	CnBr
H31_MOUSE, H32_MOUSE, H33_MOUSE	Histone H3	PKDIQLARRIRGERA	116	3.10E-06	445.5167	1778.0375	4	-0.0011	15	0	CnBr
H31_MOUSE, H32_MOUSE, H33_MOUSE	Histone H3	PKDIQLARRIRGERA	116	2.10E-03	356.6148	1778.0374	5	-0.0013	15	0	CnBr
H31_MOUSE, H32_MOUSE, H33_MOUSE	Histone H3	PKDIQLARRIRGERA	116	1.90E-06	445.5166	1778.0374	4	-0.0013	15	0	CnBr
H31_MOUSE, H32_MOUSE, H33_MOUSE	Histone H3	PKDIQLARRIRGERA	116	2.50E-07	445.5166	1778.0374	4	-0.0013	15	0	CnBr
H31_MOUSE, H32_MOUSE, H33_MOUSE	Histone H3	PKDIQLARRIRGERA	116	7.90E-03	356.6147	1778.0373	5	-0.0014	15	0	CnBr
H31_MOUSE, H32_MOUSE, H33_MOUSE	Histone H3	PKDIQLARRIRGERA	116	7.10E-06	445.5166	1778.0373	4	-0.0014	15	0	CnBr
H31_MOUSE, H32_MOUSE, H33_MOUSE	Histone H3	PKDIQLARRIRGERA	116	5.10E-06	445.5166	1778.0373	4	-0.0014	15	0	CnBr
H31_MOUSE, H32_MOUSE, H33_MOUSE	Histone H3	PKDIQLARRIRGERA	116	8.00E-03	356.6147	1778.0372	5	-0.0015	15	0	CnBr
H31_MOUSE, H32_MOUSE, H33_MOUSE	Histone H3	PKDIQLARRIRGERA	116	6.70E-03	890.0259	1778.0372	2	-0.0015	15	0	CnBr
H31_MOUSE, H32_MOUSE, H33_MOUSE	Histone H3	PKDIQLARRIRGERA	116	5.40E-05	593.6863	1778.0371	3	-0.0016	15	0	CnBr
H31_MOUSE, H32_MOUSE, H33_MOUSE	Histone H3	PKDIQLARRIRGERA	116	1.00E-05	445.5165	1778.0371	4	-0.0016	15	0	CnBr
H31_MOUSE, H32_MOUSE, H33_MOUSE	Histone H3	PKDIQLARRIRGERA	116	5.20E-03	356.6146	1778.0369	5	-0.0018	15	0	CnBr
H31_MOUSE, H32_MOUSE, H33_MOUSE	Histone H3	PKDIQLARRIRGERA	116	5.10E-07	445.5165	1778.0369	4	-0.0018	15	0	CnBr
H31_MOUSE, H32_MOUSE, H33_MOUSE	Histone H3	PKDIQLARRIRGERA	116	6.30E-04	356.6146	1778.0368	5	-0.0019	15	0	CnBr
H31_MOUSE, H32_MOUSE, H33_MOUSE	Histone H3	EIRRYQKSTELLIR	2	1.80E-06	602.3507	1804.0303	3	-0.0015	14	3	Semi-tryptic
H31_MOUSE, H32_MOUSE, H33_MOUSE	Histone H3	EIRRYQKSTELLIR	2	5.80E-08	602.3507	1804.0303	3	-0.0015	14	3	Semi-tryptic
H31_MOUSE, H32_MOUSE, H33_MOUSE	Histone H3	RVTIMPKDIQLARR	5	1.20E-03	566.3383	1695.9932	3	0.0002	14	3	Semi-tryptic
H31_MOUSE, H32_MOUSE, H33_MOUSE	Histone H3	RVTIMPKDIQLARR	5	8.30E-05	425.0055	1695.9930	4	0.0000	14	3	Semi-tryptic
H31_MOUSE, H32_MOUSE, H33_MOUSE	Histone H3	RVTIMPKDIQLARR	5	4.30E-06	425.0055	1695.9930	4	0.0000	14	3	Semi-tryptic
H31_MOUSE, H32_MOUSE, H33_MOUSE	Histone H3	RVTIMPKDIQLARR	5	8.60E-03	425.0053	1695.9922	4	-0.0008	14	3	Semi-tryptic
H31_MOUSE, H32_MOUSE, H33_MOUSE	Histone H3	RVTIMPKDIQLARR	5	5.70E-03	425.0051	1695.9914	4	-0.0016	14	3	Semi-tryptic
H31_MOUSE, H32_MOUSE, H33_MOUSE	Histone H3	LVREIAQDFKTDLR	13	3.30E-08	852.4746	1702.9346	2	-0.0020	14	2	Semi-tryptic
H31_MOUSE, H32_MOUSE, H33_MOUSE	Histone H3	LLIRKLPFQRLVRE	111	3.50E-05	594.3799	1780.1179	3	-0.0020	14	0	GluC
H31_MOUSE, H32_MOUSE, H33_MOUSE	Histone H3	LLIRKLPFQRLVRE	111	2.90E-03	446.0367	1780.1175	4	-0.0024	14	0	GluC
H31_MOUSE, H32_MOUSE, H33_MOUSE	Histone H3	LVREIAQDFKTDLR	13	5.00E-03	568.652	1702.9341	3	-0.0025	14	2	Semi-tryptic
H31_MOUSE, H32_MOUSE, H33_MOUSE	Histone H3	LLIRKLPFQRLVRE	111	9.90E-03	446.0365	1780.1169	4	-0.0030	14	0	GluC
H31_MOUSE, H32_MOUSE, H33_MOUSE	Histone H3	LLIRKLPFQRLVRE	111	4.90E-04	594.3793	1780.1161	3	-0.0038	14	0	GluC
H31_MOUSE, H32_MOUSE, H33_MOUSE	Histone H3	LLIRKLPFQRLVRE	111	6.70E-05	594.3793	1780.1161	3	-0.0038	14	0	GluC
H31_MOUSE, H32_MOUSE, H33_MOUSE	Histone H3	LVREIAQDFKTDLR	13	2.30E-04	568.6514	1702.9323	3	-0.0043	14	2	Semi-tryptic
H31_MOUSE, H32_MOUSE, H33_MOUSE	Histone H3	LLIRKLPFQRLVRE	111	9.30E-04	594.3784	1780.1133	3	-0.0066	14	0	GluC
H31_MOUSE, H32_MOUSE, H33_MOUSE	Histone H3	LVREIAQDFKTDLR	13	4.70E-07	852.4781	1702.9417	2	0.0051	14	2	Semi-tryptic
H31_MOUSE, H32_MOUSE, H33_MOUSE	Histone H3	LVREIAQDFKTDLR	13	5.60E-04	568.6533	1702.9380	3	0.0014	14	2	Semi-tryptic
H31_MOUSE, H32_MOUSE, H33_MOUSE	Histone H3	LVREIAQDFKTDLR	13	2.20E-05	568.6533	1702.9380	3	0.0014	14	2	Semi-tryptic
H31_MOUSE, H32_MOUSE, H33_MOUSE	Histone H3	LVREIAQDFKTDLR	13	2.40E-03	568.6531	1702.9376	3	0.0010	14	2	Semi-tryptic
H31_MOUSE, H32_MOUSE, H33_MOUSE	Histone H3	LVREIAQDFKTDLR	13	5.60E-04	568.6528	1702.9365	3	-0.0001	14	2	Semi-tryptic
H31_MOUSE, H32_MOUSE, H33_MOUSE	Histone H3	LVREIAQDFKTDLR	13	1.90E-05	568.6528	1702.9365	3	-0.0001	14	2	Semi-tryptic
H31_MOUSE, H32_MOUSE, H33_MOUSE	Histone H3	LVREIAQDFKTDLR	13	2.60E-03	568.6526	1702.9360	3	-0.0006	14	2	Semi-tryptic
H31_MOUSE, H32_MOUSE, H33_MOUSE	Histone H3	LVREIAQDFKTDLR	13	1.60E-03	568.6526	1702.9358	3	-0.0008	14	2	Semi-tryptic
H31_MOUSE, H32_MOUSE, H33_MOUSE	Histone H3	LVREIAQDFKTDLR	13	2.30E-03	568.6524	1702.9355	3	-0.0011	14	2	Semi-tryptic
H31_MOUSE, H32_MOUSE, H33_MOUSE	Histone H3	LVREIAQDFKTDLR	13	4.40E-03	568.6524	1702.9354	3	-0.0012	14	2	Semi-tryptic
H31_MOUSE, H32_MOUSE, H33_MOUSE	Histone H3	LLIRKLPFQRLVRE	111	1.50E-05	594.3851	1780.1336	3	0.0137	14	0	GluC
H31_MOUSE, H32_MOUSE, H33_MOUSE	Histone H3	LLIRKLPFQRLVRE	111	1.50E-06	446.0407	1780.1336	4	0.0137	14	0	GluC
H31_MOUSE, H32_MOUSE, H33_MOUSE	Histone H3	LLIRKLPFQRLVRE	111	2.10E-07	594.3851	1780.1336	3	0.0137	14	0	GluC
H31_MOUSE, H32_MOUSE, H33_MOUSE	Histone H3	LLIRKLPFQRLVRE	111	1.40E-03	594.3851	1780.1334	3	0.0134	14	0	GluC
H31_MOUSE, H32_MOUSE, H33_MOUSE	Histone H3	LLIRKLPFQRLVRE	111	1.40E-06	446.0406	1780.1332	4	0.0133	14	0	GluC
H31_MOUSE, H32_MOUSE, H33_MOUSE	Histone H3	LLIRKLPFQRLVRE	111	1.40E-05	594.3847	1780.1323	3	0.0124	14	0	GluC
H31_MOUSE, H32_MOUSE, H33_MOUSE	Histone H3	LLIRKLPFQRLVRE	111	7.10E-06	594.3847	1780.1323	3	0.0124	14	0	GluC
H31_MOUSE, H32_MOUSE, H33_MOUSE	Histone H3	LLIRKLPFQRLVRE	111	2.70E-05	446.0403	1780.1322	4	0.0123	14	0	GluC
H31_MOUSE, H32_MOUSE, H33_MOUSE	Histone H3	LLIRKLPFQRLVRE	111	7.60E-06	446.0402	1780.1318	4	0.0119	14	0	GluC
H31_MOUSE, H32_MOUSE, H33_MOUSE	Histone H3	LLIRKLPFQRLVRE	111	3.80E-06	594.3845	1780.1316	3	0.0117	14	0	GluC
H31_MOUSE, H32_MOUSE, H33_MOUSE	Histone H3	LLIRKLPFQRLVRE	111	8.30E-08	594.3843	1780.1312	3	0.0113	14	0	GluC
H31_MOUSE, H32_MOUSE, H33_MOUSE	Histone H3	LLIRKLPFQRLVRE	111	3.00E-03	891.0727	1780.1309	2	0.0110	14	0	GluC
H31_MOUSE, H32_MOUSE, H33_MOUSE	Histone H3	LLIRKLPFQRLVRE	111	1.60E-07	594.3842	1780.1307	3	0.0108	14	0	GluC
H31_MOUSE, H32_MOUSE, H33_MOUSE	Histone H3	LLIRKLPFQRLVRE	111	6.00E-03	891.0725	1780.1305	2	0.0106	14	0	GluC
H31_MOUSE, H32_MOUSE, H33_MOUSE	Histone H3	LLIRKLPFQRLVRE	111	1.20E-06	446.0399	1780.1305	4	0.0106	14	0	GluC
H31_MOUSE, H32_MOUSE, H33_MOUSE	Histone H3	LLIRKLPFQRLVRE	111	7.70E-07	446.0399	1780.1303	4	0.0104	14	0	GluC

H31_MOUSE, H32_MOUSE, H33_MOUSE	Histone H3	LLIRKLPFQRLVRE	111	8.30E-05	594.3839	1780.1300	3	0.0101	14	0	GluC
H31_MOUSE, H32_MOUSE, H33_MOUSE	Histone H3	LLIRKLPFQRLVRE	111	5.80E-05	446.0397	1780.1297	4	0.0098	14	0	GluC
H31_MOUSE, H32_MOUSE, H33_MOUSE	Histone H3	LLIRKLPFQRLVRE	111	3.80E-05	594.3839	1780.1298	3	0.0098	14	0	GluC
H31_MOUSE, H32_MOUSE, H33_MOUSE	Histone H3	LLIRKLPFQRLVRE	111	1.30E-04	446.0396	1780.1293	4	0.0094	14	0	GluC
H31_MOUSE, H32_MOUSE, H33_MOUSE	Histone H3	LLIRKLPFQRLVRE	111	6.20E-04	446.0395	1780.1291	4	0.0092	14	0	GluC
H31_MOUSE, H32_MOUSE, H33_MOUSE	Histone H3	LLIRKLPFQRLVRE	111	5.60E-06	594.3836	1780.1291	3	0.0092	14	0	GluC
H31_MOUSE, H32_MOUSE, H33_MOUSE	Histone H3	LLIRKLPFQRLVRE	111	5.90E-06	446.0394	1780.1284	4	0.0085	14	0	GluC
H31_MOUSE, H32_MOUSE, H33_MOUSE	Histone H3	LLIRKLPFQRLVRE	111	3.80E-06	594.3834	1780.1283	3	0.0084	14	0	GluC
H31_MOUSE, H32_MOUSE, H33_MOUSE	Histone H3	LLIRKLPFQRLVRE	111	4.60E-05	594.3833	1780.1282	3	0.0083	14	0	GluC
H31_MOUSE, H32_MOUSE, H33_MOUSE	Histone H3	LLIRKLPFQRLVRE	111	1.20E-05	594.3834	1780.1283	3	0.0083	14	0	GluC
H31_MOUSE, H32_MOUSE, H33_MOUSE	Histone H3	LLIRKLPFQRLVRE	111	2.30E-04	594.3832	1780.1278	3	0.0079	14	0	GluC
H31_MOUSE, H32_MOUSE, H33_MOUSE	Histone H3	LLIRKLPFQRLVRE	111	4.50E-07	446.0392	1780.1278	4	0.0079	14	0	GluC
H31_MOUSE, H32_MOUSE, H33_MOUSE	Histone H3	LLIRKLPFQRLVRE	111	1.50E-07	594.383	1780.1271	3	0.0072	14	0	GluC
H31_MOUSE, H32_MOUSE, H33_MOUSE	Histone H3	LLIRKLPFQRLVRE	111	1.80E-03	446.039	1780.1269	4	0.0070	14	0	GluC
H31_MOUSE, H32_MOUSE, H33_MOUSE	Histone H3	LLIRKLPFQRLVRE	111	2.00E-03	446.039	1780.1268	4	0.0069	14	0	GluC
H31_MOUSE, H32_MOUSE, H33_MOUSE	Histone H3	LLIRKLPFQRLVRE	111	7.20E-06	594.3827	1780.1264	3	0.0065	14	0	GluC
H31_MOUSE, H32_MOUSE, H33_MOUSE	Histone H3	LLIRKLPFQRLVRE	111	6.70E-03	357.0325	1780.1263	5	0.0064	14	0	GluC
H31_MOUSE, H32_MOUSE, H33_MOUSE	Histone H3	LLIRKLPFQRLVRE	111	4.30E-04	594.3827	1780.1263	3	0.0064	14	0	GluC
H31_MOUSE, H32_MOUSE, H33_MOUSE	Histone H3	LLIRKLPFQRLVRE	111	7.00E-05	594.3827	1780.1261	3	0.0062	14	0	GluC
H31_MOUSE, H32_MOUSE, H33_MOUSE	Histone H3	LLIRKLPFQRLVRE	111	2.70E-03	594.3826	1780.1260	3	0.0061	14	0	GluC
H31_MOUSE, H32_MOUSE, H33_MOUSE	Histone H3	LLIRKLPFQRLVRE	111	1.40E-03	594.3826	1780.1259	3	0.0060	14	0	GluC
H31_MOUSE, H32_MOUSE, H33_MOUSE	Histone H3	LLIRKLPFQRLVRE	111	2.20E-04	594.3825	1780.1257	3	0.0058	14	0	GluC
H31_MOUSE, H32_MOUSE, H33_MOUSE	Histone H3	LLIRKLPFQRLVRE	111	3.90E-03	594.3825	1780.1256	3	0.0057	14	0	GluC
H31_MOUSE, H32_MOUSE, H33_MOUSE	Histone H3	LLIRKLPFQRLVRE	111	8.20E-05	594.3824	1780.1254	3	0.0055	14	0	GluC
H31_MOUSE, H32_MOUSE, H33_MOUSE	Histone H3	LLIRKLPFQRLVRE	111	2.90E-03	594.3824	1780.1253	3	0.0054	14	0	GluC
H31_MOUSE, H32_MOUSE, H33_MOUSE	Histone H3	LLIRKLPFQRLVRE	111	1.10E-05	594.3823	1780.1252	3	0.0053	14	0	GluC
H31_MOUSE, H32_MOUSE, H33_MOUSE	Histone H3	LLIRKLPFQRLVRE	111	5.00E-03	446.0385	1780.1249	4	0.0050	14	0	GluC
H31_MOUSE, H32_MOUSE, H33_MOUSE	Histone H3	LLIRKLPFQRLVRE	111	1.50E-03	446.0385	1780.1249	4	0.0050	14	0	GluC
H31_MOUSE, H32_MOUSE, H33_MOUSE	Histone H3	LLIRKLPFQRLVRE	111	1.00E-03	446.0384	1780.1246	4	0.0047	14	0	GluC
H31_MOUSE, H32_MOUSE, H33_MOUSE	Histone H3	LLIRKLPFQRLVRE	111	4.40E-05	446.0384	1780.1245	4	0.0046	14	0	GluC
H31_MOUSE, H32_MOUSE, H33_MOUSE	Histone H3	LLIRKLPFQRLVRE	111	4.90E-03	357.0321	1780.1243	5	0.0044	14	0	GluC
H31_MOUSE, H32_MOUSE, H33_MOUSE	Histone H3	LLIRKLPFQRLVRE	111	1.60E-03	446.0383	1780.1243	4	0.0044	14	0	GluC
H31_MOUSE, H32_MOUSE, H33_MOUSE	Histone H3	LLIRKLPFQRLVRE	111	1.30E-05	594.382	1780.1243	3	0.0044	14	0	GluC
H31_MOUSE, H32_MOUSE, H33_MOUSE	Histone H3	LLIRKLPFQRLVRE	111	6.10E-04	594.382	1780.1241	3	0.0042	14	0	GluC
H31_MOUSE, H32_MOUSE, H33_MOUSE	Histone H3	LLIRKLPFQRLVRE	111	9.20E-06	594.382	1780.1241	3	0.0042	14	0	GluC
H31_MOUSE, H32_MOUSE, H33_MOUSE	Histone H3	LLIRKLPFQRLVRE	111	1.70E-04	594.3819	1780.1239	3	0.0040	14	0	GluC
H31_MOUSE, H32_MOUSE, H33_MOUSE	Histone H3	LLIRKLPFQRLVRE	111	5.90E-05	594.3819	1780.1239	3	0.0040	14	0	GluC
H31_MOUSE, H32_MOUSE, H33_MOUSE	Histone H3	LLIRKLPFQRLVRE	111	2.60E-04	594.3819	1780.1238	3	0.0039	14	0	GluC
H31_MOUSE, H32_MOUSE, H33_MOUSE	Histone H3	LLIRKLPFQRLVRE	111	1.50E-05	594.3818	1780.1237	3	0.0038	14	0	GluC
H31_MOUSE, H32_MOUSE, H33_MOUSE	Histone H3	LLIRKLPFQRLVRE	111	1.60E-05	594.3818	1780.1236	3	0.0037	14	0	GluC
H31_MOUSE, H32_MOUSE, H33_MOUSE	Histone H3	LLIRKLPFQRLVRE	111	4.60E-06	594.3818	1780.1236	3	0.0037	14	0	GluC
H31_MOUSE, H32_MOUSE, H33_MOUSE	Histone H3	LLIRKLPFQRLVRE	111	5.20E-04	594.3817	1780.1233	3	0.0034	14	0	GluC
H31_MOUSE, H32_MOUSE, H33_MOUSE	Histone H3	LLIRKLPFQRLVRE	111	9.40E-03	446.038	1780.1229	4	0.0030	14	0	GluC
H31_MOUSE, H32_MOUSE, H33_MOUSE	Histone H3	LLIRKLPFQRLVRE	111	2.70E-03	446.038	1780.1229	4	0.0030	14	0	GluC
H31_MOUSE, H32_MOUSE, H33_MOUSE	Histone H3	LLIRKLPFQRLVRE	111	5.70E-04	594.3816	1780.1229	3	0.0030	14	0	GluC
H31_MOUSE, H32_MOUSE, H33_MOUSE	Histone H3	LLIRKLPFQRLVRE	111	7.70E-04	594.3815	1780.1227	3	0.0028	14	0	GluC
H31_MOUSE, H32_MOUSE, H33_MOUSE	Histone H3	LLIRKLPFQRLVRE	111	4.50E-03	446.0379	1780.1224	4	0.0025	14	0	GluC
H31_MOUSE, H32_MOUSE, H33_MOUSE	Histone H3	LLIRKLPFQRLVRE	111	6.00E-05	594.3814	1780.1224	3	0.0025	14	0	GluC
H31_MOUSE, H32_MOUSE, H33_MOUSE	Histone H3	LLIRKLPFQRLVRE	111	3.60E-03	446.0378	1780.1221	4	0.0022	14	0	GluC
H31_MOUSE, H32_MOUSE, H33_MOUSE	Histone H3	LLIRKLPFQRLVRE	111	5.00E-04	594.3813	1780.1221	3	0.0022	14	0	GluC
H31_MOUSE, H32_MOUSE, H33_MOUSE	Histone H3	LLIRKLPFQRLVRE	111	4.50E-05	594.3813	1780.1220	3	0.0021	14	0	GluC
H31_MOUSE, H32_MOUSE, H33_MOUSE	Histone H3	LLIRKLPFQRLVRE	111	4.10E-03	446.0377	1780.1219	4	0.0020	14	0	GluC
H31_MOUSE, H32_MOUSE, H33_MOUSE	Histone H3	LLIRKLPFQRLVRE	111	8.50E-04	446.0378	1780.1219	4	0.0020	14	0	GluC
H31_MOUSE, H32_MOUSE, H33_MOUSE	Histone H3	LLIRKLPFQRLVRE	111	2.00E-04	594.3812	1780.1219	3	0.0020	14	0	GluC
H31_MOUSE, H32_MOUSE, H33_MOUSE	Histone H3	LLIRKLPFQRLVRE	111	2.20E-04	446.0377	1780.1217	4	0.0018	14	0	GluC
H31_MOUSE, H32_MOUSE, H33_MOUSE	Histone H3	LLIRKLPFQRLVRE	111	2.00E-04	446.0377	1780.1217	4	0.0018	14	0	GluC
H31_MOUSE, H32_MOUSE, H33_MOUSE	Histone H3	LLIRKLPFQRLVRE	111	2.60E-05	594.3811	1780.1216	3	0.0017	14	0	GluC
H31_MOUSE, H32_MOUSE, H33_MOUSE	Histone H3	LLIRKLPFQRLVRE	111	2.70E-08	594.3811	1780.1215	3	0.0016	14	0	GluC
H31_MOUSE, H32_MOUSE, H33_MOUSE	Histone H3	LLIRKLPFQRLVRE	111	1.70E-08	594.381	1780.1213	3	0.0014	14	0	GluC
H31_MOUSE, H32_MOUSE, H33_MOUSE	Histone H3	LLIRKLPFQRLVRE	111	2.20E-04	594.381	1780.1211	3	0.0012	14	0	GluC
H31_MOUSE, H32_MOUSE, H33_MOUSE	Histone H3	LLIRKLPFQRLVRE	111	4.50E-04	594.3809	1780.1210	3	0.0011	14	0	GluC
H31_MOUSE, H32_MOUSE, H33_MOUSE	Histone H3	LLIRKLPFQRLVRE	111	5.00E-04	594.3809	1780.1208	3	0.0009	14	0	GluC
H31_MOUSE, H32_MOUSE, H33_MOUSE	Histone H3	LLIRKLPFQRLVRE	111	3.50E-08	594.3809	1780.1208	3	0.0009	14	0	GluC
H31_MOUSE, H32_MOUSE, H33_MOUSE	Histone H3	LLIRKLPFQRLVRE	111	1.90E-03	446.0375	1780.1207	4	0.0008	14	0	GluC
H31_MOUSE, H32_MOUSE, H33_MOUSE	Histone H3	LLIRKLPFQRLVRE	111	3.60E-03	446.0374	1780.1205	4	0.0006	14	0	GluC
H31_MOUSE, H32_MOUSE, H33_MOUSE	Histone H3	LLIRKLPFQRLVRE	111	7.50E-04	446.0374	1780.1205	4	0.0006	14	0	GluC
H31_MOUSE, H32_MOUSE, H33_MOUSE	Histone H3	LLIRKLPFQRLVRE	111	8.20E-04	594.3808	1780.1205	3	0.0005	14	0	GluC
H31_MOUSE, H32_MOUSE, H33_MOUSE	Histone H3	LLIRKLPFQRLVRE	111	1.90E-05	594.3807	1780.1203	3	0.0004	14	0	GluC
H31_MOUSE, H32_MOUSE, H33_MOUSE	Histone H3	LLIRKLPFQRLVRE	111	6.60E-03	446.0373	1780.1200	4	0.0001	14	0	GluC
H31_MOUSE, H32_MOUSE, H33_MOUSE	Histone H3	LLIRKLPFQRLVRE	111	7.20E-05	446.0373	1780.1200	4	0.0001	14	0	GluC
H31_MOUSE, H32_MOUSE, H33_MOUSE	Histone H3	LLIRKLPFQRLVRE	111	5.10E-03	594.3806	1780.1199	3	0.0000	14	0	GluC
H31_MOUSE, H32_MOUSE, H33_MOUSE	Histone H3	LLIRKLPFQRLVRE	111	1.30E-03	357.0312	1780.1199	5	0.0000	14	0	GluC
H31_MOUSE, H32_MOUSE, H33_MOUSE	Histone H3	LLIRKLPFQRLVRE	111	8.80E-03	446.0372	1780.1198	4	-0.0001	14	0	GluC
H31_MOUSE, H32_MOUSE, H33_MOUSE	Histone H3	LLIRKLPFQRLVRE	111	5.00E-03	446.0372	1780.1197	4	-0.0002	14	0	GluC

H31_MOUSE, H32_MOUSE, H33_MOUSE	Histone H3	LLIRKLPFQRLVRE	111	2.40E-05	446.0372	1780.1197	4	-0.0002	14	0	GluC
H31_MOUSE, H32_MOUSE, H33_MOUSE	Histone H3	LLIRKLPFQRLVRE	111	8.80E-03	594.3805	1780.1196	3	-0.0003	14	0	GluC
H31_MOUSE, H32_MOUSE, H33_MOUSE	Histone H3	LLIRKLPFQRLVRE	111	1.80E-03	446.0372	1780.1195	4	-0.0004	14	0	GluC
H31_MOUSE, H32_MOUSE, H33_MOUSE	Histone H3	LLIRKLPFQRLVRE	111	6.10E-06	594.3805	1780.1196	3	-0.0004	14	0	GluC
H31_MOUSE, H32_MOUSE, H33_MOUSE	Histone H3	LLIRKLPFQRLVRE	111	3.00E-06	446.0371	1780.1195	4	-0.0004	14	0	GluC
H31_MOUSE, H32_MOUSE, H33_MOUSE	Histone H3	LLIRKLPFQRLVRE	111	2.90E-03	446.0371	1780.1194	4	-0.0005	14	0	GluC
H31_MOUSE, H32_MOUSE, H33_MOUSE	Histone H3	LLIRKLPFQRLVRE	111	7.40E-04	594.3804	1780.1194	3	-0.0005	14	0	GluC
H31_MOUSE, H32_MOUSE, H33_MOUSE	Histone H3	LLIRKLPFQRLVRE	111	1.80E-05	446.0371	1780.1193	4	-0.0006	14	0	GluC
H31_MOUSE, H32_MOUSE, H33_MOUSE	Histone H3	LLIRKLPFQRLVRE	111	3.20E-07	594.3804	1780.1193	3	-0.0006	14	0	GluC
H31_MOUSE, H32_MOUSE, H33_MOUSE	Histone H3	LLIRKLPFQRLVRE	111	7.20E-03	357.031	1780.1189	5	-0.0010	14	0	GluC
H31_MOUSE, H32_MOUSE, H33_MOUSE	Histone H3	LLIRKLPFQRLVRE	111	5.00E-04	594.3802	1780.1188	3	-0.0011	14	0	GluC
H31_MOUSE, H32_MOUSE, H33_MOUSE	Histone H3	LLIRKLPFQRLVRE	111	5.90E-04	594.3802	1780.1187	3	-0.0012	14	0	GluC
H31_MOUSE, H32_MOUSE, H33_MOUSE	Histone H3	LLIRKLPFQRLVRE	111	7.40E-06	446.037	1780.1187	4	-0.0012	14	0	GluC
H31_MOUSE, H32_MOUSE, H33_MOUSE	Histone H3	LLIRKLPFQRLVRE	111	1.00E-03	594.3801	1780.1185	3	-0.0014	14	0	GluC
H31_MOUSE, H32_MOUSE, H33_MOUSE	Histone H3	LLIRKLPFQRLVRE	111	1.60E-03	594.38	1780.1183	3	-0.0016	14	0	GluC
H31_MOUSE, H32_MOUSE, H33_MOUSE	Histone H3	KPHRYRPGTVALR	2	2.90E-03	388.4814	1549.8965	4	0.0012	13	1	Semi-tryptic
H31_MOUSE, H32_MOUSE, H33_MOUSE	Histone H3	KPHRYRPGTVALR	2	6.10E-05	388.4814	1549.8965	4	0.0012	13	1	Semi-tryptic
H31_MOUSE, H32_MOUSE, H33_MOUSE	Histone H3	VTIMPKDIQLARR	2	4.10E-03	514.3049	1539.8929	3	0.0010	13	2	Semi-tryptic
H31_MOUSE, H32_MOUSE, H33_MOUSE	Histone H3	RPGTVALREIRRY	4	4.30E-05	397.4864	1585.9166	4	0.0001	13	1	Chymotrypsin
H31_MOUSE, H32_MOUSE, H33_MOUSE	Histone H3	RPGTVALREIRRY	4	9.60E-03	397.4863	1585.9163	4	-0.0002	13	1	Chymotrypsin
H31_MOUSE, H32_MOUSE, H33_MOUSE	Histone H3	RPGTVALREIRRY	4	1.40E-04	529.646	1585.9162	3	-0.0003	13	1	Chymotrypsin
H31_MOUSE, H32_MOUSE, H33_MOUSE	Histone H3	RPGTVALREIRRY	4	4.50E-04	529.6459	1585.9160	3	-0.0005	13	1	Chymotrypsin
H31_MOUSE, H32_MOUSE, H33_MOUSE	Histone H3	YRPGTVALREIRR	8	8.80E-03	397.4871	1585.9195	4	0.0030	13	2	Semi-tryptic
H31_MOUSE, H32_MOUSE, H33_MOUSE	Histone H3	STELLIRKLPFQR	8	6.20E-03	800.9804	1599.9463	2	0.0003	13	2	Semi-tryptic
H31_MOUSE, H32_MOUSE, H33_MOUSE	Histone H3	STELLIRKLPFQR	8	1.40E-03	534.3227	1599.9462	3	0.0002	13	2	Semi-tryptic
H31_MOUSE, H32_MOUSE, H33_MOUSE	Histone H3	STELLIRKLPFQR	8	3.80E-05	534.3227	1599.9462	3	0.0002	13	2	Semi-tryptic
H31_MOUSE, H32_MOUSE, H33_MOUSE	Histone H3	STELLIRKLPFQR	8	5.70E-04	534.3226	1599.9461	3	0.0000	13	2	Semi-tryptic
H31_MOUSE, H32_MOUSE, H33_MOUSE	Histone H3	STELLIRKLPFQR	8	3.90E-05	534.3226	1599.9459	3	-0.0001	13	2	Semi-tryptic
H31_MOUSE, H32_MOUSE, H33_MOUSE	Histone H3	STELLIRKLPFQR	8	6.50E-04	534.3224	1599.9453	3	-0.0007	13	2	Semi-tryptic
H31_MOUSE, H32_MOUSE, H33_MOUSE	Histone H3	STELLIRKLPFQR	8	2.40E-06	534.3224	1599.9453	3	-0.0007	13	2	Semi-tryptic
H31_MOUSE, H32_MOUSE, H33_MOUSE	Histone H3	YRPGTVALREIRR	8	8.80E-03	529.6458	1585.9156	3	-0.0009	13	2	Semi-tryptic
H31_MOUSE, H32_MOUSE, H33_MOUSE	Histone H3	YRPGTVALREIRR	8	3.80E-04	529.6458	1585.9156	3	-0.0009	13	2	Semi-tryptic
H31_MOUSE, H32_MOUSE, H33_MOUSE	Histone H3	YRPGTVALREIRR	8	7.70E-03	397.4861	1585.9154	4	-0.0011	13	2	Semi-tryptic
H31_MOUSE, H32_MOUSE, H33_MOUSE	Histone H3	YRPGTVALREIRR	8	4.30E-04	397.4861	1585.9153	4	-0.0011	13	2	Semi-tryptic
H31_MOUSE, H32_MOUSE, H33_MOUSE	Histone H3	YRPGTVALREIRR	8	8.90E-05	397.4861	1585.9154	4	-0.0011	13	2	Semi-tryptic
H31_MOUSE, H32_MOUSE, H33_MOUSE	Histone H3	RVTIMPKDIQLAR	10	3.30E-03	770.9539	1539.8932	2	0.0013	13	2	Semi-tryptic
H31_MOUSE, H32_MOUSE, H33_MOUSE	Histone H3	RVTIMPKDIQLAR	10	1.40E-05	514.3048	1539.8926	3	0.0007	13	2	Semi-tryptic
H31_MOUSE, H32_MOUSE, H33_MOUSE	Histone H3	RVTIMPKDIQLAR	10	4.70E-07	514.3048	1539.8926	3	0.0007	13	2	Semi-tryptic
H31_MOUSE, H32_MOUSE, H33_MOUSE	Histone H3	RVTIMPKDIQLAR	10	2.30E-08	514.3048	1539.8926	3	0.0007	13	2	Semi-tryptic
H31_MOUSE, H32_MOUSE, H33_MOUSE	Histone H3	RVTIMPKDIQLAR	10	1.20E-06	514.3047	1539.8922	3	0.0003	13	2	Semi-tryptic
H31_MOUSE, H32_MOUSE, H33_MOUSE	Histone H3	RVTIMPKDIQLAR	10	2.50E-07	514.3045	1539.8918	3	-0.0001	13	2	Semi-tryptic
H31_MOUSE, H32_MOUSE, H33_MOUSE	Histone H3	RVTIMPKDIQLAR	10	9.50E-04	385.9801	1539.8913	4	-0.0006	13	2	Semi-tryptic
H31_MOUSE, H32_MOUSE, H33_MOUSE	Histone H3	RVTIMPKDIQLAR	10	4.30E-07	385.9801	1539.8913	4	-0.0006	13	2	Semi-tryptic
H31_MOUSE, H32_MOUSE, H33_MOUSE	Histone H3	RVTIMPKDIQLAR	10	8.50E-06	514.3043	1539.8912	3	-0.0007	13	2	Semi-tryptic
H31_MOUSE, H32_MOUSE, H33_MOUSE	Histone H3	RVTIMPKDIQLAR	10	4.60E-06	514.3042	1539.8907	3	-0.0012	13	2	Semi-tryptic
H31_MOUSE, H32_MOUSE, H33_MOUSE	Histone H3	DIQLARRIRGERA	70	9.90E-06	389.2294	1552.8885	4	-0.0024	13	0	AspN
H31_MOUSE, H32_MOUSE, H33_MOUSE	Histone H3	YRPGTVALREIRR	8	2.00E-06	529.6452	1585.9138	3	-0.0027	13	2	Semi-tryptic
H31_MOUSE, H32_MOUSE, H33_MOUSE	Histone H3	YRPGTVALREIRR	8	2.80E-07	529.6452	1585.9138	3	-0.0027	13	2	Semi-tryptic
H31_MOUSE, H32_MOUSE, H33_MOUSE	Histone H3	DIQLARRIRGERA	70	9.80E-06	389.2293	1552.8881	4	-0.0028	13	0	AspN
H31_MOUSE, H32_MOUSE, H33_MOUSE	Histone H3	STELLIRKLPFQR	8	5.80E-05	534.3215	1599.9426	3	-0.0034	13	2	Semi-tryptic
H31_MOUSE, H32_MOUSE, H33_MOUSE	Histone H3	VTIMPKDIQLARR	2	5.70E-03	514.3033	1539.8880	3	-0.0039	13	2	Semi-tryptic
H31_MOUSE, H32_MOUSE, H33_MOUSE	Histone H3	DIQLARRIRGERA	70	2.10E-04	389.2285	1552.8847	4	-0.0062	13	0	AspN
H31_MOUSE, H32_MOUSE, H33_MOUSE	Histone H3	DIQLARRIRGERA	70	9.30E-06	518.6354	1552.8845	3	-0.0065	13	0	AspN
H31_MOUSE, H32_MOUSE, H33_MOUSE	Histone H3	DIQLARRIRGERA	70	1.50E-07	518.6353	1552.8840	3	-0.0070	13	0	AspN
H31_MOUSE, H32_MOUSE, H33_MOUSE	Histone H3	DIQLARRIRGERA	70	1.30E-03	777.4585	1552.9025	2	0.0115	13	0	AspN
H31_MOUSE, H32_MOUSE, H33_MOUSE	Histone H3	DIQLARRIRGERA	70	1.50E-06	389.2325	1552.9009	4	0.0100	13	0	AspN
H31_MOUSE, H32_MOUSE, H33_MOUSE	Histone H3	DIQLARRIRGERA	70	5.70E-06	518.6409	1552.9007	3	0.0098	13	0	AspN
H31_MOUSE, H32_MOUSE, H33_MOUSE	Histone H3	DIQLARRIRGERA	70	2.70E-05	389.2322	1552.8998	4	0.0089	13	0	AspN
H31_MOUSE, H32_MOUSE, H33_MOUSE	Histone H3	DIQLARRIRGERA	70	1.40E-05	389.2313	1552.8961	4	0.0052	13	0	AspN
H31_MOUSE, H32_MOUSE, H33_MOUSE	Histone H3	DIQLARRIRGERA	70	1.70E-04	518.6393	1552.8961	3	0.0051	13	0	AspN
H31_MOUSE, H32_MOUSE, H33_MOUSE	Histone H3	DIQLARRIRGERA	70	2.60E-05	518.6391	1552.8954	3	0.0045	13	0	AspN
H31_MOUSE, H32_MOUSE, H33_MOUSE	Histone H3	DIQLARRIRGERA	70	3.90E-03	777.4547	1552.8949	2	0.0039	13	0	AspN
H31_MOUSE, H32_MOUSE, H33_MOUSE	Histone H3	DIQLARRIRGERA	70	4.10E-03	777.4547	1552.8947	2	0.0038	13	0	AspN
H31_MOUSE, H32_MOUSE, H33_MOUSE	Histone H3	DIQLARRIRGERA	70	4.00E-04	777.4547	1552.8947	2	0.0038	13	0	AspN
H31_MOUSE, H32_MOUSE, H33_MOUSE	Histone H3	DIQLARRIRGERA	70	2.60E-07	518.6389	1552.8948	3	0.0038	13	0	AspN
H31_MOUSE, H32_MOUSE, H33_MOUSE	Histone H3	DIQLARRIRGERA	70	2.40E-03	777.4546	1552.8947	2	0.0037	13	0	AspN
H31_MOUSE, H32_MOUSE, H33_MOUSE	Histone H3	DIQLARRIRGERA	70	5.30E-06	518.6388	1552.8947	3	0.0037	13	0	AspN
H31_MOUSE, H32_MOUSE, H33_MOUSE	Histone H3	DIQLARRIRGERA	70	2.40E-06	777.4546	1552.8947	2	0.0037	13	0	AspN
H31_MOUSE, H32_MOUSE, H33_MOUSE	Histone H3	DIQLARRIRGERA	70	1.60E-05	389.2309	1552.8945	4	0.0035	13	0	AspN
H31_MOUSE, H32_MOUSE, H33_MOUSE	Histone H3	DIQLARRIRGERA	70	2.30E-03	389.2309	1552.8944	4	0.0034	13	0	AspN
H31_MOUSE, H32_MOUSE, H33_MOUSE	Histone H3	DIQLARRIRGERA	70	1.40E-03	518.6387	1552.8942	3	0.0033	13	0	AspN
H31_MOUSE, H32_MOUSE, H33_MOUSE	Histone H3	DIQLARRIRGERA	70	7.40E-04	777.4544	1552.8942	2	0.0032	13	0	AspN
H31_MOUSE, H32_MOUSE, H33_MOUSE	Histone H3	DIQLARRIRGERA	70	2.40E-06	777.4544	1552.8942	2	0.0032	13	0	AspN
H31_MOUSE, H32_MOUSE, H33_MOUSE	Histone H3	DIQLARRIRGERA	70	2.10E-03	389.2308	1552.8941	4	0.0031	13	0	AspN

H31_MOUSE, H32_MOUSE, H33_MOUSE	Histone H3	DIQLARRIRGERA	70	3.10E-04	518.6386	1552.8940	3	0.0031	13	0	AspN
H31_MOUSE, H32_MOUSE, H33_MOUSE	Histone H3	DIQLARRIRGERA	70	4.30E-06	518.6386	1552.8939	3	0.0030	13	0	AspN
H31_MOUSE, H32_MOUSE, H33_MOUSE	Histone H3	DIQLARRIRGERA	70	2.80E-03	389.2308	1552.8939	4	0.0029	13	0	AspN
H31_MOUSE, H32_MOUSE, H33_MOUSE	Histone H3	DIQLARRIRGERA	70	1.10E-03	777.4541	1552.8937	2	0.0028	13	0	AspN
H31_MOUSE, H32_MOUSE, H33_MOUSE	Histone H3	DIQLARRIRGERA	70	4.40E-03	389.2307	1552.8936	4	0.0027	13	0	AspN
H31_MOUSE, H32_MOUSE, H33_MOUSE	Histone H3	DIQLARRIRGERA	70	1.20E-06	518.6385	1552.8936	3	0.0027	13	0	AspN
H31_MOUSE, H32_MOUSE, H33_MOUSE	Histone H3	DIQLARRIRGERA	70	3.80E-04	389.2307	1552.8935	4	0.0026	13	0	AspN
H31_MOUSE, H32_MOUSE, H33_MOUSE	Histone H3	DIQLARRIRGERA	70	2.30E-03	518.6384	1552.8934	3	0.0024	13	0	AspN
H31_MOUSE, H32_MOUSE, H33_MOUSE	Histone H3	DIQLARRIRGERA	70	1.00E-05	518.6384	1552.8933	3	0.0024	13	0	AspN
H31_MOUSE, H32_MOUSE, H33_MOUSE	Histone H3	DIQLARRIRGERA	70	3.30E-05	389.2306	1552.8933	4	0.0023	13	0	AspN
H31_MOUSE, H32_MOUSE, H33_MOUSE	Histone H3	DIQLARRIRGERA	70	4.50E-06	389.2306	1552.8933	4	0.0023	13	0	AspN
H31_MOUSE, H32_MOUSE, H33_MOUSE	Histone H3	DIQLARRIRGERA	70	1.70E-07	518.6384	1552.8933	3	0.0023	13	0	AspN
H31_MOUSE, H32_MOUSE, H33_MOUSE	Histone H3	DIQLARRIRGERA	70	1.40E-05	389.2306	1552.8931	4	0.0022	13	0	AspN
H31_MOUSE, H32_MOUSE, H33_MOUSE	Histone H3	DIQLARRIRGERA	70	2.40E-03	777.4538	1552.8931	2	0.0021	13	0	AspN
H31_MOUSE, H32_MOUSE, H33_MOUSE	Histone H3	DIQLARRIRGERA	70	1.40E-04	389.2305	1552.8931	4	0.0021	13	0	AspN
H31_MOUSE, H32_MOUSE, H33_MOUSE	Histone H3	DIQLARRIRGERA	70	1.40E-07	518.6383	1552.8930	3	0.0020	13	0	AspN
H31_MOUSE, H32_MOUSE, H33_MOUSE	Histone H3	DIQLARRIRGERA	70	7.80E-03	389.2305	1552.8929	4	0.0019	13	0	AspN
H31_MOUSE, H32_MOUSE, H33_MOUSE	Histone H3	DIQLARRIRGERA	70	5.40E-06	389.2305	1552.8928	4	0.0019	13	0	AspN
H31_MOUSE, H32_MOUSE, H33_MOUSE	Histone H3	DIQLARRIRGERA	70	8.70E-06	389.2305	1552.8928	4	0.0018	13	0	AspN
H31_MOUSE, H32_MOUSE, H33_MOUSE	Histone H3	DIQLARRIRGERA	70	5.80E-07	518.6382	1552.8927	3	0.0018	13	0	AspN
H31_MOUSE, H32_MOUSE, H33_MOUSE	Histone H3	DIQLARRIRGERA	70	3.70E-07	518.6382	1552.8928	3	0.0018	13	0	AspN
H31_MOUSE, H32_MOUSE, H33_MOUSE	Histone H3	DIQLARRIRGERA	70	9.20E-05	777.4536	1552.8926	2	0.0016	13	0	AspN
H31_MOUSE, H32_MOUSE, H33_MOUSE	Histone H3	DIQLARRIRGERA	70	5.40E-05	518.6381	1552.8926	3	0.0016	13	0	AspN
H31_MOUSE, H32_MOUSE, H33_MOUSE	Histone H3	DIQLARRIRGERA	70	3.90E-04	389.2304	1552.8924	4	0.0015	13	0	AspN
H31_MOUSE, H32_MOUSE, H33_MOUSE	Histone H3	DIQLARRIRGERA	70	1.30E-05	389.2304	1552.8924	4	0.0014	13	0	AspN
H31_MOUSE, H32_MOUSE, H33_MOUSE	Histone H3	DIQLARRIRGERA	70	2.70E-05	389.2303	1552.8923	4	0.0013	13	0	AspN
H31_MOUSE, H32_MOUSE, H33_MOUSE	Histone H3	DIQLARRIRGERA	70	2.10E-06	518.638	1552.8922	3	0.0012	13	0	AspN
H31_MOUSE, H32_MOUSE, H33_MOUSE	Histone H3	DIQLARRIRGERA	70	3.90E-03	777.4532	1552.8918	2	0.0009	13	0	AspN
H31_MOUSE, H32_MOUSE, H33_MOUSE	Histone H3	DIQLARRIRGERA	70	2.10E-03	777.4532	1552.8918	2	0.0009	13	0	AspN
H31_MOUSE, H32_MOUSE, H33_MOUSE	Histone H3	DIQLARRIRGERA	70	2.70E-04	518.6378	1552.8917	3	0.0007	13	0	AspN
H31_MOUSE, H32_MOUSE, H33_MOUSE	Histone H3	DIQLARRIRGERA	70	3.20E-06	518.6378	1552.8915	3	0.0006	13	0	AspN
H31_MOUSE, H32_MOUSE, H33_MOUSE	Histone H3	DIQLARRIRGERA	70	5.00E-07	518.6378	1552.8915	3	0.0005	13	0	AspN
H31_MOUSE, H32_MOUSE, H33_MOUSE	Histone H3	DIQLARRIRGERA	70	4.00E-05	389.2301	1552.8913	4	0.0004	13	0	AspN
H31_MOUSE, H32_MOUSE, H33_MOUSE	Histone H3	DIQLARRIRGERA	70	1.10E-06	518.6377	1552.8913	3	0.0004	13	0	AspN
H31_MOUSE, H32_MOUSE, H33_MOUSE	Histone H3	DIQLARRIRGERA	70	3.40E-03	389.2301	1552.8913	4	0.0003	13	0	AspN
H31_MOUSE, H32_MOUSE, H33_MOUSE	Histone H3	DIQLARRIRGERA	70	8.50E-07	518.6376	1552.8910	3	0.0001	13	0	AspN
H31_MOUSE, H32_MOUSE, H33_MOUSE	Histone H3	DIQLARRIRGERA	70	1.10E-07	518.6376	1552.8911	3	0.0001	13	0	AspN
H31_MOUSE, H32_MOUSE, H33_MOUSE	Histone H3	DIQLARRIRGERA	70	5.50E-04	777.4528	1552.8910	2	0.0000	13	0	AspN
H31_MOUSE, H32_MOUSE, H33_MOUSE	Histone H3	DIQLARRIRGERA	70	4.20E-04	777.4528	1552.8910	2	0.0000	13	0	AspN
H31_MOUSE, H32_MOUSE, H33_MOUSE	Histone H3	DIQLARRIRGERA	70	7.30E-06	389.23	1552.8910	4	0.0000	13	0	AspN
H31_MOUSE, H32_MOUSE, H33_MOUSE	Histone H3	DIQLARRIRGERA	70	3.60E-06	389.23	1552.8908	4	-0.0002	13	0	AspN
H31_MOUSE, H32_MOUSE, H33_MOUSE	Histone H3	DIQLARRIRGERA	70	1.10E-05	389.2299	1552.8904	4	-0.0005	13	0	AspN
H31_MOUSE, H32_MOUSE, H33_MOUSE	Histone H3	DIQLARRIRGERA	70	1.10E-03	518.6374	1552.8904	3	-0.0006	13	0	AspN
H31_MOUSE, H32_MOUSE, H33_MOUSE	Histone H3	DIQLARRIRGERA	70	2.40E-06	518.6374	1552.8903	3	-0.0007	13	0	AspN
H31_MOUSE, H32_MOUSE, H33_MOUSE	Histone H3	DIQLARRIRGERA	70	2.90E-05	389.2298	1552.8901	4	-0.0009	13	0	AspN
H31_MOUSE, H32_MOUSE, H33_MOUSE	Histone H3	REIRRYQKSTEL	2	8.90E-03	526.9623	1577.8650	3	0.0012	12	1	Chymotrypsin
H31_MOUSE, H32_MOUSE, H33_MOUSE	Histone H3	REIRRYQKSTEL	2	4.60E-03	395.473	1577.8628	4	-0.0009	12	1	Chymotrypsin
H31_MOUSE, H32_MOUSE, H33_MOUSE	Histone H3	VREIAQDFKTDL	3	3.40E-04	478.925	1433.7531	3	0.0016	12	1	Chymotrypsin
H31_MOUSE, H32_MOUSE, H33_MOUSE	Histone H3	VREIAQDFKTDL	3	7.90E-03	478.9247	1433.7524	3	0.0009	12	1	Chymotrypsin
H31_MOUSE, H32_MOUSE, H33_MOUSE	Histone H3	VREIAQDFKTDL	3	2.90E-05	478.9241	1433.7506	3	-0.0008	12	1	Chymotrypsin
H31_MOUSE, H32_MOUSE, H33_MOUSE	Histone H3	RYQKSTELLIRK	7	5.30E-04	512.3074	1533.9003	3	0.0013	12	3	Semi-tryptic
H31_MOUSE, H32_MOUSE, H33_MOUSE	Histone H3	RYQKSTELLIRK	7	1.90E-06	512.3074	1533.9003	3	0.0013	12	3	Semi-tryptic
H31_MOUSE, H32_MOUSE, H33_MOUSE	Histone H3	RYQKSTELLIRK	7	8.50E-03	384.4822	1533.8997	4	0.0007	12	3	Semi-tryptic
H31_MOUSE, H32_MOUSE, H33_MOUSE	Histone H3	RYQKSTELLIRK	7	1.00E-06	384.4821	1533.8995	4	0.0004	12	3	Semi-tryptic
H31_MOUSE, H32_MOUSE, H33_MOUSE	Histone H3	RYQKSTELLIRK	7	5.40E-08	384.4821	1533.8995	4	0.0004	12	3	Semi-tryptic
H31_MOUSE, H32_MOUSE, H33_MOUSE	Histone H3	RYQKSTELLIRK	7	9.70E-03	512.307	1533.8992	3	0.0002	12	3	Semi-tryptic
H31_MOUSE, H32_MOUSE, H33_MOUSE	Histone H3	IQLARRIRGERA	10	1.00E-02	360.4747	1437.8695	4	0.0055	12	1	GluC
H31_MOUSE, H32_MOUSE, H33_MOUSE	Histone H3	IQLARRIRGERA	10	3.00E-03	360.4745	1437.8691	4	0.0050	12	1	GluC
H31_MOUSE, H32_MOUSE, H33_MOUSE	Histone H3	IQLARRIRGERA	10	7.00E-06	480.2958	1437.8657	3	0.0017	12	1	GluC
H31_MOUSE, H32_MOUSE, H33_MOUSE	Histone H3	IQLARRIRGERA	10	1.50E-03	719.9401	1437.8656	2	0.0016	12	1	GluC
H31_MOUSE, H32_MOUSE, H33_MOUSE	Histone H3	IQLARRIRGERA	10	2.90E-04	360.4736	1437.8655	4	0.0014	12	1	GluC
H31_MOUSE, H32_MOUSE, H33_MOUSE	Histone H3	IQLARRIRGERA	10	1.60E-04	360.4736	1437.8653	4	0.0013	12	1	GluC
H31_MOUSE, H32_MOUSE, H33_MOUSE	Histone H3	IQLARRIRGERA	10	5.70E-06	360.4734	1437.8644	4	0.0004	12	1	GluC
H31_MOUSE, H32_MOUSE, H33_MOUSE	Histone H3	IQLARRIRGERA	10	1.80E-06	480.2951	1437.8636	3	-0.0004	12	1	GluC
H31_MOUSE, H32_MOUSE, H33_MOUSE	Histone H3	IQLARRIRGERA	10	4.00E-06	480.295	1437.8631	3	-0.0009	12	1	GluC
H31_MOUSE, H32_MOUSE, H33_MOUSE	Histone H3	YRPGTVALREIR	23	3.50E-04	477.6117	1429.8134	3	-0.0020	12	1	Semi-tryptic
H31_MOUSE, H32_MOUSE, H33_MOUSE	Histone H3	YRPGTVALREIR	23	1.30E-04	477.6117	1429.8134	3	-0.0020	12	1	Semi-tryptic
H31_MOUSE, H32_MOUSE, H33_MOUSE	Histone H3	IQLARRIRGERA	10	1.30E-04	360.4727	1437.8616	4	-0.0024	12	1	GluC
H31_MOUSE, H32_MOUSE, H33_MOUSE	Histone H3	RYQKSTELLIRK	7	1.40E-03	384.4814	1533.8966	4	-0.0025	12	3	Semi-tryptic
H31_MOUSE, H32_MOUSE, H33_MOUSE	Histone H3	VTIMPKDIQLAR	13	2.80E-03	462.2722	1383.7949	3	0.0041	12	1	Semi-tryptic
H31_MOUSE, H32_MOUSE, H33_MOUSE	Histone H3	VTIMPKDIQLAR	13	5.40E-04	462.2722	1383.7948	3	0.0040	12	1	Semi-tryptic
H31_MOUSE, H32_MOUSE, H33_MOUSE	Histone H3	VTIMPKDIQLAR	13	6.10E-06	692.9033	1383.7921	2	0.0013	12	1	Semi-tryptic
H31_MOUSE, H32_MOUSE, H33_MOUSE	Histone H3	VTIMPKDIQLAR	13	5.40E-03	462.2713	1383.7920	3	0.0012	12	1	Semi-tryptic
H31_MOUSE, H32_MOUSE, H33_MOUSE	Histone H3	VTIMPKDIQLAR	13	2.80E-04	462.2713	1383.7920	3	0.0012	12	1	Semi-tryptic

H31_MOUSE, H32_MOUSE, H33_MOUSE	Histone H3	VTIMPKDIQLAR	13	3.50E-06	692.9027	1383.7908	2	0.0001	12	1	Semi-tryptic
H31_MOUSE, H32_MOUSE, H33_MOUSE	Histone H3	VTIMPKDIQLAR	13	3.40E-06	692.9027	1383.7908	2	0.0001	12	1	Semi-tryptic
H31_MOUSE, H32_MOUSE, H33_MOUSE	Histone H3	VTIMPKDIQLAR	13	6.10E-04	462.2708	1383.7904	3	-0.0004	12	1	Semi-tryptic
H31_MOUSE, H32_MOUSE, H33_MOUSE	Histone H3	VTIMPKDIQLAR	13	9.50E-04	462.2707	1383.7903	3	-0.0005	12	1	Semi-tryptic
H31_MOUSE, H32_MOUSE, H33_MOUSE	Histone H3	VTIMPKDIQLAR	13	2.40E-04	462.2707	1383.7903	3	-0.0005	12	1	Semi-tryptic
H31_MOUSE, H32_MOUSE, H33_MOUSE	Histone H3	VTIMPKDIQLAR	13	2.20E-04	462.2707	1383.7902	3	-0.0006	12	1	Semi-tryptic
H31_MOUSE, H32_MOUSE, H33_MOUSE	Histone H3	VTIMPKDIQLAR	13	5.00E-03	692.902	1383.7895	2	-0.0013	12	1	Semi-tryptic
H31_MOUSE, H32_MOUSE, H33_MOUSE	Histone H3	VTIMPKDIQLAR	13	2.20E-04	692.902	1383.7895	2	-0.0013	12	1	Semi-tryptic
H31_MOUSE, H32_MOUSE, H33_MOUSE	Histone H3	YRPGTVALREIR	23	2.70E-03	477.6146	1429.8218	3	0.0065	12	1	Semi-tryptic
H31_MOUSE, H32_MOUSE, H33_MOUSE	Histone H3	YRPGTVALREIR	23	9.40E-04	358.4615	1429.8168	4	0.0015	12	1	Semi-tryptic
H31_MOUSE, H32_MOUSE, H33_MOUSE	Histone H3	YRPGTVALREIR	23	7.50E-04	358.4615	1429.8167	4	0.0014	12	1	Semi-tryptic
H31_MOUSE, H32_MOUSE, H33_MOUSE	Histone H3	YRPGTVALREIR	23	6.70E-03	358.4614	1429.8167	4	0.0013	12	1	Semi-tryptic
H31_MOUSE, H32_MOUSE, H33_MOUSE	Histone H3	YRPGTVALREIR	23	4.20E-03	715.9156	1429.8166	2	0.0013	12	1	Semi-tryptic
H31_MOUSE, H32_MOUSE, H33_MOUSE	Histone H3	YRPGTVALREIR	23	1.30E-04	477.6128	1429.8167	3	0.0013	12	1	Semi-tryptic
H31_MOUSE, H32_MOUSE, H33_MOUSE	Histone H3	YRPGTVALREIR	23	3.80E-04	477.6128	1429.8166	3	0.0012	12	1	Semi-tryptic
H31_MOUSE, H32_MOUSE, H33_MOUSE	Histone H3	YRPGTVALREIR	23	2.10E-04	358.4614	1429.8165	4	0.0012	12	1	Semi-tryptic
H31_MOUSE, H32_MOUSE, H33_MOUSE	Histone H3	YRPGTVALREIR	23	4.90E-06	358.4614	1429.8165	4	0.0012	12	1	Semi-tryptic
H31_MOUSE, H32_MOUSE, H33_MOUSE	Histone H3	YRPGTVALREIR	23	5.70E-03	358.4614	1429.8163	4	0.0010	12	1	Semi-tryptic
H31_MOUSE, H32_MOUSE, H33_MOUSE	Histone H3	YRPGTVALREIR	23	7.10E-04	477.6127	1429.8164	3	0.0010	12	1	Semi-tryptic
H31_MOUSE, H32_MOUSE, H33_MOUSE	Histone H3	YRPGTVALREIR	23	2.10E-03	477.6126	1429.8161	3	0.0008	12	1	Semi-tryptic
H31_MOUSE, H32_MOUSE, H33_MOUSE	Histone H3	YRPGTVALREIR	23	1.80E-03	477.6126	1429.8161	3	0.0007	12	1	Semi-tryptic
H31_MOUSE, H32_MOUSE, H33_MOUSE	Histone H3	YRPGTVALREIR	23	4.00E-05	715.9153	1429.8161	2	0.0007	12	1	Semi-tryptic
H31_MOUSE, H32_MOUSE, H33_MOUSE	Histone H3	YRPGTVALREIR	23	3.40E-04	477.6125	1429.8157	3	0.0003	12	1	Semi-tryptic
H31_MOUSE, H32_MOUSE, H33_MOUSE	Histone H3	YRPGTVALREIR	23	2.80E-03	715.9151	1429.8156	2	0.0002	12	1	Semi-tryptic
H31_MOUSE, H32_MOUSE, H33_MOUSE	Histone H3	YRPGTVALREIR	23	1.20E-03	477.6125	1429.8156	3	0.0002	12	1	Semi-tryptic
H31_MOUSE, H32_MOUSE, H33_MOUSE	Histone H3	YRPGTVALREIR	23	1.70E-03	477.6124	1429.8154	3	0.0000	12	1	Semi-tryptic
H31_MOUSE, H32_MOUSE, H33_MOUSE	Histone H3	YRPGTVALREIR	23	7.50E-03	715.9149	1429.8152	2	-0.0001	12	1	Semi-tryptic
H31_MOUSE, H32_MOUSE, H33_MOUSE	Histone H3	YRPGTVALREIR	23	2.60E-04	477.6122	1429.8149	3	-0.0005	12	1	Semi-tryptic
H31_MOUSE, H32_MOUSE, H33_MOUSE	Histone H3	YRPGTVALREIR	23	1.30E-05	477.6122	1429.8149	3	-0.0005	12	1	Semi-tryptic
H31_MOUSE, H32_MOUSE, H33_MOUSE	Histone H3	YQKSTELLIRK	4	1.20E-04	460.2731	1377.7976	3	-0.0004	11	2	Semi-tryptic
H31_MOUSE, H32_MOUSE, H33_MOUSE	Histone H3	YQKSTELLIRK	4	7.80E-04	460.2731	1377.7973	3	-0.0006	11	2	Semi-tryptic
H31_MOUSE, H32_MOUSE, H33_MOUSE	Histone H3	QRLVREIAQDF	7	3.90E-04	458.9231	1373.7475	3	0.0060	11	1	Chymotrypsin
H31_MOUSE, H32_MOUSE, H33_MOUSE	Histone H3	QRLVREIAQDF	7	1.30E-06	458.9225	1373.7455	3	0.0040	11	1	Chymotrypsin
H31_MOUSE, H32_MOUSE, H33_MOUSE	Histone H3	QRLVREIAQDF	7	6.90E-04	458.9214	1373.7423	3	0.0008	11	1	Chymotrypsin
H31_MOUSE, H32_MOUSE, H33_MOUSE	Histone H3	QRLVREIAQDF	7	1.50E-03	458.9212	1373.7417	3	0.0002	11	1	Chymotrypsin
H31_MOUSE, H32_MOUSE, H33_MOUSE	Histone H3	QRLVREIAQDF	7	2.90E-04	458.921	1373.7411	3	-0.0004	11	1	Chymotrypsin
H31_MOUSE, H32_MOUSE, H33_MOUSE	Histone H3	QRLVREIAQDF	7	2.10E-05	458.9209	1373.7408	3	-0.0007	11	1	Chymotrypsin
H31_MOUSE, H32_MOUSE, H33_MOUSE	Histone H3	QRLVREIAQDF	7	3.10E-04	458.9208	1373.7405	3	-0.0010	11	1	Chymotrypsin
H31_MOUSE, H32_MOUSE, H33_MOUSE	Histone H3	YQKSTELLIRK	4	1.90E-04	460.2724	1377.7954	3	-0.0026	11	2	Semi-tryptic
H31_MOUSE, H32_MOUSE, H33_MOUSE	Histone H3	YQKSTELLIRK	4	4.90E-06	460.2724	1377.7954	3	-0.0026	11	2	Semi-tryptic
H31_MOUSE, H32_MOUSE, H33_MOUSE	Histone H3	RYQKSTELLIR	16	1.90E-04	703.9101	1405.8056	2	0.0015	11	2	Semi-tryptic
H31_MOUSE, H32_MOUSE, H33_MOUSE	Histone H3	RYQKSTELLIR	16	3.10E-05	703.9101	1405.8056	2	0.0015	11	2	Semi-tryptic
H31_MOUSE, H32_MOUSE, H33_MOUSE	Histone H3	RYQKSTELLIR	16	1.50E-03	469.6091	1405.8054	3	0.0013	11	2	Semi-tryptic
H31_MOUSE, H32_MOUSE, H33_MOUSE	Histone H3	RYQKSTELLIR	16	2.50E-04	703.9098	1405.8050	2	0.0009	11	2	Semi-tryptic
H31_MOUSE, H32_MOUSE, H33_MOUSE	Histone H3	RYQKSTELLIR	16	1.40E-04	469.6088	1405.8047	3	0.0006	11	2	Semi-tryptic
H31_MOUSE, H32_MOUSE, H33_MOUSE	Histone H3	RYQKSTELLIR	16	4.90E-04	469.6087	1405.8044	3	0.0003	11	2	Semi-tryptic
H31_MOUSE, H32_MOUSE, H33_MOUSE	Histone H3	RYQKSTELLIR	16	3.30E-04	469.6087	1405.8044	3	0.0003	11	2	Semi-tryptic
H31_MOUSE, H32_MOUSE, H33_MOUSE	Histone H3	RYQKSTELLIR	16	3.80E-04	469.6087	1405.8042	3	0.0001	11	2	Semi-tryptic
H31_MOUSE, H32_MOUSE, H33_MOUSE	Histone H3	RYQKSTELLIR	16	2.60E-04	469.6087	1405.8042	3	0.0001	11	2	Semi-tryptic
H31_MOUSE, H32_MOUSE, H33_MOUSE	Histone H3	RYQKSTELLIR	16	1.00E-04	469.6086	1405.8040	3	-0.0001	11	2	Semi-tryptic
H31_MOUSE, H32_MOUSE, H33_MOUSE	Histone H3	RYQKSTELLIR	16	8.60E-05	469.6086	1405.8040	3	-0.0001	11	2	Semi-tryptic
H31_MOUSE, H32_MOUSE, H33_MOUSE	Histone H3	RYQKSTELLIR	16	6.50E-06	469.6086	1405.8040	3	-0.0001	11	2	Semi-tryptic
H31_MOUSE, H32_MOUSE, H33_MOUSE	Histone H3	RYQKSTELLIR	16	2.50E-03	469.6085	1405.8038	3	-0.0003	11	2	Semi-tryptic
H31_MOUSE, H32_MOUSE, H33_MOUSE	Histone H3	RYQKSTELLIR	16	5.40E-04	469.6085	1405.8036	3	-0.0005	11	2	Semi-tryptic
H31_MOUSE, H32_MOUSE, H33_MOUSE	Histone H3	RYQKSTELLIR	16	2.60E-05	469.6082	1405.8028	3	-0.0013	11	2	Semi-tryptic
H31_MOUSE, H32_MOUSE, H33_MOUSE	Histone H3	RYQKSTELLIR	16	1.60E-06	469.6082	1405.8028	3	-0.0013	11	2	Semi-tryptic
H31_MOUSE, H32_MOUSE, H33_MOUSE	Histone H3	EIAQDFKTDLR	22	1.90E-06	668.3517	1334.6888	2	0.0058	11	1	Semi-tryptic
H31_MOUSE, H32_MOUSE, H33_MOUSE	Histone H3	EIAQDFKTDLR	22	4.50E-03	668.3499	1334.6853	2	0.0023	11	1	Semi-tryptic
H31_MOUSE, H32_MOUSE, H33_MOUSE	Histone H3	EIAQDFKTDLR	22	1.20E-03	668.3498	1334.6851	2	0.0021	11	1	Semi-tryptic
H31_MOUSE, H32_MOUSE, H33_MOUSE	Histone H3	EIAQDFKTDLR	22	8.10E-03	668.3496	1334.6846	2	0.0016	11	1	Semi-tryptic
H31_MOUSE, H32_MOUSE, H33_MOUSE	Histone H3	EIAQDFKTDLR	22	1.40E-03	668.3495	1334.6845	2	0.0015	11	1	Semi-tryptic
H31_MOUSE, H32_MOUSE, H33_MOUSE	Histone H3	EIAQDFKTDLR	22	2.60E-03	668.3495	1334.6844	2	0.0014	11	1	Semi-tryptic
H31_MOUSE, H32_MOUSE, H33_MOUSE	Histone H3	EIAQDFKTDLR	22	7.30E-04	668.3495	1334.6844	2	0.0014	11	1	Semi-tryptic
H31_MOUSE, H32_MOUSE, H33_MOUSE	Histone H3	EIAQDFKTDLR	22	2.10E-03	668.3494	1334.6843	2	0.0013	11	1	Semi-tryptic
H31_MOUSE, H32_MOUSE, H33_MOUSE	Histone H3	EIAQDFKTDLR	22	1.80E-04	668.3494	1334.6842	2	0.0012	11	1	Semi-tryptic
H31_MOUSE, H32_MOUSE, H33_MOUSE	Histone H3	EIAQDFKTDLR	22	5.70E-06	668.3494	1334.6842	2	0.0012	11	1	Semi-tryptic
H31_MOUSE, H32_MOUSE, H33_MOUSE	Histone H3	EIAQDFKTDLR	22	2.40E-04	668.3492	1334.6839	2	0.0009	11	1	Semi-tryptic
H31_MOUSE, H32_MOUSE, H33_MOUSE	Histone H3	EIAQDFKTDLR	22	1.10E-04	668.3492	1334.6838	2	0.0008	11	1	Semi-tryptic
H31_MOUSE, H32_MOUSE, H33_MOUSE	Histone H3	EIAQDFKTDLR	22	6.00E-04	668.3491	1334.6836	2	0.0006	11	1	Semi-tryptic
H31_MOUSE, H32_MOUSE, H33_MOUSE	Histone H3	EIAQDFKTDLR	22	1.90E-04	445.9018	1334.6836	3	0.0006	11	1	Semi-tryptic
H31_MOUSE, H32_MOUSE, H33_MOUSE	Histone H3	EIAQDFKTDLR	22	8.90E-06	445.9018	1334.6836	3	0.0006	11	1	Semi-tryptic
H31_MOUSE, H32_MOUSE, H33_MOUSE	Histone H3	EIAQDFKTDLR	22	2.40E-03	668.349	1334.6834	2	0.0004	11	1	Semi-tryptic
H31_MOUSE, H32_MOUSE, H33_MOUSE	Histone H3	EIAQDFKTDLR	22	3.40E-04	668.3489	1334.6832	2	0.0002	11	1	Semi-tryptic
H31_MOUSE, H32_MOUSE, H33_MOUSE	Histone H3	EIAQDFKTDLR	22	8.20E-05	668.3487	1334.6829	2	-0.0001	11	1	Semi-tryptic

H31_MOUSE, H32_MOUSE, H33_MOUSE	Histone H3	EIAQDFKTDLR	22	7.90E-04	668.3486	1334.6826	2	-0.0004	11	1	Semi-tryptic
H31_MOUSE, H32_MOUSE, H33_MOUSE	Histone H3	EIAQDFKTDLR	22	2.80E-03	445.9013	1334.6820	3	-0.0010	11	1	Semi-tryptic
H31_MOUSE, H32_MOUSE, H33_MOUSE	Histone H3	EIAQDFKTDLR	22	5.30E-05	445.9013	1334.6820	3	-0.0010	11	1	Semi-tryptic
H31_MOUSE, H32_MOUSE, H33_MOUSE	Histone H3	EIAQDFKTDLR	22	5.40E-03	668.3482	1334.6819	2	-0.0011	11	1	Semi-tryptic
H31_MOUSE, H32_MOUSE, H33_MOUSE	Histone H3	LIRKLPFQRL	5	3.40E-04	428.6162	1282.8269	3	0.0032	10	2	Chymotrypsin
H31_MOUSE, H32_MOUSE, H33_MOUSE	Histone H3	LIRKLPFQRL	5	1.00E-02	428.6154	1282.8245	3	0.0008	10	2	Chymotrypsin
H31_MOUSE, H32_MOUSE, H33_MOUSE	Histone H3	LIRKLPFQRL	5	5.30E-03	642.4196	1282.8246	2	0.0008	10	2	Chymotrypsin
H31_MOUSE, H32_MOUSE, H33_MOUSE	Histone H3	LIRKLPFQRL	5	1.40E-03	428.6154	1282.8245	3	0.0008	10	2	Chymotrypsin
H31_MOUSE, H32_MOUSE, H33_MOUSE	Histone H3	LIRKLPFQRL	5	2.10E-04	428.6155	1282.8246	3	0.0008	10	2	Chymotrypsin
H31_MOUSE, H32_MOUSE, H33_MOUSE	Histone H3	IQLARRIRGE	24	3.10E-03	303.6882	1210.7235	4	-0.0023	10	0	GluC
H31_MOUSE, H32_MOUSE, H33_MOUSE	Histone H3	IQLARRIRGE	24	3.70E-04	404.5817	1210.7232	3	-0.0026	10	0	GluC
H31_MOUSE, H32_MOUSE, H33_MOUSE	Histone H3	IQLARRIRGE	24	1.50E-03	404.5815	1210.7228	3	-0.0030	10	0	GluC
H31_MOUSE, H32_MOUSE, H33_MOUSE	Histone H3	LVREIAQDFK	12	2.90E-03	406.9005	1217.6798	3	0.0030	10	1	Semi-tryptic
H31_MOUSE, H32_MOUSE, H33_MOUSE	Histone H3	LVREIAQDFK	12	7.30E-05	609.8465	1217.6784	2	0.0016	10	1	Semi-tryptic
H31_MOUSE, H32_MOUSE, H33_MOUSE	Histone H3	LVREIAQDFK	12	7.60E-03	406.8998	1217.6777	3	0.0009	10	1	Semi-tryptic
H31_MOUSE, H32_MOUSE, H33_MOUSE	Histone H3	LVREIAQDFK	12	3.20E-03	406.8999	1217.6778	3	0.0009	10	1	Semi-tryptic
H31_MOUSE, H32_MOUSE, H33_MOUSE	Histone H3	LVREIAQDFK	12	2.90E-03	406.8997	1217.6773	3	0.0005	10	1	Semi-tryptic
H31_MOUSE, H32_MOUSE, H33_MOUSE	Histone H3	LVREIAQDFK	12	3.10E-04	609.8459	1217.6773	2	0.0005	10	1	Semi-tryptic
H31_MOUSE, H32_MOUSE, H33_MOUSE	Histone H3	LVREIAQDFK	12	1.00E-04	609.8459	1217.6773	2	0.0005	10	1	Semi-tryptic
H31_MOUSE, H32_MOUSE, H33_MOUSE	Histone H3	LVREIAQDFK	12	2.90E-04	609.8459	1217.6772	2	0.0004	10	1	Semi-tryptic
H31_MOUSE, H32_MOUSE, H33_MOUSE	Histone H3	LVREIAQDFK	12	9.90E-03	406.8996	1217.6771	3	0.0003	10	1	Semi-tryptic
H31_MOUSE, H32_MOUSE, H33_MOUSE	Histone H3	LVREIAQDFK	12	1.30E-03	406.8996	1217.6771	3	0.0003	10	1	Semi-tryptic
H31_MOUSE, H32_MOUSE, H33_MOUSE	Histone H3	LVREIAQDFK	12	4.40E-03	609.8457	1217.6769	2	0.0001	10	1	Semi-tryptic
H31_MOUSE, H32_MOUSE, H33_MOUSE	Histone H3	LVREIAQDFK	12	9.90E-05	609.8455	1217.6765	2	-0.0003	10	1	Semi-tryptic
H31_MOUSE, H32_MOUSE, H33_MOUSE	Histone H3	YQKSTELLIR	17	4.90E-04	417.5774	1249.7103	3	0.0073	10	1	Semi-tryptic
H31_MOUSE, H32_MOUSE, H33_MOUSE	Histone H3	YQKSTELLIR	17	5.20E-03	417.5762	1249.7067	3	0.0037	10	1	Semi-tryptic
H31_MOUSE, H32_MOUSE, H33_MOUSE	Histone H3	YQKSTELLIR	17	1.60E-03	417.5759	1249.7060	3	0.0030	10	1	Semi-tryptic
H31_MOUSE, H32_MOUSE, H33_MOUSE	Histone H3	YQKSTELLIR	17	1.40E-04	417.5759	1249.7060	3	0.0030	10	1	Semi-tryptic
H31_MOUSE, H32_MOUSE, H33_MOUSE	Histone H3	YQKSTELLIR	17	7.90E-07	625.8599	1249.7051	2	0.0021	10	1	Semi-tryptic
H31_MOUSE, H32_MOUSE, H33_MOUSE	Histone H3	YQKSTELLIR	17	1.50E-04	417.5755	1249.7046	3	0.0016	10	1	Semi-tryptic
H31_MOUSE, H32_MOUSE, H33_MOUSE	Histone H3	YQKSTELLIR	17	4.50E-03	625.8595	1249.7045	2	0.0015	10	1	Semi-tryptic
H31_MOUSE, H32_MOUSE, H33_MOUSE	Histone H3	YQKSTELLIR	17	3.00E-05	417.5754	1249.7045	3	0.0015	10	1	Semi-tryptic
H31_MOUSE, H32_MOUSE, H33_MOUSE	Histone H3	YQKSTELLIR	17	1.20E-06	625.8594	1249.7042	2	0.0012	10	1	Semi-tryptic
H31_MOUSE, H32_MOUSE, H33_MOUSE	Histone H3	YQKSTELLIR	17	1.40E-06	625.8593	1249.7041	2	0.0011	10	1	Semi-tryptic
H31_MOUSE, H32_MOUSE, H33_MOUSE	Histone H3	YQKSTELLIR	17	1.10E-04	417.5752	1249.7039	3	0.0009	10	1	Semi-tryptic
H31_MOUSE, H32_MOUSE, H33_MOUSE	Histone H3	YQKSTELLIR	17	2.10E-06	625.8592	1249.7039	2	0.0009	10	1	Semi-tryptic
H31_MOUSE, H32_MOUSE, H33_MOUSE	Histone H3	YQKSTELLIR	17	1.00E-04	417.5748	1249.7026	3	-0.0004	10	1	Semi-tryptic
H31_MOUSE, H32_MOUSE, H33_MOUSE	Histone H3	YQKSTELLIR	17	7.00E-05	625.8586	1249.7026	2	-0.0004	10	1	Semi-tryptic
H31_MOUSE, H32_MOUSE, H33_MOUSE	Histone H3	YQKSTELLIR	17	7.80E-06	417.5748	1249.7026	3	-0.0004	10	1	Semi-tryptic
H31_MOUSE, H32_MOUSE, H33_MOUSE	Histone H3	YQKSTELLIR	17	3.00E-05	625.8585	1249.7025	2	-0.0005	10	1	Semi-tryptic
H31_MOUSE, H32_MOUSE, H33_MOUSE	Histone H3	YQKSTELLIR	17	7.10E-03	625.8584	1249.7023	2	-0.0007	10	1	Semi-tryptic
H31_MOUSE, H32_MOUSE, H33_MOUSE	Histone H3	IQLARRIRGE	24	6.90E-03	303.6899	1210.7305	4	0.0047	10	0	GluC
H31_MOUSE, H32_MOUSE, H33_MOUSE	Histone H3	IQLARRIRGE	24	3.40E-03	404.584	1210.7301	3	0.0043	10	0	GluC
H31_MOUSE, H32_MOUSE, H33_MOUSE	Histone H3	IQLARRIRGE	24	8.10E-03	303.6894	1210.7285	4	0.0027	10	0	GluC
H31_MOUSE, H32_MOUSE, H33_MOUSE	Histone H3	IQLARRIRGE	24	3.60E-03	404.5834	1210.7283	3	0.0025	10	0	GluC
H31_MOUSE, H32_MOUSE, H33_MOUSE	Histone H3	IQLARRIRGE	24	1.80E-03	404.5834	1210.7283	3	0.0025	10	0	GluC
H31_MOUSE, H32_MOUSE, H33_MOUSE	Histone H3	IQLARRIRGE	24	6.40E-03	303.6893	1210.7282	4	0.0024	10	0	GluC
H31_MOUSE, H32_MOUSE, H33_MOUSE	Histone H3	IQLARRIRGE	24	7.40E-03	303.6891	1210.7275	4	0.0017	10	0	GluC
H31_MOUSE, H32_MOUSE, H33_MOUSE	Histone H3	IQLARRIRGE	24	2.30E-03	303.6891	1210.7275	4	0.0017	10	0	GluC
H31_MOUSE, H32_MOUSE, H33_MOUSE	Histone H3	IQLARRIRGE	24	1.90E-03	404.583	1210.7273	3	0.0015	10	0	GluC
H31_MOUSE, H32_MOUSE, H33_MOUSE	Histone H3	IQLARRIRGE	24	9.20E-03	303.6891	1210.7272	4	0.0014	10	0	GluC
H31_MOUSE, H32_MOUSE, H33_MOUSE	Histone H3	IQLARRIRGE	24	1.20E-03	404.583	1210.7271	3	0.0013	10	0	GluC
H31_MOUSE, H32_MOUSE, H33_MOUSE	Histone H3	IQLARRIRGE	24	3.00E-03	404.5829	1210.7270	3	0.0012	10	0	GluC
H31_MOUSE, H32_MOUSE, H33_MOUSE	Histone H3	IQLARRIRGE	24	5.50E-03	404.5828	1210.7265	3	0.0007	10	0	GluC
H31_MOUSE, H32_MOUSE, H33_MOUSE	Histone H3	IQLARRIRGE	24	8.80E-03	303.6888	1210.7263	4	0.0005	10	0	GluC
H31_MOUSE, H32_MOUSE, H33_MOUSE	Histone H3	IQLARRIRGE	24	9.90E-04	404.5827	1210.7262	3	0.0004	10	0	GluC
H31_MOUSE, H32_MOUSE, H33_MOUSE	Histone H3	IQLARRIRGE	24	9.90E-03	404.5826	1210.7259	3	0.0001	10	0	GluC
H31_MOUSE, H32_MOUSE, H33_MOUSE	Histone H3	IQLARRIRGE	24	3.60E-03	404.5826	1210.7258	3	0.0000	10	0	GluC
H31_MOUSE, H32_MOUSE, H33_MOUSE	Histone H3	IQLARRIRGE	24	3.70E-04	404.5825	1210.7258	3	0.0000	10	0	GluC
H31_MOUSE, H32_MOUSE, H33_MOUSE	Histone H3	IQLARRIRGE	24	1.00E-02	404.5824	1210.7254	3	-0.0004	10	0	GluC
H31_MOUSE, H32_MOUSE, H33_MOUSE	Histone H3	IQLARRIRGE	24	9.30E-03	303.6886	1210.7251	4	-0.0007	10	0	GluC
H31_MOUSE, H32_MOUSE, H33_MOUSE	Histone H3	IQLARRIRGE	24	1.10E-03	404.5821	1210.7244	3	-0.0014	10	0	GluC
H31_MOUSE, H32_MOUSE, H33_MOUSE	Histone H3	KLPFQRLVR	2	8.40E-03	386.2487	1155.7244	3	0.0003	9	2	Semi-tryptic
H31_MOUSE, H32_MOUSE, H33_MOUSE	Histone H3	KLPFQRLVR	2	3.70E-04	386.2487	1155.7244	3	0.0003	9	2	Semi-tryptic
H31_MOUSE, H32_MOUSE, H33_MOUSE	Histone H3	KQLATKAAR	13	2.50E-07	493.8075	985.6005	2	-0.0027	9	2	Semi-tryptic
H31_MOUSE, H32_MOUSE, H33_MOUSE	Histone H3	IRRYQKSTE	58	1.20E-04	394.218	1179.6323	3	-0.0037	9	0	GluC
H31_MOUSE, H32_MOUSE, H33_MOUSE	Histone H3	KQLATKAAR	13	1.40E-03	493.8064	985.5982	2	-0.0050	9	2	Semi-tryptic
H31_MOUSE, H32_MOUSE, H33_MOUSE	Histone H3	KQLATKAAR	13	4.50E-03	329.5424	985.6055	3	0.0022	9	2	Semi-tryptic
H31_MOUSE, H32_MOUSE, H33_MOUSE	Histone H3	KQLATKAAR	13	1.30E-04	329.5423	985.6050	3	0.0018	9	2	Semi-tryptic
H31_MOUSE, H32_MOUSE, H33_MOUSE	Histone H3	KQLATKAAR	13	9.30E-06	329.5423	985.6050	3	0.0018	9	2	Semi-tryptic
H31_MOUSE, H32_MOUSE, H33_MOUSE	Histone H3	KQLATKAAR	13	3.40E-03	329.5421	985.6045	3	0.0013	9	2	Semi-tryptic
H31_MOUSE, H32_MOUSE, H33_MOUSE	Histone H3	KQLATKAAR	13	5.20E-04	329.542	985.6043	3	0.0010	9	2	Semi-tryptic
H31_MOUSE, H32_MOUSE, H33_MOUSE	Histone H3	KQLATKAAR	13	2.70E-05	493.8088	985.6031	2	-0.0001	9	2	Semi-tryptic
H31_MOUSE, H32_MOUSE, H33_MOUSE	Histone H3	KQLATKAAR	13	1.40E-06	493.8088	985.6031	2	-0.0001	9	2	Semi-tryptic

H31_MOUSE, H32_MOUSE, H33_MOUSE	Histone H3	KQLATKAAR	13	3.20E-05	329.5416	985.6029	3	-0.0004	9	2	Semi-tryptic
H31_MOUSE, H32_MOUSE, H33_MOUSE	Histone H3	KQLATKAAR	13	4.80E-04	493.8086	985.6026	2	-0.0006	9	2	Semi-tryptic
H31_MOUSE, H32_MOUSE, H33_MOUSE	Histone H3	KQLATKAAR	13	1.50E-04	329.5415	985.6026	3	-0.0006	9	2	Semi-tryptic
H31_MOUSE, H32_MOUSE, H33_MOUSE	Histone H3	KQLATKAAR	13	5.80E-04	493.8081	985.6017	2	-0.0015	9	2	Semi-tryptic
H31_MOUSE, H32_MOUSE, H33_MOUSE	Histone H3	YRPGTVALR	18	6.20E-04	516.8029	1031.5912	2	0.0036	9	0	Semi-tryptic
H31_MOUSE, H32_MOUSE, H33_MOUSE	Histone H3	YRPGTVALR	18	4.80E-04	516.8028	1031.5910	2	0.0034	9	0	Semi-tryptic
H31_MOUSE, H32_MOUSE, H33_MOUSE	Histone H3	YRPGTVALR	18	8.90E-04	516.8025	1031.5905	2	0.0029	9	0	Semi-tryptic
H31_MOUSE, H32_MOUSE, H33_MOUSE	Histone H3	YRPGTVALR	18	1.10E-03	516.8022	1031.5899	2	0.0023	9	0	Semi-tryptic
H31_MOUSE, H32_MOUSE, H33_MOUSE	Histone H3	YRPGTVALR	18	7.20E-03	516.8019	1031.5893	2	0.0017	9	0	Semi-tryptic
H31_MOUSE, H32_MOUSE, H33_MOUSE	Histone H3	YRPGTVALR	18	5.90E-03	516.8019	1031.5893	2	0.0017	9	0	Semi-tryptic
H31_MOUSE, H32_MOUSE, H33_MOUSE	Histone H3	YRPGTVALR	18	3.20E-03	344.8702	1031.5889	3	0.0013	9	0	Semi-tryptic
H31_MOUSE, H32_MOUSE, H33_MOUSE	Histone H3	YRPGTVALR	18	6.70E-04	344.8702	1031.5889	3	0.0013	9	0	Semi-tryptic
H31_MOUSE, H32_MOUSE, H33_MOUSE	Histone H3	YRPGTVALR	18	3.90E-03	516.8014	1031.5883	2	0.0007	9	0	Semi-tryptic
H31_MOUSE, H32_MOUSE, H33_MOUSE	Histone H3	YRPGTVALR	18	8.80E-04	516.8014	1031.5883	2	0.0007	9	0	Semi-tryptic
H31_MOUSE, H32_MOUSE, H33_MOUSE	Histone H3	YRPGTVALR	18	9.90E-04	516.8014	1031.5882	2	0.0006	9	0	Semi-tryptic
H31_MOUSE, H32_MOUSE, H33_MOUSE	Histone H3	YRPGTVALR	18	7.80E-03	344.87	1031.5881	3	0.0005	9	0	Semi-tryptic
H31_MOUSE, H32_MOUSE, H33_MOUSE	Histone H3	YRPGTVALR	18	2.20E-03	344.87	1031.5881	3	0.0005	9	0	Semi-tryptic
H31_MOUSE, H32_MOUSE, H33_MOUSE	Histone H3	YRPGTVALR	18	7.80E-03	516.8012	1031.5879	2	0.0003	9	0	Semi-tryptic
H31_MOUSE, H32_MOUSE, H33_MOUSE	Histone H3	YRPGTVALR	18	2.10E-03	516.8011	1031.5877	2	0.0001	9	0	Semi-tryptic
H31_MOUSE, H32_MOUSE, H33_MOUSE	Histone H3	YRPGTVALR	18	8.80E-03	516.801	1031.5874	2	-0.0002	9	0	Semi-tryptic
H31_MOUSE, H32_MOUSE, H33_MOUSE	Histone H3	YRPGTVALR	18	2.40E-03	516.8009	1031.5873	2	-0.0003	9	0	Semi-tryptic
H31_MOUSE, H32_MOUSE, H33_MOUSE	Histone H3	YRPGTVALR	18	7.70E-04	516.8009	1031.5872	2	-0.0004	9	0	Semi-tryptic
H31_MOUSE, H32_MOUSE, H33_MOUSE	Histone H3	IRRYQKSTE	58	6.50E-03	394.2211	1179.6414	3	0.0054	9	0	GluC
H31_MOUSE, H32_MOUSE, H33_MOUSE	Histone H3	IRRYQKSTE	58	3.60E-04	394.2208	1179.6406	3	0.0046	9	0	GluC
H31_MOUSE, H32_MOUSE, H33_MOUSE	Histone H3	IRRYQKSTE	58	5.80E-05	394.2207	1179.6403	3	0.0043	9	0	GluC
H31_MOUSE, H32_MOUSE, H33_MOUSE	Histone H3	IRRYQKSTE	58	1.90E-03	394.2207	1179.6402	3	0.0042	9	0	GluC
H31_MOUSE, H32_MOUSE, H33_MOUSE	Histone H3	IRRYQKSTE	58	1.60E-03	394.2207	1179.6402	3	0.0042	9	0	GluC
H31_MOUSE, H32_MOUSE, H33_MOUSE	Histone H3	IRRYQKSTE	58	3.80E-04	394.2206	1179.6398	3	0.0038	9	0	GluC
H31_MOUSE, H32_MOUSE, H33_MOUSE	Histone H3	IRRYQKSTE	58	5.30E-05	394.2205	1179.6397	3	0.0037	9	0	GluC
H31_MOUSE, H32_MOUSE, H33_MOUSE	Histone H3	IRRYQKSTE	58	2.50E-04	394.2204	1179.6394	3	0.0034	9	0	GluC
H31_MOUSE, H32_MOUSE, H33_MOUSE	Histone H3	IRRYQKSTE	58	7.90E-04	394.2202	1179.6387	3	0.0027	9	0	GluC
H31_MOUSE, H32_MOUSE, H33_MOUSE	Histone H3	IRRYQKSTE	58	3.50E-05	394.2201	1179.6385	3	0.0025	9	0	GluC
H31_MOUSE, H32_MOUSE, H33_MOUSE	Histone H3	IRRYQKSTE	58	4.50E-04	394.22	1179.6383	3	0.0023	9	0	GluC
H31_MOUSE, H32_MOUSE, H33_MOUSE	Histone H3	IRRYQKSTE	58	6.90E-04	394.2199	1179.6377	3	0.0017	9	0	GluC
H31_MOUSE, H32_MOUSE, H33_MOUSE	Histone H3	IRRYQKSTE	58	1.00E-02	394.2198	1179.6375	3	0.0016	9	0	GluC
H31_MOUSE, H32_MOUSE, H33_MOUSE	Histone H3	IRRYQKSTE	58	7.80E-03	394.2198	1179.6376	3	0.0016	9	0	GluC
H31_MOUSE, H32_MOUSE, H33_MOUSE	Histone H3	IRRYQKSTE	58	2.60E-04	394.2198	1179.6375	3	0.0015	9	0	GluC
H31_MOUSE, H32_MOUSE, H33_MOUSE	Histone H3	IRRYQKSTE	58	3.70E-03	394.2196	1179.6371	3	0.0011	9	0	GluC
H31_MOUSE, H32_MOUSE, H33_MOUSE	Histone H3	IRRYQKSTE	58	3.00E-04	394.2196	1179.6370	3	0.0010	9	0	GluC
H31_MOUSE, H32_MOUSE, H33_MOUSE	Histone H3	IRRYQKSTE	58	1.70E-03	394.2196	1179.6369	3	0.0009	9	0	GluC
H31_MOUSE, H32_MOUSE, H33_MOUSE	Histone H3	IRRYQKSTE	58	8.50E-03	394.2196	1179.6368	3	0.0008	9	0	GluC
H31_MOUSE, H32_MOUSE, H33_MOUSE	Histone H3	IRRYQKSTE	58	3.40E-03	394.2196	1179.6368	3	0.0008	9	0	GluC
H31_MOUSE, H32_MOUSE, H33_MOUSE	Histone H3	IRRYQKSTE	58	4.60E-05	394.2195	1179.6368	3	0.0008	9	0	GluC
H31_MOUSE, H32_MOUSE, H33_MOUSE	Histone H3	IRRYQKSTE	58	8.70E-05	394.2195	1179.6367	3	0.0007	9	0	GluC
H31_MOUSE, H32_MOUSE, H33_MOUSE	Histone H3	IRRYQKSTE	58	5.40E-04	394.2195	1179.6366	3	0.0006	9	0	GluC
H31_MOUSE, H32_MOUSE, H33_MOUSE	Histone H3	IRRYQKSTE	58	8.50E-03	394.2194	1179.6365	3	0.0005	9	0	GluC
H31_MOUSE, H32_MOUSE, H33_MOUSE	Histone H3	IRRYQKSTE	58	5.30E-03	394.2194	1179.6365	3	0.0005	9	0	GluC
H31_MOUSE, H32_MOUSE, H33_MOUSE	Histone H3	IRRYQKSTE	58	5.20E-04	394.2194	1179.6365	3	0.0005	9	0	GluC
H31_MOUSE, H32_MOUSE, H33_MOUSE	Histone H3	IRRYQKSTE	58	8.10E-03	394.2194	1179.6364	3	0.0004	9	0	GluC
H31_MOUSE, H32_MOUSE, H33_MOUSE	Histone H3	IRRYQKSTE	58	1.90E-04	394.2194	1179.6363	3	0.0004	9	0	GluC
H31_MOUSE, H32_MOUSE, H33_MOUSE	Histone H3	IRRYQKSTE	58	7.50E-05	394.2194	1179.6364	3	0.0004	9	0	GluC
H31_MOUSE, H32_MOUSE, H33_MOUSE	Histone H3	IRRYQKSTE	58	2.70E-05	394.2194	1179.6363	3	0.0003	9	0	GluC
H31_MOUSE, H32_MOUSE, H33_MOUSE	Histone H3	IRRYQKSTE	58	1.10E-05	394.2194	1179.6363	3	0.0003	9	0	GluC
H31_MOUSE, H32_MOUSE, H33_MOUSE	Histone H3	IRRYQKSTE	58	2.40E-03	394.2194	1179.6362	3	0.0002	9	0	GluC
H31_MOUSE, H32_MOUSE, H33_MOUSE	Histone H3	IRRYQKSTE	58	2.40E-03	394.2193	1179.6361	3	0.0001	9	0	GluC
H31_MOUSE, H32_MOUSE, H33_MOUSE	Histone H3	IRRYQKSTE	58	1.40E-03	394.2193	1179.6361	3	0.0001	9	0	GluC
H31_MOUSE, H32_MOUSE, H33_MOUSE	Histone H3	IRRYQKSTE	58	5.20E-04	394.2193	1179.6361	3	0.0001	9	0	GluC
H31_MOUSE, H32_MOUSE, H33_MOUSE	Histone H3	IRRYQKSTE	58	2.00E-04	394.2193	1179.6361	3	0.0001	9	0	GluC
H31_MOUSE, H32_MOUSE, H33_MOUSE	Histone H3	IRRYQKSTE	58	1.40E-04	394.2193	1179.6361	3	0.0001	9	0	GluC
H31_MOUSE, H32_MOUSE, H33_MOUSE	Histone H3	IRRYQKSTE	58	6.90E-05	394.2193	1179.6360	3	0.0001	9	0	GluC
H31_MOUSE, H32_MOUSE, H33_MOUSE	Histone H3	IRRYQKSTE	58	3.90E-05	394.2193	1179.6361	3	0.0001	9	0	GluC
H31_MOUSE, H32_MOUSE, H33_MOUSE	Histone H3	IRRYQKSTE	58	8.80E-03	394.2193	1179.6360	3	0.0000	9	0	GluC
H31_MOUSE, H32_MOUSE, H33_MOUSE	Histone H3	IRRYQKSTE	58	2.70E-04	394.2192	1179.6359	3	-0.0001	9	0	GluC
H31_MOUSE, H32_MOUSE, H33_MOUSE	Histone H3	IRRYQKSTE	58	8.50E-03	394.2192	1179.6358	3	-0.0002	9	0	GluC
H31_MOUSE, H32_MOUSE, H33_MOUSE	Histone H3	IRRYQKSTE	58	6.20E-03	394.2192	1179.6358	3	-0.0002	9	0	GluC
H31_MOUSE, H32_MOUSE, H33_MOUSE	Histone H3	IRRYQKSTE	58	2.50E-03	394.2192	1179.6358	3	-0.0002	9	0	GluC
H31_MOUSE, H32_MOUSE, H33_MOUSE	Histone H3	IRRYQKSTE	58	7.00E-04	394.2192	1179.6358	3	-0.0002	9	0	GluC
H31_MOUSE, H32_MOUSE, H33_MOUSE	Histone H3	IRRYQKSTE	58	5.70E-04	394.2192	1179.6358	3	-0.0002	9	0	GluC
H31_MOUSE, H32_MOUSE, H33_MOUSE	Histone H3	IRRYQKSTE	58	8.10E-03	394.2191	1179.6356	3	-0.0004	9	0	GluC
H31_MOUSE, H32_MOUSE, H33_MOUSE	Histone H3	IRRYQKSTE	58	3.20E-03	394.2191	1179.6355	3	-0.0005	9	0	GluC
H31_MOUSE, H32_MOUSE, H33_MOUSE	Histone H3	IRRYQKSTE	58	7.20E-05	394.2191	1179.6355	3	-0.0005	9	0	GluC
H31_MOUSE, H32_MOUSE, H33_MOUSE	Histone H3	IRRYQKSTE	58	4.40E-03	394.2191	1179.6354	3	-0.0006	9	0	GluC
H31_MOUSE, H32_MOUSE, H33_MOUSE	Histone H3	IRRYQKSTE	58	4.30E-03	394.219	1179.6353	3	-0.0007	9	0	GluC
H31_MOUSE, H32_MOUSE, H33_MOUSE	Histone H3	IRRYQKSTE	58	7.50E-04	394.219	1179.6352	3	-0.0008	9	0	GluC

H31_MOUSE, H32_MOUSE, H33_MOUSE	Histone H3	IRRYQKSTE	58	2.70E-04	394.219	1179.6351	3	-0.0008	9	0	GluC
H31_MOUSE, H32_MOUSE, H33_MOUSE	Histone H3	IRRYQKSTE	58	6.10E-03	394.219	1179.6351	3	-0.0009	9	0	GluC
H31_MOUSE, H32_MOUSE, H33_MOUSE	Histone H3	IRRYQKSTE	58	3.50E-03	394.2189	1179.6350	3	-0.0010	9	0	GluC
H31_MOUSE, H32_MOUSE, H33_MOUSE	Histone H3	IRRYQKSTE	58	7.90E-03	394.2188	1179.6347	3	-0.0013	9	0	GluC
H31_MOUSE, H32_MOUSE, H33_MOUSE	Histone H3	IRRYQKSTE	58	2.50E-03	394.2188	1179.6345	3	-0.0015	9	0	GluC
H31_MOUSE, H32_MOUSE, H33_MOUSE	Histone H3	STGGKAPR	2	9.70E-06	387.217	772.4195	2	0.0004	8	1	Semi-tryptic
H31_MOUSE, H32_MOUSE, H33_MOUSE	Histone H3	STGGKAPR	2	1.70E-04	387.217	772.4195	2	0.0003	8	1	Semi-tryptic
H31_MOUSE, H32_MOUSE, H33_MOUSE	Histone H3	STELLIRK	4	1.90E-03	480.298	958.5814	2	0.0003	8	1	Semi-tryptic
H31_MOUSE, H32_MOUSE, H33_MOUSE	Histone H3	STELLIRK	4	1.40E-04	480.2978	958.5810	2	-0.0001	8	1	Semi-tryptic
H31_MOUSE, H32_MOUSE, H33_MOUSE	Histone H3	STELLIRK	4	1.30E-04	480.2976	958.5807	2	-0.0004	8	1	Semi-tryptic
H31_MOUSE, H32_MOUSE, H33_MOUSE	Histone H3	STELLIRK	4	7.80E-04	480.2962	958.5779	2	-0.0032	8	1	Semi-tryptic
H31_MOUSE, H32_MOUSE, H33_MOUSE	Histone H3	VREIAQDF	13	1.40E-04	489.2573	976.5000	2	0.0023	8	0	Chymotrypsin
H31_MOUSE, H32_MOUSE, H33_MOUSE	Histone H3	VREIAQDF	13	1.60E-03	489.2571	976.4997	2	0.0019	8	0	Chymotrypsin
H31_MOUSE, H32_MOUSE, H33_MOUSE	Histone H3	VREIAQDF	13	1.90E-03	489.257	976.4994	2	0.0016	8	0	Chymotrypsin
H31_MOUSE, H32_MOUSE, H33_MOUSE	Histone H3	VREIAQDF	13	5.90E-05	489.2568	976.4990	2	0.0012	8	0	Chymotrypsin
H31_MOUSE, H32_MOUSE, H33_MOUSE	Histone H3	VREIAQDF	13	2.80E-04	489.2567	976.4989	2	0.0011	8	0	Chymotrypsin
H31_MOUSE, H32_MOUSE, H33_MOUSE	Histone H3	VREIAQDF	13	8.60E-04	489.2565	976.4985	2	0.0007	8	0	Chymotrypsin
H31_MOUSE, H32_MOUSE, H33_MOUSE	Histone H3	VREIAQDF	13	9.60E-04	489.2564	976.4982	2	0.0004	8	0	Chymotrypsin
H31_MOUSE, H32_MOUSE, H33_MOUSE	Histone H3	VREIAQDF	13	8.60E-04	489.2564	976.4982	2	0.0004	8	0	Chymotrypsin
H31_MOUSE, H32_MOUSE, H33_MOUSE	Histone H3	VREIAQDF	13	7.40E-03	489.2563	976.4980	2	0.0002	8	0	Chymotrypsin
H31_MOUSE, H32_MOUSE, H33_MOUSE	Histone H3	VREIAQDF	13	7.40E-03	489.2562	976.4978	2	0.0001	8	0	Chymotrypsin
H31_MOUSE, H32_MOUSE, H33_MOUSE	Histone H3	VREIAQDF	13	2.10E-03	489.2562	976.4978	2	0.0001	8	0	Chymotrypsin
H31_MOUSE, H32_MOUSE, H33_MOUSE	Histone H3	VREIAQDF	13	8.70E-05	489.2562	976.4978	2	0.0000	8	0	Chymotrypsin
H31_MOUSE, H32_MOUSE, H33_MOUSE	Histone H3	VREIAQDF	13	4.70E-04	489.2561	976.4976	2	-0.0002	8	0	Chymotrypsin
H31_MOUSE, H32_MOUSE, H33_MOUSE	Histone H3	IAQDFKTD	69	4.00E-04	469.2369	936.4593	2	0.0040	8	1	GluC
H31_MOUSE, H32_MOUSE, H33_MOUSE	Histone H3	IAQDFKTD	69	3.20E-03	469.2366	936.4586	2	0.0033	8	1	GluC
H31_MOUSE, H32_MOUSE, H33_MOUSE	Histone H3	IAQDFKTD	69	2.90E-03	469.2364	936.4582	2	0.0030	8	1	GluC
H31_MOUSE, H32_MOUSE, H33_MOUSE	Histone H3	IAQDFKTD	69	2.00E-03	469.2362	936.4578	2	0.0026	8	1	GluC
H31_MOUSE, H32_MOUSE, H33_MOUSE	Histone H3	IAQDFKTD	69	5.40E-05	469.2362	936.4578	2	0.0025	8	1	GluC
H31_MOUSE, H32_MOUSE, H33_MOUSE	Histone H3	IAQDFKTD	69	4.90E-03	469.2361	936.4577	2	0.0024	8	1	GluC
H31_MOUSE, H32_MOUSE, H33_MOUSE	Histone H3	IAQDFKTD	69	4.20E-05	469.2361	936.4577	2	0.0024	8	1	GluC
H31_MOUSE, H32_MOUSE, H33_MOUSE	Histone H3	IAQDFKTD	69	4.10E-03	469.2361	936.4576	2	0.0023	8	1	GluC
H31_MOUSE, H32_MOUSE, H33_MOUSE	Histone H3	IAQDFKTD	69	5.60E-05	469.236	936.4575	2	0.0023	8	1	GluC
H31_MOUSE, H32_MOUSE, H33_MOUSE	Histone H3	IAQDFKTD	69	1.40E-03	469.236	936.4574	2	0.0022	8	1	GluC
H31_MOUSE, H32_MOUSE, H33_MOUSE	Histone H3	IAQDFKTD	69	1.10E-04	469.2359	936.4572	2	0.0020	8	1	GluC
H31_MOUSE, H32_MOUSE, H33_MOUSE	Histone H3	IAQDFKTD	69	1.10E-03	469.2359	936.4572	2	0.0019	8	1	GluC
H31_MOUSE, H32_MOUSE, H33_MOUSE	Histone H3	IAQDFKTD	69	5.50E-03	469.2358	936.4570	2	0.0018	8	1	GluC
H31_MOUSE, H32_MOUSE, H33_MOUSE	Histone H3	IAQDFKTD	69	2.10E-04	469.2358	936.4570	2	0.0018	8	1	GluC
H31_MOUSE, H32_MOUSE, H33_MOUSE	Histone H3	IAQDFKTD	69	5.20E-03	469.2357	936.4569	2	0.0016	8	1	GluC
H31_MOUSE, H32_MOUSE, H33_MOUSE	Histone H3	IAQDFKTD	69	3.80E-03	469.2357	936.4569	2	0.0016	8	1	GluC
H31_MOUSE, H32_MOUSE, H33_MOUSE	Histone H3	IAQDFKTD	69	1.70E-05	469.2357	936.4568	2	0.0015	8	1	GluC
H31_MOUSE, H32_MOUSE, H33_MOUSE	Histone H3	IAQDFKTD	69	9.70E-06	469.2357	936.4568	2	0.0015	8	1	GluC
H31_MOUSE, H32_MOUSE, H33_MOUSE	Histone H3	IAQDFKTD	69	5.50E-06	469.2356	936.4567	2	0.0015	8	1	GluC
H31_MOUSE, H32_MOUSE, H33_MOUSE	Histone H3	IAQDFKTD	69	9.80E-04	469.2356	936.4566	2	0.0014	8	1	GluC
H31_MOUSE, H32_MOUSE, H33_MOUSE	Histone H3	IAQDFKTD	69	5.40E-05	469.2356	936.4567	2	0.0014	8	1	GluC
H31_MOUSE, H32_MOUSE, H33_MOUSE	Histone H3	IAQDFKTD	69	3.50E-03	469.2355	936.4565	2	0.0012	8	1	GluC
H31_MOUSE, H32_MOUSE, H33_MOUSE	Histone H3	IAQDFKTD	69	2.30E-04	469.2354	936.4563	2	0.0010	8	1	GluC
H31_MOUSE, H32_MOUSE, H33_MOUSE	Histone H3	IAQDFKTD	69	9.70E-03	469.2354	936.4562	2	0.0009	8	1	GluC
H31_MOUSE, H32_MOUSE, H33_MOUSE	Histone H3	IAQDFKTD	69	1.70E-03	469.2353	936.4561	2	0.0009	8	1	GluC
H31_MOUSE, H32_MOUSE, H33_MOUSE	Histone H3	IAQDFKTD	69	1.40E-05	469.2354	936.4562	2	0.0009	8	1	GluC
H31_MOUSE, H32_MOUSE, H33_MOUSE	Histone H3	IAQDFKTD	69	3.70E-05	469.2353	936.4560	2	0.0007	8	1	GluC
H31_MOUSE, H32_MOUSE, H33_MOUSE	Histone H3	IAQDFKTD	69	9.10E-04	469.2352	936.4558	2	0.0005	8	1	GluC
H31_MOUSE, H32_MOUSE, H33_MOUSE	Histone H3	IAQDFKTD	69	1.80E-04	469.2352	936.4558	2	0.0005	8	1	GluC
H31_MOUSE, H32_MOUSE, H33_MOUSE	Histone H3	IAQDFKTD	69	2.20E-04	469.2351	936.4557	2	0.0004	8	1	GluC
H31_MOUSE, H32_MOUSE, H33_MOUSE	Histone H3	IAQDFKTD	69	1.40E-05	469.2351	936.4556	2	0.0004	8	1	GluC
H31_MOUSE, H32_MOUSE, H33_MOUSE	Histone H3	IAQDFKTD	69	3.10E-05	469.2351	936.4556	2	0.0003	8	1	GluC
H31_MOUSE, H32_MOUSE, H33_MOUSE	Histone H3	IAQDFKTD	69	7.40E-06	469.2351	936.4555	2	0.0003	8	1	GluC
H31_MOUSE, H32_MOUSE, H33_MOUSE	Histone H3	IAQDFKTD	69	2.00E-03	469.235	936.4555	2	0.0002	8	1	GluC
H31_MOUSE, H32_MOUSE, H33_MOUSE	Histone H3	IAQDFKTD	69	5.40E-04	469.235	936.4554	2	0.0002	8	1	GluC
H31_MOUSE, H32_MOUSE, H33_MOUSE	Histone H3	IAQDFKTD	69	9.10E-03	469.235	936.4554	2	0.0001	8	1	GluC
H31_MOUSE, H32_MOUSE, H33_MOUSE	Histone H3	IAQDFKTD	69	1.70E-03	469.235	936.4553	2	0.0001	8	1	GluC
H31_MOUSE, H32_MOUSE, H33_MOUSE	Histone H3	IAQDFKTD	69	8.20E-06	469.2349	936.4553	2	0.0001	8	1	GluC
H31_MOUSE, H32_MOUSE, H33_MOUSE	Histone H3	IAQDFKTD	69	7.10E-06	469.235	936.4554	2	0.0001	8	1	GluC
H31_MOUSE, H32_MOUSE, H33_MOUSE	Histone H3	IAQDFKTD	69	9.40E-03	469.2349	936.4552	2	0.0000	8	1	GluC
H31_MOUSE, H32_MOUSE, H33_MOUSE	Histone H3	IAQDFKTD	69	7.60E-03	469.2349	936.4553	2	0.0000	8	1	GluC
H31_MOUSE, H32_MOUSE, H33_MOUSE	Histone H3	IAQDFKTD	69	1.10E-03	469.2349	936.4553	2	0.0000	8	1	GluC
H31_MOUSE, H32_MOUSE, H33_MOUSE	Histone H3	IAQDFKTD	69	6.70E-04	469.2349	936.4553	2	0.0000	8	1	GluC
H31_MOUSE, H32_MOUSE, H33_MOUSE	Histone H3	IAQDFKTD	69	2.30E-05	469.2349	936.4553	2	0.0000	8	1	GluC
H31_MOUSE, H32_MOUSE, H33_MOUSE	Histone H3	IAQDFKTD	69	2.20E-03	469.2348	936.4551	2	-0.0001	8	1	GluC
H31_MOUSE, H32_MOUSE, H33_MOUSE	Histone H3	IAQDFKTD	69	9.70E-05	469.2348	936.4551	2	-0.0001	8	1	GluC
H31_MOUSE, H32_MOUSE, H33_MOUSE	Histone H3	IAQDFKTD	69	3.10E-05	469.2349	936.4552	2	-0.0001	8	1	GluC
H31_MOUSE, H32_MOUSE, H33_MOUSE	Histone H3	IAQDFKTD	69	2.70E-06	469.2349	936.4552	2	-0.0001	8	1	GluC
H31_MOUSE, H32_MOUSE, H33_MOUSE	Histone H3	IAQDFKTD	69	6.90E-03	469.2348	936.4551	2	-0.0002	8	1	GluC
H31_MOUSE, H32_MOUSE, H33_MOUSE	Histone H3	IAQDFKTD	69	6.90E-03	469.2348	936.4551	2	-0.0002	8	1	GluC

H31_MOUSE, H32_MOUSE, H33_MOUSE	Histone H3	IAQDFKTD	69	1.50E-03	469.2348	936.4550	2	-0.0002	8	1	GluC
H31_MOUSE, H32_MOUSE, H33_MOUSE	Histone H3	IAQDFKTD	69	1.70E-04	469.2348	936.4551	2	-0.0002	8	1	GluC
H31_MOUSE, H32_MOUSE, H33_MOUSE	Histone H3	IAQDFKTD	69	1.50E-04	469.2348	936.4551	2	-0.0002	8	1	GluC
H31_MOUSE, H32_MOUSE, H33_MOUSE	Histone H3	IAQDFKTD	69	7.90E-05	469.2348	936.4551	2	-0.0002	8	1	GluC
H31_MOUSE, H32_MOUSE, H33_MOUSE	Histone H3	IAQDFKTD	69	2.90E-05	469.2348	936.4551	2	-0.0002	8	1	GluC
H31_MOUSE, H32_MOUSE, H33_MOUSE	Histone H3	IAQDFKTD	69	3.30E-05	469.2347	936.4549	2	-0.0003	8	1	GluC
H31_MOUSE, H32_MOUSE, H33_MOUSE	Histone H3	IAQDFKTD	69	3.80E-03	469.2347	936.4548	2	-0.0004	8	1	GluC
H31_MOUSE, H32_MOUSE, H33_MOUSE	Histone H3	IAQDFKTD	69	5.50E-05	469.2347	936.4549	2	-0.0004	8	1	GluC
H31_MOUSE, H32_MOUSE, H33_MOUSE	Histone H3	IAQDFKTD	69	7.60E-06	469.2347	936.4548	2	-0.0004	8	1	GluC
H31_MOUSE, H32_MOUSE, H33_MOUSE	Histone H3	IAQDFKTD	69	2.90E-03	469.2347	936.4548	2	-0.0005	8	1	GluC
H31_MOUSE, H32_MOUSE, H33_MOUSE	Histone H3	IAQDFKTD	69	1.50E-04	469.2346	936.4547	2	-0.0005	8	1	GluC
H31_MOUSE, H32_MOUSE, H33_MOUSE	Histone H3	IAQDFKTD	69	7.20E-03	469.2346	936.4547	2	-0.0006	8	1	GluC
H31_MOUSE, H32_MOUSE, H33_MOUSE	Histone H3	IAQDFKTD	69	3.20E-05	469.2346	936.4547	2	-0.0006	8	1	GluC
H31_MOUSE, H32_MOUSE, H33_MOUSE	Histone H3	IAQDFKTD	69	2.90E-05	469.2346	936.4546	2	-0.0006	8	1	GluC
H31_MOUSE, H32_MOUSE, H33_MOUSE	Histone H3	IAQDFKTD	69	7.60E-03	469.2345	936.4545	2	-0.0008	8	1	GluC
H31_MOUSE, H32_MOUSE, H33_MOUSE	Histone H3	IAQDFKTD	69	3.60E-03	469.2345	936.4544	2	-0.0008	8	1	GluC
H31_MOUSE, H32_MOUSE, H33_MOUSE	Histone H3	IAQDFKTD	69	7.50E-04	469.2345	936.4545	2	-0.0008	8	1	GluC
H31_MOUSE, H32_MOUSE, H33_MOUSE	Histone H3	IAQDFKTD	69	3.90E-03	469.2345	936.4544	2	-0.0009	8	1	GluC
H31_MOUSE, H32_MOUSE, H33_MOUSE	Histone H3	IAQDFKTD	69	3.60E-05	469.2343	936.4541	2	-0.0012	8	1	GluC
H31_MOUSE, H32_MOUSE, H33_MOUSE	Histone H3	EIRRYQK	3	8.10E-03	331.5261	991.5565	3	0.0003	7	2	Semi-tryptic
H31_MOUSE, H32_MOUSE, H33_MOUSE	Histone H3	EIRRYQK	3	7.70E-04	331.5261	991.5565	3	0.0003	7	2	Semi-tryptic
H31_MOUSE, H32_MOUSE, H33_MOUSE	Histone H3	EIRRYQK	3	7.20E-03	496.7847	991.5548	2	-0.0014	7	2	Semi-tryptic
H31_MOUSE, H32_MOUSE, H33_MOUSE	Histone H3	RVTIMPK	4	6.20E-03	422.7584	843.5022	2	0.0022	7	1	Semi-tryptic
H31_MOUSE, H32_MOUSE, H33_MOUSE	Histone H3	RVTIMPK	4	7.30E-03	422.7578	843.5010	2	0.0010	7	1	Semi-tryptic
H31_MOUSE, H32_MOUSE, H33_MOUSE	Histone H3	RVTIMPK	4	7.40E-03	422.7576	843.5007	2	0.0007	7	1	Semi-tryptic
H31_MOUSE, H32_MOUSE, H33_MOUSE	Histone H3	RVTIMPK	4	9.30E-03	422.7572	843.4999	2	-0.0001	7	1	Semi-tryptic
H31_MOUSE, H32_MOUSE, H33_MOUSE	Histone H3	DIQLARR	5	7.70E-03	436.2595	870.5045	2	0.0010	7	1	Semi-tryptic
H31_MOUSE, H32_MOUSE, H33_MOUSE	Histone H3	DIQLARR	5	8.30E-03	436.2591	870.5037	2	0.0001	7	1	Semi-tryptic
H31_MOUSE, H32_MOUSE, H33_MOUSE	Histone H3	DIQLARR	5	2.80E-03	436.259	870.5035	2	0.0000	7	1	Semi-tryptic
H31_MOUSE, H32_MOUSE, H33_MOUSE	Histone H3	DIQLARR	5	3.20E-03	436.2589	870.5033	2	-0.0002	7	1	Semi-tryptic
H31_MOUSE, H32_MOUSE, H33_MOUSE	Histone H3	DIQLARR	5	4.30E-03	436.2587	870.5029	2	-0.0006	7	1	Semi-tryptic
H31_MOUSE, H32_MOUSE, H33_MOUSE	Histone H3	LIRKLPF	6	5.20E-03	443.7974	885.5802	2	0.0002	7	1	Chymotrypsin
H31_MOUSE, H32_MOUSE, H33_MOUSE	Histone H3	LIRKLPF	6	7.60E-03	443.7972	885.5799	2	-0.0001	7	1	Chymotrypsin
H31_MOUSE, H32_MOUSE, H33_MOUSE	Histone H3	LIRKLPF	6	5.20E-03	443.7972	885.5798	2	-0.0002	7	1	Chymotrypsin
H31_MOUSE, H32_MOUSE, H33_MOUSE	Histone H3	LIRKLPF	6	5.20E-03	443.7971	885.5796	2	-0.0003	7	1	Chymotrypsin
H31_MOUSE, H32_MOUSE, H33_MOUSE	Histone H3	LIRKLPF	6	5.20E-03	443.7968	885.5791	2	-0.0009	7	1	Chymotrypsin
H31_MOUSE, H32_MOUSE, H33_MOUSE	Histone H3	LIRKLPF	6	5.30E-03	443.7967	885.5789	2	-0.0011	7	1	Chymotrypsin
H31_MOUSE, H32_MOUSE, H33_MOUSE	Histone H3	EIAQDFK	8	1.80E-03	425.7202	849.4259	2	0.0027	7	0	Semi-tryptic
H31_MOUSE, H32_MOUSE, H33_MOUSE	Histone H3	EIAQDFK	8	2.10E-03	425.7191	849.4237	2	0.0005	7	0	Semi-tryptic
H31_MOUSE, H32_MOUSE, H33_MOUSE	Histone H3	EIAQDFK	8	9.90E-03	425.7191	849.4236	2	0.0004	7	0	Semi-tryptic
H31_MOUSE, H32_MOUSE, H33_MOUSE	Histone H3	EIAQDFK	8	5.30E-03	425.7191	849.4236	2	0.0004	7	0	Semi-tryptic
H31_MOUSE, H32_MOUSE, H33_MOUSE	Histone H3	EIAQDFK	8	6.70E-03	425.7189	849.4233	2	0.0001	7	0	Semi-tryptic
H31_MOUSE, H32_MOUSE, H33_MOUSE	Histone H3	EIAQDFK	8	2.20E-03	425.7188	849.4231	2	-0.0001	7	0	Semi-tryptic
H31_MOUSE, H32_MOUSE, H33_MOUSE	Histone H3	EIAQDFK	8	9.10E-03	425.7178	849.4210	2	-0.0022	7	0	Semi-tryptic
H31_MOUSE, H32_MOUSE, H33_MOUSE	Histone H3	EIAQDFK	8	2.40E-03	425.7178	849.4210	2	-0.0022	7	0	Semi-tryptic
H31_MOUSE, H32_MOUSE, H33_MOUSE	Histone H3	RPGTVAL	18	9.90E-03	357.2172	712.4198	2	-0.0034	7	0	Chymotrypsin
H31_MOUSE, H32_MOUSE, H33_MOUSE	Histone H3	RPGTVAL	18	1.30E-03	357.2199	712.4253	2	0.0022	7	0	Chymotrypsin
H31_MOUSE, H32_MOUSE, H33_MOUSE	Histone H3	RPGTVAL	18	3.80E-03	357.2192	712.4238	2	0.0006	7	0	Chymotrypsin
H31_MOUSE, H32_MOUSE, H33_MOUSE	Histone H3	RPGTVAL	18	1.30E-03	357.2192	712.4238	2	0.0006	7	0	Chymotrypsin
H31_MOUSE, H32_MOUSE, H33_MOUSE	Histone H3	RPGTVAL	18	2.50E-03	357.219	712.4234	2	0.0003	7	0	Chymotrypsin
H31_MOUSE, H32_MOUSE, H33_MOUSE	Histone H3	RPGTVAL	18	4.40E-03	357.2189	712.4232	2	0.0001	7	0	Chymotrypsin
H31_MOUSE, H32_MOUSE, H33_MOUSE	Histone H3	RPGTVAL	18	3.40E-03	357.2189	712.4232	2	0.0001	7	0	Chymotrypsin
H31_MOUSE, H32_MOUSE, H33_MOUSE	Histone H3	RPGTVAL	18	3.40E-03	357.2189	712.4233	2	0.0001	7	0	GluC
H31_MOUSE, H32_MOUSE, H33_MOUSE	Histone H3	RPGTVAL	18	9.30E-03	357.2188	712.4231	2	0.0000	7	0	Chymotrypsin
H31_MOUSE, H32_MOUSE, H33_MOUSE	Histone H3	RPGTVAL	18	8.10E-03	357.2189	712.4232	2	0.0000	7	0	Chymotrypsin
H31_MOUSE, H32_MOUSE, H33_MOUSE	Histone H3	RPGTVAL	18	4.40E-03	357.2189	712.4232	2	0.0000	7	0	Chymotrypsin
H31_MOUSE, H32_MOUSE, H33_MOUSE	Histone H3	RPGTVAL	18	3.60E-03	357.2189	712.4232	2	0.0000	7	0	Chymotrypsin
H31_MOUSE, H32_MOUSE, H33_MOUSE	Histone H3	RPGTVAL	18	3.40E-03	357.2189	712.4232	2	0.0000	7	0	Chymotrypsin
H31_MOUSE, H32_MOUSE, H33_MOUSE	Histone H3	RPGTVAL	18	3.30E-03	357.2188	712.4231	2	0.0000	7	0	Chymotrypsin
H31_MOUSE, H32_MOUSE, H33_MOUSE	Histone H3	RPGTVAL	18	1.30E-03	357.2187	712.4229	2	-0.0002	7	0	Chymotrypsin
H31_MOUSE, H32_MOUSE, H33_MOUSE	Histone H3	RPGTVAL	18	1.00E-03	357.2187	712.4228	2	-0.0003	7	0	Chymotrypsin
H31_MOUSE, H32_MOUSE, H33_MOUSE	Histone H3	RPGTVAL	18	7.80E-04	357.2187	712.4228	2	-0.0003	7	0	Chymotrypsin
H31_MOUSE, H32_MOUSE, H33_MOUSE	Histone H3	RPGTVAL	18	2.60E-03	357.2186	712.4226	2	-0.0006	7	0	Chymotrypsin
H31_MOUSE, H32_MOUSE, H33_MOUSE	Histone H3	STELLIR	30	4.70E-04	416.2525	830.4905	2	0.0044	7	0	Semi-tryptic
H31_MOUSE, H32_MOUSE, H33_MOUSE	Histone H3	STELLIR	30	4.80E-04	416.2522	830.4898	2	0.0037	7	0	Semi-tryptic
H31_MOUSE, H32_MOUSE, H33_MOUSE	Histone H3	STELLIR	30	3.70E-05	416.2522	830.4899	2	0.0037	7	0	Semi-tryptic
H31_MOUSE, H32_MOUSE, H33_MOUSE	Histone H3	STELLIR	30	3.60E-05	416.2518	830.4890	2	0.0028	7	0	Semi-tryptic
H31_MOUSE, H32_MOUSE, H33_MOUSE	Histone H3	STELLIR	30	3.00E-03	416.2517	830.4889	2	0.0027	7	0	Semi-tryptic
H31_MOUSE, H32_MOUSE, H33_MOUSE	Histone H3	STELLIR	30	4.50E-04	416.2517	830.4889	2	0.0027	7	0	Semi-tryptic
H31_MOUSE, H32_MOUSE, H33_MOUSE	Histone H3	STELLIR	30	3.40E-05	416.2516	830.4885	2	0.0024	7	0	Semi-tryptic
H31_MOUSE, H32_MOUSE, H33_MOUSE	Histone H3	STELLIR	30	3.70E-04	416.2513	830.4881	2	0.0019	7	0	Semi-tryptic
H31_MOUSE, H32_MOUSE, H33_MOUSE	Histone H3	STELLIR	30	5.00E-05	416.2513	830.4880	2	0.0019	7	0	Semi-tryptic
H31_MOUSE, H32_MOUSE, H33_MOUSE	Histone H3	STELLIR	30	5.00E-05	416.2512	830.4878	2	0.0017	7	0	Semi-tryptic
H31_MOUSE, H32_MOUSE, H33_MOUSE	Histone H3	STELLIR	30	1.50E-04	416.2511	830.4875	2	0.0014	7	0	Semi-tryptic

H31_MOUSE, H32_MOUSE, H33_MOUSE	Histone H3	STELLIR	30	2.20E-05	416.2511	830.4875	2	0.0014	7	0	Semi-tryptic
H31_MOUSE, H32_MOUSE, H33_MOUSE	Histone H3	STELLIR	30	5.00E-05	416.2509	830.4873	2	0.0012	7	0	Semi-tryptic
H31_MOUSE, H32_MOUSE, H33_MOUSE	Histone H3	STELLIR	30	3.90E-04	416.2508	830.4871	2	0.0009	7	0	Semi-tryptic
H31_MOUSE, H32_MOUSE, H33_MOUSE	Histone H3	STELLIR	30	1.40E-04	416.2507	830.4869	2	0.0008	7	0	Semi-tryptic
H31_MOUSE, H32_MOUSE, H33_MOUSE	Histone H3	STELLIR	30	5.20E-05	416.2506	830.4867	2	0.0006	7	0	Semi-tryptic
H31_MOUSE, H32_MOUSE, H33_MOUSE	Histone H3	STELLIR	30	1.30E-03	416.2506	830.4866	2	0.0005	7	0	Semi-tryptic
H31_MOUSE, H32_MOUSE, H33_MOUSE	Histone H3	STELLIR	30	1.50E-04	416.2506	830.4866	2	0.0005	7	0	Semi-tryptic
H31_MOUSE, H32_MOUSE, H33_MOUSE	Histone H3	STELLIR	30	5.10E-05	416.2506	830.4867	2	0.0005	7	0	Semi-tryptic
H31_MOUSE, H32_MOUSE, H33_MOUSE	Histone H3	STELLIR	30	5.10E-05	416.2506	830.4867	2	0.0005	7	0	Semi-tryptic
H31_MOUSE, H32_MOUSE, H33_MOUSE	Histone H3	STELLIR	30	1.60E-04	416.2505	830.4865	2	0.0004	7	0	Semi-tryptic
H31_MOUSE, H32_MOUSE, H33_MOUSE	Histone H3	STELLIR	30	5.30E-05	416.2505	830.4865	2	0.0004	7	0	Semi-tryptic
H31_MOUSE, H32_MOUSE, H33_MOUSE	Histone H3	STELLIR	30	6.10E-03	416.2505	830.4865	2	0.0003	7	0	Semi-tryptic
H31_MOUSE, H32_MOUSE, H33_MOUSE	Histone H3	STELLIR	30	9.10E-04	416.2505	830.4865	2	0.0003	7	0	Semi-tryptic
H31_MOUSE, H32_MOUSE, H33_MOUSE	Histone H3	STELLIR	30	5.10E-05	416.2505	830.4864	2	0.0002	7	0	Semi-tryptic
H31_MOUSE, H32_MOUSE, H33_MOUSE	Histone H3	STELLIR	30	5.20E-05	416.2503	830.4861	2	0.0000	7	0	Semi-tryptic
H31_MOUSE, H32_MOUSE, H33_MOUSE	Histone H3	STELLIR	30	8.90E-03	416.2503	830.4860	2	-0.0001	7	0	Semi-tryptic
H31_MOUSE, H32_MOUSE, H33_MOUSE	Histone H3	STELLIR	30	6.10E-04	416.2503	830.4860	2	-0.0001	7	0	Semi-tryptic
H31_MOUSE, H32_MOUSE, H33_MOUSE	Histone H3	STELLIR	30	4.60E-05	416.2501	830.4856	2	-0.0005	7	0	Semi-tryptic
H31_MOUSE, H32_MOUSE, H33_MOUSE	Histone H3	STELLIR	30	4.70E-05	416.25	830.4855	2	-0.0006	7	0	Semi-tryptic
H31_MOUSE, H32_MOUSE, H33_MOUSE	Histone H3	DIQLAR	2	7.00E-03	358.209	714.4034	2	0.0010	6	0	Semi-tryptic
H31_MOUSE, H32_MOUSE, H33_MOUSE	Histone H3	DIQLAR	2	4.00E-03	358.2087	714.4029	2	0.0005	6	0	Semi-tryptic
H31_MOUSE, H32_MOUSE, H33_MOUSE	Histone H3	KLPFQR	8	8.10E-03	394.744	787.4735	2	0.0030	6	1	Semi-tryptic
H31_MOUSE, H32_MOUSE, H33_MOUSE	Histone H3	KLPFQR	8	8.70E-03	394.7438	787.4731	2	0.0027	6	1	Semi-tryptic
H31_MOUSE, H32_MOUSE, H33_MOUSE	Histone H3	KLPFQR	8	8.30E-03	394.7437	787.4729	2	0.0025	6	1	Semi-tryptic
H31_MOUSE, H32_MOUSE, H33_MOUSE	Histone H3	KLPFQR	8	4.20E-03	394.7437	787.4728	2	0.0023	6	1	Semi-tryptic
H31_MOUSE, H32_MOUSE, H33_MOUSE	Histone H3	KLPFQR	8	4.30E-03	394.7436	787.4726	2	0.0022	6	1	Semi-tryptic
H31_MOUSE, H32_MOUSE, H33_MOUSE	Histone H3	KLPFQR	8	4.20E-03	394.7434	787.4722	2	0.0018	6	1	Semi-tryptic
H31_MOUSE, H32_MOUSE, H33_MOUSE	Histone H3	KLPFQR	8	5.70E-03	394.743	787.4713	2	0.0009	6	1	Semi-tryptic
H31_MOUSE, H32_MOUSE, H33_MOUSE	Histone H3	KLPFQR	8	5.10E-03	394.7428	787.4711	2	0.0007	6	1	Semi-tryptic
H32_MOUSE, H33_MOUSE	Histone H3	ALQEASEAYLVGLFEDTNLCAIHAKRVTIM	5	2.10E-04	1102.8961	3305.6665	3	-0.0123	30	0	CnBr
H32_MOUSE, H33_MOUSE	Histone H3	ALQEASEAYLVGLFEDTNLCAIHAKRVTIM	5	2.70E-05	1102.8959	3305.6659	3	-0.0129	30	0	CnBr
H32_MOUSE, H33_MOUSE	Histone H3	ALQEASEAYLVGLFEDTNLCAIHAKRVTIM	5	7.50E-10	1102.8951	3305.6635	3	-0.0153	30	0	CnBr
H32_MOUSE, H33_MOUSE	Histone H3	ALQEASEAYLVGLFEDTNLCAIHAKRVTIM	5	3.50E-03	827.4226	3305.6615	4	-0.0173	30	0	CnBr
H32_MOUSE, H33_MOUSE	Histone H3	ALQEASEAYLVGLFEDTNLCAIHAKRVTIM	5	8.40E-04	827.4226	3305.6615	4	-0.0173	30	0	CnBr
H33_MOUSE	Histone H3	IAQDFKTDLRFQSAAIGALQE	75	1.10E-04	774.747	2321.2192	3	0.0176	21	2	GluC
H33_MOUSE	Histone H3	IAQDFKTDLRFQSAAIGALQE	75	5.20E-04	774.7464	2321.2173	3	0.0157	21	2	GluC
H33_MOUSE	Histone H3	IAQDFKTDLRFQSAAIGALQE	75	1.30E-06	774.7462	2321.2167	3	0.0152	21	2	GluC
H33_MOUSE	Histone H3	IAQDFKTDLRFQSAAIGALQE	75	4.90E-06	774.7455	2321.2145	3	0.0130	21	2	GluC
H33_MOUSE	Histone H3	IAQDFKTDLRFQSAAIGALQE	75	4.50E-06	774.7455	2321.2145	3	0.0130	21	2	GluC
H33_MOUSE	Histone H3	IAQDFKTDLRFQSAAIGALQE	75	3.10E-04	774.745	2321.2133	3	0.0117	21	2	GluC
H33_MOUSE	Histone H3	IAQDFKTDLRFQSAAIGALQE	75	1.00E-03	774.745	2321.2131	3	0.0116	21	2	GluC
H33_MOUSE	Histone H3	IAQDFKTDLRFQSAAIGALQE	75	2.50E-05	774.7448	2321.2126	3	0.0110	21	2	GluC
H33_MOUSE	Histone H3	IAQDFKTDLRFQSAAIGALQE	75	2.00E-03	774.7442	2321.2109	3	0.0093	21	2	GluC
H33_MOUSE	Histone H3	IAQDFKTDLRFQSAAIGALQE	75	2.30E-09	1161.6123	2321.2100	2	0.0085	21	2	GluC
H33_MOUSE	Histone H3	IAQDFKTDLRFQSAAIGALQE	75	1.50E-09	1161.6119	2321.2092	2	0.0077	21	2	GluC
H33_MOUSE	Histone H3	IAQDFKTDLRFQSAAIGALQE	75	8.00E-07	774.7434	2321.2085	3	0.0069	21	2	GluC
H33_MOUSE	Histone H3	IAQDFKTDLRFQSAAIGALQE	75	2.70E-08	1161.6113	2321.2080	2	0.0065	21	2	GluC
H33_MOUSE	Histone H3	IAQDFKTDLRFQSAAIGALQE	75	6.50E-09	1161.6111	2321.2076	2	0.0061	21	2	GluC
H33_MOUSE	Histone H3	IAQDFKTDLRFQSAAIGALQE	75	4.60E-05	774.7431	2321.2073	3	0.0058	21	2	GluC
H33_MOUSE	Histone H3	IAQDFKTDLRFQSAAIGALQE	75	3.90E-05	774.743	2321.2071	3	0.0056	21	2	GluC
H33_MOUSE	Histone H3	IAQDFKTDLRFQSAAIGALQE	75	3.50E-05	774.743	2321.2072	3	0.0056	21	2	GluC
H33_MOUSE	Histone H3	IAQDFKTDLRFQSAAIGALQE	75	2.80E-05	774.743	2321.2071	3	0.0056	21	2	GluC
H33_MOUSE	Histone H3	IAQDFKTDLRFQSAAIGALQE	75	1.80E-05	774.743	2321.2072	3	0.0056	21	2	GluC
H33_MOUSE	Histone H3	IAQDFKTDLRFQSAAIGALQE	75	4.40E-04	774.7429	2321.2069	3	0.0054	21	2	GluC
H33_MOUSE	Histone H3	IAQDFKTDLRFQSAAIGALQE	75	1.30E-05	774.7429	2321.2069	3	0.0054	21	2	GluC
H33_MOUSE	Histone H3	IAQDFKTDLRFQSAAIGALQE	75	1.60E-03	774.7429	2321.2068	3	0.0053	21	2	GluC
H33_MOUSE	Histone H3	IAQDFKTDLRFQSAAIGALQE	75	1.10E-05	774.7429	2321.2069	3	0.0053	21	2	GluC
H33_MOUSE	Histone H3	IAQDFKTDLRFQSAAIGALQE	75	2.50E-03	774.7428	2321.2067	3	0.0052	21	2	GluC
H33_MOUSE	Histone H3	IAQDFKTDLRFQSAAIGALQE	75	9.60E-03	774.7428	2321.2066	3	0.0051	21	2	GluC
H33_MOUSE	Histone H3	IAQDFKTDLRFQSAAIGALQE	75	9.80E-04	774.7428	2321.2066	3	0.0050	21	2	GluC
H33_MOUSE	Histone H3	IAQDFKTDLRFQSAAIGALQE	75	2.30E-05	774.7428	2321.2066	3	0.0050	21	2	GluC
H33_MOUSE	Histone H3	IAQDFKTDLRFQSAAIGALQE	75	8.00E-03	774.7427	2321.2064	3	0.0048	21	2	GluC
H33_MOUSE	Histone H3	IAQDFKTDLRFQSAAIGALQE	75	1.10E-03	774.7427	2321.2064	3	0.0048	21	2	GluC
H33_MOUSE	Histone H3	IAQDFKTDLRFQSAAIGALQE	75	8.80E-04	774.7426	2321.2060	3	0.0045	21	2	GluC
H33_MOUSE	Histone H3	IAQDFKTDLRFQSAAIGALQE	75	9.60E-05	774.7426	2321.2061	3	0.0045	21	2	GluC
H33_MOUSE	Histone H3	IAQDFKTDLRFQSAAIGALQE	75	4.70E-05	774.7426	2321.2060	3	0.0045	21	2	GluC
H33_MOUSE	Histone H3	IAQDFKTDLRFQSAAIGALQE	75	4.60E-05	774.7426	2321.2060	3	0.0045	21	2	GluC
H33_MOUSE	Histone H3	IAQDFKTDLRFQSAAIGALQE	75	1.30E-05	774.7426	2321.2060	3	0.0045	21	2	GluC
H33_MOUSE	Histone H3	IAQDFKTDLRFQSAAIGALQE	75	7.60E-06	1161.6103	2321.2060	2	0.0045	21	2	GluC
H33_MOUSE	Histone H3	IAQDFKTDLRFQSAAIGALQE	75	1.90E-04	774.7426	2321.2060	3	0.0044	21	2	GluC
H33_MOUSE	Histone H3	IAQDFKTDLRFQSAAIGALQE	75	8.10E-04	774.7426	2321.2059	3	0.0043	21	2	GluC
H33_MOUSE	Histone H3	IAQDFKTDLRFQSAAIGALQE	75	7.80E-04	774.7426	2321.2059	3	0.0043	21	2	GluC
H33_MOUSE	Histone H3	IAQDFKTDLRFQSAAIGALQE	75	5.80E-05	774.7425	2321.2057	3	0.0042	21	2	GluC
H33_MOUSE	Histone H3	IAQDFKTDLRFQSAAIGALQE	75	2.20E-03	774.7425	2321.2056	3	0.0041	21	2	GluC

H33_MOUSE	Histone H3	IAQDFKTDLRFQSAAIGALQE	75	8.00E-05	774.7425	2321.2056	3	0.0041	21	2	GluC
H33_MOUSE	Histone H3	IAQDFKTDLRFQSAAIGALQE	75	3.30E-04	774.7425	2321.2056	3	0.0040	21	2	GluC
H33_MOUSE	Histone H3	IAQDFKTDLRFQSAAIGALQE	75	1.20E-05	774.7424	2321.2055	3	0.0040	21	2	GluC
H33_MOUSE	Histone H3	IAQDFKTDLRFQSAAIGALQE	75	1.00E-02	774.7424	2321.2055	3	0.0039	21	2	GluC
H33_MOUSE	Histone H3	IAQDFKTDLRFQSAAIGALQE	75	1.60E-05	774.7424	2321.2054	3	0.0038	21	2	GluC
H33_MOUSE	Histone H3	IAQDFKTDLRFQSAAIGALQE	75	5.60E-06	774.7424	2321.2054	3	0.0038	21	2	GluC
H33_MOUSE	Histone H3	IAQDFKTDLRFQSAAIGALQE	75	2.10E-03	774.7423	2321.2052	3	0.0037	21	2	GluC
H33_MOUSE	Histone H3	IAQDFKTDLRFQSAAIGALQE	75	1.10E-03	1161.6099	2321.2052	2	0.0037	21	2	GluC
H33_MOUSE	Histone H3	IAQDFKTDLRFQSAAIGALQE	75	6.70E-04	774.7423	2321.2052	3	0.0037	21	2	GluC
H33_MOUSE	Histone H3	IAQDFKTDLRFQSAAIGALQE	75	3.10E-04	774.7423	2321.2052	3	0.0037	21	2	GluC
H33_MOUSE	Histone H3	IAQDFKTDLRFQSAAIGALQE	75	1.80E-05	774.7423	2321.2052	3	0.0037	21	2	GluC
H33_MOUSE	Histone H3	IAQDFKTDLRFQSAAIGALQE	75	2.00E-03	774.7423	2321.2051	3	0.0036	21	2	GluC
H33_MOUSE	Histone H3	IAQDFKTDLRFQSAAIGALQE	75	1.30E-03	774.7423	2321.2051	3	0.0036	21	2	GluC
H33_MOUSE	Histone H3	IAQDFKTDLRFQSAAIGALQE	75	4.10E-06	774.7423	2321.2051	3	0.0036	21	2	GluC
H33_MOUSE	Histone H3	IAQDFKTDLRFQSAAIGALQE	75	1.50E-03	774.7423	2321.2049	3	0.0034	21	2	GluC
H33_MOUSE	Histone H3	IAQDFKTDLRFQSAAIGALQE	75	3.40E-06	774.7422	2321.2049	3	0.0034	21	2	GluC
H33_MOUSE	Histone H3	IAQDFKTDLRFQSAAIGALQE	75	1.90E-03	774.7422	2321.2048	3	0.0033	21	2	GluC
H33_MOUSE	Histone H3	IAQDFKTDLRFQSAAIGALQE	75	2.80E-04	774.7422	2321.2048	3	0.0033	21	2	GluC
H33_MOUSE	Histone H3	IAQDFKTDLRFQSAAIGALQE	75	6.00E-05	774.7422	2321.2048	3	0.0033	21	2	GluC
H33_MOUSE	Histone H3	IAQDFKTDLRFQSAAIGALQE	75	3.00E-04	774.7421	2321.2046	3	0.0031	21	2	GluC
H33_MOUSE	Histone H3	IAQDFKTDLRFQSAAIGALQE	75	2.20E-04	774.7421	2321.2044	3	0.0029	21	2	GluC
H33_MOUSE	Histone H3	IAQDFKTDLRFQSAAIGALQE	75	5.00E-06	774.7421	2321.2044	3	0.0029	21	2	GluC
H33_MOUSE	Histone H3	IAQDFKTDLRFQSAAIGALQE	75	6.70E-06	774.7421	2321.2044	3	0.0028	21	2	GluC
H33_MOUSE	Histone H3	IAQDFKTDLRFQSAAIGALQE	75	1.50E-05	774.742	2321.2041	3	0.0025	21	2	GluC
H33_MOUSE	Histone H3	IAQDFKTDLRFQSAAIGALQE	75	4.10E-06	774.742	2321.2041	3	0.0025	21	2	GluC
H33_MOUSE	Histone H3	IAQDFKTDLRFQSAAIGALQE	75	2.30E-06	774.742	2321.2041	3	0.0025	21	2	GluC
H33_MOUSE	Histone H3	IAQDFKTDLRFQSAAIGALQE	75	1.70E-05	774.7419	2321.2040	3	0.0024	21	2	GluC
H33_MOUSE	Histone H3	IAQDFKTDLRFQSAAIGALQE	75	1.60E-05	774.7419	2321.2039	3	0.0024	21	2	GluC
H33_MOUSE	Histone H3	IAQDFKTDLRFQSAAIGALQE	75	1.80E-06	774.7419	2321.2039	3	0.0024	21	2	GluC
H33_MOUSE	Histone H3	IAQDFKTDLRFQSAAIGALQE	75	3.40E-07	774.7418	2321.2035	3	0.0019	21	2	GluC
H33_MOUSE	Histone H3	IAQDFKTDLRFQSAAIGALQE	75	3.20E-04	774.7417	2321.2033	3	0.0018	21	2	GluC
H33_MOUSE	Histone H3	IAQDFKTDLRFQSAAIGALQE	75	7.20E-07	774.7417	2321.2032	3	0.0017	21	2	GluC
H33_MOUSE	Histone H3	IAQDFKTDLRFQSAAIGALQE	75	5.10E-03	774.7416	2321.2029	3	0.0014	21	2	GluC
H33_MOUSE	Histone H3	IAQDFKTDLRFQSAAIGALQE	75	2.10E-05	774.7416	2321.2029	3	0.0014	21	2	GluC
H33_MOUSE	Histone H3	IAQDFKTDLRFQSAAIGALQE	75	7.10E-05	774.7414	2321.2022	3	0.0007	21	2	GluC
H33_MOUSE	Histone H3	FKTDLRFQSAAIGALQE	4	1.10E-05	632.3412	1894.0018	3	0.0070	17	1	GluC
H33_MOUSE	Histone H3	FKTDLRFQSAAIGALQE	4	7.30E-03	632.3407	1894.0002	3	0.0054	17	1	GluC
H33_MOUSE	Histone H3	FKTDLRFQSAAIGALQE	4	3.80E-06	632.3393	1893.9961	3	0.0012	17	1	GluC
H33_MOUSE	Histone H3	FKTDLRFQSAAIGALQE	4	6.50E-03	632.3391	1893.9954	3	0.0005	17	1	GluC
H33_MOUSE	Histone H3	RFQSAAIGALQEASEAY	111	3.20E-03	906.4482	1810.8819	2	-0.0030	17	2	Chymotrypsin
H33_MOUSE	Histone H3	RFQSAAIGALQEASEAY	111	6.80E-07	906.4482	1810.8819	2	-0.0030	17	2	Chymotrypsin
H33_MOUSE	Histone H3	RFQSAAIGALQEASEAY	111	5.80E-04	906.4461	1810.8775	2	-0.0074	17	2	Chymotrypsin
H33_MOUSE	Histone H3	RFQSAAIGALQEASEAY	111	1.30E-09	906.4461	1810.8775	2	-0.0074	17	2	Chymotrypsin
H33_MOUSE	Histone H3	RFQSAAIGALQEASEAY	111	1.70E-06	906.4458	1810.8770	2	-0.0079	17	2	Chymotrypsin
H33_MOUSE	Histone H3	RFQSAAIGALQEASEAY	111	7.00E-03	906.4556	1810.8967	2	0.0118	17	2	Chymotrypsin
H33_MOUSE	Histone H3	RFQSAAIGALQEASEAY	111	6.40E-03	906.4529	1810.8913	2	0.0063	17	2	Chymotrypsin
H33_MOUSE	Histone H3	RFQSAAIGALQEASEAY	111	7.20E-04	906.4527	1810.8908	2	0.0059	17	2	Chymotrypsin
H33_MOUSE	Histone H3	RFQSAAIGALQEASEAY	111	1.70E-03	906.4526	1810.8907	2	0.0058	17	2	Chymotrypsin
H33_MOUSE	Histone H3	RFQSAAIGALQEASEAY	111	1.50E-07	906.4526	1810.8907	2	0.0058	17	2	Chymotrypsin
H33_MOUSE	Histone H3	RFQSAAIGALQEASEAY	111	4.40E-07	906.4526	1810.8907	2	0.0057	17	2	Chymotrypsin
H33_MOUSE	Histone H3	RFQSAAIGALQEASEAY	111	6.10E-05	906.4524	1810.8902	2	0.0053	17	2	Chymotrypsin
H33_MOUSE	Histone H3	RFQSAAIGALQEASEAY	111	3.00E-07	906.4524	1810.8902	2	0.0053	17	2	Chymotrypsin
H33_MOUSE	Histone H3	RFQSAAIGALQEASEAY	111	5.60E-06	906.4523	1810.8900	2	0.0051	17	2	Chymotrypsin
H33_MOUSE	Histone H3	RFQSAAIGALQEASEAY	111	1.60E-07	906.4523	1810.8900	2	0.0051	17	2	Chymotrypsin
H33_MOUSE	Histone H3	RFQSAAIGALQEASEAY	111	3.40E-07	906.4522	1810.8899	2	0.0050	17	2	Chymotrypsin
H33_MOUSE	Histone H3	RFQSAAIGALQEASEAY	111	3.00E-05	906.4522	1810.8898	2	0.0048	17	2	Chymotrypsin
H33_MOUSE	Histone H3	RFQSAAIGALQEASEAY	111	1.70E-05	906.4521	1810.8896	2	0.0047	17	2	Chymotrypsin
H33_MOUSE	Histone H3	RFQSAAIGALQEASEAY	111	6.80E-06	906.4521	1810.8896	2	0.0047	17	2	Chymotrypsin
H33_MOUSE	Histone H3	RFQSAAIGALQEASEAY	111	7.70E-07	906.452	1810.8895	2	0.0046	17	2	Chymotrypsin
H33_MOUSE	Histone H3	RFQSAAIGALQEASEAY	111	3.40E-07	906.452	1810.8895	2	0.0046	17	2	Chymotrypsin
H33_MOUSE	Histone H3	RFQSAAIGALQEASEAY	111	4.40E-06	906.4519	1810.8893	2	0.0044	17	2	Chymotrypsin
H33_MOUSE	Histone H3	RFQSAAIGALQEASEAY	111	1.60E-05	906.4519	1810.8892	2	0.0042	17	2	Chymotrypsin
H33_MOUSE	Histone H3	RFQSAAIGALQEASEAY	111	4.50E-07	906.4518	1810.8890	2	0.0041	17	2	Chymotrypsin
H33_MOUSE	Histone H3	RFQSAAIGALQEASEAY	111	1.70E-06	906.4518	1810.8890	2	0.0040	17	2	Chymotrypsin
H33_MOUSE	Histone H3	RFQSAAIGALQEASEAY	111	8.20E-07	906.4517	1810.8889	2	0.0040	17	2	Chymotrypsin
H33_MOUSE	Histone H3	RFQSAAIGALQEASEAY	111	9.30E-03	906.4516	1810.8887	2	0.0038	17	2	Chymotrypsin
H33_MOUSE	Histone H3	RFQSAAIGALQEASEAY	111	6.00E-06	906.4516	1810.8887	2	0.0038	17	2	Chymotrypsin
H33_MOUSE	Histone H3	RFQSAAIGALQEASEAY	111	4.70E-06	906.4516	1810.8887	2	0.0038	17	2	Chymotrypsin
H33_MOUSE	Histone H3	RFQSAAIGALQEASEAY	111	2.30E-03	906.4516	1810.8886	2	0.0037	17	2	Chymotrypsin
H33_MOUSE	Histone H3	RFQSAAIGALQEASEAY	111	1.60E-04	906.4516	1810.8886	2	0.0037	17	2	Chymotrypsin
H33_MOUSE	Histone H3	RFQSAAIGALQEASEAY	111	1.50E-06	906.4516	1810.8886	2	0.0037	17	2	Chymotrypsin
H33_MOUSE	Histone H3	RFQSAAIGALQEASEAY	111	3.90E-07	906.4515	1810.8885	2	0.0036	17	2	Chymotrypsin
H33_MOUSE	Histone H3	RFQSAAIGALQEASEAY	111	3.70E-07	906.4515	1810.8885	2	0.0036	17	2	Chymotrypsin
H33_MOUSE	Histone H3	RFQSAAIGALQEASEAY	111	1.60E-07	906.4515	1810.8885	2	0.0036	17	2	Chymotrypsin

H33_MOUSE	Histone H3	RFQSAAIGALQEASEAY	111	6.70E-08	906.4516	1810.8886	2	0.0036	17	2	Chymotrypsin
H33_MOUSE	Histone H3	RFQSAAIGALQEASEAY	111	7.00E-06	906.4515	1810.8884	2	0.0035	17	2	Chymotrypsin
H33_MOUSE	Histone H3	RFQSAAIGALQEASEAY	111	2.30E-06	906.4515	1810.8884	2	0.0035	17	2	Chymotrypsin
H33_MOUSE	Histone H3	RFQSAAIGALQEASEAY	111	1.20E-06	906.4515	1810.8884	2	0.0035	17	2	Chymotrypsin
H33_MOUSE	Histone H3	RFQSAAIGALQEASEAY	111	2.20E-05	906.4514	1810.8883	2	0.0034	17	2	Chymotrypsin
H33_MOUSE	Histone H3	RFQSAAIGALQEASEAY	111	2.20E-06	604.6367	1810.8883	3	0.0034	17	2	Chymotrypsin
H33_MOUSE	Histone H3	RFQSAAIGALQEASEAY	111	3.20E-07	906.4515	1810.8883	2	0.0034	17	2	Chymotrypsin
H33_MOUSE	Histone H3	RFQSAAIGALQEASEAY	111	1.80E-08	906.4514	1810.8883	2	0.0034	17	2	Chymotrypsin
H33_MOUSE	Histone H3	RFQSAAIGALQEASEAY	111	5.20E-06	906.4514	1810.8883	2	0.0033	17	2	Chymotrypsin
H33_MOUSE	Histone H3	RFQSAAIGALQEASEAY	111	6.20E-03	906.4513	1810.8881	2	0.0032	17	2	Chymotrypsin
H33_MOUSE	Histone H3	RFQSAAIGALQEASEAY	111	1.70E-06	906.4513	1810.8881	2	0.0032	17	2	Chymotrypsin
H33_MOUSE	Histone H3	RFQSAAIGALQEASEAY	111	1.60E-06	906.4514	1810.8881	2	0.0032	17	2	Chymotrypsin
H33_MOUSE	Histone H3	RFQSAAIGALQEASEAY	111	8.40E-07	906.4513	1810.8881	2	0.0032	17	2	Chymotrypsin
H33_MOUSE	Histone H3	RFQSAAIGALQEASEAY	111	2.20E-07	906.4513	1810.8881	2	0.0032	17	2	Chymotrypsin
H33_MOUSE	Histone H3	RFQSAAIGALQEASEAY	111	7.70E-04	906.4513	1810.8880	2	0.0031	17	2	Chymotrypsin
H33_MOUSE	Histone H3	RFQSAAIGALQEASEAY	111	1.40E-06	906.4512	1810.8879	2	0.0030	17	2	Chymotrypsin
H33_MOUSE	Histone H3	RFQSAAIGALQEASEAY	111	2.50E-06	906.4512	1810.8878	2	0.0029	17	2	Chymotrypsin
H33_MOUSE	Histone H3	RFQSAAIGALQEASEAY	111	3.50E-07	906.4512	1810.8878	2	0.0029	17	2	Chymotrypsin
H33_MOUSE	Histone H3	RFQSAAIGALQEASEAY	111	3.40E-07	906.4512	1810.8878	2	0.0029	17	2	Chymotrypsin
H33_MOUSE	Histone H3	RFQSAAIGALQEASEAY	111	5.60E-05	906.4511	1810.8877	2	0.0028	17	2	Chymotrypsin
H33_MOUSE	Histone H3	RFQSAAIGALQEASEAY	111	2.60E-05	906.4512	1810.8878	2	0.0028	17	2	Chymotrypsin
H33_MOUSE	Histone H3	RFQSAAIGALQEASEAY	111	5.90E-07	906.4511	1810.8877	2	0.0028	17	2	Chymotrypsin
H33_MOUSE	Histone H3	RFQSAAIGALQEASEAY	111	5.50E-07	906.4511	1810.8877	2	0.0028	17	2	Chymotrypsin
H33_MOUSE	Histone H3	RFQSAAIGALQEASEAY	111	3.40E-07	906.4511	1810.8877	2	0.0028	17	2	Chymotrypsin
H33_MOUSE	Histone H3	RFQSAAIGALQEASEAY	111	1.70E-07	906.4511	1810.8876	2	0.0027	17	2	Chymotrypsin
H33_MOUSE	Histone H3	RFQSAAIGALQEASEAY	111	1.40E-03	906.451	1810.8875	2	0.0026	17	2	Chymotrypsin
H33_MOUSE	Histone H3	RFQSAAIGALQEASEAY	111	4.20E-06	906.451	1810.8875	2	0.0026	17	2	Chymotrypsin
H33_MOUSE	Histone H3	RFQSAAIGALQEASEAY	111	1.10E-06	906.451	1810.8875	2	0.0026	17	2	Chymotrypsin
H33_MOUSE	Histone H3	RFQSAAIGALQEASEAY	111	1.10E-06	906.451	1810.8875	2	0.0026	17	2	Chymotrypsin
H33_MOUSE	Histone H3	RFQSAAIGALQEASEAY	111	1.50E-07	906.4511	1810.8876	2	0.0026	17	2	Chymotrypsin
H33_MOUSE	Histone H3	RFQSAAIGALQEASEAY	111	7.90E-07	906.451	1810.8874	2	0.0025	17	2	Chymotrypsin
H33_MOUSE	Histone H3	RFQSAAIGALQEASEAY	111	1.40E-07	906.451	1810.8874	2	0.0025	17	2	Chymotrypsin
H33_MOUSE	Histone H3	RFQSAAIGALQEASEAY	111	8.90E-07	906.451	1810.8874	2	0.0024	17	2	Chymotrypsin
H33_MOUSE	Histone H3	RFQSAAIGALQEASEAY	111	2.00E-07	906.4509	1810.8873	2	0.0024	17	2	Chymotrypsin
H33_MOUSE	Histone H3	RFQSAAIGALQEASEAY	111	1.70E-07	906.4509	1810.8873	2	0.0024	17	2	Chymotrypsin
H33_MOUSE	Histone H3	RFQSAAIGALQEASEAY	111	4.00E-07	906.4509	1810.8872	2	0.0023	17	2	Chymotrypsin
H33_MOUSE	Histone H3	RFQSAAIGALQEASEAY	111	6.50E-06	906.4508	1810.8871	2	0.0022	17	2	Chymotrypsin
H33_MOUSE	Histone H3	RFQSAAIGALQEASEAY	111	8.00E-07	906.4508	1810.8871	2	0.0022	17	2	Chymotrypsin
H33_MOUSE	Histone H3	RFQSAAIGALQEASEAY	111	1.30E-03	906.4508	1810.8871	2	0.0021	17	2	Chymotrypsin
H33_MOUSE	Histone H3	RFQSAAIGALQEASEAY	111	4.00E-04	906.4508	1810.8870	2	0.0021	17	2	Chymotrypsin
H33_MOUSE	Histone H3	RFQSAAIGALQEASEAY	111	2.70E-05	906.4508	1810.8870	2	0.0021	17	2	Chymotrypsin
H33_MOUSE	Histone H3	RFQSAAIGALQEASEAY	111	6.90E-06	906.4508	1810.8871	2	0.0021	17	2	Chymotrypsin
H33_MOUSE	Histone H3	RFQSAAIGALQEASEAY	111	1.60E-07	906.4508	1810.8870	2	0.0021	17	2	Chymotrypsin
H33_MOUSE	Histone H3	RFQSAAIGALQEASEAY	111	3.10E-03	906.4507	1810.8869	2	0.0020	17	2	Chymotrypsin
H33_MOUSE	Histone H3	RFQSAAIGALQEASEAY	111	1.50E-05	906.4507	1810.8869	2	0.0020	17	2	Chymotrypsin
H33_MOUSE	Histone H3	RFQSAAIGALQEASEAY	111	1.40E-05	906.4507	1810.8869	2	0.0020	17	2	Chymotrypsin
H33_MOUSE	Histone H3	RFQSAAIGALQEASEAY	111	4.60E-07	906.4507	1810.8869	2	0.0020	17	2	Chymotrypsin
H33_MOUSE	Histone H3	RFQSAAIGALQEASEAY	111	5.60E-06	906.4506	1810.8867	2	0.0018	17	2	Chymotrypsin
H33_MOUSE	Histone H3	RFQSAAIGALQEASEAY	111	3.70E-07	906.4506	1810.8867	2	0.0018	17	2	Chymotrypsin
H33_MOUSE	Histone H3	RFQSAAIGALQEASEAY	111	1.50E-04	604.6362	1810.8867	3	0.0017	17	2	Chymotrypsin
H33_MOUSE	Histone H3	RFQSAAIGALQEASEAY	111	9.20E-05	604.6362	1810.8867	3	0.0017	17	2	Chymotrypsin
H33_MOUSE	Histone H3	RFQSAAIGALQEASEAY	111	2.10E-05	906.4506	1810.8867	2	0.0017	17	2	Chymotrypsin
H33_MOUSE	Histone H3	RFQSAAIGALQEASEAY	111	7.70E-07	906.4506	1810.8866	2	0.0017	17	2	Chymotrypsin
H33_MOUSE	Histone H3	RFQSAAIGALQEASEAY	111	2.30E-04	604.6361	1810.8865	3	0.0016	17	2	Chymotrypsin
H33_MOUSE	Histone H3	RFQSAAIGALQEASEAY	111	5.40E-06	906.4505	1810.8865	2	0.0016	17	2	Chymotrypsin
H33_MOUSE	Histone H3	RFQSAAIGALQEASEAY	111	2.00E-06	604.6361	1810.8865	3	0.0016	17	2	Chymotrypsin
H33_MOUSE	Histone H3	RFQSAAIGALQEASEAY	111	1.00E-06	906.4505	1810.8865	2	0.0016	17	2	Chymotrypsin
H33_MOUSE	Histone H3	RFQSAAIGALQEASEAY	111	2.30E-07	906.4505	1810.8864	2	0.0015	17	2	Chymotrypsin
H33_MOUSE	Histone H3	RFQSAAIGALQEASEAY	111	1.10E-04	906.4504	1810.8863	2	0.0014	17	2	Chymotrypsin
H33_MOUSE	Histone H3	RFQSAAIGALQEASEAY	111	7.70E-07	906.4505	1810.8863	2	0.0014	17	2	Chymotrypsin
H33_MOUSE	Histone H3	RFQSAAIGALQEASEAY	111	7.50E-09	906.4504	1810.8863	2	0.0014	17	2	Chymotrypsin
H33_MOUSE	Histone H3	RFQSAAIGALQEASEAY	111	2.00E-03	604.6359	1810.8860	3	0.0011	17	2	Chymotrypsin
H33_MOUSE	Histone H3	RFQSAAIGALQEASEAY	111	4.50E-04	604.636	1810.8861	3	0.0011	17	2	Chymotrypsin
H33_MOUSE	Histone H3	RFQSAAIGALQEASEAY	111	4.90E-07	604.6359	1810.8860	3	0.0011	17	2	Chymotrypsin
H33_MOUSE	Histone H3	RFQSAAIGALQEASEAY	111	1.60E-06	906.4501	1810.8857	2	0.0008	17	2	Chymotrypsin
H33_MOUSE	Histone H3	RFQSAAIGALQEASEAY	111	3.40E-07	906.4501	1810.8856	2	0.0007	17	2	Chymotrypsin
H33_MOUSE	Histone H3	RFQSAAIGALQEASEAY	111	3.30E-04	604.6357	1810.8853	3	0.0004	17	2	Chymotrypsin
H33_MOUSE	Histone H3	RFQSAAIGALQEASEAY	111	2.70E-04	604.6357	1810.8853	3	0.0004	17	2	Chymotrypsin
H33_MOUSE	Histone H3	RFQSAAIGALQEASEAY	111	1.30E-06	604.6357	1810.8853	3	0.0004	17	2	Chymotrypsin
H33_MOUSE	Histone H3	RFQSAAIGALQEASEAY	111	2.70E-08	604.6357	1810.8853	3	0.0004	17	2	Chymotrypsin
H33_MOUSE	Histone H3	RFQSAAIGALQEASEAY	111	3.30E-07	906.4499	1810.8852	2	0.0003	17	2	Chymotrypsin
H33_MOUSE	Histone H3	RFQSAAIGALQEASEAY	111	2.60E-03	906.4498	1810.8850	2	0.0001	17	2	Chymotrypsin
H33_MOUSE	Histone H3	RFQSAAIGALQEASEAY	111	7.70E-07	906.4498	1810.8850	2	0.0001	17	2	Chymotrypsin
H33_MOUSE	Histone H3	RFQSAAIGALQEASEAY	111	7.60E-09	906.4493	1810.8840	2	-0.0010	17	2	Chymotrypsin

H33_MOUSE	Histone H3	RFQSAAIGALQEASEAY	111	4.00E-03	906.4488	1810.8830	2	-0.0019	17	2	Chymotrypsin
H33_MOUSE	Histone H3	RFQSAAIGALQEASEAY	111	4.80E-05	906.4488	1810.8830	2	-0.0019	17	2	Chymotrypsin
H33_MOUSE	Histone H3	QSAAIGALQEASEAY	5	4.50E-03	754.8702	1507.7259	2	0.0105	15	1	Chymotrypsin
H33_MOUSE	Histone H3	QSAAIGALQEASEAY	5	2.50E-07	754.8659	1507.7171	2	0.0017	15	1	Chymotrypsin
H33_MOUSE	Histone H3	QSAAIGALQEASEAY	5	1.80E-03	754.8658	1507.7170	2	0.0016	15	1	Chymotrypsin
H33_MOUSE	Histone H3	QSAAIGALQEASEAY	5	5.30E-03	754.8655	1507.7164	2	0.0010	15	1	Chymotrypsin
H33_MOUSE	Histone H3	QSAAIGALQEASEAY	5	2.30E-07	754.8653	1507.7160	2	0.0006	15	1	Chymotrypsin
H33_MOUSE	Histone H3	LRFQSAAIGALQE	76	1.80E-03	702.3824	1402.7502	2	-0.0066	13	0	GluC
H33_MOUSE	Histone H3	LRFQSAAIGALQE	76	5.90E-06	702.3909	1402.7673	2	0.0105	13	0	GluC
H33_MOUSE	Histone H3	LRFQSAAIGALQE	76	4.50E-03	702.3892	1402.7639	2	0.0071	13	0	GluC
H33_MOUSE	Histone H3	LRFQSAAIGALQE	76	3.40E-03	702.3892	1402.7638	2	0.0070	13	0	GluC
H33_MOUSE	Histone H3	LRFQSAAIGALQE	76	4.60E-04	702.388	1402.7615	2	0.0047	13	0	GluC
H33_MOUSE	Histone H3	LRFQSAAIGALQE	76	2.00E-04	702.3879	1402.7612	2	0.0044	13	0	GluC
H33_MOUSE	Histone H3	LRFQSAAIGALQE	76	3.00E-04	702.3878	1402.7611	2	0.0043	13	0	GluC
H33_MOUSE	Histone H3	LRFQSAAIGALQE	76	1.60E-04	702.3877	1402.7609	2	0.0041	13	0	GluC
H33_MOUSE	Histone H3	LRFQSAAIGALQE	76	1.60E-04	702.3877	1402.7609	2	0.0040	13	0	GluC
H33_MOUSE	Histone H3	LRFQSAAIGALQE	76	4.80E-05	702.3877	1402.7608	2	0.0040	13	0	GluC
H33_MOUSE	Histone H3	LRFQSAAIGALQE	76	3.70E-04	702.3876	1402.7607	2	0.0039	13	0	GluC
H33_MOUSE	Histone H3	LRFQSAAIGALQE	76	1.30E-03	702.3875	1402.7605	2	0.0037	13	0	GluC
H33_MOUSE	Histone H3	LRFQSAAIGALQE	76	8.30E-06	702.3876	1402.7606	2	0.0037	13	0	GluC
H33_MOUSE	Histone H3	LRFQSAAIGALQE	76	1.60E-05	702.3875	1402.7604	2	0.0036	13	0	GluC
H33_MOUSE	Histone H3	LRFQSAAIGALQE	76	1.30E-05	702.3874	1402.7603	2	0.0035	13	0	GluC
H33_MOUSE	Histone H3	LRFQSAAIGALQE	76	1.10E-03	702.3874	1402.7603	2	0.0034	13	0	GluC
H33_MOUSE	Histone H3	LRFQSAAIGALQE	76	8.30E-03	702.3873	1402.7601	2	0.0033	13	0	GluC
H33_MOUSE	Histone H3	LRFQSAAIGALQE	76	5.40E-03	702.3873	1402.7601	2	0.0033	13	0	GluC
H33_MOUSE	Histone H3	LRFQSAAIGALQE	76	2.50E-03	702.3874	1402.7602	2	0.0033	13	0	GluC
H33_MOUSE	Histone H3	LRFQSAAIGALQE	76	1.00E-03	702.3873	1402.7601	2	0.0033	13	0	GluC
H33_MOUSE	Histone H3	LRFQSAAIGALQE	76	1.00E-05	702.3874	1402.7602	2	0.0033	13	0	GluC
H33_MOUSE	Histone H3	LRFQSAAIGALQE	76	6.20E-03	702.3873	1402.7600	2	0.0032	13	0	GluC
H33_MOUSE	Histone H3	LRFQSAAIGALQE	76	1.80E-04	702.3873	1402.7600	2	0.0032	13	0	GluC
H33_MOUSE	Histone H3	LRFQSAAIGALQE	76	1.90E-06	702.3873	1402.7600	2	0.0032	13	0	GluC
H33_MOUSE	Histone H3	LRFQSAAIGALQE	76	7.00E-05	702.3871	1402.7597	2	0.0029	13	0	GluC
H33_MOUSE	Histone H3	LRFQSAAIGALQE	76	4.90E-05	702.3871	1402.7596	2	0.0028	13	0	GluC
H33_MOUSE	Histone H3	LRFQSAAIGALQE	76	1.90E-05	702.387	1402.7595	2	0.0027	13	0	GluC
H33_MOUSE	Histone H3	LRFQSAAIGALQE	76	3.70E-03	702.387	1402.7594	2	0.0026	13	0	GluC
H33_MOUSE	Histone H3	LRFQSAAIGALQE	76	2.30E-04	702.3869	1402.7592	2	0.0024	13	0	GluC
H33_MOUSE	Histone H3	LRFQSAAIGALQE	76	1.60E-05	702.3868	1402.7591	2	0.0023	13	0	GluC
H33_MOUSE	Histone H3	LRFQSAAIGALQE	76	8.20E-06	702.3868	1402.7591	2	0.0023	13	0	GluC
H33_MOUSE	Histone H3	LRFQSAAIGALQE	76	4.50E-04	702.3868	1402.7590	2	0.0022	13	0	GluC
H33_MOUSE	Histone H3	LRFQSAAIGALQE	76	2.00E-03	702.3868	1402.7590	2	0.0021	13	0	GluC
H33_MOUSE	Histone H3	LRFQSAAIGALQE	76	3.50E-04	702.3867	1402.7588	2	0.0020	13	0	GluC
H33_MOUSE	Histone H3	LRFQSAAIGALQE	76	2.40E-04	702.3866	1402.7587	2	0.0019	13	0	GluC
H33_MOUSE	Histone H3	LRFQSAAIGALQE	76	2.00E-04	702.3867	1402.7587	2	0.0019	13	0	GluC
H33_MOUSE	Histone H3	LRFQSAAIGALQE	76	9.90E-03	702.3866	1402.7586	2	0.0018	13	0	GluC
H33_MOUSE	Histone H3	LRFQSAAIGALQE	76	2.00E-04	702.3866	1402.7586	2	0.0018	13	0	GluC
H33_MOUSE	Histone H3	LRFQSAAIGALQE	76	8.00E-06	702.3866	1402.7586	2	0.0018	13	0	GluC
H33_MOUSE	Histone H3	LRFQSAAIGALQE	76	3.00E-03	702.3865	1402.7585	2	0.0017	13	0	GluC
H33_MOUSE	Histone H3	LRFQSAAIGALQE	76	1.30E-04	702.3865	1402.7585	2	0.0017	13	0	GluC
H33_MOUSE	Histone H3	LRFQSAAIGALQE	76	4.10E-03	702.3865	1402.7584	2	0.0016	13	0	GluC
H33_MOUSE	Histone H3	LRFQSAAIGALQE	76	3.60E-03	702.3865	1402.7584	2	0.0016	13	0	GluC
H33_MOUSE	Histone H3	LRFQSAAIGALQE	76	1.80E-03	702.3865	1402.7585	2	0.0016	13	0	GluC
H33_MOUSE	Histone H3	LRFQSAAIGALQE	76	1.80E-04	702.3865	1402.7584	2	0.0016	13	0	GluC
H33_MOUSE	Histone H3	LRFQSAAIGALQE	76	2.10E-05	702.3865	1402.7584	2	0.0016	13	0	GluC
H33_MOUSE	Histone H3	LRFQSAAIGALQE	76	9.60E-06	702.3865	1402.7584	2	0.0016	13	0	GluC
H33_MOUSE	Histone H3	LRFQSAAIGALQE	76	6.50E-03	702.3865	1402.7584	2	0.0015	13	0	GluC
H33_MOUSE	Histone H3	LRFQSAAIGALQE	76	9.30E-04	702.3864	1402.7583	2	0.0015	13	0	GluC
H33_MOUSE	Histone H3	LRFQSAAIGALQE	76	6.50E-06	702.3864	1402.7582	2	0.0014	13	0	GluC
H33_MOUSE	Histone H3	LRFQSAAIGALQE	76	3.20E-05	468.5933	1402.7582	3	0.0013	13	0	GluC
H33_MOUSE	Histone H3	LRFQSAAIGALQE	76	2.60E-06	702.3863	1402.7581	2	0.0013	13	0	GluC
H33_MOUSE	Histone H3	LRFQSAAIGALQE	76	2.50E-03	702.3863	1402.7580	2	0.0011	13	0	GluC
H33_MOUSE	Histone H3	LRFQSAAIGALQE	76	1.80E-04	702.3862	1402.7579	2	0.0011	13	0	GluC
H33_MOUSE	Histone H3	LRFQSAAIGALQE	76	5.70E-05	468.5933	1402.7579	3	0.0011	13	0	GluC
H33_MOUSE	Histone H3	LRFQSAAIGALQE	76	5.40E-06	702.3862	1402.7579	2	0.0011	13	0	GluC
H33_MOUSE	Histone H3	LRFQSAAIGALQE	76	8.90E-05	702.3862	1402.7578	2	0.0010	13	0	GluC
H33_MOUSE	Histone H3	LRFQSAAIGALQE	76	4.10E-05	702.3862	1402.7578	2	0.0010	13	0	GluC
H33_MOUSE	Histone H3	LRFQSAAIGALQE	76	3.80E-03	702.3861	1402.7577	2	0.0009	13	0	GluC
H33_MOUSE	Histone H3	LRFQSAAIGALQE	76	1.70E-03	702.3861	1402.7577	2	0.0009	13	0	GluC
H33_MOUSE	Histone H3	LRFQSAAIGALQE	76	5.60E-04	702.3862	1402.7578	2	0.0009	13	0	GluC
H33_MOUSE	Histone H3	LRFQSAAIGALQE	76	5.40E-04	702.3861	1402.7577	2	0.0009	13	0	GluC
H33_MOUSE	Histone H3	LRFQSAAIGALQE	76	3.50E-04	702.3862	1402.7578	2	0.0009	13	0	GluC
H33_MOUSE	Histone H3	LRFQSAAIGALQE	76	8.80E-05	702.3861	1402.7577	2	0.0009	13	0	GluC
H33_MOUSE	Histone H3	LRFQSAAIGALQE	76	9.10E-04	702.386	1402.7575	2	0.0007	13	0	GluC
H33_MOUSE	Histone H3	LRFQSAAIGALQE	76	2.90E-04	468.5931	1402.7575	3	0.0007	13	0	GluC
H33_MOUSE	Histone H3	LRFQSAAIGALQE	76	1.80E-05	702.386	1402.7575	2	0.0006	13	0	GluC

H33_MOUSE	Histone H3	LRFQSAAIGALQE	76	5.80E-05	702.386	1402.7574	2	0.0005	13	0	GluC
H33_MOUSE	Histone H3	LRFQSAAIGALQE	76	4.80E-03	702.3859	1402.7572	2	0.0004	13	0	GluC
H33_MOUSE	Histone H3	LRFQSAAIGALQE	76	7.60E-05	468.593	1402.7572	3	0.0004	13	0	GluC
H33_MOUSE	Histone H3	LRFQSAAIGALQE	76	8.60E-04	702.3857	1402.7569	2	0.0001	13	0	GluC
H33_MOUSE	Histone H3	LRFQSAAIGALQE	76	3.90E-05	702.3858	1402.7570	2	0.0001	13	0	GluC
H33_MOUSE	Histone H3	LRFQSAAIGALQE	76	5.80E-07	702.3858	1402.7569	2	0.0001	13	0	GluC
H33_MOUSE	Histone H3	LRFQSAAIGALQE	76	2.30E-03	702.3857	1402.7568	2	0.0000	13	0	GluC
H33_MOUSE	Histone H3	LRFQSAAIGALQE	76	8.80E-04	702.3857	1402.7568	2	0.0000	13	0	GluC
H33_MOUSE	Histone H3	LRFQSAAIGALQE	76	5.40E-06	702.3855	1402.7564	2	-0.0004	13	0	GluC
H3L_MOUSE	Histone H3	KQLATKAASK	2	7.10E-04	523.3244	1044.6342	2	0.0051	10	2	Semi-tryptic
H3L_MOUSE	Histone H3	KQLATKAASK	2	3.80E-04	523.3236	1044.6326	2	0.0035	10	2	Semi-tryptic
H4_MOUSE	Histone H4	DAVTYTEHAKRKTVTAMDVVYALKRQGRTLYGFGG	5	3.10E-03	781.4164	3902.0454	5	0.0193	35	1	AspN
H4_MOUSE	Histone H4	DAVTYTEHAKRKTVTAMDVVYALKRQGRTLYGFGG	5	1.90E-04	781.4137	3902.0320	5	0.0058	35	1	AspN
H4_MOUSE	Histone H4	DAVTYTEHAKRKTVTAMDVVYALKRQGRTLYGFGG	5	6.70E-04	976.5149	3902.0304	4	0.0043	35	1	AspN
H4_MOUSE	Histone H4	DAVTYTEHAKRKTVTAMDVVYALKRQGRTLYGFGG	5	5.30E-03	781.4133	3902.0302	5	0.0041	35	1	AspN
H4_MOUSE	Histone H4	DAVTYTEHAKRKTVTAMDVVYALKRQGRTLYGFGG	5	1.90E-05	781.4133	3902.0300	5	0.0038	35	1	AspN
H4_MOUSE	Histone H4	AVTYTEHAKRKTVTAMDVVYALKRQGRTLYGFGG	10	2.30E-05	947.7621	3787.0191	4	0.0199	34	2	GluC
H4_MOUSE	Histone H4	AVTYTEHAKRKTVTAMDVVYALKRQGRTLYGFGG	10	2.30E-03	758.4109	3787.0183	5	0.0191	34	2	GluC
H4_MOUSE	Histone H4	AVTYTEHAKRKTVTAMDVVYALKRQGRTLYGFGG	10	6.30E-06	758.4109	3787.0183	5	0.0191	34	2	GluC
H4_MOUSE	Histone H4	AVTYTEHAKRKTVTAMDVVYALKRQGRTLYGFGG	10	1.60E-03	947.7617	3787.0177	4	0.0186	34	2	GluC
H4_MOUSE	Histone H4	AVTYTEHAKRKTVTAMDVVYALKRQGRTLYGFGG	10	5.20E-03	1263.3461	3787.0165	3	0.0173	34	2	GluC
H4_MOUSE	Histone H4	AVTYTEHAKRKTVTAMDVVYALKRQGRTLYGFGG	10	6.20E-03	758.4105	3787.0161	5	0.0169	34	2	GluC
H4_MOUSE	Histone H4	AVTYTEHAKRKTVTAMDVVYALKRQGRTLYGFGG	10	1.70E-05	758.4104	3787.0156	5	0.0164	34	2	GluC
H4_MOUSE	Histone H4	AVTYTEHAKRKTVTAMDVVYALKRQGRTLYGFGG	10	3.20E-03	947.7597	3787.0098	4	0.0106	34	2	GluC
H4_MOUSE	Histone H4	AVTYTEHAKRKTVTAMDVVYALKRQGRTLYGFGG	10	2.60E-03	758.4079	3787.0029	5	0.0037	34	2	GluC
H4_MOUSE	Histone H4	AVTYTEHAKRKTVTAMDVVYALKRQGRTLYGFGG	10	5.90E-03	758.4077	3787.0020	5	0.0028	34	2	GluC
H4_MOUSE	Histone H4	ISGLIYEETRGVLKVFLENVIRDAVTYTEHAK	4	7.30E-03	916.7508	3662.9743	4	0.0071	32	3	Semi-tryptic
H4_MOUSE	Histone H4	ISGLIYEETRGVLKVFLENVIRDAVTYTEHAK	4	1.40E-03	916.7506	3662.9733	4	0.0061	32	3	Semi-tryptic
H4_MOUSE	Histone H4	ISGLIYEETRGVLKVFLENVIRDAVTYTEHAK	4	1.20E-03	916.7505	3662.9729	4	0.0057	32	3	Semi-tryptic
H4_MOUSE	Histone H4	ISGLIYEETRGVLKVFLENVIRDAVTYTEHAK	4	1.10E-03	916.7501	3662.9713	4	0.0041	32	3	Semi-tryptic
H4_MOUSE	Histone H4	NIQGITKPAIRRLARRGGVKRISGLIYEE	21	4.00E-04	816.982	3263.8991	4	-0.0063	29	1	GluC
H4_MOUSE	Histone H4	NIQGITKPAIRRLARRGGVKRISGLIYEE	21	6.50E-03	653.787	3263.8984	5	-0.0070	29	1	GluC
H4_MOUSE	Histone H4	NIQGITKPAIRRLARRGGVKRISGLIYEE	21	1.00E-03	816.9886	3263.9253	4	0.0199	29	1	GluC
H4_MOUSE	Histone H4	NIQGITKPAIRRLARRGGVKRISGLIYEE	21	2.40E-03	816.9883	3263.9239	4	0.0185	29	1	GluC
H4_MOUSE	Histone H4	NIQGITKPAIRRLARRGGVKRISGLIYEE	21	2.20E-03	653.7919	3263.9233	5	0.0179	29	1	GluC
H4_MOUSE	Histone H4	NIQGITKPAIRRLARRGGVKRISGLIYEE	21	7.30E-03	816.988	3263.9228	4	0.0174	29	1	GluC
H4_MOUSE	Histone H4	NIQGITKPAIRRLARRGGVKRISGLIYEE	21	4.90E-04	816.9877	3263.9219	4	0.0164	29	1	GluC
H4_MOUSE	Histone H4	NIQGITKPAIRRLARRGGVKRISGLIYEE	21	4.50E-03	816.9874	3263.9204	4	0.0150	29	1	GluC
H4_MOUSE	Histone H4	NIQGITKPAIRRLARRGGVKRISGLIYEE	21	1.40E-03	816.9873	3263.9200	4	0.0146	29	1	GluC
H4_MOUSE	Histone H4	NIQGITKPAIRRLARRGGVKRISGLIYEE	21	5.00E-04	816.987	3263.9188	4	0.0134	29	1	GluC
H4_MOUSE	Histone H4	NIQGITKPAIRRLARRGGVKRISGLIYEE	21	5.20E-04	816.9865	3263.9170	4	0.0116	29	1	GluC
H4_MOUSE	Histone H4	NIQGITKPAIRRLARRGGVKRISGLIYEE	21	3.50E-03	816.9862	3263.9158	4	0.0104	29	1	GluC
H4_MOUSE	Histone H4	NIQGITKPAIRRLARRGGVKRISGLIYEE	21	6.10E-03	816.9859	3263.9145	4	0.0091	29	1	GluC
H4_MOUSE	Histone H4	NIQGITKPAIRRLARRGGVKRISGLIYEE	21	6.20E-03	816.9854	3263.9124	4	0.0070	29	1	GluC
H4_MOUSE	Histone H4	NIQGITKPAIRRLARRGGVKRISGLIYEE	21	6.90E-03	816.985	3263.9108	4	0.0054	29	1	GluC
H4_MOUSE	Histone H4	NIQGITKPAIRRLARRGGVKRISGLIYEE	21	1.00E-02	816.9848	3263.9101	4	0.0046	29	1	GluC
H4_MOUSE	Histone H4	NIQGITKPAIRRLARRGGVKRISGLIYEE	21	4.30E-04	816.9847	3263.9097	4	0.0042	29	1	GluC
H4_MOUSE	Histone H4	NIQGITKPAIRRLARRGGVKRISGLIYEE	21	1.00E-03	816.9845	3263.9089	4	0.0035	29	1	GluC
H4_MOUSE	Histone H4	NIQGITKPAIRRLARRGGVKRISGLIYEE	21	3.20E-03	816.9844	3263.9085	4	0.0031	29	1	GluC
H4_MOUSE	Histone H4	NIQGITKPAIRRLARRGGVKRISGLIYEE	21	5.30E-03	816.9842	3263.9076	4	0.0022	29	1	GluC
H4_MOUSE	Histone H4	NIQGITKPAIRRLARRGGVKRISGLIYEE	21	3.40E-03	816.9837	3263.9059	4	0.0005	29	1	GluC
H4_MOUSE	Histone H4	HAKRKTVTAMDVVYALKRQGRTLYGFGG	132	1.40E-04	781.6799	3122.6905	4	-0.0019	28	1	GluC
H4_MOUSE	Histone H4	HAKRKTVTAMDVVYALKRQGRTLYGFGG	132	1.40E-04	781.6799	3122.6905	4	-0.0019	28	1	GluC
H4_MOUSE	Histone H4	NIQGITKPAIRRLARRGGVKRISGLIYE	35	2.10E-03	784.7225	3134.8608	4	-0.0020	28	0	GluC
H4_MOUSE	Histone H4	HAKRKTVTAMDVVYALKRQGRTLYGFGG	132	2.70E-03	781.6798	3122.6903	4	-0.0021	28	1	GluC
H4_MOUSE	Histone H4	HAKRKTVTAMDVVYALKRQGRTLYGFGG	132	1.50E-04	781.6798	3122.6903	4	-0.0021	28	1	GluC
H4_MOUSE	Histone H4	HAKRKTVTAMDVVYALKRQGRTLYGFGG	132	1.40E-04	521.4555	3122.6891	6	-0.0033	28	1	GluC
H4_MOUSE	Histone H4	NIQGITKPAIRRLARRGGVKRISGLIYE	35	2.90E-03	784.7221	3134.8591	4	-0.0037	28	0	GluC
H4_MOUSE	Histone H4	HAKRKTVTAMDVVYALKRQGRTLYGFGG	132	6.60E-06	521.4552	3122.6878	6	-0.0045	28	1	GluC
H4_MOUSE	Histone H4	NIQGITKPAIRRLARRGGVKRISGLIYE	35	9.70E-04	784.7217	3134.8578	4	-0.0050	28	0	GluC
H4_MOUSE	Histone H4	HAKRKTVTAMDVVYALKRQGRTLYGFGG	132	1.20E-03	521.4551	3122.6870	6	-0.0054	28	1	GluC
H4_MOUSE	Histone H4	NIQGITKPAIRRLARRGGVKRISGLIYE	35	2.00E-05	784.7215	3134.8568	4	-0.0060	28	0	GluC
H4_MOUSE	Histone H4	NIQGITKPAIRRLARRGGVKRISGLIYE	35	1.30E-04	784.7278	3134.8821	4	0.0193	28	0	GluC
H4_MOUSE	Histone H4	NIQGITKPAIRRLARRGGVKRISGLIYE	35	6.40E-04	784.7276	3134.8813	4	0.0185	28	0	GluC
H4_MOUSE	Histone H4	NIQGITKPAIRRLARRGGVKRISGLIYE	35	4.10E-03	1045.9677	3134.8813	3	0.0184	28	0	GluC
H4_MOUSE	Histone H4	NIQGITKPAIRRLARRGGVKRISGLIYE	35	2.50E-04	784.7276	3134.8812	4	0.0184	28	0	GluC
H4_MOUSE	Histone H4	NIQGITKPAIRRLARRGGVKRISGLIYE	35	7.50E-03	784.7272	3134.8796	4	0.0168	28	0	GluC
H4_MOUSE	Histone H4	NIQGITKPAIRRLARRGGVKRISGLIYE	35	4.20E-03	627.983	3134.8788	5	0.0159	28	0	GluC
H4_MOUSE	Histone H4	NIQGITKPAIRRLARRGGVKRISGLIYE	35	6.10E-06	784.7266	3134.8774	4	0.0146	28	0	GluC
H4_MOUSE	Histone H4	NIQGITKPAIRRLARRGGVKRISGLIYE	35	4.80E-03	784.7264	3134.8767	4	0.0138	28	0	GluC
H4_MOUSE	Histone H4	NIQGITKPAIRRLARRGGVKRISGLIYE	35	2.90E-03	784.7264	3134.8766	4	0.0138	28	0	GluC
H4_MOUSE	Histone H4	NIQGITKPAIRRLARRGGVKRISGLIYE	35	2.10E-03	784.7263	3134.8762	4	0.0134	28	0	GluC
H4_MOUSE	Histone H4	NIQGITKPAIRRLARRGGVKRISGLIYE	35	1.60E-03	627.9825	3134.8762	5	0.0133	28	0	GluC
H4_MOUSE	Histone H4	NIQGITKPAIRRLARRGGVKRISGLIYE	35	3.00E-03	784.7262	3134.8759	4	0.0130	28	0	GluC

H4_MOUSE	Histone H4	NIQGITKPAIRRLARRGGVKRISGLIYE	35	8.90E-05	784.7259	3134.8747	4	0.0118	28	0	GluC
H4_MOUSE	Histone H4	NIQGITKPAIRRLARRGGVKRISGLIYE	35	1.00E-04	784.7258	3134.8740	4	0.0112	28	0	GluC
H4_MOUSE	Histone H4	NIQGITKPAIRRLARRGGVKRISGLIYE	35	4.00E-05	784.7256	3134.8731	4	0.0103	28	0	GluC
H4_MOUSE	Histone H4	NIQGITKPAIRRLARRGGVKRISGLIYE	35	1.60E-04	784.7251	3134.8714	4	0.0086	28	0	GluC
H4_MOUSE	Histone H4	NIQGITKPAIRRLARRGGVKRISGLIYE	35	9.00E-03	784.725	3134.8709	4	0.0080	28	0	GluC
H4_MOUSE	Histone H4	NIQGITKPAIRRLARRGGVKRISGLIYE	35	8.80E-04	784.7249	3134.8705	4	0.0077	28	0	GluC
H4_MOUSE	Histone H4	NIQGITKPAIRRLARRGGVKRISGLIYE	35	2.60E-03	784.7247	3134.8699	4	0.0070	28	0	GluC
H4_MOUSE	Histone H4	NIQGITKPAIRRLARRGGVKRISGLIYE	35	2.00E-05	784.7246	3134.8691	4	0.0063	28	0	GluC
H4_MOUSE	Histone H4	NIQGITKPAIRRLARRGGVKRISGLIYE	35	1.80E-03	784.7244	3134.8685	4	0.0057	28	0	GluC
H4_MOUSE	Histone H4	NIQGITKPAIRRLARRGGVKRISGLIYE	35	5.90E-03	784.7244	3134.8684	4	0.0056	28	0	GluC
H4_MOUSE	Histone H4	NIQGITKPAIRRLARRGGVKRISGLIYE	35	2.30E-05	784.7241	3134.8675	4	0.0046	28	0	GluC
H4_MOUSE	Histone H4	NIQGITKPAIRRLARRGGVKRISGLIYE	35	5.30E-03	784.7241	3134.8672	4	0.0044	28	0	GluC
H4_MOUSE	Histone H4	NIQGITKPAIRRLARRGGVKRISGLIYE	35	3.40E-03	784.7239	3134.8665	4	0.0037	28	0	GluC
H4_MOUSE	Histone H4	NIQGITKPAIRRLARRGGVKRISGLIYE	35	5.00E-03	784.7237	3134.8656	4	0.0028	28	0	GluC
H4_MOUSE	Histone H4	NIQGITKPAIRRLARRGGVKRISGLIYE	35	2.10E-03	784.7234	3134.8645	4	0.0017	28	0	GluC
H4_MOUSE	Histone H4	NIQGITKPAIRRLARRGGVKRISGLIYE	35	1.20E-03	784.7229	3134.8625	4	-0.0003	28	0	GluC
H4_MOUSE	Histone H4	NIQGITKPAIRRLARRGGVKRISGLIYE	35	2.30E-03	784.7229	3134.8623	4	-0.0005	28	0	GluC
H4_MOUSE	Histone H4	NIQGITKPAIRRLARRGGVKRISGLIYE	35	1.30E-03	627.9797	3134.8620	5	-0.0008	28	0	GluC
H4_MOUSE	Histone H4	NIQGITKPAIRRLARRGGVKRISGLIYE	35	3.10E-05	784.7226	3134.8613	4	-0.0015	28	0	GluC
H4_MOUSE	Histone H4	HAKRKTVTAMDVVYALKRQGRTLYGFGG	132	4.40E-05	781.6852	3122.7117	4	0.0193	28	1	GluC
H4_MOUSE	Histone H4	HAKRKTVTAMDVVYALKRQGRTLYGFGG	132	4.70E-06	781.6852	3122.7117	4	0.0193	28	1	GluC
H4_MOUSE	Histone H4	HAKRKTVTAMDVVYALKRQGRTLYGFGG	132	3.30E-04	781.6852	3122.7116	4	0.0192	28	1	GluC
H4_MOUSE	Histone H4	HAKRKTVTAMDVVYALKRQGRTLYGFGG	132	1.00E-04	781.6852	3122.7116	4	0.0192	28	1	GluC
H4_MOUSE	Histone H4	HAKRKTVTAMDVVYALKRQGRTLYGFGG	132	1.00E-04	625.5495	3122.7114	5	0.0190	28	1	GluC
H4_MOUSE	Histone H4	HAKRKTVTAMDVVYALKRQGRTLYGFGG	132	7.40E-05	521.4591	3122.7111	6	0.0188	28	1	GluC
H4_MOUSE	Histone H4	HAKRKTVTAMDVVYALKRQGRTLYGFGG	132	7.80E-03	1041.9109	3122.7109	3	0.0185	28	1	GluC
H4_MOUSE	Histone H4	HAKRKTVTAMDVVYALKRQGRTLYGFGG	132	4.20E-03	1041.9109	3122.7109	3	0.0185	28	1	GluC
H4_MOUSE	Histone H4	HAKRKTVTAMDVVYALKRQGRTLYGFGG	132	6.30E-05	625.5494	3122.7109	5	0.0185	28	1	GluC
H4_MOUSE	Histone H4	HAKRKTVTAMDVVYALKRQGRTLYGFGG	132	2.00E-06	781.685	3122.7109	4	0.0185	28	1	GluC
H4_MOUSE	Histone H4	HAKRKTVTAMDVVYALKRQGRTLYGFGG	132	4.80E-07	781.6849	3122.7105	4	0.0181	28	1	GluC
H4_MOUSE	Histone H4	HAKRKTVTAMDVVYALKRQGRTLYGFGG	132	1.50E-08	521.4589	3122.7100	6	0.0176	28	1	GluC
H4_MOUSE	Histone H4	HAKRKTVTAMDVVYALKRQGRTLYGFGG	132	2.00E-05	625.5492	3122.7096	5	0.0173	28	1	GluC
H4_MOUSE	Histone H4	HAKRKTVTAMDVVYALKRQGRTLYGFGG	132	1.10E-05	781.6846	3122.7093	4	0.0169	28	1	GluC
H4_MOUSE	Histone H4	HAKRKTVTAMDVVYALKRQGRTLYGFGG	132	4.10E-03	781.6846	3122.7092	4	0.0168	28	1	GluC
H4_MOUSE	Histone H4	HAKRKTVTAMDVVYALKRQGRTLYGFGG	132	1.40E-04	781.6846	3122.7092	4	0.0168	28	1	GluC
H4_MOUSE	Histone H4	HAKRKTVTAMDVVYALKRQGRTLYGFGG	132	5.90E-05	625.5487	3122.7072	5	0.0149	28	1	GluC
H4_MOUSE	Histone H4	HAKRKTVTAMDVVYALKRQGRTLYGFGG	132	4.00E-03	625.5487	3122.7069	5	0.0146	28	1	GluC
H4_MOUSE	Histone H4	HAKRKTVTAMDVVYALKRQGRTLYGFGG	132	2.80E-06	521.4584	3122.7067	6	0.0143	28	1	GluC
H4_MOUSE	Histone H4	HAKRKTVTAMDVVYALKRQGRTLYGFGG	132	1.10E-06	781.6839	3122.7067	4	0.0143	28	1	GluC
H4_MOUSE	Histone H4	HAKRKTVTAMDVVYALKRQGRTLYGFGG	132	5.00E-04	625.5486	3122.7064	5	0.0141	28	1	GluC
H4_MOUSE	Histone H4	HAKRKTVTAMDVVYALKRQGRTLYGFGG	132	1.20E-05	781.6836	3122.7055	4	0.0131	28	1	GluC
H4_MOUSE	Histone H4	HAKRKTVTAMDVVYALKRQGRTLYGFGG	132	4.50E-03	521.4581	3122.7049	6	0.0125	28	1	GluC
H4_MOUSE	Histone H4	HAKRKTVTAMDVVYALKRQGRTLYGFGG	132	2.90E-07	781.6831	3122.7031	4	0.0108	28	1	GluC
H4_MOUSE	Histone H4	HAKRKTVTAMDVVYALKRQGRTLYGFGG	132	4.40E-05	625.5478	3122.7024	5	0.0101	28	1	GluC
H4_MOUSE	Histone H4	HAKRKTVTAMDVVYALKRQGRTLYGFGG	132	1.40E-03	625.5475	3122.7011	5	0.0087	28	1	GluC
H4_MOUSE	Histone H4	HAKRKTVTAMDVVYALKRQGRTLYGFGG	132	6.60E-06	625.5475	3122.7009	5	0.0085	28	1	GluC
H4_MOUSE	Histone H4	HAKRKTVTAMDVVYALKRQGRTLYGFGG	132	8.50E-03	1041.9075	3122.7007	3	0.0083	28	1	GluC
H4_MOUSE	Histone H4	HAKRKTVTAMDVVYALKRQGRTLYGFGG	132	2.60E-04	781.6824	3122.7007	4	0.0083	28	1	GluC
H4_MOUSE	Histone H4	HAKRKTVTAMDVVYALKRQGRTLYGFGG	132	1.90E-03	781.6821	3122.6995	4	0.0071	28	1	GluC
H4_MOUSE	Histone H4	HAKRKTVTAMDVVYALKRQGRTLYGFGG	132	9.00E-06	781.6821	3122.6995	4	0.0071	28	1	GluC
H4_MOUSE	Histone H4	HAKRKTVTAMDVVYALKRQGRTLYGFGG	132	8.50E-03	781.6821	3122.6994	4	0.0070	28	1	GluC
H4_MOUSE	Histone H4	HAKRKTVTAMDVVYALKRQGRTLYGFGG	132	1.60E-03	781.682	3122.6987	4	0.0063	28	1	GluC
H4_MOUSE	Histone H4	HAKRKTVTAMDVVYALKRQGRTLYGFGG	132	9.40E-05	781.682	3122.6987	4	0.0063	28	1	GluC
H4_MOUSE	Histone H4	HAKRKTVTAMDVVYALKRQGRTLYGFGG	132	1.20E-03	1041.9068	3122.6986	3	0.0062	28	1	GluC
H4_MOUSE	Histone H4	HAKRKTVTAMDVVYALKRQGRTLYGFGG	132	2.00E-04	781.6819	3122.6983	4	0.0060	28	1	GluC
H4_MOUSE	Histone H4	HAKRKTVTAMDVVYALKRQGRTLYGFGG	132	4.80E-08	781.6819	3122.6983	4	0.0060	28	1	GluC
H4_MOUSE	Histone H4	HAKRKTVTAMDVVYALKRQGRTLYGFGG	132	1.30E-04	1041.9067	3122.6983	3	0.0059	28	1	GluC
H4_MOUSE	Histone H4	HAKRKTVTAMDVVYALKRQGRTLYGFGG	132	1.60E-04	781.6818	3122.6980	4	0.0056	28	1	GluC
H4_MOUSE	Histone H4	HAKRKTVTAMDVVYALKRQGRTLYGFGG	132	1.20E-03	1041.9065	3122.6977	3	0.0053	28	1	GluC
H4_MOUSE	Histone H4	HAKRKTVTAMDVVYALKRQGRTLYGFGG	132	1.40E-03	781.6816	3122.6974	4	0.0050	28	1	GluC
H4_MOUSE	Histone H4	HAKRKTVTAMDVVYALKRQGRTLYGFGG	132	3.60E-04	781.6816	3122.6974	4	0.0050	28	1	GluC
H4_MOUSE	Histone H4	HAKRKTVTAMDVVYALKRQGRTLYGFGG	132	6.50E-04	781.6816	3122.6973	4	0.0049	28	1	GluC
H4_MOUSE	Histone H4	HAKRKTVTAMDVVYALKRQGRTLYGFGG	132	5.80E-04	781.6816	3122.6973	4	0.0049	28	1	GluC
H4_MOUSE	Histone H4	HAKRKTVTAMDVVYALKRQGRTLYGFGG	132	2.70E-04	781.6815	3122.6968	4	0.0044	28	1	GluC
H4_MOUSE	Histone H4	HAKRKTVTAMDVVYALKRQGRTLYGFGG	132	2.50E-05	781.6815	3122.6968	4	0.0044	28	1	GluC
H4_MOUSE	Histone H4	HAKRKTVTAMDVVYALKRQGRTLYGFGG	132	2.60E-07	781.6815	3122.6968	4	0.0044	28	1	GluC
H4_MOUSE	Histone H4	HAKRKTVTAMDVVYALKRQGRTLYGFGG	132	1.30E-03	781.6814	3122.6967	4	0.0043	28	1	GluC
H4_MOUSE	Histone H4	HAKRKTVTAMDVVYALKRQGRTLYGFGG	132	4.90E-05	781.6814	3122.6967	4	0.0043	28	1	GluC
H4_MOUSE	Histone H4	HAKRKTVTAMDVVYALKRQGRTLYGFGG	132	7.00E-04	781.6814	3122.6966	4	0.0042	28	1	GluC
H4_MOUSE	Histone H4	HAKRKTVTAMDVVYALKRQGRTLYGFGG	132	4.20E-05	781.6814	3122.6966	4	0.0042	28	1	GluC
H4_MOUSE	Histone H4	HAKRKTVTAMDVVYALKRQGRTLYGFGG	132	7.40E-04	781.6813	3122.6962	4	0.0038	28	1	GluC
H4_MOUSE	Histone H4	HAKRKTVTAMDVVYALKRQGRTLYGFGG	132	7.20E-05	781.6813	3122.6962	4	0.0038	28	1	GluC
H4_MOUSE	Histone H4	HAKRKTVTAMDVVYALKRQGRTLYGFGG	132	3.00E-04	625.5465	3122.6960	5	0.0037	28	1	GluC
H4_MOUSE	Histone H4	HAKRKTVTAMDVVYALKRQGRTLYGFGG	132	1.90E-03	781.6813	3122.6959	4	0.0036	28	1	GluC

H4_MOUSE	Histone H4	HAKRKTVTAMDVVYALKRQGRTLYGFGG	132	1.20E-04	781.6813	3122.6959	4	0.0036	28	1	GluC
H4_MOUSE	Histone H4	HAKRKTVTAMDVVYALKRQGRTLYGFGG	132	2.70E-04	625.5464	3122.6958	5	0.0034	28	1	GluC
H4_MOUSE	Histone H4	HAKRKTVTAMDVVYALKRQGRTLYGFGG	132	3.40E-05	781.6812	3122.6957	4	0.0034	28	1	GluC
H4_MOUSE	Histone H4	HAKRKTVTAMDVVYALKRQGRTLYGFGG	132	2.80E-05	781.6812	3122.6957	4	0.0034	28	1	GluC
H4_MOUSE	Histone H4	HAKRKTVTAMDVVYALKRQGRTLYGFGG	132	9.40E-03	781.6812	3122.6957	4	0.0033	28	1	GluC
H4_MOUSE	Histone H4	HAKRKTVTAMDVVYALKRQGRTLYGFGG	132	6.80E-03	781.6812	3122.6957	4	0.0033	28	1	GluC
H4_MOUSE	Histone H4	HAKRKTVTAMDVVYALKRQGRTLYGFGG	132	5.60E-03	781.6812	3122.6957	4	0.0033	28	1	GluC
H4_MOUSE	Histone H4	HAKRKTVTAMDVVYALKRQGRTLYGFGG	132	1.20E-03	781.6812	3122.6957	4	0.0033	28	1	GluC
H4_MOUSE	Histone H4	HAKRKTVTAMDVVYALKRQGRTLYGFGG	132	7.20E-04	781.6812	3122.6957	4	0.0033	28	1	GluC
H4_MOUSE	Histone H4	HAKRKTVTAMDVVYALKRQGRTLYGFGG	132	6.20E-04	781.6812	3122.6957	4	0.0033	28	1	GluC
H4_MOUSE	Histone H4	HAKRKTVTAMDVVYALKRQGRTLYGFGG	132	4.50E-05	781.6812	3122.6957	4	0.0033	28	1	GluC
H4_MOUSE	Histone H4	HAKRKTVTAMDVVYALKRQGRTLYGFGG	132	3.20E-05	781.6812	3122.6957	4	0.0033	28	1	GluC
H4_MOUSE	Histone H4	HAKRKTVTAMDVVYALKRQGRTLYGFGG	132	4.30E-03	781.6811	3122.6955	4	0.0031	28	1	GluC
H4_MOUSE	Histone H4	HAKRKTVTAMDVVYALKRQGRTLYGFGG	132	1.50E-03	781.6811	3122.6955	4	0.0031	28	1	GluC
H4_MOUSE	Histone H4	HAKRKTVTAMDVVYALKRQGRTLYGFGG	132	1.10E-03	625.5464	3122.6955	5	0.0031	28	1	GluC
H4_MOUSE	Histone H4	HAKRKTVTAMDVVYALKRQGRTLYGFGG	132	2.20E-04	781.6811	3122.6955	4	0.0031	28	1	GluC
H4_MOUSE	Histone H4	HAKRKTVTAMDVVYALKRQGRTLYGFGG	132	1.70E-03	781.6811	3122.6953	4	0.0029	28	1	GluC
H4_MOUSE	Histone H4	HAKRKTVTAMDVVYALKRQGRTLYGFGG	132	6.90E-05	781.6811	3122.6953	4	0.0029	28	1	GluC
H4_MOUSE	Histone H4	HAKRKTVTAMDVVYALKRQGRTLYGFGG	132	1.30E-05	625.5463	3122.6950	5	0.0026	28	1	GluC
H4_MOUSE	Histone H4	HAKRKTVTAMDVVYALKRQGRTLYGFGG	132	6.10E-03	781.681	3122.6949	4	0.0025	28	1	GluC
H4_MOUSE	Histone H4	HAKRKTVTAMDVVYALKRQGRTLYGFGG	132	1.10E-03	521.4564	3122.6949	6	0.0025	28	1	GluC
H4_MOUSE	Histone H4	HAKRKTVTAMDVVYALKRQGRTLYGFGG	132	4.40E-06	781.681	3122.6949	4	0.0025	28	1	GluC
H4_MOUSE	Histone H4	HAKRKTVTAMDVVYALKRQGRTLYGFGG	132	5.00E-04	781.681	3122.6947	4	0.0024	28	1	GluC
H4_MOUSE	Histone H4	HAKRKTVTAMDVVYALKRQGRTLYGFGG	132	9.70E-05	781.681	3122.6948	4	0.0024	28	1	GluC
H4_MOUSE	Histone H4	HAKRKTVTAMDVVYALKRQGRTLYGFGG	132	9.90E-04	781.681	3122.6947	4	0.0023	28	1	GluC
H4_MOUSE	Histone H4	HAKRKTVTAMDVVYALKRQGRTLYGFGG	132	5.50E-04	781.681	3122.6947	4	0.0023	28	1	GluC
H4_MOUSE	Histone H4	HAKRKTVTAMDVVYALKRQGRTLYGFGG	132	7.60E-05	781.681	3122.6947	4	0.0023	28	1	GluC
H4_MOUSE	Histone H4	HAKRKTVTAMDVVYALKRQGRTLYGFGG	132	1.50E-03	625.5462	3122.6945	5	0.0022	28	1	GluC
H4_MOUSE	Histone H4	HAKRKTVTAMDVVYALKRQGRTLYGFGG	132	1.60E-03	781.6809	3122.6945	4	0.0021	28	1	GluC
H4_MOUSE	Histone H4	HAKRKTVTAMDVVYALKRQGRTLYGFGG	132	8.30E-05	781.6809	3122.6945	4	0.0021	28	1	GluC
H4_MOUSE	Histone H4	HAKRKTVTAMDVVYALKRQGRTLYGFGG	132	6.60E-05	625.5461	3122.6943	5	0.0019	28	1	GluC
H4_MOUSE	Histone H4	HAKRKTVTAMDVVYALKRQGRTLYGFGG	132	5.80E-03	625.5461	3122.6940	5	0.0017	28	1	GluC
H4_MOUSE	Histone H4	HAKRKTVTAMDVVYALKRQGRTLYGFGG	132	4.10E-03	521.4563	3122.6940	6	0.0017	28	1	GluC
H4_MOUSE	Histone H4	HAKRKTVTAMDVVYALKRQGRTLYGFGG	132	2.40E-04	625.5461	3122.6940	5	0.0017	28	1	GluC
H4_MOUSE	Histone H4	HAKRKTVTAMDVVYALKRQGRTLYGFGG	132	1.00E-04	521.4563	3122.6940	6	0.0017	28	1	GluC
H4_MOUSE	Histone H4	HAKRKTVTAMDVVYALKRQGRTLYGFGG	132	3.70E-05	625.5461	3122.6940	5	0.0017	28	1	GluC
H4_MOUSE	Histone H4	HAKRKTVTAMDVVYALKRQGRTLYGFGG	132	1.00E-02	781.6807	3122.6939	4	0.0015	28	1	GluC
H4_MOUSE	Histone H4	HAKRKTVTAMDVVYALKRQGRTLYGFGG	132	2.20E-03	521.4562	3122.6938	6	0.0014	28	1	GluC
H4_MOUSE	Histone H4	HAKRKTVTAMDVVYALKRQGRTLYGFGG	132	4.00E-04	781.6807	3122.6938	4	0.0014	28	1	GluC
H4_MOUSE	Histone H4	HAKRKTVTAMDVVYALKRQGRTLYGFGG	132	6.70E-06	781.6807	3122.6938	4	0.0014	28	1	GluC
H4_MOUSE	Histone H4	HAKRKTVTAMDVVYALKRQGRTLYGFGG	132	5.00E-06	781.6807	3122.6938	4	0.0014	28	1	GluC
H4_MOUSE	Histone H4	HAKRKTVTAMDVVYALKRQGRTLYGFGG	132	3.80E-04	781.6807	3122.6937	4	0.0013	28	1	GluC
H4_MOUSE	Histone H4	HAKRKTVTAMDVVYALKRQGRTLYGFGG	132	6.30E-07	521.4562	3122.6937	6	0.0013	28	1	GluC
H4_MOUSE	Histone H4	HAKRKTVTAMDVVYALKRQGRTLYGFGG	132	6.10E-04	781.6807	3122.6936	4	0.0012	28	1	GluC
H4_MOUSE	Histone H4	HAKRKTVTAMDVVYALKRQGRTLYGFGG	132	1.20E-04	781.6807	3122.6936	4	0.0012	28	1	GluC
H4_MOUSE	Histone H4	HAKRKTVTAMDVVYALKRQGRTLYGFGG	132	1.10E-04	521.4562	3122.6935	6	0.0012	28	1	GluC
H4_MOUSE	Histone H4	HAKRKTVTAMDVVYALKRQGRTLYGFGG	132	1.90E-05	781.6806	3122.6933	4	0.0010	28	1	GluC
H4_MOUSE	Histone H4	HAKRKTVTAMDVVYALKRQGRTLYGFGG	132	1.00E-05	781.6806	3122.6933	4	0.0010	28	1	GluC
H4_MOUSE	Histone H4	HAKRKTVTAMDVVYALKRQGRTLYGFGG	132	7.10E-04	625.5459	3122.6932	5	0.0009	28	1	GluC
H4_MOUSE	Histone H4	HAKRKTVTAMDVVYALKRQGRTLYGFGG	132	2.80E-04	781.6806	3122.6932	4	0.0008	28	1	GluC
H4_MOUSE	Histone H4	HAKRKTVTAMDVVYALKRQGRTLYGFGG	132	1.60E-04	781.6806	3122.6932	4	0.0008	28	1	GluC
H4_MOUSE	Histone H4	HAKRKTVTAMDVVYALKRQGRTLYGFGG	132	6.30E-06	521.4561	3122.6930	6	0.0006	28	1	GluC
H4_MOUSE	Histone H4	HAKRKTVTAMDVVYALKRQGRTLYGFGG	132	3.40E-04	781.6805	3122.6929	4	0.0005	28	1	GluC
H4_MOUSE	Histone H4	HAKRKTVTAMDVVYALKRQGRTLYGFGG	132	1.90E-03	781.6804	3122.6927	4	0.0003	28	1	GluC
H4_MOUSE	Histone H4	HAKRKTVTAMDVVYALKRQGRTLYGFGG	132	1.50E-03	781.6804	3122.6927	4	0.0003	28	1	GluC
H4_MOUSE	Histone H4	HAKRKTVTAMDVVYALKRQGRTLYGFGG	132	1.90E-03	781.6804	3122.6925	4	0.0002	28	1	GluC
H4_MOUSE	Histone H4	HAKRKTVTAMDVVYALKRQGRTLYGFGG	132	4.50E-04	781.6804	3122.6924	4	0.0000	28	1	GluC
H4_MOUSE	Histone H4	HAKRKTVTAMDVVYALKRQGRTLYGFGG	132	3.30E-04	781.6803	3122.6921	4	-0.0003	28	1	GluC
H4_MOUSE	Histone H4	HAKRKTVTAMDVVYALKRQGRTLYGFGG	132	1.80E-03	781.6803	3122.6920	4	-0.0004	28	1	GluC
H4_MOUSE	Histone H4	HAKRKTVTAMDVVYALKRQGRTLYGFGG	132	5.40E-05	781.6803	3122.6920	4	-0.0004	28	1	GluC
H4_MOUSE	Histone H4	HAKRKTVTAMDVVYALKRQGRTLYGFGG	132	5.50E-03	521.4559	3122.6917	6	-0.0007	28	1	GluC
H4_MOUSE	Histone H4	HAKRKTVTAMDVVYALKRQGRTLYGFGG	132	6.60E-05	625.5456	3122.6915	5	-0.0008	28	1	GluC
H4_MOUSE	Histone H4	HAKRKTVTAMDVVYALKRQGRTLYGFGG	132	6.40E-04	781.6801	3122.6915	4	-0.0009	28	1	GluC
H4_MOUSE	Histone H4	HAKRKTVTAMDVVYALKRQGRTLYGFGG	132	1.80E-05	521.4558	3122.6911	6	-0.0012	28	1	GluC
H4_MOUSE	Histone H4	HAKRKTVTAMDVVYALKRQGRTLYGFGG	132	7.60E-04	521.4558	3122.6911	6	-0.0013	28	1	GluC
H4_MOUSE	Histone H4	HAKRKTVTAMDVVYALKRQGRTLYGFGG	132	1.00E-04	521.4558	3122.6910	6	-0.0013	28	1	GluC
H4_MOUSE	Histone H4	HAKRKTVTAMDVVYALKRQGRTLYGFGG	132	4.70E-05	781.68	3122.6910	4	-0.0014	28	1	GluC
H4_MOUSE	Histone H4	HAKRKTVTAMDVVYALKRQGRTLYGFGG	132	5.30E-04	781.6799	3122.6905	4	-0.0018	28	1	GluC
H4_MOUSE	Histone H4	HAKRKTVTAMDVVYALKRQGRTLYGFGG	132	2.20E-04	781.6799	3122.6905	4	-0.0018	28	1	GluC
H4_MOUSE	Histone H4	HAKRKTVTAMDVVYALKRQGRTLYGFGG	132	6.70E-03	625.5454	3122.6904	5	-0.0019	28	1	GluC
H4_MOUSE	Histone H4	GVLKVFLENVIRDAVTYTEHAKR	3	2.50E-07	886.8303	2657.4690	3	0.0037	23	3	Semi-tryptic
H4_MOUSE	Histone H4	GVLKVFLENVIRDAVTYTEHAKR	3	3.30E-04	665.3742	2657.4677	4	0.0024	23	3	Semi-tryptic
H4_MOUSE	Histone H4	GVLKVFLENVIRDAVTYTEHAKR	3	3.00E-03	665.3741	2657.4673	4	0.0020	23	3	Semi-tryptic
H4_MOUSE	Histone H4	ISGLIYEETRGVLKVFLENVIR	6	1.30E-05	1274.7315	2547.4484	2	0.0060	22	2	Semi-tryptic

H4_MOUSE	Histone H4	ISGLIYEETRGVLKVFLENVIR	6	1.70E-06	850.1566	2547.4479	3	0.0055	22	2	Semi-tryptic
H4_MOUSE	Histone H4	ISGLIYEETRGVLKVFLENVIR	6	5.00E-07	850.1566	2547.4479	3	0.0055	22	2	Semi-tryptic
H4_MOUSE	Histone H4	ISGLIYEETRGVLKVFLENVIR	6	9.60E-09	850.1565	2547.4477	3	0.0053	22	2	Semi-tryptic
H4_MOUSE	Histone H4	ISGLIYEETRGVLKVFLENVIR	6	9.40E-08	850.1563	2547.4470	3	0.0046	22	2	Semi-tryptic
H4_MOUSE	Histone H4	ISGLIYEETRGVLKVFLENVIR	6	3.10E-06	850.1562	2547.4468	3	0.0044	22	2	Semi-tryptic
H4_MOUSE	Histone H4	GVLKVFLENVIRDAVTYTEHAK	7	1.50E-07	834.7969	2501.3689	3	0.0047	22	2	Semi-tryptic
H4_MOUSE	Histone H4	GVLKVFLENVIRDAVTYTEHAK	7	2.30E-08	834.7962	2501.3667	3	0.0025	22	2	Semi-tryptic
H4_MOUSE	Histone H4	GVLKVFLENVIRDAVTYTEHAK	7	2.10E-07	834.7961	2501.3665	3	0.0023	22	2	Semi-tryptic
H4_MOUSE	Histone H4	GVLKVFLENVIRDAVTYTEHAK	7	2.60E-04	626.3487	2501.3656	4	0.0014	22	2	Semi-tryptic
H4_MOUSE	Histone H4	GVLKVFLENVIRDAVTYTEHAK	7	7.80E-03	626.3487	2501.3655	4	0.0013	22	2	Semi-tryptic
H4_MOUSE	Histone H4	GVLKVFLENVIRDAVTYTEHAK	7	1.60E-05	626.3486	2501.3654	4	0.0012	22	2	Semi-tryptic
H4_MOUSE	Histone H4	GVLKVFLENVIRDAVTYTEHAK	7	1.60E-03	626.3485	2501.3648	4	0.0006	22	2	Semi-tryptic
H4_MOUSE	Histone H4	ARRGGVKRISGLIYEETRGVL	4	3.70E-05	777.4577	2329.3512	3	0.0169	21	2	Chymotrypsin
H4_MOUSE	Histone H4	ARRGGVKRISGLIYEETRGVL	4	2.40E-05	777.4558	2329.3456	3	0.0114	21	2	Chymotrypsin
H4_MOUSE	Histone H4	ARRGGVKRISGLIYEETRGVL	4	3.00E-12	583.3434	2329.3445	4	0.0103	21	2	Chymotrypsin
H4_MOUSE	Histone H4	ARRGGVKRISGLIYEETRGVL	4	1.60E-08	583.3422	2329.3398	4	0.0056	21	2	Chymotrypsin
H4_MOUSE	Histone H4	VFLENVIRDAVTYTEHAKRK	3	7.00E-04	598.0816	2388.2972	4	0.0059	20	3	Semi-tryptic
H4_MOUSE	Histone H4	VFLENVIRDAVTYTEHAKRK	3	5.00E-07	598.0816	2388.2972	4	0.0059	20	3	Semi-tryptic
H4_MOUSE	Histone H4	VFLENVIRDAVTYTEHAKRK	3	6.30E-05	797.1055	2388.2948	3	0.0034	20	3	Semi-tryptic
H4_MOUSE	Histone H4	VFLENVIRDAVTYTEHAKR	3	7.70E-05	566.0575	2260.2009	4	0.0045	19	2	Semi-tryptic
H4_MOUSE	Histone H4	VFLENVIRDAVTYTEHAKR	3	4.00E-07	566.0575	2260.2009	4	0.0045	19	2	Semi-tryptic
H4_MOUSE	Histone H4	VFLENVIRDAVTYTEHAKR	3	1.30E-08	754.4059	2260.1960	3	-0.0004	19	2	Semi-tryptic
H4_MOUSE	Histone H4	DVVYALKRQGRTLYGFGG	107	3.40E-07	500.7728	1999.0620	4	-0.0019	18	0	CnBr
H4_MOUSE	Histone H4	DVVYALKRQGRTLYGFGG	107	2.50E-07	667.3611	1999.0614	3	-0.0025	18	0	CnBr
H4_MOUSE	Histone H4	VFLENVIRDAVTYTEHAK	17	1.60E-04	702.375	2104.1031	3	0.0078	18	1	Semi-tryptic
H4_MOUSE	Histone H4	VFLENVIRDAVTYTEHAK	17	2.00E-05	702.375	2104.1031	3	0.0078	18	1	Semi-tryptic
H4_MOUSE	Histone H4	VFLENVIRDAVTYTEHAK	17	7.20E-05	702.3738	2104.0997	3	0.0044	18	1	Semi-tryptic
H4_MOUSE	Histone H4	VFLENVIRDAVTYTEHAK	17	4.20E-06	1053.0564	2104.0982	2	0.0030	18	1	Semi-tryptic
H4_MOUSE	Histone H4	VFLENVIRDAVTYTEHAK	17	7.00E-07	702.3734	2104.0983	3	0.0030	18	1	Semi-tryptic
H4_MOUSE	Histone H4	VFLENVIRDAVTYTEHAK	17	3.80E-07	702.3732	2104.0978	3	0.0025	18	1	Semi-tryptic
H4_MOUSE	Histone H4	VFLENVIRDAVTYTEHAK	17	9.80E-07	702.3732	2104.0977	3	0.0024	18	1	Semi-tryptic
H4_MOUSE	Histone H4	VFLENVIRDAVTYTEHAK	17	8.00E-07	702.3732	2104.0977	3	0.0024	18	1	Semi-tryptic
H4_MOUSE	Histone H4	VFLENVIRDAVTYTEHAK	17	1.20E-07	702.3732	2104.0977	3	0.0024	18	1	Semi-tryptic
H4_MOUSE	Histone H4	VFLENVIRDAVTYTEHAK	17	3.20E-04	702.3731	2104.0976	3	0.0023	18	1	Semi-tryptic
H4_MOUSE	Histone H4	VFLENVIRDAVTYTEHAK	17	5.60E-07	702.3731	2104.0975	3	0.0022	18	1	Semi-tryptic
H4_MOUSE	Histone H4	VFLENVIRDAVTYTEHAK	17	2.60E-06	702.373	2104.0973	3	0.0020	18	1	Semi-tryptic
H4_MOUSE	Histone H4	VFLENVIRDAVTYTEHAK	17	2.30E-07	702.373	2104.0973	3	0.0020	18	1	Semi-tryptic
H4_MOUSE	Histone H4	VFLENVIRDAVTYTEHAK	17	1.10E-04	702.373	2104.0972	3	0.0019	18	1	Semi-tryptic
H4_MOUSE	Histone H4	VFLENVIRDAVTYTEHAK	17	4.20E-04	1053.0558	2104.0970	2	0.0018	18	1	Semi-tryptic
H4_MOUSE	Histone H4	VFLENVIRDAVTYTEHAK	17	2.70E-04	702.3729	2104.0969	3	0.0016	18	1	Semi-tryptic
H4_MOUSE	Histone H4	VFLENVIRDAVTYTEHAK	17	1.10E-05	702.3729	2104.0969	3	0.0016	18	1	Semi-tryptic
H4_MOUSE	Histone H4	DVVYALKRQGRTLYGFGG	107	2.10E-05	667.3663	1999.0770	3	0.0131	18	0	AspN
H4_MOUSE	Histone H4	DVVYALKRQGRTLYGFGG	107	7.00E-04	667.3658	1999.0755	3	0.0115	18	0	AspN
H4_MOUSE	Histone H4	DVVYALKRQGRTLYGFGG	107	1.80E-06	667.3657	1999.0752	3	0.0113	18	0	AspN
H4_MOUSE	Histone H4	DVVYALKRQGRTLYGFGG	107	7.90E-03	1000.5424	1999.0702	2	0.0063	18	0	AspN
H4_MOUSE	Histone H4	DVVYALKRQGRTLYGFGG	107	5.90E-06	667.3638	1999.0697	3	0.0057	18	0	AspN
H4_MOUSE	Histone H4	DVVYALKRQGRTLYGFGG	107	9.90E-07	667.3638	1999.0697	3	0.0057	18	0	AspN
H4_MOUSE	Histone H4	DVVYALKRQGRTLYGFGG	107	4.00E-04	667.3636	1999.0690	3	0.0050	18	0	CnBr
H4_MOUSE	Histone H4	DVVYALKRQGRTLYGFGG	107	5.70E-03	667.3635	1999.0686	3	0.0046	18	0	CnBr
H4_MOUSE	Histone H4	DVVYALKRQGRTLYGFGG	107	1.10E-03	667.3635	1999.0686	3	0.0046	18	0	CnBr
H4_MOUSE	Histone H4	DVVYALKRQGRTLYGFGG	107	7.80E-03	1000.5413	1999.0680	2	0.0041	18	0	CnBr
H4_MOUSE	Histone H4	DVVYALKRQGRTLYGFGG	107	1.20E-05	667.3631	1999.0676	3	0.0036	18	0	CnBr
H4_MOUSE	Histone H4	DVVYALKRQGRTLYGFGG	107	1.00E-02	1000.5409	1999.0672	2	0.0033	18	0	AspN
H4_MOUSE	Histone H4	DVVYALKRQGRTLYGFGG	107	8.50E-04	667.363	1999.0671	3	0.0031	18	0	CnBr
H4_MOUSE	Histone H4	DVVYALKRQGRTLYGFGG	107	8.20E-03	667.3629	1999.0668	3	0.0029	18	0	CnBr
H4_MOUSE	Histone H4	DVVYALKRQGRTLYGFGG	107	6.90E-03	1000.5407	1999.0668	2	0.0029	18	0	CnBr
H4_MOUSE	Histone H4	DVVYALKRQGRTLYGFGG	107	6.10E-03	1000.5407	1999.0668	2	0.0029	18	0	CnBr
H4_MOUSE	Histone H4	DVVYALKRQGRTLYGFGG	107	4.70E-05	667.3629	1999.0668	3	0.0029	18	0	CnBr
H4_MOUSE	Histone H4	DVVYALKRQGRTLYGFGG	107	3.40E-06	667.3629	1999.0668	3	0.0028	18	0	CnBr
H4_MOUSE	Histone H4	DVVYALKRQGRTLYGFGG	107	6.30E-03	667.3628	1999.0666	3	0.0027	18	0	CnBr
H4_MOUSE	Histone H4	DVVYALKRQGRTLYGFGG	107	1.80E-03	667.3628	1999.0665	3	0.0026	18	0	AspN
H4_MOUSE	Histone H4	DVVYALKRQGRTLYGFGG	107	3.80E-04	667.3628	1999.0666	3	0.0026	18	0	CnBr
H4_MOUSE	Histone H4	DVVYALKRQGRTLYGFGG	107	7.70E-03	667.3628	1999.0664	3	0.0025	18	0	CnBr
H4_MOUSE	Histone H4	DVVYALKRQGRTLYGFGG	107	5.80E-03	1000.5405	1999.0664	2	0.0025	18	0	CnBr
H4_MOUSE	Histone H4	DVVYALKRQGRTLYGFGG	107	5.40E-03	1000.5405	1999.0664	2	0.0025	18	0	CnBr
H4_MOUSE	Histone H4	DVVYALKRQGRTLYGFGG	107	6.30E-03	667.3627	1999.0663	3	0.0024	18	0	AspN
H4_MOUSE	Histone H4	DVVYALKRQGRTLYGFGG	107	1.00E-02	667.3627	1999.0662	3	0.0023	18	0	CnBr
H4_MOUSE	Histone H4	DVVYALKRQGRTLYGFGG	107	6.70E-03	667.3627	1999.0662	3	0.0023	18	0	CnBr
H4_MOUSE	Histone H4	DVVYALKRQGRTLYGFGG	107	6.40E-03	1000.5404	1999.0662	2	0.0023	18	0	CnBr
H4_MOUSE	Histone H4	DVVYALKRQGRTLYGFGG	107	4.80E-03	1000.5404	1999.0662	2	0.0023	18	0	AspN
H4_MOUSE	Histone H4	DVVYALKRQGRTLYGFGG	107	9.80E-04	667.3627	1999.0662	3	0.0023	18	0	CnBr
H4_MOUSE	Histone H4	DVVYALKRQGRTLYGFGG	107	5.50E-04	667.3627	1999.0662	3	0.0023	18	0	CnBr
H4_MOUSE	Histone H4	DVVYALKRQGRTLYGFGG	107	1.80E-05	667.3627	1999.0662	3	0.0022	18	0	CnBr
H4_MOUSE	Histone H4	DVVYALKRQGRTLYGFGG	107	5.90E-06	667.3626	1999.0661	3	0.0021	18	0	AspN

H4_MOUSE	Histone H4	DVVYALKRQGRTLYGFGG	107	3.20E-06	667.3626	1999.0660	3	0.0021	18	0	CnBr
H4_MOUSE	Histone H4	DVVYALKRQGRTLYGFGG	107	6.70E-03	667.3626	1999.0659	3	0.0020	18	0	CnBr
H4_MOUSE	Histone H4	DVVYALKRQGRTLYGFGG	107	1.10E-05	667.3626	1999.0659	3	0.0020	18	0	CnBr
H4_MOUSE	Histone H4	DVVYALKRQGRTLYGFGG	107	7.60E-03	667.3626	1999.0659	3	0.0019	18	0	AspN
H4_MOUSE	Histone H4	DVVYALKRQGRTLYGFGG	107	4.70E-04	667.3626	1999.0659	3	0.0019	18	0	CnBr
H4_MOUSE	Histone H4	DVVYALKRQGRTLYGFGG	107	7.20E-06	667.3626	1999.0659	3	0.0019	18	0	AspN
H4_MOUSE	Histone H4	DVVYALKRQGRTLYGFGG	107	9.90E-03	667.3625	1999.0658	3	0.0018	18	0	CnBr
H4_MOUSE	Histone H4	DVVYALKRQGRTLYGFGG	107	9.10E-03	667.3625	1999.0658	3	0.0018	18	0	AspN
H4_MOUSE	Histone H4	DVVYALKRQGRTLYGFGG	107	3.30E-03	667.3625	1999.0658	3	0.0018	18	0	AspN
H4_MOUSE	Histone H4	DVVYALKRQGRTLYGFGG	107	9.70E-05	667.3625	1999.0658	3	0.0018	18	0	CnBr
H4_MOUSE	Histone H4	DVVYALKRQGRTLYGFGG	107	5.10E-03	667.3625	1999.0656	3	0.0017	18	0	AspN
H4_MOUSE	Histone H4	DVVYALKRQGRTLYGFGG	107	3.80E-03	1000.5401	1999.0656	2	0.0017	18	0	AspN
H4_MOUSE	Histone H4	DVVYALKRQGRTLYGFGG	107	9.00E-07	667.3625	1999.0656	3	0.0017	18	0	AspN
H4_MOUSE	Histone H4	DVVYALKRQGRTLYGFGG	107	1.00E-03	667.3624	1999.0655	3	0.0016	18	0	CnBr
H4_MOUSE	Histone H4	DVVYALKRQGRTLYGFGG	107	2.00E-05	667.3625	1999.0656	3	0.0016	18	0	AspN
H4_MOUSE	Histone H4	DVVYALKRQGRTLYGFGG	107	9.10E-03	667.3624	1999.0655	3	0.0015	18	0	CnBr
H4_MOUSE	Histone H4	DVVYALKRQGRTLYGFGG	107	1.80E-03	500.7736	1999.0655	4	0.0015	18	0	CnBr
H4_MOUSE	Histone H4	DVVYALKRQGRTLYGFGG	107	1.60E-05	667.3624	1999.0655	3	0.0015	18	0	CnBr
H4_MOUSE	Histone H4	DVVYALKRQGRTLYGFGG	107	2.70E-06	667.3624	1999.0653	3	0.0014	18	0	CnBr
H4_MOUSE	Histone H4	DVVYALKRQGRTLYGFGG	107	2.80E-06	667.3624	1999.0653	3	0.0013	18	0	CnBr
H4_MOUSE	Histone H4	DVVYALKRQGRTLYGFGG	107	1.00E-06	667.3624	1999.0653	3	0.0013	18	0	AspN
H4_MOUSE	Histone H4	DVVYALKRQGRTLYGFGG	107	9.90E-03	667.3623	1999.0651	3	0.0012	18	0	CnBr
H4_MOUSE	Histone H4	DVVYALKRQGRTLYGFGG	107	3.40E-05	667.3623	1999.0651	3	0.0012	18	0	CnBr
H4_MOUSE	Histone H4	DVVYALKRQGRTLYGFGG	107	8.40E-03	667.3623	1999.0650	3	0.0010	18	0	CnBr
H4_MOUSE	Histone H4	DVVYALKRQGRTLYGFGG	107	8.00E-03	667.3623	1999.0650	3	0.0010	18	0	CnBr
H4_MOUSE	Histone H4	DVVYALKRQGRTLYGFGG	107	7.50E-03	667.3623	1999.0650	3	0.0010	18	0	CnBr
H4_MOUSE	Histone H4	DVVYALKRQGRTLYGFGG	107	7.10E-04	667.3623	1999.0650	3	0.0010	18	0	AspN
H4_MOUSE	Histone H4	DVVYALKRQGRTLYGFGG	107	7.30E-05	667.3623	1999.0650	3	0.0010	18	0	CnBr
H4_MOUSE	Histone H4	DVVYALKRQGRTLYGFGG	107	6.80E-06	667.3623	1999.0650	3	0.0010	18	0	CnBr
H4_MOUSE	Histone H4	DVVYALKRQGRTLYGFGG	107	4.50E-06	667.3623	1999.0650	3	0.0010	18	0	CnBr
H4_MOUSE	Histone H4	DVVYALKRQGRTLYGFGG	107	2.70E-03	667.3622	1999.0649	3	0.0009	18	0	AspN
H4_MOUSE	Histone H4	DVVYALKRQGRTLYGFGG	107	9.80E-06	667.3622	1999.0649	3	0.0009	18	0	CnBr
H4_MOUSE	Histone H4	DVVYALKRQGRTLYGFGG	107	7.80E-06	667.3622	1999.0648	3	0.0009	18	0	CnBr
H4_MOUSE	Histone H4	DVVYALKRQGRTLYGFGG	107	2.70E-06	667.3622	1999.0649	3	0.0009	18	0	AspN
H4_MOUSE	Histone H4	DVVYALKRQGRTLYGFGG	107	1.50E-07	500.7735	1999.0649	4	0.0009	18	0	CnBr
H4_MOUSE	Histone H4	DVVYALKRQGRTLYGFGG	107	4.30E-03	667.3622	1999.0648	3	0.0008	18	0	CnBr
H4_MOUSE	Histone H4	DVVYALKRQGRTLYGFGG	107	1.90E-03	667.3622	1999.0647	3	0.0008	18	0	CnBr
H4_MOUSE	Histone H4	DVVYALKRQGRTLYGFGG	107	1.60E-03	667.3622	1999.0647	3	0.0008	18	0	CnBr
H4_MOUSE	Histone H4	DVVYALKRQGRTLYGFGG	107	1.60E-04	667.3622	1999.0648	3	0.0008	18	0	CnBr
H4_MOUSE	Histone H4	DVVYALKRQGRTLYGFGG	107	3.10E-05	667.3622	1999.0648	3	0.0008	18	0	CnBr
H4_MOUSE	Histone H4	DVVYALKRQGRTLYGFGG	107	2.20E-06	500.7734	1999.0646	4	0.0006	18	0	CnBr
H4_MOUSE	Histone H4	DVVYALKRQGRTLYGFGG	107	4.00E-03	667.3621	1999.0644	3	0.0005	18	0	AspN
H4_MOUSE	Histone H4	DVVYALKRQGRTLYGFGG	107	1.10E-04	667.3621	1999.0644	3	0.0005	18	0	AspN
H4_MOUSE	Histone H4	DVVYALKRQGRTLYGFGG	107	2.90E-06	667.3621	1999.0645	3	0.0005	18	0	CnBr
H4_MOUSE	Histone H4	DVVYALKRQGRTLYGFGG	107	9.30E-03	667.3621	1999.0644	3	0.0004	18	0	CnBr
H4_MOUSE	Histone H4	DVVYALKRQGRTLYGFGG	107	4.20E-05	500.7734	1999.0643	4	0.0004	18	0	CnBr
H4_MOUSE	Histone H4	DVVYALKRQGRTLYGFGG	107	2.10E-03	500.7733	1999.0643	4	0.0003	18	0	CnBr
H4_MOUSE	Histone H4	DVVYALKRQGRTLYGFGG	107	2.20E-04	667.362	1999.0642	3	0.0003	18	0	CnBr
H4_MOUSE	Histone H4	DVVYALKRQGRTLYGFGG	107	2.10E-05	667.362	1999.0643	3	0.0003	18	0	CnBr
H4_MOUSE	Histone H4	DVVYALKRQGRTLYGFGG	107	4.50E-06	500.7733	1999.0642	4	0.0003	18	0	CnBr
H4_MOUSE	Histone H4	DVVYALKRQGRTLYGFGG	107	1.50E-06	667.362	1999.0643	3	0.0003	18	0	CnBr
H4_MOUSE	Histone H4	DVVYALKRQGRTLYGFGG	107	1.90E-08	500.7733	1999.0643	4	0.0003	18	0	AspN
H4_MOUSE	Histone H4	DVVYALKRQGRTLYGFGG	107	1.80E-08	500.7733	1999.0643	4	0.0003	18	0	CnBr
H4_MOUSE	Histone H4	DVVYALKRQGRTLYGFGG	107	1.90E-05	667.362	1999.0641	3	0.0001	18	0	AspN
H4_MOUSE	Histone H4	DVVYALKRQGRTLYGFGG	107	4.40E-08	500.7733	1999.0641	4	0.0001	18	0	CnBr
H4_MOUSE	Histone H4	DVVYALKRQGRTLYGFGG	107	5.80E-05	667.3619	1999.0639	3	0.0000	18	0	AspN
H4_MOUSE	Histone H4	DVVYALKRQGRTLYGFGG	107	3.70E-06	500.7733	1999.0640	4	0.0000	18	0	CnBr
H4_MOUSE	Histone H4	DVVYALKRQGRTLYGFGG	107	4.20E-03	667.3619	1999.0638	3	-0.0001	18	0	AspN
H4_MOUSE	Histone H4	DVVYALKRQGRTLYGFGG	107	1.60E-06	667.3619	1999.0638	3	-0.0001	18	0	AspN
H4_MOUSE	Histone H4	DVVYALKRQGRTLYGFGG	107	3.10E-08	500.7733	1999.0639	4	-0.0001	18	0	CnBr
H4_MOUSE	Histone H4	DVVYALKRQGRTLYGFGG	107	7.70E-03	667.3619	1999.0638	3	-0.0002	18	0	CnBr
H4_MOUSE	Histone H4	DVVYALKRQGRTLYGFGG	107	2.00E-05	667.3619	1999.0638	3	-0.0002	18	0	CnBr
H4_MOUSE	Histone H4	DVVYALKRQGRTLYGFGG	107	1.10E-05	667.3619	1999.0638	3	-0.0002	18	0	CnBr
H4_MOUSE	Histone H4	DVVYALKRQGRTLYGFGG	107	8.60E-03	667.3618	1999.0637	3	-0.0003	18	0	CnBr
H4_MOUSE	Histone H4	DVVYALKRQGRTLYGFGG	107	4.20E-07	500.7732	1999.0637	4	-0.0003	18	0	CnBr
H4_MOUSE	Histone H4	DVVYALKRQGRTLYGFGG	107	6.90E-07	500.7732	1999.0636	4	-0.0004	18	0	CnBr
H4_MOUSE	Histone H4	DVVYALKRQGRTLYGFGG	107	1.50E-05	500.7731	1999.0634	4	-0.0006	18	0	CnBr
H4_MOUSE	Histone H4	DVVYALKRQGRTLYGFGG	107	9.20E-04	500.7731	1999.0632	4	-0.0007	18	0	CnBr
H4_MOUSE	Histone H4	DVVYALKRQGRTLYGFGG	107	4.70E-07	500.7731	1999.0633	4	-0.0007	18	0	CnBr
H4_MOUSE	Histone H4	DVVYALKRQGRTLYGFGG	107	3.00E-03	500.7731	1999.0631	4	-0.0009	18	0	CnBr
H4_MOUSE	Histone H4	DVVYALKRQGRTLYGFGG	107	1.20E-04	500.773	1999.0631	4	-0.0009	18	0	CnBr
H4_MOUSE	Histone H4	DVVYALKRQGRTLYGFGG	107	1.80E-06	667.3615	1999.0628	3	-0.0011	18	0	AspN
H4_MOUSE	Histone H4	VVYALKRQGRTLYGFGG	67	5.30E-03	943.0247	1884.0348	2	-0.0022	17	0	GluC
H4_MOUSE	Histone H4	VVYALKRQGRTLYGFGG	67	2.00E-03	472.016	1884.0348	4	-0.0022	17	0	GluC

H4_MOUSE	Histone H4	VVYALKRQGRTLYGFGG	67	1.20E-03	629.0187	1884.0344	3	-0.0026	17	0	GluC
H4_MOUSE	Histone H4	DAVTYTEHAKRKTVTAM	34	1.10E-07	641.3306	1920.9700	3	-0.0028	17	0	AspN
H4_MOUSE	Histone H4	VVYALKRQGRTLYGFGG	67	6.20E-04	629.0186	1884.0341	3	-0.0029	17	0	GluC
H4_MOUSE	Histone H4	DAVTYTEHAKRKTVTAM	34	1.10E-07	481.2495	1920.9691	4	-0.0036	17	0	AspN
H4_MOUSE	Histone H4	DAVTYTEHAKRKTVTAM	34	1.30E-03	961.5011	1920.9876	2	0.0149	17	0	AspN
H4_MOUSE	Histone H4	DAVTYTEHAKRKTVTAM	34	2.70E-03	961.4963	1920.9781	2	0.0054	17	0	AspN
H4_MOUSE	Histone H4	DAVTYTEHAKRKTVTAM	34	4.20E-05	961.4963	1920.9781	2	0.0054	17	0	AspN
H4_MOUSE	Histone H4	DAVTYTEHAKRKTVTAM	34	2.50E-05	961.4963	1920.9781	2	0.0054	17	0	AspN
H4_MOUSE	Histone H4	DAVTYTEHAKRKTVTAM	34	1.00E-05	961.4958	1920.9770	2	0.0043	17	0	AspN
H4_MOUSE	Histone H4	DAVTYTEHAKRKTVTAM	34	2.60E-03	961.4957	1920.9768	2	0.0041	17	0	AspN
H4_MOUSE	Histone H4	DAVTYTEHAKRKTVTAM	34	5.40E-03	641.3328	1920.9765	3	0.0037	17	0	AspN
H4_MOUSE	Histone H4	DAVTYTEHAKRKTVTAM	34	1.80E-07	641.3328	1920.9765	3	0.0037	17	0	AspN
H4_MOUSE	Histone H4	DAVTYTEHAKRKTVTAM	34	4.80E-06	641.3327	1920.9762	3	0.0034	17	0	AspN
H4_MOUSE	Histone H4	DAVTYTEHAKRKTVTAM	34	9.90E-03	961.4953	1920.9761	2	0.0033	17	0	AspN
H4_MOUSE	Histone H4	DAVTYTEHAKRKTVTAM	34	8.50E-09	641.3325	1920.9756	3	0.0028	17	0	AspN
H4_MOUSE	Histone H4	DAVTYTEHAKRKTVTAM	34	8.30E-10	481.2511	1920.9755	4	0.0028	17	0	AspN
H4_MOUSE	Histone H4	DAVTYTEHAKRKTVTAM	34	2.30E-04	961.495	1920.9754	2	0.0027	17	0	AspN
H4_MOUSE	Histone H4	DAVTYTEHAKRKTVTAM	34	7.00E-05	961.495	1920.9754	2	0.0027	17	0	AspN
H4_MOUSE	Histone H4	DAVTYTEHAKRKTVTAM	34	9.30E-04	641.3324	1920.9753	3	0.0026	17	0	AspN
H4_MOUSE	Histone H4	DAVTYTEHAKRKTVTAM	34	5.20E-03	961.4949	1920.9752	2	0.0025	17	0	AspN
H4_MOUSE	Histone H4	DAVTYTEHAKRKTVTAM	34	4.30E-07	961.4949	1920.9752	2	0.0025	17	0	AspN
H4_MOUSE	Histone H4	DAVTYTEHAKRKTVTAM	34	2.90E-07	481.2511	1920.9752	4	0.0025	17	0	AspN
H4_MOUSE	Histone H4	DAVTYTEHAKRKTVTAM	34	8.90E-06	641.3323	1920.9750	3	0.0023	17	0	AspN
H4_MOUSE	Histone H4	DAVTYTEHAKRKTVTAM	34	2.60E-03	641.3321	1920.9745	3	0.0018	17	0	AspN
H4_MOUSE	Histone H4	DAVTYTEHAKRKTVTAM	34	1.10E-07	641.3321	1920.9745	3	0.0018	17	0	AspN
H4_MOUSE	Histone H4	DAVTYTEHAKRKTVTAM	34	1.90E-08	641.3321	1920.9745	3	0.0018	17	0	AspN
H4_MOUSE	Histone H4	DAVTYTEHAKRKTVTAM	34	8.30E-10	481.2508	1920.9741	4	0.0014	17	0	AspN
H4_MOUSE	Histone H4	DAVTYTEHAKRKTVTAM	34	1.10E-08	481.2508	1920.9740	4	0.0013	17	0	AspN
H4_MOUSE	Histone H4	DAVTYTEHAKRKTVTAM	34	4.10E-04	481.2507	1920.9739	4	0.0011	17	0	AspN
H4_MOUSE	Histone H4	DAVTYTEHAKRKTVTAM	34	9.80E-03	641.3319	1920.9737	3	0.0010	17	0	AspN
H4_MOUSE	Histone H4	DAVTYTEHAKRKTVTAM	34	8.90E-07	641.3319	1920.9737	3	0.0010	17	0	AspN
H4_MOUSE	Histone H4	DAVTYTEHAKRKTVTAM	34	6.50E-08	481.2507	1920.9735	4	0.0008	17	0	AspN
H4_MOUSE	Histone H4	DAVTYTEHAKRKTVTAM	34	5.30E-03	641.3316	1920.9731	3	0.0004	17	0	AspN
H4_MOUSE	Histone H4	DAVTYTEHAKRKTVTAM	34	1.20E-05	961.4938	1920.9731	2	0.0004	17	0	AspN
H4_MOUSE	Histone H4	DAVTYTEHAKRKTVTAM	34	1.40E-09	481.2505	1920.9731	4	0.0003	17	0	AspN
H4_MOUSE	Histone H4	DAVTYTEHAKRKTVTAM	34	9.60E-03	481.2504	1920.9725	4	-0.0002	17	0	AspN
H4_MOUSE	Histone H4	VVYALKRQGRTLYGFGG	67	1.00E-02	943.0319	1884.0493	2	0.0123	17	0	GluC
H4_MOUSE	Histone H4	VVYALKRQGRTLYGFGG	67	7.10E-03	943.0305	1884.0465	2	0.0095	17	0	GluC
H4_MOUSE	Histone H4	VVYALKRQGRTLYGFGG	67	5.60E-04	629.0227	1884.0462	3	0.0092	17	0	GluC
H4_MOUSE	Histone H4	VVYALKRQGRTLYGFGG	67	1.50E-03	943.0303	1884.0461	2	0.0091	17	0	GluC
H4_MOUSE	Histone H4	VVYALKRQGRTLYGFGG	67	2.80E-09	629.0226	1884.0458	3	0.0088	17	0	GluC
H4_MOUSE	Histone H4	VVYALKRQGRTLYGFGG	67	8.70E-03	943.0301	1884.0457	2	0.0087	17	0	GluC
H4_MOUSE	Histone H4	VVYALKRQGRTLYGFGG	67	3.80E-03	629.0221	1884.0446	3	0.0076	17	0	GluC
H4_MOUSE	Histone H4	VVYALKRQGRTLYGFGG	67	4.60E-11	629.0221	1884.0446	3	0.0076	17	0	GluC
H4_MOUSE	Histone H4	VVYALKRQGRTLYGFGG	67	3.10E-09	629.0221	1884.0444	3	0.0074	17	0	GluC
H4_MOUSE	Histone H4	VVYALKRQGRTLYGFGG	67	4.10E-07	629.022	1884.0442	3	0.0072	17	0	GluC
H4_MOUSE	Histone H4	VVYALKRQGRTLYGFGG	67	4.00E-05	629.0219	1884.0439	3	0.0069	17	0	GluC
H4_MOUSE	Histone H4	VVYALKRQGRTLYGFGG	67	1.70E-10	472.0182	1884.0437	4	0.0067	17	0	GluC
H4_MOUSE	Histone H4	VVYALKRQGRTLYGFGG	67	6.10E-06	629.0218	1884.0436	3	0.0066	17	0	GluC
H4_MOUSE	Histone H4	VVYALKRQGRTLYGFGG	67	4.90E-04	472.0181	1884.0432	4	0.0062	17	0	GluC
H4_MOUSE	Histone H4	VVYALKRQGRTLYGFGG	67	7.60E-06	629.0215	1884.0426	3	0.0056	17	0	GluC
H4_MOUSE	Histone H4	VVYALKRQGRTLYGFGG	67	1.00E-07	629.0215	1884.0426	3	0.0055	17	0	GluC
H4_MOUSE	Histone H4	VVYALKRQGRTLYGFGG	67	5.70E-08	629.0213	1884.0421	3	0.0051	17	0	GluC
H4_MOUSE	Histone H4	VVYALKRQGRTLYGFGG	67	2.10E-04	629.0212	1884.0416	3	0.0046	17	0	GluC
H4_MOUSE	Histone H4	VVYALKRQGRTLYGFGG	67	1.00E-10	629.0212	1884.0416	3	0.0046	17	0	GluC
H4_MOUSE	Histone H4	VVYALKRQGRTLYGFGG	67	3.60E-03	943.0278	1884.0410	2	0.0040	17	0	GluC
H4_MOUSE	Histone H4	VVYALKRQGRTLYGFGG	67	1.30E-03	943.0278	1884.0410	2	0.0040	17	0	GluC
H4_MOUSE	Histone H4	VVYALKRQGRTLYGFGG	67	3.20E-04	472.0174	1884.0406	4	0.0036	17	0	GluC
H4_MOUSE	Histone H4	VVYALKRQGRTLYGFGG	67	2.30E-06	629.0208	1884.0406	3	0.0036	17	0	GluC
H4_MOUSE	Histone H4	VVYALKRQGRTLYGFGG	67	2.50E-07	629.0208	1884.0406	3	0.0036	17	0	GluC
H4_MOUSE	Histone H4	VVYALKRQGRTLYGFGG	67	5.40E-06	472.0174	1884.0405	4	0.0035	17	0	GluC
H4_MOUSE	Histone H4	VVYALKRQGRTLYGFGG	67	1.20E-07	472.0173	1884.0401	4	0.0031	17	0	GluC
H4_MOUSE	Histone H4	VVYALKRQGRTLYGFGG	67	1.10E-09	629.0205	1884.0398	3	0.0028	17	0	GluC
H4_MOUSE	Histone H4	VVYALKRQGRTLYGFGG	67	1.20E-04	629.0203	1884.0391	3	0.0021	17	0	GluC
H4_MOUSE	Histone H4	VVYALKRQGRTLYGFGG	67	3.80E-05	629.0203	1884.0391	3	0.0021	17	0	GluC
H4_MOUSE	Histone H4	VVYALKRQGRTLYGFGG	67	3.30E-08	629.0203	1884.0390	3	0.0020	17	0	GluC
H4_MOUSE	Histone H4	VVYALKRQGRTLYGFGG	67	2.70E-06	629.0203	1884.0390	3	0.0019	17	0	GluC
H4_MOUSE	Histone H4	VVYALKRQGRTLYGFGG	67	2.00E-11	629.0203	1884.0389	3	0.0019	17	0	GluC
H4_MOUSE	Histone H4	VVYALKRQGRTLYGFGG	67	4.20E-04	629.0201	1884.0384	3	0.0014	17	0	GluC
H4_MOUSE	Histone H4	VVYALKRQGRTLYGFGG	67	1.60E-04	629.02	1884.0382	3	0.0012	17	0	GluC
H4_MOUSE	Histone H4	VVYALKRQGRTLYGFGG	67	8.50E-03	629.02	1884.0381	3	0.0011	17	0	GluC
H4_MOUSE	Histone H4	VVYALKRQGRTLYGFGG	67	6.30E-03	629.02	1884.0381	3	0.0011	17	0	GluC
H4_MOUSE	Histone H4	VVYALKRQGRTLYGFGG	67	2.00E-06	472.0168	1884.0381	4	0.0011	17	0	GluC
H4_MOUSE	Histone H4	VVYALKRQGRTLYGFGG	67	4.00E-09	629.02	1884.0381	3	0.0011	17	0	GluC

H4_MOUSE	Histone H4	VVYALKRQGRTLYGFGG	67	2.90E-03	943.0263	1884.0380	2	0.0010	17	0	GluC
H4_MOUSE	Histone H4	VVYALKRQGRTLYGFGG	67	7.40E-04	472.0168	1884.0380	4	0.0010	17	0	GluC
H4_MOUSE	Histone H4	VVYALKRQGRTLYGFGG	67	3.20E-05	472.0167	1884.0379	4	0.0009	17	0	GluC
H4_MOUSE	Histone H4	VVYALKRQGRTLYGFGG	67	1.50E-05	472.0168	1884.0379	4	0.0009	17	0	GluC
H4_MOUSE	Histone H4	VVYALKRQGRTLYGFGG	67	5.90E-06	629.0199	1884.0379	3	0.0009	17	0	GluC
H4_MOUSE	Histone H4	VVYALKRQGRTLYGFGG	67	5.60E-06	472.0168	1884.0379	4	0.0009	17	0	GluC
H4_MOUSE	Histone H4	VVYALKRQGRTLYGFGG	67	7.10E-04	472.0167	1884.0378	4	0.0008	17	0	GluC
H4_MOUSE	Histone H4	VVYALKRQGRTLYGFGG	67	3.90E-03	472.0167	1884.0375	4	0.0005	17	0	GluC
H4_MOUSE	Histone H4	VVYALKRQGRTLYGFGG	67	6.10E-07	629.0198	1884.0375	3	0.0005	17	0	GluC
H4_MOUSE	Histone H4	VVYALKRQGRTLYGFGG	67	5.00E-04	629.0198	1884.0374	3	0.0004	17	0	GluC
H4_MOUSE	Histone H4	VVYALKRQGRTLYGFGG	67	9.80E-05	472.0166	1884.0371	4	0.0001	17	0	GluC
H4_MOUSE	Histone H4	VVYALKRQGRTLYGFGG	67	1.30E-06	629.0197	1884.0372	3	0.0001	17	0	GluC
H4_MOUSE	Histone H4	VVYALKRQGRTLYGFGG	67	7.20E-03	472.0165	1884.0368	4	-0.0002	17	0	GluC
H4_MOUSE	Histone H4	VVYALKRQGRTLYGFGG	67	2.10E-07	629.0196	1884.0369	3	-0.0002	17	0	GluC
H4_MOUSE	Histone H4	VVYALKRQGRTLYGFGG	67	4.30E-10	629.0196	1884.0369	3	-0.0002	17	0	GluC
H4_MOUSE	Histone H4	VVYALKRQGRTLYGFGG	67	4.80E-04	629.0195	1884.0367	3	-0.0003	17	0	GluC
H4_MOUSE	Histone H4	VVYALKRQGRTLYGFGG	67	8.70E-08	629.0195	1884.0366	3	-0.0004	17	0	GluC
H4_MOUSE	Histone H4	VVYALKRQGRTLYGFGG	67	8.10E-03	472.0164	1884.0364	4	-0.0006	17	0	GluC
H4_MOUSE	Histone H4	VVYALKRQGRTLYGFGG	67	1.80E-03	629.0193	1884.0361	3	-0.0009	17	0	GluC
H4_MOUSE	Histone H4	VVYALKRQGRTLYGFGG	67	3.20E-08	472.0163	1884.0361	4	-0.0009	17	0	GluC
H4_MOUSE	Histone H4	VVYALKRQGRTLYGFGG	67	3.50E-09	629.0193	1884.0361	3	-0.0009	17	0	GluC
H4_MOUSE	Histone H4	VVYALKRQGRTLYGFGG	67	1.80E-06	629.0193	1884.0360	3	-0.0010	17	0	GluC
H4_MOUSE	Histone H4	VVYALKRQGRTLYGFGG	67	3.30E-06	629.0192	1884.0359	3	-0.0011	17	0	GluC
H4_MOUSE	Histone H4	VVYALKRQGRTLYGFGG	67	8.90E-03	472.0162	1884.0357	4	-0.0013	17	0	GluC
H4_MOUSE	Histone H4	VVYALKRQGRTLYGFGG	67	6.20E-09	629.019	1884.0353	3	-0.0017	17	0	GluC
H4_MOUSE	Histone H4	TVTAMDVVYALKRQGR	3	4.90E-03	603.3335	1806.9787	3	0.0013	16	2	Semi-tryptic
H4_MOUSE	Histone H4	TVTAMDVVYALKRQGR	3	6.00E-03	603.3334	1806.9784	3	0.0010	16	2	Semi-tryptic
H4_MOUSE	Histone H4	TVTAMDVVYALKRQGR	3	6.50E-03	452.7511	1806.9755	4	-0.0019	16	2	Semi-tryptic
H4_MOUSE	Histone H4	TEHAKRKTVTAMDVVY	11	6.40E-03	924.9872	1847.9599	2	0.0036	16	0	Chymotrypsin
H4_MOUSE	Histone H4	TEHAKRKTVTAMDVVY	11	1.70E-03	924.9866	1847.9587	2	0.0023	16	0	Chymotrypsin
H4_MOUSE	Histone H4	TEHAKRKTVTAMDVVY	11	3.20E-08	616.9935	1847.9587	3	0.0023	16	0	Chymotrypsin
H4_MOUSE	Histone H4	TEHAKRKTVTAMDVVY	11	8.90E-03	924.9862	1847.9579	2	0.0015	16	0	GluC
H4_MOUSE	Histone H4	TEHAKRKTVTAMDVVY	11	9.40E-05	616.9932	1847.9578	3	0.0014	16	0	Chymotrypsin
H4_MOUSE	Histone H4	TEHAKRKTVTAMDVVY	11	2.30E-03	616.9931	1847.9575	3	0.0011	16	0	Chymotrypsin
H4_MOUSE	Histone H4	TEHAKRKTVTAMDVVY	11	2.90E-06	616.993	1847.9572	3	0.0009	16	0	GluC
H4_MOUSE	Histone H4	TEHAKRKTVTAMDVVY	11	2.50E-03	616.9929	1847.9569	3	0.0006	16	0	Chymotrypsin
H4_MOUSE	Histone H4	TEHAKRKTVTAMDVVY	11	2.10E-05	616.9928	1847.9566	3	0.0002	16	0	Chymotrypsin
H4_MOUSE	Histone H4	TEHAKRKTVTAMDVVY	11	2.20E-05	616.9928	1847.9565	3	0.0001	16	0	Chymotrypsin
H4_MOUSE	Histone H4	TEHAKRKTVTAMDVVY	11	2.50E-05	462.9961	1847.9554	4	-0.0010	16	0	Chymotrypsin
H4_MOUSE	Histone H4	RISGLIYEETRGVLK	8	3.30E-05	867.5	1732.9855	2	0.0019	15	2	Semi-tryptic
H4_MOUSE	Histone H4	RISGLIYEETRGVLK	8	2.30E-08	578.6685	1732.9837	3	0.0002	15	2	Semi-tryptic
H4_MOUSE	Histone H4	RISGLIYEETRGVLK	8	1.80E-09	578.6685	1732.9837	3	0.0002	15	2	Semi-tryptic
H4_MOUSE	Histone H4	RISGLIYEETRGVLK	8	2.70E-04	578.6684	1732.9833	3	-0.0003	15	2	Semi-tryptic
H4_MOUSE	Histone H4	RDNIQGITKPAIRRL	9	1.50E-06	438.5174	1750.0405	4	0.0079	15	0	Chymotrypsin
H4_MOUSE	Histone H4	RDNIQGITKPAIRRL	9	5.20E-09	584.3541	1750.0404	3	0.0079	15	0	Chymotrypsin
H4_MOUSE	Histone H4	RDNIQGITKPAIRRL	9	9.40E-09	584.3532	1750.0377	3	0.0051	15	0	Chymotrypsin
H4_MOUSE	Histone H4	RDNIQGITKPAIRRL	9	8.90E-03	438.5163	1750.0360	4	0.0034	15	0	Chymotrypsin
H4_MOUSE	Histone H4	RDNIQGITKPAIRRL	9	5.00E-08	584.3524	1750.0355	3	0.0029	15	0	Chymotrypsin
H4_MOUSE	Histone H4	RDNIQGITKPAIRRL	9	1.20E-03	438.5156	1750.0333	4	0.0007	15	0	Chymotrypsin
H4_MOUSE	Histone H4	RDNIQGITKPAIRRL	9	1.00E-03	584.3517	1750.0332	3	0.0006	15	0	Chymotrypsin
H4_MOUSE	Histone H4	RDNIQGITKPAIRRL	9	2.90E-03	438.5154	1750.0324	4	-0.0001	15	0	GluC
H4_MOUSE	Histone H4	RKTVTAMDVVYALKR	10	4.30E-07	438.5061	1749.9951	4	0.0028	15	3	Semi-tryptic
H4_MOUSE	Histone H4	RKTVTAMDVVYALKR	10	1.50E-08	438.5061	1749.9951	4	0.0028	15	3	Semi-tryptic
H4_MOUSE	Histone H4	RKTVTAMDVVYALKR	10	5.30E-07	438.5054	1749.9925	4	0.0001	15	3	Semi-tryptic
H4_MOUSE	Histone H4	RKTVTAMDVVYALKR	10	1.30E-08	438.5054	1749.9925	4	0.0001	15	3	Semi-tryptic
H4_MOUSE	Histone H4	RKTVTAMDVVYALKR	10	6.40E-04	438.5052	1749.9915	4	-0.0008	15	3	Semi-tryptic
H4_MOUSE	Histone H4	RKTVTAMDVVYALKR	10	3.20E-03	438.5051	1749.9914	4	-0.0010	15	3	Semi-tryptic
H4_MOUSE	Histone H4	RKTVTAMDVVYALKR	10	3.90E-07	438.505	1749.9909	4	-0.0014	15	3	Semi-tryptic
H4_MOUSE	Histone H4	RKTVTAMDVVYALKR	10	1.20E-05	438.5047	1749.9898	4	-0.0025	15	3	Semi-tryptic
H4_MOUSE	Histone H4	RISGLIYEETRGVLK	8	1.30E-04	578.6675	1732.9807	3	-0.0028	15	2	Semi-tryptic
H4_MOUSE	Histone H4	RISGLIYEETRGVLK	8	1.70E-04	578.6674	1732.9802	3	-0.0033	15	2	Semi-tryptic
H4_MOUSE	Histone H4	RISGLIYEETRGVLK	8	1.30E-05	578.6668	1732.9786	3	-0.0050	15	2	Semi-tryptic
H4_MOUSE	Histone H4	RISGLIYEETRGVLK	8	4.10E-05	578.6663	1732.9771	3	-0.0064	15	2	Semi-tryptic
H4_MOUSE	Histone H4	RDNIQGITKPAIRRL	9	8.60E-05	584.3491	1750.0256	3	-0.0070	15	0	Chymotrypsin
H4_MOUSE	Histone H4	RKTVTAMDVVYALKR	10	1.40E-07	584.3356	1749.9851	3	-0.0072	15	3	Semi-tryptic
H4_MOUSE	Histone H4	RKTVTAMDVVYALKR	10	2.50E-07	438.5029	1749.9823	4	-0.0100	15	3	Semi-tryptic
H4_MOUSE	Histone H4	RKTVTAMDVVYALK	2	1.60E-04	532.3059	1593.8958	3	0.0045	14	2	Semi-tryptic
H4_MOUSE	Histone H4	RKTVTAMDVVYALK	2	6.80E-06	532.3059	1593.8958	3	0.0045	14	2	Semi-tryptic
H4_MOUSE	Histone H4	ARRGGVKRISGLIY	4	1.70E-08	387.239	1544.9269	4	0.0006	14	1	Chymotrypsin
H4_MOUSE	Histone H4	ARRGGVKRISGLIY	4	6.30E-04	387.2387	1544.9255	4	-0.0008	14	1	GluC
H4_MOUSE	Histone H4	ARRGGVKRISGLIY	4	1.20E-08	515.9824	1544.9253	3	-0.0010	14	1	Chymotrypsin
H4_MOUSE	Histone H4	KTVTAMDVVYALKR	5	3.90E-04	532.3057	1593.8953	3	0.0041	14	2	Semi-tryptic
H4_MOUSE	Histone H4	KTVTAMDVVYALKR	5	2.50E-06	532.3045	1593.8917	3	0.0004	14	2	Semi-tryptic
H4_MOUSE	Histone H4	ISGLIYEETRGVLK	59	1.70E-06	789.4475	1576.8805	2	-0.0019	14	1	Semi-tryptic

H4_MOUSE	Histone H4	KTVTAMDVVYALKR	5	4.70E-10	532.3037	1593.8892	3	-0.0020	14	2	Semi-tryptic
H4_MOUSE	Histone H4	KTVTAMDVVYALKR	5	5.60E-03	532.3036	1593.8891	3	-0.0021	14	2	Semi-tryptic
H4_MOUSE	Histone H4	ARRGGVKRISGLIY	4	1.00E-02	387.2383	1544.9240	4	-0.0023	14	1	Chymotrypsin
H4_MOUSE	Histone H4	ISGLIYEETRGVLK	59	1.90E-03	526.6338	1576.8797	3	-0.0027	14	1	Semi-tryptic
H4_MOUSE	Histone H4	ISGLIYEETRGVLK	59	2.40E-03	526.6338	1576.8797	3	-0.0028	14	1	Semi-tryptic
H4_MOUSE	Histone H4	ISGLIYEETRGVLK	59	3.30E-04	526.6337	1576.8793	3	-0.0031	14	1	Semi-tryptic
H4_MOUSE	Histone H4	KVFLENVIRDAVTY	26	4.30E-04	833.9601	1665.9055	2	-0.0034	14	2	Chymotrypsin
H4_MOUSE	Histone H4	ISGLIYEETRGVLK	59	1.40E-05	789.4468	1576.8790	2	-0.0034	14	1	Semi-tryptic
H4_MOUSE	Histone H4	ISGLIYEETRGVLK	59	5.10E-07	789.4467	1576.8789	2	-0.0036	14	1	Semi-tryptic
H4_MOUSE	Histone H4	KTVTAMDVVYALKR	5	8.70E-09	532.303	1593.8873	3	-0.0039	14	2	Semi-tryptic
H4_MOUSE	Histone H4	ISGLIYEETRGVLK	59	7.50E-03	526.6334	1576.8783	3	-0.0041	14	1	Semi-tryptic
H4_MOUSE	Histone H4	KVFLENVIRDAVTY	26	1.90E-05	833.9671	1665.9197	2	0.0108	14	2	Chymotrypsin
H4_MOUSE	Histone H4	KVFLENVIRDAVTY	26	3.90E-04	833.9652	1665.9159	2	0.0070	14	2	Chymotrypsin
H4_MOUSE	Histone H4	KVFLENVIRDAVTY	26	2.50E-04	833.9639	1665.9133	2	0.0043	14	2	Chymotrypsin
H4_MOUSE	Histone H4	KVFLENVIRDAVTY	26	1.10E-03	833.9636	1665.9127	2	0.0037	14	2	Chymotrypsin
H4_MOUSE	Histone H4	KVFLENVIRDAVTY	26	3.30E-03	833.9635	1665.9124	2	0.0035	14	2	Chymotrypsin
H4_MOUSE	Histone H4	KVFLENVIRDAVTY	26	4.30E-06	556.3114	1665.9123	3	0.0033	14	2	Chymotrypsin
H4_MOUSE	Histone H4	KVFLENVIRDAVTY	26	2.10E-03	833.9633	1665.9121	2	0.0031	14	2	Chymotrypsin
H4_MOUSE	Histone H4	KVFLENVIRDAVTY	26	1.10E-03	833.963	1665.9114	2	0.0024	14	2	Chymotrypsin
H4_MOUSE	Histone H4	KVFLENVIRDAVTY	26	1.60E-03	833.9629	1665.9113	2	0.0023	14	2	Chymotrypsin
H4_MOUSE	Histone H4	KVFLENVIRDAVTY	26	1.60E-04	833.9629	1665.9113	2	0.0023	14	2	Chymotrypsin
H4_MOUSE	Histone H4	KVFLENVIRDAVTY	26	4.50E-03	833.9629	1665.9111	2	0.0022	14	2	Chymotrypsin
H4_MOUSE	Histone H4	KVFLENVIRDAVTY	26	7.80E-08	556.311	1665.9111	3	0.0021	14	2	Chymotrypsin
H4_MOUSE	Histone H4	KVFLENVIRDAVTY	26	1.60E-03	833.9628	1665.9110	2	0.0020	14	2	Chymotrypsin
H4_MOUSE	Histone H4	KVFLENVIRDAVTY	26	1.70E-04	833.9628	1665.9110	2	0.0020	14	2	Chymotrypsin
H4_MOUSE	Histone H4	KVFLENVIRDAVTY	26	2.90E-04	833.9626	1665.9106	2	0.0016	14	2	Chymotrypsin
H4_MOUSE	Histone H4	KVFLENVIRDAVTY	26	1.70E-03	833.9625	1665.9104	2	0.0014	14	2	Chymotrypsin
H4_MOUSE	Histone H4	KVFLENVIRDAVTY	26	8.90E-03	833.9624	1665.9103	2	0.0013	14	2	Chymotrypsin
H4_MOUSE	Histone H4	KVFLENVIRDAVTY	26	7.00E-03	556.3105	1665.9097	3	0.0007	14	2	Chymotrypsin
H4_MOUSE	Histone H4	KVFLENVIRDAVTY	26	8.30E-04	833.9621	1665.9097	2	0.0007	14	2	Chymotrypsin
H4_MOUSE	Histone H4	KVFLENVIRDAVTY	26	5.10E-04	833.9621	1665.9097	2	0.0007	14	2	Chymotrypsin
H4_MOUSE	Histone H4	KVFLENVIRDAVTY	26	9.10E-03	556.3105	1665.9096	3	0.0006	14	2	Chymotrypsin
H4_MOUSE	Histone H4	KVFLENVIRDAVTY	26	2.40E-04	833.9621	1665.9096	2	0.0006	14	2	Chymotrypsin
H4_MOUSE	Histone H4	KVFLENVIRDAVTY	26	2.30E-04	556.3104	1665.9094	3	0.0004	14	2	Chymotrypsin
H4_MOUSE	Histone H4	KVFLENVIRDAVTY	26	2.50E-07	556.3103	1665.9092	3	0.0002	14	2	Chymotrypsin
H4_MOUSE	Histone H4	KVFLENVIRDAVTY	26	1.40E-07	556.3099	1665.9080	3	-0.0010	14	2	Chymotrypsin
H4_MOUSE	Histone H4	ISGLIYEETRGVLK	59	2.60E-06	789.4498	1576.8851	2	0.0026	14	1	Semi-tryptic
H4_MOUSE	Histone H4	ISGLIYEETRGVLK	59	8.00E-03	789.4496	1576.8847	2	0.0023	14	1	Semi-tryptic
H4_MOUSE	Histone H4	ISGLIYEETRGVLK	59	6.50E-05	789.4496	1576.8846	2	0.0022	14	1	Semi-tryptic
H4_MOUSE	Histone H4	ISGLIYEETRGVLK	59	6.00E-04	789.4495	1576.8845	2	0.0021	14	1	Semi-tryptic
H4_MOUSE	Histone H4	ISGLIYEETRGVLK	59	2.40E-05	789.4495	1576.8845	2	0.0021	14	1	Semi-tryptic
H4_MOUSE	Histone H4	ISGLIYEETRGVLK	59	2.60E-03	789.4495	1576.8845	2	0.0020	14	1	Semi-tryptic
H4_MOUSE	Histone H4	ISGLIYEETRGVLK	59	7.30E-06	789.4495	1576.8844	2	0.0020	14	1	Semi-tryptic
H4_MOUSE	Histone H4	ISGLIYEETRGVLK	59	6.90E-06	789.4494	1576.8843	2	0.0019	14	1	Semi-tryptic
H4_MOUSE	Histone H4	ISGLIYEETRGVLK	59	1.40E-03	526.6353	1576.8841	3	0.0017	14	1	Semi-tryptic
H4_MOUSE	Histone H4	ISGLIYEETRGVLK	59	6.20E-05	789.4494	1576.8842	2	0.0017	14	1	Semi-tryptic
H4_MOUSE	Histone H4	ISGLIYEETRGVLK	59	3.10E-05	789.4494	1576.8842	2	0.0017	14	1	Semi-tryptic
H4_MOUSE	Histone H4	ISGLIYEETRGVLK	59	1.50E-04	789.4492	1576.8839	2	0.0015	14	1	Semi-tryptic
H4_MOUSE	Histone H4	ISGLIYEETRGVLK	59	6.30E-05	789.4492	1576.8839	2	0.0015	14	1	Semi-tryptic
H4_MOUSE	Histone H4	ISGLIYEETRGVLK	59	6.10E-05	789.4492	1576.8839	2	0.0015	14	1	Semi-tryptic
H4_MOUSE	Histone H4	ISGLIYEETRGVLK	59	1.30E-05	789.4493	1576.8840	2	0.0015	14	1	Semi-tryptic
H4_MOUSE	Histone H4	ISGLIYEETRGVLK	59	4.20E-06	789.4492	1576.8839	2	0.0015	14	1	Semi-tryptic
H4_MOUSE	Histone H4	ISGLIYEETRGVLK	59	4.50E-07	789.4492	1576.8839	2	0.0015	14	1	Semi-tryptic
H4_MOUSE	Histone H4	ISGLIYEETRGVLK	59	1.10E-03	526.6352	1576.8838	3	0.0014	14	1	Semi-tryptic
H4_MOUSE	Histone H4	ISGLIYEETRGVLK	59	1.30E-04	526.6352	1576.8838	3	0.0014	14	1	Semi-tryptic
H4_MOUSE	Histone H4	ISGLIYEETRGVLK	59	2.50E-03	526.6352	1576.8837	3	0.0012	14	1	Semi-tryptic
H4_MOUSE	Histone H4	ISGLIYEETRGVLK	59	2.90E-06	526.635	1576.8833	3	0.0008	14	1	Semi-tryptic
H4_MOUSE	Histone H4	ISGLIYEETRGVLK	59	1.30E-06	789.4489	1576.8833	2	0.0008	14	1	Semi-tryptic
H4_MOUSE	Histone H4	ISGLIYEETRGVLK	59	7.70E-08	526.635	1576.8833	3	0.0008	14	1	Semi-tryptic
H4_MOUSE	Histone H4	ISGLIYEETRGVLK	59	3.70E-05	789.4488	1576.8831	2	0.0007	14	1	Semi-tryptic
H4_MOUSE	Histone H4	ISGLIYEETRGVLK	59	7.80E-06	789.4488	1576.8830	2	0.0005	14	1	Semi-tryptic
H4_MOUSE	Histone H4	ISGLIYEETRGVLK	59	2.90E-04	526.6349	1576.8828	3	0.0004	14	1	Semi-tryptic
H4_MOUSE	Histone H4	ISGLIYEETRGVLK	59	2.20E-04	526.6349	1576.8828	3	0.0004	14	1	Semi-tryptic
H4_MOUSE	Histone H4	ISGLIYEETRGVLK	59	3.70E-07	789.4486	1576.8827	2	0.0003	14	1	Semi-tryptic
H4_MOUSE	Histone H4	ISGLIYEETRGVLK	59	6.80E-05	526.6348	1576.8826	3	0.0002	14	1	Semi-tryptic
H4_MOUSE	Histone H4	ISGLIYEETRGVLK	59	6.40E-05	789.4486	1576.8827	2	0.0002	14	1	Semi-tryptic
H4_MOUSE	Histone H4	ISGLIYEETRGVLK	59	2.10E-06	789.4486	1576.8826	2	0.0002	14	1	Semi-tryptic
H4_MOUSE	Histone H4	ISGLIYEETRGVLK	59	6.70E-05	526.6347	1576.8824	3	0.0000	14	1	Semi-tryptic
H4_MOUSE	Histone H4	ISGLIYEETRGVLK	59	9.50E-04	526.6347	1576.8823	3	-0.0001	14	1	Semi-tryptic
H4_MOUSE	Histone H4	ISGLIYEETRGVLK	59	3.30E-03	526.6347	1576.8822	3	-0.0002	14	1	Semi-tryptic
H4_MOUSE	Histone H4	ISGLIYEETRGVLK	59	2.40E-03	526.6347	1576.8822	3	-0.0002	14	1	Semi-tryptic
H4_MOUSE	Histone H4	ISGLIYEETRGVLK	59	1.10E-03	526.6347	1576.8822	3	-0.0002	14	1	Semi-tryptic
H4_MOUSE	Histone H4	ISGLIYEETRGVLK	59	1.10E-03	526.6347	1576.8822	3	-0.0002	14	1	Semi-tryptic
H4_MOUSE	Histone H4	ISGLIYEETRGVLK	59	2.30E-03	526.6346	1576.8821	3	-0.0003	14	1	Semi-tryptic

H4_MOUSE	Histone H4	ISGLIYEETRGVLK	59	1.30E-03	526.6346	1576.8821	3	-0.0003	14	1	Semi-tryptic
H4_MOUSE	Histone H4	ISGLIYEETRGVLK	59	4.40E-04	526.6346	1576.8821	3	-0.0003	14	1	Semi-tryptic
H4_MOUSE	Histone H4	ISGLIYEETRGVLK	59	4.00E-04	526.6347	1576.8822	3	-0.0003	14	1	Semi-tryptic
H4_MOUSE	Histone H4	ISGLIYEETRGVLK	59	1.80E-03	526.6346	1576.8821	3	-0.0004	14	1	Semi-tryptic
H4_MOUSE	Histone H4	ISGLIYEETRGVLK	59	9.30E-04	789.4483	1576.8821	2	-0.0004	14	1	Semi-tryptic
H4_MOUSE	Histone H4	ISGLIYEETRGVLK	59	1.00E-03	526.6346	1576.8819	3	-0.0005	14	1	Semi-tryptic
H4_MOUSE	Histone H4	ISGLIYEETRGVLK	59	2.50E-04	526.6345	1576.8818	3	-0.0007	14	1	Semi-tryptic
H4_MOUSE	Histone H4	ISGLIYEETRGVLK	59	2.60E-04	526.6345	1576.8816	3	-0.0008	14	1	Semi-tryptic
H4_MOUSE	Histone H4	ISGLIYEETRGVLK	59	1.40E-06	789.448	1576.8814	2	-0.0010	14	1	Semi-tryptic
H4_MOUSE	Histone H4	ISGLIYEETRGVLK	59	4.00E-04	526.6344	1576.8813	3	-0.0011	14	1	Semi-tryptic
H4_MOUSE	Histone H4	ISGLIYEETRGVLK	59	1.90E-04	526.6343	1576.8810	3	-0.0014	14	1	Semi-tryptic
H4_MOUSE	Histone H4	ISGLIYEETRGVLK	59	8.10E-08	789.4478	1576.8810	2	-0.0014	14	1	Semi-tryptic
H4_MOUSE	Histone H4	ISGLIYEETRGVLK	59	1.50E-03	526.6342	1576.8807	3	-0.0018	14	1	Semi-tryptic
H4_MOUSE	Histone H4	ISGLIYEETRGVLK	59	5.20E-03	526.6341	1576.8805	3	-0.0019	14	1	Semi-tryptic
H4_MOUSE	Histone H4	KTVTAMDVVYALK	8	1.90E-05	480.2721	1437.7944	3	0.0043	13	1	Semi-tryptic
H4_MOUSE	Histone H4	KTVTAMDVVYALK	8	4.70E-07	480.2721	1437.7944	3	0.0043	13	1	Semi-tryptic
H4_MOUSE	Histone H4	KTVTAMDVVYALK	8	4.20E-03	719.9043	1437.7941	2	0.0040	13	1	Semi-tryptic
H4_MOUSE	Histone H4	KTVTAMDVVYALK	8	1.10E-05	480.2716	1437.7929	3	0.0027	13	1	Semi-tryptic
H4_MOUSE	Histone H4	KTVTAMDVVYALK	8	4.20E-07	480.2716	1437.7929	3	0.0027	13	1	Semi-tryptic
H4_MOUSE	Histone H4	KTVTAMDVVYALK	8	3.40E-04	719.9026	1437.7907	2	0.0006	13	1	Semi-tryptic
H4_MOUSE	Histone H4	KTVTAMDVVYALK	8	4.50E-06	480.2705	1437.7896	3	-0.0005	13	1	Semi-tryptic
H4_MOUSE	Histone H4	KTVTAMDVVYALK	8	2.70E-07	480.2705	1437.7896	3	-0.0005	13	1	Semi-tryptic
H4_MOUSE	Histone H4	DNIQGITKPAIRR	18	5.40E-03	494.6222	1480.8447	3	-0.0027	13	1	Semi-tryptic
H4_MOUSE	Histone H4	TVTAMDVVYALKR	17	2.90E-06	489.6072	1465.7999	3	0.0036	13	1	Semi-tryptic
H4_MOUSE	Histone H4	TVTAMDVVYALKR	17	5.40E-07	489.6072	1465.7999	3	0.0036	13	1	Semi-tryptic
H4_MOUSE	Histone H4	TVTAMDVVYALKR	17	7.90E-09	733.9065	1465.7985	2	0.0022	13	1	Semi-tryptic
H4_MOUSE	Histone H4	TVTAMDVVYALKR	17	2.50E-05	733.9062	1465.7978	2	0.0015	13	1	Semi-tryptic
H4_MOUSE	Histone H4	TVTAMDVVYALKR	17	9.60E-09	733.9059	1465.7973	2	0.0010	13	1	Semi-tryptic
H4_MOUSE	Histone H4	TVTAMDVVYALKR	17	9.50E-09	733.9059	1465.7973	2	0.0010	13	1	Semi-tryptic
H4_MOUSE	Histone H4	TVTAMDVVYALKR	17	4.40E-09	733.9059	1465.7973	2	0.0010	13	1	Semi-tryptic
H4_MOUSE	Histone H4	TVTAMDVVYALKR	17	1.50E-08	733.9058	1465.7971	2	0.0009	13	1	Semi-tryptic
H4_MOUSE	Histone H4	TVTAMDVVYALKR	17	2.50E-03	489.6063	1465.7970	3	0.0007	13	1	Semi-tryptic
H4_MOUSE	Histone H4	TVTAMDVVYALKR	17	1.70E-04	489.6063	1465.7970	3	0.0007	13	1	Semi-tryptic
H4_MOUSE	Histone H4	TVTAMDVVYALKR	17	1.50E-06	733.9058	1465.7970	2	0.0007	13	1	Semi-tryptic
H4_MOUSE	Histone H4	TVTAMDVVYALKR	17	4.00E-04	733.9056	1465.7967	2	0.0004	13	1	Semi-tryptic
H4_MOUSE	Histone H4	TVTAMDVVYALKR	17	1.00E-02	733.9052	1465.7958	2	-0.0005	13	1	Semi-tryptic
H4_MOUSE	Histone H4	TVTAMDVVYALKR	17	4.40E-03	733.9051	1465.7957	2	-0.0006	13	1	Semi-tryptic
H4_MOUSE	Histone H4	TVTAMDVVYALKR	17	3.60E-03	489.6058	1465.7956	3	-0.0007	13	1	Semi-tryptic
H4_MOUSE	Histone H4	TVTAMDVVYALKR	17	6.40E-03	489.6057	1465.7952	3	-0.0010	13	1	Semi-tryptic
H4_MOUSE	Histone H4	TVTAMDVVYALKR	17	4.40E-04	489.6057	1465.7952	3	-0.0010	13	1	Semi-tryptic
H4_MOUSE	Histone H4	DNIQGITKPAIRR	18	3.40E-05	371.2196	1480.8493	4	0.0019	13	1	Semi-tryptic
H4_MOUSE	Histone H4	DNIQGITKPAIRR	18	1.30E-06	371.2196	1480.8493	4	0.0019	13	1	Semi-tryptic
H4_MOUSE	Histone H4	DNIQGITKPAIRR	18	1.40E-03	741.4318	1480.8491	2	0.0017	13	1	Semi-tryptic
H4_MOUSE	Histone H4	DNIQGITKPAIRR	18	2.90E-03	494.6234	1480.8485	3	0.0011	13	1	Semi-tryptic
H4_MOUSE	Histone H4	DNIQGITKPAIRR	18	1.60E-04	494.6234	1480.8485	3	0.0011	13	1	Semi-tryptic
H4_MOUSE	Histone H4	DNIQGITKPAIRR	18	2.60E-03	494.6232	1480.8479	3	0.0005	13	1	Semi-tryptic
H4_MOUSE	Histone H4	DNIQGITKPAIRR	18	3.00E-03	494.6232	1480.8476	3	0.0002	13	1	Semi-tryptic
H4_MOUSE	Histone H4	DNIQGITKPAIRR	18	7.70E-04	371.2191	1480.8474	4	0.0000	13	1	Semi-tryptic
H4_MOUSE	Histone H4	DNIQGITKPAIRR	18	4.30E-06	371.2191	1480.8474	4	0.0000	13	1	Semi-tryptic
H4_MOUSE	Histone H4	DNIQGITKPAIRR	18	2.20E-03	494.623	1480.8473	3	-0.0001	13	1	Semi-tryptic
H4_MOUSE	Histone H4	DNIQGITKPAIRR	18	1.50E-03	494.6229	1480.8468	3	-0.0005	13	1	Semi-tryptic
H4_MOUSE	Histone H4	DNIQGITKPAIRR	18	5.40E-03	494.6229	1480.8468	3	-0.0006	13	1	Semi-tryptic
H4_MOUSE	Histone H4	DNIQGITKPAIRR	18	3.00E-03	494.6229	1480.8468	3	-0.0006	13	1	Semi-tryptic
H4_MOUSE	Histone H4	DNIQGITKPAIRR	18	4.20E-03	494.6228	1480.8467	3	-0.0007	13	1	Semi-tryptic
H4_MOUSE	Histone H4	DNIQGITKPAIRR	18	5.10E-03	494.6228	1480.8466	3	-0.0008	13	1	Semi-tryptic
H4_MOUSE	Histone H4	DNIQGITKPAIRR	18	3.20E-03	494.6228	1480.8465	3	-0.0008	13	1	Semi-tryptic
H4_MOUSE	Histone H4	DNIQGITKPAIRR	18	1.20E-03	494.6228	1480.8466	3	-0.0008	13	1	Semi-tryptic
H4_MOUSE	Histone H4	DAVTYTEHAKRK	3	9.10E-05	709.8734	1417.7322	2	0.0008	12	2	Semi-tryptic
H4_MOUSE	Histone H4	DAVTYTEHAKRK	3	1.20E-04	473.5846	1417.7319	3	0.0005	12	2	Semi-tryptic
H4_MOUSE	Histone H4	DAVTYTEHAKRK	3	1.70E-05	473.5846	1417.7319	3	0.0005	12	2	Semi-tryptic
H4_MOUSE	Histone H4	ARRGGVKRISGL	7	1.50E-04	318.2031	1268.7833	4	0.0044	12	0	Chymotrypsin
H4_MOUSE	Histone H4	ARRGGVKRISGL	7	1.70E-08	423.9347	1268.7823	3	0.0034	12	0	Chymotrypsin
H4_MOUSE	Histone H4	ARRGGVKRISGL	7	3.00E-04	318.2024	1268.7805	4	0.0016	12	0	Chymotrypsin
H4_MOUSE	Histone H4	ARRGGVKRISGL	7	9.70E-03	318.2023	1268.7801	4	0.0011	12	0	Chymotrypsin
H4_MOUSE	Histone H4	ARRGGVKRISGL	7	1.90E-03	423.9337	1268.7791	3	0.0002	12	0	Chymotrypsin
H4_MOUSE	Histone H4	ARRGGVKRISGL	7	2.30E-03	423.9334	1268.7783	3	-0.0006	12	0	Chymotrypsin
H4_MOUSE	Histone H4	TVTAMDVVYALK	8	1.40E-05	655.8556	1309.6967	2	0.0015	12	0	Semi-tryptic
H4_MOUSE	Histone H4	TVTAMDVVYALK	8	1.80E-08	655.8555	1309.6964	2	0.0012	12	0	Semi-tryptic
H4_MOUSE	Histone H4	TVTAMDVVYALK	8	9.00E-08	655.8554	1309.6963	2	0.0011	12	0	Semi-tryptic
H4_MOUSE	Histone H4	TVTAMDVVYALK	8	1.20E-06	655.8554	1309.6962	2	0.0010	12	0	Semi-tryptic
H4_MOUSE	Histone H4	TVTAMDVVYALK	8	3.40E-03	655.8553	1309.6960	2	0.0008	12	0	Semi-tryptic
H4_MOUSE	Histone H4	TVTAMDVVYALK	8	2.20E-09	655.8552	1309.6958	2	0.0006	12	0	Semi-tryptic
H4_MOUSE	Histone H4	TVTAMDVVYALK	8	9.10E-04	655.854	1309.6934	2	-0.0018	12	0	Semi-tryptic
H4_MOUSE	Histone H4	TVTAMDVVYALK	8	6.90E-05	655.854	1309.6934	2	-0.0018	12	0	Semi-tryptic

H4_MOUSE	Histone H4	ARRGGVKRISGL	7	3.90E-06	423.9328	1268.7765	3	-0.0025	12	0	Chymotrypsin
H4_MOUSE	Histone H4	GVLKVFLENVIR	11	6.90E-05	693.929	1385.8434	2	0.0039	12	1	Semi-tryptic
H4_MOUSE	Histone H4	GVLKVFLENVIR	11	2.90E-06	693.929	1385.8434	2	0.0039	12	1	Semi-tryptic
H4_MOUSE	Histone H4	GVLKVFLENVIR	11	3.10E-06	462.9547	1385.8423	3	0.0028	12	1	Semi-tryptic
H4_MOUSE	Histone H4	GVLKVFLENVIR	11	4.50E-08	462.9547	1385.8423	3	0.0028	12	1	Semi-tryptic
H4_MOUSE	Histone H4	GVLKVFLENVIR	11	1.10E-08	693.928	1385.8414	2	0.0019	12	1	Semi-tryptic
H4_MOUSE	Histone H4	GVLKVFLENVIR	11	2.20E-04	693.9277	1385.8409	2	0.0015	12	1	Semi-tryptic
H4_MOUSE	Histone H4	GVLKVFLENVIR	11	1.30E-09	693.9277	1385.8408	2	0.0013	12	1	Semi-tryptic
H4_MOUSE	Histone H4	GVLKVFLENVIR	11	7.30E-07	693.9276	1385.8406	2	0.0011	12	1	Semi-tryptic
H4_MOUSE	Histone H4	GVLKVFLENVIR	11	6.10E-05	693.9273	1385.8401	2	0.0006	12	1	Semi-tryptic
H4_MOUSE	Histone H4	GVLKVFLENVIR	11	1.00E-05	462.9536	1385.8390	3	-0.0005	12	1	Semi-tryptic
H4_MOUSE	Histone H4	GVLKVFLENVIR	11	1.90E-03	462.9535	1385.8386	3	-0.0009	12	1	Semi-tryptic
H4_MOUSE	Histone H4	GGKGLGKGGAKR	12	1.80E-05	543.3323	1084.6500	2	0.0035	12	3	Semi-tryptic
H4_MOUSE	Histone H4	GGKGLGKGGAKR	12	1.10E-04	362.5573	1084.6499	3	0.0034	12	3	Semi-tryptic
H4_MOUSE	Histone H4	GGKGLGKGGAKR	12	1.80E-03	362.557	1084.6492	3	0.0027	12	3	Semi-tryptic
H4_MOUSE	Histone H4	GGKGLGKGGAKR	12	2.70E-06	543.3313	1084.6480	2	0.0015	12	3	Semi-tryptic
H4_MOUSE	Histone H4	GGKGLGKGGAKR	12	8.10E-06	362.5566	1084.6479	3	0.0013	12	3	Semi-tryptic
H4_MOUSE	Histone H4	GGKGLGKGGAKR	12	1.10E-05	362.5564	1084.6475	3	0.0009	12	3	Semi-tryptic
H4_MOUSE	Histone H4	GGKGLGKGGAKR	12	1.50E-04	362.5563	1084.6471	3	0.0006	12	3	Semi-tryptic
H4_MOUSE	Histone H4	GGKGLGKGGAKR	12	5.30E-03	543.3307	1084.6469	2	0.0004	12	3	Semi-tryptic
H4_MOUSE	Histone H4	GGKGLGKGGAKR	12	2.00E-03	543.3305	1084.6465	2	0.0000	12	3	Semi-tryptic
H4_MOUSE	Histone H4	GGKGLGKGGAKR	12	5.30E-05	362.5561	1084.6464	3	-0.0001	12	3	Semi-tryptic
H4_MOUSE	Histone H4	GGKGLGKGGAKR	12	2.30E-03	362.556	1084.6463	3	-0.0002	12	3	Semi-tryptic
H4_MOUSE	Histone H4	GGKGLGKGGAKR	12	3.20E-06	362.556	1084.6462	3	-0.0003	12	3	Semi-tryptic
H4_MOUSE	Histone H4	DNIQGITKPAIR	58	9.70E-04	663.383	1324.7515	2	0.0053	12	0	Semi-tryptic
H4_MOUSE	Histone H4	DNIQGITKPAIR	58	5.40E-06	663.3828	1324.7511	2	0.0048	12	0	Semi-tryptic
H4_MOUSE	Histone H4	DNIQGITKPAIR	58	4.20E-05	663.3824	1324.7502	2	0.0040	12	0	Semi-tryptic
H4_MOUSE	Histone H4	DNIQGITKPAIR	58	1.70E-04	442.5906	1324.7499	3	0.0037	12	0	Semi-tryptic
H4_MOUSE	Histone H4	DNIQGITKPAIR	58	1.70E-05	663.3821	1324.7497	2	0.0034	12	0	Semi-tryptic
H4_MOUSE	Histone H4	DNIQGITKPAIR	58	7.80E-05	442.5904	1324.7494	3	0.0031	12	0	Semi-tryptic
H4_MOUSE	Histone H4	DNIQGITKPAIR	58	4.90E-04	442.5904	1324.7493	3	0.0030	12	0	Semi-tryptic
H4_MOUSE	Histone H4	DNIQGITKPAIR	58	7.70E-06	442.5904	1324.7493	3	0.0030	12	0	Semi-tryptic
H4_MOUSE	Histone H4	DNIQGITKPAIR	58	3.70E-05	663.3818	1324.7489	2	0.0027	12	0	Semi-tryptic
H4_MOUSE	Histone H4	DNIQGITKPAIR	58	9.90E-07	663.3818	1324.7490	2	0.0027	12	0	Semi-tryptic
H4_MOUSE	Histone H4	DNIQGITKPAIR	58	1.10E-05	663.3816	1324.7487	2	0.0024	12	0	Semi-tryptic
H4_MOUSE	Histone H4	DNIQGITKPAIR	58	6.80E-06	663.3816	1324.7487	2	0.0024	12	0	Semi-tryptic
H4_MOUSE	Histone H4	DNIQGITKPAIR	58	1.60E-06	663.3816	1324.7487	2	0.0024	12	0	Semi-tryptic
H4_MOUSE	Histone H4	DNIQGITKPAIR	58	1.20E-05	663.3816	1324.7486	2	0.0023	12	0	Semi-tryptic
H4_MOUSE	Histone H4	DNIQGITKPAIR	58	1.30E-05	663.3815	1324.7484	2	0.0022	12	0	Semi-tryptic
H4_MOUSE	Histone H4	DNIQGITKPAIR	58	6.20E-06	663.3815	1324.7484	2	0.0021	12	0	Semi-tryptic
H4_MOUSE	Histone H4	DNIQGITKPAIR	58	6.30E-03	442.59	1324.7481	3	0.0018	12	0	Semi-tryptic
H4_MOUSE	Histone H4	DNIQGITKPAIR	58	1.30E-03	442.59	1324.7481	3	0.0018	12	0	Semi-tryptic
H4_MOUSE	Histone H4	DNIQGITKPAIR	58	1.40E-05	663.3813	1324.7481	2	0.0018	12	0	Semi-tryptic
H4_MOUSE	Histone H4	DNIQGITKPAIR	58	3.60E-06	663.3813	1324.7481	2	0.0018	12	0	Semi-tryptic
H4_MOUSE	Histone H4	DNIQGITKPAIR	58	1.00E-03	663.3812	1324.7479	2	0.0017	12	0	Semi-tryptic
H4_MOUSE	Histone H4	DNIQGITKPAIR	58	8.40E-05	663.3813	1324.7480	2	0.0017	12	0	Semi-tryptic
H4_MOUSE	Histone H4	DNIQGITKPAIR	58	1.60E-05	442.5899	1324.7480	3	0.0017	12	0	Semi-tryptic
H4_MOUSE	Histone H4	DNIQGITKPAIR	58	1.00E-05	442.5899	1324.7480	3	0.0017	12	0	Semi-tryptic
H4_MOUSE	Histone H4	DNIQGITKPAIR	58	1.50E-03	442.5899	1324.7478	3	0.0015	12	0	Semi-tryptic
H4_MOUSE	Histone H4	DNIQGITKPAIR	58	1.60E-05	663.3812	1324.7478	2	0.0015	12	0	Semi-tryptic
H4_MOUSE	Histone H4	DNIQGITKPAIR	58	9.10E-04	442.5898	1324.7475	3	0.0012	12	0	Semi-tryptic
H4_MOUSE	Histone H4	DNIQGITKPAIR	58	1.00E-02	663.381	1324.7474	2	0.0011	12	0	Semi-tryptic
H4_MOUSE	Histone H4	DNIQGITKPAIR	58	6.90E-04	663.381	1324.7474	2	0.0011	12	0	Semi-tryptic
H4_MOUSE	Histone H4	DNIQGITKPAIR	58	8.70E-05	663.381	1324.7474	2	0.0011	12	0	Semi-tryptic
H4_MOUSE	Histone H4	DNIQGITKPAIR	58	1.00E-05	442.5897	1324.7474	3	0.0011	12	0	Semi-tryptic
H4_MOUSE	Histone H4	DNIQGITKPAIR	58	8.30E-06	442.5897	1324.7473	3	0.0011	12	0	Semi-tryptic
H4_MOUSE	Histone H4	DNIQGITKPAIR	58	3.50E-06	663.3809	1324.7473	2	0.0010	12	0	Semi-tryptic
H4_MOUSE	Histone H4	DNIQGITKPAIR	58	2.80E-05	442.5896	1324.7471	3	0.0008	12	0	Semi-tryptic
H4_MOUSE	Histone H4	DNIQGITKPAIR	58	5.80E-03	663.3808	1324.7470	2	0.0007	12	0	Semi-tryptic
H4_MOUSE	Histone H4	DNIQGITKPAIR	58	1.10E-03	442.5896	1324.7469	3	0.0007	12	0	Semi-tryptic
H4_MOUSE	Histone H4	DNIQGITKPAIR	58	1.50E-04	442.5896	1324.7469	3	0.0007	12	0	Semi-tryptic
H4_MOUSE	Histone H4	DNIQGITKPAIR	58	1.60E-04	442.5896	1324.7469	3	0.0006	12	0	Semi-tryptic
H4_MOUSE	Histone H4	DNIQGITKPAIR	58	2.90E-05	442.5895	1324.7468	3	0.0005	12	0	Semi-tryptic
H4_MOUSE	Histone H4	DNIQGITKPAIR	58	2.30E-04	442.5895	1324.7466	3	0.0004	12	0	Semi-tryptic
H4_MOUSE	Histone H4	DNIQGITKPAIR	58	1.80E-05	442.5895	1324.7466	3	0.0004	12	0	Semi-tryptic
H4_MOUSE	Histone H4	DNIQGITKPAIR	58	5.40E-04	442.5894	1324.7465	3	0.0002	12	0	Semi-tryptic
H4_MOUSE	Histone H4	DNIQGITKPAIR	58	1.90E-05	442.5894	1324.7465	3	0.0002	12	0	Semi-tryptic
H4_MOUSE	Histone H4	DNIQGITKPAIR	58	9.50E-06	663.3805	1324.7465	2	0.0002	12	0	Semi-tryptic
H4_MOUSE	Histone H4	DNIQGITKPAIR	58	3.60E-06	663.3805	1324.7464	2	0.0002	12	0	Semi-tryptic
H4_MOUSE	Histone H4	DNIQGITKPAIR	58	2.70E-05	442.5894	1324.7463	3	0.0000	12	0	Semi-tryptic
H4_MOUSE	Histone H4	DNIQGITKPAIR	58	3.90E-05	442.5894	1324.7462	3	-0.0001	12	0	Semi-tryptic
H4_MOUSE	Histone H4	DNIQGITKPAIR	58	4.50E-06	663.3803	1324.7460	2	-0.0002	12	0	Semi-tryptic
H4_MOUSE	Histone H4	DNIQGITKPAIR	58	1.60E-03	442.5893	1324.7460	3	-0.0003	12	0	Semi-tryptic
H4_MOUSE	Histone H4	DNIQGITKPAIR	58	3.50E-04	442.5893	1324.7460	3	-0.0003	12	0	Semi-tryptic

H4_MOUSE	Histone H4	DNIQGITKPAIR	58	8.40E-05	442.5893	1324.7460	3	-0.0003	12	0	Semi-tryptic
H4_MOUSE	Histone H4	DNIQGITKPAIR	58	8.10E-05	442.5893	1324.7460	3	-0.0003	12	0	Semi-tryptic
H4_MOUSE	Histone H4	DNIQGITKPAIR	58	3.10E-05	442.5893	1324.7460	3	-0.0003	12	0	Semi-tryptic
H4_MOUSE	Histone H4	DNIQGITKPAIR	58	3.20E-04	663.3801	1324.7457	2	-0.0005	12	0	Semi-tryptic
H4_MOUSE	Histone H4	DNIQGITKPAIR	58	1.40E-04	442.589	1324.7452	3	-0.0011	12	0	Semi-tryptic
H4_MOUSE	Histone H4	DNIQGITKPAIR	58	4.40E-06	663.3798	1324.7451	2	-0.0012	12	0	Semi-tryptic
H4_MOUSE	Histone H4	DNIQGITKPAIR	58	3.90E-06	663.3797	1324.7449	2	-0.0013	12	0	Semi-tryptic
H4_MOUSE	Histone H4	DNIQGITKPAIR	58	2.20E-04	442.5888	1324.7446	3	-0.0016	12	0	Semi-tryptic
H4_MOUSE	Histone H4	RISGLIYEETR	5	2.70E-04	668.8677	1335.7208	2	0.0062	11	1	Semi-tryptic
H4_MOUSE	Histone H4	RISGLIYEETR	5	7.20E-03	446.2472	1335.7197	3	0.0050	11	1	Semi-tryptic
H4_MOUSE	Histone H4	RISGLIYEETR	5	1.50E-04	668.8667	1335.7188	2	0.0042	11	1	Semi-tryptic
H4_MOUSE	Histone H4	RISGLIYEETR	5	5.90E-05	446.2457	1335.7154	3	0.0008	11	1	Semi-tryptic
H4_MOUSE	Histone H4	RISGLIYEETR	5	5.30E-05	668.8643	1335.7141	2	-0.0005	11	1	Semi-tryptic
H4_MOUSE	Histone H4	HAKRKTVTAMD	30	8.20E-06	419.8953	1256.6640	3	-0.0020	11	0	GluC
H4_MOUSE	Histone H4	NVIRDAVTYTE	175	8.60E-03	640.8266	1279.6387	2	-0.0021	11	1	GluC
H4_MOUSE	Histone H4	NVIRDAVTYTE	175	1.70E-03	640.8266	1279.6386	2	-0.0022	11	1	GluC
H4_MOUSE	Histone H4	NVIRDAVTYTE	175	7.00E-04	640.8266	1279.6386	2	-0.0022	11	1	GluC
H4_MOUSE	Histone H4	ETRGVLKVFLE	92	4.10E-05	430.9178	1289.7316	3	-0.0027	11	1	GluC
H4_MOUSE	Histone H4	HAKRKTVTAMD	30	1.20E-03	315.173	1256.6630	4	-0.0029	11	0	GluC
H4_MOUSE	Histone H4	NVIRDAVTYTE	175	4.40E-05	640.8262	1279.6378	2	-0.0030	11	1	GluC
H4_MOUSE	Histone H4	NVIRDAVTYTE	175	4.60E-04	640.826	1279.6374	2	-0.0034	11	1	GluC
H4_MOUSE	Histone H4	NVIRDAVTYTE	175	2.50E-04	640.826	1279.6374	2	-0.0034	11	1	GluC
H4_MOUSE	Histone H4	ETRGVLKVFLE	92	8.40E-04	645.8726	1289.7306	2	-0.0037	11	1	GluC
H4_MOUSE	Histone H4	LENVIRDAVTY	61	1.70E-05	646.8433	1291.6720	2	-0.0052	11	1	Chymotrypsin
H4_MOUSE	Histone H4	LENVIRDAVTY	61	1.40E-04	646.8432	1291.6718	2	-0.0054	11	1	Chymotrypsin
H4_MOUSE	Histone H4	ETRGVLKVFLE	92	6.30E-03	430.9168	1289.7286	3	-0.0057	11	1	GluC
H4_MOUSE	Histone H4	NVIRDAVTYTE	175	4.30E-05	640.8247	1279.6349	2	-0.0060	11	1	GluC
H4_MOUSE	Histone H4	ETRGVLKVFLE	92	1.40E-05	430.9163	1289.7272	3	-0.0071	11	1	GluC
H4_MOUSE	Histone H4	ETRGVLKVFLE	92	1.40E-04	645.87	1289.7255	2	-0.0088	11	1	GluC
H4_MOUSE	Histone H4	HAKRKTVTAMD	30	5.10E-05	419.8977	1256.6713	3	0.0054	11	0	GluC
H4_MOUSE	Histone H4	HAKRKTVTAMD	30	4.40E-04	419.8975	1256.6707	3	0.0048	11	0	GluC
H4_MOUSE	Histone H4	HAKRKTVTAMD	30	5.40E-04	419.8974	1256.6703	3	0.0044	11	0	GluC
H4_MOUSE	Histone H4	HAKRKTVTAMD	30	2.00E-06	419.8974	1256.6703	3	0.0044	11	0	GluC
H4_MOUSE	Histone H4	HAKRKTVTAMD	30	2.10E-06	419.8973	1256.6701	3	0.0042	11	0	GluC
H4_MOUSE	Histone H4	HAKRKTVTAMD	30	7.90E-03	629.3422	1256.6699	2	0.0039	11	0	GluC
H4_MOUSE	Histone H4	HAKRKTVTAMD	30	2.70E-08	419.8972	1256.6699	3	0.0039	11	0	GluC
H4_MOUSE	Histone H4	HAKRKTVTAMD	30	3.50E-06	419.8971	1256.6695	3	0.0036	11	0	GluC
H4_MOUSE	Histone H4	HAKRKTVTAMD	30	3.70E-04	419.8969	1256.6688	3	0.0029	11	0	GluC
H4_MOUSE	Histone H4	HAKRKTVTAMD	30	2.30E-06	419.8967	1256.6682	3	0.0023	11	0	GluC
H4_MOUSE	Histone H4	HAKRKTVTAMD	30	3.40E-03	419.8966	1256.6681	3	0.0022	11	0	GluC
H4_MOUSE	Histone H4	HAKRKTVTAMD	30	1.10E-06	419.8967	1256.6682	3	0.0022	11	0	GluC
H4_MOUSE	Histone H4	HAKRKTVTAMD	30	1.40E-04	419.8964	1256.6673	3	0.0014	11	0	GluC
H4_MOUSE	Histone H4	HAKRKTVTAMD	30	1.30E-06	419.8963	1256.6672	3	0.0013	11	0	GluC
H4_MOUSE	Histone H4	HAKRKTVTAMD	30	1.20E-06	419.8963	1256.6672	3	0.0013	11	0	GluC
H4_MOUSE	Histone H4	HAKRKTVTAMD	30	4.90E-05	419.8963	1256.6670	3	0.0011	11	0	GluC
H4_MOUSE	Histone H4	HAKRKTVTAMD	30	2.60E-06	419.8961	1256.6664	3	0.0005	11	0	GluC
H4_MOUSE	Histone H4	HAKRKTVTAMD	30	3.10E-03	419.896	1256.6663	3	0.0004	11	0	GluC
H4_MOUSE	Histone H4	HAKRKTVTAMD	30	1.30E-05	419.8961	1256.6664	3	0.0004	11	0	GluC
H4_MOUSE	Histone H4	HAKRKTVTAMD	30	1.10E-06	419.896	1256.6661	3	0.0001	11	0	GluC
H4_MOUSE	Histone H4	HAKRKTVTAMD	30	7.70E-04	419.8959	1256.6658	3	-0.0002	11	0	GluC
H4_MOUSE	Histone H4	HAKRKTVTAMD	30	2.50E-05	419.8958	1256.6656	3	-0.0003	11	0	GluC
H4_MOUSE	Histone H4	HAKRKTVTAMD	30	5.70E-06	419.8958	1256.6656	3	-0.0004	11	0	GluC
H4_MOUSE	Histone H4	HAKRKTVTAMD	30	5.50E-05	419.8957	1256.6654	3	-0.0005	11	0	GluC
H4_MOUSE	Histone H4	HAKRKTVTAMD	30	9.60E-03	629.3398	1256.6651	2	-0.0008	11	0	GluC
H4_MOUSE	Histone H4	HAKRKTVTAMD	30	1.20E-07	419.8954	1256.6644	3	-0.0016	11	0	GluC
H4_MOUSE	Histone H4	HAKRKTVTAMD	30	2.00E-06	419.8954	1256.6642	3	-0.0017	11	0	GluC
H4_MOUSE	Histone H4	HAKRKTVTAMD	30	8.30E-04	419.8953	1256.6641	3	-0.0018	11	0	GluC
H4_MOUSE	Histone H4	LENVIRDAVTY	61	4.90E-05	646.8505	1291.6865	2	0.0093	11	1	Chymotrypsin
H4_MOUSE	Histone H4	LENVIRDAVTY	61	1.90E-04	646.8499	1291.6853	2	0.0081	11	1	Chymotrypsin
H4_MOUSE	Histone H4	LENVIRDAVTY	61	8.00E-03	646.8497	1291.6849	2	0.0077	11	1	Chymotrypsin
H4_MOUSE	Histone H4	LENVIRDAVTY	61	4.30E-04	646.8497	1291.6848	2	0.0076	11	1	Chymotrypsin
H4_MOUSE	Histone H4	LENVIRDAVTY	61	6.50E-03	646.8491	1291.6837	2	0.0065	11	1	Chymotrypsin
H4_MOUSE	Histone H4	LENVIRDAVTY	61	1.00E-04	646.849	1291.6835	2	0.0063	11	1	Chymotrypsin
H4_MOUSE	Histone H4	LENVIRDAVTY	61	1.60E-04	646.849	1291.6834	2	0.0062	11	1	Chymotrypsin
H4_MOUSE	Histone H4	LENVIRDAVTY	61	1.20E-04	646.849	1291.6834	2	0.0062	11	1	Chymotrypsin
H4_MOUSE	Histone H4	LENVIRDAVTY	61	4.50E-05	646.8487	1291.6828	2	0.0056	11	1	Chymotrypsin
H4_MOUSE	Histone H4	LENVIRDAVTY	61	1.70E-04	646.8484	1291.6823	2	0.0051	11	1	Chymotrypsin
H4_MOUSE	Histone H4	LENVIRDAVTY	61	8.00E-05	646.8484	1291.6822	2	0.0050	11	1	Chymotrypsin
H4_MOUSE	Histone H4	LENVIRDAVTY	61	1.50E-04	646.8479	1291.6812	2	0.0040	11	1	Chymotrypsin
H4_MOUSE	Histone H4	LENVIRDAVTY	61	4.30E-05	646.8475	1291.6805	2	0.0033	11	1	Chymotrypsin
H4_MOUSE	Histone H4	LENVIRDAVTY	61	7.80E-04	646.8474	1291.6801	2	0.0029	11	1	Chymotrypsin
H4_MOUSE	Histone H4	LENVIRDAVTY	61	2.30E-04	646.847	1291.6795	2	0.0023	11	1	Chymotrypsin
H4_MOUSE	Histone H4	LENVIRDAVTY	61	3.70E-05	646.847	1291.6795	2	0.0023	11	1	Chymotrypsin
H4_MOUSE	Histone H4	LENVIRDAVTY	61	1.50E-03	646.8469	1291.6793	2	0.0021	11	1	Chymotrypsin

H4_MOUSE	Histone H4	LENVIRDAVTY	61	8.40E-03	646.8468	1291.6791	2	0.0019	11	1	Chymotrypsin
H4_MOUSE	Histone H4	LENVIRDAVTY	61	4.70E-05	646.8468	1291.6789	2	0.0017	11	1	Chymotrypsin
H4_MOUSE	Histone H4	LENVIRDAVTY	61	8.00E-03	646.8467	1291.6788	2	0.0016	11	1	Chymotrypsin
H4_MOUSE	Histone H4	LENVIRDAVTY	61	5.00E-03	646.8467	1291.6788	2	0.0016	11	1	Chymotrypsin
H4_MOUSE	Histone H4	LENVIRDAVTY	61	1.80E-03	646.8467	1291.6788	2	0.0016	11	1	Chymotrypsin
H4_MOUSE	Histone H4	LENVIRDAVTY	61	9.10E-03	646.8466	1291.6787	2	0.0015	11	1	Chymotrypsin
H4_MOUSE	Histone H4	LENVIRDAVTY	61	3.80E-04	646.8466	1291.6787	2	0.0015	11	1	Chymotrypsin
H4_MOUSE	Histone H4	LENVIRDAVTY	61	3.00E-03	646.8466	1291.6786	2	0.0014	11	1	Chymotrypsin
H4_MOUSE	Histone H4	LENVIRDAVTY	61	2.70E-03	646.8466	1291.6786	2	0.0014	11	1	Chymotrypsin
H4_MOUSE	Histone H4	LENVIRDAVTY	61	9.00E-03	646.8465	1291.6785	2	0.0013	11	1	Chymotrypsin
H4_MOUSE	Histone H4	LENVIRDAVTY	61	4.50E-03	646.8465	1291.6785	2	0.0013	11	1	Chymotrypsin
H4_MOUSE	Histone H4	LENVIRDAVTY	61	1.90E-03	646.8465	1291.6785	2	0.0013	11	1	Chymotrypsin
H4_MOUSE	Histone H4	LENVIRDAVTY	61	1.20E-03	646.8465	1291.6785	2	0.0013	11	1	Chymotrypsin
H4_MOUSE	Histone H4	LENVIRDAVTY	61	2.90E-04	646.8465	1291.6784	2	0.0012	11	1	Chymotrypsin
H4_MOUSE	Histone H4	LENVIRDAVTY	61	2.40E-03	646.8464	1291.6783	2	0.0011	11	1	Chymotrypsin
H4_MOUSE	Histone H4	LENVIRDAVTY	61	1.40E-05	646.8464	1291.6783	2	0.0011	11	1	Chymotrypsin
H4_MOUSE	Histone H4	LENVIRDAVTY	61	1.20E-03	646.8464	1291.6782	2	0.0010	11	1	Chymotrypsin
H4_MOUSE	Histone H4	LENVIRDAVTY	61	8.70E-04	646.8464	1291.6782	2	0.0010	11	1	Chymotrypsin
H4_MOUSE	Histone H4	LENVIRDAVTY	61	1.30E-04	646.8464	1291.6782	2	0.0010	11	1	Chymotrypsin
H4_MOUSE	Histone H4	LENVIRDAVTY	61	1.50E-04	646.8463	1291.6781	2	0.0009	11	1	Chymotrypsin
H4_MOUSE	Histone H4	LENVIRDAVTY	61	3.60E-05	646.8463	1291.6781	2	0.0009	11	1	Chymotrypsin
H4_MOUSE	Histone H4	LENVIRDAVTY	61	9.40E-03	646.8462	1291.6779	2	0.0007	11	1	Chymotrypsin
H4_MOUSE	Histone H4	LENVIRDAVTY	61	7.50E-03	646.8462	1291.6779	2	0.0007	11	1	Chymotrypsin
H4_MOUSE	Histone H4	LENVIRDAVTY	61	3.80E-03	646.8461	1291.6777	2	0.0005	11	1	Chymotrypsin
H4_MOUSE	Histone H4	LENVIRDAVTY	61	3.20E-03	646.8461	1291.6777	2	0.0005	11	1	Chymotrypsin
H4_MOUSE	Histone H4	LENVIRDAVTY	61	2.30E-03	646.8461	1291.6777	2	0.0005	11	1	Chymotrypsin
H4_MOUSE	Histone H4	LENVIRDAVTY	61	1.20E-03	646.8461	1291.6777	2	0.0005	11	1	Chymotrypsin
H4_MOUSE	Histone H4	LENVIRDAVTY	61	4.50E-05	646.8461	1291.6777	2	0.0005	11	1	Chymotrypsin
H4_MOUSE	Histone H4	LENVIRDAVTY	61	2.10E-04	646.8461	1291.6776	2	0.0004	11	1	Chymotrypsin
H4_MOUSE	Histone H4	LENVIRDAVTY	61	1.70E-04	646.8461	1291.6776	2	0.0004	11	1	Chymotrypsin
H4_MOUSE	Histone H4	LENVIRDAVTY	61	1.30E-05	646.8461	1291.6776	2	0.0004	11	1	Chymotrypsin
H4_MOUSE	Histone H4	LENVIRDAVTY	61	1.70E-03	646.846	1291.6775	2	0.0003	11	1	Chymotrypsin
H4_MOUSE	Histone H4	LENVIRDAVTY	61	5.30E-04	646.846	1291.6775	2	0.0003	11	1	Chymotrypsin
H4_MOUSE	Histone H4	LENVIRDAVTY	61	1.10E-04	646.846	1291.6775	2	0.0003	11	1	Chymotrypsin
H4_MOUSE	Histone H4	LENVIRDAVTY	61	2.00E-03	646.846	1291.6773	2	0.0001	11	1	Chymotrypsin
H4_MOUSE	Histone H4	LENVIRDAVTY	61	3.30E-04	646.846	1291.6773	2	0.0001	11	1	Chymotrypsin
H4_MOUSE	Histone H4	LENVIRDAVTY	61	4.90E-03	646.8459	1291.6772	2	0.0000	11	1	Chymotrypsin
H4_MOUSE	Histone H4	LENVIRDAVTY	61	6.80E-04	646.8459	1291.6772	2	0.0000	11	1	Chymotrypsin
H4_MOUSE	Histone H4	LENVIRDAVTY	61	6.30E-03	646.8457	1291.6769	2	-0.0003	11	1	Chymotrypsin
H4_MOUSE	Histone H4	LENVIRDAVTY	61	4.00E-05	646.8457	1291.6769	2	-0.0003	11	1	Chymotrypsin
H4_MOUSE	Histone H4	LENVIRDAVTY	61	4.00E-04	646.8455	1291.6765	2	-0.0007	11	1	GluC
H4_MOUSE	Histone H4	LENVIRDAVTY	61	4.30E-05	646.845	1291.6754	2	-0.0018	11	1	Chymotrypsin
H4_MOUSE	Histone H4	ETRGVLKVFLE	92	7.40E-05	645.8789	1289.7432	2	0.0089	11	1	GluC
H4_MOUSE	Histone H4	ETRGVLKVFLE	92	4.60E-04	645.8789	1289.7431	2	0.0088	11	1	GluC
H4_MOUSE	Histone H4	ETRGVLKVFLE	92	5.00E-03	645.8786	1289.7427	2	0.0084	11	1	GluC
H4_MOUSE	Histone H4	ETRGVLKVFLE	92	2.00E-05	430.9215	1289.7426	3	0.0083	11	1	GluC
H4_MOUSE	Histone H4	ETRGVLKVFLE	92	1.80E-03	645.8785	1289.7425	2	0.0082	11	1	GluC
H4_MOUSE	Histone H4	ETRGVLKVFLE	92	9.60E-04	645.8785	1289.7424	2	0.0081	11	1	GluC
H4_MOUSE	Histone H4	ETRGVLKVFLE	92	7.70E-03	430.9214	1289.7423	3	0.0080	11	1	GluC
H4_MOUSE	Histone H4	ETRGVLKVFLE	92	1.80E-03	430.921	1289.7411	3	0.0068	11	1	GluC
H4_MOUSE	Histone H4	ETRGVLKVFLE	92	3.50E-03	645.8777	1289.7408	2	0.0065	11	1	GluC
H4_MOUSE	Histone H4	ETRGVLKVFLE	92	5.50E-06	430.9209	1289.7409	3	0.0065	11	1	GluC
H4_MOUSE	Histone H4	ETRGVLKVFLE	92	2.70E-05	645.8777	1289.7407	2	0.0064	11	1	GluC
H4_MOUSE	Histone H4	ETRGVLKVFLE	92	1.10E-03	430.9208	1289.7406	3	0.0063	11	1	GluC
H4_MOUSE	Histone H4	ETRGVLKVFLE	92	8.70E-04	645.8773	1289.7399	2	0.0056	11	1	GluC
H4_MOUSE	Histone H4	ETRGVLKVFLE	92	6.50E-04	645.8772	1289.7398	2	0.0055	11	1	GluC
H4_MOUSE	Histone H4	ETRGVLKVFLE	92	1.10E-03	645.8766	1289.7386	2	0.0043	11	1	GluC
H4_MOUSE	Histone H4	ETRGVLKVFLE	92	3.30E-05	430.9197	1289.7373	3	0.0030	11	1	GluC
H4_MOUSE	Histone H4	ETRGVLKVFLE	92	5.30E-04	430.9197	1289.7372	3	0.0029	11	1	GluC
H4_MOUSE	Histone H4	ETRGVLKVFLE	92	1.10E-03	430.9197	1289.7372	3	0.0028	11	1	GluC
H4_MOUSE	Histone H4	ETRGVLKVFLE	92	1.00E-05	430.9196	1289.7370	3	0.0027	11	1	GluC
H4_MOUSE	Histone H4	ETRGVLKVFLE	92	4.40E-03	645.8758	1289.7369	2	0.0026	11	1	GluC
H4_MOUSE	Histone H4	ETRGVLKVFLE	92	5.00E-03	645.8757	1289.7368	2	0.0025	11	1	GluC
H4_MOUSE	Histone H4	ETRGVLKVFLE	92	6.30E-03	645.8756	1289.7367	2	0.0024	11	1	GluC
H4_MOUSE	Histone H4	ETRGVLKVFLE	92	5.30E-04	430.9195	1289.7366	3	0.0023	11	1	GluC
H4_MOUSE	Histone H4	ETRGVLKVFLE	92	4.90E-03	645.8755	1289.7365	2	0.0022	11	1	GluC
H4_MOUSE	Histone H4	ETRGVLKVFLE	92	1.00E-03	645.8755	1289.7365	2	0.0022	11	1	GluC
H4_MOUSE	Histone H4	ETRGVLKVFLE	92	7.40E-03	645.8755	1289.7364	2	0.0021	11	1	GluC
H4_MOUSE	Histone H4	ETRGVLKVFLE	92	3.40E-05	645.8755	1289.7364	2	0.0021	11	1	GluC
H4_MOUSE	Histone H4	ETRGVLKVFLE	92	9.10E-03	645.8754	1289.7362	2	0.0019	11	1	GluC
H4_MOUSE	Histone H4	ETRGVLKVFLE	92	7.30E-03	645.8754	1289.7362	2	0.0019	11	1	GluC
H4_MOUSE	Histone H4	ETRGVLKVFLE	92	6.40E-03	645.8754	1289.7362	2	0.0018	11	1	GluC
H4_MOUSE	Histone H4	ETRGVLKVFLE	92	4.80E-03	645.8753	1289.7360	2	0.0017	11	1	GluC
H4_MOUSE	Histone H4	ETRGVLKVFLE	92	1.30E-03	645.8753	1289.7360	2	0.0017	11	1	GluC

H4_MOUSE	Histone H4	ETRGVLKVFLE	92	6.70E-05	645.8753	1289.7359	2	0.0016	11	1	GluC
H4_MOUSE	Histone H4	ETRGVLKVFLE	92	7.20E-03	645.8752	1289.7358	2	0.0015	11	1	GluC
H4_MOUSE	Histone H4	ETRGVLKVFLE	92	2.30E-03	430.9192	1289.7359	3	0.0015	11	1	GluC
H4_MOUSE	Histone H4	ETRGVLKVFLE	92	2.40E-03	645.8751	1289.7357	2	0.0014	11	1	GluC
H4_MOUSE	Histone H4	ETRGVLKVFLE	92	3.70E-04	645.8751	1289.7357	2	0.0014	11	1	GluC
H4_MOUSE	Histone H4	ETRGVLKVFLE	92	1.20E-04	430.9191	1289.7356	3	0.0012	11	1	GluC
H4_MOUSE	Histone H4	ETRGVLKVFLE	92	8.70E-03	645.875	1289.7354	2	0.0011	11	1	GluC
H4_MOUSE	Histone H4	ETRGVLKVFLE	92	3.90E-03	645.875	1289.7354	2	0.0011	11	1	GluC
H4_MOUSE	Histone H4	ETRGVLKVFLE	92	6.40E-03	645.8749	1289.7352	2	0.0009	11	1	GluC
H4_MOUSE	Histone H4	ETRGVLKVFLE	92	7.10E-04	645.8749	1289.7352	2	0.0009	11	1	GluC
H4_MOUSE	Histone H4	ETRGVLKVFLE	92	2.90E-04	645.8749	1289.7353	2	0.0009	11	1	GluC
H4_MOUSE	Histone H4	ETRGVLKVFLE	92	5.90E-05	645.8749	1289.7352	2	0.0009	11	1	GluC
H4_MOUSE	Histone H4	ETRGVLKVFLE	92	2.80E-06	430.919	1289.7351	3	0.0008	11	1	GluC
H4_MOUSE	Histone H4	ETRGVLKVFLE	92	4.80E-03	430.9189	1289.7350	3	0.0007	11	1	GluC
H4_MOUSE	Histone H4	ETRGVLKVFLE	92	3.20E-03	645.8748	1289.7350	2	0.0007	11	1	GluC
H4_MOUSE	Histone H4	ETRGVLKVFLE	92	1.40E-04	645.8748	1289.7350	2	0.0007	11	1	GluC
H4_MOUSE	Histone H4	ETRGVLKVFLE	92	2.50E-05	645.8748	1289.7350	2	0.0007	11	1	GluC
H4_MOUSE	Histone H4	ETRGVLKVFLE	92	9.10E-06	645.8748	1289.7350	2	0.0007	11	1	GluC
H4_MOUSE	Histone H4	ETRGVLKVFLE	92	6.70E-03	645.8747	1289.7348	2	0.0005	11	1	GluC
H4_MOUSE	Histone H4	ETRGVLKVFLE	92	4.70E-03	645.8747	1289.7348	2	0.0005	11	1	GluC
H4_MOUSE	Histone H4	ETRGVLKVFLE	92	3.60E-03	430.9189	1289.7349	3	0.0005	11	1	GluC
H4_MOUSE	Histone H4	ETRGVLKVFLE	92	2.10E-03	430.9189	1289.7348	3	0.0005	11	1	GluC
H4_MOUSE	Histone H4	ETRGVLKVFLE	92	1.50E-03	645.8747	1289.7348	2	0.0005	11	1	GluC
H4_MOUSE	Histone H4	ETRGVLKVFLE	92	1.00E-04	430.9189	1289.7348	3	0.0005	11	1	GluC
H4_MOUSE	Histone H4	ETRGVLKVFLE	92	5.10E-05	645.8747	1289.7348	2	0.0005	11	1	GluC
H4_MOUSE	Histone H4	ETRGVLKVFLE	92	7.90E-06	430.9189	1289.7348	3	0.0005	11	1	GluC
H4_MOUSE	Histone H4	ETRGVLKVFLE	92	2.40E-07	430.9189	1289.7349	3	0.0005	11	1	GluC
H4_MOUSE	Histone H4	ETRGVLKVFLE	92	2.30E-05	430.9189	1289.7348	3	0.0004	11	1	GluC
H4_MOUSE	Histone H4	ETRGVLKVFLE	92	7.60E-03	645.8746	1289.7346	2	0.0003	11	1	GluC
H4_MOUSE	Histone H4	ETRGVLKVFLE	92	1.20E-03	430.9188	1289.7346	3	0.0003	11	1	GluC
H4_MOUSE	Histone H4	ETRGVLKVFLE	92	1.50E-03	645.8745	1289.7345	2	0.0002	11	1	GluC
H4_MOUSE	Histone H4	ETRGVLKVFLE	92	2.60E-04	430.9188	1289.7346	3	0.0002	11	1	GluC
H4_MOUSE	Histone H4	ETRGVLKVFLE	92	2.40E-05	430.9188	1289.7345	3	0.0002	11	1	GluC
H4_MOUSE	Histone H4	ETRGVLKVFLE	92	1.10E-05	430.9188	1289.7345	3	0.0002	11	1	GluC
H4_MOUSE	Histone H4	ETRGVLKVFLE	92	3.50E-05	430.9187	1289.7344	3	0.0001	11	1	GluC
H4_MOUSE	Histone H4	ETRGVLKVFLE	92	5.90E-03	430.9187	1289.7343	3	0.0000	11	1	GluC
H4_MOUSE	Histone H4	ETRGVLKVFLE	92	4.90E-04	645.8744	1289.7343	2	0.0000	11	1	GluC
H4_MOUSE	Histone H4	ETRGVLKVFLE	92	1.80E-04	430.9187	1289.7343	3	0.0000	11	1	GluC
H4_MOUSE	Histone H4	ETRGVLKVFLE	92	1.20E-05	430.9187	1289.7344	3	0.0000	11	1	GluC
H4_MOUSE	Histone H4	ETRGVLKVFLE	92	2.60E-07	430.9187	1289.7344	3	0.0000	11	1	GluC
H4_MOUSE	Histone H4	ETRGVLKVFLE	92	9.10E-03	645.8744	1289.7343	2	-0.0001	11	1	GluC
H4_MOUSE	Histone H4	ETRGVLKVFLE	92	5.70E-03	430.9187	1289.7342	3	-0.0001	11	1	GluC
H4_MOUSE	Histone H4	ETRGVLKVFLE	92	1.00E-03	430.9187	1289.7342	3	-0.0001	11	1	GluC
H4_MOUSE	Histone H4	ETRGVLKVFLE	92	1.30E-05	430.9187	1289.7342	3	-0.0001	11	1	GluC
H4_MOUSE	Histone H4	ETRGVLKVFLE	92	6.20E-03	430.9187	1289.7341	3	-0.0002	11	1	GluC
H4_MOUSE	Histone H4	ETRGVLKVFLE	92	1.50E-04	430.9186	1289.7339	3	-0.0004	11	1	GluC
H4_MOUSE	Histone H4	ETRGVLKVFLE	92	4.90E-05	430.9186	1289.7339	3	-0.0004	11	1	GluC
H4_MOUSE	Histone H4	ETRGVLKVFLE	92	1.50E-03	645.8742	1289.7339	2	-0.0005	11	1	GluC
H4_MOUSE	Histone H4	ETRGVLKVFLE	92	7.50E-05	430.9185	1289.7338	3	-0.0005	11	1	GluC
H4_MOUSE	Histone H4	ETRGVLKVFLE	92	2.40E-04	430.9185	1289.7337	3	-0.0006	11	1	GluC
H4_MOUSE	Histone H4	ETRGVLKVFLE	92	1.00E-02	645.874	1289.7335	2	-0.0008	11	1	GluC
H4_MOUSE	Histone H4	ETRGVLKVFLE	92	8.20E-06	430.9184	1289.7334	3	-0.0009	11	1	GluC
H4_MOUSE	Histone H4	ETRGVLKVFLE	92	2.60E-06	430.9184	1289.7334	3	-0.0009	11	1	GluC
H4_MOUSE	Histone H4	ETRGVLKVFLE	92	4.40E-04	430.9184	1289.7333	3	-0.0010	11	1	GluC
H4_MOUSE	Histone H4	ETRGVLKVFLE	92	4.70E-03	430.9182	1289.7327	3	-0.0016	11	1	GluC
H4_MOUSE	Histone H4	NVIRDAVTYTE	175	1.50E-04	640.8321	1279.6496	2	0.0088	11	1	GluC
H4_MOUSE	Histone H4	NVIRDAVTYTE	175	3.10E-03	640.8317	1279.6489	2	0.0081	11	1	GluC
H4_MOUSE	Histone H4	NVIRDAVTYTE	175	3.20E-04	640.8312	1279.6478	2	0.0069	11	1	GluC
H4_MOUSE	Histone H4	NVIRDAVTYTE	175	5.80E-05	640.8307	1279.6468	2	0.0060	11	1	GluC
H4_MOUSE	Histone H4	NVIRDAVTYTE	175	4.50E-05	640.8306	1279.6467	2	0.0058	11	1	GluC
H4_MOUSE	Histone H4	NVIRDAVTYTE	175	1.10E-03	640.8305	1279.6464	2	0.0056	11	1	GluC
H4_MOUSE	Histone H4	NVIRDAVTYTE	175	1.40E-04	640.8302	1279.6458	2	0.0049	11	1	GluC
H4_MOUSE	Histone H4	NVIRDAVTYTE	175	4.30E-05	640.8301	1279.6457	2	0.0049	11	1	GluC
H4_MOUSE	Histone H4	NVIRDAVTYTE	175	7.60E-04	640.8301	1279.6456	2	0.0048	11	1	GluC
H4_MOUSE	Histone H4	NVIRDAVTYTE	175	2.10E-03	640.83	1279.6455	2	0.0047	11	1	GluC
H4_MOUSE	Histone H4	NVIRDAVTYTE	175	1.40E-04	640.8301	1279.6455	2	0.0047	11	1	GluC
H4_MOUSE	Histone H4	NVIRDAVTYTE	175	6.20E-05	640.83	1279.6455	2	0.0047	11	1	GluC
H4_MOUSE	Histone H4	NVIRDAVTYTE	175	2.10E-04	640.83	1279.6453	2	0.0045	11	1	GluC
H4_MOUSE	Histone H4	NVIRDAVTYTE	175	1.30E-04	640.8299	1279.6452	2	0.0044	11	1	GluC
H4_MOUSE	Histone H4	NVIRDAVTYTE	175	6.10E-03	640.8298	1279.6451	2	0.0043	11	1	GluC
H4_MOUSE	Histone H4	NVIRDAVTYTE	175	5.90E-03	640.8298	1279.6451	2	0.0043	11	1	GluC
H4_MOUSE	Histone H4	NVIRDAVTYTE	175	1.50E-04	640.8297	1279.6448	2	0.0040	11	1	GluC
H4_MOUSE	Histone H4	NVIRDAVTYTE	175	6.00E-03	640.8297	1279.6448	2	0.0039	11	1	GluC
H4_MOUSE	Histone H4	NVIRDAVTYTE	175	3.20E-03	640.8296	1279.6447	2	0.0039	11	1	GluC

H4_MOUSE	Histone H4	NVIRDAVTYTE	175	6.20E-03	640.8296	1279.6445	2	0.0037	11	1	GluC
H4_MOUSE	Histone H4	NVIRDAVTYTE	175	2.20E-03	640.8295	1279.6444	2	0.0036	11	1	GluC
H4_MOUSE	Histone H4	NVIRDAVTYTE	175	5.40E-03	640.8294	1279.6443	2	0.0035	11	1	GluC
H4_MOUSE	Histone H4	NVIRDAVTYTE	175	3.00E-04	640.8294	1279.6443	2	0.0035	11	1	GluC
H4_MOUSE	Histone H4	NVIRDAVTYTE	175	1.80E-04	640.8294	1279.6443	2	0.0035	11	1	GluC
H4_MOUSE	Histone H4	NVIRDAVTYTE	175	3.10E-04	640.8294	1279.6442	2	0.0034	11	1	GluC
H4_MOUSE	Histone H4	NVIRDAVTYTE	175	3.60E-05	640.8293	1279.6441	2	0.0032	11	1	GluC
H4_MOUSE	Histone H4	NVIRDAVTYTE	175	9.30E-03	640.8293	1279.6440	2	0.0031	11	1	GluC
H4_MOUSE	Histone H4	NVIRDAVTYTE	175	7.00E-06	640.8293	1279.6440	2	0.0031	11	1	GluC
H4_MOUSE	Histone H4	NVIRDAVTYTE	175	6.10E-03	640.8291	1279.6435	2	0.0027	11	1	GluC
H4_MOUSE	Histone H4	NVIRDAVTYTE	175	4.30E-05	640.8291	1279.6435	2	0.0027	11	1	GluC
H4_MOUSE	Histone H4	NVIRDAVTYTE	175	1.30E-03	640.8289	1279.6433	2	0.0025	11	1	GluC
H4_MOUSE	Histone H4	NVIRDAVTYTE	175	1.90E-04	640.8289	1279.6433	2	0.0025	11	1	GluC
H4_MOUSE	Histone H4	NVIRDAVTYTE	175	3.70E-04	640.8289	1279.6432	2	0.0024	11	1	GluC
H4_MOUSE	Histone H4	NVIRDAVTYTE	175	1.30E-05	640.8289	1279.6432	2	0.0024	11	1	GluC
H4_MOUSE	Histone H4	NVIRDAVTYTE	175	8.30E-03	640.8288	1279.6431	2	0.0023	11	1	GluC
H4_MOUSE	Histone H4	NVIRDAVTYTE	175	9.70E-04	640.8288	1279.6431	2	0.0023	11	1	GluC
H4_MOUSE	Histone H4	NVIRDAVTYTE	175	1.90E-05	640.8288	1279.6431	2	0.0023	11	1	GluC
H4_MOUSE	Histone H4	NVIRDAVTYTE	175	5.20E-06	640.8288	1279.6430	2	0.0022	11	1	GluC
H4_MOUSE	Histone H4	NVIRDAVTYTE	175	3.40E-03	640.8288	1279.6429	2	0.0021	11	1	GluC
H4_MOUSE	Histone H4	NVIRDAVTYTE	175	1.20E-03	640.8288	1279.6429	2	0.0021	11	1	GluC
H4_MOUSE	Histone H4	NVIRDAVTYTE	175	6.00E-04	640.8287	1279.6429	2	0.0021	11	1	GluC
H4_MOUSE	Histone H4	NVIRDAVTYTE	175	1.30E-04	640.8288	1279.6429	2	0.0021	11	1	GluC
H4_MOUSE	Histone H4	NVIRDAVTYTE	175	4.70E-03	640.8287	1279.6428	2	0.0020	11	1	GluC
H4_MOUSE	Histone H4	NVIRDAVTYTE	175	7.10E-03	640.8286	1279.6427	2	0.0019	11	1	GluC
H4_MOUSE	Histone H4	NVIRDAVTYTE	175	2.80E-04	640.8286	1279.6427	2	0.0019	11	1	GluC
H4_MOUSE	Histone H4	NVIRDAVTYTE	175	4.40E-03	640.8286	1279.6427	2	0.0018	11	1	GluC
H4_MOUSE	Histone H4	NVIRDAVTYTE	175	1.80E-04	640.8286	1279.6426	2	0.0018	11	1	GluC
H4_MOUSE	Histone H4	NVIRDAVTYTE	175	7.90E-05	640.8285	1279.6425	2	0.0017	11	1	GluC
H4_MOUSE	Histone H4	NVIRDAVTYTE	175	3.80E-05	640.8285	1279.6425	2	0.0017	11	1	GluC
H4_MOUSE	Histone H4	NVIRDAVTYTE	175	1.90E-05	640.8285	1279.6425	2	0.0017	11	1	GluC
H4_MOUSE	Histone H4	NVIRDAVTYTE	175	3.20E-03	640.8285	1279.6424	2	0.0016	11	1	GluC
H4_MOUSE	Histone H4	NVIRDAVTYTE	175	2.10E-03	640.8285	1279.6424	2	0.0016	11	1	GluC
H4_MOUSE	Histone H4	NVIRDAVTYTE	175	4.30E-05	640.8285	1279.6424	2	0.0016	11	1	GluC
H4_MOUSE	Histone H4	NVIRDAVTYTE	175	5.70E-03	640.8284	1279.6423	2	0.0015	11	1	GluC
H4_MOUSE	Histone H4	NVIRDAVTYTE	175	4.40E-04	640.8284	1279.6423	2	0.0015	11	1	GluC
H4_MOUSE	Histone H4	NVIRDAVTYTE	175	2.70E-04	640.8284	1279.6423	2	0.0015	11	1	GluC
H4_MOUSE	Histone H4	NVIRDAVTYTE	175	2.30E-04	640.8284	1279.6423	2	0.0015	11	1	GluC
H4_MOUSE	Histone H4	NVIRDAVTYTE	175	3.70E-03	640.8284	1279.6422	2	0.0014	11	1	GluC
H4_MOUSE	Histone H4	NVIRDAVTYTE	175	2.70E-04	640.8284	1279.6423	2	0.0014	11	1	GluC
H4_MOUSE	Histone H4	NVIRDAVTYTE	175	5.50E-05	640.8284	1279.6422	2	0.0014	11	1	GluC
H4_MOUSE	Histone H4	NVIRDAVTYTE	175	4.40E-03	640.8283	1279.6421	2	0.0013	11	1	GluC
H4_MOUSE	Histone H4	NVIRDAVTYTE	175	1.00E-03	640.8283	1279.6421	2	0.0013	11	1	GluC
H4_MOUSE	Histone H4	NVIRDAVTYTE	175	1.20E-04	640.8283	1279.6421	2	0.0013	11	1	GluC
H4_MOUSE	Histone H4	NVIRDAVTYTE	175	1.20E-04	640.8284	1279.6421	2	0.0013	11	1	GluC
H4_MOUSE	Histone H4	NVIRDAVTYTE	175	4.10E-06	640.8283	1279.6421	2	0.0013	11	1	GluC
H4_MOUSE	Histone H4	NVIRDAVTYTE	175	9.00E-03	640.8283	1279.6421	2	0.0012	11	1	GluC
H4_MOUSE	Histone H4	NVIRDAVTYTE	175	6.50E-03	640.8283	1279.6421	2	0.0012	11	1	GluC
H4_MOUSE	Histone H4	NVIRDAVTYTE	175	1.80E-03	640.8283	1279.6420	2	0.0012	11	1	GluC
H4_MOUSE	Histone H4	NVIRDAVTYTE	175	1.30E-03	640.8283	1279.6421	2	0.0012	11	1	GluC
H4_MOUSE	Histone H4	NVIRDAVTYTE	175	8.20E-04	640.8283	1279.6420	2	0.0012	11	1	GluC
H4_MOUSE	Histone H4	NVIRDAVTYTE	175	3.50E-04	640.8283	1279.6421	2	0.0012	11	1	GluC
H4_MOUSE	Histone H4	NVIRDAVTYTE	175	1.40E-04	640.8283	1279.6420	2	0.0012	11	1	GluC
H4_MOUSE	Histone H4	NVIRDAVTYTE	175	9.10E-05	640.8283	1279.6420	2	0.0012	11	1	GluC
H4_MOUSE	Histone H4	NVIRDAVTYTE	175	4.40E-05	640.8283	1279.6421	2	0.0012	11	1	GluC
H4_MOUSE	Histone H4	NVIRDAVTYTE	175	2.00E-05	640.8283	1279.6420	2	0.0012	11	1	GluC
H4_MOUSE	Histone H4	NVIRDAVTYTE	175	1.70E-05	640.8283	1279.6420	2	0.0012	11	1	GluC
H4_MOUSE	Histone H4	NVIRDAVTYTE	175	2.60E-03	640.8283	1279.6419	2	0.0011	11	1	GluC
H4_MOUSE	Histone H4	NVIRDAVTYTE	175	1.90E-03	640.8282	1279.6419	2	0.0011	11	1	GluC
H4_MOUSE	Histone H4	NVIRDAVTYTE	175	1.90E-03	640.8282	1279.6419	2	0.0011	11	1	GluC
H4_MOUSE	Histone H4	NVIRDAVTYTE	175	9.40E-04	640.8283	1279.6419	2	0.0011	11	1	GluC
H4_MOUSE	Histone H4	NVIRDAVTYTE	175	5.90E-04	640.8283	1279.6419	2	0.0011	11	1	GluC
H4_MOUSE	Histone H4	NVIRDAVTYTE	175	3.10E-04	640.8282	1279.6419	2	0.0011	11	1	GluC
H4_MOUSE	Histone H4	NVIRDAVTYTE	175	8.40E-05	640.8282	1279.6419	2	0.0011	11	1	GluC
H4_MOUSE	Histone H4	NVIRDAVTYTE	175	7.10E-05	640.8283	1279.6419	2	0.0011	11	1	GluC
H4_MOUSE	Histone H4	NVIRDAVTYTE	175	6.90E-05	640.8282	1279.6419	2	0.0011	11	1	GluC
H4_MOUSE	Histone H4	NVIRDAVTYTE	175	6.80E-05	640.8283	1279.6420	2	0.0011	11	1	GluC
H4_MOUSE	Histone H4	NVIRDAVTYTE	175	4.00E-05	640.8282	1279.6419	2	0.0011	11	1	GluC
H4_MOUSE	Histone H4	NVIRDAVTYTE	175	3.30E-05	640.8283	1279.6419	2	0.0011	11	1	GluC
H4_MOUSE	Histone H4	NVIRDAVTYTE	175	6.60E-03	640.8282	1279.6419	2	0.0010	11	1	GluC
H4_MOUSE	Histone H4	NVIRDAVTYTE	175	3.00E-03	640.8282	1279.6418	2	0.0010	11	1	GluC
H4_MOUSE	Histone H4	NVIRDAVTYTE	175	1.90E-03	640.8282	1279.6419	2	0.0010	11	1	GluC
H4_MOUSE	Histone H4	NVIRDAVTYTE	175	1.30E-03	640.8282	1279.6419	2	0.0010	11	1	GluC
H4_MOUSE	Histone H4	NVIRDAVTYTE	175	9.20E-04	640.8282	1279.6418	2	0.0010	11	1	GluC

H4_MOUSE	Histone H4	NVIRDAVTYTE	175	1.80E-04	640.8282	1279.6418	2	0.0010	11	1	GluC
H4_MOUSE	Histone H4	NVIRDAVTYTE	175	1.20E-04	640.8282	1279.6418	2	0.0010	11	1	GluC
H4_MOUSE	Histone H4	NVIRDAVTYTE	175	8.80E-05	640.8282	1279.6418	2	0.0010	11	1	GluC
H4_MOUSE	Histone H4	NVIRDAVTYTE	175	1.60E-05	640.8282	1279.6419	2	0.0010	11	1	GluC
H4_MOUSE	Histone H4	NVIRDAVTYTE	175	9.80E-06	640.8282	1279.6418	2	0.0010	11	1	GluC
H4_MOUSE	Histone H4	NVIRDAVTYTE	175	3.10E-03	640.8282	1279.6418	2	0.0009	11	1	GluC
H4_MOUSE	Histone H4	NVIRDAVTYTE	175	2.70E-03	640.8281	1279.6417	2	0.0009	11	1	GluC
H4_MOUSE	Histone H4	NVIRDAVTYTE	175	2.40E-03	640.8281	1279.6417	2	0.0009	11	1	GluC
H4_MOUSE	Histone H4	NVIRDAVTYTE	175	2.30E-03	640.8281	1279.6417	2	0.0009	11	1	GluC
H4_MOUSE	Histone H4	NVIRDAVTYTE	175	1.40E-03	640.8281	1279.6417	2	0.0009	11	1	GluC
H4_MOUSE	Histone H4	NVIRDAVTYTE	175	1.40E-03	640.8282	1279.6418	2	0.0009	11	1	GluC
H4_MOUSE	Histone H4	NVIRDAVTYTE	175	1.30E-03	640.8281	1279.6417	2	0.0009	11	1	GluC
H4_MOUSE	Histone H4	NVIRDAVTYTE	175	4.80E-05	640.8281	1279.6417	2	0.0009	11	1	GluC
H4_MOUSE	Histone H4	NVIRDAVTYTE	175	1.00E-05	640.8281	1279.6417	2	0.0009	11	1	GluC
H4_MOUSE	Histone H4	NVIRDAVTYTE	175	3.70E-03	640.8281	1279.6416	2	0.0008	11	1	GluC
H4_MOUSE	Histone H4	NVIRDAVTYTE	175	9.30E-04	640.8281	1279.6416	2	0.0008	11	1	GluC
H4_MOUSE	Histone H4	NVIRDAVTYTE	175	8.80E-06	640.8281	1279.6416	2	0.0008	11	1	GluC
H4_MOUSE	Histone H4	NVIRDAVTYTE	175	6.70E-06	640.8281	1279.6417	2	0.0008	11	1	GluC
H4_MOUSE	Histone H4	NVIRDAVTYTE	175	2.70E-06	640.8281	1279.6416	2	0.0008	11	1	GluC
H4_MOUSE	Histone H4	NVIRDAVTYTE	175	9.00E-04	640.828	1279.6415	2	0.0007	11	1	GluC
H4_MOUSE	Histone H4	NVIRDAVTYTE	175	9.10E-05	640.828	1279.6415	2	0.0007	11	1	GluC
H4_MOUSE	Histone H4	NVIRDAVTYTE	175	1.60E-05	640.828	1279.6415	2	0.0007	11	1	GluC
H4_MOUSE	Histone H4	NVIRDAVTYTE	175	2.10E-03	640.828	1279.6414	2	0.0006	11	1	GluC
H4_MOUSE	Histone H4	NVIRDAVTYTE	175	2.00E-03	640.828	1279.6414	2	0.0006	11	1	GluC
H4_MOUSE	Histone H4	NVIRDAVTYTE	175	3.40E-05	640.828	1279.6414	2	0.0006	11	1	GluC
H4_MOUSE	Histone H4	NVIRDAVTYTE	175	5.50E-03	640.8279	1279.6413	2	0.0005	11	1	GluC
H4_MOUSE	Histone H4	NVIRDAVTYTE	175	9.40E-04	640.8279	1279.6413	2	0.0005	11	1	GluC
H4_MOUSE	Histone H4	NVIRDAVTYTE	175	3.80E-05	640.8279	1279.6413	2	0.0005	11	1	GluC
H4_MOUSE	Histone H4	NVIRDAVTYTE	175	6.40E-07	640.828	1279.6414	2	0.0005	11	1	GluC
H4_MOUSE	Histone H4	NVIRDAVTYTE	175	6.10E-03	640.8279	1279.6412	2	0.0004	11	1	GluC
H4_MOUSE	Histone H4	NVIRDAVTYTE	175	4.80E-05	640.8279	1279.6412	2	0.0004	11	1	GluC
H4_MOUSE	Histone H4	NVIRDAVTYTE	175	4.60E-06	640.8279	1279.6412	2	0.0004	11	1	GluC
H4_MOUSE	Histone H4	NVIRDAVTYTE	175	6.70E-03	640.8278	1279.6411	2	0.0003	11	1	GluC
H4_MOUSE	Histone H4	NVIRDAVTYTE	175	1.40E-03	640.8278	1279.6411	2	0.0003	11	1	GluC
H4_MOUSE	Histone H4	NVIRDAVTYTE	175	1.60E-04	640.8279	1279.6412	2	0.0003	11	1	GluC
H4_MOUSE	Histone H4	NVIRDAVTYTE	175	7.70E-03	640.8278	1279.6410	2	0.0002	11	1	GluC
H4_MOUSE	Histone H4	NVIRDAVTYTE	175	2.30E-03	640.8278	1279.6410	2	0.0002	11	1	GluC
H4_MOUSE	Histone H4	NVIRDAVTYTE	175	2.10E-03	640.8278	1279.6410	2	0.0002	11	1	GluC
H4_MOUSE	Histone H4	NVIRDAVTYTE	175	1.90E-03	640.8278	1279.6410	2	0.0002	11	1	GluC
H4_MOUSE	Histone H4	NVIRDAVTYTE	175	7.50E-04	640.8278	1279.6410	2	0.0002	11	1	GluC
H4_MOUSE	Histone H4	NVIRDAVTYTE	175	1.10E-05	640.8278	1279.6410	2	0.0002	11	1	GluC
H4_MOUSE	Histone H4	NVIRDAVTYTE	175	1.20E-06	640.8278	1279.6410	2	0.0002	11	1	GluC
H4_MOUSE	Histone H4	NVIRDAVTYTE	175	2.20E-04	640.8277	1279.6409	2	0.0001	11	1	GluC
H4_MOUSE	Histone H4	NVIRDAVTYTE	175	9.20E-05	640.8278	1279.6410	2	0.0001	11	1	GluC
H4_MOUSE	Histone H4	NVIRDAVTYTE	175	3.80E-05	640.8278	1279.6410	2	0.0001	11	1	GluC
H4_MOUSE	Histone H4	NVIRDAVTYTE	175	3.10E-04	640.8277	1279.6409	2	0.0000	11	1	GluC
H4_MOUSE	Histone H4	NVIRDAVTYTE	175	4.50E-07	640.8277	1279.6408	2	0.0000	11	1	GluC
H4_MOUSE	Histone H4	NVIRDAVTYTE	175	8.80E-03	640.8276	1279.6407	2	-0.0002	11	1	GluC
H4_MOUSE	Histone H4	NVIRDAVTYTE	175	8.20E-03	640.8276	1279.6406	2	-0.0002	11	1	GluC
H4_MOUSE	Histone H4	NVIRDAVTYTE	175	2.10E-03	640.8276	1279.6406	2	-0.0002	11	1	GluC
H4_MOUSE	Histone H4	NVIRDAVTYTE	175	1.20E-03	640.8276	1279.6407	2	-0.0002	11	1	GluC
H4_MOUSE	Histone H4	NVIRDAVTYTE	175	5.10E-04	640.8276	1279.6406	2	-0.0002	11	1	GluC
H4_MOUSE	Histone H4	NVIRDAVTYTE	175	6.10E-04	640.8275	1279.6405	2	-0.0003	11	1	GluC
H4_MOUSE	Histone H4	NVIRDAVTYTE	175	3.80E-04	640.8275	1279.6405	2	-0.0003	11	1	GluC
H4_MOUSE	Histone H4	NVIRDAVTYTE	175	1.60E-04	640.8275	1279.6405	2	-0.0003	11	1	GluC
H4_MOUSE	Histone H4	NVIRDAVTYTE	175	6.40E-05	640.8275	1279.6405	2	-0.0003	11	1	GluC
H4_MOUSE	Histone H4	NVIRDAVTYTE	175	5.00E-05	640.8275	1279.6405	2	-0.0003	11	1	GluC
H4_MOUSE	Histone H4	NVIRDAVTYTE	175	7.40E-03	640.8275	1279.6404	2	-0.0004	11	1	GluC
H4_MOUSE	Histone H4	NVIRDAVTYTE	175	1.80E-06	640.8275	1279.6404	2	-0.0004	11	1	GluC
H4_MOUSE	Histone H4	NVIRDAVTYTE	175	1.60E-04	640.8274	1279.6403	2	-0.0005	11	1	GluC
H4_MOUSE	Histone H4	NVIRDAVTYTE	175	7.90E-05	640.8274	1279.6402	2	-0.0006	11	1	GluC
H4_MOUSE	Histone H4	NVIRDAVTYTE	175	7.70E-06	640.8274	1279.6402	2	-0.0006	11	1	GluC
H4_MOUSE	Histone H4	NVIRDAVTYTE	175	2.70E-03	640.8274	1279.6401	2	-0.0007	11	1	GluC
H4_MOUSE	Histone H4	NVIRDAVTYTE	175	1.90E-03	640.8274	1279.6402	2	-0.0007	11	1	GluC
H4_MOUSE	Histone H4	NVIRDAVTYTE	175	1.60E-04	640.8273	1279.6401	2	-0.0007	11	1	GluC
H4_MOUSE	Histone H4	NVIRDAVTYTE	175	1.80E-03	640.8273	1279.6400	2	-0.0008	11	1	GluC
H4_MOUSE	Histone H4	NVIRDAVTYTE	175	1.40E-03	640.8273	1279.6400	2	-0.0008	11	1	GluC
H4_MOUSE	Histone H4	NVIRDAVTYTE	175	2.10E-04	640.8273	1279.6400	2	-0.0009	11	1	GluC
H4_MOUSE	Histone H4	NVIRDAVTYTE	175	1.20E-05	640.8273	1279.6399	2	-0.0009	11	1	GluC
H4_MOUSE	Histone H4	NVIRDAVTYTE	175	5.80E-03	640.8272	1279.6398	2	-0.0010	11	1	GluC
H4_MOUSE	Histone H4	NVIRDAVTYTE	175	7.40E-06	640.8272	1279.6398	2	-0.0010	11	1	GluC
H4_MOUSE	Histone H4	NVIRDAVTYTE	175	6.20E-03	640.827	1279.6395	2	-0.0013	11	1	GluC
H4_MOUSE	Histone H4	NVIRDAVTYTE	175	3.30E-06	640.827	1279.6395	2	-0.0013	11	1	GluC
H4_MOUSE	Histone H4	NVIRDAVTYTE	175	2.20E-04	640.8268	1279.6390	2	-0.0018	11	1	GluC

H4_MOUSE	Histone H4	NVIRDAVTYTE	175	7.40E-03	640.8267	1279.6389	2	-0.0019	11	1	GluC
H4_MOUSE	Histone H4	QGRTLYGFGG	3	4.30E-03	528.2683	1054.5220	2	0.0024	10	1	Semi-tryptic
H4_MOUSE	Histone H4	QGRTLYGFGG	3	5.50E-04	528.2683	1054.5220	2	0.0024	10	1	Semi-tryptic
H4_MOUSE	Histone H4	ALKRQGRTLY	3	3.60E-04	402.5761	1204.7063	3	0.0023	10	2	Chymotrypsin
H4_MOUSE	Histone H4	QGRTLYGFGG	3	2.30E-03	528.2675	1054.5204	2	0.0008	10	1	Semi-tryptic
H4_MOUSE	Histone H4	ALKRQGRTLY	3	2.40E-03	402.5754	1204.7042	3	0.0002	10	2	Chymotrypsin
H4_MOUSE	Histone H4	ALKRQGRTLY	3	5.50E-05	402.575	1204.7033	3	-0.0007	10	2	Chymotrypsin
H4_MOUSE	Histone H4	ENVIRDAVTY	4	2.30E-03	590.3049	1178.5952	2	0.0020	10	0	Chymotrypsin
H4_MOUSE	Histone H4	ENVIRDAVTY	4	2.00E-04	590.3045	1178.5945	2	0.0014	10	0	Chymotrypsin
H4_MOUSE	Histone H4	ENVIRDAVTY	4	9.30E-04	590.3045	1178.5944	2	0.0012	10	0	Chymotrypsin
H4_MOUSE	Histone H4	ENVIRDAVTY	4	2.80E-04	590.3045	1178.5944	2	0.0012	10	0	Chymotrypsin
H4_MOUSE	Histone H4	TRGVLKVFLE	633	3.20E-03	387.9038	1160.6896	3	-0.0022	10	0	GluC
H4_MOUSE	Histone H4	TRGVLKVFLE	633	1.50E-04	387.9038	1160.6896	3	-0.0022	10	0	GluC
H4_MOUSE	Histone H4	TRGVLKVFLE	633	8.40E-04	387.9037	1160.6892	3	-0.0026	10	0	GluC
H4_MOUSE	Histone H4	TRGVLKVFLE	633	4.60E-03	387.9036	1160.6891	3	-0.0027	10	0	GluC
H4_MOUSE	Histone H4	TRGVLKVFLE	633	7.00E-04	581.3518	1160.6890	2	-0.0027	10	0	GluC
H4_MOUSE	Histone H4	TRGVLKVFLE	633	2.10E-03	387.9036	1160.6890	3	-0.0028	10	0	GluC
H4_MOUSE	Histone H4	TRGVLKVFLE	633	4.20E-03	581.3513	1160.6881	2	-0.0036	10	0	GluC
H4_MOUSE	Histone H4	TRGVLKVFLE	633	2.00E-04	581.3513	1160.6881	2	-0.0036	10	0	GluC
H4_MOUSE	Histone H4	TRGVLKVFLE	633	8.00E-05	387.9033	1160.6882	3	-0.0036	10	0	GluC
H4_MOUSE	Histone H4	TRGVLKVFLE	633	8.90E-03	387.9033	1160.6880	3	-0.0038	10	0	GluC
H4_MOUSE	Histone H4	TRGVLKVFLE	633	1.90E-03	387.9032	1160.6877	3	-0.0040	10	0	GluC
H4_MOUSE	Histone H4	TRGVLKVFLE	633	3.00E-04	387.9032	1160.6878	3	-0.0040	10	0	GluC
H4_MOUSE	Histone H4	TRGVLKVFLE	633	9.20E-06	387.9032	1160.6877	3	-0.0040	10	0	GluC
H4_MOUSE	Histone H4	TRGVLKVFLE	633	2.50E-06	387.9032	1160.6877	3	-0.0040	10	0	GluC
H4_MOUSE	Histone H4	TRGVLKVFLE	633	4.00E-04	387.9031	1160.6875	3	-0.0042	10	0	GluC
H4_MOUSE	Histone H4	TRGVLKVFLE	633	1.40E-04	387.9031	1160.6875	3	-0.0042	10	0	GluC
H4_MOUSE	Histone H4	TRGVLKVFLE	633	1.60E-03	387.9028	1160.6866	3	-0.0051	10	0	GluC
H4_MOUSE	Histone H4	DAVTYTEHAK	34	9.10E-03	567.7757	1133.5368	2	0.0015	10	0	Semi-tryptic
H4_MOUSE	Histone H4	DAVTYTEHAK	34	1.80E-03	378.8529	1133.5368	3	0.0015	10	0	Semi-tryptic
H4_MOUSE	Histone H4	DAVTYTEHAK	34	1.40E-03	567.7757	1133.5368	2	0.0015	10	0	Semi-tryptic
H4_MOUSE	Histone H4	DAVTYTEHAK	34	6.50E-06	378.8529	1133.5368	3	0.0015	10	0	Semi-tryptic
H4_MOUSE	Histone H4	DAVTYTEHAK	34	1.40E-05	567.7756	1133.5366	2	0.0014	10	0	Semi-tryptic
H4_MOUSE	Histone H4	DAVTYTEHAK	34	1.80E-03	567.7755	1133.5365	2	0.0013	10	0	Semi-tryptic
H4_MOUSE	Histone H4	DAVTYTEHAK	34	5.80E-04	378.8528	1133.5365	3	0.0012	10	0	Semi-tryptic
H4_MOUSE	Histone H4	DAVTYTEHAK	34	2.00E-04	378.8527	1133.5363	3	0.0010	10	0	Semi-tryptic
H4_MOUSE	Histone H4	DAVTYTEHAK	34	5.80E-05	378.8527	1133.5363	3	0.0010	10	0	Semi-tryptic
H4_MOUSE	Histone H4	DAVTYTEHAK	34	5.10E-03	378.8527	1133.5362	3	0.0009	10	0	Semi-tryptic
H4_MOUSE	Histone H4	DAVTYTEHAK	34	1.30E-04	378.8527	1133.5362	3	0.0009	10	0	Semi-tryptic
H4_MOUSE	Histone H4	DAVTYTEHAK	34	8.20E-06	567.7754	1133.5362	2	0.0009	10	0	Semi-tryptic
H4_MOUSE	Histone H4	DAVTYTEHAK	34	5.80E-07	567.7753	1133.5360	2	0.0007	10	0	Semi-tryptic
H4_MOUSE	Histone H4	DAVTYTEHAK	34	4.00E-03	567.7752	1133.5359	2	0.0006	10	0	Semi-tryptic
H4_MOUSE	Histone H4	DAVTYTEHAK	34	7.90E-06	567.7752	1133.5358	2	0.0006	10	0	Semi-tryptic
H4_MOUSE	Histone H4	DAVTYTEHAK	34	6.10E-04	567.7752	1133.5358	2	0.0005	10	0	Semi-tryptic
H4_MOUSE	Histone H4	DAVTYTEHAK	34	8.50E-05	567.7752	1133.5358	2	0.0005	10	0	Semi-tryptic
H4_MOUSE	Histone H4	DAVTYTEHAK	34	7.60E-03	378.8525	1133.5357	3	0.0004	10	0	Semi-tryptic
H4_MOUSE	Histone H4	DAVTYTEHAK	34	8.90E-04	378.8525	1133.5357	3	0.0004	10	0	Semi-tryptic
H4_MOUSE	Histone H4	DAVTYTEHAK	34	1.50E-06	567.7751	1133.5356	2	0.0003	10	0	Semi-tryptic
H4_MOUSE	Histone H4	DAVTYTEHAK	34	2.30E-04	567.7749	1133.5353	2	0.0001	10	0	Semi-tryptic
H4_MOUSE	Histone H4	DAVTYTEHAK	34	7.90E-06	567.7749	1133.5353	2	0.0001	10	0	Semi-tryptic
H4_MOUSE	Histone H4	DAVTYTEHAK	34	2.20E-06	567.7749	1133.5353	2	0.0001	10	0	Semi-tryptic
H4_MOUSE	Histone H4	DAVTYTEHAK	34	9.50E-07	567.775	1133.5354	2	0.0001	10	0	Semi-tryptic
H4_MOUSE	Histone H4	DAVTYTEHAK	34	2.30E-05	567.7749	1133.5353	2	0.0000	10	0	Semi-tryptic
H4_MOUSE	Histone H4	DAVTYTEHAK	34	1.60E-06	567.7749	1133.5351	2	-0.0001	10	0	Semi-tryptic
H4_MOUSE	Histone H4	DAVTYTEHAK	34	1.20E-05	567.7748	1133.5351	2	-0.0002	10	0	Semi-tryptic
H4_MOUSE	Histone H4	DAVTYTEHAK	34	1.50E-04	567.7748	1133.5350	2	-0.0003	10	0	Semi-tryptic
H4_MOUSE	Histone H4	DAVTYTEHAK	34	2.10E-06	567.7748	1133.5350	2	-0.0003	10	0	Semi-tryptic
H4_MOUSE	Histone H4	DAVTYTEHAK	34	2.80E-03	567.7747	1133.5349	2	-0.0004	10	0	Semi-tryptic
H4_MOUSE	Histone H4	DAVTYTEHAK	34	2.80E-04	567.7747	1133.5349	2	-0.0004	10	0	Semi-tryptic
H4_MOUSE	Histone H4	DAVTYTEHAK	34	5.20E-03	567.7746	1133.5346	2	-0.0007	10	0	Semi-tryptic
H4_MOUSE	Histone H4	DAVTYTEHAK	34	7.00E-04	567.7746	1133.5346	2	-0.0007	10	0	Semi-tryptic
H4_MOUSE	Histone H4	DAVTYTEHAK	34	2.30E-06	567.7744	1133.5343	2	-0.0010	10	0	Semi-tryptic
H4_MOUSE	Histone H4	ISGLIYEETR	35	1.60E-07	590.8166	1179.6186	2	0.0051	10	0	Semi-tryptic
H4_MOUSE	Histone H4	ISGLIYEETR	35	9.60E-06	590.8163	1179.6180	2	0.0045	10	0	Semi-tryptic
H4_MOUSE	Histone H4	ISGLIYEETR	35	2.10E-03	590.8157	1179.6168	2	0.0032	10	0	Semi-tryptic
H4_MOUSE	Histone H4	ISGLIYEETR	35	2.30E-04	590.8157	1179.6168	2	0.0032	10	0	Semi-tryptic
H4_MOUSE	Histone H4	ISGLIYEETR	35	4.10E-04	590.8155	1179.6164	2	0.0029	10	0	Semi-tryptic
H4_MOUSE	Histone H4	ISGLIYEETR	35	2.40E-03	590.8152	1179.6159	2	0.0024	10	0	Semi-tryptic
H4_MOUSE	Histone H4	ISGLIYEETR	35	2.50E-04	590.8152	1179.6159	2	0.0024	10	0	Semi-tryptic
H4_MOUSE	Histone H4	ISGLIYEETR	35	9.00E-06	590.8147	1179.6149	2	0.0014	10	0	GluC
H4_MOUSE	Histone H4	ISGLIYEETR	35	3.60E-03	590.8146	1179.6146	2	0.0011	10	0	GluC
H4_MOUSE	Histone H4	ISGLIYEETR	35	7.50E-07	590.8146	1179.6145	2	0.0010	10	0	Semi-tryptic
H4_MOUSE	Histone H4	ISGLIYEETR	35	1.30E-07	590.8144	1179.6143	2	0.0008	10	0	Semi-tryptic
H4_MOUSE	Histone H4	ISGLIYEETR	35	1.20E-07	590.8144	1179.6143	2	0.0008	10	0	Semi-tryptic

H4_MOUSE	Histone H4	ISGLIYEETR	35	8.60E-05	590.8144	1179.6143	2	0.0007	10	0	GluC
H4_MOUSE	Histone H4	ISGLIYEETR	35	2.80E-06	590.8144	1179.6142	2	0.0007	10	0	Semi-tryptic
H4_MOUSE	Histone H4	ISGLIYEETR	35	2.80E-03	590.8143	1179.6140	2	0.0005	10	0	Semi-tryptic
H4_MOUSE	Histone H4	ISGLIYEETR	35	3.20E-07	590.8143	1179.6140	2	0.0005	10	0	Semi-tryptic
H4_MOUSE	Histone H4	ISGLIYEETR	35	6.80E-04	590.8143	1179.6139	2	0.0004	10	0	GluC
H4_MOUSE	Histone H4	ISGLIYEETR	35	8.70E-08	590.8142	1179.6138	2	0.0003	10	0	Semi-tryptic
H4_MOUSE	Histone H4	ISGLIYEETR	35	3.80E-03	590.8141	1179.6137	2	0.0002	10	0	Semi-tryptic
H4_MOUSE	Histone H4	ISGLIYEETR	35	1.80E-04	590.8141	1179.6137	2	0.0002	10	0	Semi-tryptic
H4_MOUSE	Histone H4	ISGLIYEETR	35	3.00E-07	590.8141	1179.6137	2	0.0002	10	0	Semi-tryptic
H4_MOUSE	Histone H4	ISGLIYEETR	35	1.60E-05	590.8141	1179.6136	2	0.0000	10	0	GluC
H4_MOUSE	Histone H4	ISGLIYEETR	35	1.30E-07	590.814	1179.6135	2	0.0000	10	0	Semi-tryptic
H4_MOUSE	Histone H4	ISGLIYEETR	35	5.80E-07	590.814	1179.6134	2	-0.0001	10	0	Semi-tryptic
H4_MOUSE	Histone H4	ISGLIYEETR	35	8.70E-08	590.814	1179.6134	2	-0.0001	10	0	Semi-tryptic
H4_MOUSE	Histone H4	ISGLIYEETR	35	2.00E-07	590.8139	1179.6133	2	-0.0002	10	0	Semi-tryptic
H4_MOUSE	Histone H4	ISGLIYEETR	35	3.20E-03	590.8139	1179.6133	2	-0.0003	10	0	GluC
H4_MOUSE	Histone H4	ISGLIYEETR	35	1.30E-07	590.8139	1179.6132	2	-0.0003	10	0	Semi-tryptic
H4_MOUSE	Histone H4	ISGLIYEETR	35	1.40E-06	590.8138	1179.6130	2	-0.0005	10	0	Semi-tryptic
H4_MOUSE	Histone H4	ISGLIYEETR	35	1.40E-07	590.8138	1179.6130	2	-0.0005	10	0	Semi-tryptic
H4_MOUSE	Histone H4	ISGLIYEETR	35	1.40E-07	590.8137	1179.6128	2	-0.0007	10	0	Semi-tryptic
H4_MOUSE	Histone H4	ISGLIYEETR	35	6.50E-07	590.8137	1179.6128	2	-0.0008	10	0	Semi-tryptic
H4_MOUSE	Histone H4	ISGLIYEETR	35	5.20E-07	590.8135	1179.6125	2	-0.0010	10	0	Semi-tryptic
H4_MOUSE	Histone H4	ISGLIYEETR	35	2.80E-07	590.8135	1179.6124	2	-0.0011	10	0	Semi-tryptic
H4_MOUSE	Histone H4	ISGLIYEETR	35	1.40E-07	590.8133	1179.6121	2	-0.0015	10	0	Semi-tryptic
H4_MOUSE	Histone H4	TRGVLKVFLE	633	3.50E-06	581.3576	1160.7007	2	0.0090	10	0	GluC
H4_MOUSE	Histone H4	TRGVLKVFLE	633	4.00E-04	581.3576	1160.7007	2	0.0089	10	0	GluC
H4_MOUSE	Histone H4	TRGVLKVFLE	633	1.20E-04	581.3574	1160.7003	2	0.0085	10	0	GluC
H4_MOUSE	Histone H4	TRGVLKVFLE	633	1.30E-06	581.3573	1160.7001	2	0.0084	10	0	GluC
H4_MOUSE	Histone H4	TRGVLKVFLE	633	9.70E-07	387.9073	1160.7001	3	0.0084	10	0	GluC
H4_MOUSE	Histone H4	TRGVLKVFLE	633	2.90E-07	387.9073	1160.7001	3	0.0084	10	0	GluC
H4_MOUSE	Histone H4	TRGVLKVFLE	633	3.40E-06	581.3572	1160.6999	2	0.0082	10	0	GluC
H4_MOUSE	Histone H4	TRGVLKVFLE	633	4.30E-07	387.9072	1160.6999	3	0.0082	10	0	GluC
H4_MOUSE	Histone H4	TRGVLKVFLE	633	3.40E-04	581.3571	1160.6997	2	0.0080	10	0	GluC
H4_MOUSE	Histone H4	TRGVLKVFLE	633	6.80E-06	581.3572	1160.6998	2	0.0080	10	0	GluC
H4_MOUSE	Histone H4	TRGVLKVFLE	633	1.60E-06	581.3571	1160.6997	2	0.0079	10	0	GluC
H4_MOUSE	Histone H4	TRGVLKVFLE	633	2.60E-05	581.357	1160.6994	2	0.0077	10	0	GluC
H4_MOUSE	Histone H4	TRGVLKVFLE	633	2.90E-07	387.9071	1160.6994	3	0.0077	10	0	GluC
H4_MOUSE	Histone H4	TRGVLKVFLE	633	3.40E-03	387.9071	1160.6994	3	0.0076	10	0	GluC
H4_MOUSE	Histone H4	TRGVLKVFLE	633	6.30E-06	581.3569	1160.6993	2	0.0076	10	0	GluC
H4_MOUSE	Histone H4	TRGVLKVFLE	633	4.80E-07	387.907	1160.6993	3	0.0076	10	0	GluC
H4_MOUSE	Histone H4	TRGVLKVFLE	633	6.10E-04	581.3569	1160.6992	2	0.0075	10	0	GluC
H4_MOUSE	Histone H4	TRGVLKVFLE	633	3.50E-06	581.3569	1160.6992	2	0.0075	10	0	GluC
H4_MOUSE	Histone H4	TRGVLKVFLE	633	2.90E-07	387.907	1160.6993	3	0.0075	10	0	GluC
H4_MOUSE	Histone H4	TRGVLKVFLE	633	6.20E-06	581.3569	1160.6992	2	0.0074	10	0	GluC
H4_MOUSE	Histone H4	TRGVLKVFLE	633	2.10E-03	581.3568	1160.6991	2	0.0073	10	0	GluC
H4_MOUSE	Histone H4	TRGVLKVFLE	633	1.00E-03	581.3568	1160.6990	2	0.0073	10	0	GluC
H4_MOUSE	Histone H4	TRGVLKVFLE	633	1.70E-06	581.3568	1160.6990	2	0.0073	10	0	GluC
H4_MOUSE	Histone H4	TRGVLKVFLE	633	5.00E-07	387.9069	1160.6990	3	0.0073	10	0	GluC
H4_MOUSE	Histone H4	TRGVLKVFLE	633	5.90E-05	581.3567	1160.6989	2	0.0071	10	0	GluC
H4_MOUSE	Histone H4	TRGVLKVFLE	633	3.40E-06	581.3567	1160.6988	2	0.0071	10	0	GluC
H4_MOUSE	Histone H4	TRGVLKVFLE	633	8.80E-05	581.3566	1160.6987	2	0.0070	10	0	GluC
H4_MOUSE	Histone H4	TRGVLKVFLE	633	4.70E-05	581.3567	1160.6988	2	0.0070	10	0	GluC
H4_MOUSE	Histone H4	TRGVLKVFLE	633	3.10E-06	387.9069	1160.6988	3	0.0070	10	0	GluC
H4_MOUSE	Histone H4	TRGVLKVFLE	633	3.40E-06	581.3566	1160.6987	2	0.0069	10	0	GluC
H4_MOUSE	Histone H4	TRGVLKVFLE	633	9.70E-07	387.9068	1160.6986	3	0.0069	10	0	GluC
H4_MOUSE	Histone H4	TRGVLKVFLE	633	1.50E-03	581.3565	1160.6985	2	0.0068	10	0	GluC
H4_MOUSE	Histone H4	TRGVLKVFLE	633	2.50E-07	387.9068	1160.6985	3	0.0067	10	0	GluC
H4_MOUSE	Histone H4	TRGVLKVFLE	633	1.40E-03	581.3564	1160.6983	2	0.0066	10	0	GluC
H4_MOUSE	Histone H4	TRGVLKVFLE	633	2.30E-04	581.3564	1160.6983	2	0.0066	10	0	GluC
H4_MOUSE	Histone H4	TRGVLKVFLE	633	9.20E-05	581.3564	1160.6983	2	0.0066	10	0	GluC
H4_MOUSE	Histone H4	TRGVLKVFLE	633	6.00E-06	581.3565	1160.6984	2	0.0066	10	0	GluC
H4_MOUSE	Histone H4	TRGVLKVFLE	633	1.40E-06	581.3565	1160.6984	2	0.0066	10	0	GluC
H4_MOUSE	Histone H4	TRGVLKVFLE	633	4.20E-03	581.3564	1160.6982	2	0.0065	10	0	GluC
H4_MOUSE	Histone H4	TRGVLKVFLE	633	2.10E-03	581.3564	1160.6983	2	0.0065	10	0	GluC
H4_MOUSE	Histone H4	TRGVLKVFLE	633	3.70E-06	581.3563	1160.6980	2	0.0063	10	0	GluC
H4_MOUSE	Histone H4	TRGVLKVFLE	633	1.00E-04	581.3563	1160.6980	2	0.0062	10	0	GluC
H4_MOUSE	Histone H4	TRGVLKVFLE	633	5.00E-07	387.9065	1160.6978	3	0.0060	10	0	GluC
H4_MOUSE	Histone H4	TRGVLKVFLE	633	7.60E-04	581.3561	1160.6976	2	0.0059	10	0	GluC
H4_MOUSE	Histone H4	TRGVLKVFLE	633	3.70E-06	581.3559	1160.6973	2	0.0056	10	0	GluC
H4_MOUSE	Histone H4	TRGVLKVFLE	633	2.40E-06	387.9064	1160.6973	3	0.0056	10	0	GluC
H4_MOUSE	Histone H4	TRGVLKVFLE	633	6.40E-06	581.3559	1160.6972	2	0.0055	10	0	GluC
H4_MOUSE	Histone H4	TRGVLKVFLE	633	7.80E-07	387.9063	1160.6972	3	0.0055	10	0	GluC
H4_MOUSE	Histone H4	TRGVLKVFLE	633	7.30E-07	387.9063	1160.6972	3	0.0055	10	0	GluC
H4_MOUSE	Histone H4	TRGVLKVFLE	633	2.80E-06	387.9063	1160.6971	3	0.0054	10	0	GluC
H4_MOUSE	Histone H4	TRGVLKVFLE	633	3.00E-07	387.9063	1160.6971	3	0.0054	10	0	GluC

H4_MOUSE	Histone H4	TRGVLKVFLE	633	5.90E-03	581.3557	1160.6969	2	0.0052	10	0	GluC
H4_MOUSE	Histone H4	TRGVLKVFLE	633	6.80E-05	581.3558	1160.6970	2	0.0052	10	0	GluC
H4_MOUSE	Histone H4	TRGVLKVFLE	633	3.00E-03	581.3556	1160.6967	2	0.0050	10	0	GluC
H4_MOUSE	Histone H4	TRGVLKVFLE	633	9.00E-07	387.9062	1160.6968	3	0.0050	10	0	GluC
H4_MOUSE	Histone H4	TRGVLKVFLE	633	7.10E-04	581.3556	1160.6966	2	0.0049	10	0	GluC
H4_MOUSE	Histone H4	TRGVLKVFLE	633	1.60E-06	387.9062	1160.6967	3	0.0049	10	0	GluC
H4_MOUSE	Histone H4	TRGVLKVFLE	633	8.50E-03	581.3556	1160.6966	2	0.0048	10	0	GluC
H4_MOUSE	Histone H4	TRGVLKVFLE	633	2.60E-04	581.3556	1160.6966	2	0.0048	10	0	GluC
H4_MOUSE	Histone H4	TRGVLKVFLE	633	1.00E-04	581.3556	1160.6966	2	0.0048	10	0	GluC
H4_MOUSE	Histone H4	TRGVLKVFLE	633	5.60E-03	387.9061	1160.6965	3	0.0047	10	0	GluC
H4_MOUSE	Histone H4	TRGVLKVFLE	633	9.00E-06	581.3555	1160.6964	2	0.0047	10	0	GluC
H4_MOUSE	Histone H4	TRGVLKVFLE	633	6.40E-03	581.3554	1160.6962	2	0.0045	10	0	GluC
H4_MOUSE	Histone H4	TRGVLKVFLE	633	2.20E-05	387.906	1160.6963	3	0.0045	10	0	GluC
H4_MOUSE	Histone H4	TRGVLKVFLE	633	2.40E-06	387.906	1160.6962	3	0.0045	10	0	GluC
H4_MOUSE	Histone H4	TRGVLKVFLE	633	6.80E-03	581.3553	1160.6960	2	0.0043	10	0	GluC
H4_MOUSE	Histone H4	TRGVLKVFLE	633	6.80E-03	581.3553	1160.6960	2	0.0042	10	0	GluC
H4_MOUSE	Histone H4	TRGVLKVFLE	633	6.80E-03	581.3552	1160.6959	2	0.0042	10	0	GluC
H4_MOUSE	Histone H4	TRGVLKVFLE	633	6.60E-03	581.3552	1160.6959	2	0.0042	10	0	GluC
H4_MOUSE	Histone H4	TRGVLKVFLE	633	1.40E-03	581.3552	1160.6958	2	0.0041	10	0	GluC
H4_MOUSE	Histone H4	TRGVLKVFLE	633	6.80E-05	387.9059	1160.6958	3	0.0041	10	0	GluC
H4_MOUSE	Histone H4	TRGVLKVFLE	633	6.60E-03	581.3551	1160.6956	2	0.0039	10	0	GluC
H4_MOUSE	Histone H4	TRGVLKVFLE	633	3.90E-04	581.3551	1160.6956	2	0.0039	10	0	GluC
H4_MOUSE	Histone H4	TRGVLKVFLE	633	4.80E-07	387.9058	1160.6957	3	0.0039	10	0	GluC
H4_MOUSE	Histone H4	TRGVLKVFLE	633	1.70E-03	387.9058	1160.6955	3	0.0038	10	0	GluC
H4_MOUSE	Histone H4	TRGVLKVFLE	633	2.30E-05	387.9058	1160.6955	3	0.0038	10	0	GluC
H4_MOUSE	Histone H4	TRGVLKVFLE	633	2.40E-03	581.355	1160.6954	2	0.0037	10	0	GluC
H4_MOUSE	Histone H4	TRGVLKVFLE	633	9.40E-04	387.9057	1160.6954	3	0.0037	10	0	GluC
H4_MOUSE	Histone H4	TRGVLKVFLE	633	4.50E-04	581.355	1160.6954	2	0.0037	10	0	GluC
H4_MOUSE	Histone H4	TRGVLKVFLE	633	3.40E-03	387.9057	1160.6954	3	0.0036	10	0	GluC
H4_MOUSE	Histone H4	TRGVLKVFLE	633	1.80E-06	387.9057	1160.6954	3	0.0036	10	0	GluC
H4_MOUSE	Histone H4	TRGVLKVFLE	633	1.60E-07	387.9057	1160.6953	3	0.0036	10	0	GluC
H4_MOUSE	Histone H4	TRGVLKVFLE	633	6.60E-03	581.3549	1160.6953	2	0.0035	10	0	GluC
H4_MOUSE	Histone H4	TRGVLKVFLE	633	5.20E-04	581.3549	1160.6952	2	0.0035	10	0	GluC
H4_MOUSE	Histone H4	TRGVLKVFLE	633	2.90E-04	581.3549	1160.6953	2	0.0035	10	0	GluC
H4_MOUSE	Histone H4	TRGVLKVFLE	633	6.80E-03	387.9057	1160.6952	3	0.0034	10	0	GluC
H4_MOUSE	Histone H4	TRGVLKVFLE	633	6.60E-03	387.9057	1160.6952	3	0.0034	10	0	GluC
H4_MOUSE	Histone H4	TRGVLKVFLE	633	9.10E-04	581.3548	1160.6951	2	0.0034	10	0	GluC
H4_MOUSE	Histone H4	TRGVLKVFLE	633	3.20E-04	581.3548	1160.6951	2	0.0034	10	0	GluC
H4_MOUSE	Histone H4	TRGVLKVFLE	633	1.80E-04	387.9056	1160.6951	3	0.0033	10	0	GluC
H4_MOUSE	Histone H4	TRGVLKVFLE	633	6.80E-06	581.3548	1160.6951	2	0.0033	10	0	GluC
H4_MOUSE	Histone H4	TRGVLKVFLE	633	1.30E-03	581.3547	1160.6949	2	0.0032	10	0	GluC
H4_MOUSE	Histone H4	TRGVLKVFLE	633	2.00E-04	581.3548	1160.6950	2	0.0032	10	0	GluC
H4_MOUSE	Histone H4	TRGVLKVFLE	633	4.50E-06	387.9056	1160.6950	3	0.0032	10	0	GluC
H4_MOUSE	Histone H4	TRGVLKVFLE	633	4.00E-06	387.9056	1160.6949	3	0.0032	10	0	GluC
H4_MOUSE	Histone H4	TRGVLKVFLE	633	9.30E-03	581.3547	1160.6949	2	0.0031	10	0	GluC
H4_MOUSE	Histone H4	TRGVLKVFLE	633	8.00E-03	581.3547	1160.6948	2	0.0031	10	0	GluC
H4_MOUSE	Histone H4	TRGVLKVFLE	633	6.00E-03	581.3547	1160.6948	2	0.0031	10	0	GluC
H4_MOUSE	Histone H4	TRGVLKVFLE	633	3.10E-04	581.3547	1160.6948	2	0.0031	10	0	GluC
H4_MOUSE	Histone H4	TRGVLKVFLE	633	2.90E-04	581.3547	1160.6949	2	0.0031	10	0	GluC
H4_MOUSE	Histone H4	TRGVLKVFLE	633	1.80E-04	581.3547	1160.6948	2	0.0031	10	0	GluC
H4_MOUSE	Histone H4	TRGVLKVFLE	633	1.30E-04	387.9056	1160.6949	3	0.0031	10	0	GluC
H4_MOUSE	Histone H4	TRGVLKVFLE	633	3.80E-03	581.3546	1160.6947	2	0.0030	10	0	GluC
H4_MOUSE	Histone H4	TRGVLKVFLE	633	2.60E-03	581.3546	1160.6947	2	0.0030	10	0	GluC
H4_MOUSE	Histone H4	TRGVLKVFLE	633	1.30E-03	581.3547	1160.6948	2	0.0030	10	0	GluC
H4_MOUSE	Histone H4	TRGVLKVFLE	633	5.30E-04	581.3547	1160.6948	2	0.0030	10	0	GluC
H4_MOUSE	Histone H4	TRGVLKVFLE	633	6.00E-05	387.9055	1160.6947	3	0.0030	10	0	GluC
H4_MOUSE	Histone H4	TRGVLKVFLE	633	3.80E-05	387.9055	1160.6948	3	0.0030	10	0	GluC
H4_MOUSE	Histone H4	TRGVLKVFLE	633	6.80E-06	581.3546	1160.6947	2	0.0030	10	0	GluC
H4_MOUSE	Histone H4	TRGVLKVFLE	633	8.40E-03	581.3546	1160.6946	2	0.0029	10	0	GluC
H4_MOUSE	Histone H4	TRGVLKVFLE	633	5.50E-04	387.9055	1160.6946	3	0.0029	10	0	GluC
H4_MOUSE	Histone H4	TRGVLKVFLE	633	1.30E-04	387.9055	1160.6946	3	0.0029	10	0	GluC
H4_MOUSE	Histone H4	TRGVLKVFLE	633	1.40E-06	387.9055	1160.6946	3	0.0029	10	0	GluC
H4_MOUSE	Histone H4	TRGVLKVFLE	633	1.90E-07	387.9055	1160.6946	3	0.0029	10	0	GluC
H4_MOUSE	Histone H4	TRGVLKVFLE	633	8.60E-03	581.3546	1160.6946	2	0.0028	10	0	GluC
H4_MOUSE	Histone H4	TRGVLKVFLE	633	6.40E-03	581.3545	1160.6945	2	0.0028	10	0	GluC
H4_MOUSE	Histone H4	TRGVLKVFLE	633	6.40E-03	581.3546	1160.6946	2	0.0028	10	0	GluC
H4_MOUSE	Histone H4	TRGVLKVFLE	633	4.90E-03	581.3545	1160.6945	2	0.0028	10	0	GluC
H4_MOUSE	Histone H4	TRGVLKVFLE	633	3.50E-03	581.3545	1160.6945	2	0.0028	10	0	GluC
H4_MOUSE	Histone H4	TRGVLKVFLE	633	1.00E-03	581.3546	1160.6945	2	0.0028	10	0	GluC
H4_MOUSE	Histone H4	TRGVLKVFLE	633	6.30E-04	581.3546	1160.6946	2	0.0028	10	0	GluC
H4_MOUSE	Histone H4	TRGVLKVFLE	633	3.40E-04	581.3545	1160.6945	2	0.0028	10	0	GluC
H4_MOUSE	Histone H4	TRGVLKVFLE	633	9.10E-05	581.3546	1160.6945	2	0.0028	10	0	GluC
H4_MOUSE	Histone H4	TRGVLKVFLE	633	4.10E-03	581.3545	1160.6944	2	0.0027	10	0	GluC
H4_MOUSE	Histone H4	TRGVLKVFLE	633	3.00E-03	581.3545	1160.6944	2	0.0027	10	0	GluC

H4_MOUSE	Histone H4	TRGVLKVFLE	633	3.10E-04	581.3545	1160.6944	2	0.0027	10	0	GluC
H4_MOUSE	Histone H4	TRGVLKVFLE	633	4.20E-05	581.3545	1160.6944	2	0.0027	10	0	GluC
H4_MOUSE	Histone H4	TRGVLKVFLE	633	2.40E-05	387.9054	1160.6944	3	0.0027	10	0	GluC
H4_MOUSE	Histone H4	TRGVLKVFLE	633	4.50E-04	387.9054	1160.6943	3	0.0026	10	0	GluC
H4_MOUSE	Histone H4	TRGVLKVFLE	633	2.60E-05	387.9054	1160.6944	3	0.0026	10	0	GluC
H4_MOUSE	Histone H4	TRGVLKVFLE	633	1.00E-02	581.3544	1160.6942	2	0.0025	10	0	GluC
H4_MOUSE	Histone H4	TRGVLKVFLE	633	7.80E-03	581.3544	1160.6942	2	0.0025	10	0	GluC
H4_MOUSE	Histone H4	TRGVLKVFLE	633	5.10E-03	581.3544	1160.6942	2	0.0025	10	0	GluC
H4_MOUSE	Histone H4	TRGVLKVFLE	633	2.70E-03	581.3544	1160.6942	2	0.0025	10	0	GluC
H4_MOUSE	Histone H4	TRGVLKVFLE	633	1.50E-04	581.3544	1160.6942	2	0.0025	10	0	GluC
H4_MOUSE	Histone H4	TRGVLKVFLE	633	7.60E-03	581.3543	1160.6941	2	0.0024	10	0	GluC
H4_MOUSE	Histone H4	TRGVLKVFLE	633	6.30E-03	581.3543	1160.6941	2	0.0024	10	0	GluC
H4_MOUSE	Histone H4	TRGVLKVFLE	633	4.60E-03	581.3544	1160.6942	2	0.0024	10	0	GluC
H4_MOUSE	Histone H4	TRGVLKVFLE	633	4.10E-03	581.3543	1160.6941	2	0.0024	10	0	GluC
H4_MOUSE	Histone H4	TRGVLKVFLE	633	1.30E-03	581.3544	1160.6942	2	0.0024	10	0	GluC
H4_MOUSE	Histone H4	TRGVLKVFLE	633	6.30E-03	581.3543	1160.6940	2	0.0023	10	0	GluC
H4_MOUSE	Histone H4	TRGVLKVFLE	633	6.10E-03	581.3543	1160.6940	2	0.0023	10	0	GluC
H4_MOUSE	Histone H4	TRGVLKVFLE	633	1.70E-04	387.9053	1160.6940	3	0.0023	10	0	GluC
H4_MOUSE	Histone H4	TRGVLKVFLE	633	1.30E-05	387.9053	1160.6940	3	0.0023	10	0	GluC
H4_MOUSE	Histone H4	TRGVLKVFLE	633	3.40E-04	581.3542	1160.6939	2	0.0022	10	0	GluC
H4_MOUSE	Histone H4	TRGVLKVFLE	633	9.20E-03	581.3542	1160.6938	2	0.0021	10	0	GluC
H4_MOUSE	Histone H4	TRGVLKVFLE	633	1.30E-04	387.9052	1160.6939	3	0.0021	10	0	GluC
H4_MOUSE	Histone H4	TRGVLKVFLE	633	7.20E-06	387.9052	1160.6939	3	0.0021	10	0	GluC
H4_MOUSE	Histone H4	TRGVLKVFLE	633	9.10E-03	581.3542	1160.6938	2	0.0020	10	0	GluC
H4_MOUSE	Histone H4	TRGVLKVFLE	633	3.00E-03	581.3541	1160.6937	2	0.0020	10	0	GluC
H4_MOUSE	Histone H4	TRGVLKVFLE	633	1.50E-04	581.3541	1160.6937	2	0.0020	10	0	GluC
H4_MOUSE	Histone H4	TRGVLKVFLE	633	4.90E-03	581.3541	1160.6936	2	0.0019	10	0	GluC
H4_MOUSE	Histone H4	TRGVLKVFLE	633	4.30E-03	581.3541	1160.6936	2	0.0019	10	0	GluC
H4_MOUSE	Histone H4	TRGVLKVFLE	633	3.60E-03	581.3541	1160.6936	2	0.0019	10	0	GluC
H4_MOUSE	Histone H4	TRGVLKVFLE	633	3.10E-03	387.9051	1160.6936	3	0.0019	10	0	GluC
H4_MOUSE	Histone H4	TRGVLKVFLE	633	2.30E-03	581.3541	1160.6937	2	0.0019	10	0	GluC
H4_MOUSE	Histone H4	TRGVLKVFLE	633	2.00E-03	581.3541	1160.6936	2	0.0019	10	0	GluC
H4_MOUSE	Histone H4	TRGVLKVFLE	633	4.30E-05	581.3541	1160.6936	2	0.0019	10	0	GluC
H4_MOUSE	Histone H4	TRGVLKVFLE	633	3.40E-05	581.3541	1160.6936	2	0.0019	10	0	GluC
H4_MOUSE	Histone H4	TRGVLKVFLE	633	9.20E-03	387.9051	1160.6936	3	0.0018	10	0	GluC
H4_MOUSE	Histone H4	TRGVLKVFLE	633	8.80E-03	581.3541	1160.6936	2	0.0018	10	0	GluC
H4_MOUSE	Histone H4	TRGVLKVFLE	633	5.90E-03	581.354	1160.6935	2	0.0018	10	0	GluC
H4_MOUSE	Histone H4	TRGVLKVFLE	633	4.40E-03	581.3541	1160.6935	2	0.0018	10	0	GluC
H4_MOUSE	Histone H4	TRGVLKVFLE	633	1.90E-03	387.9051	1160.6936	3	0.0018	10	0	GluC
H4_MOUSE	Histone H4	TRGVLKVFLE	633	3.40E-04	581.3541	1160.6936	2	0.0018	10	0	GluC
H4_MOUSE	Histone H4	TRGVLKVFLE	633	3.10E-04	581.354	1160.6935	2	0.0018	10	0	GluC
H4_MOUSE	Histone H4	TRGVLKVFLE	633	3.10E-04	581.3541	1160.6935	2	0.0018	10	0	GluC
H4_MOUSE	Histone H4	TRGVLKVFLE	633	7.00E-03	387.9051	1160.6935	3	0.0017	10	0	GluC
H4_MOUSE	Histone H4	TRGVLKVFLE	633	3.50E-03	581.354	1160.6934	2	0.0017	10	0	GluC
H4_MOUSE	Histone H4	TRGVLKVFLE	633	2.70E-03	581.354	1160.6934	2	0.0017	10	0	GluC
H4_MOUSE	Histone H4	TRGVLKVFLE	633	2.00E-03	581.354	1160.6934	2	0.0017	10	0	GluC
H4_MOUSE	Histone H4	TRGVLKVFLE	633	1.80E-03	581.354	1160.6934	2	0.0017	10	0	GluC
H4_MOUSE	Histone H4	TRGVLKVFLE	633	1.20E-03	387.9051	1160.6934	3	0.0017	10	0	GluC
H4_MOUSE	Histone H4	TRGVLKVFLE	633	2.30E-04	581.354	1160.6934	2	0.0017	10	0	GluC
H4_MOUSE	Histone H4	TRGVLKVFLE	633	1.70E-04	581.354	1160.6934	2	0.0017	10	0	GluC
H4_MOUSE	Histone H4	TRGVLKVFLE	633	1.50E-04	581.354	1160.6934	2	0.0017	10	0	GluC
H4_MOUSE	Histone H4	TRGVLKVFLE	633	1.40E-04	387.9051	1160.6934	3	0.0017	10	0	GluC
H4_MOUSE	Histone H4	TRGVLKVFLE	633	1.50E-05	387.9051	1160.6934	3	0.0017	10	0	GluC
H4_MOUSE	Histone H4	TRGVLKVFLE	633	3.60E-06	387.9051	1160.6935	3	0.0017	10	0	GluC
H4_MOUSE	Histone H4	TRGVLKVFLE	633	1.00E-02	581.3539	1160.6933	2	0.0016	10	0	GluC
H4_MOUSE	Histone H4	TRGVLKVFLE	633	8.80E-03	581.3539	1160.6933	2	0.0016	10	0	GluC
H4_MOUSE	Histone H4	TRGVLKVFLE	633	6.30E-03	581.354	1160.6933	2	0.0016	10	0	GluC
H4_MOUSE	Histone H4	TRGVLKVFLE	633	5.40E-03	581.3539	1160.6933	2	0.0016	10	0	GluC
H4_MOUSE	Histone H4	TRGVLKVFLE	633	4.00E-03	581.3539	1160.6933	2	0.0016	10	0	GluC
H4_MOUSE	Histone H4	TRGVLKVFLE	633	9.10E-04	581.3539	1160.6933	2	0.0016	10	0	GluC
H4_MOUSE	Histone H4	TRGVLKVFLE	633	5.00E-04	581.3539	1160.6933	2	0.0016	10	0	GluC
H4_MOUSE	Histone H4	TRGVLKVFLE	633	3.30E-04	581.354	1160.6933	2	0.0016	10	0	GluC
H4_MOUSE	Histone H4	TRGVLKVFLE	633	1.80E-04	581.3539	1160.6933	2	0.0016	10	0	GluC
H4_MOUSE	Histone H4	TRGVLKVFLE	633	1.60E-04	581.3539	1160.6933	2	0.0016	10	0	GluC
H4_MOUSE	Histone H4	TRGVLKVFLE	633	8.20E-05	581.3539	1160.6933	2	0.0016	10	0	GluC
H4_MOUSE	Histone H4	TRGVLKVFLE	633	2.40E-05	387.9051	1160.6934	3	0.0016	10	0	GluC
H4_MOUSE	Histone H4	TRGVLKVFLE	633	2.90E-03	581.3539	1160.6933	2	0.0015	10	0	GluC
H4_MOUSE	Histone H4	TRGVLKVFLE	633	2.40E-03	581.3539	1160.6932	2	0.0015	10	0	GluC
H4_MOUSE	Histone H4	TRGVLKVFLE	633	2.10E-03	581.3539	1160.6932	2	0.0015	10	0	GluC
H4_MOUSE	Histone H4	TRGVLKVFLE	633	1.40E-03	581.3539	1160.6933	2	0.0015	10	0	GluC
H4_MOUSE	Histone H4	TRGVLKVFLE	633	9.80E-04	581.3539	1160.6933	2	0.0015	10	0	GluC
H4_MOUSE	Histone H4	TRGVLKVFLE	633	9.20E-04	581.3539	1160.6932	2	0.0015	10	0	GluC
H4_MOUSE	Histone H4	TRGVLKVFLE	633	8.90E-04	581.3539	1160.6932	2	0.0015	10	0	GluC
H4_MOUSE	Histone H4	TRGVLKVFLE	633	3.20E-04	581.3539	1160.6932	2	0.0015	10	0	GluC

H4_MOUSE	Histone H4	TRGVLKVFLE	633	3.20E-04	581.3539	1160.6933	2	0.0015	10	0	GluC
H4_MOUSE	Histone H4	TRGVLKVFLE	633	8.60E-05	581.3539	1160.6933	2	0.0015	10	0	GluC
H4_MOUSE	Histone H4	TRGVLKVFLE	633	8.10E-05	581.3539	1160.6932	2	0.0015	10	0	GluC
H4_MOUSE	Histone H4	TRGVLKVFLE	633	7.00E-03	581.3539	1160.6932	2	0.0014	10	0	GluC
H4_MOUSE	Histone H4	TRGVLKVFLE	633	6.30E-03	387.905	1160.6931	3	0.0014	10	0	GluC
H4_MOUSE	Histone H4	TRGVLKVFLE	633	5.40E-03	581.3538	1160.6931	2	0.0014	10	0	GluC
H4_MOUSE	Histone H4	TRGVLKVFLE	633	5.40E-03	387.905	1160.6932	3	0.0014	10	0	GluC
H4_MOUSE	Histone H4	TRGVLKVFLE	633	4.70E-03	581.3538	1160.6931	2	0.0014	10	0	GluC
H4_MOUSE	Histone H4	TRGVLKVFLE	633	4.00E-03	581.3539	1160.6932	2	0.0014	10	0	GluC
H4_MOUSE	Histone H4	TRGVLKVFLE	633	1.00E-03	387.905	1160.6931	3	0.0014	10	0	GluC
H4_MOUSE	Histone H4	TRGVLKVFLE	633	6.80E-04	581.3538	1160.6931	2	0.0014	10	0	GluC
H4_MOUSE	Histone H4	TRGVLKVFLE	633	6.40E-04	581.3538	1160.6931	2	0.0014	10	0	GluC
H4_MOUSE	Histone H4	TRGVLKVFLE	633	5.40E-04	387.905	1160.6931	3	0.0014	10	0	GluC
H4_MOUSE	Histone H4	TRGVLKVFLE	633	2.90E-04	387.905	1160.6931	3	0.0014	10	0	GluC
H4_MOUSE	Histone H4	TRGVLKVFLE	633	2.90E-04	581.3539	1160.6932	2	0.0014	10	0	GluC
H4_MOUSE	Histone H4	TRGVLKVFLE	633	1.40E-04	581.3539	1160.6932	2	0.0014	10	0	GluC
H4_MOUSE	Histone H4	TRGVLKVFLE	633	1.20E-04	387.905	1160.6931	3	0.0014	10	0	GluC
H4_MOUSE	Histone H4	TRGVLKVFLE	633	1.50E-05	387.905	1160.6932	3	0.0014	10	0	GluC
H4_MOUSE	Histone H4	TRGVLKVFLE	633	1.50E-05	387.905	1160.6931	3	0.0014	10	0	GluC
H4_MOUSE	Histone H4	TRGVLKVFLE	633	1.00E-02	581.3538	1160.6930	2	0.0013	10	0	GluC
H4_MOUSE	Histone H4	TRGVLKVFLE	633	1.00E-02	387.905	1160.6931	3	0.0013	10	0	GluC
H4_MOUSE	Histone H4	TRGVLKVFLE	633	8.80E-03	581.3538	1160.6930	2	0.0013	10	0	GluC
H4_MOUSE	Histone H4	TRGVLKVFLE	633	7.70E-03	581.3538	1160.6930	2	0.0013	10	0	GluC
H4_MOUSE	Histone H4	TRGVLKVFLE	633	7.30E-03	581.3538	1160.6931	2	0.0013	10	0	GluC
H4_MOUSE	Histone H4	TRGVLKVFLE	633	2.70E-03	581.3538	1160.6930	2	0.0013	10	0	GluC
H4_MOUSE	Histone H4	TRGVLKVFLE	633	1.20E-03	581.3538	1160.6930	2	0.0013	10	0	GluC
H4_MOUSE	Histone H4	TRGVLKVFLE	633	1.50E-04	581.3538	1160.6930	2	0.0013	10	0	GluC
H4_MOUSE	Histone H4	TRGVLKVFLE	633	1.40E-04	581.3538	1160.6931	2	0.0013	10	0	GluC
H4_MOUSE	Histone H4	TRGVLKVFLE	633	8.70E-05	581.3538	1160.6930	2	0.0013	10	0	GluC
H4_MOUSE	Histone H4	TRGVLKVFLE	633	8.20E-06	581.3538	1160.6931	2	0.0013	10	0	GluC
H4_MOUSE	Histone H4	TRGVLKVFLE	633	6.40E-03	387.9049	1160.6929	3	0.0012	10	0	GluC
H4_MOUSE	Histone H4	TRGVLKVFLE	633	5.20E-03	387.9049	1160.6930	3	0.0012	10	0	GluC
H4_MOUSE	Histone H4	TRGVLKVFLE	633	3.20E-03	581.3537	1160.6929	2	0.0012	10	0	GluC
H4_MOUSE	Histone H4	TRGVLKVFLE	633	2.10E-03	581.3537	1160.6929	2	0.0012	10	0	GluC
H4_MOUSE	Histone H4	TRGVLKVFLE	633	3.90E-04	581.3537	1160.6929	2	0.0012	10	0	GluC
H4_MOUSE	Histone H4	TRGVLKVFLE	633	2.90E-04	387.9049	1160.6929	3	0.0012	10	0	GluC
H4_MOUSE	Histone H4	TRGVLKVFLE	633	1.90E-04	581.3537	1160.6929	2	0.0012	10	0	GluC
H4_MOUSE	Histone H4	TRGVLKVFLE	633	9.20E-05	581.3538	1160.6930	2	0.0012	10	0	GluC
H4_MOUSE	Histone H4	TRGVLKVFLE	633	4.90E-05	387.9049	1160.6930	3	0.0012	10	0	GluC
H4_MOUSE	Histone H4	TRGVLKVFLE	633	3.80E-05	387.9049	1160.6930	3	0.0012	10	0	GluC
H4_MOUSE	Histone H4	TRGVLKVFLE	633	3.30E-05	387.9049	1160.6929	3	0.0012	10	0	GluC
H4_MOUSE	Histone H4	TRGVLKVFLE	633	1.50E-05	387.9049	1160.6930	3	0.0012	10	0	GluC
H4_MOUSE	Histone H4	TRGVLKVFLE	633	1.40E-05	387.9049	1160.6929	3	0.0012	10	0	GluC
H4_MOUSE	Histone H4	TRGVLKVFLE	633	7.30E-06	387.9049	1160.6929	3	0.0012	10	0	GluC
H4_MOUSE	Histone H4	TRGVLKVFLE	633	7.50E-03	581.3537	1160.6929	2	0.0011	10	0	GluC
H4_MOUSE	Histone H4	TRGVLKVFLE	633	5.30E-03	387.9049	1160.6928	3	0.0011	10	0	GluC
H4_MOUSE	Histone H4	TRGVLKVFLE	633	4.80E-03	581.3537	1160.6929	2	0.0011	10	0	GluC
H4_MOUSE	Histone H4	TRGVLKVFLE	633	3.90E-03	387.9049	1160.6929	3	0.0011	10	0	GluC
H4_MOUSE	Histone H4	TRGVLKVFLE	633	3.70E-03	581.3537	1160.6929	2	0.0011	10	0	GluC
H4_MOUSE	Histone H4	TRGVLKVFLE	633	2.50E-03	581.3537	1160.6928	2	0.0011	10	0	GluC
H4_MOUSE	Histone H4	TRGVLKVFLE	633	2.50E-03	581.3537	1160.6928	2	0.0011	10	0	GluC
H4_MOUSE	Histone H4	TRGVLKVFLE	633	1.30E-03	581.3537	1160.6929	2	0.0011	10	0	GluC
H4_MOUSE	Histone H4	TRGVLKVFLE	633	1.20E-03	581.3537	1160.6928	2	0.0011	10	0	GluC
H4_MOUSE	Histone H4	TRGVLKVFLE	633	6.80E-04	387.9049	1160.6929	3	0.0011	10	0	GluC
H4_MOUSE	Histone H4	TRGVLKVFLE	633	6.10E-04	581.3537	1160.6929	2	0.0011	10	0	GluC
H4_MOUSE	Histone H4	TRGVLKVFLE	633	5.30E-04	581.3537	1160.6928	2	0.0011	10	0	GluC
H4_MOUSE	Histone H4	TRGVLKVFLE	633	5.20E-04	581.3537	1160.6928	2	0.0011	10	0	GluC
H4_MOUSE	Histone H4	TRGVLKVFLE	633	3.30E-04	581.3537	1160.6929	2	0.0011	10	0	GluC
H4_MOUSE	Histone H4	TRGVLKVFLE	633	3.10E-04	581.3537	1160.6928	2	0.0011	10	0	GluC
H4_MOUSE	Histone H4	TRGVLKVFLE	633	1.00E-04	581.3537	1160.6928	2	0.0011	10	0	GluC
H4_MOUSE	Histone H4	TRGVLKVFLE	633	3.90E-05	387.9049	1160.6929	3	0.0011	10	0	GluC
H4_MOUSE	Histone H4	TRGVLKVFLE	633	5.30E-06	581.3537	1160.6928	2	0.0011	10	0	GluC
H4_MOUSE	Histone H4	TRGVLKVFLE	633	4.40E-06	387.9049	1160.6928	3	0.0011	10	0	GluC
H4_MOUSE	Histone H4	TRGVLKVFLE	633	3.30E-07	387.9049	1160.6929	3	0.0011	10	0	GluC
H4_MOUSE	Histone H4	TRGVLKVFLE	633	1.00E-02	581.3536	1160.6927	2	0.0010	10	0	GluC
H4_MOUSE	Histone H4	TRGVLKVFLE	633	6.40E-03	581.3537	1160.6928	2	0.0010	10	0	GluC
H4_MOUSE	Histone H4	TRGVLKVFLE	633	3.60E-03	581.3536	1160.6927	2	0.0010	10	0	GluC
H4_MOUSE	Histone H4	TRGVLKVFLE	633	3.00E-03	581.3536	1160.6927	2	0.0010	10	0	GluC
H4_MOUSE	Histone H4	TRGVLKVFLE	633	2.80E-03	387.9049	1160.6928	3	0.0010	10	0	GluC
H4_MOUSE	Histone H4	TRGVLKVFLE	633	1.20E-03	581.3537	1160.6927	2	0.0010	10	0	GluC
H4_MOUSE	Histone H4	TRGVLKVFLE	633	9.70E-04	581.3537	1160.6928	2	0.0010	10	0	GluC
H4_MOUSE	Histone H4	TRGVLKVFLE	633	8.90E-04	581.3536	1160.6927	2	0.0010	10	0	GluC
H4_MOUSE	Histone H4	TRGVLKVFLE	633	3.60E-04	387.9049	1160.6928	3	0.0010	10	0	GluC
H4_MOUSE	Histone H4	TRGVLKVFLE	633	3.20E-04	581.3536	1160.6927	2	0.0010	10	0	GluC

H4_MOUSE	Histone H4	TRGVLKVFLE	633	2.20E-04	581.3536	1160.6927	2	0.0010	10	0	GluC
H4_MOUSE	Histone H4	TRGVLKVFLE	633	1.90E-04	581.3537	1160.6928	2	0.0010	10	0	GluC
H4_MOUSE	Histone H4	TRGVLKVFLE	633	1.60E-04	581.3536	1160.6927	2	0.0010	10	0	GluC
H4_MOUSE	Histone H4	TRGVLKVFLE	633	7.60E-05	581.3537	1160.6927	2	0.0010	10	0	GluC
H4_MOUSE	Histone H4	TRGVLKVFLE	633	4.00E-05	387.9049	1160.6928	3	0.0010	10	0	GluC
H4_MOUSE	Histone H4	TRGVLKVFLE	633	2.90E-05	387.9049	1160.6927	3	0.0010	10	0	GluC
H4_MOUSE	Histone H4	TRGVLKVFLE	633	4.00E-06	581.3536	1160.6927	2	0.0010	10	0	GluC
H4_MOUSE	Histone H4	TRGVLKVFLE	633	8.50E-03	581.3536	1160.6926	2	0.0009	10	0	GluC
H4_MOUSE	Histone H4	TRGVLKVFLE	633	7.90E-03	581.3536	1160.6927	2	0.0009	10	0	GluC
H4_MOUSE	Histone H4	TRGVLKVFLE	633	7.70E-03	581.3536	1160.6926	2	0.0009	10	0	GluC
H4_MOUSE	Histone H4	TRGVLKVFLE	633	7.60E-03	581.3536	1160.6926	2	0.0009	10	0	GluC
H4_MOUSE	Histone H4	TRGVLKVFLE	633	5.80E-03	581.3536	1160.6926	2	0.0009	10	0	GluC
H4_MOUSE	Histone H4	TRGVLKVFLE	633	5.80E-03	387.9048	1160.6926	3	0.0009	10	0	GluC
H4_MOUSE	Histone H4	TRGVLKVFLE	633	5.50E-03	581.3536	1160.6927	2	0.0009	10	0	GluC
H4_MOUSE	Histone H4	TRGVLKVFLE	633	5.30E-03	581.3536	1160.6926	2	0.0009	10	0	GluC
H4_MOUSE	Histone H4	TRGVLKVFLE	633	5.00E-03	581.3536	1160.6927	2	0.0009	10	0	GluC
H4_MOUSE	Histone H4	TRGVLKVFLE	633	4.40E-03	581.3536	1160.6926	2	0.0009	10	0	GluC
H4_MOUSE	Histone H4	TRGVLKVFLE	633	4.40E-03	581.3536	1160.6927	2	0.0009	10	0	GluC
H4_MOUSE	Histone H4	TRGVLKVFLE	633	3.10E-03	387.9048	1160.6926	3	0.0009	10	0	GluC
H4_MOUSE	Histone H4	TRGVLKVFLE	633	2.90E-03	581.3536	1160.6926	2	0.0009	10	0	GluC
H4_MOUSE	Histone H4	TRGVLKVFLE	633	2.70E-03	581.3536	1160.6926	2	0.0009	10	0	GluC
H4_MOUSE	Histone H4	TRGVLKVFLE	633	2.20E-03	581.3536	1160.6926	2	0.0009	10	0	GluC
H4_MOUSE	Histone H4	TRGVLKVFLE	633	1.90E-03	581.3536	1160.6926	2	0.0009	10	0	GluC
H4_MOUSE	Histone H4	TRGVLKVFLE	633	1.80E-03	387.9048	1160.6926	3	0.0009	10	0	GluC
H4_MOUSE	Histone H4	TRGVLKVFLE	633	1.40E-03	387.9048	1160.6926	3	0.0009	10	0	GluC
H4_MOUSE	Histone H4	TRGVLKVFLE	633	8.90E-04	581.3536	1160.6926	2	0.0009	10	0	GluC
H4_MOUSE	Histone H4	TRGVLKVFLE	633	5.70E-04	581.3536	1160.6926	2	0.0009	10	0	GluC
H4_MOUSE	Histone H4	TRGVLKVFLE	633	3.50E-04	581.3536	1160.6926	2	0.0009	10	0	GluC
H4_MOUSE	Histone H4	TRGVLKVFLE	633	1.50E-04	581.3536	1160.6926	2	0.0009	10	0	GluC
H4_MOUSE	Histone H4	TRGVLKVFLE	633	1.40E-04	581.3536	1160.6927	2	0.0009	10	0	GluC
H4_MOUSE	Histone H4	TRGVLKVFLE	633	8.00E-05	581.3536	1160.6926	2	0.0009	10	0	GluC
H4_MOUSE	Histone H4	TRGVLKVFLE	633	6.00E-05	387.9048	1160.6926	3	0.0009	10	0	GluC
H4_MOUSE	Histone H4	TRGVLKVFLE	633	3.20E-05	387.9048	1160.6926	3	0.0009	10	0	GluC
H4_MOUSE	Histone H4	TRGVLKVFLE	633	2.70E-05	387.9048	1160.6926	3	0.0009	10	0	GluC
H4_MOUSE	Histone H4	TRGVLKVFLE	633	1.10E-05	387.9048	1160.6927	3	0.0009	10	0	GluC
H4_MOUSE	Histone H4	TRGVLKVFLE	633	8.00E-03	581.3536	1160.6926	2	0.0008	10	0	GluC
H4_MOUSE	Histone H4	TRGVLKVFLE	633	7.90E-03	387.9048	1160.6926	3	0.0008	10	0	GluC
H4_MOUSE	Histone H4	TRGVLKVFLE	633	6.50E-03	581.3536	1160.6926	2	0.0008	10	0	GluC
H4_MOUSE	Histone H4	TRGVLKVFLE	633	6.00E-03	581.3536	1160.6926	2	0.0008	10	0	GluC
H4_MOUSE	Histone H4	TRGVLKVFLE	633	4.30E-03	581.3535	1160.6925	2	0.0008	10	0	GluC
H4_MOUSE	Histone H4	TRGVLKVFLE	633	4.10E-03	387.9048	1160.6926	3	0.0008	10	0	GluC
H4_MOUSE	Histone H4	TRGVLKVFLE	633	1.40E-03	581.3535	1160.6925	2	0.0008	10	0	GluC
H4_MOUSE	Histone H4	TRGVLKVFLE	633	9.90E-04	581.3536	1160.6926	2	0.0008	10	0	GluC
H4_MOUSE	Histone H4	TRGVLKVFLE	633	9.40E-04	581.3535	1160.6925	2	0.0008	10	0	GluC
H4_MOUSE	Histone H4	TRGVLKVFLE	633	5.60E-04	387.9048	1160.6926	3	0.0008	10	0	GluC
H4_MOUSE	Histone H4	TRGVLKVFLE	633	2.90E-04	581.3535	1160.6925	2	0.0008	10	0	GluC
H4_MOUSE	Histone H4	TRGVLKVFLE	633	1.60E-04	581.3535	1160.6925	2	0.0008	10	0	GluC
H4_MOUSE	Histone H4	TRGVLKVFLE	633	8.90E-05	581.3535	1160.6925	2	0.0008	10	0	GluC
H4_MOUSE	Histone H4	TRGVLKVFLE	633	8.60E-05	581.3535	1160.6925	2	0.0008	10	0	GluC
H4_MOUSE	Histone H4	TRGVLKVFLE	633	7.40E-05	581.3535	1160.6925	2	0.0008	10	0	GluC
H4_MOUSE	Histone H4	TRGVLKVFLE	633	4.90E-05	581.3536	1160.6925	2	0.0008	10	0	GluC
H4_MOUSE	Histone H4	TRGVLKVFLE	633	2.20E-05	581.3536	1160.6926	2	0.0008	10	0	GluC
H4_MOUSE	Histone H4	TRGVLKVFLE	633	1.80E-05	387.9048	1160.6926	3	0.0008	10	0	GluC
H4_MOUSE	Histone H4	TRGVLKVFLE	633	3.60E-06	581.3536	1160.6925	2	0.0008	10	0	GluC
H4_MOUSE	Histone H4	TRGVLKVFLE	633	3.30E-06	387.9048	1160.6925	3	0.0008	10	0	GluC
H4_MOUSE	Histone H4	TRGVLKVFLE	633	1.00E-02	581.3535	1160.6925	2	0.0007	10	0	GluC
H4_MOUSE	Histone H4	TRGVLKVFLE	633	9.50E-03	387.9048	1160.6925	3	0.0007	10	0	GluC
H4_MOUSE	Histone H4	TRGVLKVFLE	633	7.30E-03	581.3535	1160.6925	2	0.0007	10	0	GluC
H4_MOUSE	Histone H4	TRGVLKVFLE	633	6.40E-03	581.3535	1160.6924	2	0.0007	10	0	GluC
H4_MOUSE	Histone H4	TRGVLKVFLE	633	5.80E-03	581.3535	1160.6925	2	0.0007	10	0	GluC
H4_MOUSE	Histone H4	TRGVLKVFLE	633	5.60E-03	581.3535	1160.6924	2	0.0007	10	0	GluC
H4_MOUSE	Histone H4	TRGVLKVFLE	633	4.80E-03	581.3535	1160.6925	2	0.0007	10	0	GluC
H4_MOUSE	Histone H4	TRGVLKVFLE	633	4.30E-03	581.3535	1160.6924	2	0.0007	10	0	GluC
H4_MOUSE	Histone H4	TRGVLKVFLE	633	2.50E-03	581.3535	1160.6924	2	0.0007	10	0	GluC
H4_MOUSE	Histone H4	TRGVLKVFLE	633	2.40E-03	581.3535	1160.6924	2	0.0007	10	0	GluC
H4_MOUSE	Histone H4	TRGVLKVFLE	633	2.30E-03	581.3535	1160.6924	2	0.0007	10	0	GluC
H4_MOUSE	Histone H4	TRGVLKVFLE	633	2.20E-03	581.3535	1160.6924	2	0.0007	10	0	GluC
H4_MOUSE	Histone H4	TRGVLKVFLE	633	2.10E-03	581.3535	1160.6925	2	0.0007	10	0	GluC
H4_MOUSE	Histone H4	TRGVLKVFLE	633	1.90E-03	581.3535	1160.6925	2	0.0007	10	0	GluC
H4_MOUSE	Histone H4	TRGVLKVFLE	633	1.70E-03	581.3535	1160.6924	2	0.0007	10	0	GluC
H4_MOUSE	Histone H4	TRGVLKVFLE	633	1.40E-03	581.3535	1160.6925	2	0.0007	10	0	GluC
H4_MOUSE	Histone H4	TRGVLKVFLE	633	1.10E-03	581.3535	1160.6924	2	0.0007	10	0	GluC
H4_MOUSE	Histone H4	TRGVLKVFLE	633	1.10E-03	581.3535	1160.6925	2	0.0007	10	0	GluC
H4_MOUSE	Histone H4	TRGVLKVFLE	633	9.60E-04	581.3535	1160.6924	2	0.0007	10	0	GluC

H4_MOUSE	Histone H4	TRGVLKVFLE	633	7.10E-04	387.9048	1160.6925	3	0.0007	10	0	GluC
H4_MOUSE	Histone H4	TRGVLKVFLE	633	7.00E-04	581.3535	1160.6924	2	0.0007	10	0	GluC
H4_MOUSE	Histone H4	TRGVLKVFLE	633	3.40E-04	581.3535	1160.6925	2	0.0007	10	0	GluC
H4_MOUSE	Histone H4	TRGVLKVFLE	633	1.50E-04	581.3535	1160.6924	2	0.0007	10	0	GluC
H4_MOUSE	Histone H4	TRGVLKVFLE	633	9.20E-05	387.9048	1160.6925	3	0.0007	10	0	GluC
H4_MOUSE	Histone H4	TRGVLKVFLE	633	8.20E-05	581.3535	1160.6924	2	0.0007	10	0	GluC
H4_MOUSE	Histone H4	TRGVLKVFLE	633	7.50E-05	581.3535	1160.6924	2	0.0007	10	0	GluC
H4_MOUSE	Histone H4	TRGVLKVFLE	633	3.90E-05	387.9048	1160.6925	3	0.0007	10	0	GluC
H4_MOUSE	Histone H4	TRGVLKVFLE	633	3.20E-05	387.9048	1160.6925	3	0.0007	10	0	GluC
H4_MOUSE	Histone H4	TRGVLKVFLE	633	5.10E-07	387.9048	1160.6925	3	0.0007	10	0	GluC
H4_MOUSE	Histone H4	TRGVLKVFLE	633	9.80E-03	581.3535	1160.6924	2	0.0006	10	0	GluC
H4_MOUSE	Histone H4	TRGVLKVFLE	633	6.50E-03	581.3534	1160.6923	2	0.0006	10	0	GluC
H4_MOUSE	Histone H4	TRGVLKVFLE	633	4.90E-03	581.3534	1160.6923	2	0.0006	10	0	GluC
H4_MOUSE	Histone H4	TRGVLKVFLE	633	4.20E-03	581.3534	1160.6923	2	0.0006	10	0	GluC
H4_MOUSE	Histone H4	TRGVLKVFLE	633	3.90E-03	581.3535	1160.6924	2	0.0006	10	0	GluC
H4_MOUSE	Histone H4	TRGVLKVFLE	633	1.30E-03	387.9047	1160.6924	3	0.0006	10	0	GluC
H4_MOUSE	Histone H4	TRGVLKVFLE	633	8.20E-04	581.3534	1160.6923	2	0.0006	10	0	GluC
H4_MOUSE	Histone H4	TRGVLKVFLE	633	6.10E-04	581.3535	1160.6924	2	0.0006	10	0	GluC
H4_MOUSE	Histone H4	TRGVLKVFLE	633	4.00E-04	581.3535	1160.6924	2	0.0006	10	0	GluC
H4_MOUSE	Histone H4	TRGVLKVFLE	633	3.40E-04	581.3535	1160.6924	2	0.0006	10	0	GluC
H4_MOUSE	Histone H4	TRGVLKVFLE	633	8.70E-05	581.3535	1160.6924	2	0.0006	10	0	GluC
H4_MOUSE	Histone H4	TRGVLKVFLE	633	7.10E-06	387.9047	1160.6923	3	0.0006	10	0	GluC
H4_MOUSE	Histone H4	TRGVLKVFLE	633	1.00E-02	581.3534	1160.6923	2	0.0005	10	0	GluC
H4_MOUSE	Histone H4	TRGVLKVFLE	633	8.90E-03	581.3534	1160.6923	2	0.0005	10	0	GluC
H4_MOUSE	Histone H4	TRGVLKVFLE	633	8.00E-03	581.3534	1160.6922	2	0.0005	10	0	GluC
H4_MOUSE	Histone H4	TRGVLKVFLE	633	6.40E-03	581.3534	1160.6923	2	0.0005	10	0	GluC
H4_MOUSE	Histone H4	TRGVLKVFLE	633	6.00E-03	581.3534	1160.6923	2	0.0005	10	0	GluC
H4_MOUSE	Histone H4	TRGVLKVFLE	633	5.00E-03	581.3534	1160.6922	2	0.0005	10	0	GluC
H4_MOUSE	Histone H4	TRGVLKVFLE	633	3.60E-03	581.3534	1160.6923	2	0.0005	10	0	GluC
H4_MOUSE	Histone H4	TRGVLKVFLE	633	3.10E-03	581.3534	1160.6923	2	0.0005	10	0	GluC
H4_MOUSE	Histone H4	TRGVLKVFLE	633	2.20E-03	581.3534	1160.6923	2	0.0005	10	0	GluC
H4_MOUSE	Histone H4	TRGVLKVFLE	633	2.20E-03	387.9047	1160.6923	3	0.0005	10	0	GluC
H4_MOUSE	Histone H4	TRGVLKVFLE	633	2.10E-03	387.9047	1160.6923	3	0.0005	10	0	GluC
H4_MOUSE	Histone H4	TRGVLKVFLE	633	1.90E-03	581.3534	1160.6923	2	0.0005	10	0	GluC
H4_MOUSE	Histone H4	TRGVLKVFLE	633	6.70E-04	581.3534	1160.6922	2	0.0005	10	0	GluC
H4_MOUSE	Histone H4	TRGVLKVFLE	633	6.00E-04	581.3534	1160.6922	2	0.0005	10	0	GluC
H4_MOUSE	Histone H4	TRGVLKVFLE	633	4.10E-04	581.3534	1160.6922	2	0.0005	10	0	GluC
H4_MOUSE	Histone H4	TRGVLKVFLE	633	3.40E-04	581.3534	1160.6923	2	0.0005	10	0	GluC
H4_MOUSE	Histone H4	TRGVLKVFLE	633	3.20E-04	581.3534	1160.6923	2	0.0005	10	0	GluC
H4_MOUSE	Histone H4	TRGVLKVFLE	633	2.90E-04	387.9047	1160.6922	3	0.0005	10	0	GluC
H4_MOUSE	Histone H4	TRGVLKVFLE	633	2.00E-04	581.3534	1160.6922	2	0.0005	10	0	GluC
H4_MOUSE	Histone H4	TRGVLKVFLE	633	1.00E-04	387.9047	1160.6923	3	0.0005	10	0	GluC
H4_MOUSE	Histone H4	TRGVLKVFLE	633	8.00E-05	581.3534	1160.6923	2	0.0005	10	0	GluC
H4_MOUSE	Histone H4	TRGVLKVFLE	633	4.80E-05	581.3534	1160.6923	2	0.0005	10	0	GluC
H4_MOUSE	Histone H4	TRGVLKVFLE	633	2.30E-05	581.3534	1160.6922	2	0.0005	10	0	GluC
H4_MOUSE	Histone H4	TRGVLKVFLE	633	8.50E-03	581.3534	1160.6922	2	0.0004	10	0	GluC
H4_MOUSE	Histone H4	TRGVLKVFLE	633	6.00E-03	581.3534	1160.6922	2	0.0004	10	0	GluC
H4_MOUSE	Histone H4	TRGVLKVFLE	633	5.90E-03	387.9047	1160.6922	3	0.0004	10	0	GluC
H4_MOUSE	Histone H4	TRGVLKVFLE	633	5.30E-03	387.9047	1160.6922	3	0.0004	10	0	GluC
H4_MOUSE	Histone H4	TRGVLKVFLE	633	4.70E-03	581.3533	1160.6921	2	0.0004	10	0	GluC
H4_MOUSE	Histone H4	TRGVLKVFLE	633	2.50E-03	581.3534	1160.6922	2	0.0004	10	0	GluC
H4_MOUSE	Histone H4	TRGVLKVFLE	633	2.30E-03	581.3534	1160.6922	2	0.0004	10	0	GluC
H4_MOUSE	Histone H4	TRGVLKVFLE	633	8.40E-04	387.9047	1160.6921	3	0.0004	10	0	GluC
H4_MOUSE	Histone H4	TRGVLKVFLE	633	7.00E-04	581.3534	1160.6922	2	0.0004	10	0	GluC
H4_MOUSE	Histone H4	TRGVLKVFLE	633	6.10E-04	581.3534	1160.6922	2	0.0004	10	0	GluC
H4_MOUSE	Histone H4	TRGVLKVFLE	633	5.80E-04	581.3534	1160.6922	2	0.0004	10	0	GluC
H4_MOUSE	Histone H4	TRGVLKVFLE	633	4.90E-04	581.3534	1160.6922	2	0.0004	10	0	GluC
H4_MOUSE	Histone H4	TRGVLKVFLE	633	1.50E-04	581.3534	1160.6922	2	0.0004	10	0	GluC
H4_MOUSE	Histone H4	TRGVLKVFLE	633	9.50E-05	387.9047	1160.6922	3	0.0004	10	0	GluC
H4_MOUSE	Histone H4	TRGVLKVFLE	633	7.40E-05	581.3534	1160.6922	2	0.0004	10	0	GluC
H4_MOUSE	Histone H4	TRGVLKVFLE	633	4.60E-05	581.3533	1160.6921	2	0.0004	10	0	GluC
H4_MOUSE	Histone H4	TRGVLKVFLE	633	2.30E-05	387.9047	1160.6921	3	0.0004	10	0	GluC
H4_MOUSE	Histone H4	TRGVLKVFLE	633	1.10E-05	387.9047	1160.6922	3	0.0004	10	0	GluC
H4_MOUSE	Histone H4	TRGVLKVFLE	633	9.50E-03	581.3533	1160.6920	2	0.0003	10	0	GluC
H4_MOUSE	Histone H4	TRGVLKVFLE	633	9.20E-03	387.9046	1160.6921	3	0.0003	10	0	GluC
H4_MOUSE	Histone H4	TRGVLKVFLE	633	8.50E-03	581.3533	1160.6920	2	0.0003	10	0	GluC
H4_MOUSE	Histone H4	TRGVLKVFLE	633	8.20E-03	387.9046	1160.6921	3	0.0003	10	0	GluC
H4_MOUSE	Histone H4	TRGVLKVFLE	633	7.70E-03	581.3533	1160.6920	2	0.0003	10	0	GluC
H4_MOUSE	Histone H4	TRGVLKVFLE	633	4.90E-03	581.3533	1160.6920	2	0.0003	10	0	GluC
H4_MOUSE	Histone H4	TRGVLKVFLE	633	4.30E-03	581.3533	1160.6920	2	0.0003	10	0	GluC
H4_MOUSE	Histone H4	TRGVLKVFLE	633	2.70E-03	581.3533	1160.6921	2	0.0003	10	0	GluC
H4_MOUSE	Histone H4	TRGVLKVFLE	633	2.30E-03	581.3533	1160.6920	2	0.0003	10	0	GluC
H4_MOUSE	Histone H4	TRGVLKVFLE	633	2.30E-03	581.3533	1160.6921	2	0.0003	10	0	GluC
H4_MOUSE	Histone H4	TRGVLKVFLE	633	1.20E-03	581.3533	1160.6920	2	0.0003	10	0	GluC

H4_MOUSE	Histone H4	TRGVLKVFLE	633	1.10E-03	387.9046	1160.6921	3	0.0003	10	0	GluC
H4_MOUSE	Histone H4	TRGVLKVFLE	633	4.20E-04	581.3533	1160.6920	2	0.0003	10	0	GluC
H4_MOUSE	Histone H4	TRGVLKVFLE	633	3.70E-04	581.3533	1160.6921	2	0.0003	10	0	GluC
H4_MOUSE	Histone H4	TRGVLKVFLE	633	3.20E-04	581.3533	1160.6920	2	0.0003	10	0	GluC
H4_MOUSE	Histone H4	TRGVLKVFLE	633	2.20E-04	387.9046	1160.6920	3	0.0003	10	0	GluC
H4_MOUSE	Histone H4	TRGVLKVFLE	633	1.90E-04	581.3533	1160.6920	2	0.0003	10	0	GluC
H4_MOUSE	Histone H4	TRGVLKVFLE	633	1.80E-04	581.3533	1160.6921	2	0.0003	10	0	GluC
H4_MOUSE	Histone H4	TRGVLKVFLE	633	1.60E-04	581.3533	1160.6921	2	0.0003	10	0	GluC
H4_MOUSE	Histone H4	TRGVLKVFLE	633	1.30E-04	581.3533	1160.6920	2	0.0003	10	0	GluC
H4_MOUSE	Histone H4	TRGVLKVFLE	633	9.50E-05	387.9046	1160.6920	3	0.0003	10	0	GluC
H4_MOUSE	Histone H4	TRGVLKVFLE	633	9.30E-05	387.9046	1160.6921	3	0.0003	10	0	GluC
H4_MOUSE	Histone H4	TRGVLKVFLE	633	6.20E-05	387.9046	1160.6920	3	0.0003	10	0	GluC
H4_MOUSE	Histone H4	TRGVLKVFLE	633	5.80E-05	387.9046	1160.6921	3	0.0003	10	0	GluC
H4_MOUSE	Histone H4	TRGVLKVFLE	633	5.20E-05	581.3533	1160.6921	2	0.0003	10	0	GluC
H4_MOUSE	Histone H4	TRGVLKVFLE	633	5.10E-05	387.9046	1160.6921	3	0.0003	10	0	GluC
H4_MOUSE	Histone H4	TRGVLKVFLE	633	1.60E-05	581.3533	1160.6921	2	0.0003	10	0	GluC
H4_MOUSE	Histone H4	TRGVLKVFLE	633	1.50E-05	387.9046	1160.6921	3	0.0003	10	0	GluC
H4_MOUSE	Histone H4	TRGVLKVFLE	633	3.50E-06	387.9046	1160.6920	3	0.0003	10	0	GluC
H4_MOUSE	Histone H4	TRGVLKVFLE	633	9.20E-03	387.9046	1160.6919	3	0.0002	10	0	GluC
H4_MOUSE	Histone H4	TRGVLKVFLE	633	8.00E-03	387.9046	1160.6919	3	0.0002	10	0	GluC
H4_MOUSE	Histone H4	TRGVLKVFLE	633	7.80E-03	581.3533	1160.6919	2	0.0002	10	0	GluC
H4_MOUSE	Histone H4	TRGVLKVFLE	633	6.40E-03	387.9046	1160.6920	3	0.0002	10	0	GluC
H4_MOUSE	Histone H4	TRGVLKVFLE	633	5.30E-03	581.3532	1160.6919	2	0.0002	10	0	GluC
H4_MOUSE	Histone H4	TRGVLKVFLE	633	5.30E-03	581.3533	1160.6920	2	0.0002	10	0	GluC
H4_MOUSE	Histone H4	TRGVLKVFLE	633	5.10E-03	581.3533	1160.6919	2	0.0002	10	0	GluC
H4_MOUSE	Histone H4	TRGVLKVFLE	633	4.40E-03	581.3533	1160.6919	2	0.0002	10	0	GluC
H4_MOUSE	Histone H4	TRGVLKVFLE	633	3.30E-03	581.3533	1160.6919	2	0.0002	10	0	GluC
H4_MOUSE	Histone H4	TRGVLKVFLE	633	1.70E-03	581.3533	1160.6919	2	0.0002	10	0	GluC
H4_MOUSE	Histone H4	TRGVLKVFLE	633	1.50E-03	581.3533	1160.6919	2	0.0002	10	0	GluC
H4_MOUSE	Histone H4	TRGVLKVFLE	633	9.30E-04	581.3533	1160.6920	2	0.0002	10	0	GluC
H4_MOUSE	Histone H4	TRGVLKVFLE	633	3.50E-04	581.3533	1160.6920	2	0.0002	10	0	GluC
H4_MOUSE	Histone H4	TRGVLKVFLE	633	2.90E-04	581.3532	1160.6919	2	0.0002	10	0	GluC
H4_MOUSE	Histone H4	TRGVLKVFLE	633	1.60E-04	581.3532	1160.6919	2	0.0002	10	0	GluC
H4_MOUSE	Histone H4	TRGVLKVFLE	633	1.20E-04	387.9046	1160.6920	3	0.0002	10	0	GluC
H4_MOUSE	Histone H4	TRGVLKVFLE	633	1.00E-04	387.9046	1160.6920	3	0.0002	10	0	GluC
H4_MOUSE	Histone H4	TRGVLKVFLE	633	9.30E-05	387.9046	1160.6919	3	0.0002	10	0	GluC
H4_MOUSE	Histone H4	TRGVLKVFLE	633	7.80E-05	581.3533	1160.6919	2	0.0002	10	0	GluC
H4_MOUSE	Histone H4	TRGVLKVFLE	633	6.40E-05	387.9046	1160.6919	3	0.0002	10	0	GluC
H4_MOUSE	Histone H4	TRGVLKVFLE	633	6.30E-05	387.9046	1160.6920	3	0.0002	10	0	GluC
H4_MOUSE	Histone H4	TRGVLKVFLE	633	4.50E-05	387.9046	1160.6919	3	0.0002	10	0	GluC
H4_MOUSE	Histone H4	TRGVLKVFLE	633	3.70E-05	387.9046	1160.6919	3	0.0002	10	0	GluC
H4_MOUSE	Histone H4	TRGVLKVFLE	633	1.50E-05	387.9046	1160.6920	3	0.0002	10	0	GluC
H4_MOUSE	Histone H4	TRGVLKVFLE	633	4.40E-03	581.3532	1160.6919	2	0.0001	10	0	GluC
H4_MOUSE	Histone H4	TRGVLKVFLE	633	4.40E-03	387.9046	1160.6919	3	0.0001	10	0	GluC
H4_MOUSE	Histone H4	TRGVLKVFLE	633	2.90E-03	387.9045	1160.6918	3	0.0001	10	0	GluC
H4_MOUSE	Histone H4	TRGVLKVFLE	633	1.50E-03	387.9045	1160.6918	3	0.0001	10	0	GluC
H4_MOUSE	Histone H4	TRGVLKVFLE	633	1.20E-03	581.3532	1160.6919	2	0.0001	10	0	GluC
H4_MOUSE	Histone H4	TRGVLKVFLE	633	8.60E-04	581.3532	1160.6919	2	0.0001	10	0	GluC
H4_MOUSE	Histone H4	TRGVLKVFLE	633	6.00E-04	581.3532	1160.6919	2	0.0001	10	0	GluC
H4_MOUSE	Histone H4	TRGVLKVFLE	633	1.90E-04	387.9046	1160.6919	3	0.0001	10	0	GluC
H4_MOUSE	Histone H4	TRGVLKVFLE	633	1.70E-04	581.3532	1160.6918	2	0.0001	10	0	GluC
H4_MOUSE	Histone H4	TRGVLKVFLE	633	1.60E-04	387.9045	1160.6918	3	0.0001	10	0	GluC
H4_MOUSE	Histone H4	TRGVLKVFLE	633	1.30E-04	387.9045	1160.6918	3	0.0001	10	0	GluC
H4_MOUSE	Histone H4	TRGVLKVFLE	633	5.20E-05	387.9046	1160.6919	3	0.0001	10	0	GluC
H4_MOUSE	Histone H4	TRGVLKVFLE	633	5.20E-05	387.9046	1160.6919	3	0.0001	10	0	GluC
H4_MOUSE	Histone H4	TRGVLKVFLE	633	4.50E-05	581.3532	1160.6919	2	0.0001	10	0	GluC
H4_MOUSE	Histone H4	TRGVLKVFLE	633	3.90E-05	387.9046	1160.6919	3	0.0001	10	0	GluC
H4_MOUSE	Histone H4	TRGVLKVFLE	633	1.40E-05	387.9045	1160.6918	3	0.0001	10	0	GluC
H4_MOUSE	Histone H4	TRGVLKVFLE	633	7.00E-06	387.9045	1160.6918	3	0.0001	10	0	GluC
H4_MOUSE	Histone H4	TRGVLKVFLE	633	8.80E-03	387.9045	1160.6918	3	0.0000	10	0	GluC
H4_MOUSE	Histone H4	TRGVLKVFLE	633	8.50E-03	581.3532	1160.6917	2	0.0000	10	0	GluC
H4_MOUSE	Histone H4	TRGVLKVFLE	633	8.00E-03	581.3531	1160.6917	2	0.0000	10	0	GluC
H4_MOUSE	Histone H4	TRGVLKVFLE	633	6.30E-03	387.9045	1160.6918	3	0.0000	10	0	GluC
H4_MOUSE	Histone H4	TRGVLKVFLE	633	6.10E-03	387.9045	1160.6917	3	0.0000	10	0	GluC
H4_MOUSE	Histone H4	TRGVLKVFLE	633	5.40E-03	581.3532	1160.6918	2	0.0000	10	0	GluC
H4_MOUSE	Histone H4	TRGVLKVFLE	633	5.30E-03	387.9045	1160.6918	3	0.0000	10	0	GluC
H4_MOUSE	Histone H4	TRGVLKVFLE	633	3.70E-03	581.3532	1160.6917	2	0.0000	10	0	GluC
H4_MOUSE	Histone H4	TRGVLKVFLE	633	1.60E-03	387.9045	1160.6918	3	0.0000	10	0	GluC
H4_MOUSE	Histone H4	TRGVLKVFLE	633	8.40E-04	581.3532	1160.6917	2	0.0000	10	0	GluC
H4_MOUSE	Histone H4	TRGVLKVFLE	633	4.10E-04	581.3532	1160.6918	2	0.0000	10	0	GluC
H4_MOUSE	Histone H4	TRGVLKVFLE	633	2.50E-05	387.9045	1160.6917	3	0.0000	10	0	GluC
H4_MOUSE	Histone H4	TRGVLKVFLE	633	1.10E-05	387.9045	1160.6918	3	0.0000	10	0	GluC
H4_MOUSE	Histone H4	TRGVLKVFLE	633	9.60E-03	581.3531	1160.6916	2	-0.0001	10	0	GluC
H4_MOUSE	Histone H4	TRGVLKVFLE	633	9.00E-03	387.9045	1160.6916	3	-0.0001	10	0	GluC

H4_MOUSE	Histone H4	TRGVLKVFLE	633	7.40E-03	581.3531	1160.6916	2	-0.0001	10	0	GluC
H4_MOUSE	Histone H4	TRGVLKVFLE	633	7.20E-03	387.9045	1160.6916	3	-0.0001	10	0	GluC
H4_MOUSE	Histone H4	TRGVLKVFLE	633	5.50E-03	387.9045	1160.6916	3	-0.0001	10	0	GluC
H4_MOUSE	Histone H4	TRGVLKVFLE	633	4.60E-03	387.9045	1160.6917	3	-0.0001	10	0	GluC
H4_MOUSE	Histone H4	TRGVLKVFLE	633	7.70E-04	581.3531	1160.6916	2	-0.0001	10	0	GluC
H4_MOUSE	Histone H4	TRGVLKVFLE	633	7.40E-04	387.9045	1160.6916	3	-0.0001	10	0	GluC
H4_MOUSE	Histone H4	TRGVLKVFLE	633	7.20E-04	581.3531	1160.6917	2	-0.0001	10	0	GluC
H4_MOUSE	Histone H4	TRGVLKVFLE	633	6.50E-04	581.3531	1160.6917	2	-0.0001	10	0	GluC
H4_MOUSE	Histone H4	TRGVLKVFLE	633	4.20E-04	581.3531	1160.6917	2	-0.0001	10	0	GluC
H4_MOUSE	Histone H4	TRGVLKVFLE	633	2.90E-04	387.9045	1160.6917	3	-0.0001	10	0	GluC
H4_MOUSE	Histone H4	TRGVLKVFLE	633	2.20E-04	581.3531	1160.6917	2	-0.0001	10	0	GluC
H4_MOUSE	Histone H4	TRGVLKVFLE	633	1.40E-04	387.9045	1160.6916	3	-0.0001	10	0	GluC
H4_MOUSE	Histone H4	TRGVLKVFLE	633	7.70E-05	581.3531	1160.6917	2	-0.0001	10	0	GluC
H4_MOUSE	Histone H4	TRGVLKVFLE	633	5.80E-05	387.9045	1160.6916	3	-0.0001	10	0	GluC
H4_MOUSE	Histone H4	TRGVLKVFLE	633	4.90E-05	387.9045	1160.6916	3	-0.0001	10	0	GluC
H4_MOUSE	Histone H4	TRGVLKVFLE	633	3.80E-05	387.9045	1160.6916	3	-0.0001	10	0	GluC
H4_MOUSE	Histone H4	TRGVLKVFLE	633	1.80E-05	387.9045	1160.6916	3	-0.0001	10	0	GluC
H4_MOUSE	Histone H4	TRGVLKVFLE	633	1.50E-05	387.9045	1160.6916	3	-0.0001	10	0	GluC
H4_MOUSE	Histone H4	TRGVLKVFLE	633	6.80E-06	387.9045	1160.6916	3	-0.0001	10	0	GluC
H4_MOUSE	Histone H4	TRGVLKVFLE	633	6.30E-06	387.9045	1160.6917	3	-0.0001	10	0	GluC
H4_MOUSE	Histone H4	TRGVLKVFLE	633	5.20E-06	387.9045	1160.6916	3	-0.0001	10	0	GluC
H4_MOUSE	Histone H4	TRGVLKVFLE	633	9.90E-03	387.9045	1160.6916	3	-0.0002	10	0	GluC
H4_MOUSE	Histone H4	TRGVLKVFLE	633	7.10E-03	581.353	1160.6915	2	-0.0002	10	0	GluC
H4_MOUSE	Histone H4	TRGVLKVFLE	633	3.70E-03	387.9045	1160.6916	3	-0.0002	10	0	GluC
H4_MOUSE	Histone H4	TRGVLKVFLE	633	3.00E-03	387.9045	1160.6916	3	-0.0002	10	0	GluC
H4_MOUSE	Histone H4	TRGVLKVFLE	633	2.70E-03	581.3531	1160.6916	2	-0.0002	10	0	GluC
H4_MOUSE	Histone H4	TRGVLKVFLE	633	2.10E-03	581.3531	1160.6916	2	-0.0002	10	0	GluC
H4_MOUSE	Histone H4	TRGVLKVFLE	633	1.40E-03	387.9045	1160.6916	3	-0.0002	10	0	GluC
H4_MOUSE	Histone H4	TRGVLKVFLE	633	1.10E-03	387.9045	1160.6916	3	-0.0002	10	0	GluC
H4_MOUSE	Histone H4	TRGVLKVFLE	633	3.30E-04	581.3531	1160.6916	2	-0.0002	10	0	GluC
H4_MOUSE	Histone H4	TRGVLKVFLE	633	8.40E-05	387.9044	1160.6915	3	-0.0002	10	0	GluC
H4_MOUSE	Histone H4	TRGVLKVFLE	633	5.60E-05	581.3531	1160.6916	2	-0.0002	10	0	GluC
H4_MOUSE	Histone H4	TRGVLKVFLE	633	4.40E-05	387.9045	1160.6916	3	-0.0002	10	0	GluC
H4_MOUSE	Histone H4	TRGVLKVFLE	633	1.90E-05	581.3531	1160.6916	2	-0.0002	10	0	GluC
H4_MOUSE	Histone H4	TRGVLKVFLE	633	5.60E-06	387.9045	1160.6916	3	-0.0002	10	0	GluC
H4_MOUSE	Histone H4	TRGVLKVFLE	633	9.00E-03	581.353	1160.6914	2	-0.0003	10	0	GluC
H4_MOUSE	Histone H4	TRGVLKVFLE	633	6.70E-03	581.353	1160.6914	2	-0.0003	10	0	GluC
H4_MOUSE	Histone H4	TRGVLKVFLE	633	4.50E-03	387.9044	1160.6915	3	-0.0003	10	0	GluC
H4_MOUSE	Histone H4	TRGVLKVFLE	633	1.30E-03	387.9044	1160.6915	3	-0.0003	10	0	GluC
H4_MOUSE	Histone H4	TRGVLKVFLE	633	8.30E-04	581.353	1160.6914	2	-0.0003	10	0	GluC
H4_MOUSE	Histone H4	TRGVLKVFLE	633	3.00E-04	387.9044	1160.6914	3	-0.0003	10	0	GluC
H4_MOUSE	Histone H4	TRGVLKVFLE	633	7.60E-05	581.353	1160.6915	2	-0.0003	10	0	GluC
H4_MOUSE	Histone H4	TRGVLKVFLE	633	6.20E-05	387.9044	1160.6915	3	-0.0003	10	0	GluC
H4_MOUSE	Histone H4	TRGVLKVFLE	633	4.00E-05	387.9044	1160.6914	3	-0.0003	10	0	GluC
H4_MOUSE	Histone H4	TRGVLKVFLE	633	3.10E-05	387.9044	1160.6915	3	-0.0003	10	0	GluC
H4_MOUSE	Histone H4	TRGVLKVFLE	633	5.60E-03	581.353	1160.6914	2	-0.0004	10	0	GluC
H4_MOUSE	Histone H4	TRGVLKVFLE	633	4.00E-03	581.3529	1160.6913	2	-0.0004	10	0	GluC
H4_MOUSE	Histone H4	TRGVLKVFLE	633	1.10E-03	581.353	1160.6914	2	-0.0004	10	0	GluC
H4_MOUSE	Histone H4	TRGVLKVFLE	633	8.80E-04	387.9044	1160.6914	3	-0.0004	10	0	GluC
H4_MOUSE	Histone H4	TRGVLKVFLE	633	6.50E-04	581.3529	1160.6913	2	-0.0004	10	0	GluC
H4_MOUSE	Histone H4	TRGVLKVFLE	633	6.10E-04	387.9044	1160.6913	3	-0.0004	10	0	GluC
H4_MOUSE	Histone H4	TRGVLKVFLE	633	3.40E-04	581.353	1160.6914	2	-0.0004	10	0	GluC
H4_MOUSE	Histone H4	TRGVLKVFLE	633	6.80E-05	387.9044	1160.6913	3	-0.0004	10	0	GluC
H4_MOUSE	Histone H4	TRGVLKVFLE	633	2.70E-05	387.9044	1160.6914	3	-0.0004	10	0	GluC
H4_MOUSE	Histone H4	TRGVLKVFLE	633	5.00E-03	387.9044	1160.6913	3	-0.0005	10	0	GluC
H4_MOUSE	Histone H4	TRGVLKVFLE	633	3.90E-03	387.9044	1160.6912	3	-0.0005	10	0	GluC
H4_MOUSE	Histone H4	TRGVLKVFLE	633	2.00E-03	581.3529	1160.6913	2	-0.0005	10	0	GluC
H4_MOUSE	Histone H4	TRGVLKVFLE	633	9.50E-04	387.9044	1160.6912	3	-0.0005	10	0	GluC
H4_MOUSE	Histone H4	TRGVLKVFLE	633	9.40E-05	581.3529	1160.6912	2	-0.0005	10	0	GluC
H4_MOUSE	Histone H4	TRGVLKVFLE	633	2.60E-05	387.9044	1160.6913	3	-0.0005	10	0	GluC
H4_MOUSE	Histone H4	TRGVLKVFLE	633	2.30E-05	581.3529	1160.6912	2	-0.0005	10	0	GluC
H4_MOUSE	Histone H4	TRGVLKVFLE	633	1.00E-02	387.9043	1160.6912	3	-0.0006	10	0	GluC
H4_MOUSE	Histone H4	TRGVLKVFLE	633	2.90E-03	581.3528	1160.6911	2	-0.0006	10	0	GluC
H4_MOUSE	Histone H4	TRGVLKVFLE	633	1.70E-03	581.3528	1160.6911	2	-0.0006	10	0	GluC
H4_MOUSE	Histone H4	TRGVLKVFLE	633	1.10E-03	387.9043	1160.6911	3	-0.0006	10	0	GluC
H4_MOUSE	Histone H4	TRGVLKVFLE	633	1.00E-03	387.9043	1160.6911	3	-0.0006	10	0	GluC
H4_MOUSE	Histone H4	TRGVLKVFLE	633	6.70E-04	581.3528	1160.6911	2	-0.0006	10	0	GluC
H4_MOUSE	Histone H4	TRGVLKVFLE	633	3.30E-04	387.9043	1160.6911	3	-0.0006	10	0	GluC
H4_MOUSE	Histone H4	TRGVLKVFLE	633	1.70E-04	581.3529	1160.6912	2	-0.0006	10	0	GluC
H4_MOUSE	Histone H4	TRGVLKVFLE	633	1.50E-04	581.3528	1160.6911	2	-0.0006	10	0	GluC
H4_MOUSE	Histone H4	TRGVLKVFLE	633	5.80E-06	387.9043	1160.6912	3	-0.0006	10	0	GluC
H4_MOUSE	Histone H4	TRGVLKVFLE	633	8.70E-03	581.3528	1160.6911	2	-0.0007	10	0	GluC
H4_MOUSE	Histone H4	TRGVLKVFLE	633	4.50E-03	581.3528	1160.6910	2	-0.0007	10	0	GluC
H4_MOUSE	Histone H4	TRGVLKVFLE	633	2.20E-03	387.9043	1160.6910	3	-0.0007	10	0	GluC

H4_MOUSE	Histone H4	TRGVLKVFLE	633	1.90E-03	581.3528	1160.6911	2	-0.0007	10	0	GluC
H4_MOUSE	Histone H4	TRGVLKVFLE	633	8.10E-04	581.3528	1160.6911	2	-0.0007	10	0	GluC
H4_MOUSE	Histone H4	TRGVLKVFLE	633	3.90E-04	581.3528	1160.6910	2	-0.0007	10	0	GluC
H4_MOUSE	Histone H4	TRGVLKVFLE	633	1.70E-04	581.3528	1160.6910	2	-0.0007	10	0	GluC
H4_MOUSE	Histone H4	TRGVLKVFLE	633	1.50E-04	581.3528	1160.6911	2	-0.0007	10	0	GluC
H4_MOUSE	Histone H4	TRGVLKVFLE	633	4.10E-03	387.9043	1160.6910	3	-0.0008	10	0	GluC
H4_MOUSE	Histone H4	TRGVLKVFLE	633	3.50E-03	581.3527	1160.6909	2	-0.0008	10	0	GluC
H4_MOUSE	Histone H4	TRGVLKVFLE	633	2.10E-03	581.3528	1160.6910	2	-0.0008	10	0	GluC
H4_MOUSE	Histone H4	TRGVLKVFLE	633	1.00E-03	387.9043	1160.6910	3	-0.0008	10	0	GluC
H4_MOUSE	Histone H4	TRGVLKVFLE	633	3.00E-04	387.9043	1160.6910	3	-0.0008	10	0	GluC
H4_MOUSE	Histone H4	TRGVLKVFLE	633	7.20E-05	581.3527	1160.6909	2	-0.0008	10	0	GluC
H4_MOUSE	Histone H4	TRGVLKVFLE	633	1.60E-06	387.9043	1160.6910	3	-0.0008	10	0	GluC
H4_MOUSE	Histone H4	TRGVLKVFLE	633	1.00E-02	387.9042	1160.6908	3	-0.0009	10	0	GluC
H4_MOUSE	Histone H4	TRGVLKVFLE	633	8.10E-03	387.9042	1160.6908	3	-0.0009	10	0	GluC
H4_MOUSE	Histone H4	TRGVLKVFLE	633	3.40E-03	387.9042	1160.6909	3	-0.0009	10	0	GluC
H4_MOUSE	Histone H4	TRGVLKVFLE	633	1.90E-03	387.9042	1160.6908	3	-0.0009	10	0	GluC
H4_MOUSE	Histone H4	TRGVLKVFLE	633	1.30E-03	387.9042	1160.6908	3	-0.0009	10	0	GluC
H4_MOUSE	Histone H4	TRGVLKVFLE	633	1.20E-03	581.3527	1160.6908	2	-0.0009	10	0	GluC
H4_MOUSE	Histone H4	TRGVLKVFLE	633	5.40E-04	387.9042	1160.6908	3	-0.0009	10	0	GluC
H4_MOUSE	Histone H4	TRGVLKVFLE	633	2.90E-04	387.9042	1160.6908	3	-0.0009	10	0	GluC
H4_MOUSE	Histone H4	TRGVLKVFLE	633	1.60E-04	387.9042	1160.6908	3	-0.0009	10	0	GluC
H4_MOUSE	Histone H4	TRGVLKVFLE	633	4.20E-05	581.3527	1160.6908	2	-0.0009	10	0	GluC
H4_MOUSE	Histone H4	TRGVLKVFLE	633	6.10E-03	581.3526	1160.6907	2	-0.0010	10	0	GluC
H4_MOUSE	Histone H4	TRGVLKVFLE	633	5.80E-03	581.3526	1160.6907	2	-0.0011	10	0	GluC
H4_MOUSE	Histone H4	TRGVLKVFLE	633	5.20E-03	581.3526	1160.6907	2	-0.0011	10	0	GluC
H4_MOUSE	Histone H4	TRGVLKVFLE	633	3.90E-03	387.9042	1160.6907	3	-0.0011	10	0	GluC
H4_MOUSE	Histone H4	TRGVLKVFLE	633	2.80E-04	581.3526	1160.6907	2	-0.0011	10	0	GluC
H4_MOUSE	Histone H4	TRGVLKVFLE	633	2.20E-04	387.9042	1160.6906	3	-0.0011	10	0	GluC
H4_MOUSE	Histone H4	TRGVLKVFLE	633	6.40E-03	387.9041	1160.6906	3	-0.0012	10	0	GluC
H4_MOUSE	Histone H4	TRGVLKVFLE	633	2.00E-04	581.3525	1160.6905	2	-0.0012	10	0	GluC
H4_MOUSE	Histone H4	TRGVLKVFLE	633	1.40E-04	387.9041	1160.6905	3	-0.0012	10	0	GluC
H4_MOUSE	Histone H4	TRGVLKVFLE	633	2.20E-05	387.9041	1160.6906	3	-0.0012	10	0	GluC
H4_MOUSE	Histone H4	TRGVLKVFLE	633	8.90E-06	387.9041	1160.6906	3	-0.0012	10	0	GluC
H4_MOUSE	Histone H4	TRGVLKVFLE	633	6.10E-03	581.3525	1160.6904	2	-0.0013	10	0	GluC
H4_MOUSE	Histone H4	TRGVLKVFLE	633	3.60E-04	581.3525	1160.6904	2	-0.0013	10	0	GluC
H4_MOUSE	Histone H4	TRGVLKVFLE	633	4.80E-03	387.904	1160.6903	3	-0.0014	10	0	GluC
H4_MOUSE	Histone H4	TRGVLKVFLE	633	1.00E-04	581.3524	1160.6903	2	-0.0014	10	0	GluC
H4_MOUSE	Histone H4	TRGVLKVFLE	633	1.30E-05	387.904	1160.6903	3	-0.0014	10	0	GluC
H4_MOUSE	Histone H4	TRGVLKVFLE	633	4.30E-04	387.904	1160.6901	3	-0.0016	10	0	GluC
H4_MOUSE	Histone H4	TRGVLKVFLE	633	6.70E-03	581.3523	1160.6900	2	-0.0017	10	0	GluC
H4_MOUSE	Histone H4	TRGVLKVFLE	633	3.30E-04	581.3523	1160.6900	2	-0.0017	10	0	GluC
H4_MOUSE	Histone H4	TRGVLKVFLE	633	2.70E-04	387.904	1160.6901	3	-0.0017	10	0	GluC
H4_MOUSE	Histone H4	TRGVLKVFLE	633	1.20E-04	581.3523	1160.6900	2	-0.0017	10	0	GluC
H4_MOUSE	Histone H4	TRGVLKVFLE	633	1.30E-05	387.904	1160.6900	3	-0.0017	10	0	GluC
H4_MOUSE	Histone H4	TRGVLKVFLE	633	3.70E-06	581.3523	1160.6900	2	-0.0017	10	0	GluC
H4_MOUSE	Histone H4	TRGVLKVFLE	633	3.80E-03	387.9039	1160.6899	3	-0.0018	10	0	GluC
H4_MOUSE	Histone H4	TRGVLKVFLE	633	1.90E-05	387.9039	1160.6899	3	-0.0018	10	0	GluC
H4_MOUSE	Histone H4	GLGKGGAKR	4	9.70E-04	422.2619	842.5092	2	0.0006	9	2	Semi-tryptic
H4_MOUSE	Histone H4	GLGKGGAKR	4	8.20E-04	422.2618	842.5090	2	0.0003	9	2	Semi-tryptic
H4_MOUSE	Histone H4	GLGKGGAKR	4	7.80E-04	422.2617	842.5089	2	0.0002	9	2	Semi-tryptic
H4_MOUSE	Histone H4	GLGKGGAKR	4	2.50E-03	422.2612	842.5079	2	-0.0008	9	2	Semi-tryptic
H4_MOUSE	Histone H4	IYEETRGVL	15	7.90E-03	540.2934	1078.5723	2	0.0064	9	1	Chymotrypsin
H4_MOUSE	Histone H4	IYEETRGVL	15	3.30E-03	540.2931	1078.5716	2	0.0058	9	1	Chymotrypsin
H4_MOUSE	Histone H4	IYEETRGVL	15	5.40E-03	540.2922	1078.5699	2	0.0041	9	1	Chymotrypsin
H4_MOUSE	Histone H4	IYEETRGVL	15	3.70E-03	540.292	1078.5695	2	0.0036	9	1	Chymotrypsin
H4_MOUSE	Histone H4	IYEETRGVL	15	3.40E-03	540.292	1078.5695	2	0.0036	9	1	Chymotrypsin
H4_MOUSE	Histone H4	IYEETRGVL	15	6.30E-03	540.2917	1078.5688	2	0.0030	9	1	Chymotrypsin
H4_MOUSE	Histone H4	IYEETRGVL	15	1.00E-03	540.2917	1078.5689	2	0.0030	9	1	Chymotrypsin
H4_MOUSE	Histone H4	IYEETRGVL	15	4.30E-04	540.2917	1078.5689	2	0.0030	9	1	Chymotrypsin
H4_MOUSE	Histone H4	IYEETRGVL	15	2.40E-04	540.2916	1078.5686	2	0.0028	9	1	Chymotrypsin
H4_MOUSE	Histone H4	IYEETRGVL	15	9.40E-03	540.2916	1078.5686	2	0.0027	9	1	Chymotrypsin
H4_MOUSE	Histone H4	IYEETRGVL	15	2.60E-03	540.2908	1078.5671	2	0.0013	9	1	Chymotrypsin
H4_MOUSE	Histone H4	IYEETRGVL	15	1.80E-03	540.2904	1078.5663	2	0.0004	9	1	Chymotrypsin
H4_MOUSE	Histone H4	IYEETRGVL	15	2.00E-03	540.2902	1078.5659	2	0.0000	9	1	Chymotrypsin
H4_MOUSE	Histone H4	IYEETRGVL	15	3.40E-04	540.2902	1078.5659	2	0.0000	9	1	Chymotrypsin
H4_MOUSE	Histone H4	IYEETRGVL	15	1.10E-03	540.2901	1078.5656	2	-0.0002	9	1	Chymotrypsin
H4_MOUSE	Histone H4	VFLENVIR	20	4.70E-07	495.291	988.5675	2	-0.0030	8	0	Semi-tryptic
H4_MOUSE	Histone H4	VFLENVIR	20	2.60E-04	495.2949	988.5752	2	0.0047	8	0	Semi-tryptic
H4_MOUSE	Histone H4	VFLENVIR	20	4.70E-03	495.2939	988.5732	2	0.0027	8	0	Semi-tryptic
H4_MOUSE	Histone H4	VFLENVIR	20	2.10E-06	495.2928	988.5710	2	0.0005	8	0	Semi-tryptic
H4_MOUSE	Histone H4	VFLENVIR	20	5.10E-07	495.2927	988.5709	2	0.0004	8	0	Semi-tryptic
H4_MOUSE	Histone H4	VFLENVIR	20	4.60E-07	495.2928	988.5710	2	0.0004	8	0	Semi-tryptic
H4_MOUSE	Histone H4	VFLENVIR	20	4.60E-07	495.2926	988.5706	2	0.0001	8	0	Semi-tryptic
H4_MOUSE	Histone H4	VFLENVIR	20	3.60E-06	495.2926	988.5705	2	0.0000	8	0	Semi-tryptic

H4_MOUSE	Histone H4	VFLENVIR	20	4.60E-07	495.2925	988.5705	2	0.0000	8	0	Semi-tryptic
H4_MOUSE	Histone H4	VFLENVIR	20	3.30E-04	495.2925	988.5705	2	-0.0001	8	0	Semi-tryptic
H4_MOUSE	Histone H4	VFLENVIR	20	4.30E-07	495.2925	988.5704	2	-0.0001	8	0	Semi-tryptic
H4_MOUSE	Histone H4	VFLENVIR	20	3.10E-05	495.2925	988.5704	2	-0.0002	8	0	Semi-tryptic
H4_MOUSE	Histone H4	VFLENVIR	20	1.00E-06	495.2923	988.5701	2	-0.0004	8	0	Semi-tryptic
H4_MOUSE	Histone H4	VFLENVIR	20	9.00E-07	495.2923	988.5700	2	-0.0005	8	0	Semi-tryptic
H4_MOUSE	Histone H4	VFLENVIR	20	1.10E-06	495.2922	988.5699	2	-0.0006	8	0	Semi-tryptic
H4_MOUSE	Histone H4	VFLENVIR	20	6.30E-06	495.2922	988.5698	2	-0.0007	8	0	Semi-tryptic
H4_MOUSE	Histone H4	VFLENVIR	20	1.10E-06	495.2922	988.5699	2	-0.0007	8	0	Semi-tryptic
H4_MOUSE	Histone H4	VFLENVIR	20	1.00E-06	495.2922	988.5699	2	-0.0007	8	0	Semi-tryptic
H4_MOUSE	Histone H4	VFLENVIR	20	5.00E-07	495.2921	988.5696	2	-0.0009	8	0	Semi-tryptic
H4_MOUSE	Histone H4	VFLENVIR	20	5.70E-06	495.2921	988.5696	2	-0.0010	8	0	Semi-tryptic
