# Supplementary material for: Identification of Combinatorial Patterns of Post-Translational Modifications on Individual Histones in the Mouse Brain
Source: PLoS One. 2012 May 31;7(5):e36980. doi: 10.1371/journal.pone.0036980 (PMC3365036; doi:10.1371/journal.pone.0036980)
Supplement: Figure S5 — MS/MS spectra and ion tables of all modified peptides, i.e. those with one or more PTMs, obtained from histone digests across all experiments. The type of modification is indicated after the residue modified e.g. Sphospho for a phosphorylated serine. N-terminal acetylation is indicated by square brackets e.g. [Acetyl]. Ambiguous residues are indicated by parentheses e.g. (acetyl). (PDF) [file pone.0036980.s005.pdf]

sp|P15864|H12_MOUSE

AS $^{Acetyl}_{42.01}$ GPPVSELITK

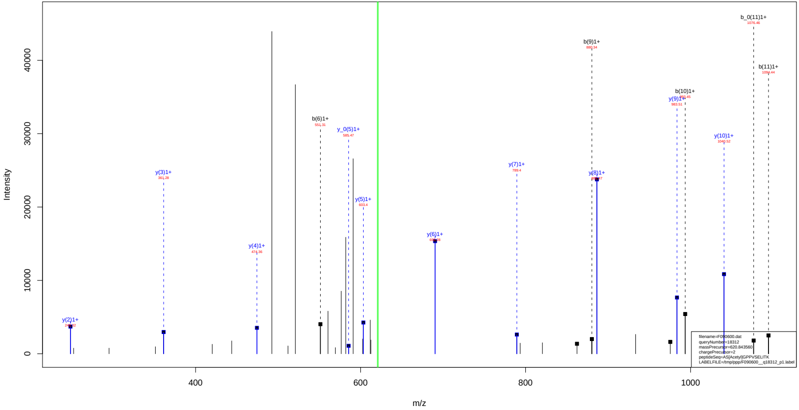

sp|P15864|H12_MOUSE

AS $^{Acetyl}_{42.01}$ GPPVSELITK

- fragmentation table for charge state 1+
- specType=cid
- score=73.84
- F090600.dat
- query=q18312_p1
- precursor=620.843560
- chargePrecursor=2
- itol=0.5

AA	a	a*	a_0	b	b*	b_0	y	y*	y_0	AA
A[1]	44.049	0.000	0.000	72.044	0.000	0.000	1240.678	1223.652	1222.668	A[12]
S[2]	173.092	0.000	155.082	201.087	0.000	183.076	1169.641	1152.615	1151.631	S[11]
G[3]	230.114	0.000	212.103	258.108	0.000	240.098	**1040.599**	1023.572	1022.588	G[10]
P[4]	327.166	0.000	309.156	355.161	0.000	337.151	**983.577**	966.551	965.567	P[9]
P[5]	424.219	0.000	406.208	452.214	0.000	434.203	**886.524**	869.498	868.514	P[8]
V[6]	523.287	0.000	505.277	**551.282**	0.000	533.272	**789.472**	772.445	771.461	V[7]
S[7]	610.320	0.000	592.309	638.314	0.000	620.304	**690.403**	673.377	672.393	S[6]
E[8]	739.362	0.000	721.352	767.357	0.000	749.346	**603.371**	586.345	**585.361**	E[5]
L[9]	852.446	0.000	834.436	**880.441**	0.000	**862.431**	**474.329**	457.302	456.318	L[4]
I[10]	965.530	0.000	947.520	**993.525**	0.000	**975.515**	**361.245**	344.218	343.234	I[3]
T[11]	1066.578	0.000	1048.567	**1094.573**	0.000	**1076.562**	**248.160**	231.134	230.150	T[2]
K[12]	1194.673	1177.646	1176.662	1222.668	1205.641	1204.657	147.113	130.086	0.000	K[1]

sp|P15864|H12_MOUSE

KASGPPVS $^{\text{Acetyl}}_{42.01}$ ELITK

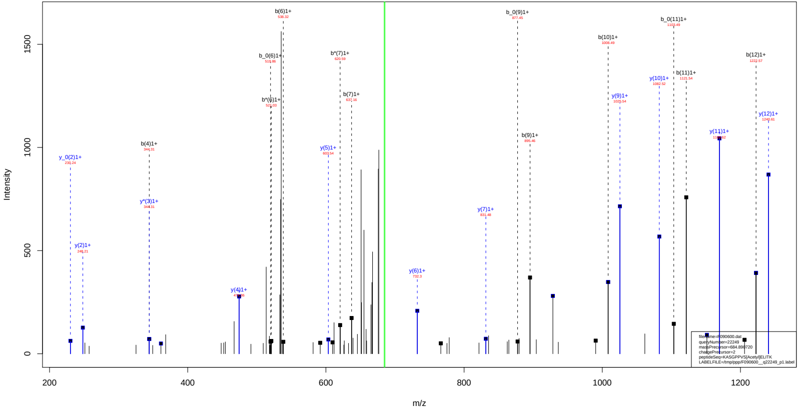

sp|P15864|H12_MOUSE

KASGPPVS $^{\text{Acetyl}}_{42.01}$ ELITK

- fragmentation table for charge state 1+
- specType=cid
- score=81.97
- F090600.dat
- query=q22249_p1
- precursor=684.890720
- chargePrecursor=2
- itol=0.5

AA	a	a*	a_0	b	b*	b_0	y	y*	y_0	AA
K[1]	101.107	84.081	0.000	129.102	112.076	0.000	1368.773	1351.747	1350.763	K[13]
A[2]	172.144	155.118	0.000	200.139	183.113	0.000	**1240.678**	1223.652	**1222.668**	A[12]
S[3]	259.176	242.150	241.166	287.171	270.145	269.161	**1169.641**	1152.615	**1151.631**	S[11]
G[4]	316.198	299.171	298.187	**344.193**	327.166	326.182	**1082.609**	1065.583	1064.599	G[10]
P[5]	413.251	396.224	395.240	441.246	424.219	423.235	**1025.588**	**1008.561**	1007.577	P[9]
P[6]	510.303	493.277	492.293	**538.298**	**521.272**	**520.288**	**928.535**	911.508	910.524	P[8]
V[7]	**609.372**	592.345	**591.361**	**637.367**	**620.340**	619.356	**831.482**	814.456	813.472	V[7]
S[8]	738.414	721.388	720.404	**766.409**	749.383	748.399	**732.414**	715.387	714.403	S[6]
E[9]	867.457	850.431	849.446	**895.452**	878.425	**877.441**	**603.371**	586.345	585.361	E[5]
L[10]	980.541	963.515	962.531	**1008.536**	991.509	**990.525**	**474.329**	457.302	456.318	L[4]
I[11]	1093.625	1076.599	1075.615	**1121.620**	1104.594	**1103.610**	**361.245**	**344.218**	343.234	I[3]
T[12]	1194.673	1177.646	1176.662	**1222.668**	**1205.641**	1204.657	**248.160**	231.134	**230.150**	T[2]
K[13]	1322.768	1305.741	1304.757	1350.763	1333.736	1332.752	147.113	130.086	0.000	K[1]

sp|P70696|H2B1A_MOUSE

HAVS $^{\text{Acetyl}}_{42.01}$ EGTK

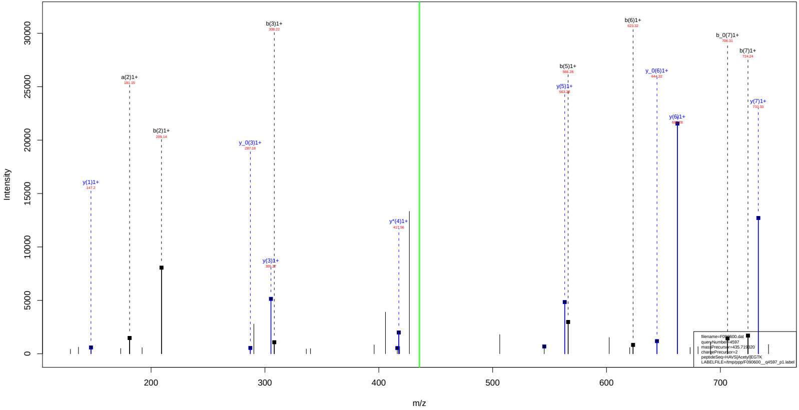

sp|P70696|H2B1A_MOUSE

HAVS $^{\text{Acetyl}}_{42.01}$ EGTK

- fragmentation table for charge state 1+
- specType=cid
- score=40.15
- F090600.dat
- query=q4597_p1
- precursor=435.719320
- chargePrecursor=2
- itol=0.5

AA	a	a*	a_0	b	b*	b_0	y	y*	y_0	AA
H[1]	110.071	0.000	0.000	138.066	0.000	0.000	870.432	853.405	852.421	H[8]
A[2]	**181.108**	0.000	0.000	**209.103**	0.000	0.000	**733.373**	716.346	715.362	A[7]
V[3]	280.177	0.000	0.000	**308.172**	0.000	0.000	**662.336**	645.309	**644.325**	V[6]
S[4]	409.219	0.000	391.209	437.214	0.000	419.204	**563.267**	546.241	**545.257**	S[5]
E[5]	538.262	0.000	520.251	**566.257**	0.000	548.246	434.225	**417.198**	**416.214**	E[4]
G[6]	595.283	0.000	577.273	**623.278**	0.000	605.268	**305.182**	288.155	**287.171**	G[3]
T[7]	696.331	0.000	678.321	**724.326**	0.000	**706.315**	248.160	231.134	230.150	T[2]
K[8]	824.426	807.400	806.416	852.421	835.394	834.410	**147.113**	130.086	0.000	K[1]

sp|P27661|H2AX_MOUSE

AS $^{\text{Acetyl}}_{42.01}$ QASQEY

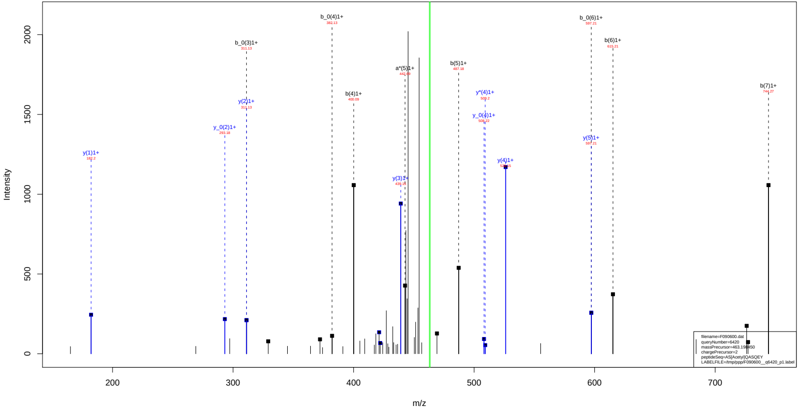

sp|P27661|H2AX_MOUSE

AS $^{Acetyl}_{42.01}$ QASQEY

- fragmentation table for charge state 1+
- specType=cid
- score=44.68
- F090600.dat
- query=q6420_p1
- precursor=463.198450
- chargePrecursor=2
- itol=0.5

AA	a	a*	a_0	b	b*	b_0	y	y*	y_0	AA
A[1]	44.049	0.000	0.000	72.044	0.000	0.000	925.390	908.363	907.379	A[8]
S[2]	173.092	0.000	155.082	201.087	0.000	183.076	854.353	837.326	836.342	S[7]
Q[3]	301.151	284.124	283.140	**329.146**	312.119	**311.135**	725.310	708.284	707.299	Q[6]
A[4]	**372.188**	355.161	354.177	**400.183**	383.156	**382.172**	**597.251**	580.225	579.241	A[5]
S[5]	459.220	**442.193**	441.209	**487.215**	470.188	**469.204**	**526.214**	**509.188**	**508.204**	S[4]
Q[6]	587.278	570.252	569.268	**615.273**	598.247	**597.263**	**439.182**	**422.156**	**421.172**	Q[3]
E[7]	716.321	699.294	698.310	**744.316**	**727.289**	**726.305**	**311.124**	0.000	**293.113**	E[2]
Y[8]	879.384	862.358	861.374	907.379	890.353	889.369	**182.081**	0.000	0.000	Y[1]

sp|P10922|H10_MOUSE

[Acetyl]MTENSTSAPAAKPK

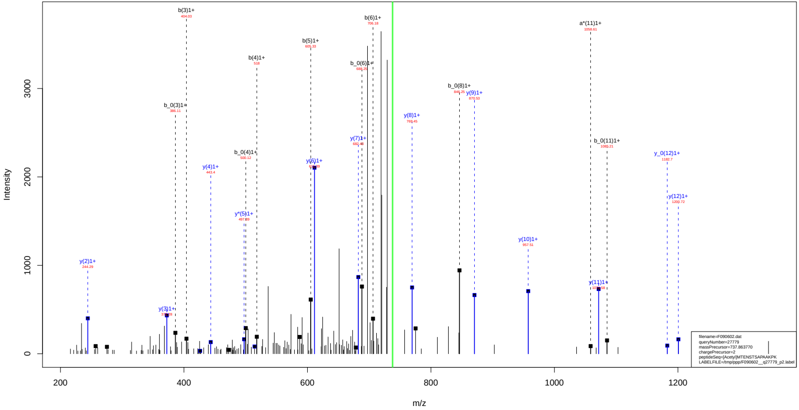

sp|P10922|H10_MOUSE

[Acetyl]MTENSTSAPAAKPK

- fragmentation table for charge state 1+
- specType=cid
- score=59.60
- F090602.dat
- query=q27779_p2
- precursor=737.863770
- chargePrecursor=2
- itol=0.5

AA	a	a*	a_0	b	b*	b_0	y	y*	y_0	AA
M[1]	146.063	0.000	0.000	174.058	0.000	0.000	1474.721	1457.694	1456.710	M[14]
T[2]	247.111	0.000	229.101	**275.106**	0.000	**257.095**	1301.670	1284.643	1283.659	T[13]
E[3]	376.154	0.000	358.143	**404.149**	0.000	**386.138**	**1200.622**	1183.595	**1182.611**	E[12]
N[4]	490.197	473.170	**472.186**	**518.192**	501.165	**500.181**	**1071.579**	1054.553	1053.569	N[11]
S[5]	577.229	560.202	559.218	**605.224**	588.197	**587.213**	**957.536**	940.510	939.526	S[10]
T[6]	**678.276**	661.250	660.266	**706.271**	689.245	**688.261**	**870.504**	853.478	852.494	T[9]
S[7]	765.308	748.282	747.298	793.303	776.277	**775.293**	**769.457**	752.430	751.446	S[8]
A[8]	836.345	819.319	818.335	864.340	847.314	**846.330**	**682.425**	665.398	0.000	A[7]
P[9]	933.398	916.372	915.388	961.393	944.367	943.383	**611.388**	594.361	0.000	P[6]
A[10]	1004.435	987.409	986.425	1032.430	1015.404	1014.420	**514.335**	**497.308**	0.000	A[5]
A[11]	1075.472	**1058.446**	1057.462	1103.467	1086.441	**1085.457**	**443.298**	**426.271**	0.000	A[4]
K[12]	1203.567	1186.541	1185.557	1231.562	1214.536	1213.552	**372.261**	355.234	0.000	K[3]
P[13]	1300.620	1283.594	1282.610	1328.615	1311.589	1310.605	**244.166**	227.139	0.000	P[2]
K[14]	1428.715	1411.689	1410.705	1456.710	1439.684	1438.699	147.113	130.086	0.000	K[1]

sp|P43277|H13_MOUSE

[Acetyl]S $^{Acetyl}_{42.01}$ ETAPAAPAAPAPVEKTPVK

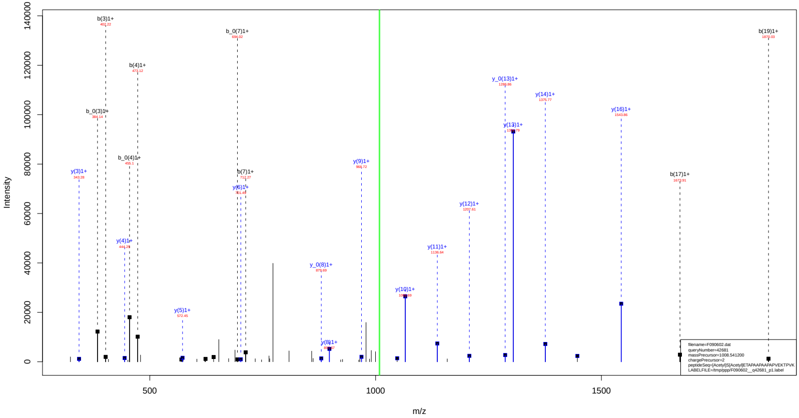

sp|P43277|H13_MOUSE

[Acetyl]S $^{Acetyl}_{42.01}$ ETAPAAPAAPAPVEKTPVK

- fragmentation table for charge state 1+
- specType=cid
- score=105.69
- F090602.dat
- query=q42681_p1
- precursor=1008.541200
- chargePrecursor=2
- itol=0.5

AA	a	a*	a_0	b	b*	b_0	y	y*	y_0	AA
S[1]	144.066	0.000	126.055	172.060	0.000	154.050	2016.065	1999.038	1998.054	S[20]
E[2]	273.108	0.000	255.098	301.103	0.000	283.092	1845.012	1827.985	1827.001	E[19]
T[3]	374.156	0.000	356.145	**402.151**	0.000	**384.140**	1715.969	1698.943	1697.958	T[18]
A[4]	445.193	0.000	427.182	**473.188**	0.000	**455.177**	1614.921	1597.895	1596.911	A[17]
P[5]	542.246	0.000	524.235	**570.241**	0.000	552.230	**1543.884**	1526.858	1525.874	P[16]
A[6]	613.283	0.000	595.272	**641.278**	0.000	**623.267**	**1446.831**	1429.805	1428.821	A[15]
A[7]	684.320	0.000	666.309	**712.315**	0.000	**694.304**	**1375.794**	1358.768	1357.784	A[14]
P[8]	781.373	0.000	763.362	809.368	0.000	791.357	**1304.757**	1287.731	**1286.747**	P[13]
A[9]	852.410	0.000	834.399	880.405	0.000	862.394	**1207.705**	1190.678	1189.694	A[12]
A[10]	923.447	0.000	905.436	951.442	0.000	933.431	**1136.667**	1119.641	1118.657	A[11]
P[11]	1020.500	0.000	1002.489	1048.495	0.000	1030.484	**1065.630**	1048.604	**1047.620**	P[10]
A[12]	1091.537	0.000	1073.526	1119.532	0.000	1101.521	**968.578**	951.551	950.567	A[9]
P[13]	1188.590	0.000	1170.579	1216.584	0.000	1198.574	**897.540**	880.514	**879.530**	P[8]
V[14]	1287.658	0.000	1269.647	1315.653	0.000	1297.642	800.488	783.461	782.477	V[7]
E[15]	1416.701	0.000	1398.690	1444.695	0.000	1426.685	**701.419**	684.393	683.409	E[6]
K[16]	1544.796	1527.769	1526.785	1572.790	1555.764	1554.780	**572.377**	555.350	554.366	K[5]
T[17]	1645.843	1628.817	1627.833	**1673.838**	1656.812	1655.828	**444.282**	427.255	426.271	T[4]
P[18]	1742.896	1725.869	1724.885	1770.891	1753.864	1752.880	**343.234**	326.207	0.000	P[3]
V[19]	1841.964	1824.938	1823.954	**1869.959**	1852.933	1851.949	246.181	229.155	0.000	V[2]
K[20]	1970.059	1953.033	1952.049	1998.054	1981.028	1980.044	147.113	130.086	0.000	K[1]

sp|P27661|H2AX_MOUSE

KAS $^{\text{Phospho}}_{79.97}$ QASQEY

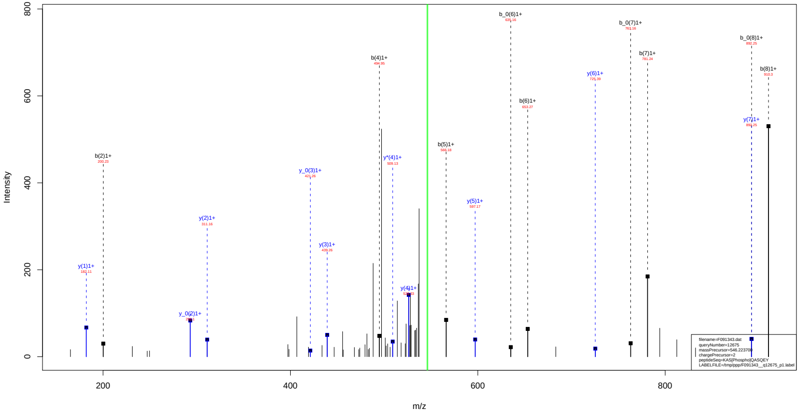

sp|P27661|H2AX_MOUSE

KAS $^{Phospho}_{79.97}$ QASQEY

- fragmentation table for charge state 1+
- specType=cid
- score=59.60
- F091343.dat
- query=q12675_p1
- precursor=546.223700
- chargePrecursor=2
- itol=0.5

AA	a	a*	a_0	b	b*	b_0	y	y*	y_0	AA
K[1]	101.107	84.081	0.000	129.102	112.076	0.000	1091.440	1074.414	1073.430	K[9]
A[2]	172.144	155.118	0.000	**200.139**	183.113	0.000	963.346	946.319	945.335	A[8]
S[3]	339.143	322.116	321.132	367.138	350.111	349.127	**892.308**	875.282	874.298	S[7]
Q[4]	467.201	450.175	449.191	**495.196**	478.170	477.186	**725.310**	708.284	707.299	Q[6]
A[5]	538.238	521.212	520.228	**566.233**	549.207	548.223	**597.251**	580.225	579.241	A[5]
S[6]	625.271	608.244	607.260	**653.265**	636.239	**635.255**	**526.214**	**509.188**	508.204	S[4]
Q[7]	753.329	736.303	735.319	**781.324**	764.297	**763.313**	**439.182**	422.156	**421.172**	Q[3]
E[8]	882.372	865.345	864.361	**910.367**	893.340	**892.356**	**311.124**	0.000	**293.113**	E[2]
Y[9]	1045.435	1028.408	1027.424	1073.430	1056.403	1055.419	**182.081**	0.000	0.000	Y[1]

sp|P27661|H2AX_MOUSE

KASQAS $^{\text{Phospho}}_{79.97}$ QEY

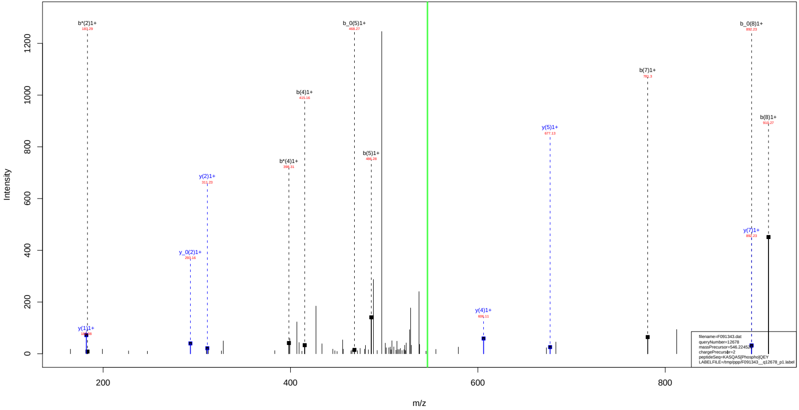

sp|P27661|H2AX_MOUSE

KASQAS $^{Phospho}_{79.97}$ QEY

- fragmentation table for charge state 1+
- specType=cid
- score=35.79
- F091343.dat
- query=q12678_p1
- precursor=546.224520
- chargePrecursor=2
- itol=0.5

AA	a	a*	a_0	b	b*	b_0	y	y*	y_0	AA
K[1]	101.107	84.081	0.000	129.102	112.076	0.000	1091.440	1074.414	1073.430	K[9]
A[2]	172.144	155.118	0.000	200.139	**183.113**	0.000	963.346	946.319	945.335	A[8]
S[3]	259.176	242.150	241.166	287.171	270.145	269.161	**892.308**	875.282	874.298	S[7]
Q[4]	387.235	370.208	369.224	**415.230**	**398.203**	397.219	805.276	788.250	787.266	Q[6]
A[5]	458.272	441.246	440.262	**486.267**	469.241	**468.257**	**677.218**	660.191	659.207	A[5]
S[6]	625.271	608.244	607.260	653.265	636.239	635.255	**606.181**	589.154	588.170	S[4]
Q[7]	753.329	736.303	735.319	**781.324**	764.297	763.313	439.182	422.156	421.172	Q[3]
E[8]	882.372	865.345	864.361	**910.367**	893.340	**892.356**	**311.124**	0.000	**293.113**	E[2]
Y[9]	1045.435	1028.408	1027.424	1073.430	1056.403	1055.419	**182.081**	0.000	0.000	Y[1]

sp|P43274|H14_MOUSE

KAPKS $^{\text{Phospho}}_{79.97}$ PAKAK

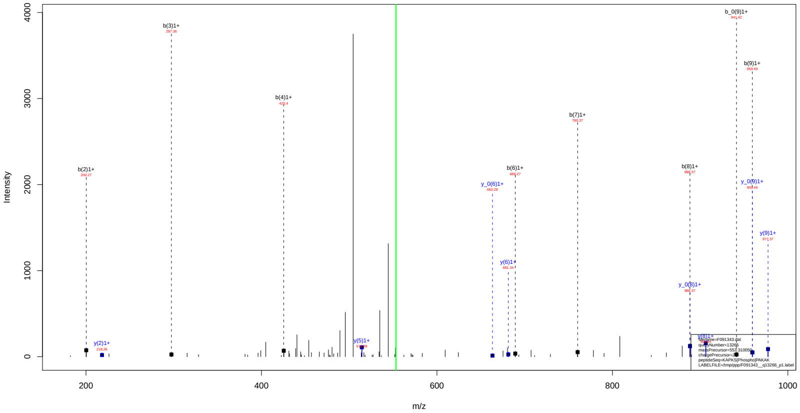

sp|P43274|H14_MOUSE

KAPKS $^{Phospho}_{79.97}$ PAKAK

- fragmentation table for charge state 1+
- specType=cid
- score=47.55
- F091343.dat
- query=q13266_p1
- precursor=553.310050
- chargePrecursor=2
- itol=0.5

AA	a	a*	a_0	b	b*	b_0	y	y*	y_0	AA
K[1]	101.107	84.081	0.000	129.102	112.076	0.000	1105.613	1088.586	1087.602	K[10]
A[2]	172.144	155.118	0.000	**200.139**	183.113	0.000	**977.518**	960.491	**959.507**	A[9]
P[3]	269.197	252.171	0.000	**297.192**	280.166	0.000	**906.481**	889.454	**888.470**	P[8]
K[4]	397.292	380.266	0.000	**425.287**	408.261	0.000	809.428	792.402	791.418	K[7]
S[5]	564.291	547.264	546.280	592.285	575.259	574.275	**681.333**	664.307	**663.323**	S[6]
P[6]	661.343	644.317	643.333	**689.338**	672.312	671.328	**514.335**	497.308	0.000	P[5]
A[7]	732.380	715.354	714.370	**760.375**	743.349	742.365	417.282	400.255	0.000	A[4]
K[8]	860.475	843.449	842.465	**888.470**	871.444	870.460	346.245	329.218	0.000	K[3]
A[9]	931.512	914.486	913.502	**959.507**	942.481	**941.497**	**218.150**	201.123	0.000	A[2]
K[10]	1059.607	1042.581	1041.597	1087.602	1070.576	1069.592	147.113	130.086	0.000	K[1]

sp|P43274|H14_MOUSE

AKKAPKS$^{\text{Phospho}}_{79.97}$PAK

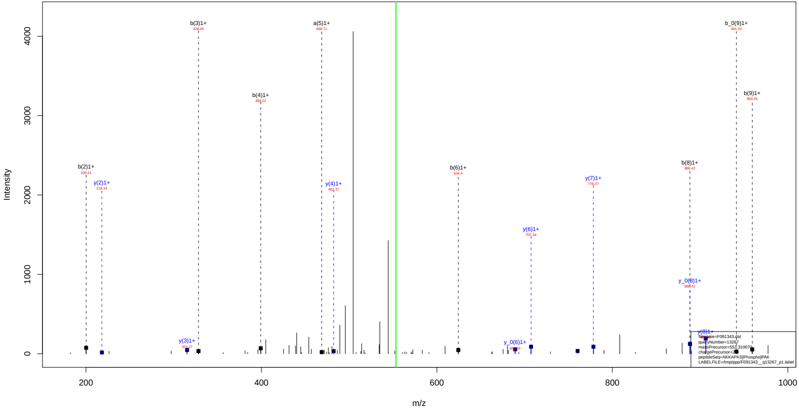

sp|P43274|H14_MOUSE

AKKAPKS $^{Phospho}_{79.97}$ PAK

- fragmentation table for charge state 1+
- specType=cid
- score=44.95
- F091343.dat
- query=q13267_p1
- precursor=553.310070
- chargePrecursor=2
- itol=0.5

AA	a	a*	a_0	b	b*	b_0	y	y*	y_0	AA
A[1]	44.049	0.000	0.000	72.044	0.000	0.000	1105.613	1088.586	1087.602	A[10]
K[2]	172.144	155.118	0.000	**200.139**	183.113	0.000	1034.576	1017.549	1016.565	K[9]
K[3]	300.239	283.213	0.000	**328.234**	311.208	0.000	**906.481**	889.454	**888.470**	K[8]
A[4]	371.277	354.250	0.000	**399.271**	382.245	0.000	**778.386**	761.359	**760.375**	A[7]
P[5]	**468.329**	451.303	0.000	496.324	479.298	0.000	**707.349**	690.322	**689.338**	P[6]
K[6]	596.424	579.398	0.000	**624.419**	607.393	0.000	610.296	593.269	592.285	K[5]
S[7]	763.423	746.396	745.412	791.418	774.391	773.407	**482.201**	465.174	464.190	S[4]
P[8]	860.475	843.449	842.465	**888.470**	871.444	870.460	**315.203**	298.176	0.000	P[3]
A[9]	931.512	914.486	913.502	**959.507**	942.481	**941.497**	**218.150**	201.123	0.000	A[2]
K[10]	1059.607	1042.581	1041.597	1087.602	1070.576	1069.592	147.113	130.086	0.000	K[1]

sp|P43274|H14_MOUSE

KAPKS $^{Phospho}_{79.97}$ PAKAK

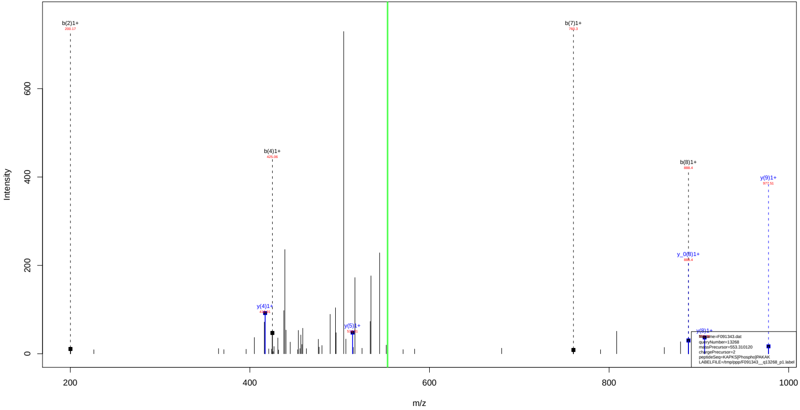

sp|P43274|H14_MOUSE

KAPKS $^{\text{Phospho}}_{79.97}$ PAKAK

- fragmentation table for charge state 1+
- specType=cid
- score=25.93
- F091343.dat
- query=q13268_p1
- precursor=553.310120
- chargePrecursor=2
- itol=0.5

AA	a	a*	a_0	b	b*	b_0	y	y*	y_0	AA
K[1]	101.107	84.081	0.000	129.102	112.076	0.000	1105.613	1088.586	1087.602	K[10]
A[2]	172.144	155.118	0.000	**200.139**	183.113	0.000	**977.518**	960.491	959.507	A[9]
P[3]	269.197	252.171	0.000	297.192	280.166	0.000	**906.481**	889.454	**888.470**	P[8]
K[4]	397.292	380.266	0.000	**425.287**	408.261	0.000	809.428	792.402	791.418	K[7]
S[5]	564.291	547.264	546.280	592.285	575.259	574.275	681.333	664.307	663.323	S[6]
P[6]	661.343	644.317	643.333	689.338	672.312	671.328	**514.335**	497.308	0.000	P[5]
A[7]	732.380	715.354	714.370	**760.375**	743.349	742.365	**417.282**	400.255	0.000	A[4]
K[8]	860.475	843.449	842.465	**888.470**	871.444	870.460	346.245	329.218	0.000	K[3]
A[9]	931.512	914.486	913.502	959.507	942.481	941.497	218.150	201.123	0.000	A[2]
K[10]	1059.607	1042.581	1041.597	1087.602	1070.576	1069.592	147.113	130.086	0.000	K[1]

sp|P43274|H14_MOUSE

KAPKS $^{Phospho}_{79.97}$ PAKAK

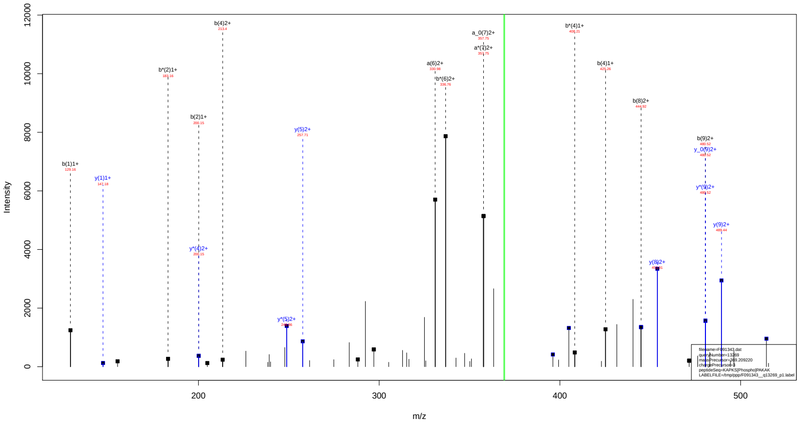

sp|P43274|H14_MOUSE

KAPKS $^{\text{Phospho}}_{79.97}$ PAKAK

- fragmentation table for charge state 1+
- specType=cid
- score=37.29
- F091343.dat
- query=q13269_p1
- precursor=369.209220
- chargePrecursor=3
- itol=0.5

AA	a	a*	a_0	b	b*	b_0	y	y*	y_0	AA
K[1]	101.107	84.081	0.000	**129.102**	112.076	0.000	1105.613	1088.586	1087.602	K[10]
A[2]	172.144	**155.118**	0.000	**200.139**	**183.113**	0.000	977.518	960.491	959.507	A[9]
P[3]	269.197	252.171	0.000	**297.192**	280.166	0.000	906.481	889.454	888.470	P[8]
K[4]	397.292	380.266	0.000	**425.287**	**408.261**	0.000	809.428	792.402	791.418	K[7]
S[5]	564.291	547.264	546.280	592.285	575.259	574.275	681.333	664.307	663.323	S[6]
P[6]	661.343	644.317	643.333	689.338	672.312	671.328	**514.335**	497.308	0.000	P[5]
A[7]	732.380	715.354	714.370	760.375	743.349	742.365	417.282	400.255	0.000	A[4]
K[8]	860.475	843.449	842.465	888.470	871.444	870.460	346.245	329.218	0.000	K[3]
A[9]	931.512	914.486	913.502	959.507	942.481	941.497	218.150	201.123	0.000	A[2]
K[10]	1059.607	1042.581	1041.597	1087.602	1070.576	1069.592	**147.113**	130.086	0.000	K[1]

sp|P43274|H14_MOUSE

KAPKS $^{\text{Phospho}}_{79.97}$ PAKAK

- fragmentation table for charge state 2+
- specType=cid
- score=37.29
- F091343.dat
- query=q13269_p1
- precursor=369.209220
- chargePrecursor=3
- itol=0.5

AA	a	a*	a_0	b	b*	b_0	y	y*	y_0	AA
K[1]	51.057	42.544	0.504	65.055	56.541	0.504	553.310	544.797	544.305	K[10]
A[2]	86.576	78.063	0.504	100.573	92.060	0.504	**489.263**	**480.749**	**480.257**	A[9]
P[3]	135.102	126.589	0.504	149.100	140.586	0.504	**453.744**	**445.231**	**444.739**	P[8]
K[4]	199.150	190.636	0.504	**213.147**	**204.634**	0.504	**405.218**	396.704	**396.212**	K[7]
S[5]	282.649	274.136	273.644	**296.646**	**288.133**	287.641	341.170	332.657	332.165	S[6]
P[6]	**331.175**	322.662	322.170	345.173	**336.659**	336.167	**257.671**	**249.158**	0.504	P[5]
A[7]	366.694	**358.181**	**357.689**	380.691	372.178	371.686	209.145	**200.631**	0.504	A[4]
K[8]	430.741	422.228	421.736	**444.739**	436.226	435.733	173.626	165.113	0.504	K[3]
A[9]	466.260	457.747	457.255	**480.257**	**471.744**	**471.252**	109.579	101.065	0.504	A[2]
K[10]	530.307	521.794	521.302	544.305	535.792	535.300	74.060	65.547	0.504	K[1]

sp|P43274|H14_MOUSE

AKKAPKS $^{\text{Phospho}}_{79.97}$ PAK

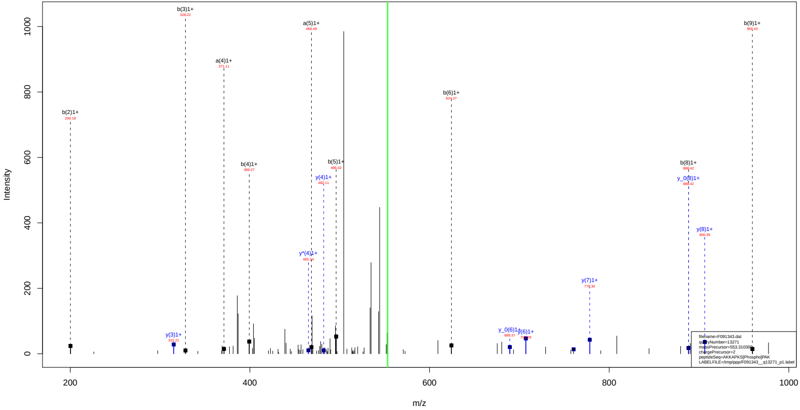

sp|P43274|H14_MOUSE

AKKAPKS $^{Phospho}_{79.97}$ PAK

- fragmentation table for charge state 1+
- specType=cid
- score=40.95
- F091343.dat
- query=q13271_p1
- precursor=553.310300
- chargePrecursor=2
- itol=0.5

AA	a	a*	a_0	b	b*	b_0	y	y*	y_0	AA
A[1]	44.049	0.000	0.000	72.044	0.000	0.000	1105.613	1088.586	1087.602	A[10]
K[2]	172.144	155.118	0.000	**200.139**	183.113	0.000	1034.576	1017.549	1016.565	K[9]
K[3]	300.239	283.213	0.000	**328.234**	311.208	0.000	**906.481**	889.454	**888.470**	K[8]
A[4]	**371.277**	354.250	0.000	**399.271**	382.245	0.000	**778.386**	761.359	**760.375**	A[7]
P[5]	**468.329**	451.303	0.000	**496.324**	479.298	0.000	**707.349**	690.322	**689.338**	P[6]
K[6]	596.424	579.398	0.000	**624.419**	607.393	0.000	610.296	593.269	592.285	K[5]
S[7]	763.423	746.396	745.412	791.418	774.391	773.407	**482.201**	**465.174**	464.190	S[4]
P[8]	860.475	843.449	842.465	**888.470**	871.444	870.460	**315.203**	298.176	0.000	P[3]
A[9]	931.512	914.486	913.502	**959.507**	942.481	941.497	218.150	201.123	0.000	A[2]
K[10]	1059.607	1042.581	1041.597	1087.602	1070.576	1069.592	147.113	130.086	0.000	K[1]

sp|P43274|H14_MOUSE

KAPKS $^{Phospho}_{79.97}$ PAKAK

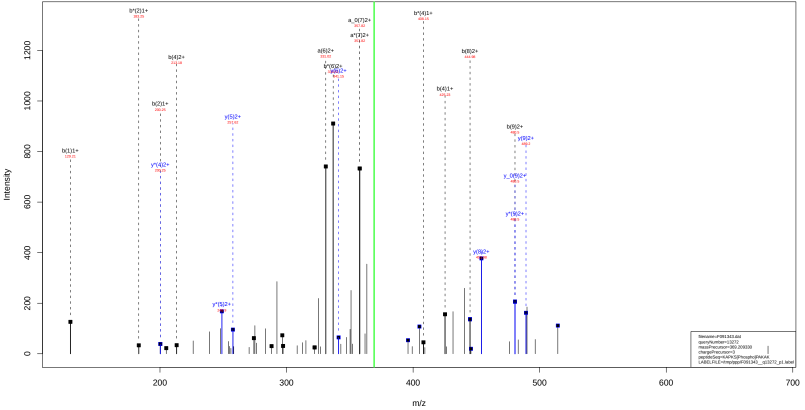

sp|P43274|H14_MOUSE

KAPKS $^{Phospho}_{79.97}$ PAKAK

- fragmentation table for charge state 1+
- specType=cid
- score=25.03
- F091343.dat
- query=q13272_p1
- precursor=369.209330
- chargePrecursor=3
- itol=0.5

AA	a	a*	a_0	b	b*	b_0	y	y*	y_0	AA
K[1]	101.107	84.081	0.000	**129.102**	112.076	0.000	1105.613	1088.586	1087.602	K[10]
A[2]	172.144	155.118	0.000	**200.139**	**183.113**	0.000	977.518	960.491	959.507	A[9]
P[3]	269.197	252.171	0.000	**297.192**	280.166	0.000	906.481	889.454	888.470	P[8]
K[4]	397.292	380.266	0.000	**425.287**	**408.261**	0.000	809.428	792.402	791.418	K[7]
S[5]	564.291	547.264	546.280	592.285	575.259	574.275	681.333	664.307	663.323	S[6]
P[6]	661.343	644.317	643.333	689.338	672.312	671.328	**514.335**	497.308	0.000	P[5]
A[7]	732.380	715.354	714.370	760.375	743.349	742.365	417.282	400.255	0.000	A[4]
K[8]	860.475	843.449	842.465	888.470	871.444	870.460	346.245	329.218	0.000	K[3]
A[9]	931.512	914.486	913.502	959.507	942.481	941.497	218.150	201.123	0.000	A[2]
K[10]	1059.607	1042.581	1041.597	1087.602	1070.576	1069.592	147.113	130.086	0.000	K[1]

sp|P43274|H14_MOUSE

KAPKS $^{Phospho}_{79.97}$ PAKAK

- fragmentation table for charge state 2+
- specType=cid
- score=25.03
- F091343.dat
- query=q13272_p1
- precursor=369.209330
- chargePrecursor=3
- itol=0.5

AA	a	a*	a_0	b	b*	b_0	y	y*	y_0	AA
K[1]	51.057	42.544	0.504	65.055	56.541	0.504	553.310	544.797	544.305	K[10]
A[2]	86.576	78.063	0.504	100.573	92.060	0.504	**489.263**	**480.749**	**480.257**	A[9]
P[3]	135.102	126.589	0.504	149.100	140.586	0.504	**453.744**	**445.231**	**444.739**	P[8]
K[4]	199.150	190.636	0.504	**213.147**	**204.634**	0.504	**405.218**	396.704	**396.212**	K[7]
S[5]	282.649	**274.136**	273.644	**296.646**	**288.133**	287.641	**341.170**	332.657	332.165	S[6]
P[6]	**331.175**	**322.662**	**322.170**	345.173	**336.659**	336.167	**257.671**	**249.158**	0.504	P[5]
A[7]	366.694	**358.181**	**357.689**	380.691	372.178	371.686	209.145	**200.631**	0.504	A[4]
K[8]	430.741	422.228	421.736	**444.739**	436.226	435.733	173.626	165.113	0.504	K[3]
A[9]	466.260	457.747	457.255	**480.257**	471.744	471.252	109.579	101.065	0.504	A[2]
K[10]	530.307	521.794	521.302	544.305	535.792	535.300	74.060	65.547	0.504	K[1]

sp|P43274|H14_MOUSE

KAPKS $^{\text{Phospho}}_{79.97}$ PAKAK

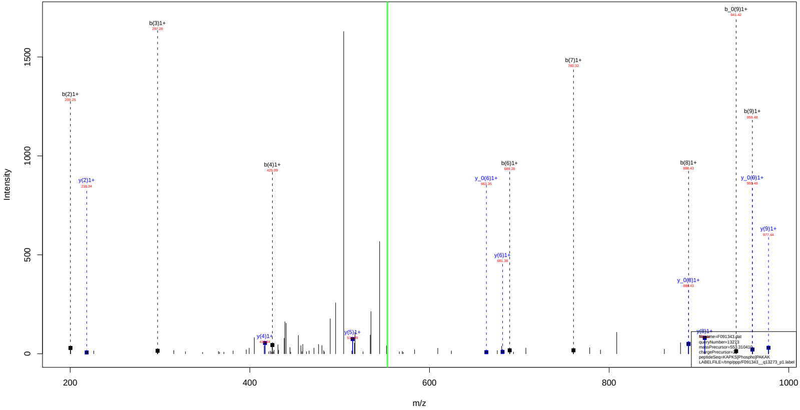

sp|P43274|H14_MOUSE

KAPKS $^{Phospho}_{79.97}$ PAKAK

- fragmentation table for charge state 1+
- specType=cid
- score=45.71
- F091343.dat
- query=q13273_p1
- precursor=553.310410
- chargePrecursor=2
- itol=0.5

AA	a	a*	a_0	b	b*	b_0	y	y*	y_0	AA
K[1]	101.107	84.081	0.000	129.102	112.076	0.000	1105.613	1088.586	1087.602	K[10]
A[2]	172.144	155.118	0.000	**200.139**	183.113	0.000	**977.518**	960.491	**959.507**	A[9]
P[3]	269.197	252.171	0.000	**297.192**	280.166	0.000	**906.481**	889.454	**888.470**	P[8]
K[4]	397.292	380.266	0.000	**425.287**	408.261	0.000	809.428	792.402	791.418	K[7]
S[5]	564.291	547.264	546.280	592.285	575.259	574.275	**681.333**	664.307	**663.323**	S[6]
P[6]	661.343	644.317	643.333	**689.338**	672.312	671.328	**514.335**	497.308	0.000	P[5]
A[7]	732.380	715.354	714.370	**760.375**	743.349	742.365	**417.282**	400.255	0.000	A[4]
K[8]	860.475	843.449	842.465	**888.470**	871.444	870.460	346.245	329.218	0.000	K[3]
A[9]	931.512	914.486	913.502	**959.507**	942.481	**941.497**	**218.150**	201.123	0.000	A[2]
K[10]	1059.607	1042.581	1041.597	1087.602	1070.576	1069.592	147.113	130.086	0.000	K[1]

sp|P43274|H14_MOUSE

KAPKS $^{Phospho}_{79.97}$ PAKAK

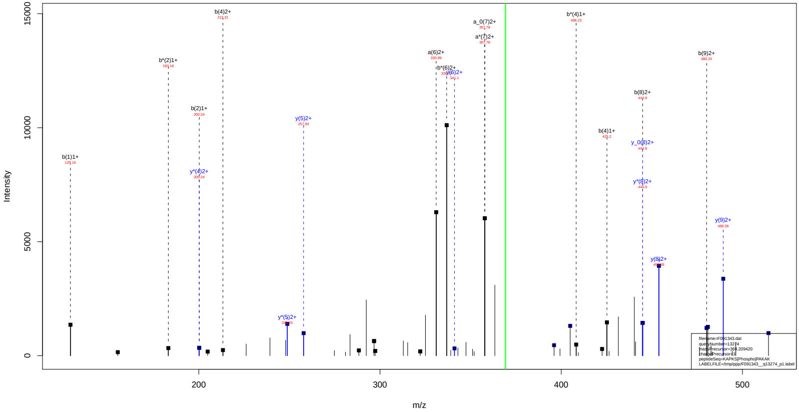

sp|P43274|H14_MOUSE

KAPKS$^{Phospho}_{79.97}$PAKAK

- fragmentation table for charge state 1+
- specType=cid
- score=26.65
- F091343.dat
- query=q13274_p1
- precursor=369.209420
- chargePrecursor=3
- itol=0.5

AA	a	a*	a_0	b	b*	b_0	y	y*	y_0	AA
K[1]	101.107	84.081	0.000	**129.102**	112.076	0.000	1105.613	1088.586	1087.602	K[10]
A[2]	172.144	**155.118**	0.000	**200.139**	**183.113**	0.000	977.518	960.491	959.507	A[9]
P[3]	269.197	252.171	0.000	**297.192**	280.166	0.000	906.481	889.454	888.470	P[8]
K[4]	397.292	380.266	0.000	**425.287**	**408.261**	0.000	809.428	792.402	791.418	K[7]
S[5]	564.291	547.264	546.280	592.285	575.259	574.275	681.333	664.307	663.323	S[6]
P[6]	661.343	644.317	643.333	689.338	672.312	671.328	**514.335**	497.308	0.000	P[5]
A[7]	732.380	715.354	714.370	760.375	743.349	742.365	417.282	400.255	0.000	A[4]
K[8]	860.475	843.449	842.465	888.470	871.444	870.460	346.245	329.218	0.000	K[3]
A[9]	931.512	914.486	913.502	959.507	942.481	941.497	218.150	201.123	0.000	A[2]
K[10]	1059.607	1042.581	1041.597	1087.602	1070.576	1069.592	147.113	130.086	0.000	K[1]

sp|P43274|H14_MOUSE

KAPKS$^{\text{Phospho}}_{79.97}$PAKAK

- fragmentation table for charge state 2+
- specType=cid
- score=26.65
- F091343.dat
- query=q13274_p1
- precursor=369.209420
- chargePrecursor=3
- itol=0.5

AA	a	a*	a_0	b	b*	b_0	y	y*	y_0	AA
K[1]	51.057	42.544	0.504	65.055	56.541	0.504	553.310	544.797	544.305	K[10]
A[2]	86.576	78.063	0.504	100.573	92.060	0.504	**489.263**	**480.749**	**480.257**	A[9]
P[3]	135.102	126.589	0.504	149.100	140.586	0.504	**453.744**	**445.231**	**444.739**	P[8]
K[4]	199.150	190.636	0.504	**213.147**	**204.634**	0.504	**405.218**	396.704	**396.212**	K[7]
S[5]	282.649	274.136	273.644	**296.646**	**288.133**	287.641	**341.170**	332.657	332.165	S[6]
P[6]	**331.175**	**322.662**	**322.170**	345.173	**336.659**	336.167	**257.671**	**249.158**	0.504	P[5]
A[7]	366.694	**358.181**	**357.689**	380.691	372.178	371.686	209.145	**200.631**	0.504	A[4]
K[8]	430.741	**422.228**	421.736	**444.739**	436.226	435.733	173.626	165.113	0.504	K[3]
A[9]	466.260	457.747	457.255	**480.257**	471.744	471.252	109.579	101.065	0.504	A[2]
K[10]	530.307	521.794	521.302	544.305	535.792	535.300	74.060	65.547	0.504	K[1]

sp|Q6ZWY9|H2B1C_MOUSE

PEPAKS $^{\text{Phospho}}_{79.97}$ APAPK

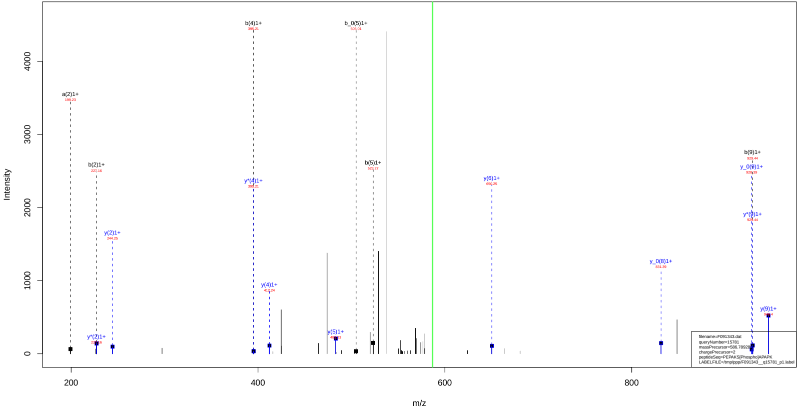

sp|Q6ZWY9|H2B1C_MOUSE

PEPAKS $^{Phospho}_{79.97}$ APAPK

- fragmentation table for charge state 1+
- specType=cid
- score=41.29
- F091343.dat
- query=q15781_p1
- precursor=586.789280
- chargePrecursor=2
- itol=0.5

AA	a	a*	a_0	b	b*	b_0	y	y*	y_0	AA
P[1]	70.065	0.000	0.000	98.060	0.000	0.000	1172.571	1155.545	1154.561	P[11]
E[2]	**199.108**	0.000	181.097	**227.103**	0.000	209.092	1075.518	1058.492	1057.508	E[10]
P[3]	296.160	0.000	278.150	324.155	0.000	306.145	**946.476**	**929.449**	**928.465**	P[9]
A[4]	367.198	0.000	349.187	**395.193**	0.000	377.182	849.423	832.396	**831.412**	A[8]
K[5]	495.293	478.266	477.282	**523.287**	506.261	**505.277**	778.386	761.359	760.375	K[7]
S[6]	662.291	645.264	644.280	690.286	673.259	672.275	**650.291**	633.264	632.280	S[6]
A[7]	733.328	716.301	715.317	761.323	744.296	743.312	**483.293**	466.266	0.000	A[5]
P[8]	830.381	813.354	812.370	858.376	841.349	840.365	**412.255**	**395.229**	0.000	P[4]
A[9]	901.418	884.391	883.407	**929.413**	912.386	911.402	315.203	298.176	0.000	A[3]
P[10]	998.471	981.444	980.460	1026.466	1009.439	1008.455	**244.166**	**227.139**	0.000	P[2]
K[11]	1126.566	1109.539	1108.555	1154.561	1137.534	1136.550	147.113	130.086	0.000	K[1]

sp|Q6ZWY9|H2B1C_MOUSE

PEPAKS $^{Phospho}_{79.97}$ APAPK

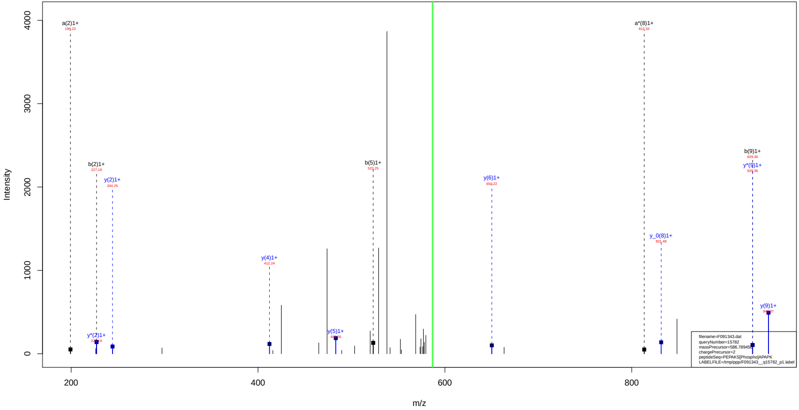

sp|Q6ZWY9|H2B1C_MOUSE

PEPAKS $^{Phospho}_{79.97}$ APAPK

- fragmentation table for charge state 1+
- specType=cid
- score=38.67
- F091343.dat
- query=q15782_p1
- precursor=586.789450
- chargePrecursor=2
- itol=0.5

AA	a	a*	a_0	b	b*	b_0	y	y*	y_0	AA
P[1]	70.065	0.000	0.000	98.060	0.000	0.000	1172.571	1155.545	1154.561	P[11]
E[2]	**199.108**	0.000	181.097	**227.103**	0.000	209.092	1075.518	1058.492	1057.508	E[10]
P[3]	296.160	0.000	278.150	324.155	0.000	306.145	**946.476**	**929.449**	928.465	P[9]
A[4]	367.198	0.000	349.187	395.193	0.000	377.182	849.423	832.396	**831.412**	A[8]
K[5]	495.293	478.266	477.282	**523.287**	506.261	505.277	778.386	761.359	760.375	K[7]
S[6]	662.291	645.264	644.280	690.286	673.259	672.275	**650.291**	633.264	632.280	S[6]
A[7]	733.328	716.301	715.317	761.323	744.296	743.312	**483.293**	466.266	0.000	A[5]
P[8]	830.381	**813.354**	812.370	858.376	841.349	840.365	**412.255**	395.229	0.000	P[4]
A[9]	901.418	884.391	883.407	**929.413**	912.386	911.402	315.203	298.176	0.000	A[3]
P[10]	998.471	981.444	980.460	1026.466	1009.439	1008.455	**244.166**	**227.139**	0.000	P[2]
K[11]	1126.566	1109.539	1108.555	1154.561	1137.534	1136.550	147.113	130.086	0.000	K[1]

sp|Q9D2U9|H2B3A_MOUSE

PEPSRS $^{\text{Phospho}}_{79.97}$ TPAPK

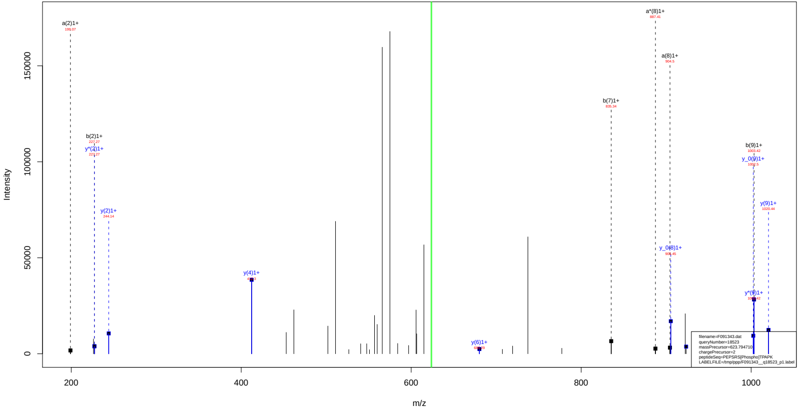

sp|Q9D2U9|H2B3A_MOUSE

PEPSRS $^{\text{Phospho}}_{79.97}$ TPAPK

- fragmentation table for charge state 1+
- specType=cid
- score=32.51
- F091343.dat
- query=q18523_p1
- precursor=623.794710
- chargePrecursor=2
- itol=0.5

AA	a	a*	a_0	b	b*	b_0	y	y*	y_0	AA
P[1]	70.065	0.000	0.000	98.060	0.000	0.000	1246.583	1229.556	1228.572	P[11]
E[2]	**199.108**	0.000	181.097	**227.103**	0.000	209.092	1149.530	1132.503	1131.519	E[10]
P[3]	296.160	0.000	278.150	324.155	0.000	306.145	**1020.487**	**1003.461**	**1002.477**	P[9]
S[4]	383.193	0.000	365.182	411.187	0.000	393.177	**923.435**	906.408	**905.424**	S[8]
R[5]	539.294	522.267	521.283	567.289	550.262	549.278	836.403	819.376	818.392	R[7]
S[6]	706.292	689.265	688.281	734.287	717.260	716.276	**680.301**	663.275	662.291	S[6]
T[7]	807.340	790.313	789.329	**835.335**	818.308	817.324	513.303	496.277	495.293	T[5]
P[8]	**904.392**	**887.366**	886.382	932.387	915.361	914.377	**412.255**	395.229	0.000	P[4]
A[9]	975.430	958.403	957.419	**1003.424**	986.398	985.414	315.203	298.176	0.000	A[3]
P[10]	1072.482	1055.456	1054.472	1100.477	1083.451	1082.467	**244.166**	**227.139**	0.000	P[2]
K[11]	1200.577	1183.551	1182.567	1228.572	1211.546	1210.562	147.113	130.086	0.000	K[1]

sp|Q9D2U9|H2B3A_MOUSE

PEPSRST $^{Phospho}_{79.97}$ PAPK

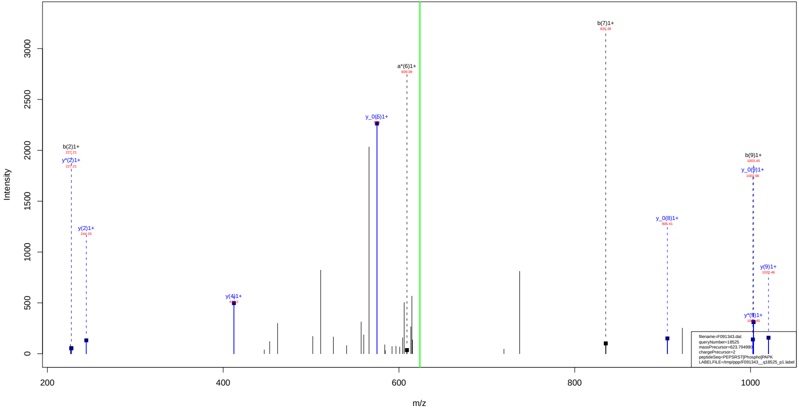

sp|Q9D2U9|H2B3A_MOUSE

PEPSRST $^{\text{Phospho}}_{79.97}$ PAPK

- fragmentation table for charge state 1+
- specType=cid
- score=34.46
- F091343.dat
- query=q18525_p1
- precursor=623.794990
- chargePrecursor=2
- itol=0.5

AA	a	a*	a_0	b	b*	b_0	y	y*	y_0	AA
P[1]	70.065	0.000	0.000	98.060	0.000	0.000	1246.583	1229.556	1228.572	P[11]
E[2]	199.108	0.000	181.097	**227.103**	0.000	209.092	1149.530	1132.503	1131.519	E[10]
P[3]	296.160	0.000	278.150	324.155	0.000	306.145	**1020.487**	**1003.461**	**1002.477**	P[9]
S[4]	383.193	0.000	365.182	411.187	0.000	393.177	923.435	906.408	**905.424**	S[8]
R[5]	539.294	522.267	521.283	567.289	550.262	549.278	836.403	819.376	818.392	R[7]
S[6]	626.326	**609.299**	608.315	654.321	637.294	636.310	680.301	663.275	662.291	S[6]
T[7]	807.340	790.313	789.329	**835.335**	818.308	817.324	593.269	576.243	**575.259**	T[5]
P[8]	904.392	887.366	886.382	932.387	915.361	914.377	**412.255**	395.229	0.000	P[4]
A[9]	975.430	958.403	957.419	**1003.424**	986.398	985.414	315.203	298.176	0.000	A[3]
P[10]	1072.482	1055.456	1054.472	1100.477	1083.451	1082.467	**244.166**	**227.139**	0.000	P[2]
K[11]	1200.577	1183.551	1182.567	1228.572	1211.546	1210.562	147.113	130.086	0.000	K[1]

sp|Q9D2U9|H2B3A_MOUSE

PEPSRS $^{\text{Phospho}}_{79.97}$ TPAPK

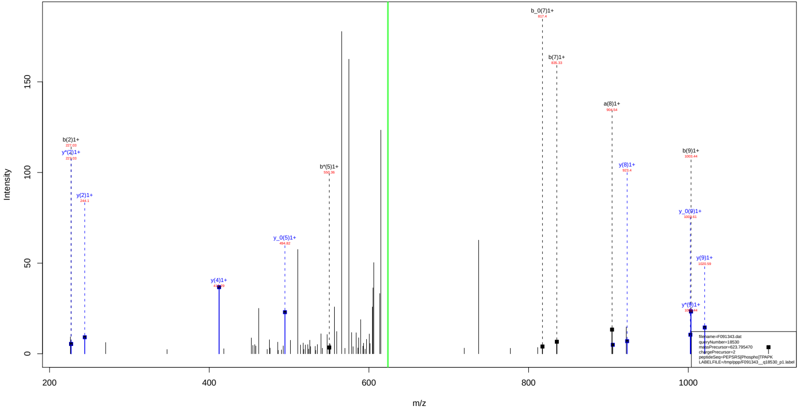

sp|Q9D2U9|H2B3A_MOUSE

PEPSRS $^{Phospho}_{79.97}$ TPAPK

- fragmentation table for charge state 1+
- specType=cid
- score=31.78
- F091343.dat
- query=q18530_p1
- precursor=623.795470
- chargePrecursor=2
- itol=0.5

AA	a	a*	a_0	b	b*	b_0	y	y*	y_0	AA
P[1]	70.065	0.000	0.000	98.060	0.000	0.000	1246.583	1229.556	1228.572	P[11]
E[2]	199.108	0.000	181.097	**227.103**	0.000	209.092	1149.530	1132.503	1131.519	E[10]
P[3]	296.160	0.000	278.150	324.155	0.000	306.145	**1020.487**	**1003.461**	**1002.477**	P[9]
S[4]	383.193	0.000	365.182	411.187	0.000	393.177	**923.435**	906.408	**905.424**	S[8]
R[5]	539.294	522.267	521.283	567.289	**550.262**	549.278	836.403	819.376	818.392	R[7]
S[6]	706.292	689.265	688.281	734.287	717.260	716.276	680.301	663.275	662.291	S[6]
T[7]	807.340	790.313	789.329	**835.335**	818.308	**817.324**	513.303	496.277	**495.293**	T[5]
P[8]	**904.392**	887.366	886.382	932.387	915.361	914.377	**412.255**	395.229	0.000	P[4]
A[9]	975.430	958.403	957.419	**1003.424**	986.398	985.414	315.203	298.176	0.000	A[3]
P[10]	1072.482	1055.456	1054.472	**1100.477**	1083.451	1082.467	**244.166**	**227.139**	0.000	P[2]
K[11]	1200.577	1183.551	1182.567	1228.572	1211.546	1210.562	147.113	130.086	0.000	K[1]

sp|Q9D2U9|H2B3A_MOUSE

PEPSRS $^{\text{Phospho}}_{79.97}$ TPAPK

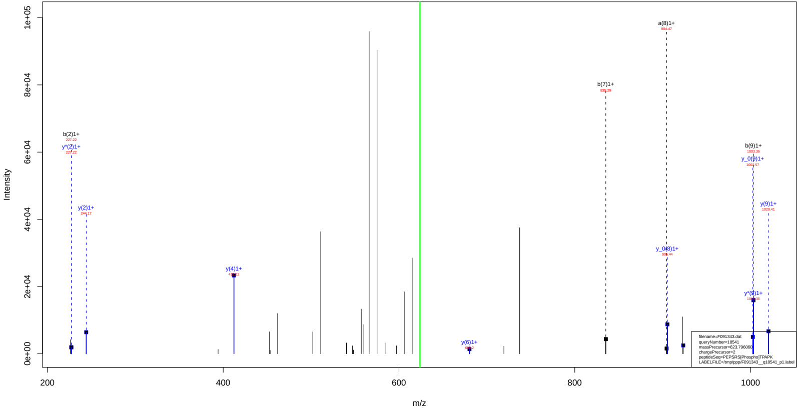

sp|Q9D2U9|H2B3A_MOUSE

PEPSRS $^{Phospho}_{79.97}$ TPAPK

- fragmentation table for charge state 1+
- specType=cid
- score=40.78
- F091343.dat
- query=q18541_p1
- precursor=623.796060
- chargePrecursor=2
- itol=0.5

AA	a	a*	a_0	b	b*	b_0	y	y*	y_0	AA
P[1]	70.065	0.000	0.000	98.060	0.000	0.000	1246.583	1229.556	1228.572	P[11]
E[2]	199.108	0.000	181.097	**227.103**	0.000	209.092	1149.530	1132.503	1131.519	E[10]
P[3]	296.160	0.000	278.150	324.155	0.000	306.145	**1020.487**	**1003.461**	**1002.477**	P[9]
S[4]	383.193	0.000	365.182	411.187	0.000	393.177	**923.435**	906.408	**905.424**	S[8]
R[5]	539.294	522.267	521.283	567.289	550.262	549.278	836.403	819.376	818.392	R[7]
S[6]	706.292	689.265	688.281	734.287	717.260	716.276	**680.301**	663.275	662.291	S[6]
T[7]	807.340	790.313	789.329	**835.335**	818.308	817.324	513.303	496.277	495.293	T[5]
P[8]	**904.392**	887.366	886.382	932.387	915.361	914.377	**412.255**	395.229	0.000	P[4]
A[9]	975.430	958.403	957.419	**1003.424**	986.398	985.414	315.203	298.176	0.000	A[3]
P[10]	1072.482	1055.456	1054.472	1100.477	1083.451	1082.467	**244.166**	**227.139**	0.000	P[2]
K[11]	1200.577	1183.551	1182.567	1228.572	1211.546	1210.562	147.113	130.086	0.000	K[1]

sp|P70696|H2B1A_MOUSE

HAVSEGT $^{\text{Phospho}}_{79.97}$ KAVTK

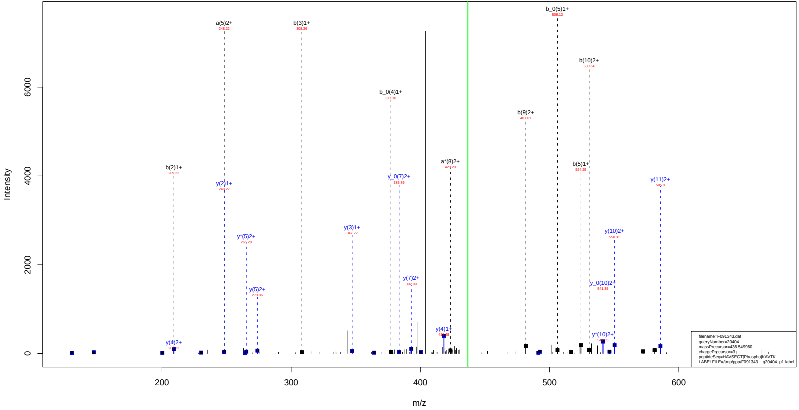

sp|P70696|H2B1A_MOUSE

HAVSEGT $^{Phospho}_{79.97}$ KAVTK

- fragmentation table for charge state 1+
- specType=cid
- score=37.84
- F091343.dat
- query=q20404_p1
- precursor=436.549960
- chargePrecursor=3
- itol=0.5

AA	a	a*	a_0	b	b*	b_0	y	y*	y_0	AA
H[1]	110.071	0.000	0.000	138.066	0.000	0.000	1307.636	1290.609	1289.625	H[12]
A[2]	181.108	0.000	0.000	**209.103**	0.000	0.000	1170.577	1153.550	1152.566	A[11]
V[3]	280.177	0.000	0.000	**308.172**	0.000	0.000	1099.539	1082.513	1081.529	V[10]
S[4]	367.209	0.000	349.198	395.204	0.000	**377.193**	1000.471	983.445	982.461	S[9]
E[5]	496.251	0.000	478.241	**524.246**	0.000	**506.236**	913.439	896.412	895.428	E[8]
G[6]	553.273	0.000	535.262	**581.268**	0.000	563.257	784.396	767.370	766.386	G[7]
T[7]	734.287	0.000	716.276	762.282	0.000	744.271	727.375	710.348	709.364	T[6]
K[8]	862.382	845.355	844.371	890.377	873.350	872.366	**546.361**	529.334	528.350	K[5]
A[9]	933.419	916.392	915.408	961.414	944.387	943.403	**418.266**	401.239	**400.255**	A[4]
V[10]	1032.487	1015.461	1014.477	1060.482	1043.456	1042.472	**347.229**	330.202	329.218	V[3]
T[11]	1133.535	1116.509	1115.525	1161.530	1144.503	1143.519	**248.160**	231.134	**230.150**	T[2]
K[12]	1261.630	1244.603	1243.619	1289.625	1272.598	1271.614	**147.113**	**130.086**	0.000	K[1]

sp|P70696|H2B1A_MOUSE

HAVSEGT $^{Phospho}_{79.97}$ KAVTK

- fragmentation table for charge state 2+
- specType=cid
- score=37.84
- F091343.dat
- query=q20404_p1
- precursor=436.549960
- chargePrecursor=3
- itol=0.5

AA	a	a*	a_0	b	b*	b_0	y	y*	y_0	AA
H[1]	55.539	0.504	0.504	69.537	0.504	0.504	654.321	645.808	645.316	H[12]
A[2]	91.058	0.504	0.504	105.055	0.504	0.504	**585.792**	577.279	576.787	A[11]
V[3]	140.592	0.504	0.504	154.589	0.504	0.504	**550.273**	**541.760**	**541.268**	V[10]
S[4]	184.108	0.504	175.103	198.106	0.504	189.100	500.739	**492.226**	**491.734**	S[9]
E[5]	**248.629**	0.504	239.624	262.627	0.504	253.622	457.223	448.710	448.218	E[8]
G[6]	277.140	0.504	268.135	291.138	0.504	282.132	**392.702**	384.189	**383.697**	G[7]
T[7]	367.647	0.504	358.642	381.645	0.504	372.639	**364.191**	355.678	355.186	T[6]
K[8]	431.695	**423.181**	422.689	445.692	437.179	436.687	**273.684**	**265.171**	**264.679**	K[5]
A[9]	467.213	458.700	458.208	**481.211**	472.697	472.205	**209.637**	201.123	**200.631**	A[4]
V[10]	**516.747**	508.234	507.742	**530.745**	522.232	521.740	174.118	165.605	165.113	V[3]
T[11]	567.271	558.758	558.266	**581.269**	**572.755**	**572.263**	124.584	116.071	115.579	T[2]
K[12]	631.319	622.805	622.313	645.316	636.803	636.311	74.060	65.547	0.504	K[1]

sp|P68433|H31_MOUSE

Y $^{Phospho}_{79.97}$ QKSTELLIR

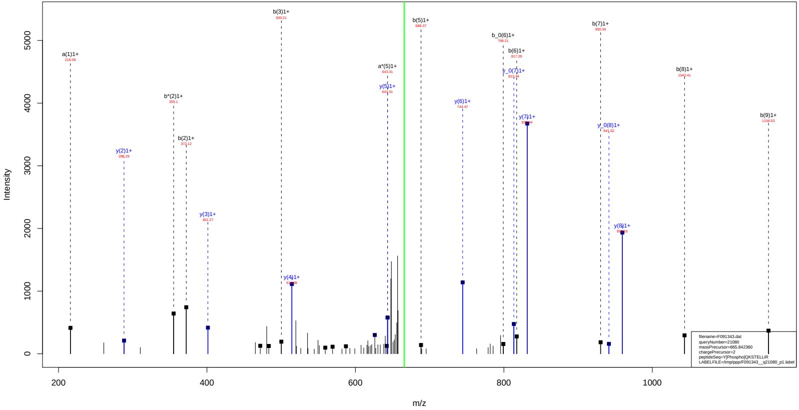

sp|P68433|H31_MOUSE

Y $^{Phospho}_{79.97}$ QKSTELLIR

- fragmentation table for charge state 1+
- specType=cid
- score=49.73
- F091343.dat
- query=q21080_p1
- precursor=665.842360
- chargePrecursor=2
- itol=0.5

AA	a	a*	a_0	b	b*	b_0	y	y*	y_0	AA
Y[1]	**216.042**	0.000	0.000	244.037	0.000	0.000	1330.677	1313.650	1312.666	Y[10]
Q[2]	344.101	327.074	0.000	**372.096**	**355.069**	0.000	1087.647	1070.620	1069.636	Q[9]
K[3]	**472.196**	455.169	0.000	**500.190**	**483.164**	0.000	**959.588**	942.562	**941.578**	K[8]
S[4]	**559.228**	542.201	541.217	**587.223**	570.196	**569.212**	**831.493**	814.467	**813.483**	S[7]
T[5]	660.275	**643.249**	**642.265**	**688.270**	671.244	670.260	**744.461**	727.435	726.451	T[6]
E[6]	789.318	772.291	771.307	**817.313**	800.286	**799.302**	**643.414**	**626.387**	625.403	E[5]
L[7]	902.402	885.375	884.391	**930.397**	913.370	912.386	**514.371**	497.345	0.000	L[4]
L[8]	1015.486	998.459	997.475	**1043.481**	1026.454	1025.470	**401.287**	384.261	0.000	L[3]
I[9]	1128.570	1111.544	1110.559	**1156.565**	1139.538	1138.554	**288.203**	271.176	0.000	I[2]
R[10]	1284.671	1267.645	1266.661	1312.666	1295.640	1294.656	175.119	158.092	0.000	R[1]

sp|P68433|H31_MOUSE

YQKST $^{\text{Phospho}}_{79.97}$ ELLIR

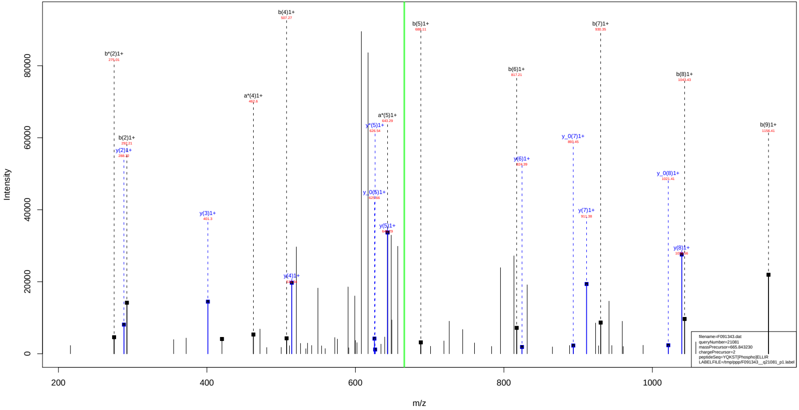

sp|P68433|H31_MOUSE

YQKST $^{Phospho}_{79.97}$ ELLIR

- fragmentation table for charge state 1+
- specType=cid
- score=56.00
- F091343.dat
- query=q21081_p1
- precursor=665.843230
- chargePrecursor=2
- itol=0.5

AA	a	a*	a_0	b	b*	b_0	y	y*	y_0	AA
Y[1]	136.076	0.000	0.000	164.071	0.000	0.000	1330.677	1313.650	1312.666	Y[10]
Q[2]	264.134	247.108	0.000	**292.129**	**275.103**	0.000	1167.613	1150.587	1149.603	Q[9]
K[3]	392.229	375.203	0.000	**420.224**	403.198	0.000	**1039.555**	1022.528	**1021.544**	K[8]
S[4]	479.261	**462.235**	461.251	**507.256**	490.230	489.246	**911.460**	894.433	**893.449**	S[7]
T[5]	660.275	**643.249**	642.265	**688.270**	671.244	670.260	**824.428**	807.401	806.417	T[6]
E[6]	789.318	772.291	771.307	**817.313**	800.286	799.302	**643.414**	**626.387**	**625.403**	E[5]
L[7]	902.402	885.375	884.391	**930.397**	913.370	912.386	**514.371**	497.345	0.000	L[4]
L[8]	1015.486	998.459	997.475	**1043.481**	1026.454	1025.470	**401.287**	384.261	0.000	L[3]
I[9]	1128.570	1111.544	1110.559	**1156.565**	1139.538	1138.554	**288.203**	271.176	0.000	I[2]
R[10]	1284.671	1267.645	1266.661	1312.666	1295.640	1294.656	175.119	158.092	0.000	R[1]

sp|Q64475|H2B1B_MOUSE

KES $^{Phospho}_{79.97}$ YSVYVYK

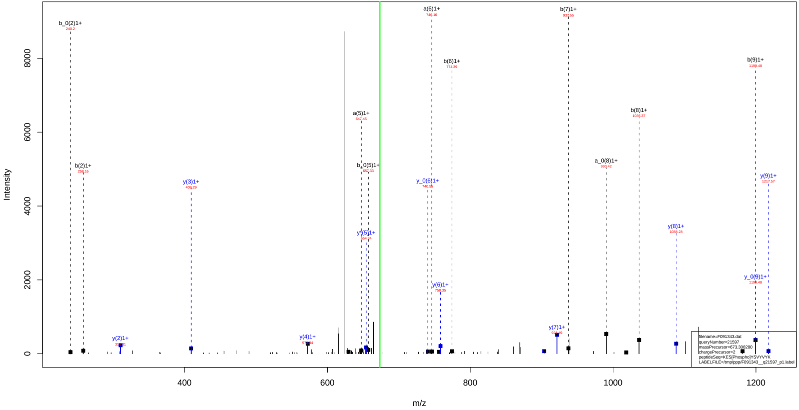

sp|Q64475|H2B1B_MOUSE

KES $^{\text{Phospho}}_{79.97}$ YSVYVYK

- fragmentation table for charge state 1+
- specType=cid
- score=46.16
- F091343.dat
- query=q21597_p1
- precursor=673.308280
- chargePrecursor=2
- itol=0.5

AA	a	a*	a_0	b	b*	b_0	y	y*	y_0	AA
K[1]	101.107	84.081	0.000	129.102	112.076	0.000	1345.608	1328.581	1327.597	K[10]
E[2]	230.150	213.123	212.139	**258.145**	241.118	**240.134**	**1217.513**	1200.486	**1199.502**	E[9]
S[3]	397.148	380.122	379.138	425.143	408.117	407.133	**1088.470**	1071.443	1070.459	S[8]
Y[4]	560.212	543.185	542.201	588.207	571.180	570.196	**921.472**	904.445	**903.461**	Y[7]
S[5]	**647.244**	630.217	**629.233**	675.239	658.212	**657.228**	**758.408**	741.382	**740.398**	S[6]
V[6]	**746.312**	729.285	728.301	**774.307**	757.280	**756.296**	671.376	**654.350**	0.000	V[5]
Y[7]	909.375	892.349	891.365	**937.370**	920.344	919.360	**572.308**	555.281	0.000	Y[4]
V[8]	1008.444	991.417	**990.433**	**1036.439**	1019.412	**1018.428**	**409.245**	392.218	0.000	V[3]
Y[9]	1171.507	1154.481	1153.497	**1199.502**	1182.475	**1181.491**	**310.176**	293.150	0.000	Y[2]
K[10]	1299.602	1282.576	1281.592	1327.597	1310.570	1309.586	147.113	130.086	0.000	K[1]

sp|P15864|H12_MOUSE

GTGAS $^{\text{Phospho}}_{79.97}$ GSFKLNKK

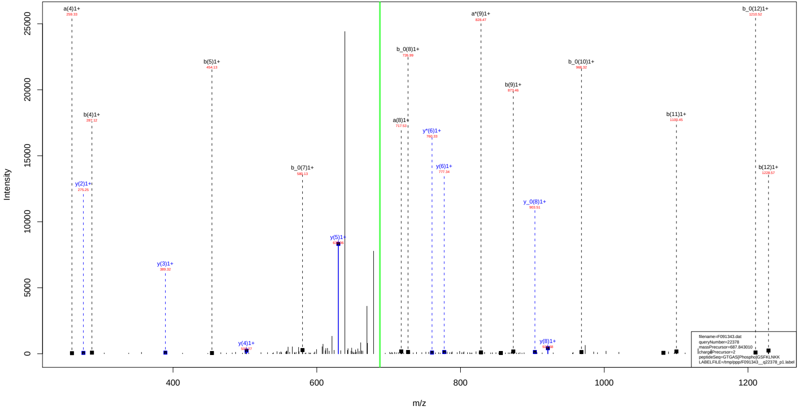

sp|P15864|H12_MOUSE

GTGAS $^{\text{Phospho}}_{79.97}$ GSFKLNKK

- fragmentation table for charge state 1+
- specType=cid
- score=37.74
- F091343.dat
- query=q22378_p1
- precursor=687.843010
- chargePrecursor=2
- itol=0.5

AA	a	a*	a_0	b	b*	b_0	y	y*	y_0	AA
G[1]	30.034	0.000	0.000	58.029	0.000	0.000	1374.678	1357.651	1356.667	G[13]
T[2]	131.082	0.000	113.071	159.076	0.000	141.066	1317.656	1300.630	1299.646	T[12]
G[3]	188.103	0.000	170.092	216.098	0.000	198.087	1216.609	1199.582	1198.598	G[11]
A[4]	**259.140**	0.000	241.130	**287.135**	0.000	269.124	1159.587	1142.561	1141.577	A[10]
S[5]	426.138	0.000	408.128	**454.133**	0.000	436.123	1088.550	1071.523	1070.539	S[9]
G[6]	483.160	0.000	465.149	511.155	0.000	493.144	**921.552**	904.525	**903.541**	G[8]
S[7]	570.192	0.000	552.181	598.187	0.000	**580.176**	864.530	847.504	846.520	S[7]
F[8]	**717.260**	0.000	699.250	745.255	0.000	**727.245**	**777.498**	**760.472**	0.000	F[6]
K[9]	845.355	**828.329**	827.345	**873.350**	**856.324**	855.340	**630.430**	613.403	0.000	K[5]
L[10]	958.439	941.413	940.429	986.434	969.408	**968.424**	**502.335**	485.308	0.000	L[4]
N[11]	1072.482	1055.456	1054.472	**1100.477**	1083.451	**1082.467**	**389.251**	372.224	0.000	N[3]
K[12]	1200.577	1183.551	1182.567	**1228.572**	1211.546	**1210.562**	**275.208**	258.181	0.000	K[2]
K[13]	1328.672	1311.646	1310.662	1356.667	1339.641	1338.657	147.113	130.086	0.000	K[1]

sp|P15864|H12_MOUSE

KAS $^{Phospho}_{79.97}$ GPPVSELITK

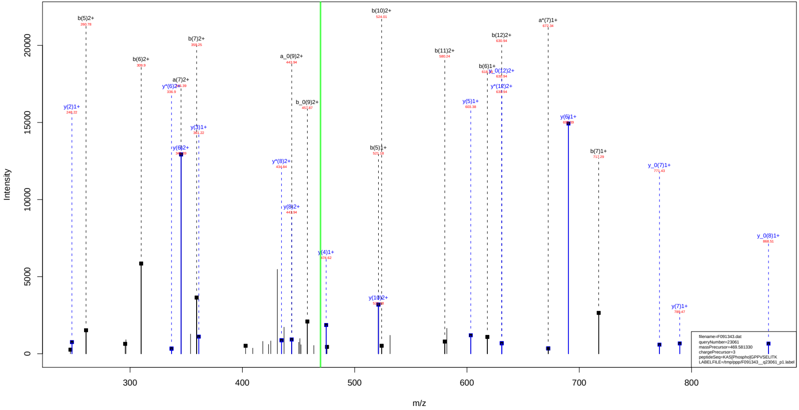

sp|P15864|H12_MOUSE

KAS $^{Phospho}_{79.97}$ GPPVSELITK

- fragmentation table for charge state 1+
- specType=cid
- score=54.88
- F091343.dat
- query=q23061_p1
- precursor=469.581330
- chargePrecursor=3
- itol=0.5

AA	a	a*	a_0	b	b*	b_0	y	y*	y_0	AA
K[1]	101.107	84.081	0.000	129.102	112.076	0.000	1406.729	1389.703	1388.719	K[13]
A[2]	172.144	155.118	0.000	200.139	183.113	0.000	1278.634	1261.608	1260.624	A[12]
S[3]	339.143	322.116	321.132	367.138	350.111	349.127	1207.597	1190.570	1189.586	S[11]
G[4]	396.164	379.138	378.154	424.159	407.133	406.149	1040.599	1023.572	1022.588	G[10]
P[5]	493.217	476.190	**475.206**	**521.212**	504.185	503.201	983.577	966.551	965.567	P[9]
P[6]	590.270	573.243	572.259	**618.265**	601.238	600.254	886.524	869.498	**868.514**	P[8]
V[7]	689.338	**672.312**	671.328	**717.333**	700.307	699.323	**789.472**	772.445	**771.461**	V[7]
S[8]	776.370	759.344	758.360	804.365	787.339	786.355	**690.403**	673.377	**672.393**	S[6]
E[9]	905.413	888.386	887.402	933.408	916.381	915.397	**603.371**	586.345	585.361	E[5]
L[10]	1018.497	1001.470	1000.486	1046.492	1029.465	1028.481	**474.329**	457.302	456.318	L[4]
I[11]	1131.581	1114.554	1113.570	1159.576	1142.549	1141.565	**361.245**	344.218	343.234	I[3]
T[12]	1232.629	1215.602	1214.618	1260.624	1243.597	1242.613	**248.160**	231.134	230.150	T[2]
K[13]	1360.724	1343.697	1342.713	1388.719	1371.692	1370.708	147.113	130.086	0.000	K[1]

sp|P15864|H12_MOUSE

KAS $^{Phospho}_{79.97}$ GPPVSELITK

- fragmentation table for charge state 2+
- specType=cid
- score=54.88
- F091343.dat
- query=q23061_p1
- precursor=469.581330
- chargePrecursor=3
- itol=0.5

AA	a	a*	a_0	b	b*	b_0	y	y*	y_0	AA
K[1]	51.057	42.544	0.504	65.055	56.541	0.504	703.868	695.355	694.863	K[13]
A[2]	86.576	78.063	0.504	100.573	92.060	0.504	639.821	**631.307**	**630.815**	A[12]
S[3]	170.075	161.562	161.070	184.072	175.559	175.067	604.302	595.789	595.297	S[11]
G[4]	198.586	190.072	189.580	212.583	204.070	203.578	**520.803**	512.290	511.798	G[10]
P[5]	**247.112**	238.599	238.107	**261.110**	252.596	252.104	492.292	483.779	483.287	P[9]
P[6]	**295.639**	287.125	286.633	**309.636**	301.123	300.631	**443.766**	**435.253**	**434.761**	P[8]
V[7]	**345.173**	**336.659**	336.167	**359.170**	350.657	350.165	395.239	386.726	386.234	V[7]
S[8]	388.689	380.175	379.683	**402.686**	394.173	393.681	**345.705**	**337.192**	**336.700**	S[6]
E[9]	453.210	444.697	**444.205**	467.208	458.694	**458.202**	302.189	293.676	293.184	E[5]
L[10]	509.752	501.239	500.747	**523.750**	515.236	514.744	237.668	229.155	228.663	L[4]
I[11]	566.294	557.781	557.289	**580.292**	571.778	571.286	181.126	172.613	172.121	I[3]
T[12]	616.818	608.305	607.813	**630.815**	622.302	621.810	124.584	116.071	115.579	T[2]
K[13]	680.865	**672.352**	**671.860**	694.863	686.350	685.858	74.060	65.547	0.504	K[1]

sp|P15864|H12_MOUSE

KAS $^{Phospho}_{79.97}$ GPPVSELITK

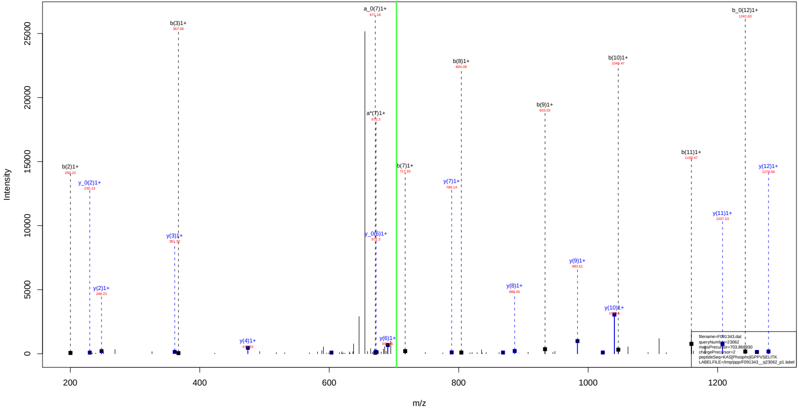

sp|P15864|H12_MOUSE

KAS $^{Phospho}_{79.97}$ GPPVSELITK

- fragmentation table for charge state 1+
- specType=cid
- score=82.96
- F091343.dat
- query=q23062_p1
- precursor=703.868930
- chargePrecursor=2
- itol=0.5

AA	a	a*	a_0	b	b*	b_0	y	y*	y_0	AA
K[1]	101.107	84.081	0.000	129.102	112.076	0.000	1406.729	1389.703	1388.719	K[13]
A[2]	172.144	155.118	0.000	**200.139**	183.113	0.000	**1278.634**	1261.608	**1260.624**	A[12]
S[3]	339.143	322.116	321.132	**367.138**	350.111	349.127	**1207.597**	1190.570	1189.586	S[11]
G[4]	396.164	379.138	378.154	424.159	407.133	406.149	**1040.599**	1023.572	**1022.588**	G[10]
P[5]	493.217	476.190	475.206	521.212	504.185	503.201	**983.577**	966.551	965.567	P[9]
P[6]	590.270	573.243	572.259	618.265	601.238	600.254	**886.524**	869.498	**868.514**	P[8]
V[7]	689.338	**672.312**	**671.328**	**717.333**	700.307	699.323	**789.472**	772.445	771.461	V[7]
S[8]	776.370	759.344	758.360	**804.365**	787.339	786.355	**690.403**	673.377	**672.393**	S[6]
E[9]	905.413	888.386	887.402	**933.408**	916.381	915.397	**603.371**	586.345	585.361	E[5]
L[10]	1018.497	1001.470	1000.486	**1046.492**	1029.465	1028.481	**474.329**	457.302	456.318	L[4]
I[11]	1131.581	1114.554	1113.570	**1159.576**	1142.549	1141.565	**361.245**	344.218	343.234	I[3]
T[12]	1232.629	1215.602	1214.618	**1260.624**	1243.597	**1242.613**	**248.160**	231.134	**230.150**	T[2]
K[13]	1360.724	1343.697	1342.713	1388.719	1371.692	1370.708	147.113	130.086	0.000	K[1]

sp|P68433|H31_MOUSE

EIAQDFKT $^{Phospho}_{79.97}$ DLR

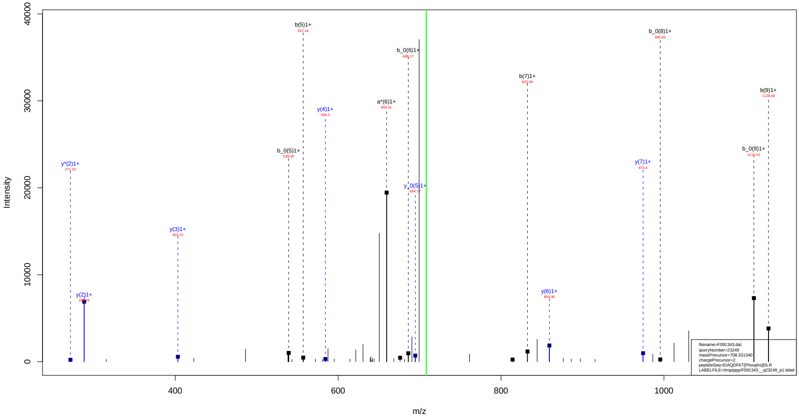

sp|P68433|H31_MOUSE

EIAQDFKT $^{Phospho}_{79.97}$ DLR

- fragmentation table for charge state 1+
- specType=cid
- score=30.41
- F091343.dat
- query=q23249_p1
- precursor=708.331340
- chargePrecursor=2
- itol=0.5

AA	a	a*	a_0	b	b*	b_0	y	y*	y_0	AA
E[1]	102.055	0.000	84.044	130.050	0.000	112.039	1415.657	1398.630	1397.646	E[11]
I[2]	215.139	0.000	197.128	243.134	0.000	225.123	1286.614	1269.587	1268.603	I[10]
A[3]	286.176	0.000	268.166	314.171	0.000	296.160	1173.530	1156.503	1155.519	A[9]
Q[4]	414.235	397.208	396.224	442.230	425.203	424.219	1102.493	1085.466	1084.482	Q[8]
D[5]	529.262	512.235	511.251	**557.257**	540.230	**539.246**	**974.434**	957.408	956.424	D[7]
F[6]	**676.330**	**659.304**	658.320	704.325	687.298	**686.314**	**859.407**	842.381	841.397	F[6]
K[7]	804.425	787.398	786.414	**832.420**	815.393	**814.409**	712.339	695.312	**694.328**	K[5]
T[8]	985.439	968.412	967.428	1013.434	996.407	**995.423**	**584.244**	567.217	566.233	T[4]
D[9]	1100.466	1083.439	1082.455	**1128.461**	1111.434	**1110.450**	**403.230**	386.203	385.219	D[3]
L[10]	1213.550	1196.523	1195.539	1241.545	1224.518	1223.534	**288.203**	**271.176**	0.000	L[2]
R[11]	1369.651	1352.625	1351.641	1397.646	1380.620	1379.636	175.119	158.092	0.000	R[1]

sp|P68433|H31_MOUSE

EIAQDFKT $^{\text{Phospho}}_{79.97}$ DLR

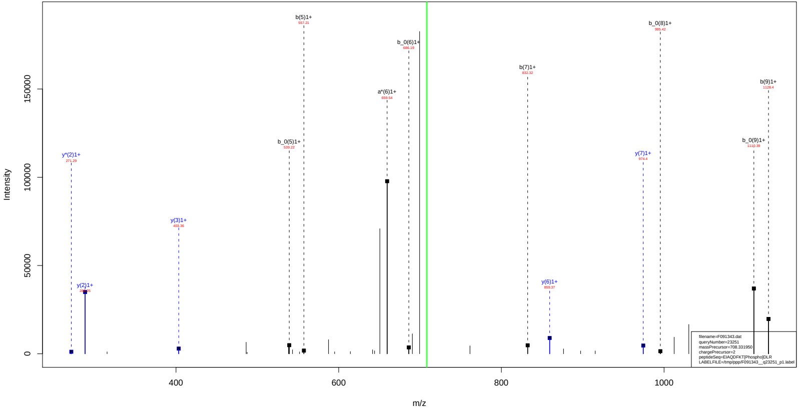

sp|P68433|H31_MOUSE

EIAQDFKT$^{\text{Phospho}}_{79.97}$DLR

- fragmentation table for charge state 1+
- specType=cid
- score=47.14
- F091343.dat
- query=q23251_p1
- precursor=708.331950
- chargePrecursor=2
- itol=0.5

AA	a	a*	a_0	b	b*	b_0	y	y*	y_0	AA
E[1]	102.055	0.000	84.044	130.050	0.000	112.039	1415.657	1398.630	1397.646	E[11]
I[2]	215.139	0.000	197.128	243.134	0.000	225.123	1286.614	1269.587	1268.603	I[10]
A[3]	286.176	0.000	268.166	314.171	0.000	296.160	1173.530	1156.503	1155.519	A[9]
Q[4]	414.235	397.208	396.224	442.230	425.203	424.219	1102.493	1085.466	1084.482	Q[8]
D[5]	529.262	512.235	511.251	**557.257**	540.230	**539.246**	**974.434**	957.408	956.424	D[7]
F[6]	676.330	**659.304**	658.320	704.325	687.298	**686.314**	**859.407**	842.381	841.397	F[6]
K[7]	804.425	787.398	786.414	**832.420**	815.393	814.409	712.339	695.312	694.328	K[5]
T[8]	985.439	968.412	967.428	1013.434	996.407	**995.423**	584.244	567.217	566.233	T[4]
D[9]	1100.466	1083.439	1082.455	**1128.461**	1111.434	**1110.450**	**403.230**	386.203	385.219	D[3]
L[10]	1213.550	1196.523	1195.539	1241.545	1224.518	1223.534	**288.203**	**271.176**	0.000	L[2]
R[11]	1369.651	1352.625	1351.641	1397.646	1380.620	1379.636	175.119	158.092	0.000	R[1]

sp|P62806|H4_MOUSE

RIS $^{\text{Phospho}}_{79.97}$ GLIYEETR

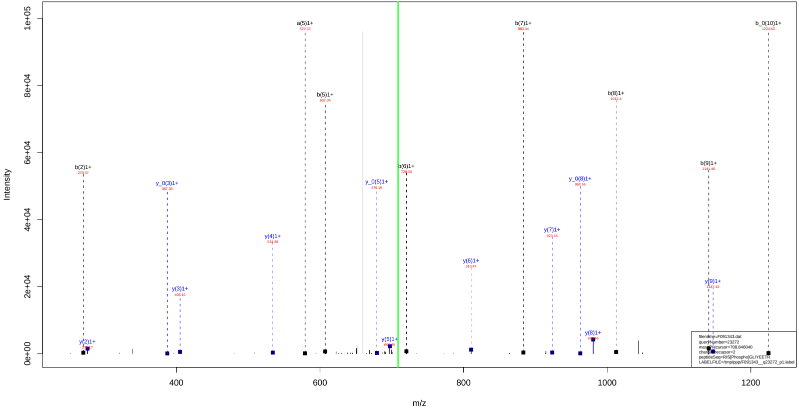

sp|P62806|H4_MOUSE

RIS $^{Phospho}_{79.97}$ GLIYEETR

- fragmentation table for charge state 1+
- specType=cid
- score=78.91
- F091343.dat
- query=q23272_p1
- precursor=708.846040
- chargePrecursor=2
- itol=0.5

AA	a	a*	a_0	b	b*	b_0	y	y*	y_0	AA
R[1]	129.113	112.087	0.000	157.108	140.082	0.000	1416.688	1399.662	1398.678	R[11]
I[2]	242.198	225.171	0.000	**270.192**	253.166	0.000	1260.587	1243.561	1242.577	I[10]
S[3]	409.196	392.169	391.185	437.191	420.164	419.180	**1147.503**	1130.477	1129.493	S[9]
G[4]	466.217	449.191	448.207	494.212	477.186	476.202	**980.505**	963.478	**962.494**	G[8]
L[5]	**579.301**	562.275	561.291	**607.296**	590.270	589.286	**923.483**	906.457	905.473	L[7]
I[6]	692.385	675.359	674.375	**720.380**	703.354	702.370	**810.399**	793.373	792.389	I[6]
Y[7]	855.449	838.422	837.438	**883.444**	866.417	865.433	**697.315**	680.289	**679.305**	Y[5]
E[8]	984.491	967.465	966.481	**1012.486**	995.460	994.476	**534.252**	517.225	516.241	E[4]
E[9]	1113.534	1096.507	1095.523	**1141.529**	1124.502	1123.518	**405.209**	388.183	**387.199**	E[3]
T[10]	1214.582	1197.555	1196.571	1242.577	1225.550	**1224.566**	**276.167**	259.140	258.156	T[2]
R[11]	1370.683	1353.656	1352.672	1398.678	1381.651	1380.667	175.119	158.092	0.000	R[1]

sp|P62806|H4_MOUSE

RIS $^{Phospho}_{79.97}$ GLIYEETR

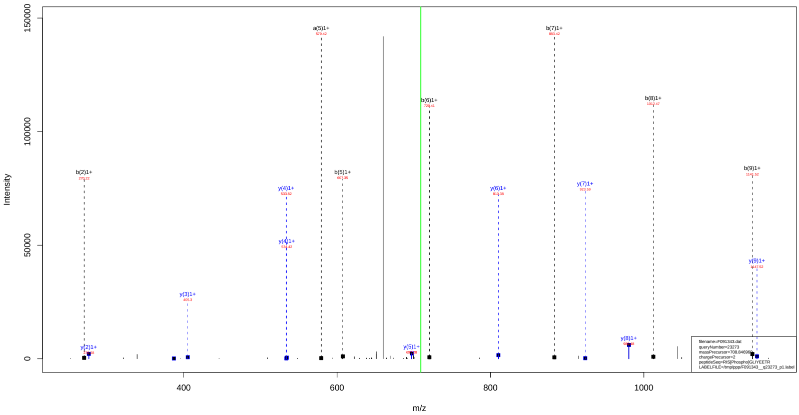

sp|P62806|H4_MOUSE

RIS $^{Phospho}_{79.97}$ GLIYEETR

- fragmentation table for charge state 1+
- specType=cid
- score=75.45
- F091343.dat
- query=q23273_p1
- precursor=708.846960
- chargePrecursor=2
- itol=0.5

AA	a	a*	a_0	b	b*	b_0	y	y*	y_0	AA
R[1]	129.113	112.087	0.000	157.108	140.082	0.000	1416.688	1399.662	1398.678	R[11]
I[2]	242.198	225.171	0.000	**270.192**	253.166	0.000	1260.587	1243.561	1242.577	I[10]
S[3]	409.196	392.169	391.185	437.191	420.164	419.180	**1147.503**	1130.477	1129.493	S[9]
G[4]	466.217	449.191	448.207	494.212	477.186	476.202	**980.505**	963.478	962.494	G[8]
L[5]	**579.301**	562.275	561.291	**607.296**	590.270	589.286	**923.483**	906.457	905.473	L[7]
I[6]	692.385	675.359	674.375	**720.380**	703.354	702.370	**810.399**	793.373	792.389	I[6]
Y[7]	855.449	838.422	837.438	**883.444**	866.417	865.433	**697.315**	680.289	679.305	Y[5]
E[8]	984.491	967.465	966.481	**1012.486**	995.460	994.476	**534.252**	517.225	516.241	E[4]
E[9]	1113.534	1096.507	1095.523	**1141.529**	1124.502	1123.518	**405.209**	388.183	**387.199**	E[3]
T[10]	1214.582	1197.555	1196.571	1242.577	1225.550	1224.566	**276.167**	259.140	258.156	T[2]
R[11]	1370.683	1353.656	1352.672	1398.678	1381.651	1380.667	175.119	158.092	0.000	R[1]

sp|P43274|H14_MOUSE

KTS $^{\text{Phospho}}_{79.97}$ GPPVSELITK

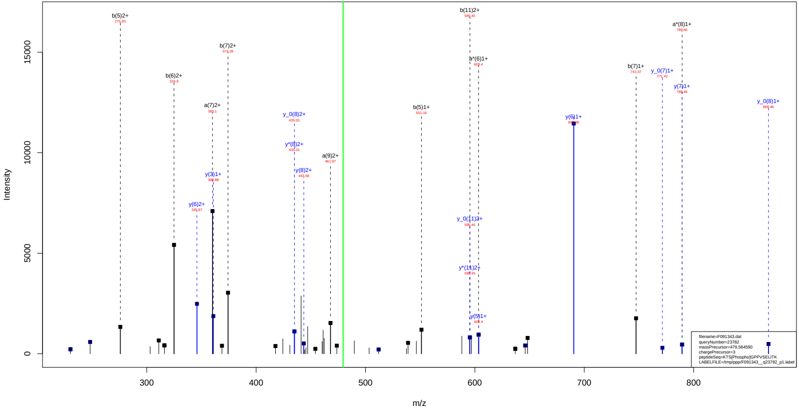

sp|P43274|H14_MOUSE

KTS $^{\text{Phospho}}_{79.97}$ GPPVSELITK

- fragmentation table for charge state 1+
- specType=cid
- score=46.84
- F091343.dat
- query=q23782_p1
- precursor=479.584590
- chargePrecursor=3
- itol=0.5

AA	a	a*	a_0	b	b*	b_0	y	y*	y_0	AA
K[1]	101.107	84.081	0.000	129.102	112.076	0.000	1436.740	1419.713	1418.729	K[13]
T[2]	202.155	185.128	184.144	**230.150**	213.123	212.139	1308.645	1291.618	1290.634	T[12]
S[3]	**369.153**	352.127	351.143	397.148	380.122	379.138	1207.597	1190.570	1189.586	S[11]
G[4]	426.175	409.148	408.164	**454.170**	437.143	436.159	1040.599	1023.572	1022.588	G[10]
P[5]	523.228	506.201	505.217	**551.223**	534.196	533.212	983.577	966.551	965.567	P[9]
P[6]	620.280	**603.254**	602.270	**648.275**	631.249	630.265	886.524	869.498	**868.514**	P[8]
V[7]	719.349	702.322	701.338	**747.344**	730.317	729.333	**789.472**	772.445	**771.461**	V[7]
S[8]	806.381	**789.354**	788.370	834.376	817.349	816.365	**690.403**	673.377	672.393	S[6]
E[9]	935.423	918.397	917.413	963.418	946.392	945.408	**603.371**	586.345	585.361	E[5]
L[10]	1048.507	1031.481	1030.497	1076.502	1059.476	1058.492	474.329	457.302	456.318	L[4]
I[11]	1161.592	1144.565	1143.581	1189.586	1172.560	1171.576	**361.245**	344.218	343.234	I[3]
T[12]	1262.639	1245.613	1244.629	1290.634	1273.608	1272.624	**248.160**	231.134	**230.150**	T[2]
K[13]	1390.734	1373.708	1372.724	1418.729	1401.703	1400.719	147.113	130.086	0.000	K[1]

sp|P43274|H14_MOUSE

KTS $^{Phospho}_{79.97}$ GPPVSELITK

- fragmentation table for charge state 2+
- specType=cid
- score=46.84
- F091343.dat
- query=q23782_p1
- precursor=479.584590
- chargePrecursor=3
- itol=0.5

AA	a	a*	a_0	b	b*	b_0	y	y*	y_0	AA
K[1]	51.057	42.544	0.504	65.055	56.541	0.504	718.873	710.360	709.868	K[13]
T[2]	101.581	93.068	92.576	115.579	107.065	106.573	654.826	**646.313**	**645.821**	T[12]
S[3]	185.080	176.567	176.075	199.078	190.565	190.072	604.302	**595.789**	**595.297**	S[11]
G[4]	213.591	205.078	204.586	227.589	219.075	218.583	520.803	**512.290**	**511.798**	G[10]
P[5]	262.117	253.604	253.112	**276.115**	267.602	267.110	492.292	483.779	483.287	P[9]
P[6]	**310.644**	302.131	301.639	**324.641**	**316.128**	**315.636**	**443.766**	**435.253**	**434.761**	P[8]
V[7]	**360.178**	351.665	351.173	**374.175**	365.662	365.170	395.239	386.726	386.234	V[7]
S[8]	403.694	395.181	394.689	**417.691**	409.178	408.686	**345.705**	337.192	336.700	S[6]
E[9]	**468.215**	459.702	459.210	482.213	**473.700**	473.208	302.189	293.676	293.184	E[5]
L[10]	524.757	516.244	515.752	**538.755**	530.242	529.750	237.668	229.155	228.663	L[4]
I[11]	581.299	572.786	572.294	**595.297**	586.784	586.292	181.126	172.613	172.121	I[3]
T[12]	631.823	623.310	622.818	**645.821**	**637.307**	**636.815**	124.584	116.071	115.579	T[2]
K[13]	695.871	687.357	686.865	709.868	701.355	700.863	74.060	65.547	0.504	K[1]

sp|P43274|H14_MOUSE

KTS $^{Phospho}_{79.97}$ GPPVSELITK

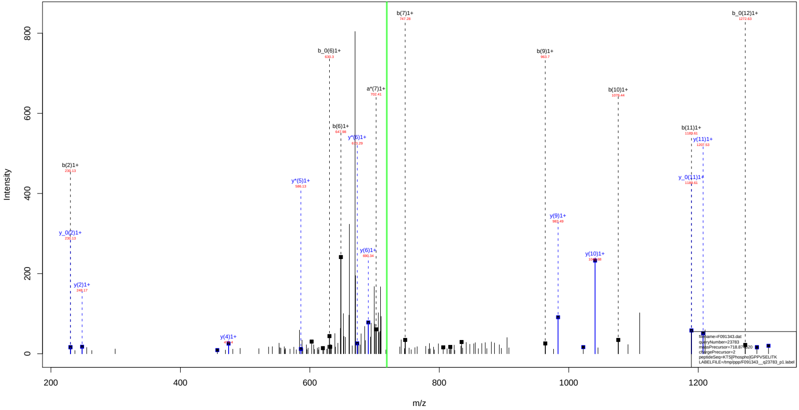

sp|P43274|H14_MOUSE

KTS $^{Phospho}_{79.97}$ GPPVSELITK

- fragmentation table for charge state 1+
- specType=cid
- score=70.19
- F091343.dat
- query=q23783_p1
- precursor=718.874620
- chargePrecursor=2
- itol=0.5

AA	a	a*	a_0	b	b*	b_0	y	y*	y_0	AA
K[1]	101.107	84.081	0.000	129.102	112.076	0.000	1436.740	1419.713	1418.729	K[13]
T[2]	202.155	185.128	184.144	**230.150**	213.123	212.139	**1308.645**	1291.618	**1290.634**	T[12]
S[3]	369.153	352.127	351.143	397.148	380.122	379.138	**1207.597**	1190.570	**1189.586**	S[11]
G[4]	426.175	409.148	408.164	454.170	437.143	436.159	**1040.599**	1023.572	**1022.588**	G[10]
P[5]	523.228	506.201	505.217	551.223	534.196	533.212	**983.577**	966.551	965.567	P[9]
P[6]	**620.280**	603.254	**602.270**	**648.275**	**631.249**	**630.265**	886.524	869.498	868.514	P[8]
V[7]	719.349	**702.322**	701.338	**747.344**	730.317	729.333	789.472	772.445	771.461	V[7]
S[8]	**806.381**	789.354	788.370	**834.376**	**817.349**	816.365	**690.403**	**673.377**	672.393	S[6]
E[9]	935.423	918.397	917.413	**963.418**	946.392	945.408	603.371	**586.345**	585.361	E[5]
L[10]	1048.507	1031.481	1030.497	**1076.502**	1059.476	1058.492	**474.329**	**457.302**	456.318	L[4]
I[11]	1161.592	1144.565	1143.581	**1189.586**	1172.560	1171.576	361.245	344.218	343.234	I[3]
T[12]	1262.639	1245.613	1244.629	**1290.634**	1273.608	**1272.624**	**248.160**	231.134	**230.150**	T[2]
K[13]	1390.734	1373.708	1372.724	1418.729	1401.703	1400.719	147.113	130.086	0.000	K[1]

sp|P70696|H2B1A_MOUSE

IAS $^{Phospho}_{79.97}$ EASR

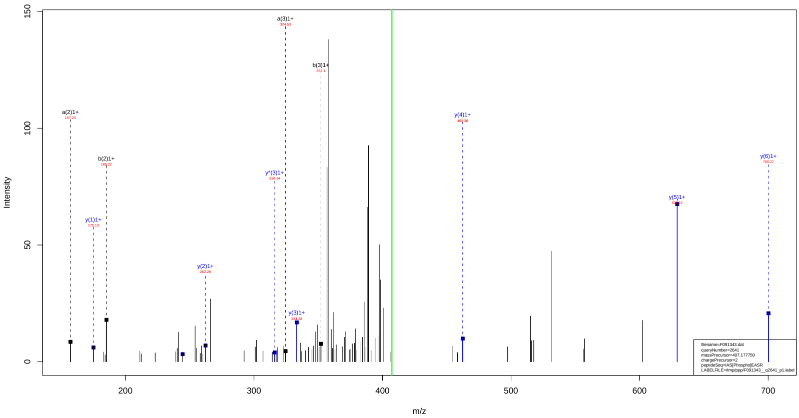

sp|P70696|H2B1A_MOUSE

IAS $^{Phospho}_{79.97}$ EASR

- fragmentation table for charge state 1+
- specType=cid
- score=32.32
- F091343.dat
- query=q2641_p1
- precursor=407.177750
- chargePrecursor=2
- itol=0.5

AA	a	a*	a_0	b	b*	b_0	y	y*	y_0	AA
I[1]	86.096	0.000	0.000	114.091	0.000	0.000	813.350	796.324	795.340	I[7]
A[2]	**157.134**	0.000	0.000	**185.128**	0.000	0.000	**700.266**	683.240	682.256	A[6]
S[3]	**324.132**	0.000	306.121	**352.127**	0.000	334.116	**629.229**	612.202	611.218	S[5]
E[4]	453.174	0.000	435.164	481.169	0.000	463.159	**462.231**	445.204	444.220	E[4]
A[5]	524.212	0.000	506.201	552.207	0.000	534.196	**333.188**	**316.162**	315.178	A[3]
S[6]	611.244	0.000	593.233	639.239	0.000	621.228	**262.151**	245.124	**244.140**	S[2]
R[7]	767.345	750.318	749.334	795.340	778.313	777.329	**175.119**	158.092	0.000	R[1]

sp|P70696|H2B1A_MOUSE

IAS $^{Phospho}_{79.97}$ EASR

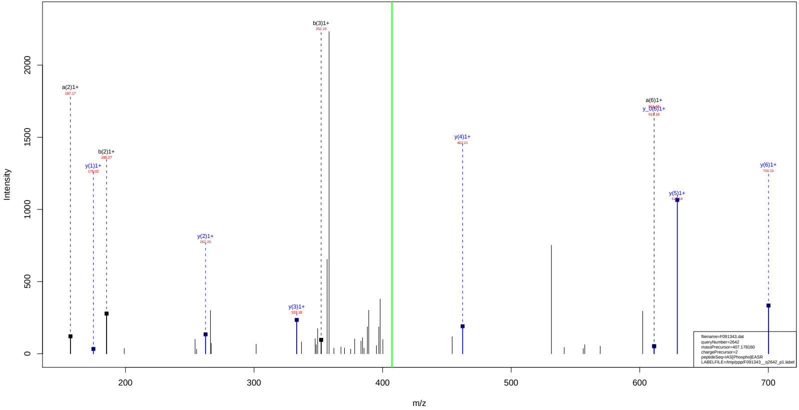

sp|P70696|H2B1A_MOUSE

IAS $^{\text{Phospho}}_{79.97}$ EASR

- fragmentation table for charge state 1+
- specType=cid
- score=44.48
- F091343.dat
- query=q2642_p1
- precursor=407.178160
- chargePrecursor=2
- itol=0.5

AA	a	a*	a_0	b	b*	b_0	y	y*	y_0	AA
I[1]	86.096	0.000	0.000	114.091	0.000	0.000	813.350	796.324	795.340	I[7]
A[2]	**157.134**	0.000	0.000	**185.128**	0.000	0.000	**700.266**	683.240	682.256	A[6]
S[3]	324.132	0.000	306.121	**352.127**	0.000	334.116	**629.229**	612.202	**611.218**	S[5]
E[4]	453.174	0.000	435.164	481.169	0.000	463.159	**462.231**	445.204	444.220	E[4]
A[5]	524.212	0.000	506.201	552.207	0.000	534.196	**333.188**	316.162	315.178	A[3]
S[6]	**611.244**	0.000	593.233	639.239	0.000	621.228	**262.151**	245.124	244.140	S[2]
R[7]	767.345	750.318	749.334	795.340	778.313	777.329	**175.119**	158.092	0.000	R[1]

sp|P70696|H2B1A_MOUSE

STITSR $^{(Phospho)}_{(79.97)}$ EIQTAVR

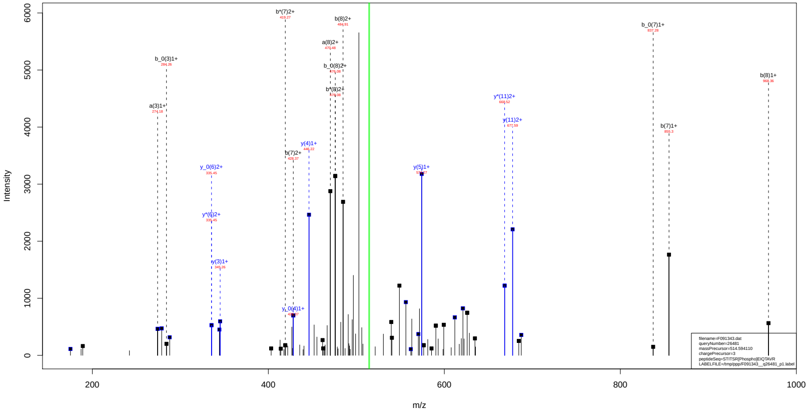

sp|P70696|H2B1A_MOUSE

STITSR $^{(Phospho)}_{(79.97)}$ EIQTAVR

- fragmentation table for charge state 1+
- specType=cid
- score=49.54
- F091343.dat
- query=q26481_p1
- precursor=514.594110
- chargePrecursor=3
- itol=0.5

AA	a	a*	a_0	b	b*	b_0	y	y*	y_0	AA
S[1]	60.044	0.000	42.034	88.039	0.000	70.029	1541.768	1524.742	1523.758	S[13]
T[2]	161.092	0.000	143.082	**189.087**	0.000	171.076	1454.736	1437.710	1436.726	T[12]
I[3]	**274.176**	0.000	256.166	302.171	0.000	**284.160**	1353.689	1336.662	1335.678	I[11]
T[4]	375.224	0.000	357.213	**403.219**	0.000	385.208	1240.605	1223.578	1222.594	T[10]
S[5]	**462.256**	0.000	444.245	490.251	0.000	472.240	1139.557	1122.530	1121.546	S[9]
R[6]	698.323	681.297	680.313	726.318	709.292	708.308	1052.525	1035.498	1034.514	R[8]
E[7]	827.366	810.339	809.355	**855.361**	838.334	**837.350**	816.457	799.431	798.447	E[7]
I[8]	940.450	923.423	922.439	**968.445**	951.418	950.434	**687.415**	670.388	669.404	I[6]
Q[9]	1068.509	1051.482	1050.498	1096.503	1079.477	1078.493	**574.331**	557.304	**556.320**	Q[5]
T[10]	1169.556	1152.530	1151.546	1197.551	1180.525	1179.541	**446.272**	429.246	**428.262**	T[4]
A[11]	1240.593	1223.567	1222.583	1268.588	1251.562	1250.578	**345.224**	328.198	0.000	A[3]
V[12]	1339.662	1322.635	1321.651	1367.657	1350.630	1349.646	**274.187**	257.161	0.000	V[2]
R[13]	1495.763	1478.736	1477.752	1523.758	1506.731	1505.747	**175.119**	158.092	0.000	R[1]

sp|P70696|H2B1A_MOUSE

STITSR$^{(Phospho)}_{(79.97)}$EIQTAVR

- fragmentation table for charge state 2+
- specType=cid
- score=49.54
- F091343.dat
- query=q26481_p1
- precursor=514.594110
- chargePrecursor=3
- itol=0.5

AA	a	a*	a_0	b	b*	b_0	y	y*	y_0	AA
S[1]	30.526	0.504	21.521	44.523	0.504	35.518	771.388	762.875	762.383	S[13]
T[2]	81.050	0.504	72.044	95.047	0.504	86.042	727.872	719.359	718.866	T[12]
I[3]	137.592	0.504	128.586	151.589	0.504	142.584	**677.348**	**668.835**	**668.343**	I[11]
T[4]	188.116	0.504	179.110	202.113	0.504	193.108	**620.806**	**612.293**	**611.801**	T[10]
S[5]	231.632	0.504	222.626	245.629	0.504	236.624	**570.282**	**561.769**	561.277	S[9]
R[6]	349.665	341.152	340.660	363.663	355.149	354.657	526.766	518.253	517.761	R[8]
E[7]	**414.187**	405.673	405.181	**428.184**	**419.671**	**419.179**	408.732	400.219	399.727	E[7]
I[8]	**470.729**	**462.215**	**461.723**	**484.726**	**476.213**	**475.721**	**344.211**	**335.698**	**335.206**	I[6]
Q[9]	534.758	526.245	525.753	**548.755**	**540.242**	**539.750**	**287.669**	**279.156**	**278.664**	Q[5]
T[10]	**585.282**	**576.768**	**576.276**	**599.279**	**590.766**	**590.274**	223.640	215.126	214.634	T[4]
A[11]	**620.800**	**612.287**	**611.795**	**634.798**	**626.284**	**625.792**	173.116	164.603	0.504	A[3]
V[12]	670.334	661.821	661.329	**684.332**	675.819	675.327	137.597	129.084	0.504	V[2]
R[13]	748.385	739.872	739.380	762.383	753.869	753.377	88.063	79.550	0.504	R[1]

sp|P70696|H2B1A_MOUSE

STITS$^{(Phospho)}_{(79.97)}$REIQTAVR

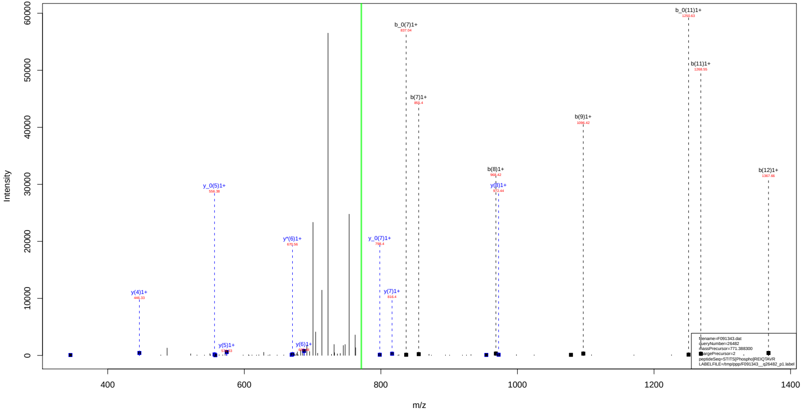

sp|P70696|H2B1A_MOUSE

STITS$^{(Phospho)}_{(79.97)}$REIQTAVR

- fragmentation table for charge state 1+
- specType=cid
- score=38.58
- F091343.dat
- query=q26482_p1
- precursor=771.388300
- chargePrecursor=2
- itol=0.5

AA	a	a*	a_0	b	b*	b_0	y	y*	y_0	AA
S[1]	60.044	0.000	42.034	88.039	0.000	70.029	1541.768	1524.742	1523.758	S[13]
T[2]	161.092	0.000	143.082	189.087	0.000	171.076	1454.736	1437.710	1436.726	T[12]
I[3]	274.176	0.000	256.166	302.171	0.000	284.160	1353.689	1336.662	1335.678	I[11]
T[4]	375.224	0.000	357.213	403.219	0.000	385.208	1240.605	1223.578	1222.594	T[10]
S[5]	542.222	0.000	524.212	570.217	0.000	552.207	1139.557	1122.530	1121.546	S[9]
R[6]	698.323	681.297	680.313	726.318	709.292	708.308	**972.559**	955.532	**954.548**	R[8]
E[7]	827.366	810.339	809.355	**855.361**	838.334	**837.350**	**816.457**	799.431	**798.447**	E[7]
I[8]	940.450	923.423	922.439	**968.445**	951.418	950.434	**687.415**	**670.388**	**669.404**	I[6]
Q[9]	1068.509	1051.482	1050.498	**1096.503**	1079.477	**1078.493**	**574.331**	**557.304**	**556.320**	Q[5]
T[10]	1169.556	1152.530	1151.546	1197.551	1180.525	1179.541	**446.272**	429.246	428.262	T[4]
A[11]	1240.593	1223.567	1222.583	**1268.588**	1251.562	**1250.578**	**345.224**	328.198	0.000	A[3]
V[12]	1339.662	1322.635	1321.651	**1367.657**	1350.630	1349.646	274.187	257.161	0.000	V[2]
R[13]	1495.763	1478.736	1477.752	1523.758	1506.731	1505.747	175.119	158.092	0.000	R[1]

sp|P43274|H14_MOUSE

S $^{Phospho}_{79.97}$ ETAPAAPAAPAPAEK

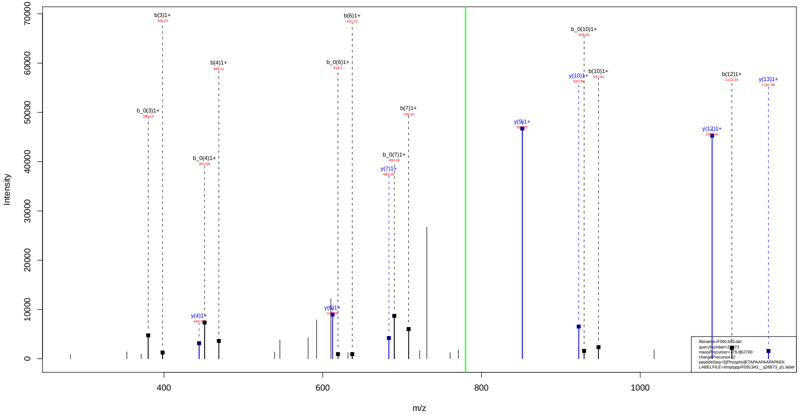

sp|P43274|H14_MOUSE

$S^{Phospho}_{79.97}$ETAPAAPAAPAPAEK

- fragmentation table for charge state 1+
- specType=cid
- score=59.59
- F091343.dat
- query=q26873_p1
- precursor=779.862700
- chargePrecursor=2
- itol=0.5

AA	a	a*	a_0	b	b*	b_0	y	y*	y_0	AA
S[1]	140.011	0.000	122.000	168.006	0.000	149.995	1558.715	1541.688	1540.704	S[16]
E[2]	269.053	0.000	251.043	297.048	0.000	279.038	1391.717	1374.690	1373.706	E[15]
T[3]	370.101	0.000	352.090	**398.096**	0.000	**380.085**	1262.674	1245.647	1244.663	T[14]
A[4]	441.138	0.000	423.128	**469.133**	0.000	**451.122**	**1161.626**	1144.600	1143.616	A[13]
P[5]	538.191	0.000	520.180	566.186	0.000	548.175	**1090.589**	1073.563	1072.579	P[12]
A[6]	609.228	0.000	591.217	**637.223**	0.000	**619.212**	993.536	976.510	975.526	A[11]
A[7]	680.265	0.000	662.255	**708.260**	0.000	**690.249**	**922.499**	905.473	904.489	A[10]
P[8]	777.318	0.000	759.307	805.313	0.000	787.302	**851.462**	834.436	833.452	P[9]
A[9]	848.355	0.000	830.344	876.350	0.000	858.339	754.409	737.383	736.399	A[8]
A[10]	919.392	0.000	901.382	**947.387**	0.000	**929.376**	**683.372**	666.346	665.362	A[7]
P[11]	1016.445	0.000	998.434	1044.440	0.000	1026.429	**612.335**	595.309	594.325	P[6]
A[12]	1087.482	0.000	1069.471	**1115.477**	0.000	1097.466	515.282	498.256	497.272	A[5]
P[13]	1184.535	0.000	1166.524	1212.530	0.000	1194.519	**444.245**	427.219	426.235	P[4]
A[14]	1255.572	0.000	1237.561	1283.567	0.000	1265.556	347.193	330.166	329.182	A[3]
E[15]	1384.614	0.000	1366.604	1412.609	0.000	1394.599	276.155	259.129	258.145	E[2]
K[16]	1512.709	1495.683	1494.699	1540.704	1523.678	1522.694	147.113	130.086	0.000	K[1]

sp|P15864|H12_MOUSE

RKAS $^{Phospho}_{79.97}$ GPPVSELITK

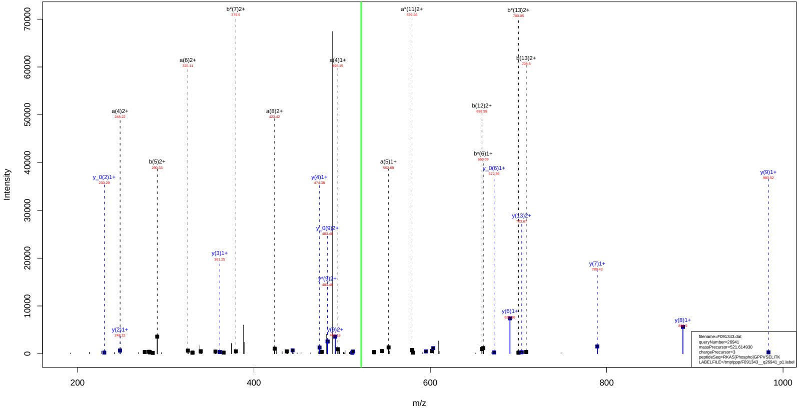

sp|P15864|H12_MOUSE

RKAS $^{Phospho}_{79.97}$ GPPVSELITK

- fragmentation table for charge state 1+
- specType=cid
- score=59.08
- F091343.dat
- query=q26941_p1
- precursor=521.614930
- chargePrecursor=3
- itol=0.5

AA	a	a*	a_0	b	b*	b_0	y	y*	y_0	AA
R[1]	129.113	112.087	0.000	157.108	140.082	0.000	1562.830	1545.804	1544.820	R[14]
K[2]	257.208	240.182	0.000	**285.203**	268.177	0.000	1406.729	1389.703	1388.719	K[13]
A[3]	328.246	311.219	0.000	**356.240**	**339.214**	0.000	1278.634	1261.608	1260.624	A[12]
S[4]	**495.244**	478.217	**477.233**	523.239	506.212	505.228	1207.597	1190.570	1189.586	S[11]
G[5]	**552.265**	535.239	534.255	**580.260**	563.234	562.250	1040.599	1023.572	1022.588	G[10]
P[6]	649.318	632.292	631.308	677.313	**660.287**	659.302	**983.577**	966.551	965.567	P[9]
P[7]	746.371	729.344	728.360	774.366	757.339	756.355	**886.524**	869.498	868.514	P[8]
V[8]	845.439	828.413	827.429	873.434	856.408	855.424	**789.472**	772.445	771.461	V[7]
S[9]	932.471	915.445	914.461	960.466	943.440	942.456	**690.403**	673.377	**672.393**	S[6]
E[10]	1061.514	1044.487	1043.503	1089.509	1072.482	1071.498	**603.371**	586.345	585.361	E[5]
L[11]	1174.598	1157.571	1156.587	1202.593	1185.566	1184.582	**474.329**	457.302	456.318	L[4]
I[12]	1287.682	1270.656	1269.671	1315.677	1298.650	1297.666	**361.245**	344.218	343.234	I[3]
T[13]	1388.730	1371.703	1370.719	1416.725	1399.698	1398.714	**248.160**	231.134	**230.150**	T[2]
K[14]	1516.825	1499.798	1498.814	1544.820	1527.793	1526.809	147.113	130.086	0.000	K[1]

sp|P15864|H12_MOUSE

RKAS $^{Phospho}_{79.97}$ GPPVSELITK

- fragmentation table for charge state 2+
- specType=cid
- score=59.08
- F091343.dat
- query=q26941_p1
- precursor=521.614930
- chargePrecursor=3
- itol=0.5

AA	a	a*	a_0	b	b*	b_0	y	y*	y_0	AA
R[1]	65.060	56.547	0.504	79.058	70.545	0.504	781.919	773.405	772.913	R[14]
K[2]	129.108	120.595	0.504	143.105	134.592	0.504	**703.868**	695.355	694.863	K[13]
A[3]	164.626	156.113	0.504	178.624	170.111	0.504	639.821	631.307	630.815	A[12]
S[4]	**248.126**	239.612	239.120	262.123	253.610	253.118	604.302	595.789	**595.297**	S[11]
G[5]	**276.636**	268.123	267.631	**290.634**	**282.121**	**281.628**	520.803	**512.290**	**511.798**	G[10]
P[6]	**325.163**	316.649	316.157	**339.160**	**330.647**	**330.155**	**492.292**	**483.779**	**483.287**	P[9]
P[7]	373.689	**365.176**	364.684	387.687	**379.173**	378.681	**443.766**	435.253	434.761	P[8]
V[8]	**423.223**	414.710	414.218	**437.221**	428.707	428.215	395.239	386.726	386.234	V[7]
S[9]	466.739	458.226	457.734	480.737	472.223	471.731	345.705	337.192	336.700	S[6]
E[10]	531.261	522.747	522.255	**545.258**	**536.745**	**536.253**	302.189	293.676	293.184	E[5]
L[11]	587.803	**579.289**	**578.797**	**601.800**	593.287	592.795	237.668	229.155	228.663	L[4]
I[12]	644.345	635.831	635.339	**658.342**	649.829	649.337	181.126	172.613	172.121	I[3]
T[13]	694.869	686.355	685.863	**708.866**	**700.353**	**699.861**	124.584	116.071	115.579	T[2]
K[14]	758.916	750.403	749.911	772.913	764.400	763.908	74.060	65.547	0.504	K[1]

sp|P15864|H12_MOUSE

RKAS $^{Phospho}_{79.97}$ GPPVSELITK

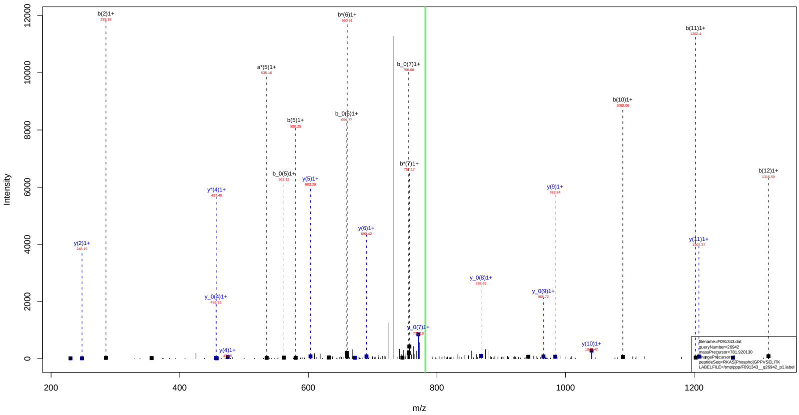

sp|P15864|H12_MOUSE

RKAS $^{Phospho}_{79.97}$ GPPVSELITK

- fragmentation table for charge state 1+
- specType=cid
- score=40.66
- F091343.dat
- query=q26942_p1
- precursor=781.920130
- chargePrecursor=2
- itol=0.5

AA	a	a*	a_0	b	b*	b_0	y	y*	y_0	AA
R[1]	129.113	112.087	0.000	157.108	140.082	0.000	1562.830	1545.804	1544.820	R[14]
K[2]	257.208	240.182	0.000	**285.203**	268.177	0.000	1406.729	1389.703	1388.719	K[13]
A[3]	328.246	311.219	0.000	**356.240**	339.214	0.000	1278.634	1261.608	**1260.624**	A[12]
S[4]	495.244	478.217	477.233	523.239	506.212	505.228	**1207.597**	1190.570	1189.586	S[11]
G[5]	552.265	**535.239**	534.255	**580.260**	563.234	**562.250**	**1040.599**	1023.572	1022.588	G[10]
P[6]	649.318	**632.292**	**631.308**	677.313	**660.287**	**659.302**	**983.577**	966.551	**965.567**	P[9]
P[7]	**746.371**	729.344	728.360	774.366	**757.339**	**756.355**	886.524	869.498	**868.514**	P[8]
V[8]	845.439	828.413	827.429	873.434	856.408	855.424	789.472	772.445	**771.461**	V[7]
S[9]	932.471	915.445	914.461	960.466	943.440	**942.456**	**690.403**	673.377	**672.393**	S[6]
E[10]	1061.514	1044.487	1043.503	**1089.509**	1072.482	1071.498	**603.371**	586.345	585.361	E[5]
L[11]	1174.598	1157.571	1156.587	**1202.593**	1185.566	1184.582	**474.329**	**457.302**	**456.318**	L[4]
I[12]	1287.682	1270.656	1269.671	**1315.677**	1298.650	1297.666	361.245	344.218	343.234	I[3]
T[13]	1388.730	1371.703	1370.719	1416.725	1399.698	1398.714	**248.160**	231.134	**230.150**	T[2]
K[14]	1516.825	1499.798	1498.814	1544.820	1527.793	1526.809	147.113	130.086	0.000	K[1]

sp|P43277|H13_MOUSE

S $^{Phospho}_{79.97}$ ETAPAAPAAPAPVEK

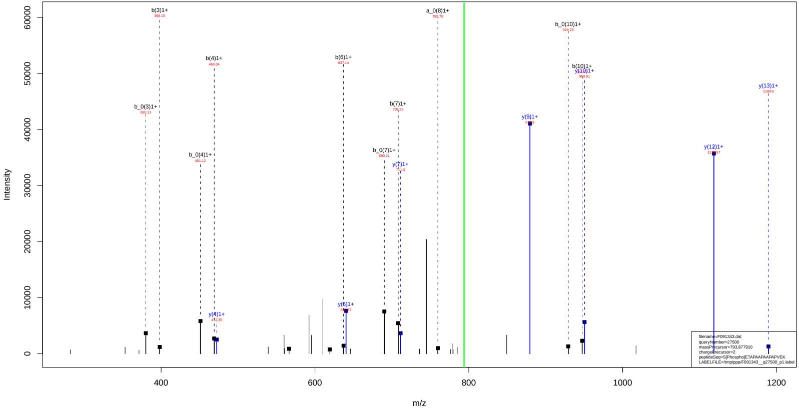

sp|P43277|H13_MOUSE

S $^{Phospho}_{79.97}$ ETAPAAPAAPAPVEK

- fragmentation table for charge state 1+
- specType=cid
- score=60.10
- F091343.dat
- query=q27500_p1
- precursor=793.877910
- chargePrecursor=2
- itol=0.5

AA	a	a*	a_0	b	b*	b_0	y	y*	y_0	AA
S[1]	140.011	0.000	122.000	168.006	0.000	149.995	1586.746	1569.720	1568.736	S[16]
E[2]	269.053	0.000	251.043	297.048	0.000	279.038	1419.748	1402.721	1401.737	E[15]
T[3]	370.101	0.000	352.090	**398.096**	0.000	**380.085**	1290.705	1273.679	1272.695	T[14]
A[4]	441.138	0.000	423.128	**469.133**	0.000	**451.122**	**1189.658**	1172.631	1171.647	A[13]
P[5]	538.191	0.000	520.180	**566.186**	0.000	548.175	**1118.620**	1101.594	1100.610	P[12]
A[6]	609.228	0.000	591.217	**637.223**	0.000	**619.212**	1021.568	1004.541	1003.557	A[11]
A[7]	680.265	0.000	662.255	**708.260**	0.000	**690.249**	**950.531**	933.504	932.520	A[10]
P[8]	777.318	0.000	**759.307**	805.313	0.000	787.302	**879.493**	862.467	861.483	P[9]
A[9]	848.355	0.000	830.344	876.350	0.000	858.339	782.441	765.414	764.430	A[8]
A[10]	919.392	0.000	901.382	**947.387**	0.000	**929.376**	**711.404**	694.377	693.393	A[7]
P[11]	1016.445	0.000	998.434	1044.440	0.000	1026.429	**640.366**	623.340	622.356	P[6]
A[12]	1087.482	0.000	1069.471	1115.477	0.000	1097.466	543.314	526.287	525.303	A[5]
P[13]	1184.535	0.000	1166.524	1212.530	0.000	1194.519	**472.277**	455.250	454.266	P[4]
V[14]	1283.603	0.000	1265.593	1311.598	0.000	1293.587	375.224	358.197	357.213	V[3]
E[15]	1412.646	0.000	1394.635	1440.641	0.000	1422.630	276.155	259.129	258.145	E[2]
K[16]	1540.741	1523.714	1522.730	1568.736	1551.709	1550.725	147.113	130.086	0.000	K[1]

sp|P43274|H14_MOUSE

RKT $^{Phospho}_{79.97}$ SGPPVSELITK

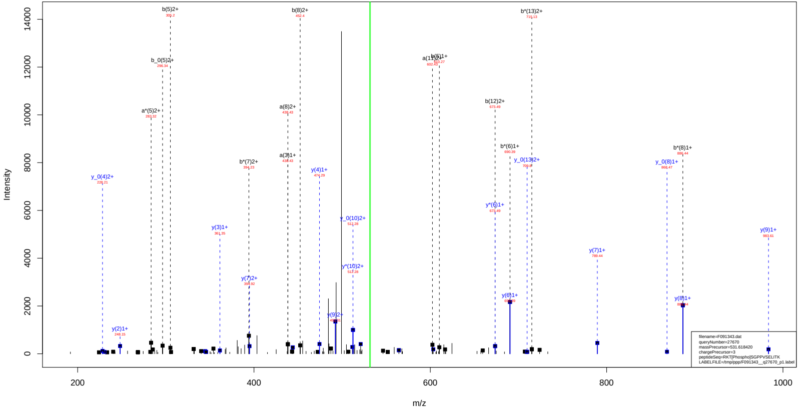

sp|P43274|H14_MOUSE

RKT $^{Phospho}_{79.97}$ SGPPVSELITK

- fragmentation table for charge state 1+
- specType=cid
- score=41.45
- F091343.dat
- query=q27670_p1
- precursor=531.618420
- chargePrecursor=3
- itol=0.5

AA	a	a*	a_0	b	b*	b_0	y	y*	y_0	AA
R[1]	129.113	112.087	0.000	157.108	140.082	0.000	1592.841	1575.814	1574.830	R[14]
K[2]	257.208	**240.182**	0.000	**285.203**	**268.177**	0.000	1436.740	1419.713	1418.729	K[13]
T[3]	**438.222**	421.196	420.212	466.217	449.191	448.207	1308.645	1291.618	1290.634	T[12]
S[4]	525.254	508.228	**507.244**	553.249	536.223	535.239	1127.631	1110.604	1109.620	S[11]
G[5]	582.276	565.249	**564.265**	**610.271**	593.244	592.260	1040.599	1023.572	1022.588	G[10]
P[6]	679.329	662.302	661.318	**707.324**	**690.297**	689.313	**983.577**	966.551	965.567	P[9]
P[7]	776.381	759.355	758.371	804.376	787.350	786.366	**886.524**	869.498	**868.514**	P[8]
V[8]	875.450	858.423	857.439	903.445	**886.418**	885.434	**789.472**	772.445	771.461	V[7]
S[9]	962.482	945.455	944.471	990.477	973.450	972.466	**690.403**	**673.377**	672.393	S[6]
E[10]	1091.524	1074.498	1073.514	1119.519	1102.493	1101.509	**603.371**	586.345	585.361	E[5]
L[11]	1204.609	1187.582	1186.598	1232.603	1215.577	1214.593	**474.329**	457.302	456.318	L[4]
I[12]	1317.693	1300.666	1299.682	1345.688	1328.661	1327.677	**361.245**	**344.218**	343.234	I[3]
T[13]	1418.740	1401.714	1400.730	1446.735	1429.709	1428.725	**248.160**	231.134	**230.150**	T[2]
K[14]	1546.835	1529.809	1528.825	1574.830	1557.804	1556.820	147.113	130.086	0.000	K[1]

sp|P43274|H14_MOUSE

RKT $^{Phospho}_{79.97}$ SGPPVSELITK

- fragmentation table for charge state 2+
- specType=cid
- score=41.45
- F091343.dat
- query=q27670_p1
- precursor=531.618420
- chargePrecursor=3
- itol=0.5

AA	a	a*	a_0	b	b*	b_0	y	y*	y_0	AA
R[1]	65.060	56.547	0.504	79.058	70.545	0.504	796.924	788.411	787.919	R[14]
K[2]	129.108	120.595	0.504	143.105	134.592	0.504	718.873	710.360	**709.868**	K[13]
T[3]	219.615	211.102	210.610	**233.612**	225.099	**224.607**	654.826	646.313	645.821	T[12]
S[4]	263.131	254.618	254.126	277.128	**268.615**	**268.123**	**564.319**	555.806	555.314	S[11]
G[5]	291.642	**283.128**	**282.636**	**305.639**	297.126	**296.634**	**520.803**	**512.290**	**511.798**	G[10]
P[6]	**340.168**	**331.655**	**331.163**	**354.165**	**345.652**	**345.160**	**492.292**	483.779	483.287	P[9]
P[7]	388.694	380.181	379.689	402.692	**394.179**	393.687	**443.766**	435.253	434.761	P[8]
V[8]	**438.229**	429.715	429.223	**452.226**	**443.713**	**443.221**	**395.239**	386.726	386.234	V[7]
S[9]	481.745	473.231	**472.739**	495.742	**487.229**	**486.737**	**345.705**	337.192	336.700	S[6]
E[10]	**546.266**	537.753	537.261	560.263	**551.750**	**551.258**	302.189	293.676	293.184	E[5]
L[11]	**602.808**	594.295	593.803	**616.805**	608.292	607.800	237.668	229.155	**228.663**	L[4]
I[12]	**659.350**	650.837	650.345	**673.347**	664.834	664.342	181.126	172.613	172.121	I[3]
T[13]	**709.874**	701.361	700.869	**723.871**	**715.358**	**714.866**	124.584	116.071	115.579	T[2]
K[14]	773.921	765.408	764.916	787.919	779.405	778.913	74.060	65.547	0.504	K[1]

sp|P43274|H14_MOUSE

RKT $^{\text{Phospho}}_{79.97}$ SGPPVSELITK

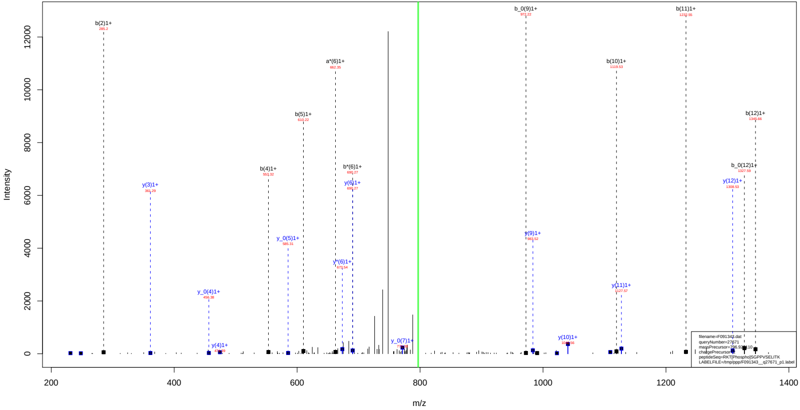

sp|P43274|H14_MOUSE

RKT $^{Phospho}_{79.97}$ SGPPVSELITK

- fragmentation table for charge state 1+
- specType=cid
- score=42.92
- F091343.dat
- query=q27671_p1
- precursor=796.925110
- chargePrecursor=2
- itol=0.5

AA	a	a*	a_0	b	b*	b_0	y	y*	y_0	AA
R[1]	129.113	112.087	0.000	157.108	140.082	0.000	1592.841	1575.814	1574.830	R[14]
K[2]	257.208	240.182	0.000	**285.203**	268.177	0.000	1436.740	1419.713	1418.729	K[13]
T[3]	438.222	421.196	420.212	466.217	449.191	448.207	**1308.645**	1291.618	1290.634	T[12]
S[4]	525.254	508.228	507.244	**553.249**	536.223	535.239	**1127.631**	1110.604	**1109.620**	S[11]
G[5]	582.276	565.249	564.265	**610.271**	593.244	592.260	**1040.599**	1023.572	**1022.588**	G[10]
P[6]	679.329	**662.302**	661.318	707.324	**690.297**	689.313	**983.577**	966.551	965.567	P[9]
P[7]	776.381	759.355	758.371	804.376	787.350	786.366	886.524	869.498	868.514	P[8]
V[8]	875.450	858.423	857.439	903.445	886.418	885.434	789.472	772.445	**771.461**	V[7]
S[9]	962.482	945.455	944.471	**990.477**	973.450	**972.466**	**690.403**	**673.377**	672.393	S[6]
E[10]	1091.524	1074.498	1073.514	**1119.519**	1102.493	1101.509	603.371	586.345	**585.361**	E[5]
L[11]	1204.609	1187.582	1186.598	**1232.603**	1215.577	1214.593	**474.329**	457.302	**456.318**	L[4]
I[12]	1317.693	1300.666	1299.682	**1345.688**	1328.661	**1327.677**	**361.245**	344.218	343.234	I[3]
T[13]	1418.740	1401.714	1400.730	1446.735	1429.709	1428.725	**248.160**	**231.134**	230.150	T[2]
K[14]	1546.835	1529.809	1528.825	1574.830	1557.804	1556.820	147.113	130.086	0.000	K[1]

sp|P27661|H2AX_MOUSE

APAVGKKAS $^{Phospho}_{79.97}$ QASQEY

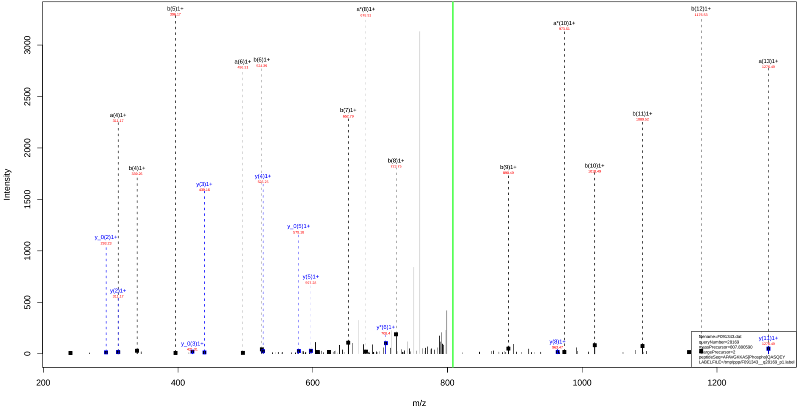

sp|P27661|H2AX_MOUSE

APAVGKKAS $^{Phospho}_{79.97}$ QASQEY

- fragmentation table for charge state 1+
- specType=cid
- score=71.15
- F091343.dat
- query=q28169_p1
- precursor=807.880590
- chargePrecursor=2
- itol=0.5

AA	a	a*	a_0	b	b*	b_0	y	y*	y_0	AA
A[1]	44.049	0.000	0.000	72.044	0.000	0.000	1614.752	1597.726	1596.742	A[15]
P[2]	141.102	0.000	0.000	169.097	0.000	0.000	1543.715	1526.689	1525.705	P[14]
A[3]	212.139	0.000	0.000	**240.134**	0.000	0.000	1446.662	1429.636	1428.652	A[13]
V[4]	**311.208**	0.000	0.000	**339.203**	0.000	0.000	1375.625	1358.599	1357.615	V[12]
G[5]	368.229	0.000	0.000	**396.224**	0.000	0.000	**1276.557**	1259.530	1258.546	G[11]
K[6]	**496.324**	479.298	0.000	**524.319**	507.293	0.000	1219.535	1202.509	1201.525	K[10]
K[7]	**624.419**	**607.393**	0.000	**652.414**	635.388	0.000	1091.440	1074.414	1073.430	K[9]
A[8]	695.456	**678.430**	0.000	**723.451**	706.425	0.000	**963.346**	946.319	945.335	A[8]
S[9]	862.455	845.428	844.444	**890.450**	873.423	872.439	892.308	875.282	874.298	S[7]
Q[10]	990.513	**973.487**	972.503	**1018.508**	1001.482	1000.498	725.310	**708.284**	707.299	Q[6]
A[11]	1061.550	1044.524	1043.540	**1089.545**	1072.519	1071.535	**597.251**	580.225	**579.241**	A[5]
S[12]	1148.582	1131.556	1130.572	**1176.577**	1159.551	**1158.567**	**526.214**	509.188	508.204	S[4]
Q[13]	**1276.641**	1259.614	1258.630	1304.636	1287.609	1286.625	**439.182**	422.156	**421.172**	Q[3]
E[14]	1405.684	1388.657	1387.673	1433.678	1416.652	1415.668	**311.124**	0.000	**293.113**	E[2]
Y[15]	1568.747	1551.720	1550.736	1596.742	1579.715	1578.731	182.081	0.000	0.000	Y[1]

sp|P43274|H14_MOUSE

S $^{Phospho}_{79.97}$ ET $^{Phospho}_{79.97}$ APAAPAAPAPAEK

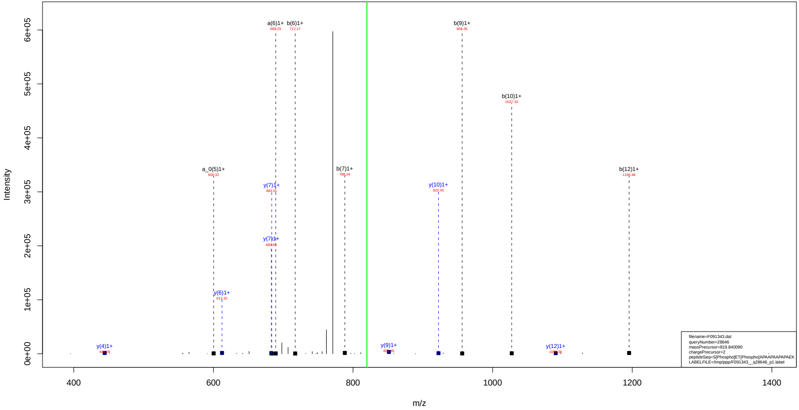

sp|P43274|H14_MOUSE

S $^{Phospho}_{79.97}$ ET $^{Phospho}_{79.97}$ APAAPAAPAPAEK

- fragmentation table for charge state 1+
- specType=cid
- score=34.27
- F091343.dat
- query=q28646_p1
- precursor=819.840090
- chargePrecursor=2
- itol=0.5

AA	a	a*	a_0	b	b*	b_0	y	y*	y_0	AA
S[1]	140.011	0.000	122.000	168.006	0.000	149.995	1638.681	1621.655	1620.671	S[16]
E[2]	269.053	0.000	251.043	297.048	0.000	279.038	1471.683	1454.656	1453.672	E[15]
T[3]	450.067	0.000	432.057	478.062	0.000	460.052	1342.640	1325.614	1324.630	T[14]
A[4]	521.104	0.000	503.094	549.099	0.000	531.089	1161.626	1144.600	1143.616	A[13]
P[5]	618.157	0.000	**600.147**	646.152	0.000	628.142	**1090.589**	1073.563	1072.579	P[12]
A[6]	**689.194**	0.000	671.184	**717.189**	0.000	699.179	993.536	976.510	975.526	A[11]
A[7]	760.231	0.000	742.221	**788.226**	0.000	770.216	**922.499**	905.473	904.489	A[10]
P[8]	857.284	0.000	839.274	885.279	0.000	867.269	**851.462**	834.436	833.452	P[9]
A[9]	928.321	0.000	910.311	**956.316**	0.000	938.306	754.409	737.383	736.399	A[8]
A[10]	999.358	0.000	981.348	**1027.353**	0.000	1009.343	**683.372**	666.346	665.362	A[7]
P[11]	1096.411	0.000	1078.401	1124.406	0.000	1106.396	**612.335**	595.309	594.325	P[6]
A[12]	1167.448	0.000	1149.438	**1195.443**	0.000	1177.433	515.282	498.256	497.272	A[5]
P[13]	1264.501	0.000	1246.490	1292.496	0.000	1274.485	**444.245**	427.219	426.235	P[4]
A[14]	1335.538	0.000	1317.528	1363.533	0.000	1345.523	347.193	330.166	329.182	A[3]
E[15]	1464.581	0.000	1446.570	1492.576	0.000	1474.565	276.155	259.129	258.145	E[2]
K[16]	1592.676	1575.649	1574.665	1620.671	1603.644	1602.660	147.113	130.086	0.000	K[1]

sp|P43274|H14_MOUSE

S $^{Phospho}_{79.97}$ ET $^{Phospho}_{79.97}$ APAAPAAPAPAEK

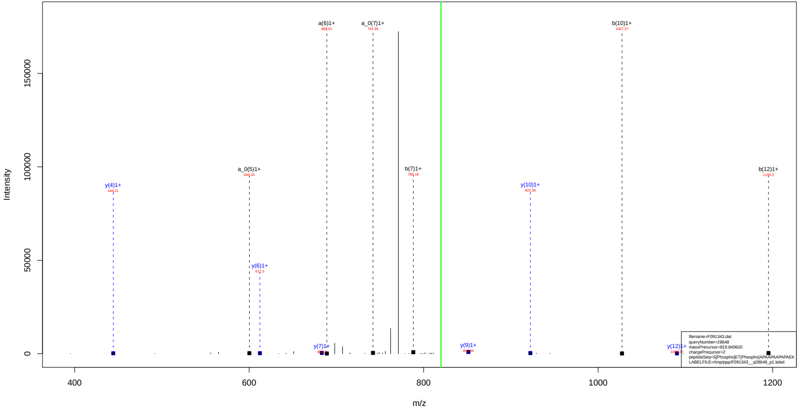

sp|P43274|H14_MOUSE

S $^{Phospho}_{79.97}$ ET $^{Phospho}_{79.97}$ APAAPAAPAPAEK

- fragmentation table for charge state 1+
- specType=cid
- score=32.59
- F091343.dat
- query=q28648_p1
- precursor=819.840620
- chargePrecursor=2
- itol=0.5

AA	a	a*	a_0	b	b*	b_0	y	y*	y_0	AA
S[1]	140.011	0.000	122.000	168.006	0.000	149.995	1638.681	1621.655	1620.671	S[16]
E[2]	269.053	0.000	251.043	297.048	0.000	279.038	1471.683	1454.656	1453.672	E[15]
T[3]	450.067	0.000	432.057	478.062	0.000	460.052	1342.640	1325.614	1324.630	T[14]
A[4]	521.104	0.000	503.094	549.099	0.000	531.089	1161.626	1144.600	1143.616	A[13]
P[5]	618.157	0.000	**600.147**	646.152	0.000	628.142	**1090.589**	1073.563	1072.579	P[12]
A[6]	**689.194**	0.000	671.184	717.189	0.000	699.179	993.536	976.510	975.526	A[11]
A[7]	760.231	0.000	**742.221**	**788.226**	0.000	770.216	**922.499**	905.473	904.489	A[10]
P[8]	857.284	0.000	839.274	885.279	0.000	867.269	**851.462**	834.436	833.452	P[9]
A[9]	928.321	0.000	910.311	956.316	0.000	938.306	754.409	737.383	736.399	A[8]
A[10]	999.358	0.000	981.348	**1027.353**	0.000	1009.343	**683.372**	666.346	665.362	A[7]
P[11]	1096.411	0.000	1078.401	1124.406	0.000	1106.396	**612.335**	595.309	594.325	P[6]
A[12]	1167.448	0.000	1149.438	**1195.443**	0.000	1177.433	515.282	498.256	497.272	A[5]
P[13]	1264.501	0.000	1246.490	1292.496	0.000	1274.485	**444.245**	427.219	426.235	P[4]
A[14]	1335.538	0.000	1317.528	1363.533	0.000	1345.523	347.193	330.166	329.182	A[3]
E[15]	1464.581	0.000	1446.570	1492.576	0.000	1474.565	276.155	259.129	258.145	E[2]
K[16]	1592.676	1575.649	1574.665	1620.671	1603.644	1602.660	147.113	130.086	0.000	K[1]

sp|P43274|H14_MOUSE

S $^{Phospho}_{79.97}$ ET $^{Phospho}_{79.97}$ APAAPAAPAPAEK

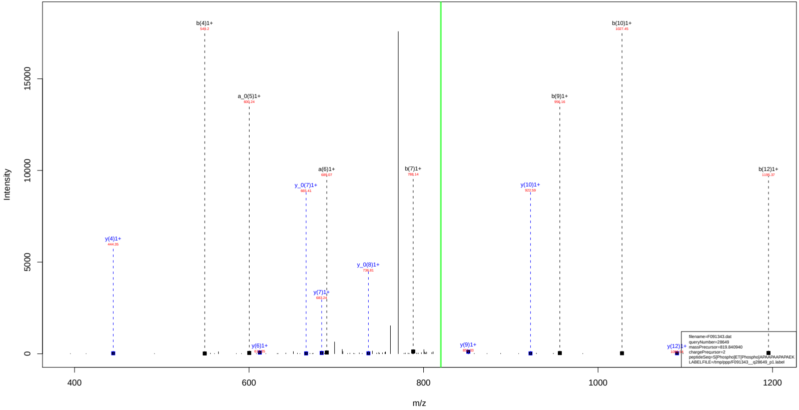

sp|P43274|H14_MOUSE

S $^{Phospho}_{79.97}$ ET $^{Phospho}_{79.97}$ APAAPAAPAPAEK

- fragmentation table for charge state 1+
- specType=cid
- score=32.71
- F091343.dat
- query=q28649_p1
- precursor=819.840940
- chargePrecursor=2
- itol=0.5

AA	a	a*	a_0	b	b*	b_0	y	y*	y_0	AA
S[1]	140.011	0.000	122.000	168.006	0.000	149.995	1638.681	1621.655	1620.671	S[16]
E[2]	269.053	0.000	251.043	297.048	0.000	279.038	1471.683	1454.656	1453.672	E[15]
T[3]	450.067	0.000	432.057	478.062	0.000	460.052	1342.640	1325.614	1324.630	T[14]
A[4]	521.104	0.000	503.094	**549.099**	0.000	531.089	1161.626	1144.600	1143.616	A[13]
P[5]	618.157	0.000	**600.147**	646.152	0.000	628.142	**1090.589**	1073.563	1072.579	P[12]
A[6]	**689.194**	0.000	671.184	717.189	0.000	699.179	993.536	976.510	975.526	A[11]
A[7]	760.231	0.000	742.221	**788.226**	0.000	770.216	**922.499**	905.473	904.489	A[10]
P[8]	857.284	0.000	839.274	885.279	0.000	867.269	**851.462**	834.436	833.452	P[9]
A[9]	928.321	0.000	910.311	**956.316**	0.000	938.306	754.409	737.383	**736.399**	A[8]
A[10]	999.358	0.000	981.348	**1027.353**	0.000	1009.343	**683.372**	666.346	**665.362**	A[7]
P[11]	1096.411	0.000	1078.401	1124.406	0.000	1106.396	**612.335**	595.309	594.325	P[6]
A[12]	1167.448	0.000	1149.438	**1195.443**	0.000	1177.433	515.282	498.256	497.272	A[5]
P[13]	1264.501	0.000	1246.490	1292.496	0.000	1274.485	**444.245**	427.219	426.235	P[4]
A[14]	1335.538	0.000	1317.528	1363.533	0.000	1345.523	347.193	330.166	329.182	A[3]
E[15]	1464.581	0.000	1446.570	1492.576	0.000	1474.565	276.155	259.129	258.145	E[2]
K[16]	1592.676	1575.649	1574.665	1620.671	1603.644	1602.660	147.113	130.086	0.000	K[1]

sp|P43276|H15_MOUSE

$S^{(Phospho)}_{(79.97)}$ ETAPAETAAPAPVEK

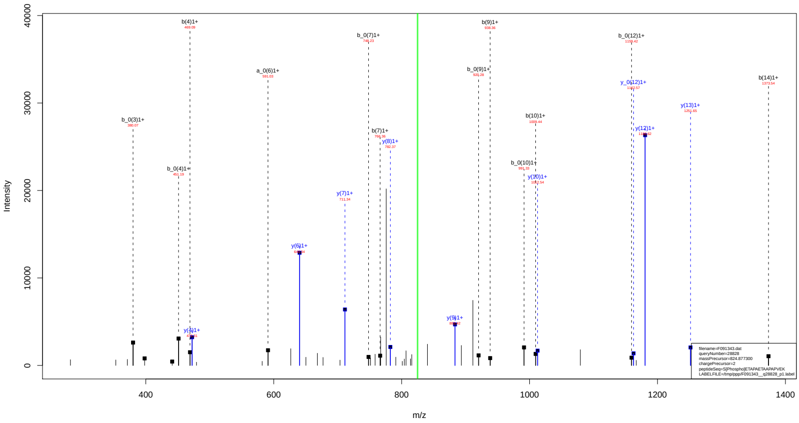

sp|P43276|H15_MOUSE

$S^{(Phospho)}_{(79.97)}$ETAPAETAAPAPVEK

- fragmentation table for charge state 1+
- specType=cid
- score=73.84
- F091343.dat
- query=q28828_p1
- precursor=824.877300
- chargePrecursor=2
- itol=0.5

AA	a	a*	a_0	b	b*	b_0	y	y*	y_0	AA
S[1]	140.011	0.000	122.000	168.006	0.000	149.995	1648.747	1631.720	1630.736	S[16]
E[2]	269.053	0.000	251.043	297.048	0.000	279.038	1481.748	1464.722	1463.738	E[15]
T[3]	370.101	0.000	352.090	**398.096**	0.000	**380.085**	1352.706	1335.679	1334.695	T[14]
A[4]	**441.138**	0.000	423.128	**469.133**	0.000	**451.122**	**1251.658**	1234.631	1233.647	A[13]
P[5]	538.191	0.000	520.180	566.186	0.000	548.175	**1180.621**	1163.594	**1162.610**	P[12]
A[6]	609.228	0.000	**591.217**	637.223	0.000	619.212	1083.568	1066.542	1065.558	A[11]
E[7]	738.271	0.000	720.260	**766.265**	0.000	**748.255**	**1012.531**	995.504	994.520	E[10]
T[8]	839.318	0.000	821.308	867.313	0.000	849.303	**883.488**	866.462	865.478	T[9]
A[9]	910.355	0.000	892.345	**938.350**	0.000	**920.340**	**782.441**	765.414	764.430	A[8]
A[10]	981.392	0.000	963.382	**1009.387**	0.000	**991.377**	**711.404**	694.377	693.393	A[7]
P[11]	1078.445	0.000	1060.435	1106.440	0.000	1088.430	**640.366**	623.340	622.356	P[6]
A[12]	1149.482	0.000	1131.472	1177.477	0.000	**1159.467**	543.314	526.287	525.303	A[5]
P[13]	1246.535	0.000	1228.525	1274.530	0.000	1256.519	**472.277**	455.250	454.266	P[4]
V[14]	1345.604	0.000	1327.593	**1373.598**	0.000	1355.588	375.224	358.197	357.213	V[3]
E[15]	1474.646	0.000	1456.636	1502.641	0.000	1484.630	276.155	259.129	258.145	E[2]
K[16]	1602.741	1585.715	1584.731	1630.736	1613.709	1612.725	147.113	130.086	0.000	K[1]

sp|P62806|H4_MOUSE

RIS $^{Phospho}_{79.97}$ GLIYEETRGVLK

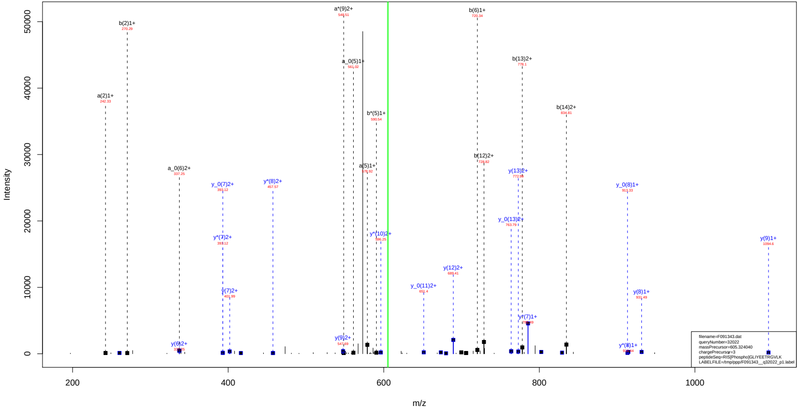

sp|P62806|H4_MOUSE

RIS $^{Phospho}_{79.97}$ GLIYEETRGVLK

- fragmentation table for charge state 1+
- specType=cid
- score=44.32
- F091343.dat
- query=q32022_p1
- precursor=605.324040
- chargePrecursor=3
- itol=0.5

AA	a	a*	a_0	b	b*	b_0	y	y*	y_0	AA
R[1]	129.113	112.087	0.000	157.108	140.082	0.000	1813.957	1796.931	1795.947	R[15]
I[2]	**242.198**	225.171	0.000	**270.192**	253.166	0.000	1657.856	1640.830	1639.846	I[14]
S[3]	409.196	392.169	391.185	437.191	420.164	419.180	1544.772	1527.745	1526.761	S[13]
G[4]	466.217	449.191	448.207	494.212	477.186	476.202	1377.774	1360.747	1359.763	G[12]
L[5]	**579.301**	562.275	**561.291**	607.296	**590.270**	589.286	1320.752	1303.726	1302.742	L[11]
I[6]	692.385	675.359	674.375	**720.380**	703.354	702.370	1207.668	1190.642	1189.658	I[10]
Y[7]	855.449	838.422	837.438	883.444	866.417	865.433	**1094.584**	1077.558	1076.573	Y[9]
E[8]	984.491	967.465	966.481	1012.486	995.460	994.476	**931.521**	**914.494**	**913.510**	E[8]
E[9]	1113.534	1096.507	1095.523	1141.529	1124.502	1123.518	**802.478**	**785.452**	784.468	E[7]
T[10]	1214.582	1197.555	1196.571	1242.577	1225.550	1224.566	**673.436**	656.409	655.425	T[6]
R[11]	1370.683	1353.656	1352.672	1398.678	1381.651	1380.667	572.388	555.361	0.000	R[5]
G[12]	1427.704	1410.678	1409.694	1455.699	1438.673	1437.689	**416.287**	399.260	0.000	G[4]
V[13]	1526.773	1509.746	1508.762	1554.768	1537.741	1536.757	359.265	342.239	0.000	V[3]
L[14]	1639.857	1622.830	1621.846	1667.852	1650.825	1649.841	**260.197**	243.170	0.000	L[2]
K[15]	1767.952	1750.925	1749.941	1795.947	1778.920	1777.936	147.113	130.086	0.000	K[1]

sp|P62806|H4_MOUSE

RIS $^{\text{Phospho}}_{79.97}$ GLIYEETRGVLK

- fragmentation table for charge state 2+
- specType=cid
- score=44.32
- F091343.dat
- query=q32022_p1
- precursor=605.324040
- chargePrecursor=3
- itol=0.5

AA	a	a*	a_0	b	b*	b_0	y	y*	y_0	AA
R[1]	65.060	56.547	0.504	79.058	70.545	0.504	907.482	898.969	898.477	R[15]
I[2]	121.602	113.089	0.504	135.600	127.087	0.504	**829.432**	820.918	820.426	I[14]
S[3]	205.102	196.588	196.096	219.099	210.586	210.094	**772.890**	764.376	**763.884**	S[13]
G[4]	233.612	225.099	224.607	247.610	239.097	238.604	**689.390**	680.877	**680.385**	G[12]
L[5]	290.154	281.641	281.149	304.152	295.639	295.147	660.880	652.366	**651.874**	L[11]
I[6]	346.696	338.183	**337.691**	360.694	352.181	351.689	604.338	**595.824**	595.332	I[10]
Y[7]	428.228	419.715	419.223	442.226	433.712	433.220	**547.796**	539.282	538.790	Y[9]
E[8]	492.749	484.236	483.744	506.747	498.234	497.742	466.264	**457.751**	**457.259**	E[8]
E[9]	557.271	**548.757**	**548.265**	571.268	562.755	562.263	**401.743**	**393.229**	**392.737**	E[7]
T[10]	607.794	599.281	598.789	621.792	613.279	612.787	**337.221**	328.708	328.216	T[6]
R[11]	685.845	677.332	676.840	**699.842**	691.329	690.837	286.698	278.184	0.504	R[5]
G[12]	714.356	**705.842**	**705.350**	**728.353**	719.840	719.348	208.647	200.134	0.504	G[4]
V[13]	**763.890**	755.377	754.885	**777.887**	769.374	768.882	180.136	171.623	0.504	V[3]
L[14]	820.432	811.919	811.427	**834.429**	825.916	825.424	130.602	122.089	0.504	L[2]
K[15]	884.479	875.966	875.474	898.477	889.964	889.472	74.060	65.547	0.504	K[1]

sp|P70696|H2B1A_MOUSE

HAVSEGT $^{\text{Phospho}}_{79.97}$ KAVTKYTSSK

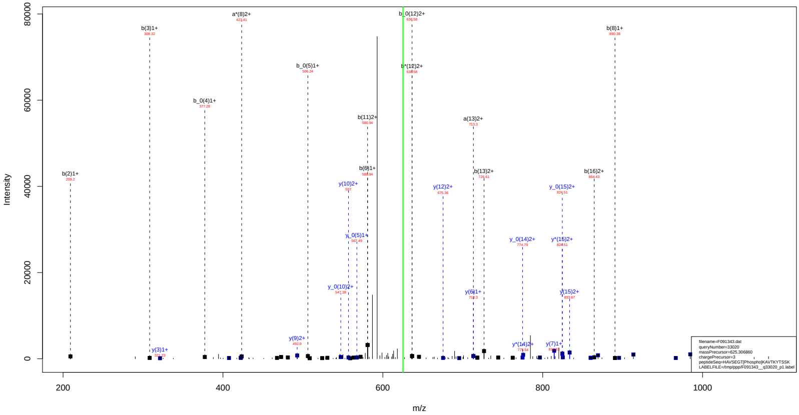

sp|P70696|H2B1A_MOUSE

HAVSEGT $^{Phospho}_{79.97}$ KAVTKYTSSK

- fragmentation table for charge state 1+
- specType=cid
- score=33.74
- F091343.dat
- query=q33020_p1
- precursor=625.306860
- chargePrecursor=3
- itol=0.5

AA	a	a*	a_0	b	b*	b_0	y	y*	y_0	AA
H[1]	110.071	0.000	0.000	138.066	0.000	0.000	1873.906	1856.879	1855.895	H[17]
A[2]	181.108	0.000	0.000	**209.103**	0.000	0.000	1736.847	1719.820	1718.836	A[16]
V[3]	280.177	0.000	0.000	**308.172**	0.000	0.000	1665.810	1648.783	1647.799	V[15]
S[4]	367.209	0.000	349.198	395.204	0.000	**377.193**	1566.741	1549.715	1548.731	S[14]
E[5]	496.251	0.000	478.241	**524.246**	0.000	**506.236**	1479.709	1462.683	1461.699	E[13]
G[6]	553.273	0.000	535.262	**581.268**	0.000	**563.257**	1350.666	1333.640	1332.656	G[12]
T[7]	734.287	0.000	716.276	**762.282**	0.000	**744.271**	1293.645	1276.618	1275.634	T[11]
K[8]	862.382	845.355	844.371	**890.377**	873.350	872.366	1112.631	1095.604	1094.620	K[10]
A[9]	933.419	916.392	915.408	961.414	944.387	943.403	**984.536**	967.509	**966.525**	A[9]
V[10]	1032.487	1015.461	1014.477	1060.482	1043.456	1042.472	**913.499**	896.472	**895.488**	V[8]
T[11]	1133.535	1116.509	1115.525	1161.530	1144.503	1143.519	**814.431**	797.404	**796.420**	T[7]
K[12]	1261.630	1244.603	1243.619	1289.625	1272.598	1271.614	**713.383**	696.356	**695.372**	K[6]
Y[13]	1424.693	1407.667	1406.683	1452.688	1435.662	1434.678	585.288	568.261	**567.277**	Y[5]
T[14]	1525.741	1508.714	1507.730	1553.736	1536.709	1535.725	**422.225**	405.198	404.214	T[4]
S[15]	1612.773	1595.747	1594.763	1640.768	1623.741	1622.757	**321.177**	304.150	303.166	S[3]
S[16]	1699.805	1682.779	1681.795	1727.800	1710.773	1709.789	234.145	217.118	216.134	S[2]
K[17]	1827.900	1810.874	1809.889	1855.895	1838.868	1837.884	147.113	130.086	0.000	K[1]

sp|P70696|H2B1A_MOUSE

HAVSEGT $^{Phospho}_{79.97}$ KAVTKYTSSK

- fragmentation table for charge state 2+
- specType=cid
- score=33.74
- F091343.dat
- query=q33020_p1
- precursor=625.306860
- chargePrecursor=3
- itol=0.5

AA	a	a*	a_0	b	b*	b_0	y	y*	y_0	AA
H[1]	55.539	0.504	0.504	69.537	0.504	0.504	937.456	928.943	928.451	H[17]
A[2]	91.058	0.504	0.504	105.055	0.504	0.504	**868.927**	**860.414**	**859.922**	A[16]
V[3]	140.592	0.504	0.504	154.589	0.504	0.504	**833.408**	**824.895**	**824.403**	V[15]
S[4]	184.108	0.504	175.103	198.106	0.504	189.100	783.874	**775.361**	**774.869**	S[14]
E[5]	248.629	0.504	239.624	262.627	0.504	253.622	740.358	731.845	731.353	E[13]
G[6]	277.140	0.504	268.135	291.138	0.504	282.132	**675.837**	667.324	666.832	G[12]
T[7]	367.647	0.504	358.642	381.645	0.504	372.639	647.326	638.813	638.321	T[11]
K[8]	431.695	**423.181**	**422.689**	445.692	437.179	436.687	**556.819**	548.306	**547.814**	K[10]
A[9]	**467.213**	458.700	458.208	**481.211**	**472.697**	472.205	**492.772**	484.258	483.766	A[9]
V[10]	516.747	**508.234**	507.742	**530.745**	522.232	521.740	457.253	448.740	448.248	V[8]
T[11]	**567.271**	**558.758**	558.266	**581.269**	572.755	**572.263**	**407.719**	399.206	398.714	T[7]
K[12]	631.319	622.805	622.313	**645.316**	**636.803**	**636.311**	357.195	348.682	348.190	K[6]
Y[13]	**712.850**	704.337	703.845	**726.848**	**718.335**	**717.842**	293.148	284.634	284.142	Y[5]
T[14]	763.374	754.861	754.369	777.372	768.858	768.366	211.616	203.103	202.611	T[4]
S[15]	806.890	798.377	797.885	820.888	812.374	811.882	161.092	152.579	152.087	S[3]
S[16]	850.406	841.893	841.401	**864.404**	855.890	855.398	117.576	109.063	108.571	S[2]
K[17]	914.454	905.940	905.448	928.451	919.938	919.446	74.060	65.547	0.504	K[1]

sp|P43274|H14_MOUSE

S$^{Phospho}_{79.97}$ETAPAAPAAPAPAEKTPVK

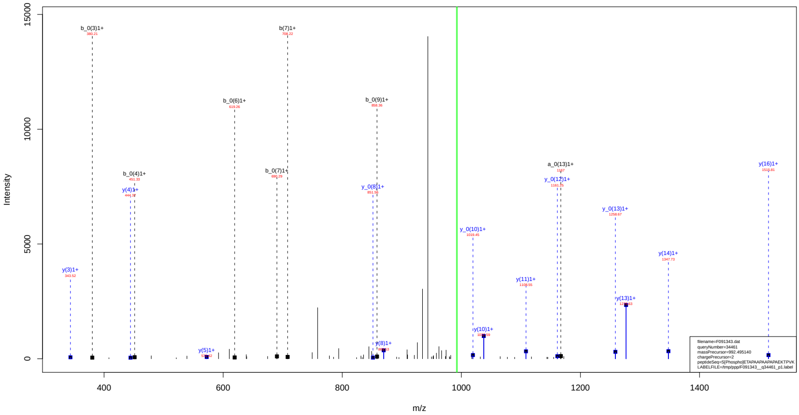

sp|P43274|H14_MOUSE

S $^{Phospho}_{79.97}$ ETAPAAPAAPAPAEKTPVK

- fragmentation table for charge state 1+
- specType=cid
- score=55.80
- F091343.dat
- query=q34461_p1
- precursor=992.495140
- chargePrecursor=2
- itol=0.5

AA	a	a*	a_0	b	b*	b_0	y	y*	y_0	AA
S[1]	140.011	0.000	122.000	168.006	0.000	149.995	1983.979	1966.952	1965.968	S[20]
E[2]	269.053	0.000	251.043	297.048	0.000	279.038	1816.980	1799.954	1798.970	E[19]
T[3]	370.101	0.000	352.090	398.096	0.000	**380.085**	1687.938	1670.911	1669.927	T[18]
A[4]	441.138	0.000	423.128	469.133	0.000	**451.122**	1586.890	1569.864	1568.880	A[17]
P[5]	538.191	0.000	520.180	566.186	0.000	548.175	**1515.853**	1498.826	1497.842	P[16]
A[6]	609.228	0.000	591.217	637.223	0.000	**619.212**	1418.800	1401.774	1400.790	A[15]
A[7]	680.265	0.000	662.255	**708.260**	0.000	**690.249**	**1347.763**	1330.737	1329.753	A[14]
P[8]	777.318	0.000	759.307	805.313	0.000	787.302	**1276.726**	1259.699	**1258.715**	P[13]
A[9]	848.355	0.000	830.344	876.350	0.000	**858.339**	1179.673	1162.647	**1161.663**	A[12]
A[10]	919.392	0.000	901.382	947.387	0.000	929.376	**1108.636**	1091.610	1090.626	A[11]
P[11]	1016.445	0.000	998.434	1044.440	0.000	1026.429	**1037.599**	1020.572	**1019.588**	P[10]
A[12]	1087.482	0.000	1069.471	1115.477	0.000	1097.466	940.546	923.520	922.536	A[9]
P[13]	1184.535	0.000	**1166.524**	1212.530	0.000	1194.519	**869.509**	852.483	**851.499**	P[8]
A[14]	1255.572	0.000	1237.561	1283.567	0.000	1265.556	772.456	755.430	754.446	A[7]
E[15]	1384.614	0.000	1366.604	1412.609	0.000	1394.599	701.419	684.393	683.409	E[6]
K[16]	1512.709	1495.683	1494.699	1540.704	1523.678	1522.694	**572.377**	555.350	554.366	K[5]
T[17]	1613.757	1596.731	1595.747	1641.752	1624.725	1623.741	**444.282**	427.255	426.271	T[4]
P[18]	1710.810	1693.783	1692.799	1738.805	1721.778	1720.794	**343.234**	326.207	0.000	P[3]
V[19]	1809.878	1792.852	1791.868	1837.873	1820.847	1819.863	246.181	229.155	0.000	V[2]
K[20]	1937.973	1920.947	1919.963	1965.968	1948.942	1947.958	147.113	130.086	0.000	K[1]

sp|P43276|H15_MOUSE

SETAPAETAAPAPVEKS $^{Phospho}_{79.97}$ PAK

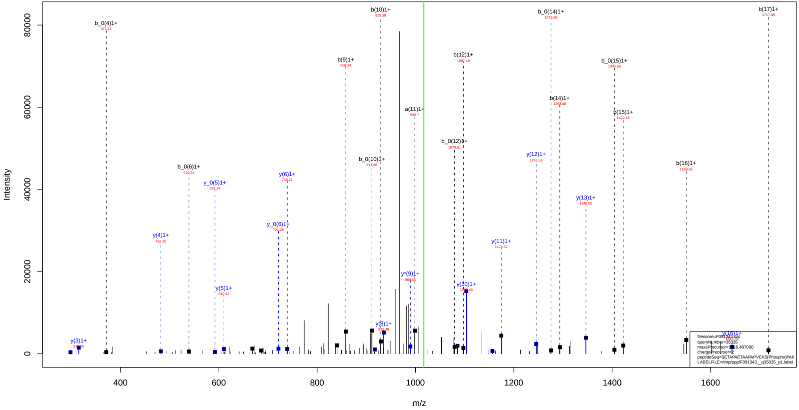

sp|P43276|H15_MOUSE

SETAPAETAAPAPVEKS $^{Phospho}_{79.97}$ PAK

- fragmentation table for charge state 1+
- specType=cid
- score=64.11
- F091343.dat
- query=q35035_p1
- precursor=1016.487000
- chargePrecursor=2
- itol=0.5

AA	a	a*	a_0	b	b*	b_0	y	y*	y_0	AA
S[1]	60.044	0.000	42.034	88.039	0.000	70.029	2031.963	2014.937	2013.953	S[20]
E[2]	189.087	0.000	171.076	217.082	0.000	199.071	1944.931	1927.905	1926.921	E[19]
T[3]	290.135	0.000	272.124	318.130	0.000	300.119	1815.889	1798.862	1797.878	T[18]
A[4]	361.172	0.000	343.161	389.167	0.000	**371.156**	1714.841	1697.815	1696.831	A[17]
P[5]	458.225	0.000	440.214	486.219	0.000	468.209	**1643.804**	1626.777	1625.793	P[16]
A[6]	529.262	0.000	511.251	557.257	0.000	**539.246**	1546.751	1529.725	1528.741	A[15]
E[7]	658.304	0.000	640.294	**686.299**	0.000	**668.289**	1475.714	1458.688	1457.704	E[14]
T[8]	759.352	0.000	741.341	787.347	0.000	769.336	**1346.672**	1329.645	1328.661	T[13]
A[9]	830.389	0.000	812.378	**858.384**	0.000	**840.373**	**1245.624**	1228.597	1227.613	A[12]
A[10]	901.426	0.000	883.416	**929.421**	0.000	**911.411**	**1174.587**	1157.560	**1156.576**	A[11]
P[11]	**998.479**	0.000	980.468	1026.474	0.000	1008.463	**1103.550**	1086.523	**1085.539**	P[10]
A[12]	1069.516	0.000	1051.505	**1097.511**	0.000	**1079.500**	1006.497	**989.470**	988.486	A[9]
P[13]	1166.569	0.000	1148.558	1194.564	0.000	1176.553	**935.460**	918.433	**917.449**	P[8]
V[14]	1265.637	0.000	1247.627	**1293.632**	0.000	**1275.622**	838.407	821.380	820.396	V[7]
E[15]	1394.680	0.000	1376.669	**1422.675**	0.000	**1404.664**	**739.339**	722.312	**721.328**	E[6]
K[16]	1522.775	1505.748	1504.764	**1550.770**	1533.743	1532.759	**610.296**	593.269	**592.285**	K[5]
S[17]	1689.773	1672.747	1671.763	**1717.768**	1700.741	1699.757	**482.201**	465.174	464.190	S[4]
P[18]	1786.826	1769.799	1768.815	1814.821	1797.794	1796.810	**315.203**	**298.176**	0.000	P[3]
A[19]	1857.863	1840.836	1839.852	1885.858	1868.831	1867.847	218.150	201.123	0.000	A[2]
K[20]	1985.958	1968.931	1967.947	2013.953	1996.926	1995.942	147.113	130.086	0.000	K[1]

sp|P43276|H15_MOUSE

SETAPAETAAPAPVEKS $^{\text{Phospho}}_{79.97}$ PAK

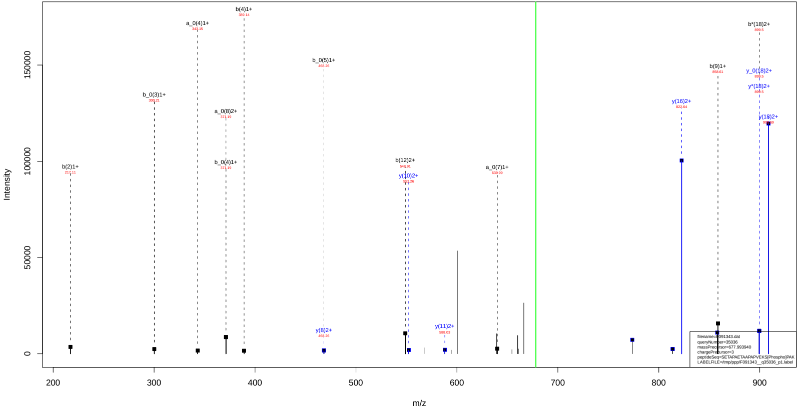

sp|P43276|H15_MOUSE

SETAPAETAAPAPVEKS $^{Phospho}_{79.97}$ PAK

- fragmentation table for charge state 1+
- specType=cid
- score=38.38
- F091343.dat
- query=q35036_p1
- precursor=677.993940
- chargePrecursor=3
- itol=0.5

AA	a	a*	a_0	b	b*	b_0	y	y*	y_0	AA
S[1]	60.044	0.000	42.034	88.039	0.000	70.029	2031.963	2014.937	2013.953	S[20]
E[2]	189.087	0.000	171.076	**217.082**	0.000	199.071	1944.931	1927.905	1926.921	E[19]
T[3]	290.135	0.000	272.124	318.130	0.000	**300.119**	1815.889	1798.862	1797.878	T[18]
A[4]	361.172	0.000	**343.161**	**389.167**	0.000	**371.156**	1714.841	1697.815	1696.831	A[17]
P[5]	458.225	0.000	440.214	486.219	0.000	**468.209**	1643.804	1626.777	1625.793	P[16]
A[6]	529.262	0.000	511.251	557.257	0.000	539.246	1546.751	1529.725	1528.741	A[15]
E[7]	658.304	0.000	**640.294**	686.299	0.000	668.289	1475.714	1458.688	1457.704	E[14]
T[8]	759.352	0.000	741.341	787.347	0.000	769.336	1346.672	1329.645	1328.661	T[13]
A[9]	830.389	0.000	812.378	**858.384**	0.000	840.373	1245.624	1228.597	1227.613	A[12]
A[10]	901.426	0.000	883.416	929.421	0.000	911.411	1174.587	1157.560	1156.576	A[11]
P[11]	998.479	0.000	980.468	1026.474	0.000	1008.463	1103.550	1086.523	1085.539	P[10]
A[12]	1069.516	0.000	1051.505	1097.511	0.000	1079.500	1006.497	989.470	988.486	A[9]
P[13]	1166.569	0.000	1148.558	1194.564	0.000	1176.553	935.460	918.433	917.449	P[8]
V[14]	1265.637	0.000	1247.627	1293.632	0.000	1275.622	838.407	821.380	820.396	V[7]
E[15]	1394.680	0.000	1376.669	1422.675	0.000	1404.664	739.339	722.312	721.328	E[6]
K[16]	1522.775	1505.748	1504.764	1550.770	1533.743	1532.759	610.296	593.269	592.285	K[5]
S[17]	1689.773	1672.747	1671.763	1717.768	1700.741	1699.757	482.201	465.174	464.190	S[4]
P[18]	1786.826	1769.799	1768.815	1814.821	1797.794	1796.810	315.203	298.176	0.000	P[3]
A[19]	1857.863	1840.836	1839.852	1885.858	1868.831	1867.847	218.150	201.123	0.000	A[2]
K[20]	1985.958	1968.931	1967.947	2013.953	1996.926	1995.942	147.113	130.086	0.000	K[1]

sp|P43276|H15_MOUSE

SETAPAETAAPAPVEKS $^{Phospho}_{79.97}$ PAK

- fragmentation table for charge state 2+
- specType=cid
- score=38.38
- F091343.dat
- query=q35036_p1
- precursor=677.993940
- chargePrecursor=3
- itol=0.5

AA	a	a*	a_0	b	b*	b_0	y	y*	y_0	AA
S[1]	30.526	0.504	21.521	44.523	0.504	35.518	1016.485	1007.972	1007.480	S[20]
E[2]	95.047	0.504	86.042	109.045	0.504	100.039	972.969	964.456	963.964	E[19]
T[3]	145.571	0.504	136.566	159.568	0.504	150.563	**908.448**	**899.935**	**899.443**	T[18]
A[4]	181.090	0.504	172.084	195.087	0.504	186.082	**857.924**	849.411	848.919	A[17]
P[5]	229.616	0.504	220.611	243.613	0.504	234.608	**822.406**	**813.892**	**813.400**	P[16]
A[6]	265.134	0.504	256.129	279.132	0.504	270.127	**773.879**	765.366	764.874	A[15]
E[7]	329.656	0.504	320.650	343.653	0.504	334.648	738.361	729.847	729.355	E[14]
T[8]	380.180	0.504	**371.174**	394.177	0.504	385.172	673.839	665.326	664.834	T[13]
A[9]	415.698	0.504	406.693	429.696	0.504	420.690	623.316	614.802	614.310	A[12]
A[10]	451.217	0.504	442.211	465.214	0.504	456.209	**587.797**	579.284	578.792	A[11]
P[11]	499.743	0.504	490.738	513.741	0.504	504.735	**552.278**	543.765	543.273	P[10]
A[12]	535.262	0.504	526.256	**549.259**	0.504	540.254	503.752	495.239	494.747	A[9]
P[13]	583.788	0.504	574.783	597.785	0.504	588.780	**468.234**	459.720	459.228	P[8]
V[14]	633.322	0.504	624.317	647.320	0.504	638.314	419.707	411.194	410.702	V[7]
E[15]	697.844	0.504	688.838	711.841	0.504	702.836	370.173	361.660	361.168	E[6]
K[16]	761.891	753.378	752.886	775.888	767.375	766.883	305.652	297.138	296.646	K[5]
S[17]	845.390	836.877	836.385	859.388	850.874	850.382	241.604	233.091	232.599	S[4]
P[18]	893.917	885.403	884.911	907.914	**899.401**	898.909	158.105	149.592	0.504	P[3]
A[19]	929.435	920.922	920.430	943.433	934.919	934.427	109.579	101.065	0.504	A[2]
K[20]	993.483	984.969	984.477	1007.480	998.967	998.475	74.060	65.547	0.504	K[1]

sp|P43274|H14_MOUSE

S Phospho 79.97 ET Phospho 79.97 APAAPAAPAPAEKTPVK

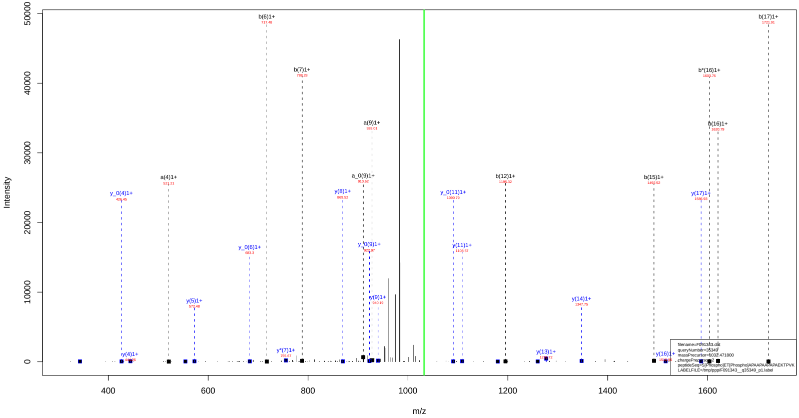

sp|P43274|H14_MOUSE

S $^{Phospho}_{79.97}$ ET $^{Phospho}_{79.97}$ APAAPAAPAPAEKTPVK

- fragmentation table for charge state 1+
- specType=cid
- score=38.12
- F091343.dat
- query=q35349_p1
- precursor=1032.471800
- chargePrecursor=2
- itol=0.5

AA	a	a*	a_0	b	b*	b_0	y	y*	y_0	AA
S[1]	140.011	0.000	122.000	168.006	0.000	149.995	2063.945	2046.918	2045.934	S[20]
E[2]	269.053	0.000	251.043	297.048	0.000	279.038	1896.947	1879.920	1878.936	E[19]
T[3]	450.067	0.000	432.057	478.062	0.000	460.052	1767.904	1750.878	1749.894	T[18]
A[4]	**521.104**	0.000	503.094	549.099	0.000	531.089	**1586.890**	1569.864	1568.880	A[17]
P[5]	618.157	0.000	600.147	646.152	0.000	628.142	**1515.853**	1498.826	1497.842	P[16]
A[6]	689.194	0.000	671.184	**717.189**	0.000	699.179	1418.800	1401.774	1400.790	A[15]
A[7]	760.231	0.000	742.221	**788.226**	0.000	770.216	**1347.763**	1330.737	1329.753	A[14]
P[8]	857.284	0.000	839.274	885.279	0.000	867.269	**1276.726**	**1259.699**	1258.715	P[13]
A[9]	**928.321**	0.000	**910.311**	956.316	0.000	938.306	**1179.673**	1162.647	1161.663	A[12]
A[10]	999.358	0.000	981.348	1027.353	0.000	1009.343	**1108.636**	1091.610	**1090.626**	A[11]
P[11]	1096.411	0.000	1078.401	1124.406	0.000	1106.396	1037.599	1020.572	1019.588	P[10]
A[12]	1167.448	0.000	1149.438	**1195.443**	0.000	1177.433	**940.546**	923.520	**922.536**	A[9]
P[13]	1264.501	0.000	1246.490	1292.496	0.000	1274.485	**869.509**	852.483	851.499	P[8]
A[14]	1335.538	0.000	1317.528	1363.533	0.000	1345.523	772.456	**755.430**	754.446	A[7]
E[15]	1464.581	0.000	1446.570	**1492.576**	0.000	1474.565	701.419	684.393	**683.409**	E[6]
K[16]	1592.676	1575.649	1574.665	**1620.671**	**1603.644**	1602.660	**572.377**	555.350	**554.366**	K[5]
T[17]	1693.723	1676.697	1675.713	**1721.718**	1704.692	1703.708	**444.282**	427.255	**426.271**	T[4]
P[18]	1790.776	1773.750	1772.766	1818.771	1801.745	1800.761	**343.234**	326.207	0.000	P[3]
V[19]	1889.845	1872.818	1871.834	1917.840	1900.813	1899.829	246.181	229.155	0.000	V[2]
K[20]	2017.940	2000.913	1999.929	2045.934	2028.908	2027.924	147.113	130.086	0.000	K[1]

sp|P43277|H13_MOUSE

S $^{Phospho}_{79.97}$ ET $^{Phospho}_{79.97}$ APAAPAAPAPVEKTPVK

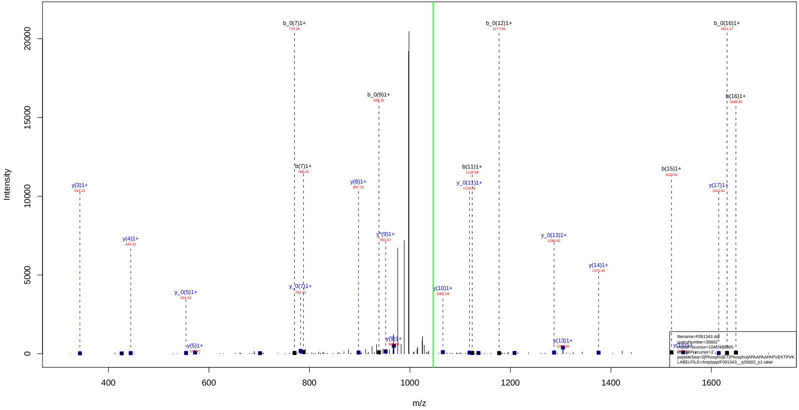

sp|P43277|H13_MOUSE

S $^{Phospho}_{79.97}$ ET $^{Phospho}_{79.97}$ APAAPAAPAPVEKTPVK

- fragmentation table for charge state 1+
- specType=cid
- score=55.02
- F091343.dat
- query=q35602_p1
- precursor=1046.488900
- chargePrecursor=2
- itol=0.5

AA	a	a*	a_0	b	b*	b_0	y	y*	y_0	AA
S[1]	140.011	0.000	122.000	168.006	0.000	149.995	2091.976	2074.950	2073.966	S[20]
E[2]	269.053	0.000	251.043	297.048	0.000	279.038	1924.978	1907.951	1906.967	E[19]
T[3]	450.067	0.000	432.057	478.062	0.000	460.052	1795.935	1778.909	1777.925	T[18]
A[4]	521.104	0.000	503.094	549.099	0.000	531.089	**1614.921**	1597.895	1596.911	A[17]
P[5]	618.157	0.000	600.147	646.152	0.000	628.142	**1543.884**	1526.858	1525.874	P[16]
A[6]	689.194	0.000	671.184	717.189	0.000	699.179	1446.831	1429.805	1428.821	A[15]
A[7]	760.231	0.000	742.221	**788.226**	0.000	**770.216**	**1375.794**	1358.768	1357.784	A[14]
P[8]	857.284	0.000	839.274	885.279	0.000	867.269	**1304.757**	1287.731	**1286.747**	P[13]
A[9]	928.321	0.000	910.311	956.316	0.000	**938.306**	**1207.705**	1190.678	1189.694	A[12]
A[10]	999.358	0.000	981.348	1027.353	0.000	1009.343	**1136.667**	1119.641	**1118.657**	A[11]
P[11]	1096.411	0.000	1078.401	**1124.406**	0.000	1106.396	**1065.630**	1048.604	1047.620	P[10]
A[12]	1167.448	0.000	1149.438	1195.443	0.000	**1177.433**	**968.578**	**951.551**	950.567	A[9]
P[13]	1264.501	0.000	1246.490	1292.496	0.000	1274.485	**897.540**	880.514	879.530	P[8]
V[14]	1363.569	0.000	1345.559	1391.564	0.000	1373.554	800.488	783.461	**782.477**	V[7]
E[15]	1492.612	0.000	1474.602	**1520.607**	0.000	1502.596	**701.419**	684.393	683.409	E[6]
K[16]	1620.707	1603.680	1602.696	**1648.702**	1631.675	**1630.691**	**572.377**	555.350	**554.366**	K[5]
T[17]	1721.755	1704.728	1703.744	1749.750	1732.723	1731.739	**444.282**	427.255	**426.271**	T[4]
P[18]	1818.807	1801.781	1800.797	1846.802	1829.776	1828.792	**343.234**	326.207	0.000	P[3]
V[19]	1917.876	1900.849	1899.865	1945.871	1928.844	1927.860	246.181	229.155	0.000	V[2]
K[20]	2045.971	2028.944	2027.960	2073.966	2056.939	2055.955	147.113	130.086	0.000	K[1]

sp|P43276|H15_MOUSE

S $^{Phospho}_{79.97}$ ET $^{Phospho}_{79.97}$ APAETAAPAPVEKSPAK

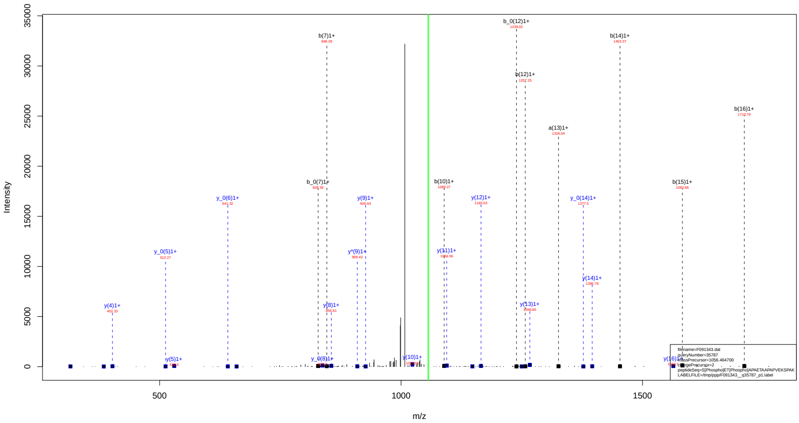

sp|P43276|H15_MOUSE

S $^{Phospho}_{79.97}$ ET $^{Phospho}_{79.97}$ APAETAAPAPVEKSPAK

- fragmentation table for charge state 1+
- specType=cid
- score=40.68
- F091343.dat
- query=q35787_p1
- precursor=1056.464700
- chargePrecursor=2
- itol=0.5

AA	a	a*	a_0	b	b*	b_0	y	y*	y_0	AA
S[1]	140.011	0.000	122.000	168.006	0.000	149.995	2111.930	2094.903	2093.919	S[20]
E[2]	269.053	0.000	251.043	297.048	0.000	279.038	1944.931	1927.905	1926.921	E[19]
T[3]	450.067	0.000	432.057	478.062	0.000	460.052	1815.889	1798.862	1797.878	T[18]
A[4]	521.104	0.000	503.094	549.099	0.000	531.089	1634.875	1617.848	1616.864	A[17]
P[5]	618.157	0.000	600.147	646.152	0.000	628.142	**1563.838**	1546.811	1545.827	P[16]
A[6]	689.194	0.000	671.184	717.189	0.000	699.179	1466.785	1449.758	1448.774	A[15]
E[7]	818.237	0.000	800.226	**846.232**	0.000	**828.221**	**1395.748**	1378.721	**1377.737**	E[14]
T[8]	919.285	0.000	901.274	947.280	0.000	929.269	**1266.705**	**1249.679**	1248.695	T[13]
A[9]	990.322	0.000	972.311	1018.317	0.000	1000.306	**1165.658**	1148.631	**1147.647**	A[12]
A[10]	1061.359	0.000	1043.348	**1089.354**	0.000	1071.343	**1094.620**	1077.594	1076.610	A[11]
P[11]	1158.412	0.000	1140.401	1186.406	0.000	1168.396	**1023.583**	1006.557	1005.573	P[10]
A[12]	1229.449	0.000	1211.438	**1257.444**	0.000	**1239.433**	**926.531**	**909.504**	908.520	A[9]
P[13]	**1326.501**	0.000	1308.491	1354.496	0.000	1336.486	**855.493**	838.467	**837.483**	P[8]
V[14]	1425.570	0.000	1407.559	**1453.565**	0.000	1435.554	758.441	741.414	740.430	V[7]
E[15]	1554.612	0.000	1536.602	**1582.607**	0.000	1564.597	**659.372**	642.346	**641.362**	E[6]
K[16]	1682.707	1665.681	1664.697	**1710.702**	1693.676	1692.692	**530.330**	513.303	**512.319**	K[5]
S[17]	1769.739	1752.713	1751.729	1797.734	1780.708	1779.724	**402.235**	385.208	**384.224**	S[4]
P[18]	1866.792	1849.766	1848.782	1894.787	1877.761	1876.777	**315.203**	298.176	0.000	P[3]
A[19]	1937.829	1920.803	1919.819	1965.824	1948.798	1947.814	218.150	201.123	0.000	A[2]
K[20]	2065.924	2048.898	2047.914	2093.919	2076.893	2075.909	147.113	130.086	0.000	K[1]

sp|P43274|H14_MOUSE

S $^{Phospho}_{79.97}$ ETAPAAPAAPAPAEKTPVKK

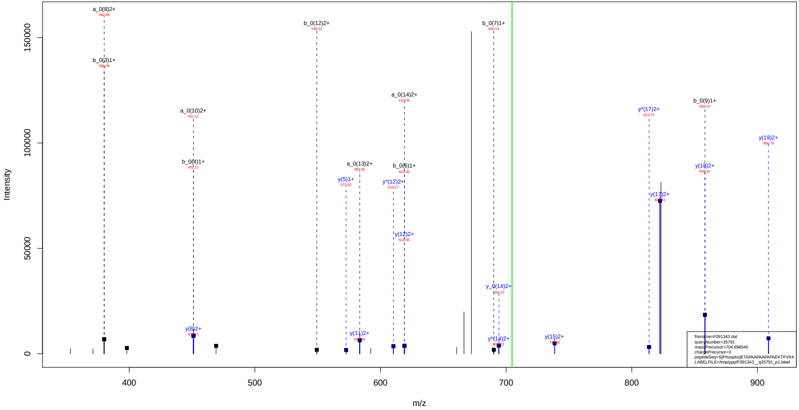

sp|P43274|H14_MOUSE

S $^{Phospho}_{79.97}$ ETAPAAPAAPAPAEKTPVKK

- fragmentation table for charge state 1+
- specType=cid
- score=46.18
- F091343.dat
- query=q35791_p1
- precursor=704.696540
- chargePrecursor=3
- itol=0.5

AA	a	a*	a_0	b	b*	b_0	y	y*	y_0	AA
S[1]	140.011	0.000	122.000	168.006	0.000	149.995	2112.074	2095.047	2094.063	S[21]
E[2]	269.053	0.000	251.043	297.048	0.000	279.038	1945.075	1928.049	1927.065	E[20]
T[3]	370.101	0.000	352.090	**398.096**	0.000	**380.085**	1816.033	1799.006	1798.022	T[19]
A[4]	441.138	0.000	423.128	**469.133**	0.000	**451.122**	1714.985	1697.958	1696.974	A[18]
P[5]	538.191	0.000	520.180	566.186	0.000	548.175	1643.948	1626.921	1625.937	P[17]
A[6]	609.228	0.000	591.217	637.223	0.000	**619.212**	1546.895	1529.869	1528.885	A[16]
A[7]	680.265	0.000	662.255	708.260	0.000	**690.249**	1475.858	1458.831	1457.847	A[15]
P[8]	777.318	0.000	759.307	805.313	0.000	787.302	1404.821	1387.794	1386.810	P[14]
A[9]	848.355	0.000	830.344	876.350	0.000	**858.339**	1307.768	1290.742	1289.758	A[13]
A[10]	919.392	0.000	901.382	947.387	0.000	929.376	1236.731	1219.705	1218.720	A[12]
P[11]	1016.445	0.000	998.434	1044.440	0.000	1026.429	1165.694	1148.667	1147.683	P[11]
A[12]	1087.482	0.000	1069.471	1115.477	0.000	1097.466	1068.641	1051.615	1050.631	A[10]
P[13]	1184.535	0.000	1166.524	1212.530	0.000	1194.519	997.604	980.578	979.593	P[9]
A[14]	1255.572	0.000	1237.561	1283.567	0.000	1265.556	900.551	883.525	882.541	A[8]
E[15]	1384.614	0.000	1366.604	1412.609	0.000	1394.599	829.514	812.488	811.504	E[7]
K[16]	1512.709	1495.683	1494.699	1540.704	1523.678	1522.694	700.472	683.445	682.461	K[6]
T[17]	1613.757	1596.731	1595.747	1641.752	1624.725	1623.741	**572.377**	555.350	554.366	T[5]
P[18]	1710.810	1693.783	1692.799	1738.805	1721.778	1720.794	471.329	454.302	0.000	P[4]
V[19]	1809.878	1792.852	1791.868	1837.873	1820.847	1819.863	374.276	357.250	0.000	V[3]
K[20]	1937.973	1920.947	1919.963	1965.968	1948.942	1947.958	275.208	258.181	0.000	K[2]
K[21]	2066.068	2049.042	2048.058	2094.063	2077.037	2076.053	147.113	130.086	0.000	K[1]

sp|P43274|H14_MOUSE

S $^{Phospho}_{79.97}$ ETAPAAPAAPAPAEKTPVKK

- fragmentation table for charge state 2+
- specType=cid
- score=46.18
- F091343.dat
- query=q35791_p1
- precursor=704.696540
- chargePrecursor=3
- itol=0.5

AA	a	a*	a_0	b	b*	b_0	y	y*	y_0	AA
S[1]	70.509	0.504	61.504	84.506	0.504	75.501	1056.540	1048.027	1047.535	S[21]
E[2]	135.030	0.504	126.025	149.028	0.504	140.022	973.041	964.528	964.036	E[20]
T[3]	185.554	0.504	176.549	199.552	0.504	190.546	**908.520**	900.007	899.515	T[19]
A[4]	221.073	0.504	212.067	235.070	0.504	226.065	**857.996**	849.483	848.991	A[18]
P[5]	269.599	0.504	260.594	283.597	0.504	274.591	**822.478**	**813.964**	**813.472**	P[17]
A[6]	305.118	0.504	296.112	319.115	0.504	310.110	773.951	765.438	764.946	A[16]
A[7]	340.636	0.504	331.631	354.634	0.504	345.628	**738.433**	729.919	729.427	A[15]
P[8]	389.163	0.504	**380.157**	403.160	0.504	394.155	702.914	**694.401**	**693.909**	P[14]
A[9]	424.681	0.504	415.676	438.679	0.504	429.673	654.388	645.874	645.382	A[13]
A[10]	460.200	0.504	**451.194**	474.197	0.504	465.192	**618.869**	**610.356**	**609.864**	A[12]
P[11]	508.726	0.504	499.721	522.724	0.504	513.718	**583.351**	574.837	574.345	P[11]
A[12]	544.245	0.504	535.239	558.242	0.504	**549.237**	534.824	526.311	525.819	A[10]
P[13]	592.771	0.504	**583.766**	606.768	0.504	597.763	499.306	490.792	490.300	P[9]
A[14]	628.290	0.504	**619.284**	642.287	0.504	633.282	**450.779**	442.266	441.774	A[8]
E[15]	692.811	0.504	683.806	706.808	0.504	697.803	415.261	406.747	406.255	E[7]
K[16]	756.858	748.345	747.853	770.856	762.343	761.851	350.739	342.226	341.734	K[6]
T[17]	807.382	798.869	798.377	821.380	812.866	812.374	286.692	278.179	277.687	T[5]
P[18]	855.909	847.395	846.903	869.906	861.393	860.901	236.168	227.655	0.504	P[4]
V[19]	905.443	896.929	896.437	919.440	910.927	910.435	187.642	179.128	0.504	V[3]
K[20]	969.490	960.977	960.485	983.488	974.974	974.482	138.108	129.594	0.504	K[2]
K[21]	1033.538	1025.024	1024.532	1047.535	1039.022	1038.530	74.060	65.547	0.504	K[1]

sp|P43276|H15_MOUSE

SETAPAETAAPAPVEKS $^{Phospho}_{79.97}$ PAKK

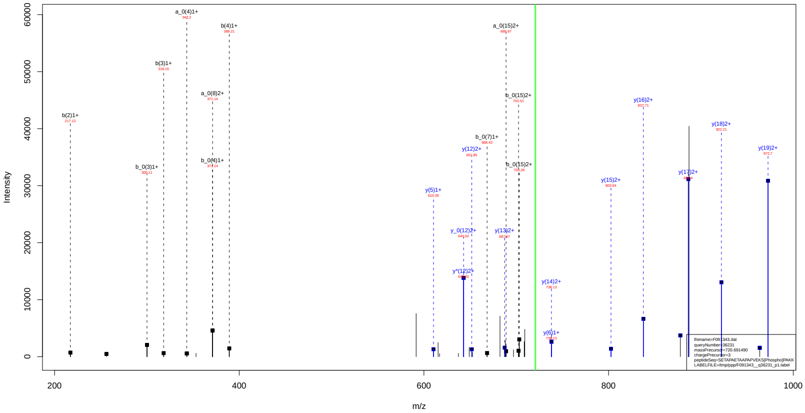

sp|P43276|H15_MOUSE

SETAPAETAAPAPVEKS $^{Phospho}_{79.97}$ PAKK

- fragmentation table for charge state 1+
- specType=cid
- score=46.26
- F091343.dat
- query=q36231_p1
- precursor=720.691490
- chargePrecursor=3
- itol=0.5

AA	a	a*	a_0	b	b*	b_0	y	y*	y_0	AA
S[1]	60.044	0.000	42.034	88.039	0.000	70.029	2160.058	2143.032	2142.048	S[21]
E[2]	189.087	0.000	171.076	**217.082**	0.000	199.071	2073.026	2056.000	2055.016	E[20]
T[3]	290.135	0.000	272.124	**318.130**	0.000	**300.119**	1943.984	1926.957	1925.973	T[19]
A[4]	361.172	0.000	**343.161**	**389.167**	0.000	**371.156**	1842.936	1825.910	1824.926	A[18]
P[5]	458.225	0.000	440.214	486.219	0.000	468.209	1771.899	1754.872	1753.888	P[17]
A[6]	529.262	0.000	511.251	557.257	0.000	539.246	1674.846	1657.820	1656.836	A[16]
E[7]	658.304	0.000	640.294	686.299	0.000	**668.289**	1603.809	1586.783	1585.799	E[15]
T[8]	759.352	0.000	741.341	787.347	0.000	769.336	1474.767	1457.740	1456.756	T[14]
A[9]	830.389	0.000	812.378	858.384	0.000	840.373	1373.719	1356.692	1355.708	A[13]
A[10]	901.426	0.000	883.416	929.421	0.000	911.411	1302.682	1285.655	1284.671	A[12]
P[11]	998.479	0.000	980.468	1026.474	0.000	1008.463	1231.645	1214.618	1213.634	P[11]
A[12]	1069.516	0.000	1051.505	1097.511	0.000	1079.500	1134.592	1117.565	1116.581	A[10]
P[13]	1166.569	0.000	1148.558	1194.564	0.000	1176.553	1063.555	1046.528	1045.544	P[9]
V[14]	1265.637	0.000	1247.627	1293.632	0.000	1275.622	966.502	949.475	948.491	V[8]
E[15]	1394.680	0.000	1376.669	1422.675	0.000	1404.664	867.434	850.407	849.423	E[7]
K[16]	1522.775	1505.748	1504.764	1550.770	1533.743	1532.759	**738.391**	721.364	720.380	K[6]
S[17]	1689.773	1672.747	1671.763	1717.768	1700.741	1699.757	**610.296**	593.269	592.285	S[5]
P[18]	1786.826	1769.799	1768.815	1814.821	1797.794	1796.810	443.298	426.271	0.000	P[4]
A[19]	1857.863	1840.836	1839.852	1885.858	1868.831	1867.847	346.245	329.218	0.000	A[3]
K[20]	1985.958	1968.931	1967.947	2013.953	1996.926	1995.942	275.208	258.181	0.000	K[2]
K[21]	2114.053	2097.026	2096.042	2142.048	2125.021	2124.037	147.113	130.086	0.000	K[1]

sp|P43276|H15_MOUSE

SETAPAETAAPAPVEKS $^{\text{Phospho}}_{79.97}$ PAKK

- fragmentation table for charge state 2+
- specType=cid
- score=46.26
- F091343.dat
- query=q36231_p1
- precursor=720.691490
- chargePrecursor=3
- itol=0.5

AA	a	a*	a_0	b	b*	b_0	y	y*	y_0	AA
S[1]	30.526	0.504	21.521	44.523	0.504	35.518	1080.533	1072.020	1071.528	S[21]
E[2]	95.047	0.504	86.042	109.045	0.504	100.039	1037.017	1028.504	1028.012	E[20]
T[3]	145.571	0.504	136.566	159.568	0.504	150.563	**972.496**	**963.982**	**963.490**	T[19]
A[4]	181.090	0.504	172.084	195.087	0.504	186.082	**921.972**	913.458	912.966	A[18]
P[5]	229.616	0.504	220.611	243.613	0.504	234.608	**886.453**	**877.940**	**877.448**	P[17]
A[6]	265.134	0.504	**256.129**	279.132	0.504	270.127	**837.927**	829.413	828.921	A[16]
E[7]	329.656	0.504	320.650	**343.653**	0.504	334.648	**802.408**	793.895	793.403	E[15]
T[8]	380.180	0.504	**371.174**	394.177	0.504	385.172	**737.887**	729.374	728.882	T[14]
A[9]	415.698	0.504	406.693	429.696	0.504	420.690	**687.363**	678.850	678.358	A[13]
A[10]	451.217	0.504	442.211	465.214	0.504	456.209	**651.845**	**643.331**	**642.839**	A[12]
P[11]	499.743	0.504	490.738	513.741	0.504	504.735	616.326	607.813	607.321	P[11]
A[12]	535.262	0.504	526.256	549.259	0.504	540.254	567.800	559.286	558.794	A[10]
P[13]	583.788	0.504	574.783	597.785	0.504	588.780	532.281	523.768	523.276	P[9]
V[14]	633.322	0.504	624.317	647.320	0.504	638.314	483.755	475.241	474.749	V[8]
E[15]	697.844	0.504	**688.838**	711.841	0.504	**702.836**	434.220	425.707	425.215	E[7]
K[16]	761.891	753.378	752.886	775.888	767.375	766.883	369.699	361.186	360.694	K[6]
S[17]	845.390	836.877	836.385	859.388	850.874	850.382	305.652	297.138	296.646	S[5]
P[18]	893.917	885.403	884.911	907.914	899.401	898.909	222.152	213.639	0.504	P[4]
A[19]	929.435	920.922	920.430	943.433	934.919	934.427	173.626	165.113	0.504	A[3]
K[20]	993.483	984.969	984.477	1007.480	998.967	998.475	138.108	129.594	0.504	K[2]
K[21]	1057.530	1049.017	1048.525	1071.528	1063.014	1062.522	74.060	65.547	0.504	K[1]

sp|Q9QZQ8-1|H2AY_MOUSE

AASADSTTEGT $^{Phospho}_{79.97}$ PTDGFTVLSTK

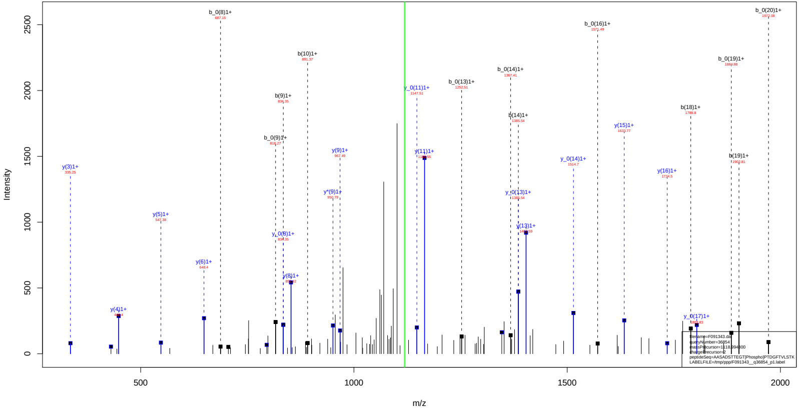

sp|Q9QZQ8-1|H2AY_MOUSE

AASADSTTEGT $^{Phospho}_{79.97}$ PTDGFTVLSTK

- fragmentation table for charge state 1+
- specType=cid
- score=48.04
- F091343.dat
- query=q36854_p1
- precursor=1118.994800
- chargePrecursor=2
- itol=0.5

AA	a	a*	a_0	b	b*	b_0	y	y*	y_0	AA
A[1]	44.049	0.000	0.000	72.044	0.000	0.000	2236.986	2219.959	2218.975	A[22]
A[2]	115.087	0.000	0.000	143.082	0.000	0.000	2165.949	2148.922	2147.938	A[21]
S[3]	202.119	0.000	184.108	230.114	0.000	212.103	2094.911	2077.885	2076.901	S[20]
A[4]	273.156	0.000	255.145	301.151	0.000	283.140	2007.879	1990.853	1989.869	A[19]
D[5]	388.183	0.000	370.172	416.178	0.000	398.167	1936.842	1919.816	1918.832	D[18]
S[6]	475.215	0.000	457.204	503.210	0.000	485.199	1821.815	1804.789	**1803.805**	S[17]
T[7]	576.262	0.000	558.252	604.257	0.000	586.247	**1734.783**	1717.757	1716.773	T[16]
T[8]	677.310	0.000	659.299	**705.305**	0.000	**687.294**	**1633.736**	1616.709	1615.725	T[15]
E[9]	806.353	0.000	788.342	**834.348**	0.000	**816.337**	1532.688	1515.661	**1514.677**	E[14]
G[10]	863.374	0.000	845.364	**891.369**	0.000	873.358	**1403.645**	1386.619	**1385.635**	G[13]
T[11]	1044.388	0.000	1026.378	1072.383	0.000	1054.372	**1346.624**	1329.597	1328.613	T[12]
P[12]	1141.441	0.000	1123.430	1169.436	0.000	1151.425	**1165.610**	1148.583	**1147.599**	P[11]
T[13]	1242.489	0.000	1224.478	1270.483	0.000	**1252.473**	1068.557	1051.531	1050.547	T[10]
D[14]	1357.516	0.000	1339.505	**1385.510**	0.000	**1367.500**	**967.509**	**950.483**	949.499	D[9]
G[15]	1414.537	0.000	1396.526	1442.532	0.000	1424.521	**852.483**	835.456	**834.472**	G[8]
F[16]	1561.605	0.000	1543.595	1589.600	0.000	**1571.590**	**795.461**	778.435	777.451	F[7]
T[17]	1662.653	0.000	1644.643	1690.648	0.000	1672.637	**648.393**	631.366	630.382	T[6]
V[18]	1761.721	0.000	1743.711	**1789.716**	0.000	1771.706	**547.345**	530.318	529.334	V[5]
L[19]	1874.806	0.000	1856.795	**1902.800**	0.000	**1884.790**	**448.277**	431.250	**430.266**	L[4]
S[20]	1961.838	0.000	1943.827	1989.832	0.000	**1971.822**	**335.193**	318.166	317.182	S[3]
T[21]	2062.885	0.000	2044.875	2090.880	0.000	2072.870	248.160	231.134	230.150	T[2]
K[22]	2190.980	2173.954	2172.970	2218.975	2201.949	2200.965	147.113	130.086	0.000	K[1]

sp|P43276|H15_MOUSE

S Phospho 79.97 ETAPAETAAPAPVEKS Phospho 79.97 PAKK

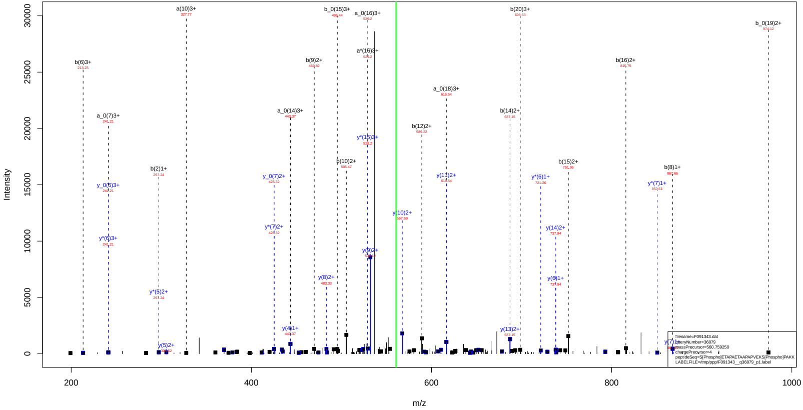

sp|P43276|H15_MOUSE

S $^{Phospho}_{79.97}$ ETAPAETAAPAPVEKS $^{Phospho}_{79.97}$ PAKK

- fragmentation table for charge state 1+
- specType=cid
- score=38.95
- F091343.dat
- query=q36879_p1
- precursor=560.759250
- chargePrecursor=4
- itol=0.5

AA	a	a*	a_0	b	b*	b_0	y	y*	y_0	AA
S[1]	140.011	0.000	122.000	168.006	0.000	149.995	2240.025	2222.998	2222.014	S[21]
E[2]	269.053	0.000	251.043	**297.048**	0.000	279.038	2073.026	2056.000	2055.016	E[20]
T[3]	**370.101**	0.000	352.090	**398.096**	0.000	380.085	1943.984	1926.957	1925.973	T[19]
A[4]	441.138	0.000	423.128	469.133	0.000	451.122	1842.936	1825.910	1824.926	A[18]
P[5]	538.191	0.000	**520.180**	566.186	0.000	548.175	1771.899	1754.872	1753.888	P[17]
A[6]	609.228	0.000	591.217	637.223	0.000	619.212	1674.846	1657.820	1656.836	A[16]
E[7]	**738.271**	0.000	720.260	766.265	0.000	**748.255**	1603.809	1586.783	1585.799	E[15]
T[8]	839.318	0.000	821.308	**867.313**	0.000	849.303	1474.767	1457.740	1456.756	T[14]
A[9]	910.355	0.000	892.345	938.350	0.000	920.340	1373.719	1356.692	1355.708	A[13]
A[10]	981.392	0.000	963.382	1009.387	0.000	991.377	1302.682	1285.655	1284.671	A[12]
P[11]	1078.445	0.000	1060.435	1106.440	0.000	1088.430	1231.645	1214.618	1213.634	P[11]
A[12]	1149.482	0.000	1131.472	1177.477	0.000	1159.467	1134.592	1117.565	1116.581	A[10]
P[13]	1246.535	0.000	1228.525	1274.530	0.000	1256.519	1063.555	1046.528	1045.544	P[9]
V[14]	1345.604	0.000	1327.593	1373.598	0.000	1355.588	966.502	949.475	948.491	V[8]
E[15]	1474.646	0.000	1456.636	1502.641	0.000	1484.630	**867.434**	**850.407**	849.423	E[7]
K[16]	1602.741	1585.715	1584.731	1630.736	1613.709	1612.725	**738.391**	**721.364**	720.380	K[6]
S[17]	1769.739	1752.713	1751.729	1797.734	1780.708	1779.724	**610.296**	593.269	**592.285**	S[5]
P[18]	1866.792	1849.766	1848.782	1894.787	1877.761	1876.777	**443.298**	426.271	0.000	P[4]
A[19]	1937.829	1920.803	1919.819	1965.824	1948.798	1947.814	346.245	329.218	0.000	A[3]
K[20]	2065.924	2048.898	2047.914	2093.919	2076.893	2075.909	275.208	258.181	0.000	K[2]
K[21]	2194.019	2176.993	2176.009	2222.014	2204.988	2204.004	147.113	130.086	0.000	K[1]

sp|P43276|H15_MOUSE

S $^{Phospho}_{79.97}$ ETAPAETAAPAPVEKS $^{Phospho}_{79.97}$ PAKK

- fragmentation table for charge state 2+
- specType=cid
- score=38.95
- F091343.dat
- query=q36879_p1
- precursor=560.759250
- chargePrecursor=4
- itol=0.5

AA	a	a*	a_0	b	b*	b_0	y	y*	y_0	AA
S[1]	70.509	0.504	61.504	84.506	0.504	75.501	1120.516	1112.003	1111.511	S[21]
E[2]	135.030	0.504	126.025	149.028	0.504	140.022	1037.017	1028.504	1028.012	E[20]
T[3]	185.554	0.504	176.549	**199.552**	0.504	190.546	972.496	963.982	963.490	T[19]
A[4]	221.073	0.504	212.067	235.070	0.504	226.065	921.972	913.458	912.966	A[18]
P[5]	269.599	0.504	260.594	**283.597**	0.504	274.591	886.453	877.940	877.448	P[17]
A[6]	305.118	0.504	296.112	319.115	0.504	310.110	837.927	829.413	828.921	A[16]
E[7]	**369.639**	0.504	360.634	**383.636**	0.504	**374.631**	802.408	793.895	**793.403**	E[15]
T[8]	**420.163**	0.504	**411.157**	**434.160**	0.504	**425.155**	**737.887**	729.374	**728.882**	T[14]
A[9]	**455.681**	0.504	446.676	**469.679**	0.504	**460.673**	**687.363**	678.850	**678.358**	A[13]
A[10]	**491.200**	0.504	482.195	**505.197**	0.504	**496.192**	**651.845**	**643.331**	**642.839**	A[12]
P[11]	539.726	0.504	530.721	**553.724**	0.504	544.718	**616.326**	607.813	**607.321**	P[11]
A[12]	**575.245**	0.504	566.240	**589.242**	0.504	**580.237**	**567.800**	559.286	558.794	A[10]
P[13]	623.771	0.504	614.766	**637.769**	0.504	628.763	**532.281**	**523.768**	**523.276**	P[9]
V[14]	673.305	0.504	664.300	**687.303**	0.504	**678.298**	**483.755**	475.241	**474.749**	V[8]
E[15]	**737.827**	0.504	**728.821**	**751.824**	0.504	**742.819**	**434.220**	**425.707**	**425.215**	E[7]
K[16]	801.874	**793.361**	**792.869**	**815.872**	**807.358**	**806.866**	**369.699**	361.186	360.694	K[6]
S[17]	885.373	876.860	876.368	899.371	890.858	890.366	**305.652**	**297.138**	296.646	S[5]
P[18]	933.900	925.386	924.894	947.897	939.384	938.892	222.152	**213.639**	0.504	P[4]
A[19]	969.418	960.905	960.413	983.416	974.902	**974.410**	173.626	165.113	0.504	A[3]
K[20]	1033.466	1024.953	1024.461	1047.463	1038.950	1038.458	138.108	129.594	0.504	K[2]
K[21]	1097.513	1089.000	1088.508	1111.511	1102.997	1102.505	74.060	65.547	0.504	K[1]

sp|P43276|H15_MOUSE

S $^{Phospho}_{79.97}$ ETAPAETAAPAPVEKS $^{Phospho}_{79.97}$ PAKK

- fragmentation table for charge state 3+
- specType=cid
- score=38.95
- F091343.dat
- query=q36879_p1
- precursor=560.759250
- chargePrecursor=4
- itol=0.5

AA	a	a*	a_0	b	b*	b_0	y	y*	y_0	AA
S[1]	47.342	0.672	41.338	56.673	0.672	50.670	747.346	741.671	741.343	S[21]
E[2]	90.356	0.672	84.352	99.688	0.672	93.684	691.680	686.005	685.677	E[20]
T[3]	124.039	0.672	118.035	133.370	0.672	127.367	648.666	**642.991**	**642.663**	T[19]
A[4]	147.718	0.672	141.714	157.049	0.672	151.046	614.984	609.308	608.980	A[18]
P[5]	180.068	0.672	174.065	189.400	0.672	183.397	591.305	585.629	585.301	P[17]
A[6]	203.748	0.672	197.744	**213.079**	0.672	207.076	558.954	553.278	552.950	A[16]
E[7]	246.762	0.672	**240.758**	256.093	0.672	250.090	535.275	**529.599**	**529.271**	E[15]
T[8]	280.444	0.672	274.441	289.776	0.672	283.772	492.260	486.585	486.257	T[14]
A[9]	304.123	0.672	298.120	313.455	0.672	307.451	458.578	**452.902**	**452.574**	A[13]
A[10]	**327.802**	0.672	321.799	337.134	0.672	331.130	**434.899**	429.223	428.895	A[12]
P[11]	**360.153**	0.672	354.150	**369.485**	0.672	363.481	**411.220**	405.544	405.216	P[11]
A[12]	**383.832**	0.672	377.829	393.164	0.672	387.160	**378.869**	373.193	372.865	A[10]
P[13]	416.183	0.672	410.180	**425.515**	0.672	419.511	355.190	349.514	349.186	P[9]
V[14]	449.206	0.672	**443.203**	458.538	0.672	**452.534**	322.839	317.163	316.835	V[8]
E[15]	492.220	0.672	486.217	501.552	0.672	**495.548**	289.816	284.141	283.813	E[7]
K[16]	534.919	**529.243**	**528.915**	**544.250**	538.575	538.247	246.802	**241.126**	**240.798**	K[6]
S[17]	590.585	584.909	584.581	599.916	**594.241**	**593.913**	204.104	198.428	198.100	S[5]
P[18]	**622.936**	617.260	**616.932**	632.267	**626.592**	**626.264**	148.437	142.762	0.672	P[4]
A[19]	**646.615**	640.939	640.611	**655.946**	650.271	**649.943**	116.086	110.411	0.672	A[3]
K[20]	**689.313**	683.637	683.309	**698.645**	**692.969**	**692.641**	92.407	86.732	0.672	K[2]
K[21]	732.011	726.336	726.008	741.343	735.667	735.339	49.709	44.034	0.672	K[1]

sp|P43274|H14_MOUSE

SETAPAAPAAPAPAEKT $^{Phospho}_{79.97}$ PVKKK

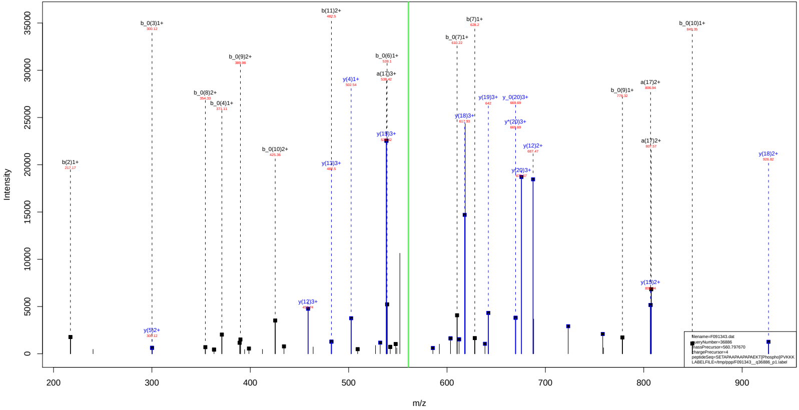

sp|P43274|H14_MOUSE

SETAPAAPAAPAPAEKT $^{Phospho}_{79.97}$ PVKKK

- fragmentation table for charge state 1+
- specType=cid
- score=35.68
- F091343.dat
- query=q36886_p1
- precursor=560.797670
- chargePrecursor=4
- itol=0.5

AA	a	a*	a_0	b	b*	b_0	y	y*	y_0	AA
S[1]	60.044	0.000	42.034	88.039	0.000	70.029	2240.169	2223.142	2222.158	S[22]
E[2]	189.087	0.000	171.076	**217.082**	0.000	199.071	2153.137	2136.110	2135.126	E[21]
T[3]	290.135	0.000	272.124	318.130	0.000	**300.119**	2024.094	2007.067	2006.083	T[20]
A[4]	361.172	0.000	343.161	**389.167**	0.000	**371.156**	1923.046	1906.020	1905.036	A[19]
P[5]	458.225	0.000	440.214	486.219	0.000	468.209	1852.009	1834.983	1833.999	P[18]
A[6]	529.262	0.000	511.251	557.257	0.000	**539.246**	1754.956	1737.930	1736.946	A[17]
A[7]	600.299	0.000	582.288	**628.294**	0.000	**610.283**	1683.919	1666.893	1665.909	A[16]
P[8]	697.352	0.000	679.341	725.346	0.000	707.336	1612.882	1595.856	1594.872	P[15]
A[9]	768.389	0.000	750.378	796.384	0.000	**778.373**	1515.829	1498.803	1497.819	A[14]
A[10]	839.426	0.000	821.415	867.421	0.000	**849.410**	1444.792	1427.766	1426.782	A[13]
P[11]	936.479	0.000	918.468	964.473	0.000	946.463	1373.755	1356.729	1355.745	P[12]
A[12]	1007.516	0.000	989.505	1035.511	0.000	1017.500	1276.702	1259.676	1258.692	A[11]
P[13]	1104.568	0.000	1086.558	1132.563	0.000	1114.553	1205.665	1188.639	1187.655	P[10]
A[14]	1175.606	0.000	1157.595	1203.600	0.000	1185.590	1108.613	1091.586	1090.602	A[9]
E[15]	1304.648	0.000	1286.638	1332.643	0.000	1314.632	1037.575	1020.549	1019.565	E[8]
K[16]	1432.743	1415.717	1414.733	1460.738	1443.711	1442.727	908.533	891.506	890.522	K[7]
T[17]	1613.757	1596.731	1595.747	1641.752	1624.725	1623.741	780.438	763.411	762.427	T[6]
P[18]	1710.810	1693.783	1692.799	1738.805	1721.778	1720.794	599.424	582.397	0.000	P[5]
V[19]	1809.878	1792.852	1791.868	1837.873	1820.847	1819.863	**502.371**	485.345	0.000	V[4]
K[20]	1937.973	1920.947	1919.963	1965.968	1948.942	1947.958	403.303	386.276	0.000	K[3]
K[21]	2066.068	2049.042	2048.058	2094.063	2077.037	2076.053	275.208	258.181	0.000	K[2]
K[22]	2194.163	2177.137	2176.153	2222.158	2205.132	2204.147	147.113	130.086	0.000	K[1]

sp|P43274|H14_MOUSE

SETAPAAPAAPAPAEKT $^{Phospho}_{79.97}$ PVKKK

- fragmentation table for charge state 2+
- specType=cid
- score=35.68
- F091343.dat
- query=q36886_p1
- precursor=560.797670
- chargePrecursor=4
- itol=0.5

AA	a	a*	a_0	b	b*	b_0	y	y*	y_0	AA
S[1]	30.526	0.504	21.521	44.523	0.504	35.518	1120.588	1112.075	1111.583	S[22]
E[2]	95.047	0.504	86.042	109.045	0.504	100.039	1077.072	1068.559	1068.067	E[21]
T[3]	145.571	0.504	136.566	159.568	0.504	150.563	1012.551	1004.037	1003.545	T[20]
A[4]	181.090	0.504	172.084	195.087	0.504	186.082	962.027	953.514	953.022	A[19]
P[5]	229.616	0.504	220.611	243.613	0.504	234.608	**926.508**	917.995	917.503	P[18]
A[6]	265.134	0.504	256.129	279.132	0.504	270.127	877.982	869.469	868.977	A[17]
A[7]	300.653	0.504	291.648	314.650	0.504	305.645	842.463	833.950	833.458	A[16]
P[8]	349.179	0.504	340.174	**363.177**	0.504	**354.172**	**806.945**	798.431	797.939	P[15]
A[9]	384.698	0.504	375.693	**398.695**	0.504	**389.690**	**758.418**	749.905	749.413	A[14]
A[10]	420.217	0.504	411.211	**434.214**	0.504	**425.209**	**722.900**	714.387	713.895	A[13]
P[11]	468.743	0.504	459.738	**482.740**	0.504	473.735	**687.381**	678.868	678.376	P[12]
A[12]	504.261	0.504	495.256	518.259	0.504	**509.254**	**638.855**	630.342	629.850	A[11]
P[13]	552.788	0.504	543.783	566.785	0.504	557.780	**603.336**	594.823	594.331	P[10]
A[14]	588.306	0.504	579.301	602.304	0.504	593.299	554.810	546.297	545.805	A[9]
E[15]	652.828	0.504	643.822	666.825	0.504	657.820	519.291	510.778	510.286	E[8]
K[16]	716.875	708.362	707.870	730.873	722.359	721.867	454.770	446.257	445.765	K[7]
T[17]	**807.382**	798.869	798.377	821.380	812.866	812.374	390.723	382.209	381.717	T[6]
P[18]	855.909	847.395	846.903	869.906	861.393	860.901	**300.216**	291.702	0.504	P[5]
V[19]	905.443	896.929	896.437	919.440	910.927	910.435	251.689	243.176	0.504	V[4]
K[20]	969.490	960.977	960.485	983.488	974.974	974.482	202.155	193.642	0.504	K[3]
K[21]	1033.538	1025.024	1024.532	1047.535	1039.022	1038.530	138.108	129.594	0.504	K[2]
K[22]	1097.585	1089.072	1088.580	1111.583	1103.069	1102.577	74.060	65.547	0.504	K[1]

sp|P43274|H14_MOUSE

SETAPAAPAAPAPAEKT $^{Phospho}_{79.97}$ PVKKK

- fragmentation table for charge state 3+
- specType=cid
- score=35.68
- F091343.dat
- query=q36886_p1
- precursor=560.797670
- chargePrecursor=4
- itol=0.5

AA	a	a*	a_0	b	b*	b_0	y	y*	y_0	AA
S[1]	20.686	0.672	14.683	30.018	0.672	24.014	747.394	741.719	741.391	S[22]
E[2]	63.701	0.672	57.697	73.032	0.672	67.029	718.384	712.708	712.380	E[21]
T[3]	97.383	0.672	91.380	106.715	0.672	100.711	**675.370**	**669.694**	**669.366**	T[20]
A[4]	121.062	0.672	115.059	130.394	0.672	124.390	**641.687**	636.011	635.683	A[19]
P[5]	153.413	0.672	147.410	162.745	0.672	156.741	**618.008**	**612.332**	**612.004**	P[18]
A[6]	177.092	0.672	171.089	186.424	0.672	180.420	**585.657**	579.981	579.653	A[17]
A[7]	200.771	0.672	194.768	210.103	0.672	204.099	561.978	556.302	555.974	A[16]
P[8]	233.122	0.672	227.119	242.454	0.672	236.450	**538.299**	532.623	**532.295**	P[15]
A[9]	256.801	0.672	250.798	266.133	0.672	260.129	505.948	500.272	499.944	A[14]
A[10]	280.480	0.672	274.477	289.812	0.672	283.808	**482.269**	476.593	476.265	A[13]
P[11]	312.831	0.672	306.828	322.163	0.672	316.159	**458.590**	452.914	452.586	P[12]
A[12]	336.510	0.672	330.507	345.842	0.672	339.838	426.239	420.563	420.235	A[11]
P[13]	368.861	0.672	**362.857**	378.193	0.672	372.189	402.560	396.884	396.556	P[10]
A[14]	392.540	0.672	386.537	401.872	0.672	395.868	370.209	364.534	364.206	A[9]
E[15]	435.554	0.672	429.551	444.886	0.672	438.882	346.530	340.854	340.526	E[8]
K[16]	478.253	472.577	472.249	487.584	481.909	481.581	303.516	297.840	297.512	K[7]
T[17]	**538.591**	532.915	532.587	**547.922**	**542.247**	**541.919**	260.817	255.142	254.814	T[6]
P[18]	570.941	565.266	564.938	580.273	574.598	574.270	200.479	194.804	0.672	P[5]
V[19]	**603.964**	598.289	597.961	613.296	607.620	607.292	168.129	162.453	0.672	V[4]
K[20]	646.663	640.987	640.659	655.994	650.319	649.991	135.106	129.430	0.672	K[3]
K[21]	689.361	683.685	683.357	698.693	693.017	692.689	92.407	86.732	0.672	K[2]
K[22]	732.059	726.384	726.056	741.391	735.715	735.387	49.709	44.034	0.672	K[1]

sp|P43274|H14_MOUSE

SETAPAAPAAPAPAEKT $^{Phospho}_{79.97}$ PVKKK

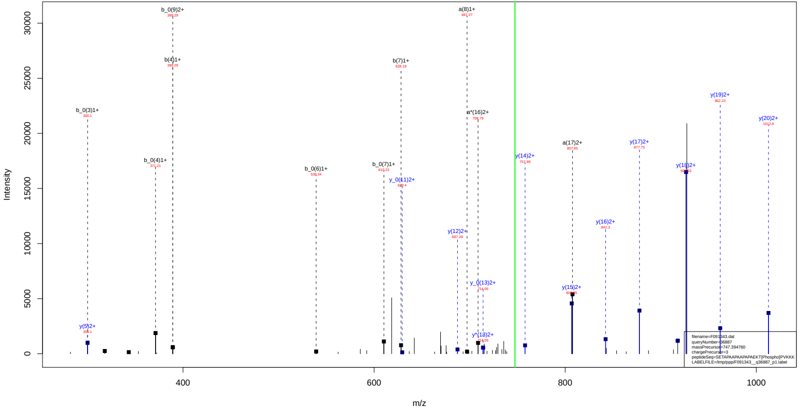

sp|P43274|H14_MOUSE

SETAPAAPAAPAPAEKT $^{Phospho}_{79.97}$ PVKKK

- fragmentation table for charge state 1+
- specType=cid
- score=36.54
- F091343.dat
- query=q36887_p1
- precursor=747.394760
- chargePrecursor=3
- itol=0.5

AA	a	a*	a_0	b	b*	b_0	y	y*	y_0	AA
S[1]	60.044	0.000	42.034	88.039	0.000	70.029	2240.169	2223.142	2222.158	S[22]
E[2]	189.087	0.000	171.076	217.082	0.000	199.071	2153.137	2136.110	2135.126	E[21]
T[3]	290.135	0.000	272.124	**318.130**	0.000	**300.119**	2024.094	2007.067	2006.083	T[20]
A[4]	361.172	0.000	**343.161**	**389.167**	0.000	**371.156**	1923.046	1906.020	1905.036	A[19]
P[5]	458.225	0.000	440.214	486.219	0.000	468.209	1852.009	1834.983	1833.999	P[18]
A[6]	529.262	0.000	511.251	557.257	0.000	**539.246**	1754.956	1737.930	1736.946	A[17]
A[7]	600.299	0.000	582.288	**628.294**	0.000	**610.283**	1683.919	1666.893	1665.909	A[16]
P[8]	**697.352**	0.000	679.341	725.346	0.000	707.336	1612.882	1595.856	1594.872	P[15]
A[9]	768.389	0.000	750.378	796.384	0.000	778.373	1515.829	1498.803	1497.819	A[14]
A[10]	839.426	0.000	821.415	867.421	0.000	849.410	1444.792	1427.766	1426.782	A[13]
P[11]	936.479	0.000	918.468	964.473	0.000	946.463	1373.755	1356.729	1355.745	P[12]
A[12]	1007.516	0.000	989.505	1035.511	0.000	1017.500	1276.702	1259.676	1258.692	A[11]
P[13]	1104.568	0.000	1086.558	1132.563	0.000	1114.553	1205.665	1188.639	1187.655	P[10]
A[14]	1175.606	0.000	1157.595	1203.600	0.000	1185.590	1108.613	1091.586	1090.602	A[9]
E[15]	1304.648	0.000	1286.638	1332.643	0.000	1314.632	1037.575	1020.549	1019.565	E[8]
K[16]	1432.743	1415.717	1414.733	1460.738	1443.711	1442.727	908.533	891.506	890.522	K[7]
T[17]	1613.757	1596.731	1595.747	1641.752	1624.725	1623.741	780.438	763.411	762.427	T[6]
P[18]	1710.810	1693.783	1692.799	1738.805	1721.778	1720.794	599.424	582.397	0.000	P[5]
V[19]	1809.878	1792.852	1791.868	1837.873	1820.847	1819.863	502.371	485.345	0.000	V[4]
K[20]	1937.973	1920.947	1919.963	1965.968	1948.942	1947.958	403.303	386.276	0.000	K[3]
K[21]	2066.068	2049.042	2048.058	2094.063	2077.037	2076.053	275.208	258.181	0.000	K[2]
K[22]	2194.163	2177.137	2176.153	2222.158	2205.132	2204.147	147.113	130.086	0.000	K[1]

sp|P43274|H14_MOUSE

SETAPAAPAAPAPAEKT $^{Phospho}_{79.97}$ PVKKK

- fragmentation table for charge state 2+
- specType=cid
- score=36.54
- F091343.dat
- query=q36887_p1
- precursor=747.394760
- chargePrecursor=3
- itol=0.5

AA	a	a*	a_0	b	b*	b_0	y	y*	y_0	AA
S[1]	30.526	0.504	21.521	44.523	0.504	35.518	1120.588	1112.075	1111.583	S[22]
E[2]	95.047	0.504	86.042	109.045	0.504	100.039	1077.072	1068.559	1068.067	E[21]
T[3]	145.571	0.504	136.566	159.568	0.504	150.563	**1012.551**	1004.037	1003.545	T[20]
A[4]	181.090	0.504	172.084	195.087	0.504	186.082	**962.027**	953.514	953.022	A[19]
P[5]	229.616	0.504	220.611	243.613	0.504	234.608	**926.508**	**917.995**	**917.503**	P[18]
A[6]	265.134	0.504	256.129	279.132	0.504	270.127	**877.982**	869.469	868.977	A[17]
A[7]	300.653	0.504	291.648	314.650	0.504	305.645	**842.463**	833.950	833.458	A[16]
P[8]	349.179	0.504	340.174	363.177	0.504	354.172	**806.945**	798.431	797.939	P[15]
A[9]	384.698	0.504	375.693	398.695	0.504	**389.690**	**758.418**	749.905	749.413	A[14]
A[10]	420.217	0.504	411.211	434.214	0.504	425.209	722.900	**714.387**	**713.895**	A[13]
P[11]	468.743	0.504	459.738	482.740	0.504	473.735	**687.381**	678.868	678.376	P[12]
A[12]	504.261	0.504	495.256	518.259	0.504	509.254	638.855	630.342	**629.850**	A[11]
P[13]	552.788	0.504	543.783	566.785	0.504	557.780	603.336	594.823	594.331	P[10]
A[14]	588.306	0.504	579.301	602.304	0.504	593.299	554.810	546.297	545.805	A[9]
E[15]	652.828	0.504	643.822	666.825	0.504	657.820	519.291	510.778	510.286	E[8]
K[16]	716.875	**708.362**	707.870	730.873	722.359	721.867	454.770	446.257	445.765	K[7]
T[17]	**807.382**	798.869	798.377	821.380	812.866	812.374	390.723	382.209	381.717	T[6]
P[18]	855.909	847.395	846.903	869.906	861.393	860.901	**300.216**	291.702	0.504	P[5]
V[19]	905.443	896.929	896.437	919.440	910.927	910.435	251.689	243.176	0.504	V[4]
K[20]	969.490	960.977	960.485	983.488	974.974	974.482	202.155	193.642	0.504	K[3]
K[21]	1033.538	1025.024	1024.532	1047.535	1039.022	1038.530	138.108	129.594	0.504	K[2]
K[22]	1097.585	1089.072	1088.580	1111.583	1103.069	1102.577	74.060	65.547	0.504	K[1]

sp|P43276|H15_MOUSE

SETAPAETAAPAPVEKS $^{\text{Phospho}}_{79.97}$ PAKKK

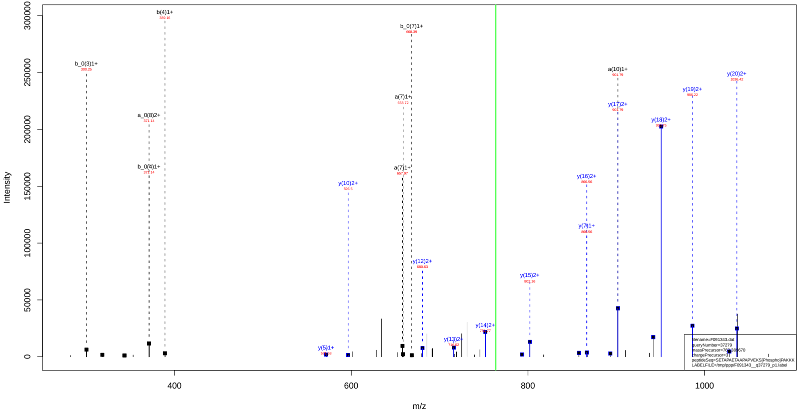

sp|P43276|H15_MOUSE

SETAPAETAAPAPVEKS $^{Phospho}_{79.97}$ PAKKK

- fragmentation table for charge state 1+
- specType=cid
- score=56.09
- F091343.dat
- query=q37279_p1
- precursor=763.389670
- chargePrecursor=3
- itol=0.5

AA	a	a*	a_0	b	b*	b_0	y	y*	y_0	AA
S[1]	60.044	0.000	42.034	88.039	0.000	70.029	2288.153	2271.127	2270.143	S[22]
E[2]	189.087	0.000	171.076	217.082	0.000	199.071	2201.121	2184.095	2183.111	E[21]
T[3]	290.135	0.000	272.124	**318.130**	0.000	**300.119**	2072.079	2055.052	2054.068	T[20]
A[4]	361.172	0.000	**343.161**	**389.167**	0.000	**371.156**	1971.031	1954.005	1953.021	A[19]
P[5]	458.225	0.000	440.214	486.219	0.000	468.209	1899.994	1882.967	1881.983	P[18]
A[6]	529.262	0.000	511.251	557.257	0.000	539.246	1802.941	1785.915	1784.931	A[17]
E[7]	**658.304**	0.000	640.294	686.299	0.000	**668.289**	1731.904	1714.878	1713.894	E[16]
T[8]	759.352	0.000	741.341	787.347	0.000	769.336	1602.861	1585.835	1584.851	T[15]
A[9]	830.389	0.000	812.378	858.384	0.000	840.373	1501.814	1484.787	1483.803	A[14]
A[10]	**901.426**	0.000	883.416	929.421	0.000	911.411	1430.777	1413.750	1412.766	A[13]
P[11]	998.479	0.000	980.468	1026.474	0.000	1008.463	1359.740	1342.713	1341.729	P[12]
A[12]	1069.516	0.000	1051.505	1097.511	0.000	1079.500	1262.687	1245.660	1244.676	A[11]
P[13]	1166.569	0.000	1148.558	1194.564	0.000	1176.553	1191.650	1174.623	1173.639	P[10]
V[14]	1265.637	0.000	1247.627	1293.632	0.000	1275.622	1094.597	1077.570	1076.586	V[9]
E[15]	1394.680	0.000	1376.669	1422.675	0.000	1404.664	995.529	978.502	977.518	E[8]
K[16]	1522.775	1505.748	1504.764	1550.770	1533.743	1532.759	**866.486**	849.459	848.475	K[7]
S[17]	1689.773	1672.747	1671.763	1717.768	1700.741	1699.757	738.391	721.364	720.380	S[6]
P[18]	1786.826	1769.799	1768.815	1814.821	1797.794	1796.810	**571.393**	554.366	0.000	P[5]
A[19]	1857.863	1840.836	1839.852	1885.858	1868.831	1867.847	474.340	457.313	0.000	A[4]
K[20]	1985.958	1968.931	1967.947	2013.953	1996.926	1995.942	403.303	386.276	0.000	K[3]
K[21]	2114.053	2097.026	2096.042	2142.048	2125.021	2124.037	275.208	258.181	0.000	K[2]
K[22]	2242.148	2225.121	2224.137	2270.143	2253.116	2252.132	147.113	130.086	0.000	K[1]

sp|P43276|H15_MOUSE

SETAPAETAAPAPVEKS $^{Phospho}_{79.97}$ PAKKK

- fragmentation table for charge state 2+
- specType=cid
- score=56.09
- F091343.dat
- query=q37279_p1
- precursor=763.389670
- chargePrecursor=3
- itol=0.5

AA	a	a*	a_0	b	b*	b_0	y	y*	y_0	AA
S[1]	30.526	0.504	21.521	44.523	0.504	35.518	1144.580	1136.067	1135.575	S[22]
E[2]	95.047	0.504	86.042	109.045	0.504	100.039	1101.064	1092.551	1092.059	E[21]
T[3]	145.571	0.504	136.566	159.568	0.504	150.563	**1036.543**	**1028.030**	**1027.538**	T[20]
A[4]	181.090	0.504	172.084	195.087	0.504	186.082	**986.019**	977.506	977.014	A[19]
P[5]	229.616	0.504	220.611	243.613	0.504	234.608	**950.501**	**941.987**	**941.495**	P[18]
A[6]	265.134	0.504	256.129	279.132	0.504	270.127	**901.974**	**893.461**	**892.969**	A[17]
E[7]	329.656	0.504	320.650	**343.653**	0.504	334.648	**866.456**	**857.942**	**857.450**	E[16]
T[8]	380.180	0.504	**371.174**	394.177	0.504	385.172	**801.934**	**793.421**	**792.929**	T[15]
A[9]	415.698	0.504	406.693	429.696	0.504	420.690	**751.411**	742.897	742.405	A[14]
A[10]	451.217	0.504	442.211	465.214	0.504	456.209	**715.892**	707.379	706.887	A[13]
P[11]	499.743	0.504	490.738	513.741	0.504	504.735	**680.373**	671.860	671.368	P[12]
A[12]	535.262	0.504	526.256	549.259	0.504	540.254	631.847	623.334	622.842	A[11]
P[13]	583.788	0.504	574.783	597.785	0.504	588.780	**596.328**	587.815	587.323	P[10]
V[14]	633.322	0.504	624.317	647.320	0.504	638.314	547.802	539.289	538.797	V[9]
E[15]	697.844	0.504	688.838	711.841	0.504	702.836	498.268	489.755	489.263	E[8]
K[16]	761.891	753.378	752.886	775.888	767.375	766.883	433.747	425.233	424.741	K[7]
S[17]	845.390	836.877	836.385	859.388	850.874	850.382	369.699	361.186	360.694	S[6]
P[18]	893.917	885.403	884.911	907.914	899.401	898.909	286.200	277.687	0.504	P[5]
A[19]	929.435	920.922	920.430	943.433	934.919	934.427	237.674	229.160	0.504	A[4]
K[20]	993.483	984.969	984.477	1007.480	998.967	998.475	202.155	193.642	0.504	K[3]
K[21]	1057.530	1049.017	1048.525	1071.528	1063.014	1062.522	138.108	129.594	0.504	K[2]
K[22]	1121.578	1113.064	1112.572	1135.575	1127.062	1126.570	74.060	65.547	0.504	K[1]

sp|P15864|H12_MOUSE

GTGAGSGS $^{(\mathrm{Phospho})}_{(79.97)}$ FK

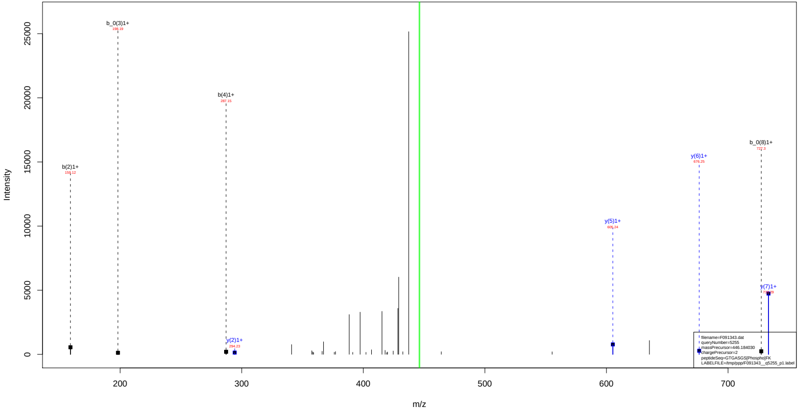

sp|P15864|H12_MOUSE

GTGASGS$^{(Phospho)}_{(79.97)}$FK

- fragmentation table for charge state 1+
- specType=cid
- score=29.16
- F091343.dat
- query=q5255_p1
- precursor=446.184030
- chargePrecursor=2
- itol=0.5

AA	a	a*	a_0	b	b*	b_0	y	y*	y_0	AA
G[1]	30.034	0.000	0.000	58.029	0.000	0.000	891.361	874.334	873.350	G[9]
T[2]	131.082	0.000	113.071	**159.076**	0.000	141.066	834.339	817.313	816.329	T[8]
G[3]	188.103	0.000	170.092	216.098	0.000	**198.087**	**733.292**	716.265	715.281	G[7]
A[4]	259.140	0.000	241.130	**287.135**	0.000	269.124	**676.270**	659.244	658.260	A[6]
S[5]	346.172	0.000	328.162	374.167	0.000	356.156	**605.233**	588.207	587.223	S[5]
G[6]	403.194	0.000	385.183	431.188	0.000	413.178	518.201	501.174	500.190	G[4]
S[7]	570.192	0.000	552.181	598.187	0.000	580.176	461.180	444.153	443.169	S[3]
F[8]	717.260	0.000	699.250	745.255	0.000	**727.245**	**294.181**	277.155	0.000	F[2]
K[9]	845.355	828.329	827.345	873.350	856.324	855.340	147.113	130.086	0.000	K[1]

sp|P43274|H14_MOUSE

KAPKS $^{\text{Phospho}}_{79.97}$ PAK

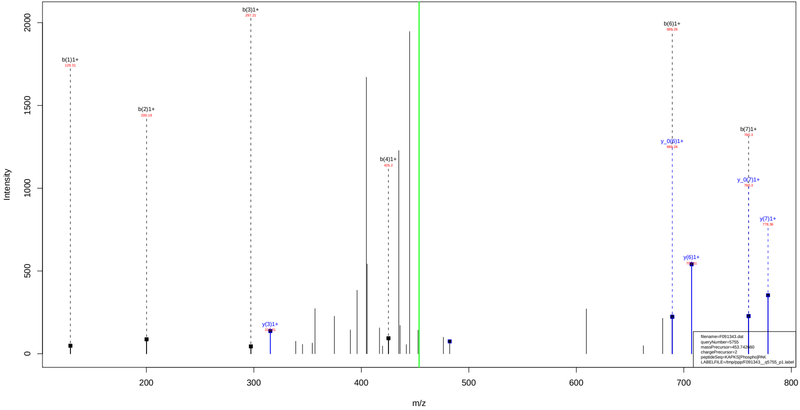

sp|P43274|H14_MOUSE

KAPKS $^{Phospho}_{79.97}$ PAK

- fragmentation table for charge state 1+
- specType=cid
- score=45.10
- F091343.dat
- query=q5755_p1
- precursor=453.742660
- chargePrecursor=2
- itol=0.5

AA	a	a*	a_0	b	b*	b_0	y	y*	y_0	AA
K[1]	101.107	84.081	0.000	**129.102**	112.076	0.000	906.481	889.454	888.470	K[8]
A[2]	172.144	155.118	0.000	**200.139**	183.113	0.000	**778.386**	761.359	**760.375**	A[7]
P[3]	269.197	252.171	0.000	**297.192**	280.166	0.000	**707.349**	690.322	**689.338**	P[6]
K[4]	397.292	380.266	0.000	**425.287**	408.261	0.000	610.296	593.269	592.285	K[5]
S[5]	564.291	547.264	546.280	592.285	575.259	574.275	**482.201**	465.174	464.190	S[4]
P[6]	661.343	644.317	643.333	**689.338**	672.312	671.328	**315.203**	298.176	0.000	P[3]
A[7]	732.380	715.354	714.370	**760.375**	743.349	742.365	218.150	201.123	0.000	A[2]
K[8]	860.475	843.449	842.465	888.470	871.444	870.460	147.113	130.086	0.000	K[1]

sp|P43274|H14_MOUSE

KAPKS $^{Phospho}_{79.97}$ PAK

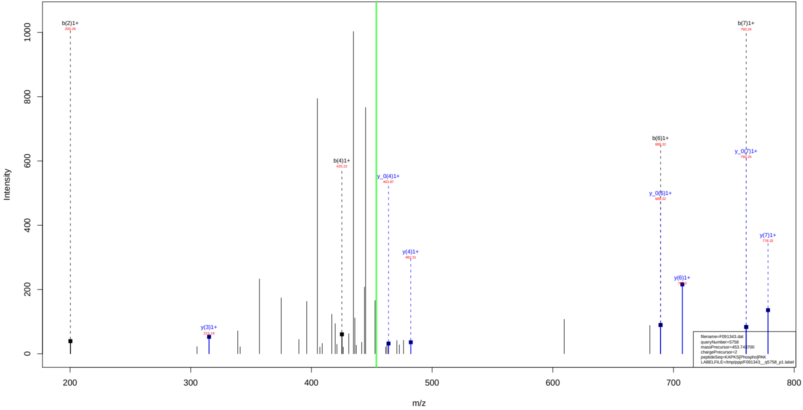

sp|P43274|H14_MOUSE

KAPKS $^{Phospho}_{79.97}$ PAK

- fragmentation table for charge state 1+
- specType=cid
- score=27.72
- F091343.dat
- query=q5758_p1
- precursor=453.743700
- chargePrecursor=2
- itol=0.5

AA	a	a*	a_0	b	b*	b_0	y	y*	y_0	AA
K[1]	101.107	84.081	0.000	129.102	112.076	0.000	906.481	889.454	888.470	K[8]
A[2]	172.144	155.118	0.000	**200.139**	183.113	0.000	**778.386**	761.359	**760.375**	A[7]
P[3]	269.197	252.171	0.000	297.192	280.166	0.000	**707.349**	690.322	**689.338**	P[6]
K[4]	397.292	380.266	0.000	**425.287**	408.261	0.000	610.296	593.269	592.285	K[5]
S[5]	564.291	547.264	546.280	592.285	575.259	574.275	**482.201**	465.174	**464.190**	S[4]
P[6]	661.343	644.317	643.333	**689.338**	672.312	671.328	**315.203**	298.176	0.000	P[3]
A[7]	732.380	715.354	714.370	**760.375**	743.349	742.365	218.150	201.123	0.000	A[2]
K[8]	860.475	843.449	842.465	888.470	871.444	870.460	147.113	130.086	0.000	K[1]

sp|P43274|H14_MOUSE

KAPKS $^{\text{Phospho}}_{79.97}$ PAK

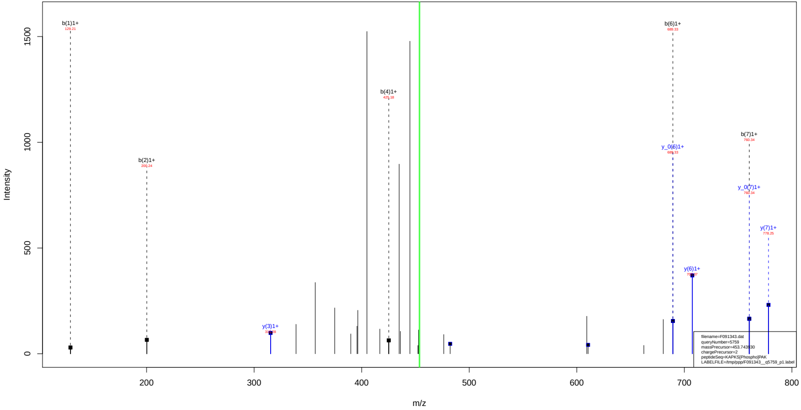

sp|P43274|H14_MOUSE

KAPKS $^{Phospho}_{79.97}$ PAK

- fragmentation table for charge state 1+
- specType=cid
- score=41.67
- F091343.dat
- query=q5759_p1
- precursor=453.743930
- chargePrecursor=2
- itol=0.5

AA	a	a*	a_0	b	b*	b_0	y	y*	y_0	AA
K[1]	101.107	84.081	0.000	**129.102**	112.076	0.000	906.481	889.454	888.470	K[8]
A[2]	172.144	155.118	0.000	**200.139**	183.113	0.000	**778.386**	761.359	**760.375**	A[7]
P[3]	269.197	252.171	0.000	297.192	280.166	0.000	**707.349**	690.322	**689.338**	P[6]
K[4]	397.292	380.266	0.000	**425.287**	408.261	0.000	**610.296**	593.269	592.285	K[5]
S[5]	564.291	547.264	546.280	592.285	575.259	574.275	**482.201**	465.174	464.190	S[4]
P[6]	661.343	644.317	643.333	**689.338**	672.312	671.328	**315.203**	298.176	0.000	P[3]
A[7]	732.380	715.354	714.370	**760.375**	743.349	742.365	218.150	201.123	0.000	A[2]
K[8]	860.475	843.449	842.465	888.470	871.444	870.460	147.113	130.086	0.000	K[1]

sp|P70696|H2B1A_MOUSE

HAVS $^{\text{Phospho}}_{79.97}$ EGTK

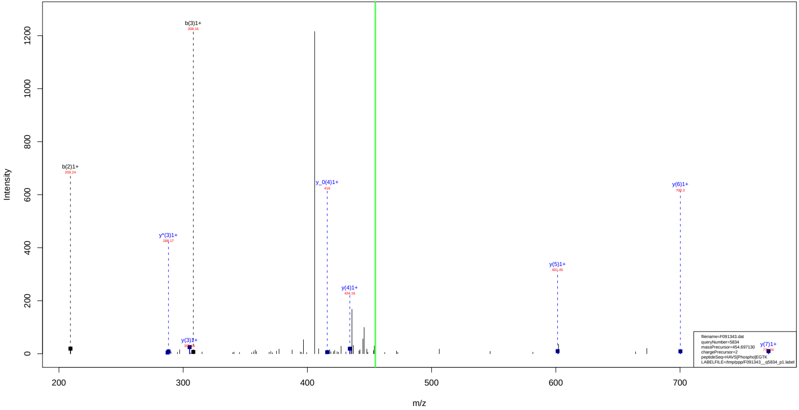

sp|P70696|H2B1A_MOUSE

HAVS $^{\text{Phospho}}_{79.97}$ EGTK

- fragmentation table for charge state 1+
- specType=cid
- score=33.15
- F091343.dat
- query=q5834_p1
- precursor=454.697130
- chargePrecursor=2
- itol=0.5

AA	a	a*	a_0	b	b*	b_0	y	y*	y_0	AA
H[1]	110.071	0.000	0.000	138.066	0.000	0.000	908.387	891.361	890.377	H[8]
A[2]	181.108	0.000	0.000	**209.103**	0.000	0.000	**771.328**	754.302	753.318	A[7]
V[3]	280.177	0.000	0.000	**308.172**	0.000	0.000	**700.291**	683.265	682.281	V[6]
S[4]	447.175	0.000	429.165	475.170	0.000	457.160	**601.223**	584.196	583.212	S[5]
E[5]	576.218	0.000	558.207	604.213	0.000	586.202	**434.225**	417.198	**416.214**	E[4]
G[6]	633.239	0.000	615.229	661.234	0.000	643.224	**305.182**	**288.155**	**287.171**	G[3]
T[7]	734.287	0.000	716.276	762.282	0.000	744.271	248.160	231.134	230.150	T[2]
K[8]	862.382	845.355	844.371	890.377	873.350	872.366	147.113	130.086	0.000	K[1]

sp|P70696|H2B1A_MOUSE

HAVS $^{Phospho}_{79.97}$ EGTK

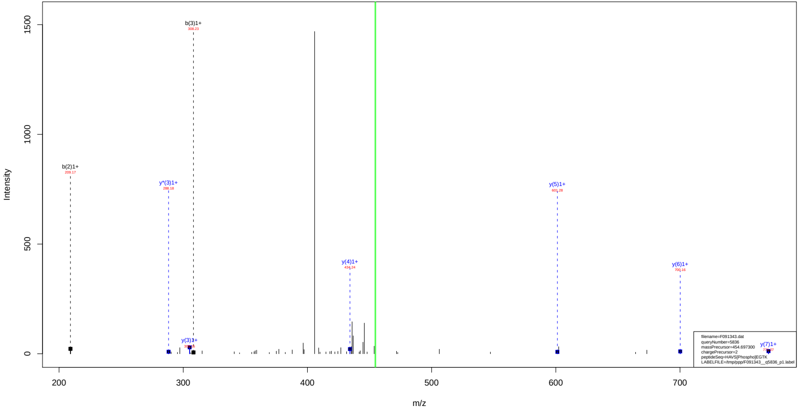

sp|P70696|H2B1A_MOUSE

HAVS $^{Phospho}_{79.97}$ EGTK

- fragmentation table for charge state 1+
- specType=cid
- score=30.50
- F091343.dat
- query=q5836_p1
- precursor=454.697300
- chargePrecursor=2
- itol=0.5

AA	a	a*	a_0	b	b*	b_0	y	y*	y_0	AA
H[1]	110.071	0.000	0.000	138.066	0.000	0.000	908.387	891.361	890.377	H[8]
A[2]	181.108	0.000	0.000	**209.103**	0.000	0.000	**771.328**	754.302	753.318	A[7]
V[3]	280.177	0.000	0.000	**308.172**	0.000	0.000	**700.291**	683.265	682.281	V[6]
S[4]	447.175	0.000	429.165	475.170	0.000	457.160	**601.223**	584.196	583.212	S[5]
E[5]	576.218	0.000	558.207	604.213	0.000	586.202	**434.225**	417.198	416.214	E[4]
G[6]	633.239	0.000	615.229	661.234	0.000	643.224	**305.182**	**288.155**	287.171	G[3]
T[7]	734.287	0.000	716.276	762.282	0.000	744.271	248.160	231.134	230.150	T[2]
K[8]	862.382	845.355	844.371	890.377	873.350	872.366	147.113	130.086	0.000	K[1]

sp|P70696|H2B1A_MOUSE

HAVS $^{Phospho}_{79.97}$ EGTK

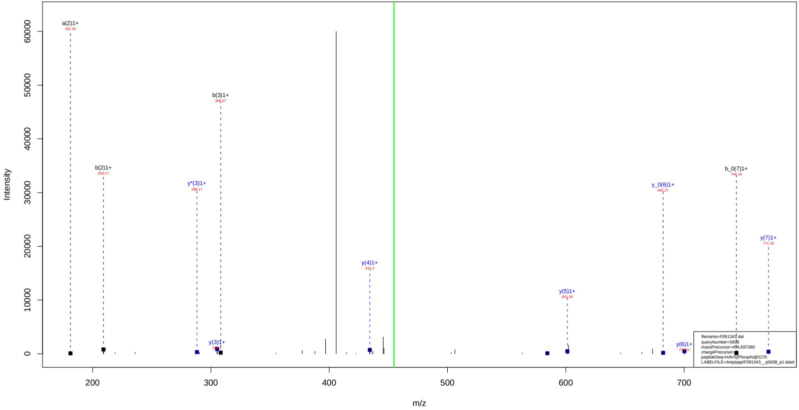

sp|P70696|H2B1A_MOUSE

HAVS $^{Phospho}_{79.97}$ EGTK

- fragmentation table for charge state 1+
- specType=cid
- score=42.55
- F091343.dat
- query=q5838_p1
- precursor=454.697360
- chargePrecursor=2
- itol=0.5

AA	a	a*	a_0	b	b*	b_0	y	y*	y_0	AA
H[1]	110.071	0.000	0.000	138.066	0.000	0.000	908.387	891.361	890.377	H[8]
A[2]	**181.108**	0.000	0.000	**209.103**	0.000	0.000	**771.328**	754.302	753.318	A[7]
V[3]	280.177	0.000	0.000	**308.172**	0.000	0.000	**700.291**	683.265	**682.281**	V[6]
S[4]	447.175	0.000	429.165	475.170	0.000	457.160	**601.223**	**584.196**	583.212	S[5]
E[5]	576.218	0.000	558.207	604.213	0.000	586.202	**434.225**	417.198	416.214	E[4]
G[6]	633.239	0.000	615.229	661.234	0.000	643.224	**305.182**	**288.155**	287.171	G[3]
T[7]	734.287	0.000	716.276	762.282	0.000	**744.271**	248.160	231.134	230.150	T[2]
K[8]	862.382	845.355	844.371	890.377	873.350	872.366	147.113	130.086	0.000	K[1]

sp|P43274|H14_MOUSE

APKS $^{Phospho}_{79.97}$ PAKAK

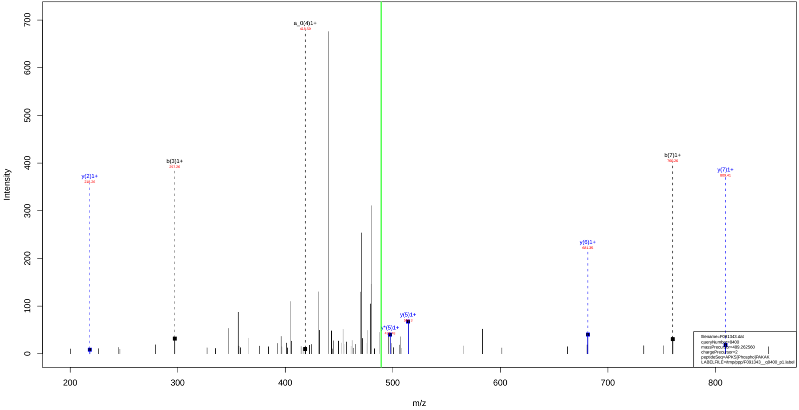

sp|P43274|H14_MOUSE

APKS $^{Phospho}_{79.97}$ PAKAK

- fragmentation table for charge state 1+
- specType=cid
- score=27.56
- F091343.dat
- query=q8400_p1
- precursor=489.262560
- chargePrecursor=2
- itol=0.5

AA	a	a*	a_0	b	b*	b_0	y	y*	y_0	AA
A[1]	44.049	0.000	0.000	72.044	0.000	0.000	977.518	960.491	959.507	A[9]
P[2]	141.102	0.000	0.000	169.097	0.000	0.000	906.481	889.454	888.470	P[8]
K[3]	269.197	252.171	0.000	**297.192**	280.166	0.000	**809.428**	792.402	791.418	K[7]
S[4]	436.196	419.169	**418.185**	464.190	447.164	446.180	**681.333**	664.307	663.323	S[6]
P[5]	533.248	516.222	515.238	561.243	544.217	543.233	**514.335**	**497.308**	0.000	P[5]
A[6]	604.285	587.259	586.275	632.280	615.254	614.270	417.282	400.255	0.000	A[4]
K[7]	732.380	715.354	714.370	**760.375**	743.349	742.365	346.245	329.218	0.000	K[3]
A[8]	803.418	786.391	785.407	831.412	814.386	813.402	**218.150**	201.123	0.000	A[2]
K[9]	931.512	914.486	913.502	959.507	942.481	941.497	147.113	130.086	0.000	K[1]

sp|Q6GSS7|H2A2A_MOUSE

LNKLLGK $^{\text{Dimethyl}}_{28.03}$ VTIAQGGVLPNIQAVLLPKK $^{\text{Methyl}}_{14.02}$ TE

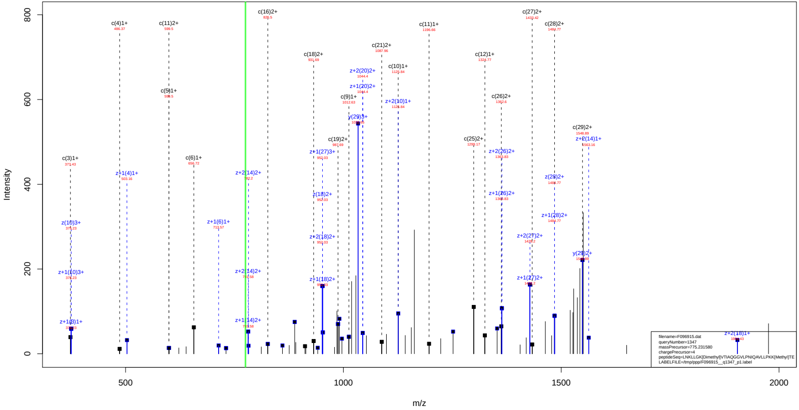

sp|Q6GSS7|H2A2A_MOUSE

LNKLLGK $^{\text{Dimethyl}}_{28.03}$ VTIAQGGVLPNIQAVLLPKK $^{\text{Methyl}}_{14.02}$ TE

- fragmentation table for charge state 1+
- specType=etd
- score=49.66
- F096915.dat
- query=q1347_p1
- precursor=775.231580
- chargePrecursor=4
- itol=0.6

AA	c	y	z+1	z+2	z	AA
L[1]	131.118	3097.908	3081.889	0.000	3080.881	L[29]
N[2]	245.161	2984.823	2968.805	2969.813	2967.797	N[28]
K[3]	**373.256**	2870.781	2854.762	2855.770	2853.754	K[27]
L[4]	**486.340**	2742.686	2726.667	2727.675	2725.659	L[26]
L[5]	**599.424**	2629.601	2613.583	2614.591	2612.575	L[25]
G[6]	**656.445**	2516.517	2500.499	2501.507	2499.491	G[24]
K[7]	812.572	2459.496	2443.477	2444.485	2442.469	K[23]
V[8]	**911.640**	2303.370	2287.351	2288.359	2286.343	V[22]
T[9]	**1012.688**	2204.301	2188.283	2189.290	2187.275	T[21]
I[10]	**1125.772**	2103.254	2087.235	2088.243	2086.227	I[20]
A[11]	**1196.809**	1990.170	1974.151	1975.159	1973.143	A[19]
Q[12]	**1324.867**	1919.132	1903.114	**1904.122**	1902.106	Q[18]
G[13]	1381.889	1791.074	1775.055	1776.063	1774.047	G[17]
G[14]	1438.910	1734.052	1718.034	1719.041	1717.026	G[16]
V[15]	1537.979	1677.031	1661.012	1662.020	1660.004	V[15]
L[16]	1651.063	1577.963	1561.944	**1562.952**	1560.936	L[14]
P[17]	1748.116	1464.878	1448.860	1449.868	1447.852	P[13]
N[18]	1862.159	1367.826	1351.807	**1352.815**	1350.799	N[12]
I[19]	1975.243	1253.783	1237.764	1238.772	1236.756	I[11]
Q[20]	2103.301	1140.699	1124.680	**1125.688**	1123.672	Q[10]
A[21]	2174.338	**1012.640**	**996.621**	997.629	995.614	A[9]
V[22]	2273.407	**941.603**	925.584	926.592	924.576	V[8]
L[23]	2386.491	842.535	**826.516**	827.524	825.508	L[7]
L[24]	2499.575	729.451	**713.432**	714.440	712.424	L[6]
P[25]	2596.628	616.366	600.348	601.356	**599.340**	P[5]
K[26]	2724.723	519.314	**503.295**	504.303	502.287	K[4]
K[27]	2866.833	391.219	**375.200**	376.208	374.192	K[3]
T[28]	2967.881	249.108	233.089	234.097	232.082	T[2]
E[29]	3096.923	148.060	132.042	133.050	131.034	E[1]

sp|Q6GSS7|H2A2A_MOUSE

LNKLLGK $^{\text{Dimethyl}}_{28.03}$ VTIAQGGVLPNIQAVLLPKK $^{\text{Methyl}}_{14.02}$ TE

- fragmentation table for charge state 2+
- specType=etd
- score=49.66
- F096915.dat
- query=q1347_p1
- precursor=775.231580
- chargePrecursor=4
- itol=0.6

AA	c	y	z+1	z+2	z	AA
L[1]	66.063	**1549.457**	1541.448	0.504	1540.944	L[29]
N[2]	123.084	1492.915	**1484.906**	1485.410	**1484.402**	N[28]
K[3]	187.132	1435.894	**1427.885**	**1428.388**	1427.381	K[27]
L[4]	243.674	1371.846	**1363.837**	**1364.341**	**1363.333**	L[26]
L[5]	300.216	1315.304	1307.295	1307.799	1306.791	L[25]
G[6]	328.726	1258.762	1250.753	**1251.257**	1250.249	G[24]
K[7]	406.789	1230.252	1222.242	1222.746	1221.738	K[23]
V[8]	456.324	1152.188	1144.179	1144.683	1143.675	V[22]
T[9]	506.848	1102.654	1094.645	1095.149	1094.141	T[21]
I[10]	563.390	1052.130	**1044.121**	**1044.625**	1043.617	I[20]
A[11]	**598.908**	995.588	**987.579**	**988.083**	987.075	A[19]
Q[12]	662.937	960.070	**952.060**	**952.564**	**951.557**	Q[18]
G[13]	691.448	896.041	**888.031**	**888.535**	887.527	G[17]
G[14]	719.959	867.530	859.520	**860.024**	859.017	G[16]
V[15]	769.493	839.019	831.010	831.514	830.506	V[15]
L[16]	**826.035**	789.485	**781.476**	**781.979**	780.972	L[14]
P[17]	874.561	732.943	724.933	725.437	724.430	P[13]
N[18]	**931.583**	684.416	676.407	676.911	675.903	N[12]
I[19]	**988.125**	627.395	619.386	619.890	618.882	I[11]
Q[20]	1052.154	570.853	562.844	563.348	562.340	Q[10]
A[21]	**1087.673**	506.824	498.814	499.318	498.310	A[9]
V[22]	1137.207	471.305	463.296	463.800	462.792	V[8]
L[23]	1193.749	421.771	413.762	414.265	413.258	L[7]
L[24]	1250.291	365.229	357.220	357.723	356.716	L[6]
P[25]	**1298.817**	308.687	300.678	301.181	300.174	P[5]
K[26]	**1362.865**	260.160	252.151	252.655	251.647	K[4]
K[27]	**1433.920**	196.113	188.104	188.608	187.600	K[3]
T[28]	**1484.444**	125.058	117.048	117.552	116.544	T[2]
E[29]	**1548.965**	74.534	66.524	67.028	66.021	E[1]

sp|Q6GSS7|H2A2A_MOUSE

LNKLLGK $^{Dimethyl}_{28.03}$ VTIAQGGVLPNIQAVLLPKK $^{Methyl}_{14.02}$ TE

- fragmentation table for charge state 3+
- specType=etd
- score=49.66
- F096915.dat
- query=q1347_p1
- precursor=775.231580
- chargePrecursor=4
- itol=0.6

AA	c	y	z+1	z+2	z	AA
L[1]	44.377	**1033.307**	1027.968	0.672	1027.632	L[29]
N[2]	82.392	995.613	990.273	**990.609**	989.937	N[28]
K[3]	125.090	957.598	**952.259**	**952.595**	**951.923**	K[27]
L[4]	162.785	914.900	909.560	909.896	909.225	L[26]
L[5]	200.479	877.205	871.866	872.202	871.530	L[25]
G[6]	219.487	839.511	834.171	834.507	833.835	G[24]
K[7]	271.529	820.504	815.164	815.500	814.828	K[23]
V[8]	304.552	768.461	763.122	763.458	762.786	V[22]
T[9]	338.234	735.439	**730.099**	**730.435**	729.763	T[21]
I[10]	375.929	701.756	696.416	696.752	696.081	I[20]
A[11]	399.608	664.061	658.722	659.058	658.386	A[19]
Q[12]	442.294	640.382	635.043	635.379	634.707	Q[18]
G[13]	461.301	597.696	592.357	592.693	592.021	G[17]
G[14]	480.308	578.689	573.349	573.685	573.013	G[16]
V[15]	513.331	559.682	554.342	554.678	554.006	V[15]
L[16]	551.026	526.659	521.319	521.655	520.984	L[14]
P[17]	583.377	488.964	483.625	483.961	483.289	P[13]
N[18]	621.391	456.613	451.274	451.610	450.938	N[12]
I[19]	659.086	418.599	413.260	413.595	412.924	I[11]
Q[20]	701.772	380.904	**375.565**	375.901	**375.229**	Q[10]
A[21]	725.451	338.218	332.879	333.215	332.543	A[9]
V[22]	758.474	314.539	309.200	309.536	308.864	V[8]
L[23]	796.168	281.516	276.177	276.513	275.841	L[7]
L[24]	833.863	243.822	238.482	238.818	238.146	L[6]
P[25]	866.214	206.127	200.787	201.123	200.451	P[5]
K[26]	908.912	173.776	168.437	168.772	168.101	K[4]
K[27]	956.283	131.078	125.738	126.074	125.402	K[3]
T[28]	989.965	83.708	78.368	78.704	78.032	T[2]
E[29]	1032.979	50.025	44.685	45.021	44.349	E[1]

sp|P43277|H13_MOUSE

KNNSRIKLGLKSLVSKGTLVQTKGTGASGSFKLNKK $^{\text{Dimethyl}}_{28.03}$ AAS $^{\text{Acetyl}}_{42.01}$ GE

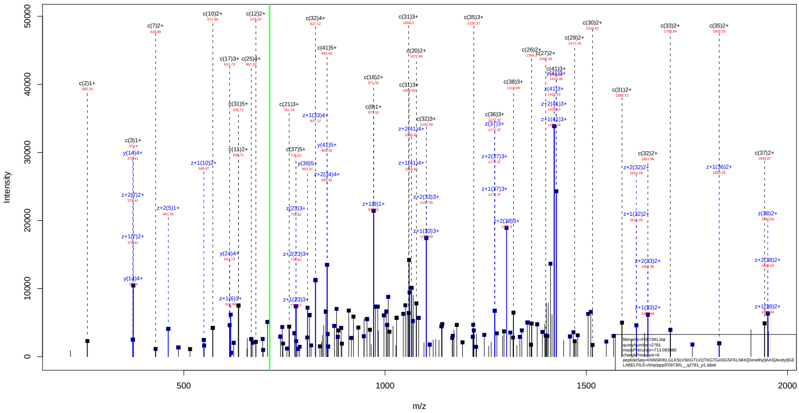

sp|P43277|H13_MOUSE

KNNSRIKLGLKSLVSKGTLVQTKGTGASGSFKLNKK$^{\text{Dimethyl}}_{28.03}AAS^{\text{Acetyl}}_{42.01}$GE

- fragmentation table for charge state 1+
- specType=etd
- score=116.85
- F097391.dat
- query=q2781_p1
- precursor=713.083680
- chargePrecursor=6
- itol=0.8

AA	c	y	z+1	z+2	z	AA
K[1]	146.129	4273.435	4257.417	4258.425	4256.409	K[41]
N[2]	**260.172**	4145.340	4129.322	4130.330	4128.314	N[40]
N[3]	**374.215**	4031.298	4015.279	4016.287	4014.271	N[39]
S[4]	**461.247**	3917.255	3901.236	3902.244	3900.228	S[38]
R[5]	**617.348**	3830.223	3814.204	3815.212	3813.196	R[37]
I[6]	730.432	3674.121	3658.103	3659.111	3657.095	I[36]
K[7]	**858.527**	3561.037	3545.019	3546.027	3544.011	K[35]
L[8]	**971.611**	3432.942	3416.924	3417.932	3415.916	L[34]
G[9]	**1028.632**	3319.858	3303.840	3304.847	3302.832	G[33]
L[10]	**1141.716**	3262.837	3246.818	3247.826	3245.810	L[32]
K[11]	1269.811	3149.753	3133.734	3134.742	3132.726	K[31]
S[12]	1356.843	3021.658	3005.639	3006.647	3004.631	S[30]
L[13]	1469.927	2934.626	2918.607	2919.615	2917.599	L[29]
V[14]	1568.996	2821.542	2805.523	2806.531	2804.515	V[28]
S[15]	1656.028	2722.473	2706.455	2707.462	2705.447	S[27]
K[16]	1784.123	2635.441	2619.423	2620.430	2618.415	K[26]
G[17]	1841.144	2507.346	2491.328	2492.335	2490.320	G[25]
T[18]	1942.192	2450.325	2434.306	2435.314	2433.298	T[24]
L[19]	2055.276	2349.277	2333.259	2334.266	2332.251	L[23]
V[20]	2154.344	2236.193	2220.174	2221.182	2219.167	V[22]
Q[21]	2282.403	2137.125	2121.106	2122.114	2120.098	Q[21]
T[22]	2383.451	2009.066	1993.047	1994.055	1992.040	T[20]
K[23]	2511.546	1908.019	1892.000	1893.008	1890.992	K[19]
G[24]	2568.567	1779.924	**1763.905**	1764.913	**1762.897**	G[18]
T[25]	2669.615	1722.902	1706.883	1707.891	1705.876	T[17]
G[26]	2726.636	1621.854	1605.836	1606.844	1604.828	G[16]
A[27]	2797.673	1564.833	1548.814	**1549.822**	1547.806	A[15]
S[28]	2884.705	1493.796	1477.777	**1478.785**	1476.769	S[14]
G[29]	2941.727	1406.764	**1390.745**	1391.753	1389.737	G[13]
S[30]	3028.759	1349.742	1333.724	**1334.731**	1332.716	S[12]
F[31]	3175.827	1262.710	**1246.692**	1247.699	1245.684	F[11]
K[32]	3303.922	1115.642	1099.623	1100.631	1098.615	K[10]
L[33]	3417.006	987.547	**971.528**	972.536	970.520	L[9]
N[34]	3531.049	**874.463**	**858.444**	859.452	**857.436**	N[8]
K[35]	3659.144	760.420	**744.401**	745.409	743.393	K[7]
K[36]	3815.271	632.325	**616.306**	**617.314**	615.298	K[6]
A[37]	3886.308	476.199	460.180	**461.188**	459.172	A[5]
A[38]	3957.345	405.162	389.143	390.151	388.135	A[4]
S[39]	4086.387	334.124	318.106	319.114	317.098	S[3]
G[40]	4143.409	205.082	189.063	190.071	188.055	G[2]
E[41]	4272.451	148.060	132.042	133.050	131.034	E[1]

sp|P43277|H13_MOUSE

KNNSRIKLGLKSLVSKGTLVQTKGTGASGSFKLNKK $^{\text{Dimethyl}}_{28.03}$ AAS $^{\text{Acetyl}}_{42.01}$ GE

- fragmentation table for charge state 2+
- specType=etd
- score=116.85
- F097391.dat
- query=q2781_p1
- precursor=713.083680
- chargePrecursor=6
- itol=0.8

AA	c	y	z+1	z+2	z	AA
K[1]	73.568	2137.221	2129.212	2129.716	2128.708	K[41]
N[2]	130.589	2073.174	2065.165	2065.668	2064.661	N[40]
N[3]	187.611	2016.152	2008.143	2008.647	2007.639	N[39]
S[4]	231.127	1959.131	**1951.122**	**1951.625**	**1950.618**	S[38]
R[5]	309.178	1915.615	1907.606	1908.109	1907.102	R[37]
I[6]	365.720	1837.564	**1829.555**	**1830.059**	1829.051	I[36]
K[7]	**429.767**	1781.022	1773.013	1773.517	1772.509	K[35]
L[8]	**486.309**	1716.975	**1708.965**	**1709.469**	**1708.462**	L[34]
G[9]	**514.820**	1660.433	**1652.423**	**1652.927**	1651.920	G[33]
L[10]	**571.362**	1631.922	**1623.913**	**1624.417**	1623.409	L[32]
K[11]	**635.409**	1575.380	**1567.371**	**1567.875**	1566.867	K[31]
S[12]	**678.925**	**1511.333**	1503.323	**1503.827**	1502.819	S[30]
L[13]	735.467	**1467.817**	**1459.807**	**1460.311**	**1459.303**	L[29]
V[14]	785.002	**1411.275**	**1403.265**	1403.769	**1402.761**	V[28]
S[15]	828.518	1361.740	**1353.731**	**1354.235**	**1353.227**	S[27]
K[16]	**892.565**	**1318.224**	1310.215	**1310.719**	1309.711	K[26]
G[17]	**921.076**	1254.177	**1246.167**	**1246.671**	1245.664	G[25]
T[18]	**971.600**	**1225.666**	**1217.657**	**1218.161**	1217.153	T[24]
L[19]	**1028.142**	1175.142	**1167.133**	**1167.637**	**1166.629**	L[23]
V[20]	**1077.676**	1118.600	**1110.591**	**1111.095**	**1110.087**	V[22]
Q[21]	**1141.705**	**1069.066**	**1061.057**	**1061.561**	**1060.553**	Q[21]
T[22]	**1192.229**	**1005.037**	**997.027**	**997.531**	**996.523**	T[20]
K[23]	1256.276	**954.513**	**946.504**	**947.007**	946.000	K[19]
G[24]	1284.787	**890.465**	**882.456**	**882.960**	**881.952**	G[18]
T[25]	**1335.311**	861.955	853.945	854.449	**853.441**	T[17]
G[26]	**1363.822**	811.431	803.421	803.925	802.918	G[16]
A[27]	**1399.340**	**782.920**	**774.911**	**775.415**	**774.407**	A[15]
S[28]	1442.856	747.402	**739.392**	**739.896**	738.888	S[14]
G[29]	**1471.367**	703.886	**695.876**	**696.380**	**695.372**	G[13]
S[30]	**1514.883**	675.375	**667.365**	**667.869**	**666.862**	S[12]
F[31]	**1588.417**	631.859	**623.849**	**624.353**	**623.346**	F[11]
K[32]	**1652.465**	558.325	**550.315**	**550.819**	**549.811**	K[10]
L[33]	**1709.007**	494.277	**486.268**	**486.772**	**485.764**	L[9]
N[34]	1766.028	437.735	**429.726**	**430.230**	**429.222**	N[8]
K[35]	**1830.076**	380.714	**372.704**	**373.208**	372.200	K[7]
K[36]	1908.139	316.666	308.657	309.161	308.153	K[6]
A[37]	**1943.657**	238.603	230.594	231.098	230.090	A[5]
A[38]	1979.176	203.084	195.075	195.579	194.571	A[4]
S[39]	2043.697	167.566	159.557	160.060	159.053	S[3]
G[40]	2072.208	103.045	95.035	95.539	94.531	G[2]
E[41]	2136.729	74.534	66.524	67.028	66.021	E[1]

sp|P43277|H13_MOUSE

KNNSRIKLGLKSLVSKGTLVQTKGTGASGSFKLNKK $^{\text{Dimethyl}}_{28.03}$ AAS $^{\text{Acetyl}}_{42.01}$ GE

- fragmentation table for charge state 3+
- specType=etd
- score=116.85
- F097391.dat
- query=q2781_p1
- precursor=713.083680
- chargePrecursor=6
- itol=0.8

AA	c	y	z+1	z+2	z	AA
K[1]	49.381	**1425.150**	**1419.810**	**1420.146**	**1419.474**	K[41]
N[2]	87.395	1382.452	**1377.112**	**1377.448**	1376.776	N[40]
N[3]	125.410	1344.437	**1339.098**	**1339.434**	1338.762	N[39]
S[4]	154.420	1306.423	1301.083	**1301.419**	1300.748	S[38]
R[5]	206.454	**1277.412**	**1272.073**	**1272.409**	**1271.737**	R[37]
I[6]	244.149	**1225.379**	**1220.039**	**1220.375**	**1219.703**	I[36]
K[7]	286.847	1187.684	1182.344	1182.680	1182.008	K[35]
L[8]	324.542	1144.986	**1139.646**	**1139.982**	1139.310	L[34]
G[9]	343.549	1107.291	**1101.951**	**1102.287**	1101.615	G[33]
L[10]	381.244	1088.284	**1082.944**	**1083.280**	1082.608	L[32]
K[11]	423.942	**1050.589**	**1045.250**	**1045.586**	**1044.914**	K[31]
S[12]	452.953	**1007.891**	**1002.551**	**1002.887**	**1002.215**	S[30]
L[13]	490.647	978.880	973.541	973.877	973.205	L[29]
V[14]	523.670	941.185	935.846	936.182	935.510	V[28]
S[15]	552.681	908.163	902.823	903.159	902.487	S[27]
K[16]	595.379	**879.152**	**873.812**	**874.148**	**873.476**	K[26]
G[17]	**614.386**	836.454	831.114	831.450	830.778	G[25]
T[18]	648.069	817.446	**812.107**	**812.443**	**811.771**	T[24]
L[19]	685.764	**783.764**	**778.424**	**778.760**	**778.088**	L[23]
V[20]	718.786	**746.069**	**740.730**	741.066	**740.394**	V[22]
Q[21]	**761.473**	713.046	**707.707**	**708.043**	**707.371**	Q[21]
T[22]	795.155	**670.360**	665.021	665.357	664.685	T[20]
K[23]	**837.853**	636.678	631.338	631.674	631.002	K[19]
G[24]	**856.861**	593.979	588.640	588.976	588.304	G[18]
T[25]	**890.543**	574.972	569.633	569.969	569.297	T[17]
G[26]	**909.550**	541.290	535.950	536.286	535.614	G[16]
A[27]	**933.229**	522.282	516.943	517.279	516.607	A[15]
S[28]	**962.240**	498.603	493.264	493.600	492.928	S[14]
G[29]	**981.247**	469.593	464.253	464.589	463.917	G[13]
S[30]	**1010.258**	450.586	445.246	445.582	444.910	S[12]
F[31]	**1059.281**	421.575	416.235	416.571	415.899	F[11]
K[32]	**1101.979**	372.552	367.213	367.549	366.877	K[10]
L[33]	**1139.674**	329.854	324.514	324.850	324.178	L[9]
N[34]	**1177.688**	292.159	286.820	287.156	286.484	N[8]
K[35]	**1220.386**	254.145	248.805	249.141	248.469	K[7]
K[36]	**1272.428**	211.447	206.107	206.443	205.771	K[6]
A[37]	**1296.107**	159.404	154.065	154.401	153.729	A[5]
A[38]	**1319.786**	135.725	130.386	130.722	130.050	A[4]
S[39]	**1362.801**	112.046	106.707	107.043	106.371	S[3]
G[40]	1381.808	69.032	63.693	64.029	63.357	G[2]
E[41]	**1424.822**	50.025	44.685	45.021	44.349	E[1]

sp|P43277|H13_MOUSE

KNNSRIKLGLKSLVSKGTLVQTKGTGASGSFKLNKK $^{Dimethyl}_{28.03}$ AAS $^{Acetyl}_{42.01}$ GE

- fragmentation table for charge state 4+
- specType=etd
- score=116.85
- F097391.dat
- query=q2781_p1
- precursor=713.083680
- chargePrecursor=6
- itol=0.8

AA	c	y	z+1	z+2	z	AA
K[1]	37.288	**1069.114**	**1065.110**	**1065.362**	**1064.858**	K[41]
N[2]	65.798	1037.091	1033.086	1033.338	1032.834	N[40]
N[3]	94.309	**1008.580**	**1004.575**	**1004.827**	**1004.323**	N[39]
S[4]	116.067	980.069	**976.064**	**976.316**	**975.812**	S[38]
R[5]	155.092	958.311	**954.306**	**954.558**	954.054	R[37]
I[6]	183.363	919.286	915.281	**915.533**	915.029	I[36]
K[7]	215.387	**891.015**	887.010	887.262	886.758	K[35]
L[8]	243.658	**858.991**	854.986	**855.238**	854.734	L[34]
G[9]	257.914	830.720	**826.715**	**826.967**	**826.463**	G[33]
L[10]	286.185	**816.465**	**812.460**	**812.712**	**812.208**	L[32]
K[11]	318.208	**788.194**	784.189	784.441	**783.937**	K[31]
S[12]	339.966	**756.170**	752.165	752.417	751.913	S[30]
L[13]	368.237	734.412	730.407	730.659	730.155	L[29]
V[14]	393.004	706.141	702.136	702.388	701.884	V[28]
S[15]	414.762	681.374	677.369	677.621	677.117	S[27]
K[16]	446.786	659.616	655.611	655.863	655.359	K[26]
G[17]	**461.042**	627.592	**623.587**	**623.839**	**623.335**	G[25]
T[18]	**486.303**	**613.337**	609.332	609.584	609.080	T[24]
L[19]	514.574	588.075	584.070	584.322	583.818	L[23]
V[20]	539.342	559.804	555.799	556.051	555.547	V[22]
Q[21]	**571.356**	535.037	531.032	531.284	530.780	Q[21]
T[22]	596.618	503.022	499.017	499.269	498.765	T[20]
K[23]	628.642	477.760	473.755	474.007	473.503	K[19]
G[24]	642.897	445.736	441.732	441.984	441.480	G[18]
T[25]	**668.159**	431.481	427.476	427.728	427.224	T[17]
G[26]	682.415	406.219	402.214	402.466	401.962	G[16]
A[27]	700.174	391.964	387.959	388.211	387.707	A[15]
S[28]	721.932	**374.204**	370.200	370.452	369.948	S[14]
G[29]	736.187	352.446	348.442	348.694	348.190	G[13]
S[30]	757.945	338.191	334.186	334.438	333.934	S[12]
F[31]	794.712	316.433	312.428	312.680	312.176	F[11]
K[32]	**826.736**	279.666	275.661	275.913	275.409	K[10]
L[33]	855.007	247.642	243.638	243.889	243.386	L[9]
N[34]	**883.518**	219.371	215.366	215.618	215.115	N[8]
K[35]	**915.542**	190.860	186.856	187.108	186.604	K[7]
K[36]	**954.573**	158.837	154.832	155.084	154.580	K[6]
A[37]	972.332	119.805	115.800	116.052	115.548	A[5]
A[38]	990.092	102.046	98.041	98.293	97.789	A[4]
S[39]	1022.352	84.287	80.282	80.534	80.030	S[3]
G[40]	1036.608	52.026	48.021	48.273	47.769	G[2]
E[41]	**1068.868**	37.771	33.766	34.018	33.514	E[1]

sp|P43277|H13_MOUSE

KNNSRIKLGLKSLVSKGTLVQTKGTGASGSFKLNKK $^{Dimethyl}_{28.03}$ AAS $^{Acetyl}_{42.01}$ GE

- fragmentation table for charge state 5+
- specType=etd
- score=116.85
- F097391.dat
- query=q2781_p1
- precursor=713.083680
- chargePrecursor=6
- itol=0.8

AA	c	y	z+1	z+2	z	AA
K[1]	30.032	**855.493**	**852.289**	**852.491**	**852.088**	K[41]
N[2]	52.840	829.874	**826.670**	**826.872**	**826.469**	N[40]
N[3]	75.649	**807.065**	803.862	804.063	803.660	N[39]
S[4]	93.055	784.257	781.053	781.255	780.851	S[38]
R[5]	124.275	766.850	763.647	763.848	763.445	R[37]
I[6]	146.892	735.630	732.426	732.628	732.225	I[36]
K[7]	172.511	713.013	709.810	710.011	709.608	K[35]
L[8]	195.128	687.394	684.191	684.392	683.989	L[34]
G[9]	206.532	664.777	661.574	661.775	661.372	G[33]
L[10]	229.149	653.373	650.169	650.371	649.968	L[32]
K[11]	254.768	630.756	627.553	627.754	627.351	K[31]
S[12]	272.174	605.137	601.934	602.135	601.732	S[30]
L[13]	294.791	587.731	584.527	584.729	584.326	L[29]
V[14]	314.605	565.114	561.910	562.112	561.709	V[28]
S[15]	332.011	545.300	542.097	542.298	541.895	S[27]
K[16]	357.630	527.894	524.690	524.892	524.489	K[26]
G[17]	369.035	502.275	499.071	499.273	498.870	G[25]
T[18]	389.244	490.871	487.667	487.869	487.465	T[24]
L[19]	411.861	470.661	467.458	467.659	467.256	L[23]
V[20]	431.675	448.044	444.841	445.042	444.639	V[22]
Q[21]	457.286	428.231	425.027	425.229	424.825	Q[21]
T[22]	477.496	402.619	399.415	399.617	399.214	T[20]
K[23]	503.115	382.410	379.206	379.407	379.004	K[19]
G[24]	514.519	356.791	353.587	353.788	353.385	G[18]
T[25]	534.729	345.386	342.182	342.384	341.981	T[17]
G[26]	546.133	325.177	321.973	322.175	321.771	G[16]
A[27]	560.341	313.772	310.569	310.770	310.367	A[15]
S[28]	577.747	299.565	296.361	296.563	296.160	S[14]
G[29]	589.151	282.159	278.955	279.156	278.753	G[13]
S[30]	606.558	270.754	267.551	267.752	267.349	S[12]
F[31]	**635.971**	253.348	250.144	250.346	249.943	F[11]
K[32]	661.590	223.934	220.730	220.932	220.529	K[10]
L[33]	684.207	198.315	195.111	195.313	194.910	L[9]
N[34]	707.016	175.698	172.495	172.696	172.293	N[8]
K[35]	732.635	152.890	149.686	149.888	149.484	K[7]
K[36]	763.860	127.271	124.067	124.269	123.866	K[6]
A[37]	**778.067**	96.046	92.842	93.043	92.640	A[5]
A[38]	792.275	81.838	78.634	78.836	78.433	A[4]
S[39]	818.083	67.631	64.427	64.629	64.225	S[3]
G[40]	829.488	41.822	38.618	38.820	38.417	G[2]
E[41]	**855.296**	30.418	27.214	27.416	27.013	E[1]

sp|P43277|H13_MOUSE

KNNSRIKLGLKSLVSKGTLVQTKGTGASGSFKLNKK $^{\text{Dimethyl}}_{28.03}$ AAS $^{\text{Acetyl}}_{42.01}$ GE

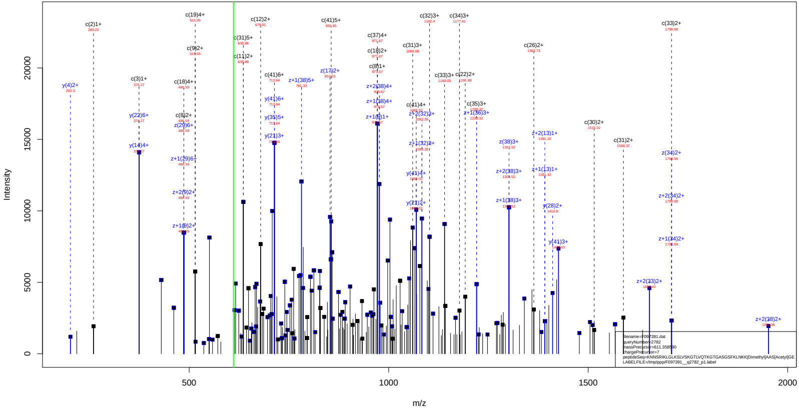

sp|P43277|H13_MOUSE

KNNSRIKLGLKSLVSKGTLVQTKGTGASGSFKLNKK $^{\text{Dimethyl}}_{28.03}$ AAS $^{\text{Acetyl}}_{42.01}$ GE

- fragmentation table for charge state 1+
- specType=etd
- score=57.60
- F097391.dat
- query=q2782_p1
- precursor=611.358590
- chargePrecursor=7
- itol=0.8

AA	c	y	z+1	z+2	z	AA
K[1]	146.129	4273.435	4257.417	4258.425	4256.409	K[41]
N[2]	**260.172**	4145.340	4129.322	4130.330	4128.314	N[40]
N[3]	**374.215**	4031.298	4015.279	4016.287	4014.271	N[39]
S[4]	**461.247**	3917.255	3901.236	3902.244	3900.228	S[38]
R[5]	617.348	3830.223	3814.204	3815.212	3813.196	R[37]
I[6]	**730.432**	3674.121	3658.103	3659.111	3657.095	I[36]
K[7]	**858.527**	3561.037	3545.019	3546.027	3544.011	K[35]
L[8]	**971.611**	3432.942	3416.924	3417.932	3415.916	L[34]
G[9]	**1028.632**	3319.858	3303.840	3304.847	3302.832	G[33]
L[10]	**1141.716**	3262.837	3246.818	3247.826	3245.810	L[32]
K[11]	**1269.811**	3149.753	3133.734	3134.742	3132.726	K[31]
S[12]	1356.843	3021.658	3005.639	3006.647	3004.631	S[30]
L[13]	1469.927	2934.626	2918.607	2919.615	2917.599	L[29]
V[14]	1568.996	2821.542	2805.523	2806.531	2804.515	V[28]
S[15]	1656.028	2722.473	2706.455	2707.462	2705.447	S[27]
K[16]	1784.123	2635.441	2619.423	2620.430	2618.415	K[26]
G[17]	1841.144	2507.346	2491.328	2492.335	2490.320	G[25]
T[18]	1942.192	2450.325	2434.306	2435.314	2433.298	T[24]
L[19]	2055.276	2349.277	2333.259	2334.266	2332.251	L[23]
V[20]	2154.344	2236.193	2220.174	2221.182	2219.167	V[22]
Q[21]	2282.403	2137.125	2121.106	2122.114	2120.098	Q[21]
T[22]	2383.451	2009.066	1993.047	1994.055	1992.040	T[20]
K[23]	2511.546	1908.019	1892.000	1893.008	1890.992	K[19]
G[24]	2568.567	1779.924	1763.905	1764.913	1762.897	G[18]
T[25]	2669.615	1722.902	1706.883	1707.891	1705.876	T[17]
G[26]	2726.636	1621.854	1605.836	1606.844	1604.828	G[16]
A[27]	2797.673	1564.833	1548.814	1549.822	1547.806	A[15]
S[28]	2884.705	1493.796	**1477.777**	1478.785	1476.769	S[14]
G[29]	2941.727	1406.764	**1390.745**	**1391.753**	1389.737	G[13]
S[30]	3028.759	1349.742	1333.724	1334.731	1332.716	S[12]
F[31]	3175.827	1262.710	1246.692	**1247.699**	1245.684	F[11]
K[32]	3303.922	1115.642	**1099.623**	1100.631	1098.615	K[10]
L[33]	3417.006	**987.547**	**971.528**	972.536	970.520	L[9]
N[34]	3531.049	**874.463**	**858.444**	**859.452**	857.436	N[8]
K[35]	3659.144	760.420	**744.401**	745.409	743.393	K[7]
K[36]	3815.271	632.325	**616.306**	617.314	615.298	K[6]
A[37]	3886.308	476.199	460.180	**461.188**	459.172	A[5]
A[38]	3957.345	405.162	389.143	390.151	388.135	A[4]
S[39]	4086.387	334.124	318.106	319.114	317.098	S[3]
G[40]	4143.409	205.082	189.063	190.071	188.055	G[2]
E[41]	4272.451	148.060	132.042	133.050	131.034	E[1]

sp|P43277|H13_MOUSE

KNNSRIKLGLKSLVSKGTLVQTKGTGASGSFKLNKK $^{\text{Dimethyl}}_{28.03}$ AAS $^{\text{Acetyl}}_{42.01}$ GE

- fragmentation table for charge state 2+
- specType=etd
- score=57.60
- F097391.dat
- query=q2782_p1
- precursor=611.358590
- chargePrecursor=7
- itol=0.8

AA	c	y	z+1	z+2	z	AA
K[1]	73.568	2137.221	2129.212	2129.716	2128.708	K[41]
N[2]	130.589	2073.174	2065.165	2065.668	2064.661	N[40]
N[3]	187.611	2016.152	2008.143	2008.647	2007.639	N[39]
S[4]	231.127	1959.131	1951.122	**1951.625**	1950.618	S[38]
R[5]	309.178	1915.615	1907.606	1908.109	1907.102	R[37]
I[6]	365.720	1837.564	1829.555	1830.059	1829.051	I[36]
K[7]	**429.767**	1781.022	1773.013	1773.517	1772.509	K[35]
L[8]	**486.309**	1716.975	**1708.965**	**1709.469**	**1708.462**	L[34]
G[9]	**514.820**	1660.433	1652.423	**1652.927**	1651.920	G[33]
L[10]	**571.362**	1631.922	1623.913	1624.417	1623.409	L[32]
K[11]	**635.409**	1575.380	**1567.371**	**1567.875**	**1566.867**	K[31]
S[12]	**678.925**	**1511.333**	1503.323	**1503.827**	1502.819	S[30]
L[13]	735.467	1467.817	1459.807	1460.311	1459.303	L[29]
V[14]	**785.002**	**1411.275**	1403.265	1403.769	1402.761	V[28]
S[15]	**828.518**	1361.740	1353.731	1354.235	1353.227	S[27]
K[16]	892.565	1318.224	1310.215	1310.719	1309.711	K[26]
G[17]	**921.076**	1254.177	1246.167	1246.671	1245.664	G[25]
T[18]	**971.600**	**1225.666**	1217.657	1218.161	1217.153	T[24]
L[19]	**1028.142**	1175.142	**1167.133**	**1167.637**	1166.629	L[23]
V[20]	**1077.676**	1118.600	**1110.591**	**1111.095**	1110.087	V[22]
Q[21]	**1141.705**	**1069.066**	1061.057	1061.561	**1060.553**	Q[21]
T[22]	**1192.229**	**1005.037**	**997.027**	**997.531**	996.523	T[20]
K[23]	1256.276	954.513	**946.504**	**947.007**	**946.000**	K[19]
G[24]	**1284.787**	**890.465**	882.456	882.960	881.952	G[18]
T[25]	1335.311	861.955	853.945	**854.449**	**853.441**	T[17]
G[26]	**1363.822**	811.431	**803.421**	**803.925**	802.918	G[16]
A[27]	1399.340	782.920	**774.911**	**775.415**	774.407	A[15]
S[28]	1442.856	747.402	**739.392**	**739.896**	**738.888**	S[14]
G[29]	1471.367	**703.886**	**695.876**	**696.380**	695.372	G[13]
S[30]	**1514.883**	675.375	**667.365**	**667.869**	**666.862**	S[12]
F[31]	**1588.417**	**631.859**	**623.849**	**624.353**	623.346	F[11]
K[32]	1652.465	**558.325**	**550.315**	**550.819**	**549.811**	K[10]
L[33]	**1709.007**	494.277	**486.268**	**486.772**	485.764	L[9]
N[34]	1766.028	437.735	**429.726**	**430.230**	**429.222**	N[8]
K[35]	1830.076	380.714	372.704	373.208	372.200	K[7]
K[36]	1908.139	316.666	308.657	309.161	308.153	K[6]
A[37]	1943.657	238.603	230.594	231.098	230.090	A[5]
A[38]	1979.176	**203.084**	195.075	195.579	194.571	A[4]
S[39]	2043.697	167.566	159.557	160.060	159.053	S[3]
G[40]	2072.208	103.045	95.035	95.539	94.531	G[2]
E[41]	2136.729	74.534	66.524	67.028	66.021	E[1]

sp|P43277|H13_MOUSE

KNNSRIKLGLKSLVSKGTLVQTKGTGASGSFKLNKK $^{Dimethyl}_{28.03}$ AAS $^{Acetyl}_{42.01}$ GE

- fragmentation table for charge state 3+
- specType=etd
- score=57.60
- F097391.dat
- query=q2782_p1
- precursor=611.358590
- chargePrecursor=7
- itol=0.8

AA	c	y	z+1	z+2	z	AA
K[1]	49.381	**1425.150**	1419.810	1420.146	1419.474	K[41]
N[2]	87.395	**1382.452**	1377.112	1377.448	1376.776	N[40]
N[3]	125.410	1344.437	1339.098	**1339.434**	1338.762	N[39]
S[4]	154.420	1306.423	**1301.083**	**1301.419**	**1300.748**	S[38]
R[5]	206.454	1277.412	**1272.073**	**1272.409**	**1271.737**	R[37]
I[6]	244.149	**1225.379**	**1220.039**	**1220.375**	**1219.703**	I[36]
K[7]	286.847	1187.684	1182.344	1182.680	1182.008	K[35]
L[8]	324.542	1144.986	**1139.646**	**1139.982**	**1139.310**	L[34]
G[9]	343.549	1107.291	**1101.951**	**1102.287**	**1101.615**	G[33]
L[10]	381.244	1088.284	**1082.944**	**1083.280**	**1082.608**	L[32]
K[11]	423.942	**1050.589**	**1045.250**	**1045.586**	**1044.914**	K[31]
S[12]	452.953	**1007.891**	**1002.551**	**1002.887**	1002.215	S[30]
L[13]	490.647	**978.880**	973.541	973.877	973.205	L[29]
V[14]	523.670	941.185	935.846	936.182	935.510	V[28]
S[15]	552.681	908.163	**902.823**	**903.159**	**902.487**	S[27]
K[16]	595.379	**879.152**	**873.812**	**874.148**	**873.476**	K[26]
G[17]	614.386	836.454	831.114	831.450	830.778	G[25]
T[18]	**648.069**	817.446	**812.107**	**812.443**	**811.771**	T[24]
L[19]	**685.764**	783.764	**778.424**	**778.760**	778.088	L[23]
V[20]	718.786	**746.069**	**740.730**	**741.066**	**740.394**	V[22]
Q[21]	**761.473**	**713.046**	**707.707**	**708.043**	**707.371**	Q[21]
T[22]	**795.155**	670.360	**665.021**	**665.357**	**664.685**	T[20]
K[23]	**837.853**	636.678	**631.338**	**631.674**	**631.002**	K[19]
G[24]	856.861	593.979	588.640	588.976	588.304	G[18]
T[25]	**890.543**	574.972	569.633	569.969	569.297	T[17]
G[26]	**909.550**	541.290	**535.950**	**536.286**	535.614	G[16]
A[27]	**933.229**	522.282	516.943	517.279	**516.607**	A[15]
S[28]	**962.240**	498.603	493.264	493.600	492.928	S[14]
G[29]	**981.247**	469.593	464.253	464.589	463.917	G[13]
S[30]	**1010.258**	450.586	445.246	445.582	444.910	S[12]
F[31]	**1059.281**	421.575	416.235	416.571	415.899	F[11]
K[32]	**1101.979**	372.552	367.213	367.549	366.877	K[10]
L[33]	**1139.674**	329.854	324.514	324.850	324.178	L[9]
N[34]	**1177.688**	292.159	286.820	287.156	286.484	N[8]
K[35]	**1220.386**	254.145	248.805	249.141	248.469	K[7]
K[36]	**1272.428**	211.447	206.107	206.443	205.771	K[6]
A[37]	1296.107	159.404	154.065	154.401	153.729	A[5]
A[38]	1319.786	135.725	130.386	130.722	130.050	A[4]
S[39]	1362.801	112.046	106.707	107.043	106.371	S[3]
G[40]	1381.808	69.032	63.693	64.029	63.357	G[2]
E[41]	1424.822	50.025	44.685	45.021	44.349	E[1]

sp|P43277|H13_MOUSE

KNNSRIKLGLKSLVSKGTLVQTKGTGASGSFKLNKK $^{\text{Dimethyl}}_{28.03}$ AAS $^{\text{Acetyl}}_{42.01}$ GE

- fragmentation table for charge state 4+
- specType=etd
- score=57.60
- F097391.dat
- query=q2782_p1
- precursor=611.358590
- chargePrecursor=7
- itol=0.8

AA	c	y	z+1	z+2	z	AA
K[1]	37.288	**1069.114**	**1065.110**	**1065.362**	**1064.858**	K[41]
N[2]	65.798	1037.091	1033.086	**1033.338**	1032.834	N[40]
N[3]	94.309	**1008.580**	**1004.575**	**1004.827**	**1004.323**	N[39]
S[4]	116.067	980.069	**976.064**	**976.316**	975.812	S[38]
R[5]	155.092	**958.311**	954.306	**954.558**	954.054	R[37]
I[6]	183.363	919.286	915.281	915.533	915.029	I[36]
K[7]	215.387	**891.015**	887.010	887.262	886.758	K[35]
L[8]	243.658	**858.991**	**854.986**	**855.238**	**854.734**	L[34]
G[9]	257.914	830.720	**826.715**	**826.967**	**826.463**	G[33]
L[10]	286.185	**816.465**	**812.460**	**812.712**	**812.208**	L[32]
K[11]	318.208	788.194	784.189	**784.441**	783.937	K[31]
S[12]	339.966	**756.170**	**752.165**	**752.417**	**751.913**	S[30]
L[13]	368.237	734.412	**730.407**	**730.659**	**730.155**	L[29]
V[14]	393.004	**706.141**	**702.136**	**702.388**	**701.884**	V[28]
S[15]	414.762	681.374	**677.369**	**677.621**	**677.117**	S[27]
K[16]	446.786	659.616	**655.611**	**655.863**	**655.359**	K[26]
G[17]	**461.042**	627.592	**623.587**	**623.839**	623.335	G[25]
T[18]	**486.303**	**613.337**	609.332	609.584	609.080	T[24]
L[19]	**514.574**	588.075	584.070	584.322	583.818	L[23]
V[20]	539.342	559.804	555.799	556.051	555.547	V[22]
Q[21]	**571.356**	535.037	531.032	531.284	530.780	Q[21]
T[22]	596.618	503.022	499.017	499.269	498.765	T[20]
K[23]	628.642	477.760	473.755	474.007	473.503	K[19]
G[24]	**642.897**	445.736	441.732	441.984	441.480	G[18]
T[25]	**668.159**	431.481	427.476	427.728	427.224	T[17]
G[26]	**682.415**	406.219	402.214	402.466	401.962	G[16]
A[27]	700.174	391.964	387.959	388.211	387.707	A[15]
S[28]	**721.932**	**374.204**	370.200	370.452	369.948	S[14]
G[29]	736.187	352.446	348.442	348.694	348.190	G[13]
S[30]	**757.945**	338.191	334.186	334.438	333.934	S[12]
F[31]	**794.712**	316.433	312.428	312.680	312.176	F[11]
K[32]	**826.736**	279.666	275.661	275.913	275.409	K[10]
L[33]	**855.007**	247.642	243.638	243.889	243.386	L[9]
N[34]	**883.518**	219.371	215.366	215.618	215.115	N[8]
K[35]	915.542	190.860	186.856	187.108	186.604	K[7]
K[36]	**954.573**	158.837	154.832	155.084	154.580	K[6]
A[37]	**972.332**	119.805	115.800	116.052	115.548	A[5]
A[38]	990.092	102.046	98.041	98.293	97.789	A[4]
S[39]	1022.352	84.287	80.282	80.534	80.030	S[3]
G[40]	1036.608	52.026	48.021	48.273	47.769	G[2]
E[41]	**1068.868**	37.771	33.766	34.018	33.514	E[1]

sp|P43277|H13_MOUSE

KNNSRIKLGLKSLVSKGTLVQTKGTGASGSFKLNKK $^{\text{Dimethyl}}_{28.03}$ AAS $^{\text{Acetyl}}_{42.01}$ GE

- fragmentation table for charge state 5+
- specType=etd
- score=57.60
- F097391.dat
- query=q2782_p1
- precursor=611.358590
- chargePrecursor=7
- itol=0.8

AA	c	y	z+1	z+2	z	AA
K[1]	30.032	**855.493**	**852.289**	**852.491**	852.088	K[41]
N[2]	52.840	829.874	**826.670**	**826.872**	**826.469**	N[40]
N[3]	75.649	**807.065**	**803.862**	**804.063**	**803.660**	N[39]
S[4]	93.055	784.257	**781.053**	**781.255**	**780.851**	S[38]
R[5]	124.275	766.850	**763.647**	**763.848**	**763.445**	R[37]
I[6]	146.892	735.630	**732.426**	**732.628**	**732.225**	I[36]
K[7]	172.511	**713.013**	709.810	710.011	709.608	K[35]
L[8]	195.128	687.394	684.191	684.392	683.989	L[34]
G[9]	206.532	**664.777**	**661.574**	**661.775**	661.372	G[33]
L[10]	229.149	653.373	650.169	650.371	649.968	L[32]
K[11]	254.768	**630.756**	627.553	627.754	627.351	K[31]
S[12]	272.174	605.137	601.934	602.135	601.732	S[30]
L[13]	294.791	587.731	584.527	584.729	584.326	L[29]
V[14]	314.605	565.114	561.910	562.112	561.709	V[28]
S[15]	332.011	545.300	542.097	542.298	541.895	S[27]
K[16]	357.630	527.894	524.690	524.892	524.489	K[26]
G[17]	369.035	502.275	499.071	499.273	498.870	G[25]
T[18]	389.244	490.871	487.667	487.869	487.465	T[24]
L[19]	411.861	470.661	467.458	467.659	467.256	L[23]
V[20]	431.675	448.044	444.841	445.042	444.639	V[22]
Q[21]	457.286	428.231	425.027	425.229	424.825	Q[21]
T[22]	477.496	402.619	399.415	399.617	399.214	T[20]
K[23]	503.115	382.410	379.206	379.407	379.004	K[19]
G[24]	**514.519**	356.791	353.587	353.788	353.385	G[18]
T[25]	534.729	345.386	342.182	342.384	341.981	T[17]
G[26]	546.133	325.177	321.973	322.175	321.771	G[16]
A[27]	560.341	313.772	310.569	310.770	310.367	A[15]
S[28]	577.747	299.565	296.361	296.563	296.160	S[14]
G[29]	589.151	282.159	278.955	279.156	278.753	G[13]
S[30]	606.558	270.754	267.551	267.752	267.349	S[12]
F[31]	**635.971**	253.348	250.144	250.346	249.943	F[11]
K[32]	**661.590**	223.934	220.730	220.932	220.529	K[10]
L[33]	684.207	198.315	195.111	195.313	194.910	L[9]
N[34]	**707.016**	175.698	172.495	172.696	172.293	N[8]
K[35]	**732.635**	152.890	149.686	149.888	149.484	K[7]
K[36]	**763.860**	127.271	124.067	124.269	123.866	K[6]
A[37]	778.067	96.046	92.842	93.043	92.640	A[5]
A[38]	792.275	81.838	78.634	78.836	78.433	A[4]
S[39]	818.083	67.631	64.427	64.629	64.225	S[3]
G[40]	**829.488**	41.822	38.618	38.820	38.417	G[2]
E[41]	**855.296**	30.418	27.214	27.416	27.013	E[1]

sp|P43277|H13_MOUSE

KNNSRIKLGLKSLVSKGTLVQTKGTGASGSFKLNKK $^{\text{Dimethyl}}_{28.03}$ AAS $^{\text{Acetyl}}_{42.01}$ GE

- fragmentation table for charge state 6+
- specType=etd
- score=57.60
- F097391.dat
- query=q2782_p1
- precursor=611.358590
- chargePrecursor=7
- itol=0.8

AA	c	y	z+1	z+2	z	AA
K[1]	25.194	**713.079**	710.409	710.577	710.241	K[41]
N[2]	44.201	691.729	689.060	689.228	688.892	N[40]
N[3]	63.209	672.722	670.053	670.221	669.885	N[39]
S[4]	77.714	653.715	651.045	**651.213**	650.877	S[38]
R[5]	103.731	639.210	636.540	636.708	**636.372**	R[37]
I[6]	122.578	**613.193**	610.523	610.691	610.355	I[36]
K[7]	143.927	594.346	591.676	591.844	591.508	K[35]
L[8]	162.775	572.996	570.327	570.495	570.159	L[34]
G[9]	172.278	554.149	551.479	551.647	**551.311**	G[33]
L[10]	191.125	544.646	541.976	542.144	541.808	L[32]
K[11]	212.475	525.798	523.128	523.296	522.960	K[31]
S[12]	226.980	504.449	501.779	501.947	501.611	S[30]
L[13]	245.827	489.944	**487.274**	487.442	**487.106**	L[29]
V[14]	262.339	471.096	468.427	468.595	468.259	V[28]
S[15]	276.844	454.585	451.915	452.083	451.747	S[27]
K[16]	298.193	440.080	437.410	437.578	437.242	K[26]
G[17]	307.697	418.730	416.061	416.229	415.893	G[25]
T[18]	324.538	409.227	406.557	406.725	406.389	T[24]
L[19]	343.385	392.386	389.716	389.884	389.548	L[23]
V[20]	359.897	**373.538**	370.868	371.036	370.701	V[22]
Q[21]	381.240	357.027	354.357	354.525	354.189	Q[21]
T[22]	398.081	335.684	333.014	333.182	332.846	T[20]
K[23]	419.430	318.842	316.173	316.341	316.005	K[19]
G[24]	428.934	297.493	294.824	294.992	294.656	G[18]
T[25]	445.775	287.990	285.320	285.488	285.152	T[17]
G[26]	455.279	271.148	268.479	268.647	268.311	G[16]
A[27]	467.118	261.645	258.975	259.143	258.807	A[15]
S[28]	481.624	249.805	247.136	247.304	246.968	S[14]
G[29]	491.127	235.300	232.630	232.798	232.462	G[13]
S[30]	505.633	225.796	223.127	223.295	222.959	S[12]
F[31]	530.144	211.291	208.621	208.789	208.453	F[11]
K[32]	551.493	186.780	184.110	184.278	183.942	K[10]
L[33]	570.340	165.431	162.761	162.929	162.593	L[9]
N[34]	589.348	146.583	143.913	144.081	143.745	N[8]
K[35]	610.697	127.576	124.906	125.074	124.738	K[7]
K[36]	636.718	106.227	103.557	103.725	103.389	K[6]
A[37]	**648.557**	80.206	77.536	77.704	77.368	A[5]
A[38]	660.397	68.366	65.697	65.865	65.529	A[4]
S[39]	681.904	56.527	53.857	54.025	53.689	S[3]
G[40]	691.408	35.020	32.350	32.518	32.182	G[2]
E[41]	**712.915**	25.516	22.846	23.014	22.678	E[1]

sp|P43277|H13_MOUSE

KNNSRIKLGLKSLVSKGTLVQTKGTGASGSFKLNK $^{(\mathrm{Dimethyl})}_{(28.03)}$ K $^{(\mathrm{Trimethyl})}_{(42.05)}$ AASGE

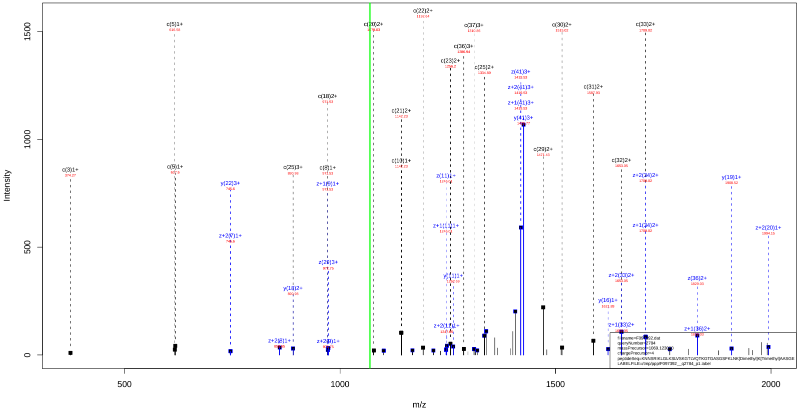

sp|P43277|H13_MOUSE

KNNSRIKLGLKSLVSKGTLVQTKGTGASGSFKLNK $^{(\text{Dimethyl})}_{(28.03)}$ K $^{(\text{Trimethyl})}_{(42.05)}$ AASGE

- fragmentation table for charge state 1+
- specType=etd
- score=53.36
- F097392.dat
- query=q2784_p1
- precursor=1069.123000
- chargePrecursor=4
- itol=0.8

AA	c	y	z+1	z+2	z	AA
K[1]	146.129	4273.472	4257.453	4258.461	4256.445	K[41]
N[2]	260.172	4145.377	4129.358	4130.366	4128.350	N[40]
N[3]	**374.215**	4031.334	4015.315	4016.323	4014.307	N[39]
S[4]	461.247	3917.291	3901.272	3902.280	3900.264	S[38]
R[5]	**617.348**	3830.259	3814.240	3815.248	3813.232	R[37]
I[6]	730.432	3674.158	3658.139	3659.147	3657.131	I[36]
K[7]	858.527	3561.074	3545.055	3546.063	3544.047	K[35]
L[8]	**971.611**	3432.979	3416.960	3417.968	3415.952	L[34]
G[9]	1028.632	3319.895	3303.876	3304.884	3302.868	G[33]
L[10]	**1141.716**	3262.873	3246.855	3247.862	3245.847	L[32]
K[11]	1269.811	3149.789	3133.771	3134.778	3132.763	K[31]
S[12]	1356.843	3021.694	3005.676	3006.683	3004.668	S[30]
L[13]	1469.927	2934.662	2918.644	2919.651	2917.636	L[29]
V[14]	1568.996	2821.578	2805.559	2806.567	2804.552	V[28]
S[15]	1656.028	2722.510	2706.491	2707.499	2705.483	S[27]
K[16]	1784.123	2635.478	2619.459	2620.467	2618.451	K[26]
G[17]	1841.144	2507.383	2491.364	2492.372	2490.356	G[25]
T[18]	1942.192	2450.361	2434.343	2435.350	2433.335	T[24]
L[19]	2055.276	2349.314	2333.295	2334.303	2332.287	L[23]
V[20]	2154.344	2236.230	2220.211	2221.219	2219.203	V[22]
Q[21]	2282.403	2137.161	2121.142	2122.150	2120.135	Q[21]
T[22]	2383.451	2009.103	1993.084	**1994.092**	1992.076	T[20]
K[23]	2511.546	**1908.055**	1892.036	1893.044	1891.028	K[19]
G[24]	2568.567	1779.960	1763.941	**1764.949**	1762.933	G[18]
T[25]	2669.615	1722.938	1706.920	1707.928	1705.912	T[17]
G[26]	2726.636	**1621.891**	1605.872	1606.880	1604.864	G[16]
A[27]	2797.673	1564.869	1548.851	1549.858	1547.843	A[15]
S[28]	2884.705	1493.832	1477.813	1478.821	1476.806	S[14]
G[29]	2941.727	**1406.800**	1390.781	1391.789	1389.774	G[13]
S[30]	3028.759	1349.779	1333.760	**1334.768**	1332.752	S[12]
F[31]	3175.827	**1262.747**	**1246.728**	**1247.736**	**1245.720**	F[11]
K[32]	3303.922	1115.678	1099.660	**1100.667**	1098.652	K[10]
L[33]	3417.006	987.583	**971.565**	**972.572**	970.557	L[9]
N[34]	3531.049	874.499	858.481	**859.488**	857.473	N[8]
K[35]	3687.176	760.456	744.438	**745.445**	743.430	K[7]
K[36]	3857.317	604.330	588.311	589.319	587.304	K[6]
A[37]	3928.355	434.188	418.169	419.177	417.162	A[5]
A[38]	3999.392	363.151	347.132	348.140	346.124	A[4]
S[39]	4086.424	292.114	276.095	277.103	275.087	S[3]
G[40]	4143.445	205.082	189.063	190.071	188.055	G[2]
E[41]	4272.488	148.060	132.042	133.050	131.034	E[1]

sp|P43277|H13_MOUSE

KNNSRIKLGLKSLVSKGTLVQTKGTGASGSFKLNK $^{(Dimethyl)}_{(28.03)}$ K $^{(Trimethyl)}_{(42.05)}$ AASGE

- fragmentation table for charge state 2+
- specType=etd
- score=53.36
- F097392.dat
- query=q2784_p1
- precursor=1069.123000
- chargePrecursor=4
- itol=0.8

AA	c	y	z+1	z+2	z	AA
K[1]	73.568	2137.240	2129.230	2129.734	2128.726	K[41]
N[2]	130.589	2073.192	2065.183	2065.687	2064.679	N[40]
N[3]	187.611	2016.171	2008.161	2008.665	2007.657	N[39]
S[4]	231.127	1959.149	1951.140	1951.644	1950.636	S[38]
R[5]	309.178	1915.633	1907.624	**1908.128**	1907.120	R[37]
I[6]	365.720	1837.583	**1829.573**	1830.077	**1829.069**	I[36]
K[7]	429.767	1781.041	1773.031	1773.535	1772.527	K[35]
L[8]	486.309	1716.993	**1708.984**	**1709.488**	**1708.480**	L[34]
G[9]	514.820	1660.451	**1652.442**	**1652.946**	1651.938	G[33]
L[10]	571.362	1631.940	1623.931	1624.435	1623.427	L[32]
K[11]	635.409	1575.398	1567.389	1567.893	1566.885	K[31]
S[12]	678.925	1511.351	1503.341	1503.845	1502.838	S[30]
L[13]	735.467	1467.835	1459.825	1460.329	1459.321	L[29]
V[14]	785.002	1411.293	1403.283	1403.787	1402.779	V[28]
S[15]	828.518	1361.759	1353.749	1354.253	1353.245	S[27]
K[16]	892.565	**1318.243**	**1310.233**	**1310.737**	1309.729	K[26]
G[17]	921.076	1254.195	**1246.186**	**1246.690**	**1245.682**	G[25]
T[18]	**971.600**	1225.684	1217.675	1218.179	**1217.171**	T[24]
L[19]	1028.142	1175.160	1167.151	**1167.655**	1166.647	L[23]
V[20]	**1077.676**	1118.618	1110.609	1111.113	1110.105	V[22]
Q[21]	**1141.705**	1069.084	1061.075	1061.579	1060.571	Q[21]
T[22]	**1192.229**	1005.055	997.046	997.549	996.542	T[20]
K[23]	**1256.276**	954.531	946.522	947.026	946.018	K[19]
G[24]	1284.787	**890.484**	882.474	882.978	881.970	G[18]
T[25]	**1335.311**	861.973	853.964	854.467	853.460	T[17]
G[26]	1363.822	811.449	803.440	803.944	802.936	G[16]
A[27]	1399.340	782.938	774.929	775.433	774.425	A[15]
S[28]	1442.856	747.420	739.410	739.914	738.906	S[14]
G[29]	**1471.367**	703.904	695.894	696.398	695.390	G[13]
S[30]	**1514.883**	675.393	667.384	667.888	666.880	S[12]
F[31]	**1588.417**	631.877	623.868	624.372	623.364	F[11]
K[32]	**1652.465**	558.343	550.333	550.837	549.830	K[10]
L[33]	**1709.007**	494.295	486.286	486.790	485.782	L[9]
N[34]	1766.028	437.753	429.744	430.248	429.240	N[8]
K[35]	1844.091	380.732	372.722	373.226	372.219	K[7]
K[36]	1929.162	302.669	294.659	295.163	294.155	K[6]
A[37]	1964.681	217.598	209.588	210.092	209.084	A[5]
A[38]	2000.199	182.079	174.070	174.574	173.566	A[4]
S[39]	2043.716	146.561	138.551	139.055	138.047	S[3]
G[40]	2072.226	103.045	95.035	95.539	94.531	G[2]
E[41]	2136.748	74.534	66.524	67.028	66.021	E[1]

sp|P43277|H13_MOUSE

KNNSRIKLGLKSLVSKGTLVQTKGTGASGSFKLNK $^{(Dimethyl)}_{(28.03)}$ K $^{(Trimethyl)}_{(42.05)}$ AASGE

- fragmentation table for charge state 3+
- specType=etd
- score=53.36
- F097392.dat
- query=q2784_p1
- precursor=1069.123000
- chargePrecursor=4
- itol=0.8

AA	c	y	z+1	z+2	z	AA
K[1]	49.381	**1425.162**	**1419.823**	**1420.158**	**1419.487**	K[41]
N[2]	87.395	1382.464	1377.124	1377.460	1376.788	N[40]
N[3]	125.410	1344.449	**1339.110**	**1339.446**	**1338.774**	N[39]
S[4]	154.420	1306.435	1301.096	1301.432	1300.760	S[38]
R[5]	206.454	1277.425	1272.085	1272.421	1271.749	R[37]
I[6]	244.149	1225.391	1220.051	1220.387	1219.715	I[36]
K[7]	286.847	1187.696	1182.357	1182.692	1182.021	K[35]
L[8]	324.542	1144.998	1139.658	1139.994	1139.322	L[34]
G[9]	343.549	1107.303	1101.964	1102.299	**1101.628**	G[33]
L[10]	381.244	1088.296	1082.956	1083.292	1082.620	L[32]
K[11]	423.942	1050.601	1045.262	1045.598	1044.926	K[31]
S[12]	452.953	1007.903	1002.563	1002.899	1002.227	S[30]
L[13]	490.647	978.892	973.553	973.889	**973.217**	L[29]
V[14]	523.670	941.198	935.858	936.194	935.522	V[28]
S[15]	552.681	908.175	902.835	903.171	902.499	S[27]
K[16]	595.379	879.164	873.825	874.160	873.489	K[26]
G[17]	614.386	836.466	831.126	831.462	830.790	G[25]
T[18]	648.069	817.459	812.119	812.455	811.783	T[24]
L[19]	685.764	783.776	778.436	778.772	778.101	L[23]
V[20]	718.786	**746.081**	740.742	741.078	740.406	V[22]
Q[21]	761.473	713.059	707.719	708.055	707.383	Q[21]
T[22]	795.155	670.372	665.033	665.369	664.697	T[20]
K[23]	837.853	636.690	631.350	631.686	631.014	K[19]
G[24]	856.861	593.991	588.652	588.988	588.316	G[18]
T[25]	**890.543**	574.984	569.645	569.981	569.309	T[17]
G[26]	909.550	541.302	535.962	536.298	535.626	G[16]
A[27]	933.229	522.295	516.955	517.291	516.619	A[15]
S[28]	962.240	498.616	493.276	493.612	492.940	S[14]
G[29]	981.247	469.605	464.265	464.601	463.929	G[13]
S[30]	1010.258	450.598	445.258	445.594	444.922	S[12]
F[31]	1059.281	421.587	416.248	416.583	415.912	F[11]
K[32]	1101.979	372.564	367.225	367.561	366.889	K[10]
L[33]	1139.674	329.866	324.526	324.862	324.190	L[9]
N[34]	1177.688	292.171	286.832	287.168	286.496	N[8]
K[35]	1229.730	254.157	248.817	249.153	248.481	K[7]
K[36]	**1286.444**	202.115	196.775	197.111	196.439	K[6]
A[37]	**1310.123**	145.401	140.061	140.397	139.725	A[5]
A[38]	1333.802	121.722	116.382	116.718	116.046	A[4]
S[39]	1362.813	98.043	92.703	93.039	92.367	S[3]
G[40]	1381.820	69.032	63.693	64.029	63.357	G[2]
E[41]	1424.834	50.025	44.685	45.021	44.349	E[1]

sp|P62806|H4_MOUSE

[Acetyl]S $^{Acetyl}_{42.01}$ GRGKGGKGLGKGGAK $^{Acetyl}_{42.01}$ RHRK $^{Dimethyl}_{28.03}$ VLRD

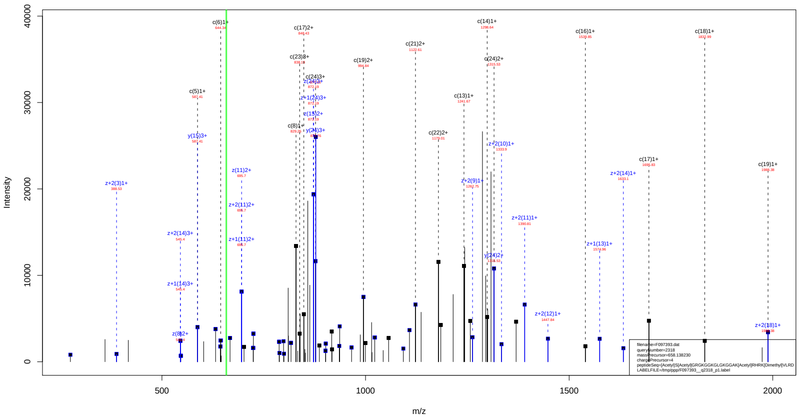

sp|P62806|H4_MOUSE

[Acetyl]S $^{Acetyl}_{42.01}$ GRGKGGKGLGKGGAK $^{Acetyl}_{42.01}$ RHRK $^{Dimethyl}_{28.03}$ VLRD

- fragmentation table for charge state 1+
- specType=etd
- score=92.84
- F097393.dat
- query=q2318_p1
- precursor=658.138230
- chargePrecursor=4
- itol=0.8

AA	c	y	z+1	z+2	z	AA
S[1]	189.087	2629.523	2613.505	0.000	2612.497	S[24]
G[2]	246.108	2458.470	2442.451	0.000	2441.444	G[23]
R[3]	402.210	2401.449	2385.430	2386.438	2384.422	R[22]
G[4]	459.231	2245.348	2229.329	2230.337	2228.321	G[21]
K[5]	**587.326**	2188.326	2172.307	2173.315	2171.300	K[20]
G[6]	**644.347**	2060.231	2044.212	2045.220	2043.205	G[19]
G[7]	**701.369**	2003.210	1987.191	**1988.199**	1986.183	G[18]
K[8]	**829.464**	1946.188	1930.170	1931.177	1929.162	K[17]
G[9]	**886.485**	1818.093	1802.075	1803.082	1801.067	G[16]
L[10]	**999.569**	1761.072	1745.053	1746.061	1744.045	L[15]
G[11]	**1056.591**	1647.988	1631.969	**1632.977**	1630.961	G[14]
K[12]	**1184.686**	1590.966	**1574.948**	1575.955	1573.940	K[13]
G[13]	**1241.707**	1462.871	1446.853	**1447.860**	1445.845	G[12]
G[14]	**1298.729**	1405.850	1389.831	**1390.839**	1388.823	G[11]
A[15]	**1369.766**	1348.828	1332.810	**1333.818**	1331.802	A[10]
K[16]	**1539.871**	1277.791	1261.773	**1262.780**	1260.765	K[9]
R[17]	**1695.973**	**1107.686**	1091.667	**1092.675**	1090.659	R[8]
H[18]	**1833.031**	951.585	**935.566**	**936.574**	934.558	H[7]
R[19]	**1989.133**	814.526	**798.507**	**799.515**	797.499	R[6]
K[20]	2145.259	658.425	642.406	**643.414**	641.398	K[5]
V[21]	2244.327	502.298	486.280	487.287	485.272	V[4]
L[22]	2357.411	403.230	387.211	**388.219**	386.203	L[3]
R[23]	2513.512	290.146	274.127	**275.135**	273.119	R[2]
D[24]	2628.539	134.045	118.026	119.034	117.018	D[1]

sp|P62806|H4_MOUSE

[Acetyl]S $^{Acetyl}_{42.01}$ GRGKGGKGLGKGGAK $^{Acetyl}_{42.01}$ RHRK $^{Dimethyl}_{28.03}$ VLRD

- fragmentation table for charge state 2+
- specType=etd
- score=92.84
- F097393.dat
- query=q2318_p1
- precursor=658.138230
- chargePrecursor=4
- itol=0.8

AA	c	y	z+1	z+2	z	AA
S[1]	95.047	**1315.265**	1307.256	0.504	1306.752	S[24]
G[2]	123.558	1229.739	1221.729	0.504	1221.225	G[23]
R[3]	201.608	1201.228	1193.219	1193.723	1192.715	R[22]
G[4]	230.119	**1123.177**	1115.168	1115.672	1114.664	G[21]
K[5]	294.167	1094.667	1086.657	1087.161	1086.153	K[20]
G[6]	322.677	1030.619	**1022.610**	**1023.114**	**1022.106**	G[19]
G[7]	351.188	1002.108	**994.099**	**994.603**	993.595	G[18]
K[8]	415.236	973.598	**965.588**	**966.092**	**965.084**	K[17]
G[9]	443.746	909.550	**901.541**	**902.045**	**901.037**	G[16]
L[10]	500.288	881.040	873.030	873.534	**872.526**	L[15]
G[11]	528.799	824.498	**816.488**	**816.992**	815.984	G[14]
K[12]	592.847	795.987	**787.977**	**788.481**	**787.474**	K[13]
G[13]	621.357	731.939	**723.930**	**724.434**	**723.426**	G[12]
G[14]	649.868	703.429	**695.419**	**695.923**	**694.915**	G[11]
A[15]	685.387	674.918	**666.908**	**667.412**	666.405	A[10]
K[16]	770.439	639.399	**631.390**	**631.894**	630.886	K[9]
R[17]	**848.490**	554.347	**546.337**	**546.841**	**545.833**	R[8]
H[18]	**917.019**	476.296	468.287	468.791	467.783	H[7]
R[19]	**995.070**	407.767	399.757	400.261	399.253	R[6]
K[20]	1073.133	329.716	321.707	322.211	321.203	K[5]
V[21]	**1122.667**	251.653	243.643	244.147	243.140	V[4]
L[22]	**1179.209**	202.119	194.109	194.613	193.605	L[3]
R[23]	**1257.260**	145.577	137.567	138.071	137.063	R[2]
D[24]	**1314.773**	67.526	59.517	60.021	59.013	D[1]

sp|P62806|H4_MOUSE

[Acetyl]S $^{Acetyl}_{42.01}$ GRGKGGKGLGKGGAK $^{Acetyl}_{42.01}$ RHRK $^{Dimethyl}_{28.03}$ VLRD

- fragmentation table for charge state 3+
- specType=etd
- score=92.84
- F097393.dat
- query=q2318_p1
- precursor=658.138230
- chargePrecursor=4
- itol=0.8

AA	c	y	z+1	z+2	z	AA
S[1]	63.701	**877.179**	**871.840**	0.672	**871.504**	S[24]
G[2]	82.708	820.162	814.822	0.672	814.486	G[23]
R[3]	134.741	801.154	795.815	796.151	795.479	R[22]
G[4]	153.749	749.121	743.781	744.117	743.445	G[21]
K[5]	196.447	730.114	**724.774**	**725.110**	**724.438**	K[20]
G[6]	215.454	687.415	682.076	682.412	681.740	G[19]
G[7]	234.461	668.408	663.069	663.404	662.733	G[18]
K[8]	277.159	649.401	**644.061**	**644.397**	**643.725**	K[17]
G[9]	296.167	606.703	601.363	601.699	601.027	G[16]
L[10]	333.861	**587.695**	582.356	582.692	582.020	L[15]
G[11]	352.868	550.001	**544.661**	**544.997**	544.325	G[14]
K[12]	395.567	530.994	525.654	525.990	525.318	K[13]
G[13]	414.574	488.295	482.956	483.292	482.620	G[12]
G[14]	433.581	469.288	463.949	464.285	463.613	G[11]
A[15]	457.260	450.281	444.941	445.277	444.605	A[10]
K[16]	513.962	426.602	421.262	421.598	420.926	K[9]
R[17]	565.996	369.900	364.561	364.896	364.225	R[8]
H[18]	611.682	317.866	312.527	312.863	312.191	H[7]
R[19]	663.716	272.180	266.841	267.176	266.505	R[6]
K[20]	715.758	220.146	214.807	215.143	214.471	K[5]
V[21]	748.781	168.104	162.765	163.101	162.429	V[4]
L[22]	786.475	135.082	129.742	130.078	129.406	L[3]
R[23]	**838.509**	97.387	92.047	92.383	91.711	R[2]
D[24]	**876.851**	45.353	40.014	40.349	39.678	D[1]

sp|Q64525|H2B2B_MOUSE

PDPAKSAPAPKKGS $^{Acetyl}_{42.01}$ KKAVTK $^{Acetyl}_{42.01}$ VQKKD

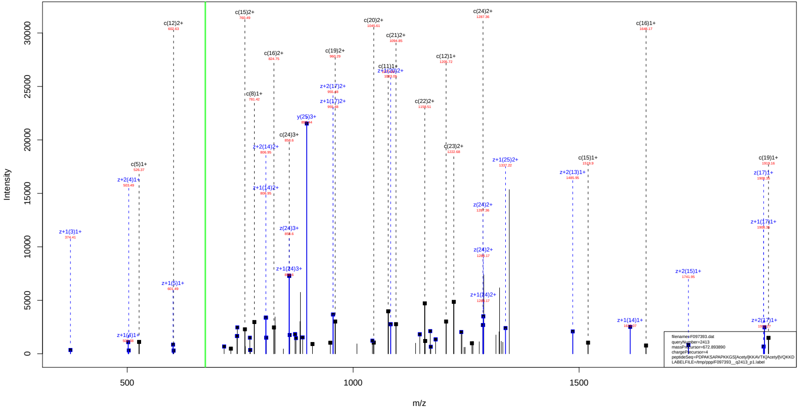

sp|Q64525|H2B2B_MOUSE

PDPAKSAPAPKKGS $^{Acetyl}_{42.01}$ KKAVTK $^{Acetyl}_{42.01}$ VQKKD

- fragmentation table for charge state 1+
- specType=etd
- score=93.99
- F097393.dat
- query=q2413_p1
- precursor=672.893890
- chargePrecursor=4
- itol=0.8

AA	c	y	z+1	z+2	z	AA
P[1]	115.087	2688.541	2672.522	0.000	2671.514	P[25]
D[2]	230.114	2591.488	2575.469	0.000	2574.461	D[24]
P[3]	327.166	2476.461	2460.442	0.000	2459.434	P[23]
A[4]	398.203	2379.408	2363.389	0.000	2362.382	A[22]
K[5]	**526.298**	2308.371	2292.352	2293.360	2291.345	K[21]
S[6]	613.330	2180.276	2164.257	2165.265	2163.250	S[20]
A[7]	684.368	2093.244	2077.225	2078.233	2076.218	A[19]
P[8]	**781.420**	2022.207	2006.188	2007.196	2005.180	P[18]
A[9]	852.457	1925.154	**1909.135**	**1910.143**	**1908.128**	A[17]
P[10]	**949.510**	1854.117	1838.098	1839.106	1837.091	P[16]
K[11]	**1077.605**	1757.064	1741.046	**1742.053**	1740.038	K[15]
K[12]	**1205.700**	1628.969	**1612.951**	1613.958	1611.943	K[14]
G[13]	**1262.722**	1500.874	1484.856	**1485.864**	1483.848	G[13]
S[14]	1391.764	1443.853	1427.834	1428.842	1426.826	S[12]
K[15]	**1519.859**	1314.810	1298.792	1299.799	1297.784	K[11]
K[16]	**1647.954**	1186.715	**1170.697**	**1171.705**	1169.689	K[10]
A[17]	1718.991	1058.620	**1042.602**	1043.610	1041.594	A[9]
V[18]	1818.060	987.583	971.565	972.572	970.557	V[8]
T[19]	**1919.107**	**888.515**	872.496	**873.504**	**871.488**	T[7]
K[20]	2089.213	787.467	**771.449**	**772.456**	770.441	K[6]
V[21]	2188.281	617.362	**601.343**	**602.351**	600.335	V[5]
Q[22]	2316.340	518.293	**502.275**	**503.282**	501.267	Q[4]
K[23]	2444.435	390.235	**374.216**	375.224	373.208	K[3]
K[24]	2572.530	262.140	246.121	247.129	245.113	K[2]
D[25]	2687.557	134.045	118.026	119.034	117.018	D[1]

sp|Q64525|H2B2B_MOUSE

PDPAKSAPAPKKGS $^{Acetyl}_{42.01}$ KKAVTK $^{Acetyl}_{42.01}$ VQKKD

- fragmentation table for charge state 2+
- specType=etd
- score=93.99
- F097393.dat
- query=q2413_p1
- precursor=672.893890
- chargePrecursor=4
- itol=0.8

AA	c	y	z+1	z+2	z	AA
P[1]	58.047	1344.774	**1336.765**	0.504	1336.261	P[25]
D[2]	115.560	1296.248	**1288.238**	0.504	**1287.734**	D[24]
P[3]	164.087	**1238.734**	1230.725	0.504	1230.221	P[23]
A[4]	199.605	1190.208	**1182.198**	0.504	**1181.694**	A[22]
K[5]	263.653	1154.689	**1146.680**	**1147.184**	1146.176	K[21]
S[6]	307.169	1090.642	**1082.632**	**1083.136**	1082.128	S[20]
A[7]	342.687	1047.126	1039.116	1039.620	1038.612	A[19]
P[8]	391.214	1011.607	1003.598	1004.102	1003.094	P[18]
A[9]	426.732	963.081	**955.071**	**955.575**	954.567	A[17]
P[10]	475.259	927.562	919.553	920.057	919.049	P[16]
K[11]	539.306	879.036	**871.026**	**871.530**	870.523	K[15]
K[12]	**603.354**	814.988	**806.979**	**807.483**	**806.475**	K[14]
G[13]	631.864	750.941	**742.931**	**743.435**	**742.428**	G[13]
S[14]	696.386	722.430	**714.421**	**714.925**	**713.917**	S[12]
K[15]	**760.433**	657.909	649.899	650.403	649.396	K[11]
K[16]	**824.481**	593.861	585.852	586.356	585.348	K[10]
A[17]	**859.999**	529.814	521.804	522.308	521.301	A[9]
V[18]	**909.533**	494.295	486.286	486.790	485.782	V[8]
T[19]	**960.057**	444.761	436.752	437.256	436.248	T[7]
K[20]	**1045.110**	394.237	386.228	386.732	385.724	K[6]
V[21]	**1094.644**	309.184	301.175	301.679	300.671	V[5]
Q[22]	**1158.674**	259.650	251.641	252.145	251.137	Q[4]
K[23]	**1222.721**	195.621	187.612	188.116	187.108	K[3]
K[24]	**1286.768**	131.574	123.564	124.068	123.060	K[2]
D[25]	1344.282	67.526	59.517	60.021	59.013	D[1]

sp|Q64525|H2B2B_MOUSE

PDPAKSAPAPKKGS $^{\text{Acetyl}}_{42.01}$ KKAVTK $^{\text{Acetyl}}_{42.01}$ VQKKD

- fragmentation table for charge state 3+
- specType=etd
- score=93.99
- F097393.dat
- query=q2413_p1
- precursor=672.893890
- chargePrecursor=4
- itol=0.8

AA	c	y	z+1	z+2	z	AA
P[1]	39.034	**896.852**	891.512	0.672	891.176	P[25]
D[2]	77.376	864.501	**859.161**	0.672	**858.825**	D[24]
P[3]	109.727	826.159	820.819	0.672	820.483	P[23]
A[4]	133.406	793.808	788.468	0.672	788.132	A[22]
K[5]	176.104	770.129	764.789	765.125	764.453	K[21]
S[6]	205.115	727.430	722.091	722.427	721.755	S[20]
A[7]	228.794	698.420	693.080	693.416	692.744	A[19]
P[8]	261.145	674.741	669.401	669.737	669.065	P[18]
A[9]	284.824	642.390	637.050	637.386	636.714	A[17]
P[10]	317.175	618.711	613.371	613.707	613.035	P[16]
K[11]	359.873	586.360	581.020	581.356	580.684	K[15]
K[12]	402.572	543.661	538.322	538.658	537.986	K[14]
G[13]	421.579	500.963	495.623	495.959	495.287	G[13]
S[14]	464.593	481.956	476.616	476.952	476.280	S[12]
K[15]	507.291	438.942	433.602	433.938	433.266	K[11]
K[16]	549.990	396.243	390.904	391.240	390.568	K[10]
A[17]	573.669	353.545	348.205	348.541	347.869	A[9]
V[18]	606.691	329.866	324.526	324.862	324.190	V[8]
T[19]	640.374	296.843	291.504	291.840	291.168	T[7]
K[20]	697.076	263.161	257.821	258.157	257.485	K[6]
V[21]	**730.099**	206.459	201.119	201.455	200.783	V[5]
Q[22]	**772.785**	173.436	168.096	168.432	167.760	Q[4]
K[23]	815.483	130.750	125.410	125.746	125.074	K[3]
K[24]	**858.181**	88.051	82.712	83.048	82.376	K[2]
D[25]	896.524	45.353	40.014	40.349	39.678	D[1]

sp|P70696|H2B1A_MOUSE

LAK $^{Acetyl}_{42.01}$ HAVSE

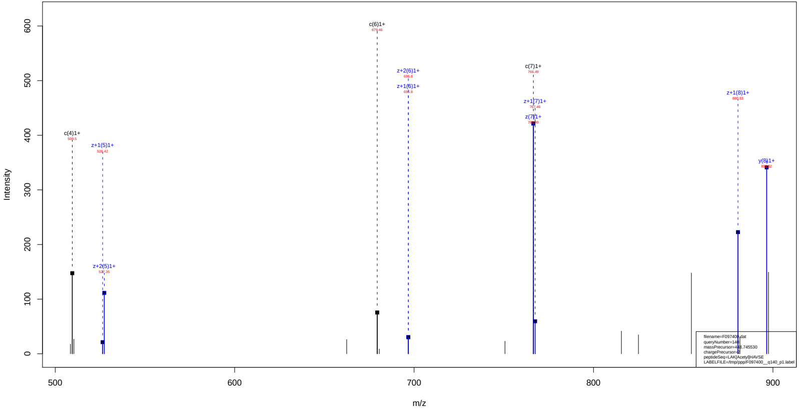

sp|P70696|H2B1A_MOUSE

LAK $^{Acetyl}_{42.01}$ HAVSE

- fragmentation table for charge state 1+
- specType=etd
- score=31.20
- F097400.dat
- query=q140_p1
- precursor=448.745530
- chargePrecursor=2
- itol=0.8

AA	c	y	z+1	z+2	z	AA
L[1]	131.118	**896.484**	**880.465**	0.000	879.457	L[8]
A[2]	202.155	783.400	**767.381**	0.000	**766.373**	A[7]
K[3]	372.261	712.362	**696.344**	**697.352**	695.336	K[6]
H[4]	**509.319**	542.257	**526.238**	**527.246**	525.230	H[5]
A[5]	580.357	405.198	389.179	390.187	388.171	A[4]
V[6]	**679.425**	334.161	318.142	319.150	317.134	V[3]
S[7]	**766.457**	235.092	219.074	220.082	218.066	S[2]
E[8]	895.500	148.060	132.042	133.050	131.034	E[1]

sp|Q64525|H2B2B_MOUSE

PDPAKSAPAPKKGS $^{\text{Acetyl}}_{42.01}$ KKAVTK $^{\text{Acetyl}}_{42.01}$ VQKKD

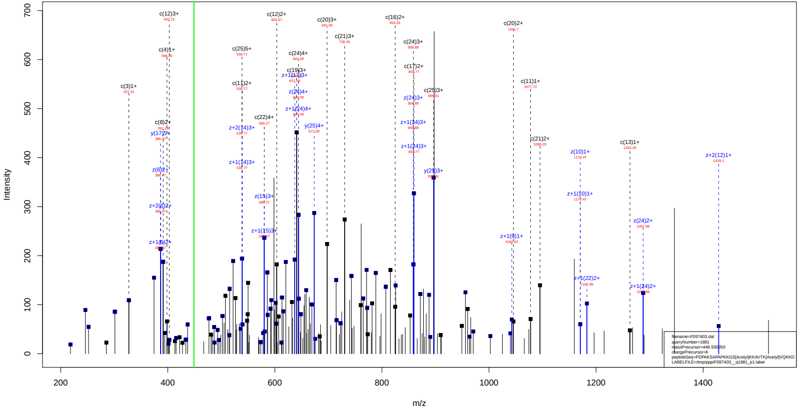

sp|Q64525|H2B2B_MOUSE

PDPAKSAPAPKKGS $^{Acetyl}_{42.01}$ KKAVTK $^{Acetyl}_{42.01}$ VQKKD

- fragmentation table for charge state 1+
- specType=etd
- score=70.44
- F097403.dat
- query=q1881_p1
- precursor=448.930050
- chargePrecursor=6
- itol=0.8

AA	c	y	z+1	z+2	z	AA
P[1]	115.087	2688.541	2672.522	0.000	2671.514	P[25]
D[2]	230.114	2591.488	2575.469	0.000	2574.461	D[24]
P[3]	**327.166**	2476.461	2460.442	0.000	2459.434	P[23]
A[4]	**398.203**	2379.408	2363.389	0.000	2362.382	A[22]
K[5]	**526.298**	2308.371	2292.352	2293.360	2291.345	K[21]
S[6]	**613.330**	2180.276	2164.257	2165.265	2163.250	S[20]
A[7]	**684.368**	2093.244	2077.225	2078.233	2076.218	A[19]
P[8]	**781.420**	2022.207	2006.188	2007.196	2005.180	P[18]
A[9]	**852.457**	1925.154	1909.135	1910.143	1908.128	A[17]
P[10]	**949.510**	1854.117	1838.098	1839.106	1837.091	P[16]
K[11]	**1077.605**	1757.064	1741.046	1742.053	1740.038	K[15]
K[12]	1205.700	1628.969	1612.951	1613.958	1611.943	K[14]
G[13]	**1262.722**	1500.874	1484.856	1485.864	1483.848	G[13]
S[14]	1391.764	1443.853	1427.834	**1428.842**	1426.826	S[12]
K[15]	1519.859	1314.810	1298.792	1299.799	1297.784	K[11]
K[16]	1647.954	1186.715	**1170.697**	1171.705	**1169.689**	K[10]
A[17]	1718.991	1058.620	**1042.602**	1043.610	1041.594	A[9]
V[18]	1818.060	987.583	971.565	972.572	**970.557**	V[8]
T[19]	1919.107	**888.515**	**872.496**	873.504	**871.488**	T[7]
K[20]	2089.213	787.467	**771.449**	**772.456**	770.441	K[6]
V[21]	2188.281	617.362	**601.343**	**602.351**	600.335	V[5]
Q[22]	2316.340	518.293	**502.275**	503.282	501.267	Q[4]
K[23]	2444.435	390.235	**374.216**	375.224	373.208	K[3]
K[24]	2572.530	262.140	**246.121**	247.129	245.113	K[2]
D[25]	2687.557	134.045	118.026	119.034	117.018	D[1]

sp|Q64525|H2B2B_MOUSE

PDPAKSAPAPKKGS $^{Acetyl}_{42.01}$ KKAVTK $^{Acetyl}_{42.01}$ VQKKD

- fragmentation table for charge state 2+
- specType=etd
- score=70.44
- F097403.dat
- query=q1881_p1
- precursor=448.930050
- chargePrecursor=6
- itol=0.8

AA	c	y	z+1	z+2	z	AA
P[1]	58.047	1344.774	1336.765	0.504	1336.261	P[25]
D[2]	115.560	1296.248	**1288.238**	0.504	**1287.734**	D[24]
P[3]	164.087	1238.734	1230.725	0.504	1230.221	P[23]
A[4]	199.605	1190.208	**1182.198**	0.504	1181.694	A[22]
K[5]	263.653	1154.689	1146.680	1147.184	1146.176	K[21]
S[6]	307.169	1090.642	1082.632	1083.136	1082.128	S[20]
A[7]	342.687	1047.126	1039.116	**1039.620**	1038.612	A[19]
P[8]	**391.214**	1011.607	1003.598	1004.102	**1003.094**	P[18]
A[9]	**426.732**	**963.081**	955.071	**955.575**	954.567	A[17]
P[10]	475.259	927.562	919.553	920.057	919.049	P[16]
K[11]	**539.306**	879.036	**871.026**	**871.530**	870.523	K[15]
K[12]	**603.354**	814.988	**806.979**	**807.483**	**806.475**	K[14]
G[13]	**631.864**	750.941	**742.931**	**743.435**	**742.428**	G[13]
S[14]	696.386	**722.430**	**714.421**	**714.925**	**713.917**	S[12]
K[15]	**760.433**	**657.909**	649.899	650.403	**649.396**	K[11]
K[16]	**824.481**	**593.861**	**585.852**	**586.356**	**585.348**	K[10]
A[17]	**859.999**	529.814	**521.804**	**522.308**	**521.301**	A[9]
V[18]	**909.533**	494.295	**486.286**	**486.790**	**485.782**	V[8]
T[19]	**960.057**	444.761	**436.752**	**437.256**	436.248	T[7]
K[20]	**1045.110**	**394.237**	**386.228**	**386.732**	**385.724**	K[6]
V[21]	**1094.644**	309.184	**301.175**	**301.679**	**300.671**	V[5]
Q[22]	1158.674	259.650	**251.641**	**252.145**	251.137	Q[4]
K[23]	1222.721	195.621	187.612	188.116	187.108	K[3]
K[24]	1286.768	131.574	123.564	124.068	123.060	K[2]
D[25]	1344.282	67.526	59.517	60.021	59.013	D[1]

sp|Q64525|H2B2B_MOUSE

PDPAKSAPAPKKGS $^{Acetyl}_{42.01}$ KKAVTK $^{Acetyl}_{42.01}$ VQKKD

- fragmentation table for charge state 3+
- specType=etd
- score=70.44
- F097403.dat
- query=q1881_p1
- precursor=448.930050
- chargePrecursor=6
- itol=0.8

AA	c	y	z+1	z+2	z	AA
P[1]	39.034	**896.852**	891.512	0.672	**891.176**	P[25]
D[2]	77.376	864.501	**859.161**	0.672	**858.825**	D[24]
P[3]	109.727	**826.159**	820.819	0.672	820.483	P[23]
A[4]	133.406	793.808	**788.468**	0.672	**788.132**	A[22]
K[5]	176.104	770.129	**764.789**	**765.125**	**764.453**	K[21]
S[6]	205.115	727.430	**722.091**	**722.427**	721.755	S[20]
A[7]	228.794	698.420	693.080	693.416	692.744	A[19]
P[8]	261.145	**674.741**	**669.401**	**669.737**	**669.065**	P[18]
A[9]	**284.824**	642.390	**637.050**	**637.386**	**636.714**	A[17]
P[10]	317.175	618.711	**613.371**	**613.707**	**613.035**	P[16]
K[11]	359.873	**586.360**	**581.020**	**581.356**	**580.684**	K[15]
K[12]	**402.572**	543.661	**538.322**	**538.658**	**537.986**	K[14]
G[13]	**421.579**	500.963	**495.623**	**495.959**	**495.287**	G[13]
S[14]	464.593	481.956	**476.616**	**476.952**	**476.280**	S[12]
K[15]	**507.291**	438.942	**433.602**	**433.938**	**433.266**	K[11]
K[16]	**549.990**	396.243	**390.904**	**391.240**	390.568	K[10]
A[17]	**573.669**	353.545	348.205	348.541	347.869	A[9]
V[18]	**606.691**	329.866	324.526	324.862	324.190	V[8]
T[19]	**640.374**	296.843	291.504	291.840	291.168	T[7]
K[20]	**697.076**	263.161	257.821	258.157	257.485	K[6]
V[21]	**730.099**	206.459	201.119	201.455	200.783	V[5]
Q[22]	**772.785**	173.436	168.096	168.432	167.760	Q[4]
K[23]	**815.483**	130.750	125.410	125.746	125.074	K[3]
K[24]	**858.181**	88.051	82.712	83.048	82.376	K[2]
D[25]	**896.524**	45.353	40.014	40.349	39.678	D[1]

sp|Q64525|H2B2B_MOUSE

PDPAKSAPAPKKGS $^{Acetyl}_{42.01}$ KKAVTK $^{Acetyl}_{42.01}$ VQKKD

- fragmentation table for charge state 4+
- specType=etd
- score=70.44
- F097403.dat
- query=q1881_p1
- precursor=448.930050
- chargePrecursor=6
- itol=0.8

AA	c	y	z+1	z+2	z	AA
P[1]	29.527	**672.891**	**668.886**	0.755	**668.634**	P[25]
D[2]	58.284	**648.627**	**644.623**	0.755	**644.371**	D[24]
P[3]	82.547	**619.871**	**615.866**	0.755	**615.614**	P[23]
A[4]	100.306	595.608	**591.603**	0.755	**591.351**	A[22]
K[5]	132.330	**577.848**	**573.844**	**574.096**	**573.592**	K[21]
S[6]	154.088	545.824	541.820	542.072	541.568	S[20]
A[7]	171.847	524.066	520.062	520.314	519.810	A[19]
P[8]	196.111	506.307	**502.303**	**502.554**	**502.051**	P[18]
A[9]	213.870	482.044	478.039	478.291	477.787	A[17]
P[10]	238.133	464.285	460.280	460.532	460.028	P[16]
K[11]	270.157	440.022	436.017	**436.269**	435.765	K[15]
K[12]	302.180	407.998	403.993	404.245	403.741	K[14]
G[13]	316.436	375.974	371.969	372.221	371.717	G[13]
S[14]	348.696	361.719	357.714	357.966	357.462	S[12]
K[15]	380.720	329.458	325.453	325.705	325.201	K[11]
K[16]	**412.744**	297.434	293.430	293.682	293.178	K[10]
A[17]	430.503	265.411	261.406	261.658	261.154	A[9]
V[18]	455.270	247.651	243.647	243.899	243.395	V[8]
T[19]	**480.532**	222.884	**218.880**	219.131	**218.628**	T[7]
K[20]	523.059	197.622	193.618	193.870	193.366	K[6]
V[21]	**547.826**	155.096	151.091	151.343	150.839	V[5]
Q[22]	**579.840**	130.329	126.324	126.576	126.072	Q[4]
K[23]	**611.864**	98.314	94.309	94.561	94.057	K[3]
K[24]	**643.888**	66.290	62.286	62.538	62.034	K[2]
D[25]	672.645	34.267	30.262	30.514	30.010	D[1]

sp|Q64525|H2B2B_MOUSE

PDPAKSAPAPKKGS $^{Acetyl}_{42.01}$ KKAVTK $^{Acetyl}_{42.01}$ VQKKD

- fragmentation table for charge state 5+
- specType=etd
- score=70.44
- F097403.dat
- query=q1881_p1
- precursor=448.930050
- chargePrecursor=6
- itol=0.8

AA	c	y	z+1	z+2	z	AA
P[1]	23.823	**538.514**	**535.310**	0.806	535.109	P[25]
D[2]	46.829	519.103	**515.900**	0.806	**515.698**	D[24]
P[3]	66.239	**496.098**	**492.894**	0.806	**492.693**	P[23]
A[4]	80.447	**476.687**	473.484	0.806	473.282	A[22]
K[5]	106.065	462.480	459.276	459.478	459.075	K[21]
S[6]	123.472	**436.861**	**433.657**	**433.859**	**433.456**	S[20]
A[7]	137.679	419.455	**416.251**	416.452	**416.049**	A[19]
P[8]	157.090	405.247	**402.043**	**402.245**	**401.842**	P[18]
A[9]	171.297	**385.837**	382.633	382.834	382.431	A[17]
P[10]	190.708	371.629	368.425	368.627	368.224	P[16]
K[11]	216.327	352.219	349.015	349.217	348.813	K[15]
K[12]	241.946	**326.600**	323.396	323.598	323.194	K[14]
G[13]	253.350	**300.981**	297.777	297.979	297.575	G[13]
S[14]	279.159	289.576	286.373	286.574	286.171	S[12]
K[15]	304.778	263.768	260.564	260.766	260.363	K[11]
K[16]	330.397	238.149	234.945	235.147	234.744	K[10]
A[17]	344.604	212.530	209.326	209.528	209.125	A[9]
V[18]	364.418	198.322	195.119	195.320	194.917	V[8]
T[19]	384.627	178.509	175.305	175.507	175.103	T[7]
K[20]	418.648	158.299	155.096	155.297	154.894	K[6]
V[21]	438.462	124.278	121.074	121.276	120.873	V[5]
Q[22]	464.074	104.464	101.261	101.462	101.059	Q[4]
K[23]	489.693	78.853	75.649	75.851	75.447	K[3]
K[24]	**515.312**	53.234	50.030	50.232	49.828	K[2]
D[25]	**538.317**	27.615	24.411	24.613	24.209	D[1]

sp|Q64525|H2B2B_MOUSE

PDPAKSAPAPKKGS $^{Acetyl}_{42.01}$ KKAVTK $^{Acetyl}_{42.01}$ VQKKD

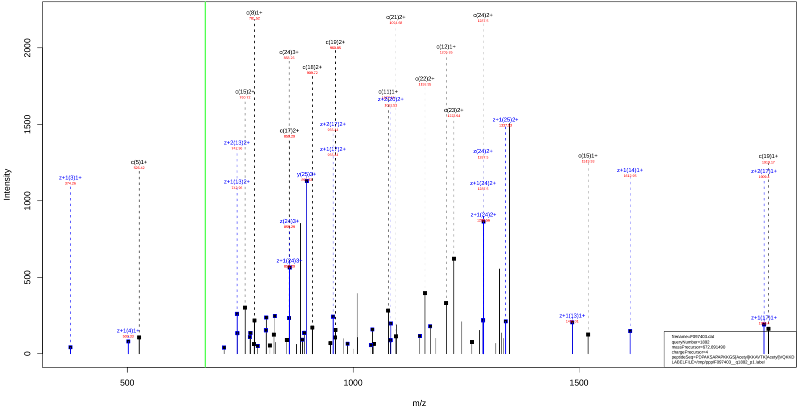

sp|Q64525|H2B2B_MOUSE

PDPAKSAPAPKKGS $^{Acetyl}_{42.01}$ KKAVTK $^{Acetyl}_{42.01}$ VQKKD

- fragmentation table for charge state 1+
- specType=etd
- score=80.75
- F097403.dat
- query=q1882_p1
- precursor=672.891490
- chargePrecursor=4
- itol=0.8

AA	c	y	z+1	z+2	z	AA
P[1]	115.087	2688.541	2672.522	0.000	2671.514	P[25]
D[2]	230.114	2591.488	2575.469	0.000	2574.461	D[24]
P[3]	327.166	2476.461	2460.442	0.000	2459.434	P[23]
A[4]	398.203	2379.408	2363.389	0.000	2362.382	A[22]
K[5]	**526.298**	2308.371	2292.352	2293.360	2291.345	K[21]
S[6]	613.330	2180.276	2164.257	2165.265	2163.250	S[20]
A[7]	684.368	2093.244	2077.225	2078.233	2076.218	A[19]
P[8]	**781.420**	2022.207	2006.188	2007.196	2005.180	P[18]
A[9]	**852.457**	1925.154	**1909.135**	**1910.143**	1908.128	A[17]
P[10]	**949.510**	1854.117	1838.098	1839.106	1837.091	P[16]
K[11]	**1077.605**	1757.064	1741.046	1742.053	1740.038	K[15]
K[12]	**1205.700**	1628.969	**1612.951**	1613.958	1611.943	K[14]
G[13]	**1262.722**	1500.874	**1484.856**	1485.864	1483.848	G[13]
S[14]	1391.764	1443.853	1427.834	1428.842	1426.826	S[12]
K[15]	**1519.859**	1314.810	1298.792	1299.799	1297.784	K[11]
K[16]	1647.954	1186.715	**1170.697**	1171.705	1169.689	K[10]
A[17]	1718.991	1058.620	**1042.602**	1043.610	1041.594	A[9]
V[18]	1818.060	**987.583**	971.565	972.572	970.557	V[8]
T[19]	**1919.107**	**888.515**	872.496	873.504	871.488	T[7]
K[20]	2089.213	787.467	**771.449**	**772.456**	770.441	K[6]
V[21]	2188.281	617.362	601.343	602.351	600.335	V[5]
Q[22]	2316.340	518.293	**502.275**	503.282	501.267	Q[4]
K[23]	2444.435	390.235	**374.216**	375.224	373.208	K[3]
K[24]	2572.530	262.140	246.121	247.129	245.113	K[2]
D[25]	2687.557	134.045	118.026	119.034	117.018	D[1]

sp|Q64525|H2B2B_MOUSE

PDPAKSAPAPKKGS $^{Acetyl}_{42.01}$ KKAVTK $^{Acetyl}_{42.01}$ VQKKD

- fragmentation table for charge state 2+
- specType=etd
- score=80.75
- F097403.dat
- query=q1882_p1
- precursor=672.891490
- chargePrecursor=4
- itol=0.8

AA	c	y	z+1	z+2	z	AA
P[1]	58.047	1344.774	**1336.765**	0.504	1336.261	P[25]
D[2]	115.560	1296.248	**1288.238**	0.504	**1287.734**	D[24]
P[3]	164.087	1238.734	1230.725	0.504	1230.221	P[23]
A[4]	199.605	1190.208	1182.198	0.504	1181.694	A[22]
K[5]	263.653	1154.689	**1146.680**	**1147.184**	1146.176	K[21]
S[6]	307.169	1090.642	**1082.632**	**1083.136**	**1082.128**	S[20]
A[7]	342.687	1047.126	**1039.116**	**1039.620**	1038.612	A[19]
P[8]	391.214	1011.607	1003.598	1004.102	1003.094	P[18]
A[9]	426.732	963.081	**955.071**	**955.575**	954.567	A[17]
P[10]	475.259	927.562	919.553	920.057	919.049	P[16]
K[11]	539.306	879.036	871.026	871.530	870.523	K[15]
K[12]	603.354	814.988	**806.979**	**807.483**	**806.475**	K[14]
G[13]	631.864	750.941	**742.931**	**743.435**	**742.428**	G[13]
S[14]	696.386	722.430	**714.421**	**714.925**	**713.917**	S[12]
K[15]	**760.433**	657.909	649.899	650.403	649.396	K[11]
K[16]	**824.481**	593.861	585.852	586.356	585.348	K[10]
A[17]	**859.999**	529.814	521.804	522.308	521.301	A[9]
V[18]	**909.533**	494.295	486.286	486.790	485.782	V[8]
T[19]	**960.057**	444.761	436.752	437.256	436.248	T[7]
K[20]	**1045.110**	394.237	386.228	386.732	385.724	K[6]
V[21]	**1094.644**	309.184	301.175	301.679	300.671	V[5]
Q[22]	**1158.674**	259.650	251.641	252.145	251.137	Q[4]
K[23]	**1222.721**	195.621	187.612	188.116	187.108	K[3]
K[24]	**1286.768**	131.574	123.564	124.068	123.060	K[2]
D[25]	1344.282	67.526	59.517	60.021	59.013	D[1]

sp|Q64525|H2B2B_MOUSE

PDPAKSAPAPKKGS $^{\text{Acetyl}}_{42.01}$ KKAVTK $^{\text{Acetyl}}_{42.01}$ VQKKD

- fragmentation table for charge state 3+
- specType=etd
- score=80.75
- F097403.dat
- query=q1882_p1
- precursor=672.891490
- chargePrecursor=4
- itol=0.8

AA	c	y	z+1	z+2	z	AA
P[1]	39.034	**896.852**	**891.512**	0.672	**891.176**	P[25]
D[2]	77.376	864.501	**859.161**	0.672	**858.825**	D[24]
P[3]	109.727	**826.159**	820.819	0.672	820.483	P[23]
A[4]	133.406	793.808	**788.468**	0.672	**788.132**	A[22]
K[5]	176.104	770.129	764.789	765.125	764.453	K[21]
S[6]	205.115	727.430	722.091	722.427	721.755	S[20]
A[7]	228.794	698.420	693.080	693.416	692.744	A[19]
P[8]	261.145	674.741	669.401	669.737	669.065	P[18]
A[9]	284.824	642.390	637.050	637.386	636.714	A[17]
P[10]	317.175	618.711	613.371	613.707	613.035	P[16]
K[11]	359.873	586.360	581.020	581.356	580.684	K[15]
K[12]	402.572	543.661	538.322	538.658	537.986	K[14]
G[13]	421.579	500.963	495.623	495.959	495.287	G[13]
S[14]	464.593	481.956	476.616	476.952	476.280	S[12]
K[15]	507.291	438.942	433.602	433.938	433.266	K[11]
K[16]	549.990	396.243	390.904	391.240	390.568	K[10]
A[17]	573.669	353.545	348.205	348.541	347.869	A[9]
V[18]	606.691	329.866	324.526	324.862	324.190	V[8]
T[19]	640.374	296.843	291.504	291.840	291.168	T[7]
K[20]	697.076	263.161	257.821	258.157	257.485	K[6]
V[21]	730.099	206.459	201.119	201.455	200.783	V[5]
Q[22]	**772.785**	173.436	168.096	168.432	167.760	Q[4]
K[23]	**815.483**	130.750	125.410	125.746	125.074	K[3]
K[24]	**858.181**	88.051	82.712	83.048	82.376	K[2]
D[25]	896.524	45.353	40.014	40.349	39.678	D[1]

sp|P62806|H4_MOUSE

[Acetyl]SGRGKGGKGL

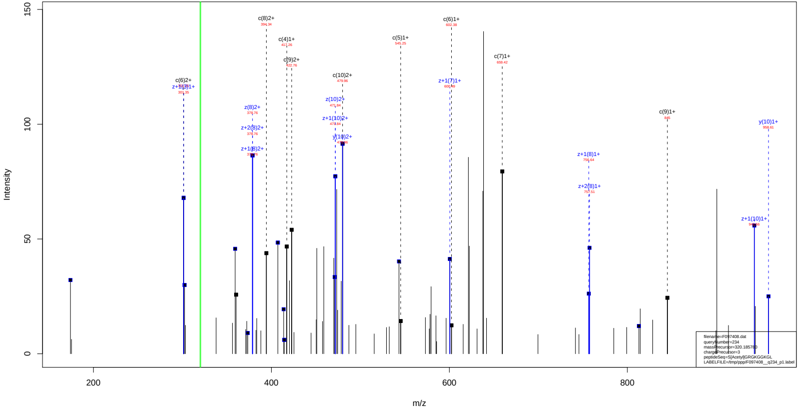

sp|P62806|H4_MOUSE

[Acetyl]SGRGKGGKGL

- fragmentation table for charge state 1+
- specType=etd
- score=36.06
- F097408.dat
- query=q234_p1
- precursor=320.185780
- chargePrecursor=3
- itol=0.8

AA	c	y	z+1	z+2	z	AA
S[1]	147.076	**958.543**	**942.524**	0.000	941.516	S[10]
G[2]	204.098	829.500	**813.482**	0.000	**812.474**	G[9]
R[3]	**360.199**	772.479	**756.460**	**757.468**	755.452	R[8]
G[4]	**417.220**	616.378	**600.359**	601.367	599.351	G[7]
K[5]	**545.315**	559.356	**543.337**	544.345	542.330	K[6]
G[6]	**602.337**	431.261	**415.243**	416.250	**414.235**	G[5]
G[7]	**659.358**	**374.240**	358.221	**359.229**	357.213	G[4]
K[8]	787.453	317.218	**301.200**	**302.207**	300.192	K[3]
G[9]	**844.475**	189.123	173.105	**174.112**	172.097	G[2]
L[10]	957.559	132.102	116.083	117.091	115.075	L[1]

sp|P62806|H4_MOUSE

[Acetyl]SGRGKGGKGL

- fragmentation table for charge state 2+
- specType=etd
- score=36.06
- F097408.dat
- query=q234_p1
- precursor=320.185780
- chargePrecursor=3
- itol=0.8

AA	c	y	z+1	z+2	z	AA
S[1]	74.042	**479.775**	**471.766**	0.504	**471.262**	S[10]
G[2]	102.553	415.254	**407.244**	0.504	**406.740**	G[9]
R[3]	180.603	386.743	**378.734**	**379.238**	**378.230**	R[8]
G[4]	209.114	308.692	**300.683**	**301.187**	300.179	G[7]
K[5]	273.161	280.182	272.172	272.676	271.668	K[6]
G[6]	**301.672**	216.134	208.125	208.629	207.621	G[5]
G[7]	330.183	187.624	179.614	180.118	179.110	G[4]
K[8]	**394.230**	159.113	151.103	151.607	150.600	K[3]
G[9]	**422.741**	95.065	87.056	87.560	86.552	G[2]
L[10]	**479.283**	66.555	58.545	59.049	58.041	L[1]

sp|P15864|H12_MOUSE

[Acetyl]SEAAPAAPAAAPPAEKAPAKKKAAKKPAGVRRKASGPPVSEL

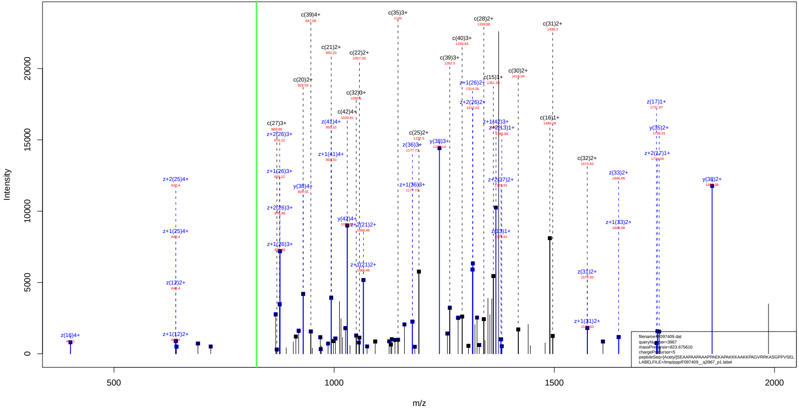

sp|P15864|H12_MOUSE

[Acetyl]SEAAPAAPAAAPPAEKAPAKKKAAKKPAGVRRKASGPPVSEL

- fragmentation table for charge state 1+
- specType=etd
- score=37.87
- F097409.dat
- query=q3967_p1
- precursor=823.675610
- chargePrecursor=5
- itol=0.8

AA	c	y	z+1	z+2	z	AA
S[1]	147.076	4114.325	4098.306	0.000	4097.298	S[42]
E[2]	276.119	3985.282	3969.263	0.000	3968.256	E[41]
A[3]	347.156	3856.240	3840.221	0.000	3839.213	A[40]
A[4]	418.193	3785.202	3769.184	0.000	3768.176	A[39]
P[5]	515.246	3714.165	3698.147	0.000	3697.139	P[38]
A[6]	586.283	3617.113	3601.094	0.000	3600.086	A[37]
A[7]	657.320	3546.075	3530.057	0.000	3529.049	A[36]
P[8]	754.373	3475.038	3459.020	0.000	3458.012	P[35]
A[9]	825.410	3377.986	3361.967	0.000	3360.959	A[34]
A[10]	896.447	3306.948	3290.930	0.000	3289.922	A[33]
A[11]	967.484	3235.911	3219.893	0.000	3218.885	A[32]
P[12]	1064.537	3164.874	3148.856	0.000	3147.848	P[31]
P[13]	1161.590	3067.821	3051.803	0.000	3050.795	P[30]
A[14]	1232.627	2970.769	2954.750	0.000	2953.742	A[29]
E[15]	**1361.670**	2899.732	2883.713	0.000	2882.705	E[28]
K[16]	**1489.765**	2770.689	2754.670	2755.678	2753.662	K[27]
A[17]	1560.802	2642.594	2626.575	2627.583	2625.568	A[26]
P[18]	1657.854	2571.557	2555.538	2556.546	2554.530	P[25]
A[19]	1728.892	2474.504	2458.485	2459.493	2457.478	A[24]
K[20]	1856.986	2403.467	2387.448	2388.456	2386.441	K[23]
K[21]	1985.081	2275.372	2259.353	2260.361	2258.346	K[22]
K[22]	2113.176	2147.277	2131.258	2132.266	2130.251	K[21]
A[23]	2184.214	2019.182	2003.163	2004.171	2002.156	A[20]
A[24]	2255.251	1948.145	1932.126	1933.134	1931.119	A[19]
K[25]	2383.346	1877.108	1861.089	1862.097	1860.081	K[18]
K[26]	2511.441	1749.013	1732.994	**1734.002**	**1731.986**	K[17]
P[27]	2608.493	1620.918	1604.899	1605.907	1603.891	P[16]
A[28]	2679.530	1523.865	1507.847	1508.854	1506.839	A[15]
G[29]	2736.552	1452.828	1436.809	1437.817	1435.802	G[14]
V[30]	2835.620	1395.807	1379.788	**1380.796**	**1378.780**	V[13]
R[31]	2991.721	1296.738	**1280.720**	**1281.727**	1279.712	R[12]
R[32]	3147.823	1140.637	**1124.618**	1125.626	1123.611	R[11]
K[33]	3275.918	984.536	**968.517**	**969.525**	967.509	K[10]
A[34]	3346.955	856.441	840.422	841.430	839.415	A[9]
S[35]	3433.987	785.404	769.385	770.393	768.377	S[8]
G[36]	3491.008	698.372	682.353	683.361	681.345	G[7]
P[37]	3588.061	**641.350**	625.332	626.340	624.324	P[6]
P[38]	3685.114	544.298	528.279	529.287	527.271	P[5]
V[39]	3784.182	447.245	431.226	432.234	430.218	V[4]
S[40]	3871.214	348.177	332.158	333.166	331.150	S[3]
E[41]	4000.257	261.144	245.126	246.134	244.118	E[2]
L[42]	4113.341	132.102	116.083	117.091	115.075	L[1]

sp|P15864|H12_MOUSE

[Acetyl]SEAAPAAPAAAPPAEKAPAKKKAAKKPAGVRRKASGPPVSEL

- fragmentation table for charge state 2+
- specType=etd
- score=37.87
- F097409.dat
- query=q3967_p1
- precursor=823.675610
- chargePrecursor=5
- itol=0.8

AA	c	y	z+1	z+2	z	AA
S[1]	74.042	2057.666	2049.657	0.504	2049.153	S[42]
E[2]	138.563	1993.145	1985.135	0.504	1984.631	E[41]
A[3]	174.082	1928.623	1920.614	0.504	1920.110	A[40]
A[4]	209.600	1893.105	1885.096	0.504	1884.592	A[39]
P[5]	258.127	**1857.586**	1849.577	0.504	1849.073	P[38]
A[6]	293.645	1809.060	1801.051	0.504	1800.547	A[37]
A[7]	329.164	1773.541	1765.532	0.504	1765.028	A[36]
P[8]	377.690	**1738.023**	1730.013	0.504	1729.510	P[35]
A[9]	413.209	1689.496	1681.487	0.504	1680.983	A[34]
A[10]	448.727	1653.978	**1645.969**	0.504	**1645.465**	A[33]
A[11]	484.246	1618.459	**1610.450**	0.504	**1609.946**	A[32]
P[12]	532.772	1582.941	**1574.931**	0.504	**1574.427**	P[31]
P[13]	581.299	1534.414	1526.405	0.504	1525.901	P[30]
A[14]	616.817	1485.888	1477.879	0.504	1477.375	A[29]
E[15]	681.338	1450.369	1442.360	0.504	1441.856	E[28]
K[16]	745.386	1385.848	1377.839	**1378.343**	1377.335	K[27]
A[17]	780.904	1321.801	**1313.791**	**1314.295**	1313.287	A[26]
P[18]	829.431	1286.282	1278.273	1278.777	1277.769	P[25]
A[19]	864.949	1237.756	1229.746	1230.250	1229.242	A[24]
K[20]	**928.997**	1202.237	1194.228	1194.732	1193.724	K[23]
K[21]	**993.044**	**1138.190**	**1130.180**	**1130.684**	1129.676	K[22]
K[22]	**1057.092**	**1074.142**	**1066.133**	**1066.637**	1065.629	K[21]
A[23]	**1092.610**	1010.095	**1002.085**	**1002.589**	1001.581	A[20]
A[24]	**1128.129**	974.576	966.567	967.071	966.063	A[19]
K[25]	**1192.176**	939.058	931.048	931.552	930.544	K[18]
K[26]	1256.224	875.010	**867.001**	**867.505**	**866.497**	K[17]
P[27]	**1304.750**	810.963	802.953	803.457	802.449	P[16]
A[28]	**1340.269**	762.436	754.427	754.931	753.923	A[15]
G[29]	1368.780	726.918	718.908	**719.412**	718.404	G[14]
V[30]	**1418.314**	698.407	**690.398**	**690.902**	**689.894**	V[13]
R[31]	**1496.364**	648.873	**640.863**	**641.367**	**640.359**	R[12]
R[32]	**1574.415**	570.822	562.813	563.317	562.309	R[11]
K[33]	1638.462	492.772	484.762	485.266	484.258	K[10]
A[34]	1673.981	428.724	420.715	421.219	420.211	A[9]
S[35]	1717.497	393.206	385.196	385.700	384.692	S[8]
G[36]	1746.008	349.690	341.680	342.184	341.176	G[7]
P[37]	1794.534	321.179	313.170	313.673	312.666	P[6]
P[38]	1843.060	272.652	264.643	265.147	264.139	P[5]
V[39]	1892.595	224.126	216.117	216.621	215.613	V[4]
S[40]	1936.111	174.592	166.583	167.086	166.079	S[3]
E[41]	2000.632	131.076	123.067	123.570	122.563	E[2]
L[42]	2057.174	66.555	58.545	59.049	58.041	L[1]

sp|P15864|H12_MOUSE

[Acetyl]SEAAPAAPAAAPPAEKAPAKKKAAKKPAGVRRKASGPPVSEL

- fragmentation table for charge state 3+
- specType=etd
- score=37.87
- F097409.dat
- query=q3967_p1
- precursor=823.675610
- chargePrecursor=5
- itol=0.8

AA	c	y	z+1	z+2	z	AA
S[1]	49.697	1372.113	**1366.774**	0.672	1366.438	S[42]
E[2]	92.711	**1329.099**	**1323.759**	0.672	1323.423	E[41]
A[3]	116.390	1286.085	**1280.745**	0.672	1280.409	A[40]
A[4]	140.069	**1262.406**	**1257.066**	0.672	**1256.730**	A[39]
P[5]	172.420	**1238.727**	1233.387	0.672	1233.051	P[38]
A[6]	196.099	1206.376	1201.036	0.672	1200.700	A[37]
A[7]	219.778	**1182.697**	**1177.357**	0.672	**1177.021**	A[36]
P[8]	252.129	**1159.018**	1153.678	0.672	1153.342	P[35]
A[9]	275.808	1126.667	1121.327	0.672	1120.991	A[34]
A[10]	299.487	1102.988	1097.648	0.672	1097.312	A[33]
A[11]	323.166	1079.309	1073.969	0.672	1073.633	A[32]
P[12]	355.517	**1055.630**	**1050.290**	0.672	**1049.954**	P[31]
P[13]	387.868	1023.279	1017.939	0.672	1017.603	P[30]
A[14]	411.547	990.928	**985.588**	0.672	985.252	A[29]
E[15]	454.561	967.249	961.909	0.672	961.573	E[28]
K[16]	497.260	924.235	**918.895**	**919.231**	**918.559**	K[27]
A[17]	520.939	881.536	**876.197**	**876.533**	**875.861**	A[26]
P[18]	553.290	857.857	852.518	852.854	852.182	P[25]
A[19]	576.969	825.506	820.167	820.503	819.831	A[24]
K[20]	619.667	801.827	796.488	796.824	796.152	K[23]
K[21]	662.365	759.129	753.789	754.125	753.453	K[22]
K[22]	705.064	716.431	711.091	711.427	710.755	K[21]
A[23]	728.743	673.732	668.393	668.729	668.057	A[20]
A[24]	752.422	650.053	644.714	645.050	644.378	A[19]
K[25]	795.120	626.374	621.035	621.371	620.699	K[18]
K[26]	837.818	583.676	578.336	578.672	578.000	K[17]
P[27]	**870.169**	540.978	535.638	535.974	535.302	P[16]
A[28]	893.848	508.627	503.287	503.623	502.951	A[15]
G[29]	**912.855**	484.948	479.608	479.944	479.272	G[14]
V[30]	945.878	465.940	460.601	460.937	460.265	V[13]
R[31]	**997.912**	432.918	427.578	427.914	427.242	R[12]
R[32]	**1049.946**	380.884	375.544	375.880	375.208	R[11]
K[33]	**1092.644**	328.850	323.511	323.847	323.175	K[10]
A[34]	1116.323	286.152	280.812	281.148	280.476	A[9]
S[35]	**1145.334**	262.473	257.133	257.469	256.797	S[8]
G[36]	1164.341	233.462	228.123	228.459	227.787	G[7]
P[37]	1196.692	214.455	209.115	209.451	208.779	P[6]
P[38]	1229.043	182.104	176.765	177.100	176.429	P[5]
V[39]	**1262.066**	149.753	144.414	144.750	144.078	V[4]
S[40]	**1291.076**	116.730	111.391	111.727	111.055	S[3]
E[41]	1334.090	87.720	82.380	82.716	82.044	E[2]
L[42]	1371.785	44.705	39.366	39.702	39.030	L[1]

sp|P15864|H12_MOUSE

[Acetyl]SEAAPAAPAAAPPAEKAPAKKKAAKKPAGVRRKASGPPVSEL

- fragmentation table for charge state 4+
- specType=etd
- score=37.87
- F097409.dat
- query=q3967_p1
- precursor=823.675610
- chargePrecursor=5
- itol=0.8

AA	c	y	z+1	z+2	z	AA
S[1]	37.525	**1029.337**	**1025.332**	0.755	**1025.080**	S[42]
E[2]	69.785	997.076	**993.071**	0.755	**992.819**	E[41]
A[3]	87.544	964.815	960.811	0.755	960.559	A[40]
A[4]	105.304	**947.056**	943.051	0.755	942.799	A[39]
P[5]	129.567	**929.297**	925.292	0.755	925.040	P[38]
A[6]	147.326	905.034	901.029	0.755	900.777	A[37]
A[7]	165.086	887.274	883.270	0.755	883.018	A[36]
P[8]	189.349	**869.515**	865.510	0.755	865.258	P[35]
A[9]	207.108	845.252	841.247	0.755	840.995	A[34]
A[10]	224.867	827.493	823.488	0.755	823.236	A[33]
A[11]	242.627	809.733	805.729	0.755	805.477	A[32]
P[12]	266.890	791.974	787.969	0.755	787.717	P[31]
P[13]	291.153	767.711	763.706	0.755	763.454	P[30]
A[14]	308.912	743.448	739.443	0.755	739.191	A[29]
E[15]	341.173	725.688	721.684	0.755	721.432	E[28]
K[16]	373.197	693.428	689.423	689.675	689.171	K[27]
A[17]	390.956	661.404	657.399	657.651	657.147	A[26]
P[18]	415.219	643.645	**639.640**	**639.892**	639.388	P[25]
A[19]	432.978	619.381	615.377	615.629	615.125	A[24]
K[20]	465.002	601.622	597.618	597.869	597.366	K[23]
K[21]	497.026	569.598	565.594	565.846	565.342	K[22]
K[22]	529.050	537.575	533.570	533.822	533.318	K[21]
A[23]	546.809	505.551	501.546	501.798	501.294	A[20]
A[24]	564.568	487.792	483.787	484.039	483.535	A[19]
K[25]	596.592	470.032	466.028	466.280	465.776	K[18]
K[26]	628.616	438.009	434.004	434.256	433.752	K[17]
P[27]	652.879	405.985	401.980	402.232	**401.728**	P[16]
A[28]	670.638	381.722	377.717	377.969	377.465	A[15]
G[29]	684.893	363.962	359.958	360.210	359.706	G[14]
V[30]	709.661	349.707	345.702	345.954	345.450	V[13]
R[31]	748.686	324.940	320.935	321.187	320.683	R[12]
R[32]	787.711	285.915	281.910	282.162	281.658	R[11]
K[33]	819.735	246.889	242.885	243.137	242.633	K[10]
A[34]	837.494	214.866	210.861	211.113	210.609	A[9]
S[35]	859.252	197.106	193.102	193.354	192.850	S[8]
G[36]	873.507	175.348	171.344	171.596	171.092	G[7]
P[37]	897.771	161.093	157.088	157.340	156.836	P[6]
P[38]	922.034	136.830	132.825	133.077	132.573	P[5]
V[39]	**946.801**	112.567	108.562	108.814	108.310	V[4]
S[40]	**968.559**	87.800	83.795	84.047	83.543	S[3]
E[41]	1000.820	66.042	62.037	62.289	61.785	E[2]
L[42]	**1029.091**	33.781	29.776	30.028	29.524	L[1]

sp|P62806|H4_MOUSE

GKGGAK $^{Acetyl}_{42.01}$ RHRK $^{Dimethyl}_{28.03}$ VL

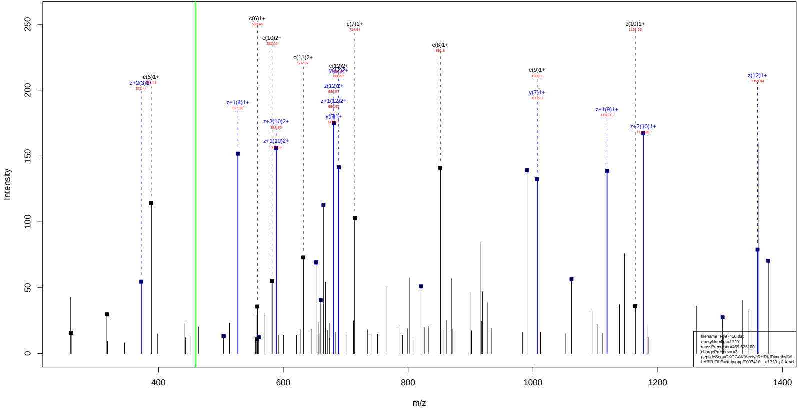

sp|P62806|H4_MOUSE

GKGGAK $^{\text{Acetyl}}_{42.01}$ RHRK $^{\text{Dimethyl}}_{28.03}$ VL

- fragmentation table for charge state 1+
- specType=etd
- score=31.96
- F097410.dat
- query=q1729_p1
- precursor=459.625100
- chargePrecursor=3
- itol=0.8

AA	c	y	z+1	z+2	z	AA
G[1]	75.055	**1376.860**	1360.841	0.000	**1359.833**	G[12]
K[2]	203.150	1319.838	**1303.820**	1304.827	1302.812	K[11]
G[3]	**260.172**	1191.743	1175.725	**1176.732**	1174.717	G[10]
G[4]	**317.193**	1134.722	**1118.703**	1119.711	1117.695	G[9]
A[5]	**388.230**	1077.700	**1061.682**	1062.689	1060.674	A[8]
K[6]	**558.336**	**1006.663**	**990.645**	991.652	989.637	K[7]
R[7]	**714.437**	836.558	**820.539**	821.547	819.531	R[6]
H[8]	**851.496**	**680.457**	**664.438**	665.446	663.430	H[5]
R[9]	**1007.597**	543.398	**527.379**	528.387	526.371	R[4]
K[10]	**1163.723**	387.297	371.278	**372.286**	370.270	K[3]
V[11]	1262.792	231.170	215.152	216.159	214.144	V[2]
L[12]	1375.876	132.102	116.083	117.091	115.075	L[1]

sp|P62806|H4_MOUSE

GKGGAK $^{\text{Acetyl}}_{42.01}$ RHRK $^{\text{Dimethyl}}_{28.03}$ VL

- fragmentation table for charge state 2+
- specType=etd
- score=31.96
- F097410.dat
- query=q1729_p1
- precursor=459.625100
- chargePrecursor=3
- itol=0.8

AA	c	y	z+1	z+2	z	AA
G[1]	38.031	**688.933**	**680.924**	0.504	**680.420**	G[12]
K[2]	102.079	**660.423**	**652.413**	**652.917**	**651.909**	K[11]
G[3]	130.589	596.375	**588.366**	**588.870**	587.862	G[10]
G[4]	159.100	567.865	**559.855**	**560.359**	559.351	G[9]
A[5]	194.619	539.354	531.344	531.848	530.841	A[8]
K[6]	279.672	**503.835**	495.826	496.330	495.322	K[7]
R[7]	357.722	418.782	410.773	411.277	410.269	R[6]
H[8]	426.252	340.732	332.723	333.226	332.219	H[5]
R[9]	**504.302**	272.202	264.193	264.697	263.689	R[4]
K[10]	**582.365**	194.152	186.143	186.646	185.639	K[3]
V[11]	**631.899**	116.089	108.079	108.583	107.576	V[2]
L[12]	**688.441**	66.555	58.545	59.049	58.041	L[1]

sp|P62806|H4_MOUSE

[Acetyl]SGRGK $^{Acetyl}_{42.01}$ GGKGL

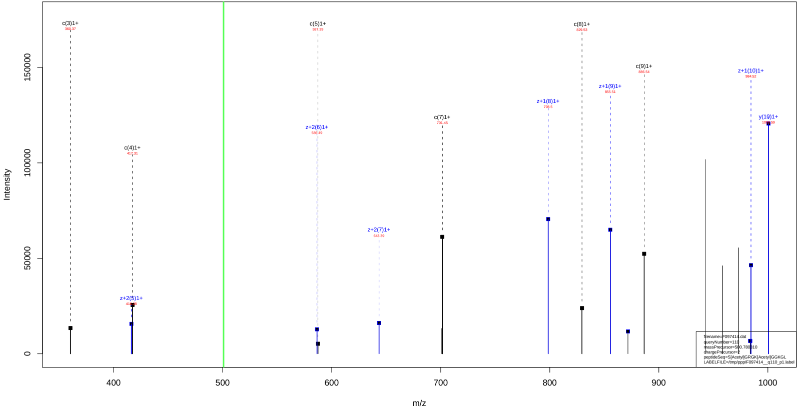

sp|P62806|H4_MOUSE

[Acetyl]SGRGK $^{Acetyl}_{42.01}$ GGKGL

- fragmentation table for charge state 1+
- specType=etd
- score=49.48
- F097414.dat
- query=q110_p1
- precursor=500.780310
- chargePrecursor=2
- itol=0.8

AA	c	y	z+1	z+2	z	AA
S[1]	147.076	**1000.553**	**984.535**	0.000	**983.527**	S[10]
G[2]	204.098	**871.511**	**855.492**	0.000	854.484	G[9]
R[3]	**360.199**	814.489	**798.471**	799.478	797.463	R[8]
G[4]	**417.220**	658.388	642.370	**643.377**	641.362	G[7]
K[5]	**587.326**	601.367	585.348	**586.356**	584.340	K[6]
G[6]	644.347	431.261	415.243	**416.250**	414.235	G[5]
G[7]	**701.369**	374.240	358.221	359.229	357.213	G[4]
K[8]	**829.464**	317.218	301.200	302.207	300.192	K[3]
G[9]	**886.485**	189.123	173.105	174.112	172.097	G[2]
L[10]	999.569	132.102	116.083	117.091	115.075	L[1]

sp|P62806|H4_MOUSE

[Acetyl]SGRGKGGKGLGKGGAKRHRK $^{\text{Dimethyl}}_{28.03}$ VLR

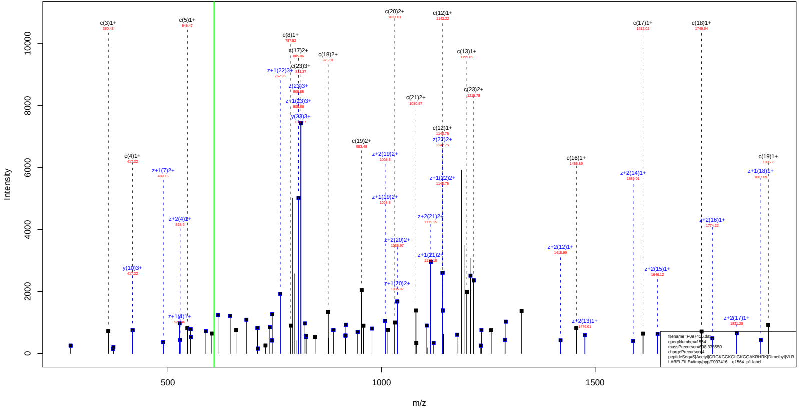

sp|P62806|H4_MOUSE

[Acetyl]SGRGKGGKGLGKGGAKRHRK $^{\text{Dimethyl}}_{28.03}$ VLR

- fragmentation table for charge state 1+
- specType=etd
- score=148.32
- F097416.dat
- query=q1564_p1
- precursor=608.378550
- chargePrecursor=4
- itol=0.8

AA	c	y	z+1	z+2	z	AA
S[1]	147.076	2430.475	2414.457	0.000	2413.449	S[23]
G[2]	204.098	2301.433	2285.414	0.000	2284.406	G[22]
R[3]	**360.199**	2244.411	2228.392	2229.400	2227.385	R[21]
G[4]	**417.220**	2088.310	2072.291	2073.299	2071.284	G[20]
K[5]	**545.315**	2031.289	2015.270	2016.278	2014.262	K[19]
G[6]	**602.337**	1903.194	**1887.175**	**1888.183**	1886.167	G[18]
G[7]	**659.358**	1846.172	1830.153	**1831.161**	1829.146	G[17]
K[8]	**787.453**	1789.151	1773.132	**1774.140**	1772.124	K[16]
G[9]	**844.475**	1661.056	1645.037	**1646.045**	1644.029	G[15]
L[10]	**957.559**	1604.034	1588.016	**1589.023**	1587.008	L[14]
G[11]	**1014.580**	1490.950	1474.932	**1475.939**	1473.924	G[13]
K[12]	**1142.675**	1433.929	1417.910	**1418.918**	1416.902	K[12]
G[13]	**1199.697**	1305.834	1289.815	**1290.823**	**1288.807**	G[11]
G[14]	**1256.718**	1248.812	**1232.794**	**1233.801**	**1231.786**	G[10]
A[15]	**1327.755**	1191.791	1175.772	**1176.780**	1174.764	A[9]
K[16]	**1455.850**	1120.754	1104.735	**1105.743**	1103.727	K[8]
R[17]	**1611.951**	992.659	976.640	**977.648**	975.632	R[7]
H[18]	**1749.010**	836.558	**820.539**	821.547	819.531	H[6]
R[19]	**1905.111**	699.499	**683.480**	684.488	682.472	R[5]
K[20]	2061.238	543.398	**527.379**	**528.387**	526.371	K[4]
V[21]	2160.306	387.271	**371.253**	**372.261**	370.245	V[3]
L[22]	2273.390	288.203	**272.184**	273.192	271.176	L[2]
R[23]	2429.491	175.119	159.100	160.108	158.092	R[1]

sp|P62806|H4_MOUSE

[Acetyl]SGRGKGGKGLGKGGAKRHRK$^{\text{Dimethyl}}_{28.03}$VLR

- fragmentation table for charge state 2+
- specType=etd
- score=148.32
- F097416.dat
- query=q1564_p1
- precursor=608.378550
- chargePrecursor=4
- itol=0.8

AA	c	y	z+1	z+2	z	AA
S[1]	74.042	**1215.741**	**1207.732**	0.504	1207.228	S[23]
G[2]	102.553	1151.220	**1143.211**	0.504	**1142.707**	G[22]
R[3]	180.603	**1122.709**	**1114.700**	**1115.204**	1114.196	R[21]
G[4]	209.114	1044.659	**1036.649**	**1037.153**	1036.145	G[20]
K[5]	273.161	1016.148	**1008.139**	**1008.643**	1007.635	K[19]
G[6]	301.672	952.100	**944.091**	**944.595**	**943.587**	G[18]
G[7]	330.183	923.590	**915.580**	**916.084**	**915.076**	G[17]
K[8]	394.230	895.079	**887.070**	**887.574**	**886.566**	K[16]
G[9]	422.741	831.032	**823.022**	**823.526**	**822.518**	G[15]
L[10]	479.283	802.521	794.511	795.015	794.008	L[14]
G[11]	507.794	745.979	**737.969**	**738.473**	737.465	G[13]
K[12]	571.841	717.468	**709.459**	**709.963**	**708.955**	K[12]
G[13]	600.352	653.421	**645.411**	**645.915**	644.907	G[11]
G[14]	628.863	624.910	**616.900**	**617.404**	616.397	G[10]
A[15]	664.381	596.399	**588.390**	**588.894**	**587.886**	A[9]
K[16]	**728.429**	560.881	**552.871**	**553.375**	**552.367**	K[8]
R[17]	**806.479**	496.833	**488.824**	**489.328**	488.320	R[7]
H[18]	**875.009**	418.782	410.773	411.277	410.269	H[6]
R[19]	**953.059**	350.253	342.244	342.748	341.740	R[5]
K[20]	**1031.122**	**272.202**	264.193	264.697	263.689	K[4]
V[21]	**1080.657**	194.139	186.130	186.634	185.626	V[3]
L[22]	1137.199	144.605	136.596	137.100	136.092	L[2]
R[23]	**1215.249**	88.063	80.054	80.558	79.550	R[1]

sp|P62806|H4_MOUSE

[Acetyl]SGRGKGGKGLGKGGAKRHRK $^{Dimethyl}_{28.03}$ VLR

- fragmentation table for charge state 3+
- specType=etd
- score=148.32
- F097416.dat
- query=q1564_p1
- precursor=608.378550
- chargePrecursor=4
- itol=0.8

AA	c	y	z+1	z+2	z	AA
S[1]	49.697	**810.830**	**805.490**	0.672	**805.154**	S[23]
G[2]	68.704	767.816	**762.476**	0.672	762.140	G[22]
R[3]	120.738	748.809	**743.469**	**743.805**	**743.133**	R[21]
G[4]	139.745	696.775	691.435	691.771	691.099	G[20]
K[5]	182.443	677.768	672.428	672.764	672.092	K[19]
G[6]	201.450	635.069	629.730	630.066	629.394	G[18]
G[7]	220.458	616.062	610.723	611.059	610.387	G[17]
K[8]	263.156	597.055	591.716	592.051	591.380	K[16]
G[9]	282.163	**554.357**	549.017	549.353	548.681	G[15]
L[10]	319.858	535.350	530.010	530.346	529.674	L[14]
G[11]	338.865	497.655	492.315	492.651	491.979	G[13]
K[12]	381.563	478.648	473.308	473.644	472.972	K[12]
G[13]	400.570	435.949	430.610	430.946	430.274	G[11]
G[14]	419.578	**416.942**	411.603	411.939	411.267	G[10]
A[15]	443.257	397.935	392.596	392.932	392.260	A[9]
K[16]	485.955	374.256	368.917	369.252	368.581	K[8]
R[17]	537.989	331.558	326.218	326.554	325.882	R[7]
H[18]	583.675	279.524	274.185	274.520	273.849	H[6]
R[19]	635.709	233.838	228.498	228.834	228.162	R[5]
K[20]	687.751	181.804	176.465	176.800	176.129	K[4]
V[21]	720.774	129.762	124.422	124.758	124.086	V[3]
L[22]	758.468	96.739	91.400	91.736	91.064	L[2]
R[23]	**810.502**	59.045	53.705	54.041	53.369	R[1]

sp|Q6ZWY9|H2B1C_MOUSE

PEPAKSAPAPKKGS $^{Acetyl}_{42.01}$ K $^{Acetyl}_{42.01}$ KAVT $^{Acetyl}_{42.01}$ KAQKK

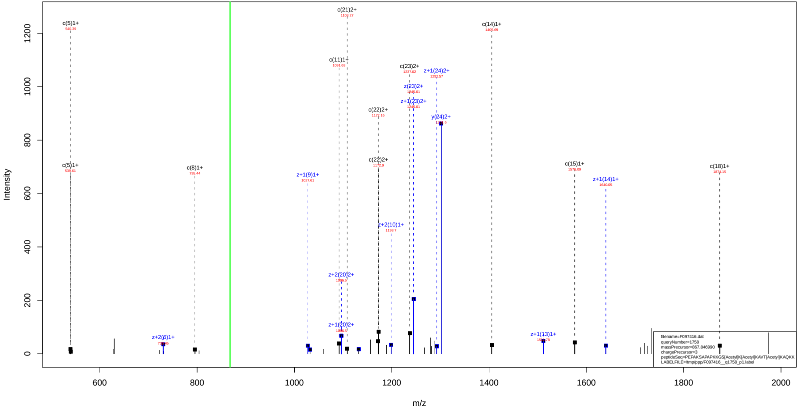

sp|Q6ZWY9|H2B1C_MOUSE

PEPAKSAPAPKKGS $^{Acetyl}_{42.01}$ K $^{Acetyl}_{42.01}$ KAVT $^{Acetyl}_{42.01}$ KAQKK

- fragmentation table for charge state 1+
- specType=etd
- score=30.31
- F097416.dat
- query=q1758_p1
- precursor=867.846990
- chargePrecursor=3
- itol=0.8

AA	c	y	z+1	z+2	z	AA
P[1]	115.087	2601.509	2585.490	0.000	2584.482	P[24]
E[2]	244.129	2504.456	2488.437	0.000	2487.429	E[23]
P[3]	341.182	2375.413	2359.395	0.000	2358.387	P[22]
A[4]	412.219	2278.361	2262.342	0.000	2261.334	A[21]
K[5]	**540.314**	2207.323	2191.305	2192.313	2190.297	K[20]
S[6]	627.346	2079.228	2063.210	2064.218	2062.202	S[19]
A[7]	698.383	1992.196	1976.178	1977.186	1975.170	A[18]
P[8]	**795.436**	1921.159	1905.141	1906.148	1904.133	P[17]
A[9]	866.473	1824.107	1808.088	1809.096	1807.080	A[16]
P[10]	963.526	1753.069	1737.051	1738.059	1736.043	P[15]
K[11]	**1091.621**	1656.017	**1639.998**	1641.006	1638.990	K[14]
K[12]	1219.716	1527.922	**1511.903**	1512.911	1510.895	K[13]
G[13]	1276.737	1399.827	1383.808	1384.816	1382.800	G[12]
S[14]	**1405.780**	1342.805	1326.787	1327.794	1325.779	S[11]
K[15]	**1575.885**	1213.763	1197.744	**1198.752**	1196.736	K[10]
K[16]	1703.980	1043.657	**1027.638**	1028.646	1026.631	K[9]
A[17]	1775.017	915.562	899.543	900.551	898.536	A[8]
V[18]	**1874.086**	844.525	828.506	829.514	827.499	V[7]
T[19]	2017.144	745.457	729.438	**730.446**	728.430	T[6]
K[20]	2145.239	602.398	586.380	587.388	585.372	K[5]
A[21]	2216.276	474.303	458.285	459.293	457.277	A[4]
Q[22]	2344.335	403.266	387.248	388.255	386.240	Q[3]
K[23]	2472.430	275.208	259.189	260.197	258.181	K[2]
K[24]	2600.525	147.113	131.094	132.102	130.086	K[1]

sp|Q6ZWY9|H2B1C_MOUSE

PEPAKSAPAPKKGS $^{Acetyl}_{42.01}$ K $^{Acetyl}_{42.01}$ KAVT $^{Acetyl}_{42.01}$ KAQKK

- fragmentation table for charge state 2+
- specType=etd
- score=30.31
- F097416.dat
- query=q1758_p1
- precursor=867.846990
- chargePrecursor=3
- itol=0.8

AA	c	y	z+1	z+2	z	AA
P[1]	58.047	**1301.258**	**1293.249**	0.504	**1292.745**	P[24]
E[2]	122.568	1252.732	**1244.722**	0.504	**1244.218**	E[23]
P[3]	171.095	1188.210	1180.201	0.504	1179.697	P[22]
A[4]	206.613	1139.684	**1131.675**	0.504	**1131.171**	A[21]
K[5]	270.661	1104.165	**1096.156**	**1096.660**	1095.652	K[20]
S[6]	314.177	1040.118	**1032.109**	**1032.612**	**1031.605**	S[19]
A[7]	349.695	996.602	988.592	989.096	988.089	A[18]
P[8]	398.222	961.083	953.074	953.578	952.570	P[17]
A[9]	433.740	912.557	904.548	905.051	904.044	A[16]
P[10]	482.267	877.038	869.029	869.533	868.525	P[15]
K[11]	546.314	828.512	820.503	821.007	819.999	K[14]
K[12]	610.362	764.464	756.455	756.959	755.951	K[13]
G[13]	638.872	700.417	692.408	692.912	691.904	G[12]
S[14]	703.394	671.906	663.897	664.401	663.393	S[11]
K[15]	788.446	607.385	599.376	599.880	598.872	K[10]
K[16]	852.494	522.332	514.323	514.827	513.819	K[9]
A[17]	888.012	458.285	450.275	450.779	449.771	A[8]
V[18]	937.547	422.766	414.757	415.261	414.253	V[7]
T[19]	1009.076	373.232	365.223	365.727	364.719	T[6]
K[20]	1073.123	301.703	293.693	294.197	293.190	K[5]
A[21]	**1108.642**	237.655	229.646	230.150	229.142	A[4]
Q[22]	**1172.671**	202.137	194.127	194.631	193.624	Q[3]
K[23]	**1236.718**	138.108	130.098	130.602	129.594	K[2]
K[24]	1300.766	74.060	66.051	66.555	65.547	K[1]

sp|Q8R1M2|H2AJ_MOUSE

DEELNKLLGRVTIAQGGVLPNIQAVLLPKKTESQKVKSK $^{\text{Acetyl}}_{42.01}$

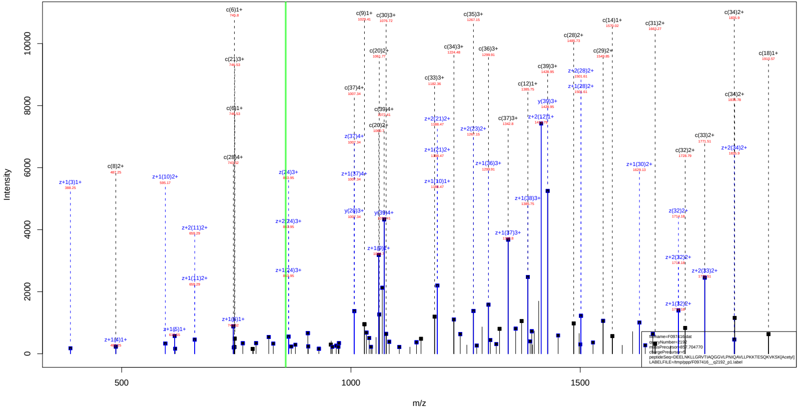

sp|Q8R1M2|H2AJ_MOUSE

DEELNKLLGRVTIAQGGVLPNIQAVLLPKKTESQKVKSK $^{Acetyl}_{42.01}$

- fragmentation table for charge state 1+
- specType=etd
- score=97.18
- F097416.dat
- query=q2192_p1
- precursor=857.704770
- chargePrecursor=5
- itol=0.8

AA	c	y	z+1	z+2	z	AA
D[1]	133.061	4284.465	4268.447	0.000	4267.439	D[39]
E[2]	262.103	4169.438	4153.420	0.000	4152.412	E[38]
E[3]	391.146	4040.396	4024.377	0.000	4023.369	E[37]
L[4]	504.230	3911.353	3895.334	0.000	3894.327	L[36]
N[5]	618.273	3798.269	3782.250	3783.258	3781.243	N[35]
K[6]	**746.368**	3684.226	3668.207	3669.215	3667.200	K[34]
L[7]	859.452	3556.131	3540.113	3541.120	3539.105	L[33]
L[8]	**972.536**	3443.047	3427.028	3428.036	3426.021	L[32]
G[9]	**1029.558**	3329.963	3313.944	3314.952	3312.937	G[31]
R[10]	1185.659	3272.942	3256.923	3257.931	3255.915	R[30]
V[11]	1284.727	3116.841	3100.822	3101.830	3099.814	V[29]
T[12]	**1385.775**	3017.772	3001.753	3002.761	3000.746	T[28]
I[13]	1498.859	2916.724	2900.706	2901.714	2899.698	I[27]
A[14]	**1569.896**	2803.640	2787.622	2788.629	2786.614	A[26]
Q[15]	1697.954	2732.603	2716.585	2717.592	2715.577	Q[25]
G[16]	1754.976	2604.545	2588.526	2589.534	2587.518	G[24]
G[17]	1811.997	2547.523	2531.505	2532.512	2530.497	G[23]
V[18]	**1911.066**	2490.502	2474.483	2475.491	2473.475	V[22]
L[19]	2024.150	2391.433	2375.415	2376.422	2374.407	L[21]
P[20]	2121.203	2278.349	2262.331	2263.338	2261.323	P[20]
N[21]	2235.246	2181.297	2165.278	2166.286	2164.270	N[19]
I[22]	2348.330	2067.254	2051.235	2052.243	2050.227	I[18]
Q[23]	2476.388	1954.170	1938.151	1939.159	1937.143	Q[17]
A[24]	2547.425	1826.111	1810.092	1811.100	1809.084	A[16]
V[25]	2646.494	1755.074	1739.055	1740.063	1738.047	V[15]
L[26]	2759.578	1656.005	1639.987	**1640.995**	1638.979	L[14]
L[27]	2872.662	1542.921	1526.903	**1527.910**	1525.895	L[13]
P[28]	2969.715	1429.837	1413.819	**1414.826**	1412.811	P[12]
K[29]	3097.810	1332.785	**1316.766**	1317.774	1315.758	K[11]
K[30]	3225.905	1204.690	**1188.671**	1189.679	1187.663	K[10]
T[31]	3326.952	**1076.595**	**1060.576**	**1061.584**	1059.568	T[9]
E[32]	3455.995	975.547	**959.528**	960.536	958.520	E[8]
S[33]	3543.027	846.504	**830.486**	831.493	829.478	S[7]
Q[34]	3671.085	759.472	**743.454**	**744.461**	742.446	Q[6]
K[35]	3799.180	631.414	**615.395**	**616.403**	614.387	K[5]
V[36]	3898.249	503.319	**487.300**	488.308	486.292	V[4]
K[37]	4026.344	404.250	**388.232**	389.239	387.224	K[3]
S[38]	4113.376	276.155	260.137	261.144	259.129	S[2]
K[39]	4283.481	189.123	173.105	174.112	172.097	K[1]

sp|Q8R1M2|H2AJ_MOUSE

DEELNKLLGRVTIAQGGVLPNIQAVLLPKKTESQKVKSK $^{Acetyl}_{42.01}$

- fragmentation table for charge state 2+
- specType=etd
- score=97.18
- F097416.dat
- query=q2192_p1
- precursor=857.704770
- chargePrecursor=5
- itol=0.8

AA	c	y	z+1	z+2	z	AA
D[1]	67.034	2142.736	2134.727	0.504	2134.223	D[39]
E[2]	131.555	2085.223	2077.213	0.504	2076.710	E[38]
E[3]	196.077	2020.702	2012.692	0.504	2012.188	E[37]
L[4]	252.619	1956.180	1948.171	0.504	1947.667	L[36]
N[5]	309.640	1899.638	1891.629	1892.133	1891.125	N[35]
K[6]	373.688	1842.617	1834.607	**1835.111**	1834.103	K[34]
L[7]	430.230	1778.569	1770.560	**1771.064**	1770.056	L[33]
L[8]	**486.772**	1722.027	**1714.018**	**1714.522**	**1713.514**	L[32]
G[9]	515.282	1665.485	**1657.476**	**1657.980**	1656.972	G[31]
R[10]	593.333	1636.974	**1628.965**	**1629.469**	**1628.461**	R[30]
V[11]	642.867	1558.924	1550.915	1551.418	**1550.411**	V[29]
T[12]	693.391	1509.390	**1501.380**	**1501.884**	**1500.876**	T[28]
I[13]	749.933	1458.866	1450.857	**1451.360**	1450.353	I[27]
A[14]	**785.452**	1402.324	**1394.314**	**1394.818**	**1393.811**	A[26]
Q[15]	849.481	1366.805	**1358.796**	**1359.300**	1358.292	Q[25]
G[16]	877.992	1302.776	1294.767	1295.271	1294.263	G[24]
G[17]	**906.502**	**1274.265**	1266.256	**1266.760**	1265.752	G[23]
V[18]	**956.037**	1245.755	**1237.745**	**1238.249**	1237.241	V[22]
L[19]	1012.579	1196.220	**1188.211**	**1188.715**	**1187.707**	L[21]
P[20]	**1061.105**	1139.678	1131.669	1132.173	1131.165	P[20]
N[21]	1118.126	1091.152	**1083.143**	**1083.646**	**1082.639**	N[19]
I[22]	1174.668	**1034.130**	1026.121	1026.625	1025.617	I[18]
Q[23]	**1238.698**	977.588	969.579	970.083	969.075	Q[17]
A[24]	**1274.216**	913.559	**905.550**	**906.054**	905.046	A[16]
V[25]	**1323.751**	**878.041**	**870.031**	**870.535**	**869.527**	V[15]
L[26]	1380.293	828.506	**820.497**	**821.001**	**819.993**	L[14]
L[27]	1436.835	771.964	**763.955**	**764.459**	**763.451**	L[13]
P[28]	**1485.361**	715.422	707.413	707.917	706.909	P[12]
K[29]	**1549.408**	666.896	**658.887**	**659.390**	658.383	K[11]
K[30]	1613.456	602.848	**594.839**	**595.343**	594.335	K[10]
T[31]	**1663.980**	538.801	530.792	531.295	530.288	T[9]
E[32]	**1728.501**	488.277	480.268	480.772	479.764	E[8]
S[33]	**1772.017**	423.756	415.746	416.250	415.243	S[7]
Q[34]	**1836.046**	380.240	372.230	372.734	371.727	Q[6]
K[35]	1900.094	316.211	308.201	308.705	307.697	K[5]
V[36]	1949.628	252.163	244.154	244.658	243.650	V[4]
K[37]	2013.676	202.629	194.619	195.123	194.116	K[3]
S[38]	2057.192	138.581	130.572	131.076	130.068	S[2]
K[39]	2142.244	95.065	87.056	87.560	86.552	K[1]

sp|Q8R1M2|H2AJ_MOUSE

DEELNKLLGRVTIAQGGVLPNIQAVLLPKKTESQKVKSK $^{\text{Acetyl}}_{42.01}$

- fragmentation table for charge state 3+
- specType=etd
- score=97.18
- F097416.dat
- query=q2192_p1
- precursor=857.704770
- chargePrecursor=5
- itol=0.8

AA	c	y	z+1	z+2	z	AA
D[1]	45.025	**1428.827**	1423.487	0.672	1423.151	D[39]
E[2]	88.039	**1390.484**	**1385.145**	0.672	1384.809	E[38]
E[3]	131.054	1347.470	**1342.131**	0.672	1341.795	E[37]
L[4]	168.748	**1304.456**	**1299.116**	0.672	1298.780	L[36]
N[5]	206.762	**1266.761**	1261.422	1261.758	1261.086	N[35]
K[6]	249.461	1228.747	1223.407	**1223.743**	1223.071	K[34]
L[7]	287.156	1186.049	1180.709	1181.045	1180.373	L[33]
L[8]	324.850	1148.354	**1143.014**	**1143.350**	**1142.678**	L[32]
G[9]	343.857	1110.659	**1105.320**	**1105.656**	**1104.984**	G[31]
R[10]	395.891	1091.652	1086.312	1086.648	1085.977	R[30]
V[11]	428.914	**1039.618**	**1034.279**	1034.615	**1033.943**	V[29]
T[12]	462.596	**1006.596**	1001.256	1001.592	1000.920	T[28]
I[13]	500.291	**972.913**	**967.573**	**967.909**	**967.237**	I[27]
A[14]	523.970	935.218	**929.879**	**930.215**	**929.543**	A[26]
Q[15]	566.656	911.539	**906.200**	**906.536**	**905.864**	Q[25]
G[16]	585.663	868.853	**863.514**	**863.849**	**863.178**	G[24]
G[17]	604.671	849.846	844.506	844.842	844.170	G[23]
V[18]	637.693	**830.839**	825.499	825.835	825.163	V[22]
L[19]	675.388	797.816	**792.476**	**792.812**	792.140	L[21]
P[20]	707.739	760.121	754.782	755.118	754.446	P[20]
N[21]	**745.753**	727.770	722.431	722.767	722.095	N[19]
I[22]	783.448	689.756	684.416	684.752	684.081	I[18]
Q[23]	826.134	652.061	646.722	647.058	646.386	Q[17]
A[24]	849.813	609.375	604.036	604.372	603.700	A[16]
V[25]	882.836	585.696	580.357	580.692	580.021	V[15]
L[26]	920.531	552.673	547.334	547.670	546.998	L[14]
L[27]	958.225	514.979	509.639	509.975	509.303	L[13]
P[28]	990.576	477.284	471.944	472.280	471.608	P[12]
K[29]	**1033.275**	444.933	439.593	439.929	439.258	K[11]
K[30]	**1075.973**	402.235	396.895	397.231	396.559	K[10]
T[31]	1109.656	359.536	354.197	354.533	353.861	T[9]
E[32]	**1152.670**	325.854	320.514	320.850	320.178	E[8]
S[33]	**1181.680**	282.840	277.500	277.836	277.164	S[7]
Q[34]	**1224.367**	253.829	248.489	248.825	248.153	Q[6]
K[35]	**1267.065**	211.143	205.803	206.139	205.467	K[5]
V[36]	**1300.088**	168.444	163.105	163.441	162.769	V[4]
K[37]	**1342.786**	135.422	130.082	130.418	129.746	K[3]
S[38]	**1371.797**	92.723	87.384	87.720	87.048	S[2]
K[39]	**1428.499**	63.713	58.373	58.709	58.037	K[1]

sp|Q8R1M2|H2AJ_MOUSE

DEELNKLLGRVTIAQGGVLPNIQAVLLPKKTESQKVKSK $^{\text{Acetyl}}_{42.01}$

- fragmentation table for charge state 4+
- specType=etd
- score=97.18
- F097416.dat
- query=q2192_p1
- precursor=857.704770
- chargePrecursor=5
- itol=0.8

AA	c	y	z+1	z+2	z	AA
D[1]	34.021	**1071.872**	**1067.867**	0.755	**1067.615**	D[39]
E[2]	66.281	**1043.115**	**1039.110**	0.755	1038.858	E[38]
E[3]	98.542	1010.854	**1006.850**	0.755	**1006.598**	E[37]
L[4]	126.813	978.594	**974.589**	0.755	**974.337**	L[36]
N[5]	155.324	950.323	946.318	946.570	946.066	N[35]
K[6]	187.347	921.812	917.807	918.059	917.555	K[34]
L[7]	215.618	889.788	885.784	886.036	885.532	L[33]
L[8]	243.889	861.517	857.513	857.765	857.261	L[32]
G[9]	258.145	833.246	829.242	829.494	828.990	G[31]
R[10]	297.170	818.991	814.986	815.238	814.734	R[30]
V[11]	321.937	779.966	775.961	776.213	775.709	V[29]
T[12]	347.199	755.198	751.194	751.446	750.942	T[28]
I[13]	375.470	729.937	725.932	726.184	725.680	I[27]
A[14]	393.229	701.666	697.661	697.913	697.409	A[26]
Q[15]	425.244	683.906	679.902	680.154	679.650	Q[25]
G[16]	439.499	651.892	647.887	648.139	647.635	G[24]
G[17]	453.755	637.636	633.632	633.884	633.380	G[23]
V[18]	478.522	623.381	619.376	619.628	619.124	V[22]
L[19]	506.793	598.614	**594.609**	**594.861**	594.357	L[21]
P[20]	531.056	570.343	566.338	566.590	566.086	P[20]
N[21]	559.567	546.080	542.075	542.327	541.823	N[19]
I[22]	587.838	517.569	513.564	513.816	513.312	I[18]
Q[23]	619.853	489.298	485.293	485.545	485.041	Q[17]
A[24]	637.612	457.283	453.279	453.530	453.027	A[16]
V[25]	662.379	439.524	435.519	435.771	435.267	V[15]
L[26]	690.650	414.757	410.752	411.004	410.500	L[14]
L[27]	718.921	386.486	382.481	382.733	382.229	L[13]
P[28]	**743.184**	358.215	354.210	354.462	353.958	P[12]
K[29]	775.208	333.952	329.947	330.199	329.695	K[11]
K[30]	807.232	301.928	297.923	298.175	297.671	K[10]
T[31]	832.494	269.904	265.899	266.151	265.647	T[9]
E[32]	864.754	244.642	240.638	240.889	240.386	E[8]
S[33]	886.512	212.382	208.377	208.629	208.125	S[7]
Q[34]	918.527	190.624	186.619	186.871	186.367	Q[6]
K[35]	950.551	158.609	154.604	154.856	154.352	K[5]
V[36]	975.318	126.585	122.580	122.832	122.329	V[4]
K[37]	**1007.341**	101.818	97.813	98.065	97.561	K[3]
S[38]	**1029.099**	69.794	65.790	66.042	65.538	S[2]
K[39]	**1071.626**	48.036	44.032	44.284	43.780	K[1]

sp|Q6GSS7|H2A2A_MOUSE

DEELNKLLGK Dimethyl 28.03 VTIAQGGVLPNIQAVLLPKKTESHHKAKGK

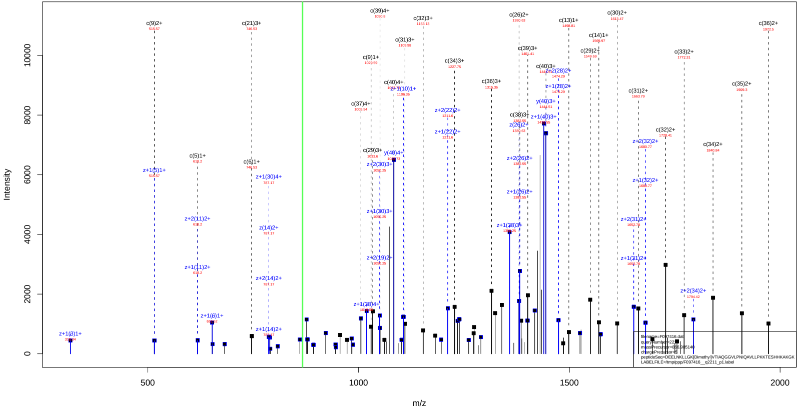

sp|Q6GSS7|H2A2A_MOUSE

DEELNKLLGK $^{Dimethyl}_{28.03}$ VTIAQGGVLPNIQAVLLPKKTESHHKAKGK

- fragmentation table for charge state 1+
- specType=etd
- score=100.64
- F097416.dat
- query=q2211_p1
- precursor=866.905140
- chargePrecursor=5
- itol=0.8

AA	c	y	z+1	z+2	z	AA
D[1]	133.061	4330.497	4314.479	0.000	4313.471	D[40]
E[2]	262.103	4215.470	4199.452	0.000	4198.444	E[39]
E[3]	391.146	4086.428	4070.409	0.000	4069.401	E[38]
L[4]	504.230	3957.385	3941.366	0.000	3940.359	L[37]
N[5]	**618.273**	3844.301	3828.282	3829.290	3827.275	N[36]
K[6]	**746.368**	3730.258	3714.239	3715.247	3713.232	K[35]
L[7]	859.452	3602.163	3586.144	3587.152	3585.137	L[34]
L[8]	**972.536**	3489.079	3473.060	3474.068	3472.053	L[33]
G[9]	**1029.558**	3375.995	3359.976	3360.984	3358.969	G[32]
K[10]	1185.684	3318.974	3302.955	3303.963	3301.947	K[31]
V[11]	1284.752	3162.847	3146.829	3147.836	3145.821	V[30]
T[12]	**1385.800**	3063.779	3047.760	3048.768	3046.752	T[29]
I[13]	**1498.884**	2962.731	2946.713	2947.720	2945.705	I[28]
A[14]	**1569.921**	2849.647	2833.628	2834.636	2832.621	A[27]
Q[15]	**1697.980**	2778.610	2762.591	2763.599	2761.584	Q[26]
G[16]	**1755.001**	2650.552	2634.533	2635.541	2633.525	G[25]
G[17]	1812.023	2593.530	2577.511	2578.519	2576.504	G[24]
V[18]	1911.091	2536.509	2520.490	2521.498	2519.482	V[23]
L[19]	2024.175	2437.440	2421.421	2422.429	2420.414	L[22]
P[20]	2121.228	2324.356	2308.337	2309.345	2307.330	P[21]
N[21]	2235.271	2227.303	2211.285	2212.292	2210.277	N[20]
I[22]	2348.355	2113.260	2097.242	2098.250	2096.234	I[19]
Q[23]	2476.413	2000.176	1984.158	1985.165	1983.150	Q[18]
A[24]	2547.450	1872.118	1856.099	1857.107	1855.091	A[17]
V[25]	2646.519	1801.081	1785.062	1786.070	1784.054	V[16]
L[26]	2759.603	1702.012	1685.994	1687.001	1684.986	L[15]
L[27]	2872.687	1588.928	1572.909	**1573.917**	1571.902	L[14]
P[28]	2969.740	1475.844	1459.825	1460.833	1458.818	P[13]
K[29]	3097.835	1378.791	1362.773	1363.780	1361.765	K[12]
K[30]	3225.930	1250.696	**1234.678**	1235.685	1233.670	K[11]
T[31]	3326.977	1122.601	**1106.583**	1107.591	**1105.575**	T[10]
E[32]	3456.020	1021.554	**1005.535**	1006.543	1004.527	E[9]
S[33]	3543.052	**892.511**	**876.492**	877.500	875.485	S[8]
H[34]	3680.111	805.479	**789.460**	**790.468**	788.453	H[7]
H[35]	3817.170	668.420	**652.401**	**653.409**	651.394	H[6]
K[36]	3945.265	531.361	**515.343**	**516.350**	514.335	K[5]
A[37]	4016.302	403.266	387.248	388.255	386.240	A[4]
K[38]	4144.397	332.229	**316.211**	317.218	315.203	K[3]
G[39]	4201.418	204.134	188.116	189.123	187.108	G[2]
K[40]	4329.513	147.113	131.094	132.102	130.086	K[1]

sp|Q6GSS7|H2A2A_MOUSE

DEELNKLLGK $^{\text{Dimethyl}}_{28.03}$ VTIAQGGVLPNIQAVLLPKKTESHHKAKGK

- fragmentation table for charge state 2+
- specType=etd
- score=100.64
- F097416.dat
- query=q2211_p1
- precursor=866.905140
- chargePrecursor=5
- itol=0.8

AA	c	y	z+1	z+2	z	AA
D[1]	67.034	2165.752	2157.743	0.504	2157.239	D[40]
E[2]	131.555	2108.239	2100.229	0.504	2099.726	E[39]
E[3]	196.077	2043.718	2035.708	0.504	2035.204	E[38]
L[4]	252.619	1979.196	1971.187	0.504	1970.683	L[37]
N[5]	309.640	1922.654	1914.645	1915.149	1914.141	N[36]
K[6]	373.688	1865.633	1857.623	1858.127	1857.119	K[35]
L[7]	430.230	1801.585	1793.576	**1794.080**	1793.072	L[34]
L[8]	486.772	1745.043	1737.034	1737.538	1736.530	L[33]
G[9]	**515.282**	1688.501	**1680.492**	**1680.996**	**1679.988**	G[32]
K[10]	593.346	1659.990	**1651.981**	**1652.485**	1651.477	K[31]
V[11]	642.880	1581.927	**1573.918**	**1574.422**	**1573.414**	V[30]
T[12]	693.404	1532.393	**1524.384**	**1524.888**	1523.880	T[29]
I[13]	749.946	1481.869	**1473.860**	**1474.364**	1473.356	I[28]
A[14]	785.464	1425.327	**1417.318**	**1417.822**	1416.814	A[27]
Q[15]	849.493	1389.809	**1381.799**	**1382.303**	**1381.295**	Q[26]
G[16]	**878.004**	1325.779	1317.770	1318.274	1317.266	G[25]
G[17]	906.515	1297.269	1289.259	**1289.763**	1288.755	G[24]
V[18]	**956.049**	1268.758	**1260.749**	**1261.252**	1260.245	V[23]
L[19]	1012.591	1219.224	**1211.214**	**1211.718**	1210.710	L[22]
P[20]	**1061.118**	1162.682	1154.672	1155.176	1154.168	P[21]
N[21]	1118.139	1114.155	**1106.146**	**1106.650**	**1105.642**	N[20]
I[22]	1174.681	1057.134	1049.124	**1049.628**	1048.621	I[19]
Q[23]	**1238.710**	1000.592	992.582	993.086	992.079	Q[18]
A[24]	**1274.229**	936.563	928.553	929.057	928.049	A[17]
V[25]	**1323.763**	901.044	**893.035**	**893.539**	**892.531**	V[16]
L[26]	**1380.305**	851.510	843.500	844.004	842.996	L[15]
L[27]	1436.847	794.968	**786.958**	**787.462**	**786.454**	L[14]
P[28]	**1485.374**	738.426	730.416	730.920	729.912	P[13]
K[29]	**1549.421**	689.899	**681.890**	**682.394**	**681.386**	K[12]
K[30]	**1613.468**	625.852	**617.842**	**618.346**	617.339	K[11]
T[31]	**1663.992**	561.804	553.795	554.299	553.291	T[10]
E[32]	**1728.514**	511.281	503.271	503.775	502.767	E[9]
S[33]	**1772.030**	446.759	438.750	439.254	438.246	S[8]
H[34]	**1840.559**	403.243	395.234	395.738	394.730	H[7]
H[35]	**1909.089**	334.714	326.704	327.208	326.200	H[6]
K[36]	**1973.136**	266.184	258.175	258.679	257.671	K[5]
A[37]	2008.655	202.137	194.127	194.631	193.624	A[4]
K[38]	2072.702	166.618	158.609	159.113	158.105	K[3]
G[39]	2101.213	102.571	94.561	95.065	94.057	G[2]
K[40]	2165.260	74.060	66.051	66.555	65.547	K[1]

sp|Q6GSS7|H2A2A_MOUSE

DEELNKLLGK $^{Dimethyl}_{28.03}$ VTIAQGGVLPNIQAVLLPKKTESHHKAKGK

- fragmentation table for charge state 3+
- specType=etd
- score=100.64
- F097416.dat
- query=q2211_p1
- precursor=866.905140
- chargePrecursor=5
- itol=0.8

AA	c	y	z+1	z+2	z	AA
D[1]	45.025	**1444.171**	**1438.831**	0.672	1438.495	D[40]
E[2]	88.039	1405.828	**1400.489**	0.672	**1400.153**	E[39]
E[3]	131.054	1362.814	**1357.475**	0.672	1357.139	E[38]
L[4]	168.748	1319.800	1314.460	0.672	1314.124	L[37]
N[5]	206.762	1282.105	1276.766	1277.102	1276.430	N[36]
K[6]	249.461	1244.091	**1238.751**	**1239.087**	**1238.415**	K[35]
L[7]	287.156	1201.393	**1196.053**	**1196.389**	**1195.717**	L[34]
L[8]	324.850	1163.698	1158.358	1158.694	1158.022	L[33]
G[9]	343.857	1126.003	1120.664	1121.000	1120.328	G[32]
K[10]	395.899	**1106.996**	**1101.656**	**1101.992**	**1101.321**	K[31]
V[11]	428.922	1054.954	**1049.614**	**1049.950**	1049.278	V[30]
T[12]	462.605	1021.931	1016.592	1016.928	1016.256	T[29]
I[13]	500.299	988.249	**982.909**	**983.245**	982.573	I[28]
A[14]	523.979	950.554	**945.214**	**945.550**	**944.878**	A[27]
Q[15]	566.665	926.875	**921.535**	**921.871**	**921.199**	Q[26]
G[16]	585.672	884.189	**878.849**	**879.185**	**878.513**	G[25]
G[17]	604.679	865.182	**859.842**	**860.178**	859.506	G[24]
V[18]	637.702	846.174	840.835	841.171	840.499	V[23]
L[19]	675.397	813.152	**807.812**	**808.148**	**807.476**	L[22]
P[20]	707.747	775.457	770.117	770.453	769.781	P[21]
N[21]	**745.762**	743.106	737.766	738.102	737.430	N[20]
I[22]	783.456	705.092	699.752	700.088	699.416	I[19]
Q[23]	826.143	667.397	662.057	662.393	661.721	Q[18]
A[24]	849.822	624.711	619.371	619.707	619.035	A[17]
V[25]	882.844	601.032	595.692	596.028	595.356	V[16]
L[26]	920.539	568.009	562.669	563.005	562.333	L[15]
L[27]	958.234	530.314	524.975	525.311	524.639	L[14]
P[28]	990.585	492.620	487.280	487.616	486.944	P[13]
K[29]	**1033.283**	460.269	454.929	455.265	454.593	K[12]
K[30]	1075.981	417.570	412.231	412.567	411.895	K[11]
T[31]	**1109.664**	374.872	369.532	369.868	369.196	T[10]
E[32]	**1152.678**	341.189	335.850	336.186	335.514	E[9]
S[33]	**1181.689**	298.175	292.836	293.172	292.500	S[8]
H[34]	**1227.375**	269.165	263.825	264.161	263.489	H[7]
H[35]	**1273.061**	223.478	218.139	218.475	217.803	H[6]
K[36]	**1315.760**	177.792	172.452	172.788	172.116	K[5]
A[37]	**1339.439**	135.094	129.754	130.090	129.418	A[4]
K[38]	**1382.137**	111.415	106.075	106.411	105.739	K[3]
G[39]	**1401.144**	68.716	63.377	63.713	63.041	G[2]
K[40]	**1443.843**	49.709	44.370	44.705	44.034	K[1]

sp|Q6GSS7|H2A2A_MOUSE

DEELNKLLGK $^{\text{Dimethyl}}_{28.03}$ VTIAQGGVLPNIQAVLLPKKTESHHKAKGK

- fragmentation table for charge state 4+
- specType=etd
- score=100.64
- F097416.dat
- query=q2211_p1
- precursor=866.905140
- chargePrecursor=5
- itol=0.8

AA	c	y	z+1	z+2	z	AA
D[1]	34.021	**1083.380**	1079.375	0.755	1079.123	D[40]
E[2]	66.281	1054.623	**1050.618**	0.755	**1050.366**	E[39]
E[3]	98.542	1022.362	**1018.358**	0.755	1018.106	E[38]
L[4]	126.813	990.102	**986.097**	0.755	**985.845**	L[37]
N[5]	155.324	961.831	957.826	958.078	957.574	N[36]
K[6]	187.347	933.320	929.315	929.567	929.063	K[35]
L[7]	215.618	901.296	897.292	897.544	897.040	L[34]
L[8]	243.889	873.025	869.021	869.273	868.769	L[33]
G[9]	258.145	844.754	840.750	841.002	840.498	G[32]
K[10]	297.176	830.499	826.494	826.746	826.242	K[31]
V[11]	321.944	791.467	**787.463**	**787.715**	**787.211**	V[30]
T[12]	347.205	766.700	762.696	762.947	762.444	T[29]
I[13]	375.476	741.438	737.434	737.686	737.182	I[28]
A[14]	393.236	713.167	709.163	709.415	708.911	A[27]
Q[15]	425.250	695.408	691.403	691.655	691.151	Q[26]
G[16]	439.506	663.393	659.389	659.641	659.137	G[25]
G[17]	453.761	649.138	645.133	645.385	644.881	G[24]
V[18]	478.528	634.883	630.878	631.130	630.626	V[23]
L[19]	506.799	610.116	606.111	606.363	605.859	L[22]
P[20]	531.062	581.844	577.840	578.092	577.588	P[21]
N[21]	559.573	557.581	553.577	553.829	553.325	N[20]
I[22]	587.844	529.071	525.066	525.318	524.814	I[19]
Q[23]	619.859	500.800	496.795	497.047	496.543	Q[18]
A[24]	637.618	468.785	464.780	465.032	464.528	A[17]
V[25]	662.385	451.026	447.021	447.273	446.769	V[16]
L[26]	690.656	426.259	422.254	422.506	422.002	L[15]
L[27]	718.927	397.988	393.983	394.235	393.731	L[14]
P[28]	743.190	369.716	365.712	365.964	365.460	P[13]
K[29]	775.214	345.453	341.449	341.701	341.197	K[12]
K[30]	807.238	313.430	309.425	309.677	309.173	K[11]
T[31]	832.500	281.406	277.401	277.653	277.149	T[10]
E[32]	864.760	256.144	252.139	252.391	251.887	E[9]
S[33]	886.518	223.883	219.879	220.131	219.627	S[8]
H[34]	920.783	202.125	198.121	198.373	197.869	H[7]
H[35]	955.048	167.861	163.856	164.108	163.604	H[6]
K[36]	**987.072**	133.596	129.591	129.843	129.339	K[5]
A[37]	**1004.831**	101.572	97.567	97.819	97.315	A[4]
K[38]	1036.855	83.813	79.808	80.060	79.556	K[3]
G[39]	**1051.110**	51.789	47.784	48.036	47.532	G[2]
K[40]	**1083.134**	37.534	33.529	33.781	33.277	K[1]

sp|Q6GSS7|H2A2A_MOUSE

DEELNKLLGK Dimethyl 28.03 VTIAQGGVLPNIQAVLLPKKTESHHKAKGK

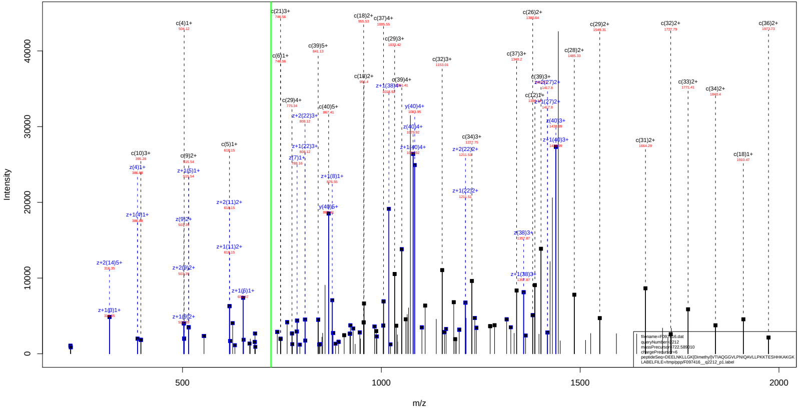

sp|Q6GSS7|H2A2A_MOUSE

DEELNKLLGK $^{\text{Dimethyl}}_{28.03}$ VTIAQGGVLPNIQAVLLPKKTESHHKAKGK

- fragmentation table for charge state 1+
- specType=etd
- score=47.59
- F097416.dat
- query=q2212_p1
- precursor=722.589010
- chargePrecursor=6
- itol=0.8

AA	c	y	z+1	z+2	z	AA
D[1]	133.061	4330.497	4314.479	0.000	4313.471	D[40]
E[2]	262.103	4215.470	4199.452	0.000	4198.444	E[39]
E[3]	391.146	4086.428	4070.409	0.000	4069.401	E[38]
L[4]	**504.230**	3957.385	3941.366	0.000	3940.359	L[37]
N[5]	**618.273**	3844.301	3828.282	3829.290	3827.275	N[36]
K[6]	**746.368**	3730.258	3714.239	3715.247	3713.232	K[35]
L[7]	859.452	3602.163	3586.144	3587.152	3585.137	L[34]
L[8]	972.536	3489.079	3473.060	3474.068	3472.053	L[33]
G[9]	1029.558	3375.995	3359.976	3360.984	3358.969	G[32]
K[10]	**1185.684**	3318.974	3302.955	3303.963	3301.947	K[31]
V[11]	**1284.752**	3162.847	3146.829	3147.836	3145.821	V[30]
T[12]	**1385.800**	3063.779	3047.760	3048.768	3046.752	T[29]
I[13]	1498.884	2962.731	2946.713	2947.720	2945.705	I[28]
A[14]	1569.921	2849.647	2833.628	2834.636	2832.621	A[27]
Q[15]	1697.980	2778.610	2762.591	2763.599	2761.584	Q[26]
G[16]	1755.001	2650.552	2634.533	2635.541	2633.525	G[25]
G[17]	1812.023	2593.530	2577.511	2578.519	2576.504	G[24]
V[18]	**1911.091**	2536.509	2520.490	2521.498	2519.482	V[23]
L[19]	2024.175	2437.440	2421.421	2422.429	2420.414	L[22]
P[20]	2121.228	2324.356	2308.337	2309.345	2307.330	P[21]
N[21]	2235.271	2227.303	2211.285	2212.292	2210.277	N[20]
I[22]	2348.355	2113.260	2097.242	2098.250	2096.234	I[19]
Q[23]	2476.413	2000.176	1984.158	1985.165	1983.150	Q[18]
A[24]	2547.450	1872.118	1856.099	1857.107	1855.091	A[17]
V[25]	2646.519	1801.081	1785.062	1786.070	1784.054	V[16]
L[26]	2759.603	1702.012	1685.994	1687.001	1684.986	L[15]
L[27]	2872.687	1588.928	1572.909	1573.917	1571.902	L[14]
P[28]	2969.740	1475.844	1459.825	1460.833	1458.818	P[13]
K[29]	3097.835	1378.791	**1362.773**	1363.780	1361.765	K[12]
K[30]	3225.930	1250.696	**1234.678**	**1235.685**	1233.670	K[11]
T[31]	3326.977	1122.601	1106.583	1107.591	1105.575	T[10]
E[32]	3456.020	1021.554	**1005.535**	1006.543	**1004.527**	E[9]
S[33]	3543.052	**892.511**	**876.492**	877.500	875.485	S[8]
H[34]	3680.111	805.479	789.460	790.468	**788.453**	H[7]
H[35]	3817.170	**668.420**	**652.401**	**653.409**	651.394	H[6]
K[36]	3945.265	531.361	**515.343**	516.350	514.335	K[5]
A[37]	4016.302	403.266	**387.248**	388.255	**386.240**	A[4]
K[38]	4144.397	332.229	**316.211**	317.218	315.203	K[3]
G[39]	4201.418	204.134	188.116	189.123	187.108	G[2]
K[40]	4329.513	147.113	131.094	132.102	130.086	K[1]

sp|Q6GSS7|H2A2A_MOUSE

DEELNKLLGK $^{Dimethyl}_{28.03}$ VTIAQGGVLPNIQAVLLPKKTESHHKAKGK

- fragmentation table for charge state 2+
- specType=etd
- score=47.59
- F097416.dat
- query=q2212_p1
- precursor=722.589010
- chargePrecursor=6
- itol=0.8

AA	c	y	z+1	z+2	z	AA
D[1]	67.034	2165.752	2157.743	0.504	2157.239	D[40]
E[2]	131.555	2108.239	2100.229	0.504	2099.726	E[39]
E[3]	196.077	2043.718	2035.708	0.504	2035.204	E[38]
L[4]	252.619	1979.196	1971.187	0.504	1970.683	L[37]
N[5]	309.640	1922.654	1914.645	1915.149	1914.141	N[36]
K[6]	373.688	1865.633	1857.623	1858.127	1857.119	K[35]
L[7]	430.230	1801.585	1793.576	1794.080	1793.072	L[34]
L[8]	486.772	1745.043	1737.034	1737.538	1736.530	L[33]
G[9]	**515.282**	1688.501	1680.492	1680.996	1679.988	G[32]
K[10]	593.346	1659.990	1651.981	1652.485	1651.477	K[31]
V[11]	642.880	1581.927	1573.918	1574.422	1573.414	V[30]
T[12]	693.404	1532.393	1524.384	1524.888	1523.880	T[29]
I[13]	749.946	1481.869	1473.860	1474.364	1473.356	I[28]
A[14]	785.464	1425.327	**1417.318**	**1417.822**	1416.814	A[27]
Q[15]	849.493	1389.809	1381.799	1382.303	**1381.295**	Q[26]
G[16]	**878.004**	**1325.779**	1317.770	1318.274	1317.266	G[25]
G[17]	**906.515**	1297.269	1289.259	1289.763	1288.755	G[24]
V[18]	**956.049**	1268.758	1260.749	1261.252	1260.245	V[23]
L[19]	1012.591	1219.224	**1211.214**	**1211.718**	**1210.710**	L[22]
P[20]	**1061.118**	**1162.682**	1154.672	1155.176	1154.168	P[21]
N[21]	1118.139	1114.155	1106.146	1106.650	1105.642	N[20]
I[22]	1174.681	1057.134	1049.124	1049.628	1048.621	I[19]
Q[23]	**1238.710**	1000.592	992.582	993.086	992.079	Q[18]
A[24]	**1274.229**	936.563	**928.553**	**929.057**	928.049	A[17]
V[25]	1323.763	901.044	**893.035**	893.539	**892.531**	V[16]
L[26]	**1380.305**	851.510	**843.500**	**844.004**	842.996	L[15]
L[27]	1436.847	**794.968**	**786.958**	**787.462**	786.454	L[14]
P[28]	**1485.374**	**738.426**	730.416	730.920	729.912	P[13]
K[29]	**1549.421**	689.899	**681.890**	**682.394**	**681.386**	K[12]
K[30]	1613.468	**625.852**	**617.842**	**618.346**	617.339	K[11]
T[31]	**1663.992**	561.804	**553.795**	**554.299**	**553.291**	T[10]
E[32]	**1728.514**	511.281	**503.271**	**503.775**	**502.767**	E[9]
S[33]	**1772.030**	446.759	438.750	439.254	438.246	S[8]
H[34]	**1840.559**	403.243	**395.234**	**395.738**	**394.730**	H[7]
H[35]	1909.089	334.714	326.704	327.208	326.200	H[6]
K[36]	**1973.136**	266.184	258.175	258.679	257.671	K[5]
A[37]	2008.655	202.137	194.127	194.631	193.624	A[4]
K[38]	2072.702	166.618	158.609	159.113	158.105	K[3]
G[39]	2101.213	102.571	94.561	95.065	94.057	G[2]
K[40]	2165.260	74.060	66.051	66.555	65.547	K[1]

sp|Q6GSS7|H2A2A_MOUSE

DEELNKLLGK $^{\text{Dimethyl}}_{28.03}$ VTIAQGGVLPNIQAVLLPKKTESHHKAKGK

- fragmentation table for charge state 3+
- specType=etd
- score=47.59
- F097416.dat
- query=q2212_p1
- precursor=722.589010
- chargePrecursor=6
- itol=0.8

AA	c	y	z+1	z+2	z	AA
D[1]	45.025	1444.171	**1438.831**	0.672	**1438.495**	D[40]
E[2]	88.039	1405.828	1400.489	0.672	1400.153	E[39]
E[3]	131.054	**1362.814**	**1357.475**	0.672	**1357.139**	E[38]
L[4]	168.748	1319.800	**1314.460**	0.672	1314.124	L[37]
N[5]	206.762	1282.105	1276.766	1277.102	1276.430	N[36]
K[6]	249.461	1244.091	**1238.751**	**1239.087**	**1238.415**	K[35]
L[7]	287.156	1201.393	**1196.053**	**1196.389**	**1195.717**	L[34]
L[8]	324.850	1163.698	**1158.358**	**1158.694**	**1158.022**	L[33]
G[9]	343.857	1126.003	1120.664	1121.000	1120.328	G[32]
K[10]	**395.899**	1106.996	**1101.656**	**1101.992**	**1101.321**	K[31]
V[11]	428.922	1054.954	1049.614	1049.950	1049.278	V[30]
T[12]	462.605	1021.931	1016.592	1016.928	1016.256	T[29]
I[13]	500.299	**988.249**	**982.909**	**983.245**	**982.573**	I[28]
A[14]	523.979	950.554	**945.214**	**945.550**	944.878	A[27]
Q[15]	566.665	926.875	**921.535**	**921.871**	**921.199**	Q[26]
G[16]	585.672	**884.189**	**878.849**	**879.185**	**878.513**	G[25]
G[17]	604.679	865.182	859.842	860.178	859.506	G[24]
V[18]	637.702	846.174	**840.835**	**841.171**	**840.499**	V[23]
L[19]	675.397	813.152	**807.812**	**808.148**	**807.476**	L[22]
P[20]	707.747	**775.457**	770.117	770.453	769.781	P[21]
N[21]	**745.762**	743.106	**737.766**	**738.102**	737.430	N[20]
I[22]	783.456	705.092	699.752	700.088	699.416	I[19]
Q[23]	826.143	667.397	662.057	662.393	661.721	Q[18]
A[24]	849.822	624.711	**619.371**	**619.707**	**619.035**	A[17]
V[25]	882.844	601.032	595.692	596.028	595.356	V[16]
L[26]	**920.539**	568.009	562.669	563.005	562.333	L[15]
L[27]	958.234	530.314	524.975	525.311	524.639	L[14]
P[28]	990.585	492.620	487.280	487.616	486.944	P[13]
K[29]	**1033.283**	460.269	454.929	455.265	454.593	K[12]
K[30]	1075.981	417.570	412.231	412.567	411.895	K[11]
T[31]	**1109.664**	374.872	369.532	369.868	369.196	T[10]
E[32]	**1152.678**	341.189	335.850	336.186	335.514	E[9]
S[33]	**1181.689**	298.175	292.836	293.172	292.500	S[8]
H[34]	**1227.375**	269.165	263.825	264.161	263.489	H[7]
H[35]	**1273.061**	223.478	**218.139**	**218.475**	**217.803**	H[6]
K[36]	**1315.760**	177.792	172.452	172.788	172.116	K[5]
A[37]	**1339.439**	135.094	129.754	130.090	129.418	A[4]
K[38]	1382.137	111.415	106.075	106.411	105.739	K[3]
G[39]	**1401.144**	68.716	63.377	63.713	63.041	G[2]
K[40]	1443.843	49.709	44.370	44.705	44.034	K[1]

sp|Q6GSS7|H2A2A_MOUSE

DEELNKLLGK $^{\text{Dimethyl}}_{28.03}$ VTIAQGGVLPNIQAVLLPKKTESHHKAKGK

- fragmentation table for charge state 4+
- specType=etd
- score=47.59
- F097416.dat
- query=q2212_p1
- precursor=722.589010
- chargePrecursor=6
- itol=0.8

AA	c	y	z+1	z+2	z	AA
D[1]	34.021	**1083.380**	**1079.375**	0.755	**1079.123**	D[40]
E[2]	66.281	1054.623	**1050.618**	0.755	1050.366	E[39]
E[3]	98.542	**1022.362**	**1018.358**	0.755	1018.106	E[38]
L[4]	126.813	990.102	986.097	0.755	985.845	L[37]
N[5]	155.324	961.831	957.826	958.078	957.574	N[36]
K[6]	187.347	933.320	**929.315**	**929.567**	**929.063**	K[35]
L[7]	215.618	901.296	897.292	897.544	897.040	L[34]
L[8]	243.889	873.025	869.021	869.273	868.769	L[33]
G[9]	258.145	**844.754**	**840.750**	**841.002**	**840.498**	G[32]
K[10]	297.176	830.499	826.494	826.746	826.242	K[31]
V[11]	321.944	791.467	**787.463**	**787.715**	**787.211**	V[30]
T[12]	347.205	766.700	**762.696**	**762.947**	**762.444**	T[29]
I[13]	375.476	741.438	737.434	**737.686**	737.182	I[28]
A[14]	393.236	713.167	709.163	709.415	708.911	A[27]
Q[15]	425.250	695.408	691.403	691.655	691.151	Q[26]
G[16]	439.506	663.393	659.389	659.641	659.137	G[25]
G[17]	453.761	649.138	645.133	645.385	644.881	G[24]
V[18]	478.528	634.883	630.878	**631.130**	630.626	V[23]
L[19]	506.799	610.116	606.111	606.363	605.859	L[22]
P[20]	531.062	581.844	577.840	578.092	577.588	P[21]
N[21]	559.573	557.581	**553.577**	**553.829**	**553.325**	N[20]
I[22]	587.844	529.071	525.066	525.318	524.814	I[19]
Q[23]	**619.859**	500.800	496.795	497.047	496.543	Q[18]
A[24]	637.618	468.785	464.780	465.032	464.528	A[17]
V[25]	662.385	451.026	447.021	447.273	446.769	V[16]
L[26]	690.656	426.259	422.254	422.506	422.002	L[15]
L[27]	718.927	397.988	393.983	394.235	393.731	L[14]
P[28]	743.190	369.716	365.712	365.964	365.460	P[13]
K[29]	**775.214**	345.453	341.449	341.701	341.197	K[12]
K[30]	807.238	313.430	309.425	309.677	309.173	K[11]
T[31]	832.500	281.406	277.401	277.653	277.149	T[10]
E[32]	864.760	256.144	252.139	252.391	251.887	E[9]
S[33]	886.518	223.883	219.879	220.131	**219.627**	S[8]
H[34]	**920.783**	202.125	198.121	198.373	197.869	H[7]
H[35]	**955.048**	167.861	163.856	164.108	163.604	H[6]
K[36]	**987.072**	133.596	129.591	129.843	129.339	K[5]
A[37]	**1004.831**	101.572	97.567	97.819	97.315	A[4]
K[38]	**1036.855**	83.813	79.808	80.060	79.556	K[3]
G[39]	**1051.110**	51.789	47.784	48.036	47.532	G[2]
K[40]	1083.134	37.534	33.529	33.781	33.277	K[1]

sp|Q6GSS7|H2A2A_MOUSE

DEELNKLLGK $^{Dimethyl}_{28.03}$ VTIAQGGVLPNIQAVLLPKKTESHHKAKGK

- fragmentation table for charge state 5+
- specType=etd
- score=47.59
- F097416.dat
- query=q2212_p1
- precursor=722.589010
- chargePrecursor=6
- itol=0.8

AA	c	y	z+1	z+2	z	AA
D[1]	27.418	**866.905**	863.702	0.806	863.500	D[40]
E[2]	53.226	**843.900**	**840.696**	0.806	**840.495**	E[39]
E[3]	79.035	818.091	814.888	0.806	814.686	E[38]
L[4]	101.652	792.283	789.079	0.806	**788.878**	L[37]
N[5]	124.460	769.666	766.462	766.664	766.261	N[36]
K[6]	150.079	**746.857**	743.654	743.855	743.452	K[35]
L[7]	172.696	721.238	718.035	718.236	717.833	L[34]
L[8]	195.313	698.622	695.418	695.619	695.216	L[33]
G[9]	206.717	676.005	672.801	673.003	672.600	G[32]
K[10]	237.943	664.601	661.397	661.598	661.195	K[31]
V[11]	257.756	633.375	630.172	630.373	629.970	V[30]
T[12]	277.966	613.562	610.358	610.559	610.156	T[29]
I[13]	300.583	593.352	590.148	590.350	589.947	I[28]
A[14]	314.790	570.735	567.532	567.733	567.330	A[27]
Q[15]	340.402	556.528	**553.324**	**553.526**	553.123	Q[26]
G[16]	351.806	530.916	527.712	527.914	527.511	G[25]
G[17]	363.210	519.512	**516.308**	516.510	**516.107**	G[24]
V[18]	383.024	508.108	**504.904**	505.105	**504.702**	V[23]
L[19]	405.641	488.294	485.090	485.292	484.889	L[22]
P[20]	425.051	465.677	462.473	462.675	462.272	P[21]
N[21]	447.860	446.266	443.063	443.264	442.861	N[20]
I[22]	470.477	423.458	420.254	420.456	420.053	I[19]
Q[23]	496.088	400.841	397.637	397.839	397.436	Q[18]
A[24]	510.296	375.229	372.026	372.227	371.824	A[17]
V[25]	530.110	361.022	357.818	358.020	357.617	V[16]
L[26]	552.726	341.208	338.005	338.206	337.803	L[15]
L[27]	575.343	318.591	315.388	**315.589**	315.186	L[14]
P[28]	594.754	295.975	292.771	292.972	292.569	P[13]
K[29]	**620.373**	276.564	273.360	273.562	273.159	K[12]
K[30]	645.992	250.945	247.741	247.943	247.540	K[11]
T[31]	666.201	225.326	222.122	222.324	221.921	T[10]
E[32]	692.010	205.117	201.913	202.114	201.711	E[9]
S[33]	709.416	179.308	176.104	176.306	175.903	S[8]
H[34]	736.828	161.902	158.698	158.899	158.496	H[7]
H[35]	764.240	134.490	131.286	131.488	131.085	H[6]
K[36]	789.859	107.078	103.874	104.076	103.673	K[5]
A[37]	804.066	81.459	78.255	78.457	78.054	A[4]
K[38]	829.685	67.252	64.048	64.249	63.846	K[3]
G[39]	**841.089**	41.633	38.429	38.630	38.227	G[2]
K[40]	**866.708**	30.228	27.025	27.226	26.823	K[1]

sp|Q6GSS7|H2A2A_MOUSE

DEELNKLLGK $^{\text{Dimethyl}}_{28.03}$ VTIAQGGVLPNIQAVLLPKKTESHHKAKGK

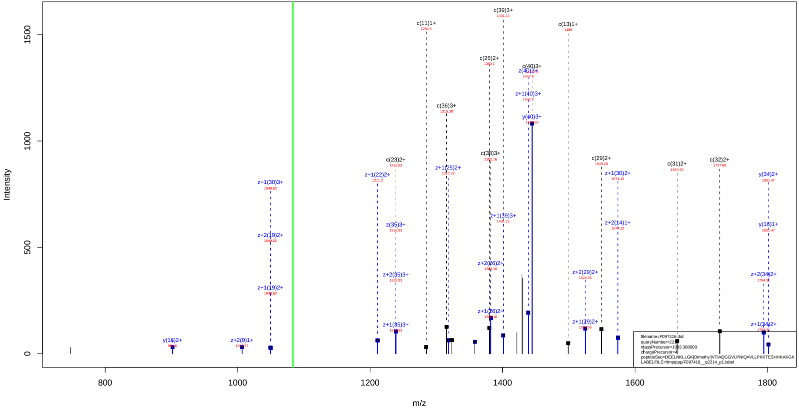

sp|Q6GSS7|H2A2A_MOUSE

DEELNKLLGK $^{\text{Dimethyl}}_{28.03}$ VTIAQGGVLPNIQAVLLPKKTESHHKAKGK

- fragmentation table for charge state 1+
- specType=etd
- score=47.35
- F097416.dat
- query=q2214_p1
- precursor=1083.380000
- chargePrecursor=4
- itol=0.8

AA	c	y	z+1	z+2	z	AA
D[1]	133.061	4330.497	4314.479	0.000	4313.471	D[40]
E[2]	262.103	4215.470	4199.452	0.000	4198.444	E[39]
E[3]	391.146	4086.428	4070.409	0.000	4069.401	E[38]
L[4]	504.230	3957.385	3941.366	0.000	3940.359	L[37]
N[5]	618.273	3844.301	3828.282	3829.290	3827.275	N[36]
K[6]	746.368	3730.258	3714.239	3715.247	3713.232	K[35]
L[7]	859.452	3602.163	3586.144	3587.152	3585.137	L[34]
L[8]	972.536	3489.079	3473.060	3474.068	3472.053	L[33]
G[9]	1029.558	3375.995	3359.976	3360.984	3358.969	G[32]
K[10]	1185.684	3318.974	3302.955	3303.963	3301.947	K[31]
V[11]	**1284.752**	3162.847	3146.829	3147.836	3145.821	V[30]
T[12]	1385.800	3063.779	3047.760	3048.768	3046.752	T[29]
I[13]	**1498.884**	2962.731	2946.713	2947.720	2945.705	I[28]
A[14]	1569.921	2849.647	2833.628	2834.636	2832.621	A[27]
Q[15]	1697.980	2778.610	2762.591	2763.599	2761.584	Q[26]
G[16]	1755.001	2650.552	2634.533	2635.541	2633.525	G[25]
G[17]	1812.023	2593.530	2577.511	2578.519	2576.504	G[24]
V[18]	1911.091	2536.509	2520.490	2521.498	2519.482	V[23]
L[19]	2024.175	2437.440	2421.421	2422.429	2420.414	L[22]
P[20]	2121.228	2324.356	2308.337	2309.345	2307.330	P[21]
N[21]	2235.271	2227.303	2211.285	2212.292	2210.277	N[20]
I[22]	2348.355	2113.260	2097.242	2098.250	2096.234	I[19]
Q[23]	2476.413	2000.176	1984.158	1985.165	1983.150	Q[18]
A[24]	2547.450	1872.118	1856.099	1857.107	1855.091	A[17]
V[25]	2646.519	**1801.081**	1785.062	1786.070	1784.054	V[16]
L[26]	2759.603	1702.012	1685.994	1687.001	1684.986	L[15]
L[27]	2872.687	1588.928	1572.909	**1573.917**	1571.902	L[14]
P[28]	2969.740	1475.844	1459.825	1460.833	1458.818	P[13]
K[29]	3097.835	1378.791	1362.773	1363.780	1361.765	K[12]
K[30]	3225.930	1250.696	1234.678	1235.685	1233.670	K[11]
T[31]	3326.977	1122.601	1106.583	1107.591	1105.575	T[10]
E[32]	3456.020	1021.554	1005.535	**1006.543**	1004.527	E[9]
S[33]	3543.052	892.511	876.492	877.500	875.485	S[8]
H[34]	3680.111	805.479	789.460	790.468	788.453	H[7]
H[35]	3817.170	668.420	652.401	653.409	651.394	H[6]
K[36]	3945.265	531.361	515.343	516.350	514.335	K[5]
A[37]	4016.302	403.266	387.248	388.255	386.240	A[4]
K[38]	4144.397	332.229	316.211	317.218	315.203	K[3]
G[39]	4201.418	204.134	188.116	189.123	187.108	G[2]
K[40]	4329.513	147.113	131.094	132.102	130.086	K[1]

sp|Q6GSS7|H2A2A_MOUSE

DEELNKLLGK $^{Dimethyl}_{28.03}$ VTIAQGGVLPNIQAVLLPKKTESHHKAKGK

- fragmentation table for charge state 2+
- specType=etd
- score=47.35
- F097416.dat
- query=q2214_p1
- precursor=1083.380000
- chargePrecursor=4
- itol=0.8

AA	c	y	z+1	z+2	z	AA
D[1]	67.034	2165.752	2157.743	0.504	2157.239	D[40]
E[2]	131.555	2108.239	2100.229	0.504	2099.726	E[39]
E[3]	196.077	2043.718	2035.708	0.504	2035.204	E[38]
L[4]	252.619	1979.196	1971.187	0.504	1970.683	L[37]
N[5]	309.640	1922.654	1914.645	1915.149	1914.141	N[36]
K[6]	373.688	1865.633	1857.623	1858.127	1857.119	K[35]
L[7]	430.230	**1801.585**	**1793.576**	**1794.080**	1793.072	L[34]
L[8]	486.772	1745.043	1737.034	1737.538	1736.530	L[33]
G[9]	515.282	1688.501	1680.492	1680.996	1679.988	G[32]
K[10]	593.346	1659.990	1651.981	1652.485	1651.477	K[31]
V[11]	642.880	1581.927	**1573.918**	**1574.422**	**1573.414**	V[30]
T[12]	693.404	1532.393	**1524.384**	**1524.888**	1523.880	T[29]
I[13]	749.946	1481.869	1473.860	1474.364	1473.356	I[28]
A[14]	785.464	1425.327	1417.318	1417.822	1416.814	A[27]
Q[15]	849.493	1389.809	**1381.799**	**1382.303**	1381.295	Q[26]
G[16]	878.004	1325.779	**1317.770**	**1318.274**	**1317.266**	G[25]
G[17]	906.515	1297.269	1289.259	1289.763	1288.755	G[24]
V[18]	956.049	1268.758	1260.749	1261.252	1260.245	V[23]
L[19]	1012.591	1219.224	**1211.214**	**1211.718**	**1210.710**	L[22]
P[20]	1061.118	1162.682	1154.672	1155.176	1154.168	P[21]
N[21]	1118.139	1114.155	1106.146	1106.650	1105.642	N[20]
I[22]	1174.681	1057.134	**1049.124**	**1049.628**	1048.621	I[19]
Q[23]	**1238.710**	1000.592	992.582	993.086	992.079	Q[18]
A[24]	1274.229	936.563	928.553	929.057	928.049	A[17]
V[25]	**1323.763**	**901.044**	893.035	893.539	892.531	V[16]
L[26]	**1380.305**	851.510	843.500	844.004	842.996	L[15]
L[27]	1436.847	794.968	786.958	787.462	786.454	L[14]
P[28]	1485.374	738.426	730.416	730.920	729.912	P[13]
K[29]	**1549.421**	689.899	681.890	682.394	681.386	K[12]
K[30]	1613.468	625.852	617.842	618.346	617.339	K[11]
T[31]	**1663.992**	561.804	553.795	554.299	553.291	T[10]
E[32]	**1728.514**	511.281	503.271	503.775	502.767	E[9]
S[33]	1772.030	446.759	438.750	439.254	438.246	S[8]
H[34]	1840.559	403.243	395.234	395.738	394.730	H[7]
H[35]	1909.089	334.714	326.704	327.208	326.200	H[6]
K[36]	1973.136	266.184	258.175	258.679	257.671	K[5]
A[37]	2008.655	202.137	194.127	194.631	193.624	A[4]
K[38]	2072.702	166.618	158.609	159.113	158.105	K[3]
G[39]	2101.213	102.571	94.561	95.065	94.057	G[2]
K[40]	2165.260	74.060	66.051	66.555	65.547	K[1]

sp|Q6GSS7|H2A2A_MOUSE

DEELNKLLGK $^{\text{Dimethyl}}_{28.03}$ VTIAQGGVLPNIQAVLLPKKTESHHKAKGK

- fragmentation table for charge state 3+
- specType=etd
- score=47.35
- F097416.dat
- query=q2214_p1
- precursor=1083.380000
- chargePrecursor=4
- itol=0.8

AA	c	y	z+1	z+2	z	AA
D[1]	45.025	**1444.171**	**1438.831**	0.672	**1438.495**	D[40]
E[2]	88.039	1405.828	**1400.489**	0.672	1400.153	E[39]
E[3]	131.054	1362.814	**1357.475**	0.672	1357.139	E[38]
L[4]	168.748	1319.800	1314.460	0.672	1314.124	L[37]
N[5]	206.762	1282.105	1276.766	1277.102	1276.430	N[36]
K[6]	249.461	1244.091	**1238.751**	**1239.087**	**1238.415**	K[35]
L[7]	287.156	1201.393	1196.053	1196.389	1195.717	L[34]
L[8]	324.850	1163.698	1158.358	1158.694	1158.022	L[33]
G[9]	343.857	1126.003	1120.664	1121.000	1120.328	G[32]
K[10]	395.899	1106.996	1101.656	1101.992	1101.321	K[31]
V[11]	428.922	1054.954	**1049.614**	**1049.950**	**1049.278**	V[30]
T[12]	462.605	1021.931	1016.592	1016.928	1016.256	T[29]
I[13]	500.299	988.249	982.909	983.245	982.573	I[28]
A[14]	523.979	950.554	945.214	945.550	944.878	A[27]
Q[15]	566.665	926.875	921.535	921.871	921.199	Q[26]
G[16]	585.672	884.189	878.849	879.185	878.513	G[25]
G[17]	604.679	865.182	859.842	860.178	859.506	G[24]
V[18]	637.702	846.174	840.835	841.171	840.499	V[23]
L[19]	675.397	813.152	807.812	808.148	807.476	L[22]
P[20]	707.747	775.457	770.117	770.453	769.781	P[21]
N[21]	745.762	743.106	737.766	738.102	737.430	N[20]
I[22]	783.456	705.092	699.752	700.088	699.416	I[19]
Q[23]	826.143	667.397	662.057	662.393	661.721	Q[18]
A[24]	849.822	624.711	619.371	619.707	619.035	A[17]
V[25]	882.844	601.032	595.692	596.028	595.356	V[16]
L[26]	920.539	568.009	562.669	563.005	562.333	L[15]
L[27]	958.234	530.314	524.975	525.311	524.639	L[14]
P[28]	990.585	492.620	487.280	487.616	486.944	P[13]
K[29]	1033.283	460.269	454.929	455.265	454.593	K[12]
K[30]	1075.981	417.570	412.231	412.567	411.895	K[11]
T[31]	1109.664	374.872	369.532	369.868	369.196	T[10]
E[32]	1152.678	341.189	335.850	336.186	335.514	E[9]
S[33]	1181.689	298.175	292.836	293.172	292.500	S[8]
H[34]	1227.375	269.165	263.825	264.161	263.489	H[7]
H[35]	1273.061	223.478	218.139	218.475	217.803	H[6]
K[36]	**1315.760**	177.792	172.452	172.788	172.116	K[5]
A[37]	1339.439	135.094	129.754	130.090	129.418	A[4]
K[38]	**1382.137**	111.415	106.075	106.411	105.739	K[3]
G[39]	**1401.144**	68.716	63.377	63.713	63.041	G[2]
K[40]	**1443.843**	49.709	44.370	44.705	44.034	K[1]

sp|Q6GSS7|H2A2A_MOUSE

DEELNKLLGK $^{\text{Dimethyl}}_{28.03}$ VTIAQGGVLPNIQAVLLPKKTESHHKAKGK

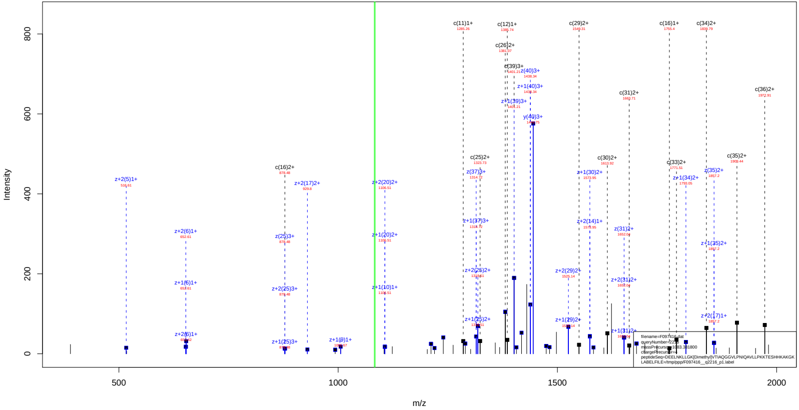

sp|Q6GSS7|H2A2A_MOUSE

DEELNKLLGK $^{Dimethyl}_{28.03}$ VTIAQGGVLPNIQAVLLPKKTESHHKAKGK

- fragmentation table for charge state 1+
- specType=etd
- score=75.08
- F097416.dat
- query=q2216_p1
- precursor=1083.381800
- chargePrecursor=4
- itol=0.8

AA	c	y	z+1	z+2	z	AA
D[1]	133.061	4330.497	4314.479	0.000	4313.471	D[40]
E[2]	262.103	4215.470	4199.452	0.000	4198.444	E[39]
E[3]	391.146	4086.428	4070.409	0.000	4069.401	E[38]
L[4]	504.230	3957.385	3941.366	0.000	3940.359	L[37]
N[5]	618.273	3844.301	3828.282	3829.290	3827.275	N[36]
K[6]	746.368	3730.258	3714.239	3715.247	3713.232	K[35]
L[7]	859.452	3602.163	3586.144	3587.152	3585.137	L[34]
L[8]	972.536	3489.079	3473.060	3474.068	3472.053	L[33]
G[9]	1029.558	3375.995	3359.976	3360.984	3358.969	G[32]
K[10]	1185.684	3318.974	3302.955	3303.963	3301.947	K[31]
V[11]	**1284.752**	3162.847	3146.829	3147.836	3145.821	V[30]
T[12]	**1385.800**	3063.779	3047.760	3048.768	3046.752	T[29]
I[13]	1498.884	2962.731	2946.713	2947.720	2945.705	I[28]
A[14]	1569.921	2849.647	2833.628	2834.636	2832.621	A[27]
Q[15]	1697.980	2778.610	2762.591	2763.599	2761.584	Q[26]
G[16]	**1755.001**	2650.552	2634.533	2635.541	2633.525	G[25]
G[17]	1812.023	2593.530	2577.511	2578.519	2576.504	G[24]
V[18]	1911.091	2536.509	2520.490	2521.498	2519.482	V[23]
L[19]	2024.175	2437.440	2421.421	2422.429	2420.414	L[22]
P[20]	2121.228	2324.356	2308.337	2309.345	2307.330	P[21]
N[21]	2235.271	2227.303	2211.285	2212.292	2210.277	N[20]
I[22]	2348.355	2113.260	2097.242	2098.250	2096.234	I[19]
Q[23]	2476.413	2000.176	1984.158	1985.165	1983.150	Q[18]
A[24]	2547.450	1872.118	1856.099	**1857.107**	1855.091	A[17]
V[25]	2646.519	1801.081	1785.062	1786.070	1784.054	V[16]
L[26]	2759.603	1702.012	1685.994	1687.001	1684.986	L[15]
L[27]	2872.687	1588.928	1572.909	**1573.917**	1571.902	L[14]
P[28]	2969.740	1475.844	1459.825	1460.833	1458.818	P[13]
K[29]	3097.835	1378.791	1362.773	1363.780	1361.765	K[12]
K[30]	3225.930	1250.696	1234.678	1235.685	1233.670	K[11]
T[31]	3326.977	1122.601	**1106.583**	1107.591	1105.575	T[10]
E[32]	3456.020	1021.554	**1005.535**	1006.543	1004.527	E[9]
S[33]	3543.052	892.511	876.492	877.500	875.485	S[8]
H[34]	3680.111	805.479	789.460	790.468	788.453	H[7]
H[35]	3817.170	668.420	**652.401**	**653.409**	651.394	H[6]
K[36]	3945.265	531.361	515.343	**516.350**	514.335	K[5]
A[37]	4016.302	403.266	387.248	388.255	386.240	A[4]
K[38]	4144.397	332.229	316.211	317.218	315.203	K[3]
G[39]	4201.418	204.134	188.116	189.123	187.108	G[2]
K[40]	4329.513	147.113	131.094	132.102	130.086	K[1]

sp|Q6GSS7|H2A2A_MOUSE

DEELNKLLGK $^{\text{Dimethyl}}_{28.03}$ VTIAQGGVLPNIQAVLLPKKTESHHKAKGK

- fragmentation table for charge state 2+
- specType=etd
- score=75.08
- F097416.dat
- query=q2216_p1
- precursor=1083.381800
- chargePrecursor=4
- itol=0.8

AA	c	y	z+1	z+2	z	AA
D[1]	67.034	2165.752	2157.743	0.504	2157.239	D[40]
E[2]	131.555	2108.239	2100.229	0.504	2099.726	E[39]
E[3]	196.077	2043.718	2035.708	0.504	2035.204	E[38]
L[4]	252.619	1979.196	1971.187	0.504	1970.683	L[37]
N[5]	309.640	1922.654	1914.645	1915.149	1914.141	N[36]
K[6]	373.688	1865.633	**1857.623**	1858.127	**1857.119**	K[35]
L[7]	430.230	1801.585	**1793.576**	1794.080	**1793.072**	L[34]
L[8]	486.772	1745.043	1737.034	1737.538	1736.530	L[33]
G[9]	515.282	1688.501	**1680.492**	**1680.996**	**1679.988**	G[32]
K[10]	593.346	1659.990	**1651.981**	**1652.485**	**1651.477**	K[31]
V[11]	642.880	**1581.927**	**1573.918**	**1574.422**	**1573.414**	V[30]
T[12]	693.404	1532.393	**1524.384**	**1524.888**	1523.880	T[29]
I[13]	749.946	**1481.869**	**1473.860**	**1474.364**	1473.356	I[28]
A[14]	785.464	1425.327	1417.318	**1417.822**	1416.814	A[27]
Q[15]	849.493	1389.809	**1381.799**	1382.303	**1381.295**	Q[26]
G[16]	**878.004**	1325.779	**1317.770**	**1318.274**	1317.266	G[25]
G[17]	906.515	1297.269	**1289.259**	**1289.763**	1288.755	G[24]
V[18]	956.049	1268.758	1260.749	1261.252	1260.245	V[23]
L[19]	1012.591	**1219.224**	**1211.214**	**1211.718**	1210.710	L[22]
P[20]	1061.118	1162.682	1154.672	1155.176	1154.168	P[21]
N[21]	1118.139	1114.155	**1106.146**	**1106.650**	1105.642	N[20]
I[22]	1174.681	1057.134	1049.124	1049.628	1048.621	I[19]
Q[23]	1238.710	1000.592	**992.582**	**993.086**	992.079	Q[18]
A[24]	1274.229	936.563	928.553	**929.057**	928.049	A[17]
V[25]	**1323.763**	901.044	893.035	893.539	892.531	V[16]
L[26]	**1380.305**	851.510	843.500	844.004	842.996	L[15]
L[27]	1436.847	794.968	786.958	787.462	786.454	L[14]
P[28]	1485.374	738.426	730.416	730.920	729.912	P[13]
K[29]	**1549.421**	689.899	681.890	682.394	681.386	K[12]
K[30]	**1613.468**	625.852	617.842	618.346	617.339	K[11]
T[31]	**1663.992**	561.804	553.795	554.299	553.291	T[10]
E[32]	1728.514	511.281	503.271	503.775	502.767	E[9]
S[33]	**1772.030**	446.759	438.750	439.254	438.246	S[8]
H[34]	**1840.559**	403.243	395.234	395.738	394.730	H[7]
H[35]	**1909.089**	334.714	326.704	327.208	326.200	H[6]
K[36]	**1973.136**	266.184	258.175	258.679	257.671	K[5]
A[37]	2008.655	202.137	194.127	194.631	193.624	A[4]
K[38]	2072.702	166.618	158.609	159.113	158.105	K[3]
G[39]	2101.213	102.571	94.561	95.065	94.057	G[2]
K[40]	2165.260	74.060	66.051	66.555	65.547	K[1]

sp|Q6GSS7|H2A2A_MOUSE

DEELNKLLGK $^{\text{Dimethyl}}_{28.03}$ VTIAQGGVLPNIQAVLLPKKTESHHKAKGK

- fragmentation table for charge state 3+
- specType=etd
- score=75.08
- F097416.dat
- query=q2216_p1
- precursor=1083.381800
- chargePrecursor=4
- itol=0.8

AA	c	y	z+1	z+2	z	AA
D[1]	45.025	**1444.171**	**1438.831**	0.672	**1438.495**	D[40]
E[2]	88.039	**1405.828**	**1400.489**	0.672	1400.153	E[39]
E[3]	131.054	1362.814	1357.475	0.672	1357.139	E[38]
L[4]	168.748	1319.800	**1314.460**	0.672	**1314.124**	L[37]
N[5]	206.762	1282.105	1276.766	1277.102	1276.430	N[36]
K[6]	249.461	1244.091	1238.751	**1239.087**	1238.415	K[35]
L[7]	287.156	1201.393	1196.053	1196.389	1195.717	L[34]
L[8]	324.850	1163.698	1158.358	1158.694	1158.022	L[33]
G[9]	343.857	1126.003	1120.664	1121.000	1120.328	G[32]
K[10]	395.899	**1106.996**	1101.656	1101.992	1101.321	K[31]
V[11]	428.922	1054.954	1049.614	1049.950	1049.278	V[30]
T[12]	462.605	1021.931	1016.592	1016.928	1016.256	T[29]
I[13]	500.299	988.249	982.909	983.245	982.573	I[28]
A[14]	523.979	950.554	945.214	945.550	944.878	A[27]
Q[15]	566.665	926.875	921.535	921.871	921.199	Q[26]
G[16]	585.672	884.189	**878.849**	**879.185**	**878.513**	G[25]
G[17]	604.679	865.182	859.842	860.178	859.506	G[24]
V[18]	637.702	846.174	840.835	841.171	840.499	V[23]
L[19]	675.397	813.152	807.812	808.148	807.476	L[22]
P[20]	707.747	775.457	770.117	770.453	769.781	P[21]
N[21]	745.762	743.106	737.766	738.102	737.430	N[20]
I[22]	783.456	705.092	699.752	700.088	699.416	I[19]
Q[23]	826.143	667.397	662.057	662.393	661.721	Q[18]
A[24]	849.822	624.711	619.371	619.707	619.035	A[17]
V[25]	882.844	601.032	595.692	596.028	595.356	V[16]
L[26]	920.539	568.009	562.669	563.005	562.333	L[15]
L[27]	958.234	530.314	524.975	525.311	524.639	L[14]
P[28]	990.585	492.620	487.280	487.616	486.944	P[13]
K[29]	1033.283	460.269	454.929	455.265	454.593	K[12]
K[30]	1075.981	417.570	412.231	412.567	411.895	K[11]
T[31]	1109.664	374.872	369.532	369.868	369.196	T[10]
E[32]	1152.678	341.189	335.850	336.186	335.514	E[9]
S[33]	1181.689	298.175	292.836	293.172	292.500	S[8]
H[34]	1227.375	269.165	263.825	264.161	263.489	H[7]
H[35]	1273.061	223.478	218.139	218.475	217.803	H[6]
K[36]	1315.760	177.792	172.452	172.788	172.116	K[5]
A[37]	1339.439	135.094	129.754	130.090	129.418	A[4]
K[38]	1382.137	111.415	106.075	106.411	105.739	K[3]
G[39]	**1401.144**	68.716	63.377	63.713	63.041	G[2]
K[40]	1443.843	49.709	44.370	44.705	44.034	K[1]

sp|Q6GSS7|H2A2A_MOUSE

DEELNKLLGK Dimethyl 28.03 VTIAQGGVLPNIQAVLLPKKTESHHKAKGK Acetyl 42.01

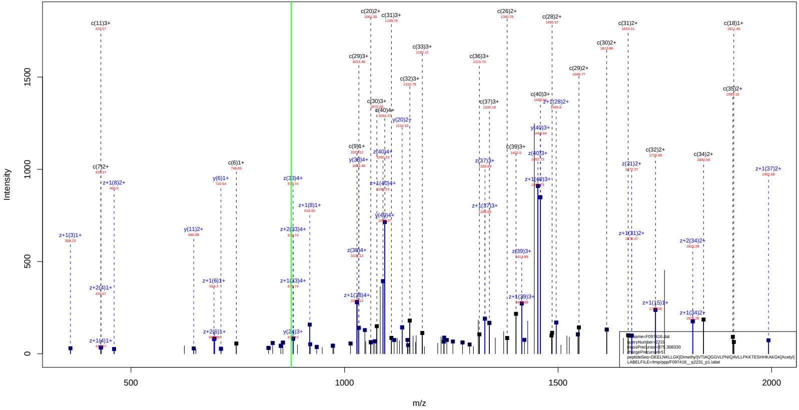

sp|Q6GSS7|H2A2A_MOUSE

DEELNKLLGK $^{\text{Dimethyl}}_{28.03}$ VTIAQGGVLPNIQAVLLPKKTESHHKAKGK $^{\text{Acetyl}}_{42.01}$

- fragmentation table for charge state 1+
- specType=etd
- score=43.77
- F097416.dat
- query=q2231_p1
- precursor=875.308330
- chargePrecursor=5
- itol=0.8

AA	c	y	z+1	z+2	z	AA
D[1]	133.061	4372.508	4356.489	0.000	4355.481	D[40]
E[2]	262.103	4257.481	4241.462	0.000	4240.454	E[39]
E[3]	391.146	4128.438	4112.420	0.000	4111.412	E[38]
L[4]	504.230	3999.396	3983.377	0.000	3982.369	L[37]
N[5]	618.273	3886.312	3870.293	3871.301	3869.285	N[36]
K[6]	**746.368**	3772.269	3756.250	3757.258	3755.242	K[35]
L[7]	859.452	3644.174	3628.155	3629.163	3627.147	L[34]
L[8]	**972.536**	3531.090	3515.071	3516.079	3514.063	L[33]
G[9]	**1029.558**	3418.006	3401.987	3402.995	3400.979	G[32]
K[10]	1185.684	3360.984	3344.965	3345.973	3343.958	K[31]
V[11]	1284.752	3204.858	3188.839	3189.847	3187.831	V[30]
T[12]	1385.800	3105.790	3089.771	3090.779	3088.763	T[29]
I[13]	1498.884	3004.742	2988.723	2989.731	2987.715	I[28]
A[14]	1569.921	2891.658	2875.639	2876.647	2874.631	A[27]
Q[15]	1697.980	2820.621	2804.602	2805.610	2803.594	Q[26]
G[16]	1755.001	2692.562	2676.543	2677.551	2675.536	G[25]
G[17]	1812.023	2635.541	2619.522	2620.530	2618.514	G[24]
V[18]	**1911.091**	2578.519	2562.500	2563.508	2561.493	V[23]
L[19]	2024.175	2479.451	2463.432	2464.440	2462.424	L[22]
P[20]	2121.228	2366.367	2350.348	2351.356	2349.340	P[21]
N[21]	2235.271	2269.314	2253.295	2254.303	2252.287	N[20]
I[22]	2348.355	2155.271	2139.252	2140.260	2138.244	I[19]
Q[23]	2476.413	2042.187	2026.168	2027.176	2025.160	Q[18]
A[24]	2547.450	1914.128	1898.110	1899.117	1897.102	A[17]
V[25]	2646.519	1843.091	1827.073	1828.080	1826.065	V[16]
L[26]	2759.603	1744.023	**1728.004**	1729.012	1726.996	L[15]
L[27]	2872.687	1630.939	1614.920	1615.928	**1613.912**	L[14]
P[28]	2969.740	1517.855	1501.836	1502.844	1500.828	P[13]
K[29]	3097.835	**1420.802**	1404.783	1405.791	1403.775	K[12]
K[30]	3225.930	**1292.707**	**1276.688**	1277.696	**1275.680**	K[11]
T[31]	3326.977	1164.612	**1148.593**	1149.601	**1147.585**	T[10]
E[32]	3456.020	1063.564	**1047.546**	1048.553	1046.538	E[9]
S[33]	3543.052	**934.522**	**918.503**	**919.511**	917.495	S[8]
H[34]	3680.111	847.490	**831.471**	**832.479**	830.463	H[7]
H[35]	3817.170	**710.431**	**694.412**	**695.420**	693.404	H[6]
K[36]	3945.265	573.372	557.353	558.361	556.345	K[5]
A[37]	4016.302	445.277	**429.258**	**430.266**	428.250	A[4]
K[38]	4144.397	374.240	**358.221**	359.229	357.213	K[3]
G[39]	4201.418	246.145	230.126	231.134	229.118	G[2]
K[40]	4371.524	189.123	173.105	174.112	172.097	K[1]

sp|Q6GSS7|H2A2A_MOUSE

DEELNKLLGK $^{\text{Dimethyl}}_{28.03}$ VTIAQGGVLPNIQAVLLPKKTESHHKAKGK $^{\text{Acetyl}}_{42.01}$

- fragmentation table for charge state 2+
- specType=etd
- score=43.77
- F097416.dat
- query=q2231_p1
- precursor=875.308330
- chargePrecursor=5
- itol=0.8

AA	c	y	z+1	z+2	z	AA
D[1]	67.034	2186.758	2178.748	0.504	2178.244	D[40]
E[2]	131.555	2129.244	2121.235	0.504	2120.731	E[39]
E[3]	196.077	2064.723	2056.713	0.504	2056.210	E[38]
L[4]	252.619	2000.202	**1992.192**	0.504	1991.688	L[37]
N[5]	309.640	1943.659	1935.650	1936.154	1935.146	N[36]
K[6]	373.688	1886.638	1878.629	1879.133	1878.125	K[35]
L[7]	**430.230**	1822.591	**1814.581**	**1815.085**	1814.077	L[34]
L[8]	486.772	1766.048	1758.039	1758.543	1757.535	L[33]
G[9]	515.282	1709.506	1701.497	1702.001	1700.993	G[32]
K[10]	593.346	1680.996	**1672.986**	1673.490	**1672.482**	K[31]
V[11]	642.880	1602.933	1594.923	1595.427	1594.419	V[30]
T[12]	693.404	1553.398	**1545.389**	**1545.893**	1544.885	T[29]
I[13]	749.946	1502.875	**1494.865**	**1495.369**	1494.361	I[28]
A[14]	785.464	1446.333	1438.323	1438.827	1437.819	A[27]
Q[15]	849.493	1410.814	1402.805	1403.309	1402.301	Q[26]
G[16]	878.004	1346.785	**1338.775**	**1339.279**	1338.271	G[25]
G[17]	906.515	1318.274	1310.265	1310.768	1309.761	G[24]
V[18]	956.049	1289.763	1281.754	1282.258	1281.250	V[23]
L[19]	1012.591	**1240.229**	**1232.220**	**1232.724**	**1231.716**	L[22]
P[20]	**1061.118**	1183.687	1175.678	1176.182	1175.174	P[21]
N[21]	1118.139	**1135.161**	1127.151	1127.655	1126.647	N[20]
I[22]	1174.681	1078.139	**1070.130**	**1070.634**	1069.626	I[19]
Q[23]	**1238.710**	1021.597	**1013.588**	**1014.092**	1013.084	Q[18]
A[24]	1274.229	957.568	949.558	950.062	949.055	A[17]
V[25]	1323.763	922.049	914.040	914.544	913.536	V[16]
L[26]	**1380.305**	872.515	864.506	865.010	864.002	L[15]
L[27]	1436.847	815.973	807.964	808.468	807.460	L[14]
P[28]	**1485.374**	759.431	751.422	751.926	750.918	P[13]
K[29]	**1549.421**	**710.905**	702.895	703.399	702.391	K[12]
K[30]	**1613.468**	**646.857**	638.848	639.352	638.344	K[11]
T[31]	**1663.992**	582.810	574.800	575.304	574.296	T[10]
E[32]	**1728.514**	532.286	524.276	524.780	523.773	E[9]
S[33]	1772.030	467.765	**459.755**	**460.259**	459.251	S[8]
H[34]	**1840.559**	424.248	416.239	416.743	415.735	H[7]
H[35]	**1909.089**	355.719	347.710	348.214	347.206	H[6]
K[36]	1973.136	287.190	279.180	279.684	278.676	K[5]
A[37]	2008.655	223.142	215.133	215.637	214.629	A[4]
K[38]	2072.702	187.624	179.614	180.118	179.110	K[3]
G[39]	2101.213	123.576	115.567	116.071	115.063	G[2]
K[40]	2186.266	95.065	87.056	87.560	86.552	K[1]

sp|Q6GSS7|H2A2A_MOUSE

DEELNKLLGK $^{Dimethyl}_{28.03}$ VTIAQGGVLPNIQAVLLPKKTESHHKAKGK $^{Acetyl}_{42.01}$

- fragmentation table for charge state 3+
- specType=etd
- score=43.77
- F097416.dat
- query=q2231_p1
- precursor=875.308330
- chargePrecursor=5
- itol=0.8

AA	c	y	z+1	z+2	z	AA
D[1]	45.025	**1458.174**	**1452.835**	0.672	**1452.499**	D[40]
E[2]	88.039	1419.832	**1414.492**	0.672	**1414.156**	E[39]
E[3]	131.054	1376.818	1371.478	0.672	1371.142	E[38]
L[4]	168.748	1333.803	**1328.464**	0.672	**1328.128**	L[37]
N[5]	206.762	1296.109	1290.769	1291.105	1290.433	N[36]
K[6]	249.461	1258.094	1252.755	**1253.091**	1252.419	K[35]
L[7]	287.156	1215.396	1210.057	1210.392	1209.721	L[34]
L[8]	324.850	1177.701	1172.362	1172.698	1172.026	L[33]
G[9]	343.857	1140.007	**1134.667**	**1135.003**	**1134.331**	G[32]
K[10]	395.899	1121.000	**1115.660**	**1115.996**	1115.324	K[31]
V[11]	**428.922**	1068.957	1063.618	1063.954	1063.282	V[30]
T[12]	462.605	1035.935	1030.595	1030.931	1030.259	T[29]
I[13]	500.299	1002.252	996.913	997.248	996.577	I[28]
A[14]	523.979	964.557	959.218	959.554	958.882	A[27]
Q[15]	566.665	940.878	**935.539**	935.875	**935.203**	Q[26]
G[16]	585.672	898.192	892.853	893.189	892.517	G[25]
G[17]	604.679	**879.185**	873.845	874.181	873.510	G[24]
V[18]	637.702	860.178	854.838	**855.174**	854.502	V[23]
L[19]	675.397	827.155	**821.816**	**822.151**	**821.480**	L[22]
P[20]	707.747	789.460	784.121	784.457	783.785	P[21]
N[21]	745.762	757.109	751.770	752.106	751.434	N[20]
I[22]	783.456	719.095	713.756	714.092	713.420	I[19]
Q[23]	826.143	681.400	676.061	676.397	675.725	Q[18]
A[24]	849.822	638.714	633.375	633.711	633.039	A[17]
V[25]	882.844	615.035	609.696	610.032	609.360	V[16]
L[26]	920.539	582.012	576.673	577.009	576.337	L[15]
L[27]	958.234	544.318	538.978	539.314	538.642	L[14]
P[28]	990.585	506.623	501.284	501.619	500.948	P[13]
K[29]	**1033.283**	474.272	468.933	469.269	468.597	K[12]
K[30]	**1075.981**	431.574	426.234	426.570	425.898	K[11]
T[31]	**1109.664**	388.876	383.536	383.872	383.200	T[10]
E[32]	**1152.678**	355.193	349.853	350.189	349.517	E[9]
S[33]	**1181.689**	312.179	306.839	307.175	306.503	S[8]
H[34]	1227.375	283.168	277.829	278.164	277.493	H[7]
H[35]	1273.061	237.482	232.142	232.478	231.806	H[6]
K[36]	**1315.760**	191.795	186.456	186.792	186.120	K[5]
A[37]	**1339.439**	149.097	143.758	144.094	143.422	A[4]
K[38]	1382.137	125.418	120.079	120.414	119.743	K[3]
G[39]	**1401.144**	82.720	77.380	77.716	77.044	G[2]
K[40]	**1457.846**	63.713	58.373	58.709	58.037	K[1]

sp|Q6GSS7|H2A2A_MOUSE

DEELNKLLGK $^{\text{Dimethyl}}_{28.03}$ VTIAQGGVLPNIQAVLLPKKTESHHKAKGK $^{\text{Acetyl}}_{42.01}$

- fragmentation table for charge state 4+
- specType=etd
- score=43.77
- F097416.dat
- query=q2231_p1
- precursor=875.308330
- chargePrecursor=5
- itol=0.8

AA	c	y	z+1	z+2	z	AA
D[1]	34.021	**1093.882**	**1089.878**	0.755	**1089.626**	D[40]
E[2]	66.281	1065.126	**1061.121**	0.755	**1060.869**	E[39]
E[3]	98.542	**1032.865**	**1028.860**	0.755	**1028.608**	E[38]
L[4]	126.813	1000.604	996.600	0.755	996.348	L[37]
N[5]	155.324	**972.333**	968.329	968.581	968.077	N[36]
K[6]	187.347	943.823	939.818	940.070	939.566	K[35]
L[7]	215.618	911.799	907.794	908.046	907.542	L[34]
L[8]	243.889	883.528	**879.523**	**879.775**	**879.271**	L[33]
G[9]	258.145	**855.257**	**851.252**	**851.504**	**851.000**	G[32]
K[10]	297.176	841.002	836.997	837.249	836.745	K[31]
V[11]	321.944	801.970	797.965	798.217	797.713	V[30]
T[12]	347.205	777.203	773.198	773.450	772.946	T[29]
I[13]	375.476	751.941	747.936	748.188	747.684	I[28]
A[14]	393.236	723.670	719.665	719.917	719.413	A[27]
Q[15]	425.250	705.911	701.906	702.158	701.654	Q[26]
G[16]	439.506	673.896	669.891	670.143	669.639	G[25]
G[17]	453.761	659.641	655.636	655.888	655.384	G[24]
V[18]	478.528	645.385	641.381	641.633	641.129	V[23]
L[19]	506.799	620.618	616.613	616.865	616.362	L[22]
P[20]	531.062	592.347	588.342	588.594	588.090	P[21]
N[21]	559.573	568.084	564.079	564.331	563.827	N[20]
I[22]	587.844	539.573	535.569	535.820	535.317	I[19]
Q[23]	619.859	511.302	507.298	507.549	507.046	Q[18]
A[24]	637.618	479.288	475.283	475.535	475.031	A[17]
V[25]	662.385	461.528	457.524	457.776	457.272	V[16]
L[26]	690.656	436.761	432.756	433.008	432.505	L[15]
L[27]	718.927	408.490	404.485	404.737	404.234	L[14]
P[28]	743.190	380.219	376.214	376.466	375.962	P[13]
K[29]	775.214	355.956	351.951	352.203	351.699	K[12]
K[30]	807.238	323.932	319.928	320.179	319.676	K[11]
T[31]	832.500	291.908	287.904	288.156	287.652	T[10]
E[32]	864.760	266.647	262.642	262.894	262.390	E[9]
S[33]	886.518	234.386	230.381	230.633	230.129	S[8]
H[34]	920.783	212.628	208.623	208.875	208.371	H[7]
H[35]	955.048	178.363	174.358	174.610	174.107	H[6]
K[36]	987.072	144.098	140.094	140.346	139.842	K[5]
A[37]	1004.831	112.075	108.070	108.322	107.818	A[4]
K[38]	1036.855	94.315	90.311	90.563	90.059	K[3]
G[39]	1051.110	62.292	58.287	58.539	58.035	G[2]
K[40]	**1093.636**	48.036	44.032	44.284	43.780	K[1]

sp|P62806|H4_MOUSE

[Acetyl]SGRGKGGKGLGKGGAKRHRK Trimethyl 42.05 VLRD

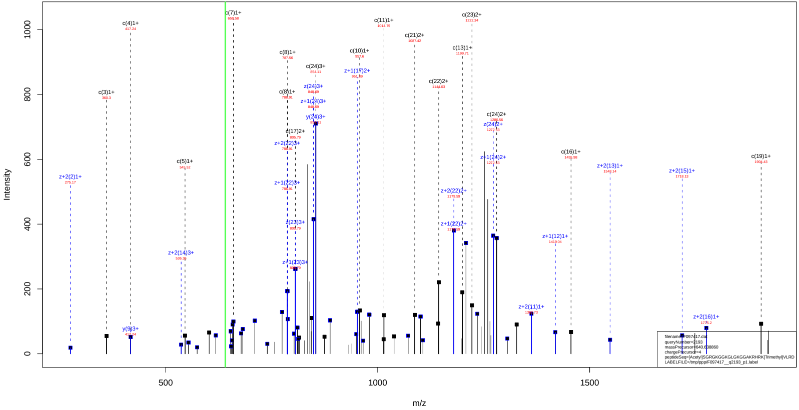

sp|P62806|H4_MOUSE

[Acetyl]SGRGKGGKGLGKGGAKRHRK $^{Trimethyl}_{42.05}$ VLRD

- fragmentation table for charge state 1+
- specType=etd
- score=96.08
- F097417.dat
- query=q2193_p1
- precursor=640.638860
- chargePrecursor=4
- itol=0.8

AA	c	y	z+1	z+2	z	AA
S[1]	147.076	2559.518	2543.499	0.000	2542.491	S[24]
G[2]	204.098	2430.475	2414.457	0.000	2413.449	G[23]
R[3]	**360.199**	2373.454	2357.435	2358.443	2356.427	R[22]
G[4]	**417.220**	2217.353	2201.334	2202.342	2200.326	G[21]
K[5]	**545.315**	2160.331	2144.313	2145.320	2143.305	K[20]
G[6]	**602.337**	2032.236	2016.218	2017.225	2015.210	G[19]
G[7]	**659.358**	1975.215	1959.196	1960.204	1958.188	G[18]
K[8]	**787.453**	1918.193	1902.175	1903.182	1901.167	K[17]
G[9]	**844.475**	1790.098	1774.080	**1775.087**	1773.072	G[16]
L[10]	**957.559**	1733.077	1717.058	**1718.066**	1716.050	L[15]
G[11]	**1014.580**	1619.993	1603.974	1604.982	1602.966	G[14]
K[12]	**1142.675**	1562.971	1546.953	**1547.960**	1545.945	K[13]
G[13]	**1199.697**	1434.876	**1418.858**	1419.866	1417.850	G[12]
G[14]	1256.718	1377.855	1361.836	**1362.844**	1360.828	G[11]
A[15]	**1327.755**	1320.833	1304.815	**1305.823**	1303.807	A[10]
K[16]	**1455.850**	1249.796	1233.778	**1234.785**	1232.770	K[9]
R[17]	1611.951	1121.701	1105.683	**1106.691**	1104.675	R[8]
H[18]	1749.010	**965.600**	**949.582**	950.589	948.574	H[7]
R[19]	**1905.111**	828.541	**812.523**	813.530	811.515	R[6]
K[20]	2075.253	672.440	**656.422**	**657.429**	655.414	K[5]
V[21]	2174.322	502.298	486.280	487.287	485.272	V[4]
L[22]	2287.406	403.230	387.211	388.219	386.203	L[3]
R[23]	2443.507	290.146	274.127	**275.135**	273.119	R[2]
D[24]	2558.534	134.045	118.026	119.034	117.018	D[1]

sp|P62806|H4_MOUSE

[Acetyl]SGRGKGGKGLGKGGAKRHRK $^{\text{Trimethyl}}_{42.05}$ VLRD

- fragmentation table for charge state 2+
- specType=etd
- score=96.08
- F097417.dat
- query=q2193_p1
- precursor=640.638860
- chargePrecursor=4
- itol=0.8

AA	c	y	z+1	z+2	z	AA
S[1]	74.042	**1280.263**	**1272.253**	0.504	**1271.749**	S[24]
G[2]	102.553	1215.741	**1207.732**	0.504	1207.228	G[23]
R[3]	180.603	1187.231	**1179.221**	**1179.725**	1178.717	R[22]
G[4]	209.114	1109.180	**1101.171**	**1101.675**	**1100.667**	G[21]
K[5]	273.161	1080.669	1072.660	1073.164	**1072.156**	K[20]
G[6]	301.672	1016.622	1008.612	1009.116	1008.108	G[19]
G[7]	330.183	988.111	**980.102**	**980.606**	**979.598**	G[18]
K[8]	394.230	959.600	**951.591**	**952.095**	**951.087**	K[17]
G[9]	422.741	895.553	**887.543**	**888.047**	**887.040**	G[16]
L[10]	479.283	867.042	859.033	859.537	858.529	L[15]
G[11]	507.794	**810.500**	**802.491**	**802.995**	801.987	G[14]
K[12]	571.841	781.989	**773.980**	**774.484**	773.476	K[13]
G[13]	600.352	717.942	**709.932**	**710.436**	**709.429**	G[12]
G[14]	628.863	689.431	**681.422**	**681.926**	680.918	G[11]
A[15]	664.381	660.920	**652.911**	**653.415**	**652.407**	A[10]
K[16]	728.429	625.402	**617.392**	**617.896**	616.889	K[9]
R[17]	**806.479**	561.354	**553.345**	**553.849**	**552.841**	R[8]
H[18]	**875.009**	483.304	475.294	475.798	474.791	H[7]
R[19]	953.059	414.774	406.765	407.269	406.261	R[6]
K[20]	**1038.130**	336.724	328.714	329.218	328.211	K[5]
V[21]	**1087.665**	251.653	243.643	244.147	243.140	V[4]
L[22]	**1144.207**	202.119	194.109	194.613	193.605	L[3]
R[23]	**1222.257**	145.577	137.567	138.071	137.063	R[2]
D[24]	**1279.771**	67.526	59.517	60.021	59.013	D[1]

sp|P62806|H4_MOUSE

[Acetyl]SGRGKGGKGLGKGGAKRHRK $^{\text{Trimethyl}}_{42.05}$ VLRD

- fragmentation table for charge state 3+
- specType=etd
- score=96.08
- F097417.dat
- query=q2193_p1
- precursor=640.638860
- chargePrecursor=4
- itol=0.8

AA	c	y	z+1	z+2	z	AA
S[1]	49.697	**853.844**	**848.505**	0.672	**848.169**	S[24]
G[2]	68.704	**810.830**	**805.490**	0.672	**805.154**	G[23]
R[3]	120.738	791.823	**786.483**	**786.819**	**786.147**	R[22]
G[4]	139.745	**739.789**	734.450	734.785	734.114	G[21]
K[5]	182.443	720.782	715.442	715.778	715.106	K[20]
G[6]	201.450	**678.084**	672.744	673.080	672.408	G[19]
G[7]	220.458	**659.076**	**653.737**	**654.073**	**653.401**	G[18]
K[8]	263.156	640.069	634.730	635.066	634.394	K[17]
G[9]	282.163	597.371	592.031	592.367	591.695	G[16]
L[10]	319.858	578.364	573.024	**573.360**	572.688	L[15]
G[11]	338.865	540.669	535.330	**535.666**	534.994	G[14]
K[12]	381.563	521.662	516.322	516.658	515.986	K[13]
G[13]	400.570	478.964	473.624	473.960	473.288	G[12]
G[14]	419.578	459.957	454.617	454.953	454.281	G[11]
A[15]	443.257	440.949	435.610	435.946	435.274	A[10]
K[16]	485.955	**417.270**	411.931	412.267	411.595	K[9]
R[17]	537.989	374.572	369.232	369.568	368.896	R[8]
H[18]	583.675	322.538	317.199	317.535	316.863	H[7]
R[19]	635.709	276.852	271.512	271.848	271.176	R[6]
K[20]	692.423	224.818	219.479	219.815	219.143	K[5]
V[21]	725.445	168.104	162.765	163.101	162.429	V[4]
L[22]	763.140	135.082	129.742	130.078	129.406	L[3]
R[23]	**815.174**	97.387	92.047	92.383	91.711	R[2]
D[24]	**853.516**	45.353	40.014	40.349	39.678	D[1]

sp|P62806|H4_MOUSE

SGR $^{\text{Trimethyl}}_{42.05}$ GKGGKGLGKGGAK $^{\text{Acetyl}}_{42.01}$ RHRK $^{\text{Acetyl}}_{42.01}$ VLRD

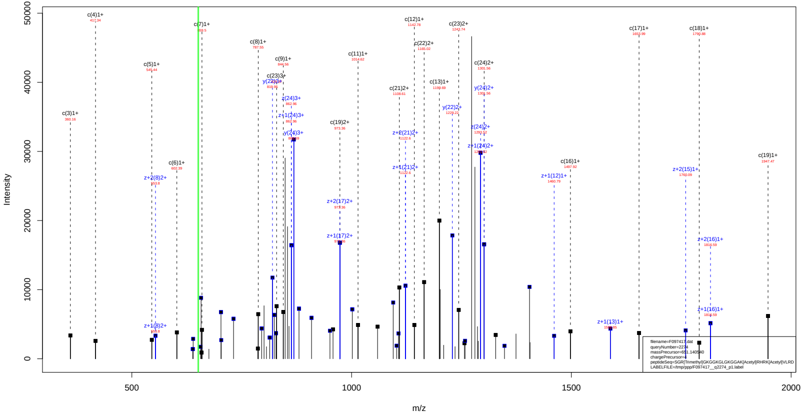

sp|P62806|H4_MOUSE

SGR $^{\text{Trimethyl}}_{42.05}$ GKGGKGLGKGGAK $^{\text{Acetyl}}_{42.01}$ RHRK $^{\text{Acetyl}}_{42.01}$ VLRD

- fragmentation table for charge state 1+
- specType=etd
- score=137.05
- F097417.dat
- query=q2274_p1
- precursor=651.140540
- chargePrecursor=4
- itol=0.8

AA	c	y	z+1	z+2	z	AA
S[1]	105.066	2601.528	2585.510	0.000	2584.502	S[24]
G[2]	162.087	2514.496	2498.478	0.000	2497.470	G[23]
R[3]	**360.235**	2457.475	2441.456	2442.464	2440.448	R[22]
G[4]	**417.257**	2259.327	2243.308	2244.316	2242.300	G[21]
K[5]	**545.352**	2202.305	2186.287	2187.295	2185.279	K[20]
G[6]	**602.373**	2074.210	2058.192	2059.200	2057.184	G[19]
G[7]	**659.395**	2017.189	2001.170	2002.178	2000.162	G[18]
K[8]	**787.490**	1960.168	1944.149	1945.157	1943.141	K[17]
G[9]	**844.511**	1832.073	**1816.054**	**1817.062**	1815.046	G[16]
L[10]	**957.595**	1775.051	1759.032	**1760.040**	1758.025	L[15]
G[11]	**1014.617**	1661.967	1645.948	1646.956	1644.940	G[14]
K[12]	**1142.712**	1604.946	**1588.927**	1589.935	1587.919	K[13]
G[13]	**1199.733**	1476.851	**1460.832**	1461.840	1459.824	G[12]
G[14]	**1256.755**	1419.829	1403.810	**1404.818**	1402.803	G[11]
A[15]	**1327.792**	1362.808	1346.789	**1347.797**	1345.781	A[10]
K[16]	**1497.897**	1291.771	1275.752	1276.760	1274.744	K[9]
R[17]	**1653.998**	1121.665	1105.646	**1106.654**	1104.638	R[8]
H[18]	**1791.057**	965.564	949.545	**950.553**	948.537	H[7]
R[19]	**1947.158**	**828.505**	812.486	**813.494**	811.478	R[6]
K[20]	2117.264	672.404	**656.385**	**657.393**	655.377	K[5]
V[21]	2216.332	502.298	486.280	487.287	485.272	V[4]
L[22]	2329.416	403.230	387.211	388.219	386.203	L[3]
R[23]	2485.517	290.146	274.127	275.135	273.119	R[2]
D[24]	2600.544	134.045	118.026	119.034	117.018	D[1]

sp|P62806|H4_MOUSE

SGR $^{\text{Trimethyl}}_{42.05}$ GKGGKGLGKGGAK $^{\text{Acetyl}}_{42.01}$ RHRK $^{\text{Acetyl}}_{42.01}$ VLRD

- fragmentation table for charge state 2+
- specType=etd
- score=137.05
- F097417.dat
- query=q2274_p1
- precursor=651.140540
- chargePrecursor=4
- itol=0.8

AA	c	y	z+1	z+2	z	AA
S[1]	53.037	**1301.268**	**1293.258**	0.504	**1292.755**	S[24]
G[2]	81.547	**1257.752**	1249.742	0.504	1249.239	G[23]
R[3]	180.621	**1229.241**	1221.232	1221.736	1220.728	R[22]
G[4]	209.132	1130.167	**1122.158**	**1122.662**	1121.654	G[21]
K[5]	273.180	**1101.656**	1093.647	**1094.151**	1093.143	K[20]
G[6]	301.690	1037.609	1029.600	1030.103	1029.096	G[19]
G[7]	330.201	1009.098	**1001.089**	**1001.593**	1000.585	G[18]
K[8]	394.248	980.587	**972.578**	**973.082**	972.074	K[17]
G[9]	422.759	916.540	**908.531**	**909.034**	908.027	G[16]
L[10]	479.301	888.029	**880.020**	**880.524**	**879.516**	L[15]
G[11]	507.812	831.487	**823.478**	**823.982**	822.974	G[14]
K[12]	571.859	802.976	**794.967**	**795.471**	794.463	K[13]
G[13]	600.370	738.929	**730.920**	**731.423**	730.416	G[12]
G[14]	628.881	710.418	**702.409**	**702.913**	701.905	G[11]
A[15]	664.399	681.907	673.898	674.402	673.394	A[10]
K[16]	749.452	646.389	**638.380**	**638.883**	637.876	K[9]
R[17]	**827.503**	561.336	**553.327**	**553.831**	552.823	R[8]
H[18]	896.032	483.286	475.276	475.780	474.772	H[7]
R[19]	**974.083**	414.756	406.747	407.251	406.243	R[6]
K[20]	**1059.136**	336.706	328.696	329.200	328.192	K[5]
V[21]	**1108.670**	251.653	243.643	244.147	243.140	V[4]
L[22]	**1165.212**	202.119	194.109	194.613	193.605	L[3]
R[23]	**1243.262**	145.577	137.567	138.071	137.063	R[2]
D[24]	**1300.776**	67.526	59.517	60.021	59.013	D[1]

sp|P62806|H4_MOUSE

SGR $^{Trimethyl}_{42.05}$ GKGGKGLGKGGAK $^{Acetyl}_{42.01}$ RHRK $^{Acetyl}_{42.01}$ VLRD

- fragmentation table for charge state 3+
- specType=etd
- score=137.05
- F097417.dat
- query=q2274_p1
- precursor=651.140540
- chargePrecursor=4
- itol=0.8

AA	c	y	z+1	z+2	z	AA
S[1]	35.693	**867.848**	**862.508**	0.672	**862.172**	S[24]
G[2]	54.701	838.837	833.497	0.672	833.161	G[23]
R[3]	120.750	**819.830**	**814.490**	814.826	**814.154**	R[22]
G[4]	139.757	753.780	748.441	748.777	748.105	G[21]
K[5]	182.455	734.773	729.434	729.770	729.098	K[20]
G[6]	201.463	692.075	686.735	687.071	686.399	G[19]
G[7]	220.470	673.068	667.728	668.064	667.392	G[18]
K[8]	263.168	654.061	648.721	649.057	648.385	K[17]
G[9]	282.175	611.362	606.023	606.359	605.687	G[16]
L[10]	319.870	592.355	587.016	587.352	586.680	L[15]
G[11]	338.877	554.661	549.321	549.657	548.985	G[14]
K[12]	381.575	535.653	530.314	530.650	529.978	K[13]
G[13]	400.583	492.955	487.615	487.951	487.280	G[12]
G[14]	419.590	473.948	468.608	468.944	468.272	G[11]
A[15]	443.269	454.941	449.601	449.937	449.265	A[10]
K[16]	499.971	431.262	425.922	426.258	425.586	K[9]
R[17]	552.004	374.560	369.220	369.556	368.884	R[8]
H[18]	597.691	322.526	317.187	317.523	316.851	H[7]
R[19]	649.724	276.840	271.500	271.836	271.164	R[6]
K[20]	706.426	224.806	219.467	219.803	219.131	K[5]
V[21]	739.449	168.104	162.765	163.101	162.429	V[4]
L[22]	777.144	135.082	129.742	130.078	129.406	L[3]
R[23]	**829.177**	97.387	92.047	92.383	91.711	R[2]
D[24]	867.520	45.353	40.014	40.349	39.678	D[1]

sp|P62806|H4_MOUSE

[Acetyl]SGRGKGGK $^{\text{Acetyl}}_{42.01}$ GLGK $^{\text{Acetyl}}_{42.01}$ GGAK $^{\text{Acetyl}}_{42.01}$ RHRK $^{\text{Methyl}}_{14.02}$ VLRD

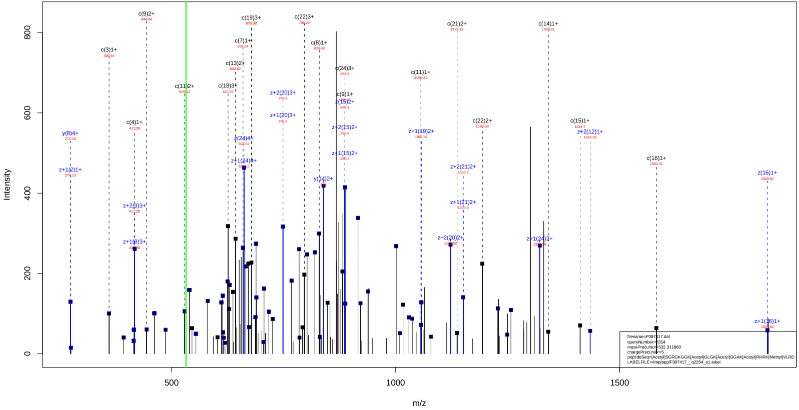

sp|P62806|H4_MOUSE

[Acetyl]SGRGKGGK $^{\text{Acetyl}}_{42.01}$ GLGK $^{\text{Acetyl}}_{42.01}$ GGAK $^{\text{Acetyl}}_{42.01}$ RHRK $^{\text{Methyl}}_{14.02}$ VLRD

- fragmentation table for charge state 1+
- specType=etd
- score=92.99
- F097417.dat
- query=q2354_p1
- precursor=532.311860
- chargePrecursor=5
- itol=0.8

AA	c	y	z+1	z+2	z	AA
S[1]	147.076	2657.518	2641.500	0.000	2640.492	S[24]
G[2]	204.098	2528.476	2512.457	0.000	2511.449	G[23]
R[3]	**360.199**	2471.454	2455.435	2456.443	2454.428	R[22]
G[4]	**417.220**	2315.353	2299.334	2300.342	2298.327	G[21]
K[5]	**545.315**	2258.332	2242.313	2243.321	2241.305	K[20]
G[6]	**602.337**	2130.237	2114.218	2115.226	2113.210	G[19]
G[7]	**659.358**	2073.215	2057.196	2058.204	2056.189	G[18]
K[8]	**829.464**	2016.194	2000.175	2001.183	1999.167	K[17]
G[9]	**886.485**	1846.088	**1830.069**	1831.077	**1829.062**	G[16]
L[10]	999.569	1789.067	1773.048	1774.056	1772.040	L[15]
G[11]	**1056.591**	1675.983	1659.964	1660.972	1658.956	G[14]
K[12]	1226.696	1618.961	1602.942	1603.950	1601.935	K[13]
G[13]	1283.718	1448.856	1432.837	**1433.845**	1431.829	G[12]
G[14]	**1340.739**	1391.834	1375.816	1376.823	1374.808	G[11]
A[15]	**1411.776**	1334.813	1318.794	1319.802	1317.786	A[10]
K[16]	**1581.882**	1263.776	1247.757	**1248.765**	1246.749	K[9]
R[17]	1737.983	1093.670	1077.651	**1078.659**	1076.644	R[8]
H[18]	1875.042	**937.569**	**921.550**	922.558	920.542	H[7]
R[19]	2031.143	800.510	**784.491**	**785.499**	783.484	R[6]
K[20]	2173.254	644.409	**628.390**	**629.398**	627.382	K[5]
V[21]	2272.322	502.298	**486.280**	487.287	485.272	V[4]
L[22]	2385.406	403.230	387.211	388.219	386.203	L[3]
R[23]	2541.507	290.146	**274.127**	**275.135**	273.119	R[2]
D[24]	2656.534	134.045	118.026	119.034	117.018	D[1]

sp|P62806|H4_MOUSE

[Acetyl]SGRGKGGK $^{Acetyl}_{42.01}$ GLGK $^{Acetyl}_{42.01}$ GGAK $^{Acetyl}_{42.01}$ RHRK $^{Methyl}_{14.02}$ VLRD

- fragmentation table for charge state 2+
- specType=etd
- score=92.99
- F097417.dat
- query=q2354_p1
- precursor=532.311860
- chargePrecursor=5
- itol=0.8

AA	c	y	z+1	z+2	z	AA
S[1]	74.042	1329.263	**1321.253**	0.504	1320.749	S[24]
G[2]	102.553	1264.741	**1256.732**	0.504	1256.228	G[23]
R[3]	180.603	1236.231	**1228.221**	1228.725	**1227.717**	R[22]
G[4]	209.114	1158.180	**1150.171**	**1150.675**	1149.667	G[21]
K[5]	273.161	1129.669	1121.660	**1122.164**	1121.156	K[20]
G[6]	301.672	1065.622	**1057.613**	**1058.117**	**1057.109**	G[19]
G[7]	330.183	**1037.111**	**1029.102**	**1029.606**	1028.598	G[18]
K[8]	**415.236**	**1008.601**	**1000.591**	**1001.095**	1000.087	K[17]
G[9]	**443.746**	923.548	**915.538**	**916.042**	915.034	G[16]
L[10]	500.288	895.037	**887.028**	**887.532**	**886.524**	L[15]
G[11]	**528.799**	**838.495**	**830.486**	**830.990**	**829.982**	G[14]
K[12]	**613.852**	809.984	**801.975**	**802.479**	801.471	K[13]
G[13]	**642.363**	**724.931**	**716.922**	**717.426**	**716.418**	G[12]
G[14]	**670.873**	696.421	**688.411**	**688.915**	**687.907**	G[11]
A[15]	**706.392**	667.910	**659.901**	660.405	**659.397**	A[10]
K[16]	**791.445**	632.391	**624.382**	**624.886**	623.878	K[9]
R[17]	869.495	547.339	**539.329**	**539.833**	**538.825**	R[8]
H[18]	**938.025**	469.288	**461.279**	**461.783**	**460.775**	H[7]
R[19]	**1016.075**	400.759	**392.749**	**393.253**	**392.245**	R[6]
K[20]	1087.130	322.708	314.699	315.203	314.195	K[5]
V[21]	**1136.665**	251.653	243.643	244.147	243.140	V[4]
L[22]	**1193.207**	202.119	194.109	194.613	193.605	L[3]
R[23]	1271.257	145.577	137.567	138.071	137.063	R[2]
D[24]	1328.771	67.526	59.517	60.021	59.013	D[1]

sp|P62806|H4_MOUSE

[Acetyl]SGRGKGGK $^{\text{Acetyl}}_{42.01}$ GLGK $^{\text{Acetyl}}_{42.01}$ GGAK $^{\text{Acetyl}}_{42.01}$ RHRK $^{\text{Methyl}}_{14.02}$ VLRD

- fragmentation table for charge state 3+
- specType=etd
- score=92.99
- F097417.dat
- query=q2354_p1
- precursor=532.311860
- chargePrecursor=5
- itol=0.8

AA	c	y	z+1	z+2	z	AA
S[1]	49.697	**886.511**	**881.171**	0.672	**880.835**	S[24]
G[2]	68.704	843.497	838.157	0.672	837.821	G[23]
R[3]	120.738	824.490	**819.150**	**819.486**	818.814	R[22]
G[4]	139.745	772.456	**767.116**	**767.452**	766.780	G[21]
K[5]	182.443	753.449	**748.109**	**748.445**	**747.773**	K[20]
G[6]	201.450	710.750	**705.411**	**705.747**	**705.075**	G[19]
G[7]	220.458	691.743	**686.404**	**686.740**	686.068	G[18]
K[8]	277.159	**672.736**	667.397	667.732	667.061	K[17]
G[9]	296.167	**616.034**	**610.695**	**611.031**	**610.359**	G[16]
L[10]	333.861	597.027	591.688	592.023	591.352	L[15]
G[11]	352.868	559.332	**553.993**	**554.329**	**553.657**	G[14]
K[12]	409.570	**540.325**	534.986	535.322	534.650	K[13]
G[13]	428.577	483.623	478.284	478.620	477.948	G[12]
G[14]	447.585	464.616	459.277	459.613	458.941	G[11]
A[15]	471.264	445.609	440.270	440.605	439.934	A[10]
K[16]	527.966	421.930	**416.590**	**416.926**	**416.255**	K[9]
R[17]	**579.999**	365.228	**359.889**	**360.225**	**359.553**	R[8]
H[18]	**625.686**	313.195	307.855	308.191	307.519	H[7]
R[19]	**677.719**	267.508	262.169	262.505	261.833	R[6]
K[20]	**725.089**	215.475	210.135	210.471	209.799	K[5]
V[21]	758.112	168.104	162.765	163.101	162.429	V[4]
L[22]	**795.807**	135.082	129.742	130.078	129.406	L[3]
R[23]	**847.841**	97.387	92.047	92.383	91.711	R[2]
D[24]	**886.183**	45.353	40.014	40.349	39.678	D[1]

sp|P62806|H4_MOUSE

[Acetyl]SGRGKGGK $^{Acetyl}_{42.01}$ GLGK $^{Acetyl}_{42.01}$ GGAK $^{Acetyl}_{42.01}$ RHRK $^{Methyl}_{14.02}$ VLRD

- fragmentation table for charge state 4+
- specType=etd
- score=92.99
- F097417.dat
- query=q2354_p1
- precursor=532.311860
- chargePrecursor=5
- itol=0.8

AA	c	y	z+1	z+2	z	AA
S[1]	37.525	**665.135**	**661.130**	0.755	**660.878**	S[24]
G[2]	51.780	632.874	**628.870**	0.755	**628.618**	G[23]
R[3]	90.805	**618.619**	**614.614**	**614.866**	**614.362**	R[22]
G[4]	105.061	**579.594**	575.589	575.841	575.337	G[21]
K[5]	137.084	565.338	561.334	561.586	561.082	K[20]
G[6]	151.340	533.315	**529.310**	**529.562**	**529.058**	G[19]
G[7]	165.595	519.059	515.055	515.307	514.803	G[18]
K[8]	208.121	504.804	500.799	501.051	500.547	K[17]
G[9]	222.377	**462.278**	458.273	458.525	458.021	G[16]
L[10]	250.648	448.022	**444.017**	**444.269**	**443.766**	L[15]
G[11]	264.903	419.751	**415.746**	**415.998**	**415.494**	G[14]
K[12]	307.430	405.496	401.491	401.743	401.239	K[13]
G[13]	321.685	362.969	358.965	359.217	358.713	G[12]
G[14]	335.940	348.714	344.709	344.961	344.457	G[11]
A[15]	353.700	334.459	330.454	330.706	330.202	A[10]
K[16]	396.226	316.699	312.695	312.947	312.443	K[9]
R[17]	435.251	**274.173**	270.168	270.420	269.916	R[8]
H[18]	469.516	235.148	231.143	231.395	230.891	H[7]
R[19]	508.541	200.883	196.878	197.130	196.626	R[6]
K[20]	544.069	161.858	157.853	158.105	157.601	K[5]
V[21]	568.836	126.330	122.325	122.577	122.073	V[4]
L[22]	597.107	101.563	97.558	97.810	97.306	L[3]
R[23]	**636.132**	73.292	69.287	69.539	69.035	R[2]
D[24]	**664.889**	34.267	30.262	30.514	30.010	D[1]

sp|P62806|H4_MOUSE

[Acetyl]SGRGKGGKGLGKGGAKRHRK Methyl 14.02 VLRD

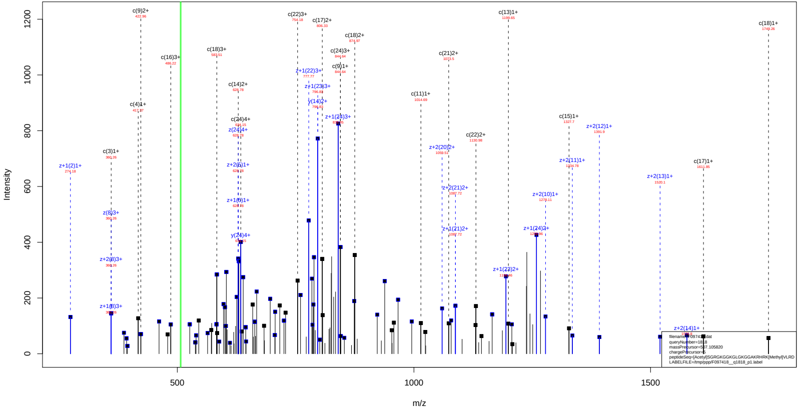

sp|P62806|H4_MOUSE

[Acetyl]SGRGKGGKGLGKGGAKRHRK $^{Methyl}_{14.02}$ VLRD

- fragmentation table for charge state 1+
- specType=etd
- score=119.64
- F097418.dat
- query=q1818_p1
- precursor=507.105820
- chargePrecursor=5
- itol=0.8

AA	c	y	z+1	z+2	z	AA
S[1]	147.076	2531.487	2515.468	0.000	2514.460	S[24]
G[2]	204.098	2402.444	2386.425	0.000	2385.417	G[23]
R[3]	**360.199**	2345.423	2329.404	2330.412	2328.396	R[22]
G[4]	**417.220**	2189.321	2173.303	2174.310	2172.295	G[21]
K[5]	**545.315**	2132.300	2116.281	2117.289	2115.273	K[20]
G[6]	**602.337**	2004.205	1988.186	1989.194	1987.178	G[19]
G[7]	**659.358**	1947.184	1931.165	1932.173	1930.157	G[18]
K[8]	**787.453**	1890.162	1874.143	1875.151	1873.135	K[17]
G[9]	**844.475**	1762.067	1746.048	1747.056	1745.041	G[16]
L[10]	**957.559**	1705.046	1689.027	1690.035	1688.019	L[15]
G[11]	**1014.580**	1591.962	1575.943	**1576.951**	1574.935	G[14]
K[12]	**1142.675**	1534.940	1518.921	**1519.929**	1517.914	K[13]
G[13]	**1199.697**	1406.845	1390.826	**1391.834**	1389.819	G[12]
G[14]	1256.718	1349.824	1333.805	**1334.813**	1332.797	G[11]
A[15]	**1327.755**	1292.802	1276.783	**1277.791**	1275.776	A[10]
K[16]	1455.850	1221.765	1205.746	**1206.754**	1204.739	K[9]
R[17]	**1611.951**	1093.670	1077.651	**1078.659**	1076.644	R[8]
H[18]	**1749.010**	**937.569**	921.550	**922.558**	920.542	H[7]
R[19]	1905.111	**800.510**	**784.491**	**785.499**	783.484	R[6]
K[20]	2047.222	**644.409**	**628.390**	**629.398**	627.382	K[5]
V[21]	2146.290	502.298	**486.280**	487.287	485.272	V[4]
L[22]	2259.374	403.230	**387.211**	388.219	386.203	L[3]
R[23]	2415.476	290.146	**274.127**	275.135	273.119	R[2]
D[24]	2530.503	134.045	118.026	119.034	117.018	D[1]

sp|P62806|H4_MOUSE

[Acetyl]SGRGKGGKGLGKGGAKRHRK$^{\text{Methyl}}_{14.02}$VLRD

- fragmentation table for charge state 2+
- specType=etd
- score=119.64
- F097418.dat
- query=q1818_p1
- precursor=507.105820
- chargePrecursor=5
- itol=0.8

AA	c	y	z+1	z+2	z	AA
S[1]	74.042	1266.247	**1258.238**	0.504	1257.734	S[24]
G[2]	102.553	1201.726	**1193.716**	0.504	1193.212	G[23]
R[3]	180.603	1173.215	**1165.206**	**1165.709**	**1164.702**	R[22]
G[4]	209.114	1095.164	**1087.155**	**1087.659**	1086.651	G[21]
K[5]	273.161	1066.654	1058.644	**1059.148**	1058.140	K[20]
G[6]	301.672	1002.606	**994.597**	**995.101**	994.093	G[19]
G[7]	330.183	974.095	**966.086**	**966.590**	965.582	G[18]
K[8]	**394.230**	945.585	**937.575**	**938.079**	937.071	K[17]
G[9]	**422.741**	881.537	**873.528**	**874.032**	873.024	G[16]
L[10]	**479.283**	**853.026**	**845.017**	**845.521**	**844.513**	L[15]
G[11]	507.794	**796.484**	**788.475**	**788.979**	**787.971**	G[14]
K[12]	**571.841**	767.974	**759.964**	**760.468**	759.460	K[13]
G[13]	**600.352**	703.926	**695.917**	**696.421**	695.413	G[12]
G[14]	**628.863**	675.415	**667.406**	**667.910**	666.902	G[11]
A[15]	**664.381**	646.905	**638.895**	**639.399**	638.391	A[10]
K[16]	**728.429**	**611.386**	**603.377**	**603.881**	**602.873**	K[9]
R[17]	**806.479**	547.339	**539.329**	**539.833**	**538.825**	R[8]
H[18]	**875.009**	469.288	**461.279**	**461.783**	**460.775**	H[7]
R[19]	**953.059**	400.759	**392.749**	**393.253**	**392.245**	R[6]
K[20]	**1024.115**	322.708	314.699	315.203	314.195	K[5]
V[21]	**1073.649**	251.653	243.643	244.147	243.140	V[4]
L[22]	**1130.191**	202.119	194.109	194.613	193.605	L[3]
R[23]	**1208.241**	145.577	137.567	138.071	137.063	R[2]
D[24]	1265.755	67.526	59.517	60.021	59.013	D[1]

sp|P62806|H4_MOUSE

[Acetyl]SGRGKGGKGLGKGGAKRHRK$^{Methyl}_{14.02}$VLRD

- fragmentation table for charge state 3+
- specType=etd
- score=119.64
- F097418.dat
- query=q1818_p1
- precursor=507.105820
- chargePrecursor=5
- itol=0.8

AA	c	y	z+1	z+2	z	AA
S[1]	49.697	**844.500**	**839.161**	0.672	838.825	S[24]
G[2]	68.704	**801.486**	**796.147**	0.672	795.811	G[23]
R[3]	120.738	782.479	**777.139**	**777.475**	776.804	R[22]
G[4]	139.745	730.445	**725.106**	**725.442**	**724.770**	G[21]
K[5]	182.443	711.438	**706.099**	**706.435**	**705.763**	K[20]
G[6]	201.450	668.740	**663.400**	**663.736**	**663.064**	G[19]
G[7]	220.458	649.733	**644.393**	**644.729**	**644.057**	G[18]
K[8]	263.156	**630.726**	**625.386**	**625.722**	**625.050**	K[17]
G[9]	282.163	**588.027**	**582.688**	**583.024**	**582.352**	G[16]
L[10]	319.858	569.020	**563.680**	**564.016**	**563.345**	L[15]
G[11]	338.865	531.325	**525.986**	**526.322**	**525.650**	G[14]
K[12]	381.563	512.318	506.979	507.315	506.643	K[13]
G[13]	400.570	469.620	464.280	464.616	463.944	G[12]
G[14]	419.578	450.613	445.273	445.609	444.937	G[11]
A[15]	443.257	431.606	426.266	426.602	425.930	A[10]
K[16]	**485.955**	407.927	402.587	402.923	402.251	K[9]
R[17]	**537.989**	365.228	**359.889**	**360.225**	**359.553**	R[8]
H[18]	**583.675**	313.195	307.855	308.191	307.519	H[7]
R[19]	**635.709**	267.508	262.169	262.505	261.833	R[6]
K[20]	**683.079**	215.475	210.135	210.471	209.799	K[5]
V[21]	**716.102**	168.104	162.765	163.101	162.429	V[4]
L[22]	**753.796**	135.082	129.742	130.078	129.406	L[3]
R[23]	**805.830**	97.387	92.047	92.383	91.711	R[2]
D[24]	**844.172**	45.353	40.014	40.349	39.678	D[1]

sp|P62806|H4_MOUSE

[Acetyl]SGRGKGGKGLGKGGAKRHRK$^{Methyl}_{14.02}$VLRD

- fragmentation table for charge state 4+
- specType=etd
- score=119.64
- F097418.dat
- query=q1818_p1
- precursor=507.105820
- chargePrecursor=5
- itol=0.8

AA	c	y	z+1	z+2	z	AA
S[1]	37.525	**633.627**	**629.622**	0.755	**629.370**	S[24]
G[2]	51.780	**601.366**	**597.362**	0.755	**597.110**	G[23]
R[3]	90.805	587.111	**583.106**	**583.358**	**582.854**	R[22]
G[4]	105.061	548.086	544.081	544.333	543.829	G[21]
K[5]	137.084	533.830	529.826	530.078	529.574	K[20]
G[6]	151.340	501.807	497.802	498.054	497.550	G[19]
G[7]	165.595	487.551	483.547	483.799	483.295	G[18]
K[8]	197.619	473.296	469.291	469.543	469.039	K[17]
G[9]	211.874	441.272	437.268	437.520	437.016	G[16]
L[10]	240.145	427.017	**423.012**	**423.264**	**422.760**	L[15]
G[11]	254.401	398.746	**394.741**	**394.993**	**394.489**	G[14]
K[12]	286.424	384.490	380.486	380.738	380.234	K[13]
G[13]	300.680	352.467	348.462	348.714	348.210	G[12]
G[14]	314.935	338.211	334.207	334.459	333.955	G[11]
A[15]	332.694	323.956	319.951	320.203	319.699	A[10]
K[16]	364.718	306.197	302.192	302.444	301.940	K[9]
R[17]	403.743	**274.173**	270.168	270.420	269.916	R[8]
H[18]	438.008	235.148	231.143	231.395	230.891	H[7]
R[19]	477.033	200.883	196.878	197.130	196.626	R[6]
K[20]	512.561	161.858	157.853	158.105	157.601	K[5]
V[21]	**537.328**	126.330	122.325	122.577	122.073	V[4]
L[22]	565.599	101.563	97.558	97.810	97.306	L[3]
R[23]	604.624	73.292	69.287	69.539	69.035	R[2]
D[24]	**633.381**	34.267	30.262	30.514	30.010	D[1]

sp|P62806|H4_MOUSE

[Acetyl]SGRGKGGKGLGKGGAKRHRK $^{Methyl}_{14.02}$ VLRD

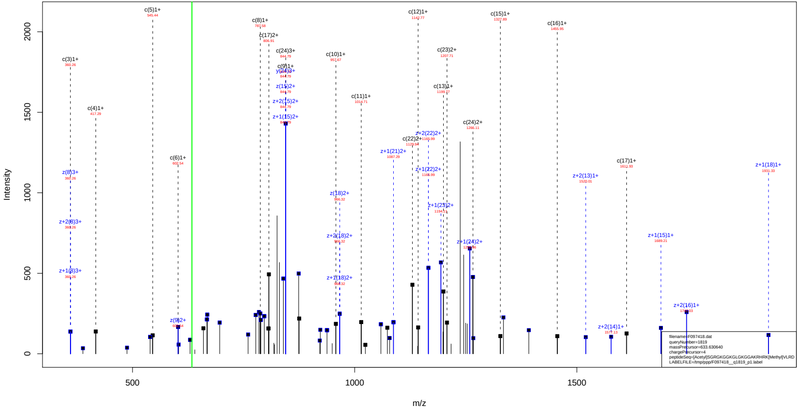

sp|P62806|H4_MOUSE

[Acetyl]SGRGKGGKGLGKGGAKRHRK $^{\text{Methyl}}_{14.02}$ VLRD

- fragmentation table for charge state 1+
- specType=etd
- score=128.04
- F097418.dat
- query=q1819_p1
- precursor=633.630640
- chargePrecursor=4
- itol=0.8

AA	c	y	z+1	z+2	z	AA
S[1]	147.076	2531.487	2515.468	0.000	2514.460	S[24]
G[2]	204.098	2402.444	2386.425	0.000	2385.417	G[23]
R[3]	**360.199**	2345.423	2329.404	2330.412	2328.396	R[22]
G[4]	**417.220**	2189.321	2173.303	2174.310	2172.295	G[21]
K[5]	**545.315**	2132.300	2116.281	2117.289	2115.273	K[20]
G[6]	**602.337**	2004.205	1988.186	1989.194	1987.178	G[19]
G[7]	**659.358**	1947.184	**1931.165**	1932.173	1930.157	G[18]
K[8]	**787.453**	1890.162	1874.143	1875.151	1873.135	K[17]
G[9]	**844.475**	1762.067	1746.048	**1747.056**	1745.041	G[16]
L[10]	**957.559**	1705.046	**1689.027**	1690.035	1688.019	L[15]
G[11]	**1014.580**	1591.962	1575.943	**1576.951**	1574.935	G[14]
K[12]	**1142.675**	1534.940	1518.921	**1519.929**	1517.914	K[13]
G[13]	**1199.697**	1406.845	1390.826	**1391.834**	1389.819	G[12]
G[14]	1256.718	1349.824	1333.805	**1334.813**	1332.797	G[11]
A[15]	**1327.755**	1292.802	1276.783	1277.791	1275.776	A[10]
K[16]	**1455.850**	1221.765	1205.746	1206.754	1204.739	K[9]
R[17]	**1611.951**	1093.670	1077.651	**1078.659**	1076.644	R[8]
H[18]	1749.010	**937.569**	**921.550**	**922.558**	920.542	H[7]
R[19]	1905.111	800.510	**784.491**	785.499	783.484	R[6]
K[20]	2047.222	644.409	628.390	**629.398**	627.382	K[5]
V[21]	2146.290	502.298	486.280	**487.287**	485.272	V[4]
L[22]	2259.374	403.230	387.211	**388.219**	386.203	L[3]
R[23]	2415.476	290.146	274.127	275.135	273.119	R[2]
D[24]	2530.503	134.045	118.026	119.034	117.018	D[1]

sp|P62806|H4_MOUSE

[Acetyl]SGRGKGGKGLGKGGAKRHRK$^{Methyl}_{14.02}$VLRD

- fragmentation table for charge state 2+
- specType=etd
- score=128.04
- F097418.dat
- query=q1819_p1
- precursor=633.630640
- chargePrecursor=4
- itol=0.8

AA	c	y	z+1	z+2	z	AA
S[1]	74.042	**1266.247**	**1258.238**	0.504	1257.734	S[24]
G[2]	102.553	1201.726	**1193.716**	0.504	1193.212	G[23]
R[3]	180.603	1173.215	**1165.206**	**1165.709**	1164.702	R[22]
G[4]	209.114	1095.164	**1087.155**	**1087.659**	**1086.651**	G[21]
K[5]	273.161	1066.654	**1058.644**	**1059.148**	1058.140	K[20]
G[6]	301.672	1002.606	994.597	995.101	994.093	G[19]
G[7]	330.183	974.095	**966.086**	**966.590**	**965.582**	G[18]
K[8]	394.230	945.585	**937.575**	**938.079**	**937.071**	K[17]
G[9]	422.741	881.537	**873.528**	**874.032**	873.024	G[16]
L[10]	479.283	853.026	**845.017**	**845.521**	**844.513**	L[15]
G[11]	507.794	**796.484**	**788.475**	**788.979**	**787.971**	G[14]
K[12]	571.841	767.974	**759.964**	**760.468**	**759.460**	K[13]
G[13]	600.352	703.926	**695.917**	**696.421**	695.413	G[12]
G[14]	**628.863**	675.415	**667.406**	**667.910**	**666.902**	G[11]
A[15]	664.381	646.905	638.895	639.399	638.391	A[10]
K[16]	728.429	611.386	**603.377**	**603.881**	**602.873**	K[9]
R[17]	**806.479**	547.339	**539.329**	**539.833**	**538.825**	R[8]
H[18]	**875.009**	469.288	461.279	461.783	460.775	H[7]
R[19]	953.059	400.759	392.749	393.253	392.245	R[6]
K[20]	**1024.115**	322.708	314.699	315.203	314.195	K[5]
V[21]	**1073.649**	251.653	243.643	244.147	243.140	V[4]
L[22]	**1130.191**	202.119	194.109	194.613	193.605	L[3]
R[23]	**1208.241**	145.577	137.567	138.071	137.063	R[2]
D[24]	**1265.755**	67.526	59.517	60.021	59.013	D[1]

sp|P62806|H4_MOUSE

[Acetyl]SGRGKGGKGLGKGGAKRHRK$^{\text{Methyl}}_{14.02}$VLRD

- fragmentation table for charge state 3+
- specType=etd
- score=128.04
- F097418.dat
- query=q1819_p1
- precursor=633.630640
- chargePrecursor=4
- itol=0.8

AA	c	y	z+1	z+2	z	AA
S[1]	49.697	**844.500**	**839.161**	0.672	**838.825**	S[24]
G[2]	68.704	801.486	**796.147**	0.672	**795.811**	G[23]
R[3]	120.738	782.479	**777.139**	**777.475**	**776.804**	R[22]
G[4]	139.745	730.445	725.106	725.442	724.770	G[21]
K[5]	182.443	711.438	706.099	706.435	705.763	K[20]
G[6]	201.450	**668.740**	663.400	663.736	663.064	G[19]
G[7]	220.458	649.733	644.393	644.729	644.057	G[18]
K[8]	263.156	630.726	625.386	625.722	625.050	K[17]
G[9]	282.163	588.027	582.688	583.024	582.352	G[16]
L[10]	319.858	569.020	563.680	564.016	563.345	L[15]
G[11]	338.865	531.325	525.986	526.322	525.650	G[14]
K[12]	381.563	512.318	506.979	507.315	506.643	K[13]
G[13]	400.570	469.620	464.280	464.616	463.944	G[12]
G[14]	419.578	450.613	445.273	445.609	444.937	G[11]
A[15]	443.257	431.606	426.266	426.602	425.930	A[10]
K[16]	485.955	407.927	402.587	402.923	402.251	K[9]
R[17]	537.989	365.228	**359.889**	**360.225**	**359.553**	R[8]
H[18]	583.675	313.195	307.855	308.191	307.519	H[7]
R[19]	635.709	267.508	262.169	262.505	261.833	R[6]
K[20]	683.079	215.475	210.135	210.471	209.799	K[5]
V[21]	716.102	168.104	162.765	163.101	162.429	V[4]
L[22]	753.796	135.082	129.742	130.078	129.406	L[3]
R[23]	**805.830**	97.387	92.047	92.383	91.711	R[2]
D[24]	**844.172**	45.353	40.014	40.349	39.678	D[1]

sp|P62806|H4_MOUSE

[Acetyl]SGR $^{\text{Methyl}}_{14.02}$ GKGGKGLGKGGAKRHR $^{\text{Dimethyl}}_{28.03}$ KVLRD

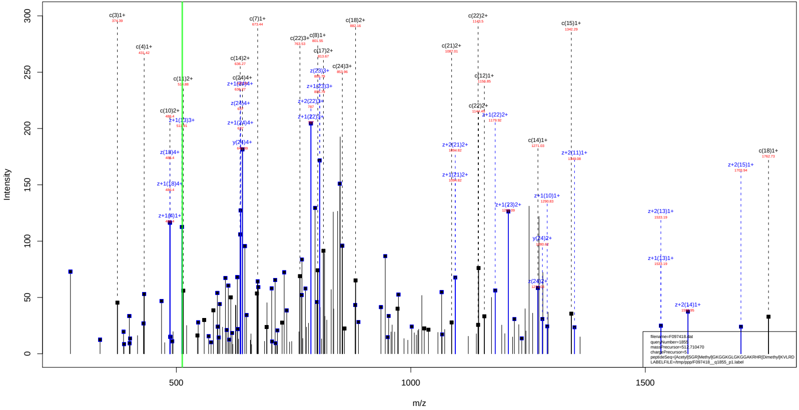

sp|P62806|H4_MOUSE

[Acetyl]SGR $^{Methyl}_{14.02}$ GKGGKGLGKGGAKRHR $^{Dimethyl}_{28.03}$ KVLRD

- fragmentation table for charge state 1+
- specType=etd
- score=108.76
- F097418.dat
- query=q1855_p1
- precursor=512.710470
- chargePrecursor=5
- itol=0.8

AA	c	y	z+1	z+2	z	AA
S[1]	147.076	2559.518	2543.499	0.000	2542.491	S[24]
G[2]	204.098	2430.475	2414.457	0.000	2413.449	G[23]
R[3]	**374.215**	2373.454	2357.435	2358.443	2356.427	R[22]
G[4]	**431.236**	2203.337	2187.318	2188.326	2186.310	G[21]
K[5]	**559.331**	2146.316	2130.297	2131.305	2129.289	K[20]
G[6]	**616.353**	2018.221	2002.202	2003.210	2001.194	G[19]
G[7]	**673.374**	1961.199	1945.180	1946.188	1944.173	G[18]
K[8]	**801.469**	1904.178	1888.159	1889.167	1887.151	K[17]
G[9]	**858.490**	1776.083	1760.064	1761.072	1759.056	G[16]
L[10]	**971.574**	1719.061	1703.043	**1704.050**	1702.035	L[15]
G[11]	**1028.596**	1605.977	1589.958	**1590.966**	1588.951	G[14]
K[12]	**1156.691**	1548.956	**1532.937**	**1533.945**	1531.929	K[13]
G[13]	1213.712	1420.861	1404.842	1405.850	1403.834	G[12]
G[14]	**1270.734**	1363.839	1347.821	**1348.828**	1346.813	G[11]
A[15]	**1341.771**	1306.818	**1290.799**	1291.807	1289.791	A[10]
K[16]	1469.866	**1235.781**	1219.762	**1220.770**	1218.754	K[9]
R[17]	1625.967	1107.686	1091.667	1092.675	1090.659	R[8]
H[18]	**1763.026**	**951.585**	**935.566**	**936.574**	934.558	H[7]
R[19]	1947.158	814.526	798.507	**799.515**	797.499	R[6]
K[20]	2075.253	**630.393**	614.375	615.382	**613.367**	K[5]
V[21]	2174.322	502.298	**486.280**	**487.287**	485.272	V[4]
L[22]	2287.406	403.230	**387.211**	**388.219**	386.203	L[3]
R[23]	2443.507	290.146	**274.127**	275.135	273.119	R[2]
D[24]	2558.534	134.045	118.026	119.034	117.018	D[1]

sp|P62806|H4_MOUSE

[Acetyl]SGR $^{\text{Methyl}}_{14.02}$ GKGGKGLGKGGAKRHR $^{\text{Dimethyl}}_{28.03}$ KVLRD

- fragmentation table for charge state 2+
- specType=etd
- score=108.76
- F097418.dat
- query=q1855_p1
- precursor=512.710470
- chargePrecursor=5
- itol=0.8

AA	c	y	z+1	z+2	z	AA
S[1]	74.042	**1280.263**	1272.253	0.504	**1271.749**	S[24]
G[2]	102.553	1215.741	**1207.732**	0.504	1207.228	G[23]
R[3]	187.611	1187.231	**1179.221**	**1179.725**	1178.717	R[22]
G[4]	216.122	1102.172	**1094.163**	**1094.667**	1093.659	G[21]
K[5]	280.169	1073.661	**1065.652**	**1066.156**	**1065.148**	K[20]
G[6]	308.680	1009.614	**1001.605**	**1002.108**	1001.101	G[19]
G[7]	**337.191**	981.103	**973.094**	**973.598**	972.590	G[18]
K[8]	**401.238**	**952.592**	944.583	**945.087**	944.079	K[17]
G[9]	**429.749**	**888.545**	880.536	**881.040**	880.032	G[16]
L[10]	**486.291**	860.034	852.025	852.529	851.521	L[15]
G[11]	**514.802**	803.492	**795.483**	**795.987**	794.979	G[14]
K[12]	**578.849**	**774.982**	**766.972**	**767.476**	**766.468**	K[13]
G[13]	**607.360**	**710.934**	**702.925**	**703.429**	702.421	G[12]
G[14]	**635.871**	682.423	**674.414**	**674.918**	**673.910**	G[11]
A[15]	**671.389**	653.913	**645.903**	**646.407**	**645.399**	A[10]
K[16]	**735.437**	**618.394**	**610.385**	**610.889**	609.881	K[9]
R[17]	**813.487**	554.347	**546.337**	**546.841**	**545.833**	R[8]
H[18]	**882.017**	476.296	**468.287**	**468.791**	**467.783**	H[7]
R[19]	**974.083**	407.767	**399.757**	**400.261**	**399.253**	R[6]
K[20]	**1038.130**	315.700	307.691	308.195	307.187	K[5]
V[21]	**1087.665**	251.653	243.643	244.147	243.140	V[4]
L[22]	**1144.207**	202.119	194.109	194.613	193.605	L[3]
R[23]	1222.257	145.577	137.567	138.071	137.063	R[2]
D[24]	1279.771	67.526	59.517	60.021	59.013	D[1]

sp|P62806|H4_MOUSE

[Acetyl]SGR $^{\text{Methyl}}_{14.02}$ GKGGKGLGKGGAKRHR $^{\text{Dimethyl}}_{28.03}$ KVLRD

- fragmentation table for charge state 3+
- specType=etd
- score=108.76
- F097418.dat
- query=q1855_p1
- precursor=512.710470
- chargePrecursor=5
- itol=0.8

AA	c	y	z+1	z+2	z	AA
S[1]	49.697	**853.844**	**848.505**	0.672	**848.169**	S[24]
G[2]	68.704	810.830	**805.490**	0.672	**805.154**	G[23]
R[3]	125.410	791.823	**786.483**	**786.819**	786.147	R[22]
G[4]	144.417	**735.117**	**729.778**	**730.114**	**729.442**	G[21]
K[5]	187.115	**716.110**	**710.770**	**711.106**	**710.435**	K[20]
G[6]	206.122	**673.412**	668.072	668.408	667.736	G[19]
G[7]	225.130	654.405	**649.065**	**649.401**	648.729	G[18]
K[8]	267.828	635.397	**630.058**	**630.394**	**629.722**	K[17]
G[9]	286.835	**592.699**	**587.360**	**587.695**	**587.024**	G[16]
L[10]	324.530	**573.692**	**568.352**	**568.688**	568.016	L[15]
G[11]	343.537	535.997	530.658	530.994	530.322	G[14]
K[12]	386.235	516.990	**511.651**	**511.986**	**511.315**	K[13]
G[13]	405.242	474.292	**468.952**	469.288	**468.616**	G[12]
G[14]	424.249	455.285	449.945	450.281	449.609	G[11]
A[15]	447.929	436.277	**430.938**	**431.274**	**430.602**	A[10]
K[16]	**490.627**	412.598	407.259	407.595	406.923	K[9]
R[17]	542.661	369.900	364.561	364.896	364.225	R[8]
H[18]	**588.347**	317.866	312.527	312.863	312.191	H[7]
R[19]	**649.724**	272.180	266.841	267.176	266.505	R[6]
K[20]	**692.423**	210.803	205.463	205.799	205.127	K[5]
V[21]	**725.445**	168.104	162.765	163.101	162.429	V[4]
L[22]	**763.140**	135.082	129.742	130.078	129.406	L[3]
R[23]	815.174	97.387	92.047	92.383	91.711	R[2]
D[24]	**853.516**	45.353	40.014	40.349	39.678	D[1]

sp|P62806|H4_MOUSE

[Acetyl]SGR $^{\text{Methyl}}_{14.02}$ GKGGKGLGKGGAKRHR $^{\text{Dimethyl}}_{28.03}$ KVLRD

- fragmentation table for charge state 4+
- specType=etd
- score=108.76
- F097418.dat
- query=q1855_p1
- precursor=512.710470
- chargePrecursor=5
- itol=0.8

AA	c	y	z+1	z+2	z	AA
S[1]	37.525	**640.635**	**636.630**	0.755	**636.378**	S[24]
G[2]	51.780	**608.374**	**604.370**	0.755	**604.118**	G[23]
R[3]	94.309	594.119	**590.114**	**590.366**	**589.862**	R[22]
G[4]	108.564	551.590	547.585	547.837	**547.333**	G[21]
K[5]	140.588	537.334	533.330	533.582	533.078	K[20]
G[6]	154.844	505.311	501.306	501.558	501.054	G[19]
G[7]	169.099	**491.055**	**487.051**	**487.303**	**486.799**	G[18]
K[8]	201.123	476.800	472.795	473.047	472.543	K[17]
G[9]	215.378	444.776	440.771	441.023	440.520	G[16]
L[10]	243.649	**430.521**	426.516	426.768	426.264	L[15]
G[11]	257.904	**402.250**	398.245	398.497	397.993	G[14]
K[12]	289.928	**387.994**	383.990	384.242	383.738	K[13]
G[13]	304.184	355.971	351.966	352.218	351.714	G[12]
G[14]	318.439	341.715	**337.711**	**337.963**	**337.459**	G[11]
A[15]	336.198	327.460	323.455	323.707	323.203	A[10]
K[16]	368.222	309.701	305.696	305.948	305.444	K[9]
R[17]	407.247	277.677	**273.672**	**273.924**	**273.420**	R[8]
H[18]	441.512	238.652	234.647	234.899	234.395	H[7]
R[19]	**487.545**	204.387	200.382	200.634	200.130	R[6]
K[20]	519.569	158.354	154.349	154.601	154.097	K[5]
V[21]	**544.336**	126.330	122.325	122.577	122.073	V[4]
L[22]	572.607	101.563	97.558	97.810	97.306	L[3]
R[23]	**611.632**	73.292	69.287	69.539	69.035	R[2]
D[24]	**640.389**	34.267	30.262	30.514	30.010	D[1]

sp|P62806|H4_MOUSE

[Acetyl]SGR $^{Methyl}_{14.02}$ GKGGKGLGKGGAKRHRK $^{Dimethyl}_{28.03}$ VLRD

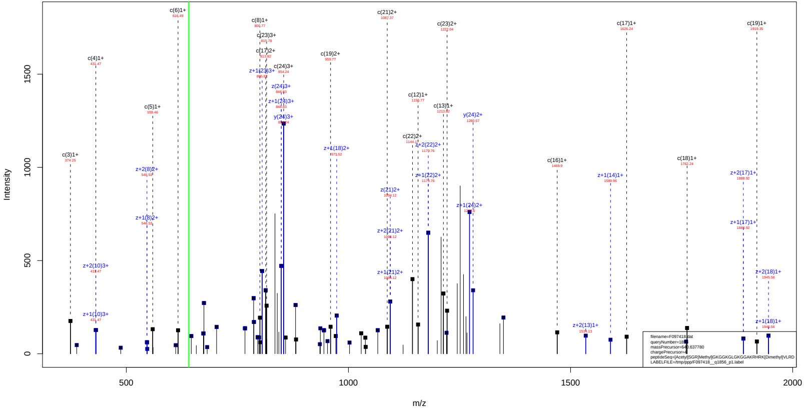

sp|P62806|H4_MOUSE

[Acetyl]SGR $^{\text{Methyl}}_{14.02}$ GKGGKGLGKGGAKRHRK $^{\text{Dimethyl}}_{28.03}$ VLRD

- fragmentation table for charge state 1+
- specType=etd
- score=105.94
- F097418.dat
- query=q1856_p1
- precursor=640.637780
- chargePrecursor=4
- itol=0.8

AA	c	y	z+1	z+2	z	AA
S[1]	147.076	2559.518	2543.499	0.000	2542.491	S[24]
G[2]	204.098	2430.475	2414.457	0.000	2413.449	G[23]
R[3]	**374.215**	2373.454	2357.435	2358.443	2356.427	R[22]
G[4]	**431.236**	2203.337	2187.318	2188.326	2186.310	G[21]
K[5]	**559.331**	2146.316	2130.297	2131.305	2129.289	K[20]
G[6]	**616.353**	2018.221	2002.202	2003.210	2001.194	G[19]
G[7]	**673.374**	1961.199	**1945.180**	**1946.188**	1944.173	G[18]
K[8]	**801.469**	1904.178	**1888.159**	**1889.167**	1887.151	K[17]
G[9]	**858.490**	1776.083	**1760.064**	1761.072	1759.056	G[16]
L[10]	**971.574**	1719.061	1703.043	1704.050	1702.035	L[15]
G[11]	**1028.596**	1605.977	**1589.958**	1590.966	1588.951	G[14]
K[12]	**1156.691**	1548.956	1532.937	**1533.945**	1531.929	K[13]
G[13]	**1213.712**	1420.861	1404.842	1405.850	1403.834	G[12]
G[14]	1270.734	1363.839	1347.821	**1348.828**	1346.813	G[11]
A[15]	1341.771	1306.818	1290.799	1291.807	1289.791	A[10]
K[16]	**1469.866**	1235.781	1219.762	**1220.770**	1218.754	K[9]
R[17]	**1625.967**	1107.686	1091.667	1092.675	1090.659	R[8]
H[18]	**1763.026**	951.585	**935.566**	**936.574**	934.558	H[7]
R[19]	**1919.127**	**814.526**	**798.507**	**799.515**	797.499	R[6]
K[20]	2075.253	658.425	642.406	643.414	641.398	K[5]
V[21]	2174.322	502.298	486.280	**487.287**	485.272	V[4]
L[22]	2287.406	403.230	387.211	**388.219**	386.203	L[3]
R[23]	2443.507	290.146	274.127	275.135	273.119	R[2]
D[24]	2558.534	134.045	118.026	119.034	117.018	D[1]

sp|P62806|H4_MOUSE

[Acetyl]SGR $^{\text{Methyl}}_{14.02}$ GKGGKGLGKGGAKRHRK $^{\text{Dimethyl}}_{28.03}$ VLRD

- fragmentation table for charge state 2+
- specType=etd
- score=105.94
- F097418.dat
- query=q1856_p1
- precursor=640.637780
- chargePrecursor=4
- itol=0.8

AA	c	y	z+1	z+2	z	AA
S[1]	74.042	**1280.263**	**1272.253**	0.504	1271.749	S[24]
G[2]	102.553	1215.741	1207.732	0.504	1207.228	G[23]
R[3]	187.611	1187.231	**1179.221**	**1179.725**	1178.717	R[22]
G[4]	216.122	1102.172	**1094.163**	**1094.667**	**1093.659**	G[21]
K[5]	280.169	1073.661	**1065.652**	**1066.156**	1065.148	K[20]
G[6]	308.680	1009.614	1001.605	**1002.108**	1001.101	G[19]
G[7]	337.191	981.103	**973.094**	**973.598**	972.590	G[18]
K[8]	401.238	**952.592**	**944.583**	**945.087**	**944.079**	K[17]
G[9]	429.749	888.545	**880.536**	**881.040**	880.032	G[16]
L[10]	486.291	860.034	852.025	852.529	851.521	L[15]
G[11]	514.802	803.492	**795.483**	**795.987**	**794.979**	G[14]
K[12]	578.849	774.982	**766.972**	**767.476**	**766.468**	K[13]
G[13]	607.360	710.934	**702.925**	**703.429**	702.421	G[12]
G[14]	635.871	**682.423**	**674.414**	**674.918**	**673.910**	G[11]
A[15]	671.389	653.913	**645.903**	**646.407**	645.399	A[10]
K[16]	735.437	618.394	610.385	**610.889**	609.881	K[9]
R[17]	**813.487**	554.347	**546.337**	**546.841**	**545.833**	R[8]
H[18]	**882.017**	476.296	468.287	468.791	467.783	H[7]
R[19]	**960.067**	407.767	399.757	400.261	399.253	R[6]
K[20]	**1038.130**	329.716	321.707	322.211	321.203	K[5]
V[21]	**1087.665**	251.653	243.643	244.147	243.140	V[4]
L[22]	**1144.207**	202.119	194.109	194.613	193.605	L[3]
R[23]	**1222.257**	145.577	137.567	138.071	137.063	R[2]
D[24]	1279.771	67.526	59.517	60.021	59.013	D[1]

sp|P62806|H4_MOUSE

[Acetyl]SGR $^{Methyl}_{14.02}$ GKGGKGLGKGGAKRHRK $^{Dimethyl}_{28.03}$ VLRD

- fragmentation table for charge state 3+
- specType=etd
- score=105.94
- F097418.dat
- query=q1856_p1
- precursor=640.637780
- chargePrecursor=4
- itol=0.8

AA	c	y	z+1	z+2	z	AA
S[1]	49.697	**853.844**	**848.505**	0.672	**848.169**	S[24]
G[2]	68.704	810.830	**805.490**	0.672	**805.154**	G[23]
R[3]	125.410	791.823	**786.483**	**786.819**	**786.147**	R[22]
G[4]	144.417	735.117	729.778	730.114	729.442	G[21]
K[5]	187.115	716.110	710.770	711.106	710.435	K[20]
G[6]	206.122	**673.412**	668.072	668.408	667.736	G[19]
G[7]	225.130	654.405	649.065	649.401	648.729	G[18]
K[8]	267.828	635.397	630.058	630.394	629.722	K[17]
G[9]	286.835	592.699	587.360	587.695	587.024	G[16]
L[10]	324.530	573.692	568.352	568.688	568.016	L[15]
G[11]	343.537	535.997	530.658	530.994	530.322	G[14]
K[12]	386.235	516.990	511.651	511.986	511.315	K[13]
G[13]	405.242	474.292	468.952	469.288	468.616	G[12]
G[14]	424.249	455.285	449.945	450.281	449.609	G[11]
A[15]	447.929	436.277	**430.938**	**431.274**	430.602	A[10]
K[16]	490.627	412.598	407.259	407.595	406.923	K[9]
R[17]	542.661	369.900	364.561	364.896	364.225	R[8]
H[18]	588.347	317.866	312.527	312.863	312.191	H[7]
R[19]	640.381	272.180	266.841	267.176	266.505	R[6]
K[20]	692.423	220.146	214.807	215.143	214.471	K[5]
V[21]	725.445	168.104	162.765	163.101	162.429	V[4]
L[22]	763.140	135.082	129.742	130.078	129.406	L[3]
R[23]	**815.174**	97.387	92.047	92.383	91.711	R[2]
D[24]	**853.516**	45.353	40.014	40.349	39.678	D[1]

sp|P62806|H4_MOUSE

[Acetyl]SGRGKGGKGLGKGGAK $^{\text{Acetyl}}_{42.01}$ RHRK $^{\text{Methyl}}_{14.02}$ VLRD

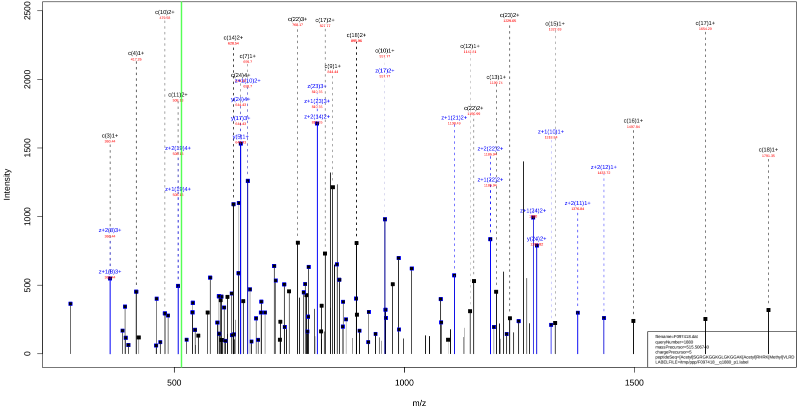

sp|P62806|H4_MOUSE

[Acetyl]SGRGKGGKGLGKGGAK $^{Acetyl}_{42.01}$ RHRK $^{Methyl}_{14.02}$ VLRD

- fragmentation table for charge state 1+
- specType=etd
- score=112.71
- F097418.dat
- query=q1880_p1
- precursor=515.506740
- chargePrecursor=5
- itol=0.8

AA	c	y	z+1	z+2	z	AA
S[1]	147.076	2573.497	2557.478	0.000	2556.471	S[24]
G[2]	204.098	2444.455	2428.436	0.000	2427.428	G[23]
R[3]	**360.199**	2387.433	2371.414	2372.422	2370.407	R[22]
G[4]	**417.220**	2231.332	2215.313	2216.321	2214.305	G[21]
K[5]	**545.315**	2174.310	2158.292	2159.300	2157.284	K[20]
G[6]	**602.337**	2046.216	2030.197	2031.205	2029.189	G[19]
G[7]	**659.358**	1989.194	1973.175	1974.183	1972.168	G[18]
K[8]	**787.453**	1932.173	1916.154	1917.162	1915.146	K[17]
G[9]	**844.475**	1804.078	1788.059	1789.067	1787.051	G[16]
L[10]	**957.559**	1747.056	1731.037	1732.045	1730.030	L[15]
G[11]	1014.580	1633.972	1617.953	1618.961	1616.946	G[14]
K[12]	**1142.675**	1576.951	1560.932	1561.940	1559.924	K[13]
G[13]	**1199.697**	1448.856	1432.837	**1433.845**	1431.829	G[12]
G[14]	1256.718	1391.834	1375.816	**1376.823**	1374.808	G[11]
A[15]	**1327.755**	1334.813	**1318.794**	1319.802	1317.786	A[10]
K[16]	**1497.861**	1263.776	**1247.757**	**1248.765**	1246.749	K[9]
R[17]	**1653.962**	1093.670	1077.651	**1078.659**	1076.644	R[8]
H[18]	**1791.021**	**937.569**	**921.550**	**922.558**	920.542	H[7]
R[19]	1947.122	800.510	**784.491**	785.499	783.484	R[6]
K[20]	2089.233	**644.409**	**628.390**	**629.398**	627.382	K[5]
V[21]	2188.301	502.298	**486.280**	487.287	485.272	V[4]
L[22]	2301.385	403.230	**387.211**	388.219	386.203	L[3]
R[23]	2457.486	290.146	**274.127**	275.135	273.119	R[2]
D[24]	2572.513	134.045	118.026	119.034	117.018	D[1]

sp|P62806|H4_MOUSE

[Acetyl]SGRGKGGKGLGKGGAK $^{Acetyl}_{42.01}$ RHRK $^{Methyl}_{14.02}$ VLRD

- fragmentation table for charge state 2+
- specType=etd
- score=112.71
- F097418.dat
- query=q1880_p1
- precursor=515.506740
- chargePrecursor=5
- itol=0.8

AA	c	y	z+1	z+2	z	AA
S[1]	74.042	**1287.252**	**1279.243**	0.504	1278.739	S[24]
G[2]	102.553	**1222.731**	1214.722	0.504	1214.218	G[23]
R[3]	180.603	**1194.220**	**1186.211**	**1186.715**	1185.707	R[22]
G[4]	209.114	1116.170	**1108.160**	**1108.664**	1107.656	G[21]
K[5]	273.161	1087.659	**1079.650**	**1080.153**	**1079.146**	K[20]
G[6]	301.672	1023.611	**1015.602**	**1016.106**	**1015.098**	G[19]
G[7]	330.183	995.101	**987.091**	**987.595**	986.587	G[18]
K[8]	**394.230**	966.590	**958.581**	**959.084**	**958.077**	K[17]
G[9]	**422.741**	**902.542**	**894.533**	**895.037**	894.029	G[16]
L[10]	**479.283**	**874.032**	**866.022**	**866.526**	**865.518**	L[15]
G[11]	**507.794**	817.490	809.480	**809.984**	808.976	G[14]
K[12]	**571.841**	**788.979**	**780.970**	**781.474**	**780.466**	K[13]
G[13]	**600.352**	724.931	**716.922**	**717.426**	**716.418**	G[12]
G[14]	**628.863**	**696.421**	**688.411**	**688.915**	**687.907**	G[11]
A[15]	**664.381**	**667.910**	**659.901**	**660.405**	**659.397**	A[10]
K[16]	**749.434**	632.391	**624.382**	**624.886**	**623.878**	K[9]
R[17]	**827.485**	547.339	**539.329**	**539.833**	**538.825**	R[8]
H[18]	**896.014**	**469.288**	**461.279**	**461.783**	**460.775**	H[7]
R[19]	**974.065**	**400.759**	**392.749**	**393.253**	**392.245**	R[6]
K[20]	1045.120	322.708	314.699	315.203	314.195	K[5]
V[21]	**1094.654**	251.653	243.643	244.147	243.140	V[4]
L[22]	**1151.196**	202.119	194.109	194.613	193.605	L[3]
R[23]	**1229.247**	145.577	137.567	138.071	137.063	R[2]
D[24]	1286.760	67.526	59.517	60.021	59.013	D[1]

sp|P62806|H4_MOUSE

[Acetyl]SGRGKGGKGLGKGGAK $^{Acetyl}_{42.01}$ RHRK $^{Methyl}_{14.02}$ VLRD

- fragmentation table for charge state 3+
- specType=etd
- score=112.71
- F097418.dat
- query=q1880_p1
- precursor=515.506740
- chargePrecursor=5
- itol=0.8

AA	c	y	z+1	z+2	z	AA
S[1]	49.697	**858.504**	**853.164**	0.672	**852.828**	S[24]
G[2]	68.704	815.490	**810.150**	0.672	**809.814**	G[23]
R[3]	120.738	796.483	**791.143**	**791.479**	**790.807**	R[22]
G[4]	139.745	744.449	**739.109**	**739.445**	**738.773**	G[21]
K[5]	182.443	725.442	**720.102**	**720.438**	**719.766**	K[20]
G[6]	201.450	**682.743**	**677.404**	**677.740**	**677.068**	G[19]
G[7]	220.458	**663.736**	658.397	658.733	658.061	G[18]
K[8]	263.156	**644.729**	**639.389**	**639.725**	**639.054**	K[17]
G[9]	282.163	**602.031**	**596.691**	**597.027**	**596.355**	G[16]
L[10]	319.858	583.024	**577.684**	**578.020**	**577.348**	L[15]
G[11]	338.865	**545.329**	**539.989**	**540.325**	**539.653**	G[14]
K[12]	381.563	**526.322**	520.982	521.318	520.646	K[13]
G[13]	**400.570**	483.623	478.284	478.620	477.948	G[12]
G[14]	419.578	464.616	459.277	459.613	458.941	G[11]
A[15]	443.257	445.609	440.270	440.605	439.934	A[10]
K[16]	499.958	421.930	**416.590**	**416.926**	416.255	K[9]
R[17]	**551.992**	365.228	**359.889**	**360.225**	359.553	R[8]
H[18]	**597.678**	313.195	307.855	308.191	307.519	H[7]
R[19]	**649.712**	267.508	262.169	262.505	261.833	R[6]
K[20]	**697.082**	215.475	210.135	210.471	209.799	K[5]
V[21]	**730.105**	168.104	162.765	163.101	162.429	V[4]
L[22]	**767.800**	135.082	129.742	130.078	129.406	L[3]
R[23]	**819.834**	97.387	92.047	92.383	91.711	R[2]
D[24]	**858.176**	45.353	40.014	40.349	39.678	D[1]

sp|P62806|H4_MOUSE

[Acetyl]SGRGKGGKGLGKGGAK $^{Acetyl}_{42.01}$ RHRK $^{Methyl}_{14.02}$ VLRD

- fragmentation table for charge state 4+
- specType=etd
- score=112.71
- F097418.dat
- query=q1880_p1
- precursor=515.506740
- chargePrecursor=5
- itol=0.8

AA	c	y	z+1	z+2	z	AA
S[1]	37.525	**644.130**	**640.125**	0.755	**639.873**	S[24]
G[2]	51.780	**611.869**	**607.864**	0.755	**607.612**	G[23]
R[3]	90.805	**597.614**	**593.609**	**593.861**	**593.357**	R[22]
G[4]	105.061	558.588	554.584	554.836	554.332	G[21]
K[5]	137.084	544.333	**540.328**	**540.580**	**540.076**	K[20]
G[6]	151.340	512.309	**508.305**	**508.557**	**508.053**	G[19]
G[7]	165.595	498.054	494.049	494.301	493.797	G[18]
K[8]	197.619	483.799	**479.794**	**480.046**	**479.542**	K[17]
G[9]	211.874	451.775	447.770	448.022	447.518	G[16]
L[10]	240.145	437.520	433.515	433.767	433.263	L[15]
G[11]	254.401	409.248	405.244	405.496	404.992	G[14]
K[12]	286.424	**394.993**	390.988	391.240	390.736	K[13]
G[13]	300.680	362.969	358.965	359.217	358.713	G[12]
G[14]	314.935	348.714	344.709	344.961	344.457	G[11]
A[15]	332.694	334.459	330.454	330.706	330.202	A[10]
K[16]	375.221	316.699	312.695	312.947	312.443	K[9]
R[17]	414.246	**274.173**	270.168	270.420	269.916	R[8]
H[18]	448.511	235.148	231.143	231.395	230.891	H[7]
R[19]	487.536	200.883	196.878	197.130	196.626	R[6]
K[20]	523.064	161.858	157.853	158.105	157.601	K[5]
V[21]	547.831	126.330	122.325	122.577	122.073	V[4]
L[22]	576.102	101.563	97.558	97.810	97.306	L[3]
R[23]	**615.127**	73.292	69.287	69.539	69.035	R[2]
D[24]	**643.884**	34.267	30.262	30.514	30.010	D[1]

sp|P62806|H4_MOUSE

[Acetyl]SGR $^{\text{Methyl}}_{14.02}$ GKGGKGLGKGGAK $^{\text{Acetyl}}_{42.01}$ RHRK $^{\text{(Dimethyl)}}_{(28.03)}$ VLRD

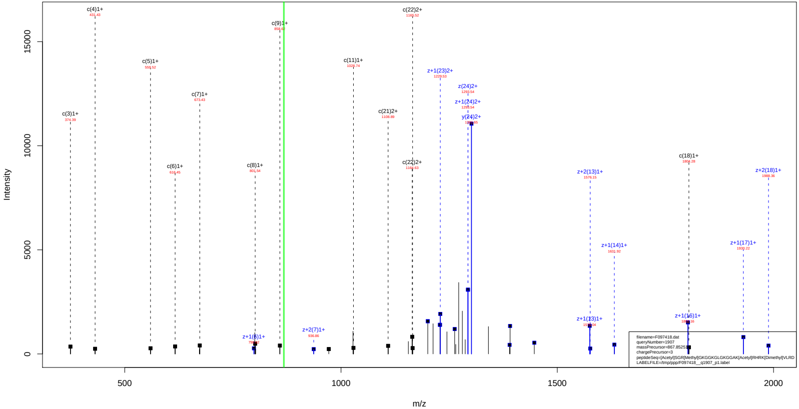

sp|P62806|H4_MOUSE

[Acetyl]SGR$^{\text{Methyl}}_{14.02}$GKGGKGLGKGGAK$^{\text{Acetyl}}_{42.01}$RHRK$^{\text{(Dimethyl)}}_{(28.03)}$VLRD

- fragmentation table for charge state 1+
- specType=etd
- score=58.22
- F097418.dat
- query=q1907_p1
- precursor=867.852510
- chargePrecursor=3
- itol=0.8

AA	c	y	z+1	z+2	z	AA
S[1]	147.076	2601.528	2585.510	0.000	2584.502	S[24]
G[2]	204.098	2472.486	2456.467	0.000	2455.459	G[23]
R[3]	**374.215**	2415.464	2399.446	2400.453	2398.438	R[22]
G[4]	**431.236**	2245.348	2229.329	2230.337	2228.321	G[21]
K[5]	**559.331**	2188.326	2172.307	2173.315	2171.300	K[20]
G[6]	**616.353**	2060.231	2044.212	2045.220	2043.205	G[19]
G[7]	**673.374**	2003.210	1987.191	**1988.199**	1986.183	G[18]
K[8]	**801.469**	1946.188	**1930.170**	1931.177	1929.162	K[17]
G[9]	**858.490**	1818.093	**1802.075**	1803.082	1801.067	G[16]
L[10]	**971.574**	1761.072	1745.053	1746.061	1744.045	L[15]
G[11]	**1028.596**	1647.988	**1631.969**	1632.977	1630.961	G[14]
K[12]	1156.691	1590.966	**1574.948**	**1575.955**	1573.940	K[13]
G[13]	1213.712	1462.871	**1446.853**	1447.860	1445.845	G[12]
G[14]	1270.734	1405.850	**1389.831**	**1390.839**	1388.823	G[11]
A[15]	1341.771	1348.828	1332.810	1333.818	1331.802	A[10]
K[16]	1511.876	1277.791	1261.773	**1262.780**	1260.765	K[9]
R[17]	1667.978	1107.686	1091.667	1092.675	1090.659	R[8]
H[18]	**1805.037**	951.585	935.566	**936.574**	934.558	H[7]
R[19]	1961.138	814.526	**798.507**	799.515	797.499	R[6]
K[20]	2117.264	658.425	642.406	643.414	641.398	K[5]
V[21]	2216.332	502.298	486.280	487.287	485.272	V[4]
L[22]	2329.416	403.230	387.211	388.219	386.203	L[3]
R[23]	2485.517	290.146	274.127	275.135	273.119	R[2]
D[24]	2600.544	134.045	118.026	119.034	117.018	D[1]

sp|P62806|H4_MOUSE

[Acetyl]SGR $^{\text{Methyl}}_{14.02}$ GKGGKGLGKGGAK $^{\text{Acetyl}}_{42.01}$ RHRK $^{\text{Dimethyl}}_{28.03}$ VLRD

- fragmentation table for charge state 2+
- specType=etd
- score=58.22
- F097418.dat
- query=q1907_p1
- precursor=867.852510
- chargePrecursor=3
- itol=0.8

AA	c	y	z+1	z+2	z	AA
S[1]	74.042	**1301.268**	**1293.258**	0.504	**1292.755**	S[24]
G[2]	102.553	1236.747	**1228.737**	0.504	**1228.233**	G[23]
R[3]	187.611	1208.236	**1200.226**	**1200.730**	1199.723	R[22]
G[4]	216.122	1123.177	1115.168	1115.672	1114.664	G[21]
K[5]	280.169	1094.667	1086.657	1087.161	1086.153	K[20]
G[6]	308.680	1030.619	1022.610	1023.114	1022.106	G[19]
G[7]	337.191	1002.108	994.099	994.603	993.595	G[18]
K[8]	401.238	973.598	965.588	966.092	965.084	K[17]
G[9]	429.749	909.550	901.541	902.045	901.037	G[16]
L[10]	486.291	881.040	873.030	873.534	872.526	L[15]
G[11]	514.802	824.498	816.488	816.992	815.984	G[14]
K[12]	578.849	795.987	787.977	788.481	787.474	K[13]
G[13]	607.360	731.939	723.930	724.434	723.426	G[12]
G[14]	635.871	703.429	695.419	695.923	694.915	G[11]
A[15]	671.389	674.918	666.908	667.412	666.405	A[10]
K[16]	756.442	639.399	631.390	631.894	630.886	K[9]
R[17]	834.492	554.347	546.337	546.841	545.833	R[8]
H[18]	903.022	476.296	468.287	468.791	467.783	H[7]
R[19]	981.072	407.767	399.757	400.261	399.253	R[6]
K[20]	1059.136	329.716	321.707	322.211	321.203	K[5]
V[21]	**1108.670**	251.653	243.643	244.147	243.140	V[4]
L[22]	**1165.212**	202.119	194.109	194.613	193.605	L[3]
R[23]	1243.262	145.577	137.567	138.071	137.063	R[2]
D[24]	1300.776	67.526	59.517	60.021	59.013	D[1]

sp|P62806|H4_MOUSE

[Acetyl]SGR $^{\text{Methyl}}_{14.02}$ GKGGKGLGKGGAK $^{\text{Acetyl}}_{42.01}$ RHRK $^{\text{Trimethyl}}_{42.05}$ VLRD

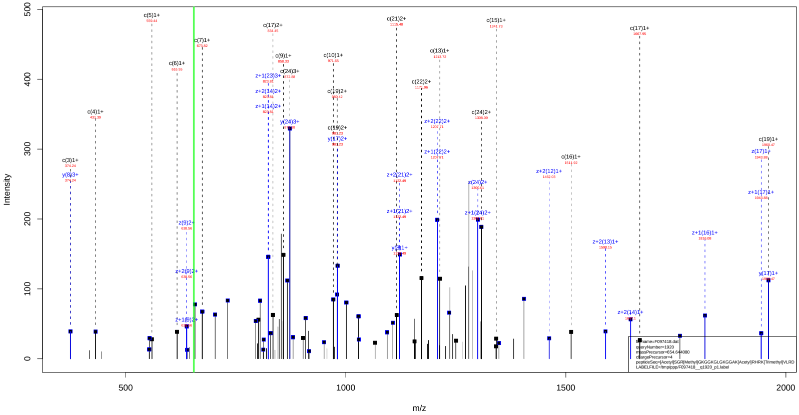

sp|P62806|H4_MOUSE

[Acetyl]SGR $^{\text{Methyl}}_{14.02}$ GKGGKGLGKGGAK $^{\text{Acetyl}}_{42.01}$ RHRK $^{\text{Trimethyl}}_{42.05}$ VLRD

- fragmentation table for charge state 1+
- specType=etd
- score=101.54
- F097418.dat
- query=q1920_p1
- precursor=654.644080
- chargePrecursor=4
- itol=0.8

AA	c	y	z+1	z+2	z	AA
S[1]	147.076	2615.544	2599.525	0.000	2598.518	S[24]
G[2]	204.098	2486.501	2470.483	0.000	2469.475	G[23]
R[3]	**374.215**	2429.480	2413.461	2414.469	2412.453	R[22]
G[4]	**431.236**	2259.363	2243.345	2244.352	2242.337	G[21]
K[5]	**559.331**	2202.342	2186.323	2187.331	2185.315	K[20]
G[6]	**616.353**	2074.247	2058.228	2059.236	2057.220	G[19]
G[7]	**673.374**	2017.225	2001.207	2002.214	2000.199	G[18]
K[8]	**801.469**	**1960.204**	**1944.185**	1945.193	**1943.177**	K[17]
G[9]	**858.490**	1832.109	**1816.090**	1817.098	1815.082	G[16]
L[10]	**971.574**	1775.087	**1759.069**	1760.077	1758.061	L[15]
G[11]	**1028.596**	1662.003	1645.985	**1646.993**	1644.977	G[14]
K[12]	**1156.691**	1604.982	1588.963	**1589.971**	1587.955	K[13]
G[13]	**1213.712**	1476.887	1460.868	**1461.876**	1459.860	G[12]
G[14]	1270.734	1419.866	1403.847	**1404.855**	1402.839	G[11]
A[15]	**1341.771**	1362.844	1346.825	**1347.833**	1345.818	A[10]
K[16]	**1511.876**	1291.807	1275.788	1276.796	1274.780	K[9]
R[17]	**1667.978**	**1121.701**	1105.683	**1106.691**	1104.675	R[8]
H[18]	1805.037	965.600	949.582	**950.589**	948.574	H[7]
R[19]	**1961.138**	**828.541**	**812.523**	**813.530**	811.515	R[6]
K[20]	2131.280	672.440	656.422	**657.429**	655.414	K[5]
V[21]	2230.348	502.298	486.280	487.287	485.272	V[4]
L[22]	2343.432	403.230	387.211	388.219	386.203	L[3]
R[23]	2499.533	290.146	274.127	275.135	273.119	R[2]
D[24]	2614.560	134.045	118.026	119.034	117.018	D[1]

sp|P62806|H4_MOUSE

[Acetyl]SGR $^{\text{Methyl}}_{14.02}$ GKGGKGLGKGGAK $^{\text{Acetyl}}_{42.01}$ RHRK $^{\text{Trimethyl}}_{42.05}$ VLRD

- fragmentation table for charge state 2+
- specType=etd
- score=101.54
- F097418.dat
- query=q1920_p1
- precursor=654.644080
- chargePrecursor=4
- itol=0.8

AA	c	y	z+1	z+2	z	AA
S[1]	74.042	**1308.276**	**1300.266**	0.504	**1299.762**	S[24]
G[2]	102.553	1243.754	1235.745	0.504	**1235.241**	G[23]
R[3]	187.611	1215.244	**1207.234**	**1207.738**	1206.730	R[22]
G[4]	216.122	1130.185	**1122.176**	**1122.680**	1121.672	G[21]
K[5]	280.169	1101.675	**1093.665**	**1094.169**	**1093.161**	K[20]
G[6]	308.680	1037.627	**1029.618**	**1030.122**	**1029.114**	G[19]
G[7]	337.191	1009.116	**1001.107**	**1001.611**	**1000.603**	G[18]
K[8]	401.238	**980.606**	972.596	973.100	**972.092**	K[17]
G[9]	429.749	**916.558**	**908.549**	**909.053**	**908.045**	G[16]
L[10]	486.291	888.047	**880.038**	**880.542**	**879.534**	L[15]
G[11]	514.802	831.505	**823.496**	**824.000**	822.992	G[14]
K[12]	578.849	802.995	**794.985**	**795.489**	794.481	K[13]
G[13]	607.360	738.947	**730.938**	**731.442**	730.434	G[12]
G[14]	635.871	710.436	**702.427**	**702.931**	701.923	G[11]
A[15]	671.389	681.926	**673.916**	**674.420**	**673.412**	A[10]
K[16]	756.442	646.407	**638.398**	**638.902**	**637.894**	K[9]
R[17]	**834.492**	561.354	**553.345**	**553.849**	**552.841**	R[8]
H[18]	**903.022**	483.304	475.294	475.798	474.791	H[7]
R[19]	**981.072**	414.774	406.765	407.269	406.261	R[6]
K[20]	**1066.143**	336.724	328.714	329.218	328.211	K[5]
V[21]	**1115.678**	251.653	243.643	244.147	243.140	V[4]
L[22]	**1172.220**	202.119	194.109	194.613	193.605	L[3]
R[23]	**1250.270**	145.577	137.567	138.071	137.063	R[2]
D[24]	**1307.784**	67.526	59.517	60.021	59.013	D[1]

sp|P62806|H4_MOUSE

[Acetyl]SGR $^{Methyl}_{14.02}$ GKGGKGLGKGGAK $^{Acetyl}_{42.01}$ RHRK $^{Trimethyl}_{42.05}$ VLRD

- fragmentation table for charge state 3+
- specType=etd
- score=101.54
- F097418.dat
- query=q1920_p1
- precursor=654.644080
- chargePrecursor=4
- itol=0.8

AA	c	y	z+1	z+2	z	AA
S[1]	49.697	**872.520**	**867.180**	0.672	**866.844**	S[24]
G[2]	68.704	**829.505**	**824.166**	0.672	**823.830**	G[23]
R[3]	125.410	810.498	**805.159**	**805.495**	**804.823**	R[22]
G[4]	144.417	753.793	748.453	748.789	748.117	G[21]
K[5]	187.115	734.785	729.446	729.782	729.110	K[20]
G[6]	206.122	692.087	686.748	687.083	686.412	G[19]
G[7]	225.130	**673.080**	667.740	668.076	667.404	G[18]
K[8]	267.828	654.073	648.733	649.069	648.397	K[17]
G[9]	286.835	611.374	606.035	606.371	605.699	G[16]
L[10]	324.530	592.367	587.028	587.364	586.692	L[15]
G[11]	343.537	**554.673**	549.333	549.669	548.997	G[14]
K[12]	386.235	535.666	530.326	530.662	529.990	K[13]
G[13]	405.242	492.967	487.628	487.964	487.292	G[12]
G[14]	424.249	473.960	468.620	468.956	468.285	G[11]
A[15]	447.929	454.953	449.613	449.949	449.277	A[10]
K[16]	504.630	**431.274**	425.934	426.270	425.598	K[9]
R[17]	556.664	**374.572**	369.232	369.568	368.896	R[8]
H[18]	602.350	322.538	317.199	317.535	316.863	H[7]
R[19]	654.384	276.852	271.512	271.848	271.176	R[6]
K[20]	711.098	224.818	219.479	219.815	219.143	K[5]
V[21]	744.121	168.104	162.765	163.101	162.429	V[4]
L[22]	781.816	135.082	129.742	130.078	129.406	L[3]
R[23]	**833.849**	97.387	92.047	92.383	91.711	R[2]
D[24]	**872.192**	45.353	40.014	40.349	39.678	D[1]

sp|P62806|H4_MOUSE

[Acetyl]SGRGKGGKGLGKGGAKRHRK $^{\text{Trimethyl}}_{42.05}$ VLRD

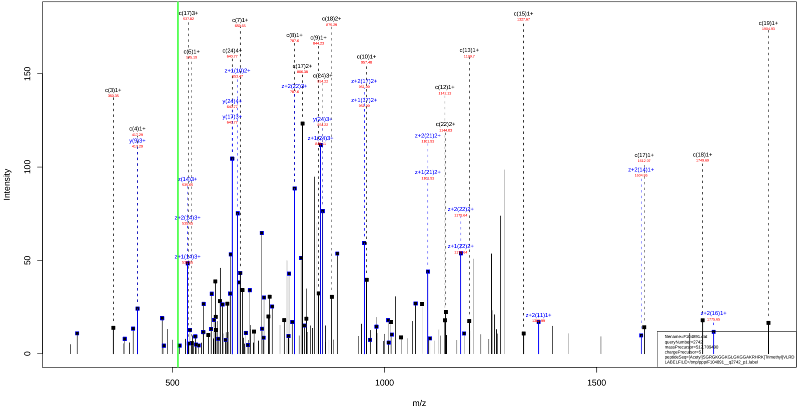

sp|P62806|H4_MOUSE

[Acetyl]SGRGKGGKGLGKGGAKRHRK$^{\text{Trimethyl}}_{42.05}$VLRD

- fragmentation table for charge state 1+
- specType=etd
- score=76.65
- F104891.dat
- query=q2742_p1
- precursor=512.709490
- chargePrecursor=5
- itol=0.8

AA	c	y	z+1	z+2	z	AA
S[1]	147.076	2559.518	2543.499	0.000	2542.491	S[24]
G[2]	204.098	2430.475	2414.457	0.000	2413.449	G[23]
R[3]	**360.199**	2373.454	2357.435	2358.443	2356.427	R[22]
G[4]	**417.220**	2217.353	2201.334	2202.342	2200.326	G[21]
K[5]	**545.315**	2160.331	2144.313	2145.320	2143.305	K[20]
G[6]	**602.337**	2032.236	2016.218	2017.225	2015.210	G[19]
G[7]	**659.358**	1975.215	1959.196	1960.204	1958.188	G[18]
K[8]	**787.453**	1918.193	1902.175	1903.182	1901.167	K[17]
G[9]	**844.475**	1790.098	1774.080	**1775.087**	1773.072	G[16]
L[10]	**957.559**	1733.077	1717.058	1718.066	1716.050	L[15]
G[11]	**1014.580**	1619.993	1603.974	**1604.982**	1602.966	G[14]
K[12]	**1142.675**	1562.971	1546.953	1547.960	1545.945	K[13]
G[13]	**1199.697**	1434.876	1418.858	1419.866	1417.850	G[12]
G[14]	1256.718	1377.855	1361.836	**1362.844**	1360.828	G[11]
A[15]	**1327.755**	1320.833	1304.815	1305.823	1303.807	A[10]
K[16]	1455.850	1249.796	1233.778	1234.785	1232.770	K[9]
R[17]	**1611.951**	1121.701	1105.683	**1106.691**	1104.675	R[8]
H[18]	**1749.010**	**965.600**	949.582	950.589	948.574	H[7]
R[19]	**1905.111**	828.541	812.523	813.530	811.515	R[6]
K[20]	2075.253	**672.440**	**656.422**	657.429	655.414	K[5]
V[21]	2174.322	502.298	486.280	487.287	485.272	V[4]
L[22]	2287.406	403.230	**387.211**	388.219	386.203	L[3]
R[23]	2443.507	290.146	274.127	**275.135**	273.119	R[2]
D[24]	2558.534	134.045	118.026	119.034	117.018	D[1]

sp|P62806|H4_MOUSE

[Acetyl]SGRGKGGKGLGKGGAKRHRK$^{\text{Trimethyl}}_{42.05}$VLRD

- fragmentation table for charge state 2+
- specType=etd
- score=76.65
- F104891.dat
- query=q2742_p1
- precursor=512.709490
- chargePrecursor=5
- itol=0.8

AA	c	y	z+1	z+2	z	AA
S[1]	74.042	1280.263	1272.253	0.504	1271.749	S[24]
G[2]	102.553	1215.741	1207.732	0.504	1207.228	G[23]
R[3]	180.603	**1187.231**	**1179.221**	**1179.725**	1178.717	R[22]
G[4]	209.114	1109.180	**1101.171**	**1101.675**	1100.667	G[21]
K[5]	273.161	1080.669	**1072.660**	**1073.164**	**1072.156**	K[20]
G[6]	301.672	**1016.622**	**1008.612**	**1009.116**	**1008.108**	G[19]
G[7]	330.183	988.111	**980.102**	**980.606**	979.598	G[18]
K[8]	394.230	959.600	**951.591**	**952.095**	951.087	K[17]
G[9]	422.741	895.553	887.543	**888.047**	887.040	G[16]
L[10]	**479.283**	867.042	859.033	859.537	858.529	L[15]
G[11]	507.794	**810.500**	**802.491**	**802.995**	801.987	G[14]
K[12]	**571.841**	**781.989**	**773.980**	**774.484**	**773.476**	K[13]
G[13]	**600.352**	717.942	**709.932**	**710.436**	709.429	G[12]
G[14]	**628.863**	689.431	**681.422**	**681.926**	680.918	G[11]
A[15]	**664.381**	660.920	**652.911**	**653.415**	652.407	A[10]
K[16]	**728.429**	**625.402**	**617.392**	**617.896**	**616.889**	K[9]
R[17]	**806.479**	**561.354**	**553.345**	**553.849**	**552.841**	R[8]
H[18]	**875.009**	483.304	**475.294**	**475.798**	**474.791**	H[7]
R[19]	953.059	414.774	**406.765**	**407.269**	406.261	R[6]
K[20]	**1038.130**	336.724	328.714	329.218	328.211	K[5]
V[21]	**1087.665**	251.653	243.643	244.147	243.140	V[4]
L[22]	**1144.207**	202.119	194.109	194.613	193.605	L[3]
R[23]	1222.257	145.577	137.567	138.071	137.063	R[2]
D[24]	1279.771	67.526	59.517	60.021	59.013	D[1]

sp|P62806|H4_MOUSE

[Acetyl]SGRGKGGKGLGKGGAKRHRK$^{\text{Trimethyl}}_{42.05}$VLRD

- fragmentation table for charge state 3+
- specType=etd
- score=76.65
- F104891.dat
- query=q2742_p1
- precursor=512.709490
- chargePrecursor=5
- itol=0.8

AA	c	y	z+1	z+2	z	AA
S[1]	49.697	**853.844**	**848.505**	0.672	848.169	S[24]
G[2]	68.704	**810.830**	805.490	0.672	805.154	G[23]
R[3]	120.738	791.823	786.483	**786.819**	786.147	R[22]
G[4]	139.745	739.789	**734.450**	**734.785**	734.114	G[21]
K[5]	182.443	720.782	**715.442**	**715.778**	**715.106**	K[20]
G[6]	201.450	**678.084**	**672.744**	**673.080**	**672.408**	G[19]
G[7]	220.458	**659.076**	**653.737**	**654.073**	**653.401**	G[18]
K[8]	263.156	**640.069**	634.730	**635.066**	634.394	K[17]
G[9]	282.163	**597.371**	**592.031**	**592.367**	**591.695**	G[16]
L[10]	319.858	578.364	**573.024**	**573.360**	**572.688**	L[15]
G[11]	338.865	**540.669**	**535.330**	**535.666**	**534.994**	G[14]
K[12]	381.563	521.662	**516.322**	**516.658**	**515.986**	K[13]
G[13]	400.570	**478.964**	473.624	473.960	473.288	G[12]
G[14]	419.578	459.957	454.617	454.953	454.281	G[11]
A[15]	443.257	440.949	435.610	435.946	435.274	A[10]
K[16]	485.955	**417.270**	411.931	412.267	411.595	K[9]
R[17]	**537.989**	374.572	369.232	369.568	368.896	R[8]
H[18]	**583.675**	322.538	317.199	317.535	316.863	H[7]
R[19]	**635.709**	276.852	271.512	271.848	271.176	R[6]
K[20]	**692.423**	224.818	219.479	219.815	219.143	K[5]
V[21]	**725.445**	168.104	162.765	163.101	162.429	V[4]
L[22]	**763.140**	135.082	129.742	130.078	129.406	L[3]
R[23]	**815.174**	97.387	92.047	92.383	91.711	R[2]
D[24]	**853.516**	45.353	40.014	40.349	39.678	D[1]

sp|P62806|H4_MOUSE

[Acetyl]SGRGKGGKGLGKGGAKRHRK $^{\text{Trimethyl}}_{42.05}$ VLRD

- fragmentation table for charge state 4+
- specType=etd
- score=76.65
- F104891.dat
- query=q2742_p1
- precursor=512.709490
- chargePrecursor=5
- itol=0.8

AA	c	y	z+1	z+2	z	AA
S[1]	37.525	**640.635**	**636.630**	0.755	**636.378**	S[24]
G[2]	51.780	**608.374**	604.370	0.755	604.118	G[23]
R[3]	90.805	594.119	**590.114**	**590.366**	589.862	R[22]
G[4]	105.061	**555.094**	551.089	551.341	550.837	G[21]
K[5]	137.084	**540.838**	536.834	**537.086**	536.582	K[20]
G[6]	151.340	508.815	504.810	505.062	504.558	G[19]
G[7]	165.595	494.559	490.554	490.806	490.303	G[18]
K[8]	197.619	**480.304**	**476.299**	476.551	**476.047**	K[17]
G[9]	211.874	448.280	444.275	444.527	444.023	G[16]
L[10]	240.145	434.025	430.020	430.272	429.768	L[15]
G[11]	254.401	405.754	401.749	402.001	401.497	G[14]
K[12]	286.424	391.498	**387.494**	**387.746**	**387.242**	K[13]
G[13]	300.680	359.475	355.470	355.722	355.218	G[12]
G[14]	314.935	345.219	341.215	341.466	340.963	G[11]
A[15]	332.694	330.964	326.959	327.211	326.707	A[10]
K[16]	364.718	313.205	309.200	309.452	308.948	K[9]
R[17]	403.743	281.181	277.176	277.428	276.924	R[8]
H[18]	438.008	242.156	238.151	238.403	237.899	H[7]
R[19]	477.033	207.891	203.886	204.138	203.634	R[6]
K[20]	519.569	168.866	164.861	165.113	164.609	K[5]
V[21]	544.336	126.330	122.325	122.577	122.073	V[4]
L[22]	**572.607**	101.563	97.558	97.810	97.306	L[3]
R[23]	**611.632**	73.292	69.287	69.539	69.035	R[2]
D[24]	**640.389**	34.267	30.262	30.514	30.010	D[1]

sp|P62806|H4_MOUSE

[Acetyl]SGR $^{Methyl}_{14.02}$ GK $^{Acetyl}_{42.01}$ GGKGL

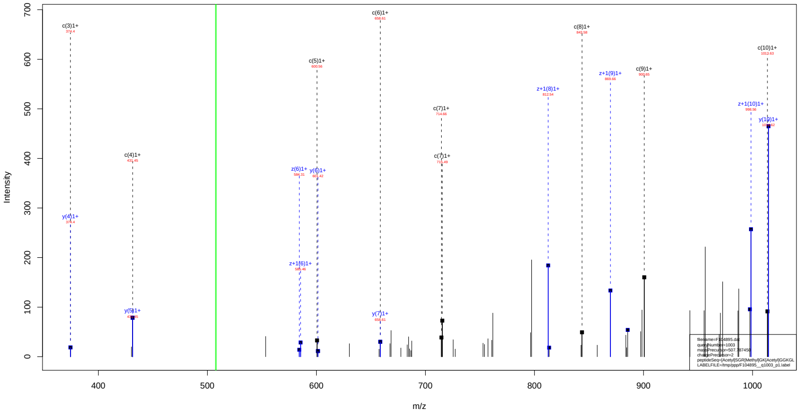

sp|P62806|H4_MOUSE

[Acetyl]SGR$^{\text{Methyl}}_{14.02}$GK$^{\text{Acetyl}}_{42.01}$GGKGL

- fragmentation table for charge state 1+
- specType=etd
- score=45.40
- F104895.dat
- query=q1003_p1
- precursor=507.787450
- chargePrecursor=2
- itol=0.8

AA	c	y	z+1	z+2	z	AA
S[1]	147.076	**1014.569**	**998.550**	0.000	**997.543**	S[10]
G[2]	204.098	**885.526**	**869.508**	0.000	868.500	G[9]
R[3]	**374.215**	828.505	**812.486**	**813.494**	811.478	R[8]
G[4]	**431.236**	**658.388**	642.370	643.377	641.362	G[7]
K[5]	**601.342**	**601.367**	**585.348**	586.356	**584.340**	K[6]
G[6]	**658.363**	**431.261**	415.243	416.250	414.235	G[5]
G[7]	**715.385**	**374.240**	358.221	359.229	357.213	G[4]
K[8]	**843.480**	317.218	301.200	302.207	300.192	K[3]
G[9]	**900.501**	189.123	173.105	174.112	172.097	G[2]
L[10]	**1013.585**	132.102	116.083	117.091	115.075	L[1]

sp|P62806|H4_MOUSE

[Acetyl]SGR $^{\text{Methyl}}_{14.02}$ GKGGKGL

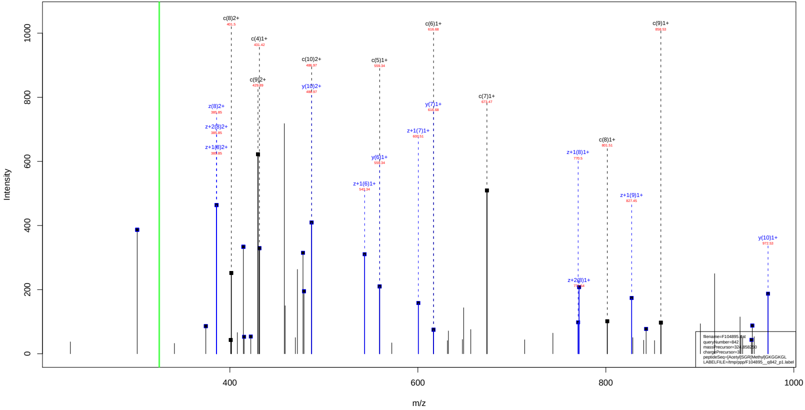

sp|P62806|H4_MOUSE

[Acetyl]SGR $^{\text{Methyl}}_{14.02}$ GKGGKGL

- fragmentation table for charge state 1+
- specType=etd
- score=44.07
- F104895.dat
- query=q842_p1
- precursor=324.858250
- chargePrecursor=3
- itol=0.8

AA	c	y	z+1	z+2	z	AA
S[1]	147.076	**972.559**	**956.540**	0.000	**955.532**	S[10]
G[2]	204.098	**843.516**	**827.497**	0.000	826.489	G[9]
R[3]	**374.215**	786.494	**770.476**	**771.484**	769.468	R[8]
G[4]	**431.236**	**616.378**	**600.359**	601.367	599.351	G[7]
K[5]	**559.331**	**559.356**	**543.337**	544.345	542.330	K[6]
G[6]	**616.353**	**431.261**	**415.243**	416.250	**414.235**	G[5]
G[7]	**673.374**	**374.240**	358.221	359.229	357.213	G[4]
K[8]	**801.469**	317.218	**301.200**	302.207	300.192	K[3]
G[9]	**858.490**	189.123	173.105	174.112	172.097	G[2]
L[10]	971.574	132.102	116.083	117.091	115.075	L[1]

sp|P62806|H4_MOUSE

[Acetyl]SGR $^{Methyl}_{14.02}$ GKGGKGL

- fragmentation table for charge state 2+
- specType=etd
- score=44.07
- F104895.dat
- query=q842_p1
- precursor=324.858250
- chargePrecursor=3
- itol=0.8

AA	c	y	z+1	z+2	z	AA
S[1]	74.042	**486.783**	**478.774**	0.504	**478.270**	S[10]
G[2]	102.553	**422.262**	**414.252**	0.504	**413.748**	G[9]
R[3]	187.611	393.751	**385.742**	**386.245**	**385.238**	R[8]
G[4]	216.122	308.692	**300.683**	**301.187**	300.179	G[7]
K[5]	280.169	280.182	272.172	272.676	271.668	K[6]
G[6]	308.680	216.134	208.125	208.629	207.621	G[5]
G[7]	337.191	187.624	179.614	180.118	179.110	G[4]
K[8]	**401.238**	159.113	151.103	151.607	150.600	K[3]
G[9]	**429.749**	95.065	87.056	87.560	86.552	G[2]
L[10]	**486.291**	66.555	58.545	59.049	58.041	L[1]

sp|P62806|H4_MOUSE

[Acetyl]SGR $^{\text{Methyl}}_{14.02}$ GKGGK $^{\text{Acetyl}}_{42.01}$ GL

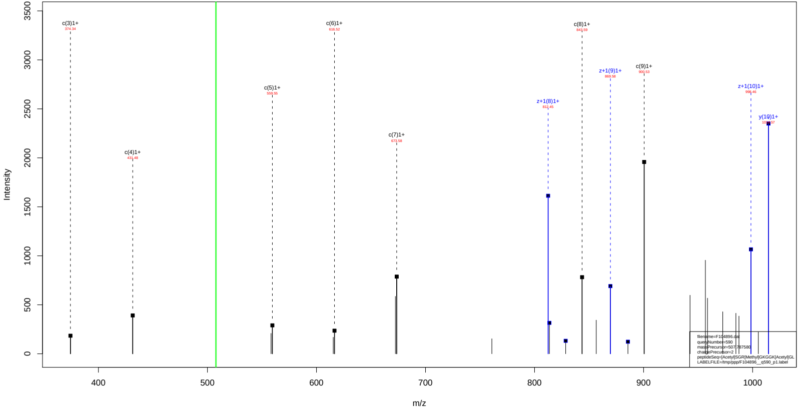

sp|P62806|H4_MOUSE

[Acetyl]SGR $^{Methyl}_{14.02}$ GKGGK $^{Acetyl}_{42.01}$ GL

- fragmentation table for charge state 1+
- specType=etd
- score=52.77
- F104896.dat
- query=q590_p1
- precursor=507.787580
- chargePrecursor=2
- itol=0.8

AA	c	y	z+1	z+2	z	AA
S[1]	147.076	**1014.569**	**998.550**	0.000	997.543	S[10]
G[2]	204.098	**885.526**	**869.508**	0.000	868.500	G[9]
R[3]	**374.215**	**828.505**	**812.486**	**813.494**	811.478	R[8]
G[4]	**431.236**	658.388	642.370	643.377	641.362	G[7]
K[5]	**559.331**	601.367	585.348	586.356	584.340	K[6]
G[6]	**616.353**	473.272	457.253	458.261	456.245	G[5]
G[7]	**673.374**	416.250	400.232	401.239	399.224	G[4]
K[8]	**843.480**	359.229	343.210	344.218	342.202	K[3]
G[9]	**900.501**	189.123	173.105	174.112	172.097	G[2]
L[10]	1013.585	132.102	116.083	117.091	115.075	L[1]

sp|P62806|H4_MOUSE

[Acetyl]SGRGKGGKGLGKGGAKRHRK $^{\text{Trimethyl}}_{42.05}$ VLR

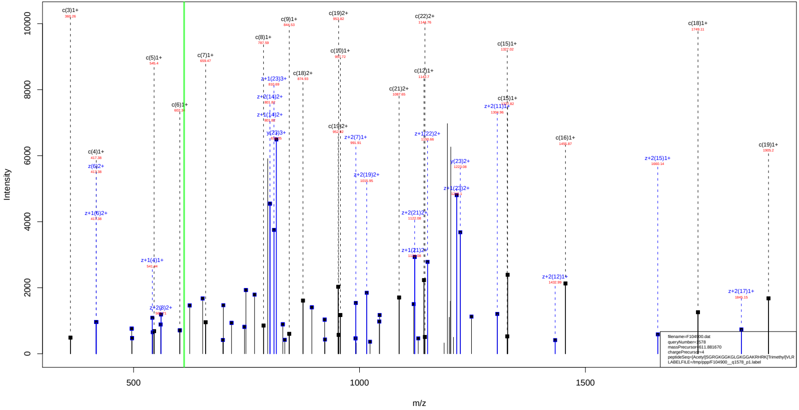

sp|P62806|H4_MOUSE

[Acetyl]SGRGKGGKGLGKGGAKRHRK $^{Trimethyl}_{42.05}$ VLR

- fragmentation table for charge state 1+
- specType=etd
- score=119.32
- F104900.dat
- query=q1578_p1
- precursor=611.881670
- chargePrecursor=4
- itol=0.8

AA	c	y	z+1	z+2	z	AA
S[1]	147.076	2444.491	2428.472	0.000	2427.464	S[23]
G[2]	204.098	2315.448	2299.430	0.000	2298.422	G[22]
R[3]	**360.199**	2258.427	2242.408	2243.416	2241.400	R[21]
G[4]	**417.220**	2102.326	2086.307	2087.315	2085.299	G[20]
K[5]	**545.315**	2045.304	2029.286	2030.293	2028.278	K[19]
G[6]	**602.337**	1917.209	1901.191	1902.198	1900.183	G[18]
G[7]	**659.358**	1860.188	1844.169	**1845.177**	1843.161	G[17]
K[8]	**787.453**	1803.166	1787.148	1788.156	1786.140	K[16]
G[9]	**844.475**	1675.071	1659.053	**1660.061**	1658.045	G[15]
L[10]	**957.559**	1618.050	1602.031	1603.039	1601.023	L[14]
G[11]	1014.580	1504.966	1488.947	1489.955	1487.939	G[13]
K[12]	**1142.675**	1447.944	1431.926	**1432.934**	1430.918	K[12]
G[13]	1199.697	1319.849	1303.831	**1304.839**	1302.823	G[11]
G[14]	1256.718	1262.828	1246.809	**1247.817**	1245.801	G[10]
A[15]	**1327.755**	1205.807	1189.788	1190.796	1188.780	A[9]
K[16]	**1455.850**	1134.769	1118.751	**1119.759**	1117.743	K[8]
R[17]	1611.951	1006.674	**990.656**	**991.664**	989.648	R[7]
H[18]	**1749.010**	850.573	**834.555**	835.562	833.547	H[6]
R[19]	**1905.111**	713.514	**697.496**	**698.504**	696.488	R[5]
K[20]	2075.253	557.413	**541.395**	**542.402**	540.387	K[4]
V[21]	2174.322	387.271	371.253	372.261	370.245	V[3]
L[22]	2287.406	288.203	272.184	273.192	271.176	L[2]
R[23]	2443.507	175.119	159.100	160.108	158.092	R[1]

sp|P62806|H4_MOUSE

[Acetyl]SGRGKGGKGLGKGGAKRHRK$^{\text{Trimethyl}}_{42.05}$VLR

- fragmentation table for charge state 2+
- specType=etd
- score=119.32
- F104900.dat
- query=q1578_p1
- precursor=611.881670
- chargePrecursor=4
- itol=0.8

AA	c	y	z+1	z+2	z	AA
S[1]	74.042	**1222.749**	**1214.740**	0.504	1214.236	S[23]
G[2]	102.553	1158.228	**1150.218**	0.504	1149.715	G[22]
R[3]	180.603	**1129.717**	**1121.708**	**1122.212**	1121.204	R[21]
G[4]	209.114	1051.667	**1043.657**	**1044.161**	**1043.153**	G[20]
K[5]	273.161	**1023.156**	1015.146	**1015.650**	1014.643	K[19]
G[6]	301.672	959.108	951.099	951.603	950.595	G[18]
G[7]	330.183	930.598	**922.588**	**923.092**	**922.084**	G[17]
K[8]	394.230	902.087	**894.077**	**894.581**	893.574	K[16]
G[9]	422.741	838.039	**830.030**	**830.534**	**829.526**	G[15]
L[10]	479.283	809.529	**801.519**	**802.023**	801.015	L[14]
G[11]	507.794	752.987	**744.977**	**745.481**	744.473	G[13]
K[12]	571.841	724.476	**716.466**	**716.970**	715.963	K[12]
G[13]	600.352	660.428	**652.419**	**652.923**	651.915	G[11]
G[14]	628.863	631.918	**623.908**	**624.412**	623.404	G[10]
A[15]	664.381	603.407	595.398	595.901	594.894	A[9]
K[16]	728.429	567.888	**559.879**	**560.383**	**559.375**	K[8]
R[17]	806.479	503.841	**495.832**	**496.335**	**495.328**	R[7]
H[18]	**875.009**	425.790	**417.781**	418.285	**417.277**	H[6]
R[19]	**953.059**	357.261	349.252	349.755	348.748	R[5]
K[20]	1038.130	279.210	271.201	271.705	270.697	K[4]
V[21]	**1087.665**	194.139	186.130	186.634	185.626	V[3]
L[22]	**1144.207**	144.605	136.596	137.100	136.092	L[2]
R[23]	1222.257	88.063	80.054	80.558	79.550	R[1]

sp|P62806|H4_MOUSE

[Acetyl]SGRGKGGKGLGKGGAKRHRK $^{\text{Trimethyl}}_{42.05}$ VLR

- fragmentation table for charge state 3+
- specType=etd
- score=119.32
- F104900.dat
- query=q1578_p1
- precursor=611.881670
- chargePrecursor=4
- itol=0.8

AA	c	y	z+1	z+2	z	AA
S[1]	49.697	**815.502**	**810.162**	0.672	809.826	S[23]
G[2]	68.704	772.488	**767.148**	0.672	766.812	G[22]
R[3]	120.738	753.480	**748.141**	**748.477**	747.805	R[21]
G[4]	139.745	701.447	696.107	696.443	695.771	G[20]
K[5]	182.443	682.440	677.100	677.436	676.764	K[19]
G[6]	201.450	639.741	634.402	634.738	634.066	G[18]
G[7]	220.458	620.734	615.395	615.731	615.059	G[17]
K[8]	263.156	**601.727**	596.387	596.723	596.051	K[16]
G[9]	282.163	559.029	553.689	554.025	553.353	G[15]
L[10]	319.858	540.022	534.682	535.018	534.346	L[14]
G[11]	338.865	502.327	**496.987**	**497.323**	**496.651**	G[13]
K[12]	381.563	483.320	477.980	478.316	477.644	K[12]
G[13]	400.570	440.621	435.282	435.618	434.946	G[11]
G[14]	419.578	421.614	416.275	**416.611**	415.939	G[10]
A[15]	443.257	402.607	397.267	397.603	396.932	A[9]
K[16]	485.955	378.928	373.588	373.924	373.252	K[8]
R[17]	537.989	336.230	330.890	331.226	330.554	R[7]
H[18]	583.675	284.196	278.856	279.192	278.520	H[6]
R[19]	635.709	238.510	233.170	233.506	232.834	R[5]
K[20]	692.423	186.476	181.136	181.472	180.800	K[4]
V[21]	725.445	129.762	124.422	124.758	124.086	V[3]
L[22]	763.140	96.739	91.400	91.736	91.064	L[2]
R[23]	815.174	59.045	53.705	54.041	53.369	R[1]

sp|P62806|H4_MOUSE

[Acetyl]SGR $^{\text{Methyl}}_{14.02}$ GKGGKGLGKGGAKRHRK $^{\text{Trimethyl}}_{42.05}$ VLR

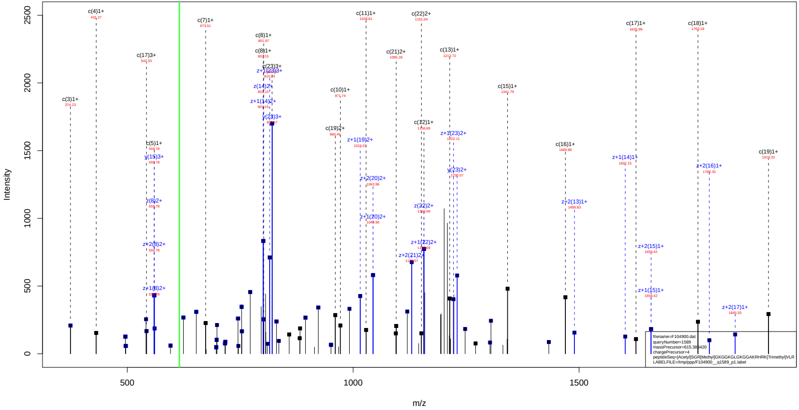

sp|P62806|H4_MOUSE

[Acetyl]SGR $^{\text{Methyl}}_{14.02}$ GKGGKGLGKGGAKRHRK $^{\text{Trimethyl}}_{42.05}$ VLR

- fragmentation table for charge state 1+
- specType=etd
- score=130.12
- F104900.dat
- query=q1589_p1
- precursor=615.386420
- chargePrecursor=4
- itol=0.8

AA	c	y	z+1	z+2	z	AA
S[1]	147.076	2458.507	2442.488	0.000	2441.480	S[23]
G[2]	204.098	2329.464	2313.445	0.000	2312.437	G[22]
R[3]	**374.215**	2272.443	2256.424	2257.432	2255.416	R[21]
G[4]	**431.236**	2102.326	2086.307	2087.315	2085.299	G[20]
K[5]	**559.331**	2045.304	2029.286	2030.293	2028.278	K[19]
G[6]	616.353	1917.209	1901.191	1902.198	1900.183	G[18]
G[7]	**673.374**	1860.188	1844.169	**1845.177**	1843.161	G[17]
K[8]	**801.469**	1803.166	1787.148	**1788.156**	1786.140	K[16]
G[9]	**858.490**	1675.071	**1659.053**	**1660.061**	1658.045	G[15]
L[10]	**971.574**	1618.050	**1602.031**	1603.039	1601.023	L[14]
G[11]	**1028.596**	1504.966	1488.947	**1489.955**	1487.939	G[13]
K[12]	**1156.691**	1447.944	1431.926	**1432.934**	1430.918	K[12]
G[13]	**1213.712**	1319.849	1303.831	**1304.839**	**1302.823**	G[11]
G[14]	**1270.734**	1262.828	1246.809	**1247.817**	1245.801	G[10]
A[15]	**1341.771**	1205.807	1189.788	1190.796	1188.780	A[9]
K[16]	**1469.866**	1134.769	1118.751	**1119.759**	1117.743	K[8]
R[17]	**1625.967**	1006.674	990.656	**991.664**	989.648	R[7]
H[18]	**1763.026**	850.573	834.555	**835.562**	833.547	H[6]
R[19]	**1919.127**	713.514	**697.496**	**698.504**	**696.488**	R[5]
K[20]	2089.269	557.413	**541.395**	**542.402**	540.387	K[4]
V[21]	2188.337	387.271	371.253	372.261	370.245	V[3]
L[22]	2301.421	288.203	272.184	273.192	271.176	L[2]
R[23]	2457.523	175.119	159.100	160.108	158.092	R[1]

sp|P62806|H4_MOUSE

[Acetyl]SGR $^{\text{Methyl}}_{14.02}$ GKGGKGLGKGGAKRHRK $^{\text{Trimethyl}}_{42.05}$ VLR

- fragmentation table for charge state 2+
- specType=etd
- score=130.12
- F104900.dat
- query=q1589_p1
- precursor=615.386420
- chargePrecursor=4
- itol=0.8

AA	c	y	z+1	z+2	z	AA
S[1]	74.042	**1229.757**	**1221.748**	0.504	1221.244	S[23]
G[2]	102.553	1165.236	**1157.226**	0.504	**1156.722**	G[22]
R[3]	187.611	1136.725	1128.716	**1129.219**	1128.212	R[21]
G[4]	216.122	1051.667	**1043.657**	**1044.161**	1043.153	G[20]
K[5]	280.169	1023.156	**1015.146**	**1015.650**	1014.643	K[19]
G[6]	308.680	959.108	**951.099**	**951.603**	**950.595**	G[18]
G[7]	337.191	930.598	**922.588**	**923.092**	**922.084**	G[17]
K[8]	401.238	902.087	**894.077**	**894.581**	893.574	K[16]
G[9]	429.749	838.039	**830.030**	**830.534**	**829.526**	G[15]
L[10]	486.291	**809.529**	**801.519**	**802.023**	**801.015**	L[14]
G[11]	514.802	**752.987**	**744.977**	**745.481**	**744.473**	G[13]
K[12]	578.849	724.476	**716.466**	**716.970**	**715.963**	K[12]
G[13]	607.360	660.428	**652.419**	**652.923**	651.915	G[11]
G[14]	635.871	631.918	**623.908**	**624.412**	623.404	G[10]
A[15]	671.389	603.407	**595.398**	**595.901**	594.894	A[9]
K[16]	735.437	567.888	**559.879**	**560.383**	**559.375**	K[8]
R[17]	813.487	503.841	**495.832**	**496.335**	**495.328**	R[7]
H[18]	**882.017**	425.790	417.781	418.285	417.277	H[6]
R[19]	**960.067**	357.261	349.252	349.755	348.748	R[5]
K[20]	1045.138	279.210	271.201	271.705	270.697	K[4]
V[21]	**1094.672**	194.139	186.130	186.634	185.626	V[3]
L[22]	**1151.214**	144.605	136.596	137.100	136.092	L[2]
R[23]	1229.265	88.063	80.054	80.558	79.550	R[1]

sp|P62806|H4_MOUSE

[Acetyl]SGR $^{\text{Methyl}}_{14.02}$ GKGGKGLGKGGAKRHRK $^{\text{Trimethyl}}_{42.05}$ VLR

- fragmentation table for charge state 3+
- specType=etd
- score=130.12
- F104900.dat
- query=q1589_p1
- precursor=615.386420
- chargePrecursor=4
- itol=0.8

AA	c	y	z+1	z+2	z	AA
S[1]	49.697	**820.174**	**814.834**	0.672	**814.498**	S[23]
G[2]	68.704	777.160	**771.820**	0.672	**771.484**	G[22]
R[3]	125.410	758.152	**752.813**	**753.149**	**752.477**	R[21]
G[4]	144.417	701.447	696.107	**696.443**	695.771	G[20]
K[5]	187.115	682.440	677.100	677.436	676.764	K[19]
G[6]	206.122	639.741	634.402	634.738	634.066	G[18]
G[7]	225.130	620.734	615.395	615.731	615.059	G[17]
K[8]	267.828	601.727	**596.387**	596.723	**596.051**	K[16]
G[9]	286.835	**559.029**	553.689	554.025	553.353	G[15]
L[10]	324.530	540.022	534.682	535.018	534.346	L[14]
G[11]	343.537	502.327	**496.987**	**497.323**	**496.651**	G[13]
K[12]	386.235	483.320	477.980	478.316	477.644	K[12]
G[13]	405.242	440.621	435.282	435.618	434.946	G[11]
G[14]	424.249	421.614	416.275	416.611	415.939	G[10]
A[15]	447.929	402.607	397.267	397.603	396.932	A[9]
K[16]	490.627	378.928	**373.588**	**373.924**	373.252	K[8]
R[17]	**542.661**	336.230	330.890	331.226	330.554	R[7]
H[18]	588.347	284.196	278.856	279.192	278.520	H[6]
R[19]	640.381	238.510	233.170	233.506	232.834	R[5]
K[20]	**697.095**	186.476	181.136	181.472	180.800	K[4]
V[21]	730.117	129.762	124.422	124.758	124.086	V[3]
L[22]	767.812	96.739	91.400	91.736	91.064	L[2]
R[23]	**819.846**	59.045	53.705	54.041	53.369	R[1]

sp|P62806|H4_MOUSE

[Acetyl]SGR $^{Methyl}_{14.02}$ GKGGKGLGKGGAK $^{Acetyl}_{42.01}$ RHRK $^{Trimethyl}_{42.05}$ VLR

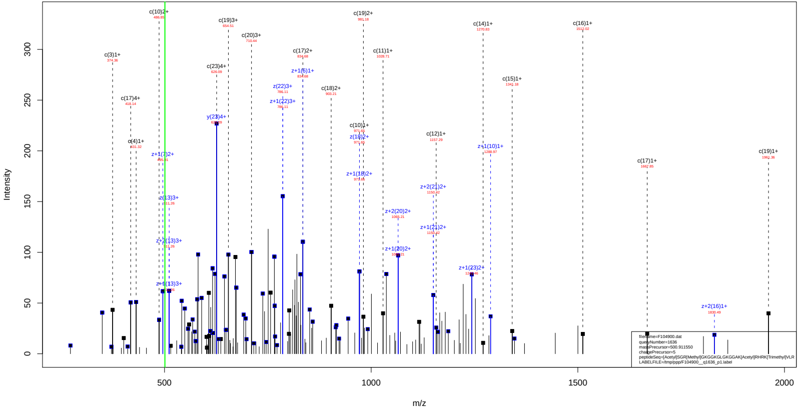

sp|P62806|H4_MOUSE

[Acetyl]SGR $^{\text{Methyl}}_{14.02}$ GKGGKGLGKGGAK $^{\text{Acetyl}}_{42.01}$ RHRK $^{\text{Trimethyl}}_{42.05}$ VLR

- fragmentation table for charge state 1+
- specType=etd
- score=94.51
- F104900.dat
- query=q1636_p1
- precursor=500.911550
- chargePrecursor=5
- itol=0.8

AA	c	y	z+1	z+2	z	AA
S[1]	147.076	2500.517	2484.498	0.000	2483.491	S[23]
G[2]	204.098	2371.475	2355.456	0.000	2354.448	G[22]
R[3]	**374.215**	2314.453	2298.434	2299.442	2297.427	R[21]
G[4]	**431.236**	2144.336	2128.318	2129.325	2127.310	G[20]
K[5]	**559.331**	2087.315	2071.296	2072.304	2070.288	K[19]
G[6]	**616.353**	1959.220	1943.201	1944.209	1942.193	G[18]
G[7]	**673.374**	1902.198	1886.180	1887.188	1885.172	G[17]
K[8]	**801.469**	1845.177	1829.158	**1830.166**	1828.150	K[16]
G[9]	**858.490**	1717.082	1701.063	1702.071	1700.055	G[15]
L[10]	**971.574**	1660.061	1644.042	1645.050	1643.034	L[14]
G[11]	**1028.596**	1546.976	1530.958	1531.966	1529.950	G[13]
K[12]	**1156.691**	1489.955	1473.936	1474.944	1472.928	K[12]
G[13]	1213.712	1361.860	1345.841	**1346.849**	1344.833	G[11]
G[14]	**1270.734**	1304.839	**1288.820**	1289.828	1287.812	G[10]
A[15]	**1341.771**	1247.817	1231.798	1232.806	1230.791	A[9]
K[16]	**1511.876**	1176.780	**1160.761**	**1161.769**	1159.753	K[8]
R[17]	**1667.978**	1006.674	990.656	**991.664**	989.648	R[7]
H[18]	1805.037	**850.573**	**834.555**	835.562	833.547	H[6]
R[19]	**1961.138**	713.514	697.496	**698.504**	**696.488**	R[5]
K[20]	2131.280	**557.413**	**541.395**	**542.402**	**540.387**	K[4]
V[21]	2230.348	387.271	**371.253**	**372.261**	370.245	V[3]
L[22]	2343.432	288.203	**272.184**	273.192	271.176	L[2]
R[23]	2499.533	175.119	159.100	160.108	158.092	R[1]

sp|P62806|H4_MOUSE

[Acetyl]SGR$^{\text{Methyl}}_{14.02}$GKGGKGLGKGGAK$^{\text{Acetyl}}_{42.01}$RHRK$^{\text{Trimethyl}}_{42.05}$VLR

- fragmentation table for charge state 2+
- specType=etd
- score=94.51
- F104900.dat
- query=q1636_p1
- precursor=500.911550
- chargePrecursor=5
- itol=0.8

AA	c	y	z+1	z+2	z	AA
S[1]	74.042	1250.762	**1242.753**	0.504	1242.249	S[23]
G[2]	102.553	**1186.241**	1178.232	0.504	1177.728	G[22]
R[3]	187.611	**1157.730**	**1149.721**	**1150.225**	1149.217	R[21]
G[4]	216.122	1072.672	**1064.662**	**1065.166**	1064.159	G[20]
K[5]	280.169	1044.161	**1036.152**	**1036.656**	1035.648	K[19]
G[6]	308.680	980.114	**972.104**	972.608	**971.600**	G[18]
G[7]	337.191	951.603	**943.593**	**944.097**	943.090	G[17]
K[8]	**401.238**	**923.092**	**915.083**	**915.587**	**914.579**	K[16]
G[9]	429.749	**859.045**	**851.035**	**851.539**	**850.531**	G[15]
L[10]	**486.291**	830.534	822.525	823.028	822.021	L[14]
G[11]	**514.802**	773.992	**765.983**	**766.486**	**765.479**	G[13]
K[12]	**578.849**	**745.481**	**737.472**	**737.976**	**736.968**	K[12]
G[13]	**607.360**	681.434	**673.424**	**673.928**	**672.920**	G[11]
G[14]	**635.871**	652.923	**644.914**	**645.417**	**644.410**	G[10]
A[15]	**671.389**	624.412	**616.403**	**616.907**	**615.899**	A[9]
K[16]	**756.442**	588.894	**580.884**	**581.388**	580.380	K[8]
R[17]	**834.492**	503.841	**495.832**	**496.335**	**495.328**	R[7]
H[18]	**903.022**	425.790	**417.781**	**418.285**	417.277	H[6]
R[19]	**981.072**	357.261	**349.252**	**349.755**	**348.748**	R[5]
K[20]	1066.143	279.210	271.201	**271.705**	270.697	K[4]
V[21]	**1115.678**	194.139	186.130	186.634	185.626	V[3]
L[22]	1172.220	144.605	136.596	137.100	136.092	L[2]
R[23]	1250.270	88.063	80.054	80.558	79.550	R[1]

sp|P62806|H4_MOUSE

[Acetyl]SGR $^{\text{Methyl}}_{14.02}$ GKGGKGLGKGGAK $^{\text{Acetyl}}_{42.01}$ RHRK $^{\text{Trimethyl}}_{42.05}$ VLR

- fragmentation table for charge state 3+
- specType=etd
- score=94.51
- F104900.dat
- query=q1636_p1
- precursor=500.911550
- chargePrecursor=5
- itol=0.8

AA	c	y	z+1	z+2	z	AA
S[1]	49.697	**834.177**	**828.838**	0.672	**828.502**	S[23]
G[2]	68.704	791.163	**785.823**	0.672	**785.488**	G[22]
R[3]	125.410	**772.156**	**766.816**	**767.152**	**766.480**	R[21]
G[4]	144.417	**715.450**	**710.111**	**710.447**	**709.775**	G[20]
K[5]	187.115	**696.443**	**691.104**	**691.440**	**690.768**	K[19]
G[6]	206.122	**653.745**	**648.405**	**648.741**	**648.069**	G[18]
G[7]	225.130	634.738	629.398	**629.734**	629.062	G[17]
K[8]	267.828	**615.731**	**610.391**	**610.727**	**610.055**	K[16]
G[9]	286.835	**573.032**	**567.693**	**568.029**	**567.357**	G[15]
L[10]	324.530	554.025	**548.685**	**549.021**	**548.350**	L[14]
G[11]	343.537	516.330	**510.991**	**511.327**	**510.655**	G[13]
K[12]	386.235	497.323	491.984	492.320	491.648	K[12]
G[13]	405.242	454.625	449.285	449.621	448.949	G[11]
G[14]	424.249	435.618	430.278	**430.614**	429.942	G[10]
A[15]	447.929	416.611	**411.271**	**411.607**	**410.935**	A[9]
K[16]	504.630	392.932	387.592	387.928	387.256	K[8]
R[17]	**556.664**	336.230	330.890	331.226	330.554	R[7]
H[18]	**602.350**	284.196	278.856	279.192	278.520	H[6]
R[19]	**654.384**	238.510	233.170	233.506	232.834	R[5]
K[20]	**711.098**	186.476	181.136	181.472	180.800	K[4]
V[21]	744.121	129.762	124.422	124.758	124.086	V[3]
L[22]	781.816	96.739	91.400	91.736	91.064	L[2]
R[23]	833.849	59.045	53.705	54.041	53.369	R[1]

sp|P62806|H4_MOUSE

[Acetyl]SGR $^{\text{Methyl}}_{14.02}$ GKGGKGLGKGGAK $^{\text{Acetyl}}_{42.01}$ RHRK $^{\text{Trimethyl}}_{42.05}$ VLR

- fragmentation table for charge state 4+
- specType=etd
- score=94.51
- F104900.dat
- query=q1636_p1
- precursor=500.911550
- chargePrecursor=5
- itol=0.8

AA	c	y	z+1	z+2	z	AA
S[1]	37.525	**625.885**	**621.880**	0.755	**621.628**	S[23]
G[2]	51.780	593.624	**589.619**	0.755	**589.367**	G[22]
R[3]	94.309	**579.369**	**575.364**	**575.616**	**575.112**	R[21]
G[4]	108.564	536.840	532.835	533.087	532.583	G[20]
K[5]	140.588	522.584	518.579	518.831	518.328	K[19]
G[6]	154.844	490.560	**486.556**	**486.808**	**486.304**	G[18]
G[7]	169.099	476.305	472.300	472.552	472.048	G[17]
K[8]	201.123	462.050	458.045	458.297	457.793	K[16]
G[9]	215.378	430.026	426.021	426.273	425.769	G[15]
L[10]	243.649	415.771	411.766	412.018	**411.514**	L[14]
G[11]	257.904	387.500	383.495	383.747	383.243	G[13]
K[12]	289.928	373.244	369.240	369.491	368.988	K[12]
G[13]	304.184	341.220	337.216	337.468	336.964	G[11]
G[14]	318.439	326.965	322.960	323.212	322.708	G[10]
A[15]	336.198	312.710	308.705	308.957	308.453	A[9]
K[16]	378.725	294.950	290.946	291.198	290.694	K[8]
R[17]	**417.750**	252.424	248.419	248.671	248.167	R[7]
H[18]	452.015	213.399	209.394	209.646	209.142	H[6]
R[19]	491.040	179.134	175.129	175.381	174.877	R[5]
K[20]	533.575	140.109	136.104	136.356	135.852	K[4]
V[21]	558.342	97.573	93.569	93.821	93.317	V[3]
L[22]	586.613	72.806	68.802	69.053	68.550	L[2]
R[23]	**625.639**	44.535	40.531	40.782	40.279	R[1]

sp|P62806|H4_MOUSE

[Acetyl]SGR $^{\text{Methyl}}_{14.02}$ GKGGKGLGKGGAK $^{\text{Acetyl}}_{42.01}$ RHRK $^{\text{Methyl}}_{14.02}$ VLR $^{\text{Dimethyl}}_{28.03}$

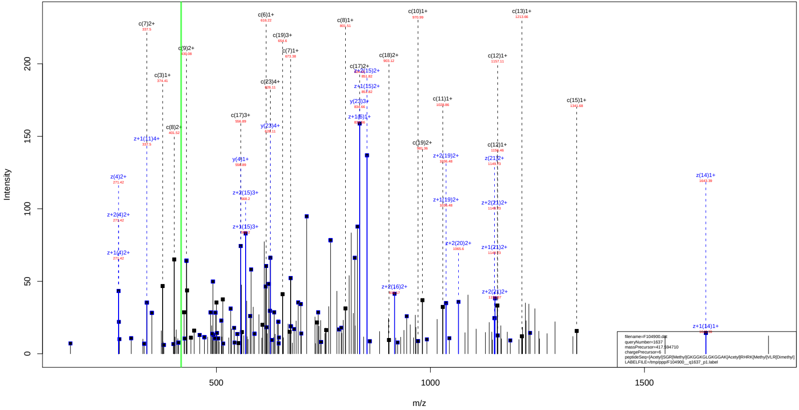

sp|P62806|H4_MOUSE

[Acetyl]SGR $^{Methyl}_{14.02}$ GKGGKGLGKGGAK $^{Acetyl}_{42.01}$ RHRK $^{Methyl}_{14.02}$ VLR $^{Dimethyl}_{28.03}$

- fragmentation table for charge state 1+
- specType=etd
- score=68.83
- F104900.dat
- query=q1637_p1
- precursor=417.594710
- chargePrecursor=6
- itol=0.8

AA	c	y	z+1	z+2	z	AA
S[1]	147.076	2500.517	2484.498	0.000	2483.491	S[23]
G[2]	204.098	2371.475	2355.456	0.000	2354.448	G[22]
R[3]	**374.215**	2314.453	2298.434	2299.442	2297.427	R[21]
G[4]	**431.236**	2144.336	2128.318	2129.325	2127.310	G[20]
K[5]	**559.331**	2087.315	2071.296	2072.304	2070.288	K[19]
G[6]	**616.353**	1959.220	1943.201	1944.209	1942.193	G[18]
G[7]	**673.374**	1902.198	1886.180	1887.188	1885.172	G[17]
K[8]	**801.469**	1845.177	1829.158	1830.166	1828.150	K[16]
G[9]	**858.490**	1717.082	1701.063	1702.071	1700.055	G[15]
L[10]	**971.574**	1660.061	**1644.042**	1645.050	**1643.034**	L[14]
G[11]	**1028.596**	1546.976	1530.958	1531.966	1529.950	G[13]
K[12]	**1156.691**	1489.955	1473.936	1474.944	1472.928	K[12]
G[13]	**1213.712**	1361.860	1345.841	1346.849	1344.833	G[11]
G[14]	1270.734	1304.839	1288.820	1289.828	1287.812	G[10]
A[15]	**1341.771**	1247.817	1231.798	**1232.806**	1230.791	A[9]
K[16]	1511.876	1176.780	1160.761	1161.769	1159.753	K[8]
R[17]	1667.978	1006.674	990.656	**991.664**	989.648	R[7]
H[18]	1805.037	850.573	**834.555**	835.562	833.547	H[6]
R[19]	1961.138	713.514	**697.496**	**698.504**	**696.488**	R[5]
K[20]	2103.248	**557.413**	**541.395**	**542.402**	540.387	K[4]
V[21]	2202.317	415.303	**399.284**	400.292	398.276	V[3]
L[22]	2315.401	316.234	**300.216**	**301.223**	299.208	L[2]
R[23]	2499.533	203.150	187.132	188.139	186.124	R[1]

sp|P62806|H4_MOUSE

[Acetyl]SGR$^{\text{Methyl}}_{14.02}$GKGGKGLGKGGAK$^{\text{Acetyl}}_{42.01}$RHRK$^{\text{Methyl}}_{14.02}$VLR$^{\text{Dimethyl}}_{28.03}$

- fragmentation table for charge state 2+
- specType=etd
- score=68.83
- F104900.dat
- query=q1637_p1
- precursor=417.594710
- chargePrecursor=6
- itol=0.8

AA	c	y	z+1	z+2	z	AA
S[1]	74.042	1250.762	1242.753	0.504	1242.249	S[23]
G[2]	102.553	**1186.241**	1178.232	0.504	1177.728	G[22]
R[3]	187.611	**1157.730**	**1149.721**	**1150.225**	**1149.217**	R[21]
G[4]	216.122	1072.672	1064.662	**1065.166**	1064.159	G[20]
K[5]	280.169	**1044.161**	**1036.152**	**1036.656**	1035.648	K[19]
G[6]	308.680	980.114	972.104	972.608	**971.600**	G[18]
G[7]	**337.191**	951.603	943.593	**944.097**	943.090	G[17]
K[8]	**401.238**	**923.092**	915.083	**915.587**	914.579	K[16]
G[9]	**429.749**	**859.045**	**851.035**	**851.539**	850.531	G[15]
L[10]	**486.291**	830.534	**822.525**	**823.028**	822.021	L[14]
G[11]	**514.802**	773.992	**765.983**	**766.486**	765.479	G[13]
K[12]	**578.849**	**745.481**	**737.472**	**737.976**	736.968	K[12]
G[13]	**607.360**	**681.434**	**673.424**	**673.928**	**672.920**	G[11]
G[14]	**635.871**	652.923	**644.914**	**645.417**	**644.410**	G[10]
A[15]	**671.389**	**624.412**	**616.403**	**616.907**	**615.899**	A[9]
K[16]	**756.442**	**588.894**	**580.884**	**581.388**	**580.380**	K[8]
R[17]	**834.492**	**503.841**	**495.832**	**496.335**	495.328	R[7]
H[18]	**903.022**	**425.790**	417.781	418.285	417.277	H[6]
R[19]	**981.072**	357.261	**349.252**	**349.755**	**348.748**	R[5]
K[20]	1052.128	279.210	**271.201**	**271.705**	**270.697**	K[4]
V[21]	1101.662	208.155	200.146	200.650	199.642	V[3]
L[22]	1158.204	**158.621**	150.611	151.115	150.108	L[2]
R[23]	1250.270	102.079	94.069	94.573	93.565	R[1]

sp|P62806|H4_MOUSE

[Acetyl]SGR $^{\text{Methyl}}_{14.02}$ GKGGKGLGKGGAK $^{\text{Acetyl}}_{42.01}$ RHRK $^{\text{Methyl}}_{14.02}$ VLR $^{\text{Dimethyl}}_{28.03}$

- fragmentation table for charge state 3+
- specType=etd
- score=68.83
- F104900.dat
- query=q1637_p1
- precursor=417.594710
- chargePrecursor=6
- itol=0.8

AA	c	y	z+1	z+2	z	AA
S[1]	49.697	**834.177**	**828.838**	0.672	828.502	S[23]
G[2]	68.704	**791.163**	**785.823**	0.672	785.488	G[22]
R[3]	125.410	772.156	**766.816**	**767.152**	**766.480**	R[21]
G[4]	144.417	715.450	**710.111**	**710.447**	709.775	G[20]
K[5]	187.115	**696.443**	**691.104**	**691.440**	**690.768**	K[19]
G[6]	206.122	653.745	648.405	648.741	648.069	G[18]
G[7]	225.130	**634.738**	**629.398**	**629.734**	**629.062**	G[17]
K[8]	267.828	**615.731**	610.391	610.727	610.055	K[16]
G[9]	286.835	573.032	**567.693**	**568.029**	567.357	G[15]
L[10]	324.530	554.025	**548.685**	**549.021**	**548.350**	L[14]
G[11]	343.537	**516.330**	**510.991**	**511.327**	510.655	G[13]
K[12]	386.235	**497.323**	**491.984**	**492.320**	**491.648**	K[12]
G[13]	405.242	454.625	449.285	449.621	448.949	G[11]
G[14]	**424.249**	435.618	**430.278**	**430.614**	**429.942**	G[10]
A[15]	**447.929**	416.611	**411.271**	**411.607**	**410.935**	A[9]
K[16]	**504.630**	392.932	387.592	387.928	387.256	K[8]
R[17]	**556.664**	336.230	**330.890**	**331.226**	**330.554**	R[7]
H[18]	602.350	284.196	278.856	279.192	278.520	H[6]
R[19]	**654.384**	238.510	233.170	233.506	232.834	R[5]
K[20]	701.754	186.476	181.136	181.472	180.800	K[4]
V[21]	**734.777**	139.106	133.766	134.102	133.430	V[3]
L[22]	772.472	106.083	100.743	101.079	100.407	L[2]
R[23]	833.849	68.388	63.049	63.385	62.713	R[1]

sp|P62806|H4_MOUSE

[Acetyl]SGR $^{Methyl}_{14.02}$ GKGGKGLGKGGAK $^{Acetyl}_{42.01}$ RHRK $^{Methyl}_{14.02}$ VLR $^{Dimethyl}_{28.03}$

- fragmentation table for charge state 4+
- specType=etd
- score=68.83
- F104900.dat
- query=q1637_p1
- precursor=417.594710
- chargePrecursor=6
- itol=0.8

AA	c	y	z+1	z+2	z	AA
S[1]	37.525	**625.885**	**621.880**	0.755	**621.628**	S[23]
G[2]	51.780	593.624	**589.619**	0.755	**589.367**	G[22]
R[3]	94.309	**579.369**	575.364	575.616	575.112	R[21]
G[4]	108.564	536.840	**532.835**	**533.087**	**532.583**	G[20]
K[5]	140.588	522.584	518.579	518.831	518.328	K[19]
G[6]	154.844	**490.560**	**486.556**	**486.808**	**486.304**	G[18]
G[7]	169.099	476.305	**472.300**	**472.552**	**472.048**	G[17]
K[8]	201.123	462.050	458.045	458.297	457.793	K[16]
G[9]	215.378	**430.026**	**426.021**	**426.273**	**425.769**	G[15]
L[10]	243.649	415.771	**411.766**	**412.018**	**411.514**	L[14]
G[11]	257.904	387.500	383.495	383.747	383.243	G[13]
K[12]	289.928	373.244	369.240	369.491	368.988	K[12]
G[13]	304.184	341.220	**337.216**	**337.468**	**336.964**	G[11]
G[14]	318.439	326.965	322.960	323.212	322.708	G[10]
A[15]	336.198	312.710	308.705	308.957	308.453	A[9]
K[16]	378.725	294.950	290.946	291.198	290.694	K[8]
R[17]	417.750	252.424	248.419	248.671	248.167	R[7]
H[18]	452.015	213.399	209.394	209.646	209.142	H[6]
R[19]	491.040	179.134	175.129	175.381	174.877	R[5]
K[20]	526.568	140.109	136.104	136.356	135.852	K[4]
V[21]	**551.335**	104.581	100.576	100.828	100.325	V[3]
L[22]	**579.606**	79.814	75.809	76.061	75.557	L[2]
R[23]	**625.639**	51.543	47.538	47.790	47.286	R[1]

sp|P62806|H4_MOUSE

[Acetyl]SGR $^{\text{Methyl}}_{14.02}$ GKGGKGLGKGGAK $^{\text{Acetyl}}_{42.01}$ RHRK $^{\text{Methyl}}_{14.02}$ VLR $^{\text{Dimethyl}}_{28.03}$

- fragmentation table for charge state 5+
- specType=etd
- score=68.83
- F104900.dat
- query=q1637_p1
- precursor=417.594710
- chargePrecursor=6
- itol=0.8

AA	c	y	z+1	z+2	z	AA
S[1]	30.221	**500.909**	**497.706**	0.806	**497.504**	S[23]
G[2]	41.625	475.101	**471.897**	0.806	**471.695**	G[22]
R[3]	75.649	463.696	**460.493**	**460.694**	**460.291**	R[21]
G[4]	87.053	**429.673**	**426.469**	426.671	**426.268**	G[20]
K[5]	112.672	418.269	415.065	415.267	414.863	K[19]
G[6]	124.076	392.650	389.446	389.648	389.244	G[18]
G[7]	135.481	381.246	**378.042**	378.243	**377.840**	G[17]
K[8]	161.100	369.841	366.637	366.839	366.436	K[16]
G[9]	172.504	344.222	341.018	341.220	340.817	G[15]
L[10]	195.121	332.818	329.614	329.816	329.413	L[14]
G[11]	206.525	310.201	306.997	307.199	306.796	G[13]
K[12]	232.144	298.797	295.593	295.795	295.392	K[12]
G[13]	243.548	**273.178**	269.974	270.176	269.773	G[11]
G[14]	254.953	261.774	258.570	258.771	258.368	G[10]
A[15]	269.160	250.369	247.165	247.367	246.964	A[9]
K[16]	303.181	236.162	232.958	233.160	232.757	K[8]
R[17]	334.401	202.141	198.937	199.139	198.735	R[7]
H[18]	361.813	170.920	167.717	167.918	167.515	H[6]
R[19]	393.033	143.509	140.305	140.507	140.103	R[5]
K[20]	421.455	112.288	109.085	109.286	108.883	K[4]
V[21]	**441.269**	83.866	80.663	80.864	80.461	V[3]
L[22]	463.886	64.053	60.849	61.051	60.647	L[2]
R[23]	**500.712**	41.436	38.232	38.434	38.031	R[1]

sp|P62806|H4_MOUSE

[Acetyl]SGRGKGGK $^{\text{Acetyl}}_{42.01}$ GLGKGGAK $^{\text{Trimethyl}}_{42.05}$ RHRK $^{\text{Dimethyl}}_{28.03}$ VLR

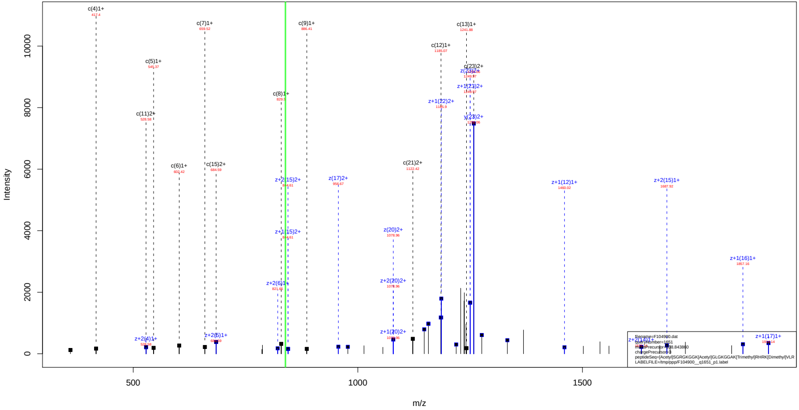

sp|P62806|H4_MOUSE

[Acetyl]SGRGKGGK $^{\text{Acetyl}}_{42.01}$ GLGKGGAK $^{\text{Trimethyl}}_{42.05}$ RHRK $^{\text{Dimethyl}}_{28.03}$ VLR

- fragmentation table for charge state 1+
- specType=etd
- score=70.98
- F104900.dat
- query=q1651_p1
- precursor=838.843860
- chargePrecursor=3
- itol=0.8

AA	c	y	z+1	z+2	z	AA
S[1]	147.076	2514.533	2498.514	0.000	2497.506	S[23]
G[2]	204.098	2385.490	2369.471	0.000	2368.464	G[22]
R[3]	**360.199**	2328.469	2312.450	2313.458	2311.442	R[21]
G[4]	**417.220**	2172.368	2156.349	2157.357	2155.341	G[20]
K[5]	**545.315**	2115.346	2099.327	2100.335	2098.320	K[19]
G[6]	**602.337**	1987.251	1971.232	1972.240	1970.225	G[18]
G[7]	**659.358**	1930.230	**1914.211**	1915.219	1913.203	G[17]
K[8]	**829.464**	1873.208	**1857.190**	1858.197	1856.182	K[16]
G[9]	**886.485**	1703.103	1687.084	**1688.092**	1686.076	G[15]
L[10]	999.569	1646.081	1630.063	**1631.070**	1629.055	L[14]
G[11]	1056.591	1532.997	1516.978	1517.986	1515.971	G[13]
K[12]	**1184.686**	1475.976	**1459.957**	1460.965	1458.949	K[12]
G[13]	**1241.707**	1347.881	1331.862	**1332.870**	1330.854	G[11]
G[14]	1298.729	1290.859	1274.841	**1275.848**	1273.833	G[10]
A[15]	1369.766	1233.838	1217.819	**1218.827**	1216.811	A[9]
K[16]	1539.908	1162.801	1146.782	**1147.790**	1145.774	K[8]
R[17]	1696.009	992.659	976.640	**977.648**	975.632	R[7]
H[18]	1833.068	836.558	820.539	**821.547**	819.531	H[6]
R[19]	1989.169	699.499	683.480	**684.488**	682.472	R[5]
K[20]	2145.295	543.398	527.379	**528.387**	526.371	K[4]
V[21]	2244.364	387.271	371.253	372.261	370.245	V[3]
L[22]	2357.448	288.203	272.184	273.192	271.176	L[2]
R[23]	2513.549	175.119	159.100	160.108	158.092	R[1]

sp|P62806|H4_MOUSE

[Acetyl]SGRGKGGK $^{Acetyl}_{42.01}$ GLGKGGAK $^{Trimethyl}_{42.05}$ RHRK $^{Dimethyl}_{28.03}$ VLR

- fragmentation table for charge state 2+
- specType=etd
- score=70.98
- F104900.dat
- query=q1651_p1
- precursor=838.843860
- chargePrecursor=3
- itol=0.8

AA	c	y	z+1	z+2	z	AA
S[1]	74.042	**1257.770**	**1249.761**	0.504	**1249.257**	S[23]
G[2]	102.553	1193.249	**1185.239**	0.504	**1184.735**	G[22]
R[3]	180.603	1164.738	**1156.729**	**1157.233**	1156.225	R[21]
G[4]	209.114	1086.687	**1078.678**	**1079.182**	**1078.174**	G[20]
K[5]	273.161	1058.177	1050.167	1050.671	1049.663	K[19]
G[6]	301.672	994.129	986.120	986.624	985.616	G[18]
G[7]	330.183	965.619	957.609	958.113	**957.105**	G[17]
K[8]	415.236	937.108	929.098	929.602	928.594	K[16]
G[9]	443.746	852.055	**844.046**	**844.550**	843.542	G[15]
L[10]	500.288	823.544	815.535	816.039	815.031	L[14]
G[11]	**528.799**	767.002	758.993	759.497	758.489	G[13]
K[12]	592.847	738.492	730.482	730.986	729.978	K[12]
G[13]	621.357	674.444	666.435	666.939	665.931	G[11]
G[14]	649.868	645.933	637.924	638.428	637.420	G[10]
A[15]	**685.387**	617.423	609.413	609.917	608.909	A[9]
K[16]	770.458	581.904	573.895	574.399	573.391	K[8]
R[17]	848.508	496.833	488.824	489.328	488.320	R[7]
H[18]	917.038	418.782	410.773	411.277	410.269	H[6]
R[19]	995.088	350.253	342.244	342.748	341.740	R[5]
K[20]	1073.151	272.202	264.193	264.697	263.689	K[4]
V[21]	**1122.685**	194.139	186.130	186.634	185.626	V[3]
L[22]	1179.227	144.605	136.596	137.100	136.092	L[2]
R[23]	**1257.278**	88.063	80.054	80.558	79.550	R[1]

sp|P62806|H4_MOUSE

[Acetyl]SGRGKGGKGLGK $^{\text{Acetyl}}_{42.01}$ GGAK $^{\text{Trimethyl}}_{42.05}$ RHRK $^{\text{Dimethyl}}_{28.03}$ VLR

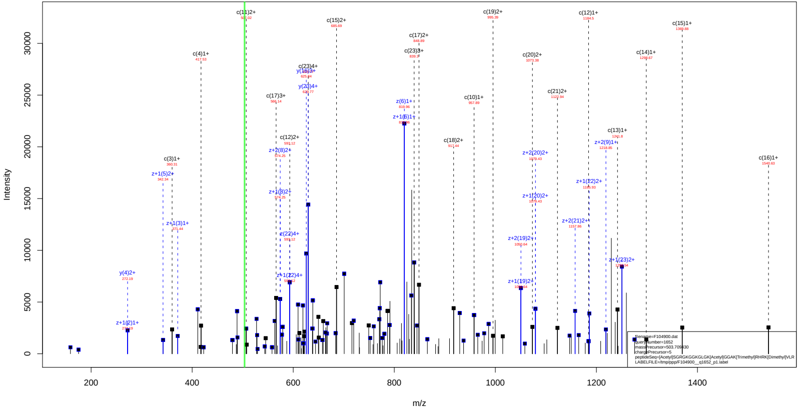

sp|P62806|H4_MOUSE

[Acetyl]SGRGKGGKGLGK $^{Acetyl}_{42.01}$ GGAK $^{Trimethyl}_{42.05}$ RHRK $^{Dimethyl}_{28.03}$ VLR

- fragmentation table for charge state 1+
- specType=etd
- score=100.07
- F104900.dat
- query=q1652_p1
- precursor=503.709430
- chargePrecursor=5
- itol=0.8

AA	c	y	z+1	z+2	z	AA
S[1]	147.076	2514.533	2498.514	0.000	2497.506	S[23]
G[2]	204.098	2385.490	2369.471	0.000	2368.464	G[22]
R[3]	**360.199**	2328.469	2312.450	2313.458	2311.442	R[21]
G[4]	**417.220**	2172.368	2156.349	2157.357	2155.341	G[20]
K[5]	**545.315**	2115.346	2099.327	2100.335	2098.320	K[19]
G[6]	602.337	1987.251	1971.232	1972.240	1970.225	G[18]
G[7]	**659.358**	1930.230	1914.211	1915.219	1913.203	G[17]
K[8]	**787.453**	1873.208	1857.190	1858.197	1856.182	K[16]
G[9]	**844.475**	1745.113	1729.095	1730.102	1728.087	G[15]
L[10]	**957.559**	1688.092	1672.073	1673.081	1671.065	L[14]
G[11]	**1014.580**	1575.008	1558.989	1559.997	1557.981	G[13]
K[12]	**1184.686**	1517.986	1501.968	1502.975	1500.960	K[12]
G[13]	**1241.707**	1347.881	1331.862	1332.870	1330.854	G[11]
G[14]	**1298.729**	1290.859	**1274.841**	**1275.848**	1273.833	G[10]
A[15]	**1369.766**	1233.838	1217.819	**1218.827**	1216.811	A[9]
K[16]	**1539.908**	1162.801	**1146.782**	1147.790	1145.774	K[8]
R[17]	1696.009	992.659	976.640	**977.648**	975.632	R[7]
H[18]	1833.068	836.558	**820.539**	821.547	**819.531**	H[6]
R[19]	1989.169	699.499	**683.480**	**684.488**	682.472	R[5]
K[20]	2145.295	**543.398**	**527.379**	**528.387**	526.371	K[4]
V[21]	2244.364	387.271	**371.253**	372.261	370.245	V[3]
L[22]	2357.448	288.203	**272.184**	273.192	271.176	L[2]
R[23]	2513.549	**175.119**	**159.100**	160.108	158.092	R[1]

sp|P62806|H4_MOUSE

[Acetyl]SGRGKGGKGLGK $^{\text{Acetyl}}_{42.01}$ GGAK $^{\text{Trimethyl}}_{42.05}$ RHRK $^{\text{Dimethyl}}_{28.03}$ VLR

- fragmentation table for charge state 2+
- specType=etd
- score=100.07
- F104900.dat
- query=q1652_p1
- precursor=503.709430
- chargePrecursor=5
- itol=0.8

AA	c	y	z+1	z+2	z	AA
S[1]	74.042	1257.770	**1249.761**	0.504	1249.257	S[23]
G[2]	102.553	1193.249	**1185.239**	0.504	**1184.735**	G[22]
R[3]	180.603	**1164.738**	1156.729	**1157.233**	1156.225	R[21]
G[4]	209.114	1086.687	**1078.678**	**1079.182**	1078.174	G[20]
K[5]	273.161	**1058.177**	**1050.167**	**1050.671**	1049.663	K[19]
G[6]	301.672	994.129	**986.120**	**986.624**	985.616	G[18]
G[7]	330.183	**965.619**	**957.609**	**958.113**	**957.105**	G[17]
K[8]	394.230	**937.108**	**929.098**	**929.602**	928.594	K[16]
G[9]	**422.741**	873.060	**865.051**	**865.555**	864.547	G[15]
L[10]	**479.283**	**844.550**	836.540	837.044	836.036	L[14]
G[11]	**507.794**	788.008	**779.998**	**780.502**	779.494	G[13]
K[12]	**592.847**	**759.497**	**751.487**	**751.991**	750.984	K[12]
G[13]	**621.357**	674.444	**666.435**	**666.939**	**665.931**	G[11]
G[14]	**649.868**	645.933	**637.924**	**638.428**	**637.420**	G[10]
A[15]	**685.387**	617.423	**609.413**	**609.917**	**608.909**	A[9]
K[16]	**770.458**	581.904	**573.895**	**574.399**	573.391	K[8]
R[17]	**848.508**	496.833	**488.824**	**489.328**	**488.320**	R[7]
H[18]	**917.038**	418.782	**410.773**	**411.277**	410.269	H[6]
R[19]	**995.088**	350.253	**342.244**	**342.748**	**341.740**	R[5]
K[20]	**1073.151**	**272.202**	264.193	264.697	263.689	K[4]
V[21]	**1122.685**	194.139	186.130	186.634	185.626	V[3]
L[22]	1179.227	144.605	136.596	137.100	136.092	L[2]
R[23]	1257.278	88.063	80.054	80.558	79.550	R[1]

sp|P62806|H4_MOUSE

[Acetyl]SGRGKGGKGLGK $^{Acetyl}_{42.01}$ GGAK $^{Trimethyl}_{42.05}$ RHRK $^{Dimethyl}_{28.03}$ VLR

- fragmentation table for charge state 3+
- specType=etd
- score=100.07
- F104900.dat
- query=q1652_p1
- precursor=503.709430
- chargePrecursor=5
- itol=0.8

AA	c	y	z+1	z+2	z	AA
S[1]	49.697	**838.849**	**833.510**	0.672	**833.174**	S[23]
G[2]	68.704	795.835	**790.495**	0.672	**790.159**	G[22]
R[3]	120.738	**776.828**	**771.488**	**771.824**	**771.152**	R[21]
G[4]	139.745	724.794	**719.454**	**719.790**	**719.119**	G[20]
K[5]	182.443	705.787	**700.447**	**700.783**	**700.111**	K[19]
G[6]	201.450	**663.089**	**657.749**	**658.085**	**657.413**	G[18]
G[7]	220.458	**644.081**	**638.742**	**639.078**	**638.406**	G[17]
K[8]	263.156	**625.074**	**619.735**	**620.071**	**619.399**	K[16]
G[9]	282.163	582.376	**577.036**	**577.372**	**576.700**	G[15]
L[10]	319.858	**563.369**	**558.029**	**558.365**	**557.693**	L[14]
G[11]	338.865	525.674	520.335	520.670	519.999	G[13]
K[12]	395.567	**506.667**	501.327	501.663	500.991	K[12]
G[13]	414.574	449.965	444.626	444.961	444.290	G[11]
G[14]	433.581	430.958	425.618	425.954	425.282	G[10]
A[15]	457.260	411.951	406.611	406.947	406.275	A[9]
K[16]	513.974	388.272	382.932	383.268	382.596	K[8]
R[17]	**566.008**	331.558	326.218	326.554	325.882	R[7]
H[18]	**611.694**	279.524	274.185	274.520	273.849	H[6]
R[19]	**663.728**	233.838	228.498	228.834	228.162	R[5]
K[20]	**715.770**	181.804	176.465	176.800	176.129	K[4]
V[21]	**748.793**	129.762	124.422	124.758	124.086	V[3]
L[22]	**786.487**	96.739	91.400	91.736	91.064	L[2]
R[23]	**838.521**	59.045	53.705	54.041	53.369	R[1]

sp|P62806|H4_MOUSE

[Acetyl]SGRGKGGKGLGK $^{Acetyl}_{42.01}$ GGAK $^{Trimethyl}_{42.05}$ RHRK $^{Dimethyl}_{28.03}$ VLR

- fragmentation table for charge state 4+
- specType=etd
- score=100.07
- F104900.dat
- query=q1652_p1
- precursor=503.709430
- chargePrecursor=5
- itol=0.8

AA	c	y	z+1	z+2	z	AA
S[1]	37.525	**629.389**	**625.384**	0.755	**625.132**	S[23]
G[2]	51.780	597.128	**593.123**	0.755	**592.871**	G[22]
R[3]	90.805	582.873	**578.868**	**579.120**	**578.616**	R[21]
G[4]	105.061	**543.847**	539.843	540.095	539.591	G[20]
K[5]	137.084	**529.592**	525.587	525.839	525.335	K[19]
G[6]	151.340	497.568	493.564	493.816	493.312	G[18]
G[7]	165.595	483.313	**479.308**	**479.560**	**479.056**	G[17]
K[8]	197.619	469.058	465.053	465.305	464.801	K[16]
G[9]	211.874	437.034	433.029	433.281	432.777	G[15]
L[10]	240.145	**422.778**	418.774	419.026	418.522	L[14]
G[11]	254.401	394.507	390.503	390.755	390.251	G[13]
K[12]	296.927	380.252	376.247	376.499	375.995	K[12]
G[13]	311.182	337.726	333.721	333.973	333.469	G[11]
G[14]	325.438	323.470	319.466	319.718	319.214	G[10]
A[15]	343.197	309.215	305.210	305.462	304.958	A[9]
K[16]	385.732	291.456	287.451	287.703	287.199	K[8]
R[17]	424.758	248.920	244.915	245.167	244.664	R[7]
H[18]	459.022	209.895	205.890	206.142	205.638	H[6]
R[19]	498.048	**175.630**	171.625	171.877	171.374	R[5]
K[20]	537.079	136.605	132.600	132.852	132.348	K[4]
V[21]	561.846	97.573	93.569	93.821	93.317	V[3]
L[22]	590.117	72.806	68.802	69.053	68.550	L[2]
R[23]	**629.143**	44.535	40.531	40.782	40.279	R[1]

sp|P62806|H4_MOUSE

[Acetyl]SGRGKGGK $^{Acetyl}_{42.01}$ GLGKGGAK $^{Trimethyl}_{42.05}$ RHRK $^{Dimethyl}_{28.03}$ VLR

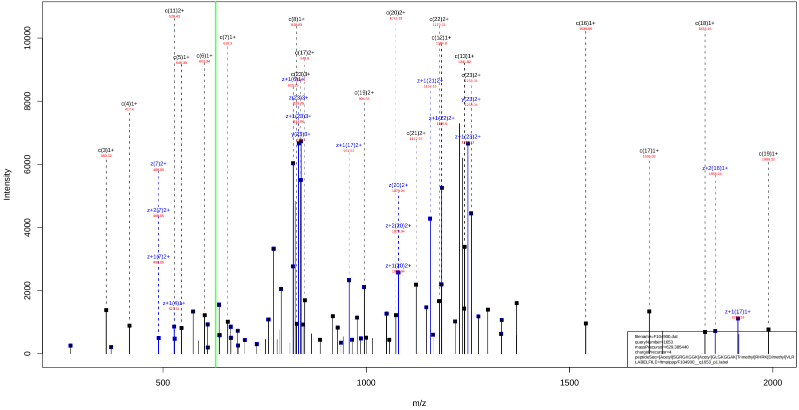

sp|P62806|H4_MOUSE

[Acetyl]SGRGKGGK $^{Acetyl}_{42.01}$ GLGKGGAK $^{Trimethyl}_{42.05}$ RHRK $^{Dimethyl}_{28.03}$ VLR

- fragmentation table for charge state 1+
- specType=etd
- score=139.19
- F104900.dat
- query=q1653_p1
- precursor=629.385440
- chargePrecursor=4
- itol=0.8

AA	c	y	z+1	z+2	z	AA
S[1]	147.076	2514.533	2498.514	0.000	2497.506	S[23]
G[2]	204.098	2385.490	2369.471	0.000	2368.464	G[22]
R[3]	**360.199**	2328.469	2312.450	2313.458	2311.442	R[21]
G[4]	**417.220**	2172.368	2156.349	2157.357	2155.341	G[20]
K[5]	**545.315**	2115.346	2099.327	2100.335	2098.320	K[19]
G[6]	**602.337**	1987.251	1971.232	1972.240	1970.225	G[18]
G[7]	**659.358**	1930.230	**1914.211**	1915.219	1913.203	G[17]
K[8]	**829.464**	1873.208	1857.190	**1858.197**	1856.182	K[16]
G[9]	**886.485**	1703.103	1687.084	1688.092	1686.076	G[15]
L[10]	**999.569**	1646.081	1630.063	1631.070	1629.055	L[14]
G[11]	**1056.591**	1532.997	1516.978	1517.986	1515.971	G[13]
K[12]	**1184.686**	1475.976	1459.957	1460.965	1458.949	K[12]
G[13]	**1241.707**	1347.881	**1331.862**	**1332.870**	**1330.854**	G[11]
G[14]	**1298.729**	1290.859	1274.841	**1275.848**	1273.833	G[10]
A[15]	**1369.766**	1233.838	1217.819	**1218.827**	1216.811	A[9]
K[16]	**1539.908**	1162.801	1146.782	**1147.790**	1145.774	K[8]
R[17]	**1696.009**	992.659	976.640	**977.648**	975.632	R[7]
H[18]	**1833.068**	836.558	**820.539**	821.547	**819.531**	H[6]
R[19]	**1989.169**	699.499	**683.480**	**684.488**	682.472	R[5]
K[20]	2145.295	543.398	**527.379**	**528.387**	526.371	K[4]
V[21]	2244.364	387.271	371.253	**372.261**	370.245	V[3]
L[22]	2357.448	288.203	**272.184**	273.192	271.176	L[2]
R[23]	2513.549	175.119	159.100	160.108	158.092	R[1]

sp|P62806|H4_MOUSE

[Acetyl]SGRGKGGK $^{Acetyl}_{42.01}$ GLGKGGAK $^{Trimethyl}_{42.05}$ RHRK $^{Dimethyl}_{28.03}$ VLR

- fragmentation table for charge state 2+
- specType=etd
- score=139.19
- F104900.dat
- query=q1653_p1
- precursor=629.385440
- chargePrecursor=4
- itol=0.8

AA	c	y	z+1	z+2	z	AA
S[1]	74.042	**1257.770**	**1249.761**	0.504	1249.257	S[23]
G[2]	102.553	1193.249	**1185.239**	0.504	**1184.735**	G[22]
R[3]	180.603	**1164.738**	**1156.729**	**1157.233**	1156.225	R[21]
G[4]	209.114	1086.687	**1078.678**	**1079.182**	**1078.174**	G[20]
K[5]	273.161	1058.177	**1050.167**	1050.671	**1049.663**	K[19]
G[6]	301.672	**994.129**	**986.120**	**986.624**	**985.616**	G[18]
G[7]	330.183	**965.619**	**957.609**	**958.113**	**957.105**	G[17]
K[8]	415.236	**937.108**	**929.098**	**929.602**	928.594	K[16]
G[9]	443.746	852.055	**844.046**	**844.550**	843.542	G[15]
L[10]	500.288	823.544	815.535	816.039	815.031	L[14]
G[11]	**528.799**	767.002	**758.993**	**759.497**	**758.489**	G[13]
K[12]	592.847	738.492	**730.482**	**730.986**	**729.978**	K[12]
G[13]	621.357	674.444	**666.435**	**666.939**	**665.931**	G[11]
G[14]	649.868	645.933	**637.924**	**638.428**	**637.420**	G[10]
A[15]	**685.387**	617.423	**609.413**	**609.917**	**608.909**	A[9]
K[16]	770.458	581.904	**573.895**	**574.399**	**573.391**	K[8]
R[17]	**848.508**	496.833	**488.824**	**489.328**	**488.320**	R[7]
H[18]	**917.038**	418.782	410.773	411.277	410.269	H[6]
R[19]	**995.088**	350.253	342.244	342.748	341.740	R[5]
K[20]	**1073.151**	**272.202**	264.193	264.697	263.689	K[4]
V[21]	**1122.685**	194.139	186.130	186.634	185.626	V[3]
L[22]	**1179.227**	144.605	136.596	137.100	136.092	L[2]
R[23]	**1257.278**	88.063	80.054	80.558	79.550	R[1]

sp|P62806|H4_MOUSE

[Acetyl]SGRGKGGK $^{Acetyl}_{42.01}$ GLGKGGAK $^{Trimethyl}_{42.05}$ RHRK $^{Dimethyl}_{28.03}$ VLR

- fragmentation table for charge state 3+
- specType=etd
- score=139.19
- F104900.dat
- query=q1653_p1
- precursor=629.385440
- chargePrecursor=4
- itol=0.8

AA	c	y	z+1	z+2	z	AA
S[1]	49.697	**838.849**	**833.510**	0.672	**833.174**	S[23]
G[2]	68.704	795.835	**790.495**	0.672	**790.159**	G[22]
R[3]	120.738	776.828	**771.488**	**771.824**	**771.152**	R[21]
G[4]	139.745	724.794	719.454	719.790	719.119	G[20]
K[5]	182.443	705.787	700.447	**700.783**	700.111	K[19]
G[6]	201.450	663.089	657.749	658.085	657.413	G[18]
G[7]	220.458	644.081	**638.742**	**639.078**	**638.406**	G[17]
K[8]	277.159	625.074	619.735	620.071	619.399	K[16]
G[9]	296.167	568.372	563.033	563.369	562.697	G[15]
L[10]	333.861	549.365	544.026	544.362	543.690	L[14]
G[11]	352.868	511.671	506.331	506.667	505.995	G[13]
K[12]	395.567	492.663	487.324	487.660	486.988	K[12]
G[13]	414.574	449.965	444.626	444.961	444.290	G[11]
G[14]	433.581	430.958	425.618	425.954	425.282	G[10]
A[15]	457.260	411.951	406.611	406.947	406.275	A[9]
K[16]	513.974	388.272	382.932	383.268	382.596	K[8]
R[17]	566.008	331.558	326.218	326.554	325.882	R[7]
H[18]	611.694	279.524	274.185	274.520	273.849	H[6]
R[19]	663.728	233.838	228.498	228.834	228.162	R[5]
K[20]	715.770	181.804	176.465	176.800	176.129	K[4]
V[21]	748.793	129.762	124.422	124.758	124.086	V[3]
L[22]	786.487	96.739	91.400	91.736	91.064	L[2]
R[23]	**838.521**	59.045	53.705	54.041	53.369	R[1]

sp|P62806|H4_MOUSE

[Acetyl]SGRGK $^{\text{Trimethyl}}_{42.05}$ GGKGLGK $^{\text{Acetyl}}_{42.01}$ GGAK $^{\text{Acetyl}}_{42.01}$ RHRK $^{\text{Methyl}}_{14.02}$ VLR

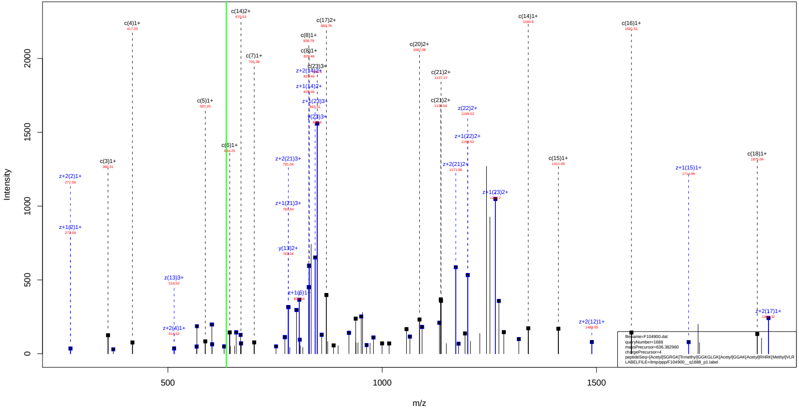

sp|P62806|H4_MOUSE

[Acetyl]SGRGK $^{Trimethyl}_{42.05}$ GGKGLGK $^{Acetyl}_{42.01}$ GGAK $^{Acetyl}_{42.01}$ RHRK $^{Methyl}_{14.02}$ VLR

- fragmentation table for charge state 1+
- specType=etd
- score=101.67
- F104900.dat
- query=q1688_p1
- precursor=636.382960
- chargePrecursor=4
- itol=0.8

AA	c	y	z+1	z+2	z	AA
S[1]	147.076	2542.528	2526.509	0.000	2525.501	S[23]
G[2]	204.098	2413.485	2397.466	0.000	2396.459	G[22]
R[3]	**360.199**	2356.464	2340.445	2341.453	2339.437	R[21]
G[4]	**417.220**	2200.363	2184.344	2185.352	2183.336	G[20]
K[5]	**587.362**	2143.341	2127.322	2128.330	2126.315	K[19]
G[6]	**644.384**	1973.199	1957.180	1958.188	1956.173	G[18]
G[7]	**701.405**	1916.178	1900.159	**1901.167**	1899.151	G[17]
K[8]	**829.500**	1859.156	1843.138	1844.145	1842.130	K[16]
G[9]	**886.522**	1731.061	**1715.043**	1716.050	1714.035	G[15]
L[10]	**999.606**	1674.040	1658.021	1659.029	1657.013	L[14]
G[11]	**1056.627**	1560.956	1544.937	1545.945	1543.929	G[13]
K[12]	1226.733	1503.934	1487.916	**1488.923**	1486.908	K[12]
G[13]	**1283.754**	1333.829	1317.810	**1318.818**	1316.802	G[11]
G[14]	**1340.776**	1276.807	1260.789	1261.796	1259.781	G[10]
A[15]	**1411.813**	1219.786	1203.767	1204.775	1202.759	A[9]
K[16]	**1581.918**	1148.749	**1132.730**	**1133.738**	1131.722	K[8]
R[17]	1738.019	978.643	962.624	**963.632**	961.617	R[7]
H[18]	**1875.078**	822.542	**806.523**	**807.531**	805.516	H[6]
R[19]	2031.179	685.483	**669.464**	**670.472**	668.457	R[5]
K[20]	2173.290	529.382	513.363	**514.371**	512.355	K[4]
V[21]	2272.359	387.271	371.253	**372.261**	370.245	V[3]
L[22]	2385.443	288.203	**272.184**	**273.192**	271.176	L[2]
R[23]	2541.544	175.119	159.100	160.108	158.092	R[1]

sp|P62806|H4_MOUSE

[Acetyl]SGRGK $^{\text{Trimethyl}}_{42.05}$ GGKGLGK $^{\text{Acetyl}}_{42.01}$ GGAK $^{\text{Acetyl}}_{42.01}$ RHRK $^{\text{Methyl}}_{14.02}$ VLR

- fragmentation table for charge state 2+
- specType=etd
- score=101.67
- F104900.dat
- query=q1688_p1
- precursor=636.382960
- chargePrecursor=4
- itol=0.8

AA	c	y	z+1	z+2	z	AA
S[1]	74.042	**1271.767**	**1263.758**	0.504	1263.254	S[23]
G[2]	102.553	1207.246	**1199.237**	0.504	**1198.733**	G[22]
R[3]	180.603	**1178.735**	1170.726	**1171.230**	1170.222	R[21]
G[4]	209.114	1100.685	**1092.676**	**1093.179**	1092.172	G[20]
K[5]	294.185	1072.174	**1064.165**	**1064.669**	1063.661	K[19]
G[6]	322.696	987.103	**979.094**	**979.598**	978.590	G[18]
G[7]	351.206	958.592	**950.583**	**951.087**	950.079	G[17]
K[8]	415.254	930.082	**922.072**	**922.576**	921.568	K[16]
G[9]	443.765	866.034	**858.025**	**858.529**	857.521	G[15]
L[10]	500.307	837.524	**829.514**	**830.018**	**829.010**	L[14]
G[11]	528.817	**780.982**	**772.972**	**773.476**	**772.468**	G[13]
K[12]	613.870	**752.471**	744.461	744.965	743.958	K[12]
G[13]	642.381	667.418	**659.409**	**659.913**	**658.905**	G[11]
G[14]	**670.891**	638.907	**630.898**	**631.402**	630.394	G[10]
A[15]	706.410	610.397	**602.387**	**602.891**	**601.883**	A[9]
K[16]	791.463	574.878	**566.869**	**567.373**	**566.365**	K[8]
R[17]	**869.513**	489.825	481.816	482.320	481.312	R[7]
H[18]	**938.043**	411.775	403.765	404.269	403.261	H[6]
R[19]	**1016.093**	343.245	335.236	335.740	334.732	R[5]
K[20]	**1087.149**	265.195	257.185	257.689	256.681	K[4]
V[21]	**1136.683**	194.139	186.130	186.634	185.626	V[3]
L[22]	**1193.225**	144.605	136.596	137.100	136.092	L[2]
R[23]	1271.275	88.063	80.054	80.558	79.550	R[1]

sp|P62806|H4_MOUSE

[Acetyl]SGRGK $^{\text{Trimethyl}}_{42.05}$ GGKGLGK $^{\text{Acetyl}}_{42.01}$ GGAK $^{\text{Acetyl}}_{42.01}$ RHRK $^{\text{Methyl}}_{14.02}$ VLR

- fragmentation table for charge state 3+
- specType=etd
- score=101.67
- F104900.dat
- query=q1688_p1
- precursor=636.382960
- chargePrecursor=4
- itol=0.8

AA	c	y	z+1	z+2	z	AA
S[1]	49.697	**848.181**	**842.841**	0.672	842.505	S[23]
G[2]	68.704	805.167	**799.827**	0.672	**799.491**	G[22]
R[3]	120.738	786.159	**780.820**	**781.156**	**780.484**	R[21]
G[4]	139.745	734.126	728.786	729.122	728.450	G[20]
K[5]	196.459	715.119	709.779	710.115	709.443	K[19]
G[6]	215.466	658.405	653.065	653.401	652.729	G[18]
G[7]	234.473	639.397	634.058	634.394	633.722	G[17]
K[8]	277.172	620.390	615.051	615.387	614.715	K[16]
G[9]	296.179	577.692	572.352	572.688	572.016	G[15]
L[10]	333.873	558.685	553.345	553.681	553.009	L[14]
G[11]	352.881	520.990	515.651	515.986	**515.315**	G[13]
K[12]	409.582	501.983	496.643	496.979	496.307	K[12]
G[13]	428.590	445.281	439.942	440.277	439.606	G[11]
G[14]	447.597	426.274	420.934	421.270	420.598	G[10]
A[15]	471.276	407.267	401.927	402.263	401.591	A[9]
K[16]	527.978	383.588	378.248	378.584	377.912	K[8]
R[17]	580.011	326.886	321.546	321.882	321.210	R[7]
H[18]	625.698	274.852	269.513	269.849	269.177	H[6]
R[19]	677.731	229.166	223.826	224.162	223.490	R[5]
K[20]	725.102	177.132	171.793	172.129	171.457	K[4]
V[21]	758.124	129.762	124.422	124.758	124.086	V[3]
L[22]	795.819	96.739	91.400	91.736	91.064	L[2]
R[23]	**847.853**	59.045	53.705	54.041	53.369	R[1]

sp|Q6GSS7|H2A2A_MOUSE

LNKLLGK $^{\text{Dimethyl}}_{28.03}$ VTIAQGGVLPNIQAVLLPKKTE

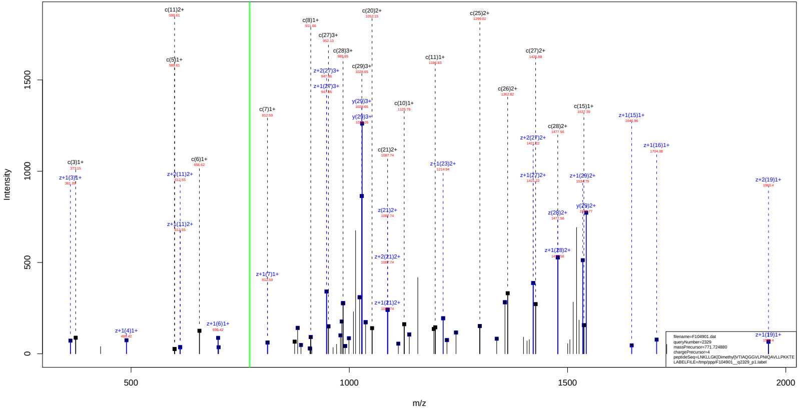

sp|Q6GSS7|H2A2A_MOUSE

LNKLLGK $^{Dimethyl}_{28.03}$ VTIAQGGVLPNIQAVLLPKKTE

- fragmentation table for charge state 1+
- specType=etd
- score=83.51
- F104901.dat
- query=q2329_p1
- precursor=771.724880
- chargePrecursor=4
- itol=0.8

AA	c	y	z+1	z+2	z	AA
L[1]	131.118	3083.892	3067.873	0.000	3066.865	L[29]
N[2]	245.161	2970.808	2954.789	2955.797	2953.781	N[28]
K[3]	**373.256**	2856.765	2840.746	2841.754	2839.738	K[27]
L[4]	486.340	2728.670	2712.651	2713.659	2711.643	L[26]
L[5]	**599.424**	2615.586	2599.567	2600.575	2598.559	L[25]
G[6]	**656.445**	2502.502	2486.483	2487.491	2485.475	G[24]
K[7]	**812.572**	2445.480	2429.462	2430.469	2428.454	K[23]
V[8]	**911.640**	2289.354	2273.335	2274.343	2272.327	V[22]
T[9]	1012.688	2190.286	2174.267	2175.275	2173.259	T[21]
I[10]	**1125.772**	2089.238	2073.219	2074.227	2072.211	I[20]
A[11]	**1196.809**	1976.154	**1960.135**	**1961.143**	1959.127	A[19]
Q[12]	1324.867	1905.117	1889.098	1890.106	1888.090	Q[18]
G[13]	1381.889	1777.058	1761.039	1762.047	1760.032	G[17]
G[14]	1438.910	1720.037	**1704.018**	1705.026	1703.010	G[16]
V[15]	**1537.979**	1663.015	**1646.997**	1648.004	1645.989	V[15]
L[16]	1651.063	1563.947	1547.928	1548.936	1546.920	L[14]
P[17]	1748.116	1450.863	1434.844	1435.852	1433.836	P[13]
N[18]	1862.159	1353.810	**1337.791**	1338.799	1336.783	N[12]
I[19]	1975.243	1239.767	**1223.748**	1224.756	1222.741	I[11]
Q[20]	2103.301	1126.683	1110.664	**1111.672**	1109.656	Q[10]
A[21]	2174.338	**998.624**	**982.606**	983.614	981.598	A[9]
V[22]	2273.407	927.587	**911.569**	912.576	910.561	V[8]
L[23]	2386.491	828.519	**812.500**	813.508	811.492	L[7]
L[24]	2499.575	715.435	**699.416**	**700.424**	698.408	L[6]
P[25]	2596.628	602.351	586.332	587.340	585.324	P[5]
K[26]	2724.723	505.298	**489.279**	490.287	488.271	K[4]
K[27]	2852.818	377.203	**361.184**	362.192	360.177	K[3]
T[28]	2953.865	249.108	233.089	234.097	232.082	T[2]
E[29]	3082.908	148.060	132.042	133.050	131.034	E[1]

sp|Q6GSS7|H2A2A_MOUSE

LNKLLGK $^{\text{Dimethyl}}_{28.03}$ VTIAQGGVLPNIQAVLLPKKTE

- fragmentation table for charge state 2+
- specType=etd
- score=83.51
- F104901.dat
- query=q2329_p1
- precursor=771.724880
- chargePrecursor=4
- itol=0.8

AA	c	y	z+1	z+2	z	AA
L[1]	66.063	**1542.450**	**1534.440**	0.504	1533.936	L[29]
N[2]	123.084	1485.908	**1477.898**	1478.402	**1477.394**	N[28]
K[3]	187.132	1428.886	**1420.877**	**1421.381**	1420.373	K[27]
L[4]	243.674	1364.839	**1356.829**	**1357.333**	**1356.325**	L[26]
L[5]	300.216	1308.297	1300.287	1300.791	**1299.783**	L[25]
G[6]	328.726	1251.755	**1243.745**	**1244.249**	1243.241	G[24]
K[7]	406.789	**1223.244**	**1215.234**	1215.738	**1214.731**	K[23]
V[8]	456.324	1145.181	**1137.171**	**1137.675**	**1136.667**	V[22]
T[9]	506.848	1095.646	**1087.637**	**1088.141**	**1087.133**	T[21]
I[10]	563.390	1045.123	**1037.113**	**1037.617**	**1036.609**	I[20]
A[11]	**598.908**	988.581	980.571	981.075	**980.067**	A[19]
Q[12]	662.937	953.062	945.053	945.557	944.549	Q[18]
G[13]	691.448	**889.033**	**881.023**	**881.527**	880.519	G[17]
G[14]	719.959	860.522	852.513	853.017	852.009	G[16]
V[15]	769.493	832.011	824.002	824.506	823.498	V[15]
L[16]	826.035	782.477	774.468	774.972	773.964	L[14]
P[17]	**874.561**	725.935	717.926	718.430	717.422	P[13]
N[18]	931.583	677.409	669.399	669.903	668.895	N[12]
I[19]	988.125	620.387	**612.378**	**612.882**	**611.874**	I[11]
Q[20]	**1052.154**	563.845	555.836	556.340	555.332	Q[10]
A[21]	**1087.673**	499.816	491.807	492.310	491.303	A[9]
V[22]	**1137.207**	464.297	456.288	456.792	455.784	V[8]
L[23]	**1193.749**	414.763	406.754	407.258	406.250	L[7]
L[24]	1250.291	358.221	350.212	350.716	349.708	L[6]
P[25]	**1298.817**	301.679	293.670	294.174	293.166	P[5]
K[26]	**1362.865**	253.153	245.143	245.647	244.639	K[4]
K[27]	**1426.912**	189.105	181.096	181.600	180.592	K[3]
T[28]	**1477.436**	125.058	117.048	117.552	116.544	T[2]
E[29]	1541.958	74.534	66.524	67.028	66.021	E[1]

sp|Q6GSS7|H2A2A_MOUSE

LNKLLGK $^{\text{Dimethyl}}_{28.03}$ VTIAQGGVLPNIQAVLLPKKTE

- fragmentation table for charge state 3+
- specType=etd
- score=83.51
- F104901.dat
- query=q2329_p1
- precursor=771.724880
- chargePrecursor=4
- itol=0.8

AA	c	y	z+1	z+2	z	AA
L[1]	44.377	**1028.635**	**1023.296**	0.672	**1022.960**	L[29]
N[2]	82.392	**990.941**	**985.601**	**985.937**	**985.265**	N[28]
K[3]	125.090	**952.926**	**947.587**	**947.923**	**947.251**	K[27]
L[4]	162.785	**910.228**	904.889	905.225	904.553	L[26]
L[5]	200.479	872.533	867.194	867.530	866.858	L[25]
G[6]	219.487	834.839	829.499	829.835	829.163	G[24]
K[7]	271.529	815.832	810.492	810.828	810.156	K[23]
V[8]	304.552	763.790	758.450	758.786	758.114	V[22]
T[9]	338.234	730.767	725.427	725.763	725.091	T[21]
I[10]	375.929	697.084	691.745	692.081	691.409	I[20]
A[11]	399.608	659.389	654.050	654.386	653.714	A[19]
Q[12]	442.294	635.710	630.371	630.707	630.035	Q[18]
G[13]	461.301	593.024	587.685	588.021	587.349	G[17]
G[14]	480.308	574.017	568.678	569.013	568.342	G[16]
V[15]	513.331	555.010	549.670	550.006	549.334	V[15]
L[16]	551.026	521.987	516.648	516.984	516.312	L[14]
P[17]	583.377	484.292	478.953	479.289	478.617	P[13]
N[18]	621.391	451.942	446.602	446.938	446.266	N[12]
I[19]	659.086	413.927	408.588	408.924	408.252	I[11]
Q[20]	701.772	376.233	370.893	371.229	370.557	Q[10]
A[21]	725.451	333.546	328.207	328.543	327.871	A[9]
V[22]	758.474	309.867	304.528	304.864	304.192	V[8]
L[23]	796.168	276.844	271.505	271.841	271.169	L[7]
L[24]	833.863	239.150	233.810	234.146	233.474	L[6]
P[25]	866.214	201.455	196.116	196.451	195.780	P[5]
K[26]	**908.912**	169.104	163.765	164.101	163.429	K[4]
K[27]	**951.611**	126.406	121.066	121.402	120.730	K[3]
T[28]	**985.293**	83.708	78.368	78.704	78.032	T[2]
E[29]	**1028.307**	50.025	44.685	45.021	44.349	E[1]

sp|Q6GSS7|H2A2A_MOUSE

LNKLLGK $^{\text{Dimethyl}}_{28.03}$ VTIAQGGVLPNIQAVLLPKKTE

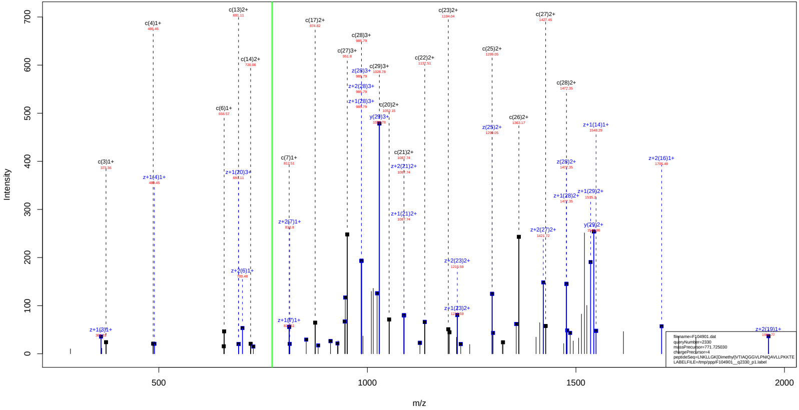

sp|Q6GSS7|H2A2A_MOUSE

LNKLLGK $^{\text{Dimethyl}}_{28.03}$ VTIAQGGVLPNIQAVLLPKKTE

- fragmentation table for charge state 1+
- specType=etd
- score=64.26
- F104901.dat
- query=q2330_p1
- precursor=771.725030
- chargePrecursor=4
- itol=0.8

AA	c	y	z+1	z+2	z	AA
L[1]	131.118	3083.892	3067.873	0.000	3066.865	L[29]
N[2]	245.161	2970.808	2954.789	2955.797	2953.781	N[28]
K[3]	**373.256**	2856.765	2840.746	2841.754	2839.738	K[27]
L[4]	**486.340**	2728.670	2712.651	2713.659	2711.643	L[26]
L[5]	599.424	2615.586	2599.567	2600.575	2598.559	L[25]
G[6]	**656.445**	2502.502	2486.483	2487.491	2485.475	G[24]
K[7]	**812.572**	2445.480	2429.462	2430.469	2428.454	K[23]
V[8]	**911.640**	2289.354	2273.335	2274.343	2272.327	V[22]
T[9]	1012.688	2190.286	2174.267	2175.275	2173.259	T[21]
I[10]	**1125.772**	2089.238	2073.219	2074.227	2072.211	I[20]
A[11]	**1196.809**	1976.154	1960.135	**1961.143**	1959.127	A[19]
Q[12]	**1324.867**	1905.117	1889.098	1890.106	1888.090	Q[18]
G[13]	1381.889	1777.058	1761.039	1762.047	1760.032	G[17]
G[14]	1438.910	1720.037	1704.018	**1705.026**	1703.010	G[16]
V[15]	1537.979	1663.015	1646.997	1648.004	1645.989	V[15]
L[16]	1651.063	1563.947	**1547.928**	**1548.936**	1546.920	L[14]
P[17]	1748.116	1450.863	1434.844	1435.852	1433.836	P[13]
N[18]	1862.159	1353.810	1337.791	1338.799	1336.783	N[12]
I[19]	1975.243	1239.767	**1223.748**	1224.756	1222.741	I[11]
Q[20]	2103.301	**1126.683**	1110.664	1111.672	1109.656	Q[10]
A[21]	2174.338	998.624	982.606	983.614	981.598	A[9]
V[22]	2273.407	**927.587**	**911.569**	912.576	910.561	V[8]
L[23]	2386.491	828.519	**812.500**	**813.508**	811.492	L[7]
L[24]	2499.575	715.435	699.416	**700.424**	698.408	L[6]
P[25]	2596.628	602.351	586.332	587.340	585.324	P[5]
K[26]	2724.723	505.298	**489.279**	490.287	488.271	K[4]
K[27]	2852.818	377.203	**361.184**	362.192	360.177	K[3]
T[28]	2953.865	249.108	233.089	234.097	232.082	T[2]
E[29]	3082.908	148.060	132.042	133.050	131.034	E[1]

sp|Q6GSS7|H2A2A_MOUSE

LNKLLGK $^{Dimethyl}_{28.03}$ VTIAQGGVLPNIQAVLLPKKTE

- fragmentation table for charge state 2+
- specType=etd
- score=64.26
- F104901.dat
- query=q2330_p1
- precursor=771.725030
- chargePrecursor=4
- itol=0.8

AA	c	y	z+1	z+2	z	AA
L[1]	66.063	**1542.450**	**1534.440**	0.504	1533.936	L[29]
N[2]	123.084	**1485.908**	**1477.898**	**1478.402**	**1477.394**	N[28]
K[3]	187.132	1428.886	1420.877	**1421.381**	1420.373	K[27]
L[4]	243.674	1364.839	**1356.829**	**1357.333**	**1356.325**	L[26]
L[5]	300.216	1308.297	1300.287	**1300.791**	**1299.783**	L[25]
G[6]	328.726	1251.755	1243.745	1244.249	1243.241	G[24]
K[7]	406.789	**1223.244**	**1215.234**	**1215.738**	1214.731	K[23]
V[8]	456.324	1145.181	**1137.171**	**1137.675**	1136.667	V[22]
T[9]	506.848	1095.646	**1087.637**	**1088.141**	**1087.133**	T[21]
I[10]	563.390	1045.123	1037.113	1037.617	1036.609	I[20]
A[11]	598.908	988.581	980.571	981.075	980.067	A[19]
Q[12]	662.937	953.062	**945.053**	**945.557**	944.549	Q[18]
G[13]	**691.448**	889.033	881.023	**881.527**	880.519	G[17]
G[14]	**719.959**	860.522	852.513	**853.017**	852.009	G[16]
V[15]	769.493	832.011	824.002	824.506	823.498	V[15]
L[16]	826.035	782.477	774.468	774.972	773.964	L[14]
P[17]	**874.561**	**725.935**	717.926	718.430	717.422	P[13]
N[18]	931.583	677.409	669.399	669.903	668.895	N[12]
I[19]	988.125	620.387	612.378	612.882	611.874	I[11]
Q[20]	**1052.154**	563.845	555.836	556.340	555.332	Q[10]
A[21]	**1087.673**	499.816	491.807	492.310	491.303	A[9]
V[22]	**1137.207**	464.297	456.288	456.792	455.784	V[8]
L[23]	**1193.749**	414.763	406.754	407.258	406.250	L[7]
L[24]	1250.291	358.221	350.212	350.716	349.708	L[6]
P[25]	**1298.817**	301.679	293.670	294.174	293.166	P[5]
K[26]	**1362.865**	253.153	245.143	245.647	244.639	K[4]
K[27]	**1426.912**	189.105	181.096	181.600	180.592	K[3]
T[28]	**1477.436**	125.058	117.048	117.552	116.544	T[2]
E[29]	1541.958	74.534	66.524	67.028	66.021	E[1]

sp|Q6GSS7|H2A2A_MOUSE

LNKLLGK $^{Dimethyl}_{28.03}$ VTIAQGGVLPNIQAVLLPKKTE

- fragmentation table for charge state 3+
- specType=etd
- score=64.26
- F104901.dat
- query=q2330_p1
- precursor=771.725030
- chargePrecursor=4
- itol=0.8

AA	c	y	z+1	z+2	z	AA
L[1]	44.377	**1028.635**	**1023.296**	0.672	**1022.960**	L[29]
N[2]	82.392	990.941	**985.601**	**985.937**	**985.265**	N[28]
K[3]	125.090	952.926	**947.587**	947.923	**947.251**	K[27]
L[4]	162.785	910.228	904.889	905.225	904.553	L[26]
L[5]	200.479	872.533	867.194	867.530	866.858	L[25]
G[6]	219.487	834.839	829.499	829.835	829.163	G[24]
K[7]	271.529	815.832	810.492	810.828	810.156	K[23]
V[8]	304.552	763.790	758.450	758.786	758.114	V[22]
T[9]	338.234	730.767	725.427	725.763	725.091	T[21]
I[10]	375.929	697.084	**691.745**	692.081	**691.409**	I[20]
A[11]	399.608	659.389	654.050	654.386	653.714	A[19]
Q[12]	442.294	635.710	630.371	630.707	630.035	Q[18]
G[13]	461.301	593.024	587.685	588.021	587.349	G[17]
G[14]	480.308	574.017	568.678	569.013	568.342	G[16]
V[15]	513.331	555.010	549.670	550.006	549.334	V[15]
L[16]	551.026	521.987	516.648	516.984	516.312	L[14]
P[17]	583.377	484.292	478.953	479.289	478.617	P[13]
N[18]	621.391	451.942	446.602	446.938	446.266	N[12]
I[19]	659.086	413.927	408.588	408.924	408.252	I[11]
Q[20]	701.772	376.233	370.893	371.229	370.557	Q[10]
A[21]	725.451	333.546	328.207	328.543	327.871	A[9]
V[22]	758.474	309.867	304.528	304.864	304.192	V[8]
L[23]	796.168	276.844	271.505	271.841	271.169	L[7]
L[24]	833.863	239.150	233.810	234.146	233.474	L[6]
P[25]	866.214	201.455	196.116	196.451	195.780	P[5]
K[26]	908.912	169.104	163.765	164.101	163.429	K[4]
K[27]	**951.611**	126.406	121.066	121.402	120.730	K[3]
T[28]	**985.293**	83.708	78.368	78.704	78.032	T[2]
E[29]	**1028.307**	50.025	44.685	45.021	44.349	E[1]

sp|Q6GSS7|H2A2A_MOUSE

LNKLLGK $^{\text{Dimethyl}}_{28.03}$ VTIAQGGVLPNIQAVLLPKKTE

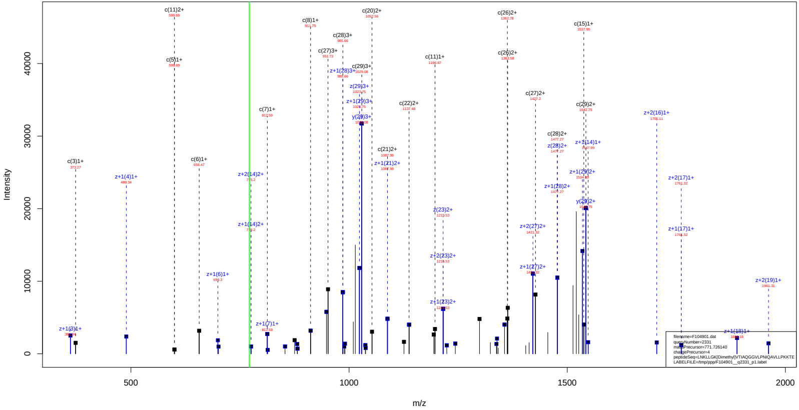

sp|Q6GSS7|H2A2A_MOUSE

LNKLLGK $^{\text{Dimethyl}}_{28.03}$ VTIAQGGVLPNIQAVLLPKKTE

- fragmentation table for charge state 1+
- specType=etd
- score=89.67
- F104901.dat
- query=q2331_p1
- precursor=771.726140
- chargePrecursor=4
- itol=0.8

AA	c	y	z+1	z+2	z	AA
L[1]	131.118	3083.892	3067.873	0.000	3066.865	L[29]
N[2]	245.161	2970.808	2954.789	2955.797	2953.781	N[28]
K[3]	**373.256**	2856.765	2840.746	2841.754	2839.738	K[27]
L[4]	486.340	2728.670	2712.651	2713.659	2711.643	L[26]
L[5]	**599.424**	2615.586	2599.567	2600.575	2598.559	L[25]
G[6]	**656.445**	2502.502	2486.483	2487.491	2485.475	G[24]
K[7]	**812.572**	2445.480	2429.462	2430.469	2428.454	K[23]
V[8]	**911.640**	2289.354	2273.335	2274.343	2272.327	V[22]
T[9]	1012.688	2190.286	2174.267	2175.275	2173.259	T[21]
I[10]	**1125.772**	2089.238	2073.219	2074.227	2072.211	I[20]
A[11]	**1196.809**	1976.154	1960.135	**1961.143**	1959.127	A[19]
Q[12]	1324.867	1905.117	**1889.098**	1890.106	1888.090	Q[18]
G[13]	1381.889	1777.058	**1761.039**	**1762.047**	1760.032	G[17]
G[14]	1438.910	1720.037	1704.018	**1705.026**	1703.010	G[16]
V[15]	**1537.979**	1663.015	1646.997	1648.004	1645.989	V[15]
L[16]	1651.063	1563.947	**1547.928**	1548.936	1546.920	L[14]
P[17]	1748.116	1450.863	1434.844	1435.852	1433.836	P[13]
N[18]	1862.159	1353.810	**1337.791**	**1338.799**	1336.783	N[12]
I[19]	1975.243	1239.767	**1223.748**	1224.756	1222.741	I[11]
Q[20]	2103.301	1126.683	1110.664	1111.672	1109.656	Q[10]
A[21]	2174.338	998.624	982.606	983.614	981.598	A[9]
V[22]	2273.407	927.587	**911.569**	912.576	910.561	V[8]
L[23]	2386.491	828.519	**812.500**	**813.508**	811.492	L[7]
L[24]	2499.575	715.435	**699.416**	**700.424**	698.408	L[6]
P[25]	2596.628	602.351	586.332	587.340	585.324	P[5]
K[26]	2724.723	505.298	**489.279**	490.287	488.271	K[4]
K[27]	2852.818	377.203	**361.184**	362.192	360.177	K[3]
T[28]	2953.865	249.108	233.089	234.097	232.082	T[2]
E[29]	3082.908	148.060	132.042	133.050	131.034	E[1]

sp|Q6GSS7|H2A2A_MOUSE

LNKLLGK $^{Dimethyl}_{28.03}$ VTIAQGGVLPNIQAVLLPKKTE

- fragmentation table for charge state 2+
- specType=etd
- score=89.67
- F104901.dat
- query=q2331_p1
- precursor=771.726140
- chargePrecursor=4
- itol=0.8

AA	c	y	z+1	z+2	z	AA
L[1]	66.063	**1542.450**	**1534.440**	0.504	1533.936	L[29]
N[2]	123.084	1485.908	**1477.898**	1478.402	**1477.394**	N[28]
K[3]	187.132	1428.886	**1420.877**	**1421.381**	1420.373	K[27]
L[4]	243.674	1364.839	**1356.829**	1357.333	**1356.325**	L[26]
L[5]	300.216	1308.297	1300.287	1300.791	1299.783	L[25]
G[6]	328.726	1251.755	**1243.745**	**1244.249**	**1243.241**	G[24]
K[7]	406.789	**1223.244**	**1215.234**	**1215.738**	**1214.731**	K[23]
V[8]	456.324	1145.181	**1137.171**	**1137.675**	1136.667	V[22]
T[9]	506.848	1095.646	**1087.637**	**1088.141**	1087.133	T[21]
I[10]	563.390	1045.123	**1037.113**	**1037.617**	**1036.609**	I[20]
A[11]	**598.908**	**988.581**	980.571	981.075	980.067	A[19]
Q[12]	662.937	953.062	945.053	945.557	944.549	Q[18]
G[13]	691.448	889.033	**881.023**	**881.527**	**880.519**	G[17]
G[14]	719.959	860.522	**852.513**	**853.017**	852.009	G[16]
V[15]	769.493	832.011	824.002	824.506	823.498	V[15]
L[16]	826.035	782.477	**774.468**	**774.972**	773.964	L[14]
P[17]	**874.561**	725.935	717.926	718.430	717.422	P[13]
N[18]	931.583	677.409	669.399	669.903	668.895	N[12]
I[19]	**988.125**	620.387	612.378	612.882	611.874	I[11]
Q[20]	**1052.154**	563.845	555.836	556.340	555.332	Q[10]
A[21]	**1087.673**	499.816	491.807	492.310	491.303	A[9]
V[22]	**1137.207**	464.297	456.288	456.792	455.784	V[8]
L[23]	**1193.749**	414.763	406.754	407.258	406.250	L[7]
L[24]	1250.291	358.221	350.212	350.716	349.708	L[6]
P[25]	**1298.817**	301.679	293.670	294.174	293.166	P[5]
K[26]	**1362.865**	253.153	245.143	245.647	244.639	K[4]
K[27]	**1426.912**	189.105	181.096	181.600	180.592	K[3]
T[28]	**1477.436**	125.058	117.048	117.552	116.544	T[2]
E[29]	**1541.958**	74.534	66.524	67.028	66.021	E[1]

sp|Q6GSS7|H2A2A_MOUSE

LNKLLGK $^{Dimethyl}_{28.03}$ VTIAQGGVLPNIQAVLLPKKTE

- fragmentation table for charge state 3+
- specType=etd
- score=89.67
- F104901.dat
- query=q2331_p1
- precursor=771.726140
- chargePrecursor=4
- itol=0.8

AA	c	y	z+1	z+2	z	AA
L[1]	44.377	**1028.635**	**1023.296**	0.672	**1022.960**	L[29]
N[2]	82.392	**990.941**	**985.601**	**985.937**	**985.265**	N[28]
K[3]	125.090	952.926	**947.587**	**947.923**	947.251	K[27]
L[4]	162.785	910.228	904.889	905.225	904.553	L[26]
L[5]	200.479	872.533	867.194	867.530	866.858	L[25]
G[6]	219.487	834.839	829.499	829.835	829.163	G[24]
K[7]	271.529	815.832	810.492	810.828	810.156	K[23]
V[8]	304.552	763.790	758.450	758.786	758.114	V[22]
T[9]	338.234	730.767	725.427	725.763	725.091	T[21]
I[10]	375.929	697.084	691.745	692.081	691.409	I[20]
A[11]	399.608	659.389	654.050	654.386	653.714	A[19]
Q[12]	442.294	635.710	630.371	630.707	630.035	Q[18]
G[13]	461.301	593.024	587.685	588.021	587.349	G[17]
G[14]	480.308	574.017	568.678	569.013	568.342	G[16]
V[15]	513.331	555.010	549.670	550.006	549.334	V[15]
L[16]	551.026	521.987	516.648	516.984	516.312	L[14]
P[17]	583.377	484.292	478.953	479.289	478.617	P[13]
N[18]	621.391	451.942	446.602	446.938	446.266	N[12]
I[19]	659.086	413.927	408.588	408.924	408.252	I[11]
Q[20]	701.772	376.233	370.893	371.229	370.557	Q[10]
A[21]	725.451	333.546	328.207	328.543	327.871	A[9]
V[22]	758.474	309.867	304.528	304.864	304.192	V[8]
L[23]	796.168	276.844	271.505	271.841	271.169	L[7]
L[24]	833.863	239.150	233.810	234.146	233.474	L[6]
P[25]	866.214	201.455	196.116	196.451	195.780	P[5]
K[26]	908.912	169.104	163.765	164.101	163.429	K[4]
K[27]	**951.611**	126.406	121.066	121.402	120.730	K[3]
T[28]	**985.293**	83.708	78.368	78.704	78.032	T[2]
E[29]	**1028.307**	50.025	44.685	45.021	44.349	E[1]

sp|Q6GSS7|H2A2A_MOUSE

LNKLLGK $^{\text{Dimethyl}}_{28.03}$ VTIAQGGVLPNIQAVLLPKKTE

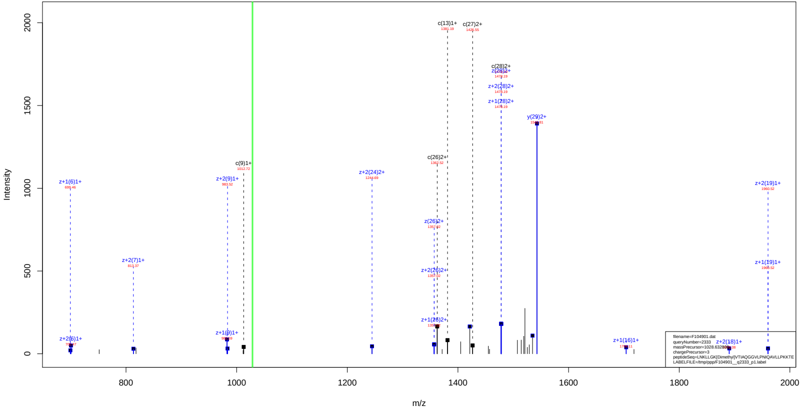

sp|Q6GSS7|H2A2A_MOUSE

LNKLLGK $^{\text{Dimethyl}}_{28.03}$ VTIAQGGVLPNIQAVLLPKKTE

- fragmentation table for charge state 1+
- specType=etd
- score=28.57
- F104901.dat
- query=q2333_p1
- precursor=1028.632800
- chargePrecursor=3
- itol=0.8

AA	c	y	z+1	z+2	z	AA
L[1]	131.118	3083.892	3067.873	0.000	3066.865	L[29]
N[2]	245.161	2970.808	2954.789	2955.797	2953.781	N[28]
K[3]	373.256	2856.765	2840.746	2841.754	2839.738	K[27]
L[4]	486.340	2728.670	2712.651	2713.659	2711.643	L[26]
L[5]	599.424	2615.586	2599.567	2600.575	2598.559	L[25]
G[6]	656.445	2502.502	2486.483	2487.491	2485.475	G[24]
K[7]	812.572	2445.480	2429.462	2430.469	2428.454	K[23]
V[8]	911.640	2289.354	2273.335	2274.343	2272.327	V[22]
T[9]	**1012.688**	2190.286	2174.267	2175.275	2173.259	T[21]
I[10]	1125.772	2089.238	2073.219	2074.227	2072.211	I[20]
A[11]	1196.809	1976.154	**1960.135**	**1961.143**	1959.127	A[19]
Q[12]	1324.867	1905.117	1889.098	**1890.106**	1888.090	Q[18]
G[13]	**1381.889**	1777.058	1761.039	1762.047	1760.032	G[17]
G[14]	1438.910	1720.037	**1704.018**	1705.026	1703.010	G[16]
V[15]	1537.979	1663.015	1646.997	1648.004	1645.989	V[15]
L[16]	1651.063	1563.947	1547.928	1548.936	1546.920	L[14]
P[17]	1748.116	1450.863	1434.844	1435.852	1433.836	P[13]
N[18]	1862.159	1353.810	1337.791	1338.799	1336.783	N[12]
I[19]	1975.243	1239.767	1223.748	1224.756	1222.741	I[11]
Q[20]	2103.301	1126.683	1110.664	1111.672	1109.656	Q[10]
A[21]	2174.338	998.624	**982.606**	**983.614**	981.598	A[9]
V[22]	2273.407	927.587	911.569	912.576	910.561	V[8]
L[23]	2386.491	828.519	812.500	**813.508**	811.492	L[7]
L[24]	2499.575	715.435	**699.416**	**700.424**	698.408	L[6]
P[25]	2596.628	602.351	586.332	587.340	585.324	P[5]
K[26]	2724.723	505.298	489.279	490.287	488.271	K[4]
K[27]	2852.818	377.203	361.184	362.192	360.177	K[3]
T[28]	2953.865	249.108	233.089	234.097	232.082	T[2]
E[29]	3082.908	148.060	132.042	133.050	131.034	E[1]

sp|Q6GSS7|H2A2A_MOUSE

LNKLLGK $^{\text{Dimethyl}}_{28.03}$ VTIAQGGVLPNIQAVLLPKKTE

- fragmentation table for charge state 2+
- specType=etd
- score=28.57
- F104901.dat
- query=q2333_p1
- precursor=1028.632800
- chargePrecursor=3
- itol=0.8

AA	c	y	z+1	z+2	z	AA
L[1]	66.063	**1542.450**	**1534.440**	0.504	1533.936	L[29]
N[2]	123.084	1485.908	**1477.898**	**1478.402**	**1477.394**	N[28]
K[3]	187.132	1428.886	**1420.877**	**1421.381**	1420.373	K[27]
L[4]	243.674	1364.839	**1356.829**	**1357.333**	**1356.325**	L[26]
L[5]	300.216	1308.297	1300.287	1300.791	1299.783	L[25]
G[6]	328.726	1251.755	1243.745	**1244.249**	1243.241	G[24]
K[7]	406.789	1223.244	1215.234	1215.738	1214.731	K[23]
V[8]	456.324	1145.181	1137.171	1137.675	1136.667	V[22]
T[9]	506.848	1095.646	1087.637	1088.141	1087.133	T[21]
I[10]	563.390	1045.123	1037.113	1037.617	1036.609	I[20]
A[11]	598.908	988.581	980.571	981.075	980.067	A[19]
Q[12]	662.937	953.062	945.053	945.557	944.549	Q[18]
G[13]	691.448	889.033	881.023	881.527	880.519	G[17]
G[14]	719.959	860.522	852.513	853.017	852.009	G[16]
V[15]	769.493	832.011	824.002	824.506	823.498	V[15]
L[16]	826.035	782.477	774.468	774.972	773.964	L[14]
P[17]	874.561	725.935	717.926	718.430	717.422	P[13]
N[18]	931.583	677.409	669.399	669.903	668.895	N[12]
I[19]	988.125	620.387	612.378	612.882	611.874	I[11]
Q[20]	1052.154	563.845	555.836	556.340	555.332	Q[10]
A[21]	1087.673	499.816	491.807	492.310	491.303	A[9]
V[22]	1137.207	464.297	456.288	456.792	455.784	V[8]
L[23]	1193.749	414.763	406.754	407.258	406.250	L[7]
L[24]	1250.291	358.221	350.212	350.716	349.708	L[6]
P[25]	1298.817	301.679	293.670	294.174	293.166	P[5]
K[26]	**1362.865**	253.153	245.143	245.647	244.639	K[4]
K[27]	**1426.912**	189.105	181.096	181.600	180.592	K[3]
T[28]	**1477.436**	125.058	117.048	117.552	116.544	T[2]
E[29]	1541.958	74.534	66.524	67.028	66.021	E[1]

sp|Q6GSS7|H2A2A_MOUSE

LNKLLGK $^{\text{Dimethyl}}_{28.03}$ VTIAQGGVLPNIQAVLLPKKTE

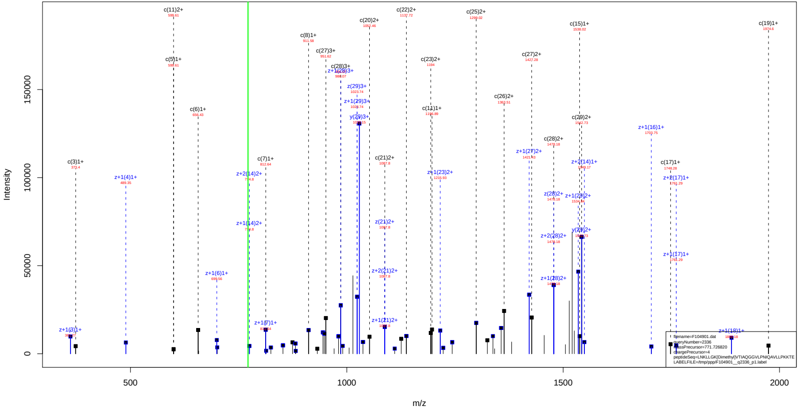

sp|Q6GSS7|H2A2A_MOUSE

LNKLLGK $^{\text{Dimethyl}}_{28.03}$ VTIAQGGVLPNIQAVLLPKKTE

- fragmentation table for charge state 1+
- specType=etd
- score=112.07
- F104901.dat
- query=q2336_p1
- precursor=771.726820
- chargePrecursor=4
- itol=0.8

AA	c	y	z+1	z+2	z	AA
L[1]	131.118	3083.892	3067.873	0.000	3066.865	L[29]
N[2]	245.161	2970.808	2954.789	2955.797	2953.781	N[28]
K[3]	**373.256**	2856.765	2840.746	2841.754	2839.738	K[27]
L[4]	486.340	2728.670	2712.651	2713.659	2711.643	L[26]
L[5]	**599.424**	2615.586	2599.567	2600.575	2598.559	L[25]
G[6]	**656.445**	2502.502	2486.483	2487.491	2485.475	G[24]
K[7]	**812.572**	2445.480	2429.462	2430.469	2428.454	K[23]
V[8]	**911.640**	2289.354	2273.335	2274.343	2272.327	V[22]
T[9]	1012.688	2190.286	2174.267	2175.275	2173.259	T[21]
I[10]	**1125.772**	2089.238	2073.219	2074.227	2072.211	I[20]
A[11]	**1196.809**	1976.154	1960.135	1961.143	1959.127	A[19]
Q[12]	**1324.867**	1905.117	**1889.098**	1890.106	1888.090	Q[18]
G[13]	1381.889	1777.058	**1761.039**	**1762.047**	1760.032	G[17]
G[14]	1438.910	1720.037	**1704.018**	1705.026	**1703.010**	G[16]
V[15]	**1537.979**	1663.015	1646.997	1648.004	1645.989	V[15]
L[16]	1651.063	1563.947	1547.928	**1548.936**	1546.920	L[14]
P[17]	**1748.116**	1450.863	1434.844	1435.852	1433.836	P[13]
N[18]	1862.159	1353.810	**1337.791**	1338.799	1336.783	N[12]
I[19]	**1975.243**	1239.767	1223.748	1224.756	**1222.741**	I[11]
Q[20]	2103.301	1126.683	**1110.664**	1111.672	1109.656	Q[10]
A[21]	2174.338	998.624	982.606	983.614	**981.598**	A[9]
V[22]	2273.407	927.587	**911.569**	912.576	910.561	V[8]
L[23]	2386.491	828.519	**812.500**	**813.508**	811.492	L[7]
L[24]	2499.575	715.435	**699.416**	**700.424**	698.408	L[6]
P[25]	2596.628	602.351	586.332	587.340	585.324	P[5]
K[26]	2724.723	505.298	**489.279**	490.287	488.271	K[4]
K[27]	2852.818	377.203	**361.184**	362.192	360.177	K[3]
T[28]	2953.865	249.108	233.089	234.097	232.082	T[2]
E[29]	3082.908	148.060	132.042	133.050	131.034	E[1]

sp|Q6GSS7|H2A2A_MOUSE

LNKLLGK $^{Dimethyl}_{28.03}$ VTIAQGGVLPNIQAVLLPKKTE

- fragmentation table for charge state 2+
- specType=etd
- score=112.07
- F104901.dat
- query=q2336_p1
- precursor=771.726820
- chargePrecursor=4
- itol=0.8

AA	c	y	z+1	z+2	z	AA
L[1]	66.063	**1542.450**	**1534.440**	0.504	1533.936	L[29]
N[2]	123.084	1485.908	**1477.898**	**1478.402**	**1477.394**	N[28]
K[3]	187.132	1428.886	**1420.877**	**1421.381**	1420.373	K[27]
L[4]	243.674	1364.839	**1356.829**	**1357.333**	**1356.325**	L[26]
L[5]	300.216	1308.297	1300.287	1300.791	**1299.783**	L[25]
G[6]	328.726	1251.755	**1243.745**	**1244.249**	**1243.241**	G[24]
K[7]	406.789	**1223.244**	**1215.234**	**1215.738**	1214.731	K[23]
V[8]	456.324	1145.181	**1137.171**	**1137.675**	1136.667	V[22]
T[9]	506.848	1095.646	**1087.637**	**1088.141**	**1087.133**	T[21]
I[10]	563.390	1045.123	**1037.113**	**1037.617**	**1036.609**	I[20]
A[11]	**598.908**	988.581	**980.571**	**981.075**	**980.067**	A[19]
Q[12]	662.937	953.062	**945.053**	**945.557**	**944.549**	Q[18]
G[13]	691.448	889.033	**881.023**	**881.527**	**880.519**	G[17]
G[14]	719.959	860.522	**852.513**	**853.017**	**852.009**	G[16]
V[15]	769.493	832.011	**824.002**	**824.506**	823.498	V[15]
L[16]	826.035	782.477	**774.468**	**774.972**	773.964	L[14]
P[17]	**874.561**	725.935	717.926	718.430	717.422	P[13]
N[18]	**931.583**	677.409	669.399	669.903	668.895	N[12]
I[19]	988.125	620.387	612.378	612.882	611.874	I[11]
Q[20]	**1052.154**	563.845	555.836	556.340	555.332	Q[10]
A[21]	**1087.673**	499.816	491.807	492.310	491.303	A[9]
V[22]	**1137.207**	464.297	456.288	456.792	455.784	V[8]
L[23]	**1193.749**	414.763	406.754	407.258	406.250	L[7]
L[24]	1250.291	358.221	350.212	350.716	349.708	L[6]
P[25]	**1298.817**	301.679	293.670	294.174	293.166	P[5]
K[26]	**1362.865**	253.153	245.143	245.647	244.639	K[4]
K[27]	**1426.912**	189.105	181.096	181.600	180.592	K[3]
T[28]	**1477.436**	125.058	117.048	117.552	116.544	T[2]
E[29]	**1541.958**	74.534	66.524	67.028	66.021	E[1]

sp|Q6GSS7|H2A2A_MOUSE

LNKLLGK $^{\text{Dimethyl}}_{28.03}$ VTIAQGGVLPNIQAVLLPKKTE

- fragmentation table for charge state 3+
- specType=etd
- score=112.07
- F104901.dat
- query=q2336_p1
- precursor=771.726820
- chargePrecursor=4
- itol=0.8

AA	c	y	z+1	z+2	z	AA
L[1]	44.377	**1028.635**	**1023.296**	0.672	**1022.960**	L[29]
N[2]	82.392	**990.941**	**985.601**	**985.937**	985.265	N[28]
K[3]	125.090	952.926	**947.587**	**947.923**	**947.251**	K[27]
L[4]	162.785	910.228	904.889	905.225	904.553	L[26]
L[5]	200.479	872.533	867.194	867.530	866.858	L[25]
G[6]	219.487	834.839	829.499	829.835	829.163	G[24]
K[7]	271.529	815.832	810.492	810.828	810.156	K[23]
V[8]	304.552	763.790	758.450	758.786	758.114	V[22]
T[9]	338.234	730.767	725.427	725.763	725.091	T[21]
I[10]	375.929	697.084	691.745	692.081	691.409	I[20]
A[11]	399.608	659.389	654.050	654.386	653.714	A[19]
Q[12]	442.294	635.710	630.371	630.707	630.035	Q[18]
G[13]	461.301	593.024	587.685	588.021	587.349	G[17]
G[14]	480.308	574.017	568.678	569.013	568.342	G[16]
V[15]	513.331	555.010	549.670	550.006	549.334	V[15]
L[16]	551.026	521.987	516.648	516.984	516.312	L[14]
P[17]	583.377	484.292	478.953	479.289	478.617	P[13]
N[18]	621.391	451.942	446.602	446.938	446.266	N[12]
I[19]	659.086	413.927	408.588	408.924	408.252	I[11]
Q[20]	701.772	376.233	370.893	371.229	370.557	Q[10]
A[21]	725.451	333.546	328.207	328.543	327.871	A[9]
V[22]	758.474	309.867	304.528	304.864	304.192	V[8]
L[23]	796.168	276.844	271.505	271.841	271.169	L[7]
L[24]	833.863	239.150	233.810	234.146	233.474	L[6]
P[25]	866.214	201.455	196.116	196.451	195.780	P[5]
K[26]	908.912	169.104	163.765	164.101	163.429	K[4]
K[27]	**951.611**	126.406	121.066	121.402	120.730	K[3]
T[28]	**985.293**	83.708	78.368	78.704	78.032	T[2]
E[29]	1028.307	50.025	44.685	45.021	44.349	E[1]

sp|Q6GSS7|H2A2A_MOUSE

LNKLLGK $^{\text{Dimethyl}}_{28.03}$ VTIAQGGVLPNIQAVLLPKKTE

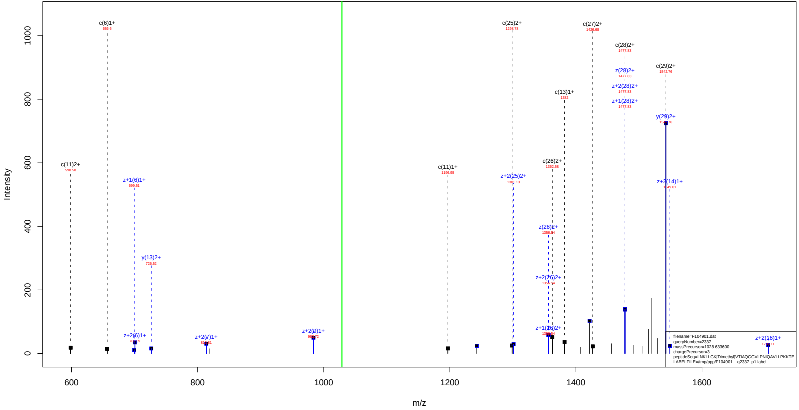

sp|Q6GSS7|H2A2A_MOUSE

LNKLLGK $^{Dimethyl}_{28.03}$ VTIAQGGVLPNIQAVLLPKKTE

- fragmentation table for charge state 1+
- specType=etd
- score=35.15
- F104901.dat
- query=q2337_p1
- precursor=1028.633600
- chargePrecursor=3
- itol=0.8

AA	c	y	z+1	z+2	z	AA
L[1]	131.118	3083.892	3067.873	0.000	3066.865	L[29]
N[2]	245.161	2970.808	2954.789	2955.797	2953.781	N[28]
K[3]	373.256	2856.765	2840.746	2841.754	2839.738	K[27]
L[4]	486.340	2728.670	2712.651	2713.659	2711.643	L[26]
L[5]	599.424	2615.586	2599.567	2600.575	2598.559	L[25]
G[6]	**656.445**	2502.502	2486.483	2487.491	2485.475	G[24]
K[7]	812.572	2445.480	2429.462	2430.469	2428.454	K[23]
V[8]	911.640	2289.354	2273.335	2274.343	2272.327	V[22]
T[9]	1012.688	2190.286	2174.267	2175.275	2173.259	T[21]
I[10]	1125.772	2089.238	2073.219	2074.227	2072.211	I[20]
A[11]	**1196.809**	1976.154	1960.135	1961.143	1959.127	A[19]
Q[12]	1324.867	1905.117	1889.098	1890.106	1888.090	Q[18]
G[13]	**1381.889**	1777.058	1761.039	1762.047	1760.032	G[17]
G[14]	1438.910	1720.037	1704.018	**1705.026**	1703.010	G[16]
V[15]	1537.979	1663.015	1646.997	1648.004	1645.989	V[15]
L[16]	1651.063	1563.947	1547.928	**1548.936**	1546.920	L[14]
P[17]	1748.116	1450.863	1434.844	1435.852	1433.836	P[13]
N[18]	1862.159	1353.810	1337.791	1338.799	1336.783	N[12]
I[19]	1975.243	1239.767	1223.748	1224.756	1222.741	I[11]
Q[20]	2103.301	1126.683	1110.664	1111.672	1109.656	Q[10]
A[21]	2174.338	998.624	982.606	**983.614**	981.598	A[9]
V[22]	2273.407	927.587	911.569	912.576	910.561	V[8]
L[23]	2386.491	828.519	812.500	**813.508**	811.492	L[7]
L[24]	2499.575	715.435	**699.416**	**700.424**	698.408	L[6]
P[25]	2596.628	602.351	586.332	587.340	585.324	P[5]
K[26]	2724.723	505.298	489.279	490.287	488.271	K[4]
K[27]	2852.818	377.203	361.184	362.192	360.177	K[3]
T[28]	2953.865	249.108	233.089	234.097	232.082	T[2]
E[29]	3082.908	148.060	132.042	133.050	131.034	E[1]

sp|Q6GSS7|H2A2A_MOUSE

LNKLLGK $^{\text{Dimethyl}}_{28.03}$ VTIAQGGVLPNIQAVLLPKKTE

- fragmentation table for charge state 2+
- specType=etd
- score=35.15
- F104901.dat
- query=q2337_p1
- precursor=1028.633600
- chargePrecursor=3
- itol=0.8

AA	c	y	z+1	z+2	z	AA
L[1]	66.063	**1542.450**	1534.440	0.504	1533.936	L[29]
N[2]	123.084	1485.908	**1477.898**	**1478.402**	**1477.394**	N[28]
K[3]	187.132	1428.886	1420.877	**1421.381**	1420.373	K[27]
L[4]	243.674	1364.839	**1356.829**	**1357.333**	**1356.325**	L[26]
L[5]	300.216	1308.297	1300.287	**1300.791**	1299.783	L[25]
G[6]	328.726	1251.755	1243.745	1244.249	**1243.241**	G[24]
K[7]	406.789	1223.244	1215.234	1215.738	1214.731	K[23]
V[8]	456.324	1145.181	1137.171	1137.675	1136.667	V[22]
T[9]	506.848	1095.646	1087.637	1088.141	1087.133	T[21]
I[10]	563.390	1045.123	1037.113	1037.617	1036.609	I[20]
A[11]	**598.908**	988.581	980.571	981.075	980.067	A[19]
Q[12]	662.937	953.062	945.053	945.557	944.549	Q[18]
G[13]	691.448	889.033	881.023	881.527	880.519	G[17]
G[14]	719.959	860.522	852.513	853.017	852.009	G[16]
V[15]	769.493	832.011	824.002	824.506	823.498	V[15]
L[16]	826.035	782.477	774.468	774.972	773.964	L[14]
P[17]	874.561	**725.935**	717.926	718.430	717.422	P[13]
N[18]	931.583	677.409	669.399	669.903	668.895	N[12]
I[19]	988.125	620.387	612.378	612.882	611.874	I[11]
Q[20]	1052.154	563.845	555.836	556.340	555.332	Q[10]
A[21]	1087.673	499.816	491.807	492.310	491.303	A[9]
V[22]	1137.207	464.297	456.288	456.792	455.784	V[8]
L[23]	1193.749	414.763	406.754	407.258	406.250	L[7]
L[24]	1250.291	358.221	350.212	350.716	349.708	L[6]
P[25]	**1298.817**	301.679	293.670	294.174	293.166	P[5]
K[26]	**1362.865**	253.153	245.143	245.647	244.639	K[4]
K[27]	**1426.912**	189.105	181.096	181.600	180.592	K[3]
T[28]	**1477.436**	125.058	117.048	117.552	116.544	T[2]
E[29]	**1541.958**	74.534	66.524	67.028	66.021	E[1]

sp|Q6GSS7|H2A2A_MOUSE

LNKLLGK $^{\text{Dimethyl}}_{28.03}$ VTIAQGGVLPNIQAVLLPKKTE

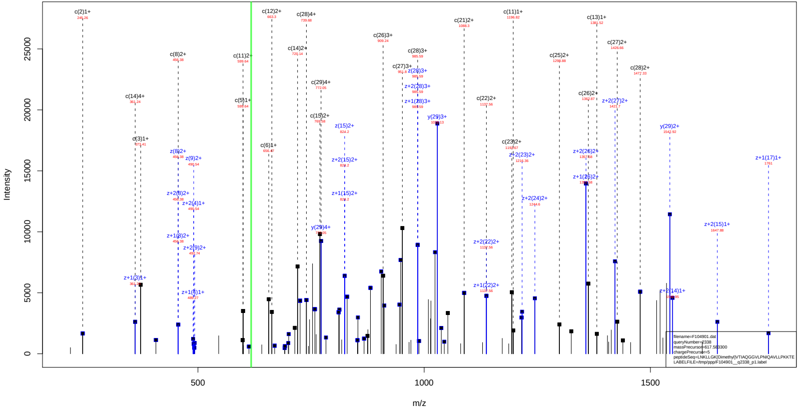

sp|Q6GSS7|H2A2A_MOUSE

LNKLLGK $^{\text{Dimethyl}}_{28.03}$ VTIAQGGVLPNIQAVLLPKKTE

- fragmentation table for charge state 1+
- specType=etd
- score=106.53
- F104901.dat
- query=q2338_p1
- precursor=617.583300
- chargePrecursor=5
- itol=0.8

AA	c	y	z+1	z+2	z	AA
L[1]	131.118	3083.892	3067.873	0.000	3066.865	L[29]
N[2]	**245.161**	2970.808	2954.789	2955.797	2953.781	N[28]
K[3]	**373.256**	2856.765	2840.746	2841.754	2839.738	K[27]
L[4]	486.340	2728.670	2712.651	2713.659	2711.643	L[26]
L[5]	**599.424**	2615.586	2599.567	2600.575	2598.559	L[25]
G[6]	**656.445**	2502.502	2486.483	2487.491	2485.475	G[24]
K[7]	**812.572**	2445.480	2429.462	2430.469	2428.454	K[23]
V[8]	**911.640**	2289.354	2273.335	2274.343	2272.327	V[22]
T[9]	1012.688	2190.286	2174.267	2175.275	2173.259	T[21]
I[10]	1125.772	2089.238	2073.219	2074.227	2072.211	I[20]
A[11]	**1196.809**	1976.154	1960.135	1961.143	1959.127	A[19]
Q[12]	**1324.867**	1905.117	1889.098	1890.106	1888.090	Q[18]
G[13]	**1381.889**	1777.058	**1761.039**	1762.047	1760.032	G[17]
G[14]	**1438.910**	1720.037	1704.018	1705.026	1703.010	G[16]
V[15]	1537.979	1663.015	1646.997	**1648.004**	1645.989	V[15]
L[16]	1651.063	1563.947	1547.928	**1548.936**	1546.920	L[14]
P[17]	1748.116	1450.863	1434.844	1435.852	1433.836	P[13]
N[18]	1862.159	1353.810	1337.791	1338.799	1336.783	N[12]
I[19]	1975.243	1239.767	1223.748	1224.756	1222.741	I[11]
Q[20]	2103.301	1126.683	1110.664	1111.672	1109.656	Q[10]
A[21]	2174.338	998.624	982.606	983.614	981.598	A[9]
V[22]	2273.407	927.587	**911.569**	912.576	910.561	V[8]
L[23]	2386.491	828.519	**812.500**	813.508	**811.492**	L[7]
L[24]	2499.575	715.435	**699.416**	**700.424**	698.408	L[6]
P[25]	2596.628	602.351	586.332	587.340	585.324	P[5]
K[26]	2724.723	505.298	**489.279**	**490.287**	488.271	K[4]
K[27]	2852.818	377.203	**361.184**	362.192	360.177	K[3]
T[28]	2953.865	249.108	233.089	234.097	232.082	T[2]
E[29]	3082.908	148.060	132.042	133.050	131.034	E[1]

sp|Q6GSS7|H2A2A_MOUSE

LNKLLGK $^{Dimethyl}_{28.03}$ VTIAQGGVLPNIQAVLLPKKTE

- fragmentation table for charge state 2+
- specType=etd
- score=106.53
- F104901.dat
- query=q2338_p1
- precursor=617.583300
- chargePrecursor=5
- itol=0.8

AA	c	y	z+1	z+2	z	AA
L[1]	66.063	**1542.450**	1534.440	0.504	1533.936	L[29]
N[2]	123.084	1485.908	**1477.898**	1478.402	**1477.394**	N[28]
K[3]	187.132	1428.886	1420.877	**1421.381**	1420.373	K[27]
L[4]	243.674	1364.839	**1356.829**	**1357.333**	1356.325	L[26]
L[5]	300.216	1308.297	1300.287	1300.791	1299.783	L[25]
G[6]	328.726	1251.755	1243.745	**1244.249**	1243.241	G[24]
K[7]	**406.789**	1223.244	**1215.234**	**1215.738**	**1214.731**	K[23]
V[8]	**456.324**	1145.181	**1137.171**	**1137.675**	1136.667	V[22]
T[9]	506.848	1095.646	**1087.637**	**1088.141**	1087.133	T[21]
I[10]	563.390	**1045.123**	**1037.113**	**1037.617**	1036.609	I[20]
A[11]	**598.908**	**988.581**	980.571	981.075	980.067	A[19]
Q[12]	**662.937**	953.062	**945.053**	**945.557**	944.549	Q[18]
G[13]	**691.448**	889.033	**881.023**	**881.527**	**880.519**	G[17]
G[14]	**719.959**	860.522	**852.513**	**853.017**	**852.009**	G[16]
V[15]	**769.493**	832.011	**824.002**	**824.506**	**823.498**	V[15]
L[16]	826.035	**782.477**	774.468	774.972	773.964	L[14]
P[17]	**874.561**	**725.935**	717.926	718.430	717.422	P[13]
N[18]	931.583	677.409	**669.399**	**669.903**	**668.895**	N[12]
I[19]	**988.125**	620.387	**612.378**	**612.882**	611.874	I[11]
Q[20]	**1052.154**	563.845	555.836	556.340	555.332	Q[10]
A[21]	**1087.673**	499.816	**491.807**	**492.310**	**491.303**	A[9]
V[22]	**1137.207**	464.297	**456.288**	**456.792**	**455.784**	V[8]
L[23]	**1193.749**	414.763	**406.754**	**407.258**	406.250	L[7]
L[24]	1250.291	358.221	350.212	350.716	349.708	L[6]
P[25]	**1298.817**	301.679	293.670	294.174	293.166	P[5]
K[26]	**1362.865**	253.153	**245.143**	**245.647**	**244.639**	K[4]
K[27]	**1426.912**	189.105	181.096	181.600	180.592	K[3]
T[28]	**1477.436**	125.058	117.048	117.552	116.544	T[2]
E[29]	1541.958	74.534	66.524	67.028	66.021	E[1]

sp|Q6GSS7|H2A2A_MOUSE

LNKLLGK $^{Dimethyl}_{28.03}$ VTIAQGGVLPNIQAVLLPKKTE

- fragmentation table for charge state 3+
- specType=etd
- score=106.53
- F104901.dat
- query=q2338_p1
- precursor=617.583300
- chargePrecursor=5
- itol=0.8

AA	c	y	z+1	z+2	z	AA
L[1]	44.377	**1028.635**	**1023.296**	0.672	1022.960	L[29]
N[2]	82.392	990.941	**985.601**	**985.937**	**985.265**	N[28]
K[3]	125.090	952.926	**947.587**	**947.923**	**947.251**	K[27]
L[4]	162.785	910.228	**904.889**	**905.225**	**904.553**	L[26]
L[5]	200.479	872.533	**867.194**	**867.530**	**866.858**	L[25]
G[6]	219.487	834.839	**829.499**	**829.835**	**829.163**	G[24]
K[7]	271.529	815.832	**810.492**	**810.828**	**810.156**	K[23]
V[8]	304.552	763.790	**758.450**	**758.786**	**758.114**	V[22]
T[9]	338.234	730.767	**725.427**	**725.763**	**725.091**	T[21]
I[10]	375.929	697.084	**691.745**	**692.081**	**691.409**	I[20]
A[11]	399.608	659.389	654.050	654.386	653.714	A[19]
Q[12]	442.294	635.710	630.371	630.707	630.035	Q[18]
G[13]	461.301	593.024	587.685	588.021	587.349	G[17]
G[14]	480.308	574.017	568.678	569.013	568.342	G[16]
V[15]	513.331	555.010	549.670	550.006	549.334	V[15]
L[16]	551.026	521.987	516.648	516.984	516.312	L[14]
P[17]	583.377	484.292	478.953	479.289	478.617	P[13]
N[18]	621.391	451.942	446.602	446.938	446.266	N[12]
I[19]	659.086	413.927	408.588	408.924	408.252	I[11]
Q[20]	701.772	376.233	370.893	371.229	370.557	Q[10]
A[21]	**725.451**	333.546	328.207	328.543	327.871	A[9]
V[22]	**758.474**	309.867	304.528	304.864	304.192	V[8]
L[23]	796.168	276.844	271.505	271.841	271.169	L[7]
L[24]	833.863	239.150	233.810	234.146	233.474	L[6]
P[25]	866.214	201.455	196.116	196.451	195.780	P[5]
K[26]	**908.912**	169.104	163.765	164.101	163.429	K[4]
K[27]	**951.611**	126.406	121.066	121.402	120.730	K[3]
T[28]	**985.293**	83.708	78.368	78.704	78.032	T[2]
E[29]	1028.307	50.025	44.685	45.021	44.349	E[1]

sp|Q6GSS7|H2A2A_MOUSE

LNKLLGK $^{\text{Dimethyl}}_{28.03}$ VTIAQGGVLPNIQAVLLPKKTE

- fragmentation table for charge state 4+
- specType=etd
- score=106.53
- F104901.dat
- query=q2338_p1
- precursor=617.583300
- chargePrecursor=5
- itol=0.8

AA	c	y	z+1	z+2	z	AA
L[1]	33.535	**771.728**	767.724	0.755	767.472	L[29]
N[2]	62.046	743.457	**739.453**	**739.705**	**739.201**	N[28]
K[3]	94.069	714.947	710.942	711.194	710.690	K[27]
L[4]	122.340	682.923	678.918	679.170	678.666	L[26]
L[5]	150.611	654.652	650.647	650.899	650.395	L[25]
G[6]	164.867	626.381	622.376	622.628	622.124	G[24]
K[7]	203.898	**612.126**	608.121	608.373	607.869	K[23]
V[8]	228.665	573.094	569.089	569.341	568.837	V[22]
T[9]	253.927	548.327	544.322	544.574	544.070	T[21]
I[10]	282.198	523.065	519.060	519.312	518.808	I[20]
A[11]	299.958	494.794	**490.789**	**491.041**	**490.537**	A[19]
Q[12]	331.972	477.035	473.030	473.282	472.778	Q[18]
G[13]	346.228	445.020	441.015	441.267	440.763	G[17]
G[14]	**360.483**	430.765	426.760	427.012	426.508	G[16]
V[15]	385.250	416.509	412.505	412.757	412.253	V[15]
L[16]	413.521	391.742	387.737	387.989	387.486	L[14]
P[17]	437.784	363.471	359.466	359.718	359.215	P[13]
N[18]	466.295	339.208	335.203	335.455	334.951	N[12]
I[19]	494.566	310.697	306.693	306.945	306.441	I[11]
Q[20]	526.581	282.426	278.422	278.673	278.170	Q[10]
A[21]	544.340	250.412	246.407	246.659	246.155	A[9]
V[22]	569.107	232.652	228.648	228.900	228.396	V[8]
L[23]	597.378	207.885	203.881	204.132	203.629	L[7]
L[24]	625.649	179.614	175.609	175.861	175.358	L[6]
P[25]	649.912	151.343	147.338	147.590	147.087	P[5]
K[26]	681.936	127.080	123.075	123.327	122.823	K[4]
K[27]	**713.960**	95.056	91.052	91.304	90.800	K[3]
T[28]	**739.222**	63.032	59.028	59.280	58.776	T[2]
E[29]	**771.482**	37.771	33.766	34.018	33.514	E[1]

sp|Q6GSS7|H2A2A_MOUSE

LNKLLGK $^{\text{Dimethyl}}_{28.03}$ VTIAQGGVLPNIQAVLLPKKTE

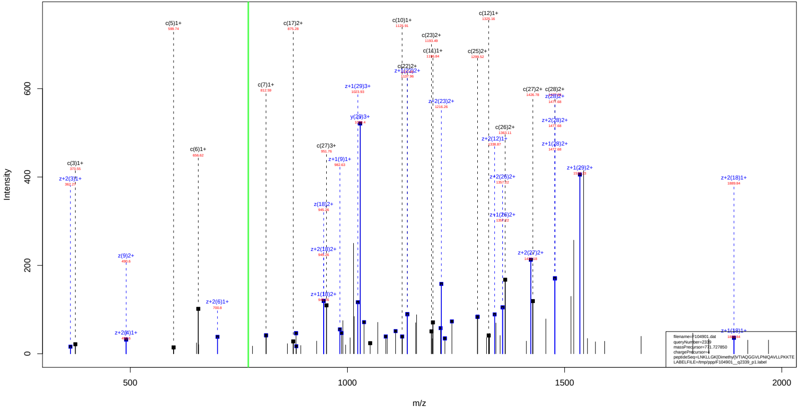

sp|Q6GSS7|H2A2A_MOUSE

LNKLLGK $^{\text{Dimethyl}}_{28.03}$ VTIAQGGVLPNIQAVLLPKKTE

- fragmentation table for charge state 1+
- specType=etd
- score=39.21
- F104901.dat
- query=q2339_p1
- precursor=771.727850
- chargePrecursor=4
- itol=0.8

AA	c	y	z+1	z+2	z	AA
L[1]	131.118	3083.892	3067.873	0.000	3066.865	L[29]
N[2]	245.161	2970.808	2954.789	2955.797	2953.781	N[28]
K[3]	**373.256**	2856.765	2840.746	2841.754	2839.738	K[27]
L[4]	486.340	2728.670	2712.651	2713.659	2711.643	L[26]
L[5]	**599.424**	2615.586	2599.567	2600.575	2598.559	L[25]
G[6]	**656.445**	2502.502	2486.483	2487.491	2485.475	G[24]
K[7]	**812.572**	2445.480	2429.462	2430.469	2428.454	K[23]
V[8]	911.640	2289.354	2273.335	2274.343	2272.327	V[22]
T[9]	1012.688	2190.286	2174.267	2175.275	2173.259	T[21]
I[10]	**1125.772**	2089.238	2073.219	2074.227	2072.211	I[20]
A[11]	**1196.809**	1976.154	1960.135	1961.143	1959.127	A[19]
Q[12]	**1324.867**	1905.117	**1889.098**	**1890.106**	1888.090	Q[18]
G[13]	1381.889	1777.058	1761.039	1762.047	1760.032	G[17]
G[14]	1438.910	1720.037	1704.018	1705.026	1703.010	G[16]
V[15]	1537.979	1663.015	1646.997	1648.004	1645.989	V[15]
L[16]	1651.063	1563.947	1547.928	1548.936	1546.920	L[14]
P[17]	1748.116	1450.863	1434.844	1435.852	1433.836	P[13]
N[18]	1862.159	1353.810	1337.791	**1338.799**	1336.783	N[12]
I[19]	1975.243	**1239.767**	**1223.748**	**1224.756**	1222.741	I[11]
Q[20]	2103.301	**1126.683**	**1110.664**	1111.672	1109.656	Q[10]
A[21]	2174.338	998.624	**982.606**	983.614	981.598	A[9]
V[22]	2273.407	927.587	911.569	912.576	910.561	V[8]
L[23]	2386.491	828.519	**812.500**	813.508	811.492	L[7]
L[24]	2499.575	715.435	699.416	**700.424**	698.408	L[6]
P[25]	2596.628	602.351	586.332	587.340	585.324	P[5]
K[26]	2724.723	505.298	489.279	**490.287**	488.271	K[4]
K[27]	2852.818	377.203	361.184	**362.192**	360.177	K[3]
T[28]	2953.865	249.108	233.089	234.097	232.082	T[2]
E[29]	3082.908	148.060	132.042	133.050	131.034	E[1]

sp|Q6GSS7|H2A2A_MOUSE

LNKLLGK $^{Dimethyl}_{28.03}$ VTIAQGGVLPNIQAVLLPKKTE

- fragmentation table for charge state 2+
- specType=etd
- score=39.21
- F104901.dat
- query=q2339_p1
- precursor=771.727850
- chargePrecursor=4
- itol=0.8

AA	c	y	z+1	z+2	z	AA
L[1]	66.063	1542.450	**1534.440**	0.504	1533.936	L[29]
N[2]	123.084	1485.908	**1477.898**	**1478.402**	**1477.394**	N[28]
K[3]	187.132	1428.886	1420.877	**1421.381**	1420.373	K[27]
L[4]	243.674	1364.839	**1356.829**	**1357.333**	1356.325	L[26]
L[5]	300.216	1308.297	**1300.287**	1300.791	**1299.783**	L[25]
G[6]	328.726	1251.755	1243.745	1244.249	1243.241	G[24]
K[7]	406.789	1223.244	1215.234	**1215.738**	**1214.731**	K[23]
V[8]	456.324	1145.181	**1137.171**	**1137.675**	1136.667	V[22]
T[9]	506.848	1095.646	**1087.637**	**1088.141**	1087.133	T[21]
I[10]	563.390	1045.123	1037.113	**1037.617**	1036.609	I[20]
A[11]	598.908	988.581	980.571	981.075	980.067	A[19]
Q[12]	662.937	953.062	**945.053**	**945.557**	**944.549**	Q[18]
G[13]	691.448	889.033	**881.023**	**881.527**	880.519	G[17]
G[14]	719.959	860.522	852.513	853.017	852.009	G[16]
V[15]	769.493	832.011	824.002	824.506	823.498	V[15]
L[16]	826.035	782.477	774.468	774.972	773.964	L[14]
P[17]	**874.561**	725.935	717.926	718.430	717.422	P[13]
N[18]	931.583	677.409	669.399	669.903	668.895	N[12]
I[19]	988.125	620.387	612.378	612.882	611.874	I[11]
Q[20]	**1052.154**	563.845	555.836	556.340	555.332	Q[10]
A[21]	**1087.673**	499.816	491.807	492.310	**491.303**	A[9]
V[22]	**1137.207**	464.297	456.288	456.792	455.784	V[8]
L[23]	**1193.749**	414.763	406.754	407.258	406.250	L[7]
L[24]	1250.291	358.221	350.212	350.716	349.708	L[6]
P[25]	**1298.817**	301.679	293.670	294.174	293.166	P[5]
K[26]	**1362.865**	253.153	245.143	245.647	244.639	K[4]
K[27]	**1426.912**	189.105	181.096	181.600	180.592	K[3]
T[28]	**1477.436**	125.058	117.048	117.552	116.544	T[2]
E[29]	1541.958	74.534	66.524	67.028	66.021	E[1]

sp|Q6GSS7|H2A2A_MOUSE

LNKLLGK $^{\text{Dimethyl}}_{28.03}$ VTIAQGGVLPNIQAVLLPKKTE

- fragmentation table for charge state 3+
- specType=etd
- score=39.21
- F104901.dat
- query=q2339_p1
- precursor=771.727850
- chargePrecursor=4
- itol=0.8

AA	c	y	z+1	z+2	z	AA
L[1]	44.377	**1028.635**	**1023.296**	0.672	1022.960	L[29]
N[2]	82.392	990.941	985.601	**985.937**	985.265	N[28]
K[3]	125.090	952.926	947.587	947.923	947.251	K[27]
L[4]	162.785	910.228	904.889	905.225	904.553	L[26]
L[5]	200.479	872.533	867.194	867.530	866.858	L[25]
G[6]	219.487	834.839	829.499	829.835	829.163	G[24]
K[7]	271.529	815.832	810.492	810.828	810.156	K[23]
V[8]	304.552	763.790	758.450	758.786	758.114	V[22]
T[9]	338.234	730.767	725.427	725.763	725.091	T[21]
I[10]	375.929	697.084	691.745	692.081	691.409	I[20]
A[11]	399.608	659.389	654.050	654.386	653.714	A[19]
Q[12]	442.294	635.710	630.371	630.707	630.035	Q[18]
G[13]	461.301	593.024	587.685	588.021	587.349	G[17]
G[14]	480.308	574.017	568.678	569.013	568.342	G[16]
V[15]	513.331	555.010	549.670	550.006	549.334	V[15]
L[16]	551.026	521.987	516.648	516.984	516.312	L[14]
P[17]	583.377	484.292	478.953	479.289	478.617	P[13]
N[18]	621.391	451.942	446.602	446.938	446.266	N[12]
I[19]	659.086	413.927	408.588	408.924	408.252	I[11]
Q[20]	701.772	376.233	370.893	371.229	370.557	Q[10]
A[21]	725.451	333.546	328.207	328.543	327.871	A[9]
V[22]	758.474	309.867	304.528	304.864	304.192	V[8]
L[23]	796.168	276.844	271.505	271.841	271.169	L[7]
L[24]	833.863	239.150	233.810	234.146	233.474	L[6]
P[25]	866.214	201.455	196.116	196.451	195.780	P[5]
K[26]	908.912	169.104	163.765	164.101	163.429	K[4]
K[27]	**951.611**	126.406	121.066	121.402	120.730	K[3]
T[28]	985.293	83.708	78.368	78.704	78.032	T[2]
E[29]	1028.307	50.025	44.685	45.021	44.349	E[1]

sp|P43276|H15_MOUSE

TAAPAPVEKS $^{Phospho}_{79.97}$ PAKKKTTKKAGAAKRKATGPPVSE

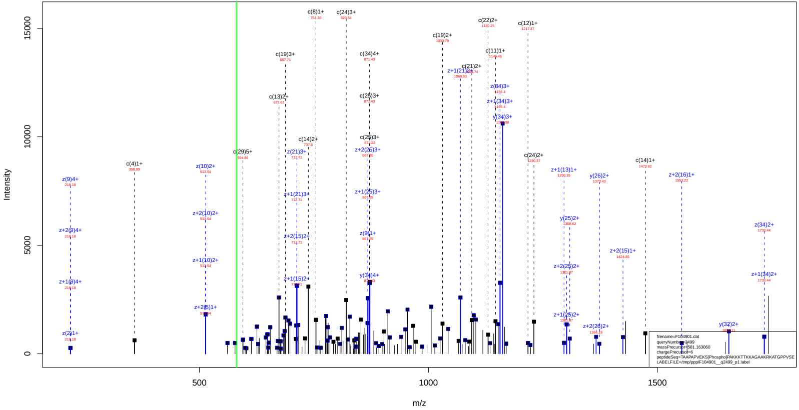

sp|P43276|H15_MOUSE

TAAPAPVEKS $^{Phospho}_{79.97}$ PAKKKTTKKAGAAKRKATGPPVSE

- fragmentation table for charge state 1+
- specType=etd
- score=51.45
- F104901.dat
- query=q2499_p1
- precursor=581.163060
- chargePrecursor=6
- itol=0.8

AA	c	y	z+1	z+2	z	AA
T[1]	119.082	3481.925	3465.907	0.000	3464.899	T[34]
A[2]	190.119	3380.878	3364.859	0.000	3363.851	A[33]
A[3]	261.156	3309.841	3293.822	0.000	3292.814	A[32]
P[4]	**358.208**	3238.804	3222.785	0.000	3221.777	P[31]
A[5]	429.246	3141.751	3125.732	0.000	3124.724	A[30]
P[6]	526.298	3070.714	3054.695	0.000	3053.687	P[29]
V[7]	**625.367**	2973.661	2957.642	0.000	2956.634	V[28]
E[8]	**754.409**	2874.592	2858.574	0.000	2857.566	E[27]
K[9]	882.504	2745.550	2729.531	2730.539	2728.523	K[26]
S[10]	1049.503	2617.455	2601.436	2602.444	2600.428	S[25]
P[11]	**1146.555**	2450.457	2434.438	2435.446	2433.430	P[24]
A[12]	**1217.593**	2353.404	2337.385	2338.393	2336.377	A[23]
K[13]	1345.688	2282.367	2266.348	2267.356	2265.340	K[22]
K[14]	**1473.783**	2154.272	2138.253	2139.261	2137.245	K[21]
K[15]	1601.877	2026.177	2010.158	2011.166	2009.150	K[20]
T[16]	1702.925	1898.082	1882.063	1883.071	1881.055	T[19]
T[17]	1803.973	1797.034	1781.015	1782.023	1780.008	T[18]
K[18]	1932.068	1695.986	1679.968	1680.976	1678.960	K[17]
K[19]	2060.163	1567.891	1551.873	**1552.881**	1550.865	K[16]
A[20]	2131.200	1439.797	1423.778	**1424.786**	1422.770	A[15]
G[21]	2188.221	1368.759	1352.741	1353.748	1351.733	G[14]
A[22]	2259.258	1311.738	**1295.719**	**1296.727**	1294.711	A[13]
A[23]	2330.296	1240.701	1224.682	1225.690	**1223.674**	A[12]
K[24]	2458.391	**1169.664**	1153.645	1154.653	**1152.637**	K[11]
R[25]	2614.492	1041.569	**1025.550**	1026.558	1024.542	R[10]
K[26]	2742.587	**885.468**	869.449	870.457	**868.441**	K[9]
A[27]	2813.624	**757.373**	741.354	742.362	740.346	A[8]
T[28]	2914.671	**686.336**	670.317	**671.325**	**669.309**	T[7]
G[29]	2971.693	585.288	569.269	570.277	568.261	G[6]
P[30]	3068.746	528.266	512.248	**513.256**	511.240	P[5]
P[31]	3165.798	431.214	415.195	416.203	414.187	P[4]
V[32]	3264.867	334.161	318.142	319.150	317.134	V[3]
S[33]	3351.899	235.092	219.074	220.082	**218.066**	S[2]
E[34]	3480.941	148.060	132.042	133.050	131.034	E[1]

sp|P43276|H15_MOUSE

TAAPAPVEKS $^{\text{Phospho}}_{79.97}$ PAKKKTTKKAGAAKRKATGPPVSE

- fragmentation table for charge state 2+
- specType=etd
- score=51.45
- F104901.dat
- query=q2499_p1
- precursor=581.163060
- chargePrecursor=6
- itol=0.8

AA	c	y	z+1	z+2	z	AA
T[1]	60.044	1741.466	**1733.457**	0.504	**1732.953**	T[34]
A[2]	95.563	1690.943	1682.933	0.504	1682.429	A[33]
A[3]	131.082	**1655.424**	1647.415	0.504	1646.911	A[32]
P[4]	179.608	1619.905	1611.896	0.504	1611.392	P[31]
A[5]	215.126	1571.379	1563.370	0.504	1562.866	A[30]
P[6]	263.653	1535.860	1527.851	0.504	1527.347	P[29]
V[7]	313.187	1487.334	1479.325	0.504	1478.821	V[28]
E[8]	377.708	1437.800	1429.791	0.504	1429.287	E[27]
K[9]	441.756	**1373.279**	1365.269	**1365.773**	1364.765	K[26]
S[10]	525.255	**1309.231**	**1301.222**	**1301.726**	1300.718	S[25]
P[11]	573.781	1225.732	**1217.723**	**1218.226**	**1217.219**	P[24]
A[12]	609.300	1177.206	**1169.196**	**1169.700**	1168.692	A[23]
K[13]	**673.347**	1141.687	1133.678	**1134.182**	1133.174	K[22]
K[14]	**737.395**	1077.639	**1069.630**	**1070.134**	1069.126	K[21]
K[15]	**801.442**	**1013.592**	**1005.583**	**1006.087**	1005.079	K[20]
T[16]	**851.966**	**949.545**	941.535	942.039	**941.031**	T[19]
T[17]	**902.490**	**899.021**	**891.011**	**891.515**	890.507	T[18]
K[18]	**966.538**	848.497	840.487	**840.991**	839.984	K[17]
K[19]	**1030.585**	**784.449**	**776.440**	**776.944**	**775.936**	K[16]
A[20]	**1066.104**	720.402	**712.393**	**712.896**	**711.889**	A[15]
G[21]	**1094.614**	684.883	**676.874**	**677.378**	**676.370**	G[14]
A[22]	**1130.133**	656.373	**648.363**	**648.867**	**647.859**	A[13]
A[23]	1165.651	620.854	**612.845**	**613.349**	612.341	A[12]
K[24]	**1229.699**	585.335	**577.326**	**577.830**	576.822	K[11]
R[25]	1307.749	521.288	**513.279**	**513.783**	**512.775**	R[10]
K[26]	1371.797	443.237	435.228	435.732	434.724	K[9]
A[27]	1407.315	379.190	371.181	371.685	370.677	A[8]
T[28]	1457.839	343.671	335.662	336.166	335.158	T[7]
G[29]	1486.350	293.148	285.138	285.642	284.634	G[6]
P[30]	1534.876	264.637	256.627	257.131	256.124	P[5]
P[31]	1583.403	216.110	208.101	208.605	207.597	P[4]
V[32]	1632.937	167.584	159.575	160.079	159.071	V[3]
S[33]	1676.453	118.050	110.041	110.544	109.537	S[2]
E[34]	1740.974	74.534	66.524	67.028	66.021	E[1]

sp|P43276|H15_MOUSE

TAAPAPVEKS $^{Phospho}_{79.97}$ PAKKKTTKKAGAAKRKATGPPVSE

- fragmentation table for charge state 3+
- specType=etd
- score=51.45
- F104901.dat
- query=q2499_p1
- precursor=581.163060
- chargePrecursor=6
- itol=0.8

AA	c	y	z+1	z+2	z	AA
T[1]	40.365	**1161.313**	**1155.974**	0.672	**1155.638**	T[34]
A[2]	64.044	1127.631	1122.291	0.672	1121.955	A[33]
A[3]	87.723	**1103.952**	**1098.612**	0.672	**1098.276**	A[32]
P[4]	120.074	**1080.273**	1074.933	0.672	1074.597	P[31]
A[5]	143.753	1047.922	**1042.582**	0.672	1042.246	A[30]
P[6]	176.104	1024.243	1018.903	0.672	1018.567	P[29]
V[7]	209.127	991.892	**986.552**	0.672	**986.216**	V[28]
E[8]	252.141	**958.869**	**953.529**	0.672	953.193	E[27]
K[9]	294.840	**915.855**	910.515	**910.851**	910.179	K[26]
S[10]	350.506	873.156	**867.817**	**868.153**	**867.481**	S[25]
P[11]	382.857	817.490	812.151	812.487	811.815	P[24]
A[12]	406.536	**785.139**	**779.800**	**780.136**	**779.464**	A[23]
K[13]	449.234	761.460	**756.121**	**756.457**	755.785	K[22]
K[14]	491.932	718.762	**713.423**	713.758	**713.087**	K[21]
K[15]	534.631	676.064	**670.724**	**671.060**	670.388	K[20]
T[16]	568.313	633.365	**628.026**	**628.362**	**627.690**	T[19]
T[17]	**601.996**	**599.683**	**594.343**	**594.679**	594.007	T[18]
K[18]	**644.694**	566.000	**560.661**	**560.997**	560.325	K[17]
K[19]	**687.392**	523.302	517.962	518.298	517.626	K[16]
A[20]	**711.071**	480.604	475.264	475.600	474.928	A[15]
G[21]	**730.079**	456.925	451.585	451.921	451.249	G[14]
A[22]	**753.758**	437.917	432.578	432.914	432.242	A[13]
A[23]	777.437	414.238	408.899	409.235	408.563	A[12]
K[24]	**820.135**	390.559	385.220	385.556	384.884	K[11]
R[25]	**872.169**	347.861	342.522	342.857	342.186	R[10]
K[26]	**914.867**	295.827	290.488	290.824	290.152	K[9]
A[27]	938.546	253.129	247.789	248.125	247.454	A[8]
T[28]	**972.229**	229.450	224.110	224.446	223.775	T[7]
G[29]	991.236	195.767	190.428	190.764	190.092	G[6]
P[30]	1023.587	176.760	171.421	171.757	171.085	P[5]
P[31]	1055.938	144.409	139.070	139.406	138.734	P[4]
V[32]	**1088.960**	112.058	106.719	107.055	106.383	V[3]
S[33]	1117.971	79.036	73.696	74.032	73.360	S[2]
E[34]	1160.985	50.025	44.685	45.021	44.349	E[1]

sp|P43276|H15_MOUSE

TAAPAPVEKS $^{Phospho}_{79.97}$ PAKKKTTKKAGAAKRKATGPPVSE

- fragmentation table for charge state 4+
- specType=etd
- score=51.45
- F104901.dat
- query=q2499_p1
- precursor=581.163060
- chargePrecursor=6
- itol=0.8

AA	c	y	z+1	z+2	z	AA
T[1]	30.526	**871.237**	**867.232**	0.755	**866.980**	T[34]
A[2]	48.285	845.975	**841.970**	0.755	**841.718**	A[33]
A[3]	66.044	**828.216**	**824.211**	0.755	**823.959**	A[32]
P[4]	90.308	**810.456**	**806.452**	0.755	806.200	P[31]
A[5]	108.067	786.193	782.188	0.755	781.937	A[30]
P[6]	132.330	768.434	**764.429**	0.755	**764.177**	P[29]
V[7]	157.097	744.171	740.166	0.755	739.914	V[28]
E[8]	189.358	719.404	**715.399**	0.755	**715.147**	E[27]
K[9]	221.382	**687.143**	**683.138**	**683.390**	**682.886**	K[26]
S[10]	263.131	**655.119**	**651.115**	**651.366**	**650.863**	S[25]
P[11]	287.394	**613.370**	609.365	609.617	609.113	P[24]
A[12]	305.154	589.106	585.102	585.354	584.850	A[23]
K[13]	337.177	571.347	567.342	567.594	567.090	K[22]
K[14]	369.201	539.323	535.319	535.571	535.067	K[21]
K[15]	401.225	507.300	503.295	503.547	503.043	K[20]
T[16]	426.487	475.276	471.271	471.523	471.019	T[19]
T[17]	451.749	450.014	446.009	446.261	445.757	T[18]
K[18]	483.772	424.752	420.747	420.999	420.495	K[17]
K[19]	515.796	392.728	388.724	388.976	388.472	K[16]
A[20]	533.555	360.705	356.700	356.952	356.448	A[15]
G[21]	547.811	342.945	338.941	339.193	338.689	G[14]
A[22]	565.570	328.690	324.685	324.937	324.433	A[13]
A[23]	583.329	310.931	306.926	307.178	306.674	A[12]
K[24]	615.353	293.171	289.167	289.419	288.915	K[11]
R[25]	**654.378**	261.148	257.143	257.395	256.891	R[10]
K[26]	**686.402**	222.122	**218.118**	**218.370**	**217.866**	K[9]
A[27]	704.161	190.099	186.094	186.346	185.842	A[8]
T[28]	729.423	172.339	168.335	168.587	168.083	T[7]
G[29]	743.679	147.077	143.073	143.325	142.821	G[6]
P[30]	767.942	132.822	128.817	129.069	128.565	P[5]
P[31]	**792.205**	108.559	104.554	104.806	104.302	P[4]
V[32]	816.972	84.296	80.291	80.543	80.039	V[3]
S[33]	**838.730**	59.529	55.524	55.776	55.272	S[2]
E[34]	**870.991**	37.771	33.766	34.018	33.514	E[1]

sp|P43276|H15_MOUSE

TAAPAPVEKS $^{Phospho}_{79.97}$ PAKKKTTKKAGAAKRKATGPPVSE

- fragmentation table for charge state 5+
- specType=etd
- score=51.45
- F104901.dat
- query=q2499_p1
- precursor=581.163060
- chargePrecursor=6
- itol=0.8

AA	c	y	z+1	z+2	z	AA
T[1]	24.622	**697.191**	**693.987**	0.806	**693.786**	T[34]
A[2]	38.830	**676.981**	**673.778**	0.806	**673.576**	A[33]
A[3]	53.037	662.774	659.570	0.806	659.369	A[32]
P[4]	72.448	**648.567**	**645.363**	0.806	**645.161**	P[31]
A[5]	86.655	629.156	**625.952**	0.806	**625.751**	A[30]
P[6]	106.065	614.949	611.745	0.806	611.543	P[29]
V[7]	125.879	**595.538**	592.334	0.806	592.133	V[28]
E[8]	151.688	575.724	572.521	0.806	572.319	E[27]
K[9]	177.307	549.916	546.712	546.914	546.510	K[26]
S[10]	210.706	524.297	521.093	521.295	520.891	S[25]
P[11]	230.117	490.897	487.693	487.895	487.492	P[24]
A[12]	244.324	471.487	468.283	468.484	468.081	A[23]
K[13]	269.943	457.279	454.075	454.277	453.874	K[22]
K[14]	295.562	431.660	428.456	428.658	428.255	K[21]
K[15]	321.181	406.041	402.837	403.039	402.636	K[20]
T[16]	341.391	380.422	377.218	377.420	377.017	T[19]
T[17]	361.600	360.213	357.009	357.210	356.807	T[18]
K[18]	387.219	340.003	336.799	337.001	336.598	K[17]
K[19]	412.838	314.384	311.180	311.382	310.979	K[16]
A[20]	427.046	288.765	285.561	285.763	285.360	A[15]
G[21]	438.450	274.558	271.354	271.556	271.152	G[14]
A[22]	452.658	263.153	259.950	260.151	259.748	A[13]
A[23]	466.865	248.946	245.742	245.944	245.541	A[12]
K[24]	492.484	234.739	231.535	231.736	231.333	K[11]
R[25]	523.704	209.120	205.916	206.117	205.714	R[10]
K[26]	549.323	177.899	174.696	174.897	174.494	K[9]
A[27]	563.531	152.280	149.077	149.278	148.875	A[8]
T[28]	583.740	138.073	134.869	135.071	134.668	T[7]
G[29]	**595.144**	117.863	114.660	114.861	114.458	G[6]
P[30]	614.555	106.459	103.255	103.457	103.054	P[5]
P[31]	633.965	87.049	83.845	84.046	83.643	P[4]
V[32]	653.779	67.638	64.434	64.636	64.233	V[3]
S[33]	**671.186**	47.824	44.621	44.822	44.419	S[2]
E[34]	**696.994**	30.418	27.214	27.416	27.013	E[1]

sp|P43276|H15_MOUSE

TAAPAPVEKS $^{\text{Phospho}}_{79.97}$ PAKKKTTKKAGAAKRKATGPPVSE

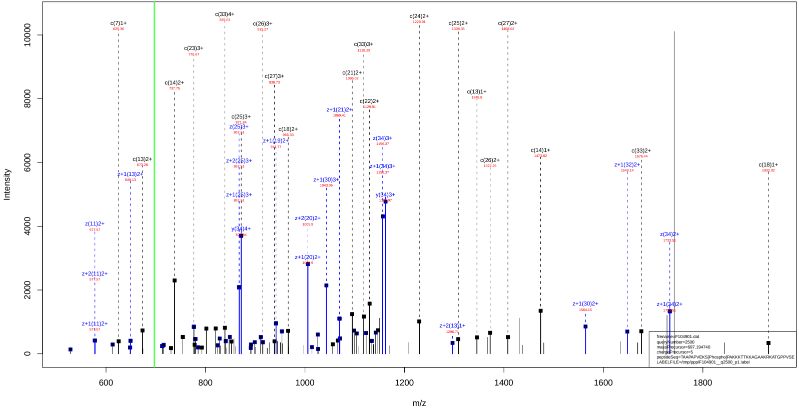

sp|P43276|H15_MOUSE

TAAPAPVEKS $^{\text{Phospho}}_{79.97}$ PAKKKTTKKAGAAKRKATGPPVSE

- fragmentation table for charge state 1+
- specType=etd
- score=68.06
- F104901.dat
- query=q2500_p1
- precursor=697.194740
- chargePrecursor=5
- itol=0.8

AA	c	y	z+1	z+2	z	AA
T[1]	119.082	3481.925	3465.907	0.000	3464.899	T[34]
A[2]	190.119	3380.878	3364.859	0.000	3363.851	A[33]
A[3]	261.156	3309.841	3293.822	0.000	3292.814	A[32]
P[4]	358.208	3238.804	3222.785	0.000	3221.777	P[31]
A[5]	429.246	3141.751	3125.732	0.000	3124.724	A[30]
P[6]	526.298	3070.714	3054.695	0.000	3053.687	P[29]
V[7]	**625.367**	2973.661	2957.642	0.000	2956.634	V[28]
E[8]	**754.409**	2874.592	2858.574	0.000	2857.566	E[27]
K[9]	882.504	2745.550	2729.531	2730.539	2728.523	K[26]
S[10]	1049.503	2617.455	2601.436	2602.444	2600.428	S[25]
P[11]	**1146.555**	2450.457	2434.438	2435.446	2433.430	P[24]
A[12]	1217.593	2353.404	2337.385	2338.393	2336.377	A[23]
K[13]	**1345.688**	2282.367	2266.348	2267.356	2265.340	K[22]
K[14]	**1473.783**	2154.272	2138.253	2139.261	2137.245	K[21]
K[15]	1601.877	2026.177	2010.158	2011.166	2009.150	K[20]
T[16]	1702.925	1898.082	1882.063	1883.071	1881.055	T[19]
T[17]	1803.973	1797.034	1781.015	1782.023	1780.008	T[18]
K[18]	**1932.068**	1695.986	1679.968	1680.976	1678.960	K[17]
K[19]	2060.163	1567.891	1551.873	1552.881	1550.865	K[16]
A[20]	2131.200	1439.797	1423.778	1424.786	1422.770	A[15]
G[21]	2188.221	1368.759	1352.741	1353.748	1351.733	G[14]
A[22]	2259.258	1311.738	1295.719	**1296.727**	1294.711	A[13]
A[23]	2330.296	1240.701	1224.682	1225.690	1223.674	A[12]
K[24]	2458.391	1169.664	1153.645	1154.653	1152.637	K[11]
R[25]	2614.492	1041.569	**1025.550**	**1026.558**	1024.542	R[10]
K[26]	2742.587	885.468	869.449	870.457	868.441	K[9]
A[27]	2813.624	757.373	741.354	742.362	740.346	A[8]
T[28]	2914.671	686.336	670.317	671.325	669.309	T[7]
G[29]	2971.693	585.288	569.269	570.277	568.261	G[6]
P[30]	3068.746	**528.266**	512.248	513.256	511.240	P[5]
P[31]	3165.798	431.214	415.195	416.203	414.187	P[4]
V[32]	3264.867	334.161	318.142	319.150	317.134	V[3]
S[33]	3351.899	235.092	219.074	220.082	218.066	S[2]
E[34]	3480.941	148.060	132.042	133.050	131.034	E[1]

sp|P43276|H15_MOUSE

TAAPAPVEKS $^{Phospho}_{79.97}$ PAKKKTTKKAGAAKRKATGPPVSE

- fragmentation table for charge state 2+
- specType=etd
- score=68.06
- F104901.dat
- query=q2500_p1
- precursor=697.194740
- chargePrecursor=5
- itol=0.8

AA	c	y	z+1	z+2	z	AA
T[1]	60.044	1741.466	**1733.457**	0.504	**1732.953**	T[34]
A[2]	95.563	1690.943	1682.933	0.504	1682.429	A[33]
A[3]	131.082	1655.424	**1647.415**	0.504	1646.911	A[32]
P[4]	179.608	1619.905	1611.896	0.504	1611.392	P[31]
A[5]	215.126	1571.379	**1563.370**	0.504	1562.866	A[30]
P[6]	263.653	1535.860	1527.851	0.504	1527.347	P[29]
V[7]	313.187	1487.334	1479.325	0.504	1478.821	V[28]
E[8]	377.708	1437.800	1429.791	0.504	1429.287	E[27]
K[9]	441.756	1373.279	1365.269	1365.773	1364.765	K[26]
S[10]	525.255	1309.231	1301.222	1301.726	1300.718	S[25]
P[11]	573.781	1225.732	1217.723	1218.226	1217.219	P[24]
A[12]	609.300	1177.206	1169.196	1169.700	1168.692	A[23]
K[13]	**673.347**	**1141.687**	**1133.678**	**1134.182**	1133.174	K[22]
K[14]	**737.395**	1077.639	**1069.630**	**1070.134**	**1069.126**	K[21]
K[15]	**801.442**	**1013.592**	**1005.583**	**1006.087**	1005.079	K[20]
T[16]	**851.966**	949.545	**941.535**	**942.039**	**941.031**	T[19]
T[17]	902.490	**899.021**	**891.011**	**891.515**	**890.507**	T[18]
K[18]	**966.538**	**848.497**	**840.487**	**840.991**	**839.984**	K[17]
K[19]	1030.585	**784.449**	**776.440**	**776.944**	**775.936**	K[16]
A[20]	**1066.104**	720.402	712.393	**712.896**	711.889	A[15]
G[21]	**1094.614**	684.883	676.874	677.378	676.370	G[14]
A[22]	**1130.133**	656.373	**648.363**	**648.867**	**647.859**	A[13]
A[23]	1165.651	620.854	**612.845**	**613.349**	612.341	A[12]
K[24]	**1229.699**	585.335	**577.326**	**577.830**	**576.822**	K[11]
R[25]	**1307.749**	521.288	513.279	513.783	512.775	R[10]
K[26]	**1371.797**	443.237	435.228	435.732	434.724	K[9]
A[27]	**1407.315**	379.190	371.181	371.685	370.677	A[8]
T[28]	1457.839	343.671	335.662	336.166	335.158	T[7]
G[29]	1486.350	293.148	285.138	285.642	284.634	G[6]
P[30]	1534.876	264.637	256.627	257.131	256.124	P[5]
P[31]	1583.403	216.110	208.101	208.605	207.597	P[4]
V[32]	1632.937	167.584	159.575	160.079	159.071	V[3]
S[33]	**1676.453**	118.050	110.041	110.544	109.537	S[2]
E[34]	1740.974	74.534	66.524	67.028	66.021	E[1]

sp|P43276|H15_MOUSE

TAAPAPVEKS $^{Phospho}_{79.97}$ PAKKKTTKKAGAAKRKATGPPVSE

- fragmentation table for charge state 3+
- specType=etd
- score=68.06
- F104901.dat
- query=q2500_p1
- precursor=697.194740
- chargePrecursor=5
- itol=0.8

AA	c	y	z+1	z+2	z	AA
T[1]	40.365	**1161.313**	**1155.974**	0.672	**1155.638**	T[34]
A[2]	64.044	1127.631	**1122.291**	0.672	1121.955	A[33]
A[3]	87.723	**1103.952**	**1098.612**	0.672	1098.276	A[32]
P[4]	120.074	1080.273	1074.933	0.672	1074.597	P[31]
A[5]	143.753	1047.922	**1042.582**	0.672	1042.246	A[30]
P[6]	176.104	1024.243	1018.903	0.672	1018.567	P[29]
V[7]	209.127	991.892	986.552	0.672	986.216	V[28]
E[8]	252.141	958.869	**953.529**	0.672	**953.193**	E[27]
K[9]	294.840	**915.855**	**910.515**	**910.851**	**910.179**	K[26]
S[10]	350.506	873.156	**867.817**	**868.153**	**867.481**	S[25]
P[11]	382.857	817.490	812.151	812.487	811.815	P[24]
A[12]	406.536	**785.139**	**779.800**	**780.136**	**779.464**	A[23]
K[13]	449.234	761.460	756.121	756.457	755.785	K[22]
K[14]	491.932	718.762	**713.423**	**713.758**	**713.087**	K[21]
K[15]	534.631	676.064	670.724	671.060	670.388	K[20]
T[16]	568.313	633.365	628.026	628.362	627.690	T[19]
T[17]	601.996	599.683	594.343	594.679	594.007	T[18]
K[18]	644.694	566.000	560.661	560.997	560.325	K[17]
K[19]	687.392	523.302	517.962	518.298	517.626	K[16]
A[20]	711.071	480.604	475.264	475.600	474.928	A[15]
G[21]	**730.079**	456.925	451.585	451.921	451.249	G[14]
A[22]	**753.758**	437.917	432.578	432.914	432.242	A[13]
A[23]	**777.437**	414.238	408.899	409.235	408.563	A[12]
K[24]	**820.135**	390.559	385.220	385.556	384.884	K[11]
R[25]	**872.169**	347.861	342.522	342.857	342.186	R[10]
K[26]	**914.867**	295.827	290.488	290.824	290.152	K[9]
A[27]	**938.546**	253.129	247.789	248.125	247.454	A[8]
T[28]	972.229	229.450	224.110	224.446	223.775	T[7]
G[29]	991.236	195.767	190.428	190.764	190.092	G[6]
P[30]	1023.587	176.760	171.421	171.757	171.085	P[5]
P[31]	**1055.938**	144.409	139.070	139.406	138.734	P[4]
V[32]	1088.960	112.058	106.719	107.055	106.383	V[3]
S[33]	**1117.971**	79.036	73.696	74.032	73.360	S[2]
E[34]	1160.985	50.025	44.685	45.021	44.349	E[1]

sp|P43276|H15_MOUSE

TAAPAPVEKS $^{Phospho}_{79.97}$ PAKKKTTKKAGAAKRKATGPPVSE

- fragmentation table for charge state 4+
- specType=etd
- score=68.06
- F104901.dat
- query=q2500_p1
- precursor=697.194740
- chargePrecursor=5
- itol=0.8

AA	c	y	z+1	z+2	z	AA
T[1]	30.526	**871.237**	**867.232**	0.755	**866.980**	T[34]
A[2]	48.285	845.975	841.970	0.755	841.718	A[33]
A[3]	66.044	**828.216**	**824.211**	0.755	**823.959**	A[32]
P[4]	90.308	810.456	806.452	0.755	806.200	P[31]
A[5]	108.067	786.193	782.188	0.755	781.937	A[30]
P[6]	132.330	768.434	764.429	0.755	764.177	P[29]
V[7]	157.097	744.171	740.166	0.755	739.914	V[28]
E[8]	189.358	719.404	**715.399**	0.755	**715.147**	E[27]
K[9]	221.382	687.143	683.138	683.390	682.886	K[26]
S[10]	263.131	655.119	651.115	651.366	650.863	S[25]
P[11]	287.394	**613.370**	609.365	609.617	609.113	P[24]
A[12]	305.154	589.106	585.102	585.354	584.850	A[23]
K[13]	337.177	571.347	567.342	567.594	567.090	K[22]
K[14]	369.201	539.323	535.319	535.571	535.067	K[21]
K[15]	401.225	507.300	503.295	503.547	503.043	K[20]
T[16]	426.487	475.276	471.271	471.523	471.019	T[19]
T[17]	451.749	450.014	446.009	446.261	445.757	T[18]
K[18]	483.772	424.752	420.747	420.999	420.495	K[17]
K[19]	515.796	392.728	388.724	388.976	388.472	K[16]
A[20]	533.555	360.705	356.700	356.952	356.448	A[15]
G[21]	547.811	342.945	338.941	339.193	338.689	G[14]
A[22]	565.570	328.690	324.685	324.937	324.433	A[13]
A[23]	583.329	310.931	306.926	307.178	306.674	A[12]
K[24]	615.353	293.171	289.167	289.419	288.915	K[11]
R[25]	654.378	261.148	257.143	257.395	256.891	R[10]
K[26]	686.402	222.122	218.118	218.370	217.866	K[9]
A[27]	704.161	190.099	186.094	186.346	185.842	A[8]
T[28]	729.423	172.339	168.335	168.587	168.083	T[7]
G[29]	743.679	147.077	143.073	143.325	142.821	G[6]
P[30]	767.942	132.822	128.817	129.069	128.565	P[5]
P[31]	**792.205**	108.559	104.554	104.806	104.302	P[4]
V[32]	816.972	84.296	80.291	80.543	80.039	V[3]
S[33]	**838.730**	59.529	55.524	55.776	55.272	S[2]
E[34]	870.991	37.771	33.766	34.018	33.514	E[1]

sp|P43276|H15_MOUSE

TAAPAPVEKS $^{\text{Phospho}}_{79.97}$ PAKKKTTKKAGAAKRKATGPPVSE

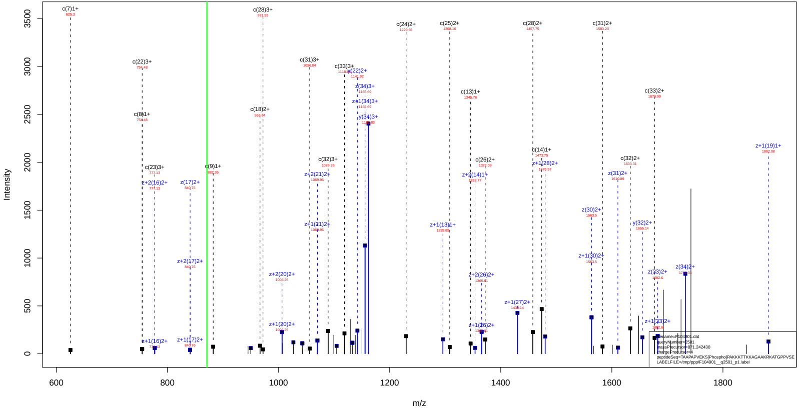

sp|P43276|H15_MOUSE

TAAPAPVEKS $^{Phospho}_{79.97}$ PAKKKTTKKAGAAKRKATGPPVSE

- fragmentation table for charge state 1+
- specType=etd
- score=50.70
- F104901.dat
- query=q2501_p1
- precursor=871.242430
- chargePrecursor=4
- itol=0.8

AA	c	y	z+1	z+2	z	AA
T[1]	119.082	3481.925	3465.907	0.000	3464.899	T[34]
A[2]	190.119	3380.878	3364.859	0.000	3363.851	A[33]
A[3]	261.156	3309.841	3293.822	0.000	3292.814	A[32]
P[4]	358.208	3238.804	3222.785	0.000	3221.777	P[31]
A[5]	429.246	3141.751	3125.732	0.000	3124.724	A[30]
P[6]	526.298	3070.714	3054.695	0.000	3053.687	P[29]
V[7]	**625.367**	2973.661	2957.642	0.000	2956.634	V[28]
E[8]	**754.409**	2874.592	2858.574	0.000	2857.566	E[27]
K[9]	**882.504**	2745.550	2729.531	2730.539	2728.523	K[26]
S[10]	1049.503	2617.455	2601.436	2602.444	2600.428	S[25]
P[11]	1146.555	2450.457	2434.438	2435.446	2433.430	P[24]
A[12]	1217.593	2353.404	2337.385	2338.393	2336.377	A[23]
K[13]	**1345.688**	2282.367	2266.348	2267.356	2265.340	K[22]
K[14]	**1473.783**	2154.272	2138.253	2139.261	2137.245	K[21]
K[15]	1601.877	2026.177	2010.158	2011.166	2009.150	K[20]
T[16]	1702.925	1898.082	**1882.063**	1883.071	1881.055	T[19]
T[17]	1803.973	1797.034	1781.015	1782.023	1780.008	T[18]
K[18]	1932.068	1695.986	1679.968	1680.976	1678.960	K[17]
K[19]	2060.163	1567.891	1551.873	1552.881	1550.865	K[16]
A[20]	2131.200	1439.797	1423.778	1424.786	1422.770	A[15]
G[21]	2188.221	1368.759	1352.741	**1353.748**	1351.733	G[14]
A[22]	2259.258	1311.738	**1295.719**	1296.727	1294.711	A[13]
A[23]	2330.296	1240.701	1224.682	1225.690	1223.674	A[12]
K[24]	2458.391	1169.664	1153.645	1154.653	1152.637	K[11]
R[25]	2614.492	1041.569	1025.550	**1026.558**	1024.542	R[10]
K[26]	2742.587	885.468	869.449	870.457	868.441	K[9]
A[27]	2813.624	757.373	741.354	742.362	740.346	A[8]
T[28]	2914.671	686.336	670.317	671.325	669.309	T[7]
G[29]	2971.693	585.288	569.269	570.277	568.261	G[6]
P[30]	3068.746	528.266	512.248	513.256	511.240	P[5]
P[31]	3165.798	431.214	415.195	416.203	414.187	P[4]
V[32]	3264.867	334.161	318.142	319.150	317.134	V[3]
S[33]	3351.899	235.092	219.074	220.082	218.066	S[2]
E[34]	3480.941	148.060	132.042	133.050	131.034	E[1]

sp|P43276|H15_MOUSE

TAAPAPVEKS $^{Phospho}_{79.97}$ PAKKKTTKKAGAAKRKATGPPVSE

- fragmentation table for charge state 2+
- specType=etd
- score=50.70
- F104901.dat
- query=q2501_p1
- precursor=871.242430
- chargePrecursor=4
- itol=0.8

AA	c	y	z+1	z+2	z	AA
T[1]	60.044	1741.466	1733.457	0.504	**1732.953**	T[34]
A[2]	95.563	1690.943	**1682.933**	0.504	**1682.429**	A[33]
A[3]	131.082	**1655.424**	1647.415	0.504	1646.911	A[32]
P[4]	179.608	1619.905	1611.896	0.504	**1611.392**	P[31]
A[5]	215.126	1571.379	**1563.370**	0.504	**1562.866**	A[30]
P[6]	263.653	1535.860	1527.851	0.504	1527.347	P[29]
V[7]	313.187	1487.334	**1479.325**	0.504	1478.821	V[28]
E[8]	377.708	1437.800	**1429.791**	0.504	1429.287	E[27]
K[9]	441.756	1373.279	**1365.269**	**1365.773**	1364.765	K[26]
S[10]	525.255	1309.231	1301.222	1301.726	1300.718	S[25]
P[11]	573.781	1225.732	1217.723	1218.226	1217.219	P[24]
A[12]	609.300	1177.206	1169.196	1169.700	1168.692	A[23]
K[13]	673.347	**1141.687**	**1133.678**	1134.182	**1133.174**	K[22]
K[14]	737.395	1077.639	**1069.630**	**1070.134**	1069.126	K[21]
K[15]	801.442	1013.592	**1005.583**	**1006.087**	1005.079	K[20]
T[16]	851.966	**949.545**	941.535	942.039	941.031	T[19]
T[17]	902.490	899.021	891.011	891.515	890.507	T[18]
K[18]	**966.538**	848.497	**840.487**	**840.991**	**839.984**	K[17]
K[19]	1030.585	784.449	**776.440**	**776.944**	775.936	K[16]
A[20]	1066.104	720.402	712.393	712.896	711.889	A[15]
G[21]	1094.614	684.883	676.874	677.378	676.370	G[14]
A[22]	1130.133	656.373	648.363	648.867	647.859	A[13]
A[23]	1165.651	620.854	612.845	613.349	612.341	A[12]
K[24]	**1229.699**	585.335	577.326	577.830	576.822	K[11]
R[25]	**1307.749**	521.288	513.279	513.783	512.775	R[10]
K[26]	**1371.797**	443.237	435.228	435.732	434.724	K[9]
A[27]	1407.315	379.190	371.181	371.685	370.677	A[8]
T[28]	**1457.839**	343.671	335.662	336.166	335.158	T[7]
G[29]	1486.350	293.148	285.138	285.642	284.634	G[6]
P[30]	1534.876	264.637	256.627	257.131	256.124	P[5]
P[31]	**1583.403**	216.110	208.101	208.605	207.597	P[4]
V[32]	**1632.937**	167.584	159.575	160.079	159.071	V[3]
S[33]	**1676.453**	118.050	110.041	110.544	109.537	S[2]
E[34]	1740.974	74.534	66.524	67.028	66.021	E[1]

sp|P43276|H15_MOUSE

TAAPAPVEKS $^{Phospho}_{79.97}$ PAKKKTTKKAGAAKRKATGPPVSE

- fragmentation table for charge state 3+
- specType=etd
- score=50.70
- F104901.dat
- query=q2501_p1
- precursor=871.242430
- chargePrecursor=4
- itol=0.8

AA	c	y	z+1	z+2	z	AA
T[1]	40.365	**1161.313**	**1155.974**	0.672	**1155.638**	T[34]
A[2]	64.044	1127.631	1122.291	0.672	1121.955	A[33]
A[3]	87.723	**1103.952**	1098.612	0.672	1098.276	A[32]
P[4]	120.074	1080.273	1074.933	0.672	1074.597	P[31]
A[5]	143.753	1047.922	**1042.582**	0.672	**1042.246**	A[30]
P[6]	176.104	1024.243	1018.903	0.672	1018.567	P[29]
V[7]	209.127	991.892	986.552	0.672	986.216	V[28]
E[8]	252.141	958.869	953.529	0.672	953.193	E[27]
K[9]	294.840	915.855	910.515	910.851	910.179	K[26]
S[10]	350.506	873.156	867.817	868.153	867.481	S[25]
P[11]	382.857	817.490	812.151	812.487	811.815	P[24]
A[12]	406.536	785.139	779.800	780.136	779.464	A[23]
K[13]	449.234	761.460	756.121	756.457	755.785	K[22]
K[14]	491.932	718.762	713.423	713.758	713.087	K[21]
K[15]	534.631	676.064	670.724	671.060	670.388	K[20]
T[16]	568.313	633.365	628.026	628.362	627.690	T[19]
T[17]	601.996	599.683	594.343	594.679	594.007	T[18]
K[18]	644.694	566.000	560.661	560.997	560.325	K[17]
K[19]	687.392	523.302	517.962	518.298	517.626	K[16]
A[20]	711.071	480.604	475.264	475.600	474.928	A[15]
G[21]	730.079	456.925	451.585	451.921	451.249	G[14]
A[22]	**753.758**	437.917	432.578	432.914	432.242	A[13]
A[23]	**777.437**	414.238	408.899	409.235	408.563	A[12]
K[24]	820.135	390.559	385.220	385.556	384.884	K[11]
R[25]	872.169	347.861	342.522	342.857	342.186	R[10]
K[26]	914.867	295.827	290.488	290.824	290.152	K[9]
A[27]	938.546	253.129	247.789	248.125	247.454	A[8]
T[28]	**972.229**	229.450	224.110	224.446	223.775	T[7]
G[29]	991.236	195.767	190.428	190.764	190.092	G[6]
P[30]	1023.587	176.760	171.421	171.757	171.085	P[5]
P[31]	**1055.938**	144.409	139.070	139.406	138.734	P[4]
V[32]	**1088.960**	112.058	106.719	107.055	106.383	V[3]
S[33]	**1117.971**	79.036	73.696	74.032	73.360	S[2]
E[34]	1160.985	50.025	44.685	45.021	44.349	E[1]

sp|P43274|H14_MOUSE

[Acetyl]SETAPAAPAAPAPAEKTPVKKKARKAAGGAKRKTSGPPVSE

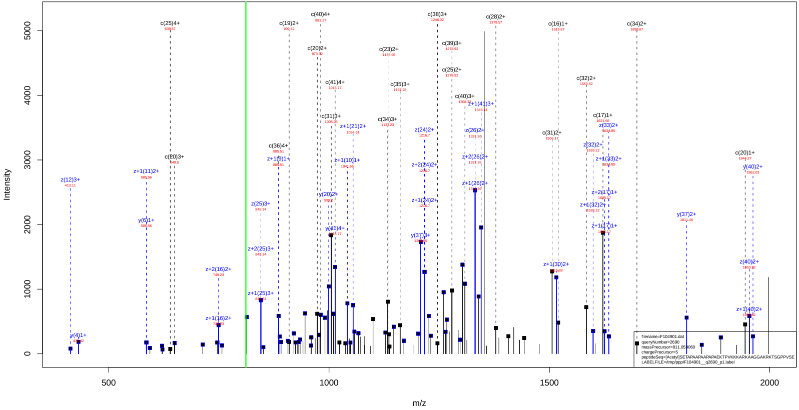

sp|P43274|H14_MOUSE

[Acetyl]SETAPAAPAAPAPAEKTPVKKKARKAAGGAKRKTSGPPVSE

- fragmentation table for charge state 1+
- specType=etd
- score=80.43
- F104901.dat
- query=q2690_p1
- precursor=811.059060
- chargePrecursor=5
- itol=0.8

AA	c	y	z+1	z+2	z	AA
S[1]	147.076	4051.241	4035.222	0.000	4034.215	S[41]
E[2]	276.119	3922.198	3906.180	0.000	3905.172	E[40]
T[3]	377.167	3793.156	3777.137	0.000	3776.129	T[39]
A[4]	448.204	3692.108	3676.090	0.000	3675.082	A[38]
P[5]	545.257	3621.071	3605.052	0.000	3604.045	P[37]
A[6]	616.294	3524.018	3508.000	0.000	3506.992	A[36]
A[7]	687.331	3452.981	3436.963	0.000	3435.955	A[35]
P[8]	784.384	3381.944	3365.925	0.000	3364.918	P[34]
A[9]	855.421	3284.891	3268.873	0.000	3267.865	A[33]
A[10]	926.458	3213.854	3197.836	0.000	3196.828	A[32]
P[11]	**1023.511**	3142.817	3126.798	0.000	3125.791	P[31]
A[12]	1094.548	3045.764	3029.746	0.000	3028.738	A[30]
P[13]	1191.600	2974.727	2958.709	0.000	2957.701	P[29]
A[14]	1262.638	2877.674	2861.656	0.000	2860.648	A[28]
E[15]	1391.680	2806.637	2790.619	0.000	2789.611	E[27]
K[16]	**1519.775**	2677.595	2661.576	2662.584	2660.568	K[26]
T[17]	**1620.823**	2549.500	2533.481	2534.489	2532.473	T[25]
P[18]	1717.876	2448.452	2432.433	2433.441	2431.426	P[24]
V[19]	1816.944	2351.399	2335.381	2336.388	2334.373	V[23]
K[20]	**1945.039**	2252.331	2236.312	2237.320	2235.304	K[22]
K[21]	2073.134	2124.236	2108.217	2109.225	2107.209	K[21]
K[22]	2201.229	1996.141	1980.122	1981.130	1979.114	K[20]
A[23]	2272.266	1868.046	1852.027	1853.035	1851.020	A[19]
R[24]	2428.367	1797.009	1780.990	1781.998	1779.982	R[18]
K[25]	2556.462	1640.908	**1624.889**	**1625.897**	1623.881	K[17]
A[26]	2627.499	1512.813	1496.794	1497.802	1495.786	A[16]
A[27]	2698.536	1441.776	1425.757	1426.765	1424.749	A[15]
G[28]	2755.558	1370.739	1354.720	1355.728	1353.712	G[14]
G[29]	2812.579	1313.717	**1297.698**	1298.706	**1296.691**	G[13]
A[30]	2883.616	1256.696	1240.677	1241.685	1239.669	A[12]
K[31]	3011.711	1185.659	**1169.640**	1170.648	1168.632	K[11]
R[32]	3167.812	**1057.564**	**1041.545**	1042.553	1040.537	R[10]
K[33]	3295.907	901.463	**885.444**	886.452	884.436	K[9]
T[34]	3396.955	773.368	**757.349**	758.357	**756.341**	T[8]
S[35]	3483.987	672.320	656.301	657.309	655.293	S[7]
G[36]	3541.009	**585.288**	569.269	570.277	568.261	G[6]
P[37]	3638.061	528.266	512.248	513.256	511.240	P[5]
P[38]	3735.114	**431.214**	415.195	416.203	414.187	P[4]
V[39]	3834.182	334.161	318.142	319.150	317.134	V[3]
S[40]	3921.214	235.092	219.074	220.082	218.066	S[2]
E[41]	4050.257	148.060	132.042	133.050	131.034	E[1]

sp|P43274|H14_MOUSE

[Acetyl]SETAPAAPAAPAPAEKTPVKKKARKAAGGAKRKTSGPPVSE

- fragmentation table for charge state 2+
- specType=etd
- score=80.43
- F104901.dat
- query=q2690_p1
- precursor=811.059060
- chargePrecursor=5
- itol=0.8

AA	c	y	z+1	z+2	z	AA
S[1]	74.042	2026.124	2018.115	0.504	2017.611	S[41]
E[2]	138.563	**1961.603**	**1953.594**	0.504	**1953.090**	E[40]
T[3]	189.087	1897.082	**1889.072**	0.504	**1888.568**	T[39]
A[4]	224.606	**1846.558**	1838.548	0.504	1838.044	A[38]
P[5]	273.132	**1811.039**	1803.030	0.504	1802.526	P[37]
A[6]	308.650	1762.513	1754.503	0.504	1754.000	A[36]
A[7]	344.169	1726.994	1718.985	0.504	1718.481	A[35]
P[8]	392.695	1691.476	1683.466	0.504	1682.962	P[34]
A[9]	428.214	1642.949	**1634.940**	0.504	**1634.436**	A[33]
A[10]	463.733	1607.431	**1599.421**	0.504	**1598.917**	A[32]
P[11]	512.259	1571.912	1563.903	0.504	1563.399	P[31]
A[12]	547.777	1523.386	**1515.376**	0.504	1514.873	A[30]
P[13]	596.304	1487.867	1479.858	0.504	1479.354	P[29]
A[14]	631.822	1439.341	1431.332	0.504	1430.828	A[28]
E[15]	696.344	1403.822	1395.813	0.504	1395.309	E[27]
K[16]	760.391	**1339.301**	**1331.292**	**1331.796**	**1330.788**	K[26]
T[17]	810.915	1275.254	**1267.244**	**1267.748**	**1266.740**	T[25]
P[18]	859.441	1224.730	**1216.720**	**1217.224**	**1216.216**	P[24]
V[19]	**908.976**	1176.203	1168.194	1168.698	1167.690	V[23]
K[20]	**973.023**	1126.669	1118.660	1119.164	1118.156	K[22]
K[21]	**1037.071**	1062.622	**1054.612**	**1055.116**	**1054.108**	K[21]
K[22]	1101.118	**998.574**	**990.565**	**991.069**	**990.061**	K[20]
A[23]	**1136.637**	**934.527**	926.517	927.021	926.013	A[19]
R[24]	1214.687	899.008	**890.999**	**891.503**	**890.495**	R[18]
K[25]	**1278.735**	820.958	**812.948**	**813.452**	812.444	K[17]
A[26]	1314.253	**756.910**	**748.901**	**749.405**	748.397	A[16]
A[27]	1349.772	721.392	**713.382**	**713.886**	**712.878**	A[15]
G[28]	**1378.283**	685.873	677.864	678.368	677.360	G[14]
G[29]	**1406.793**	657.362	**649.353**	**649.857**	**648.849**	G[13]
A[30]	**1442.312**	628.852	**620.842**	**621.346**	620.338	A[12]
K[31]	**1506.359**	**593.333**	**585.324**	**585.827**	**584.820**	K[11]
R[32]	**1584.410**	529.285	521.276	521.780	520.772	R[10]
K[33]	1648.457	451.235	443.226	443.729	442.722	K[9]
T[34]	**1698.981**	387.187	379.178	379.682	378.674	T[8]
S[35]	1742.497	336.664	328.654	329.158	328.150	S[7]
G[36]	1771.008	293.148	285.138	285.642	284.634	G[6]
P[37]	1819.534	264.637	256.627	257.131	256.124	P[5]
P[38]	1868.061	216.110	208.101	208.605	207.597	P[4]
V[39]	1917.595	167.584	159.575	160.079	159.071	V[3]
S[40]	1961.111	118.050	110.041	110.544	109.537	S[2]
E[41]	2025.632	74.534	66.524	67.028	66.021	E[1]

sp|P43274|H14_MOUSE

[Acetyl]SETAPAAPAAPAPAEKTPVKKKARKAAGGAKRKTSGPPVSE

- fragmentation table for charge state 3+
- specType=etd
- score=80.43
- F104901.dat
- query=q2690_p1
- precursor=811.059060
- chargePrecursor=5
- itol=0.8

AA	c	y	z+1	z+2	z	AA
S[1]	49.697	1351.085	**1345.746**	0.672	**1345.410**	S[41]
E[2]	92.711	**1308.071**	**1302.731**	0.672	**1302.396**	E[40]
T[3]	126.394	**1265.057**	**1259.717**	0.672	**1259.381**	T[39]
A[4]	150.073	**1231.374**	**1226.035**	0.672	1225.699	A[38]
P[5]	182.424	**1207.695**	**1202.356**	0.672	**1202.020**	P[37]
A[6]	206.103	1175.344	**1170.005**	0.672	**1169.669**	A[36]
A[7]	229.782	1151.665	**1146.326**	0.672	**1145.990**	A[35]
P[8]	262.133	**1127.986**	1122.647	0.672	1122.311	P[34]
A[9]	285.812	1095.635	1090.296	0.672	1089.960	A[33]
A[10]	309.491	1071.956	**1066.617**	0.672	**1066.281**	A[32]
P[11]	341.842	**1048.277**	1042.938	0.672	1042.602	P[31]
A[12]	365.521	1015.926	**1010.587**	0.672	**1010.251**	A[30]
P[13]	397.872	992.247	986.908	0.672	986.572	P[29]
A[14]	421.551	**959.896**	954.557	0.672	954.221	A[28]
E[15]	464.565	936.217	**930.878**	0.672	**930.542**	E[27]
K[16]	507.263	893.203	**887.864**	**888.199**	**887.528**	K[26]
T[17]	540.946	**850.505**	**845.165**	**845.501**	**844.829**	T[25]
P[18]	573.297	816.822	811.483	811.819	811.147	P[24]
V[19]	606.320	784.471	779.132	779.468	778.796	V[23]
K[20]	**649.018**	751.449	**746.109**	**746.445**	**745.773**	K[22]
K[21]	691.716	708.750	703.411	703.747	703.075	K[21]
K[22]	734.414	666.052	660.712	661.048	660.376	K[20]
A[23]	758.094	623.354	618.014	618.350	617.678	A[19]
R[24]	810.127	599.675	594.335	594.671	**593.999**	R[18]
K[25]	852.826	547.641	542.301	542.637	541.965	K[17]
A[26]	876.505	504.942	499.603	499.939	499.267	A[16]
A[27]	900.184	481.263	475.924	476.260	475.588	A[15]
G[28]	919.191	457.584	452.245	452.581	451.909	G[14]
G[29]	938.198	438.577	433.238	433.574	432.902	G[13]
A[30]	961.877	419.570	414.231	414.566	**413.895**	A[12]
K[31]	**1004.575**	395.891	390.551	390.887	390.216	K[11]
R[32]	1056.609	353.193	347.853	348.189	347.517	R[10]
K[33]	**1099.307**	301.159	295.819	296.155	295.484	K[9]
T[34]	**1132.990**	258.461	253.121	253.457	252.785	T[8]
S[35]	**1162.001**	224.778	219.439	219.775	219.103	S[7]
G[36]	1181.008	195.767	190.428	190.764	190.092	G[6]
P[37]	1213.359	176.760	171.421	171.757	171.085	P[5]
P[38]	**1245.710**	144.409	139.070	139.406	138.734	P[4]
V[39]	**1278.732**	112.058	106.719	107.055	106.383	V[3]
S[40]	**1307.743**	79.036	73.696	74.032	73.360	S[2]
E[41]	1350.757	50.025	44.685	45.021	44.349	E[1]

sp|P43274|H14_MOUSE

[Acetyl]SETAPAAPAAPAPAEKTPVKKKARKAAGGAKRKTSGPPVSE

- fragmentation table for charge state 4+
- specType=etd
- score=80.43
- F104901.dat
- query=q2690_p1
- precursor=811.059060
- chargePrecursor=5
- itol=0.8

AA	c	y	z+1	z+2	z	AA
S[1]	37.525	**1013.566**	**1009.561**	0.755	**1009.309**	S[41]
E[2]	69.785	**981.305**	**977.300**	0.755	**977.048**	E[40]
T[3]	95.047	949.044	**945.040**	0.755	**944.788**	T[39]
A[4]	112.806	**923.783**	**919.778**	0.755	**919.526**	A[38]
P[5]	137.070	**906.023**	902.019	0.755	901.767	P[37]
A[6]	154.829	881.760	877.755	0.755	877.503	A[36]
A[7]	172.588	864.001	859.996	0.755	859.744	A[35]
P[8]	196.851	846.241	842.237	0.755	841.985	P[34]
A[9]	214.611	821.978	817.974	0.755	817.722	A[33]
A[10]	232.370	804.219	800.214	0.755	799.962	A[32]
P[11]	256.633	786.460	782.455	0.755	782.203	P[31]
A[12]	274.392	762.197	758.192	0.755	757.940	A[30]
P[13]	298.656	744.437	740.433	0.755	740.181	P[29]
A[14]	316.415	720.174	716.169	0.755	715.917	A[28]
E[15]	348.675	702.415	698.410	0.755	698.158	E[27]
K[16]	380.699	670.154	666.149	666.401	665.898	K[26]
T[17]	405.961	638.130	634.126	634.378	633.874	T[25]
P[18]	430.224	612.868	608.864	609.116	608.612	P[24]
V[19]	454.991	588.605	584.601	**584.853**	584.349	V[23]
K[20]	487.015	563.838	559.834	560.085	559.582	K[22]
K[21]	519.039	531.814	527.810	528.062	527.558	K[21]
K[22]	551.063	499.791	495.786	496.038	495.534	K[20]
A[23]	568.822	467.767	463.762	464.014	463.510	A[19]
R[24]	607.847	450.008	446.003	446.255	445.751	R[18]
K[25]	**639.871**	410.982	406.978	407.230	406.726	K[17]
A[26]	657.630	378.959	374.954	375.206	374.702	A[16]
A[27]	675.390	361.199	357.195	357.447	356.943	A[15]
G[28]	689.645	343.440	339.435	339.687	339.183	G[14]
G[29]	703.900	329.185	325.180	325.432	324.928	G[13]
A[30]	721.660	314.929	310.925	311.177	310.673	A[12]
K[31]	753.683	297.170	293.165	293.417	292.913	K[11]
R[32]	792.709	265.146	261.142	261.394	260.890	R[10]
K[33]	824.732	226.121	222.116	222.368	221.864	K[9]
T[34]	849.994	194.097	190.093	190.345	189.841	T[8]
S[35]	871.752	168.835	164.831	165.083	164.579	S[7]
G[36]	**886.008**	147.077	143.073	143.325	142.821	G[6]
P[37]	910.271	132.822	128.817	129.069	128.565	P[5]
P[38]	**934.534**	108.559	104.554	104.806	104.302	P[4]
V[39]	**959.301**	84.296	80.291	80.543	80.039	V[3]
S[40]	**981.059**	59.529	55.524	55.776	55.272	S[2]
E[41]	**1013.320**	37.771	33.766	34.018	33.514	E[1]

sp|P43277|H13_MOUSE

[Acetyl]SETAPAAPAAPAPVEKTPVKKKAKKTGAAAGKRKASGPPVSE

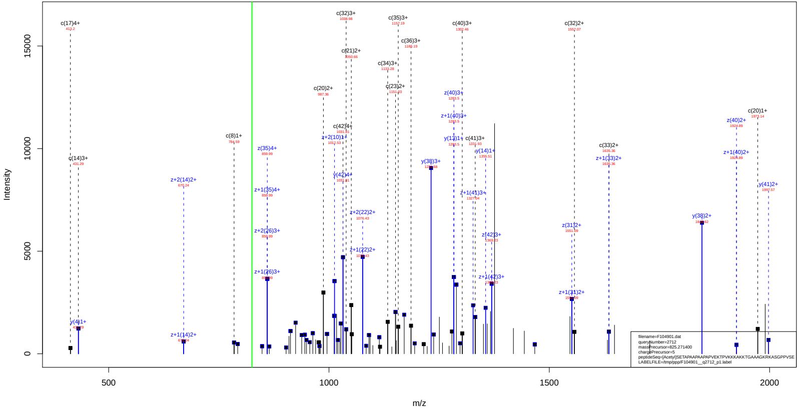

sp|P43277|H13_MOUSE

[Acetyl]SETAPAAPAAPAPVEKTPVKKAKKTGAAAGKRKASGPPVSE

- fragmentation table for charge state 1+
- specType=etd
- score=58.42
- F104901.dat
- query=q2712_p1
- precursor=825.271400
- chargePrecursor=5
- itol=0.8

AA	c	y	z+1	z+2	z	AA
S[1]	147.076	4122.303	4106.285	0.000	4105.277	S[42]
E[2]	276.119	3993.261	3977.242	0.000	3976.234	E[41]
T[3]	377.167	3864.218	3848.199	0.000	3847.192	T[40]
A[4]	448.204	3763.170	3747.152	0.000	3746.144	A[39]
P[5]	545.257	3692.133	3676.115	0.000	3675.107	P[38]
A[6]	616.294	3595.081	3579.062	0.000	3578.054	A[37]
A[7]	687.331	3524.044	3508.025	0.000	3507.017	A[36]
P[8]	**784.384**	3453.006	3436.988	0.000	3435.980	P[35]
A[9]	855.421	3355.954	3339.935	0.000	3338.927	A[34]
A[10]	926.458	3284.917	3268.898	0.000	3267.890	A[33]
P[11]	1023.511	3213.879	3197.861	0.000	3196.853	P[32]
A[12]	1094.548	3116.827	3100.808	0.000	3099.800	A[31]
P[13]	1191.600	3045.790	3029.771	0.000	3028.763	P[30]
V[14]	1290.669	2948.737	2932.718	0.000	2931.710	V[29]
E[15]	1419.711	2849.668	2833.650	0.000	2832.642	E[28]
K[16]	1547.806	2720.626	2704.607	2705.615	2703.599	K[27]
T[17]	1648.854	2592.531	2576.512	2577.520	2575.504	T[26]
P[18]	1745.907	2491.483	2475.464	2476.472	2474.457	P[25]
V[19]	1844.975	2394.430	2378.412	2379.419	2377.404	V[24]
K[20]	**1973.070**	2295.362	2279.343	2280.351	2278.335	K[23]
K[21]	2101.165	2167.267	2151.248	2152.256	2150.240	K[22]
K[22]	2229.260	2039.172	2023.153	2024.161	2022.145	K[21]
A[23]	2300.297	1911.077	1895.058	1896.066	1894.050	A[20]
K[24]	2428.392	1840.040	1824.021	1825.029	1823.013	K[19]
K[25]	2556.487	1711.945	1695.926	1696.934	1694.918	K[18]
T[26]	2657.535	1583.850	1567.831	1568.839	1566.823	T[17]
G[27]	2714.556	1482.802	**1466.784**	**1467.791**	1465.776	G[16]
A[28]	2785.593	1425.781	1409.762	1410.770	1408.754	A[15]
A[29]	2856.631	**1354.744**	1338.725	1339.733	1337.717	A[14]
A[30]	2927.668	**1283.707**	1267.688	1268.696	1266.680	A[13]
G[31]	2984.689	1212.670	1196.651	1197.659	1195.643	G[12]
K[32]	3112.784	1155.648	1139.629	1140.637	1138.621	K[11]
R[33]	3268.885	**1027.553**	**1011.534**	**1012.542**	1010.527	R[10]
K[34]	3396.980	871.452	855.433	856.441	854.425	K[9]
A[35]	3468.017	743.357	727.338	728.346	726.330	A[8]
S[36]	3555.049	672.320	656.301	657.309	655.293	S[7]
G[37]	3612.071	585.288	569.269	570.277	568.261	G[6]
P[38]	3709.124	528.266	512.248	513.256	511.240	P[5]
P[39]	3806.176	**431.214**	415.195	416.203	414.187	P[4]
V[40]	3905.245	334.161	318.142	319.150	317.134	V[3]
S[41]	3992.277	235.092	219.074	220.082	218.066	S[2]
E[42]	4121.319	148.060	132.042	133.050	131.034	E[1]

sp|P43277|H13_MOUSE

[Acetyl]SETAPAAPAAPAPVEKTPVKKAKKTGAAAGKRKASGPPVSE

- fragmentation table for charge state 2+
- specType=etd
- score=58.42
- F104901.dat
- query=q2712_p1
- precursor=825.271400
- chargePrecursor=5
- itol=0.8

AA	c	y	z+1	z+2	z	AA
S[1]	74.042	2061.655	2053.646	0.504	2053.142	S[42]
E[2]	138.563	**1997.134**	1989.125	0.504	1988.621	E[41]
T[3]	189.087	1932.613	**1924.603**	0.504	**1924.099**	T[40]
A[4]	224.606	1882.089	1874.080	0.504	1873.576	A[39]
P[5]	273.132	**1846.570**	1838.561	0.504	1838.057	P[38]
A[6]	308.650	1798.044	1790.035	0.504	1789.531	A[37]
A[7]	344.169	1762.525	1754.516	0.504	1754.012	A[36]
P[8]	392.695	1727.007	1718.997	0.504	1718.494	P[35]
A[9]	428.214	1678.480	1670.471	0.504	1669.967	A[34]
A[10]	463.733	1642.962	**1634.953**	0.504	1634.449	A[33]
P[11]	512.259	1607.443	1599.434	0.504	1598.930	P[32]
A[12]	547.777	1558.917	**1550.908**	0.504	**1550.404**	A[31]
P[13]	596.304	1523.398	1515.389	0.504	1514.885	P[30]
V[14]	645.838	1474.872	**1466.863**	0.504	**1466.359**	V[29]
E[15]	710.359	1425.338	1417.328	0.504	1416.825	E[28]
K[16]	774.407	1360.817	1352.807	1353.311	1352.303	K[27]
T[17]	824.931	**1296.769**	**1288.760**	**1289.264**	1288.256	T[26]
P[18]	873.457	1246.245	**1238.236**	1238.740	**1237.732**	P[25]
V[19]	922.991	1197.719	1189.709	1190.213	1189.206	V[24]
K[20]	**987.039**	1148.185	1140.175	1140.679	1139.671	K[23]
K[21]	**1051.086**	**1084.137**	**1076.128**	**1076.632**	1075.624	K[22]
K[22]	**1115.134**	**1020.090**	**1012.080**	**1012.584**	**1011.576**	K[21]
A[23]	**1150.652**	**956.042**	948.033	**948.537**	947.529	A[20]
K[24]	**1214.700**	920.524	**912.514**	**913.018**	**912.010**	K[19]
K[25]	**1278.747**	856.476	**848.467**	**848.971**	**847.963**	K[18]
T[26]	1329.271	**792.429**	**784.419**	**784.923**	**783.915**	T[17]
G[27]	1357.782	741.905	733.895	734.399	733.392	G[16]
A[28]	1393.300	713.394	705.385	705.889	704.881	A[15]
A[29]	1428.819	677.876	**669.866**	**670.370**	669.362	A[14]
A[30]	1464.337	642.357	634.348	634.852	633.844	A[13]
G[31]	1492.848	606.838	598.829	599.333	598.325	G[12]
K[32]	**1556.896**	578.328	570.318	570.822	569.814	K[11]
R[33]	**1634.946**	514.280	506.271	506.775	505.767	R[10]
K[34]	1698.994	436.230	428.220	428.724	427.716	K[9]
A[35]	1734.512	372.182	364.173	364.677	363.669	A[8]
S[36]	1778.028	336.664	328.654	329.158	328.150	S[7]
G[37]	1806.539	293.148	285.138	285.642	284.634	G[6]
P[38]	1855.065	264.637	256.627	257.131	256.124	P[5]
P[39]	1903.592	216.110	208.101	208.605	207.597	P[4]
V[40]	1953.126	167.584	159.575	160.079	159.071	V[3]
S[41]	1996.642	118.050	110.041	110.544	109.537	S[2]
E[42]	2061.163	74.534	66.524	67.028	66.021	E[1]

sp|P43277|H13_MOUSE

[Acetyl]SETAPAAPAAPAPVEKTPVKKAKKTGAAAGKRKASGPPVSE

- fragmentation table for charge state 3+
- specType=etd
- score=58.42
- F104901.dat
- query=q2712_p1
- precursor=825.271400
- chargePrecursor=5
- itol=0.8

AA	c	y	z+1	z+2	z	AA
S[1]	49.697	1374.773	**1369.433**	0.672	**1369.097**	S[42]
E[2]	92.711	**1331.758**	**1326.419**	0.672	1326.083	E[41]
T[3]	126.394	**1288.744**	**1283.405**	0.672	**1283.069**	T[40]
A[4]	150.073	1255.062	1249.722	0.672	1249.386	A[39]
P[5]	182.424	**1231.383**	1226.043	0.672	1225.707	P[38]
A[6]	206.103	1199.032	**1193.692**	0.672	1193.356	A[37]
A[7]	229.782	1175.353	**1170.013**	0.672	1169.677	A[36]
P[8]	262.133	**1151.674**	1146.334	0.672	1145.998	P[35]
A[9]	285.812	1119.323	**1113.983**	0.672	**1113.647**	A[34]
A[10]	309.491	1095.644	**1090.304**	0.672	1089.968	A[33]
P[11]	341.842	1071.965	1066.625	0.672	1066.289	P[32]
A[12]	365.521	**1039.614**	1034.274	0.672	1033.938	A[31]
P[13]	397.872	1015.935	1010.595	0.672	1010.259	P[30]
V[14]	**430.894**	983.584	**978.244**	0.672	**977.908**	V[29]
E[15]	473.909	950.561	**945.221**	0.672	**944.885**	E[28]
K[16]	516.607	907.547	**902.207**	**902.543**	901.871	K[27]
T[17]	550.290	**864.848**	**859.509**	**859.845**	859.173	T[26]
P[18]	582.640	831.166	825.826	826.162	825.490	P[25]
V[19]	615.663	798.815	**793.475**	793.811	**793.139**	V[24]
K[20]	658.362	765.792	760.453	760.789	760.117	K[23]
K[21]	701.060	723.094	717.754	718.090	717.418	K[22]
K[22]	743.758	680.396	675.056	675.392	674.720	K[21]
A[23]	767.437	637.697	632.358	632.694	632.022	A[20]
K[24]	810.136	614.018	608.679	609.015	608.343	K[19]
K[25]	852.834	571.320	565.980	566.316	565.644	K[18]
T[26]	886.516	528.622	523.282	523.618	522.946	T[17]
G[27]	905.524	494.939	489.599	489.935	489.263	G[16]
A[28]	929.203	475.932	470.592	470.928	470.256	A[15]
A[29]	952.882	452.253	446.913	447.249	446.577	A[14]
A[30]	**976.561**	428.574	423.234	423.570	422.898	A[13]
G[31]	**995.568**	404.895	399.555	399.891	399.219	G[12]
K[32]	**1038.266**	385.888	380.548	380.884	380.212	K[11]
R[33]	**1090.300**	343.189	337.850	338.186	337.514	R[10]
K[34]	**1132.998**	291.156	285.816	286.152	285.480	K[9]
A[35]	**1156.677**	248.457	243.118	243.454	242.782	A[8]
S[36]	**1185.688**	224.778	219.439	219.775	219.103	S[7]
G[37]	1204.695	195.767	190.428	190.764	190.092	G[6]
P[38]	**1237.046**	176.760	171.421	171.757	171.085	P[5]
P[39]	1269.397	144.409	139.070	139.406	138.734	P[4]
V[40]	**1302.420**	112.058	106.719	107.055	106.383	V[3]
S[41]	**1331.430**	79.036	73.696	74.032	73.360	S[2]
E[42]	1374.445	50.025	44.685	45.021	44.349	E[1]

sp|P43277|H13_MOUSE

[Acetyl]SETAPAAPAAPAPVEKTPVKKKAKKTGAAAGKRKASGPPVSE

- fragmentation table for charge state 4+
- specType=etd
- score=58.42
- F104901.dat
- query=q2712_p1
- precursor=825.271400
- chargePrecursor=5
- itol=0.8

AA	c	y	z+1	z+2	z	AA
S[1]	37.525	**1031.331**	**1027.327**	0.755	**1027.075**	S[42]
E[2]	69.785	999.071	**995.066**	0.755	**994.814**	E[41]
T[3]	95.047	966.810	**962.805**	0.755	**962.553**	T[40]
A[4]	112.806	941.548	**937.543**	0.755	937.291	A[39]
P[5]	137.070	**923.789**	919.784	0.755	919.532	P[38]
A[6]	154.829	899.526	895.521	0.755	895.269	A[37]
A[7]	172.588	881.766	877.762	0.755	877.510	A[36]
P[8]	196.851	**864.007**	**860.002**	0.755	**859.750**	P[35]
A[9]	214.611	839.744	835.739	0.755	835.487	A[34]
A[10]	232.370	821.985	817.980	0.755	817.728	A[33]
P[11]	256.633	804.225	800.221	0.755	799.969	P[32]
A[12]	274.392	779.962	775.957	0.755	775.705	A[31]
P[13]	298.656	762.203	758.198	0.755	757.946	P[30]
V[14]	323.423	737.940	733.935	0.755	733.683	V[29]
E[15]	355.683	713.173	709.168	0.755	708.916	E[28]
K[16]	387.707	680.912	676.907	677.159	676.655	K[27]
T[17]	**412.969**	648.888	644.883	645.135	644.632	T[26]
P[18]	437.232	623.626	619.622	619.874	619.370	P[25]
V[19]	461.999	599.363	595.358	595.610	595.106	V[24]
K[20]	494.023	574.596	570.591	570.843	570.339	K[23]
K[21]	526.047	542.572	538.568	538.819	538.316	K[22]
K[22]	558.070	510.548	506.544	506.796	506.292	K[21]
A[23]	575.830	478.525	474.520	474.772	474.268	A[20]
K[24]	607.854	460.765	456.761	457.013	456.509	K[19]
K[25]	639.877	428.742	424.737	424.989	424.485	K[18]
T[26]	665.139	396.718	392.713	392.965	392.461	T[17]
G[27]	679.395	371.456	367.451	367.703	367.199	G[16]
A[28]	697.154	357.201	353.196	353.448	352.944	A[15]
A[29]	714.913	339.441	335.437	335.689	335.185	A[14]
A[30]	732.672	321.682	317.677	317.929	317.425	A[13]
G[31]	746.928	303.923	299.918	300.170	299.666	G[12]
K[32]	778.951	289.667	285.663	285.915	285.411	K[11]
R[33]	817.977	257.644	253.639	253.891	253.387	R[10]
K[34]	850.001	218.618	214.614	214.866	214.362	K[9]
A[35]	867.760	186.595	182.590	182.842	182.338	A[8]
S[36]	889.518	168.835	164.831	165.083	164.579	S[7]
G[37]	903.773	147.077	143.073	143.325	142.821	G[6]
P[38]	928.036	132.822	128.817	129.069	128.565	P[5]
P[39]	952.300	108.559	104.554	104.806	104.302	P[4]
V[40]	**977.067**	84.296	80.291	80.543	80.039	V[3]
S[41]	998.825	59.529	55.524	55.776	55.272	S[2]
E[42]	**1031.085**	37.771	33.766	34.018	33.514	E[1]

sp|P22752|H2A1_MOUSE

SHHKAK $^{Acetyl}_{42.01}$ GK

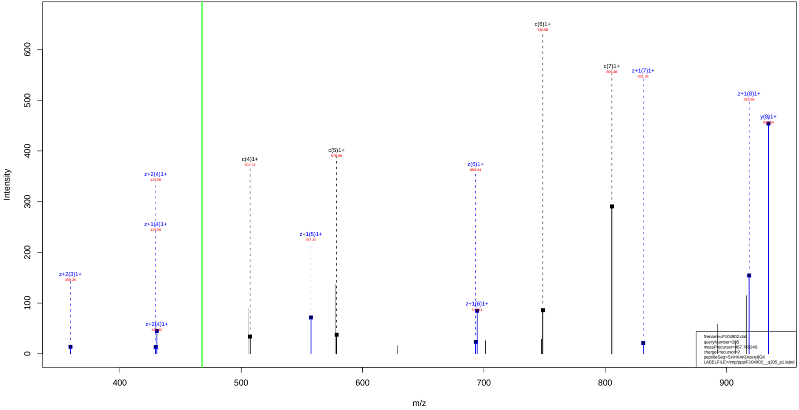

sp|P22752|H2A1_MOUSE

SHHKAK $^{\text{Acetyl}}_{42.01}$ GK

- fragmentation table for charge state 1+
- specType=etd
- score=38.20
- F104902.dat
- query=q205_p1
- precursor=467.765240
- chargePrecursor=2
- itol=0.8

AA	c	y	z+1	z+2	z	AA
S[1]	105.066	**934.522**	**918.503**	0.000	917.495	S[8]
H[2]	242.125	847.490	**831.471**	0.000	830.463	H[7]
H[3]	379.184	710.431	**694.412**	0.000	**693.404**	H[6]
K[4]	**507.279**	573.372	**557.353**	558.361	556.345	K[5]
A[5]	**578.316**	445.277	**429.258**	**430.266**	428.250	A[4]
K[6]	**748.421**	374.240	358.221	**359.229**	357.213	K[3]
G[7]	**805.443**	204.134	188.116	189.123	187.108	G[2]
K[8]	933.538	147.113	131.094	132.102	130.086	K[1]

sp|P62806|H4_MOUSE

[Acetyl]SGRGKGGKGLGKGGAKRHRK $^{Dimethyl}_{28.03}$ VLRD

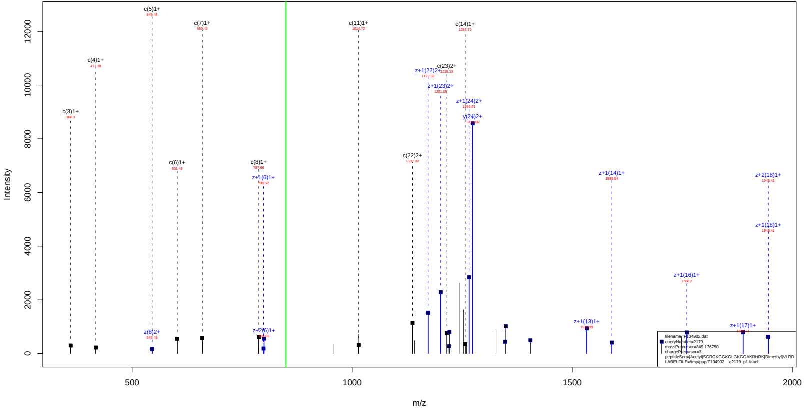

sp|P62806|H4_MOUSE

[Acetyl]SGRGKGGKGLGKGGAKRHRK $^{\text{Dimethyl}}_{28.03}$ VLRD

- fragmentation table for charge state 1+
- specType=etd
- score=65.65
- F104902.dat
- query=q2179_p1
- precursor=849.176750
- chargePrecursor=3
- itol=0.8

AA	c	y	z+1	z+2	z	AA
S[1]	147.076	2545.502	2529.483	0.000	2528.476	S[24]
G[2]	204.098	2416.460	2400.441	0.000	2399.433	G[23]
R[3]	**360.199**	2359.438	2343.419	2344.427	2342.412	R[22]
G[4]	**417.220**	2203.337	2187.318	2188.326	2186.310	G[21]
K[5]	**545.315**	2146.316	2130.297	2131.305	2129.289	K[20]
G[6]	**602.337**	2018.221	2002.202	2003.210	2001.194	G[19]
G[7]	**659.358**	1961.199	**1945.180**	**1946.188**	1944.173	G[18]
K[8]	**787.453**	1904.178	**1888.159**	1889.167	1887.151	K[17]
G[9]	844.475	1776.083	**1760.064**	1761.072	1759.056	G[16]
L[10]	957.559	1719.061	**1703.043**	1704.050	1702.035	L[15]
G[11]	**1014.580**	1605.977	**1589.958**	1590.966	1588.951	G[14]
K[12]	1142.675	1548.956	**1532.937**	1533.945	1531.929	K[13]
G[13]	1199.697	1420.861	**1404.842**	1405.850	1403.834	G[12]
G[14]	**1256.718**	1363.839	**1347.821**	**1348.828**	1346.813	G[11]
A[15]	1327.755	1306.818	1290.799	1291.807	1289.791	A[10]
K[16]	1455.850	1235.781	**1219.762**	**1220.770**	1218.754	K[9]
R[17]	1611.951	1107.686	1091.667	1092.675	1090.659	R[8]
H[18]	1749.010	951.585	935.566	936.574	934.558	H[7]
R[19]	1905.111	814.526	**798.507**	**799.515**	797.499	R[6]
K[20]	2061.238	658.425	642.406	643.414	641.398	K[5]
V[21]	2160.306	502.298	486.280	487.287	485.272	V[4]
L[22]	2273.390	403.230	387.211	388.219	386.203	L[3]
R[23]	2429.491	290.146	274.127	275.135	273.119	R[2]
D[24]	2544.518	134.045	118.026	119.034	117.018	D[1]

sp|P62806|H4_MOUSE

[Acetyl]SGRGKGGKGLGKGGAKRHRK $^{\text{Dimethyl}}_{28.03}$ VLRD

- fragmentation table for charge state 2+
- specType=etd
- score=65.65
- F104902.dat
- query=q2179_p1
- precursor=849.176750
- chargePrecursor=3
- itol=0.8

AA	c	y	z+1	z+2	z	AA
S[1]	74.042	**1273.255**	**1265.245**	0.504	1264.741	S[24]
G[2]	102.553	1208.733	**1200.724**	0.504	1200.220	G[23]
R[3]	180.603	1180.223	**1172.213**	**1172.717**	1171.709	R[22]
G[4]	209.114	1102.172	1094.163	1094.667	1093.659	G[21]
K[5]	273.161	1073.661	1065.652	1066.156	1065.148	K[20]
G[6]	301.672	1009.614	1001.605	1002.108	1001.101	G[19]
G[7]	330.183	981.103	973.094	973.598	972.590	G[18]
K[8]	394.230	952.592	944.583	945.087	944.079	K[17]
G[9]	422.741	888.545	880.536	881.040	880.032	G[16]
L[10]	479.283	860.034	852.025	852.529	851.521	L[15]
G[11]	507.794	803.492	795.483	795.987	794.979	G[14]
K[12]	571.841	774.982	766.972	767.476	766.468	K[13]
G[13]	600.352	710.934	702.925	703.429	702.421	G[12]
G[14]	628.863	682.423	674.414	674.918	673.910	G[11]
A[15]	664.381	653.913	645.903	646.407	645.399	A[10]
K[16]	728.429	618.394	610.385	610.889	609.881	K[9]
R[17]	806.479	554.347	546.337	546.841	**545.833**	R[8]
H[18]	875.009	476.296	468.287	468.791	467.783	H[7]
R[19]	953.059	407.767	399.757	400.261	399.253	R[6]
K[20]	1031.122	329.716	321.707	322.211	321.203	K[5]
V[21]	1080.657	251.653	243.643	244.147	243.140	V[4]
L[22]	**1137.199**	202.119	194.109	194.613	193.605	L[3]
R[23]	**1215.249**	145.577	137.567	138.071	137.063	R[2]
D[24]	1272.763	67.526	59.517	60.021	59.013	D[1]

sp|P62806|H4_MOUSE

[Acetyl]SGRGKGGKGLGKGGAK $^{\text{Acetyl}}_{42.01}$ RHRK $^{\text{Dimethyl}}_{28.03}$ VLRD

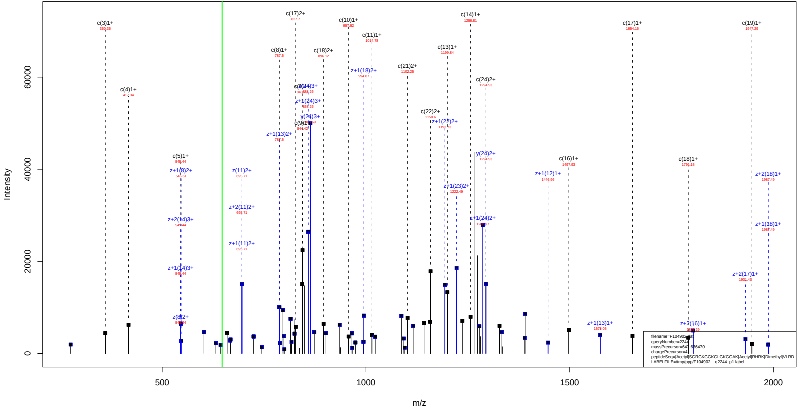

sp|P62806|H4_MOUSE

[Acetyl]SGRGKGGKGLGKGGAK $^{Acetyl}_{42.01}$ RHRK $^{Dimethyl}_{28.03}$ VLRD

- fragmentation table for charge state 1+
- specType=etd
- score=152.26
- F104902.dat
- query=q2244_p1
- precursor=647.636470
- chargePrecursor=4
- itol=0.8

AA	c	y	z+1	z+2	z	AA
S[1]	147.076	2587.513	2571.494	0.000	2570.486	S[24]
G[2]	204.098	2458.470	2442.451	0.000	2441.444	G[23]
R[3]	**360.199**	2401.449	2385.430	2386.438	2384.422	R[22]
G[4]	**417.220**	2245.348	2229.329	2230.337	2228.321	G[21]
K[5]	**545.315**	2188.326	2172.307	2173.315	2171.300	K[20]
G[6]	**602.337**	2060.231	2044.212	2045.220	2043.205	G[19]
G[7]	**659.358**	2003.210	**1987.191**	**1988.199**	1986.183	G[18]
K[8]	**787.453**	1946.188	1930.170	**1931.177**	1929.162	K[17]
G[9]	**844.475**	1818.093	1802.075	**1803.082**	1801.067	G[16]
L[10]	**957.559**	1761.072	1745.053	1746.061	1744.045	L[15]
G[11]	**1014.580**	1647.988	1631.969	1632.977	1630.961	G[14]
K[12]	**1142.675**	1590.966	**1574.948**	1575.955	1573.940	K[13]
G[13]	**1199.697**	1462.871	**1446.853**	1447.860	1445.845	G[12]
G[14]	**1256.718**	1405.850	**1389.831**	**1390.839**	1388.823	G[11]
A[15]	**1327.755**	1348.828	1332.810	**1333.818**	1331.802	A[10]
K[16]	**1497.861**	**1277.791**	1261.773	1262.780	1260.765	K[9]
R[17]	**1653.962**	1107.686	1091.667	**1092.675**	1090.659	R[8]
H[18]	**1791.021**	951.585	**935.566**	936.574	934.558	H[7]
R[19]	**1947.122**	**814.526**	**798.507**	**799.515**	797.499	R[6]
K[20]	2103.248	658.425	642.406	**643.414**	641.398	K[5]
V[21]	2202.317	502.298	486.280	487.287	485.272	V[4]
L[22]	2315.401	403.230	387.211	388.219	386.203	L[3]
R[23]	2471.502	290.146	274.127	**275.135**	273.119	R[2]
D[24]	2586.529	134.045	118.026	119.034	117.018	D[1]

sp|P62806|H4_MOUSE

[Acetyl]SGRGKGGKGLGKGGAK$^{Acetyl}_{42.01}$RHRK$^{Dimethyl}_{28.03}$VLRD

- fragmentation table for charge state 2+
- specType=etd
- score=152.26
- F104902.dat
- query=q2244_p1
- precursor=647.636470
- chargePrecursor=4
- itol=0.8

AA	c	y	z+1	z+2	z	AA
S[1]	74.042	**1294.260**	**1286.251**	0.504	1285.747	S[24]
G[2]	102.553	1229.739	**1221.729**	0.504	1221.225	G[23]
R[3]	180.603	1201.228	**1193.219**	**1193.723**	1192.715	R[22]
G[4]	209.114	1123.177	1115.168	**1115.672**	1114.664	G[21]
K[5]	273.161	**1094.667**	**1086.657**	**1087.161**	1086.153	K[20]
G[6]	301.672	1030.619	**1022.610**	**1023.114**	1022.106	G[19]
G[7]	330.183	1002.108	**994.099**	**994.603**	**993.595**	G[18]
K[8]	394.230	**973.598**	**965.588**	**966.092**	**965.084**	K[17]
G[9]	422.741	909.550	**901.541**	**902.045**	**901.037**	G[16]
L[10]	479.283	881.040	**873.030**	**873.534**	**872.526**	L[15]
G[11]	507.794	**824.498**	816.488	**816.992**	815.984	G[14]
K[12]	571.841	**795.987**	**787.977**	**788.481**	**787.474**	K[13]
G[13]	600.352	731.939	**723.930**	**724.434**	723.426	G[12]
G[14]	628.863	703.429	**695.419**	**695.923**	**694.915**	G[11]
A[15]	664.381	674.918	**666.908**	**667.412**	**666.405**	A[10]
K[16]	749.434	639.399	**631.390**	**631.894**	**630.886**	K[9]
R[17]	**827.485**	554.347	**546.337**	**546.841**	**545.833**	R[8]
H[18]	**896.014**	476.296	468.287	468.791	467.783	H[7]
R[19]	**974.065**	407.767	399.757	400.261	399.253	R[6]
K[20]	1052.128	329.716	321.707	322.211	321.203	K[5]
V[21]	**1101.662**	251.653	243.643	244.147	243.140	V[4]
L[22]	**1158.204**	202.119	194.109	194.613	193.605	L[3]
R[23]	**1236.255**	145.577	137.567	138.071	137.063	R[2]
D[24]	**1293.768**	67.526	59.517	60.021	59.013	D[1]

sp|P62806|H4_MOUSE

[Acetyl]SGRGKGGKGLGKGGAK $^{Acetyl}_{42.01}$ RHRK $^{Dimethyl}_{28.03}$ VLRD

- fragmentation table for charge state 3+
- specType=etd
- score=152.26
- F104902.dat
- query=q2244_p1
- precursor=647.636470
- chargePrecursor=4
- itol=0.8

AA	c	y	z+1	z+2	z	AA
S[1]	49.697	**863.176**	**857.836**	0.672	**857.500**	S[24]
G[2]	68.704	820.162	**814.822**	0.672	**814.486**	G[23]
R[3]	120.738	801.154	**795.815**	**796.151**	**795.479**	R[22]
G[4]	139.745	749.121	**743.781**	**744.117**	743.445	G[21]
K[5]	182.443	730.114	**724.774**	725.110	**724.438**	K[20]
G[6]	201.450	687.415	682.076	682.412	681.740	G[19]
G[7]	220.458	**668.408**	663.069	663.404	662.733	G[18]
K[8]	263.156	649.401	**644.061**	644.397	**643.725**	K[17]
G[9]	282.163	606.703	601.363	**601.699**	601.027	G[16]
L[10]	319.858	587.695	582.356	582.692	582.020	L[15]
G[11]	338.865	550.001	**544.661**	**544.997**	544.325	G[14]
K[12]	381.563	530.994	525.654	525.990	525.318	K[13]
G[13]	400.570	488.295	482.956	483.292	482.620	G[12]
G[14]	419.578	469.288	463.949	464.285	463.613	G[11]
A[15]	443.257	450.281	444.941	445.277	444.605	A[10]
K[16]	499.958	426.602	421.262	421.598	420.926	K[9]
R[17]	551.992	369.900	364.561	364.896	364.225	R[8]
H[18]	597.678	317.866	312.527	312.863	312.191	H[7]
R[19]	649.712	272.180	266.841	267.176	266.505	R[6]
K[20]	701.754	220.146	214.807	215.143	214.471	K[5]
V[21]	734.777	168.104	162.765	163.101	162.429	V[4]
L[22]	772.472	135.082	129.742	130.078	129.406	L[3]
R[23]	**824.505**	97.387	92.047	92.383	91.711	R[2]
D[24]	862.848	45.353	40.014	40.349	39.678	D[1]

sp|P62806|H4_MOUSE

[Acetyl]SGRGKGGKGLGKGGAKRHR $^{(\text{Dimethyl})}_{(28.03)}$ K $^{(\text{Acetyl})}_{(42.01)}$ VLRD

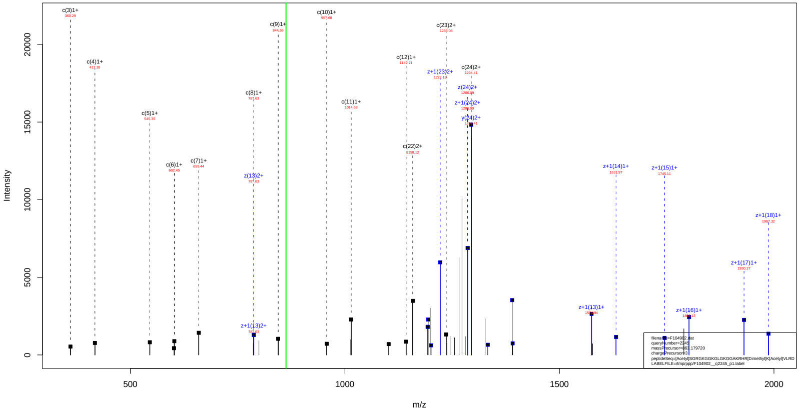

sp|P62806|H4_MOUSE

[Acetyl]SGRGKGGKGLGKGGAKRHR $^{(Dimethyl)}_{(28.03)}$ K $^{(Acetyl)}_{(42.01)}$ VLRD

- fragmentation table for charge state 1+
- specType=etd
- score=66.49
- F104902.dat
- query=q2245_p1
- precursor=863.179720
- chargePrecursor=3
- itol=0.8

AA	c	y	z+1	z+2	z	AA
S[1]	147.076	2587.513	2571.494	0.000	2570.486	S[24]
G[2]	204.098	2458.470	2442.451	0.000	2441.444	G[23]
R[3]	**360.199**	2401.449	2385.430	2386.438	2384.422	R[22]
G[4]	**417.220**	2245.348	2229.329	2230.337	2228.321	G[21]
K[5]	**545.315**	2188.326	2172.307	2173.315	2171.300	K[20]
G[6]	**602.337**	2060.231	2044.212	2045.220	2043.205	G[19]
G[7]	**659.358**	2003.210	**1987.191**	1988.199	1986.183	G[18]
K[8]	**787.453**	1946.188	**1930.170**	1931.177	1929.162	K[17]
G[9]	**844.475**	1818.093	**1802.075**	1803.082	1801.067	G[16]
L[10]	**957.559**	1761.072	**1745.053**	1746.061	1744.045	L[15]
G[11]	**1014.580**	1647.988	**1631.969**	1632.977	1630.961	G[14]
K[12]	**1142.675**	1590.966	**1574.948**	1575.955	1573.940	K[13]
G[13]	1199.697	1462.871	1446.853	1447.860	1445.845	G[12]
G[14]	1256.718	1405.850	**1389.831**	**1390.839**	1388.823	G[11]
A[15]	1327.755	1348.828	**1332.810**	1333.818	1331.802	A[10]
K[16]	1455.850	1277.791	1261.773	1262.780	1260.765	K[9]
R[17]	1611.951	1149.696	1133.678	1134.685	1132.670	R[8]
H[18]	1749.010	993.595	977.577	978.584	976.569	H[7]
R[19]	1933.143	856.536	840.518	841.525	839.510	R[6]
K[20]	2103.248	672.404	656.385	657.393	655.377	K[5]
V[21]	2202.317	502.298	486.280	487.287	485.272	V[4]
L[22]	2315.401	403.230	387.211	388.219	386.203	L[3]
R[23]	2471.502	290.146	274.127	275.135	273.119	R[2]
D[24]	2586.529	134.045	118.026	119.034	117.018	D[1]

sp|P62806|H4_MOUSE

[Acetyl]SGRGKGGKGLGKGGAKRHR $^{(Dimethyl)}_{(28.03)}$ K $^{(Acetyl)}_{(42.01)}$ VLRD

- fragmentation table for charge state 2+
- specType=etd
- score=66.49
- F104902.dat
- query=q2245_p1
- precursor=863.179720
- chargePrecursor=3
- itol=0.8

AA	c	y	z+1	z+2	z	AA
S[1]	74.042	**1294.260**	**1286.251**	0.504	**1285.747**	S[24]
G[2]	102.553	1229.739	**1221.729**	0.504	1221.225	G[23]
R[3]	180.603	**1201.228**	**1193.219**	**1193.723**	**1192.715**	R[22]
G[4]	209.114	1123.177	1115.168	1115.672	1114.664	G[21]
K[5]	273.161	1094.667	1086.657	1087.161	1086.153	K[20]
G[6]	301.672	1030.619	1022.610	1023.114	1022.106	G[19]
G[7]	330.183	1002.108	994.099	994.603	993.595	G[18]
K[8]	394.230	973.598	965.588	966.092	965.084	K[17]
G[9]	422.741	909.550	901.541	902.045	901.037	G[16]
L[10]	479.283	881.040	873.030	873.534	872.526	L[15]
G[11]	507.794	824.498	816.488	816.992	815.984	G[14]
K[12]	571.841	795.987	**787.977**	788.481	**787.474**	K[13]
G[13]	600.352	731.939	723.930	724.434	723.426	G[12]
G[14]	628.863	703.429	695.419	695.923	694.915	G[11]
A[15]	664.381	674.918	666.908	667.412	666.405	A[10]
K[16]	728.429	639.399	631.390	631.894	630.886	K[9]
R[17]	806.479	575.352	567.342	567.846	566.839	R[8]
H[18]	875.009	497.301	489.292	489.796	488.788	H[7]
R[19]	967.075	428.772	420.762	421.266	420.259	R[6]
K[20]	1052.128	336.706	328.696	329.200	328.192	K[5]
V[21]	**1101.662**	251.653	243.643	244.147	243.140	V[4]
L[22]	**1158.204**	202.119	194.109	194.613	193.605	L[3]
R[23]	**1236.255**	145.577	137.567	138.071	137.063	R[2]
D[24]	**1293.768**	67.526	59.517	60.021	59.013	D[1]

sp|P62806|H4_MOUSE

[Acetyl]SGR $^{\text{Dimethyl}}_{28.03}$ GKGGKGLGKGGAK $^{\text{Acetyl}}_{42.01}$ RHRK $^{\text{Dimethyl}}_{28.03}$ VLRD

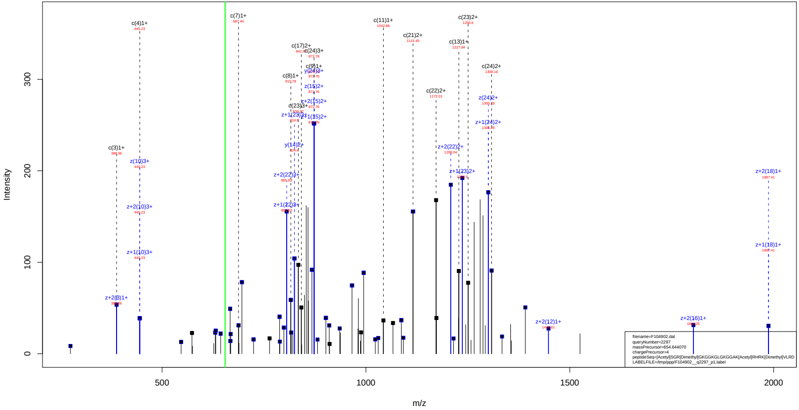

sp|P62806|H4_MOUSE

[Acetyl]SGR $^{\text{Dimethyl}}_{28.03}$ GKGGKGLGKGGAK $^{\text{Acetyl}}_{42.01}$ RHRK $^{\text{Dimethyl}}_{28.03}$ VLRD

- fragmentation table for charge state 1+
- specType=etd
- score=94.91
- F104902.dat
- query=q2297_p1
- precursor=654.644070
- chargePrecursor=4
- itol=0.8

AA	c	y	z+1	z+2	z	AA
S[1]	147.076	2615.544	2599.525	0.000	2598.518	S[24]
G[2]	204.098	2486.501	2470.483	0.000	2469.475	G[23]
R[3]	**388.230**	2429.480	2413.461	2414.469	2412.453	R[22]
G[4]	**445.252**	2245.348	2229.329	2230.337	2228.321	G[21]
K[5]	**573.347**	2188.326	2172.307	2173.315	2171.300	K[20]
G[6]	**630.368**	2060.231	2044.212	2045.220	2043.205	G[19]
G[7]	**687.390**	2003.210	**1987.191**	**1988.199**	1986.183	G[18]
K[8]	**815.485**	1946.188	1930.170	1931.177	1929.162	K[17]
G[9]	**872.506**	1818.093	1802.075	**1803.082**	1801.067	G[16]
L[10]	985.590	1761.072	1745.053	1746.061	1744.045	L[15]
G[11]	**1042.612**	1647.988	1631.969	1632.977	1630.961	G[14]
K[12]	1170.707	1590.966	1574.948	1575.955	1573.940	K[13]
G[13]	**1227.728**	1462.871	1446.853	**1447.860**	1445.845	G[12]
G[14]	1284.749	1405.850	1389.831	**1390.839**	1388.823	G[11]
A[15]	1355.787	1348.828	1332.810	**1333.818**	1331.802	A[10]
K[16]	1525.892	1277.791	1261.773	1262.780	1260.765	K[9]
R[17]	1681.993	1107.686	**1091.667**	1092.675	1090.659	R[8]
H[18]	1819.052	951.585	**935.566**	936.574	934.558	H[7]
R[19]	1975.153	814.526	**798.507**	**799.515**	797.499	R[6]
K[20]	2131.280	658.425	642.406	**643.414**	641.398	K[5]
V[21]	2230.348	502.298	486.280	487.287	485.272	V[4]
L[22]	2343.432	403.230	387.211	**388.219**	386.203	L[3]
R[23]	2499.533	290.146	274.127	**275.135**	273.119	R[2]
D[24]	2614.560	134.045	118.026	119.034	117.018	D[1]

sp|P62806|H4_MOUSE

[Acetyl]SGR $^{\text{Dimethyl}}_{28.03}$ GKGGKGLGKGGAK $^{\text{Acetyl}}_{42.01}$ RHRK $^{\text{Dimethyl}}_{28.03}$ VLRD

- fragmentation table for charge state 2+
- specType=etd
- score=94.91
- F104902.dat
- query=q2297_p1
- precursor=654.644070
- chargePrecursor=4
- itol=0.8

AA	c	y	z+1	z+2	z	AA
S[1]	74.042	**1308.276**	**1300.266**	0.504	**1299.762**	S[24]
G[2]	102.553	1243.754	**1235.745**	0.504	1235.241	G[23]
R[3]	194.619	**1215.244**	1207.234	**1207.738**	1206.730	R[22]
G[4]	223.130	1123.177	**1115.168**	**1115.672**	**1114.664**	G[21]
K[5]	287.177	1094.667	**1086.657**	**1087.161**	**1086.153**	K[20]
G[6]	315.688	**1030.619**	**1022.610**	**1023.114**	1022.106	G[19]
G[7]	344.198	1002.108	**994.099**	**994.603**	**993.595**	G[18]
K[8]	408.246	973.598	**965.588**	**966.092**	**965.084**	K[17]
G[9]	436.757	**909.550**	**901.541**	**902.045**	**901.037**	G[16]
L[10]	493.299	**881.040**	**873.030**	**873.534**	**872.526**	L[15]
G[11]	521.809	**824.498**	**816.488**	**816.992**	**815.984**	G[14]
K[12]	585.857	795.987	**787.977**	**788.481**	**787.474**	K[13]
G[13]	614.368	731.939	**723.930**	**724.434**	723.426	G[12]
G[14]	**642.878**	703.429	**695.419**	**695.923**	694.915	G[11]
A[15]	678.397	674.918	**666.908**	**667.412**	**666.405**	A[10]
K[16]	**763.450**	639.399	**631.390**	**631.894**	**630.886**	K[9]
R[17]	**841.500**	554.347	**546.337**	**546.841**	545.833	R[8]
H[18]	**910.030**	476.296	468.287	468.791	467.783	H[7]
R[19]	**988.080**	407.767	399.757	400.261	399.253	R[6]
K[20]	**1066.143**	329.716	321.707	322.211	321.203	K[5]
V[21]	**1115.678**	251.653	243.643	244.147	243.140	V[4]
L[22]	**1172.220**	202.119	194.109	194.613	193.605	L[3]
R[23]	**1250.270**	145.577	137.567	138.071	137.063	R[2]
D[24]	**1307.784**	67.526	59.517	60.021	59.013	D[1]

sp|P62806|H4_MOUSE

[Acetyl]SGR $^{\text{Dimethyl}}_{28.03}$ GKGGKGLGKGGAK $^{\text{Acetyl}}_{42.01}$ RHRK $^{\text{Dimethyl}}_{28.03}$ VLRD

- fragmentation table for charge state 3+
- specType=etd
- score=94.91
- F104902.dat
- query=q2297_p1
- precursor=654.644070
- chargePrecursor=4
- itol=0.8

AA	c	y	z+1	z+2	z	AA
S[1]	49.697	**872.520**	**867.180**	0.672	**866.844**	S[24]
G[2]	68.704	829.505	**824.166**	0.672	**823.830**	G[23]
R[3]	130.082	810.498	**805.159**	**805.495**	804.823	R[22]
G[4]	149.089	749.121	743.781	744.117	743.445	G[21]
K[5]	191.787	730.114	**724.774**	**725.110**	**724.438**	K[20]
G[6]	210.794	**687.415**	682.076	682.412	681.740	G[19]
G[7]	229.801	**668.408**	663.069	663.404	662.733	G[18]
K[8]	272.500	649.401	**644.061**	**644.397**	**643.725**	K[17]
G[9]	291.507	606.703	601.363	601.699	601.027	G[16]
L[10]	329.202	587.695	582.356	582.692	582.020	L[15]
G[11]	348.209	550.001	544.661	544.997	544.325	G[14]
K[12]	390.907	530.994	525.654	525.990	525.318	K[13]
G[13]	409.914	488.295	482.956	483.292	482.620	G[12]
G[14]	428.921	469.288	463.949	464.285	463.613	G[11]
A[15]	452.600	450.281	**444.941**	**445.277**	**444.605**	A[10]
K[16]	509.302	426.602	421.262	421.598	420.926	K[9]
R[17]	561.336	369.900	364.561	364.896	364.225	R[8]
H[18]	607.022	317.866	312.527	312.863	312.191	H[7]
R[19]	659.056	272.180	266.841	267.176	266.505	R[6]
K[20]	711.098	220.146	214.807	215.143	214.471	K[5]
V[21]	744.121	168.104	162.765	163.101	162.429	V[4]
L[22]	781.816	135.082	129.742	130.078	129.406	L[3]
R[23]	**833.849**	97.387	92.047	92.383	91.711	R[2]
D[24]	**872.192**	45.353	40.014	40.349	39.678	D[1]

sp|Q6ZWY9|H2B1C_MOUSE

PEPAKSAPAPKK $^{Acetyl}_{42.01}$ GSKKAVTKAQKKD

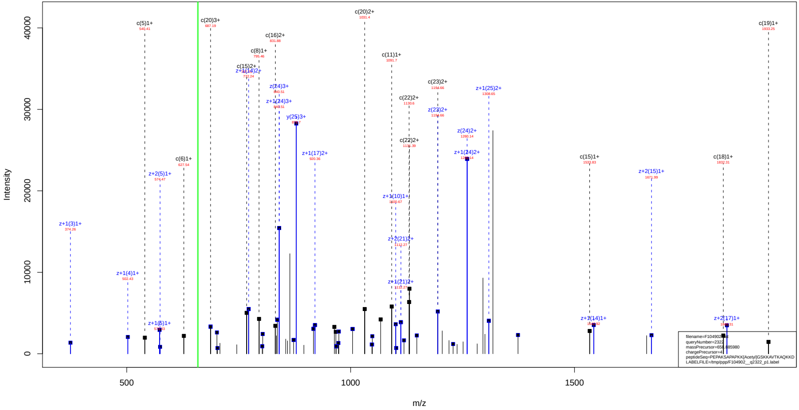

sp|Q6ZWY9|H2B1C_MOUSE

PEPAKSAPAPKK $^{Acetyl}_{42.01}$ GSKKAVTKAQKKD

- fragmentation table for charge state 1+
- specType=etd
- score=82.24
- F104902.dat
- query=q2322_p1
- precursor=658.885980
- chargePrecursor=4
- itol=0.8

AA	c	y	z+1	z+2	z	AA
P[1]	115.087	2632.514	2616.496	0.000	2615.488	P[25]
E[2]	244.129	2535.462	2519.443	0.000	2518.435	E[24]
P[3]	341.182	2406.419	2390.400	0.000	2389.393	P[23]
A[4]	412.219	2309.366	2293.348	0.000	2292.340	A[22]
K[5]	**540.314**	2238.329	2222.311	2223.318	2221.303	K[21]
S[6]	**627.346**	2110.234	2094.216	2095.223	2093.208	S[20]
A[7]	698.383	2023.202	2007.184	2008.191	2006.176	A[19]
P[8]	**795.436**	1952.165	1936.146	1937.154	1935.139	P[18]
A[9]	866.473	1855.112	1839.094	**1840.101**	1838.086	A[17]
P[10]	**963.526**	1784.075	1768.057	1769.064	1767.049	P[16]
K[11]	**1091.621**	1687.022	1671.004	**1672.012**	1669.996	K[15]
K[12]	1261.726	1558.928	**1542.909**	1543.917	1541.901	K[14]
G[13]	1318.748	1388.822	1372.803	**1373.811**	1371.795	G[13]
S[14]	1405.780	1331.801	1315.782	1316.790	1314.774	S[12]
K[15]	**1533.875**	1244.768	**1228.750**	1229.758	1227.742	K[11]
K[16]	1661.970	1116.674	**1100.655**	**1101.663**	1099.647	K[10]
A[17]	1733.007	988.579	**972.560**	**973.568**	971.552	A[9]
V[18]	**1832.075**	**917.541**	901.523	902.531	900.515	V[8]
T[19]	**1933.123**	818.473	**802.454**	**803.462**	801.446	T[7]
K[20]	2061.218	717.425	**701.407**	**702.414**	700.399	K[6]
A[21]	2132.255	589.330	**573.312**	**574.320**	572.304	A[5]
Q[22]	2260.314	518.293	**502.275**	503.282	501.267	Q[4]
K[23]	2388.409	390.235	**374.216**	375.224	373.208	K[3]
K[24]	2516.504	262.140	246.121	247.129	245.113	K[2]
D[25]	2631.530	134.045	118.026	119.034	117.018	D[1]

sp|Q6ZWY9|H2B1C_MOUSE

PEPAKSAPAPKK $^{Acetyl}_{42.01}$ GSKKAVTKAQKKD

- fragmentation table for charge state 2+
- specType=etd
- score=82.24
- F104902.dat
- query=q2322_p1
- precursor=658.885980
- chargePrecursor=4
- itol=0.8

AA	c	y	z+1	z+2	z	AA
P[1]	58.047	1316.761	**1308.752**	0.504	**1308.248**	P[25]
E[2]	122.568	1268.234	**1260.225**	0.504	**1259.721**	E[24]
P[3]	171.095	1203.713	1195.704	0.504	**1195.200**	P[23]
A[4]	206.613	1155.187	**1147.177**	0.504	1146.674	A[22]
K[5]	270.661	**1119.668**	**1111.659**	**1112.163**	1111.155	K[21]
S[6]	314.177	1055.621	**1047.611**	**1048.115**	**1047.107**	S[20]
A[7]	349.695	1012.105	**1004.095**	**1004.599**	**1003.591**	A[19]
P[8]	398.222	976.586	**968.577**	969.081	**968.073**	P[18]
A[9]	433.740	928.060	**920.050**	**920.554**	919.547	A[17]
P[10]	482.267	892.541	884.532	885.036	884.028	P[16]
K[11]	546.314	844.015	**836.006**	**836.509**	**835.502**	K[15]
K[12]	631.367	779.967	**771.958**	**772.462**	**771.454**	K[14]
G[13]	659.878	694.915	**686.905**	**687.409**	**686.401**	G[13]
S[14]	703.394	666.404	658.395	658.898	657.891	S[12]
K[15]	**767.441**	622.888	614.879	615.382	614.375	K[11]
K[16]	**831.488**	558.840	550.831	551.335	550.327	K[10]
A[17]	867.007	494.793	486.784	487.287	486.280	A[9]
V[18]	**916.541**	459.274	451.265	451.769	450.761	V[8]
T[19]	**967.065**	409.740	401.731	402.235	401.227	T[7]
K[20]	**1031.113**	359.216	351.207	351.711	350.703	K[6]
A[21]	**1066.631**	295.169	287.159	287.663	286.656	A[5]
Q[22]	**1130.660**	259.650	251.641	252.145	251.137	Q[4]
K[23]	**1194.708**	195.621	187.612	188.116	187.108	K[3]
K[24]	1258.755	131.574	123.564	124.068	123.060	K[2]
D[25]	1316.269	67.526	59.517	60.021	59.013	D[1]

sp|Q6ZWY9|H2B1C_MOUSE

PEPAKSAPAPKK $^{Acetyl}_{42.01}$ GSKKAVTKAQKKD

- fragmentation table for charge state 3+
- specType=etd
- score=82.24
- F104902.dat
- query=q2322_p1
- precursor=658.885980
- chargePrecursor=4
- itol=0.8

AA	c	y	z+1	z+2	z	AA
P[1]	39.034	**878.176**	**872.837**	0.672	**872.501**	P[25]
E[2]	82.048	845.825	**840.486**	0.672	**840.150**	E[24]
P[3]	114.399	**802.811**	797.472	0.672	797.136	P[23]
A[4]	138.078	770.460	765.121	0.672	764.785	A[22]
K[5]	180.776	746.781	741.442	741.778	741.106	K[21]
S[6]	209.787	704.083	698.743	699.079	698.407	S[20]
A[7]	233.466	675.072	669.733	670.069	669.397	A[19]
P[8]	265.817	651.393	646.054	646.390	645.718	P[18]
A[9]	289.496	619.042	613.703	614.039	613.367	A[17]
P[10]	321.847	595.363	590.024	590.360	589.688	P[16]
K[11]	364.545	563.012	557.673	558.009	557.337	K[15]
K[12]	421.247	520.314	514.974	515.310	514.639	K[14]
G[13]	440.254	463.612	458.273	458.609	457.937	G[13]
S[14]	469.265	444.605	439.265	439.601	438.930	S[12]
K[15]	511.963	415.594	410.255	410.591	409.919	K[11]
K[16]	554.661	372.896	367.556	367.892	367.221	K[10]
A[17]	578.340	330.198	324.858	325.194	324.522	A[9]
V[18]	611.363	306.519	301.179	301.515	300.843	V[8]
T[19]	645.046	273.496	268.156	268.492	267.820	T[7]
K[20]	**687.744**	239.813	234.474	234.810	234.138	K[6]
A[21]	711.423	197.115	191.775	192.111	191.439	A[5]
Q[22]	754.109	173.436	168.096	168.432	167.760	Q[4]
K[23]	796.808	130.750	125.410	125.746	125.074	K[3]
K[24]	839.506	88.051	82.712	83.048	82.376	K[2]
D[25]	877.848	45.353	40.014	40.349	39.678	D[1]

sp|Q6ZWY9|H2B1C_MOUSE

PEPAKSAPAPKK $^{\text{Acetyl}}_{42.01}$ GSKKAVTKAQKKD

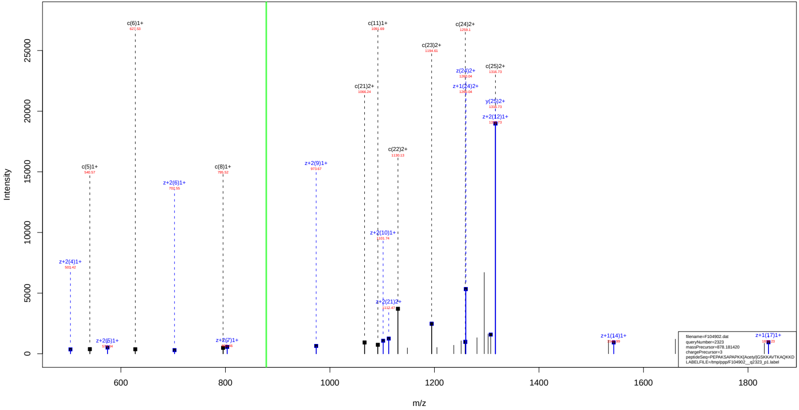

sp|Q6ZWY9|H2B1C_MOUSE

PEPAKSAPAPKK $^{Acetyl}_{42.01}$ GSKKAVTKAQKKD

- fragmentation table for charge state 1+
- specType=etd
- score=46.32
- F104902.dat
- query=q2323_p1
- precursor=878.181420
- chargePrecursor=3
- itol=0.8

AA	c	y	z+1	z+2	z	AA
P[1]	115.087	2632.514	2616.496	0.000	2615.488	P[25]
E[2]	244.129	2535.462	2519.443	0.000	2518.435	E[24]
P[3]	341.182	2406.419	2390.400	0.000	2389.393	P[23]
A[4]	412.219	2309.366	2293.348	0.000	2292.340	A[22]
K[5]	**540.314**	2238.329	2222.311	2223.318	2221.303	K[21]
S[6]	**627.346**	2110.234	2094.216	2095.223	2093.208	S[20]
A[7]	698.383	2023.202	2007.184	2008.191	2006.176	A[19]
P[8]	**795.436**	1952.165	1936.146	1937.154	1935.139	P[18]
A[9]	866.473	1855.112	**1839.094**	1840.101	1838.086	A[17]
P[10]	963.526	1784.075	1768.057	1769.064	1767.049	P[16]
K[11]	**1091.621**	1687.022	1671.004	1672.012	1669.996	K[15]
K[12]	1261.726	1558.928	**1542.909**	1543.917	1541.901	K[14]
G[13]	1318.748	1388.822	1372.803	1373.811	1371.795	G[13]
S[14]	1405.780	1331.801	1315.782	**1316.790**	1314.774	S[12]
K[15]	1533.875	1244.768	1228.750	1229.758	1227.742	K[11]
K[16]	1661.970	1116.674	1100.655	**1101.663**	1099.647	K[10]
A[17]	1733.007	988.579	972.560	**973.568**	971.552	A[9]
V[18]	1832.075	917.541	901.523	902.531	900.515	V[8]
T[19]	1933.123	818.473	802.454	**803.462**	801.446	T[7]
K[20]	2061.218	717.425	701.407	**702.414**	700.399	K[6]
A[21]	2132.255	589.330	573.312	**574.320**	572.304	A[5]
Q[22]	2260.314	518.293	502.275	**503.282**	501.267	Q[4]
K[23]	2388.409	390.235	374.216	375.224	373.208	K[3]
K[24]	2516.504	262.140	246.121	247.129	245.113	K[2]
D[25]	2631.530	134.045	118.026	119.034	117.018	D[1]

sp|Q6ZWY9|H2B1C_MOUSE

PEPAKSAPAPKK $^{Acetyl}_{42.01}$ GSKKAVTKAQKKD

- fragmentation table for charge state 2+
- specType=etd
- score=46.32
- F104902.dat
- query=q2323_p1
- precursor=878.181420
- chargePrecursor=3
- itol=0.8

AA	c	y	z+1	z+2	z	AA
P[1]	58.047	**1316.761**	1308.752	0.504	**1308.248**	P[25]
E[2]	122.568	1268.234	**1260.225**	0.504	**1259.721**	E[24]
P[3]	171.095	1203.713	1195.704	0.504	**1195.200**	P[23]
A[4]	206.613	1155.187	1147.177	0.504	1146.674	A[22]
K[5]	270.661	1119.668	1111.659	**1112.163**	1111.155	K[21]
S[6]	314.177	1055.621	1047.611	1048.115	1047.107	S[20]
A[7]	349.695	1012.105	1004.095	1004.599	1003.591	A[19]
P[8]	398.222	976.586	968.577	969.081	968.073	P[18]
A[9]	433.740	928.060	920.050	920.554	919.547	A[17]
P[10]	482.267	892.541	884.532	885.036	884.028	P[16]
K[11]	546.314	844.015	836.006	836.509	835.502	K[15]
K[12]	631.367	779.967	771.958	772.462	771.454	K[14]
G[13]	659.878	694.915	686.905	687.409	686.401	G[13]
S[14]	703.394	666.404	658.395	658.898	657.891	S[12]
K[15]	767.441	622.888	614.879	615.382	614.375	K[11]
K[16]	831.488	558.840	550.831	551.335	550.327	K[10]
A[17]	867.007	494.793	486.784	487.287	486.280	A[9]
V[18]	916.541	459.274	451.265	451.769	450.761	V[8]
T[19]	967.065	409.740	401.731	402.235	401.227	T[7]
K[20]	1031.113	359.216	351.207	351.711	350.703	K[6]
A[21]	**1066.631**	295.169	287.159	287.663	286.656	A[5]
Q[22]	**1130.660**	259.650	251.641	252.145	251.137	Q[4]
K[23]	**1194.708**	195.621	187.612	188.116	187.108	K[3]
K[24]	**1258.755**	131.574	123.564	124.068	123.060	K[2]
D[25]	**1316.269**	67.526	59.517	60.021	59.013	D[1]

sp|Q64525|H2B2B_MOUSE

PDPAKSAPAPKKGSKKAVTK $^{Acetyl}_{42.01}$ VQKKD

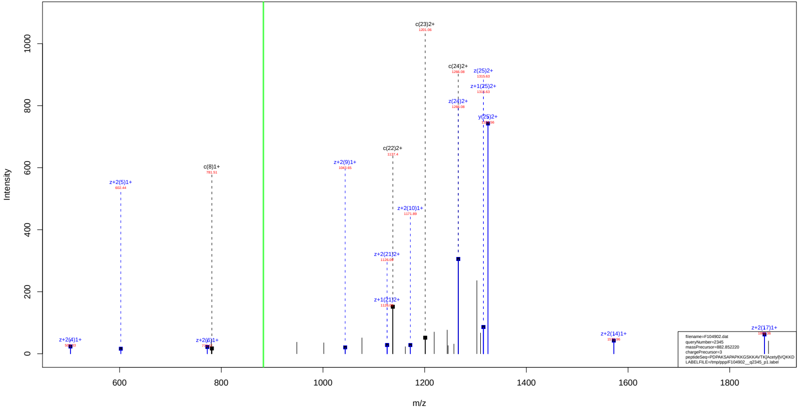

sp|Q64525|H2B2B_MOUSE

PDPAKSAPAPKKGSKKAVTK $^{Acetyl}_{42.01}$ VQKKD

- fragmentation table for charge state 1+
- specType=etd
- score=32.43
- F104902.dat
- query=q2345_p1
- precursor=882.852220
- chargePrecursor=3
- itol=0.8

AA	c	y	z+1	z+2	z	AA
P[1]	115.087	2646.530	2630.511	0.000	2629.504	P[25]
D[2]	230.114	2549.477	2533.459	0.000	2532.451	D[24]
P[3]	327.166	2434.450	2418.432	0.000	2417.424	P[23]
A[4]	398.203	2337.398	2321.379	0.000	2320.371	A[22]
K[5]	526.298	2266.361	2250.342	2251.350	2249.334	K[21]
S[6]	613.330	2138.266	2122.247	2123.255	2121.239	S[20]
A[7]	684.368	2051.234	2035.215	2036.223	2034.207	A[19]
P[8]	**781.420**	1980.196	1964.178	1965.186	1963.170	P[18]
A[9]	852.457	1883.144	1867.125	**1868.133**	1866.117	A[17]
P[10]	949.510	1812.107	1796.088	1797.096	1795.080	P[16]
K[11]	1077.605	1715.054	1699.035	1700.043	1698.027	K[15]
K[12]	1205.700	1586.959	1570.940	**1571.948**	1569.932	K[14]
G[13]	1262.722	1458.864	1442.845	1443.853	1441.837	G[13]
S[14]	1349.754	1401.842	1385.824	1386.831	1384.816	S[12]
K[15]	1477.849	1314.810	1298.792	1299.799	1297.784	K[11]
K[16]	1605.944	1186.715	1170.697	**1171.705**	1169.689	K[10]
A[17]	1676.981	1058.620	1042.602	**1043.610**	1041.594	A[9]
V[18]	1776.049	987.583	971.565	972.572	970.557	V[8]
T[19]	1877.097	888.515	872.496	873.504	871.488	T[7]
K[20]	2047.202	787.467	771.449	**772.456**	770.441	K[6]
V[21]	2146.271	617.362	601.343	**602.351**	600.335	V[5]
Q[22]	2274.329	518.293	502.275	**503.282**	501.267	Q[4]
K[23]	2402.424	390.235	374.216	375.224	373.208	K[3]
K[24]	2530.519	262.140	246.121	247.129	245.113	K[2]
D[25]	2645.546	134.045	118.026	119.034	117.018	D[1]

sp|Q64525|H2B2B_MOUSE

PDPAKSAPAPKKGSKKAVTK $^{Acetyl}_{42.01}$ VQKKD

- fragmentation table for charge state 2+
- specType=etd
- score=32.43
- F104902.dat
- query=q2345_p1
- precursor=882.852220
- chargePrecursor=3
- itol=0.8

AA	c	y	z+1	z+2	z	AA
P[1]	58.047	**1323.769**	**1315.759**	0.504	**1315.255**	P[25]
D[2]	115.560	1275.242	1267.233	0.504	**1266.729**	D[24]
P[3]	164.087	1217.729	1209.719	0.504	1209.216	P[23]
A[4]	199.605	1169.202	1161.193	0.504	1160.689	A[22]
K[5]	263.653	1133.684	**1125.675**	**1126.178**	1125.171	K[21]
S[6]	307.169	1069.636	1061.627	1062.131	1061.123	S[20]
A[7]	342.687	1026.120	1018.111	1018.615	1017.607	A[19]
P[8]	391.214	990.602	982.592	983.096	982.089	P[18]
A[9]	426.732	942.075	934.066	934.570	933.562	A[17]
P[10]	475.259	906.557	898.548	899.051	898.044	P[16]
K[11]	539.306	858.031	850.021	850.525	849.517	K[15]
K[12]	603.354	793.983	785.974	786.478	785.470	K[14]
G[13]	631.864	729.936	721.926	722.430	721.422	G[13]
S[14]	675.380	701.425	693.415	693.919	692.912	S[12]
K[15]	739.428	657.909	649.899	650.403	649.396	K[11]
K[16]	803.475	593.861	585.852	586.356	585.348	K[10]
A[17]	838.994	529.814	521.804	522.308	521.301	A[9]
V[18]	888.528	494.295	486.286	486.790	485.782	V[8]
T[19]	939.052	444.761	436.752	437.256	436.248	T[7]
K[20]	1024.105	394.237	386.228	386.732	385.724	K[6]
V[21]	1073.639	309.184	301.175	301.679	300.671	V[5]
Q[22]	**1137.668**	259.650	251.641	252.145	251.137	Q[4]
K[23]	**1201.716**	195.621	187.612	188.116	187.108	K[3]
K[24]	**1265.763**	131.574	123.564	124.068	123.060	K[2]
D[25]	1323.277	67.526	59.517	60.021	59.013	D[1]

sp|P62806|H4_MOUSE

[Acetyl]S $^{Phospho}_{79.97}$ GRGKGGKGLGKGGAK $^{Acetyl}_{42.01}$ RHRK $^{Dimethyl}_{28.03}$ VLRD

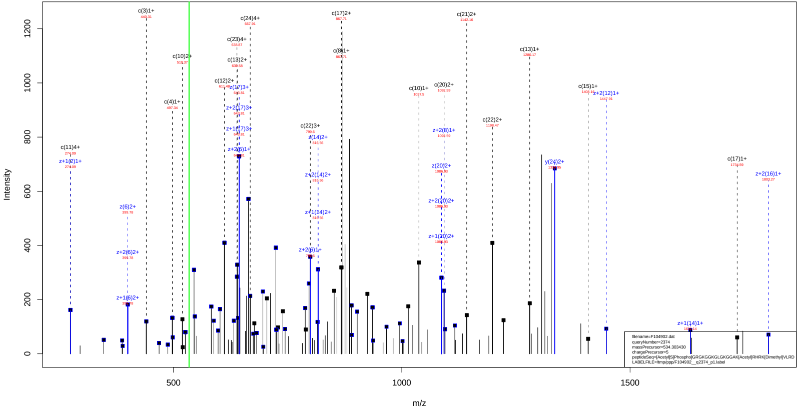

sp|P62806|H4_MOUSE

[Acetyl]S $^{Phospho}_{79.97}$ GRGKGGKGLGKGGAK $^{Acetyl}_{42.01}$ RHRK $^{Dimethyl}_{28.03}$ VLRD

- fragmentation table for charge state 1+
- specType=etd
- score=74.22
- F104902.dat
- query=q2374_p1
- precursor=534.303430
- chargePrecursor=5
- itol=0.8

AA	c	y	z+1	z+2	z	AA
S[1]	227.043	2667.479	2651.460	0.000	2650.453	S[24]
G[2]	284.064	2458.470	2442.451	0.000	2441.444	G[23]
R[3]	**440.165**	2401.449	2385.430	2386.438	2384.422	R[22]
G[4]	**497.187**	2245.348	2229.329	2230.337	2228.321	G[21]
K[5]	625.282	2188.326	2172.307	2173.315	2171.300	K[20]
G[6]	**682.303**	2060.231	2044.212	2045.220	2043.205	G[19]
G[7]	**739.325**	2003.210	1987.191	1988.199	1986.183	G[18]
K[8]	**867.420**	1946.188	1930.170	1931.177	1929.162	K[17]
G[9]	**924.441**	1818.093	1802.075	**1803.082**	1801.067	G[16]
L[10]	**1037.525**	1761.072	1745.053	1746.061	1744.045	L[15]
G[11]	**1094.547**	1647.988	**1631.969**	1632.977	1630.961	G[14]
K[12]	**1222.642**	1590.966	1574.948	1575.955	1573.940	K[13]
G[13]	**1279.663**	1462.871	1446.853	**1447.860**	1445.845	G[12]
G[14]	1336.685	1405.850	1389.831	1390.839	1388.823	G[11]
A[15]	**1407.722**	1348.828	1332.810	1333.818	1331.802	A[10]
K[16]	1577.827	1277.791	1261.773	1262.780	1260.765	K[9]
R[17]	**1733.928**	1107.686	1091.667	**1092.675**	1090.659	R[8]
H[18]	1870.987	951.585	**935.566**	**936.574**	934.558	H[7]
R[19]	2027.088	814.526	798.507	**799.515**	**797.499**	R[6]
K[20]	2183.215	658.425	642.406	**643.414**	**641.398**	K[5]
V[21]	2282.283	502.298	486.280	**487.287**	485.272	V[4]
L[22]	2395.367	403.230	**387.211**	**388.219**	386.203	L[3]
R[23]	2551.468	290.146	**274.127**	275.135	273.119	R[2]
D[24]	2666.495	134.045	118.026	119.034	117.018	D[1]

sp|P62806|H4_MOUSE

[Acetyl]S $^{Phospho}_{79.97}$ GRGKGGKGLGKGGAK $^{Acetyl}_{42.01}$ RHRK $^{Dimethyl}_{28.03}$ VLRD

- fragmentation table for charge state 2+
- specType=etd
- score=74.22
- F104902.dat
- query=q2374_p1
- precursor=534.303430
- chargePrecursor=5
- itol=0.8

AA	c	y	z+1	z+2	z	AA
S[1]	114.025	**1334.243**	1326.234	0.504	1325.730	S[24]
G[2]	142.536	1229.739	1221.729	0.504	1221.225	G[23]
R[3]	220.586	1201.228	1193.219	1193.723	1192.715	R[22]
G[4]	249.097	1123.177	1115.168	**1115.672**	1114.664	G[21]
K[5]	313.145	**1094.667**	**1086.657**	**1087.161**	**1086.153**	K[20]
G[6]	341.655	1030.619	1022.610	1023.114	1022.106	G[19]
G[7]	370.166	**1002.108**	994.099	**994.603**	993.595	G[18]
K[8]	434.213	973.598	965.588	**966.092**	965.084	K[17]
G[9]	462.724	909.550	**901.541**	**902.045**	901.037	G[16]
L[10]	**519.266**	881.040	873.030	873.534	872.526	L[15]
G[11]	547.777	824.498	**816.488**	**816.992**	**815.984**	G[14]
K[12]	**611.824**	**795.987**	**787.977**	**788.481**	787.474	K[13]
G[13]	**640.335**	731.939	**723.930**	**724.434**	**723.426**	G[12]
G[14]	668.846	703.429	**695.419**	**695.923**	694.915	G[11]
A[15]	**704.364**	**674.918**	666.908	**667.412**	666.405	A[10]
K[16]	**789.417**	**639.399**	**631.390**	**631.894**	630.886	K[9]
R[17]	**867.468**	554.347	**546.337**	**546.841**	545.833	R[8]
H[18]	**935.997**	476.296	**468.287**	**468.791**	**467.783**	H[7]
R[19]	**1014.048**	407.767	**399.757**	**400.261**	**399.253**	R[6]
K[20]	**1092.111**	329.716	321.707	322.211	321.203	K[5]
V[21]	**1141.645**	251.653	243.643	244.147	243.140	V[4]
L[22]	**1198.187**	202.119	194.109	194.613	193.605	L[3]
R[23]	1276.238	145.577	137.567	138.071	137.063	R[2]
D[24]	1333.751	67.526	59.517	60.021	59.013	D[1]

sp|P62806|H4_MOUSE

[Acetyl]S $^{Phospho}_{79.97}$ GRGKGGKGLGKGGAK $^{Acetyl}_{42.01}$ RHRK $^{Dimethyl}_{28.03}$ VLRD

- fragmentation table for charge state 3+
- specType=etd
- score=74.22
- F104902.dat
- query=q2374_p1
- precursor=534.303430
- chargePrecursor=5
- itol=0.8

AA	c	y	z+1	z+2	z	AA
S[1]	76.352	**889.831**	884.492	0.672	884.156	S[24]
G[2]	95.360	820.162	**814.822**	0.672	814.486	G[23]
R[3]	147.393	801.154	795.815	**796.151**	795.479	R[22]
G[4]	166.400	749.121	**743.781**	**744.117**	743.445	G[21]
K[5]	209.099	730.114	**724.774**	**725.110**	**724.438**	K[20]
G[6]	228.106	687.415	**682.076**	**682.412**	**681.740**	G[19]
G[7]	247.113	**668.408**	663.069	**663.404**	662.733	G[18]
K[8]	289.811	649.401	**644.061**	**644.397**	**643.725**	K[17]
G[9]	308.819	606.703	**601.363**	**601.699**	**601.027**	G[16]
L[10]	**346.513**	**587.695**	**582.356**	**582.692**	**582.020**	L[15]
G[11]	365.520	550.001	**544.661**	**544.997**	**544.325**	G[14]
K[12]	408.219	530.994	**525.654**	**525.990**	**525.318**	K[13]
G[13]	427.226	**488.295**	482.956	483.292	482.620	G[12]
G[14]	446.233	469.288	463.949	464.285	463.613	G[11]
A[15]	469.912	450.281	444.941	445.277	444.605	A[10]
K[16]	**526.614**	426.602	421.262	421.598	420.926	K[9]
R[17]	578.648	369.900	364.561	364.896	364.225	R[8]
H[18]	624.334	317.866	312.527	312.863	312.191	H[7]
R[19]	**676.368**	272.180	266.841	267.176	266.505	R[6]
K[20]	**728.410**	220.146	214.807	215.143	214.471	K[5]
V[21]	761.433	168.104	162.765	163.101	162.429	V[4]
L[22]	**799.127**	135.082	129.742	130.078	129.406	L[3]
R[23]	**851.161**	97.387	92.047	92.383	91.711	R[2]
D[24]	**889.503**	45.353	40.014	40.349	39.678	D[1]

sp|P62806|H4_MOUSE

[Acetyl]S $^{\text{Phospho}}_{79.97}$ GRGKGGKGLGKGGAK $^{\text{Acetyl}}_{42.01}$ RHRK $^{\text{Dimethyl}}_{28.03}$ VLRD

- fragmentation table for charge state 4+
- specType=etd
- score=74.22
- F104902.dat
- query=q2374_p1
- precursor=534.303430
- chargePrecursor=5
- itol=0.8

AA	c	y	z+1	z+2	z	AA
S[1]	57.516	**667.625**	**663.621**	0.755	**663.369**	S[24]
G[2]	71.772	615.373	**611.368**	0.755	**611.116**	G[23]
R[3]	110.797	**601.118**	**597.113**	**597.365**	**596.861**	R[22]
G[4]	125.052	562.092	558.088	558.340	557.836	G[21]
K[5]	157.076	547.837	543.832	**544.084**	543.580	K[20]
G[6]	171.331	515.813	511.809	512.061	511.557	G[19]
G[7]	185.587	501.558	**497.553**	**497.805**	**497.301**	G[18]
K[8]	217.610	**487.303**	483.298	483.550	483.046	K[17]
G[9]	231.866	455.279	451.274	451.526	451.022	G[16]
L[10]	260.137	**441.023**	437.019	437.271	436.767	L[15]
G[11]	**274.392**	412.752	408.748	409.000	408.496	G[14]
K[12]	306.416	398.497	394.492	394.744	394.240	K[13]
G[13]	320.671	366.473	362.469	362.721	362.217	G[12]
G[14]	334.927	352.218	348.213	348.465	**347.961**	G[11]
A[15]	352.686	337.963	333.958	334.210	333.706	A[10]
K[16]	395.212	320.203	316.199	316.451	315.947	K[9]
R[17]	434.238	277.677	**273.672**	**273.924**	**273.420**	R[8]
H[18]	**468.502**	238.652	234.647	234.899	234.395	H[7]
R[19]	507.528	204.387	200.382	200.634	200.130	R[6]
K[20]	**546.559**	165.362	161.357	161.609	161.105	K[5]
V[21]	571.326	126.330	122.325	122.577	122.073	V[4]
L[22]	599.597	101.563	97.558	97.810	97.306	L[3]
R[23]	**638.622**	73.292	69.287	69.539	69.035	R[2]
D[24]	**667.379**	34.267	30.262	30.514	30.010	D[1]

sp|P62806|H4_MOUSE

[Acetyl]SGRGK $^{Acetyl}_{42.01}$ GGK $^{Acetyl}_{42.01}$ GLGK $^{Acetyl}_{42.01}$ GGAKRHRK $^{Dimethyl}_{28.03}$ VLRD

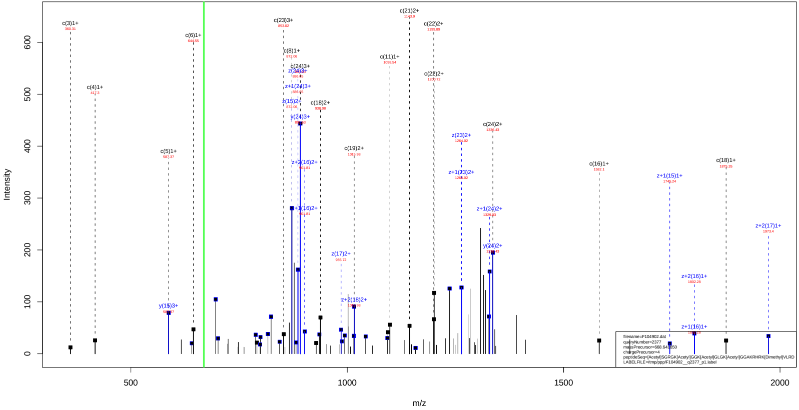

sp|P62806|H4_MOUSE

[Acetyl]SGRGK $^{Acetyl}_{42.01}$ GGK $^{Acetyl}_{42.01}$ GLGK $^{Acetyl}_{42.01}$ GGAKRHRK $^{Dimethyl}_{28.03}$ VLRD

- fragmentation table for charge state 1+
- specType=etd
- score=53.17
- F104902.dat
- query=q2377_p1
- precursor=668.641650
- chargePrecursor=4
- itol=0.8

AA	c	y	z+1	z+2	z	AA
S[1]	147.076	2671.534	2655.515	0.000	2654.507	S[24]
G[2]	204.098	2542.491	2526.473	0.000	2525.465	G[23]
R[3]	**360.199**	2485.470	2469.451	2470.459	2468.443	R[22]
G[4]	**417.220**	2329.369	2313.350	2314.358	2312.342	G[21]
K[5]	**587.326**	2272.347	2256.329	2257.336	2255.321	K[20]
G[6]	**644.347**	2102.242	2086.223	2087.231	2085.215	G[19]
G[7]	**701.369**	2045.220	2029.202	2030.209	2028.194	G[18]
K[8]	**871.474**	1988.199	1972.180	**1973.188**	1971.172	K[17]
G[9]	**928.496**	1818.093	**1802.075**	**1803.082**	1801.067	G[16]
L[10]	1041.580	1761.072	**1745.053**	1746.061	1744.045	L[15]
G[11]	**1098.601**	1647.988	1631.969	1632.977	1630.961	G[14]
K[12]	1268.707	1590.966	1574.948	1575.955	1573.940	K[13]
G[13]	1325.728	1420.861	1404.842	1405.850	1403.834	G[12]
G[14]	1382.750	1363.839	1347.821	1348.828	1346.813	G[11]
A[15]	1453.787	1306.818	1290.799	1291.807	1289.791	A[10]
K[16]	**1581.882**	**1235.781**	1219.762	1220.770	1218.754	K[9]
R[17]	1737.983	1107.686	1091.667	**1092.675**	1090.659	R[8]
H[18]	**1875.042**	951.585	**935.566**	936.574	934.558	H[7]
R[19]	2031.143	814.526	**798.507**	**799.515**	797.499	R[6]
K[20]	2187.269	658.425	642.406	643.414	**641.398**	K[5]
V[21]	2286.338	502.298	486.280	487.287	485.272	V[4]
L[22]	2399.422	403.230	387.211	388.219	386.203	L[3]
R[23]	2555.523	290.146	274.127	275.135	273.119	R[2]
D[24]	2670.550	134.045	118.026	119.034	117.018	D[1]

sp|P62806|H4_MOUSE

[Acetyl]SGRGK $^{\text{Acetyl}}_{42.01}$ GGK $^{\text{Acetyl}}_{42.01}$ GLGK $^{\text{Acetyl}}_{42.01}$ GGAKRHRK $^{\text{Dimethyl}}_{28.03}$ VLRD

- fragmentation table for charge state 2+
- specType=etd
- score=53.17
- F104902.dat
- query=q2377_p1
- precursor=668.641650
- chargePrecursor=4
- itol=0.8

AA	c	y	z+1	z+2	z	AA
S[1]	74.042	**1336.271**	**1328.261**	0.504	**1327.757**	S[24]
G[2]	102.553	1271.749	**1263.740**	0.504	**1263.236**	G[23]
R[3]	180.603	1243.239	1235.229	**1235.733**	1234.725	R[22]
G[4]	209.114	1165.188	**1157.179**	**1157.683**	1156.675	G[21]
K[5]	294.167	1136.677	1128.668	1129.172	1128.164	K[20]
G[6]	322.677	1051.625	1043.615	1044.119	**1043.111**	G[19]
G[7]	351.188	1023.114	**1015.104**	**1015.608**	**1014.601**	G[18]
K[8]	436.241	**994.603**	986.594	**987.098**	**986.090**	K[17]
G[9]	464.752	909.550	**901.541**	**902.045**	**901.037**	G[16]
L[10]	521.294	**881.040**	873.030	873.534	**872.526**	L[15]
G[11]	549.804	**824.498**	**816.488**	**816.992**	**815.984**	G[14]
K[12]	634.857	795.987	**787.977**	**788.481**	787.474	K[13]
G[13]	663.368	710.934	702.925	703.429	702.421	G[12]
G[14]	691.879	682.423	674.414	674.918	673.910	G[11]
A[15]	727.397	653.913	645.903	646.407	645.399	A[10]
K[16]	**791.445**	618.394	610.385	610.889	609.881	K[9]
R[17]	869.495	554.347	546.337	546.841	545.833	R[8]
H[18]	**938.025**	476.296	468.287	468.791	467.783	H[7]
R[19]	**1016.075**	407.767	399.757	400.261	399.253	R[6]
K[20]	**1094.138**	329.716	321.707	322.211	321.203	K[5]
V[21]	**1143.673**	251.653	243.643	244.147	243.140	V[4]
L[22]	**1200.215**	202.119	194.109	194.613	193.605	L[3]
R[23]	1278.265	145.577	137.567	138.071	137.063	R[2]
D[24]	**1335.779**	67.526	59.517	60.021	59.013	D[1]

sp|P62806|H4_MOUSE

[Acetyl]SGRGK $^{Acetyl}_{42.01}$ GGK $^{Acetyl}_{42.01}$ GLGK $^{Acetyl}_{42.01}$ GGAKRHRK $^{Dimethyl}_{28.03}$ VLRD

- fragmentation table for charge state 3+
- specType=etd
- score=53.17
- F104902.dat
- query=q2377_p1
- precursor=668.641650
- chargePrecursor=4
- itol=0.8

AA	c	y	z+1	z+2	z	AA
S[1]	49.697	**891.183**	**885.843**	0.672	**885.507**	S[24]
G[2]	68.704	848.169	**842.829**	0.672	842.493	G[23]
R[3]	120.738	829.161	**823.822**	**824.158**	**823.486**	R[22]
G[4]	139.745	777.128	771.788	772.124	771.452	G[21]
K[5]	196.447	758.121	752.781	753.117	752.445	K[20]
G[6]	215.454	**701.419**	**696.079**	**696.415**	**695.743**	G[19]
G[7]	234.461	682.412	677.072	677.408	676.736	G[18]
K[8]	291.163	663.404	658.065	658.401	657.729	K[17]
G[9]	310.170	606.703	601.363	601.699	601.027	G[16]
L[10]	347.865	**587.695**	582.356	582.692	582.020	L[15]
G[11]	366.872	550.001	544.661	544.997	544.325	G[14]
K[12]	423.574	530.994	525.654	525.990	525.318	K[13]
G[13]	442.581	474.292	468.952	469.288	468.616	G[12]
G[14]	461.588	455.285	449.945	450.281	449.609	G[11]
A[15]	485.267	436.277	430.938	431.274	430.602	A[10]
K[16]	527.966	412.598	407.259	407.595	406.923	K[9]
R[17]	579.999	369.900	364.561	364.896	364.225	R[8]
H[18]	625.686	317.866	312.527	312.863	312.191	H[7]
R[19]	677.719	272.180	266.841	267.176	266.505	R[6]
K[20]	729.761	220.146	214.807	215.143	214.471	K[5]
V[21]	762.784	168.104	162.765	163.101	162.429	V[4]
L[22]	800.479	135.082	129.742	130.078	129.406	L[3]
R[23]	**852.513**	97.387	92.047	92.383	91.711	R[2]
D[24]	**890.855**	45.353	40.014	40.349	39.678	D[1]

sp|P62806|H4_MOUSE

[Acetyl]SGRGKGGK $^{\text{Acetyl}}_{42.01}$ GLGK $^{\text{Acetyl}}_{42.01}$ GGAK $^{\text{Acetyl}}_{42.01}$ RHRK $^{\text{Dimethyl}}_{28.03}$ VLRD

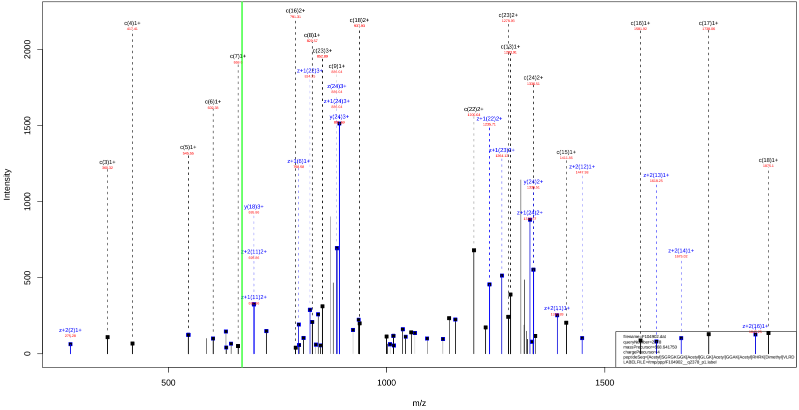

sp|P62806|H4_MOUSE

[Acetyl]SGRGKGGK $^{\text{Acetyl}}_{42.01}$ GLGK $^{\text{Acetyl}}_{42.01}$ GGAK $^{\text{Acetyl}}_{42.01}$ RHRK $^{\text{Dimethyl}}_{28.03}$ VLRD

- fragmentation table for charge state 1+
- specType=etd
- score=123.52
- F104902.dat
- query=q2378_p1
- precursor=668.641750
- chargePrecursor=4
- itol=0.8

AA	c	y	z+1	z+2	z	AA
S[1]	147.076	2671.534	2655.515	0.000	2654.507	S[24]
G[2]	204.098	2542.491	2526.473	0.000	2525.465	G[23]
R[3]	**360.199**	2485.470	2469.451	2470.459	2468.443	R[22]
G[4]	**417.220**	2329.369	2313.350	2314.358	2312.342	G[21]
K[5]	**545.315**	2272.347	2256.329	2257.336	2255.321	K[20]
G[6]	**602.337**	2144.252	2128.234	2129.241	2127.226	G[19]
G[7]	**659.358**	2087.231	2071.212	2072.220	2070.204	G[18]
K[8]	**829.464**	2030.209	2014.191	2015.198	2013.183	K[17]
G[9]	**886.485**	1860.104	1844.085	**1845.093**	1843.077	G[16]
L[10]	**999.569**	1803.082	1787.064	1788.071	1786.056	L[15]
G[11]	**1056.591**	1689.998	1673.980	**1674.987**	1672.972	G[14]
K[12]	**1226.696**	1632.977	1616.958	**1617.966**	1615.950	K[13]
G[13]	**1283.718**	1462.871	1446.853	**1447.860**	1445.845	G[12]
G[14]	**1340.739**	1405.850	1389.831	**1390.839**	1388.823	G[11]
A[15]	**1411.776**	1348.828	**1332.810**	**1333.818**	1331.802	A[10]
K[16]	**1581.882**	1277.791	1261.773	1262.780	1260.765	K[9]
R[17]	**1737.983**	1107.686	1091.667	**1092.675**	1090.659	R[8]
H[18]	**1875.042**	951.585	**935.566**	936.574	934.558	H[7]
R[19]	2031.143	814.526	**798.507**	**799.515**	797.499	R[6]
K[20]	2187.269	658.425	642.406	**643.414**	641.398	K[5]
V[21]	2286.338	502.298	486.280	487.287	485.272	V[4]
L[22]	2399.422	403.230	387.211	388.219	386.203	L[3]
R[23]	2555.523	290.146	274.127	**275.135**	273.119	R[2]
D[24]	2670.550	134.045	118.026	119.034	117.018	D[1]

sp|P62806|H4_MOUSE

[Acetyl]SGRGKGGK $^{Acetyl}_{42.01}$ GLGK $^{Acetyl}_{42.01}$ GGAK $^{Acetyl}_{42.01}$ RHRK $^{Dimethyl}_{28.03}$ VLRD

- fragmentation table for charge state 2+
- specType=etd
- score=123.52
- F104902.dat
- query=q2378_p1
- precursor=668.641750
- chargePrecursor=4
- itol=0.8

AA	c	y	z+1	z+2	z	AA
S[1]	74.042	**1336.271**	**1328.261**	0.504	1327.757	S[24]
G[2]	102.553	1271.749	**1263.740**	0.504	1263.236	G[23]
R[3]	180.603	1243.239	**1235.229**	**1235.733**	1234.725	R[22]
G[4]	209.114	1165.188	**1157.179**	**1157.683**	1156.675	G[21]
K[5]	273.161	1136.677	**1128.668**	**1129.172**	1128.164	K[20]
G[6]	301.672	1072.630	**1064.620**	**1065.124**	**1064.117**	G[19]
G[7]	330.183	**1044.119**	**1036.110**	**1036.614**	1035.606	G[18]
K[8]	415.236	**1015.608**	**1007.599**	**1008.103**	**1007.095**	K[17]
G[9]	443.746	930.556	**922.546**	**923.050**	922.042	G[16]
L[10]	500.288	902.045	894.035	894.539	893.532	L[15]
G[11]	528.799	845.503	**837.493**	**837.997**	836.990	G[14]
K[12]	613.852	816.992	**808.983**	**809.487**	808.479	K[13]
G[13]	642.363	731.939	**723.930**	**724.434**	723.426	G[12]
G[14]	670.873	703.429	**695.419**	**695.923**	694.915	G[11]
A[15]	706.392	674.918	666.908	667.412	666.405	A[10]
K[16]	**791.445**	639.399	**631.390**	**631.894**	630.886	K[9]
R[17]	869.495	554.347	**546.337**	546.841	**545.833**	R[8]
H[18]	**938.025**	476.296	468.287	468.791	467.783	H[7]
R[19]	**1016.075**	407.767	399.757	400.261	399.253	R[6]
K[20]	1094.138	329.716	321.707	322.211	321.203	K[5]
V[21]	**1143.673**	251.653	243.643	244.147	243.140	V[4]
L[22]	**1200.215**	202.119	194.109	194.613	193.605	L[3]
R[23]	**1278.265**	145.577	137.567	138.071	137.063	R[2]
D[24]	**1335.779**	67.526	59.517	60.021	59.013	D[1]

sp|P62806|H4_MOUSE

[Acetyl]SGRGKGGK $^{Acetyl}_{42.01}$ GLGK $^{Acetyl}_{42.01}$ GGAK $^{Acetyl}_{42.01}$ RHRK $^{Dimethyl}_{28.03}$ VLRD

- fragmentation table for charge state 3+
- specType=etd
- score=123.52
- F104902.dat
- query=q2378_p1
- precursor=668.641750
- chargePrecursor=4
- itol=0.8

AA	c	y	z+1	z+2	z	AA
S[1]	49.697	**891.183**	**885.843**	0.672	**885.507**	S[24]
G[2]	68.704	**848.169**	**842.829**	0.672	**842.493**	G[23]
R[3]	120.738	**829.161**	**823.822**	**824.158**	**823.486**	R[22]
G[4]	139.745	777.128	771.788	772.124	771.452	G[21]
K[5]	182.443	758.121	752.781	753.117	752.445	K[20]
G[6]	201.450	715.422	710.083	710.419	709.747	G[19]
G[7]	220.458	**696.415**	691.076	691.412	690.740	G[18]
K[8]	277.159	677.408	672.068	672.404	671.732	K[17]
G[9]	296.167	620.706	615.367	615.703	615.031	G[16]
L[10]	333.861	**601.699**	596.359	596.695	596.023	L[15]
G[11]	352.868	564.004	558.665	559.001	558.329	G[14]
K[12]	409.570	**544.997**	539.658	539.994	539.322	K[13]
G[13]	428.577	488.295	482.956	483.292	482.620	G[12]
G[14]	447.585	469.288	463.949	464.285	463.613	G[11]
A[15]	471.264	450.281	444.941	445.277	444.605	A[10]
K[16]	527.966	426.602	421.262	421.598	420.926	K[9]
R[17]	579.999	369.900	364.561	364.896	364.225	R[8]
H[18]	625.686	317.866	312.527	312.863	312.191	H[7]
R[19]	677.719	272.180	266.841	267.176	266.505	R[6]
K[20]	729.761	220.146	214.807	215.143	214.471	K[5]
V[21]	762.784	168.104	162.765	163.101	162.429	V[4]
L[22]	800.479	135.082	129.742	130.078	129.406	L[3]
R[23]	**852.513**	97.387	92.047	92.383	91.711	R[2]
D[24]	890.855	45.353	40.014	40.349	39.678	D[1]

sp|Q6ZWY9|H2B1C_MOUSE

PEPAKSAPAPKK $^{Acetyl}_{42.01}$ GSK $^{Acetyl}_{42.01}$ KAVTKAQKKD

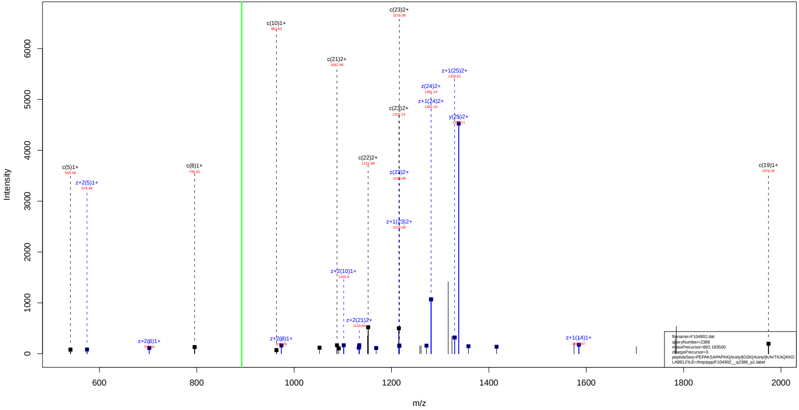

sp|Q6ZWY9|H2B1C_MOUSE

PEPAKSAPAPKK $^{Acetyl}_{42.01}$ GSK $^{Acetyl}_{42.01}$ KAVTKAQKKD

- fragmentation table for charge state 1+
- specType=etd
- score=47.35
- F104902.dat
- query=q2386_p1
- precursor=892.183500
- chargePrecursor=3
- itol=0.8

AA	c	y	z+1	z+2	z	AA
P[1]	115.087	2674.525	2658.506	0.000	2657.498	P[25]
E[2]	244.129	2577.472	2561.454	0.000	2560.446	E[24]
P[3]	341.182	2448.430	2432.411	0.000	2431.403	P[23]
A[4]	412.219	2351.377	2335.358	0.000	2334.350	A[22]
K[5]	**540.314**	2280.340	2264.321	2265.329	2263.313	K[21]
S[6]	627.346	2152.245	2136.226	2137.234	2135.218	S[20]
A[7]	698.383	2065.213	2049.194	2050.202	2048.186	A[19]
P[8]	**795.436**	1994.176	1978.157	1979.165	1977.149	P[18]
A[9]	866.473	1897.123	1881.104	1882.112	1880.096	A[17]
P[10]	**963.526**	1826.086	1810.067	1811.075	1809.059	P[16]
K[11]	**1091.621**	1729.033	1713.014	1714.022	1712.006	K[15]
K[12]	1261.726	1600.938	**1584.919**	1585.927	1583.912	K[14]
G[13]	1318.748	1430.833	1414.814	**1415.822**	1413.806	G[13]
S[14]	1405.780	1373.811	**1357.792**	1358.800	1356.785	S[12]
K[15]	1575.885	1286.779	1270.760	**1271.768**	1269.753	K[11]
K[16]	1703.980	1116.674	1100.655	**1101.663**	1099.647	K[10]
A[17]	1775.017	988.579	972.560	**973.568**	971.552	A[9]
V[18]	1874.086	917.541	901.523	902.531	900.515	V[8]
T[19]	**1975.133**	818.473	802.454	803.462	801.446	T[7]
K[20]	2103.228	717.425	701.407	**702.414**	700.399	K[6]
A[21]	2174.266	589.330	573.312	**574.320**	572.304	A[5]
Q[22]	2302.324	518.293	502.275	503.282	501.267	Q[4]
K[23]	2430.419	390.235	374.216	375.224	373.208	K[3]
K[24]	2558.514	262.140	246.121	247.129	245.113	K[2]
D[25]	2673.541	134.045	118.026	119.034	117.018	D[1]

sp|Q6ZWY9|H2B1C_MOUSE

PEPAKSAPAPKK $^{Acetyl}_{42.01}$ GSK $^{Acetyl}_{42.01}$ KAVTKAQKKD

- fragmentation table for charge state 2+
- specType=etd
- score=47.35
- F104902.dat
- query=q2386_p1
- precursor=892.183500
- chargePrecursor=3
- itol=0.8

AA	c	y	z+1	z+2	z	AA
P[1]	58.047	**1337.766**	**1329.757**	0.504	**1329.253**	P[25]
E[2]	122.568	1289.240	**1281.230**	0.504	**1280.726**	E[24]
P[3]	171.095	1224.718	**1216.709**	0.504	**1216.205**	P[23]
A[4]	206.613	1176.192	**1168.183**	0.504	1167.679	A[22]
K[5]	270.661	1140.674	**1132.664**	**1133.168**	**1132.160**	K[21]
S[6]	314.177	1076.626	1068.617	1069.121	1068.113	S[20]
A[7]	349.695	1033.110	1025.101	1025.605	1024.597	A[19]
P[8]	398.222	997.591	989.582	990.086	989.078	P[18]
A[9]	433.740	949.065	941.056	941.560	940.552	A[17]
P[10]	482.267	913.547	905.537	906.041	905.033	P[16]
K[11]	546.314	865.020	857.011	857.515	856.507	K[15]
K[12]	631.367	800.973	792.963	793.467	792.459	K[14]
G[13]	659.878	715.920	707.911	708.414	707.407	G[13]
S[14]	703.394	687.409	679.400	679.904	678.896	S[12]
K[15]	788.446	643.893	635.884	636.388	635.380	K[11]
K[16]	852.494	558.840	550.831	551.335	550.327	K[10]
A[17]	888.012	494.793	486.784	487.287	486.280	A[9]
V[18]	937.547	459.274	451.265	451.769	450.761	V[8]
T[19]	988.070	409.740	401.731	402.235	401.227	T[7]
K[20]	**1052.118**	359.216	351.207	351.711	350.703	K[6]
A[21]	**1087.636**	295.169	287.159	287.663	286.656	A[5]
Q[22]	**1151.666**	259.650	251.641	252.145	251.137	Q[4]
K[23]	**1215.713**	195.621	187.612	188.116	187.108	K[3]
K[24]	1279.761	131.574	123.564	124.068	123.060	K[2]
D[25]	1337.274	67.526	59.517	60.021	59.013	D[1]

sp|P62806|H4_MOUSE

[Acetyl]SGRGK Acetyl 42.01 GGK Acetyl 42.01 GLGK Acetyl 42.01 GGAK Acetyl 42.01 RHR Dimethyl 28.03 KVLRD

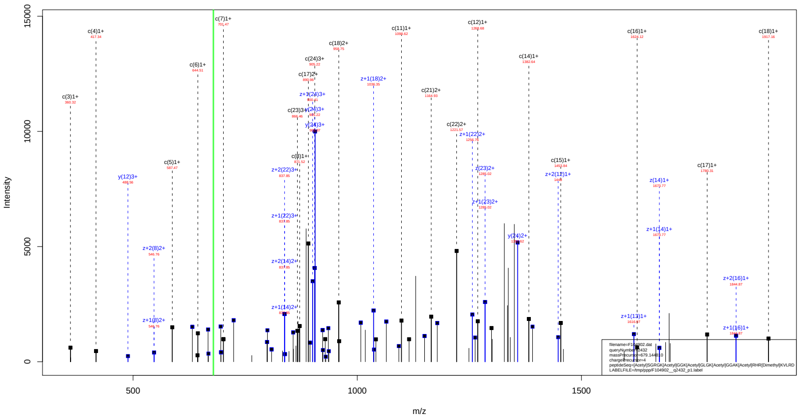

sp|P62806|H4_MOUSE

[Acetyl]SGRGK $^{Acetyl}_{42.01}$ GGK $^{Acetyl}_{42.01}$ GLGK $^{Acetyl}_{42.01}$ GGAK $^{Acetyl}_{42.01}$ RHR $^{Dimethyl}_{28.03}$ KVLRD

- fragmentation table for charge state 1+
- specType=etd
- score=99.92
- F104902.dat
- query=q2432_p1
- precursor=679.144810
- chargePrecursor=4
- itol=0.8

AA	c	y	z+1	z+2	z	AA
S[1]	147.076	2713.544	2697.526	0.000	2696.518	S[24]
G[2]	204.098	2584.502	2568.483	0.000	2567.475	G[23]
R[3]	**360.199**	2527.480	2511.462	2512.470	2510.454	R[22]
G[4]	**417.220**	2371.379	2355.361	2356.368	2354.353	G[21]
K[5]	**587.326**	2314.358	2298.339	2299.347	2297.331	K[20]
G[6]	**644.347**	2144.252	2128.234	2129.241	2127.226	G[19]
G[7]	**701.369**	2087.231	2071.212	2072.220	2070.204	G[18]
K[8]	**871.474**	2030.209	2014.191	2015.198	2013.183	K[17]
G[9]	**928.496**	1860.104	**1844.085**	**1845.093**	1843.077	G[16]
L[10]	**1041.580**	1803.082	1787.064	1788.071	1786.056	L[15]
G[11]	**1098.601**	1689.998	**1673.980**	1674.987	**1672.972**	G[14]
K[12]	**1268.707**	1632.977	**1616.958**	1617.966	1615.950	K[13]
G[13]	1325.728	1462.871	1446.853	**1447.860**	1445.845	G[12]
G[14]	**1382.750**	1405.850	1389.831	**1390.839**	1388.823	G[11]
A[15]	**1453.787**	1348.828	1332.810	1333.818	1331.802	A[10]
K[16]	**1623.893**	1277.791	1261.773	**1262.780**	1260.765	K[9]
R[17]	**1779.994**	1107.686	1091.667	**1092.675**	1090.659	R[8]
H[18]	**1917.053**	951.585	**935.566**	**936.574**	934.558	H[7]
R[19]	2101.185	814.526	**798.507**	**799.515**	797.499	R[6]
K[20]	2229.280	630.393	614.375	615.382	613.367	K[5]
V[21]	2328.348	502.298	486.280	487.287	485.272	V[4]
L[22]	2441.432	403.230	387.211	388.219	386.203	L[3]
R[23]	2597.534	290.146	274.127	275.135	273.119	R[2]
D[24]	2712.560	134.045	118.026	119.034	117.018	D[1]

sp|P62806|H4_MOUSE

[Acetyl]SGRGK $^{Acetyl}_{42.01}$ GGK $^{Acetyl}_{42.01}$ GLGK $^{Acetyl}_{42.01}$ GGAK $^{Acetyl}_{42.01}$ RHR $^{Dimethyl}_{28.03}$ KVLRD

- fragmentation table for charge state 2+
- specType=etd
- score=99.92
- F104902.dat
- query=q2432_p1
- precursor=679.144810
- chargePrecursor=4
- itol=0.8

AA	c	y	z+1	z+2	z	AA
S[1]	74.042	**1357.276**	1349.267	0.504	1348.763	S[24]
G[2]	102.553	1292.755	**1284.745**	0.504	**1284.241**	G[23]
R[3]	180.603	1264.244	**1256.234**	**1256.738**	1255.731	R[22]
G[4]	209.114	1186.193	**1178.184**	**1178.688**	**1177.680**	G[21]
K[5]	294.167	1157.683	**1149.673**	**1150.177**	1149.169	K[20]
G[6]	322.677	1072.630	**1064.620**	**1065.124**	**1064.117**	G[19]
G[7]	351.188	1044.119	**1036.110**	**1036.614**	**1035.606**	G[18]
K[8]	436.241	1015.608	**1007.599**	**1008.103**	**1007.095**	K[17]
G[9]	464.752	**930.556**	**922.546**	**923.050**	**922.042**	G[16]
L[10]	521.294	902.045	**894.035**	**894.539**	893.532	L[15]
G[11]	549.804	845.503	**837.493**	**837.997**	836.990	G[14]
K[12]	634.857	816.992	**808.983**	**809.487**	**808.479**	K[13]
G[13]	663.368	731.939	**723.930**	**724.434**	723.426	G[12]
G[14]	691.879	703.429	**695.419**	**695.923**	**694.915**	G[11]
A[15]	727.397	674.918	**666.908**	**667.412**	666.405	A[10]
K[16]	812.450	639.399	**631.390**	**631.894**	630.886	K[9]
R[17]	**890.500**	554.347	**546.337**	**546.841**	545.833	R[8]
H[18]	**959.030**	476.296	468.287	468.791	467.783	H[7]
R[19]	1051.096	407.767	399.757	400.261	399.253	R[6]
K[20]	**1115.144**	315.700	307.691	308.195	307.187	K[5]
V[21]	**1164.678**	251.653	243.643	244.147	243.140	V[4]
L[22]	**1221.220**	202.119	194.109	194.613	193.605	L[3]
R[23]	**1299.270**	145.577	137.567	138.071	137.063	R[2]
D[24]	1356.784	67.526	59.517	60.021	59.013	D[1]

sp|P62806|H4_MOUSE

[Acetyl]SGRGK $^{\text{Acetyl}}_{42.01}$ GGK $^{\text{Acetyl}}_{42.01}$ GLGK $^{\text{Acetyl}}_{42.01}$ GGAK $^{\text{Acetyl}}_{42.01}$ RHR $^{\text{Dimethyl}}_{28.03}$ KVLRD

- fragmentation table for charge state 3+
- specType=etd
- score=99.92
- F104902.dat
- query=q2432_p1
- precursor=679.144810
- chargePrecursor=4
- itol=0.8

AA	c	y	z+1	z+2	z	AA
S[1]	49.697	**905.186**	**899.847**	0.672	899.511	S[24]
G[2]	68.704	862.172	**856.833**	0.672	**856.497**	G[23]
R[3]	120.738	843.165	**837.825**	**838.161**	**837.489**	R[22]
G[4]	139.745	791.131	785.792	786.128	785.456	G[21]
K[5]	196.447	772.124	766.785	767.120	766.449	K[20]
G[6]	215.454	715.422	710.083	710.419	709.747	G[19]
G[7]	234.461	**696.415**	691.076	691.412	690.740	G[18]
K[8]	291.163	677.408	672.068	672.404	671.732	K[17]
G[9]	310.170	620.706	615.367	615.703	615.031	G[16]
L[10]	347.865	601.699	596.359	596.695	596.023	L[15]
G[11]	366.872	564.004	558.665	559.001	558.329	G[14]
K[12]	423.574	544.997	539.658	539.994	539.322	K[13]
G[13]	442.581	**488.295**	482.956	483.292	482.620	G[12]
G[14]	461.588	469.288	463.949	464.285	463.613	G[11]
A[15]	485.267	450.281	444.941	445.277	444.605	A[10]
K[16]	541.969	426.602	421.262	421.598	420.926	K[9]
R[17]	594.003	369.900	364.561	364.896	364.225	R[8]
H[18]	639.689	317.866	312.527	312.863	312.191	H[7]
R[19]	**701.067**	272.180	266.841	267.176	266.505	R[6]
K[20]	743.765	210.803	205.463	205.799	205.127	K[5]
V[21]	776.788	168.104	162.765	163.101	162.429	V[4]
L[22]	814.482	135.082	129.742	130.078	129.406	L[3]
R[23]	**866.516**	97.387	92.047	92.383	91.711	R[2]
D[24]	**904.858**	45.353	40.014	40.349	39.678	D[1]

sp|Q6GSS7|H2A2A_MOUSE

LNKLLGK Dimethyl 28.03 VTIAQGGVLPNIQAVLLPKKTE

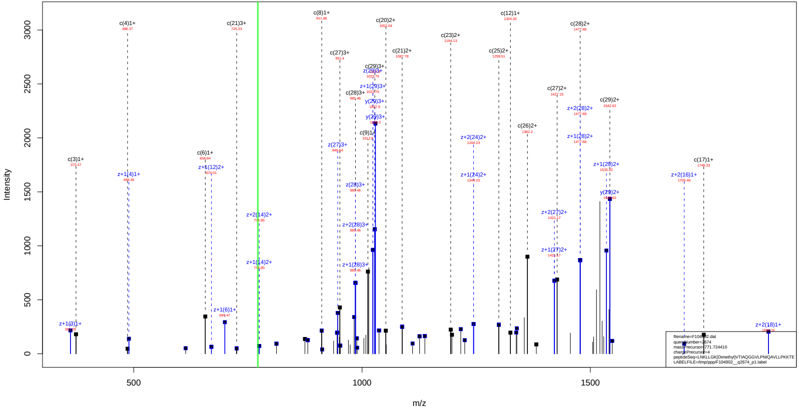

sp|Q6GSS7|H2A2A_MOUSE

LNKLLGK $^{\text{Dimethyl}}_{28.03}$ VTIAQGGVLPNIQAVLLPKKTE

- fragmentation table for charge state 1+
- specType=etd
- score=81.34
- F104902.dat
- query=q2674_p1
- precursor=771.724410
- chargePrecursor=4
- itol=0.8

AA	c	y	z+1	z+2	z	AA
L[1]	131.118	3083.892	3067.873	0.000	3066.865	L[29]
N[2]	245.161	2970.808	2954.789	2955.797	2953.781	N[28]
K[3]	**373.256**	2856.765	2840.746	2841.754	2839.738	K[27]
L[4]	**486.340**	2728.670	2712.651	2713.659	2711.643	L[26]
L[5]	599.424	2615.586	2599.567	2600.575	2598.559	L[25]
G[6]	**656.445**	2502.502	2486.483	2487.491	2485.475	G[24]
K[7]	**812.572**	2445.480	2429.462	2430.469	2428.454	K[23]
V[8]	**911.640**	2289.354	2273.335	2274.343	2272.327	V[22]
T[9]	**1012.688**	2190.286	2174.267	2175.275	2173.259	T[21]
I[10]	**1125.772**	2089.238	2073.219	2074.227	2072.211	I[20]
A[11]	**1196.809**	1976.154	1960.135	1961.143	1959.127	A[19]
Q[12]	**1324.867**	1905.117	1889.098	**1890.106**	1888.090	Q[18]
G[13]	**1381.889**	1777.058	1761.039	1762.047	1760.032	G[17]
G[14]	1438.910	1720.037	1704.018	**1705.026**	1703.010	G[16]
V[15]	1537.979	1663.015	1646.997	1648.004	1645.989	V[15]
L[16]	1651.063	1563.947	**1547.928**	1548.936	1546.920	L[14]
P[17]	**1748.116**	1450.863	1434.844	1435.852	1433.836	P[13]
N[18]	1862.159	1353.810	**1337.791**	**1338.799**	1336.783	N[12]
I[19]	1975.243	1239.767	1223.748	**1224.756**	1222.741	I[11]
Q[20]	2103.301	**1126.683**	**1110.664**	1111.672	1109.656	Q[10]
A[21]	2174.338	998.624	**982.606**	983.614	981.598	A[9]
V[22]	2273.407	927.587	**911.569**	**912.576**	910.561	V[8]
L[23]	2386.491	828.519	**812.500**	813.508	811.492	L[7]
L[24]	2499.575	715.435	**699.416**	700.424	698.408	L[6]
P[25]	2596.628	602.351	586.332	587.340	585.324	P[5]
K[26]	2724.723	505.298	**489.279**	490.287	488.271	K[4]
K[27]	2852.818	377.203	**361.184**	362.192	360.177	K[3]
T[28]	2953.865	249.108	233.089	234.097	232.082	T[2]
E[29]	3082.908	148.060	132.042	133.050	131.034	E[1]

sp|Q6GSS7|H2A2A_MOUSE

LNKLLGK $^{\text{Dimethyl}}_{28.03}$ VTIAQGGVLPNIQAVLLPKKTE

- fragmentation table for charge state 2+
- specType=etd
- score=81.34
- F104902.dat
- query=q2674_p1
- precursor=771.724410
- chargePrecursor=4
- itol=0.8

AA	c	y	z+1	z+2	z	AA
L[1]	66.063	**1542.450**	**1534.440**	0.504	1533.936	L[29]
N[2]	123.084	1485.908	**1477.898**	**1478.402**	**1477.394**	N[28]
K[3]	187.132	1428.886	**1420.877**	**1421.381**	1420.373	K[27]
L[4]	243.674	1364.839	1356.829	1357.333	1356.325	L[26]
L[5]	300.216	1308.297	**1300.287**	1300.791	**1299.783**	L[25]
G[6]	328.726	1251.755	**1243.745**	**1244.249**	1243.241	G[24]
K[7]	406.789	1223.244	1215.234	**1215.738**	1214.731	K[23]
V[8]	456.324	1145.181	**1137.171**	**1137.675**	**1136.667**	V[22]
T[9]	506.848	1095.646	**1087.637**	**1088.141**	**1087.133**	T[21]
I[10]	563.390	1045.123	**1037.113**	**1037.617**	**1036.609**	I[20]
A[11]	598.908	**988.581**	980.571	981.075	980.067	A[19]
Q[12]	662.937	**953.062**	**945.053**	**945.557**	**944.549**	Q[18]
G[13]	691.448	889.033	**881.023**	**881.527**	**880.519**	G[17]
G[14]	719.959	860.522	852.513	853.017	852.009	G[16]
V[15]	769.493	832.011	824.002	824.506	823.498	V[15]
L[16]	826.035	782.477	**774.468**	**774.972**	773.964	L[14]
P[17]	**874.561**	**725.935**	717.926	718.430	717.422	P[13]
N[18]	931.583	677.409	**669.399**	**669.903**	668.895	N[12]
I[19]	**988.125**	620.387	612.378	**612.882**	611.874	I[11]
Q[20]	**1052.154**	563.845	555.836	556.340	555.332	Q[10]
A[21]	**1087.673**	499.816	491.807	492.310	491.303	A[9]
V[22]	**1137.207**	464.297	456.288	456.792	455.784	V[8]
L[23]	**1193.749**	414.763	406.754	407.258	406.250	L[7]
L[24]	1250.291	358.221	350.212	350.716	349.708	L[6]
P[25]	**1298.817**	301.679	293.670	294.174	293.166	P[5]
K[26]	**1362.865**	253.153	245.143	245.647	244.639	K[4]
K[27]	**1426.912**	189.105	181.096	181.600	180.592	K[3]
T[28]	**1477.436**	125.058	117.048	117.552	116.544	T[2]
E[29]	**1541.958**	74.534	66.524	67.028	66.021	E[1]

sp|Q6GSS7|H2A2A_MOUSE

LNKLLGK $^{\text{Dimethyl}}_{28.03}$ VTIAQGGVLPNIQAVLLPKKTE

- fragmentation table for charge state 3+
- specType=etd
- score=81.34
- F104902.dat
- query=q2674_p1
- precursor=771.724410
- chargePrecursor=4
- itol=0.8

AA	c	y	z+1	z+2	z	AA
L[1]	44.377	**1028.635**	**1023.296**	0.672	**1022.960**	L[29]
N[2]	82.392	990.941	**985.601**	**985.937**	**985.265**	N[28]
K[3]	125.090	**952.926**	947.587	947.923	**947.251**	K[27]
L[4]	162.785	910.228	904.889	905.225	904.553	L[26]
L[5]	200.479	872.533	867.194	867.530	866.858	L[25]
G[6]	219.487	834.839	829.499	829.835	829.163	G[24]
K[7]	271.529	815.832	810.492	810.828	810.156	K[23]
V[8]	304.552	763.790	758.450	758.786	758.114	V[22]
T[9]	338.234	730.767	**725.427**	**725.763**	**725.091**	T[21]
I[10]	375.929	697.084	691.745	692.081	691.409	I[20]
A[11]	399.608	659.389	654.050	654.386	653.714	A[19]
Q[12]	442.294	635.710	630.371	630.707	630.035	Q[18]
G[13]	461.301	593.024	587.685	588.021	587.349	G[17]
G[14]	480.308	574.017	568.678	569.013	568.342	G[16]
V[15]	513.331	555.010	549.670	550.006	549.334	V[15]
L[16]	551.026	521.987	516.648	516.984	516.312	L[14]
P[17]	583.377	484.292	478.953	479.289	478.617	P[13]
N[18]	621.391	451.942	446.602	446.938	446.266	N[12]
I[19]	659.086	413.927	408.588	408.924	408.252	I[11]
Q[20]	701.772	376.233	370.893	371.229	370.557	Q[10]
A[21]	**725.451**	333.546	328.207	328.543	327.871	A[9]
V[22]	758.474	309.867	304.528	304.864	304.192	V[8]
L[23]	796.168	276.844	271.505	271.841	271.169	L[7]
L[24]	833.863	239.150	233.810	234.146	233.474	L[6]
P[25]	866.214	201.455	196.116	196.451	195.780	P[5]
K[26]	908.912	169.104	163.765	164.101	163.429	K[4]
K[27]	**951.611**	126.406	121.066	121.402	120.730	K[3]
T[28]	**985.293**	83.708	78.368	78.704	78.032	T[2]
E[29]	**1028.307**	50.025	44.685	45.021	44.349	E[1]

sp|Q6GSS7|H2A2A_MOUSE

LNK $^{Dimethyl}_{28.03}$ LLGKVTIAQGGVLPNIQAVLLPKKTE

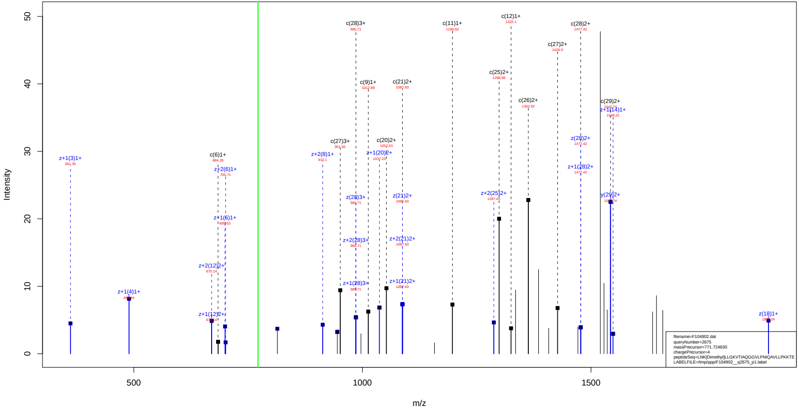

sp|Q6GSS7|H2A2A_MOUSE

LNK $^{Dimethyl}_{28.03}$ LLGKVTIAQGGVLPNIQAVLLPKKTE

- fragmentation table for charge state 1+
- specType=etd
- score=32.11
- F104902.dat
- query=q2675_p1
- precursor=771.724630
- chargePrecursor=4
- itol=0.8

AA	c	y	z+1	z+2	z	AA
L[1]	131.118	3083.892	3067.873	0.000	3066.865	L[29]
N[2]	245.161	2970.808	2954.789	2955.797	2953.781	N[28]
K[3]	401.287	2856.765	2840.746	2841.754	2839.738	K[27]
L[4]	514.371	2700.639	2684.620	2685.628	2683.612	L[26]
L[5]	627.455	2587.555	2571.536	2572.544	2570.528	L[25]
G[6]	**684.477**	2474.470	2458.452	2459.460	2457.444	G[24]
K[7]	812.572	2417.449	2401.430	2402.438	2400.422	K[23]
V[8]	911.640	2289.354	2273.335	2274.343	2272.327	V[22]
T[9]	**1012.688**	2190.286	2174.267	2175.275	2173.259	T[21]
I[10]	1125.772	2089.238	2073.219	2074.227	2072.211	I[20]
A[11]	**1196.809**	1976.154	1960.135	1961.143	1959.127	A[19]
Q[12]	**1324.867**	1905.117	1889.098	1890.106	**1888.090**	Q[18]
G[13]	1381.889	1777.058	1761.039	1762.047	1760.032	G[17]
G[14]	1438.910	1720.037	1704.018	1705.026	1703.010	G[16]
V[15]	1537.979	1663.015	1646.997	1648.004	1645.989	V[15]
L[16]	1651.063	1563.947	**1547.928**	**1548.936**	1546.920	L[14]
P[17]	1748.116	1450.863	1434.844	1435.852	1433.836	P[13]
N[18]	1862.159	1353.810	1337.791	1338.799	1336.783	N[12]
I[19]	1975.243	1239.767	1223.748	1224.756	1222.741	I[11]
Q[20]	2103.301	1126.683	1110.664	1111.672	1109.656	Q[10]
A[21]	2174.338	998.624	982.606	983.614	981.598	A[9]
V[22]	2273.407	927.587	911.569	**912.576**	910.561	V[8]
L[23]	2386.491	828.519	812.500	**813.508**	811.492	L[7]
L[24]	2499.575	715.435	**699.416**	**700.424**	698.408	L[6]
P[25]	2596.628	602.351	586.332	587.340	585.324	P[5]
K[26]	2724.723	505.298	**489.279**	490.287	488.271	K[4]
K[27]	2852.818	377.203	**361.184**	362.192	360.177	K[3]
T[28]	2953.865	249.108	233.089	234.097	232.082	T[2]
E[29]	3082.908	148.060	132.042	133.050	131.034	E[1]

sp|Q6GSS7|H2A2A_MOUSE

LNK $^{\text{Dimethyl}}_{28.03}$ LLGKVTIAQGGVLPNIQAVLLPKKTE

- fragmentation table for charge state 2+
- specType=etd
- score=32.11
- F104902.dat
- query=q2675_p1
- precursor=771.724630
- chargePrecursor=4
- itol=0.8

AA	c	y	z+1	z+2	z	AA
L[1]	66.063	**1542.450**	1534.440	0.504	1533.936	L[29]
N[2]	123.084	1485.908	**1477.898**	1478.402	**1477.394**	N[28]
K[3]	201.147	1428.886	1420.877	1421.381	1420.373	K[27]
L[4]	257.689	1350.823	1342.814	1343.317	1342.310	L[26]
L[5]	314.231	1294.281	1286.272	**1286.775**	1285.768	L[25]
G[6]	342.742	1237.739	1229.730	1230.233	1229.226	G[24]
K[7]	406.789	1209.228	1201.219	1201.723	1200.715	K[23]
V[8]	456.324	1145.181	1137.171	1137.675	1136.667	V[22]
T[9]	506.848	1095.646	**1087.637**	**1088.141**	**1087.133**	T[21]
I[10]	563.390	1045.123	**1037.113**	**1037.617**	**1036.609**	I[20]
A[11]	598.908	988.581	980.571	981.075	980.067	A[19]
Q[12]	662.937	953.062	**945.053**	**945.557**	**944.549**	Q[18]
G[13]	691.448	889.033	881.023	881.527	880.519	G[17]
G[14]	719.959	860.522	852.513	853.017	852.009	G[16]
V[15]	769.493	832.011	824.002	824.506	823.498	V[15]
L[16]	826.035	782.477	774.468	774.972	773.964	L[14]
P[17]	874.561	725.935	717.926	718.430	717.422	P[13]
N[18]	931.583	677.409	**669.399**	**669.903**	668.895	N[12]
I[19]	988.125	620.387	612.378	612.882	611.874	I[11]
Q[20]	**1052.154**	563.845	555.836	556.340	555.332	Q[10]
A[21]	**1087.673**	499.816	491.807	492.310	491.303	A[9]
V[22]	1137.207	464.297	456.288	456.792	455.784	V[8]
L[23]	1193.749	414.763	406.754	407.258	406.250	L[7]
L[24]	1250.291	358.221	350.212	350.716	349.708	L[6]
P[25]	**1298.817**	301.679	293.670	294.174	293.166	P[5]
K[26]	**1362.865**	253.153	245.143	245.647	244.639	K[4]
K[27]	**1426.912**	189.105	181.096	181.600	180.592	K[3]
T[28]	**1477.436**	125.058	117.048	117.552	116.544	T[2]
E[29]	**1541.958**	74.534	66.524	67.028	66.021	E[1]

sp|Q6GSS7|H2A2A_MOUSE

LNK $^{Dimethyl}_{28.03}$ LLGKVTIAQGGVLPNIQAVLLPKKTE

- fragmentation table for charge state 3+
- specType=etd
- score=32.11
- F104902.dat
- query=q2675_p1
- precursor=771.724630
- chargePrecursor=4
- itol=0.8

AA	c	y	z+1	z+2	z	AA
L[1]	44.377	1028.635	1023.296	0.672	1022.960	L[29]
N[2]	82.392	990.941	**985.601**	**985.937**	**985.265**	N[28]
K[3]	134.434	952.926	947.587	947.923	947.251	K[27]
L[4]	172.129	900.884	895.545	895.881	895.209	L[26]
L[5]	209.823	863.190	857.850	858.186	857.514	L[25]
G[6]	228.830	825.495	820.155	820.491	819.819	G[24]
K[7]	271.529	806.488	801.148	801.484	800.812	K[23]
V[8]	304.552	763.790	758.450	758.786	758.114	V[22]
T[9]	338.234	730.767	725.427	725.763	725.091	T[21]
I[10]	375.929	697.084	691.745	692.081	691.409	I[20]
A[11]	399.608	659.389	654.050	654.386	653.714	A[19]
Q[12]	442.294	635.710	630.371	630.707	630.035	Q[18]
G[13]	461.301	593.024	587.685	588.021	587.349	G[17]
G[14]	480.308	574.017	568.678	569.013	568.342	G[16]
V[15]	513.331	555.010	549.670	550.006	549.334	V[15]
L[16]	551.026	521.987	516.648	516.984	516.312	L[14]
P[17]	583.377	484.292	478.953	479.289	478.617	P[13]
N[18]	621.391	451.942	446.602	446.938	446.266	N[12]
I[19]	659.086	413.927	408.588	408.924	408.252	I[11]
Q[20]	701.772	376.233	370.893	371.229	370.557	Q[10]
A[21]	725.451	333.546	328.207	328.543	327.871	A[9]
V[22]	758.474	309.867	304.528	304.864	304.192	V[8]
L[23]	796.168	276.844	271.505	271.841	271.169	L[7]
L[24]	833.863	239.150	233.810	234.146	233.474	L[6]
P[25]	866.214	201.455	196.116	196.451	195.780	P[5]
K[26]	908.912	169.104	163.765	164.101	163.429	K[4]
K[27]	**951.611**	126.406	121.066	121.402	120.730	K[3]
T[28]	**985.293**	83.708	78.368	78.704	78.032	T[2]
E[29]	1028.307	50.025	44.685	45.021	44.349	E[1]

sp|Q8BFU2|H2A3_MOUSE

[Acetyl]SGRGKQGGKARAKAKSRSSRAGLQFPVGRVHRLLRKGNYSE

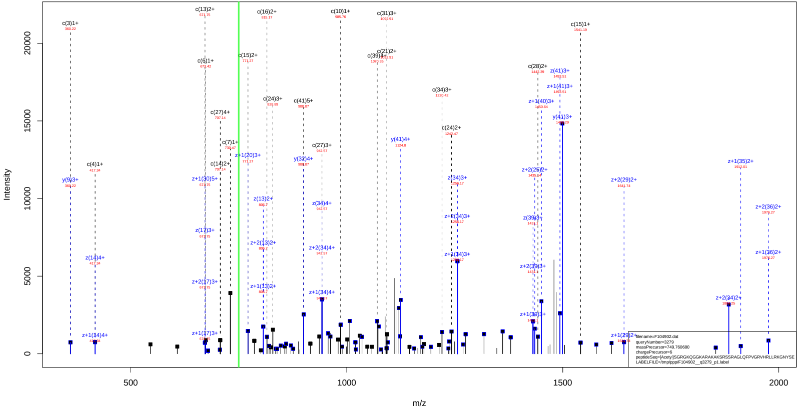

sp|Q8BFU2|H2A3_MOUSE

[Acetyl]SGRGKQGGKARAKAKSRSSRAGLQFPVGRVHRLLRKGNYSE

- fragmentation table for charge state 1+
- specType=etd
- score=42.65
- F104902.dat
- query=q3279_p1
- precursor=749.760680
- chargePrecursor=6
- itol=0.8

AA	c	y	z+1	z+2	z	AA
S[1]	147.076	4493.505	4477.486	0.000	4476.478	S[41]
G[2]	204.098	4364.462	4348.444	0.000	4347.436	G[40]
R[3]	**360.199**	4307.441	4291.422	4292.430	4290.414	R[39]
G[4]	**417.220**	4151.340	4135.321	4136.329	4134.313	G[38]
K[5]	**545.315**	4094.318	4078.300	4079.307	4077.292	K[37]
Q[6]	**673.374**	3966.223	3950.205	3951.212	3949.197	Q[36]
G[7]	**730.395**	3838.165	3822.146	3823.154	3821.138	G[35]
G[8]	787.417	3781.143	3765.125	3766.132	3764.117	G[34]
K[9]	**915.512**	3724.122	3708.103	3709.111	3707.095	K[33]
A[10]	**986.549**	3596.027	3580.008	3581.016	3579.000	A[32]
R[11]	1142.650	3524.990	3508.971	3509.979	3507.963	R[31]
A[12]	**1213.687**	3368.889	3352.870	3353.878	3351.862	A[30]
K[13]	1341.782	3297.852	3281.833	3282.841	3280.825	K[29]
A[14]	1412.819	3169.757	3153.738	3154.746	3152.730	A[28]
K[15]	**1540.914**	3098.719	3082.701	3083.709	3081.693	K[27]
S[16]	1627.946	2970.625	2954.606	2955.614	2953.598	S[26]
R[17]	1784.047	2883.592	2867.574	2868.582	2866.566	R[25]
S[18]	1871.079	2727.491	2711.473	2712.480	2710.465	S[24]
S[19]	1958.111	2640.459	2624.441	2625.448	2623.433	S[23]
R[20]	2114.213	2553.427	2537.409	2538.416	2536.401	R[22]
A[21]	2185.250	2397.326	2381.307	2382.315	2380.300	A[21]
G[22]	2242.271	2326.289	2310.270	2311.278	2309.263	G[20]
L[23]	2355.355	2269.268	2253.249	2254.257	2252.241	L[19]
Q[24]	2483.414	2156.184	2140.165	2141.173	2139.157	Q[18]
F[25]	2630.482	2028.125	2012.106	2013.114	2011.098	F[17]
P[26]	2727.535	1881.057	1865.038	1866.046	1864.030	P[16]
V[27]	2826.603	1784.004	1767.985	1768.993	1766.977	V[15]
G[28]	2883.625	1684.935	1668.917	1669.924	1667.909	G[14]
R[29]	3039.726	1627.914	1611.895	**1612.903**	1610.887	R[13]
V[30]	3138.794	1471.813	1455.794	1456.802	1454.786	V[12]
H[31]	3275.853	1372.744	1356.726	1357.734	1355.718	H[11]
R[32]	3431.954	**1235.685**	**1219.667**	**1220.675**	1218.659	R[10]
L[33]	3545.038	**1079.584**	1063.566	1064.573	1062.558	L[9]
L[34]	3658.123	966.500	950.482	951.489	949.474	L[8]
R[35]	3814.224	853.416	837.398	**838.405**	836.390	R[7]
K[36]	3942.319	697.315	681.296	682.304	680.289	K[6]
G[37]	3999.340	569.220	553.201	554.209	552.194	G[5]
N[38]	4113.383	512.199	496.180	497.188	495.172	N[4]
Y[39]	4276.446	398.156	382.137	383.145	381.129	Y[3]
S[40]	4363.478	235.092	219.074	220.082	218.066	S[2]
E[41]	4492.521	148.060	132.042	133.050	131.034	E[1]

sp|Q8BFU2|H2A3_MOUSE

[Acetyl]SGRGKQGGKARAKAKSRSSRAGLQFPVGRVHRLLRKGNYSE

- fragmentation table for charge state 2+
- specType=etd
- score=42.65
- F104902.dat
- query=q3279_p1
- precursor=749.760680
- chargePrecursor=6
- itol=0.8

AA	c	y	z+1	z+2	z	AA
S[1]	74.042	2247.256	2239.247	0.504	2238.743	S[41]
G[2]	102.553	2182.735	2174.725	0.504	2174.222	G[40]
R[3]	180.603	2154.224	2146.215	2146.719	2145.711	R[39]
G[4]	209.114	2076.174	2068.164	2068.668	2067.660	G[38]
K[5]	273.161	2047.663	2039.653	2040.157	2039.150	K[37]
Q[6]	337.191	1983.615	**1975.606**	**1976.110**	1975.102	Q[36]
G[7]	365.701	1919.586	**1911.577**	**1912.081**	1911.073	G[35]
G[8]	394.212	1891.075	1883.066	**1883.570**	1882.562	G[34]
K[9]	458.260	1862.565	**1854.555**	1855.059	**1854.051**	K[33]
A[10]	493.778	1798.517	1790.508	1791.012	1790.004	A[32]
R[11]	571.829	1762.999	1754.989	1755.493	1754.485	R[31]
A[12]	**607.347**	1684.948	1676.939	1677.443	1676.435	A[30]
K[13]	**671.395**	1649.429	**1641.420**	**1641.924**	1640.916	K[29]
A[14]	**706.913**	1585.382	**1577.373**	**1577.876**	**1576.869**	A[28]
K[15]	**770.961**	1549.863	**1541.854**	1542.358	**1541.350**	K[27]
S[16]	**814.477**	1485.816	1477.807	1478.310	1477.303	S[26]
R[17]	892.527	**1442.300**	1434.291	**1434.794**	1433.787	R[25]
S[18]	**936.043**	1364.249	1356.240	1356.744	1355.736	S[24]
S[19]	**979.559**	1320.733	1312.724	1313.228	1312.220	S[23]
R[20]	**1057.610**	1277.217	**1269.208**	**1269.712**	**1268.704**	R[22]
A[21]	**1093.128**	1199.167	1191.157	1191.661	1190.653	A[21]
G[22]	1121.639	1163.648	**1155.639**	**1156.143**	1155.135	G[20]
L[23]	**1178.181**	1135.137	1127.128	1127.632	1126.624	L[19]
Q[24]	**1242.211**	**1078.595**	**1070.586**	**1071.090**	**1070.082**	Q[18]
F[25]	1315.745	1014.566	**1006.557**	**1007.061**	1006.053	F[17]
P[26]	1364.271	941.032	933.023	933.526	932.519	P[16]
V[27]	1413.805	892.506	884.496	885.000	883.992	V[15]
G[28]	**1442.316**	842.971	**834.962**	**835.466**	834.458	G[14]
R[29]	1520.367	**814.461**	**806.451**	**806.955**	**805.947**	R[13]
V[30]	1569.901	736.410	728.401	728.905	727.897	V[12]
H[31]	1638.430	686.876	**678.866**	**679.370**	**678.363**	H[11]
R[32]	1716.481	618.346	610.337	610.841	609.833	R[10]
L[33]	1773.023	540.296	532.286	532.790	531.783	L[9]
L[34]	1829.565	483.754	475.744	476.248	475.241	L[8]
R[35]	1907.615	427.212	419.202	419.706	418.698	R[7]
K[36]	1971.663	349.161	341.152	341.656	340.648	K[6]
G[37]	2000.174	285.114	277.104	277.608	276.600	G[5]
N[38]	2057.195	256.603	248.594	249.098	248.090	N[4]
Y[39]	2138.727	199.582	191.572	192.076	191.068	Y[3]
S[40]	2182.243	118.050	110.041	110.544	109.537	S[2]
E[41]	2246.764	74.534	66.524	67.028	66.021	E[1]

sp|Q8BFU2|H2A3_MOUSE

[Acetyl]SGRGKQGGKARAKAKSRSSRAGLQFPVGRVHRLLRKGNYSE

- fragmentation table for charge state 3+
- specType=etd
- score=42.65
- F104902.dat
- query=q3279_p1
- precursor=749.760680
- chargePrecursor=6
- itol=0.8

AA	c	y	z+1	z+2	z	AA
S[1]	49.697	**1498.507**	**1493.167**	0.672	**1492.831**	S[41]
G[2]	68.704	1455.492	**1450.153**	0.672	1449.817	G[40]
R[3]	120.738	1436.485	**1431.146**	**1431.482**	**1430.810**	R[39]
G[4]	139.745	1384.451	**1379.112**	**1379.448**	**1378.776**	G[38]
K[5]	182.443	1365.444	**1360.105**	**1360.441**	1359.769	K[37]
Q[6]	225.130	1322.746	**1317.406**	**1317.742**	**1317.070**	Q[36]
G[7]	244.137	1280.060	**1274.720**	**1275.056**	**1274.384**	G[35]
G[8]	263.144	1261.053	**1255.713**	**1256.049**	**1255.377**	G[34]
K[9]	305.842	**1242.045**	**1236.706**	**1237.042**	**1236.370**	K[33]
A[10]	329.521	1199.347	**1194.008**	**1194.344**	1193.672	A[32]
R[11]	381.555	**1175.668**	1170.329	**1170.664**	1169.993	R[31]
A[12]	405.234	**1123.634**	1118.295	1118.631	1117.959	A[30]
K[13]	447.932	1099.955	**1094.616**	**1094.952**	1094.280	K[29]
A[14]	471.611	1057.257	1051.917	1052.253	1051.582	A[28]
K[15]	514.310	1033.578	1028.238	1028.574	1027.902	K[27]
S[16]	543.320	990.880	**985.540**	**985.876**	**985.204**	S[26]
R[17]	595.354	**961.869**	**956.529**	**956.865**	**956.193**	R[25]
S[18]	624.365	909.835	904.496	904.832	904.160	S[24]
S[19]	653.375	880.825	**875.485**	**875.821**	875.149	S[23]
R[20]	705.409	851.814	**846.474**	**846.810**	846.138	R[22]
A[21]	729.088	799.780	794.441	794.777	794.105	A[21]
G[22]	748.095	776.101	**770.762**	**771.098**	770.426	G[20]
L[23]	**785.790**	757.094	751.754	752.090	751.419	L[19]
Q[24]	**828.476**	719.399	714.060	714.396	713.724	Q[18]
F[25]	877.499	676.713	**671.374**	**671.710**	**671.038**	F[17]
P[26]	909.850	627.690	622.351	622.687	622.015	P[16]
V[27]	**942.873**	595.339	590.000	590.336	589.664	V[15]
G[28]	**961.880**	562.317	556.977	557.313	556.641	G[14]
R[29]	1013.914	543.309	537.970	538.306	537.634	R[13]
V[30]	**1046.936**	491.276	485.936	486.272	485.600	V[12]
H[31]	**1092.623**	458.253	452.913	453.249	452.577	H[11]
R[32]	**1144.656**	412.567	407.227	407.563	406.891	R[10]
L[33]	1182.351	**360.533**	355.193	355.529	354.857	L[9]
L[34]	**1220.046**	322.838	317.499	317.835	317.163	L[8]
R[35]	1272.079	285.144	279.804	280.140	279.468	R[7]
K[36]	1314.778	233.110	227.770	228.106	227.434	K[6]
G[37]	1333.785	190.412	185.072	185.408	184.736	G[5]
N[38]	1371.799	171.404	166.065	166.401	165.729	N[4]
Y[39]	1426.154	133.390	128.051	128.386	127.715	Y[3]
S[40]	1455.164	79.036	73.696	74.032	73.360	S[2]
E[41]	1498.178	50.025	44.685	45.021	44.349	E[1]

sp|Q8BFU2|H2A3_MOUSE

[Acetyl]SGRGKQGGKARAKAKSRSSRAGLQFPVGRVHRLLRKGNYSE

- fragmentation table for charge state 4+
- specType=etd
- score=42.65
- F104902.dat
- query=q3279_p1
- precursor=749.760680
- chargePrecursor=6
- itol=0.8

AA	c	y	z+1	z+2	z	AA
S[1]	37.525	**1124.132**	**1120.127**	0.755	**1119.875**	S[41]
G[2]	51.780	**1091.871**	1087.866	0.755	1087.614	G[40]
R[3]	90.805	1077.616	**1073.611**	**1073.863**	1073.359	R[39]
G[4]	105.061	1038.590	**1034.586**	**1034.838**	1034.334	G[38]
K[5]	137.084	1024.335	**1020.330**	**1020.582**	**1020.078**	K[37]
Q[6]	169.099	992.311	988.307	**988.559**	988.055	Q[36]
G[7]	183.354	960.297	**956.292**	**956.544**	**956.040**	G[35]
G[8]	197.610	946.041	**942.037**	**942.289**	**941.785**	G[34]
K[9]	229.633	931.786	927.781	928.033	927.529	K[33]
A[10]	247.393	**899.762**	895.757	896.009	895.506	A[32]
R[11]	286.418	882.003	877.998	878.250	877.746	R[31]
A[12]	304.177	842.978	**838.973**	**839.225**	**838.721**	A[30]
K[13]	336.201	825.218	821.214	821.466	820.962	K[29]
A[14]	353.960	793.195	789.190	789.442	788.938	A[28]
K[15]	385.984	775.435	**771.431**	**771.683**	**771.179**	K[27]
S[16]	407.742	743.412	739.407	739.659	739.155	S[26]
R[17]	446.767	721.654	717.649	717.901	717.397	R[25]
S[18]	468.525	682.628	**678.624**	**678.876**	**678.372**	S[24]
S[19]	490.283	660.870	656.866	657.118	656.614	S[23]
R[20]	529.309	639.112	635.108	635.360	634.856	R[22]
A[21]	547.068	600.087	596.082	596.334	595.830	A[21]
G[22]	561.323	582.328	578.323	578.575	578.071	G[20]
L[23]	589.594	568.072	564.068	564.320	563.816	L[19]
Q[24]	621.609	539.801	535.797	536.049	535.545	Q[18]
F[25]	658.376	507.787	503.782	504.034	503.530	F[17]
P[26]	682.639	471.020	467.015	467.267	466.763	P[16]
V[27]	**707.406**	446.756	442.752	443.004	442.500	V[15]
G[28]	721.662	421.989	**417.985**	418.237	**417.733**	G[14]
R[29]	760.687	407.734	403.729	403.981	403.477	R[13]
V[30]	**785.454**	368.709	364.704	364.956	364.452	V[12]
H[31]	**819.719**	343.942	339.937	340.189	339.685	H[11]
R[32]	**858.744**	309.677	305.672	305.924	305.420	R[10]
L[33]	887.015	270.652	266.647	266.899	266.395	L[9]
L[34]	**915.286**	242.381	238.376	238.628	238.124	L[8]
R[35]	954.311	214.110	210.105	210.357	209.853	R[7]
K[36]	**986.335**	175.084	171.080	171.332	170.828	K[6]
G[37]	**1000.590**	143.061	139.056	139.308	138.804	G[5]
N[38]	**1029.101**	128.805	124.800	125.052	124.548	N[4]
Y[39]	**1069.867**	100.294	96.290	96.542	96.038	Y[3]
S[40]	**1091.625**	59.529	55.524	55.776	55.272	S[2]
E[41]	**1123.886**	37.771	33.766	34.018	33.514	E[1]

sp|Q8BFU2|H2A3_MOUSE

[Acetyl]SGRGKQGGKARAKAKSRSSRAGLQFPVGRVHRLLRKGNYSE

- fragmentation table for charge state 5+
- specType=etd
- score=42.65
- F104902.dat
- query=q3279_p1
- precursor=749.760680
- chargePrecursor=6
- itol=0.8

AA	c	y	z+1	z+2	z	AA
S[1]	30.221	**899.507**	896.303	0.806	896.102	S[41]
G[2]	41.625	873.698	**870.495**	0.806	**870.293**	G[40]
R[3]	72.846	862.294	**859.090**	**859.292**	**858.889**	R[39]
G[4]	84.250	831.074	827.870	828.072	827.668	G[38]
K[5]	109.869	**819.669**	816.466	816.667	816.264	K[37]
Q[6]	135.481	794.050	790.847	791.048	790.645	Q[36]
G[7]	146.885	768.439	765.235	765.437	765.033	G[35]
G[8]	158.289	757.034	753.831	754.032	753.629	G[34]
K[9]	183.908	745.630	742.426	742.628	742.225	K[33]
A[10]	198.116	720.011	716.807	717.009	716.606	A[32]
R[11]	229.336	**705.804**	702.600	702.802	702.398	R[31]
A[12]	243.543	674.584	**671.380**	**671.581**	**671.178**	A[30]
K[13]	269.162	660.376	657.172	657.374	656.971	K[29]
A[14]	283.370	634.757	631.553	631.755	631.352	A[28]
K[15]	308.989	620.550	617.346	617.548	617.144	K[27]
S[16]	326.395	594.931	591.727	591.929	591.525	S[26]
R[17]	357.615	577.524	574.321	574.522	574.119	R[25]
S[18]	375.022	546.304	543.100	543.302	542.899	S[24]
S[19]	392.428	528.898	525.694	525.896	525.492	S[23]
R[20]	423.648	511.491	508.288	508.489	508.086	R[22]
A[21]	437.856	480.271	477.067	477.269	476.866	A[21]
G[22]	449.260	466.064	462.860	463.061	462.658	G[20]
L[23]	471.877	454.659	451.456	451.657	451.254	L[19]
Q[24]	497.489	432.043	428.839	429.040	428.637	Q[18]
F[25]	526.902	406.431	403.227	403.429	403.026	F[17]
P[26]	546.313	377.017	373.813	374.015	373.612	P[16]
V[27]	566.126	357.607	354.403	354.604	354.201	V[15]
G[28]	577.531	337.793	334.589	334.791	334.388	G[14]
R[29]	608.751	326.389	323.185	323.386	322.983	R[13]
V[30]	628.565	295.168	291.965	292.166	291.763	V[12]
H[31]	655.976	275.355	272.151	272.353	271.949	H[11]
R[32]	687.197	247.943	244.739	244.941	244.538	R[10]
L[33]	709.814	216.723	213.519	213.721	213.317	L[9]
L[34]	732.430	194.106	190.902	191.104	190.701	L[8]
R[35]	763.651	171.489	168.285	168.487	168.084	R[7]
K[36]	789.270	140.269	137.065	137.267	136.864	K[6]
G[37]	**800.674**	114.650	111.446	111.648	111.245	G[5]
N[38]	**823.482**	103.246	100.042	100.243	99.840	N[4]
Y[39]	**856.095**	80.437	77.233	77.435	77.032	Y[3]
S[40]	873.501	47.824	44.621	44.822	44.419	S[2]
E[41]	**899.310**	30.418	27.214	27.416	27.013	E[1]

sp|Q8CGP5|H2A1F_MOUSE

[Acetyl]SGRGKQGGKARAKAKTRSSRAGLQFPVGRVHRLLRKGNYSE

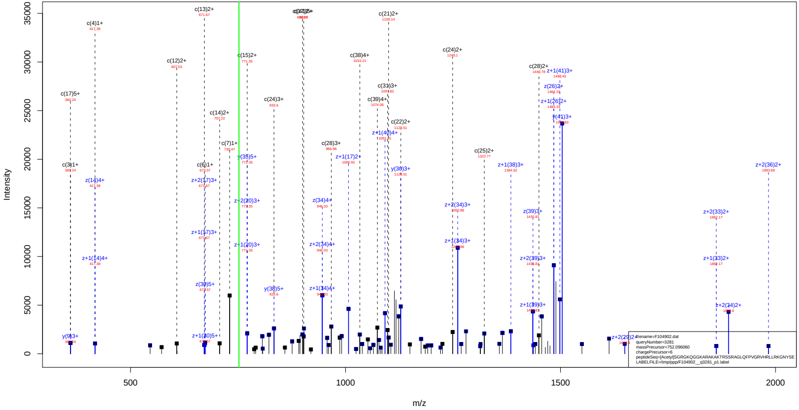

sp|Q8CGP5|H2A1F_MOUSE

[Acetyl]SGRGKQGGKARAKAKTRSSRAGLQFPVGRVHRLLRKGNYSE

- fragmentation table for charge state 1+
- specType=etd
- score=54.81
- F104902.dat
- query=q3281_p1
- precursor=752.096060
- chargePrecursor=6
- itol=0.8

AA	c	y	z+1	z+2	z	AA
S[1]	147.076	4507.521	4491.502	0.000	4490.494	S[41]
G[2]	204.098	4378.478	4362.459	0.000	4361.451	G[40]
R[3]	**360.199**	4321.457	4305.438	4306.446	4304.430	R[39]
G[4]	**417.220**	4165.355	4149.337	4150.345	4148.329	G[38]
K[5]	**545.315**	4108.334	4092.315	4093.323	4091.307	K[37]
Q[6]	**673.374**	3980.239	3964.220	3965.228	3963.212	Q[36]
G[7]	**730.395**	3852.180	3836.162	3837.170	3835.154	G[35]
G[8]	**787.417**	3795.159	3779.140	3780.148	3778.132	G[34]
K[9]	915.512	3738.138	3722.119	3723.127	3721.111	K[33]
A[10]	**986.549**	3610.043	3594.024	3595.032	3593.016	A[32]
R[11]	1142.650	3539.005	3522.987	3523.995	3521.979	R[31]
A[12]	1213.687	3382.904	3366.886	3367.893	3365.878	A[30]
K[13]	1341.782	3311.867	3295.848	3296.856	3294.841	K[29]
A[14]	1412.819	3183.772	3167.754	3168.761	3166.746	A[28]
K[15]	1540.914	3112.735	3096.716	3097.724	3095.709	K[27]
T[16]	1641.962	2984.640	2968.621	2969.629	2967.614	T[26]
R[17]	1798.063	2883.592	2867.574	2868.582	2866.566	R[25]
S[18]	1885.095	2727.491	2711.473	2712.480	2710.465	S[24]
S[19]	1972.127	2640.459	2624.441	2625.448	2623.433	S[23]
R[20]	2128.228	2553.427	2537.409	2538.416	2536.401	R[22]
A[21]	2199.265	2397.326	2381.307	2382.315	2380.300	A[21]
G[22]	2256.287	2326.289	2310.270	2311.278	2309.263	G[20]
L[23]	2369.371	2269.268	2253.249	2254.257	2252.241	L[19]
Q[24]	2497.429	2156.184	2140.165	2141.173	2139.157	Q[18]
F[25]	2644.498	2028.125	2012.106	2013.114	2011.098	F[17]
P[26]	2741.551	1881.057	1865.038	1866.046	1864.030	P[16]
V[27]	2840.619	1784.004	1767.985	1768.993	1766.977	V[15]
G[28]	2897.641	1684.935	1668.917	1669.924	1667.909	G[14]
R[29]	3053.742	1627.914	1611.895	**1612.903**	1610.887	R[13]
V[30]	3152.810	1471.813	**1455.794**	**1456.802**	1454.786	V[12]
H[31]	3289.869	1372.744	1356.726	**1357.734**	1355.718	H[11]
R[32]	3445.970	1235.685	1219.667	**1220.675**	1218.659	R[10]
L[33]	3559.054	1079.584	1063.566	**1064.573**	1062.558	L[9]
L[34]	3672.138	**966.500**	950.482	951.489	949.474	L[8]
R[35]	3828.239	853.416	837.398	838.405	836.390	R[7]
K[36]	3956.334	697.315	681.296	682.304	680.289	K[6]
G[37]	4013.356	569.220	553.201	554.209	552.194	G[5]
N[38]	4127.399	512.199	496.180	497.188	495.172	N[4]
Y[39]	4290.462	398.156	382.137	383.145	381.129	Y[3]
S[40]	4377.494	235.092	219.074	220.082	218.066	S[2]
E[41]	4506.537	148.060	132.042	133.050	131.034	E[1]

sp|Q8CGP5|H2A1F_MOUSE

[Acetyl]SGRGKQGGKARAKAKTRSSRAGLQFPVGRVHRLLRKGNYSE

- fragmentation table for charge state 2+
- specType=etd
- score=54.81
- F104902.dat
- query=q3281_p1
- precursor=752.096060
- chargePrecursor=6
- itol=0.8

AA	c	y	z+1	z+2	z	AA
S[1]	74.042	2254.264	2246.255	0.504	2245.751	S[41]
G[2]	102.553	2189.743	2181.733	0.504	2181.229	G[40]
R[3]	180.603	2161.232	2153.223	2153.726	2152.719	R[39]
G[4]	209.114	2083.181	2075.172	2075.676	2074.668	G[38]
K[5]	273.161	2054.671	2046.661	2047.165	2046.157	K[37]
Q[6]	337.191	1990.623	1982.614	**1983.118**	1982.110	Q[36]
G[7]	365.701	1926.594	1918.584	1919.088	1918.081	G[35]
G[8]	394.212	1898.083	1890.074	**1890.578**	1889.570	G[34]
K[9]	458.260	1869.572	**1861.563**	**1862.067**	1861.059	K[33]
A[10]	493.778	1805.525	1797.516	1798.019	1797.012	A[32]
R[11]	**571.829**	1770.006	1761.997	1762.501	1761.493	R[31]
A[12]	**607.347**	1691.956	1683.946	1684.450	1683.443	A[30]
K[13]	**671.395**	1656.437	1648.428	**1648.932**	1647.924	K[29]
A[14]	**706.913**	1592.390	1584.380	1584.884	1583.876	A[28]
K[15]	**770.961**	1556.871	1548.862	**1549.366**	1548.358	K[27]
T[16]	**821.485**	1492.824	**1484.814**	1485.318	**1484.310**	T[26]
R[17]	**899.535**	1442.300	1434.291	1434.794	1433.787	R[25]
S[18]	943.051	**1364.249**	1356.240	1356.744	1355.736	S[24]
S[19]	**986.567**	1320.733	**1312.724**	**1313.228**	1312.220	S[23]
R[20]	**1064.618**	1277.217	**1269.208**	**1269.712**	**1268.704**	R[22]
A[21]	**1100.136**	**1199.167**	**1191.157**	**1191.661**	**1190.653**	A[21]
G[22]	**1128.647**	1163.648	1155.639	1156.143	1155.135	G[20]
L[23]	**1185.189**	1135.137	1127.128	1127.632	1126.624	L[19]
Q[24]	**1249.218**	1078.595	1070.586	1071.090	1070.082	Q[18]
F[25]	**1322.753**	1014.566	**1006.557**	**1007.061**	1006.053	F[17]
P[26]	1371.279	941.032	933.023	933.526	932.519	P[16]
V[27]	1420.813	892.506	884.496	885.000	883.992	V[15]
G[28]	**1449.324**	842.971	834.962	835.466	834.458	G[14]
R[29]	1527.374	814.461	**806.451**	**806.955**	**805.947**	R[13]
V[30]	1576.909	736.410	728.401	728.905	727.897	V[12]
H[31]	1645.438	686.876	678.866	679.370	678.363	H[11]
R[32]	1723.489	618.346	610.337	610.841	609.833	R[10]
L[33]	1780.031	540.296	532.286	532.790	531.783	L[9]
L[34]	1836.573	483.754	475.744	476.248	475.241	L[8]
R[35]	1914.623	427.212	419.202	419.706	418.698	R[7]
K[36]	1978.671	349.161	341.152	341.656	340.648	K[6]
G[37]	2007.181	285.114	277.104	277.608	276.600	G[5]
N[38]	2064.203	256.603	248.594	249.098	248.090	N[4]
Y[39]	2145.735	199.582	191.572	192.076	191.068	Y[3]
S[40]	2189.251	118.050	110.041	110.544	109.537	S[2]
E[41]	2253.772	74.534	66.524	67.028	66.021	E[1]

sp|Q8CGP5|H2A1F_MOUSE

[Acetyl]SGRGKQGGKARAKAKTRSSRAGLQFPVGRVHRLLRKGNYSE

- fragmentation table for charge state 3+
- specType=etd
- score=54.81
- F104902.dat
- query=q3281_p1
- precursor=752.096060
- chargePrecursor=6
- itol=0.8

AA	c	y	z+1	z+2	z	AA
S[1]	49.697	**1503.178**	**1497.839**	0.672	1497.503	S[41]
G[2]	68.704	1460.164	1454.825	0.672	1454.489	G[40]
R[3]	120.738	**1441.157**	**1435.817**	**1436.153**	**1435.482**	R[39]
G[4]	139.745	1389.123	**1383.784**	**1384.120**	1383.448	G[38]
K[5]	182.443	1370.116	**1364.777**	**1365.113**	**1364.441**	K[37]
Q[6]	225.130	1327.418	**1322.078**	**1322.414**	1321.742	Q[36]
G[7]	244.137	1284.732	1279.392	**1279.728**	1279.056	G[35]
G[8]	263.144	1265.725	**1260.385**	**1260.721**	1260.049	G[34]
K[9]	305.842	1246.717	1241.378	1241.714	1241.042	K[33]
A[10]	329.521	1204.019	**1198.679**	**1199.015**	1198.344	A[32]
R[11]	381.555	1180.340	**1175.000**	**1175.336**	1174.664	R[31]
A[12]	405.234	**1128.306**	**1122.967**	**1123.303**	1122.631	A[30]
K[13]	447.932	**1104.627**	1099.288	**1099.624**	1098.952	K[29]
A[14]	471.611	1061.929	1056.589	**1056.925**	1056.253	A[28]
K[15]	514.310	**1038.250**	**1032.910**	**1033.246**	**1032.574**	K[27]
T[16]	547.992	995.552	**990.212**	**990.548**	989.876	T[26]
R[17]	600.026	961.869	**956.529**	**956.865**	956.193	R[25]
S[18]	629.037	909.835	904.496	904.832	904.160	S[24]
S[19]	658.047	880.825	**875.485**	**875.821**	**875.149**	S[23]
R[20]	710.081	851.814	846.474	846.810	846.138	R[22]
A[21]	733.760	799.780	794.441	794.777	794.105	A[21]
G[22]	752.767	776.101	**770.762**	**771.098**	770.426	G[20]
L[23]	**790.462**	757.094	751.754	752.090	751.419	L[19]
Q[24]	**833.148**	719.399	714.060	714.396	713.724	Q[18]
F[25]	882.171	676.713	**671.374**	**671.710**	**671.038**	F[17]
P[26]	914.522	627.690	622.351	622.687	622.015	P[16]
V[27]	947.545	595.339	590.000	590.336	589.664	V[15]
G[28]	**966.552**	562.317	556.977	557.313	556.641	G[14]
R[29]	1018.585	543.309	537.970	538.306	537.634	R[13]
V[30]	**1051.608**	491.276	485.936	486.272	485.600	V[12]
H[31]	**1097.294**	458.253	452.913	453.249	452.577	H[11]
R[32]	**1149.328**	412.567	407.227	407.563	406.891	R[10]
L[33]	1187.023	**360.533**	355.193	355.529	354.857	L[9]
L[34]	**1224.718**	322.838	317.499	317.835	317.163	L[8]
R[35]	1276.751	285.144	279.804	280.140	279.468	R[7]
K[36]	1319.450	233.110	227.770	228.106	227.434	K[6]
G[37]	1338.457	190.412	185.072	185.408	184.736	G[5]
N[38]	1376.471	171.404	166.065	166.401	165.729	N[4]
Y[39]	1430.826	133.390	128.051	128.386	127.715	Y[3]
S[40]	1459.836	79.036	73.696	74.032	73.360	S[2]
E[41]	1502.850	50.025	44.685	45.021	44.349	E[1]

sp|Q8CGP5|H2A1F_MOUSE

[Acetyl]SGRGKQGGKARAKAKTRSSRAGLQFPVGRVHRLLRKGNYSE

- fragmentation table for charge state 4+
- specType=etd
- score=54.81
- F104902.dat
- query=q3281_p1
- precursor=752.096060
- chargePrecursor=6
- itol=0.8

AA	c	y	z+1	z+2	z	AA
S[1]	37.525	1127.636	**1123.631**	0.755	**1123.379**	S[41]
G[2]	51.780	1095.375	**1091.370**	0.755	**1091.118**	G[40]
R[3]	90.805	**1081.120**	**1077.115**	**1077.367**	**1076.863**	R[39]
G[4]	105.061	1042.094	1038.090	**1038.342**	1037.838	G[38]
K[5]	137.084	1027.839	**1023.834**	**1024.086**	1023.582	K[37]
Q[6]	169.099	995.815	991.811	992.062	**991.559**	Q[36]
G[7]	183.354	963.801	**959.796**	**960.048**	959.544	G[35]
G[8]	197.610	949.545	**945.541**	**945.792**	**945.289**	G[34]
K[9]	229.633	935.290	931.285	931.537	931.033	K[33]
A[10]	247.393	**903.266**	**899.261**	**899.513**	899.009	A[32]
R[11]	286.418	885.507	881.502	881.754	881.250	R[31]
A[12]	304.177	846.482	842.477	842.729	842.225	A[30]
K[13]	336.201	828.722	824.718	824.970	824.466	K[29]
A[14]	353.960	796.699	792.694	792.946	792.442	A[28]
K[15]	385.984	778.939	774.935	775.187	774.683	K[27]
T[16]	411.246	746.915	742.911	743.163	742.659	T[26]
R[17]	450.271	721.654	717.649	717.901	717.397	R[25]
S[18]	472.029	682.628	678.624	678.876	678.372	S[24]
S[19]	493.787	660.870	656.866	657.118	656.614	S[23]
R[20]	532.813	639.112	635.108	635.360	634.856	R[22]
A[21]	550.572	600.087	596.082	596.334	595.830	A[21]
G[22]	564.827	582.328	578.323	578.575	578.071	G[20]
L[23]	593.098	568.072	564.068	564.320	563.816	L[19]
Q[24]	625.113	539.801	535.797	536.049	535.545	Q[18]
F[25]	661.880	507.787	503.782	504.034	503.530	F[17]
P[26]	686.143	471.020	467.015	467.267	466.763	P[16]
V[27]	710.910	446.756	442.752	443.004	442.500	V[15]
G[28]	725.166	421.989	**417.985**	418.237	**417.733**	G[14]
R[29]	764.191	407.734	403.729	403.981	403.477	R[13]
V[30]	788.958	368.709	364.704	364.956	364.452	V[12]
H[31]	823.223	343.942	339.937	340.189	339.685	H[11]
R[32]	862.248	309.677	305.672	305.924	305.420	R[10]
L[33]	**890.519**	270.652	266.647	266.899	266.395	L[9]
L[34]	**918.790**	242.381	238.376	238.628	238.124	L[8]
R[35]	**957.815**	214.110	210.105	210.357	209.853	R[7]
K[36]	989.839	175.084	171.080	171.332	170.828	K[6]
G[37]	1004.094	143.061	139.056	139.308	138.804	G[5]
N[38]	**1032.605**	128.805	124.800	125.052	124.548	N[4]
Y[39]	**1073.371**	100.294	96.290	96.542	96.038	Y[3]
S[40]	1095.129	59.529	55.524	55.776	55.272	S[2]
E[41]	1127.390	37.771	33.766	34.018	33.514	E[1]

sp|Q8CGP5|H2A1F_MOUSE

[Acetyl]SGRGKQGGKARAKAKTRSSRAGLQFPVGRVHRLLRKGNYSE

- fragmentation table for charge state 5+
- specType=etd
- score=54.81
- F104902.dat
- query=q3281_p1
- precursor=752.096060
- chargePrecursor=6
- itol=0.8

AA	c	y	z+1	z+2	z	AA
S[1]	30.221	**902.310**	899.106	0.806	898.905	S[41]
G[2]	41.625	**876.501**	873.298	0.806	873.096	G[40]
R[3]	72.846	865.097	861.893	862.095	861.692	R[39]
G[4]	84.250	**833.877**	830.673	830.875	830.472	G[38]
K[5]	109.869	**822.473**	819.269	819.470	819.067	K[37]
Q[6]	135.481	796.854	793.650	793.851	793.448	Q[36]
G[7]	146.885	**771.242**	768.038	768.240	767.837	G[35]
G[8]	158.289	759.838	756.634	756.835	756.432	G[34]
K[9]	183.908	748.433	745.230	745.431	745.028	K[33]
A[10]	198.116	722.814	719.611	719.812	719.409	A[32]
R[11]	229.336	708.607	705.403	705.605	705.202	R[31]
A[12]	243.543	677.387	**674.183**	674.385	**673.981**	A[30]
K[13]	269.162	663.179	659.976	660.177	659.774	K[29]
A[14]	283.370	637.560	634.357	634.558	634.155	A[28]
K[15]	308.989	623.353	620.149	620.351	619.948	K[27]
T[16]	329.198	597.734	594.530	594.732	594.329	T[26]
R[17]	**360.418**	577.524	574.321	574.522	574.119	R[25]
S[18]	377.825	**546.304**	543.100	543.302	542.899	S[24]
S[19]	395.231	528.898	525.694	525.896	525.492	S[23]
R[20]	426.451	511.491	508.288	508.489	508.086	R[22]
A[21]	440.659	480.271	477.067	477.269	476.866	A[21]
G[22]	452.063	466.064	462.860	463.061	462.658	G[20]
L[23]	474.680	454.659	451.456	451.657	451.254	L[19]
Q[24]	500.292	432.043	428.839	429.040	428.637	Q[18]
F[25]	529.705	406.431	403.227	403.429	403.026	F[17]
P[26]	549.116	377.017	373.813	374.015	373.612	P[16]
V[27]	568.930	357.607	354.403	354.604	354.201	V[15]
G[28]	580.334	337.793	334.589	334.791	334.388	G[14]
R[29]	611.554	326.389	323.185	323.386	322.983	R[13]
V[30]	631.368	295.168	291.965	292.166	291.763	V[12]
H[31]	658.780	275.355	272.151	272.353	271.949	H[11]
R[32]	690.000	247.943	244.739	244.941	244.538	R[10]
L[33]	712.617	216.723	213.519	213.721	213.317	L[9]
L[34]	735.233	194.106	190.902	191.104	190.701	L[8]
R[35]	766.454	171.489	168.285	168.487	168.084	R[7]
K[36]	792.073	140.269	137.065	137.267	136.864	K[6]
G[37]	803.477	114.650	111.446	111.648	111.245	G[5]
N[38]	826.286	103.246	100.042	100.243	99.840	N[4]
Y[39]	**858.898**	80.437	77.233	77.435	77.032	Y[3]
S[40]	**876.305**	47.824	44.621	44.822	44.419	S[2]
E[41]	**902.113**	30.418	27.214	27.416	27.013	E[1]

sp|P62806|H4_MOUSE

[Acetyl]SGRGKGGKGLGKGGAKRHRK $^{\text{Dimethyl}}_{28.03}$ VLRD

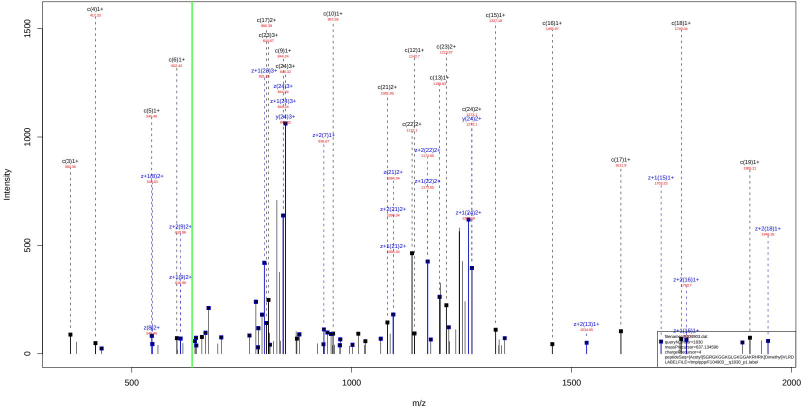

sp|P62806|H4_MOUSE

[Acetyl]SGRGKGGKGLGKGGAKRHRK$^{\text{Dimethyl}}_{28.03}$VLRD

- fragmentation table for charge state 1+
- specType=etd
- score=103.88
- F104903.dat
- query=q1830_p1
- precursor=637.134590
- chargePrecursor=4
- itol=0.8

AA	c	y	z+1	z+2	z	AA
S[1]	147.076	2545.502	2529.483	0.000	2528.476	S[24]
G[2]	204.098	2416.460	2400.441	0.000	2399.433	G[23]
R[3]	**360.199**	2359.438	2343.419	2344.427	2342.412	R[22]
G[4]	**417.220**	2203.337	2187.318	2188.326	2186.310	G[21]
K[5]	**545.315**	2146.316	2130.297	2131.305	2129.289	K[20]
G[6]	**602.337**	2018.221	2002.202	2003.210	2001.194	G[19]
G[7]	**659.358**	1961.199	1945.180	**1946.188**	1944.173	G[18]
K[8]	**787.453**	1904.178	**1888.159**	1889.167	1887.151	K[17]
G[9]	**844.475**	1776.083	**1760.064**	**1761.072**	1759.056	G[16]
L[10]	**957.559**	1719.061	**1703.043**	1704.050	1702.035	L[15]
G[11]	**1014.580**	1605.977	1589.958	1590.966	1588.951	G[14]
K[12]	**1142.675**	1548.956	1532.937	**1533.945**	1531.929	K[13]
G[13]	**1199.697**	1420.861	1404.842	1405.850	1403.834	G[12]
G[14]	1256.718	1363.839	**1347.821**	1348.828	1346.813	G[11]
A[15]	**1327.755**	1306.818	1290.799	1291.807	1289.791	A[10]
K[16]	**1455.850**	1235.781	1219.762	**1220.770**	1218.754	K[9]
R[17]	**1611.951**	1107.686	1091.667	1092.675	1090.659	R[8]
H[18]	**1749.010**	951.585	**935.566**	**936.574**	934.558	H[7]
R[19]	**1905.111**	**814.526**	798.507	799.515	797.499	R[6]
K[20]	2061.238	658.425	642.406	**643.414**	641.398	K[5]
V[21]	2160.306	502.298	486.280	487.287	485.272	V[4]
L[22]	2273.390	403.230	387.211	388.219	386.203	L[3]
R[23]	2429.491	290.146	274.127	275.135	273.119	R[2]
D[24]	2544.518	134.045	118.026	119.034	117.018	D[1]

sp|P62806|H4_MOUSE

[Acetyl]SGRGKGGKGLGKGGAKRHRK $^{\text{Dimethyl}}_{28.03}$ VLRD

- fragmentation table for charge state 2+
- specType=etd
- score=103.88
- F104903.dat
- query=q1830_p1
- precursor=637.134590
- chargePrecursor=4
- itol=0.8

AA	c	y	z+1	z+2	z	AA
S[1]	74.042	**1273.255**	**1265.245**	0.504	1264.741	S[24]
G[2]	102.553	1208.733	1200.724	0.504	**1200.220**	G[23]
R[3]	180.603	**1180.223**	**1172.213**	**1172.717**	1171.709	R[22]
G[4]	209.114	1102.172	**1094.163**	**1094.667**	**1093.659**	G[21]
K[5]	273.161	1073.661	**1065.652**	**1066.156**	1065.148	K[20]
G[6]	301.672	1009.614	**1001.605**	**1002.108**	**1001.101**	G[19]
G[7]	330.183	981.103	**973.094**	**973.598**	**972.590**	G[18]
K[8]	394.230	**952.592**	**944.583**	**945.087**	**944.079**	K[17]
G[9]	422.741	888.545	**880.536**	**881.040**	**880.032**	G[16]
L[10]	479.283	860.034	852.025	852.529	851.521	L[15]
G[11]	507.794	803.492	**795.483**	**795.987**	794.979	G[14]
K[12]	571.841	774.982	**766.972**	**767.476**	766.468	K[13]
G[13]	600.352	710.934	**702.925**	**703.429**	702.421	G[12]
G[14]	628.863	682.423	**674.414**	**674.918**	**673.910**	G[11]
A[15]	664.381	653.913	**645.903**	**646.407**	**645.399**	A[10]
K[16]	728.429	618.394	**610.385**	**610.889**	609.881	K[9]
R[17]	**806.479**	554.347	**546.337**	**546.841**	**545.833**	R[8]
H[18]	**875.009**	476.296	468.287	468.791	467.783	H[7]
R[19]	**953.059**	407.767	399.757	400.261	399.253	R[6]
K[20]	**1031.122**	329.716	321.707	322.211	321.203	K[5]
V[21]	**1080.657**	251.653	243.643	244.147	243.140	V[4]
L[22]	**1137.199**	202.119	194.109	194.613	193.605	L[3]
R[23]	**1215.249**	145.577	137.567	138.071	137.063	R[2]
D[24]	**1272.763**	67.526	59.517	60.021	59.013	D[1]

sp|P62806|H4_MOUSE

[Acetyl]SGRGKGGKGLGKGGAKRHRK $^{Dimethyl}_{28.03}$ VLRD

- fragmentation table for charge state 3+
- specType=etd
- score=103.88
- F104903.dat
- query=q1830_p1
- precursor=637.134590
- chargePrecursor=4
- itol=0.8

AA	c	y	z+1	z+2	z	AA
S[1]	49.697	**849.172**	**843.833**	0.672	**843.497**	S[24]
G[2]	68.704	**806.158**	**800.818**	0.672	**800.483**	G[23]
R[3]	120.738	**787.151**	**781.811**	**782.147**	**781.475**	R[22]
G[4]	139.745	735.117	729.778	730.114	729.442	G[21]
K[5]	182.443	716.110	710.770	711.106	710.435	K[20]
G[6]	201.450	673.412	**668.072**	**668.408**	**667.736**	G[19]
G[7]	220.458	654.405	649.065	649.401	648.729	G[18]
K[8]	263.156	635.397	630.058	630.394	629.722	K[17]
G[9]	282.163	592.699	587.360	587.695	587.024	G[16]
L[10]	319.858	573.692	568.352	568.688	568.016	L[15]
G[11]	338.865	535.997	530.658	530.994	530.322	G[14]
K[12]	381.563	516.990	511.651	511.986	511.315	K[13]
G[13]	400.570	474.292	468.952	469.288	468.616	G[12]
G[14]	419.578	455.285	449.945	450.281	449.609	G[11]
A[15]	443.257	436.277	**430.938**	**431.274**	430.602	A[10]
K[16]	485.955	412.598	407.259	407.595	406.923	K[9]
R[17]	537.989	369.900	364.561	364.896	364.225	R[8]
H[18]	583.675	317.866	312.527	312.863	312.191	H[7]
R[19]	635.709	272.180	266.841	267.176	266.505	R[6]
K[20]	687.751	220.146	214.807	215.143	214.471	K[5]
V[21]	720.774	168.104	162.765	163.101	162.429	V[4]
L[22]	758.468	135.082	129.742	130.078	129.406	L[3]
R[23]	**810.502**	97.387	92.047	92.383	91.711	R[2]
D[24]	**848.844**	45.353	40.014	40.349	39.678	D[1]

sp|P62806|H4_MOUSE

[Acetyl]SGRGKGGKGLGKGGAKRHRK $^{\text{Dimethyl}}_{28.03}$ VLRD

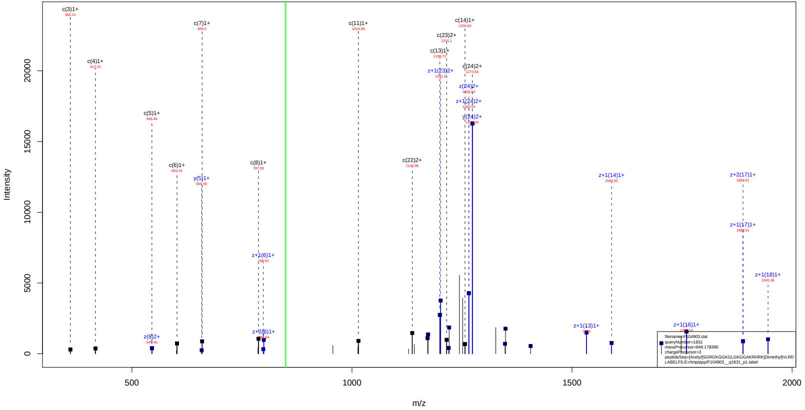

sp|P62806|H4_MOUSE

[Acetyl]SGRGKGGKGLGKGGAKRHRK $^{\text{Dimethyl}}_{28.03}$ VLRD

- fragmentation table for charge state 1+
- specType=etd
- score=57.75
- F104903.dat
- query=q1831_p1
- precursor=849.178390
- chargePrecursor=3
- itol=0.8

AA	c	y	z+1	z+2	z	AA
S[1]	147.076	2545.502	2529.483	0.000	2528.476	S[24]
G[2]	204.098	2416.460	2400.441	0.000	2399.433	G[23]
R[3]	**360.199**	2359.438	2343.419	2344.427	2342.412	R[22]
G[4]	**417.220**	2203.337	2187.318	2188.326	2186.310	G[21]
K[5]	**545.315**	2146.316	2130.297	2131.305	2129.289	K[20]
G[6]	**602.337**	2018.221	2002.202	2003.210	2001.194	G[19]
G[7]	**659.358**	1961.199	**1945.180**	1946.188	1944.173	G[18]
K[8]	**787.453**	1904.178	**1888.159**	**1889.167**	1887.151	K[17]
G[9]	844.475	1776.083	**1760.064**	1761.072	1759.056	G[16]
L[10]	957.559	1719.061	**1703.043**	1704.050	1702.035	L[15]
G[11]	**1014.580**	1605.977	**1589.958**	1590.966	1588.951	G[14]
K[12]	1142.675	1548.956	**1532.937**	1533.945	1531.929	K[13]
G[13]	**1199.697**	1420.861	1404.842	**1405.850**	1403.834	G[12]
G[14]	**1256.718**	1363.839	**1347.821**	**1348.828**	1346.813	G[11]
A[15]	1327.755	1306.818	1290.799	1291.807	1289.791	A[10]
K[16]	1455.850	1235.781	**1219.762**	**1220.770**	1218.754	K[9]
R[17]	1611.951	1107.686	1091.667	1092.675	1090.659	R[8]
H[18]	1749.010	951.585	935.566	936.574	934.558	H[7]
R[19]	1905.111	814.526	**798.507**	**799.515**	797.499	R[6]
K[20]	2061.238	**658.425**	642.406	643.414	641.398	K[5]
V[21]	2160.306	502.298	486.280	487.287	485.272	V[4]
L[22]	2273.390	403.230	387.211	388.219	386.203	L[3]
R[23]	2429.491	290.146	274.127	275.135	273.119	R[2]
D[24]	2544.518	134.045	118.026	119.034	117.018	D[1]

sp|P62806|H4_MOUSE

[Acetyl]SGRGKGGKGLGKGGAKRHRK $^{Dimethyl}_{28.03}$ VLRD

- fragmentation table for charge state 2+
- specType=etd
- score=57.75
- F104903.dat
- query=q1831_p1
- precursor=849.178390
- chargePrecursor=3
- itol=0.8

AA	c	y	z+1	z+2	z	AA
S[1]	74.042	**1273.255**	**1265.245**	0.504	**1264.741**	S[24]
G[2]	102.553	1208.733	**1200.724**	0.504	**1200.220**	G[23]
R[3]	180.603	1180.223	**1172.213**	**1172.717**	**1171.709**	R[22]
G[4]	209.114	1102.172	1094.163	1094.667	1093.659	G[21]
K[5]	273.161	1073.661	1065.652	1066.156	1065.148	K[20]
G[6]	301.672	1009.614	1001.605	1002.108	1001.101	G[19]
G[7]	330.183	981.103	973.094	973.598	972.590	G[18]
K[8]	394.230	952.592	944.583	945.087	944.079	K[17]
G[9]	422.741	888.545	880.536	881.040	880.032	G[16]
L[10]	479.283	860.034	852.025	852.529	851.521	L[15]
G[11]	507.794	803.492	795.483	795.987	794.979	G[14]
K[12]	571.841	774.982	766.972	767.476	766.468	K[13]
G[13]	600.352	710.934	702.925	703.429	702.421	G[12]
G[14]	628.863	682.423	674.414	674.918	673.910	G[11]
A[15]	664.381	653.913	645.903	646.407	645.399	A[10]
K[16]	728.429	618.394	610.385	610.889	609.881	K[9]
R[17]	806.479	554.347	546.337	546.841	**545.833**	R[8]
H[18]	875.009	476.296	468.287	468.791	467.783	H[7]
R[19]	953.059	407.767	399.757	400.261	399.253	R[6]
K[20]	1031.122	329.716	321.707	322.211	321.203	K[5]
V[21]	1080.657	251.653	243.643	244.147	243.140	V[4]
L[22]	**1137.199**	202.119	194.109	194.613	193.605	L[3]
R[23]	**1215.249**	145.577	137.567	138.071	137.063	R[2]
D[24]	**1272.763**	67.526	59.517	60.021	59.013	D[1]

sp|P62806|H4_MOUSE

[Acetyl]SGRGKGGKGLGKGGAKRHRK$^{Acetyl}_{42.01}$VLRD

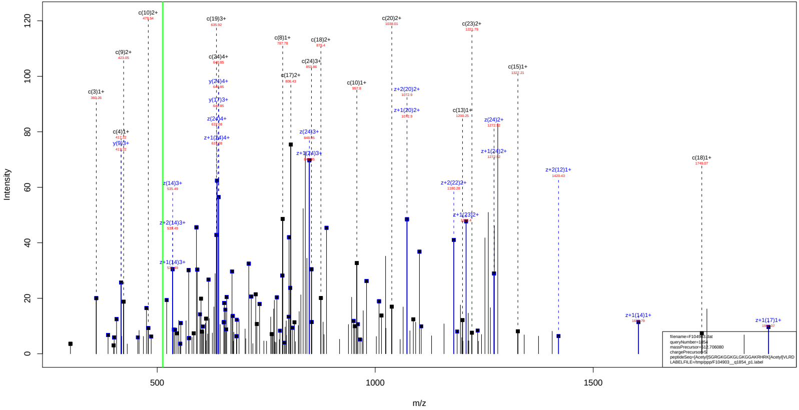

sp|P62806|H4_MOUSE

[Acetyl]SGRGKGGKGLGKGGAKRHRK $^{Acetyl}_{42.01}$ VLRD

- fragmentation table for charge state 1+
- specType=etd
- score=62.39
- F104903.dat
- query=q1854_p1
- precursor=512.706080
- chargePrecursor=5
- itol=0.8

AA	c	y	z+1	z+2	z	AA
S[1]	147.076	2559.481	2543.463	0.000	2542.455	S[24]
G[2]	204.098	2430.439	2414.420	0.000	2413.412	G[23]
R[3]	**360.199**	2373.417	2357.399	2358.407	2356.391	R[22]
G[4]	**417.220**	2217.316	2201.298	2202.305	2200.290	G[21]
K[5]	**545.315**	2160.295	2144.276	2145.284	2143.268	K[20]
G[6]	**602.337**	2032.200	2016.181	2017.189	2015.173	G[19]
G[7]	**659.358**	1975.178	1959.160	1960.168	1958.152	G[18]
K[8]	**787.453**	1918.157	**1902.138**	1903.146	1901.130	K[17]
G[9]	844.475	1790.062	1774.043	1775.051	1773.035	G[16]
L[10]	**957.559**	1733.041	1717.022	1718.030	1716.014	L[15]
G[11]	**1014.580**	1619.956	**1603.938**	1604.946	1602.930	G[14]
K[12]	1142.675	1562.935	1546.916	1547.924	1545.908	K[13]
G[13]	**1199.697**	1434.840	1418.821	**1419.829**	1417.813	G[12]
G[14]	1256.718	1377.819	1361.800	1362.808	1360.792	G[11]
A[15]	**1327.755**	1320.797	1304.778	1305.786	1303.771	A[10]
K[16]	1455.850	1249.760	1233.741	**1234.749**	1232.733	K[9]
R[17]	1611.951	1121.665	**1105.646**	1106.654	1104.638	R[8]
H[18]	**1749.010**	**965.564**	949.545	**950.553**	948.537	H[7]
R[19]	1905.111	828.505	812.486	813.494	811.478	R[6]
K[20]	2075.217	**672.404**	**656.385**	**657.393**	655.377	K[5]
V[21]	2174.285	502.298	**486.280**	487.287	485.272	V[4]
L[22]	2287.369	403.230	**387.211**	388.219	386.203	L[3]
R[23]	2443.471	290.146	274.127	275.135	273.119	R[2]
D[24]	2558.497	134.045	118.026	119.034	117.018	D[1]

sp|P62806|H4_MOUSE

[Acetyl]SGRGKGGKGLGKGGAKRHRK$^{Acetyl}_{42.01}$VLRD

- fragmentation table for charge state 2+
- specType=etd
- score=62.39
- F104903.dat
- query=q1854_p1
- precursor=512.706080
- chargePrecursor=5
- itol=0.8

AA	c	y	z+1	z+2	z	AA
S[1]	74.042	1280.244	**1272.235**	0.504	**1271.731**	S[24]
G[2]	102.553	1215.723	**1207.714**	0.504	1207.210	G[23]
R[3]	180.603	**1187.212**	1179.203	**1179.707**	1178.699	R[22]
G[4]	209.114	1109.162	**1101.152**	**1101.656**	1100.649	G[21]
K[5]	273.161	1080.651	**1072.642**	**1073.146**	**1072.138**	K[20]
G[6]	**301.672**	1016.604	**1008.594**	**1009.098**	**1008.090**	G[19]
G[7]	330.183	988.093	**980.083**	**980.587**	**979.580**	G[18]
K[8]	394.230	**959.582**	951.573	952.077	**951.069**	K[17]
G[9]	**422.741**	895.535	**887.525**	**888.029**	887.021	G[16]
L[10]	**479.283**	867.024	859.015	859.518	858.511	L[15]
G[11]	507.794	**810.482**	**802.473**	**802.976**	**801.969**	G[14]
K[12]	**571.841**	**781.971**	**773.962**	**774.466**	773.458	K[13]
G[13]	**600.352**	717.924	**709.914**	**710.418**	**709.410**	G[12]
G[14]	628.863	689.413	**681.404**	**681.907**	680.900	G[11]
A[15]	664.381	660.902	**652.893**	**653.397**	**652.389**	A[10]
K[16]	**728.429**	625.384	**617.374**	**617.878**	616.870	K[9]
R[17]	**806.479**	561.336	**553.327**	**553.831**	**552.823**	R[8]
H[18]	**875.009**	483.286	**475.276**	**475.780**	**474.772**	H[7]
R[19]	**953.059**	414.756	**406.747**	**407.251**	**406.243**	R[6]
K[20]	**1038.112**	336.706	328.696	329.200	328.192	K[5]
V[21]	**1087.646**	251.653	243.643	244.147	243.140	V[4]
L[22]	1144.188	202.119	194.109	194.613	193.605	L[3]
R[23]	**1222.239**	145.577	137.567	138.071	137.063	R[2]
D[24]	1279.752	67.526	59.517	60.021	59.013	D[1]

sp|P62806|H4_MOUSE

[Acetyl]SGRGKGGKGLGKGGAKRHRK$^{\text{Acetyl}}_{42.01}$VLRD

- fragmentation table for charge state 3+
- specType=etd
- score=62.39
- F104903.dat
- query=q1854_p1
- precursor=512.706080
- chargePrecursor=5
- itol=0.8

AA	c	y	z+1	z+2	z	AA
S[1]	49.697	**853.832**	**848.492**	0.672	**848.156**	S[24]
G[2]	68.704	**810.818**	**805.478**	0.672	**805.142**	G[23]
R[3]	120.738	**791.811**	**786.471**	**786.807**	786.135	R[22]
G[4]	139.745	739.777	**734.437**	**734.773**	**734.101**	G[21]
K[5]	182.443	720.770	**715.430**	**715.766**	**715.094**	K[20]
G[6]	201.450	678.071	**672.732**	**673.068**	**672.396**	G[19]
G[7]	220.458	**659.064**	**653.725**	**654.061**	**653.389**	G[18]
K[8]	263.156	**640.057**	634.718	635.054	634.382	K[17]
G[9]	282.163	**597.359**	**592.019**	**592.355**	**591.683**	G[16]
L[10]	319.858	578.352	**573.012**	**573.348**	**572.676**	L[15]
G[11]	338.865	**540.657**	**535.317**	**535.653**	**534.981**	G[14]
K[12]	381.563	**521.650**	516.310	516.646	515.974	K[13]
G[13]	**400.570**	**478.952**	473.612	473.948	473.276	G[12]
G[14]	419.578	459.944	454.605	**454.941**	454.269	G[11]
A[15]	443.257	440.937	435.598	435.934	435.262	A[10]
K[16]	**485.955**	**417.258**	411.919	412.255	411.583	K[9]
R[17]	**537.989**	374.560	369.220	369.556	368.884	R[8]
H[18]	**583.675**	322.526	317.187	317.523	316.851	H[7]
R[19]	**635.709**	276.840	271.500	271.836	271.164	R[6]
K[20]	692.410	224.806	219.467	219.803	219.131	K[5]
V[21]	**725.433**	168.104	162.765	163.101	162.429	V[4]
L[22]	**763.128**	135.082	129.742	130.078	129.406	L[3]
R[23]	**815.162**	97.387	92.047	92.383	91.711	R[2]
D[24]	**853.504**	45.353	40.014	40.349	39.678	D[1]

sp|P62806|H4_MOUSE

[Acetyl]SGRGKGGKGLGKGGAKRHRK$^{\text{Acetyl}}_{42.01}$VLRD

- fragmentation table for charge state 4+
- specType=etd
- score=62.39
- F104903.dat
- query=q1854_p1
- precursor=512.706080
- chargePrecursor=5
- itol=0.8

AA	c	y	z+1	z+2	z	AA
S[1]	37.525	**640.626**	**636.621**	0.755	**636.369**	S[24]
G[2]	51.780	608.365	**604.360**	0.755	**604.109**	G[23]
R[3]	90.805	594.110	**590.105**	**590.357**	**589.853**	R[22]
G[4]	105.061	555.085	551.080	551.332	550.828	G[21]
K[5]	137.084	**540.829**	536.824	537.076	536.573	K[20]
G[6]	151.340	508.805	504.801	505.053	504.549	G[19]
G[7]	165.595	494.550	490.545	490.797	490.293	G[18]
K[8]	197.619	**480.295**	476.290	476.542	**476.038**	K[17]
G[9]	211.874	448.271	444.266	444.518	444.014	G[16]
L[10]	240.145	434.016	430.011	430.263	429.759	L[15]
G[11]	254.401	405.745	**401.740**	**401.992**	**401.488**	G[14]
K[12]	286.424	391.489	**387.485**	**387.736**	**387.233**	K[13]
G[13]	**300.680**	**359.465**	355.461	355.713	355.209	G[12]
G[14]	314.935	345.210	341.205	341.457	340.953	G[11]
A[15]	332.694	330.955	326.950	327.202	326.698	A[10]
K[16]	364.718	313.195	309.191	309.443	308.939	K[9]
R[17]	403.743	281.172	277.167	277.419	276.915	R[8]
H[18]	438.008	242.146	238.142	238.394	237.890	H[7]
R[19]	477.033	207.882	203.877	204.129	203.625	R[6]
K[20]	519.560	168.856	164.852	165.104	164.600	K[5]
V[21]	544.327	126.330	122.325	122.577	122.073	V[4]
L[22]	**572.598**	101.563	97.558	97.810	97.306	L[3]
R[23]	**611.623**	73.292	69.287	69.539	69.035	R[2]
D[24]	**640.380**	34.267	30.262	30.514	30.010	D[1]

sp|P22752|H2A1_MOUSE

SHHKAKGK $^{\text{Acetyl}}_{42.01}$

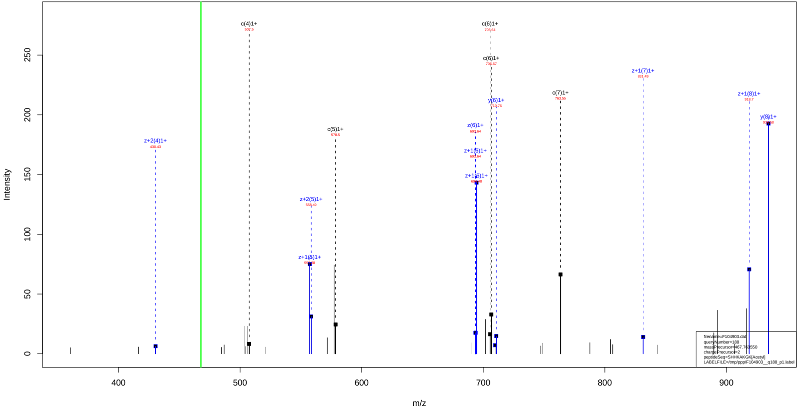

sp|P22752|H2A1_MOUSE

SHHKAKGK $^{Acetyl}_{42.01}$

- fragmentation table for charge state 1+
- specType=etd
- score=31.77
- F104903.dat
- query=q188_p1
- precursor=467.763550
- chargePrecursor=2
- itol=0.8

AA	c	y	z+1	z+2	z	AA
S[1]	105.066	**934.522**	**918.503**	0.000	917.495	S[8]
H[2]	242.125	847.490	**831.471**	0.000	830.463	H[7]
H[3]	379.184	**710.431**	**694.412**	0.000	**693.404**	H[6]
K[4]	**507.279**	573.372	**557.353**	**558.361**	556.345	K[5]
A[5]	**578.316**	445.277	429.258	**430.266**	428.250	A[4]
K[6]	**706.411**	374.240	358.221	359.229	357.213	K[3]
G[7]	**763.432**	246.145	230.126	231.134	229.118	G[2]
K[8]	933.538	189.123	173.105	174.112	172.097	K[1]

sp|P62806|H4_MOUSE

[Acetyl]SGRGKGGKGLGKGGAK $^{\text{Acetyl}}_{42.01}$ RHRK $^{\text{Dimethyl}}_{28.03}$ VLRD

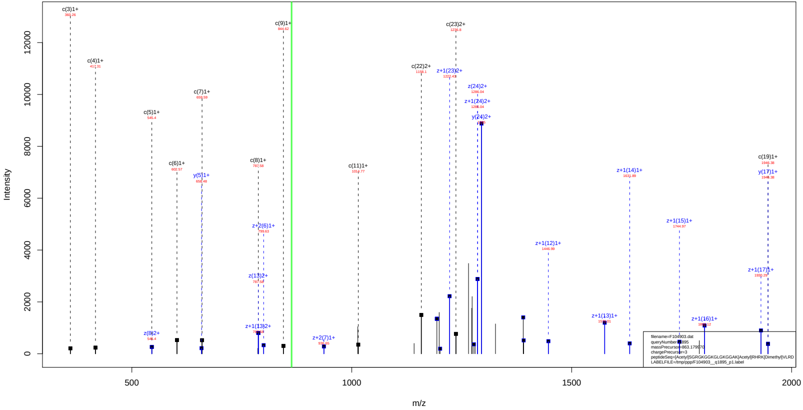

sp|P62806|H4_MOUSE

[Acetyl]SGRGKGGKGLGKGGAK $^{\text{Acetyl}}_{42.01}$ RHRK $^{\text{Dimethyl}}_{28.03}$ VLRD

- fragmentation table for charge state 1+
- specType=etd
- score=61.25
- F104903.dat
- query=q1895_p1
- precursor=863.179970
- chargePrecursor=3
- itol=0.8

AA	c	y	z+1	z+2	z	AA
S[1]	147.076	2587.513	2571.494	0.000	2570.486	S[24]
G[2]	204.098	2458.470	2442.451	0.000	2441.444	G[23]
R[3]	**360.199**	2401.449	2385.430	2386.438	2384.422	R[22]
G[4]	**417.220**	2245.348	2229.329	2230.337	2228.321	G[21]
K[5]	**545.315**	2188.326	2172.307	2173.315	2171.300	K[20]
G[6]	**602.337**	2060.231	2044.212	2045.220	2043.205	G[19]
G[7]	**659.358**	2003.210	1987.191	1988.199	1986.183	G[18]
K[8]	**787.453**	**1946.188**	**1930.170**	1931.177	1929.162	K[17]
G[9]	**844.475**	1818.093	**1802.075**	1803.082	1801.067	G[16]
L[10]	957.559	1761.072	**1745.053**	1746.061	1744.045	L[15]
G[11]	**1014.580**	1647.988	**1631.969**	1632.977	1630.961	G[14]
K[12]	1142.675	1590.966	**1574.948**	1575.955	1573.940	K[13]
G[13]	1199.697	1462.871	**1446.853**	1447.860	1445.845	G[12]
G[14]	1256.718	1405.850	**1389.831**	**1390.839**	1388.823	G[11]
A[15]	1327.755	1348.828	1332.810	1333.818	1331.802	A[10]
K[16]	1497.861	**1277.791**	1261.773	1262.780	1260.765	K[9]
R[17]	1653.962	1107.686	1091.667	1092.675	1090.659	R[8]
H[18]	1791.021	951.585	935.566	**936.574**	934.558	H[7]
R[19]	**1947.122**	814.526	798.507	**799.515**	797.499	R[6]
K[20]	2103.248	**658.425**	642.406	643.414	641.398	K[5]
V[21]	2202.317	502.298	486.280	487.287	485.272	V[4]
L[22]	2315.401	403.230	387.211	388.219	386.203	L[3]
R[23]	2471.502	290.146	274.127	275.135	273.119	R[2]
D[24]	2586.529	134.045	118.026	119.034	117.018	D[1]

sp|P62806|H4_MOUSE

[Acetyl]SGRGKGGKGLGKGGAK $^{Acetyl}_{42.01}$ RHRK $^{Dimethyl}_{28.03}$ VLRD

- fragmentation table for charge state 2+
- specType=etd
- score=61.25
- F104903.dat
- query=q1895_p1
- precursor=863.179970
- chargePrecursor=3
- itol=0.8

AA	c	y	z+1	z+2	z	AA
S[1]	74.042	**1294.260**	**1286.251**	0.504	**1285.747**	S[24]
G[2]	102.553	1229.739	**1221.729**	0.504	1221.225	G[23]
R[3]	180.603	**1201.228**	**1193.219**	**1193.723**	**1192.715**	R[22]
G[4]	209.114	1123.177	1115.168	1115.672	1114.664	G[21]
K[5]	273.161	1094.667	1086.657	1087.161	1086.153	K[20]
G[6]	301.672	1030.619	1022.610	1023.114	1022.106	G[19]
G[7]	330.183	1002.108	994.099	994.603	993.595	G[18]
K[8]	394.230	973.598	965.588	966.092	965.084	K[17]
G[9]	422.741	909.550	901.541	902.045	901.037	G[16]
L[10]	479.283	881.040	873.030	873.534	872.526	L[15]
G[11]	507.794	824.498	816.488	816.992	815.984	G[14]
K[12]	571.841	795.987	**787.977**	788.481	**787.474**	K[13]
G[13]	600.352	731.939	723.930	724.434	723.426	G[12]
G[14]	628.863	703.429	695.419	695.923	694.915	G[11]
A[15]	664.381	674.918	666.908	667.412	666.405	A[10]
K[16]	749.434	639.399	631.390	631.894	630.886	K[9]
R[17]	827.485	554.347	546.337	546.841	**545.833**	R[8]
H[18]	896.014	476.296	468.287	468.791	467.783	H[7]
R[19]	974.065	407.767	399.757	400.261	399.253	R[6]
K[20]	1052.128	329.716	321.707	322.211	321.203	K[5]
V[21]	1101.662	251.653	243.643	244.147	243.140	V[4]
L[22]	**1158.204**	202.119	194.109	194.613	193.605	L[3]
R[23]	**1236.255**	145.577	137.567	138.071	137.063	R[2]
D[24]	1293.768	67.526	59.517	60.021	59.013	D[1]

sp|P62806|H4_MOUSE

[Acetyl]SGRGKGGKGLGKGGAK $^{Acetyl}_{42.01}$ RHRK $^{Dimethyl}_{28.03}$ VLRD

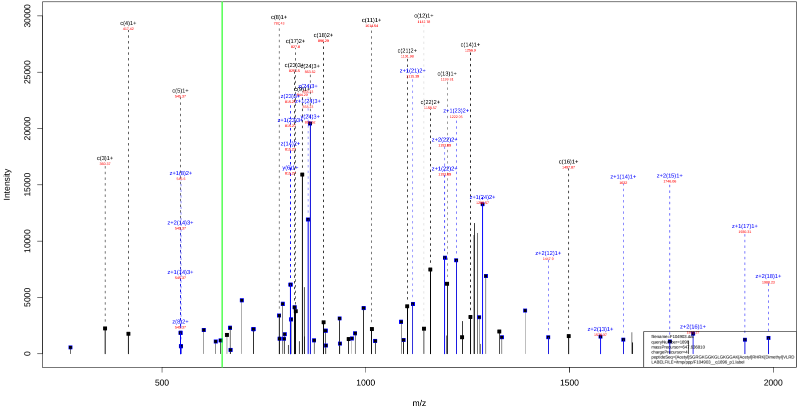

sp|P62806|H4_MOUSE

[Acetyl]SGRGKGGKGLGKGGAK $^{\text{Acetyl}}_{42.01}$ RHRK $^{\text{Dimethyl}}_{28.03}$ VLRD

- fragmentation table for charge state 1+
- specType=etd
- score=115.26
- F104903.dat
- query=q1896_p1
- precursor=647.636810
- chargePrecursor=4
- itol=0.8

AA	c	y	z+1	z+2	z	AA
S[1]	147.076	2587.513	2571.494	0.000	2570.486	S[24]
G[2]	204.098	2458.470	2442.451	0.000	2441.444	G[23]
R[3]	**360.199**	2401.449	2385.430	2386.438	2384.422	R[22]
G[4]	**417.220**	2245.348	2229.329	2230.337	2228.321	G[21]
K[5]	**545.315**	2188.326	2172.307	2173.315	2171.300	K[20]
G[6]	**602.337**	2060.231	2044.212	2045.220	2043.205	G[19]
G[7]	**659.358**	2003.210	1987.191	**1988.199**	1986.183	G[18]
K[8]	**787.453**	1946.188	**1930.170**	1931.177	1929.162	K[17]
G[9]	**844.475**	1818.093	1802.075	**1803.082**	1801.067	G[16]
L[10]	**957.559**	1761.072	1745.053	**1746.061**	1744.045	L[15]
G[11]	**1014.580**	1647.988	**1631.969**	1632.977	1630.961	G[14]
K[12]	**1142.675**	1590.966	1574.948	**1575.955**	1573.940	K[13]
G[13]	**1199.697**	1462.871	1446.853	**1447.860**	1445.845	G[12]
G[14]	**1256.718**	1405.850	1389.831	**1390.839**	1388.823	G[11]
A[15]	**1327.755**	1348.828	1332.810	**1333.818**	1331.802	A[10]
K[16]	**1497.861**	**1277.791**	1261.773	1262.780	1260.765	K[9]
R[17]	1653.962	1107.686	1091.667	**1092.675**	1090.659	R[8]
H[18]	1791.021	951.585	**935.566**	**936.574**	934.558	H[7]
R[19]	1947.122	**814.526**	798.507	**799.515**	797.499	R[6]
K[20]	2103.248	658.425	642.406	**643.414**	641.398	K[5]
V[21]	2202.317	502.298	486.280	487.287	485.272	V[4]
L[22]	2315.401	403.230	387.211	388.219	386.203	L[3]
R[23]	2471.502	290.146	274.127	**275.135**	273.119	R[2]
D[24]	2586.529	134.045	118.026	119.034	117.018	D[1]

sp|P62806|H4_MOUSE

[Acetyl]SGRGKGGKGLGKGGAK $^{Acetyl}_{42.01}$ RHRK $^{Dimethyl}_{28.03}$ VLRD

- fragmentation table for charge state 2+
- specType=etd
- score=115.26
- F104903.dat
- query=q1896_p1
- precursor=647.636810
- chargePrecursor=4
- itol=0.8

AA	c	y	z+1	z+2	z	AA
S[1]	74.042	**1294.260**	**1286.251**	0.504	1285.747	S[24]
G[2]	102.553	1229.739	**1221.729**	0.504	1221.225	G[23]
R[3]	180.603	1201.228	**1193.219**	**1193.723**	1192.715	R[22]
G[4]	209.114	1123.177	**1115.168**	**1115.672**	**1114.664**	G[21]
K[5]	273.161	1094.667	**1086.657**	**1087.161**	**1086.153**	K[20]
G[6]	301.672	1030.619	**1022.610**	**1023.114**	1022.106	G[19]
G[7]	330.183	1002.108	**994.099**	**994.603**	993.595	G[18]
K[8]	394.230	**973.598**	**965.588**	**966.092**	965.084	K[17]
G[9]	422.741	909.550	**901.541**	**902.045**	**901.037**	G[16]
L[10]	479.283	881.040	**873.030**	**873.534**	872.526	L[15]
G[11]	507.794	**824.498**	**816.488**	**816.992**	**815.984**	G[14]
K[12]	571.841	**795.987**	**787.977**	**788.481**	**787.474**	K[13]
G[13]	600.352	731.939	**723.930**	**724.434**	723.426	G[12]
G[14]	628.863	703.429	**695.419**	**695.923**	694.915	G[11]
A[15]	664.381	674.918	**666.908**	**667.412**	**666.405**	A[10]
K[16]	749.434	639.399	**631.390**	**631.894**	630.886	K[9]
R[17]	**827.485**	554.347	**546.337**	**546.841**	**545.833**	R[8]
H[18]	**896.014**	476.296	468.287	468.791	467.783	H[7]
R[19]	**974.065**	407.767	399.757	400.261	399.253	R[6]
K[20]	1052.128	329.716	321.707	322.211	321.203	K[5]
V[21]	**1101.662**	251.653	243.643	244.147	243.140	V[4]
L[22]	**1158.204**	202.119	194.109	194.613	193.605	L[3]
R[23]	**1236.255**	145.577	137.567	138.071	137.063	R[2]
D[24]	1293.768	67.526	59.517	60.021	59.013	D[1]

sp|P62806|H4_MOUSE

[Acetyl]SGRGKGGKGLGKGGAK $^{Acetyl}_{42.01}$ RHRK $^{Dimethyl}_{28.03}$ VLRD

- fragmentation table for charge state 3+
- specType=etd
- score=115.26
- F104903.dat
- query=q1896_p1
- precursor=647.636810
- chargePrecursor=4
- itol=0.8

AA	c	y	z+1	z+2	z	AA
S[1]	49.697	**863.176**	**857.836**	0.672	**857.500**	S[24]
G[2]	68.704	820.162	**814.822**	0.672	**814.486**	G[23]
R[3]	120.738	**801.154**	**795.815**	**796.151**	**795.479**	R[22]
G[4]	139.745	749.121	743.781	744.117	743.445	G[21]
K[5]	182.443	730.114	**724.774**	**725.110**	**724.438**	K[20]
G[6]	201.450	687.415	682.076	682.412	681.740	G[19]
G[7]	220.458	**668.408**	663.069	663.404	662.733	G[18]
K[8]	263.156	649.401	**644.061**	**644.397**	**643.725**	K[17]
G[9]	282.163	606.703	601.363	**601.699**	601.027	G[16]
L[10]	319.858	587.695	582.356	582.692	582.020	L[15]
G[11]	338.865	550.001	**544.661**	**544.997**	544.325	G[14]
K[12]	381.563	530.994	525.654	525.990	525.318	K[13]
G[13]	400.570	488.295	482.956	483.292	482.620	G[12]
G[14]	419.578	469.288	463.949	464.285	463.613	G[11]
A[15]	443.257	450.281	444.941	445.277	444.605	A[10]
K[16]	499.958	426.602	421.262	421.598	420.926	K[9]
R[17]	551.992	369.900	364.561	364.896	364.225	R[8]
H[18]	597.678	317.866	312.527	312.863	312.191	H[7]
R[19]	649.712	272.180	266.841	267.176	266.505	R[6]
K[20]	701.754	220.146	214.807	215.143	214.471	K[5]
V[21]	734.777	168.104	162.765	163.101	162.429	V[4]
L[22]	772.472	135.082	129.742	130.078	129.406	L[3]
R[23]	**824.505**	97.387	92.047	92.383	91.711	R[2]
D[24]	**862.848**	45.353	40.014	40.349	39.678	D[1]

sp|P22752|H2A1_MOUSE

SHHKAKGK $^{\text{Acetyl}}_{42.01}$

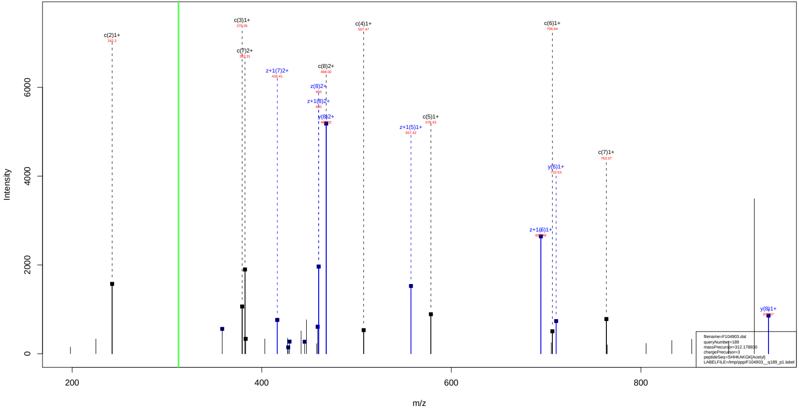

sp|P22752|H2A1_MOUSE

SHHKAKGK $^{Acetyl}_{42.01}$

- fragmentation table for charge state 1+
- specType=etd
- score=37.63
- F104903.dat
- query=q189_p1
- precursor=312.178930
- chargePrecursor=3
- itol=0.8

AA	c	y	z+1	z+2	z	AA
S[1]	105.066	**934.522**	918.503	0.000	917.495	S[8]
H[2]	**242.125**	847.490	831.471	0.000	830.463	H[7]
H[3]	**379.184**	**710.431**	**694.412**	0.000	693.404	H[6]
K[4]	**507.279**	573.372	**557.353**	558.361	556.345	K[5]
A[5]	**578.316**	**445.277**	**429.258**	430.266	**428.250**	A[4]
K[6]	**706.411**	374.240	**358.221**	359.229	357.213	K[3]
G[7]	**763.432**	246.145	230.126	231.134	229.118	G[2]
K[8]	933.538	189.123	173.105	174.112	172.097	K[1]

sp|P22752|H2A1_MOUSE

SHHKAKGK $^{\text{Acetyl}}_{42.01}$

- fragmentation table for charge state 2+
- specType=etd
- score=37.63
- F104903.dat
- query=q189_p1
- precursor=312.178930
- chargePrecursor=3
- itol=0.8

AA	c	y	z+1	z+2	z	AA
S[1]	53.037	**467.765**	**459.755**	0.504	**459.251**	S[8]
H[2]	121.566	424.248	**416.239**	0.504	**415.735**	H[7]
H[3]	190.095	355.719	347.710	0.504	347.206	H[6]
K[4]	254.143	287.190	279.180	279.684	278.676	K[5]
A[5]	289.662	223.142	215.133	215.637	214.629	A[4]
K[6]	353.709	187.624	179.614	180.118	179.110	K[3]
G[7]	**382.220**	123.576	115.567	116.071	115.063	G[2]
K[8]	**467.272**	95.065	87.056	87.560	86.552	K[1]

sp|P22752|H2A1_MOUSE

SHHKAK $^{\text{Acetyl}}_{42.01}$ GK

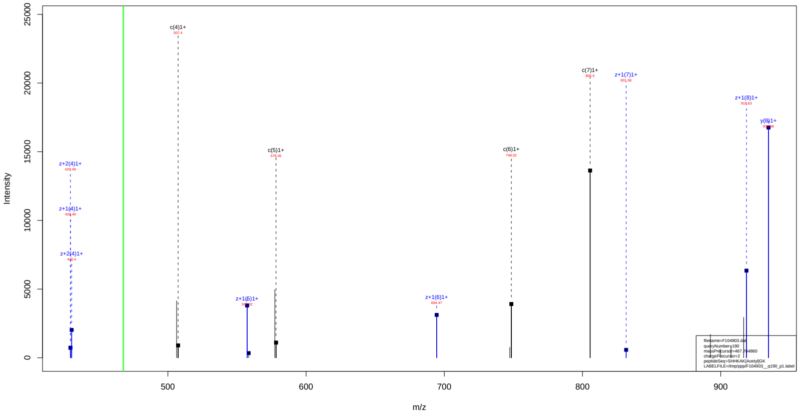

sp|P22752|H2A1_MOUSE

SHHKAK $^{\text{Acetyl}}_{42.01}$ GK

- fragmentation table for charge state 1+
- specType=etd
- score=36.98
- F104903.dat
- query=q190_p1
- precursor=467.764860
- chargePrecursor=2
- itol=0.8

AA	c	y	z+1	z+2	z	AA
S[1]	105.066	**934.522**	**918.503**	0.000	917.495	S[8]
H[2]	242.125	847.490	**831.471**	0.000	830.463	H[7]
H[3]	379.184	710.431	**694.412**	0.000	693.404	H[6]
K[4]	**507.279**	573.372	**557.353**	**558.361**	556.345	K[5]
A[5]	**578.316**	445.277	**429.258**	**430.266**	428.250	A[4]
K[6]	**748.421**	374.240	358.221	359.229	357.213	K[3]
G[7]	**805.443**	204.134	188.116	189.123	187.108	G[2]
K[8]	933.538	147.113	131.094	132.102	130.086	K[1]

sp|P22752|H2A1_MOUSE

SHHKAK $^{\text{Acetyl}}_{42.01}$ GK

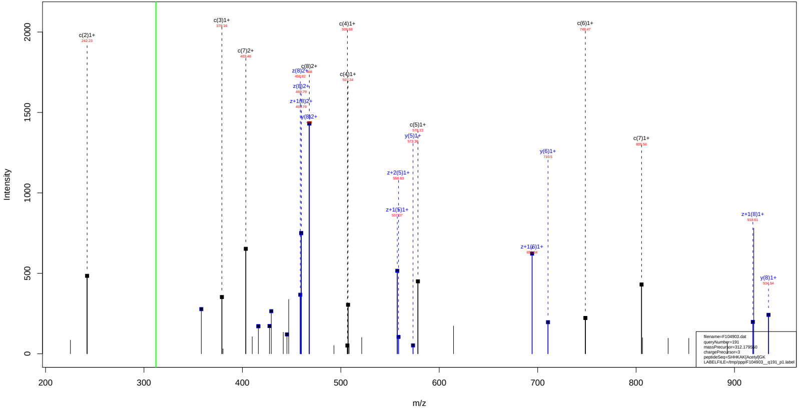

sp|P22752|H2A1_MOUSE

SHHKAK$^{Acetyl}_{42.01}$GK

- fragmentation table for charge state 1+
- specType=etd
- score=34.21
- F104903.dat
- query=q191_p1
- precursor=312.179550
- chargePrecursor=3
- itol=0.8

AA	c	y	z+1	z+2	z	AA
S[1]	105.066	**934.522**	**918.503**	0.000	917.495	S[8]
H[2]	**242.125**	847.490	831.471	0.000	830.463	H[7]
H[3]	**379.184**	**710.431**	**694.412**	0.000	693.404	H[6]
K[4]	**507.279**	**573.372**	**557.353**	**558.361**	556.345	K[5]
A[5]	**578.316**	**445.277**	**429.258**	430.266	**428.250**	A[4]
K[6]	**748.421**	374.240	**358.221**	359.229	357.213	K[3]
G[7]	**805.443**	204.134	188.116	189.123	187.108	G[2]
K[8]	933.538	147.113	131.094	132.102	130.086	K[1]

sp|P22752|H2A1_MOUSE

SHHKAK $^{\text{Acetyl}}_{42.01}$ GK

- fragmentation table for charge state 2+
- specType=etd
- score=34.21
- F104903.dat
- query=q191_p1
- precursor=312.179550
- chargePrecursor=3
- itol=0.8

AA	c	y	z+1	z+2	z	AA
S[1]	53.037	**467.765**	**459.755**	0.504	**459.251**	S[8]
H[2]	121.566	424.248	**416.239**	0.504	**415.735**	H[7]
H[3]	190.095	355.719	347.710	0.504	347.206	H[6]
K[4]	254.143	287.190	279.180	279.684	278.676	K[5]
A[5]	289.662	223.142	215.133	215.637	214.629	A[4]
K[6]	374.714	187.624	179.614	180.118	179.110	K[3]
G[7]	**403.225**	102.571	94.561	95.065	94.057	G[2]
K[8]	**467.272**	74.060	66.051	66.555	65.547	K[1]

sp|P62806|H4_MOUSE

[Acetyl]SGRGKGGKGLGK $^{Acetyl}_{42.01}$ GGAK $^{Acetyl}_{42.01}$ RHRK $^{Dimethyl}_{28.03}$ VLRD

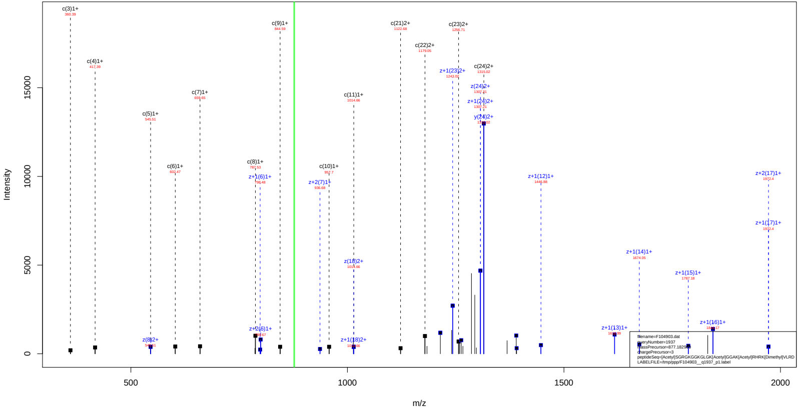

sp|P62806|H4_MOUSE

[Acetyl]SGRGKGGKGLGK $^{Acetyl}_{42.01}$ GGAK $^{Acetyl}_{42.01}$ RHRK $^{Dimethyl}_{28.03}$ VLRD

- fragmentation table for charge state 1+
- specType=etd
- score=69.41
- F104903.dat
- query=q1937_p1
- precursor=877.182900
- chargePrecursor=3
- itol=0.8

AA	c	y	z+1	z+2	z	AA
S[1]	147.076	2629.523	2613.505	0.000	2612.497	S[24]
G[2]	204.098	2500.481	2484.462	0.000	2483.454	G[23]
R[3]	**360.199**	2443.459	2427.441	2428.448	2426.433	R[22]
G[4]	**417.220**	2287.358	2271.339	2272.347	2270.332	G[21]
K[5]	**545.315**	2230.337	2214.318	2215.326	2213.310	K[20]
G[6]	**602.337**	2102.242	2086.223	2087.231	2085.215	G[19]
G[7]	**659.358**	2045.220	2029.202	2030.209	2028.194	G[18]
K[8]	**787.453**	1988.199	**1972.180**	**1973.188**	1971.172	K[17]
G[9]	**844.475**	1860.104	**1844.085**	1845.093	1843.077	G[16]
L[10]	**957.559**	1803.082	**1787.064**	1788.071	1786.056	L[15]
G[11]	**1014.580**	1689.998	**1673.980**	1674.987	1672.972	G[14]
K[12]	1184.686	1632.977	**1616.958**	1617.966	1615.950	K[13]
G[13]	1241.707	1462.871	**1446.853**	1447.860	1445.845	G[12]
G[14]	1298.729	1405.850	**1389.831**	**1390.839**	1388.823	G[11]
A[15]	1369.766	1348.828	1332.810	1333.818	1331.802	A[10]
K[16]	1539.871	1277.791	1261.773	**1262.780**	1260.765	K[9]
R[17]	1695.973	1107.686	1091.667	1092.675	1090.659	R[8]
H[18]	1833.031	951.585	935.566	**936.574**	934.558	H[7]
R[19]	1989.133	814.526	**798.507**	**799.515**	797.499	R[6]
K[20]	2145.259	658.425	642.406	643.414	641.398	K[5]
V[21]	2244.327	502.298	486.280	487.287	485.272	V[4]
L[22]	2357.411	403.230	387.211	388.219	386.203	L[3]
R[23]	2513.512	290.146	274.127	275.135	273.119	R[2]
D[24]	2628.539	134.045	118.026	119.034	117.018	D[1]

sp|P62806|H4_MOUSE

[Acetyl]SGRGKGGKGLGK $^{\text{Acetyl}}_{42.01}$ GGAK $^{\text{Acetyl}}_{42.01}$ RHRK $^{\text{Dimethyl}}_{28.03}$ VLRD

- fragmentation table for charge state 2+
- specType=etd
- score=69.41
- F104903.dat
- query=q1937_p1
- precursor=877.182900
- chargePrecursor=3
- itol=0.8

AA	c	y	z+1	z+2	z	AA
S[1]	74.042	**1315.265**	**1307.256**	0.504	**1306.752**	S[24]
G[2]	102.553	1250.744	**1242.735**	0.504	**1242.231**	G[23]
R[3]	180.603	1222.233	**1214.224**	**1214.728**	1213.720	R[22]
G[4]	209.114	1144.183	1136.173	1136.677	1135.669	G[21]
K[5]	273.161	1115.672	1107.663	1108.167	1107.159	K[20]
G[6]	301.672	1051.625	1043.615	1044.119	1043.111	G[19]
G[7]	330.183	1023.114	**1015.104**	1015.608	**1014.601**	G[18]
K[8]	394.230	994.603	986.594	987.098	986.090	K[17]
G[9]	422.741	930.556	922.546	923.050	922.042	G[16]
L[10]	479.283	902.045	894.035	894.539	893.532	L[15]
G[11]	507.794	845.503	837.493	837.997	836.990	G[14]
K[12]	592.847	816.992	808.983	809.487	808.479	K[13]
G[13]	621.357	731.939	723.930	724.434	723.426	G[12]
G[14]	649.868	703.429	695.419	695.923	694.915	G[11]
A[15]	685.387	674.918	666.908	667.412	666.405	A[10]
K[16]	770.439	639.399	631.390	631.894	630.886	K[9]
R[17]	848.490	554.347	546.337	546.841	**545.833**	R[8]
H[18]	917.019	476.296	468.287	468.791	467.783	H[7]
R[19]	995.070	407.767	399.757	400.261	399.253	R[6]
K[20]	1073.133	329.716	321.707	322.211	321.203	K[5]
V[21]	**1122.667**	251.653	243.643	244.147	243.140	V[4]
L[22]	**1179.209**	202.119	194.109	194.613	193.605	L[3]
R[23]	**1257.260**	145.577	137.567	138.071	137.063	R[2]
D[24]	**1314.773**	67.526	59.517	60.021	59.013	D[1]

sp|P62806|H4_MOUSE

[Acetyl]SGRGKGGKGLGK $^{Acetyl}_{42.01}$ GGAK $^{Acetyl}_{42.01}$ RHRK $^{Dimethyl}_{28.03}$ VLRD

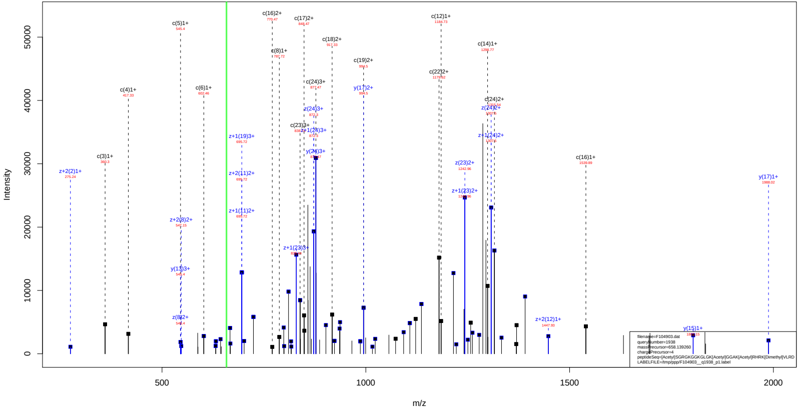

sp|P62806|H4_MOUSE

[Acetyl]SGRGKGGKGLGK $^{Acetyl}_{42.01}$ GGAK $^{Acetyl}_{42.01}$ RHRK $^{Dimethyl}_{28.03}$ VLRD

- fragmentation table for charge state 1+
- specType=etd
- score=77.58
- F104903.dat
- query=q1938_p1
- precursor=658.139260
- chargePrecursor=4
- itol=0.8

AA	c	y	z+1	z+2	z	AA
S[1]	147.076	2629.523	2613.505	0.000	2612.497	S[24]
G[2]	204.098	2500.481	2484.462	0.000	2483.454	G[23]
R[3]	**360.199**	2443.459	2427.441	2428.448	2426.433	R[22]
G[4]	**417.220**	2287.358	2271.339	2272.347	2270.332	G[21]
K[5]	**545.315**	2230.337	2214.318	2215.326	2213.310	K[20]
G[6]	**602.337**	2102.242	2086.223	2087.231	2085.215	G[19]
G[7]	659.358	2045.220	2029.202	2030.209	2028.194	G[18]
K[8]	**787.453**	**1988.199**	1972.180	1973.188	1971.172	K[17]
G[9]	844.475	1860.104	1844.085	1845.093	1843.077	G[16]
L[10]	957.559	**1803.082**	1787.064	1788.071	1786.056	L[15]
G[11]	1014.580	1689.998	1673.980	1674.987	1672.972	G[14]
K[12]	**1184.686**	1632.977	1616.958	1617.966	1615.950	K[13]
G[13]	1241.707	1462.871	1446.853	**1447.860**	1445.845	G[12]
G[14]	**1298.729**	1405.850	1389.831	**1390.839**	1388.823	G[11]
A[15]	**1369.766**	1348.828	**1332.810**	1333.818	1331.802	A[10]
K[16]	**1539.871**	**1277.791**	**1261.773**	1262.780	1260.765	K[9]
R[17]	1695.973	**1107.686**	1091.667	**1092.675**	1090.659	R[8]
H[18]	1833.031	951.585	**935.566**	**936.574**	934.558	H[7]
R[19]	1989.133	814.526	**798.507**	**799.515**	797.499	R[6]
K[20]	2145.259	658.425	642.406	**643.414**	641.398	K[5]
V[21]	2244.327	502.298	486.280	487.287	485.272	V[4]
L[22]	2357.411	403.230	387.211	388.219	386.203	L[3]
R[23]	2513.512	290.146	274.127	**275.135**	273.119	R[2]
D[24]	2628.539	134.045	118.026	119.034	117.018	D[1]

sp|P62806|H4_MOUSE

[Acetyl]SGRGKGGKGLGK $^{Acetyl}_{42.01}$ GGAK $^{Acetyl}_{42.01}$ RHRK $^{Dimethyl}_{28.03}$ VLRD

- fragmentation table for charge state 2+
- specType=etd
- score=77.58
- F104903.dat
- query=q1938_p1
- precursor=658.139260
- chargePrecursor=4
- itol=0.8

AA	c	y	z+1	z+2	z	AA
S[1]	74.042	**1315.265**	**1307.256**	0.504	**1306.752**	S[24]
G[2]	102.553	**1250.744**	**1242.735**	0.504	**1242.231**	G[23]
R[3]	180.603	**1222.233**	1214.224	**1214.728**	1213.720	R[22]
G[4]	209.114	1144.183	**1136.173**	**1136.677**	1135.669	G[21]
K[5]	273.161	1115.672	**1107.663**	**1108.167**	1107.159	K[20]
G[6]	301.672	1051.625	1043.615	1044.119	1043.111	G[19]
G[7]	330.183	**1023.114**	1015.104	**1015.608**	1014.601	G[18]
K[8]	394.230	**994.603**	**986.594**	**987.098**	**986.090**	K[17]
G[9]	422.741	930.556	**922.546**	**923.050**	922.042	G[16]
L[10]	479.283	**902.045**	894.035	894.539	893.532	L[15]
G[11]	507.794	845.503	837.493	**837.997**	836.990	G[14]
K[12]	592.847	**816.992**	808.983	809.487	808.479	K[13]
G[13]	621.357	731.939	**723.930**	**724.434**	**723.426**	G[12]
G[14]	649.868	703.429	**695.419**	**695.923**	694.915	G[11]
A[15]	685.387	674.918	**666.908**	**667.412**	**666.405**	A[10]
K[16]	**770.439**	639.399	**631.390**	**631.894**	**630.886**	K[9]
R[17]	**848.490**	554.347	546.337	**546.841**	**545.833**	R[8]
H[18]	**917.019**	476.296	468.287	468.791	467.783	H[7]
R[19]	**995.070**	407.767	399.757	400.261	399.253	R[6]
K[20]	**1073.133**	329.716	321.707	322.211	321.203	K[5]
V[21]	**1122.667**	251.653	243.643	244.147	243.140	V[4]
L[22]	**1179.209**	202.119	194.109	194.613	193.605	L[3]
R[23]	**1257.260**	145.577	137.567	138.071	137.063	R[2]
D[24]	**1314.773**	67.526	59.517	60.021	59.013	D[1]

sp|P62806|H4_MOUSE

[Acetyl]SGRGKGGKGLGK $^{\text{Acetyl}}_{42.01}$ GGAK $^{\text{Acetyl}}_{42.01}$ RHRK $^{\text{Dimethyl}}_{28.03}$ VLRD

- fragmentation table for charge state 3+
- specType=etd
- score=77.58
- F104903.dat
- query=q1938_p1
- precursor=658.139260
- chargePrecursor=4
- itol=0.8

AA	c	y	z+1	z+2	z	AA
S[1]	49.697	**877.179**	**871.840**	0.672	**871.504**	S[24]
G[2]	68.704	834.165	**828.826**	0.672	828.490	G[23]
R[3]	120.738	815.158	**809.818**	**810.154**	809.482	R[22]
G[4]	139.745	763.124	757.785	758.121	757.449	G[21]
K[5]	182.443	744.117	738.778	739.113	738.442	K[20]
G[6]	201.450	**701.419**	**696.079**	**696.415**	**695.743**	G[19]
G[7]	220.458	682.412	677.072	677.408	676.736	G[18]
K[8]	263.156	663.404	658.065	658.401	657.729	K[17]
G[9]	282.163	620.706	615.367	615.703	615.031	G[16]
L[10]	319.858	**601.699**	596.359	596.695	596.023	L[15]
G[11]	338.865	564.004	558.665	559.001	558.329	G[14]
K[12]	395.567	**544.997**	539.658	539.994	539.322	K[13]
G[13]	414.574	488.295	482.956	483.292	482.620	G[12]
G[14]	433.581	469.288	463.949	464.285	463.613	G[11]
A[15]	457.260	450.281	444.941	445.277	444.605	A[10]
K[16]	513.962	426.602	421.262	421.598	420.926	K[9]
R[17]	565.996	369.900	364.561	364.896	364.225	R[8]
H[18]	611.682	317.866	312.527	312.863	312.191	H[7]
R[19]	663.716	272.180	266.841	267.176	266.505	R[6]
K[20]	715.758	220.146	214.807	215.143	214.471	K[5]
V[21]	748.781	168.104	162.765	163.101	162.429	V[4]
L[22]	786.475	135.082	129.742	130.078	129.406	L[3]
R[23]	**838.509**	97.387	92.047	92.383	91.711	R[2]
D[24]	**876.851**	45.353	40.014	40.349	39.678	D[1]

sp|P62806|H4_MOUSE

[Acetyl]S $^{\text{Phospho}}_{79.97}$ GRGKGGKGLGKGGAK $^{\text{Acetyl}}_{42.01}$ RHRK $^{\text{Dimethyl}}_{28.03}$ VLRD

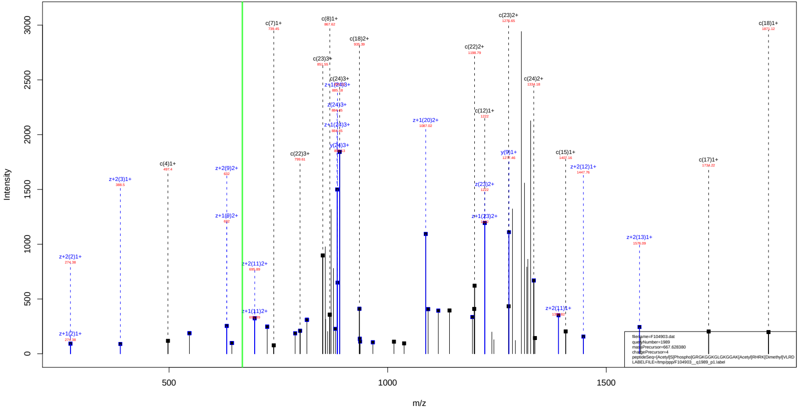

sp|P62806|H4_MOUSE

[Acetyl]S $^{\text{Phospho}}_{79.97}$ GRGKGGKGLGKGGAK $^{\text{Acetyl}}_{42.01}$ RHRK $^{\text{Dimethyl}}_{28.03}$ VLRD

- fragmentation table for charge state 1+
- specType=etd
- score=78.08
- F104903.dat
- query=q1989_p1
- precursor=667.628380
- chargePrecursor=4
- itol=0.8

AA	c	y	z+1	z+2	z	AA
S[1]	227.043	2667.479	2651.460	0.000	2650.453	S[24]
G[2]	284.064	2458.470	2442.451	0.000	2441.444	G[23]
R[3]	440.165	2401.449	2385.430	2386.438	2384.422	R[22]
G[4]	**497.187**	2245.348	2229.329	2230.337	2228.321	G[21]
K[5]	625.282	2188.326	2172.307	2173.315	2171.300	K[20]
G[6]	682.303	2060.231	2044.212	2045.220	2043.205	G[19]
G[7]	**739.325**	2003.210	1987.191	1988.199	1986.183	G[18]
K[8]	**867.420**	1946.188	1930.170	1931.177	1929.162	K[17]
G[9]	924.441	1818.093	1802.075	1803.082	1801.067	G[16]
L[10]	**1037.525**	1761.072	1745.053	1746.061	1744.045	L[15]
G[11]	1094.547	1647.988	1631.969	1632.977	1630.961	G[14]
K[12]	**1222.642**	1590.966	1574.948	**1575.955**	1573.940	K[13]
G[13]	1279.663	1462.871	1446.853	**1447.860**	1445.845	G[12]
G[14]	**1336.685**	1405.850	1389.831	**1390.839**	1388.823	G[11]
A[15]	**1407.722**	1348.828	1332.810	**1333.818**	1331.802	A[10]
K[16]	1577.827	**1277.791**	1261.773	1262.780	1260.765	K[9]
R[17]	**1733.928**	1107.686	1091.667	**1092.675**	1090.659	R[8]
H[18]	**1870.987**	951.585	**935.566**	**936.574**	934.558	H[7]
R[19]	2027.088	**814.526**	798.507	**799.515**	797.499	R[6]
K[20]	2183.215	658.425	642.406	**643.414**	641.398	K[5]
V[21]	2282.283	502.298	486.280	487.287	485.272	V[4]
L[22]	2395.367	403.230	387.211	**388.219**	386.203	L[3]
R[23]	2551.468	290.146	**274.127**	**275.135**	273.119	R[2]
D[24]	2666.495	134.045	118.026	119.034	117.018	D[1]

sp|P62806|H4_MOUSE

[Acetyl]S $^{Phospho}_{79.97}$ GRGKGGKGLGKGGAK $^{Acetyl}_{42.01}$ RHRK $^{Dimethyl}_{28.03}$ VLRD

- fragmentation table for charge state 2+
- specType=etd
- score=78.08
- F104903.dat
- query=q1989_p1
- precursor=667.628380
- chargePrecursor=4
- itol=0.8

AA	c	y	z+1	z+2	z	AA
S[1]	114.025	**1334.243**	1326.234	0.504	1325.730	S[24]
G[2]	142.536	1229.739	**1221.729**	0.504	**1221.225**	G[23]
R[3]	220.586	1201.228	**1193.219**	**1193.723**	1192.715	R[22]
G[4]	249.097	1123.177	**1115.168**	**1115.672**	1114.664	G[21]
K[5]	313.145	1094.667	**1086.657**	**1087.161**	1086.153	K[20]
G[6]	341.655	1030.619	1022.610	1023.114	1022.106	G[19]
G[7]	370.166	1002.108	994.099	994.603	993.595	G[18]
K[8]	434.213	973.598	**965.588**	**966.092**	965.084	K[17]
G[9]	462.724	909.550	901.541	902.045	901.037	G[16]
L[10]	519.266	**881.040**	873.030	873.534	872.526	L[15]
G[11]	547.777	824.498	816.488	816.992	**815.984**	G[14]
K[12]	611.824	795.987	**787.977**	**788.481**	787.474	K[13]
G[13]	640.335	731.939	**723.930**	**724.434**	723.426	G[12]
G[14]	668.846	703.429	**695.419**	**695.923**	694.915	G[11]
A[15]	704.364	674.918	666.908	667.412	666.405	A[10]
K[16]	789.417	639.399	**631.390**	**631.894**	630.886	K[9]
R[17]	**867.468**	554.347	**546.337**	**546.841**	**545.833**	R[8]
H[18]	**935.997**	476.296	468.287	468.791	467.783	H[7]
R[19]	**1014.048**	407.767	399.757	400.261	399.253	R[6]
K[20]	**1092.111**	329.716	321.707	322.211	321.203	K[5]
V[21]	**1141.645**	251.653	243.643	244.147	243.140	V[4]
L[22]	**1198.187**	202.119	194.109	194.613	193.605	L[3]
R[23]	**1276.238**	145.577	137.567	138.071	137.063	R[2]
D[24]	**1333.751**	67.526	59.517	60.021	59.013	D[1]

sp|P62806|H4_MOUSE

[Acetyl]S $^{\text{Phospho}}_{79.97}$ GRGKGGKGLGKGGAK $^{\text{Acetyl}}_{42.01}$ RHRK $^{\text{Dimethyl}}_{28.03}$ VLRD

- fragmentation table for charge state 3+
- specType=etd
- score=78.08
- F104903.dat
- query=q1989_p1
- precursor=667.628380
- chargePrecursor=4
- itol=0.8

AA	c	y	z+1	z+2	z	AA
S[1]	76.352	**889.831**	**884.492**	0.672	**884.156**	S[24]
G[2]	95.360	820.162	**814.822**	0.672	**814.486**	G[23]
R[3]	147.393	801.154	795.815	796.151	795.479	R[22]
G[4]	166.400	749.121	743.781	744.117	743.445	G[21]
K[5]	209.099	730.114	**724.774**	**725.110**	**724.438**	K[20]
G[6]	228.106	687.415	682.076	682.412	681.740	G[19]
G[7]	247.113	668.408	663.069	663.404	662.733	G[18]
K[8]	289.811	649.401	**644.061**	**644.397**	**643.725**	K[17]
G[9]	308.819	606.703	601.363	601.699	601.027	G[16]
L[10]	346.513	587.695	582.356	582.692	582.020	L[15]
G[11]	365.520	550.001	544.661	544.997	544.325	G[14]
K[12]	408.219	530.994	525.654	525.990	525.318	K[13]
G[13]	427.226	488.295	482.956	483.292	482.620	G[12]
G[14]	446.233	469.288	463.949	464.285	463.613	G[11]
A[15]	469.912	450.281	444.941	445.277	444.605	A[10]
K[16]	526.614	426.602	421.262	421.598	420.926	K[9]
R[17]	578.648	369.900	364.561	364.896	364.225	R[8]
H[18]	624.334	317.866	312.527	312.863	312.191	H[7]
R[19]	676.368	272.180	266.841	267.176	266.505	R[6]
K[20]	728.410	220.146	214.807	215.143	214.471	K[5]
V[21]	761.433	168.104	162.765	163.101	162.429	V[4]
L[22]	**799.127**	135.082	129.742	130.078	129.406	L[3]
R[23]	**851.161**	97.387	92.047	92.383	91.711	R[2]
D[24]	**889.503**	45.353	40.014	40.349	39.678	D[1]

sp|P62806|H4_MOUSE

[Acetyl]SGRGKGGK $^{\text{Acetyl}}_{42.01}$ GLGK $^{\text{Acetyl}}_{42.01}$ GGAK $^{\text{Acetyl}}_{42.01}$ RHRK $^{\text{Dimethyl}}_{28.03}$ VLRD

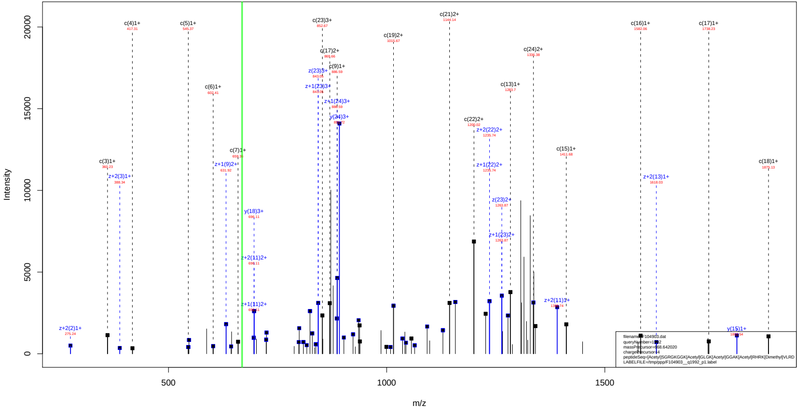

sp|P62806|H4_MOUSE

[Acetyl]SGRGKGGK $^{\text{Acetyl}}_{42.01}$ GLGK $^{\text{Acetyl}}_{42.01}$ GGAK $^{\text{Acetyl}}_{42.01}$ RHRK $^{\text{Dimethyl}}_{28.03}$ VLRD

- fragmentation table for charge state 1+
- specType=etd
- score=100.83
- F104903.dat
- query=q1992_p1
- precursor=668.642020
- chargePrecursor=4
- itol=0.8

AA	c	y	z+1	z+2	z	AA
S[1]	147.076	2671.534	2655.515	0.000	2654.507	S[24]
G[2]	204.098	2542.491	2526.473	0.000	2525.465	G[23]
R[3]	**360.199**	2485.470	2469.451	2470.459	2468.443	R[22]
G[4]	**417.220**	2329.369	2313.350	2314.358	2312.342	G[21]
K[5]	**545.315**	2272.347	2256.329	2257.336	2255.321	K[20]
G[6]	**602.337**	2144.252	2128.234	2129.241	2127.226	G[19]
G[7]	**659.358**	2087.231	2071.212	2072.220	2070.204	G[18]
K[8]	**829.464**	2030.209	2014.191	2015.198	2013.183	K[17]
G[9]	**886.485**	1860.104	1844.085	1845.093	1843.077	G[16]
L[10]	**999.569**	**1803.082**	1787.064	1788.071	1786.056	L[15]
G[11]	**1056.591**	1689.998	1673.980	1674.987	1672.972	G[14]
K[12]	**1226.696**	1632.977	1616.958	**1617.966**	1615.950	K[13]
G[13]	**1283.718**	1462.871	1446.853	1447.860	1445.845	G[12]
G[14]	**1340.739**	1405.850	1389.831	**1390.839**	1388.823	G[11]
A[15]	**1411.776**	1348.828	1332.810	1333.818	1331.802	A[10]
K[16]	**1581.882**	**1277.791**	1261.773	1262.780	1260.765	K[9]
R[17]	**1737.983**	1107.686	1091.667	**1092.675**	1090.659	R[8]
H[18]	**1875.042**	951.585	**935.566**	936.574	934.558	H[7]
R[19]	2031.143	814.526	**798.507**	**799.515**	797.499	R[6]
K[20]	2187.269	658.425	642.406	**643.414**	641.398	K[5]
V[21]	2286.338	502.298	486.280	487.287	485.272	V[4]
L[22]	2399.422	403.230	387.211	**388.219**	386.203	L[3]
R[23]	2555.523	290.146	274.127	**275.135**	273.119	R[2]
D[24]	2670.550	134.045	118.026	119.034	117.018	D[1]

sp|P62806|H4_MOUSE

[Acetyl]SGRGKGGK $^{Acetyl}_{42.01}$ GLGK $^{Acetyl}_{42.01}$ GGAK $^{Acetyl}_{42.01}$ RHRK $^{Dimethyl}_{28.03}$ VLRD

- fragmentation table for charge state 2+
- specType=etd
- score=100.83
- F104903.dat
- query=q1992_p1
- precursor=668.642020
- chargePrecursor=4
- itol=0.8

AA	c	y	z+1	z+2	z	AA
S[1]	74.042	**1336.271**	1328.261	0.504	1327.757	S[24]
G[2]	102.553	1271.749	**1263.740**	0.504	**1263.236**	G[23]
R[3]	180.603	1243.239	**1235.229**	**1235.733**	1234.725	R[22]
G[4]	209.114	1165.188	**1157.179**	**1157.683**	**1156.675**	G[21]
K[5]	273.161	1136.677	**1128.668**	**1129.172**	**1128.164**	K[20]
G[6]	301.672	1072.630	**1064.620**	1065.124	**1064.117**	G[19]
G[7]	330.183	**1044.119**	**1036.110**	**1036.614**	**1035.606**	G[18]
K[8]	415.236	**1015.608**	1007.599	**1008.103**	1007.095	K[17]
G[9]	443.746	930.556	**922.546**	**923.050**	922.042	G[16]
L[10]	500.288	**902.045**	894.035	894.539	893.532	L[15]
G[11]	528.799	845.503	**837.493**	**837.997**	**836.990**	G[14]
K[12]	613.852	**816.992**	**808.983**	**809.487**	**808.479**	K[13]
G[13]	642.363	731.939	**723.930**	**724.434**	**723.426**	G[12]
G[14]	670.873	703.429	**695.419**	**695.923**	**694.915**	G[11]
A[15]	706.392	674.918	666.908	667.412	666.405	A[10]
K[16]	791.445	639.399	**631.390**	**631.894**	630.886	K[9]
R[17]	**869.495**	554.347	**546.337**	**546.841**	**545.833**	R[8]
H[18]	**938.025**	476.296	468.287	468.791	467.783	H[7]
R[19]	**1016.075**	407.767	399.757	400.261	399.253	R[6]
K[20]	1094.138	329.716	321.707	322.211	321.203	K[5]
V[21]	**1143.673**	251.653	243.643	244.147	243.140	V[4]
L[22]	**1200.215**	202.119	194.109	194.613	193.605	L[3]
R[23]	**1278.265**	145.577	137.567	138.071	137.063	R[2]
D[24]	**1335.779**	67.526	59.517	60.021	59.013	D[1]

sp|P62806|H4_MOUSE

[Acetyl]SGRGKGGK $^{Acetyl}_{42.01}$ GLGK $^{Acetyl}_{42.01}$ GGAK $^{Acetyl}_{42.01}$ RHRK $^{Dimethyl}_{28.03}$ VLRD

- fragmentation table for charge state 3+
- specType=etd
- score=100.83
- F104903.dat
- query=q1992_p1
- precursor=668.642020
- chargePrecursor=4
- itol=0.8

AA	c	y	z+1	z+2	z	AA
S[1]	49.697	**891.183**	**885.843**	0.672	**885.507**	S[24]
G[2]	68.704	848.169	**842.829**	0.672	**842.493**	G[23]
R[3]	120.738	**829.161**	**823.822**	**824.158**	**823.486**	R[22]
G[4]	139.745	777.128	771.788	772.124	771.452	G[21]
K[5]	182.443	758.121	752.781	753.117	752.445	K[20]
G[6]	201.450	715.422	710.083	710.419	709.747	G[19]
G[7]	220.458	**696.415**	691.076	691.412	690.740	G[18]
K[8]	277.159	677.408	672.068	672.404	671.732	K[17]
G[9]	296.167	620.706	615.367	615.703	615.031	G[16]
L[10]	333.861	**601.699**	596.359	596.695	596.023	L[15]
G[11]	352.868	564.004	558.665	559.001	558.329	G[14]
K[12]	409.570	**544.997**	539.658	539.994	539.322	K[13]
G[13]	428.577	488.295	482.956	483.292	482.620	G[12]
G[14]	447.585	469.288	463.949	464.285	463.613	G[11]
A[15]	471.264	450.281	444.941	445.277	444.605	A[10]
K[16]	527.966	426.602	421.262	421.598	420.926	K[9]
R[17]	579.999	369.900	364.561	364.896	364.225	R[8]
H[18]	625.686	317.866	312.527	312.863	312.191	H[7]
R[19]	677.719	272.180	266.841	267.176	266.505	R[6]
K[20]	729.761	220.146	214.807	215.143	214.471	K[5]
V[21]	762.784	168.104	162.765	163.101	162.429	V[4]
L[22]	800.479	135.082	129.742	130.078	129.406	L[3]
R[23]	**852.513**	97.387	92.047	92.383	91.711	R[2]
D[24]	890.855	45.353	40.014	40.349	39.678	D[1]

sp|P62806|H4_MOUSE

[Acetyl]SGRGKGGK Acetyl 42.01 GLGK Acetyl 42.01 GGAK Acetyl 42.01 RHRK Dimethyl 28.03 VLRD

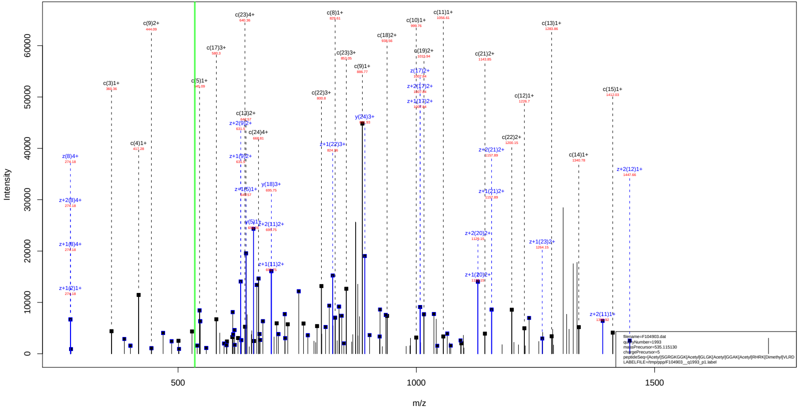

sp|P62806|H4_MOUSE

[Acetyl]SGRGKGGK $^{Acetyl}_{42.01}$ GLGK $^{Acetyl}_{42.01}$ GGAK $^{Acetyl}_{42.01}$ RHRK $^{Dimethyl}_{28.03}$ VLRD

- fragmentation table for charge state 1+
- specType=etd
- score=92.29
- F104903.dat
- query=q1993_p1
- precursor=535.115130
- chargePrecursor=5
- itol=0.8

AA	c	y	z+1	z+2	z	AA
S[1]	147.076	2671.534	2655.515	0.000	2654.507	S[24]
G[2]	204.098	2542.491	2526.473	0.000	2525.465	G[23]
R[3]	**360.199**	2485.470	2469.451	2470.459	2468.443	R[22]
G[4]	**417.220**	2329.369	2313.350	2314.358	2312.342	G[21]
K[5]	**545.315**	2272.347	2256.329	2257.336	2255.321	K[20]
G[6]	**602.337**	2144.252	2128.234	2129.241	2127.226	G[19]
G[7]	**659.358**	2087.231	2071.212	2072.220	2070.204	G[18]
K[8]	**829.464**	2030.209	2014.191	2015.198	2013.183	K[17]
G[9]	**886.485**	1860.104	1844.085	1845.093	1843.077	G[16]
L[10]	**999.569**	1803.082	1787.064	1788.071	1786.056	L[15]
G[11]	**1056.591**	1689.998	1673.980	1674.987	1672.972	G[14]
K[12]	**1226.696**	1632.977	1616.958	1617.966	1615.950	K[13]
G[13]	**1283.718**	1462.871	1446.853	**1447.860**	1445.845	G[12]
G[14]	**1340.739**	1405.850	1389.831	**1390.839**	1388.823	G[11]
A[15]	**1411.776**	1348.828	1332.810	1333.818	1331.802	A[10]
K[16]	1581.882	1277.791	1261.773	1262.780	1260.765	K[9]
R[17]	1737.983	1107.686	1091.667	**1092.675**	1090.659	R[8]
H[18]	1875.042	951.585	**935.566**	**936.574**	934.558	H[7]
R[19]	2031.143	814.526	798.507	799.515	797.499	R[6]
K[20]	2187.269	**658.425**	**642.406**	643.414	641.398	K[5]
V[21]	2286.338	**502.298**	**486.280**	**487.287**	485.272	V[4]
L[22]	2399.422	403.230	**387.211**	388.219	386.203	L[3]
R[23]	2555.523	290.146	**274.127**	**275.135**	273.119	R[2]
D[24]	2670.550	134.045	118.026	119.034	117.018	D[1]

sp|P62806|H4_MOUSE

[Acetyl]SGRGKGGK $^{Acetyl}_{42.01}$ GLGK $^{Acetyl}_{42.01}$ GGAK $^{Acetyl}_{42.01}$ RHRK $^{Dimethyl}_{28.03}$ VLRD

- fragmentation table for charge state 2+
- specType=etd
- score=92.29
- F104903.dat
- query=q1993_p1
- precursor=535.115130
- chargePrecursor=5
- itol=0.8

AA	c	y	z+1	z+2	z	AA
S[1]	74.042	1336.271	1328.261	0.504	1327.757	S[24]
G[2]	102.553	1271.749	**1263.740**	0.504	1263.236	G[23]
R[3]	180.603	1243.239	1235.229	**1235.733**	1234.725	R[22]
G[4]	209.114	1165.188	**1157.179**	**1157.683**	1156.675	G[21]
K[5]	273.161	1136.677	**1128.668**	**1129.172**	1128.164	K[20]
G[6]	301.672	**1072.630**	**1064.620**	**1065.124**	**1064.117**	G[19]
G[7]	330.183	**1044.119**	**1036.110**	**1036.614**	1035.606	G[18]
K[8]	415.236	**1015.608**	**1007.599**	**1008.103**	**1007.095**	K[17]
G[9]	**443.746**	930.556	**922.546**	**923.050**	922.042	G[16]
L[10]	**500.288**	**902.045**	894.035	894.539	893.532	L[15]
G[11]	**528.799**	845.503	**837.493**	**837.997**	**836.990**	G[14]
K[12]	**613.852**	**816.992**	**808.983**	**809.487**	808.479	K[13]
G[13]	**642.363**	731.939	**723.930**	**724.434**	**723.426**	G[12]
G[14]	**670.873**	703.429	**695.419**	**695.923**	694.915	G[11]
A[15]	**706.392**	674.918	666.908	667.412	666.405	A[10]
K[16]	**791.445**	639.399	**631.390**	**631.894**	**630.886**	K[9]
R[17]	869.495	554.347	**546.337**	**546.841**	**545.833**	R[8]
H[18]	**938.025**	476.296	**468.287**	**468.791**	467.783	H[7]
R[19]	**1016.075**	407.767	**399.757**	**400.261**	**399.253**	R[6]
K[20]	**1094.138**	329.716	321.707	322.211	321.203	K[5]
V[21]	**1143.673**	251.653	243.643	244.147	243.140	V[4]
L[22]	**1200.215**	202.119	194.109	194.613	193.605	L[3]
R[23]	1278.265	145.577	137.567	138.071	137.063	R[2]
D[24]	1335.779	67.526	59.517	60.021	59.013	D[1]

sp|P62806|H4_MOUSE

[Acetyl]SGRGKGGK $^{\text{Acetyl}}_{42.01}$ GLGK $^{\text{Acetyl}}_{42.01}$ GGAK $^{\text{Acetyl}}_{42.01}$ RHRK $^{\text{Dimethyl}}_{28.03}$ VLRD

- fragmentation table for charge state 3+
- specType=etd
- score=92.29
- F104903.dat
- query=q1993_p1
- precursor=535.115130
- chargePrecursor=5
- itol=0.8

AA	c	y	z+1	z+2	z	AA
S[1]	49.697	**891.183**	885.843	0.672	885.507	S[24]
G[2]	68.704	**848.169**	**842.829**	0.672	842.493	G[23]
R[3]	120.738	**829.161**	**823.822**	**824.158**	823.486	R[22]
G[4]	139.745	777.128	**771.788**	**772.124**	771.452	G[21]
K[5]	182.443	758.121	**752.781**	**753.117**	752.445	K[20]
G[6]	201.450	715.422	**710.083**	**710.419**	**709.747**	G[19]
G[7]	220.458	**696.415**	691.076	691.412	690.740	G[18]
K[8]	277.159	**677.408**	**672.068**	**672.404**	**671.732**	K[17]
G[9]	296.167	620.706	**615.367**	**615.703**	**615.031**	G[16]
L[10]	333.861	**601.699**	**596.359**	**596.695**	**596.023**	L[15]
G[11]	352.868	564.004	**558.665**	**559.001**	558.329	G[14]
K[12]	409.570	**544.997**	**539.658**	**539.994**	539.322	K[13]
G[13]	428.577	488.295	482.956	483.292	482.620	G[12]
G[14]	447.585	**469.288**	463.949	464.285	463.613	G[11]
A[15]	471.264	450.281	444.941	445.277	**444.605**	A[10]
K[16]	527.966	426.602	421.262	421.598	420.926	K[9]
R[17]	**579.999**	369.900	364.561	364.896	364.225	R[8]
H[18]	**625.686**	317.866	312.527	312.863	312.191	H[7]
R[19]	**677.719**	272.180	266.841	267.176	266.505	R[6]
K[20]	**729.761**	220.146	214.807	215.143	214.471	K[5]
V[21]	**762.784**	168.104	162.765	163.101	162.429	V[4]
L[22]	**800.479**	135.082	129.742	130.078	129.406	L[3]
R[23]	**852.513**	97.387	92.047	92.383	91.711	R[2]
D[24]	890.855	45.353	40.014	40.349	39.678	D[1]

sp|P62806|H4_MOUSE

[Acetyl]SGRGKGGK $^{Acetyl}_{42.01}$ GLGK $^{Acetyl}_{42.01}$ GGAK $^{Acetyl}_{42.01}$ RHRK $^{Dimethyl}_{28.03}$ VLRD

- fragmentation table for charge state 4+
- specType=etd
- score=92.29
- F104903.dat
- query=q1993_p1
- precursor=535.115130
- chargePrecursor=5
- itol=0.8

AA	c	y	z+1	z+2	z	AA
S[1]	37.525	**668.639**	**664.634**	0.755	**664.382**	S[24]
G[2]	51.780	636.378	**632.374**	0.755	**632.122**	G[23]
R[3]	90.805	622.123	**618.118**	**618.370**	**617.866**	R[22]
G[4]	105.061	583.098	579.093	579.345	578.841	G[21]
K[5]	137.084	568.842	564.838	565.090	564.586	K[20]
G[6]	151.340	536.819	532.814	533.066	532.562	G[19]
G[7]	165.595	522.563	518.558	518.810	518.307	G[18]
K[8]	208.121	508.308	504.303	504.555	504.051	K[17]
G[9]	222.377	465.781	461.777	462.029	461.525	G[16]
L[10]	250.648	451.526	447.521	447.773	447.269	L[15]
G[11]	264.903	423.255	419.250	419.502	418.998	G[14]
K[12]	307.430	409.000	404.995	405.247	404.743	K[13]
G[13]	321.685	366.473	362.469	362.721	362.217	G[12]
G[14]	335.940	352.218	348.213	348.465	347.961	G[11]
A[15]	353.700	337.963	333.958	334.210	333.706	A[10]
K[16]	396.226	320.203	316.199	316.451	315.947	K[9]
R[17]	435.251	277.677	**273.672**	**273.924**	**273.420**	R[8]
H[18]	469.516	238.652	234.647	234.899	234.395	H[7]
R[19]	508.541	204.387	200.382	200.634	200.130	R[6]
K[20]	547.573	165.362	161.357	161.609	161.105	K[5]
V[21]	572.340	126.330	122.325	122.577	122.073	V[4]
L[22]	600.611	101.563	97.558	97.810	97.306	L[3]
R[23]	**639.636**	73.292	69.287	69.539	69.035	R[2]
D[24]	**668.393**	34.267	30.262	30.514	30.010	D[1]

sp|Q6GSS7|H2A2A_MOUSE

LNKLLGK $^{\text{Dimethyl}}_{28.03}$ VTIAQGGVLPNIQAVLLPKKTE

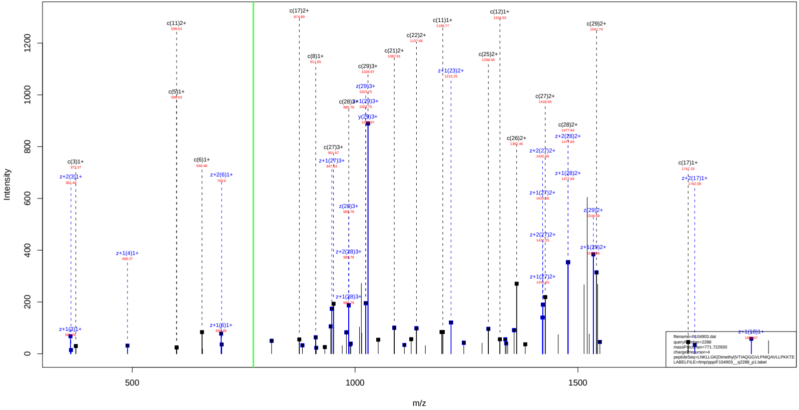

sp|Q6GSS7|H2A2A_MOUSE

LNKLLGK $^{\text{Dimethyl}}_{28.03}$ VTIAQGGVLPNIQAVLLPKKTE

- fragmentation table for charge state 1+
- specType=etd
- score=84.66
- F104903.dat
- query=q2288_p1
- precursor=771.722930
- chargePrecursor=4
- itol=0.8

AA	c	y	z+1	z+2	z	AA
L[1]	131.118	3083.892	3067.873	0.000	3066.865	L[29]
N[2]	245.161	2970.808	2954.789	2955.797	2953.781	N[28]
K[3]	**373.256**	2856.765	2840.746	2841.754	2839.738	K[27]
L[4]	486.340	2728.670	2712.651	2713.659	2711.643	L[26]
L[5]	**599.424**	2615.586	2599.567	2600.575	2598.559	L[25]
G[6]	**656.445**	2502.502	2486.483	2487.491	2485.475	G[24]
K[7]	**812.572**	2445.480	2429.462	2430.469	2428.454	K[23]
V[8]	**911.640**	2289.354	2273.335	2274.343	2272.327	V[22]
T[9]	1012.688	2190.286	2174.267	2175.275	2173.259	T[21]
I[10]	**1125.772**	2089.238	2073.219	2074.227	2072.211	I[20]
A[11]	**1196.809**	1976.154	1960.135	1961.143	1959.127	A[19]
Q[12]	**1324.867**	1905.117	**1889.098**	1890.106	1888.090	Q[18]
G[13]	**1381.889**	1777.058	1761.039	**1762.047**	1760.032	G[17]
G[14]	1438.910	1720.037	1704.018	1705.026	1703.010	G[16]
V[15]	1537.979	1663.015	1646.997	1648.004	1645.989	V[15]
L[16]	1651.063	1563.947	1547.928	**1548.936**	1546.920	L[14]
P[17]	**1748.116**	1450.863	1434.844	1435.852	1433.836	P[13]
N[18]	1862.159	1353.810	**1337.791**	**1338.799**	**1336.783**	N[12]
I[19]	1975.243	1239.767	1223.748	1224.756	1222.741	I[11]
Q[20]	2103.301	1126.683	**1110.664**	1111.672	1109.656	Q[10]
A[21]	2174.338	998.624	982.606	983.614	981.598	A[9]
V[22]	2273.407	927.587	**911.569**	**912.576**	910.561	V[8]
L[23]	2386.491	828.519	**812.500**	813.508	811.492	L[7]
L[24]	2499.575	715.435	**699.416**	**700.424**	698.408	L[6]
P[25]	2596.628	602.351	586.332	587.340	585.324	P[5]
K[26]	2724.723	505.298	**489.279**	490.287	488.271	K[4]
K[27]	2852.818	377.203	**361.184**	**362.192**	360.177	K[3]
T[28]	2953.865	249.108	233.089	234.097	232.082	T[2]
E[29]	3082.908	148.060	132.042	133.050	131.034	E[1]

sp|Q6GSS7|H2A2A_MOUSE

LNKLLGK $^{\text{Dimethyl}}_{28.03}$ VTIAQGGVLPNIQAVLLPKKTE

- fragmentation table for charge state 2+
- specType=etd
- score=84.66
- F104903.dat
- query=q2288_p1
- precursor=771.722930
- chargePrecursor=4
- itol=0.8

AA	c	y	z+1	z+2	z	AA
L[1]	66.063	**1542.450**	**1534.440**	0.504	**1533.936**	L[29]
N[2]	123.084	1485.908	**1477.898**	**1478.402**	**1477.394**	N[28]
K[3]	187.132	1428.886	**1420.877**	**1421.381**	**1420.373**	K[27]
L[4]	243.674	1364.839	**1356.829**	**1357.333**	**1356.325**	L[26]
L[5]	300.216	1308.297	1300.287	1300.791	**1299.783**	L[25]
G[6]	328.726	1251.755	**1243.745**	**1244.249**	**1243.241**	G[24]
K[7]	406.789	1223.244	**1215.234**	**1215.738**	**1214.731**	K[23]
V[8]	456.324	1145.181	**1137.171**	**1137.675**	1136.667	V[22]
T[9]	506.848	1095.646	**1087.637**	**1088.141**	**1087.133**	T[21]
I[10]	563.390	1045.123	1037.113	1037.617	1036.609	I[20]
A[11]	**598.908**	**988.581**	**980.571**	**981.075**	**980.067**	A[19]
Q[12]	662.937	953.062	**945.053**	**945.557**	**944.549**	Q[18]
G[13]	691.448	889.033	**881.023**	**881.527**	880.519	G[17]
G[14]	719.959	860.522	852.513	853.017	852.009	G[16]
V[15]	769.493	832.011	824.002	824.506	823.498	V[15]
L[16]	826.035	782.477	774.468	774.972	773.964	L[14]
P[17]	**874.561**	725.935	717.926	718.430	717.422	P[13]
N[18]	**931.583**	677.409	669.399	669.903	668.895	N[12]
I[19]	**988.125**	620.387	612.378	612.882	611.874	I[11]
Q[20]	**1052.154**	563.845	555.836	556.340	555.332	Q[10]
A[21]	**1087.673**	499.816	491.807	492.310	491.303	A[9]
V[22]	**1137.207**	464.297	456.288	456.792	455.784	V[8]
L[23]	**1193.749**	414.763	406.754	407.258	406.250	L[7]
L[24]	1250.291	358.221	350.212	350.716	349.708	L[6]
P[25]	**1298.817**	301.679	293.670	294.174	293.166	P[5]
K[26]	**1362.865**	253.153	245.143	245.647	244.639	K[4]
K[27]	**1426.912**	189.105	181.096	181.600	180.592	K[3]
T[28]	**1477.436**	125.058	117.048	117.552	116.544	T[2]
E[29]	**1541.958**	74.534	66.524	67.028	66.021	E[1]

sp|Q6GSS7|H2A2A_MOUSE

LNKLLGK $^{Dimethyl}_{28.03}$ VTIAQGGVLPNIQAVLLPKKTE

- fragmentation table for charge state 3+
- specType=etd
- score=84.66
- F104903.dat
- query=q2288_p1
- precursor=771.722930
- chargePrecursor=4
- itol=0.8

AA	c	y	z+1	z+2	z	AA
L[1]	44.377	**1028.635**	**1023.296**	0.672	**1022.960**	L[29]
N[2]	82.392	**990.941**	**985.601**	**985.937**	**985.265**	N[28]
K[3]	125.090	952.926	**947.587**	**947.923**	**947.251**	K[27]
L[4]	162.785	910.228	904.889	905.225	904.553	L[26]
L[5]	200.479	872.533	867.194	867.530	866.858	L[25]
G[6]	219.487	834.839	829.499	829.835	829.163	G[24]
K[7]	271.529	815.832	810.492	810.828	810.156	K[23]
V[8]	304.552	763.790	758.450	758.786	758.114	V[22]
T[9]	338.234	730.767	725.427	725.763	725.091	T[21]
I[10]	375.929	697.084	691.745	692.081	691.409	I[20]
A[11]	399.608	659.389	654.050	654.386	653.714	A[19]
Q[12]	442.294	635.710	630.371	630.707	630.035	Q[18]
G[13]	461.301	593.024	587.685	588.021	587.349	G[17]
G[14]	480.308	574.017	568.678	569.013	568.342	G[16]
V[15]	513.331	555.010	549.670	550.006	549.334	V[15]
L[16]	551.026	521.987	516.648	516.984	516.312	L[14]
P[17]	583.377	484.292	478.953	479.289	478.617	P[13]
N[18]	621.391	451.942	446.602	446.938	446.266	N[12]
I[19]	659.086	413.927	408.588	408.924	408.252	I[11]
Q[20]	701.772	376.233	370.893	371.229	370.557	Q[10]
A[21]	725.451	333.546	328.207	328.543	327.871	A[9]
V[22]	758.474	309.867	304.528	304.864	304.192	V[8]
L[23]	796.168	276.844	271.505	271.841	271.169	L[7]
L[24]	833.863	239.150	233.810	234.146	233.474	L[6]
P[25]	866.214	201.455	196.116	196.451	195.780	P[5]
K[26]	908.912	169.104	163.765	164.101	163.429	K[4]
K[27]	**951.611**	126.406	121.066	121.402	120.730	K[3]
T[28]	**985.293**	83.708	78.368	78.704	78.032	T[2]
E[29]	**1028.307**	50.025	44.685	45.021	44.349	E[1]

sp|Q6GSS7|H2A2A_MOUSE

LNKLLGK $^{Dimethyl}_{28.03}$ VTIAQGGVLPNIQAVLLPKKTE

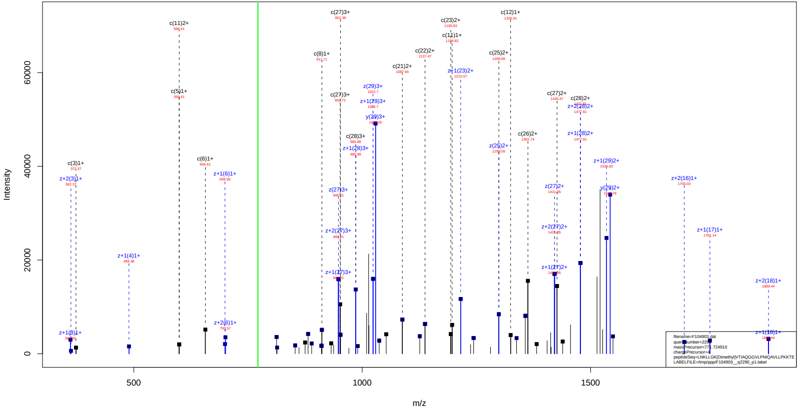

sp|Q6GSS7|H2A2A_MOUSE

LNKLLGK $^{\text{Dimethyl}}_{28.03}$ VTIAQGGVLPNIQAVLLPKKTE

- fragmentation table for charge state 1+
- specType=etd
- score=78.65
- F104903.dat
- query=q2290_p1
- precursor=771.724910
- chargePrecursor=4
- itol=0.8

AA	c	y	z+1	z+2	z	AA
L[1]	131.118	3083.892	3067.873	0.000	3066.865	L[29]
N[2]	245.161	2970.808	2954.789	2955.797	2953.781	N[28]
K[3]	**373.256**	2856.765	2840.746	2841.754	2839.738	K[27]
L[4]	486.340	2728.670	2712.651	2713.659	2711.643	L[26]
L[5]	**599.424**	2615.586	2599.567	2600.575	2598.559	L[25]
G[6]	**656.445**	2502.502	2486.483	2487.491	2485.475	G[24]
K[7]	**812.572**	2445.480	2429.462	2430.469	2428.454	K[23]
V[8]	**911.640**	2289.354	2273.335	2274.343	2272.327	V[22]
T[9]	1012.688	2190.286	2174.267	2175.275	2173.259	T[21]
I[10]	**1125.772**	2089.238	2073.219	2074.227	2072.211	I[20]
A[11]	**1196.809**	1976.154	1960.135	1961.143	1959.127	A[19]
Q[12]	**1324.867**	1905.117	**1889.098**	**1890.106**	1888.090	Q[18]
G[13]	**1381.889**	1777.058	**1761.039**	1762.047	1760.032	G[17]
G[14]	**1438.910**	1720.037	1704.018	**1705.026**	1703.010	G[16]
V[15]	1537.979	1663.015	1646.997	1648.004	1645.989	V[15]
L[16]	1651.063	1563.947	1547.928	**1548.936**	1546.920	L[14]
P[17]	1748.116	1450.863	1434.844	1435.852	1433.836	P[13]
N[18]	1862.159	1353.810	**1337.791**	1338.799	1336.783	N[12]
I[19]	1975.243	1239.767	1223.748	1224.756	1222.741	I[11]
Q[20]	2103.301	**1126.683**	1110.664	1111.672	1109.656	Q[10]
A[21]	2174.338	998.624	982.606	983.614	981.598	A[9]
V[22]	2273.407	927.587	**911.569**	912.576	**910.561**	V[8]
L[23]	2386.491	828.519	**812.500**	**813.508**	811.492	L[7]
L[24]	2499.575	715.435	**699.416**	**700.424**	698.408	L[6]
P[25]	2596.628	602.351	586.332	587.340	585.324	P[5]
K[26]	2724.723	505.298	**489.279**	490.287	488.271	K[4]
K[27]	2852.818	377.203	**361.184**	**362.192**	360.177	K[3]
T[28]	2953.865	249.108	233.089	234.097	232.082	T[2]
E[29]	3082.908	148.060	132.042	133.050	131.034	E[1]

sp|Q6GSS7|H2A2A_MOUSE

LNKLLGK $^{\text{Dimethyl}}_{28.03}$ VTIAQGGVLPNIQAVLLPKKTE

- fragmentation table for charge state 2+
- specType=etd
- score=78.65
- F104903.dat
- query=q2290_p1
- precursor=771.724910
- chargePrecursor=4
- itol=0.8

AA	c	y	z+1	z+2	z	AA
L[1]	66.063	**1542.450**	**1534.440**	0.504	1533.936	L[29]
N[2]	123.084	1485.908	**1477.898**	**1478.402**	**1477.394**	N[28]
K[3]	187.132	1428.886	**1420.877**	**1421.381**	**1420.373**	K[27]
L[4]	243.674	1364.839	**1356.829**	**1357.333**	**1356.325**	L[26]
L[5]	300.216	1308.297	1300.287	1300.791	**1299.783**	L[25]
G[6]	328.726	1251.755	**1243.745**	**1244.249**	**1243.241**	G[24]
K[7]	406.789	1223.244	**1215.234**	**1215.738**	1214.731	K[23]
V[8]	456.324	1145.181	**1137.171**	**1137.675**	1136.667	V[22]
T[9]	506.848	1095.646	**1087.637**	**1088.141**	1087.133	T[21]
I[10]	563.390	1045.123	**1037.113**	**1037.617**	**1036.609**	I[20]
A[11]	**598.908**	988.581	980.571	981.075	980.067	A[19]
Q[12]	662.937	**953.062**	945.053	945.557	944.549	Q[18]
G[13]	691.448	**889.033**	**881.023**	**881.527**	880.519	G[17]
G[14]	719.959	860.522	**852.513**	**853.017**	852.009	G[16]
V[15]	769.493	832.011	824.002	824.506	823.498	V[15]
L[16]	826.035	782.477	774.468	774.972	773.964	L[14]
P[17]	**874.561**	725.935	717.926	718.430	717.422	P[13]
N[18]	**931.583**	677.409	669.399	669.903	668.895	N[12]
I[19]	988.125	620.387	612.378	612.882	611.874	I[11]
Q[20]	**1052.154**	563.845	555.836	556.340	555.332	Q[10]
A[21]	**1087.673**	499.816	491.807	492.310	491.303	A[9]
V[22]	**1137.207**	464.297	456.288	456.792	455.784	V[8]
L[23]	**1193.749**	414.763	406.754	407.258	406.250	L[7]
L[24]	1250.291	358.221	350.212	350.716	349.708	L[6]
P[25]	**1298.817**	301.679	293.670	294.174	293.166	P[5]
K[26]	**1362.865**	253.153	245.143	245.647	244.639	K[4]
K[27]	**1426.912**	189.105	181.096	181.600	180.592	K[3]
T[28]	**1477.436**	125.058	117.048	117.552	116.544	T[2]
E[29]	1541.958	74.534	66.524	67.028	66.021	E[1]

sp|Q6GSS7|H2A2A_MOUSE

LNKLLGK $^{Dimethyl}_{28.03}$ VTIAQGGVLPNIQAVLLPKKTE

- fragmentation table for charge state 3+
- specType=etd
- score=78.65
- F104903.dat
- query=q2290_p1
- precursor=771.724910
- chargePrecursor=4
- itol=0.8

AA	c	y	z+1	z+2	z	AA
L[1]	44.377	**1028.635**	**1023.296**	0.672	**1022.960**	L[29]
N[2]	82.392	**990.941**	**985.601**	**985.937**	**985.265**	N[28]
K[3]	125.090	**952.926**	**947.587**	**947.923**	**947.251**	K[27]
L[4]	162.785	**910.228**	904.889	905.225	904.553	L[26]
L[5]	200.479	872.533	867.194	867.530	866.858	L[25]
G[6]	219.487	834.839	829.499	829.835	829.163	G[24]
K[7]	271.529	815.832	810.492	810.828	810.156	K[23]
V[8]	304.552	763.790	758.450	758.786	758.114	V[22]
T[9]	338.234	730.767	725.427	725.763	725.091	T[21]
I[10]	375.929	697.084	691.745	692.081	691.409	I[20]
A[11]	399.608	659.389	654.050	654.386	653.714	A[19]
Q[12]	442.294	635.710	630.371	630.707	630.035	Q[18]
G[13]	461.301	593.024	587.685	588.021	587.349	G[17]
G[14]	480.308	574.017	568.678	569.013	568.342	G[16]
V[15]	513.331	555.010	549.670	550.006	549.334	V[15]
L[16]	551.026	521.987	516.648	516.984	516.312	L[14]
P[17]	583.377	484.292	478.953	479.289	478.617	P[13]
N[18]	621.391	451.942	446.602	446.938	446.266	N[12]
I[19]	659.086	413.927	408.588	408.924	408.252	I[11]
Q[20]	701.772	376.233	370.893	371.229	370.557	Q[10]
A[21]	725.451	333.546	328.207	328.543	327.871	A[9]
V[22]	758.474	309.867	304.528	304.864	304.192	V[8]
L[23]	796.168	276.844	271.505	271.841	271.169	L[7]
L[24]	833.863	239.150	233.810	234.146	233.474	L[6]
P[25]	866.214	201.455	196.116	196.451	195.780	P[5]
K[26]	908.912	169.104	163.765	164.101	163.429	K[4]
K[27]	**951.611**	126.406	121.066	121.402	120.730	K[3]
T[28]	**985.293**	83.708	78.368	78.704	78.032	T[2]
E[29]	1028.307	50.025	44.685	45.021	44.349	E[1]

sp|Q6GSS7|H2A2A_MOUSE

LNKLLGK Dimethyl 28.03 VTIAQGGVLPNIQAVLLPKKTE

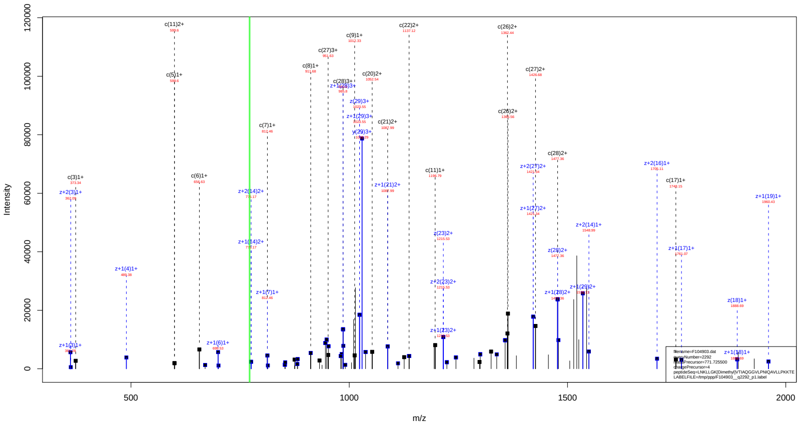

sp|Q6GSS7|H2A2A_MOUSE

LNKLLGK $^{\text{Dimethyl}}_{28.03}$ VTIAQGGVLPNIQAVLLPKKTE

- fragmentation table for charge state 1+
- specType=etd
- score=103.71
- F104903.dat
- query=q2292_p1
- precursor=771.725500
- chargePrecursor=4
- itol=0.8

AA	c	y	z+1	z+2	z	AA
L[1]	131.118	3083.892	3067.873	0.000	3066.865	L[29]
N[2]	245.161	2970.808	2954.789	2955.797	2953.781	N[28]
K[3]	**373.256**	2856.765	2840.746	2841.754	2839.738	K[27]
L[4]	486.340	2728.670	2712.651	2713.659	2711.643	L[26]
L[5]	**599.424**	2615.586	2599.567	2600.575	2598.559	L[25]
G[6]	**656.445**	2502.502	2486.483	2487.491	2485.475	G[24]
K[7]	**812.572**	2445.480	2429.462	2430.469	2428.454	K[23]
V[8]	**911.640**	2289.354	2273.335	2274.343	2272.327	V[22]
T[9]	**1012.688**	2190.286	2174.267	2175.275	2173.259	T[21]
I[10]	**1125.772**	2089.238	2073.219	2074.227	2072.211	I[20]
A[11]	**1196.809**	1976.154	**1960.135**	**1961.143**	1959.127	A[19]
Q[12]	**1324.867**	1905.117	**1889.098**	1890.106	**1888.090**	Q[18]
G[13]	1381.889	1777.058	**1761.039**	1762.047	1760.032	G[17]
G[14]	1438.910	1720.037	1704.018	**1705.026**	1703.010	G[16]
V[15]	1537.979	1663.015	1646.997	1648.004	1645.989	V[15]
L[16]	1651.063	1563.947	1547.928	**1548.936**	1546.920	L[14]
P[17]	**1748.116**	1450.863	1434.844	1435.852	1433.836	P[13]
N[18]	1862.159	1353.810	**1337.791**	1338.799	1336.783	N[12]
I[19]	1975.243	1239.767	**1223.748**	1224.756	1222.741	I[11]
Q[20]	2103.301	1126.683	1110.664	**1111.672**	1109.656	Q[10]
A[21]	2174.338	998.624	**982.606**	983.614	981.598	A[9]
V[22]	2273.407	927.587	**911.569**	912.576	910.561	V[8]
L[23]	2386.491	828.519	**812.500**	**813.508**	811.492	L[7]
L[24]	2499.575	715.435	**699.416**	**700.424**	698.408	L[6]
P[25]	2596.628	602.351	586.332	587.340	585.324	P[5]
K[26]	2724.723	505.298	**489.279**	490.287	488.271	K[4]
K[27]	2852.818	377.203	**361.184**	**362.192**	360.177	K[3]
T[28]	2953.865	249.108	233.089	234.097	232.082	T[2]
E[29]	3082.908	148.060	132.042	133.050	131.034	E[1]

sp|Q6GSS7|H2A2A_MOUSE

LNKLLGK $^{Dimethyl}_{28.03}$ VTIAQGGVLPNIQAVLLPKKTE

- fragmentation table for charge state 2+
- specType=etd
- score=103.71
- F104903.dat
- query=q2292_p1
- precursor=771.725500
- chargePrecursor=4
- itol=0.8

AA	c	y	z+1	z+2	z	AA
L[1]	66.063	1542.450	**1534.440**	0.504	1533.936	L[29]
N[2]	123.084	1485.908	**1477.898**	**1478.402**	**1477.394**	N[28]
K[3]	187.132	1428.886	**1420.877**	**1421.381**	1420.373	K[27]
L[4]	243.674	1364.839	**1356.829**	**1357.333**	**1356.325**	L[26]
L[5]	300.216	1308.297	**1300.287**	**1300.791**	**1299.783**	L[25]
G[6]	328.726	1251.755	**1243.745**	**1244.249**	1243.241	G[24]
K[7]	406.789	**1223.244**	**1215.234**	**1215.738**	**1214.731**	K[23]
V[8]	456.324	1145.181	**1137.171**	**1137.675**	**1136.667**	V[22]
T[9]	506.848	1095.646	**1087.637**	**1088.141**	1087.133	T[21]
I[10]	563.390	1045.123	**1037.113**	**1037.617**	**1036.609**	I[20]
A[11]	**598.908**	988.581	**980.571**	**981.075**	**980.067**	A[19]
Q[12]	662.937	**953.062**	**945.053**	**945.557**	**944.549**	Q[18]
G[13]	691.448	889.033	**881.023**	**881.527**	**880.519**	G[17]
G[14]	719.959	860.522	**852.513**	**853.017**	**852.009**	G[16]
V[15]	769.493	832.011	824.002	824.506	823.498	V[15]
L[16]	826.035	782.477	**774.468**	**774.972**	773.964	L[14]
P[17]	**874.561**	725.935	717.926	718.430	717.422	P[13]
N[18]	**931.583**	677.409	**669.399**	**669.903**	668.895	N[12]
I[19]	988.125	620.387	612.378	612.882	611.874	I[11]
Q[20]	**1052.154**	563.845	555.836	556.340	555.332	Q[10]
A[21]	**1087.673**	499.816	491.807	492.310	491.303	A[9]
V[22]	**1137.207**	464.297	456.288	456.792	455.784	V[8]
L[23]	1193.749	414.763	406.754	407.258	406.250	L[7]
L[24]	1250.291	358.221	350.212	350.716	349.708	L[6]
P[25]	**1298.817**	301.679	293.670	294.174	293.166	P[5]
K[26]	**1362.865**	253.153	245.143	245.647	244.639	K[4]
K[27]	**1426.912**	189.105	181.096	181.600	180.592	K[3]
T[28]	**1477.436**	125.058	117.048	117.552	116.544	T[2]
E[29]	1541.958	74.534	66.524	67.028	66.021	E[1]

sp|Q6GSS7|H2A2A_MOUSE

LNKLLGK $^{\text{Dimethyl}}_{28.03}$ VTIAQGGVLPNIQAVLLPKKTE

- fragmentation table for charge state 3+
- specType=etd
- score=103.71
- F104903.dat
- query=q2292_p1
- precursor=771.725500
- chargePrecursor=4
- itol=0.8

AA	c	y	z+1	z+2	z	AA
L[1]	44.377	**1028.635**	**1023.296**	0.672	**1022.960**	L[29]
N[2]	82.392	**990.941**	**985.601**	**985.937**	**985.265**	N[28]
K[3]	125.090	**952.926**	**947.587**	**947.923**	947.251	K[27]
L[4]	162.785	910.228	904.889	905.225	904.553	L[26]
L[5]	200.479	872.533	867.194	867.530	866.858	L[25]
G[6]	219.487	834.839	829.499	829.835	829.163	G[24]
K[7]	271.529	815.832	810.492	810.828	810.156	K[23]
V[8]	304.552	763.790	758.450	758.786	758.114	V[22]
T[9]	338.234	730.767	725.427	725.763	725.091	T[21]
I[10]	375.929	697.084	691.745	692.081	691.409	I[20]
A[11]	399.608	659.389	654.050	654.386	653.714	A[19]
Q[12]	442.294	635.710	630.371	630.707	630.035	Q[18]
G[13]	461.301	593.024	587.685	588.021	587.349	G[17]
G[14]	480.308	574.017	568.678	569.013	568.342	G[16]
V[15]	513.331	555.010	549.670	550.006	549.334	V[15]
L[16]	551.026	521.987	516.648	516.984	516.312	L[14]
P[17]	583.377	484.292	478.953	479.289	478.617	P[13]
N[18]	621.391	451.942	446.602	446.938	446.266	N[12]
I[19]	659.086	413.927	408.588	408.924	408.252	I[11]
Q[20]	701.772	376.233	370.893	371.229	370.557	Q[10]
A[21]	725.451	333.546	328.207	328.543	327.871	A[9]
V[22]	758.474	309.867	304.528	304.864	304.192	V[8]
L[23]	796.168	276.844	271.505	271.841	271.169	L[7]
L[24]	833.863	239.150	233.810	234.146	233.474	L[6]
P[25]	866.214	201.455	196.116	196.451	195.780	P[5]
K[26]	908.912	169.104	163.765	164.101	163.429	K[4]
K[27]	**951.611**	126.406	121.066	121.402	120.730	K[3]
T[28]	**985.293**	83.708	78.368	78.704	78.032	T[2]
E[29]	1028.307	50.025	44.685	45.021	44.349	E[1]

sp|Q6GSS7|H2A2A_MOUSE

LNKLLGK $^{\text{Dimethyl}}_{28.03}$ VTIAQGGVLPNIQAVLLPKKTE

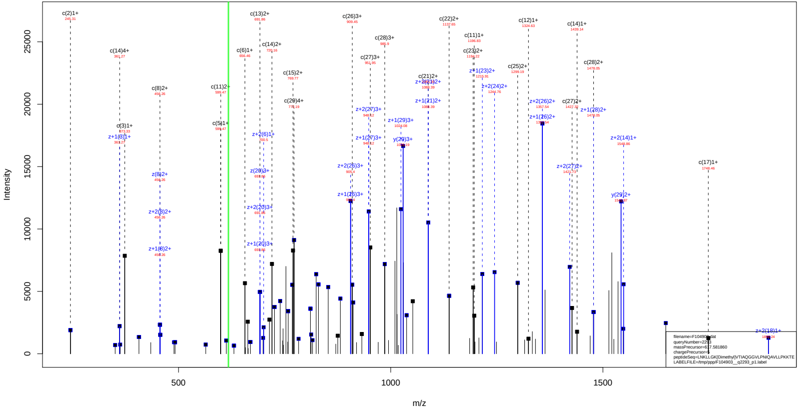

sp|Q6GSS7|H2A2A_MOUSE

LNKLLGK $^{Dimethyl}_{28.03}$ VTIAQGGVLPNIQAVLLPKKTE

- fragmentation table for charge state 1+
- specType=etd
- score=103.32
- F104903.dat
- query=q2293_p1
- precursor=617.581860
- chargePrecursor=5
- itol=0.8

AA	c	y	z+1	z+2	z	AA
L[1]	131.118	3083.892	3067.873	0.000	3066.865	L[29]
N[2]	**245.161**	2970.808	2954.789	2955.797	2953.781	N[28]
K[3]	**373.256**	2856.765	2840.746	2841.754	2839.738	K[27]
L[4]	486.340	2728.670	2712.651	2713.659	2711.643	L[26]
L[5]	**599.424**	2615.586	2599.567	2600.575	2598.559	L[25]
G[6]	**656.445**	2502.502	2486.483	2487.491	2485.475	G[24]
K[7]	**812.572**	2445.480	2429.462	2430.469	2428.454	K[23]
V[8]	**911.640**	2289.354	2273.335	2274.343	2272.327	V[22]
T[9]	1012.688	2190.286	2174.267	2175.275	2173.259	T[21]
I[10]	1125.772	2089.238	2073.219	2074.227	2072.211	I[20]
A[11]	**1196.809**	1976.154	1960.135	1961.143	1959.127	A[19]
Q[12]	**1324.867**	1905.117	1889.098	**1890.106**	1888.090	Q[18]
G[13]	1381.889	1777.058	1761.039	1762.047	1760.032	G[17]
G[14]	**1438.910**	1720.037	1704.018	1705.026	1703.010	G[16]
V[15]	1537.979	1663.015	1646.997	**1648.004**	1645.989	V[15]
L[16]	1651.063	1563.947	**1547.928**	**1548.936**	1546.920	L[14]
P[17]	**1748.116**	1450.863	1434.844	1435.852	1433.836	P[13]
N[18]	1862.159	1353.810	1337.791	1338.799	1336.783	N[12]
I[19]	1975.243	1239.767	1223.748	1224.756	1222.741	I[11]
Q[20]	2103.301	1126.683	1110.664	1111.672	1109.656	Q[10]
A[21]	2174.338	998.624	982.606	983.614	981.598	A[9]
V[22]	2273.407	927.587	**911.569**	912.576	910.561	V[8]
L[23]	2386.491	828.519	**812.500**	813.508	**811.492**	L[7]
L[24]	2499.575	715.435	**699.416**	**700.424**	698.408	L[6]
P[25]	2596.628	602.351	586.332	587.340	585.324	P[5]
K[26]	2724.723	505.298	**489.279**	490.287	488.271	K[4]
K[27]	2852.818	377.203	**361.184**	**362.192**	360.177	K[3]
T[28]	2953.865	249.108	233.089	234.097	232.082	T[2]
E[29]	3082.908	148.060	132.042	133.050	131.034	E[1]

sp|Q6GSS7|H2A2A_MOUSE

LNKLLGK $^{Dimethyl}_{28.03}$ VTIAQGGVLPNIQAVLLPKKTE

- fragmentation table for charge state 2+
- specType=etd
- score=103.32
- F104903.dat
- query=q2293_p1
- precursor=617.581860
- chargePrecursor=5
- itol=0.8

AA	c	y	z+1	z+2	z	AA
L[1]	66.063	**1542.450**	1534.440	0.504	1533.936	L[29]
N[2]	123.084	1485.908	**1477.898**	**1478.402**	**1477.394**	N[28]
K[3]	187.132	1428.886	1420.877	**1421.381**	1420.373	K[27]
L[4]	243.674	1364.839	**1356.829**	**1357.333**	1356.325	L[26]
L[5]	300.216	1308.297	1300.287	1300.791	**1299.783**	L[25]
G[6]	328.726	1251.755	1243.745	**1244.249**	1243.241	G[24]
K[7]	**406.789**	1223.244	**1215.234**	**1215.738**	1214.731	K[23]
V[8]	**456.324**	1145.181	**1137.171**	**1137.675**	1136.667	V[22]
T[9]	506.848	1095.646	**1087.637**	**1088.141**	1087.133	T[21]
I[10]	**563.390**	1045.123	**1037.113**	**1037.617**	1036.609	I[20]
A[11]	**598.908**	988.581	980.571	981.075	980.067	A[19]
Q[12]	**662.937**	953.062	945.053	945.557	944.549	Q[18]
G[13]	**691.448**	889.033	**881.023**	**881.527**	880.519	G[17]
G[14]	**719.959**	860.522	**852.513**	**853.017**	**852.009**	G[16]
V[15]	**769.493**	832.011	**824.002**	**824.506**	823.498	V[15]
L[16]	826.035	**782.477**	774.468	774.972	773.964	L[14]
P[17]	**874.561**	**725.935**	717.926	718.430	717.422	P[13]
N[18]	**931.583**	677.409	**669.399**	**669.903**	**668.895**	N[12]
I[19]	988.125	620.387	**612.378**	**612.882**	**611.874**	I[11]
Q[20]	**1052.154**	**563.845**	555.836	556.340	555.332	Q[10]
A[21]	**1087.673**	499.816	**491.807**	**492.310**	**491.303**	A[9]
V[22]	**1137.207**	464.297	**456.288**	**456.792**	**455.784**	V[8]
L[23]	**1193.749**	414.763	**406.754**	**407.258**	**406.250**	L[7]
L[24]	1250.291	358.221	**350.212**	**350.716**	349.708	L[6]
P[25]	**1298.817**	301.679	293.670	294.174	293.166	P[5]
K[26]	1362.865	253.153	**245.143**	**245.647**	**244.639**	K[4]
K[27]	**1426.912**	189.105	181.096	181.600	180.592	K[3]
T[28]	**1477.436**	125.058	117.048	117.552	116.544	T[2]
E[29]	1541.958	74.534	66.524	67.028	66.021	E[1]

sp|Q6GSS7|H2A2A_MOUSE

LNKLLGK $^{\text{Dimethyl}}_{28.03}$ VTIAQGGVLPNIQAVLLPKKTE

- fragmentation table for charge state 3+
- specType=etd
- score=103.32
- F104903.dat
- query=q2293_p1
- precursor=617.581860
- chargePrecursor=5
- itol=0.8

AA	c	y	z+1	z+2	z	AA
L[1]	44.377	**1028.635**	**1023.296**	0.672	1022.960	L[29]
N[2]	82.392	990.941	**985.601**	**985.937**	**985.265**	N[28]
K[3]	125.090	952.926	**947.587**	**947.923**	947.251	K[27]
L[4]	162.785	**910.228**	**904.889**	**905.225**	904.553	L[26]
L[5]	200.479	872.533	867.194	867.530	866.858	L[25]
G[6]	219.487	834.839	**829.499**	**829.835**	**829.163**	G[24]
K[7]	271.529	**815.832**	**810.492**	**810.828**	**810.156**	K[23]
V[8]	304.552	763.790	**758.450**	**758.786**	**758.114**	V[22]
T[9]	338.234	730.767	**725.427**	**725.763**	725.091	T[21]
I[10]	375.929	697.084	**691.745**	**692.081**	**691.409**	I[20]
A[11]	399.608	659.389	654.050	654.386	653.714	A[19]
Q[12]	442.294	635.710	**630.371**	**630.707**	**630.035**	Q[18]
G[13]	461.301	593.024	587.685	588.021	587.349	G[17]
G[14]	480.308	574.017	568.678	569.013	568.342	G[16]
V[15]	513.331	555.010	549.670	550.006	549.334	V[15]
L[16]	551.026	521.987	516.648	516.984	516.312	L[14]
P[17]	583.377	484.292	478.953	479.289	478.617	P[13]
N[18]	621.391	451.942	446.602	446.938	446.266	N[12]
I[19]	659.086	413.927	408.588	408.924	408.252	I[11]
Q[20]	701.772	376.233	370.893	371.229	370.557	Q[10]
A[21]	**725.451**	333.546	328.207	328.543	327.871	A[9]
V[22]	**758.474**	309.867	304.528	304.864	304.192	V[8]
L[23]	796.168	276.844	271.505	271.841	271.169	L[7]
L[24]	833.863	239.150	233.810	234.146	233.474	L[6]
P[25]	866.214	201.455	196.116	196.451	195.780	P[5]
K[26]	**908.912**	169.104	163.765	164.101	163.429	K[4]
K[27]	**951.611**	126.406	121.066	121.402	120.730	K[3]
T[28]	**985.293**	83.708	78.368	78.704	78.032	T[2]
E[29]	1028.307	50.025	44.685	45.021	44.349	E[1]

sp|Q6GSS7|H2A2A_MOUSE

LNKLLGK $^{\text{Dimethyl}}_{28.03}$ VTIAQGGVLPNIQAVLLPKKTE

- fragmentation table for charge state 4+
- specType=etd
- score=103.32
- F104903.dat
- query=q2293_p1
- precursor=617.581860
- chargePrecursor=5
- itol=0.8

AA	c	y	z+1	z+2	z	AA
L[1]	33.535	**771.728**	**767.724**	0.755	**767.472**	L[29]
N[2]	62.046	743.457	**739.453**	**739.705**	**739.201**	N[28]
K[3]	94.069	714.947	710.942	711.194	710.690	K[27]
L[4]	122.340	682.923	678.918	679.170	678.666	L[26]
L[5]	150.611	654.652	650.647	650.899	650.395	L[25]
G[6]	164.867	626.381	622.376	622.628	622.124	G[24]
K[7]	203.898	**612.126**	608.121	608.373	607.869	K[23]
V[8]	228.665	573.094	569.089	569.341	568.837	V[22]
T[9]	253.927	548.327	544.322	544.574	544.070	T[21]
I[10]	282.198	523.065	519.060	519.312	518.808	I[20]
A[11]	299.958	494.794	490.789	491.041	490.537	A[19]
Q[12]	331.972	477.035	473.030	473.282	472.778	Q[18]
G[13]	346.228	445.020	441.015	441.267	440.763	G[17]
G[14]	**360.483**	430.765	426.760	427.012	426.508	G[16]
V[15]	385.250	416.509	412.505	412.757	412.253	V[15]
L[16]	413.521	391.742	387.737	387.989	387.486	L[14]
P[17]	437.784	363.471	359.466	359.718	359.215	P[13]
N[18]	466.295	339.208	335.203	335.455	334.951	N[12]
I[19]	494.566	310.697	306.693	306.945	306.441	I[11]
Q[20]	526.581	282.426	278.422	278.673	278.170	Q[10]
A[21]	544.340	250.412	246.407	246.659	246.155	A[9]
V[22]	569.107	232.652	228.648	228.900	228.396	V[8]
L[23]	597.378	207.885	203.881	204.132	203.629	L[7]
L[24]	625.649	179.614	175.609	175.861	175.358	L[6]
P[25]	649.912	151.343	147.338	147.590	147.087	P[5]
K[26]	681.936	127.080	123.075	123.327	122.823	K[4]
K[27]	**713.960**	95.056	91.052	91.304	90.800	K[3]
T[28]	**739.222**	63.032	59.028	59.280	58.776	T[2]
E[29]	**771.482**	37.771	33.766	34.018	33.514	E[1]

sp|Q6GSS7|H2A2A_MOUSE

LNKLLGK $^{Dimethyl}_{28.03}$ VTIAQGGVLPNIQAVLLPKKTE

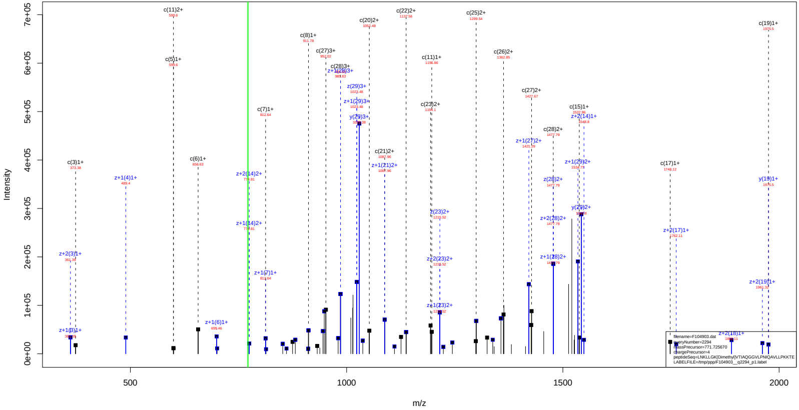

sp|Q6GSS7|H2A2A_MOUSE

LNKLLGK $^{\text{Dimethyl}}_{28.03}$ VTIAQGGVLPNIQAVLLPKKTE

- fragmentation table for charge state 1+
- specType=etd
- score=103.61
- F104903.dat
- query=q2294_p1
- precursor=771.725670
- chargePrecursor=4
- itol=0.8

AA	c	y	z+1	z+2	z	AA
L[1]	131.118	3083.892	3067.873	0.000	3066.865	L[29]
N[2]	245.161	2970.808	2954.789	2955.797	2953.781	N[28]
K[3]	**373.256**	2856.765	2840.746	2841.754	2839.738	K[27]
L[4]	486.340	2728.670	2712.651	2713.659	2711.643	L[26]
L[5]	**599.424**	2615.586	2599.567	2600.575	2598.559	L[25]
G[6]	**656.445**	2502.502	2486.483	2487.491	2485.475	G[24]
K[7]	**812.572**	2445.480	2429.462	2430.469	2428.454	K[23]
V[8]	**911.640**	2289.354	2273.335	2274.343	2272.327	V[22]
T[9]	1012.688	2190.286	2174.267	2175.275	2173.259	T[21]
I[10]	**1125.772**	2089.238	2073.219	2074.227	2072.211	I[20]
A[11]	**1196.809**	**1976.154**	1960.135	**1961.143**	1959.127	A[19]
Q[12]	**1324.867**	1905.117	1889.098	**1890.106**	1888.090	Q[18]
G[13]	1381.889	1777.058	1761.039	**1762.047**	1760.032	G[17]
G[14]	1438.910	1720.037	1704.018	1705.026	1703.010	G[16]
V[15]	**1537.979**	1663.015	1646.997	1648.004	1645.989	V[15]
L[16]	1651.063	1563.947	1547.928	**1548.936**	1546.920	L[14]
P[17]	**1748.116**	1450.863	1434.844	1435.852	1433.836	P[13]
N[18]	1862.159	1353.810	**1337.791**	1338.799	1336.783	N[12]
I[19]	**1975.243**	1239.767	**1223.748**	1224.756	1222.741	I[11]
Q[20]	2103.301	1126.683	**1110.664**	1111.672	1109.656	Q[10]
A[21]	2174.338	998.624	982.606	983.614	981.598	A[9]
V[22]	2273.407	927.587	**911.569**	**912.576**	**910.561**	V[8]
L[23]	2386.491	828.519	**812.500**	**813.508**	811.492	L[7]
L[24]	2499.575	715.435	**699.416**	**700.424**	698.408	L[6]
P[25]	2596.628	602.351	586.332	587.340	585.324	P[5]
K[26]	2724.723	505.298	**489.279**	490.287	488.271	K[4]
K[27]	2852.818	377.203	**361.184**	**362.192**	360.177	K[3]
T[28]	2953.865	249.108	233.089	234.097	232.082	T[2]
E[29]	3082.908	148.060	132.042	133.050	131.034	E[1]

sp|Q6GSS7|H2A2A_MOUSE

LNKLLGK $^{\text{Dimethyl}}_{28.03}$ VTIAQGGVLPNIQAVLLPKKTE

- fragmentation table for charge state 2+
- specType=etd
- score=103.61
- F104903.dat
- query=q2294_p1
- precursor=771.725670
- chargePrecursor=4
- itol=0.8

AA	c	y	z+1	z+2	z	AA
L[1]	66.063	**1542.450**	**1534.440**	0.504	1533.936	L[29]
N[2]	123.084	1485.908	**1477.898**	**1478.402**	**1477.394**	N[28]
K[3]	187.132	1428.886	**1420.877**	**1421.381**	1420.373	K[27]
L[4]	243.674	1364.839	**1356.829**	**1357.333**	**1356.325**	L[26]
L[5]	300.216	1308.297	**1300.287**	1300.791	**1299.783**	L[25]
G[6]	328.726	1251.755	**1243.745**	**1244.249**	1243.241	G[24]
K[7]	406.789	**1223.244**	**1215.234**	**1215.738**	**1214.731**	K[23]
V[8]	456.324	1145.181	**1137.171**	**1137.675**	1136.667	V[22]
T[9]	506.848	1095.646	**1087.637**	**1088.141**	1087.133	T[21]
I[10]	563.390	1045.123	**1037.113**	**1037.617**	**1036.609**	I[20]
A[11]	**598.908**	988.581	**980.571**	**981.075**	**980.067**	A[19]
Q[12]	662.937	953.062	**945.053**	**945.557**	**944.549**	Q[18]
G[13]	691.448	889.033	**881.023**	**881.527**	880.519	G[17]
G[14]	719.959	**860.522**	**852.513**	**853.017**	**852.009**	G[16]
V[15]	769.493	832.011	824.002	824.506	823.498	V[15]
L[16]	826.035	782.477	**774.468**	**774.972**	773.964	L[14]
P[17]	**874.561**	725.935	717.926	718.430	717.422	P[13]
N[18]	**931.583**	677.409	669.399	669.903	668.895	N[12]
I[19]	988.125	620.387	612.378	612.882	611.874	I[11]
Q[20]	**1052.154**	563.845	555.836	556.340	555.332	Q[10]
A[21]	**1087.673**	499.816	491.807	492.310	491.303	A[9]
V[22]	**1137.207**	464.297	456.288	456.792	455.784	V[8]
L[23]	**1193.749**	414.763	406.754	407.258	406.250	L[7]
L[24]	1250.291	358.221	350.212	350.716	349.708	L[6]
P[25]	**1298.817**	301.679	293.670	294.174	293.166	P[5]
K[26]	**1362.865**	253.153	245.143	245.647	244.639	K[4]
K[27]	**1426.912**	189.105	181.096	181.600	180.592	K[3]
T[28]	**1477.436**	125.058	117.048	117.552	116.544	T[2]
E[29]	1541.958	74.534	66.524	67.028	66.021	E[1]

sp|Q6GSS7|H2A2A_MOUSE

LNKLLGK $^{Dimethyl}_{28.03}$ VTIAQGGVLPNIQAVLLPKKTE

- fragmentation table for charge state 3+
- specType=etd
- score=103.61
- F104903.dat
- query=q2294_p1
- precursor=771.725670
- chargePrecursor=4
- itol=0.8

AA	c	y	z+1	z+2	z	AA
L[1]	44.377	**1028.635**	**1023.296**	0.672	**1022.960**	L[29]
N[2]	82.392	990.941	**985.601**	**985.937**	**985.265**	N[28]
K[3]	125.090	952.926	**947.587**	**947.923**	947.251	K[27]
L[4]	162.785	910.228	904.889	905.225	904.553	L[26]
L[5]	200.479	872.533	867.194	867.530	866.858	L[25]
G[6]	219.487	834.839	829.499	829.835	829.163	G[24]
K[7]	271.529	815.832	810.492	810.828	810.156	K[23]
V[8]	304.552	763.790	758.450	758.786	758.114	V[22]
T[9]	338.234	730.767	725.427	725.763	725.091	T[21]
I[10]	375.929	697.084	691.745	692.081	691.409	I[20]
A[11]	399.608	659.389	654.050	654.386	653.714	A[19]
Q[12]	442.294	635.710	630.371	630.707	630.035	Q[18]
G[13]	461.301	593.024	587.685	588.021	587.349	G[17]
G[14]	480.308	574.017	568.678	569.013	568.342	G[16]
V[15]	513.331	555.010	549.670	550.006	549.334	V[15]
L[16]	551.026	521.987	516.648	516.984	516.312	L[14]
P[17]	583.377	484.292	478.953	479.289	478.617	P[13]
N[18]	621.391	451.942	446.602	446.938	446.266	N[12]
I[19]	659.086	413.927	408.588	408.924	408.252	I[11]
Q[20]	701.772	376.233	370.893	371.229	370.557	Q[10]
A[21]	725.451	333.546	328.207	328.543	327.871	A[9]
V[22]	758.474	309.867	304.528	304.864	304.192	V[8]
L[23]	796.168	276.844	271.505	271.841	271.169	L[7]
L[24]	833.863	239.150	233.810	234.146	233.474	L[6]
P[25]	866.214	201.455	196.116	196.451	195.780	P[5]
K[26]	908.912	169.104	163.765	164.101	163.429	K[4]
K[27]	**951.611**	126.406	121.066	121.402	120.730	K[3]
T[28]	**985.293**	83.708	78.368	78.704	78.032	T[2]
E[29]	1028.307	50.025	44.685	45.021	44.349	E[1]

sp|Q6GSS7|H2A2A_MOUSE

LNKLLGK $^{Dimethyl}_{28.03}$ VTIAQGGVLPNIQAVLLPKKTE

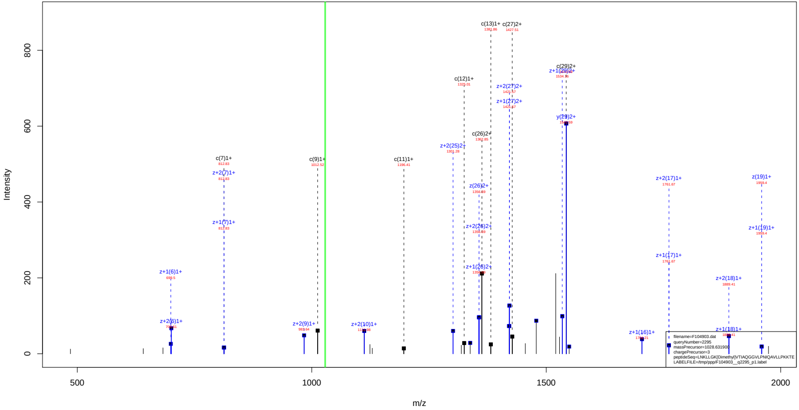

sp|Q6GSS7|H2A2A_MOUSE

LNKLLGK $^{\text{Dimethyl}}_{28.03}$ VTIAQGGVLPNIQAVLLPKKTE

- fragmentation table for charge state 1+
- specType=etd
- score=44.60
- F104903.dat
- query=q2295_p1
- precursor=1028.631900
- chargePrecursor=3
- itol=0.8

AA	c	y	z+1	z+2	z	AA
L[1]	131.118	3083.892	3067.873	0.000	3066.865	L[29]
N[2]	245.161	2970.808	2954.789	2955.797	2953.781	N[28]
K[3]	373.256	2856.765	2840.746	2841.754	2839.738	K[27]
L[4]	486.340	2728.670	2712.651	2713.659	2711.643	L[26]
L[5]	599.424	2615.586	2599.567	2600.575	2598.559	L[25]
G[6]	656.445	2502.502	2486.483	2487.491	2485.475	G[24]
K[7]	**812.572**	2445.480	2429.462	2430.469	2428.454	K[23]
V[8]	911.640	2289.354	2273.335	2274.343	2272.327	V[22]
T[9]	**1012.688**	2190.286	2174.267	2175.275	2173.259	T[21]
I[10]	1125.772	2089.238	2073.219	2074.227	2072.211	I[20]
A[11]	**1196.809**	1976.154	**1960.135**	1961.143	**1959.127**	A[19]
Q[12]	**1324.867**	1905.117	**1889.098**	**1890.106**	1888.090	Q[18]
G[13]	**1381.889**	1777.058	**1761.039**	**1762.047**	1760.032	G[17]
G[14]	1438.910	1720.037	**1704.018**	1705.026	1703.010	G[16]
V[15]	1537.979	1663.015	1646.997	1648.004	1645.989	V[15]
L[16]	1651.063	1563.947	1547.928	**1548.936**	1546.920	L[14]
P[17]	1748.116	1450.863	1434.844	1435.852	1433.836	P[13]
N[18]	1862.159	1353.810	**1337.791**	1338.799	1336.783	N[12]
I[19]	1975.243	1239.767	1223.748	1224.756	1222.741	I[11]
Q[20]	2103.301	1126.683	1110.664	**1111.672**	1109.656	Q[10]
A[21]	2174.338	998.624	982.606	**983.614**	981.598	A[9]
V[22]	2273.407	927.587	911.569	912.576	910.561	V[8]
L[23]	2386.491	828.519	**812.500**	**813.508**	811.492	L[7]
L[24]	2499.575	715.435	**699.416**	**700.424**	698.408	L[6]
P[25]	2596.628	602.351	586.332	587.340	585.324	P[5]
K[26]	2724.723	505.298	489.279	490.287	488.271	K[4]
K[27]	2852.818	377.203	361.184	362.192	360.177	K[3]
T[28]	2953.865	249.108	233.089	234.097	232.082	T[2]
E[29]	3082.908	148.060	132.042	133.050	131.034	E[1]

sp|Q6GSS7|H2A2A_MOUSE

LNKLLGK $^{\text{Dimethyl}}_{28.03}$ VTIAQGGVLPNIQAVLLPKKTE

- fragmentation table for charge state 2+
- specType=etd
- score=44.60
- F104903.dat
- query=q2295_p1
- precursor=1028.631900
- chargePrecursor=3
- itol=0.8

AA	c	y	z+1	z+2	z	AA
L[1]	66.063	**1542.450**	**1534.440**	0.504	**1533.936**	L[29]
N[2]	123.084	1485.908	1477.898	**1478.402**	1477.394	N[28]
K[3]	187.132	1428.886	**1420.877**	**1421.381**	**1420.373**	K[27]
L[4]	243.674	1364.839	**1356.829**	**1357.333**	**1356.325**	L[26]
L[5]	300.216	1308.297	1300.287	**1300.791**	1299.783	L[25]
G[6]	328.726	1251.755	1243.745	1244.249	1243.241	G[24]
K[7]	406.789	1223.244	1215.234	1215.738	1214.731	K[23]
V[8]	456.324	1145.181	1137.171	1137.675	1136.667	V[22]
T[9]	506.848	1095.646	1087.637	1088.141	1087.133	T[21]
I[10]	563.390	1045.123	1037.113	1037.617	1036.609	I[20]
A[11]	598.908	988.581	980.571	981.075	980.067	A[19]
Q[12]	662.937	953.062	945.053	945.557	944.549	Q[18]
G[13]	691.448	889.033	881.023	881.527	880.519	G[17]
G[14]	719.959	860.522	852.513	853.017	852.009	G[16]
V[15]	769.493	832.011	824.002	824.506	823.498	V[15]
L[16]	826.035	782.477	774.468	774.972	773.964	L[14]
P[17]	874.561	725.935	717.926	718.430	717.422	P[13]
N[18]	931.583	677.409	669.399	669.903	668.895	N[12]
I[19]	988.125	620.387	612.378	612.882	611.874	I[11]
Q[20]	1052.154	563.845	555.836	556.340	555.332	Q[10]
A[21]	1087.673	499.816	491.807	492.310	491.303	A[9]
V[22]	1137.207	464.297	456.288	456.792	455.784	V[8]
L[23]	1193.749	414.763	406.754	407.258	406.250	L[7]
L[24]	1250.291	358.221	350.212	350.716	349.708	L[6]
P[25]	1298.817	301.679	293.670	294.174	293.166	P[5]
K[26]	**1362.865**	253.153	245.143	245.647	244.639	K[4]
K[27]	**1426.912**	189.105	181.096	181.600	180.592	K[3]
T[28]	1477.436	125.058	117.048	117.552	116.544	T[2]
E[29]	**1541.958**	74.534	66.524	67.028	66.021	E[1]

sp|Q6GSS7|H2A2A_MOUSE

LNKLLGK Dimethyl 28.03 VTIAQGGVLPNIQAVLLPKKTE

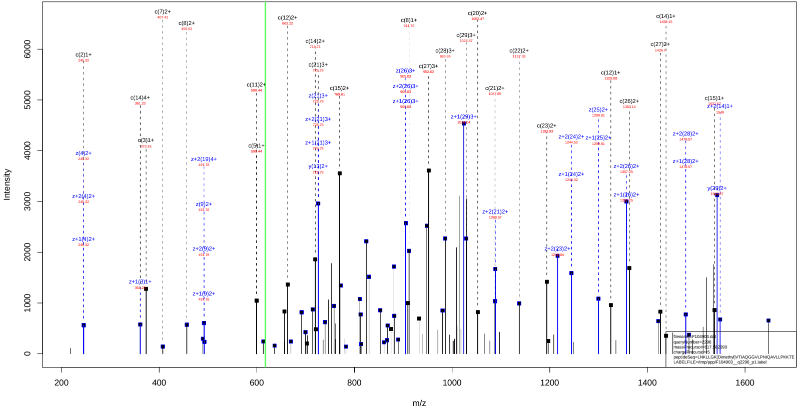

sp|Q6GSS7|H2A2A_MOUSE

LNKLLGK $^{Dimethyl}_{28.03}$ VTIAQGGVLPNIQAVLLPKKTE

- fragmentation table for charge state 1+
- specType=etd
- score=98.64
- F104903.dat
- query=q2296_p1
- precursor=617.582090
- chargePrecursor=5
- itol=0.8

AA	c	y	z+1	z+2	z	AA
L[1]	131.118	3083.892	3067.873	0.000	3066.865	L[29]
N[2]	**245.161**	2970.808	2954.789	2955.797	2953.781	N[28]
K[3]	**373.256**	2856.765	2840.746	2841.754	2839.738	K[27]
L[4]	486.340	2728.670	2712.651	2713.659	2711.643	L[26]
L[5]	**599.424**	2615.586	2599.567	2600.575	2598.559	L[25]
G[6]	**656.445**	2502.502	2486.483	2487.491	2485.475	G[24]
K[7]	**812.572**	2445.480	2429.462	2430.469	2428.454	K[23]
V[8]	**911.640**	2289.354	2273.335	2274.343	2272.327	V[22]
T[9]	1012.688	2190.286	2174.267	2175.275	2173.259	T[21]
I[10]	1125.772	2089.238	2073.219	2074.227	2072.211	I[20]
A[11]	**1196.809**	1976.154	1960.135	1961.143	1959.127	A[19]
Q[12]	**1324.867**	1905.117	1889.098	1890.106	1888.090	Q[18]
G[13]	1381.889	1777.058	1761.039	1762.047	1760.032	G[17]
G[14]	**1438.910**	1720.037	1704.018	1705.026	1703.010	G[16]
V[15]	**1537.979**	1663.015	1646.997	**1648.004**	1645.989	V[15]
L[16]	1651.063	1563.947	1547.928	**1548.936**	1546.920	L[14]
P[17]	1748.116	1450.863	1434.844	1435.852	1433.836	P[13]
N[18]	1862.159	1353.810	1337.791	1338.799	1336.783	N[12]
I[19]	1975.243	1239.767	1223.748	1224.756	1222.741	I[11]
Q[20]	2103.301	1126.683	1110.664	1111.672	1109.656	Q[10]
A[21]	2174.338	998.624	982.606	983.614	981.598	A[9]
V[22]	2273.407	927.587	**911.569**	912.576	910.561	V[8]
L[23]	2386.491	828.519	**812.500**	**813.508**	**811.492**	L[7]
L[24]	2499.575	715.435	**699.416**	**700.424**	698.408	L[6]
P[25]	2596.628	602.351	586.332	587.340	585.324	P[5]
K[26]	2724.723	505.298	**489.279**	490.287	488.271	K[4]
K[27]	2852.818	377.203	**361.184**	362.192	360.177	K[3]
T[28]	2953.865	249.108	233.089	234.097	232.082	T[2]
E[29]	3082.908	148.060	132.042	133.050	131.034	E[1]

sp|Q6GSS7|H2A2A_MOUSE

LNKLLGK $^{\text{Dimethyl}}_{28.03}$ VTIAQGGVLPNIQAVLLPKKTE

- fragmentation table for charge state 2+
- specType=etd
- score=98.64
- F104903.dat
- query=q2296_p1
- precursor=617.582090
- chargePrecursor=5
- itol=0.8

AA	c	y	z+1	z+2	z	AA
L[1]	66.063	**1542.450**	1534.440	0.504	1533.936	L[29]
N[2]	123.084	**1485.908**	**1477.898**	**1478.402**	1477.394	N[28]
K[3]	187.132	1428.886	1420.877	**1421.381**	1420.373	K[27]
L[4]	243.674	1364.839	**1356.829**	**1357.333**	1356.325	L[26]
L[5]	300.216	1308.297	**1300.287**	1300.791	**1299.783**	L[25]
G[6]	328.726	1251.755	**1243.745**	**1244.249**	1243.241	G[24]
K[7]	**406.789**	1223.244	1215.234	**1215.738**	1214.731	K[23]
V[8]	**456.324**	1145.181	**1137.171**	**1137.675**	**1136.667**	V[22]
T[9]	506.848	1095.646	**1087.637**	**1088.141**	**1087.133**	T[21]
I[10]	563.390	1045.123	1037.113	1037.617	1036.609	I[20]
A[11]	**598.908**	988.581	**980.571**	**981.075**	**980.067**	A[19]
Q[12]	**662.937**	953.062	945.053	945.557	944.549	Q[18]
G[13]	**691.448**	**889.033**	**881.023**	**881.527**	**880.519**	G[17]
G[14]	**719.959**	**860.522**	**852.513**	**853.017**	852.009	G[16]
V[15]	**769.493**	832.011	**824.002**	**824.506**	823.498	V[15]
L[16]	826.035	**782.477**	774.468	774.972	773.964	L[14]
P[17]	**874.561**	**725.935**	717.926	718.430	717.422	P[13]
N[18]	**931.583**	677.409	**669.399**	**669.903**	668.895	N[12]
I[19]	988.125	620.387	612.378	**612.882**	611.874	I[11]
Q[20]	**1052.154**	563.845	555.836	556.340	555.332	Q[10]
A[21]	**1087.673**	499.816	**491.807**	**492.310**	**491.303**	A[9]
V[22]	**1137.207**	464.297	**456.288**	**456.792**	455.784	V[8]
L[23]	**1193.749**	414.763	**406.754**	**407.258**	406.250	L[7]
L[24]	1250.291	358.221	350.212	350.716	349.708	L[6]
P[25]	1298.817	301.679	293.670	294.174	293.166	P[5]
K[26]	**1362.865**	253.153	**245.143**	**245.647**	**244.639**	K[4]
K[27]	**1426.912**	189.105	181.096	181.600	180.592	K[3]
T[28]	1477.436	125.058	117.048	117.552	116.544	T[2]
E[29]	1541.958	74.534	66.524	67.028	66.021	E[1]

sp|Q6GSS7|H2A2A_MOUSE

LNKLLGK $^{\text{Dimethyl}}_{28.03}$ VTIAQGGVLPNIQAVLLPKKTE

- fragmentation table for charge state 3+
- specType=etd
- score=98.64
- F104903.dat
- query=q2296_p1
- precursor=617.582090
- chargePrecursor=5
- itol=0.8

AA	c	y	z+1	z+2	z	AA
L[1]	44.377	**1028.635**	**1023.296**	0.672	1022.960	L[29]
N[2]	82.392	990.941	**985.601**	**985.937**	**985.265**	N[28]
K[3]	125.090	952.926	**947.587**	**947.923**	947.251	K[27]
L[4]	162.785	910.228	**904.889**	**905.225**	**904.553**	L[26]
L[5]	200.479	872.533	**867.194**	**867.530**	**866.858**	L[25]
G[6]	219.487	834.839	**829.499**	**829.835**	**829.163**	G[24]
K[7]	271.529	815.832	**810.492**	**810.828**	810.156	K[23]
V[8]	304.552	763.790	**758.450**	**758.786**	**758.114**	V[22]
T[9]	338.234	730.767	**725.427**	**725.763**	**725.091**	T[21]
I[10]	375.929	697.084	**691.745**	**692.081**	**691.409**	I[20]
A[11]	399.608	659.389	654.050	654.386	653.714	A[19]
Q[12]	442.294	**635.710**	630.371	630.707	630.035	Q[18]
G[13]	461.301	593.024	587.685	588.021	587.349	G[17]
G[14]	480.308	574.017	568.678	569.013	568.342	G[16]
V[15]	513.331	555.010	549.670	550.006	549.334	V[15]
L[16]	551.026	521.987	516.648	516.984	516.312	L[14]
P[17]	583.377	484.292	478.953	479.289	478.617	P[13]
N[18]	621.391	451.942	446.602	446.938	446.266	N[12]
I[19]	659.086	413.927	408.588	408.924	408.252	I[11]
Q[20]	**701.772**	376.233	370.893	371.229	370.557	Q[10]
A[21]	**725.451**	333.546	328.207	328.543	327.871	A[9]
V[22]	**758.474**	309.867	304.528	304.864	304.192	V[8]
L[23]	796.168	276.844	271.505	271.841	271.169	L[7]
L[24]	833.863	239.150	233.810	234.146	233.474	L[6]
P[25]	**866.214**	201.455	196.116	196.451	195.780	P[5]
K[26]	**908.912**	169.104	163.765	164.101	163.429	K[4]
K[27]	**951.611**	126.406	121.066	121.402	120.730	K[3]
T[28]	**985.293**	83.708	78.368	78.704	78.032	T[2]
E[29]	**1028.307**	50.025	44.685	45.021	44.349	E[1]

sp|Q6GSS7|H2A2A_MOUSE

LNKLLGK $^{Dimethyl}_{28.03}$ VTIAQGGVLPNIQAVLLPKKTE

- fragmentation table for charge state 4+
- specType=etd
- score=98.64
- F104903.dat
- query=q2296_p1
- precursor=617.582090
- chargePrecursor=5
- itol=0.8

AA	c	y	z+1	z+2	z	AA
L[1]	33.535	**771.728**	767.724	0.755	767.472	L[29]
N[2]	62.046	743.457	**739.453**	**739.705**	**739.201**	N[28]
K[3]	94.069	**714.947**	710.942	711.194	710.690	K[27]
L[4]	122.340	682.923	678.918	679.170	678.666	L[26]
L[5]	150.611	654.652	650.647	650.899	650.395	L[25]
G[6]	164.867	626.381	622.376	622.628	622.124	G[24]
K[7]	203.898	612.126	608.121	608.373	607.869	K[23]
V[8]	228.665	573.094	569.089	569.341	568.837	V[22]
T[9]	253.927	548.327	544.322	544.574	544.070	T[21]
I[10]	282.198	523.065	519.060	519.312	518.808	I[20]
A[11]	299.958	494.794	490.789	**491.041**	490.537	A[19]
Q[12]	331.972	477.035	473.030	473.282	472.778	Q[18]
G[13]	346.228	445.020	441.015	441.267	440.763	G[17]
G[14]	**360.483**	430.765	426.760	427.012	426.508	G[16]
V[15]	385.250	416.509	412.505	412.757	412.253	V[15]
L[16]	413.521	391.742	387.737	387.989	387.486	L[14]
P[17]	437.784	363.471	359.466	359.718	359.215	P[13]
N[18]	466.295	339.208	335.203	335.455	334.951	N[12]
I[19]	494.566	310.697	306.693	306.945	306.441	I[11]
Q[20]	526.581	282.426	278.422	278.673	278.170	Q[10]
A[21]	544.340	250.412	246.407	246.659	246.155	A[9]
V[22]	569.107	232.652	228.648	228.900	228.396	V[8]
L[23]	597.378	207.885	203.881	204.132	203.629	L[7]
L[24]	625.649	179.614	175.609	175.861	175.358	L[6]
P[25]	649.912	151.343	147.338	147.590	147.087	P[5]
K[26]	681.936	127.080	123.075	123.327	122.823	K[4]
K[27]	**713.960**	95.056	91.052	91.304	90.800	K[3]
T[28]	**739.222**	63.032	59.028	59.280	58.776	T[2]
E[29]	**771.482**	37.771	33.766	34.018	33.514	E[1]

sp|Q6GSS7|H2A2A_MOUSE

LNKLLGK $^{Dimethyl}_{28.03}$ VTIAQGGVLPNIQAVLLPKKTE

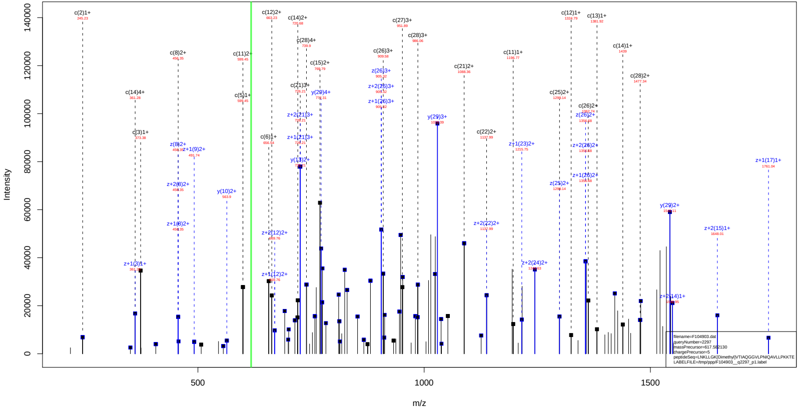

sp|Q6GSS7|H2A2A_MOUSE

LNKLLGK $^{\text{Dimethyl}}_{28.03}$ VTIAQGGVLPNIQAVLLPKKTE

- fragmentation table for charge state 1+
- specType=etd
- score=136.68
- F104903.dat
- query=q2297_p1
- precursor=617.582130
- chargePrecursor=5
- itol=0.8

AA	c	y	z+1	z+2	z	AA
L[1]	131.118	3083.892	3067.873	0.000	3066.865	L[29]
N[2]	**245.161**	2970.808	2954.789	2955.797	2953.781	N[28]
K[3]	**373.256**	2856.765	2840.746	2841.754	2839.738	K[27]
L[4]	486.340	2728.670	2712.651	2713.659	2711.643	L[26]
L[5]	**599.424**	2615.586	2599.567	2600.575	2598.559	L[25]
G[6]	**656.445**	2502.502	2486.483	2487.491	2485.475	G[24]
K[7]	**812.572**	2445.480	2429.462	2430.469	2428.454	K[23]
V[8]	**911.640**	2289.354	2273.335	2274.343	2272.327	V[22]
T[9]	1012.688	2190.286	2174.267	2175.275	2173.259	T[21]
I[10]	**1125.772**	2089.238	2073.219	2074.227	2072.211	I[20]
A[11]	**1196.809**	1976.154	1960.135	1961.143	1959.127	A[19]
Q[12]	**1324.867**	1905.117	1889.098	1890.106	1888.090	Q[18]
G[13]	**1381.889**	1777.058	**1761.039**	1762.047	1760.032	G[17]
G[14]	**1438.910**	1720.037	1704.018	1705.026	1703.010	G[16]
V[15]	1537.979	1663.015	1646.997	**1648.004**	1645.989	V[15]
L[16]	1651.063	1563.947	1547.928	**1548.936**	1546.920	L[14]
P[17]	1748.116	1450.863	1434.844	1435.852	1433.836	P[13]
N[18]	1862.159	1353.810	1337.791	1338.799	1336.783	N[12]
I[19]	1975.243	1239.767	1223.748	1224.756	1222.741	I[11]
Q[20]	2103.301	**1126.683**	1110.664	1111.672	1109.656	Q[10]
A[21]	2174.338	998.624	982.606	983.614	981.598	A[9]
V[22]	2273.407	927.587	**911.569**	**912.576**	910.561	V[8]
L[23]	2386.491	828.519	**812.500**	**813.508**	**811.492**	L[7]
L[24]	2499.575	715.435	**699.416**	**700.424**	698.408	L[6]
P[25]	2596.628	602.351	586.332	587.340	585.324	P[5]
K[26]	2724.723	505.298	489.279	490.287	488.271	K[4]
K[27]	2852.818	377.203	**361.184**	362.192	360.177	K[3]
T[28]	2953.865	249.108	233.089	234.097	232.082	T[2]
E[29]	3082.908	148.060	132.042	133.050	131.034	E[1]

sp|Q6GSS7|H2A2A_MOUSE

LNKLLGK $^{Dimethyl}_{28.03}$ VTIAQGGVLPNIQAVLLPKKTE

- fragmentation table for charge state 2+
- specType=etd
- score=136.68
- F104903.dat
- query=q2297_p1
- precursor=617.582130
- chargePrecursor=5
- itol=0.8

AA	c	y	z+1	z+2	z	AA
L[1]	66.063	**1542.450**	1534.440	0.504	1533.936	L[29]
N[2]	123.084	1485.908	**1477.898**	**1478.402**	**1477.394**	N[28]
K[3]	187.132	1428.886	**1420.877**	**1421.381**	1420.373	K[27]
L[4]	243.674	1364.839	**1356.829**	**1357.333**	**1356.325**	L[26]
L[5]	300.216	1308.297	1300.287	1300.791	**1299.783**	L[25]
G[6]	328.726	1251.755	1243.745	**1244.249**	1243.241	G[24]
K[7]	**406.789**	1223.244	**1215.234**	**1215.738**	1214.731	K[23]
V[8]	**456.324**	1145.181	1137.171	**1137.675**	1136.667	V[22]
T[9]	**506.848**	1095.646	**1087.637**	**1088.141**	1087.133	T[21]
I[10]	**563.390**	1045.123	**1037.113**	**1037.617**	1036.609	I[20]
A[11]	**598.908**	988.581	**980.571**	**981.075**	**980.067**	A[19]
Q[12]	**662.937**	**953.062**	**945.053**	**945.557**	**944.549**	Q[18]
G[13]	**691.448**	889.033	**881.023**	**881.527**	**880.519**	G[17]
G[14]	**719.959**	860.522	**852.513**	**853.017**	**852.009**	G[16]
V[15]	**769.493**	832.011	**824.002**	**824.506**	823.498	V[15]
L[16]	826.035	**782.477**	**774.468**	**774.972**	**773.964**	L[14]
P[17]	**874.561**	**725.935**	717.926	718.430	717.422	P[13]
N[18]	**931.583**	677.409	**669.399**	**669.903**	668.895	N[12]
I[19]	988.125	620.387	612.378	612.882	611.874	I[11]
Q[20]	**1052.154**	**563.845**	**555.836**	**556.340**	**555.332**	Q[10]
A[21]	**1087.673**	499.816	**491.807**	**492.310**	**491.303**	A[9]
V[22]	**1137.207**	464.297	**456.288**	**456.792**	**455.784**	V[8]
L[23]	1193.749	414.763	**406.754**	**407.258**	**406.250**	L[7]
L[24]	1250.291	358.221	**350.212**	**350.716**	349.708	L[6]
P[25]	**1298.817**	301.679	293.670	294.174	293.166	P[5]
K[26]	**1362.865**	253.153	**245.143**	**245.647**	**244.639**	K[4]
K[27]	1426.912	189.105	181.096	181.600	180.592	K[3]
T[28]	**1477.436**	125.058	117.048	117.552	116.544	T[2]
E[29]	1541.958	74.534	66.524	67.028	66.021	E[1]

sp|Q6GSS7|H2A2A_MOUSE

LNKLLGK $^{Dimethyl}_{28.03}$ VTIAQGGVLPNIQAVLLPKKTE

- fragmentation table for charge state 3+
- specType=etd
- score=136.68
- F104903.dat
- query=q2297_p1
- precursor=617.582130
- chargePrecursor=5
- itol=0.8

AA	c	y	z+1	z+2	z	AA
L[1]	44.377	**1028.635**	**1023.296**	0.672	1022.960	L[29]
N[2]	82.392	990.941	**985.601**	**985.937**	**985.265**	N[28]
K[3]	125.090	**952.926**	**947.587**	**947.923**	**947.251**	K[27]
L[4]	162.785	**910.228**	**904.889**	**905.225**	**904.553**	L[26]
L[5]	200.479	872.533	**867.194**	867.530	**866.858**	L[25]
G[6]	219.487	834.839	**829.499**	**829.835**	**829.163**	G[24]
K[7]	271.529	815.832	**810.492**	**810.828**	810.156	K[23]
V[8]	304.552	763.790	**758.450**	**758.786**	**758.114**	V[22]
T[9]	338.234	730.767	**725.427**	**725.763**	725.091	T[21]
I[10]	375.929	697.084	**691.745**	**692.081**	**691.409**	I[20]
A[11]	399.608	659.389	654.050	654.386	653.714	A[19]
Q[12]	442.294	635.710	630.371	630.707	630.035	Q[18]
G[13]	461.301	593.024	587.685	588.021	587.349	G[17]
G[14]	480.308	574.017	568.678	569.013	568.342	G[16]
V[15]	513.331	555.010	549.670	550.006	549.334	V[15]
L[16]	551.026	521.987	516.648	516.984	516.312	L[14]
P[17]	583.377	484.292	478.953	479.289	478.617	P[13]
N[18]	621.391	451.942	446.602	446.938	446.266	N[12]
I[19]	659.086	413.927	408.588	408.924	408.252	I[11]
Q[20]	701.772	376.233	370.893	371.229	370.557	Q[10]
A[21]	**725.451**	333.546	328.207	328.543	327.871	A[9]
V[22]	**758.474**	309.867	304.528	304.864	304.192	V[8]
L[23]	796.168	276.844	271.505	271.841	271.169	L[7]
L[24]	833.863	239.150	233.810	234.146	233.474	L[6]
P[25]	**866.214**	201.455	196.116	196.451	195.780	P[5]
K[26]	**908.912**	169.104	163.765	164.101	163.429	K[4]
K[27]	**951.611**	126.406	121.066	121.402	120.730	K[3]
T[28]	**985.293**	83.708	78.368	78.704	78.032	T[2]
E[29]	1028.307	50.025	44.685	45.021	44.349	E[1]

sp|Q6GSS7|H2A2A_MOUSE

LNKLLGK $^{\text{Dimethyl}}_{28.03}$ VTIAQGGVLPNIQAVLLPKKTE

- fragmentation table for charge state 4+
- specType=etd
- score=136.68
- F104903.dat
- query=q2297_p1
- precursor=617.582130
- chargePrecursor=5
- itol=0.8

AA	c	y	z+1	z+2	z	AA
L[1]	33.535	**771.728**	767.724	0.755	767.472	L[29]
N[2]	62.046	743.457	**739.453**	**739.705**	**739.201**	N[28]
K[3]	94.069	**714.947**	710.942	711.194	710.690	K[27]
L[4]	122.340	682.923	678.918	679.170	678.666	L[26]
L[5]	150.611	654.652	650.647	650.899	650.395	L[25]
G[6]	164.867	626.381	622.376	622.628	622.124	G[24]
K[7]	203.898	612.126	608.121	608.373	607.869	K[23]
V[8]	228.665	573.094	569.089	569.341	568.837	V[22]
T[9]	253.927	548.327	544.322	544.574	544.070	T[21]
I[10]	282.198	523.065	519.060	519.312	518.808	I[20]
A[11]	299.958	494.794	490.789	**491.041**	490.537	A[19]
Q[12]	331.972	477.035	473.030	473.282	472.778	Q[18]
G[13]	346.228	445.020	441.015	441.267	440.763	G[17]
G[14]	**360.483**	430.765	426.760	427.012	426.508	G[16]
V[15]	385.250	416.509	412.505	412.757	412.253	V[15]
L[16]	413.521	391.742	387.737	387.989	387.486	L[14]
P[17]	437.784	363.471	359.466	359.718	359.215	P[13]
N[18]	466.295	339.208	335.203	335.455	334.951	N[12]
I[19]	494.566	310.697	306.693	306.945	306.441	I[11]
Q[20]	526.581	282.426	278.422	278.673	278.170	Q[10]
A[21]	544.340	250.412	246.407	246.659	246.155	A[9]
V[22]	569.107	232.652	228.648	228.900	228.396	V[8]
L[23]	597.378	207.885	203.881	204.132	203.629	L[7]
L[24]	625.649	179.614	175.609	175.861	175.358	L[6]
P[25]	649.912	151.343	147.338	147.590	147.087	P[5]
K[26]	681.936	127.080	123.075	123.327	122.823	K[4]
K[27]	**713.960**	95.056	91.052	91.304	90.800	K[3]
T[28]	**739.222**	63.032	59.028	59.280	58.776	T[2]
E[29]	771.482	37.771	33.766	34.018	33.514	E[1]

sp|Q6GSS7|H2A2A_MOUSE

LNKLLGK $^{Dimethyl}_{28.03}$ VTIAQGGVLPNIQAVLLPKKTE

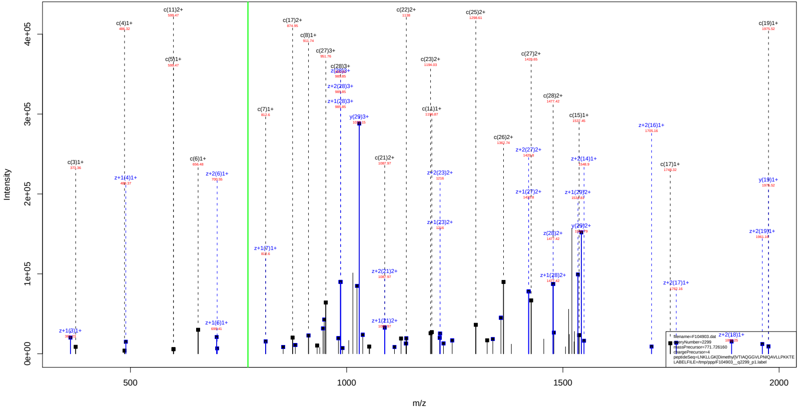

sp|Q6GSS7|H2A2A_MOUSE

LNKLLGK $^{\text{Dimethyl}}_{28.03}$ VTIAQGGVLPNIQAVLLPKKTE

- fragmentation table for charge state 1+
- specType=etd
- score=110.04
- F104903.dat
- query=q2299_p1
- precursor=771.726160
- chargePrecursor=4
- itol=0.8

AA	c	y	z+1	z+2	z	AA
L[1]	131.118	3083.892	3067.873	0.000	3066.865	L[29]
N[2]	245.161	2970.808	2954.789	2955.797	2953.781	N[28]
K[3]	**373.256**	2856.765	2840.746	2841.754	2839.738	K[27]
L[4]	**486.340**	2728.670	2712.651	2713.659	2711.643	L[26]
L[5]	**599.424**	2615.586	2599.567	2600.575	2598.559	L[25]
G[6]	**656.445**	2502.502	2486.483	2487.491	2485.475	G[24]
K[7]	**812.572**	2445.480	2429.462	2430.469	2428.454	K[23]
V[8]	**911.640**	2289.354	2273.335	2274.343	2272.327	V[22]
T[9]	1012.688	2190.286	2174.267	2175.275	2173.259	T[21]
I[10]	**1125.772**	2089.238	2073.219	2074.227	2072.211	I[20]
A[11]	**1196.809**	**1976.154**	1960.135	**1961.143**	1959.127	A[19]
Q[12]	**1324.867**	1905.117	1889.098	**1890.106**	1888.090	Q[18]
G[13]	1381.889	1777.058	1761.039	**1762.047**	1760.032	G[17]
G[14]	1438.910	1720.037	1704.018	**1705.026**	1703.010	G[16]
V[15]	**1537.979**	1663.015	1646.997	1648.004	1645.989	V[15]
L[16]	1651.063	1563.947	1547.928	**1548.936**	1546.920	L[14]
P[17]	**1748.116**	1450.863	1434.844	1435.852	1433.836	P[13]
N[18]	1862.159	1353.810	**1337.791**	1338.799	1336.783	N[12]
I[19]	**1975.243**	1239.767	**1223.748**	1224.756	1222.741	I[11]
Q[20]	2103.301	1126.683	**1110.664**	1111.672	1109.656	Q[10]
A[21]	2174.338	998.624	982.606	983.614	**981.598**	A[9]
V[22]	2273.407	927.587	**911.569**	912.576	910.561	V[8]
L[23]	2386.491	828.519	**812.500**	813.508	811.492	L[7]
L[24]	2499.575	715.435	**699.416**	**700.424**	698.408	L[6]
P[25]	2596.628	602.351	586.332	587.340	585.324	P[5]
K[26]	2724.723	505.298	**489.279**	490.287	488.271	K[4]
K[27]	2852.818	377.203	**361.184**	362.192	360.177	K[3]
T[28]	2953.865	249.108	233.089	234.097	232.082	T[2]
E[29]	3082.908	148.060	132.042	133.050	131.034	E[1]

sp|Q6GSS7|H2A2A_MOUSE

LNKLLGK $^{\text{Dimethyl}}_{28.03}$ VTIAQGGVLPNIQAVLLPKKTE

- fragmentation table for charge state 2+
- specType=etd
- score=110.04
- F104903.dat
- query=q2299_p1
- precursor=771.726160
- chargePrecursor=4
- itol=0.8

AA	c	y	z+1	z+2	z	AA
L[1]	66.063	**1542.450**	**1534.440**	0.504	1533.936	L[29]
N[2]	123.084	1485.908	**1477.898**	**1478.402**	**1477.394**	N[28]
K[3]	187.132	1428.886	**1420.877**	**1421.381**	**1420.373**	K[27]
L[4]	243.674	1364.839	**1356.829**	**1357.333**	**1356.325**	L[26]
L[5]	300.216	1308.297	1300.287	1300.791	1299.783	L[25]
G[6]	328.726	1251.755	**1243.745**	**1244.249**	**1243.241**	G[24]
K[7]	406.789	**1223.244**	**1215.234**	**1215.738**	**1214.731**	K[23]
V[8]	456.324	1145.181	**1137.171**	**1137.675**	**1136.667**	V[22]
T[9]	506.848	1095.646	**1087.637**	**1088.141**	1087.133	T[21]
I[10]	563.390	1045.123	**1037.113**	**1037.617**	**1036.609**	I[20]
A[11]	**598.908**	988.581	**980.571**	**981.075**	980.067	A[19]
Q[12]	662.937	953.062	**945.053**	**945.557**	944.549	Q[18]
G[13]	691.448	889.033	**881.023**	**881.527**	**880.519**	G[17]
G[14]	719.959	860.522	**852.513**	**853.017**	852.009	G[16]
V[15]	769.493	832.011	824.002	824.506	823.498	V[15]
L[16]	826.035	782.477	774.468	774.972	773.964	L[14]
P[17]	**874.561**	725.935	717.926	718.430	717.422	P[13]
N[18]	**931.583**	677.409	669.399	669.903	668.895	N[12]
I[19]	988.125	620.387	612.378	612.882	611.874	I[11]
Q[20]	**1052.154**	563.845	555.836	556.340	555.332	Q[10]
A[21]	**1087.673**	499.816	491.807	492.310	491.303	A[9]
V[22]	**1137.207**	464.297	456.288	456.792	455.784	V[8]
L[23]	**1193.749**	414.763	406.754	407.258	406.250	L[7]
L[24]	1250.291	358.221	350.212	350.716	349.708	L[6]
P[25]	**1298.817**	301.679	293.670	294.174	293.166	P[5]
K[26]	**1362.865**	253.153	245.143	245.647	244.639	K[4]
K[27]	**1426.912**	189.105	181.096	181.600	180.592	K[3]
T[28]	**1477.436**	125.058	117.048	117.552	116.544	T[2]
E[29]	1541.958	74.534	66.524	67.028	66.021	E[1]

sp|Q6GSS7|H2A2A_MOUSE

LNKLLGK $^{Dimethyl}_{28.03}$ VTIAQGGVLPNIQAVLLPKKTE

- fragmentation table for charge state 3+
- specType=etd
- score=110.04
- F104903.dat
- query=q2299_p1
- precursor=771.726160
- chargePrecursor=4
- itol=0.8

AA	c	y	z+1	z+2	z	AA
L[1]	44.377	**1028.635**	**1023.296**	0.672	1022.960	L[29]
N[2]	82.392	**990.941**	**985.601**	**985.937**	**985.265**	N[28]
K[3]	125.090	952.926	**947.587**	**947.923**	947.251	K[27]
L[4]	162.785	910.228	904.889	905.225	904.553	L[26]
L[5]	200.479	872.533	867.194	867.530	866.858	L[25]
G[6]	219.487	834.839	829.499	829.835	829.163	G[24]
K[7]	271.529	815.832	810.492	810.828	810.156	K[23]
V[8]	304.552	763.790	758.450	758.786	758.114	V[22]
T[9]	338.234	730.767	725.427	725.763	725.091	T[21]
I[10]	375.929	697.084	691.745	692.081	691.409	I[20]
A[11]	399.608	659.389	654.050	654.386	653.714	A[19]
Q[12]	442.294	635.710	630.371	630.707	630.035	Q[18]
G[13]	461.301	593.024	587.685	588.021	587.349	G[17]
G[14]	480.308	574.017	568.678	569.013	568.342	G[16]
V[15]	513.331	555.010	549.670	550.006	549.334	V[15]
L[16]	551.026	521.987	516.648	516.984	516.312	L[14]
P[17]	583.377	484.292	478.953	479.289	478.617	P[13]
N[18]	621.391	451.942	446.602	446.938	446.266	N[12]
I[19]	659.086	413.927	408.588	408.924	408.252	I[11]
Q[20]	701.772	376.233	370.893	371.229	370.557	Q[10]
A[21]	725.451	333.546	328.207	328.543	327.871	A[9]
V[22]	758.474	309.867	304.528	304.864	304.192	V[8]
L[23]	796.168	276.844	271.505	271.841	271.169	L[7]
L[24]	833.863	239.150	233.810	234.146	233.474	L[6]
P[25]	866.214	201.455	196.116	196.451	195.780	P[5]
K[26]	908.912	169.104	163.765	164.101	163.429	K[4]
K[27]	**951.611**	126.406	121.066	121.402	120.730	K[3]
T[28]	**985.293**	83.708	78.368	78.704	78.032	T[2]
E[29]	1028.307	50.025	44.685	45.021	44.349	E[1]

sp|Q6GSS7|H2A2A_MOUSE

LNKLLGK $^{\text{Dimethyl}}_{28.03}$ VTIAQGGVLPNIQAVLLPKKTE

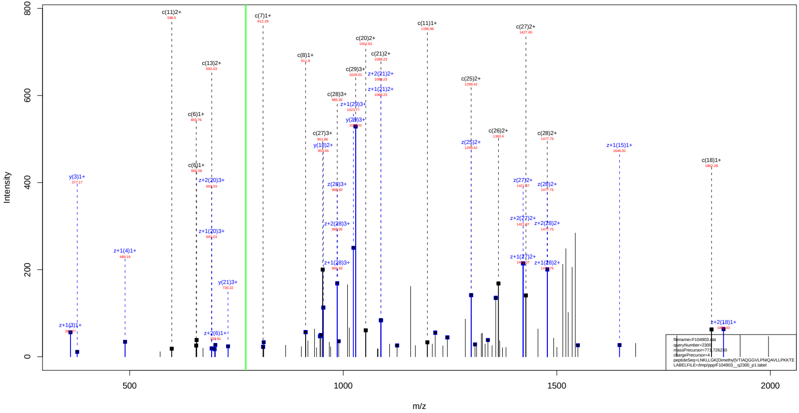

sp|Q6GSS7|H2A2A_MOUSE

LNKLLGK $^{\text{Dimethyl}}_{28.03}$ VTIAQGGVLPNIQAVLLPKKTE

- fragmentation table for charge state 1+
- specType=etd
- score=33.04
- F104903.dat
- query=q2300_p1
- precursor=771.726230
- chargePrecursor=4
- itol=0.8

AA	c	y	z+1	z+2	z	AA
L[1]	131.118	3083.892	3067.873	0.000	3066.865	L[29]
N[2]	245.161	2970.808	2954.789	2955.797	2953.781	N[28]
K[3]	373.256	2856.765	2840.746	2841.754	2839.738	K[27]
L[4]	486.340	2728.670	2712.651	2713.659	2711.643	L[26]
L[5]	599.424	2615.586	2599.567	2600.575	2598.559	L[25]
G[6]	**656.445**	2502.502	2486.483	2487.491	2485.475	G[24]
K[7]	**812.572**	2445.480	2429.462	2430.469	2428.454	K[23]
V[8]	**911.640**	2289.354	2273.335	2274.343	2272.327	V[22]
T[9]	1012.688	2190.286	2174.267	2175.275	2173.259	T[21]
I[10]	**1125.772**	2089.238	2073.219	2074.227	2072.211	I[20]
A[11]	**1196.809**	1976.154	1960.135	1961.143	1959.127	A[19]
Q[12]	1324.867	1905.117	1889.098	**1890.106**	1888.090	Q[18]
G[13]	1381.889	1777.058	1761.039	1762.047	1760.032	G[17]
G[14]	1438.910	1720.037	1704.018	1705.026	1703.010	G[16]
V[15]	1537.979	1663.015	**1646.997**	1648.004	1645.989	V[15]
L[16]	1651.063	1563.947	1547.928	**1548.936**	1546.920	L[14]
P[17]	1748.116	1450.863	1434.844	1435.852	1433.836	P[13]
N[18]	**1862.159**	1353.810	1337.791	**1338.799**	1336.783	N[12]
I[19]	1975.243	1239.767	1223.748	1224.756	1222.741	I[11]
Q[20]	2103.301	**1126.683**	1110.664	1111.672	1109.656	Q[10]
A[21]	2174.338	998.624	982.606	983.614	981.598	A[9]
V[22]	2273.407	927.587	**911.569**	**912.576**	910.561	V[8]
L[23]	2386.491	828.519	**812.500**	**813.508**	811.492	L[7]
L[24]	2499.575	715.435	**699.416**	**700.424**	**698.408**	L[6]
P[25]	2596.628	602.351	586.332	587.340	585.324	P[5]
K[26]	2724.723	505.298	**489.279**	490.287	488.271	K[4]
K[27]	2852.818	**377.203**	**361.184**	362.192	360.177	K[3]
T[28]	2953.865	249.108	233.089	234.097	232.082	T[2]
E[29]	3082.908	148.060	132.042	133.050	131.034	E[1]

sp|Q6GSS7|H2A2A_MOUSE

LNKLLGK $^{Dimethyl}_{28.03}$ VTIAQGGVLPNIQAVLLPKKTE

- fragmentation table for charge state 2+
- specType=etd
- score=33.04
- F104903.dat
- query=q2300_p1
- precursor=771.726230
- chargePrecursor=4
- itol=0.8

AA	c	y	z+1	z+2	z	AA
L[1]	66.063	1542.450	1534.440	0.504	1533.936	L[29]
N[2]	123.084	1485.908	**1477.898**	**1478.402**	**1477.394**	N[28]
K[3]	187.132	1428.886	**1420.877**	**1421.381**	**1420.373**	K[27]
L[4]	243.674	1364.839	**1356.829**	**1357.333**	**1356.325**	L[26]
L[5]	300.216	**1308.297**	1300.287	1300.791	**1299.783**	L[25]
G[6]	328.726	1251.755	**1243.745**	**1244.249**	**1243.241**	G[24]
K[7]	406.789	1223.244	**1215.234**	**1215.738**	**1214.731**	K[23]
V[8]	456.324	1145.181	1137.171	1137.675	1136.667	V[22]
T[9]	506.848	1095.646	**1087.637**	**1088.141**	1087.133	T[21]
I[10]	563.390	1045.123	1037.113	1037.617	1036.609	I[20]
A[11]	**598.908**	**988.581**	980.571	981.075	980.067	A[19]
Q[12]	662.937	**953.062**	**945.053**	**945.557**	944.549	Q[18]
G[13]	**691.448**	889.033	881.023	881.527	880.519	G[17]
G[14]	719.959	860.522	852.513	853.017	852.009	G[16]
V[15]	769.493	832.011	824.002	824.506	823.498	V[15]
L[16]	826.035	782.477	774.468	774.972	773.964	L[14]
P[17]	874.561	725.935	717.926	718.430	717.422	P[13]
N[18]	931.583	677.409	669.399	669.903	668.895	N[12]
I[19]	988.125	620.387	612.378	612.882	611.874	I[11]
Q[20]	**1052.154**	563.845	555.836	556.340	555.332	Q[10]
A[21]	**1087.673**	499.816	491.807	492.310	491.303	A[9]
V[22]	1137.207	464.297	456.288	456.792	455.784	V[8]
L[23]	1193.749	414.763	406.754	407.258	406.250	L[7]
L[24]	1250.291	358.221	350.212	350.716	349.708	L[6]
P[25]	**1298.817**	301.679	293.670	294.174	293.166	P[5]
K[26]	**1362.865**	253.153	245.143	245.647	244.639	K[4]
K[27]	**1426.912**	189.105	181.096	181.600	180.592	K[3]
T[28]	**1477.436**	125.058	117.048	117.552	116.544	T[2]
E[29]	1541.958	74.534	66.524	67.028	66.021	E[1]

sp|Q6GSS7|H2A2A_MOUSE

LNKLLGK $^{\text{Dimethyl}}_{28.03}$ VTIAQGGVLPNIQAVLLPKKTE

- fragmentation table for charge state 3+
- specType=etd
- score=33.04
- F104903.dat
- query=q2300_p1
- precursor=771.726230
- chargePrecursor=4
- itol=0.8

AA	c	y	z+1	z+2	z	AA
L[1]	44.377	**1028.635**	**1023.296**	0.672	1022.960	L[29]
N[2]	82.392	990.941	**985.601**	**985.937**	**985.265**	N[28]
K[3]	125.090	**952.926**	**947.587**	**947.923**	**947.251**	K[27]
L[4]	162.785	910.228	904.889	905.225	904.553	L[26]
L[5]	200.479	872.533	867.194	867.530	866.858	L[25]
G[6]	219.487	834.839	829.499	829.835	829.163	G[24]
K[7]	271.529	815.832	810.492	810.828	810.156	K[23]
V[8]	304.552	763.790	758.450	758.786	758.114	V[22]
T[9]	338.234	**730.767**	725.427	725.763	725.091	T[21]
I[10]	375.929	697.084	**691.745**	**692.081**	**691.409**	I[20]
A[11]	399.608	659.389	654.050	654.386	653.714	A[19]
Q[12]	442.294	635.710	630.371	630.707	630.035	Q[18]
G[13]	461.301	593.024	587.685	588.021	587.349	G[17]
G[14]	480.308	574.017	568.678	569.013	568.342	G[16]
V[15]	513.331	555.010	549.670	550.006	549.334	V[15]
L[16]	551.026	521.987	516.648	516.984	516.312	L[14]
P[17]	583.377	484.292	478.953	479.289	478.617	P[13]
N[18]	621.391	451.942	446.602	446.938	446.266	N[12]
I[19]	659.086	413.927	408.588	408.924	408.252	I[11]
Q[20]	701.772	376.233	370.893	371.229	370.557	Q[10]
A[21]	725.451	333.546	328.207	328.543	327.871	A[9]
V[22]	758.474	309.867	304.528	304.864	304.192	V[8]
L[23]	796.168	276.844	271.505	271.841	271.169	L[7]
L[24]	833.863	239.150	233.810	234.146	233.474	L[6]
P[25]	866.214	201.455	196.116	196.451	195.780	P[5]
K[26]	908.912	169.104	163.765	164.101	163.429	K[4]
K[27]	**951.611**	126.406	121.066	121.402	120.730	K[3]
T[28]	**985.293**	83.708	78.368	78.704	78.032	T[2]
E[29]	**1028.307**	50.025	44.685	45.021	44.349	E[1]

sp|Q6GSS7|H2A2A_MOUSE

LNKLLGK Dimethyl 28.03 VTIAQGGVLPNIQAVLLPKKTE

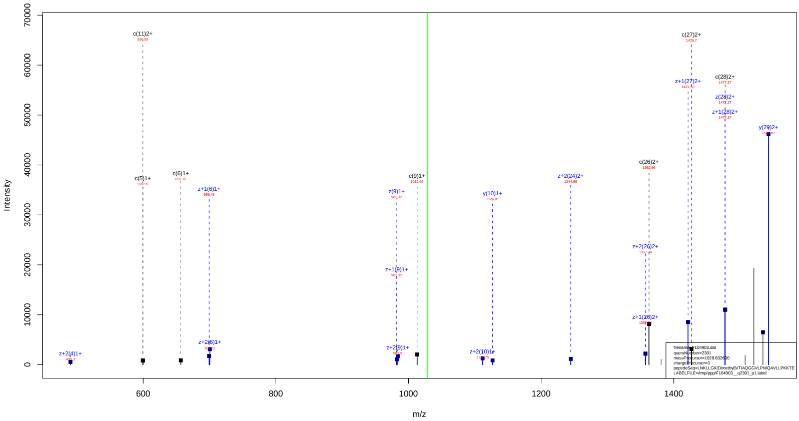

sp|Q6GSS7|H2A2A_MOUSE

LNKLLGK $^{Dimethyl}_{28.03}$ VTIAQGGVLPNIQAVLLPKKTE

- fragmentation table for charge state 1+
- specType=etd
- score=29.21
- F104903.dat
- query=q2301_p1
- precursor=1028.632600
- chargePrecursor=3
- itol=0.8

AA	c	y	z+1	z+2	z	AA
L[1]	131.118	3083.892	3067.873	0.000	3066.865	L[29]
N[2]	245.161	2970.808	2954.789	2955.797	2953.781	N[28]
K[3]	373.256	2856.765	2840.746	2841.754	2839.738	K[27]
L[4]	486.340	2728.670	2712.651	2713.659	2711.643	L[26]
L[5]	**599.424**	2615.586	2599.567	2600.575	2598.559	L[25]
G[6]	**656.445**	2502.502	2486.483	2487.491	2485.475	G[24]
K[7]	812.572	2445.480	2429.462	2430.469	2428.454	K[23]
V[8]	911.640	2289.354	2273.335	2274.343	2272.327	V[22]
T[9]	**1012.688**	2190.286	2174.267	2175.275	2173.259	T[21]
I[10]	1125.772	2089.238	2073.219	2074.227	2072.211	I[20]
A[11]	1196.809	1976.154	1960.135	1961.143	1959.127	A[19]
Q[12]	1324.867	1905.117	1889.098	1890.106	1888.090	Q[18]
G[13]	1381.889	1777.058	1761.039	1762.047	1760.032	G[17]
G[14]	1438.910	1720.037	1704.018	1705.026	1703.010	G[16]
V[15]	1537.979	1663.015	1646.997	1648.004	1645.989	V[15]
L[16]	1651.063	1563.947	1547.928	1548.936	1546.920	L[14]
P[17]	1748.116	1450.863	1434.844	1435.852	1433.836	P[13]
N[18]	1862.159	1353.810	1337.791	1338.799	1336.783	N[12]
I[19]	1975.243	1239.767	1223.748	1224.756	1222.741	I[11]
Q[20]	2103.301	**1126.683**	1110.664	**1111.672**	1109.656	Q[10]
A[21]	2174.338	998.624	**982.606**	**983.614**	**981.598**	A[9]
V[22]	2273.407	927.587	911.569	912.576	910.561	V[8]
L[23]	2386.491	828.519	812.500	813.508	811.492	L[7]
L[24]	2499.575	715.435	**699.416**	**700.424**	698.408	L[6]
P[25]	2596.628	602.351	586.332	587.340	585.324	P[5]
K[26]	2724.723	505.298	489.279	**490.287**	488.271	K[4]
K[27]	2852.818	377.203	361.184	362.192	360.177	K[3]
T[28]	2953.865	249.108	233.089	234.097	232.082	T[2]
E[29]	3082.908	148.060	132.042	133.050	131.034	E[1]

sp|Q6GSS7|H2A2A_MOUSE

LNKLLGK $^{\text{Dimethyl}}_{28.03}$ VTIAQGGVLPNIQAVLLPKKTE

- fragmentation table for charge state 2+
- specType=etd
- score=29.21
- F104903.dat
- query=q2301_p1
- precursor=1028.632600
- chargePrecursor=3
- itol=0.8

AA	c	y	z+1	z+2	z	AA
L[1]	66.063	**1542.450**	**1534.440**	0.504	**1533.936**	L[29]
N[2]	123.084	1485.908	**1477.898**	1478.402	**1477.394**	N[28]
K[3]	187.132	1428.886	**1420.877**	**1421.381**	1420.373	K[27]
L[4]	243.674	1364.839	**1356.829**	**1357.333**	1356.325	L[26]
L[5]	300.216	1308.297	1300.287	1300.791	1299.783	L[25]
G[6]	328.726	1251.755	1243.745	**1244.249**	1243.241	G[24]
K[7]	406.789	1223.244	1215.234	1215.738	1214.731	K[23]
V[8]	456.324	1145.181	1137.171	1137.675	1136.667	V[22]
T[9]	506.848	1095.646	1087.637	1088.141	1087.133	T[21]
I[10]	563.390	1045.123	1037.113	1037.617	1036.609	I[20]
A[11]	**598.908**	988.581	980.571	981.075	980.067	A[19]
Q[12]	662.937	953.062	945.053	945.557	944.549	Q[18]
G[13]	691.448	889.033	881.023	881.527	880.519	G[17]
G[14]	719.959	860.522	852.513	853.017	852.009	G[16]
V[15]	769.493	832.011	824.002	824.506	823.498	V[15]
L[16]	826.035	782.477	774.468	774.972	773.964	L[14]
P[17]	874.561	725.935	717.926	718.430	717.422	P[13]
N[18]	931.583	677.409	669.399	669.903	668.895	N[12]
I[19]	988.125	620.387	612.378	612.882	611.874	I[11]
Q[20]	1052.154	563.845	555.836	556.340	555.332	Q[10]
A[21]	1087.673	499.816	491.807	492.310	491.303	A[9]
V[22]	1137.207	464.297	456.288	456.792	455.784	V[8]
L[23]	1193.749	414.763	406.754	407.258	406.250	L[7]
L[24]	1250.291	358.221	350.212	350.716	349.708	L[6]
P[25]	1298.817	301.679	293.670	294.174	293.166	P[5]
K[26]	**1362.865**	253.153	245.143	245.647	244.639	K[4]
K[27]	**1426.912**	189.105	181.096	181.600	180.592	K[3]
T[28]	**1477.436**	125.058	117.048	117.552	116.544	T[2]
E[29]	1541.958	74.534	66.524	67.028	66.021	E[1]

sp|Q6GSS7|H2A2A_MOUSE

LNKLLGK $^{\text{Dimethyl}}_{28.03}$ VTIAQGGVLPNIQAVLLPKKTE

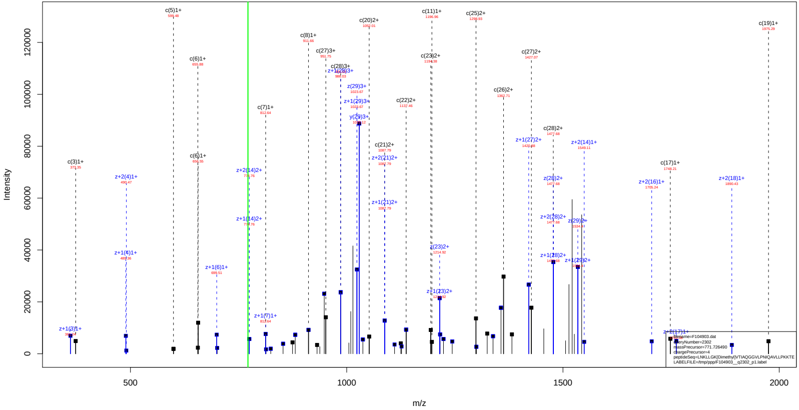

sp|Q6GSS7|H2A2A_MOUSE

LNKLLGK $^{\text{Dimethyl}}_{28.03}$ VTIAQGGVLPNIQAVLLPKKTE

- fragmentation table for charge state 1+
- specType=etd
- score=108.80
- F104903.dat
- query=q2302_p1
- precursor=771.726490
- chargePrecursor=4
- itol=0.8

AA	c	y	z+1	z+2	z	AA
L[1]	131.118	3083.892	3067.873	0.000	3066.865	L[29]
N[2]	245.161	2970.808	2954.789	2955.797	2953.781	N[28]
K[3]	**373.256**	2856.765	2840.746	2841.754	2839.738	K[27]
L[4]	486.340	2728.670	2712.651	2713.659	2711.643	L[26]
L[5]	**599.424**	2615.586	2599.567	2600.575	2598.559	L[25]
G[6]	**656.445**	2502.502	2486.483	2487.491	2485.475	G[24]
K[7]	**812.572**	2445.480	2429.462	2430.469	2428.454	K[23]
V[8]	**911.640**	2289.354	2273.335	2274.343	2272.327	V[22]
T[9]	1012.688	2190.286	2174.267	2175.275	2173.259	T[21]
I[10]	**1125.772**	2089.238	2073.219	2074.227	2072.211	I[20]
A[11]	**1196.809**	1976.154	1960.135	1961.143	1959.127	A[19]
Q[12]	**1324.867**	1905.117	1889.098	**1890.106**	1888.090	Q[18]
G[13]	**1381.889**	1777.058	1761.039	**1762.047**	1760.032	G[17]
G[14]	1438.910	1720.037	1704.018	**1705.026**	1703.010	G[16]
V[15]	1537.979	1663.015	1646.997	1648.004	1645.989	V[15]
L[16]	1651.063	1563.947	1547.928	**1548.936**	1546.920	L[14]
P[17]	**1748.116**	1450.863	1434.844	1435.852	1433.836	P[13]
N[18]	1862.159	1353.810	**1337.791**	**1338.799**	1336.783	N[12]
I[19]	**1975.243**	1239.767	**1223.748**	1224.756	1222.741	I[11]
Q[20]	2103.301	**1126.683**	**1110.664**	1111.672	1109.656	Q[10]
A[21]	2174.338	998.624	982.606	983.614	981.598	A[9]
V[22]	2273.407	927.587	**911.569**	912.576	910.561	V[8]
L[23]	2386.491	828.519	**812.500**	**813.508**	811.492	L[7]
L[24]	2499.575	715.435	**699.416**	**700.424**	698.408	L[6]
P[25]	2596.628	602.351	586.332	587.340	585.324	P[5]
K[26]	2724.723	505.298	**489.279**	**490.287**	488.271	K[4]
K[27]	2852.818	377.203	**361.184**	362.192	360.177	K[3]
T[28]	2953.865	249.108	233.089	234.097	232.082	T[2]
E[29]	3082.908	148.060	132.042	133.050	131.034	E[1]

sp|Q6GSS7|H2A2A_MOUSE

LNKLLGK $^{\text{Dimethyl}}_{28.03}$ VTIAQGGVLPNIQAVLLPKKTE

- fragmentation table for charge state 2+
- specType=etd
- score=108.80
- F104903.dat
- query=q2302_p1
- precursor=771.726490
- chargePrecursor=4
- itol=0.8

AA	c	y	z+1	z+2	z	AA
L[1]	66.063	1542.450	**1534.440**	0.504	**1533.936**	L[29]
N[2]	123.084	1485.908	**1477.898**	**1478.402**	**1477.394**	N[28]
K[3]	187.132	1428.886	**1420.877**	**1421.381**	**1420.373**	K[27]
L[4]	243.674	1364.839	**1356.829**	**1357.333**	**1356.325**	L[26]
L[5]	300.216	1308.297	**1300.287**	1300.791	**1299.783**	L[25]
G[6]	328.726	1251.755	**1243.745**	**1244.249**	**1243.241**	G[24]
K[7]	406.789	**1223.244**	**1215.234**	**1215.738**	**1214.731**	K[23]
V[8]	456.324	1145.181	**1137.171**	**1137.675**	**1136.667**	V[22]
T[9]	506.848	1095.646	**1087.637**	**1088.141**	**1087.133**	T[21]
I[10]	563.390	1045.123	**1037.113**	**1037.617**	**1036.609**	I[20]
A[11]	**598.908**	988.581	980.571	981.075	980.067	A[19]
Q[12]	662.937	953.062	945.053	945.557	944.549	Q[18]
G[13]	691.448	889.033	**881.023**	**881.527**	**880.519**	G[17]
G[14]	719.959	860.522	**852.513**	**853.017**	852.009	G[16]
V[15]	769.493	832.011	**824.002**	**824.506**	823.498	V[15]
L[16]	826.035	782.477	**774.468**	**774.972**	**773.964**	L[14]
P[17]	**874.561**	725.935	717.926	718.430	717.422	P[13]
N[18]	**931.583**	677.409	669.399	669.903	668.895	N[12]
I[19]	988.125	620.387	612.378	612.882	611.874	I[11]
Q[20]	**1052.154**	563.845	555.836	556.340	555.332	Q[10]
A[21]	**1087.673**	499.816	491.807	492.310	491.303	A[9]
V[22]	**1137.207**	464.297	456.288	456.792	455.784	V[8]
L[23]	**1193.749**	414.763	406.754	407.258	406.250	L[7]
L[24]	1250.291	358.221	350.212	350.716	349.708	L[6]
P[25]	**1298.817**	301.679	293.670	294.174	293.166	P[5]
K[26]	**1362.865**	253.153	245.143	245.647	244.639	K[4]
K[27]	**1426.912**	189.105	181.096	181.600	180.592	K[3]
T[28]	**1477.436**	125.058	117.048	117.552	116.544	T[2]
E[29]	1541.958	74.534	66.524	67.028	66.021	E[1]

sp|Q6GSS7|H2A2A_MOUSE

LNKLLGK $^{Dimethyl}_{28.03}$ VTIAQGGVLPNIQAVLLPKKTE

- fragmentation table for charge state 3+
- specType=etd
- score=108.80
- F104903.dat
- query=q2302_p1
- precursor=771.726490
- chargePrecursor=4
- itol=0.8

AA	c	y	z+1	z+2	z	AA
L[1]	44.377	**1028.635**	**1023.296**	0.672	**1022.960**	L[29]
N[2]	82.392	990.941	**985.601**	**985.937**	**985.265**	N[28]
K[3]	125.090	952.926	**947.587**	**947.923**	947.251	K[27]
L[4]	162.785	910.228	904.889	905.225	904.553	L[26]
L[5]	200.479	872.533	867.194	867.530	866.858	L[25]
G[6]	219.487	834.839	829.499	829.835	829.163	G[24]
K[7]	271.529	815.832	810.492	810.828	810.156	K[23]
V[8]	304.552	763.790	758.450	758.786	758.114	V[22]
T[9]	338.234	730.767	725.427	725.763	725.091	T[21]
I[10]	375.929	697.084	691.745	692.081	691.409	I[20]
A[11]	399.608	659.389	654.050	654.386	653.714	A[19]
Q[12]	442.294	635.710	630.371	630.707	630.035	Q[18]
G[13]	461.301	593.024	587.685	588.021	587.349	G[17]
G[14]	480.308	574.017	568.678	569.013	568.342	G[16]
V[15]	513.331	555.010	549.670	550.006	549.334	V[15]
L[16]	551.026	521.987	516.648	516.984	516.312	L[14]
P[17]	583.377	484.292	478.953	479.289	478.617	P[13]
N[18]	621.391	451.942	446.602	446.938	446.266	N[12]
I[19]	659.086	413.927	408.588	408.924	408.252	I[11]
Q[20]	701.772	376.233	370.893	371.229	370.557	Q[10]
A[21]	725.451	333.546	328.207	328.543	327.871	A[9]
V[22]	758.474	309.867	304.528	304.864	304.192	V[8]
L[23]	796.168	276.844	271.505	271.841	271.169	L[7]
L[24]	833.863	239.150	233.810	234.146	233.474	L[6]
P[25]	866.214	201.455	196.116	196.451	195.780	P[5]
K[26]	908.912	169.104	163.765	164.101	163.429	K[4]
K[27]	**951.611**	126.406	121.066	121.402	120.730	K[3]
T[28]	**985.293**	83.708	78.368	78.704	78.032	T[2]
E[29]	1028.307	50.025	44.685	45.021	44.349	E[1]

sp|Q6GSS7|H2A2A_MOUSE

LNKLLGK $^{\text{Dimethyl}}_{28.03}$ VTIAQGGVLPNIQAVLLPKKTE

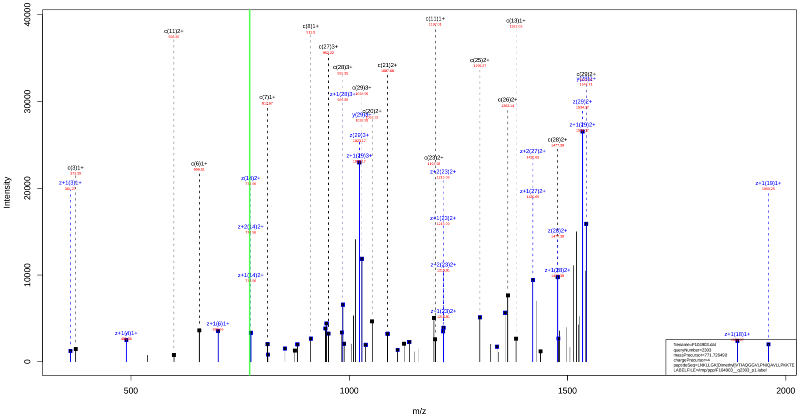

sp|Q6GSS7|H2A2A_MOUSE

LNKLLGK $^{\text{Dimethyl}}_{28.03}$ VTIAQGGVLPNIQAVLLPKKTE

- fragmentation table for charge state 1+
- specType=etd
- score=78.21
- F104903.dat
- query=q2303_p1
- precursor=771.726490
- chargePrecursor=4
- itol=0.8

AA	c	y	z+1	z+2	z	AA
L[1]	131.118	3083.892	3067.873	0.000	3066.865	L[29]
N[2]	245.161	2970.808	2954.789	2955.797	2953.781	N[28]
K[3]	**373.256**	2856.765	2840.746	2841.754	2839.738	K[27]
L[4]	486.340	2728.670	2712.651	2713.659	2711.643	L[26]
L[5]	599.424	2615.586	2599.567	2600.575	2598.559	L[25]
G[6]	**656.445**	2502.502	2486.483	2487.491	2485.475	G[24]
K[7]	**812.572**	2445.480	2429.462	2430.469	2428.454	K[23]
V[8]	**911.640**	2289.354	2273.335	2274.343	2272.327	V[22]
T[9]	1012.688	2190.286	2174.267	2175.275	2173.259	T[21]
I[10]	**1125.772**	2089.238	2073.219	2074.227	2072.211	I[20]
A[11]	**1196.809**	1976.154	**1960.135**	1961.143	1959.127	A[19]
Q[12]	1324.867	1905.117	**1889.098**	1890.106	1888.090	Q[18]
G[13]	**1381.889**	1777.058	1761.039	1762.047	1760.032	G[17]
G[14]	**1438.910**	1720.037	1704.018	1705.026	1703.010	G[16]
V[15]	1537.979	1663.015	1646.997	1648.004	1645.989	V[15]
L[16]	1651.063	1563.947	1547.928	1548.936	1546.920	L[14]
P[17]	1748.116	1450.863	1434.844	1435.852	1433.836	P[13]
N[18]	1862.159	1353.810	**1337.791**	**1338.799**	1336.783	N[12]
I[19]	1975.243	1239.767	1223.748	1224.756	1222.741	I[11]
Q[20]	2103.301	1126.683	**1110.664**	1111.672	1109.656	Q[10]
A[21]	2174.338	998.624	**982.606**	983.614	981.598	A[9]
V[22]	2273.407	927.587	**911.569**	**912.576**	910.561	V[8]
L[23]	2386.491	828.519	**812.500**	**813.508**	811.492	L[7]
L[24]	2499.575	715.435	**699.416**	700.424	698.408	L[6]
P[25]	2596.628	602.351	586.332	587.340	585.324	P[5]
K[26]	2724.723	505.298	**489.279**	490.287	488.271	K[4]
K[27]	2852.818	377.203	**361.184**	362.192	360.177	K[3]
T[28]	2953.865	249.108	233.089	234.097	232.082	T[2]
E[29]	3082.908	148.060	132.042	133.050	131.034	E[1]

sp|Q6GSS7|H2A2A_MOUSE

LNKLLGK $^{\text{Dimethyl}}_{28.03}$ VTIAQGGVLPNIQAVLLPKKTE

- fragmentation table for charge state 2+
- specType=etd
- score=78.21
- F104903.dat
- query=q2303_p1
- precursor=771.726490
- chargePrecursor=4
- itol=0.8

AA	c	y	z+1	z+2	z	AA
L[1]	66.063	**1542.450**	**1534.440**	0.504	**1533.936**	L[29]
N[2]	123.084	1485.908	**1477.898**	**1478.402**	**1477.394**	N[28]
K[3]	187.132	1428.886	**1420.877**	**1421.381**	**1420.373**	K[27]
L[4]	243.674	1364.839	**1356.829**	**1357.333**	**1356.325**	L[26]
L[5]	300.216	1308.297	1300.287	1300.791	**1299.783**	L[25]
G[6]	328.726	1251.755	1243.745	1244.249	1243.241	G[24]
K[7]	406.789	1223.244	**1215.234**	**1215.738**	**1214.731**	K[23]
V[8]	456.324	1145.181	**1137.171**	**1137.675**	1136.667	V[22]
T[9]	506.848	1095.646	**1087.637**	**1088.141**	**1087.133**	T[21]
I[10]	563.390	1045.123	**1037.113**	**1037.617**	**1036.609**	I[20]
A[11]	**598.908**	**988.581**	980.571	981.075	980.067	A[19]
Q[12]	662.937	953.062	**945.053**	**945.557**	944.549	Q[18]
G[13]	691.448	889.033	**881.023**	**881.527**	**880.519**	G[17]
G[14]	719.959	860.522	**852.513**	**853.017**	**852.009**	G[16]
V[15]	769.493	832.011	824.002	824.506	823.498	V[15]
L[16]	826.035	782.477	**774.468**	**774.972**	**773.964**	L[14]
P[17]	**874.561**	725.935	717.926	718.430	717.422	P[13]
N[18]	931.583	677.409	669.399	669.903	668.895	N[12]
I[19]	**988.125**	620.387	612.378	612.882	611.874	I[11]
Q[20]	**1052.154**	563.845	555.836	556.340	555.332	Q[10]
A[21]	**1087.673**	499.816	491.807	492.310	491.303	A[9]
V[22]	**1137.207**	464.297	456.288	456.792	455.784	V[8]
L[23]	**1193.749**	414.763	406.754	407.258	406.250	L[7]
L[24]	1250.291	358.221	350.212	350.716	349.708	L[6]
P[25]	**1298.817**	301.679	293.670	294.174	293.166	P[5]
K[26]	**1362.865**	253.153	245.143	245.647	244.639	K[4]
K[27]	1426.912	189.105	181.096	181.600	180.592	K[3]
T[28]	**1477.436**	125.058	117.048	117.552	116.544	T[2]
E[29]	**1541.958**	74.534	66.524	67.028	66.021	E[1]

sp|Q6GSS7|H2A2A_MOUSE

LNKLLGK $^{Dimethyl}_{28.03}$ VTIAQGGVLPNIQAVLLPKKTE

- fragmentation table for charge state 3+
- specType=etd
- score=78.21
- F104903.dat
- query=q2303_p1
- precursor=771.726490
- chargePrecursor=4
- itol=0.8

AA	c	y	z+1	z+2	z	AA
L[1]	44.377	**1028.635**	**1023.296**	0.672	**1022.960**	L[29]
N[2]	82.392	990.941	**985.601**	**985.937**	**985.265**	N[28]
K[3]	125.090	**952.926**	**947.587**	**947.923**	**947.251**	K[27]
L[4]	162.785	910.228	904.889	905.225	904.553	L[26]
L[5]	200.479	872.533	867.194	867.530	866.858	L[25]
G[6]	219.487	834.839	829.499	829.835	829.163	G[24]
K[7]	271.529	815.832	810.492	810.828	810.156	K[23]
V[8]	304.552	763.790	758.450	758.786	758.114	V[22]
T[9]	338.234	730.767	725.427	725.763	725.091	T[21]
I[10]	375.929	697.084	691.745	692.081	691.409	I[20]
A[11]	399.608	659.389	654.050	654.386	653.714	A[19]
Q[12]	442.294	635.710	630.371	630.707	630.035	Q[18]
G[13]	461.301	593.024	587.685	588.021	587.349	G[17]
G[14]	480.308	574.017	568.678	569.013	568.342	G[16]
V[15]	513.331	555.010	549.670	550.006	549.334	V[15]
L[16]	551.026	521.987	516.648	516.984	516.312	L[14]
P[17]	583.377	484.292	478.953	479.289	478.617	P[13]
N[18]	621.391	451.942	446.602	446.938	446.266	N[12]
I[19]	659.086	413.927	408.588	408.924	408.252	I[11]
Q[20]	701.772	376.233	370.893	371.229	370.557	Q[10]
A[21]	725.451	333.546	328.207	328.543	327.871	A[9]
V[22]	758.474	309.867	304.528	304.864	304.192	V[8]
L[23]	796.168	276.844	271.505	271.841	271.169	L[7]
L[24]	833.863	239.150	233.810	234.146	233.474	L[6]
P[25]	866.214	201.455	196.116	196.451	195.780	P[5]
K[26]	908.912	169.104	163.765	164.101	163.429	K[4]
K[27]	**951.611**	126.406	121.066	121.402	120.730	K[3]
T[28]	**985.293**	83.708	78.368	78.704	78.032	T[2]
E[29]	**1028.307**	50.025	44.685	45.021	44.349	E[1]

sp|Q6GSS7|H2A2A_MOUSE

LNKLLGK $^{\text{Dimethyl}}_{28.03}$ VTIAQGGVLPNIQAVLLPKKTE

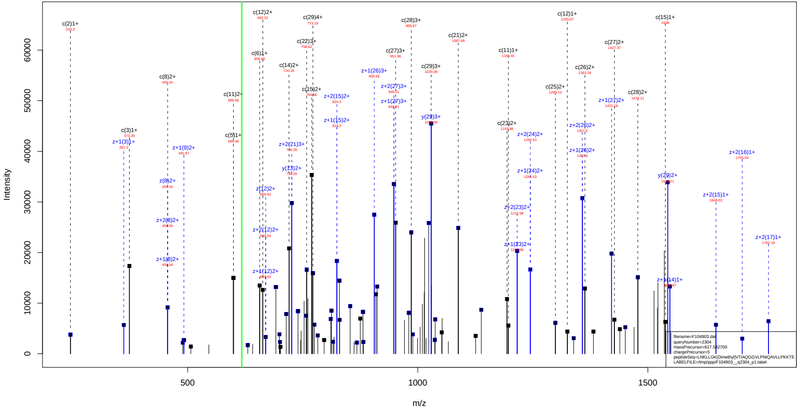

sp|Q6GSS7|H2A2A_MOUSE

LNKLLGK $^{\text{Dimethyl}}_{28.03}$ VTIAQGGVLPNIQAVLLPKKTE

- fragmentation table for charge state 1+
- specType=etd
- score=95.23
- F104903.dat
- query=q2304_p1
- precursor=617.582700
- chargePrecursor=5
- itol=0.8

AA	c	y	z+1	z+2	z	AA
L[1]	131.118	3083.892	3067.873	0.000	3066.865	L[29]
N[2]	**245.161**	2970.808	2954.789	2955.797	2953.781	N[28]
K[3]	**373.256**	2856.765	2840.746	2841.754	2839.738	K[27]
L[4]	486.340	2728.670	2712.651	2713.659	2711.643	L[26]
L[5]	**599.424**	2615.586	2599.567	2600.575	2598.559	L[25]
G[6]	**656.445**	2502.502	2486.483	2487.491	2485.475	G[24]
K[7]	**812.572**	2445.480	2429.462	2430.469	2428.454	K[23]
V[8]	**911.640**	2289.354	2273.335	2274.343	2272.327	V[22]
T[9]	1012.688	2190.286	2174.267	2175.275	2173.259	T[21]
I[10]	**1125.772**	2089.238	2073.219	2074.227	2072.211	I[20]
A[11]	**1196.809**	1976.154	1960.135	1961.143	1959.127	A[19]
Q[12]	**1324.867**	1905.117	1889.098	1890.106	1888.090	Q[18]
G[13]	**1381.889**	1777.058	1761.039	**1762.047**	1760.032	G[17]
G[14]	**1438.910**	1720.037	1704.018	**1705.026**	1703.010	G[16]
V[15]	**1537.979**	1663.015	1646.997	**1648.004**	1645.989	V[15]
L[16]	1651.063	1563.947	**1547.928**	1548.936	1546.920	L[14]
P[17]	1748.116	**1450.863**	1434.844	1435.852	1433.836	P[13]
N[18]	1862.159	1353.810	1337.791	**1338.799**	1336.783	N[12]
I[19]	1975.243	1239.767	1223.748	1224.756	1222.741	I[11]
Q[20]	2103.301	1126.683	1110.664	1111.672	1109.656	Q[10]
A[21]	2174.338	998.624	982.606	983.614	981.598	A[9]
V[22]	2273.407	927.587	**911.569**	912.576	910.561	V[8]
L[23]	2386.491	828.519	**812.500**	813.508	**811.492**	L[7]
L[24]	2499.575	715.435	**699.416**	**700.424**	698.408	L[6]
P[25]	2596.628	602.351	586.332	587.340	585.324	P[5]
K[26]	2724.723	505.298	**489.279**	490.287	488.271	K[4]
K[27]	2852.818	377.203	**361.184**	362.192	360.177	K[3]
T[28]	2953.865	249.108	233.089	234.097	232.082	T[2]
E[29]	3082.908	148.060	132.042	133.050	131.034	E[1]

sp|Q6GSS7|H2A2A_MOUSE

LNKLLGK $^{\text{Dimethyl}}_{28.03}$ VTIAQGGVLPNIQAVLLPKKTE

- fragmentation table for charge state 2+
- specType=etd
- score=95.23
- F104903.dat
- query=q2304_p1
- precursor=617.582700
- chargePrecursor=5
- itol=0.8

AA	c	y	z+1	z+2	z	AA
L[1]	66.063	**1542.450**	1534.440	0.504	1533.936	L[29]
N[2]	123.084	1485.908	**1477.898**	**1478.402**	1477.394	N[28]
K[3]	187.132	1428.886	**1420.877**	**1421.381**	1420.373	K[27]
L[4]	243.674	1364.839	**1356.829**	**1357.333**	1356.325	L[26]
L[5]	300.216	1308.297	1300.287	1300.791	**1299.783**	L[25]
G[6]	328.726	1251.755	**1243.745**	**1244.249**	1243.241	G[24]
K[7]	406.789	1223.244	**1215.234**	**1215.738**	1214.731	K[23]
V[8]	**456.324**	1145.181	1137.171	**1137.675**	1136.667	V[22]
T[9]	**506.848**	1095.646	**1087.637**	**1088.141**	1087.133	T[21]
I[10]	563.390	1045.123	**1037.113**	**1037.617**	**1036.609**	I[20]
A[11]	**598.908**	**988.581**	**980.571**	**981.075**	**980.067**	A[19]
Q[12]	**662.937**	953.062	945.053	945.557	944.549	Q[18]
G[13]	**691.448**	889.033	**881.023**	**881.527**	**880.519**	G[17]
G[14]	**719.959**	860.522	**852.513**	**853.017**	852.009	G[16]
V[15]	**769.493**	832.011	**824.002**	**824.506**	823.498	V[15]
L[16]	826.035	**782.477**	774.468	**774.972**	773.964	L[14]
P[17]	**874.561**	**725.935**	717.926	718.430	717.422	P[13]
N[18]	931.583	677.409	**669.399**	**669.903**	**668.895**	N[12]
I[19]	988.125	620.387	612.378	612.882	611.874	I[11]
Q[20]	**1052.154**	563.845	555.836	556.340	555.332	Q[10]
A[21]	**1087.673**	499.816	**491.807**	**492.310**	**491.303**	A[9]
V[22]	1137.207	464.297	**456.288**	**456.792**	**455.784**	V[8]
L[23]	**1193.749**	414.763	406.754	407.258	406.250	L[7]
L[24]	1250.291	358.221	350.212	350.716	349.708	L[6]
P[25]	**1298.817**	301.679	293.670	294.174	293.166	P[5]
K[26]	**1362.865**	253.153	**245.143**	**245.647**	**244.639**	K[4]
K[27]	**1426.912**	189.105	181.096	181.600	180.592	K[3]
T[28]	**1477.436**	125.058	117.048	117.552	116.544	T[2]
E[29]	1541.958	74.534	66.524	67.028	66.021	E[1]

sp|Q6GSS7|H2A2A_MOUSE

LNKLLGK $^{Dimethyl}_{28.03}$ VTIAQGGVLPNIQAVLLPKKTE

- fragmentation table for charge state 3+
- specType=etd
- score=95.23
- F104903.dat
- query=q2304_p1
- precursor=617.582700
- chargePrecursor=5
- itol=0.8

AA	c	y	z+1	z+2	z	AA
L[1]	44.377	**1028.635**	**1023.296**	0.672	1022.960	L[29]
N[2]	82.392	990.941	**985.601**	**985.937**	**985.265**	N[28]
K[3]	125.090	952.926	**947.587**	**947.923**	947.251	K[27]
L[4]	162.785	910.228	**904.889**	**905.225**	904.553	L[26]
L[5]	200.479	872.533	**867.194**	**867.530**	866.858	L[25]
G[6]	219.487	834.839	**829.499**	**829.835**	**829.163**	G[24]
K[7]	271.529	**815.832**	**810.492**	**810.828**	**810.156**	K[23]
V[8]	304.552	763.790	**758.450**	**758.786**	**758.114**	V[22]
T[9]	338.234	730.767	725.427	**725.763**	725.091	T[21]
I[10]	375.929	697.084	**691.745**	**692.081**	**691.409**	I[20]
A[11]	399.608	659.389	654.050	654.386	653.714	A[19]
Q[12]	442.294	635.710	**630.371**	**630.707**	**630.035**	Q[18]
G[13]	461.301	593.024	587.685	588.021	587.349	G[17]
G[14]	480.308	574.017	568.678	569.013	568.342	G[16]
V[15]	513.331	555.010	549.670	550.006	549.334	V[15]
L[16]	551.026	521.987	516.648	516.984	516.312	L[14]
P[17]	583.377	484.292	478.953	479.289	478.617	P[13]
N[18]	621.391	451.942	446.602	446.938	446.266	N[12]
I[19]	659.086	413.927	408.588	408.924	408.252	I[11]
Q[20]	**701.772**	376.233	370.893	371.229	370.557	Q[10]
A[21]	725.451	333.546	328.207	328.543	327.871	A[9]
V[22]	**758.474**	309.867	304.528	304.864	304.192	V[8]
L[23]	**796.168**	276.844	271.505	271.841	271.169	L[7]
L[24]	833.863	239.150	233.810	234.146	233.474	L[6]
P[25]	866.214	201.455	196.116	196.451	195.780	P[5]
K[26]	**908.912**	169.104	163.765	164.101	163.429	K[4]
K[27]	**951.611**	126.406	121.066	121.402	120.730	K[3]
T[28]	**985.293**	83.708	78.368	78.704	78.032	T[2]
E[29]	**1028.307**	50.025	44.685	45.021	44.349	E[1]

sp|Q6GSS7|H2A2A_MOUSE

LNKLLGK $^{Dimethyl}_{28.03}$ VTIAQGGVLPNIQAVLLPKKTE

- fragmentation table for charge state 4+
- specType=etd
- score=95.23
- F104903.dat
- query=q2304_p1
- precursor=617.582700
- chargePrecursor=5
- itol=0.8

AA	c	y	z+1	z+2	z	AA
L[1]	33.535	**771.728**	767.724	0.755	767.472	L[29]
N[2]	62.046	743.457	**739.453**	**739.705**	**739.201**	N[28]
K[3]	94.069	**714.947**	710.942	711.194	710.690	K[27]
L[4]	122.340	682.923	678.918	679.170	678.666	L[26]
L[5]	150.611	654.652	650.647	650.899	650.395	L[25]
G[6]	164.867	626.381	622.376	622.628	622.124	G[24]
K[7]	203.898	612.126	608.121	608.373	607.869	K[23]
V[8]	228.665	573.094	569.089	569.341	568.837	V[22]
T[9]	253.927	548.327	544.322	544.574	544.070	T[21]
I[10]	282.198	523.065	519.060	519.312	518.808	I[20]
A[11]	299.958	494.794	490.789	491.041	490.537	A[19]
Q[12]	331.972	477.035	473.030	473.282	472.778	Q[18]
G[13]	346.228	445.020	441.015	441.267	440.763	G[17]
G[14]	360.483	430.765	426.760	427.012	426.508	G[16]
V[15]	385.250	416.509	412.505	412.757	412.253	V[15]
L[16]	413.521	391.742	387.737	387.989	387.486	L[14]
P[17]	437.784	363.471	359.466	359.718	359.215	P[13]
N[18]	466.295	339.208	335.203	335.455	334.951	N[12]
I[19]	494.566	310.697	306.693	306.945	306.441	I[11]
Q[20]	526.581	282.426	278.422	278.673	278.170	Q[10]
A[21]	544.340	250.412	246.407	246.659	246.155	A[9]
V[22]	569.107	232.652	228.648	228.900	228.396	V[8]
L[23]	597.378	207.885	203.881	204.132	203.629	L[7]
L[24]	625.649	179.614	175.609	175.861	175.358	L[6]
P[25]	649.912	151.343	147.338	147.590	147.087	P[5]
K[26]	681.936	127.080	123.075	123.327	122.823	K[4]
K[27]	**713.960**	95.056	91.052	91.304	90.800	K[3]
T[28]	**739.222**	63.032	59.028	59.280	58.776	T[2]
E[29]	**771.482**	37.771	33.766	34.018	33.514	E[1]

sp|Q6GSS7|H2A2A_MOUSE

LNKLLGK $^{\text{Dimethyl}}_{28.03}$ VTIAQGGVLPNIQAVLLPKKTE

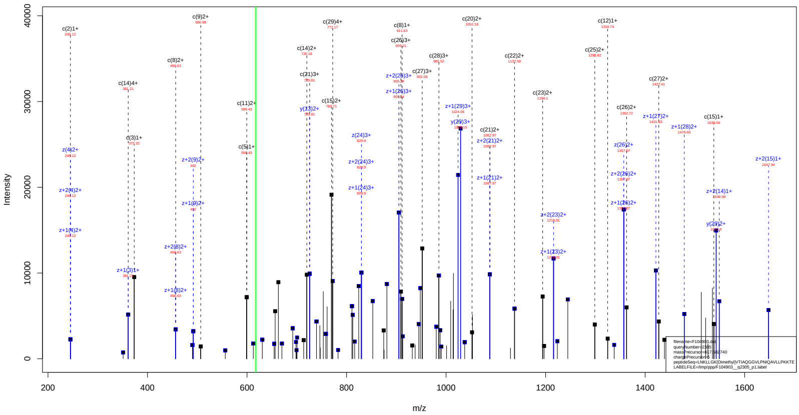

sp|Q6GSS7|H2A2A_MOUSE

LNKLLGK $^{Dimethyl}_{28.03}$ VTIAQGGVLPNIQAVLLPKKTE

- fragmentation table for charge state 1+
- specType=etd
- score=128.74
- F104903.dat
- query=q2305_p1
- precursor=617.582740
- chargePrecursor=5
- itol=0.8

AA	c	y	z+1	z+2	z	AA
L[1]	131.118	3083.892	3067.873	0.000	3066.865	L[29]
N[2]	**245.161**	2970.808	2954.789	2955.797	2953.781	N[28]
K[3]	**373.256**	2856.765	2840.746	2841.754	2839.738	K[27]
L[4]	486.340	2728.670	2712.651	2713.659	2711.643	L[26]
L[5]	**599.424**	2615.586	2599.567	2600.575	2598.559	L[25]
G[6]	**656.445**	2502.502	2486.483	2487.491	2485.475	G[24]
K[7]	**812.572**	2445.480	2429.462	2430.469	2428.454	K[23]
V[8]	**911.640**	2289.354	2273.335	2274.343	2272.327	V[22]
T[9]	1012.688	2190.286	2174.267	2175.275	2173.259	T[21]
I[10]	1125.772	2089.238	2073.219	2074.227	2072.211	I[20]
A[11]	**1196.809**	1976.154	1960.135	1961.143	1959.127	A[19]
Q[12]	**1324.867**	1905.117	1889.098	1890.106	1888.090	Q[18]
G[13]	1381.889	1777.058	1761.039	1762.047	1760.032	G[17]
G[14]	**1438.910**	1720.037	1704.018	1705.026	1703.010	G[16]
V[15]	**1537.979**	1663.015	1646.997	**1648.004**	1645.989	V[15]
L[16]	1651.063	1563.947	1547.928	**1548.936**	1546.920	L[14]
P[17]	1748.116	1450.863	1434.844	1435.852	1433.836	P[13]
N[18]	1862.159	1353.810	**1337.791**	1338.799	1336.783	N[12]
I[19]	1975.243	1239.767	**1223.748**	1224.756	1222.741	I[11]
Q[20]	2103.301	1126.683	1110.664	1111.672	1109.656	Q[10]
A[21]	2174.338	998.624	982.606	983.614	981.598	A[9]
V[22]	2273.407	927.587	**911.569**	**912.576**	910.561	V[8]
L[23]	2386.491	828.519	**812.500**	813.508	**811.492**	L[7]
L[24]	2499.575	715.435	**699.416**	**700.424**	**698.408**	L[6]
P[25]	2596.628	602.351	586.332	587.340	585.324	P[5]
K[26]	2724.723	505.298	489.279	**490.287**	488.271	K[4]
K[27]	2852.818	377.203	**361.184**	362.192	360.177	K[3]
T[28]	2953.865	249.108	233.089	234.097	232.082	T[2]
E[29]	3082.908	148.060	132.042	133.050	131.034	E[1]

sp|Q6GSS7|H2A2A_MOUSE

LNKLLGK $^{\text{Dimethyl}}_{28.03}$ VTIAQGGVLPNIQAVLLPKKTE

- fragmentation table for charge state 2+
- specType=etd
- score=128.74
- F104903.dat
- query=q2305_p1
- precursor=617.582740
- chargePrecursor=5
- itol=0.8

AA	c	y	z+1	z+2	z	AA
L[1]	66.063	**1542.450**	1534.440	0.504	1533.936	L[29]
N[2]	123.084	1485.908	**1477.898**	**1478.402**	1477.394	N[28]
K[3]	187.132	1428.886	**1420.877**	**1421.381**	1420.373	K[27]
L[4]	243.674	1364.839	**1356.829**	**1357.333**	**1356.325**	L[26]
L[5]	300.216	1308.297	1300.287	1300.791	1299.783	L[25]
G[6]	328.726	1251.755	1243.745	**1244.249**	1243.241	G[24]
K[7]	406.789	**1223.244**	**1215.234**	**1215.738**	1214.731	K[23]
V[8]	**456.324**	1145.181	**1137.171**	**1137.675**	1136.667	V[22]
T[9]	**506.848**	1095.646	**1087.637**	**1088.141**	1087.133	T[21]
I[10]	563.390	1045.123	**1037.113**	**1037.617**	**1036.609**	I[20]
A[11]	**598.908**	**988.581**	**980.571**	981.075	**980.067**	A[19]
Q[12]	**662.937**	953.062	**945.053**	**945.557**	**944.549**	Q[18]
G[13]	**691.448**	889.033	**881.023**	**881.527**	**880.519**	G[17]
G[14]	**719.959**	860.522	**852.513**	**853.017**	**852.009**	G[16]
V[15]	**769.493**	832.011	**824.002**	**824.506**	823.498	V[15]
L[16]	826.035	**782.477**	774.468	774.972	773.964	L[14]
P[17]	**874.561**	**725.935**	717.926	718.430	717.422	P[13]
N[18]	**931.583**	677.409	**669.399**	**669.903**	668.895	N[12]
I[19]	**988.125**	620.387	**612.378**	**612.882**	**611.874**	I[11]
Q[20]	**1052.154**	563.845	**555.836**	**556.340**	555.332	Q[10]
A[21]	**1087.673**	499.816	**491.807**	**492.310**	**491.303**	A[9]
V[22]	**1137.207**	464.297	**456.288**	**456.792**	455.784	V[8]
L[23]	**1193.749**	414.763	406.754	407.258	406.250	L[7]
L[24]	1250.291	358.221	**350.212**	**350.716**	349.708	L[6]
P[25]	**1298.817**	301.679	293.670	294.174	293.166	P[5]
K[26]	**1362.865**	253.153	**245.143**	**245.647**	**244.639**	K[4]
K[27]	**1426.912**	189.105	181.096	181.600	180.592	K[3]
T[28]	1477.436	125.058	117.048	117.552	116.544	T[2]
E[29]	1541.958	74.534	66.524	67.028	66.021	E[1]

sp|Q6GSS7|H2A2A_MOUSE

LNKLLGK $^{\text{Dimethyl}}_{28.03}$ VTIAQGGVLPNIQAVLLPKKTE

- fragmentation table for charge state 3+
- specType=etd
- score=128.74
- F104903.dat
- query=q2305_p1
- precursor=617.582740
- chargePrecursor=5
- itol=0.8

AA	c	y	z+1	z+2	z	AA
L[1]	44.377	**1028.635**	**1023.296**	0.672	1022.960	L[29]
N[2]	82.392	**990.941**	**985.601**	**985.937**	**985.265**	N[28]
K[3]	125.090	952.926	**947.587**	**947.923**	947.251	K[27]
L[4]	162.785	910.228	**904.889**	**905.225**	**904.553**	L[26]
L[5]	200.479	872.533	867.194	867.530	866.858	L[25]
G[6]	219.487	834.839	**829.499**	**829.835**	**829.163**	G[24]
K[7]	271.529	**815.832**	**810.492**	**810.828**	**810.156**	K[23]
V[8]	304.552	763.790	**758.450**	**758.786**	**758.114**	V[22]
T[9]	338.234	730.767	**725.427**	**725.763**	**725.091**	T[21]
I[10]	375.929	697.084	**691.745**	**692.081**	**691.409**	I[20]
A[11]	399.608	659.389	**654.050**	**654.386**	**653.714**	A[19]
Q[12]	442.294	635.710	**630.371**	**630.707**	**630.035**	Q[18]
G[13]	461.301	593.024	587.685	588.021	587.349	G[17]
G[14]	480.308	574.017	568.678	569.013	568.342	G[16]
V[15]	513.331	555.010	549.670	550.006	549.334	V[15]
L[16]	551.026	521.987	516.648	516.984	516.312	L[14]
P[17]	583.377	484.292	478.953	479.289	478.617	P[13]
N[18]	621.391	451.942	446.602	446.938	446.266	N[12]
I[19]	659.086	413.927	408.588	408.924	408.252	I[11]
Q[20]	701.772	376.233	370.893	371.229	370.557	Q[10]
A[21]	**725.451**	333.546	328.207	328.543	327.871	A[9]
V[22]	**758.474**	309.867	304.528	304.864	304.192	V[8]
L[23]	796.168	276.844	271.505	271.841	271.169	L[7]
L[24]	833.863	239.150	233.810	234.146	233.474	L[6]
P[25]	866.214	201.455	196.116	196.451	195.780	P[5]
K[26]	**908.912**	169.104	163.765	164.101	163.429	K[4]
K[27]	**951.611**	126.406	121.066	121.402	120.730	K[3]
T[28]	**985.293**	83.708	78.368	78.704	78.032	T[2]
E[29]	1028.307	50.025	44.685	45.021	44.349	E[1]

sp|Q6GSS7|H2A2A_MOUSE

LNKLLGK $^{Dimethyl}_{28.03}$ VTIAQGGVLPNIQAVLLPKKTE

- fragmentation table for charge state 4+
- specType=etd
- score=128.74
- F104903.dat
- query=q2305_p1
- precursor=617.582740
- chargePrecursor=5
- itol=0.8

AA	c	y	z+1	z+2	z	AA
L[1]	33.535	**771.728**	767.724	0.755	767.472	L[29]
N[2]	62.046	743.457	**739.453**	**739.705**	**739.201**	N[28]
K[3]	94.069	714.947	710.942	711.194	710.690	K[27]
L[4]	122.340	682.923	678.918	679.170	678.666	L[26]
L[5]	150.611	**654.652**	650.647	650.899	650.395	L[25]
G[6]	164.867	626.381	622.376	622.628	622.124	G[24]
K[7]	203.898	**612.126**	608.121	608.373	607.869	K[23]
V[8]	228.665	573.094	569.089	569.341	568.837	V[22]
T[9]	253.927	548.327	544.322	544.574	544.070	T[21]
I[10]	282.198	523.065	519.060	519.312	518.808	I[20]
A[11]	299.958	494.794	**490.789**	**491.041**	**490.537**	A[19]
Q[12]	331.972	477.035	473.030	473.282	472.778	Q[18]
G[13]	346.228	445.020	441.015	441.267	440.763	G[17]
G[14]	**360.483**	430.765	426.760	427.012	426.508	G[16]
V[15]	385.250	416.509	412.505	412.757	412.253	V[15]
L[16]	413.521	391.742	387.737	387.989	387.486	L[14]
P[17]	437.784	363.471	359.466	359.718	359.215	P[13]
N[18]	466.295	339.208	335.203	335.455	334.951	N[12]
I[19]	494.566	310.697	306.693	306.945	306.441	I[11]
Q[20]	526.581	282.426	278.422	278.673	278.170	Q[10]
A[21]	544.340	250.412	246.407	246.659	246.155	A[9]
V[22]	569.107	232.652	228.648	228.900	228.396	V[8]
L[23]	597.378	207.885	203.881	204.132	203.629	L[7]
L[24]	625.649	179.614	175.609	175.861	175.358	L[6]
P[25]	649.912	151.343	147.338	147.590	147.087	P[5]
K[26]	681.936	127.080	123.075	123.327	122.823	K[4]
K[27]	**713.960**	95.056	91.052	91.304	90.800	K[3]
T[28]	**739.222**	63.032	59.028	59.280	58.776	T[2]
E[29]	**771.482**	37.771	33.766	34.018	33.514	E[1]

sp|Q6GSS7|H2A2A_MOUSE

LNKLLGK $^{Dimethyl}_{28.03}$ VTIAQGGVLPNIQAVLLPKKTE

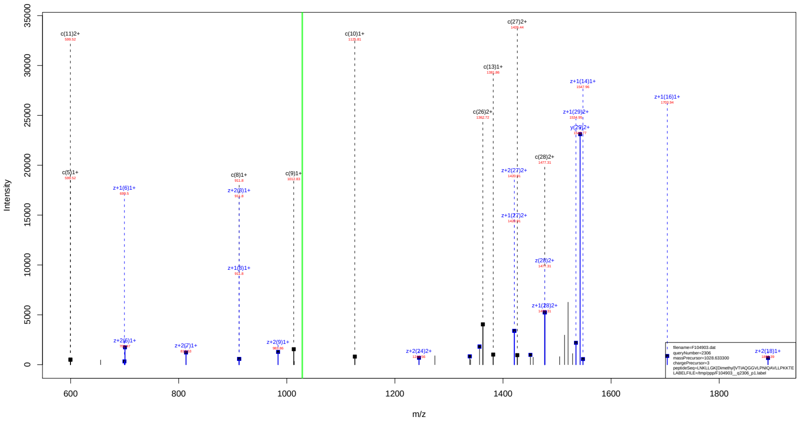

sp|Q6GSS7|H2A2A_MOUSE

LNKLLGK $^{Dimethyl}_{28.03}$ VTIAQGGVLPNIQAVLLPKKTE

- fragmentation table for charge state 1+
- specType=etd
- score=39.89
- F104903.dat
- query=q2306_p1
- precursor=1028.633300
- chargePrecursor=3
- itol=0.8

AA	c	y	z+1	z+2	z	AA
L[1]	131.118	3083.892	3067.873	0.000	3066.865	L[29]
N[2]	245.161	2970.808	2954.789	2955.797	2953.781	N[28]
K[3]	373.256	2856.765	2840.746	2841.754	2839.738	K[27]
L[4]	486.340	2728.670	2712.651	2713.659	2711.643	L[26]
L[5]	**599.424**	2615.586	2599.567	2600.575	2598.559	L[25]
G[6]	656.445	2502.502	2486.483	2487.491	2485.475	G[24]
K[7]	812.572	2445.480	2429.462	2430.469	2428.454	K[23]
V[8]	**911.640**	2289.354	2273.335	2274.343	2272.327	V[22]
T[9]	**1012.688**	2190.286	2174.267	2175.275	2173.259	T[21]
I[10]	**1125.772**	2089.238	2073.219	2074.227	2072.211	I[20]
A[11]	1196.809	1976.154	1960.135	1961.143	1959.127	A[19]
Q[12]	1324.867	1905.117	1889.098	**1890.106**	1888.090	Q[18]
G[13]	**1381.889**	1777.058	1761.039	1762.047	1760.032	G[17]
G[14]	1438.910	1720.037	**1704.018**	1705.026	1703.010	G[16]
V[15]	1537.979	1663.015	1646.997	1648.004	1645.989	V[15]
L[16]	1651.063	1563.947	**1547.928**	1548.936	1546.920	L[14]
P[17]	1748.116	**1450.863**	1434.844	1435.852	1433.836	P[13]
N[18]	1862.159	1353.810	**1337.791**	**1338.799**	1336.783	N[12]
I[19]	1975.243	1239.767	1223.748	1224.756	1222.741	I[11]
Q[20]	2103.301	1126.683	1110.664	1111.672	1109.656	Q[10]
A[21]	2174.338	998.624	982.606	**983.614**	981.598	A[9]
V[22]	2273.407	927.587	**911.569**	**912.576**	910.561	V[8]
L[23]	2386.491	828.519	812.500	**813.508**	811.492	L[7]
L[24]	2499.575	715.435	**699.416**	**700.424**	698.408	L[6]
P[25]	2596.628	602.351	586.332	587.340	585.324	P[5]
K[26]	2724.723	505.298	489.279	490.287	488.271	K[4]
K[27]	2852.818	377.203	361.184	362.192	360.177	K[3]
T[28]	2953.865	249.108	233.089	234.097	232.082	T[2]
E[29]	3082.908	148.060	132.042	133.050	131.034	E[1]

sp|Q6GSS7|H2A2A_MOUSE

LNKLLGK $^{\text{Dimethyl}}_{28.03}$ VTIAQGGVLPNIQAVLLPKKTE

- fragmentation table for charge state 2+
- specType=etd
- score=39.89
- F104903.dat
- query=q2306_p1
- precursor=1028.633300
- chargePrecursor=3
- itol=0.8

AA	c	y	z+1	z+2	z	AA
L[1]	66.063	**1542.450**	**1534.440**	0.504	1533.936	L[29]
N[2]	123.084	1485.908	**1477.898**	1478.402	**1477.394**	N[28]
K[3]	187.132	1428.886	**1420.877**	**1421.381**	**1420.373**	K[27]
L[4]	243.674	1364.839	**1356.829**	**1357.333**	**1356.325**	L[26]
L[5]	300.216	1308.297	1300.287	1300.791	1299.783	L[25]
G[6]	328.726	1251.755	1243.745	**1244.249**	1243.241	G[24]
K[7]	406.789	1223.244	1215.234	1215.738	1214.731	K[23]
V[8]	456.324	1145.181	1137.171	1137.675	1136.667	V[22]
T[9]	506.848	1095.646	1087.637	1088.141	1087.133	T[21]
I[10]	563.390	1045.123	1037.113	1037.617	1036.609	I[20]
A[11]	**598.908**	988.581	980.571	981.075	980.067	A[19]
Q[12]	662.937	953.062	945.053	945.557	944.549	Q[18]
G[13]	691.448	889.033	881.023	881.527	880.519	G[17]
G[14]	719.959	860.522	852.513	853.017	852.009	G[16]
V[15]	769.493	832.011	824.002	824.506	823.498	V[15]
L[16]	826.035	782.477	774.468	774.972	773.964	L[14]
P[17]	874.561	725.935	717.926	718.430	717.422	P[13]
N[18]	931.583	677.409	669.399	669.903	668.895	N[12]
I[19]	988.125	620.387	612.378	612.882	611.874	I[11]
Q[20]	1052.154	563.845	555.836	556.340	555.332	Q[10]
A[21]	1087.673	499.816	491.807	492.310	491.303	A[9]
V[22]	1137.207	464.297	456.288	456.792	455.784	V[8]
L[23]	1193.749	414.763	406.754	407.258	406.250	L[7]
L[24]	1250.291	358.221	350.212	350.716	349.708	L[6]
P[25]	1298.817	301.679	293.670	294.174	293.166	P[5]
K[26]	**1362.865**	253.153	245.143	245.647	244.639	K[4]
K[27]	**1426.912**	189.105	181.096	181.600	180.592	K[3]
T[28]	**1477.436**	125.058	117.048	117.552	116.544	T[2]
E[29]	1541.958	74.534	66.524	67.028	66.021	E[1]

sp|Q8CGP5|H2A1F_MOUSE

LNK $^{\text{Dimethyl}}_{28.03}$ LLGRVTIAQGGVLPNIQAVLLPKKTE

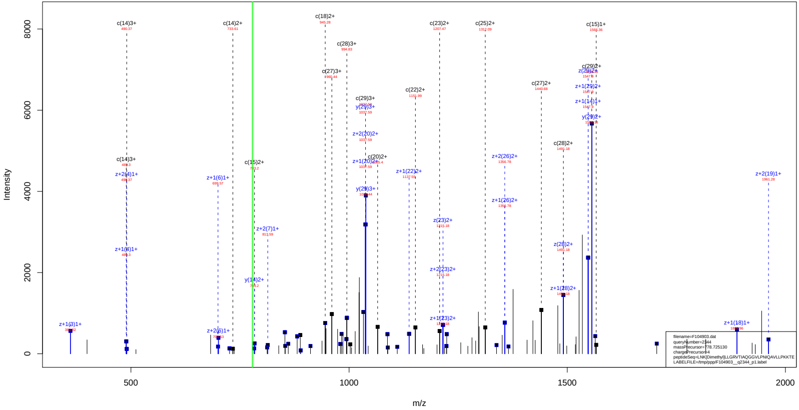

sp|Q8CGP5|H2A1F_MOUSE

LNK $^{\text{Dimethyl}}_{28.03}$ LLGRVTIAQGGVLPNIQAVLLPKKTE

- fragmentation table for charge state 1+
- specType=etd
- score=59.85
- F104903.dat
- query=q2344_p1
- precursor=778.725130
- chargePrecursor=4
- itol=0.8

AA	c	y	z+1	z+2	z	AA
L[1]	131.118	3111.898	3095.879	0.000	3094.871	L[29]
N[2]	245.161	2998.814	2982.795	2983.803	2981.787	N[28]
K[3]	401.287	2884.771	2868.752	2869.760	2867.744	K[27]
L[4]	514.371	2728.645	2712.626	2713.634	2711.618	L[26]
L[5]	627.455	2615.561	2599.542	2600.550	2598.534	L[25]
G[6]	684.477	2502.477	2486.458	2487.466	2485.450	G[24]
R[7]	840.578	2445.455	2429.436	2430.444	2428.429	R[23]
V[8]	939.646	2289.354	2273.335	2274.343	2272.327	V[22]
T[9]	1040.694	2190.286	2174.267	2175.275	2173.259	T[21]
I[10]	1153.778	2089.238	2073.219	2074.227	2072.211	I[20]
A[11]	1224.815	1976.154	1960.135	**1961.143**	1959.127	A[19]
Q[12]	1352.874	1905.117	**1889.098**	1890.106	1888.090	Q[18]
G[13]	1409.895	1777.058	1761.039	1762.047	1760.032	G[17]
G[14]	1466.917	1720.037	1704.018	**1705.026**	1703.010	G[16]
V[15]	**1565.985**	1663.015	1646.997	1648.004	1645.989	V[15]
L[16]	1679.069	**1563.947**	**1547.928**	1548.936	1546.920	L[14]
P[17]	1776.122	1450.863	1434.844	1435.852	1433.836	P[13]
N[18]	1890.165	1353.810	**1337.791**	1338.799	1336.783	N[12]
I[19]	2003.249	1239.767	**1223.748**	1224.756	**1222.741**	I[11]
Q[20]	2131.307	1126.683	**1110.664**	1111.672	1109.656	Q[10]
A[21]	2202.344	998.624	**982.606**	983.614	981.598	A[9]
V[22]	2301.413	927.587	**911.569**	912.576	910.561	V[8]
L[23]	2414.497	828.519	**812.500**	**813.508**	**811.492**	L[7]
L[24]	2527.581	715.435	**699.416**	**700.424**	698.408	L[6]
P[25]	2624.634	602.351	586.332	587.340	585.324	P[5]
K[26]	2752.729	505.298	**489.279**	**490.287**	488.271	K[4]
K[27]	2880.824	377.203	**361.184**	362.192	360.177	K[3]
T[28]	2981.871	249.108	233.089	234.097	232.082	T[2]
E[29]	3110.914	148.060	132.042	133.050	131.034	E[1]

sp|Q8CGP5|H2A1F_MOUSE

LNK $^{Dimethyl}_{28.03}$ LLGRVTIAQGGVLPNIQAVLLPKKTE

- fragmentation table for charge state 2+
- specType=etd
- score=59.85
- F104903.dat
- query=q2344_p1
- precursor=778.725130
- chargePrecursor=4
- itol=0.8

AA	c	y	z+1	z+2	z	AA
L[1]	66.063	**1556.453**	**1548.443**	0.504	**1547.939**	L[29]
N[2]	123.084	1499.911	**1491.901**	1492.405	**1491.397**	N[28]
K[3]	201.147	1442.889	1434.880	1435.384	1434.376	K[27]
L[4]	257.689	**1364.826**	**1356.817**	**1357.321**	**1356.313**	L[26]
L[5]	314.231	1308.284	1300.275	1300.779	1299.771	L[25]
G[6]	342.742	1251.742	1243.733	1244.236	1243.229	G[24]
R[7]	420.793	**1223.231**	**1215.222**	**1215.726**	**1214.718**	R[23]
V[8]	470.327	1145.181	**1137.171**	**1137.675**	1136.667	V[22]
T[9]	520.851	1095.646	**1087.637**	**1088.141**	1087.133	T[21]
I[10]	577.393	1045.123	**1037.113**	**1037.617**	1036.609	I[20]
A[11]	612.911	988.581	**980.571**	981.075	**980.067**	A[19]
Q[12]	676.940	953.062	**945.053**	**945.557**	**944.549**	Q[18]
G[13]	705.451	**889.033**	**881.023**	**881.527**	**880.519**	G[17]
G[14]	**733.962**	**860.522**	**852.513**	**853.017**	852.009	G[16]
V[15]	**783.496**	832.011	824.002	824.506	823.498	V[15]
L[16]	840.038	**782.477**	774.468	774.972	773.964	L[14]
P[17]	**888.565**	**725.935**	717.926	718.430	717.422	P[13]
N[18]	**945.586**	677.409	669.399	669.903	668.895	N[12]
I[19]	**1002.128**	620.387	612.378	612.882	611.874	I[11]
Q[20]	**1066.157**	563.845	555.836	556.340	555.332	Q[10]
A[21]	1101.676	499.816	491.807	492.310	491.303	A[9]
V[22]	**1151.210**	464.297	456.288	456.792	455.784	V[8]
L[23]	**1207.752**	414.763	406.754	407.258	406.250	L[7]
L[24]	1264.294	358.221	350.212	350.716	349.708	L[6]
P[25]	**1312.821**	301.679	293.670	294.174	293.166	P[5]
K[26]	1376.868	253.153	245.143	245.647	244.639	K[4]
K[27]	**1440.915**	189.105	181.096	181.600	180.592	K[3]
T[28]	**1491.439**	125.058	117.048	117.552	116.544	T[2]
E[29]	**1555.961**	74.534	66.524	67.028	66.021	E[1]

sp|Q8CGP5|H2A1F_MOUSE

LNK $^{Dimethyl}_{28.03}$ LLGRVTIAQGGVLPNIQAVLLPKKTE

- fragmentation table for charge state 3+
- specType=etd
- score=59.85
- F104903.dat
- query=q2344_p1
- precursor=778.725130
- chargePrecursor=4
- itol=0.8

AA	c	y	z+1	z+2	z	AA
L[1]	44.377	**1037.971**	**1032.631**	0.672	**1032.295**	L[29]
N[2]	82.392	1000.276	**994.937**	**995.273**	**994.601**	N[28]
K[3]	134.434	962.262	956.922	957.258	956.586	K[27]
L[4]	172.129	910.220	904.880	905.216	904.544	L[26]
L[5]	209.823	872.525	867.186	867.521	866.850	L[25]
G[6]	228.830	834.830	829.491	829.827	829.155	G[24]
R[7]	280.864	815.823	810.484	810.820	810.148	R[23]
V[8]	313.887	763.790	758.450	758.786	758.114	V[22]
T[9]	347.569	730.767	**725.427**	**725.763**	725.091	T[21]
I[10]	385.264	697.084	691.745	692.081	691.409	I[20]
A[11]	408.943	659.389	654.050	654.386	653.714	A[19]
Q[12]	451.629	635.710	630.371	630.707	630.035	Q[18]
G[13]	470.637	593.024	587.685	588.021	587.349	G[17]
G[14]	**489.644**	574.017	568.678	569.013	568.342	G[16]
V[15]	522.667	555.010	549.670	550.006	549.334	V[15]
L[16]	560.361	521.987	516.648	516.984	516.312	L[14]
P[17]	592.712	484.292	478.953	479.289	478.617	P[13]
N[18]	630.726	451.942	446.602	446.938	446.266	N[12]
I[19]	668.421	413.927	408.588	408.924	408.252	I[11]
Q[20]	711.107	376.233	370.893	371.229	370.557	Q[10]
A[21]	734.786	333.546	328.207	328.543	327.871	A[9]
V[22]	767.809	309.867	304.528	304.864	304.192	V[8]
L[23]	805.504	276.844	271.505	271.841	271.169	L[7]
L[24]	843.199	239.150	233.810	234.146	233.474	L[6]
P[25]	875.549	201.455	196.116	196.451	195.780	P[5]
K[26]	918.248	169.104	163.765	164.101	163.429	K[4]
K[27]	**960.946**	126.406	121.066	121.402	120.730	K[3]
T[28]	**994.629**	83.708	78.368	78.704	78.032	T[2]
E[29]	**1037.643**	50.025	44.685	45.021	44.349	E[1]

sp|P68433|H31_MOUSE

IAQDFK $^{\text{Dimethyl}}_{28.03}$ TD

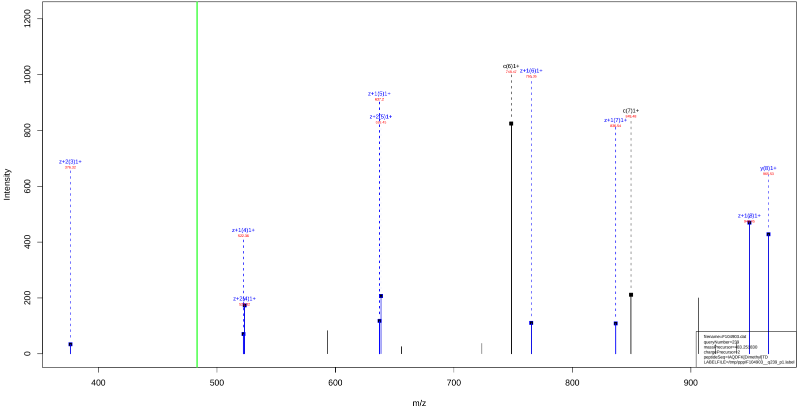

sp|P68433|H31_MOUSE

IAQDFK $^{Dimethyl}_{28.03}$ TD

- fragmentation table for charge state 1+
- specType=etd
- score=41.87
- F104903.dat
- query=q239_p1
- precursor=483.251830
- chargePrecursor=2
- itol=0.8

AA	c	y	z+1	z+2	z	AA
I[1]	131.118	**965.494**	**949.475**	0.000	948.467	I[8]
A[2]	202.155	852.410	**836.391**	0.000	835.383	A[7]
Q[3]	330.214	781.373	**765.354**	766.362	764.346	Q[6]
D[4]	445.241	653.314	**637.295**	**638.303**	636.288	D[5]
F[5]	592.309	538.287	**522.268**	**523.276**	521.261	F[4]
K[6]	**748.435**	391.219	375.200	**376.208**	374.192	K[3]
T[7]	**849.483**	235.092	219.074	220.082	218.066	T[2]
D[8]	964.510	134.045	118.026	119.034	117.018	D[1]

sp|Q6GSS7|H2A2A_MOUSE

ELNKLLGK $^{\text{Dimethyl}}_{28.03}$ VTIAQGGVLPNIQAVLLPKKTE

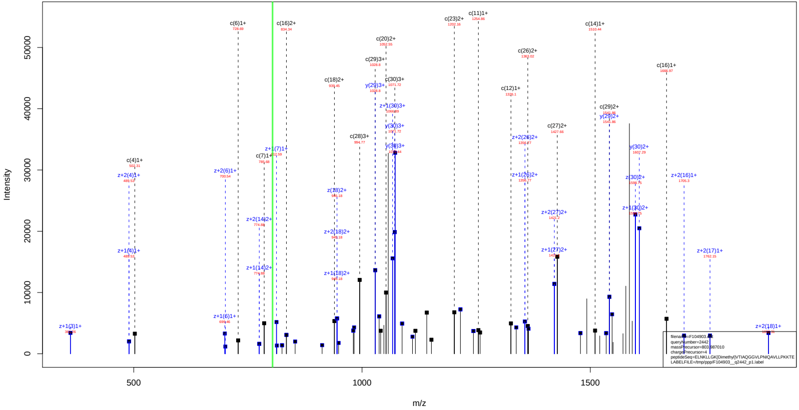

sp|Q6GSS7|H2A2A_MOUSE

ELNKLLGK $^{Dimethyl}_{28.03}$ VTIAQGGVLPNIQAVLLPKKTE

- fragmentation table for charge state 1+
- specType=etd
- score=102.99
- F104903.dat
- query=q2442_p1
- precursor=803.987010
- chargePrecursor=4
- itol=0.8

AA	c	y	z+1	z+2	z	AA
E[1]	147.076	3212.934	3196.916	0.000	3195.908	E[30]
L[2]	260.160	3083.892	3067.873	0.000	3066.865	L[29]
N[3]	374.203	2970.808	2954.789	2955.797	2953.781	N[28]
K[4]	**502.298**	2856.765	2840.746	2841.754	2839.738	K[27]
L[5]	615.382	2728.670	2712.651	2713.659	2711.643	L[26]
L[6]	**728.466**	2615.586	2599.567	2600.575	2598.559	L[25]
G[7]	**785.488**	2502.502	2486.483	2487.491	2485.475	G[24]
K[8]	941.614	2445.480	2429.462	2430.469	2428.454	K[23]
V[9]	**1040.683**	2289.354	2273.335	2274.343	2272.327	V[22]
T[10]	**1141.730**	2190.286	2174.267	2175.275	2173.259	T[21]
I[11]	**1254.814**	2089.238	2073.219	2074.227	2072.211	I[20]
A[12]	**1325.851**	1976.154	1960.135	1961.143	1959.127	A[19]
Q[13]	1453.910	1905.117	1889.098	**1890.106**	1888.090	Q[18]
G[14]	**1510.932**	1777.058	1761.039	**1762.047**	1760.032	G[17]
G[15]	1567.953	1720.037	1704.018	**1705.026**	1703.010	G[16]
V[16]	**1667.021**	1663.015	1646.997	1648.004	1645.989	V[15]
L[17]	1780.105	1563.947	**1547.928**	1548.936	1546.920	L[14]
P[18]	1877.158	1450.863	1434.844	1435.852	1433.836	P[13]
N[19]	1991.201	1353.810	**1337.791**	1338.799	1336.783	N[12]
I[20]	2104.285	1239.767	1223.748	1224.756	1222.741	I[11]
Q[21]	2232.344	1126.683	**1110.664**	1111.672	1109.656	Q[10]
A[22]	2303.381	998.624	**982.606**	983.614	**981.598**	A[9]
V[23]	2402.449	927.587	911.569	**912.576**	910.561	V[8]
L[24]	2515.533	828.519	**812.500**	**813.508**	811.492	L[7]
L[25]	2628.617	715.435	**699.416**	**700.424**	698.408	L[6]
P[26]	2725.670	602.351	586.332	587.340	585.324	P[5]
K[27]	2853.765	505.298	**489.279**	**490.287**	488.271	K[4]
K[28]	2981.860	377.203	**361.184**	362.192	360.177	K[3]
T[29]	3082.908	249.108	233.089	234.097	232.082	T[2]
E[30]	3211.950	148.060	132.042	133.050	131.034	E[1]

sp|Q6GSS7|H2A2A_MOUSE

ELNKLLGK $^{\text{Dimethyl}}_{28.03}$ VTIAQGGVLPNIQAVLLPKKTE

- fragmentation table for charge state 2+
- specType=etd
- score=102.99
- F104903.dat
- query=q2442_p1
- precursor=803.987010
- chargePrecursor=4
- itol=0.8

AA	c	y	z+1	z+2	z	AA
E[1]	74.042	**1606.971**	**1598.962**	0.504	**1598.458**	E[30]
L[2]	130.584	**1542.450**	**1534.440**	0.504	**1533.936**	L[29]
N[3]	187.605	1485.908	**1477.898**	**1478.402**	1477.394	N[28]
K[4]	251.653	1428.886	**1420.877**	**1421.381**	1420.373	K[27]
L[5]	308.195	**1364.839**	**1356.829**	**1357.333**	**1356.325**	L[26]
L[6]	364.737	1308.297	1300.287	1300.791	1299.783	L[25]
G[7]	393.248	1251.755	**1243.745**	**1244.249**	**1243.241**	G[24]
K[8]	471.311	1223.244	**1215.234**	**1215.738**	1214.731	K[23]
V[9]	520.845	1145.181	1137.171	1137.675	1136.667	V[22]
T[10]	571.369	1095.646	**1087.637**	**1088.141**	**1087.133**	T[21]
I[11]	627.911	1045.123	**1037.113**	**1037.617**	1036.609	I[20]
A[12]	663.429	988.581	**980.571**	**981.075**	980.067	A[19]
Q[13]	727.459	953.062	**945.053**	**945.557**	**944.549**	Q[18]
G[14]	755.969	889.033	881.023	881.527	880.519	G[17]
G[15]	784.480	860.522	852.513	**853.017**	852.009	G[16]
V[16]	**834.014**	832.011	**824.002**	**824.506**	823.498	V[15]
L[17]	890.556	782.477	**774.468**	**774.972**	773.964	L[14]
P[18]	**939.083**	725.935	717.926	718.430	717.422	P[13]
N[19]	996.104	677.409	669.399	669.903	668.895	N[12]
I[20]	**1052.646**	620.387	612.378	612.882	611.874	I[11]
Q[21]	**1116.676**	563.845	555.836	556.340	555.332	Q[10]
A[22]	**1152.194**	499.816	491.807	492.310	491.303	A[9]
V[23]	**1201.728**	464.297	456.288	456.792	455.784	V[8]
L[24]	**1258.270**	414.763	406.754	407.258	406.250	L[7]
L[25]	1314.812	358.221	350.212	350.716	349.708	L[6]
P[26]	**1363.339**	301.679	293.670	294.174	293.166	P[5]
K[27]	**1427.386**	253.153	245.143	245.647	244.639	K[4]
K[28]	1491.434	189.105	181.096	181.600	180.592	K[3]
T[29]	**1541.958**	125.058	117.048	117.552	116.544	T[2]
E[30]	1606.479	74.534	66.524	67.028	66.021	E[1]

sp|Q6GSS7|H2A2A_MOUSE

ELNKLLGK $^{Dimethyl}_{28.03}$ VTIAQGGVLPNIQAVLLPKKTE

- fragmentation table for charge state 3+
- specType=etd
- score=102.99
- F104903.dat
- query=q2442_p1
- precursor=803.987010
- chargePrecursor=4
- itol=0.8

AA	c	y	z+1	z+2	z	AA
E[1]	49.697	**1071.650**	**1066.310**	0.672	1065.974	E[30]
L[2]	87.392	**1028.635**	1023.296	0.672	1022.960	L[29]
N[3]	125.406	990.941	985.601	985.937	985.265	N[28]
K[4]	168.104	952.926	947.587	**947.923**	947.251	K[27]
L[5]	205.799	910.228	904.889	905.225	904.553	L[26]
L[6]	243.494	872.533	867.194	867.530	866.858	L[25]
G[7]	262.501	**834.839**	829.499	829.835	829.163	G[24]
K[8]	314.543	815.832	810.492	810.828	810.156	K[23]
V[9]	347.566	763.790	758.450	758.786	758.114	V[22]
T[10]	381.248	730.767	725.427	725.763	725.091	T[21]
I[11]	418.943	697.084	691.745	692.081	691.409	I[20]
A[12]	442.622	659.389	654.050	654.386	653.714	A[19]
Q[13]	485.308	635.710	630.371	630.707	630.035	Q[18]
G[14]	504.315	593.024	587.685	588.021	587.349	G[17]
G[15]	523.323	574.017	568.678	569.013	568.342	G[16]
V[16]	556.345	555.010	549.670	550.006	549.334	V[15]
L[17]	594.040	521.987	516.648	516.984	516.312	L[14]
P[18]	626.391	484.292	478.953	479.289	478.617	P[13]
N[19]	664.405	451.942	446.602	446.938	446.266	N[12]
I[20]	702.100	413.927	408.588	408.924	408.252	I[11]
Q[21]	744.786	376.233	370.893	371.229	370.557	Q[10]
A[22]	768.465	333.546	328.207	328.543	327.871	A[9]
V[23]	801.488	309.867	304.528	304.864	304.192	V[8]
L[24]	839.183	276.844	271.505	271.841	271.169	L[7]
L[25]	876.877	239.150	233.810	234.146	233.474	L[6]
P[26]	909.228	201.455	196.116	196.451	195.780	P[5]
K[27]	951.927	169.104	163.765	164.101	163.429	K[4]
K[28]	**994.625**	126.406	121.066	121.402	120.730	K[3]
T[29]	**1028.307**	83.708	78.368	78.704	78.032	T[2]
E[30]	**1071.322**	50.025	44.685	45.021	44.349	E[1]

sp|Q6GSS7|H2A2A_MOUSE

ELNKLLGK $^{Dimethyl}_{28.03}$ VTIAQGGVLPNIQAVLLPKKTE

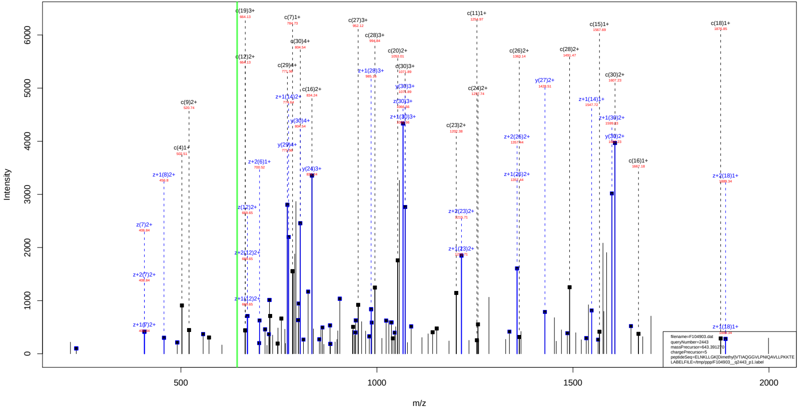

sp|Q6GSS7|H2A2A_MOUSE

ELNKLLGK $^{Dimethyl}_{28.03}$ VTIAQGGVLPNIQAVLLPKKTE

- fragmentation table for charge state 1+
- specType=etd
- score=62.62
- F104903.dat
- query=q2443_p1
- precursor=643.391270
- chargePrecursor=5
- itol=0.8

AA	c	y	z+1	z+2	z	AA
E[1]	147.076	3212.934	3196.916	0.000	3195.908	E[30]
L[2]	260.160	3083.892	3067.873	0.000	3066.865	L[29]
N[3]	374.203	2970.808	2954.789	2955.797	2953.781	N[28]
K[4]	**502.298**	2856.765	2840.746	2841.754	2839.738	K[27]
L[5]	615.382	2728.670	2712.651	2713.659	2711.643	L[26]
L[6]	728.466	2615.586	2599.567	2600.575	2598.559	L[25]
G[7]	**785.488**	2502.502	2486.483	2487.491	2485.475	G[24]
K[8]	941.614	2445.480	2429.462	2430.469	2428.454	K[23]
V[9]	**1040.683**	2289.354	2273.335	2274.343	2272.327	V[22]
T[10]	**1141.730**	2190.286	2174.267	2175.275	2173.259	T[21]
I[11]	**1254.814**	2089.238	2073.219	2074.227	2072.211	I[20]
A[12]	1325.851	1976.154	1960.135	1961.143	1959.127	A[19]
Q[13]	1453.910	1905.117	**1889.098**	**1890.106**	1888.090	Q[18]
G[14]	1510.932	1777.058	1761.039	1762.047	1760.032	G[17]
G[15]	**1567.953**	1720.037	1704.018	1705.026	1703.010	G[16]
V[16]	**1667.021**	1663.015	1646.997	**1648.004**	1645.989	V[15]
L[17]	1780.105	**1563.947**	**1547.928**	1548.936	1546.920	L[14]
P[18]	**1877.158**	1450.863	1434.844	1435.852	1433.836	P[13]
N[19]	1991.201	1353.810	**1337.791**	1338.799	1336.783	N[12]
I[20]	2104.285	1239.767	1223.748	1224.756	1222.741	I[11]
Q[21]	2232.344	1126.683	1110.664	1111.672	1109.656	Q[10]
A[22]	2303.381	998.624	982.606	983.614	981.598	A[9]
V[23]	2402.449	927.587	911.569	912.576	910.561	V[8]
L[24]	2515.533	828.519	**812.500**	813.508	811.492	L[7]
L[25]	2628.617	715.435	**699.416**	**700.424**	698.408	L[6]
P[26]	2725.670	602.351	586.332	587.340	585.324	P[5]
K[27]	2853.765	505.298	489.279	**490.287**	488.271	K[4]
K[28]	2981.860	377.203	361.184	362.192	360.177	K[3]
T[29]	3082.908	249.108	**233.089**	234.097	232.082	T[2]
E[30]	3211.950	148.060	132.042	133.050	131.034	E[1]

sp|Q6GSS7|H2A2A_MOUSE

ELNKLLGK $^{Dimethyl}_{28.03}$ VTIAQGGVLPNIQAVLLPKKTE

- fragmentation table for charge state 2+
- specType=etd
- score=62.62
- F104903.dat
- query=q2443_p1
- precursor=643.391270
- chargePrecursor=5
- itol=0.8

AA	c	y	z+1	z+2	z	AA
E[1]	74.042	**1606.971**	**1598.962**	0.504	1598.458	E[30]
L[2]	130.584	1542.450	**1534.440**	0.504	**1533.936**	L[29]
N[3]	187.605	**1485.908**	1477.898	1478.402	1477.394	N[28]
K[4]	251.653	**1428.886**	1420.877	1421.381	1420.373	K[27]
L[5]	308.195	1364.839	**1356.829**	**1357.333**	1356.325	L[26]
L[6]	364.737	1308.297	1300.287	1300.791	1299.783	L[25]
G[7]	393.248	1251.755	1243.745	1244.249	1243.241	G[24]
K[8]	471.311	1223.244	**1215.234**	**1215.738**	1214.731	K[23]
V[9]	**520.845**	1145.181	1137.171	1137.675	1136.667	V[22]
T[10]	**571.369**	1095.646	**1087.637**	**1088.141**	**1087.133**	T[21]
I[11]	627.911	**1045.123**	**1037.113**	**1037.617**	**1036.609**	I[20]
A[12]	**663.429**	988.581	**980.571**	**981.075**	**980.067**	A[19]
Q[13]	**727.459**	953.062	**945.053**	**945.557**	**944.549**	Q[18]
G[14]	**755.969**	889.033	**881.023**	**881.527**	**880.519**	G[17]
G[15]	**784.480**	**860.522**	**852.513**	**853.017**	852.009	G[16]
V[16]	**834.014**	832.011	**824.002**	**824.506**	823.498	V[15]
L[17]	890.556	782.477	**774.468**	**774.972**	**773.964**	L[14]
P[18]	**939.083**	**725.935**	717.926	718.430	717.422	P[13]
N[19]	996.104	677.409	**669.399**	**669.903**	**668.895**	N[12]
I[20]	**1052.646**	620.387	612.378	612.882	611.874	I[11]
Q[21]	1116.676	563.845	555.836	**556.340**	555.332	Q[10]
A[22]	**1152.194**	499.816	491.807	492.310	**491.303**	A[9]
V[23]	**1201.728**	464.297	**456.288**	**456.792**	455.784	V[8]
L[24]	**1258.270**	414.763	**406.754**	**407.258**	**406.250**	L[7]
L[25]	1314.812	358.221	350.212	350.716	349.708	L[6]
P[26]	**1363.339**	301.679	293.670	294.174	293.166	P[5]
K[27]	1427.386	253.153	245.143	245.647	244.639	K[4]
K[28]	**1491.434**	189.105	181.096	181.600	180.592	K[3]
T[29]	1541.958	125.058	117.048	117.552	116.544	T[2]
E[30]	**1606.479**	74.534	66.524	67.028	66.021	E[1]

sp|Q6GSS7|H2A2A_MOUSE

ELNKLLGK $^{Dimethyl}_{28.03}$ VTIAQGGVLPNIQAVLLPKKTE

- fragmentation table for charge state 3+
- specType=etd
- score=62.62
- F104903.dat
- query=q2443_p1
- precursor=643.391270
- chargePrecursor=5
- itol=0.8

AA	c	y	z+1	z+2	z	AA
E[1]	49.697	**1071.650**	**1066.310**	0.672	**1065.974**	E[30]
L[2]	87.392	1028.635	**1023.296**	0.672	**1022.960**	L[29]
N[3]	125.406	990.941	**985.601**	**985.937**	**985.265**	N[28]
K[4]	168.104	952.926	947.587	947.923	947.251	K[27]
L[5]	205.799	910.228	**904.889**	**905.225**	**904.553**	L[26]
L[6]	243.494	872.533	867.194	867.530	866.858	L[25]
G[7]	262.501	**834.839**	829.499	829.835	829.163	G[24]
K[8]	314.543	815.832	810.492	810.828	810.156	K[23]
V[9]	347.566	763.790	758.450	758.786	758.114	V[22]
T[10]	381.248	730.767	**725.427**	**725.763**	**725.091**	T[21]
I[11]	418.943	697.084	691.745	692.081	691.409	I[20]
A[12]	442.622	659.389	654.050	654.386	653.714	A[19]
Q[13]	485.308	635.710	630.371	630.707	630.035	Q[18]
G[14]	504.315	593.024	587.685	588.021	587.349	G[17]
G[15]	523.323	574.017	568.678	569.013	568.342	G[16]
V[16]	**556.345**	555.010	549.670	550.006	549.334	V[15]
L[17]	594.040	521.987	516.648	516.984	516.312	L[14]
P[18]	626.391	484.292	478.953	479.289	478.617	P[13]
N[19]	**664.405**	451.942	446.602	446.938	446.266	N[12]
I[20]	702.100	413.927	408.588	408.924	408.252	I[11]
Q[21]	744.786	376.233	370.893	371.229	370.557	Q[10]
A[22]	768.465	333.546	328.207	328.543	327.871	A[9]
V[23]	801.488	309.867	304.528	304.864	304.192	V[8]
L[24]	839.183	276.844	271.505	271.841	271.169	L[7]
L[25]	876.877	239.150	**233.810**	234.146	**233.474**	L[6]
P[26]	909.228	201.455	196.116	196.451	195.780	P[5]
K[27]	**951.927**	169.104	163.765	164.101	163.429	K[4]
K[28]	**994.625**	126.406	121.066	121.402	120.730	K[3]
T[29]	1028.307	83.708	78.368	78.704	78.032	T[2]
E[30]	**1071.322**	50.025	44.685	45.021	44.349	E[1]

sp|Q6GSS7|H2A2A_MOUSE

ELNKLLGK $^{\text{Dimethyl}}_{28.03}$ VTIAQGGVLPNIQAVLLPKKTE

- fragmentation table for charge state 4+
- specType=etd
- score=62.62
- F104903.dat
- query=q2443_p1
- precursor=643.391270
- chargePrecursor=5
- itol=0.8

AA	c	y	z+1	z+2	z	AA
E[1]	37.525	**803.989**	**799.984**	0.755	**799.732**	E[30]
L[2]	65.796	**771.728**	767.724	0.755	767.472	L[29]
N[3]	94.306	743.457	739.453	739.705	739.201	N[28]
K[4]	126.330	**714.947**	710.942	711.194	710.690	K[27]
L[5]	154.601	682.923	678.918	679.170	678.666	L[26]
L[6]	182.872	654.652	650.647	650.899	650.395	L[25]
G[7]	197.127	626.381	622.376	622.628	622.124	G[24]
K[8]	236.159	612.126	608.121	608.373	607.869	K[23]
V[9]	260.926	573.094	569.089	569.341	568.837	V[22]
T[10]	286.188	548.327	544.322	544.574	544.070	T[21]
I[11]	314.459	523.065	519.060	519.312	518.808	I[20]
A[12]	332.218	494.794	**490.789**	**491.041**	**490.537**	A[19]
Q[13]	364.233	477.035	473.030	473.282	472.778	Q[18]
G[14]	378.488	445.020	441.015	441.267	440.763	G[17]
G[15]	392.744	430.765	426.760	427.012	426.508	G[16]
V[16]	417.511	416.509	412.505	412.757	412.253	V[15]
L[17]	445.782	391.742	387.737	387.989	387.486	L[14]
P[18]	470.045	363.471	359.466	359.718	359.215	P[13]
N[19]	498.556	339.208	335.203	335.455	334.951	N[12]
I[20]	526.827	310.697	306.693	306.945	306.441	I[11]
Q[21]	558.841	282.426	278.422	278.673	278.170	Q[10]
A[22]	576.601	250.412	246.407	246.659	246.155	A[9]
V[23]	601.368	**232.652**	228.648	228.900	228.396	V[8]
L[24]	629.639	207.885	203.881	204.132	203.629	L[7]
L[25]	657.910	179.614	175.609	175.861	175.358	L[6]
P[26]	682.173	151.343	147.338	147.590	147.087	P[5]
K[27]	**714.197**	127.080	123.075	123.327	122.823	K[4]
K[28]	**746.220**	95.056	91.052	91.304	90.800	K[3]
T[29]	**771.482**	63.032	59.028	59.280	58.776	T[2]
E[30]	**803.743**	37.771	33.766	34.018	33.514	E[1]

sp|Q6GSS7|H2A2A_MOUSE

ELNKLLGK $^{\text{Dimethyl}}_{28.03}$ VTIAQGGVLPNIQAVLLPKKTE

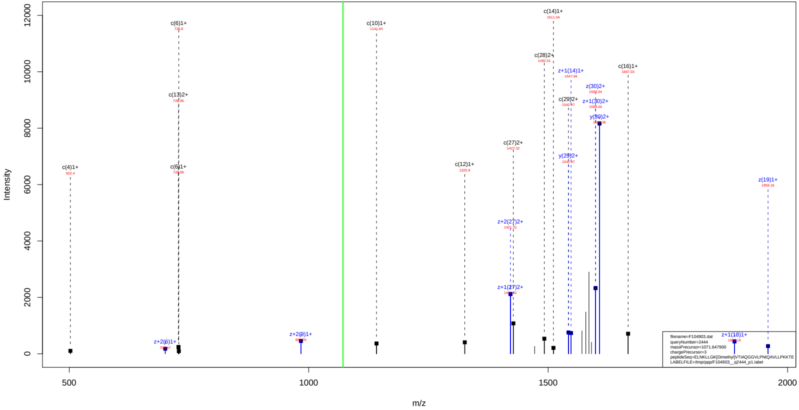

sp|Q6GSS7|H2A2A_MOUSE

ELNKLLGK $^{\text{Dimethyl}}_{28.03}$ VTIAQGGVLPNIQAVLLPKKTE

- fragmentation table for charge state 1+
- specType=etd
- score=32.85
- F104903.dat
- query=q2444_p1
- precursor=1071.647900
- chargePrecursor=3
- itol=0.8

AA	c	y	z+1	z+2	z	AA
E[1]	147.076	3212.934	3196.916	0.000	3195.908	E[30]
L[2]	260.160	3083.892	3067.873	0.000	3066.865	L[29]
N[3]	374.203	2970.808	2954.789	2955.797	2953.781	N[28]
K[4]	**502.298**	2856.765	2840.746	2841.754	2839.738	K[27]
L[5]	615.382	2728.670	2712.651	2713.659	2711.643	L[26]
L[6]	**728.466**	2615.586	2599.567	2600.575	2598.559	L[25]
G[7]	785.488	2502.502	2486.483	2487.491	2485.475	G[24]
K[8]	941.614	2445.480	2429.462	2430.469	2428.454	K[23]
V[9]	1040.683	2289.354	2273.335	2274.343	2272.327	V[22]
T[10]	**1141.730**	2190.286	2174.267	2175.275	2173.259	T[21]
I[11]	1254.814	2089.238	2073.219	2074.227	2072.211	I[20]
A[12]	**1325.851**	1976.154	1960.135	1961.143	**1959.127**	A[19]
Q[13]	1453.910	1905.117	**1889.098**	1890.106	1888.090	Q[18]
G[14]	**1510.932**	1777.058	1761.039	1762.047	1760.032	G[17]
G[15]	1567.953	1720.037	1704.018	1705.026	1703.010	G[16]
V[16]	**1667.021**	1663.015	1646.997	1648.004	1645.989	V[15]
L[17]	1780.105	1563.947	**1547.928**	1548.936	1546.920	L[14]
P[18]	1877.158	1450.863	1434.844	1435.852	1433.836	P[13]
N[19]	1991.201	1353.810	1337.791	1338.799	1336.783	N[12]
I[20]	2104.285	1239.767	1223.748	1224.756	1222.741	I[11]
Q[21]	2232.344	1126.683	1110.664	1111.672	1109.656	Q[10]
A[22]	2303.381	998.624	982.606	**983.614**	981.598	A[9]
V[23]	2402.449	927.587	911.569	912.576	910.561	V[8]
L[24]	2515.533	828.519	812.500	813.508	811.492	L[7]
L[25]	2628.617	715.435	699.416	**700.424**	698.408	L[6]
P[26]	2725.670	602.351	586.332	587.340	585.324	P[5]
K[27]	2853.765	505.298	489.279	490.287	488.271	K[4]
K[28]	2981.860	377.203	361.184	362.192	360.177	K[3]
T[29]	3082.908	249.108	233.089	234.097	232.082	T[2]
E[30]	3211.950	148.060	132.042	133.050	131.034	E[1]

sp|Q6GSS7|H2A2A_MOUSE

ELNKLLGK $^{Dimethyl}_{28.03}$ VTIAQGGVLPNIQAVLLPKKTE

- fragmentation table for charge state 2+
- specType=etd
- score=32.85
- F104903.dat
- query=q2444_p1
- precursor=1071.647900
- chargePrecursor=3
- itol=0.8

AA	c	y	z+1	z+2	z	AA
E[1]	74.042	**1606.971**	**1598.962**	0.504	**1598.458**	E[30]
L[2]	130.584	**1542.450**	1534.440	0.504	1533.936	L[29]
N[3]	187.605	1485.908	1477.898	1478.402	1477.394	N[28]
K[4]	251.653	1428.886	**1420.877**	**1421.381**	1420.373	K[27]
L[5]	308.195	1364.839	1356.829	1357.333	1356.325	L[26]
L[6]	364.737	1308.297	1300.287	1300.791	1299.783	L[25]
G[7]	393.248	1251.755	1243.745	1244.249	1243.241	G[24]
K[8]	471.311	1223.244	1215.234	1215.738	1214.731	K[23]
V[9]	520.845	1145.181	1137.171	1137.675	1136.667	V[22]
T[10]	571.369	1095.646	1087.637	1088.141	1087.133	T[21]
I[11]	627.911	1045.123	1037.113	1037.617	1036.609	I[20]
A[12]	663.429	988.581	980.571	981.075	980.067	A[19]
Q[13]	**727.459**	953.062	945.053	945.557	944.549	Q[18]
G[14]	755.969	889.033	881.023	881.527	880.519	G[17]
G[15]	784.480	860.522	852.513	853.017	852.009	G[16]
V[16]	834.014	832.011	824.002	824.506	823.498	V[15]
L[17]	890.556	782.477	774.468	774.972	773.964	L[14]
P[18]	939.083	725.935	717.926	718.430	717.422	P[13]
N[19]	996.104	677.409	669.399	669.903	668.895	N[12]
I[20]	1052.646	620.387	612.378	612.882	611.874	I[11]
Q[21]	1116.676	563.845	555.836	556.340	555.332	Q[10]
A[22]	1152.194	499.816	491.807	492.310	491.303	A[9]
V[23]	1201.728	464.297	456.288	456.792	455.784	V[8]
L[24]	1258.270	414.763	406.754	407.258	406.250	L[7]
L[25]	1314.812	358.221	350.212	350.716	349.708	L[6]
P[26]	1363.339	301.679	293.670	294.174	293.166	P[5]
K[27]	**1427.386**	253.153	245.143	245.647	244.639	K[4]
K[28]	**1491.434**	189.105	181.096	181.600	180.592	K[3]
T[29]	**1541.958**	125.058	117.048	117.552	116.544	T[2]
E[30]	1606.479	74.534	66.524	67.028	66.021	E[1]

sp|Q8CGP5|H2A1F_MOUSE

[Acetyl]SGRGKQGGKARAKAKTRSSRAGLQFPVGRVHRLLRKGNYSE

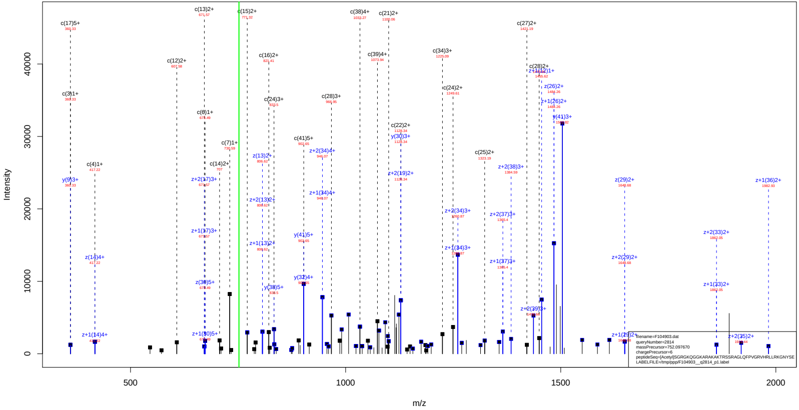

sp|Q8CGP5|H2A1F_MOUSE

[Acetyl]SGRGKQGGKARAKAKTRSSRAGLQFPVGRVHRLLRKGNYSE

- fragmentation table for charge state 1+
- specType=etd
- score=61.55
- F104903.dat
- query=q2814_p1
- precursor=752.097670
- chargePrecursor=6
- itol=0.8

AA	c	y	z+1	z+2	z	AA
S[1]	147.076	4507.521	4491.502	0.000	4490.494	S[41]
G[2]	204.098	4378.478	4362.459	0.000	4361.451	G[40]
R[3]	**360.199**	4321.457	4305.438	4306.446	4304.430	R[39]
G[4]	**417.220**	4165.355	4149.337	4150.345	4148.329	G[38]
K[5]	**545.315**	4108.334	4092.315	4093.323	4091.307	K[37]
Q[6]	**673.374**	3980.239	3964.220	3965.228	3963.212	Q[36]
G[7]	**730.395**	3852.180	3836.162	3837.170	3835.154	G[35]
G[8]	**787.417**	3795.159	3779.140	3780.148	3778.132	G[34]
K[9]	**915.512**	3738.138	3722.119	3723.127	3721.111	K[33]
A[10]	**986.549**	3610.043	3594.024	3595.032	3593.016	A[32]
R[11]	**1142.650**	3539.005	3522.987	3523.995	3521.979	R[31]
A[12]	1213.687	3382.904	3366.886	3367.893	3365.878	A[30]
K[13]	1341.782	3311.867	3295.848	3296.856	3294.841	K[29]
A[14]	1412.819	3183.772	3167.754	3168.761	3166.746	A[28]
K[15]	1540.914	3112.735	3096.716	3097.724	3095.709	K[27]
T[16]	1641.962	2984.640	2968.621	2969.629	2967.614	T[26]
R[17]	1798.063	2883.592	2867.574	2868.582	2866.566	R[25]
S[18]	1885.095	2727.491	2711.473	2712.480	2710.465	S[24]
S[19]	1972.127	2640.459	2624.441	2625.448	2623.433	S[23]
R[20]	2128.228	2553.427	2537.409	2538.416	2536.401	R[22]
A[21]	2199.265	2397.326	2381.307	2382.315	2380.300	A[21]
G[22]	2256.287	2326.289	2310.270	2311.278	2309.263	G[20]
L[23]	2369.371	2269.268	2253.249	2254.257	2252.241	L[19]
Q[24]	2497.429	2156.184	2140.165	2141.173	2139.157	Q[18]
F[25]	2644.498	2028.125	2012.106	2013.114	2011.098	F[17]
P[26]	2741.551	1881.057	1865.038	1866.046	1864.030	P[16]
V[27]	2840.619	1784.004	1767.985	1768.993	1766.977	V[15]
G[28]	2897.641	1684.935	1668.917	1669.924	1667.909	G[14]
R[29]	3053.742	1627.914	1611.895	**1612.903**	1610.887	R[13]
V[30]	3152.810	1471.813	**1455.794**	1456.802	1454.786	V[12]
H[31]	3289.869	1372.744	**1356.726**	1357.734	1355.718	H[11]
R[32]	3445.970	1235.685	1219.667	1220.675	1218.659	R[10]
L[33]	3559.054	1079.584	1063.566	1064.573	1062.558	L[9]
L[34]	3672.138	**966.500**	950.482	951.489	949.474	L[8]
R[35]	3828.239	853.416	837.398	**838.405**	836.390	R[7]
K[36]	3956.334	697.315	681.296	682.304	680.289	K[6]
G[37]	4013.356	569.220	553.201	554.209	552.194	G[5]
N[38]	4127.399	512.199	496.180	497.188	495.172	N[4]
Y[39]	4290.462	398.156	382.137	383.145	381.129	Y[3]
S[40]	4377.494	235.092	219.074	220.082	218.066	S[2]
E[41]	4506.537	148.060	132.042	133.050	131.034	E[1]

sp|Q8CGP5|H2A1F_MOUSE

[Acetyl]SGRGKQGGKARAKAKTRSSRAGLQFPVGRVHRLLRKGNYSE

- fragmentation table for charge state 2+
- specType=etd
- score=61.55
- F104903.dat
- query=q2814_p1
- precursor=752.097670
- chargePrecursor=6
- itol=0.8

AA	c	y	z+1	z+2	z	AA
S[1]	74.042	2254.264	2246.255	0.504	2245.751	S[41]
G[2]	102.553	2189.743	2181.733	0.504	2181.229	G[40]
R[3]	180.603	2161.232	2153.223	2153.726	2152.719	R[39]
G[4]	209.114	2083.181	2075.172	2075.676	2074.668	G[38]
K[5]	273.161	2054.671	2046.661	2047.165	2046.157	K[37]
Q[6]	337.191	1990.623	**1982.614**	**1983.118**	1982.110	Q[36]
G[7]	365.701	1926.594	1918.584	**1919.088**	1918.081	G[35]
G[8]	394.212	1898.083	1890.074	1890.578	1889.570	G[34]
K[9]	458.260	1869.572	**1861.563**	**1862.067**	1861.059	K[33]
A[10]	493.778	1805.525	1797.516	1798.019	1797.012	A[32]
R[11]	**571.829**	1770.006	1761.997	1762.501	1761.493	R[31]
A[12]	**607.347**	1691.956	1683.946	1684.450	1683.443	A[30]
K[13]	**671.395**	1656.437	**1648.428**	**1648.932**	**1647.924**	K[29]
A[14]	**706.913**	1592.390	1584.380	**1584.884**	1583.876	A[28]
K[15]	**770.961**	1556.871	1548.862	**1549.366**	1548.358	K[27]
T[16]	**821.485**	1492.824	**1484.814**	1485.318	**1484.310**	T[26]
R[17]	899.535	1442.300	1434.291	1434.794	1433.787	R[25]
S[18]	943.051	1364.249	**1356.240**	**1356.744**	1355.736	S[24]
S[19]	**986.567**	1320.733	1312.724	**1313.228**	1312.220	S[23]
R[20]	1064.618	1277.217	**1269.208**	**1269.712**	1268.704	R[22]
A[21]	**1100.136**	**1199.167**	**1191.157**	**1191.661**	**1190.653**	A[21]
G[22]	**1128.647**	1163.648	**1155.639**	**1156.143**	1155.135	G[20]
L[23]	**1185.189**	1135.137	1127.128	**1127.632**	1126.624	L[19]
Q[24]	**1249.218**	1078.595	1070.586	1071.090	1070.082	Q[18]
F[25]	**1322.753**	1014.566	**1006.557**	**1007.061**	1006.053	F[17]
P[26]	1371.279	941.032	933.023	933.526	932.519	P[16]
V[27]	**1420.813**	892.506	884.496	885.000	883.992	V[15]
G[28]	**1449.324**	842.971	**834.962**	835.466	**834.458**	G[14]
R[29]	1527.374	814.461	**806.451**	**806.955**	**805.947**	R[13]
V[30]	1576.909	736.410	728.401	728.905	727.897	V[12]
H[31]	1645.438	686.876	678.866	679.370	678.363	H[11]
R[32]	1723.489	618.346	610.337	610.841	609.833	R[10]
L[33]	1780.031	540.296	532.286	532.790	531.783	L[9]
L[34]	1836.573	483.754	475.744	476.248	475.241	L[8]
R[35]	1914.623	427.212	419.202	419.706	418.698	R[7]
K[36]	1978.671	349.161	341.152	341.656	340.648	K[6]
G[37]	2007.181	285.114	277.104	277.608	276.600	G[5]
N[38]	2064.203	256.603	248.594	249.098	248.090	N[4]
Y[39]	2145.735	199.582	191.572	192.076	191.068	Y[3]
S[40]	2189.251	118.050	110.041	110.544	109.537	S[2]
E[41]	2253.772	74.534	66.524	67.028	66.021	E[1]

sp|Q8CGP5|H2A1F_MOUSE

[Acetyl]SGRGKQGGKARAKAKTRSSRAGLQFPVGRVHRLLRKGNYSE

- fragmentation table for charge state 3+
- specType=etd
- score=61.55
- F104903.dat
- query=q2814_p1
- precursor=752.097670
- chargePrecursor=6
- itol=0.8

AA	c	y	z+1	z+2	z	AA
S[1]	49.697	**1503.178**	1497.839	0.672	1497.503	S[41]
G[2]	68.704	1460.164	**1454.825**	0.672	1454.489	G[40]
R[3]	120.738	1441.157	1435.817	**1436.153**	1435.482	R[39]
G[4]	139.745	1389.123	1383.784	**1384.120**	1383.448	G[38]
K[5]	182.443	1370.116	**1364.777**	**1365.113**	1364.441	K[37]
Q[6]	225.130	1327.418	1322.078	**1322.414**	1321.742	Q[36]
G[7]	244.137	1284.732	1279.392	1279.728	1279.056	G[35]
G[8]	263.144	1265.725	**1260.385**	**1260.721**	1260.049	G[34]
K[9]	305.842	1246.717	1241.378	1241.714	1241.042	K[33]
A[10]	329.521	1204.019	**1198.679**	**1199.015**	**1198.344**	A[32]
R[11]	381.555	1180.340	**1175.000**	**1175.336**	1174.664	R[31]
A[12]	405.234	**1128.306**	1122.967	**1123.303**	1122.631	A[30]
K[13]	447.932	1104.627	**1099.288**	**1099.624**	**1098.952**	K[29]
A[14]	471.611	1061.929	**1056.589**	**1056.925**	1056.253	A[28]
K[15]	514.310	**1038.250**	**1032.910**	**1033.246**	**1032.574**	K[27]
T[16]	547.992	995.552	**990.212**	**990.548**	989.876	T[26]
R[17]	600.026	961.869	**956.529**	**956.865**	**956.193**	R[25]
S[18]	629.037	909.835	904.496	904.832	904.160	S[24]
S[19]	658.047	880.825	**875.485**	**875.821**	**875.149**	S[23]
R[20]	**710.081**	851.814	846.474	846.810	846.138	R[22]
A[21]	**733.760**	799.780	794.441	794.777	794.105	A[21]
G[22]	752.767	776.101	**770.762**	**771.098**	770.426	G[20]
L[23]	**790.462**	757.094	751.754	752.090	751.419	L[19]
Q[24]	**833.148**	719.399	714.060	714.396	713.724	Q[18]
F[25]	882.171	676.713	**671.374**	**671.710**	**671.038**	F[17]
P[26]	914.522	627.690	622.351	622.687	622.015	P[16]
V[27]	947.545	595.339	590.000	590.336	589.664	V[15]
G[28]	**966.552**	562.317	556.977	557.313	556.641	G[14]
R[29]	1018.585	543.309	537.970	538.306	537.634	R[13]
V[30]	**1051.608**	491.276	485.936	486.272	485.600	V[12]
H[31]	**1097.294**	458.253	452.913	453.249	452.577	H[11]
R[32]	**1149.328**	412.567	407.227	407.563	406.891	R[10]
L[33]	**1187.023**	**360.533**	355.193	355.529	354.857	L[9]
L[34]	**1224.718**	322.838	317.499	317.835	317.163	L[8]
R[35]	1276.751	285.144	279.804	280.140	279.468	R[7]
K[36]	1319.450	233.110	227.770	228.106	227.434	K[6]
G[37]	1338.457	190.412	185.072	185.408	184.736	G[5]
N[38]	1376.471	171.404	166.065	166.401	165.729	N[4]
Y[39]	1430.826	133.390	128.051	128.386	127.715	Y[3]
S[40]	1459.836	79.036	73.696	74.032	73.360	S[2]
E[41]	1502.850	50.025	44.685	45.021	44.349	E[1]

sp|Q8CGP5|H2A1F_MOUSE

[Acetyl]SGRGKQGGKARAKAKTRSSRAGLQFPVGRVHRLLRKGNYSE

- fragmentation table for charge state 4+
- specType=etd
- score=61.55
- F104903.dat
- query=q2814_p1
- precursor=752.097670
- chargePrecursor=6
- itol=0.8

AA	c	y	z+1	z+2	z	AA
S[1]	37.525	**1127.636**	**1123.631**	0.755	**1123.379**	S[41]
G[2]	51.780	1095.375	**1091.370**	0.755	1091.118	G[40]
R[3]	90.805	1081.120	**1077.115**	**1077.367**	**1076.863**	R[39]
G[4]	105.061	1042.094	**1038.090**	**1038.342**	**1037.838**	G[38]
K[5]	137.084	1027.839	**1023.834**	**1024.086**	**1023.582**	K[37]
Q[6]	169.099	995.815	991.811	992.062	991.559	Q[36]
G[7]	183.354	963.801	**959.796**	**960.048**	**959.544**	G[35]
G[8]	197.610	949.545	**945.541**	**945.792**	**945.289**	G[34]
K[9]	229.633	935.290	931.285	931.537	931.033	K[33]
A[10]	247.393	**903.266**	899.261	899.513	899.009	A[32]
R[11]	286.418	885.507	881.502	881.754	881.250	R[31]
A[12]	304.177	846.482	842.477	842.729	842.225	A[30]
K[13]	336.201	828.722	824.718	824.970	824.466	K[29]
A[14]	353.960	796.699	792.694	792.946	792.442	A[28]
K[15]	385.984	778.939	774.935	775.187	774.683	K[27]
T[16]	411.246	746.915	742.911	743.163	742.659	T[26]
R[17]	450.271	721.654	717.649	717.901	717.397	R[25]
S[18]	472.029	682.628	678.624	678.876	678.372	S[24]
S[19]	493.787	660.870	656.866	657.118	656.614	S[23]
R[20]	532.813	639.112	635.108	635.360	634.856	R[22]
A[21]	550.572	600.087	596.082	596.334	595.830	A[21]
G[22]	564.827	582.328	578.323	578.575	578.071	G[20]
L[23]	593.098	568.072	564.068	564.320	563.816	L[19]
Q[24]	625.113	539.801	535.797	536.049	535.545	Q[18]
F[25]	661.880	507.787	503.782	504.034	503.530	F[17]
P[26]	686.143	471.020	467.015	467.267	466.763	P[16]
V[27]	**710.910**	446.756	442.752	443.004	442.500	V[15]
G[28]	725.166	421.989	**417.985**	418.237	**417.733**	G[14]
R[29]	764.191	407.734	403.729	403.981	403.477	R[13]
V[30]	788.958	368.709	364.704	364.956	364.452	V[12]
H[31]	**823.223**	343.942	339.937	340.189	339.685	H[11]
R[32]	862.248	309.677	305.672	305.924	305.420	R[10]
L[33]	**890.519**	270.652	266.647	266.899	266.395	L[9]
L[34]	918.790	242.381	238.376	238.628	238.124	L[8]
R[35]	957.815	214.110	210.105	210.357	209.853	R[7]
K[36]	989.839	175.084	171.080	171.332	170.828	K[6]
G[37]	1004.094	143.061	139.056	139.308	138.804	G[5]
N[38]	**1032.605**	128.805	124.800	125.052	124.548	N[4]
Y[39]	**1073.371**	100.294	96.290	96.542	96.038	Y[3]
S[40]	1095.129	59.529	55.524	55.776	55.272	S[2]
E[41]	1127.390	37.771	33.766	34.018	33.514	E[1]

sp|Q8CGP5|H2A1F_MOUSE

[Acetyl]SGRGKQGGKARAKAKTRSSRAGLQFPVGRVHRLLRKGNYSE

- fragmentation table for charge state 5+
- specType=etd
- score=61.55
- F104903.dat
- query=q2814_p1
- precursor=752.097670
- chargePrecursor=6
- itol=0.8

AA	c	y	z+1	z+2	z	AA
S[1]	30.221	**902.310**	899.106	0.806	898.905	S[41]
G[2]	41.625	**876.501**	**873.298**	0.806	**873.096**	G[40]
R[3]	72.846	865.097	861.893	862.095	861.692	R[39]
G[4]	84.250	**833.877**	830.673	830.875	830.472	G[38]
K[5]	109.869	822.473	819.269	819.470	819.067	K[37]
Q[6]	135.481	796.854	793.650	793.851	793.448	Q[36]
G[7]	146.885	**771.242**	768.038	768.240	767.837	G[35]
G[8]	158.289	759.838	756.634	756.835	756.432	G[34]
K[9]	183.908	748.433	745.230	745.431	745.028	K[33]
A[10]	198.116	722.814	719.611	719.812	719.409	A[32]
R[11]	229.336	708.607	705.403	705.605	705.202	R[31]
A[12]	243.543	677.387	**674.183**	674.385	**673.981**	A[30]
K[13]	269.162	663.179	659.976	660.177	659.774	K[29]
A[14]	283.370	637.560	634.357	634.558	634.155	A[28]
K[15]	308.989	623.353	620.149	620.351	619.948	K[27]
T[16]	329.198	597.734	594.530	594.732	594.329	T[26]
R[17]	**360.418**	577.524	574.321	574.522	574.119	R[25]
S[18]	377.825	546.304	543.100	543.302	542.899	S[24]
S[19]	395.231	528.898	525.694	525.896	525.492	S[23]
R[20]	426.451	511.491	508.288	508.489	508.086	R[22]
A[21]	440.659	480.271	477.067	477.269	476.866	A[21]
G[22]	452.063	466.064	462.860	463.061	462.658	G[20]
L[23]	474.680	454.659	451.456	451.657	451.254	L[19]
Q[24]	500.292	432.043	428.839	429.040	428.637	Q[18]
F[25]	529.705	406.431	403.227	403.429	403.026	F[17]
P[26]	549.116	377.017	373.813	374.015	373.612	P[16]
V[27]	568.930	357.607	354.403	354.604	354.201	V[15]
G[28]	580.334	337.793	334.589	334.791	334.388	G[14]
R[29]	611.554	326.389	323.185	323.386	322.983	R[13]
V[30]	631.368	295.168	291.965	292.166	291.763	V[12]
H[31]	658.780	275.355	272.151	272.353	271.949	H[11]
R[32]	690.000	247.943	244.739	244.941	244.538	R[10]
L[33]	712.617	216.723	213.519	213.721	213.317	L[9]
L[34]	735.233	194.106	190.902	191.104	190.701	L[8]
R[35]	766.454	171.489	168.285	168.487	168.084	R[7]
K[36]	792.073	140.269	137.065	137.267	136.864	K[6]
G[37]	803.477	114.650	111.446	111.648	111.245	G[5]
N[38]	826.286	103.246	100.042	100.243	99.840	N[4]
Y[39]	858.898	80.437	77.233	77.435	77.032	Y[3]
S[40]	**876.305**	47.824	44.621	44.822	44.419	S[2]
E[41]	**902.113**	30.418	27.214	27.416	27.013	E[1]

sp|Q8CGP5|H2A1F_MOUSE

[Acetyl]SGRGKQGGKARAKAKTRSSRAGLQFPVGRVHRLLRKGNYSE

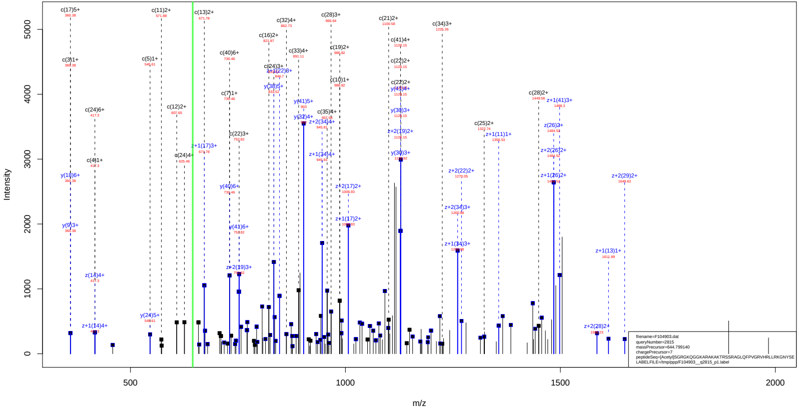

sp|Q8CGP5|H2A1F_MOUSE

[Acetyl]SGRGKQGGKARAKAKTRSSRAGLQFPVGRVHRLLRKGNYSE

- fragmentation table for charge state 1+
- specType=etd
- score=38.34
- F104903.dat
- query=q2815_p1
- precursor=644.799140
- chargePrecursor=7
- itol=0.8

AA	c	y	z+1	z+2	z	AA
S[1]	147.076	4507.521	4491.502	0.000	4490.494	S[41]
G[2]	204.098	4378.478	4362.459	0.000	4361.451	G[40]
R[3]	**360.199**	4321.457	4305.438	4306.446	4304.430	R[39]
G[4]	**417.220**	4165.355	4149.337	4150.345	4148.329	G[38]
K[5]	**545.315**	4108.334	4092.315	4093.323	4091.307	K[37]
Q[6]	**673.374**	3980.239	3964.220	3965.228	3963.212	Q[36]
G[7]	**730.395**	3852.180	3836.162	3837.170	3835.154	G[35]
G[8]	**787.417**	3795.159	3779.140	3780.148	3778.132	G[34]
K[9]	915.512	3738.138	3722.119	3723.127	3721.111	K[33]
A[10]	**986.549**	3610.043	3594.024	3595.032	3593.016	A[32]
R[11]	**1142.650**	3539.005	3522.987	3523.995	3521.979	R[31]
A[12]	1213.687	3382.904	3366.886	3367.893	3365.878	A[30]
K[13]	1341.782	3311.867	3295.848	3296.856	3294.841	K[29]
A[14]	1412.819	3183.772	3167.754	3168.761	3166.746	A[28]
K[15]	1540.914	3112.735	3096.716	3097.724	3095.709	K[27]
T[16]	1641.962	2984.640	2968.621	2969.629	2967.614	T[26]
R[17]	1798.063	2883.592	2867.574	2868.582	2866.566	R[25]
S[18]	1885.095	2727.491	2711.473	2712.480	2710.465	S[24]
S[19]	1972.127	2640.459	2624.441	2625.448	2623.433	S[23]
R[20]	2128.228	2553.427	2537.409	2538.416	2536.401	R[22]
A[21]	2199.265	2397.326	2381.307	2382.315	2380.300	A[21]
G[22]	2256.287	2326.289	2310.270	2311.278	2309.263	G[20]
L[23]	2369.371	2269.268	2253.249	2254.257	2252.241	L[19]
Q[24]	2497.429	2156.184	2140.165	2141.173	2139.157	Q[18]
F[25]	2644.498	2028.125	2012.106	2013.114	2011.098	F[17]
P[26]	2741.551	1881.057	1865.038	1866.046	1864.030	P[16]
V[27]	2840.619	1784.004	1767.985	1768.993	1766.977	V[15]
G[28]	2897.641	1684.935	1668.917	1669.924	1667.909	G[14]
R[29]	3053.742	1627.914	**1611.895**	1612.903	1610.887	R[13]
V[30]	3152.810	1471.813	**1455.794**	**1456.802**	1454.786	V[12]
H[31]	3289.869	1372.744	**1356.726**	1357.734	1355.718	H[11]
R[32]	3445.970	1235.685	**1219.667**	**1220.675**	1218.659	R[10]
L[33]	3559.054	1079.584	1063.566	**1064.573**	1062.558	L[9]
L[34]	3672.138	**966.500**	**950.482**	951.489	949.474	L[8]
R[35]	3828.239	853.416	**837.398**	**838.405**	836.390	R[7]
K[36]	3956.334	697.315	681.296	682.304	680.289	K[6]
G[37]	4013.356	569.220	553.201	554.209	552.194	G[5]
N[38]	4127.399	512.199	496.180	497.188	495.172	N[4]
Y[39]	4290.462	398.156	382.137	383.145	381.129	Y[3]
S[40]	4377.494	235.092	219.074	220.082	218.066	S[2]
E[41]	4506.537	148.060	132.042	133.050	131.034	E[1]

sp|Q8CGP5|H2A1F_MOUSE

[Acetyl]SGRGKQGGKARAKAKTRSSRAGLQFPVGRVHRLLRKGNYSE

- fragmentation table for charge state 2+
- specType=etd
- score=38.34
- F104903.dat
- query=q2815_p1
- precursor=644.799140
- chargePrecursor=7
- itol=0.8

AA	c	y	z+1	z+2	z	AA
S[1]	74.042	2254.264	2246.255	0.504	2245.751	S[41]
G[2]	102.553	2189.743	2181.733	0.504	2181.229	G[40]
R[3]	180.603	2161.232	2153.223	2153.726	2152.719	R[39]
G[4]	209.114	2083.181	2075.172	2075.676	2074.668	G[38]
K[5]	273.161	2054.671	2046.661	2047.165	2046.157	K[37]
Q[6]	337.191	1990.623	1982.614	1983.118	1982.110	Q[36]
G[7]	365.701	1926.594	1918.584	1919.088	1918.081	G[35]
G[8]	394.212	1898.083	1890.074	1890.578	1889.570	G[34]
K[9]	**458.260**	1869.572	1861.563	1862.067	1861.059	K[33]
A[10]	493.778	1805.525	1797.516	1798.019	1797.012	A[32]
R[11]	**571.829**	1770.006	1761.997	1762.501	1761.493	R[31]
A[12]	**607.347**	1691.956	1683.946	1684.450	1683.443	A[30]
K[13]	**671.395**	1656.437	1648.428	**1648.932**	1647.924	K[29]
A[14]	**706.913**	1592.390	1584.380	**1584.884**	1583.876	A[28]
K[15]	**770.961**	1556.871	1548.862	1549.366	1548.358	K[27]
T[16]	**821.485**	1492.824	**1484.814**	**1485.318**	**1484.310**	T[26]
R[17]	899.535	1442.300	1434.291	1434.794	1433.787	R[25]
S[18]	**943.051**	1364.249	**1356.240**	**1356.744**	**1355.736**	S[24]
S[19]	**986.567**	1320.733	1312.724	**1313.228**	1312.220	S[23]
R[20]	**1064.618**	1277.217	1269.208	**1269.712**	1268.704	R[22]
A[21]	**1100.136**	**1199.167**	**1191.157**	**1191.661**	1190.653	A[21]
G[22]	**1128.647**	1163.648	**1155.639**	**1156.143**	1155.135	G[20]
L[23]	1185.189	1135.137	1127.128	**1127.632**	1126.624	L[19]
Q[24]	1249.218	1078.595	**1070.586**	**1071.090**	**1070.082**	Q[18]
F[25]	**1322.753**	1014.566	**1006.557**	**1007.061**	1006.053	F[17]
P[26]	1371.279	**941.032**	933.023	933.526	**932.519**	P[16]
V[27]	1420.813	892.506	884.496	**885.000**	883.992	V[15]
G[28]	**1449.324**	842.971	**834.962**	**835.466**	834.458	G[14]
R[29]	1527.374	**814.461**	**806.451**	**806.955**	**805.947**	R[13]
V[30]	1576.909	736.410	728.401	728.905	727.897	V[12]
H[31]	1645.438	686.876	**678.866**	**679.370**	678.363	H[11]
R[32]	1723.489	618.346	610.337	610.841	609.833	R[10]
L[33]	1780.031	540.296	532.286	532.790	531.783	L[9]
L[34]	1836.573	483.754	475.744	476.248	475.241	L[8]
R[35]	1914.623	427.212	419.202	419.706	418.698	R[7]
K[36]	1978.671	349.161	341.152	341.656	340.648	K[6]
G[37]	2007.181	285.114	277.104	277.608	276.600	G[5]
N[38]	2064.203	256.603	248.594	249.098	248.090	N[4]
Y[39]	2145.735	199.582	191.572	192.076	191.068	Y[3]
S[40]	2189.251	118.050	110.041	110.544	109.537	S[2]
E[41]	2253.772	74.534	66.524	67.028	66.021	E[1]

sp|Q8CGP5|H2A1F_MOUSE

[Acetyl]SGRGKQGGKARAKAKTRSSRAGLQFPVGRVHRLLRKGNYSE

- fragmentation table for charge state 3+
- specType=etd
- score=38.34
- F104903.dat
- query=q2815_p1
- precursor=644.799140
- chargePrecursor=7
- itol=0.8

AA	c	y	z+1	z+2	z	AA
S[1]	49.697	1503.178	**1497.839**	0.672	**1497.503**	S[41]
G[2]	68.704	1460.164	1454.825	0.672	1454.489	G[40]
R[3]	120.738	**1441.157**	**1435.817**	**1436.153**	**1435.482**	R[39]
G[4]	139.745	1389.123	1383.784	**1384.120**	1383.448	G[38]
K[5]	182.443	1370.116	1364.777	**1365.113**	1364.441	K[37]
Q[6]	225.130	1327.418	**1322.078**	**1322.414**	1321.742	Q[36]
G[7]	244.137	1284.732	1279.392	1279.728	1279.056	G[35]
G[8]	263.144	1265.725	**1260.385**	**1260.721**	1260.049	G[34]
K[9]	305.842	1246.717	1241.378	1241.714	1241.042	K[33]
A[10]	329.521	1204.019	**1198.679**	**1199.015**	1198.344	A[32]
R[11]	381.555	1180.340	**1175.000**	1175.336	**1174.664**	R[31]
A[12]	405.234	**1128.306**	1122.967	1123.303	1122.631	A[30]
K[13]	447.932	1104.627	**1099.288**	**1099.624**	**1098.952**	K[29]
A[14]	471.611	1061.929	**1056.589**	**1056.925**	**1056.253**	A[28]
K[15]	514.310	**1038.250**	**1032.910**	**1033.246**	1032.574	K[27]
T[16]	547.992	995.552	**990.212**	**990.548**	**989.876**	T[26]
R[17]	600.026	**961.869**	956.529	**956.865**	956.193	R[25]
S[18]	629.037	909.835	904.496	904.832	904.160	S[24]
S[19]	**658.047**	880.825	**875.485**	**875.821**	875.149	S[23]
R[20]	**710.081**	851.814	**846.474**	**846.810**	**846.138**	R[22]
A[21]	**733.760**	799.780	794.441	**794.777**	**794.105**	A[21]
G[22]	**752.767**	776.101	**770.762**	**771.098**	**770.426**	G[20]
L[23]	790.462	**757.094**	**751.754**	**752.090**	**751.419**	L[19]
Q[24]	**833.148**	719.399	714.060	714.396	713.724	Q[18]
F[25]	882.171	676.713	**671.374**	**671.710**	**671.038**	F[17]
P[26]	**914.522**	627.690	622.351	622.687	622.015	P[16]
V[27]	947.545	595.339	590.000	590.336	589.664	V[15]
G[28]	**966.552**	562.317	556.977	557.313	556.641	G[14]
R[29]	1018.585	543.309	537.970	538.306	537.634	R[13]
V[30]	**1051.608**	491.276	485.936	486.272	485.600	V[12]
H[31]	1097.294	**458.253**	452.913	453.249	452.577	H[11]
R[32]	**1149.328**	412.567	407.227	407.563	406.891	R[10]
L[33]	1187.023	**360.533**	355.193	355.529	354.857	L[9]
L[34]	**1224.718**	322.838	317.499	317.835	317.163	L[8]
R[35]	1276.751	285.144	279.804	280.140	279.468	R[7]
K[36]	1319.450	233.110	227.770	228.106	227.434	K[6]
G[37]	1338.457	190.412	185.072	185.408	184.736	G[5]
N[38]	1376.471	171.404	166.065	166.401	165.729	N[4]
Y[39]	1430.826	133.390	128.051	128.386	127.715	Y[3]
S[40]	1459.836	79.036	73.696	74.032	73.360	S[2]
E[41]	1502.850	50.025	44.685	45.021	44.349	E[1]

sp|Q8CGP5|H2A1F_MOUSE

[Acetyl]SGRGKQGGKARAKAKTRSSRAGLQFPVGRVHRLLRKGNYSE

- fragmentation table for charge state 4+
- specType=etd
- score=38.34
- F104903.dat
- query=q2815_p1
- precursor=644.799140
- chargePrecursor=7
- itol=0.8

AA	c	y	z+1	z+2	z	AA
S[1]	37.525	**1127.636**	1123.631	0.755	1123.379	S[41]
G[2]	51.780	1095.375	**1091.370**	0.755	**1091.118**	G[40]
R[3]	90.805	**1081.120**	**1077.115**	**1077.367**	1076.863	R[39]
G[4]	105.061	1042.094	**1038.090**	**1038.342**	1037.838	G[38]
K[5]	137.084	1027.839	**1023.834**	**1024.086**	**1023.582**	K[37]
Q[6]	169.099	995.815	**991.811**	992.062	**991.559**	Q[36]
G[7]	183.354	963.801	**959.796**	**960.048**	**959.544**	G[35]
G[8]	197.610	949.545	**945.541**	**945.792**	**945.289**	G[34]
K[9]	229.633	**935.290**	**931.285**	**931.537**	931.033	K[33]
A[10]	247.393	**903.266**	899.261	899.513	899.009	A[32]
R[11]	286.418	**885.507**	881.502	881.754	881.250	R[31]
A[12]	304.177	**846.482**	842.477	842.729	842.225	A[30]
K[13]	336.201	828.722	**824.718**	**824.970**	**824.466**	K[29]
A[14]	353.960	796.699	**792.694**	**792.946**	792.442	A[28]
K[15]	385.984	778.939	774.935	775.187	774.683	K[27]
T[16]	411.246	746.915	**742.911**	**743.163**	**742.659**	T[26]
R[17]	450.271	721.654	**717.649**	**717.901**	**717.397**	R[25]
S[18]	472.029	682.628	**678.624**	**678.876**	678.372	S[24]
S[19]	493.787	660.870	656.866	657.118	656.614	S[23]
R[20]	532.813	639.112	635.108	635.360	634.856	R[22]
A[21]	550.572	600.087	596.082	596.334	595.830	A[21]
G[22]	564.827	582.328	578.323	578.575	578.071	G[20]
L[23]	593.098	568.072	564.068	564.320	563.816	L[19]
Q[24]	**625.113**	539.801	535.797	536.049	535.545	Q[18]
F[25]	661.880	507.787	503.782	504.034	503.530	F[17]
P[26]	686.143	471.020	467.015	467.267	466.763	P[16]
V[27]	**710.910**	446.756	442.752	443.004	442.500	V[15]
G[28]	**725.166**	421.989	**417.985**	418.237	**417.733**	G[14]
R[29]	764.191	407.734	403.729	403.981	403.477	R[13]
V[30]	**788.958**	368.709	364.704	364.956	364.452	V[12]
H[31]	823.223	343.942	339.937	340.189	339.685	H[11]
R[32]	**862.248**	309.677	305.672	305.924	305.420	R[10]
L[33]	**890.519**	270.652	266.647	266.899	266.395	L[9]
L[34]	**918.790**	242.381	238.376	238.628	238.124	L[8]
R[35]	**957.815**	214.110	210.105	210.357	209.853	R[7]
K[36]	**989.839**	175.084	171.080	171.332	170.828	K[6]
G[37]	1004.094	143.061	139.056	139.308	138.804	G[5]
N[38]	1032.605	128.805	124.800	125.052	124.548	N[4]
Y[39]	1073.371	100.294	96.290	96.542	96.038	Y[3]
S[40]	1095.129	59.529	55.524	55.776	55.272	S[2]
E[41]	**1127.390**	37.771	33.766	34.018	33.514	E[1]

sp|Q8CGP5|H2A1F_MOUSE

[Acetyl]SGRGKQGGKARAKAKTRSSRAGLQFPVGRVHRLLRKGNYSE

- fragmentation table for charge state 5+
- specType=etd
- score=38.34
- F104903.dat
- query=q2815_p1
- precursor=644.799140
- chargePrecursor=7
- itol=0.8

AA	c	y	z+1	z+2	z	AA
S[1]	30.221	**902.310**	899.106	0.806	898.905	S[41]
G[2]	41.625	**876.501**	**873.298**	0.806	**873.096**	G[40]
R[3]	72.846	865.097	861.893	**862.095**	861.692	R[39]
G[4]	84.250	**833.877**	830.673	830.875	830.472	G[38]
K[5]	109.869	**822.473**	819.269	819.470	819.067	K[37]
Q[6]	135.481	796.854	**793.650**	**793.851**	**793.448**	Q[36]
G[7]	146.885	**771.242**	768.038	768.240	767.837	G[35]
G[8]	158.289	759.838	**756.634**	**756.835**	756.432	G[34]
K[9]	183.908	748.433	**745.230**	**745.431**	**745.028**	K[33]
A[10]	198.116	722.814	719.611	719.812	719.409	A[32]
R[11]	229.336	708.607	705.403	705.605	705.202	R[31]
A[12]	243.543	677.387	**674.183**	674.385	**673.981**	A[30]
K[13]	269.162	663.179	**659.976**	**660.177**	**659.774**	K[29]
A[14]	283.370	637.560	634.357	634.558	634.155	A[28]
K[15]	308.989	623.353	620.149	620.351	619.948	K[27]
T[16]	329.198	597.734	594.530	594.732	594.329	T[26]
R[17]	**360.418**	577.524	574.321	574.522	574.119	R[25]
S[18]	377.825	**546.304**	543.100	543.302	542.899	S[24]
S[19]	395.231	528.898	525.694	525.896	525.492	S[23]
R[20]	426.451	511.491	508.288	508.489	508.086	R[22]
A[21]	440.659	480.271	477.067	477.269	476.866	A[21]
G[22]	452.063	466.064	462.860	463.061	462.658	G[20]
L[23]	474.680	454.659	451.456	451.657	451.254	L[19]
Q[24]	500.292	432.043	428.839	429.040	428.637	Q[18]
F[25]	529.705	406.431	403.227	403.429	403.026	F[17]
P[26]	549.116	377.017	373.813	374.015	373.612	P[16]
V[27]	568.930	357.607	354.403	354.604	354.201	V[15]
G[28]	580.334	337.793	334.589	334.791	334.388	G[14]
R[29]	611.554	326.389	323.185	323.386	322.983	R[13]
V[30]	631.368	295.168	291.965	292.166	291.763	V[12]
H[31]	**658.780**	275.355	272.151	272.353	271.949	H[11]
R[32]	690.000	247.943	244.739	244.941	244.538	R[10]
L[33]	712.617	216.723	213.519	213.721	213.317	L[9]
L[34]	735.233	194.106	190.902	191.104	190.701	L[8]
R[35]	766.454	171.489	168.285	168.487	168.084	R[7]
K[36]	792.073	140.269	137.065	137.267	136.864	K[6]
G[37]	803.477	114.650	111.446	111.648	111.245	G[5]
N[38]	826.286	103.246	100.042	100.243	99.840	N[4]
Y[39]	858.898	80.437	77.233	77.435	77.032	Y[3]
S[40]	**876.305**	47.824	44.621	44.822	44.419	S[2]
E[41]	902.113	30.418	27.214	27.416	27.013	E[1]

sp|Q8CGP5|H2A1F_MOUSE

[Acetyl]SGRGKQGGKARAKAKTRSSRAGLQFPVGRVHRLLRKGNYSE

- fragmentation table for charge state 6+
- specType=etd
- score=38.34
- F104903.dat
- query=q2815_p1
- precursor=644.799140
- chargePrecursor=7
- itol=0.8

AA	c	y	z+1	z+2	z	AA
S[1]	25.352	**752.093**	749.423	0.839	749.255	S[41]
G[2]	34.856	**730.586**	727.916	0.839	727.748	G[40]
R[3]	60.873	721.082	**718.412**	**718.580**	**718.244**	R[39]
G[4]	70.376	695.065	692.396	692.563	692.228	G[38]
K[5]	91.725	685.562	682.892	683.060	682.724	K[37]
Q[6]	113.068	664.213	661.543	661.711	661.375	Q[36]
G[7]	122.572	642.869	640.200	640.368	640.032	G[35]
G[8]	132.076	633.366	630.696	630.864	630.528	G[34]
K[9]	153.425	623.862	621.193	621.360	621.025	K[33]
A[10]	165.264	602.513	599.843	600.011	599.675	A[32]
R[11]	191.281	590.674	588.004	588.172	587.836	R[31]
A[12]	203.121	564.657	561.987	562.155	561.819	A[30]
K[13]	224.470	552.817	550.147	550.315	549.980	K[29]
A[14]	236.309	531.468	528.798	528.966	528.630	A[28]
K[15]	257.658	519.629	516.959	517.127	516.791	K[27]
T[16]	274.500	498.279	495.610	495.778	495.442	T[26]
R[17]	300.517	481.438	478.768	478.936	478.600	R[25]
S[18]	315.022	455.421	452.752	452.919	452.584	S[24]
S[19]	329.527	440.916	438.246	438.414	438.078	S[23]
R[20]	355.544	426.411	423.741	423.909	423.573	R[22]
A[21]	367.384	400.394	397.724	397.892	397.556	A[21]
G[22]	376.887	388.554	385.884	386.052	385.716	G[20]
L[23]	395.735	379.051	376.381	376.549	376.213	L[19]
Q[24]	**417.078**	**360.203**	357.534	357.702	357.366	Q[18]
F[25]	441.589	338.860	336.190	336.358	336.022	F[17]
P[26]	457.765	314.349	311.679	311.847	311.511	P[16]
V[27]	474.276	298.173	295.504	295.672	295.336	V[15]
G[28]	483.779	281.662	278.992	279.160	278.824	G[14]
R[29]	509.796	272.158	269.489	269.657	269.321	R[13]
V[30]	526.308	246.142	243.472	243.640	243.304	V[12]
H[31]	549.151	229.630	226.960	227.128	226.792	H[11]
R[32]	575.168	206.787	204.117	204.285	203.949	R[10]
L[33]	594.015	180.770	178.100	178.268	177.932	L[9]
L[34]	612.862	161.923	159.253	159.421	159.085	L[8]
R[35]	638.879	143.075	140.406	140.574	140.238	R[7]
K[36]	**660.228**	117.059	114.389	114.557	114.221	K[6]
G[37]	669.732	95.709	93.040	93.208	92.872	G[5]
N[38]	688.739	86.206	83.536	83.704	83.368	N[4]
Y[39]	715.916	67.199	64.529	64.697	64.361	Y[3]
S[40]	**730.422**	40.021	37.352	37.520	37.184	S[2]
E[41]	**751.929**	25.516	22.846	23.014	22.678	E[1]

sp|Q8BFU2|H2A3_MOUSE

[Acetyl]SGRGK $^{Acetyl}_{42.01}$ QGGKARAKAKSRSSRAGLQFPVGRVHRLLRKGNYSE

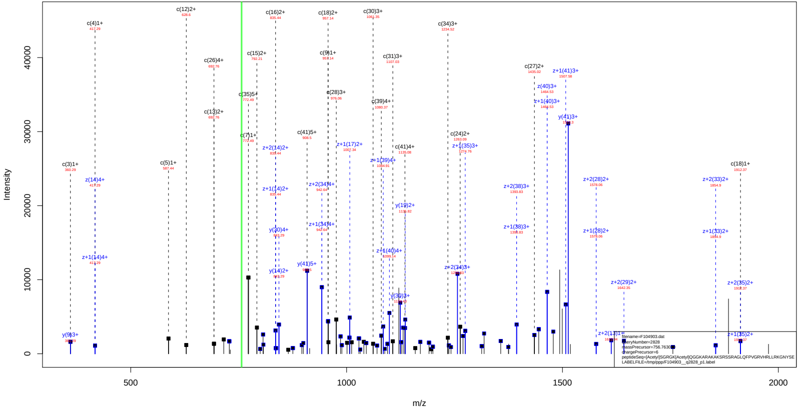

sp|Q8BFU2|H2A3_MOUSE

[Acetyl]SGRGK $^{\text{Acetyl}}_{42.01}$ QGGKARAKAKSRSSRAGLQFPVGRVHRLLRKGNYSE

- fragmentation table for charge state 1+
- specType=etd
- score=62.47
- F104903.dat
- query=q2828_p1
- precursor=756.763030
- chargePrecursor=6
- itol=0.8

AA	c	y	z+1	z+2	z	AA
S[1]	147.076	4535.516	4519.497	0.000	4518.489	S[41]
G[2]	204.098	4406.473	4390.454	0.000	4389.446	G[40]
R[3]	**360.199**	4349.451	4333.433	4334.441	4332.425	R[39]
G[4]	**417.220**	4193.350	4177.332	4178.339	4176.324	G[38]
K[5]	**587.326**	4136.329	4120.310	4121.318	4119.302	K[37]
Q[6]	**715.385**	3966.223	3950.205	3951.212	3949.197	Q[36]
G[7]	**772.406**	3838.165	3822.146	3823.154	3821.138	G[35]
G[8]	829.427	3781.143	3765.125	3766.132	3764.117	G[34]
K[9]	**957.522**	3724.122	3708.103	3709.111	3707.095	K[33]
A[10]	**1028.560**	3596.027	3580.008	3581.016	3579.000	A[32]
R[11]	1184.661	3524.990	3508.971	3509.979	3507.963	R[31]
A[12]	1255.698	3368.889	3352.870	3353.878	3351.862	A[30]
K[13]	1383.793	3297.852	3281.833	3282.841	3280.825	K[29]
A[14]	1454.830	3169.757	3153.738	3154.746	3152.730	A[28]
K[15]	1582.925	3098.719	3082.701	3083.709	3081.693	K[27]
S[16]	1669.957	2970.625	2954.606	2955.614	2953.598	S[26]
R[17]	1826.058	2883.592	2867.574	2868.582	2866.566	R[25]
S[18]	**1913.090**	2727.491	2711.473	2712.480	2710.465	S[24]
S[19]	2000.122	2640.459	2624.441	2625.448	2623.433	S[23]
R[20]	2156.223	2553.427	2537.409	2538.416	2536.401	R[22]
A[21]	2227.260	2397.326	2381.307	2382.315	2380.300	A[21]
G[22]	2284.282	2326.289	2310.270	2311.278	2309.263	G[20]
L[23]	2397.366	2269.268	2253.249	2254.257	2252.241	L[19]
Q[24]	2525.424	2156.184	2140.165	2141.173	2139.157	Q[18]
F[25]	2672.493	2028.125	2012.106	2013.114	2011.098	F[17]
P[26]	2769.546	1881.057	1865.038	1866.046	1864.030	P[16]
V[27]	2868.614	1784.004	1767.985	1768.993	1766.977	V[15]
G[28]	2925.635	1684.935	1668.917	1669.924	1667.909	G[14]
R[29]	3081.737	1627.914	1611.895	**1612.903**	1610.887	R[13]
V[30]	3180.805	1471.813	1455.794	1456.802	1454.786	V[12]
H[31]	3317.864	1372.744	**1356.726**	**1357.734**	1355.718	H[11]
R[32]	3473.965	1235.685	1219.667	1220.675	1218.659	R[10]
L[33]	3587.049	**1079.584**	1063.566	1064.573	1062.558	L[9]
L[34]	3700.133	966.500	950.482	951.489	949.474	L[8]
R[35]	3856.234	853.416	837.398	838.405	**836.390**	R[7]
K[36]	3984.329	697.315	681.296	682.304	680.289	K[6]
G[37]	4041.351	569.220	553.201	554.209	552.194	G[5]
N[38]	4155.394	512.199	496.180	497.188	495.172	N[4]
Y[39]	4318.457	398.156	382.137	383.145	381.129	Y[3]
S[40]	4405.489	235.092	219.074	220.082	218.066	S[2]
E[41]	4534.532	148.060	132.042	133.050	131.034	E[1]

sp|Q8BFU2|H2A3_MOUSE

[Acetyl]SGRGK $^{Acetyl}_{42.01}$ QGGKARAKAKSRSSRAGLQFPVGRVHRLLRKGNYSE

- fragmentation table for charge state 2+
- specType=etd
- score=62.47
- F104903.dat
- query=q2828_p1
- precursor=756.763030
- chargePrecursor=6
- itol=0.8

AA	c	y	z+1	z+2	z	AA
S[1]	74.042	2268.261	2260.252	0.504	2259.748	S[41]
G[2]	102.553	2203.740	2195.731	0.504	2195.227	G[40]
R[3]	180.603	2175.229	2167.220	2167.724	2166.716	R[39]
G[4]	209.114	2097.179	2089.169	2089.673	2088.666	G[38]
K[5]	294.167	2068.668	2060.659	2061.163	2060.155	K[37]
Q[6]	358.196	1983.615	1975.606	1976.110	1975.102	Q[36]
G[7]	386.707	1919.586	**1911.577**	**1912.081**	1911.073	G[35]
G[8]	415.217	1891.075	1883.066	1883.570	1882.562	G[34]
K[9]	479.265	1862.565	**1854.555**	**1855.059**	1854.051	K[33]
A[10]	514.783	1798.517	1790.508	1791.012	1790.004	A[32]
R[11]	592.834	1762.999	**1754.989**	**1755.493**	1754.485	R[31]
A[12]	**628.353**	1684.948	1676.939	1677.443	1676.435	A[30]
K[13]	**692.400**	1649.429	1641.420	**1641.924**	1640.916	K[29]
A[14]	**727.919**	1585.382	**1577.373**	**1577.876**	1576.869	A[28]
K[15]	**791.966**	1549.863	1541.854	1542.358	1541.350	K[27]
S[16]	**835.482**	1485.816	1477.807	**1478.310**	1477.303	S[26]
R[17]	913.533	1442.300	**1434.291**	**1434.794**	1433.787	R[25]
S[18]	**957.049**	1364.249	**1356.240**	**1356.744**	1355.736	S[24]
S[19]	**1000.565**	1320.733	**1312.724**	**1313.228**	**1312.220**	S[23]
R[20]	1078.615	1277.217	**1269.208**	**1269.712**	1268.704	R[22]
A[21]	1114.134	**1199.167**	**1191.157**	**1191.661**	**1190.653**	A[21]
G[22]	1142.644	1163.648	1155.639	1156.143	1155.135	G[20]
L[23]	**1199.187**	**1135.137**	**1127.128**	**1127.632**	**1126.624**	L[19]
Q[24]	**1263.216**	1078.595	**1070.586**	**1071.090**	**1070.082**	Q[18]
F[25]	1336.750	1014.566	**1006.557**	**1007.061**	**1006.053**	F[17]
P[26]	1385.276	941.032	933.023	933.526	932.519	P[16]
V[27]	**1434.811**	892.506	884.496	885.000	883.992	V[15]
G[28]	1463.321	**842.971**	**834.962**	**835.466**	834.458	G[14]
R[29]	1541.372	814.461	**806.451**	**806.955**	**805.947**	R[13]
V[30]	1590.906	736.410	**728.401**	**728.905**	**727.897**	V[12]
H[31]	1659.436	686.876	678.866	679.370	678.363	H[11]
R[32]	1737.486	618.346	610.337	610.841	609.833	R[10]
L[33]	1794.028	540.296	532.286	532.790	531.783	L[9]
L[34]	1850.570	483.754	475.744	476.248	475.241	L[8]
R[35]	1928.621	427.212	419.202	419.706	418.698	R[7]
K[36]	1992.668	349.161	341.152	341.656	340.648	K[6]
G[37]	2021.179	285.114	277.104	277.608	276.600	G[5]
N[38]	2078.200	256.603	248.594	249.098	248.090	N[4]
Y[39]	2159.732	199.582	191.572	192.076	191.068	Y[3]
S[40]	2203.248	118.050	110.041	110.544	109.537	S[2]
E[41]	2267.769	74.534	66.524	67.028	66.021	E[1]

sp|Q8BFU2|H2A3_MOUSE

[Acetyl]SGRGK $^{\text{Acetyl}}_{42.01}$ QGGKARAKAKSRSSRAGLQFPVGRVHRLLRKGNYSE

- fragmentation table for charge state 3+
- specType=etd
- score=62.47
- F104903.dat
- query=q2828_p1
- precursor=756.763030
- chargePrecursor=6
- itol=0.8

AA	c	y	z+1	z+2	z	AA
S[1]	49.697	**1512.510**	**1507.170**	0.672	**1506.835**	S[41]
G[2]	68.704	1469.496	**1464.156**	0.672	**1463.820**	G[40]
R[3]	120.738	1450.489	**1445.149**	**1445.485**	**1444.813**	R[39]
G[4]	139.745	1398.455	**1393.115**	**1393.451**	1392.779	G[38]
K[5]	196.447	1379.448	**1374.108**	**1374.444**	1373.772	K[37]
Q[6]	239.133	1322.746	1317.406	**1317.742**	1317.070	Q[36]
G[7]	258.140	1280.060	**1274.720**	**1275.056**	**1274.384**	G[35]
G[8]	277.147	1261.053	1255.713	**1256.049**	1255.377	G[34]
K[9]	319.846	**1242.045**	**1236.706**	**1237.042**	**1236.370**	K[33]
A[10]	343.525	**1199.347**	1194.008	1194.344	1193.672	A[32]
R[11]	395.558	1175.668	**1170.329**	**1170.664**	1169.993	R[31]
A[12]	419.237	**1123.634**	1118.295	1118.631	1117.959	A[30]
K[13]	461.936	1099.955	**1094.616**	**1094.952**	**1094.280**	K[29]
A[14]	485.615	1057.257	1051.917	1052.253	1051.582	A[28]
K[15]	528.313	1033.578	**1028.238**	**1028.574**	**1027.902**	K[27]
S[16]	557.324	990.880	**985.540**	**985.876**	**985.204**	S[26]
R[17]	609.358	961.869	**956.529**	**956.865**	956.193	R[25]
S[18]	638.368	909.835	904.496	904.832	904.160	S[24]
S[19]	667.379	880.825	**875.485**	**875.821**	**875.149**	S[23]
R[20]	719.413	851.814	846.474	846.810	846.138	R[22]
A[21]	743.092	**799.780**	794.441	794.777	794.105	A[21]
G[22]	762.099	776.101	770.762	771.098	770.426	G[20]
L[23]	**799.793**	757.094	751.754	752.090	751.419	L[19]
Q[24]	842.480	719.399	714.060	714.396	713.724	Q[18]
F[25]	891.502	676.713	671.374	671.710	671.038	F[17]
P[26]	923.853	627.690	622.351	622.687	622.015	P[16]
V[27]	**956.876**	595.339	590.000	590.336	589.664	V[15]
G[28]	**975.883**	562.317	556.977	557.313	556.641	G[14]
R[29]	**1027.917**	543.309	537.970	538.306	537.634	R[13]
V[30]	**1060.940**	491.276	485.936	486.272	485.600	V[12]
H[31]	**1106.626**	458.253	452.913	453.249	452.577	H[11]
R[32]	**1158.660**	412.567	407.227	407.563	406.891	R[10]
L[33]	**1196.355**	**360.533**	355.193	355.529	354.857	L[9]
L[34]	**1234.049**	322.838	317.499	317.835	317.163	L[8]
R[35]	1286.083	285.144	279.804	280.140	279.468	R[7]
K[36]	1328.781	233.110	227.770	228.106	227.434	K[6]
G[37]	1347.788	190.412	185.072	185.408	184.736	G[5]
N[38]	1385.803	171.404	166.065	166.401	165.729	N[4]
Y[39]	1440.157	133.390	128.051	128.386	127.715	Y[3]
S[40]	1469.168	79.036	73.696	74.032	73.360	S[2]
E[41]	1512.182	50.025	44.685	45.021	44.349	E[1]

sp|Q8BFU2|H2A3_MOUSE

[Acetyl]SGRGK$^{Acetyl}_{42.01}$QGGKARAKAKSRSSRAGLQFPVGRVHRLLRKGNYSE

- fragmentation table for charge state 4+
- specType=etd
- score=62.47
- F104903.dat
- query=q2828_p1
- precursor=756.763030
- chargePrecursor=6
- itol=0.8

AA	c	y	z+1	z+2	z	AA
S[1]	37.525	**1134.634**	**1130.630**	0.755	**1130.378**	S[41]
G[2]	51.780	1102.374	**1098.369**	0.755	1098.117	G[40]
R[3]	90.805	**1088.118**	**1084.114**	**1084.366**	1083.862	R[39]
G[4]	105.061	1049.093	**1045.088**	**1045.340**	**1044.836**	G[38]
K[5]	147.587	1034.838	**1030.833**	**1031.085**	1030.581	K[37]
Q[6]	179.602	992.311	**988.307**	**988.559**	988.055	Q[36]
G[7]	193.857	960.297	956.292	**956.544**	956.040	G[35]
G[8]	208.112	946.041	**942.037**	**942.289**	941.785	G[34]
K[9]	240.136	931.786	927.781	928.033	927.529	K[33]
A[10]	257.895	**899.762**	**895.757**	**896.009**	895.506	A[32]
R[11]	296.921	882.003	877.998	878.250	877.746	R[31]
A[12]	314.680	**842.978**	838.973	839.225	838.721	A[30]
K[13]	346.704	825.218	821.214	821.466	820.962	K[29]
A[14]	364.463	793.195	789.190	789.442	788.938	A[28]
K[15]	396.487	775.435	771.431	771.683	771.179	K[27]
S[16]	418.245	743.412	739.407	739.659	739.155	S[26]
R[17]	457.270	721.654	717.649	717.901	717.397	R[25]
S[18]	479.028	682.628	678.624	678.876	678.372	S[24]
S[19]	500.786	660.870	656.866	657.118	656.614	S[23]
R[20]	539.811	639.112	635.108	635.360	634.856	R[22]
A[21]	557.571	600.087	596.082	596.334	595.830	A[21]
G[22]	571.826	582.328	578.323	578.575	578.071	G[20]
L[23]	600.097	568.072	564.068	564.320	563.816	L[19]
Q[24]	632.112	539.801	535.797	536.049	535.545	Q[18]
F[25]	668.879	507.787	503.782	504.034	503.530	F[17]
P[26]	**693.142**	471.020	467.015	467.267	466.763	P[16]
V[27]	717.909	446.756	442.752	443.004	442.500	V[15]
G[28]	732.164	421.989	**417.985**	418.237	**417.733**	G[14]
R[29]	771.190	407.734	403.729	403.981	403.477	R[13]
V[30]	795.957	368.709	364.704	364.956	364.452	V[12]
H[31]	830.221	343.942	339.937	340.189	339.685	H[11]
R[32]	869.247	309.677	305.672	305.924	305.420	R[10]
L[33]	897.518	270.652	266.647	266.899	266.395	L[9]
L[34]	925.789	242.381	238.376	238.628	238.124	L[8]
R[35]	964.814	214.110	210.105	210.357	209.853	R[7]
K[36]	996.838	175.084	171.080	171.332	170.828	K[6]
G[37]	**1011.093**	143.061	139.056	139.308	138.804	G[5]
N[38]	**1039.604**	128.805	124.800	125.052	124.548	N[4]
Y[39]	**1080.370**	100.294	96.290	96.542	96.038	Y[3]
S[40]	1102.128	59.529	55.524	55.776	55.272	S[2]
E[41]	**1134.388**	37.771	33.766	34.018	33.514	E[1]

sp|Q8BFU2|H2A3_MOUSE

[Acetyl]SGRGK $^{Acetyl}_{42.01}$ QGGKARAKAKSRSSRAGLQFPVGRVHRLLRKGNYSE

- fragmentation table for charge state 5+
- specType=etd
- score=62.47
- F104903.dat
- query=q2828_p1
- precursor=756.763030
- chargePrecursor=6
- itol=0.8

AA	c	y	z+1	z+2	z	AA
S[1]	30.221	**907.909**	904.705	0.806	904.504	S[41]
G[2]	41.625	882.100	878.897	0.806	878.695	G[40]
R[3]	72.846	870.696	867.492	867.694	867.291	R[39]
G[4]	84.250	839.476	**836.272**	**836.474**	**836.071**	G[38]
K[5]	118.271	828.072	824.868	825.069	824.666	K[37]
Q[6]	143.883	794.050	790.847	791.048	790.645	Q[36]
G[7]	155.287	768.439	765.235	765.437	765.033	G[35]
G[8]	166.691	757.034	753.831	754.032	753.629	G[34]
K[9]	192.310	745.630	742.426	742.628	742.225	K[33]
A[10]	206.518	720.011	716.807	717.009	716.606	A[32]
R[11]	237.738	705.804	702.600	702.802	702.398	R[31]
A[12]	251.945	674.584	671.380	671.581	671.178	A[30]
K[13]	277.564	660.376	657.172	657.374	656.971	K[29]
A[14]	291.772	634.757	631.553	631.755	631.352	A[28]
K[15]	317.391	620.550	617.346	617.548	617.144	K[27]
S[16]	334.797	594.931	591.727	591.929	591.525	S[26]
R[17]	366.017	577.524	574.321	574.522	574.119	R[25]
S[18]	383.424	546.304	543.100	543.302	542.899	S[24]
S[19]	400.830	528.898	525.694	525.896	525.492	S[23]
R[20]	432.050	511.491	508.288	508.489	508.086	R[22]
A[21]	446.258	480.271	477.067	477.269	476.866	A[21]
G[22]	457.662	466.064	462.860	463.061	462.658	G[20]
L[23]	480.279	454.659	451.456	451.657	451.254	L[19]
Q[24]	505.891	432.043	428.839	429.040	428.637	Q[18]
F[25]	535.304	406.431	403.227	403.429	403.026	F[17]
P[26]	554.715	377.017	373.813	374.015	373.612	P[16]
V[27]	574.529	357.607	354.403	354.604	354.201	V[15]
G[28]	585.933	337.793	334.589	334.791	334.388	G[14]
R[29]	617.153	326.389	323.185	323.386	322.983	R[13]
V[30]	636.967	295.168	291.965	292.166	291.763	V[12]
H[31]	664.379	275.355	272.151	272.353	271.949	H[11]
R[32]	695.599	247.943	244.739	244.941	244.538	R[10]
L[33]	718.216	216.723	213.519	213.721	213.317	L[9]
L[34]	740.832	194.106	190.902	191.104	190.701	L[8]
R[35]	**772.053**	171.489	168.285	168.487	168.084	R[7]
K[36]	797.672	140.269	137.065	137.267	136.864	K[6]
G[37]	809.076	114.650	111.446	111.648	111.245	G[5]
N[38]	831.885	103.246	100.042	100.243	99.840	N[4]
Y[39]	**864.497**	80.437	77.233	77.435	77.032	Y[3]
S[40]	881.904	47.824	44.621	44.822	44.419	S[2]
E[41]	**907.712**	30.418	27.214	27.416	27.013	E[1]

sp|Q6GSS7|H2A2A_MOUSE

[Acetyl]SGRGK $^{\text{Acetyl}}_{42.01}$ QGGK $^{\text{Dimethyl}}_{28.03}$ ARAKAKSRSSRAGLQFPVGRVHRLLRKGNYAE

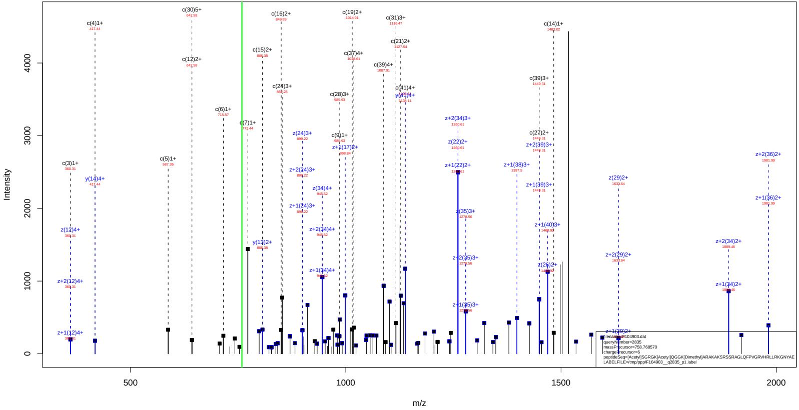

sp|Q6GSS7|H2A2A_MOUSE

[Acetyl]SGRGK $^{\text{Acetyl}}_{42.01}$ QGGK $^{\text{Dimethyl}}_{28.03}$ ARAKAKSRSSRAGLQFPVGRVHRLLRKGNYAE

- fragmentation table for charge state 1+
- specType=etd
- score=57.66
- F104903.dat
- query=q2835_p1
- precursor=758.768570
- chargePrecursor=6
- itol=0.8

AA	c	y	z+1	z+2	z	AA
S[1]	147.076	4547.552	4531.533	0.000	4530.525	S[41]
G[2]	204.098	4418.509	4402.491	0.000	4401.483	G[40]
R[3]	**360.199**	4361.488	4345.469	4346.477	4344.461	R[39]
G[4]	**417.220**	4205.387	4189.368	4190.376	4188.360	G[38]
K[5]	**587.326**	4148.365	4132.347	4133.354	4131.339	K[37]
Q[6]	**715.385**	3978.260	3962.241	3963.249	3961.233	Q[36]
G[7]	**772.406**	3850.201	3834.182	3835.190	3833.175	G[35]
G[8]	829.427	3793.180	3777.161	3778.169	3776.153	G[34]
K[9]	**985.554**	3736.158	3720.140	3721.147	3719.132	K[33]
A[10]	**1056.591**	3580.032	3564.013	3565.021	3563.005	A[32]
R[11]	**1212.692**	3508.995	3492.976	3493.984	3491.968	R[31]
A[12]	1283.729	3352.894	3336.875	3337.883	3335.867	A[30]
K[13]	1411.824	3281.857	3265.838	3266.846	3264.830	K[29]
A[14]	**1482.861**	3153.762	3137.743	3138.751	3136.735	A[28]
K[15]	1610.956	3082.725	3066.706	3067.714	3065.698	K[27]
S[16]	1697.988	2954.630	2938.611	2939.619	2937.603	S[26]
R[17]	1854.089	2867.598	2851.579	2852.587	2850.571	R[25]
S[18]	1941.121	2711.496	2695.478	2696.486	2694.470	S[24]
S[19]	2028.153	2624.464	2608.446	2609.454	2607.438	S[23]
R[20]	2184.254	2537.432	2521.414	2522.422	2520.406	R[22]
A[21]	2255.292	2381.331	2365.313	2366.320	2364.305	A[21]
G[22]	2312.313	2310.294	2294.275	2295.283	2293.268	G[20]
L[23]	2425.397	2253.273	2237.254	2238.262	2236.246	L[19]
Q[24]	2553.456	2140.189	2124.170	2125.178	2123.162	Q[18]
F[25]	2700.524	2012.130	1996.111	1997.119	1995.104	F[17]
P[26]	2797.577	1865.062	1849.043	1850.051	1848.035	P[16]
V[27]	2896.645	1768.009	1751.990	1752.998	1750.982	V[15]
G[28]	2953.667	1668.940	1652.922	1653.930	1651.914	G[14]
R[29]	3109.768	1611.919	**1595.900**	**1596.908**	1594.892	R[13]
V[30]	3208.836	1455.818	1439.799	1440.807	1438.791	V[12]
H[31]	3345.895	1356.749	1340.731	**1341.739**	1339.723	H[11]
R[32]	3501.996	1219.691	1203.672	**1204.680**	1202.664	R[10]
L[33]	3615.080	**1063.589**	**1047.571**	**1048.579**	1046.563	L[9]
L[34]	3728.164	950.505	934.487	935.495	**933.479**	L[8]
R[35]	3884.266	**837.421**	**821.403**	822.410	820.395	R[7]
K[36]	4012.360	681.320	665.302	666.309	664.294	K[6]
G[37]	4069.382	553.225	537.207	538.214	536.199	G[5]
N[38]	4183.425	496.204	480.185	481.193	479.177	N[4]
Y[39]	4346.488	382.161	366.142	367.150	365.134	Y[3]
A[40]	4417.525	219.098	203.079	204.087	202.071	A[2]
E[41]	4546.568	148.060	132.042	133.050	131.034	E[1]

sp|Q6GSS7|H2A2A_MOUSE

[Acetyl]SGRGK $^{\text{Acetyl}}_{42.01}$ QGGK $^{\text{Dimethyl}}_{28.03}$ ARAKAKSRSSRAGLQFPVGRVHRLLRKGNYAE

- fragmentation table for charge state 2+
- specType=etd
- score=57.66
- F104903.dat
- query=q2835_p1
- precursor=758.768570
- chargePrecursor=6
- itol=0.8

AA	c	y	z+1	z+2	z	AA
S[1]	74.042	2274.280	2266.270	0.504	2265.766	S[41]
G[2]	102.553	2209.758	2201.749	0.504	2201.245	G[40]
R[3]	180.603	2181.248	2173.238	2173.742	2172.734	R[39]
G[4]	209.114	2103.197	2095.188	2095.692	2094.684	G[38]
K[5]	294.167	2074.686	2066.677	2067.181	2066.173	K[37]
Q[6]	358.196	1989.634	**1981.624**	**1982.128**	1981.120	Q[36]
G[7]	386.707	1925.604	1917.595	**1918.099**	1917.091	G[35]
G[8]	415.217	1897.093	**1889.084**	**1889.588**	1888.580	G[34]
K[9]	493.281	1868.583	1860.573	1861.077	1860.069	K[33]
A[10]	528.799	1790.520	1782.510	1783.014	1782.006	A[32]
R[11]	606.850	1755.001	1746.992	1747.496	1746.488	R[31]
A[12]	**642.368**	1676.951	1668.941	1669.445	1668.437	A[30]
K[13]	**706.416**	1641.432	**1633.423**	**1633.927**	**1632.919**	K[29]
A[14]	**741.934**	1577.384	1569.375	**1569.879**	1568.871	A[28]
K[15]	**805.982**	1541.866	1533.857	**1534.360**	1533.353	K[27]
S[16]	**849.498**	1477.818	1469.809	1470.313	**1469.305**	S[26]
R[17]	**927.548**	1434.302	**1426.293**	**1426.797**	**1425.789**	R[25]
S[18]	**971.064**	1356.252	**1348.243**	**1348.746**	**1347.739**	S[24]
S[19]	**1014.580**	1312.736	**1304.726**	**1305.230**	1304.223	S[23]
R[20]	**1092.631**	1269.220	**1261.210**	1261.714	**1260.707**	R[22]
A[21]	**1128.149**	1191.169	1183.160	**1183.664**	1182.656	A[21]
G[22]	1156.660	1155.651	1147.641	1148.145	1147.137	G[20]
L[23]	**1213.202**	**1127.140**	1119.131	1119.635	1118.627	L[19]
Q[24]	1277.231	**1070.598**	**1062.589**	**1063.093**	1062.085	Q[18]
F[25]	1350.766	1006.569	**998.559**	**999.063**	**998.055**	F[17]
P[26]	1399.292	**933.034**	925.025	925.529	924.521	P[16]
V[27]	**1448.826**	884.508	876.499	877.003	875.995	V[15]
G[28]	1477.337	834.974	**826.965**	**827.468**	**826.461**	G[14]
R[29]	1555.388	**806.463**	**798.454**	**798.958**	797.950	R[13]
V[30]	1604.922	728.413	720.403	720.907	719.899	V[12]
H[31]	1673.451	678.878	670.869	671.373	670.365	H[11]
R[32]	1751.502	610.349	602.340	602.843	601.836	R[10]
L[33]	1808.044	532.298	524.289	524.793	523.785	L[9]
L[34]	1864.586	475.756	467.747	468.251	467.243	L[8]
R[35]	1942.636	419.214	411.205	411.709	410.701	R[7]
K[36]	2006.684	341.164	333.154	333.658	332.650	K[6]
G[37]	2035.195	277.116	269.107	269.611	268.603	G[5]
N[38]	2092.216	248.606	240.596	241.100	240.092	N[4]
Y[39]	2173.748	191.584	183.575	184.079	183.071	Y[3]
A[40]	2209.266	110.052	102.043	102.547	101.539	A[2]
E[41]	2273.788	74.534	66.524	67.028	66.021	E[1]

sp|Q6GSS7|H2A2A_MOUSE

[Acetyl]SGRGK $^{\text{Acetyl}}_{42.01}$ QGGK $^{\text{Dimethyl}}_{28.03}$ ARAKAKSRSSRAGLQFPVGRVHRLLRKGNYAE

- fragmentation table for charge state 3+
- specType=etd
- score=57.66
- F104903.dat
- query=q2835_p1
- precursor=758.768570
- chargePrecursor=6
- itol=0.8

AA	c	y	z+1	z+2	z	AA
S[1]	49.697	1516.522	1511.183	0.672	1510.847	S[41]
G[2]	68.704	1473.508	**1468.168**	0.672	1467.832	G[40]
R[3]	120.738	**1454.501**	**1449.161**	**1449.497**	**1448.825**	R[39]
G[4]	139.745	1402.467	**1397.128**	**1397.463**	**1396.792**	G[38]
K[5]	196.447	1383.460	**1378.120**	**1378.456**	1377.784	K[37]
Q[6]	239.133	1326.758	**1321.419**	**1321.754**	1321.083	Q[36]
G[7]	258.140	1284.072	**1278.732**	**1279.068**	**1278.396**	G[35]
G[8]	277.147	1265.065	1259.725	**1260.061**	1259.389	G[34]
K[9]	329.189	1246.058	**1240.718**	**1241.054**	**1240.382**	K[33]
A[10]	352.868	1194.016	1188.676	1189.012	1188.340	A[32]
R[11]	404.902	1170.336	1164.997	**1165.333**	1164.661	R[31]
A[12]	428.581	1118.303	1112.963	1113.299	1112.627	A[30]
K[13]	471.280	1094.624	1089.284	1089.620	1088.948	K[29]
A[14]	494.959	1051.925	1046.586	**1046.922**	1046.250	A[28]
K[15]	537.657	1028.246	1022.907	**1023.243**	1022.571	K[27]
S[16]	566.668	**985.548**	**980.208**	**980.544**	**979.873**	S[26]
R[17]	618.701	956.537	951.198	**951.534**	950.862	R[25]
S[18]	647.712	904.504	**899.164**	**899.500**	**898.828**	S[24]
S[19]	676.723	875.493	**870.153**	**870.489**	**869.817**	S[23]
R[20]	728.756	846.482	**841.143**	**841.479**	840.807	R[22]
A[21]	**752.435**	794.449	789.109	789.445	788.773	A[21]
G[22]	771.443	770.770	765.430	765.766	765.094	G[20]
L[23]	809.137	751.762	746.423	746.759	746.087	L[19]
Q[24]	**851.823**	714.068	708.728	709.064	708.392	Q[18]
F[25]	900.846	671.382	666.042	666.378	665.706	F[17]
P[26]	**933.197**	622.359	617.019	617.355	616.683	P[16]
V[27]	966.220	590.008	584.668	585.004	584.332	V[15]
G[28]	**985.227**	556.985	551.645	551.981	551.309	G[14]
R[29]	1037.261	537.978	532.638	532.974	532.302	R[13]
V[30]	1070.284	485.944	480.605	480.941	480.269	V[12]
H[31]	**1115.970**	452.921	447.582	447.918	447.246	H[11]
R[32]	**1168.004**	407.235	401.895	402.231	401.560	R[10]
L[33]	1205.698	355.201	349.862	350.198	349.526	L[9]
L[34]	**1243.393**	317.507	312.167	312.503	311.831	L[8]
R[35]	1295.427	279.812	274.472	274.808	274.136	R[7]
K[36]	1338.125	227.778	222.439	222.775	222.103	K[6]
G[37]	1357.132	185.080	179.740	180.076	179.404	G[5]
N[38]	1395.146	166.073	160.733	161.069	160.397	N[4]
Y[39]	**1449.501**	128.058	122.719	123.055	122.383	Y[3]
A[40]	1473.180	73.704	68.364	68.700	68.029	A[2]
E[41]	1516.194	50.025	44.685	45.021	44.349	E[1]

sp|Q6GSS7|H2A2A_MOUSE

[Acetyl]SGRGK $^{Acetyl}_{42.01}$ QGGK $^{Dimethyl}_{28.03}$ ARAKAKSRSSRAGLQFPVGRVHRLLRKGNYAE

- fragmentation table for charge state 4+
- specType=etd
- score=57.66
- F104903.dat
- query=q2835_p1
- precursor=758.768570
- chargePrecursor=6
- itol=0.8

AA	c	y	z+1	z+2	z	AA
S[1]	37.525	**1137.643**	**1133.639**	0.755	1133.387	S[41]
G[2]	51.780	**1105.383**	**1101.378**	0.755	**1101.126**	G[40]
R[3]	90.805	1091.127	**1087.123**	**1087.375**	1086.871	R[39]
G[4]	105.061	1052.102	**1048.097**	**1048.349**	**1047.846**	G[38]
K[5]	147.587	1037.847	1033.842	1034.094	1033.590	K[37]
Q[6]	179.602	995.320	**991.316**	**991.568**	**991.064**	Q[36]
G[7]	193.857	963.306	**959.301**	**959.553**	**959.049**	G[35]
G[8]	208.112	949.050	**945.046**	**945.298**	**944.794**	G[34]
K[9]	247.144	934.795	930.790	931.042	930.538	K[33]
A[10]	264.903	895.763	891.759	892.011	891.507	A[32]
R[11]	303.928	878.004	873.999	874.251	873.748	R[31]
A[12]	321.688	838.979	834.974	835.226	834.722	A[30]
K[13]	353.711	**821.220**	817.215	817.467	816.963	K[29]
A[14]	371.471	789.196	785.191	785.443	784.939	A[28]
K[15]	403.494	771.437	767.432	767.684	767.180	K[27]
S[16]	425.252	739.413	735.408	735.660	735.156	S[26]
R[17]	464.278	717.655	713.650	713.902	713.398	R[25]
S[18]	486.036	678.630	674.625	674.877	674.373	S[24]
S[19]	507.794	656.872	652.867	653.119	652.615	S[23]
R[20]	546.819	635.114	631.109	631.361	630.857	R[22]
A[21]	564.578	596.088	592.084	592.336	591.832	A[21]
G[22]	578.834	578.329	574.324	574.576	574.072	G[20]
L[23]	607.105	564.074	560.069	560.321	559.817	L[19]
Q[24]	639.119	535.803	531.798	532.050	531.546	Q[18]
F[25]	675.886	503.788	499.783	500.035	499.531	F[17]
P[26]	700.150	467.021	463.016	463.268	462.764	P[16]
V[27]	724.917	442.758	438.753	439.005	438.501	V[15]
G[28]	739.172	**417.991**	413.986	414.238	413.734	G[14]
R[29]	778.197	403.735	399.731	399.982	399.479	R[13]
V[30]	802.965	364.710	**360.705**	**360.957**	**360.453**	V[12]
H[31]	**837.229**	339.943	335.938	336.190	335.686	H[11]
R[32]	876.255	305.678	301.673	301.925	301.421	R[10]
L[33]	904.526	266.653	262.648	262.900	262.396	L[9]
L[34]	**932.797**	238.382	234.377	234.629	234.125	L[8]
R[35]	971.822	210.111	206.106	206.358	205.854	R[7]
K[36]	1003.846	171.086	167.081	167.333	166.829	K[6]
G[37]	**1018.101**	139.062	135.057	135.309	134.805	G[5]
N[38]	1046.612	124.806	120.802	121.054	120.550	N[4]
Y[39]	**1087.378**	96.296	92.291	92.543	92.039	Y[3]
A[40]	**1105.137**	55.530	51.525	51.777	51.273	A[2]
E[41]	**1137.397**	37.771	33.766	34.018	33.514	E[1]

sp|Q6GSS7|H2A2A_MOUSE

[Acetyl]SGRGK $^{Acetyl}_{42.01}$ QGGK $^{Dimethyl}_{28.03}$ ARAKAKSRSSRAGLQFPVGRVHRLLRKGNYAE

- fragmentation table for charge state 5+
- specType=etd
- score=57.66
- F104903.dat
- query=q2835_p1
- precursor=758.768570
- chargePrecursor=6
- itol=0.8

AA	c	y	z+1	z+2	z	AA
S[1]	30.221	**910.316**	907.112	0.806	906.911	S[41]
G[2]	41.625	884.508	**881.304**	0.806	881.102	G[40]
R[3]	72.846	873.103	**869.900**	**870.101**	869.698	R[39]
G[4]	84.250	**841.883**	838.679	838.881	838.478	G[38]
K[5]	118.271	830.479	**827.275**	**827.477**	**827.074**	K[37]
Q[6]	143.883	796.458	793.254	793.456	793.052	Q[36]
G[7]	155.287	770.846	767.642	767.844	767.441	G[35]
G[8]	166.691	759.442	756.238	756.440	756.036	G[34]
K[9]	197.917	748.037	744.834	745.035	744.632	K[33]
A[10]	212.124	716.812	713.608	713.810	713.407	A[32]
R[11]	243.344	702.605	699.401	699.603	699.199	R[31]
A[12]	257.552	671.385	668.181	668.382	667.979	A[30]
K[13]	283.171	657.177	653.973	654.175	653.772	K[29]
A[14]	297.378	631.558	628.354	628.556	628.153	A[28]
K[15]	322.997	617.351	614.147	614.349	613.945	K[27]
S[16]	340.403	591.732	588.528	588.730	588.326	S[26]
R[17]	371.624	574.325	571.122	571.323	570.920	R[25]
S[18]	389.030	543.105	539.901	540.103	539.700	S[24]
S[19]	406.436	525.699	522.495	522.697	522.293	S[23]
R[20]	437.657	508.292	505.089	505.290	504.887	R[22]
A[21]	451.864	477.072	473.868	474.070	473.667	A[21]
G[22]	463.268	462.865	459.661	459.862	459.459	G[20]
L[23]	485.885	451.460	448.257	448.458	448.055	L[19]
Q[24]	511.497	428.844	425.640	425.841	425.438	Q[18]
F[25]	540.911	403.232	400.028	400.230	399.827	F[17]
P[26]	560.321	373.818	370.614	370.816	370.413	P[16]
V[27]	580.135	354.408	351.204	351.405	351.002	V[15]
G[28]	591.539	334.594	331.390	331.592	331.189	G[14]
R[29]	622.759	323.190	319.986	320.187	319.784	R[13]
V[30]	**642.573**	291.969	288.766	288.967	288.564	V[12]
H[31]	669.985	272.156	268.952	269.154	268.750	H[11]
R[32]	701.205	244.744	241.540	241.742	241.339	R[10]
L[33]	723.822	213.524	210.320	210.522	210.118	L[9]
L[34]	746.439	190.907	187.703	187.905	187.502	L[8]
R[35]	777.659	168.290	165.086	165.288	164.885	R[7]
K[36]	803.278	137.070	133.866	134.068	133.665	K[6]
G[37]	814.682	111.451	108.247	108.449	108.046	G[5]
N[38]	**837.491**	100.047	96.843	97.044	96.641	N[4]
Y[39]	**870.103**	77.238	74.034	74.236	73.833	Y[3]
A[40]	884.311	44.625	41.422	41.623	41.220	A[2]
E[41]	910.119	30.418	27.214	27.416	27.013	E[1]

sp|Q8CGP5|H2A1F_MOUSE

[Acetyl]SGRGK $^{Acetyl}_{42.01}$ QGGKARAKAKTRSSRAGLQFPVGRVHRLLRKGNYSE

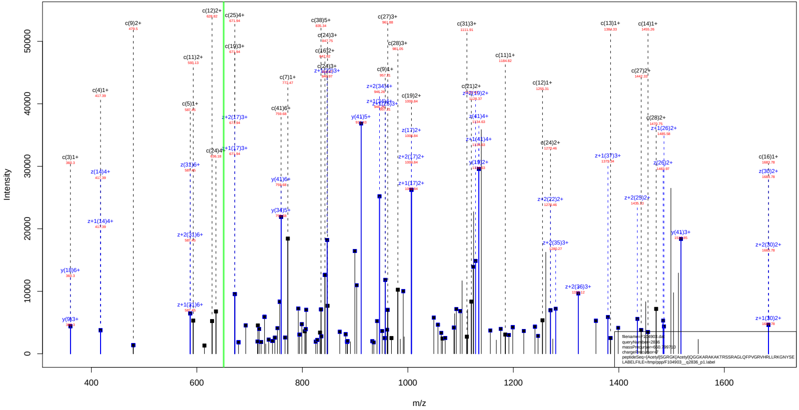

sp|Q8CGP5|H2A1F_MOUSE

[Acetyl]SGRGK $^{Acetyl}_{42.01}$ QGGKARAKAKTRSSRAGLQFPVGRVHRLLRKGNYSE

- fragmentation table for charge state 1+
- specType=etd
- score=46.71
- F104903.dat
- query=q2836_p1
- precursor=650.799710
- chargePrecursor=7
- itol=0.8

AA	c	y	z+1	z+2	z	AA
S[1]	147.076	4549.531	4533.512	0.000	4532.505	S[41]
G[2]	204.098	4420.489	4404.470	0.000	4403.462	G[40]
R[3]	**360.199**	4363.467	4347.448	4348.456	4346.441	R[39]
G[4]	**417.220**	4207.366	4191.347	4192.355	4190.339	G[38]
K[5]	**587.326**	4150.345	4134.326	4135.334	4133.318	K[37]
Q[6]	**715.385**	3980.239	3964.220	3965.228	3963.212	Q[36]
G[7]	**772.406**	3852.180	3836.162	3837.170	3835.154	G[35]
G[8]	829.427	3795.159	3779.140	3780.148	3778.132	G[34]
K[9]	**957.522**	3738.138	3722.119	3723.127	3721.111	K[33]
A[10]	1028.560	3610.043	3594.024	3595.032	3593.016	A[32]
R[11]	**1184.661**	3539.005	3522.987	3523.995	3521.979	R[31]
A[12]	**1255.698**	3382.904	3366.886	3367.893	3365.878	A[30]
K[13]	**1383.793**	3311.867	3295.848	3296.856	3294.841	K[29]
A[14]	**1454.830**	3183.772	3167.754	3168.761	3166.746	A[28]
K[15]	1582.925	3112.735	3096.716	3097.724	3095.709	K[27]
T[16]	**1683.973**	2984.640	2968.621	2969.629	2967.614	T[26]
R[17]	1840.074	2883.592	2867.574	2868.582	2866.566	R[25]
S[18]	1927.106	2727.491	2711.473	2712.480	2710.465	S[24]
S[19]	2014.138	2640.459	2624.441	2625.448	2623.433	S[23]
R[20]	2170.239	2553.427	2537.409	2538.416	2536.401	R[22]
A[21]	2241.276	2397.326	2381.307	2382.315	2380.300	A[21]
G[22]	2298.297	2326.289	2310.270	2311.278	2309.263	G[20]
L[23]	2411.381	2269.268	2253.249	2254.257	2252.241	L[19]
Q[24]	2539.440	2156.184	2140.165	2141.173	2139.157	Q[18]
F[25]	2686.508	2028.125	2012.106	2013.114	2011.098	F[17]
P[26]	2783.561	1881.057	1865.038	1866.046	1864.030	P[16]
V[27]	2882.630	1784.004	1767.985	1768.993	1766.977	V[15]
G[28]	2939.651	1684.935	1668.917	1669.924	1667.909	G[14]
R[29]	3095.752	1627.914	1611.895	1612.903	1610.887	R[13]
V[30]	3194.821	1471.813	**1455.794**	1456.802	**1454.786**	V[12]
H[31]	3331.880	1372.744	**1356.726**	1357.734	1355.718	H[11]
R[32]	3487.981	1235.685	**1219.667**	1220.675	1218.659	R[10]
L[33]	3601.065	1079.584	**1063.566**	1064.573	1062.558	L[9]
L[34]	3714.149	966.500	950.482	**951.489**	949.474	L[8]
R[35]	3870.250	853.416	837.398	838.405	**836.390**	R[7]
K[36]	3998.345	697.315	681.296	682.304	680.289	K[6]
G[37]	4055.366	569.220	553.201	554.209	552.194	G[5]
N[38]	4169.409	512.199	496.180	497.188	495.172	N[4]
Y[39]	4332.473	398.156	382.137	383.145	381.129	Y[3]
S[40]	4419.505	235.092	219.074	220.082	218.066	S[2]
E[41]	4548.547	148.060	132.042	133.050	131.034	E[1]

sp|Q8CGP5|H2A1F_MOUSE

[Acetyl]SGRGK $^{Acetyl}_{42.01}$ QGGKARAKAKTRSSRAGLQFPVGRVHRLLRKGNYSE

- fragmentation table for charge state 2+
- specType=etd
- score=46.71
- F104903.dat
- query=q2836_p1
- precursor=650.799710
- chargePrecursor=7
- itol=0.8

AA	c	y	z+1	z+2	z	AA
S[1]	74.042	2275.269	2267.260	0.504	2266.756	S[41]
G[2]	102.553	2210.748	2202.739	0.504	2202.235	G[40]
R[3]	180.603	2182.237	2174.228	2174.732	2173.724	R[39]
G[4]	209.114	2104.187	2096.177	2096.681	2095.673	G[38]
K[5]	294.167	2075.676	2067.667	2068.170	2067.163	K[37]
Q[6]	358.196	1990.623	1982.614	1983.118	1982.110	Q[36]
G[7]	386.707	1926.594	1918.584	1919.088	1918.081	G[35]
G[8]	415.217	1898.083	1890.074	1890.578	1889.570	G[34]
K[9]	**479.265**	1869.572	1861.563	1862.067	1861.059	K[33]
A[10]	514.783	1805.525	1797.516	1798.019	1797.012	A[32]
R[11]	**592.834**	1770.006	1761.997	1762.501	1761.493	R[31]
A[12]	**628.353**	1691.956	**1683.946**	**1684.450**	**1683.443**	A[30]
K[13]	**692.400**	1656.437	1648.428	1648.932	1647.924	K[29]
A[14]	**727.919**	1592.390	1584.380	1584.884	1583.876	A[28]
K[15]	**791.966**	1556.871	1548.862	1549.366	1548.358	K[27]
T[16]	**842.490**	1492.824	**1484.814**	**1485.318**	**1484.310**	T[26]
R[17]	920.540	**1442.300**	1434.291	**1434.794**	1433.787	R[25]
S[18]	964.056	1364.249	**1356.240**	**1356.744**	1355.736	S[24]
S[19]	**1007.572**	1320.733	1312.724	1313.228	1312.220	S[23]
R[20]	1085.623	1277.217	1269.208	**1269.712**	1268.704	R[22]
A[21]	**1121.142**	**1199.167**	**1191.157**	**1191.661**	1190.653	A[21]
G[22]	1149.652	1163.648	**1155.639**	**1156.143**	1155.135	G[20]
L[23]	1206.194	**1135.137**	1127.128	**1127.632**	1126.624	L[19]
Q[24]	**1270.224**	1078.595	**1070.586**	**1071.090**	1070.082	Q[18]
F[25]	1343.758	1014.566	**1006.557**	**1007.061**	**1006.053**	F[17]
P[26]	1392.284	**941.032**	**933.023**	933.526	**932.519**	P[16]
V[27]	**1441.818**	892.506	**884.496**	**885.000**	883.992	V[15]
G[28]	**1470.329**	**842.971**	**834.962**	**835.466**	834.458	G[14]
R[29]	1548.380	814.461	**806.451**	**806.955**	**805.947**	R[13]
V[30]	1597.914	**736.410**	**728.401**	**728.905**	**727.897**	V[12]
H[31]	1666.443	686.876	**678.866**	**679.370**	**678.363**	H[11]
R[32]	1744.494	618.346	610.337	610.841	609.833	R[10]
L[33]	1801.036	540.296	532.286	532.790	531.783	L[9]
L[34]	1857.578	483.754	475.744	476.248	475.241	L[8]
R[35]	1935.629	427.212	419.202	419.706	418.698	R[7]
K[36]	1999.676	349.161	341.152	341.656	340.648	K[6]
G[37]	2028.187	285.114	277.104	277.608	276.600	G[5]
N[38]	2085.208	256.603	248.594	249.098	248.090	N[4]
Y[39]	2166.740	199.582	191.572	192.076	191.068	Y[3]
S[40]	2210.256	118.050	110.041	110.544	109.537	S[2]
E[41]	2274.777	74.534	66.524	67.028	66.021	E[1]

sp|Q8CGP5|H2A1F_MOUSE

[Acetyl]SGRGK $^{Acetyl}_{42.01}$ QGGKARAKAKTRSSRAGLQFPVGRVHRLLRKGNYSE

- fragmentation table for charge state 3+
- specType=etd
- score=46.71
- F104903.dat
- query=q2836_p1
- precursor=650.799710
- chargePrecursor=7
- itol=0.8

AA	c	y	z+1	z+2	z	AA
S[1]	49.697	**1517.182**	1511.842	0.672	1511.506	S[41]
G[2]	68.704	1474.168	1468.828	0.672	1468.492	G[40]
R[3]	120.738	**1455.161**	1449.821	1450.157	1449.485	R[39]
G[4]	139.745	1403.127	1397.787	**1398.123**	1397.451	G[38]
K[5]	196.447	**1384.120**	**1378.780**	**1379.116**	1378.444	K[37]
Q[6]	239.133	1327.418	1322.078	**1322.414**	1321.742	Q[36]
G[7]	258.140	1284.732	1279.392	**1279.728**	1279.056	G[35]
G[8]	277.147	1265.725	1260.385	1260.721	1260.049	G[34]
K[9]	319.846	**1246.717**	**1241.378**	**1241.714**	**1241.042**	K[33]
A[10]	343.525	1204.019	**1198.679**	**1199.015**	**1198.344**	A[32]
R[11]	395.558	1180.340	1175.000	**1175.336**	1174.664	R[31]
A[12]	419.237	**1128.306**	1122.967	**1123.303**	1122.631	A[30]
K[13]	461.936	1104.627	**1099.288**	**1099.624**	**1098.952**	K[29]
A[14]	485.615	1061.929	**1056.589**	**1056.925**	1056.253	A[28]
K[15]	528.313	1038.250	1032.910	1033.246	1032.574	K[27]
T[16]	561.996	995.552	990.212	**990.548**	989.876	T[26]
R[17]	**614.029**	**961.869**	**956.529**	**956.865**	**956.193**	R[25]
S[18]	643.040	909.835	904.496	904.832	904.160	S[24]
S[19]	**672.051**	880.825	875.485	875.821	875.149	S[23]
R[20]	724.084	851.814	**846.474**	**846.810**	846.138	R[22]
A[21]	**747.763**	**799.780**	**794.441**	**794.777**	**794.105**	A[21]
G[22]	**766.771**	776.101	770.762	771.098	770.426	G[20]
L[23]	**804.465**	**757.094**	751.754	**752.090**	751.419	L[19]
Q[24]	**847.152**	719.399	714.060	**714.396**	713.724	Q[18]
F[25]	896.174	676.713	**671.374**	**671.710**	671.038	F[17]
P[26]	928.525	627.690	622.351	622.687	622.015	P[16]
V[27]	**961.548**	595.339	590.000	590.336	589.664	V[15]
G[28]	**980.555**	562.317	556.977	557.313	556.641	G[14]
R[29]	1032.589	543.309	537.970	538.306	537.634	R[13]
V[30]	**1065.612**	491.276	485.936	486.272	485.600	V[12]
H[31]	**1111.298**	458.253	452.913	453.249	452.577	H[11]
R[32]	1163.332	412.567	407.227	407.563	406.891	R[10]
L[33]	1201.026	**360.533**	355.193	355.529	354.857	L[9]
L[34]	1238.721	322.838	317.499	317.835	317.163	L[8]
R[35]	1290.755	285.144	279.804	280.140	279.468	R[7]
K[36]	1333.453	233.110	227.770	228.106	227.434	K[6]
G[37]	1352.460	190.412	185.072	185.408	184.736	G[5]
N[38]	1390.475	171.404	166.065	166.401	165.729	N[4]
Y[39]	1444.829	133.390	128.051	128.386	127.715	Y[3]
S[40]	1473.840	79.036	73.696	74.032	73.360	S[2]
E[41]	1516.854	50.025	44.685	45.021	44.349	E[1]

sp|Q8CGP5|H2A1F_MOUSE

[Acetyl]SGRGK $^{Acetyl}_{42.01}$ QGGKARAKAKTRSSRAGLQFPVGRVHRLLRKGNYSE

- fragmentation table for charge state 4+
- specType=etd
- score=46.71
- F104903.dat
- query=q2836_p1
- precursor=650.799710
- chargePrecursor=7
- itol=0.8

AA	c	y	z+1	z+2	z	AA
S[1]	37.525	1138.138	**1134.134**	0.755	**1133.882**	S[41]
G[2]	51.780	1105.878	1101.873	0.755	1101.621	G[40]
R[3]	90.805	**1091.622**	**1087.618**	**1087.870**	**1087.366**	R[39]
G[4]	105.061	1052.597	**1048.592**	**1048.844**	1048.340	G[38]
K[5]	147.587	1038.342	1034.337	1034.589	1034.085	K[37]
Q[6]	179.602	995.815	**991.811**	992.062	**991.559**	Q[36]
G[7]	193.857	963.801	959.796	**960.048**	959.544	G[35]
G[8]	208.112	949.545	**945.541**	**945.792**	945.289	G[34]
K[9]	240.136	**935.290**	931.285	931.537	931.033	K[33]
A[10]	257.895	**903.266**	**899.261**	**899.513**	**899.009**	A[32]
R[11]	296.921	**885.507**	**881.502**	**881.754**	881.250	R[31]
A[12]	314.680	**846.482**	**842.477**	**842.729**	**842.225**	A[30]
K[13]	346.704	**828.722**	**824.718**	**824.970**	824.466	K[29]
A[14]	364.463	796.699	**792.694**	**792.946**	**792.442**	A[28]
K[15]	396.487	778.939	774.935	775.187	774.683	K[27]
T[16]	421.749	746.915	**742.911**	**743.163**	**742.659**	T[26]
R[17]	460.774	**721.654**	**717.649**	**717.901**	**717.397**	R[25]
S[18]	482.532	682.628	**678.624**	**678.876**	**678.372**	S[24]
S[19]	504.290	660.870	656.866	657.118	656.614	S[23]
R[20]	543.315	639.112	635.108	635.360	634.856	R[22]
A[21]	561.074	600.087	596.082	596.334	595.830	A[21]
G[22]	575.330	582.328	578.323	578.575	578.071	G[20]
L[23]	603.601	568.072	564.068	564.320	563.816	L[19]
Q[24]	**635.615**	539.801	535.797	536.049	535.545	Q[18]
F[25]	**672.383**	507.787	503.782	504.034	503.530	F[17]
P[26]	696.646	471.020	467.015	467.267	466.763	P[16]
V[27]	**721.413**	446.756	442.752	443.004	442.500	V[15]
G[28]	**735.668**	421.989	**417.985**	418.237	**417.733**	G[14]
R[29]	774.694	407.734	403.729	403.981	403.477	R[13]
V[30]	**799.461**	368.709	364.704	364.956	364.452	V[12]
H[31]	**833.725**	343.942	339.937	340.189	339.685	H[11]
R[32]	872.751	309.677	305.672	305.924	305.420	R[10]
L[33]	901.022	270.652	266.647	266.899	266.395	L[9]
L[34]	929.293	242.381	238.376	238.628	238.124	L[8]
R[35]	**968.318**	214.110	210.105	210.357	209.853	R[7]
K[36]	1000.342	175.084	171.080	171.332	170.828	K[6]
G[37]	1014.597	143.061	139.056	139.308	138.804	G[5]
N[38]	1043.108	128.805	124.800	125.052	124.548	N[4]
Y[39]	1083.874	100.294	96.290	96.542	96.038	Y[3]
S[40]	1105.632	59.529	55.524	55.776	55.272	S[2]
E[41]	1137.892	37.771	33.766	34.018	33.514	E[1]

sp|Q8CGP5|H2A1F_MOUSE

[Acetyl]SGRGK $^{Acetyl}_{42.01}$ QGGKARAKAKTRSSRAGLQFPVGRVHRLLRKGNYSE

- fragmentation table for charge state 5+
- specType=etd
- score=46.71
- F104903.dat
- query=q2836_p1
- precursor=650.799710
- chargePrecursor=7
- itol=0.8

AA	c	y	z+1	z+2	z	AA
S[1]	30.221	**910.712**	907.508	0.806	907.307	S[41]
G[2]	41.625	**884.904**	**881.700**	0.806	**881.498**	G[40]
R[3]	72.846	873.499	**870.295**	**870.497**	**870.094**	R[39]
G[4]	84.250	**842.279**	839.075	839.277	838.874	G[38]
K[5]	118.271	830.875	**827.671**	**827.873**	827.469	K[37]
Q[6]	143.883	796.854	793.650	**793.851**	793.448	Q[36]
G[7]	155.287	771.242	768.038	768.240	**767.837**	G[35]
G[8]	166.691	**759.838**	**756.634**	**756.835**	**756.432**	G[34]
K[9]	192.310	**748.433**	745.230	745.431	745.028	K[33]
A[10]	206.518	722.814	719.611	719.812	719.409	A[32]
R[11]	237.738	708.607	705.403	705.605	705.202	R[31]
A[12]	251.945	677.387	674.183	674.385	673.981	A[30]
K[13]	277.564	663.179	659.976	660.177	659.774	K[29]
A[14]	291.772	637.560	634.357	634.558	634.155	A[28]
K[15]	317.391	623.353	620.149	620.351	619.948	K[27]
T[16]	337.600	597.734	594.530	594.732	594.329	T[26]
R[17]	368.821	577.524	574.321	574.522	574.119	R[25]
S[18]	386.227	546.304	543.100	543.302	542.899	S[24]
S[19]	403.633	528.898	525.694	525.896	525.492	S[23]
R[20]	434.854	511.491	508.288	508.489	508.086	R[22]
A[21]	449.061	**480.271**	477.067	477.269	476.866	A[21]
G[22]	460.465	466.064	462.860	463.061	462.658	G[20]
L[23]	483.082	454.659	451.456	451.657	451.254	L[19]
Q[24]	508.694	432.043	428.839	429.040	428.637	Q[18]
F[25]	538.108	406.431	403.227	403.429	403.026	F[17]
P[26]	557.518	377.017	373.813	374.015	373.612	P[16]
V[27]	577.332	357.607	354.403	354.604	354.201	V[15]
G[28]	588.736	337.793	334.589	334.791	334.388	G[14]
R[29]	619.956	326.389	323.185	323.386	322.983	R[13]
V[30]	639.770	295.168	291.965	292.166	291.763	V[12]
H[31]	667.182	275.355	272.151	272.353	271.949	H[11]
R[32]	698.402	247.943	244.739	244.941	244.538	R[10]
L[33]	**721.019**	216.723	213.519	213.721	213.317	L[9]
L[34]	**743.636**	194.106	190.902	191.104	190.701	L[8]
R[35]	774.856	171.489	168.285	168.487	168.084	R[7]
K[36]	800.475	140.269	137.065	137.267	136.864	K[6]
G[37]	811.879	114.650	111.446	111.648	111.245	G[5]
N[38]	**834.688**	103.246	100.042	100.243	99.840	N[4]
Y[39]	867.300	80.437	77.233	77.435	77.032	Y[3]
S[40]	**884.707**	47.824	44.621	44.822	44.419	S[2]
E[41]	910.515	30.418	27.214	27.416	27.013	E[1]

sp|Q8CGP5|H2A1F_MOUSE

[Acetyl]SGRGK $^{Acetyl}_{42.01}$ QGGKARAKAKTRSSRAGLQFPVGRVHRLLRKGNYSE

- fragmentation table for charge state 6+
- specType=etd
- score=46.71
- F104903.dat
- query=q2836_p1
- precursor=650.799710
- chargePrecursor=7
- itol=0.8

AA	c	y	z+1	z+2	z	AA
S[1]	25.352	**759.095**	**756.425**	0.839	**756.257**	S[41]
G[2]	34.856	737.587	734.918	0.839	734.750	G[40]
R[3]	60.873	**728.084**	725.414	725.582	725.246	R[39]
G[4]	70.376	702.067	699.397	699.565	699.229	G[38]
K[5]	98.727	**692.563**	689.894	690.062	689.726	K[37]
Q[6]	120.070	664.213	661.543	661.711	661.375	Q[36]
G[7]	129.574	642.869	640.200	640.368	640.032	G[35]
G[8]	139.077	633.366	630.696	630.864	630.528	G[34]
K[9]	160.426	623.862	621.193	621.360	621.025	K[33]
A[10]	172.266	602.513	599.843	600.011	599.675	A[32]
R[11]	198.283	590.674	**588.004**	**588.172**	**587.836**	R[31]
A[12]	210.122	564.657	561.987	562.155	561.819	A[30]
K[13]	231.472	552.817	550.147	550.315	549.980	K[29]
A[14]	243.311	531.468	528.798	528.966	528.630	A[28]
K[15]	264.660	519.629	516.959	517.127	516.791	K[27]
T[16]	281.501	498.279	495.610	495.778	495.442	T[26]
R[17]	307.518	481.438	**478.768**	**478.936**	478.600	R[25]
S[18]	322.024	455.421	452.752	452.919	452.584	S[24]
S[19]	336.529	440.916	438.246	438.414	438.078	S[23]
R[20]	362.546	426.411	423.741	423.909	423.573	R[22]
A[21]	374.385	400.394	397.724	397.892	397.556	A[21]
G[22]	383.889	388.554	385.884	386.052	385.716	G[20]
L[23]	402.736	379.051	376.381	376.549	376.213	L[19]
Q[24]	424.079	**360.203**	357.534	357.702	357.366	Q[18]
F[25]	448.591	338.860	336.190	336.358	336.022	F[17]
P[26]	464.766	314.349	311.679	311.847	311.511	P[16]
V[27]	481.278	298.173	295.504	295.672	295.336	V[15]
G[28]	490.781	281.662	278.992	279.160	278.824	G[14]
R[29]	516.798	272.158	269.489	269.657	269.321	R[13]
V[30]	533.309	246.142	243.472	243.640	243.304	V[12]
H[31]	556.153	229.630	226.960	227.128	226.792	H[11]
R[32]	582.169	206.787	204.117	204.285	203.949	R[10]
L[33]	601.017	180.770	178.100	178.268	177.932	L[9]
L[34]	619.864	161.923	159.253	159.421	159.085	L[8]
R[35]	645.881	143.075	140.406	140.574	140.238	R[7]
K[36]	667.230	117.059	114.389	114.557	114.221	K[6]
G[37]	676.734	95.709	93.040	93.208	92.872	G[5]
N[38]	695.741	86.206	83.536	83.704	83.368	N[4]
Y[39]	722.918	67.199	64.529	64.697	64.361	Y[3]
S[40]	737.423	40.021	37.352	37.520	37.184	S[2]
E[41]	**758.931**	25.516	22.846	23.014	22.678	E[1]

sp|Q8CGP5|H2A1F_MOUSE

[Acetyl]SGRGK $^{Acetyl}_{42.01}$ QGGKARAKAKTRSSRAGLQFPVGRVHRLLRKGNYSE

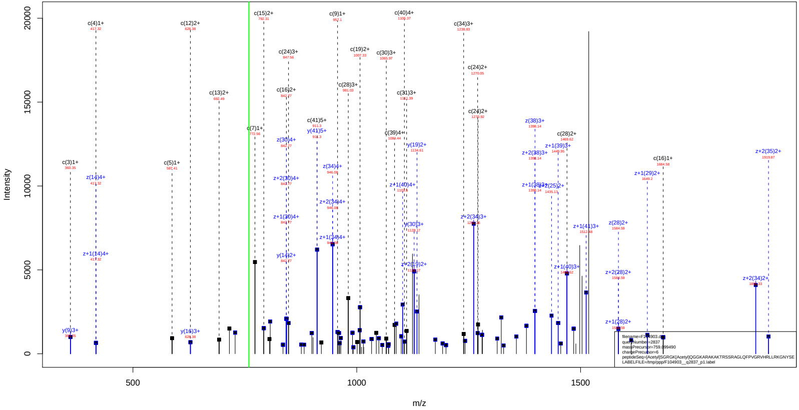

sp|Q8CGP5|H2A1F_MOUSE

[Acetyl]SGRGK $^{Acetyl}_{42.01}$ QGGKARAKAKTRSSRAGLQFPVGRVHRLLRKGNYSE

- fragmentation table for charge state 1+
- specType=etd
- score=62.93
- F104903.dat
- query=q2837_p1
- precursor=759.099490
- chargePrecursor=6
- itol=0.8

AA	c	y	z+1	z+2	z	AA
S[1]	147.076	4549.531	4533.512	0.000	4532.505	S[41]
G[2]	204.098	4420.489	4404.470	0.000	4403.462	G[40]
R[3]	**360.199**	4363.467	4347.448	4348.456	4346.441	R[39]
G[4]	**417.220**	4207.366	4191.347	4192.355	4190.339	G[38]
K[5]	**587.326**	4150.345	4134.326	4135.334	4133.318	K[37]
Q[6]	**715.385**	3980.239	3964.220	3965.228	3963.212	Q[36]
G[7]	**772.406**	3852.180	3836.162	3837.170	3835.154	G[35]
G[8]	829.427	3795.159	3779.140	3780.148	3778.132	G[34]
K[9]	**957.522**	3738.138	3722.119	3723.127	3721.111	K[33]
A[10]	1028.560	3610.043	3594.024	3595.032	3593.016	A[32]
R[11]	1184.661	3539.005	3522.987	3523.995	3521.979	R[31]
A[12]	1255.698	3382.904	3366.886	3367.893	3365.878	A[30]
K[13]	1383.793	3311.867	3295.848	3296.856	3294.841	K[29]
A[14]	1454.830	3183.772	3167.754	3168.761	3166.746	A[28]
K[15]	1582.925	3112.735	3096.716	3097.724	3095.709	K[27]
T[16]	**1683.973**	2984.640	2968.621	2969.629	2967.614	T[26]
R[17]	1840.074	2883.592	2867.574	2868.582	2866.566	R[25]
S[18]	1927.106	2727.491	2711.473	2712.480	2710.465	S[24]
S[19]	2014.138	2640.459	2624.441	2625.448	2623.433	S[23]
R[20]	2170.239	2553.427	2537.409	2538.416	2536.401	R[22]
A[21]	2241.276	2397.326	2381.307	2382.315	2380.300	A[21]
G[22]	2298.297	2326.289	2310.270	2311.278	2309.263	G[20]
L[23]	2411.381	2269.268	2253.249	2254.257	2252.241	L[19]
Q[24]	2539.440	2156.184	2140.165	2141.173	2139.157	Q[18]
F[25]	2686.508	2028.125	2012.106	2013.114	2011.098	F[17]
P[26]	2783.561	1881.057	1865.038	1866.046	1864.030	P[16]
V[27]	2882.630	1784.004	1767.985	1768.993	1766.977	V[15]
G[28]	2939.651	**1684.935**	1668.917	1669.924	1667.909	G[14]
R[29]	3095.752	1627.914	1611.895	**1612.903**	1610.887	R[13]
V[30]	3194.821	1471.813	**1455.794**	1456.802	1454.786	V[12]
H[31]	3331.880	1372.744	**1356.726**	1357.734	1355.718	H[11]
R[32]	3487.981	1235.685	1219.667	1220.675	1218.659	R[10]
L[33]	3601.065	1079.584	1063.566	1064.573	1062.558	L[9]
L[34]	3714.149	966.500	950.482	951.489	949.474	L[8]
R[35]	3870.250	853.416	837.398	838.405	836.390	R[7]
K[36]	3998.345	697.315	681.296	682.304	680.289	K[6]
G[37]	4055.366	569.220	553.201	554.209	552.194	G[5]
N[38]	4169.409	512.199	496.180	497.188	495.172	N[4]
Y[39]	4332.473	398.156	382.137	383.145	381.129	Y[3]
S[40]	4419.505	235.092	219.074	220.082	218.066	S[2]
E[41]	4548.547	148.060	132.042	133.050	131.034	E[1]

sp|Q8CGP5|H2A1F_MOUSE

[Acetyl]SGRGK $^{Acetyl}_{42.01}$ QGGKARAKAKTRSSRAGLQFPVGRVHRLLRKGNYSE

- fragmentation table for charge state 2+
- specType=etd
- score=62.93
- F104903.dat
- query=q2837_p1
- precursor=759.099490
- chargePrecursor=6
- itol=0.8

AA	c	y	z+1	z+2	z	AA
S[1]	74.042	2275.269	2267.260	0.504	2266.756	S[41]
G[2]	102.553	2210.748	2202.739	0.504	2202.235	G[40]
R[3]	180.603	2182.237	2174.228	2174.732	2173.724	R[39]
G[4]	209.114	2104.187	2096.177	2096.681	2095.673	G[38]
K[5]	294.167	2075.676	2067.667	2068.170	2067.163	K[37]
Q[6]	358.196	1990.623	1982.614	1983.118	1982.110	Q[36]
G[7]	386.707	1926.594	1918.584	**1919.088**	1918.081	G[35]
G[8]	415.217	1898.083	1890.074	**1890.578**	1889.570	G[34]
K[9]	479.265	1869.572	1861.563	1862.067	1861.059	K[33]
A[10]	514.783	1805.525	1797.516	1798.019	1797.012	A[32]
R[11]	592.834	1770.006	1761.997	1762.501	1761.493	R[31]
A[12]	**628.353**	1691.956	**1683.946**	**1684.450**	1683.443	A[30]
K[13]	**692.400**	1656.437	**1648.428**	**1648.932**	1647.924	K[29]
A[14]	**727.919**	1592.390	**1584.380**	**1584.884**	**1583.876**	A[28]
K[15]	**791.966**	1556.871	1548.862	1549.366	1548.358	K[27]
T[16]	**842.490**	1492.824	**1484.814**	**1485.318**	**1484.310**	T[26]
R[17]	**920.540**	1442.300	1434.291	**1434.794**	1433.787	R[25]
S[18]	**964.056**	1364.249	**1356.240**	**1356.744**	1355.736	S[24]
S[19]	**1007.572**	1320.733	1312.724	**1313.228**	1312.220	S[23]
R[20]	1085.623	1277.217	1269.208	**1269.712**	1268.704	R[22]
A[21]	1121.142	**1199.167**	**1191.157**	**1191.661**	1190.653	A[21]
G[22]	1149.652	1163.648	1155.639	1156.143	1155.135	G[20]
L[23]	1206.194	**1135.137**	1127.128	**1127.632**	1126.624	L[19]
Q[24]	**1270.224**	1078.595	**1070.586**	**1071.090**	**1070.082**	Q[18]
F[25]	1343.758	**1014.566**	**1006.557**	**1007.061**	**1006.053**	F[17]
P[26]	1392.284	941.032	933.023	933.526	932.519	P[16]
V[27]	1441.818	892.506	884.496	885.000	883.992	V[15]
G[28]	**1470.329**	**842.971**	**834.962**	**835.466**	**834.458**	G[14]
R[29]	1548.380	814.461	**806.451**	**806.955**	**805.947**	R[13]
V[30]	1597.914	736.410	**728.401**	**728.905**	**727.897**	V[12]
H[31]	1666.443	686.876	678.866	679.370	678.363	H[11]
R[32]	1744.494	618.346	610.337	610.841	609.833	R[10]
L[33]	1801.036	540.296	532.286	532.790	531.783	L[9]
L[34]	1857.578	483.754	475.744	476.248	475.241	L[8]
R[35]	1935.629	427.212	419.202	419.706	418.698	R[7]
K[36]	1999.676	349.161	341.152	341.656	340.648	K[6]
G[37]	2028.187	285.114	277.104	277.608	276.600	G[5]
N[38]	2085.208	256.603	248.594	249.098	248.090	N[4]
Y[39]	2166.740	199.582	191.572	192.076	191.068	Y[3]
S[40]	2210.256	118.050	110.041	110.544	109.537	S[2]
E[41]	2274.777	74.534	66.524	67.028	66.021	E[1]

sp|Q8CGP5|H2A1F_MOUSE

[Acetyl]SGRGK $^{Acetyl}_{42.01}$ QGGKARAKAKTRSSRAGLQFPVGRVHRLLRKGNYSE

- fragmentation table for charge state 3+
- specType=etd
- score=62.93
- F104903.dat
- query=q2837_p1
- precursor=759.099490
- chargePrecursor=6
- itol=0.8

AA	c	y	z+1	z+2	z	AA
S[1]	49.697	1517.182	**1511.842**	0.672	1511.506	S[41]
G[2]	68.704	1474.168	**1468.828**	0.672	1468.492	G[40]
R[3]	120.738	**1455.161**	**1449.821**	**1450.157**	**1449.485**	R[39]
G[4]	139.745	1403.127	**1397.787**	**1398.123**	**1397.451**	G[38]
K[5]	196.447	1384.120	**1378.780**	**1379.116**	**1378.444**	K[37]
Q[6]	239.133	**1327.418**	**1322.078**	**1322.414**	1321.742	Q[36]
G[7]	258.140	1284.732	**1279.392**	**1279.728**	**1279.056**	G[35]
G[8]	277.147	1265.725	1260.385	**1260.721**	1260.049	G[34]
K[9]	319.846	1246.717	**1241.378**	**1241.714**	1241.042	K[33]
A[10]	343.525	1204.019	**1198.679**	**1199.015**	1198.344	A[32]
R[11]	395.558	1180.340	**1175.000**	**1175.336**	1174.664	R[31]
A[12]	419.237	**1128.306**	1122.967	1123.303	1122.631	A[30]
K[13]	461.936	1104.627	**1099.288**	**1099.624**	**1098.952**	K[29]
A[14]	485.615	1061.929	**1056.589**	**1056.925**	1056.253	A[28]
K[15]	528.313	1038.250	**1032.910**	**1033.246**	**1032.574**	K[27]
T[16]	561.996	995.552	**990.212**	**990.548**	**989.876**	T[26]
R[17]	614.029	**961.869**	**956.529**	**956.865**	956.193	R[25]
S[18]	643.040	909.835	904.496	904.832	904.160	S[24]
S[19]	672.051	880.825	**875.485**	**875.821**	875.149	S[23]
R[20]	724.084	851.814	846.474	**846.810**	846.138	R[22]
A[21]	747.763	799.780	794.441	794.777	794.105	A[21]
G[22]	766.771	776.101	770.762	771.098	770.426	G[20]
L[23]	**804.465**	757.094	751.754	752.090	751.419	L[19]
Q[24]	**847.152**	719.399	714.060	714.396	713.724	Q[18]
F[25]	896.174	676.713	671.374	671.710	671.038	F[17]
P[26]	928.525	**627.690**	622.351	622.687	622.015	P[16]
V[27]	**961.548**	595.339	590.000	590.336	589.664	V[15]
G[28]	**980.555**	562.317	556.977	557.313	556.641	G[14]
R[29]	**1032.589**	543.309	537.970	538.306	537.634	R[13]
V[30]	**1065.612**	491.276	485.936	486.272	485.600	V[12]
H[31]	**1111.298**	458.253	452.913	453.249	452.577	H[11]
R[32]	1163.332	412.567	407.227	407.563	406.891	R[10]
L[33]	1201.026	**360.533**	355.193	355.529	354.857	L[9]
L[34]	**1238.721**	322.838	317.499	317.835	317.163	L[8]
R[35]	1290.755	285.144	279.804	280.140	279.468	R[7]
K[36]	1333.453	233.110	227.770	228.106	227.434	K[6]
G[37]	1352.460	190.412	185.072	185.408	184.736	G[5]
N[38]	1390.475	171.404	166.065	166.401	165.729	N[4]
Y[39]	1444.829	133.390	128.051	128.386	127.715	Y[3]
S[40]	1473.840	79.036	73.696	74.032	73.360	S[2]
E[41]	1516.854	50.025	44.685	45.021	44.349	E[1]

sp|Q8CGP5|H2A1F_MOUSE

[Acetyl]SGRGK $^{Acetyl}_{42.01}$ QGGKARAKAKTRSSRAGLQFPVGRVHRLLRKGNYSE

- fragmentation table for charge state 4+
- specType=etd
- score=62.93
- F104903.dat
- query=q2837_p1
- precursor=759.099490
- chargePrecursor=6
- itol=0.8

AA	c	y	z+1	z+2	z	AA
S[1]	37.525	1138.138	**1134.134**	0.755	**1133.882**	S[41]
G[2]	51.780	**1105.878**	**1101.873**	0.755	1101.621	G[40]
R[3]	90.805	1091.622	**1087.618**	**1087.870**	1087.366	R[39]
G[4]	105.061	1052.597	**1048.592**	**1048.844**	1048.340	G[38]
K[5]	147.587	1038.342	1034.337	1034.589	1034.085	K[37]
Q[6]	179.602	995.815	**991.811**	**992.062**	991.559	Q[36]
G[7]	193.857	**963.801**	**959.796**	**960.048**	**959.544**	G[35]
G[8]	208.112	949.545	**945.541**	**945.792**	**945.289**	G[34]
K[9]	240.136	935.290	931.285	931.537	931.033	K[33]
A[10]	257.895	903.266	**899.261**	**899.513**	**899.009**	A[32]
R[11]	296.921	885.507	881.502	**881.754**	881.250	R[31]
A[12]	314.680	846.482	**842.477**	**842.729**	**842.225**	A[30]
K[13]	346.704	828.722	824.718	824.970	824.466	K[29]
A[14]	364.463	796.699	**792.694**	**792.946**	**792.442**	A[28]
K[15]	396.487	778.939	774.935	775.187	774.683	K[27]
T[16]	421.749	746.915	742.911	743.163	742.659	T[26]
R[17]	460.774	721.654	717.649	717.901	717.397	R[25]
S[18]	482.532	682.628	678.624	678.876	678.372	S[24]
S[19]	504.290	660.870	656.866	657.118	656.614	S[23]
R[20]	543.315	639.112	635.108	635.360	634.856	R[22]
A[21]	561.074	600.087	596.082	596.334	595.830	A[21]
G[22]	575.330	582.328	578.323	578.575	578.071	G[20]
L[23]	603.601	568.072	564.068	564.320	563.816	L[19]
Q[24]	635.615	539.801	535.797	536.049	535.545	Q[18]
F[25]	672.383	507.787	503.782	504.034	503.530	F[17]
P[26]	696.646	471.020	467.015	467.267	466.763	P[16]
V[27]	721.413	446.756	442.752	443.004	442.500	V[15]
G[28]	735.668	421.989	**417.985**	418.237	**417.733**	G[14]
R[29]	774.694	407.734	403.729	403.981	403.477	R[13]
V[30]	799.461	368.709	364.704	364.956	364.452	V[12]
H[31]	833.725	343.942	339.937	340.189	339.685	H[11]
R[32]	872.751	309.677	305.672	305.924	305.420	R[10]
L[33]	901.022	270.652	266.647	266.899	266.395	L[9]
L[34]	929.293	242.381	238.376	238.628	238.124	L[8]
R[35]	968.318	214.110	210.105	210.357	209.853	R[7]
K[36]	**1000.342**	175.084	171.080	171.332	170.828	K[6]
G[37]	**1014.597**	143.061	139.056	139.308	138.804	G[5]
N[38]	**1043.108**	128.805	124.800	125.052	124.548	N[4]
Y[39]	**1083.874**	100.294	96.290	96.542	96.038	Y[3]
S[40]	**1105.632**	59.529	55.524	55.776	55.272	S[2]
E[41]	1137.892	37.771	33.766	34.018	33.514	E[1]

sp|Q8CGP5|H2A1F_MOUSE

[Acetyl]SGRGK $^{Acetyl}_{42.01}$ QGGKARAKAKTRSSRAGLQFPVGRVHRLLRKGNYSE

- fragmentation table for charge state 5+
- specType=etd
- score=62.93
- F104903.dat
- query=q2837_p1
- precursor=759.099490
- chargePrecursor=6
- itol=0.8

AA	c	y	z+1	z+2	z	AA
S[1]	30.221	**910.712**	907.508	0.806	907.307	S[41]
G[2]	41.625	884.904	881.700	0.806	881.498	G[40]
R[3]	72.846	873.499	870.295	870.497	870.094	R[39]
G[4]	84.250	**842.279**	839.075	839.277	838.874	G[38]
K[5]	118.271	830.875	827.671	827.873	827.469	K[37]
Q[6]	143.883	796.854	793.650	793.851	793.448	Q[36]
G[7]	155.287	771.242	768.038	768.240	767.837	G[35]
G[8]	166.691	759.838	756.634	756.835	756.432	G[34]
K[9]	192.310	748.433	745.230	745.431	745.028	K[33]
A[10]	206.518	722.814	719.611	719.812	719.409	A[32]
R[11]	237.738	708.607	705.403	705.605	705.202	R[31]
A[12]	251.945	677.387	674.183	674.385	673.981	A[30]
K[13]	277.564	663.179	659.976	660.177	659.774	K[29]
A[14]	291.772	637.560	634.357	634.558	634.155	A[28]
K[15]	317.391	623.353	620.149	620.351	619.948	K[27]
T[16]	337.600	597.734	594.530	594.732	594.329	T[26]
R[17]	368.821	577.524	574.321	574.522	574.119	R[25]
S[18]	386.227	546.304	543.100	543.302	542.899	S[24]
S[19]	403.633	528.898	525.694	525.896	525.492	S[23]
R[20]	434.854	511.491	508.288	508.489	508.086	R[22]
A[21]	449.061	480.271	477.067	477.269	476.866	A[21]
G[22]	460.465	466.064	462.860	463.061	462.658	G[20]
L[23]	483.082	454.659	451.456	451.657	451.254	L[19]
Q[24]	508.694	432.043	428.839	429.040	428.637	Q[18]
F[25]	538.108	406.431	403.227	403.429	403.026	F[17]
P[26]	557.518	377.017	373.813	374.015	373.612	P[16]
V[27]	577.332	357.607	354.403	354.604	354.201	V[15]
G[28]	588.736	337.793	334.589	334.791	334.388	G[14]
R[29]	619.956	326.389	323.185	323.386	322.983	R[13]
V[30]	639.770	295.168	291.965	292.166	291.763	V[12]
H[31]	667.182	275.355	272.151	272.353	271.949	H[11]
R[32]	698.402	247.943	244.739	244.941	244.538	R[10]
L[33]	721.019	216.723	213.519	213.721	213.317	L[9]
L[34]	743.636	194.106	190.902	191.104	190.701	L[8]
R[35]	774.856	171.489	168.285	168.487	168.084	R[7]
K[36]	800.475	140.269	137.065	137.267	136.864	K[6]
G[37]	811.879	114.650	111.446	111.648	111.245	G[5]
N[38]	**834.688**	103.246	100.042	100.243	99.840	N[4]
Y[39]	867.300	80.437	77.233	77.435	77.032	Y[3]
S[40]	884.707	47.824	44.621	44.822	44.419	S[2]
E[41]	**910.515**	30.418	27.214	27.416	27.013	E[1]

sp|Q6GSS7|H2A2A_MOUSE

S $^{\text{Phospho}}_{79.97}$ GRGKQGGK $^{\text{Acetyl}}_{42.01}$ ARAKAKSRSSRAGLQFPVGRVHRLLRKGNYAE

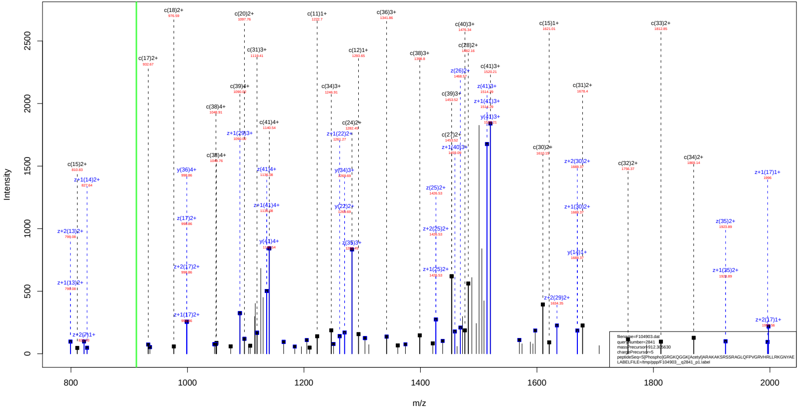

sp|Q6GSS7|H2A2A_MOUSE

S $^{Phospho}_{79.97}$ GRGKQGGK $^{Acetyl}_{42.01}$ ARAKAKSRSSRAGLQFPVGRVHRLLRKGNYAE

- fragmentation table for charge state 1+
- specType=etd
- score=59.84
- F104903.dat
- query=q2841_p1
- precursor=912.305630
- chargePrecursor=5
- itol=0.8

AA	c	y	z+1	z+2	z	AA
S[1]	185.032	4557.476	4541.458	0.000	4540.450	S[41]
G[2]	242.054	4390.478	4374.459	0.000	4373.451	G[40]
R[3]	398.155	4333.457	4317.438	4318.446	4316.430	R[39]
G[4]	455.176	4177.355	4161.337	4162.345	4160.329	G[38]
K[5]	583.271	4120.334	4104.315	4105.323	4103.307	K[37]
Q[6]	711.330	3992.239	3976.220	3977.228	3975.212	Q[36]
G[7]	768.351	3864.180	3848.162	3849.170	3847.154	G[35]
G[8]	825.373	3807.159	3791.140	3792.148	3790.132	G[34]
K[9]	995.478	3750.138	3734.119	3735.127	3733.111	K[33]
A[10]	1066.515	3580.032	3564.013	3565.021	3563.005	A[32]
R[11]	**1222.616**	3508.995	3492.976	3493.984	3491.968	R[31]
A[12]	**1293.654**	3352.894	3336.875	3337.883	3335.867	A[30]
K[13]	**1421.749**	3281.857	3265.838	3266.846	3264.830	K[29]
A[14]	1492.786	3153.762	3137.743	3138.751	3136.735	A[28]
K[15]	**1620.881**	3082.725	3066.706	3067.714	3065.698	K[27]
S[16]	1707.913	2954.630	2938.611	2939.619	2937.603	S[26]
R[17]	1864.014	2867.598	2851.579	2852.587	2850.571	R[25]
S[18]	1951.046	2711.496	2695.478	2696.486	2694.470	S[24]
S[19]	2038.078	2624.464	2608.446	2609.454	2607.438	S[23]
R[20]	2194.179	2537.432	2521.414	2522.422	2520.406	R[22]
A[21]	2265.216	2381.331	2365.313	2366.320	2364.305	A[21]
G[22]	2322.237	2310.294	2294.275	2295.283	2293.268	G[20]
L[23]	2435.322	2253.273	2237.254	2238.262	2236.246	L[19]
Q[24]	2563.380	2140.189	2124.170	2125.178	2123.162	Q[18]
F[25]	2710.449	2012.130	**1996.111**	**1997.119**	1995.104	F[17]
P[26]	2807.501	1865.062	1849.043	1850.051	1848.035	P[16]
V[27]	2906.570	1768.009	1751.990	1752.998	1750.982	V[15]
G[28]	2963.591	**1668.940**	1652.922	1653.930	1651.914	G[14]
R[29]	3119.692	1611.919	1595.900	**1596.908**	1594.892	R[13]
V[30]	3218.761	1455.818	1439.799	1440.807	**1438.791**	V[12]
H[31]	3355.820	1356.749	1340.731	**1341.739**	1339.723	H[11]
R[32]	3511.921	1219.691	1203.672	**1204.680**	1202.664	R[10]
L[33]	3625.005	1063.589	1047.571	**1048.579**	**1046.563**	L[9]
L[34]	3738.089	950.505	934.487	**935.495**	933.479	L[8]
R[35]	3894.190	837.421	821.403	**822.410**	820.395	R[7]
K[36]	4022.285	681.320	665.302	666.309	664.294	K[6]
G[37]	4079.306	553.225	537.207	538.214	536.199	G[5]
N[38]	4193.349	496.204	480.185	481.193	479.177	N[4]
Y[39]	4356.413	382.161	366.142	367.150	365.134	Y[3]
A[40]	4427.450	219.098	203.079	204.087	202.071	A[2]
E[41]	4556.492	148.060	132.042	133.050	131.034	E[1]

sp|Q6GSS7|H2A2A_MOUSE

S $^{Phospho}_{79.97}$ GRGKQGGK $^{Acetyl}_{42.01}$ ARAKAKSRSSRAGLQFPVGRVHRLLRKGNYAE

- fragmentation table for charge state 2+
- specType=etd
- score=59.84
- F104903.dat
- query=q2841_p1
- precursor=912.305630
- chargePrecursor=5
- itol=0.8

AA	c	y	z+1	z+2	z	AA
S[1]	93.020	2279.242	2271.232	0.504	2270.729	S[41]
G[2]	121.530	2195.743	2187.733	0.504	2187.229	G[40]
R[3]	199.581	2167.232	2159.223	2159.726	2158.719	R[39]
G[4]	228.092	2089.181	2081.172	2081.676	2080.668	G[38]
K[5]	292.139	2060.671	2052.661	2053.165	2052.157	K[37]
Q[6]	356.169	**1996.623**	1988.614	1989.118	1988.110	Q[36]
G[7]	384.679	1932.594	**1924.584**	1925.088	**1924.081**	G[35]
G[8]	413.190	1904.083	1896.074	1896.578	1895.570	G[34]
K[9]	498.243	1875.572	1867.563	1868.067	1867.059	K[33]
A[10]	533.761	1790.520	1782.510	1783.014	1782.006	A[32]
R[11]	611.812	1755.001	1746.992	1747.496	1746.488	R[31]
A[12]	647.330	1676.951	**1668.941**	**1669.445**	1668.437	A[30]
K[13]	711.378	1641.432	1633.423	**1633.927**	1632.919	K[29]
A[14]	746.896	1577.384	**1569.375**	**1569.879**	1568.871	A[28]
K[15]	**810.944**	1541.866	1533.857	1534.360	1533.353	K[27]
S[16]	854.460	1477.818	1469.809	1470.313	**1469.305**	S[26]
R[17]	**932.511**	1434.302	**1426.293**	**1426.797**	**1425.789**	R[25]
S[18]	**976.027**	1356.252	1348.243	1348.746	1347.739	S[24]
S[19]	1019.543	1312.736	**1304.726**	**1305.230**	**1304.223**	S[23]
R[20]	**1097.593**	**1269.220**	**1261.210**	**1261.714**	**1260.707**	R[22]
A[21]	1133.112	1191.169	1183.160	**1183.664**	1182.656	A[21]
G[22]	1161.622	1155.651	1147.641	1148.145	1147.137	G[20]
L[23]	1218.164	1127.140	**1119.131**	**1119.635**	**1118.627**	L[19]
Q[24]	**1282.194**	1070.598	1062.589	1063.093	1062.085	Q[18]
F[25]	1355.728	1006.569	**998.559**	**999.063**	**998.055**	F[17]
P[26]	1404.254	**933.034**	925.025	925.529	924.521	P[16]
V[27]	**1453.788**	884.508	876.499	877.003	875.995	V[15]
G[28]	**1482.299**	834.974	**826.965**	**827.468**	826.461	G[14]
R[29]	1560.350	806.463	**798.454**	**798.958**	797.950	R[13]
V[30]	**1609.884**	728.413	720.403	720.907	719.899	V[12]
H[31]	**1678.413**	678.878	670.869	671.373	670.365	H[11]
R[32]	**1756.464**	610.349	602.340	602.843	601.836	R[10]
L[33]	**1813.006**	532.298	524.289	524.793	523.785	L[9]
L[34]	**1869.548**	475.756	467.747	468.251	467.243	L[8]
R[35]	1947.599	419.214	411.205	411.709	410.701	R[7]
K[36]	2011.646	341.164	333.154	333.658	332.650	K[6]
G[37]	2040.157	277.116	269.107	269.611	268.603	G[5]
N[38]	2097.178	248.606	240.596	241.100	240.092	N[4]
Y[39]	2178.710	191.584	183.575	184.079	183.071	Y[3]
A[40]	2214.229	110.052	102.043	102.547	101.539	A[2]
E[41]	2278.750	74.534	66.524	67.028	66.021	E[1]

sp|Q6GSS7|H2A2A_MOUSE

S $^{\text{Phospho}}_{79.97}$ GRGKQGGK $^{\text{Acetyl}}_{42.01}$ ARAKAKSRSSRAGLQFPVGRVHRLLRKGNYAE

- fragmentation table for charge state 3+
- specType=etd
- score=59.84
- F104903.dat
- query=q2841_p1
- precursor=912.305630
- chargePrecursor=5
- itol=0.8

AA	c	y	z+1	z+2	z	AA
S[1]	62.349	**1519.830**	**1514.491**	0.672	**1514.155**	S[41]
G[2]	81.356	1464.164	**1458.825**	0.672	**1458.489**	G[40]
R[3]	133.390	1445.157	1439.817	1440.153	1439.482	R[39]
G[4]	152.397	1393.123	1387.784	1388.120	1387.448	G[38]
K[5]	195.095	**1374.116**	1368.777	1369.113	1368.441	K[37]
Q[6]	237.781	1331.418	1326.078	1326.414	1325.742	Q[36]
G[7]	256.789	1288.732	1283.392	1283.728	**1283.056**	G[35]
G[8]	275.796	**1269.725**	1264.385	1264.721	1264.049	G[34]
K[9]	332.498	**1250.717**	1245.378	1245.714	1245.042	K[33]
A[10]	356.177	1194.016	1188.676	1189.012	1188.340	A[32]
R[11]	408.210	1170.336	**1164.997**	**1165.333**	**1164.661**	R[31]
A[12]	431.889	1118.303	1112.963	1113.299	1112.627	A[30]
K[13]	474.588	1094.624	**1089.284**	**1089.620**	1088.948	K[29]
A[14]	498.267	1051.925	**1046.586**	**1046.922**	**1046.250**	A[28]
K[15]	540.965	1028.246	1022.907	1023.243	1022.571	K[27]
S[16]	569.976	985.548	980.208	980.544	979.873	S[26]
R[17]	622.009	956.537	951.198	951.534	950.862	R[25]
S[18]	651.020	904.504	899.164	899.500	898.828	S[24]
S[19]	680.031	875.493	870.153	870.489	869.817	S[23]
R[20]	732.064	846.482	841.143	841.479	840.807	R[22]
A[21]	755.744	794.449	789.109	789.445	788.773	A[21]
G[22]	774.751	770.770	765.430	765.766	765.094	G[20]
L[23]	812.445	751.762	746.423	746.759	746.087	L[19]
Q[24]	855.132	714.068	708.728	709.064	708.392	Q[18]
F[25]	904.154	671.382	666.042	666.378	665.706	F[17]
P[26]	936.505	622.359	617.019	617.355	616.683	P[16]
V[27]	969.528	590.008	584.668	585.004	584.332	V[15]
G[28]	988.535	556.985	551.645	551.981	551.309	G[14]
R[29]	1040.569	537.978	532.638	532.974	532.302	R[13]
V[30]	**1073.592**	485.944	480.605	480.941	480.269	V[12]
H[31]	**1119.278**	452.921	447.582	447.918	447.246	H[11]
R[32]	1171.312	407.235	401.895	402.231	401.560	R[10]
L[33]	**1209.006**	355.201	349.862	350.198	349.526	L[9]
L[34]	**1246.701**	317.507	312.167	312.503	311.831	L[8]
R[35]	1298.735	279.812	274.472	274.808	274.136	R[7]
K[36]	**1341.433**	227.778	222.439	222.775	222.103	K[6]
G[37]	**1360.440**	185.080	179.740	180.076	179.404	G[5]
N[38]	**1398.455**	166.073	160.733	161.069	160.397	N[4]
Y[39]	**1452.809**	128.058	122.719	123.055	122.383	Y[3]
A[40]	**1476.488**	73.704	68.364	68.700	68.029	A[2]
E[41]	**1519.502**	50.025	44.685	45.021	44.349	E[1]

sp|Q6GSS7|H2A2A_MOUSE

S $^{Phospho}_{79.97}$ GRGKQGGK $^{Acetyl}_{42.01}$ ARAKAKSRSSRAGLQFPVGRVHRLLRKGNYAE

- fragmentation table for charge state 4+
- specType=etd
- score=59.84
- F104903.dat
- query=q2841_p1
- precursor=912.305630
- chargePrecursor=5
- itol=0.8

AA	c	y	z+1	z+2	z	AA
S[1]	47.014	**1140.125**	**1136.120**	0.755	**1135.868**	S[41]
G[2]	61.269	**1098.375**	1094.370	0.755	1094.118	G[40]
R[3]	100.294	1084.120	1080.115	1080.367	1079.863	R[39]
G[4]	114.550	1045.094	1041.090	1041.342	1040.838	G[38]
K[5]	146.573	1030.839	1026.834	1027.086	1026.582	K[37]
Q[6]	178.588	**998.815**	994.811	995.062	994.559	Q[36]
G[7]	192.843	966.801	962.796	963.048	962.544	G[35]
G[8]	207.099	952.545	948.541	948.792	948.289	G[34]
K[9]	249.625	938.290	934.285	934.537	934.033	K[33]
A[10]	267.384	895.763	891.759	892.011	891.507	A[32]
R[11]	306.410	878.004	873.999	874.251	873.748	R[31]
A[12]	324.169	838.979	834.974	835.226	834.722	A[30]
K[13]	356.193	821.220	817.215	817.467	816.963	K[29]
A[14]	373.952	789.196	785.191	785.443	784.939	A[28]
K[15]	405.976	771.437	767.432	767.684	767.180	K[27]
S[16]	427.734	739.413	735.408	735.660	735.156	S[26]
R[17]	466.759	717.655	713.650	713.902	713.398	R[25]
S[18]	488.517	678.630	674.625	674.877	674.373	S[24]
S[19]	510.275	656.872	652.867	653.119	652.615	S[23]
R[20]	549.300	635.114	631.109	631.361	630.857	R[22]
A[21]	567.059	596.088	592.084	592.336	591.832	A[21]
G[22]	581.315	578.329	574.324	574.576	574.072	G[20]
L[23]	609.586	564.074	560.069	560.321	559.817	L[19]
Q[24]	641.600	535.803	531.798	532.050	531.546	Q[18]
F[25]	678.368	503.788	499.783	500.035	499.531	F[17]
P[26]	702.631	467.021	463.016	463.268	462.764	P[16]
V[27]	727.398	442.758	438.753	439.005	438.501	V[15]
G[28]	741.653	417.991	413.986	414.238	413.734	G[14]
R[29]	780.679	403.735	399.731	399.982	399.479	R[13]
V[30]	805.446	364.710	360.705	360.957	360.453	V[12]
H[31]	839.710	339.943	335.938	336.190	335.686	H[11]
R[32]	878.736	305.678	301.673	301.925	301.421	R[10]
L[33]	907.007	266.653	262.648	262.900	262.396	L[9]
L[34]	**935.278**	238.382	234.377	234.629	234.125	L[8]
R[35]	974.303	210.111	206.106	206.358	205.854	R[7]
K[36]	1006.327	171.086	167.081	167.333	166.829	K[6]
G[37]	1020.582	139.062	135.057	135.309	134.805	G[5]
N[38]	**1049.093**	124.806	120.802	121.054	120.550	N[4]
Y[39]	**1089.859**	96.296	92.291	92.543	92.039	Y[3]
A[40]	**1107.618**	55.530	51.525	51.777	51.273	A[2]
E[41]	**1139.879**	37.771	33.766	34.018	33.514	E[1]

sp|Q6GSS7|H2A2A_MOUSE

[Acetyl]S $^{\text{Phospho}}_{79.97}$ GRGKQGGKARAKAKSRSSRAGLQFPVGRVHRLLRKGNYAE

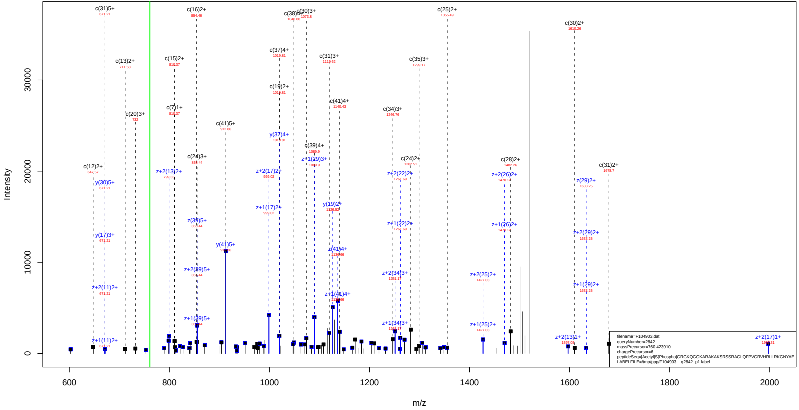

sp|Q6GSS7|H2A2A_MOUSE

[Acetyl]S $^{Phospho}_{79.97}$ GRGKQGGKARAKAKSRSSRAGLQFPVGRVHRLLRKGNYAE

- fragmentation table for charge state 1+
- specType=etd
- score=53.15
- F104903.dat
- query=q2842_p1
- precursor=760.423910
- chargePrecursor=6
- itol=0.8

AA	c	y	z+1	z+2	z	AA
S[1]	227.043	4557.476	4541.458	0.000	4540.450	S[41]
G[2]	284.064	4348.467	4332.449	0.000	4331.441	G[40]
R[3]	440.165	4291.446	4275.427	4276.435	4274.419	R[39]
G[4]	497.187	4135.345	4119.326	4120.334	4118.318	G[38]
K[5]	625.282	4078.323	4062.305	4063.313	4061.297	K[37]
Q[6]	**753.340**	3950.228	3934.210	3935.218	3933.202	Q[36]
G[7]	**810.362**	3822.170	3806.151	3807.159	3805.143	G[35]
G[8]	867.383	3765.148	3749.130	3750.138	3748.122	G[34]
K[9]	995.478	3708.127	3692.108	3693.116	3691.100	K[33]
A[10]	1066.515	3580.032	3564.013	3565.021	3563.005	A[32]
R[11]	1222.616	3508.995	3492.976	3493.984	3491.968	R[31]
A[12]	**1293.654**	3352.894	3336.875	3337.883	3335.867	A[30]
K[13]	1421.749	3281.857	3265.838	3266.846	3264.830	K[29]
A[14]	1492.786	3153.762	3137.743	3138.751	3136.735	A[28]
K[15]	1620.881	3082.725	3066.706	3067.714	3065.698	K[27]
S[16]	1707.913	2954.630	2938.611	2939.619	2937.603	S[26]
R[17]	1864.014	2867.598	2851.579	2852.587	2850.571	R[25]
S[18]	1951.046	2711.496	2695.478	2696.486	2694.470	S[24]
S[19]	2038.078	2624.464	2608.446	2609.454	2607.438	S[23]
R[20]	2194.179	2537.432	2521.414	2522.422	2520.406	R[22]
A[21]	2265.216	2381.331	2365.313	2366.320	2364.305	A[21]
G[22]	2322.237	2310.294	2294.275	2295.283	2293.268	G[20]
L[23]	2435.322	2253.273	2237.254	2238.262	2236.246	L[19]
Q[24]	2563.380	2140.189	2124.170	2125.178	2123.162	Q[18]
F[25]	2710.449	2012.130	1996.111	**1997.119**	1995.104	F[17]
P[26]	2807.501	1865.062	1849.043	1850.051	1848.035	P[16]
V[27]	2906.570	1768.009	1751.990	1752.998	1750.982	V[15]
G[28]	2963.591	1668.940	1652.922	1653.930	1651.914	G[14]
R[29]	3119.692	1611.919	1595.900	**1596.908**	1594.892	R[13]
V[30]	3218.761	1455.818	1439.799	1440.807	1438.791	V[12]
H[31]	3355.820	1356.749	1340.731	**1341.739**	1339.723	H[11]
R[32]	3511.921	**1219.691**	**1203.672**	1204.680	1202.664	R[10]
L[33]	3625.005	**1063.589**	**1047.571**	**1048.579**	**1046.563**	L[9]
L[34]	3738.089	950.505	**934.487**	**935.495**	**933.479**	L[8]
R[35]	3894.190	837.421	**821.403**	822.410	820.395	R[7]
K[36]	4022.285	681.320	665.302	666.309	664.294	K[6]
G[37]	4079.306	553.225	537.207	538.214	536.199	G[5]
N[38]	4193.349	496.204	480.185	481.193	479.177	N[4]
Y[39]	4356.413	382.161	366.142	367.150	365.134	Y[3]
A[40]	4427.450	219.098	203.079	204.087	202.071	A[2]
E[41]	4556.492	148.060	132.042	133.050	131.034	E[1]

sp|Q6GSS7|H2A2A_MOUSE

[Acetyl]S $^{Phospho}_{79.97}$ GRGKQGGKARAKAKSRSSRAGLQFPVGRVHRLLRKGNYAE

- fragmentation table for charge state 2+
- specType=etd
- score=53.15
- F104903.dat
- query=q2842_p1
- precursor=760.423910
- chargePrecursor=6
- itol=0.8

AA	c	y	z+1	z+2	z	AA
S[1]	114.025	2279.242	2271.232	0.504	2270.729	S[41]
G[2]	142.536	2174.737	2166.728	0.504	2166.224	G[40]
R[3]	220.586	2146.227	2138.217	2138.721	2137.713	R[39]
G[4]	249.097	2068.176	2060.167	2060.671	2059.663	G[38]
K[5]	313.145	2039.665	2031.656	2032.160	2031.152	K[37]
Q[6]	377.174	1975.618	1967.608	1968.112	1967.105	Q[36]
G[7]	405.685	1911.589	1903.579	1904.083	1903.075	G[35]
G[8]	434.195	1883.078	1875.068	1875.572	1874.565	G[34]
K[9]	498.243	1854.567	1846.558	1847.062	1846.054	K[33]
A[10]	533.761	1790.520	1782.510	1783.014	1782.006	A[32]
R[11]	611.812	1755.001	1746.992	1747.496	1746.488	R[31]
A[12]	**647.330**	1676.951	1668.941	1669.445	1668.437	A[30]
K[13]	**711.378**	1641.432	**1633.423**	**1633.927**	**1632.919**	K[29]
A[14]	746.896	1577.384	1569.375	1569.879	1568.871	A[28]
K[15]	**810.944**	1541.866	1533.857	1534.360	1533.353	K[27]
S[16]	**854.460**	1477.818	**1469.809**	**1470.313**	1469.305	S[26]
R[17]	**932.511**	1434.302	**1426.293**	**1426.797**	1425.789	R[25]
S[18]	**976.027**	**1356.252**	**1348.243**	**1348.746**	1347.739	S[24]
S[19]	**1019.543**	**1312.736**	1304.726	**1305.230**	1304.223	S[23]
R[20]	**1097.593**	1269.220	**1261.210**	**1261.714**	**1260.707**	R[22]
A[21]	1133.112	1191.169	1183.160	**1183.664**	1182.656	A[21]
G[22]	1161.622	1155.651	1147.641	**1148.145**	1147.137	G[20]
L[23]	1218.164	**1127.140**	**1119.131**	**1119.635**	1118.627	L[19]
Q[24]	**1282.194**	**1070.598**	**1062.589**	**1063.093**	1062.085	Q[18]
F[25]	**1355.728**	1006.569	**998.559**	**999.063**	998.055	F[17]
P[26]	1404.254	**933.034**	925.025	925.529	924.521	P[16]
V[27]	1453.788	884.508	876.499	877.003	875.995	V[15]
G[28]	**1482.299**	834.974	**826.965**	**827.468**	826.461	G[14]
R[29]	1560.350	806.463	**798.454**	**798.958**	**797.950**	R[13]
V[30]	**1609.884**	728.413	720.403	720.907	719.899	V[12]
H[31]	**1678.413**	678.878	**670.869**	**671.373**	670.365	H[11]
R[32]	1756.464	610.349	**602.340**	**602.843**	**601.836**	R[10]
L[33]	1813.006	532.298	524.289	524.793	523.785	L[9]
L[34]	1869.548	475.756	467.747	468.251	467.243	L[8]
R[35]	1947.599	419.214	411.205	411.709	410.701	R[7]
K[36]	2011.646	341.164	333.154	333.658	332.650	K[6]
G[37]	2040.157	277.116	269.107	269.611	268.603	G[5]
N[38]	2097.178	248.606	240.596	241.100	240.092	N[4]
Y[39]	2178.710	191.584	183.575	184.079	183.071	Y[3]
A[40]	2214.229	110.052	102.043	102.547	101.539	A[2]
E[41]	2278.750	74.534	66.524	67.028	66.021	E[1]

sp|Q6GSS7|H2A2A_MOUSE

[Acetyl]S $^{Phospho}_{79.97}$ GRGKQGGKARAKAKSRSSRAGLQFPVGRVHRLLRKGNYAE

- fragmentation table for charge state 3+
- specType=etd
- score=53.15
- F104903.dat
- query=q2842_p1
- precursor=760.423910
- chargePrecursor=6
- itol=0.8

AA	c	y	z+1	z+2	z	AA
S[1]	76.352	1519.830	1514.491	0.672	1514.155	S[41]
G[2]	95.360	1450.161	1444.821	0.672	1444.485	G[40]
R[3]	147.393	1431.154	1425.814	1426.150	1425.478	R[39]
G[4]	166.400	1379.120	1373.780	1374.116	1373.444	G[38]
K[5]	209.099	1360.113	**1354.773**	**1355.109**	1354.437	K[37]
Q[6]	251.785	1317.414	**1312.075**	**1312.411**	**1311.739**	Q[36]
G[7]	270.792	1274.728	**1269.389**	**1269.725**	1269.053	G[35]
G[8]	289.799	1255.721	**1250.381**	**1250.717**	1250.045	G[34]
K[9]	332.498	1236.714	**1231.374**	**1231.710**	1231.038	K[33]
A[10]	356.177	1194.016	1188.676	1189.012	1188.340	A[32]
R[11]	408.210	1170.336	**1164.997**	**1165.333**	1164.661	R[31]
A[12]	431.889	1118.303	1112.963	1113.299	1112.627	A[30]
K[13]	474.588	1094.624	**1089.284**	**1089.620**	1088.948	K[29]
A[14]	498.267	1051.925	**1046.586**	**1046.922**	1046.250	A[28]
K[15]	540.965	1028.246	1022.907	1023.243	1022.571	K[27]
S[16]	569.976	985.548	**980.208**	**980.544**	**979.873**	S[26]
R[17]	622.009	956.537	**951.198**	**951.534**	**950.862**	R[25]
S[18]	651.020	**904.504**	899.164	899.500	898.828	S[24]
S[19]	680.031	875.493	**870.153**	**870.489**	**869.817**	S[23]
R[20]	**732.064**	846.482	**841.143**	**841.479**	**840.807**	R[22]
A[21]	755.744	794.449	**789.109**	**789.445**	788.773	A[21]
G[22]	774.751	770.770	765.430	765.766	765.094	G[20]
L[23]	**812.445**	751.762	746.423	746.759	746.087	L[19]
Q[24]	**855.132**	714.068	708.728	709.064	708.392	Q[18]
F[25]	**904.154**	**671.382**	666.042	666.378	665.706	F[17]
P[26]	936.505	622.359	617.019	617.355	616.683	P[16]
V[27]	**969.528**	590.008	584.668	585.004	584.332	V[15]
G[28]	**988.535**	556.985	551.645	551.981	551.309	G[14]
R[29]	1040.569	537.978	532.638	532.974	532.302	R[13]
V[30]	**1073.592**	485.944	480.605	480.941	480.269	V[12]
H[31]	**1119.278**	452.921	447.582	447.918	447.246	H[11]
R[32]	**1171.312**	407.235	401.895	402.231	401.560	R[10]
L[33]	**1209.006**	355.201	349.862	350.198	349.526	L[9]
L[34]	**1246.701**	317.507	312.167	312.503	311.831	L[8]
R[35]	**1298.735**	279.812	274.472	274.808	274.136	R[7]
K[36]	**1341.433**	227.778	222.439	222.775	222.103	K[6]
G[37]	1360.440	185.080	179.740	180.076	179.404	G[5]
N[38]	1398.455	166.073	160.733	161.069	160.397	N[4]
Y[39]	1452.809	128.058	122.719	123.055	122.383	Y[3]
A[40]	1476.488	73.704	68.364	68.700	68.029	A[2]
E[41]	1519.502	50.025	44.685	45.021	44.349	E[1]

sp|Q6GSS7|H2A2A_MOUSE

[Acetyl]S $^{Phospho}_{79.97}$ GRGKQGGKARAKAKSRSSRAGLQFPVGRVHRLLRKGNYAE

- fragmentation table for charge state 4+
- specType=etd
- score=53.15
- F104903.dat
- query=q2842_p1
- precursor=760.423910
- chargePrecursor=6
- itol=0.8

AA	c	y	z+1	z+2	z	AA
S[1]	57.516	**1140.125**	**1136.120**	0.755	**1135.868**	S[41]
G[2]	71.772	1087.872	**1083.868**	0.755	**1083.616**	G[40]
R[3]	110.797	**1073.617**	1069.612	1069.864	1069.360	R[39]
G[4]	125.052	1034.592	1030.587	1030.839	1030.335	G[38]
K[5]	157.076	**1020.336**	1016.332	1016.584	1016.080	K[37]
Q[6]	189.091	**988.313**	984.308	984.560	984.056	Q[36]
G[7]	203.346	956.298	952.293	952.545	**952.041**	G[35]
G[8]	217.601	942.043	938.038	938.290	937.786	G[34]
K[9]	249.625	927.787	923.783	924.034	923.531	K[33]
A[10]	267.384	895.763	891.759	892.011	891.507	A[32]
R[11]	306.410	878.004	873.999	874.251	873.748	R[31]
A[12]	324.169	838.979	834.974	835.226	834.722	A[30]
K[13]	356.193	**821.220**	817.215	817.467	816.963	K[29]
A[14]	373.952	**789.196**	785.191	785.443	784.939	A[28]
K[15]	405.976	771.437	767.432	767.684	767.180	K[27]
S[16]	427.734	739.413	735.408	735.660	735.156	S[26]
R[17]	466.759	717.655	713.650	713.902	713.398	R[25]
S[18]	488.517	678.630	674.625	674.877	674.373	S[24]
S[19]	510.275	656.872	652.867	653.119	652.615	S[23]
R[20]	549.300	635.114	631.109	631.361	630.857	R[22]
A[21]	567.059	596.088	592.084	592.336	591.832	A[21]
G[22]	581.315	578.329	574.324	574.576	574.072	G[20]
L[23]	609.586	564.074	560.069	560.321	559.817	L[19]
Q[24]	641.600	535.803	531.798	532.050	531.546	Q[18]
F[25]	678.368	503.788	499.783	500.035	499.531	F[17]
P[26]	702.631	467.021	463.016	463.268	462.764	P[16]
V[27]	727.398	442.758	438.753	439.005	438.501	V[15]
G[28]	741.653	417.991	413.986	414.238	413.734	G[14]
R[29]	780.679	403.735	399.731	399.982	399.479	R[13]
V[30]	805.446	364.710	360.705	360.957	360.453	V[12]
H[31]	**839.710**	339.943	335.938	336.190	335.686	H[11]
R[32]	878.736	305.678	301.673	301.925	301.421	R[10]
L[33]	907.007	266.653	262.648	262.900	262.396	L[9]
L[34]	**935.278**	238.382	234.377	234.629	234.125	L[8]
R[35]	974.303	210.111	206.106	206.358	205.854	R[7]
K[36]	1006.327	171.086	167.081	167.333	166.829	K[6]
G[37]	**1020.582**	139.062	135.057	135.309	134.805	G[5]
N[38]	**1049.093**	124.806	120.802	121.054	120.550	N[4]
Y[39]	**1089.859**	96.296	92.291	92.543	92.039	Y[3]
A[40]	**1107.618**	55.530	51.525	51.777	51.273	A[2]
E[41]	**1139.879**	37.771	33.766	34.018	33.514	E[1]

sp|Q6GSS7|H2A2A_MOUSE

[Acetyl]S $^{Phospho}_{79.97}$ GRGKQGGKARAKAKSRSSRAGLQFPVGRVHRLLRKGNYAE

- fragmentation table for charge state 5+
- specType=etd
- score=53.15
- F104903.dat
- query=q2842_p1
- precursor=760.423910
- chargePrecursor=6
- itol=0.8

AA	c	y	z+1	z+2	z	AA
S[1]	46.214	**912.301**	909.097	0.806	908.896	S[41]
G[2]	57.619	**870.499**	867.296	0.806	867.094	G[40]
R[3]	88.839	859.095	**855.891**	**856.093**	**855.690**	R[39]
G[4]	100.243	**827.875**	824.671	824.873	824.469	G[38]
K[5]	125.862	816.471	**813.267**	**813.468**	**813.065**	K[37]
Q[6]	151.474	790.852	787.648	787.849	787.446	Q[36]
G[7]	162.878	765.240	762.036	762.238	761.834	G[35]
G[8]	174.282	**753.836**	750.632	750.833	750.430	G[34]
K[9]	199.901	742.431	739.227	739.429	739.026	K[33]
A[10]	214.109	716.812	713.608	713.810	713.407	A[32]
R[11]	245.329	702.605	699.401	699.603	699.199	R[31]
A[12]	259.537	**671.385**	668.181	668.382	667.979	A[30]
K[13]	285.156	657.177	653.973	654.175	653.772	K[29]
A[14]	299.363	631.558	628.354	628.556	628.153	A[28]
K[15]	324.982	617.351	614.147	614.349	613.945	K[27]
S[16]	342.388	591.732	588.528	588.730	588.326	S[26]
R[17]	373.609	574.325	571.122	571.323	570.920	R[25]
S[18]	391.015	543.105	539.901	540.103	539.700	S[24]
S[19]	408.421	525.699	522.495	522.697	522.293	S[23]
R[20]	439.642	508.292	505.089	505.290	504.887	R[22]
A[21]	453.849	477.072	473.868	474.070	473.667	A[21]
G[22]	465.253	462.865	459.661	459.862	459.459	G[20]
L[23]	487.870	451.460	448.257	448.458	448.055	L[19]
Q[24]	513.482	428.844	425.640	425.841	425.438	Q[18]
F[25]	542.896	403.232	400.028	400.230	399.827	F[17]
P[26]	562.306	373.818	370.614	370.816	370.413	P[16]
V[27]	582.120	354.408	351.204	351.405	351.002	V[15]
G[28]	593.524	334.594	331.390	331.592	331.189	G[14]
R[29]	624.744	323.190	319.986	320.187	319.784	R[13]
V[30]	644.558	291.969	288.766	288.967	288.564	V[12]
H[31]	**671.970**	272.156	268.952	269.154	268.750	H[11]
R[32]	703.190	244.744	241.540	241.742	241.339	R[10]
L[33]	725.807	213.524	210.320	210.522	210.118	L[9]
L[34]	748.424	190.907	187.703	187.905	187.502	L[8]
R[35]	779.644	168.290	165.086	165.288	164.885	R[7]
K[36]	805.263	137.070	133.866	134.068	133.665	K[6]
G[37]	816.667	111.451	108.247	108.449	108.046	G[5]
N[38]	**839.476**	100.047	96.843	97.044	96.641	N[4]
Y[39]	872.088	77.238	74.034	74.236	73.833	Y[3]
A[40]	886.296	44.625	41.422	41.623	41.220	A[2]
E[41]	**912.104**	30.418	27.214	27.416	27.013	E[1]

sp|Q6GSS7|H2A2A_MOUSE

[Acetyl]S $^{Phospho}_{79.97}$ GRGKQGGK $^{Dimethyl}_{28.03}$ ARAKAKSRSSRAGLQFPVGRVHRLLRKGNYAE

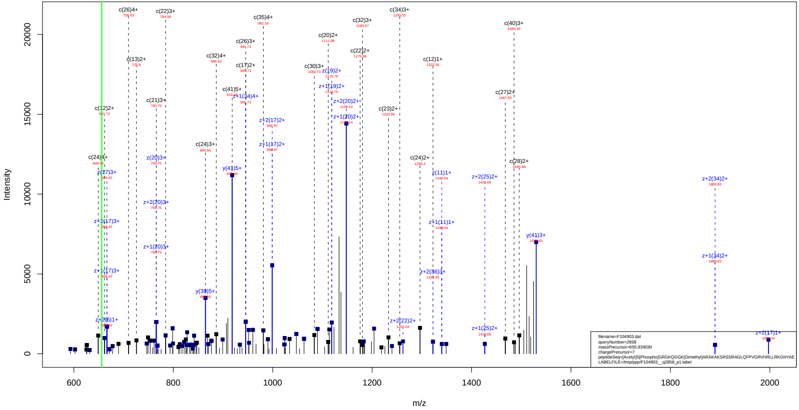

sp|Q6GSS7|H2A2A_MOUSE

[Acetyl]S $^{Phospho}_{79.97}$ GRGKQGGK $^{Dimethyl}_{28.03}$ ARAKAKSRSSRAGLQFPVGRVHRLLRKGNYAE

- fragmentation table for charge state 1+
- specType=etd
- score=61.45
- F104903.dat
- query=q2858_p1
- precursor=655.939030
- chargePrecursor=7
- itol=0.8

AA	c	y	z+1	z+2	z	AA
S[1]	227.043	4585.508	4569.489	0.000	4568.481	S[41]
G[2]	284.064	4376.499	4360.480	0.000	4359.472	G[40]
R[3]	440.165	4319.477	4303.459	4304.466	4302.451	R[39]
G[4]	497.187	4163.376	4147.357	4148.365	4146.350	G[38]
K[5]	**625.282**	4106.355	4090.336	4091.344	4089.328	K[37]
Q[6]	**753.340**	3978.260	3962.241	3963.249	3961.233	Q[36]
G[7]	**810.362**	3850.201	3834.182	3835.190	3833.175	G[35]
G[8]	867.383	3793.180	3777.161	3778.169	3776.153	G[34]
K[9]	**1023.510**	3736.158	3720.140	3721.147	3719.132	K[33]
A[10]	1094.547	3580.032	3564.013	3565.021	3563.005	A[32]
R[11]	1250.648	3508.995	3492.976	3493.984	3491.968	R[31]
A[12]	**1321.685**	3352.894	3336.875	3337.883	3335.867	A[30]
K[13]	1449.780	3281.857	3265.838	3266.846	3264.830	K[29]
A[14]	1520.817	3153.762	3137.743	3138.751	3136.735	A[28]
K[15]	1648.912	3082.725	3066.706	3067.714	3065.698	K[27]
S[16]	1735.944	2954.630	2938.611	2939.619	2937.603	S[26]
R[17]	1892.045	2867.598	2851.579	2852.587	2850.571	R[25]
S[18]	1979.077	2711.496	2695.478	2696.486	2694.470	S[24]
S[19]	2066.109	2624.464	2608.446	2609.454	2607.438	S[23]
R[20]	2222.210	2537.432	2521.414	2522.422	2520.406	R[22]
A[21]	2293.247	2381.331	2365.313	2366.320	2364.305	A[21]
G[22]	2350.269	2310.294	2294.275	2295.283	2293.268	G[20]
L[23]	2463.353	2253.273	2237.254	2238.262	2236.246	L[19]
Q[24]	2591.411	2140.189	2124.170	2125.178	2123.162	Q[18]
F[25]	2738.480	2012.130	1996.111	**1997.119**	1995.104	F[17]
P[26]	2835.533	1865.062	1849.043	1850.051	1848.035	P[16]
V[27]	2934.601	1768.009	1751.990	1752.998	1750.982	V[15]
G[28]	2991.622	1668.940	1652.922	1653.930	1651.914	G[14]
R[29]	3147.724	1611.919	1595.900	1596.908	1594.892	R[13]
V[30]	3246.792	1455.818	1439.799	1440.807	1438.791	V[12]
H[31]	3383.851	1356.749	**1340.731**	1341.739	**1339.723**	H[11]
R[32]	3539.952	1219.691	**1203.672**	1204.680	1202.664	R[10]
L[33]	3653.036	**1063.589**	**1047.571**	1048.579	1046.563	L[9]
L[34]	3766.120	950.505	**934.487**	935.495	**933.479**	L[8]
R[35]	3922.221	837.421	**821.403**	**822.410**	820.395	R[7]
K[36]	4050.316	681.320	665.302	**666.309**	664.294	K[6]
G[37]	4107.338	553.225	537.207	538.214	536.199	G[5]
N[38]	4221.381	496.204	480.185	481.193	479.177	N[4]
Y[39]	4384.444	382.161	366.142	367.150	365.134	Y[3]
A[40]	4455.481	219.098	203.079	204.087	202.071	A[2]
E[41]	4584.524	148.060	132.042	133.050	131.034	E[1]

sp|Q6GSS7|H2A2A_MOUSE

[Acetyl]S $^{Phospho}_{79.97}$ GRGKQGGK $^{Dimethyl}_{28.03}$ ARAKAKSRSSRAGLQFPVGRVHRLLRKGNYAE

- fragmentation table for charge state 2+
- specType=etd
- score=61.45
- F104903.dat
- query=q2858_p1
- precursor=655.939030
- chargePrecursor=7
- itol=0.8

AA	c	y	z+1	z+2	z	AA
S[1]	114.025	2293.257	2285.248	0.504	2284.744	S[41]
G[2]	142.536	2188.753	2180.744	0.504	2180.240	G[40]
R[3]	220.586	2160.242	2152.233	2152.737	2151.729	R[39]
G[4]	249.097	2082.192	2074.182	2074.686	2073.678	G[38]
K[5]	313.145	2053.681	2045.672	2046.176	2045.168	K[37]
Q[6]	377.174	1989.634	1981.624	1982.128	1981.120	Q[36]
G[7]	405.685	1925.604	1917.595	1918.099	1917.091	G[35]
G[8]	434.195	1897.093	**1889.084**	**1889.588**	1888.580	G[34]
K[9]	512.258	1868.583	1860.573	1861.077	1860.069	K[33]
A[10]	547.777	1790.520	1782.510	1783.014	1782.006	A[32]
R[11]	**625.828**	1755.001	1746.992	1747.496	1746.488	R[31]
A[12]	**661.346**	1676.951	1668.941	1669.445	1668.437	A[30]
K[13]	**725.394**	1641.432	1633.423	1633.927	1632.919	K[29]
A[14]	**760.912**	1577.384	1569.375	1569.879	1568.871	A[28]
K[15]	**824.960**	1541.866	1533.857	1534.360	1533.353	K[27]
S[16]	**868.476**	1477.818	1469.809	1470.313	1469.305	S[26]
R[17]	**946.526**	1434.302	**1426.293**	**1426.797**	1425.789	R[25]
S[18]	**990.042**	1356.252	**1348.243**	**1348.746**	1347.739	S[24]
S[19]	**1033.558**	1312.736	1304.726	1305.230	1304.223	S[23]
R[20]	**1111.609**	1269.220	1261.210	**1261.714**	1260.707	R[22]
A[21]	1147.127	1191.169	**1183.160**	**1183.664**	**1182.656**	A[21]
G[22]	**1175.638**	1155.651	**1147.641**	**1148.145**	1147.137	G[20]
L[23]	**1232.180**	1127.140	**1119.131**	1119.635	**1118.627**	L[19]
Q[24]	**1296.209**	1070.598	**1062.589**	**1063.093**	1062.085	Q[18]
F[25]	1369.744	1006.569	**998.559**	**999.063**	998.055	F[17]
P[26]	1418.270	933.034	925.025	925.529	924.521	P[16]
V[27]	**1467.804**	884.508	**876.499**	**877.003**	**875.995**	V[15]
G[28]	**1496.315**	**834.974**	**826.965**	**827.468**	**826.461**	G[14]
R[29]	1574.365	806.463	**798.454**	**798.958**	**797.950**	R[13]
V[30]	1623.900	728.413	720.403	720.907	719.899	V[12]
H[31]	1692.429	678.878	**670.869**	**671.373**	670.365	H[11]
R[32]	1770.480	610.349	**602.340**	**602.843**	**601.836**	R[10]
L[33]	1827.022	532.298	524.289	524.793	523.785	L[9]
L[34]	1883.564	475.756	467.747	468.251	467.243	L[8]
R[35]	1961.614	419.214	411.205	411.709	410.701	R[7]
K[36]	2025.662	341.164	333.154	333.658	332.650	K[6]
G[37]	2054.172	277.116	269.107	269.611	268.603	G[5]
N[38]	2111.194	248.606	240.596	241.100	240.092	N[4]
Y[39]	2192.726	191.584	183.575	184.079	183.071	Y[3]
A[40]	2228.244	110.052	102.043	102.547	101.539	A[2]
E[41]	2292.765	74.534	66.524	67.028	66.021	E[1]

sp|Q6GSS7|H2A2A_MOUSE

[Acetyl]S $^{Phospho}_{79.97}$ GRGKQGGK $^{Dimethyl}_{28.03}$ ARAKAKSRSSRAGLQFPVGRVHRLLRKGNYAE

- fragmentation table for charge state 3+
- specType=etd
- score=61.45
- F104903.dat
- query=q2858_p1
- precursor=655.939030
- chargePrecursor=7
- itol=0.8

AA	c	y	z+1	z+2	z	AA
S[1]	76.352	**1529.174**	1523.835	0.672	1523.499	S[41]
G[2]	95.360	1459.504	1454.165	0.672	1453.829	G[40]
R[3]	147.393	1440.497	1435.158	1435.494	1434.822	R[39]
G[4]	166.400	1388.464	1383.124	1383.460	1382.788	G[38]
K[5]	209.099	1369.456	1364.117	1364.453	1363.781	K[37]
Q[6]	251.785	1326.758	1321.419	**1321.754**	1321.083	Q[36]
G[7]	270.792	1284.072	1278.732	1279.068	1278.396	G[35]
G[8]	289.799	1265.065	1259.725	1260.061	1259.389	G[34]
K[9]	341.841	1246.058	1240.718	1241.054	**1240.382**	K[33]
A[10]	365.520	1194.016	1188.676	1189.012	1188.340	A[32]
R[11]	417.554	1170.336	1164.997	1165.333	1164.661	R[31]
A[12]	441.233	**1118.303**	1112.963	**1113.299**	1112.627	A[30]
K[13]	483.931	1094.624	**1089.284**	**1089.620**	1088.948	K[29]
A[14]	507.610	1051.925	1046.586	**1046.922**	1046.250	A[28]
K[15]	550.309	1028.246	**1022.907**	**1023.243**	1022.571	K[27]
S[16]	579.319	985.548	980.208	**980.544**	979.873	S[26]
R[17]	**631.353**	956.537	**951.198**	**951.534**	**950.862**	R[25]
S[18]	660.364	904.504	**899.164**	**899.500**	**898.828**	S[24]
S[19]	**689.375**	875.493	**870.153**	**870.489**	**869.817**	S[23]
R[20]	741.408	846.482	**841.143**	**841.479**	**840.807**	R[22]
A[21]	**765.087**	794.449	789.109	789.445	788.773	A[21]
G[22]	**784.094**	770.770	**765.430**	**765.766**	**765.094**	G[20]
L[23]	**821.789**	751.762	**746.423**	**746.759**	**746.087**	L[19]
Q[24]	**864.475**	714.068	708.728	709.064	708.392	Q[18]
F[25]	913.498	**671.382**	**666.042**	**666.378**	**665.706**	F[17]
P[26]	**945.849**	622.359	617.019	617.355	616.683	P[16]
V[27]	978.872	590.008	584.668	585.004	584.332	V[15]
G[28]	997.879	556.985	551.645	551.981	551.309	G[14]
R[29]	1049.913	537.978	532.638	532.974	532.302	R[13]
V[30]	**1082.936**	485.944	480.605	480.941	480.269	V[12]
H[31]	1128.622	452.921	447.582	447.918	447.246	H[11]
R[32]	**1180.656**	407.235	401.895	402.231	401.560	R[10]
L[33]	**1218.350**	355.201	349.862	350.198	349.526	L[9]
L[34]	**1256.045**	317.507	312.167	312.503	311.831	L[8]
R[35]	1308.079	279.812	274.472	274.808	274.136	R[7]
K[36]	1350.777	227.778	222.439	222.775	222.103	K[6]
G[37]	1369.784	185.080	179.740	180.076	179.404	G[5]
N[38]	1407.798	166.073	160.733	161.069	160.397	N[4]
Y[39]	1462.153	128.058	122.719	123.055	122.383	Y[3]
A[40]	**1485.832**	73.704	68.364	68.700	68.029	A[2]
E[41]	1528.846	50.025	44.685	45.021	44.349	E[1]

sp|Q6GSS7|H2A2A_MOUSE

[Acetyl]S $^{Phospho}_{79.97}$ GRGKQGGK $^{Dimethyl}_{28.03}$ ARAKAKSRSSRAGLQFPVGRVHRLLRKGNYAE

- fragmentation table for charge state 4+
- specType=etd
- score=61.45
- F104903.dat
- query=q2858_p1
- precursor=655.939030
- chargePrecursor=7
- itol=0.8

AA	c	y	z+1	z+2	z	AA
S[1]	57.516	1147.132	1143.128	0.755	1142.876	S[41]
G[2]	71.772	1094.880	1090.875	0.755	**1090.624**	G[40]
R[3]	110.797	1080.625	1076.620	1076.872	1076.368	R[39]
G[4]	125.052	1041.600	1037.595	1037.847	1037.343	G[38]
K[5]	157.076	1027.344	**1023.339**	**1023.591**	**1023.087**	K[37]
Q[6]	189.091	995.320	991.316	991.568	**991.064**	Q[36]
G[7]	203.346	963.306	**959.301**	**959.553**	959.049	G[35]
G[8]	217.601	949.050	**945.046**	**945.298**	944.794	G[34]
K[9]	256.633	934.795	930.790	931.042	930.538	K[33]
A[10]	274.392	895.763	891.759	892.011	891.507	A[32]
R[11]	313.417	878.004	873.999	874.251	873.748	R[31]
A[12]	331.177	**838.979**	**834.974**	**835.226**	**834.722**	A[30]
K[13]	363.200	**821.220**	**817.215**	**817.467**	**816.963**	K[29]
A[14]	380.960	789.196	**785.191**	785.443	**784.939**	A[28]
K[15]	412.983	771.437	**767.432**	**767.684**	**767.180**	K[27]
S[16]	434.741	739.413	735.408	735.660	735.156	S[26]
R[17]	473.767	717.655	713.650	713.902	713.398	R[25]
S[18]	495.525	**678.630**	674.625	674.877	674.373	S[24]
S[19]	517.283	656.872	652.867	653.119	652.615	S[23]
R[20]	556.308	635.114	**631.109**	**631.361**	630.857	R[22]
A[21]	574.067	596.088	592.084	**592.336**	591.832	A[21]
G[22]	588.323	578.329	574.324	574.576	574.072	G[20]
L[23]	616.594	564.074	560.069	560.321	559.817	L[19]
Q[24]	**648.608**	535.803	531.798	532.050	531.546	Q[18]
F[25]	685.375	503.788	499.783	500.035	499.531	F[17]
P[26]	**709.639**	467.021	463.016	463.268	462.764	P[16]
V[27]	734.406	442.758	438.753	439.005	438.501	V[15]
G[28]	**748.661**	417.991	413.986	414.238	413.734	G[14]
R[29]	787.686	403.735	399.731	399.982	399.479	R[13]
V[30]	**812.453**	364.710	360.705	360.957	360.453	V[12]
H[31]	**846.718**	339.943	335.938	336.190	335.686	H[11]
R[32]	**885.743**	305.678	301.673	301.925	301.421	R[10]
L[33]	914.014	266.653	262.648	262.900	262.396	L[9]
L[34]	942.285	238.382	234.377	234.629	234.125	L[8]
R[35]	**981.311**	210.111	206.106	206.358	205.854	R[7]
K[36]	1013.335	171.086	167.081	167.333	166.829	K[6]
G[37]	1027.590	139.062	135.057	135.309	134.805	G[5]
N[38]	1056.101	124.806	120.802	121.054	120.550	N[4]
Y[39]	1096.866	96.296	92.291	92.543	92.039	Y[3]
A[40]	1114.626	55.530	51.525	51.777	51.273	A[2]
E[41]	1146.886	37.771	33.766	34.018	33.514	E[1]

sp|Q6GSS7|H2A2A_MOUSE

[Acetyl]S $^{Phospho}_{79.97}$ GRGKQGGK $^{Dimethyl}_{28.03}$ ARAKAKSRSSRAGLQFPVGRVHRLLRKGNYAE

- fragmentation table for charge state 5+
- specType=etd
- score=61.45
- F104903.dat
- query=q2858_p1
- precursor=655.939030
- chargePrecursor=7
- itol=0.8

AA	c	y	z+1	z+2	z	AA
S[1]	46.214	**917.907**	914.704	0.806	914.502	S[41]
G[2]	57.619	**876.106**	872.902	0.806	872.700	G[40]
R[3]	88.839	**864.701**	861.498	861.699	861.296	R[39]
G[4]	100.243	**833.481**	830.277	830.479	830.076	G[38]
K[5]	125.862	**822.077**	818.873	819.075	818.671	K[37]
Q[6]	151.474	796.458	**793.254**	**793.456**	**793.052**	Q[36]
G[7]	162.878	770.846	**767.642**	**767.844**	**767.441**	G[35]
G[8]	174.282	759.442	756.238	756.440	756.036	G[34]
K[9]	205.508	748.037	744.834	745.035	744.632	K[33]
A[10]	219.715	716.812	713.608	713.810	713.407	A[32]
R[11]	250.935	702.605	699.401	699.603	699.199	R[31]
A[12]	265.143	**671.385**	668.181	668.382	667.979	A[30]
K[13]	290.762	657.177	653.973	654.175	653.772	K[29]
A[14]	304.969	**631.558**	628.354	628.556	628.153	A[28]
K[15]	330.588	617.351	614.147	614.349	613.945	K[27]
S[16]	347.995	591.732	588.528	588.730	588.326	S[26]
R[17]	379.215	574.325	571.122	571.323	570.920	R[25]
S[18]	396.621	543.105	539.901	540.103	539.700	S[24]
S[19]	414.028	525.699	522.495	522.697	522.293	S[23]
R[20]	445.248	508.292	505.089	505.290	504.887	R[22]
A[21]	459.455	477.072	473.868	474.070	473.667	A[21]
G[22]	470.860	462.865	459.661	459.862	459.459	G[20]
L[23]	493.476	451.460	448.257	448.458	448.055	L[19]
Q[24]	519.088	428.844	425.640	425.841	425.438	Q[18]
F[25]	548.502	403.232	400.028	400.230	399.827	F[17]
P[26]	567.912	373.818	370.614	370.816	370.413	P[16]
V[27]	587.726	354.408	351.204	351.405	351.002	V[15]
G[28]	599.130	334.594	331.390	331.592	331.189	G[14]
R[29]	630.351	323.190	319.986	320.187	319.784	R[13]
V[30]	650.164	291.969	288.766	288.967	288.564	V[12]
H[31]	**677.576**	272.156	268.952	269.154	268.750	H[11]
R[32]	708.796	244.744	241.540	241.742	241.339	R[10]
L[33]	731.413	213.524	210.320	210.522	210.118	L[9]
L[34]	**754.030**	190.907	187.703	187.905	187.502	L[8]
R[35]	**785.250**	168.290	165.086	165.288	164.885	R[7]
K[36]	**810.869**	137.070	133.866	134.068	133.665	K[6]
G[37]	**822.273**	111.451	108.247	108.449	108.046	G[5]
N[38]	**845.082**	100.047	96.843	97.044	96.641	N[4]
Y[39]	877.695	77.238	74.034	74.236	73.833	Y[3]
A[40]	891.902	44.625	41.422	41.623	41.220	A[2]
E[41]	**917.711**	30.418	27.214	27.416	27.013	E[1]

sp|Q6GSS7|H2A2A_MOUSE

[Acetyl]S $^{\text{Phospho}}_{79.97}$ GRGKQGGK $^{\text{Dimethyl}}_{28.03}$ ARAKAKSRSSRAGLQFPVGRVHRLLRKGNYAE

- fragmentation table for charge state 6+
- specType=etd
- score=61.45
- F104903.dat
- query=q2858_p1
- precursor=655.939030
- chargePrecursor=7
- itol=0.8

AA	c	y	z+1	z+2	z	AA
S[1]	38.680	**765.091**	762.421	0.839	**762.253**	S[41]
G[2]	48.183	730.256	727.586	0.839	727.418	G[40]
R[3]	74.200	720.752	718.082	718.250	717.915	R[39]
G[4]	83.704	694.735	692.066	692.234	691.898	G[38]
K[5]	105.053	685.232	682.562	682.730	682.394	K[37]
Q[6]	126.396	663.883	**661.213**	**661.381**	**661.045**	Q[36]
G[7]	135.900	642.540	639.870	640.038	639.702	G[35]
G[8]	145.403	633.036	630.366	630.534	630.198	G[34]
K[9]	171.424	623.532	620.863	621.031	620.695	K[33]
A[10]	183.264	597.511	594.842	595.010	594.674	A[32]
R[11]	209.281	585.672	583.002	583.170	582.834	R[31]
A[12]	221.120	559.655	556.985	557.153	556.817	A[30]
K[13]	242.469	547.816	545.146	545.314	544.978	K[29]
A[14]	254.309	526.466	523.797	523.965	523.629	A[28]
K[15]	275.658	514.627	511.957	512.125	511.789	K[27]
S[16]	290.163	493.278	490.608	490.776	490.440	S[26]
R[17]	316.180	478.772	476.103	476.271	475.935	R[25]
S[18]	330.686	452.755	450.086	450.254	449.918	S[24]
S[19]	345.191	438.250	435.580	435.748	435.412	S[23]
R[20]	371.208	423.745	421.075	421.243	420.907	R[22]
A[21]	383.047	397.728	395.058	395.226	394.890	A[21]
G[22]	392.551	385.888	383.219	383.387	383.051	G[20]
L[23]	411.398	376.385	373.715	373.883	373.547	L[19]
Q[24]	432.741	357.538	354.868	355.036	354.700	Q[18]
F[25]	457.253	336.194	333.525	333.693	333.357	F[17]
P[26]	473.428	311.683	309.013	309.181	308.845	P[16]
V[27]	489.940	295.508	292.838	293.006	292.670	V[15]
G[28]	499.443	278.996	276.326	276.494	276.158	G[14]
R[29]	525.460	269.493	266.823	266.991	266.655	R[13]
V[30]	541.971	243.476	240.806	240.974	240.638	V[12]
H[31]	564.815	226.964	224.295	224.462	224.127	H[11]
R[32]	590.831	204.121	201.451	201.619	201.283	R[10]
L[33]	609.679	178.104	175.435	175.602	175.267	L[9]
L[34]	628.526	159.257	156.587	156.755	156.419	L[8]
R[35]	654.543	140.410	137.740	137.908	137.572	R[7]
K[36]	675.892	114.393	111.723	111.891	111.555	K[6]
G[37]	685.396	93.044	90.374	90.542	90.206	G[5]
N[38]	704.403	83.540	80.870	81.038	80.702	N[4]
Y[39]	731.580	64.533	61.863	62.031	61.695	Y[3]
A[40]	743.420	37.356	34.686	34.854	34.518	A[2]
E[41]	764.927	25.516	22.846	23.014	22.678	E[1]

sp|Q64523|H2A2C_MOUSE

SHKAK $^{Acetyl}_{42.01}$ SK

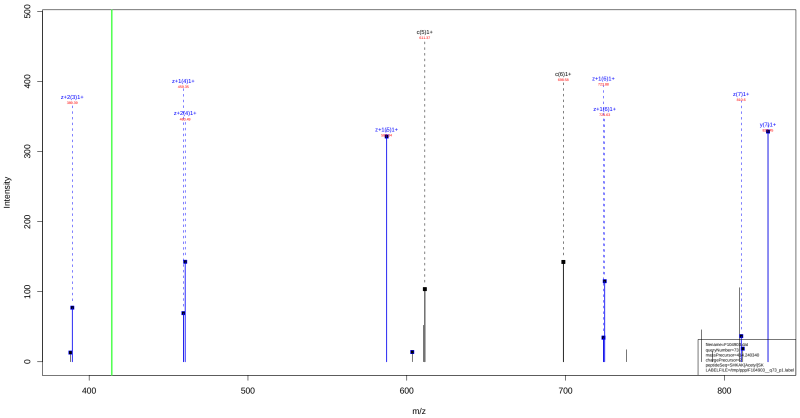

sp|Q64523|H2A2C_MOUSE

SHKAK $^{Acetyl}_{42.01}$ SK

- fragmentation table for charge state 1+
- specType=etd
- score=30.06
- F104903.dat
- query=q73_p1
- precursor=414.240340
- chargePrecursor=2
- itol=0.8

AA	c	y	z+1	z+2	z	AA
S[1]	105.066	**827.473**	**811.455**	0.000	**810.447**	S[7]
H[2]	242.125	740.441	**724.423**	0.000	**723.415**	H[6]
K[3]	370.220	**603.382**	**587.364**	588.372	586.356	K[5]
A[4]	441.257	475.287	**459.269**	**460.277**	458.261	A[4]
K[5]	**611.362**	404.250	**388.232**	**389.239**	387.224	K[3]
S[6]	**698.394**	234.145	218.126	219.134	217.118	S[2]
K[7]	826.489	147.113	131.094	132.102	130.086	K[1]

sp|P22752|H2A1_MOUSE

SHHKAKGK Acetyl 42.01

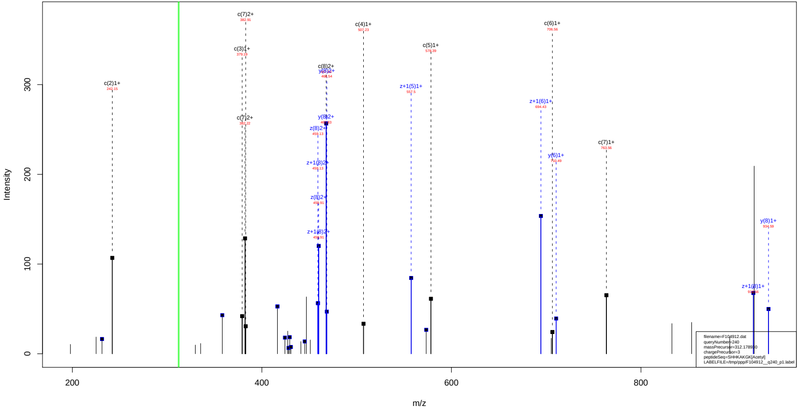

sp|P22752|H2A1_MOUSE

SHHKAKGK $^{\text{Acetyl}}_{42.01}$

- fragmentation table for charge state 1+
- specType=etd
- score=44.06
- F104912.dat
- query=q240_p1
- precursor=312.178930
- chargePrecursor=3
- itol=0.8

AA	c	y	z+1	z+2	z	AA
S[1]	105.066	**934.522**	**918.503**	0.000	917.495	S[8]
H[2]	**242.125**	847.490	831.471	0.000	830.463	H[7]
H[3]	**379.184**	**710.431**	**694.412**	0.000	693.404	H[6]
K[4]	**507.279**	**573.372**	**557.353**	558.361	556.345	K[5]
A[5]	**578.316**	**445.277**	**429.258**	**430.266**	**428.250**	A[4]
K[6]	**706.411**	374.240	**358.221**	359.229	357.213	K[3]
G[7]	**763.432**	246.145	230.126	**231.134**	229.118	G[2]
K[8]	933.538	189.123	173.105	174.112	172.097	K[1]

sp|P22752|H2A1_MOUSE

SHHKAKGK $^{\text{Acetyl}}_{42.01}$

- fragmentation table for charge state 2+
- specType=etd
- score=44.06
- F104912.dat
- query=q240_p1
- precursor=312.178930
- chargePrecursor=3
- itol=0.8

AA	c	y	z+1	z+2	z	AA
S[1]	53.037	**467.765**	**459.755**	0.504	**459.251**	S[8]
H[2]	121.566	**424.248**	**416.239**	0.504	**415.735**	H[7]
H[3]	190.095	355.719	347.710	0.504	347.206	H[6]
K[4]	254.143	287.190	279.180	279.684	278.676	K[5]
A[5]	289.662	223.142	215.133	215.637	214.629	A[4]
K[6]	353.709	187.624	179.614	180.118	179.110	K[3]
G[7]	**382.220**	123.576	115.567	116.071	115.063	G[2]
K[8]	**467.272**	95.065	87.056	87.560	86.552	K[1]

sp|P22752|H2A1_MOUSE

SHHKAKGK $^{Acetyl}_{42.01}$

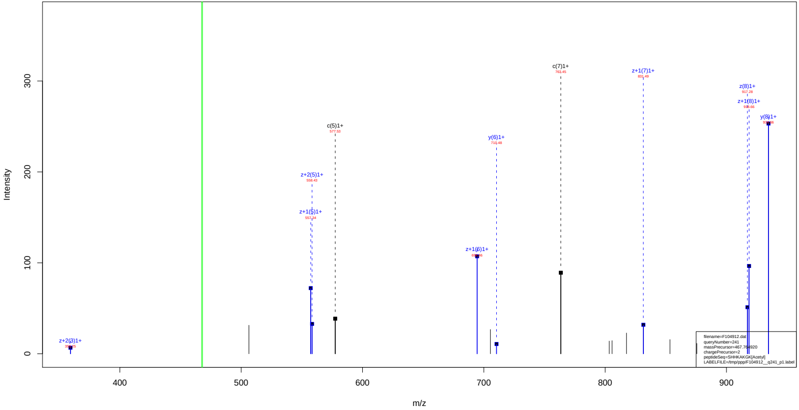

sp|P22752|H2A1_MOUSE

SHHKAKGK $^{Acetyl}_{42.01}$

- fragmentation table for charge state 1+
- specType=etd
- score=27.43
- F104912.dat
- query=q241_p1
- precursor=467.764920
- chargePrecursor=2
- itol=0.8

AA	c	y	z+1	z+2	z	AA
S[1]	105.066	**934.522**	**918.503**	0.000	**917.495**	S[8]
H[2]	242.125	847.490	**831.471**	0.000	830.463	H[7]
H[3]	379.184	**710.431**	**694.412**	0.000	693.404	H[6]
K[4]	507.279	573.372	**557.353**	**558.361**	556.345	K[5]
A[5]	**578.316**	445.277	429.258	430.266	428.250	A[4]
K[6]	706.411	374.240	358.221	**359.229**	357.213	K[3]
G[7]	**763.432**	246.145	230.126	231.134	229.118	G[2]
K[8]	933.538	189.123	173.105	174.112	172.097	K[1]

sp|P62806|H4_MOUSE

[Acetyl]SGRGKGGKGLGKGGAKRHRK $^{\text{Dimethyl}}_{28.03}$ VLRD

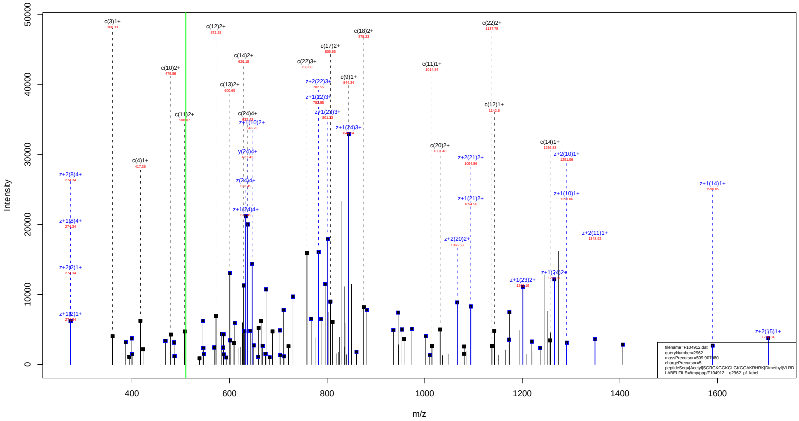

sp|P62806|H4_MOUSE

[Acetyl]SGRGKGGKGLGKGGAKRHRK $^{\text{Dimethyl}}_{28.03}$ VLRD

- fragmentation table for charge state 1+
- specType=etd
- score=119.56
- F104912.dat
- query=q2962_p1
- precursor=509.907080
- chargePrecursor=5
- itol=0.8

AA	c	y	z+1	z+2	z	AA
S[1]	147.076	2545.502	2529.483	0.000	2528.476	S[24]
G[2]	204.098	2416.460	2400.441	0.000	2399.433	G[23]
R[3]	**360.199**	2359.438	2343.419	2344.427	2342.412	R[22]
G[4]	**417.220**	2203.337	2187.318	2188.326	2186.310	G[21]
K[5]	**545.315**	2146.316	2130.297	2131.305	2129.289	K[20]
G[6]	602.337	2018.221	2002.202	2003.210	2001.194	G[19]
G[7]	**659.358**	1961.199	1945.180	1946.188	1944.173	G[18]
K[8]	**787.453**	1904.178	1888.159	1889.167	1887.151	K[17]
G[9]	**844.475**	1776.083	1760.064	1761.072	1759.056	G[16]
L[10]	**957.559**	1719.061	1703.043	**1704.050**	1702.035	L[15]
G[11]	**1014.580**	1605.977	**1589.958**	1590.966	1588.951	G[14]
K[12]	**1142.675**	1548.956	1532.937	1533.945	1531.929	K[13]
G[13]	1199.697	1420.861	1404.842	**1405.850**	1403.834	G[12]
G[14]	**1256.718**	1363.839	1347.821	**1348.828**	1346.813	G[11]
A[15]	1327.755	1306.818	**1290.799**	**1291.807**	1289.791	A[10]
K[16]	1455.850	**1235.781**	**1219.762**	1220.770	**1218.754**	K[9]
R[17]	1611.951	1107.686	1091.667	1092.675	1090.659	R[8]
H[18]	1749.010	951.585	**935.566**	**936.574**	934.558	H[7]
R[19]	1905.111	814.526	798.507	799.515	797.499	R[6]
K[20]	2061.238	**658.425**	**642.406**	643.414	641.398	K[5]
V[21]	2160.306	502.298	**486.280**	**487.287**	485.272	V[4]
L[22]	2273.390	403.230	**387.211**	388.219	386.203	L[3]
R[23]	2429.491	290.146	**274.127**	**275.135**	273.119	R[2]
D[24]	2544.518	134.045	118.026	119.034	117.018	D[1]

sp|P62806|H4_MOUSE

[Acetyl]SGRGKGGKGLGKGGAKRHRK $^{\text{Dimethyl}}_{28.03}$ VLRD

- fragmentation table for charge state 2+
- specType=etd
- score=119.56
- F104912.dat
- query=q2962_p1
- precursor=509.907080
- chargePrecursor=5
- itol=0.8

AA	c	y	z+1	z+2	z	AA
S[1]	74.042	1273.255	**1265.245**	0.504	1264.741	S[24]
G[2]	102.553	1208.733	**1200.724**	0.504	1200.220	G[23]
R[3]	180.603	1180.223	**1172.213**	**1172.717**	1171.709	R[22]
G[4]	209.114	1102.172	**1094.163**	**1094.667**	1093.659	G[21]
K[5]	273.161	1073.661	1065.652	**1066.156**	1065.148	K[20]
G[6]	301.672	**1009.614**	**1001.605**	**1002.108**	1001.101	G[19]
G[7]	330.183	981.103	**973.094**	**973.598**	972.590	G[18]
K[8]	**394.230**	**952.592**	944.583	**945.087**	944.079	K[17]
G[9]	**422.741**	888.545	880.536	**881.040**	880.032	G[16]
L[10]	**479.283**	**860.034**	852.025	852.529	851.521	L[15]
G[11]	**507.794**	803.492	**795.483**	**795.987**	794.979	G[14]
K[12]	**571.841**	774.982	**766.972**	**767.476**	766.468	K[13]
G[13]	**600.352**	**710.934**	**702.925**	**703.429**	702.421	G[12]
G[14]	**628.863**	**682.423**	**674.414**	**674.918**	**673.910**	G[11]
A[15]	**664.381**	653.913	**645.903**	**646.407**	645.399	A[10]
K[16]	728.429	618.394	**610.385**	**610.889**	609.881	K[9]
R[17]	**806.479**	554.347	**546.337**	**546.841**	**545.833**	R[8]
H[18]	**875.009**	476.296	**468.287**	**468.791**	**467.783**	H[7]
R[19]	**953.059**	407.767	**399.757**	**400.261**	**399.253**	R[6]
K[20]	**1031.122**	329.716	321.707	322.211	321.203	K[5]
V[21]	**1080.657**	251.653	243.643	244.147	243.140	V[4]
L[22]	**1137.199**	202.119	194.109	194.613	193.605	L[3]
R[23]	1215.249	145.577	137.567	138.071	137.063	R[2]
D[24]	1272.763	67.526	59.517	60.021	59.013	D[1]

sp|P62806|H4_MOUSE

[Acetyl]SGRGKGGKGLGKGGAKRHRK$^{\text{Dimethyl}}_{28.03}$VLRD

- fragmentation table for charge state 3+
- specType=etd
- score=119.56
- F104912.dat
- query=q2962_p1
- precursor=509.907080
- chargePrecursor=5
- itol=0.8

AA	c	y	z+1	z+2	z	AA
S[1]	49.697	849.172	**843.833**	0.672	843.497	S[24]
G[2]	68.704	**806.158**	**800.818**	0.672	800.483	G[23]
R[3]	120.738	**787.151**	**781.811**	**782.147**	781.475	R[22]
G[4]	139.745	735.117	**729.778**	**730.114**	**729.442**	G[21]
K[5]	182.443	716.110	**710.770**	**711.106**	**710.435**	K[20]
G[6]	201.450	**673.412**	**668.072**	**668.408**	**667.736**	G[19]
G[7]	220.458	654.405	**649.065**	**649.401**	648.729	G[18]
K[8]	263.156	635.397	**630.058**	**630.394**	**629.722**	K[17]
G[9]	282.163	**592.699**	**587.360**	**587.695**	**587.024**	G[16]
L[10]	319.858	573.692	**568.352**	**568.688**	**568.016**	L[15]
G[11]	338.865	535.997	530.658	530.994	530.322	G[14]
K[12]	381.563	516.990	511.651	511.986	511.315	K[13]
G[13]	**400.570**	474.292	**468.952**	**469.288**	**468.616**	G[12]
G[14]	419.578	455.285	449.945	450.281	449.609	G[11]
A[15]	443.257	436.277	430.938	431.274	430.602	A[10]
K[16]	**485.955**	412.598	407.259	407.595	406.923	K[9]
R[17]	**537.989**	369.900	364.561	364.896	364.225	R[8]
H[18]	**583.675**	317.866	312.527	312.863	312.191	H[7]
R[19]	635.709	272.180	266.841	267.176	266.505	R[6]
K[20]	**687.751**	220.146	214.807	215.143	214.471	K[5]
V[21]	**720.774**	168.104	162.765	163.101	162.429	V[4]
L[22]	**758.468**	135.082	129.742	130.078	129.406	L[3]
R[23]	**810.502**	97.387	92.047	92.383	91.711	R[2]
D[24]	848.844	45.353	40.014	40.349	39.678	D[1]

sp|P62806|H4_MOUSE

[Acetyl]SGRGKGGKGLGKGGAKRHRK $^{\text{Dimethyl}}_{28.03}$ VLRD

- fragmentation table for charge state 4+
- specType=etd
- score=119.56
- F104912.dat
- query=q2962_p1
- precursor=509.907080
- chargePrecursor=5
- itol=0.8

AA	c	y	z+1	z+2	z	AA
S[1]	37.525	**637.131**	**633.126**	0.755	**632.874**	S[24]
G[2]	51.780	604.870	**600.866**	0.755	**600.614**	G[23]
R[3]	90.805	590.615	**586.610**	**586.862**	**586.358**	R[22]
G[4]	105.061	551.590	**547.585**	**547.837**	**547.333**	G[21]
K[5]	137.084	537.334	533.330	533.582	533.078	K[20]
G[6]	151.340	505.311	501.306	501.558	501.054	G[19]
G[7]	165.595	491.055	**487.051**	**487.303**	**486.799**	G[18]
K[8]	197.619	476.800	472.795	473.047	472.543	K[17]
G[9]	211.874	444.776	440.771	441.023	440.520	G[16]
L[10]	240.145	430.521	426.516	426.768	426.264	L[15]
G[11]	254.401	402.250	398.245	398.497	397.993	G[14]
K[12]	286.424	**387.994**	383.990	384.242	383.738	K[13]
G[13]	300.680	355.971	351.966	352.218	351.714	G[12]
G[14]	314.935	341.715	337.711	337.963	337.459	G[11]
A[15]	332.694	327.460	323.455	323.707	323.203	A[10]
K[16]	364.718	309.701	305.696	305.948	305.444	K[9]
R[17]	403.743	277.677	**273.672**	**273.924**	273.420	R[8]
H[18]	438.008	238.652	234.647	234.899	234.395	H[7]
R[19]	477.033	204.387	200.382	200.634	200.130	R[6]
K[20]	516.065	165.362	161.357	161.609	161.105	K[5]
V[21]	540.832	126.330	122.325	122.577	122.073	V[4]
L[22]	**569.103**	101.563	97.558	97.810	97.306	L[3]
R[23]	**608.128**	73.292	69.287	69.539	69.035	R[2]
D[24]	**636.885**	34.267	30.262	30.514	30.010	D[1]

sp|P62806|H4_MOUSE

[Acetyl]SGRGKGGKGLGKGGAKRHRK $^{\text{Dimethyl}}_{28.03}$ VLRD

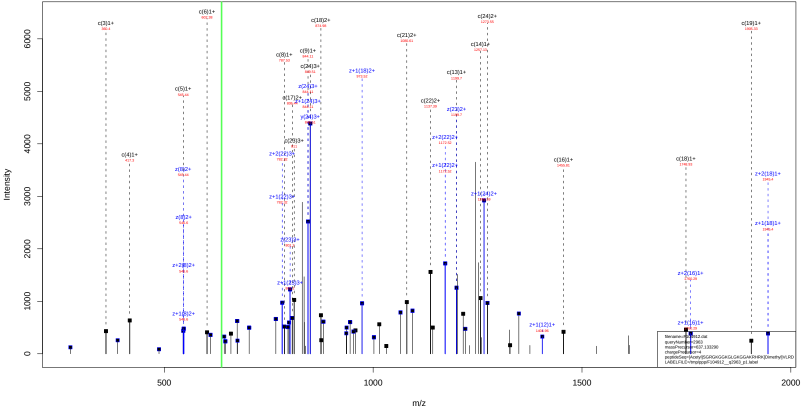

sp|P62806|H4_MOUSE

[Acetyl]SGRGKGGKGLGKGGAKRHRK$^{\text{Dimethyl}}_{28.03}$VLRD

- fragmentation table for charge state 1+
- specType=etd
- score=119.38
- F104912.dat
- query=q2963_p1
- precursor=637.133290
- chargePrecursor=4
- itol=0.8

AA	c	y	z+1	z+2	z	AA
S[1]	147.076	2545.502	2529.483	0.000	2528.476	S[24]
G[2]	204.098	2416.460	2400.441	0.000	2399.433	G[23]
R[3]	**360.199**	2359.438	2343.419	2344.427	2342.412	R[22]
G[4]	**417.220**	2203.337	2187.318	2188.326	2186.310	G[21]
K[5]	**545.315**	2146.316	2130.297	2131.305	2129.289	K[20]
G[6]	**602.337**	2018.221	2002.202	2003.210	2001.194	G[19]
G[7]	**659.358**	1961.199	**1945.180**	**1946.188**	1944.173	G[18]
K[8]	**787.453**	1904.178	1888.159	1889.167	1887.151	K[17]
G[9]	**844.475**	1776.083	**1760.064**	**1761.072**	1759.056	G[16]
L[10]	**957.559**	1719.061	1703.043	1704.050	1702.035	L[15]
G[11]	**1014.580**	1605.977	1589.958	1590.966	1588.951	G[14]
K[12]	**1142.675**	1548.956	1532.937	1533.945	1531.929	K[13]
G[13]	**1199.697**	1420.861	**1404.842**	1405.850	1403.834	G[12]
G[14]	**1256.718**	1363.839	1347.821	**1348.828**	1346.813	G[11]
A[15]	**1327.755**	1306.818	1290.799	1291.807	1289.791	A[10]
K[16]	**1455.850**	1235.781	1219.762	**1220.770**	1218.754	K[9]
R[17]	1611.951	1107.686	1091.667	1092.675	1090.659	R[8]
H[18]	**1749.010**	951.585	**935.566**	**936.574**	934.558	H[7]
R[19]	**1905.111**	814.526	**798.507**	799.515	797.499	R[6]
K[20]	2061.238	658.425	642.406	**643.414**	641.398	K[5]
V[21]	2160.306	502.298	486.280	**487.287**	485.272	V[4]
L[22]	2273.390	403.230	387.211	**388.219**	386.203	L[3]
R[23]	2429.491	290.146	274.127	**275.135**	273.119	R[2]
D[24]	2544.518	134.045	118.026	119.034	117.018	D[1]

sp|P62806|H4_MOUSE

[Acetyl]SGRGKGGKGLGKGGAKRHRK$^{\text{Dimethyl}}_{28.03}$VLRD

- fragmentation table for charge state 2+
- specType=etd
- score=119.38
- F104912.dat
- query=q2963_p1
- precursor=637.133290
- chargePrecursor=4
- itol=0.8

AA	c	y	z+1	z+2	z	AA
S[1]	74.042	**1273.255**	**1265.245**	0.504	1264.741	S[24]
G[2]	102.553	1208.733	1200.724	0.504	**1200.220**	G[23]
R[3]	180.603	1180.223	**1172.213**	**1172.717**	1171.709	R[22]
G[4]	209.114	1102.172	**1094.163**	**1094.667**	**1093.659**	G[21]
K[5]	273.161	1073.661	**1065.652**	**1066.156**	**1065.148**	K[20]
G[6]	301.672	1009.614	**1001.605**	**1002.108**	**1001.101**	G[19]
G[7]	330.183	981.103	**973.094**	**973.598**	972.590	G[18]
K[8]	394.230	**952.592**	**944.583**	**945.087**	**944.079**	K[17]
G[9]	422.741	888.545	**880.536**	**881.040**	880.032	G[16]
L[10]	479.283	860.034	852.025	852.529	851.521	L[15]
G[11]	507.794	803.492	**795.483**	**795.987**	794.979	G[14]
K[12]	571.841	774.982	**766.972**	**767.476**	**766.468**	K[13]
G[13]	600.352	710.934	**702.925**	**703.429**	**702.421**	G[12]
G[14]	628.863	682.423	**674.414**	**674.918**	**673.910**	G[11]
A[15]	664.381	653.913	**645.903**	**646.407**	**645.399**	A[10]
K[16]	728.429	618.394	**610.385**	**610.889**	609.881	K[9]
R[17]	**806.479**	554.347	**546.337**	**546.841**	**545.833**	R[8]
H[18]	**875.009**	476.296	468.287	468.791	467.783	H[7]
R[19]	**953.059**	407.767	399.757	400.261	399.253	R[6]
K[20]	**1031.122**	329.716	321.707	322.211	321.203	K[5]
V[21]	**1080.657**	251.653	243.643	244.147	243.140	V[4]
L[22]	**1137.199**	202.119	194.109	194.613	193.605	L[3]
R[23]	**1215.249**	145.577	137.567	138.071	137.063	R[2]
D[24]	**1272.763**	67.526	59.517	60.021	59.013	D[1]

sp|P62806|H4_MOUSE

[Acetyl]SGRGKGGKGLGKGGAKRHRK $^{\text{Dimethyl}}_{28.03}$ VLRD

- fragmentation table for charge state 3+
- specType=etd
- score=119.38
- F104912.dat
- query=q2963_p1
- precursor=637.133290
- chargePrecursor=4
- itol=0.8

AA	c	y	z+1	z+2	z	AA
S[1]	49.697	**849.172**	**843.833**	0.672	**843.497**	S[24]
G[2]	68.704	**806.158**	**800.818**	0.672	**800.483**	G[23]
R[3]	120.738	**787.151**	**781.811**	**782.147**	781.475	R[22]
G[4]	139.745	735.117	729.778	730.114	729.442	G[21]
K[5]	182.443	716.110	710.770	711.106	710.435	K[20]
G[6]	201.450	673.412	668.072	668.408	667.736	G[19]
G[7]	220.458	654.405	649.065	649.401	648.729	G[18]
K[8]	263.156	635.397	630.058	630.394	629.722	K[17]
G[9]	282.163	592.699	587.360	587.695	587.024	G[16]
L[10]	319.858	573.692	568.352	568.688	568.016	L[15]
G[11]	338.865	535.997	530.658	530.994	530.322	G[14]
K[12]	381.563	516.990	511.651	511.986	511.315	K[13]
G[13]	400.570	474.292	468.952	469.288	468.616	G[12]
G[14]	419.578	455.285	449.945	450.281	449.609	G[11]
A[15]	443.257	436.277	430.938	431.274	430.602	A[10]
K[16]	485.955	412.598	407.259	407.595	406.923	K[9]
R[17]	537.989	369.900	364.561	364.896	364.225	R[8]
H[18]	583.675	317.866	312.527	312.863	312.191	H[7]
R[19]	635.709	272.180	266.841	267.176	266.505	R[6]
K[20]	687.751	220.146	214.807	215.143	214.471	K[5]
V[21]	720.774	168.104	162.765	163.101	162.429	V[4]
L[22]	758.468	135.082	129.742	130.078	129.406	L[3]
R[23]	**810.502**	97.387	92.047	92.383	91.711	R[2]
D[24]	**848.844**	45.353	40.014	40.349	39.678	D[1]

sp|Q6GSS7|H2A2A_MOUSE

LNKLLGK $^{\text{Dimethyl}}_{28.03}$ VTIAQGGVLPNIQAVLLPKKTE

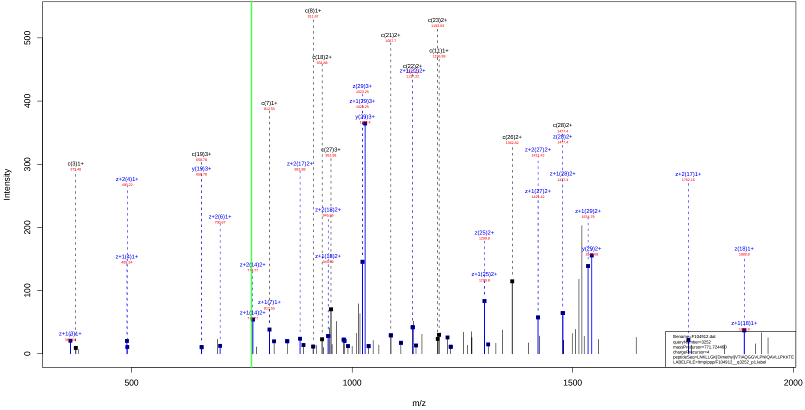

sp|Q6GSS7|H2A2A_MOUSE

LNKLLGK $^{\text{Dimethyl}}_{28.03}$ VTIAQGGVLPNIQAVLLPKKTE

- fragmentation table for charge state 1+
- specType=etd
- score=38.13
- F104912.dat
- query=q3252_p1
- precursor=771.724460
- chargePrecursor=4
- itol=0.8

AA	c	y	z+1	z+2	z	AA
L[1]	131.118	3083.892	3067.873	0.000	3066.865	L[29]
N[2]	245.161	2970.808	2954.789	2955.797	2953.781	N[28]
K[3]	**373.256**	2856.765	2840.746	2841.754	2839.738	K[27]
L[4]	486.340	2728.670	2712.651	2713.659	2711.643	L[26]
L[5]	599.424	2615.586	2599.567	2600.575	2598.559	L[25]
G[6]	656.445	2502.502	2486.483	2487.491	2485.475	G[24]
K[7]	**812.572**	2445.480	2429.462	2430.469	2428.454	K[23]
V[8]	**911.640**	2289.354	2273.335	2274.343	2272.327	V[22]
T[9]	1012.688	2190.286	2174.267	2175.275	2173.259	T[21]
I[10]	1125.772	2089.238	2073.219	2074.227	2072.211	I[20]
A[11]	**1196.809**	1976.154	1960.135	1961.143	1959.127	A[19]
Q[12]	1324.867	1905.117	**1889.098**	1890.106	**1888.090**	Q[18]
G[13]	1381.889	1777.058	1761.039	**1762.047**	1760.032	G[17]
G[14]	1438.910	1720.037	1704.018	1705.026	1703.010	G[16]
V[15]	1537.979	1663.015	1646.997	1648.004	1645.989	V[15]
L[16]	1651.063	1563.947	1547.928	1548.936	1546.920	L[14]
P[17]	1748.116	1450.863	1434.844	1435.852	1433.836	P[13]
N[18]	1862.159	1353.810	1337.791	1338.799	1336.783	N[12]
I[19]	1975.243	1239.767	**1223.748**	1224.756	**1222.741**	I[11]
Q[20]	2103.301	1126.683	**1110.664**	1111.672	**1109.656**	Q[10]
A[21]	2174.338	998.624	**982.606**	**983.614**	**981.598**	A[9]
V[22]	2273.407	927.587	**911.569**	912.576	910.561	V[8]
L[23]	2386.491	828.519	**812.500**	813.508	811.492	L[7]
L[24]	2499.575	715.435	699.416	**700.424**	698.408	L[6]
P[25]	2596.628	602.351	586.332	587.340	585.324	P[5]
K[26]	2724.723	505.298	**489.279**	**490.287**	488.271	K[4]
K[27]	2852.818	377.203	**361.184**	362.192	360.177	K[3]
T[28]	2953.865	249.108	233.089	234.097	232.082	T[2]
E[29]	3082.908	148.060	132.042	133.050	131.034	E[1]

sp|Q6GSS7|H2A2A_MOUSE

LNKLLGK $^{Dimethyl}_{28.03}$ VTIAQGGVLPNIQAVLLPKKTE

- fragmentation table for charge state 2+
- specType=etd
- score=38.13
- F104912.dat
- query=q3252_p1
- precursor=771.724460
- chargePrecursor=4
- itol=0.8

AA	c	y	z+1	z+2	z	AA
L[1]	66.063	**1542.450**	**1534.440**	0.504	1533.936	L[29]
N[2]	123.084	1485.908	**1477.898**	1478.402	**1477.394**	N[28]
K[3]	187.132	1428.886	**1420.877**	**1421.381**	1420.373	K[27]
L[4]	243.674	1364.839	1356.829	1357.333	1356.325	L[26]
L[5]	300.216	**1308.297**	**1300.287**	1300.791	**1299.783**	L[25]
G[6]	328.726	1251.755	1243.745	1244.249	1243.241	G[24]
K[7]	406.789	**1223.244**	1215.234	**1215.738**	1214.731	K[23]
V[8]	456.324	**1145.181**	**1137.171**	**1137.675**	**1136.667**	V[22]
T[9]	506.848	1095.646	**1087.637**	**1088.141**	**1087.133**	T[21]
I[10]	563.390	1045.123	**1037.113**	**1037.617**	**1036.609**	I[20]
A[11]	598.908	988.581	**980.571**	**981.075**	**980.067**	A[19]
Q[12]	662.937	953.062	**945.053**	**945.557**	944.549	Q[18]
G[13]	691.448	**889.033**	881.023	**881.527**	880.519	G[17]
G[14]	719.959	860.522	**852.513**	**853.017**	**852.009**	G[16]
V[15]	769.493	832.011	824.002	824.506	**823.498**	V[15]
L[16]	826.035	782.477	**774.468**	**774.972**	773.964	L[14]
P[17]	874.561	725.935	717.926	718.430	717.422	P[13]
N[18]	**931.583**	677.409	669.399	669.903	668.895	N[12]
I[19]	988.125	620.387	612.378	612.882	611.874	I[11]
Q[20]	1052.154	563.845	555.836	556.340	555.332	Q[10]
A[21]	**1087.673**	499.816	491.807	492.310	491.303	A[9]
V[22]	**1137.207**	464.297	456.288	456.792	455.784	V[8]
L[23]	**1193.749**	414.763	406.754	407.258	406.250	L[7]
L[24]	1250.291	358.221	350.212	350.716	349.708	L[6]
P[25]	1298.817	301.679	293.670	294.174	293.166	P[5]
K[26]	**1362.865**	253.153	245.143	245.647	244.639	K[4]
K[27]	1426.912	189.105	181.096	181.600	180.592	K[3]
T[28]	**1477.436**	125.058	117.048	117.552	116.544	T[2]
E[29]	1541.958	74.534	66.524	67.028	66.021	E[1]

sp|Q6GSS7|H2A2A_MOUSE

LNKLLGK $^{\text{Dimethyl}}_{28.03}$ VTIAQGGVLPNIQAVLLPKKTE

- fragmentation table for charge state 3+
- specType=etd
- score=38.13
- F104912.dat
- query=q3252_p1
- precursor=771.724460
- chargePrecursor=4
- itol=0.8

AA	c	y	z+1	z+2	z	AA
L[1]	44.377	**1028.635**	**1023.296**	0.672	**1022.960**	L[29]
N[2]	82.392	**990.941**	985.601	985.937	985.265	N[28]
K[3]	125.090	952.926	947.587	947.923	947.251	K[27]
L[4]	162.785	910.228	904.889	905.225	904.553	L[26]
L[5]	200.479	872.533	867.194	867.530	866.858	L[25]
G[6]	219.487	834.839	829.499	829.835	829.163	G[24]
K[7]	271.529	815.832	810.492	810.828	810.156	K[23]
V[8]	304.552	763.790	758.450	758.786	758.114	V[22]
T[9]	338.234	730.767	725.427	725.763	725.091	T[21]
I[10]	375.929	697.084	691.745	692.081	691.409	I[20]
A[11]	399.608	**659.389**	654.050	654.386	653.714	A[19]
Q[12]	442.294	635.710	630.371	630.707	630.035	Q[18]
G[13]	461.301	593.024	587.685	588.021	587.349	G[17]
G[14]	480.308	574.017	568.678	569.013	568.342	G[16]
V[15]	513.331	555.010	549.670	550.006	549.334	V[15]
L[16]	551.026	521.987	516.648	516.984	516.312	L[14]
P[17]	583.377	484.292	478.953	479.289	478.617	P[13]
N[18]	621.391	451.942	446.602	446.938	446.266	N[12]
I[19]	**659.086**	413.927	408.588	408.924	408.252	I[11]
Q[20]	701.772	376.233	370.893	371.229	370.557	Q[10]
A[21]	725.451	333.546	328.207	328.543	327.871	A[9]
V[22]	758.474	309.867	304.528	304.864	304.192	V[8]
L[23]	796.168	276.844	271.505	271.841	271.169	L[7]
L[24]	833.863	239.150	233.810	234.146	233.474	L[6]
P[25]	866.214	201.455	196.116	196.451	195.780	P[5]
K[26]	908.912	169.104	163.765	164.101	163.429	K[4]
K[27]	**951.611**	126.406	121.066	121.402	120.730	K[3]
T[28]	985.293	83.708	78.368	78.704	78.032	T[2]
E[29]	1028.307	50.025	44.685	45.021	44.349	E[1]

sp|Q6GSS7|H2A2A_MOUSE

LNKLLGK $^{\text{Dimethyl}}_{28.03}$ VTIAQGGVLPNIQAVLLPKKTE

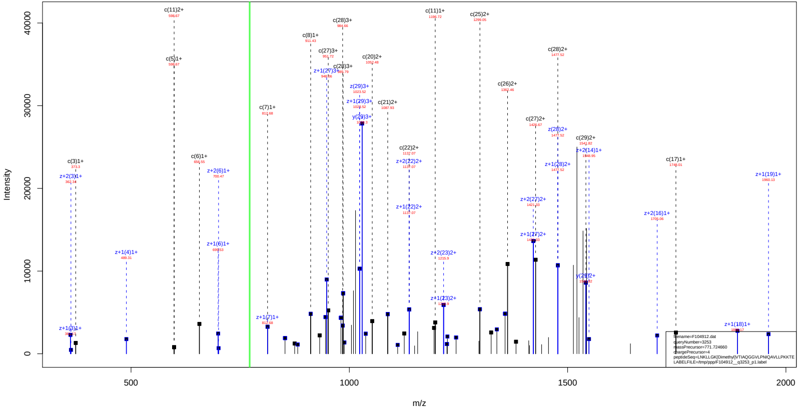

sp|Q6GSS7|H2A2A_MOUSE

LNKLLGK $^{Dimethyl}_{28.03}$ VTIAQGGVLPNIQAVLLPKKTE

- fragmentation table for charge state 1+
- specType=etd
- score=89.03
- F104912.dat
- query=q3253_p1
- precursor=771.724660
- chargePrecursor=4
- itol=0.8

AA	c	y	z+1	z+2	z	AA
L[1]	131.118	3083.892	3067.873	0.000	3066.865	L[29]
N[2]	245.161	2970.808	2954.789	2955.797	2953.781	N[28]
K[3]	**373.256**	2856.765	2840.746	2841.754	2839.738	K[27]
L[4]	486.340	2728.670	2712.651	2713.659	2711.643	L[26]
L[5]	**599.424**	2615.586	2599.567	2600.575	2598.559	L[25]
G[6]	**656.445**	2502.502	2486.483	2487.491	2485.475	G[24]
K[7]	**812.572**	2445.480	2429.462	2430.469	2428.454	K[23]
V[8]	**911.640**	2289.354	2273.335	2274.343	2272.327	V[22]
T[9]	1012.688	2190.286	2174.267	2175.275	2173.259	T[21]
I[10]	**1125.772**	2089.238	2073.219	2074.227	2072.211	I[20]
A[11]	**1196.809**	1976.154	**1960.135**	1961.143	1959.127	A[19]
Q[12]	**1324.867**	1905.117	**1889.098**	1890.106	1888.090	Q[18]
G[13]	**1381.889**	1777.058	1761.039	1762.047	1760.032	G[17]
G[14]	1438.910	1720.037	1704.018	**1705.026**	1703.010	G[16]
V[15]	1537.979	1663.015	1646.997	1648.004	1645.989	V[15]
L[16]	1651.063	1563.947	1547.928	**1548.936**	1546.920	L[14]
P[17]	**1748.116**	1450.863	1434.844	1435.852	1433.836	P[13]
N[18]	1862.159	1353.810	**1337.791**	1338.799	1336.783	N[12]
I[19]	1975.243	1239.767	**1223.748**	**1224.756**	**1222.741**	I[11]
Q[20]	2103.301	1126.683	**1110.664**	1111.672	1109.656	Q[10]
A[21]	2174.338	998.624	982.606	983.614	**981.598**	A[9]
V[22]	2273.407	927.587	**911.569**	912.576	910.561	V[8]
L[23]	2386.491	828.519	**812.500**	813.508	811.492	L[7]
L[24]	2499.575	715.435	**699.416**	**700.424**	698.408	L[6]
P[25]	2596.628	602.351	586.332	587.340	585.324	P[5]
K[26]	2724.723	505.298	**489.279**	490.287	488.271	K[4]
K[27]	2852.818	377.203	**361.184**	**362.192**	360.177	K[3]
T[28]	2953.865	249.108	233.089	234.097	232.082	T[2]
E[29]	3082.908	148.060	132.042	133.050	131.034	E[1]

sp|Q6GSS7|H2A2A_MOUSE

LNKLLGK $^{Dimethyl}_{28.03}$ VTIAQGGVLPNIQAVLLPKKTE

- fragmentation table for charge state 2+
- specType=etd
- score=89.03
- F104912.dat
- query=q3253_p1
- precursor=771.724660
- chargePrecursor=4
- itol=0.8

AA	c	y	z+1	z+2	z	AA
L[1]	66.063	**1542.450**	1534.440	0.504	1533.936	L[29]
N[2]	123.084	1485.908	**1477.898**	1478.402	**1477.394**	N[28]
K[3]	187.132	1428.886	**1420.877**	**1421.381**	1420.373	K[27]
L[4]	243.674	1364.839	**1356.829**	**1357.333**	**1356.325**	L[26]
L[5]	300.216	1308.297	1300.287	1300.791	**1299.783**	L[25]
G[6]	328.726	1251.755	**1243.745**	**1244.249**	1243.241	G[24]
K[7]	406.789	**1223.244**	**1215.234**	**1215.738**	1214.731	K[23]
V[8]	456.324	1145.181	**1137.171**	**1137.675**	**1136.667**	V[22]
T[9]	506.848	1095.646	**1087.637**	**1088.141**	**1087.133**	T[21]
I[10]	563.390	1045.123	**1037.113**	**1037.617**	1036.609	I[20]
A[11]	**598.908**	**988.581**	**980.571**	**981.075**	**980.067**	A[19]
Q[12]	662.937	953.062	**945.053**	**945.557**	944.549	Q[18]
G[13]	691.448	889.033	**881.023**	**881.527**	880.519	G[17]
G[14]	719.959	860.522	**852.513**	**853.017**	**852.009**	G[16]
V[15]	769.493	832.011	824.002	824.506	823.498	V[15]
L[16]	826.035	782.477	774.468	774.972	773.964	L[14]
P[17]	**874.561**	725.935	717.926	718.430	717.422	P[13]
N[18]	**931.583**	677.409	669.399	669.903	668.895	N[12]
I[19]	**988.125**	620.387	612.378	612.882	611.874	I[11]
Q[20]	**1052.154**	563.845	555.836	556.340	555.332	Q[10]
A[21]	**1087.673**	499.816	491.807	492.310	491.303	A[9]
V[22]	**1137.207**	464.297	456.288	456.792	455.784	V[8]
L[23]	**1193.749**	414.763	406.754	407.258	406.250	L[7]
L[24]	1250.291	358.221	350.212	350.716	349.708	L[6]
P[25]	**1298.817**	301.679	293.670	294.174	293.166	P[5]
K[26]	**1362.865**	253.153	245.143	245.647	244.639	K[4]
K[27]	**1426.912**	189.105	181.096	181.600	180.592	K[3]
T[28]	**1477.436**	125.058	117.048	117.552	116.544	T[2]
E[29]	**1541.958**	74.534	66.524	67.028	66.021	E[1]

sp|Q6GSS7|H2A2A_MOUSE

LNKLLGK $^{Dimethyl}_{28.03}$ VTIAQGGVLPNIQAVLLPKKTE

- fragmentation table for charge state 3+
- specType=etd
- score=89.03
- F104912.dat
- query=q3253_p1
- precursor=771.724660
- chargePrecursor=4
- itol=0.8

AA	c	y	z+1	z+2	z	AA
L[1]	44.377	**1028.635**	**1023.296**	0.672	**1022.960**	L[29]
N[2]	82.392	990.941	**985.601**	**985.937**	**985.265**	N[28]
K[3]	125.090	952.926	**947.587**	**947.923**	947.251	K[27]
L[4]	162.785	910.228	904.889	905.225	904.553	L[26]
L[5]	200.479	872.533	867.194	867.530	866.858	L[25]
G[6]	219.487	834.839	829.499	829.835	829.163	G[24]
K[7]	271.529	815.832	810.492	810.828	810.156	K[23]
V[8]	304.552	763.790	758.450	758.786	758.114	V[22]
T[9]	338.234	730.767	725.427	725.763	725.091	T[21]
I[10]	375.929	697.084	691.745	692.081	691.409	I[20]
A[11]	399.608	659.389	654.050	654.386	653.714	A[19]
Q[12]	442.294	635.710	630.371	630.707	630.035	Q[18]
G[13]	461.301	593.024	587.685	588.021	587.349	G[17]
G[14]	480.308	574.017	568.678	569.013	568.342	G[16]
V[15]	513.331	555.010	549.670	550.006	549.334	V[15]
L[16]	551.026	521.987	516.648	516.984	516.312	L[14]
P[17]	583.377	484.292	478.953	479.289	478.617	P[13]
N[18]	621.391	451.942	446.602	446.938	446.266	N[12]
I[19]	659.086	413.927	408.588	408.924	408.252	I[11]
Q[20]	701.772	376.233	370.893	371.229	370.557	Q[10]
A[21]	725.451	333.546	328.207	328.543	327.871	A[9]
V[22]	758.474	309.867	304.528	304.864	304.192	V[8]
L[23]	796.168	276.844	271.505	271.841	271.169	L[7]
L[24]	833.863	239.150	233.810	234.146	233.474	L[6]
P[25]	866.214	201.455	196.116	196.451	195.780	P[5]
K[26]	908.912	169.104	163.765	164.101	163.429	K[4]
K[27]	**951.611**	126.406	121.066	121.402	120.730	K[3]
T[28]	**985.293**	83.708	78.368	78.704	78.032	T[2]
E[29]	1028.307	50.025	44.685	45.021	44.349	E[1]

sp|Q6GSS7|H2A2A_MOUSE

LNKLLGK $^{\text{Dimethyl}}_{28.03}$ VTIAQGGVLPNIQAVLLPKKTE

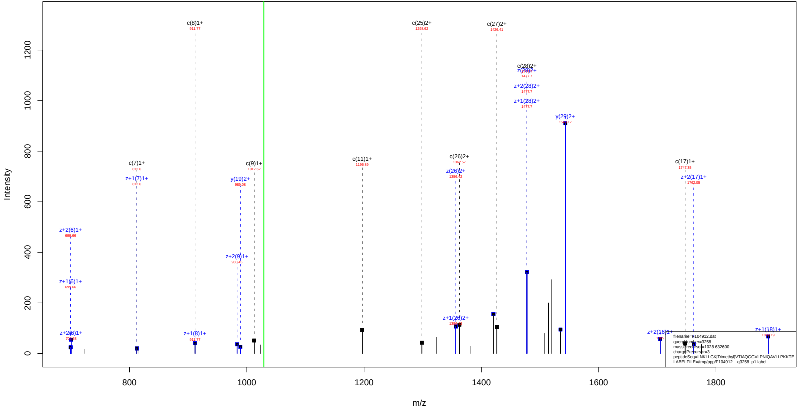

sp|Q6GSS7|H2A2A_MOUSE

LNKLLGK $^{Dimethyl}_{28.03}$ VTIAQGGVLPNIQAVLLPKKTE

- fragmentation table for charge state 1+
- specType=etd
- score=36.59
- F104912.dat
- query=q3258_p1
- precursor=1028.632600
- chargePrecursor=3
- itol=0.8

AA	c	y	z+1	z+2	z	AA
L[1]	131.118	3083.892	3067.873	0.000	3066.865	L[29]
N[2]	245.161	2970.808	2954.789	2955.797	2953.781	N[28]
K[3]	373.256	2856.765	2840.746	2841.754	2839.738	K[27]
L[4]	486.340	2728.670	2712.651	2713.659	2711.643	L[26]
L[5]	599.424	2615.586	2599.567	2600.575	2598.559	L[25]
G[6]	656.445	2502.502	2486.483	2487.491	2485.475	G[24]
K[7]	**812.572**	2445.480	2429.462	2430.469	2428.454	K[23]
V[8]	**911.640**	2289.354	2273.335	2274.343	2272.327	V[22]
T[9]	**1012.688**	2190.286	2174.267	2175.275	2173.259	T[21]
I[10]	1125.772	2089.238	2073.219	2074.227	2072.211	I[20]
A[11]	**1196.809**	1976.154	1960.135	1961.143	1959.127	A[19]
Q[12]	1324.867	1905.117	**1889.098**	1890.106	1888.090	Q[18]
G[13]	1381.889	1777.058	1761.039	**1762.047**	1760.032	G[17]
G[14]	1438.910	1720.037	1704.018	**1705.026**	1703.010	G[16]
V[15]	1537.979	1663.015	1646.997	1648.004	1645.989	V[15]
L[16]	1651.063	1563.947	1547.928	1548.936	1546.920	L[14]
P[17]	**1748.116**	1450.863	1434.844	1435.852	1433.836	P[13]
N[18]	1862.159	1353.810	1337.791	1338.799	1336.783	N[12]
I[19]	1975.243	1239.767	1223.748	1224.756	1222.741	I[11]
Q[20]	2103.301	1126.683	1110.664	1111.672	1109.656	Q[10]
A[21]	2174.338	998.624	982.606	**983.614**	981.598	A[9]
V[22]	2273.407	927.587	**911.569**	912.576	910.561	V[8]
L[23]	2386.491	828.519	**812.500**	813.508	811.492	L[7]
L[24]	2499.575	715.435	**699.416**	**700.424**	698.408	L[6]
P[25]	2596.628	602.351	586.332	587.340	585.324	P[5]
K[26]	2724.723	505.298	489.279	490.287	488.271	K[4]
K[27]	2852.818	377.203	361.184	362.192	360.177	K[3]
T[28]	2953.865	249.108	233.089	234.097	232.082	T[2]
E[29]	3082.908	148.060	132.042	133.050	131.034	E[1]

sp|Q6GSS7|H2A2A_MOUSE

LNKLLGK $^{\text{Dimethyl}}_{28.03}$ VTIAQGGVLPNIQAVLLPKKTE

- fragmentation table for charge state 2+
- specType=etd
- score=36.59
- F104912.dat
- query=q3258_p1
- precursor=1028.632600
- chargePrecursor=3
- itol=0.8

AA	c	y	z+1	z+2	z	AA
L[1]	66.063	**1542.450**	**1534.440**	0.504	1533.936	L[29]
N[2]	123.084	1485.908	**1477.898**	**1478.402**	**1477.394**	N[28]
K[3]	187.132	1428.886	**1420.877**	1421.381	**1420.373**	K[27]
L[4]	243.674	1364.839	**1356.829**	1357.333	**1356.325**	L[26]
L[5]	300.216	1308.297	1300.287	1300.791	1299.783	L[25]
G[6]	328.726	1251.755	1243.745	1244.249	1243.241	G[24]
K[7]	406.789	1223.244	1215.234	1215.738	1214.731	K[23]
V[8]	456.324	1145.181	1137.171	1137.675	1136.667	V[22]
T[9]	506.848	1095.646	1087.637	1088.141	1087.133	T[21]
I[10]	563.390	1045.123	1037.113	1037.617	1036.609	I[20]
A[11]	598.908	**988.581**	980.571	981.075	980.067	A[19]
Q[12]	662.937	953.062	945.053	945.557	944.549	Q[18]
G[13]	691.448	889.033	881.023	881.527	880.519	G[17]
G[14]	719.959	860.522	852.513	853.017	852.009	G[16]
V[15]	769.493	832.011	824.002	824.506	823.498	V[15]
L[16]	826.035	782.477	774.468	774.972	773.964	L[14]
P[17]	874.561	725.935	717.926	718.430	717.422	P[13]
N[18]	931.583	677.409	669.399	669.903	668.895	N[12]
I[19]	988.125	620.387	612.378	612.882	611.874	I[11]
Q[20]	1052.154	563.845	555.836	556.340	555.332	Q[10]
A[21]	1087.673	499.816	491.807	492.310	491.303	A[9]
V[22]	1137.207	464.297	456.288	456.792	455.784	V[8]
L[23]	1193.749	414.763	406.754	407.258	406.250	L[7]
L[24]	1250.291	358.221	350.212	350.716	349.708	L[6]
P[25]	**1298.817**	301.679	293.670	294.174	293.166	P[5]
K[26]	**1362.865**	253.153	245.143	245.647	244.639	K[4]
K[27]	**1426.912**	189.105	181.096	181.600	180.592	K[3]
T[28]	**1477.436**	125.058	117.048	117.552	116.544	T[2]
E[29]	1541.958	74.534	66.524	67.028	66.021	E[1]

sp|Q6GSS7|H2A2A_MOUSE

LNKLLGK $^{\text{Dimethyl}}_{28.03}$ VTIAQGGVLPNIQAVLLPKKTE

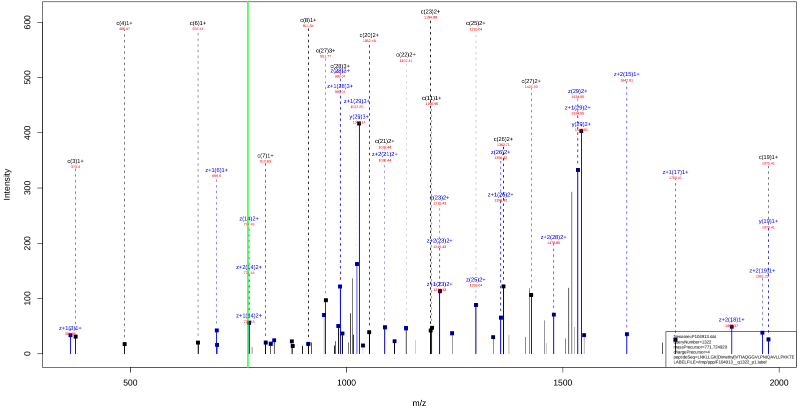

sp|Q6GSS7|H2A2A_MOUSE

LNKLLGK$^{\text{Dimethyl}}_{28.03}$VTIAQGGVLPNIQAVLLPKKTE

- fragmentation table for charge state 1+
- specType=etd
- score=48.66
- F104913.dat
- query=q1322_p1
- precursor=771.724920
- chargePrecursor=4
- itol=0.8

AA	c	y	z+1	z+2	z	AA
L[1]	131.118	3083.892	3067.873	0.000	3066.865	L[29]
N[2]	245.161	2970.808	2954.789	2955.797	2953.781	N[28]
K[3]	**373.256**	2856.765	2840.746	2841.754	2839.738	K[27]
L[4]	**486.340**	2728.670	2712.651	2713.659	2711.643	L[26]
L[5]	599.424	2615.586	2599.567	2600.575	2598.559	L[25]
G[6]	**656.445**	2502.502	2486.483	2487.491	2485.475	G[24]
K[7]	**812.572**	2445.480	2429.462	2430.469	2428.454	K[23]
V[8]	**911.640**	2289.354	2273.335	2274.343	2272.327	V[22]
T[9]	1012.688	2190.286	2174.267	2175.275	2173.259	T[21]
I[10]	1125.772	2089.238	2073.219	2074.227	2072.211	I[20]
A[11]	**1196.809**	**1976.154**	1960.135	**1961.143**	1959.127	A[19]
Q[12]	1324.867	1905.117	1889.098	**1890.106**	1888.090	Q[18]
G[13]	1381.889	1777.058	**1761.039**	1762.047	**1760.032**	G[17]
G[14]	1438.910	1720.037	1704.018	1705.026	1703.010	G[16]
V[15]	1537.979	1663.015	1646.997	**1648.004**	1645.989	V[15]
L[16]	1651.063	1563.947	1547.928	**1548.936**	1546.920	L[14]
P[17]	1748.116	1450.863	1434.844	1435.852	1433.836	P[13]
N[18]	1862.159	1353.810	1337.791	**1338.799**	1336.783	N[12]
I[19]	**1975.243**	1239.767	1223.748	1224.756	1222.741	I[11]
Q[20]	2103.301	1126.683	**1110.664**	1111.672	1109.656	Q[10]
A[21]	2174.338	998.624	982.606	983.614	**981.598**	A[9]
V[22]	2273.407	927.587	**911.569**	912.576	910.561	V[8]
L[23]	2386.491	828.519	**812.500**	813.508	811.492	L[7]
L[24]	2499.575	715.435	**699.416**	**700.424**	698.408	L[6]
P[25]	2596.628	602.351	586.332	587.340	585.324	P[5]
K[26]	2724.723	505.298	489.279	490.287	488.271	K[4]
K[27]	2852.818	377.203	**361.184**	362.192	360.177	K[3]
T[28]	2953.865	249.108	233.089	234.097	232.082	T[2]
E[29]	3082.908	148.060	132.042	133.050	131.034	E[1]

sp|Q6GSS7|H2A2A_MOUSE

LNKLLGK $^{Dimethyl}_{28.03}$ VTIAQGGVLPNIQAVLLPKKTE

- fragmentation table for charge state 2+
- specType=etd
- score=48.66
- F104913.dat
- query=q1322_p1
- precursor=771.724920
- chargePrecursor=4
- itol=0.8

AA	c	y	z+1	z+2	z	AA
L[1]	66.063	**1542.450**	**1534.440**	0.504	**1533.936**	L[29]
N[2]	123.084	1485.908	1477.898	**1478.402**	1477.394	N[28]
K[3]	187.132	1428.886	1420.877	1421.381	1420.373	K[27]
L[4]	243.674	1364.839	**1356.829**	1357.333	**1356.325**	L[26]
L[5]	300.216	1308.297	1300.287	1300.791	**1299.783**	L[25]
G[6]	328.726	1251.755	**1243.745**	**1244.249**	1243.241	G[24]
K[7]	406.789	1223.244	**1215.234**	**1215.738**	**1214.731**	K[23]
V[8]	456.324	1145.181	**1137.171**	**1137.675**	**1136.667**	V[22]
T[9]	506.848	1095.646	1087.637	**1088.141**	1087.133	T[21]
I[10]	563.390	1045.123	**1037.113**	**1037.617**	1036.609	I[20]
A[11]	598.908	988.581	**980.571**	**981.075**	980.067	A[19]
Q[12]	662.937	953.062	945.053	945.557	944.549	Q[18]
G[13]	691.448	889.033	881.023	881.527	880.519	G[17]
G[14]	719.959	860.522	852.513	853.017	852.009	G[16]
V[15]	769.493	**832.011**	**824.002**	**824.506**	**823.498**	V[15]
L[16]	826.035	782.477	**774.468**	**774.972**	**773.964**	L[14]
P[17]	**874.561**	725.935	717.926	718.430	717.422	P[13]
N[18]	931.583	677.409	669.399	669.903	668.895	N[12]
I[19]	988.125	620.387	612.378	612.882	611.874	I[11]
Q[20]	**1052.154**	563.845	555.836	556.340	555.332	Q[10]
A[21]	**1087.673**	499.816	491.807	492.310	491.303	A[9]
V[22]	**1137.207**	464.297	456.288	456.792	455.784	V[8]
L[23]	**1193.749**	414.763	406.754	407.258	406.250	L[7]
L[24]	1250.291	358.221	350.212	350.716	349.708	L[6]
P[25]	**1298.817**	301.679	293.670	294.174	293.166	P[5]
K[26]	**1362.865**	253.153	245.143	245.647	244.639	K[4]
K[27]	**1426.912**	189.105	181.096	181.600	180.592	K[3]
T[28]	1477.436	125.058	117.048	117.552	116.544	T[2]
E[29]	1541.958	74.534	66.524	67.028	66.021	E[1]

sp|Q6GSS7|H2A2A_MOUSE

LNKLLGK $^{Dimethyl}_{28.03}$ VTIAQGGVLPNIQAVLLPKKTE

- fragmentation table for charge state 3+
- specType=etd
- score=48.66
- F104913.dat
- query=q1322_p1
- precursor=771.724920
- chargePrecursor=4
- itol=0.8

AA	c	y	z+1	z+2	z	AA
L[1]	44.377	**1028.635**	**1023.296**	0.672	1022.960	L[29]
N[2]	82.392	**990.941**	**985.601**	985.937	**985.265**	N[28]
K[3]	125.090	952.926	**947.587**	**947.923**	**947.251**	K[27]
L[4]	162.785	910.228	904.889	905.225	904.553	L[26]
L[5]	200.479	**872.533**	867.194	867.530	866.858	L[25]
G[6]	219.487	834.839	829.499	829.835	829.163	G[24]
K[7]	271.529	815.832	810.492	810.828	810.156	K[23]
V[8]	304.552	763.790	758.450	758.786	758.114	V[22]
T[9]	338.234	730.767	725.427	725.763	725.091	T[21]
I[10]	375.929	697.084	691.745	692.081	691.409	I[20]
A[11]	399.608	659.389	654.050	654.386	653.714	A[19]
Q[12]	442.294	635.710	630.371	630.707	630.035	Q[18]
G[13]	461.301	593.024	587.685	588.021	587.349	G[17]
G[14]	480.308	574.017	568.678	569.013	568.342	G[16]
V[15]	513.331	555.010	549.670	550.006	549.334	V[15]
L[16]	551.026	521.987	516.648	516.984	516.312	L[14]
P[17]	583.377	484.292	478.953	479.289	478.617	P[13]
N[18]	621.391	451.942	446.602	446.938	446.266	N[12]
I[19]	659.086	413.927	408.588	408.924	408.252	I[11]
Q[20]	701.772	376.233	370.893	371.229	370.557	Q[10]
A[21]	725.451	333.546	328.207	328.543	327.871	A[9]
V[22]	758.474	309.867	304.528	304.864	304.192	V[8]
L[23]	796.168	276.844	271.505	271.841	271.169	L[7]
L[24]	833.863	239.150	233.810	234.146	233.474	L[6]
P[25]	866.214	201.455	196.116	196.451	195.780	P[5]
K[26]	908.912	169.104	163.765	164.101	163.429	K[4]
K[27]	**951.611**	126.406	121.066	121.402	120.730	K[3]
T[28]	**985.293**	83.708	78.368	78.704	78.032	T[2]
E[29]	1028.307	50.025	44.685	45.021	44.349	E[1]

sp|Q6GSS7|H2A2A_MOUSE

LNKLLGK $^{Dimethyl}_{28.03}$ VTIAQGGVLPNIQAVLLPKKTE

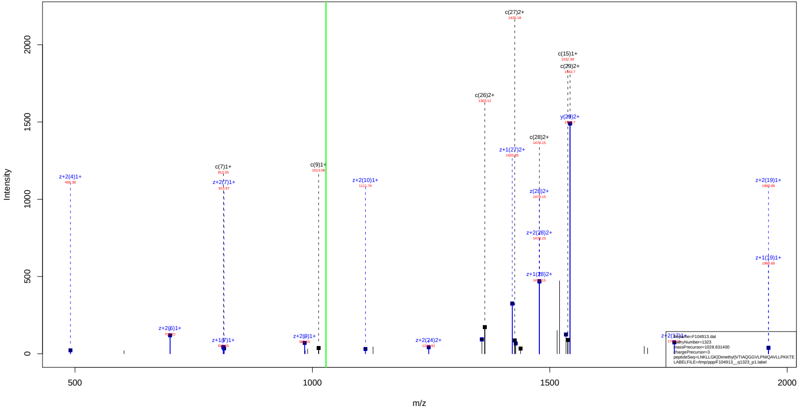

sp|Q6GSS7|H2A2A_MOUSE

LNKLLGK $^{\text{Dimethyl}}_{28.03}$ VTIAQGGVLPNIQAVLLPKKTE

- fragmentation table for charge state 1+
- specType=etd
- score=40.22
- F104913.dat
- query=q1323_p1
- precursor=1028.631400
- chargePrecursor=3
- itol=0.8

AA	c	y	z+1	z+2	z	AA
L[1]	131.118	3083.892	3067.873	0.000	3066.865	L[29]
N[2]	245.161	2970.808	2954.789	2955.797	2953.781	N[28]
K[3]	373.256	2856.765	2840.746	2841.754	2839.738	K[27]
L[4]	486.340	2728.670	2712.651	2713.659	2711.643	L[26]
L[5]	599.424	2615.586	2599.567	2600.575	2598.559	L[25]
G[6]	656.445	2502.502	2486.483	2487.491	2485.475	G[24]
K[7]	**812.572**	2445.480	2429.462	2430.469	2428.454	K[23]
V[8]	911.640	2289.354	2273.335	2274.343	2272.327	V[22]
T[9]	**1012.688**	2190.286	2174.267	2175.275	2173.259	T[21]
I[10]	1125.772	2089.238	2073.219	2074.227	2072.211	I[20]
A[11]	1196.809	1976.154	**1960.135**	**1961.143**	1959.127	A[19]
Q[12]	1324.867	1905.117	1889.098	1890.106	1888.090	Q[18]
G[13]	1381.889	1777.058	1761.039	**1762.047**	1760.032	G[17]
G[14]	**1438.910**	1720.037	1704.018	1705.026	1703.010	G[16]
V[15]	**1537.979**	1663.015	1646.997	1648.004	1645.989	V[15]
L[16]	1651.063	1563.947	1547.928	1548.936	1546.920	L[14]
P[17]	1748.116	1450.863	1434.844	1435.852	1433.836	P[13]
N[18]	1862.159	1353.810	1337.791	1338.799	1336.783	N[12]
I[19]	1975.243	1239.767	1223.748	1224.756	1222.741	I[11]
Q[20]	2103.301	1126.683	1110.664	**1111.672**	1109.656	Q[10]
A[21]	2174.338	998.624	982.606	**983.614**	981.598	A[9]
V[22]	2273.407	927.587	911.569	912.576	910.561	V[8]
L[23]	2386.491	828.519	**812.500**	**813.508**	811.492	L[7]
L[24]	2499.575	715.435	699.416	**700.424**	698.408	L[6]
P[25]	2596.628	602.351	586.332	587.340	585.324	P[5]
K[26]	2724.723	505.298	489.279	**490.287**	488.271	K[4]
K[27]	2852.818	377.203	361.184	362.192	360.177	K[3]
T[28]	2953.865	249.108	233.089	234.097	232.082	T[2]
E[29]	3082.908	148.060	132.042	133.050	131.034	E[1]

sp|Q6GSS7|H2A2A_MOUSE

LNKLLGK $^{\text{Dimethyl}}_{28.03}$ VTIAQGGVLPNIQAVLLPKKTE

- fragmentation table for charge state 2+
- specType=etd
- score=40.22
- F104913.dat
- query=q1323_p1
- precursor=1028.631400
- chargePrecursor=3
- itol=0.8

AA	c	y	z+1	z+2	z	AA
L[1]	66.063	**1542.450**	**1534.440**	0.504	**1533.936**	L[29]
N[2]	123.084	1485.908	**1477.898**	**1478.402**	**1477.394**	N[28]
K[3]	187.132	**1428.886**	**1420.877**	**1421.381**	**1420.373**	K[27]
L[4]	243.674	1364.839	**1356.829**	**1357.333**	**1356.325**	L[26]
L[5]	300.216	1308.297	1300.287	1300.791	1299.783	L[25]
G[6]	328.726	1251.755	1243.745	**1244.249**	1243.241	G[24]
K[7]	406.789	1223.244	1215.234	1215.738	1214.731	K[23]
V[8]	456.324	1145.181	1137.171	1137.675	1136.667	V[22]
T[9]	506.848	1095.646	1087.637	1088.141	1087.133	T[21]
I[10]	563.390	1045.123	1037.113	1037.617	1036.609	I[20]
A[11]	598.908	988.581	980.571	981.075	980.067	A[19]
Q[12]	662.937	953.062	945.053	945.557	944.549	Q[18]
G[13]	691.448	889.033	881.023	881.527	880.519	G[17]
G[14]	719.959	860.522	852.513	853.017	852.009	G[16]
V[15]	769.493	832.011	824.002	824.506	823.498	V[15]
L[16]	826.035	782.477	774.468	774.972	773.964	L[14]
P[17]	874.561	725.935	717.926	718.430	717.422	P[13]
N[18]	931.583	677.409	669.399	669.903	668.895	N[12]
I[19]	988.125	620.387	612.378	612.882	611.874	I[11]
Q[20]	1052.154	563.845	555.836	556.340	555.332	Q[10]
A[21]	1087.673	499.816	491.807	492.310	491.303	A[9]
V[22]	1137.207	464.297	456.288	456.792	455.784	V[8]
L[23]	1193.749	414.763	406.754	407.258	406.250	L[7]
L[24]	1250.291	358.221	350.212	350.716	349.708	L[6]
P[25]	1298.817	301.679	293.670	294.174	293.166	P[5]
K[26]	**1362.865**	253.153	245.143	245.647	244.639	K[4]
K[27]	**1426.912**	189.105	181.096	181.600	180.592	K[3]
T[28]	**1477.436**	125.058	117.048	117.552	116.544	T[2]
E[29]	**1541.958**	74.534	66.524	67.028	66.021	E[1]

sp|Q6GSS7|H2A2A_MOUSE

LNKLLGK $^{\text{Dimethyl}}_{28.03}$ VTIAQGGVLPNIQAVLLPKKTE

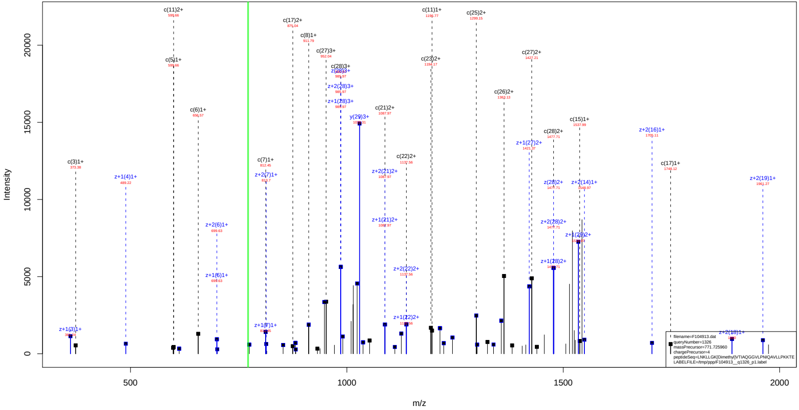

sp|Q6GSS7|H2A2A_MOUSE

LNKLLGK $^{Dimethyl}_{28.03}$ VTIAQGGVLPNIQAVLLPKKTE

- fragmentation table for charge state 1+
- specType=etd
- score=95.34
- F104913.dat
- query=q1326_p1
- precursor=771.725960
- chargePrecursor=4
- itol=0.8

AA	c	y	z+1	z+2	z	AA
L[1]	131.118	3083.892	3067.873	0.000	3066.865	L[29]
N[2]	245.161	2970.808	2954.789	2955.797	2953.781	N[28]
K[3]	**373.256**	2856.765	2840.746	2841.754	2839.738	K[27]
L[4]	486.340	2728.670	2712.651	2713.659	2711.643	L[26]
L[5]	**599.424**	2615.586	2599.567	2600.575	2598.559	L[25]
G[6]	**656.445**	2502.502	2486.483	2487.491	2485.475	G[24]
K[7]	**812.572**	2445.480	2429.462	2430.469	2428.454	K[23]
V[8]	**911.640**	2289.354	2273.335	2274.343	2272.327	V[22]
T[9]	1012.688	2190.286	2174.267	2175.275	2173.259	T[21]
I[10]	**1125.772**	2089.238	2073.219	2074.227	2072.211	I[20]
A[11]	**1196.809**	1976.154	1960.135	**1961.143**	1959.127	A[19]
Q[12]	**1324.867**	1905.117	1889.098	**1890.106**	1888.090	Q[18]
G[13]	**1381.889**	1777.058	1761.039	1762.047	1760.032	G[17]
G[14]	**1438.910**	1720.037	1704.018	**1705.026**	1703.010	G[16]
V[15]	**1537.979**	1663.015	1646.997	1648.004	1645.989	V[15]
L[16]	1651.063	1563.947	1547.928	**1548.936**	1546.920	L[14]
P[17]	**1748.116**	1450.863	1434.844	1435.852	1433.836	P[13]
N[18]	1862.159	1353.810	1337.791	**1338.799**	1336.783	N[12]
I[19]	1975.243	1239.767	**1223.748**	1224.756	1222.741	I[11]
Q[20]	2103.301	**1126.683**	**1110.664**	1111.672	1109.656	Q[10]
A[21]	2174.338	998.624	982.606	983.614	981.598	A[9]
V[22]	2273.407	927.587	**911.569**	**912.576**	910.561	V[8]
L[23]	2386.491	828.519	**812.500**	**813.508**	811.492	L[7]
L[24]	2499.575	715.435	**699.416**	**700.424**	698.408	L[6]
P[25]	2596.628	602.351	586.332	587.340	585.324	P[5]
K[26]	2724.723	505.298	**489.279**	490.287	488.271	K[4]
K[27]	2852.818	377.203	**361.184**	362.192	360.177	K[3]
T[28]	2953.865	249.108	233.089	234.097	232.082	T[2]
E[29]	3082.908	148.060	132.042	133.050	131.034	E[1]

sp|Q6GSS7|H2A2A_MOUSE

LNKLLGK $^{Dimethyl}_{28.03}$ VTIAQGGVLPNIQAVLLPKKTE

- fragmentation table for charge state 2+
- specType=etd
- score=95.34
- F104913.dat
- query=q1326_p1
- precursor=771.725960
- chargePrecursor=4
- itol=0.8

AA	c	y	z+1	z+2	z	AA
L[1]	66.063	1542.450	**1534.440**	0.504	1533.936	L[29]
N[2]	123.084	1485.908	**1477.898**	**1478.402**	**1477.394**	N[28]
K[3]	187.132	1428.886	**1420.877**	**1421.381**	1420.373	K[27]
L[4]	243.674	1364.839	**1356.829**	**1357.333**	**1356.325**	L[26]
L[5]	300.216	1308.297	**1300.287**	**1300.791**	**1299.783**	L[25]
G[6]	328.726	1251.755	**1243.745**	**1244.249**	1243.241	G[24]
K[7]	406.789	**1223.244**	**1215.234**	**1215.738**	**1214.731**	K[23]
V[8]	456.324	1145.181	**1137.171**	**1137.675**	1136.667	V[22]
T[9]	506.848	1095.646	**1087.637**	**1088.141**	1087.133	T[21]
I[10]	563.390	1045.123	**1037.113**	**1037.617**	**1036.609**	I[20]
A[11]	**598.908**	988.581	980.571	981.075	980.067	A[19]
Q[12]	662.937	953.062	945.053	945.557	944.549	Q[18]
G[13]	691.448	889.033	**881.023**	**881.527**	**880.519**	G[17]
G[14]	719.959	860.522	**852.513**	**853.017**	**852.009**	G[16]
V[15]	769.493	832.011	824.002	824.506	823.498	V[15]
L[16]	826.035	782.477	**774.468**	**774.972**	**773.964**	L[14]
P[17]	**874.561**	725.935	717.926	718.430	717.422	P[13]
N[18]	**931.583**	677.409	669.399	669.903	668.895	N[12]
I[19]	988.125	620.387	**612.378**	**612.882**	**611.874**	I[11]
Q[20]	**1052.154**	563.845	555.836	556.340	555.332	Q[10]
A[21]	**1087.673**	499.816	491.807	492.310	491.303	A[9]
V[22]	**1137.207**	464.297	456.288	456.792	455.784	V[8]
L[23]	**1193.749**	414.763	406.754	407.258	406.250	L[7]
L[24]	1250.291	358.221	350.212	350.716	349.708	L[6]
P[25]	**1298.817**	301.679	293.670	294.174	293.166	P[5]
K[26]	**1362.865**	253.153	245.143	245.647	244.639	K[4]
K[27]	**1426.912**	189.105	181.096	181.600	180.592	K[3]
T[28]	**1477.436**	125.058	117.048	117.552	116.544	T[2]
E[29]	1541.958	74.534	66.524	67.028	66.021	E[1]

sp|Q6GSS7|H2A2A_MOUSE

LNKLLGK $^{Dimethyl}_{28.03}$ VTIAQGGVLPNIQAVLLPKKTE

- fragmentation table for charge state 3+
- specType=etd
- score=95.34
- F104913.dat
- query=q1326_p1
- precursor=771.725960
- chargePrecursor=4
- itol=0.8

AA	c	y	z+1	z+2	z	AA
L[1]	44.377	**1028.635**	**1023.296**	0.672	**1022.960**	L[29]
N[2]	82.392	**990.941**	**985.601**	**985.937**	**985.265**	N[28]
K[3]	125.090	952.926	**947.587**	**947.923**	947.251	K[27]
L[4]	162.785	910.228	904.889	905.225	904.553	L[26]
L[5]	200.479	872.533	867.194	867.530	866.858	L[25]
G[6]	219.487	834.839	829.499	829.835	829.163	G[24]
K[7]	271.529	815.832	810.492	810.828	810.156	K[23]
V[8]	304.552	763.790	758.450	758.786	758.114	V[22]
T[9]	338.234	730.767	725.427	725.763	725.091	T[21]
I[10]	375.929	697.084	691.745	692.081	691.409	I[20]
A[11]	399.608	659.389	654.050	654.386	653.714	A[19]
Q[12]	442.294	635.710	630.371	630.707	630.035	Q[18]
G[13]	461.301	593.024	587.685	588.021	587.349	G[17]
G[14]	480.308	574.017	568.678	569.013	568.342	G[16]
V[15]	513.331	555.010	549.670	550.006	549.334	V[15]
L[16]	551.026	521.987	516.648	516.984	516.312	L[14]
P[17]	583.377	484.292	478.953	479.289	478.617	P[13]
N[18]	621.391	451.942	446.602	446.938	446.266	N[12]
I[19]	659.086	413.927	408.588	408.924	408.252	I[11]
Q[20]	701.772	376.233	370.893	371.229	370.557	Q[10]
A[21]	725.451	333.546	328.207	328.543	327.871	A[9]
V[22]	758.474	309.867	304.528	304.864	304.192	V[8]
L[23]	796.168	276.844	271.505	271.841	271.169	L[7]
L[24]	833.863	239.150	233.810	234.146	233.474	L[6]
P[25]	866.214	201.455	196.116	196.451	195.780	P[5]
K[26]	908.912	169.104	163.765	164.101	163.429	K[4]
K[27]	**951.611**	126.406	121.066	121.402	120.730	K[3]
T[28]	**985.293**	83.708	78.368	78.704	78.032	T[2]
E[29]	1028.307	50.025	44.685	45.021	44.349	E[1]

sp|Q6GSS7|H2A2A_MOUSE

LNKLLGK $^{\text{Dimethyl}}_{28.03}$ VTIAQGGVLPNIQAVLLPKKTE

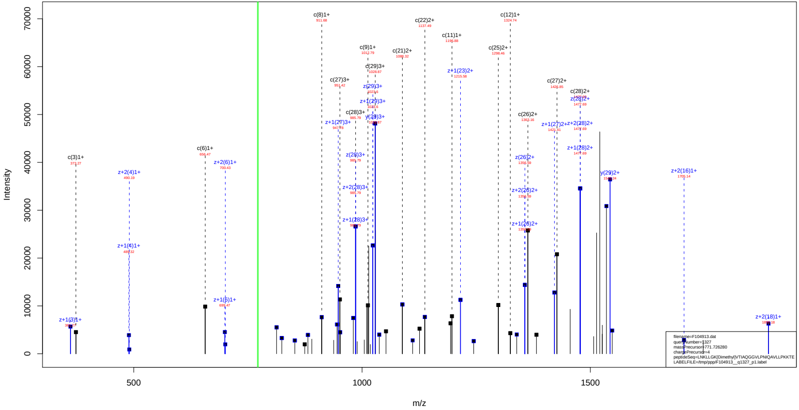

sp|Q6GSS7|H2A2A_MOUSE

LNKLLGK $^{\text{Dimethyl}}_{28.03}$ VTIAQGGVLPNIQAVLLPKKTE

- fragmentation table for charge state 1+
- specType=etd
- score=87.04
- F104913.dat
- query=q1327_p1
- precursor=771.726280
- chargePrecursor=4
- itol=0.8

AA	c	y	z+1	z+2	z	AA
L[1]	131.118	3083.892	3067.873	0.000	3066.865	L[29]
N[2]	245.161	2970.808	2954.789	2955.797	2953.781	N[28]
K[3]	**373.256**	2856.765	2840.746	2841.754	2839.738	K[27]
L[4]	486.340	2728.670	2712.651	2713.659	2711.643	L[26]
L[5]	599.424	2615.586	2599.567	2600.575	2598.559	L[25]
G[6]	**656.445**	2502.502	2486.483	2487.491	2485.475	G[24]
K[7]	**812.572**	2445.480	2429.462	2430.469	2428.454	K[23]
V[8]	**911.640**	2289.354	2273.335	2274.343	2272.327	V[22]
T[9]	**1012.688**	2190.286	2174.267	2175.275	2173.259	T[21]
I[10]	**1125.772**	2089.238	2073.219	2074.227	2072.211	I[20]
A[11]	**1196.809**	1976.154	1960.135	1961.143	1959.127	A[19]
Q[12]	**1324.867**	1905.117	1889.098	**1890.106**	1888.090	Q[18]
G[13]	**1381.889**	1777.058	1761.039	1762.047	1760.032	G[17]
G[14]	1438.910	1720.037	1704.018	**1705.026**	1703.010	G[16]
V[15]	1537.979	1663.015	1646.997	1648.004	1645.989	V[15]
L[16]	1651.063	1563.947	**1547.928**	1548.936	1546.920	L[14]
P[17]	1748.116	1450.863	1434.844	1435.852	1433.836	P[13]
N[18]	1862.159	1353.810	1337.791	**1338.799**	1336.783	N[12]
I[19]	1975.243	1239.767	1223.748	1224.756	1222.741	I[11]
Q[20]	2103.301	1126.683	**1110.664**	1111.672	1109.656	Q[10]
A[21]	2174.338	998.624	982.606	983.614	981.598	A[9]
V[22]	2273.407	927.587	**911.569**	912.576	910.561	V[8]
L[23]	2386.491	828.519	**812.500**	813.508	811.492	L[7]
L[24]	2499.575	715.435	**699.416**	**700.424**	698.408	L[6]
P[25]	2596.628	602.351	586.332	587.340	585.324	P[5]
K[26]	2724.723	505.298	**489.279**	**490.287**	488.271	K[4]
K[27]	2852.818	377.203	**361.184**	362.192	360.177	K[3]
T[28]	2953.865	249.108	233.089	234.097	232.082	T[2]
E[29]	3082.908	148.060	132.042	133.050	131.034	E[1]

sp|Q6GSS7|H2A2A_MOUSE

LNKLLGK $^{\text{Dimethyl}}_{28.03}$ VTIAQGGVLPNIQAVLLPKKTE

- fragmentation table for charge state 2+
- specType=etd
- score=87.04
- F104913.dat
- query=q1327_p1
- precursor=771.726280
- chargePrecursor=4
- itol=0.8

AA	c	y	z+1	z+2	z	AA
L[1]	66.063	**1542.450**	**1534.440**	0.504	1533.936	L[29]
N[2]	123.084	1485.908	**1477.898**	**1478.402**	**1477.394**	N[28]
K[3]	187.132	1428.886	**1420.877**	**1421.381**	1420.373	K[27]
L[4]	243.674	1364.839	**1356.829**	**1357.333**	**1356.325**	L[26]
L[5]	300.216	1308.297	1300.287	1300.791	1299.783	L[25]
G[6]	328.726	1251.755	**1243.745**	**1244.249**	1243.241	G[24]
K[7]	406.789	1223.244	**1215.234**	**1215.738**	1214.731	K[23]
V[8]	456.324	1145.181	**1137.171**	**1137.675**	1136.667	V[22]
T[9]	506.848	1095.646	**1087.637**	**1088.141**	1087.133	T[21]
I[10]	563.390	1045.123	**1037.113**	**1037.617**	1036.609	I[20]
A[11]	598.908	988.581	**980.571**	**981.075**	**980.067**	A[19]
Q[12]	662.937	953.062	**945.053**	**945.557**	**944.549**	Q[18]
G[13]	691.448	889.033	**881.023**	**881.527**	880.519	G[17]
G[14]	719.959	860.522	**852.513**	**853.017**	**852.009**	G[16]
V[15]	769.493	832.011	**824.002**	**824.506**	**823.498**	V[15]
L[16]	826.035	782.477	774.468	774.972	773.964	L[14]
P[17]	**874.561**	725.935	717.926	718.430	717.422	P[13]
N[18]	931.583	677.409	669.399	669.903	668.895	N[12]
I[19]	988.125	620.387	612.378	612.882	611.874	I[11]
Q[20]	**1052.154**	563.845	555.836	556.340	555.332	Q[10]
A[21]	**1087.673**	499.816	491.807	492.310	491.303	A[9]
V[22]	**1137.207**	464.297	456.288	456.792	455.784	V[8]
L[23]	**1193.749**	414.763	406.754	407.258	406.250	L[7]
L[24]	1250.291	358.221	350.212	350.716	349.708	L[6]
P[25]	**1298.817**	301.679	293.670	294.174	293.166	P[5]
K[26]	**1362.865**	253.153	245.143	245.647	244.639	K[4]
K[27]	**1426.912**	189.105	181.096	181.600	180.592	K[3]
T[28]	**1477.436**	125.058	117.048	117.552	116.544	T[2]
E[29]	1541.958	74.534	66.524	67.028	66.021	E[1]

sp|Q6GSS7|H2A2A_MOUSE

LNKLLGK $^{\text{Dimethyl}}_{28.03}$ VTIAQGGVLPNIQAVLLPKKTE

- fragmentation table for charge state 3+
- specType=etd
- score=87.04
- F104913.dat
- query=q1327_p1
- precursor=771.726280
- chargePrecursor=4
- itol=0.8

AA	c	y	z+1	z+2	z	AA
L[1]	44.377	**1028.635**	**1023.296**	0.672	**1022.960**	L[29]
N[2]	82.392	990.941	**985.601**	**985.937**	**985.265**	N[28]
K[3]	125.090	**952.926**	**947.587**	**947.923**	**947.251**	K[27]
L[4]	162.785	910.228	904.889	905.225	904.553	L[26]
L[5]	200.479	872.533	867.194	867.530	866.858	L[25]
G[6]	219.487	834.839	829.499	829.835	829.163	G[24]
K[7]	271.529	815.832	810.492	810.828	810.156	K[23]
V[8]	304.552	763.790	758.450	758.786	758.114	V[22]
T[9]	338.234	730.767	725.427	725.763	725.091	T[21]
I[10]	375.929	697.084	691.745	692.081	691.409	I[20]
A[11]	399.608	659.389	654.050	654.386	653.714	A[19]
Q[12]	442.294	635.710	630.371	630.707	630.035	Q[18]
G[13]	461.301	593.024	587.685	588.021	587.349	G[17]
G[14]	480.308	574.017	568.678	569.013	568.342	G[16]
V[15]	513.331	555.010	549.670	550.006	549.334	V[15]
L[16]	551.026	521.987	516.648	516.984	516.312	L[14]
P[17]	583.377	484.292	478.953	479.289	478.617	P[13]
N[18]	621.391	451.942	446.602	446.938	446.266	N[12]
I[19]	659.086	413.927	408.588	408.924	408.252	I[11]
Q[20]	701.772	376.233	370.893	371.229	370.557	Q[10]
A[21]	725.451	333.546	328.207	328.543	327.871	A[9]
V[22]	758.474	309.867	304.528	304.864	304.192	V[8]
L[23]	796.168	276.844	271.505	271.841	271.169	L[7]
L[24]	833.863	239.150	233.810	234.146	233.474	L[6]
P[25]	866.214	201.455	196.116	196.451	195.780	P[5]
K[26]	908.912	169.104	163.765	164.101	163.429	K[4]
K[27]	**951.611**	126.406	121.066	121.402	120.730	K[3]
T[28]	**985.293**	83.708	78.368	78.704	78.032	T[2]
E[29]	**1028.307**	50.025	44.685	45.021	44.349	E[1]

sp|Q6GSS7|H2A2A_MOUSE

LNKLLGK $^{Dimethyl}_{28.03}$ VTIAQGGVLPNIQAVLLPKKTE

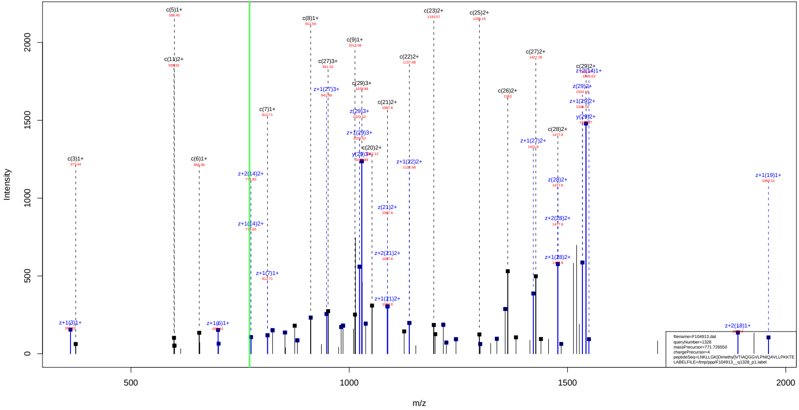

sp|Q6GSS7|H2A2A_MOUSE

LNKLLGK $^{\text{Dimethyl}}_{28.03}$ VTIAQGGVLPNIQAVLLPKKTE

- fragmentation table for charge state 1+
- specType=etd
- score=94.58
- F104913.dat
- query=q1328_p1
- precursor=771.726550
- chargePrecursor=4
- itol=0.8

AA	c	y	z+1	z+2	z	AA
L[1]	131.118	3083.892	3067.873	0.000	3066.865	L[29]
N[2]	245.161	2970.808	2954.789	2955.797	2953.781	N[28]
K[3]	**373.256**	2856.765	2840.746	2841.754	2839.738	K[27]
L[4]	486.340	2728.670	2712.651	2713.659	2711.643	L[26]
L[5]	**599.424**	2615.586	2599.567	2600.575	2598.559	L[25]
G[6]	**656.445**	2502.502	2486.483	2487.491	2485.475	G[24]
K[7]	**812.572**	2445.480	2429.462	2430.469	2428.454	K[23]
V[8]	**911.640**	2289.354	2273.335	2274.343	2272.327	V[22]
T[9]	**1012.688**	2190.286	2174.267	2175.275	2173.259	T[21]
I[10]	**1125.772**	2089.238	2073.219	2074.227	2072.211	I[20]
A[11]	**1196.809**	1976.154	**1960.135**	1961.143	1959.127	A[19]
Q[12]	1324.867	1905.117	1889.098	**1890.106**	1888.090	Q[18]
G[13]	**1381.889**	1777.058	1761.039	1762.047	1760.032	G[17]
G[14]	**1438.910**	1720.037	1704.018	1705.026	1703.010	G[16]
V[15]	1537.979	1663.015	1646.997	1648.004	1645.989	V[15]
L[16]	1651.063	1563.947	1547.928	**1548.936**	1546.920	L[14]
P[17]	1748.116	1450.863	1434.844	1435.852	1433.836	P[13]
N[18]	1862.159	1353.810	**1337.791**	**1338.799**	1336.783	N[12]
I[19]	1975.243	1239.767	1223.748	1224.756	**1222.741**	I[11]
Q[20]	2103.301	1126.683	1110.664	1111.672	1109.656	Q[10]
A[21]	2174.338	998.624	982.606	983.614	**981.598**	A[9]
V[22]	2273.407	927.587	**911.569**	912.576	910.561	V[8]
L[23]	2386.491	828.519	**812.500**	813.508	811.492	L[7]
L[24]	2499.575	715.435	**699.416**	**700.424**	698.408	L[6]
P[25]	2596.628	602.351	586.332	587.340	585.324	P[5]
K[26]	2724.723	505.298	489.279	490.287	488.271	K[4]
K[27]	2852.818	377.203	**361.184**	362.192	360.177	K[3]
T[28]	2953.865	249.108	233.089	234.097	232.082	T[2]
E[29]	3082.908	148.060	132.042	133.050	131.034	E[1]

sp|Q6GSS7|H2A2A_MOUSE

LNKLLGK $^{\text{Dimethyl}}_{28.03}$ VTIAQGGVLPNIQAVLLPKKTE

- fragmentation table for charge state 2+
- specType=etd
- score=94.58
- F104913.dat
- query=q1328_p1
- precursor=771.726550
- chargePrecursor=4
- itol=0.8

AA	c	y	z+1	z+2	z	AA
L[1]	66.063	**1542.450**	**1534.440**	0.504	**1533.936**	L[29]
N[2]	123.084	**1485.908**	**1477.898**	**1478.402**	**1477.394**	N[28]
K[3]	187.132	1428.886	**1420.877**	**1421.381**	1420.373	K[27]
L[4]	243.674	1364.839	**1356.829**	**1357.333**	1356.325	L[26]
L[5]	300.216	1308.297	**1300.287**	1300.791	**1299.783**	L[25]
G[6]	328.726	1251.755	**1243.745**	**1244.249**	1243.241	G[24]
K[7]	406.789	**1223.244**	**1215.234**	**1215.738**	**1214.731**	K[23]
V[8]	456.324	1145.181	**1137.171**	**1137.675**	1136.667	V[22]
T[9]	506.848	1095.646	**1087.637**	**1088.141**	**1087.133**	T[21]
I[10]	563.390	1045.123	**1037.113**	**1037.617**	1036.609	I[20]
A[11]	**598.908**	988.581	980.571	**981.075**	980.067	A[19]
Q[12]	662.937	953.062	945.053	945.557	944.549	Q[18]
G[13]	691.448	889.033	**881.023**	**881.527**	**880.519**	G[17]
G[14]	719.959	860.522	**852.513**	**853.017**	**852.009**	G[16]
V[15]	769.493	832.011	**824.002**	**824.506**	**823.498**	V[15]
L[16]	826.035	782.477	**774.468**	**774.972**	773.964	L[14]
P[17]	**874.561**	725.935	717.926	718.430	717.422	P[13]
N[18]	931.583	677.409	669.399	669.903	668.895	N[12]
I[19]	988.125	620.387	612.378	612.882	611.874	I[11]
Q[20]	**1052.154**	563.845	555.836	556.340	555.332	Q[10]
A[21]	**1087.673**	499.816	491.807	492.310	491.303	A[9]
V[22]	**1137.207**	464.297	456.288	456.792	455.784	V[8]
L[23]	**1193.749**	414.763	406.754	407.258	406.250	L[7]
L[24]	1250.291	358.221	350.212	350.716	349.708	L[6]
P[25]	**1298.817**	301.679	293.670	294.174	293.166	P[5]
K[26]	**1362.865**	253.153	245.143	245.647	244.639	K[4]
K[27]	**1426.912**	189.105	181.096	181.600	180.592	K[3]
T[28]	**1477.436**	125.058	117.048	117.552	116.544	T[2]
E[29]	**1541.958**	74.534	66.524	67.028	66.021	E[1]

sp|Q6GSS7|H2A2A_MOUSE

LNKLLGK $^{Dimethyl}_{28.03}$ VTIAQGGVLPNIQAVLLPKKTE

- fragmentation table for charge state 3+
- specType=etd
- score=94.58
- F104913.dat
- query=q1328_p1
- precursor=771.726550
- chargePrecursor=4
- itol=0.8

AA	c	y	z+1	z+2	z	AA
L[1]	44.377	**1028.635**	**1023.296**	0.672	**1022.960**	L[29]
N[2]	82.392	990.941	**985.601**	**985.937**	**985.265**	N[28]
K[3]	125.090	952.926	**947.587**	**947.923**	**947.251**	K[27]
L[4]	162.785	910.228	904.889	905.225	904.553	L[26]
L[5]	200.479	872.533	867.194	867.530	866.858	L[25]
G[6]	219.487	834.839	829.499	829.835	829.163	G[24]
K[7]	271.529	815.832	810.492	810.828	810.156	K[23]
V[8]	304.552	763.790	758.450	758.786	758.114	V[22]
T[9]	338.234	730.767	725.427	725.763	725.091	T[21]
I[10]	375.929	697.084	691.745	692.081	691.409	I[20]
A[11]	399.608	659.389	654.050	654.386	653.714	A[19]
Q[12]	442.294	635.710	630.371	630.707	630.035	Q[18]
G[13]	461.301	593.024	587.685	588.021	587.349	G[17]
G[14]	480.308	574.017	568.678	569.013	568.342	G[16]
V[15]	513.331	555.010	549.670	550.006	549.334	V[15]
L[16]	551.026	521.987	516.648	516.984	516.312	L[14]
P[17]	583.377	484.292	478.953	479.289	478.617	P[13]
N[18]	621.391	451.942	446.602	446.938	446.266	N[12]
I[19]	659.086	413.927	408.588	408.924	408.252	I[11]
Q[20]	701.772	376.233	370.893	371.229	370.557	Q[10]
A[21]	725.451	333.546	328.207	328.543	327.871	A[9]
V[22]	758.474	309.867	304.528	304.864	304.192	V[8]
L[23]	796.168	276.844	271.505	271.841	271.169	L[7]
L[24]	833.863	239.150	233.810	234.146	233.474	L[6]
P[25]	866.214	201.455	196.116	196.451	195.780	P[5]
K[26]	908.912	169.104	163.765	164.101	163.429	K[4]
K[27]	**951.611**	126.406	121.066	121.402	120.730	K[3]
T[28]	**985.293**	83.708	78.368	78.704	78.032	T[2]
E[29]	**1028.307**	50.025	44.685	45.021	44.349	E[1]

sp|Q6GSS7|H2A2A_MOUSE

LNKLLGK $^{\text{Dimethyl}}_{28.03}$ VTIAQGGVLPNIQAVLLPKKTE

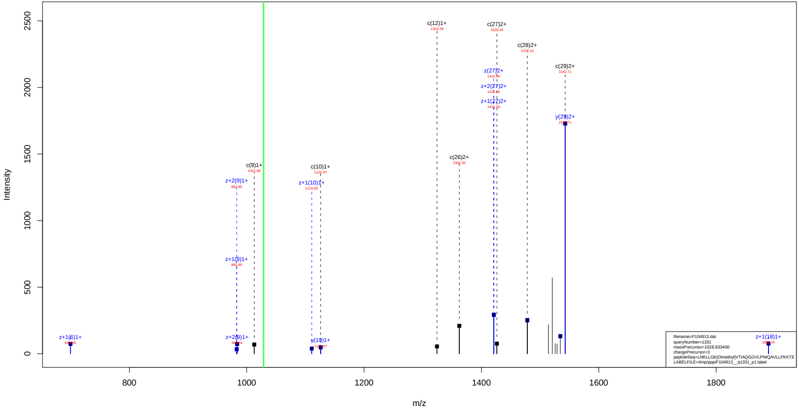

sp|Q6GSS7|H2A2A_MOUSE

LNKLLGK $^{Dimethyl}_{28.03}$ VTIAQGGVLPNIQAVLLPKKTE

- fragmentation table for charge state 1+
- specType=etd
- score=31.52
- F104913.dat
- query=q1331_p1
- precursor=1028.633400
- chargePrecursor=3
- itol=0.8

AA	c	y	z+1	z+2	z	AA
L[1]	131.118	3083.892	3067.873	0.000	3066.865	L[29]
N[2]	245.161	2970.808	2954.789	2955.797	2953.781	N[28]
K[3]	373.256	2856.765	2840.746	2841.754	2839.738	K[27]
L[4]	486.340	2728.670	2712.651	2713.659	2711.643	L[26]
L[5]	599.424	2615.586	2599.567	2600.575	2598.559	L[25]
G[6]	656.445	2502.502	2486.483	2487.491	2485.475	G[24]
K[7]	812.572	2445.480	2429.462	2430.469	2428.454	K[23]
V[8]	911.640	2289.354	2273.335	2274.343	2272.327	V[22]
T[9]	**1012.688**	2190.286	2174.267	2175.275	2173.259	T[21]
I[10]	**1125.772**	2089.238	2073.219	2074.227	2072.211	I[20]
A[11]	1196.809	1976.154	1960.135	1961.143	1959.127	A[19]
Q[12]	**1324.867**	1905.117	**1889.098**	1890.106	1888.090	Q[18]
G[13]	1381.889	1777.058	1761.039	1762.047	1760.032	G[17]
G[14]	1438.910	1720.037	1704.018	1705.026	1703.010	G[16]
V[15]	1537.979	1663.015	1646.997	1648.004	1645.989	V[15]
L[16]	1651.063	1563.947	1547.928	1548.936	1546.920	L[14]
P[17]	1748.116	1450.863	1434.844	1435.852	1433.836	P[13]
N[18]	1862.159	1353.810	1337.791	1338.799	1336.783	N[12]
I[19]	1975.243	1239.767	1223.748	1224.756	1222.741	I[11]
Q[20]	2103.301	**1126.683**	**1110.664**	1111.672	1109.656	Q[10]
A[21]	2174.338	998.624	**982.606**	**983.614**	981.598	A[9]
V[22]	2273.407	927.587	911.569	912.576	910.561	V[8]
L[23]	2386.491	828.519	812.500	813.508	811.492	L[7]
L[24]	2499.575	715.435	**699.416**	700.424	698.408	L[6]
P[25]	2596.628	602.351	586.332	587.340	585.324	P[5]
K[26]	2724.723	505.298	489.279	490.287	488.271	K[4]
K[27]	2852.818	377.203	361.184	362.192	360.177	K[3]
T[28]	2953.865	249.108	233.089	234.097	232.082	T[2]
E[29]	3082.908	148.060	132.042	133.050	131.034	E[1]

sp|Q6GSS7|H2A2A_MOUSE

LNKLLGK $^{\text{Dimethyl}}_{28.03}$ VTIAQGGVLPNIQAVLLPKKTE

- fragmentation table for charge state 2+
- specType=etd
- score=31.52
- F104913.dat
- query=q1331_p1
- precursor=1028.633400
- chargePrecursor=3
- itol=0.8

AA	c	y	z+1	z+2	z	AA
L[1]	66.063	**1542.450**	**1534.440**	0.504	**1533.936**	L[29]
N[2]	123.084	1485.908	**1477.898**	**1478.402**	**1477.394**	N[28]
K[3]	187.132	1428.886	**1420.877**	**1421.381**	**1420.373**	K[27]
L[4]	243.674	1364.839	1356.829	1357.333	1356.325	L[26]
L[5]	300.216	1308.297	1300.287	1300.791	1299.783	L[25]
G[6]	328.726	1251.755	1243.745	1244.249	1243.241	G[24]
K[7]	406.789	1223.244	1215.234	1215.738	1214.731	K[23]
V[8]	456.324	1145.181	1137.171	1137.675	1136.667	V[22]
T[9]	506.848	1095.646	1087.637	1088.141	1087.133	T[21]
I[10]	563.390	1045.123	1037.113	1037.617	1036.609	I[20]
A[11]	598.908	988.581	980.571	981.075	980.067	A[19]
Q[12]	662.937	953.062	945.053	945.557	944.549	Q[18]
G[13]	691.448	889.033	881.023	881.527	880.519	G[17]
G[14]	719.959	860.522	852.513	853.017	852.009	G[16]
V[15]	769.493	832.011	824.002	824.506	823.498	V[15]
L[16]	826.035	782.477	774.468	774.972	773.964	L[14]
P[17]	874.561	725.935	717.926	718.430	717.422	P[13]
N[18]	931.583	677.409	669.399	669.903	668.895	N[12]
I[19]	988.125	620.387	612.378	612.882	611.874	I[11]
Q[20]	1052.154	563.845	555.836	556.340	555.332	Q[10]
A[21]	1087.673	499.816	491.807	492.310	491.303	A[9]
V[22]	1137.207	464.297	456.288	456.792	455.784	V[8]
L[23]	1193.749	414.763	406.754	407.258	406.250	L[7]
L[24]	1250.291	358.221	350.212	350.716	349.708	L[6]
P[25]	1298.817	301.679	293.670	294.174	293.166	P[5]
K[26]	**1362.865**	253.153	245.143	245.647	244.639	K[4]
K[27]	**1426.912**	189.105	181.096	181.600	180.592	K[3]
T[28]	**1477.436**	125.058	117.048	117.552	116.544	T[2]
E[29]	**1541.958**	74.534	66.524	67.028	66.021	E[1]

sp|Q6GSS7|H2A2A_MOUSE

LNKLLGK $^{\text{Dimethyl}}_{28.03}$ VTIAQGGVLPNIQAVLLPKKTE

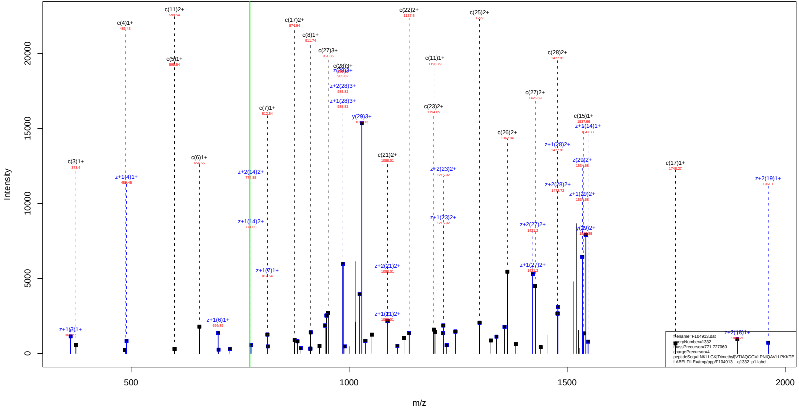

sp|Q6GSS7|H2A2A_MOUSE

LNKLLGK $^{\text{Dimethyl}}_{28.03}$ VTIAQGGVLPNIQAVLLPKKTE

- fragmentation table for charge state 1+
- specType=etd
- score=106.94
- F104913.dat
- query=q1332_p1
- precursor=771.727060
- chargePrecursor=4
- itol=0.8

AA	c	y	z+1	z+2	z	AA
L[1]	131.118	3083.892	3067.873	0.000	3066.865	L[29]
N[2]	245.161	2970.808	2954.789	2955.797	2953.781	N[28]
K[3]	**373.256**	2856.765	2840.746	2841.754	2839.738	K[27]
L[4]	**486.340**	2728.670	2712.651	2713.659	2711.643	L[26]
L[5]	**599.424**	2615.586	2599.567	2600.575	2598.559	L[25]
G[6]	**656.445**	2502.502	2486.483	2487.491	2485.475	G[24]
K[7]	**812.572**	2445.480	2429.462	2430.469	2428.454	K[23]
V[8]	**911.640**	2289.354	2273.335	2274.343	2272.327	V[22]
T[9]	1012.688	2190.286	2174.267	2175.275	2173.259	T[21]
I[10]	**1125.772**	2089.238	2073.219	2074.227	2072.211	I[20]
A[11]	**1196.809**	1976.154	1960.135	**1961.143**	1959.127	A[19]
Q[12]	**1324.867**	1905.117	1889.098	**1890.106**	1888.090	Q[18]
G[13]	**1381.889**	1777.058	1761.039	1762.047	1760.032	G[17]
G[14]	**1438.910**	1720.037	1704.018	1705.026	1703.010	G[16]
V[15]	**1537.979**	1663.015	1646.997	1648.004	1645.989	V[15]
L[16]	1651.063	1563.947	**1547.928**	1548.936	1546.920	L[14]
P[17]	**1748.116**	1450.863	1434.844	1435.852	1433.836	P[13]
N[18]	1862.159	1353.810	**1337.791**	1338.799	1336.783	N[12]
I[19]	1975.243	1239.767	**1223.748**	1224.756	**1222.741**	I[11]
Q[20]	2103.301	1126.683	**1110.664**	1111.672	1109.656	Q[10]
A[21]	2174.338	998.624	982.606	983.614	981.598	A[9]
V[22]	2273.407	927.587	**911.569**	912.576	**910.561**	V[8]
L[23]	2386.491	828.519	**812.500**	**813.508**	811.492	L[7]
L[24]	2499.575	715.435	**699.416**	**700.424**	698.408	L[6]
P[25]	2596.628	602.351	586.332	587.340	585.324	P[5]
K[26]	2724.723	505.298	**489.279**	490.287	488.271	K[4]
K[27]	2852.818	377.203	**361.184**	362.192	360.177	K[3]
T[28]	2953.865	249.108	233.089	234.097	232.082	T[2]
E[29]	3082.908	148.060	132.042	133.050	131.034	E[1]

sp|Q6GSS7|H2A2A_MOUSE

LNKLLGK $^{Dimethyl}_{28.03}$ VTIAQGGVLPNIQAVLLPKKTE

- fragmentation table for charge state 2+
- specType=etd
- score=106.94
- F104913.dat
- query=q1332_p1
- precursor=771.727060
- chargePrecursor=4
- itol=0.8

AA	c	y	z+1	z+2	z	AA
L[1]	66.063	**1542.450**	**1534.440**	0.504	**1533.936**	L[29]
N[2]	123.084	1485.908	**1477.898**	**1478.402**	**1477.394**	N[28]
K[3]	187.132	1428.886	**1420.877**	**1421.381**	1420.373	K[27]
L[4]	243.674	1364.839	**1356.829**	1357.333	**1356.325**	L[26]
L[5]	300.216	1308.297	1300.287	1300.791	**1299.783**	L[25]
G[6]	328.726	1251.755	**1243.745**	**1244.249**	**1243.241**	G[24]
K[7]	406.789	**1223.244**	**1215.234**	**1215.738**	**1214.731**	K[23]
V[8]	456.324	1145.181	**1137.171**	**1137.675**	1136.667	V[22]
T[9]	506.848	1095.646	**1087.637**	**1088.141**	1087.133	T[21]
I[10]	563.390	1045.123	**1037.113**	**1037.617**	**1036.609**	I[20]
A[11]	**598.908**	988.581	980.571	981.075	980.067	A[19]
Q[12]	662.937	953.062	**945.053**	**945.557**	**944.549**	Q[18]
G[13]	691.448	**889.033**	**881.023**	**881.527**	880.519	G[17]
G[14]	719.959	860.522	852.513	853.017	852.009	G[16]
V[15]	769.493	832.011	824.002	824.506	823.498	V[15]
L[16]	826.035	782.477	**774.468**	**774.972**	773.964	L[14]
P[17]	**874.561**	**725.935**	717.926	718.430	717.422	P[13]
N[18]	**931.583**	677.409	669.399	669.903	668.895	N[12]
I[19]	988.125	620.387	612.378	612.882	611.874	I[11]
Q[20]	**1052.154**	563.845	555.836	556.340	555.332	Q[10]
A[21]	**1087.673**	499.816	491.807	492.310	491.303	A[9]
V[22]	**1137.207**	464.297	456.288	456.792	455.784	V[8]
L[23]	**1193.749**	414.763	406.754	407.258	406.250	L[7]
L[24]	1250.291	358.221	350.212	350.716	349.708	L[6]
P[25]	**1298.817**	301.679	293.670	294.174	293.166	P[5]
K[26]	**1362.865**	253.153	245.143	245.647	244.639	K[4]
K[27]	**1426.912**	189.105	181.096	181.600	180.592	K[3]
T[28]	**1477.436**	125.058	117.048	117.552	116.544	T[2]
E[29]	1541.958	74.534	66.524	67.028	66.021	E[1]

sp|Q6GSS7|H2A2A_MOUSE

LNKLLGK $^{\text{Dimethyl}}_{28.03}$ VTIAQGGVLPNIQAVLLPKKTE

- fragmentation table for charge state 3+
- specType=etd
- score=106.94
- F104913.dat
- query=q1332_p1
- precursor=771.727060
- chargePrecursor=4
- itol=0.8

AA	c	y	z+1	z+2	z	AA
L[1]	44.377	**1028.635**	**1023.296**	0.672	1022.960	L[29]
N[2]	82.392	**990.941**	**985.601**	**985.937**	**985.265**	N[28]
K[3]	125.090	952.926	**947.587**	**947.923**	**947.251**	K[27]
L[4]	162.785	910.228	904.889	905.225	904.553	L[26]
L[5]	200.479	872.533	867.194	867.530	866.858	L[25]
G[6]	219.487	834.839	829.499	829.835	829.163	G[24]
K[7]	271.529	815.832	810.492	810.828	810.156	K[23]
V[8]	304.552	763.790	758.450	758.786	758.114	V[22]
T[9]	338.234	730.767	725.427	**725.763**	725.091	T[21]
I[10]	375.929	697.084	691.745	692.081	691.409	I[20]
A[11]	399.608	659.389	654.050	654.386	653.714	A[19]
Q[12]	442.294	635.710	630.371	630.707	630.035	Q[18]
G[13]	461.301	593.024	587.685	588.021	587.349	G[17]
G[14]	480.308	574.017	568.678	569.013	568.342	G[16]
V[15]	513.331	555.010	549.670	550.006	549.334	V[15]
L[16]	551.026	521.987	516.648	516.984	516.312	L[14]
P[17]	583.377	484.292	478.953	479.289	478.617	P[13]
N[18]	621.391	451.942	446.602	446.938	446.266	N[12]
I[19]	659.086	413.927	408.588	408.924	408.252	I[11]
Q[20]	701.772	376.233	370.893	371.229	370.557	Q[10]
A[21]	725.451	333.546	328.207	328.543	327.871	A[9]
V[22]	758.474	309.867	304.528	304.864	304.192	V[8]
L[23]	796.168	276.844	271.505	271.841	271.169	L[7]
L[24]	833.863	239.150	233.810	234.146	233.474	L[6]
P[25]	866.214	201.455	196.116	196.451	195.780	P[5]
K[26]	908.912	169.104	163.765	164.101	163.429	K[4]
K[27]	**951.611**	126.406	121.066	121.402	120.730	K[3]
T[28]	**985.293**	83.708	78.368	78.704	78.032	T[2]
E[29]	1028.307	50.025	44.685	45.021	44.349	E[1]

sp|Q6GSS7|H2A2A_MOUSE

LNKLLGK $^{\text{Dimethyl}}_{28.03}$ VTIAQGGVLPNIQAVLLPKKTE

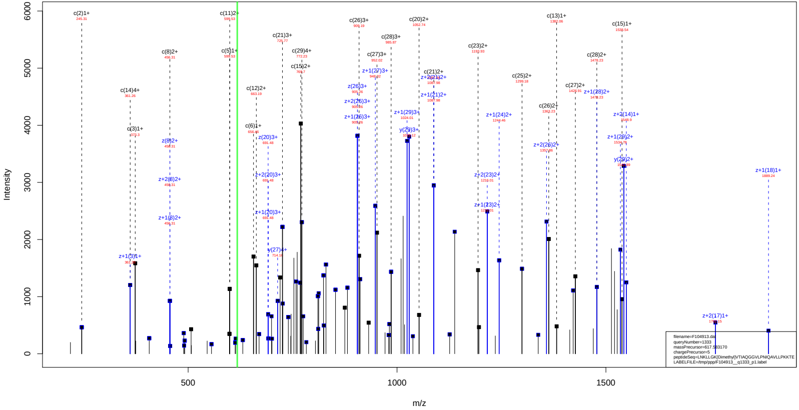

sp|Q6GSS7|H2A2A_MOUSE

LNKLLGK $^{Dimethyl}_{28.03}$ VTIAQGGVLPNIQAVLLPKKTE

- fragmentation table for charge state 1+
- specType=etd
- score=128.04
- F104913.dat
- query=q1333_p1
- precursor=617.583170
- chargePrecursor=5
- itol=0.8

AA	c	y	z+1	z+2	z	AA
L[1]	131.118	3083.892	3067.873	0.000	3066.865	L[29]
N[2]	**245.161**	2970.808	2954.789	2955.797	2953.781	N[28]
K[3]	**373.256**	2856.765	2840.746	2841.754	2839.738	K[27]
L[4]	486.340	2728.670	2712.651	2713.659	2711.643	L[26]
L[5]	**599.424**	2615.586	2599.567	2600.575	2598.559	L[25]
G[6]	**656.445**	2502.502	2486.483	2487.491	2485.475	G[24]
K[7]	**812.572**	2445.480	2429.462	2430.469	2428.454	K[23]
V[8]	**911.640**	2289.354	2273.335	2274.343	2272.327	V[22]
T[9]	1012.688	2190.286	2174.267	2175.275	2173.259	T[21]
I[10]	**1125.772**	2089.238	2073.219	2074.227	2072.211	I[20]
A[11]	**1196.809**	1976.154	1960.135	1961.143	1959.127	A[19]
Q[12]	1324.867	1905.117	**1889.098**	1890.106	1888.090	Q[18]
G[13]	**1381.889**	1777.058	1761.039	**1762.047**	1760.032	G[17]
G[14]	1438.910	1720.037	1704.018	1705.026	1703.010	G[16]
V[15]	**1537.979**	1663.015	1646.997	1648.004	1645.989	V[15]
L[16]	1651.063	1563.947	1547.928	**1548.936**	1546.920	L[14]
P[17]	1748.116	1450.863	1434.844	1435.852	1433.836	P[13]
N[18]	1862.159	1353.810	**1337.791**	1338.799	1336.783	N[12]
I[19]	1975.243	1239.767	1223.748	1224.756	1222.741	I[11]
Q[20]	2103.301	**1126.683**	1110.664	1111.672	1109.656	Q[10]
A[21]	2174.338	998.624	982.606	983.614	**981.598**	A[9]
V[22]	2273.407	927.587	**911.569**	912.576	910.561	V[8]
L[23]	2386.491	828.519	**812.500**	813.508	**811.492**	L[7]
L[24]	2499.575	715.435	**699.416**	**700.424**	698.408	L[6]
P[25]	2596.628	602.351	586.332	587.340	585.324	P[5]
K[26]	2724.723	505.298	**489.279**	**490.287**	488.271	K[4]
K[27]	2852.818	377.203	**361.184**	362.192	360.177	K[3]
T[28]	2953.865	249.108	233.089	234.097	232.082	T[2]
E[29]	3082.908	148.060	132.042	133.050	131.034	E[1]

sp|Q6GSS7|H2A2A_MOUSE

LNKLLGK $^{\text{Dimethyl}}_{28.03}$ VTIAQGGVLPNIQAVLLPKKTE

- fragmentation table for charge state 2+
- specType=etd
- score=128.04
- F104913.dat
- query=q1333_p1
- precursor=617.583170
- chargePrecursor=5
- itol=0.8

AA	c	y	z+1	z+2	z	AA
L[1]	66.063	**1542.450**	**1534.440**	0.504	1533.936	L[29]
N[2]	123.084	1485.908	**1477.898**	**1478.402**	1477.394	N[28]
K[3]	187.132	1428.886	**1420.877**	**1421.381**	1420.373	K[27]
L[4]	243.674	1364.839	1356.829	**1357.333**	1356.325	L[26]
L[5]	300.216	1308.297	1300.287	1300.791	**1299.783**	L[25]
G[6]	328.726	1251.755	**1243.745**	**1244.249**	1243.241	G[24]
K[7]	**406.789**	1223.244	**1215.234**	**1215.738**	1214.731	K[23]
V[8]	**456.324**	1145.181	1137.171	**1137.675**	1136.667	V[22]
T[9]	**506.848**	1095.646	**1087.637**	**1088.141**	1087.133	T[21]
I[10]	563.390	1045.123	1037.113	**1037.617**	1036.609	I[20]
A[11]	**598.908**	988.581	**980.571**	**981.075**	**980.067**	A[19]
Q[12]	**662.937**	953.062	945.053	945.557	944.549	Q[18]
G[13]	**691.448**	889.033	**881.023**	**881.527**	**880.519**	G[17]
G[14]	**719.959**	860.522	**852.513**	**853.017**	**852.009**	G[16]
V[15]	**769.493**	832.011	**824.002**	**824.506**	**823.498**	V[15]
L[16]	826.035	**782.477**	774.468	**774.972**	773.964	L[14]
P[17]	**874.561**	**725.935**	717.926	718.430	717.422	P[13]
N[18]	**931.583**	677.409	**669.399**	**669.903**	668.895	N[12]
I[19]	988.125	620.387	**612.378**	**612.882**	**611.874**	I[11]
Q[20]	**1052.154**	563.845	**555.836**	**556.340**	**555.332**	Q[10]
A[21]	**1087.673**	499.816	**491.807**	**492.310**	**491.303**	A[9]
V[22]	1137.207	464.297	**456.288**	**456.792**	**455.784**	V[8]
L[23]	**1193.749**	414.763	**406.754**	**407.258**	**406.250**	L[7]
L[24]	1250.291	358.221	350.212	350.716	349.708	L[6]
P[25]	**1298.817**	301.679	293.670	294.174	293.166	P[5]
K[26]	**1362.865**	253.153	**245.143**	**245.647**	**244.639**	K[4]
K[27]	**1426.912**	189.105	181.096	181.600	180.592	K[3]
T[28]	**1477.436**	125.058	117.048	117.552	116.544	T[2]
E[29]	1541.958	74.534	66.524	67.028	66.021	E[1]

sp|Q6GSS7|H2A2A_MOUSE

LNKLLGK $^{\text{Dimethyl}}_{28.03}$ VTIAQGGVLPNIQAVLLPKKTE

- fragmentation table for charge state 3+
- specType=etd
- score=128.04
- F104913.dat
- query=q1333_p1
- precursor=617.583170
- chargePrecursor=5
- itol=0.8

AA	c	y	z+1	z+2	z	AA
L[1]	44.377	**1028.635**	**1023.296**	0.672	1022.960	L[29]
N[2]	82.392	990.941	**985.601**	**985.937**	**985.265**	N[28]
K[3]	125.090	952.926	**947.587**	**947.923**	**947.251**	K[27]
L[4]	162.785	910.228	**904.889**	**905.225**	**904.553**	L[26]
L[5]	200.479	872.533	867.194	867.530	866.858	L[25]
G[6]	219.487	834.839	**829.499**	**829.835**	**829.163**	G[24]
K[7]	271.529	815.832	**810.492**	**810.828**	**810.156**	K[23]
V[8]	304.552	763.790	**758.450**	**758.786**	**758.114**	V[22]
T[9]	338.234	730.767	**725.427**	**725.763**	**725.091**	T[21]
I[10]	375.929	697.084	**691.745**	**692.081**	**691.409**	I[20]
A[11]	399.608	659.389	654.050	654.386	653.714	A[19]
Q[12]	442.294	635.710	**630.371**	**630.707**	**630.035**	Q[18]
G[13]	461.301	593.024	587.685	588.021	587.349	G[17]
G[14]	480.308	574.017	568.678	569.013	568.342	G[16]
V[15]	513.331	555.010	549.670	550.006	549.334	V[15]
L[16]	551.026	521.987	516.648	516.984	516.312	L[14]
P[17]	583.377	484.292	478.953	479.289	478.617	P[13]
N[18]	621.391	451.942	446.602	446.938	446.266	N[12]
I[19]	659.086	413.927	408.588	408.924	408.252	I[11]
Q[20]	701.772	376.233	370.893	371.229	370.557	Q[10]
A[21]	**725.451**	333.546	328.207	328.543	327.871	A[9]
V[22]	**758.474**	309.867	304.528	304.864	304.192	V[8]
L[23]	796.168	276.844	271.505	271.841	271.169	L[7]
L[24]	833.863	239.150	233.810	234.146	233.474	L[6]
P[25]	866.214	201.455	196.116	196.451	195.780	P[5]
K[26]	**908.912**	169.104	163.765	164.101	163.429	K[4]
K[27]	**951.611**	126.406	121.066	121.402	120.730	K[3]
T[28]	**985.293**	83.708	78.368	78.704	78.032	T[2]
E[29]	1028.307	50.025	44.685	45.021	44.349	E[1]

sp|Q6GSS7|H2A2A_MOUSE

LNKLLGK Dimethyl 28.03 VTIAQGGVLPNIQAVLLPKKTE

- fragmentation table for charge state 4+
- specType=etd
- score=128.04
- F104913.dat
- query=q1333_p1
- precursor=617.583170
- chargePrecursor=5
- itol=0.8

AA	c	y	z+1	z+2	z	AA
L[1]	33.535	**771.728**	**767.724**	0.755	**767.472**	L[29]
N[2]	62.046	743.457	**739.453**	**739.705**	**739.201**	N[28]
K[3]	94.069	**714.947**	710.942	711.194	710.690	K[27]
L[4]	122.340	682.923	678.918	679.170	678.666	L[26]
L[5]	150.611	654.652	650.647	650.899	650.395	L[25]
G[6]	164.867	626.381	622.376	622.628	622.124	G[24]
K[7]	203.898	**612.126**	608.121	608.373	607.869	K[23]
V[8]	228.665	573.094	569.089	569.341	568.837	V[22]
T[9]	253.927	548.327	544.322	544.574	544.070	T[21]
I[10]	282.198	523.065	519.060	519.312	518.808	I[20]
A[11]	299.958	494.794	**490.789**	**491.041**	**490.537**	A[19]
Q[12]	331.972	477.035	473.030	473.282	472.778	Q[18]
G[13]	346.228	445.020	441.015	441.267	440.763	G[17]
G[14]	**360.483**	430.765	426.760	427.012	426.508	G[16]
V[15]	385.250	416.509	412.505	412.757	412.253	V[15]
L[16]	413.521	391.742	387.737	387.989	387.486	L[14]
P[17]	437.784	363.471	359.466	359.718	359.215	P[13]
N[18]	466.295	339.208	335.203	335.455	334.951	N[12]
I[19]	494.566	310.697	306.693	306.945	306.441	I[11]
Q[20]	526.581	282.426	278.422	278.673	278.170	Q[10]
A[21]	544.340	250.412	246.407	246.659	246.155	A[9]
V[22]	569.107	232.652	228.648	228.900	228.396	V[8]
L[23]	597.378	207.885	203.881	204.132	203.629	L[7]
L[24]	625.649	179.614	175.609	175.861	175.358	L[6]
P[25]	649.912	151.343	147.338	147.590	147.087	P[5]
K[26]	681.936	127.080	123.075	123.327	122.823	K[4]
K[27]	**713.960**	95.056	91.052	91.304	90.800	K[3]
T[28]	**739.222**	63.032	59.028	59.280	58.776	T[2]
E[29]	**771.482**	37.771	33.766	34.018	33.514	E[1]

sp|Q6GSS7|H2A2A_MOUSE

LNKLLGK Dimethyl 28.03 VTIAQGGVLPNIQAVLLPKKTE

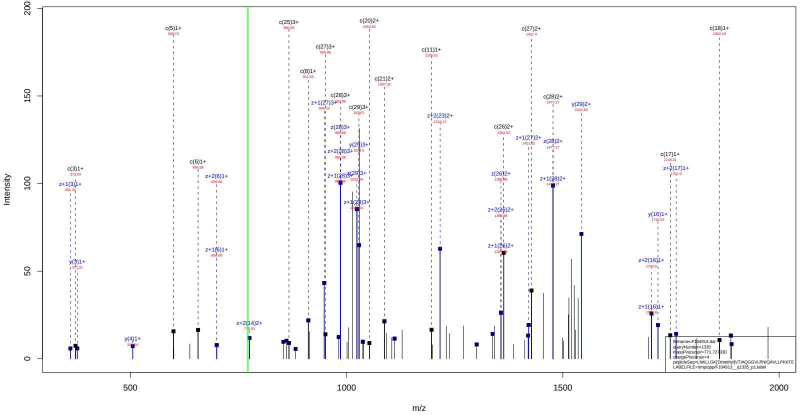

sp|Q6GSS7|H2A2A_MOUSE

LNKLLGK $^{\text{Dimethyl}}_{28.03}$ VTIAQGGVLPNIQAVLLPKKTE

- fragmentation table for charge state 1+
- specType=etd
- score=33.66
- F104913.dat
- query=q1335_p1
- precursor=771.727330
- chargePrecursor=4
- itol=0.8

AA	c	y	z+1	z+2	z	AA
L[1]	131.118	3083.892	3067.873	0.000	3066.865	L[29]
N[2]	245.161	2970.808	2954.789	2955.797	2953.781	N[28]
K[3]	**373.256**	2856.765	2840.746	2841.754	2839.738	K[27]
L[4]	486.340	2728.670	2712.651	2713.659	2711.643	L[26]
L[5]	**599.424**	2615.586	2599.567	2600.575	2598.559	L[25]
G[6]	**656.445**	2502.502	2486.483	2487.491	2485.475	G[24]
K[7]	812.572	2445.480	2429.462	2430.469	2428.454	K[23]
V[8]	**911.640**	2289.354	2273.335	2274.343	2272.327	V[22]
T[9]	1012.688	2190.286	2174.267	2175.275	2173.259	T[21]
I[10]	1125.772	2089.238	2073.219	2074.227	2072.211	I[20]
A[11]	**1196.809**	1976.154	1960.135	1961.143	1959.127	A[19]
Q[12]	1324.867	1905.117	**1889.098**	**1890.106**	**1888.090**	Q[18]
G[13]	1381.889	1777.058	1761.039	**1762.047**	1760.032	G[17]
G[14]	1438.910	**1720.037**	**1704.018**	**1705.026**	1703.010	G[16]
V[15]	1537.979	1663.015	1646.997	1648.004	1645.989	V[15]
L[16]	1651.063	1563.947	1547.928	1548.936	1546.920	L[14]
P[17]	**1748.116**	1450.863	1434.844	1435.852	1433.836	P[13]
N[18]	**1862.159**	1353.810	**1337.791**	1338.799	1336.783	N[12]
I[19]	1975.243	1239.767	1223.748	1224.756	1222.741	I[11]
Q[20]	2103.301	1126.683	**1110.664**	**1111.672**	1109.656	Q[10]
A[21]	2174.338	998.624	**982.606**	983.614	**981.598**	A[9]
V[22]	2273.407	927.587	**911.569**	912.576	910.561	V[8]
L[23]	2386.491	828.519	812.500	813.508	811.492	L[7]
L[24]	2499.575	715.435	**699.416**	**700.424**	698.408	L[6]
P[25]	2596.628	602.351	586.332	587.340	585.324	P[5]
K[26]	2724.723	**505.298**	489.279	490.287	488.271	K[4]
K[27]	2852.818	**377.203**	**361.184**	362.192	360.177	K[3]
T[28]	2953.865	249.108	233.089	234.097	232.082	T[2]
E[29]	3082.908	148.060	132.042	133.050	131.034	E[1]

sp|Q6GSS7|H2A2A_MOUSE

LNKLLGK $^{\text{Dimethyl}}_{28.03}$ VTIAQGGVLPNIQAVLLPKKTE

- fragmentation table for charge state 2+
- specType=etd
- score=33.66
- F104913.dat
- query=q1335_p1
- precursor=771.727330
- chargePrecursor=4
- itol=0.8

AA	c	y	z+1	z+2	z	AA
L[1]	66.063	**1542.450**	1534.440	0.504	1533.936	L[29]
N[2]	123.084	1485.908	**1477.898**	1478.402	**1477.394**	N[28]
K[3]	187.132	1428.886	**1420.877**	**1421.381**	**1420.373**	K[27]
L[4]	243.674	1364.839	**1356.829**	**1357.333**	**1356.325**	L[26]
L[5]	300.216	1308.297	**1300.287**	**1300.791**	1299.783	L[25]
G[6]	328.726	1251.755	1243.745	1244.249	1243.241	G[24]
K[7]	406.789	1223.244	1215.234	**1215.738**	1214.731	K[23]
V[8]	456.324	1145.181	1137.171	1137.675	1136.667	V[22]
T[9]	506.848	1095.646	**1087.637**	1088.141	**1087.133**	T[21]
I[10]	563.390	1045.123	**1037.113**	**1037.617**	1036.609	I[20]
A[11]	598.908	988.581	980.571	981.075	980.067	A[19]
Q[12]	662.937	953.062	945.053	945.557	944.549	Q[18]
G[13]	691.448	889.033	881.023	**881.527**	880.519	G[17]
G[14]	719.959	**860.522**	852.513	**853.017**	852.009	G[16]
V[15]	769.493	832.011	824.002	824.506	823.498	V[15]
L[16]	826.035	782.477	774.468	**774.972**	773.964	L[14]
P[17]	874.561	725.935	717.926	718.430	717.422	P[13]
N[18]	931.583	677.409	669.399	669.903	668.895	N[12]
I[19]	988.125	620.387	612.378	612.882	611.874	I[11]
Q[20]	**1052.154**	563.845	555.836	556.340	555.332	Q[10]
A[21]	**1087.673**	499.816	491.807	492.310	491.303	A[9]
V[22]	1137.207	464.297	456.288	456.792	455.784	V[8]
L[23]	1193.749	414.763	406.754	407.258	406.250	L[7]
L[24]	1250.291	358.221	350.212	350.716	349.708	L[6]
P[25]	1298.817	301.679	293.670	294.174	293.166	P[5]
K[26]	**1362.865**	253.153	245.143	245.647	244.639	K[4]
K[27]	**1426.912**	189.105	181.096	181.600	180.592	K[3]
T[28]	**1477.436**	125.058	117.048	117.552	116.544	T[2]
E[29]	1541.958	74.534	66.524	67.028	66.021	E[1]

sp|Q6GSS7|H2A2A_MOUSE

LNKLLGK $^{Dimethyl}_{28.03}$ VTIAQGGVLPNIQAVLLPKKTE

- fragmentation table for charge state 3+
- specType=etd
- score=33.66
- F104913.dat
- query=q1335_p1
- precursor=771.727330
- chargePrecursor=4
- itol=0.8

AA	c	y	z+1	z+2	z	AA
L[1]	44.377	**1028.635**	**1023.296**	0.672	**1022.960**	L[29]
N[2]	82.392	990.941	**985.601**	**985.937**	**985.265**	N[28]
K[3]	125.090	952.926	**947.587**	**947.923**	947.251	K[27]
L[4]	162.785	910.228	904.889	905.225	904.553	L[26]
L[5]	200.479	872.533	**867.194**	867.530	**866.858**	L[25]
G[6]	219.487	834.839	829.499	829.835	829.163	G[24]
K[7]	271.529	815.832	810.492	810.828	810.156	K[23]
V[8]	304.552	763.790	758.450	758.786	758.114	V[22]
T[9]	338.234	730.767	725.427	725.763	725.091	T[21]
I[10]	375.929	697.084	691.745	692.081	691.409	I[20]
A[11]	399.608	659.389	654.050	654.386	653.714	A[19]
Q[12]	442.294	635.710	630.371	630.707	630.035	Q[18]
G[13]	461.301	593.024	587.685	588.021	587.349	G[17]
G[14]	480.308	574.017	568.678	569.013	568.342	G[16]
V[15]	513.331	555.010	549.670	550.006	549.334	V[15]
L[16]	551.026	521.987	516.648	516.984	516.312	L[14]
P[17]	583.377	484.292	478.953	479.289	478.617	P[13]
N[18]	621.391	451.942	446.602	446.938	446.266	N[12]
I[19]	659.086	413.927	408.588	408.924	408.252	I[11]
Q[20]	701.772	376.233	370.893	371.229	370.557	Q[10]
A[21]	725.451	333.546	328.207	328.543	327.871	A[9]
V[22]	758.474	309.867	304.528	304.864	304.192	V[8]
L[23]	796.168	276.844	271.505	271.841	271.169	L[7]
L[24]	833.863	239.150	233.810	234.146	233.474	L[6]
P[25]	**866.214**	201.455	196.116	196.451	195.780	P[5]
K[26]	908.912	169.104	163.765	164.101	163.429	K[4]
K[27]	**951.611**	126.406	121.066	121.402	120.730	K[3]
T[28]	**985.293**	83.708	78.368	78.704	78.032	T[2]
E[29]	**1028.307**	50.025	44.685	45.021	44.349	E[1]

sp|Q6GSS7|H2A2A_MOUSE

LNKLLGK $^{\text{Dimethyl}}_{28.03}$ VTIAQGGVLPNIQAVLLPKKTE

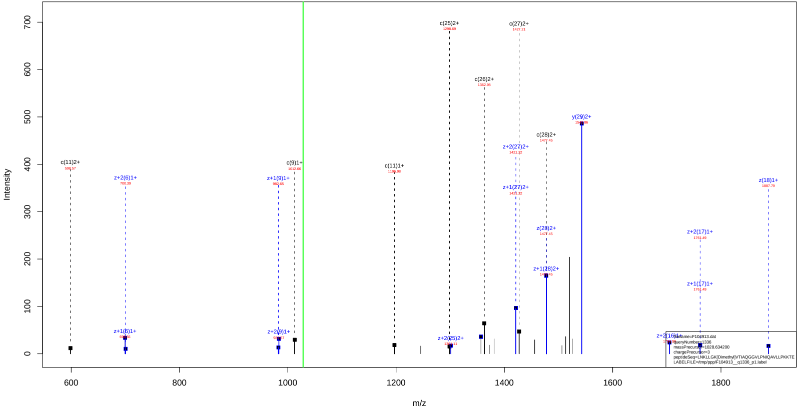

sp|Q6GSS7|H2A2A_MOUSE

LNKLLGK $^{Dimethyl}_{28.03}$ VTIAQGGVLPNIQAVLLPKKTE

- fragmentation table for charge state 1+
- specType=etd
- score=28.04
- F104913.dat
- query=q1336_p1
- precursor=1028.634200
- chargePrecursor=3
- itol=0.8

AA	c	y	z+1	z+2	z	AA
L[1]	131.118	3083.892	3067.873	0.000	3066.865	L[29]
N[2]	245.161	2970.808	2954.789	2955.797	2953.781	N[28]
K[3]	373.256	2856.765	2840.746	2841.754	2839.738	K[27]
L[4]	486.340	2728.670	2712.651	2713.659	2711.643	L[26]
L[5]	599.424	2615.586	2599.567	2600.575	2598.559	L[25]
G[6]	656.445	2502.502	2486.483	2487.491	2485.475	G[24]
K[7]	812.572	2445.480	2429.462	2430.469	2428.454	K[23]
V[8]	911.640	2289.354	2273.335	2274.343	2272.327	V[22]
T[9]	**1012.688**	2190.286	2174.267	2175.275	2173.259	T[21]
I[10]	1125.772	2089.238	2073.219	2074.227	2072.211	I[20]
A[11]	**1196.809**	1976.154	1960.135	1961.143	1959.127	A[19]
Q[12]	1324.867	1905.117	1889.098	1890.106	**1888.090**	Q[18]
G[13]	1381.889	1777.058	**1761.039**	**1762.047**	1760.032	G[17]
G[14]	1438.910	1720.037	1704.018	**1705.026**	1703.010	G[16]
V[15]	1537.979	1663.015	1646.997	1648.004	1645.989	V[15]
L[16]	1651.063	1563.947	1547.928	1548.936	1546.920	L[14]
P[17]	1748.116	1450.863	1434.844	1435.852	1433.836	P[13]
N[18]	1862.159	1353.810	1337.791	1338.799	1336.783	N[12]
I[19]	1975.243	1239.767	1223.748	1224.756	1222.741	I[11]
Q[20]	2103.301	1126.683	1110.664	1111.672	1109.656	Q[10]
A[21]	2174.338	998.624	**982.606**	**983.614**	981.598	A[9]
V[22]	2273.407	927.587	911.569	912.576	910.561	V[8]
L[23]	2386.491	828.519	812.500	813.508	811.492	L[7]
L[24]	2499.575	715.435	**699.416**	**700.424**	698.408	L[6]
P[25]	2596.628	602.351	586.332	587.340	585.324	P[5]
K[26]	2724.723	505.298	489.279	490.287	488.271	K[4]
K[27]	2852.818	377.203	361.184	362.192	360.177	K[3]
T[28]	2953.865	249.108	233.089	234.097	232.082	T[2]
E[29]	3082.908	148.060	132.042	133.050	131.034	E[1]

sp|Q6GSS7|H2A2A_MOUSE

LNKLLGK $^{Dimethyl}_{28.03}$ VTIAQGGVLPNIQAVLLPKKTE

- fragmentation table for charge state 2+
- specType=etd
- score=28.04
- F104913.dat
- query=q1336_p1
- precursor=1028.634200
- chargePrecursor=3
- itol=0.8

AA	c	y	z+1	z+2	z	AA
L[1]	66.063	**1542.450**	1534.440	0.504	1533.936	L[29]
N[2]	123.084	1485.908	**1477.898**	1478.402	**1477.394**	N[28]
K[3]	187.132	1428.886	**1420.877**	**1421.381**	1420.373	K[27]
L[4]	243.674	1364.839	**1356.829**	**1357.333**	**1356.325**	L[26]
L[5]	300.216	1308.297	1300.287	**1300.791**	1299.783	L[25]
G[6]	328.726	1251.755	1243.745	1244.249	1243.241	G[24]
K[7]	406.789	1223.244	1215.234	1215.738	1214.731	K[23]
V[8]	456.324	1145.181	1137.171	1137.675	1136.667	V[22]
T[9]	506.848	1095.646	1087.637	1088.141	1087.133	T[21]
I[10]	563.390	1045.123	1037.113	1037.617	1036.609	I[20]
A[11]	**598.908**	988.581	980.571	981.075	980.067	A[19]
Q[12]	662.937	953.062	945.053	945.557	944.549	Q[18]
G[13]	691.448	889.033	881.023	881.527	880.519	G[17]
G[14]	719.959	860.522	852.513	853.017	852.009	G[16]
V[15]	769.493	832.011	824.002	824.506	823.498	V[15]
L[16]	826.035	782.477	774.468	774.972	773.964	L[14]
P[17]	874.561	725.935	717.926	718.430	717.422	P[13]
N[18]	931.583	677.409	669.399	669.903	668.895	N[12]
I[19]	988.125	620.387	612.378	612.882	611.874	I[11]
Q[20]	1052.154	563.845	555.836	556.340	555.332	Q[10]
A[21]	1087.673	499.816	491.807	492.310	491.303	A[9]
V[22]	1137.207	464.297	456.288	456.792	455.784	V[8]
L[23]	1193.749	414.763	406.754	407.258	406.250	L[7]
L[24]	1250.291	358.221	350.212	350.716	349.708	L[6]
P[25]	**1298.817**	301.679	293.670	294.174	293.166	P[5]
K[26]	**1362.865**	253.153	245.143	245.647	244.639	K[4]
K[27]	**1426.912**	189.105	181.096	181.600	180.592	K[3]
T[28]	**1477.436**	125.058	117.048	117.552	116.544	T[2]
E[29]	1541.958	74.534	66.524	67.028	66.021	E[1]

sp|Q6GSS7|H2A2A_MOUSE

LNKLLGK $^{\text{Dimethyl}}_{28.03}$ VTIAQGGVLPNIQAVLLPKKTE

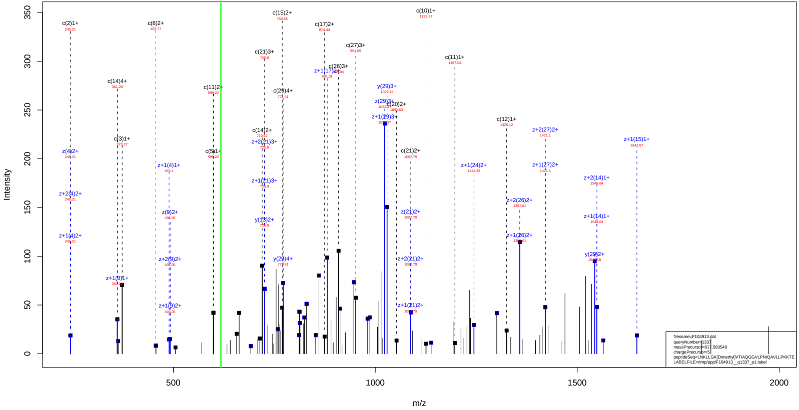

sp|Q6GSS7|H2A2A_MOUSE

LNKLLGK $^{\text{Dimethyl}}_{28.03}$ VTIAQGGVLPNIQAVLLPKKTE

- fragmentation table for charge state 1+
- specType=etd
- score=42.99
- F104913.dat
- query=q1337_p1
- precursor=617.583540
- chargePrecursor=5
- itol=0.8

AA	c	y	z+1	z+2	z	AA
L[1]	131.118	3083.892	3067.873	0.000	3066.865	L[29]
N[2]	**245.161**	2970.808	2954.789	2955.797	2953.781	N[28]
K[3]	**373.256**	2856.765	2840.746	2841.754	2839.738	K[27]
L[4]	486.340	2728.670	2712.651	2713.659	2711.643	L[26]
L[5]	**599.424**	2615.586	2599.567	2600.575	2598.559	L[25]
G[6]	**656.445**	2502.502	2486.483	2487.491	2485.475	G[24]
K[7]	**812.572**	2445.480	2429.462	2430.469	2428.454	K[23]
V[8]	911.640	2289.354	2273.335	2274.343	2272.327	V[22]
T[9]	1012.688	2190.286	2174.267	2175.275	2173.259	T[21]
I[10]	**1125.772**	2089.238	2073.219	2074.227	2072.211	I[20]
A[11]	**1196.809**	1976.154	1960.135	1961.143	1959.127	A[19]
Q[12]	**1324.867**	1905.117	1889.098	1890.106	1888.090	Q[18]
G[13]	1381.889	1777.058	1761.039	1762.047	1760.032	G[17]
G[14]	1438.910	1720.037	1704.018	1705.026	1703.010	G[16]
V[15]	1537.979	1663.015	**1646.997**	**1648.004**	1645.989	V[15]
L[16]	1651.063	**1563.947**	**1547.928**	**1548.936**	1546.920	L[14]
P[17]	1748.116	1450.863	1434.844	1435.852	1433.836	P[13]
N[18]	1862.159	1353.810	1337.791	1338.799	1336.783	N[12]
I[19]	1975.243	1239.767	1223.748	1224.756	1222.741	I[11]
Q[20]	2103.301	1126.683	1110.664	1111.672	1109.656	Q[10]
A[21]	2174.338	998.624	982.606	983.614	**981.598**	A[9]
V[22]	2273.407	927.587	911.569	**912.576**	910.561	V[8]
L[23]	2386.491	828.519	**812.500**	**813.508**	**811.492**	L[7]
L[24]	2499.575	715.435	699.416	700.424	698.408	L[6]
P[25]	2596.628	602.351	586.332	587.340	585.324	P[5]
K[26]	2724.723	**505.298**	**489.279**	490.287	488.271	K[4]
K[27]	2852.818	377.203	**361.184**	362.192	360.177	K[3]
T[28]	2953.865	249.108	233.089	234.097	232.082	T[2]
E[29]	3082.908	148.060	132.042	133.050	131.034	E[1]

sp|Q6GSS7|H2A2A_MOUSE

LNKLLGK $^{\text{Dimethyl}}_{28.03}$ VTIAQGGVLPNIQAVLLPKKTE

- fragmentation table for charge state 2+
- specType=etd
- score=42.99
- F104913.dat
- query=q1337_p1
- precursor=617.583540
- chargePrecursor=5
- itol=0.8

AA	c	y	z+1	z+2	z	AA
L[1]	66.063	**1542.450**	1534.440	0.504	1533.936	L[29]
N[2]	123.084	1485.908	1477.898	1478.402	1477.394	N[28]
K[3]	187.132	1428.886	**1420.877**	**1421.381**	**1420.373**	K[27]
L[4]	243.674	1364.839	**1356.829**	**1357.333**	1356.325	L[26]
L[5]	300.216	1308.297	**1300.287**	**1300.791**	1299.783	L[25]
G[6]	328.726	1251.755	**1243.745**	**1244.249**	1243.241	G[24]
K[7]	406.789	1223.244	1215.234	1215.738	1214.731	K[23]
V[8]	**456.324**	1145.181	1137.171	**1137.675**	1136.667	V[22]
T[9]	506.848	1095.646	**1087.637**	**1088.141**	**1087.133**	T[21]
I[10]	563.390	1045.123	1037.113	1037.617	1036.609	I[20]
A[11]	**598.908**	988.581	**980.571**	**981.075**	980.067	A[19]
Q[12]	**662.937**	953.062	945.053	945.557	944.549	Q[18]
G[13]	**691.448**	889.033	**881.023**	**881.527**	**880.519**	G[17]
G[14]	**719.959**	**860.522**	**852.513**	**853.017**	**852.009**	G[16]
V[15]	**769.493**	832.011	**824.002**	**824.506**	823.498	V[15]
L[16]	826.035	782.477	774.468	774.972	773.964	L[14]
P[17]	**874.561**	**725.935**	717.926	718.430	717.422	P[13]
N[18]	931.583	677.409	669.399	669.903	668.895	N[12]
I[19]	988.125	620.387	612.378	612.882	611.874	I[11]
Q[20]	**1052.154**	563.845	555.836	556.340	555.332	Q[10]
A[21]	**1087.673**	499.816	**491.807**	**492.310**	**491.303**	A[9]
V[22]	1137.207	464.297	**456.288**	**456.792**	455.784	V[8]
L[23]	1193.749	414.763	406.754	407.258	406.250	L[7]
L[24]	1250.291	358.221	350.212	350.716	349.708	L[6]
P[25]	1298.817	301.679	293.670	294.174	293.166	P[5]
K[26]	1362.865	253.153	**245.143**	**245.647**	**244.639**	K[4]
K[27]	1426.912	189.105	181.096	181.600	180.592	K[3]
T[28]	1477.436	125.058	117.048	117.552	116.544	T[2]
E[29]	1541.958	74.534	66.524	67.028	66.021	E[1]

sp|Q6GSS7|H2A2A_MOUSE

LNKLLGK $^{Dimethyl}_{28.03}$ VTIAQGGVLPNIQAVLLPKKTE

- fragmentation table for charge state 3+
- specType=etd
- score=42.99
- F104913.dat
- query=q1337_p1
- precursor=617.583540
- chargePrecursor=5
- itol=0.8

AA	c	y	z+1	z+2	z	AA
L[1]	44.377	**1028.635**	**1023.296**	0.672	**1022.960**	L[29]
N[2]	82.392	990.941	985.601	**985.937**	985.265	N[28]
K[3]	125.090	952.926	**947.587**	947.923	**947.251**	K[27]
L[4]	162.785	910.228	904.889	905.225	904.553	L[26]
L[5]	200.479	872.533	867.194	867.530	866.858	L[25]
G[6]	219.487	834.839	**829.499**	**829.835**	**829.163**	G[24]
K[7]	271.529	815.832	**810.492**	**810.828**	810.156	K[23]
V[8]	304.552	763.790	**758.450**	**758.786**	**758.114**	V[22]
T[9]	338.234	730.767	**725.427**	**725.763**	725.091	T[21]
I[10]	375.929	697.084	**691.745**	**692.081**	**691.409**	I[20]
A[11]	399.608	659.389	654.050	654.386	653.714	A[19]
Q[12]	442.294	635.710	630.371	630.707	630.035	Q[18]
G[13]	461.301	593.024	587.685	588.021	587.349	G[17]
G[14]	480.308	574.017	568.678	569.013	568.342	G[16]
V[15]	513.331	555.010	549.670	550.006	549.334	V[15]
L[16]	551.026	521.987	516.648	516.984	516.312	L[14]
P[17]	583.377	484.292	478.953	479.289	478.617	P[13]
N[18]	621.391	451.942	446.602	446.938	446.266	N[12]
I[19]	659.086	413.927	408.588	408.924	408.252	I[11]
Q[20]	701.772	376.233	370.893	371.229	370.557	Q[10]
A[21]	**725.451**	333.546	328.207	328.543	327.871	A[9]
V[22]	**758.474**	309.867	304.528	304.864	304.192	V[8]
L[23]	796.168	276.844	271.505	271.841	271.169	L[7]
L[24]	833.863	239.150	233.810	234.146	233.474	L[6]
P[25]	866.214	201.455	196.116	196.451	195.780	P[5]
K[26]	**908.912**	169.104	163.765	164.101	163.429	K[4]
K[27]	**951.611**	126.406	121.066	121.402	120.730	K[3]
T[28]	985.293	83.708	78.368	78.704	78.032	T[2]
E[29]	1028.307	50.025	44.685	45.021	44.349	E[1]

sp|Q6GSS7|H2A2A_MOUSE

LNKLLGK $^{Dimethyl}_{28.03}$ VTIAQGGVLPNIQAVLLPKKTE

- fragmentation table for charge state 4+
- specType=etd
- score=42.99
- F104913.dat
- query=q1337_p1
- precursor=617.583540
- chargePrecursor=5
- itol=0.8

AA	c	y	z+1	z+2	z	AA
L[1]	33.535	**771.728**	767.724	0.755	767.472	L[29]
N[2]	62.046	743.457	739.453	739.705	739.201	N[28]
K[3]	94.069	714.947	710.942	711.194	710.690	K[27]
L[4]	122.340	682.923	678.918	679.170	678.666	L[26]
L[5]	150.611	654.652	650.647	650.899	650.395	L[25]
G[6]	164.867	626.381	622.376	622.628	622.124	G[24]
K[7]	203.898	612.126	608.121	608.373	607.869	K[23]
V[8]	228.665	573.094	569.089	569.341	568.837	V[22]
T[9]	253.927	548.327	544.322	544.574	544.070	T[21]
I[10]	282.198	523.065	519.060	519.312	518.808	I[20]
A[11]	299.958	494.794	490.789	491.041	490.537	A[19]
Q[12]	331.972	477.035	473.030	473.282	472.778	Q[18]
G[13]	346.228	445.020	441.015	441.267	440.763	G[17]
G[14]	**360.483**	430.765	426.760	427.012	426.508	G[16]
V[15]	385.250	416.509	412.505	412.757	412.253	V[15]
L[16]	413.521	391.742	387.737	387.989	387.486	L[14]
P[17]	437.784	**363.471**	359.466	359.718	359.215	P[13]
N[18]	466.295	339.208	335.203	335.455	334.951	N[12]
I[19]	494.566	310.697	306.693	306.945	306.441	I[11]
Q[20]	526.581	282.426	278.422	278.673	278.170	Q[10]
A[21]	544.340	250.412	246.407	246.659	246.155	A[9]
V[22]	569.107	232.652	228.648	228.900	228.396	V[8]
L[23]	597.378	207.885	203.881	204.132	203.629	L[7]
L[24]	625.649	179.614	175.609	175.861	175.358	L[6]
P[25]	649.912	151.343	147.338	147.590	147.087	P[5]
K[26]	681.936	127.080	123.075	123.327	122.823	K[4]
K[27]	**713.960**	95.056	91.052	91.304	90.800	K[3]
T[28]	739.222	63.032	59.028	59.280	58.776	T[2]
E[29]	**771.482**	37.771	33.766	34.018	33.514	E[1]

sp|Q6GSS7|H2A2A_MOUSE

LNKLLGK $^{\text{Dimethyl}}_{28.03}$ VTIAQGGVLPNIQAVLLPKKTE

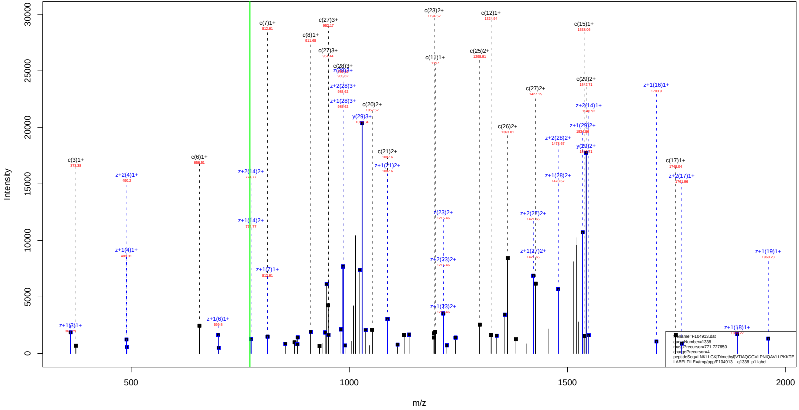

sp|Q6GSS7|H2A2A_MOUSE

LNKLLGK $^{Dimethyl}_{28.03}$ VTIAQGGVLPNIQAVLLPKKTE

- fragmentation table for charge state 1+
- specType=etd
- score=97.43
- F104913.dat
- query=q1338_p1
- precursor=771.727650
- chargePrecursor=4
- itol=0.8

AA	c	y	z+1	z+2	z	AA
L[1]	131.118	3083.892	3067.873	0.000	3066.865	L[29]
N[2]	245.161	2970.808	2954.789	2955.797	2953.781	N[28]
K[3]	**373.256**	2856.765	2840.746	2841.754	2839.738	K[27]
L[4]	486.340	2728.670	2712.651	2713.659	2711.643	L[26]
L[5]	599.424	2615.586	2599.567	2600.575	2598.559	L[25]
G[6]	**656.445**	2502.502	2486.483	2487.491	2485.475	G[24]
K[7]	**812.572**	2445.480	2429.462	2430.469	2428.454	K[23]
V[8]	**911.640**	2289.354	2273.335	2274.343	2272.327	V[22]
T[9]	1012.688	2190.286	2174.267	2175.275	2173.259	T[21]
I[10]	**1125.772**	2089.238	2073.219	2074.227	2072.211	I[20]
A[11]	**1196.809**	1976.154	**1960.135**	1961.143	1959.127	A[19]
Q[12]	**1324.867**	1905.117	**1889.098**	1890.106	1888.090	Q[18]
G[13]	**1381.889**	1777.058	1761.039	**1762.047**	1760.032	G[17]
G[14]	1438.910	1720.037	**1704.018**	1705.026	1703.010	G[16]
V[15]	**1537.979**	1663.015	1646.997	1648.004	1645.989	V[15]
L[16]	1651.063	1563.947	1547.928	**1548.936**	1546.920	L[14]
P[17]	**1748.116**	1450.863	1434.844	1435.852	1433.836	P[13]
N[18]	1862.159	1353.810	**1337.791**	1338.799	1336.783	N[12]
I[19]	1975.243	1239.767	**1223.748**	1224.756	1222.741	I[11]
Q[20]	2103.301	1126.683	**1110.664**	1111.672	1109.656	Q[10]
A[21]	2174.338	998.624	982.606	983.614	**981.598**	A[9]
V[22]	2273.407	927.587	**911.569**	912.576	910.561	V[8]
L[23]	2386.491	828.519	**812.500**	813.508	811.492	L[7]
L[24]	2499.575	715.435	**699.416**	**700.424**	698.408	L[6]
P[25]	2596.628	602.351	586.332	587.340	585.324	P[5]
K[26]	2724.723	505.298	**489.279**	**490.287**	488.271	K[4]
K[27]	2852.818	377.203	**361.184**	362.192	360.177	K[3]
T[28]	2953.865	249.108	233.089	234.097	232.082	T[2]
E[29]	3082.908	148.060	132.042	133.050	131.034	E[1]

sp|Q6GSS7|H2A2A_MOUSE

LNKLLGK $^{Dimethyl}_{28.03}$ VTIAQGGVLPNIQAVLLPKKTE

- fragmentation table for charge state 2+
- specType=etd
- score=97.43
- F104913.dat
- query=q1338_p1
- precursor=771.727650
- chargePrecursor=4
- itol=0.8

AA	c	y	z+1	z+2	z	AA
L[1]	66.063	**1542.450**	**1534.440**	0.504	1533.936	L[29]
N[2]	123.084	1485.908	**1477.898**	**1478.402**	1477.394	N[28]
K[3]	187.132	1428.886	**1420.877**	**1421.381**	1420.373	K[27]
L[4]	243.674	1364.839	**1356.829**	1357.333	**1356.325**	L[26]
L[5]	300.216	1308.297	1300.287	1300.791	1299.783	L[25]
G[6]	328.726	1251.755	**1243.745**	**1244.249**	**1243.241**	G[24]
K[7]	406.789	**1223.244**	**1215.234**	**1215.738**	**1214.731**	K[23]
V[8]	456.324	1145.181	**1137.171**	**1137.675**	**1136.667**	V[22]
T[9]	506.848	1095.646	**1087.637**	**1088.141**	**1087.133**	T[21]
I[10]	563.390	1045.123	**1037.113**	**1037.617**	1036.609	I[20]
A[11]	598.908	988.581	**980.571**	**981.075**	**980.067**	A[19]
Q[12]	662.937	953.062	**945.053**	**945.557**	**944.549**	Q[18]
G[13]	691.448	889.033	**881.023**	**881.527**	**880.519**	G[17]
G[14]	719.959	860.522	852.513	**853.017**	852.009	G[16]
V[15]	769.493	832.011	824.002	824.506	823.498	V[15]
L[16]	826.035	782.477	**774.468**	**774.972**	773.964	L[14]
P[17]	**874.561**	725.935	717.926	718.430	717.422	P[13]
N[18]	**931.583**	677.409	669.399	669.903	668.895	N[12]
I[19]	988.125	620.387	612.378	612.882	611.874	I[11]
Q[20]	**1052.154**	563.845	555.836	556.340	555.332	Q[10]
A[21]	**1087.673**	499.816	491.807	492.310	491.303	A[9]
V[22]	**1137.207**	464.297	456.288	456.792	455.784	V[8]
L[23]	**1193.749**	414.763	406.754	407.258	406.250	L[7]
L[24]	1250.291	358.221	350.212	350.716	349.708	L[6]
P[25]	**1298.817**	301.679	293.670	294.174	293.166	P[5]
K[26]	**1362.865**	253.153	245.143	245.647	244.639	K[4]
K[27]	**1426.912**	189.105	181.096	181.600	180.592	K[3]
T[28]	1477.436	125.058	117.048	117.552	116.544	T[2]
E[29]	**1541.958**	74.534	66.524	67.028	66.021	E[1]

sp|Q6GSS7|H2A2A_MOUSE

LNKLLGK $^{\text{Dimethyl}}_{28.03}$ VTIAQGGVLPNIQAVLLPKKTE

- fragmentation table for charge state 3+
- specType=etd
- score=97.43
- F104913.dat
- query=q1338_p1
- precursor=771.727650
- chargePrecursor=4
- itol=0.8

AA	c	y	z+1	z+2	z	AA
L[1]	44.377	**1028.635**	**1023.296**	0.672	1022.960	L[29]
N[2]	82.392	**990.941**	**985.601**	**985.937**	**985.265**	N[28]
K[3]	125.090	**952.926**	**947.587**	**947.923**	947.251	K[27]
L[4]	162.785	910.228	904.889	905.225	904.553	L[26]
L[5]	200.479	872.533	867.194	867.530	866.858	L[25]
G[6]	219.487	834.839	829.499	829.835	829.163	G[24]
K[7]	271.529	815.832	810.492	810.828	810.156	K[23]
V[8]	304.552	763.790	758.450	758.786	758.114	V[22]
T[9]	338.234	730.767	725.427	725.763	725.091	T[21]
I[10]	375.929	697.084	691.745	692.081	691.409	I[20]
A[11]	399.608	659.389	654.050	654.386	653.714	A[19]
Q[12]	442.294	635.710	630.371	630.707	630.035	Q[18]
G[13]	461.301	593.024	587.685	588.021	587.349	G[17]
G[14]	480.308	574.017	568.678	569.013	568.342	G[16]
V[15]	513.331	555.010	549.670	550.006	549.334	V[15]
L[16]	551.026	521.987	516.648	516.984	516.312	L[14]
P[17]	583.377	484.292	478.953	479.289	478.617	P[13]
N[18]	621.391	451.942	446.602	446.938	446.266	N[12]
I[19]	659.086	413.927	408.588	408.924	408.252	I[11]
Q[20]	701.772	376.233	370.893	371.229	370.557	Q[10]
A[21]	725.451	333.546	328.207	328.543	327.871	A[9]
V[22]	758.474	309.867	304.528	304.864	304.192	V[8]
L[23]	796.168	276.844	271.505	271.841	271.169	L[7]
L[24]	833.863	239.150	233.810	234.146	233.474	L[6]
P[25]	866.214	201.455	196.116	196.451	195.780	P[5]
K[26]	908.912	169.104	163.765	164.101	163.429	K[4]
K[27]	**951.611**	126.406	121.066	121.402	120.730	K[3]
T[28]	**985.293**	83.708	78.368	78.704	78.032	T[2]
E[29]	1028.307	50.025	44.685	45.021	44.349	E[1]

sp|Q6GSS7|H2A2A_MOUSE

LNKLLGK $^{\text{Dimethyl}}_{28.03}$ VTIAQGGVLPNIQAVLLPKKTE

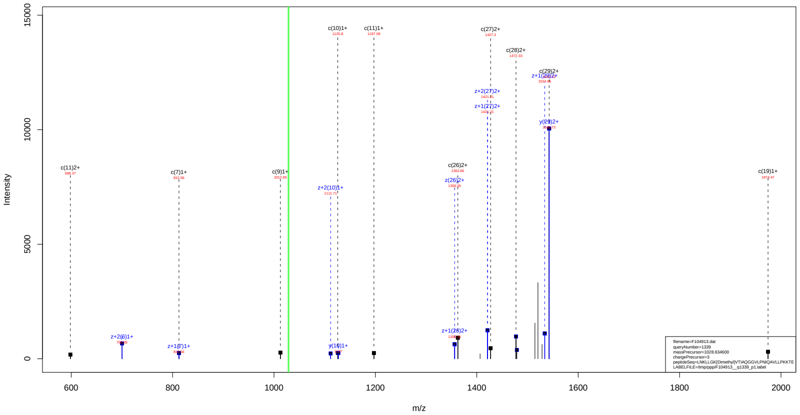

sp|Q6GSS7|H2A2A_MOUSE

LNKLLGK $^{Dimethyl}_{28.03}$ VTIAQGGVLPNIQAVLLPKKTE

- fragmentation table for charge state 1+
- specType=etd
- score=32.90
- F104913.dat
- query=q1339_p1
- precursor=1028.634600
- chargePrecursor=3
- itol=0.8

AA	c	y	z+1	z+2	z	AA
L[1]	131.118	3083.892	3067.873	0.000	3066.865	L[29]
N[2]	245.161	2970.808	2954.789	2955.797	2953.781	N[28]
K[3]	373.256	2856.765	2840.746	2841.754	2839.738	K[27]
L[4]	486.340	2728.670	2712.651	2713.659	2711.643	L[26]
L[5]	599.424	2615.586	2599.567	2600.575	2598.559	L[25]
G[6]	656.445	2502.502	2486.483	2487.491	2485.475	G[24]
K[7]	**812.572**	2445.480	2429.462	2430.469	2428.454	K[23]
V[8]	911.640	2289.354	2273.335	2274.343	2272.327	V[22]
T[9]	**1012.688**	2190.286	2174.267	2175.275	2173.259	T[21]
I[10]	**1125.772**	2089.238	2073.219	2074.227	2072.211	I[20]
A[11]	**1196.809**	1976.154	1960.135	1961.143	1959.127	A[19]
Q[12]	1324.867	1905.117	1889.098	1890.106	1888.090	Q[18]
G[13]	1381.889	1777.058	1761.039	1762.047	1760.032	G[17]
G[14]	1438.910	1720.037	1704.018	1705.026	1703.010	G[16]
V[15]	1537.979	1663.015	1646.997	1648.004	1645.989	V[15]
L[16]	1651.063	1563.947	1547.928	1548.936	1546.920	L[14]
P[17]	1748.116	1450.863	1434.844	1435.852	1433.836	P[13]
N[18]	1862.159	1353.810	1337.791	1338.799	1336.783	N[12]
I[19]	**1975.243**	1239.767	1223.748	1224.756	1222.741	I[11]
Q[20]	2103.301	**1126.683**	1110.664	**1111.672**	1109.656	Q[10]
A[21]	2174.338	998.624	982.606	983.614	981.598	A[9]
V[22]	2273.407	927.587	911.569	912.576	910.561	V[8]
L[23]	2386.491	828.519	**812.500**	813.508	811.492	L[7]
L[24]	2499.575	715.435	699.416	**700.424**	698.408	L[6]
P[25]	2596.628	602.351	586.332	587.340	585.324	P[5]
K[26]	2724.723	505.298	489.279	490.287	488.271	K[4]
K[27]	2852.818	377.203	361.184	362.192	360.177	K[3]
T[28]	2953.865	249.108	233.089	234.097	232.082	T[2]
E[29]	3082.908	148.060	132.042	133.050	131.034	E[1]

sp|Q6GSS7|H2A2A_MOUSE

LNKLLGK $^{\text{Dimethyl}}_{28.03}$ VTIAQGGVLPNIQAVLLPKKTE

- fragmentation table for charge state 2+
- specType=etd
- score=32.90
- F104913.dat
- query=q1339_p1
- precursor=1028.634600
- chargePrecursor=3
- itol=0.8

AA	c	y	z+1	z+2	z	AA
L[1]	66.063	**1542.450**	**1534.440**	0.504	**1533.936**	L[29]
N[2]	123.084	1485.908	**1477.898**	**1478.402**	**1477.394**	N[28]
K[3]	187.132	1428.886	**1420.877**	**1421.381**	1420.373	K[27]
L[4]	243.674	1364.839	**1356.829**	1357.333	**1356.325**	L[26]
L[5]	300.216	1308.297	1300.287	1300.791	1299.783	L[25]
G[6]	328.726	1251.755	1243.745	1244.249	1243.241	G[24]
K[7]	406.789	1223.244	1215.234	1215.738	1214.731	K[23]
V[8]	456.324	1145.181	1137.171	1137.675	1136.667	V[22]
T[9]	506.848	1095.646	1087.637	1088.141	1087.133	T[21]
I[10]	563.390	1045.123	1037.113	1037.617	1036.609	I[20]
A[11]	**598.908**	988.581	980.571	981.075	980.067	A[19]
Q[12]	662.937	953.062	945.053	945.557	944.549	Q[18]
G[13]	691.448	889.033	881.023	881.527	880.519	G[17]
G[14]	719.959	860.522	852.513	853.017	852.009	G[16]
V[15]	769.493	832.011	824.002	824.506	823.498	V[15]
L[16]	826.035	782.477	774.468	774.972	773.964	L[14]
P[17]	874.561	725.935	717.926	718.430	717.422	P[13]
N[18]	931.583	677.409	669.399	669.903	668.895	N[12]
I[19]	988.125	620.387	612.378	612.882	611.874	I[11]
Q[20]	1052.154	563.845	555.836	556.340	555.332	Q[10]
A[21]	1087.673	499.816	491.807	492.310	491.303	A[9]
V[22]	1137.207	464.297	456.288	456.792	455.784	V[8]
L[23]	1193.749	414.763	406.754	407.258	406.250	L[7]
L[24]	1250.291	358.221	350.212	350.716	349.708	L[6]
P[25]	1298.817	301.679	293.670	294.174	293.166	P[5]
K[26]	**1362.865**	253.153	245.143	245.647	244.639	K[4]
K[27]	**1426.912**	189.105	181.096	181.600	180.592	K[3]
T[28]	**1477.436**	125.058	117.048	117.552	116.544	T[2]
E[29]	**1541.958**	74.534	66.524	67.028	66.021	E[1]

sp|Q6GSS7|H2A2A_MOUSE

LNKLLGK $^{\text{Dimethyl}}_{28.03}$ VTIAQGGVLPNIQAVLLPKKTE

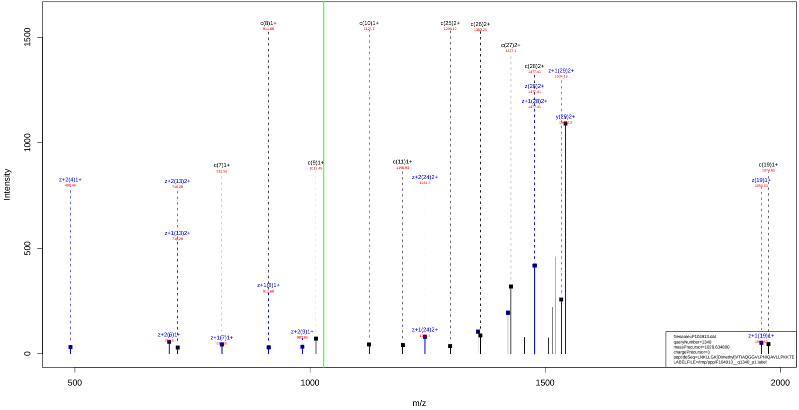

sp|Q6GSS7|H2A2A_MOUSE

LNKLLGK $^{\text{Dimethyl}}_{28.03}$ VTIAQGGVLPNIQAVLLPKKTE

- fragmentation table for charge state 1+
- specType=etd
- score=56.59
- F104913.dat
- query=q1340_p1
- precursor=1028.634800
- chargePrecursor=3
- itol=0.8

AA	c	y	z+1	z+2	z	AA
L[1]	131.118	3083.892	3067.873	0.000	3066.865	L[29]
N[2]	245.161	2970.808	2954.789	2955.797	2953.781	N[28]
K[3]	373.256	2856.765	2840.746	2841.754	2839.738	K[27]
L[4]	486.340	2728.670	2712.651	2713.659	2711.643	L[26]
L[5]	599.424	2615.586	2599.567	2600.575	2598.559	L[25]
G[6]	656.445	2502.502	2486.483	2487.491	2485.475	G[24]
K[7]	**812.572**	2445.480	2429.462	2430.469	2428.454	K[23]
V[8]	**911.640**	2289.354	2273.335	2274.343	2272.327	V[22]
T[9]	**1012.688**	2190.286	2174.267	2175.275	2173.259	T[21]
I[10]	**1125.772**	2089.238	2073.219	2074.227	2072.211	I[20]
A[11]	**1196.809**	1976.154	**1960.135**	1961.143	**1959.127**	A[19]
Q[12]	1324.867	1905.117	1889.098	1890.106	1888.090	Q[18]
G[13]	1381.889	1777.058	1761.039	1762.047	1760.032	G[17]
G[14]	1438.910	1720.037	1704.018	1705.026	1703.010	G[16]
V[15]	1537.979	1663.015	1646.997	1648.004	1645.989	V[15]
L[16]	1651.063	1563.947	1547.928	1548.936	1546.920	L[14]
P[17]	1748.116	1450.863	1434.844	1435.852	1433.836	P[13]
N[18]	1862.159	1353.810	1337.791	1338.799	1336.783	N[12]
I[19]	**1975.243**	1239.767	1223.748	1224.756	1222.741	I[11]
Q[20]	2103.301	1126.683	1110.664	1111.672	1109.656	Q[10]
A[21]	2174.338	998.624	982.606	**983.614**	981.598	A[9]
V[22]	2273.407	927.587	**911.569**	912.576	910.561	V[8]
L[23]	2386.491	828.519	**812.500**	813.508	811.492	L[7]
L[24]	2499.575	715.435	699.416	**700.424**	698.408	L[6]
P[25]	2596.628	602.351	586.332	587.340	585.324	P[5]
K[26]	2724.723	505.298	489.279	**490.287**	488.271	K[4]
K[27]	2852.818	377.203	361.184	362.192	360.177	K[3]
T[28]	2953.865	249.108	233.089	234.097	232.082	T[2]
E[29]	3082.908	148.060	132.042	133.050	131.034	E[1]

sp|Q6GSS7|H2A2A_MOUSE

LNKLLGK $^{\text{Dimethyl}}_{28.03}$ VTIAQGGVLPNIQAVLLPKKTE

- fragmentation table for charge state 2+
- specType=etd
- score=56.59
- F104913.dat
- query=q1340_p1
- precursor=1028.634800
- chargePrecursor=3
- itol=0.8

AA	c	y	z+1	z+2	z	AA
L[1]	66.063	**1542.450**	**1534.440**	0.504	**1533.936**	L[29]
N[2]	123.084	1485.908	**1477.898**	1478.402	**1477.394**	N[28]
K[3]	187.132	1428.886	**1420.877**	**1421.381**	**1420.373**	K[27]
L[4]	243.674	1364.839	**1356.829**	**1357.333**	**1356.325**	L[26]
L[5]	300.216	1308.297	1300.287	1300.791	1299.783	L[25]
G[6]	328.726	1251.755	**1243.745**	**1244.249**	1243.241	G[24]
K[7]	406.789	1223.244	1215.234	1215.738	1214.731	K[23]
V[8]	456.324	1145.181	1137.171	1137.675	1136.667	V[22]
T[9]	506.848	1095.646	1087.637	1088.141	1087.133	T[21]
I[10]	563.390	1045.123	1037.113	1037.617	1036.609	I[20]
A[11]	598.908	988.581	980.571	981.075	980.067	A[19]
Q[12]	662.937	953.062	945.053	945.557	944.549	Q[18]
G[13]	691.448	889.033	881.023	881.527	880.519	G[17]
G[14]	719.959	860.522	852.513	853.017	852.009	G[16]
V[15]	769.493	832.011	824.002	824.506	823.498	V[15]
L[16]	826.035	782.477	774.468	774.972	773.964	L[14]
P[17]	874.561	725.935	**717.926**	**718.430**	717.422	P[13]
N[18]	931.583	677.409	669.399	669.903	668.895	N[12]
I[19]	988.125	620.387	612.378	612.882	611.874	I[11]
Q[20]	1052.154	563.845	555.836	556.340	555.332	Q[10]
A[21]	1087.673	499.816	491.807	492.310	491.303	A[9]
V[22]	1137.207	464.297	456.288	456.792	455.784	V[8]
L[23]	1193.749	414.763	406.754	407.258	406.250	L[7]
L[24]	1250.291	358.221	350.212	350.716	349.708	L[6]
P[25]	**1298.817**	301.679	293.670	294.174	293.166	P[5]
K[26]	**1362.865**	253.153	245.143	245.647	244.639	K[4]
K[27]	**1426.912**	189.105	181.096	181.600	180.592	K[3]
T[28]	**1477.436**	125.058	117.048	117.552	116.544	T[2]
E[29]	1541.958	74.534	66.524	67.028	66.021	E[1]

sp|Q6GSS7|H2A2A_MOUSE

LNK $^{Dimethyl}_{28.03}$ LLGKVTIAQGGVLPNIQAVLLPKKTE

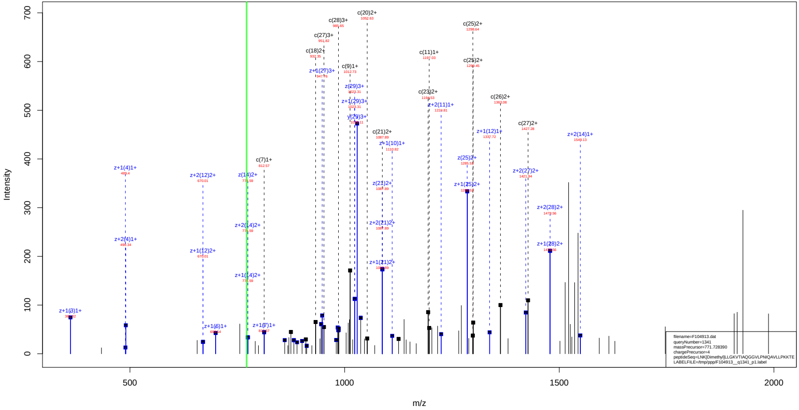

sp|Q6GSS7|H2A2A_MOUSE

LNK $^{Dimethyl}_{28.03}$ LLGKVTIAQGGVLPNIQAVLLPKKTE

- fragmentation table for charge state 1+
- specType=etd
- score=28.18
- F104913.dat
- query=q1341_p1
- precursor=771.728390
- chargePrecursor=4
- itol=0.8

AA	c	y	z+1	z+2	z	AA
L[1]	131.118	3083.892	3067.873	0.000	3066.865	L[29]
N[2]	245.161	2970.808	2954.789	2955.797	2953.781	N[28]
K[3]	401.287	2856.765	2840.746	2841.754	2839.738	K[27]
L[4]	514.371	2700.639	2684.620	2685.628	2683.612	L[26]
L[5]	627.455	2587.555	2571.536	2572.544	2570.528	L[25]
G[6]	684.477	2474.470	2458.452	2459.460	2457.444	G[24]
K[7]	**812.572**	2417.449	2401.430	2402.438	2400.422	K[23]
V[8]	**911.640**	2289.354	2273.335	2274.343	2272.327	V[22]
T[9]	**1012.688**	2190.286	2174.267	2175.275	2173.259	T[21]
I[10]	**1125.772**	2089.238	2073.219	2074.227	2072.211	I[20]
A[11]	**1196.809**	1976.154	1960.135	1961.143	1959.127	A[19]
Q[12]	1324.867	1905.117	1889.098	1890.106	1888.090	Q[18]
G[13]	1381.889	1777.058	1761.039	1762.047	1760.032	G[17]
G[14]	1438.910	1720.037	1704.018	1705.026	1703.010	G[16]
V[15]	1537.979	1663.015	1646.997	1648.004	1645.989	V[15]
L[16]	1651.063	1563.947	1547.928	**1548.936**	1546.920	L[14]
P[17]	1748.116	1450.863	1434.844	1435.852	1433.836	P[13]
N[18]	1862.159	1353.810	**1337.791**	1338.799	1336.783	N[12]
I[19]	1975.243	1239.767	1223.748	**1224.756**	1222.741	I[11]
Q[20]	2103.301	1126.683	**1110.664**	1111.672	1109.656	Q[10]
A[21]	2174.338	998.624	982.606	**983.614**	981.598	A[9]
V[22]	2273.407	927.587	**911.569**	912.576	910.561	V[8]
L[23]	2386.491	828.519	**812.500**	813.508	811.492	L[7]
L[24]	2499.575	715.435	**699.416**	700.424	698.408	L[6]
P[25]	2596.628	602.351	586.332	587.340	585.324	P[5]
K[26]	2724.723	505.298	**489.279**	**490.287**	488.271	K[4]
K[27]	2852.818	377.203	**361.184**	362.192	360.177	K[3]
T[28]	2953.865	249.108	233.089	234.097	232.082	T[2]
E[29]	3082.908	148.060	132.042	133.050	131.034	E[1]

sp|Q6GSS7|H2A2A_MOUSE

LNK $^{\text{Dimethyl}}_{28.03}$ LLGKVTIAQGGVLPNIQAVLLPKKTE

- fragmentation table for charge state 2+
- specType=etd
- score=28.18
- F104913.dat
- query=q1341_p1
- precursor=771.728390
- chargePrecursor=4
- itol=0.8

AA	c	y	z+1	z+2	z	AA
L[1]	66.063	1542.450	1534.440	0.504	1533.936	L[29]
N[2]	123.084	1485.908	**1477.898**	**1478.402**	1477.394	N[28]
K[3]	201.147	1428.886	1420.877	**1421.381**	1420.373	K[27]
L[4]	257.689	1350.823	1342.814	1343.317	1342.310	L[26]
L[5]	314.231	1294.281	**1286.272**	1286.775	**1285.768**	L[25]
G[6]	342.742	1237.739	1229.730	1230.233	1229.226	G[24]
K[7]	406.789	1209.228	1201.219	1201.723	1200.715	K[23]
V[8]	456.324	1145.181	1137.171	1137.675	1136.667	V[22]
T[9]	506.848	1095.646	**1087.637**	**1088.141**	**1087.133**	T[21]
I[10]	563.390	1045.123	**1037.113**	**1037.617**	**1036.609**	I[20]
A[11]	598.908	988.581	**980.571**	**981.075**	**980.067**	A[19]
Q[12]	662.937	953.062	**945.053**	**945.557**	**944.549**	Q[18]
G[13]	691.448	**889.033**	**881.023**	**881.527**	880.519	G[17]
G[14]	719.959	**860.522**	852.513	853.017	852.009	G[16]
V[15]	769.493	832.011	824.002	824.506	823.498	V[15]
L[16]	826.035	782.477	**774.468**	**774.972**	**773.964**	L[14]
P[17]	**874.561**	725.935	717.926	718.430	717.422	P[13]
N[18]	**931.583**	677.409	**669.399**	**669.903**	668.895	N[12]
I[19]	988.125	620.387	612.378	612.882	611.874	I[11]
Q[20]	**1052.154**	563.845	555.836	556.340	555.332	Q[10]
A[21]	**1087.673**	499.816	491.807	492.310	491.303	A[9]
V[22]	1137.207	464.297	456.288	456.792	455.784	V[8]
L[23]	**1193.749**	414.763	406.754	407.258	406.250	L[7]
L[24]	1250.291	358.221	350.212	350.716	349.708	L[6]
P[25]	**1298.817**	301.679	293.670	294.174	293.166	P[5]
K[26]	**1362.865**	253.153	245.143	245.647	244.639	K[4]
K[27]	**1426.912**	189.105	181.096	181.600	180.592	K[3]
T[28]	1477.436	125.058	117.048	117.552	116.544	T[2]
E[29]	1541.958	74.534	66.524	67.028	66.021	E[1]

sp|Q6GSS7|H2A2A_MOUSE

LNK $^{Dimethyl}_{28.03}$ LLGKVTIAQGGVLPNIQAVLLPKKTE

- fragmentation table for charge state 3+
- specType=etd
- score=28.18
- F104913.dat
- query=q1341_p1
- precursor=771.728390
- chargePrecursor=4
- itol=0.8

AA	c	y	z+1	z+2	z	AA
L[1]	44.377	**1028.635**	**1023.296**	0.672	**1022.960**	L[29]
N[2]	82.392	990.941	**985.601**	**985.937**	**985.265**	N[28]
K[3]	134.434	952.926	**947.587**	**947.923**	**947.251**	K[27]
L[4]	172.129	**900.884**	895.545	895.881	895.209	L[26]
L[5]	209.823	863.190	857.850	858.186	857.514	L[25]
G[6]	228.830	825.495	820.155	820.491	819.819	G[24]
K[7]	271.529	806.488	801.148	801.484	800.812	K[23]
V[8]	304.552	763.790	758.450	758.786	758.114	V[22]
T[9]	338.234	730.767	725.427	725.763	725.091	T[21]
I[10]	375.929	697.084	691.745	692.081	691.409	I[20]
A[11]	399.608	659.389	654.050	654.386	653.714	A[19]
Q[12]	442.294	635.710	630.371	630.707	630.035	Q[18]
G[13]	461.301	593.024	587.685	588.021	587.349	G[17]
G[14]	480.308	574.017	568.678	569.013	568.342	G[16]
V[15]	513.331	555.010	549.670	550.006	549.334	V[15]
L[16]	551.026	521.987	516.648	516.984	516.312	L[14]
P[17]	583.377	484.292	478.953	479.289	478.617	P[13]
N[18]	621.391	451.942	446.602	446.938	446.266	N[12]
I[19]	659.086	413.927	408.588	408.924	408.252	I[11]
Q[20]	701.772	376.233	370.893	371.229	370.557	Q[10]
A[21]	725.451	333.546	328.207	328.543	327.871	A[9]
V[22]	758.474	309.867	304.528	304.864	304.192	V[8]
L[23]	796.168	276.844	271.505	271.841	271.169	L[7]
L[24]	833.863	239.150	233.810	234.146	233.474	L[6]
P[25]	866.214	201.455	196.116	196.451	195.780	P[5]
K[26]	**908.912**	169.104	163.765	164.101	163.429	K[4]
K[27]	**951.611**	126.406	121.066	121.402	120.730	K[3]
T[28]	**985.293**	83.708	78.368	78.704	78.032	T[2]
E[29]	1028.307	50.025	44.685	45.021	44.349	E[1]

sp|Q6GSS7|H2A2A_MOUSE

ELNKLLGK $^{\text{Dimethyl}}_{28.03}$ VTIAQGGVLPNIQAVLLPKKTE

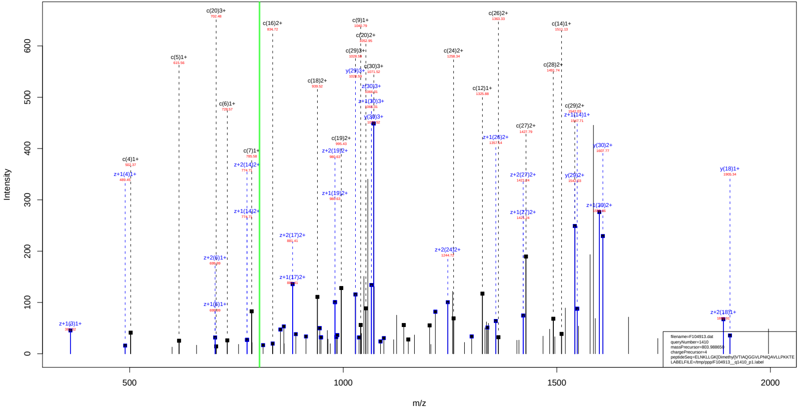

sp|Q6GSS7|H2A2A_MOUSE

ELNKLLGK $^{Dimethyl}_{28.03}$ VTIAQGGVLPNIQAVLLPKKTE

- fragmentation table for charge state 1+
- specType=etd
- score=66.59
- F104913.dat
- query=q1410_p1
- precursor=803.988650
- chargePrecursor=4
- itol=0.8

AA	c	y	z+1	z+2	z	AA
E[1]	147.076	3212.934	3196.916	0.000	3195.908	E[30]
L[2]	260.160	3083.892	3067.873	0.000	3066.865	L[29]
N[3]	374.203	2970.808	2954.789	2955.797	2953.781	N[28]
K[4]	**502.298**	2856.765	2840.746	2841.754	2839.738	K[27]
L[5]	**615.382**	2728.670	2712.651	2713.659	2711.643	L[26]
L[6]	**728.466**	2615.586	2599.567	2600.575	2598.559	L[25]
G[7]	**785.488**	2502.502	2486.483	2487.491	2485.475	G[24]
K[8]	941.614	2445.480	2429.462	2430.469	2428.454	K[23]
V[9]	**1040.683**	2289.354	2273.335	2274.343	2272.327	V[22]
T[10]	**1141.730**	2190.286	2174.267	2175.275	2173.259	T[21]
I[11]	1254.814	2089.238	2073.219	2074.227	2072.211	I[20]
A[12]	**1325.851**	1976.154	1960.135	1961.143	1959.127	A[19]
Q[13]	1453.910	**1905.117**	1889.098	**1890.106**	1888.090	Q[18]
G[14]	**1510.932**	1777.058	1761.039	1762.047	1760.032	G[17]
G[15]	1567.953	1720.037	1704.018	1705.026	1703.010	G[16]
V[16]	1667.021	1663.015	1646.997	1648.004	1645.989	V[15]
L[17]	1780.105	1563.947	**1547.928**	1548.936	**1546.920**	L[14]
P[18]	1877.158	1450.863	1434.844	1435.852	1433.836	P[13]
N[19]	1991.201	1353.810	**1337.791**	1338.799	1336.783	N[12]
I[20]	2104.285	1239.767	1223.748	1224.756	1222.741	I[11]
Q[21]	2232.344	1126.683	1110.664	1111.672	1109.656	Q[10]
A[22]	2303.381	998.624	982.606	**983.614**	981.598	A[9]
V[23]	2402.449	927.587	911.569	**912.576**	910.561	V[8]
L[24]	2515.533	828.519	**812.500**	813.508	811.492	L[7]
L[25]	2628.617	715.435	**699.416**	**700.424**	698.408	L[6]
P[26]	2725.670	602.351	586.332	587.340	585.324	P[5]
K[27]	2853.765	505.298	**489.279**	490.287	488.271	K[4]
K[28]	2981.860	377.203	**361.184**	362.192	360.177	K[3]
T[29]	3082.908	249.108	233.089	234.097	232.082	T[2]
E[30]	3211.950	148.060	132.042	133.050	131.034	E[1]

sp|Q6GSS7|H2A2A_MOUSE

ELNKLLGK $^{\text{Dimethyl}}_{28.03}$ VTIAQGGVLPNIQAVLLPKKTE

- fragmentation table for charge state 2+
- specType=etd
- score=66.59
- F104913.dat
- query=q1410_p1
- precursor=803.988650
- chargePrecursor=4
- itol=0.8

AA	c	y	z+1	z+2	z	AA
E[1]	74.042	**1606.971**	**1598.962**	0.504	1598.458	E[30]
L[2]	130.584	**1542.450**	1534.440	0.504	1533.936	L[29]
N[3]	187.605	1485.908	1477.898	1478.402	1477.394	N[28]
K[4]	251.653	1428.886	**1420.877**	**1421.381**	1420.373	K[27]
L[5]	308.195	1364.839	**1356.829**	**1357.333**	1356.325	L[26]
L[6]	364.737	1308.297	**1300.287**	**1300.791**	1299.783	L[25]
G[7]	393.248	1251.755	1243.745	**1244.249**	1243.241	G[24]
K[8]	471.311	1223.244	**1215.234**	**1215.738**	1214.731	K[23]
V[9]	520.845	1145.181	1137.171	1137.675	1136.667	V[22]
T[10]	571.369	**1095.646**	**1087.637**	1088.141	**1087.133**	T[21]
I[11]	627.911	1045.123	**1037.113**	**1037.617**	**1036.609**	I[20]
A[12]	663.429	988.581	**980.571**	**981.075**	**980.067**	A[19]
Q[13]	727.459	953.062	**945.053**	**945.557**	**944.549**	Q[18]
G[14]	755.969	**889.033**	**881.023**	**881.527**	880.519	G[17]
G[15]	784.480	**860.522**	**852.513**	**853.017**	852.009	G[16]
V[16]	**834.014**	832.011	824.002	824.506	823.498	V[15]
L[17]	890.556	782.477	**774.468**	**774.972**	**773.964**	L[14]
P[18]	**939.083**	725.935	717.926	718.430	717.422	P[13]
N[19]	**996.104**	677.409	669.399	669.903	668.895	N[12]
I[20]	**1052.646**	620.387	612.378	612.882	611.874	I[11]
Q[21]	1116.676	563.845	555.836	556.340	555.332	Q[10]
A[22]	**1152.194**	499.816	491.807	492.310	491.303	A[9]
V[23]	**1201.728**	464.297	456.288	456.792	455.784	V[8]
L[24]	**1258.270**	414.763	406.754	407.258	406.250	L[7]
L[25]	1314.812	358.221	350.212	350.716	349.708	L[6]
P[26]	**1363.339**	301.679	293.670	294.174	293.166	P[5]
K[27]	**1427.386**	253.153	245.143	245.647	244.639	K[4]
K[28]	**1491.434**	189.105	181.096	181.600	180.592	K[3]
T[29]	**1541.958**	125.058	117.048	117.552	116.544	T[2]
E[30]	1606.479	74.534	66.524	67.028	66.021	E[1]

sp|Q6GSS7|H2A2A_MOUSE

ELNKLLGK $^{\text{Dimethyl}}_{28.03}$ VTIAQGGVLPNIQAVLLPKKTE

- fragmentation table for charge state 3+
- specType=etd
- score=66.59
- F104913.dat
- query=q1410_p1
- precursor=803.988650
- chargePrecursor=4
- itol=0.8

AA	c	y	z+1	z+2	z	AA
E[1]	49.697	**1071.650**	**1066.310**	0.672	**1065.974**	E[30]
L[2]	87.392	**1028.635**	1023.296	0.672	1022.960	L[29]
N[3]	125.406	990.941	**985.601**	**985.937**	985.265	N[28]
K[4]	168.104	952.926	**947.587**	**947.923**	**947.251**	K[27]
L[5]	205.799	910.228	904.889	905.225	904.553	L[26]
L[6]	243.494	872.533	867.194	867.530	866.858	L[25]
G[7]	262.501	**834.839**	829.499	829.835	829.163	G[24]
K[8]	314.543	815.832	810.492	810.828	810.156	K[23]
V[9]	347.566	763.790	758.450	758.786	758.114	V[22]
T[10]	381.248	730.767	725.427	725.763	725.091	T[21]
I[11]	418.943	697.084	691.745	692.081	691.409	I[20]
A[12]	442.622	659.389	654.050	654.386	653.714	A[19]
Q[13]	485.308	635.710	630.371	630.707	630.035	Q[18]
G[14]	504.315	593.024	587.685	588.021	587.349	G[17]
G[15]	523.323	574.017	568.678	569.013	568.342	G[16]
V[16]	556.345	555.010	549.670	550.006	549.334	V[15]
L[17]	594.040	521.987	516.648	516.984	516.312	L[14]
P[18]	626.391	484.292	478.953	479.289	478.617	P[13]
N[19]	664.405	451.942	446.602	446.938	446.266	N[12]
I[20]	**702.100**	413.927	408.588	408.924	408.252	I[11]
Q[21]	744.786	376.233	370.893	371.229	370.557	Q[10]
A[22]	768.465	333.546	328.207	328.543	327.871	A[9]
V[23]	801.488	309.867	304.528	304.864	304.192	V[8]
L[24]	839.183	276.844	271.505	271.841	271.169	L[7]
L[25]	876.877	239.150	233.810	234.146	233.474	L[6]
P[26]	909.228	201.455	196.116	196.451	195.780	P[5]
K[27]	951.927	169.104	163.765	164.101	163.429	K[4]
K[28]	994.625	126.406	121.066	121.402	120.730	K[3]
T[29]	**1028.307**	83.708	78.368	78.704	78.032	T[2]
E[30]	**1071.322**	50.025	44.685	45.021	44.349	E[1]

sp|P70696|H2B1A_MOUSE

ASRLAHYNKRSTITS $^{\text{Phospho}}_{79.97}$ RE

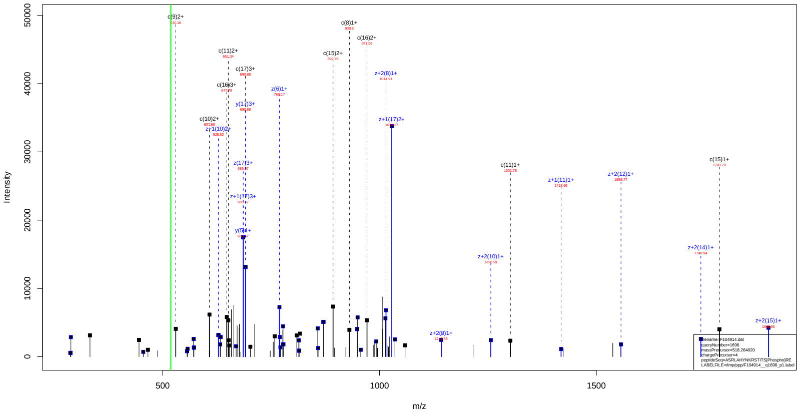

sp|P70696|H2B1A_MOUSE

ASRLAHYNKRSTITS$^{\text{Phospho}}_{79.97}$RE

- fragmentation table for charge state 1+
- specType=etd
- score=54.28
- F104914.dat
- query=q1696_p1
- precursor=518.264020
- chargePrecursor=4
- itol=0.8

AA	c	y	z+1	z+2	z	AA
A[1]	89.071	2070.024	2054.005	0.000	2052.997	A[17]
S[2]	176.103	1998.987	1982.968	0.000	1981.960	S[16]
R[3]	**332.204**	1911.955	1895.936	**1896.944**	1894.928	R[15]
L[4]	**445.288**	1755.854	1739.835	**1740.843**	1738.827	L[14]
A[5]	516.325	1642.770	1626.751	1627.759	1625.743	A[13]
H[6]	**653.384**	1571.733	1555.714	**1556.722**	1554.706	H[12]
Y[7]	**816.447**	1434.674	**1418.655**	1419.663	1417.647	Y[11]
N[8]	**930.490**	1271.610	1255.592	**1256.599**	1254.584	N[10]
K[9]	**1058.585**	1157.567	1141.549	**1142.557**	1140.541	K[9]
R[10]	1214.686	1029.472	**1013.454**	**1014.462**	1012.446	R[8]
S[11]	**1301.719**	873.371	**857.353**	**858.360**	856.345	S[7]
T[12]	1402.766	786.339	**770.321**	**771.328**	**769.313**	T[6]
I[13]	1515.850	**685.292**	**669.273**	670.281	668.265	I[5]
T[14]	1616.898	**572.208**	**556.189**	**557.197**	555.181	T[4]
S[15]	**1783.896**	471.160	**455.141**	456.149	454.133	S[3]
R[16]	1939.997	304.162	**288.143**	289.151	**287.135**	R[2]
E[17]	2069.040	148.060	132.042	133.050	131.034	E[1]

sp|P70696|H2B1A_MOUSE

ASRLAHYNKRSTITS$^{Phospho}_{79.97}$RE

- fragmentation table for charge state 2+
- specType=etd
- score=54.28
- F104914.dat
- query=q1696_p1
- precursor=518.264020
- chargePrecursor=4
- itol=0.8

AA	c	y	z+1	z+2	z	AA
A[1]	45.039	**1035.516**	**1027.506**	0.504	1027.002	A[17]
S[2]	88.555	999.997	**991.988**	0.504	991.484	S[16]
R[3]	166.606	**956.481**	**948.472**	**948.976**	**947.968**	R[15]
L[4]	223.148	878.431	**870.421**	**870.925**	**869.917**	L[14]
A[5]	258.666	821.888	**813.879**	**814.383**	**813.375**	A[13]
H[6]	327.196	786.370	**778.361**	**778.864**	**777.857**	H[12]
Y[7]	408.727	717.840	709.831	710.335	709.327	Y[11]
N[8]	**465.749**	636.309	**628.299**	**628.803**	**627.796**	N[10]
K[9]	**529.796**	579.287	**571.278**	**571.782**	**570.774**	K[9]
R[10]	**607.847**	515.240	507.231	507.734	506.727	R[8]
S[11]	**651.363**	437.189	429.180	429.684	428.676	S[7]
T[12]	**701.887**	393.673	385.664	386.168	385.160	T[6]
I[13]	**758.429**	343.149	335.140	335.644	334.636	I[5]
T[14]	**808.953**	**286.607**	278.598	279.102	278.094	T[4]
S[15]	**892.452**	236.084	228.074	228.578	227.570	S[3]
R[16]	**970.502**	152.584	144.575	145.079	144.071	R[2]
E[17]	**1035.024**	74.534	66.524	67.028	66.021	E[1]

sp|P70696|H2B1A_MOUSE

ASRLAHYNKRSTITS$^{Phospho}_{79.97}$RE

- fragmentation table for charge state 3+
- specType=etd
- score=54.28
- F104914.dat
- query=q1696_p1
- precursor=518.264020
- chargePrecursor=4
- itol=0.8

AA	c	y	z+1	z+2	z	AA
A[1]	30.362	**690.680**	**685.340**	0.672	**685.004**	A[17]
S[2]	59.373	667.000	661.661	0.672	661.325	S[16]
R[3]	111.406	637.990	**632.650**	**632.986**	**632.314**	R[15]
L[4]	149.101	585.956	580.617	580.952	580.281	L[14]
A[5]	172.780	548.261	542.922	543.258	542.586	A[13]
H[6]	218.466	524.582	519.243	519.579	518.907	H[12]
Y[7]	272.821	478.896	473.557	473.892	473.221	Y[11]
N[8]	310.835	424.542	419.202	419.538	418.866	N[10]
K[9]	353.533	386.527	381.188	381.524	380.852	K[9]
R[10]	405.567	343.829	338.489	338.825	338.153	R[8]
S[11]	434.578	291.795	**286.456**	**286.792**	286.120	S[7]
T[12]	468.260	262.785	257.445	257.781	257.109	T[6]
I[13]	505.955	229.102	223.762	224.098	223.427	I[5]
T[14]	539.637	191.407	186.068	186.404	185.732	T[4]
S[15]	595.304	157.725	152.385	152.721	152.049	S[3]
R[16]	**647.337**	102.059	96.719	97.055	96.383	R[2]
E[17]	**690.352**	50.025	44.685	45.021	44.349	E[1]

sp|P70696|H2B1A_MOUSE

ASRLAHYNKRSTIT$^{(\mathrm{Phospho})}_{(79.97)}$SRE

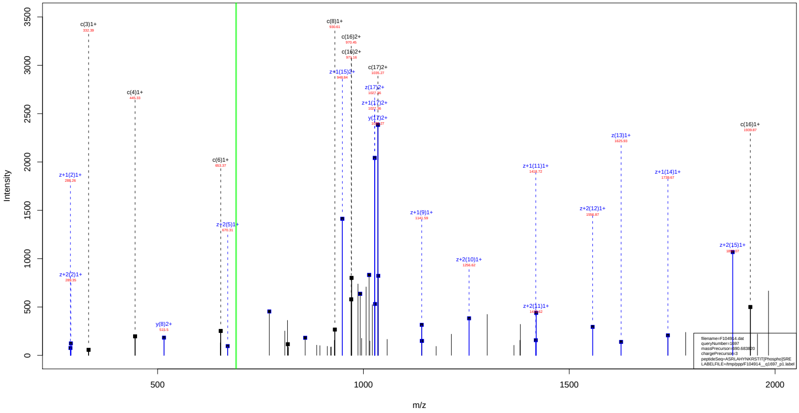

sp|P70696|H2B1A_MOUSE

ASRLAHYNKRSTIT$^{(Phospho)}_{(79.97)}$SRE

- fragmentation table for charge state 1+
- specType=etd
- score=74.57
- F104914.dat
- query=q1697_p1
- precursor=690.683820
- chargePrecursor=3
- itol=0.8

AA	c	y	z+1	z+2	z	AA
A[1]	89.071	2070.024	2054.005	0.000	2052.997	A[17]
S[2]	176.103	1998.987	1982.968	0.000	1981.960	S[16]
R[3]	**332.204**	1911.955	1895.936	**1896.944**	1894.928	R[15]
L[4]	**445.288**	1755.854	**1739.835**	1740.843	1738.827	L[14]
A[5]	516.325	1642.770	1626.751	1627.759	**1625.743**	A[13]
H[6]	**653.384**	1571.733	1555.714	**1556.722**	1554.706	H[12]
Y[7]	**816.447**	1434.674	**1418.655**	**1419.663**	1417.647	Y[11]
N[8]	**930.490**	1271.610	1255.592	**1256.599**	1254.584	N[10]
K[9]	1058.585	1157.567	**1141.549**	**1142.557**	1140.541	K[9]
R[10]	1214.686	1029.472	**1013.454**	**1014.462**	1012.446	R[8]
S[11]	1301.719	873.371	857.353	**858.360**	856.345	S[7]
T[12]	1402.766	786.339	770.321	**771.328**	769.313	T[6]
I[13]	1515.850	685.292	669.273	**670.281**	668.265	I[5]
T[14]	1696.864	572.208	556.189	557.197	555.181	T[4]
S[15]	1783.896	391.194	375.175	376.183	374.167	S[3]
R[16]	**1939.997**	304.162	**288.143**	**289.151**	287.135	R[2]
E[17]	2069.040	148.060	132.042	133.050	131.034	E[1]

sp|P70696|H2B1A_MOUSE

ASRLAHYNKRSTIT $^{(Phospho)}_{(79.97)}$ SRE

- fragmentation table for charge state 2+
- specType=etd
- score=74.57
- F104914.dat
- query=q1697_p1
- precursor=690.683820
- chargePrecursor=3
- itol=0.8

AA	c	y	z+1	z+2	z	AA
A[1]	45.039	**1035.516**	**1027.506**	0.504	**1027.002**	A[17]
S[2]	88.555	999.997	**991.988**	0.504	**991.484**	S[16]
R[3]	166.606	956.481	**948.472**	**948.976**	947.968	R[15]
L[4]	223.148	878.431	870.421	870.925	869.917	L[14]
A[5]	258.666	821.888	813.879	814.383	813.375	A[13]
H[6]	327.196	786.370	778.361	778.864	777.857	H[12]
Y[7]	408.727	717.840	709.831	710.335	709.327	Y[11]
N[8]	465.749	636.309	628.299	628.803	627.796	N[10]
K[9]	529.796	579.287	571.278	571.782	570.774	K[9]
R[10]	607.847	**515.240**	507.231	507.734	506.727	R[8]
S[11]	651.363	437.189	429.180	429.684	428.676	S[7]
T[12]	701.887	393.673	385.664	386.168	385.160	T[6]
I[13]	758.429	343.149	335.140	335.644	334.636	I[5]
T[14]	848.936	286.607	278.598	279.102	278.094	T[4]
S[15]	892.452	196.100	188.091	188.595	187.587	S[3]
R[16]	**970.502**	152.584	144.575	145.079	144.071	R[2]
E[17]	**1035.024**	74.534	66.524	67.028	66.021	E[1]

tr|Q80ZM5|Q80ZM5_MOUSE

RRGASAASS $^{\text{Phospho}}_{79.97}$ PAPKARTAAADRTPARPQPE

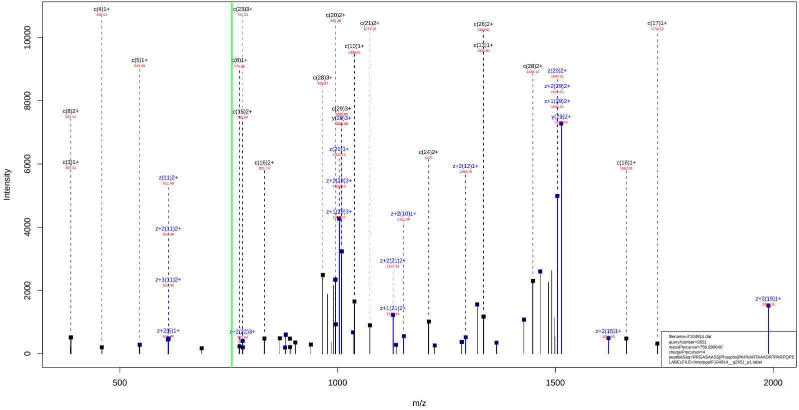

tr|Q80ZM5|Q80ZM5_MOUSE

RRGASAASS $^{Phospho}_{79.97}$ PAPKARTAAADRTPARPQPE

- fragmentation table for charge state 1+
- specType=etd
- score=64.55
- F104914.dat
- query=q2651_p1
- precursor=756.890640
- chargePrecursor=4
- itol=0.8

AA	c	y	z+1	z+2	z	AA
R[1]	174.135	3024.524	3008.505	3009.513	3007.497	R[29]
R[2]	330.236	2868.422	2852.404	2853.412	2851.396	R[28]
G[3]	**387.258**	2712.321	2696.303	2697.310	2695.295	G[27]
A[4]	**458.295**	2655.300	2639.281	2640.289	2638.273	A[26]
S[5]	**545.327**	2584.263	2568.244	2569.252	2567.236	S[25]
A[6]	616.364	2497.231	2481.212	2482.220	2480.204	A[24]
A[7]	**687.401**	2426.194	2410.175	2411.183	2409.167	A[23]
S[8]	**774.433**	2355.156	2339.138	2340.146	2338.130	S[22]
S[9]	941.431	2268.124	2252.106	2253.114	2251.098	S[21]
P[10]	**1038.484**	2101.126	2085.107	2086.115	2084.100	P[20]
A[11]	1109.521	2004.073	1988.055	**1989.062**	1987.047	A[19]
P[12]	1206.574	1933.036	1917.018	1918.025	1916.010	P[18]
K[13]	**1334.669**	1835.983	1819.965	1820.973	1818.957	K[17]
A[14]	1405.706	1707.889	1691.870	1692.878	1690.862	A[16]
R[15]	1561.807	1636.851	1620.833	**1621.840**	1619.825	R[15]
T[16]	**1662.855**	1480.750	**1464.732**	**1465.739**	1463.724	T[14]
A[17]	**1733.892**	1379.703	1363.684	**1364.692**	1362.676	A[13]
A[18]	1804.929	1308.665	1292.647	**1293.655**	1291.639	A[12]
A[19]	1875.966	1237.628	1221.610	**1222.617**	1220.602	A[11]
D[20]	1990.993	1166.591	1150.573	**1151.580**	1149.565	D[10]
R[21]	2147.094	1051.564	**1035.546**	1036.553	1034.538	R[9]
T[22]	2248.142	895.463	**879.444**	**880.452**	878.437	T[8]
P[23]	2345.195	794.416	778.397	779.405	777.389	P[7]
A[24]	2416.232	697.363	681.344	682.352	680.336	A[6]
R[25]	2572.333	626.326	**610.307**	**611.315**	609.299	R[5]
P[26]	2669.386	470.225	454.206	455.214	453.198	P[4]
Q[27]	2797.444	373.172	357.153	358.161	356.145	Q[3]
P[28]	2894.497	245.113	229.094	230.102	228.087	P[2]
E[29]	3023.540	148.060	132.042	133.050	131.034	E[1]

tr|Q80ZM5|Q80ZM5_MOUSE

RRGASAASS $^{\text{Phospho}}_{79.97}$ PAPKARTAAADRTPARPQPE

- fragmentation table for charge state 2+
- specType=etd
- score=64.55
- F104914.dat
- query=q2651_p1
- precursor=756.890640
- chargePrecursor=4
- itol=0.8

AA	c	y	z+1	z+2	z	AA
R[1]	87.571	**1512.765**	**1504.756**	**1505.260**	**1504.252**	R[29]
R[2]	165.622	1434.715	**1426.705**	**1427.209**	1426.202	R[28]
G[3]	194.132	1356.664	1348.655	1349.159	1348.151	G[27]
A[4]	229.651	1328.154	**1320.144**	**1320.648**	1319.640	A[26]
S[5]	273.167	1292.635	**1284.626**	**1285.130**	**1284.122**	S[25]
A[6]	308.686	1249.119	1241.110	1241.614	1240.606	A[24]
A[7]	344.204	1213.600	1205.591	1206.095	1205.087	A[23]
S[8]	**387.720**	1178.082	1170.073	1170.576	1169.569	S[22]
S[9]	471.219	**1134.566**	**1126.557**	**1127.060**	1126.053	S[21]
P[10]	519.746	1051.067	1043.057	1043.561	1042.553	P[20]
A[11]	555.264	1002.540	**994.531**	**995.035**	**994.027**	A[19]
P[12]	603.791	967.022	959.012	959.516	958.508	P[18]
K[13]	667.838	918.495	910.486	910.990	909.982	K[17]
A[14]	703.357	854.448	846.439	846.942	845.935	A[16]
R[15]	**781.407**	818.929	810.920	811.424	810.416	R[15]
T[16]	**831.931**	740.879	732.869	733.373	732.366	T[14]
A[17]	**867.450**	690.355	682.346	682.849	681.842	A[13]
A[18]	**902.968**	654.836	646.827	647.331	646.323	A[12]
A[19]	**938.487**	619.318	**611.308**	**611.812**	**610.805**	A[11]
D[20]	**996.000**	583.799	575.790	576.294	575.286	D[10]
R[21]	**1074.051**	526.286	518.276	518.780	517.773	R[9]
T[22]	1124.575	448.235	440.226	440.730	439.722	T[8]
P[23]	1173.101	397.711	389.702	390.206	389.198	P[7]
A[24]	**1208.620**	349.185	341.176	341.680	340.672	A[6]
R[25]	1286.670	313.666	305.657	306.161	305.153	R[5]
P[26]	**1335.196**	235.616	227.607	228.110	227.103	P[4]
Q[27]	1399.226	187.090	179.080	179.584	178.576	Q[3]
P[28]	**1447.752**	123.060	115.051	115.555	114.547	P[2]
E[29]	1512.273	74.534	66.524	67.028	66.021	E[1]

tr|Q80ZM5|Q80ZM5_MOUSE

RRGASAASS $^{Phospho}_{79.97}$ PAPKARTAAADRTPARPQPE

- fragmentation table for charge state 3+
- specType=etd
- score=64.55
- F104914.dat
- query=q2651_p1
- precursor=756.890640
- chargePrecursor=4
- itol=0.8

AA	c	y	z+1	z+2	z	AA
R[1]	58.716	**1008.846**	**1003.506**	**1003.842**	**1003.171**	R[29]
R[2]	110.750	956.812	951.473	951.809	951.137	R[28]
G[3]	129.757	904.779	899.439	899.775	899.103	G[27]
A[4]	153.436	885.771	**880.432**	**880.768**	**880.096**	A[26]
S[5]	182.447	862.092	856.753	857.089	856.417	S[25]
A[6]	206.126	833.082	827.742	828.078	827.406	A[24]
A[7]	229.805	809.403	804.063	804.399	803.727	A[23]
S[8]	258.816	785.724	780.384	**780.720**	780.048	S[22]
S[9]	314.482	756.713	751.373	751.709	751.037	S[21]
P[10]	346.833	701.047	695.707	696.043	695.371	P[20]
A[11]	370.512	668.696	663.356	663.692	663.020	A[19]
P[12]	402.863	645.017	639.677	640.013	639.341	P[18]
K[13]	445.561	612.666	607.326	607.662	606.990	K[17]
A[14]	469.240	569.968	564.628	564.964	564.292	A[16]
R[15]	521.274	**546.289**	540.949	541.285	540.613	R[15]
T[16]	554.956	494.255	488.915	489.251	488.579	T[14]
A[17]	578.635	460.572	455.233	455.569	454.897	A[13]
A[18]	602.315	436.893	431.554	431.890	431.218	A[12]
A[19]	625.994	413.214	407.875	408.211	407.539	A[11]
D[20]	664.336	389.535	384.196	384.532	383.860	D[10]
R[21]	716.370	351.193	345.853	346.189	345.517	R[9]
T[22]	750.052	299.159	293.820	294.156	293.484	T[8]
P[23]	**782.403**	265.477	260.137	260.473	259.801	P[7]
A[24]	806.082	233.126	227.786	228.122	227.450	A[6]
R[25]	858.116	209.447	204.107	204.443	203.771	R[5]
P[26]	**890.467**	157.413	152.073	152.409	151.738	P[4]
Q[27]	933.153	125.062	119.723	120.058	119.387	Q[3]
P[28]	**965.504**	82.376	77.036	77.372	76.700	P[2]
E[29]	**1008.518**	50.025	44.685	45.021	44.349	E[1]

sp|Q6GSS7|H2A2A_MOUSE

LNKLLGK $^{\text{Dimethyl}}_{28.03}$ VTIAQGGVLPNIQAVLLPKKTE

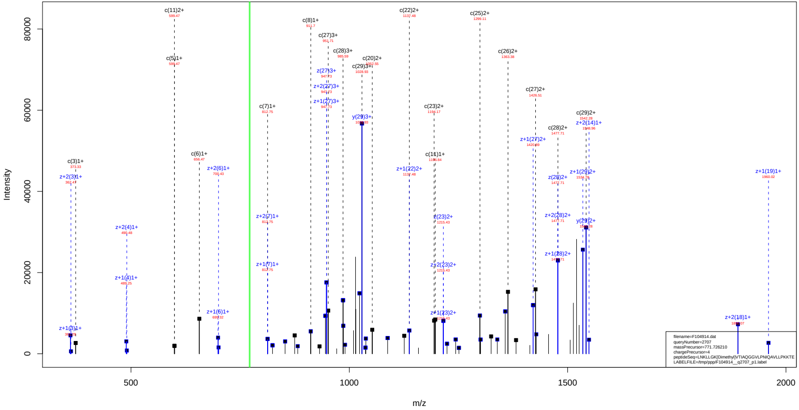

sp|Q6GSS7|H2A2A_MOUSE

LNKLLGK $^{\text{Dimethyl}}_{28.03}$ VTIAQGGVLPNIQAVLLPKKTE

- fragmentation table for charge state 1+
- specType=etd
- score=107.04
- F104914.dat
- query=q2707_p1
- precursor=771.726210
- chargePrecursor=4
- itol=0.8

AA	c	y	z+1	z+2	z	AA
L[1]	131.118	3083.892	3067.873	0.000	3066.865	L[29]
N[2]	245.161	2970.808	2954.789	2955.797	2953.781	N[28]
K[3]	**373.256**	2856.765	2840.746	2841.754	2839.738	K[27]
L[4]	486.340	2728.670	2712.651	2713.659	2711.643	L[26]
L[5]	**599.424**	2615.586	2599.567	2600.575	2598.559	L[25]
G[6]	**656.445**	2502.502	2486.483	2487.491	2485.475	G[24]
K[7]	**812.572**	2445.480	2429.462	2430.469	2428.454	K[23]
V[8]	**911.640**	2289.354	2273.335	2274.343	2272.327	V[22]
T[9]	1012.688	2190.286	2174.267	2175.275	2173.259	T[21]
I[10]	**1125.772**	2089.238	2073.219	2074.227	2072.211	I[20]
A[11]	**1196.809**	1976.154	**1960.135**	1961.143	1959.127	A[19]
Q[12]	**1324.867**	1905.117	1889.098	**1890.106**	1888.090	Q[18]
G[13]	**1381.889**	1777.058	1761.039	1762.047	1760.032	G[17]
G[14]	1438.910	1720.037	1704.018	1705.026	1703.010	G[16]
V[15]	1537.979	1663.015	1646.997	1648.004	1645.989	V[15]
L[16]	1651.063	1563.947	1547.928	**1548.936**	1546.920	L[14]
P[17]	1748.116	1450.863	1434.844	1435.852	1433.836	P[13]
N[18]	1862.159	1353.810	1337.791	**1338.799**	1336.783	N[12]
I[19]	1975.243	1239.767	**1223.748**	1224.756	1222.741	I[11]
Q[20]	2103.301	1126.683	1110.664	1111.672	1109.656	Q[10]
A[21]	2174.338	998.624	982.606	983.614	981.598	A[9]
V[22]	2273.407	927.587	**911.569**	912.576	910.561	V[8]
L[23]	2386.491	828.519	**812.500**	**813.508**	811.492	L[7]
L[24]	2499.575	715.435	**699.416**	**700.424**	698.408	L[6]
P[25]	2596.628	602.351	586.332	587.340	585.324	P[5]
K[26]	2724.723	505.298	**489.279**	**490.287**	488.271	K[4]
K[27]	2852.818	377.203	**361.184**	**362.192**	360.177	K[3]
T[28]	2953.865	249.108	233.089	234.097	232.082	T[2]
E[29]	3082.908	148.060	132.042	133.050	131.034	E[1]

sp|Q6GSS7|H2A2A_MOUSE

LNKLLGK $^{\text{Dimethyl}}_{28.03}$ VTIAQGGVLPNIQAVLLPKKTE

- fragmentation table for charge state 2+
- specType=etd
- score=107.04
- F104914.dat
- query=q2707_p1
- precursor=771.726210
- chargePrecursor=4
- itol=0.8

AA	c	y	z+1	z+2	z	AA
L[1]	66.063	**1542.450**	**1534.440**	0.504	1533.936	L[29]
N[2]	123.084	1485.908	**1477.898**	**1478.402**	**1477.394**	N[28]
K[3]	187.132	**1428.886**	**1420.877**	**1421.381**	**1420.373**	K[27]
L[4]	243.674	1364.839	**1356.829**	**1357.333**	**1356.325**	L[26]
L[5]	300.216	1308.297	**1300.287**	**1300.791**	**1299.783**	L[25]
G[6]	328.726	**1251.755**	**1243.745**	**1244.249**	**1243.241**	G[24]
K[7]	406.789	**1223.244**	**1215.234**	**1215.738**	**1214.731**	K[23]
V[8]	456.324	1145.181	**1137.171**	**1137.675**	1136.667	V[22]
T[9]	506.848	1095.646	**1087.637**	**1088.141**	**1087.133**	T[21]
I[10]	563.390	1045.123	**1037.113**	**1037.617**	**1036.609**	I[20]
A[11]	**598.908**	988.581	980.571	981.075	980.067	A[19]
Q[12]	662.937	953.062	**945.053**	**945.557**	**944.549**	Q[18]
G[13]	691.448	889.033	881.023	**881.527**	880.519	G[17]
G[14]	719.959	860.522	**852.513**	**853.017**	852.009	G[16]
V[15]	769.493	832.011	**824.002**	**824.506**	**823.498**	V[15]
L[16]	826.035	782.477	774.468	774.972	773.964	L[14]
P[17]	**874.561**	725.935	717.926	718.430	717.422	P[13]
N[18]	**931.583**	677.409	669.399	669.903	668.895	N[12]
I[19]	988.125	620.387	612.378	612.882	611.874	I[11]
Q[20]	**1052.154**	563.845	555.836	556.340	555.332	Q[10]
A[21]	**1087.673**	499.816	491.807	492.310	491.303	A[9]
V[22]	**1137.207**	464.297	456.288	456.792	455.784	V[8]
L[23]	**1193.749**	414.763	406.754	407.258	406.250	L[7]
L[24]	**1250.291**	358.221	350.212	350.716	349.708	L[6]
P[25]	**1298.817**	301.679	293.670	294.174	293.166	P[5]
K[26]	**1362.865**	253.153	245.143	245.647	244.639	K[4]
K[27]	**1426.912**	189.105	181.096	181.600	180.592	K[3]
T[28]	**1477.436**	125.058	117.048	117.552	116.544	T[2]
E[29]	**1541.958**	74.534	66.524	67.028	66.021	E[1]

sp|Q6GSS7|H2A2A_MOUSE

LNKLLGK $^{Dimethyl}_{28.03}$ VTIAQGGVLPNIQAVLLPKKTE

- fragmentation table for charge state 3+
- specType=etd
- score=107.04
- F104914.dat
- query=q2707_p1
- precursor=771.726210
- chargePrecursor=4
- itol=0.8

AA	c	y	z+1	z+2	z	AA
L[1]	44.377	**1028.635**	**1023.296**	0.672	**1022.960**	L[29]
N[2]	82.392	**990.941**	**985.601**	**985.937**	**985.265**	N[28]
K[3]	125.090	952.926	**947.587**	**947.923**	**947.251**	K[27]
L[4]	162.785	910.228	904.889	905.225	904.553	L[26]
L[5]	200.479	872.533	867.194	867.530	866.858	L[25]
G[6]	219.487	834.839	829.499	829.835	829.163	G[24]
K[7]	271.529	815.832	810.492	810.828	810.156	K[23]
V[8]	304.552	763.790	758.450	758.786	758.114	V[22]
T[9]	338.234	730.767	725.427	725.763	725.091	T[21]
I[10]	375.929	697.084	691.745	692.081	691.409	I[20]
A[11]	399.608	659.389	654.050	654.386	653.714	A[19]
Q[12]	442.294	635.710	630.371	630.707	630.035	Q[18]
G[13]	461.301	593.024	587.685	588.021	587.349	G[17]
G[14]	480.308	574.017	568.678	569.013	568.342	G[16]
V[15]	513.331	555.010	549.670	550.006	549.334	V[15]
L[16]	551.026	521.987	516.648	516.984	516.312	L[14]
P[17]	583.377	484.292	478.953	479.289	478.617	P[13]
N[18]	621.391	451.942	446.602	446.938	446.266	N[12]
I[19]	659.086	413.927	408.588	408.924	408.252	I[11]
Q[20]	701.772	376.233	370.893	371.229	370.557	Q[10]
A[21]	725.451	333.546	328.207	328.543	327.871	A[9]
V[22]	758.474	309.867	304.528	304.864	304.192	V[8]
L[23]	796.168	276.844	271.505	271.841	271.169	L[7]
L[24]	833.863	239.150	233.810	234.146	233.474	L[6]
P[25]	866.214	201.455	196.116	196.451	195.780	P[5]
K[26]	908.912	169.104	163.765	164.101	163.429	K[4]
K[27]	**951.611**	126.406	121.066	121.402	120.730	K[3]
T[28]	**985.293**	83.708	78.368	78.704	78.032	T[2]
E[29]	**1028.307**	50.025	44.685	45.021	44.349	E[1]

tr|Q80ZM5|Q80ZM5_MOUSE

GGAERRGASAAS $^{Phospho}_{79.97}$ SPAPKARTAAADRTPARPQPE

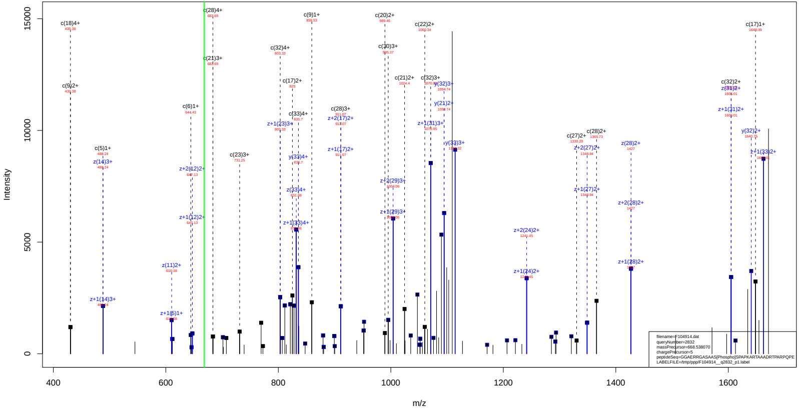

tr|Q80ZM5|Q80ZM5_MOUSE

GGAERRGASAAS $^{Phospho}_{79.97}$ SPAPKARTAAADRTPARPQPE

- fragmentation table for charge state 1+
- specType=etd
- score=73.57
- F104914.dat
- query=q2832_p1
- precursor=668.538070
- chargePrecursor=5
- itol=0.8

AA	c	y	z+1	z+2	z	AA
G[1]	75.055	3338.646	3322.627	0.000	3321.620	G[33]
G[2]	132.077	3281.625	3265.606	0.000	3264.598	G[32]
A[3]	203.114	3224.603	3208.585	0.000	3207.577	A[31]
E[4]	332.156	3153.566	3137.547	0.000	3136.540	E[30]
R[5]	**488.258**	3024.524	3008.505	3009.513	3007.497	R[29]
R[6]	**644.359**	2868.422	2852.404	2853.412	2851.396	R[28]
G[7]	**701.380**	2712.321	2696.303	2697.310	2695.295	G[27]
A[8]	**772.417**	2655.300	2639.281	2640.289	2638.273	A[26]
S[9]	**859.449**	2584.263	2568.244	2569.252	2567.236	S[25]
A[10]	930.486	2497.231	2481.212	2482.220	2480.204	A[24]
A[11]	1001.524	2426.194	2410.175	2411.183	2409.167	A[23]
S[12]	1168.522	2355.157	2339.138	2340.146	2338.130	S[22]
S[13]	1255.554	2188.158	2172.139	2173.147	2171.132	S[21]
P[14]	1352.607	2101.126	2085.107	2086.115	2084.100	P[20]
A[15]	1423.644	2004.073	1988.055	1989.062	1987.047	A[19]
P[16]	1520.697	1933.036	1917.018	1918.025	1916.010	P[18]
K[17]	**1648.792**	1835.983	1819.965	1820.973	1818.957	K[17]
A[18]	1719.829	1707.889	1691.870	1692.878	1690.862	A[16]
R[19]	1875.930	1636.851	1620.833	1621.840	1619.825	R[15]
T[20]	1976.977	1480.750	1464.732	1465.739	1463.724	T[14]
A[21]	2048.015	1379.703	1363.684	1364.692	1362.676	A[13]
A[22]	2119.052	1308.665	**1292.647**	**1293.655**	1291.639	A[12]
A[23]	2190.089	1237.628	**1221.610**	1222.617	1220.602	A[11]
D[24]	2305.116	1166.591	1150.573	1151.580	1149.565	D[10]
R[25]	2461.217	**1051.564**	**1035.546**	1036.553	1034.538	R[9]
T[26]	2562.264	895.463	**879.444**	**880.452**	878.437	T[8]
P[27]	2659.317	794.416	778.397	779.405	777.389	P[7]
A[28]	2730.354	697.363	681.344	682.352	680.336	A[6]
R[29]	2886.455	626.326	**610.307**	**611.315**	609.299	R[5]
P[30]	2983.508	470.225	454.206	455.214	453.198	P[4]
Q[31]	3111.567	373.172	357.153	358.161	356.145	Q[3]
P[32]	3208.620	245.113	229.094	230.102	228.087	P[2]
E[33]	3337.662	148.060	132.042	133.050	131.034	E[1]

tr|Q80ZM5|Q80ZM5_MOUSE

GGAERRGASAAS $^{Phospho}_{79.97}$ SPAPKARTAAADRTPARPQPE

- fragmentation table for charge state 2+
- specType=etd
- score=73.57
- F104914.dat
- query=q2832_p1
- precursor=668.538070
- chargePrecursor=5
- itol=0.8

AA	c	y	z+1	z+2	z	AA
G[1]	38.031	1669.827	**1661.817**	0.504	1661.313	G[33]
G[2]	66.542	**1641.316**	1633.307	0.504	1632.803	G[32]
A[3]	102.061	**1612.805**	**1604.796**	0.504	**1604.292**	A[31]
E[4]	166.582	1577.287	1569.277	0.504	1568.773	E[30]
R[5]	244.632	1512.765	1504.756	1505.260	1504.252	R[29]
R[6]	322.683	1434.715	**1426.705**	**1427.209**	**1426.202**	R[28]
G[7]	351.194	1356.664	**1348.655**	**1349.159**	**1348.151**	G[27]
A[8]	386.712	1328.154	1320.144	**1320.648**	1319.640	A[26]
S[9]	**430.228**	**1292.635**	1284.626	**1285.130**	1284.122	S[25]
A[10]	465.747	1249.119	**1241.110**	**1241.614**	1240.606	A[24]
A[11]	501.265	1213.600	1205.591	**1206.095**	1205.087	A[23]
S[12]	584.765	1178.082	1170.073	**1170.576**	1169.569	S[22]
S[13]	628.281	**1094.583**	1086.573	1087.077	1086.069	S[21]
P[14]	676.807	**1051.067**	1043.057	1043.561	1042.553	P[20]
A[15]	712.326	1002.540	994.531	**995.035**	994.027	A[19]
P[16]	760.852	967.022	959.012	959.516	958.508	P[18]
K[17]	**824.899**	918.495	**910.486**	**910.990**	909.982	K[17]
A[18]	860.418	854.448	846.439	**846.942**	845.935	A[16]
R[19]	938.469	818.929	**810.920**	**811.424**	810.416	R[15]
T[20]	**988.992**	740.879	732.869	733.373	732.366	T[14]
A[21]	**1024.511**	690.355	682.346	682.849	681.842	A[13]
A[22]	**1060.029**	654.836	**646.827**	**647.331**	**646.323**	A[12]
A[23]	1095.548	619.318	**611.308**	**611.812**	**610.805**	A[11]
D[24]	1153.061	583.799	575.790	576.294	575.286	D[10]
R[25]	1231.112	526.286	518.276	518.780	517.773	R[9]
T[26]	1281.636	448.235	440.226	440.730	439.722	T[8]
P[27]	**1330.162**	397.711	389.702	390.206	389.198	P[7]
A[28]	**1365.681**	349.185	341.176	341.680	340.672	A[6]
R[29]	1443.731	313.666	305.657	306.161	305.153	R[5]
P[30]	1492.258	235.616	227.607	228.110	227.103	P[4]
Q[31]	1556.287	187.090	179.080	179.584	178.576	Q[3]
P[32]	**1604.813**	123.060	115.051	115.555	114.547	P[2]
E[33]	1669.335	74.534	66.524	67.028	66.021	E[1]

tr|Q80ZM5|Q80ZM5_MOUSE

GGAERRGASAAS $^{Phospho}_{79.97}$ SPAPKARTAAADRTPARPQPE

- fragmentation table for charge state 3+
- specType=etd
- score=73.57
- F104914.dat
- query=q2832_p1
- precursor=668.538070
- chargePrecursor=5
- itol=0.8

AA	c	y	z+1	z+2	z	AA
G[1]	25.690	**1113.554**	1108.214	0.672	1107.878	G[33]
G[2]	44.697	**1094.546**	**1089.207**	0.672	1088.871	G[32]
A[3]	68.376	**1075.539**	**1070.200**	0.672	1069.864	A[31]
E[4]	111.390	**1051.860**	**1046.521**	0.672	1046.185	E[30]
R[5]	163.424	1008.846	**1003.506**	**1003.842**	1003.171	R[29]
R[6]	215.458	956.812	**951.473**	**951.809**	**951.137**	R[28]
G[7]	234.465	904.779	**899.439**	**899.775**	**899.103**	G[27]
A[8]	258.144	885.771	**880.432**	**880.768**	**880.096**	A[26]
S[9]	287.155	862.092	856.753	857.089	856.417	S[25]
A[10]	310.834	833.082	**827.742**	**828.078**	**827.406**	A[24]
A[11]	334.513	809.403	**804.063**	804.399	**803.727**	A[23]
S[12]	390.179	785.724	780.384	780.720	780.048	S[22]
S[13]	419.189	730.058	724.718	725.054	724.382	S[21]
P[14]	451.540	**701.047**	695.707	696.043	695.371	P[20]
A[15]	475.219	668.696	663.356	663.692	663.020	A[19]
P[16]	507.570	**645.017**	639.677	640.013	639.341	P[18]
K[17]	550.269	612.666	607.326	607.662	606.990	K[17]
A[18]	573.948	569.968	564.628	564.964	564.292	A[16]
R[19]	625.981	546.289	540.949	541.285	540.613	R[15]
T[20]	659.664	494.255	**488.915**	489.251	**488.579**	T[14]
A[21]	**683.343**	460.572	455.233	455.569	454.897	A[13]
A[22]	**707.022**	436.893	431.554	431.890	431.218	A[12]
A[23]	**730.701**	413.214	407.875	408.211	407.539	A[11]
D[24]	**769.043**	389.535	384.196	384.532	383.860	D[10]
R[25]	**821.077**	351.193	345.853	346.189	345.517	R[9]
T[26]	854.760	299.159	293.820	294.156	293.484	T[8]
P[27]	887.111	265.477	260.137	260.473	259.801	P[7]
A[28]	**910.790**	233.126	227.786	228.122	227.450	A[6]
R[29]	962.823	209.447	204.107	204.443	203.771	R[5]
P[30]	**995.174**	157.413	152.073	152.409	151.738	P[4]
Q[31]	1037.860	125.062	119.723	120.058	119.387	Q[3]
P[32]	**1070.211**	82.376	77.036	77.372	76.700	P[2]
E[33]	1113.226	50.025	44.685	45.021	44.349	E[1]

tr|Q80ZM5|Q80ZM5_MOUSE

GGAERRGASAAS $^{\text{Phospho}}_{79.97}$ SPAPKARTAAADRTPARPQPE

- fragmentation table for charge state 4+
- specType=etd
- score=73.57
- F104914.dat
- query=q2832_p1
- precursor=668.538070
- chargePrecursor=5
- itol=0.8

AA	c	y	z+1	z+2	z	AA
G[1]	19.519	**835.417**	**831.412**	0.755	**831.160**	G[33]
G[2]	33.775	**821.162**	817.157	0.755	816.905	G[32]
A[3]	51.534	**806.906**	**802.902**	0.755	**802.650**	A[31]
E[4]	83.795	789.147	785.142	0.755	784.890	E[30]
R[5]	122.820	756.886	752.882	753.134	752.630	R[29]
R[6]	161.845	717.861	713.856	714.108	713.604	R[28]
G[7]	176.100	678.836	674.831	675.083	674.579	G[27]
A[8]	193.860	664.580	660.576	660.828	660.324	A[26]
S[9]	215.618	**646.821**	642.816	643.068	642.565	S[25]
A[10]	233.377	625.063	621.058	621.310	620.807	A[24]
A[11]	251.136	607.304	603.299	603.551	603.047	A[23]
S[12]	292.886	589.545	585.540	585.792	585.288	S[22]
S[13]	314.644	547.795	543.790	544.042	543.538	S[21]
P[14]	338.907	526.037	522.032	522.284	521.780	P[20]
A[15]	356.666	501.774	497.769	498.021	497.517	A[19]
P[16]	380.930	484.015	480.010	480.262	479.758	P[18]
K[17]	412.953	459.751	455.747	455.999	455.495	K[17]
A[18]	**430.713**	427.728	423.723	423.975	423.471	A[16]
R[19]	469.738	409.968	405.964	406.216	405.712	R[15]
T[20]	495.000	370.943	366.938	367.190	366.686	T[14]
A[21]	512.759	345.681	341.676	341.928	341.424	A[13]
A[22]	530.518	327.922	323.917	324.169	323.665	A[12]
A[23]	548.278	310.163	306.158	306.410	305.906	A[11]
D[24]	577.034	292.403	288.399	288.651	288.147	D[10]
R[25]	616.060	263.647	259.642	259.894	259.390	R[9]
T[26]	641.322	224.621	220.617	220.869	220.365	T[8]
P[27]	665.585	199.359	195.355	195.607	195.103	P[7]
A[28]	**683.344**	175.096	171.091	171.343	170.840	A[6]
R[29]	722.369	157.337	153.332	153.584	153.080	R[5]
P[30]	746.633	118.312	114.307	114.559	114.055	P[4]
Q[31]	778.647	94.048	90.044	90.296	89.792	Q[3]
P[32]	**802.910**	62.034	58.029	58.281	57.777	P[2]
E[33]	**835.171**	37.771	33.766	34.018	33.514	E[1]

sp|P84244|H33_MOUSE

LRFQS $^{Phospho}_{79.97}$ AAIGALQE

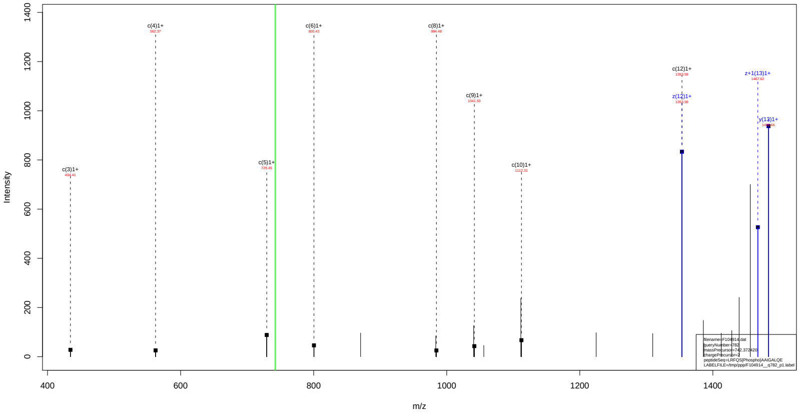

sp|P84244|H33_MOUSE

LRFQS $^{Phospho}_{79.97}$ AAIGALQE

- fragmentation table for charge state 1+
- specType=etd
- score=40.30
- F104914.dat
- query=q782_p1
- precursor=742.372420
- chargePrecursor=2
- itol=0.8

AA	c	y	z+1	z+2	z	AA
L[1]	131.118	**1483.730**	**1467.712**	0.000	1466.704	L[13]
R[2]	287.219	1370.646	1354.628	1355.636	**1353.620**	R[12]
F[3]	**434.287**	1214.545	1198.527	1199.534	1197.519	F[11]
Q[4]	**562.346**	1067.477	1051.458	1052.466	1050.450	Q[10]
S[5]	**729.344**	939.418	923.400	924.407	922.392	S[9]
A[6]	**800.381**	772.420	756.401	757.409	755.393	A[8]
A[7]	871.419	701.383	685.364	686.372	684.356	A[7]
I[8]	**984.503**	630.346	614.327	615.335	613.319	I[6]
G[9]	**1041.524**	517.262	501.243	502.251	500.235	G[5]
A[10]	**1112.561**	460.240	444.221	445.229	443.214	A[4]
L[11]	1225.645	389.203	373.184	374.192	372.177	L[3]
Q[12]	**1353.704**	276.119	260.100	261.108	259.092	Q[2]
E[13]	1482.746	148.060	132.042	133.050	131.034	E[1]

sp|P43277|H13_MOUSE

KNNSRIKLGLKSLVSKGTLVQTK Dimethyl 28.03 GTGASGSFKLNKKAASGE

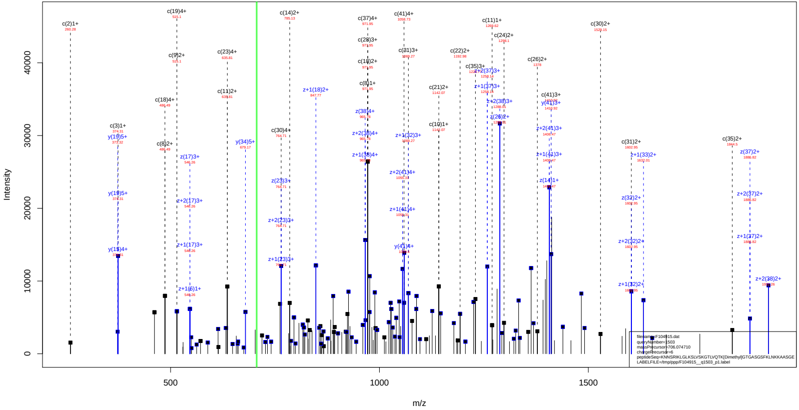

sp|P43277|H13_MOUSE

KNNSRIKLGLKSLVSKGTLVQTK $^{\text{Dimethyl}}_{28.03}$ GTGASGSFKLNKKAASGE

- fragmentation table for charge state 1+
- specType=etd
- score=71.65
- F104915.dat
- query=q1503_p1
- precursor=706.074710
- chargePrecursor=6
- itol=0.8

AA	c	y	z+1	z+2	z	AA
K[1]	146.129	4231.425	4215.406	4216.414	4214.398	K[41]
N[2]	**260.172**	4103.330	4087.311	4088.319	4086.303	N[40]
N[3]	**374.215**	3989.287	3973.268	3974.276	3972.260	N[39]
S[4]	**461.247**	3875.244	3859.225	3860.233	3858.217	S[38]
R[5]	617.348	3788.212	3772.193	3773.201	3771.185	R[37]
I[6]	730.432	3632.111	3616.092	3617.100	3615.084	I[36]
K[7]	**858.527**	3519.027	3503.008	3504.016	3502.000	K[35]
L[8]	**971.611**	3390.932	3374.913	3375.921	3373.905	L[34]
G[9]	**1028.632**	3277.848	3261.829	3262.837	3260.821	G[33]
L[10]	**1141.716**	3220.826	3204.808	3205.815	3203.800	L[32]
K[11]	**1269.811**	3107.742	3091.724	3092.731	3090.716	K[31]
S[12]	**1356.843**	2979.647	2963.629	2964.636	2962.621	S[30]
L[13]	1469.927	2892.615	2876.597	2877.604	2875.589	L[29]
V[14]	1568.996	2779.531	2763.513	2764.520	2762.505	V[28]
S[15]	1656.028	2680.463	2664.444	2665.452	2663.436	S[27]
K[16]	1784.123	2593.431	2577.412	2578.420	2576.404	K[26]
G[17]	1841.144	2465.336	2449.317	2450.325	2448.309	G[25]
T[18]	1942.192	2408.314	2392.296	2393.303	2391.288	T[24]
L[19]	2055.276	2307.267	2291.248	2292.256	2290.240	L[23]
V[20]	2154.344	2194.183	2178.164	2179.172	2177.156	V[22]
Q[21]	2282.403	2095.114	2079.095	2080.103	2078.088	Q[21]
T[22]	2383.451	1967.056	1951.037	1952.045	1950.029	T[20]
K[23]	2539.577	1866.008	1849.989	1850.997	1848.981	K[19]
G[24]	2596.598	1709.882	1693.863	1694.871	1692.855	G[18]
T[25]	2697.646	**1652.860**	1636.842	1637.849	1635.834	T[17]
G[26]	2754.668	1551.813	1535.794	1536.802	1534.786	G[16]
A[27]	2825.705	1494.791	1478.772	1479.780	1477.765	A[15]
S[28]	2912.737	1423.754	1407.735	1408.743	**1406.727**	S[14]
G[29]	2969.758	**1336.722**	1320.703	**1321.711**	1319.695	G[13]
S[30]	3056.790	1279.700	1263.682	1264.690	1262.674	S[12]
F[31]	3203.859	**1192.668**	1176.650	**1177.658**	1175.642	F[11]
K[32]	3331.954	1045.600	1029.581	1030.589	**1028.573**	K[10]
L[33]	3445.038	917.505	901.486	902.494	**900.479**	L[9]
N[34]	3559.081	804.421	**788.402**	789.410	787.394	N[8]
K[35]	3687.176	690.378	**674.359**	**675.367**	673.352	K[7]
K[36]	3815.271	**562.283**	**546.264**	547.272	545.257	K[6]
A[37]	3886.308	434.188	418.169	419.177	417.162	A[5]
A[38]	3957.345	363.151	347.132	348.140	346.124	A[4]
S[39]	4044.377	292.114	276.095	277.103	275.087	S[3]
G[40]	4101.398	205.082	189.063	190.071	188.055	G[2]
E[41]	4230.441	148.060	132.042	133.050	131.034	E[1]

sp|P43277|H13_MOUSE

KNNSRIKLGLKSLVSKGTLVQTK$^{\text{Dimethyl}}_{28.03}$GTGASGSFKLNKKAASGE

- fragmentation table for charge state 2+
- specType=etd
- score=71.65
- F104915.dat
- query=q1503_p1
- precursor=706.074710
- chargePrecursor=6
- itol=0.8

AA	c	y	z+1	z+2	z	AA
K[1]	73.568	2116.216	2108.207	2108.711	2107.703	K[41]
N[2]	130.589	2052.169	2044.159	2044.663	2043.655	N[40]
N[3]	187.611	1995.147	1987.138	1987.642	1986.634	N[39]
S[4]	231.127	1938.126	1930.116	**1930.620**	1929.612	S[38]
R[5]	309.178	1894.610	**1886.600**	**1887.104**	**1886.096**	R[37]
I[6]	365.720	1816.559	1808.550	1809.054	1808.046	I[36]
K[7]	429.767	1760.017	1752.008	1752.512	1751.504	K[35]
L[8]	**486.309**	1695.970	1687.960	1688.464	1687.456	L[34]
G[9]	**514.820**	1639.428	**1631.418**	**1631.922**	1630.914	G[33]
L[10]	**571.362**	1610.917	**1602.907**	**1603.411**	**1602.404**	L[32]
K[11]	**635.409**	1554.375	1546.365	1546.869	1545.862	K[31]
S[12]	**678.925**	**1490.327**	1482.318	**1482.822**	1481.814	S[30]
L[13]	735.467	1446.811	**1438.802**	**1439.306**	**1438.298**	L[29]
V[14]	**785.002**	1390.269	1382.260	1382.764	1381.756	V[28]
S[15]	**828.518**	1340.735	**1332.726**	**1333.230**	1332.222	S[27]
K[16]	**892.565**	1297.219	1289.210	1289.714	**1288.706**	K[26]
G[17]	**921.076**	1233.172	**1225.162**	**1225.666**	**1224.658**	G[25]
T[18]	**971.600**	1204.661	1196.651	1197.155	1196.148	T[24]
L[19]	**1028.142**	1154.137	**1146.128**	**1146.632**	1145.624	L[23]
V[20]	**1077.676**	**1097.595**	1089.586	1090.090	**1089.082**	V[22]
Q[21]	**1141.705**	**1048.061**	**1040.051**	**1040.555**	**1039.547**	Q[21]
T[22]	**1192.229**	984.031	**976.022**	**976.526**	**975.518**	T[20]
K[23]	**1270.292**	**933.508**	**925.498**	**926.002**	924.994	K[19]
G[24]	**1298.803**	**855.444**	**847.435**	**847.939**	846.931	G[18]
T[25]	1349.327	826.934	**818.924**	**819.428**	**818.420**	T[17]
G[26]	**1377.837**	776.410	768.401	768.904	767.897	G[16]
A[27]	1413.356	747.899	**739.890**	**740.394**	739.386	A[15]
S[28]	1456.872	712.381	704.371	704.875	703.867	S[14]
G[29]	1485.383	668.865	**660.855**	**661.359**	**660.351**	G[13]
S[30]	**1528.899**	640.354	**632.345**	**632.848**	**631.841**	S[12]
F[31]	**1602.433**	596.838	**588.829**	**589.332**	**588.325**	F[11]
K[32]	1666.480	523.304	**515.294**	**515.798**	**514.790**	K[10]
L[33]	1723.022	459.256	451.247	451.751	450.743	L[9]
N[34]	1780.044	402.714	394.705	395.209	394.201	N[8]
K[35]	**1844.091**	345.693	337.683	338.187	337.179	K[7]
K[36]	1908.139	281.645	273.636	274.140	273.132	K[6]
A[37]	1943.657	217.598	209.588	210.092	209.084	A[5]
A[38]	1979.176	182.079	174.070	174.574	173.566	A[4]
S[39]	2022.692	146.561	138.551	139.055	138.047	S[3]
G[40]	2051.203	103.045	95.035	95.539	94.531	G[2]
E[41]	2115.724	74.534	66.524	67.028	66.021	E[1]

sp|P43277|H13_MOUSE

KNNSRIKLGLKSLVSKGTLVQTK $^{\text{Dimethyl}}_{28.03}$ GTGASGSFKLNKKAASGE

- fragmentation table for charge state 3+
- specType=etd
- score=71.65
- F104915.dat
- query=q1503_p1
- precursor=706.074710
- chargePrecursor=6
- itol=0.8

AA	c	y	z+1	z+2	z	AA
K[1]	49.381	**1411.146**	**1405.807**	**1406.143**	1405.471	K[41]
N[2]	87.395	**1368.448**	**1363.109**	**1363.445**	**1362.773**	N[40]
N[3]	125.410	1330.434	1325.094	**1325.430**	1324.758	N[39]
S[4]	154.420	**1292.420**	1287.080	**1287.416**	1286.744	S[38]
R[5]	206.454	1263.409	**1258.069**	**1258.405**	**1257.733**	R[37]
I[6]	244.149	1211.375	**1206.036**	**1206.372**	**1205.700**	I[36]
K[7]	286.847	1173.680	1168.341	1168.677	1168.005	K[35]
L[8]	324.542	1130.982	**1125.643**	**1125.979**	1125.307	L[34]
G[9]	343.549	1093.287	**1087.948**	**1088.284**	**1087.612**	G[33]
L[10]	381.244	1074.280	**1068.941**	**1069.277**	**1068.605**	L[32]
K[11]	423.942	**1036.586**	**1031.246**	**1031.582**	1030.910	K[31]
S[12]	452.953	**993.887**	**988.548**	**988.884**	**988.212**	S[30]
L[13]	490.647	964.877	**959.537**	**959.873**	959.201	L[29]
V[14]	523.670	927.182	**921.842**	**922.178**	**921.506**	V[28]
S[15]	552.681	894.159	**888.820**	**889.155**	**888.484**	S[27]
K[16]	595.379	**865.148**	**859.809**	**860.145**	859.473	K[26]
G[17]	**614.386**	822.450	**817.111**	817.446	**816.775**	G[25]
T[18]	**648.069**	803.443	798.103	**798.439**	797.767	T[24]
L[19]	685.764	769.760	**764.421**	**764.757**	**764.085**	L[23]
V[20]	**718.786**	**732.066**	**726.726**	**727.062**	**726.390**	V[22]
Q[21]	**761.473**	699.043	693.703	694.039	693.367	Q[21]
T[22]	**795.155**	656.357	651.017	651.353	650.681	T[20]
K[23]	**847.197**	622.674	617.335	617.671	616.999	K[19]
G[24]	**866.204**	570.632	565.293	565.628	564.957	G[18]
T[25]	**899.887**	551.625	**546.285**	**546.621**	**545.949**	T[17]
G[26]	**918.894**	517.942	512.603	512.939	512.267	G[16]
A[27]	942.573	498.935	493.596	493.932	493.260	A[15]
S[28]	**971.584**	475.256	469.917	470.253	469.581	S[14]
G[29]	**990.591**	446.245	440.906	441.242	440.570	G[13]
S[30]	1019.602	427.238	421.899	422.235	421.563	S[12]
F[31]	**1068.624**	398.228	392.888	393.224	392.552	F[11]
K[32]	**1111.323**	349.205	343.865	344.201	343.529	K[10]
L[33]	1149.017	306.507	301.167	301.503	300.831	L[9]
N[34]	**1187.032**	268.812	263.472	263.808	263.136	N[8]
K[35]	**1229.730**	230.798	225.458	225.794	225.122	K[7]
K[36]	1272.428	188.099	182.760	183.096	182.424	K[6]
A[37]	1296.107	145.401	140.061	140.397	139.725	A[5]
A[38]	1319.786	121.722	116.382	116.718	116.046	A[4]
S[39]	1348.797	98.043	92.703	93.039	92.367	S[3]
G[40]	1367.804	69.032	63.693	64.029	63.357	G[2]
E[41]	**1410.818**	50.025	44.685	45.021	44.349	E[1]

sp|P43277|H13_MOUSE

KNNSRIKLGLKSLVSKGTLVQTK $^{Dimethyl}_{28.03}$ GTGASGSFKLNKKAASGE

- fragmentation table for charge state 4+
- specType=etd
- score=71.65
- F104915.dat
- query=q1503_p1
- precursor=706.074710
- chargePrecursor=6
- itol=0.8

AA	c	y	z+1	z+2	z	AA
K[1]	37.288	**1058.612**	**1054.607**	**1054.859**	1054.355	K[41]
N[2]	65.798	**1026.588**	**1022.583**	**1022.835**	**1022.331**	N[40]
N[3]	94.309	998.077	**994.073**	**994.324**	**993.821**	N[39]
S[4]	116.067	969.566	**965.562**	**965.814**	**965.310**	S[38]
R[5]	155.092	947.808	**943.804**	**944.056**	**943.552**	R[37]
I[6]	183.363	908.783	904.779	905.030	904.527	I[36]
K[7]	215.387	880.512	**876.507**	**876.759**	**876.256**	K[35]
L[8]	243.658	**848.488**	844.484	844.736	844.232	L[34]
G[9]	257.914	820.217	**816.213**	**816.465**	**815.961**	G[33]
L[10]	286.185	805.962	801.957	802.209	801.705	L[32]
K[11]	318.208	777.691	773.686	773.938	773.434	K[31]
S[12]	339.966	745.667	741.663	741.915	**741.411**	S[30]
L[13]	368.237	723.909	719.905	720.157	719.653	L[29]
V[14]	393.004	695.638	691.634	691.886	691.382	V[28]
S[15]	414.762	670.871	666.866	667.118	666.615	S[27]
K[16]	446.786	**649.113**	645.108	645.360	644.857	K[26]
G[17]	**461.042**	617.089	**613.085**	**613.337**	612.833	G[25]
T[18]	**486.303**	602.834	598.829	599.081	598.577	T[24]
L[19]	**514.574**	577.572	573.567	573.819	573.315	L[23]
V[20]	539.342	**549.301**	545.296	**545.548**	545.044	V[22]
Q[21]	**571.356**	524.534	520.529	520.781	520.277	Q[21]
T[22]	596.618	492.519	488.515	488.767	488.263	T[20]
K[23]	**635.650**	467.257	463.253	463.505	463.001	K[19]
G[24]	649.905	428.226	424.221	424.473	423.969	G[18]
T[25]	**675.167**	413.971	409.966	410.218	409.714	T[17]
G[26]	689.422	388.709	384.704	384.956	384.452	G[16]
A[27]	707.182	**374.453**	370.449	370.701	370.197	A[15]
S[28]	728.940	356.694	352.689	352.941	352.437	S[14]
G[29]	743.195	334.936	330.931	331.183	330.679	G[13]
S[30]	**764.953**	320.681	316.676	316.928	316.424	S[12]
F[31]	801.720	298.923	294.918	295.170	294.666	F[11]
K[32]	**833.744**	262.155	258.151	258.403	257.899	K[10]
L[33]	**862.015**	230.132	226.127	226.379	225.875	L[9]
N[34]	**890.526**	201.861	197.856	198.108	197.604	N[8]
K[35]	**922.549**	173.350	169.345	169.597	169.093	K[7]
K[36]	954.573	141.326	137.322	137.574	137.070	K[6]
A[37]	**972.332**	109.302	105.298	105.550	105.046	A[5]
A[38]	**990.092**	91.543	87.539	87.790	87.287	A[4]
S[39]	**1011.850**	73.784	69.779	70.031	69.527	S[3]
G[40]	**1026.105**	52.026	48.021	48.273	47.769	G[2]
E[41]	**1058.366**	37.771	33.766	34.018	33.514	E[1]

sp|P43277|H13_MOUSE

KNNSRIKLGLKSLVSKGTLVQTK $^{\text{Dimethyl}}_{28.03}$ GTGASGSFKLNKKAASGE

- fragmentation table for charge state 5+
- specType=etd
- score=71.65
- F104915.dat
- query=q1503_p1
- precursor=706.074710
- chargePrecursor=6
- itol=0.8

AA	c	y	z+1	z+2	z	AA
K[1]	30.032	**847.091**	843.887	844.089	843.685	K[41]
N[2]	52.840	**821.472**	818.268	**818.470**	818.066	N[40]
N[3]	75.649	**798.663**	**795.459**	**795.661**	**795.258**	N[39]
S[4]	93.055	775.855	772.651	772.852	772.449	S[38]
R[5]	124.275	758.448	755.244	755.446	755.043	R[37]
I[6]	146.892	**727.228**	724.024	724.226	723.823	I[36]
K[7]	172.511	704.611	701.407	701.609	701.206	K[35]
L[8]	195.128	**678.992**	**675.788**	675.990	**675.587**	L[34]
G[9]	206.532	656.375	653.172	653.373	652.970	G[33]
L[10]	229.149	644.971	641.767	641.969	641.566	L[32]
K[11]	254.768	622.354	619.151	619.352	618.949	K[31]
S[12]	272.174	596.735	593.532	593.733	593.330	S[30]
L[13]	294.791	579.329	576.125	576.327	575.924	L[29]
V[14]	314.605	556.712	553.508	553.710	553.307	V[28]
S[15]	332.011	536.898	533.695	533.896	533.493	S[27]
K[16]	357.630	519.492	516.288	516.490	516.087	K[26]
G[17]	369.035	493.873	490.669	490.871	490.468	G[25]
T[18]	389.244	482.469	479.265	479.467	479.063	T[24]
L[19]	411.861	462.259	459.055	459.257	458.854	L[23]
V[20]	431.675	439.642	436.439	436.640	436.237	V[22]
Q[21]	457.286	419.829	416.625	416.826	416.423	Q[21]
T[22]	477.496	394.217	391.013	391.215	390.812	T[20]
K[23]	508.721	**374.007**	370.804	371.005	370.602	K[19]
G[24]	520.126	342.782	339.578	339.780	339.377	G[18]
T[25]	540.335	331.378	328.174	328.376	327.973	T[17]
G[26]	551.739	311.168	307.965	308.166	307.763	G[16]
A[27]	565.947	299.764	296.560	296.762	296.359	A[15]
S[28]	583.353	285.557	282.353	282.554	282.151	S[14]
G[29]	594.757	268.150	264.946	265.148	264.745	G[13]
S[30]	612.164	256.746	253.542	253.744	253.341	S[12]
F[31]	641.578	239.340	236.136	236.337	235.934	F[11]
K[32]	667.197	209.926	206.722	206.924	206.521	K[10]
L[33]	689.813	184.307	181.103	181.305	180.902	L[9]
N[34]	712.622	161.690	158.486	158.688	158.285	N[8]
K[35]	738.241	138.881	135.678	135.879	135.476	K[7]
K[36]	763.860	113.262	110.059	110.260	109.857	K[6]
A[37]	778.067	87.643	84.440	84.641	84.238	A[5]
A[38]	792.275	73.436	70.232	70.434	70.031	A[4]
S[39]	809.681	59.229	56.025	56.226	55.823	S[3]
G[40]	**821.085**	41.822	38.618	38.820	38.417	G[2]
E[41]	846.894	30.418	27.214	27.416	27.013	E[1]

sp|P43277|H13_MOUSE

KNNSRIKLGLKSLVSKGTLVQTK $^{\text{Dimethyl}}_{28.03}$ GTGASGSFKLNKKAASGE

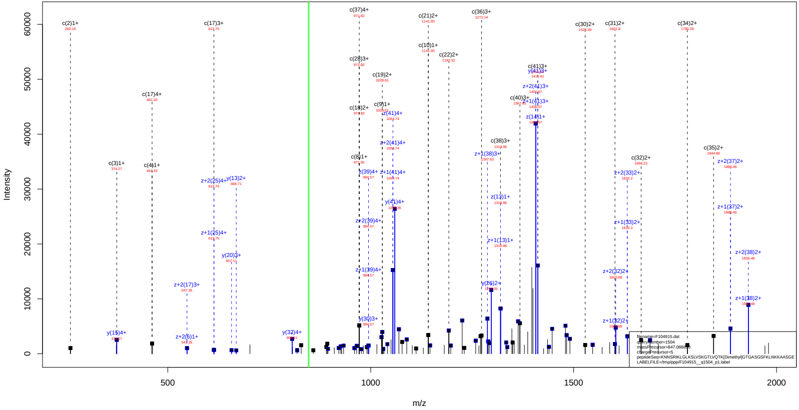

sp|P43277|H13_MOUSE

KNNSRIKLGLKSLVSKGTLVQTK $^{Dimethyl}_{28.03}$ GTGASGSFKLNKKAASGE

- fragmentation table for charge state 1+
- specType=etd
- score=75.55
- F104915.dat
- query=q1504_p1
- precursor=847.088850
- chargePrecursor=5
- itol=0.8

AA	c	y	z+1	z+2	z	AA
K[1]	146.129	4231.425	4215.406	4216.414	4214.398	K[41]
N[2]	**260.172**	4103.330	4087.311	4088.319	4086.303	N[40]
N[3]	**374.215**	3989.287	3973.268	3974.276	3972.260	N[39]
S[4]	**461.247**	3875.244	3859.225	3860.233	3858.217	S[38]
R[5]	617.348	3788.212	3772.193	3773.201	3771.185	R[37]
I[6]	730.432	3632.111	3616.092	3617.100	3615.084	I[36]
K[7]	**858.527**	3519.027	3503.008	3504.016	3502.000	K[35]
L[8]	**971.611**	3390.932	3374.913	3375.921	3373.905	L[34]
G[9]	**1028.632**	3277.848	3261.829	3262.837	3260.821	G[33]
L[10]	**1141.716**	3220.826	3204.808	3205.815	3203.800	L[32]
K[11]	1269.811	3107.742	3091.724	3092.731	3090.716	K[31]
S[12]	1356.843	2979.647	2963.629	2964.636	2962.621	S[30]
L[13]	1469.927	2892.615	2876.597	2877.604	2875.589	L[29]
V[14]	1568.996	2779.531	2763.513	2764.520	2762.505	V[28]
S[15]	1656.028	2680.463	2664.444	2665.452	2663.436	S[27]
K[16]	1784.123	2593.431	2577.412	2578.420	2576.404	K[26]
G[17]	1841.144	2465.336	2449.317	2450.325	2448.309	G[25]
T[18]	1942.192	2408.314	2392.296	2393.303	2391.288	T[24]
L[19]	2055.276	2307.267	2291.248	2292.256	2290.240	L[23]
V[20]	2154.344	2194.183	2178.164	2179.172	2177.156	V[22]
Q[21]	2282.403	2095.114	2079.095	2080.103	2078.088	Q[21]
T[22]	2383.451	1967.056	1951.037	1952.045	1950.029	T[20]
K[23]	2539.577	1866.008	1849.989	1850.997	1848.981	K[19]
G[24]	2596.598	1709.882	1693.863	1694.871	1692.855	G[18]
T[25]	2697.646	1652.860	1636.842	1637.849	1635.834	T[17]
G[26]	2754.668	1551.813	1535.794	1536.802	1534.786	G[16]
A[27]	2825.705	1494.791	1478.772	**1479.780**	1477.765	A[15]
S[28]	2912.737	1423.754	1407.735	1408.743	**1406.727**	S[14]
G[29]	2969.758	**1336.722**	**1320.703**	1321.711	**1319.695**	G[13]
S[30]	3056.790	1279.700	1263.682	1264.690	1262.674	S[12]
F[31]	3203.859	**1192.668**	1176.650	1177.658	1175.642	F[11]
K[32]	3331.954	1045.600	1029.581	**1030.589**	**1028.573**	K[10]
L[33]	3445.038	917.505	901.486	902.494	900.479	L[9]
N[34]	3559.081	804.421	788.402	789.410	787.394	N[8]
K[35]	3687.176	690.378	674.359	675.367	673.352	K[7]
K[36]	3815.271	562.283	546.264	**547.272**	545.257	K[6]
A[37]	3886.308	434.188	418.169	419.177	417.162	A[5]
A[38]	3957.345	363.151	347.132	348.140	346.124	A[4]
S[39]	4044.377	292.114	276.095	277.103	275.087	S[3]
G[40]	4101.398	205.082	189.063	190.071	188.055	G[2]
E[41]	4230.441	148.060	132.042	133.050	131.034	E[1]

sp|P43277|H13_MOUSE

KNNSRIKLGLKSLVSKGTLVQTK $^{\text{Dimethyl}}_{28.03}$ GTGASGSFKLNKKAASGE

- fragmentation table for charge state 2+
- specType=etd
- score=75.55
- F104915.dat
- query=q1504_p1
- precursor=847.088850
- chargePrecursor=5
- itol=0.8

AA	c	y	z+1	z+2	z	AA
K[1]	73.568	2116.216	2108.207	2108.711	2107.703	K[41]
N[2]	130.589	2052.169	2044.159	2044.663	2043.655	N[40]
N[3]	187.611	1995.147	1987.138	1987.642	1986.634	N[39]
S[4]	231.127	1938.126	**1930.116**	**1930.620**	1929.612	S[38]
R[5]	309.178	1894.610	**1886.600**	**1887.104**	**1886.096**	R[37]
I[6]	365.720	1816.559	1808.550	1809.054	1808.046	I[36]
K[7]	429.767	1760.017	1752.008	1752.512	1751.504	K[35]
L[8]	486.309	1695.970	**1687.960**	**1688.464**	**1687.456**	L[34]
G[9]	514.820	1639.428	**1631.418**	**1631.922**	1630.914	G[33]
L[10]	571.362	1610.917	**1602.907**	**1603.411**	**1602.404**	L[32]
K[11]	635.409	1554.375	**1546.365**	**1546.869**	1545.862	K[31]
S[12]	678.925	**1490.327**	**1482.318**	**1482.822**	1481.814	S[30]
L[13]	735.467	**1446.811**	**1438.802**	**1439.306**	1438.298	L[29]
V[14]	785.002	1390.269	1382.260	1382.764	1381.756	V[28]
S[15]	**828.518**	1340.735	1332.726	**1333.230**	1332.222	S[27]
K[16]	**892.565**	**1297.219**	**1289.210**	**1289.714**	**1288.706**	K[26]
G[17]	**921.076**	1233.172	**1225.162**	**1225.666**	**1224.658**	G[25]
T[18]	**971.600**	1204.661	**1196.651**	**1197.155**	1196.148	T[24]
L[19]	**1028.142**	1154.137	**1146.128**	**1146.632**	**1145.624**	L[23]
V[20]	**1077.676**	1097.595	**1089.586**	1090.090	**1089.082**	V[22]
Q[21]	**1141.705**	1048.061	1040.051	**1040.555**	1039.547	Q[21]
T[22]	**1192.229**	984.031	976.022	**976.526**	975.518	T[20]
K[23]	**1270.292**	**933.508**	925.498	**926.002**	924.994	K[19]
G[24]	1298.803	855.444	847.435	847.939	846.931	G[18]
T[25]	**1349.327**	826.934	**818.924**	**819.428**	**818.420**	T[17]
G[26]	1377.837	776.410	768.401	768.904	767.897	G[16]
A[27]	1413.356	747.899	739.890	740.394	739.386	A[15]
S[28]	1456.872	712.381	704.371	704.875	703.867	S[14]
G[29]	1485.383	**668.865**	660.855	661.359	660.351	G[13]
S[30]	**1528.899**	640.354	632.345	632.848	631.841	S[12]
F[31]	**1602.433**	596.838	588.829	589.332	588.325	F[11]
K[32]	**1666.480**	523.304	515.294	515.798	514.790	K[10]
L[33]	1723.022	459.256	451.247	451.751	450.743	L[9]
N[34]	**1780.044**	402.714	394.705	395.209	394.201	N[8]
K[35]	**1844.091**	345.693	337.683	338.187	337.179	K[7]
K[36]	1908.139	281.645	273.636	274.140	273.132	K[6]
A[37]	1943.657	217.598	209.588	210.092	209.084	A[5]
A[38]	1979.176	182.079	174.070	174.574	173.566	A[4]
S[39]	2022.692	146.561	138.551	139.055	138.047	S[3]
G[40]	2051.203	103.045	95.035	95.539	94.531	G[2]
E[41]	2115.724	74.534	66.524	67.028	66.021	E[1]

sp|P43277|H13_MOUSE

KNNSRIKLGLKSLVSKGTLVQTK $^{Dimethyl}_{28.03}$ GTGASGSFKLNKKAASGE

- fragmentation table for charge state 3+
- specType=etd
- score=75.55
- F104915.dat
- query=q1504_p1
- precursor=847.088850
- chargePrecursor=5
- itol=0.8

AA	c	y	z+1	z+2	z	AA
K[1]	49.381	**1411.146**	**1405.807**	**1406.143**	1405.471	K[41]
N[2]	87.395	1368.448	**1363.109**	**1363.445**	**1362.773**	N[40]
N[3]	125.410	1330.434	1325.094	1325.430	1324.758	N[39]
S[4]	154.420	**1292.420**	**1287.080**	**1287.416**	1286.744	S[38]
R[5]	206.454	1263.409	**1258.069**	**1258.405**	1257.733	R[37]
I[6]	244.149	1211.375	1206.036	1206.372	1205.700	I[36]
K[7]	286.847	1173.680	1168.341	1168.677	1168.005	K[35]
L[8]	324.542	1130.982	1125.643	1125.979	1125.307	L[34]
G[9]	343.549	1093.287	1087.948	**1088.284**	1087.612	G[33]
L[10]	381.244	1074.280	**1068.941**	**1069.277**	1068.605	L[32]
K[11]	423.942	1036.586	**1031.246**	1031.582	**1030.910**	K[31]
S[12]	452.953	**993.887**	988.548	988.884	988.212	S[30]
L[13]	490.647	**964.877**	**959.537**	**959.873**	**959.201**	L[29]
V[14]	523.670	927.182	**921.842**	**922.178**	**921.506**	V[28]
S[15]	552.681	**894.159**	888.820	889.155	888.484	S[27]
K[16]	595.379	865.148	859.809	860.145	859.473	K[26]
G[17]	**614.386**	822.450	817.111	817.446	816.775	G[25]
T[18]	648.069	803.443	798.103	798.439	797.767	T[24]
L[19]	685.764	769.760	764.421	764.757	764.085	L[23]
V[20]	718.786	732.066	726.726	727.062	726.390	V[22]
Q[21]	761.473	699.043	693.703	694.039	693.367	Q[21]
T[22]	795.155	**656.357**	651.017	651.353	650.681	T[20]
K[23]	847.197	622.674	617.335	617.671	616.999	K[19]
G[24]	866.204	570.632	565.293	565.628	564.957	G[18]
T[25]	899.887	551.625	546.285	**546.621**	545.949	T[17]
G[26]	918.894	517.942	512.603	512.939	512.267	G[16]
A[27]	942.573	498.935	493.596	493.932	493.260	A[15]
S[28]	**971.584**	475.256	469.917	470.253	469.581	S[14]
G[29]	**990.591**	446.245	440.906	441.242	440.570	G[13]
S[30]	1019.602	427.238	421.899	422.235	421.563	S[12]
F[31]	1068.624	398.228	392.888	393.224	392.552	F[11]
K[32]	**1111.323**	349.205	343.865	344.201	343.529	K[10]
L[33]	1149.017	306.507	301.167	301.503	300.831	L[9]
N[34]	1187.032	268.812	263.472	263.808	263.136	N[8]
K[35]	**1229.730**	230.798	225.458	225.794	225.122	K[7]
K[36]	**1272.428**	188.099	182.760	183.096	182.424	K[6]
A[37]	1296.107	145.401	140.061	140.397	139.725	A[5]
A[38]	**1319.786**	121.722	116.382	116.718	116.046	A[4]
S[39]	1348.797	98.043	92.703	93.039	92.367	S[3]
G[40]	**1367.804**	69.032	63.693	64.029	63.357	G[2]
E[41]	**1410.818**	50.025	44.685	45.021	44.349	E[1]

sp|P43277|H13_MOUSE

KNNSRIKLGLKSLVSKGTLVQTK $^{Dimethyl}_{28.03}$ GTGASGSFKLNKKAASGE

- fragmentation table for charge state 4+
- specType=etd
- score=75.55
- F104915.dat
- query=q1504_p1
- precursor=847.088850
- chargePrecursor=5
- itol=0.8

AA	c	y	z+1	z+2	z	AA
K[1]	37.288	**1058.612**	**1054.607**	**1054.859**	**1054.355**	K[41]
N[2]	65.798	**1026.588**	1022.583	1022.835	1022.331	N[40]
N[3]	94.309	998.077	**994.073**	**994.324**	**993.821**	N[39]
S[4]	116.067	969.566	**965.562**	**965.814**	**965.310**	S[38]
R[5]	155.092	947.808	943.804	944.056	943.552	R[37]
I[6]	183.363	908.783	904.779	905.030	904.527	I[36]
K[7]	215.387	880.512	876.507	876.759	876.256	K[35]
L[8]	243.658	848.488	844.484	844.736	844.232	L[34]
G[9]	257.914	820.217	816.213	816.465	815.961	G[33]
L[10]	286.185	**805.962**	801.957	802.209	801.705	L[32]
K[11]	318.208	777.691	773.686	773.938	773.434	K[31]
S[12]	339.966	745.667	741.663	741.915	741.411	S[30]
L[13]	368.237	723.909	719.905	720.157	719.653	L[29]
V[14]	393.004	695.638	691.634	691.886	691.382	V[28]
S[15]	414.762	670.871	666.866	667.118	666.615	S[27]
K[16]	446.786	649.113	645.108	645.360	644.857	K[26]
G[17]	**461.042**	617.089	**613.085**	**613.337**	612.833	G[25]
T[18]	486.303	602.834	598.829	599.081	598.577	T[24]
L[19]	514.574	577.572	573.567	573.819	573.315	L[23]
V[20]	539.342	549.301	545.296	545.548	545.044	V[22]
Q[21]	571.356	524.534	520.529	520.781	520.277	Q[21]
T[22]	596.618	492.519	488.515	488.767	488.263	T[20]
K[23]	635.650	467.257	463.253	463.505	463.001	K[19]
G[24]	649.905	428.226	424.221	424.473	423.969	G[18]
T[25]	675.167	413.971	409.966	410.218	409.714	T[17]
G[26]	689.422	388.709	384.704	384.956	384.452	G[16]
A[27]	707.182	**374.453**	370.449	370.701	370.197	A[15]
S[28]	728.940	356.694	352.689	352.941	352.437	S[14]
G[29]	743.195	334.936	330.931	331.183	330.679	G[13]
S[30]	764.953	320.681	316.676	316.928	316.424	S[12]
F[31]	801.720	298.923	294.918	295.170	294.666	F[11]
K[32]	833.744	262.155	258.151	258.403	257.899	K[10]
L[33]	862.015	230.132	226.127	226.379	225.875	L[9]
N[34]	**890.526**	201.861	197.856	198.108	197.604	N[8]
K[35]	922.549	173.350	169.345	169.597	169.093	K[7]
K[36]	954.573	141.326	137.322	137.574	137.070	K[6]
A[37]	**972.332**	109.302	105.298	105.550	105.046	A[5]
A[38]	**990.092**	91.543	87.539	87.790	87.287	A[4]
S[39]	1011.850	73.784	69.779	70.031	69.527	S[3]
G[40]	**1026.105**	52.026	48.021	48.273	47.769	G[2]
E[41]	1058.366	37.771	33.766	34.018	33.514	E[1]

sp|Q6GSS7|H2A2A_MOUSE

LNKLLGK $^{\text{Dimethyl}}_{28.03}$ VTIAQGGVLPNIQAVLLPKKTE

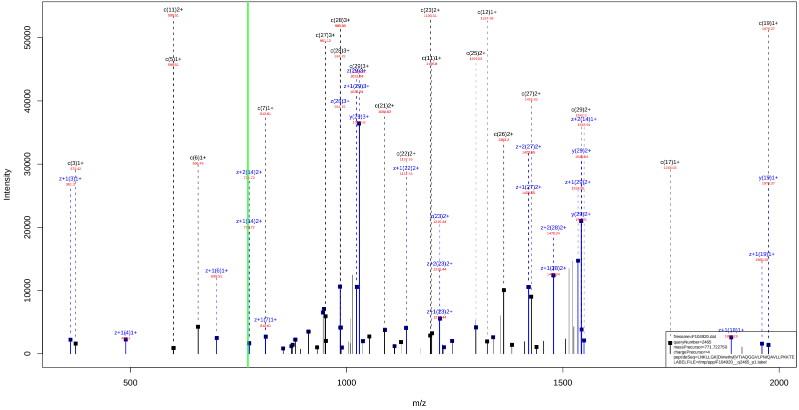

sp|Q6GSS7|H2A2A_MOUSE

LNKLLGK $^{\text{Dimethyl}}_{28.03}$ VTIAQGGVLPNIQAVLLPKKTE

- fragmentation table for charge state 1+
- specType=etd
- score=77.22
- F104920.dat
- query=q2465_p1
- precursor=771.722750
- chargePrecursor=4
- itol=0.8

AA	c	y	z+1	z+2	z	AA
L[1]	131.118	3083.892	3067.873	0.000	3066.865	L[29]
N[2]	245.161	2970.808	2954.789	2955.797	2953.781	N[28]
K[3]	**373.256**	2856.765	2840.746	2841.754	2839.738	K[27]
L[4]	486.340	2728.670	2712.651	2713.659	2711.643	L[26]
L[5]	**599.424**	2615.586	2599.567	2600.575	2598.559	L[25]
G[6]	**656.445**	2502.502	2486.483	2487.491	2485.475	G[24]
K[7]	**812.572**	2445.480	2429.462	2430.469	2428.454	K[23]
V[8]	**911.640**	2289.354	2273.335	2274.343	2272.327	V[22]
T[9]	1012.688	2190.286	2174.267	2175.275	2173.259	T[21]
I[10]	**1125.772**	2089.238	2073.219	2074.227	2072.211	I[20]
A[11]	**1196.809**	**1976.154**	**1960.135**	1961.143	1959.127	A[19]
Q[12]	**1324.867**	1905.117	**1889.098**	1890.106	1888.090	Q[18]
G[13]	**1381.889**	1777.058	1761.039	1762.047	1760.032	G[17]
G[14]	**1438.910**	1720.037	1704.018	1705.026	1703.010	G[16]
V[15]	1537.979	1663.015	1646.997	1648.004	1645.989	V[15]
L[16]	1651.063	1563.947	1547.928	**1548.936**	1546.920	L[14]
P[17]	**1748.116**	1450.863	1434.844	1435.852	1433.836	P[13]
N[18]	1862.159	1353.810	1337.791	**1338.799**	1336.783	N[12]
I[19]	**1975.243**	1239.767	1223.748	**1224.756**	1222.741	I[11]
Q[20]	2103.301	1126.683	**1110.664**	1111.672	1109.656	Q[10]
A[21]	2174.338	998.624	982.606	983.614	981.598	A[9]
V[22]	2273.407	927.587	**911.569**	912.576	910.561	V[8]
L[23]	2386.491	828.519	**812.500**	813.508	811.492	L[7]
L[24]	2499.575	715.435	**699.416**	700.424	698.408	L[6]
P[25]	2596.628	602.351	586.332	587.340	585.324	P[5]
K[26]	2724.723	505.298	**489.279**	490.287	488.271	K[4]
K[27]	2852.818	377.203	**361.184**	362.192	360.177	K[3]
T[28]	2953.865	249.108	233.089	234.097	232.082	T[2]
E[29]	3082.908	148.060	132.042	133.050	131.034	E[1]

sp|Q6GSS7|H2A2A_MOUSE

LNKLLGK $^{Dimethyl}_{28.03}$ VTIAQGGVLPNIQAVLLPKKTE

- fragmentation table for charge state 2+
- specType=etd
- score=77.22
- F104920.dat
- query=q2465_p1
- precursor=771.722750
- chargePrecursor=4
- itol=0.8

AA	c	y	z+1	z+2	z	AA
L[1]	66.063	**1542.450**	**1534.440**	0.504	1533.936	L[29]
N[2]	123.084	1485.908	**1477.898**	**1478.402**	1477.394	N[28]
K[3]	187.132	1428.886	**1420.877**	**1421.381**	**1420.373**	K[27]
L[4]	243.674	1364.839	1356.829	1357.333	1356.325	L[26]
L[5]	300.216	1308.297	1300.287	1300.791	**1299.783**	L[25]
G[6]	328.726	1251.755	**1243.745**	**1244.249**	1243.241	G[24]
K[7]	406.789	1223.244	**1215.234**	**1215.738**	**1214.731**	K[23]
V[8]	456.324	1145.181	**1137.171**	**1137.675**	1136.667	V[22]
T[9]	506.848	1095.646	**1087.637**	**1088.141**	1087.133	T[21]
I[10]	563.390	1045.123	**1037.113**	**1037.617**	1036.609	I[20]
A[11]	**598.908**	**988.581**	980.571	981.075	980.067	A[19]
Q[12]	662.937	953.062	**945.053**	**945.557**	944.549	Q[18]
G[13]	691.448	889.033	**881.023**	**881.527**	880.519	G[17]
G[14]	719.959	860.522	852.513	**853.017**	852.009	G[16]
V[15]	769.493	832.011	824.002	824.506	823.498	V[15]
L[16]	826.035	782.477	**774.468**	**774.972**	**773.964**	L[14]
P[17]	**874.561**	725.935	717.926	718.430	717.422	P[13]
N[18]	**931.583**	677.409	669.399	669.903	668.895	N[12]
I[19]	**988.125**	620.387	612.378	612.882	611.874	I[11]
Q[20]	**1052.154**	563.845	555.836	556.340	555.332	Q[10]
A[21]	**1087.673**	499.816	491.807	492.310	491.303	A[9]
V[22]	**1137.207**	464.297	456.288	456.792	455.784	V[8]
L[23]	**1193.749**	414.763	406.754	407.258	406.250	L[7]
L[24]	1250.291	358.221	350.212	350.716	349.708	L[6]
P[25]	**1298.817**	301.679	293.670	294.174	293.166	P[5]
K[26]	**1362.865**	253.153	245.143	245.647	244.639	K[4]
K[27]	**1426.912**	189.105	181.096	181.600	180.592	K[3]
T[28]	1477.436	125.058	117.048	117.552	116.544	T[2]
E[29]	**1541.958**	74.534	66.524	67.028	66.021	E[1]

sp|Q6GSS7|H2A2A_MOUSE

LNKLLGK $^{\text{Dimethyl}}_{28.03}$ VTIAQGGVLPNIQAVLLPKKTE

- fragmentation table for charge state 3+
- specType=etd
- score=77.22
- F104920.dat
- query=q2465_p1
- precursor=771.722750
- chargePrecursor=4
- itol=0.8

AA	c	y	z+1	z+2	z	AA
L[1]	44.377	**1028.635**	**1023.296**	0.672	**1022.960**	L[29]
N[2]	82.392	990.941	**985.601**	**985.937**	**985.265**	N[28]
K[3]	125.090	952.926	**947.587**	**947.923**	**947.251**	K[27]
L[4]	162.785	910.228	904.889	905.225	904.553	L[26]
L[5]	200.479	**872.533**	867.194	867.530	866.858	L[25]
G[6]	219.487	834.839	829.499	829.835	829.163	G[24]
K[7]	271.529	815.832	810.492	810.828	810.156	K[23]
V[8]	304.552	763.790	758.450	758.786	758.114	V[22]
T[9]	338.234	730.767	725.427	725.763	725.091	T[21]
I[10]	375.929	697.084	691.745	692.081	691.409	I[20]
A[11]	399.608	659.389	654.050	654.386	653.714	A[19]
Q[12]	442.294	635.710	630.371	630.707	630.035	Q[18]
G[13]	461.301	593.024	587.685	588.021	587.349	G[17]
G[14]	480.308	574.017	568.678	569.013	568.342	G[16]
V[15]	513.331	555.010	549.670	550.006	549.334	V[15]
L[16]	551.026	521.987	516.648	516.984	516.312	L[14]
P[17]	583.377	484.292	478.953	479.289	478.617	P[13]
N[18]	621.391	451.942	446.602	446.938	446.266	N[12]
I[19]	659.086	413.927	408.588	408.924	408.252	I[11]
Q[20]	701.772	376.233	370.893	371.229	370.557	Q[10]
A[21]	725.451	333.546	328.207	328.543	327.871	A[9]
V[22]	758.474	309.867	304.528	304.864	304.192	V[8]
L[23]	796.168	276.844	271.505	271.841	271.169	L[7]
L[24]	833.863	239.150	233.810	234.146	233.474	L[6]
P[25]	866.214	201.455	196.116	196.451	195.780	P[5]
K[26]	908.912	169.104	163.765	164.101	163.429	K[4]
K[27]	**951.611**	126.406	121.066	121.402	120.730	K[3]
T[28]	**985.293**	83.708	78.368	78.704	78.032	T[2]
E[29]	**1028.307**	50.025	44.685	45.021	44.349	E[1]

sp|Q6GSS7|H2A2A_MOUSE

LNKLLGK $^{\text{Dimethyl}}_{28.03}$ VTIAQGGVLPNIQAVLLPKKTE

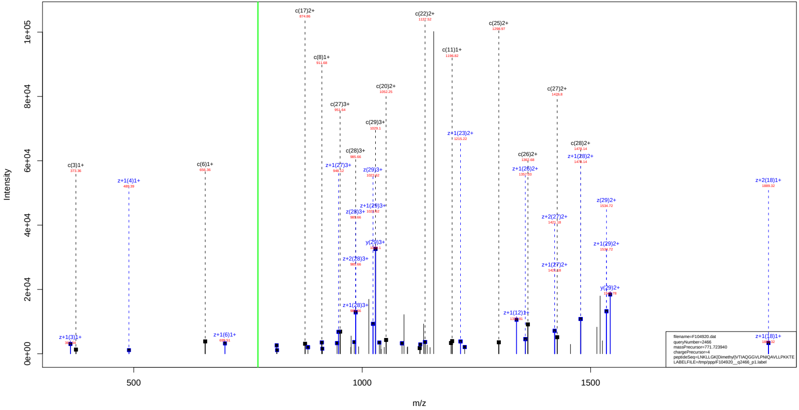

sp|Q6GSS7|H2A2A_MOUSE

LNKLLGK $^{Dimethyl}_{28.03}$ VTIAQGGVLPNIQAVLLPKKTE

- fragmentation table for charge state 1+
- specType=etd
- score=63.27
- F104920.dat
- query=q2466_p1
- precursor=771.723940
- chargePrecursor=4
- itol=0.8

AA	c	y	z+1	z+2	z	AA
L[1]	131.118	3083.892	3067.873	0.000	3066.865	L[29]
N[2]	245.161	2970.808	2954.789	2955.797	2953.781	N[28]
K[3]	**373.256**	2856.765	2840.746	2841.754	2839.738	K[27]
L[4]	486.340	2728.670	2712.651	2713.659	2711.643	L[26]
L[5]	599.424	2615.586	2599.567	2600.575	2598.559	L[25]
G[6]	**656.445**	2502.502	2486.483	2487.491	2485.475	G[24]
K[7]	**812.572**	2445.480	2429.462	2430.469	2428.454	K[23]
V[8]	**911.640**	2289.354	2273.335	2274.343	2272.327	V[22]
T[9]	1012.688	2190.286	2174.267	2175.275	2173.259	T[21]
I[10]	**1125.772**	2089.238	2073.219	2074.227	2072.211	I[20]
A[11]	**1196.809**	1976.154	1960.135	1961.143	1959.127	A[19]
Q[12]	1324.867	1905.117	**1889.098**	**1890.106**	1888.090	Q[18]
G[13]	1381.889	1777.058	1761.039	1762.047	1760.032	G[17]
G[14]	1438.910	1720.037	1704.018	1705.026	1703.010	G[16]
V[15]	1537.979	1663.015	1646.997	1648.004	1645.989	V[15]
L[16]	1651.063	1563.947	1547.928	1548.936	1546.920	L[14]
P[17]	1748.116	1450.863	1434.844	1435.852	1433.836	P[13]
N[18]	1862.159	1353.810	**1337.791**	1338.799	1336.783	N[12]
I[19]	1975.243	1239.767	**1223.748**	**1224.756**	1222.741	I[11]
Q[20]	2103.301	**1126.683**	1110.664	1111.672	1109.656	Q[10]
A[21]	2174.338	998.624	**982.606**	983.614	981.598	A[9]
V[22]	2273.407	927.587	**911.569**	**912.576**	910.561	V[8]
L[23]	2386.491	828.519	**812.500**	**813.508**	811.492	L[7]
L[24]	2499.575	715.435	**699.416**	700.424	698.408	L[6]
P[25]	2596.628	602.351	586.332	587.340	585.324	P[5]
K[26]	2724.723	505.298	**489.279**	490.287	488.271	K[4]
K[27]	2852.818	377.203	**361.184**	362.192	360.177	K[3]
T[28]	2953.865	249.108	233.089	234.097	232.082	T[2]
E[29]	3082.908	148.060	132.042	133.050	131.034	E[1]

sp|Q6GSS7|H2A2A_MOUSE

LNKLLGK $^{\text{Dimethyl}}_{28.03}$ VTIAQGGVLPNIQAVLLPKKTE

- fragmentation table for charge state 2+
- specType=etd
- score=63.27
- F104920.dat
- query=q2466_p1
- precursor=771.723940
- chargePrecursor=4
- itol=0.8

AA	c	y	z+1	z+2	z	AA
L[1]	66.063	**1542.450**	**1534.440**	0.504	**1533.936**	L[29]
N[2]	123.084	1485.908	**1477.898**	**1478.402**	**1477.394**	N[28]
K[3]	187.132	1428.886	**1420.877**	**1421.381**	1420.373	K[27]
L[4]	243.674	1364.839	**1356.829**	**1357.333**	**1356.325**	L[26]
L[5]	300.216	1308.297	1300.287	1300.791	1299.783	L[25]
G[6]	328.726	1251.755	1243.745	1244.249	1243.241	G[24]
K[7]	406.789	1223.244	**1215.234**	**1215.738**	**1214.731**	K[23]
V[8]	456.324	1145.181	**1137.171**	**1137.675**	1136.667	V[22]
T[9]	506.848	1095.646	**1087.637**	1088.141	**1087.133**	T[21]
I[10]	563.390	1045.123	**1037.113**	**1037.617**	1036.609	I[20]
A[11]	598.908	988.581	980.571	981.075	980.067	A[19]
Q[12]	662.937	953.062	**945.053**	**945.557**	**944.549**	Q[18]
G[13]	691.448	889.033	**881.023**	**881.527**	**880.519**	G[17]
G[14]	719.959	860.522	852.513	853.017	852.009	G[16]
V[15]	769.493	832.011	824.002	824.506	823.498	V[15]
L[16]	826.035	782.477	774.468	774.972	773.964	L[14]
P[17]	**874.561**	725.935	717.926	718.430	717.422	P[13]
N[18]	931.583	677.409	669.399	669.903	668.895	N[12]
I[19]	988.125	620.387	612.378	612.882	611.874	I[11]
Q[20]	**1052.154**	563.845	555.836	556.340	555.332	Q[10]
A[21]	**1087.673**	499.816	491.807	492.310	491.303	A[9]
V[22]	**1137.207**	464.297	456.288	456.792	455.784	V[8]
L[23]	**1193.749**	414.763	406.754	407.258	406.250	L[7]
L[24]	1250.291	358.221	350.212	350.716	349.708	L[6]
P[25]	**1298.817**	301.679	293.670	294.174	293.166	P[5]
K[26]	**1362.865**	253.153	245.143	245.647	244.639	K[4]
K[27]	**1426.912**	189.105	181.096	181.600	180.592	K[3]
T[28]	**1477.436**	125.058	117.048	117.552	116.544	T[2]
E[29]	1541.958	74.534	66.524	67.028	66.021	E[1]

sp|Q6GSS7|H2A2A_MOUSE

LNKLLGK $^{\text{Dimethyl}}_{28.03}$ VTIAQGGVLPNIQAVLLPKKTE

- fragmentation table for charge state 3+
- specType=etd
- score=63.27
- F104920.dat
- query=q2466_p1
- precursor=771.723940
- chargePrecursor=4
- itol=0.8

AA	c	y	z+1	z+2	z	AA
L[1]	44.377	**1028.635**	**1023.296**	0.672	**1022.960**	L[29]
N[2]	82.392	990.941	**985.601**	**985.937**	**985.265**	N[28]
K[3]	125.090	952.926	**947.587**	**947.923**	947.251	K[27]
L[4]	162.785	910.228	904.889	905.225	904.553	L[26]
L[5]	200.479	872.533	867.194	867.530	866.858	L[25]
G[6]	219.487	834.839	829.499	829.835	829.163	G[24]
K[7]	271.529	815.832	810.492	810.828	810.156	K[23]
V[8]	304.552	763.790	758.450	758.786	758.114	V[22]
T[9]	338.234	730.767	725.427	725.763	725.091	T[21]
I[10]	375.929	697.084	691.745	692.081	691.409	I[20]
A[11]	399.608	659.389	654.050	654.386	653.714	A[19]
Q[12]	442.294	635.710	630.371	630.707	630.035	Q[18]
G[13]	461.301	593.024	587.685	588.021	587.349	G[17]
G[14]	480.308	574.017	568.678	569.013	568.342	G[16]
V[15]	513.331	555.010	549.670	550.006	549.334	V[15]
L[16]	551.026	521.987	516.648	516.984	516.312	L[14]
P[17]	583.377	484.292	478.953	479.289	478.617	P[13]
N[18]	621.391	451.942	446.602	446.938	446.266	N[12]
I[19]	659.086	413.927	408.588	408.924	408.252	I[11]
Q[20]	701.772	376.233	370.893	371.229	370.557	Q[10]
A[21]	725.451	333.546	328.207	328.543	327.871	A[9]
V[22]	758.474	309.867	304.528	304.864	304.192	V[8]
L[23]	796.168	276.844	271.505	271.841	271.169	L[7]
L[24]	833.863	239.150	233.810	234.146	233.474	L[6]
P[25]	866.214	201.455	196.116	196.451	195.780	P[5]
K[26]	908.912	169.104	163.765	164.101	163.429	K[4]
K[27]	**951.611**	126.406	121.066	121.402	120.730	K[3]
T[28]	**985.293**	83.708	78.368	78.704	78.032	T[2]
E[29]	**1028.307**	50.025	44.685	45.021	44.349	E[1]

sp|Q6GSS7|H2A2A_MOUSE

LNKLLGK $^{Dimethyl}_{28.03}$ VTIAQGGVLPNIQAVLLPKKTE

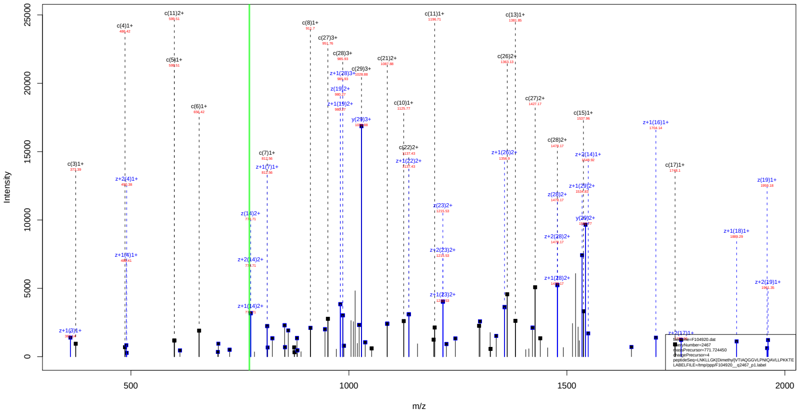

sp|Q6GSS7|H2A2A_MOUSE

LNKLLGK $^{\text{Dimethyl}}_{28.03}$ VTIAQGGVLPNIQAVLLPKKTE

- fragmentation table for charge state 1+
- specType=etd
- score=124.21
- F104920.dat
- query=q2467_p1
- precursor=771.724450
- chargePrecursor=4
- itol=0.8

AA	c	y	z+1	z+2	z	AA
L[1]	131.118	3083.892	3067.873	0.000	3066.865	L[29]
N[2]	245.161	2970.808	2954.789	2955.797	2953.781	N[28]
K[3]	**373.256**	2856.765	2840.746	2841.754	2839.738	K[27]
L[4]	**486.340**	2728.670	2712.651	2713.659	2711.643	L[26]
L[5]	**599.424**	2615.586	2599.567	2600.575	2598.559	L[25]
G[6]	**656.445**	2502.502	2486.483	2487.491	2485.475	G[24]
K[7]	**812.572**	2445.480	2429.462	2430.469	2428.454	K[23]
V[8]	**911.640**	2289.354	2273.335	2274.343	2272.327	V[22]
T[9]	1012.688	2190.286	2174.267	2175.275	2173.259	T[21]
I[10]	**1125.772**	2089.238	2073.219	2074.227	2072.211	I[20]
A[11]	**1196.809**	1976.154	1960.135	**1961.143**	**1959.127**	A[19]
Q[12]	**1324.867**	1905.117	**1889.098**	1890.106	1888.090	Q[18]
G[13]	**1381.889**	1777.058	1761.039	**1762.047**	1760.032	G[17]
G[14]	**1438.910**	1720.037	**1704.018**	1705.026	1703.010	G[16]
V[15]	**1537.979**	1663.015	1646.997	**1648.004**	1645.989	V[15]
L[16]	1651.063	1563.947	1547.928	**1548.936**	1546.920	L[14]
P[17]	**1748.116**	1450.863	1434.844	1435.852	1433.836	P[13]
N[18]	1862.159	1353.810	**1337.791**	1338.799	1336.783	N[12]
I[19]	1975.243	1239.767	**1223.748**	1224.756	1222.741	I[11]
Q[20]	2103.301	1126.683	1110.664	1111.672	1109.656	Q[10]
A[21]	2174.338	998.624	982.606	983.614	981.598	A[9]
V[22]	2273.407	927.587	**911.569**	912.576	910.561	V[8]
L[23]	2386.491	828.519	**812.500**	**813.508**	811.492	L[7]
L[24]	2499.575	715.435	**699.416**	**700.424**	698.408	L[6]
P[25]	2596.628	602.351	586.332	587.340	585.324	P[5]
K[26]	2724.723	505.298	**489.279**	**490.287**	488.271	K[4]
K[27]	2852.818	377.203	**361.184**	362.192	360.177	K[3]
T[28]	2953.865	249.108	233.089	234.097	232.082	T[2]
E[29]	3082.908	148.060	132.042	133.050	131.034	E[1]

sp|Q6GSS7|H2A2A_MOUSE

LNKLLGK $^{\text{Dimethyl}}_{28.03}$ VTIAQGGVLPNIQAVLLPKKTE

- fragmentation table for charge state 2+
- specType=etd
- score=124.21
- F104920.dat
- query=q2467_p1
- precursor=771.724450
- chargePrecursor=4
- itol=0.8

AA	c	y	z+1	z+2	z	AA
L[1]	66.063	**1542.450**	**1534.440**	0.504	1533.936	L[29]
N[2]	123.084	1485.908	**1477.898**	**1478.402**	**1477.394**	N[28]
K[3]	187.132	1428.886	**1420.877**	**1421.381**	**1420.373**	K[27]
L[4]	243.674	1364.839	**1356.829**	**1357.333**	**1356.325**	L[26]
L[5]	300.216	1308.297	**1300.287**	**1300.791**	1299.783	L[25]
G[6]	328.726	1251.755	**1243.745**	**1244.249**	1243.241	G[24]
K[7]	406.789	**1223.244**	**1215.234**	**1215.738**	**1214.731**	K[23]
V[8]	456.324	1145.181	**1137.171**	**1137.675**	**1136.667**	V[22]
T[9]	506.848	1095.646	**1087.637**	**1088.141**	**1087.133**	T[21]
I[10]	563.390	1045.123	**1037.113**	**1037.617**	**1036.609**	I[20]
A[11]	**598.908**	**988.581**	**980.571**	981.075	**980.067**	A[19]
Q[12]	662.937	953.062	**945.053**	**945.557**	**944.549**	Q[18]
G[13]	691.448	889.033	**881.023**	**881.527**	**880.519**	G[17]
G[14]	719.959	**860.522**	**852.513**	**853.017**	**852.009**	G[16]
V[15]	769.493	832.011	**824.002**	**824.506**	823.498	V[15]
L[16]	826.035	782.477	**774.468**	**774.972**	**773.964**	L[14]
P[17]	**874.561**	**725.935**	717.926	718.430	717.422	P[13]
N[18]	931.583	677.409	669.399	669.903	668.895	N[12]
I[19]	**988.125**	620.387	**612.378**	**612.882**	**611.874**	I[11]
Q[20]	**1052.154**	563.845	555.836	556.340	555.332	Q[10]
A[21]	**1087.673**	499.816	491.807	492.310	491.303	A[9]
V[22]	**1137.207**	464.297	456.288	456.792	455.784	V[8]
L[23]	**1193.749**	414.763	406.754	407.258	406.250	L[7]
L[24]	1250.291	358.221	350.212	350.716	349.708	L[6]
P[25]	**1298.817**	301.679	293.670	294.174	293.166	P[5]
K[26]	**1362.865**	253.153	245.143	245.647	244.639	K[4]
K[27]	**1426.912**	189.105	181.096	181.600	180.592	K[3]
T[28]	**1477.436**	125.058	117.048	117.552	116.544	T[2]
E[29]	1541.958	74.534	66.524	67.028	66.021	E[1]

sp|Q6GSS7|H2A2A_MOUSE

LNKLLGK $^{\text{Dimethyl}}_{28.03}$ VTIAQGGVLPNIQAVLLPKKTE

- fragmentation table for charge state 3+
- specType=etd
- score=124.21
- F104920.dat
- query=q2467_p1
- precursor=771.724450
- chargePrecursor=4
- itol=0.8

AA	c	y	z+1	z+2	z	AA
L[1]	44.377	**1028.635**	**1023.296**	0.672	**1022.960**	L[29]
N[2]	82.392	990.941	**985.601**	**985.937**	**985.265**	N[28]
K[3]	125.090	952.926	947.587	947.923	947.251	K[27]
L[4]	162.785	910.228	904.889	905.225	904.553	L[26]
L[5]	200.479	872.533	867.194	867.530	866.858	L[25]
G[6]	219.487	834.839	829.499	829.835	829.163	G[24]
K[7]	271.529	815.832	810.492	810.828	810.156	K[23]
V[8]	304.552	763.790	758.450	758.786	758.114	V[22]
T[9]	338.234	730.767	725.427	**725.763**	725.091	T[21]
I[10]	375.929	697.084	691.745	692.081	691.409	I[20]
A[11]	399.608	659.389	654.050	654.386	653.714	A[19]
Q[12]	442.294	635.710	630.371	630.707	630.035	Q[18]
G[13]	461.301	593.024	587.685	588.021	587.349	G[17]
G[14]	480.308	574.017	568.678	569.013	568.342	G[16]
V[15]	513.331	555.010	549.670	550.006	549.334	V[15]
L[16]	551.026	521.987	516.648	516.984	516.312	L[14]
P[17]	583.377	484.292	478.953	479.289	478.617	P[13]
N[18]	621.391	451.942	446.602	446.938	446.266	N[12]
I[19]	659.086	413.927	408.588	408.924	408.252	I[11]
Q[20]	701.772	376.233	370.893	371.229	370.557	Q[10]
A[21]	725.451	333.546	328.207	328.543	327.871	A[9]
V[22]	758.474	309.867	304.528	304.864	304.192	V[8]
L[23]	796.168	276.844	271.505	271.841	271.169	L[7]
L[24]	833.863	239.150	233.810	234.146	233.474	L[6]
P[25]	866.214	201.455	196.116	196.451	195.780	P[5]
K[26]	908.912	169.104	163.765	164.101	163.429	K[4]
K[27]	**951.611**	126.406	121.066	121.402	120.730	K[3]
T[28]	**985.293**	83.708	78.368	78.704	78.032	T[2]
E[29]	**1028.307**	50.025	44.685	45.021	44.349	E[1]

sp|Q6GSS7|H2A2_MOUSE

LNKLLGK $^{\text{Dimethyl}}_{28.03}$ VTIAQGGVLPNIQAVLLPKKTE

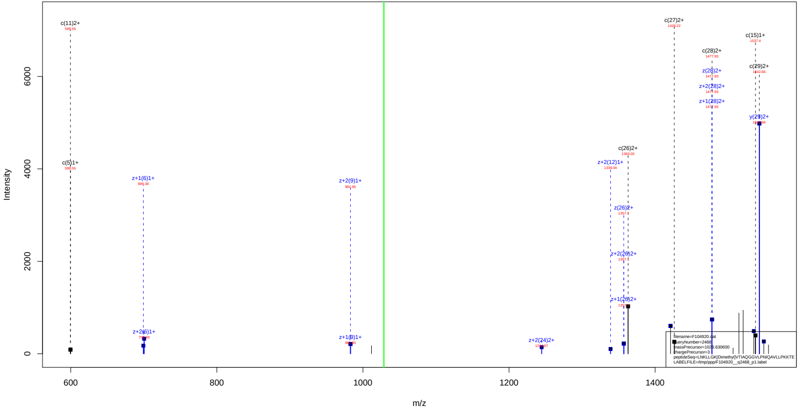

sp|Q6GSS7|H2A2A_MOUSE

LNKLLGK $^{\text{Dimethyl}}_{28.03}$ VTIAQGGVLPNIQAVLLPKKTE

- fragmentation table for charge state 1+
- specType=etd
- score=32.53
- F104920.dat
- query=q2468_p1
- precursor=1028.630600
- chargePrecursor=3
- itol=0.8

AA	c	y	z+1	z+2	z	AA
L[1]	131.118	3083.892	3067.873	0.000	3066.865	L[29]
N[2]	245.161	2970.808	2954.789	2955.797	2953.781	N[28]
K[3]	373.256	2856.765	2840.746	2841.754	2839.738	K[27]
L[4]	486.340	2728.670	2712.651	2713.659	2711.643	L[26]
L[5]	**599.424**	2615.586	2599.567	2600.575	2598.559	L[25]
G[6]	656.445	2502.502	2486.483	2487.491	2485.475	G[24]
K[7]	812.572	2445.480	2429.462	2430.469	2428.454	K[23]
V[8]	911.640	2289.354	2273.335	2274.343	2272.327	V[22]
T[9]	1012.688	2190.286	2174.267	2175.275	2173.259	T[21]
I[10]	1125.772	2089.238	2073.219	2074.227	2072.211	I[20]
A[11]	1196.809	1976.154	1960.135	1961.143	1959.127	A[19]
Q[12]	1324.867	1905.117	1889.098	1890.106	1888.090	Q[18]
G[13]	1381.889	1777.058	1761.039	1762.047	1760.032	G[17]
G[14]	1438.910	1720.037	1704.018	1705.026	1703.010	G[16]
V[15]	**1537.979**	1663.015	1646.997	1648.004	1645.989	V[15]
L[16]	1651.063	1563.947	1547.928	**1548.936**	1546.920	L[14]
P[17]	1748.116	1450.863	1434.844	1435.852	1433.836	P[13]
N[18]	1862.159	1353.810	1337.791	**1338.799**	1336.783	N[12]
I[19]	1975.243	1239.767	1223.748	1224.756	1222.741	I[11]
Q[20]	2103.301	1126.683	1110.664	1111.672	1109.656	Q[10]
A[21]	2174.338	998.624	**982.606**	**983.614**	981.598	A[9]
V[22]	2273.407	927.587	911.569	912.576	910.561	V[8]
L[23]	2386.491	828.519	812.500	813.508	811.492	L[7]
L[24]	2499.575	715.435	**699.416**	**700.424**	698.408	L[6]
P[25]	2596.628	602.351	586.332	587.340	585.324	P[5]
K[26]	2724.723	505.298	489.279	490.287	488.271	K[4]
K[27]	2852.818	377.203	361.184	362.192	360.177	K[3]
T[28]	2953.865	249.108	233.089	234.097	232.082	T[2]
E[29]	3082.908	148.060	132.042	133.050	131.034	E[1]

sp|Q6GSS7|H2A2A_MOUSE

LNKLLGK $^{Dimethyl}_{28.03}$ VTIAQGGVLPNIQAVLLPKKTE

- fragmentation table for charge state 2+
- specType=etd
- score=32.53
- F104920.dat
- query=q2468_p1
- precursor=1028.630600
- chargePrecursor=3
- itol=0.8

AA	c	y	z+1	z+2	z	AA
L[1]	66.063	**1542.450**	**1534.440**	0.504	1533.936	L[29]
N[2]	123.084	1485.908	**1477.898**	**1478.402**	**1477.394**	N[28]
K[3]	187.132	1428.886	**1420.877**	**1421.381**	**1420.373**	K[27]
L[4]	243.674	1364.839	**1356.829**	**1357.333**	**1356.325**	L[26]
L[5]	300.216	1308.297	1300.287	1300.791	1299.783	L[25]
G[6]	328.726	1251.755	1243.745	**1244.249**	1243.241	G[24]
K[7]	406.789	1223.244	1215.234	1215.738	1214.731	K[23]
V[8]	456.324	1145.181	1137.171	1137.675	1136.667	V[22]
T[9]	506.848	1095.646	1087.637	1088.141	1087.133	T[21]
I[10]	563.390	1045.123	1037.113	1037.617	1036.609	I[20]
A[11]	**598.908**	988.581	980.571	981.075	980.067	A[19]
Q[12]	662.937	953.062	945.053	945.557	944.549	Q[18]
G[13]	691.448	889.033	881.023	881.527	880.519	G[17]
G[14]	719.959	860.522	852.513	853.017	852.009	G[16]
V[15]	769.493	832.011	824.002	824.506	823.498	V[15]
L[16]	826.035	782.477	774.468	774.972	773.964	L[14]
P[17]	874.561	725.935	717.926	718.430	717.422	P[13]
N[18]	931.583	677.409	669.399	669.903	668.895	N[12]
I[19]	988.125	620.387	612.378	612.882	611.874	I[11]
Q[20]	1052.154	563.845	555.836	556.340	555.332	Q[10]
A[21]	1087.673	499.816	491.807	492.310	491.303	A[9]
V[22]	1137.207	464.297	456.288	456.792	455.784	V[8]
L[23]	1193.749	414.763	406.754	407.258	406.250	L[7]
L[24]	1250.291	358.221	350.212	350.716	349.708	L[6]
P[25]	1298.817	301.679	293.670	294.174	293.166	P[5]
K[26]	**1362.865**	253.153	245.143	245.647	244.639	K[4]
K[27]	**1426.912**	189.105	181.096	181.600	180.592	K[3]
T[28]	**1477.436**	125.058	117.048	117.552	116.544	T[2]
E[29]	**1541.958**	74.534	66.524	67.028	66.021	E[1]

sp|Q6GSS7|H2A2A_MOUSE

ELNKLLGK $^{\text{Dimethyl}}_{28.03}$ VTIAQGGVLPNIQAVLLPKKTE

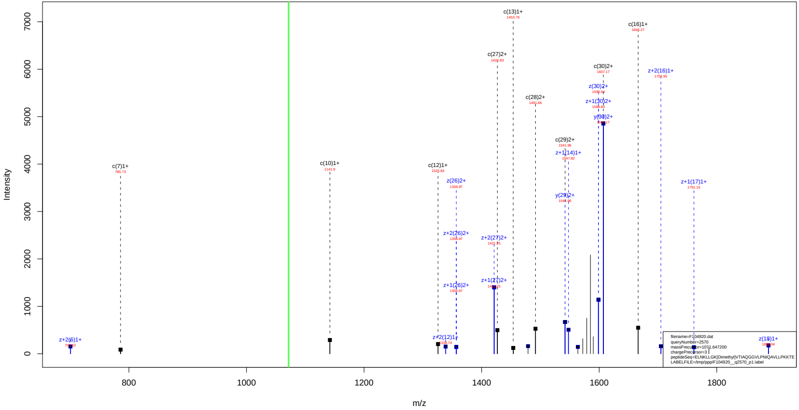

sp|Q6GSS7|H2A2A_MOUSE

ELNKLLGK $^{Dimethyl}_{28.03}$ VTIAQGGVLPNIQAVLLPKKTE

- fragmentation table for charge state 1+
- specType=etd
- score=34.48
- F104920.dat
- query=q2570_p1
- precursor=1071.647200
- chargePrecursor=3
- itol=0.8

AA	c	y	z+1	z+2	z	AA
E[1]	147.076	3212.934	3196.916	0.000	3195.908	E[30]
L[2]	260.160	3083.892	3067.873	0.000	3066.865	L[29]
N[3]	374.203	2970.808	2954.789	2955.797	2953.781	N[28]
K[4]	502.298	2856.765	2840.746	2841.754	2839.738	K[27]
L[5]	615.382	2728.670	2712.651	2713.659	2711.643	L[26]
L[6]	728.466	2615.586	2599.567	2600.575	2598.559	L[25]
G[7]	**785.488**	2502.502	2486.483	2487.491	2485.475	G[24]
K[8]	941.614	2445.480	2429.462	2430.469	2428.454	K[23]
V[9]	1040.683	2289.354	2273.335	2274.343	2272.327	V[22]
T[10]	**1141.730**	2190.286	2174.267	2175.275	2173.259	T[21]
I[11]	1254.814	2089.238	2073.219	2074.227	2072.211	I[20]
A[12]	**1325.851**	1976.154	1960.135	1961.143	1959.127	A[19]
Q[13]	**1453.910**	1905.117	1889.098	1890.106	**1888.090**	Q[18]
G[14]	1510.932	1777.058	**1761.039**	1762.047	1760.032	G[17]
G[15]	1567.953	1720.037	1704.018	**1705.026**	1703.010	G[16]
V[16]	**1667.021**	1663.015	1646.997	1648.004	1645.989	V[15]
L[17]	1780.105	**1563.947**	**1547.928**	1548.936	1546.920	L[14]
P[18]	1877.158	1450.863	1434.844	1435.852	1433.836	P[13]
N[19]	1991.201	1353.810	1337.791	**1338.799**	1336.783	N[12]
I[20]	2104.285	1239.767	1223.748	1224.756	1222.741	I[11]
Q[21]	2232.344	1126.683	1110.664	1111.672	1109.656	Q[10]
A[22]	2303.381	998.624	982.606	983.614	981.598	A[9]
V[23]	2402.449	927.587	911.569	912.576	910.561	V[8]
L[24]	2515.533	828.519	812.500	813.508	811.492	L[7]
L[25]	2628.617	715.435	699.416	**700.424**	698.408	L[6]
P[26]	2725.670	602.351	586.332	587.340	585.324	P[5]
K[27]	2853.765	505.298	489.279	490.287	488.271	K[4]
K[28]	2981.860	377.203	361.184	362.192	360.177	K[3]
T[29]	3082.908	249.108	233.089	234.097	232.082	T[2]
E[30]	3211.950	148.060	132.042	133.050	131.034	E[1]

sp|Q6GSS7|H2A2A_MOUSE

ELNKLLGK $^{\text{Dimethyl}}_{28.03}$ VTIAQGGVLPNIQAVLLPKKTE

- fragmentation table for charge state 2+
- specType=etd
- score=34.48
- F104920.dat
- query=q2570_p1
- precursor=1071.647200
- chargePrecursor=3
- itol=0.8

AA	c	y	z+1	z+2	z	AA
E[1]	74.042	**1606.971**	**1598.962**	0.504	**1598.458**	E[30]
L[2]	130.584	**1542.450**	1534.440	0.504	1533.936	L[29]
N[3]	187.605	1485.908	1477.898	**1478.402**	1477.394	N[28]
K[4]	251.653	1428.886	**1420.877**	**1421.381**	1420.373	K[27]
L[5]	308.195	1364.839	**1356.829**	**1357.333**	**1356.325**	L[26]
L[6]	364.737	1308.297	1300.287	1300.791	1299.783	L[25]
G[7]	393.248	1251.755	1243.745	1244.249	1243.241	G[24]
K[8]	471.311	1223.244	1215.234	1215.738	1214.731	K[23]
V[9]	520.845	1145.181	1137.171	1137.675	1136.667	V[22]
T[10]	571.369	1095.646	1087.637	1088.141	1087.133	T[21]
I[11]	627.911	1045.123	1037.113	1037.617	1036.609	I[20]
A[12]	663.429	988.581	980.571	981.075	980.067	A[19]
Q[13]	727.459	953.062	945.053	945.557	944.549	Q[18]
G[14]	755.969	889.033	881.023	881.527	880.519	G[17]
G[15]	784.480	860.522	852.513	853.017	852.009	G[16]
V[16]	834.014	832.011	824.002	824.506	823.498	V[15]
L[17]	890.556	782.477	774.468	774.972	773.964	L[14]
P[18]	939.083	725.935	717.926	718.430	717.422	P[13]
N[19]	996.104	677.409	669.399	669.903	668.895	N[12]
I[20]	1052.646	620.387	612.378	612.882	611.874	I[11]
Q[21]	1116.676	563.845	555.836	556.340	555.332	Q[10]
A[22]	1152.194	499.816	491.807	492.310	491.303	A[9]
V[23]	1201.728	464.297	456.288	456.792	455.784	V[8]
L[24]	1258.270	414.763	406.754	407.258	406.250	L[7]
L[25]	1314.812	358.221	350.212	350.716	349.708	L[6]
P[26]	1363.339	301.679	293.670	294.174	293.166	P[5]
K[27]	**1427.386**	253.153	245.143	245.647	244.639	K[4]
K[28]	**1491.434**	189.105	181.096	181.600	180.592	K[3]
T[29]	**1541.958**	125.058	117.048	117.552	116.544	T[2]
E[30]	**1606.479**	74.534	66.524	67.028	66.021	E[1]

sp|Q6GSS7|H2A2_MOUSE

ELNKLLGK $^{\text{Dimethyl}}_{28.03}$ VTIAQGGVLPNIQAVLLPKKTE

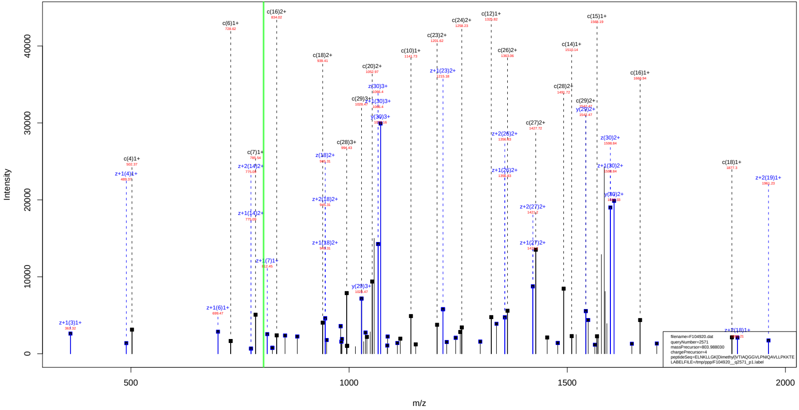

sp|Q6GSS7|H2A2A_MOUSE

ELNKLLGK $^{\text{Dimethyl}}_{28.03}$ VTIAQGGVLPNIQAVLLPKKTE

- fragmentation table for charge state 1+
- specType=etd
- score=135.22
- F104920.dat
- query=q2571_p1
- precursor=803.988030
- chargePrecursor=4
- itol=0.8

AA	c	y	z+1	z+2	z	AA
E[1]	147.076	3212.934	3196.916	0.000	3195.908	E[30]
L[2]	260.160	3083.892	3067.873	0.000	3066.865	L[29]
N[3]	374.203	2970.808	2954.789	2955.797	2953.781	N[28]
K[4]	**502.298**	2856.765	2840.746	2841.754	2839.738	K[27]
L[5]	615.382	2728.670	2712.651	2713.659	2711.643	L[26]
L[6]	**728.466**	2615.586	2599.567	2600.575	2598.559	L[25]
G[7]	**785.488**	2502.502	2486.483	2487.491	2485.475	G[24]
K[8]	941.614	2445.480	2429.462	2430.469	2428.454	K[23]
V[9]	**1040.683**	2289.354	2273.335	2274.343	2272.327	V[22]
T[10]	**1141.730**	2190.286	2174.267	2175.275	2173.259	T[21]
I[11]	**1254.814**	2089.238	2073.219	2074.227	2072.211	I[20]
A[12]	**1325.851**	1976.154	1960.135	**1961.143**	1959.127	A[19]
Q[13]	**1453.910**	1905.117	1889.098	**1890.106**	1888.090	Q[18]
G[14]	**1510.932**	1777.058	1761.039	1762.047	1760.032	G[17]
G[15]	**1567.953**	1720.037	1704.018	**1705.026**	1703.010	G[16]
V[16]	**1667.021**	1663.015	1646.997	**1648.004**	1645.989	V[15]
L[17]	1780.105	**1563.947**	**1547.928**	1548.936	1546.920	L[14]
P[18]	**1877.158**	1450.863	1434.844	1435.852	1433.836	P[13]
N[19]	1991.201	1353.810	**1337.791**	1338.799	1336.783	N[12]
I[20]	2104.285	1239.767	**1223.748**	1224.756	1222.741	I[11]
Q[21]	2232.344	1126.683	**1110.664**	1111.672	1109.656	Q[10]
A[22]	2303.381	998.624	**982.606**	**983.614**	**981.598**	A[9]
V[23]	2402.449	927.587	911.569	912.576	910.561	V[8]
L[24]	2515.533	828.519	**812.500**	813.508	811.492	L[7]
L[25]	2628.617	715.435	**699.416**	700.424	698.408	L[6]
P[26]	2725.670	602.351	586.332	587.340	585.324	P[5]
K[27]	2853.765	505.298	**489.279**	490.287	488.271	K[4]
K[28]	2981.860	377.203	**361.184**	362.192	360.177	K[3]
T[29]	3082.908	249.108	233.089	234.097	232.082	T[2]
E[30]	3211.950	148.060	132.042	133.050	131.034	E[1]

sp|Q6GSS7|H2A2A_MOUSE

ELNKLLGK $^{\text{Dimethyl}}_{28.03}$ VTIAQGGVLPNIQAVLLPKKTE

- fragmentation table for charge state 2+
- specType=etd
- score=135.22
- F104920.dat
- query=q2571_p1
- precursor=803.988030
- chargePrecursor=4
- itol=0.8

AA	c	y	z+1	z+2	z	AA
E[1]	74.042	**1606.971**	**1598.962**	0.504	**1598.458**	E[30]
L[2]	130.584	**1542.450**	1534.440	0.504	1533.936	L[29]
N[3]	187.605	1485.908	**1477.898**	**1478.402**	**1477.394**	N[28]
K[4]	251.653	1428.886	**1420.877**	**1421.381**	1420.373	K[27]
L[5]	308.195	1364.839	**1356.829**	**1357.333**	**1356.325**	L[26]
L[6]	364.737	1308.297	**1300.287**	**1300.791**	1299.783	L[25]
G[7]	393.248	1251.755	**1243.745**	**1244.249**	1243.241	G[24]
K[8]	471.311	**1223.244**	**1215.234**	**1215.738**	**1214.731**	K[23]
V[9]	520.845	1145.181	1137.171	1137.675	1136.667	V[22]
T[10]	571.369	1095.646	**1087.637**	**1088.141**	**1087.133**	T[21]
I[11]	627.911	1045.123	**1037.113**	**1037.617**	1036.609	I[20]
A[12]	663.429	988.581	**980.571**	**981.075**	**980.067**	A[19]
Q[13]	727.459	953.062	**945.053**	**945.557**	**944.549**	Q[18]
G[14]	755.969	889.033	**881.023**	**881.527**	**880.519**	G[17]
G[15]	784.480	860.522	**852.513**	**853.017**	852.009	G[16]
V[16]	**834.014**	832.011	**824.002**	**824.506**	**823.498**	V[15]
L[17]	890.556	782.477	**774.468**	**774.972**	773.964	L[14]
P[18]	**939.083**	725.935	717.926	718.430	717.422	P[13]
N[19]	**996.104**	677.409	669.399	669.903	668.895	N[12]
I[20]	**1052.646**	620.387	612.378	612.882	611.874	I[11]
Q[21]	**1116.676**	563.845	555.836	556.340	555.332	Q[10]
A[22]	**1152.194**	499.816	491.807	492.310	491.303	A[9]
V[23]	**1201.728**	464.297	456.288	456.792	455.784	V[8]
L[24]	**1258.270**	414.763	406.754	407.258	406.250	L[7]
L[25]	1314.812	358.221	350.212	350.716	349.708	L[6]
P[26]	**1363.339**	301.679	293.670	294.174	293.166	P[5]
K[27]	**1427.386**	253.153	245.143	245.647	244.639	K[4]
K[28]	**1491.434**	189.105	181.096	181.600	180.592	K[3]
T[29]	**1541.958**	125.058	117.048	117.552	116.544	T[2]
E[30]	1606.479	74.534	66.524	67.028	66.021	E[1]

sp|Q6GSS7|H2A2A_MOUSE

ELNKLLGK $^{\text{Dimethyl}}_{28.03}$ VTIAQGGVLPNIQAVLLPKKTE

- fragmentation table for charge state 3+
- specType=etd
- score=135.22
- F104920.dat
- query=q2571_p1
- precursor=803.988030
- chargePrecursor=4
- itol=0.8

AA	c	y	z+1	z+2	z	AA
E[1]	49.697	**1071.650**	**1066.310**	0.672	**1065.974**	E[30]
L[2]	87.392	**1028.635**	1023.296	0.672	1022.960	L[29]
N[3]	125.406	990.941	985.601	985.937	985.265	N[28]
K[4]	168.104	952.926	**947.587**	**947.923**	947.251	K[27]
L[5]	205.799	910.228	904.889	905.225	904.553	L[26]
L[6]	243.494	872.533	867.194	867.530	866.858	L[25]
G[7]	262.501	834.839	829.499	829.835	829.163	G[24]
K[8]	314.543	815.832	810.492	810.828	810.156	K[23]
V[9]	347.566	763.790	758.450	758.786	758.114	V[22]
T[10]	381.248	730.767	725.427	725.763	725.091	T[21]
I[11]	418.943	697.084	691.745	692.081	691.409	I[20]
A[12]	442.622	659.389	654.050	654.386	653.714	A[19]
Q[13]	485.308	635.710	630.371	630.707	630.035	Q[18]
G[14]	504.315	593.024	587.685	588.021	587.349	G[17]
G[15]	523.323	574.017	568.678	569.013	568.342	G[16]
V[16]	556.345	555.010	549.670	550.006	549.334	V[15]
L[17]	594.040	521.987	516.648	516.984	516.312	L[14]
P[18]	626.391	484.292	478.953	479.289	478.617	P[13]
N[19]	664.405	451.942	446.602	446.938	446.266	N[12]
I[20]	702.100	413.927	408.588	408.924	408.252	I[11]
Q[21]	744.786	376.233	370.893	371.229	370.557	Q[10]
A[22]	768.465	333.546	328.207	328.543	327.871	A[9]
V[23]	801.488	309.867	304.528	304.864	304.192	V[8]
L[24]	839.183	276.844	271.505	271.841	271.169	L[7]
L[25]	876.877	239.150	233.810	234.146	233.474	L[6]
P[26]	909.228	201.455	196.116	196.451	195.780	P[5]
K[27]	951.927	169.104	163.765	164.101	163.429	K[4]
K[28]	**994.625**	126.406	121.066	121.402	120.730	K[3]
T[29]	**1028.307**	83.708	78.368	78.704	78.032	T[2]
E[30]	1071.322	50.025	44.685	45.021	44.349	E[1]

sp|Q6GSS7|H2A2A_MOUSE

LNKLLGK $^{Dimethyl}_{28.03}$ VTIAQGGVLPNIQAVLLPKKTE

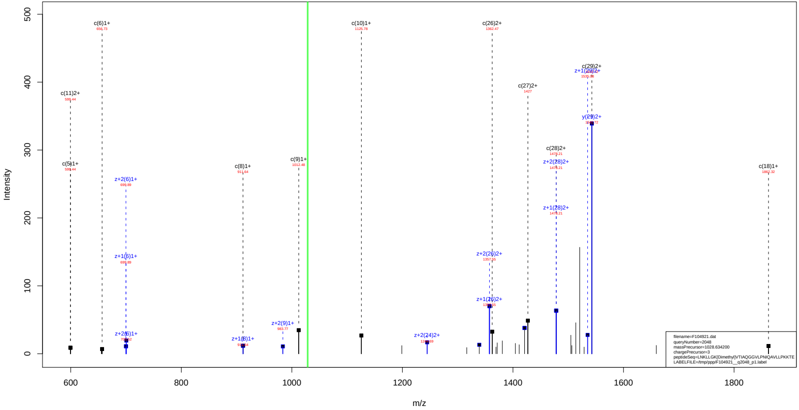

sp|Q6GSS7|H2A2A_MOUSE

LNKLLGK $^{Dimethyl}_{28.03}$ VTIAQGGVLPNIQAVLLPKKTE

- fragmentation table for charge state 1+
- specType=etd
- score=33.10
- F104921.dat
- query=q2048_p1
- precursor=1028.634200
- chargePrecursor=3
- itol=0.8

AA	c	y	z+1	z+2	z	AA
L[1]	131.118	3083.892	3067.873	0.000	3066.865	L[29]
N[2]	245.161	2970.808	2954.789	2955.797	2953.781	N[28]
K[3]	373.256	2856.765	2840.746	2841.754	2839.738	K[27]
L[4]	486.340	2728.670	2712.651	2713.659	2711.643	L[26]
L[5]	**599.424**	2615.586	2599.567	2600.575	2598.559	L[25]
G[6]	**656.445**	2502.502	2486.483	2487.491	2485.475	G[24]
K[7]	812.572	2445.480	2429.462	2430.469	2428.454	K[23]
V[8]	**911.640**	2289.354	2273.335	2274.343	2272.327	V[22]
T[9]	**1012.688**	2190.286	2174.267	2175.275	2173.259	T[21]
I[10]	**1125.772**	2089.238	2073.219	2074.227	2072.211	I[20]
A[11]	1196.809	1976.154	1960.135	1961.143	1959.127	A[19]
Q[12]	1324.867	1905.117	1889.098	1890.106	1888.090	Q[18]
G[13]	1381.889	1777.058	1761.039	1762.047	1760.032	G[17]
G[14]	1438.910	1720.037	1704.018	1705.026	1703.010	G[16]
V[15]	1537.979	1663.015	1646.997	1648.004	1645.989	V[15]
L[16]	1651.063	1563.947	1547.928	1548.936	1546.920	L[14]
P[17]	1748.116	1450.863	1434.844	1435.852	1433.836	P[13]
N[18]	**1862.159**	1353.810	1337.791	**1338.799**	1336.783	N[12]
I[19]	1975.243	1239.767	1223.748	1224.756	1222.741	I[11]
Q[20]	2103.301	1126.683	1110.664	1111.672	1109.656	Q[10]
A[21]	2174.338	998.624	982.606	**983.614**	981.598	A[9]
V[22]	2273.407	927.587	**911.569**	912.576	910.561	V[8]
L[23]	2386.491	828.519	812.500	813.508	811.492	L[7]
L[24]	2499.575	715.435	**699.416**	**700.424**	698.408	L[6]
P[25]	2596.628	602.351	586.332	587.340	585.324	P[5]
K[26]	2724.723	505.298	489.279	490.287	488.271	K[4]
K[27]	2852.818	377.203	361.184	362.192	360.177	K[3]
T[28]	2953.865	249.108	233.089	234.097	232.082	T[2]
E[29]	3082.908	148.060	132.042	133.050	131.034	E[1]

sp|Q6GSS7|H2A2A_MOUSE

LNKLLGK $^{\text{Dimethyl}}_{28.03}$ VTIAQGGVLPNIQAVLLPKKTE

- fragmentation table for charge state 2+
- specType=etd
- score=33.10
- F104921.dat
- query=q2048_p1
- precursor=1028.634200
- chargePrecursor=3
- itol=0.8

AA	c	y	z+1	z+2	z	AA
L[1]	66.063	**1542.450**	**1534.440**	0.504	1533.936	L[29]
N[2]	123.084	1485.908	**1477.898**	**1478.402**	1477.394	N[28]
K[3]	187.132	1428.886	**1420.877**	**1421.381**	**1420.373**	K[27]
L[4]	243.674	1364.839	**1356.829**	**1357.333**	1356.325	L[26]
L[5]	300.216	1308.297	1300.287	1300.791	1299.783	L[25]
G[6]	328.726	1251.755	1243.745	**1244.249**	1243.241	G[24]
K[7]	406.789	1223.244	1215.234	1215.738	1214.731	K[23]
V[8]	456.324	1145.181	1137.171	1137.675	1136.667	V[22]
T[9]	506.848	1095.646	1087.637	1088.141	1087.133	T[21]
I[10]	563.390	1045.123	1037.113	1037.617	1036.609	I[20]
A[11]	**598.908**	988.581	980.571	981.075	980.067	A[19]
Q[12]	662.937	953.062	945.053	945.557	944.549	Q[18]
G[13]	691.448	889.033	881.023	881.527	880.519	G[17]
G[14]	719.959	860.522	852.513	853.017	852.009	G[16]
V[15]	769.493	832.011	824.002	824.506	823.498	V[15]
L[16]	826.035	782.477	774.468	774.972	773.964	L[14]
P[17]	874.561	725.935	717.926	718.430	717.422	P[13]
N[18]	931.583	677.409	669.399	669.903	668.895	N[12]
I[19]	988.125	620.387	612.378	612.882	611.874	I[11]
Q[20]	1052.154	563.845	555.836	556.340	555.332	Q[10]
A[21]	1087.673	499.816	491.807	492.310	491.303	A[9]
V[22]	1137.207	464.297	456.288	456.792	455.784	V[8]
L[23]	1193.749	414.763	406.754	407.258	406.250	L[7]
L[24]	1250.291	358.221	350.212	350.716	349.708	L[6]
P[25]	1298.817	301.679	293.670	294.174	293.166	P[5]
K[26]	**1362.865**	253.153	245.143	245.647	244.639	K[4]
K[27]	**1426.912**	189.105	181.096	181.600	180.592	K[3]
T[28]	**1477.436**	125.058	117.048	117.552	116.544	T[2]
E[29]	**1541.958**	74.534	66.524	67.028	66.021	E[1]

sp|Q6GSS7|H2A2A_MOUSE

LNKLLGK $^{\text{Dimethyl}}_{28.03}$ VTIAQGGVLPNIQAVLLPKKTE

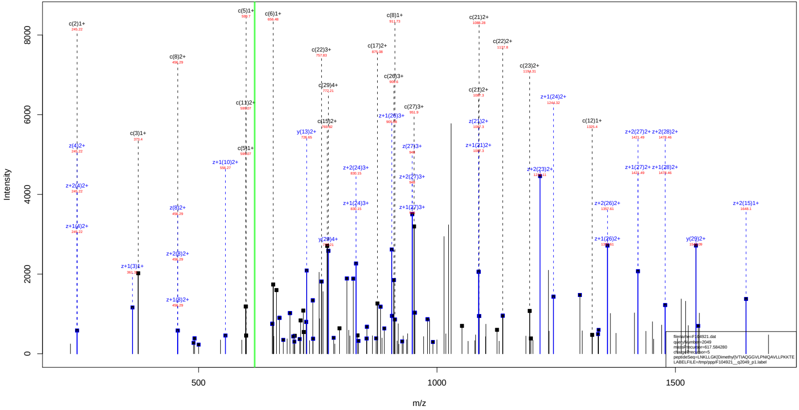

sp|Q6GSS7|H2A2A_MOUSE

LNKLLGK $^{Dimethyl}_{28.03}$ VTIAQGGVLPNIQAVLLPKKTE

- fragmentation table for charge state 1+
- specType=etd
- score=72.31
- F104921.dat
- query=q2049_p1
- precursor=617.584280
- chargePrecursor=5
- itol=0.8

AA	c	y	z+1	z+2	z	AA
L[1]	131.118	3083.892	3067.873	0.000	3066.865	L[29]
N[2]	**245.161**	2970.808	2954.789	2955.797	2953.781	N[28]
K[3]	**373.256**	2856.765	2840.746	2841.754	2839.738	K[27]
L[4]	486.340	2728.670	2712.651	2713.659	2711.643	L[26]
L[5]	**599.424**	2615.586	2599.567	2600.575	2598.559	L[25]
G[6]	**656.445**	2502.502	2486.483	2487.491	2485.475	G[24]
K[7]	812.572	2445.480	2429.462	2430.469	2428.454	K[23]
V[8]	**911.640**	2289.354	2273.335	2274.343	2272.327	V[22]
T[9]	1012.688	2190.286	2174.267	2175.275	2173.259	T[21]
I[10]	**1125.772**	2089.238	2073.219	2074.227	2072.211	I[20]
A[11]	**1196.809**	1976.154	1960.135	1961.143	1959.127	A[19]
Q[12]	**1324.867**	1905.117	1889.098	1890.106	1888.090	Q[18]
G[13]	1381.889	1777.058	1761.039	1762.047	1760.032	G[17]
G[14]	1438.910	1720.037	1704.018	1705.026	1703.010	G[16]
V[15]	1537.979	1663.015	1646.997	**1648.004**	1645.989	V[15]
L[16]	1651.063	1563.947	**1547.928**	1548.936	1546.920	L[14]
P[17]	1748.116	1450.863	1434.844	1435.852	1433.836	P[13]
N[18]	1862.159	1353.810	**1337.791**	**1338.799**	1336.783	N[12]
I[19]	1975.243	1239.767	1223.748	1224.756	1222.741	I[11]
Q[20]	2103.301	1126.683	1110.664	1111.672	1109.656	Q[10]
A[21]	2174.338	998.624	982.606	983.614	981.598	A[9]
V[22]	2273.407	**927.587**	**911.569**	912.576	910.561	V[8]
L[23]	2386.491	828.519	812.500	813.508	**811.492**	L[7]
L[24]	2499.575	715.435	699.416	**700.424**	**698.408**	L[6]
P[25]	2596.628	602.351	586.332	587.340	585.324	P[5]
K[26]	2724.723	505.298	**489.279**	490.287	488.271	K[4]
K[27]	2852.818	377.203	**361.184**	362.192	360.177	K[3]
T[28]	2953.865	249.108	233.089	234.097	232.082	T[2]
E[29]	3082.908	148.060	132.042	133.050	131.034	E[1]

sp|Q6GSS7|H2A2A_MOUSE

LNKLLGK $^{Dimethyl}_{28.03}$ VTIAQGGVLPNIQAVLLPKKTE

- fragmentation table for charge state 2+
- specType=etd
- score=72.31
- F104921.dat
- query=q2049_p1
- precursor=617.584280
- chargePrecursor=5
- itol=0.8

AA	c	y	z+1	z+2	z	AA
L[1]	66.063	**1542.450**	1534.440	0.504	1533.936	L[29]
N[2]	123.084	1485.908	**1477.898**	**1478.402**	1477.394	N[28]
K[3]	187.132	1428.886	**1420.877**	**1421.381**	1420.373	K[27]
L[4]	243.674	1364.839	**1356.829**	**1357.333**	1356.325	L[26]
L[5]	300.216	1308.297	**1300.287**	1300.791	**1299.783**	L[25]
G[6]	328.726	1251.755	**1243.745**	**1244.249**	1243.241	G[24]
K[7]	406.789	1223.244	1215.234	**1215.738**	1214.731	K[23]
V[8]	**456.324**	1145.181	**1137.171**	**1137.675**	1136.667	V[22]
T[9]	506.848	1095.646	**1087.637**	**1088.141**	**1087.133**	T[21]
I[10]	563.390	1045.123	1037.113	1037.617	1036.609	I[20]
A[11]	**598.908**	988.581	**980.571**	981.075	**980.067**	A[19]
Q[12]	**662.937**	**953.062**	945.053	945.557	944.549	Q[18]
G[13]	**691.448**	**889.033**	**881.023**	**881.527**	880.519	G[17]
G[14]	**719.959**	860.522	**852.513**	**853.017**	**852.009**	G[16]
V[15]	**769.493**	832.011	**824.002**	**824.506**	823.498	V[15]
L[16]	826.035	**782.477**	774.468	774.972	773.964	L[14]
P[17]	**874.561**	**725.935**	717.926	718.430	717.422	P[13]
N[18]	931.583	**677.409**	**669.399**	**669.903**	**668.895**	N[12]
I[19]	988.125	620.387	612.378	612.882	611.874	I[11]
Q[20]	**1052.154**	563.845	**555.836**	**556.340**	555.332	Q[10]
A[21]	**1087.673**	**499.816**	**491.807**	492.310	**491.303**	A[9]
V[22]	**1137.207**	464.297	**456.288**	**456.792**	**455.784**	V[8]
L[23]	**1193.749**	414.763	406.754	407.258	406.250	L[7]
L[24]	1250.291	358.221	350.212	350.716	349.708	L[6]
P[25]	1298.817	301.679	293.670	294.174	293.166	P[5]
K[26]	1362.865	253.153	**245.143**	**245.647**	**244.639**	K[4]
K[27]	1426.912	189.105	181.096	181.600	180.592	K[3]
T[28]	1477.436	125.058	117.048	117.552	116.544	T[2]
E[29]	1541.958	74.534	66.524	67.028	66.021	E[1]

sp|Q6GSS7|H2A2A_MOUSE

LNKLLGK $^{Dimethyl}_{28.03}$ VTIAQGGVLPNIQAVLLPKKTE

- fragmentation table for charge state 3+
- specType=etd
- score=72.31
- F104921.dat
- query=q2049_p1
- precursor=617.584280
- chargePrecursor=5
- itol=0.8

AA	c	y	z+1	z+2	z	AA
L[1]	44.377	1028.635	1023.296	0.672	1022.960	L[29]
N[2]	82.392	**990.941**	985.601	985.937	985.265	N[28]
K[3]	125.090	**952.926**	**947.587**	**947.923**	**947.251**	K[27]
L[4]	162.785	**910.228**	**904.889**	**905.225**	**904.553**	L[26]
L[5]	200.479	**872.533**	867.194	867.530	866.858	L[25]
G[6]	219.487	**834.839**	**829.499**	**829.835**	829.163	G[24]
K[7]	271.529	815.832	**810.492**	**810.828**	810.156	K[23]
V[8]	304.552	763.790	**758.450**	758.786	**758.114**	V[22]
T[9]	338.234	730.767	**725.427**	**725.763**	**725.091**	T[21]
I[10]	375.929	697.084	**691.745**	**692.081**	**691.409**	I[20]
A[11]	399.608	659.389	**654.050**	**654.386**	**653.714**	A[19]
Q[12]	442.294	635.710	630.371	630.707	630.035	Q[18]
G[13]	461.301	593.024	587.685	588.021	587.349	G[17]
G[14]	480.308	574.017	568.678	569.013	568.342	G[16]
V[15]	513.331	555.010	549.670	550.006	549.334	V[15]
L[16]	551.026	521.987	516.648	516.984	516.312	L[14]
P[17]	583.377	484.292	478.953	479.289	478.617	P[13]
N[18]	621.391	451.942	446.602	446.938	446.266	N[12]
I[19]	659.086	413.927	408.588	408.924	408.252	I[11]
Q[20]	**701.772**	376.233	370.893	371.229	370.557	Q[10]
A[21]	**725.451**	333.546	328.207	328.543	327.871	A[9]
V[22]	**758.474**	309.867	304.528	304.864	304.192	V[8]
L[23]	**796.168**	276.844	271.505	271.841	271.169	L[7]
L[24]	**833.863**	239.150	233.810	234.146	233.474	L[6]
P[25]	866.214	201.455	196.116	196.451	195.780	P[5]
K[26]	**908.912**	169.104	163.765	164.101	163.429	K[4]
K[27]	**951.611**	126.406	121.066	121.402	120.730	K[3]
T[28]	985.293	83.708	78.368	78.704	78.032	T[2]
E[29]	1028.307	50.025	44.685	45.021	44.349	E[1]

sp|Q6GSS7|H2A2A_MOUSE

LNKLLGK $^{\text{Dimethyl}}_{28.03}$ VTIAQGGVLPNIQAVLLPKKTE

- fragmentation table for charge state 4+
- specType=etd
- score=72.31
- F104921.dat
- query=q2049_p1
- precursor=617.584280
- chargePrecursor=5
- itol=0.8

AA	c	y	z+1	z+2	z	AA
L[1]	33.535	**771.728**	767.724	0.755	767.472	L[29]
N[2]	62.046	743.457	**739.453**	**739.705**	**739.201**	N[28]
K[3]	94.069	714.947	710.942	**711.194**	710.690	K[27]
L[4]	122.340	682.923	678.918	679.170	678.666	L[26]
L[5]	150.611	**654.652**	650.647	650.899	650.395	L[25]
G[6]	164.867	626.381	622.376	622.628	622.124	G[24]
K[7]	203.898	612.126	608.121	608.373	607.869	K[23]
V[8]	228.665	573.094	569.089	569.341	568.837	V[22]
T[9]	253.927	548.327	544.322	544.574	544.070	T[21]
I[10]	282.198	523.065	519.060	519.312	518.808	I[20]
A[11]	299.958	494.794	**490.789**	**491.041**	490.537	A[19]
Q[12]	331.972	477.035	473.030	473.282	472.778	Q[18]
G[13]	346.228	445.020	441.015	441.267	440.763	G[17]
G[14]	360.483	430.765	426.760	427.012	426.508	G[16]
V[15]	385.250	416.509	412.505	412.757	412.253	V[15]
L[16]	413.521	391.742	387.737	387.989	387.486	L[14]
P[17]	437.784	363.471	359.466	359.718	359.215	P[13]
N[18]	466.295	339.208	335.203	335.455	334.951	N[12]
I[19]	494.566	310.697	306.693	306.945	306.441	I[11]
Q[20]	526.581	282.426	278.422	278.673	278.170	Q[10]
A[21]	544.340	250.412	246.407	246.659	246.155	A[9]
V[22]	569.107	232.652	228.648	228.900	228.396	V[8]
L[23]	597.378	207.885	203.881	204.132	203.629	L[7]
L[24]	625.649	179.614	175.609	175.861	175.358	L[6]
P[25]	649.912	151.343	147.338	147.590	147.087	P[5]
K[26]	681.936	127.080	123.075	123.327	122.823	K[4]
K[27]	**713.960**	95.056	91.052	91.304	90.800	K[3]
T[28]	**739.222**	63.032	59.028	59.280	58.776	T[2]
E[29]	**771.482**	37.771	33.766	34.018	33.514	E[1]

sp|Q6GSS7|H2A2A_MOUSE

LNKLLGK $^{Dimethyl}_{28.03}$ VTIAQGGVLPNIQAVLLPKKTE

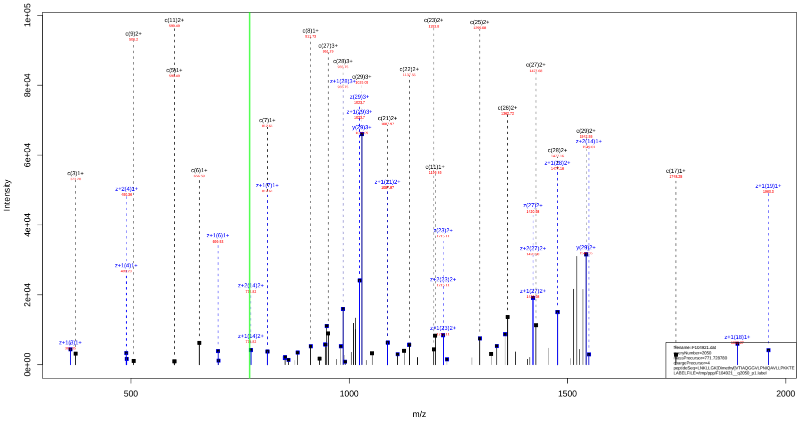

sp|Q6GSS7|H2A2A_MOUSE

LNKLLGK $^{Dimethyl}_{28.03}$ VTIAQGGVLPNIQAVLLPKKTE

- fragmentation table for charge state 1+
- specType=etd
- score=84.23
- F104921.dat
- query=q2050_p1
- precursor=771.728780
- chargePrecursor=4
- itol=0.8

AA	c	y	z+1	z+2	z	AA
L[1]	131.118	3083.892	3067.873	0.000	3066.865	L[29]
N[2]	245.161	2970.808	2954.789	2955.797	2953.781	N[28]
K[3]	**373.256**	2856.765	2840.746	2841.754	2839.738	K[27]
L[4]	486.340	2728.670	2712.651	2713.659	2711.643	L[26]
L[5]	**599.424**	2615.586	2599.567	2600.575	2598.559	L[25]
G[6]	**656.445**	2502.502	2486.483	2487.491	2485.475	G[24]
K[7]	**812.572**	2445.480	2429.462	2430.469	2428.454	K[23]
V[8]	**911.640**	2289.354	2273.335	2274.343	2272.327	V[22]
T[9]	1012.688	2190.286	2174.267	2175.275	2173.259	T[21]
I[10]	**1125.772**	2089.238	2073.219	2074.227	2072.211	I[20]
A[11]	**1196.809**	1976.154	**1960.135**	1961.143	1959.127	A[19]
Q[12]	**1324.867**	1905.117	**1889.098**	1890.106	1888.090	Q[18]
G[13]	1381.889	1777.058	1761.039	1762.047	1760.032	G[17]
G[14]	1438.910	1720.037	1704.018	1705.026	1703.010	G[16]
V[15]	1537.979	1663.015	1646.997	1648.004	1645.989	V[15]
L[16]	1651.063	1563.947	1547.928	**1548.936**	1546.920	L[14]
P[17]	**1748.116**	1450.863	1434.844	1435.852	1433.836	P[13]
N[18]	1862.159	1353.810	**1337.791**	1338.799	1336.783	N[12]
I[19]	1975.243	1239.767	**1223.748**	1224.756	1222.741	I[11]
Q[20]	2103.301	1126.683	**1110.664**	1111.672	1109.656	Q[10]
A[21]	2174.338	998.624	982.606	983.614	981.598	A[9]
V[22]	2273.407	927.587	**911.569**	912.576	910.561	V[8]
L[23]	2386.491	828.519	**812.500**	813.508	811.492	L[7]
L[24]	2499.575	715.435	**699.416**	**700.424**	698.408	L[6]
P[25]	2596.628	602.351	586.332	587.340	585.324	P[5]
K[26]	2724.723	505.298	**489.279**	**490.287**	488.271	K[4]
K[27]	2852.818	377.203	**361.184**	362.192	360.177	K[3]
T[28]	2953.865	249.108	233.089	234.097	232.082	T[2]
E[29]	3082.908	148.060	132.042	133.050	131.034	E[1]

sp|Q6GSS7|H2A2A_MOUSE

LNKLLGK Dimethyl 28.03 VTIAQGGVLPNIQAVLLPKKTE

- fragmentation table for charge state 2+
- specType=etd
- score=84.23
- F104921.dat
- query=q2050_p1
- precursor=771.728780
- chargePrecursor=4
- itol=0.8

AA	c	y	z+1	z+2	z	AA
L[1]	66.063	**1542.450**	1534.440	0.504	1533.936	L[29]
N[2]	123.084	1485.908	**1477.898**	1478.402	**1477.394**	N[28]
K[3]	187.132	1428.886	**1420.877**	**1421.381**	**1420.373**	K[27]
L[4]	243.674	1364.839	**1356.829**	**1357.333**	**1356.325**	L[26]
L[5]	300.216	1308.297	1300.287	1300.791	**1299.783**	L[25]
G[6]	328.726	1251.755	1243.745	1244.249	1243.241	G[24]
K[7]	406.789	**1223.244**	**1215.234**	**1215.738**	**1214.731**	K[23]
V[8]	456.324	1145.181	**1137.171**	**1137.675**	1136.667	V[22]
T[9]	**506.848**	1095.646	**1087.637**	**1088.141**	1087.133	T[21]
I[10]	563.390	1045.123	1037.113	1037.617	1036.609	I[20]
A[11]	**598.908**	988.581	**980.571**	**981.075**	**980.067**	A[19]
Q[12]	662.937	953.062	**945.053**	**945.557**	**944.549**	Q[18]
G[13]	691.448	889.033	**881.023**	**881.527**	880.519	G[17]
G[14]	719.959	**860.522**	**852.513**	**853.017**	**852.009**	G[16]
V[15]	769.493	832.011	824.002	824.506	823.498	V[15]
L[16]	826.035	782.477	**774.468**	**774.972**	773.964	L[14]
P[17]	874.561	725.935	717.926	718.430	717.422	P[13]
N[18]	**931.583**	677.409	669.399	669.903	668.895	N[12]
I[19]	988.125	620.387	612.378	612.882	611.874	I[11]
Q[20]	**1052.154**	563.845	555.836	556.340	555.332	Q[10]
A[21]	**1087.673**	499.816	491.807	492.310	491.303	A[9]
V[22]	**1137.207**	464.297	456.288	456.792	455.784	V[8]
L[23]	**1193.749**	414.763	406.754	407.258	406.250	L[7]
L[24]	1250.291	358.221	350.212	350.716	349.708	L[6]
P[25]	**1298.817**	301.679	293.670	294.174	293.166	P[5]
K[26]	**1362.865**	253.153	245.143	245.647	244.639	K[4]
K[27]	**1426.912**	189.105	181.096	181.600	180.592	K[3]
T[28]	**1477.436**	125.058	117.048	117.552	116.544	T[2]
E[29]	**1541.958**	74.534	66.524	67.028	66.021	E[1]

sp|Q6GSS7|H2A2A_MOUSE

LNKLLGK $^{\text{Dimethyl}}_{28.03}$ VTIAQGGVLPNIQAVLLPKKTE

- fragmentation table for charge state 3+
- specType=etd
- score=84.23
- F104921.dat
- query=q2050_p1
- precursor=771.728780
- chargePrecursor=4
- itol=0.8

AA	c	y	z+1	z+2	z	AA
L[1]	44.377	**1028.635**	**1023.296**	0.672	**1022.960**	L[29]
N[2]	82.392	**990.941**	**985.601**	**985.937**	**985.265**	N[28]
K[3]	125.090	952.926	**947.587**	**947.923**	947.251	K[27]
L[4]	162.785	910.228	904.889	905.225	904.553	L[26]
L[5]	200.479	872.533	867.194	867.530	866.858	L[25]
G[6]	219.487	834.839	829.499	829.835	829.163	G[24]
K[7]	271.529	815.832	810.492	810.828	810.156	K[23]
V[8]	304.552	763.790	758.450	758.786	758.114	V[22]
T[9]	338.234	730.767	725.427	725.763	725.091	T[21]
I[10]	375.929	697.084	691.745	692.081	691.409	I[20]
A[11]	399.608	659.389	654.050	654.386	653.714	A[19]
Q[12]	442.294	635.710	630.371	630.707	630.035	Q[18]
G[13]	461.301	593.024	587.685	588.021	587.349	G[17]
G[14]	480.308	574.017	568.678	569.013	568.342	G[16]
V[15]	513.331	555.010	549.670	550.006	549.334	V[15]
L[16]	551.026	521.987	516.648	516.984	516.312	L[14]
P[17]	583.377	484.292	478.953	479.289	478.617	P[13]
N[18]	621.391	451.942	446.602	446.938	446.266	N[12]
I[19]	659.086	413.927	408.588	408.924	408.252	I[11]
Q[20]	701.772	376.233	370.893	371.229	370.557	Q[10]
A[21]	725.451	333.546	328.207	328.543	327.871	A[9]
V[22]	758.474	309.867	304.528	304.864	304.192	V[8]
L[23]	796.168	276.844	271.505	271.841	271.169	L[7]
L[24]	833.863	239.150	233.810	234.146	233.474	L[6]
P[25]	866.214	201.455	196.116	196.451	195.780	P[5]
K[26]	908.912	169.104	163.765	164.101	163.429	K[4]
K[27]	**951.611**	126.406	121.066	121.402	120.730	K[3]
T[28]	**985.293**	83.708	78.368	78.704	78.032	T[2]
E[29]	**1028.307**	50.025	44.685	45.021	44.349	E[1]

sp|P68433|H31_MOUSE

IAQDFK $^{\text{Dimethyl}}_{28.03}$ TD

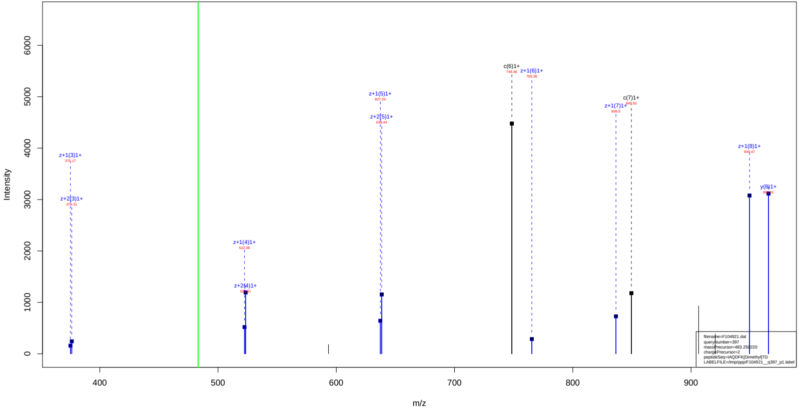

sp|P68433|H31_MOUSE

IAQDFK$^{\text{Dimethyl}}_{28.03}$TD

- fragmentation table for charge state 1+
- specType=etd
- score=49.02
- F104921.dat
- query=q397_p1
- precursor=483.250220
- chargePrecursor=2
- itol=0.8

AA	c	y	z+1	z+2	z	AA
I[1]	131.118	**965.494**	**949.475**	0.000	948.467	I[8]
A[2]	202.155	852.410	**836.391**	0.000	835.383	A[7]
Q[3]	330.214	781.373	**765.354**	766.362	764.346	Q[6]
D[4]	445.241	653.314	**637.295**	**638.303**	636.288	D[5]
F[5]	592.309	538.287	**522.268**	**523.276**	521.261	F[4]
K[6]	**748.435**	391.219	**375.200**	**376.208**	374.192	K[3]
T[7]	**849.483**	235.092	219.074	220.082	218.066	T[2]
D[8]	964.510	134.045	118.026	119.034	117.018	D[1]

sp|Q6GSS7|H2A2A_MOUSE

LNKLLGK $^{\text{Dimethyl}}_{28.03}$ VTIAQGGVLPNIQAVLLPKKTE

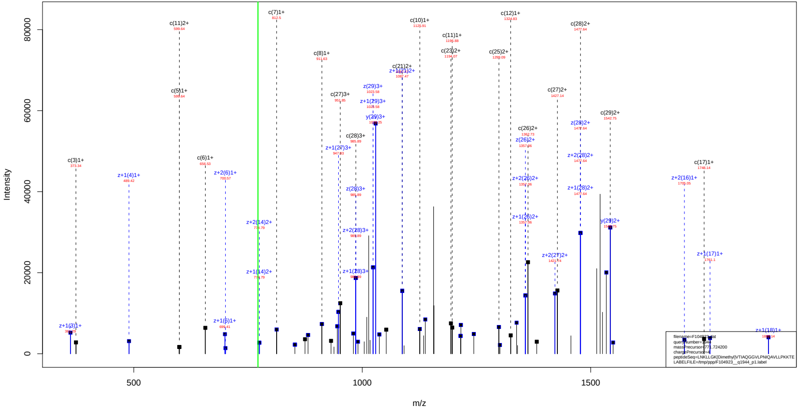

sp|Q6GSS7|H2A2A_MOUSE

LNKLLGK $^{\text{Dimethyl}}_{28.03}$ VTIAQGGVLPNIQAVLLPKKTE

- fragmentation table for charge state 1+
- specType=etd
- score=87.86
- F104923.dat
- query=q1944_p1
- precursor=771.724200
- chargePrecursor=4
- itol=0.8

AA	c	y	z+1	z+2	z	AA
L[1]	131.118	3083.892	3067.873	0.000	3066.865	L[29]
N[2]	245.161	2970.808	2954.789	2955.797	2953.781	N[28]
K[3]	**373.256**	2856.765	2840.746	2841.754	2839.738	K[27]
L[4]	486.340	2728.670	2712.651	2713.659	2711.643	L[26]
L[5]	**599.424**	2615.586	2599.567	2600.575	2598.559	L[25]
G[6]	**656.445**	2502.502	2486.483	2487.491	2485.475	G[24]
K[7]	**812.572**	2445.480	2429.462	2430.469	2428.454	K[23]
V[8]	**911.640**	2289.354	2273.335	2274.343	2272.327	V[22]
T[9]	1012.688	2190.286	2174.267	2175.275	2173.259	T[21]
I[10]	**1125.772**	2089.238	2073.219	2074.227	2072.211	I[20]
A[11]	**1196.809**	1976.154	1960.135	1961.143	1959.127	A[19]
Q[12]	**1324.867**	1905.117	**1889.098**	1890.106	1888.090	Q[18]
G[13]	**1381.889**	1777.058	**1761.039**	1762.047	1760.032	G[17]
G[14]	1438.910	1720.037	1704.018	**1705.026**	1703.010	G[16]
V[15]	1537.979	1663.015	1646.997	1648.004	1645.989	V[15]
L[16]	1651.063	1563.947	1547.928	**1548.936**	1546.920	L[14]
P[17]	**1748.116**	1450.863	1434.844	1435.852	1433.836	P[13]
N[18]	1862.159	1353.810	**1337.791**	1338.799	1336.783	N[12]
I[19]	1975.243	1239.767	1223.748	1224.756	1222.741	I[11]
Q[20]	2103.301	**1126.683**	1110.664	1111.672	1109.656	Q[10]
A[21]	2174.338	998.624	982.606	983.614	981.598	A[9]
V[22]	2273.407	927.587	**911.569**	912.576	910.561	V[8]
L[23]	2386.491	828.519	**812.500**	813.508	811.492	L[7]
L[24]	2499.575	715.435	**699.416**	**700.424**	698.408	L[6]
P[25]	2596.628	602.351	586.332	587.340	585.324	P[5]
K[26]	2724.723	505.298	**489.279**	490.287	488.271	K[4]
K[27]	2852.818	377.203	**361.184**	362.192	360.177	K[3]
T[28]	2953.865	249.108	233.089	234.097	232.082	T[2]
E[29]	3082.908	148.060	132.042	133.050	131.034	E[1]

sp|Q6GSS7|H2A2A_MOUSE

LNKLLGK $^{\text{Dimethyl}}_{28.03}$ VTIAQGGVLPNIQAVLLPKKTE

- fragmentation table for charge state 2+
- specType=etd
- score=87.86
- F104923.dat
- query=q1944_p1
- precursor=771.724200
- chargePrecursor=4
- itol=0.8

AA	c	y	z+1	z+2	z	AA
L[1]	66.063	**1542.450**	**1534.440**	0.504	**1533.936**	L[29]
N[2]	123.084	1485.908	**1477.898**	**1478.402**	**1477.394**	N[28]
K[3]	187.132	1428.886	1420.877	**1421.381**	1420.373	K[27]
L[4]	243.674	1364.839	**1356.829**	**1357.333**	**1356.325**	L[26]
L[5]	300.216	1308.297	1300.287	**1300.791**	**1299.783**	L[25]
G[6]	328.726	1251.755	**1243.745**	**1244.249**	1243.241	G[24]
K[7]	406.789	1223.244	**1215.234**	**1215.738**	**1214.731**	K[23]
V[8]	456.324	1145.181	1137.171	**1137.675**	1136.667	V[22]
T[9]	506.848	1095.646	**1087.637**	**1088.141**	**1087.133**	T[21]
I[10]	563.390	1045.123	**1037.113**	**1037.617**	1036.609	I[20]
A[11]	**598.908**	988.581	**980.571**	**981.075**	**980.067**	A[19]
Q[12]	662.937	953.062	**945.053**	**945.557**	944.549	Q[18]
G[13]	691.448	889.033	**881.023**	**881.527**	880.519	G[17]
G[14]	719.959	860.522	**852.513**	**853.017**	**852.009**	G[16]
V[15]	769.493	832.011	824.002	824.506	823.498	V[15]
L[16]	826.035	782.477	**774.468**	**774.972**	773.964	L[14]
P[17]	**874.561**	725.935	717.926	718.430	717.422	P[13]
N[18]	**931.583**	677.409	669.399	669.903	668.895	N[12]
I[19]	988.125	620.387	612.378	612.882	611.874	I[11]
Q[20]	**1052.154**	563.845	555.836	556.340	555.332	Q[10]
A[21]	**1087.673**	499.816	491.807	492.310	491.303	A[9]
V[22]	1137.207	464.297	456.288	456.792	455.784	V[8]
L[23]	**1193.749**	414.763	406.754	407.258	406.250	L[7]
L[24]	1250.291	358.221	350.212	350.716	349.708	L[6]
P[25]	**1298.817**	301.679	293.670	294.174	293.166	P[5]
K[26]	**1362.865**	253.153	245.143	245.647	244.639	K[4]
K[27]	**1426.912**	189.105	181.096	181.600	180.592	K[3]
T[28]	**1477.436**	125.058	117.048	117.552	116.544	T[2]
E[29]	**1541.958**	74.534	66.524	67.028	66.021	E[1]

sp|Q6GSS7|H2A2A_MOUSE

LNKLLGK $^{Dimethyl}_{28.03}$ VTIAQGGVLPNIQAVLLPKKTE

- fragmentation table for charge state 3+
- specType=etd
- score=87.86
- F104923.dat
- query=q1944_p1
- precursor=771.724200
- chargePrecursor=4
- itol=0.8

AA	c	y	z+1	z+2	z	AA
L[1]	44.377	**1028.635**	**1023.296**	0.672	**1022.960**	L[29]
N[2]	82.392	**990.941**	**985.601**	**985.937**	**985.265**	N[28]
K[3]	125.090	952.926	**947.587**	**947.923**	**947.251**	K[27]
L[4]	162.785	910.228	904.889	905.225	904.553	L[26]
L[5]	200.479	872.533	867.194	867.530	866.858	L[25]
G[6]	219.487	834.839	829.499	829.835	829.163	G[24]
K[7]	271.529	815.832	810.492	810.828	810.156	K[23]
V[8]	304.552	763.790	758.450	758.786	758.114	V[22]
T[9]	338.234	730.767	725.427	725.763	725.091	T[21]
I[10]	375.929	697.084	691.745	692.081	691.409	I[20]
A[11]	399.608	659.389	654.050	654.386	653.714	A[19]
Q[12]	442.294	635.710	630.371	630.707	630.035	Q[18]
G[13]	461.301	593.024	587.685	588.021	587.349	G[17]
G[14]	480.308	574.017	568.678	569.013	568.342	G[16]
V[15]	513.331	555.010	549.670	550.006	549.334	V[15]
L[16]	551.026	521.987	516.648	516.984	516.312	L[14]
P[17]	583.377	484.292	478.953	479.289	478.617	P[13]
N[18]	621.391	451.942	446.602	446.938	446.266	N[12]
I[19]	659.086	413.927	408.588	408.924	408.252	I[11]
Q[20]	701.772	376.233	370.893	371.229	370.557	Q[10]
A[21]	725.451	333.546	328.207	328.543	327.871	A[9]
V[22]	758.474	309.867	304.528	304.864	304.192	V[8]
L[23]	796.168	276.844	271.505	271.841	271.169	L[7]
L[24]	833.863	239.150	233.810	234.146	233.474	L[6]
P[25]	866.214	201.455	196.116	196.451	195.780	P[5]
K[26]	908.912	169.104	163.765	164.101	163.429	K[4]
K[27]	**951.611**	126.406	121.066	121.402	120.730	K[3]
T[28]	**985.293**	83.708	78.368	78.704	78.032	T[2]
E[29]	1028.307	50.025	44.685	45.021	44.349	E[1]

sp|Q6GSS7|H2A2A_MOUSE

LNKLLGK $^{\text{Dimethyl}}_{28.03}$ VTIAQGGVLPNIQAVLLPKKTE

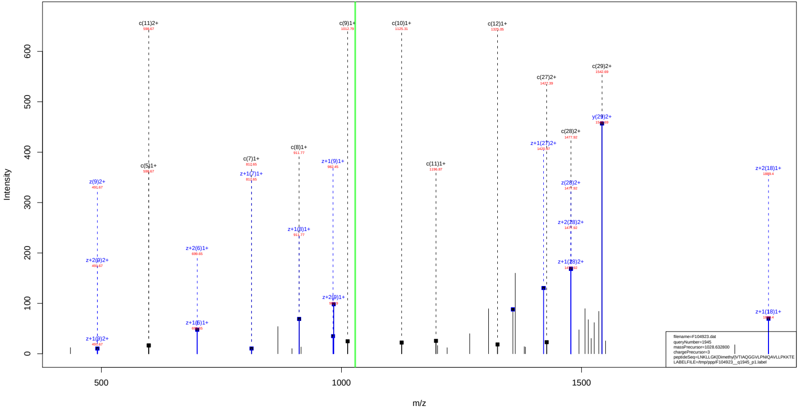

sp|Q6GSS7|H2A2A_MOUSE

LNKLLGK $^{\text{Dimethyl}}_{28.03}$ VTIAQGGVLPNIQAVLLPKKTE

- fragmentation table for charge state 1+
- specType=etd
- score=27.97
- F104923.dat
- query=q1945_p1
- precursor=1028.632800
- chargePrecursor=3
- itol=0.8

AA	c	y	z+1	z+2	z	AA
L[1]	131.118	3083.892	3067.873	0.000	3066.865	L[29]
N[2]	245.161	2970.808	2954.789	2955.797	2953.781	N[28]
K[3]	373.256	2856.765	2840.746	2841.754	2839.738	K[27]
L[4]	486.340	2728.670	2712.651	2713.659	2711.643	L[26]
L[5]	**599.424**	2615.586	2599.567	2600.575	2598.559	L[25]
G[6]	656.445	2502.502	2486.483	2487.491	2485.475	G[24]
K[7]	**812.572**	2445.480	2429.462	2430.469	2428.454	K[23]
V[8]	**911.640**	2289.354	2273.335	2274.343	2272.327	V[22]
T[9]	**1012.688**	2190.286	2174.267	2175.275	2173.259	T[21]
I[10]	**1125.772**	2089.238	2073.219	2074.227	2072.211	I[20]
A[11]	**1196.809**	1976.154	1960.135	1961.143	1959.127	A[19]
Q[12]	**1324.867**	1905.117	**1889.098**	**1890.106**	1888.090	Q[18]
G[13]	1381.889	1777.058	1761.039	1762.047	1760.032	G[17]
G[14]	1438.910	1720.037	1704.018	1705.026	1703.010	G[16]
V[15]	1537.979	1663.015	1646.997	1648.004	1645.989	V[15]
L[16]	1651.063	1563.947	1547.928	1548.936	1546.920	L[14]
P[17]	1748.116	1450.863	1434.844	1435.852	1433.836	P[13]
N[18]	1862.159	1353.810	1337.791	1338.799	1336.783	N[12]
I[19]	1975.243	1239.767	1223.748	1224.756	1222.741	I[11]
Q[20]	2103.301	1126.683	1110.664	1111.672	1109.656	Q[10]
A[21]	2174.338	998.624	**982.606**	**983.614**	981.598	A[9]
V[22]	2273.407	927.587	**911.569**	912.576	910.561	V[8]
L[23]	2386.491	828.519	**812.500**	813.508	811.492	L[7]
L[24]	2499.575	715.435	**699.416**	**700.424**	698.408	L[6]
P[25]	2596.628	602.351	586.332	587.340	585.324	P[5]
K[26]	2724.723	505.298	489.279	490.287	488.271	K[4]
K[27]	2852.818	377.203	361.184	362.192	360.177	K[3]
T[28]	2953.865	249.108	233.089	234.097	232.082	T[2]
E[29]	3082.908	148.060	132.042	133.050	131.034	E[1]

sp|Q6GSS7|H2A2A_MOUSE

LNKLLGK Dimethyl 28.03 VTIAQGGVLPNIQAVLLPKKTE

- fragmentation table for charge state 2+
- specType=etd
- score=27.97
- F104923.dat
- query=q1945_p1
- precursor=1028.632800
- chargePrecursor=3
- itol=0.8

AA	c	y	z+1	z+2	z	AA
L[1]	66.063	**1542.450**	1534.440	0.504	1533.936	L[29]
N[2]	123.084	1485.908	**1477.898**	**1478.402**	**1477.394**	N[28]
K[3]	187.132	1428.886	**1420.877**	**1421.381**	**1420.373**	K[27]
L[4]	243.674	1364.839	**1356.829**	**1357.333**	**1356.325**	L[26]
L[5]	300.216	1308.297	1300.287	1300.791	1299.783	L[25]
G[6]	328.726	1251.755	1243.745	1244.249	1243.241	G[24]
K[7]	406.789	1223.244	1215.234	1215.738	1214.731	K[23]
V[8]	456.324	1145.181	1137.171	1137.675	1136.667	V[22]
T[9]	506.848	1095.646	1087.637	1088.141	1087.133	T[21]
I[10]	563.390	1045.123	1037.113	1037.617	1036.609	I[20]
A[11]	**598.908**	988.581	980.571	981.075	980.067	A[19]
Q[12]	662.937	953.062	945.053	945.557	944.549	Q[18]
G[13]	691.448	889.033	881.023	881.527	880.519	G[17]
G[14]	719.959	860.522	852.513	853.017	852.009	G[16]
V[15]	769.493	832.011	824.002	824.506	823.498	V[15]
L[16]	826.035	782.477	774.468	774.972	773.964	L[14]
P[17]	874.561	725.935	717.926	718.430	717.422	P[13]
N[18]	931.583	677.409	669.399	669.903	668.895	N[12]
I[19]	988.125	620.387	612.378	612.882	611.874	I[11]
Q[20]	1052.154	563.845	555.836	556.340	555.332	Q[10]
A[21]	1087.673	499.816	**491.807**	**492.310**	**491.303**	A[9]
V[22]	1137.207	464.297	456.288	456.792	455.784	V[8]
L[23]	1193.749	414.763	406.754	407.258	406.250	L[7]
L[24]	1250.291	358.221	350.212	350.716	349.708	L[6]
P[25]	1298.817	301.679	293.670	294.174	293.166	P[5]
K[26]	1362.865	253.153	245.143	245.647	244.639	K[4]
K[27]	**1426.912**	189.105	181.096	181.600	180.592	K[3]
T[28]	**1477.436**	125.058	117.048	117.552	116.544	T[2]
E[29]	**1541.958**	74.534	66.524	67.028	66.021	E[1]

sp|P70696|H2B1A_MOUSE

GTK $^{Acetyl}_{42.01}$ AVTKYTSSK

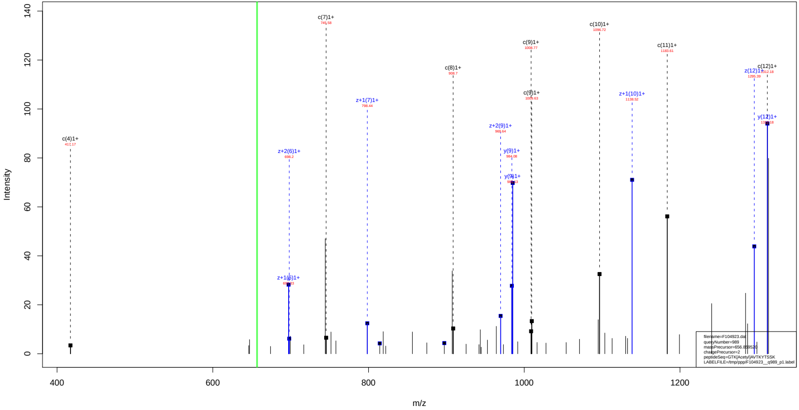

sp|P70696|H2B1A_MOUSE

GTK $^{Acetyl}_{42.01}$ AVTKYTSSK

- fragmentation table for charge state 1+
- specType=etd
- score=33.02
- F104923.dat
- query=q989_p1
- precursor=656.859520
- chargePrecursor=2
- itol=0.8

AA	c	y	z+1	z+2	z	AA
G[1]	75.055	**1312.711**	1296.692	0.000	**1295.684**	G[12]
T[2]	176.103	1255.689	1239.671	0.000	1238.663	T[11]
K[3]	346.208	1154.642	**1138.623**	1139.631	1137.615	K[10]
A[4]	**417.246**	**984.536**	968.517	**969.525**	967.509	A[9]
V[5]	516.314	913.499	**897.480**	898.488	896.472	V[8]
T[6]	617.362	**814.431**	**798.412**	799.420	797.404	T[7]
K[7]	**745.457**	713.383	**697.364**	**698.372**	696.356	K[6]
Y[8]	**908.520**	585.288	569.269	570.277	568.261	Y[5]
T[9]	**1009.568**	422.225	406.206	407.214	405.198	T[4]
S[10]	**1096.600**	321.177	305.158	306.166	304.150	S[3]
S[11]	**1183.632**	234.145	218.126	219.134	217.118	S[2]
K[12]	**1311.727**	147.113	131.094	132.102	130.086	K[1]

sp|Q6GSS7|H2A2A_MOUSE

LNKLLGK $^{Dimethyl}_{28.03}$ VTIAQGGVLPNIQAVLLPKKTE

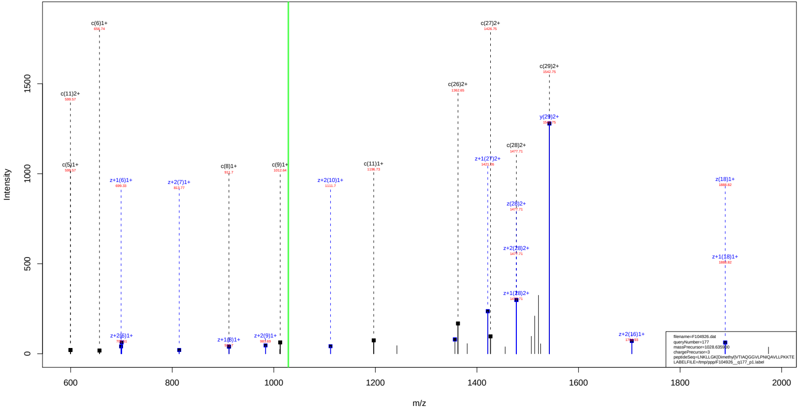

sp|Q6GSS7|H2A2A_MOUSE

LNKLLGK $^{Dimethyl}_{28.03}$ VTIAQGGVLPNIQAVLLPKKTE

- fragmentation table for charge state 1+
- specType=etd
- score=30.66
- F104926.dat
- query=q177_p1
- precursor=1028.635900
- chargePrecursor=3
- itol=0.8

AA	c	y	z+1	z+2	z	AA
L[1]	131.118	3083.892	3067.873	0.000	3066.865	L[29]
N[2]	245.161	2970.808	2954.789	2955.797	2953.781	N[28]
K[3]	373.256	2856.765	2840.746	2841.754	2839.738	K[27]
L[4]	486.340	2728.670	2712.651	2713.659	2711.643	L[26]
L[5]	**599.424**	2615.586	2599.567	2600.575	2598.559	L[25]
G[6]	**656.445**	2502.502	2486.483	2487.491	2485.475	G[24]
K[7]	812.572	2445.480	2429.462	2430.469	2428.454	K[23]
V[8]	**911.640**	2289.354	2273.335	2274.343	2272.327	V[22]
T[9]	**1012.688**	2190.286	2174.267	2175.275	2173.259	T[21]
I[10]	1125.772	2089.238	2073.219	2074.227	2072.211	I[20]
A[11]	**1196.809**	1976.154	1960.135	1961.143	1959.127	A[19]
Q[12]	1324.867	1905.117	**1889.098**	1890.106	**1888.090**	Q[18]
G[13]	1381.889	1777.058	1761.039	1762.047	1760.032	G[17]
G[14]	1438.910	1720.037	1704.018	**1705.026**	1703.010	G[16]
V[15]	1537.979	1663.015	1646.997	1648.004	1645.989	V[15]
L[16]	1651.063	1563.947	1547.928	1548.936	1546.920	L[14]
P[17]	1748.116	1450.863	1434.844	1435.852	1433.836	P[13]
N[18]	1862.159	1353.810	1337.791	1338.799	1336.783	N[12]
I[19]	1975.243	1239.767	1223.748	1224.756	1222.741	I[11]
Q[20]	2103.301	1126.683	1110.664	**1111.672**	1109.656	Q[10]
A[21]	2174.338	998.624	982.606	**983.614**	981.598	A[9]
V[22]	2273.407	927.587	**911.569**	912.576	910.561	V[8]
L[23]	2386.491	828.519	812.500	**813.508**	811.492	L[7]
L[24]	2499.575	715.435	**699.416**	**700.424**	698.408	L[6]
P[25]	2596.628	602.351	586.332	587.340	585.324	P[5]
K[26]	2724.723	505.298	489.279	490.287	488.271	K[4]
K[27]	2852.818	377.203	361.184	362.192	360.177	K[3]
T[28]	2953.865	249.108	233.089	234.097	232.082	T[2]
E[29]	3082.908	148.060	132.042	133.050	131.034	E[1]

sp|Q6GSS7|H2A2A_MOUSE

LNKLLGK $^{Dimethyl}_{28.03}$ VTIAQGGVLPNIQAVLLPKKTE

- fragmentation table for charge state 2+
- specType=etd
- score=30.66
- F104926.dat
- query=q177_p1
- precursor=1028.635900
- chargePrecursor=3
- itol=0.8

AA	c	y	z+1	z+2	z	AA
L[1]	66.063	**1542.450**	1534.440	0.504	1533.936	L[29]
N[2]	123.084	1485.908	**1477.898**	**1478.402**	**1477.394**	N[28]
K[3]	187.132	1428.886	**1420.877**	**1421.381**	1420.373	K[27]
L[4]	243.674	1364.839	**1356.829**	**1357.333**	**1356.325**	L[26]
L[5]	300.216	1308.297	1300.287	1300.791	1299.783	L[25]
G[6]	328.726	1251.755	1243.745	1244.249	1243.241	G[24]
K[7]	406.789	1223.244	1215.234	1215.738	1214.731	K[23]
V[8]	456.324	1145.181	1137.171	1137.675	1136.667	V[22]
T[9]	506.848	1095.646	1087.637	1088.141	1087.133	T[21]
I[10]	563.390	1045.123	1037.113	1037.617	1036.609	I[20]
A[11]	**598.908**	988.581	980.571	981.075	980.067	A[19]
Q[12]	662.937	953.062	945.053	945.557	944.549	Q[18]
G[13]	691.448	889.033	881.023	881.527	880.519	G[17]
G[14]	719.959	860.522	852.513	853.017	852.009	G[16]
V[15]	769.493	832.011	824.002	824.506	823.498	V[15]
L[16]	826.035	782.477	774.468	774.972	773.964	L[14]
P[17]	874.561	725.935	717.926	718.430	717.422	P[13]
N[18]	931.583	677.409	669.399	669.903	668.895	N[12]
I[19]	988.125	620.387	612.378	612.882	611.874	I[11]
Q[20]	1052.154	563.845	555.836	556.340	555.332	Q[10]
A[21]	1087.673	499.816	491.807	492.310	491.303	A[9]
V[22]	1137.207	464.297	456.288	456.792	455.784	V[8]
L[23]	1193.749	414.763	406.754	407.258	406.250	L[7]
L[24]	1250.291	358.221	350.212	350.716	349.708	L[6]
P[25]	1298.817	301.679	293.670	294.174	293.166	P[5]
K[26]	**1362.865**	253.153	245.143	245.647	244.639	K[4]
K[27]	**1426.912**	189.105	181.096	181.600	180.592	K[3]
T[28]	**1477.436**	125.058	117.048	117.552	116.544	T[2]
E[29]	**1541.958**	74.534	66.524	67.028	66.021	E[1]

sp|Q6GSS7|H2A2A_MOUSE

LNKLLGK Dimethyl 28.03 VTIAQGGVLPNIQAVLLPKKTE

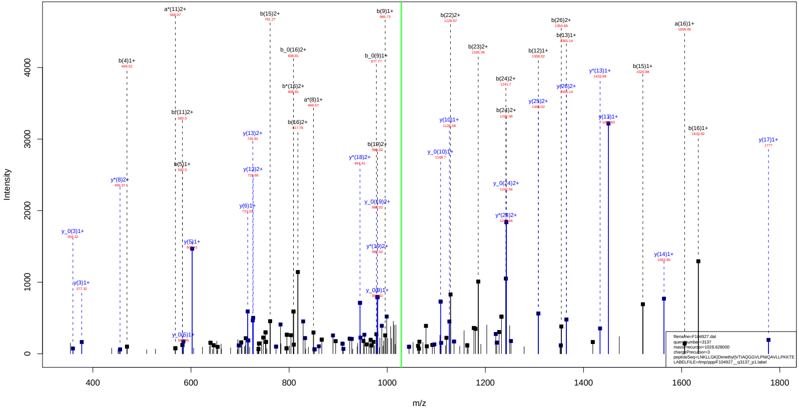

sp|Q6GSS7|H2A2A_MOUSE

LNKLLGK $^{\text{Dimethyl}}_{28.03}$ VTIAQGGVLPNIQAVLLPKKTE

- fragmentation table for charge state 1+
- specType=cid
- score=29.21
- F104927.dat
- query=q3137_p1
- precursor=1028.628000
- chargePrecursor=3
- itol=0.8

AA	a	a*	a_0	b	b*	b_0	y	y*	y_0	AA
L[1]	86.096	0.000	0.000	114.091	0.000	0.000	3083.892	3066.865	3065.881	L[29]
N[2]	200.139	183.113	0.000	228.134	211.108	0.000	2970.808	2953.781	2952.797	N[28]
K[3]	328.234	311.208	0.000	356.229	339.203	0.000	2856.765	2839.738	2838.754	K[27]
L[4]	441.318	424.292	0.000	**469.313**	452.287	0.000	2728.670	2711.643	2710.659	L[26]
L[5]	554.402	537.376	0.000	**582.397**	565.371	0.000	2615.586	2598.559	2597.575	L[25]
G[6]	611.424	594.397	0.000	**639.419**	622.392	0.000	2502.502	2485.475	2484.491	G[24]
K[7]	767.550	750.524	0.000	**795.545**	778.519	0.000	2445.480	2428.454	2427.470	K[23]
V[8]	**866.619**	**849.592**	0.000	**894.613**	877.587	0.000	2289.354	2272.327	2271.343	V[22]
T[9]	**967.666**	**950.640**	949.656	**995.661**	978.635	**977.651**	2190.286	2173.259	2172.275	T[21]
I[10]	**1080.750**	1063.724	**1062.740**	**1108.745**	**1091.719**	1090.735	2089.238	2072.211	2071.227	I[20]
A[11]	1151.787	1134.761	1133.777	**1179.782**	**1162.756**	1161.772	1976.154	1959.127	1958.143	A[19]
Q[12]	1279.846	1262.819	1261.835	**1307.841**	1290.814	1289.830	1905.117	1888.090	1887.106	Q[18]
G[13]	1336.867	1319.841	1318.857	**1364.862**	1347.836	1346.852	**1777.058**	1760.032	1759.048	G[17]
G[14]	1393.889	1376.862	1375.878	1421.884	1404.857	1403.873	1720.037	1703.010	1702.026	G[16]
V[15]	1492.957	1475.931	1474.947	**1520.952**	1503.926	1502.942	1663.015	1645.989	1645.005	V[15]
L[16]	**1606.041**	1589.015	1588.031	**1634.036**	1617.010	1616.026	**1563.947**	1546.920	1545.936	L[14]
P[17]	1703.094	1686.068	1685.084	1731.089	1714.063	1713.079	**1450.863**	**1433.836**	1432.852	P[13]
N[18]	1817.137	1800.111	1799.127	1845.132	1828.105	1827.121	**1353.810**	1336.783	1335.799	N[12]
I[19]	1930.221	1913.195	1912.211	1958.216	1941.190	1940.206	1239.767	**1222.741**	**1221.757**	I[11]
Q[20]	2058.280	2041.253	2040.269	2086.275	2069.248	2068.264	**1126.683**	**1109.656**	**1108.672**	Q[10]
A[21]	2129.317	2112.290	2111.306	2157.312	2140.285	2139.301	**998.624**	981.598	**980.614**	A[9]
V[22]	2228.385	2211.359	2210.375	2256.380	2239.354	2238.370	**927.587**	**910.561**	**909.577**	V[8]
L[23]	2341.469	2324.443	2323.459	2369.464	2352.438	2351.454	**828.519**	811.492	810.508	L[7]
L[24]	2454.553	2437.527	2436.543	2482.548	2465.522	2464.538	**715.435**	**698.408**	**697.424**	L[6]
P[25]	2551.606	2534.580	2533.596	2579.601	2562.575	2561.591	**602.351**	585.324	**584.340**	P[5]
K[26]	2679.701	2662.675	2661.691	2707.696	2690.670	2689.685	505.298	488.271	487.287	K[4]
K[27]	2807.796	2790.770	2789.786	2835.791	2818.764	2817.780	**377.203**	360.177	**359.193**	K[3]
T[28]	2908.844	2891.817	2890.833	2936.839	2919.812	2918.828	249.108	0.000	231.098	T[2]
E[29]	3037.886	3020.860	3019.876	3065.881	3048.855	3047.871	148.060	0.000	130.050	E[1]

sp|Q6GSS7|H2A2A_MOUSE

LNKLLGK $^{Dimethyl}_{28.03}$ VTIAQGGVLPNIQAVLLPKKTE

- fragmentation table for charge state 2+
- specType=cid
- score=29.21
- F104927.dat
- query=q3137_p1
- precursor=1028.628000
- chargePrecursor=3
- itol=0.8

AA	a	a*	a_0	b	b*	b_0	y	y*	y_0	AA
L[1]	43.552	0.504	0.504	57.549	0.504	0.504	1542.450	1533.936	1533.444	L[29]
N[2]	100.573	92.060	0.504	114.571	106.057	0.504	1485.908	1477.394	1476.902	N[28]
K[3]	164.621	156.108	0.504	178.618	170.105	0.504	1428.886	1420.373	1419.881	K[27]
L[4]	221.163	212.650	0.504	235.160	226.647	0.504	**1364.839**	1356.325	1355.833	L[26]
L[5]	277.705	269.192	0.504	291.702	283.189	0.504	**1308.297**	1299.783	1299.291	L[25]
G[6]	306.216	297.702	0.504	320.213	311.700	0.504	**1251.755**	**1243.241**	**1242.749**	G[24]
K[7]	384.279	375.765	0.504	398.276	389.763	0.504	**1223.244**	1214.731	1214.239	K[23]
V[8]	433.813	425.300	0.504	447.810	439.297	0.504	1145.181	**1136.667**	**1136.175**	V[22]
T[9]	484.337	475.823	475.331	498.334	489.821	489.329	**1095.646**	1087.133	1086.641	T[21]
I[10]	540.879	532.366	531.874	554.876	546.363	545.871	**1045.123**	1036.609	1036.117	I[20]
A[11]	576.397	**567.884**	**567.392**	590.395	**581.882**	581.390	**988.581**	**980.067**	**979.575**	A[19]
Q[12]	**640.427**	631.913	631.421	**654.424**	**645.911**	**645.419**	**953.062**	**944.549**	**944.057**	Q[18]
G[13]	668.937	660.424	659.932	682.935	674.422	673.930	**889.033**	880.519	880.027	G[17]
G[14]	**697.448**	688.935	688.443	**711.446**	**702.932**	**702.440**	**860.522**	**852.009**	**851.517**	G[16]
V[15]	**746.982**	**738.469**	**737.977**	**760.980**	**752.467**	**751.974**	**832.011**	823.498	823.006	V[15]
L[16]	**803.524**	**795.011**	**794.519**	**817.522**	**809.009**	**808.517**	**782.477**	**773.964**	**773.472**	L[14]
P[17]	**852.051**	843.537	843.045	**866.048**	857.535	857.043	**725.935**	**717.422**	**716.930**	P[13]
N[18]	**909.072**	900.559	900.067	**923.070**	914.556	914.064	677.409	668.895	668.403	N[12]
I[19]	**965.614**	**957.101**	956.609	**979.612**	**971.098**	970.606	620.387	611.874	611.382	I[11]
Q[20]	1029.644	1021.130	1020.638	**1043.641**	1035.128	1034.636	563.845	555.332	554.840	Q[10]
A[21]	**1065.162**	1056.649	1056.157	**1079.160**	1070.646	1070.154	499.816	491.303	490.811	A[9]
V[22]	1114.696	1106.183	1105.691	**1128.694**	**1120.180**	1119.688	464.297	**455.784**	**455.292**	V[8]
L[23]	1171.238	**1162.725**	**1162.233**	**1185.236**	**1176.722**	**1176.230**	414.763	406.250	405.758	L[7]
L[24]	**1227.780**	1219.267	1218.775	**1241.778**	**1233.265**	**1232.773**	358.221	349.708	349.216	L[6]
P[25]	1276.307	1267.793	1267.301	1290.304	1281.791	1281.299	301.679	293.166	292.674	P[5]
K[26]	1340.354	1331.841	1331.349	**1354.352**	1345.838	1345.346	253.153	244.639	244.147	K[4]
K[27]	1404.402	1395.888	1395.396	**1418.399**	1409.886	1409.394	189.105	180.592	180.100	K[3]
T[28]	1454.926	1446.412	1445.920	1468.923	1460.410	1459.918	125.058	0.504	116.052	T[2]
E[29]	1519.447	1510.934	1510.442	1533.444	1524.931	1524.439	74.534	0.504	65.529	E[1]

sp|Q6GSS7|H2A2A_MOUSE

LNKLLGK $^{\text{Dimethyl}}_{28.03}$ VTIAQGGVLPNIQAVLLPKKTE

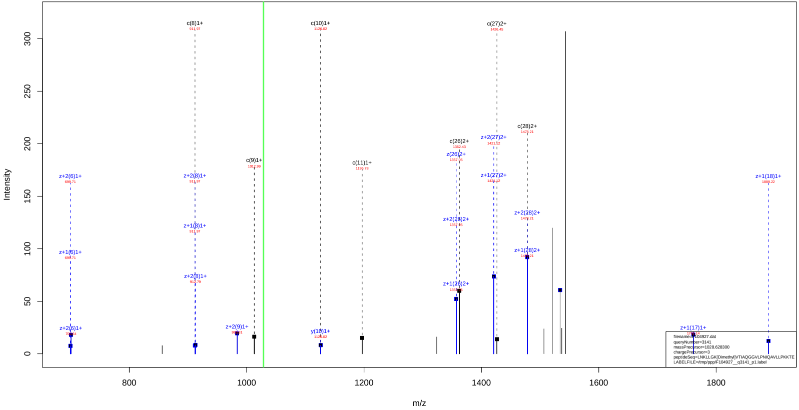

sp|Q6GSS7|H2A2A_MOUSE

LNKLLGK $^{\text{Dimethyl}}_{28.03}$ VTIAQGGVLPNIQAVLLPKKTE

- fragmentation table for charge state 1+
- specType=etd
- score=32.14
- F104927.dat
- query=q3141_p1
- precursor=1028.628300
- chargePrecursor=3
- itol=0.8

AA	c	y	z+1	z+2	z	AA
L[1]	131.118	3083.892	3067.873	0.000	3066.865	L[29]
N[2]	245.161	2970.808	2954.789	2955.797	2953.781	N[28]
K[3]	373.256	2856.765	2840.746	2841.754	2839.738	K[27]
L[4]	486.340	2728.670	2712.651	2713.659	2711.643	L[26]
L[5]	599.424	2615.586	2599.567	2600.575	2598.559	L[25]
G[6]	656.445	2502.502	2486.483	2487.491	2485.475	G[24]
K[7]	812.572	2445.480	2429.462	2430.469	2428.454	K[23]
V[8]	**911.640**	2289.354	2273.335	2274.343	2272.327	V[22]
T[9]	**1012.688**	2190.286	2174.267	2175.275	2173.259	T[21]
I[10]	**1125.772**	2089.238	2073.219	2074.227	2072.211	I[20]
A[11]	**1196.809**	1976.154	1960.135	1961.143	1959.127	A[19]
Q[12]	1324.867	1905.117	**1889.098**	1890.106	1888.090	Q[18]
G[13]	1381.889	1777.058	**1761.039**	1762.047	1760.032	G[17]
G[14]	1438.910	1720.037	1704.018	1705.026	1703.010	G[16]
V[15]	1537.979	1663.015	1646.997	1648.004	1645.989	V[15]
L[16]	1651.063	1563.947	1547.928	1548.936	1546.920	L[14]
P[17]	1748.116	1450.863	1434.844	1435.852	1433.836	P[13]
N[18]	1862.159	1353.810	1337.791	1338.799	1336.783	N[12]
I[19]	1975.243	1239.767	1223.748	1224.756	1222.741	I[11]
Q[20]	2103.301	**1126.683**	1110.664	1111.672	1109.656	Q[10]
A[21]	2174.338	998.624	982.606	**983.614**	981.598	A[9]
V[22]	2273.407	927.587	**911.569**	**912.576**	910.561	V[8]
L[23]	2386.491	828.519	812.500	813.508	811.492	L[7]
L[24]	2499.575	715.435	**699.416**	**700.424**	698.408	L[6]
P[25]	2596.628	602.351	586.332	587.340	585.324	P[5]
K[26]	2724.723	505.298	489.279	490.287	488.271	K[4]
K[27]	2852.818	377.203	361.184	362.192	360.177	K[3]
T[28]	2953.865	249.108	233.089	234.097	232.082	T[2]
E[29]	3082.908	148.060	132.042	133.050	131.034	E[1]

sp|Q6GSS7|H2A2A_MOUSE

LNKLLGK $^{\text{Dimethyl}}_{28.03}$ VTIAQGGVLPNIQAVLLPKKTE

- fragmentation table for charge state 2+
- specType=etd
- score=32.14
- F104927.dat
- query=q3141_p1
- precursor=1028.628300
- chargePrecursor=3
- itol=0.8

AA	c	y	z+1	z+2	z	AA
L[1]	66.063	1542.450	**1534.440**	0.504	**1533.936**	L[29]
N[2]	123.084	1485.908	**1477.898**	**1478.402**	1477.394	N[28]
K[3]	187.132	1428.886	**1420.877**	**1421.381**	**1420.373**	K[27]
L[4]	243.674	1364.839	**1356.829**	**1357.333**	**1356.325**	L[26]
L[5]	300.216	1308.297	1300.287	1300.791	1299.783	L[25]
G[6]	328.726	1251.755	1243.745	1244.249	1243.241	G[24]
K[7]	406.789	1223.244	1215.234	1215.738	1214.731	K[23]
V[8]	456.324	1145.181	1137.171	1137.675	1136.667	V[22]
T[9]	506.848	1095.646	1087.637	1088.141	1087.133	T[21]
I[10]	563.390	1045.123	1037.113	1037.617	1036.609	I[20]
A[11]	598.908	988.581	980.571	981.075	980.067	A[19]
Q[12]	662.937	953.062	945.053	945.557	944.549	Q[18]
G[13]	691.448	889.033	881.023	881.527	880.519	G[17]
G[14]	719.959	860.522	852.513	853.017	852.009	G[16]
V[15]	769.493	832.011	824.002	824.506	823.498	V[15]
L[16]	826.035	782.477	774.468	774.972	773.964	L[14]
P[17]	874.561	725.935	717.926	718.430	717.422	P[13]
N[18]	931.583	677.409	669.399	669.903	668.895	N[12]
I[19]	988.125	620.387	612.378	612.882	611.874	I[11]
Q[20]	1052.154	563.845	555.836	556.340	555.332	Q[10]
A[21]	1087.673	499.816	491.807	492.310	491.303	A[9]
V[22]	1137.207	464.297	456.288	456.792	455.784	V[8]
L[23]	1193.749	414.763	406.754	407.258	406.250	L[7]
L[24]	1250.291	358.221	350.212	350.716	349.708	L[6]
P[25]	1298.817	301.679	293.670	294.174	293.166	P[5]
K[26]	**1362.865**	253.153	245.143	245.647	244.639	K[4]
K[27]	**1426.912**	189.105	181.096	181.600	180.592	K[3]
T[28]	**1477.436**	125.058	117.048	117.552	116.544	T[2]
E[29]	1541.958	74.534	66.524	67.028	66.021	E[1]

sp|Q6GSS7|H2A2A_MOUSE

LNKLLGK $^{Dimethyl}_{28.03}$ VTIAQGGVLPNIQAVLLPKKTE

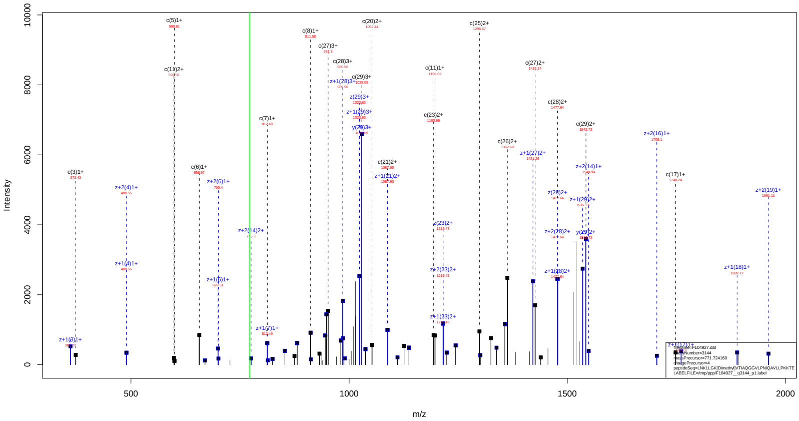

sp|Q6GSS7|H2A2A_MOUSE

LNKLLGK $^{\text{Dimethyl}}_{28.03}$ VTIAQGGVLPNIQAVLLPKKTE

- fragmentation table for charge state 1+
- specType=etd
- score=107.08
- F104927.dat
- query=q3144_p1
- precursor=771.724160
- chargePrecursor=4
- itol=0.8

AA	c	y	z+1	z+2	z	AA
L[1]	131.118	3083.892	3067.873	0.000	3066.865	L[29]
N[2]	245.161	2970.808	2954.789	2955.797	2953.781	N[28]
K[3]	**373.256**	2856.765	2840.746	2841.754	2839.738	K[27]
L[4]	486.340	2728.670	2712.651	2713.659	2711.643	L[26]
L[5]	**599.424**	2615.586	2599.567	2600.575	2598.559	L[25]
G[6]	**656.445**	2502.502	2486.483	2487.491	2485.475	G[24]
K[7]	**812.572**	2445.480	2429.462	2430.469	2428.454	K[23]
V[8]	**911.640**	2289.354	2273.335	2274.343	2272.327	V[22]
T[9]	1012.688	2190.286	2174.267	2175.275	2173.259	T[21]
I[10]	**1125.772**	2089.238	2073.219	2074.227	2072.211	I[20]
A[11]	**1196.809**	1976.154	1960.135	**1961.143**	1959.127	A[19]
Q[12]	**1324.867**	1905.117	**1889.098**	1890.106	1888.090	Q[18]
G[13]	1381.889	1777.058	**1761.039**	1762.047	1760.032	G[17]
G[14]	**1438.910**	1720.037	1704.018	**1705.026**	1703.010	G[16]
V[15]	1537.979	1663.015	1646.997	1648.004	1645.989	V[15]
L[16]	1651.063	1563.947	1547.928	**1548.936**	1546.920	L[14]
P[17]	**1748.116**	1450.863	1434.844	1435.852	1433.836	P[13]
N[18]	1862.159	1353.810	**1337.791**	1338.799	1336.783	N[12]
I[19]	1975.243	1239.767	**1223.748**	1224.756	1222.741	I[11]
Q[20]	2103.301	1126.683	**1110.664**	1111.672	1109.656	Q[10]
A[21]	2174.338	998.624	982.606	983.614	981.598	A[9]
V[22]	2273.407	927.587	**911.569**	**912.576**	910.561	V[8]
L[23]	2386.491	828.519	**812.500**	**813.508**	811.492	L[7]
L[24]	2499.575	715.435	**699.416**	**700.424**	698.408	L[6]
P[25]	2596.628	602.351	586.332	587.340	585.324	P[5]
K[26]	2724.723	505.298	**489.279**	**490.287**	488.271	K[4]
K[27]	2852.818	377.203	**361.184**	362.192	360.177	K[3]
T[28]	2953.865	249.108	233.089	234.097	232.082	T[2]
E[29]	3082.908	148.060	132.042	133.050	131.034	E[1]

sp|Q6GSS7|H2A2A_MOUSE

LNKLLGK $^{\text{Dimethyl}}_{28.03}$ VTIAQGGVLPNIQAVLLPKKTE

- fragmentation table for charge state 2+
- specType=etd
- score=107.08
- F104927.dat
- query=q3144_p1
- precursor=771.724160
- chargePrecursor=4
- itol=0.8

AA	c	y	z+1	z+2	z	AA
L[1]	66.063	**1542.450**	**1534.440**	0.504	1533.936	L[29]
N[2]	123.084	1485.908	**1477.898**	**1478.402**	**1477.394**	N[28]
K[3]	187.132	1428.886	**1420.877**	**1421.381**	1420.373	K[27]
L[4]	243.674	1364.839	**1356.829**	**1357.333**	**1356.325**	L[26]
L[5]	300.216	1308.297	**1300.287**	**1300.791**	1299.783	L[25]
G[6]	328.726	1251.755	**1243.745**	**1244.249**	1243.241	G[24]
K[7]	406.789	**1223.244**	**1215.234**	**1215.738**	**1214.731**	K[23]
V[8]	456.324	1145.181	**1137.171**	**1137.675**	**1136.667**	V[22]
T[9]	506.848	1095.646	**1087.637**	**1088.141**	1087.133	T[21]
I[10]	563.390	1045.123	**1037.113**	**1037.617**	1036.609	I[20]
A[11]	**598.908**	988.581	**980.571**	**981.075**	**980.067**	A[19]
Q[12]	662.937	953.062	**945.053**	**945.557**	944.549	Q[18]
G[13]	691.448	889.033	**881.023**	**881.527**	**880.519**	G[17]
G[14]	719.959	860.522	**852.513**	**853.017**	**852.009**	G[16]
V[15]	769.493	832.011	**824.002**	**824.506**	823.498	V[15]
L[16]	826.035	782.477	774.468	**774.972**	773.964	L[14]
P[17]	**874.561**	725.935	717.926	718.430	717.422	P[13]
N[18]	**931.583**	677.409	**669.399**	**669.903**	668.895	N[12]
I[19]	988.125	620.387	612.378	612.882	611.874	I[11]
Q[20]	**1052.154**	563.845	555.836	556.340	555.332	Q[10]
A[21]	**1087.673**	499.816	491.807	492.310	491.303	A[9]
V[22]	**1137.207**	464.297	456.288	456.792	455.784	V[8]
L[23]	**1193.749**	414.763	406.754	407.258	406.250	L[7]
L[24]	1250.291	358.221	350.212	350.716	349.708	L[6]
P[25]	**1298.817**	301.679	293.670	294.174	293.166	P[5]
K[26]	**1362.865**	253.153	245.143	245.647	244.639	K[4]
K[27]	**1426.912**	189.105	181.096	181.600	180.592	K[3]
T[28]	**1477.436**	125.058	117.048	117.552	116.544	T[2]
E[29]	**1541.958**	74.534	66.524	67.028	66.021	E[1]

sp|Q6GSS7|H2A2A_MOUSE

LNKLLGK $^{Dimethyl}_{28.03}$ VTIAQGGVLPNIQAVLLPKKTE

- fragmentation table for charge state 3+
- specType=etd
- score=107.08
- F104927.dat
- query=q3144_p1
- precursor=771.724160
- chargePrecursor=4
- itol=0.8

AA	c	y	z+1	z+2	z	AA
L[1]	44.377	**1028.635**	**1023.296**	0.672	**1022.960**	L[29]
N[2]	82.392	**990.941**	**985.601**	**985.937**	**985.265**	N[28]
K[3]	125.090	952.926	**947.587**	**947.923**	**947.251**	K[27]
L[4]	162.785	910.228	904.889	905.225	904.553	L[26]
L[5]	200.479	872.533	867.194	867.530	866.858	L[25]
G[6]	219.487	834.839	829.499	829.835	829.163	G[24]
K[7]	271.529	815.832	810.492	810.828	810.156	K[23]
V[8]	304.552	763.790	758.450	758.786	758.114	V[22]
T[9]	338.234	730.767	725.427	725.763	725.091	T[21]
I[10]	375.929	697.084	691.745	692.081	691.409	I[20]
A[11]	399.608	659.389	654.050	654.386	653.714	A[19]
Q[12]	442.294	635.710	630.371	630.707	630.035	Q[18]
G[13]	461.301	593.024	587.685	588.021	587.349	G[17]
G[14]	480.308	574.017	568.678	569.013	568.342	G[16]
V[15]	513.331	555.010	549.670	550.006	549.334	V[15]
L[16]	551.026	521.987	516.648	516.984	516.312	L[14]
P[17]	583.377	484.292	478.953	479.289	478.617	P[13]
N[18]	621.391	451.942	446.602	446.938	446.266	N[12]
I[19]	659.086	413.927	408.588	408.924	408.252	I[11]
Q[20]	701.772	376.233	370.893	371.229	370.557	Q[10]
A[21]	725.451	333.546	328.207	328.543	327.871	A[9]
V[22]	758.474	309.867	304.528	304.864	304.192	V[8]
L[23]	796.168	276.844	271.505	271.841	271.169	L[7]
L[24]	833.863	239.150	233.810	234.146	233.474	L[6]
P[25]	866.214	201.455	196.116	196.451	195.780	P[5]
K[26]	908.912	169.104	163.765	164.101	163.429	K[4]
K[27]	**951.611**	126.406	121.066	121.402	120.730	K[3]
T[28]	**985.293**	83.708	78.368	78.704	78.032	T[2]
E[29]	**1028.307**	50.025	44.685	45.021	44.349	E[1]

sp|Q6GSS7|H2A2A_MOUSE

LNKLLGK $^{\text{Dimethyl}}_{28.03}$ VTIAQGGVLPNIQAVLLPKKTE

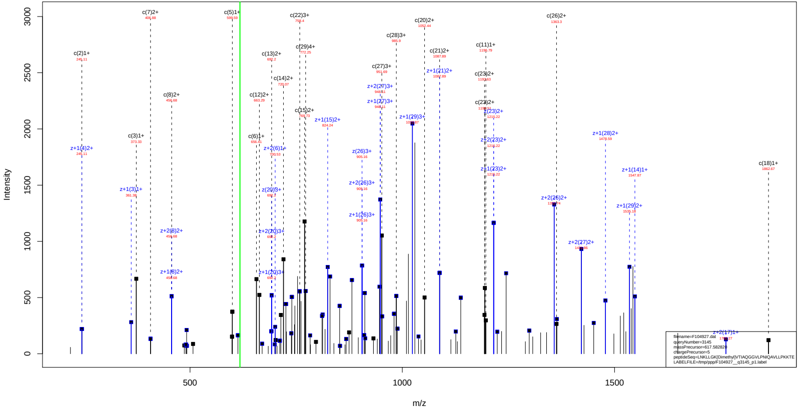

sp|Q6GSS7|H2A2A_MOUSE

LNKLLGK $^{\text{Dimethyl}}_{28.03}$ VTIAQGGVLPNIQAVLLPKKTE

- fragmentation table for charge state 1+
- specType=etd
- score=114.45
- F104927.dat
- query=q3145_p1
- precursor=617.582820
- chargePrecursor=5
- itol=0.8

AA	c	y	z+1	z+2	z	AA
L[1]	131.118	3083.892	3067.873	0.000	3066.865	L[29]
N[2]	**245.161**	2970.808	2954.789	2955.797	2953.781	N[28]
K[3]	**373.256**	2856.765	2840.746	2841.754	2839.738	K[27]
L[4]	**486.340**	2728.670	2712.651	2713.659	2711.643	L[26]
L[5]	**599.424**	2615.586	2599.567	2600.575	2598.559	L[25]
G[6]	**656.445**	2502.502	2486.483	2487.491	2485.475	G[24]
K[7]	**812.572**	2445.480	2429.462	2430.469	2428.454	K[23]
V[8]	**911.640**	2289.354	2273.335	2274.343	2272.327	V[22]
T[9]	1012.688	2190.286	2174.267	2175.275	2173.259	T[21]
I[10]	**1125.772**	2089.238	2073.219	2074.227	2072.211	I[20]
A[11]	**1196.809**	1976.154	1960.135	1961.143	1959.127	A[19]
Q[12]	1324.867	1905.117	1889.098	1890.106	1888.090	Q[18]
G[13]	1381.889	1777.058	1761.039	**1762.047**	1760.032	G[17]
G[14]	1438.910	1720.037	1704.018	1705.026	1703.010	G[16]
V[15]	1537.979	1663.015	1646.997	1648.004	1645.989	V[15]
L[16]	1651.063	1563.947	**1547.928**	1548.936	1546.920	L[14]
P[17]	1748.116	**1450.863**	1434.844	1435.852	1433.836	P[13]
N[18]	**1862.159**	1353.810	1337.791	1338.799	1336.783	N[12]
I[19]	1975.243	1239.767	**1223.748**	1224.756	1222.741	I[11]
Q[20]	2103.301	**1126.683**	1110.664	1111.672	1109.656	Q[10]
A[21]	2174.338	998.624	982.606	983.614	981.598	A[9]
V[22]	2273.407	927.587	**911.569**	**912.576**	**910.561**	V[8]
L[23]	2386.491	828.519	**812.500**	813.508	**811.492**	L[7]
L[24]	2499.575	715.435	**699.416**	**700.424**	698.408	L[6]
P[25]	2596.628	602.351	586.332	587.340	585.324	P[5]
K[26]	2724.723	505.298	489.279	**490.287**	488.271	K[4]
K[27]	2852.818	377.203	**361.184**	362.192	360.177	K[3]
T[28]	2953.865	249.108	233.089	234.097	232.082	T[2]
E[29]	3082.908	148.060	132.042	133.050	131.034	E[1]

sp|Q6GSS7|H2A2A_MOUSE

LNKLLGK $^{\text{Dimethyl}}_{28.03}$ VTIAQGGVLPNIQAVLLPKKTE

- fragmentation table for charge state 2+
- specType=etd
- score=114.45
- F104927.dat
- query=q3145_p1
- precursor=617.582820
- chargePrecursor=5
- itol=0.8

AA	c	y	z+1	z+2	z	AA
L[1]	66.063	1542.450	**1534.440**	0.504	1533.936	L[29]
N[2]	123.084	1485.908	**1477.898**	**1478.402**	1477.394	N[28]
K[3]	187.132	1428.886	1420.877	**1421.381**	1420.373	K[27]
L[4]	243.674	**1364.839**	1356.829	**1357.333**	1356.325	L[26]
L[5]	300.216	1308.297	1300.287	1300.791	**1299.783**	L[25]
G[6]	328.726	1251.755	1243.745	**1244.249**	1243.241	G[24]
K[7]	**406.789**	**1223.244**	**1215.234**	**1215.738**	**1214.731**	K[23]
V[8]	**456.324**	1145.181	**1137.171**	**1137.675**	1136.667	V[22]
T[9]	**506.848**	1095.646	**1087.637**	**1088.141**	**1087.133**	T[21]
I[10]	563.390	1045.123	1037.113	**1037.617**	1036.609	I[20]
A[11]	**598.908**	**988.581**	**980.571**	**981.075**	**980.067**	A[19]
Q[12]	**662.937**	**953.062**	945.053	**945.557**	944.549	Q[18]
G[13]	**691.448**	889.033	**881.023**	**881.527**	880.519	G[17]
G[14]	**719.959**	860.522	**852.513**	**853.017**	**852.009**	G[16]
V[15]	**769.493**	832.011	**824.002**	**824.506**	**823.498**	V[15]
L[16]	826.035	**782.477**	774.468	774.972	773.964	L[14]
P[17]	**874.561**	**725.935**	717.926	718.430	717.422	P[13]
N[18]	**931.583**	677.409	**669.399**	**669.903**	668.895	N[12]
I[19]	**988.125**	620.387	**612.378**	**612.882**	611.874	I[11]
Q[20]	**1052.154**	563.845	555.836	556.340	555.332	Q[10]
A[21]	**1087.673**	499.816	**491.807**	**492.310**	**491.303**	A[9]
V[22]	**1137.207**	464.297	**456.288**	**456.792**	455.784	V[8]
L[23]	**1193.749**	414.763	**406.754**	**407.258**	**406.250**	L[7]
L[24]	1250.291	358.221	350.212	350.716	349.708	L[6]
P[25]	**1298.817**	301.679	293.670	294.174	293.166	P[5]
K[26]	**1362.865**	253.153	**245.143**	**245.647**	**244.639**	K[4]
K[27]	1426.912	189.105	181.096	181.600	180.592	K[3]
T[28]	1477.436	125.058	117.048	117.552	116.544	T[2]
E[29]	1541.958	74.534	66.524	67.028	66.021	E[1]

sp|Q6GSS7|H2A2A_MOUSE

LNKLLGK $^{Dimethyl}_{28.03}$ VTIAQGGVLPNIQAVLLPKKTE

- fragmentation table for charge state 3+
- specType=etd
- score=114.45
- F104927.dat
- query=q3145_p1
- precursor=617.582820
- chargePrecursor=5
- itol=0.8

AA	c	y	z+1	z+2	z	AA
L[1]	44.377	1028.635	**1023.296**	0.672	1022.960	L[29]
N[2]	82.392	990.941	**985.601**	**985.937**	**985.265**	N[28]
K[3]	125.090	**952.926**	**947.587**	**947.923**	947.251	K[27]
L[4]	162.785	**910.228**	**904.889**	**905.225**	**904.553**	L[26]
L[5]	200.479	872.533	**867.194**	**867.530**	866.858	L[25]
G[6]	219.487	834.839	**829.499**	**829.835**	**829.163**	G[24]
K[7]	271.529	815.832	**810.492**	**810.828**	810.156	K[23]
V[8]	304.552	763.790	**758.450**	**758.786**	**758.114**	V[22]
T[9]	338.234	730.767	**725.427**	**725.763**	**725.091**	T[21]
I[10]	375.929	697.084	**691.745**	**692.081**	**691.409**	I[20]
A[11]	399.608	659.389	654.050	654.386	653.714	A[19]
Q[12]	442.294	635.710	630.371	630.707	630.035	Q[18]
G[13]	461.301	593.024	587.685	588.021	587.349	G[17]
G[14]	480.308	574.017	568.678	569.013	568.342	G[16]
V[15]	513.331	555.010	549.670	550.006	549.334	V[15]
L[16]	551.026	521.987	516.648	516.984	516.312	L[14]
P[17]	583.377	484.292	478.953	479.289	478.617	P[13]
N[18]	621.391	451.942	446.602	446.938	446.266	N[12]
I[19]	659.086	413.927	408.588	408.924	408.252	I[11]
Q[20]	**701.772**	376.233	370.893	371.229	370.557	Q[10]
A[21]	**725.451**	333.546	328.207	328.543	327.871	A[9]
V[22]	**758.474**	309.867	304.528	304.864	304.192	V[8]
L[23]	**796.168**	276.844	271.505	271.841	271.169	L[7]
L[24]	833.863	239.150	233.810	234.146	233.474	L[6]
P[25]	866.214	201.455	196.116	196.451	195.780	P[5]
K[26]	908.912	169.104	163.765	164.101	163.429	K[4]
K[27]	**951.611**	126.406	121.066	121.402	120.730	K[3]
T[28]	**985.293**	83.708	78.368	78.704	78.032	T[2]
E[29]	1028.307	50.025	44.685	45.021	44.349	E[1]

sp|Q6GSS7|H2A2A_MOUSE

LNKLLGK $^{\text{Dimethyl}}_{28.03}$ VTIAQGGVLPNIQAVLLPKKTE

- fragmentation table for charge state 4+
- specType=etd
- score=114.45
- F104927.dat
- query=q3145_p1
- precursor=617.582820
- chargePrecursor=5
- itol=0.8

AA	c	y	z+1	z+2	z	AA
L[1]	33.535	**771.728**	767.724	0.755	767.472	L[29]
N[2]	62.046	743.457	**739.453**	**739.705**	**739.201**	N[28]
K[3]	94.069	714.947	**710.942**	**711.194**	**710.690**	K[27]
L[4]	122.340	682.923	678.918	679.170	678.666	L[26]
L[5]	150.611	654.652	650.647	650.899	650.395	L[25]
G[6]	164.867	626.381	622.376	622.628	622.124	G[24]
K[7]	203.898	**612.126**	608.121	608.373	607.869	K[23]
V[8]	228.665	573.094	569.089	569.341	568.837	V[22]
T[9]	253.927	548.327	544.322	544.574	544.070	T[21]
I[10]	282.198	523.065	519.060	519.312	518.808	I[20]
A[11]	299.958	494.794	**490.789**	**491.041**	**490.537**	A[19]
Q[12]	331.972	477.035	473.030	473.282	472.778	Q[18]
G[13]	346.228	445.020	441.015	441.267	440.763	G[17]
G[14]	360.483	430.765	426.760	427.012	426.508	G[16]
V[15]	385.250	416.509	412.505	412.757	412.253	V[15]
L[16]	413.521	391.742	387.737	387.989	387.486	L[14]
P[17]	437.784	363.471	359.466	359.718	359.215	P[13]
N[18]	466.295	339.208	335.203	335.455	334.951	N[12]
I[19]	494.566	310.697	306.693	306.945	306.441	I[11]
Q[20]	526.581	282.426	278.422	278.673	278.170	Q[10]
A[21]	544.340	250.412	246.407	246.659	246.155	A[9]
V[22]	569.107	232.652	228.648	228.900	228.396	V[8]
L[23]	597.378	207.885	203.881	204.132	203.629	L[7]
L[24]	625.649	179.614	175.609	175.861	175.358	L[6]
P[25]	649.912	151.343	147.338	147.590	147.087	P[5]
K[26]	681.936	127.080	123.075	123.327	122.823	K[4]
K[27]	**713.960**	95.056	91.052	91.304	90.800	K[3]
T[28]	**739.222**	63.032	59.028	59.280	58.776	T[2]
E[29]	**771.482**	37.771	33.766	34.018	33.514	E[1]

sp|Q6GSS7|H2A2A_MOUSE

LNKLLGK $^{\text{Dimethyl}}_{28.03}$ VTIAQGGVLPNIQAVLLPKKTE

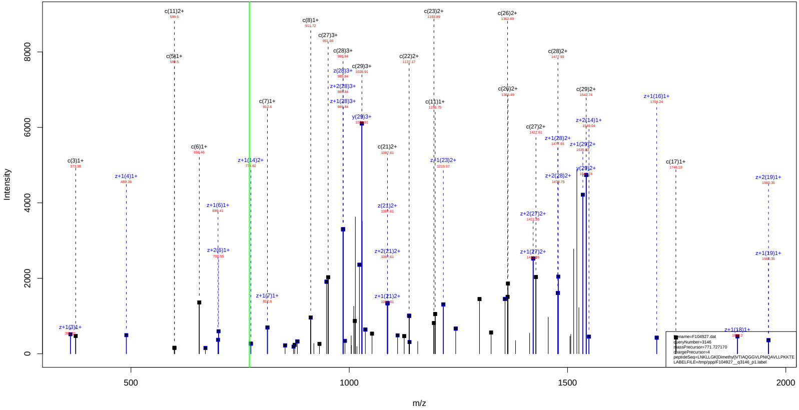

sp|Q6GSS7|H2A2A_MOUSE

LNKLLGK $^{\text{Dimethyl}}_{28.03}$ VTIAQGGVLPNIQAVLLPKKTE

- fragmentation table for charge state 1+
- specType=etd
- score=89.31
- F104927.dat
- query=q3146_p1
- precursor=771.727170
- chargePrecursor=4
- itol=0.8

AA	c	y	z+1	z+2	z	AA
L[1]	131.118	3083.892	3067.873	0.000	3066.865	L[29]
N[2]	245.161	2970.808	2954.789	2955.797	2953.781	N[28]
K[3]	**373.256**	2856.765	2840.746	2841.754	2839.738	K[27]
L[4]	486.340	2728.670	2712.651	2713.659	2711.643	L[26]
L[5]	**599.424**	2615.586	2599.567	2600.575	2598.559	L[25]
G[6]	**656.445**	2502.502	2486.483	2487.491	2485.475	G[24]
K[7]	**812.572**	2445.480	2429.462	2430.469	2428.454	K[23]
V[8]	**911.640**	2289.354	2273.335	2274.343	2272.327	V[22]
T[9]	**1012.688**	2190.286	2174.267	2175.275	2173.259	T[21]
I[10]	**1125.772**	2089.238	2073.219	2074.227	2072.211	I[20]
A[11]	**1196.809**	1976.154	**1960.135**	**1961.143**	1959.127	A[19]
Q[12]	**1324.867**	1905.117	**1889.098**	1890.106	1888.090	Q[18]
G[13]	1381.889	1777.058	1761.039	1762.047	1760.032	G[17]
G[14]	1438.910	1720.037	**1704.018**	**1705.026**	1703.010	G[16]
V[15]	1537.979	1663.015	1646.997	1648.004	1645.989	V[15]
L[16]	1651.063	1563.947	1547.928	**1548.936**	1546.920	L[14]
P[17]	**1748.116**	1450.863	1434.844	1435.852	1433.836	P[13]
N[18]	1862.159	1353.810	1337.791	1338.799	1336.783	N[12]
I[19]	1975.243	1239.767	1223.748	1224.756	1222.741	I[11]
Q[20]	2103.301	1126.683	**1110.664**	1111.672	1109.656	Q[10]
A[21]	2174.338	998.624	982.606	983.614	981.598	A[9]
V[22]	2273.407	927.587	**911.569**	912.576	910.561	V[8]
L[23]	2386.491	828.519	**812.500**	813.508	811.492	L[7]
L[24]	2499.575	715.435	**699.416**	**700.424**	698.408	L[6]
P[25]	2596.628	602.351	586.332	587.340	585.324	P[5]
K[26]	2724.723	505.298	**489.279**	490.287	488.271	K[4]
K[27]	2852.818	377.203	**361.184**	362.192	360.177	K[3]
T[28]	2953.865	249.108	233.089	234.097	232.082	T[2]
E[29]	3082.908	148.060	132.042	133.050	131.034	E[1]

sp|Q6GSS7|H2A2A_MOUSE

LNKLLGK $^{\text{Dimethyl}}_{28.03}$ VTIAQGGVLPNIQAVLLPKKTE

- fragmentation table for charge state 2+
- specType=etd
- score=89.31
- F104927.dat
- query=q3146_p1
- precursor=771.727170
- chargePrecursor=4
- itol=0.8

AA	c	y	z+1	z+2	z	AA
L[1]	66.063	**1542.450**	**1534.440**	0.504	1533.936	L[29]
N[2]	123.084	1485.908	**1477.898**	**1478.402**	**1477.394**	N[28]
K[3]	187.132	1428.886	**1420.877**	**1421.381**	1420.373	K[27]
L[4]	243.674	1364.839	**1356.829**	**1357.333**	**1356.325**	L[26]
L[5]	300.216	1308.297	1300.287	1300.791	1299.783	L[25]
G[6]	328.726	1251.755	**1243.745**	**1244.249**	**1243.241**	G[24]
K[7]	406.789	1223.244	**1215.234**	**1215.738**	1214.731	K[23]
V[8]	456.324	1145.181	**1137.171**	**1137.675**	**1136.667**	V[22]
T[9]	506.848	1095.646	**1087.637**	**1088.141**	**1087.133**	T[21]
I[10]	563.390	1045.123	**1037.113**	**1037.617**	**1036.609**	I[20]
A[11]	**598.908**	988.581	980.571	981.075	980.067	A[19]
Q[12]	662.937	953.062	945.053	945.557	944.549	Q[18]
G[13]	691.448	889.033	**881.023**	**881.527**	**880.519**	G[17]
G[14]	719.959	860.522	**852.513**	**853.017**	852.009	G[16]
V[15]	769.493	832.011	824.002	824.506	823.498	V[15]
L[16]	826.035	782.477	**774.468**	**774.972**	**773.964**	L[14]
P[17]	**874.561**	725.935	717.926	718.430	717.422	P[13]
N[18]	**931.583**	677.409	669.399	**669.903**	668.895	N[12]
I[19]	988.125	620.387	612.378	612.882	611.874	I[11]
Q[20]	**1052.154**	563.845	555.836	556.340	555.332	Q[10]
A[21]	**1087.673**	499.816	491.807	492.310	491.303	A[9]
V[22]	**1137.207**	464.297	456.288	456.792	455.784	V[8]
L[23]	**1193.749**	414.763	406.754	407.258	406.250	L[7]
L[24]	1250.291	358.221	350.212	350.716	349.708	L[6]
P[25]	**1298.817**	301.679	293.670	294.174	293.166	P[5]
K[26]	**1362.865**	253.153	245.143	245.647	244.639	K[4]
K[27]	**1426.912**	189.105	181.096	181.600	180.592	K[3]
T[28]	**1477.436**	125.058	117.048	117.552	116.544	T[2]
E[29]	**1541.958**	74.534	66.524	67.028	66.021	E[1]

sp|Q6GSS7|H2A2A_MOUSE

LNKLLGK $^{\text{Dimethyl}}_{28.03}$ VTIAQGGVLPNIQAVLLPKKTE

- fragmentation table for charge state 3+
- specType=etd
- score=89.31
- F104927.dat
- query=q3146_p1
- precursor=771.727170
- chargePrecursor=4
- itol=0.8

AA	c	y	z+1	z+2	z	AA
L[1]	44.377	**1028.635**	**1023.296**	0.672	**1022.960**	L[29]
N[2]	82.392	**990.941**	**985.601**	**985.937**	**985.265**	N[28]
K[3]	125.090	952.926	**947.587**	**947.923**	**947.251**	K[27]
L[4]	162.785	910.228	904.889	905.225	904.553	L[26]
L[5]	200.479	**872.533**	867.194	867.530	866.858	L[25]
G[6]	219.487	834.839	829.499	829.835	829.163	G[24]
K[7]	271.529	815.832	810.492	810.828	810.156	K[23]
V[8]	304.552	763.790	758.450	758.786	758.114	V[22]
T[9]	338.234	730.767	725.427	725.763	725.091	T[21]
I[10]	375.929	697.084	691.745	692.081	691.409	I[20]
A[11]	399.608	659.389	654.050	654.386	653.714	A[19]
Q[12]	442.294	635.710	630.371	630.707	630.035	Q[18]
G[13]	461.301	593.024	587.685	588.021	587.349	G[17]
G[14]	480.308	574.017	568.678	569.013	568.342	G[16]
V[15]	513.331	555.010	549.670	550.006	549.334	V[15]
L[16]	551.026	521.987	516.648	516.984	516.312	L[14]
P[17]	583.377	484.292	478.953	479.289	478.617	P[13]
N[18]	621.391	451.942	446.602	446.938	446.266	N[12]
I[19]	659.086	413.927	408.588	408.924	408.252	I[11]
Q[20]	701.772	376.233	370.893	371.229	370.557	Q[10]
A[21]	725.451	333.546	328.207	328.543	327.871	A[9]
V[22]	758.474	309.867	304.528	304.864	304.192	V[8]
L[23]	796.168	276.844	271.505	271.841	271.169	L[7]
L[24]	833.863	239.150	233.810	234.146	233.474	L[6]
P[25]	866.214	201.455	196.116	196.451	195.780	P[5]
K[26]	908.912	169.104	163.765	164.101	163.429	K[4]
K[27]	**951.611**	126.406	121.066	121.402	120.730	K[3]
T[28]	**985.293**	83.708	78.368	78.704	78.032	T[2]
E[29]	**1028.307**	50.025	44.685	45.021	44.349	E[1]

sp|Q6GSS7|H2A2A_MOUSE

ELNKLLGK $^{\text{Dimethyl}}_{28.03}$ VTIAQGGVLPNIQAVLLPKKTE

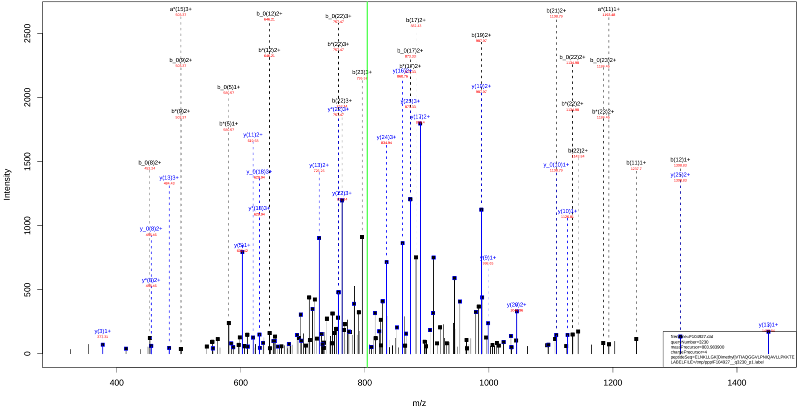

sp|Q6GSS7|H2A2A_MOUSE

ELNKLLGK $^{Dimethyl}_{28.03}$ VTIAQGGVLPNIQAVLLPKKTE

- fragmentation table for charge state 1+
- specType=cid
- score=30.71
- F104927.dat
- query=q3230_p1
- precursor=803.983900
- chargePrecursor=4
- itol=0.8

AA	a	a*	a_0	b	b*	b_0	y	y*	y_0	AA
E[1]	102.055	0.000	84.044	130.050	0.000	112.039	3212.934	3195.908	3194.924	E[30]
L[2]	215.139	0.000	197.128	243.134	0.000	225.123	3083.892	3066.865	3065.881	L[29]
N[3]	329.182	312.155	311.171	357.177	340.150	339.166	2970.808	2953.781	2952.797	N[28]
K[4]	457.277	440.250	439.266	485.272	468.245	467.261	2856.765	2839.738	2838.754	K[27]
L[5]	570.361	**553.334**	552.350	**598.356**	**581.329**	**580.345**	2728.670	2711.643	2710.659	L[26]
L[6]	683.445	666.418	**665.434**	**711.440**	694.413	**693.429**	2615.586	2598.559	2597.575	L[25]
G[7]	740.467	723.440	**722.456**	768.461	**751.435**	**750.451**	2502.502	2485.475	2484.491	G[24]
K[8]	**896.593**	879.566	878.582	924.588	907.561	906.577	2445.480	2428.454	2427.470	K[23]
V[9]	**995.661**	**978.635**	977.651	**1023.656**	1006.630	**1005.646**	2289.354	2272.327	2271.343	V[22]
T[10]	**1096.709**	1079.682	1078.698	**1124.704**	1107.677	**1106.693**	2190.286	2173.259	2172.275	T[21]
I[11]	1209.793	**1192.766**	1191.782	**1237.788**	1220.761	1219.777	2089.238	2072.211	2071.227	I[20]
A[12]	1280.830	1263.803	1262.819	**1308.825**	1291.798	1290.814	1976.154	1959.127	1958.143	A[19]
Q[13]	1408.889	1391.862	1390.878	1436.884	1419.857	1418.873	1905.117	1888.090	1887.106	Q[18]
G[14]	1465.910	1448.884	1447.900	1493.905	1476.878	1475.894	1777.058	1760.032	1759.048	G[17]
G[15]	1522.932	1505.905	1504.921	1550.926	1533.900	1532.916	1720.037	1703.010	1702.026	G[16]
V[16]	1622.000	1604.973	1603.989	1649.995	1632.968	1631.984	1663.015	1645.989	1645.005	V[15]
L[17]	1735.084	1718.057	1717.073	1763.079	1746.052	1745.068	1563.947	1546.920	1545.936	L[14]
P[18]	1832.137	1815.110	1814.126	1860.132	1843.105	1842.121	**1450.863**	1433.836	1432.852	P[13]
N[19]	1946.180	1929.153	1928.169	1974.175	1957.148	1956.164	1353.810	1336.783	1335.799	N[12]
I[20]	2059.264	2042.237	2041.253	2087.259	2070.232	2069.248	1239.767	1222.741	1221.757	I[11]
Q[21]	2187.322	2170.296	2169.312	2215.317	2198.291	2197.307	**1126.683**	1109.656	**1108.672**	Q[10]
A[22]	2258.359	2241.333	2240.349	2286.354	2269.328	2268.344	**998.624**	981.598	980.614	A[9]
V[23]	2357.428	2340.401	2339.417	2385.423	2368.396	2367.412	927.587	**910.561**	**909.577**	V[8]
L[24]	2470.512	2453.485	2452.501	2498.507	2481.480	2480.496	**828.519**	811.492	**810.508**	L[7]
L[25]	2583.596	2566.569	2565.585	2611.591	2594.564	2593.580	**715.435**	698.408	**697.424**	L[6]
P[26]	2680.649	2663.622	2662.638	2708.644	2691.617	2690.633	**602.351**	**585.324**	**584.340**	P[5]
K[27]	2808.744	2791.717	2790.733	2836.739	2819.712	2818.728	505.298	488.271	487.287	K[4]
K[28]	2936.839	2919.812	2918.828	2964.834	2947.807	2946.823	**377.203**	360.177	359.193	K[3]
T[29]	3037.886	3020.860	3019.876	3065.881	3048.855	3047.871	249.108	0.000	231.098	T[2]
E[30]	3166.929	3149.902	3148.918	3194.924	3177.897	3176.913	148.060	0.000	130.050	E[1]

sp|Q6GSS7|H2A2A_MOUSE

ELNKLLGK $^{\text{Dimethyl}}_{28.03}$ VTIAQGGVLPNIQAVLLPKKTE

- fragmentation table for charge state 2+
- specType=cid
- score=30.71
- F104927.dat
- query=q3230_p1
- precursor=803.983900
- chargePrecursor=4
- itol=0.8

AA	a	a*	a_0	b	b*	b_0	y	y*	y_0	AA
E[1]	51.531	0.504	42.526	65.529	0.504	56.523	1606.971	1598.458	1597.966	E[30]
L[2]	108.073	0.504	99.068	122.071	0.504	113.065	1542.450	1533.936	1533.444	L[29]
N[3]	165.095	156.581	156.089	179.092	170.579	170.087	1485.908	1477.394	1476.902	N[28]
K[4]	229.142	220.629	220.137	243.140	234.626	234.134	1428.886	1420.373	1419.881	K[27]
L[5]	285.684	277.171	276.679	299.682	291.168	290.676	1364.839	1356.325	1355.833	L[26]
L[6]	342.226	333.713	333.221	356.224	347.710	347.218	**1308.297**	1299.783	1299.291	L[25]
G[7]	370.737	362.224	361.732	384.734	376.221	375.729	1251.755	1243.241	1242.749	G[24]
K[8]	448.800	440.287	439.795	462.797	454.284	**453.792**	1223.244	1214.731	1214.239	K[23]
V[9]	498.334	489.821	489.329	512.332	**503.818**	**503.326**	1145.181	1136.667	1136.175	V[22]
T[10]	548.858	540.345	539.853	**562.856**	**554.342**	**553.850**	**1095.646**	1087.133	1086.641	T[21]
I[11]	**605.400**	**596.887**	**596.395**	**619.398**	**610.884**	**610.392**	**1045.123**	**1036.609**	**1036.117**	I[20]
A[12]	640.919	632.405	631.913	**654.916**	**646.403**	**645.911**	**988.581**	980.067	**979.575**	A[19]
Q[13]	**704.948**	**696.435**	**695.943**	**718.945**	**710.432**	**709.940**	**953.062**	**944.549**	**944.057**	Q[18]
G[14]	**733.459**	724.945	724.453	**747.456**	**738.943**	**738.451**	**889.033**	880.519	880.027	G[17]
G[15]	761.969	**753.456**	752.964	**775.967**	**767.454**	**766.962**	**860.522**	**852.009**	**851.517**	G[16]
V[16]	811.504	802.990	802.498	**825.501**	**816.988**	**816.496**	832.011	**823.498**	**823.006**	V[15]
L[17]	868.046	859.532	859.040	**882.043**	**873.530**	**873.038**	**782.477**	**773.964**	**773.472**	L[14]
P[18]	**916.572**	908.059	907.567	930.569	**922.056**	**921.564**	**725.935**	717.422	716.930	P[13]
N[19]	973.593	**965.080**	**964.588**	**987.591**	**979.078**	**978.586**	**677.409**	668.895	668.403	N[12]
I[20]	1030.136	1021.622	1021.130	**1044.133**	**1035.620**	**1035.128**	**620.387**	**611.874**	**611.382**	I[11]
Q[21]	**1094.165**	1085.652	1085.160	**1108.162**	1099.649	1099.157	563.845	**555.332**	**554.840**	Q[10]
A[22]	1129.683	1121.170	1120.678	**1143.681**	**1135.168**	**1134.676**	499.816	491.303	490.811	A[9]
V[23]	1179.218	1170.704	1170.212	**1193.215**	**1184.702**	**1184.210**	464.297	**455.784**	**455.292**	V[8]
L[24]	1235.760	1227.246	1226.754	1249.757	1241.244	1240.752	**414.763**	406.250	405.758	L[7]
L[25]	1292.302	1283.788	1283.296	1306.299	1297.786	1297.294	358.221	349.708	349.216	L[6]
P[26]	1340.828	1332.315	1331.823	1354.825	1346.312	1345.820	301.679	293.166	292.674	P[5]
K[27]	1404.876	1396.362	1395.870	1418.873	1410.360	1409.868	253.153	244.639	244.147	K[4]
K[28]	1468.923	1460.410	1459.918	1482.920	1474.407	1473.915	189.105	180.592	180.100	K[3]
T[29]	1519.447	1510.934	1510.442	1533.444	1524.931	1524.439	125.058	0.504	116.052	T[2]
E[30]	1583.968	1575.455	1574.963	1597.966	1589.452	1588.960	74.534	0.504	65.529	E[1]

sp|Q6GSS7|H2A2A_MOUSE

ELNKLLGK $^{Dimethyl}_{28.03}$ VTIAQGGVLPNIQAVLLPKKTE

- fragmentation table for charge state 3+
- specType=cid
- score=30.71
- F104927.dat
- query=q3230_p1
- precursor=803.983900
- chargePrecursor=4
- itol=0.8

AA	a	a*	a_0	b	b*	b_0	y	y*	y_0	AA
E[1]	34.690	0.672	28.686	44.021	0.672	38.018	1071.650	1065.974	1065.646	E[30]
L[2]	72.385	0.672	66.381	81.716	0.672	75.713	1028.635	**1022.960**	1022.632	L[29]
N[3]	110.399	104.723	104.395	119.730	114.055	113.727	990.941	985.265	984.937	N[28]
K[4]	153.097	147.422	147.094	162.429	156.753	156.425	**952.926**	947.251	946.923	K[27]
L[5]	190.792	185.116	184.788	200.123	194.448	194.120	**910.228**	**904.553**	904.225	L[26]
L[6]	228.487	222.811	222.483	237.818	232.143	231.815	**872.533**	**866.858**	**866.530**	L[25]
G[7]	247.494	241.818	241.490	256.825	251.150	250.822	**834.839**	**829.163**	**828.835**	G[24]
K[8]	299.536	293.860	293.532	308.867	303.192	302.864	**815.832**	**810.156**	**809.828**	K[23]
V[9]	332.559	326.883	326.555	341.890	336.215	335.887	**763.790**	**758.114**	**757.786**	V[22]
T[10]	366.241	360.566	360.238	375.573	369.897	369.569	**730.767**	725.091	724.763	T[21]
I[11]	403.936	398.260	397.932	413.267	407.592	407.264	**697.084**	**691.409**	**691.081**	I[20]
A[12]	427.615	421.939	421.611	436.947	431.271	430.943	**659.389**	**653.714**	**653.386**	A[19]
Q[13]	470.301	464.626	464.298	479.633	473.957	473.629	**635.710**	**630.035**	**629.707**	Q[18]
G[14]	489.308	483.633	483.305	498.640	492.964	492.636	593.024	**587.349**	**587.021**	G[17]
G[15]	508.315	**502.640**	502.312	517.647	511.971	511.643	574.017	568.342	568.014	G[16]
V[16]	541.338	535.663	535.335	550.670	**544.994**	**544.666**	**555.010**	549.334	549.006	V[15]
L[17]	579.033	573.357	573.029	**588.364**	582.689	582.361	521.987	516.312	515.984	L[14]
P[18]	**611.384**	**605.708**	**605.380**	620.715	615.040	614.712	**484.292**	478.617	478.289	P[13]
N[19]	649.398	643.723	643.395	658.730	**653.054**	**652.726**	451.942	446.266	445.938	N[12]
I[20]	687.093	681.417	681.089	**696.424**	**690.749**	**690.421**	413.927	408.252	407.924	I[11]
Q[21]	**729.779**	724.103	723.775	**739.111**	**733.435**	**733.107**	376.233	370.557	370.229	Q[10]
A[22]	**753.458**	**747.782**	**747.454**	**762.790**	**757.114**	**756.786**	333.546	327.871	327.543	A[9]
V[23]	786.481	780.805	780.477	**795.812**	**790.137**	**789.809**	309.867	304.192	303.864	V[8]
L[24]	824.175	818.500	818.172	833.507	**827.832**	827.504	276.844	271.169	270.841	L[7]
L[25]	861.870	856.195	855.867	871.202	**865.526**	**865.198**	239.150	233.474	233.146	L[6]
P[26]	894.221	888.546	888.218	903.553	**897.877**	**897.549**	201.455	195.780	195.452	P[5]
K[27]	936.919	**931.244**	930.916	946.251	940.576	940.248	169.104	163.429	163.101	K[4]
K[28]	979.618	973.942	973.614	**988.949**	**983.274**	**982.946**	126.406	120.730	120.402	K[3]
T[29]	**1013.300**	1007.625	1007.297	1022.632	1016.956	**1016.628**	83.708	0.672	77.704	T[2]
E[30]	1056.315	1050.639	1050.311	1065.646	1059.971	1059.643	50.025	0.672	44.021	E[1]

sp|P68433|H31_MOUSE

IAQDFK $^{\text{Dimethyl}}_{28.03}$ TD

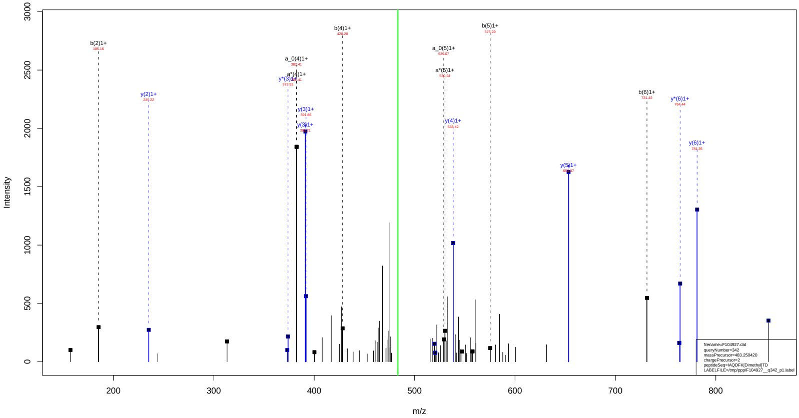

sp|P68433|H31_MOUSE

IAQDFK $^{\text{Dimethyl}}_{28.03}$ TD

- fragmentation table for charge state 1+
- specType=cid
- score=35.61
- F104927.dat
- query=q342_p1
- precursor=483.250420
- chargePrecursor=2
- itol=0.8

AA	a	a*	a_0	b	b*	b_0	y	y*	y_0	AA
I[1]	86.096	0.000	0.000	114.091	0.000	0.000	965.494	948.467	947.483	I[8]
A[2]	**157.134**	0.000	0.000	**185.128**	0.000	0.000	**852.410**	835.383	834.399	A[7]
Q[3]	285.192	268.166	0.000	**313.187**	296.160	0.000	**781.373**	**764.346**	**763.362**	Q[6]
D[4]	**400.219**	**383.193**	**382.208**	**428.214**	411.187	410.203	**653.314**	636.288	635.304	D[5]
F[5]	**547.287**	**530.261**	**529.277**	**575.282**	**558.256**	**557.272**	**538.287**	**521.261**	**520.277**	F[4]
K[6]	703.414	686.387	685.403	**731.409**	714.382	713.398	**391.219**	**374.192**	**373.208**	K[3]
T[7]	804.461	787.435	786.451	832.456	815.430	814.446	**235.092**	0.000	217.082	T[2]
D[8]	919.488	902.462	901.478	947.483	930.457	929.473	134.045	0.000	116.034	D[1]

sp|P62806|H4_MOUSE

[Acetyl]SGRGKGGKGL

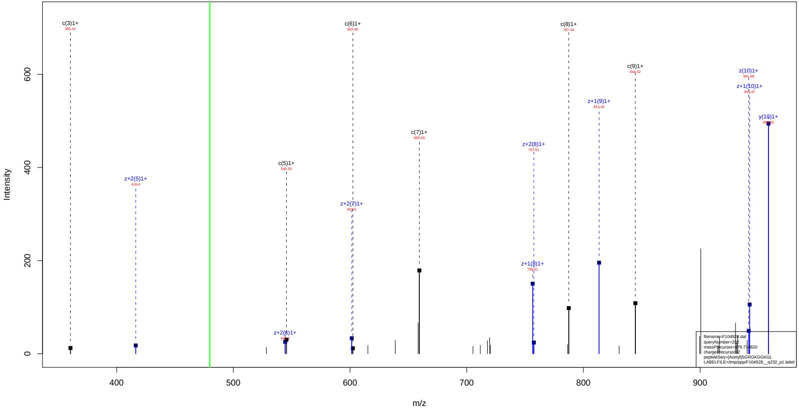

sp|P62806|H4_MOUSE

[Acetyl]SGRGKGGKGL

- fragmentation table for charge state 1+
- specType=etd
- score=35.03
- F104928.dat
- query=q232_p1
- precursor=479.774820
- chargePrecursor=2
- itol=0.8

AA	c	y	z+1	z+2	z	AA
S[1]	147.076	**958.543**	**942.524**	0.000	**941.516**	S[10]
G[2]	204.098	829.500	**813.482**	0.000	812.474	G[9]
R[3]	**360.199**	772.479	**756.460**	**757.468**	755.452	R[8]
G[4]	417.220	616.378	600.359	**601.367**	599.351	G[7]
K[5]	**545.315**	559.356	543.337	**544.345**	542.330	K[6]
G[6]	**602.337**	431.261	415.243	**416.250**	414.235	G[5]
G[7]	**659.358**	374.240	358.221	359.229	357.213	G[4]
K[8]	**787.453**	317.218	301.200	302.207	300.192	K[3]
G[9]	**844.475**	189.123	173.105	174.112	172.097	G[2]
L[10]	957.559	132.102	116.083	117.091	115.075	L[1]

sp|P62806|H4_MOUSE

[Acetyl]SGRGKGGKGL

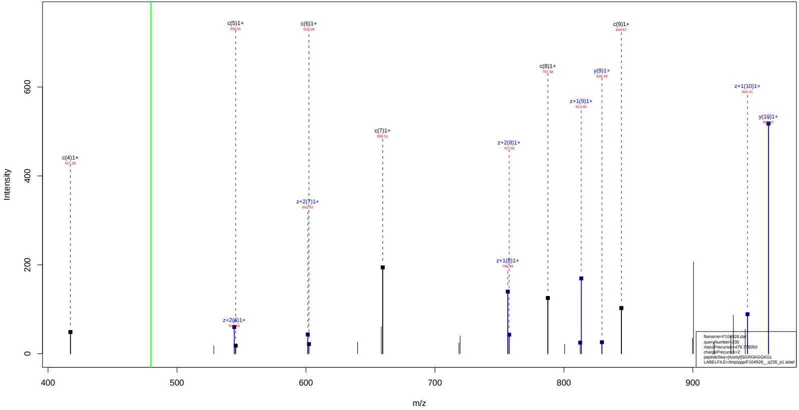

sp|P62806|H4_MOUSE

[Acetyl]SGRGKGGKGL

- fragmentation table for charge state 1+
- specType=etd
- score=44.34
- F104928.dat
- query=q235_p1
- precursor=479.775050
- chargePrecursor=2
- itol=0.8

AA	c	y	z+1	z+2	z	AA
S[1]	147.076	**958.543**	**942.524**	0.000	941.516	S[10]
G[2]	204.098	**829.500**	**813.482**	0.000	**812.474**	G[9]
R[3]	360.199	772.479	**756.460**	**757.468**	755.452	R[8]
G[4]	**417.220**	616.378	600.359	**601.367**	599.351	G[7]
K[5]	**545.315**	559.356	543.337	**544.345**	542.330	K[6]
G[6]	**602.337**	431.261	415.243	416.250	414.235	G[5]
G[7]	**659.358**	374.240	358.221	359.229	357.213	G[4]
K[8]	**787.453**	317.218	301.200	302.207	300.192	K[3]
G[9]	**844.475**	189.123	173.105	174.112	172.097	G[2]
L[10]	957.559	132.102	116.083	117.091	115.075	L[1]

sp|P62806|H4_MOUSE

[Acetyl]SGRGKGGKGL

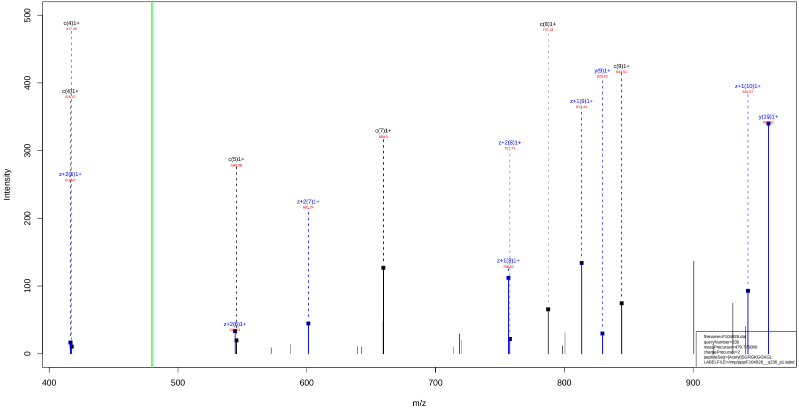

sp|P62806|H4_MOUSE
[Acetyl]SGRGKGGKGL

- fragmentation table for charge state 1+
- specType=etd
- score=34.26
- F104928.dat
- query=q236_p1
- precursor=479.775080
- chargePrecursor=2
- itol=0.8

AA	c	y	z+1	z+2	z	AA
S[1]	147.076	**958.543**	**942.524**	0.000	941.516	S[10]
G[2]	204.098	**829.500**	**813.482**	0.000	812.474	G[9]
R[3]	360.199	772.479	**756.460**	**757.468**	755.452	R[8]
G[4]	**417.220**	616.378	600.359	**601.367**	599.351	G[7]
K[5]	**545.315**	559.356	543.337	**544.345**	542.330	K[6]
G[6]	602.337	431.261	415.243	**416.250**	414.235	G[5]
G[7]	**659.358**	374.240	358.221	359.229	357.213	G[4]
K[8]	**787.453**	317.218	301.200	302.207	300.192	K[3]
G[9]	**844.475**	189.123	173.105	174.112	172.097	G[2]
L[10]	957.559	132.102	116.083	117.091	115.075	L[1]

sp|P62806|H4_MOUSE

[Acetyl]SGRGKGGKGL

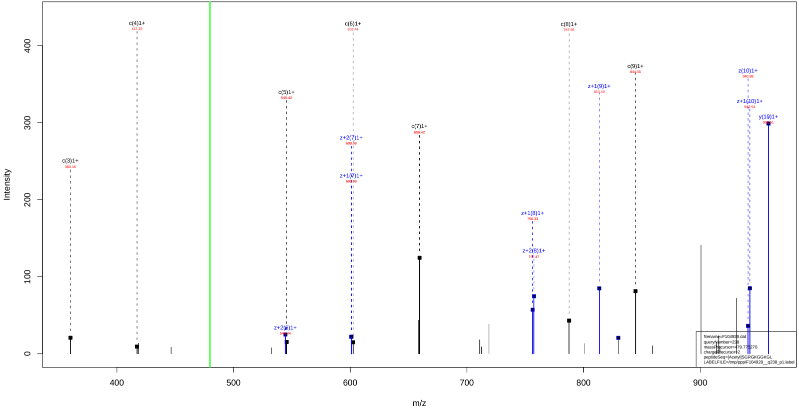

sp|P62806|H4_MOUSE

[Acetyl]SGRGKGGKGL

- fragmentation table for charge state 1+
- specType=etd
- score=49.23
- F104928.dat
- query=q238_p1
- precursor=479.775270
- chargePrecursor=2
- itol=0.8

AA	c	y	z+1	z+2	z	AA
S[1]	147.076	**958.543**	**942.524**	0.000	**941.516**	S[10]
G[2]	204.098	**829.500**	**813.482**	0.000	812.474	G[9]
R[3]	**360.199**	772.479	**756.460**	**757.468**	755.452	R[8]
G[4]	**417.220**	616.378	**600.359**	**601.367**	599.351	G[7]
K[5]	**545.315**	559.356	543.337	**544.345**	542.330	K[6]
G[6]	**602.337**	431.261	415.243	416.250	414.235	G[5]
G[7]	**659.358**	374.240	358.221	359.229	357.213	G[4]
K[8]	**787.453**	317.218	301.200	302.207	300.192	K[3]
G[9]	**844.475**	189.123	173.105	174.112	172.097	G[2]
L[10]	957.559	132.102	116.083	117.091	115.075	L[1]

sp|P62806|H4_MOUSE

[Acetyl]SGRGKGGKGL

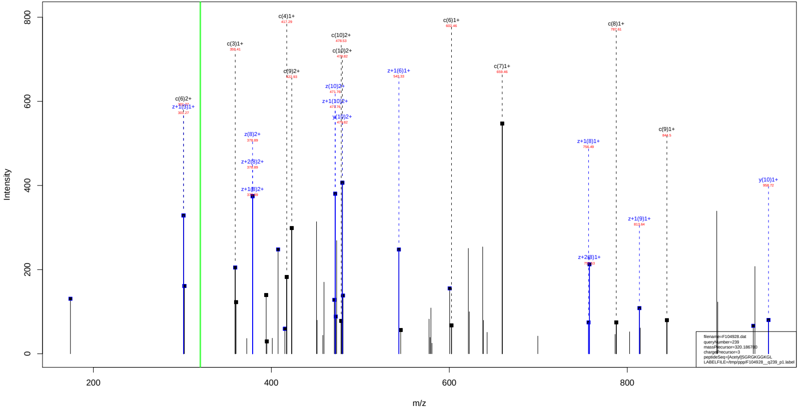

sp|P62806|H4_MOUSE

[Acetyl]SGRGKGGKGL

- fragmentation table for charge state 1+
- specType=etd
- score=46.15
- F104928.dat
- query=q239_p1
- precursor=320.186780
- chargePrecursor=3
- itol=0.8

AA	c	y	z+1	z+2	z	AA
S[1]	147.076	**958.543**	942.524	0.000	**941.516**	S[10]
G[2]	204.098	829.500	**813.482**	0.000	812.474	G[9]
R[3]	**360.199**	772.479	**756.460**	**757.468**	755.452	R[8]
G[4]	**417.220**	616.378	**600.359**	601.367	599.351	G[7]
K[5]	**545.315**	559.356	**543.337**	544.345	542.330	K[6]
G[6]	**602.337**	431.261	**415.243**	416.250	414.235	G[5]
G[7]	**659.358**	374.240	358.221	**359.229**	357.213	G[4]
K[8]	**787.453**	317.218	**301.200**	**302.207**	300.192	K[3]
G[9]	**844.475**	189.123	173.105	**174.112**	172.097	G[2]
L[10]	957.559	132.102	116.083	117.091	115.075	L[1]

sp|P62806|H4_MOUSE

[Acetyl]SGRGKGGKGL

- fragmentation table for charge state 2+
- specType=etd
- score=46.15
- F104928.dat
- query=q239_p1
- precursor=320.186780
- chargePrecursor=3
- itol=0.8

AA	c	y	z+1	z+2	z	AA
S[1]	74.042	**479.775**	**471.766**	0.504	**471.262**	S[10]
G[2]	102.553	**415.254**	**407.244**	0.504	406.740	G[9]
R[3]	180.603	386.743	**378.734**	**379.238**	**378.230**	R[8]
G[4]	209.114	308.692	**300.683**	**301.187**	300.179	G[7]
K[5]	273.161	280.182	272.172	272.676	271.668	K[6]
G[6]	**301.672**	216.134	208.125	208.629	207.621	G[5]
G[7]	330.183	187.624	179.614	180.118	179.110	G[4]
K[8]	**394.230**	159.113	151.103	151.607	150.600	K[3]
G[9]	**422.741**	95.065	87.056	87.560	86.552	G[2]
L[10]	**479.283**	66.555	58.545	59.049	58.041	L[1]

sp|P43274|H14_MOUSE

[Acetyl]SETAPAAPAAPAPAEKTPVKKKARKAAGGAKRKTSGPPVSEL

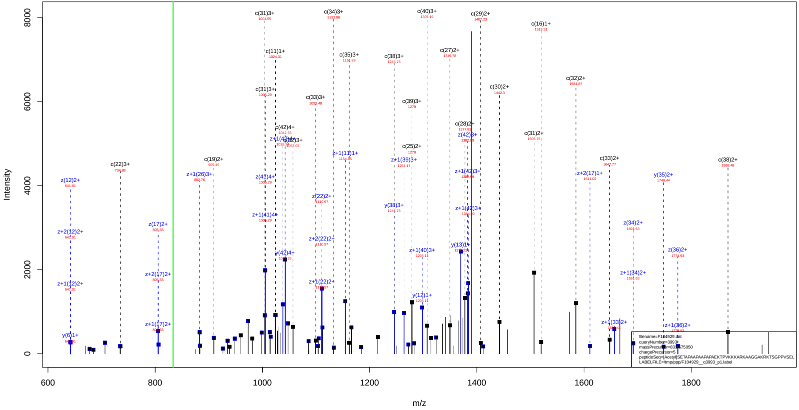

sp|P43274|H14_MOUSE

[Acetyl]SETAPAAPAAPAPAEKTPVKKKARKAAGGAKRKTSGPPVSEL

- fragmentation table for charge state 1+
- specType=etd
- score=47.24
- F104929.dat
- query=q3993_p1
- precursor=833.675050
- chargePrecursor=5
- itol=0.8

AA	c	y	z+1	z+2	z	AA
S[1]	147.076	4164.325	4148.306	0.000	4147.299	S[42]
E[2]	276.119	4035.283	4019.264	0.000	4018.256	E[41]
T[3]	377.167	3906.240	3890.221	0.000	3889.213	T[40]
A[4]	448.204	3805.192	3789.174	0.000	3788.166	A[39]
P[5]	545.257	3734.155	3718.136	0.000	3717.129	P[38]
A[6]	616.294	3637.102	3621.084	0.000	3620.076	A[37]
A[7]	687.331	3566.065	3550.047	0.000	3549.039	A[36]
P[8]	784.384	3495.028	3479.009	0.000	3478.002	P[35]
A[9]	855.421	3397.975	3381.957	0.000	3380.949	A[34]
A[10]	**926.458**	3326.938	3310.920	0.000	3309.912	A[33]
P[11]	**1023.511**	3255.901	3239.882	0.000	3238.875	P[32]
A[12]	1094.548	3158.848	3142.830	0.000	3141.822	A[31]
P[13]	1191.600	3087.811	3071.793	0.000	3070.785	P[30]
A[14]	1262.638	2990.759	2974.740	0.000	2973.732	A[29]
E[15]	1391.680	2919.721	2903.703	0.000	2902.695	E[28]
K[16]	**1519.775**	2790.679	2774.660	2775.668	2773.652	K[27]
T[17]	1620.823	2662.584	2646.565	2647.573	2645.557	T[26]
P[18]	1717.876	2561.536	2545.517	2546.525	2544.510	P[25]
V[19]	1816.944	2464.483	2448.465	2449.473	2447.457	V[24]
K[20]	1945.039	2365.415	2349.396	2350.404	2348.388	K[23]
K[21]	2073.134	2237.320	2221.301	2222.309	2220.294	K[22]
K[22]	2201.229	2109.225	2093.206	2094.214	2092.199	K[21]
A[23]	2272.266	1981.130	1965.111	1966.119	1964.104	A[20]
R[24]	2428.367	1910.093	1894.074	1895.082	1893.066	R[19]
K[25]	2556.462	1753.992	1737.973	1738.981	1736.965	K[18]
A[26]	2627.499	1625.897	1609.878	**1610.886**	1608.870	A[17]
A[27]	2698.536	1554.860	1538.841	1539.849	1537.833	A[16]
G[28]	2755.558	1483.823	1467.804	1468.812	1466.796	G[15]
G[29]	2812.579	1426.801	1410.783	**1411.790**	1409.775	G[14]
A[30]	2883.616	**1369.780**	1353.761	1354.769	1352.753	A[13]
K[31]	3011.711	**1298.743**	**1282.724**	**1283.732**	1281.716	K[12]
R[32]	3167.812	1170.648	**1154.629**	1155.637	1153.621	R[11]
K[33]	3295.907	**1014.547**	**998.528**	999.536	997.520	K[10]
T[34]	3396.955	886.452	870.433	871.441	869.425	T[9]
S[35]	3483.987	785.404	769.385	770.393	768.377	S[8]
G[36]	3541.009	698.372	682.353	683.361	681.345	G[7]
P[37]	3638.061	**641.350**	625.332	626.340	624.324	P[6]
P[38]	3735.114	544.298	528.279	529.287	527.271	P[5]
V[39]	3834.182	447.245	431.226	432.234	430.218	V[4]
S[40]	3921.214	348.177	332.158	333.166	331.150	S[3]
E[41]	4050.257	261.144	245.126	246.134	244.118	E[2]
L[42]	4163.341	132.102	116.083	117.091	115.075	L[1]

sp|P43274|H14_MOUSE

[Acetyl]SETAPAAPAAPAPAEKTPVKKKARKAAGGAKRKTSGPPVSEL

- fragmentation table for charge state 2+
- specType=etd
- score=47.24
- F104929.dat
- query=q3993_p1
- precursor=833.675050
- chargePrecursor=5
- itol=0.8

AA	c	y	z+1	z+2	z	AA
S[1]	74.042	2082.666	2074.657	0.504	2074.153	S[42]
E[2]	138.563	2018.145	2010.136	0.504	2009.632	E[41]
T[3]	189.087	1953.624	1945.614	0.504	1945.110	T[40]
A[4]	224.606	1903.100	1895.090	0.504	1894.587	A[39]
P[5]	273.132	1867.581	1859.572	0.504	1859.068	P[38]
A[6]	308.650	1819.055	1811.045	0.504	1810.542	A[37]
A[7]	344.169	1783.536	**1775.527**	0.504	**1775.023**	A[36]
P[8]	392.695	**1748.018**	1740.008	0.504	1739.504	P[35]
A[9]	428.214	1699.491	**1691.482**	0.504	**1690.978**	A[34]
A[10]	463.733	1663.973	**1655.963**	0.504	1655.460	A[33]
P[11]	512.259	1628.454	1620.445	0.504	1619.941	P[32]
A[12]	547.777	1579.928	1571.918	0.504	1571.415	A[31]
P[13]	596.304	1544.409	1536.400	0.504	1535.896	P[30]
A[14]	631.822	1495.883	1487.874	0.504	1487.370	A[29]
E[15]	696.344	1460.364	1452.355	0.504	1451.851	E[28]
K[16]	760.391	1395.843	1387.834	1388.338	1387.330	K[27]
T[17]	810.915	1331.796	**1323.786**	**1324.290**	1323.282	T[26]
P[18]	859.441	1281.272	1273.262	1273.766	**1272.758**	P[25]
V[19]	**908.976**	1232.745	1224.736	1225.240	1224.232	V[24]
K[20]	**973.023**	1183.211	1175.202	1175.706	1174.698	K[23]
K[21]	1037.071	1119.164	**1111.154**	**1111.658**	**1110.650**	K[22]
K[22]	1101.118	1055.116	**1047.107**	**1047.611**	1046.603	K[21]
A[23]	1136.637	991.069	983.059	983.563	982.555	A[20]
R[24]	**1214.687**	955.550	947.541	**948.045**	947.037	R[19]
K[25]	**1278.735**	877.500	869.490	869.994	868.986	K[18]
A[26]	**1314.253**	813.452	**805.443**	**805.947**	**804.939**	A[17]
A[27]	**1349.772**	777.934	769.924	770.428	769.420	A[16]
G[28]	**1378.283**	742.415	**734.406**	**734.910**	733.902	G[15]
G[29]	**1406.793**	713.904	**705.895**	**706.399**	705.391	G[14]
A[30]	**1442.312**	**685.394**	**677.384**	**677.888**	**676.880**	A[13]
K[31]	**1506.359**	649.875	**641.866**	**642.370**	**641.362**	K[12]
R[32]	**1584.410**	585.827	577.818	578.322	577.314	R[11]
K[33]	**1648.457**	507.777	499.768	500.271	499.264	K[10]
T[34]	1698.981	443.729	435.720	436.224	435.216	T[9]
S[35]	1742.497	393.206	385.196	385.700	384.692	S[8]
G[36]	1771.008	349.690	341.680	342.184	341.176	G[7]
P[37]	1819.534	321.179	313.170	313.673	312.666	P[6]
P[38]	**1868.061**	272.652	264.643	265.147	264.139	P[5]
V[39]	1917.595	224.126	216.117	216.621	215.613	V[4]
S[40]	1961.111	174.592	166.583	167.086	166.079	S[3]
E[41]	2025.632	131.076	123.067	123.570	122.563	E[2]
L[42]	2082.174	66.555	58.545	59.049	58.041	L[1]

sp|P43274|H14_MOUSE

[Acetyl]SETAPAAPAAPAPAEKTPVKKARKAAGGAKRKTSGPPVSEL

- fragmentation table for charge state 3+
- specType=etd
- score=47.24
- F104929.dat
- query=q3993_p1
- precursor=833.675050
- chargePrecursor=5
- itol=0.8

AA	c	y	z+1	z+2	z	AA
S[1]	49.697	1388.780	**1383.440**	0.672	**1383.104**	S[42]
E[2]	92.711	1345.766	1340.426	0.672	1340.090	E[41]
T[3]	126.394	1302.752	**1297.412**	0.672	1297.076	T[40]
A[4]	150.073	1269.069	**1263.729**	0.672	**1263.393**	A[39]
P[5]	182.424	**1245.390**	1240.050	0.672	1239.714	P[38]
A[6]	206.103	1213.039	1207.699	0.672	1207.363	A[37]
A[7]	229.782	1189.360	**1184.020**	0.672	**1183.684**	A[36]
P[8]	262.133	**1165.681**	1160.341	0.672	1160.005	P[35]
A[9]	285.812	**1133.330**	1127.990	0.672	1127.654	A[34]
A[10]	309.491	1109.651	**1104.311**	0.672	**1103.975**	A[33]
P[11]	341.842	**1085.972**	1080.632	0.672	1080.296	P[32]
A[12]	365.521	1053.621	**1048.281**	0.672	**1047.945**	A[31]
P[13]	397.872	1029.942	**1024.602**	0.672	**1024.266**	P[30]
A[14]	421.551	997.591	992.251	0.672	991.916	A[29]
E[15]	464.565	**973.912**	968.572	0.672	968.236	E[28]
K[16]	507.263	930.898	925.558	**925.894**	925.222	K[27]
T[17]	540.946	888.199	**882.860**	**883.196**	**882.524**	T[26]
P[18]	573.297	854.517	849.177	849.513	848.841	P[25]
V[19]	606.320	822.166	816.826	817.162	816.490	V[24]
K[20]	649.018	789.143	783.804	784.140	783.468	K[23]
K[21]	691.716	746.445	741.105	741.441	740.769	K[22]
K[22]	**734.414**	703.747	698.407	698.743	698.071	K[21]
A[23]	758.094	661.048	655.709	656.045	655.373	A[20]
R[24]	810.127	637.369	632.030	632.366	631.694	R[19]
K[25]	852.826	585.335	579.996	580.332	579.660	K[18]
A[26]	876.505	542.637	537.298	537.634	536.962	A[17]
A[27]	900.184	518.958	513.619	513.954	513.283	A[16]
G[28]	919.191	495.279	489.940	490.275	489.604	G[15]
G[29]	**938.198**	476.272	470.932	471.268	470.596	G[14]
A[30]	961.877	457.265	451.925	452.261	451.589	A[13]
K[31]	**1004.575**	433.586	428.246	428.582	427.910	K[12]
R[32]	**1056.609**	390.887	385.548	385.884	385.212	R[11]
K[33]	**1099.307**	338.854	333.514	333.850	333.178	K[10]
T[34]	**1132.990**	296.155	290.816	291.152	290.480	T[9]
S[35]	**1162.001**	262.473	257.133	257.469	256.797	S[8]
G[36]	1181.008	233.462	228.123	228.459	227.787	G[7]
P[37]	1213.359	214.455	209.115	209.451	208.779	P[6]
P[38]	**1245.710**	182.104	176.765	177.100	176.429	P[5]
V[39]	**1278.732**	149.753	144.414	144.750	144.078	V[4]
S[40]	**1307.743**	116.730	111.391	111.727	111.055	S[3]
E[41]	1350.757	87.720	82.380	82.716	82.044	E[2]
L[42]	1388.452	44.705	39.366	39.702	39.030	L[1]

sp|P43274|H14_MOUSE

[Acetyl]SETAPAAPAAPAPAEKTPVKKKARKAAGGAKRKTSGPPVSEL

- fragmentation table for charge state 4+
- specType=etd
- score=47.24
- F104929.dat
- query=q3993_p1
- precursor=833.675050
- chargePrecursor=5
- itol=0.8

AA	c	y	z+1	z+2	z	AA
S[1]	37.525	**1041.837**	**1037.832**	0.755	**1037.580**	S[42]
E[2]	69.785	1009.576	**1005.571**	0.755	**1005.319**	E[41]
T[3]	95.047	977.315	**973.311**	0.755	**973.059**	T[40]
A[4]	112.806	952.054	**948.049**	0.755	**947.797**	A[39]
P[5]	137.070	**934.294**	930.290	0.755	930.038	P[38]
A[6]	154.829	**910.031**	906.026	0.755	905.774	A[37]
A[7]	172.588	892.272	888.267	0.755	888.015	A[36]
P[8]	196.851	874.513	870.508	0.755	870.256	P[35]
A[9]	214.611	850.249	846.245	0.755	845.993	A[34]
A[10]	232.370	832.490	828.485	0.755	828.233	A[33]
P[11]	256.633	814.731	810.726	0.755	810.474	P[32]
A[12]	274.392	790.468	786.463	0.755	786.211	A[31]
P[13]	298.656	772.708	768.704	0.755	768.452	P[30]
A[14]	316.415	748.445	744.440	0.755	744.188	A[29]
E[15]	348.675	730.686	726.681	0.755	726.429	E[28]
K[16]	380.699	698.425	694.420	694.672	694.169	K[27]
T[17]	405.961	666.401	662.397	662.649	662.145	T[26]
P[18]	430.224	**641.140**	637.135	637.387	636.883	P[25]
V[19]	454.991	616.876	612.872	613.124	612.620	V[24]
K[20]	487.015	592.109	588.105	588.356	587.853	K[23]
K[21]	519.039	560.085	556.081	556.333	555.829	K[22]
K[22]	551.063	528.062	524.057	524.309	523.805	K[21]
A[23]	568.822	496.038	492.033	492.285	491.781	A[20]
R[24]	607.847	478.279	474.274	474.526	474.022	R[19]
K[25]	639.871	439.253	435.249	435.501	434.997	K[18]
A[26]	657.630	407.230	403.225	403.477	402.973	A[17]
A[27]	675.390	389.470	385.466	385.718	385.214	A[16]
G[28]	689.645	371.711	367.706	367.958	367.454	G[15]
G[29]	703.900	357.456	353.451	353.703	353.199	G[14]
A[30]	721.660	343.200	339.196	339.448	338.944	A[13]
K[31]	753.683	325.441	321.436	321.688	321.184	K[12]
R[32]	792.709	293.417	289.413	289.665	289.161	R[11]
K[33]	824.732	254.392	250.387	250.639	250.135	K[10]
T[34]	849.994	222.368	218.364	218.616	218.112	T[9]
S[35]	871.752	197.106	193.102	193.354	192.850	S[8]
G[36]	886.008	175.348	171.344	171.596	171.092	G[7]
P[37]	910.271	161.093	157.088	157.340	156.836	P[6]
P[38]	**934.534**	136.830	132.825	133.077	132.573	P[5]
V[39]	**959.301**	112.567	108.562	108.814	108.310	V[4]
S[40]	**981.059**	87.800	83.795	84.047	83.543	S[3]
E[41]	**1013.320**	66.042	62.037	62.289	61.785	E[2]
L[42]	**1041.591**	33.781	29.776	30.028	29.524	L[1]

sp|P43274|H14_MOUSE

S $^{(Phospho)}_{(79.97)}$ ETAPAAPAAPAPAEKTPVKKKARKAAGGAKRKTSGPPVSEL

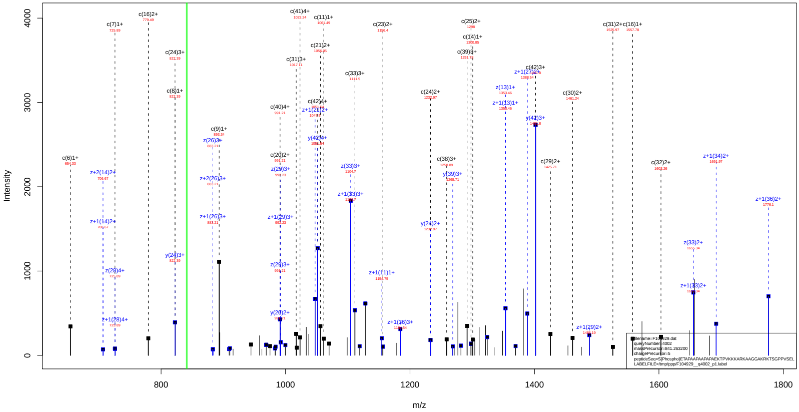

sp|P43274|H14_MOUSE

S $^{(Phospho)}_{(79.97)}$ ETAPAAPAAPAPAEKTPVKKKARKAAGGAKRKTSGPPVSEL

- fragmentation table for charge state 1+
- specType=etd
- score=46.73
- F104929.dat
- query=q4002_p1
- precursor=841.263200
- chargePrecursor=5
- itol=0.8

AA	c	y	z+1	z+2	z	AA
S[1]	185.032	4202.281	4186.262	0.000	4185.254	S[42]
E[2]	314.075	4035.283	4019.264	0.000	4018.256	E[41]
T[3]	415.122	3906.240	3890.221	0.000	3889.213	T[40]
A[4]	486.160	3805.192	3789.174	0.000	3788.166	A[39]
P[5]	583.212	3734.155	3718.136	0.000	3717.129	P[38]
A[6]	**654.249**	3637.102	3621.084	0.000	3620.076	A[37]
A[7]	**725.287**	3566.065	3550.047	0.000	3549.039	A[36]
P[8]	**822.339**	3495.028	3479.009	0.000	3478.002	P[35]
A[9]	**893.376**	3397.975	3381.957	0.000	3380.949	A[34]
A[10]	964.414	3326.938	3310.920	0.000	3309.912	A[33]
P[11]	**1061.466**	3255.901	3239.882	0.000	3238.875	P[32]
A[12]	1132.503	3158.848	3142.830	0.000	3141.822	A[31]
P[13]	1229.556	3087.811	3071.793	0.000	3070.785	P[30]
A[14]	**1300.593**	2990.759	2974.740	0.000	2973.732	A[29]
E[15]	1429.636	2919.721	2903.703	0.000	2902.695	E[28]
K[16]	**1557.731**	2790.679	2774.660	2775.668	2773.652	K[27]
T[17]	1658.779	2662.584	2646.565	2647.573	2645.557	T[26]
P[18]	1755.831	2561.536	2545.517	2546.525	2544.510	P[25]
V[19]	1854.900	2464.483	2448.465	2449.473	2447.457	V[24]
K[20]	1982.995	2365.415	2349.396	2350.404	2348.388	K[23]
K[21]	2111.090	2237.320	2221.301	2222.309	2220.294	K[22]
K[22]	2239.185	2109.225	2093.206	2094.214	2092.199	K[21]
A[23]	2310.222	1981.130	1965.111	1966.119	1964.104	A[20]
R[24]	2466.323	1910.093	1894.074	1895.082	1893.066	R[19]
K[25]	2594.418	1753.992	1737.973	1738.981	1736.965	K[18]
A[26]	2665.455	1625.897	1609.878	1610.886	1608.870	A[17]
A[27]	2736.492	1554.860	1538.841	1539.849	1537.833	A[16]
G[28]	2793.513	1483.823	1467.804	1468.812	1466.796	G[15]
G[29]	2850.535	1426.801	1410.783	1411.790	1409.775	G[14]
A[30]	2921.572	**1369.780**	**1353.761**	1354.769	**1352.753**	A[13]
K[31]	3049.667	**1298.743**	1282.724	1283.732	**1281.716**	K[12]
R[32]	3205.768	1170.648	**1154.629**	**1155.637**	1153.621	R[11]
K[33]	3333.863	1014.547	998.528	**999.536**	997.520	K[10]
T[34]	3434.911	886.452	870.433	871.441	869.425	T[9]
S[35]	3521.943	785.404	769.385	770.393	768.377	S[8]
G[36]	3578.964	698.372	682.353	683.361	681.345	G[7]
P[37]	3676.017	641.350	625.332	626.340	624.324	P[6]
P[38]	3773.070	544.298	528.279	529.287	527.271	P[5]
V[39]	3872.138	447.245	431.226	432.234	430.218	V[4]
S[40]	3959.170	348.177	332.158	333.166	331.150	S[3]
E[41]	4088.213	261.144	245.126	246.134	244.118	E[2]
L[42]	4201.297	132.102	116.083	117.091	115.075	L[1]

sp|P43274|H14_MOUSE

S $^{(Phospho)}_{(79.97)}$ ETAPAAPAAPAPAEKTPVKKKARKAAGGAKRKTSGPPVSEL

- fragmentation table for charge state 2+
- specType=etd
- score=46.73
- F104929.dat
- query=q4002_p1
- precursor=841.263200
- chargePrecursor=5
- itol=0.8

AA	c	y	z+1	z+2	z	AA
S[1]	93.020	2101.644	2093.635	0.504	2093.131	S[42]
E[2]	157.541	2018.145	2010.136	0.504	2009.632	E[41]
T[3]	208.065	1953.624	1945.614	0.504	1945.110	T[40]
A[4]	243.583	1903.100	1895.090	0.504	1894.587	A[39]
P[5]	292.110	1867.581	1859.572	0.504	1859.068	P[38]
A[6]	327.628	1819.055	1811.045	0.504	1810.542	A[37]
A[7]	363.147	1783.536	**1775.527**	0.504	1775.023	A[36]
P[8]	411.673	1748.018	1740.008	0.504	1739.504	P[35]
A[9]	447.192	1699.491	**1691.482**	0.504	1690.978	A[34]
A[10]	482.710	1663.973	**1655.963**	0.504	**1655.460**	A[33]
P[11]	531.237	1628.454	1620.445	0.504	1619.941	P[32]
A[12]	566.755	1579.928	1571.918	0.504	1571.415	A[31]
P[13]	615.282	1544.409	1536.400	0.504	1535.896	P[30]
A[14]	650.800	1495.883	**1487.874**	0.504	1487.370	A[29]
E[15]	715.322	1460.364	1452.355	0.504	1451.851	E[28]
K[16]	**779.369**	1395.843	**1387.834**	**1388.338**	1387.330	K[27]
T[17]	829.893	1331.796	**1323.786**	**1324.290**	1323.282	T[26]
P[18]	878.419	**1281.272**	1273.262	1273.766	1272.758	P[25]
V[19]	927.953	**1232.745**	1224.736	1225.240	1224.232	V[24]
K[20]	**992.001**	1183.211	1175.202	1175.706	1174.698	K[23]
K[21]	**1056.048**	**1119.164**	**1111.154**	**1111.658**	1110.650	K[22]
K[22]	1120.096	1055.116	**1047.107**	**1047.611**	1046.603	K[21]
A[23]	**1155.615**	**991.069**	**983.059**	**983.563**	**982.555**	A[20]
R[24]	**1233.665**	955.550	947.541	948.045	947.037	R[19]
K[25]	**1297.713**	877.500	869.490	869.994	868.986	K[18]
A[26]	1333.231	813.452	805.443	805.947	804.939	A[17]
A[27]	1368.750	777.934	769.924	770.428	769.420	A[16]
G[28]	1397.260	742.415	734.406	734.910	733.902	G[15]
G[29]	**1425.771**	713.904	**705.895**	**706.399**	705.391	G[14]
A[30]	**1461.290**	685.394	677.384	677.888	676.880	A[13]
K[31]	**1525.337**	649.875	641.866	642.370	641.362	K[12]
R[32]	**1603.388**	585.827	577.818	578.322	577.314	R[11]
K[33]	1667.435	507.777	499.768	500.271	499.264	K[10]
T[34]	1717.959	443.729	435.720	436.224	435.216	T[9]
S[35]	1761.475	393.206	385.196	385.700	384.692	S[8]
G[36]	1789.986	349.690	341.680	342.184	341.176	G[7]
P[37]	1838.512	321.179	313.170	313.673	312.666	P[6]
P[38]	1887.039	272.652	264.643	265.147	264.139	P[5]
V[39]	1936.573	224.126	216.117	216.621	215.613	V[4]
S[40]	1980.089	174.592	166.583	167.086	166.079	S[3]
E[41]	2044.610	131.076	123.067	123.570	122.563	E[2]
L[42]	2101.152	66.555	58.545	59.049	58.041	L[1]

sp|P43274|H14_MOUSE

S$^{(Phospho)}_{(79.97)}$ETAPAAPAAPAPAEKTPVKKKARKAAGGAKRKTSGPPVSEL

- fragmentation table for charge state 3+
- specType=etd
- score=46.73
- F104929.dat
- query=q4002_p1
- precursor=841.263200
- chargePrecursor=5
- itol=0.8

AA	c	y	z+1	z+2	z	AA
S[1]	62.349	**1401.432**	1396.092	0.672	1395.756	S[42]
E[2]	105.363	1345.766	1340.426	0.672	1340.090	E[41]
T[3]	139.046	1302.752	**1297.412**	0.672	1297.076	T[40]
A[4]	162.725	**1269.069**	1263.729	0.672	1263.393	A[39]
P[5]	195.076	1245.390	1240.050	0.672	1239.714	P[38]
A[6]	218.755	1213.039	1207.699	0.672	1207.363	A[37]
A[7]	242.434	1189.360	**1184.020**	0.672	1183.684	A[36]
P[8]	274.785	1165.681	1160.341	0.672	1160.005	P[35]
A[9]	298.464	1133.330	**1127.990**	0.672	**1127.654**	A[34]
A[10]	322.143	1109.651	**1104.311**	0.672	**1103.975**	A[33]
P[11]	354.494	1085.972	1080.632	0.672	1080.296	P[32]
A[12]	378.173	1053.621	**1048.281**	0.672	**1047.945**	A[31]
P[13]	410.524	1029.942	1024.602	0.672	1024.266	P[30]
A[14]	434.203	997.591	**992.251**	0.672	**991.916**	A[29]
E[15]	477.217	973.912	**968.572**	0.672	**968.236**	E[28]
K[16]	519.915	930.898	925.558	925.894	925.222	K[27]
T[17]	553.598	888.199	**882.860**	**883.196**	**882.524**	T[26]
P[18]	585.949	854.517	849.177	849.513	848.841	P[25]
V[19]	618.971	**822.166**	816.826	817.162	816.490	V[24]
K[20]	661.670	789.143	783.804	784.140	783.468	K[23]
K[21]	704.368	746.445	741.105	741.441	740.769	K[22]
K[22]	747.066	703.747	698.407	698.743	698.071	K[21]
A[23]	770.745	661.048	655.709	656.045	655.373	A[20]
R[24]	**822.779**	637.369	632.030	632.366	631.694	R[19]
K[25]	865.477	585.335	579.996	580.332	579.660	K[18]
A[26]	889.156	542.637	537.298	537.634	536.962	A[17]
A[27]	912.836	518.958	513.619	513.954	513.283	A[16]
G[28]	931.843	495.279	489.940	490.275	489.604	G[15]
G[29]	950.850	476.272	470.932	471.268	470.596	G[14]
A[30]	**974.529**	457.265	451.925	452.261	451.589	A[13]
K[31]	**1017.227**	433.586	428.246	428.582	427.910	K[12]
R[32]	**1069.261**	390.887	385.548	385.884	385.212	R[11]
K[33]	**1111.959**	338.854	333.514	333.850	333.178	K[10]
T[34]	1145.642	296.155	290.816	291.152	290.480	T[9]
S[35]	1174.652	262.473	257.133	257.469	256.797	S[8]
G[36]	1193.660	233.462	228.123	228.459	227.787	G[7]
P[37]	1226.011	214.455	209.115	209.451	208.779	P[6]
P[38]	**1258.361**	182.104	176.765	177.100	176.429	P[5]
V[39]	**1291.384**	149.753	144.414	144.750	144.078	V[4]
S[40]	1320.395	116.730	111.391	111.727	111.055	S[3]
E[41]	1363.409	87.720	82.380	82.716	82.044	E[2]
L[42]	**1401.104**	44.705	39.366	39.702	39.030	L[1]

sp|P43274|H14_MOUSE

S $^{(Phospho)}_{(79.97)}$ ETAPAAPAAPAPAEKTPVKKARKAAGGAKRKTSGPPVSEL

- fragmentation table for charge state 4+
- specType=etd
- score=46.73
- F104929.dat
- query=q4002_p1
- precursor=841.263200
- chargePrecursor=5
- itol=0.8

AA	c	y	z+1	z+2	z	AA
S[1]	47.014	**1051.326**	**1047.321**	0.755	**1047.069**	S[42]
E[2]	79.274	1009.576	1005.571	0.755	1005.319	E[41]
T[3]	104.536	977.315	973.311	0.755	973.059	T[40]
A[4]	122.295	952.054	948.049	0.755	947.797	A[39]
P[5]	146.559	934.294	930.290	0.755	930.038	P[38]
A[6]	164.318	**910.031**	906.026	0.755	905.774	A[37]
A[7]	182.077	892.272	888.267	0.755	888.015	A[36]
P[8]	206.340	874.513	870.508	0.755	870.256	P[35]
A[9]	224.100	850.249	846.245	0.755	845.993	A[34]
A[10]	241.859	832.490	828.485	0.755	828.233	A[33]
P[11]	266.122	814.731	810.726	0.755	810.474	P[32]
A[12]	283.881	790.468	786.463	0.755	786.211	A[31]
P[13]	308.145	772.708	768.704	0.755	768.452	P[30]
A[14]	325.904	748.445	744.440	0.755	744.188	A[29]
E[15]	358.164	730.686	**726.681**	0.755	**726.429**	E[28]
K[16]	390.188	698.425	694.420	694.672	694.169	K[27]
T[17]	415.450	666.401	662.397	662.649	662.145	T[26]
P[18]	439.713	641.140	637.135	637.387	636.883	P[25]
V[19]	464.480	616.876	612.872	613.124	612.620	V[24]
K[20]	496.504	592.109	588.105	588.356	587.853	K[23]
K[21]	528.528	560.085	556.081	556.333	555.829	K[22]
K[22]	560.552	528.062	524.057	524.309	523.805	K[21]
A[23]	578.311	496.038	492.033	492.285	491.781	A[20]
R[24]	617.336	478.279	474.274	474.526	474.022	R[19]
K[25]	649.360	439.253	435.249	435.501	434.997	K[18]
A[26]	667.119	407.230	403.225	403.477	402.973	A[17]
A[27]	684.878	389.470	385.466	385.718	385.214	A[16]
G[28]	699.134	371.711	367.706	367.958	367.454	G[15]
G[29]	713.389	357.456	353.451	353.703	353.199	G[14]
A[30]	731.148	343.200	339.196	339.448	338.944	A[13]
K[31]	763.172	325.441	321.436	321.688	321.184	K[12]
R[32]	802.197	293.417	289.413	289.665	289.161	R[11]
K[33]	834.221	254.392	250.387	250.639	250.135	K[10]
T[34]	859.483	222.368	218.364	218.616	218.112	T[9]
S[35]	881.241	197.106	193.102	193.354	192.850	S[8]
G[36]	895.497	175.348	171.344	171.596	171.092	G[7]
P[37]	919.760	161.093	157.088	157.340	156.836	P[6]
P[38]	**944.023**	136.830	132.825	133.077	132.573	P[5]
V[39]	**968.790**	112.567	108.562	108.814	108.310	V[4]
S[40]	**990.548**	87.800	83.795	84.047	83.543	S[3]
E[41]	**1022.809**	66.042	62.037	62.289	61.785	E[2]
L[42]	**1051.080**	33.781	29.776	30.028	29.524	L[1]

sp|P43277|H13_MOUSE

[Acetyl]SETAPAAPAAPAPVEKTPVKKKAKKTGAAAGKRKASGPPVSEL

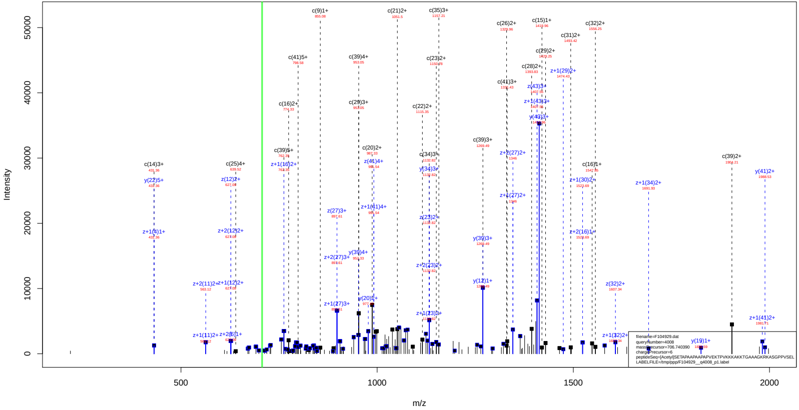

sp|P43277|H13_MOUSE

[Acetyl]SETAPAAPAAPAPVEKTPVKKAKKTGAAAGKRKASGPPVSEL

- fragmentation table for charge state 1+
- specType=etd
- score=44.16
- F104929.dat
- query=q4008_p1
- precursor=706.740390
- chargePrecursor=6
- itol=0.8

AA	c	y	z+1	z+2	z	AA
S[1]	147.076	4235.387	4219.369	0.000	4218.361	S[43]
E[2]	276.119	4106.345	4090.326	0.000	4089.318	E[42]
T[3]	377.167	3977.302	3961.284	0.000	3960.276	T[41]
A[4]	448.204	3876.255	3860.236	0.000	3859.228	A[40]
P[5]	545.257	3805.217	3789.199	0.000	3788.191	P[39]
A[6]	616.294	3708.165	3692.146	0.000	3691.138	A[38]
A[7]	687.331	3637.128	3621.109	0.000	3620.101	A[37]
P[8]	**784.384**	3566.090	3550.072	0.000	3549.064	P[36]
A[9]	**855.421**	3469.038	3453.019	0.000	3452.011	A[35]
A[10]	926.458	3398.001	3381.982	0.000	3380.974	A[34]
P[11]	**1023.511**	3326.963	3310.945	0.000	3309.937	P[33]
A[12]	1094.548	3229.911	3213.892	0.000	3212.884	A[32]
P[13]	1191.600	3158.874	3142.855	0.000	3141.847	P[31]
V[14]	1290.669	3061.821	3045.802	0.000	3044.794	V[30]
E[15]	**1419.711**	2962.752	2946.734	0.000	2945.726	E[29]
K[16]	**1547.806**	2833.710	2817.691	2818.699	2816.683	K[28]
T[17]	1648.854	2705.615	2689.596	2690.604	2688.588	T[27]
P[18]	1745.907	2604.567	2588.548	2589.556	2587.541	P[26]
V[19]	1844.975	2507.514	2491.496	2492.504	2490.488	V[25]
K[20]	1973.070	2408.446	2392.427	2393.435	2391.419	K[24]
K[21]	2101.165	2280.351	2264.332	2265.340	2263.324	K[23]
K[22]	2229.260	2152.256	2136.237	2137.245	2135.230	K[22]
A[23]	2300.297	2024.161	2008.142	2009.150	2007.135	A[21]
K[24]	2428.392	1953.124	1937.105	1938.113	1936.097	K[20]
K[25]	2556.487	**1825.029**	1809.010	1810.018	1808.002	K[19]
T[26]	2657.535	1696.934	1680.915	1681.923	1679.908	T[18]
G[27]	2714.556	1595.886	**1579.868**	1580.875	1578.860	G[17]
A[28]	2785.593	1538.865	1522.846	**1523.854**	1521.838	A[16]
A[29]	2856.631	1467.828	1451.809	1452.817	1450.801	A[15]
A[30]	2927.668	1396.791	1380.772	1381.780	1379.764	A[14]
G[31]	2984.689	1325.754	1309.735	1310.743	1308.727	G[13]
K[32]	3112.784	**1268.732**	1252.713	1253.721	1251.706	K[12]
R[33]	3268.885	**1140.637**	**1124.618**	1125.626	1123.611	R[11]
K[34]	3396.980	984.536	**968.517**	**969.525**	967.509	K[10]
A[35]	3468.017	856.441	**840.422**	**841.430**	**839.415**	A[9]
S[36]	3555.049	**785.404**	769.385	770.393	768.377	S[8]
G[37]	3612.071	**698.372**	682.353	683.361	681.345	G[7]
P[38]	3709.124	641.350	625.332	**626.340**	624.324	P[6]
P[39]	3806.176	544.298	528.279	529.287	527.271	P[5]
V[40]	3905.245	447.245	**431.226**	432.234	430.218	V[4]
S[41]	3992.277	348.177	332.158	333.166	331.150	S[3]
E[42]	4121.319	261.144	245.126	246.134	244.118	E[2]
L[43]	4234.403	132.102	116.083	117.091	115.075	L[1]

sp|P43277|H13_MOUSE

[Acetyl]SETAPAAPAAPAPVEKTPVKKKAKKTGAAAGKRKASGPPVSEL

- fragmentation table for charge state 2+
- specType=etd
- score=44.16
- F104929.dat
- query=q4008_p1
- precursor=706.740390
- chargePrecursor=6
- itol=0.8

AA	c	y	z+1	z+2	z	AA
S[1]	74.042	2118.197	2110.188	0.504	2109.684	S[43]
E[2]	138.563	2053.676	2045.667	0.504	2045.163	E[42]
T[3]	189.087	**1989.155**	**1981.145**	0.504	1980.641	T[41]
A[4]	224.606	1938.631	1930.622	0.504	1930.118	A[40]
P[5]	273.132	1903.112	1895.103	0.504	1894.599	P[39]
A[6]	308.650	1854.586	1846.577	0.504	1846.073	A[38]
A[7]	344.169	1819.067	1811.058	0.504	1810.554	A[37]
P[8]	392.695	1783.549	1775.540	0.504	1775.036	P[36]
A[9]	428.214	1735.022	1727.013	0.504	1726.509	A[35]
A[10]	463.733	1699.504	**1691.495**	0.504	1690.991	A[34]
P[11]	512.259	1663.985	1655.976	0.504	1655.472	P[33]
A[12]	547.777	1615.459	**1607.450**	0.504	**1606.946**	A[32]
P[13]	596.304	**1579.940**	1571.931	0.504	1571.427	P[31]
V[14]	645.838	1531.414	**1523.405**	0.504	**1522.901**	V[30]
E[15]	710.359	1481.880	**1473.870**	0.504	1473.367	E[29]
K[16]	**774.407**	1417.359	1409.349	1409.853	1408.845	K[28]
T[17]	**824.931**	1353.311	**1345.302**	**1345.806**	1344.798	T[27]
P[18]	873.457	1302.787	**1294.778**	1295.282	**1294.274**	P[26]
V[19]	922.991	**1254.261**	1246.251	1246.755	1245.748	V[25]
K[20]	**987.039**	1204.727	1196.717	**1197.221**	1196.213	K[24]
K[21]	**1051.086**	**1140.679**	**1132.670**	**1133.174**	**1132.166**	K[23]
K[22]	**1115.134**	1076.632	**1068.622**	**1069.126**	**1068.118**	K[22]
A[23]	**1150.652**	**1012.584**	1004.575	1005.079	1004.071	A[21]
K[24]	1214.700	**977.066**	**969.056**	**969.560**	**968.552**	K[20]
K[25]	1278.747	**913.018**	**905.009**	**905.513**	904.505	K[19]
T[26]	**1329.271**	848.971	**840.961**	**841.465**	**840.457**	T[18]
G[27]	1357.782	**798.447**	**790.437**	**790.941**	789.934	G[17]
A[28]	**1393.300**	769.936	**761.927**	**762.431**	761.423	A[16]
A[29]	**1428.819**	734.418	**726.408**	**726.912**	725.904	A[15]
A[30]	**1464.337**	**698.899**	**690.890**	**691.394**	**690.386**	A[14]
G[31]	**1492.848**	663.380	655.371	655.875	654.867	G[13]
K[32]	**1556.896**	634.870	**626.860**	**627.364**	**626.356**	K[12]
R[33]	1634.946	570.822	**562.813**	**563.317**	562.309	R[11]
K[34]	1698.994	492.772	484.762	485.266	484.258	K[10]
A[35]	1734.512	428.724	420.715	421.219	420.211	A[9]
S[36]	1778.028	393.206	385.196	385.700	384.692	S[8]
G[37]	1806.539	349.690	341.680	342.184	341.176	G[7]
P[38]	1855.065	321.179	313.170	313.673	312.666	P[6]
P[39]	**1903.592**	272.652	264.643	265.147	264.139	P[5]
V[40]	1953.126	224.126	216.117	216.621	215.613	V[4]
S[41]	1996.642	174.592	166.583	167.086	166.079	S[3]
E[42]	2061.163	131.076	123.067	123.570	122.563	E[2]
L[43]	2117.705	66.555	58.545	59.049	58.041	L[1]

sp|P43277|H13_MOUSE

[Acetyl]SETAPAAPAAPAPVEKTPVKKAKKTGAAAGKRKASGPPVSEL

- fragmentation table for charge state 3+
- specType=etd
- score=44.16
- F104929.dat
- query=q4008_p1
- precursor=706.740390
- chargePrecursor=6
- itol=0.8

AA	c	y	z+1	z+2	z	AA
S[1]	49.697	**1412.467**	**1407.128**	0.672	**1406.792**	S[43]
E[2]	92.711	1369.453	**1364.114**	0.672	**1363.778**	E[42]
T[3]	126.394	1326.439	**1321.099**	0.672	1320.763	T[41]
A[4]	150.073	1292.756	1287.417	0.672	1287.081	A[40]
P[5]	182.424	**1269.077**	**1263.738**	0.672	1263.402	P[39]
A[6]	206.103	1236.726	1231.387	0.672	1231.051	A[38]
A[7]	229.782	1213.047	1207.708	0.672	1207.372	A[37]
P[8]	262.133	1189.368	1184.029	0.672	1183.693	P[36]
A[9]	285.812	**1157.017**	1151.678	0.672	**1151.342**	A[35]
A[10]	309.491	**1133.338**	**1127.999**	0.672	**1127.663**	A[34]
P[11]	341.842	1109.659	1104.320	0.672	1103.984	P[33]
A[12]	365.521	**1077.308**	**1071.969**	0.672	1071.633	A[32]
P[13]	397.872	1053.629	**1048.290**	0.672	**1047.954**	P[31]
V[14]	**430.894**	1021.278	**1015.939**	0.672	1015.603	V[30]
E[15]	473.909	988.256	982.916	0.672	982.580	E[29]
K[16]	516.607	945.241	**939.902**	**940.238**	**939.566**	K[28]
T[17]	550.290	902.543	**897.204**	**897.539**	**896.868**	T[27]
P[18]	582.640	868.861	863.521	863.857	863.185	P[26]
V[19]	615.663	**836.510**	831.170	831.506	830.834	V[25]
K[20]	658.362	803.487	**798.147**	**798.483**	**797.811**	K[24]
K[21]	701.060	760.789	**755.449**	**755.785**	**755.113**	K[23]
K[22]	743.758	**718.090**	**712.751**	**713.087**	**712.415**	K[22]
A[23]	767.437	675.392	**670.052**	**670.388**	669.716	A[21]
K[24]	810.136	651.713	646.373	646.709	646.037	K[20]
K[25]	852.834	609.015	603.675	604.011	603.339	K[19]
T[26]	886.516	566.316	560.977	561.313	560.641	T[18]
G[27]	**905.524**	532.634	527.294	527.630	526.958	G[17]
A[28]	929.203	513.626	508.287	508.623	507.951	A[16]
A[29]	**952.882**	489.947	484.608	484.944	484.272	A[15]
A[30]	976.561	466.268	460.929	461.265	460.593	A[14]
G[31]	**995.568**	442.589	437.250	437.586	436.914	G[13]
K[32]	**1038.266**	423.582	418.243	418.579	417.907	K[12]
R[33]	**1090.300**	380.884	375.544	375.880	375.208	R[11]
K[34]	**1132.998**	328.850	323.511	323.847	323.175	K[10]
A[35]	**1156.677**	286.152	280.812	281.148	280.476	A[9]
S[36]	1185.688	262.473	257.133	257.469	256.797	S[8]
G[37]	1204.695	233.462	228.123	228.459	227.787	G[7]
P[38]	1237.046	214.455	209.115	209.451	208.779	P[6]
P[39]	**1269.397**	182.104	176.765	177.100	176.429	P[5]
V[40]	1302.420	149.753	144.414	144.750	144.078	V[4]
S[41]	**1331.430**	116.730	111.391	111.727	111.055	S[3]
E[42]	1374.445	87.720	82.380	82.716	82.044	E[2]
L[43]	1412.139	44.705	39.366	39.702	39.030	L[1]

sp|P43277|H13_MOUSE

[Acetyl]SETAPAAPAAPAPVEKTPVKKAKKTGAAAGKRKASGPPVSEL

- fragmentation table for charge state 4+
- specType=etd
- score=44.16
- F104929.dat
- query=q4008_p1
- precursor=706.740390
- chargePrecursor=6
- itol=0.8

AA	c	y	z+1	z+2	z	AA
S[1]	37.525	1059.602	**1055.598**	0.755	**1055.346**	S[43]
E[2]	69.785	1027.342	**1023.337**	0.755	**1023.085**	E[42]
T[3]	95.047	995.081	**991.076**	0.755	**990.824**	T[41]
A[4]	112.806	**969.819**	965.814	0.755	965.562	A[40]
P[5]	137.070	**952.060**	948.055	0.755	947.803	P[39]
A[6]	154.829	927.797	923.792	0.755	923.540	A[38]
A[7]	172.588	910.037	**906.033**	0.755	**905.781**	A[37]
P[8]	196.851	892.278	888.273	0.755	**888.021**	P[36]
A[9]	214.611	868.015	864.010	0.755	863.758	A[35]
A[10]	232.370	850.256	**846.251**	0.755	**845.999**	A[34]
P[11]	256.633	832.496	**828.492**	0.755	**828.240**	P[33]
A[12]	274.392	808.233	**804.228**	0.755	**803.976**	A[32]
P[13]	298.656	**790.474**	786.469	0.755	**786.217**	P[31]
V[14]	323.423	**766.211**	**762.206**	0.755	**761.954**	V[30]
E[15]	355.683	741.444	737.439	0.755	737.187	E[29]
K[16]	387.707	709.183	705.178	705.430	704.926	K[28]
T[17]	412.969	677.159	**673.154**	**673.406**	**672.903**	T[27]
P[18]	437.232	651.897	647.893	648.145	647.641	P[26]
V[19]	461.999	**627.634**	623.629	623.881	623.377	V[25]
K[20]	494.023	602.867	598.862	599.114	598.610	K[24]
K[21]	526.047	570.843	566.839	567.090	566.587	K[23]
K[22]	558.070	538.819	534.815	535.067	534.563	K[22]
A[23]	575.830	506.796	502.791	503.043	502.539	A[21]
K[24]	607.854	489.036	485.032	485.284	484.780	K[20]
K[25]	**639.877**	457.013	453.008	453.260	452.756	K[19]
T[26]	665.139	424.989	420.984	421.236	420.732	T[18]
G[27]	679.395	399.727	395.722	395.974	395.470	G[17]
A[28]	697.154	385.472	381.467	381.719	381.215	A[16]
A[29]	714.913	367.712	363.708	363.960	363.456	A[15]
A[30]	732.672	349.953	345.948	346.200	345.696	A[14]
G[31]	746.928	332.194	328.189	328.441	327.937	G[13]
K[32]	778.951	317.938	313.934	314.186	313.682	K[12]
R[33]	**817.977**	285.915	281.910	282.162	281.658	R[11]
K[34]	850.001	246.889	242.885	243.137	242.633	K[10]
A[35]	867.760	214.866	210.861	211.113	210.609	A[9]
S[36]	**889.518**	197.106	193.102	193.354	192.850	S[8]
G[37]	903.773	175.348	171.344	171.596	171.092	G[7]
P[38]	928.036	161.093	157.088	157.340	156.836	P[6]
P[39]	**952.300**	136.830	132.825	133.077	132.573	P[5]
V[40]	**977.067**	112.567	108.562	108.814	108.310	V[4]
S[41]	**998.825**	87.800	83.795	84.047	83.543	S[3]
E[42]	1031.085	66.042	62.037	62.289	61.785	E[2]
L[43]	1059.356	33.781	29.776	30.028	29.524	L[1]

sp|P43277|H13_MOUSE

[Acetyl]SETAPAAPAAPAPVEKTPVKKKAKKTGAAAGKRKASGPPVSEL

- fragmentation table for charge state 5+
- specType=etd
- score=44.16
- F104929.dat
- query=q4008_p1
- precursor=706.740390
- chargePrecursor=6
- itol=0.8

AA	c	y	z+1	z+2	z	AA
S[1]	30.221	847.883	**844.680**	0.806	**844.478**	S[43]
E[2]	56.030	822.075	**818.871**	0.806	**818.669**	E[42]
T[3]	76.239	**796.266**	**793.063**	0.806	**792.861**	T[41]
A[4]	90.447	776.057	**772.853**	0.806	**772.651**	A[40]
P[5]	109.857	**761.849**	758.646	0.806	758.444	P[39]
A[6]	124.065	742.439	739.235	0.806	739.033	A[38]
A[7]	138.272	**728.231**	725.028	0.806	724.826	A[37]
P[8]	157.683	714.024	710.820	0.806	710.619	P[36]
A[9]	171.890	694.613	**691.410**	0.806	**691.208**	A[35]
A[10]	186.097	680.406	677.202	0.806	677.001	A[34]
P[11]	205.508	666.199	662.995	0.806	662.793	P[33]
A[12]	219.715	646.788	643.584	0.806	643.383	A[32]
P[13]	239.126	632.581	629.377	0.806	629.175	P[31]
V[14]	258.940	613.170	609.966	0.806	609.765	V[30]
E[15]	284.748	593.356	590.153	0.806	589.951	E[29]
K[16]	310.367	567.548	564.344	564.546	564.142	K[28]
T[17]	330.577	541.929	538.725	538.927	538.523	T[27]
P[18]	349.987	521.719	518.516	518.717	518.314	P[26]
V[19]	369.801	502.309	499.105	499.307	498.903	V[25]
K[20]	395.420	482.495	479.291	479.493	479.090	K[24]
K[21]	421.039	456.876	453.672	453.874	453.471	K[23]
K[22]	446.658	**431.257**	428.053	428.255	427.852	K[22]
A[23]	460.865	405.638	402.434	402.636	402.233	A[21]
K[24]	486.484	391.431	388.227	388.428	388.025	K[20]
K[25]	512.103	365.812	362.608	362.809	362.406	K[19]
T[26]	532.313	340.193	336.989	337.190	336.787	T[18]
G[27]	543.717	319.983	316.779	316.981	316.578	G[17]
A[28]	557.925	308.579	305.375	305.577	305.173	A[16]
A[29]	572.132	294.371	291.168	291.369	290.966	A[15]
A[30]	586.339	280.164	276.960	277.162	276.759	A[14]
G[31]	597.744	265.957	262.753	262.954	262.551	G[13]
K[32]	623.363	254.552	251.348	251.550	251.147	K[12]
R[33]	654.583	228.933	225.730	225.931	225.528	R[11]
K[34]	680.202	197.713	194.509	194.711	194.308	K[10]
A[35]	694.409	172.094	168.890	169.092	168.689	A[9]
S[36]	711.816	157.887	154.683	154.884	154.481	S[8]
G[37]	723.220	140.480	137.276	137.478	137.075	G[7]
P[38]	742.631	129.076	125.872	126.074	125.671	P[6]
P[39]	**762.041**	109.665	106.462	106.663	106.260	P[5]
V[40]	781.855	90.255	87.051	87.253	86.849	V[4]
S[41]	**799.261**	70.441	67.237	67.439	67.036	S[3]
E[42]	**825.070**	53.035	49.831	50.033	49.629	E[2]
L[43]	847.687	27.226	24.022	24.224	23.821	L[1]

sp|P43277|H13_MOUSE

[Acetyl]SETAPAAPAAPAPVEKTPVKKKAKKTGAAAGKRKASGPPVSEL

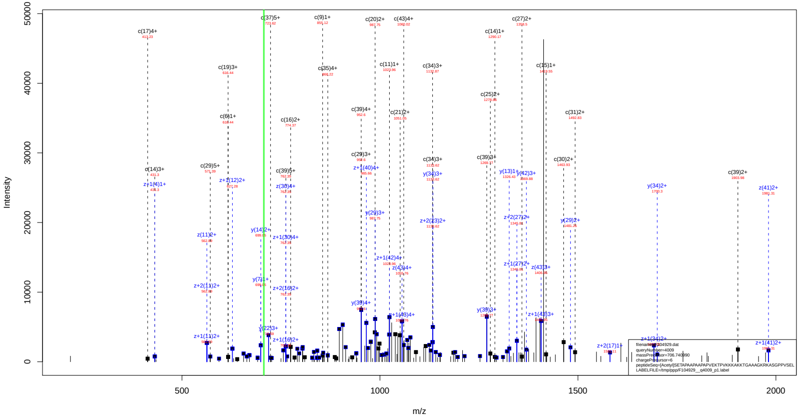

sp|P43277|H13_MOUSE

[Acetyl]SETAPAAPAAPAPVEKTPVKKKAKKTGAAAGKRKASGPPVSEL

- fragmentation table for charge state 1+
- specType=etd
- score=45.31
- F104929.dat
- query=q4009_p1
- precursor=706.740990
- chargePrecursor=6
- itol=0.8

AA	c	y	z+1	z+2	z	AA
S[1]	147.076	4235.387	4219.369	0.000	4218.361	S[43]
E[2]	276.119	4106.345	4090.326	0.000	4089.318	E[42]
T[3]	377.167	3977.302	3961.284	0.000	3960.276	T[41]
A[4]	448.204	3876.255	3860.236	0.000	3859.228	A[40]
P[5]	545.257	3805.217	3789.199	0.000	3788.191	P[39]
A[6]	**616.294**	3708.165	3692.146	0.000	3691.138	A[38]
A[7]	687.331	3637.128	3621.109	0.000	3620.101	A[37]
P[8]	**784.384**	3566.090	3550.072	0.000	3549.064	P[36]
A[9]	**855.421**	3469.038	3453.019	0.000	3452.011	A[35]
A[10]	926.458	3398.001	3381.982	0.000	3380.974	A[34]
P[11]	**1023.511**	3326.963	3310.945	0.000	3309.937	P[33]
A[12]	1094.548	3229.911	3213.892	0.000	3212.884	A[32]
P[13]	1191.600	3158.874	3142.855	0.000	3141.847	P[31]
V[14]	**1290.669**	3061.821	3045.802	0.000	3044.794	V[30]
E[15]	**1419.711**	2962.752	2946.734	0.000	2945.726	E[29]
K[16]	1547.806	2833.710	2817.691	2818.699	2816.683	K[28]
T[17]	1648.854	2705.615	2689.596	2690.604	2688.588	T[27]
P[18]	1745.907	2604.567	2588.548	2589.556	2587.541	P[26]
V[19]	1844.975	2507.514	2491.496	2492.504	2490.488	V[25]
K[20]	1973.070	2408.446	2392.427	2393.435	2391.419	K[24]
K[21]	2101.165	2280.351	2264.332	2265.340	2263.324	K[23]
K[22]	2229.260	2152.256	2136.237	2137.245	2135.230	K[22]
A[23]	2300.297	2024.161	2008.142	2009.150	2007.135	A[21]
K[24]	2428.392	1953.124	1937.105	1938.113	1936.097	K[20]
K[25]	2556.487	1825.029	1809.010	1810.018	1808.002	K[19]
T[26]	2657.535	1696.934	1680.915	1681.923	1679.908	T[18]
G[27]	2714.556	1595.886	1579.868	**1580.875**	1578.860	G[17]
A[28]	2785.593	1538.865	1522.846	1523.854	1521.838	A[16]
A[29]	2856.631	1467.828	1451.809	1452.817	1450.801	A[15]
A[30]	2927.668	1396.791	1380.772	1381.780	1379.764	A[14]
G[31]	2984.689	**1325.754**	1309.735	**1310.743**	1308.727	G[13]
K[32]	3112.784	1268.732	**1252.713**	1253.721	1251.706	K[12]
R[33]	3268.885	**1140.637**	**1124.618**	1125.626	1123.611	R[11]
K[34]	3396.980	984.536	968.517	**969.525**	967.509	K[10]
A[35]	3468.017	**856.441**	**840.422**	**841.430**	839.415	A[9]
S[36]	3555.049	785.404	769.385	770.393	768.377	S[8]
G[37]	3612.071	**698.372**	682.353	683.361	681.345	G[7]
P[38]	3709.124	641.350	625.332	626.340	624.324	P[6]
P[39]	3806.176	544.298	528.279	529.287	527.271	P[5]
V[40]	3905.245	447.245	**431.226**	432.234	430.218	V[4]
S[41]	3992.277	348.177	332.158	333.166	331.150	S[3]
E[42]	4121.319	261.144	245.126	246.134	244.118	E[2]
L[43]	4234.403	132.102	116.083	117.091	115.075	L[1]

sp|P43277|H13_MOUSE

[Acetyl]SETAPAAPAAPAPVEKTPVKKKAKKTGAAAGKRKASGPPVSEL

- fragmentation table for charge state 2+
- specType=etd
- score=45.31
- F104929.dat
- query=q4009_p1
- precursor=706.740990
- chargePrecursor=6
- itol=0.8

AA	c	y	z+1	z+2	z	AA
S[1]	74.042	2118.197	2110.188	0.504	2109.684	S[43]
E[2]	138.563	2053.676	2045.667	0.504	2045.163	E[42]
T[3]	189.087	1989.155	**1981.145**	0.504	**1980.641**	T[41]
A[4]	224.606	1938.631	1930.622	0.504	1930.118	A[40]
P[5]	273.132	1903.112	1895.103	0.504	1894.599	P[39]
A[6]	308.650	1854.586	1846.577	0.504	1846.073	A[38]
A[7]	344.169	1819.067	1811.058	0.504	1810.554	A[37]
P[8]	392.695	1783.549	1775.540	0.504	1775.036	P[36]
A[9]	428.214	1735.022	1727.013	0.504	1726.509	A[35]
A[10]	463.733	**1699.504**	**1691.495**	0.504	1690.991	A[34]
P[11]	512.259	1663.985	1655.976	0.504	1655.472	P[33]
A[12]	547.777	1615.459	1607.450	0.504	1606.946	A[32]
P[13]	596.304	1579.940	1571.931	0.504	1571.427	P[31]
V[14]	645.838	1531.414	1523.405	0.504	1522.901	V[30]
E[15]	710.359	**1481.880**	1473.870	0.504	1473.367	E[29]
K[16]	**774.407**	1417.359	1409.349	1409.853	1408.845	K[28]
T[17]	824.931	1353.311	**1345.302**	**1345.806**	1344.798	T[27]
P[18]	873.457	1302.787	**1294.778**	1295.282	**1294.274**	P[26]
V[19]	922.991	1254.261	1246.251	1246.755	1245.748	V[25]
K[20]	**987.039**	1204.727	**1196.717**	1197.221	**1196.213**	K[24]
K[21]	**1051.086**	**1140.679**	**1132.670**	**1133.174**	**1132.166**	K[23]
K[22]	**1115.134**	**1076.632**	**1068.622**	**1069.126**	**1068.118**	K[22]
A[23]	**1150.652**	**1012.584**	**1004.575**	**1005.079**	**1004.071**	A[21]
K[24]	1214.700	**977.066**	**969.056**	**969.560**	968.552	K[20]
K[25]	**1278.747**	**913.018**	905.009	**905.513**	904.505	K[19]
T[26]	1329.271	848.971	**840.961**	**841.465**	**840.457**	T[18]
G[27]	**1357.782**	798.447	790.437	**790.941**	789.934	G[17]
A[28]	1393.300	769.936	**761.927**	**762.431**	761.423	A[16]
A[29]	1428.819	734.418	**726.408**	**726.912**	**725.904**	A[15]
A[30]	**1464.337**	**698.899**	**690.890**	**691.394**	**690.386**	A[14]
G[31]	**1492.848**	**663.380**	**655.371**	**655.875**	**654.867**	G[13]
K[32]	1556.896	634.870	**626.860**	**627.364**	626.356	K[12]
R[33]	1634.946	**570.822**	**562.813**	**563.317**	**562.309**	R[11]
K[34]	1698.994	492.772	484.762	485.266	484.258	K[10]
A[35]	1734.512	428.724	420.715	421.219	420.211	A[9]
S[36]	1778.028	393.206	385.196	385.700	384.692	S[8]
G[37]	1806.539	349.690	341.680	342.184	341.176	G[7]
P[38]	1855.065	321.179	313.170	313.673	312.666	P[6]
P[39]	**1903.592**	272.652	264.643	265.147	264.139	P[5]
V[40]	1953.126	224.126	216.117	216.621	215.613	V[4]
S[41]	1996.642	174.592	166.583	167.086	166.079	S[3]
E[42]	2061.163	131.076	123.067	123.570	122.563	E[2]
L[43]	2117.705	66.555	58.545	59.049	58.041	L[1]

sp|P43277|H13_MOUSE

[Acetyl]SETAPAAPAAPAPVEKTPVKKAKKTGAAAGKRKASGPPVSEL

- fragmentation table for charge state 3+
- specType=etd
- score=45.31
- F104929.dat
- query=q4009_p1
- precursor=706.740990
- chargePrecursor=6
- itol=0.8

AA	c	y	z+1	z+2	z	AA
S[1]	49.697	1412.467	**1407.128**	0.672	**1406.792**	S[43]
E[2]	92.711	**1369.453**	1364.114	0.672	1363.778	E[42]
T[3]	126.394	**1326.439**	**1321.099**	0.672	**1320.763**	T[41]
A[4]	150.073	1292.756	1287.417	0.672	1287.081	A[40]
P[5]	182.424	**1269.077**	1263.738	0.672	1263.402	P[39]
A[6]	206.103	1236.726	1231.387	0.672	1231.051	A[38]
A[7]	229.782	**1213.047**	1207.708	0.672	1207.372	A[37]
P[8]	262.133	**1189.368**	1184.029	0.672	1183.693	P[36]
A[9]	285.812	1157.017	**1151.678**	0.672	**1151.342**	A[35]
A[10]	309.491	**1133.338**	**1127.999**	0.672	**1127.663**	A[34]
P[11]	341.842	1109.659	1104.320	0.672	1103.984	P[33]
A[12]	365.521	**1077.308**	**1071.969**	0.672	**1071.633**	A[32]
P[13]	397.872	1053.629	1048.290	0.672	1047.954	P[31]
V[14]	**430.894**	1021.278	**1015.939**	0.672	**1015.603**	V[30]
E[15]	473.909	**988.256**	982.916	0.672	982.580	E[29]
K[16]	516.607	945.241	**939.902**	**940.238**	**939.566**	K[28]
T[17]	550.290	902.543	**897.204**	**897.539**	**896.868**	T[27]
P[18]	582.640	**868.861**	863.521	863.857	863.185	P[26]
V[19]	**615.663**	836.510	831.170	831.506	830.834	V[25]
K[20]	658.362	**803.487**	798.147	798.483	797.811	K[24]
K[21]	701.060	760.789	**755.449**	**755.785**	755.113	K[23]
K[22]	743.758	**718.090**	712.751	713.087	712.415	K[22]
A[23]	767.437	675.392	**670.052**	**670.388**	**669.716**	A[21]
K[24]	**810.136**	651.713	646.373	646.709	646.037	K[20]
K[25]	852.834	609.015	603.675	604.011	603.339	K[19]
T[26]	**886.516**	566.316	560.977	561.313	560.641	T[18]
G[27]	**905.524**	532.634	527.294	527.630	526.958	G[17]
A[28]	**929.203**	513.626	508.287	508.623	507.951	A[16]
A[29]	**952.882**	489.947	484.608	484.944	484.272	A[15]
A[30]	**976.561**	466.268	460.929	461.265	460.593	A[14]
G[31]	**995.568**	442.589	437.250	437.586	436.914	G[13]
K[32]	**1038.266**	423.582	418.243	418.579	417.907	K[12]
R[33]	**1090.300**	380.884	375.544	375.880	375.208	R[11]
K[34]	**1132.998**	328.850	323.511	323.847	323.175	K[10]
A[35]	1156.677	286.152	280.812	281.148	280.476	A[9]
S[36]	**1185.688**	262.473	257.133	257.469	256.797	S[8]
G[37]	1204.695	233.462	228.123	228.459	227.787	G[7]
P[38]	1237.046	214.455	209.115	209.451	208.779	P[6]
P[39]	**1269.397**	182.104	176.765	177.100	176.429	P[5]
V[40]	1302.420	149.753	144.414	144.750	144.078	V[4]
S[41]	1331.430	116.730	111.391	111.727	111.055	S[3]
E[42]	1374.445	87.720	82.380	82.716	82.044	E[2]
L[43]	1412.139	44.705	39.366	39.702	39.030	L[1]

sp|P43277|H13_MOUSE

[Acetyl]SETAPAAPAAPAPVEKTPVKKKAKKTGAAAGKRKASGPPVSEL

- fragmentation table for charge state 4+
- specType=etd
- score=45.31
- F104929.dat
- query=q4009_p1
- precursor=706.740990
- chargePrecursor=6
- itol=0.8

AA	c	y	z+1	z+2	z	AA
S[1]	37.525	**1059.602**	**1055.598**	0.755	**1055.346**	S[43]
E[2]	69.785	1027.342	**1023.337**	0.755	**1023.085**	E[42]
T[3]	95.047	995.081	**991.076**	0.755	**990.824**	T[41]
A[4]	112.806	**969.819**	**965.814**	0.755	**965.562**	A[40]
P[5]	137.070	**952.060**	948.055	0.755	947.803	P[39]
A[6]	154.829	927.797	923.792	0.755	923.540	A[38]
A[7]	172.588	910.037	**906.033**	0.755	**905.781**	A[37]
P[8]	196.851	892.278	888.273	0.755	888.021	P[36]
A[9]	214.611	**868.015**	864.010	0.755	863.758	A[35]
A[10]	232.370	850.256	**846.251**	0.755	**845.999**	A[34]
P[11]	256.633	**832.496**	**828.492**	0.755	**828.240**	P[33]
A[12]	274.392	808.233	**804.228**	0.755	**803.976**	A[32]
P[13]	298.656	790.474	786.469	0.755	786.217	P[31]
V[14]	323.423	**766.211**	**762.206**	0.755	**761.954**	V[30]
E[15]	355.683	741.444	737.439	0.755	737.187	E[29]
K[16]	387.707	709.183	705.178	705.430	704.926	K[28]
T[17]	**412.969**	677.159	673.154	673.406	672.903	T[27]
P[18]	437.232	651.897	647.893	648.145	647.641	P[26]
V[19]	461.999	**627.634**	623.629	623.881	623.377	V[25]
K[20]	494.023	602.867	598.862	599.114	598.610	K[24]
K[21]	526.047	**570.843**	566.839	567.090	566.587	K[23]
K[22]	558.070	538.819	534.815	535.067	534.563	K[22]
A[23]	575.830	506.796	502.791	503.043	502.539	A[21]
K[24]	607.854	489.036	485.032	485.284	484.780	K[20]
K[25]	**639.877**	457.013	453.008	453.260	452.756	K[19]
T[26]	665.139	424.989	420.984	421.236	420.732	T[18]
G[27]	679.395	399.727	395.722	395.974	395.470	G[17]
A[28]	697.154	385.472	381.467	381.719	381.215	A[16]
A[29]	714.913	367.712	363.708	363.960	363.456	A[15]
A[30]	732.672	349.953	345.948	346.200	345.696	A[14]
G[31]	746.928	332.194	328.189	328.441	327.937	G[13]
K[32]	778.951	317.938	313.934	314.186	313.682	K[12]
R[33]	817.977	285.915	281.910	282.162	281.658	R[11]
K[34]	850.001	246.889	242.885	243.137	242.633	K[10]
A[35]	**867.760**	214.866	210.861	211.113	210.609	A[9]
S[36]	**889.518**	197.106	193.102	193.354	192.850	S[8]
G[37]	903.773	175.348	171.344	171.596	171.092	G[7]
P[38]	928.036	161.093	157.088	157.340	156.836	P[6]
P[39]	**952.300**	136.830	132.825	133.077	132.573	P[5]
V[40]	**977.067**	112.567	108.562	108.814	108.310	V[4]
S[41]	**998.825**	87.800	83.795	84.047	83.543	S[3]
E[42]	1031.085	66.042	62.037	62.289	61.785	E[2]
L[43]	**1059.356**	33.781	29.776	30.028	29.524	L[1]

sp|P43277|H13_MOUSE

[Acetyl]SETAPAAPAAPAPVEKTPVKKAKKTGAAAGKRKASGPPVSEL

- fragmentation table for charge state 5+
- specType=etd
- score=45.31
- F104929.dat
- query=q4009_p1
- precursor=706.740990
- chargePrecursor=6
- itol=0.8

AA	c	y	z+1	z+2	z	AA
S[1]	30.221	847.883	844.680	0.806	844.478	S[43]
E[2]	56.030	822.075	818.871	0.806	818.669	E[42]
T[3]	76.239	**796.266**	793.063	0.806	792.861	T[41]
A[4]	90.447	776.057	772.853	0.806	772.651	A[40]
P[5]	109.857	**761.849**	758.646	0.806	758.444	P[39]
A[6]	124.065	742.439	739.235	0.806	739.033	A[38]
A[7]	138.272	728.231	725.028	0.806	724.826	A[37]
P[8]	157.683	714.024	710.820	0.806	710.619	P[36]
A[9]	171.890	694.613	**691.410**	0.806	**691.208**	A[35]
A[10]	186.097	680.406	677.202	0.806	677.001	A[34]
P[11]	205.508	666.199	**662.995**	0.806	662.793	P[33]
A[12]	219.715	646.788	643.584	0.806	643.383	A[32]
P[13]	239.126	632.581	629.377	0.806	629.175	P[31]
V[14]	258.940	613.170	609.966	0.806	609.765	V[30]
E[15]	284.748	**593.356**	590.153	0.806	589.951	E[29]
K[16]	310.367	567.548	564.344	564.546	564.142	K[28]
T[17]	330.577	541.929	538.725	538.927	538.523	T[27]
P[18]	349.987	521.719	518.516	518.717	518.314	P[26]
V[19]	369.801	502.309	499.105	499.307	498.903	V[25]
K[20]	395.420	482.495	479.291	479.493	479.090	K[24]
K[21]	421.039	456.876	453.672	453.874	453.471	K[23]
K[22]	446.658	**431.257**	428.053	428.255	427.852	K[22]
A[23]	460.865	405.638	402.434	402.636	402.233	A[21]
K[24]	486.484	391.431	388.227	388.428	388.025	K[20]
K[25]	512.103	365.812	362.608	362.809	362.406	K[19]
T[26]	532.313	340.193	336.989	337.190	336.787	T[18]
G[27]	543.717	319.983	316.779	316.981	316.578	G[17]
A[28]	557.925	308.579	305.375	305.577	305.173	A[16]
A[29]	**572.132**	294.371	291.168	291.369	290.966	A[15]
A[30]	586.339	280.164	276.960	277.162	276.759	A[14]
G[31]	597.744	265.957	262.753	262.954	262.551	G[13]
K[32]	623.363	254.552	251.348	251.550	251.147	K[12]
R[33]	654.583	228.933	225.730	225.931	225.528	R[11]
K[34]	680.202	197.713	194.509	194.711	194.308	K[10]
A[35]	694.409	172.094	168.890	169.092	168.689	A[9]
S[36]	711.816	157.887	154.683	154.884	154.481	S[8]
G[37]	**723.220**	140.480	137.276	137.478	137.075	G[7]
P[38]	742.631	129.076	125.872	126.074	125.671	P[6]
P[39]	**762.041**	109.665	106.462	106.663	106.260	P[5]
V[40]	781.855	90.255	87.051	87.253	86.849	V[4]
S[41]	799.261	70.441	67.237	67.439	67.036	S[3]
E[42]	825.070	53.035	49.831	50.033	49.629	E[2]
L[43]	847.687	27.226	24.022	24.224	23.821	L[1]

sp|P43277|H13_MOUSE

[Acetyl]SETAPAAPAAPAPVEKTPVKKKAKKTGAAAGKRKASGPPVSEL

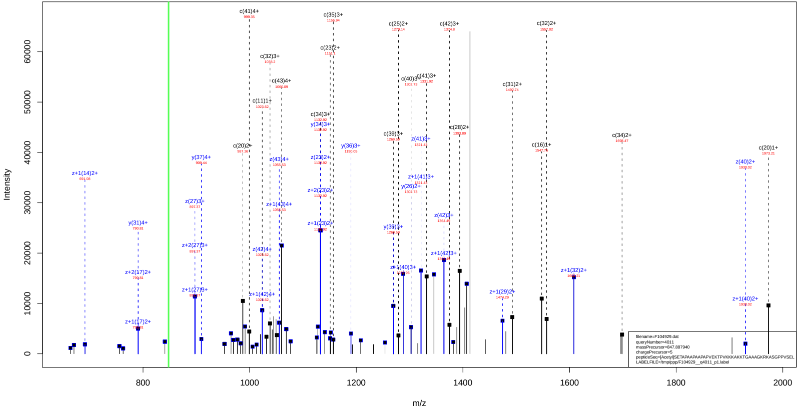

sp|P43277|H13_MOUSE

[Acetyl]SETAPAAPAAPAPVEKTPVKKAKKTGAAAGKRKASGPPVSEL

- fragmentation table for charge state 1+
- specType=etd
- score=35.60
- F104929.dat
- query=q4011_p1
- precursor=847.887940
- chargePrecursor=5
- itol=0.8

AA	c	y	z+1	z+2	z	AA
S[1]	147.076	4235.387	4219.369	0.000	4218.361	S[43]
E[2]	276.119	4106.345	4090.326	0.000	4089.318	E[42]
T[3]	377.167	3977.302	3961.284	0.000	3960.276	T[41]
A[4]	448.204	3876.255	3860.236	0.000	3859.228	A[40]
P[5]	545.257	3805.217	3789.199	0.000	3788.191	P[39]
A[6]	616.294	3708.165	3692.146	0.000	3691.138	A[38]
A[7]	687.331	3637.128	3621.109	0.000	3620.101	A[37]
P[8]	784.384	3566.090	3550.072	0.000	3549.064	P[36]
A[9]	855.421	3469.038	3453.019	0.000	3452.011	A[35]
A[10]	926.458	3398.001	3381.982	0.000	3380.974	A[34]
P[11]	**1023.511**	3326.963	3310.945	0.000	3309.937	P[33]
A[12]	1094.548	3229.911	3213.892	0.000	3212.884	A[32]
P[13]	1191.600	3158.874	3142.855	0.000	3141.847	P[31]
V[14]	1290.669	3061.821	3045.802	0.000	3044.794	V[30]
E[15]	1419.711	2962.752	2946.734	0.000	2945.726	E[29]
K[16]	**1547.806**	2833.710	2817.691	2818.699	2816.683	K[28]
T[17]	1648.854	2705.615	2689.596	2690.604	2688.588	T[27]
P[18]	1745.907	2604.567	2588.548	2589.556	2587.541	P[26]
V[19]	1844.975	2507.514	2491.496	2492.504	2490.488	V[25]
K[20]	**1973.070**	2408.446	2392.427	2393.435	2391.419	K[24]
K[21]	2101.165	2280.351	2264.332	2265.340	2263.324	K[23]
K[22]	2229.260	2152.256	2136.237	2137.245	2135.230	K[22]
A[23]	2300.297	2024.161	2008.142	2009.150	2007.135	A[21]
K[24]	2428.392	1953.124	1937.105	1938.113	1936.097	K[20]
K[25]	2556.487	1825.029	1809.010	1810.018	1808.002	K[19]
T[26]	2657.535	1696.934	1680.915	1681.923	1679.908	T[18]
G[27]	2714.556	1595.886	1579.868	1580.875	1578.860	G[17]
A[28]	2785.593	1538.865	1522.846	1523.854	1521.838	A[16]
A[29]	2856.631	1467.828	1451.809	1452.817	1450.801	A[15]
A[30]	2927.668	1396.791	1380.772	**1381.780**	1379.764	A[14]
G[31]	2984.689	1325.754	1309.735	1310.743	1308.727	G[13]
K[32]	3112.784	1268.732	1252.713	**1253.721**	1251.706	K[12]
R[33]	3268.885	**1140.637**	1124.618	**1125.626**	1123.611	R[11]
K[34]	3396.980	984.536	968.517	**969.525**	967.509	K[10]
A[35]	3468.017	856.441	**840.422**	**841.430**	839.415	A[9]
S[36]	3555.049	785.404	769.385	770.393	768.377	S[8]
G[37]	3612.071	698.372	682.353	683.361	681.345	G[7]
P[38]	3709.124	641.350	625.332	626.340	624.324	P[6]
P[39]	3806.176	544.298	528.279	529.287	527.271	P[5]
V[40]	3905.245	447.245	431.226	432.234	430.218	V[4]
S[41]	3992.277	348.177	332.158	333.166	331.150	S[3]
E[42]	4121.319	261.144	245.126	246.134	244.118	E[2]
L[43]	4234.403	132.102	116.083	117.091	115.075	L[1]

sp|P43277|H13_MOUSE

[Acetyl]SETAPAAPAAPAPVEKTPVKKAKKTGAAAGKRKASGPPVSEL

- fragmentation table for charge state 2+
- specType=etd
- score=35.60
- F104929.dat
- query=q4011_p1
- precursor=847.887940
- chargePrecursor=5
- itol=0.8

AA	c	y	z+1	z+2	z	AA
S[1]	74.042	2118.197	2110.188	0.504	2109.684	S[43]
E[2]	138.563	2053.676	2045.667	0.504	2045.163	E[42]
T[3]	189.087	1989.155	1981.145	0.504	1980.641	T[41]
A[4]	224.606	1938.631	**1930.622**	0.504	**1930.118**	A[40]
P[5]	273.132	1903.112	1895.103	0.504	1894.599	P[39]
A[6]	308.650	1854.586	1846.577	0.504	1846.073	A[38]
A[7]	344.169	1819.067	1811.058	0.504	1810.554	A[37]
P[8]	392.695	1783.549	1775.540	0.504	1775.036	P[36]
A[9]	428.214	1735.022	1727.013	0.504	1726.509	A[35]
A[10]	463.733	1699.504	1691.495	0.504	1690.991	A[34]
P[11]	512.259	1663.985	1655.976	0.504	1655.472	P[33]
A[12]	547.777	1615.459	**1607.450**	0.504	1606.946	A[32]
P[13]	596.304	1579.940	1571.931	0.504	1571.427	P[31]
V[14]	645.838	1531.414	1523.405	0.504	1522.901	V[30]
E[15]	710.359	1481.880	**1473.870**	0.504	1473.367	E[29]
K[16]	774.407	1417.359	1409.349	1409.853	1408.845	K[28]
T[17]	824.931	1353.311	**1345.302**	**1345.806**	1344.798	T[27]
P[18]	873.457	**1302.787**	1294.778	1295.282	1294.274	P[26]
V[19]	922.991	**1254.261**	1246.251	1246.755	1245.748	V[25]
K[20]	**987.039**	1204.727	1196.717	1197.221	1196.213	K[24]
K[21]	**1051.086**	**1140.679**	**1132.670**	**1133.174**	**1132.166**	K[23]
K[22]	1115.134	**1076.632**	**1068.622**	**1069.126**	**1068.118**	K[22]
A[23]	**1150.652**	**1012.584**	1004.575	**1005.079**	1004.071	A[21]
K[24]	1214.700	**977.066**	**969.056**	**969.560**	968.552	K[20]
K[25]	**1278.747**	913.018	905.009	905.513	904.505	K[19]
T[26]	1329.271	848.971	**840.961**	**841.465**	**840.457**	T[18]
G[27]	1357.782	798.447	**790.437**	**790.941**	789.934	G[17]
A[28]	**1393.300**	769.936	**761.927**	**762.431**	761.423	A[16]
A[29]	1428.819	734.418	726.408	726.912	725.904	A[15]
A[30]	1464.337	698.899	**690.890**	**691.394**	**690.386**	A[14]
G[31]	**1492.848**	**663.380**	655.371	655.875	654.867	G[13]
K[32]	**1556.896**	634.870	626.860	627.364	626.356	K[12]
R[33]	1634.946	570.822	562.813	563.317	562.309	R[11]
K[34]	**1698.994**	492.772	484.762	485.266	484.258	K[10]
A[35]	1734.512	428.724	420.715	421.219	420.211	A[9]
S[36]	1778.028	393.206	385.196	385.700	384.692	S[8]
G[37]	1806.539	349.690	341.680	342.184	341.176	G[7]
P[38]	1855.065	321.179	313.170	313.673	312.666	P[6]
P[39]	1903.592	272.652	264.643	265.147	264.139	P[5]
V[40]	1953.126	224.126	216.117	216.621	215.613	V[4]
S[41]	1996.642	174.592	166.583	167.086	166.079	S[3]
E[42]	2061.163	131.076	123.067	123.570	122.563	E[2]
L[43]	2117.705	66.555	58.545	59.049	58.041	L[1]

sp|P43277|H13_MOUSE

[Acetyl]SETAPAAPAAPAPVEKTPVKKKAKKTGAAAGKRKASGPPVSEL

- fragmentation table for charge state 3+
- specType=etd
- score=35.60
- F104929.dat
- query=q4011_p1
- precursor=847.887940
- chargePrecursor=5
- itol=0.8

AA	c	y	z+1	z+2	z	AA
S[1]	49.697	1412.467	**1407.128**	0.672	**1406.792**	S[43]
E[2]	92.711	1369.453	**1364.114**	0.672	**1363.778**	E[42]
T[3]	126.394	1326.439	**1321.099**	0.672	**1320.763**	T[41]
A[4]	150.073	1292.756	**1287.417**	0.672	1287.081	A[40]
P[5]	182.424	**1269.077**	1263.738	0.672	1263.402	P[39]
A[6]	206.103	1236.726	1231.387	0.672	1231.051	A[38]
A[7]	229.782	1213.047	**1207.708**	0.672	1207.372	A[37]
P[8]	262.133	**1189.368**	1184.029	0.672	1183.693	P[36]
A[9]	285.812	**1157.017**	**1151.678**	0.672	**1151.342**	A[35]
A[10]	309.491	**1133.338**	**1127.999**	0.672	**1127.663**	A[34]
P[11]	341.842	1109.659	1104.320	0.672	1103.984	P[33]
A[12]	365.521	**1077.308**	1071.969	0.672	1071.633	A[32]
P[13]	397.872	1053.629	1048.290	0.672	1047.954	P[31]
V[14]	430.894	1021.278	1015.939	0.672	1015.603	V[30]
E[15]	473.909	988.256	**982.916**	0.672	**982.580**	E[29]
K[16]	516.607	945.241	939.902	940.238	939.566	K[28]
T[17]	550.290	902.543	**897.204**	**897.539**	**896.868**	T[27]
P[18]	582.640	868.861	863.521	863.857	863.185	P[26]
V[19]	615.663	836.510	831.170	831.506	830.834	V[25]
K[20]	658.362	803.487	798.147	798.483	797.811	K[24]
K[21]	701.060	760.789	**755.449**	**755.785**	**755.113**	K[23]
K[22]	743.758	718.090	712.751	713.087	712.415	K[22]
A[23]	767.437	675.392	**670.052**	**670.388**	**669.716**	A[21]
K[24]	810.136	651.713	646.373	646.709	646.037	K[20]
K[25]	852.834	609.015	603.675	604.011	603.339	K[19]
T[26]	886.516	566.316	560.977	561.313	560.641	T[18]
G[27]	905.524	532.634	527.294	527.630	526.958	G[17]
A[28]	929.203	513.626	508.287	508.623	507.951	A[16]
A[29]	**952.882**	489.947	484.608	484.944	484.272	A[15]
A[30]	**976.561**	466.268	460.929	461.265	460.593	A[14]
G[31]	995.568	442.589	437.250	437.586	436.914	G[13]
K[32]	**1038.266**	423.582	418.243	418.579	417.907	K[12]
R[33]	1090.300	380.884	375.544	375.880	375.208	R[11]
K[34]	**1132.998**	328.850	323.511	323.847	323.175	K[10]
A[35]	**1156.677**	286.152	280.812	281.148	280.476	A[9]
S[36]	1185.688	262.473	257.133	257.469	256.797	S[8]
G[37]	1204.695	233.462	228.123	228.459	227.787	G[7]
P[38]	1237.046	214.455	209.115	209.451	208.779	P[6]
P[39]	**1269.397**	182.104	176.765	177.100	176.429	P[5]
V[40]	**1302.420**	149.753	144.414	144.750	144.078	V[4]
S[41]	**1331.430**	116.730	111.391	111.727	111.055	S[3]
E[42]	**1374.445**	87.720	82.380	82.716	82.044	E[2]
L[43]	1412.139	44.705	39.366	39.702	39.030	L[1]

sp|P43277|H13_MOUSE

[Acetyl]SETAPAAPAAPAPVEKTPVKKKAKKTGAAAGKRKASGPPVSEL

- fragmentation table for charge state 4+
- specType=etd
- score=35.60
- F104929.dat
- query=q4011_p1
- precursor=847.887940
- chargePrecursor=5
- itol=0.8

AA	c	y	z+1	z+2	z	AA
S[1]	37.525	**1059.602**	**1055.598**	0.755	**1055.346**	S[43]
E[2]	69.785	1027.342	**1023.337**	0.755	**1023.085**	E[42]
T[3]	95.047	995.081	**991.076**	0.755	**990.824**	T[41]
A[4]	112.806	**969.819**	**965.814**	0.755	**965.562**	A[40]
P[5]	137.070	**952.060**	948.055	0.755	947.803	P[39]
A[6]	154.829	927.797	923.792	0.755	923.540	A[38]
A[7]	172.588	**910.037**	906.033	0.755	905.781	A[37]
P[8]	196.851	892.278	888.273	0.755	888.021	P[36]
A[9]	214.611	868.015	864.010	0.755	863.758	A[35]
A[10]	232.370	850.256	846.251	0.755	845.999	A[34]
P[11]	256.633	832.496	828.492	0.755	828.240	P[33]
A[12]	274.392	808.233	804.228	0.755	803.976	A[32]
P[13]	298.656	**790.474**	786.469	0.755	786.217	P[31]
V[14]	323.423	766.211	**762.206**	0.755	**761.954**	V[30]
E[15]	355.683	741.444	737.439	0.755	737.187	E[29]
K[16]	387.707	709.183	705.178	705.430	704.926	K[28]
T[17]	412.969	677.159	673.154	673.406	672.903	T[27]
P[18]	437.232	651.897	647.893	648.145	647.641	P[26]
V[19]	461.999	627.634	623.629	623.881	623.377	V[25]
K[20]	494.023	602.867	598.862	599.114	598.610	K[24]
K[21]	526.047	570.843	566.839	567.090	566.587	K[23]
K[22]	558.070	538.819	534.815	535.067	534.563	K[22]
A[23]	575.830	506.796	502.791	503.043	502.539	A[21]
K[24]	607.854	489.036	485.032	485.284	484.780	K[20]
K[25]	639.877	457.013	453.008	453.260	452.756	K[19]
T[26]	665.139	424.989	420.984	421.236	420.732	T[18]
G[27]	679.395	399.727	395.722	395.974	395.470	G[17]
A[28]	697.154	385.472	381.467	381.719	381.215	A[16]
A[29]	714.913	367.712	363.708	363.960	363.456	A[15]
A[30]	732.672	349.953	345.948	346.200	345.696	A[14]
G[31]	746.928	332.194	328.189	328.441	327.937	G[13]
K[32]	778.951	317.938	313.934	314.186	313.682	K[12]
R[33]	817.977	285.915	281.910	282.162	281.658	R[11]
K[34]	850.001	246.889	242.885	243.137	242.633	K[10]
A[35]	867.760	214.866	210.861	211.113	210.609	A[9]
S[36]	889.518	197.106	193.102	193.354	192.850	S[8]
G[37]	903.773	175.348	171.344	171.596	171.092	G[7]
P[38]	928.036	161.093	157.088	157.340	156.836	P[6]
P[39]	**952.300**	136.830	132.825	133.077	132.573	P[5]
V[40]	**977.067**	112.567	108.562	108.814	108.310	V[4]
S[41]	**998.825**	87.800	83.795	84.047	83.543	S[3]
E[42]	**1031.085**	66.042	62.037	62.289	61.785	E[2]
L[43]	**1059.356**	33.781	29.776	30.028	29.524	L[1]

sp|P43277|H13_MOUSE

[Acetyl]SETAPAAPAAPAPVEKTPVKKKAKKTGAAAGKRKASGPPVSEL

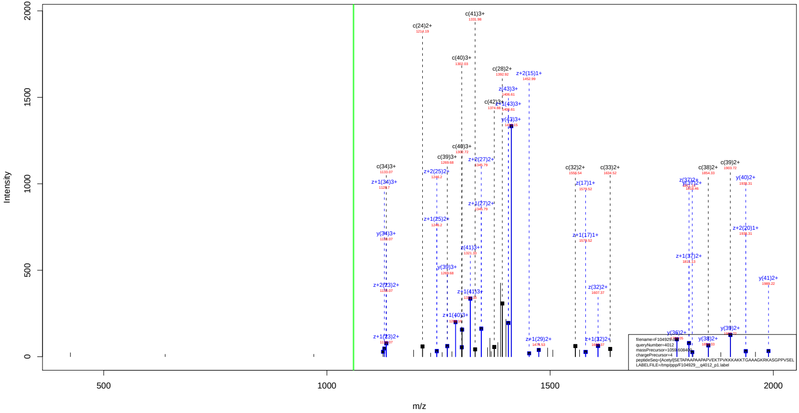

sp|P43277|H13_MOUSE

[Acetyl]SETAPAAPAAPAPVEKTPVKKKAKKTGAAAGKRKASGPPVSEL

- fragmentation table for charge state 1+
- specType=etd
- score=36.17
- F104929.dat
- query=q4012_p1
- precursor=1059.608400
- chargePrecursor=4
- itol=0.8

AA	c	y	z+1	z+2	z	AA
S[1]	147.076	4235.387	4219.369	0.000	4218.361	S[43]
E[2]	276.119	4106.345	4090.326	0.000	4089.318	E[42]
T[3]	377.167	3977.302	3961.284	0.000	3960.276	T[41]
A[4]	448.204	3876.255	3860.236	0.000	3859.228	A[40]
P[5]	545.257	3805.217	3789.199	0.000	3788.191	P[39]
A[6]	616.294	3708.165	3692.146	0.000	3691.138	A[38]
A[7]	687.331	3637.128	3621.109	0.000	3620.101	A[37]
P[8]	784.384	3566.090	3550.072	0.000	3549.064	P[36]
A[9]	855.421	3469.038	3453.019	0.000	3452.011	A[35]
A[10]	926.458	3398.001	3381.982	0.000	3380.974	A[34]
P[11]	1023.511	3326.963	3310.945	0.000	3309.937	P[33]
A[12]	1094.548	3229.911	3213.892	0.000	3212.884	A[32]
P[13]	1191.600	3158.874	3142.855	0.000	3141.847	P[31]
V[14]	1290.669	3061.821	3045.802	0.000	3044.794	V[30]
E[15]	1419.711	2962.752	2946.734	0.000	2945.726	E[29]
K[16]	1547.806	2833.710	2817.691	2818.699	2816.683	K[28]
T[17]	1648.854	2705.615	2689.596	2690.604	2688.588	T[27]
P[18]	1745.907	2604.567	2588.548	2589.556	2587.541	P[26]
V[19]	1844.975	2507.514	2491.496	2492.504	2490.488	V[25]
K[20]	1973.070	2408.446	2392.427	2393.435	2391.419	K[24]
K[21]	2101.165	2280.351	2264.332	2265.340	2263.324	K[23]
K[22]	2229.260	2152.256	2136.237	2137.245	2135.230	K[22]
A[23]	2300.297	2024.161	2008.142	2009.150	2007.135	A[21]
K[24]	2428.392	1953.124	1937.105	**1938.113**	1936.097	K[20]
K[25]	2556.487	1825.029	1809.010	1810.018	1808.002	K[19]
T[26]	2657.535	1696.934	1680.915	1681.923	1679.908	T[18]
G[27]	2714.556	1595.886	**1579.868**	1580.875	**1578.860**	G[17]
A[28]	2785.593	1538.865	1522.846	1523.854	1521.838	A[16]
A[29]	2856.631	1467.828	1451.809	**1452.817**	1450.801	A[15]
A[30]	2927.668	1396.791	1380.772	1381.780	1379.764	A[14]
G[31]	2984.689	1325.754	1309.735	1310.743	1308.727	G[13]
K[32]	3112.784	1268.732	1252.713	1253.721	1251.706	K[12]
R[33]	3268.885	1140.637	1124.618	**1125.626**	1123.611	R[11]
K[34]	3396.980	984.536	968.517	969.525	967.509	K[10]
A[35]	3468.017	856.441	840.422	841.430	839.415	A[9]
S[36]	3555.049	785.404	769.385	770.393	768.377	S[8]
G[37]	3612.071	698.372	682.353	683.361	681.345	G[7]
P[38]	3709.124	641.350	625.332	626.340	624.324	P[6]
P[39]	3806.176	544.298	528.279	529.287	527.271	P[5]
V[40]	3905.245	447.245	431.226	432.234	430.218	V[4]
S[41]	3992.277	348.177	332.158	333.166	331.150	S[3]
E[42]	4121.319	261.144	245.126	246.134	244.118	E[2]
L[43]	4234.403	132.102	116.083	117.091	115.075	L[1]

sp|P43277|H13_MOUSE

[Acetyl]SETAPAAPAAPAPVEKTPVKKKAKKTGAAAGKRKASGPPVSEL

- fragmentation table for charge state 2+
- specType=etd
- score=36.17
- F104929.dat
- query=q4012_p1
- precursor=1059.608400
- chargePrecursor=4
- itol=0.8

AA	c	y	z+1	z+2	z	AA
S[1]	74.042	2118.197	2110.188	0.504	2109.684	S[43]
E[2]	138.563	2053.676	2045.667	0.504	2045.163	E[42]
T[3]	189.087	**1989.155**	1981.145	0.504	1980.641	T[41]
A[4]	224.606	**1938.631**	1930.622	0.504	1930.118	A[40]
P[5]	273.132	**1903.112**	1895.103	0.504	1894.599	P[39]
A[6]	308.650	**1854.586**	1846.577	0.504	1846.073	A[38]
A[7]	344.169	**1819.067**	**1811.058**	0.504	**1810.554**	A[37]
P[8]	392.695	**1783.549**	1775.540	0.504	1775.036	P[36]
A[9]	428.214	1735.022	1727.013	0.504	1726.509	A[35]
A[10]	463.733	1699.504	1691.495	0.504	1690.991	A[34]
P[11]	512.259	1663.985	1655.976	0.504	1655.472	P[33]
A[12]	547.777	1615.459	**1607.450**	0.504	**1606.946**	A[32]
P[13]	596.304	**1579.940**	1571.931	0.504	1571.427	P[31]
V[14]	645.838	1531.414	1523.405	0.504	1522.901	V[30]
E[15]	710.359	1481.880	**1473.870**	0.504	1473.367	E[29]
K[16]	774.407	1417.359	1409.349	1409.853	1408.845	K[28]
T[17]	824.931	1353.311	**1345.302**	**1345.806**	1344.798	T[27]
P[18]	873.457	**1302.787**	1294.778	1295.282	1294.274	P[26]
V[19]	922.991	1254.261	**1246.251**	**1246.755**	**1245.748**	V[25]
K[20]	987.039	1204.727	1196.717	1197.221	1196.213	K[24]
K[21]	1051.086	1140.679	**1132.670**	**1133.174**	1132.166	K[23]
K[22]	1115.134	1076.632	1068.622	1069.126	1068.118	K[22]
A[23]	1150.652	1012.584	1004.575	1005.079	1004.071	A[21]
K[24]	**1214.700**	977.066	969.056	969.560	968.552	K[20]
K[25]	1278.747	913.018	905.009	905.513	904.505	K[19]
T[26]	1329.271	848.971	840.961	841.465	840.457	T[18]
G[27]	1357.782	798.447	790.437	790.941	789.934	G[17]
A[28]	**1393.300**	769.936	761.927	762.431	761.423	A[16]
A[29]	1428.819	734.418	726.408	726.912	725.904	A[15]
A[30]	1464.337	698.899	690.890	691.394	690.386	A[14]
G[31]	1492.848	663.380	655.371	655.875	654.867	G[13]
K[32]	**1556.896**	634.870	626.860	627.364	626.356	K[12]
R[33]	**1634.946**	570.822	562.813	563.317	562.309	R[11]
K[34]	1698.994	492.772	484.762	485.266	484.258	K[10]
A[35]	1734.512	428.724	420.715	421.219	420.211	A[9]
S[36]	1778.028	393.206	385.196	385.700	384.692	S[8]
G[37]	1806.539	349.690	341.680	342.184	341.176	G[7]
P[38]	**1855.065**	321.179	313.170	313.673	312.666	P[6]
P[39]	**1903.592**	272.652	264.643	265.147	264.139	P[5]
V[40]	1953.126	224.126	216.117	216.621	215.613	V[4]
S[41]	1996.642	174.592	166.583	167.086	166.079	S[3]
E[42]	2061.163	131.076	123.067	123.570	122.563	E[2]
L[43]	2117.705	66.555	58.545	59.049	58.041	L[1]

sp|P43277|H13_MOUSE

[Acetyl]SETAPAAPAAPAPVEKTPVKKAKKTGAAAGKRKASGPPVSEL

- fragmentation table for charge state 3+
- specType=etd
- score=36.17
- F104929.dat
- query=q4012_p1
- precursor=1059.608400
- chargePrecursor=4
- itol=0.8

AA	c	y	z+1	z+2	z	AA
S[1]	49.697	**1412.467**	**1407.128**	0.672	**1406.792**	S[43]
E[2]	92.711	1369.453	1364.114	0.672	1363.778	E[42]
T[3]	126.394	1326.439	**1321.099**	0.672	**1320.763**	T[41]
A[4]	150.073	1292.756	**1287.417**	0.672	1287.081	A[40]
P[5]	182.424	**1269.077**	1263.738	0.672	1263.402	P[39]
A[6]	206.103	1236.726	1231.387	0.672	1231.051	A[38]
A[7]	229.782	1213.047	1207.708	0.672	1207.372	A[37]
P[8]	262.133	1189.368	1184.029	0.672	1183.693	P[36]
A[9]	285.812	1157.017	1151.678	0.672	1151.342	A[35]
A[10]	309.491	**1133.338**	**1127.999**	0.672	1127.663	A[34]
P[11]	341.842	1109.659	1104.320	0.672	1103.984	P[33]
A[12]	365.521	1077.308	1071.969	0.672	1071.633	A[32]
P[13]	397.872	1053.629	1048.290	0.672	1047.954	P[31]
V[14]	430.894	1021.278	1015.939	0.672	1015.603	V[30]
E[15]	473.909	988.256	982.916	0.672	982.580	E[29]
K[16]	516.607	945.241	939.902	940.238	939.566	K[28]
T[17]	550.290	902.543	897.204	897.539	896.868	T[27]
P[18]	582.640	868.861	863.521	863.857	863.185	P[26]
V[19]	615.663	836.510	831.170	831.506	830.834	V[25]
K[20]	658.362	803.487	798.147	798.483	797.811	K[24]
K[21]	701.060	760.789	755.449	755.785	755.113	K[23]
K[22]	743.758	718.090	712.751	713.087	712.415	K[22]
A[23]	767.437	675.392	670.052	670.388	669.716	A[21]
K[24]	810.136	651.713	646.373	646.709	646.037	K[20]
K[25]	852.834	609.015	603.675	604.011	603.339	K[19]
T[26]	886.516	566.316	560.977	561.313	560.641	T[18]
G[27]	905.524	532.634	527.294	527.630	526.958	G[17]
A[28]	929.203	513.626	508.287	508.623	507.951	A[16]
A[29]	952.882	489.947	484.608	484.944	484.272	A[15]
A[30]	976.561	466.268	460.929	461.265	460.593	A[14]
G[31]	995.568	442.589	437.250	437.586	436.914	G[13]
K[32]	1038.266	423.582	418.243	418.579	417.907	K[12]
R[33]	1090.300	380.884	375.544	375.880	375.208	R[11]
K[34]	**1132.998**	328.850	323.511	323.847	323.175	K[10]
A[35]	1156.677	286.152	280.812	281.148	280.476	A[9]
S[36]	1185.688	262.473	257.133	257.469	256.797	S[8]
G[37]	1204.695	233.462	228.123	228.459	227.787	G[7]
P[38]	1237.046	214.455	209.115	209.451	208.779	P[6]
P[39]	**1269.397**	182.104	176.765	177.100	176.429	P[5]
V[40]	**1302.420**	149.753	144.414	144.750	144.078	V[4]
S[41]	**1331.430**	116.730	111.391	111.727	111.055	S[3]
E[42]	**1374.445**	87.720	82.380	82.716	82.044	E[2]
L[43]	1412.139	44.705	39.366	39.702	39.030	L[1]

sp|P43276|H15_MOUSE

[Acetyl]SETAPAETAAPAPVEKSPAKKKTTKKAGAAKRKATGPPVSEL

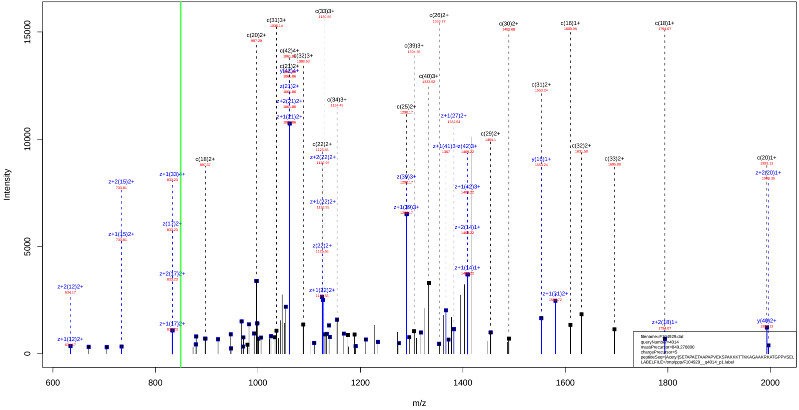

sp|P43276|H15_MOUSE

[Acetyl]SETAPAETAAPAPVEKSPAKKKTTKKAGAAKRKATGPPVSEL

- fragmentation table for charge state 1+
- specType=etd
- score=64.76
- F104929.dat
- query=q4014_p1
- precursor=849.278800
- chargePrecursor=5
- itol=0.8

AA	c	y	z+1	z+2	z	AA
S[1]	147.076	4242.346	4226.327	0.000	4225.319	S[42]
E[2]	276.119	4113.303	4097.284	0.000	4096.276	E[41]
T[3]	377.167	3984.260	3968.242	0.000	3967.234	T[40]
A[4]	448.204	3883.213	3867.194	0.000	3866.186	A[39]
P[5]	545.257	3812.176	3796.157	0.000	3795.149	P[38]
A[6]	616.294	3715.123	3699.104	0.000	3698.096	A[37]
E[7]	745.336	3644.086	3628.067	0.000	3627.059	E[36]
T[8]	846.384	3515.043	3499.024	0.000	3498.017	T[35]
A[9]	917.421	3413.995	3397.977	0.000	3396.969	A[34]
A[10]	988.458	3342.958	3326.940	0.000	3325.932	A[33]
P[11]	1085.511	3271.921	3255.903	0.000	3254.895	P[32]
A[12]	1156.548	3174.868	3158.850	0.000	3157.842	A[31]
P[13]	1253.601	3103.831	3087.813	0.000	3086.805	P[30]
V[14]	1352.669	3006.779	2990.760	0.000	2989.752	V[29]
E[15]	1481.712	2907.710	2891.691	0.000	2890.684	E[28]
K[16]	**1609.807**	2778.668	2762.649	2763.657	2761.641	K[27]
S[17]	1696.839	2650.573	2634.554	2635.562	2633.546	S[26]
P[18]	**1793.892**	2563.541	2547.522	2548.530	2546.514	P[25]
A[19]	1864.929	2466.488	2450.469	2451.477	2449.461	A[24]
K[20]	**1993.024**	2395.451	2379.432	2380.440	2378.424	K[23]
K[21]	2121.119	2267.356	2251.337	2252.345	2250.329	K[22]
K[22]	2249.214	2139.261	2123.242	2124.250	2122.234	K[21]
T[23]	2350.261	2011.166	1995.147	**1996.155**	1994.139	T[20]
T[24]	2451.309	1910.118	1894.099	1895.107	1893.092	T[19]
K[25]	2579.404	1809.070	1793.052	**1794.060**	1792.044	K[18]
K[26]	2707.499	1680.976	1664.957	1665.965	1663.949	K[17]
A[27]	2778.536	**1552.881**	1536.862	1537.870	1535.854	A[16]
G[28]	2835.557	1481.843	1465.825	1466.833	1464.817	G[15]
A[29]	2906.595	1424.822	**1408.803**	**1409.811**	1407.795	A[14]
A[30]	2977.632	**1353.785**	1337.766	1338.774	1336.758	A[13]
K[31]	3105.727	1282.748	1266.729	1267.737	1265.721	K[12]
R[32]	3261.828	**1154.653**	**1138.634**	**1139.642**	1137.626	R[11]
K[33]	3389.923	**998.552**	**982.533**	983.541	981.525	K[10]
A[34]	3460.960	870.457	854.438	855.446	853.430	A[9]
T[35]	3562.008	799.420	783.401	784.409	782.393	T[8]
G[36]	3619.029	698.372	682.353	683.361	681.345	G[7]
P[37]	3716.082	641.350	625.332	626.340	624.324	P[6]
P[38]	3813.135	544.298	528.279	529.287	527.271	P[5]
V[39]	3912.203	447.245	431.226	432.234	430.218	V[4]
S[40]	3999.235	348.177	332.158	333.166	331.150	S[3]
E[41]	4128.278	261.144	245.126	246.134	244.118	E[2]
L[42]	4241.362	132.102	116.083	117.091	115.075	L[1]

sp|P43276|H15_MOUSE

[Acetyl]SETAPAETAAPAPVEKSPAKKKTTKKAGAAKRKATGPPVSEL

- fragmentation table for charge state 2+
- specType=etd
- score=64.76
- F104929.dat
- query=q4014_p1
- precursor=849.278800
- chargePrecursor=5
- itol=0.8

AA	c	y	z+1	z+2	z	AA
S[1]	74.042	2121.676	2113.667	0.504	2113.163	S[42]
E[2]	138.563	2057.155	2049.146	0.504	2048.642	E[41]
T[3]	189.087	**1992.634**	1984.624	0.504	1984.121	T[40]
A[4]	224.606	1942.110	1934.101	0.504	1933.597	A[39]
P[5]	273.132	1906.591	1898.582	0.504	1898.078	P[38]
A[6]	308.650	1858.065	1850.056	0.504	1849.552	A[37]
E[7]	373.172	1822.547	1814.537	0.504	1814.033	E[36]
T[8]	423.696	1758.025	1750.016	0.504	1749.512	T[35]
A[9]	459.214	1707.501	1699.492	0.504	1698.988	A[34]
A[10]	494.733	1671.983	1663.973	0.504	1663.470	A[33]
P[11]	543.259	1636.464	1628.455	0.504	1627.951	P[32]
A[12]	578.778	1587.938	**1579.929**	0.504	1579.425	A[31]
P[13]	627.304	1552.419	1544.410	0.504	1543.906	P[30]
V[14]	676.838	1503.893	1495.884	0.504	1495.380	V[29]
E[15]	741.360	**1454.359**	1446.349	0.504	1445.845	E[28]
K[16]	805.407	1389.837	**1381.828**	**1382.332**	1381.324	K[27]
S[17]	848.923	1325.790	**1317.781**	**1318.285**	1317.277	S[26]
P[18]	**897.449**	1282.274	1274.265	**1274.768**	1273.761	P[25]
A[19]	932.968	**1233.748**	1225.738	1226.242	1225.234	A[24]
K[20]	**997.015**	1198.229	**1190.220**	**1190.724**	1189.716	K[23]
K[21]	**1061.063**	**1134.182**	**1126.172**	**1126.676**	**1125.668**	K[22]
K[22]	**1125.110**	1070.134	**1062.125**	**1062.629**	**1061.621**	K[21]
T[23]	**1175.634**	**1006.087**	**998.077**	**998.581**	**997.573**	T[20]
T[24]	1226.158	955.563	**947.553**	**948.057**	**947.049**	T[19]
K[25]	**1290.206**	905.039	**897.030**	**897.533**	896.526	K[18]
K[26]	**1354.253**	840.991	**832.982**	**833.486**	**832.478**	K[17]
A[27]	1389.772	776.944	768.935	769.438	768.431	A[16]
G[28]	1418.282	741.425	**733.416**	**733.920**	732.912	G[15]
A[29]	**1453.801**	712.915	**704.905**	**705.409**	**704.401**	A[14]
A[30]	**1489.319**	677.396	**669.387**	**669.891**	668.883	A[13]
K[31]	**1553.367**	641.878	**633.868**	**634.372**	633.364	K[12]
R[32]	**1631.418**	577.830	569.821	570.325	569.317	R[11]
K[33]	**1695.465**	499.779	491.770	492.274	491.266	K[10]
A[34]	1730.984	435.732	427.723	428.227	427.219	A[9]
T[35]	1781.507	400.213	392.204	392.708	391.700	T[8]
G[36]	1810.018	349.690	341.680	342.184	341.176	G[7]
P[37]	1858.545	321.179	313.170	313.673	312.666	P[6]
P[38]	1907.071	272.652	264.643	265.147	264.139	P[5]
V[39]	1956.605	224.126	216.117	216.621	215.613	V[4]
S[40]	2000.121	174.592	166.583	167.086	166.079	S[3]
E[41]	2064.642	131.076	123.067	123.570	122.563	E[2]
L[42]	2121.184	66.555	58.545	59.049	58.041	L[1]

sp|P43276|H15_MOUSE

[Acetyl]SETAPAETAAPAPVEKSPAKKKTTKKAGAAKRKATGPPVSEL

- fragmentation table for charge state 3+
- specType=etd
- score=64.76
- F104929.dat
- query=q4014_p1
- precursor=849.278800
- chargePrecursor=5
- itol=0.8

AA	c	y	z+1	z+2	z	AA
S[1]	49.697	1414.787	**1409.447**	0.672	**1409.111**	S[42]
E[2]	92.711	**1371.773**	**1366.433**	0.672	1366.097	E[41]
T[3]	126.394	1328.758	1323.419	0.672	1323.083	T[40]
A[4]	150.073	**1295.076**	**1289.736**	0.672	**1289.400**	A[39]
P[5]	182.424	1271.397	1266.057	0.672	1265.721	P[38]
A[6]	206.103	1239.046	**1233.706**	0.672	**1233.370**	A[37]
E[7]	249.117	1215.367	**1210.027**	0.672	**1209.691**	E[36]
T[8]	282.800	1172.353	**1167.013**	0.672	1166.677	T[35]
A[9]	306.479	**1138.670**	**1133.330**	0.672	1132.994	A[34]
A[10]	330.158	1114.991	**1109.651**	0.672	**1109.315**	A[33]
P[11]	362.508	1091.312	1085.972	0.672	1085.636	P[32]
A[12]	386.188	1058.961	**1053.621**	0.672	1053.285	A[31]
P[13]	418.538	1035.282	1029.942	0.672	1029.606	P[30]
V[14]	451.561	1002.931	**997.591**	0.672	**997.256**	V[29]
E[15]	494.575	969.908	964.569	0.672	964.233	E[28]
K[16]	537.274	926.894	**921.554**	**921.890**	921.219	K[27]
S[17]	566.284	884.196	**878.856**	**879.192**	**878.520**	S[26]
P[18]	598.635	855.185	849.845	850.181	849.510	P[25]
A[19]	622.314	822.834	817.495	817.831	817.159	A[24]
K[20]	665.013	799.155	793.816	794.151	793.480	K[23]
K[21]	707.711	756.457	751.117	751.453	750.781	K[22]
K[22]	750.409	713.758	708.419	708.755	708.083	K[21]
T[23]	784.092	671.060	665.721	666.057	665.385	T[20]
T[24]	817.774	637.378	632.038	632.374	631.702	T[19]
K[25]	860.473	603.695	598.355	598.691	598.019	K[18]
K[26]	903.171	560.997	555.657	555.993	555.321	K[17]
A[27]	926.850	518.298	512.959	513.295	512.623	A[16]
G[28]	945.857	494.619	489.280	489.616	488.944	G[15]
A[29]	969.536	475.612	470.273	470.609	469.937	A[14]
A[30]	**993.215**	451.933	446.594	446.930	446.258	A[13]
K[31]	**1035.914**	428.254	422.915	423.250	422.579	K[12]
R[32]	**1087.947**	385.556	380.216	380.552	379.880	R[11]
K[33]	**1130.646**	333.522	328.183	328.518	327.847	K[10]
A[34]	**1154.325**	290.824	285.484	285.820	285.148	A[9]
T[35]	**1188.007**	267.145	261.805	262.141	261.469	T[8]
G[36]	1207.015	233.462	228.123	228.459	227.787	G[7]
P[37]	1239.365	214.455	209.115	209.451	208.779	P[6]
P[38]	1271.716	182.104	176.765	177.100	176.429	P[5]
V[39]	**1304.739**	149.753	144.414	144.750	144.078	V[4]
S[40]	**1333.750**	116.730	111.391	111.727	111.055	S[3]
E[41]	1376.764	87.720	82.380	82.716	82.044	E[2]
L[42]	1414.459	44.705	39.366	39.702	39.030	L[1]

sp|P43276|H15_MOUSE

[Acetyl]SETAPAETAAPAPVEKSPAKKKTTKKAGAAKRKATGPPVSEL

- fragmentation table for charge state 4+
- specType=etd
- score=64.76
- F104929.dat
- query=q4014_p1
- precursor=849.278800
- chargePrecursor=5
- itol=0.8

AA	c	y	z+1	z+2	z	AA
S[1]	37.525	**1061.342**	1057.337	0.755	1057.085	S[42]
E[2]	69.785	1029.081	**1025.077**	0.755	**1024.825**	E[41]
T[3]	95.047	**996.821**	**992.816**	0.755	**992.564**	T[40]
A[4]	112.806	**971.559**	**967.554**	0.755	**967.302**	A[39]
P[5]	137.070	953.799	949.795	0.755	949.543	P[38]
A[6]	154.829	929.536	925.531	0.755	925.280	A[37]
E[7]	187.090	911.777	907.772	0.755	907.520	E[36]
T[8]	212.351	**879.516**	875.512	0.755	875.260	T[35]
A[9]	230.111	854.254	850.250	0.755	849.998	A[34]
A[10]	247.870	836.495	**832.490**	0.755	832.238	A[33]
P[11]	272.133	818.736	814.731	0.755	814.479	P[32]
A[12]	289.892	794.473	790.468	0.755	790.216	A[31]
P[13]	314.156	776.713	772.709	0.755	772.457	P[30]
V[14]	338.923	752.450	748.445	0.755	748.193	V[29]
E[15]	371.183	727.683	723.678	0.755	723.426	E[28]
K[16]	403.207	695.422	691.418	691.670	691.166	K[27]
S[17]	424.965	663.399	659.394	659.646	659.142	S[26]
P[18]	449.228	641.641	637.636	637.888	637.384	P[25]
A[19]	466.988	617.377	613.373	613.625	613.121	A[24]
K[20]	499.011	599.618	595.613	595.865	595.362	K[23]
K[21]	531.035	567.594	563.590	563.842	563.338	K[22]
K[22]	563.059	535.571	531.566	531.818	531.314	K[21]
T[23]	588.321	503.547	499.542	499.794	499.290	T[20]
T[24]	613.583	478.285	474.280	474.532	474.028	T[19]
K[25]	645.606	453.023	449.018	449.270	448.766	K[18]
K[26]	677.630	420.999	416.995	417.247	416.743	K[17]
A[27]	695.389	388.976	384.971	385.223	384.719	A[16]
G[28]	709.645	371.216	367.212	367.464	366.960	G[15]
A[29]	727.404	356.961	352.956	353.208	352.704	A[14]
A[30]	745.163	339.202	335.197	335.449	334.945	A[13]
K[31]	777.187	321.442	317.438	317.690	317.186	K[12]
R[32]	816.212	289.419	285.414	285.666	285.162	R[11]
K[33]	848.236	250.393	246.389	246.641	246.137	K[10]
A[34]	865.995	218.370	214.365	214.617	214.113	A[9]
T[35]	891.257	200.610	196.606	196.858	196.354	T[8]
G[36]	905.513	175.348	171.344	171.596	171.092	G[7]
P[37]	929.776	161.093	157.088	157.340	156.836	P[6]
P[38]	954.039	136.830	132.825	133.077	132.573	P[5]
V[39]	**978.806**	112.567	108.562	108.814	108.310	V[4]
S[40]	**1000.564**	87.800	83.795	84.047	83.543	S[3]
E[41]	**1032.825**	66.042	62.037	62.289	61.785	E[2]
L[42]	**1061.096**	33.781	29.776	30.028	29.524	L[1]

sp|P43274|H14_MOUSE

[Acetyl]SETAPAAPAAPAPAEKT $^{Phospho}_{79.97}$ PVKKKARKAAGGAKRKTSGPPVSEL

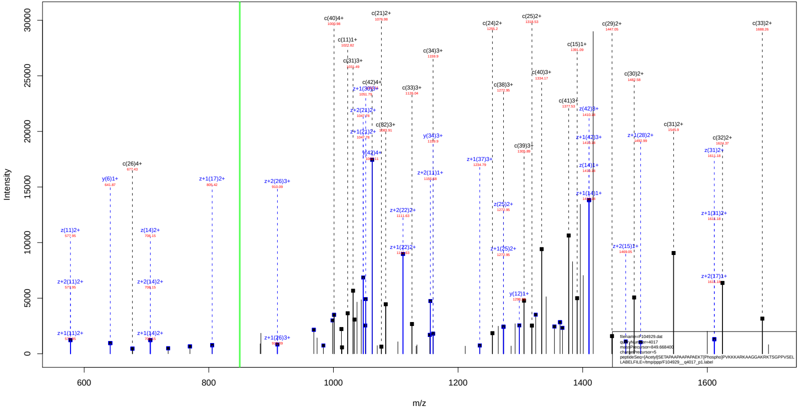

sp|P43274|H14_MOUSE

[Acetyl]SETAPAAPAAPAPAEKT $^{Phospho}_{79.97}$ PVKKKARKAAGGAKRKTSGPPVSEL

- fragmentation table for charge state 1+
- specType=etd
- score=54.41
- F104929.dat
- query=q4017_p1
- precursor=849.668400
- chargePrecursor=5
- itol=0.8

AA	c	y	z+1	z+2	z	AA
S[1]	147.076	4244.291	4228.273	0.000	4227.265	S[42]
E[2]	276.119	4115.249	4099.230	0.000	4098.222	E[41]
T[3]	377.167	3986.206	3970.188	0.000	3969.180	T[40]
A[4]	448.204	3885.159	3869.140	0.000	3868.132	A[39]
P[5]	545.257	3814.122	3798.103	0.000	3797.095	P[38]
A[6]	616.294	3717.069	3701.050	0.000	3700.042	A[37]
A[7]	687.331	3646.032	3630.013	0.000	3629.005	A[36]
P[8]	784.384	3574.995	3558.976	0.000	3557.968	P[35]
A[9]	855.421	3477.942	3461.923	0.000	3460.915	A[34]
A[10]	926.458	3406.905	3390.886	0.000	3389.878	A[33]
P[11]	**1023.511**	3335.868	3319.849	0.000	3318.841	P[32]
A[12]	1094.548	3238.815	3222.796	0.000	3221.788	A[31]
P[13]	1191.600	3167.778	3151.759	0.000	3150.751	P[30]
A[14]	1262.638	3070.725	3054.706	0.000	3053.698	A[29]
E[15]	**1391.680**	2999.688	2983.669	0.000	2982.661	E[28]
K[16]	1519.775	2870.645	2854.626	2855.634	2853.619	K[27]
T[17]	1700.789	2742.550	2726.531	2727.539	2725.524	T[26]
P[18]	1797.842	2561.536	2545.517	2546.525	2544.510	P[25]
V[19]	1896.910	2464.483	2448.465	2449.473	2447.457	V[24]
K[20]	2025.005	2365.415	2349.396	2350.404	2348.388	K[23]
K[21]	2153.100	2237.320	2221.301	2222.309	2220.294	K[22]
K[22]	2281.195	2109.225	2093.206	2094.214	2092.199	K[21]
A[23]	2352.232	1981.130	1965.111	1966.119	1964.104	A[20]
R[24]	2508.333	1910.093	1894.074	1895.082	1893.066	R[19]
K[25]	2636.428	1753.992	1737.973	1738.981	1736.965	K[18]
A[26]	2707.465	1625.897	1609.878	**1610.886**	1608.870	A[17]
A[27]	2778.503	1554.860	1538.841	1539.849	1537.833	A[16]
G[28]	2835.524	1483.823	1467.804	**1468.812**	1466.796	G[15]
G[29]	2892.546	1426.801	**1410.783**	1411.790	**1409.775**	G[14]
A[30]	2963.583	1369.780	1353.761	**1354.769**	1352.753	A[13]
K[31]	3091.678	**1298.743**	1282.724	1283.732	1281.716	K[12]
R[32]	3247.779	1170.648	**1154.629**	**1155.637**	1153.621	R[11]
K[33]	3375.874	1014.547	**998.528**	999.536	997.520	K[10]
T[34]	3476.921	886.452	870.433	871.441	869.425	T[9]
S[35]	3563.953	785.404	**769.385**	**770.393**	768.377	S[8]
G[36]	3620.975	698.372	682.353	683.361	681.345	G[7]
P[37]	3718.028	**641.350**	625.332	626.340	624.324	P[6]
P[38]	3815.080	544.298	528.279	529.287	527.271	P[5]
V[39]	3914.149	447.245	431.226	432.234	430.218	V[4]
S[40]	4001.181	348.177	332.158	333.166	331.150	S[3]
E[41]	4130.223	261.144	245.126	246.134	244.118	E[2]
L[42]	4243.307	132.102	116.083	117.091	115.075	L[1]

sp|P43274|H14_MOUSE

[Acetyl]SETAPAAPAAPAPAEKT $^{Phospho}_{79.97}$ PVKKKARKAAGGAKRKTSGPPVSEL

- fragmentation table for charge state 2+
- specType=etd
- score=54.41
- F104929.dat
- query=q4017_p1
- precursor=849.668400
- chargePrecursor=5
- itol=0.8

AA	c	y	z+1	z+2	z	AA
S[1]	74.042	2122.649	2114.640	0.504	2114.136	S[42]
E[2]	138.563	2058.128	2050.119	0.504	2049.615	E[41]
T[3]	189.087	1993.607	1985.597	0.504	1985.094	T[40]
A[4]	224.606	1943.083	1935.074	0.504	1934.570	A[39]
P[5]	273.132	1907.564	1899.555	0.504	1899.051	P[38]
A[6]	308.650	1859.038	1851.029	0.504	1850.525	A[37]
A[7]	344.169	1823.519	1815.510	0.504	1815.006	A[36]
P[8]	392.695	1788.001	1779.992	0.504	1779.488	P[35]
A[9]	428.214	1739.475	1731.465	0.504	1730.961	A[34]
A[10]	463.733	1703.956	1695.947	0.504	1695.443	A[33]
P[11]	512.259	1668.437	1660.428	0.504	1659.924	P[32]
A[12]	547.777	1619.911	**1611.902**	0.504	**1611.398**	A[31]
P[13]	596.304	1584.392	1576.383	0.504	1575.879	P[30]
A[14]	631.822	1535.866	1527.857	0.504	1527.353	A[29]
E[15]	696.344	1500.348	**1492.338**	0.504	1491.834	E[28]
K[16]	760.391	1435.826	1427.817	1428.321	1427.313	K[27]
T[17]	850.898	1371.779	**1363.769**	**1364.273**	**1363.265**	T[26]
P[18]	899.425	1281.272	**1273.262**	1273.766	**1272.758**	P[25]
V[19]	948.959	1232.745	1224.736	1225.240	1224.232	V[24]
K[20]	**1013.006**	1183.211	1175.202	1175.706	1174.698	K[23]
K[21]	**1077.054**	1119.164	**1111.154**	**1111.658**	1110.650	K[22]
K[22]	1141.101	1055.116	**1047.107**	**1047.611**	1046.603	K[21]
A[23]	1176.620	991.069	983.059	**983.563**	982.555	A[20]
R[24]	**1254.670**	955.550	947.541	948.045	947.037	R[19]
K[25]	**1318.718**	877.500	869.490	869.994	868.986	K[18]
A[26]	**1354.236**	813.452	**805.443**	**805.947**	**804.939**	A[17]
A[27]	1389.755	777.934	**769.924**	**770.428**	**769.420**	A[16]
G[28]	1418.266	742.415	**734.406**	**734.910**	733.902	G[15]
G[29]	**1446.776**	713.904	**705.895**	**706.399**	**705.391**	G[14]
A[30]	**1482.295**	685.394	**677.384**	**677.888**	**676.880**	A[13]
K[31]	**1546.342**	649.875	**641.866**	**642.370**	**641.362**	K[12]
R[32]	**1624.393**	585.827	**577.818**	**578.322**	**577.314**	R[11]
K[33]	**1688.440**	507.777	499.768	500.271	499.264	K[10]
T[34]	1738.964	443.729	435.720	436.224	435.216	T[9]
S[35]	1782.480	393.206	385.196	385.700	384.692	S[8]
G[36]	1810.991	349.690	341.680	342.184	341.176	G[7]
P[37]	1859.517	321.179	313.170	313.673	312.666	P[6]
P[38]	1908.044	272.652	264.643	265.147	264.139	P[5]
V[39]	1957.578	224.126	216.117	216.621	215.613	V[4]
S[40]	2001.094	174.592	166.583	167.086	166.079	S[3]
E[41]	2065.615	131.076	123.067	123.570	122.563	E[2]
L[42]	2122.157	66.555	58.545	59.049	58.041	L[1]

sp|P43274|H14_MOUSE

[Acetyl]SETAPAAPAAPAPAEKT $^{Phospho}_{79.97}$ PVKKKARKAAGGAKRKTSGPPVSEL

- fragmentation table for charge state 3+
- specType=etd
- score=54.41
- F104929.dat
- query=q4017_p1
- precursor=849.668400
- chargePrecursor=5
- itol=0.8

AA	c	y	z+1	z+2	z	AA
S[1]	49.697	1415.435	**1410.096**	0.672	**1409.760**	S[42]
E[2]	92.711	1372.421	**1367.082**	0.672	1366.746	E[41]
T[3]	126.394	1329.407	**1324.067**	0.672	1323.731	T[40]
A[4]	150.073	1295.724	1290.385	0.672	1290.049	A[39]
P[5]	182.424	1272.045	1266.706	0.672	1266.370	P[38]
A[6]	206.103	1239.694	**1234.355**	0.672	**1234.019**	A[37]
A[7]	229.782	1216.015	1210.676	0.672	1210.340	A[36]
P[8]	262.133	1192.336	1186.997	0.672	1186.661	P[35]
A[9]	285.812	**1159.985**	**1154.646**	0.672	**1154.310**	A[34]
A[10]	309.491	1136.306	1130.967	0.672	1130.631	A[33]
P[11]	341.842	1112.627	1107.288	0.672	1106.952	P[32]
A[12]	365.521	1080.276	1074.937	0.672	1074.601	A[31]
P[13]	397.872	1056.597	**1051.258**	0.672	**1050.922**	P[30]
A[14]	421.551	1024.246	1018.907	0.672	1018.571	A[29]
E[15]	464.565	**1000.567**	995.228	0.672	994.892	E[28]
K[16]	507.263	957.553	952.214	952.550	951.878	K[27]
T[17]	567.601	914.855	**909.515**	**909.851**	909.179	T[26]
P[18]	599.952	854.517	849.177	849.513	848.841	P[25]
V[19]	632.975	822.166	816.826	817.162	816.490	V[24]
K[20]	675.673	789.143	783.804	784.140	783.468	K[23]
K[21]	718.372	746.445	741.105	741.441	740.769	K[22]
K[22]	761.070	703.747	698.407	698.743	698.071	K[21]
A[23]	784.749	661.048	655.709	656.045	655.373	A[20]
R[24]	836.783	637.369	632.030	632.366	631.694	R[19]
K[25]	879.481	585.335	579.996	580.332	579.660	K[18]
A[26]	903.160	542.637	537.298	537.634	536.962	A[17]
A[27]	926.839	518.958	513.619	513.954	513.283	A[16]
G[28]	945.846	495.279	489.940	490.275	489.604	G[15]
G[29]	964.853	476.272	470.932	471.268	470.596	G[14]
A[30]	988.532	457.265	451.925	452.261	451.589	A[13]
K[31]	**1031.231**	433.586	428.246	428.582	427.910	K[12]
R[32]	**1083.264**	390.887	385.548	385.884	385.212	R[11]
K[33]	**1125.963**	338.854	333.514	333.850	333.178	K[10]
T[34]	**1159.645**	296.155	290.816	291.152	290.480	T[9]
S[35]	1188.656	262.473	257.133	257.469	256.797	S[8]
G[36]	1207.663	233.462	228.123	228.459	227.787	G[7]
P[37]	1240.014	214.455	209.115	209.451	208.779	P[6]
P[38]	**1272.365**	182.104	176.765	177.100	176.429	P[5]
V[39]	**1305.388**	149.753	144.414	144.750	144.078	V[4]
S[40]	**1334.398**	116.730	111.391	111.727	111.055	S[3]
E[41]	**1377.413**	87.720	82.380	82.716	82.044	E[2]
L[42]	1415.107	44.705	39.366	39.702	39.030	L[1]

sp|P43274|H14_MOUSE

[Acetyl]SETAPAAPAAPAPAEKT $^{\text{Phospho}}_{79.97}$ PVKKKARKAAGGAKRKTSGPPVSEL

- fragmentation table for charge state 4+
- specType=etd
- score=54.41
- F104929.dat
- query=q4017_p1
- precursor=849.668400
- chargePrecursor=5
- itol=0.8

AA	c	y	z+1	z+2	z	AA
S[1]	37.525	**1061.828**	1057.824	0.755	1057.572	S[42]
E[2]	69.785	1029.568	1025.563	0.755	1025.311	E[41]
T[3]	95.047	997.307	993.302	0.755	993.050	T[40]
A[4]	112.806	972.045	**968.040**	0.755	**967.788**	A[39]
P[5]	137.070	954.286	950.281	0.755	950.029	P[38]
A[6]	154.829	930.023	926.018	0.755	925.766	A[37]
A[7]	172.588	912.263	908.259	0.755	908.007	A[36]
P[8]	196.851	894.504	890.499	0.755	890.247	P[35]
A[9]	214.611	870.241	866.236	0.755	865.984	A[34]
A[10]	232.370	852.482	848.477	0.755	848.225	A[33]
P[11]	256.633	834.722	830.718	0.755	830.466	P[32]
A[12]	274.392	810.459	806.454	0.755	**806.203**	A[31]
P[13]	298.656	792.700	788.695	0.755	788.443	P[30]
A[14]	316.415	768.437	764.432	0.755	764.180	A[29]
E[15]	348.675	750.677	746.673	0.755	746.421	E[28]
K[16]	380.699	718.417	714.412	714.664	714.160	K[27]
T[17]	425.953	686.393	682.388	682.640	682.136	T[26]
P[18]	450.216	**641.140**	637.135	637.387	636.883	P[25]
V[19]	474.983	616.876	612.872	613.124	612.620	V[24]
K[20]	507.007	592.109	588.105	588.356	587.853	K[23]
K[21]	539.031	560.085	556.081	556.333	555.829	K[22]
K[22]	571.054	528.062	524.057	524.309	523.805	K[21]
A[23]	588.814	496.038	492.033	492.285	491.781	A[20]
R[24]	627.839	478.279	474.274	474.526	474.022	R[19]
K[25]	659.863	439.253	435.249	435.501	434.997	K[18]
A[26]	**677.622**	407.230	403.225	403.477	402.973	A[17]
A[27]	695.381	389.470	385.466	385.718	385.214	A[16]
G[28]	709.636	371.711	367.706	367.958	367.454	G[15]
G[29]	723.892	357.456	353.451	353.703	353.199	G[14]
A[30]	741.651	343.200	339.196	339.448	338.944	A[13]
K[31]	773.675	325.441	321.436	321.688	321.184	K[12]
R[32]	812.700	293.417	289.413	289.665	289.161	R[11]
K[33]	844.724	254.392	250.387	250.639	250.135	K[10]
T[34]	869.986	222.368	218.364	218.616	218.112	T[9]
S[35]	891.744	197.106	193.102	193.354	192.850	S[8]
G[36]	905.999	175.348	171.344	171.596	171.092	G[7]
P[37]	930.262	161.093	157.088	157.340	156.836	P[6]
P[38]	954.526	136.830	132.825	133.077	132.573	P[5]
V[39]	979.293	112.567	108.562	108.814	108.310	V[4]
S[40]	**1001.051**	87.800	83.795	84.047	83.543	S[3]
E[41]	**1033.311**	66.042	62.037	62.289	61.785	E[2]
L[42]	**1061.582**	33.781	29.776	30.028	29.524	L[1]

sp|P43277|H13_MOUSE

S (Phospho) (79.97) ETAPAAPAAPAPVEKTPVKKKAKKTGAAAGKRKASGPPVSEL

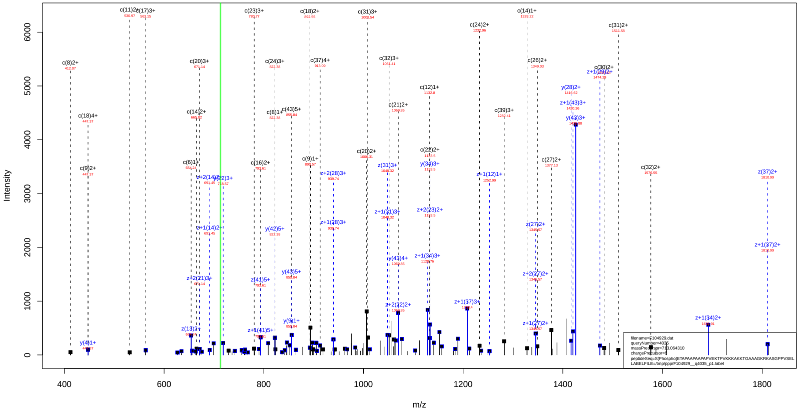

sp|P43277|H13_MOUSE

S $^{(Phospho)}_{(79.97)}$ ETAPAAPAAPAPVEKTPVKKAKKTGAAAGKRKASGPPVSEL

- fragmentation table for charge state 1+
- specType=etd
- score=55.37
- F104929.dat
- query=q4035_p1
- precursor=713.064310
- chargePrecursor=6
- itol=0.8

AA	c	y	z+1	z+2	z	AA
S[1]	185.032	4273.343	4257.324	0.000	4256.317	S[43]
E[2]	314.075	4106.345	4090.326	0.000	4089.318	E[42]
T[3]	415.122	3977.302	3961.284	0.000	3960.276	T[41]
A[4]	486.160	3876.255	3860.236	0.000	3859.228	A[40]
P[5]	583.212	3805.217	3789.199	0.000	3788.191	P[39]
A[6]	**654.249**	3708.165	3692.146	0.000	3691.138	A[38]
A[7]	725.287	3637.128	3621.109	0.000	3620.101	A[37]
P[8]	**822.339**	3566.090	3550.072	0.000	3549.064	P[36]
A[9]	**893.376**	3469.038	3453.019	0.000	3452.011	A[35]
A[10]	964.414	3398.001	3381.982	0.000	3380.974	A[34]
P[11]	**1061.466**	3326.963	3310.945	0.000	3309.937	P[33]
A[12]	**1132.503**	3229.911	3213.892	0.000	3212.884	A[32]
P[13]	1229.556	3158.874	3142.855	0.000	3141.847	P[31]
V[14]	**1328.625**	3061.821	3045.802	0.000	3044.794	V[30]
E[15]	1457.667	2962.752	2946.734	0.000	2945.726	E[29]
K[16]	1585.762	2833.710	2817.691	2818.699	2816.683	K[28]
T[17]	1686.810	2705.615	2689.596	2690.604	2688.588	T[27]
P[18]	1783.863	2604.567	2588.548	2589.556	2587.541	P[26]
V[19]	1882.931	2507.514	2491.496	2492.504	2490.488	V[25]
K[20]	2011.026	2408.446	2392.427	2393.435	2391.419	K[24]
K[21]	2139.121	2280.351	2264.332	2265.340	2263.324	K[23]
K[22]	2267.216	2152.256	2136.237	2137.245	2135.230	K[22]
A[23]	2338.253	2024.161	2008.142	2009.150	2007.135	A[21]
K[24]	2466.348	1953.124	1937.105	1938.113	1936.097	K[20]
K[25]	2594.443	1825.029	1809.010	1810.018	1808.002	K[19]
T[26]	2695.491	1696.934	1680.915	1681.923	1679.908	T[18]
G[27]	2752.512	1595.886	1579.868	1580.875	1578.860	G[17]
A[28]	2823.549	1538.865	1522.846	1523.854	1521.838	A[16]
A[29]	2894.586	1467.828	1451.809	1452.817	1450.801	A[15]
A[30]	2965.623	1396.791	1380.772	1381.780	1379.764	A[14]
G[31]	3022.645	1325.754	1309.735	1310.743	1308.727	G[13]
K[32]	3150.740	1268.732	**1252.713**	**1253.721**	1251.706	K[12]
R[33]	3306.841	**1140.637**	1124.618	1125.626	1123.611	R[11]
K[34]	3434.936	984.536	968.517	969.525	**967.509**	K[10]
A[35]	3505.973	**856.441**	840.422	**841.430**	839.415	A[9]
S[36]	3593.005	785.404	769.385	770.393	**768.377**	S[8]
G[37]	3650.027	698.372	682.353	683.361	681.345	G[7]
P[38]	3747.079	641.350	625.332	**626.340**	624.324	P[6]
P[39]	3844.132	544.298	528.279	529.287	527.271	P[5]
V[40]	3943.200	**447.245**	431.226	432.234	430.218	V[4]
S[41]	4030.233	348.177	332.158	333.166	331.150	S[3]
E[42]	4159.275	261.144	245.126	246.134	244.118	E[2]
L[43]	4272.359	132.102	116.083	117.091	115.075	L[1]

sp|P43277|H13_MOUSE

S $^{(Phospho)}_{(79.97)}$ ETAPAAPAAPAPVEKTPVKKAKKTGAAAGKRKASGPPVSEL

- fragmentation table for charge state 2+
- specType=etd
- score=55.37
- F104929.dat
- query=q4035_p1
- precursor=713.064310
- chargePrecursor=6
- itol=0.8

AA	c	y	z+1	z+2	z	AA
S[1]	93.020	2137.175	2129.166	0.504	2128.662	S[43]
E[2]	157.541	2053.676	2045.667	0.504	2045.163	E[42]
T[3]	208.065	1989.155	1981.145	0.504	1980.641	T[41]
A[4]	243.583	1938.631	1930.622	0.504	1930.118	A[40]
P[5]	292.110	1903.112	1895.103	0.504	1894.599	P[39]
A[6]	327.628	1854.586	1846.577	0.504	1846.073	A[38]
A[7]	363.147	1819.067	**1811.058**	0.504	**1810.554**	A[37]
P[8]	**411.673**	1783.549	1775.540	0.504	1775.036	P[36]
A[9]	**447.192**	1735.022	1727.013	0.504	1726.509	A[35]
A[10]	482.710	1699.504	**1691.495**	0.504	1690.991	A[34]
P[11]	**531.237**	1663.985	1655.976	0.504	1655.472	P[33]
A[12]	566.755	1615.459	1607.450	0.504	1606.946	A[32]
P[13]	615.282	1579.940	1571.931	0.504	1571.427	P[31]
V[14]	**664.816**	1531.414	1523.405	0.504	1522.901	V[30]
E[15]	**729.337**	1481.880	**1473.870**	0.504	1473.367	E[29]
K[16]	**793.385**	**1417.359**	1409.349	1409.853	1408.845	K[28]
T[17]	843.909	1353.311	**1345.302**	**1345.806**	**1344.798**	T[27]
P[18]	**892.435**	1302.787	1294.778	1295.282	1294.274	P[26]
V[19]	**941.969**	1254.261	1246.251	1246.755	1245.748	V[25]
K[20]	**1006.017**	1204.727	1196.717	1197.221	1196.213	K[24]
K[21]	**1070.064**	**1140.679**	**1132.670**	**1133.174**	**1132.166**	K[23]
K[22]	**1134.112**	**1076.632**	1068.622	**1069.126**	1068.118	K[22]
A[23]	1169.630	**1012.584**	1004.575	1005.079	1004.071	A[21]
K[24]	**1233.678**	977.066	969.056	969.560	968.552	K[20]
K[25]	1297.725	**913.018**	**905.009**	**905.513**	904.505	K[19]
T[26]	**1348.249**	848.971	840.961	**841.465**	840.457	T[18]
G[27]	**1376.760**	798.447	**790.437**	**790.941**	**789.934**	G[17]
A[28]	1412.278	769.936	**761.927**	**762.431**	**761.423**	A[16]
A[29]	1447.797	734.418	726.408	726.912	725.904	A[15]
A[30]	**1483.315**	**698.899**	**690.890**	**691.394**	690.386	A[14]
G[31]	**1511.826**	**663.380**	**655.371**	**655.875**	**654.867**	G[13]
K[32]	**1575.874**	**634.870**	**626.860**	**627.364**	**626.356**	K[12]
R[33]	1653.924	570.822	**562.813**	**563.317**	562.309	R[11]
K[34]	1717.972	492.772	484.762	485.266	484.258	K[10]
A[35]	1753.490	428.724	420.715	421.219	420.211	A[9]
S[36]	1797.006	393.206	385.196	385.700	384.692	S[8]
G[37]	1825.517	349.690	341.680	342.184	341.176	G[7]
P[38]	1874.043	321.179	313.170	313.673	312.666	P[6]
P[39]	1922.570	272.652	264.643	265.147	264.139	P[5]
V[40]	1972.104	224.126	216.117	216.621	215.613	V[4]
S[41]	2015.620	174.592	166.583	167.086	166.079	S[3]
E[42]	2080.141	131.076	123.067	123.570	122.563	E[2]
L[43]	2136.683	66.555	58.545	59.049	58.041	L[1]

sp|P43277|H13_MOUSE

S $^{(Phospho)}_{(79.97)}$ ETAPAAPAAPAPVEKTPVKKAKKTGAAAGKRKASGPPVSEL

- fragmentation table for charge state 3+
- specType=etd
- score=55.37
- F104929.dat
- query=q4035_p1
- precursor=713.064310
- chargePrecursor=6
- itol=0.8

AA	c	y	z+1	z+2	z	AA
S[1]	62.349	**1425.119**	**1419.780**	0.672	1419.444	S[43]
E[2]	105.363	1369.453	1364.114	0.672	1363.778	E[42]
T[3]	139.046	1326.439	1321.099	0.672	1320.763	T[41]
A[4]	162.725	1292.756	1287.417	0.672	1287.081	A[40]
P[5]	195.076	1269.077	1263.738	0.672	1263.402	P[39]
A[6]	218.755	**1236.726**	1231.387	0.672	1231.051	A[38]
A[7]	242.434	**1213.047**	**1207.708**	0.672	1207.372	A[37]
P[8]	274.785	**1189.368**	**1184.029**	0.672	**1183.693**	P[36]
A[9]	298.464	**1157.017**	**1151.678**	0.672	1151.342	A[35]
A[10]	322.143	**1133.338**	**1127.999**	0.672	1127.663	A[34]
P[11]	354.494	1109.659	**1104.320**	0.672	**1103.984**	P[33]
A[12]	378.173	**1077.308**	1071.969	0.672	1071.633	A[32]
P[13]	410.524	1053.629	**1048.290**	0.672	**1047.954**	P[31]
V[14]	443.546	1021.278	1015.939	0.672	1015.603	V[30]
E[15]	486.561	988.256	**982.916**	0.672	982.580	E[29]
K[16]	529.259	945.241	**939.902**	**940.238**	**939.566**	K[28]
T[17]	**562.941**	902.543	**897.204**	**897.539**	896.868	T[27]
P[18]	595.292	868.861	863.521	**863.857**	863.185	P[26]
V[19]	628.315	**836.510**	831.170	831.506	830.834	V[25]
K[20]	**671.014**	803.487	798.147	798.483	797.811	K[24]
K[21]	713.712	**760.789**	**755.449**	**755.785**	**755.113**	K[23]
K[22]	**756.410**	**718.090**	712.751	713.087	712.415	K[22]
A[23]	**780.089**	**675.392**	670.052	**670.388**	669.716	A[21]
K[24]	**822.788**	651.713	646.373	646.709	646.037	K[20]
K[25]	865.486	609.015	603.675	604.011	603.339	K[19]
T[26]	**899.168**	566.316	560.977	561.313	560.641	T[18]
G[27]	**918.176**	532.634	527.294	527.630	526.958	G[17]
A[28]	**941.855**	513.626	508.287	508.623	507.951	A[16]
A[29]	965.534	489.947	484.608	484.944	484.272	A[15]
A[30]	989.213	466.268	460.929	461.265	460.593	A[14]
G[31]	**1008.220**	442.589	437.250	437.586	436.914	G[13]
K[32]	**1050.918**	423.582	418.243	418.579	417.907	K[12]
R[33]	1102.952	380.884	375.544	375.880	375.208	R[11]
K[34]	1145.650	328.850	323.511	323.847	323.175	K[10]
A[35]	1169.329	286.152	280.812	281.148	280.476	A[9]
S[36]	1198.340	262.473	257.133	257.469	256.797	S[8]
G[37]	1217.347	233.462	228.123	228.459	227.787	G[7]
P[38]	1249.698	214.455	209.115	209.451	208.779	P[6]
P[39]	**1282.049**	182.104	176.765	177.100	176.429	P[5]
V[40]	1315.072	149.753	144.414	144.750	144.078	V[4]
S[41]	1344.082	116.730	111.391	111.727	111.055	S[3]
E[42]	1387.097	87.720	82.380	82.716	82.044	E[2]
L[43]	1424.791	44.705	39.366	39.702	39.030	L[1]

sp|P43277|H13_MOUSE

S $^{(Phospho)}_{(79.97)}$ ETAPAAPAAPAPVEKTPVKKAKKTGAAAGKRKASGPPVSEL

- fragmentation table for charge state 4+
- specType=etd
- score=55.37
- F104929.dat
- query=q4035_p1
- precursor=713.064310
- chargePrecursor=6
- itol=0.8

AA	c	y	z+1	z+2	z	AA
S[1]	47.014	**1069.091**	**1065.087**	0.755	**1064.835**	S[43]
E[2]	79.274	1027.342	1023.337	0.755	1023.085	E[42]
T[3]	104.536	995.081	991.076	0.755	990.824	T[41]
A[4]	122.295	969.819	965.814	0.755	965.562	A[40]
P[5]	146.559	952.060	948.055	0.755	947.803	P[39]
A[6]	164.318	927.797	923.792	0.755	923.540	A[38]
A[7]	182.077	910.037	**906.033**	0.755	**905.781**	A[37]
P[8]	206.340	**892.278**	888.273	0.755	888.021	P[36]
A[9]	224.100	868.015	**864.010**	0.755	**863.758**	A[35]
A[10]	241.859	850.256	**846.251**	0.755	**845.999**	A[34]
P[11]	266.122	832.496	828.492	0.755	828.240	P[33]
A[12]	283.881	**808.233**	804.228	0.755	803.976	A[32]
P[13]	308.145	**790.474**	786.469	0.755	786.217	P[31]
V[14]	332.912	766.211	**762.206**	0.755	**761.954**	V[30]
E[15]	365.172	**741.444**	737.439	0.755	737.187	E[29]
K[16]	397.196	709.183	705.178	705.430	704.926	K[28]
T[17]	422.458	677.159	673.154	673.406	672.903	T[27]
P[18]	**446.721**	651.897	647.893	648.145	647.641	P[26]
V[19]	471.488	**627.634**	623.629	623.881	623.377	V[25]
K[20]	503.512	602.867	598.862	599.114	598.610	K[24]
K[21]	535.536	570.843	566.839	567.090	566.587	K[23]
K[22]	567.559	538.819	534.815	535.067	534.563	K[22]
A[23]	585.319	506.796	502.791	503.043	502.539	A[21]
K[24]	617.342	489.036	485.032	485.284	484.780	K[20]
K[25]	649.366	457.013	453.008	453.260	452.756	K[19]
T[26]	674.628	424.989	420.984	421.236	420.732	T[18]
G[27]	688.883	399.727	395.722	395.974	395.470	G[17]
A[28]	706.643	385.472	381.467	381.719	381.215	A[16]
A[29]	724.402	367.712	363.708	363.960	363.456	A[15]
A[30]	**742.161**	349.953	345.948	346.200	345.696	A[14]
G[31]	**756.417**	332.194	328.189	328.441	327.937	G[13]
K[32]	788.440	317.938	313.934	314.186	313.682	K[12]
R[33]	827.466	285.915	281.910	282.162	281.658	R[11]
K[34]	859.489	246.889	242.885	243.137	242.633	K[10]
A[35]	877.249	214.866	210.861	211.113	210.609	A[9]
S[36]	**899.007**	197.106	193.102	193.354	192.850	S[8]
G[37]	**913.262**	175.348	171.344	171.596	171.092	G[7]
P[38]	937.525	161.093	157.088	157.340	156.836	P[6]
P[39]	**961.788**	136.830	132.825	133.077	132.573	P[5]
V[40]	986.556	112.567	108.562	108.814	108.310	V[4]
S[41]	**1008.314**	87.800	83.795	84.047	83.543	S[3]
E[42]	1040.574	66.042	62.037	62.289	61.785	E[2]
L[43]	1068.845	33.781	29.776	30.028	29.524	L[1]

sp|P43277|H13_MOUSE

S$^{(Phospho)}_{(79.97)}$ETAPAAPAAPAPVEKTPVKKAKKTGAAAGKRKASGPPVSEL

- fragmentation table for charge state 5+
- specType=etd
- score=55.37
- F104929.dat
- query=q4035_p1
- precursor=713.064310
- chargePrecursor=6
- itol=0.8

AA	c	y	z+1	z+2	z	AA
S[1]	37.812	**855.474**	**852.271**	0.806	**852.069**	S[43]
E[2]	63.621	**822.075**	818.871	0.806	818.669	E[42]
T[3]	83.830	796.266	**793.063**	0.806	**792.861**	T[41]
A[4]	98.038	776.057	772.853	0.806	772.651	A[40]
P[5]	117.448	**761.849**	758.646	0.806	758.444	P[39]
A[6]	131.656	**742.439**	739.235	0.806	739.033	A[38]
A[7]	145.863	728.231	725.028	0.806	724.826	A[37]
P[8]	165.274	714.024	710.820	0.806	710.619	P[36]
A[9]	179.481	694.613	**691.410**	0.806	**691.208**	A[35]
A[10]	193.689	680.406	677.202	0.806	677.001	A[34]
P[11]	213.099	666.199	**662.995**	0.806	**662.793**	P[33]
A[12]	227.307	646.788	643.584	0.806	643.383	A[32]
P[13]	246.717	632.581	629.377	0.806	629.175	P[31]
V[14]	266.531	613.170	609.966	0.806	609.765	V[30]
E[15]	292.339	593.356	590.153	0.806	589.951	E[29]
K[16]	317.958	567.548	564.344	564.546	564.142	K[28]
T[17]	338.168	541.929	538.725	538.927	538.523	T[27]
P[18]	357.578	521.719	518.516	518.717	518.314	P[26]
V[19]	377.392	502.309	499.105	499.307	498.903	V[25]
K[20]	403.011	482.495	479.291	479.493	479.090	K[24]
K[21]	428.630	456.876	453.672	453.874	453.471	K[23]
K[22]	454.249	431.257	428.053	428.255	427.852	K[22]
A[23]	468.456	405.638	402.434	402.636	402.233	A[21]
K[24]	494.075	391.431	388.227	388.428	388.025	K[20]
K[25]	519.694	365.812	362.608	362.809	362.406	K[19]
T[26]	539.904	340.193	336.989	337.190	336.787	T[18]
G[27]	551.308	319.983	316.779	316.981	316.578	G[17]
A[28]	565.516	308.579	305.375	305.577	305.173	A[16]
A[29]	579.723	294.371	291.168	291.369	290.966	A[15]
A[30]	593.931	280.164	276.960	277.162	276.759	A[14]
G[31]	605.335	265.957	262.753	262.954	262.551	G[13]
K[32]	630.954	254.552	251.348	251.550	251.147	K[12]
R[33]	662.174	228.933	225.730	225.931	225.528	R[11]
K[34]	687.793	197.713	194.509	194.711	194.308	K[10]
A[35]	702.000	172.094	168.890	169.092	168.689	A[9]
S[36]	719.407	157.887	154.683	154.884	154.481	S[8]
G[37]	730.811	140.480	137.276	137.478	137.075	G[7]
P[38]	750.222	129.076	125.872	126.074	125.671	P[6]
P[39]	769.632	109.665	106.462	106.663	106.260	P[5]
V[40]	789.446	90.255	87.051	87.253	86.849	V[4]
S[41]	806.852	70.441	67.237	67.439	67.036	S[3]
E[42]	832.661	53.035	49.831	50.033	49.629	E[2]
L[43]	**855.278**	27.226	24.022	24.224	23.821	L[1]

sp|P43276|H15_MOUSE

[Acetyl]SETAPAETAAPAPVEKS $^{\text{Phospho}}_{79.97}$ PAKKKTTKKAGAAKRKATGPPVSEL

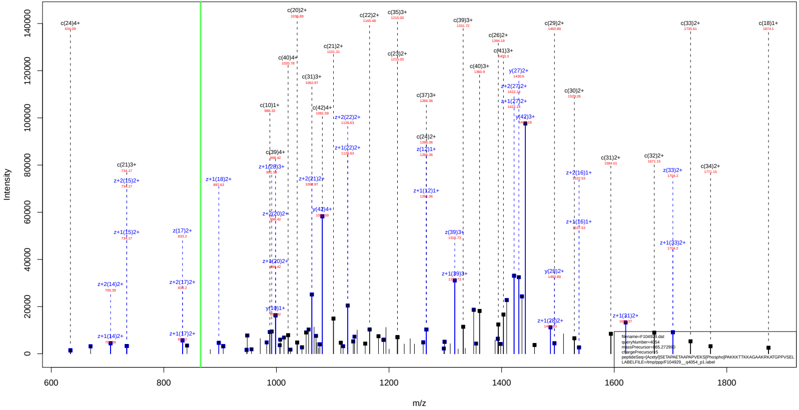

sp|P43276|H15_MOUSE

[Acetyl]SETAPAETAAPAPVEKS $^{Phospho}_{79.97}$ PAKKKTTKKAGAAKRKATGPPVSEL

- fragmentation table for charge state 1+
- specType=etd
- score=82.33
- F104929.dat
- query=q4054_p1
- precursor=865.272990
- chargePrecursor=5
- itol=0.8

AA	c	y	z+1	z+2	z	AA
S[1]	147.076	4322.312	4306.293	0.000	4305.285	S[42]
E[2]	276.119	4193.269	4177.251	0.000	4176.243	E[41]
T[3]	377.167	4064.227	4048.208	0.000	4047.200	T[40]
A[4]	448.204	3963.179	3947.160	0.000	3946.153	A[39]
P[5]	545.257	3892.142	3876.123	0.000	3875.115	P[38]
A[6]	616.294	3795.089	3779.070	0.000	3778.063	A[37]
E[7]	745.336	3724.052	3708.033	0.000	3707.026	E[36]
T[8]	846.384	3595.009	3578.991	0.000	3577.983	T[35]
A[9]	917.421	3493.962	3477.943	0.000	3476.935	A[34]
A[10]	**988.458**	3422.925	3406.906	0.000	3405.898	A[33]
P[11]	1085.511	3351.888	3335.869	0.000	3334.861	P[32]
A[12]	1156.548	3254.835	3238.816	0.000	3237.808	A[31]
P[13]	1253.601	3183.798	3167.779	0.000	3166.771	P[30]
V[14]	1352.669	3086.745	3070.726	0.000	3069.718	V[29]
E[15]	1481.712	2987.677	2971.658	0.000	2970.650	E[28]
K[16]	1609.807	2858.634	2842.615	2843.623	2841.607	K[27]
S[17]	1776.805	2730.539	2714.520	2715.528	2713.512	S[26]
P[18]	**1873.858**	2563.541	2547.522	2548.530	2546.514	P[25]
A[19]	1944.895	2466.488	2450.469	2451.477	2449.461	A[24]
K[20]	2072.990	2395.451	2379.432	2380.440	2378.424	K[23]
K[21]	2201.085	2267.356	2251.337	2252.345	2250.329	K[22]
K[22]	2329.180	2139.261	2123.242	2124.250	2122.234	K[21]
T[23]	2430.228	2011.166	1995.147	1996.155	1994.139	T[20]
T[24]	2531.275	1910.118	1894.099	1895.107	1893.092	T[19]
K[25]	2659.370	1809.070	1793.052	1794.060	1792.044	K[18]
K[26]	2787.465	1680.976	1664.957	1665.965	1663.949	K[17]
A[27]	2858.502	1552.881	**1536.862**	**1537.870**	1535.854	A[16]
G[28]	2915.524	1481.843	1465.825	1466.833	1464.817	G[15]
A[29]	2986.561	1424.822	**1408.803**	**1409.811**	1407.795	A[14]
A[30]	3057.598	1353.785	1337.766	1338.774	1336.758	A[13]
K[31]	3185.693	1282.748	**1266.729**	1267.737	**1265.721**	K[12]
R[32]	3341.794	1154.653	**1138.634**	1139.642	1137.626	R[11]
K[33]	3469.889	**998.552**	**982.533**	983.541	981.525	K[10]
A[34]	3540.926	870.457	854.438	855.446	853.430	A[9]
T[35]	3641.974	799.420	783.401	784.409	782.393	T[8]
G[36]	3698.995	698.372	682.353	683.361	681.345	G[7]
P[37]	3796.048	641.350	625.332	626.340	624.324	P[6]
P[38]	3893.101	544.298	528.279	529.287	527.271	P[5]
V[39]	3992.169	447.245	431.226	432.234	430.218	V[4]
S[40]	4079.201	348.177	332.158	333.166	331.150	S[3]
E[41]	4208.244	261.144	245.126	246.134	244.118	E[2]
L[42]	4321.328	132.102	116.083	117.091	115.075	L[1]

sp|P43276|H15_MOUSE

[Acetyl]SETAPAETAAPAPVEKS $^{Phospho}_{79.97}$ PAKKKTTKKAGAAKRKATGPPVSEL

- fragmentation table for charge state 2+
- specType=etd
- score=82.33
- F104929.dat
- query=q4054_p1
- precursor=865.272990
- chargePrecursor=5
- itol=0.8

AA	c	y	z+1	z+2	z	AA
S[1]	74.042	2161.660	2153.650	0.504	2153.146	S[42]
E[2]	138.563	2097.138	2089.129	0.504	2088.625	E[41]
T[3]	189.087	2032.617	2024.608	0.504	2024.104	T[40]
A[4]	224.606	1982.093	1974.084	0.504	1973.580	A[39]
P[5]	273.132	1946.575	1938.565	0.504	1938.061	P[38]
A[6]	308.650	1898.048	1890.039	0.504	1889.535	A[37]
E[7]	373.172	1862.530	1854.520	0.504	1854.016	E[36]
T[8]	423.696	1798.008	1789.999	0.504	1789.495	T[35]
A[9]	459.214	1747.485	1739.475	0.504	1738.971	A[34]
A[10]	494.733	1711.966	**1703.957**	0.504	**1703.453**	A[33]
P[11]	543.259	1676.447	1668.438	0.504	1667.934	P[32]
A[12]	578.778	1627.921	**1619.912**	0.504	1619.408	A[31]
P[13]	627.304	1592.402	1584.393	0.504	1583.889	P[30]
V[14]	676.838	1543.876	1535.867	0.504	1535.363	V[29]
E[15]	741.360	**1494.342**	**1486.333**	0.504	1485.829	E[28]
K[16]	805.407	**1429.821**	**1421.811**	**1422.315**	1421.307	K[27]
S[17]	888.906	1365.773	1357.764	1358.268	1357.260	S[26]
P[18]	937.433	1282.274	1274.265	1274.768	1273.761	P[25]
A[19]	972.951	1233.748	1225.738	1226.242	1225.234	A[24]
K[20]	**1036.999**	1198.229	**1190.220**	**1190.724**	**1189.716**	K[23]
K[21]	**1101.046**	1134.182	**1126.172**	**1126.676**	1125.668	K[22]
K[22]	**1165.094**	**1070.134**	1062.125	**1062.629**	1061.621	K[21]
T[23]	**1215.617**	**1006.087**	**998.077**	**998.581**	997.573	T[20]
T[24]	**1266.141**	**955.563**	**947.553**	**948.057**	**947.049**	T[19]
K[25]	1330.189	**905.039**	**897.030**	**897.533**	896.526	K[18]
K[26]	**1394.236**	**840.991**	**832.982**	**833.486**	**832.478**	K[17]
A[27]	1429.755	776.944	768.935	769.438	768.431	A[16]
G[28]	**1458.266**	741.425	**733.416**	**733.920**	732.912	G[15]
A[29]	**1493.784**	712.915	**704.905**	**705.409**	704.401	A[14]
A[30]	**1529.303**	677.396	**669.387**	**669.891**	668.883	A[13]
K[31]	**1593.350**	641.878	**633.868**	**634.372**	**633.364**	K[12]
R[32]	**1671.401**	577.830	569.821	570.325	569.317	R[11]
K[33]	**1735.448**	499.779	491.770	492.274	491.266	K[10]
A[34]	**1770.967**	435.732	427.723	428.227	427.219	A[9]
T[35]	1821.491	400.213	392.204	392.708	391.700	T[8]
G[36]	1850.001	349.690	341.680	342.184	341.176	G[7]
P[37]	1898.528	321.179	313.170	313.673	312.666	P[6]
P[38]	1947.054	272.652	264.643	265.147	264.139	P[5]
V[39]	1996.588	224.126	216.117	216.621	215.613	V[4]
S[40]	2040.104	174.592	166.583	167.086	166.079	S[3]
E[41]	2104.626	131.076	123.067	123.570	122.563	E[2]
L[42]	2161.168	66.555	58.545	59.049	58.041	L[1]

sp|P43276|H15_MOUSE

[Acetyl]SETAPAETAAPAPVEKS $^{Phospho}_{79.97}$ PAKKKTTKKAGAAKRKATGPPVSEL

- fragmentation table for charge state 3+
- specType=etd
- score=82.33
- F104929.dat
- query=q4054_p1
- precursor=865.272990
- chargePrecursor=5
- itol=0.8

AA	c	y	z+1	z+2	z	AA
S[1]	49.697	**1441.442**	**1436.103**	0.672	**1435.767**	S[42]
E[2]	92.711	**1398.428**	**1393.088**	0.672	**1392.752**	E[41]
T[3]	126.394	**1355.414**	**1350.074**	0.672	**1349.738**	T[40]
A[4]	150.073	1321.731	**1316.392**	0.672	**1316.056**	A[39]
P[5]	182.424	**1298.052**	1292.713	0.672	1292.377	P[38]
A[6]	206.103	**1265.701**	**1260.362**	0.672	**1260.026**	A[37]
E[7]	249.117	1242.022	1236.683	0.672	1236.347	E[36]
T[8]	282.800	1199.008	1193.668	0.672	1193.332	T[35]
A[9]	306.479	**1165.325**	1159.986	0.672	1159.650	A[34]
A[10]	330.158	1141.646	**1136.307**	0.672	**1135.971**	A[33]
P[11]	362.508	**1117.967**	1112.628	0.672	1112.292	P[32]
A[12]	386.188	1085.616	1080.277	0.672	1079.941	A[31]
P[13]	418.538	1061.937	**1056.598**	0.672	1056.262	P[30]
V[14]	451.561	1029.586	**1024.247**	0.672	1023.911	V[29]
E[15]	494.575	996.564	**991.224**	0.672	**990.888**	E[28]
K[16]	537.274	953.549	**948.210**	**948.546**	**947.874**	K[27]
S[17]	592.940	910.851	**905.512**	**905.848**	**905.176**	S[26]
P[18]	625.291	855.185	849.845	850.181	849.510	P[25]
A[19]	648.970	822.834	817.495	817.831	817.159	A[24]
K[20]	691.668	799.155	793.816	794.151	793.480	K[23]
K[21]	**734.367**	756.457	751.117	751.453	750.781	K[22]
K[22]	777.065	713.758	708.419	708.755	708.083	K[21]
T[23]	810.747	671.060	665.721	666.057	665.385	T[20]
T[24]	844.430	637.378	632.038	632.374	631.702	T[19]
K[25]	887.128	603.695	598.355	598.691	598.019	K[18]
K[26]	929.827	560.997	555.657	555.993	555.321	K[17]
A[27]	953.506	518.298	512.959	513.295	512.623	A[16]
G[28]	972.513	494.619	489.280	489.616	488.944	G[15]
A[29]	996.192	475.612	470.273	470.609	469.937	A[14]
A[30]	1019.871	451.933	446.594	446.930	446.258	A[13]
K[31]	**1062.569**	428.254	422.915	423.250	422.579	K[12]
R[32]	**1114.603**	385.556	380.216	380.552	379.880	R[11]
K[33]	**1157.301**	333.522	328.183	328.518	327.847	K[10]
A[34]	**1180.980**	290.824	285.484	285.820	285.148	A[9]
T[35]	**1214.663**	267.145	261.805	262.141	261.469	T[8]
G[36]	1233.670	233.462	228.123	228.459	227.787	G[7]
P[37]	**1266.021**	214.455	209.115	209.451	208.779	P[6]
P[38]	**1298.372**	182.104	176.765	177.100	176.429	P[5]
V[39]	**1331.395**	149.753	144.414	144.750	144.078	V[4]
S[40]	**1360.405**	116.730	111.391	111.727	111.055	S[3]
E[41]	**1403.419**	87.720	82.380	82.716	82.044	E[2]
L[42]	1441.114	44.705	39.366	39.702	39.030	L[1]

sp|P43276|H15_MOUSE

[Acetyl]SETAPAETAAPAPVEKS $^{\text{Phospho}}_{79.97}$ PAKKKTTKKAGAAKRKATGPPVSEL

- fragmentation table for charge state 4+
- specType=etd
- score=82.33
- F104929.dat
- query=q4054_p1
- precursor=865.272990
- chargePrecursor=5
- itol=0.8

AA	c	y	z+1	z+2	z	AA
S[1]	37.525	**1081.333**	**1077.329**	0.755	**1077.077**	S[42]
E[2]	69.785	1049.073	**1045.068**	0.755	**1044.816**	E[41]
T[3]	95.047	1016.812	**1012.807**	0.755	1012.556	T[40]
A[4]	112.806	**991.550**	**987.546**	0.755	987.294	A[39]
P[5]	137.070	973.791	969.786	0.755	969.534	P[38]
A[6]	154.829	949.528	945.523	0.755	945.271	A[37]
E[7]	187.090	931.768	927.764	0.755	927.512	E[36]
T[8]	212.351	899.508	895.503	0.755	895.251	T[35]
A[9]	230.111	874.246	870.241	0.755	869.989	A[34]
A[10]	247.870	856.487	852.482	0.755	852.230	A[33]
P[11]	272.133	838.727	834.723	0.755	834.471	P[32]
A[12]	289.892	814.464	810.459	0.755	810.208	A[31]
P[13]	314.156	796.705	792.700	0.755	792.448	P[30]
V[14]	338.923	772.442	768.437	0.755	768.185	V[29]
E[15]	371.183	747.675	743.670	0.755	743.418	E[28]
K[16]	403.207	715.414	711.409	711.661	711.157	K[27]
S[17]	444.957	683.390	679.386	679.637	679.134	S[26]
P[18]	469.220	641.641	637.636	637.888	637.384	P[25]
A[19]	486.979	617.377	613.373	613.625	613.121	A[24]
K[20]	519.003	599.618	595.613	595.865	595.362	K[23]
K[21]	551.027	567.594	563.590	563.842	563.338	K[22]
K[22]	583.050	535.571	531.566	531.818	531.314	K[21]
T[23]	608.312	503.547	499.542	499.794	499.290	T[20]
T[24]	**633.574**	478.285	474.280	474.532	474.028	T[19]
K[25]	665.598	453.023	449.018	449.270	448.766	K[18]
K[26]	697.622	420.999	416.995	417.247	416.743	K[17]
A[27]	715.381	388.976	384.971	385.223	384.719	A[16]
G[28]	729.636	371.216	367.212	367.464	366.960	G[15]
A[29]	747.396	356.961	352.956	353.208	352.704	A[14]
A[30]	765.155	339.202	335.197	335.449	334.945	A[13]
K[31]	797.179	321.442	317.438	317.690	317.186	K[12]
R[32]	836.204	289.419	285.414	285.666	285.162	R[11]
K[33]	868.228	250.393	246.389	246.641	246.137	K[10]
A[34]	885.987	218.370	214.365	214.617	214.113	A[9]
T[35]	911.249	200.610	196.606	196.858	196.354	T[8]
G[36]	925.504	175.348	171.344	171.596	171.092	G[7]
P[37]	949.767	161.093	157.088	157.340	156.836	P[6]
P[38]	974.031	136.830	132.825	133.077	132.573	P[5]
V[39]	**998.798**	112.567	108.562	108.814	108.310	V[4]
S[40]	**1020.556**	87.800	83.795	84.047	83.543	S[3]
E[41]	**1052.816**	66.042	62.037	62.289	61.785	E[2]
L[42]	**1081.087**	33.781	29.776	30.028	29.524	L[1]

sp|P43275|H11_MOUSE

[Acetyl]SETAPVAQAASTATEKPAAAKKTKKPAKAAAPRKKPAGPSVSEL

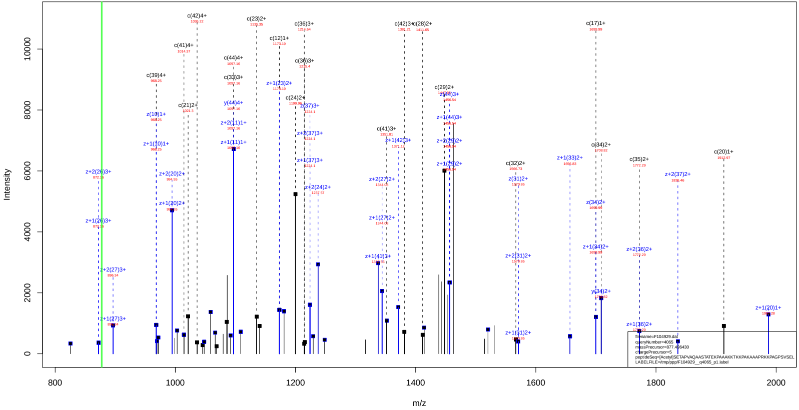

sp|P43275|H11_MOUSE

[Acetyl]SETAPVAQAASTATEKPAAAKKTKKPAKAAAPRKKPAGPSVSEL

- fragmentation table for charge state 1+
- specType=etd
- score=54.14
- F104929.dat
- query=q4065_p1
- precursor=877.496430
- chargePrecursor=5
- itol=0.8

AA	c	y	z+1	z+2	z	AA
S[1]	147.076	4383.436	4367.417	0.000	4366.409	S[44]
E[2]	276.119	4254.393	4238.375	0.000	4237.367	E[43]
T[3]	377.167	4125.351	4109.332	0.000	4108.324	T[42]
A[4]	448.204	4024.303	4008.284	0.000	4007.276	A[41]
P[5]	545.257	3953.266	3937.247	0.000	3936.239	P[40]
V[6]	644.325	3856.213	3840.194	0.000	3839.187	V[39]
A[7]	715.362	3757.145	3741.126	0.000	3740.118	A[38]
Q[8]	843.421	3686.108	3670.089	3671.097	3669.081	Q[37]
A[9]	914.458	3558.049	3542.030	3543.038	3541.022	A[36]
A[10]	985.495	3487.012	3470.993	3472.001	3469.985	A[35]
S[11]	1072.527	3415.975	3399.956	3400.964	3398.948	S[34]
T[12]	**1173.575**	3328.943	3312.924	3313.932	3311.916	T[33]
A[13]	1244.612	3227.895	3211.876	3212.884	3210.868	A[32]
T[14]	1345.659	3156.858	3140.839	3141.847	3139.831	T[31]
E[15]	1474.702	3055.810	3039.792	3040.799	3038.784	E[30]
K[16]	1602.797	2926.768	2910.749	2911.757	2909.741	K[29]
P[17]	**1699.850**	2798.673	2782.654	2783.662	2781.646	P[28]
A[18]	1770.887	2701.620	2685.601	2686.609	2684.593	A[27]
A[19]	1841.924	2630.583	2614.564	2615.572	2613.556	A[26]
A[20]	**1912.961**	2559.546	2543.527	2544.535	2542.519	A[25]
K[21]	2041.056	2488.509	2472.490	2473.498	2471.482	K[24]
K[22]	2169.151	2360.414	2344.395	2345.403	2343.387	K[23]
T[23]	2270.199	2232.319	2216.300	2217.308	2215.292	T[22]
K[24]	2398.294	2131.271	2115.252	2116.260	2114.244	K[21]
K[25]	2526.389	2003.176	**1987.157**	1988.165	1986.149	K[20]
P[26]	2623.441	1875.081	1859.062	1860.070	1858.055	P[19]
A[27]	2694.478	1778.028	1762.010	1763.017	1761.002	A[18]
K[28]	2822.573	1706.991	1690.972	1691.980	1689.965	K[17]
A[29]	2893.611	1578.896	1562.877	1563.885	1561.870	A[16]
A[30]	2964.648	1507.859	1491.840	1492.848	1490.833	A[15]
A[31]	3035.685	1436.822	1420.803	1421.811	1419.795	A[14]
P[32]	3132.738	1365.785	1349.766	1350.774	1348.758	P[13]
R[33]	3288.839	1268.732	1252.713	1253.721	1251.706	R[12]
K[34]	3416.934	1112.631	**1096.612**	**1097.620**	1095.604	K[11]
K[35]	3545.029	984.536	**968.517**	**969.525**	**967.509**	K[10]
P[36]	3642.081	856.441	840.422	841.430	839.415	P[9]
A[37]	3713.118	759.388	743.370	744.377	742.362	A[8]
G[38]	3770.140	688.351	672.332	673.340	671.325	G[7]
P[39]	3867.193	631.330	615.311	616.319	614.303	P[6]
S[40]	3954.225	534.277	518.258	519.266	517.250	S[5]
V[41]	4053.293	447.245	431.226	432.234	430.218	V[4]
S[42]	4140.325	348.177	332.158	333.166	331.150	S[3]
E[43]	4269.368	261.144	245.126	246.134	244.118	E[2]
L[44]	4382.452	132.102	116.083	117.091	115.075	L[1]

sp|P43275|H11_MOUSE

[Acetyl]SETAPVAQAASTATEKPAAAKKTKKPAKAAAPRKKPAGPSVSEL

- fragmentation table for charge state 2+
- specType=etd
- score=54.14
- F104929.dat
- query=q4065_p1
- precursor=877.496430
- chargePrecursor=5
- itol=0.8

AA	c	y	z+1	z+2	z	AA
S[1]	74.042	2192.222	2184.212	0.504	2183.708	S[44]
E[2]	138.563	2127.700	2119.691	0.504	2119.187	E[43]
T[3]	189.087	2063.179	2055.170	0.504	2054.666	T[42]
A[4]	224.606	2012.655	2004.646	0.504	2004.142	A[41]
P[5]	273.132	1977.137	1969.127	0.504	1968.623	P[40]
V[6]	322.666	1928.610	1920.601	0.504	1920.097	V[39]
A[7]	358.185	1879.076	1871.067	0.504	1870.563	A[38]
Q[8]	422.214	1843.557	1835.548	**1836.052**	1835.044	Q[37]
A[9]	457.733	1779.528	**1771.519**	**1772.023**	1771.015	A[36]
A[10]	493.251	1744.010	1736.000	1736.504	1735.496	A[35]
S[11]	536.767	**1708.491**	**1700.482**	1700.986	**1699.978**	S[34]
T[12]	587.291	1664.975	**1656.966**	**1657.470**	**1656.462**	T[33]
A[13]	622.809	1614.451	1606.442	1606.946	1605.938	A[32]
T[14]	673.333	1578.933	**1570.923**	**1571.427**	**1570.419**	T[31]
E[15]	737.855	1528.409	**1520.399**	**1520.903**	**1519.895**	E[30]
K[16]	801.902	1463.887	**1455.878**	**1456.382**	1455.374	K[29]
P[17]	850.428	1399.840	1391.831	1392.335	1391.327	P[28]
A[18]	885.947	**1351.314**	**1343.304**	**1343.808**	1342.800	A[27]
A[19]	921.466	1315.795	1307.786	1308.290	1307.282	A[26]
A[20]	956.984	1280.276	1272.267	1272.771	1271.763	A[25]
K[21]	**1021.032**	1244.758	1236.749	**1237.252**	1236.245	K[24]
K[22]	**1085.079**	**1180.710**	**1172.701**	**1173.205**	1172.197	K[23]
T[23]	**1135.603**	1116.663	**1108.654**	**1109.158**	**1108.150**	T[22]
K[24]	**1199.650**	**1066.139**	**1058.130**	**1058.634**	1057.626	K[21]
K[25]	1263.698	1002.092	**994.082**	**994.586**	993.578	K[20]
P[26]	1312.224	938.044	930.035	930.539	929.531	P[19]
A[27]	1347.743	889.518	881.508	882.012	881.005	A[18]
K[28]	**1411.790**	853.999	845.990	846.494	845.486	K[17]
A[29]	**1447.309**	789.952	781.942	782.446	781.438	A[16]
A[30]	1482.827	754.433	746.424	746.928	745.920	A[15]
A[31]	1518.346	718.915	710.905	711.409	710.401	A[14]
P[32]	**1566.872**	683.396	675.387	675.891	674.883	P[13]
R[33]	1644.923	634.870	626.860	627.364	626.356	R[12]
K[34]	**1708.970**	556.819	548.810	549.314	548.306	K[11]
K[35]	**1773.018**	492.772	484.762	485.266	484.258	K[10]
P[36]	1821.544	428.724	420.715	421.219	420.211	P[9]
A[37]	1857.063	380.198	372.188	372.692	371.685	A[8]
G[38]	1885.574	344.679	336.670	337.174	336.166	G[7]
P[39]	1934.100	316.169	308.159	308.663	307.655	P[6]
S[40]	1977.616	267.642	259.633	260.137	259.129	S[5]
V[41]	2027.150	224.126	216.117	216.621	215.613	V[4]
S[42]	2070.666	174.592	166.583	167.086	166.079	S[3]
E[43]	2135.188	131.076	123.067	123.570	122.563	E[2]
L[44]	2191.730	66.555	58.545	59.049	58.041	L[1]

sp|P43275|H11_MOUSE

[Acetyl]SETAPVAQAASTATEKPAAAKKTKKPAKAAAPRKKPAGPSVSEL

- fragmentation table for charge state 3+
- specType=etd
- score=54.14
- F104929.dat
- query=q4065_p1
- precursor=877.496430
- chargePrecursor=5
- itol=0.8

AA	c	y	z+1	z+2	z	AA
S[1]	49.697	1461.817	**1456.477**	0.672	**1456.141**	S[44]
E[2]	92.711	1418.803	**1413.463**	0.672	1413.127	E[43]
T[3]	126.394	1375.788	**1370.449**	0.672	1370.113	T[42]
A[4]	150.073	1342.106	**1336.766**	0.672	1336.430	A[41]
P[5]	182.424	1318.427	1313.087	0.672	1312.751	P[40]
V[6]	215.447	1286.076	1280.736	0.672	1280.400	V[39]
A[7]	239.126	1253.053	**1247.713**	0.672	1247.378	A[38]
Q[8]	281.812	**1229.374**	**1224.034**	**1224.370**	**1223.699**	Q[37]
A[9]	305.491	1186.688	**1181.348**	**1181.684**	**1181.012**	A[36]
A[10]	329.170	1163.009	1157.669	1158.005	1157.333	A[35]
S[11]	358.180	1139.330	1133.990	1134.326	1133.654	S[34]
T[12]	391.863	1110.319	1104.980	1105.315	1104.644	T[33]
A[13]	415.542	1076.637	1071.297	1071.633	1070.961	A[32]
T[14]	449.225	1052.957	**1047.618**	**1047.954**	**1047.282**	T[31]
E[15]	492.239	1019.275	**1013.935**	**1014.271**	**1013.599**	E[30]
K[16]	534.937	976.261	**970.921**	**971.257**	970.585	K[29]
P[17]	567.288	933.562	928.223	928.559	927.887	P[28]
A[18]	590.967	901.211	**895.872**	**896.208**	895.536	A[27]
A[19]	614.646	877.532	**872.193**	**872.529**	**871.857**	A[26]
A[20]	638.325	853.853	848.514	848.850	848.178	A[25]
K[21]	681.024	830.174	**824.835**	**825.171**	824.499	K[24]
K[22]	723.722	787.476	782.136	782.472	781.801	K[23]
T[23]	757.404	744.778	739.438	739.774	739.102	T[22]
K[24]	800.103	711.095	705.756	706.092	705.420	K[21]
K[25]	842.801	668.397	663.057	663.393	662.721	K[20]
P[26]	875.152	625.699	620.359	620.695	620.023	P[19]
A[27]	898.831	593.348	588.008	588.344	587.672	A[18]
K[28]	941.529	569.669	564.329	564.665	563.993	K[17]
A[29]	965.208	526.970	521.631	521.967	521.295	A[16]
A[30]	988.887	503.291	497.952	498.288	497.616	A[15]
A[31]	1012.566	479.612	474.273	474.609	473.937	A[14]
P[32]	**1044.917**	455.933	450.594	450.930	450.258	P[13]
R[33]	**1096.951**	423.582	418.243	418.579	417.907	R[12]
K[34]	**1139.649**	371.549	366.209	366.545	365.873	K[11]
K[35]	1182.348	328.850	323.511	323.847	323.175	K[10]
P[36]	**1214.699**	286.152	280.812	281.148	280.476	P[9]
A[37]	1238.378	253.801	248.461	248.797	248.125	A[8]
G[38]	1257.385	230.122	224.782	225.118	224.446	G[7]
P[39]	1289.736	211.115	205.775	206.111	205.439	P[6]
S[40]	1318.746	178.764	173.424	173.760	173.088	S[5]
V[41]	**1351.769**	149.753	144.414	144.750	144.078	V[4]
S[42]	**1380.780**	116.730	111.391	111.727	111.055	S[3]
E[43]	1423.794	87.720	82.380	82.716	82.044	E[2]
L[44]	1461.489	44.705	39.366	39.702	39.030	L[1]

sp|P43275|H11_MOUSE

[Acetyl]SETAPVAQAASTATEKPAAAKKTKKPAKAAAPRKKPAGPSVSEL

- fragmentation table for charge state 4+
- specType=etd
- score=54.14
- F104929.dat
- query=q4065_p1
- precursor=877.496430
- chargePrecursor=5
- itol=0.8

AA	c	y	z+1	z+2	z	AA
S[1]	37.525	**1096.614**	**1092.610**	0.755	**1092.358**	S[44]
E[2]	69.785	1064.354	1060.349	0.755	1060.097	E[43]
T[3]	95.047	1032.093	1028.088	0.755	1027.836	T[42]
A[4]	112.806	1006.831	**1002.827**	0.755	**1002.575**	A[41]
P[5]	137.070	989.072	985.067	0.755	984.815	P[40]
V[6]	161.837	964.809	960.804	0.755	960.552	V[39]
A[7]	179.596	940.042	936.037	0.755	935.785	A[38]
Q[8]	211.611	922.282	918.278	918.530	918.026	Q[37]
A[9]	229.370	890.268	886.263	886.515	886.011	A[36]
A[10]	247.129	**872.508**	868.504	868.756	868.252	A[35]
S[11]	268.887	854.749	850.744	850.996	850.493	S[34]
T[12]	294.149	832.991	828.986	829.238	828.735	T[33]
A[13]	311.908	807.729	803.725	803.976	803.473	A[32]
T[14]	337.170	789.970	785.965	786.217	785.713	T[31]
E[15]	369.431	764.708	760.703	760.955	760.451	E[30]
K[16]	401.455	732.447	728.443	728.695	728.191	K[29]
P[17]	425.718	700.424	696.419	696.671	696.167	P[28]
A[18]	443.477	676.160	672.156	672.408	671.904	A[27]
A[19]	461.236	658.401	654.396	654.648	654.145	A[26]
A[20]	478.996	640.642	636.637	636.889	636.385	A[25]
K[21]	511.019	622.883	618.878	619.130	618.626	K[24]
K[22]	543.043	590.859	586.854	587.106	586.602	K[23]
T[23]	568.305	558.835	554.830	555.082	554.578	T[22]
K[24]	600.329	533.573	529.569	529.820	529.317	K[21]
K[25]	632.353	501.549	497.545	497.797	497.293	K[20]
P[26]	656.616	469.526	465.521	465.773	465.269	P[19]
A[27]	674.375	445.263	441.258	441.510	441.006	A[18]
K[28]	706.399	427.503	423.499	423.751	423.247	K[17]
A[29]	724.158	395.480	391.475	391.727	391.223	A[16]
A[30]	741.917	377.720	373.716	373.968	373.464	A[15]
A[31]	759.677	359.961	355.956	356.208	355.704	A[14]
P[32]	783.940	342.202	338.197	338.449	337.945	P[13]
R[33]	822.965	317.938	313.934	314.186	313.682	R[12]
K[34]	854.989	278.913	274.909	275.160	274.657	K[11]
K[35]	887.013	246.889	242.885	243.137	242.633	K[10]
P[36]	911.276	214.866	210.861	211.113	210.609	P[9]
A[37]	929.035	190.603	186.598	186.850	186.346	A[8]
G[38]	943.290	172.843	168.839	169.091	168.587	G[7]
P[39]	**967.554**	158.588	154.583	154.835	154.331	P[6]
S[40]	989.312	134.325	130.320	130.572	130.068	S[5]
V[41]	**1014.079**	112.567	108.562	108.814	108.310	V[4]
S[42]	**1035.837**	87.800	83.795	84.047	83.543	S[3]
E[43]	**1068.097**	66.042	62.037	62.289	61.785	E[2]
L[44]	**1096.368**	33.781	29.776	30.028	29.524	L[1]

sp|P15864|H12_MOUSE

[Acetyl]SEAAPAAPAAAPPAEKAPAKKKAAKKPAGVRRKASGPPVSEL

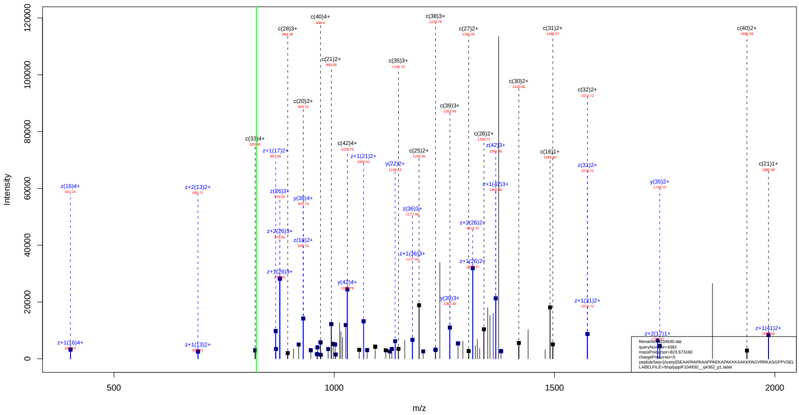

sp|P15864|H12_MOUSE

[Acetyl]SEAAPAAPAAAPPAEKAPAKKAAKKPAGVRRKASGPPVSEL

- fragmentation table for charge state 1+
- specType=etd
- score=35.51
- F104930.dat
- query=q4362_p1
- precursor=823.673160
- chargePrecursor=5
- itol=0.8

AA	c	y	z+1	z+2	z	AA
S[1]	147.076	4114.325	4098.306	0.000	4097.298	S[42]
E[2]	276.119	3985.282	3969.263	0.000	3968.256	E[41]
A[3]	347.156	3856.240	3840.221	0.000	3839.213	A[40]
A[4]	418.193	3785.202	3769.184	0.000	3768.176	A[39]
P[5]	515.246	3714.165	3698.147	0.000	3697.139	P[38]
A[6]	586.283	3617.113	3601.094	0.000	3600.086	A[37]
A[7]	657.320	3546.075	3530.057	0.000	3529.049	A[36]
P[8]	754.373	3475.038	3459.020	0.000	3458.012	P[35]
A[9]	825.410	3377.986	3361.967	0.000	3360.959	A[34]
A[10]	896.447	3306.948	3290.930	0.000	3289.922	A[33]
A[11]	967.484	3235.911	3219.893	0.000	3218.885	A[32]
P[12]	1064.537	3164.874	3148.856	0.000	3147.848	P[31]
P[13]	1161.590	3067.821	3051.803	0.000	3050.795	P[30]
A[14]	1232.627	2970.769	2954.750	0.000	2953.742	A[29]
E[15]	1361.670	2899.732	2883.713	0.000	2882.705	E[28]
K[16]	**1489.765**	2770.689	2754.670	2755.678	2753.662	K[27]
A[17]	1560.802	2642.594	2626.575	2627.583	2625.568	A[26]
P[18]	1657.854	2571.557	2555.538	2556.546	2554.530	P[25]
A[19]	1728.892	2474.504	2458.485	2459.493	2457.478	A[24]
K[20]	1856.986	2403.467	2387.448	2388.456	2386.441	K[23]
K[21]	**1985.081**	2275.372	2259.353	2260.361	2258.346	K[22]
K[22]	2113.176	2147.277	2131.258	2132.266	2130.251	K[21]
A[23]	2184.214	2019.182	2003.163	2004.171	2002.156	A[20]
A[24]	2255.251	1948.145	1932.126	1933.134	1931.119	A[19]
K[25]	2383.346	1877.108	1861.089	1862.097	1860.081	K[18]
K[26]	2511.441	1749.013	1732.994	**1734.002**	1731.986	K[17]
P[27]	2608.493	1620.918	1604.899	1605.907	1603.891	P[16]
A[28]	2679.530	1523.865	1507.847	1508.854	1506.839	A[15]
G[29]	2736.552	1452.828	1436.809	1437.817	1435.802	G[14]
V[30]	2835.620	1395.807	1379.788	1380.796	**1378.780**	V[13]
R[31]	2991.721	1296.738	**1280.720**	1281.727	1279.712	R[12]
R[32]	3147.823	1140.637	1124.618	**1125.626**	1123.611	R[11]
K[33]	3275.918	984.536	**968.517**	**969.525**	967.509	K[10]
A[34]	3346.955	856.441	840.422	841.430	839.415	A[9]
S[35]	3433.987	785.404	769.385	770.393	768.377	S[8]
G[36]	3491.008	698.372	682.353	683.361	681.345	G[7]
P[37]	3588.061	641.350	625.332	626.340	624.324	P[6]
P[38]	3685.114	544.298	528.279	529.287	527.271	P[5]
V[39]	3784.182	447.245	431.226	432.234	430.218	V[4]
S[40]	3871.214	348.177	332.158	333.166	331.150	S[3]
E[41]	4000.257	261.144	245.126	246.134	244.118	E[2]
L[42]	4113.341	132.102	116.083	117.091	115.075	L[1]

sp|P15864|H12_MOUSE

[Acetyl]SEAAPAAPAAAPPAEKAPAKKKAAKKPAGVRRKASGPPVSEL

- fragmentation table for charge state 2+
- specType=etd
- score=35.51
- F104930.dat
- query=q4362_p1
- precursor=823.673160
- chargePrecursor=5
- itol=0.8

AA	c	y	z+1	z+2	z	AA
S[1]	74.042	2057.666	2049.657	0.504	2049.153	S[42]
E[2]	138.563	1993.145	**1985.135**	0.504	1984.631	E[41]
A[3]	174.082	1928.623	1920.614	0.504	1920.110	A[40]
A[4]	209.600	1893.105	1885.096	0.504	1884.592	A[39]
P[5]	258.127	1857.586	1849.577	0.504	1849.073	P[38]
A[6]	293.645	1809.060	1801.051	0.504	1800.547	A[37]
A[7]	329.164	1773.541	1765.532	0.504	1765.028	A[36]
P[8]	377.690	**1738.023**	1730.013	0.504	1729.510	P[35]
A[9]	413.209	1689.496	1681.487	0.504	1680.983	A[34]
A[10]	448.727	1653.978	1645.969	0.504	1645.465	A[33]
A[11]	484.246	1618.459	1610.450	0.504	1609.946	A[32]
P[12]	532.772	1582.941	**1574.931**	0.504	**1574.427**	P[31]
P[13]	581.299	1534.414	1526.405	0.504	1525.901	P[30]
A[14]	616.817	1485.888	1477.879	0.504	1477.375	A[29]
E[15]	681.338	1450.369	1442.360	0.504	1441.856	E[28]
K[16]	745.386	1385.848	**1377.839**	**1378.343**	1377.335	K[27]
A[17]	780.904	1321.801	**1313.791**	**1314.295**	1313.287	A[26]
P[18]	829.431	1286.282	1278.273	1278.777	1277.769	P[25]
A[19]	864.949	1237.756	**1229.746**	**1230.250**	**1229.242**	A[24]
K[20]	**928.997**	**1202.237**	1194.228	1194.732	1193.724	K[23]
K[21]	**993.044**	**1138.190**	**1130.180**	**1130.684**	1129.676	K[22]
K[22]	**1057.092**	**1074.142**	**1066.133**	**1066.637**	1065.629	K[21]
A[23]	**1092.610**	1010.095	**1002.085**	**1002.589**	**1001.581**	A[20]
A[24]	1128.129	974.576	966.567	967.071	966.063	A[19]
K[25]	**1192.176**	939.058	931.048	931.552	**930.544**	K[18]
K[26]	1256.224	875.010	**867.001**	**867.505**	**866.497**	K[17]
P[27]	**1304.750**	810.963	802.953	803.457	802.449	P[16]
A[28]	**1340.269**	762.436	754.427	754.931	753.923	A[15]
G[29]	1368.780	726.918	718.908	719.412	718.404	G[14]
V[30]	**1418.314**	698.407	**690.398**	**690.902**	689.894	V[13]
R[31]	**1496.364**	648.873	640.863	641.367	640.359	R[12]
R[32]	**1574.415**	570.822	562.813	563.317	562.309	R[11]
K[33]	1638.462	492.772	484.762	485.266	484.258	K[10]
A[34]	1673.981	428.724	420.715	421.219	420.211	A[9]
S[35]	1717.497	393.206	385.196	385.700	384.692	S[8]
G[36]	1746.008	349.690	341.680	342.184	341.176	G[7]
P[37]	1794.534	321.179	313.170	313.673	312.666	P[6]
P[38]	1843.060	272.652	264.643	265.147	264.139	P[5]
V[39]	1892.595	224.126	216.117	216.621	215.613	V[4]
S[40]	**1936.111**	174.592	166.583	167.086	166.079	S[3]
E[41]	2000.632	131.076	123.067	123.570	122.563	E[2]
L[42]	2057.174	66.555	58.545	59.049	58.041	L[1]

sp|P15864|H12_MOUSE

[Acetyl]SEAAPAAPAAAPPAEKAPAKKKAAKKPAGVRRKASGPPVSEL

- fragmentation table for charge state 3+
- specType=etd
- score=35.51
- F104930.dat
- query=q4362_p1
- precursor=823.673160
- chargePrecursor=5
- itol=0.8

AA	c	y	z+1	z+2	z	AA
S[1]	49.697	1372.113	**1366.774**	0.672	**1366.438**	S[42]
E[2]	92.711	1329.099	1323.759	0.672	1323.423	E[41]
A[3]	116.390	1286.085	**1280.745**	0.672	**1280.409**	A[40]
A[4]	140.069	**1262.406**	1257.066	0.672	1256.730	A[39]
P[5]	172.420	1238.727	1233.387	0.672	1233.051	P[38]
A[6]	196.099	1206.376	1201.036	0.672	1200.700	A[37]
A[7]	219.778	1182.697	**1177.357**	0.672	**1177.021**	A[36]
P[8]	252.129	1159.018	1153.678	0.672	1153.342	P[35]
A[9]	275.808	1126.667	1121.327	0.672	1120.991	A[34]
A[10]	299.487	1102.988	1097.648	0.672	1097.312	A[33]
A[11]	323.166	1079.309	**1073.969**	0.672	1073.633	A[32]
P[12]	355.517	1055.630	1050.290	0.672	1049.954	P[31]
P[13]	387.868	1023.279	1017.939	0.672	1017.603	P[30]
A[14]	411.547	990.928	**985.588**	0.672	985.252	A[29]
E[15]	454.561	967.249	**961.909**	0.672	**961.573**	E[28]
K[16]	497.260	924.235	**918.895**	**919.231**	**918.559**	K[27]
A[17]	520.939	881.536	**876.197**	**876.533**	**875.861**	A[26]
P[18]	553.290	857.857	852.518	852.854	852.182	P[25]
A[19]	576.969	825.506	**820.167**	**820.503**	**819.831**	A[24]
K[20]	619.667	801.827	796.488	796.824	796.152	K[23]
K[21]	662.365	759.129	753.789	754.125	753.453	K[22]
K[22]	705.064	716.431	711.091	711.427	710.755	K[21]
A[23]	728.743	673.732	668.393	668.729	668.057	A[20]
A[24]	752.422	650.053	644.714	645.050	644.378	A[19]
K[25]	795.120	626.374	621.035	621.371	620.699	K[18]
K[26]	837.818	583.676	578.336	578.672	578.000	K[17]
P[27]	870.169	540.978	535.638	535.974	535.302	P[16]
A[28]	**893.848**	508.627	503.287	503.623	502.951	A[15]
G[29]	912.855	484.948	479.608	479.944	479.272	G[14]
V[30]	945.878	465.940	460.601	460.937	460.265	V[13]
R[31]	**997.912**	432.918	427.578	427.914	427.242	R[12]
R[32]	1049.946	380.884	375.544	375.880	375.208	R[11]
K[33]	**1092.644**	328.850	323.511	323.847	323.175	K[10]
A[34]	**1116.323**	286.152	280.812	281.148	280.476	A[9]
S[35]	**1145.334**	262.473	257.133	257.469	256.797	S[8]
G[36]	1164.341	233.462	228.123	228.459	227.787	G[7]
P[37]	1196.692	214.455	209.115	209.451	208.779	P[6]
P[38]	**1229.043**	182.104	176.765	177.100	176.429	P[5]
V[39]	**1262.066**	149.753	144.414	144.750	144.078	V[4]
S[40]	1291.076	116.730	111.391	111.727	111.055	S[3]
E[41]	1334.090	87.720	82.380	82.716	82.044	E[2]
L[42]	1371.785	44.705	39.366	39.702	39.030	L[1]

sp|P15864|H12_MOUSE

[Acetyl]SEAAPAAPAAAPPAEKAPAKKKAAKKPAGVRRKASGPPVSEL

- fragmentation table for charge state 4+
- specType=etd
- score=35.51
- F104930.dat
- query=q4362_p1
- precursor=823.673160
- chargePrecursor=5
- itol=0.8

AA	c	y	z+1	z+2	z	AA
S[1]	37.525	**1029.337**	**1025.332**	0.755	1025.080	S[42]
E[2]	69.785	**997.076**	**993.071**	0.755	**992.819**	E[41]
A[3]	87.544	964.815	**960.811**	0.755	**960.559**	A[40]
A[4]	105.304	**947.056**	943.051	0.755	942.799	A[39]
P[5]	129.567	**929.297**	925.292	0.755	925.040	P[38]
A[6]	147.326	905.034	901.029	0.755	900.777	A[37]
A[7]	165.086	887.274	883.270	0.755	883.018	A[36]
P[8]	189.349	869.515	865.510	0.755	865.258	P[35]
A[9]	207.108	845.252	841.247	0.755	840.995	A[34]
A[10]	224.867	827.493	823.488	0.755	823.236	A[33]
A[11]	242.627	809.733	805.729	0.755	805.477	A[32]
P[12]	266.890	791.974	787.969	0.755	787.717	P[31]
P[13]	291.153	767.711	763.706	0.755	763.454	P[30]
A[14]	308.912	743.448	739.443	0.755	739.191	A[29]
E[15]	341.173	725.688	721.684	0.755	721.432	E[28]
K[16]	373.197	693.428	689.423	689.675	689.171	K[27]
A[17]	390.956	661.404	657.399	657.651	657.147	A[26]
P[18]	415.219	643.645	639.640	639.892	639.388	P[25]
A[19]	432.978	619.381	615.377	615.629	615.125	A[24]
K[20]	465.002	601.622	597.618	597.869	597.366	K[23]
K[21]	497.026	569.598	565.594	565.846	565.342	K[22]
K[22]	529.050	537.575	533.570	533.822	533.318	K[21]
A[23]	546.809	505.551	501.546	501.798	501.294	A[20]
A[24]	564.568	487.792	483.787	484.039	483.535	A[19]
K[25]	596.592	470.032	466.028	466.280	465.776	K[18]
K[26]	628.616	438.009	434.004	434.256	433.752	K[17]
P[27]	652.879	405.985	**401.980**	402.232	**401.728**	P[16]
A[28]	670.638	381.722	377.717	377.969	377.465	A[15]
G[29]	684.893	363.962	359.958	360.210	359.706	G[14]
V[30]	709.661	349.707	345.702	345.954	345.450	V[13]
R[31]	748.686	324.940	320.935	321.187	320.683	R[12]
R[32]	787.711	285.915	281.910	282.162	281.658	R[11]
K[33]	**819.735**	246.889	242.885	243.137	242.633	K[10]
A[34]	837.494	214.866	210.861	211.113	210.609	A[9]
S[35]	859.252	197.106	193.102	193.354	192.850	S[8]
G[36]	873.507	175.348	171.344	171.596	171.092	G[7]
P[37]	897.771	161.093	157.088	157.340	156.836	P[6]
P[38]	922.034	136.830	132.825	133.077	132.573	P[5]
V[39]	**946.801**	112.567	108.562	108.814	108.310	V[4]
S[40]	**968.559**	87.800	83.795	84.047	83.543	S[3]
E[41]	1000.820	66.042	62.037	62.289	61.785	E[2]
L[42]	**1029.091**	33.781	29.776	30.028	29.524	L[1]

sp|P43277|H13_MOUSE

[Acetyl]SETAPAAPAAPAPVEKTPVKKKAKKTGAAAGKRKASGPPVSEL

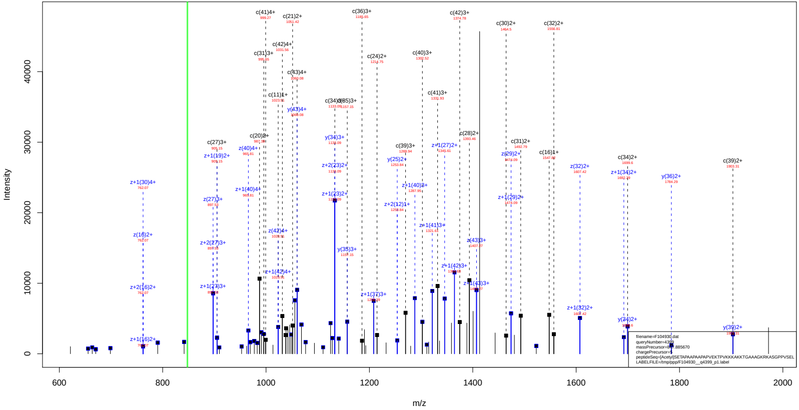

sp|P43277|H13_MOUSE

[Acetyl]SETAPAAPAAPAPVEKTPVKKKAKKTGAAAGKRKASGPPVSEL

- fragmentation table for charge state 1+
- specType=etd
- score=50.44
- F104930.dat
- query=q4399_p1
- precursor=847.885670
- chargePrecursor=5
- itol=0.8

AA	c	y	z+1	z+2	z	AA
S[1]	147.076	4235.387	4219.369	0.000	4218.361	S[43]
E[2]	276.119	4106.345	4090.326	0.000	4089.318	E[42]
T[3]	377.167	3977.302	3961.284	0.000	3960.276	T[41]
A[4]	448.204	3876.255	3860.236	0.000	3859.228	A[40]
P[5]	545.257	3805.217	3789.199	0.000	3788.191	P[39]
A[6]	616.294	3708.165	3692.146	0.000	3691.138	A[38]
A[7]	687.331	3637.128	3621.109	0.000	3620.101	A[37]
P[8]	784.384	3566.090	3550.072	0.000	3549.064	P[36]
A[9]	855.421	3469.038	3453.019	0.000	3452.011	A[35]
A[10]	926.458	3398.001	3381.982	0.000	3380.974	A[34]
P[11]	**1023.511**	3326.963	3310.945	0.000	3309.937	P[33]
A[12]	1094.548	3229.911	3213.892	0.000	3212.884	A[32]
P[13]	1191.600	3158.874	3142.855	0.000	3141.847	P[31]
V[14]	1290.669	3061.821	3045.802	0.000	3044.794	V[30]
E[15]	1419.711	2962.752	2946.734	0.000	2945.726	E[29]
K[16]	**1547.806**	2833.710	2817.691	2818.699	2816.683	K[28]
T[17]	1648.854	2705.615	2689.596	2690.604	2688.588	T[27]
P[18]	1745.907	2604.567	2588.548	2589.556	2587.541	P[26]
V[19]	1844.975	2507.514	2491.496	2492.504	2490.488	V[25]
K[20]	1973.070	2408.446	2392.427	2393.435	2391.419	K[24]
K[21]	2101.165	2280.351	2264.332	2265.340	2263.324	K[23]
K[22]	2229.260	2152.256	2136.237	2137.245	2135.230	K[22]
A[23]	2300.297	2024.161	2008.142	2009.150	2007.135	A[21]
K[24]	2428.392	1953.124	1937.105	1938.113	1936.097	K[20]
K[25]	2556.487	1825.029	1809.010	1810.018	1808.002	K[19]
T[26]	2657.535	1696.934	1680.915	1681.923	1679.908	T[18]
G[27]	2714.556	1595.886	1579.868	1580.875	1578.860	G[17]
A[28]	2785.593	1538.865	**1522.846**	1523.854	1521.838	A[16]
A[29]	2856.631	1467.828	1451.809	1452.817	1450.801	A[15]
A[30]	2927.668	1396.791	1380.772	1381.780	1379.764	A[14]
G[31]	2984.689	1325.754	1309.735	**1310.743**	1308.727	G[13]
K[32]	3112.784	1268.732	1252.713	**1253.721**	1251.706	K[12]
R[33]	3268.885	**1140.637**	**1124.618**	1125.626	1123.611	R[11]
K[34]	3396.980	984.536	968.517	**969.525**	967.509	K[10]
A[35]	3468.017	856.441	840.422	**841.430**	839.415	A[9]
S[36]	3555.049	785.404	769.385	770.393	768.377	S[8]
G[37]	3612.071	**698.372**	682.353	683.361	681.345	G[7]
P[38]	3709.124	641.350	625.332	626.340	624.324	P[6]
P[39]	3806.176	544.298	528.279	529.287	527.271	P[5]
V[40]	3905.245	447.245	431.226	432.234	430.218	V[4]
S[41]	3992.277	348.177	332.158	333.166	331.150	S[3]
E[42]	4121.319	261.144	245.126	246.134	244.118	E[2]
L[43]	4234.403	132.102	116.083	117.091	115.075	L[1]

sp|P43277|H13_MOUSE

[Acetyl]SETAPAAPAAPAPVEKTPVKKAKKTGAAAGKRKASGPPVSEL

- fragmentation table for charge state 2+
- specType=etd
- score=50.44
- F104930.dat
- query=q4399_p1
- precursor=847.885670
- chargePrecursor=5
- itol=0.8

AA	c	y	z+1	z+2	z	AA
S[1]	74.042	2118.197	2110.188	0.504	2109.684	S[43]
E[2]	138.563	2053.676	2045.667	0.504	2045.163	E[42]
T[3]	189.087	1989.155	1981.145	0.504	1980.641	T[41]
A[4]	224.606	1938.631	1930.622	0.504	1930.118	A[40]
P[5]	273.132	**1903.112**	1895.103	0.504	1894.599	P[39]
A[6]	308.650	1854.586	1846.577	0.504	1846.073	A[38]
A[7]	344.169	1819.067	1811.058	0.504	1810.554	A[37]
P[8]	392.695	**1783.549**	1775.540	0.504	1775.036	P[36]
A[9]	428.214	1735.022	1727.013	0.504	1726.509	A[35]
A[10]	463.733	**1699.504**	**1691.495**	0.504	1690.991	A[34]
P[11]	512.259	1663.985	1655.976	0.504	1655.472	P[33]
A[12]	547.777	1615.459	**1607.450**	0.504	**1606.946**	A[32]
P[13]	596.304	1579.940	1571.931	0.504	1571.427	P[31]
V[14]	645.838	1531.414	**1523.405**	0.504	**1522.901**	V[30]
E[15]	710.359	1481.880	**1473.870**	0.504	**1473.367**	E[29]
K[16]	774.407	1417.359	1409.349	1409.853	1408.845	K[28]
T[17]	824.931	1353.311	**1345.302**	**1345.806**	1344.798	T[27]
P[18]	873.457	**1302.787**	1294.778	1295.282	1294.274	P[26]
V[19]	922.991	**1254.261**	1246.251	1246.755	1245.748	V[25]
K[20]	**987.039**	1204.727	1196.717	1197.221	1196.213	K[24]
K[21]	**1051.086**	**1140.679**	**1132.670**	**1133.174**	1132.166	K[23]
K[22]	1115.134	**1076.632**	**1068.622**	**1069.126**	**1068.118**	K[22]
A[23]	1150.652	1012.584	1004.575	1005.079	1004.071	A[21]
K[24]	**1214.700**	**977.066**	**969.056**	**969.560**	968.552	K[20]
K[25]	1278.747	913.018	**905.009**	**905.513**	**904.505**	K[19]
T[26]	1329.271	848.971	**840.961**	**841.465**	840.457	T[18]
G[27]	1357.782	798.447	**790.437**	**790.941**	**789.934**	G[17]
A[28]	**1393.300**	769.936	**761.927**	**762.431**	**761.423**	A[16]
A[29]	1428.819	734.418	726.408	726.912	725.904	A[15]
A[30]	**1464.337**	**698.899**	690.890	691.394	690.386	A[14]
G[31]	**1492.848**	**663.380**	**655.371**	**655.875**	654.867	G[13]
K[32]	**1556.896**	634.870	626.860	627.364	626.356	K[12]
R[33]	1634.946	570.822	562.813	563.317	562.309	R[11]
K[34]	**1698.994**	492.772	484.762	485.266	484.258	K[10]
A[35]	1734.512	428.724	420.715	421.219	420.211	A[9]
S[36]	1778.028	393.206	385.196	385.700	384.692	S[8]
G[37]	1806.539	349.690	341.680	342.184	341.176	G[7]
P[38]	1855.065	321.179	313.170	313.673	312.666	P[6]
P[39]	**1903.592**	272.652	264.643	265.147	264.139	P[5]
V[40]	1953.126	224.126	216.117	216.621	215.613	V[4]
S[41]	1996.642	174.592	166.583	167.086	166.079	S[3]
E[42]	2061.163	131.076	123.067	123.570	122.563	E[2]
L[43]	2117.705	66.555	58.545	59.049	58.041	L[1]

sp|P43277|H13_MOUSE

[Acetyl]SETAPAAPAAPAPVEKTPVKKAKKTGAAAGKRKASGPPVSEL

- fragmentation table for charge state 3+
- specType=etd
- score=50.44
- F104930.dat
- query=q4399_p1
- precursor=847.885670
- chargePrecursor=5
- itol=0.8

AA	c	y	z+1	z+2	z	AA
S[1]	49.697	1412.467	**1407.128**	0.672	**1406.792**	S[43]
E[2]	92.711	1369.453	**1364.114**	0.672	1363.778	E[42]
T[3]	126.394	1326.439	**1321.099**	0.672	1320.763	T[41]
A[4]	150.073	1292.756	**1287.417**	0.672	1287.081	A[40]
P[5]	182.424	1269.077	1263.738	0.672	1263.402	P[39]
A[6]	206.103	1236.726	1231.387	0.672	1231.051	A[38]
A[7]	229.782	1213.047	**1207.708**	0.672	1207.372	A[37]
P[8]	262.133	1189.368	1184.029	0.672	1183.693	P[36]
A[9]	285.812	**1157.017**	1151.678	0.672	1151.342	A[35]
A[10]	309.491	**1133.338**	**1127.999**	0.672	**1127.663**	A[34]
P[11]	341.842	**1109.659**	1104.320	0.672	1103.984	P[33]
A[12]	365.521	**1077.308**	1071.969	0.672	1071.633	A[32]
P[13]	397.872	1053.629	**1048.290**	0.672	**1047.954**	P[31]
V[14]	430.894	1021.278	1015.939	0.672	1015.603	V[30]
E[15]	473.909	988.256	**982.916**	0.672	**982.580**	E[29]
K[16]	516.607	945.241	939.902	940.238	939.566	K[28]
T[17]	550.290	902.543	**897.204**	**897.539**	**896.868**	T[27]
P[18]	582.640	868.861	863.521	863.857	863.185	P[26]
V[19]	615.663	836.510	831.170	831.506	830.834	V[25]
K[20]	658.362	803.487	798.147	798.483	797.811	K[24]
K[21]	701.060	760.789	755.449	755.785	755.113	K[23]
K[22]	743.758	718.090	712.751	713.087	712.415	K[22]
A[23]	767.437	675.392	**670.052**	**670.388**	**669.716**	A[21]
K[24]	810.136	651.713	646.373	646.709	646.037	K[20]
K[25]	852.834	609.015	603.675	604.011	603.339	K[19]
T[26]	886.516	566.316	560.977	561.313	560.641	T[18]
G[27]	**905.524**	532.634	527.294	527.630	526.958	G[17]
A[28]	929.203	513.626	508.287	508.623	507.951	A[16]
A[29]	**952.882**	489.947	484.608	484.944	484.272	A[15]
A[30]	**976.561**	466.268	460.929	461.265	460.593	A[14]
G[31]	**995.568**	442.589	437.250	437.586	436.914	G[13]
K[32]	**1038.266**	423.582	418.243	418.579	417.907	K[12]
R[33]	1090.300	380.884	375.544	375.880	375.208	R[11]
K[34]	**1132.998**	328.850	323.511	323.847	323.175	K[10]
A[35]	**1156.677**	286.152	280.812	281.148	280.476	A[9]
S[36]	**1185.688**	262.473	257.133	257.469	256.797	S[8]
G[37]	1204.695	233.462	228.123	228.459	227.787	G[7]
P[38]	1237.046	214.455	209.115	209.451	208.779	P[6]
P[39]	**1269.397**	182.104	176.765	177.100	176.429	P[5]
V[40]	**1302.420**	149.753	144.414	144.750	144.078	V[4]
S[41]	**1331.430**	116.730	111.391	111.727	111.055	S[3]
E[42]	**1374.445**	87.720	82.380	82.716	82.044	E[2]
L[43]	1412.139	44.705	39.366	39.702	39.030	L[1]

sp|P43277|H13_MOUSE

[Acetyl]SETAPAAPAAPAPVEKTPVKKKAKKTGAAAGKRKASGPPVSEL

- fragmentation table for charge state 4+
- specType=etd
- score=50.44
- F104930.dat
- query=q4399_p1
- precursor=847.885670
- chargePrecursor=5
- itol=0.8

AA	c	y	z+1	z+2	z	AA
S[1]	37.525	**1059.602**	**1055.598**	0.755	**1055.346**	S[43]
E[2]	69.785	1027.342	**1023.337**	0.755	**1023.085**	E[42]
T[3]	95.047	**995.081**	**991.076**	0.755	**990.824**	T[41]
A[4]	112.806	**969.819**	**965.814**	0.755	**965.562**	A[40]
P[5]	137.070	**952.060**	948.055	0.755	947.803	P[39]
A[6]	154.829	927.797	923.792	0.755	923.540	A[38]
A[7]	172.588	**910.037**	906.033	0.755	**905.781**	A[37]
P[8]	196.851	892.278	888.273	0.755	888.021	P[36]
A[9]	214.611	868.015	864.010	0.755	863.758	A[35]
A[10]	232.370	850.256	846.251	0.755	845.999	A[34]
P[11]	256.633	832.496	828.492	0.755	828.240	P[33]
A[12]	274.392	808.233	804.228	0.755	803.976	A[32]
P[13]	298.656	**790.474**	786.469	0.755	786.217	P[31]
V[14]	323.423	766.211	**762.206**	0.755	**761.954**	V[30]
E[15]	355.683	741.444	737.439	0.755	737.187	E[29]
K[16]	387.707	709.183	705.178	705.430	704.926	K[28]
T[17]	412.969	677.159	673.154	673.406	672.903	T[27]
P[18]	437.232	651.897	647.893	648.145	647.641	P[26]
V[19]	461.999	627.634	623.629	623.881	623.377	V[25]
K[20]	494.023	602.867	598.862	599.114	598.610	K[24]
K[21]	526.047	570.843	566.839	567.090	566.587	K[23]
K[22]	558.070	538.819	534.815	535.067	534.563	K[22]
A[23]	575.830	506.796	502.791	503.043	502.539	A[21]
K[24]	607.854	489.036	485.032	485.284	484.780	K[20]
K[25]	639.877	457.013	453.008	453.260	452.756	K[19]
T[26]	665.139	424.989	420.984	421.236	420.732	T[18]
G[27]	679.395	399.727	395.722	395.974	395.470	G[17]
A[28]	697.154	385.472	381.467	381.719	381.215	A[16]
A[29]	714.913	367.712	363.708	363.960	363.456	A[15]
A[30]	732.672	349.953	345.948	346.200	345.696	A[14]
G[31]	746.928	332.194	328.189	328.441	327.937	G[13]
K[32]	778.951	317.938	313.934	314.186	313.682	K[12]
R[33]	817.977	285.915	281.910	282.162	281.658	R[11]
K[34]	850.001	246.889	242.885	243.137	242.633	K[10]
A[35]	867.760	214.866	210.861	211.113	210.609	A[9]
S[36]	889.518	197.106	193.102	193.354	192.850	S[8]
G[37]	903.773	175.348	171.344	171.596	171.092	G[7]
P[38]	928.036	161.093	157.088	157.340	156.836	P[6]
P[39]	**952.300**	136.830	132.825	133.077	132.573	P[5]
V[40]	**977.067**	112.567	108.562	108.814	108.310	V[4]
S[41]	**998.825**	87.800	83.795	84.047	83.543	S[3]
E[42]	**1031.085**	66.042	62.037	62.289	61.785	E[2]
L[43]	**1059.356**	33.781	29.776	30.028	29.524	L[1]

sp|P43277|H13_MOUSE

[Acetyl]SETAPAAPAAPAPVEKTPVKKKAKKTGAAAGKRKASGPPVSEL

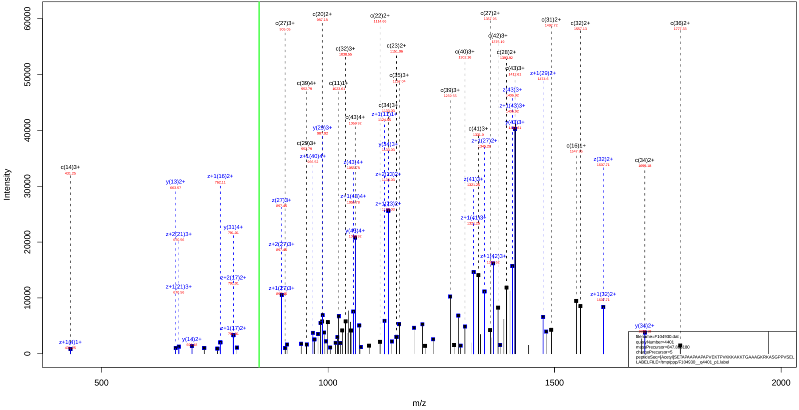

sp|P43277|H13_MOUSE

[Acetyl]SETAPAAPAAPAPVEKTPVKKKAKKTGAAAGKRKASGPPVSEL

- fragmentation table for charge state 1+
- specType=etd
- score=37.40
- F104930.dat
- query=q4401_p1
- precursor=847.886180
- chargePrecursor=5
- itol=0.8

AA	c	y	z+1	z+2	z	AA
S[1]	147.076	4235.387	4219.369	0.000	4218.361	S[43]
E[2]	276.119	4106.345	4090.326	0.000	4089.318	E[42]
T[3]	377.167	3977.302	3961.284	0.000	3960.276	T[41]
A[4]	448.204	3876.255	3860.236	0.000	3859.228	A[40]
P[5]	545.257	3805.217	3789.199	0.000	3788.191	P[39]
A[6]	616.294	3708.165	3692.146	0.000	3691.138	A[38]
A[7]	687.331	3637.128	3621.109	0.000	3620.101	A[37]
P[8]	784.384	3566.090	3550.072	0.000	3549.064	P[36]
A[9]	855.421	3469.038	3453.019	0.000	3452.011	A[35]
A[10]	926.458	3398.001	3381.982	0.000	3380.974	A[34]
P[11]	**1023.511**	3326.963	3310.945	0.000	3309.937	P[33]
A[12]	1094.548	3229.911	3213.892	0.000	3212.884	A[32]
P[13]	1191.600	3158.874	3142.855	0.000	3141.847	P[31]
V[14]	1290.669	3061.821	3045.802	0.000	3044.794	V[30]
E[15]	1419.711	2962.752	2946.734	0.000	2945.726	E[29]
K[16]	**1547.806**	2833.710	2817.691	2818.699	2816.683	K[28]
T[17]	1648.854	2705.615	2689.596	2690.604	2688.588	T[27]
P[18]	1745.907	2604.567	2588.548	2589.556	2587.541	P[26]
V[19]	1844.975	2507.514	2491.496	2492.504	2490.488	V[25]
K[20]	1973.070	2408.446	2392.427	2393.435	2391.419	K[24]
K[21]	2101.165	2280.351	2264.332	2265.340	2263.324	K[23]
K[22]	2229.260	2152.256	2136.237	2137.245	2135.230	K[22]
A[23]	2300.297	2024.161	2008.142	2009.150	2007.135	A[21]
K[24]	2428.392	1953.124	1937.105	1938.113	1936.097	K[20]
K[25]	2556.487	1825.029	1809.010	1810.018	1808.002	K[19]
T[26]	2657.535	1696.934	1680.915	1681.923	1679.908	T[18]
G[27]	2714.556	1595.886	1579.868	1580.875	1578.860	G[17]
A[28]	2785.593	1538.865	1522.846	1523.854	1521.838	A[16]
A[29]	2856.631	1467.828	1451.809	1452.817	1450.801	A[15]
A[30]	2927.668	1396.791	1380.772	1381.780	**1379.764**	A[14]
G[31]	2984.689	1325.754	1309.735	1310.743	1308.727	G[13]
K[32]	3112.784	1268.732	1252.713	1253.721	1251.706	K[12]
R[33]	3268.885	**1140.637**	**1124.618**	1125.626	1123.611	R[11]
K[34]	3396.980	984.536	968.517	**969.525**	967.509	K[10]
A[35]	3468.017	856.441	840.422	841.430	839.415	A[9]
S[36]	3555.049	785.404	769.385	770.393	768.377	S[8]
G[37]	3612.071	698.372	682.353	683.361	681.345	G[7]
P[38]	3709.124	641.350	625.332	626.340	624.324	P[6]
P[39]	3806.176	544.298	528.279	529.287	527.271	P[5]
V[40]	3905.245	447.245	**431.226**	432.234	430.218	V[4]
S[41]	3992.277	348.177	332.158	333.166	331.150	S[3]
E[42]	4121.319	261.144	245.126	246.134	244.118	E[2]
L[43]	4234.403	132.102	116.083	117.091	115.075	L[1]

sp|P43277|H13_MOUSE

[Acetyl]SETAPAAPAAPAPVEKTPVKKKAKKTGAAAGKRKASGPPVSEL

- fragmentation table for charge state 2+
- specType=etd
- score=37.40
- F104930.dat
- query=q4401_p1
- precursor=847.886180
- chargePrecursor=5
- itol=0.8

AA	c	y	z+1	z+2	z	AA
S[1]	74.042	2118.197	2110.188	0.504	2109.684	S[43]
E[2]	138.563	2053.676	2045.667	0.504	2045.163	E[42]
T[3]	189.087	1989.155	1981.145	0.504	1980.641	T[41]
A[4]	224.606	1938.631	1930.622	0.504	1930.118	A[40]
P[5]	273.132	1903.112	1895.103	0.504	1894.599	P[39]
A[6]	308.650	1854.586	1846.577	0.504	1846.073	A[38]
A[7]	344.169	1819.067	1811.058	0.504	1810.554	A[37]
P[8]	392.695	1783.549	1775.540	0.504	1775.036	P[36]
A[9]	428.214	1735.022	1727.013	0.504	1726.509	A[35]
A[10]	463.733	**1699.504**	1691.495	0.504	1690.991	A[34]
P[11]	512.259	1663.985	1655.976	0.504	1655.472	P[33]
A[12]	547.777	1615.459	**1607.450**	0.504	**1606.946**	A[32]
P[13]	596.304	1579.940	1571.931	0.504	1571.427	P[31]
V[14]	645.838	1531.414	1523.405	0.504	1522.901	V[30]
E[15]	710.359	**1481.880**	**1473.870**	0.504	1473.367	E[29]
K[16]	774.407	1417.359	1409.349	1409.853	1408.845	K[28]
T[17]	824.931	1353.311	**1345.302**	**1345.806**	**1344.798**	T[27]
P[18]	873.457	**1302.787**	1294.778	1295.282	1294.274	P[26]
V[19]	922.991	1254.261	1246.251	1246.755	1245.748	V[25]
K[20]	**987.039**	1204.727	1196.717	1197.221	1196.213	K[24]
K[21]	**1051.086**	**1140.679**	**1132.670**	**1133.174**	1132.166	K[23]
K[22]	**1115.134**	1076.632	**1068.622**	**1069.126**	**1068.118**	K[22]
A[23]	**1150.652**	1012.584	**1004.575**	**1005.079**	**1004.071**	A[21]
K[24]	**1214.700**	**977.066**	**969.056**	**969.560**	968.552	K[20]
K[25]	**1278.747**	913.018	**905.009**	**905.513**	**904.505**	K[19]
T[26]	1329.271	848.971	840.961	841.465	840.457	T[18]
G[27]	**1357.782**	**798.447**	**790.437**	**790.941**	789.934	G[17]
A[28]	**1393.300**	769.936	**761.927**	**762.431**	**761.423**	A[16]
A[29]	1428.819	734.418	**726.408**	**726.912**	**725.904**	A[15]
A[30]	1464.337	**698.899**	690.890	691.394	690.386	A[14]
G[31]	**1492.848**	**663.380**	655.371	655.875	654.867	G[13]
K[32]	**1556.896**	634.870	626.860	627.364	626.356	K[12]
R[33]	1634.946	570.822	562.813	563.317	562.309	R[11]
K[34]	**1698.994**	492.772	484.762	485.266	484.258	K[10]
A[35]	1734.512	428.724	420.715	421.219	420.211	A[9]
S[36]	**1778.028**	393.206	385.196	385.700	384.692	S[8]
G[37]	1806.539	349.690	341.680	342.184	341.176	G[7]
P[38]	1855.065	321.179	313.170	313.673	312.666	P[6]
P[39]	1903.592	272.652	264.643	265.147	264.139	P[5]
V[40]	1953.126	224.126	216.117	216.621	215.613	V[4]
S[41]	1996.642	174.592	166.583	167.086	166.079	S[3]
E[42]	2061.163	131.076	123.067	123.570	122.563	E[2]
L[43]	2117.705	66.555	58.545	59.049	58.041	L[1]

sp|P43277|H13_MOUSE

[Acetyl]SETAPAAPAAPAPVEKTPVKKAKKTGAAAGKRKASGPPVSEL

- fragmentation table for charge state 3+
- specType=etd
- score=37.40
- F104930.dat
- query=q4401_p1
- precursor=847.886180
- chargePrecursor=5
- itol=0.8

AA	c	y	z+1	z+2	z	AA
S[1]	49.697	**1412.467**	**1407.128**	0.672	**1406.792**	S[43]
E[2]	92.711	1369.453	**1364.114**	0.672	1363.778	E[42]
T[3]	126.394	1326.439	**1321.099**	0.672	**1320.763**	T[41]
A[4]	150.073	**1292.756**	**1287.417**	0.672	**1287.081**	A[40]
P[5]	182.424	**1269.077**	1263.738	0.672	1263.402	P[39]
A[6]	206.103	1236.726	**1231.387**	0.672	1231.051	A[38]
A[7]	229.782	1213.047	**1207.708**	0.672	1207.372	A[37]
P[8]	262.133	**1189.368**	1184.029	0.672	1183.693	P[36]
A[9]	285.812	**1157.017**	**1151.678**	0.672	**1151.342**	A[35]
A[10]	309.491	**1133.338**	1127.999	0.672	1127.663	A[34]
P[11]	341.842	1109.659	1104.320	0.672	1103.984	P[33]
A[12]	365.521	1077.308	**1071.969**	0.672	1071.633	A[32]
P[13]	397.872	1053.629	1048.290	0.672	1047.954	P[31]
V[14]	**430.894**	**1021.278**	**1015.939**	0.672	1015.603	V[30]
E[15]	473.909	**988.256**	**982.916**	0.672	**982.580**	E[29]
K[16]	516.607	945.241	**939.902**	**940.238**	939.566	K[28]
T[17]	550.290	902.543	**897.204**	**897.539**	**896.868**	T[27]
P[18]	582.640	868.861	863.521	863.857	863.185	P[26]
V[19]	615.663	836.510	831.170	831.506	830.834	V[25]
K[20]	658.362	803.487	**798.147**	**798.483**	797.811	K[24]
K[21]	701.060	760.789	**755.449**	**755.785**	**755.113**	K[23]
K[22]	743.758	718.090	712.751	713.087	712.415	K[22]
A[23]	767.437	675.392	**670.052**	**670.388**	669.716	A[21]
K[24]	810.136	651.713	646.373	646.709	646.037	K[20]
K[25]	852.834	609.015	603.675	604.011	603.339	K[19]
T[26]	886.516	566.316	560.977	561.313	560.641	T[18]
G[27]	**905.524**	532.634	527.294	527.630	526.958	G[17]
A[28]	929.203	513.626	508.287	508.623	507.951	A[16]
A[29]	**952.882**	489.947	484.608	484.944	484.272	A[15]
A[30]	976.561	466.268	460.929	461.265	460.593	A[14]
G[31]	**995.568**	442.589	437.250	437.586	436.914	G[13]
K[32]	**1038.266**	423.582	418.243	418.579	417.907	K[12]
R[33]	**1090.300**	380.884	375.544	375.880	375.208	R[11]
K[34]	**1132.998**	328.850	323.511	323.847	323.175	K[10]
A[35]	**1156.677**	286.152	280.812	281.148	280.476	A[9]
S[36]	1185.688	262.473	257.133	257.469	256.797	S[8]
G[37]	1204.695	233.462	228.123	228.459	227.787	G[7]
P[38]	1237.046	214.455	209.115	209.451	208.779	P[6]
P[39]	**1269.397**	182.104	176.765	177.100	176.429	P[5]
V[40]	**1302.420**	149.753	144.414	144.750	144.078	V[4]
S[41]	**1331.430**	116.730	111.391	111.727	111.055	S[3]
E[42]	**1374.445**	87.720	82.380	82.716	82.044	E[2]
L[43]	**1412.139**	44.705	39.366	39.702	39.030	L[1]

sp|P43277|H13_MOUSE

[Acetyl]SETAPAAPAAPAPVEKTPVKKKAKKTGAAAGKRKASGPPVSEL

- fragmentation table for charge state 4+
- specType=etd
- score=37.40
- F104930.dat
- query=q4401_p1
- precursor=847.886180
- chargePrecursor=5
- itol=0.8

AA	c	y	z+1	z+2	z	AA
S[1]	37.525	**1059.602**	**1055.598**	0.755	**1055.346**	S[43]
E[2]	69.785	**1027.342**	**1023.337**	0.755	**1023.085**	E[42]
T[3]	95.047	**995.081**	**991.076**	0.755	**990.824**	T[41]
A[4]	112.806	**969.819**	**965.814**	0.755	965.562	A[40]
P[5]	137.070	**952.060**	948.055	0.755	947.803	P[39]
A[6]	154.829	927.797	923.792	0.755	923.540	A[38]
A[7]	172.588	**910.037**	906.033	0.755	**905.781**	A[37]
P[8]	196.851	892.278	888.273	0.755	888.021	P[36]
A[9]	214.611	868.015	864.010	0.755	863.758	A[35]
A[10]	232.370	850.256	846.251	0.755	845.999	A[34]
P[11]	256.633	832.496	828.492	0.755	828.240	P[33]
A[12]	274.392	808.233	804.228	0.755	803.976	A[32]
P[13]	298.656	**790.474**	786.469	0.755	786.217	P[31]
V[14]	323.423	766.211	**762.206**	0.755	**761.954**	V[30]
E[15]	355.683	741.444	737.439	0.755	737.187	E[29]
K[16]	387.707	709.183	705.178	705.430	704.926	K[28]
T[17]	412.969	677.159	673.154	673.406	672.903	T[27]
P[18]	437.232	651.897	647.893	648.145	647.641	P[26]
V[19]	461.999	627.634	623.629	623.881	623.377	V[25]
K[20]	494.023	602.867	598.862	599.114	598.610	K[24]
K[21]	526.047	570.843	566.839	567.090	566.587	K[23]
K[22]	558.070	538.819	534.815	535.067	534.563	K[22]
A[23]	575.830	506.796	502.791	503.043	502.539	A[21]
K[24]	607.854	489.036	485.032	485.284	484.780	K[20]
K[25]	639.877	457.013	453.008	453.260	452.756	K[19]
T[26]	665.139	424.989	420.984	421.236	420.732	T[18]
G[27]	679.395	399.727	395.722	395.974	395.470	G[17]
A[28]	697.154	385.472	381.467	381.719	381.215	A[16]
A[29]	714.913	367.712	363.708	363.960	363.456	A[15]
A[30]	732.672	349.953	345.948	346.200	345.696	A[14]
G[31]	746.928	332.194	328.189	328.441	327.937	G[13]
K[32]	778.951	317.938	313.934	314.186	313.682	K[12]
R[33]	817.977	285.915	281.910	282.162	281.658	R[11]
K[34]	850.001	246.889	242.885	243.137	242.633	K[10]
A[35]	867.760	214.866	210.861	211.113	210.609	A[9]
S[36]	889.518	197.106	193.102	193.354	192.850	S[8]
G[37]	903.773	175.348	171.344	171.596	171.092	G[7]
P[38]	928.036	161.093	157.088	157.340	156.836	P[6]
P[39]	**952.300**	136.830	132.825	133.077	132.573	P[5]
V[40]	**977.067**	112.567	108.562	108.814	108.310	V[4]
S[41]	**998.825**	87.800	83.795	84.047	83.543	S[3]
E[42]	**1031.085**	66.042	62.037	62.289	61.785	E[2]
L[43]	**1059.356**	33.781	29.776	30.028	29.524	L[1]

sp|P43277|H13_MOUSE

[Acetyl]SETAPAAPAAPAPVEKTPVKKKAKKTGAAAGKRKASGPPVSEL

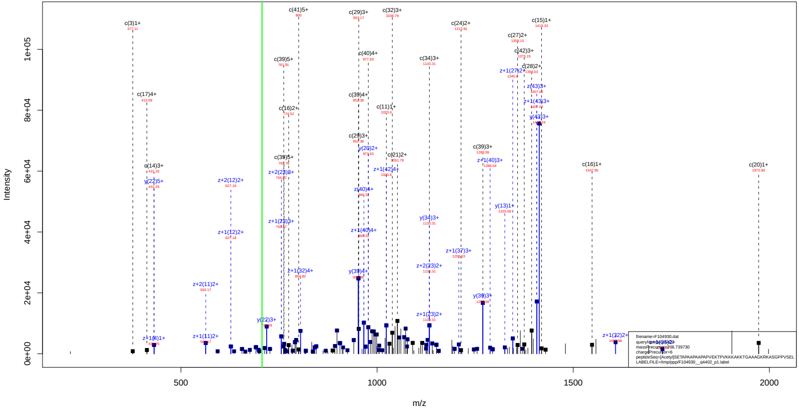

sp|P43277|H13_MOUSE

[Acetyl]SETAPAAPAAPAPVEKTPVKKAKKTGAAAGKRKASGPPVSEL

- fragmentation table for charge state 1+
- specType=etd
- score=64.25
- F104930.dat
- query=q4402_p1
- precursor=706.739730
- chargePrecursor=6
- itol=0.8

AA	c	y	z+1	z+2	z	AA
S[1]	147.076	4235.387	4219.369	0.000	4218.361	S[43]
E[2]	276.119	4106.345	4090.326	0.000	4089.318	E[42]
T[3]	**377.167**	3977.302	3961.284	0.000	3960.276	T[41]
A[4]	448.204	3876.255	3860.236	0.000	3859.228	A[40]
P[5]	545.257	3805.217	3789.199	0.000	3788.191	P[39]
A[6]	616.294	3708.165	3692.146	0.000	3691.138	A[38]
A[7]	687.331	3637.128	3621.109	0.000	3620.101	A[37]
P[8]	**784.384**	3566.090	3550.072	0.000	3549.064	P[36]
A[9]	855.421	3469.038	3453.019	0.000	3452.011	A[35]
A[10]	926.458	3398.001	3381.982	0.000	3380.974	A[34]
P[11]	**1023.511**	3326.963	3310.945	0.000	3309.937	P[33]
A[12]	1094.548	3229.911	3213.892	0.000	3212.884	A[32]
P[13]	1191.600	3158.874	3142.855	0.000	3141.847	P[31]
V[14]	1290.669	3061.821	3045.802	0.000	3044.794	V[30]
E[15]	**1419.711**	2962.752	2946.734	0.000	2945.726	E[29]
K[16]	**1547.806**	2833.710	2817.691	2818.699	2816.683	K[28]
T[17]	1648.854	2705.615	2689.596	2690.604	2688.588	T[27]
P[18]	1745.907	2604.567	2588.548	2589.556	2587.541	P[26]
V[19]	1844.975	2507.514	2491.496	2492.504	2490.488	V[25]
K[20]	**1973.070**	2408.446	2392.427	2393.435	2391.419	K[24]
K[21]	2101.165	2280.351	2264.332	2265.340	2263.324	K[23]
K[22]	2229.260	2152.256	2136.237	2137.245	2135.230	K[22]
A[23]	2300.297	2024.161	2008.142	2009.150	2007.135	A[21]
K[24]	2428.392	1953.124	1937.105	1938.113	1936.097	K[20]
K[25]	2556.487	1825.029	1809.010	1810.018	1808.002	K[19]
T[26]	2657.535	1696.934	1680.915	1681.923	1679.908	T[18]
G[27]	2714.556	1595.886	1579.868	1580.875	1578.860	G[17]
A[28]	2785.593	1538.865	1522.846	1523.854	1521.838	A[16]
A[29]	2856.631	1467.828	1451.809	1452.817	1450.801	A[15]
A[30]	2927.668	1396.791	1380.772	1381.780	1379.764	A[14]
G[31]	2984.689	**1325.754**	1309.735	1310.743	1308.727	G[13]
K[32]	3112.784	1268.732	1252.713	1253.721	1251.706	K[12]
R[33]	3268.885	**1140.637**	**1124.618**	**1125.626**	1123.611	R[11]
K[34]	3396.980	984.536	968.517	**969.525**	967.509	K[10]
A[35]	3468.017	856.441	**840.422**	**841.430**	839.415	A[9]
S[36]	3555.049	**785.404**	769.385	770.393	**768.377**	S[8]
G[37]	3612.071	**698.372**	682.353	683.361	681.345	G[7]
P[38]	3709.124	641.350	625.332	626.340	624.324	P[6]
P[39]	3806.176	544.298	528.279	529.287	527.271	P[5]
V[40]	3905.245	447.245	**431.226**	432.234	430.218	V[4]
S[41]	3992.277	348.177	332.158	333.166	331.150	S[3]
E[42]	4121.319	261.144	245.126	246.134	244.118	E[2]
L[43]	4234.403	132.102	116.083	117.091	115.075	L[1]

sp|P43277|H13_MOUSE

[Acetyl]SETAPAAPAAPAPVEKTPVKKAKKTGAAAGKRKASGPPVSEL

- fragmentation table for charge state 2+
- specType=etd
- score=64.25
- F104930.dat
- query=q4402_p1
- precursor=706.739730
- chargePrecursor=6
- itol=0.8

AA	c	y	z+1	z+2	z	AA
S[1]	74.042	2118.197	2110.188	0.504	2109.684	S[43]
E[2]	138.563	2053.676	2045.667	0.504	2045.163	E[42]
T[3]	189.087	1989.155	1981.145	0.504	1980.641	T[41]
A[4]	224.606	1938.631	1930.622	0.504	1930.118	A[40]
P[5]	273.132	1903.112	1895.103	0.504	1894.599	P[39]
A[6]	308.650	1854.586	1846.577	0.504	1846.073	A[38]
A[7]	344.169	1819.067	1811.058	0.504	1810.554	A[37]
P[8]	392.695	1783.549	1775.540	0.504	1775.036	P[36]
A[9]	428.214	1735.022	**1727.013**	0.504	1726.509	A[35]
A[10]	463.733	1699.504	1691.495	0.504	1690.991	A[34]
P[11]	512.259	1663.985	1655.976	0.504	1655.472	P[33]
A[12]	547.777	1615.459	**1607.450**	0.504	1606.946	A[32]
P[13]	596.304	1579.940	1571.931	0.504	1571.427	P[31]
V[14]	645.838	1531.414	1523.405	0.504	1522.901	V[30]
E[15]	710.359	1481.880	1473.870	0.504	1473.367	E[29]
K[16]	**774.407**	1417.359	1409.349	1409.853	1408.845	K[28]
T[17]	824.931	1353.311	**1345.302**	**1345.806**	1344.798	T[27]
P[18]	873.457	1302.787	**1294.778**	**1295.282**	**1294.274**	P[26]
V[19]	922.991	**1254.261**	**1246.251**	**1246.755**	1245.748	V[25]
K[20]	**987.039**	1204.727	**1196.717**	**1197.221**	1196.213	K[24]
K[21]	**1051.086**	**1140.679**	**1132.670**	**1133.174**	1132.166	K[23]
K[22]	**1115.134**	**1076.632**	**1068.622**	**1069.126**	1068.118	K[22]
A[23]	**1150.652**	**1012.584**	**1004.575**	**1005.079**	**1004.071**	A[21]
K[24]	**1214.700**	**977.066**	969.056	**969.560**	968.552	K[20]
K[25]	1278.747	**913.018**	**905.009**	**905.513**	**904.505**	K[19]
T[26]	1329.271	848.971	**840.961**	**841.465**	**840.457**	T[18]
G[27]	**1357.782**	798.447	**790.437**	**790.941**	789.934	G[17]
A[28]	**1393.300**	769.936	**761.927**	**762.431**	**761.423**	A[16]
A[29]	**1428.819**	734.418	**726.408**	**726.912**	725.904	A[15]
A[30]	1464.337	**698.899**	**690.890**	**691.394**	**690.386**	A[14]
G[31]	1492.848	**663.380**	**655.371**	**655.875**	**654.867**	G[13]
K[32]	1556.896	**634.870**	**626.860**	**627.364**	626.356	K[12]
R[33]	1634.946	570.822	**562.813**	**563.317**	562.309	R[11]
K[34]	1698.994	492.772	484.762	485.266	484.258	K[10]
A[35]	1734.512	428.724	420.715	421.219	420.211	A[9]
S[36]	1778.028	393.206	385.196	385.700	384.692	S[8]
G[37]	1806.539	349.690	341.680	342.184	341.176	G[7]
P[38]	1855.065	321.179	313.170	313.673	312.666	P[6]
P[39]	1903.592	272.652	264.643	265.147	264.139	P[5]
V[40]	1953.126	224.126	216.117	216.621	215.613	V[4]
S[41]	1996.642	174.592	166.583	167.086	166.079	S[3]
E[42]	2061.163	131.076	123.067	123.570	122.563	E[2]
L[43]	2117.705	66.555	58.545	59.049	58.041	L[1]

sp|P43277|H13_MOUSE

[Acetyl]SETAPAAPAAPAPVEKTPVKKKAKKTGAAAGKRKASGPPVSEL

- fragmentation table for charge state 3+
- specType=etd
- score=64.25
- F104930.dat
- query=q4402_p1
- precursor=706.739730
- chargePrecursor=6
- itol=0.8

AA	c	y	z+1	z+2	z	AA
S[1]	49.697	**1412.467**	**1407.128**	0.672	**1406.792**	S[43]
E[2]	92.711	**1369.453**	1364.114	0.672	1363.778	E[42]
T[3]	126.394	1326.439	1321.099	0.672	1320.763	T[41]
A[4]	150.073	1292.756	**1287.417**	0.672	1287.081	A[40]
P[5]	182.424	**1269.077**	1263.738	0.672	1263.402	P[39]
A[6]	206.103	1236.726	1231.387	0.672	1231.051	A[38]
A[7]	229.782	1213.047	**1207.708**	0.672	1207.372	A[37]
P[8]	262.133	1189.368	1184.029	0.672	1183.693	P[36]
A[9]	285.812	**1157.017**	**1151.678**	0.672	**1151.342**	A[35]
A[10]	309.491	**1133.338**	**1127.999**	0.672	1127.663	A[34]
P[11]	341.842	1109.659	1104.320	0.672	1103.984	P[33]
A[12]	365.521	**1077.308**	**1071.969**	0.672	1071.633	A[32]
P[13]	397.872	1053.629	1048.290	0.672	1047.954	P[31]
V[14]	**430.894**	1021.278	1015.939	0.672	1015.603	V[30]
E[15]	473.909	988.256	**982.916**	0.672	**982.580**	E[29]
K[16]	516.607	945.241	**939.902**	**940.238**	939.566	K[28]
T[17]	550.290	902.543	**897.204**	**897.539**	896.868	T[27]
P[18]	582.640	**868.861**	863.521	863.857	863.185	P[26]
V[19]	615.663	**836.510**	831.170	831.506	830.834	V[25]
K[20]	658.362	803.487	798.147	798.483	797.811	K[24]
K[21]	701.060	760.789	**755.449**	**755.785**	**755.113**	K[23]
K[22]	743.758	**718.090**	**712.751**	**713.087**	712.415	K[22]
A[23]	**767.437**	**675.392**	670.052	670.388	669.716	A[21]
K[24]	810.136	651.713	646.373	646.709	646.037	K[20]
K[25]	852.834	609.015	603.675	604.011	603.339	K[19]
T[26]	**886.516**	566.316	560.977	561.313	560.641	T[18]
G[27]	**905.524**	532.634	527.294	527.630	526.958	G[17]
A[28]	929.203	513.626	508.287	508.623	507.951	A[16]
A[29]	**952.882**	489.947	484.608	484.944	484.272	A[15]
A[30]	976.561	466.268	460.929	461.265	460.593	A[14]
G[31]	**995.568**	442.589	437.250	437.586	436.914	G[13]
K[32]	**1038.266**	423.582	418.243	418.579	417.907	K[12]
R[33]	**1090.300**	380.884	375.544	375.880	375.208	R[11]
K[34]	**1132.998**	328.850	323.511	323.847	323.175	K[10]
A[35]	**1156.677**	286.152	280.812	281.148	280.476	A[9]
S[36]	1185.688	262.473	257.133	257.469	256.797	S[8]
G[37]	1204.695	233.462	228.123	228.459	227.787	G[7]
P[38]	1237.046	214.455	209.115	209.451	208.779	P[6]
P[39]	**1269.397**	182.104	176.765	177.100	176.429	P[5]
V[40]	1302.420	149.753	144.414	144.750	144.078	V[4]
S[41]	1331.430	116.730	111.391	111.727	111.055	S[3]
E[42]	**1374.445**	87.720	82.380	82.716	82.044	E[2]
L[43]	1412.139	44.705	39.366	39.702	39.030	L[1]

sp|P43277|H13_MOUSE

[Acetyl]SETAPAAPAAPAPVEKTPVKKKAKKTGAAAGKRKASGPPVSEL

- fragmentation table for charge state 4+
- specType=etd
- score=64.25
- F104930.dat
- query=q4402_p1
- precursor=706.739730
- chargePrecursor=6
- itol=0.8

AA	c	y	z+1	z+2	z	AA
S[1]	37.525	1059.602	**1055.598**	0.755	**1055.346**	S[43]
E[2]	69.785	1027.342	**1023.337**	0.755	**1023.085**	E[42]
T[3]	95.047	**995.081**	**991.076**	0.755	**990.824**	T[41]
A[4]	112.806	**969.819**	**965.814**	0.755	**965.562**	A[40]
P[5]	137.070	**952.060**	948.055	0.755	947.803	P[39]
A[6]	154.829	927.797	**923.792**	0.755	923.540	A[38]
A[7]	172.588	910.037	906.033	0.755	**905.781**	A[37]
P[8]	196.851	**892.278**	888.273	0.755	888.021	P[36]
A[9]	214.611	868.015	864.010	0.755	863.758	A[35]
A[10]	232.370	850.256	846.251	0.755	845.999	A[34]
P[11]	256.633	832.496	828.492	0.755	828.240	P[33]
A[12]	274.392	808.233	**804.228**	0.755	803.976	A[32]
P[13]	298.656	**790.474**	786.469	0.755	786.217	P[31]
V[14]	323.423	**766.211**	**762.206**	0.755	**761.954**	V[30]
E[15]	355.683	741.444	737.439	0.755	737.187	E[29]
K[16]	387.707	709.183	705.178	705.430	704.926	K[28]
T[17]	**412.969**	677.159	673.154	673.406	672.903	T[27]
P[18]	437.232	651.897	647.893	648.145	647.641	P[26]
V[19]	461.999	**627.634**	623.629	623.881	623.377	V[25]
K[20]	494.023	602.867	598.862	599.114	598.610	K[24]
K[21]	526.047	570.843	566.839	567.090	566.587	K[23]
K[22]	558.070	538.819	534.815	535.067	534.563	K[22]
A[23]	575.830	506.796	502.791	503.043	502.539	A[21]
K[24]	607.854	489.036	485.032	485.284	484.780	K[20]
K[25]	639.877	457.013	453.008	453.260	452.756	K[19]
T[26]	665.139	424.989	420.984	421.236	420.732	T[18]
G[27]	679.395	399.727	395.722	395.974	395.470	G[17]
A[28]	697.154	385.472	381.467	381.719	381.215	A[16]
A[29]	714.913	367.712	363.708	363.960	363.456	A[15]
A[30]	732.672	349.953	345.948	346.200	345.696	A[14]
G[31]	746.928	332.194	328.189	328.441	327.937	G[13]
K[32]	778.951	317.938	313.934	314.186	313.682	K[12]
R[33]	**817.977**	285.915	281.910	282.162	281.658	R[11]
K[34]	850.001	246.889	242.885	243.137	242.633	K[10]
A[35]	867.760	214.866	210.861	211.113	210.609	A[9]
S[36]	889.518	197.106	193.102	193.354	192.850	S[8]
G[37]	903.773	175.348	171.344	171.596	171.092	G[7]
P[38]	928.036	161.093	157.088	157.340	156.836	P[6]
P[39]	**952.300**	136.830	132.825	133.077	132.573	P[5]
V[40]	**977.067**	112.567	108.562	108.814	108.310	V[4]
S[41]	**998.825**	87.800	83.795	84.047	83.543	S[3]
E[42]	**1031.085**	66.042	62.037	62.289	61.785	E[2]
L[43]	1059.356	33.781	29.776	30.028	29.524	L[1]

sp|P43277|H13_MOUSE

[Acetyl]SETAPAAPAAPAPVEKTPVKKAKKTGAAAGKRKASGPPVSEL

- fragmentation table for charge state 5+
- specType=etd
- score=64.25
- F104930.dat
- query=q4402_p1
- precursor=706.739730
- chargePrecursor=6
- itol=0.8

AA	c	y	z+1	z+2	z	AA
S[1]	30.221	847.883	**844.680**	0.806	**844.478**	S[43]
E[2]	56.030	822.075	**818.871**	0.806	**818.669**	E[42]
T[3]	76.239	796.266	**793.063**	0.806	**792.861**	T[41]
A[4]	90.447	776.057	772.853	0.806	772.651	A[40]
P[5]	109.857	**761.849**	758.646	0.806	758.444	P[39]
A[6]	124.065	742.439	739.235	0.806	739.033	A[38]
A[7]	138.272	728.231	725.028	0.806	724.826	A[37]
P[8]	157.683	**714.024**	710.820	0.806	710.619	P[36]
A[9]	171.890	694.613	**691.410**	0.806	**691.208**	A[35]
A[10]	186.097	680.406	677.202	0.806	677.001	A[34]
P[11]	205.508	666.199	662.995	0.806	662.793	P[33]
A[12]	219.715	646.788	643.584	0.806	643.383	A[32]
P[13]	239.126	632.581	629.377	0.806	629.175	P[31]
V[14]	258.940	613.170	609.966	0.806	609.765	V[30]
E[15]	284.748	**593.356**	590.153	0.806	589.951	E[29]
K[16]	310.367	567.548	564.344	564.546	564.142	K[28]
T[17]	330.577	541.929	538.725	538.927	538.523	T[27]
P[18]	349.987	521.719	518.516	518.717	518.314	P[26]
V[19]	369.801	502.309	499.105	499.307	498.903	V[25]
K[20]	395.420	482.495	479.291	479.493	479.090	K[24]
K[21]	421.039	456.876	453.672	453.874	453.471	K[23]
K[22]	446.658	**431.257**	428.053	428.255	427.852	K[22]
A[23]	460.865	405.638	402.434	402.636	402.233	A[21]
K[24]	486.484	391.431	388.227	388.428	388.025	K[20]
K[25]	512.103	365.812	362.608	362.809	362.406	K[19]
T[26]	532.313	340.193	336.989	337.190	336.787	T[18]
G[27]	543.717	319.983	316.779	316.981	316.578	G[17]
A[28]	557.925	308.579	305.375	305.577	305.173	A[16]
A[29]	572.132	294.371	291.168	291.369	290.966	A[15]
A[30]	586.339	280.164	276.960	277.162	276.759	A[14]
G[31]	597.744	265.957	262.753	262.954	262.551	G[13]
K[32]	623.363	254.552	251.348	251.550	251.147	K[12]
R[33]	654.583	228.933	225.730	225.931	225.528	R[11]
K[34]	680.202	197.713	194.509	194.711	194.308	K[10]
A[35]	694.409	172.094	168.890	169.092	168.689	A[9]
S[36]	711.816	157.887	154.683	154.884	154.481	S[8]
G[37]	723.220	140.480	137.276	137.478	137.075	G[7]
P[38]	742.631	129.076	125.872	126.074	125.671	P[6]
P[39]	**762.041**	109.665	106.462	106.663	106.260	P[5]
V[40]	781.855	90.255	87.051	87.253	86.849	V[4]
S[41]	**799.261**	70.441	67.237	67.439	67.036	S[3]
E[42]	825.070	53.035	49.831	50.033	49.629	E[2]
L[43]	847.687	27.226	24.022	24.224	23.821	L[1]

sp|P43276|H15_MOUSE

[Acetyl]SETAPAETAAPAPVEKSPAKKKTTKKAGAAKRKATGPPVSEL

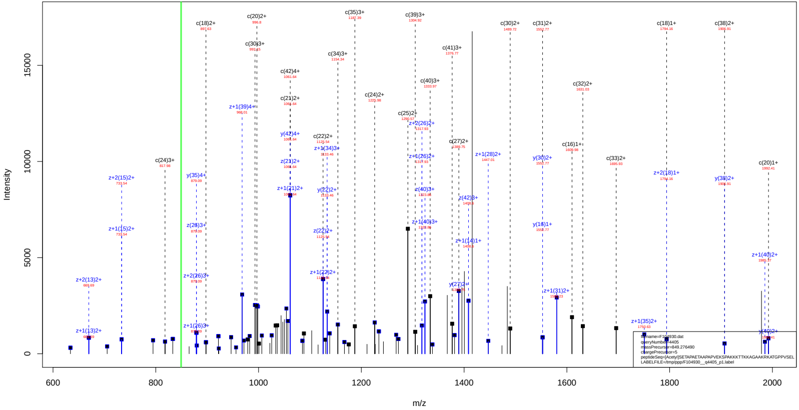

sp|P43276|H15_MOUSE

[Acetyl]SETAPAETAAPAPVEKSPAKKKTTKKAGAAKRKATGPPVSEL

- fragmentation table for charge state 1+
- specType=etd
- score=85.81
- F104930.dat
- query=q4405_p1
- precursor=849.276490
- chargePrecursor=5
- itol=0.8

AA	c	y	z+1	z+2	z	AA
S[1]	147.076	4242.346	4226.327	0.000	4225.319	S[42]
E[2]	276.119	4113.303	4097.284	0.000	4096.276	E[41]
T[3]	377.167	3984.260	3968.242	0.000	3967.234	T[40]
A[4]	448.204	3883.213	3867.194	0.000	3866.186	A[39]
P[5]	545.257	3812.176	3796.157	0.000	3795.149	P[38]
A[6]	616.294	3715.123	3699.104	0.000	3698.096	A[37]
E[7]	745.336	3644.086	3628.067	0.000	3627.059	E[36]
T[8]	846.384	3515.043	3499.024	0.000	3498.017	T[35]
A[9]	917.421	3413.995	3397.977	0.000	3396.969	A[34]
A[10]	988.458	3342.958	3326.940	0.000	3325.932	A[33]
P[11]	**1085.511**	3271.921	3255.903	0.000	3254.895	P[32]
A[12]	1156.548	3174.868	3158.850	0.000	3157.842	A[31]
P[13]	1253.601	3103.831	3087.813	0.000	3086.805	P[30]
V[14]	1352.669	3006.779	2990.760	0.000	2989.752	V[29]
E[15]	1481.712	2907.710	2891.691	0.000	2890.684	E[28]
K[16]	**1609.807**	2778.668	2762.649	2763.657	2761.641	K[27]
S[17]	1696.839	2650.573	2634.554	2635.562	2633.546	S[26]
P[18]	**1793.892**	2563.541	2547.522	2548.530	2546.514	P[25]
A[19]	1864.929	2466.488	2450.469	2451.477	2449.461	A[24]
K[20]	**1993.024**	2395.451	2379.432	2380.440	2378.424	K[23]
K[21]	2121.119	2267.356	2251.337	2252.345	2250.329	K[22]
K[22]	2249.214	2139.261	2123.242	2124.250	2122.234	K[21]
T[23]	2350.261	2011.166	1995.147	1996.155	1994.139	T[20]
T[24]	2451.309	1910.118	1894.099	1895.107	1893.092	T[19]
K[25]	2579.404	1809.070	1793.052	**1794.060**	1792.044	K[18]
K[26]	2707.499	1680.976	1664.957	1665.965	1663.949	K[17]
A[27]	2778.536	**1552.881**	1536.862	1537.870	1535.854	A[16]
G[28]	2835.557	1481.843	1465.825	1466.833	1464.817	G[15]
A[29]	2906.595	1424.822	**1408.803**	1409.811	1407.795	A[14]
A[30]	2977.632	1353.785	**1337.766**	**1338.774**	1336.758	A[13]
K[31]	3105.727	1282.748	1266.729	**1267.737**	1265.721	K[12]
R[32]	3261.828	**1154.653**	**1138.634**	1139.642	1137.626	R[11]
K[33]	3389.923	**998.552**	**982.533**	983.541	981.525	K[10]
A[34]	3460.960	870.457	854.438	855.446	853.430	A[9]
T[35]	3562.008	799.420	783.401	784.409	782.393	T[8]
G[36]	3619.029	698.372	682.353	683.361	681.345	G[7]
P[37]	3716.082	641.350	625.332	626.340	624.324	P[6]
P[38]	3813.135	544.298	528.279	529.287	527.271	P[5]
V[39]	3912.203	447.245	431.226	432.234	430.218	V[4]
S[40]	3999.235	348.177	332.158	333.166	331.150	S[3]
E[41]	4128.278	261.144	245.126	246.134	244.118	E[2]
L[42]	4241.362	132.102	116.083	117.091	115.075	L[1]

sp|P43276|H15_MOUSE

[Acetyl]SETAPAETAAPAPVEKSPAKKKTTKKAGAAKRKATGPPVSEL

- fragmentation table for charge state 2+
- specType=etd
- score=85.81
- F104930.dat
- query=q4405_p1
- precursor=849.276490
- chargePrecursor=5
- itol=0.8

AA	c	y	z+1	z+2	z	AA
S[1]	74.042	2121.676	2113.667	0.504	2113.163	S[42]
E[2]	138.563	2057.155	2049.146	0.504	2048.642	E[41]
T[3]	189.087	**1992.634**	**1984.624**	0.504	1984.121	T[40]
A[4]	224.606	1942.110	1934.101	0.504	1933.597	A[39]
P[5]	273.132	**1906.591**	1898.582	0.504	1898.078	P[38]
A[6]	308.650	1858.065	1850.056	0.504	1849.552	A[37]
E[7]	373.172	1822.547	1814.537	0.504	1814.033	E[36]
T[8]	423.696	1758.025	**1750.016**	0.504	1749.512	T[35]
A[9]	459.214	1707.501	1699.492	0.504	1698.988	A[34]
A[10]	494.733	1671.983	1663.973	0.504	1663.470	A[33]
P[11]	543.259	1636.464	1628.455	0.504	1627.951	P[32]
A[12]	578.778	1587.938	**1579.929**	0.504	1579.425	A[31]
P[13]	627.304	**1552.419**	1544.410	0.504	1543.906	P[30]
V[14]	676.838	1503.893	1495.884	0.504	1495.380	V[29]
E[15]	741.360	1454.359	**1446.349**	0.504	1445.845	E[28]
K[16]	805.407	**1389.837**	**1381.828**	1382.332	**1381.324**	K[27]
S[17]	848.923	1325.790	**1317.781**	**1318.285**	**1317.277**	S[26]
P[18]	**897.449**	1282.274	1274.265	1274.768	1273.761	P[25]
A[19]	932.968	**1233.748**	**1225.738**	**1226.242**	**1225.234**	A[24]
K[20]	**997.015**	1198.229	1190.220	1190.724	1189.716	K[23]
K[21]	**1061.063**	**1134.182**	**1126.172**	1126.676	**1125.668**	K[22]
K[22]	**1125.110**	1070.134	**1062.125**	1062.629	**1061.621**	K[21]
T[23]	**1175.634**	**1006.087**	**998.077**	**998.581**	**997.573**	T[20]
T[24]	**1226.158**	**955.563**	947.553	948.057	**947.049**	T[19]
K[25]	**1290.206**	905.039	**897.030**	**897.533**	896.526	K[18]
K[26]	1354.253	840.991	**832.982**	**833.486**	**832.478**	K[17]
A[27]	**1389.772**	776.944	768.935	769.438	768.431	A[16]
G[28]	1418.282	741.425	**733.416**	**733.920**	**732.912**	G[15]
A[29]	1453.801	712.915	**704.905**	**705.409**	704.401	A[14]
A[30]	**1489.319**	677.396	**669.387**	**669.891**	668.883	A[13]
K[31]	**1553.367**	641.878	**633.868**	**634.372**	**633.364**	K[12]
R[32]	**1631.418**	577.830	569.821	570.325	569.317	R[11]
K[33]	**1695.465**	499.779	491.770	492.274	491.266	K[10]
A[34]	1730.984	435.732	427.723	428.227	427.219	A[9]
T[35]	1781.507	400.213	392.204	392.708	391.700	T[8]
G[36]	1810.018	349.690	341.680	342.184	341.176	G[7]
P[37]	1858.545	321.179	313.170	313.673	312.666	P[6]
P[38]	**1907.071**	272.652	264.643	265.147	264.139	P[5]
V[39]	1956.605	224.126	216.117	216.621	215.613	V[4]
S[40]	2000.121	174.592	166.583	167.086	166.079	S[3]
E[41]	2064.642	131.076	123.067	123.570	122.563	E[2]
L[42]	2121.184	66.555	58.545	59.049	58.041	L[1]

sp|P43276|H15_MOUSE

[Acetyl]SETAPAETAAPAPVEKSPAKKKTTKKAGAAKRKATGPPVSEL

- fragmentation table for charge state 3+
- specType=etd
- score=85.81
- F104930.dat
- query=q4405_p1
- precursor=849.276490
- chargePrecursor=5
- itol=0.8

AA	c	y	z+1	z+2	z	AA
S[1]	49.697	1414.787	1409.447	0.672	**1409.111**	S[42]
E[2]	92.711	1371.773	1366.433	0.672	1366.097	E[41]
T[3]	126.394	1328.758	**1323.419**	0.672	**1323.083**	T[40]
A[4]	150.073	1295.076	1289.736	0.672	1289.400	A[39]
P[5]	182.424	**1271.397**	1266.057	0.672	1265.721	P[38]
A[6]	206.103	1239.046	**1233.706**	0.672	**1233.370**	A[37]
E[7]	249.117	1215.367	1210.027	0.672	1209.691	E[36]
T[8]	282.800	1172.353	**1167.013**	0.672	**1166.677**	T[35]
A[9]	306.479	**1138.670**	**1133.330**	0.672	**1132.994**	A[34]
A[10]	330.158	1114.991	1109.651	0.672	1109.315	A[33]
P[11]	362.508	1091.312	**1085.972**	0.672	**1085.636**	P[32]
A[12]	386.188	1058.961	**1053.621**	0.672	1053.285	A[31]
P[13]	418.538	1035.282	1029.942	0.672	1029.606	P[30]
V[14]	451.561	1002.931	**997.591**	0.672	**997.256**	V[29]
E[15]	494.575	969.908	964.569	0.672	964.233	E[28]
K[16]	537.274	926.894	**921.554**	**921.890**	**921.219**	K[27]
S[17]	566.284	884.196	**878.856**	**879.192**	**878.520**	S[26]
P[18]	598.635	855.185	849.845	850.181	849.510	P[25]
A[19]	622.314	822.834	**817.495**	**817.831**	817.159	A[24]
K[20]	665.013	799.155	793.816	**794.151**	793.480	K[23]
K[21]	707.711	756.457	751.117	751.453	750.781	K[22]
K[22]	750.409	713.758	708.419	708.755	708.083	K[21]
T[23]	784.092	671.060	665.721	666.057	665.385	T[20]
T[24]	**817.774**	637.378	632.038	632.374	631.702	T[19]
K[25]	860.473	603.695	598.355	598.691	598.019	K[18]
K[26]	903.171	560.997	555.657	555.993	555.321	K[17]
A[27]	926.850	518.298	512.959	513.295	512.623	A[16]
G[28]	**945.857**	494.619	489.280	489.616	488.944	G[15]
A[29]	969.536	475.612	470.273	470.609	469.937	A[14]
A[30]	**993.215**	451.933	446.594	446.930	446.258	A[13]
K[31]	**1035.914**	428.254	422.915	423.250	422.579	K[12]
R[32]	**1087.947**	385.556	380.216	380.552	379.880	R[11]
K[33]	**1130.646**	333.522	328.183	328.518	327.847	K[10]
A[34]	**1154.325**	290.824	285.484	285.820	285.148	A[9]
T[35]	**1188.007**	267.145	261.805	262.141	261.469	T[8]
G[36]	1207.015	233.462	228.123	228.459	227.787	G[7]
P[37]	1239.365	214.455	209.115	209.451	208.779	P[6]
P[38]	**1271.716**	182.104	176.765	177.100	176.429	P[5]
V[39]	**1304.739**	149.753	144.414	144.750	144.078	V[4]
S[40]	**1333.750**	116.730	111.391	111.727	111.055	S[3]
E[41]	**1376.764**	87.720	82.380	82.716	82.044	E[2]
L[42]	1414.459	44.705	39.366	39.702	39.030	L[1]

sp|P43276|H15_MOUSE

[Acetyl]SETAPAETAAPAPVEKSPAKKKTTKKAGAAKRKATGPPVSEL

- fragmentation table for charge state 4+
- specType=etd
- score=85.81
- F104930.dat
- query=q4405_p1
- precursor=849.276490
- chargePrecursor=5
- itol=0.8

AA	c	y	z+1	z+2	z	AA
S[1]	37.525	**1061.342**	**1057.337**	0.755	**1057.085**	S[42]
E[2]	69.785	1029.081	**1025.077**	0.755	**1024.825**	E[41]
T[3]	95.047	**996.821**	**992.816**	0.755	**992.564**	T[40]
A[4]	112.806	**971.559**	**967.554**	0.755	**967.302**	A[39]
P[5]	137.070	953.799	949.795	0.755	949.543	P[38]
A[6]	154.829	929.536	925.531	0.755	925.280	A[37]
E[7]	187.090	911.777	907.772	0.755	907.520	E[36]
T[8]	212.351	**879.516**	875.512	0.755	875.260	T[35]
A[9]	230.111	854.254	850.250	0.755	849.998	A[34]
A[10]	247.870	836.495	**832.490**	0.755	832.238	A[33]
P[11]	272.133	**818.736**	814.731	0.755	814.479	P[32]
A[12]	289.892	**794.473**	790.468	0.755	790.216	A[31]
P[13]	314.156	776.713	772.709	0.755	772.457	P[30]
V[14]	338.923	752.450	748.445	0.755	748.193	V[29]
E[15]	371.183	727.683	723.678	0.755	723.426	E[28]
K[16]	403.207	695.422	691.418	691.670	691.166	K[27]
S[17]	424.965	663.399	659.394	659.646	659.142	S[26]
P[18]	449.228	641.641	637.636	637.888	637.384	P[25]
A[19]	466.988	617.377	613.373	613.625	613.121	A[24]
K[20]	499.011	599.618	595.613	595.865	595.362	K[23]
K[21]	531.035	567.594	563.590	563.842	563.338	K[22]
K[22]	563.059	535.571	531.566	531.818	531.314	K[21]
T[23]	588.321	503.547	499.542	499.794	499.290	T[20]
T[24]	613.583	478.285	474.280	474.532	474.028	T[19]
K[25]	645.606	453.023	449.018	449.270	448.766	K[18]
K[26]	677.630	420.999	416.995	417.247	416.743	K[17]
A[27]	695.389	388.976	384.971	385.223	384.719	A[16]
G[28]	709.645	371.216	367.212	367.464	366.960	G[15]
A[29]	727.404	356.961	352.956	353.208	352.704	A[14]
A[30]	745.163	339.202	335.197	335.449	334.945	A[13]
K[31]	777.187	321.442	317.438	317.690	317.186	K[12]
R[32]	816.212	289.419	285.414	285.666	285.162	R[11]
K[33]	848.236	250.393	246.389	246.641	246.137	K[10]
A[34]	865.995	218.370	214.365	214.617	214.113	A[9]
T[35]	891.257	200.610	196.606	196.858	196.354	T[8]
G[36]	905.513	175.348	171.344	171.596	171.092	G[7]
P[37]	929.776	161.093	157.088	157.340	156.836	P[6]
P[38]	954.039	136.830	132.825	133.077	132.573	P[5]
V[39]	**978.806**	112.567	108.562	108.814	108.310	V[4]
S[40]	**1000.564**	87.800	83.795	84.047	83.543	S[3]
E[41]	**1032.825**	66.042	62.037	62.289	61.785	E[2]
L[42]	**1061.096**	33.781	29.776	30.028	29.524	L[1]

sp|P43276|H15_MOUSE

[Acetyl]SETAPAETAAPAPVEKSPAKKKTTKKAGAAKRKATGPPVSEL

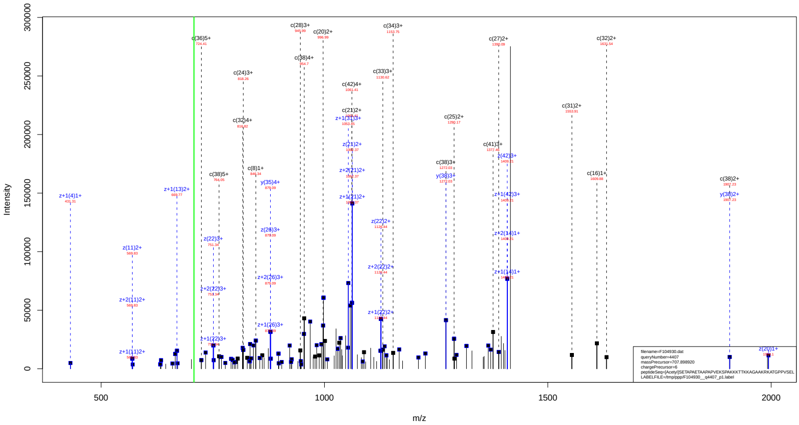

sp|P43276|H15_MOUSE

[Acetyl]SETAPAETAAPAPVEKSPAKKKTTKKAGAAKRKATGPPVSEL

- fragmentation table for charge state 1+
- specType=etd
- score=35.84
- F104930.dat
- query=q4407_p1
- precursor=707.898920
- chargePrecursor=6
- itol=0.8

AA	c	y	z+1	z+2	z	AA
S[1]	147.076	4242.346	4226.327	0.000	4225.319	S[42]
E[2]	276.119	4113.303	4097.284	0.000	4096.276	E[41]
T[3]	377.167	3984.260	3968.242	0.000	3967.234	T[40]
A[4]	448.204	3883.213	3867.194	0.000	3866.186	A[39]
P[5]	545.257	3812.176	3796.157	0.000	3795.149	P[38]
A[6]	616.294	3715.123	3699.104	0.000	3698.096	A[37]
E[7]	745.336	3644.086	3628.067	0.000	3627.059	E[36]
T[8]	**846.384**	3515.043	3499.024	0.000	3498.017	T[35]
A[9]	917.421	3413.995	3397.977	0.000	3396.969	A[34]
A[10]	**988.458**	3342.958	3326.940	0.000	3325.932	A[33]
P[11]	**1085.511**	3271.921	3255.903	0.000	3254.895	P[32]
A[12]	1156.548	3174.868	3158.850	0.000	3157.842	A[31]
P[13]	1253.601	3103.831	3087.813	0.000	3086.805	P[30]
V[14]	1352.669	3006.779	2990.760	0.000	2989.752	V[29]
E[15]	1481.712	2907.710	2891.691	0.000	2890.684	E[28]
K[16]	**1609.807**	2778.668	2762.649	2763.657	2761.641	K[27]
S[17]	1696.839	2650.573	2634.554	2635.562	2633.546	S[26]
P[18]	1793.892	2563.541	2547.522	2548.530	2546.514	P[25]
A[19]	1864.929	2466.488	2450.469	2451.477	2449.461	A[24]
K[20]	1993.024	2395.451	2379.432	2380.440	2378.424	K[23]
K[21]	2121.119	2267.356	2251.337	2252.345	2250.329	K[22]
K[22]	2249.214	2139.261	2123.242	2124.250	2122.234	K[21]
T[23]	2350.261	2011.166	1995.147	1996.155	**1994.139**	T[20]
T[24]	2451.309	1910.118	1894.099	1895.107	1893.092	T[19]
K[25]	2579.404	1809.070	1793.052	1794.060	1792.044	K[18]
K[26]	2707.499	1680.976	1664.957	1665.965	1663.949	K[17]
A[27]	2778.536	1552.881	1536.862	1537.870	1535.854	A[16]
G[28]	2835.557	1481.843	1465.825	1466.833	1464.817	G[15]
A[29]	2906.595	1424.822	**1408.803**	**1409.811**	1407.795	A[14]
A[30]	2977.632	1353.785	1337.766	1338.774	1336.758	A[13]
K[31]	3105.727	1282.748	1266.729	1267.737	1265.721	K[12]
R[32]	3261.828	1154.653	**1138.634**	1139.642	1137.626	R[11]
K[33]	3389.923	**998.552**	**982.533**	983.541	981.525	K[10]
A[34]	3460.960	870.457	**854.438**	**855.446**	853.430	A[9]
T[35]	3562.008	799.420	783.401	784.409	782.393	T[8]
G[36]	3619.029	698.372	682.353	683.361	681.345	G[7]
P[37]	3716.082	641.350	625.332	626.340	624.324	P[6]
P[38]	3813.135	544.298	528.279	529.287	527.271	P[5]
V[39]	3912.203	447.245	**431.226**	432.234	430.218	V[4]
S[40]	3999.235	348.177	332.158	333.166	331.150	S[3]
E[41]	4128.278	261.144	245.126	246.134	244.118	E[2]
L[42]	4241.362	132.102	116.083	117.091	115.075	L[1]

sp|P43276|H15_MOUSE

[Acetyl]SETAPAETAAPAPVEKSPAKKKTTKKAGAAKRKATGPPVSEL

- fragmentation table for charge state 2+
- specType=etd
- score=35.84
- F104930.dat
- query=q4407_p1
- precursor=707.898920
- chargePrecursor=6
- itol=0.8

AA	c	y	z+1	z+2	z	AA
S[1]	74.042	2121.676	2113.667	0.504	2113.163	S[42]
E[2]	138.563	2057.155	2049.146	0.504	2048.642	E[41]
T[3]	189.087	1992.634	1984.624	0.504	1984.121	T[40]
A[4]	224.606	1942.110	1934.101	0.504	1933.597	A[39]
P[5]	273.132	**1906.591**	1898.582	0.504	1898.078	P[38]
A[6]	308.650	1858.065	1850.056	0.504	1849.552	A[37]
E[7]	373.172	1822.547	1814.537	0.504	1814.033	E[36]
T[8]	423.696	1758.025	1750.016	0.504	1749.512	T[35]
A[9]	459.214	1707.501	1699.492	0.504	1698.988	A[34]
A[10]	494.733	1671.983	1663.973	0.504	1663.470	A[33]
P[11]	543.259	1636.464	1628.455	0.504	1627.951	P[32]
A[12]	578.778	1587.938	1579.929	0.504	1579.425	A[31]
P[13]	627.304	1552.419	1544.410	0.504	1543.906	P[30]
V[14]	676.838	1503.893	1495.884	0.504	1495.380	V[29]
E[15]	741.360	1454.359	1446.349	0.504	1445.845	E[28]
K[16]	**805.407**	**1389.837**	1381.828	1382.332	1381.324	K[27]
S[17]	848.923	1325.790	**1317.781**	**1318.285**	**1317.277**	S[26]
P[18]	**897.449**	1282.274	1274.265	1274.768	1273.761	P[25]
A[19]	932.968	1233.748	**1225.738**	**1226.242**	**1225.234**	A[24]
K[20]	**997.015**	1198.229	1190.220	1190.724	1189.716	K[23]
K[21]	**1061.063**	**1134.182**	**1126.172**	**1126.676**	**1125.668**	K[22]
K[22]	**1125.110**	1070.134	**1062.125**	**1062.629**	**1061.621**	K[21]
T[23]	1175.634	**1006.087**	**998.077**	**998.581**	**997.573**	T[20]
T[24]	**1226.158**	955.563	**947.553**	**948.057**	**947.049**	T[19]
K[25]	**1290.206**	**905.039**	**897.030**	**897.533**	**896.526**	K[18]
K[26]	1354.253	**840.991**	**832.982**	**833.486**	**832.478**	K[17]
A[27]	**1389.772**	776.944	**768.935**	**769.438**	768.431	A[16]
G[28]	1418.282	741.425	**733.416**	**733.920**	732.912	G[15]
A[29]	1453.801	712.915	704.905	705.409	704.401	A[14]
A[30]	1489.319	677.396	**669.387**	**669.891**	668.883	A[13]
K[31]	**1553.367**	641.878	**633.868**	**634.372**	**633.364**	K[12]
R[32]	**1631.418**	577.830	**569.821**	**570.325**	**569.317**	R[11]
K[33]	1695.465	499.779	491.770	492.274	491.266	K[10]
A[34]	1730.984	435.732	427.723	428.227	427.219	A[9]
T[35]	1781.507	400.213	392.204	392.708	391.700	T[8]
G[36]	1810.018	349.690	341.680	342.184	341.176	G[7]
P[37]	1858.545	321.179	313.170	313.673	312.666	P[6]
P[38]	**1907.071**	272.652	264.643	265.147	264.139	P[5]
V[39]	1956.605	224.126	216.117	216.621	215.613	V[4]
S[40]	2000.121	174.592	166.583	167.086	166.079	S[3]
E[41]	2064.642	131.076	123.067	123.570	122.563	E[2]
L[42]	2121.184	66.555	58.545	59.049	58.041	L[1]

sp|P43276|H15_MOUSE

[Acetyl]SETAPAETAAPAPVEKSPAKKKTTKKAGAAKRKATGPPVSEL

- fragmentation table for charge state 3+
- specType=etd
- score=35.84
- F104930.dat
- query=q4407_p1
- precursor=707.898920
- chargePrecursor=6
- itol=0.8

AA	c	y	z+1	z+2	z	AA
S[1]	49.697	1414.787	**1409.447**	0.672	**1409.111**	S[42]
E[2]	92.711	**1371.773**	**1366.433**	0.672	**1366.097**	E[41]
T[3]	126.394	1328.758	1323.419	0.672	1323.083	T[40]
A[4]	150.073	**1295.076**	**1289.736**	0.672	**1289.400**	A[39]
P[5]	182.424	**1271.397**	1266.057	0.672	1265.721	P[38]
A[6]	206.103	1239.046	1233.706	0.672	1233.370	A[37]
E[7]	249.117	1215.367	**1210.027**	0.672	**1209.691**	E[36]
T[8]	282.800	1172.353	**1167.013**	0.672	**1166.677**	T[35]
A[9]	306.479	**1138.670**	1133.330	0.672	1132.994	A[34]
A[10]	330.158	1114.991	1109.651	0.672	1109.315	A[33]
P[11]	362.508	1091.312	**1085.972**	0.672	**1085.636**	P[32]
A[12]	386.188	1058.961	**1053.621**	0.672	**1053.285**	A[31]
P[13]	418.538	**1035.282**	**1029.942**	0.672	**1029.606**	P[30]
V[14]	451.561	1002.931	**997.591**	0.672	**997.256**	V[29]
E[15]	494.575	969.908	964.569	0.672	964.233	E[28]
K[16]	537.274	**926.894**	**921.554**	**921.890**	921.219	K[27]
S[17]	566.284	884.196	**878.856**	**879.192**	**878.520**	S[26]
P[18]	598.635	**855.185**	849.845	850.181	849.510	P[25]
A[19]	622.314	822.834	**817.495**	**817.831**	**817.159**	A[24]
K[20]	665.013	799.155	**793.816**	**794.151**	793.480	K[23]
K[21]	707.711	756.457	**751.117**	**751.453**	**750.781**	K[22]
K[22]	750.409	713.758	708.419	708.755	708.083	K[21]
T[23]	784.092	**671.060**	**665.721**	**666.057**	**665.385**	T[20]
T[24]	**817.774**	637.378	**632.038**	**632.374**	631.702	T[19]
K[25]	**860.473**	603.695	598.355	598.691	598.019	K[18]
K[26]	903.171	560.997	555.657	555.993	555.321	K[17]
A[27]	**926.850**	518.298	512.959	513.295	512.623	A[16]
G[28]	**945.857**	494.619	489.280	489.616	488.944	G[15]
A[29]	969.536	475.612	470.273	470.609	469.937	A[14]
A[30]	**993.215**	451.933	446.594	446.930	446.258	A[13]
K[31]	**1035.914**	428.254	422.915	423.250	422.579	K[12]
R[32]	**1087.947**	385.556	380.216	380.552	379.880	R[11]
K[33]	**1130.646**	333.522	328.183	328.518	327.847	K[10]
A[34]	**1154.325**	290.824	285.484	285.820	285.148	A[9]
T[35]	1188.007	267.145	261.805	262.141	261.469	T[8]
G[36]	1207.015	233.462	228.123	228.459	227.787	G[7]
P[37]	1239.365	214.455	209.115	209.451	208.779	P[6]
P[38]	**1271.716**	182.104	176.765	177.100	176.429	P[5]
V[39]	1304.739	149.753	144.414	144.750	144.078	V[4]
S[40]	1333.750	116.730	111.391	111.727	111.055	S[3]
E[41]	**1376.764**	87.720	82.380	82.716	82.044	E[2]
L[42]	1414.459	44.705	39.366	39.702	39.030	L[1]

sp|P43276|H15_MOUSE

[Acetyl]SETAPAETAAPAPVEKSPAKKKTTKKAGAAKRKATGPPVSEL

- fragmentation table for charge state 4+
- specType=etd
- score=35.84
- F104930.dat
- query=q4407_p1
- precursor=707.898920
- chargePrecursor=6
- itol=0.8

AA	c	y	z+1	z+2	z	AA
S[1]	37.525	**1061.342**	**1057.337**	0.755	**1057.085**	S[42]
E[2]	69.785	**1029.081**	1025.077	0.755	1024.825	E[41]
T[3]	95.047	**996.821**	**992.816**	0.755	**992.564**	T[40]
A[4]	112.806	971.559	**967.554**	0.755	967.302	A[39]
P[5]	137.070	**953.799**	949.795	0.755	949.543	P[38]
A[6]	154.829	929.536	**925.531**	0.755	**925.280**	A[37]
E[7]	187.090	911.777	907.772	0.755	907.520	E[36]
T[8]	212.351	**879.516**	875.512	0.755	875.260	T[35]
A[9]	230.111	854.254	850.250	0.755	849.998	A[34]
A[10]	247.870	**836.495**	**832.490**	0.755	**832.238**	A[33]
P[11]	272.133	**818.736**	814.731	0.755	814.479	P[32]
A[12]	289.892	**794.473**	**790.468**	0.755	**790.216**	A[31]
P[13]	314.156	776.713	772.709	0.755	772.457	P[30]
V[14]	338.923	**752.450**	748.445	0.755	748.193	V[29]
E[15]	371.183	727.683	**723.678**	0.755	723.426	E[28]
K[16]	403.207	695.422	691.418	691.670	691.166	K[27]
S[17]	424.965	663.399	**659.394**	**659.646**	**659.142**	S[26]
P[18]	449.228	641.641	637.636	637.888	637.384	P[25]
A[19]	466.988	617.377	613.373	613.625	613.121	A[24]
K[20]	499.011	599.618	595.613	595.865	595.362	K[23]
K[21]	531.035	567.594	563.590	563.842	563.338	K[22]
K[22]	563.059	535.571	531.566	531.818	531.314	K[21]
T[23]	588.321	503.547	499.542	499.794	499.290	T[20]
T[24]	613.583	478.285	474.280	474.532	474.028	T[19]
K[25]	645.606	453.023	449.018	449.270	448.766	K[18]
K[26]	677.630	420.999	416.995	417.247	416.743	K[17]
A[27]	695.389	388.976	384.971	385.223	384.719	A[16]
G[28]	709.645	371.216	367.212	367.464	366.960	G[15]
A[29]	727.404	356.961	352.956	353.208	352.704	A[14]
A[30]	745.163	339.202	335.197	335.449	334.945	A[13]
K[31]	777.187	321.442	317.438	317.690	317.186	K[12]
R[32]	**816.212**	289.419	285.414	285.666	285.162	R[11]
K[33]	848.236	250.393	246.389	246.641	246.137	K[10]
A[34]	865.995	218.370	214.365	214.617	214.113	A[9]
T[35]	891.257	200.610	196.606	196.858	196.354	T[8]
G[36]	905.513	175.348	171.344	171.596	171.092	G[7]
P[37]	929.776	161.093	157.088	157.340	156.836	P[6]
P[38]	**954.039**	136.830	132.825	133.077	132.573	P[5]
V[39]	**978.806**	112.567	108.562	108.814	108.310	V[4]
S[40]	**1000.564**	87.800	83.795	84.047	83.543	S[3]
E[41]	**1032.825**	66.042	62.037	62.289	61.785	E[2]
L[42]	**1061.096**	33.781	29.776	30.028	29.524	L[1]

sp|P43276|H15_MOUSE

[Acetyl]SETAPAETAAPAPVEKSPAKKKTTKKAGAAKRKATGPPVSEL

- fragmentation table for charge state 5+
- specType=etd
- score=35.84
- F104930.dat
- query=q4407_p1
- precursor=707.898920
- chargePrecursor=6
- itol=0.8

AA	c	y	z+1	z+2	z	AA
S[1]	30.221	849.275	**846.071**	0.806	**845.870**	S[42]
E[2]	56.030	823.466	820.263	0.806	820.061	E[41]
T[3]	76.239	**797.658**	**794.454**	0.806	**794.253**	T[40]
A[4]	90.447	**777.448**	774.245	0.806	774.043	A[39]
P[5]	109.857	763.241	760.037	0.806	759.836	P[38]
A[6]	124.065	743.830	740.627	0.806	740.425	A[37]
E[7]	149.873	729.623	726.419	0.806	726.218	E[36]
T[8]	170.083	703.814	700.611	0.806	700.409	T[35]
A[9]	184.290	683.605	680.401	0.806	680.200	A[34]
A[10]	198.497	**669.397**	**666.194**	0.806	**665.992**	A[33]
P[11]	217.908	655.190	651.986	0.806	651.785	P[32]
A[12]	232.115	635.780	**632.576**	0.806	**632.374**	A[31]
P[13]	251.526	621.572	618.368	0.806	618.167	P[30]
V[14]	271.340	602.162	598.958	0.806	598.756	V[29]
E[15]	297.148	582.348	579.144	0.806	578.943	E[28]
K[16]	322.767	556.539	553.336	553.537	553.134	K[27]
S[17]	340.174	530.920	527.717	527.918	527.515	S[26]
P[18]	359.584	513.514	510.310	510.512	510.109	P[25]
A[19]	373.792	494.103	490.900	491.101	490.698	A[24]
K[20]	399.411	479.896	476.692	476.894	476.491	K[23]
K[21]	425.030	454.277	451.073	451.275	450.872	K[22]
K[22]	450.649	428.658	425.454	425.656	425.253	K[21]
T[23]	470.858	403.039	399.835	400.037	399.634	T[20]
T[24]	491.068	382.829	379.626	379.827	379.424	T[19]
K[25]	516.687	362.620	359.416	359.618	359.215	K[18]
K[26]	542.306	337.001	333.797	333.999	333.596	K[17]
A[27]	556.513	311.382	308.178	308.380	307.977	A[16]
G[28]	567.917	297.175	293.971	294.172	293.769	G[15]
A[29]	582.125	285.770	282.566	282.768	282.365	A[14]
A[30]	596.332	271.563	268.359	268.561	268.157	A[13]
K[31]	621.951	257.355	254.152	254.353	253.950	K[12]
R[32]	653.171	231.736	228.533	228.734	228.331	R[11]
K[33]	678.790	200.516	197.312	197.514	197.111	K[10]
A[34]	692.998	174.897	171.693	171.895	171.492	A[9]
T[35]	713.207	160.690	157.486	157.688	157.284	T[8]
G[36]	**724.612**	140.480	137.276	137.478	137.075	G[7]
P[37]	744.022	129.076	125.872	126.074	125.671	P[6]
P[38]	**763.433**	109.665	106.462	106.663	106.260	P[5]
V[39]	783.246	90.255	87.051	87.253	86.849	V[4]
S[40]	**800.653**	70.441	67.237	67.439	67.036	S[3]
E[41]	**826.461**	53.035	49.831	50.033	49.629	E[2]
L[42]	849.078	27.226	24.022	24.224	23.821	L[1]

sp|P43276|H15_MOUSE

[Acetyl]SETAPAETAAPAPVEKS $^{Phospho}_{79.97}$ PAKKKTTKKAGAAKRKATGPPVSEL

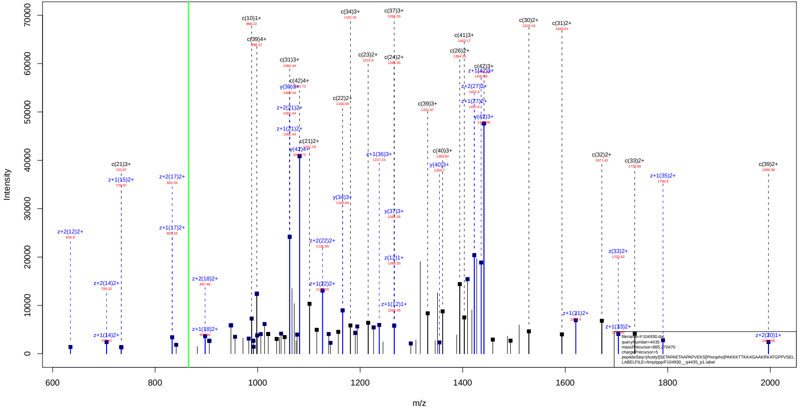

sp|P43276|H15_MOUSE

[Acetyl]SETAPAETAAPAPVEKS $^{Phospho}_{79.97}$ PAKKKTTKKAGAAKRKATGPPVSEL

- fragmentation table for charge state 1+
- specType=etd
- score=85.43
- F104930.dat
- query=q4435_p1
- precursor=865.270470
- chargePrecursor=5
- itol=0.8

AA	c	y	z+1	z+2	z	AA
S[1]	147.076	4322.312	4306.293	0.000	4305.285	S[42]
E[2]	276.119	4193.269	4177.251	0.000	4176.243	E[41]
T[3]	377.167	4064.227	4048.208	0.000	4047.200	T[40]
A[4]	448.204	3963.179	3947.160	0.000	3946.153	A[39]
P[5]	545.257	3892.142	3876.123	0.000	3875.115	P[38]
A[6]	616.294	3795.089	3779.070	0.000	3778.063	A[37]
E[7]	745.336	3724.052	3708.033	0.000	3707.026	E[36]
T[8]	846.384	3595.009	3578.991	0.000	3577.983	T[35]
A[9]	917.421	3493.962	3477.943	0.000	3476.935	A[34]
A[10]	**988.458**	3422.925	3406.906	0.000	3405.898	A[33]
P[11]	1085.511	3351.888	3335.869	0.000	3334.861	P[32]
A[12]	1156.548	3254.835	3238.816	0.000	3237.808	A[31]
P[13]	1253.601	3183.798	3167.779	0.000	3166.771	P[30]
V[14]	1352.669	3086.745	3070.726	0.000	3069.718	V[29]
E[15]	1481.712	2987.677	2971.658	0.000	2970.650	E[28]
K[16]	1609.807	2858.634	2842.615	2843.623	2841.607	K[27]
S[17]	1776.805	2730.539	2714.520	2715.528	2713.512	S[26]
P[18]	1873.858	2563.541	2547.522	2548.530	2546.514	P[25]
A[19]	1944.895	2466.488	2450.469	2451.477	2449.461	A[24]
K[20]	2072.990	2395.451	2379.432	2380.440	2378.424	K[23]
K[21]	2201.085	2267.356	2251.337	2252.345	2250.329	K[22]
K[22]	2329.180	2139.261	2123.242	2124.250	2122.234	K[21]
T[23]	2430.228	2011.166	1995.147	**1996.155**	1994.139	T[20]
T[24]	2531.275	1910.118	1894.099	1895.107	1893.092	T[19]
K[25]	2659.370	1809.070	1793.052	1794.060	1792.044	K[18]
K[26]	2787.465	1680.976	1664.957	1665.965	1663.949	K[17]
A[27]	2858.502	1552.881	1536.862	1537.870	1535.854	A[16]
G[28]	2915.524	1481.843	1465.825	1466.833	1464.817	G[15]
A[29]	2986.561	1424.822	**1408.803**	**1409.811**	1407.795	A[14]
A[30]	3057.598	1353.785	1337.766	1338.774	1336.758	A[13]
K[31]	3185.693	1282.748	**1266.729**	1267.737	**1265.721**	K[12]
R[32]	3341.794	1154.653	**1138.634**	1139.642	1137.626	R[11]
K[33]	3469.889	**998.552**	**982.533**	983.541	981.525	K[10]
A[34]	3540.926	870.457	854.438	855.446	853.430	A[9]
T[35]	3641.974	799.420	783.401	784.409	782.393	T[8]
G[36]	3698.995	698.372	682.353	683.361	681.345	G[7]
P[37]	3796.048	641.350	625.332	626.340	624.324	P[6]
P[38]	3893.101	544.298	528.279	529.287	527.271	P[5]
V[39]	3992.169	447.245	431.226	432.234	430.218	V[4]
S[40]	4079.201	348.177	332.158	333.166	331.150	S[3]
E[41]	4208.244	261.144	245.126	246.134	244.118	E[2]
L[42]	4321.328	132.102	116.083	117.091	115.075	L[1]

sp|P43276|H15_MOUSE

[Acetyl]SETAPAETAAPAPVEKS $^{Phospho}_{79.97}$ PAKKKTTKKAGAAKRKATGPPVSEL

- fragmentation table for charge state 2+
- specType=etd
- score=85.43
- F104930.dat
- query=q4435_p1
- precursor=865.270470
- chargePrecursor=5
- itol=0.8

AA	c	y	z+1	z+2	z	AA
S[1]	74.042	2161.660	2153.650	0.504	2153.146	S[42]
E[2]	138.563	2097.138	2089.129	0.504	2088.625	E[41]
T[3]	189.087	2032.617	2024.608	0.504	2024.104	T[40]
A[4]	224.606	1982.093	1974.084	0.504	1973.580	A[39]
P[5]	273.132	1946.575	1938.565	0.504	1938.061	P[38]
A[6]	308.650	1898.048	1890.039	0.504	1889.535	A[37]
E[7]	373.172	1862.530	1854.520	0.504	1854.016	E[36]
T[8]	423.696	1798.008	**1789.999**	0.504	1789.495	T[35]
A[9]	459.214	1747.485	1739.475	0.504	1738.971	A[34]
A[10]	494.733	1711.966	**1703.957**	0.504	**1703.453**	A[33]
P[11]	543.259	1676.447	1668.438	0.504	1667.934	P[32]
A[12]	578.778	1627.921	**1619.912**	0.504	1619.408	A[31]
P[13]	627.304	1592.402	1584.393	0.504	1583.889	P[30]
V[14]	676.838	1543.876	1535.867	0.504	1535.363	V[29]
E[15]	741.360	1494.342	1486.333	0.504	1485.829	E[28]
K[16]	805.407	1429.821	**1421.811**	**1422.315**	1421.307	K[27]
S[17]	888.906	1365.773	1357.764	1358.268	1357.260	S[26]
P[18]	937.433	1282.274	1274.265	1274.768	1273.761	P[25]
A[19]	972.951	1233.748	**1225.738**	**1226.242**	1225.234	A[24]
K[20]	**1036.999**	1198.229	**1190.220**	**1190.724**	**1189.716**	K[23]
K[21]	**1101.046**	1134.182	**1126.172**	**1126.676**	1125.668	K[22]
K[22]	**1165.094**	1070.134	**1062.125**	**1062.629**	1061.621	K[21]
T[23]	**1215.617**	**1006.087**	**998.077**	**998.581**	**997.573**	T[20]
T[24]	**1266.141**	**955.563**	**947.553**	**948.057**	947.049	T[19]
K[25]	1330.189	**905.039**	**897.030**	**897.533**	896.526	K[18]
K[26]	**1394.236**	**840.991**	**832.982**	**833.486**	832.478	K[17]
A[27]	1429.755	776.944	768.935	769.438	768.431	A[16]
G[28]	**1458.266**	741.425	**733.416**	**733.920**	732.912	G[15]
A[29]	**1493.784**	712.915	**704.905**	**705.409**	704.401	A[14]
A[30]	**1529.303**	677.396	669.387	669.891	668.883	A[13]
K[31]	**1593.350**	641.878	633.868	**634.372**	633.364	K[12]
R[32]	**1671.401**	577.830	569.821	570.325	569.317	R[11]
K[33]	**1735.448**	499.779	491.770	492.274	491.266	K[10]
A[34]	1770.967	435.732	427.723	428.227	427.219	A[9]
T[35]	1821.491	400.213	392.204	392.708	391.700	T[8]
G[36]	1850.001	349.690	341.680	342.184	341.176	G[7]
P[37]	1898.528	321.179	313.170	313.673	312.666	P[6]
P[38]	1947.054	272.652	264.643	265.147	264.139	P[5]
V[39]	**1996.588**	224.126	216.117	216.621	215.613	V[4]
S[40]	2040.104	174.592	166.583	167.086	166.079	S[3]
E[41]	2104.626	131.076	123.067	123.570	122.563	E[2]
L[42]	2161.168	66.555	58.545	59.049	58.041	L[1]

sp|P43276|H15_MOUSE

[Acetyl]SETAPAETAAPAPVEKS $^{\text{Phospho}}_{79.97}$ PAKKKTTKKAGAAKRKATGPPVSEL

- fragmentation table for charge state 3+
- specType=etd
- score=85.43
- F104930.dat
- query=q4435_p1
- precursor=865.270470
- chargePrecursor=5
- itol=0.8

AA	c	y	z+1	z+2	z	AA
S[1]	49.697	**1441.442**	**1436.103**	0.672	**1435.767**	S[42]
E[2]	92.711	1398.428	1393.088	0.672	1392.752	E[41]
T[3]	126.394	**1355.414**	1350.074	0.672	1349.738	T[40]
A[4]	150.073	1321.731	1316.392	0.672	1316.056	A[39]
P[5]	182.424	**1298.052**	1292.713	0.672	1292.377	P[38]
A[6]	206.103	**1265.701**	1260.362	0.672	1260.026	A[37]
E[7]	249.117	1242.022	**1236.683**	0.672	1236.347	E[36]
T[8]	282.800	1199.008	**1193.668**	0.672	1193.332	T[35]
A[9]	306.479	**1165.325**	1159.986	0.672	1159.650	A[34]
A[10]	330.158	**1141.646**	1136.307	0.672	1135.971	A[33]
P[11]	362.508	1117.967	1112.628	0.672	1112.292	P[32]
A[12]	386.188	1085.616	1080.277	0.672	1079.941	A[31]
P[13]	418.538	**1061.937**	1056.598	0.672	1056.262	P[30]
V[14]	451.561	1029.586	1024.247	0.672	1023.911	V[29]
E[15]	494.575	996.564	**991.224**	0.672	**990.888**	E[28]
K[16]	537.274	953.549	**948.210**	**948.546**	**947.874**	K[27]
S[17]	592.940	910.851	**905.512**	**905.848**	**905.176**	S[26]
P[18]	625.291	855.185	849.845	850.181	849.510	P[25]
A[19]	648.970	822.834	817.495	817.831	817.159	A[24]
K[20]	691.668	799.155	793.816	794.151	793.480	K[23]
K[21]	**734.367**	756.457	751.117	751.453	750.781	K[22]
K[22]	777.065	713.758	708.419	708.755	708.083	K[21]
T[23]	810.747	671.060	665.721	666.057	665.385	T[20]
T[24]	844.430	637.378	632.038	632.374	631.702	T[19]
K[25]	887.128	603.695	598.355	598.691	598.019	K[18]
K[26]	929.827	560.997	555.657	555.993	555.321	K[17]
A[27]	953.506	518.298	512.959	513.295	512.623	A[16]
G[28]	972.513	494.619	489.280	489.616	488.944	G[15]
A[29]	996.192	475.612	470.273	470.609	469.937	A[14]
A[30]	**1019.871**	451.933	446.594	446.930	446.258	A[13]
K[31]	**1062.569**	428.254	422.915	423.250	422.579	K[12]
R[32]	**1114.603**	385.556	380.216	380.552	379.880	R[11]
K[33]	**1157.301**	333.522	328.183	328.518	327.847	K[10]
A[34]	**1180.980**	290.824	285.484	285.820	285.148	A[9]
T[35]	1214.663	267.145	261.805	262.141	261.469	T[8]
G[36]	1233.670	233.462	228.123	228.459	227.787	G[7]
P[37]	**1266.021**	214.455	209.115	209.451	208.779	P[6]
P[38]	**1298.372**	182.104	176.765	177.100	176.429	P[5]
V[39]	**1331.395**	149.753	144.414	144.750	144.078	V[4]
S[40]	**1360.405**	116.730	111.391	111.727	111.055	S[3]
E[41]	**1403.419**	87.720	82.380	82.716	82.044	E[2]
L[42]	**1441.114**	44.705	39.366	39.702	39.030	L[1]

sp|P43276|H15_MOUSE

[Acetyl]SETAPAETAAPAPVEKS $^{Phospho}_{79.97}$ PAKKKTTKKAGAAKRKATGPPVSEL

- fragmentation table for charge state 4+
- specType=etd
- score=85.43
- F104930.dat
- query=q4435_p1
- precursor=865.270470
- chargePrecursor=5
- itol=0.8

AA	c	y	z+1	z+2	z	AA
S[1]	37.525	**1081.333**	**1077.329**	0.755	**1077.077**	S[42]
E[2]	69.785	1049.073	**1045.068**	0.755	1044.816	E[41]
T[3]	95.047	1016.812	**1012.807**	0.755	1012.556	T[40]
A[4]	112.806	**991.550**	**987.546**	0.755	987.294	A[39]
P[5]	137.070	973.791	969.786	0.755	969.534	P[38]
A[6]	154.829	949.528	945.523	0.755	945.271	A[37]
E[7]	187.090	931.768	927.764	0.755	927.512	E[36]
T[8]	212.351	899.508	895.503	0.755	895.251	T[35]
A[9]	230.111	874.246	870.241	0.755	869.989	A[34]
A[10]	247.870	856.487	852.482	0.755	852.230	A[33]
P[11]	272.133	838.727	834.723	0.755	834.471	P[32]
A[12]	289.892	814.464	810.459	0.755	810.208	A[31]
P[13]	314.156	796.705	792.700	0.755	792.448	P[30]
V[14]	338.923	772.442	768.437	0.755	768.185	V[29]
E[15]	371.183	747.675	743.670	0.755	743.418	E[28]
K[16]	403.207	715.414	711.409	711.661	711.157	K[27]
S[17]	444.957	683.390	679.386	679.637	679.134	S[26]
P[18]	469.220	641.641	637.636	637.888	637.384	P[25]
A[19]	486.979	617.377	613.373	613.625	613.121	A[24]
K[20]	519.003	599.618	595.613	595.865	595.362	K[23]
K[21]	551.027	567.594	563.590	563.842	563.338	K[22]
K[22]	583.050	535.571	531.566	531.818	531.314	K[21]
T[23]	608.312	503.547	499.542	499.794	499.290	T[20]
T[24]	633.574	478.285	474.280	474.532	474.028	T[19]
K[25]	665.598	453.023	449.018	449.270	448.766	K[18]
K[26]	697.622	420.999	416.995	417.247	416.743	K[17]
A[27]	715.381	388.976	384.971	385.223	384.719	A[16]
G[28]	729.636	371.216	367.212	367.464	366.960	G[15]
A[29]	747.396	356.961	352.956	353.208	352.704	A[14]
A[30]	765.155	339.202	335.197	335.449	334.945	A[13]
K[31]	797.179	321.442	317.438	317.690	317.186	K[12]
R[32]	836.204	289.419	285.414	285.666	285.162	R[11]
K[33]	868.228	250.393	246.389	246.641	246.137	K[10]
A[34]	885.987	218.370	214.365	214.617	214.113	A[9]
T[35]	911.249	200.610	196.606	196.858	196.354	T[8]
G[36]	925.504	175.348	171.344	171.596	171.092	G[7]
P[37]	949.767	161.093	157.088	157.340	156.836	P[6]
P[38]	974.031	136.830	132.825	133.077	132.573	P[5]
V[39]	**998.798**	112.567	108.562	108.814	108.310	V[4]
S[40]	**1020.556**	87.800	83.795	84.047	83.543	S[3]
E[41]	**1052.816**	66.042	62.037	62.289	61.785	E[2]
L[42]	**1081.087**	33.781	29.776	30.028	29.524	L[1]

sp|P43276|H15_MOUSE

[Acetyl]SETAPAETAAPAPVEKS $^{Phospho}_{79.97}$ PAKKKTTKKAGAAKRKATGPPVSEL

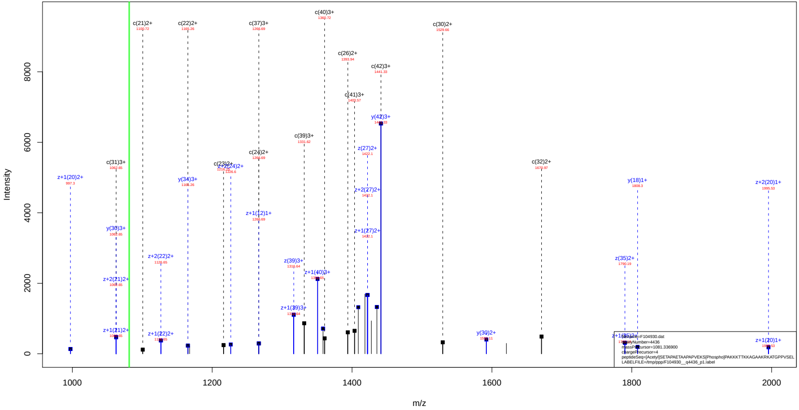

sp|P43276|H15_MOUSE

[Acetyl]SETAPAETAAPAPVEKS $^{Phospho}_{79.97}$ PAKKKTTKKAGAAKRKATGPPVSEL

- fragmentation table for charge state 1+
- specType=etd
- score=44.00
- F104930.dat
- query=q4436_p1
- precursor=1081.336900
- chargePrecursor=4
- itol=0.8

AA	c	y	z+1	z+2	z	AA
S[1]	147.076	4322.312	4306.293	0.000	4305.285	S[42]
E[2]	276.119	4193.269	4177.251	0.000	4176.243	E[41]
T[3]	377.167	4064.227	4048.208	0.000	4047.200	T[40]
A[4]	448.204	3963.179	3947.160	0.000	3946.153	A[39]
P[5]	545.257	3892.142	3876.123	0.000	3875.115	P[38]
A[6]	616.294	3795.089	3779.070	0.000	3778.063	A[37]
E[7]	745.336	3724.052	3708.033	0.000	3707.026	E[36]
T[8]	846.384	3595.009	3578.991	0.000	3577.983	T[35]
A[9]	917.421	3493.962	3477.943	0.000	3476.935	A[34]
A[10]	988.458	3422.925	3406.906	0.000	3405.898	A[33]
P[11]	1085.511	3351.888	3335.869	0.000	3334.861	P[32]
A[12]	1156.548	3254.835	3238.816	0.000	3237.808	A[31]
P[13]	1253.601	3183.798	3167.779	0.000	3166.771	P[30]
V[14]	1352.669	3086.745	3070.726	0.000	3069.718	V[29]
E[15]	1481.712	2987.677	2971.658	0.000	2970.650	E[28]
K[16]	1609.807	2858.634	2842.615	2843.623	2841.607	K[27]
S[17]	1776.805	2730.539	2714.520	2715.528	2713.512	S[26]
P[18]	1873.858	2563.541	2547.522	2548.530	2546.514	P[25]
A[19]	1944.895	2466.488	2450.469	2451.477	2449.461	A[24]
K[20]	2072.990	2395.451	2379.432	2380.440	2378.424	K[23]
K[21]	2201.085	2267.356	2251.337	2252.345	2250.329	K[22]
K[22]	2329.180	2139.261	2123.242	2124.250	2122.234	K[21]
T[23]	2430.228	2011.166	**1995.147**	**1996.155**	1994.139	T[20]
T[24]	2531.275	1910.118	1894.099	1895.107	1893.092	T[19]
K[25]	2659.370	**1809.070**	1793.052	1794.060	1792.044	K[18]
K[26]	2787.465	1680.976	1664.957	1665.965	1663.949	K[17]
A[27]	2858.502	1552.881	1536.862	1537.870	1535.854	A[16]
G[28]	2915.524	1481.843	1465.825	1466.833	1464.817	G[15]
A[29]	2986.561	1424.822	**1408.803**	1409.811	1407.795	A[14]
A[30]	3057.598	1353.785	1337.766	1338.774	1336.758	A[13]
K[31]	3185.693	1282.748	**1266.729**	1267.737	1265.721	K[12]
R[32]	3341.794	1154.653	1138.634	1139.642	1137.626	R[11]
K[33]	3469.889	998.552	982.533	983.541	981.525	K[10]
A[34]	3540.926	870.457	854.438	855.446	853.430	A[9]
T[35]	3641.974	799.420	783.401	784.409	782.393	T[8]
G[36]	3698.995	698.372	682.353	683.361	681.345	G[7]
P[37]	3796.048	641.350	625.332	626.340	624.324	P[6]
P[38]	3893.101	544.298	528.279	529.287	527.271	P[5]
V[39]	3992.169	447.245	431.226	432.234	430.218	V[4]
S[40]	4079.201	348.177	332.158	333.166	331.150	S[3]
E[41]	4208.244	261.144	245.126	246.134	244.118	E[2]
L[42]	4321.328	132.102	116.083	117.091	115.075	L[1]

sp|P43276|H15_MOUSE

[Acetyl]SETAPAETAAPAPVEKS $^{Phospho}_{79.97}$ PAKKKTTKKAGAAKRKATGPPVSEL

- fragmentation table for charge state 2+
- specType=etd
- score=44.00
- F104930.dat
- query=q4436_p1
- precursor=1081.336900
- chargePrecursor=4
- itol=0.8

AA	c	y	z+1	z+2	z	AA
S[1]	74.042	2161.660	2153.650	0.504	2153.146	S[42]
E[2]	138.563	2097.138	2089.129	0.504	2088.625	E[41]
T[3]	189.087	2032.617	2024.608	0.504	2024.104	T[40]
A[4]	224.606	1982.093	1974.084	0.504	1973.580	A[39]
P[5]	273.132	1946.575	1938.565	0.504	1938.061	P[38]
A[6]	308.650	1898.048	1890.039	0.504	1889.535	A[37]
E[7]	373.172	1862.530	1854.520	0.504	1854.016	E[36]
T[8]	423.696	1798.008	**1789.999**	0.504	**1789.495**	T[35]
A[9]	459.214	1747.485	1739.475	0.504	1738.971	A[34]
A[10]	494.733	1711.966	1703.957	0.504	1703.453	A[33]
P[11]	543.259	1676.447	1668.438	0.504	1667.934	P[32]
A[12]	578.778	1627.921	1619.912	0.504	1619.408	A[31]
P[13]	627.304	**1592.402**	1584.393	0.504	1583.889	P[30]
V[14]	676.838	1543.876	1535.867	0.504	1535.363	V[29]
E[15]	741.360	1494.342	1486.333	0.504	1485.829	E[28]
K[16]	805.407	1429.821	**1421.811**	**1422.315**	**1421.307**	K[27]
S[17]	888.906	1365.773	**1357.764**	**1358.268**	1357.260	S[26]
P[18]	937.433	1282.274	1274.265	1274.768	1273.761	P[25]
A[19]	972.951	1233.748	1225.738	**1226.242**	1225.234	A[24]
K[20]	1036.999	1198.229	1190.220	1190.724	1189.716	K[23]
K[21]	**1101.046**	1134.182	**1126.172**	**1126.676**	1125.668	K[22]
K[22]	**1165.094**	1070.134	**1062.125**	**1062.629**	1061.621	K[21]
T[23]	**1215.617**	1006.087	**998.077**	998.581	**997.573**	T[20]
T[24]	**1266.141**	955.563	947.553	948.057	947.049	T[19]
K[25]	1330.189	905.039	897.030	897.533	896.526	K[18]
K[26]	**1394.236**	840.991	832.982	833.486	832.478	K[17]
A[27]	1429.755	776.944	768.935	769.438	768.431	A[16]
G[28]	1458.266	741.425	733.416	733.920	732.912	G[15]
A[29]	1493.784	712.915	704.905	705.409	704.401	A[14]
A[30]	**1529.303**	677.396	669.387	669.891	668.883	A[13]
K[31]	1593.350	641.878	633.868	634.372	633.364	K[12]
R[32]	**1671.401**	577.830	569.821	570.325	569.317	R[11]
K[33]	1735.448	499.779	491.770	492.274	491.266	K[10]
A[34]	1770.967	435.732	427.723	428.227	427.219	A[9]
T[35]	1821.491	400.213	392.204	392.708	391.700	T[8]
G[36]	1850.001	349.690	341.680	342.184	341.176	G[7]
P[37]	1898.528	321.179	313.170	313.673	312.666	P[6]
P[38]	1947.054	272.652	264.643	265.147	264.139	P[5]
V[39]	1996.588	224.126	216.117	216.621	215.613	V[4]
S[40]	2040.104	174.592	166.583	167.086	166.079	S[3]
E[41]	2104.626	131.076	123.067	123.570	122.563	E[2]
L[42]	2161.168	66.555	58.545	59.049	58.041	L[1]

sp|P43276|H15_MOUSE

[Acetyl]SETAPAETAAPAPVEKS $^{Phospho}_{79.97}$ PAKKKTTKKAGAAKRKATGPPVSEL

- fragmentation table for charge state 3+
- specType=etd
- score=44.00
- F104930.dat
- query=q4436_p1
- precursor=1081.336900
- chargePrecursor=4
- itol=0.8

AA	c	y	z+1	z+2	z	AA
S[1]	49.697	**1441.442**	**1436.103**	0.672	**1435.767**	S[42]
E[2]	92.711	1398.428	1393.088	0.672	1392.752	E[41]
T[3]	126.394	1355.414	**1350.074**	0.672	1349.738	T[40]
A[4]	150.073	1321.731	**1316.392**	0.672	**1316.056**	A[39]
P[5]	182.424	1298.052	1292.713	0.672	1292.377	P[38]
A[6]	206.103	1265.701	1260.362	0.672	1260.026	A[37]
E[7]	249.117	1242.022	1236.683	0.672	1236.347	E[36]
T[8]	282.800	1199.008	1193.668	0.672	1193.332	T[35]
A[9]	306.479	**1165.325**	1159.986	0.672	1159.650	A[34]
A[10]	330.158	1141.646	1136.307	0.672	1135.971	A[33]
P[11]	362.508	1117.967	1112.628	0.672	1112.292	P[32]
A[12]	386.188	1085.616	1080.277	0.672	1079.941	A[31]
P[13]	418.538	**1061.937**	1056.598	0.672	1056.262	P[30]
V[14]	451.561	1029.586	1024.247	0.672	1023.911	V[29]
E[15]	494.575	**996.564**	991.224	0.672	990.888	E[28]
K[16]	537.274	953.549	948.210	948.546	947.874	K[27]
S[17]	592.940	910.851	905.512	905.848	905.176	S[26]
P[18]	625.291	855.185	849.845	850.181	849.510	P[25]
A[19]	648.970	822.834	817.495	817.831	817.159	A[24]
K[20]	691.668	799.155	793.816	794.151	793.480	K[23]
K[21]	734.367	756.457	751.117	751.453	750.781	K[22]
K[22]	777.065	713.758	708.419	708.755	708.083	K[21]
T[23]	810.747	671.060	665.721	666.057	665.385	T[20]
T[24]	844.430	637.378	632.038	632.374	631.702	T[19]
K[25]	887.128	603.695	598.355	598.691	598.019	K[18]
K[26]	929.827	560.997	555.657	555.993	555.321	K[17]
A[27]	953.506	518.298	512.959	513.295	512.623	A[16]
G[28]	972.513	494.619	489.280	489.616	488.944	G[15]
A[29]	996.192	475.612	470.273	470.609	469.937	A[14]
A[30]	1019.871	451.933	446.594	446.930	446.258	A[13]
K[31]	**1062.569**	428.254	422.915	423.250	422.579	K[12]
R[32]	1114.603	385.556	380.216	380.552	379.880	R[11]
K[33]	1157.301	333.522	328.183	328.518	327.847	K[10]
A[34]	1180.980	290.824	285.484	285.820	285.148	A[9]
T[35]	1214.663	267.145	261.805	262.141	261.469	T[8]
G[36]	1233.670	233.462	228.123	228.459	227.787	G[7]
P[37]	**1266.021**	214.455	209.115	209.451	208.779	P[6]
P[38]	1298.372	182.104	176.765	177.100	176.429	P[5]
V[39]	**1331.395**	149.753	144.414	144.750	144.078	V[4]
S[40]	**1360.405**	116.730	111.391	111.727	111.055	S[3]
E[41]	**1403.419**	87.720	82.380	82.716	82.044	E[2]
L[42]	**1441.114**	44.705	39.366	39.702	39.030	L[1]

sp|P43275|H11_MOUSE

[Acetyl]SETAPVAQAASTATEKPAAAKKTKKPAKAAAPRKKPAGPSVSEL

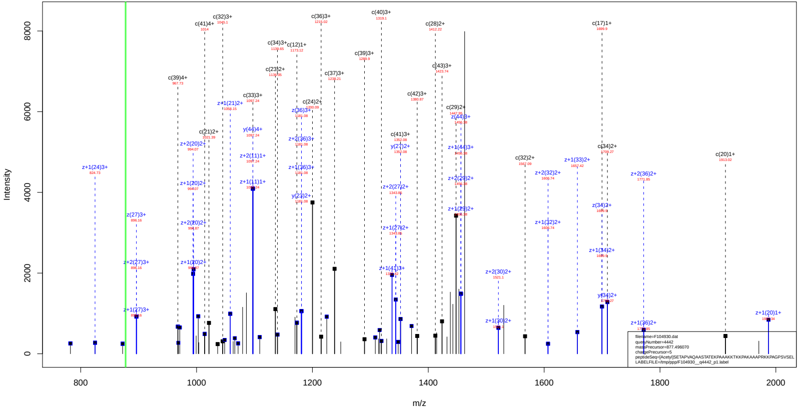

sp|P43275|H11_MOUSE

[Acetyl]SETAPVAQAASTATEKPAAAKKTKKPAKAAAPRKKPAGPSVSEL

- fragmentation table for charge state 1+
- specType=etd
- score=52.75
- F104930.dat
- query=q4442_p1
- precursor=877.496070
- chargePrecursor=5
- itol=0.8

AA	c	y	z+1	z+2	z	AA
S[1]	147.076	4383.436	4367.417	0.000	4366.409	S[44]
E[2]	276.119	4254.393	4238.375	0.000	4237.367	E[43]
T[3]	377.167	4125.351	4109.332	0.000	4108.324	T[42]
A[4]	448.204	4024.303	4008.284	0.000	4007.276	A[41]
P[5]	545.257	3953.266	3937.247	0.000	3936.239	P[40]
V[6]	644.325	3856.213	3840.194	0.000	3839.187	V[39]
A[7]	715.362	3757.145	3741.126	0.000	3740.118	A[38]
Q[8]	843.421	3686.108	3670.089	3671.097	3669.081	Q[37]
A[9]	914.458	3558.049	3542.030	3543.038	3541.022	A[36]
A[10]	985.495	3487.012	3470.993	3472.001	3469.985	A[35]
S[11]	1072.527	3415.975	3399.956	3400.964	3398.948	S[34]
T[12]	**1173.575**	3328.943	3312.924	3313.932	3311.916	T[33]
A[13]	1244.612	3227.895	3211.876	3212.884	3210.868	A[32]
T[14]	1345.659	3156.858	3140.839	3141.847	3139.831	T[31]
E[15]	1474.702	3055.810	3039.792	3040.799	3038.784	E[30]
K[16]	1602.797	2926.768	2910.749	2911.757	2909.741	K[29]
P[17]	**1699.850**	2798.673	2782.654	2783.662	2781.646	P[28]
A[18]	1770.887	2701.620	2685.601	2686.609	2684.593	A[27]
A[19]	1841.924	2630.583	2614.564	2615.572	2613.556	A[26]
A[20]	**1912.961**	2559.546	2543.527	2544.535	2542.519	A[25]
K[21]	2041.056	2488.509	2472.490	2473.498	2471.482	K[24]
K[22]	2169.151	2360.414	2344.395	2345.403	2343.387	K[23]
T[23]	2270.199	2232.319	2216.300	2217.308	2215.292	T[22]
K[24]	2398.294	2131.271	2115.252	2116.260	2114.244	K[21]
K[25]	2526.389	2003.176	**1987.157**	1988.165	1986.149	K[20]
P[26]	2623.441	1875.081	1859.062	1860.070	1858.055	P[19]
A[27]	2694.478	1778.028	1762.010	1763.017	1761.002	A[18]
K[28]	2822.573	1706.991	1690.972	1691.980	1689.965	K[17]
A[29]	2893.611	1578.896	1562.877	1563.885	1561.870	A[16]
A[30]	2964.648	1507.859	1491.840	1492.848	1490.833	A[15]
A[31]	3035.685	1436.822	1420.803	1421.811	1419.795	A[14]
P[32]	3132.738	1365.785	1349.766	1350.774	**1348.758**	P[13]
R[33]	3288.839	1268.732	1252.713	1253.721	1251.706	R[12]
K[34]	3416.934	1112.631	**1096.612**	**1097.620**	1095.604	K[11]
K[35]	3545.029	984.536	**968.517**	969.525	**967.509**	K[10]
P[36]	3642.081	856.441	840.422	841.430	839.415	P[9]
A[37]	3713.118	759.388	743.370	744.377	742.362	A[8]
G[38]	3770.140	688.351	672.332	673.340	671.325	G[7]
P[39]	3867.193	631.330	615.311	616.319	614.303	P[6]
S[40]	3954.225	534.277	518.258	519.266	517.250	S[5]
V[41]	4053.293	447.245	431.226	432.234	430.218	V[4]
S[42]	4140.325	348.177	332.158	333.166	331.150	S[3]
E[43]	4269.368	261.144	245.126	246.134	244.118	E[2]
L[44]	4382.452	132.102	116.083	117.091	115.075	L[1]

sp|P43275|H11_MOUSE

[Acetyl]SETAPVAQAASTATEKPAAAKKTKKPAKAAAPRKKPAGPSVSEL

- fragmentation table for charge state 2+
- specType=etd
- score=52.75
- F104930.dat
- query=q4442_p1
- precursor=877.496070
- chargePrecursor=5
- itol=0.8

AA	c	y	z+1	z+2	z	AA
S[1]	74.042	2192.222	2184.212	0.504	2183.708	S[44]
E[2]	138.563	2127.700	2119.691	0.504	2119.187	E[43]
T[3]	189.087	2063.179	2055.170	0.504	2054.666	T[42]
A[4]	224.606	2012.655	2004.646	0.504	2004.142	A[41]
P[5]	273.132	1977.137	1969.127	0.504	1968.623	P[40]
V[6]	322.666	1928.610	1920.601	0.504	1920.097	V[39]
A[7]	358.185	1879.076	1871.067	0.504	1870.563	A[38]
Q[8]	422.214	1843.557	1835.548	1836.052	1835.044	Q[37]
A[9]	457.733	1779.528	**1771.519**	**1772.023**	1771.015	A[36]
A[10]	493.251	1744.010	1736.000	1736.504	1735.496	A[35]
S[11]	536.767	**1708.491**	**1700.482**	1700.986	**1699.978**	S[34]
T[12]	587.291	1664.975	**1656.966**	**1657.470**	1656.462	T[33]
A[13]	622.809	1614.451	**1606.442**	**1606.946**	1605.938	A[32]
T[14]	673.333	1578.933	1570.923	1571.427	1570.419	T[31]
E[15]	737.855	1528.409	**1520.399**	**1520.903**	1519.895	E[30]
K[16]	801.902	1463.887	**1455.878**	**1456.382**	1455.374	K[29]
P[17]	850.428	1399.840	1391.831	1392.335	1391.327	P[28]
A[18]	885.947	**1351.314**	**1343.304**	**1343.808**	1342.800	A[27]
A[19]	921.466	**1315.795**	**1307.786**	**1308.290**	1307.282	A[26]
A[20]	956.984	1280.276	1272.267	1272.771	1271.763	A[25]
K[21]	**1021.032**	1244.758	1236.749	1237.252	1236.245	K[24]
K[22]	1085.079	**1180.710**	**1172.701**	**1173.205**	1172.197	K[23]
T[23]	**1135.603**	1116.663	**1108.654**	**1109.158**	1108.150	T[22]
K[24]	**1199.650**	**1066.139**	**1058.130**	**1058.634**	**1057.626**	K[21]
K[25]	1263.698	1002.092	**994.082**	**994.586**	**993.578**	K[20]
P[26]	1312.224	938.044	930.035	930.539	929.531	P[19]
A[27]	**1347.743**	889.518	881.508	882.012	881.005	A[18]
K[28]	**1411.790**	853.999	845.990	846.494	845.486	K[17]
A[29]	**1447.309**	789.952	**781.942**	**782.446**	781.438	A[16]
A[30]	1482.827	754.433	746.424	746.928	745.920	A[15]
A[31]	1518.346	718.915	710.905	711.409	710.401	A[14]
P[32]	**1566.872**	683.396	675.387	675.891	674.883	P[13]
R[33]	1644.923	634.870	626.860	627.364	626.356	R[12]
K[34]	**1708.970**	556.819	548.810	549.314	548.306	K[11]
K[35]	1773.018	492.772	484.762	485.266	484.258	K[10]
P[36]	1821.544	428.724	420.715	421.219	420.211	P[9]
A[37]	1857.063	380.198	372.188	372.692	371.685	A[8]
G[38]	1885.574	344.679	336.670	337.174	336.166	G[7]
P[39]	1934.100	316.169	308.159	308.663	307.655	P[6]
S[40]	1977.616	267.642	259.633	260.137	259.129	S[5]
V[41]	2027.150	224.126	216.117	216.621	215.613	V[4]
S[42]	2070.666	174.592	166.583	167.086	166.079	S[3]
E[43]	2135.188	131.076	123.067	123.570	122.563	E[2]
L[44]	2191.730	66.555	58.545	59.049	58.041	L[1]

sp|P43275|H11_MOUSE

[Acetyl]SETAPVAQAASTATEKPAAAKKTKKPAKAAAPRKKPAGPSVSEL

- fragmentation table for charge state 3+
- specType=etd
- score=52.75
- F104930.dat
- query=q4442_p1
- precursor=877.496070
- chargePrecursor=5
- itol=0.8

AA	c	y	z+1	z+2	z	AA
S[1]	49.697	1461.817	**1456.477**	0.672	**1456.141**	S[44]
E[2]	92.711	1418.803	1413.463	0.672	1413.127	E[43]
T[3]	126.394	1375.788	**1370.449**	0.672	1370.113	T[42]
A[4]	150.073	1342.106	**1336.766**	0.672	1336.430	A[41]
P[5]	182.424	**1318.427**	1313.087	0.672	1312.751	P[40]
V[6]	215.447	1286.076	1280.736	0.672	1280.400	V[39]
A[7]	239.126	1253.053	1247.713	0.672	1247.378	A[38]
Q[8]	281.812	1229.374	**1224.034**	**1224.370**	1223.699	Q[37]
A[9]	305.491	1186.688	**1181.348**	**1181.684**	**1181.012**	A[36]
A[10]	329.170	1163.009	1157.669	1158.005	1157.333	A[35]
S[11]	358.180	**1139.330**	1133.990	1134.326	1133.654	S[34]
T[12]	391.863	1110.319	1104.980	1105.315	1104.644	T[33]
A[13]	415.542	1076.637	**1071.297**	**1071.633**	**1070.961**	A[32]
T[14]	449.225	1052.957	1047.618	**1047.954**	1047.282	T[31]
E[15]	492.239	1019.275	**1013.935**	**1014.271**	**1013.599**	E[30]
K[16]	534.937	976.261	**970.921**	**971.257**	**970.585**	K[29]
P[17]	567.288	933.562	928.223	928.559	927.887	P[28]
A[18]	590.967	901.211	**895.872**	**896.208**	**895.536**	A[27]
A[19]	614.646	877.532	**872.193**	**872.529**	**871.857**	A[26]
A[20]	638.325	853.853	848.514	848.850	848.178	A[25]
K[21]	681.024	830.174	**824.835**	**825.171**	**824.499**	K[24]
K[22]	723.722	787.476	**782.136**	**782.472**	**781.801**	K[23]
T[23]	757.404	744.778	739.438	739.774	739.102	T[22]
K[24]	800.103	711.095	705.756	706.092	705.420	K[21]
K[25]	842.801	668.397	663.057	663.393	662.721	K[20]
P[26]	875.152	625.699	620.359	620.695	620.023	P[19]
A[27]	898.831	593.348	588.008	588.344	587.672	A[18]
K[28]	941.529	569.669	564.329	564.665	563.993	K[17]
A[29]	965.208	526.970	521.631	521.967	521.295	A[16]
A[30]	988.887	503.291	497.952	498.288	497.616	A[15]
A[31]	1012.566	479.612	474.273	474.609	473.937	A[14]
P[32]	**1044.917**	455.933	450.594	450.930	450.258	P[13]
R[33]	**1096.951**	423.582	418.243	418.579	417.907	R[12]
K[34]	**1139.649**	371.549	366.209	366.545	365.873	K[11]
K[35]	1182.348	328.850	323.511	323.847	323.175	K[10]
P[36]	**1214.699**	286.152	280.812	281.148	280.476	P[9]
A[37]	**1238.378**	253.801	248.461	248.797	248.125	A[8]
G[38]	1257.385	230.122	224.782	225.118	224.446	G[7]
P[39]	**1289.736**	211.115	205.775	206.111	205.439	P[6]
S[40]	**1318.746**	178.764	173.424	173.760	173.088	S[5]
V[41]	**1351.769**	149.753	144.414	144.750	144.078	V[4]
S[42]	**1380.780**	116.730	111.391	111.727	111.055	S[3]
E[43]	**1423.794**	87.720	82.380	82.716	82.044	E[2]
L[44]	1461.489	44.705	39.366	39.702	39.030	L[1]

sp|P43275|H11_MOUSE

[Acetyl]SETAPVAQAASTATEKPAAAKKTKKPAKAAAPRKKPAGPSVSEL

- fragmentation table for charge state 4+
- specType=etd
- score=52.75
- F104930.dat
- query=q4442_p1
- precursor=877.496070
- chargePrecursor=5
- itol=0.8

AA	c	y	z+1	z+2	z	AA
S[1]	37.525	**1096.614**	1092.610	0.755	1092.358	S[44]
E[2]	69.785	1064.354	1060.349	0.755	1060.097	E[43]
T[3]	95.047	1032.093	1028.088	0.755	1027.836	T[42]
A[4]	112.806	1006.831	**1002.827**	0.755	**1002.575**	A[41]
P[5]	137.070	989.072	985.067	0.755	984.815	P[40]
V[6]	161.837	964.809	960.804	0.755	960.552	V[39]
A[7]	179.596	940.042	936.037	0.755	935.785	A[38]
Q[8]	211.611	922.282	918.278	918.530	918.026	Q[37]
A[9]	229.370	890.268	886.263	886.515	886.011	A[36]
A[10]	247.129	**872.508**	868.504	868.756	868.252	A[35]
S[11]	268.887	854.749	850.744	850.996	850.493	S[34]
T[12]	294.149	832.991	828.986	829.238	828.735	T[33]
A[13]	311.908	807.729	803.725	803.976	803.473	A[32]
T[14]	337.170	789.970	785.965	786.217	785.713	T[31]
E[15]	369.431	764.708	760.703	760.955	760.451	E[30]
K[16]	401.455	732.447	728.443	728.695	728.191	K[29]
P[17]	425.718	700.424	696.419	696.671	696.167	P[28]
A[18]	443.477	676.160	672.156	672.408	671.904	A[27]
A[19]	461.236	658.401	654.396	654.648	654.145	A[26]
A[20]	478.996	640.642	636.637	636.889	636.385	A[25]
K[21]	511.019	622.883	618.878	619.130	618.626	K[24]
K[22]	543.043	590.859	586.854	587.106	586.602	K[23]
T[23]	568.305	558.835	554.830	555.082	554.578	T[22]
K[24]	600.329	533.573	529.569	529.820	529.317	K[21]
K[25]	632.353	501.549	497.545	497.797	497.293	K[20]
P[26]	656.616	469.526	465.521	465.773	465.269	P[19]
A[27]	674.375	445.263	441.258	441.510	441.006	A[18]
K[28]	706.399	427.503	423.499	423.751	423.247	K[17]
A[29]	724.158	395.480	391.475	391.727	391.223	A[16]
A[30]	741.917	377.720	373.716	373.968	373.464	A[15]
A[31]	759.677	359.961	355.956	356.208	355.704	A[14]
P[32]	783.940	342.202	338.197	338.449	337.945	P[13]
R[33]	822.965	317.938	313.934	314.186	313.682	R[12]
K[34]	854.989	278.913	274.909	275.160	274.657	K[11]
K[35]	887.013	246.889	242.885	243.137	242.633	K[10]
P[36]	911.276	214.866	210.861	211.113	210.609	P[9]
A[37]	929.035	190.603	186.598	186.850	186.346	A[8]
G[38]	943.290	172.843	168.839	169.091	168.587	G[7]
P[39]	**967.554**	158.588	154.583	154.835	154.331	P[6]
S[40]	989.312	134.325	130.320	130.572	130.068	S[5]
V[41]	**1014.079**	112.567	108.562	108.814	108.310	V[4]
S[42]	**1035.837**	87.800	83.795	84.047	83.543	S[3]
E[43]	1068.097	66.042	62.037	62.289	61.785	E[2]
L[44]	1096.368	33.781	29.776	30.028	29.524	L[1]

sp|P62806|H4_MOUSE

[Acetyl]SGRGKGGKGL

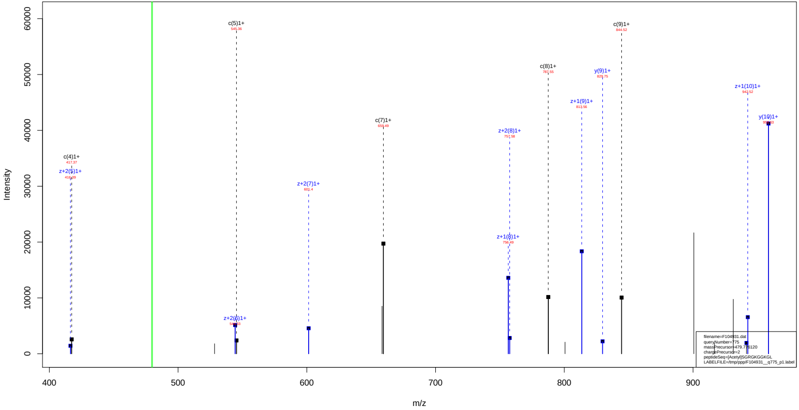

sp|P62806|H4_MOUSE

[Acetyl]SGRGKGGKGL

- fragmentation table for charge state 1+
- specType=etd
- score=39.26
- F104931.dat
- query=q775_p1
- precursor=479.776120
- chargePrecursor=2
- itol=0.8

AA	c	y	z+1	z+2	z	AA
S[1]	147.076	**958.543**	**942.524**	0.000	**941.516**	S[10]
G[2]	204.098	**829.500**	**813.482**	0.000	812.474	G[9]
R[3]	360.199	772.479	**756.460**	**757.468**	755.452	R[8]
G[4]	**417.220**	616.378	600.359	**601.367**	599.351	G[7]
K[5]	**545.315**	559.356	543.337	**544.345**	542.330	K[6]
G[6]	602.337	431.261	415.243	**416.250**	414.235	G[5]
G[7]	**659.358**	374.240	358.221	359.229	357.213	G[4]
K[8]	**787.453**	317.218	301.200	302.207	300.192	K[3]
G[9]	**844.475**	189.123	173.105	174.112	172.097	G[2]
L[10]	957.559	132.102	116.083	117.091	115.075	L[1]

sp|P62806|H4_MOUSE

[Acetyl]SGRGKGGKGL

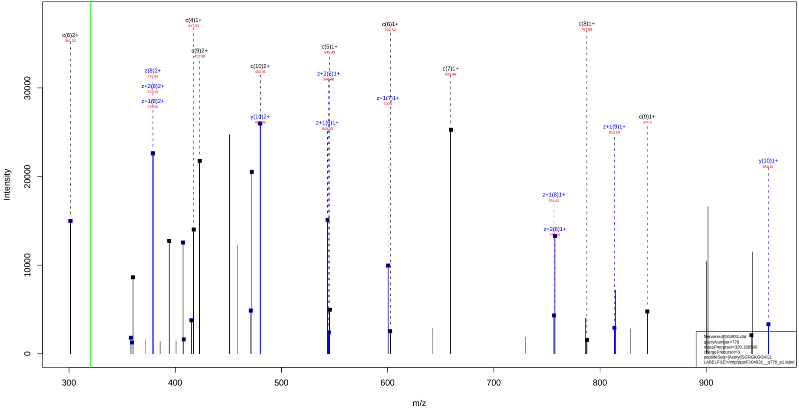

sp|P62806|H4_MOUSE

[Acetyl]SGRGKGGKGL

- fragmentation table for charge state 1+
- specType=etd
- score=59.03
- F104931.dat
- query=q776_p1
- precursor=320.186590
- chargePrecursor=3
- itol=0.8

AA	c	y	z+1	z+2	z	AA
S[1]	147.076	**958.543**	**942.524**	0.000	941.516	S[10]
G[2]	204.098	829.500	**813.482**	0.000	812.474	G[9]
R[3]	**360.199**	772.479	**756.460**	**757.468**	755.452	R[8]
G[4]	**417.220**	616.378	**600.359**	601.367	599.351	G[7]
K[5]	**545.315**	559.356	**543.337**	**544.345**	542.330	K[6]
G[6]	**602.337**	431.261	**415.243**	416.250	414.235	G[5]
G[7]	**659.358**	374.240	**358.221**	**359.229**	357.213	G[4]
K[8]	**787.453**	317.218	**301.200**	302.207	300.192	K[3]
G[9]	**844.475**	189.123	173.105	174.112	172.097	G[2]
L[10]	957.559	132.102	116.083	117.091	115.075	L[1]

sp|P62806|H4_MOUSE

[Acetyl]SGRGKGGKGL

- fragmentation table for charge state 2+
- specType=etd
- score=59.03
- F104931.dat
- query=q776_p1
- precursor=320.186590
- chargePrecursor=3
- itol=0.8

AA	c	y	z+1	z+2	z	AA
S[1]	74.042	**479.775**	**471.766**	0.504	**471.262**	S[10]
G[2]	102.553	**415.254**	**407.244**	0.504	**406.740**	G[9]
R[3]	180.603	386.743	**378.734**	**379.238**	**378.230**	R[8]
G[4]	209.114	308.692	**300.683**	**301.187**	300.179	G[7]
K[5]	273.161	280.182	272.172	272.676	271.668	K[6]
G[6]	**301.672**	216.134	208.125	208.629	207.621	G[5]
G[7]	330.183	187.624	179.614	180.118	179.110	G[4]
K[8]	**394.230**	159.113	151.103	151.607	150.600	K[3]
G[9]	**422.741**	95.065	87.056	87.560	86.552	G[2]
L[10]	**479.283**	66.555	58.545	59.049	58.041	L[1]

sp|P62806|H4_MOUSE

[Acetyl]SGRGKGGK $^{\text{Acetyl}}_{42.01}$ GL

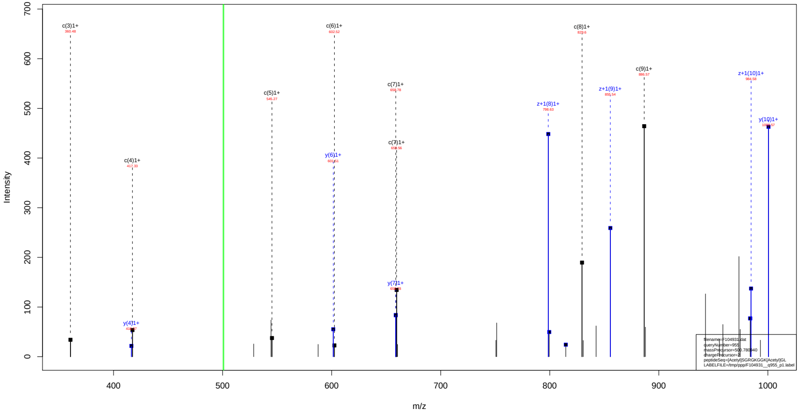

sp|P62806|H4_MOUSE

[Acetyl]SGRGKGGK$^{Acetyl}_{42.01}$GL

- fragmentation table for charge state 1+
- specType=etd
- score=43.32
- F104931.dat
- query=q955_p1
- precursor=500.780940
- chargePrecursor=2
- itol=0.8

AA	c	y	z+1	z+2	z	AA
S[1]	147.076	**1000.553**	**984.535**	0.000	**983.527**	S[10]
G[2]	204.098	871.511	**855.492**	0.000	854.484	G[9]
R[3]	**360.199**	**814.489**	**798.471**	**799.478**	797.463	R[8]
G[4]	**417.220**	**658.388**	642.370	643.377	641.362	G[7]
K[5]	**545.315**	**601.367**	585.348	586.356	584.340	K[6]
G[6]	**602.337**	473.272	457.253	458.261	456.245	G[5]
G[7]	**659.358**	**416.250**	400.232	401.239	399.224	G[4]
K[8]	**829.464**	359.229	343.210	344.218	342.202	K[3]
G[9]	**886.485**	189.123	173.105	174.112	172.097	G[2]
L[10]	999.569	132.102	116.083	117.091	115.075	L[1]

sp|P68433|H31_MOUSE

K $^{\text{Dimethyl}}_{28.03}$ TDLRF

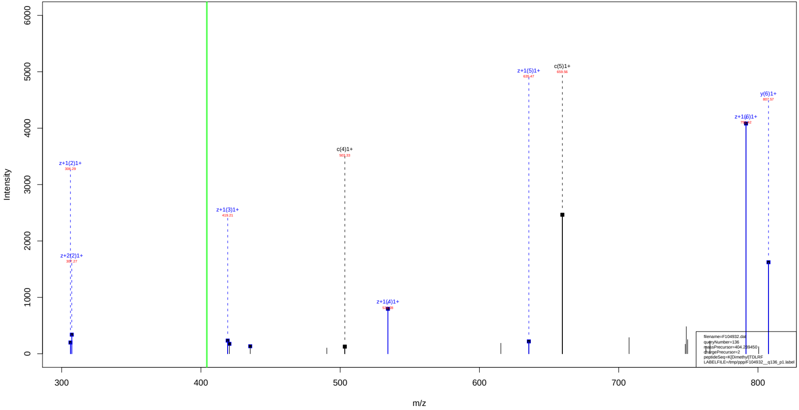

sp|P68433|H31_MOUSE

K $^{\text{Dimethyl}}_{28.03}$ TDLRF

- fragmentation table for charge state 1+
- specType=etd
- score=33.88
- F104932.dat
- query=q136_p1
- precursor=404.239450
- chargePrecursor=2
- itol=0.8

AA	c	y	z+1	z+2	z	AA
K[1]	174.160	**807.472**	**791.454**	792.461	790.446	K[6]
T[2]	275.208	651.346	**635.327**	636.335	634.320	T[5]
D[3]	390.235	550.298	**534.280**	535.287	533.272	D[4]
L[4]	**503.319**	**435.271**	**419.253**	**420.261**	418.245	L[3]
R[5]	**659.420**	322.187	**306.169**	**307.176**	305.161	R[2]
F[6]	806.488	166.086	150.068	151.075	149.060	F[1]

sp|P62806|H4_MOUSE

[Acetyl]SGRGKGGKGL

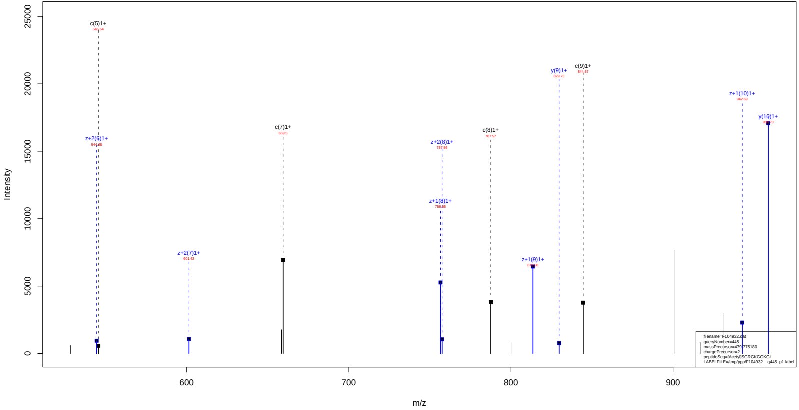

sp|P62806|H4_MOUSE

[Acetyl]SGRGKGGKGL

- fragmentation table for charge state 1+
- specType=etd
- score=34.52
- F104932.dat
- query=q445_p1
- precursor=479.775180
- chargePrecursor=2
- itol=0.8

AA	c	y	z+1	z+2	z	AA
S[1]	147.076	**958.543**	**942.524**	0.000	941.516	S[10]
G[2]	204.098	**829.500**	**813.482**	0.000	812.474	G[9]
R[3]	360.199	772.479	**756.460**	**757.468**	755.452	R[8]
G[4]	417.220	616.378	600.359	**601.367**	599.351	G[7]
K[5]	**545.315**	559.356	543.337	**544.345**	542.330	K[6]
G[6]	602.337	431.261	415.243	416.250	414.235	G[5]
G[7]	**659.358**	374.240	358.221	359.229	357.213	G[4]
K[8]	**787.453**	317.218	301.200	302.207	300.192	K[3]
G[9]	**844.475**	189.123	173.105	174.112	172.097	G[2]
L[10]	957.559	132.102	116.083	117.091	115.075	L[1]

sp|P62806|H4_MOUSE

[Acetyl]SGRGKGGKGL

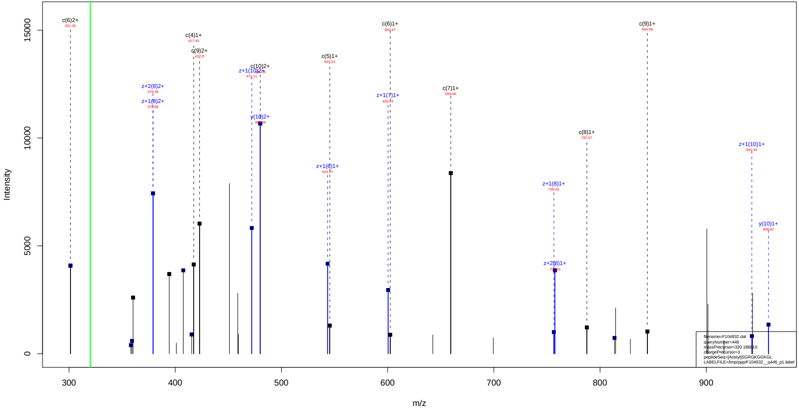

sp|P62806|H4_MOUSE

[Acetyl]SGRGKGGKGL

- fragmentation table for charge state 1+
- specType=etd
- score=60.89
- F104932.dat
- query=q446_p1
- precursor=320.186610
- chargePrecursor=3
- itol=0.8

AA	c	y	z+1	z+2	z	AA
S[1]	147.076	**958.543**	**942.524**	0.000	941.516	S[10]
G[2]	204.098	829.500	**813.482**	0.000	812.474	G[9]
R[3]	**360.199**	772.479	**756.460**	**757.468**	755.452	R[8]
G[4]	**417.220**	616.378	**600.359**	601.367	599.351	G[7]
K[5]	**545.315**	559.356	**543.337**	544.345	542.330	K[6]
G[6]	**602.337**	431.261	**415.243**	**416.250**	414.235	G[5]
G[7]	**659.358**	374.240	**358.221**	**359.229**	357.213	G[4]
K[8]	**787.453**	317.218	**301.200**	302.207	300.192	K[3]
G[9]	**844.475**	189.123	173.105	174.112	172.097	G[2]
L[10]	957.559	132.102	116.083	117.091	115.075	L[1]

sp|P62806|H4_MOUSE

[Acetyl]SGRGKGGKGL

- fragmentation table for charge state 2+
- specType=etd
- score=60.89
- F104932.dat
- query=q446_p1
- precursor=320.186610
- chargePrecursor=3
- itol=0.8

AA	c	y	z+1	z+2	z	AA
S[1]	74.042	**479.775**	**471.766**	0.504	471.262	S[10]
G[2]	102.553	**415.254**	**407.244**	0.504	406.740	G[9]
R[3]	180.603	386.743	**378.734**	**379.238**	378.230	R[8]
G[4]	209.114	308.692	**300.683**	**301.187**	300.179	G[7]
K[5]	273.161	280.182	272.172	272.676	271.668	K[6]
G[6]	**301.672**	216.134	208.125	208.629	207.621	G[5]
G[7]	330.183	187.624	179.614	180.118	179.110	G[4]
K[8]	**394.230**	159.113	151.103	151.607	150.600	K[3]
G[9]	**422.741**	95.065	87.056	87.560	86.552	G[2]
L[10]	**479.283**	66.555	58.545	59.049	58.041	L[1]

sp|P62806|H4_MOUSE

[Acetyl]SGRGK $^{\text{Acetyl}}_{42.01}$ GGKGL

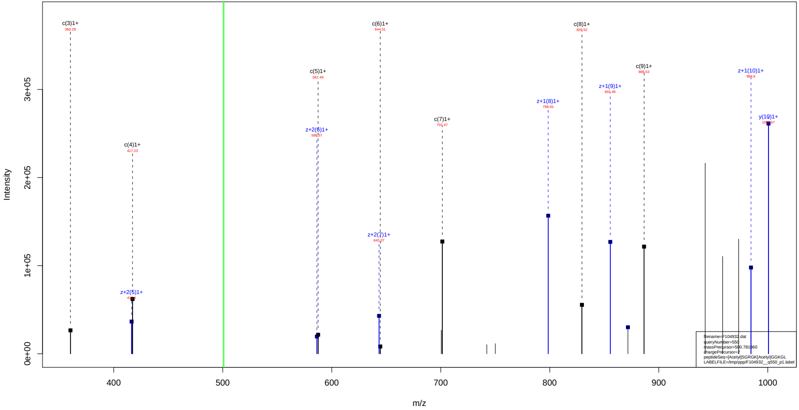

sp|P62806|H4_MOUSE

[Acetyl]SGRGK $^{Acetyl}_{42.01}$ GGKGL

- fragmentation table for charge state 1+
- specType=etd
- score=55.67
- F104932.dat
- query=q550_p1
- precursor=500.781060
- chargePrecursor=2
- itol=0.8

AA	c	y	z+1	z+2	z	AA
S[1]	147.076	**1000.553**	**984.535**	0.000	983.527	S[10]
G[2]	204.098	**871.511**	**855.492**	0.000	854.484	G[9]
R[3]	**360.199**	814.489	**798.471**	799.478	797.463	R[8]
G[4]	**417.220**	658.388	642.370	**643.377**	641.362	G[7]
K[5]	**587.326**	601.367	585.348	**586.356**	584.340	K[6]
G[6]	**644.347**	431.261	415.243	**416.250**	414.235	G[5]
G[7]	**701.369**	374.240	358.221	359.229	357.213	G[4]
K[8]	**829.464**	317.218	301.200	302.207	300.192	K[3]
G[9]	**886.485**	189.123	173.105	174.112	172.097	G[2]
L[10]	999.569	132.102	116.083	117.091	115.075	L[1]

sp|P62806|H4_MOUSE

[Acetyl]SGR $^{\text{Dimethyl}}_{28.03}$ GKGGK $^{\text{Acetyl}}_{42.01}$ GL

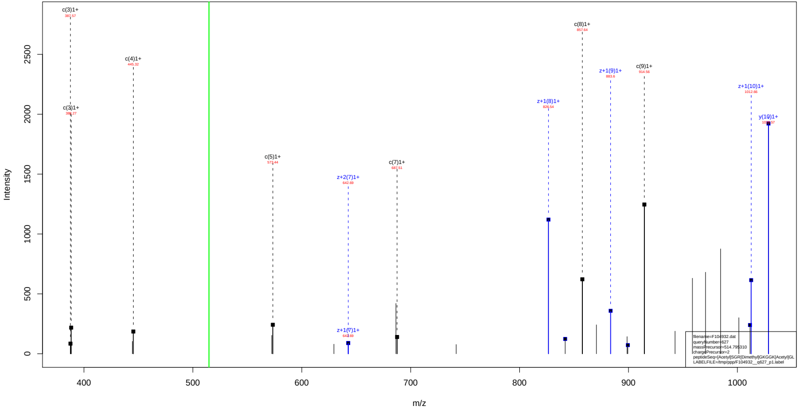

sp|P62806|H4_MOUSE

[Acetyl]SGR $^{\text{Dimethyl}}_{28.03}$ GKGGK $^{\text{Acetyl}}_{42.01}$ GL

- fragmentation table for charge state 1+
- specType=etd
- score=40.20
- F104932.dat
- query=q627_p1
- precursor=514.795310
- chargePrecursor=2
- itol=0.8

AA	c	y	z+1	z+2	z	AA
S[1]	147.076	**1028.585**	**1012.566**	0.000	**1011.558**	S[10]
G[2]	204.098	**899.542**	**883.523**	0.000	882.516	G[9]
R[3]	**388.230**	**842.521**	**826.502**	827.510	825.494	R[8]
G[4]	**445.252**	658.388	**642.370**	**643.377**	641.362	G[7]
K[5]	**573.347**	601.367	585.348	586.356	584.340	K[6]
G[6]	630.368	473.272	457.253	458.261	456.245	G[5]
G[7]	**687.390**	416.250	400.232	401.239	399.224	G[4]
K[8]	**857.495**	359.229	343.210	344.218	342.202	K[3]
G[9]	**914.517**	189.123	173.105	174.112	172.097	G[2]
L[10]	1027.601	132.102	116.083	117.091	115.075	L[1]

sp|P62806|H4_MOUSE

[Acetyl]SGRGKGGKGL

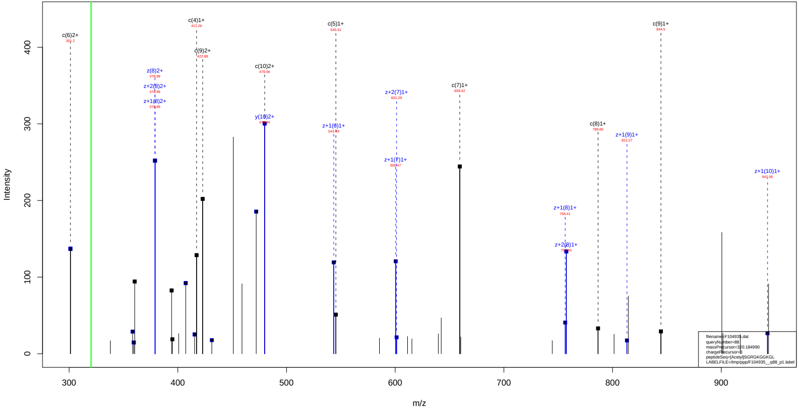

sp|P62806|H4_MOUSE

[Acetyl]SGRGKGGKGL

- fragmentation table for charge state 1+
- specType=etd
- score=51.48
- F104935.dat
- query=q88_p1
- precursor=320.184990
- chargePrecursor=3
- itol=0.8

AA	c	y	z+1	z+2	z	AA
S[1]	147.076	958.543	**942.524**	0.000	941.516	S[10]
G[2]	204.098	829.500	**813.482**	0.000	812.474	G[9]
R[3]	**360.199**	772.479	**756.460**	**757.468**	755.452	R[8]
G[4]	**417.220**	616.378	**600.359**	**601.367**	599.351	G[7]
K[5]	**545.315**	559.356	**543.337**	544.345	542.330	K[6]
G[6]	602.337	**431.261**	**415.243**	416.250	414.235	G[5]
G[7]	**659.358**	374.240	**358.221**	**359.229**	357.213	G[4]
K[8]	**787.453**	317.218	**301.200**	302.207	300.192	K[3]
G[9]	**844.475**	189.123	173.105	174.112	172.097	G[2]
L[10]	957.559	132.102	116.083	117.091	115.075	L[1]

sp|P62806|H4_MOUSE
[Acetyl]SGRGKGGKGL

- fragmentation table for charge state 2+
- specType=etd
- score=51.48
- F104935.dat
- query=q88_p1
- precursor=320.184990
- chargePrecursor=3
- itol=0.8

AA	c	y	z+1	z+2	z	AA
S[1]	74.042	**479.775**	**471.766**	0.504	471.262	S[10]
G[2]	102.553	**415.254**	**407.244**	0.504	**406.740**	G[9]
R[3]	180.603	386.743	**378.734**	**379.238**	**378.230**	R[8]
G[4]	209.114	308.692	**300.683**	**301.187**	300.179	G[7]
K[5]	273.161	280.182	272.172	272.676	271.668	K[6]
G[6]	**301.672**	216.134	208.125	208.629	207.621	G[5]
G[7]	330.183	187.624	179.614	180.118	179.110	G[4]
K[8]	**394.230**	159.113	151.103	151.607	150.600	K[3]
G[9]	**422.741**	95.065	87.056	87.560	86.552	G[2]
L[10]	**479.283**	66.555	58.545	59.049	58.041	L[1]

sp|P62806|H4_MOUSE

[Acetyl]SGRGKGGKGL

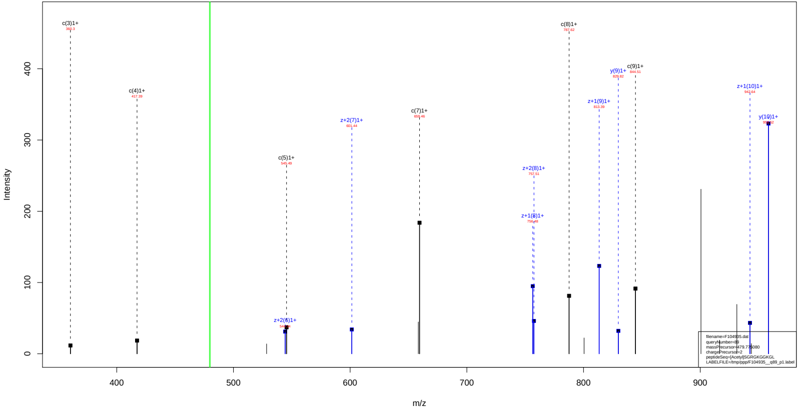

sp|P62806|H4_MOUSE

[Acetyl]SGRGKGGKGL

- fragmentation table for charge state 1+
- specType=etd
- score=50.12
- F104935.dat
- query=q89_p1
- precursor=479.775080
- chargePrecursor=2
- itol=0.8

AA	c	y	z+1	z+2	z	AA
S[1]	147.076	**958.543**	**942.524**	0.000	941.516	S[10]
G[2]	204.098	**829.500**	**813.482**	0.000	812.474	G[9]
R[3]	**360.199**	772.479	**756.460**	**757.468**	755.452	R[8]
G[4]	**417.220**	616.378	600.359	**601.367**	599.351	G[7]
K[5]	**545.315**	559.356	543.337	**544.345**	542.330	K[6]
G[6]	602.337	431.261	415.243	416.250	414.235	G[5]
G[7]	**659.358**	374.240	358.221	359.229	357.213	G[4]
K[8]	**787.453**	317.218	301.200	302.207	300.192	K[3]
G[9]	**844.475**	189.123	173.105	174.112	172.097	G[2]
L[10]	957.559	132.102	116.083	117.091	115.075	L[1]

sp|P62806|H4_MOUSE

[Acetyl]SGRGKGGKGL

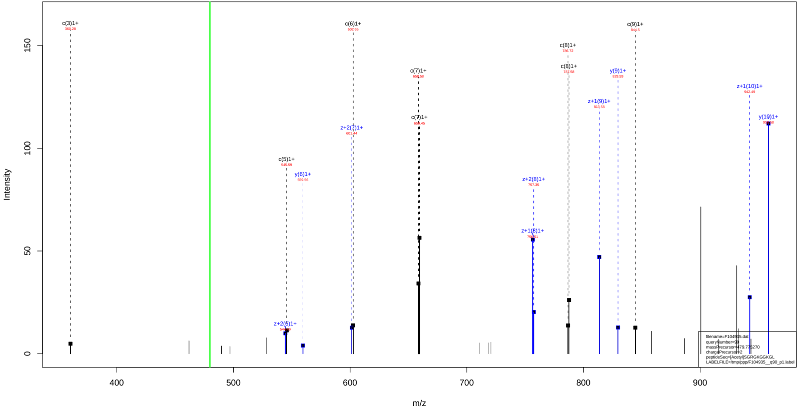

sp|P62806|H4_MOUSE

[Acetyl]SGRGKGGKGL

- fragmentation table for charge state 1+
- specType=etd
- score=42.18
- F104935.dat
- query=q90_p1
- precursor=479.775270
- chargePrecursor=2
- itol=0.8

AA	c	y	z+1	z+2	z	AA
S[1]	147.076	**958.543**	**942.524**	0.000	941.516	S[10]
G[2]	204.098	**829.500**	**813.482**	0.000	812.474	G[9]
R[3]	**360.199**	772.479	**756.460**	**757.468**	755.452	R[8]
G[4]	417.220	616.378	600.359	**601.367**	599.351	G[7]
K[5]	**545.315**	**559.356**	543.337	**544.345**	542.330	K[6]
G[6]	**602.337**	431.261	415.243	416.250	414.235	G[5]
G[7]	**659.358**	374.240	358.221	359.229	357.213	G[4]
K[8]	**787.453**	317.218	301.200	302.207	300.192	K[3]
G[9]	**844.475**	189.123	173.105	174.112	172.097	G[2]
L[10]	957.559	132.102	116.083	117.091	115.075	L[1]

sp|P62806|H4_MOUSE

[Acetyl]SGRGKGGKGL

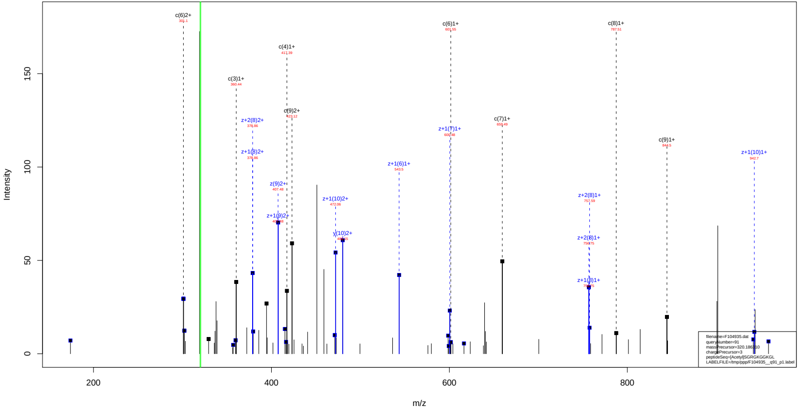

sp|P62806|H4_MOUSE
[Acetyl]SGRGKGGKGL

- fragmentation table for charge state 1+
- specType=etd
- score=33.99
- F104935.dat
- query=q91_p1
- precursor=320.186310
- chargePrecursor=3
- itol=0.8

AA	c	y	z+1	z+2	z	AA
S[1]	147.076	**958.543**	**942.524**	0.000	**941.516**	S[10]
G[2]	204.098	829.500	813.482	0.000	812.474	G[9]
R[3]	**360.199**	772.479	**756.460**	**757.468**	755.452	R[8]
G[4]	**417.220**	**616.378**	**600.359**	**601.367**	**599.351**	G[7]
K[5]	545.315	559.356	**543.337**	544.345	542.330	K[6]
G[6]	**602.337**	431.261	**415.243**	**416.250**	414.235	G[5]
G[7]	**659.358**	374.240	358.221	**359.229**	**357.213**	G[4]
K[8]	**787.453**	317.218	**301.200**	**302.207**	300.192	K[3]
G[9]	**844.475**	189.123	173.105	**174.112**	172.097	G[2]
L[10]	957.559	132.102	116.083	117.091	115.075	L[1]

sp|P62806|H4_MOUSE

[Acetyl]SGRGKGGKGL

- fragmentation table for charge state 2+
- specType=etd
- score=33.99
- F104935.dat
- query=q91_p1
- precursor=320.186310
- chargePrecursor=3
- itol=0.8

AA	c	y	z+1	z+2	z	AA
S[1]	74.042	**479.775**	**471.766**	0.504	**471.262**	S[10]
G[2]	102.553	**415.254**	**407.244**	0.504	**406.740**	G[9]
R[3]	180.603	386.743	**378.734**	**379.238**	**378.230**	R[8]
G[4]	209.114	308.692	**300.683**	**301.187**	300.179	G[7]
K[5]	273.161	280.182	272.172	272.676	271.668	K[6]
G[6]	**301.672**	216.134	208.125	208.629	207.621	G[5]
G[7]	**330.183**	187.624	179.614	180.118	179.110	G[4]
K[8]	**394.230**	159.113	151.103	151.607	150.600	K[3]
G[9]	**422.741**	95.065	87.056	87.560	86.552	G[2]
L[10]	479.283	66.555	58.545	59.049	58.041	L[1]

sp|P68433|H31_MOUSE

DTNLCAIHAK $^{Acetyl}_{42.01}$ RVTIMPK

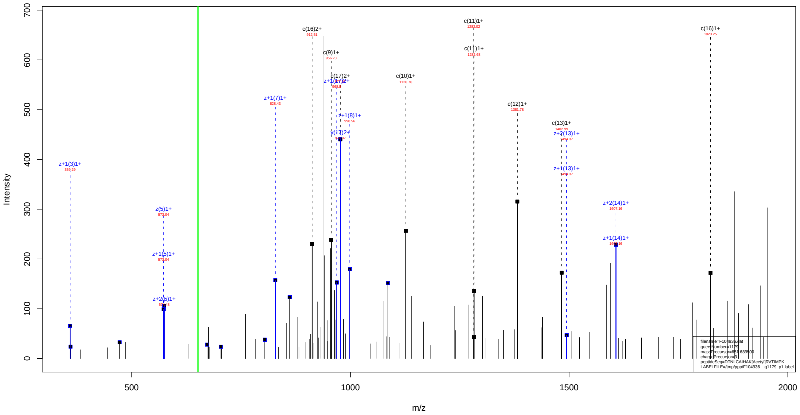

sp|P68433|H31_MOUSE

DTNLCAIHAK $^{Acetyl}_{42.01}$ RVTIMPK

- fragmentation table for charge state 1+
- specType=etd
- score=29.58
- F104936.dat
- query=q1179_p1
- precursor=651.689500
- chargePrecursor=3
- itol=0.8

AA	c	y	z+1	z+2	z	AA
D[1]	133.061	1953.041	1937.022	0.000	1936.014	D[17]
T[2]	234.108	1838.014	1821.995	0.000	1820.987	T[16]
N[3]	348.151	1736.966	1720.948	1721.955	1719.940	N[15]
L[4]	461.235	1622.923	**1606.905**	**1607.912**	1605.897	L[14]
C[5]	564.245	1509.839	**1493.821**	**1494.828**	1492.813	C[13]
A[6]	635.282	1406.830	1390.811	1391.819	1389.804	A[12]
I[7]	748.366	1335.793	1319.774	1320.782	1318.766	I[11]
H[8]	885.425	1222.709	1206.690	1207.698	1205.682	H[10]
A[9]	**956.462**	**1085.650**	1069.631	1070.639	1068.623	A[9]
K[10]	**1126.567**	1014.613	**998.594**	999.602	997.586	K[8]
R[11]	**1282.668**	844.507	**828.489**	829.496	827.481	R[7]
V[12]	**1381.737**	688.406	**672.387**	673.395	671.380	V[6]
T[13]	**1482.785**	589.338	**573.319**	**574.327**	**572.311**	T[5]
I[14]	1595.869	488.290	**472.271**	473.279	471.264	I[4]
M[15]	1726.909	375.206	**359.187**	**360.195**	358.180	M[3]
P[16]	**1823.962**	244.166	228.147	229.155	227.139	P[2]
K[17]	1952.057	147.113	131.094	132.102	130.086	K[1]

sp|P68433|H31_MOUSE

DTNLCAIHAK $^{Acetyl}_{42.01}$ RVTIMPK

- fragmentation table for charge state 2+
- specType=etd
- score=29.58
- F104936.dat
- query=q1179_p1
- precursor=651.689500
- chargePrecursor=3
- itol=0.8

AA	c	y	z+1	z+2	z	AA
D[1]	67.034	**977.024**	**969.015**	0.504	**968.511**	D[17]
T[2]	117.558	919.511	911.501	0.504	910.997	T[16]
N[3]	174.579	868.987	**860.977**	**861.481**	**860.473**	N[15]
L[4]	231.121	811.965	**803.956**	**804.460**	**803.452**	L[14]
C[5]	282.626	755.423	747.414	747.918	746.910	C[13]
A[6]	318.145	**703.919**	695.909	696.413	695.405	A[12]
I[7]	374.687	668.400	660.391	660.895	659.887	I[11]
H[8]	443.216	611.858	603.849	604.353	603.345	H[10]
A[9]	478.735	543.329	535.319	535.823	534.815	A[9]
K[10]	563.787	507.810	499.801	500.305	499.297	K[8]
R[11]	641.838	422.757	414.748	415.252	414.244	R[7]
V[12]	691.372	344.707	336.697	337.201	336.193	V[6]
T[13]	741.896	295.173	287.163	287.667	286.659	T[5]
I[14]	798.438	244.649	236.639	237.143	236.135	I[4]
M[15]	863.958	188.107	180.097	180.601	179.593	M[3]
P[16]	**912.485**	122.586	114.577	115.081	114.073	P[2]
K[17]	**976.532**	74.060	66.051	66.555	65.547	K[1]

sp|P62806|H4_MOUSE

[Acetyl]SGRGKGGKGLGKGGAK $^{\text{Acetyl}}_{42.01}$ RHRK $^{\text{Dimethyl}}_{28.03}$ VLR

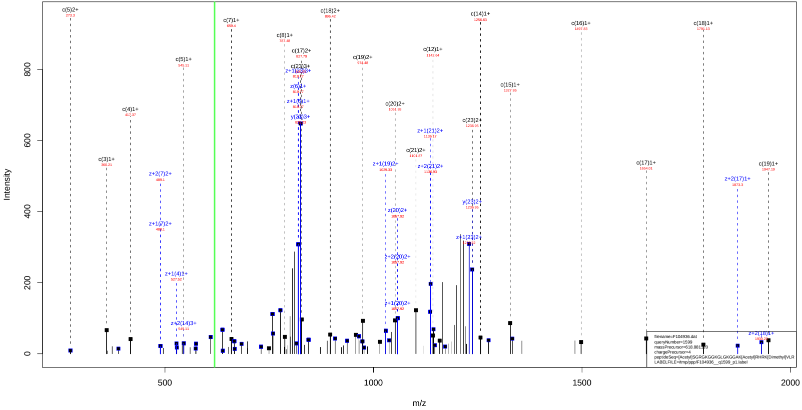

sp|P62806|H4_MOUSE

[Acetyl]SGRGKGGKGLGKGGAK$^{Acetyl}_{42.01}$RHRK$^{Dimethyl}_{28.03}$VLR

- fragmentation table for charge state 1+
- specType=etd
- score=95.30
- F104936.dat
- query=q1599_p1
- precursor=618.881120
- chargePrecursor=4
- itol=0.8

AA	c	y	z+1	z+2	z	AA
S[1]	147.076	2472.486	2456.467	0.000	2455.459	S[23]
G[2]	204.098	2343.443	2327.425	0.000	2326.417	G[22]
R[3]	**360.199**	2286.422	2270.403	2271.411	2269.395	R[21]
G[4]	**417.220**	2130.321	2114.302	2115.310	2113.294	G[20]
K[5]	**545.315**	2073.299	2057.280	2058.288	2056.273	K[19]
G[6]	602.337	1945.204	1929.186	**1930.193**	1928.178	G[18]
G[7]	**659.358**	1888.183	1872.164	**1873.172**	1871.156	G[17]
K[8]	**787.453**	1831.161	1815.143	1816.150	1814.135	K[16]
G[9]	**844.475**	1703.066	1687.048	1688.055	1686.040	G[15]
L[10]	**957.559**	1646.045	1630.026	1631.034	1629.018	L[14]
G[11]	**1014.580**	1532.961	1516.942	1517.950	1515.934	G[13]
K[12]	**1142.675**	1475.939	1459.921	1460.928	1458.913	K[12]
G[13]	1199.697	1347.844	1331.826	**1332.833**	1330.818	G[11]
G[14]	**1256.718**	1290.823	1274.804	**1275.812**	1273.796	G[10]
A[15]	**1327.755**	1233.801	1217.783	1218.791	1216.775	A[9]
K[16]	**1497.861**	1162.764	**1146.746**	1147.753	1145.738	K[8]
R[17]	**1653.962**	992.659	**976.640**	**977.648**	975.632	R[7]
H[18]	**1791.021**	836.558	**820.539**	821.547	**819.531**	H[6]
R[19]	**1947.122**	699.499	**683.480**	684.488	682.472	R[5]
K[20]	2103.248	543.398	**527.379**	**528.387**	526.371	K[4]
V[21]	2202.317	387.271	371.253	372.261	370.245	V[3]
L[22]	2315.401	288.203	272.184	**273.192**	271.176	L[2]
R[23]	2471.502	175.119	159.100	160.108	158.092	R[1]

sp|P62806|H4_MOUSE

[Acetyl]SGRGKGGKGLGKGGAK $^{Acetyl}_{42.01}$ RHRK $^{Dimethyl}_{28.03}$ VLR

- fragmentation table for charge state 2+
- specType=etd
- score=95.30
- F104936.dat
- query=q1599_p1
- precursor=618.881120
- chargePrecursor=4
- itol=0.8

AA	c	y	z+1	z+2	z	AA
S[1]	74.042	**1236.747**	**1228.737**	0.504	1228.233	S[23]
G[2]	102.553	**1172.225**	1164.216	0.504	1163.712	G[22]
R[3]	180.603	**1143.715**	**1135.705**	**1136.209**	1135.201	R[21]
G[4]	209.114	1065.664	**1057.655**	**1058.159**	**1057.151**	G[20]
K[5]	**273.161**	**1037.153**	**1029.144**	**1029.648**	**1028.640**	K[19]
G[6]	301.672	**973.106**	**965.096**	**965.600**	**964.592**	G[18]
G[7]	330.183	944.595	**936.586**	**937.090**	936.082	G[17]
K[8]	394.230	916.084	**908.075**	**908.579**	**907.571**	K[16]
G[9]	422.741	852.037	**844.027**	**844.531**	**843.524**	G[15]
L[10]	479.283	823.526	**815.517**	**816.021**	815.013	L[14]
G[11]	507.794	766.984	**758.975**	**759.479**	**758.471**	G[13]
K[12]	571.841	738.473	**730.464**	**730.968**	**729.960**	K[12]
G[13]	600.352	674.426	**666.416**	**666.920**	**665.913**	G[11]
G[14]	628.863	645.915	**637.906**	**638.410**	**637.402**	G[10]
A[15]	664.381	617.404	**609.395**	**609.899**	**608.891**	A[9]
K[16]	**749.434**	581.886	**573.876**	**574.380**	**573.373**	K[8]
R[17]	**827.485**	496.833	**488.824**	**489.328**	**488.320**	R[7]
H[18]	**896.014**	418.782	410.773	411.277	410.269	H[6]
R[19]	**974.065**	350.253	342.244	342.748	341.740	R[5]
K[20]	**1052.128**	272.202	264.193	264.697	263.689	K[4]
V[21]	**1101.662**	194.139	186.130	186.634	185.626	V[3]
L[22]	**1158.204**	144.605	136.596	137.100	136.092	L[2]
R[23]	**1236.255**	88.063	80.054	80.558	79.550	R[1]

sp|P62806|H4_MOUSE

[Acetyl]SGRGKGGKGLGKGGAK $^{Acetyl}_{42.01}$ RHRK $^{Dimethyl}_{28.03}$ VLR

- fragmentation table for charge state 3+
- specType=etd
- score=95.30
- F104936.dat
- query=q1599_p1
- precursor=618.881120
- chargePrecursor=4
- itol=0.8

AA	c	y	z+1	z+2	z	AA
S[1]	49.697	**824.833**	**819.494**	0.672	**819.158**	S[23]
G[2]	68.704	781.819	**776.480**	0.672	**776.144**	G[22]
R[3]	120.738	762.812	**757.473**	**757.808**	**757.137**	R[21]
G[4]	139.745	710.778	705.439	705.775	705.103	G[20]
K[5]	182.443	691.771	686.432	686.768	686.096	K[19]
G[6]	201.450	649.073	643.733	644.069	643.397	G[18]
G[7]	220.458	630.066	624.726	625.062	624.390	G[17]
K[8]	263.156	611.059	605.719	606.055	605.383	K[16]
G[9]	282.163	568.360	563.021	563.357	562.685	G[15]
L[10]	319.858	549.353	544.014	**544.350**	543.678	L[14]
G[11]	338.865	511.658	506.319	506.655	505.983	G[13]
K[12]	381.563	492.651	487.312	487.648	486.976	K[12]
G[13]	400.570	449.953	444.613	444.949	444.277	G[11]
G[14]	419.578	430.946	425.606	425.942	425.270	G[10]
A[15]	443.257	411.939	406.599	406.935	406.263	A[9]
K[16]	499.958	**388.260**	382.920	383.256	382.584	K[8]
R[17]	551.992	331.558	326.218	326.554	325.882	R[7]
H[18]	597.678	279.524	274.185	274.520	**273.849**	H[6]
R[19]	649.712	233.838	228.498	228.834	228.162	R[5]
K[20]	701.754	181.804	176.465	176.800	176.129	K[4]
V[21]	734.777	129.762	124.422	124.758	124.086	V[3]
L[22]	772.472	96.739	91.400	91.736	91.064	L[2]
R[23]	**824.505**	59.045	53.705	54.041	53.369	R[1]

sp|P62806|H4_MOUSE

[Acetyl]SGRGKGGKGLGKGGAK $^{\text{Acetyl}}_{42.01}$ RHRK $^{\text{Dimethyl}}_{28.03}$ VLR

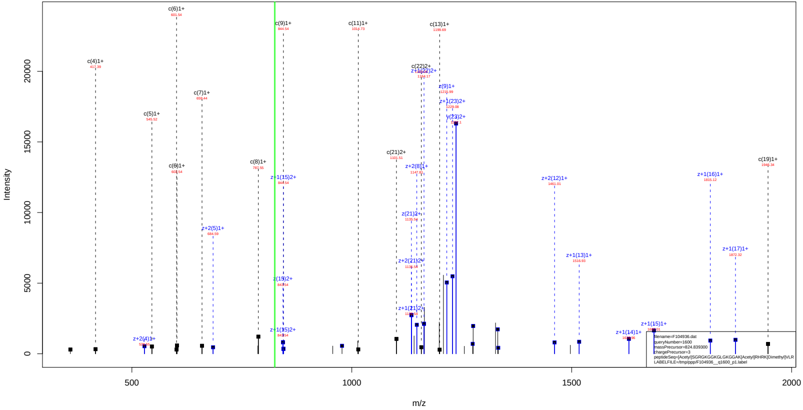

sp|P62806|H4_MOUSE

[Acetyl]SGRGKGGKGLGKGGAK $^{Acetyl}_{42.01}$ RHRK $^{Dimethyl}_{28.03}$ VLR

- fragmentation table for charge state 1+
- specType=etd
- score=69.76
- F104936.dat
- query=q1600_p1
- precursor=824.839300
- chargePrecursor=3
- itol=0.8

AA	c	y	z+1	z+2	z	AA
S[1]	147.076	2472.486	2456.467	0.000	2455.459	S[23]
G[2]	204.098	2343.443	2327.425	0.000	2326.417	G[22]
R[3]	**360.199**	2286.422	2270.403	2271.411	2269.395	R[21]
G[4]	**417.220**	2130.321	2114.302	2115.310	2113.294	G[20]
K[5]	**545.315**	2073.299	2057.280	2058.288	2056.273	K[19]
G[6]	**602.337**	1945.204	1929.186	1930.193	1928.178	G[18]
G[7]	**659.358**	1888.183	**1872.164**	1873.172	1871.156	G[17]
K[8]	**787.453**	1831.161	**1815.143**	1816.150	1814.135	K[16]
G[9]	**844.475**	1703.066	**1687.048**	1688.055	1686.040	G[15]
L[10]	957.559	1646.045	**1630.026**	1631.034	1629.018	L[14]
G[11]	**1014.580**	1532.961	**1516.942**	1517.950	1515.934	G[13]
K[12]	1142.675	1475.939	1459.921	**1460.928**	1458.913	K[12]
G[13]	**1199.697**	1347.844	**1331.826**	**1332.833**	1330.818	G[11]
G[14]	1256.718	1290.823	**1274.804**	**1275.812**	1273.796	G[10]
A[15]	1327.755	1233.801	1217.783	1218.791	**1216.775**	A[9]
K[16]	1497.861	1162.764	1146.746	**1147.753**	1145.738	K[8]
R[17]	1653.962	992.659	976.640	**977.648**	975.632	R[7]
H[18]	1791.021	836.558	820.539	821.547	819.531	H[6]
R[19]	**1947.122**	699.499	683.480	**684.488**	682.472	R[5]
K[20]	2103.248	543.398	527.379	**528.387**	526.371	K[4]
V[21]	2202.317	387.271	371.253	372.261	370.245	V[3]
L[22]	2315.401	288.203	272.184	273.192	271.176	L[2]
R[23]	2471.502	175.119	159.100	160.108	158.092	R[1]

sp|P62806|H4_MOUSE

[Acetyl]SGRGKGGKGLGKGGAK $^{Acetyl}_{42.01}$ RHRK $^{Dimethyl}_{28.03}$ VLR

- fragmentation table for charge state 2+
- specType=etd
- score=69.76
- F104936.dat
- query=q1600_p1
- precursor=824.839300
- chargePrecursor=3
- itol=0.8

AA	c	y	z+1	z+2	z	AA
S[1]	74.042	**1236.747**	**1228.737**	0.504	1228.233	S[23]
G[2]	102.553	1172.225	**1164.216**	0.504	**1163.712**	G[22]
R[3]	180.603	1143.715	**1135.705**	**1136.209**	**1135.201**	R[21]
G[4]	209.114	1065.664	1057.655	1058.159	1057.151	G[20]
K[5]	273.161	1037.153	1029.144	1029.648	1028.640	K[19]
G[6]	301.672	973.106	965.096	965.600	964.592	G[18]
G[7]	330.183	944.595	936.586	937.090	936.082	G[17]
K[8]	394.230	916.084	908.075	908.579	907.571	K[16]
G[9]	422.741	852.037	**844.027**	**844.531**	**843.524**	G[15]
L[10]	479.283	823.526	815.517	816.021	815.013	L[14]
G[11]	507.794	766.984	758.975	759.479	758.471	G[13]
K[12]	571.841	738.473	730.464	730.968	729.960	K[12]
G[13]	600.352	674.426	666.416	666.920	665.913	G[11]
G[14]	628.863	645.915	637.906	638.410	637.402	G[10]
A[15]	664.381	617.404	609.395	609.899	608.891	A[9]
K[16]	749.434	581.886	573.876	574.380	573.373	K[8]
R[17]	827.485	496.833	488.824	489.328	488.320	R[7]
H[18]	896.014	418.782	410.773	411.277	410.269	H[6]
R[19]	974.065	350.253	342.244	342.748	341.740	R[5]
K[20]	1052.128	272.202	264.193	264.697	263.689	K[4]
V[21]	**1101.662**	194.139	186.130	186.634	185.626	V[3]
L[22]	**1158.204**	144.605	136.596	137.100	136.092	L[2]
R[23]	1236.255	88.063	80.054	80.558	79.550	R[1]

sp|Q6ZWY9|H2B1C_MOUSE

PEPAKSAPAPKK $^{Acetyl}_{42.01}$ GSKKAVTKAQKK

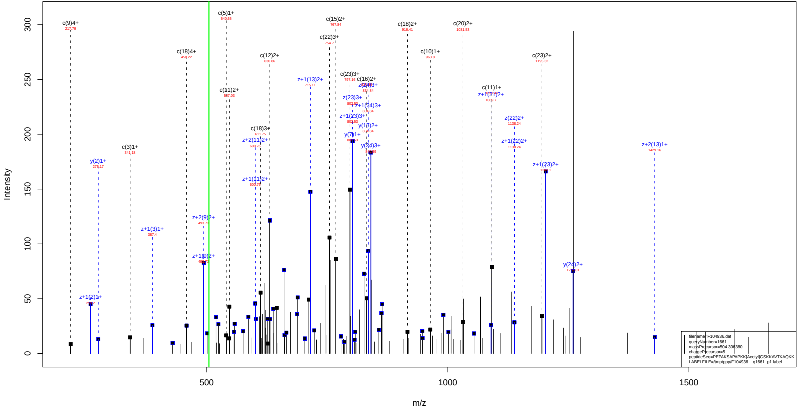

sp|Q6ZWY9|H2B1C_MOUSE

PEPAKSAPAPKK $^{Acetyl}_{42.01}$ GSKKAVTKAQKK

- fragmentation table for charge state 1+
- specType=etd
- score=50.57
- F104936.dat
- query=q1661_p1
- precursor=504.306380
- chargePrecursor=5
- itol=0.8

AA	c	y	z+1	z+2	z	AA
P[1]	115.087	2517.488	2501.469	0.000	2500.461	P[24]
E[2]	244.129	2420.435	2404.416	0.000	2403.408	E[23]
P[3]	**341.182**	2291.392	2275.373	0.000	2274.366	P[22]
A[4]	412.219	2194.339	2178.321	0.000	2177.313	A[21]
K[5]	**540.314**	2123.302	2107.284	2108.291	2106.276	K[20]
S[6]	**627.346**	1995.207	1979.189	1980.196	1978.181	S[19]
A[7]	698.383	1908.175	1892.157	1893.164	1891.149	A[18]
P[8]	795.436	1837.138	1821.119	1822.127	1820.112	P[17]
A[9]	866.473	1740.085	1724.067	1725.075	1723.059	A[16]
P[10]	**963.526**	1669.048	1653.030	1654.037	1652.022	P[15]
K[11]	**1091.621**	1571.996	1555.977	1556.985	1554.969	K[14]
K[12]	1261.726	1443.901	1427.882	**1428.890**	1426.874	K[13]
G[13]	1318.748	1273.795	1257.776	1258.784	1256.768	G[12]
S[14]	1405.780	1216.774	1200.755	1201.763	1199.747	S[11]
K[15]	1533.875	1129.742	1113.723	1114.731	1112.715	K[10]
K[16]	1661.970	**1001.647**	985.628	986.636	984.620	K[9]
A[17]	1733.007	873.552	**857.533**	858.541	**856.525**	A[8]
V[18]	1832.075	**802.515**	786.496	787.504	**785.488**	V[7]
T[19]	1933.123	**703.446**	**687.427**	**688.435**	686.420	T[6]
K[20]	2061.218	**602.398**	**586.380**	587.388	585.372	K[5]
A[21]	2132.255	474.303	**458.285**	459.293	457.277	A[4]
Q[22]	2260.314	403.266	**387.248**	388.255	386.240	Q[3]
K[23]	2388.409	**275.208**	**259.189**	260.197	258.181	K[2]
K[24]	2516.504	147.113	131.094	132.102	130.086	K[1]

sp|Q6ZWY9|H2B1C_MOUSE

PEPAKSAPAPKK $^{Acetyl}_{42.01}$ GSKKAVTKAQKK

- fragmentation table for charge state 2+
- specType=etd
- score=50.57
- F104936.dat
- query=q1661_p1
- precursor=504.306380
- chargePrecursor=5
- itol=0.8

AA	c	y	z+1	z+2	z	AA
P[1]	58.047	**1259.247**	1251.238	0.504	1250.734	P[24]
E[2]	122.568	1210.721	**1202.712**	0.504	1202.208	E[23]
P[3]	171.095	1146.200	**1138.190**	0.504	**1137.686**	P[22]
A[4]	206.613	1097.673	**1089.664**	0.504	**1089.160**	A[21]
K[5]	270.661	1062.155	**1054.145**	**1054.649**	1053.642	K[20]
S[6]	314.177	998.107	**990.098**	**990.602**	989.594	S[19]
A[7]	349.695	954.591	**946.582**	**947.086**	946.078	A[18]
P[8]	398.222	919.073	911.063	911.567	910.559	P[17]
A[9]	433.740	870.546	**862.537**	**863.041**	**862.033**	A[16]
P[10]	482.267	**835.028**	**827.018**	827.522	**826.515**	P[15]
K[11]	**546.314**	786.501	**778.492**	**778.996**	**777.988**	K[14]
K[12]	**631.367**	**722.454**	**714.445**	**714.948**	713.941	K[13]
G[13]	**659.878**	**637.401**	629.392	629.896	628.888	G[12]
S[14]	**703.394**	608.890	**600.881**	**601.385**	**600.377**	S[11]
K[15]	**767.441**	565.374	**557.365**	**557.869**	**556.861**	K[10]
K[16]	**831.488**	**501.327**	**493.318**	**493.821**	492.814	K[9]
A[17]	867.007	437.279	**429.270**	**429.774**	**428.766**	A[8]
V[18]	**916.541**	401.761	393.752	394.255	393.248	V[7]
T[19]	967.065	352.227	344.217	344.721	343.713	T[6]
K[20]	**1031.113**	301.703	293.693	294.197	293.190	K[5]
A[21]	1066.631	237.655	229.646	230.150	229.142	A[4]
Q[22]	1130.660	202.137	194.127	194.631	193.624	Q[3]
K[23]	**1194.708**	138.108	130.098	130.602	129.594	K[2]
K[24]	1258.755	74.060	66.051	66.555	65.547	K[1]

sp|Q6ZWY9|H2B1C_MOUSE

PEPAKSAPAPKK $^{\text{Acetyl}}_{42.01}$ GSKKAVTKAQKK

- fragmentation table for charge state 3+
- specType=etd
- score=50.57
- F104936.dat
- query=q1661_p1
- precursor=504.306380
- chargePrecursor=5
- itol=0.8

AA	c	y	z+1	z+2	z	AA
P[1]	39.034	**839.834**	**834.494**	0.672	**834.159**	P[24]
E[2]	82.048	**807.483**	**802.144**	0.672	**801.808**	E[23]
P[3]	114.399	764.469	759.129	0.672	758.793	P[22]
A[4]	138.078	732.118	726.778	0.672	726.442	A[21]
K[5]	180.776	708.439	**703.099**	**703.435**	**702.763**	K[20]
S[6]	209.787	**665.741**	**660.401**	**660.737**	**660.065**	S[19]
A[7]	233.466	636.730	**631.390**	**631.726**	**631.054**	A[18]
P[8]	265.817	613.051	607.711	608.047	607.375	P[17]
A[9]	289.496	580.700	**575.360**	**575.696**	**575.024**	A[16]
P[10]	321.847	**557.021**	551.681	552.017	551.345	P[15]
K[11]	364.545	**524.670**	**519.330**	**519.666**	**518.995**	K[14]
K[12]	421.247	481.972	476.632	476.968	476.296	K[13]
G[13]	440.254	425.270	419.930	420.266	419.594	G[12]
S[14]	469.265	406.263	400.923	401.259	400.587	S[11]
K[15]	511.963	377.252	371.912	372.248	371.577	K[10]
K[16]	554.661	334.554	329.214	329.550	328.878	K[9]
A[17]	578.340	291.855	286.516	286.852	286.180	A[8]
V[18]	**611.363**	268.176	262.837	263.173	262.501	V[7]
T[19]	**645.046**	235.154	229.814	230.150	229.478	T[6]
K[20]	**687.744**	201.471	196.131	196.467	195.795	K[5]
A[21]	**711.423**	158.773	153.433	153.769	153.097	A[4]
Q[22]	**754.109**	135.094	129.754	130.090	129.418	Q[3]
K[23]	**796.808**	92.407	87.068	87.404	86.732	K[2]
K[24]	839.506	49.709	44.370	44.705	44.034	K[1]

sp|Q6ZWY9|H2B1C_MOUSE

PEPAKSAPAPKK $^{Acetyl}_{42.01}$ GSKKAVTKAQKK

- fragmentation table for charge state 4+
- specType=etd
- score=50.57
- F104936.dat
- query=q1661_p1
- precursor=504.306380
- chargePrecursor=5
- itol=0.8

AA	c	y	z+1	z+2	z	AA
P[1]	29.527	**630.127**	**626.123**	0.755	**625.871**	P[24]
E[2]	61.788	605.864	**601.859**	0.755	**601.608**	E[23]
P[3]	86.051	573.603	569.599	0.755	569.347	P[22]
A[4]	103.810	549.340	545.336	0.755	545.084	A[21]
K[5]	135.834	531.581	527.576	527.828	527.324	K[20]
S[6]	157.592	499.557	495.553	495.805	495.301	S[19]
A[7]	175.351	477.799	473.795	474.047	473.543	A[18]
P[8]	199.614	460.040	456.035	456.287	455.783	P[17]
A[9]	**217.374**	435.777	431.772	432.024	431.520	A[16]
P[10]	241.637	418.018	414.013	414.265	413.761	P[15]
K[11]	273.661	393.754	389.750	390.002	389.498	K[14]
K[12]	316.187	361.731	357.726	357.978	357.474	K[13]
G[13]	330.442	319.204	315.200	315.451	314.948	G[12]
S[14]	352.200	304.949	300.944	301.196	300.692	S[11]
K[15]	384.224	283.191	279.186	279.438	278.934	K[10]
K[16]	416.248	251.167	247.162	247.414	246.910	K[9]
A[17]	434.007	219.143	215.139	215.391	214.887	A[8]
V[18]	**458.774**	201.384	197.379	197.631	197.127	V[7]
T[19]	484.036	176.617	172.612	172.864	172.360	T[6]
K[20]	516.060	151.355	147.350	147.602	147.098	K[5]
A[21]	533.819	119.331	115.327	115.579	115.075	A[4]
Q[22]	565.834	101.572	97.567	97.819	97.315	Q[3]
K[23]	597.858	69.557	65.553	65.805	65.301	K[2]
K[24]	629.881	37.534	33.529	33.781	33.277	K[1]

sp|P62806|H4_MOUSE

[Acetyl]SGRGKGGK $^{\text{Acetyl}}_{42.01}$ GLGK $^{\text{Acetyl}}_{42.01}$ GGAK $^{\text{Acetyl}}_{42.01}$ RHRK $^{\text{Dimethyl}}_{28.03}$ VLR

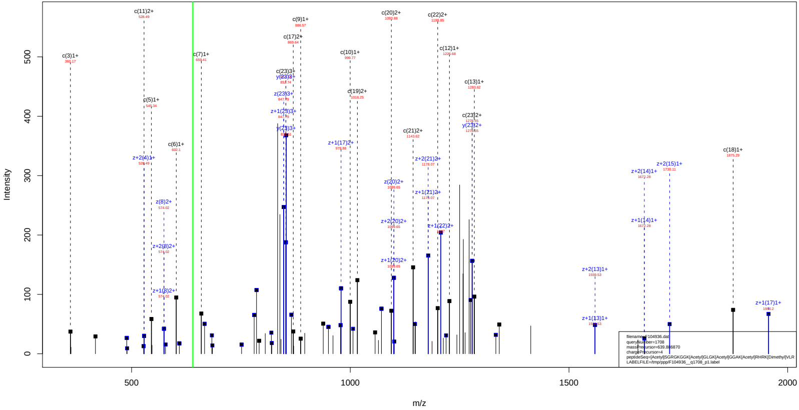

sp|P62806|H4_MOUSE

[Acetyl]SGRGKGGK $^{Acetyl}_{42.01}$ GLGK $^{Acetyl}_{42.01}$ GGAK $^{Acetyl}_{42.01}$ RHRK $^{Dimethyl}_{28.03}$ VLR

- fragmentation table for charge state 1+
- specType=etd
- score=99.07
- F104936.dat
- query=q1708_p1
- precursor=639.886870
- chargePrecursor=4
- itol=0.8

AA	c	y	z+1	z+2	z	AA
S[1]	147.076	2556.507	2540.488	0.000	2539.480	S[23]
G[2]	204.098	2427.464	2411.446	0.000	2410.438	G[22]
R[3]	**360.199**	2370.443	2354.424	2355.432	2353.416	R[21]
G[4]	**417.220**	2214.342	2198.323	2199.331	2197.315	G[20]
K[5]	**545.315**	2157.320	2141.302	2142.309	2140.294	K[19]
G[6]	**602.337**	2029.225	2013.207	2014.214	2012.199	G[18]
G[7]	**659.358**	1972.204	**1956.185**	1957.193	1955.177	G[17]
K[8]	829.464	1915.182	1899.164	1900.172	1898.156	K[16]
G[9]	**886.485**	1745.077	1729.058	**1730.066**	1728.050	G[15]
L[10]	**999.569**	1688.055	**1672.037**	**1673.045**	1671.029	L[14]
G[11]	**1056.591**	1574.971	**1558.953**	**1559.960**	1557.945	G[13]
K[12]	**1226.696**	1517.950	1501.931	1502.939	1500.923	K[12]
G[13]	**1283.718**	1347.844	1331.826	**1332.833**	1330.818	G[11]
G[14]	**1340.739**	1290.823	**1274.804**	1275.812	1273.796	G[10]
A[15]	1411.776	1233.801	1217.783	**1218.791**	1216.775	A[9]
K[16]	1581.882	1162.764	1146.746	**1147.753**	1145.738	K[8]
R[17]	1737.983	992.659	976.640	**977.648**	975.632	R[7]
H[18]	**1875.042**	836.558	**820.539**	821.547	**819.531**	H[6]
R[19]	2031.143	699.499	**683.480**	**684.488**	682.472	R[5]
K[20]	2187.269	543.398	**527.379**	**528.387**	526.371	K[4]
V[21]	2286.338	387.271	371.253	372.261	370.245	V[3]
L[22]	2399.422	288.203	272.184	273.192	271.176	L[2]
R[23]	2555.523	175.119	159.100	160.108	158.092	R[1]

sp|P62806|H4_MOUSE

[Acetyl]SGRGKGGK $^{Acetyl}_{42.01}$ GLGK $^{Acetyl}_{42.01}$ GGAK $^{Acetyl}_{42.01}$ RHRK $^{Dimethyl}_{28.03}$ VLR

- fragmentation table for charge state 2+
- specType=etd
- score=99.07
- F104936.dat
- query=q1708_p1
- precursor=639.886870
- chargePrecursor=4
- itol=0.8

AA	c	y	z+1	z+2	z	AA
S[1]	74.042	**1278.757**	1270.748	0.504	1270.244	S[23]
G[2]	102.553	1214.236	**1206.226**	0.504	1205.723	G[22]
R[3]	180.603	1185.725	**1177.716**	**1178.220**	1177.212	R[21]
G[4]	209.114	1107.675	**1099.665**	**1100.169**	**1099.161**	G[20]
K[5]	273.161	1079.164	**1071.154**	**1071.658**	**1070.651**	K[19]
G[6]	301.672	1015.116	1007.107	1007.611	**1006.603**	G[18]
G[7]	330.183	986.606	**978.596**	**979.100**	**978.092**	G[17]
K[8]	415.236	958.095	**950.085**	**950.589**	**949.582**	K[16]
G[9]	443.746	873.042	**865.033**	**865.537**	864.529	G[15]
L[10]	500.288	844.531	836.522	837.026	836.018	L[14]
G[11]	**528.799**	787.989	**779.980**	**780.484**	**779.476**	G[13]
K[12]	613.852	759.479	**751.469**	**751.973**	**750.965**	K[12]
G[13]	642.363	674.426	**666.416**	**666.920**	665.913	G[11]
G[14]	670.873	645.915	637.906	638.410	637.402	G[10]
A[15]	706.392	617.404	**609.395**	**609.899**	**608.891**	A[9]
K[16]	**791.445**	581.886	**573.876**	**574.380**	**573.373**	K[8]
R[17]	**869.495**	496.833	**488.824**	**489.328**	**488.320**	R[7]
H[18]	**938.025**	418.782	410.773	411.277	410.269	H[6]
R[19]	**1016.075**	350.253	342.244	342.748	341.740	R[5]
K[20]	**1094.138**	272.202	264.193	264.697	263.689	K[4]
V[21]	**1143.673**	194.139	186.130	186.634	185.626	V[3]
L[22]	**1200.215**	144.605	136.596	137.100	136.092	L[2]
R[23]	**1278.265**	88.063	80.054	80.558	79.550	R[1]

sp|P62806|H4_MOUSE

[Acetyl]SGRGKGGK $^{Acetyl}_{42.01}$ GLGK $^{Acetyl}_{42.01}$ GGAK $^{Acetyl}_{42.01}$ RHRK $^{Dimethyl}_{28.03}$ VLR

- fragmentation table for charge state 3+
- specType=etd
- score=99.07
- F104936.dat
- query=q1708_p1
- precursor=639.886870
- chargePrecursor=4
- itol=0.8

AA	c	y	z+1	z+2	z	AA
S[1]	49.697	**852.841**	**847.501**	0.672	**847.165**	S[23]
G[2]	68.704	809.826	804.487	0.672	804.151	G[22]
R[3]	120.738	790.819	**785.480**	**785.816**	**785.144**	R[21]
G[4]	139.745	738.785	733.446	733.782	733.110	G[20]
K[5]	182.443	719.778	714.439	714.775	714.103	K[19]
G[6]	201.450	677.080	671.740	672.076	671.404	G[18]
G[7]	220.458	658.073	652.733	653.069	652.397	G[17]
K[8]	277.159	639.066	633.726	634.062	633.390	K[16]
G[9]	296.167	582.364	**577.024**	**577.360**	576.688	G[15]
L[10]	333.861	563.357	558.017	558.353	557.681	L[14]
G[11]	352.868	525.662	520.322	520.658	519.986	G[13]
K[12]	409.570	506.655	501.315	501.651	500.979	K[12]
G[13]	428.577	449.953	444.613	444.949	444.277	G[11]
G[14]	447.585	430.946	425.606	425.942	425.270	G[10]
A[15]	471.264	411.939	406.599	406.935	406.263	A[9]
K[16]	**527.966**	388.260	382.920	383.256	382.584	K[8]
R[17]	579.999	331.558	326.218	326.554	325.882	R[7]
H[18]	625.686	279.524	274.185	274.520	273.849	H[6]
R[19]	677.719	233.838	228.498	228.834	228.162	R[5]
K[20]	729.761	181.804	176.465	176.800	176.129	K[4]
V[21]	762.784	129.762	124.422	124.758	124.086	V[3]
L[22]	800.479	96.739	91.400	91.736	91.064	L[2]
R[23]	**852.513**	59.045	53.705	54.041	53.369	R[1]

sp|P62806|H4_MOUSE

[Acetyl]SGRGK $^{\text{Acetyl}}_{42.01}$ GGK $^{\text{Acetyl}}_{42.01}$ GLGK $^{\text{Acetyl}}_{42.01}$ GGAK $^{\text{Acetyl}}_{42.01}$ RHRK $^{\text{Dimethyl}}_{28.03}$ VLR

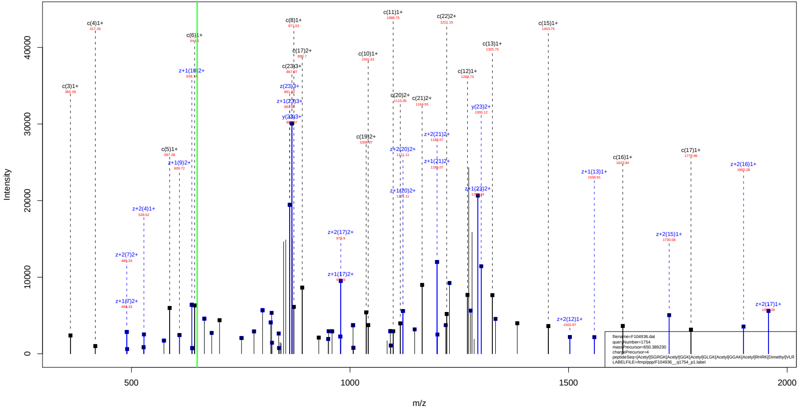

sp|P62806|H4_MOUSE

[Acetyl]SGRGK $^{\text{Acetyl}}_{42.01}$ GGK $^{\text{Acetyl}}_{42.01}$ GLGK $^{\text{Acetyl}}_{42.01}$ GGAK $^{\text{Acetyl}}_{42.01}$ RHRK $^{\text{Dimethyl}}_{28.03}$ VLR

- fragmentation table for charge state 1+
- specType=etd
- score=128.02
- F104936.dat
- query=q1754_p1
- precursor=650.389230
- chargePrecursor=4
- itol=0.8

AA	c	y	z+1	z+2	z	AA
S[1]	147.076	2598.518	2582.499	0.000	2581.491	S[23]
G[2]	204.098	2469.475	2453.456	0.000	2452.448	G[22]
R[3]	**360.199**	2412.453	2396.435	2397.443	2395.427	R[21]
G[4]	**417.220**	2256.352	2240.334	2241.341	2239.326	G[20]
K[5]	**587.326**	2199.331	2183.312	2184.320	2182.304	K[19]
G[6]	**644.347**	2029.225	2013.207	2014.214	2012.199	G[18]
G[7]	**701.369**	1972.204	1956.185	**1957.193**	1955.177	G[17]
K[8]	**871.474**	1915.182	1899.164	**1900.172**	1898.156	K[16]
G[9]	**928.496**	1745.077	1729.058	**1730.066**	1728.050	G[15]
L[10]	**1041.580**	1688.055	1672.037	1673.045	1671.029	L[14]
G[11]	**1098.601**	1574.971	**1558.953**	1559.960	1557.945	G[13]
K[12]	**1268.707**	1517.950	1501.931	**1502.939**	1500.923	K[12]
G[13]	**1325.728**	1347.844	1331.826	**1332.833**	1330.818	G[11]
G[14]	**1382.750**	1290.823	1274.804	**1275.812**	1273.796	G[10]
A[15]	**1453.787**	1233.801	1217.783	**1218.791**	1216.775	A[9]
K[16]	**1623.893**	1162.764	1146.746	**1147.753**	1145.738	K[8]
R[17]	**1779.994**	992.659	976.640	**977.648**	975.632	R[7]
H[18]	1917.053	**836.558**	**820.539**	**821.547**	819.531	H[6]
R[19]	2073.154	699.499	**683.480**	684.488	682.472	R[5]
K[20]	2229.280	543.398	**527.379**	**528.387**	526.371	K[4]
V[21]	2328.348	387.271	371.253	372.261	370.245	V[3]
L[22]	2441.432	288.203	272.184	273.192	271.176	L[2]
R[23]	2597.534	175.119	159.100	160.108	158.092	R[1]

sp|P62806|H4_MOUSE

[Acetyl]SGRGK $^{Acetyl}_{42.01}$ GGK $^{Acetyl}_{42.01}$ GLGK $^{Acetyl}_{42.01}$ GGAK $^{Acetyl}_{42.01}$ RHRK $^{Dimethyl}_{28.03}$ VLR

- fragmentation table for charge state 2+
- specType=etd
- score=128.02
- F104936.dat
- query=q1754_p1
- precursor=650.389230
- chargePrecursor=4
- itol=0.8

AA	c	y	z+1	z+2	z	AA
S[1]	74.042	**1299.762**	**1291.753**	0.504	1291.249	S[23]
G[2]	102.553	1235.241	**1227.232**	0.504	1226.728	G[22]
R[3]	180.603	1206.730	**1198.721**	**1199.225**	1198.217	R[21]
G[4]	209.114	1128.680	**1120.670**	**1121.174**	1120.167	G[20]
K[5]	294.167	1100.169	**1092.160**	**1092.664**	**1091.656**	K[19]
G[6]	322.677	1015.116	**1007.107**	**1007.611**	**1006.603**	G[18]
G[7]	351.188	986.606	**978.596**	**979.100**	**978.092**	G[17]
K[8]	436.241	**958.095**	**950.085**	**950.589**	**949.582**	K[16]
G[9]	464.752	873.042	865.033	865.537	864.529	G[15]
L[10]	521.294	844.531	**836.522**	**837.026**	**836.018**	L[14]
G[11]	549.804	787.989	**779.980**	**780.484**	779.476	G[13]
K[12]	634.857	759.479	**751.469**	**751.973**	**750.965**	K[12]
G[13]	663.368	674.426	**666.416**	**666.920**	665.913	G[11]
G[14]	691.879	645.915	**637.906**	**638.410**	**637.402**	G[10]
A[15]	727.397	617.404	**609.395**	**609.899**	608.891	A[9]
K[16]	812.450	581.886	**573.876**	**574.380**	573.373	K[8]
R[17]	**890.500**	496.833	**488.824**	**489.328**	488.320	R[7]
H[18]	**959.030**	418.782	410.773	411.277	410.269	H[6]
R[19]	**1037.080**	350.253	342.244	342.748	341.740	R[5]
K[20]	**1115.144**	272.202	264.193	264.697	263.689	K[4]
V[21]	**1164.678**	194.139	186.130	186.634	185.626	V[3]
L[22]	**1221.220**	144.605	136.596	137.100	136.092	L[2]
R[23]	1299.270	88.063	80.054	80.558	79.550	R[1]

sp|P62806|H4_MOUSE

[Acetyl]SGRGK $^{Acetyl}_{42.01}$ GGK $^{Acetyl}_{42.01}$ GLGK $^{Acetyl}_{42.01}$ GGAK $^{Acetyl}_{42.01}$ RHRK $^{Dimethyl}_{28.03}$ VLR

- fragmentation table for charge state 3+
- specType=etd
- score=128.02
- F104936.dat
- query=q1754_p1
- precursor=650.389230
- chargePrecursor=4
- itol=0.8

AA	c	y	z+1	z+2	z	AA
S[1]	49.697	**866.844**	**861.504**	0.672	**861.169**	S[23]
G[2]	68.704	823.830	**818.490**	0.672	**818.154**	G[22]
R[3]	120.738	804.823	**799.483**	**799.819**	**799.147**	R[21]
G[4]	139.745	752.789	747.449	747.785	747.113	G[20]
K[5]	196.447	733.782	728.442	728.778	728.106	K[19]
G[6]	215.454	677.080	671.740	672.076	671.404	G[18]
G[7]	234.461	658.073	652.733	653.069	652.397	G[17]
K[8]	291.163	**639.066**	633.726	634.062	633.390	K[16]
G[9]	310.170	582.364	577.024	577.360	576.688	G[15]
L[10]	347.865	563.357	558.017	558.353	557.681	L[14]
G[11]	366.872	525.662	520.322	520.658	519.986	G[13]
K[12]	423.574	506.655	501.315	501.651	500.979	K[12]
G[13]	442.581	449.953	444.613	444.949	444.277	G[11]
G[14]	461.588	430.946	425.606	425.942	425.270	G[10]
A[15]	485.267	411.939	406.599	406.935	406.263	A[9]
K[16]	541.969	388.260	382.920	383.256	382.584	K[8]
R[17]	594.003	331.558	326.218	326.554	325.882	R[7]
H[18]	**639.689**	279.524	274.185	274.520	273.849	H[6]
R[19]	691.723	233.838	228.498	228.834	228.162	R[5]
K[20]	743.765	181.804	176.465	176.800	176.129	K[4]
V[21]	776.788	129.762	124.422	124.758	124.086	V[3]
L[22]	814.482	96.739	91.400	91.736	91.064	L[2]
R[23]	**866.516**	59.045	53.705	54.041	53.369	R[1]

sp|P62806|H4_MOUSE

[Acetyl]SGRGK $^{Acetyl}_{42.01}$ GGK $^{Acetyl}_{42.01}$ GLGK $^{Acetyl}_{42.01}$ GGAK $^{Acetyl}_{42.01}$ RHRK $^{Dimethyl}_{28.03}$ VLR

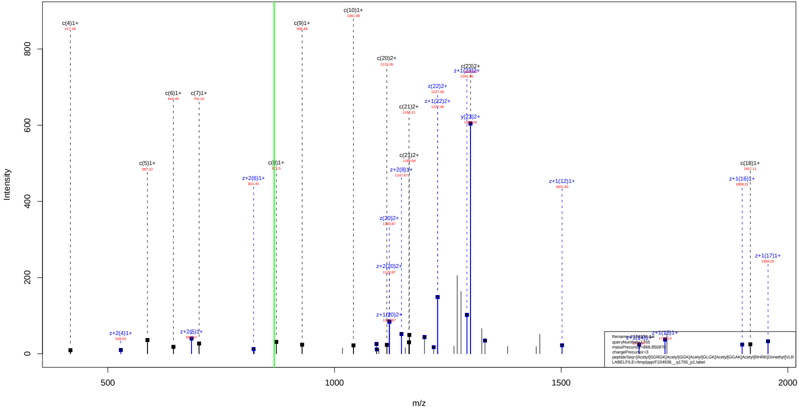

sp|P62806|H4_MOUSE

[Acetyl]SGRGK $^{Acetyl}_{42.01}$ GGK $^{Acetyl}_{42.01}$ GLGK $^{Acetyl}_{42.01}$ GGAK $^{Acetyl}_{42.01}$ RHRK $^{Dimethyl}_{28.03}$ VLR

- fragmentation table for charge state 1+
- specType=etd
- score=49.82
- F104936.dat
- query=q1755_p1
- precursor=866.850870
- chargePrecursor=3
- itol=0.8

AA	c	y	z+1	z+2	z	AA
S[1]	147.076	2598.518	2582.499	0.000	2581.491	S[23]
G[2]	204.098	2469.475	2453.456	0.000	2452.448	G[22]
R[3]	360.199	2412.453	2396.435	2397.443	2395.427	R[21]
G[4]	**417.220**	2256.352	2240.334	2241.341	2239.326	G[20]
K[5]	**587.326**	2199.331	2183.312	2184.320	2182.304	K[19]
G[6]	**644.347**	2029.225	2013.207	2014.214	2012.199	G[18]
G[7]	**701.369**	1972.204	**1956.185**	1957.193	1955.177	G[17]
K[8]	**871.474**	1915.182	**1899.164**	1900.172	1898.156	K[16]
G[9]	**928.496**	1745.077	**1729.058**	1730.066	1728.050	G[15]
L[10]	**1041.580**	1688.055	**1672.037**	1673.045	1671.029	L[14]
G[11]	1098.601	1574.971	1558.953	1559.960	1557.945	G[13]
K[12]	1268.707	1517.950	**1501.931**	1502.939	1500.923	K[12]
G[13]	1325.728	1347.844	**1331.826**	1332.833	1330.818	G[11]
G[14]	1382.750	1290.823	1274.804	1275.812	1273.796	G[10]
A[15]	1453.787	1233.801	1217.783	**1218.791**	1216.775	A[9]
K[16]	1623.893	1162.764	1146.746	**1147.753**	1145.738	K[8]
R[17]	1779.994	992.659	976.640	977.648	975.632	R[7]
H[18]	**1917.053**	836.558	820.539	**821.547**	819.531	H[6]
R[19]	2073.154	699.499	683.480	**684.488**	682.472	R[5]
K[20]	2229.280	543.398	527.379	**528.387**	526.371	K[4]
V[21]	2328.348	387.271	371.253	372.261	370.245	V[3]
L[22]	2441.432	288.203	272.184	273.192	271.176	L[2]
R[23]	2597.534	175.119	159.100	160.108	158.092	R[1]

sp|P62806|H4_MOUSE

[Acetyl]SGRGK $^{\text{Acetyl}}_{42.01}$ GGK $^{\text{Acetyl}}_{42.01}$ GLGK $^{\text{Acetyl}}_{42.01}$ GGAK $^{\text{Acetyl}}_{42.01}$ RHRK $^{\text{Dimethyl}}_{28.03}$ VLR

- fragmentation table for charge state 2+
- specType=etd
- score=49.82
- F104936.dat
- query=q1755_p1
- precursor=866.850870
- chargePrecursor=3
- itol=0.8

AA	c	y	z+1	z+2	z	AA
S[1]	74.042	**1299.762**	**1291.753**	0.504	**1291.249**	S[23]
G[2]	102.553	1235.241	**1227.232**	0.504	**1226.728**	G[22]
R[3]	180.603	1206.730	**1198.721**	1199.225	**1198.217**	R[21]
G[4]	209.114	1128.680	**1120.670**	**1121.174**	**1120.167**	G[20]
K[5]	294.167	1100.169	**1092.160**	**1092.664**	**1091.656**	K[19]
G[6]	322.677	1015.116	1007.107	1007.611	1006.603	G[18]
G[7]	351.188	986.606	978.596	979.100	978.092	G[17]
K[8]	436.241	958.095	950.085	950.589	949.582	K[16]
G[9]	464.752	873.042	865.033	865.537	864.529	G[15]
L[10]	521.294	844.531	836.522	837.026	836.018	L[14]
G[11]	549.804	787.989	779.980	780.484	779.476	G[13]
K[12]	634.857	759.479	751.469	751.973	750.965	K[12]
G[13]	663.368	674.426	666.416	666.920	665.913	G[11]
G[14]	691.879	645.915	637.906	638.410	637.402	G[10]
A[15]	727.397	617.404	609.395	609.899	608.891	A[9]
K[16]	812.450	581.886	573.876	574.380	573.373	K[8]
R[17]	890.500	496.833	488.824	489.328	488.320	R[7]
H[18]	959.030	418.782	410.773	411.277	410.269	H[6]
R[19]	1037.080	350.253	342.244	342.748	341.740	R[5]
K[20]	**1115.144**	272.202	264.193	264.697	263.689	K[4]
V[21]	**1164.678**	194.139	186.130	186.634	185.626	V[3]
L[22]	1221.220	144.605	136.596	137.100	136.092	L[2]
R[23]	**1299.270**	88.063	80.054	80.558	79.550	R[1]

sp|Q3THW5|H2AV_MOUSE

DSLIKATIAGGGVIPHIHKSLIGKKGQQK $^{\text{Dimethyl}}_{28.03}$ TA

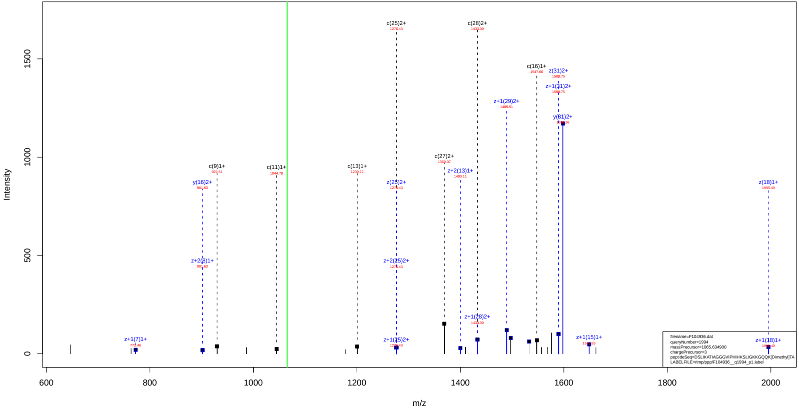

sp|Q3THW5|H2AV_MOUSE

DSLIKATIAGGGVIPHIHKSLIGKKGQQK $^{\text{Dimethyl}}_{28.03}$ TA

- fragmentation table for charge state 1+
- specType=etd
- score=25.93
- F104936.dat
- query=q1994_p1
- precursor=1065.634900
- chargePrecursor=3
- itol=0.8

AA	c	y	z+1	z+2	z	AA
D[1]	133.061	3194.874	3178.855	0.000	3177.847	D[31]
S[2]	220.093	3079.847	3063.828	0.000	3062.820	S[30]
L[3]	333.177	2992.815	2976.796	0.000	2975.788	L[29]
I[4]	446.261	2879.731	2863.712	0.000	2862.704	I[28]
K[5]	574.356	2766.646	2750.628	2751.636	2749.620	K[27]
A[6]	645.393	2638.552	2622.533	2623.541	2621.525	A[26]
T[7]	746.441	2567.514	2551.496	2552.504	2550.488	T[25]
I[8]	859.525	2466.467	2450.448	2451.456	2449.440	I[24]
A[9]	**930.562**	2353.383	2337.364	2338.372	2336.356	A[23]
G[10]	987.583	2282.346	2266.327	2267.335	2265.319	G[22]
G[11]	**1044.605**	2225.324	2209.305	2210.313	2208.298	G[21]
G[12]	1101.626	2168.303	2152.284	2153.292	2151.276	G[20]
V[13]	**1200.695**	2111.281	2095.262	2096.270	2094.255	V[19]
I[14]	1313.779	2012.213	**1996.194**	1997.202	**1995.186**	I[18]
P[15]	1410.831	1899.129	1883.110	1884.118	1882.102	P[17]
H[16]	**1547.890**	1802.076	1786.057	1787.065	1785.049	H[16]
I[17]	1660.974	1665.017	**1648.998**	1650.006	1647.990	I[15]
H[18]	1798.033	1551.933	1535.914	1536.922	1534.906	H[14]
K[19]	1926.128	1414.874	1398.855	**1399.863**	1397.847	K[13]
S[20]	2013.160	1286.779	1270.760	1271.768	1269.753	S[12]
L[21]	2126.244	1199.747	1183.728	1184.736	1182.720	L[11]
I[22]	2239.328	1086.663	1070.644	1071.652	1069.636	I[10]
G[23]	2296.350	973.579	957.560	958.568	956.552	G[9]
K[24]	2424.445	916.557	900.539	**901.547**	899.531	K[8]
K[25]	2552.540	788.462	**772.444**	773.452	771.436	K[7]
G[26]	2609.561	660.368	644.349	645.357	643.341	G[6]
Q[27]	2737.620	603.346	587.327	588.335	586.320	Q[5]
Q[28]	2865.679	475.287	459.269	460.277	458.261	Q[4]
K[29]	3021.805	347.229	331.210	332.218	330.202	K[3]
T[30]	3122.852	191.103	175.084	176.092	174.076	T[2]
A[31]	3193.890	90.055	74.036	75.044	73.028	A[1]

sp|Q3THW5|H2AV_MOUSE

DSLIKATIAGGGVIPHIHKSLIGKKGQQK $^{\text{Dimethyl}}_{28.03}$ TA

- fragmentation table for charge state 2+
- specType=etd
- score=25.93
- F104936.dat
- query=q1994_p1
- precursor=1065.634900
- chargePrecursor=3
- itol=0.8

AA	c	y	z+1	z+2	z	AA
D[1]	67.034	**1597.940**	**1589.931**	0.504	**1589.427**	D[31]
S[2]	110.550	1540.427	**1532.418**	0.504	1531.914	S[30]
L[3]	167.092	**1496.911**	**1488.902**	0.504	1488.398	L[29]
I[4]	223.634	1440.369	**1432.360**	0.504	1431.856	I[28]
K[5]	287.682	1383.827	1375.818	1376.321	1375.314	K[27]
A[6]	323.200	1319.779	1311.770	1312.274	1311.266	A[26]
T[7]	373.724	1284.261	**1276.251**	**1276.755**	**1275.748**	T[25]
I[8]	430.266	1233.737	1225.728	1226.232	1225.224	I[24]
A[9]	465.785	1177.195	1169.186	1169.690	1168.682	A[23]
G[10]	494.295	1141.676	1133.667	1134.171	1133.163	G[22]
G[11]	522.806	1113.166	1105.156	1105.660	1104.652	G[21]
G[12]	551.317	1084.655	1076.646	1077.149	1076.142	G[20]
V[13]	600.851	1056.144	1048.135	1048.639	1047.631	V[19]
I[14]	657.393	1006.610	998.601	999.105	998.097	I[18]
P[15]	705.919	950.068	942.059	942.563	941.555	P[17]
H[16]	774.449	**901.542**	893.532	894.036	893.028	H[16]
I[17]	830.991	833.012	825.003	825.507	824.499	I[15]
H[18]	899.520	776.470	768.461	768.965	767.957	H[14]
K[19]	963.568	707.941	699.931	700.435	699.427	K[13]
S[20]	1007.084	643.893	635.884	636.388	635.380	S[12]
L[21]	1063.626	600.377	592.368	592.872	591.864	L[11]
I[22]	1120.168	543.835	535.826	536.330	535.322	I[10]
G[23]	1148.679	487.293	479.284	479.788	478.780	G[9]
K[24]	1212.726	458.782	450.773	451.277	450.269	K[8]
K[25]	**1276.774**	394.735	386.726	387.229	386.222	K[7]
G[26]	1305.284	330.687	322.678	323.182	322.174	G[6]
Q[27]	**1369.314**	302.177	294.167	294.671	293.663	Q[5]
Q[28]	**1433.343**	238.147	230.138	230.642	229.634	Q[4]
K[29]	1511.406	174.118	166.109	166.613	165.605	K[3]
T[30]	1561.930	96.055	88.046	88.550	87.542	T[2]
A[31]	1597.448	45.531	37.522	38.026	37.018	A[1]

sp|Q6GSS7|H2A2A_MOUSE

DEELNKLLGK $^{\text{Dimethyl}}_{28.03}$ VTIAQGGVLPNIQAVLLPKKTESHHKAKGK

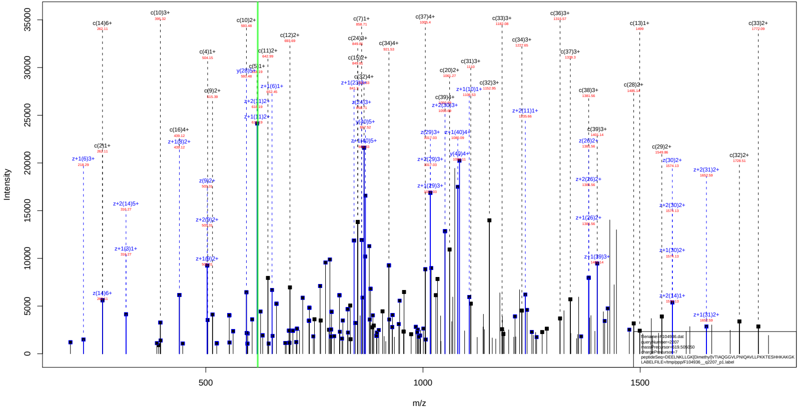

sp|Q6GSS7|H2A2A_MOUSE

DEELNKLLGK $^{\text{Dimethyl}}_{28.03}$ VTIAQGGVLPNIQAVLLPKKTESHHKAKGK

- fragmentation table for charge state 1+
- specType=etd
- score=42.48
- F104936.dat
- query=q2207_p1
- precursor=619.505050
- chargePrecursor=7
- itol=0.8

AA	c	y	z+1	z+2	z	AA
D[1]	133.061	4330.497	4314.479	0.000	4313.471	D[40]
E[2]	**262.103**	4215.470	4199.452	0.000	4198.444	E[39]
E[3]	**391.146**	4086.428	4070.409	0.000	4069.401	E[38]
L[4]	**504.230**	3957.385	3941.366	0.000	3940.359	L[37]
N[5]	**618.273**	3844.301	3828.282	3829.290	3827.275	N[36]
K[6]	746.368	3730.258	3714.239	3715.247	3713.232	K[35]
L[7]	**859.452**	3602.163	3586.144	3587.152	3585.137	L[34]
L[8]	**972.536**	3489.079	3473.060	3474.068	3472.053	L[33]
G[9]	**1029.558**	3375.995	3359.976	3360.984	3358.969	G[32]
K[10]	**1185.684**	3318.974	3302.955	3303.963	3301.947	K[31]
V[11]	**1284.752**	3162.847	3146.829	3147.836	3145.821	V[30]
T[12]	1385.800	3063.779	3047.760	3048.768	3046.752	T[29]
I[13]	**1498.884**	2962.731	2946.713	2947.720	2945.705	I[28]
A[14]	1569.921	2849.647	2833.628	2834.636	2832.621	A[27]
Q[15]	1697.980	2778.610	2762.591	2763.599	2761.584	Q[26]
G[16]	1755.001	2650.552	2634.533	2635.541	2633.525	G[25]
G[17]	1812.023	2593.530	2577.511	2578.519	2576.504	G[24]
V[18]	1911.091	2536.509	2520.490	2521.498	2519.482	V[23]
L[19]	2024.175	2437.440	2421.421	2422.429	2420.414	L[22]
P[20]	2121.228	2324.356	2308.337	2309.345	2307.330	P[21]
N[21]	2235.271	2227.303	2211.285	2212.292	2210.277	N[20]
I[22]	2348.355	2113.260	2097.242	2098.250	2096.234	I[19]
Q[23]	2476.413	2000.176	1984.158	1985.165	1983.150	Q[18]
A[24]	2547.450	1872.118	1856.099	1857.107	1855.091	A[17]
V[25]	2646.519	1801.081	1785.062	1786.070	1784.054	V[16]
L[26]	2759.603	1702.012	1685.994	1687.001	1684.986	L[15]
L[27]	2872.687	1588.928	1572.909	**1573.917**	1571.902	L[14]
P[28]	2969.740	1475.844	1459.825	1460.833	1458.818	P[13]
K[29]	3097.835	1378.791	1362.773	**1363.780**	1361.765	K[12]
K[30]	3225.930	**1250.696**	1234.678	**1235.685**	1233.670	K[11]
T[31]	3326.977	1122.601	**1106.583**	1107.591	1105.575	T[10]
E[32]	3456.020	1021.554	**1005.535**	**1006.543**	1004.527	E[9]
S[33]	3543.052	**892.511**	**876.492**	**877.500**	875.485	S[8]
H[34]	3680.111	805.479	**789.460**	790.468	**788.453**	H[7]
H[35]	3817.170	668.420	**652.401**	**653.409**	651.394	H[6]
K[36]	3945.265	531.361	**515.343**	516.350	514.335	K[5]
A[37]	4016.302	403.266	**387.248**	388.255	386.240	A[4]
K[38]	4144.397	332.229	**316.211**	317.218	315.203	K[3]
G[39]	4201.418	204.134	**188.116**	189.123	187.108	G[2]
K[40]	4329.513	147.113	131.094	132.102	130.086	K[1]

sp|Q6GSS7|H2A2A_MOUSE

DEELNKLLGK $^{Dimethyl}_{28.03}$ VTIAQGGVLPNIQAVLLPKKTESHHKAKGK

- fragmentation table for charge state 2+
- specType=etd
- score=42.48
- F104936.dat
- query=q2207_p1
- precursor=619.505050
- chargePrecursor=7
- itol=0.8

AA	c	y	z+1	z+2	z	AA
D[1]	67.034	2165.752	2157.743	0.504	2157.239	D[40]
E[2]	131.555	2108.239	2100.229	0.504	2099.726	E[39]
E[3]	196.077	2043.718	2035.708	0.504	2035.204	E[38]
L[4]	252.619	1979.196	1971.187	0.504	1970.683	L[37]
N[5]	309.640	1922.654	1914.645	1915.149	1914.141	N[36]
K[6]	373.688	1865.633	1857.623	1858.127	1857.119	K[35]
L[7]	430.230	1801.585	1793.576	1794.080	1793.072	L[34]
L[8]	486.772	1745.043	1737.034	1737.538	1736.530	L[33]
G[9]	**515.282**	1688.501	1680.492	1680.996	1679.988	G[32]
K[10]	**593.346**	1659.990	**1651.981**	**1652.485**	1651.477	K[31]
V[11]	**642.880**	1581.927	**1573.918**	**1574.422**	**1573.414**	V[30]
T[12]	**693.404**	1532.393	1524.384	1524.888	1523.880	T[29]
I[13]	**749.946**	1481.869	1473.860	**1474.364**	1473.356	I[28]
A[14]	**785.464**	**1425.327**	1417.318	**1417.822**	1416.814	A[27]
Q[15]	**849.493**	1389.809	**1381.799**	**1382.303**	**1381.295**	Q[26]
G[16]	**878.004**	1325.779	1317.770	1318.274	1317.266	G[25]
G[17]	**906.515**	1297.269	1289.259	1289.763	1288.755	G[24]
V[18]	**956.049**	1268.758	1260.749	**1261.252**	1260.245	V[23]
L[19]	1012.591	1219.224	**1211.214**	**1211.718**	1210.710	L[22]
P[20]	**1061.118**	1162.682	1154.672	1155.176	1154.168	P[21]
N[21]	1118.139	1114.155	**1106.146**	**1106.650**	1105.642	N[20]
I[22]	1174.681	1057.134	1049.124	1049.628	1048.621	I[19]
Q[23]	1238.710	**1000.592**	**992.582**	**993.086**	992.079	Q[18]
A[24]	**1274.229**	936.563	**928.553**	**929.057**	928.049	A[17]
V[25]	1323.763	901.044	**893.035**	**893.539**	**892.531**	V[16]
L[26]	1380.305	851.510	**843.500**	**844.004**	842.996	L[15]
L[27]	1436.847	**794.968**	**786.958**	**787.462**	**786.454**	L[14]
P[28]	**1485.374**	**738.426**	730.416	730.920	729.912	P[13]
K[29]	**1549.421**	689.899	681.890	**682.394**	681.386	K[12]
K[30]	1613.468	**625.852**	**617.842**	**618.346**	617.339	K[11]
T[31]	1663.992	561.804	**553.795**	**554.299**	**553.291**	T[10]
E[32]	**1728.514**	511.281	**503.271**	**503.775**	**502.767**	E[9]
S[33]	**1772.030**	**446.759**	**438.750**	**439.254**	438.246	S[8]
H[34]	1840.559	403.243	**395.234**	**395.738**	**394.730**	H[7]
H[35]	1909.089	334.714	326.704	327.208	326.200	H[6]
K[36]	1973.136	266.184	258.175	258.679	257.671	K[5]
A[37]	2008.655	202.137	194.127	194.631	193.624	A[4]
K[38]	2072.702	166.618	158.609	159.113	158.105	K[3]
G[39]	2101.213	102.571	94.561	95.065	94.057	G[2]
K[40]	2165.260	74.060	66.051	66.555	65.547	K[1]

sp|Q6GSS7|H2A2A_MOUSE

DEELNKLLGK $^{\text{Dimethyl}}_{28.03}$ VTIAQGGVLPNIQAVLLPKKTESHHKAKGK

- fragmentation table for charge state 3+
- specType=etd
- score=42.48
- F104936.dat
- query=q2207_p1
- precursor=619.505050
- chargePrecursor=7
- itol=0.8

AA	c	y	z+1	z+2	z	AA
D[1]	45.025	1444.171	1438.831	0.672	1438.495	D[40]
E[2]	88.039	1405.828	**1400.489**	0.672	1400.153	E[39]
E[3]	131.054	1362.814	1357.475	0.672	1357.139	E[38]
L[4]	168.748	1319.800	1314.460	0.672	1314.124	L[37]
N[5]	206.762	1282.105	1276.766	1277.102	1276.430	N[36]
K[6]	249.461	1244.091	1238.751	**1239.087**	1238.415	K[35]
L[7]	287.156	1201.393	1196.053	1196.389	1195.717	L[34]
L[8]	324.850	1163.698	1158.358	1158.694	1158.022	L[33]
G[9]	343.857	1126.003	1120.664	1121.000	1120.328	G[32]
K[10]	**395.899**	**1106.996**	1101.656	1101.992	1101.321	K[31]
V[11]	428.922	1054.954	1049.614	**1049.950**	1049.278	V[30]
T[12]	462.605	1021.931	**1016.592**	**1016.928**	**1016.256**	T[29]
I[13]	500.299	**988.249**	**982.909**	**983.245**	982.573	I[28]
A[14]	523.979	950.554	945.214	**945.550**	**944.878**	A[27]
Q[15]	566.665	926.875	**921.535**	**921.871**	**921.199**	Q[26]
G[16]	585.672	**884.189**	**878.849**	**879.185**	**878.513**	G[25]
G[17]	604.679	865.182	859.842	**860.178**	**859.506**	G[24]
V[18]	637.702	846.174	**840.835**	**841.171**	**840.499**	V[23]
L[19]	675.397	**813.152**	**807.812**	**808.148**	**807.476**	L[22]
P[20]	**707.747**	**775.457**	770.117	770.453	769.781	P[21]
N[21]	745.762	743.106	**737.766**	**738.102**	**737.430**	N[20]
I[22]	**783.456**	705.092	**699.752**	**700.088**	699.416	I[19]
Q[23]	**826.143**	667.397	**662.057**	**662.393**	661.721	Q[18]
A[24]	**849.822**	624.711	619.371	619.707	619.035	A[17]
V[25]	**882.844**	601.032	**595.692**	**596.028**	**595.356**	V[16]
L[26]	920.539	568.009	**562.669**	**563.005**	**562.333**	L[15]
L[27]	958.234	530.314	**524.975**	**525.311**	**524.639**	L[14]
P[28]	**990.585**	492.620	487.280	487.616	486.944	P[13]
K[29]	**1033.283**	460.269	454.929	455.265	454.593	K[12]
K[30]	1075.981	417.570	412.231	412.567	411.895	K[11]
T[31]	**1109.664**	374.872	369.532	369.868	369.196	T[10]
E[32]	**1152.678**	341.189	335.850	336.186	335.514	E[9]
S[33]	**1181.689**	298.175	292.836	293.172	292.500	S[8]
H[34]	**1227.375**	269.165	263.825	264.161	263.489	H[7]
H[35]	1273.061	223.478	**218.139**	**218.475**	**217.803**	H[6]
K[36]	**1315.760**	177.792	172.452	172.788	172.116	K[5]
A[37]	**1339.439**	135.094	129.754	130.090	129.418	A[4]
K[38]	**1382.137**	111.415	106.075	106.411	105.739	K[3]
G[39]	**1401.144**	68.716	63.377	63.713	63.041	G[2]
K[40]	1443.843	49.709	44.370	44.705	44.034	K[1]

sp|Q6GSS7|H2A2A_MOUSE

DEELNKLLGK $^{\text{Dimethyl}}_{28.03}$ VTIAQGGVLPNIQAVLLPKKTESHHKAKGK

- fragmentation table for charge state 4+
- specType=etd
- score=42.48
- F104936.dat
- query=q2207_p1
- precursor=619.505050
- chargePrecursor=7
- itol=0.8

AA	c	y	z+1	z+2	z	AA
D[1]	34.021	**1083.380**	**1079.375**	0.755	1079.123	D[40]
E[2]	66.281	1054.623	**1050.618**	0.755	**1050.366**	E[39]
E[3]	98.542	1022.362	**1018.358**	0.755	1018.106	E[38]
L[4]	126.813	**990.102**	**986.097**	0.755	985.845	L[37]
N[5]	155.324	961.831	957.826	958.078	957.574	N[36]
K[6]	187.347	933.320	**929.315**	**929.567**	**929.063**	K[35]
L[7]	215.618	901.296	897.292	**897.544**	897.040	L[34]
L[8]	243.889	873.025	869.021	869.273	868.769	L[33]
G[9]	258.145	**844.754**	**840.750**	**841.002**	**840.498**	G[32]
K[10]	297.176	830.499	**826.494**	**826.746**	**826.242**	K[31]
V[11]	321.944	791.467	**787.463**	**787.715**	**787.211**	V[30]
T[12]	347.205	766.700	**762.696**	**762.947**	**762.444**	T[29]
I[13]	375.476	741.438	**737.434**	**737.686**	**737.182**	I[28]
A[14]	393.236	713.167	**709.163**	**709.415**	**708.911**	A[27]
Q[15]	425.250	695.408	**691.403**	**691.655**	**691.151**	Q[26]
G[16]	**439.506**	**663.393**	659.389	659.641	659.137	G[25]
G[17]	453.761	649.138	**645.133**	**645.385**	**644.881**	G[24]
V[18]	478.528	634.883	**630.878**	**631.130**	**630.626**	V[23]
L[19]	506.799	610.116	**606.111**	**606.363**	605.859	L[22]
P[20]	531.062	581.844	577.840	578.092	577.588	P[21]
N[21]	559.573	557.581	**553.577**	**553.829**	**553.325**	N[20]
I[22]	587.844	529.071	**525.066**	**525.318**	**524.814**	I[19]
Q[23]	619.859	500.800	496.795	497.047	496.543	Q[18]
A[24]	637.618	468.785	464.780	465.032	464.528	A[17]
V[25]	**662.385**	451.026	**447.021**	**447.273**	**446.769**	V[16]
L[26]	690.656	426.259	422.254	422.506	422.002	L[15]
L[27]	718.927	397.988	393.983	394.235	393.731	L[14]
P[28]	743.190	369.716	365.712	365.964	365.460	P[13]
K[29]	**775.214**	345.453	341.449	341.701	341.197	K[12]
K[30]	**807.238**	313.430	309.425	309.677	309.173	K[11]
T[31]	**832.500**	281.406	277.401	277.653	277.149	T[10]
E[32]	**864.760**	256.144	252.139	252.391	251.887	E[9]
S[33]	**886.518**	223.883	219.879	220.131	219.627	S[8]
H[34]	**920.783**	202.125	198.121	198.373	197.869	H[7]
H[35]	**955.048**	167.861	163.856	164.108	163.604	H[6]
K[36]	**987.072**	133.596	129.591	129.843	129.339	K[5]
A[37]	**1004.831**	101.572	97.567	97.819	97.315	A[4]
K[38]	1036.855	83.813	79.808	80.060	79.556	K[3]
G[39]	**1051.110**	51.789	47.784	48.036	47.532	G[2]
K[40]	1083.134	37.534	33.529	33.781	33.277	K[1]

sp|Q6GSS7|H2A2A_MOUSE

DEELNKLLGK $^{Dimethyl}_{28.03}$ VTIAQGGVLPNIQAVLLPKKTESHHKAKGK

- fragmentation table for charge state 5+
- specType=etd
- score=42.48
- F104936.dat
- query=q2207_p1
- precursor=619.505050
- chargePrecursor=7
- itol=0.8

AA	c	y	z+1	z+2	z	AA
D[1]	27.418	**866.905**	**863.702**	0.806	863.500	D[40]
E[2]	53.226	**843.900**	**840.696**	0.806	840.495	E[39]
E[3]	79.035	818.091	**814.888**	0.806	**814.686**	E[38]
L[4]	101.652	792.283	**789.079**	0.806	**788.878**	L[37]
N[5]	124.460	769.666	766.462	766.664	766.261	N[36]
K[6]	150.079	**746.857**	743.654	743.855	743.452	K[35]
L[7]	172.696	721.238	718.035	718.236	717.833	L[34]
L[8]	195.313	698.622	695.418	695.619	695.216	L[33]
G[9]	206.717	676.005	672.801	673.003	672.600	G[32]
K[10]	237.943	664.601	661.397	661.598	661.195	K[31]
V[11]	257.756	633.375	**630.172**	**630.373**	629.970	V[30]
T[12]	277.966	613.562	610.358	610.559	610.156	T[29]
I[13]	300.583	**593.352**	590.148	590.350	589.947	I[28]
A[14]	314.790	570.735	567.532	567.733	567.330	A[27]
Q[15]	340.402	556.528	**553.324**	**553.526**	553.123	Q[26]
G[16]	351.806	530.916	527.712	527.914	527.511	G[25]
G[17]	363.210	519.512	516.308	516.510	**516.107**	G[24]
V[18]	383.024	508.108	**504.904**	505.105	**504.702**	V[23]
L[19]	405.641	488.294	485.090	485.292	484.889	L[22]
P[20]	425.051	465.677	462.473	462.675	462.272	P[21]
N[21]	447.860	**446.266**	443.063	443.264	442.861	N[20]
I[22]	470.477	423.458	420.254	420.456	420.053	I[19]
Q[23]	496.088	400.841	397.637	397.839	397.436	Q[18]
A[24]	510.296	375.229	372.026	372.227	371.824	A[17]
V[25]	530.110	361.022	357.818	358.020	357.617	V[16]
L[26]	552.726	341.208	338.005	338.206	337.803	L[15]
L[27]	575.343	318.591	315.388	**315.589**	315.186	L[14]
P[28]	**594.754**	295.975	292.771	292.972	292.569	P[13]
K[29]	620.373	276.564	273.360	273.562	273.159	K[12]
K[30]	645.992	250.945	247.741	247.943	247.540	K[11]
T[31]	666.201	225.326	222.122	222.324	221.921	T[10]
E[32]	**692.010**	205.117	201.913	202.114	201.711	E[9]
S[33]	**709.416**	179.308	176.104	176.306	175.903	S[8]
H[34]	**736.828**	161.902	158.698	158.899	158.496	H[7]
H[35]	**764.240**	134.490	131.286	131.488	131.085	H[6]
K[36]	**789.859**	107.078	103.874	104.076	103.673	K[5]
A[37]	804.066	81.459	78.255	78.457	78.054	A[4]
K[38]	829.685	67.252	64.048	64.249	63.846	K[3]
G[39]	**841.089**	41.633	38.429	38.630	38.227	G[2]
K[40]	**866.708**	30.228	27.025	27.226	26.823	K[1]

sp|Q6GSS7|H2A2A_MOUSE

DEELNKLLGK $^{\text{Dimethyl}}_{28.03}$ VTIAQGGVLPNIQAVLLPKKTESHHKAKGK

- fragmentation table for charge state 6+
- specType=etd
- score=42.48
- F104936.dat
- query=q2207_p1
- precursor=619.505050
- chargePrecursor=7
- itol=0.8

AA	c	y	z+1	z+2	z	AA
D[1]	23.016	**722.589**	719.919	0.839	719.751	D[40]
E[2]	44.523	703.418	**700.748**	0.839	**700.580**	E[39]
E[3]	66.030	681.911	679.241	0.839	679.073	E[38]
L[4]	84.878	660.404	657.734	0.839	657.566	L[37]
N[5]	103.885	641.556	638.886	639.054	638.718	N[36]
K[6]	125.234	622.549	619.879	620.047	619.711	K[35]
L[7]	144.081	601.200	598.530	598.698	598.362	L[34]
L[8]	162.929	582.353	579.683	579.851	579.515	L[33]
G[9]	172.432	**563.505**	560.835	561.003	560.667	G[32]
K[10]	198.453	**554.002**	551.332	551.500	551.164	K[31]
V[11]	214.965	527.981	**525.311**	**525.479**	**525.143**	V[30]
T[12]	231.806	511.469	508.799	508.967	508.631	T[29]
I[13]	250.653	494.628	491.958	492.126	491.790	I[28]
A[14]	**262.493**	475.781	473.111	473.279	472.943	A[27]
Q[15]	283.836	463.941	461.271	461.439	461.103	Q[26]
G[16]	293.340	442.598	439.928	440.096	**439.760**	G[25]
G[17]	302.843	433.094	430.425	430.593	430.257	G[24]
V[18]	319.355	423.591	420.921	421.089	420.753	V[23]
L[19]	338.202	407.079	404.410	404.578	404.242	L[22]
P[20]	354.377	388.232	385.562	385.730	385.394	P[21]
N[21]	373.385	372.057	369.387	369.555	369.219	N[20]
I[22]	392.232	353.049	350.380	350.548	350.212	I[19]
Q[23]	413.575	334.202	331.532	331.700	331.364	Q[18]
A[24]	425.414	312.859	310.189	310.357	310.021	A[17]
V[25]	441.926	301.020	298.350	298.518	298.182	V[16]
L[26]	460.773	284.508	281.838	282.006	281.670	L[15]
L[27]	479.621	265.661	262.991	263.159	**262.823**	L[14]
P[28]	495.796	246.813	244.144	244.312	243.976	P[13]
K[29]	517.145	230.638	227.968	228.136	227.800	K[12]
K[30]	538.494	209.289	206.619	206.787	206.451	K[11]
T[31]	**555.336**	**187.940**	185.270	185.438	185.102	T[10]
E[32]	576.843	171.098	168.429	168.597	168.261	E[9]
S[33]	591.348	149.591	146.921	147.089	146.753	S[8]
H[34]	614.191	135.086	132.416	132.584	132.248	H[7]
H[35]	637.034	112.243	109.573	109.741	109.405	H[6]
K[36]	658.384	89.400	86.730	86.898	86.562	K[5]
A[37]	670.223	68.050	65.381	65.549	65.213	A[4]
K[38]	**691.572**	56.211	53.541	53.709	53.373	K[3]
G[39]	**701.076**	34.862	32.192	32.360	32.024	G[2]
K[40]	**722.425**	25.358	22.688	22.856	22.520	K[1]

sp|Q6GSS7|H2A2A_MOUSE

DEELNKLLGK Dimethyl 28.03 VTIAQGGVLPNIQAVLLPKKTESHHKAKGK

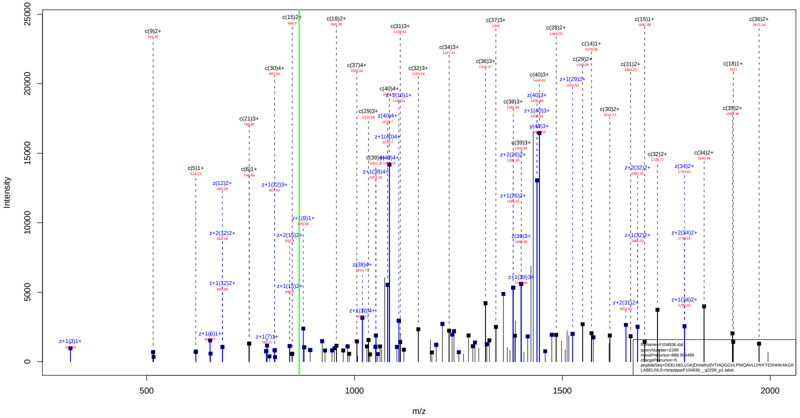

sp|Q6GSS7|H2A2A_MOUSE

DEELNKLLGK $^{Dimethyl}_{28.03}$ VTIAQGGVLPNIQAVLLPKKTESHHKAKGK

- fragmentation table for charge state 1+
- specType=etd
- score=95.75
- F104936.dat
- query=q2209_p1
- precursor=866.904480
- chargePrecursor=5
- itol=0.8

AA	c	y	z+1	z+2	z	AA
D[1]	133.061	4330.497	4314.479	0.000	4313.471	D[40]
E[2]	262.103	4215.470	4199.452	0.000	4198.444	E[39]
E[3]	391.146	4086.428	4070.409	0.000	4069.401	E[38]
L[4]	504.230	3957.385	3941.366	0.000	3940.359	L[37]
N[5]	**618.273**	3844.301	3828.282	3829.290	3827.275	N[36]
K[6]	**746.368**	3730.258	3714.239	3715.247	3713.232	K[35]
L[7]	859.452	3602.163	3586.144	3587.152	3585.137	L[34]
L[8]	**972.536**	3489.079	3473.060	3474.068	3472.053	L[33]
G[9]	**1029.558**	3375.995	3359.976	3360.984	3358.969	G[32]
K[10]	**1185.684**	3318.974	3302.955	3303.963	3301.947	K[31]
V[11]	**1284.752**	3162.847	3146.829	3147.836	3145.821	V[30]
T[12]	**1385.800**	3063.779	3047.760	3048.768	3046.752	T[29]
I[13]	1498.884	2962.731	2946.713	2947.720	2945.705	I[28]
A[14]	**1569.921**	2849.647	2833.628	2834.636	2832.621	A[27]
Q[15]	**1697.980**	2778.610	2762.591	2763.599	2761.584	Q[26]
G[16]	1755.001	2650.552	2634.533	2635.541	2633.525	G[25]
G[17]	1812.023	2593.530	2577.511	2578.519	2576.504	G[24]
V[18]	**1911.091**	2536.509	2520.490	2521.498	2519.482	V[23]
L[19]	2024.175	2437.440	2421.421	2422.429	2420.414	L[22]
P[20]	2121.228	2324.356	2308.337	2309.345	2307.330	P[21]
N[21]	2235.271	2227.303	2211.285	2212.292	2210.277	N[20]
I[22]	2348.355	2113.260	2097.242	2098.250	2096.234	I[19]
Q[23]	2476.413	2000.176	1984.158	1985.165	1983.150	Q[18]
A[24]	2547.450	1872.118	1856.099	1857.107	1855.091	A[17]
V[25]	2646.519	1801.081	1785.062	1786.070	1784.054	V[16]
L[26]	2759.603	1702.012	1685.994	1687.001	1684.986	L[15]
L[27]	2872.687	1588.928	1572.909	**1573.917**	1571.902	L[14]
P[28]	2969.740	1475.844	1459.825	1460.833	**1458.818**	P[13]
K[29]	3097.835	1378.791	1362.773	1363.780	1361.765	K[12]
K[30]	3225.930	**1250.696**	**1234.678**	1235.685	1233.670	K[11]
T[31]	3326.977	1122.601	**1106.583**	1107.591	1105.575	T[10]
E[32]	3456.020	1021.554	**1005.535**	1006.543	1004.527	E[9]
S[33]	3543.052	**892.511**	**876.492**	877.500	875.485	S[8]
H[34]	3680.111	805.479	**789.460**	790.468	788.453	H[7]
H[35]	3817.170	668.420	**652.401**	**653.409**	651.394	H[6]
K[36]	3945.265	531.361	**515.343**	**516.350**	514.335	K[5]
A[37]	4016.302	403.266	387.248	388.255	386.240	A[4]
K[38]	4144.397	332.229	**316.211**	317.218	315.203	K[3]
G[39]	4201.418	204.134	188.116	189.123	187.108	G[2]
K[40]	4329.513	147.113	131.094	132.102	130.086	K[1]

sp|Q6GSS7|H2A2A_MOUSE

DEELNKLLGK $^{\text{Dimethyl}}_{28.03}$ VTIAQGGVLPNIQAVLLPKKTESHHKAKGK

- fragmentation table for charge state 2+
- specType=etd
- score=95.75
- F104936.dat
- query=q2209_p1
- precursor=866.904480
- chargePrecursor=5
- itol=0.8

AA	c	y	z+1	z+2	z	AA
D[1]	67.034	2165.752	2157.743	0.504	2157.239	D[40]
E[2]	131.555	2108.239	2100.229	0.504	2099.726	E[39]
E[3]	196.077	2043.718	2035.708	0.504	2035.204	E[38]
L[4]	252.619	1979.196	1971.187	0.504	1970.683	L[37]
N[5]	309.640	1922.654	1914.645	1915.149	1914.141	N[36]
K[6]	373.688	1865.633	1857.623	1858.127	1857.119	K[35]
L[7]	430.230	1801.585	**1793.576**	**1794.080**	**1793.072**	L[34]
L[8]	486.772	1745.043	1737.034	1737.538	1736.530	L[33]
G[9]	**515.282**	1688.501	**1680.492**	**1680.996**	1679.988	G[32]
K[10]	593.346	1659.990	1651.981	**1652.485**	1651.477	K[31]
V[11]	642.880	1581.927	**1573.918**	**1574.422**	1573.414	V[30]
T[12]	693.404	1532.393	**1524.384**	**1524.888**	**1523.880**	T[29]
I[13]	749.946	1481.869	**1473.860**	**1474.364**	1473.356	I[28]
A[14]	785.464	1425.327	**1417.318**	1417.822	**1416.814**	A[27]
Q[15]	**849.493**	1389.809	**1381.799**	**1382.303**	**1381.295**	Q[26]
G[16]	878.004	1325.779	**1317.770**	**1318.274**	1317.266	G[25]
G[17]	906.515	1297.269	**1289.259**	**1289.763**	1288.755	G[24]
V[18]	**956.049**	1268.758	1260.749	1261.252	1260.245	V[23]
L[19]	1012.591	1219.224	**1211.214**	**1211.718**	**1210.710**	L[22]
P[20]	**1061.118**	1162.682	1154.672	1155.176	1154.168	P[21]
N[21]	**1118.139**	1114.155	**1106.146**	**1106.650**	1105.642	N[20]
I[22]	1174.681	1057.134	1049.124	**1049.628**	1048.621	I[19]
Q[23]	**1238.710**	1000.592	992.582	993.086	992.079	Q[18]
A[24]	**1274.229**	936.563	**928.553**	**929.057**	**928.049**	A[17]
V[25]	**1323.763**	901.044	**893.035**	**893.539**	**892.531**	V[16]
L[26]	1380.305	851.510	**843.500**	**844.004**	842.996	L[15]
L[27]	1436.847	**794.968**	**786.958**	**787.462**	786.454	L[14]
P[28]	**1485.374**	738.426	730.416	730.920	729.912	P[13]
K[29]	**1549.421**	689.899	**681.890**	**682.394**	**681.386**	K[12]
K[30]	**1613.468**	625.852	**617.842**	**618.346**	**617.339**	K[11]
T[31]	**1663.992**	561.804	553.795	554.299	553.291	T[10]
E[32]	**1728.514**	511.281	503.271	503.775	502.767	E[9]
S[33]	1772.030	446.759	438.750	439.254	438.246	S[8]
H[34]	**1840.559**	403.243	395.234	395.738	394.730	H[7]
H[35]	**1909.089**	334.714	326.704	327.208	326.200	H[6]
K[36]	**1973.136**	266.184	258.175	258.679	257.671	K[5]
A[37]	2008.655	202.137	194.127	194.631	193.624	A[4]
K[38]	2072.702	166.618	158.609	159.113	158.105	K[3]
G[39]	2101.213	102.571	94.561	95.065	94.057	G[2]
K[40]	2165.260	74.060	66.051	66.555	65.547	K[1]

sp|Q6GSS7|H2A2A_MOUSE

DEELNKLLGK $^{\text{Dimethyl}}_{28.03}$ VTIAQGGVLPNIQAVLLPKKTESHHKAKGK

- fragmentation table for charge state 3+
- specType=etd
- score=95.75
- F104936.dat
- query=q2209_p1
- precursor=866.904480
- chargePrecursor=5
- itol=0.8

AA	c	y	z+1	z+2	z	AA
D[1]	45.025	**1444.171**	**1438.831**	0.672	**1438.495**	D[40]
E[2]	88.039	1405.828	**1400.489**	0.672	**1400.153**	E[39]
E[3]	131.054	1362.814	**1357.475**	0.672	1357.139	E[38]
L[4]	168.748	1319.800	**1314.460**	0.672	1314.124	L[37]
N[5]	206.762	1282.105	1276.766	1277.102	1276.430	N[36]
K[6]	249.461	1244.091	**1238.751**	**1239.087**	1238.415	K[35]
L[7]	287.156	1201.393	**1196.053**	**1196.389**	**1195.717**	L[34]
L[8]	324.850	1163.698	1158.358	1158.694	1158.022	L[33]
G[9]	343.857	1126.003	1120.664	1121.000	1120.328	G[32]
K[10]	395.899	**1106.996**	**1101.656**	**1101.992**	**1101.321**	K[31]
V[11]	428.922	**1054.954**	**1049.614**	**1049.950**	1049.278	V[30]
T[12]	462.605	1021.931	1016.592	1016.928	1016.256	T[29]
I[13]	500.299	988.249	**982.909**	**983.245**	**982.573**	I[28]
A[14]	523.979	**950.554**	**945.214**	**945.550**	944.878	A[27]
Q[15]	566.665	926.875	**921.535**	**921.871**	921.199	Q[26]
G[16]	585.672	884.189	**878.849**	**879.185**	**878.513**	G[25]
G[17]	604.679	865.182	859.842	860.178	859.506	G[24]
V[18]	637.702	846.174	840.835	841.171	840.499	V[23]
L[19]	675.397	813.152	**807.812**	**808.148**	**807.476**	L[22]
P[20]	707.747	775.457	770.117	770.453	769.781	P[21]
N[21]	**745.762**	743.106	737.766	738.102	737.430	N[20]
I[22]	783.456	705.092	699.752	700.088	699.416	I[19]
Q[23]	826.143	667.397	662.057	662.393	661.721	Q[18]
A[24]	**849.822**	624.711	619.371	619.707	619.035	A[17]
V[25]	882.844	601.032	595.692	596.028	595.356	V[16]
L[26]	920.539	568.009	562.669	563.005	562.333	L[15]
L[27]	958.234	530.314	524.975	525.311	524.639	L[14]
P[28]	990.585	492.620	487.280	487.616	486.944	P[13]
K[29]	**1033.283**	460.269	454.929	455.265	454.593	K[12]
K[30]	1075.981	417.570	412.231	412.567	411.895	K[11]
T[31]	**1109.664**	374.872	369.532	369.868	369.196	T[10]
E[32]	**1152.678**	341.189	335.850	336.186	335.514	E[9]
S[33]	1181.689	298.175	292.836	293.172	292.500	S[8]
H[34]	**1227.375**	269.165	263.825	264.161	263.489	H[7]
H[35]	1273.061	223.478	218.139	218.475	217.803	H[6]
K[36]	**1315.760**	177.792	172.452	172.788	172.116	K[5]
A[37]	**1339.439**	135.094	129.754	130.090	129.418	A[4]
K[38]	**1382.137**	111.415	106.075	106.411	105.739	K[3]
G[39]	**1401.144**	68.716	63.377	63.713	63.041	G[2]
K[40]	**1443.843**	49.709	44.370	44.705	44.034	K[1]

sp|Q6GSS7|H2A2A_MOUSE

DEELNKLLGK $^{Dimethyl}_{28.03}$ VTIAQGGVLPNIQAVLLPKKTESHHKAKGK

- fragmentation table for charge state 4+
- specType=etd
- score=95.75
- F104936.dat
- query=q2209_p1
- precursor=866.904480
- chargePrecursor=5
- itol=0.8

AA	c	y	z+1	z+2	z	AA
D[1]	34.021	**1083.380**	**1079.375**	0.755	**1079.123**	D[40]
E[2]	66.281	1054.623	**1050.618**	0.755	**1050.366**	E[39]
E[3]	98.542	1022.362	**1018.358**	0.755	**1018.106**	E[38]
L[4]	126.813	990.102	986.097	0.755	985.845	L[37]
N[5]	155.324	961.831	957.826	958.078	957.574	N[36]
K[6]	187.347	933.320	**929.315**	**929.567**	**929.063**	K[35]
L[7]	215.618	901.296	897.292	897.544	897.040	L[34]
L[8]	243.889	873.025	869.021	869.273	868.769	L[33]
G[9]	258.145	844.754	840.750	841.002	840.498	G[32]
K[10]	297.176	830.499	826.494	826.746	826.242	K[31]
V[11]	321.944	791.467	**787.463**	**787.715**	**787.211**	V[30]
T[12]	347.205	766.700	762.696	762.947	762.444	T[29]
I[13]	375.476	741.438	737.434	737.686	737.182	I[28]
A[14]	393.236	713.167	709.163	709.415	708.911	A[27]
Q[15]	425.250	695.408	691.403	691.655	691.151	Q[26]
G[16]	439.506	663.393	659.389	659.641	659.137	G[25]
G[17]	453.761	649.138	645.133	645.385	644.881	G[24]
V[18]	478.528	634.883	630.878	631.130	630.626	V[23]
L[19]	506.799	610.116	606.111	606.363	605.859	L[22]
P[20]	531.062	581.844	577.840	578.092	577.588	P[21]
N[21]	559.573	557.581	553.577	553.829	553.325	N[20]
I[22]	587.844	529.071	525.066	525.318	524.814	I[19]
Q[23]	619.859	500.800	496.795	497.047	496.543	Q[18]
A[24]	637.618	468.785	464.780	465.032	464.528	A[17]
V[25]	662.385	451.026	447.021	447.273	446.769	V[16]
L[26]	690.656	426.259	422.254	422.506	422.002	L[15]
L[27]	718.927	397.988	393.983	394.235	393.731	L[14]
P[28]	743.190	369.716	365.712	365.964	365.460	P[13]
K[29]	775.214	345.453	341.449	341.701	341.197	K[12]
K[30]	**807.238**	313.430	309.425	309.677	309.173	K[11]
T[31]	832.500	281.406	277.401	277.653	277.149	T[10]
E[32]	864.760	256.144	252.139	252.391	251.887	E[9]
S[33]	886.518	223.883	219.879	220.131	219.627	S[8]
H[34]	920.783	202.125	198.121	198.373	197.869	H[7]
H[35]	955.048	167.861	163.856	164.108	163.604	H[6]
K[36]	**987.072**	133.596	129.591	129.843	129.339	K[5]
A[37]	**1004.831**	101.572	97.567	97.819	97.315	A[4]
K[38]	**1036.855**	83.813	79.808	80.060	79.556	K[3]
G[39]	**1051.110**	51.789	47.784	48.036	47.532	G[2]
K[40]	**1083.134**	37.534	33.529	33.781	33.277	K[1]

sp|Q6GSS7|H2A2A_MOUSE

DEELNKLLGK Dimethyl 28.03 VTIAQGGVLPNIQAVLLPKKTESHHKAKGK

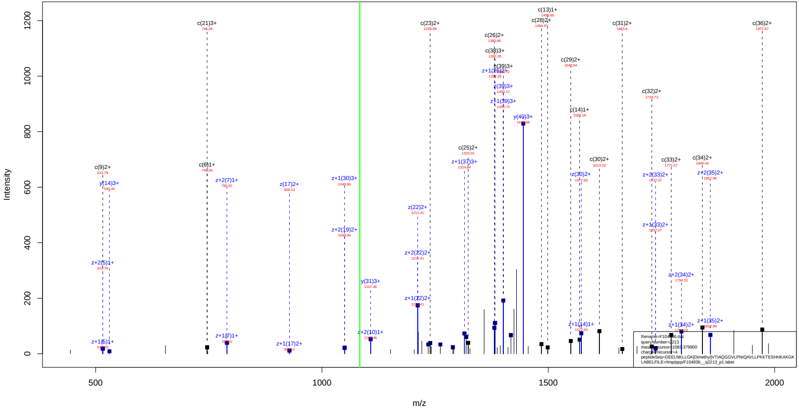

sp|Q6GSS7|H2A2A_MOUSE

DEELNKLLGK $^{\text{Dimethyl}}_{28.03}$ VTIAQGGVLPNIQAVLLPKKTESHHKAKGK

- fragmentation table for charge state 1+
- specType=etd
- score=71.51
- F104936.dat
- query=q2213_p1
- precursor=1083.379900
- chargePrecursor=4
- itol=0.8

AA	c	y	z+1	z+2	z	AA
D[1]	133.061	4330.497	4314.479	0.000	4313.471	D[40]
E[2]	262.103	4215.470	4199.452	0.000	4198.444	E[39]
E[3]	391.146	4086.428	4070.409	0.000	4069.401	E[38]
L[4]	504.230	3957.385	3941.366	0.000	3940.359	L[37]
N[5]	618.273	3844.301	3828.282	3829.290	3827.275	N[36]
K[6]	**746.368**	3730.258	3714.239	3715.247	3713.232	K[35]
L[7]	859.452	3602.163	3586.144	3587.152	3585.137	L[34]
L[8]	972.536	3489.079	3473.060	3474.068	3472.053	L[33]
G[9]	1029.558	3375.995	3359.976	3360.984	3358.969	G[32]
K[10]	1185.684	3318.974	3302.955	3303.963	3301.947	K[31]
V[11]	1284.752	3162.847	3146.829	3147.836	3145.821	V[30]
T[12]	1385.800	3063.779	3047.760	3048.768	3046.752	T[29]
I[13]	**1498.884**	2962.731	2946.713	2947.720	2945.705	I[28]
A[14]	**1569.921**	2849.647	2833.628	2834.636	2832.621	A[27]
Q[15]	1697.980	2778.610	2762.591	2763.599	2761.584	Q[26]
G[16]	1755.001	2650.552	2634.533	2635.541	2633.525	G[25]
G[17]	1812.023	2593.530	2577.511	2578.519	2576.504	G[24]
V[18]	1911.091	2536.509	2520.490	2521.498	2519.482	V[23]
L[19]	2024.175	2437.440	2421.421	2422.429	2420.414	L[22]
P[20]	2121.228	2324.356	2308.337	2309.345	2307.330	P[21]
N[21]	2235.271	2227.303	2211.285	2212.292	2210.277	N[20]
I[22]	2348.355	2113.260	2097.242	2098.250	2096.234	I[19]
Q[23]	2476.413	2000.176	1984.158	1985.165	1983.150	Q[18]
A[24]	2547.450	1872.118	1856.099	1857.107	1855.091	A[17]
V[25]	2646.519	1801.081	1785.062	1786.070	1784.054	V[16]
L[26]	2759.603	1702.012	1685.994	1687.001	1684.986	L[15]
L[27]	2872.687	1588.928	**1572.909**	1573.917	1571.902	L[14]
P[28]	2969.740	1475.844	1459.825	1460.833	1458.818	P[13]
K[29]	3097.835	1378.791	1362.773	1363.780	1361.765	K[12]
K[30]	3225.930	1250.696	**1234.678**	1235.685	1233.670	K[11]
T[31]	3326.977	1122.601	1106.583	**1107.591**	1105.575	T[10]
E[32]	3456.020	1021.554	1005.535	1006.543	1004.527	E[9]
S[33]	3543.052	892.511	876.492	877.500	875.485	S[8]
H[34]	3680.111	805.479	**789.460**	**790.468**	788.453	H[7]
H[35]	3817.170	668.420	652.401	653.409	651.394	H[6]
K[36]	3945.265	531.361	**515.343**	**516.350**	514.335	K[5]
A[37]	4016.302	403.266	387.248	388.255	386.240	A[4]
K[38]	4144.397	332.229	316.211	317.218	315.203	K[3]
G[39]	4201.418	204.134	188.116	189.123	187.108	G[2]
K[40]	4329.513	147.113	131.094	132.102	130.086	K[1]

sp|Q6GSS7|H2A2A_MOUSE

DEELNKLLGK $^{\text{Dimethyl}}_{28.03}$ VTIAQGGVLPNIQAVLLPKKTESHHKAKGK

- fragmentation table for charge state 2+
- specType=etd
- score=71.51
- F104936.dat
- query=q2213_p1
- precursor=1083.379900
- chargePrecursor=4
- itol=0.8

AA	c	y	z+1	z+2	z	AA
D[1]	67.034	2165.752	2157.743	0.504	2157.239	D[40]
E[2]	131.555	2108.239	2100.229	0.504	2099.726	E[39]
E[3]	196.077	2043.718	2035.708	0.504	2035.204	E[38]
L[4]	252.619	1979.196	1971.187	0.504	1970.683	L[37]
N[5]	309.640	1922.654	1914.645	1915.149	1914.141	N[36]
K[6]	373.688	1865.633	**1857.623**	**1858.127**	1857.119	K[35]
L[7]	430.230	1801.585	**1793.576**	**1794.080**	1793.072	L[34]
L[8]	486.772	1745.043	**1737.034**	**1737.538**	**1736.530**	L[33]
G[9]	**515.282**	1688.501	1680.492	1680.996	1679.988	G[32]
K[10]	593.346	1659.990	1651.981	1652.485	1651.477	K[31]
V[11]	642.880	1581.927	1573.918	1574.422	**1573.414**	V[30]
T[12]	693.404	1532.393	1524.384	1524.888	1523.880	T[29]
I[13]	749.946	1481.869	1473.860	1474.364	1473.356	I[28]
A[14]	785.464	1425.327	**1417.318**	**1417.822**	**1416.814**	A[27]
Q[15]	849.493	1389.809	**1381.799**	**1382.303**	**1381.295**	Q[26]
G[16]	878.004	1325.779	**1317.770**	**1318.274**	1317.266	G[25]
G[17]	906.515	1297.269	**1289.259**	**1289.763**	**1288.755**	G[24]
V[18]	956.049	1268.758	1260.749	**1261.252**	1260.245	V[23]
L[19]	1012.591	1219.224	**1211.214**	**1211.718**	**1210.710**	L[22]
P[20]	1061.118	1162.682	1154.672	1155.176	1154.168	P[21]
N[21]	1118.139	1114.155	1106.146	1106.650	1105.642	N[20]
I[22]	1174.681	1057.134	1049.124	**1049.628**	1048.621	I[19]
Q[23]	**1238.710**	1000.592	992.582	993.086	992.079	Q[18]
A[24]	1274.229	936.563	**928.553**	929.057	**928.049**	A[17]
V[25]	**1323.763**	901.044	893.035	893.539	892.531	V[16]
L[26]	**1380.305**	851.510	843.500	844.004	842.996	L[15]
L[27]	1436.847	794.968	786.958	787.462	786.454	L[14]
P[28]	**1485.374**	738.426	730.416	730.920	729.912	P[13]
K[29]	**1549.421**	689.899	681.890	682.394	681.386	K[12]
K[30]	**1613.468**	625.852	617.842	618.346	617.339	K[11]
T[31]	**1663.992**	561.804	553.795	554.299	553.291	T[10]
E[32]	**1728.514**	511.281	503.271	503.775	502.767	E[9]
S[33]	**1772.030**	446.759	438.750	439.254	438.246	S[8]
H[34]	**1840.559**	403.243	395.234	395.738	394.730	H[7]
H[35]	1909.089	334.714	326.704	327.208	326.200	H[6]
K[36]	**1973.136**	266.184	258.175	258.679	257.671	K[5]
A[37]	2008.655	202.137	194.127	194.631	193.624	A[4]
K[38]	2072.702	166.618	158.609	159.113	158.105	K[3]
G[39]	2101.213	102.571	94.561	95.065	94.057	G[2]
K[40]	2165.260	74.060	66.051	66.555	65.547	K[1]

sp|Q6GSS7|H2A2A_MOUSE

DEELNKLLGK $^{Dimethyl}_{28.03}$ VTIAQGGVLPNIQAVLLPKKTESHHKAKGK

- fragmentation table for charge state 3+
- specType=etd
- score=71.51
- F104936.dat
- query=q2213_p1
- precursor=1083.379900
- chargePrecursor=4
- itol=0.8

AA	c	y	z+1	z+2	z	AA
D[1]	45.025	**1444.171**	1438.831	0.672	1438.495	D[40]
E[2]	88.039	1405.828	**1400.489**	0.672	**1400.153**	E[39]
E[3]	131.054	1362.814	1357.475	0.672	1357.139	E[38]
L[4]	168.748	1319.800	**1314.460**	0.672	**1314.124**	L[37]
N[5]	206.762	1282.105	1276.766	1277.102	1276.430	N[36]
K[6]	249.461	1244.091	**1238.751**	**1239.087**	**1238.415**	K[35]
L[7]	287.156	1201.393	1196.053	1196.389	1195.717	L[34]
L[8]	324.850	1163.698	1158.358	1158.694	1158.022	L[33]
G[9]	343.857	1126.003	1120.664	1121.000	1120.328	G[32]
K[10]	395.899	**1106.996**	1101.656	1101.992	1101.321	K[31]
V[11]	428.922	1054.954	**1049.614**	**1049.950**	**1049.278**	V[30]
T[12]	462.605	1021.931	1016.592	1016.928	1016.256	T[29]
I[13]	500.299	988.249	982.909	983.245	982.573	I[28]
A[14]	523.979	950.554	945.214	945.550	944.878	A[27]
Q[15]	566.665	926.875	921.535	921.871	921.199	Q[26]
G[16]	585.672	884.189	878.849	879.185	878.513	G[25]
G[17]	604.679	865.182	859.842	860.178	859.506	G[24]
V[18]	637.702	846.174	840.835	841.171	840.499	V[23]
L[19]	675.397	813.152	807.812	808.148	807.476	L[22]
P[20]	707.747	775.457	770.117	770.453	769.781	P[21]
N[21]	**745.762**	743.106	737.766	738.102	737.430	N[20]
I[22]	783.456	705.092	699.752	700.088	699.416	I[19]
Q[23]	826.143	667.397	662.057	662.393	661.721	Q[18]
A[24]	849.822	624.711	619.371	619.707	619.035	A[17]
V[25]	882.844	601.032	595.692	596.028	595.356	V[16]
L[26]	920.539	568.009	562.669	563.005	562.333	L[15]
L[27]	958.234	**530.314**	524.975	525.311	524.639	L[14]
P[28]	990.585	492.620	487.280	487.616	486.944	P[13]
K[29]	1033.283	460.269	454.929	455.265	454.593	K[12]
K[30]	1075.981	417.570	412.231	412.567	411.895	K[11]
T[31]	1109.664	374.872	369.532	369.868	369.196	T[10]
E[32]	1152.678	341.189	335.850	336.186	335.514	E[9]
S[33]	1181.689	298.175	292.836	293.172	292.500	S[8]
H[34]	1227.375	269.165	263.825	264.161	263.489	H[7]
H[35]	1273.061	223.478	218.139	218.475	217.803	H[6]
K[36]	1315.760	177.792	172.452	172.788	172.116	K[5]
A[37]	1339.439	135.094	129.754	130.090	129.418	A[4]
K[38]	**1382.137**	111.415	106.075	106.411	105.739	K[3]
G[39]	**1401.144**	68.716	63.377	63.713	63.041	G[2]
K[40]	1443.843	49.709	44.370	44.705	44.034	K[1]

sp|Q6GSS7|H2A2A_MOUSE

DEELNKLLGKVTIAQGGVLPNIQAVLLPKKTESHHKAKGK (Acetyl) (42.01)

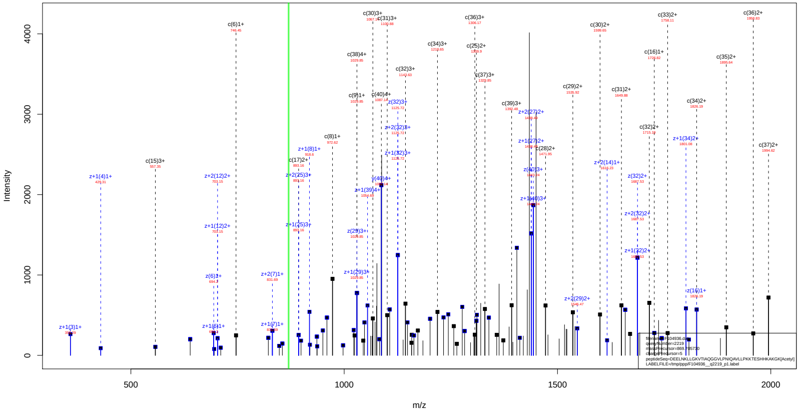

sp|Q6GSS7|H2A2A_MOUSE

DEELNKLLGKVTIAQGGVLPNIQAVLLPKKTESHHKAKGK (Acetyl) (42.01)

- fragmentation table for charge state 1+
- specType=etd
- score=63.33
- F104936.dat
- query=q2219_p1
- precursor=869.705730
- chargePrecursor=5
- itol=0.8

AA	c	y	z+1	z+2	z	AA
D[1]	133.061	4344.477	4328.458	0.000	4327.450	D[40]
E[2]	262.103	4229.450	4213.431	0.000	4212.423	E[39]
E[3]	391.146	4100.407	4084.388	0.000	4083.380	E[38]
L[4]	504.230	3971.364	3955.346	0.000	3954.338	L[37]
N[5]	618.273	3858.280	3842.262	3843.269	3841.254	N[36]
K[6]	**746.368**	3744.237	3728.219	3729.227	3727.211	K[35]
L[7]	859.452	3616.142	3600.124	3601.132	3599.116	L[34]
L[8]	**972.536**	3503.058	3487.040	3488.048	3486.032	L[33]
G[9]	**1029.558**	3389.974	3373.956	3374.963	3372.948	G[32]
K[10]	**1157.652**	3332.953	3316.934	3317.942	3315.926	K[31]
V[11]	**1256.721**	3204.858	3188.839	3189.847	3187.831	V[30]
T[12]	**1357.769**	3105.790	3089.771	3090.779	3088.763	T[29]
I[13]	1470.853	3004.742	2988.723	2989.731	2987.715	I[28]
A[14]	1541.890	2891.658	2875.639	2876.647	2874.631	A[27]
Q[15]	**1669.948**	2820.621	2804.602	2805.610	2803.594	Q[26]
G[16]	**1726.970**	2692.562	2676.543	2677.551	2675.536	G[25]
G[17]	1783.991	2635.541	2619.522	2620.530	2618.514	G[24]
V[18]	1883.060	2578.519	2562.500	2563.508	2561.493	V[23]
L[19]	1996.144	2479.451	2463.432	2464.440	2462.424	L[22]
P[20]	2093.196	2366.367	2350.348	2351.356	2349.340	P[21]
N[21]	2207.239	2269.314	2253.295	2254.303	2252.287	N[20]
I[22]	2320.323	2155.271	2139.252	2140.260	2138.244	I[19]
Q[23]	2448.382	2042.187	2026.168	2027.176	2025.160	Q[18]
A[24]	2519.419	1914.128	1898.110	1899.117	1897.102	A[17]
V[25]	2618.488	1843.091	1827.073	1828.080	**1826.065**	V[16]
L[26]	2731.572	**1744.023**	1728.004	1729.012	**1726.996**	L[15]
L[27]	2844.656	1630.939	1614.920	**1615.928**	1613.912	L[14]
P[28]	2941.708	1517.855	1501.836	1502.844	1500.828	P[13]
K[29]	3069.803	1420.802	**1404.783**	1405.791	1403.775	K[12]
K[30]	3197.898	1292.707	**1276.688**	1277.696	1275.680	K[11]
T[31]	3298.946	1164.612	**1148.593**	1149.601	1147.585	T[10]
E[32]	3427.989	1063.564	**1047.546**	1048.553	1046.538	E[9]
S[33]	3515.021	934.522	**918.503**	**919.511**	917.495	S[8]
H[34]	3652.080	**847.490**	**831.471**	**832.479**	830.463	H[7]
H[35]	3789.139	**710.431**	**694.412**	**695.420**	**693.404**	H[6]
K[36]	3917.233	573.372	**557.353**	558.361	556.345	K[5]
A[37]	3988.271	445.277	**429.258**	430.266	428.250	A[4]
K[38]	4116.366	374.240	**358.221**	359.229	357.213	K[3]
G[39]	4173.387	246.145	230.126	231.134	229.118	G[2]
K[40]	4343.493	189.123	173.105	174.112	172.097	K[1]

sp|Q6GSS7|H2A2A_MOUSE

DEELNKLLGKVTIAQGGVLPNIQAVLLPKKTESHHKAKGK (Acetyl) (42.01)

- fragmentation table for charge state 2+
- specType=etd
- score=63.33
- F104936.dat
- query=q2219_p1
- precursor=869.705730
- chargePrecursor=5
- itol=0.8

AA	c	y	z+1	z+2	z	AA
D[1]	67.034	2172.742	2164.733	0.504	2164.229	D[40]
E[2]	131.555	2115.228	2107.219	0.504	2106.715	E[39]
E[3]	196.077	2050.707	2042.698	0.504	2042.194	E[38]
L[4]	252.619	1986.186	1978.176	0.504	1977.673	L[37]
N[5]	309.640	1929.644	1921.634	1922.138	1921.131	N[36]
K[6]	373.688	1872.622	1864.613	1865.117	1864.109	K[35]
L[7]	430.230	**1808.575**	**1800.566**	**1801.069**	1800.062	L[34]
L[8]	486.772	1752.033	**1744.023**	**1744.527**	**1743.520**	L[33]
G[9]	515.282	1695.491	**1687.481**	**1687.985**	**1686.978**	G[32]
K[10]	579.330	1666.980	**1658.971**	**1659.475**	**1658.467**	K[31]
V[11]	628.864	1602.933	1594.923	1595.427	1594.419	V[30]
T[12]	679.388	1553.398	1545.389	**1545.893**	1544.885	T[29]
I[13]	735.930	1502.875	1494.865	1495.369	1494.361	I[28]
A[14]	771.449	1446.333	**1438.323**	**1438.827**	**1437.819**	A[27]
Q[15]	835.478	**1410.814**	1402.805	1403.309	1402.301	Q[26]
G[16]	863.989	1346.785	**1338.775**	**1339.279**	1338.271	G[25]
G[17]	**892.499**	1318.274	**1310.265**	**1310.768**	**1309.761**	G[24]
V[18]	942.033	1289.763	**1281.754**	**1282.258**	1281.250	V[23]
L[19]	998.575	1240.229	**1232.220**	**1232.724**	1231.716	L[22]
P[20]	**1047.102**	1183.687	1175.678	1176.182	1175.174	P[21]
N[21]	1104.123	1135.161	1127.151	1127.655	1126.647	N[20]
I[22]	1160.665	1078.139	1070.130	1070.634	1069.626	I[19]
Q[23]	1224.695	**1021.597**	1013.588	1014.092	1013.084	Q[18]
A[24]	1260.213	957.568	**949.558**	**950.062**	949.055	A[17]
V[25]	**1309.747**	922.049	914.040	914.544	913.536	V[16]
L[26]	1366.289	872.515	864.506	865.010	864.002	L[15]
L[27]	1422.831	815.973	807.964	808.468	807.460	L[14]
P[28]	**1471.358**	759.431	751.422	751.926	750.918	P[13]
K[29]	**1535.405**	**710.905**	**702.895**	**703.399**	**702.391**	K[12]
K[30]	**1599.453**	646.857	**638.848**	**639.352**	**638.344**	K[11]
T[31]	**1649.977**	582.810	574.800	575.304	574.296	T[10]
E[32]	**1714.498**	532.286	524.276	524.780	523.773	E[9]
S[33]	**1758.014**	467.765	459.755	460.259	459.251	S[8]
H[34]	**1826.543**	424.248	416.239	416.743	415.735	H[7]
H[35]	**1895.073**	355.719	347.710	348.214	347.206	H[6]
K[36]	**1959.120**	287.190	279.180	279.684	278.676	K[5]
A[37]	**1994.639**	223.142	215.133	215.637	214.629	A[4]
K[38]	2058.686	187.624	179.614	180.118	179.110	K[3]
G[39]	2087.197	123.576	115.567	116.071	115.063	G[2]
K[40]	2172.250	95.065	87.056	87.560	86.552	K[1]

sp|Q6GSS7|H2A2A_MOUSE

DEELNKLLGKVTIAQGGVLPNIQAVLLPKKTESHHKAKGK (Acetyl) (42.01)

- fragmentation table for charge state 3+
- specType=etd
- score=63.33
- F104936.dat
- query=q2219_p1
- precursor=869.705730
- chargePrecursor=5
- itol=0.8

AA	c	y	z+1	z+2	z	AA
D[1]	45.025	1448.830	**1443.491**	0.672	**1443.155**	D[40]
E[2]	88.039	1410.488	**1405.148**	0.672	**1404.813**	E[39]
E[3]	131.054	1367.474	1362.134	0.672	1361.798	E[38]
L[4]	168.748	1324.460	1319.120	0.672	1318.784	L[37]
N[5]	206.762	1286.765	1281.425	**1281.761**	1281.089	N[36]
K[6]	249.461	1248.751	**1243.411**	**1243.747**	1243.075	K[35]
L[7]	287.156	1206.052	**1200.713**	**1201.049**	1200.377	L[34]
L[8]	324.850	1168.358	**1163.018**	**1163.354**	**1162.682**	L[33]
G[9]	343.857	1130.663	**1125.323**	**1125.659**	**1124.987**	G[32]
K[10]	386.556	1111.656	**1106.316**	**1106.652**	**1105.980**	K[31]
V[11]	419.578	1068.957	1063.618	1063.954	1063.282	V[30]
T[12]	453.261	1035.935	**1030.595**	1030.931	**1030.259**	T[29]
I[13]	490.956	1002.252	**996.913**	**997.248**	996.577	I[28]
A[14]	514.635	964.557	**959.218**	**959.554**	**958.882**	A[27]
Q[15]	**557.321**	940.878	**935.539**	**935.875**	**935.203**	Q[26]
G[16]	576.328	**898.192**	**892.853**	**893.189**	**892.517**	G[25]
G[17]	595.335	879.185	873.845	874.181	873.510	G[24]
V[18]	628.358	860.178	**854.838**	**855.174**	**854.502**	V[23]
L[19]	666.053	827.155	**821.816**	**822.151**	**821.480**	L[22]
P[20]	698.404	789.460	784.121	784.457	783.785	P[21]
N[21]	736.418	757.109	751.770	752.106	751.434	N[20]
I[22]	774.113	719.095	713.756	714.092	713.420	I[19]
Q[23]	816.799	681.400	676.061	676.397	675.725	Q[18]
A[24]	840.478	**638.714**	633.375	633.711	633.039	A[17]
V[25]	873.501	615.035	609.696	610.032	609.360	V[16]
L[26]	911.195	582.012	576.673	577.009	576.337	L[15]
L[27]	948.890	544.318	538.978	539.314	538.642	L[14]
P[28]	981.241	506.623	501.284	501.619	500.948	P[13]
K[29]	**1023.939**	474.272	468.933	469.269	468.597	K[12]
K[30]	**1066.638**	431.574	426.234	426.570	425.898	K[11]
T[31]	**1100.320**	388.876	383.536	383.872	383.200	T[10]
E[32]	**1143.334**	355.193	349.853	350.189	349.517	E[9]
S[33]	**1172.345**	312.179	306.839	307.175	306.503	S[8]
H[34]	**1218.031**	283.168	277.829	278.164	277.493	H[7]
H[35]	**1263.718**	237.482	232.142	232.478	231.806	H[6]
K[36]	**1306.416**	191.795	186.456	186.792	186.120	K[5]
A[37]	**1330.095**	149.097	143.758	144.094	143.422	A[4]
K[38]	**1372.793**	125.418	120.079	120.414	119.743	K[3]
G[39]	**1391.801**	82.720	77.380	77.716	77.044	G[2]
K[40]	1448.502	63.713	58.373	58.709	58.037	K[1]

sp|Q6GSS7|H2A2A_MOUSE

DEELNKLLGKVTIAQGGVLPNIQAVLLPKKTESHHKAKGK $^{(Acetyl)}_{(42.01)}$

- fragmentation table for charge state 4+
- specType=etd
- score=63.33
- F104936.dat
- query=q2219_p1
- precursor=869.705730
- chargePrecursor=5
- itol=0.8

AA	c	y	z+1	z+2	z	AA
D[1]	34.021	**1086.875**	**1082.870**	0.755	**1082.618**	D[40]
E[2]	66.281	1058.118	**1054.113**	0.755	1053.861	E[39]
E[3]	98.542	1025.857	**1021.853**	0.755	**1021.601**	E[38]
L[4]	126.813	993.597	989.592	0.755	989.340	L[37]
N[5]	155.324	965.326	961.321	961.573	961.069	N[36]
K[6]	187.347	**936.815**	932.810	933.062	932.558	K[35]
L[7]	215.618	904.791	900.786	901.038	900.534	L[34]
L[8]	243.889	876.520	872.515	872.767	872.263	L[33]
G[9]	258.145	**848.249**	844.244	844.496	843.992	G[32]
K[10]	290.169	833.994	829.989	830.241	829.737	K[31]
V[11]	314.936	801.970	797.965	798.217	797.713	V[30]
T[12]	340.198	777.203	773.198	773.450	772.946	T[29]
I[13]	368.469	751.941	747.936	748.188	747.684	I[28]
A[14]	386.228	723.670	719.665	719.917	719.413	A[27]
Q[15]	418.243	705.911	701.906	702.158	701.654	Q[26]
G[16]	432.498	673.896	669.891	670.143	669.639	G[25]
G[17]	446.753	659.641	655.636	655.888	655.384	G[24]
V[18]	471.520	645.385	641.381	641.633	641.129	V[23]
L[19]	499.791	620.618	616.613	616.865	616.362	L[22]
P[20]	524.055	592.347	588.342	588.594	588.090	P[21]
N[21]	552.565	568.084	564.079	564.331	563.827	N[20]
I[22]	580.836	539.573	535.569	535.820	535.317	I[19]
Q[23]	612.851	511.302	507.298	507.549	507.046	Q[18]
A[24]	630.610	479.288	475.283	475.535	475.031	A[17]
V[25]	655.377	461.528	457.524	457.776	457.272	V[16]
L[26]	683.648	436.761	432.756	433.008	432.505	L[15]
L[27]	711.919	408.490	404.485	404.737	404.234	L[14]
P[28]	736.183	380.219	376.214	376.466	375.962	P[13]
K[29]	768.206	355.956	351.951	352.203	351.699	K[12]
K[30]	800.230	323.932	319.928	320.179	319.676	K[11]
T[31]	825.492	291.908	287.904	288.156	287.652	T[10]
E[32]	857.753	266.647	262.642	262.894	262.390	E[9]
S[33]	879.511	234.386	230.381	230.633	230.129	S[8]
H[34]	913.775	212.628	208.623	208.875	208.371	H[7]
H[35]	948.040	178.363	174.358	174.610	174.107	H[6]
K[36]	980.064	144.098	140.094	140.346	139.842	K[5]
A[37]	**997.823**	112.075	108.070	108.322	107.818	A[4]
K[38]	**1029.847**	94.315	90.311	90.563	90.059	K[3]
G[39]	**1044.102**	62.292	58.287	58.539	58.035	G[2]
K[40]	**1086.629**	48.036	44.032	44.284	43.780	K[1]

sp|P43276|H15_MOUSE

TAAPAPVEKSPAKK $^{Methyl}_{14.02}$ KTTKKAGAAKRKATGPPVSE

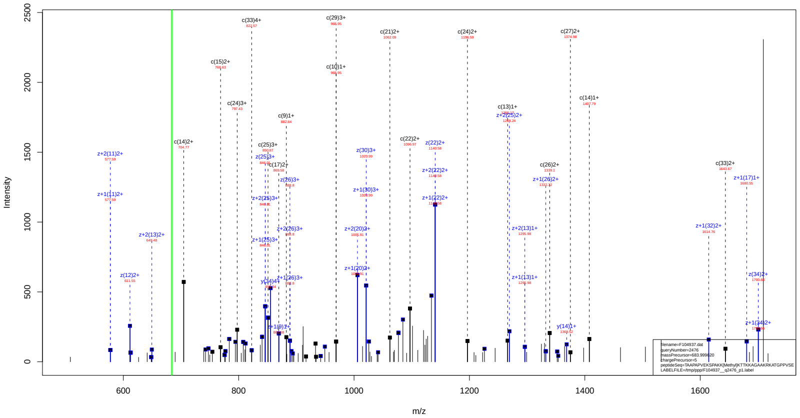

sp|P43276|H15_MOUSE

TAAPAPVEKSPAKK $^{Methyl}_{14.02}$ KTTKKAGAAKRKATGPPVSE

- fragmentation table for charge state 1+
- specType=etd
- score=40.52
- F104937.dat
- query=q2476_p1
- precursor=683.999620
- chargePrecursor=5
- itol=0.8

AA	c	y	z+1	z+2	z	AA
T[1]	119.082	3415.975	3399.956	0.000	3398.948	T[34]
A[2]	190.119	3314.927	3298.908	0.000	3297.901	A[33]
A[3]	261.156	3243.890	3227.871	0.000	3226.863	A[32]
P[4]	358.208	3172.853	3156.834	0.000	3155.826	P[31]
A[5]	429.246	3075.800	3059.781	0.000	3058.774	A[30]
P[6]	526.298	3004.763	2988.744	0.000	2987.736	P[29]
V[7]	625.367	2907.710	2891.691	0.000	2890.684	V[28]
E[8]	**754.409**	2808.642	2792.623	0.000	2791.615	E[27]
K[9]	**882.504**	2679.599	2663.580	2664.588	2662.573	K[26]
S[10]	**969.536**	2551.504	2535.486	2536.493	2534.478	S[25]
P[11]	1066.589	2464.472	2448.453	2449.461	2447.446	P[24]
A[12]	1137.626	2367.419	2351.401	2352.409	2350.393	A[23]
K[13]	**1265.721**	2296.382	2280.364	2281.371	2279.356	K[22]
K[14]	**1407.832**	2168.287	2152.269	2153.276	2151.261	K[21]
K[15]	1535.927	2026.177	2010.158	2011.166	2009.150	K[20]
T[16]	1636.974	1898.082	1882.063	1883.071	1881.055	T[19]
T[17]	1738.022	1797.034	1781.015	1782.023	1780.008	T[18]
K[18]	1866.117	1695.986	**1679.968**	**1680.976**	1678.960	K[17]
K[19]	1994.212	1567.891	1551.873	1552.881	1550.865	K[16]
A[20]	2065.249	1439.797	1423.778	1424.786	1422.770	A[15]
G[21]	2122.271	**1368.759**	**1352.741**	**1353.748**	**1351.733**	G[14]
A[22]	2193.308	1311.738	**1295.719**	**1296.727**	1294.711	A[13]
A[23]	2264.345	1240.701	1224.682	**1225.690**	1223.674	A[12]
K[24]	2392.440	1169.664	1153.645	1154.653	1152.637	K[11]
R[25]	2548.541	**1041.569**	**1025.550**	1026.558	1024.542	R[10]
K[26]	2676.636	885.468	**869.449**	870.457	868.441	K[9]
A[27]	2747.673	757.373	741.354	**742.362**	740.346	A[8]
T[28]	2848.721	686.336	670.317	671.325	669.309	T[7]
G[29]	2905.742	585.288	569.269	570.277	568.261	G[6]
P[30]	3002.795	528.266	512.248	513.256	511.240	P[5]
P[31]	3099.848	431.214	415.195	416.203	414.187	P[4]
V[32]	3198.916	334.161	318.142	319.150	317.134	V[3]
S[33]	3285.948	235.092	219.074	220.082	218.066	S[2]
E[34]	3414.991	148.060	132.042	133.050	131.034	E[1]

sp|P43276|H15_MOUSE

TAAPAPVEKSPAKK $^{Methyl}_{14.02}$ KTTKKAGAAKRKATGPPVSE

- fragmentation table for charge state 2+
- specType=etd
- score=40.52
- F104937.dat
- query=q2476_p1
- precursor=683.999620
- chargePrecursor=5
- itol=0.8

AA	c	y	z+1	z+2	z	AA
T[1]	60.044	1708.491	**1700.482**	0.504	**1699.978**	T[34]
A[2]	95.563	1657.967	1649.958	0.504	1649.454	A[33]
A[3]	131.082	1622.449	**1614.439**	0.504	1613.935	A[32]
P[4]	179.608	1586.930	1578.921	0.504	1578.417	P[31]
A[5]	215.126	1538.404	1530.394	0.504	1529.890	A[30]
P[6]	263.653	1502.885	1494.876	0.504	1494.372	P[29]
V[7]	313.187	1454.359	1446.349	0.504	1445.845	V[28]
E[8]	377.708	1404.825	1396.815	0.504	1396.311	E[27]
K[9]	441.756	1340.303	**1332.294**	**1332.798**	**1331.790**	K[26]
S[10]	485.272	1276.256	1268.246	**1268.750**	1267.742	S[25]
P[11]	533.798	1232.740	1224.730	1225.234	1224.226	P[24]
A[12]	569.317	1184.213	1176.204	1176.708	1175.700	A[23]
K[13]	633.364	1148.695	**1140.685**	**1141.189**	**1140.182**	K[22]
K[14]	**704.420**	**1084.647**	**1076.638**	**1077.142**	**1076.134**	K[21]
K[15]	**768.467**	1013.592	**1005.583**	**1006.087**	1005.079	K[20]
T[16]	818.991	**949.545**	**941.535**	**942.039**	941.031	T[19]
T[17]	**869.515**	899.021	**891.011**	**891.515**	890.507	T[18]
K[18]	**933.562**	848.497	**840.487**	**840.991**	**839.984**	K[17]
K[19]	997.610	**784.449**	**776.440**	**776.944**	**775.936**	K[16]
A[20]	1033.128	720.402	712.393	712.896	711.889	A[15]
G[21]	**1061.639**	684.883	676.874	677.378	676.370	G[14]
A[22]	**1097.158**	656.373	**648.363**	**648.867**	**647.859**	A[13]
A[23]	1132.676	620.854	**612.845**	**613.349**	**612.341**	A[12]
K[24]	**1196.724**	585.335	**577.326**	**577.830**	**576.822**	K[11]
R[25]	1274.774	521.288	513.279	513.783	512.775	R[10]
K[26]	**1338.822**	443.237	435.228	435.732	434.724	K[9]
A[27]	**1374.340**	379.190	371.181	371.685	370.677	A[8]
T[28]	1424.864	343.671	335.662	336.166	335.158	T[7]
G[29]	1453.375	293.148	285.138	285.642	284.634	G[6]
P[30]	1501.901	264.637	256.627	257.131	256.124	P[5]
P[31]	1550.427	216.110	208.101	208.605	207.597	P[4]
V[32]	1599.962	167.584	159.575	160.079	159.071	V[3]
S[33]	**1643.478**	118.050	110.041	110.544	109.537	S[2]
E[34]	1707.999	74.534	66.524	67.028	66.021	E[1]

sp|P43276|H15_MOUSE

TAAPAPVEKSPAKK $^{\text{Methyl}}_{14.02}$ KTTKKAGAAKRKATGPPVSE

- fragmentation table for charge state 3+
- specType=etd
- score=40.52
- F104937.dat
- query=q2476_p1
- precursor=683.999620
- chargePrecursor=5
- itol=0.8

AA	c	y	z+1	z+2	z	AA
T[1]	40.365	1139.330	**1133.990**	0.672	**1133.654**	T[34]
A[2]	64.044	1105.647	1100.308	0.672	1099.972	A[33]
A[3]	87.723	1081.968	**1076.629**	0.672	**1076.293**	A[32]
P[4]	120.074	1058.289	1052.950	0.672	1052.614	P[31]
A[5]	143.753	**1025.938**	**1020.599**	0.672	**1020.263**	A[30]
P[6]	176.104	1002.259	996.920	0.672	996.584	P[29]
V[7]	209.127	969.908	964.569	0.672	964.233	V[28]
E[8]	252.141	936.885	931.546	0.672	931.210	E[27]
K[9]	294.840	**893.871**	**888.532**	**888.868**	**888.196**	K[26]
S[10]	323.850	**851.173**	**845.833**	**846.169**	**845.497**	S[25]
P[11]	356.201	**822.162**	816.823	817.159	816.487	P[24]
A[12]	379.880	789.811	784.472	784.808	**784.136**	A[23]
K[13]	422.579	766.132	760.793	761.129	760.457	K[22]
K[14]	469.949	723.434	718.094	718.430	717.758	K[21]
K[15]	512.647	676.064	670.724	671.060	670.388	K[20]
T[16]	546.330	633.365	628.026	628.362	627.690	T[19]
T[17]	580.012	599.683	594.343	594.679	594.007	T[18]
K[18]	622.711	566.000	560.661	560.997	560.325	K[17]
K[19]	665.409	523.302	517.962	518.298	517.626	K[16]
A[20]	689.088	480.604	475.264	475.600	474.928	A[15]
G[21]	708.095	456.925	451.585	451.921	451.249	G[14]
A[22]	731.774	437.917	432.578	432.914	432.242	A[13]
A[23]	755.453	414.238	408.899	409.235	408.563	A[12]
K[24]	**798.151**	390.559	385.220	385.556	384.884	K[11]
R[25]	**850.185**	347.861	342.522	342.857	342.186	R[10]
K[26]	892.883	295.827	290.488	290.824	290.152	K[9]
A[27]	**916.563**	253.129	247.789	248.125	247.454	A[8]
T[28]	950.245	229.450	224.110	224.446	223.775	T[7]
G[29]	**969.252**	195.767	190.428	190.764	190.092	G[6]
P[30]	1001.603	176.760	171.421	171.757	171.085	P[5]
P[31]	1033.954	144.409	139.070	139.406	138.734	P[4]
V[32]	1066.977	112.058	106.719	107.055	106.383	V[3]
S[33]	1095.988	79.036	73.696	74.032	73.360	S[2]
E[34]	1139.002	50.025	44.685	45.021	44.349	E[1]

sp|P43276|H15_MOUSE

TAAPAPVEKSPAKK $^{Methyl}_{14.02}$ KTTKKAGAAKRKATGPPVSE

- fragmentation table for charge state 4+
- specType=etd
- score=40.52
- F104937.dat
- query=q2476_p1
- precursor=683.999620
- chargePrecursor=5
- itol=0.8

AA	c	y	z+1	z+2	z	AA
T[1]	30.526	**854.749**	**850.744**	0.755	**850.493**	T[34]
A[2]	48.285	829.487	825.483	0.755	825.231	A[33]
A[3]	66.044	**811.728**	**807.723**	0.755	**807.471**	A[32]
P[4]	90.308	**793.969**	789.964	0.755	789.712	P[31]
A[5]	108.067	769.705	765.701	0.755	765.449	A[30]
P[6]	132.330	751.946	**747.942**	0.755	**747.690**	P[29]
V[7]	157.097	727.683	723.678	0.755	723.426	V[28]
E[8]	189.358	702.916	698.911	0.755	698.659	E[27]
K[9]	221.382	670.655	666.651	666.903	666.399	K[26]
S[10]	243.140	638.632	634.627	634.879	634.375	S[25]
P[11]	267.403	616.874	**612.869**	**613.121**	**612.617**	P[24]
A[12]	285.162	592.610	588.606	588.858	588.354	A[23]
K[13]	317.186	574.851	570.846	571.098	570.594	K[22]
K[14]	352.713	542.827	538.823	539.075	538.571	K[21]
K[15]	384.737	507.300	503.295	503.547	503.043	K[20]
T[16]	409.999	475.276	471.271	471.523	471.019	T[19]
T[17]	435.261	450.014	446.009	446.261	445.757	T[18]
K[18]	467.285	424.752	420.747	420.999	420.495	K[17]
K[19]	499.308	392.728	388.724	388.976	388.472	K[16]
A[20]	517.068	360.705	356.700	356.952	356.448	A[15]
G[21]	531.323	342.945	338.941	339.193	338.689	G[14]
A[22]	549.082	328.690	324.685	324.937	324.433	A[13]
A[23]	566.842	310.931	306.926	307.178	306.674	A[12]
K[24]	598.865	293.171	289.167	289.419	288.915	K[11]
R[25]	637.891	261.148	257.143	257.395	256.891	R[10]
K[26]	669.914	222.122	218.118	218.370	217.866	K[9]
A[27]	687.674	190.099	186.094	186.346	185.842	A[8]
T[28]	712.936	172.339	168.335	168.587	168.083	T[7]
G[29]	727.191	147.077	143.073	143.325	142.821	G[6]
P[30]	751.454	132.822	128.817	129.069	128.565	P[5]
P[31]	**775.717**	108.559	104.554	104.806	104.302	P[4]
V[32]	800.484	84.296	80.291	80.543	80.039	V[3]
S[33]	**822.242**	59.529	55.524	55.776	55.272	S[2]
E[34]	854.503	37.771	33.766	34.018	33.514	E[1]

sp|P43276|H15_MOUSE

TAAPAPVEKSPAKK $^{\text{Methyl}}_{14.02}$ KTTKKAGAAKRKATGPPVSE

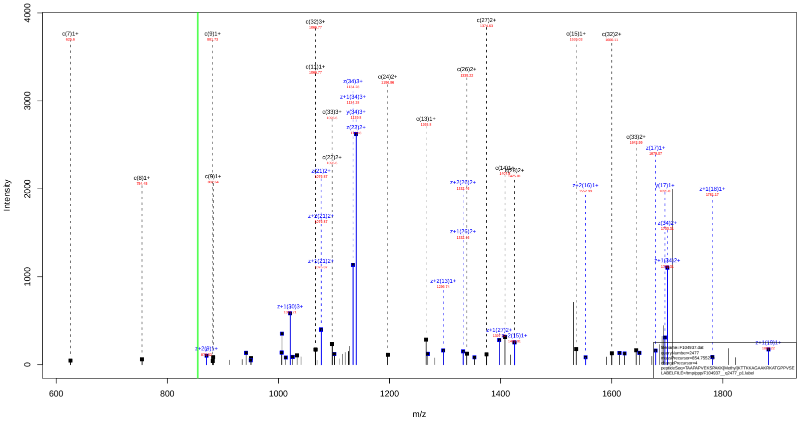

sp|P43276|H15_MOUSE

TAAPAPVEKSPAKK $^{Methyl}_{14.02}$ KTTKKAGAAKRKATGPPVSE

- fragmentation table for charge state 1+
- specType=etd
- score=48.86
- F104937.dat
- query=q2477_p1
- precursor=854.755270
- chargePrecursor=4
- itol=0.8

AA	c	y	z+1	z+2	z	AA
T[1]	119.082	3415.975	3399.956	0.000	3398.948	T[34]
A[2]	190.119	3314.927	3298.908	0.000	3297.901	A[33]
A[3]	261.156	3243.890	3227.871	0.000	3226.863	A[32]
P[4]	358.208	3172.853	3156.834	0.000	3155.826	P[31]
A[5]	429.246	3075.800	3059.781	0.000	3058.774	A[30]
P[6]	526.298	3004.763	2988.744	0.000	2987.736	P[29]
V[7]	**625.367**	2907.710	2891.691	0.000	2890.684	V[28]
E[8]	**754.409**	2808.642	2792.623	0.000	2791.615	E[27]
K[9]	**882.504**	2679.599	2663.580	2664.588	2662.573	K[26]
S[10]	969.536	2551.504	2535.486	2536.493	2534.478	S[25]
P[11]	**1066.589**	2464.472	2448.453	2449.461	2447.446	P[24]
A[12]	1137.626	2367.419	2351.401	2352.409	2350.393	A[23]
K[13]	**1265.721**	2296.382	2280.364	2281.371	2279.356	K[22]
K[14]	**1407.832**	2168.287	2152.269	2153.276	2151.261	K[21]
K[15]	**1535.927**	2026.177	2010.158	2011.166	2009.150	K[20]
T[16]	1636.974	1898.082	**1882.063**	1883.071	1881.055	T[19]
T[17]	1738.022	1797.034	**1781.015**	1782.023	1780.008	T[18]
K[18]	1866.117	**1695.986**	1679.968	1680.976	**1678.960**	K[17]
K[19]	1994.212	1567.891	1551.873	**1552.881**	1550.865	K[16]
A[20]	2065.249	1439.797	1423.778	**1424.786**	1422.770	A[15]
G[21]	2122.271	1368.759	**1352.741**	1353.748	1351.733	G[14]
A[22]	2193.308	1311.738	1295.719	**1296.727**	1294.711	A[13]
A[23]	2264.345	1240.701	1224.682	1225.690	1223.674	A[12]
K[24]	2392.440	1169.664	1153.645	1154.653	1152.637	K[11]
R[25]	2548.541	1041.569	**1025.550**	1026.558	1024.542	R[10]
K[26]	2676.636	885.468	869.449	**870.457**	868.441	K[9]
A[27]	2747.673	757.373	741.354	742.362	740.346	A[8]
T[28]	2848.721	686.336	670.317	671.325	669.309	T[7]
G[29]	2905.742	585.288	569.269	570.277	568.261	G[6]
P[30]	3002.795	528.266	512.248	513.256	511.240	P[5]
P[31]	3099.848	431.214	415.195	416.203	414.187	P[4]
V[32]	3198.916	334.161	318.142	319.150	317.134	V[3]
S[33]	3285.948	235.092	219.074	220.082	218.066	S[2]
E[34]	3414.991	148.060	132.042	133.050	131.034	E[1]

sp|P43276|H15_MOUSE

TAAPAPVEKSPAKK $^{Methyl}_{14.02}$ KTTKKAGAAKRKATGPPVSE

- fragmentation table for charge state 2+
- specType=etd
- score=48.86
- F104937.dat
- query=q2477_p1
- precursor=854.755270
- chargePrecursor=4
- itol=0.8

AA	c	y	z+1	z+2	z	AA
T[1]	60.044	1708.491	**1700.482**	0.504	**1699.978**	T[34]
A[2]	95.563	1657.967	**1649.958**	0.504	**1649.454**	A[33]
A[3]	131.082	**1622.449**	**1614.439**	0.504	**1613.935**	A[32]
P[4]	179.608	1586.930	1578.921	0.504	1578.417	P[31]
A[5]	215.126	1538.404	1530.394	0.504	1529.890	A[30]
P[6]	263.653	1502.885	1494.876	0.504	1494.372	P[29]
V[7]	313.187	1454.359	1446.349	0.504	1445.845	V[28]
E[8]	377.708	1404.825	**1396.815**	0.504	1396.311	E[27]
K[9]	441.756	1340.303	**1332.294**	**1332.798**	**1331.790**	K[26]
S[10]	485.272	1276.256	**1268.246**	**1268.750**	1267.742	S[25]
P[11]	533.798	1232.740	1224.730	1225.234	1224.226	P[24]
A[12]	569.317	1184.213	1176.204	1176.708	1175.700	A[23]
K[13]	633.364	1148.695	1140.685	1141.189	**1140.182**	K[22]
K[14]	704.420	1084.647	**1076.638**	**1077.142**	**1076.134**	K[21]
K[15]	768.467	**1013.592**	**1005.583**	**1006.087**	**1005.079**	K[20]
T[16]	818.991	**949.545**	**941.535**	**942.039**	941.031	T[19]
T[17]	869.515	899.021	891.011	891.515	890.507	T[18]
K[18]	933.562	848.497	840.487	840.991	839.984	K[17]
K[19]	997.610	784.449	776.440	776.944	775.936	K[16]
A[20]	**1033.128**	720.402	712.393	712.896	711.889	A[15]
G[21]	1061.639	684.883	676.874	677.378	676.370	G[14]
A[22]	**1097.158**	656.373	648.363	648.867	647.859	A[13]
A[23]	1132.676	620.854	612.845	613.349	612.341	A[12]
K[24]	**1196.724**	585.335	577.326	577.830	576.822	K[11]
R[25]	1274.774	521.288	513.279	513.783	512.775	R[10]
K[26]	**1338.822**	443.237	435.228	435.732	434.724	K[9]
A[27]	**1374.340**	379.190	371.181	371.685	370.677	A[8]
T[28]	**1424.864**	343.671	335.662	336.166	335.158	T[7]
G[29]	1453.375	293.148	285.138	285.642	284.634	G[6]
P[30]	1501.901	264.637	256.627	257.131	256.124	P[5]
P[31]	1550.427	216.110	208.101	208.605	207.597	P[4]
V[32]	**1599.962**	167.584	159.575	160.079	159.071	V[3]
S[33]	**1643.478**	118.050	110.041	110.544	109.537	S[2]
E[34]	1707.999	74.534	66.524	67.028	66.021	E[1]

sp|P43276|H15_MOUSE

TAAPAPVEKSPAKK $^{Methyl}_{14.02}$ KTTKKAGAAKRKATGPPVSE

- fragmentation table for charge state 3+
- specType=etd
- score=48.86
- F104937.dat
- query=q2477_p1
- precursor=854.755270
- chargePrecursor=4
- itol=0.8

AA	c	y	z+1	z+2	z	AA
T[1]	40.365	**1139.330**	**1133.990**	0.672	**1133.654**	T[34]
A[2]	64.044	1105.647	**1100.308**	0.672	1099.972	A[33]
A[3]	87.723	1081.968	**1076.629**	0.672	**1076.293**	A[32]
P[4]	120.074	1058.289	1052.950	0.672	1052.614	P[31]
A[5]	143.753	**1025.938**	**1020.599**	0.672	1020.263	A[30]
P[6]	176.104	1002.259	996.920	0.672	996.584	P[29]
V[7]	209.127	969.908	964.569	0.672	964.233	V[28]
E[8]	252.141	936.885	931.546	0.672	931.210	E[27]
K[9]	294.840	893.871	888.532	888.868	888.196	K[26]
S[10]	323.850	851.173	845.833	846.169	845.497	S[25]
P[11]	356.201	822.162	816.823	817.159	816.487	P[24]
A[12]	379.880	789.811	784.472	784.808	784.136	A[23]
K[13]	422.579	766.132	760.793	761.129	760.457	K[22]
K[14]	469.949	723.434	718.094	718.430	717.758	K[21]
K[15]	512.647	676.064	670.724	671.060	670.388	K[20]
T[16]	546.330	633.365	628.026	628.362	627.690	T[19]
T[17]	580.012	599.683	594.343	594.679	594.007	T[18]
K[18]	622.711	566.000	560.661	560.997	560.325	K[17]
K[19]	665.409	523.302	517.962	518.298	517.626	K[16]
A[20]	689.088	480.604	475.264	475.600	474.928	A[15]
G[21]	708.095	456.925	451.585	451.921	451.249	G[14]
A[22]	731.774	437.917	432.578	432.914	432.242	A[13]
A[23]	755.453	414.238	408.899	409.235	408.563	A[12]
K[24]	798.151	390.559	385.220	385.556	384.884	K[11]
R[25]	850.185	347.861	342.522	342.857	342.186	R[10]
K[26]	892.883	295.827	290.488	290.824	290.152	K[9]
A[27]	916.563	253.129	247.789	248.125	247.454	A[8]
T[28]	**950.245**	229.450	224.110	224.446	223.775	T[7]
G[29]	969.252	195.767	190.428	190.764	190.092	G[6]
P[30]	1001.603	176.760	171.421	171.757	171.085	P[5]
P[31]	**1033.954**	144.409	139.070	139.406	138.734	P[4]
V[32]	**1066.977**	112.058	106.719	107.055	106.383	V[3]
S[33]	**1095.988**	79.036	73.696	74.032	73.360	S[2]
E[34]	1139.002	50.025	44.685	45.021	44.349	E[1]

sp|P43274|H14_MOUSE

TAPAAPAAPAPAEKTPVKKKARK $^{(\text{Methyl})}_{(14.02)}$ AAGGAKRKTSGPPVSE

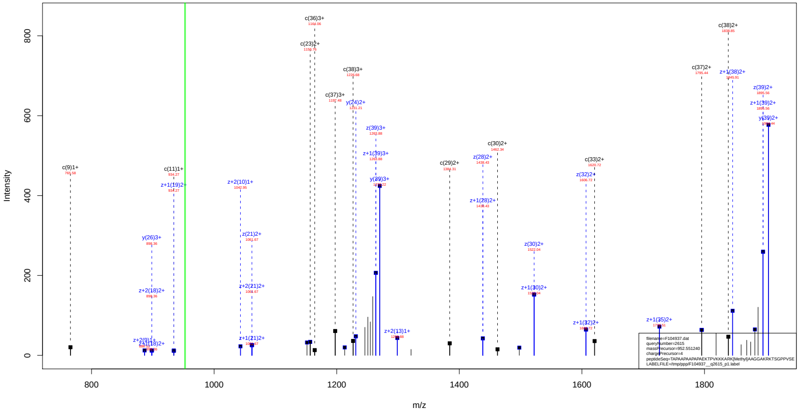

sp|P43274|H14_MOUSE

TAPAAPAAPAPAEKTPVKKKARK$^{(Methyl)}_{(14.02)}$AAGGAKRKTSGPPVSE

- fragmentation table for charge state 1+
- specType=etd
- score=44.24
- F104937.dat
- query=q2615_p1
- precursor=952.551240
- chargePrecursor=4
- itol=0.8

AA	c	y	z+1	z+2	z	AA
T[1]	119.082	3807.172	3791.153	0.000	3790.145	T[39]
A[2]	190.119	3706.124	3690.105	0.000	3689.097	A[38]
P[3]	287.171	3635.087	3619.068	0.000	3618.060	P[37]
A[4]	358.208	3538.034	3522.015	0.000	3521.007	A[36]
A[5]	429.246	3466.997	3450.978	0.000	3449.970	A[35]
P[6]	526.298	3395.960	3379.941	0.000	3378.933	P[34]
A[7]	597.335	3298.907	3282.888	0.000	3281.880	A[33]
A[8]	668.373	3227.870	3211.851	0.000	3210.843	A[32]
P[9]	**765.425**	3156.833	3140.814	0.000	3139.806	P[31]
A[10]	836.462	3059.780	3043.761	0.000	3042.753	A[30]
P[11]	**933.515**	2988.743	2972.724	0.000	2971.716	P[29]
A[12]	1004.552	2891.690	2875.671	0.000	2874.664	A[28]
E[13]	1133.595	2820.653	2804.634	0.000	2803.626	E[27]
K[14]	1261.690	2691.610	2675.592	2676.600	2674.584	K[26]
T[15]	1362.738	2563.515	2547.497	2548.505	2546.489	T[25]
P[16]	1459.790	2462.468	2446.449	2447.457	2445.441	P[24]
V[17]	1558.859	2365.415	2349.396	2350.404	2348.388	V[23]
K[18]	1686.954	2266.347	2250.328	2251.336	2249.320	K[22]
K[19]	1815.049	2138.252	2122.233	2123.241	2121.225	K[21]
K[20]	1943.144	2010.157	1994.138	1995.146	1993.130	K[20]
A[21]	2014.181	**1882.062**	1866.043	1867.051	1865.035	A[19]
R[22]	2170.282	1811.025	**1795.006**	**1796.014**	1793.998	R[18]
K[23]	2312.392	1654.923	1638.905	1639.913	1637.897	K[17]
A[24]	2383.430	1512.813	1496.794	**1497.802**	1495.786	A[16]
A[25]	2454.467	1441.776	1425.757	1426.765	1424.749	A[15]
G[26]	2511.488	1370.739	1354.720	1355.728	1353.712	G[14]
G[27]	2568.510	1313.717	1297.698	**1298.706**	1296.691	G[13]
A[28]	2639.547	1256.696	1240.677	1241.685	1239.669	A[12]
K[29]	2767.642	1185.659	1169.640	1170.648	1168.632	K[11]
R[30]	2923.743	1057.564	1041.545	**1042.553**	1040.537	R[10]
K[31]	3051.838	901.463	885.444	**886.452**	884.436	K[9]
T[32]	3152.885	773.368	757.349	758.357	756.341	T[8]
S[33]	3239.918	672.320	656.301	657.309	655.293	S[7]
G[34]	3296.939	585.288	569.269	570.277	568.261	G[6]
P[35]	3393.992	528.266	512.248	513.256	511.240	P[5]
P[36]	3491.045	431.214	415.195	416.203	414.187	P[4]
V[37]	3590.113	334.161	318.142	319.150	317.134	V[3]
S[38]	3677.145	235.092	219.074	220.082	218.066	S[2]
E[39]	3806.188	148.060	132.042	133.050	131.034	E[1]

sp|P43274|H14_MOUSE

TAPAAPAAPAPAEKTPVKKKARK $^{(Methyl)}_{(14.02)}$ AAGGAKRKTSGPPVSE

- fragmentation table for charge state 2+
- specType=etd
- score=44.24
- F104937.dat
- query=q2615_p1
- precursor=952.551240
- chargePrecursor=4
- itol=0.8

AA	c	y	z+1	z+2	z	AA
T[1]	60.044	**1904.089**	**1896.080**	0.504	**1895.576**	T[39]
A[2]	95.563	1853.566	**1845.556**	0.504	1845.052	A[38]
P[3]	144.089	1818.047	1810.038	0.504	1809.534	P[37]
A[4]	179.608	1769.521	1761.511	0.504	1761.007	A[36]
A[5]	215.126	1734.002	**1725.993**	0.504	1725.489	A[35]
P[6]	263.653	1698.484	1690.474	0.504	1689.970	P[34]
A[7]	299.171	1649.957	1641.948	0.504	1641.444	A[33]
A[8]	334.690	1614.439	**1606.429**	0.504	**1605.925**	A[32]
P[9]	383.216	1578.920	1570.911	0.504	1570.407	P[31]
A[10]	418.735	1530.394	**1522.384**	0.504	**1521.880**	A[30]
P[11]	467.261	1494.875	1486.866	0.504	1486.362	P[29]
A[12]	502.780	1446.349	**1438.339**	0.504	**1437.835**	A[28]
E[13]	567.301	1410.830	1402.821	0.504	1402.317	E[27]
K[14]	631.349	1346.309	1338.299	1338.803	1337.796	K[26]
T[15]	681.872	1282.261	1274.252	1274.756	1273.748	T[25]
P[16]	730.399	**1231.738**	1223.728	1224.232	1223.224	P[24]
V[17]	779.933	1183.211	1175.202	1175.706	1174.698	V[23]
K[18]	843.981	1133.677	1125.668	1126.171	1125.164	K[22]
K[19]	908.028	1069.629	**1061.620**	**1062.124**	**1061.116**	K[21]
K[20]	972.075	1005.582	997.573	998.077	997.069	K[20]
A[21]	1007.594	941.534	**933.525**	**934.029**	933.021	A[19]
R[22]	1085.645	906.016	**898.007**	**898.510**	897.503	R[18]
K[23]	**1156.700**	827.965	819.956	820.460	819.452	K[17]
A[24]	1192.218	756.910	748.901	749.405	748.397	A[16]
A[25]	1227.737	721.392	713.382	713.886	712.878	A[15]
G[26]	1256.248	685.873	677.864	678.368	677.360	G[14]
G[27]	1284.758	657.362	649.353	649.857	648.849	G[13]
A[28]	1320.277	628.852	620.842	621.346	620.338	A[12]
K[29]	**1384.325**	593.333	585.324	585.827	584.820	K[11]
R[30]	**1462.375**	529.285	521.276	521.780	520.772	R[10]
K[31]	1526.423	451.235	443.226	443.729	442.722	K[9]
T[32]	1576.946	387.187	379.178	379.682	378.674	T[8]
S[33]	**1620.462**	336.664	328.654	329.158	328.150	S[7]
G[34]	1648.973	293.148	285.138	285.642	284.634	G[6]
P[35]	1697.500	264.637	256.627	257.131	256.124	P[5]
P[36]	1746.026	216.110	208.101	208.605	207.597	P[4]
V[37]	**1795.560**	167.584	159.575	160.079	159.071	V[3]
S[38]	**1839.076**	118.050	110.041	110.544	109.537	S[2]
E[39]	1903.597	74.534	66.524	67.028	66.021	E[1]

sp|P43274|H14_MOUSE

TAPAAPAAPAPAEKTPVKKKARK$^{(Methyl)}_{(14.02)}$AAGGAKRKTSGPPVSE

- fragmentation table for charge state 3+
- specType=etd
- score=44.24
- F104937.dat
- query=q2615_p1
- precursor=952.551240
- chargePrecursor=4
- itol=0.8

AA	c	y	z+1	z+2	z	AA
T[1]	40.365	**1269.729**	**1264.389**	0.672	**1264.053**	T[39]
A[2]	64.044	1236.046	**1230.707**	0.672	1230.371	A[38]
P[3]	96.395	**1212.367**	1207.028	0.672	1206.692	P[37]
A[4]	120.074	1180.016	1174.677	0.672	1174.341	A[36]
A[5]	143.753	**1156.337**	**1150.998**	0.672	1150.662	A[35]
P[6]	176.104	1132.658	1127.319	0.672	1126.983	P[34]
A[7]	199.783	1100.307	1094.968	0.672	1094.632	A[33]
A[8]	223.462	1076.628	1071.289	0.672	1070.953	A[32]
P[9]	255.813	1052.949	1047.610	0.672	1047.274	P[31]
A[10]	279.492	1020.598	1015.259	0.672	1014.923	A[30]
P[11]	311.843	996.919	991.580	0.672	991.244	P[29]
A[12]	335.522	964.568	959.229	0.672	958.893	A[28]
E[13]	378.537	940.889	935.550	0.672	935.214	E[27]
K[14]	421.235	**897.875**	892.535	892.871	892.199	K[26]
T[15]	454.917	855.177	849.837	850.173	849.501	T[25]
P[16]	487.268	821.494	816.155	816.490	815.819	P[24]
V[17]	520.291	789.143	783.804	784.140	783.468	V[23]
K[18]	562.989	756.120	750.781	751.117	750.445	K[22]
K[19]	605.688	713.422	708.082	708.418	707.747	K[21]
K[20]	648.386	670.724	665.384	665.720	665.048	K[20]
A[21]	672.065	628.025	622.686	623.022	622.350	A[19]
R[22]	724.099	604.346	599.007	599.343	598.671	R[18]
K[23]	771.469	552.313	546.973	547.309	546.637	K[17]
A[24]	795.148	504.942	499.603	499.939	499.267	A[16]
A[25]	818.827	481.263	475.924	476.260	475.588	A[15]
G[26]	837.834	457.584	452.245	452.581	451.909	G[14]
G[27]	856.841	438.577	433.238	433.574	432.902	G[13]
A[28]	880.520	419.570	414.231	414.566	413.895	A[12]
K[29]	923.219	395.891	390.551	390.887	390.216	K[11]
R[30]	975.252	353.193	347.853	348.189	347.517	R[10]
K[31]	1017.951	301.159	295.819	296.155	295.484	K[9]
T[32]	1051.633	258.461	253.121	253.457	252.785	T[8]
S[33]	1080.644	224.778	219.439	219.775	219.103	S[7]
G[34]	1099.651	195.767	190.428	190.764	190.092	G[6]
P[35]	1132.002	176.760	171.421	171.757	171.085	P[5]
P[36]	**1164.353**	144.409	139.070	139.406	138.734	P[4]
V[37]	**1197.376**	112.058	106.719	107.055	106.383	V[3]
S[38]	**1226.386**	79.036	73.696	74.032	73.360	S[2]
E[39]	1269.401	50.025	44.685	45.021	44.349	E[1]

sp|P43276|H15_MOUSE

TAPAETAAPAPVEKSPAKK $^{\text{Methyl}}_{14.02}$ KTTKKAGAAKRKATGPPVSE

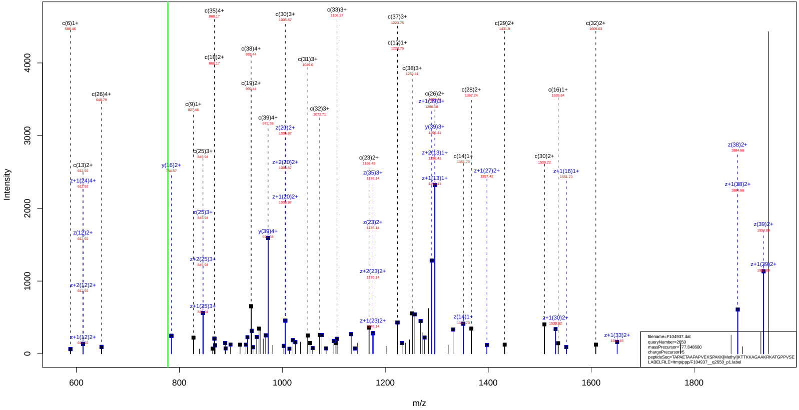

sp|P43276|H15_MOUSE

TAPAETAAPAPVEKSPAKK $^{Methyl}_{14.02}$ KTTKKAGAAKRKATGPPVSE

- fragmentation table for charge state 1+
- specType=etd
- score=80.53
- F104937.dat
- query=q2650_p1
- precursor=777.848600
- chargePrecursor=5
- itol=0.8

AA	c	y	z+1	z+2	z	AA
T[1]	119.082	3885.192	3869.173	0.000	3868.165	T[39]
A[2]	190.119	3784.144	3768.126	0.000	3767.118	A[38]
P[3]	287.171	3713.107	3697.088	0.000	3696.081	P[37]
A[4]	358.208	3616.054	3600.036	0.000	3599.028	A[36]
E[5]	487.251	3545.017	3528.999	0.000	3527.991	E[35]
T[6]	**588.299**	3415.975	3399.956	0.000	3398.948	T[34]
A[7]	659.336	3314.927	3298.908	0.000	3297.901	A[33]
A[8]	730.373	3243.890	3227.871	0.000	3226.863	A[32]
P[9]	**827.426**	3172.853	3156.834	0.000	3155.826	P[31]
A[10]	898.463	3075.800	3059.781	0.000	3058.774	A[30]
P[11]	995.516	3004.763	2988.744	0.000	2987.736	P[29]
V[12]	1094.584	2907.710	2891.691	0.000	2890.684	V[28]
E[13]	**1223.627**	2808.642	2792.623	0.000	2791.615	E[27]
K[14]	**1351.722**	2679.599	2663.580	2664.588	2662.573	K[26]
S[15]	1438.754	2551.504	2535.486	2536.493	2534.478	S[25]
P[16]	**1535.806**	2464.472	2448.453	2449.461	2447.446	P[24]
A[17]	1606.844	2367.419	2351.401	2352.409	2350.393	A[23]
K[18]	1734.938	2296.382	2280.364	2281.371	2279.356	K[22]
K[19]	1877.049	2168.287	2152.269	2153.276	2151.261	K[21]
K[20]	2005.144	2026.177	2010.158	2011.166	2009.150	K[20]
T[21]	2106.192	1898.082	1882.063	1883.071	1881.055	T[19]
T[22]	2207.239	1797.034	1781.015	1782.023	1780.008	T[18]
K[23]	2335.334	1695.986	1679.968	1680.976	1678.960	K[17]
K[24]	2463.429	1567.891	**1551.873**	1552.881	1550.865	K[16]
A[25]	2534.466	1439.797	1423.778	1424.786	1422.770	A[15]
G[26]	2591.488	1368.759	1352.741	1353.748	**1351.733**	G[14]
A[27]	2662.525	1311.738	**1295.719**	**1296.727**	1294.711	A[13]
A[28]	2733.562	1240.701	1224.682	1225.690	**1223.674**	A[12]
K[29]	2861.657	1169.664	1153.645	1154.653	1152.637	K[11]
R[30]	3017.758	1041.569	**1025.550**	1026.558	1024.542	R[10]
K[31]	3145.853	885.468	**869.449**	870.457	**868.441**	K[9]
A[32]	3216.890	757.373	741.354	742.362	740.346	A[8]
T[33]	3317.938	686.336	670.317	671.325	669.309	T[7]
G[34]	3374.959	585.288	569.269	570.277	568.261	G[6]
P[35]	3472.012	528.266	512.248	513.256	511.240	P[5]
P[36]	3569.065	431.214	415.195	416.203	414.187	P[4]
V[37]	3668.133	334.161	318.142	319.150	317.134	V[3]
S[38]	3755.165	235.092	219.074	220.082	218.066	S[2]
E[39]	3884.208	148.060	132.042	133.050	131.034	E[1]

sp|P43276|H15_MOUSE

TAPAETAAPAPVEKSPAKK $^{\text{Methyl}}_{14.02}$ KTTKKAGAAKRKATGPPVSE

- fragmentation table for charge state 2+
- specType=etd
- score=80.53
- F104937.dat
- query=q2650_p1
- precursor=777.848600
- chargePrecursor=5
- itol=0.8

AA	c	y	z+1	z+2	z	AA
T[1]	60.044	1943.100	**1935.090**	0.504	**1934.586**	T[39]
A[2]	95.563	1892.576	**1884.566**	0.504	**1884.063**	A[38]
P[3]	144.089	1857.057	1849.048	0.504	1848.544	P[37]
A[4]	179.608	1808.531	1800.522	0.504	1800.018	A[36]
E[5]	244.129	1773.012	1765.003	0.504	1764.499	E[35]
T[6]	294.653	1708.491	1700.482	0.504	1699.978	T[34]
A[7]	330.172	1657.967	**1649.958**	0.504	1649.454	A[33]
A[8]	365.690	1622.449	1614.439	0.504	1613.935	A[32]
P[9]	414.217	1586.930	1578.921	0.504	1578.417	P[31]
A[10]	449.735	1538.404	**1530.394**	0.504	1529.890	A[30]
P[11]	498.261	1502.885	1494.876	0.504	1494.372	P[29]
V[12]	547.796	1454.359	1446.349	0.504	1445.845	V[28]
E[13]	**612.317**	1404.825	**1396.815**	0.504	1396.311	E[27]
K[14]	676.364	1340.303	**1332.294**	1332.798	**1331.790**	K[26]
S[15]	719.880	**1276.256**	**1268.246**	**1268.750**	1267.742	S[25]
P[16]	768.407	**1232.740**	1224.730	1225.234	**1224.226**	P[24]
A[17]	803.925	1184.213	**1176.204**	**1176.708**	**1175.700**	A[23]
K[18]	**867.973**	1148.695	**1140.685**	**1141.189**	1140.182	K[22]
K[19]	**939.028**	**1084.647**	**1076.638**	**1077.142**	1076.134	K[21]
K[20]	**1003.076**	**1013.592**	**1005.583**	**1006.087**	**1005.079**	K[20]
T[21]	**1053.600**	**949.545**	**941.535**	942.039	**941.031**	T[19]
T[22]	**1104.123**	**899.021**	891.011	891.515	**890.507**	T[18]
K[23]	**1168.171**	848.497	840.487	840.991	839.984	K[17]
K[24]	**1232.218**	**784.449**	776.440	776.944	775.936	K[16]
A[25]	1267.737	720.402	712.393	712.896	711.889	A[15]
G[26]	**1296.248**	684.883	676.874	677.378	676.370	G[14]
A[27]	**1331.766**	656.373	**648.363**	**648.867**	647.859	A[13]
A[28]	**1367.285**	620.854	**612.845**	**613.349**	**612.341**	A[12]
K[29]	**1431.332**	585.335	577.326	577.830	576.822	K[11]
R[30]	**1509.383**	521.288	513.279	513.783	512.775	R[10]
K[31]	1573.430	443.237	435.228	435.732	434.724	K[9]
A[32]	**1608.949**	379.190	371.181	371.685	370.677	A[8]
T[33]	1659.473	343.671	335.662	336.166	335.158	T[7]
G[34]	1687.983	293.148	285.138	285.642	284.634	G[6]
P[35]	1736.510	264.637	256.627	257.131	256.124	P[5]
P[36]	1785.036	216.110	208.101	208.605	207.597	P[4]
V[37]	1834.570	167.584	159.575	160.079	159.071	V[3]
S[38]	1878.086	118.050	110.041	110.544	109.537	S[2]
E[39]	1942.608	74.534	66.524	67.028	66.021	E[1]

sp|P43276|H15_MOUSE

TAPAETAAPAPVEKSPAKK $^{Methyl}_{14.02}$ KTTKKAGAAKRKATGPPVSE

- fragmentation table for charge state 3+
- specType=etd
- score=80.53
- F104937.dat
- query=q2650_p1
- precursor=777.848600
- chargePrecursor=5
- itol=0.8

AA	c	y	z+1	z+2	z	AA
T[1]	40.365	**1295.736**	**1290.396**	0.672	**1290.060**	T[39]
A[2]	64.044	1262.053	**1256.713**	0.672	1256.377	A[38]
P[3]	96.395	1238.374	**1233.034**	0.672	**1232.698**	P[37]
A[4]	120.074	1206.023	1200.683	0.672	1200.347	A[36]
E[5]	163.089	1182.344	1177.004	0.672	**1176.668**	E[35]
T[6]	196.771	1139.330	**1133.990**	0.672	**1133.654**	T[34]
A[7]	220.450	**1105.647**	**1100.308**	0.672	**1099.972**	A[33]
A[8]	244.129	1081.968	**1076.629**	0.672	**1076.293**	A[32]
P[9]	276.480	**1058.289**	1052.950	0.672	1052.614	P[31]
A[10]	300.159	**1025.938**	**1020.599**	0.672	**1020.263**	A[30]
P[11]	332.510	**1002.259**	996.920	0.672	996.584	P[29]
V[12]	365.533	969.908	964.569	0.672	964.233	V[28]
E[13]	408.547	936.885	**931.546**	0.672	931.210	E[27]
K[14]	451.245	893.871	**888.532**	**888.868**	888.196	K[26]
S[15]	480.256	851.173	**845.833**	**846.169**	**845.497**	S[25]
P[16]	512.607	822.162	816.823	817.159	816.487	P[24]
A[17]	536.286	789.811	**784.472**	**784.808**	**784.136**	A[23]
K[18]	578.984	766.132	760.793	761.129	760.457	K[22]
K[19]	626.355	723.434	718.094	718.430	717.758	K[21]
K[20]	669.053	676.064	670.724	671.060	670.388	K[20]
T[21]	702.735	633.365	628.026	628.362	627.690	T[19]
T[22]	736.418	599.683	594.343	594.679	594.007	T[18]
K[23]	779.116	566.000	560.661	560.997	560.325	K[17]
K[24]	821.815	523.302	517.962	518.298	517.626	K[16]
A[25]	**845.494**	480.604	475.264	475.600	474.928	A[15]
G[26]	**864.501**	456.925	451.585	451.921	451.249	G[14]
A[27]	888.180	437.917	432.578	432.914	432.242	A[13]
A[28]	911.859	414.238	408.899	409.235	408.563	A[12]
K[29]	**954.557**	390.559	385.220	385.556	384.884	K[11]
R[30]	**1006.591**	347.861	342.522	342.857	342.186	R[10]
K[31]	**1049.289**	295.827	290.488	290.824	290.152	K[9]
A[32]	**1072.968**	253.129	247.789	248.125	247.454	A[8]
T[33]	**1106.651**	229.450	224.110	224.446	223.775	T[7]
G[34]	1125.658	195.767	190.428	190.764	190.092	G[6]
P[35]	1158.009	176.760	171.421	171.757	171.085	P[5]
P[36]	1190.360	144.409	139.070	139.406	138.734	P[4]
V[37]	**1223.383**	112.058	106.719	107.055	106.383	V[3]
S[38]	**1252.393**	79.036	73.696	74.032	73.360	S[2]
E[39]	1295.408	50.025	44.685	45.021	44.349	E[1]

sp|P43276|H15_MOUSE

TAPAETAAPAPVEKSPAKK $^{\text{Methyl}}_{14.02}$ KTTKKAGAAKRKATGPPVSE

- fragmentation table for charge state 4+
- specType=etd
- score=80.53
- F104937.dat
- query=q2650_p1
- precursor=777.848600
- chargePrecursor=5
- itol=0.8

AA	c	y	z+1	z+2	z	AA
T[1]	30.526	**972.053**	**968.049**	0.755	**967.797**	T[39]
A[2]	48.285	946.792	**942.787**	0.755	**942.535**	A[38]
P[3]	72.548	**929.032**	925.028	0.755	924.776	P[37]
A[4]	90.308	904.769	900.764	0.755	900.512	A[36]
E[5]	122.568	887.010	883.005	0.755	882.753	E[35]
T[6]	147.830	854.749	850.744	0.755	850.493	T[34]
A[7]	165.589	829.487	825.483	0.755	825.231	A[33]
A[8]	183.349	811.728	807.723	0.755	807.471	A[32]
P[9]	207.612	793.969	789.964	0.755	789.712	P[31]
A[10]	225.371	769.705	765.701	0.755	765.449	A[30]
P[11]	249.634	751.946	747.942	0.755	747.690	P[29]
V[12]	274.401	727.683	723.678	0.755	723.426	V[28]
E[13]	306.662	702.916	698.911	0.755	698.659	E[27]
K[14]	338.686	670.655	666.651	666.903	666.399	K[26]
S[15]	360.444	638.632	634.627	634.879	634.375	S[25]
P[16]	384.707	616.874	**612.869**	**613.121**	**612.617**	P[24]
A[17]	402.466	592.610	**588.606**	**588.858**	**588.354**	A[23]
K[18]	434.490	574.851	570.846	571.098	570.594	K[22]
K[19]	470.018	542.827	538.823	539.075	538.571	K[21]
K[20]	502.041	507.300	503.295	503.547	503.043	K[20]
T[21]	527.303	475.276	471.271	471.523	471.019	T[19]
T[22]	552.565	450.014	446.009	446.261	445.757	T[18]
K[23]	584.589	424.752	420.747	420.999	420.495	K[17]
K[24]	616.613	392.728	388.724	388.976	388.472	K[16]
A[25]	634.372	360.705	356.700	356.952	356.448	A[15]
G[26]	**648.627**	342.945	338.941	339.193	338.689	G[14]
A[27]	666.387	328.690	324.685	324.937	324.433	A[13]
A[28]	684.146	310.931	306.926	307.178	306.674	A[12]
K[29]	716.170	293.171	289.167	289.419	288.915	K[11]
R[30]	755.195	261.148	257.143	257.395	256.891	R[10]
K[31]	787.219	222.122	218.118	218.370	217.866	K[9]
A[32]	804.978	190.099	186.094	186.346	185.842	A[8]
T[33]	830.240	172.339	168.335	168.587	168.083	T[7]
G[34]	844.495	147.077	143.073	143.325	142.821	G[6]
P[35]	**868.759**	132.822	128.817	129.069	128.565	P[5]
P[36]	893.022	108.559	104.554	104.806	104.302	P[4]
V[37]	**917.789**	84.296	80.291	80.543	80.039	V[3]
S[38]	**939.547**	59.529	55.524	55.776	55.272	S[2]
E[39]	**971.807**	37.771	33.766	34.018	33.514	E[1]

sp|P68433|H31_MOUSE

IAQDFK $^{\text{Methyl}}_{14.02}$ TD

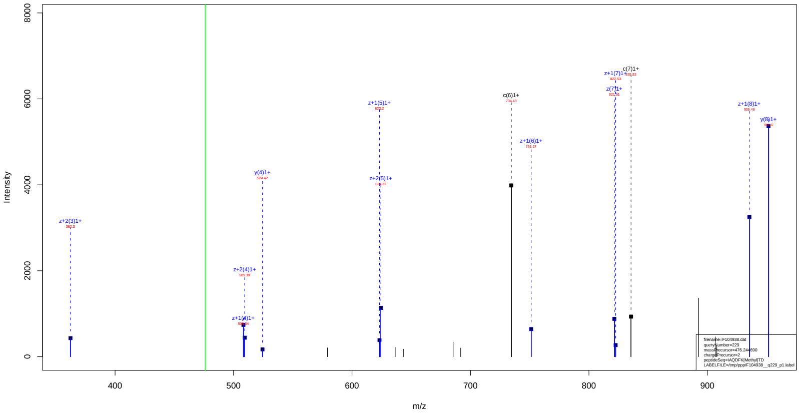

sp|P68433|H31_MOUSE

IAQDFK $^{Methyl}_{14.02}$ TD

- fragmentation table for charge state 1+
- specType=etd
- score=31.03
- F104938.dat
- query=q229_p1
- precursor=476.244690
- chargePrecursor=2
- itol=0.8

AA	c	y	z+1	z+2	z	AA
I[1]	131.118	**951.478**	**935.459**	0.000	934.452	I[8]
A[2]	202.155	838.394	**822.375**	0.000	**821.368**	A[7]
Q[3]	330.214	767.357	**751.338**	752.346	750.330	Q[6]
D[4]	445.241	639.298	**623.280**	**624.288**	622.272	D[5]
F[5]	592.309	**524.271**	**508.253**	**509.261**	507.245	F[4]
K[6]	**734.420**	377.203	361.184	**362.192**	360.177	K[3]
T[7]	**835.467**	235.092	219.074	220.082	218.066	T[2]
D[8]	950.494	134.045	118.026	119.034	117.018	D[1]

sp|Q64478|H2B1H_MOUSE

PEPAKSAPAPKKGSKKALTKAQKKDGKKRKRSRKE

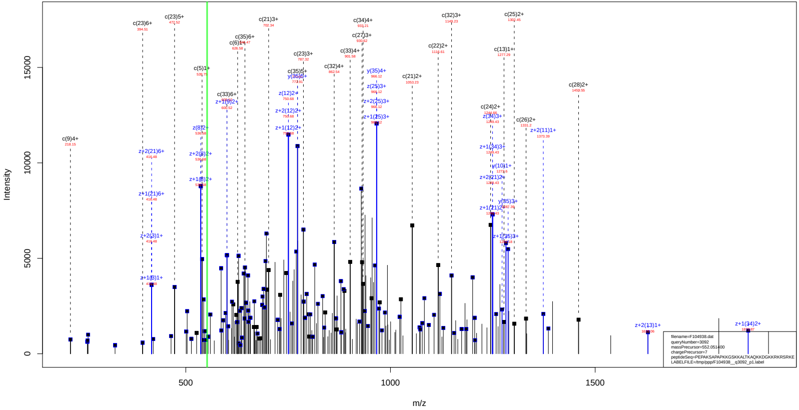

sp|Q64478|H2B1H_MOUSE

PEPAKSAPAPKKGSKKALTKAQKKDGKKRKRSRKE

- fragmentation table for charge state 1+
- specType=etd
- score=44.50
- F104938.dat
- query=q3092_p1
- precursor=552.051400
- chargePrecursor=7
- itol=0.8

AA	c	y	z+1	z+2	z	AA
P[1]	115.087	3858.299	3842.280	0.000	3841.272	P[35]
E[2]	244.129	3761.246	3745.227	0.000	3744.220	E[34]
P[3]	341.182	3632.203	3616.185	0.000	3615.177	P[33]
A[4]	412.219	3535.151	3519.132	0.000	3518.124	A[32]
K[5]	**540.314**	3464.114	3448.095	3449.103	3447.087	K[31]
S[6]	**627.346**	3336.019	3320.000	3321.008	3318.992	S[30]
A[7]	**698.383**	3248.987	3232.968	3233.976	3231.960	A[29]
P[8]	**795.436**	3177.949	3161.931	3162.939	3160.923	P[28]
A[9]	**866.473**	3080.897	3064.878	3065.886	3063.870	A[27]
P[10]	963.526	3009.860	2993.841	2994.849	2992.833	P[26]
K[11]	1091.621	2912.807	2896.788	2897.796	2895.780	K[25]
K[12]	1219.716	2784.712	2768.693	2769.701	2767.685	K[24]
G[13]	**1276.737**	2656.617	2640.598	2641.606	2639.590	G[23]
S[14]	1363.769	2599.595	2583.577	2584.585	2582.569	S[22]
K[15]	1491.864	2512.563	2496.545	2497.553	2495.537	K[21]
K[16]	1619.959	2384.468	2368.450	2369.458	2367.442	K[20]
A[17]	1690.996	2256.373	2240.355	2241.363	2239.347	A[19]
L[18]	1804.080	2185.336	2169.318	2170.325	2168.310	L[18]
T[19]	1905.128	2072.252	2056.234	2057.241	2055.226	T[17]
K[20]	2033.223	1971.205	1955.186	1956.194	1954.178	K[16]
A[21]	2104.260	1843.110	1827.091	1828.099	1826.083	A[15]
Q[22]	2232.319	1772.073	1756.054	1757.062	1755.046	Q[14]
K[23]	2360.414	1644.014	1627.995	**1629.003**	1626.987	K[13]
K[24]	2488.509	1515.919	1499.900	1500.908	1498.892	K[12]
D[25]	2603.536	1387.824	1371.805	**1372.813**	1370.798	D[11]
G[26]	2660.557	**1272.797**	1256.778	**1257.786**	1255.771	G[10]
K[27]	2788.652	1215.776	1199.757	**1200.765**	1198.749	K[9]
K[28]	2916.747	1087.681	**1071.662**	**1072.670**	**1070.654**	K[8]
R[29]	3072.848	959.586	943.567	**944.575**	942.559	R[7]
K[30]	3200.943	**803.485**	**787.466**	**788.474**	786.458	K[6]
R[31]	3357.044	**675.390**	659.371	660.379	**658.363**	R[5]
S[32]	3444.076	519.289	**503.270**	504.278	502.262	S[4]
R[33]	3600.177	432.257	**416.238**	**417.246**	415.230	R[3]
K[34]	3728.272	276.155	**260.137**	**261.144**	**259.129**	K[2]
E[35]	3857.315	148.060	132.042	133.050	131.034	E[1]

sp|Q64478|H2B1H_MOUSE

PEPAKSAPAPKKGSKKALTKAQKKDGKKRKRSRKE

- fragmentation table for charge state 2+
- specType=etd
- score=44.50
- F104938.dat
- query=q3092_p1
- precursor=552.051400
- chargePrecursor=7
- itol=0.8

AA	c	y	z+1	z+2	z	AA
P[1]	58.047	1929.653	1921.644	0.504	1921.140	P[35]
E[2]	122.568	1881.127	**1873.117**	0.504	1872.613	E[34]
P[3]	171.095	1816.605	1808.596	0.504	1808.092	P[33]
A[4]	206.613	1768.079	1760.070	0.504	1759.566	A[32]
K[5]	270.661	1732.560	1724.551	1725.055	1724.047	K[31]
S[6]	314.177	1668.513	1660.504	1661.007	1660.000	S[30]
A[7]	349.695	1624.997	1616.988	1617.491	1616.484	A[29]
P[8]	398.222	1589.478	1581.469	1581.973	1580.965	P[28]
A[9]	433.740	1540.952	1532.943	1533.447	1532.439	A[27]
P[10]	482.267	1505.433	1497.424	1497.928	1496.920	P[26]
K[11]	**546.314**	1456.907	1448.898	1449.402	1448.394	K[25]
K[12]	610.362	1392.860	1384.850	**1385.354**	1384.346	K[24]
G[13]	638.872	1328.812	1320.803	1321.307	1320.299	G[23]
S[14]	**682.388**	1300.301	1292.292	1292.796	1291.788	S[22]
K[15]	**746.436**	1256.785	**1248.776**	**1249.280**	1248.272	K[21]
K[16]	**810.483**	1192.738	**1184.729**	**1185.232**	**1184.225**	K[20]
A[17]	846.002	**1128.690**	**1120.681**	**1121.185**	**1120.177**	A[19]
L[18]	902.544	**1093.172**	1085.162	1085.666	1084.659	L[18]
T[19]	**953.068**	1036.630	1028.620	1029.124	1028.117	T[17]
K[20]	1017.115	**986.106**	978.097	**978.601**	977.593	K[16]
A[21]	**1052.634**	922.058	914.049	914.553	913.545	A[15]
Q[22]	**1116.663**	886.540	**878.531**	**879.034**	878.027	Q[14]
K[23]	1180.710	**822.511**	**814.501**	**815.005**	813.997	K[13]
K[24]	**1244.758**	**758.463**	**750.454**	**750.958**	**749.950**	K[12]
D[25]	**1302.271**	**694.416**	**686.406**	**686.910**	**685.902**	D[11]
G[26]	**1330.782**	**636.902**	**628.893**	**629.397**	**628.389**	G[10]
K[27]	1394.830	608.391	**600.382**	**600.886**	**599.878**	K[9]
K[28]	**1458.877**	**544.344**	**536.335**	**536.839**	**535.831**	K[8]
R[29]	1536.928	480.296	**472.287**	**472.791**	**471.783**	R[7]
K[30]	1600.975	402.246	**394.237**	**394.740**	**393.733**	K[6]
R[31]	1679.026	338.198	330.189	330.693	329.685	R[5]
S[32]	1722.542	**260.148**	252.139	252.642	251.635	S[4]
R[33]	1800.592	216.632	208.623	209.126	208.119	R[3]
K[34]	1864.640	138.581	130.572	131.076	130.068	K[2]
E[35]	1929.161	74.534	66.524	67.028	66.021	E[1]

sp|Q64478|H2B1H_MOUSE

PEPAKSAPAPKKGSKKALTKAQKKDGKKRKRSRKE

- fragmentation table for charge state 3+
- specType=etd
- score=44.50
- F104938.dat
- query=q3092_p1
- precursor=552.051400
- chargePrecursor=7
- itol=0.8

AA	c	y	z+1	z+2	z	AA
P[1]	39.034	**1286.771**	**1281.432**	0.672	1281.096	P[35]
E[2]	82.048	1254.420	**1249.081**	0.672	**1248.745**	E[34]
P[3]	114.399	1211.406	**1206.066**	0.672	**1205.730**	P[33]
A[4]	138.078	1179.055	**1173.716**	0.672	**1173.380**	A[32]
K[5]	180.776	**1155.376**	1150.036	1150.372	**1149.701**	K[31]
S[6]	209.787	1112.678	1107.338	1107.674	**1107.002**	S[30]
A[7]	233.466	**1083.667**	**1078.327**	1078.663	**1077.992**	A[29]
P[8]	265.817	1059.988	1054.648	1054.984	1054.312	P[28]
A[9]	289.496	1027.637	**1022.298**	**1022.633**	**1021.962**	A[27]
P[10]	321.847	1003.958	998.618	998.954	998.283	P[26]
K[11]	364.545	**971.607**	**966.268**	**966.603**	**965.932**	K[25]
K[12]	407.243	**928.909**	**923.569**	**923.905**	923.233	K[24]
G[13]	426.251	**886.210**	880.871	881.207	**880.535**	G[23]
S[14]	455.261	**867.203**	**861.864**	**862.200**	861.528	S[22]
K[15]	497.960	**838.193**	832.853	833.189	832.517	K[21]
K[16]	540.658	**795.494**	790.155	**790.491**	789.819	K[20]
A[17]	564.337	752.796	747.456	747.792	747.120	A[19]
L[18]	602.032	**729.117**	**723.777**	**724.113**	723.441	L[18]
T[19]	**635.714**	691.422	**686.083**	**686.419**	**685.747**	T[17]
K[20]	**678.413**	**657.740**	**652.400**	**652.736**	**652.064**	K[16]
A[21]	**702.092**	615.041	609.702	610.038	609.366	A[15]
Q[22]	744.778	**591.362**	**586.023**	**586.359**	**585.687**	Q[14]
K[23]	**787.476**	**548.676**	**543.337**	**543.673**	**543.001**	K[13]
K[24]	830.174	505.978	**500.638**	**500.974**	**500.302**	K[12]
D[25]	**868.517**	**463.280**	457.940	458.276	457.604	D[11]
G[26]	**887.524**	424.937	419.598	**419.934**	419.262	G[10]
K[27]	**930.222**	405.930	400.590	400.926	400.255	K[9]
K[28]	**972.920**	363.232	357.892	358.228	357.556	K[8]
R[29]	**1024.954**	320.533	315.194	315.530	314.858	R[7]
K[30]	1067.653	268.500	263.160	263.496	262.824	K[6]
R[31]	1119.686	225.801	220.462	220.798	220.126	R[5]
S[32]	**1148.697**	173.768	168.428	168.764	168.092	S[4]
R[33]	**1200.731**	144.757	139.417	139.753	139.082	R[3]
K[34]	1243.429	92.723	87.384	87.720	87.048	K[2]
E[35]	1286.443	50.025	44.685	45.021	44.349	E[1]

sp|Q64478|H2B1H_MOUSE

PEPAKSAPAPKKGSKKALTKAQKKDGKKRKRSRKE

- fragmentation table for charge state 4+
- specType=etd
- score=44.50
- F104938.dat
- query=q3092_p1
- precursor=552.051400
- chargePrecursor=7
- itol=0.8

AA	c	y	z+1	z+2	z	AA
P[1]	29.527	**965.330**	**961.325**	0.755	961.074	P[35]
E[2]	61.788	941.067	**937.062**	0.755	**936.810**	E[34]
P[3]	86.051	908.806	904.802	0.755	904.550	P[33]
A[4]	103.810	884.543	**880.538**	0.755	**880.286**	A[32]
K[5]	135.834	**866.784**	**862.779**	**863.031**	**862.527**	K[31]
S[6]	157.592	**834.760**	830.755	831.007	830.503	S[30]
A[7]	175.351	813.002	808.997	**809.249**	808.745	A[29]
P[8]	199.614	**795.243**	**791.238**	**791.490**	**790.986**	P[28]
A[9]	**217.374**	770.980	766.975	767.227	766.723	A[27]
P[10]	241.637	753.220	749.216	749.468	748.964	P[26]
K[11]	273.661	**728.957**	**724.952**	**725.204**	**724.701**	K[25]
K[12]	305.684	**696.933**	**692.929**	**693.181**	**692.677**	K[24]
G[13]	319.940	664.910	660.905	661.157	660.653	G[23]
S[14]	341.698	650.654	646.650	**646.902**	646.398	S[22]
K[15]	373.722	**628.896**	**624.892**	**625.144**	624.640	K[21]
K[16]	405.745	**596.873**	592.868	593.120	592.616	K[20]
A[17]	423.505	564.849	560.844	561.096	**560.592**	A[19]
L[18]	451.776	**547.090**	**543.085**	**543.337**	542.833	L[18]
T[19]	477.037	518.819	514.814	515.066	514.562	T[17]
K[20]	509.061	493.557	489.552	489.804	489.300	K[16]
A[21]	**526.820**	461.533	457.528	457.780	457.276	A[15]
Q[22]	558.835	443.774	439.769	440.021	439.517	Q[14]
K[23]	**590.859**	411.759	407.754	408.006	407.502	K[13]
K[24]	**622.883**	379.735	375.731	375.982	375.479	K[12]
D[25]	**651.639**	347.711	343.707	343.959	343.455	D[11]
G[26]	**665.895**	318.955	314.950	315.202	314.698	G[10]
K[27]	**697.918**	304.699	300.695	300.947	300.443	K[9]
K[28]	**729.942**	272.676	268.671	268.923	268.419	K[8]
R[29]	768.967	240.652	236.647	236.899	236.395	R[7]
K[30]	**800.991**	201.627	197.622	197.874	197.370	K[6]
R[31]	**840.016**	169.603	165.598	165.850	165.346	R[5]
S[32]	**861.774**	130.578	126.573	126.825	126.321	S[4]
R[33]	**900.800**	108.820	104.815	105.067	104.563	R[3]
K[34]	**932.824**	69.794	65.790	66.042	65.538	K[2]
E[35]	965.084	37.771	33.766	34.018	33.514	E[1]

sp|Q64478|H2B1H_MOUSE

PEPAKSAPAPKKGSKKALTKAQKKDGKKRKRSRKE

- fragmentation table for charge state 5+
- specType=etd
- score=44.50
- F104938.dat
- query=q3092_p1
- precursor=552.051400
- chargePrecursor=7
- itol=0.8

AA	c	y	z+1	z+2	z	AA
P[1]	23.823	**772.466**	**769.262**	0.806	769.060	P[35]
E[2]	49.632	753.055	749.851	0.806	749.650	E[34]
P[3]	69.042	727.247	**724.043**	0.806	**723.841**	P[33]
A[4]	83.250	707.836	704.632	0.806	704.431	A[32]
K[5]	108.869	693.629	**690.425**	**690.626**	**690.223**	K[31]
S[6]	126.275	**668.010**	664.806	665.007	664.604	S[30]
A[7]	140.482	650.603	**647.399**	**647.601**	**647.198**	A[29]
P[8]	159.893	**636.396**	**633.192**	**633.394**	**632.990**	P[28]
A[9]	174.100	616.985	613.781	613.983	**613.580**	A[27]
P[10]	193.511	602.778	599.574	**599.776**	599.372	P[26]
K[11]	219.130	583.367	580.163	580.365	579.962	K[25]
K[12]	244.749	557.748	**554.544**	**554.746**	**554.343**	K[24]
G[13]	256.153	532.129	528.925	529.127	528.724	G[23]
S[14]	273.560	520.725	517.521	517.723	517.320	S[22]
K[15]	299.179	**503.319**	500.115	**500.316**	499.913	K[21]
K[16]	324.798	477.700	474.496	474.697	474.294	K[20]
A[17]	339.005	452.081	448.877	449.078	448.675	A[19]
L[18]	361.622	437.873	434.669	434.871	434.468	L[18]
T[19]	381.831	415.256	412.053	412.254	411.851	T[17]
K[20]	407.450	**395.047**	391.843	392.045	391.641	K[16]
A[21]	421.658	369.428	366.224	366.426	366.022	A[15]
Q[22]	447.270	355.220	352.017	352.218	351.815	Q[14]
K[23]	**472.889**	329.609	**326.405**	**326.606**	326.203	K[13]
K[24]	498.508	303.990	300.786	300.987	300.584	K[12]
D[25]	521.513	278.371	275.167	275.368	274.965	D[11]
G[26]	532.917	255.365	252.161	252.363	251.960	G[10]
K[27]	558.536	243.961	240.757	240.959	240.556	K[9]
K[28]	584.155	**218.342**	215.138	215.340	214.937	K[8]
R[29]	**615.375**	192.723	189.519	189.721	189.318	R[7]
K[30]	**640.994**	161.503	158.299	158.501	158.097	K[6]
R[31]	**672.215**	135.884	132.680	132.882	132.478	R[5]
S[32]	**689.621**	104.664	101.460	101.661	101.258	S[4]
R[33]	720.841	87.257	84.053	84.255	83.852	R[3]
K[34]	**746.460**	56.037	52.833	53.035	52.632	K[2]
E[35]	**772.269**	30.418	27.214	27.416	27.013	E[1]

sp|Q64478|H2B1H_MOUSE

PEPAKSAPAPKKGSKKALTKAQKKDGKKRKRSRKE

- fragmentation table for charge state 6+
- specType=etd
- score=44.50
- F104938.dat
- query=q3092_p1
- precursor=552.051400
- chargePrecursor=7
- itol=0.8

AA	c	y	z+1	z+2	z	AA
P[1]	20.020	**643.889**	**641.219**	0.839	**641.051**	P[35]
E[2]	41.528	627.714	**625.044**	0.839	**624.876**	E[34]
P[3]	57.703	606.207	**603.537**	0.839	**603.369**	P[33]
A[4]	69.543	590.031	**587.361**	0.839	**587.193**	A[32]
K[5]	90.892	578.192	575.522	575.690	575.354	K[31]
S[6]	105.397	556.842	**554.173**	**554.341**	**554.005**	S[30]
A[7]	117.237	542.337	**539.667**	**539.835**	**539.499**	A[29]
P[8]	133.412	530.498	527.828	527.996	527.660	P[28]
A[9]	145.252	**514.322**	511.652	511.820	511.484	A[27]
P[10]	161.427	502.483	499.813	499.981	499.645	P[26]
K[11]	182.776	486.307	483.637	483.805	483.469	K[25]
K[12]	204.125	464.958	462.288	462.456	462.120	K[24]
G[13]	213.629	443.609	440.939	441.107	440.771	G[23]
S[14]	228.134	434.105	431.436	431.603	431.268	S[22]
K[15]	249.483	419.600	**416.930**	**417.098**	**416.762**	K[21]
K[16]	270.833	398.251	395.581	395.749	395.413	K[20]
A[17]	282.672	376.902	374.232	374.400	374.064	A[19]
L[18]	301.519	365.062	362.392	362.560	362.224	L[18]
T[19]	318.361	346.215	343.545	343.713	343.377	T[17]
K[20]	339.710	329.374	**326.704**	**326.872**	**326.536**	K[16]
A[21]	351.549	308.024	305.355	305.523	305.187	A[15]
Q[22]	372.893	296.185	293.515	293.683	293.347	Q[14]
K[23]	**394.242**	274.842	272.172	272.340	272.004	K[13]
K[24]	415.591	253.493	250.823	250.991	250.655	K[12]
D[25]	434.762	232.143	229.474	229.642	229.306	D[11]
G[26]	444.266	212.972	210.302	210.470	210.134	G[10]
K[27]	465.615	203.469	200.799	200.967	200.631	K[9]
K[28]	486.964	182.120	179.450	179.618	179.282	K[8]
R[29]	**512.981**	160.770	158.101	158.269	157.933	R[7]
K[30]	534.330	134.753	132.084	132.252	131.916	K[6]
R[31]	**560.347**	113.404	110.735	110.903	110.567	R[5]
S[32]	574.852	87.387	84.718	84.886	84.550	S[4]
R[33]	**600.869**	72.882	70.212	70.380	70.044	R[3]
K[34]	622.218	46.865	44.196	44.363	44.028	K[2]
E[35]	**643.725**	25.516	22.846	23.014	22.678	E[1]

sp|Q6ZWY9|H2B1C_MOUSE

PEPAKSAPAPKK $^{Trimethyl}_{42.05}$ GSKKAVTKAQKKDGKKRKRSRKE

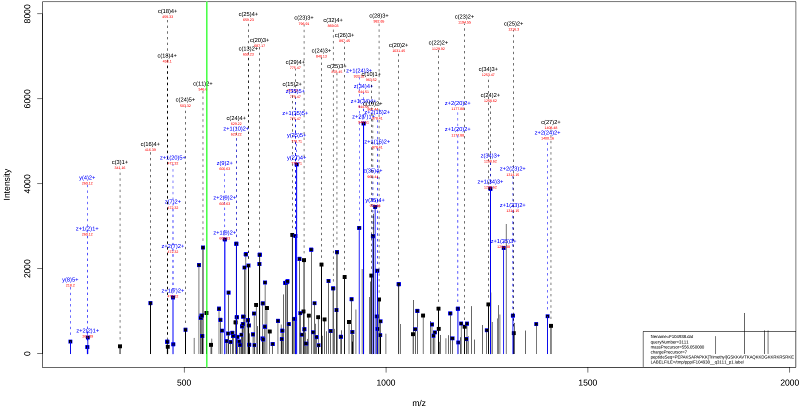

sp|Q6ZWY9|H2B1C_MOUSE

PEPAKSAPAPKK $^{Trimethyl}_{42.05}$ GSKKAVTKAQKKDGKKRKRSRKE

- fragmentation table for charge state 1+
- specType=etd
- score=63.22
- F104938.dat
- query=q3111_p1
- precursor=556.050080
- chargePrecursor=7
- itol=0.8

AA	c	y	z+1	z+2	z	AA
P[1]	115.087	3886.330	3870.311	0.000	3869.304	P[35]
E[2]	244.129	3789.277	3773.259	0.000	3772.251	E[34]
P[3]	**341.182**	3660.235	3644.216	0.000	3643.208	P[33]
A[4]	412.219	3563.182	3547.163	0.000	3546.155	A[32]
K[5]	**540.314**	3492.145	3476.126	3477.134	3475.118	K[31]
S[6]	**627.346**	3364.050	3348.031	3349.039	3347.023	S[30]
A[7]	698.383	3277.018	3260.999	3262.007	3259.991	A[29]
P[8]	**795.436**	3205.981	3189.962	3190.970	3188.954	P[28]
A[9]	866.473	3108.928	3092.909	3093.917	3091.901	A[27]
P[10]	**963.526**	3037.891	3021.872	3022.880	3020.864	P[26]
K[11]	**1091.621**	2940.838	2924.819	2925.827	2923.812	K[25]
K[12]	1261.763	2812.743	2796.724	2797.732	2795.717	K[24]
G[13]	1318.784	2642.601	2626.583	2627.590	2625.575	G[23]
S[14]	1405.816	2585.580	2569.561	2570.569	2568.553	S[22]
K[15]	1533.911	2498.548	2482.529	2483.537	2481.521	K[21]
K[16]	1662.006	2370.453	2354.434	2355.442	2353.426	K[20]
A[17]	1733.043	2242.358	2226.339	2227.347	2225.331	A[19]
V[18]	1832.112	2171.321	2155.302	2156.310	2154.294	V[18]
T[19]	1933.159	2072.252	2056.234	2057.241	2055.226	T[17]
K[20]	2061.254	1971.205	1955.186	1956.194	1954.178	K[16]
A[21]	2132.291	1843.110	1827.091	1828.099	1826.083	A[15]
Q[22]	2260.350	1772.073	1756.054	1757.062	1755.046	Q[14]
K[23]	2388.445	1644.014	1627.995	1629.003	1626.987	K[13]
K[24]	2516.540	1515.919	1499.900	1500.908	1498.892	K[12]
D[25]	2631.567	1387.824	**1371.805**	1372.813	1370.798	D[11]
G[26]	2688.588	1272.797	1256.778	1257.786	1255.771	G[10]
K[27]	2816.683	1215.776	**1199.757**	**1200.765**	**1198.749**	K[9]
K[28]	2944.778	1087.681	1071.662	**1072.670**	1070.654	K[8]
R[29]	3100.879	959.586	943.567	**944.575**	942.559	R[7]
K[30]	3228.974	803.485	**787.466**	788.474	**786.458**	K[6]
R[31]	3385.075	675.390	**659.371**	**660.379**	**658.363**	R[5]
S[32]	3472.107	519.289	**503.270**	504.278	502.262	S[4]
R[33]	3628.209	432.257	**416.238**	417.246	415.230	R[3]
K[34]	3756.304	276.155	**260.137**	**261.144**	259.129	K[2]
E[35]	3885.346	148.060	132.042	133.050	131.034	E[1]

sp|Q6ZWY9|H2B1C_MOUSE

PEPAKSAPAPKK $^{Trimethyl}_{42.05}$ GSKKAVTKAQKKDGKKRKRSRKE

- fragmentation table for charge state 2+
- specType=etd
- score=63.22
- F104938.dat
- query=q3111_p1
- precursor=556.050080
- chargePrecursor=7
- itol=0.8

AA	c	y	z+1	z+2	z	AA
P[1]	58.047	1943.669	1935.659	0.504	1935.155	P[35]
E[2]	122.568	1895.142	1887.133	0.504	1886.629	E[34]
P[3]	171.095	1830.621	1822.612	0.504	1822.108	P[33]
A[4]	206.613	1782.095	1774.085	0.504	1773.581	A[32]
K[5]	270.661	1746.576	1738.567	1739.071	1738.063	K[31]
S[6]	314.177	1682.529	1674.519	1675.023	1674.015	S[30]
A[7]	349.695	1639.013	1631.003	1631.507	1630.499	A[29]
P[8]	398.222	1603.494	1595.485	1595.989	1594.981	P[28]
A[9]	433.740	1554.968	1546.958	1547.462	1546.454	A[27]
P[10]	482.267	1519.449	1511.440	1511.944	1510.936	P[26]
K[11]	**546.314**	1470.923	1462.913	1463.417	1462.409	K[25]
K[12]	**631.385**	1406.875	1398.866	**1399.370**	1398.362	K[24]
G[13]	**659.896**	1321.804	**1313.795**	**1314.299**	1313.291	G[23]
S[14]	703.412	1293.294	1285.284	1285.788	1284.780	S[22]
K[15]	**767.459**	**1249.778**	1241.768	1242.272	1241.264	K[21]
K[16]	**831.507**	**1185.730**	**1177.721**	**1178.225**	**1177.217**	K[20]
A[17]	867.025	**1121.683**	**1113.673**	**1114.177**	**1113.169**	A[19]
V[18]	**916.559**	1086.164	**1078.155**	1078.659	**1077.651**	V[18]
T[19]	**967.083**	1036.630	1028.620	1029.124	1028.117	T[17]
K[20]	**1031.131**	**986.106**	**978.097**	**978.601**	977.593	K[16]
A[21]	**1066.649**	922.058	**914.049**	**914.553**	913.545	A[15]
Q[22]	**1130.679**	886.540	**878.531**	**879.034**	**878.027**	Q[14]
K[23]	**1194.726**	**822.511**	**814.501**	**815.005**	**813.997**	K[13]
K[24]	**1258.774**	**758.463**	**750.454**	**750.958**	**749.950**	K[12]
D[25]	**1316.287**	**694.416**	**686.406**	**686.910**	**685.902**	D[11]
G[26]	1344.798	**636.902**	**628.893**	**629.397**	628.389	G[10]
K[27]	**1408.845**	**608.391**	**600.382**	**600.886**	**599.878**	K[9]
K[28]	1472.893	**544.344**	**536.335**	**536.839**	**535.831**	K[8]
R[29]	1550.943	480.296	**472.287**	**472.791**	**471.783**	R[7]
K[30]	1614.991	402.246	394.237	394.740	393.733	K[6]
R[31]	1693.041	338.198	330.189	330.693	329.685	R[5]
S[32]	1736.557	**260.148**	252.139	252.642	251.635	S[4]
R[33]	1814.608	216.632	208.623	209.126	208.119	R[3]
K[34]	1878.655	138.581	130.572	131.076	130.068	K[2]
E[35]	1943.177	74.534	66.524	67.028	66.021	E[1]

sp|Q6ZWY9|H2B1C_MOUSE

PEPAKSAPAPKK $^{Trimethyl}_{42.05}$ GSKKAVTKAQKKDGKKRKRSRKE

- fragmentation table for charge state 3+
- specType=etd
- score=63.22
- F104938.dat
- query=q3111_p1
- precursor=556.050080
- chargePrecursor=7
- itol=0.8

AA	c	y	z+1	z+2	z	AA
P[1]	39.034	1296.115	**1290.775**	0.672	1290.439	P[35]
E[2]	82.048	1263.764	**1258.424**	0.672	**1258.088**	E[34]
P[3]	114.399	1220.750	1215.410	0.672	1215.074	P[33]
A[4]	138.078	1188.399	1183.059	0.672	1182.723	A[32]
K[5]	180.776	**1164.720**	**1159.380**	**1159.716**	**1159.044**	K[31]
S[6]	209.787	1122.021	**1116.682**	**1117.018**	1116.346	S[30]
A[7]	233.466	1093.011	1087.671	1088.007	1087.335	A[29]
P[8]	265.817	1069.332	1063.992	1064.328	1063.656	P[28]
A[9]	289.496	1036.981	**1031.641**	**1031.977**	**1031.305**	A[27]
P[10]	321.847	1013.302	1007.962	1008.298	1007.626	P[26]
K[11]	364.545	980.951	975.611	**975.947**	975.275	K[25]
K[12]	421.259	938.253	**932.913**	**933.249**	932.577	K[24]
G[13]	440.266	881.539	876.199	**876.535**	875.863	G[23]
S[14]	469.277	**862.531**	**857.192**	**857.528**	**856.856**	S[22]
K[15]	511.975	**833.521**	**828.181**	**828.517**	**827.845**	K[21]
K[16]	**554.674**	**790.822**	**785.483**	**785.819**	**785.147**	K[20]
A[17]	578.353	748.124	**742.785**	**743.120**	**742.449**	A[19]
V[18]	611.375	724.445	**719.106**	**719.441**	**718.770**	V[18]
T[19]	**645.058**	691.422	**686.083**	**686.419**	**685.747**	T[17]
K[20]	**687.756**	**657.740**	**652.400**	**652.736**	**652.064**	K[16]
A[21]	**711.435**	**615.041**	**609.702**	**610.038**	**609.366**	A[15]
Q[22]	**754.122**	591.362	**586.023**	**586.359**	**585.687**	Q[14]
K[23]	**796.820**	548.676	**543.337**	**543.673**	**543.001**	K[13]
K[24]	**839.518**	505.978	500.638	500.974	500.302	K[12]
D[25]	**877.860**	463.280	**457.940**	**458.276**	**457.604**	D[11]
G[26]	**896.868**	424.937	419.598	419.934	419.262	G[10]
K[27]	939.566	405.930	400.590	400.926	400.255	K[9]
K[28]	**982.264**	363.232	357.892	358.228	357.556	K[8]
R[29]	1034.298	320.533	315.194	315.530	314.858	R[7]
K[30]	**1076.996**	268.500	263.160	263.496	262.824	K[6]
R[31]	1129.030	225.801	220.462	220.798	220.126	R[5]
S[32]	1158.041	173.768	168.428	168.764	168.092	S[4]
R[33]	1210.074	144.757	139.417	139.753	139.082	R[3]
K[34]	**1252.773**	92.723	87.384	87.720	87.048	K[2]
E[35]	1295.787	50.025	44.685	45.021	44.349	E[1]

sp|Q6ZWY9|H2B1C_MOUSE

PEPAKSAPAPKK $^{\text{Trimethyl}}_{42.05}$ GSKKAVTKAQKKDGKKRKRSRKE

- fragmentation table for charge state 4+
- specType=etd
- score=63.22
- F104938.dat
- query=q3111_p1
- precursor=556.050080
- chargePrecursor=7
- itol=0.8

AA	c	y	z+1	z+2	z	AA
P[1]	29.527	**972.338**	968.333	0.755	**968.081**	P[35]
E[2]	61.788	948.075	**944.070**	0.755	**943.818**	E[34]
P[3]	86.051	**915.814**	911.809	0.755	911.558	P[33]
A[4]	103.810	891.551	**887.546**	0.755	**887.294**	A[32]
K[5]	135.834	873.792	**869.787**	870.039	**869.535**	K[31]
S[6]	157.592	841.768	837.763	838.015	837.511	S[30]
A[7]	175.351	**820.010**	816.005	816.257	815.753	A[29]
P[8]	199.614	**802.251**	**798.246**	**798.498**	**797.994**	P[28]
A[9]	217.374	**777.987**	**773.983**	**774.235**	**773.731**	A[27]
P[10]	241.637	760.228	**756.224**	**756.475**	**755.972**	P[26]
K[11]	273.661	735.965	**731.960**	**732.212**	**731.708**	K[25]
K[12]	316.196	703.941	**699.937**	**700.189**	**699.685**	K[24]
G[13]	330.451	**661.406**	**657.401**	**657.653**	**657.149**	G[23]
S[14]	352.210	647.150	**643.146**	**643.398**	**642.894**	S[22]
K[15]	384.233	625.392	**621.388**	**621.640**	**621.136**	K[21]
K[16]	**416.257**	**593.369**	**589.364**	**589.616**	**589.112**	K[20]
A[17]	434.016	561.345	557.340	557.592	557.088	A[19]
V[18]	**458.783**	**543.586**	**539.581**	**539.833**	539.329	V[18]
T[19]	484.045	518.819	514.814	515.066	514.562	T[17]
K[20]	516.069	493.557	489.552	489.804	489.300	K[16]
A[21]	533.828	461.533	**457.528**	**457.780**	457.276	A[15]
Q[22]	**565.843**	443.774	439.769	440.021	439.517	Q[14]
K[23]	597.867	411.759	407.754	408.006	407.502	K[13]
K[24]	**629.890**	379.735	375.731	375.982	375.479	K[12]
D[25]	**658.647**	347.711	343.707	343.959	343.455	D[11]
G[26]	**672.903**	318.955	314.950	315.202	314.698	G[10]
K[27]	**704.926**	304.699	300.695	300.947	300.443	K[9]
K[28]	736.950	272.676	268.671	268.923	268.419	K[8]
R[29]	**775.975**	240.652	236.647	236.899	236.395	R[7]
K[30]	**807.999**	201.627	197.622	197.874	197.370	K[6]
R[31]	**847.024**	169.603	165.598	165.850	165.346	R[5]
S[32]	**868.782**	130.578	126.573	126.825	126.321	S[4]
R[33]	**907.808**	108.820	104.815	105.067	104.563	R[3]
K[34]	939.831	69.794	65.790	66.042	65.538	K[2]
E[35]	972.092	37.771	33.766	34.018	33.514	E[1]

sp|Q6ZWY9|H2B1C_MOUSE

PEPAKSAPAPKK $^{Trimethyl}_{42.05}$ GSKKAVTKAQKKDGKKRKRSRKE

- fragmentation table for charge state 5+
- specType=etd
- score=63.22
- F104938.dat
- query=q3111_p1
- precursor=556.050080
- chargePrecursor=7
- itol=0.8

AA	c	y	z+1	z+2	z	AA
P[1]	23.823	**778.072**	**774.868**	0.806	**774.667**	P[35]
E[2]	49.632	**758.661**	**755.458**	0.806	**755.256**	E[34]
P[3]	69.042	**732.853**	729.649	0.806	729.447	P[33]
A[4]	83.250	713.442	710.238	0.806	710.037	A[32]
K[5]	108.869	**699.235**	**696.031**	**696.233**	**695.829**	K[31]
S[6]	126.275	**673.616**	**670.412**	**670.614**	**670.210**	S[30]
A[7]	140.482	656.209	**653.006**	**653.207**	**652.804**	A[29]
P[8]	159.893	**642.002**	**638.798**	**639.000**	**638.597**	P[28]
A[9]	174.100	622.591	**619.388**	**619.589**	**619.186**	A[27]
P[10]	193.511	**608.384**	605.180	605.382	**604.979**	P[26]
K[11]	219.130	**588.973**	**585.770**	**585.971**	**585.568**	K[25]
K[12]	253.158	563.354	560.151	560.352	559.949	K[24]
G[13]	264.563	529.326	526.122	526.324	525.921	G[23]
S[14]	281.969	517.922	514.718	514.920	514.516	S[22]
K[15]	307.588	500.515	497.312	497.513	497.110	K[21]
K[16]	333.207	474.896	**471.693**	**471.894**	471.491	K[20]
A[17]	347.414	449.277	446.074	446.275	445.872	A[19]
V[18]	367.228	435.070	431.866	432.068	431.665	V[18]
T[19]	387.438	415.256	412.053	412.254	411.851	T[17]
K[20]	413.057	395.047	391.843	392.045	391.641	K[16]
A[21]	427.264	369.428	366.224	366.426	366.022	A[15]
Q[22]	452.876	355.220	352.017	352.218	351.815	Q[14]
K[23]	478.495	329.609	326.405	326.606	326.203	K[13]
K[24]	**504.114**	303.990	300.786	300.987	300.584	K[12]
D[25]	527.119	278.371	275.167	275.368	274.965	D[11]
G[26]	538.523	255.365	252.161	252.363	251.960	G[10]
K[27]	564.142	243.961	240.757	240.959	240.556	K[9]
K[28]	**589.761**	**218.342**	215.138	215.340	214.937	K[8]
R[29]	**620.982**	192.723	189.519	189.721	189.318	R[7]
K[30]	**646.601**	161.503	158.299	158.501	158.097	K[6]
R[31]	**677.821**	135.884	132.680	132.882	132.478	R[5]
S[32]	**695.227**	104.664	101.460	101.661	101.258	S[4]
R[33]	726.448	87.257	84.053	84.255	83.852	R[3]
K[34]	752.067	56.037	52.833	53.035	52.632	K[2]
E[35]	777.875	30.418	27.214	27.416	27.013	E[1]

sp|Q6ZWY9|H2B1C_MOUSE

PEPAKSAPAPKK $^{\text{Trimethyl}}_{42.05}$ GSKKAVTKAQKKDGKKRKRSRKE

- fragmentation table for charge state 6+
- specType=etd
- score=63.22
- F104938.dat
- query=q3111_p1
- precursor=556.050080
- chargePrecursor=7
- itol=0.8

AA	c	y	z+1	z+2	z	AA
P[1]	20.020	**648.561**	**645.891**	0.839	**645.723**	P[35]
E[2]	41.528	**632.386**	**629.716**	0.839	**629.548**	E[34]
P[3]	57.703	610.879	**608.209**	0.839	**608.041**	P[33]
A[4]	69.543	594.703	592.033	0.839	591.865	A[32]
K[5]	90.892	582.864	580.194	580.362	580.026	K[31]
S[6]	105.397	561.514	558.845	559.013	558.677	S[30]
A[7]	117.237	**547.009**	**544.339**	**544.507**	**544.171**	A[29]
P[8]	133.412	535.170	532.500	532.668	532.332	P[28]
A[9]	145.252	518.994	516.324	516.492	516.156	A[27]
P[10]	161.427	507.155	504.485	504.653	504.317	P[26]
K[11]	182.776	490.979	488.309	488.477	488.141	K[25]
K[12]	211.133	469.630	466.960	467.128	466.792	K[24]
G[13]	220.637	441.273	438.603	438.771	438.435	G[23]
S[14]	235.142	431.769	429.100	429.268	428.932	S[22]
K[15]	256.491	417.264	414.594	414.762	414.426	K[21]
K[16]	277.840	395.915	393.245	393.413	393.077	K[20]
A[17]	289.680	374.566	371.896	372.064	371.728	A[19]
V[18]	306.191	362.726	360.056	360.224	359.888	V[18]
T[19]	323.033	346.215	343.545	343.713	343.377	T[17]
K[20]	344.382	329.374	326.704	326.872	326.536	K[16]
A[21]	356.221	308.024	305.355	305.523	305.187	A[15]
Q[22]	377.564	296.185	293.515	293.683	293.347	Q[14]
K[23]	398.914	274.842	272.172	272.340	272.004	K[13]
K[24]	420.263	253.493	250.823	250.991	250.655	K[12]
D[25]	439.434	232.143	229.474	229.642	229.306	D[11]
G[26]	448.937	212.972	210.302	210.470	210.134	G[10]
K[27]	470.287	203.469	200.799	200.967	200.631	K[9]
K[28]	491.636	182.120	179.450	179.618	179.282	K[8]
R[29]	517.653	160.770	158.101	158.269	157.933	R[7]
K[30]	539.002	134.753	132.084	132.252	131.916	K[6]
R[31]	565.019	113.404	110.735	110.903	110.567	R[5]
S[32]	579.524	87.387	84.718	84.886	84.550	S[4]
R[33]	605.541	72.882	70.212	70.380	70.044	R[3]
K[34]	**626.890**	46.865	44.196	44.363	44.028	K[2]
E[35]	648.397	25.516	22.846	23.014	22.678	E[1]

sp|Q6ZWY9|H2B1C_MOUSE

PEPAKSAPAPKK Trimethyl 42.05 GSKKAVTKAQKKDGKKRKRSRKE

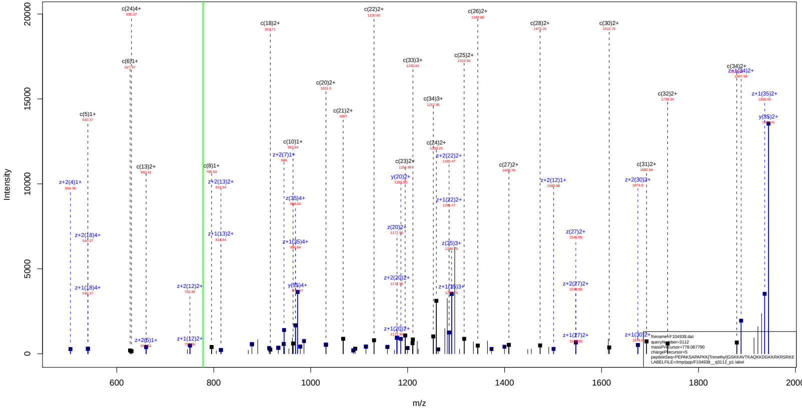

sp|Q6ZWY9|H2B1C_MOUSE

PEPAKSAPAPKK $^{\text{Trimethyl}}_{42.05}$ GSKKAVTKAQKKDGKKRKRSRKE

- fragmentation table for charge state 1+
- specType=etd
- score=75.30
- F104938.dat
- query=q3112_p1
- precursor=778.067790
- chargePrecursor=5
- itol=0.8

AA	c	y	z+1	z+2	z	AA
P[1]	115.087	3886.330	3870.311	0.000	3869.304	P[35]
E[2]	244.129	3789.277	3773.259	0.000	3772.251	E[34]
P[3]	341.182	3660.235	3644.216	0.000	3643.208	P[33]
A[4]	412.219	3563.182	3547.163	0.000	3546.155	A[32]
K[5]	**540.314**	3492.145	3476.126	3477.134	3475.118	K[31]
S[6]	**627.346**	3364.050	3348.031	3349.039	3347.023	S[30]
A[7]	698.383	3277.018	3260.999	3262.007	3259.991	A[29]
P[8]	**795.436**	3205.981	3189.962	3190.970	3188.954	P[28]
A[9]	866.473	3108.928	3092.909	3093.917	3091.901	A[27]
P[10]	**963.526**	3037.891	3021.872	3022.880	3020.864	P[26]
K[11]	**1091.621**	2940.838	2924.819	2925.827	2923.812	K[25]
K[12]	1261.763	2812.743	2796.724	2797.732	2795.717	K[24]
G[13]	1318.784	2642.601	2626.583	2627.590	2625.575	G[23]
S[14]	1405.816	2585.580	2569.561	2570.569	2568.553	S[22]
K[15]	1533.911	2498.548	2482.529	2483.537	2481.521	K[21]
K[16]	1662.006	2370.453	2354.434	2355.442	2353.426	K[20]
A[17]	1733.043	2242.358	2226.339	2227.347	2225.331	A[19]
V[18]	1832.112	2171.321	2155.302	2156.310	2154.294	V[18]
T[19]	1933.159	2072.252	2056.234	2057.241	2055.226	T[17]
K[20]	2061.254	1971.205	1955.186	1956.194	1954.178	K[16]
A[21]	2132.291	1843.110	1827.091	1828.099	1826.083	A[15]
Q[22]	2260.350	1772.073	1756.054	1757.062	1755.046	Q[14]
K[23]	2388.445	1644.014	1627.995	1629.003	1626.987	K[13]
K[24]	2516.540	1515.919	1499.900	**1500.908**	1498.892	K[12]
D[25]	2631.567	1387.824	1371.805	**1372.813**	1370.798	D[11]
G[26]	2688.588	1272.797	1256.778	1257.786	1255.771	G[10]
K[27]	2816.683	1215.776	**1199.757**	1200.765	1198.749	K[9]
K[28]	2944.778	**1087.681**	1071.662	1072.670	1070.654	K[8]
R[29]	3100.879	959.586	**943.567**	**944.575**	942.559	R[7]
K[30]	3228.974	803.485	787.466	788.474	786.458	K[6]
R[31]	3385.075	675.390	659.371	**660.379**	658.363	R[5]
S[32]	3472.107	519.289	503.270	**504.278**	502.262	S[4]
R[33]	3628.209	432.257	416.238	417.246	415.230	R[3]
K[34]	3756.304	276.155	260.137	261.144	259.129	K[2]
E[35]	3885.346	148.060	132.042	133.050	131.034	E[1]

sp|Q6ZWY9|H2B1C_MOUSE

PEPAKSAPAPKK $^{\text{Trimethyl}}_{42.05}$ GSKKAVTKAQKKDGKKRKRSRKE

- fragmentation table for charge state 2+
- specType=etd
- score=75.30
- F104938.dat
- query=q3112_p1
- precursor=778.067790
- chargePrecursor=5
- itol=0.8

AA	c	y	z+1	z+2	z	AA
P[1]	58.047	**1943.669**	**1935.659**	0.504	1935.155	P[35]
E[2]	122.568	1895.142	**1887.133**	0.504	1886.629	E[34]
P[3]	171.095	1830.621	1822.612	0.504	1822.108	P[33]
A[4]	206.613	1782.095	1774.085	0.504	1773.581	A[32]
K[5]	270.661	1746.576	1738.567	1739.071	1738.063	K[31]
S[6]	314.177	1682.529	**1674.519**	**1675.023**	1674.015	S[30]
A[7]	349.695	1639.013	1631.003	1631.507	1630.499	A[29]
P[8]	398.222	1603.494	1595.485	1595.989	1594.981	P[28]
A[9]	433.740	1554.968	**1546.958**	**1547.462**	**1546.454**	A[27]
P[10]	482.267	1519.449	1511.440	1511.944	1510.936	P[26]
K[11]	546.314	1470.923	1462.913	1463.417	1462.409	K[25]
K[12]	631.385	1406.875	1398.866	**1399.370**	1398.362	K[24]
G[13]	**659.896**	1321.804	1313.795	1314.299	1313.291	G[23]
S[14]	703.412	1293.294	**1285.284**	**1285.788**	**1284.780**	S[22]
K[15]	767.459	1249.778	1241.768	1242.272	1241.264	K[21]
K[16]	831.507	**1185.730**	**1177.721**	**1178.225**	**1177.217**	K[20]
A[17]	867.025	1121.683	**1113.673**	**1114.177**	**1113.169**	A[19]
V[18]	**916.559**	1086.164	1078.155	1078.659	1077.651	V[18]
T[19]	967.083	1036.630	1028.620	1029.124	1028.117	T[17]
K[20]	**1031.131**	**986.106**	**978.097**	**978.601**	977.593	K[16]
A[21]	**1066.649**	922.058	**914.049**	**914.553**	913.545	A[15]
Q[22]	**1130.679**	886.540	**878.531**	**879.034**	**878.027**	Q[14]
K[23]	**1194.726**	822.511	**814.501**	**815.005**	813.997	K[13]
K[24]	**1258.774**	758.463	**750.454**	**750.958**	749.950	K[12]
D[25]	**1316.287**	694.416	686.406	686.910	685.902	D[11]
G[26]	**1344.798**	636.902	628.893	629.397	628.389	G[10]
K[27]	**1408.845**	608.391	600.382	600.886	599.878	K[9]
K[28]	**1472.893**	544.344	536.335	536.839	535.831	K[8]
R[29]	1550.943	480.296	472.287	472.791	471.783	R[7]
K[30]	**1614.991**	402.246	394.237	394.740	393.733	K[6]
R[31]	**1693.041**	338.198	330.189	330.693	329.685	R[5]
S[32]	**1736.557**	260.148	252.139	252.642	251.635	S[4]
R[33]	1814.608	216.632	208.623	209.126	208.119	R[3]
K[34]	**1878.655**	138.581	130.572	131.076	130.068	K[2]
E[35]	1943.177	74.534	66.524	67.028	66.021	E[1]

sp|Q6ZWY9|H2B1C_MOUSE

PEPAKSAPAPKK $^{\text{Trimethyl}}_{42.05}$ GSKKAVTKAQKKDGKKRKRSRKE

- fragmentation table for charge state 3+
- specType=etd
- score=75.30
- F104938.dat
- query=q3112_p1
- precursor=778.067790
- chargePrecursor=5
- itol=0.8

AA	c	y	z+1	z+2	z	AA
P[1]	39.034	1296.115	**1290.775**	0.672	**1290.439**	P[35]
E[2]	82.048	**1263.764**	1258.424	0.672	1258.088	E[34]
P[3]	114.399	1220.750	1215.410	0.672	1215.074	P[33]
A[4]	138.078	1188.399	1183.059	0.672	1182.723	A[32]
K[5]	180.776	1164.720	1159.380	1159.716	**1159.044**	K[31]
S[6]	209.787	1122.021	1116.682	1117.018	1116.346	S[30]
A[7]	233.466	1093.011	**1087.671**	**1088.007**	**1087.335**	A[29]
P[8]	265.817	1069.332	1063.992	1064.328	1063.656	P[28]
A[9]	289.496	1036.981	**1031.641**	**1031.977**	**1031.305**	A[27]
P[10]	321.847	1013.302	1007.962	1008.298	1007.626	P[26]
K[11]	364.545	980.951	975.611	975.947	975.275	K[25]
K[12]	421.259	938.253	**932.913**	**933.249**	**932.577**	K[24]
G[13]	440.266	881.539	876.199	876.535	875.863	G[23]
S[14]	469.277	862.531	857.192	857.528	856.856	S[22]
K[15]	511.975	833.521	828.181	828.517	827.845	K[21]
K[16]	554.674	790.822	785.483	785.819	785.147	K[20]
A[17]	578.353	748.124	742.785	743.120	742.449	A[19]
V[18]	611.375	724.445	719.106	719.441	718.770	V[18]
T[19]	645.058	691.422	686.083	686.419	685.747	T[17]
K[20]	687.756	657.740	652.400	652.736	652.064	K[16]
A[21]	711.435	615.041	609.702	610.038	609.366	A[15]
Q[22]	754.122	591.362	586.023	586.359	585.687	Q[14]
K[23]	796.820	548.676	543.337	543.673	543.001	K[13]
K[24]	839.518	505.978	500.638	500.974	500.302	K[12]
D[25]	877.860	463.280	457.940	458.276	457.604	D[11]
G[26]	896.868	424.937	419.598	419.934	419.262	G[10]
K[27]	939.566	405.930	400.590	400.926	400.255	K[9]
K[28]	982.264	363.232	357.892	358.228	357.556	K[8]
R[29]	1034.298	320.533	315.194	315.530	314.858	R[7]
K[30]	1076.996	268.500	263.160	263.496	262.824	K[6]
R[31]	1129.030	225.801	220.462	220.798	220.126	R[5]
S[32]	**1158.041**	173.768	168.428	168.764	168.092	S[4]
R[33]	**1210.074**	144.757	139.417	139.753	139.082	R[3]
K[34]	**1252.773**	92.723	87.384	87.720	87.048	K[2]
E[35]	1295.787	50.025	44.685	45.021	44.349	E[1]

sp|Q6ZWY9|H2B1C_MOUSE

PEPAKSAPAPKK $^{\text{Trimethyl}}_{42.05}$ GSKKAVTKAQKKDGKKRKRSRKE

- fragmentation table for charge state 4+
- specType=etd
- score=75.30
- F104938.dat
- query=q3112_p1
- precursor=778.067790
- chargePrecursor=5
- itol=0.8

AA	c	y	z+1	z+2	z	AA
P[1]	29.527	**972.338**	**968.333**	0.755	**968.081**	P[35]
E[2]	61.788	948.075	**944.070**	0.755	**943.818**	E[34]
P[3]	86.051	915.814	911.809	0.755	911.558	P[33]
A[4]	103.810	891.551	887.546	0.755	887.294	A[32]
K[5]	135.834	873.792	869.787	870.039	869.535	K[31]
S[6]	157.592	841.768	837.763	838.015	837.511	S[30]
A[7]	175.351	820.010	816.005	816.257	815.753	A[29]
P[8]	199.614	802.251	798.246	798.498	797.994	P[28]
A[9]	217.374	777.987	773.983	774.235	773.731	A[27]
P[10]	241.637	760.228	756.224	756.475	755.972	P[26]
K[11]	273.661	735.965	731.960	732.212	731.708	K[25]
K[12]	316.196	703.941	699.937	700.189	699.685	K[24]
G[13]	330.451	661.406	657.401	657.653	657.149	G[23]
S[14]	352.210	647.150	643.146	643.398	642.894	S[22]
K[15]	384.233	625.392	621.388	621.640	621.136	K[21]
K[16]	416.257	593.369	589.364	589.616	589.112	K[20]
A[17]	434.016	561.345	557.340	557.592	557.088	A[19]
V[18]	458.783	543.586	**539.581**	**539.833**	539.329	V[18]
T[19]	484.045	518.819	514.814	515.066	514.562	T[17]
K[20]	516.069	493.557	489.552	489.804	489.300	K[16]
A[21]	533.828	461.533	457.528	457.780	457.276	A[15]
Q[22]	565.843	443.774	439.769	440.021	439.517	Q[14]
K[23]	597.867	411.759	407.754	408.006	407.502	K[13]
K[24]	**629.890**	379.735	375.731	375.982	375.479	K[12]
D[25]	658.647	347.711	343.707	343.959	343.455	D[11]
G[26]	672.903	318.955	314.950	315.202	314.698	G[10]
K[27]	704.926	304.699	300.695	300.947	300.443	K[9]
K[28]	736.950	272.676	268.671	268.923	268.419	K[8]
R[29]	775.975	240.652	236.647	236.899	236.395	R[7]
K[30]	807.999	201.627	197.622	197.874	197.370	K[6]
R[31]	847.024	169.603	165.598	165.850	165.346	R[5]
S[32]	868.782	130.578	126.573	126.825	126.321	S[4]
R[33]	907.808	108.820	104.815	105.067	104.563	R[3]
K[34]	939.831	69.794	65.790	66.042	65.538	K[2]
E[35]	972.092	37.771	33.766	34.018	33.514	E[1]

sp|Q6GSS7|H2A2A_MOUSE

SGR Trimethyl 42.05 GKQGGKARAKAKSRSSRAGLQFPVGRVHRLLRKGNYAE

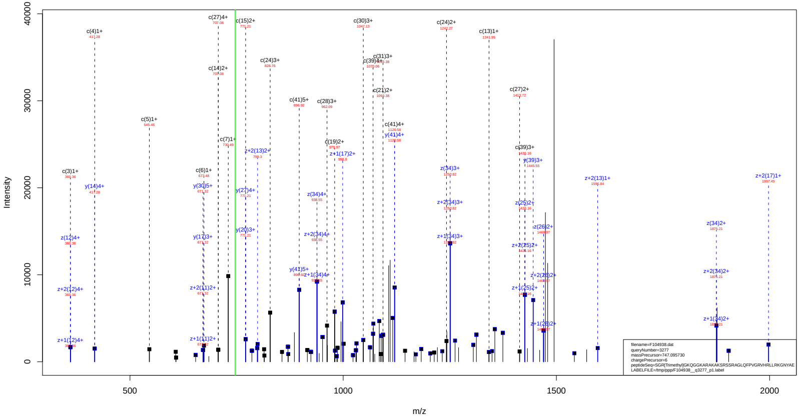

sp|Q6GSS7|H2A2A_MOUSE

SGR $^{Trimethyl}_{42.05}$ GKQGGKARAKAKSRSSRAGLQFPVGRVHRLLRKGNYAE

- fragmentation table for charge state 1+
- specType=etd
- score=48.34
- F104938.dat
- query=q3277_p1
- precursor=747.095730
- chargePrecursor=6
- itol=0.8

AA	c	y	z+1	z+2	z	AA
S[1]	105.066	4477.546	4461.528	0.000	4460.520	S[41]
G[2]	162.087	4390.514	4374.496	0.000	4373.488	G[40]
R[3]	**360.235**	4333.493	4317.474	4318.482	4316.466	R[39]
G[4]	**417.257**	4135.345	4119.326	4120.334	4118.318	G[38]
K[5]	**545.352**	4078.323	4062.305	4063.313	4061.297	K[37]
Q[6]	**673.410**	3950.228	3934.210	3935.218	3933.202	Q[36]
G[7]	**730.432**	3822.170	3806.151	3807.159	3805.143	G[35]
G[8]	787.453	3765.148	3749.130	3750.138	3748.122	G[34]
K[9]	**915.548**	3708.127	3692.108	3693.116	3691.100	K[33]
A[10]	**986.585**	3580.032	3564.013	3565.021	3563.005	A[32]
R[11]	1142.686	3508.995	3492.976	3493.984	3491.968	R[31]
A[12]	**1213.724**	3352.894	3336.875	3337.883	3335.867	A[30]
K[13]	**1341.819**	3281.857	3265.838	3266.846	3264.830	K[29]
A[14]	1412.856	3153.762	3137.743	3138.751	3136.735	A[28]
K[15]	1540.951	3082.725	3066.706	3067.714	3065.698	K[27]
S[16]	1627.983	2954.630	2938.611	2939.619	2937.603	S[26]
R[17]	1784.084	2867.598	2851.579	2852.587	2850.571	R[25]
S[18]	1871.116	2711.496	2695.478	2696.486	2694.470	S[24]
S[19]	1958.148	2624.464	2608.446	2609.454	2607.438	S[23]
R[20]	2114.249	2537.432	2521.414	2522.422	2520.406	R[22]
A[21]	2185.286	2381.331	2365.313	2366.320	2364.305	A[21]
G[22]	2242.308	2310.294	2294.275	2295.283	2293.268	G[20]
L[23]	2355.392	2253.273	2237.254	2238.262	2236.246	L[19]
Q[24]	2483.450	2140.189	2124.170	2125.178	2123.162	Q[18]
F[25]	2630.519	2012.130	1996.111	**1997.119**	1995.104	F[17]
P[26]	2727.571	1865.062	1849.043	1850.051	1848.035	P[16]
V[27]	2826.640	1768.009	1751.990	1752.998	1750.982	V[15]
G[28]	2883.661	1668.940	1652.922	1653.930	1651.914	G[14]
R[29]	3039.762	1611.919	1595.900	**1596.908**	1594.892	R[13]
V[30]	3138.831	1455.818	1439.799	1440.807	1438.791	V[12]
H[31]	3275.890	1356.749	1340.731	**1341.739**	1339.723	H[11]
R[32]	3431.991	1219.691	**1203.672**	**1204.680**	1202.664	R[10]
L[33]	3545.075	**1063.589**	**1047.571**	1048.579	**1046.563**	L[9]
L[34]	3658.159	950.505	934.487	935.495	933.479	L[8]
R[35]	3814.260	837.421	821.403	822.410	820.395	R[7]
K[36]	3942.355	681.320	665.302	666.309	664.294	K[6]
G[37]	3999.376	553.225	537.207	538.214	536.199	G[5]
N[38]	4113.419	496.204	480.185	481.193	479.177	N[4]
Y[39]	4276.483	382.161	366.142	367.150	365.134	Y[3]
A[40]	4347.520	219.098	203.079	204.087	202.071	A[2]
E[41]	4476.562	148.060	132.042	133.050	131.034	E[1]

sp|Q6GSS7|H2A2A_MOUSE

SGR $^{\text{Trimethyl}}_{42.05}$ GKQGGKARAKAKSRSSRAGLQFPVGRVHRLLRKGNYAE

- fragmentation table for charge state 2+
- specType=etd
- score=48.34
- F104938.dat
- query=q3277_p1
- precursor=747.095730
- chargePrecursor=6
- itol=0.8

AA	c	y	z+1	z+2	z	AA
S[1]	53.037	2239.277	2231.267	0.504	2230.764	S[41]
G[2]	81.547	2195.761	2187.751	0.504	2187.248	G[40]
R[3]	180.621	2167.250	2159.241	2159.745	2158.737	R[39]
G[4]	209.132	2068.176	2060.167	2060.671	2059.663	G[38]
K[5]	273.180	2039.665	2031.656	2032.160	2031.152	K[37]
Q[6]	337.209	1975.618	1967.608	1968.112	1967.105	Q[36]
G[7]	365.720	1911.589	**1903.579**	**1904.083**	1903.075	G[35]
G[8]	394.230	1883.078	**1875.068**	**1875.572**	**1874.565**	G[34]
K[9]	458.278	1854.567	1846.558	1847.062	1846.054	K[33]
A[10]	493.796	1790.520	1782.510	1783.014	1782.006	A[32]
R[11]	571.847	1755.001	1746.992	1747.496	1746.488	R[31]
A[12]	**607.365**	1676.951	1668.941	1669.445	1668.437	A[30]
K[13]	**671.413**	1641.432	1633.423	1633.927	1632.919	K[29]
A[14]	**706.931**	1577.384	1569.375	1569.879	1568.871	A[28]
K[15]	**770.979**	**1541.866**	1533.857	1534.360	1533.353	K[27]
S[16]	**814.495**	1477.818	**1469.809**	**1470.313**	**1469.305**	S[26]
R[17]	892.546	1434.302	**1426.293**	**1426.797**	**1425.789**	R[25]
S[18]	936.062	**1356.252**	1348.243	**1348.746**	1347.739	S[24]
S[19]	**979.578**	**1312.736**	**1304.726**	**1305.230**	1304.223	S[23]
R[20]	1057.628	1269.220	1261.210	**1261.714**	1260.707	R[22]
A[21]	**1093.147**	1191.169	**1183.160**	**1183.664**	**1182.656**	A[21]
G[22]	1121.657	1155.651	1147.641	1148.145	1147.137	G[20]
L[23]	1178.199	1127.140	1119.131	1119.635	1118.627	L[19]
Q[24]	**1242.229**	**1070.598**	**1062.589**	**1063.093**	1062.085	Q[18]
F[25]	1315.763	1006.569	**998.559**	**999.063**	**998.055**	F[17]
P[26]	1364.289	933.034	**925.025**	925.529	**924.521**	P[16]
V[27]	**1413.824**	884.508	876.499	877.003	875.995	V[15]
G[28]	1442.334	834.974	826.965	827.468	826.461	G[14]
R[29]	1520.385	806.463	**798.454**	**798.958**	**797.950**	R[13]
V[30]	1569.919	728.413	720.403	720.907	719.899	V[12]
H[31]	1638.448	678.878	**670.869**	**671.373**	670.365	H[11]
R[32]	1716.499	610.349	602.340	602.843	601.836	R[10]
L[33]	1773.041	532.298	524.289	524.793	523.785	L[9]
L[34]	1829.583	475.756	467.747	468.251	467.243	L[8]
R[35]	1907.634	419.214	411.205	411.709	410.701	R[7]
K[36]	1971.681	341.164	333.154	333.658	332.650	K[6]
G[37]	2000.192	277.116	269.107	269.611	268.603	G[5]
N[38]	2057.213	248.606	240.596	241.100	240.092	N[4]
Y[39]	2138.745	191.584	183.575	184.079	183.071	Y[3]
A[40]	2174.264	110.052	102.043	102.547	101.539	A[2]
E[41]	2238.785	74.534	66.524	67.028	66.021	E[1]

sp|Q6GSS7|H2A2A_MOUSE

SGR $^{Trimethyl}_{42.05}$ GKQGGKARAKAKSRSSRAGLQFPVGRVHRLLRKGNYAE

- fragmentation table for charge state 3+
- specType=etd
- score=48.34
- F104938.dat
- query=q3277_p1
- precursor=747.095730
- chargePrecursor=6
- itol=0.8

AA	c	y	z+1	z+2	z	AA
S[1]	35.693	1493.187	1487.847	0.672	1487.511	S[41]
G[2]	54.701	1464.176	1458.837	0.672	1458.501	G[40]
R[3]	120.750	**1445.169**	1439.830	1440.166	1439.494	R[39]
G[4]	139.757	1379.120	**1373.780**	**1374.116**	1373.444	G[38]
K[5]	182.455	1360.113	1354.773	**1355.109**	1354.437	K[37]
Q[6]	225.142	1317.414	**1312.075**	**1312.411**	**1311.739**	Q[36]
G[7]	244.149	1274.728	1269.389	1269.725	1269.053	G[35]
G[8]	263.156	1255.721	**1250.381**	**1250.717**	**1250.045**	G[34]
K[9]	305.854	1236.714	1231.374	**1231.710**	1231.038	K[33]
A[10]	329.533	1194.016	1188.676	1189.012	1188.340	A[32]
R[11]	381.567	**1170.336**	1164.997	1165.333	1164.661	R[31]
A[12]	405.246	1118.303	1112.963	1113.299	1112.627	A[30]
K[13]	447.944	1094.624	**1089.284**	**1089.620**	**1088.948**	K[29]
A[14]	471.623	1051.925	**1046.586**	**1046.922**	1046.250	A[28]
K[15]	514.322	1028.246	**1022.907**	**1023.243**	**1022.571**	K[27]
S[16]	543.332	**985.548**	**980.208**	**980.544**	**979.873**	S[26]
R[17]	595.366	956.537	**951.198**	**951.534**	**950.862**	R[25]
S[18]	624.377	904.504	899.164	899.500	898.828	S[24]
S[19]	**653.387**	875.493	**870.153**	**870.489**	**869.817**	S[23]
R[20]	705.421	846.482	841.143	841.479	840.807	R[22]
A[21]	729.100	794.449	789.109	789.445	788.773	A[21]
G[22]	748.107	**770.770**	765.430	765.766	765.094	G[20]
L[23]	**785.802**	751.762	746.423	746.759	746.087	L[19]
Q[24]	**828.488**	714.068	708.728	709.064	708.392	Q[18]
F[25]	877.511	**671.382**	666.042	666.378	665.706	F[17]
P[26]	909.862	622.359	617.019	617.355	616.683	P[16]
V[27]	942.885	590.008	584.668	585.004	584.332	V[15]
G[28]	**961.892**	556.985	551.645	551.981	551.309	G[14]
R[29]	1013.926	537.978	532.638	532.974	532.302	R[13]
V[30]	**1046.948**	485.944	480.605	480.941	480.269	V[12]
H[31]	**1092.635**	452.921	447.582	447.918	447.246	H[11]
R[32]	**1144.668**	407.235	401.895	402.231	401.560	R[10]
L[33]	**1182.363**	355.201	349.862	350.198	349.526	L[9]
L[34]	1220.058	317.507	312.167	312.503	311.831	L[8]
R[35]	1272.092	279.812	274.472	274.808	274.136	R[7]
K[36]	1314.790	227.778	222.439	222.775	222.103	K[6]
G[37]	1333.797	185.080	179.740	180.076	179.404	G[5]
N[38]	1371.811	166.073	160.733	161.069	160.397	N[4]
Y[39]	**1426.166**	128.058	122.719	123.055	122.383	Y[3]
A[40]	1449.845	73.704	68.364	68.700	68.029	A[2]
E[41]	1492.859	50.025	44.685	45.021	44.349	E[1]

sp|Q6GSS7|H2A2A_MOUSE

SGR $^{\text{Trimethyl}}_{42.05}$ GKQGGKARAKAKSRSSRAGLQFPVGRVHRLLRKGNYAE

- fragmentation table for charge state 4+
- specType=etd
- score=48.34
- F104938.dat
- query=q3277_p1
- precursor=747.095730
- chargePrecursor=6
- itol=0.8

AA	c	y	z+1	z+2	z	AA
S[1]	27.022	**1120.142**	**1116.137**	0.755	**1115.885**	S[41]
G[2]	41.277	1098.384	1094.379	0.755	**1094.127**	G[40]
R[3]	90.814	**1084.129**	1080.124	1080.376	1079.872	R[39]
G[4]	105.070	1034.592	**1030.587**	**1030.839**	**1030.335**	G[38]
K[5]	137.093	1020.336	1016.332	1016.584	1016.080	K[37]
Q[6]	169.108	988.313	**984.308**	**984.560**	984.056	Q[36]
G[7]	183.363	956.298	**952.293**	952.545	**952.041**	G[35]
G[8]	197.619	942.043	**938.038**	**938.290**	**937.786**	G[34]
K[9]	229.643	927.787	**923.783**	**924.034**	**923.531**	K[33]
A[10]	247.402	895.763	891.759	892.011	891.507	A[32]
R[11]	286.427	878.004	873.999	874.251	873.748	R[31]
A[12]	304.186	838.979	834.974	835.226	834.722	A[30]
K[13]	336.210	821.220	817.215	817.467	816.963	K[29]
A[14]	353.969	789.196	**785.191**	**785.443**	784.939	A[28]
K[15]	385.993	**771.437**	767.432	767.684	767.180	K[27]
S[16]	407.751	739.413	735.408	735.660	735.156	S[26]
R[17]	446.776	717.655	713.650	713.902	713.398	R[25]
S[18]	468.534	678.630	674.625	674.877	674.373	S[24]
S[19]	490.292	656.872	652.867	653.119	652.615	S[23]
R[20]	529.318	635.114	631.109	631.361	630.857	R[22]
A[21]	547.077	596.088	592.084	592.336	591.832	A[21]
G[22]	561.332	578.329	574.324	574.576	574.072	G[20]
L[23]	589.603	564.074	560.069	560.321	559.817	L[19]
Q[24]	621.618	535.803	531.798	532.050	531.546	Q[18]
F[25]	658.385	503.788	499.783	500.035	499.531	F[17]
P[26]	682.648	467.021	463.016	463.268	462.764	P[16]
V[27]	**707.415**	442.758	438.753	439.005	438.501	V[15]
G[28]	721.671	**417.991**	413.986	414.238	413.734	G[14]
R[29]	760.696	403.735	399.731	399.982	399.479	R[13]
V[30]	**785.463**	364.710	**360.705**	**360.957**	**360.453**	V[12]
H[31]	819.728	339.943	335.938	336.190	335.686	H[11]
R[32]	858.753	305.678	301.673	301.925	301.421	R[10]
L[33]	887.024	266.653	262.648	262.900	262.396	L[9]
L[34]	**915.295**	238.382	234.377	234.629	234.125	L[8]
R[35]	954.320	210.111	206.106	206.358	205.854	R[7]
K[36]	**986.344**	171.086	167.081	167.333	166.829	K[6]
G[37]	**1000.600**	139.062	135.057	135.309	134.805	G[5]
N[38]	**1029.110**	124.806	120.802	121.054	120.550	N[4]
Y[39]	**1069.876**	96.296	92.291	92.543	92.039	Y[3]
A[40]	**1087.635**	55.530	51.525	51.777	51.273	A[2]
E[41]	**1119.896**	37.771	33.766	34.018	33.514	E[1]

sp|Q6GSS7|H2A2A_MOUSE

SGR $^{\text{Trimethyl}}_{42.05}$ GKQGGKARAKAKSRSSRAGLQFPVGRVHRLLRKGNYAE

- fragmentation table for charge state 5+
- specType=etd
- score=48.34
- F104938.dat
- query=q3277_p1
- precursor=747.095730
- chargePrecursor=6
- itol=0.8

AA	c	y	z+1	z+2	z	AA
S[1]	21.819	**896.315**	893.111	0.806	892.910	S[41]
G[2]	33.223	878.909	875.705	0.806	875.503	G[40]
R[3]	72.853	867.504	864.301	864.502	864.099	R[39]
G[4]	84.257	827.875	824.671	824.873	824.469	G[38]
K[5]	109.876	816.471	813.267	813.468	813.065	K[37]
Q[6]	135.488	790.852	787.648	787.849	787.446	Q[36]
G[7]	146.892	765.240	762.036	762.238	761.834	G[35]
G[8]	158.296	753.836	750.632	750.833	750.430	G[34]
K[9]	183.915	742.431	739.227	739.429	739.026	K[33]
A[10]	198.123	716.812	713.608	713.810	713.407	A[32]
R[11]	229.343	702.605	699.401	699.603	699.199	R[31]
A[12]	243.551	**671.385**	668.181	668.382	667.979	A[30]
K[13]	269.170	657.177	**653.973**	**654.175**	**653.772**	K[29]
A[14]	283.377	631.558	628.354	628.556	628.153	A[28]
K[15]	308.996	617.351	614.147	614.349	613.945	K[27]
S[16]	326.402	591.732	588.528	588.730	588.326	S[26]
R[17]	357.623	574.325	571.122	571.323	570.920	R[25]
S[18]	375.029	543.105	539.901	540.103	539.700	S[24]
S[19]	392.435	525.699	522.495	522.697	522.293	S[23]
R[20]	423.656	508.292	505.089	505.290	504.887	R[22]
A[21]	437.863	477.072	473.868	474.070	473.667	A[21]
G[22]	449.267	462.865	459.661	459.862	459.459	G[20]
L[23]	471.884	451.460	448.257	448.458	448.055	L[19]
Q[24]	497.496	428.844	425.640	425.841	425.438	Q[18]
F[25]	526.910	403.232	400.028	400.230	399.827	F[17]
P[26]	546.320	373.818	370.614	370.816	370.413	P[16]
V[27]	566.134	354.408	351.204	351.405	351.002	V[15]
G[28]	577.538	334.594	331.390	331.592	331.189	G[14]
R[29]	**608.758**	323.190	319.986	320.187	319.784	R[13]
V[30]	628.572	291.969	288.766	288.967	288.564	V[12]
H[31]	655.984	272.156	268.952	269.154	268.750	H[11]
R[32]	687.204	244.744	241.540	241.742	241.339	R[10]
L[33]	709.821	213.524	210.320	210.522	210.118	L[9]
L[34]	732.438	190.907	187.703	187.905	187.502	L[8]
R[35]	763.658	168.290	165.086	165.288	164.885	R[7]
K[36]	789.277	137.070	133.866	134.068	133.665	K[6]
G[37]	800.681	111.451	108.247	108.449	108.046	G[5]
N[38]	823.490	100.047	96.843	97.044	96.641	N[4]
Y[39]	**856.102**	77.238	74.034	74.236	73.833	Y[3]
A[40]	**870.310**	44.625	41.422	41.623	41.220	A[2]
E[41]	**896.118**	30.418	27.214	27.416	27.013	E[1]

sp|Q6GSS7|H2A2A_MOUSE

SGR $^{\text{Trimethyl}}_{42.05}$ GKQGGKARAKAKSRSSRAGLQFPVGRVHRLLRKGNYAE

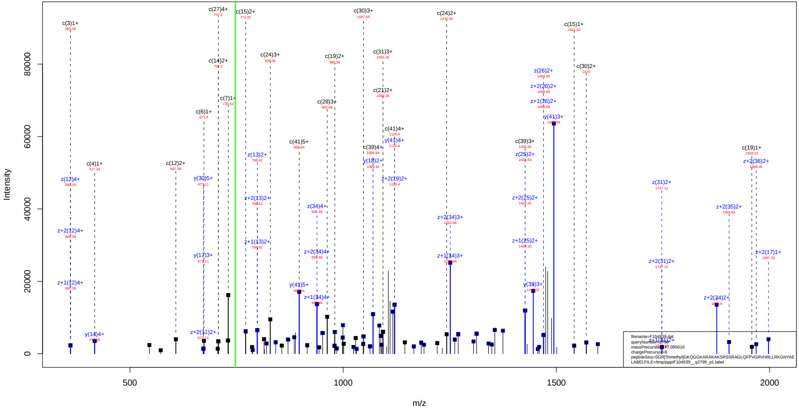

sp|Q6GSS7|H2A2A_MOUSE

SGR $^{\text{Trimethyl}}_{42.05}$ GKQGGKARAKAKSRSSRAGLQFPVGRVHRLLRKGNYAE

- fragmentation table for charge state 1+
- specType=etd
- score=64.46
- F104939.dat
- query=q2799_p1
- precursor=747.095010
- chargePrecursor=6
- itol=0.8

AA	c	y	z+1	z+2	z	AA
S[1]	105.066	4477.546	4461.528	0.000	4460.520	S[41]
G[2]	162.087	4390.514	4374.496	0.000	4373.488	G[40]
R[3]	**360.235**	4333.493	4317.474	4318.482	4316.466	R[39]
G[4]	**417.257**	4135.345	4119.326	4120.334	4118.318	G[38]
K[5]	**545.352**	4078.323	4062.305	4063.313	4061.297	K[37]
Q[6]	**673.410**	3950.228	3934.210	3935.218	3933.202	Q[36]
G[7]	**730.432**	3822.170	3806.151	3807.159	3805.143	G[35]
G[8]	**787.453**	3765.148	3749.130	3750.138	3748.122	G[34]
K[9]	**915.548**	3708.127	3692.108	3693.116	3691.100	K[33]
A[10]	986.585	3580.032	3564.013	3565.021	3563.005	A[32]
R[11]	1142.686	3508.995	3492.976	3493.984	3491.968	R[31]
A[12]	1213.724	3352.894	3336.875	3337.883	3335.867	A[30]
K[13]	1341.819	3281.857	3265.838	3266.846	3264.830	K[29]
A[14]	1412.856	3153.762	3137.743	3138.751	3136.735	A[28]
K[15]	**1540.951**	3082.725	3066.706	3067.714	3065.698	K[27]
S[16]	1627.983	2954.630	2938.611	2939.619	2937.603	S[26]
R[17]	1784.084	2867.598	2851.579	2852.587	2850.571	R[25]
S[18]	1871.116	2711.496	2695.478	2696.486	2694.470	S[24]
S[19]	**1958.148**	2624.464	2608.446	2609.454	2607.438	S[23]
R[20]	2114.249	2537.432	2521.414	2522.422	2520.406	R[22]
A[21]	2185.286	2381.331	2365.313	2366.320	2364.305	A[21]
G[22]	2242.308	2310.294	2294.275	2295.283	2293.268	G[20]
L[23]	2355.392	2253.273	2237.254	2238.262	2236.246	L[19]
Q[24]	2483.450	2140.189	2124.170	2125.178	2123.162	Q[18]
F[25]	2630.519	2012.130	1996.111	**1997.119**	1995.104	F[17]
P[26]	2727.571	1865.062	1849.043	1850.051	1848.035	P[16]
V[27]	2826.640	1768.009	1751.990	1752.998	1750.982	V[15]
G[28]	2883.661	1668.940	1652.922	1653.930	1651.914	G[14]
R[29]	3039.762	1611.919	1595.900	**1596.908**	1594.892	R[13]
V[30]	3138.831	**1455.818**	1439.799	1440.807	1438.791	V[12]
H[31]	3275.890	1356.749	**1340.731**	1341.739	1339.723	H[11]
R[32]	3431.991	1219.691	1203.672	1204.680	1202.664	R[10]
L[33]	3545.075	**1063.589**	**1047.571**	1048.579	**1046.563**	L[9]
L[34]	3658.159	950.505	934.487	935.495	933.479	L[8]
R[35]	3814.260	837.421	821.403	822.410	**820.395**	R[7]
K[36]	3942.355	681.320	665.302	666.309	664.294	K[6]
G[37]	3999.376	553.225	537.207	538.214	536.199	G[5]
N[38]	4113.419	496.204	480.185	481.193	479.177	N[4]
Y[39]	4276.483	382.161	366.142	367.150	365.134	Y[3]
A[40]	4347.520	219.098	203.079	204.087	202.071	A[2]
E[41]	4476.562	148.060	132.042	133.050	131.034	E[1]

sp|Q6GSS7|H2A2A_MOUSE

SGR $^{\text{Trimethyl}}_{42.05}$ GKQGGKARAKAKSRSSRAGLQFPVGRVHRLLRKGNYAE

- fragmentation table for charge state 2+
- specType=etd
- score=64.46
- F104939.dat
- query=q2799_p1
- precursor=747.095010
- chargePrecursor=6
- itol=0.8

AA	c	y	z+1	z+2	z	AA
S[1]	53.037	2239.277	2231.267	0.504	2230.764	S[41]
G[2]	81.547	2195.761	2187.751	0.504	2187.248	G[40]
R[3]	180.621	2167.250	2159.241	2159.745	2158.737	R[39]
G[4]	209.132	2068.176	2060.167	2060.671	2059.663	G[38]
K[5]	273.180	2039.665	2031.656	2032.160	2031.152	K[37]
Q[6]	337.209	1975.618	1967.608	**1968.112**	1967.105	Q[36]
G[7]	365.720	1911.589	1903.579	**1904.083**	1903.075	G[35]
G[8]	394.230	1883.078	1875.068	**1875.572**	1874.565	G[34]
K[9]	458.278	1854.567	1846.558	1847.062	1846.054	K[33]
A[10]	493.796	1790.520	1782.510	1783.014	1782.006	A[32]
R[11]	**571.847**	1755.001	**1746.992**	**1747.496**	**1746.488**	R[31]
A[12]	**607.365**	1676.951	1668.941	1669.445	1668.437	A[30]
K[13]	**671.413**	1641.432	1633.423	1633.927	1632.919	K[29]
A[14]	**706.931**	1577.384	**1569.375**	**1569.879**	1568.871	A[28]
K[15]	**770.979**	**1541.866**	1533.857	1534.360	1533.353	K[27]
S[16]	**814.495**	1477.818	**1469.809**	**1470.313**	**1469.305**	S[26]
R[17]	892.546	1434.302	**1426.293**	**1426.797**	**1425.789**	R[25]
S[18]	936.062	**1356.252**	**1348.243**	**1348.746**	**1347.739**	S[24]
S[19]	**979.578**	**1312.736**	1304.726	**1305.230**	1304.223	S[23]
R[20]	1057.628	**1269.220**	**1261.210**	**1261.714**	1260.707	R[22]
A[21]	**1093.147**	1191.169	**1183.160**	**1183.664**	**1182.656**	A[21]
G[22]	1121.657	1155.651	1147.641	1148.145	1147.137	G[20]
L[23]	1178.199	1127.140	1119.131	**1119.635**	1118.627	L[19]
Q[24]	**1242.229**	**1070.598**	**1062.589**	**1063.093**	1062.085	Q[18]
F[25]	1315.763	1006.569	**998.559**	**999.063**	**998.055**	F[17]
P[26]	1364.289	933.034	925.025	925.529	924.521	P[16]
V[27]	1413.824	**884.508**	876.499	877.003	875.995	V[15]
G[28]	1442.334	834.974	826.965	827.468	826.461	G[14]
R[29]	1520.385	806.463	**798.454**	**798.958**	**797.950**	R[13]
V[30]	**1569.919**	728.413	720.403	720.907	719.899	V[12]
H[31]	1638.448	678.878	670.869	**671.373**	670.365	H[11]
R[32]	1716.499	610.349	602.340	602.843	601.836	R[10]
L[33]	1773.041	532.298	524.289	524.793	523.785	L[9]
L[34]	1829.583	475.756	467.747	468.251	467.243	L[8]
R[35]	1907.634	419.214	411.205	411.709	410.701	R[7]
K[36]	1971.681	341.164	333.154	333.658	332.650	K[6]
G[37]	2000.192	277.116	269.107	269.611	268.603	G[5]
N[38]	2057.213	248.606	240.596	241.100	240.092	N[4]
Y[39]	2138.745	191.584	183.575	184.079	183.071	Y[3]
A[40]	2174.264	110.052	102.043	102.547	101.539	A[2]
E[41]	2238.785	74.534	66.524	67.028	66.021	E[1]

sp|Q6GSS7|H2A2A_MOUSE

SGR $^{Trimethyl}_{42.05}$ GKQGGKARAKAKSRSSRAGLQFPVGRVHRLLRKGNYAE

- fragmentation table for charge state 3+
- specType=etd
- score=64.46
- F104939.dat
- query=q2799_p1
- precursor=747.095010
- chargePrecursor=6
- itol=0.8

AA	c	y	z+1	z+2	z	AA
S[1]	35.693	**1493.187**	1487.847	0.672	1487.511	S[41]
G[2]	54.701	1464.176	**1458.837**	0.672	1458.501	G[40]
R[3]	120.750	**1445.169**	1439.830	1440.166	1439.494	R[39]
G[4]	139.757	1379.120	1373.780	**1374.116**	1373.444	G[38]
K[5]	182.455	1360.113	**1354.773**	**1355.109**	1354.437	K[37]
Q[6]	225.142	1317.414	**1312.075**	**1312.411**	1311.739	Q[36]
G[7]	244.149	1274.728	**1269.389**	**1269.725**	**1269.053**	G[35]
G[8]	263.156	1255.721	**1250.381**	**1250.717**	1250.045	G[34]
K[9]	305.854	1236.714	1231.374	1231.710	1231.038	K[33]
A[10]	329.533	1194.016	1188.676	**1189.012**	1188.340	A[32]
R[11]	381.567	1170.336	**1164.997**	**1165.333**	1164.661	R[31]
A[12]	405.246	1118.303	1112.963	1113.299	1112.627	A[30]
K[13]	447.944	1094.624	1089.284	**1089.620**	**1088.948**	K[29]
A[14]	471.623	1051.925	**1046.586**	**1046.922**	**1046.250**	A[28]
K[15]	514.322	1028.246	1022.907	**1023.243**	1022.571	K[27]
S[16]	543.332	**985.548**	**980.208**	**980.544**	**979.873**	S[26]
R[17]	595.366	956.537	**951.198**	**951.534**	**950.862**	R[25]
S[18]	624.377	904.504	899.164	899.500	898.828	S[24]
S[19]	653.387	875.493	**870.153**	**870.489**	869.817	S[23]
R[20]	**705.421**	846.482	**841.143**	**841.479**	840.807	R[22]
A[21]	**729.100**	794.449	789.109	789.445	788.773	A[21]
G[22]	748.107	**770.770**	765.430	765.766	765.094	G[20]
L[23]	**785.802**	751.762	746.423	746.759	746.087	L[19]
Q[24]	**828.488**	714.068	708.728	709.064	708.392	Q[18]
F[25]	877.511	**671.382**	666.042	666.378	665.706	F[17]
P[26]	909.862	622.359	617.019	617.355	616.683	P[16]
V[27]	**942.885**	590.008	584.668	585.004	584.332	V[15]
G[28]	**961.892**	556.985	551.645	551.981	551.309	G[14]
R[29]	1013.926	537.978	532.638	532.974	532.302	R[13]
V[30]	**1046.948**	485.944	480.605	480.941	480.269	V[12]
H[31]	**1092.635**	452.921	447.582	447.918	447.246	H[11]
R[32]	**1144.668**	407.235	401.895	402.231	401.560	R[10]
L[33]	**1182.363**	355.201	349.862	350.198	349.526	L[9]
L[34]	**1220.058**	317.507	312.167	312.503	311.831	L[8]
R[35]	1272.092	279.812	274.472	274.808	274.136	R[7]
K[36]	1314.790	227.778	222.439	222.775	222.103	K[6]
G[37]	1333.797	185.080	179.740	180.076	179.404	G[5]
N[38]	1371.811	166.073	160.733	161.069	160.397	N[4]
Y[39]	**1426.166**	128.058	122.719	123.055	122.383	Y[3]
A[40]	1449.845	73.704	68.364	68.700	68.029	A[2]
E[41]	1492.859	50.025	44.685	45.021	44.349	E[1]

sp|Q6GSS7|H2A2A_MOUSE

SGR $^{\text{Trimethyl}}_{42.05}$ GKQGGKARAKAKSRSSRAGLQFPVGRVHRLLRKGNYAE

- fragmentation table for charge state 4+
- specType=etd
- score=64.46
- F104939.dat
- query=q2799_p1
- precursor=747.095010
- chargePrecursor=6
- itol=0.8

AA	c	y	z+1	z+2	z	AA
S[1]	27.022	**1120.142**	**1116.137**	0.755	**1115.885**	S[41]
G[2]	41.277	1098.384	1094.379	0.755	1094.127	G[40]
R[3]	90.814	**1084.129**	1080.124	1080.376	1079.872	R[39]
G[4]	105.070	1034.592	**1030.587**	**1030.839**	1030.335	G[38]
K[5]	137.093	1020.336	1016.332	1016.584	1016.080	K[37]
Q[6]	169.108	988.313	**984.308**	**984.560**	984.056	Q[36]
G[7]	183.363	956.298	**952.293**	952.545	**952.041**	G[35]
G[8]	197.619	942.043	**938.038**	**938.290**	**937.786**	G[34]
K[9]	229.643	927.787	923.783	924.034	923.531	K[33]
A[10]	247.402	895.763	891.759	892.011	891.507	A[32]
R[11]	286.427	878.004	873.999	874.251	873.748	R[31]
A[12]	304.186	838.979	834.974	835.226	834.722	A[30]
K[13]	336.210	821.220	817.215	817.467	816.963	K[29]
A[14]	353.969	789.196	785.191	785.443	784.939	A[28]
K[15]	385.993	**771.437**	767.432	767.684	767.180	K[27]
S[16]	407.751	739.413	735.408	735.660	735.156	S[26]
R[17]	446.776	717.655	713.650	713.902	713.398	R[25]
S[18]	468.534	678.630	674.625	674.877	674.373	S[24]
S[19]	490.292	656.872	652.867	653.119	652.615	S[23]
R[20]	529.318	635.114	631.109	631.361	630.857	R[22]
A[21]	547.077	596.088	592.084	592.336	591.832	A[21]
G[22]	561.332	578.329	574.324	574.576	574.072	G[20]
L[23]	589.603	564.074	560.069	560.321	559.817	L[19]
Q[24]	621.618	535.803	531.798	532.050	531.546	Q[18]
F[25]	658.385	503.788	499.783	500.035	499.531	F[17]
P[26]	682.648	467.021	463.016	463.268	462.764	P[16]
V[27]	**707.415**	442.758	438.753	439.005	438.501	V[15]
G[28]	721.671	**417.991**	413.986	414.238	413.734	G[14]
R[29]	760.696	403.735	399.731	399.982	399.479	R[13]
V[30]	785.463	364.710	**360.705**	**360.957**	**360.453**	V[12]
H[31]	**819.728**	339.943	335.938	336.190	335.686	H[11]
R[32]	858.753	305.678	301.673	301.925	301.421	R[10]
L[33]	887.024	266.653	262.648	262.900	262.396	L[9]
L[34]	**915.295**	238.382	234.377	234.629	234.125	L[8]
R[35]	954.320	210.111	206.106	206.358	205.854	R[7]
K[36]	986.344	171.086	167.081	167.333	166.829	K[6]
G[37]	**1000.600**	139.062	135.057	135.309	134.805	G[5]
N[38]	**1029.110**	124.806	120.802	121.054	120.550	N[4]
Y[39]	**1069.876**	96.296	92.291	92.543	92.039	Y[3]
A[40]	**1087.635**	55.530	51.525	51.777	51.273	A[2]
E[41]	**1119.896**	37.771	33.766	34.018	33.514	E[1]

sp|Q6GSS7|H2A2A_MOUSE

SGR $^{\text{Trimethyl}}_{42.05}$ GKQGGKARAKAKSRSSRAGLQFPVGRVHRLLRKGNYAE

- fragmentation table for charge state 5+
- specType=etd
- score=64.46
- F104939.dat
- query=q2799_p1
- precursor=747.095010
- chargePrecursor=6
- itol=0.8

AA	c	y	z+1	z+2	z	AA
S[1]	21.819	**896.315**	893.111	0.806	892.910	S[41]
G[2]	33.223	878.909	875.705	0.806	875.503	G[40]
R[3]	72.853	867.504	864.301	864.502	864.099	R[39]
G[4]	84.257	827.875	824.671	824.873	824.469	G[38]
K[5]	109.876	816.471	813.267	813.468	813.065	K[37]
Q[6]	135.488	790.852	**787.648**	**787.849**	**787.446**	Q[36]
G[7]	146.892	765.240	762.036	762.238	761.834	G[35]
G[8]	158.296	753.836	750.632	750.833	750.430	G[34]
K[9]	183.915	742.431	739.227	739.429	739.026	K[33]
A[10]	198.123	716.812	713.608	713.810	713.407	A[32]
R[11]	229.343	702.605	699.401	699.603	699.199	R[31]
A[12]	243.551	**671.385**	668.181	668.382	667.979	A[30]
K[13]	269.170	657.177	653.973	654.175	653.772	K[29]
A[14]	283.377	631.558	628.354	628.556	628.153	A[28]
K[15]	308.996	617.351	614.147	614.349	613.945	K[27]
S[16]	326.402	591.732	588.528	588.730	588.326	S[26]
R[17]	357.623	574.325	571.122	571.323	570.920	R[25]
S[18]	375.029	543.105	539.901	540.103	539.700	S[24]
S[19]	392.435	525.699	522.495	522.697	522.293	S[23]
R[20]	423.656	508.292	505.089	505.290	504.887	R[22]
A[21]	437.863	477.072	473.868	474.070	473.667	A[21]
G[22]	449.267	462.865	459.661	459.862	459.459	G[20]
L[23]	471.884	451.460	448.257	448.458	448.055	L[19]
Q[24]	497.496	428.844	425.640	425.841	425.438	Q[18]
F[25]	526.910	403.232	400.028	400.230	399.827	F[17]
P[26]	546.320	373.818	370.614	370.816	370.413	P[16]
V[27]	566.134	354.408	351.204	351.405	351.002	V[15]
G[28]	577.538	334.594	331.390	331.592	331.189	G[14]
R[29]	608.758	323.190	319.986	320.187	319.784	R[13]
V[30]	628.572	291.969	288.766	288.967	288.564	V[12]
H[31]	655.984	272.156	268.952	269.154	268.750	H[11]
R[32]	687.204	244.744	241.540	241.742	241.339	R[10]
L[33]	709.821	213.524	210.320	210.522	210.118	L[9]
L[34]	732.438	190.907	187.703	187.905	187.502	L[8]
R[35]	763.658	168.290	165.086	165.288	164.885	R[7]
K[36]	789.277	137.070	133.866	134.068	133.665	K[6]
G[37]	800.681	111.451	108.247	108.449	108.046	G[5]
N[38]	823.490	100.047	96.843	97.044	96.641	N[4]
Y[39]	**856.102**	77.238	74.034	74.236	73.833	Y[3]
A[40]	**870.310**	44.625	41.422	41.623	41.220	A[2]
E[41]	**896.118**	30.418	27.214	27.416	27.013	E[1]

sp|Q8BFU2|H2A3_MOUSE

SGR $^{\text{Trimethyl}}_{42.05}$ GKQGGKARAKAKSRSSRAGLQFPVGRVHRLLRKGNYSE

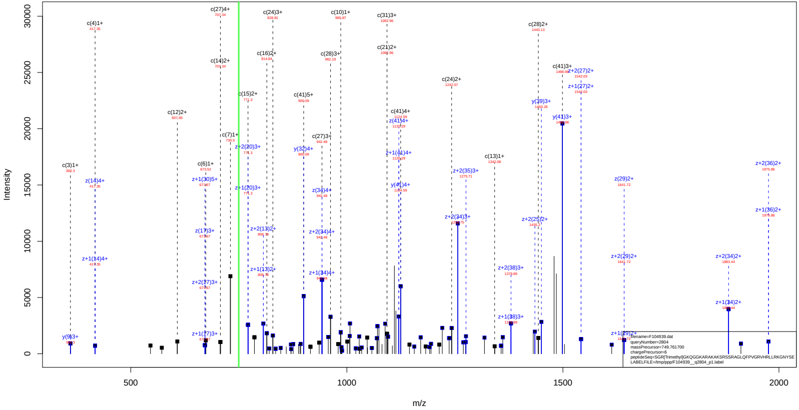

sp|Q8BFU2|H2A3_MOUSE

SGR $^{Trimethyl}_{42.05}$ GKQGGKARAKAKSRSSRAGLQFPVGRVHRLLRKGNYSE

- fragmentation table for charge state 1+
- specType=etd
- score=50.34
- F104939.dat
- query=q2804_p1
- precursor=749.761700
- chargePrecursor=6
- itol=0.8

AA	c	y	z+1	z+2	z	AA
S[1]	105.066	4493.541	4477.523	0.000	4476.515	S[41]
G[2]	162.087	4406.509	4390.491	0.000	4389.483	G[40]
R[3]	**360.235**	4349.488	4333.469	4334.477	4332.461	R[39]
G[4]	**417.257**	4151.340	4135.321	4136.329	4134.313	G[38]
K[5]	**545.352**	4094.318	4078.300	4079.307	4077.292	K[37]
Q[6]	**673.410**	3966.223	3950.205	3951.212	3949.197	Q[36]
G[7]	**730.432**	3838.165	3822.146	3823.154	3821.138	G[35]
G[8]	787.453	3781.143	3765.125	3766.132	3764.117	G[34]
K[9]	**915.548**	3724.122	3708.103	3709.111	3707.095	K[33]
A[10]	**986.585**	3596.027	3580.008	3581.016	3579.000	A[32]
R[11]	1142.686	3524.990	3508.971	3509.979	3507.963	R[31]
A[12]	**1213.724**	3368.889	3352.870	3353.878	3351.862	A[30]
K[13]	**1341.819**	3297.852	3281.833	3282.841	3280.825	K[29]
A[14]	1412.856	3169.757	3153.738	3154.746	3152.730	A[28]
K[15]	1540.951	3098.719	3082.701	3083.709	3081.693	K[27]
S[16]	1627.983	2970.625	2954.606	2955.614	2953.598	S[26]
R[17]	1784.084	2883.592	2867.574	2868.582	2866.566	R[25]
S[18]	1871.116	2727.491	2711.473	2712.480	2710.465	S[24]
S[19]	1958.148	2640.459	2624.441	2625.448	2623.433	S[23]
R[20]	2114.249	2553.427	2537.409	2538.416	2536.401	R[22]
A[21]	2185.286	2397.326	2381.307	2382.315	2380.300	A[21]
G[22]	2242.308	2326.289	2310.270	2311.278	2309.263	G[20]
L[23]	2355.392	2269.268	2253.249	2254.257	2252.241	L[19]
Q[24]	2483.450	2156.184	2140.165	2141.173	2139.157	Q[18]
F[25]	2630.519	2028.125	2012.106	2013.114	2011.098	F[17]
P[26]	2727.571	1881.057	1865.038	1866.046	1864.030	P[16]
V[27]	2826.640	1784.004	1767.985	1768.993	1766.977	V[15]
G[28]	2883.661	1684.935	1668.917	1669.924	1667.909	G[14]
R[29]	3039.762	1627.914	1611.895	**1612.903**	1610.887	R[13]
V[30]	3138.831	1471.813	1455.794	1456.802	1454.786	V[12]
H[31]	3275.890	1372.744	**1356.726**	**1357.734**	1355.718	H[11]
R[32]	3431.991	1235.685	1219.667	**1220.675**	1218.659	R[10]
L[33]	3545.075	1079.584	1063.566	1064.573	1062.558	L[9]
L[34]	3658.159	966.500	950.482	951.489	949.474	L[8]
R[35]	3814.260	853.416	837.398	838.405	836.390	R[7]
K[36]	3942.355	697.315	681.296	682.304	680.289	K[6]
G[37]	3999.376	569.220	553.201	554.209	552.194	G[5]
N[38]	4113.419	512.199	496.180	497.188	495.172	N[4]
Y[39]	4276.483	398.156	382.137	383.145	381.129	Y[3]
S[40]	4363.515	235.092	219.074	220.082	218.066	S[2]
E[41]	4492.557	148.060	132.042	133.050	131.034	E[1]

sp|Q8BFU2|H2A3_MOUSE

SGR $^{\text{Trimethyl}}_{42.05}$ GKQGGKARAKAKSRSSRAGLQFPVGRVHRLLRKGNYSE

- fragmentation table for charge state 2+
- specType=etd
- score=50.34
- F104939.dat
- query=q2804_p1
- precursor=749.761700
- chargePrecursor=6
- itol=0.8

AA	c	y	z+1	z+2	z	AA
S[1]	53.037	2247.274	2239.265	0.504	2238.761	S[41]
G[2]	81.547	2203.758	2195.749	0.504	2195.245	G[40]
R[3]	180.621	2175.248	2167.238	2167.742	2166.734	R[39]
G[4]	209.132	2076.174	2068.164	2068.668	2067.660	G[38]
K[5]	273.180	2047.663	2039.653	2040.157	2039.150	K[37]
Q[6]	337.209	1983.615	**1975.606**	**1976.110**	**1975.102**	Q[36]
G[7]	365.720	1919.586	**1911.577**	**1912.081**	1911.073	G[35]
G[8]	394.230	1891.075	**1883.066**	**1883.570**	1882.562	G[34]
K[9]	458.278	1862.565	1854.555	1855.059	1854.051	K[33]
A[10]	493.796	1798.517	1790.508	1791.012	1790.004	A[32]
R[11]	**571.847**	1762.999	1754.989	1755.493	1754.485	R[31]
A[12]	**607.365**	1684.948	1676.939	1677.443	1676.435	A[30]
K[13]	**671.413**	1649.429	**1641.420**	**1641.924**	**1640.916**	K[29]
A[14]	**706.931**	1585.382	1577.373	1577.876	1576.869	A[28]
K[15]	**770.979**	1549.863	**1541.854**	**1542.358**	**1541.350**	K[27]
S[16]	**814.495**	1485.816	1477.807	1478.310	1477.303	S[26]
R[17]	**892.546**	1442.300	1434.291	**1434.794**	1433.787	R[25]
S[18]	**936.062**	1364.249	**1356.240**	**1356.744**	1355.736	S[24]
S[19]	**979.578**	1320.733	1312.724	1313.228	1312.220	S[23]
R[20]	**1057.628**	1277.217	**1269.208**	**1269.712**	1268.704	R[22]
A[21]	**1093.147**	1199.167	**1191.157**	**1191.661**	1190.653	A[21]
G[22]	1121.657	1163.648	**1155.639**	**1156.143**	1155.135	G[20]
L[23]	1178.199	1135.137	1127.128	1127.632	1126.624	L[19]
Q[24]	**1242.229**	1078.595	**1070.586**	**1071.090**	**1070.082**	Q[18]
F[25]	1315.763	1014.566	**1006.557**	**1007.061**	**1006.053**	F[17]
P[26]	1364.289	941.032	933.023	933.526	932.519	P[16]
V[27]	1413.824	**892.506**	884.496	885.000	883.992	V[15]
G[28]	**1442.334**	842.971	**834.962**	**835.466**	**834.458**	G[14]
R[29]	1520.385	**814.461**	**806.451**	**806.955**	805.947	R[13]
V[30]	1569.919	736.410	728.401	728.905	727.897	V[12]
H[31]	1638.448	686.876	678.866	679.370	678.363	H[11]
R[32]	1716.499	618.346	610.337	610.841	609.833	R[10]
L[33]	1773.041	540.296	532.286	532.790	531.783	L[9]
L[34]	1829.583	483.754	475.744	476.248	475.241	L[8]
R[35]	1907.634	427.212	419.202	419.706	418.698	R[7]
K[36]	1971.681	349.161	341.152	341.656	340.648	K[6]
G[37]	2000.192	285.114	277.104	277.608	276.600	G[5]
N[38]	2057.213	256.603	248.594	249.098	248.090	N[4]
Y[39]	2138.745	199.582	191.572	192.076	191.068	Y[3]
S[40]	2182.261	118.050	110.041	110.544	109.537	S[2]
E[41]	2246.782	74.534	66.524	67.028	66.021	E[1]

sp|Q8BFU2|H2A3_MOUSE

SGR $^{\text{Trimethyl}}_{42.05}$ GKQGGKARAKAKSRSSRAGLQFPVGRVHRLLRKGNYSE

- fragmentation table for charge state 3+
- specType=etd
- score=50.34
- F104939.dat
- query=q2804_p1
- precursor=749.761700
- chargePrecursor=6
- itol=0.8

AA	c	y	z+1	z+2	z	AA
S[1]	35.693	**1498.519**	1493.179	0.672	1492.843	S[41]
G[2]	54.701	1469.508	1464.168	0.672	1463.832	G[40]
R[3]	120.750	**1450.501**	1445.161	1445.497	1444.825	R[39]
G[4]	139.757	1384.451	**1379.112**	**1379.448**	1378.776	G[38]
K[5]	182.455	1365.444	**1360.105**	**1360.441**	1359.769	K[37]
Q[6]	225.142	1322.746	1317.406	**1317.742**	1317.070	Q[36]
G[7]	244.149	1280.060	**1274.720**	**1275.056**	**1274.384**	G[35]
G[8]	263.156	1261.053	1255.713	**1256.049**	1255.377	G[34]
K[9]	305.854	**1242.045**	**1236.706**	**1237.042**	**1236.370**	K[33]
A[10]	329.533	1199.347	**1194.008**	**1194.344**	1193.672	A[32]
R[11]	381.567	1175.668	**1170.329**	**1170.664**	1169.993	R[31]
A[12]	405.246	1123.634	1118.295	1118.631	1117.959	A[30]
K[13]	447.944	1099.955	**1094.616**	**1094.952**	1094.280	K[29]
A[14]	471.623	**1057.257**	1051.917	1052.253	1051.582	A[28]
K[15]	514.322	1033.578	**1028.238**	**1028.574**	**1027.902**	K[27]
S[16]	543.332	990.880	**985.540**	**985.876**	**985.204**	S[26]
R[17]	595.366	**961.869**	**956.529**	**956.865**	956.193	R[25]
S[18]	624.377	909.835	904.496	904.832	904.160	S[24]
S[19]	653.387	880.825	**875.485**	**875.821**	**875.149**	S[23]
R[20]	705.421	851.814	**846.474**	**846.810**	**846.138**	R[22]
A[21]	729.100	799.780	794.441	794.777	794.105	A[21]
G[22]	748.107	776.101	**770.762**	**771.098**	770.426	G[20]
L[23]	**785.802**	757.094	751.754	752.090	751.419	L[19]
Q[24]	**828.488**	719.399	714.060	714.396	713.724	Q[18]
F[25]	877.511	676.713	**671.374**	**671.710**	**671.038**	F[17]
P[26]	909.862	627.690	622.351	622.687	622.015	P[16]
V[27]	**942.885**	595.339	590.000	590.336	589.664	V[15]
G[28]	**961.892**	562.317	556.977	557.313	556.641	G[14]
R[29]	1013.926	543.309	537.970	538.306	537.634	R[13]
V[30]	**1046.948**	491.276	485.936	486.272	485.600	V[12]
H[31]	**1092.635**	458.253	452.913	453.249	452.577	H[11]
R[32]	**1144.668**	412.567	407.227	407.563	406.891	R[10]
L[33]	**1182.363**	**360.533**	355.193	355.529	354.857	L[9]
L[34]	1220.058	322.838	317.499	317.835	317.163	L[8]
R[35]	1272.092	285.144	279.804	280.140	279.468	R[7]
K[36]	1314.790	233.110	227.770	228.106	227.434	K[6]
G[37]	1333.797	190.412	185.072	185.408	184.736	G[5]
N[38]	1371.811	171.404	166.065	166.401	165.729	N[4]
Y[39]	1426.166	133.390	128.051	128.386	127.715	Y[3]
S[40]	1455.176	79.036	73.696	74.032	73.360	S[2]
E[41]	**1498.191**	50.025	44.685	45.021	44.349	E[1]

sp|Q8BFU2|H2A3_MOUSE

SGR $^{\text{Trimethyl}}_{42.05}$ GKQGGKARAKAKSRSSRAGLQFPVGRVHRLLRKGNYSE

- fragmentation table for charge state 4+
- specType=etd
- score=50.34
- F104939.dat
- query=q2804_p1
- precursor=749.761700
- chargePrecursor=6
- itol=0.8

AA	c	y	z+1	z+2	z	AA
S[1]	27.022	**1124.141**	**1120.136**	0.755	**1119.884**	S[41]
G[2]	41.277	1102.383	1098.378	0.755	1098.126	G[40]
R[3]	90.814	**1088.127**	1084.123	1084.375	1083.871	R[39]
G[4]	105.070	1038.590	**1034.586**	**1034.838**	**1034.334**	G[38]
K[5]	137.093	1024.335	**1020.330**	**1020.582**	**1020.078**	K[37]
Q[6]	169.108	992.311	**988.307**	**988.559**	**988.055**	Q[36]
G[7]	183.363	960.297	**956.292**	**956.544**	956.040	G[35]
G[8]	197.619	946.041	**942.037**	**942.289**	**941.785**	G[34]
K[9]	229.643	931.786	927.781	928.033	927.529	K[33]
A[10]	247.402	**899.762**	895.757	896.009	895.506	A[32]
R[11]	286.427	882.003	877.998	878.250	877.746	R[31]
A[12]	304.186	842.978	838.973	839.225	838.721	A[30]
K[13]	336.210	825.218	**821.214**	821.466	**820.962**	K[29]
A[14]	353.969	793.195	789.190	789.442	788.938	A[28]
K[15]	385.993	775.435	**771.431**	**771.683**	**771.179**	K[27]
S[16]	407.751	743.412	739.407	739.659	739.155	S[26]
R[17]	446.776	721.654	717.649	717.901	717.397	R[25]
S[18]	468.534	682.628	678.624	678.876	678.372	S[24]
[truncated: 346,243 more chars]
